# Decreased Steroid Hormone Receptor *NR4A2* Expression in Kawasaki Disease Before IVIG Treatment

**DOI:** 10.3389/fped.2019.00007

**Published:** 2019-02-04

**Authors:** Ying-Hsien Huang, Kuang-Den Chen, Mao-Hung Lo, Xin-Yuan Cai, Ho-Chang Kuo

**Affiliations:** ^1^Department of Pediatrics, Kaohsiung Chang Gung Memorial Hospital, Chang Gung University College of Medicine, Kaohsiung, Taiwan; ^2^Kawasaki Disease Center, Kaohsiung Chang Gung Memorial Hospital, Kaohsiung, Taiwan; ^3^Department of Surgery, Liver Transplantation Center, Institute for Translational Research in Biomedicine, Kaohsiung Chang Gung Memorial Hospital, Chang Gung University College of Medicine, Kaohsiung, Taiwan

**Keywords:** Kawasaki disease (KD), steroid receptor, Nr4a2, immunology, IVIG = intravenous immunoglobulin

## Abstract

Kawasaki disease (KD) is anacute febrile coronary vasculitis disease in children. In general, this disease can be treated with a single dose of 2 g/kg intravenous immunoglobulin (IVIG). However, the best timing for administering steroid treatment in acute-stage KD is still under debate. In this study, we recruited 174 participants to survey the transcript levels of steroid hormone receptors in KD patients. The chip studies consisted of 18 KD patients that were analyzed before IVIG treatment and at least 3 weeks after IVIG administration, as well as 36 control subjects, using GeneChip® HTA 2.0. Another cohort consisting of 120 subjects was analyzed to validate qRT-PCR. Our microarray study demonstrated significant downregulated expressions of the mRNA levels of NR1A2, RORA, NR4A1-3, THRA, and PPARD in KD patients in comparision to the controls. However, these genes increased considerably in KD patients after IVIG administration. After PCR validation, our data only revealed decreased NR4A2 mRNA expression in the KD patients compared to those of the controls, which increased after they received IVIG treatment. Our study is the first to report the potential effective utilization of steroid treatment in KD. Prior to IVIG treatment, decreased steroid receptors allowed for the reduced treatment role of steroids. However, after IVIG treatment, increased steroid receptors indicate that steroids are effective as a supplementary treatment for KD.

## Introduction

Kawasaki disease (KD), an acute inflammatory conronary vasculitis, has an unknown etiology but involves numerous organs and influences children <5 years old. In 1974, Dr. Tomisaki Kawasaki first published in English his observations on 50 cases of KD (1). KD presents with the following symptoms: sustained fever for more than 5 days, conjunctivitis without discharge, oral, and throat mucosal inflammatory change, non-suppurative lymphadenopathy, macular-papular erythematous skin rashes, and desquamation of the fingertips t (2). Furthermore, KD mainly affects the coronary arteries (2). The most important known complication of KD is coronary artery lesions (CAL) and coronary artery aneurysms (CAA) (3–5). Of all the untreated children, 20% develop sequelae of vasculitis with coronary artery aneurysm (6).

The generally accepted standard treatment of KD consists of high-dose intravenous immunoglobulin (IVIG) with a combination of high-dose aspirin, which can decrease CAA complications from 20–25% to 3–5% (3). Our research team has reported that, like other autoimmune diseases, KD was related to higher levels of interleukin (IL)-17A and IL-6 cytokine profiles (7). Although intravenous prednisolone has been extensively used to treat other autoimmune diseases suffered by children, the use of steroids for treating KD is still being debated. As reported by randomized trial studies of KD, when using corticosteroids in the beginning of treatment, the coronary dimensions and development of CAL did not differ (8–10). However, in addition to IVIG therapy, corticosteroids can quickly reduce serum cytokine levels and systemic inflammation in KD (9, 11). The combination of intravenous prednisolone plus a second course of IVIG therapy has been effective in patients of KD who were initially resistant to IVIG treatment alone (12, 13). In the current consensus, clinicians have postponed using steroid therapy in primary treatment but will often use it in the second course of IVIG administration or in IVIG-resistant KD patients. In fact, glucocorticoids belong to steroid hormones that interact with the DNA binding domain and initiate signal transduction after binding to steroid hormones (14). Although steroid hormones play a crucial role in inhibiting inflammation, the gene expressions of steroid hormones have not been thoroughly studied in KD patients. Therefore, in this study, we have comprehensively studied the mRNA expressions of steroid hormone receptors in different stages of KD patients and control subjects, as well as the outcome of the disease.

## Materials and Methods

### Patients

KD patients were required to meet the KD diagnosis criteria of the American Heart Association (15, 16) and had to be treated with a single dosage of IVIG treatment (2 g/kg) over 12 h in our hospital. In this study, we adopted Affymetrix GeneChip® Human Transcriptome Array 2.0 to quantify and compare the gene expressions of steroid hormone receptors (Supplementary Table 3) in 18 KD patients (both before and at least 3 weeks after IVIG administration), as well as in 18 healthy and 18 febrile controls. Then, we validated the mRNA levels of genes in a separate cohort (Supplementary Table 1) of 48 KD patients, 48 healthy controls, and 24 febrile subjects using real-time quantitative PCR. The subjects in the febrile controls had been diagnosed with acute pharyngitis, tonsillitis, bronchopneumonia, pneumonia, or urinary tract infection. We further collected peripheral blood samples from patients of KD both before receiving IVIG treatment (pre-IVIG) and then again at least 3 days after completing IVIG treatment, as previously described in another study (17, 18). CAL was identified through echocardiography and defined as previously described (19, 20). This study was ratified by the Institutional Review Board of Chang Gung Memorial Hospital, and we obtained written informed consent from the parents or guardians of all participants. The enrolled children could withdraw from the study at any time during the study period, and we anonymized all experimental results prior to analysis.

### Experiment Design

First, we collected whole blood samples from the participants, which were then subjected to white blood cell (WBC) enrichment, as described in one of our previous studies (21). We used an isolation kit (mirVana™ miRNA Isolation Kit, Catalog number: AM1560, Life Technologies, Carlsbad, CA) as the manufacturer's instructions, as previously described (22).

### Gene Expression Profiling With Microarray

To obtain strong and unbiased results, we developed pooled RNA libraries by evenly pooling six RNA samples to create three pooled healthy controls, three fever controls, three pre-IVIG, and three post-IVIG libraries, as previously described (18). We executed a microarray assay study to establish gene expression profiles with GeneChip® Human Transcriptome Array 2.0 (HTA 2.0, Affymetrix, Santa Clara). Following the Affymetrix instruction manual, the HTA 2.0 chips' raw data were subjected to quality control, as previously described (18).

### Real-Time Quantitative RT-PCR

To quantify mRNA levels of interest, we adopted the LightCycler® 480 Real-Time PCR System (Roche Molecular Systems, Inc. Indiana, USA) to perform qRT- PCR according to the manufacturer's instructions and performed PCR using a SYBR Green PCR Master Mix containing 10 μM with specific forward and reverse primers (Supplementary Table 2). The relative quantification of gene expressions was based on the comparative threshold cycle (C_T_) method, thus enabling us to determine the target amount as 2^−(Δ*CTtarget*−Δ*CTcalibrator*)^ or 2^−ΔΔ*CT*^ (23). We carried out all experiments twice to verify the efficiency of amplification.

### Statistical Analysis

All data are presented as mean ± standard error. Chips were evaluated with Partek (Partek, St. Louis), which is commercial software specifically designed to analyze microarray data. Either student's *t*-test, paired sample *t-*test or one-way ANOVA, as appropriate, was adopted to assess the quantitative data. We used SPSS version 12.0 for Windows XP (SPSS, Inc., Chicago, USA) for all statistical analyses. Two-sided *p* < 0.05 was considered statistically significant.

## Results

### Significantly Altered mRNA Expressions of Steroid Hormone Receptors in KD Patients and Controls as Well as Changes Following IVIG Treatment

**Figure 1:**
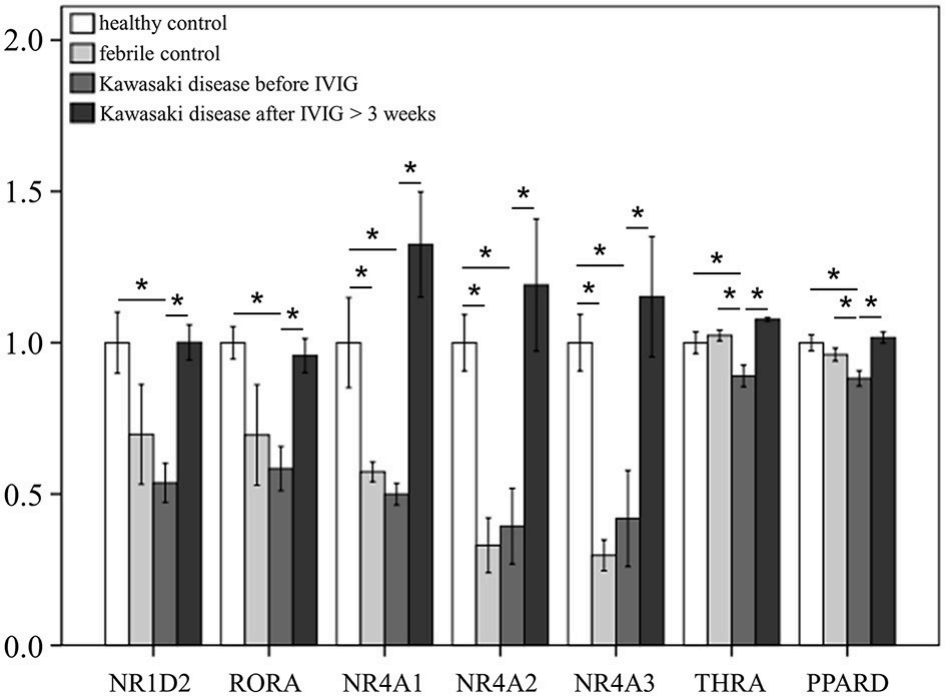
mRNA expressions of steroid hormone receptors by GeneChip® Human Transcriptome Array 2.0 between patients of Kawasaki disease (KD) and control subjects. Data are expressed as mean ± standard error for the three replications. ^*^Significance (*p* < 0.05).

We surveyed 57 steroid hormone receptors in 174 cases, including 66 KD patients, 42 fever controls, and 66 non-fever controls. At the start of this study, we employed the GeneChip® Human Transcriptome Array 2.0 (Data Sheet 1) to determine the gene expression profiling of steroid hormone receptors in both KD patients and control subjects and all data from the microarray was submitted to NCBI GEO (please refer to accession number GSE109351). We further conducted GO analysis on the genes that were downregulated in KD and found that the steroid hormone receptor pathway was significant. (Enrichment Score = 12.1597, *p* = 0.00000523719). As shown in Supplementary Table 3 and Figure 1, we observed differential expressions of NR1A2, RORA, and NR4A1-3 in KD patients in comparison to both the febrile and healthy controls. The mRNA levels of NR1A2, RORA, NR4A1-3, THRA, and PPARD were significantly lower in acute-stage KD patients than in the healthy controls (*p* < 0.05). These gene expression levels significantly increased in KD patients after they underwent IVIG treatment (KD3 vs. KD1 in Supplementary Table 3). Nevertheless, we found no significant differences in these genes between KD patients and the febrile controls.

### Significantly Decreased NR4A2 Expressions in the WBCs of KD Patients and Controls

**Figure 2:**
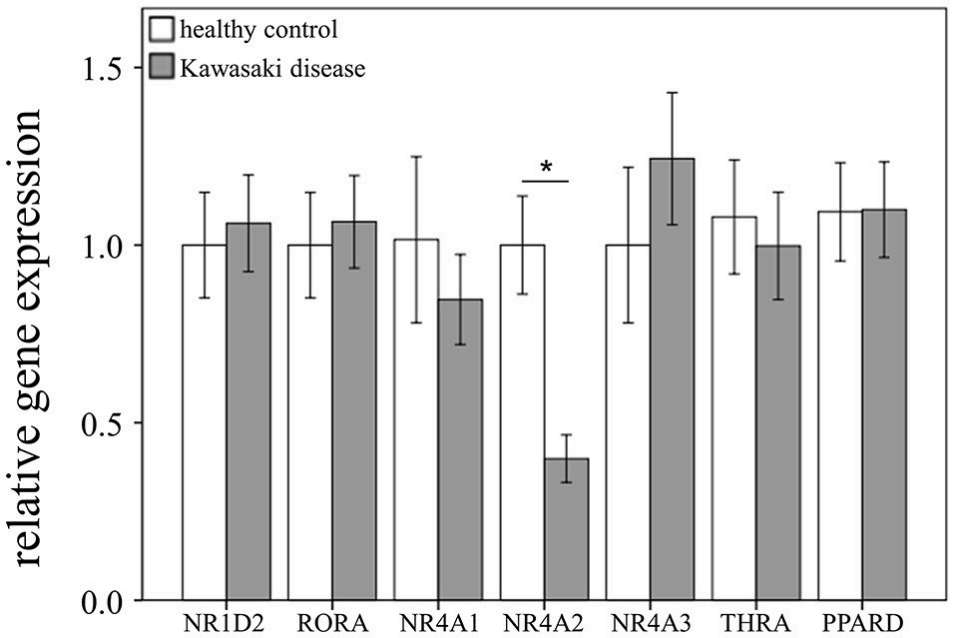
Analyses of mRNA expression of steroid hormone receptors in the peripheral white blood cells of 48 patients with KD and 48 healthy controls using qRT-PCR. Data are expressed as mean ± standard error. ^*^Significance (*p* < 0.05) between the groups.

**Figure 3:**
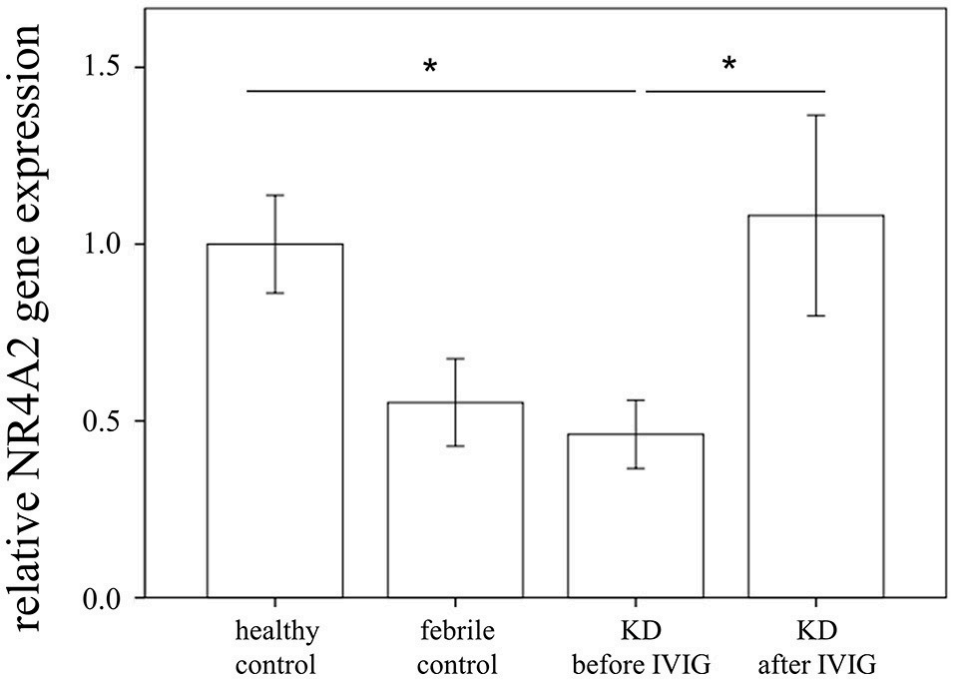
Analyses of NR4A2 mRNA in the peripheral white blood cells of 48 patients with KD before and after intravenous immunoglobin administration, as well as 48 healthy and 24 febrile controls using qRT-PCR. Data are expressed as mean ± standard error. ^*^Significance (*p* < 0.05) between the groups.

**Figure 4:**
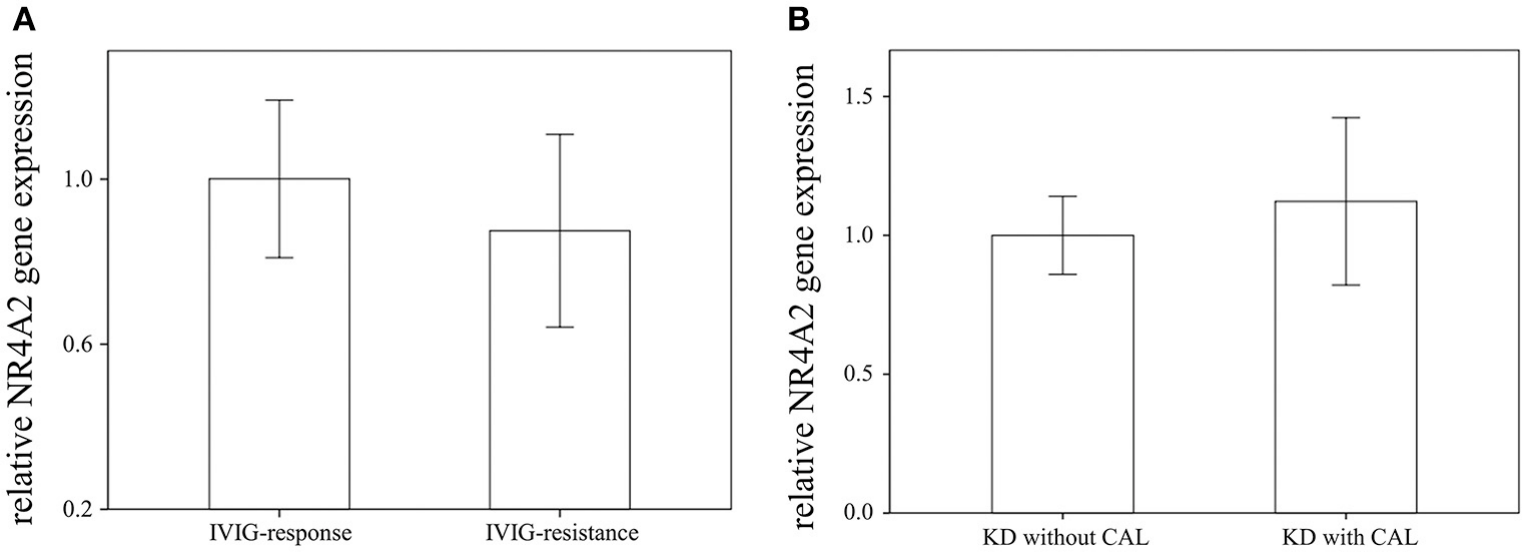
Comparison of NR4A2 mRNA in KD patients **(A)** with intravenous immunoglobulin (IVIG) (*n* = 6) resistance and without (*n* = 42), as **(B)** well as with (*n* = 28) without (*n* = 20) coronary artery lesion (CAL). Data are presented as mean ± standard error.

Through qRTPCR, we examined the mRNA levels of NR1A2, RORA, NR4A1-3, THRA, and PPARD in a separate cohort of 48 KD patients and 48 healthy controls. Our data showed decreased NR4A2 mRNA levels (*p* < 0.001) in KD patients in comparison to those of the healthy control subjects (Figure 2). The mRNA level of NR4A2 also increased following IVIG treatment (*p* = 0.018) (Figure 3). However, we observed no remarkable difference in the NR4A2 mRNA levels between patients of KD with IVIG resistance or CAL and those who did not (*p* = 0.73 and *p* = 0.29, respectively) (Figure 4).

## Discussion

To our knowledge, we were the first to perform a comprehensive survey of transcripts of 57 steroid hormone receptors between KD patients and control subjects. Our important findings include that KD patients demonstrated a differential expression of NR1A2, RORA, NR4A1-3, THRA, and PPARD when compared to control subjects. The mRNA levels of NR1A2, RORA, NR4A1-3, THRA, and PPARD were significantly decreased in acute-stage KD than in the healthy controls. These gene expression values significantly increased in KD patients who were administered IVIG. However, we found no remarkable differences in these genes between acute-stage KD patients and febrile controls. In addition, the expression of steroid hormone receptors demonstrated no difference between KD patients and fever controls, thus indicating that steroids may have no treatment role in children with fever from infectious diseases and acute-stage KD prior to IVIG treatment. While the expression of relative steroid hormone receptors was lower in the fever controls than in the healthy controls, it did not reach statistical significance (Figure 3).

Performing qRT-PCR, we also found decreased NR4A2 mRNA levels in the KD patients compared to the controls, which increased following IVIG treatment, thus agreeing with the results of Transcriptome Array 2.0. Regarding nuclear steroid receptors, the orphan NR4A subfamily, which includes NR4A1, NR4A2, and NR4A3, has recently been investigated as a therapeutic target for treating inflammatory diseases (24). Although the underlying mechanisms remain unclear, a negative feedback mechanism that maintains inflammatory homeostasis is involved (24). In one recent study, the NR4A subfamily was shown to regulate the development of T-regulatory cells by activating Foxp3 (25). Interestingly, the overexpression of NR4A2 inhibited the proliferation of vascular smooth muscle cells, while NR4A2 silencing enhanced their growth (26).

Even though administering a single high dose of IVIG plus aspirin is standard for treating children with KD, 10–30% of patients continue to have persistent or recurrent fever after the first course of IVIG treatment (4). Such IVIG non-responders are at a higher risk for the development of CAL and may require supplemental or alternative anti-inflammation therapy (27). In the comparison of intravenous methylprednisolone (IVMP) combined with IVIG and IVIG therapy alone, it was found to not significantly improve KD outcome in primary treatment for patients (28). Although IVMP seems not to have beneficial value in primary treatment and the mechanism remains unknown, IVMP therapy appears to benefit IVIG-resistant KD patients (29). A study from Japan, known as the Randomized controlled trial to Assess Immunoglobulin plus Steroid Efficacy for KD (RAISE), found that prednisolone with initial IVIG can reduce the prevalence of CAL formation (13, 27). Moderate evidence has indicated that using steroids in primary treatment for KD may be associated with a decreased duration of clinical symptoms, length of hospital stay, and improvement regarding the development of CAL (30).

Discovered in 1967 in Japan, the first report on KD was published in English in 1974. IVIG has been used to treat KD since 1983 (31). Prior to establishing IVIG treatment, many other therapies, including steroids, antibiotics, and anti-inflammation medications, were prescribed for KD patients but did not demonstrate significant effectiveness in reducing the formation of aneurysm. Only high-dose IVIG treatment can significantly reduce the aneurysm formation rate from 25 to 3–5%. Overall, steroids only have a treatment role in KD when combined with IVIG or after IVIG treatment but not when administered without IVIG. Our study suggests that this finding might due to decreased steroid receptor expression in KD prior to IVIG treatment. This study has some limitations. First, all of our KD patients belonged to the Asian population, so this result would need to be investigated in other populations with KD. Secondly, the febrile controls were children with infectious diseases, and the febrile control results need further investigation besides infectious disease. Finally, this study still needs more cases before a final conclusion can be reached about steroid receptors in KD or fever controls.

## Conclusion

This report is the first to explore the effectiveness of steroid treatment in KD. Prior to IVIG treatment, decreased steroid receptors reduced the treatment role of steroids. Following IVIG treatment, increased steroid receptors make steroids effective as a supplementary treatment for KD in regard to preventing coronary artery lesions.

## Author Contributions

Y-HH and H-CK conceptualized and designed the study, drafted the initial manuscript, and reviewed and revised the manuscript. X-YC, M-HL, and K-DC designed the data collection instruments, collected data, carried out the initial analyses, and reviewed and revised the manuscript. H-CK conceptualized and designed the study, coordinated and supervised data collection, and critically reviewed the manuscript for important intellectual content. All authors approved the final manuscript as submitted and agree to be accountable for all aspects of the work.

### Conflict of Interest Statement

The authors declare that the research was conducted in the absence of any commercial or financial relationships that could be construed as a potential conflict of interest.
