# Supplementary material for: Decreased Steroid Hormone Receptor NR4A2 Expression in Kawasaki Disease Before IVIG Treatment
Source: Front Pediatr. 2019 Feb 4;7:7. doi: 10.3389/fped.2019.00007 (PMC6369254; doi:10.3389/fped.2019.00007)
Supplement: Supplementary file 1 [file Table_1.docx]

Supplementary Table 1

Basal characteristics of patients with KD and control subjects

| Characteristic | Healthy controls  (n=48) | Febrile controls  (n=24) | Patients with KD  (n=48) |
| --- | --- | --- | --- |
| Male gender, n (%) | 32 (66.7) | 9 (37.5) | 37 (77.1) |
| Mean (SD), age (y) | 6.7±4.5 | 2.9±1.2 | 2.1±3 |
| Age range (y) | 0-19 | 0-5 | 0-18 |
| CAL formation |  |  | 28 (58.3%) |
| IVIG resistance |  |  | 6 (12.5%) |

CAL, coronary artery lesion; IVIG, intravenous immunoglobulin; KD, Kawasaki disease.
