# Supplementary material for: Decreased Steroid Hormone Receptor NR4A2 Expression in Kawasaki Disease Before IVIG Treatment
Source: Front Pediatr. 2019 Feb 4;7:7. doi: 10.3389/fped.2019.00007 (PMC6369254; doi:10.3389/fped.2019.00007)
Supplement: Supplementary file 2 [file Table_2.docx]

Supplementary Table 2

| Gene symbol | Accession number | Hybridization | Primers (5’ to 3’) |
| --- | --- | --- | --- |
| RNA18S5 | NR_003286.2 | forward | GTAACCCGTTGAACCCCATT |
|  |  | reverse | CCATCCAATCGGTAGTAGCG |
| NR1D2 | NM_001145425.1 | forward  reverse | GAGGAGGTGTGATTGCCTATATC  AGGAGGAGGACTGGAAACTAT |
| RORA | NM_002943.3 | forward | TTTGGTTGTTCTTTATAGTGTGGAT |
|  |  | reverse | TAGTAGCCTAGAAGCGGT |
| NR4A1 | NM_001202233.1 | forward | GTACATCTGCCTGGCTAAC |
|  |  | reverse | CAGGGAAGTGAGGAGATTG |
| NR4A2 | NM_006186.3 | forward | GTTACCACTCTTCGGGAGAATAC |
|  |  | reverse | AGAAGTGGTGGCAGTGATTT |
| NR4A3 | NM_006981.3 | forward | GAGGCAGTCATGTTAGCAA |
|  |  | reverse | TAATCCAGTTTATAATCTGATCCACTT |
| THRA | NM_001190918 | forward  reverse | CTCTGCCTGGCAACATCTTA  CCAAGGAAGCACAGACAACTA |
| PPARD | NM_006238 | forward | CCGCAAACCCTTCAGTGATA |
|  |  | reverse | GAATGATGGCCGCAATGAATAG |
