# Supplementary material for: Decreased Steroid Hormone Receptor NR4A2 Expression in Kawasaki Disease Before IVIG Treatment
Source: Front Pediatr. 2019 Feb 4;7:7. doi: 10.3389/fped.2019.00007 (PMC6369254; doi:10.3389/fped.2019.00007)
Supplement: Supplementary file 3 [file Table_3.doc]

Supplementary Table 3: Transcripts expressions of hormone receptors between Kawasaki disease patients and control subjects

| **Symbol** | **Annotated Term** | **RefSeq** | **Fold-Change**  **(KD1 vs. HC)** | **p value**  **(KD1 vs. HC)** | **Fold-Change**  **(KD1 vs. FC)** | **p value**  **(KD1 vs. FC)** | **Fold-Change**  **(KD3 vs. KD1)** | **p value**  **(KD3 vs. KD1)** |
| --- | --- | --- | --- | --- | --- | --- | --- | --- |
| ABHD2 | steroid hormone receptor activity | NM_007011 | 1.321 | 0.071 | 1.453 | 0.024* | -1.170 | 0.275 |
| AR | androgen receptor activity | NM_000044 | 1.030 | 0.525 | 1.012 | 0.789 | 1.024 | 0.617 |
| ESR1 | estrogen receptor activity | NM_000125 | 1.008 | 0.881 | -1.066 | 0.272 | 1.042 | 0.469 |
|  | RNA polymerase II transcription factor activity, estrogen-activated sequence-specific DNA binding |  |  |  |  |  |  |  |
|  | steroid hormone receptor activity |  |  |  |  |  |  |  |
| ESR2 | estrogen receptor activity | NM_001040275 | 1.033 | 0.478 | -1.018 | 0.692 | -1.000 | 0.991 |
|  | RNA polymerase II transcription factor activity, estrogen-activated sequence-specific DNA binding |  |  |  |  |  |  |  |
|  | steroid hormone receptor activity |  |  |  |  |  |  |  |
| ESRRA | steroid hormone receptor activity | NM_004451 | 1.041 | 0.606 | -1.069 | 0.403 | 1.007 | 0.927 |
| ESRRB | steroid hormone receptor activity | NM_004452 | -1.037 | 0.705 | -1.003 | 0.973 | 1.120 | 0.257 |
| ESRRG | steroid hormone receptor activity | NM_001134285 | 1.008 | 0.892 | -1.080 | 0.189 | 1.035 | 0.542 |
| GPER1 | estrogen receptor activity | NM_001039966 | 1.039 | 0.725 | -1.086 | 0.460 | 1.007 | 0.951 |
|  | mineralocorticoid receptor activity |  |  |  |  |  |  |  |
|  | steroid hormone receptor activity |  |  |  |  |  |  |  |
| HNF4A | steroid hormone receptor activity | NM_178849 | -1.033 | 0.688 | -1.053 | 0.526 | 1.051 | 0.546 |
| HNF4G | steroid hormone receptor activity | NM_004133 | -1.005 | 0.915 | -1.065 | 0.172 | 1.068 | 0.156 |
| LEF1 | estrogen receptor activity | NM_001130714 | -1.613 | 0.007* | -1.255 | 0.121 | 1.584 | 0.008* |
| NKX3-1 | androgen receptor activity | NM_001256339 | 1.054 | 0.630 | 1.050 | 0.657 | -1.039 | 0.728 |
|  | estrogen receptor activity |  |  |  |  |  |  |  |
| NR0B1 | steroid hormone receptor activity | NM_000475 | 1.065 | 0.492 | -1.059 | 0.532 | 1.001 | 0.989 |
| NR0B2 | steroid hormone receptor activity | NM_021969 | -1.125 | 0.209 | -1.078 | 0.412 | 1.236 | 0.040* |
| **NR1D1** | **steroid hormone receptor activity** | **NM_021724** | **-1.292** | **0.001*** | **-1.268** | **0.002*** | **1.338** | **0.001*** |
| NR1D2 | steroid hormone receptor activity | NM_001145425 | -1.873 | 0.013* | -1.254 | 0.284 | 1.887 | 0.012* |
| NR1H2 | steroid hormone receptor activity | NM_007121 | 1.030 | 0.674 | -1.068 | 0.354 | 1.008 | 0.910 |
| NR1H3 | steroid hormone receptor activity | NM_001130101 | -1.028 | 0.738 | -1.039 | 0.647 | 1.076 | 0.389 |
| NR1H4 | steroid hormone receptor activity | NM_001206977 | 1.009 | 0.860 | -1.023 | 0.674 | 1.038 | 0.487 |
| NR1I2 | steroid hormone receptor activity | NM_022002 | -1.016 | 0.830 | -1.083 | 0.304 | 1.067 | 0.397 |
| NR1I3 | steroid hormone receptor activity | NM_001077482 | 1.031 | 0.694 | 1.042 | 0.598 | 1.048 | 0.552 |
| NR2C1 | steroid hormone receptor activity | NM_001032287 | -1.273 | 0.189 | -1.075 | 0.677 | 1.147 | 0.438 |
| NR2C2 | steroid hormone receptor activity | NM_003298 | 1.027 | 0.729 | -1.018 | 0.814 | -1.016 | 0.841 |
| NR2E1 | steroid hormone receptor activity | NM_003269 | 1.175 | 0.124 | 1.052 | 0.600 | -1.070 | 0.491 |
| NR2E3 | steroid hormone receptor activity | NM_014249 | -1.020 | 0.869 | -1.030 | 0.809 | 1.082 | 0.516 |
| NR2F1 | steroid hormone receptor activity | NM_005654 | -1.010 | 0.936 | -1.026 | 0.840 | 1.078 | 0.567 |
| NR2F2 | steroid hormone receptor activity | NM_001145156 | 1.009 | 0.912 | -1.092 | 0.275 | 1.046 | 0.568 |
| NR2F6 | steroid hormone receptor activity | NM_005234 | -1.034 | 0.763 | -1.135 | 0.269 | 1.003 | 0.978 |
| NR3C1 | glucocorticoid receptor activity | NM_001018077 | -1.037 | 0.679 | 1.123 | 0.205 | -1.005 | 0.951 |
|  | RNA polymerase II transcription factor activity, glucocorticoid-activated sequence-specific DNA binding |  |  |  |  |  |  |  |
|  | steroid hormone receptor activity |  |  |  |  |  |  |  |
| NR3C2 | mineralocorticoid receptor activity | NM_000901 | -1.299 | 0.009* | -1.128 | 0.154 | 1.274 | 0.013* |
|  | steroid hormone receptor activity |  |  |  |  |  |  |  |
| **NR4A1** | **steroid hormone receptor activity** | **NM_001202233** | **-1.961** | **0.003*** | **-1.149** | **0.418** | **2.617** | **0.000*** |
| **NR4A2** | **steroid hormone receptor activity** | **NM_006186** | **-2.801** | **0.013*** | **1.147** | **0.684** | **3.260** | **0.007*** |
| **NR4A3** | **steroid hormone receptor activity** | **NM_173199** | **-2.712** | **0.013*** | **1.262** | **0.479** | **3.055** | **0.007*** |
| NR5A1 | steroid hormone receptor activity | NM_004959 | -1.009 | 0.939 | 1.010 | 0.934 | 1.088 | 0.486 |
| NR5A2 | steroid hormone receptor activity | NM_003822 | 1.030 | 0.556 | -1.041 | 0.430 | 1.007 | 0.884 |
| NR6A1 | steroid hormone receptor activity | NM_001489 | 1.264 | 0.012* | 1.170 | 0.064 | -1.204 | 0.034* |
| OR51E2 | steroid hormone receptor activity | NM_030774 | 1.010 | 0.911 | -1.085 | 0.351 | 1.037 | 0.672 |
| PAQR5 | steroid hormone receptor activity | NM_001104554 | -1.049 | 0.461 | -1.013 | 0.840 | 1.137 | 0.071 |
| PAQR6 | steroid hormone receptor activity | NM_198406 | 1.041 | 0.367 | 1.008 | 0.865 | -1.014 | 0.745 |
| PAQR7 | steroid hormone receptor activity | NM_178422 | -1.040 | 0.703 | -1.185 | 0.122 | 1.088 | 0.414 |
| PAQR8 | steroid hormone receptor activity | NM_133367 | 1.038 | 0.476 | -1.027 | 0.607 | -1.103 | 0.085 |
| PAQR9 | steroid hormone receptor activity | NM_198504 | 1.074 | 0.117 | -1.041 | 0.354 | 1.023 | 0.599 |
| PGR | steroid hormone receptor activity | NM_000926 | 1.013 | 0.788 | -1.069 | 0.183 | 1.061 | 0.232 |
| PPARA | steroid hormone receptor activity | NM_001001928 | -1.005 | 0.837 | -1.028 | 0.279 | 1.036 | 0.170 |
| PPARD | steroid hormone receptor activity | NM_006238 | -1.090 | 0.037* | -1.134 | 0.006* | 1.153 | 0.003* |
| PPARG | steroid hormone receptor activity | NM_005037 | 1.197 | 0.004* | 1.106 | 0.059 | -1.132 | 0.027* |
| RARA | steroid hormone receptor activity | NM_000964 | 1.243 | 0.091 | 1.202 | 0.141 | -1.165 | 0.214 |
| RARB | steroid hormone receptor activity | NM_000965 | 1.061 | 0.255 | -1.056 | 0.289 | 1.007 | 0.891 |
| RARG | steroid hormone receptor activity | NM_000966 | -1.010 | 0.883 | -1.083 | 0.276 | 1.062 | 0.406 |
| **RORA** | **steroid hormone receptor activity** | **NM_002943** | **-1.734** | **0.020*** | **-1.148** | **0.488** | **1.659** | **0.029*** |
| RORB | steroid hormone receptor activity | NM_006914 | 1.005 | 0.913 | -1.065 | 0.164 | 1.019 | 0.665 |
| RORC | steroid hormone receptor activity | NM_001001523 | -1.072 | 0.466 | -1.147 | 0.168 | 1.122 | 0.237 |
| RXRA | steroid hormone receptor activity | NM_002957 | 1.210 | 0.018* | 1.019 | 0.777 | -1.207 | 0.019* |
| RXRB | steroid hormone receptor activity | NM_021976 | -1.018 | 0.744 | -1.093 | 0.136 | 1.015 | 0.789 |
| RXRG | steroid hormone receptor activity | NM_006917 | 1.006 | 0.926 | -1.019 | 0.777 | 1.115 | 0.128 |
| THRA | steroid hormone receptor activity | NM_001190918 | -1.152 | 0.008* | -1.124 | 0.019* | 1.212 | 0.001* |
| THRB | steroid hormone receptor activity | NM_000461 | 1.045 | 0.392 | -1.065 | 0.237 | 1.008 | 0.872 |
| VDR | steroid hormone receptor activity | NM_000376 | 1.183 | 0.152 | 1.022 | 0.840 | -1.068 | 0.554 |

KD1: Kawasaki disease before IVIG treatment; KD3: Kawasaki disease > 3 weeks after IVIG treatment; FC: febrile control; HC: healthy control.
