# Supplementary material for: Decreased Steroid Hormone Receptor NR4A2 Expression in Kawasaki Disease Before IVIG Treatment
Source: Front Pediatr. 2019 Feb 4;7:7. doi: 10.3389/fped.2019.00007 (PMC6369254; doi:10.3389/fped.2019.00007)
Supplement: Supplementary file 4 [file Data_Sheet_1.PDF]

ID	Gene Symbol	febrile control (mean±SD)	healthy control (mean±SD)	Kawasaki disease before IVIG (mean±SD)	Kawasaki disease after IVIG>3 weeks (mean±SD)
TC01001820.hg.1	43160	7.78 ± 0.06	7.38 ± 0.09	7.93 ± 0.29	7.22 ± 0.08
TC04001700.hg.1	43160	7.79 ± 0.11	7.68 ± 0.12	7.83 ± 0.16	7.57 ± 0.05
TC01001819.hg.1	43161	6.25 ± 0.20	6.22 ± 0.11	6.23 ± 0.10	6.27 ± 0.02
TC19000150.hg.1	43161	9.14 ± 0.12	8.72 ± 0.06	9.17 ± 0.27	8.79 ± 0.11
TC05001746.hg.1	43162	7.78 ± 0.05	7.78 ± 0.01	7.72 ± 0.03	7.86 ± 0.02
TC02002754.hg.1	43163	7.55 ± 0.19	7.50 ± 0.19	7.45 ± 0.11	7.61 ± 0.09
TC10000657.hg.1	43164	8.27 ± 0.18	8.18 ± 0.18	8.40 ± 0.30	8.12 ± 0.13
TC05000074.hg.1	43165	9.37 ± 0.42	9.64 ± 0.19	9.45 ± 0.35	9.69 ± 0.05
TC02000967.hg.1	43166	9.53 ± 0.57	9.92 ± 0.33	10.11 ± 0.50	9.86 ± 0.23
TC10001224.hg.1	43167	8.84 ± 0.03	8.87 ± 0.11	8.73 ± 0.16	8.84 ± 0.05
TC12000543.hg.1	43168	8.09 ± 0.11	8.01 ± 0.05	7.84 ± 0.13	8.00 ± 0.02
TC17001778.hg.1	43169	6.32 ± 0.17	6.35 ± 0.16	6.32 ± 0.10	6.38 ± 0.01
TC05001179.hg.1	43170	6.23 ± 0.23	6.15 ± 0.10	6.17 ± 0.10	6.29 ± 0.15
TC16001029.hg.1	43344	10.64 ± 0.10	10.57 ± 0.10	10.22 ± 0.10	10.48 ± 0.03
TC02001477.hg.1	43345	9.27 ± 0.09	9.32 ± 0.01	9.30 ± 0.06	9.37 ± 0.06
TC22000350.hg.1	43346	7.17 ± 0.19	7.13 ± 0.10	7.10 ± 0.16	7.18 ± 0.02
TC17001735.hg.1	43347	7.51 ± 0.34	7.27 ± 0.15	7.46 ± 0.29	7.26 ± 0.09
TC0X001312.hg.1	43349	10.29 ± 0.10	10.33 ± 0.04	9.99 ± 0.13	10.26 ± 0.06
TC07000228.hg.1	43350	8.18 ± 0.51	8.59 ± 0.12	8.38 ± 0.37	8.51 ± 0.08
TC05001772.hg.1	43351	7.13 ± 0.11	7.08 ± 0.13	7.03 ± 0.12	7.14 ± 0.04
TC17000885.hg.1	43352	9.51 ± 0.09	9.52 ± 0.06	9.26 ± 0.11	9.50 ± 0.03
TC02002187.hg.1	43353	6.00 ± 0.11	5.93 ± 0.04	6.01 ± 0.03	6.01 ± 0.02
TC04000430.hg.1	43354	7.64 ± 0.25	7.58 ± 0.11	7.60 ± 0.11	7.51 ± 0.04
TC16000835.hg.1	43355	7.22 ± 0.15	7.19 ± 0.18	7.15 ± 0.20	7.31 ± 0.03
TC07001405.hg.1	43357	5.37 ± 0.12	5.26 ± 0.01	5.39 ± 0.09	5.25 ± 0.03
TC01002836.hg.1	43358	9.61 ± 0.22	9.82 ± 0.17	9.71 ± 0.18	9.66 ± 0.04

TC09000588.hg.1	43435	6.13 ± 0.11	6.06 ± 0.10	6.05 ± 0.08	6.18 ± 0.08
TC19002723.hg.1	A1BG	8.20 ± 0.18	8.23 ± 0.15	8.17 ± 0.12	8.34 ± 0.07
TC19000960.hg.1	A1BG-AS1	7.77 ± 0.25	7.74 ± 0.19	7.76 ± 0.13	7.81 ± 0.12
TC10001292.hg.1	A1CF	5.30 ± 0.09	5.22 ± 0.10	5.25 ± 0.11	5.29 ± 0.08
TC12001187.hg.1	A2M	5.88 ± 0.09	5.85 ± 0.10	5.80 ± 0.06	5.83 ± 0.07
TC12000139.hg.1	A2M-AS1	7.60 ± 0.20	7.81 ± 0.08	7.55 ± 0.21	7.74 ± 0.06
TC12000136.hg.1	A2ML1	5.83 ± 0.18	5.76 ± 0.09	5.75 ± 0.12	5.83 ± 0.04
TC12001182.hg.1	A2ML1-AS1	4.28 ± 0.20	4.04 ± 0.09	4.24 ± 0.21	4.11 ± 0.11
TC12001183.hg.1	A2ML1-AS2	7.87 ± 0.14	7.81 ± 0.07	7.80 ± 0.03	7.89 ± 0.06
TC12001189.hg.1	A2MP1	5.40 ± 0.20	5.21 ± 0.07	5.28 ± 0.10	5.29 ± 0.17
TC12001191.hg.1	A2MP1	4.41 ± 0.11	4.20 ± 0.04	4.28 ± 0.07	4.22 ± 0.07
TC22000852.hg.1	A4GALT	7.92 ± 0.27	7.93 ± 0.24	7.90 ± 0.23	8.05 ± 0.03
TC03001820.hg.1	A4GNT	6.19 ± 0.08	6.12 ± 0.02	6.09 ± 0.06	6.06 ± 0.04
TC17001356.hg.1	AA06	5.47 ± 0.11	5.29 ± 0.17	5.48 ± 0.08	5.33 ± 0.13
TC12001547.hg.1	AAAS	8.78 ± 0.13	8.53 ± 0.06	8.47 ± 0.07	8.34 ± 0.06
TC12001003.hg.1	AACS	7.45 ± 0.13	7.42 ± 0.08	7.39 ± 0.09	7.46 ± 0.04
TC05002127.hg.1	AACSP1	7.26 ± 0.22	7.23 ± 0.10	7.18 ± 0.10	7.28 ± 0.04
TC03000830.hg.1	AADAC	5.01 ± 0.12	4.92 ± 0.03	4.98 ± 0.14	4.90 ± 0.08
TC03000827.hg.1	AADACL2	5.46 ± 0.09	5.29 ± 0.07	5.39 ± 0.08	5.38 ± 0.02
TC01000160.hg.1	AADACL3	6.43 ± 0.23	6.31 ± 0.17	6.18 ± 0.30	6.35 ± 0.07
TC01000159.hg.1	AADACL4	5.52 ± 0.22	5.70 ± 0.24	5.65 ± 0.15	5.90 ± 0.13
TC04001730.hg.1	AADAT	5.90 ± 0.18	5.84 ± 0.12	5.82 ± 0.09	5.88 ± 0.03
TC09001377.hg.1	AAED1	7.95 ± 0.02	7.95 ± 0.12	7.88 ± 0.15	7.94 ± 0.17
TC15001587.hg.1	AAGAB	7.76 ± 0.06	7.62 ± 0.10	7.62 ± 0.13	7.50 ± 0.05
TC02001950.hg.1	AAK1	9.55 ± 0.31	9.72 ± 0.05	9.39 ± 0.31	9.67 ± 0.11
TC11000832.hg.1	AAMDC	6.99 ± 0.12	6.94 ± 0.06	6.97 ± 0.04	6.99 ± 0.04
TC02002769.hg.1	AAMP	8.28 ± 0.12	8.19 ± 0.08	8.11 ± 0.17	8.18 ± 0.02
TC17000873.hg.1	AANAT	7.54 ± 0.17	7.42 ± 0.13	7.41 ± 0.13	7.48 ± 0.06
TC20000266.hg.1	AAR2	7.26 ± 0.29	7.31 ± 0.07	7.09 ± 0.20	7.27 ± 0.02
TC08000684.hg.1	AARD	8.00 ± 0.25	7.94 ± 0.21	7.92 ± 0.20	8.05 ± 0.04
TC16001234.hg.1	AARS	8.78 ± 0.25	8.31 ± 0.05	8.31 ± 0.25	8.03 ± 0.13

TC06001764.hg.1	AARS2	8.04 ± 0.11	8.00 ± 0.10	7.83 ± 0.17	8.07 ± 0.04
TC04001219.hg.1	AASDH	6.87 ± 0.30	6.87 ± 0.22	6.84 ± 0.28	6.66 ± 0.08
TC11000970.hg.1	AASDHPPT	7.91 ± 0.28	7.95 ± 0.09	7.95 ± 0.29	7.78 ± 0.15
TC07001811.hg.1	AASS	5.67 ± 0.09	5.67 ± 0.03	5.62 ± 0.05	5.66 ± 0.13
TC17000444.hg.1	AATF	9.98 ± 0.24	9.96 ± 0.05	9.94 ± 0.09	9.80 ± 0.22
TC17001941.hg.1	AATK	8.73 ± 0.24	8.60 ± 0.15	8.80 ± 0.13	8.69 ± 0.09
TC17000926.hg.1	AATK-AS1	8.33 ± 0.16	8.28 ± 0.21	8.29 ± 0.20	8.36 ± 0.02
TC16000148.hg.1	ABAT	8.28 ± 0.13	8.05 ± 0.06	8.34 ± 0.01	8.03 ± 0.03
TC09001434.hg.1	ABCA1	8.48 ± 0.48	8.08 ± 0.10	8.78 ± 0.25	8.13 ± 0.08
TC17001829.hg.1	ABCA10	4.92 ± 0.08	4.85 ± 0.02	4.89 ± 0.05	4.88 ± 0.03
TC02002746.hg.1	ABCA12	4.82 ± 0.08	4.74 ± 0.10	4.77 ± 0.04	4.80 ± 0.05
TC07000308.hg.1	ABCA13	6.25 ± 0.23	5.84 ± 0.03	6.37 ± 0.32	5.90 ± 0.04
TC16000071.hg.1	ABCA17P	6.14 ± 0.16	6.09 ± 0.07	6.02 ± 0.06	6.13 ± 0.06
TC09001749.hg.1	ABCA2	8.89 ± 0.04	8.85 ± 0.12	8.97 ± 0.05	8.85 ± 0.06
TC16000783.hg.1	ABCA3	7.83 ± 0.13	7.78 ± 0.15	7.76 ± 0.19	7.86 ± 0.03
TC01002883.hg.1	ABCA4	6.45 ± 0.15	6.41 ± 0.13	6.39 ± 0.10	6.50 ± 0.06
TC17001830.hg.1	ABCA5	6.37 ± 0.58	6.83 ± 0.32	6.44 ± 0.46	6.69 ± 0.18
TC17001827.hg.1	ABCA6	4.60 ± 0.11	4.50 ± 0.02	4.51 ± 0.05	4.54 ± 0.08
TC19000028.hg.1	ABCA7	9.02 ± 0.06	8.93 ± 0.03	8.99 ± 0.07	8.86 ± 0.06
TC17001825.hg.1	ABCA8	4.70 ± 0.13	4.60 ± 0.05	4.64 ± 0.03	4.63 ± 0.03
TC17001826.hg.1	ABCA9	4.83 ± 0.10	4.69 ± 0.06	4.78 ± 0.05	4.74 ± 0.05
TC17000805.hg.1	ABCA9-AS1	5.63 ± 0.15	5.57 ± 0.16	5.47 ± 0.14	5.64 ± 0.20
TC07001579.hg.1	ABCB1	6.40 ± 0.25	6.45 ± 0.10	6.12 ± 0.07	6.44 ± 0.05
TC01003949.hg.1	ABCB10	7.99 ± 0.14	8.03 ± 0.10	7.94 ± 0.20	7.94 ± 0.08
TC02002508.hg.1	ABCB11	5.49 ± 0.10	5.41 ± 0.08	5.47 ± 0.07	5.50 ± 0.06
TC07001578.hg.1	ABCB4	6.28 ± 0.13	6.44 ± 0.11	6.22 ± 0.05	6.37 ± 0.03
TC07000127.hg.1	ABCB5	4.41 ± 0.09	4.28 ± 0.04	4.35 ± 0.04	4.33 ± 0.02
TC02005056.hg.1	ABCB6	7.63 ± 0.16	7.48 ± 0.11	7.45 ± 0.14	7.53 ± 0.03
TC0X001164.hg.1	ABCB7	8.04 ± 0.18	8.14 ± 0.22	7.77 ± 0.25	7.84 ± 0.08
TC07001010.hg.1	ABCB8	8.13 ± 0.14	8.05 ± 0.11	8.00 ± 0.12	8.10 ± 0.06
TC12002081.hg.1	ABCB9	7.78 ± 0.19	7.78 ± 0.18	7.68 ± 0.14	7.83 ± 0.02

TC16000193.hg.1	ABCC1	8.97 ± 0.10	8.90 ± 0.05	8.75 ± 0.07	8.95 ± 0.05
TC06000600.hg.1	ABCC10	8.28 ± 0.03	8.12 ± 0.02	8.12 ± 0.05	8.13 ± 0.06
TC16001094.hg.1	ABCC11	6.74 ± 0.15	6.74 ± 0.15	6.71 ± 0.15	6.82 ± 0.08
TC16001093.hg.1	ABCC12	6.03 ± 0.15	5.97 ± 0.13	5.96 ± 0.10	6.04 ± 0.06
TC21000026.hg.1	ABCC13	4.41 ± 0.09	4.24 ± 0.05	4.31 ± 0.06	4.29 ± 0.06
TC10000715.hg.1	ABCC2	6.29 ± 0.04	6.25 ± 0.01	6.41 ± 0.20	6.23 ± 0.04
TC17000672.hg.1	ABCC3	8.26 ± 0.15	8.00 ± 0.12	8.32 ± 0.17	8.10 ± 0.09
TC13000799.hg.1	ABCC4	7.31 ± 0.19	7.09 ± 0.08	7.51 ± 0.24	7.10 ± 0.09
TC03002064.hg.1	ABCC5	7.69 ± 0.15	7.63 ± 0.05	7.63 ± 0.02	7.57 ± 0.02
TC03000988.hg.1	ABCC5-AS1	5.42 ± 0.24	5.36 ± 0.07	5.37 ± 0.05	5.39 ± 0.10
TC16000901.hg.1	ABCC6	7.92 ± 0.23	7.91 ± 0.21	7.87 ± 0.15	8.02 ± 0.05
TC16000206.hg.1	ABCC6P1	7.54 ± 0.22	7.51 ± 0.16	7.48 ± 0.14	7.62 ± 0.07
TC16000886.hg.1	ABCC6P2	7.74 ± 0.34	7.67 ± 0.14	7.72 ± 0.33	7.69 ± 0.09
TC11001457.hg.1	ABCC8	7.54 ± 0.19	7.51 ± 0.19	7.46 ± 0.22	7.61 ± 0.06
TC12001300.hg.1	ABCC9	4.99 ± 0.11	4.89 ± 0.05	4.90 ± 0.05	4.94 ± 0.06
TC0X000755.hg.1	ABCD1	8.77 ± 0.11	8.66 ± 0.08	8.71 ± 0.07	8.65 ± 0.04
TC12001392.hg.1	ABCD2	7.08 ± 0.54	7.56 ± 0.27	7.02 ± 0.38	7.20 ± 0.24
TC01000872.hg.1	ABCD3	7.15 ± 0.42	7.26 ± 0.24	7.23 ± 0.38	7.03 ± 0.09
TC14001304.hg.1	ABCD4	8.15 ± 0.03	8.09 ± 0.04	7.98 ± 0.09	8.10 ± 0.07
TC04000709.hg.1	ABCE1	8.11 ± 0.51	8.51 ± 0.22	7.92 ± 0.42	8.41 ± 0.05
TC06000341.hg.1	ABCF1	8.98 ± 0.07	8.99 ± 0.11	8.69 ± 0.09	9.00 ± 0.07
TC6_cox_hap2000039.hg.1	ABCF1	8.93 ± 0.05	8.94 ± 0.11	8.64 ± 0.10	8.94 ± 0.07
TC6_dbb_hap3000032.hg.1	ABCF1	8.93 ± 0.05	8.94 ± 0.11	8.64 ± 0.10	8.94 ± 0.07
TC6_mann_hap4000034.hg.1	ABCF1	8.93 ± 0.05	8.94 ± 0.11	8.64 ± 0.10	8.94 ± 0.07
TC6_mcf_hap5000026.hg.1	ABCF1	8.93 ± 0.05	8.94 ± 0.11	8.64 ± 0.10	8.94 ± 0.07
TC6_qbl_hap6000032.hg.1	ABCF1	8.93 ± 0.05	8.94 ± 0.11	8.64 ± 0.10	8.94 ± 0.07
TC6_ssto_hap7000032.hg.1	ABCF1	8.93 ± 0.05	8.94 ± 0.11	8.64 ± 0.10	8.94 ± 0.07
TC07002034.hg.1	ABCF2	8.93 ± 0.17	8.58 ± 0.06	8.69 ± 0.17	8.40 ± 0.18
TC03000995.hg.1	ABCF3	8.81 ± 0.01	8.72 ± 0.03	8.66 ± 0.05	8.76 ± 0.02
TC21000198.hg.1	ABCG1	8.28 ± 0.09	8.29 ± 0.11	8.19 ± 0.07	8.45 ± 0.07
TC04001370.hg.1	ABCG2	5.16 ± 0.09	5.10 ± 0.06	5.10 ± 0.07	5.18 ± 0.02

TC11001086.hg.1	ABCG4	7.50 ± 0.24	7.45 ± 0.20	7.43 ± 0.15	7.61 ± 0.07
TC02001794.hg.1	ABCG5	6.53 ± 0.18	6.50 ± 0.15	6.50 ± 0.09	6.56 ± 0.03
TC02000272.hg.1	ABCG8	7.30 ± 0.20	7.24 ± 0.11	7.22 ± 0.17	7.29 ± 0.07
TC02000165.hg.1	ABHD1	6.73 ± 0.16	6.71 ± 0.15	6.63 ± 0.13	6.80 ± 0.06
TC03000566.hg.1	ABHD10	7.31 ± 0.26	7.20 ± 0.13	7.28 ± 0.33	7.10 ± 0.06
TC07001505.hg.1	ABHD11	8.16 ± 0.10	8.07 ± 0.07	8.10 ± 0.12	8.11 ± 0.01
TC20000732.hg.1	ABHD12	8.56 ± 0.18	8.37 ± 0.09	8.32 ± 0.19	8.33 ± 0.02
TC14000291.hg.1	ABHD12B	5.68 ± 0.11	5.59 ± 0.10	5.71 ± 0.08	5.65 ± 0.03
TC13000382.hg.1	ABHD13	8.86 ± 0.17	9.13 ± 0.08	9.14 ± 0.22	9.09 ± 0.05
TC03003357.hg.1	ABHD14A	8.03 ± 0.12	7.92 ± 0.09	7.89 ± 0.08	7.96 ± 0.01
TC03003388.hg.1	ABHD14B	8.46 ± 0.12	8.50 ± 0.11	8.29 ± 0.11	8.48 ± 0.04
TC17001319.hg.1	ABHD15	8.18 ± 0.10	8.19 ± 0.03	8.08 ± 0.05	8.15 ± 0.06
TC06004116.hg.1	ABHD16A	8.55 ± 0.12	8.44 ± 0.03	8.60 ± 0.17	8.44 ± 0.01
TC6_cox_hap2000230.hg.1	ABHD16A	8.65 ± 0.13	8.52 ± 0.03	8.69 ± 0.19	8.52 ± 0.01
TC6_dbb_hap3000233.hg.1	ABHD16A	8.65 ± 0.13	8.52 ± 0.03	8.69 ± 0.19	8.52 ± 0.01
TC6_mann_hap4000203.hg.1	ABHD16A	8.65 ± 0.13	8.52 ± 0.03	8.69 ± 0.19	8.52 ± 0.01
TC6_mcf_hap5000218.hg.1	ABHD16A	8.65 ± 0.13	8.52 ± 0.03	8.69 ± 0.19	8.52 ± 0.01
TC6_qbl_hap6000212.hg.1	ABHD16A	8.64 ± 0.13	8.52 ± 0.03	8.69 ± 0.19	8.52 ± 0.01
TC6_ssto_hap7000206.hg.1	ABHD16A	8.65 ± 0.13	8.53 ± 0.03	8.70 ± 0.19	8.53 ± 0.01
TC20000525.hg.1	ABHD16B	8.24 ± 0.39	8.13 ± 0.21	8.17 ± 0.29	8.21 ± 0.07
TC19001016.hg.1	ABHD17A	9.65 ± 0.09	9.52 ± 0.09	9.52 ± 0.07	9.46 ± 0.01
TC09001205.hg.1	ABHD17B	8.84 ± 0.08	8.89 ± 0.09	8.70 ± 0.10	8.86 ± 0.03
TC15000751.hg.1	ABHD17C	6.36 ± 0.09	6.35 ± 0.06	6.40 ± 0.04	6.37 ± 0.03
TC15000844.hg.1	ABHD2	9.36 ± 0.24	9.49 ± 0.18	9.90 ± 0.24	9.67 ± 0.28
TC18000402.hg.1	ABHD3	8.73 ± 0.24	8.82 ± 0.19	9.16 ± 0.45	8.50 ± 0.18
TC14000130.hg.1	ABHD4	8.48 ± 0.13	8.17 ± 0.09	8.46 ± 0.26	8.25 ± 0.15
TC03000231.hg.1	ABHD5	8.47 ± 0.07	8.48 ± 0.22	8.81 ± 0.29	8.43 ± 0.17
TC03000376.hg.1	ABHD6	7.69 ± 0.14	7.48 ± 0.09	7.53 ± 0.08	7.49 ± 0.04
TC19001272.hg.1	ABHD8	8.37 ± 0.28	8.24 ± 0.18	8.24 ± 0.19	8.24 ± 0.02
TC10001120.hg.1	ABI1	9.55 ± 0.27	9.69 ± 0.08	9.81 ± 0.29	9.66 ± 0.16
TC02001198.hg.1	ABI2	7.16 ± 0.13	7.11 ± 0.04	7.00 ± 0.07	7.03 ± 0.03

TC17000640.hg.1	ABI3	9.01 ± 0.30	8.53 ± 0.14	8.70 ± 0.14	8.40 ± 0.10
TC03001612.hg.1	ABI3BP	5.42 ± 0.13	5.35 ± 0.11	5.39 ± 0.06	5.47 ± 0.09
TC09000741.hg.1	ABL1	8.75 ± 0.08	8.69 ± 0.08	8.54 ± 0.05	8.59 ± 0.03
TC01003566.hg.1	ABL2	7.48 ± 0.11	7.43 ± 0.13	7.53 ± 0.17	7.39 ± 0.01
TC10001676.hg.1	ABLIM1	8.60 ± 0.16	8.72 ± 0.03	8.27 ± 0.08	8.72 ± 0.02
TC04001015.hg.1	ABLIM2	7.54 ± 0.20	7.51 ± 0.17	7.45 ± 0.19	7.61 ± 0.06
TC05000816.hg.1	ABLIM3	7.87 ± 0.05	7.38 ± 0.07	7.90 ± 0.17	7.57 ± 0.08
TC09001683.hg.1	ABO	7.33 ± 0.19	7.14 ± 0.06	7.32 ± 0.11	7.34 ± 0.06
TC07001008.hg.1	ABP1	7.56 ± 0.16	7.40 ± 0.13	7.57 ± 0.16	7.51 ± 0.11
TC17000970.hg.1	ABR	9.50 ± 0.25	9.23 ± 0.04	9.52 ± 0.12	9.15 ± 0.12
TC08001520.hg.1	ABRA	5.77 ± 0.10	5.85 ± 0.11	5.82 ± 0.06	5.94 ± 0.09
TC06001034.hg.1	ABRACL	8.23 ± 0.20	8.27 ± 0.07	8.32 ± 0.12	8.12 ± 0.12
TC06000205.hg.1	ABT1	8.58 ± 0.15	8.48 ± 0.19	8.19 ± 0.22	8.35 ± 0.05
TC03000667.hg.1	ABTB1	9.32 ± 0.18	9.11 ± 0.01	9.36 ± 0.24	9.14 ± 0.08
TC11001544.hg.1	ABTB2	7.75 ± 0.11	7.70 ± 0.11	7.87 ± 0.20	7.83 ± 0.09
TC03001293.hg.1	ACAA1	8.42 ± 0.10	8.22 ± 0.01	8.43 ± 0.11	8.22 ± 0.02
TC18000506.hg.1	ACAA2	8.81 ± 0.16	8.87 ± 0.17	8.76 ± 0.05	8.64 ± 0.11
TC17001406.hg.1	ACACA	7.14 ± 0.03	7.07 ± 0.12	6.99 ± 0.02	7.04 ± 0.02
TC12000846.hg.1	ACACB	7.43 ± 0.05	7.42 ± 0.04	7.43 ± 0.04	7.49 ± 0.06
TC12003211.hg.1	ACAD10	7.81 ± 0.05	7.63 ± 0.01	7.66 ± 0.05	7.62 ± 0.03
TC03003341.hg.1	ACAD11	7.09 ± 0.46	7.43 ± 0.15	7.07 ± 0.33	7.34 ± 0.14
TC11001215.hg.1	ACAD8	7.81 ± 0.08	7.74 ± 0.02	7.70 ± 0.11	7.72 ± 0.03
TC03000684.hg.1	ACAD9	8.06 ± 0.01	7.90 ± 0.05	7.86 ± 0.07	7.87 ± 0.05
TC02002731.hg.1	ACADL	5.45 ± 0.28	5.31 ± 0.07	5.33 ± 0.12	5.40 ± 0.07
TC01000776.hg.1	ACADM	7.42 ± 0.20	7.35 ± 0.10	7.34 ± 0.22	7.14 ± 0.11
TC12000951.hg.1	ACADS	8.22 ± 0.14	8.17 ± 0.23	8.12 ± 0.19	8.24 ± 0.05
TC10000896.hg.1	ACADSB	7.34 ± 0.45	7.77 ± 0.25	7.36 ± 0.28	7.62 ± 0.05
TC17000085.hg.1	ACADVL	9.03 ± 0.04	8.97 ± 0.12	8.99 ± 0.06	8.95 ± 0.08
TC15000842.hg.1	ACAN	7.42 ± 0.21	7.38 ± 0.22	7.41 ± 0.16	7.48 ± 0.03
TC17000089.hg.1	ACAP1	10.05 ± 0.22	9.87 ± 0.06	10.00 ± 0.28	9.69 ± 0.06
TC03002141.hg.1	ACAP2	9.12 ± 0.36	9.39 ± 0.23	9.54 ± 0.43	9.25 ± 0.18

TC03002142.hg.1	ACAP2-IT1	7.06 ± 0.47	7.03 ± 0.39	7.44 ± 0.72	6.81 ± 0.31
TC01002090.hg.1	ACAP3	8.60 ± 0.24	8.58 ± 0.15	8.49 ± 0.20	8.62 ± 0.03
TC11000979.hg.1	ACAT1	7.21 ± 0.07	7.01 ± 0.15	7.03 ± 0.09	6.74 ± 0.08
TC06001147.hg.1	ACAT2	7.53 ± 0.09	7.73 ± 0.19	7.38 ± 0.01	7.53 ± 0.12
TC01003897.hg.1	ACBD3	9.19 ± 0.29	9.38 ± 0.16	9.18 ± 0.28	9.26 ± 0.05
TC17000579.hg.1	ACBD4	7.95 ± 0.18	7.87 ± 0.11	7.85 ± 0.12	7.94 ± 0.05
TC10001128.hg.1	ACBD5	8.11 ± 0.26	8.23 ± 0.11	8.24 ± 0.19	8.15 ± 0.03
TC01006385.hg.1	ACBD6	7.91 ± 0.10	7.84 ± 0.11	7.74 ± 0.05	7.78 ± 0.06
TC10002933.hg.1	ACBD7	6.76 ± 0.06	6.75 ± 0.07	6.81 ± 0.02	6.80 ± 0.04
TC11003445.hg.1	ACCS	7.99 ± 0.24	8.02 ± 0.14	7.93 ± 0.10	8.04 ± 0.15
TC11000370.hg.1	ACCSL	6.39 ± 0.08	6.22 ± 0.13	6.32 ± 0.05	6.32 ± 0.06
TC16001193.hg.1	ACD	8.77 ± 0.20	8.51 ± 0.08	8.51 ± 0.14	8.44 ± 0.01
TC17000766.hg.1	ACE	7.33 ± 0.19	7.27 ± 0.16	7.24 ± 0.17	7.32 ± 0.06
TC0X000878.hg.1	ACE2	4.53 ± 0.11	4.50 ± 0.10	4.50 ± 0.08	4.54 ± 0.03
TC19001092.hg.1	ACER1	7.18 ± 0.15	7.17 ± 0.20	7.11 ± 0.10	7.24 ± 0.03
TC09000094.hg.1	ACER2	7.43 ± 0.08	7.20 ± 0.06	7.43 ± 0.10	7.29 ± 0.06
TC11000824.hg.1	ACER3	7.07 ± 0.22	6.99 ± 0.17	7.73 ± 0.29	6.87 ± 0.10
TC07001680.hg.1	ACHE	8.55 ± 0.26	8.50 ± 0.22	8.48 ± 0.20	8.60 ± 0.03
TC14000948.hg.1	ACIN1	8.98 ± 0.14	8.93 ± 0.08	8.97 ± 0.15	8.82 ± 0.05
TC17001520.hg.1	ACLY	9.63 ± 0.24	9.54 ± 0.08	9.48 ± 0.14	9.30 ± 0.10
TC02000877.hg.1	ACMSD	5.81 ± 0.14	5.80 ± 0.09	5.79 ± 0.08	5.89 ± 0.08
TC07000578.hg.1	ACN9	6.28 ± 0.07	6.30 ± 0.11	6.40 ± 0.19	6.12 ± 0.04
TC09000128.hg.1	ACO1	7.32 ± 0.04	7.20 ± 0.07	7.22 ± 0.09	7.18 ± 0.04
TC22000337.hg.1	ACO2	9.48 ± 0.28	9.16 ± 0.08	9.14 ± 0.11	9.09 ± 0.08
TC14002199.hg.1	ACOT1	7.46 ± 0.17	7.40 ± 0.15	7.21 ± 0.20	7.38 ± 0.06
TC01000668.hg.1	ACOT11	7.76 ± 0.18	7.70 ± 0.19	7.70 ± 0.16	7.80 ± 0.07
TC05001544.hg.1	ACOT12	4.95 ± 0.15	4.87 ± 0.08	4.84 ± 0.10	4.88 ± 0.06
TC06000147.hg.1	ACOT13	7.37 ± 0.30	6.99 ± 0.18	7.43 ± 0.16	6.73 ± 0.23
TC14002200.hg.1	ACOT2	7.86 ± 0.14	7.76 ± 0.03	7.63 ± 0.09	7.77 ± 0.01
TC14000446.hg.1	ACOT4	5.90 ± 0.21	5.78 ± 0.09	5.84 ± 0.13	5.72 ± 0.05
TC14000447.hg.1	ACOT6	5.16 ± 0.16	4.94 ± 0.02	5.14 ± 0.05	5.17 ± 0.05

TC01002149.hg.1	ACOT7	8.26 ± 0.23	8.15 ± 0.16	8.17 ± 0.28	8.10 ± 0.02
TC20000890.hg.1	ACOT8	8.00 ± 0.17	7.81 ± 0.04	7.87 ± 0.15	7.80 ± 0.05
TC0X000921.hg.1	ACOT9	7.88 ± 0.30	7.79 ± 0.19	8.10 ± 0.46	7.79 ± 0.06
TC17001890.hg.1	ACOX1	9.35 ± 0.25	8.91 ± 0.14	9.67 ± 0.38	8.70 ± 0.30
TC03001500.hg.1	ACOX2	6.77 ± 0.15	6.72 ± 0.17	6.71 ± 0.15	6.84 ± 0.08
TC04001018.hg.1	ACOX3	7.85 ± 0.06	7.70 ± 0.03	7.75 ± 0.08	7.70 ± 0.03
TC02000694.hg.1	ACOXL	6.50 ± 0.19	6.35 ± 0.10	6.39 ± 0.09	6.41 ± 0.02
TC02000002.hg.1	ACP1	8.20 ± 0.27	8.40 ± 0.16	7.99 ± 0.22	8.36 ± 0.04
TC11001733.hg.1	ACP2	8.38 ± 0.22	7.99 ± 0.13	8.21 ± 0.09	8.01 ± 0.04
TC19001188.hg.1	ACP5	8.43 ± 0.16	8.31 ± 0.08	8.28 ± 0.13	8.29 ± 0.04
TC01003128.hg.1	ACP6	7.00 ± 0.09	6.88 ± 0.04	6.96 ± 0.11	6.88 ± 0.06
TC03000759.hg.1	ACPL2	6.95 ± 0.07	6.90 ± 0.02	6.87 ± 0.05	6.91 ± 0.07
TC03000714.hg.1	ACPP	7.32 ± 0.21	6.96 ± 0.19	7.90 ± 0.16	6.87 ± 0.26
TC19000768.hg.1	ACPT	8.30 ± 0.27	8.25 ± 0.16	8.24 ± 0.25	8.31 ± 0.03
TC22000436.hg.1	ACR	6.96 ± 0.14	6.90 ± 0.12	6.87 ± 0.10	6.99 ± 0.03
TC12003270.hg.1	ACRBP	8.61 ± 0.11	8.13 ± 0.15	8.48 ± 0.16	8.16 ± 0.08
TC0X000389.hg.1	ACRC	6.89 ± 0.13	6.87 ± 0.05	7.09 ± 0.20	6.89 ± 0.10
TC11002422.hg.1	ACRV1	5.48 ± 0.16	5.37 ± 0.12	5.42 ± 0.07	5.42 ± 0.07
TC15001701.hg.1	ACSBG1	7.01 ± 0.14	6.94 ± 0.17	6.87 ± 0.16	7.05 ± 0.04
TC19000111.hg.1	ACSBG2	6.23 ± 0.12	6.12 ± 0.12	6.13 ± 0.11	6.18 ± 0.03
TC17000667.hg.1	ACSF2	7.74 ± 0.13	7.68 ± 0.11	7.64 ± 0.13	7.74 ± 0.06
TC16000689.hg.1	ACSF3	8.52 ± 0.09	8.41 ± 0.10	8.41 ± 0.13	8.43 ± 0.03
TC04001809.hg.1	ACSL1	10.90 ± 0.35	10.80 ± 0.28	11.79 ± 0.35	10.69 ± 0.43
TC02001350.hg.1	ACSL3	8.15 ± 0.26	8.32 ± 0.11	8.48 ± 0.50	8.11 ± 0.18
TC0X002337.hg.1	ACSL4	8.59 ± 0.35	8.57 ± 0.34	9.50 ± 0.53	8.53 ± 0.36
TC10000813.hg.1	ACSL5	9.17 ± 0.24	9.03 ± 0.14	9.02 ± 0.13	8.63 ± 0.17
TC05001761.hg.1	ACSL6	6.65 ± 0.07	6.68 ± 0.11	6.63 ± 0.08	6.68 ± 0.05
TC16000929.hg.1	ACSM1	6.68 ± 0.14	6.57 ± 0.04	6.62 ± 0.05	6.64 ± 0.09
TC16000219.hg.1	ACSM2A	6.19 ± 0.18	6.15 ± 0.08	6.15 ± 0.10	6.27 ± 0.06
TC16000927.hg.1	ACSM2B	6.18 ± 0.16	6.11 ± 0.13	6.11 ± 0.10	6.23 ± 0.08
TC16000222.hg.1	ACSM3	6.91 ± 0.17	6.93 ± 0.17	6.95 ± 0.07	7.00 ± 0.09

TC16000223.hg.1	ACSM3	5.75 ± 0.22	5.75 ± 0.12	5.67 ± 0.15	5.72 ± 0.07
TC12000110.hg.1	ACSM4	5.90 ± 0.11	5.89 ± 0.04	5.87 ± 0.04	5.96 ± 0.07
TC16000218.hg.1	ACSM5	7.00 ± 0.15	6.92 ± 0.12	6.91 ± 0.19	6.99 ± 0.08
TC20000727.hg.1	ACSS1	8.59 ± 0.07	8.39 ± 0.02	8.27 ± 0.15	8.30 ± 0.05
TC20000245.hg.1	ACSS2	7.66 ± 0.22	7.37 ± 0.01	7.67 ± 0.16	7.34 ± 0.03
TC12000672.hg.1	ACSS3	5.37 ± 0.08	5.31 ± 0.08	5.37 ± 0.04	5.37 ± 0.08
TC01003945.hg.1	ACTA1	9.26 ± 0.13	9.09 ± 0.07	9.25 ± 0.21	9.08 ± 0.17
TC10001498.hg.1	ACTA2	8.19 ± 0.23	7.84 ± 0.13	8.08 ± 0.11	7.86 ± 0.01
TC10000633.hg.1	ACTA2-AS1	6.64 ± 0.10	6.58 ± 0.03	6.58 ± 0.11	6.51 ± 0.12
TC07001120.hg.1	ACTB	11.86 ± 0.10	11.81 ± 0.01	12.00 ± 0.07	11.78 ± 0.08
TC05001381.hg.1	ACTBL2	4.76 ± 0.09	4.72 ± 0.05	4.64 ± 0.04	4.70 ± 0.08
TC15001201.hg.1	ACTC1	7.74 ± 0.16	7.62 ± 0.10	7.72 ± 0.24	7.68 ± 0.06
TC17001952.hg.1	ACTG1	13.07 ± 0.20	12.97 ± 0.04	13.16 ± 0.10	12.87 ± 0.18
TC02000448.hg.1	ACTG2	6.82 ± 0.19	6.69 ± 0.13	6.80 ± 0.14	6.78 ± 0.03
TC20000233.hg.1	ACTL10	7.76 ± 0.20	7.80 ± 0.14	7.69 ± 0.22	7.97 ± 0.12
TC03000955.hg.1	ACTL6A	7.42 ± 0.10	7.40 ± 0.17	7.26 ± 0.09	7.21 ± 0.01
TC07001675.hg.1	ACTL6B	7.67 ± 0.19	7.60 ± 0.15	7.61 ± 0.17	7.65 ± 0.01
TC09000547.hg.1	ACTL7A	7.29 ± 0.10	7.33 ± 0.03	7.47 ± 0.10	7.32 ± 0.08
TC09001454.hg.1	ACTL7B	7.15 ± 0.08	7.01 ± 0.20	6.98 ± 0.41	7.11 ± 0.11
TC01000238.hg.1	ACTL8	7.25 ± 0.17	7.40 ± 0.12	7.29 ± 0.15	7.47 ± 0.08
TC19001137.hg.1	ACTL9	7.49 ± 0.22	7.45 ± 0.17	7.45 ± 0.07	7.49 ± 0.15
TC14001255.hg.1	ACTN1	10.71 ± 0.19	10.64 ± 0.07	11.00 ± 0.17	10.70 ± 0.16
TC14000412.hg.1	ACTN1-AS1	7.00 ± 0.13	6.93 ± 0.15	6.92 ± 0.10	7.05 ± 0.07
TC01001955.hg.1	ACTN2	6.34 ± 0.18	6.27 ± 0.10	6.32 ± 0.12	6.32 ± 0.05
TC11000671.hg.1	ACTN3	8.10 ± 0.11	8.01 ± 0.10	8.06 ± 0.11	8.03 ± 0.05
TC19000532.hg.1	ACTN4	11.27 ± 0.13	11.13 ± 0.08	11.35 ± 0.12	11.04 ± 0.24
TC14000345.hg.1	ACTR10	8.44 ± 0.13	8.37 ± 0.12	8.49 ± 0.31	8.18 ± 0.14
TC10001623.hg.1	ACTR1A	9.42 ± 0.22	8.97 ± 0.16	9.41 ± 0.23	8.76 ± 0.30
TC02002121.hg.1	ACTR1B	9.06 ± 0.17	8.99 ± 0.06	8.84 ± 0.12	8.99 ± 0.04
TC02000378.hg.1	ACTR2	10.56 ± 0.25	10.74 ± 0.17	10.97 ± 0.33	10.67 ± 0.17
TC02000735.hg.1	ACTR3	11.08 ± 0.32	11.30 ± 0.15	11.51 ± 0.27	11.21 ± 0.25

TC07001031.hg.1	ACTR3B	7.75 ± 0.08	7.66 ± 0.09	7.69 ± 0.07	7.64 ± 0.05
TC02000565.hg.1	ACTR3BP2	4.94 ± 0.27	4.84 ± 0.08	5.02 ± 0.12	5.03 ± 0.34
TC04000530.hg.1	ACTR3BP4	4.54 ± 0.09	4.37 ± 0.07	4.36 ± 0.32	4.52 ± 0.18
TC10000260.hg.1	ACTR3BP5	5.44 ± 0.14	5.27 ± 0.06	5.28 ± 0.13	5.53 ± 0.27
TC22000011.hg.1	ACTR3BP6	5.55 ± 0.29	5.34 ± 0.03	5.45 ± 0.13	5.49 ± 0.23
TC07002014.hg.1	ACTR3C	7.34 ± 0.15	7.34 ± 0.01	7.35 ± 0.04	7.28 ± 0.04
TC20000289.hg.1	ACTR5	7.37 ± 0.05	7.44 ± 0.07	7.15 ± 0.08	7.40 ± 0.09
TC12000774.hg.1	ACTR6	7.46 ± 0.34	7.47 ± 0.20	7.42 ± 0.33	7.37 ± 0.13
TC03001473.hg.1	ACTR8	7.96 ± 0.12	7.84 ± 0.12	7.88 ± 0.17	7.66 ± 0.14
TC0X001350.hg.1	ACTRT1	5.46 ± 0.37	5.55 ± 0.25	5.34 ± 0.12	5.45 ± 0.11
TC01000066.hg.1	ACTRT2	7.59 ± 0.20	7.65 ± 0.24	7.55 ± 0.23	7.74 ± 0.04
TC03001983.hg.1	ACTRT3	5.21 ± 0.08	5.27 ± 0.20	5.32 ± 0.11	5.35 ± 0.02
TC02002455.hg.1	ACVR1	7.95 ± 0.04	7.95 ± 0.03	7.94 ± 0.05	7.96 ± 0.06
TC12000411.hg.1	ACVR1B	7.95 ± 0.08	7.80 ± 0.05	8.03 ± 0.16	7.91 ± 0.10
TC02002453.hg.1	ACVR1C	6.24 ± 0.03	6.30 ± 0.06	6.21 ± 0.03	6.35 ± 0.06
TC02000923.hg.1	ACVR2A	6.62 ± 0.19	6.58 ± 0.11	6.52 ± 0.09	6.56 ± 0.04
TC03000192.hg.1	ACVR2B	7.60 ± 0.18	7.63 ± 0.16	7.50 ± 0.18	7.63 ± 0.08
TC03001295.hg.1	ACVR2B-AS1	7.12 ± 0.09	7.14 ± 0.06	7.06 ± 0.19	7.15 ± 0.03
TC12000410.hg.1	ACVRL1	7.99 ± 0.27	7.88 ± 0.24	7.86 ± 0.19	8.00 ± 0.06
TC03003358.hg.1	ACY1	8.22 ± 0.23	8.12 ± 0.16	8.10 ± 0.21	8.20 ± 0.08
TC11001994.hg.1	ACY3	7.91 ± 0.17	7.90 ± 0.17	7.82 ± 0.08	7.90 ± 0.04
TC14001316.hg.1	ACYP1	7.10 ± 0.15	7.19 ± 0.13	7.08 ± 0.05	7.07 ± 0.09
TC02004966.hg.1	ACYP2	6.18 ± 0.10	6.08 ± 0.04	6.06 ± 0.04	6.06 ± 0.02
TC20000868.hg.1	ADA	8.50 ± 0.24	8.22 ± 0.14	8.22 ± 0.21	8.12 ± 0.01
TC04000627.hg.1	ADAD1	5.48 ± 0.12	5.44 ± 0.06	5.42 ± 0.11	5.43 ± 0.01
TC16000655.hg.1	ADAD2	8.17 ± 0.32	8.25 ± 0.27	8.14 ± 0.23	8.34 ± 0.06
TC15000327.hg.1	ADAL	6.13 ± 0.06	5.97 ± 0.16	5.98 ± 0.08	5.84 ± 0.07
TC15001484.hg.1	ADAM10	9.41 ± 0.32	9.56 ± 0.20	9.75 ± 0.34	9.37 ± 0.11
TC17000574.hg.1	ADAM11	8.16 ± 0.26	8.12 ± 0.15	8.10 ± 0.20	8.19 ± 0.04
TC10001744.hg.1	ADAM12	6.70 ± 0.20	6.74 ± 0.17	6.59 ± 0.09	6.75 ± 0.07
TC01006331.hg.1	ADAM15	8.43 ± 0.13	8.29 ± 0.09	8.35 ± 0.06	8.35 ± 0.02

TC02001558.hg.1	ADAM17	8.97 ± 0.12	8.72 ± 0.20	9.23 ± 0.34	8.61 ± 0.30
TC08000304.hg.1	ADAM18	4.67 ± 0.07	4.52 ± 0.01	4.56 ± 0.05	4.54 ± 0.04
TC05001986.hg.1	ADAM19	9.14 ± 0.06	8.91 ± 0.10	9.10 ± 0.17	8.80 ± 0.04
TC12000873.hg.1	ADAM1A	7.82 ± 0.19	7.58 ± 0.16	7.56 ± 0.15	7.36 ± 0.10
TC08001159.hg.1	ADAM2	4.49 ± 0.10	4.39 ± 0.03	4.45 ± 0.07	4.45 ± 0.05
TC14001270.hg.1	ADAM20	4.95 ± 0.25	4.93 ± 0.09	4.92 ± 0.08	4.83 ± 0.07
TC14001269.hg.1	ADAM20P1	4.44 ± 0.49	4.43 ± 0.15	4.51 ± 0.25	4.44 ± 0.15
TC14000424.hg.1	ADAM21	4.60 ± 0.14	4.47 ± 0.06	4.48 ± 0.21	4.50 ± 0.09
TC14001266.hg.1	ADAM21P1	4.73 ± 0.18	4.83 ± 0.15	4.74 ± 0.05	4.99 ± 0.23
TC07000525.hg.1	ADAM22	6.09 ± 0.09	6.03 ± 0.03	5.99 ± 0.02	6.04 ± 0.05
TC02001218.hg.1	ADAM23	6.73 ± 0.07	6.86 ± 0.08	6.56 ± 0.11	6.83 ± 0.16
TC08000190.hg.1	ADAM28	7.44 ± 0.57	8.02 ± 0.25	7.41 ± 0.44	8.10 ± 0.10
TC04000863.hg.1	ADAM29	4.67 ± 0.10	4.57 ± 0.08	4.59 ± 0.02	4.67 ± 0.10
TC01003053.hg.1	ADAM30	5.05 ± 0.12	4.92 ± 0.08	4.99 ± 0.06	5.06 ± 0.17
TC08000299.hg.1	ADAM32	4.92 ± 0.12	4.78 ± 0.02	4.83 ± 0.06	4.85 ± 0.05
TC20000574.hg.1	ADAM33	8.44 ± 0.20	8.42 ± 0.18	8.39 ± 0.16	8.51 ± 0.04
TC08001158.hg.1	ADAM3A	4.60 ± 0.11	4.45 ± 0.03	4.53 ± 0.04	4.51 ± 0.06
TC08000300.hg.1	ADAM5	4.77 ± 0.04	4.72 ± 0.02	4.73 ± 0.01	4.79 ± 0.05
TC14002244.hg.1	ADAM6	6.91 ± 0.15	6.75 ± 0.09	6.79 ± 0.02	6.87 ± 0.15
TC08000192.hg.1	ADAM7	4.68 ± 0.10	4.57 ± 0.05	4.64 ± 0.05	4.63 ± 0.01
TC10001783.hg.1	ADAM8	9.83 ± 0.22	9.65 ± 0.04	10.00 ± 0.19	9.74 ± 0.03
TC08000298.hg.1	ADAM9	7.63 ± 0.28	7.52 ± 0.10	8.24 ± 0.42	7.69 ± 0.23
TC08000191.hg.1	ADAMDEC1	5.00 ± 0.06	4.92 ± 0.04	4.97 ± 0.08	4.94 ± 0.06
TC21000345.hg.1	ADAMTS1	6.24 ± 0.14	6.16 ± 0.08	6.20 ± 0.07	6.29 ± 0.16
TC19001136.hg.1	ADAMTS10	8.34 ± 0.09	8.37 ± 0.10	8.19 ± 0.15	8.47 ± 0.07
TC05001243.hg.1	ADAMTS12	6.37 ± 0.15	6.36 ± 0.13	6.38 ± 0.09	6.45 ± 0.07
TC09000775.hg.1	ADAMTS13	7.86 ± 0.20	7.88 ± 0.22	7.80 ± 0.19	7.96 ± 0.06
TC10000433.hg.1	ADAMTS14	7.74 ± 0.17	7.69 ± 0.21	7.64 ± 0.17	7.81 ± 0.05
TC11001197.hg.1	ADAMTS15	7.63 ± 0.22	7.67 ± 0.19	7.64 ± 0.25	7.77 ± 0.02
TC05000039.hg.1	ADAMTS16	7.07 ± 0.21	7.01 ± 0.15	7.00 ± 0.15	7.12 ± 0.06
TC15001992.hg.1	ADAMTS17	7.61 ± 0.15	7.57 ± 0.13	7.49 ± 0.18	7.63 ± 0.04

TC16001288.hg.1	ADAMTS18	6.44 ± 0.13	6.41 ± 0.15	6.44 ± 0.11	6.53 ± 0.06
TC05000617.hg.1	ADAMTS19	5.52 ± 0.13	5.45 ± 0.10	5.44 ± 0.09	5.53 ± 0.04
TC05002130.hg.1	ADAMTS2	7.98 ± 0.33	7.81 ± 0.22	7.91 ± 0.24	7.92 ± 0.01
TC12001410.hg.1	ADAMTS20	5.11 ± 0.11	4.98 ± 0.03	4.98 ± 0.05	5.02 ± 0.05
TC04001277.hg.1	ADAMTS3	5.35 ± 0.12	5.26 ± 0.07	5.37 ± 0.11	5.36 ± 0.13
TC01003421.hg.1	ADAMTS4	7.15 ± 0.09	7.11 ± 0.19	7.10 ± 0.15	7.23 ± 0.03
TC21000346.hg.1	ADAMTS5	6.70 ± 0.09	6.61 ± 0.07	6.61 ± 0.06	6.67 ± 0.04
TC05001406.hg.1	ADAMTS6	6.15 ± 0.11	6.13 ± 0.03	6.05 ± 0.05	6.11 ± 0.05
TC15001711.hg.1	ADAMTS7	8.19 ± 0.24	8.21 ± 0.21	8.22 ± 0.19	8.31 ± 0.06
TC11002452.hg.1	ADAMTS8	7.69 ± 0.19	7.64 ± 0.22	7.58 ± 0.20	7.72 ± 0.06
TC03001527.hg.1	ADAMTS9	5.87 ± 0.16	5.80 ± 0.11	5.79 ± 0.08	5.89 ± 0.09
TC03000401.hg.1	ADAMTS9-AS1	5.14 ± 0.13	5.05 ± 0.15	5.14 ± 0.02	5.17 ± 0.14
TC03000402.hg.1	ADAMTS9-AS2	6.19 ± 0.20	6.13 ± 0.04	6.03 ± 0.13	6.24 ± 0.19
TC09000086.hg.1	ADAMTSL1	6.62 ± 0.16	6.59 ± 0.15	6.58 ± 0.10	6.69 ± 0.08
TC09000777.hg.1	ADAMTSL2	7.99 ± 0.28	7.94 ± 0.23	7.93 ± 0.24	8.04 ± 0.06
TC15000800.hg.1	ADAMTSL3	6.27 ± 0.11	6.26 ± 0.18	6.21 ± 0.14	6.39 ± 0.12
TC01001190.hg.1	ADAMTSL4	8.47 ± 0.19	8.31 ± 0.15	8.55 ± 0.06	8.32 ± 0.05
TC19001006.hg.1	ADAMTSL5	8.13 ± 0.19	8.13 ± 0.22	8.08 ± 0.18	8.21 ± 0.06
TC07003348.hg.1	ADAP1	8.42 ± 0.09	8.33 ± 0.05	8.39 ± 0.11	8.32 ± 0.01
TC17000357.hg.1	ADAP2	7.80 ± 0.29	7.44 ± 0.07	7.76 ± 0.05	7.48 ± 0.19
TC01003295.hg.1	ADAR	10.30 ± 0.32	10.10 ± 0.19	10.39 ± 0.36	9.96 ± 0.17
TC21000241.hg.1	ADARB1	7.76 ± 0.02	7.71 ± 0.04	7.63 ± 0.03	7.66 ± 0.08
TC10000969.hg.1	ADARB2	7.94 ± 0.14	7.90 ± 0.15	7.96 ± 0.06	8.00 ± 0.07
TC10000013.hg.1	ADARB2-AS1	7.03 ± 0.25	7.07 ± 0.21	6.91 ± 0.17	7.27 ± 0.12
TC16001283.hg.1	ADAT1	8.01 ± 0.09	7.91 ± 0.13	7.81 ± 0.10	7.74 ± 0.04
TC06002186.hg.1	ADAT2	7.56 ± 0.43	7.71 ± 0.15	7.37 ± 0.12	7.69 ± 0.11
TC19000046.hg.1	ADAT3	8.17 ± 0.14	8.14 ± 0.12	8.05 ± 0.11	8.16 ± 0.01
TC01000439.hg.1	ADC	7.44 ± 0.14	7.47 ± 0.20	7.38 ± 0.14	7.56 ± 0.04
TC14000494.hg.1	ADCK1	7.40 ± 0.09	7.30 ± 0.08	7.30 ± 0.05	7.28 ± 0.01
TC07003343.hg.1	ADCK2	7.11 ± 0.11	7.08 ± 0.11	6.90 ± 0.16	7.13 ± 0.07
TC01001866.hg.1	ADCK3	8.45 ± 0.13	8.40 ± 0.08	8.28 ± 0.16	8.48 ± 0.06

TC19001544.hg.1	ADCK4	8.33 ± 0.20	8.12 ± 0.10	8.25 ± 0.11	8.13 ± 0.03
TC08000851.hg.1	ADCK5	8.19 ± 0.24	8.15 ± 0.19	8.09 ± 0.19	8.25 ± 0.06
TC07000283.hg.1	ADCY1	7.36 ± 0.16	7.33 ± 0.22	7.30 ± 0.13	7.44 ± 0.03
TC01003483.hg.1	ADCY10	5.34 ± 0.08	5.28 ± 0.05	5.31 ± 0.04	5.32 ± 0.07
TC06001711.hg.1	ADCY10P1	5.11 ± 0.15	5.08 ± 0.08	5.10 ± 0.15	5.09 ± 0.09
TC05000054.hg.1	ADCY2	6.28 ± 0.13	6.20 ± 0.12	6.24 ± 0.12	6.26 ± 0.05
TC05000055.hg.1	ADCY2	5.92 ± 0.33	6.08 ± 0.26	5.79 ± 0.23	6.19 ± 0.07
TC02001653.hg.1	ADCY3	8.46 ± 0.38	8.15 ± 0.07	8.41 ± 0.06	8.19 ± 0.01
TC14002316.hg.1	ADCY4	7.95 ± 0.12	7.81 ± 0.13	7.95 ± 0.05	7.89 ± 0.01
TC03001710.hg.1	ADCY5	7.24 ± 0.16	7.23 ± 0.20	7.21 ± 0.18	7.33 ± 0.06
TC12001457.hg.1	ADCY6	7.55 ± 0.17	7.54 ± 0.18	7.53 ± 0.13	7.64 ± 0.05
TC16000439.hg.1	ADCY7	9.59 ± 0.21	9.44 ± 0.05	9.52 ± 0.18	9.32 ± 0.08
TC08001646.hg.1	ADCY8	6.30 ± 0.15	6.24 ± 0.16	6.27 ± 0.10	6.33 ± 0.06
TC16000824.hg.1	ADCY9	7.16 ± 0.18	7.02 ± 0.05	6.98 ± 0.07	6.92 ± 0.06
TC18000007.hg.1	ADCYAP1	7.15 ± 0.12	7.18 ± 0.14	7.12 ± 0.15	7.23 ± 0.04
TC07000201.hg.1	ADCYAP1R1	7.27 ± 0.10	7.23 ± 0.18	7.21 ± 0.11	7.32 ± 0.05
TC04000039.hg.1	ADD1	9.43 ± 0.05	9.36 ± 0.10	9.29 ± 0.13	9.23 ± 0.05
TC02001965.hg.1	ADD2	7.27 ± 0.13	7.25 ± 0.07	7.17 ± 0.10	7.27 ± 0.05
TC10000797.hg.1	ADD3	9.17 ± 0.54	9.48 ± 0.16	9.34 ± 0.28	9.39 ± 0.05
TC10001653.hg.1	ADD3-AS1	7.45 ± 0.11	7.41 ± 0.02	7.42 ± 0.09	7.51 ± 0.07
TC06001067.hg.1	ADGB	4.71 ± 0.11	4.57 ± 0.04	4.65 ± 0.09	4.62 ± 0.04
TC04001409.hg.1	ADH1A	5.60 ± 0.15	5.55 ± 0.12	5.56 ± 0.09	5.73 ± 0.17
TC04001410.hg.1	ADH1B	5.56 ± 0.14	5.44 ± 0.11	5.46 ± 0.11	5.56 ± 0.11
TC04001411.hg.1	ADH1C	5.69 ± 0.06	5.61 ± 0.02	5.65 ± 0.03	5.71 ± 0.09
TC04001406.hg.1	ADH4	4.74 ± 0.11	4.66 ± 0.03	4.72 ± 0.04	4.70 ± 0.07
TC04001405.hg.1	ADH5	7.84 ± 0.15	7.85 ± 0.18	7.57 ± 0.12	7.77 ± 0.12
TC06001847.hg.1	ADH5P4	8.06 ± 0.14	7.97 ± 0.07	7.62 ± 0.03	7.98 ± 0.14
TC04001408.hg.1	ADH6	4.95 ± 0.17	4.79 ± 0.04	4.87 ± 0.01	4.85 ± 0.03
TC04001412.hg.1	ADH7	5.25 ± 0.15	5.16 ± 0.07	5.17 ± 0.14	5.20 ± 0.09
TC08002604.hg.1	ADHFE1	7.35 ± 0.24	7.35 ± 0.10	7.35 ± 0.23	7.30 ± 0.18
TC02001524.hg.1	ADI1	7.91 ± 0.04	7.89 ± 0.07	7.90 ± 0.18	7.79 ± 0.05

TC20000286.hg.1	ADIG	7.10 ± 0.15	7.10 ± 0.19	7.09 ± 0.11	7.31 ± 0.08
TC03001027.hg.1	ADIPOQ	5.75 ± 0.11	5.57 ± 0.07	5.58 ± 0.02	5.68 ± 0.08
TC03002090.hg.1	ADIPOQ-AS1	5.52 ± 0.21	5.37 ± 0.14	5.50 ± 0.10	5.62 ± 0.35
TC01003711.hg.1	ADIPOR1	10.57 ± 0.12	10.42 ± 0.04	10.81 ± 0.13	10.63 ± 0.26
TC12000018.hg.1	ADIPOR2	8.41 ± 0.16	8.03 ± 0.19	8.33 ± 0.19	7.96 ± 0.15
TC10002938.hg.1	ADIRF	8.25 ± 0.27	8.25 ± 0.31	8.13 ± 0.26	8.36 ± 0.06
TC10000479.hg.1	ADK	7.98 ± 0.13	8.22 ± 0.20	7.71 ± 0.28	8.03 ± 0.10
TC11000182.hg.1	ADM	8.83 ± 0.47	8.55 ± 0.18	9.54 ± 0.21	8.56 ± 0.29
TC22000426.hg.1	ADM2	8.51 ± 0.32	8.51 ± 0.31	8.40 ± 0.24	8.60 ± 0.09
TC19002647.hg.1	ADM5	7.73 ± 0.06	7.75 ± 0.11	7.71 ± 0.09	7.79 ± 0.04
TC20000939.hg.1	ADNP	8.81 ± 0.19	8.91 ± 0.18	8.74 ± 0.18	8.81 ± 0.08
TC18000268.hg.1	ADNP2	7.88 ± 0.20	8.35 ± 0.28	7.78 ± 0.26	8.52 ± 0.13
TC10000383.hg.1	ADO	7.73 ± 0.20	7.65 ± 0.05	7.71 ± 0.09	7.63 ± 0.10
TC01001683.hg.1	ADORA1	7.68 ± 0.17	7.68 ± 0.23	7.61 ± 0.18	7.79 ± 0.03
TC22001457.hg.1	ADORA2A	8.81 ± 0.15	8.77 ± 0.08	8.74 ± 0.09	8.90 ± 0.03
TC22000588.hg.1	ADORA2A-AS1	7.33 ± 0.19	7.34 ± 0.09	7.32 ± 0.14	7.44 ± 0.06
TC17000180.hg.1	ADORA2B	8.89 ± 0.26	8.80 ± 0.18	8.82 ± 0.30	8.91 ± 0.03
TC01002986.hg.1	ADORA3	6.89 ± 0.18	6.73 ± 0.07	6.94 ± 0.26	6.83 ± 0.07
TC15001630.hg.1	ADPGK	8.96 ± 0.15	8.96 ± 0.14	8.90 ± 0.21	8.94 ± 0.02
TC15000663.hg.1	ADPGK-AS1	7.62 ± 0.21	7.57 ± 0.10	7.49 ± 0.03	7.75 ± 0.15
TC03000610.hg.1	ADPRH	7.60 ± 0.19	7.33 ± 0.07	7.53 ± 0.13	7.32 ± 0.04
TC13000894.hg.1	ADPRHL1	7.87 ± 0.16	7.82 ± 0.12	7.81 ± 0.16	7.88 ± 0.07
TC01000467.hg.1	ADPRHL2	8.12 ± 0.17	8.09 ± 0.13	7.95 ± 0.22	8.16 ± 0.08
TC17000151.hg.1	ADPRM	7.07 ± 0.27	7.03 ± 0.21	6.75 ± 0.31	6.97 ± 0.11
TC08001076.hg.1	ADRA1A	6.39 ± 0.14	6.29 ± 0.11	6.30 ± 0.11	6.39 ± 0.10
TC05000889.hg.1	ADRA1B	8.22 ± 0.23	8.27 ± 0.16	8.13 ± 0.23	8.37 ± 0.12
TC20000584.hg.1	ADRA1D	7.28 ± 0.23	7.39 ± 0.20	7.16 ± 0.22	7.47 ± 0.06
TC10000811.hg.1	ADRA2A	6.59 ± 0.16	6.57 ± 0.19	6.65 ± 0.27	6.72 ± 0.11
TC02002100.hg.1	ADRA2B	7.78 ± 0.13	7.72 ± 0.06	7.73 ± 0.13	7.75 ± 0.03
TC04000052.hg.1	ADRA2C	8.74 ± 0.31	8.62 ± 0.19	8.63 ± 0.26	8.69 ± 0.03
TC10000829.hg.1	ADRB1	7.71 ± 0.22	7.78 ± 0.14	7.56 ± 0.22	7.77 ± 0.04

TC05000814.hg.1	ADRB2	8.14 ± 0.40	7.78 ± 0.13	8.22 ± 0.33	7.60 ± 0.10
TC08001140.hg.1	ADRB3	7.12 ± 0.27	7.15 ± 0.19	7.09 ± 0.18	7.21 ± 0.07
TC11000682.hg.1	ADRBK1	10.99 ± 0.14	10.88 ± 0.02	10.97 ± 0.09	10.85 ± 0.01
TC22000165.hg.1	ADRBK2	8.84 ± 0.11	8.75 ± 0.23	8.91 ± 0.10	8.65 ± 0.18
TC20000487.hg.1	ADRM1	9.27 ± 0.19	9.04 ± 0.08	9.07 ± 0.16	8.99 ± 0.05
TC22001472.hg.1	ADSL	8.53 ± 0.08	8.47 ± 0.17	8.29 ± 0.14	8.37 ± 0.05
TC01004046.hg.1	ADSS	8.69 ± 0.29	8.81 ± 0.16	8.80 ± 0.21	8.76 ± 0.08
TC14000819.hg.1	ADSSL1	7.19 ± 0.19	7.14 ± 0.14	7.10 ± 0.14	7.19 ± 0.06
TC06001275.hg.1	ADTRP	6.29 ± 0.04	6.20 ± 0.03	6.23 ± 0.01	6.27 ± 0.11
TC07000273.hg.1	AEBP1	8.33 ± 0.14	8.31 ± 0.11	8.15 ± 0.15	8.32 ± 0.06
TC12000222.hg.1	AEBP2	8.76 ± 0.25	8.95 ± 0.11	8.71 ± 0.21	8.88 ± 0.10
TC15000840.hg.1	AEN	8.17 ± 0.12	8.08 ± 0.07	7.92 ± 0.17	8.08 ± 0.03
TC19001041.hg.1	AES	11.52 ± 0.09	11.71 ± 0.11	11.01 ± 0.08	11.64 ± 0.06
TC04001014.hg.1	AFAP1	7.01 ± 0.10	7.02 ± 0.15	6.96 ± 0.12	7.06 ± 0.04
TC04000083.hg.1	AFAP1-AS1	6.34 ± 0.41	6.36 ± 0.07	6.20 ± 0.13	6.65 ± 0.21
TC05000817.hg.1	AFAP1L1	7.36 ± 0.22	7.37 ± 0.21	7.33 ± 0.24	7.50 ± 0.05
TC10001675.hg.1	AFAP1L2	7.15 ± 0.14	7.03 ± 0.16	7.11 ± 0.18	7.11 ± 0.03
TC04000470.hg.1	AFF1	9.85 ± 0.23	9.79 ± 0.18	10.16 ± 0.30	9.74 ± 0.12
TC0X000708.hg.1	AFF2	7.25 ± 0.05	7.25 ± 0.03	7.23 ± 0.08	7.29 ± 0.04
TC0X000709.hg.1	AFF2-IT1	4.91 ± 0.06	4.89 ± 0.03	4.89 ± 0.10	4.84 ± 0.08
TC02002136.hg.1	AFF3	8.58 ± 0.13	8.78 ± 0.17	8.38 ± 0.13	8.64 ± 0.21
TC05001776.hg.1	AFF4	8.88 ± 0.24	9.08 ± 0.15	8.91 ± 0.36	9.11 ± 0.09
TC16000710.hg.1	AFG3L1P	7.45 ± 0.12	7.38 ± 0.06	7.34 ± 0.07	7.41 ± 0.01
TC18000373.hg.1	AFG3L2	8.09 ± 0.12	8.12 ± 0.22	7.73 ± 0.11	8.05 ± 0.10
TC04000405.hg.1	AFM	4.11 ± 0.10	4.08 ± 0.03	4.13 ± 0.11	4.12 ± 0.04
TC17000898.hg.1	AFMID	7.53 ± 0.07	7.44 ± 0.08	7.50 ± 0.06	7.51 ± 0.02
TC04000404.hg.1	AFP	4.67 ± 0.08	4.54 ± 0.02	4.59 ± 0.06	4.58 ± 0.03
TC02000368.hg.1	AFTPH	9.33 ± 0.35	9.47 ± 0.19	9.73 ± 0.48	9.32 ± 0.26
TC04001769.hg.1	AGA	7.96 ± 0.17	7.74 ± 0.15	7.85 ± 0.25	7.64 ± 0.03
TC02001430.hg.1	AGAP1	7.19 ± 0.09	7.19 ± 0.09	7.05 ± 0.19	7.16 ± 0.08
TC10002939.hg.1	AGAP11	6.66 ± 0.16	6.54 ± 0.09	6.54 ± 0.09	6.56 ± 0.05

TC02001431.hg.1	AGAP1-IT1	6.98 ± 0.49	6.90 ± 0.27	6.87 ± 0.32	7.01 ± 0.14
TC12001632.hg.1	AGAP2	9.05 ± 0.15	8.79 ± 0.02	8.95 ± 0.11	8.65 ± 0.08
TC12000540.hg.1	AGAP2-AS1	8.28 ± 0.49	8.15 ± 0.29	8.33 ± 0.26	8.17 ± 0.07
TC07001013.hg.1	AGAP3	8.08 ± 0.15	8.05 ± 0.15	8.01 ± 0.13	8.11 ± 0.04
TC10001228.hg.1	AGAP4	8.99 ± 0.45	9.12 ± 0.17	8.95 ± 0.36	9.13 ± 0.08
TC10001416.hg.1	AGAP5	8.76 ± 0.37	8.69 ± 0.16	8.59 ± 0.28	8.66 ± 0.04
TC10000340.hg.1	AGAP6	9.47 ± 0.55	9.57 ± 0.20	9.39 ± 0.42	9.56 ± 0.04
TC10001281.hg.1	AGAP7	9.63 ± 0.57	9.78 ± 0.25	9.59 ± 0.42	9.80 ± 0.06
TC10001279.hg.1	AGAP8	8.12 ± 0.33	8.13 ± 0.18	8.01 ± 0.26	8.05 ± 0.05
TC10000312.hg.1	AGAP9	8.53 ± 0.44	8.59 ± 0.22	8.42 ± 0.35	8.65 ± 0.04
TC10001240.hg.1	AGAP9	8.52 ± 0.42	8.56 ± 0.21	8.38 ± 0.35	8.62 ± 0.05
TC15000832.hg.1	AGBL1	5.98 ± 0.13	6.01 ± 0.19	5.99 ± 0.10	6.14 ± 0.07
TC15001810.hg.1	AGBL1-AS1	5.20 ± 0.03	4.97 ± 0.13	5.04 ± 0.11	5.12 ± 0.08
TC11001747.hg.1	AGBL2	6.11 ± 0.14	6.02 ± 0.10	5.99 ± 0.07	5.96 ± 0.05
TC07000839.hg.1	AGBL3	5.02 ± 0.06	4.98 ± 0.01	4.96 ± 0.05	5.02 ± 0.01
TC01002638.hg.1	AGBL4	6.30 ± 0.09	6.28 ± 0.13	6.26 ± 0.08	6.39 ± 0.12
TC01002640.hg.1	AGBL4-IT1	4.81 ± 0.18	4.48 ± 0.04	4.59 ± 0.05	4.50 ± 0.14
TC02000159.hg.1	AGBL5	7.49 ± 0.11	7.33 ± 0.05	7.33 ± 0.11	7.33 ± 0.06
TC02001672.hg.1	AGBL5-AS1	8.43 ± 0.32	8.36 ± 0.22	8.36 ± 0.24	8.47 ± 0.09
TC02000162.hg.1	AGBL5-IT1	7.50 ± 0.22	7.51 ± 0.08	7.41 ± 0.05	7.68 ± 0.14
TC06004049.hg.1	AGER	8.52 ± 0.03	8.40 ± 0.05	8.60 ± 0.03	8.46 ± 0.08
TC6_apd_hap1000095.hg.1	AGER	8.31 ± 0.05	8.17 ± 0.06	8.36 ± 0.03	8.24 ± 0.10
TC6_cox_hap2000187.hg.1	AGER	8.34 ± 0.05	8.21 ± 0.05	8.40 ± 0.04	8.28 ± 0.10
TC6_mann_hap4000152.hg.1	AGER	8.34 ± 0.05	8.21 ± 0.05	8.40 ± 0.04	8.28 ± 0.10
TC6_mcf_hap5000162.hg.1	AGER	8.31 ± 0.05	8.17 ± 0.06	8.36 ± 0.03	8.24 ± 0.10
TC6_qbl_hap6000176.hg.1	AGER	8.34 ± 0.05	8.21 ± 0.05	8.40 ± 0.04	8.28 ± 0.10
TC6_ssto_hap7000156.hg.1	AGER	8.34 ± 0.05	8.21 ± 0.05	8.40 ± 0.04	8.28 ± 0.10
TC02001363.hg.1	AGFG1	9.46 ± 0.21	9.30 ± 0.11	9.96 ± 0.34	9.34 ± 0.15
TC07000625.hg.1	AGFG2	8.41 ± 0.14	8.24 ± 0.10	8.18 ± 0.17	8.11 ± 0.03
TC05000377.hg.1	AGGF1	8.38 ± 0.27	8.33 ± 0.16	8.59 ± 0.34	8.17 ± 0.16
TC07000889.hg.1	AGK	8.11 ± 0.11	8.01 ± 0.13	7.89 ± 0.02	7.78 ± 0.02

TC01000895.hg.1	AGL	7.04 ± 0.52	7.30 ± 0.28	7.11 ± 0.45	7.02 ± 0.12
TC01002246.hg.1	AGMAT	7.58 ± 0.09	7.50 ± 0.09	7.29 ± 0.19	7.35 ± 0.05
TC07001155.hg.1	AGMO	4.46 ± 0.08	4.37 ± 0.03	4.39 ± 0.08	4.40 ± 0.05
TC01000463.hg.1	AGO1	8.96 ± 0.16	8.88 ± 0.13	8.90 ± 0.11	8.88 ± 0.07
TC08001684.hg.1	AGO2	9.15 ± 0.07	9.12 ± 0.12	9.16 ± 0.08	9.28 ± 0.22
TC01000465.hg.1	AGO3	8.46 ± 0.35	8.59 ± 0.22	8.52 ± 0.33	8.41 ± 0.18
TC01000462.hg.1	AGO4	9.90 ± 0.24	9.79 ± 0.30	10.47 ± 0.33	9.71 ± 0.25
TC06001561.hg.1	AGPAT1	8.58 ± 0.09	8.26 ± 0.05	8.50 ± 0.12	8.09 ± 0.06
TC6_apd_hap1000094.hg.1	AGPAT1	8.53 ± 0.09	8.19 ± 0.04	8.46 ± 0.12	8.03 ± 0.04
TC6_cox_hap2000186.hg.1	AGPAT1	8.53 ± 0.08	8.19 ± 0.04	8.46 ± 0.12	8.03 ± 0.04
TC6_mann_hap4000151.hg.1	AGPAT1	8.52 ± 0.08	8.18 ± 0.04	8.44 ± 0.12	8.02 ± 0.05
TC6_mcf_hap5000161.hg.1	AGPAT1	8.53 ± 0.08	8.19 ± 0.04	8.46 ± 0.12	8.03 ± 0.04
TC6_qbl_hap6000175.hg.1	AGPAT1	8.53 ± 0.08	8.19 ± 0.04	8.46 ± 0.12	8.03 ± 0.04
TC6_ssto_hap7000155.hg.1	AGPAT1	8.53 ± 0.09	8.19 ± 0.04	8.46 ± 0.12	8.03 ± 0.04
TC09001738.hg.1	AGPAT2	8.86 ± 0.24	8.74 ± 0.14	8.83 ± 0.20	8.79 ± 0.05
TC21000215.hg.1	AGPAT3	8.45 ± 0.12	8.34 ± 0.06	8.34 ± 0.13	8.31 ± 0.03
TC06002284.hg.1	AGPAT4	7.38 ± 0.07	7.28 ± 0.06	7.48 ± 0.10	7.33 ± 0.05
TC06002285.hg.1	AGPAT4-IT1	6.90 ± 0.40	6.70 ± 0.27	6.72 ± 0.20	6.62 ± 0.12
TC08000022.hg.1	AGPAT5	7.97 ± 0.13	7.98 ± 0.17	7.91 ± 0.18	7.95 ± 0.09
TC08000319.hg.1	AGPAT6	8.40 ± 0.06	8.39 ± 0.16	8.28 ± 0.15	8.46 ± 0.15
TC04000460.hg.1	AGPAT9	8.45 ± 0.17	8.11 ± 0.08	8.96 ± 0.38	8.08 ± 0.24
TC02001065.hg.1	AGPS	8.33 ± 0.33	8.48 ± 0.20	8.37 ± 0.34	8.26 ± 0.10
TC07001163.hg.1	AGR2	4.71 ± 0.08	4.62 ± 0.07	4.67 ± 0.05	4.72 ± 0.06
TC07001164.hg.1	AGR3	3.93 ± 0.10	3.79 ± 0.01	3.88 ± 0.04	3.83 ± 0.02
TC01000024.hg.1	AGRN	8.55 ± 0.20	8.53 ± 0.18	8.47 ± 0.19	8.58 ± 0.03
TC16001191.hg.1	AGRP	7.66 ± 0.10	7.67 ± 0.16	7.61 ± 0.18	7.73 ± 0.16
TC01006281.hg.1	AGT	7.16 ± 0.16	7.12 ± 0.22	7.03 ± 0.16	7.25 ± 0.09
TC09001287.hg.1	AGTPBP1	9.04 ± 0.42	9.20 ± 0.26	9.69 ± 0.43	9.14 ± 0.17
TC03000798.hg.1	AGTR1	6.00 ± 0.22	5.96 ± 0.15	5.99 ± 0.13	5.97 ± 0.04
TC0X000570.hg.1	AGTR2	4.50 ± 0.06	4.27 ± 0.07	4.35 ± 0.03	4.36 ± 0.11
TC01000147.hg.1	AGTRAP	9.67 ± 0.49	8.98 ± 0.07	9.84 ± 0.27	8.90 ± 0.11

TC02001471.hg.1	AGXT	8.14 ± 0.22	8.17 ± 0.23	8.08 ± 0.21	8.25 ± 0.05
TC05001260.hg.1	AGXT2	5.67 ± 0.17	5.58 ± 0.11	5.57 ± 0.13	5.67 ± 0.08
TC01004062.hg.1	AHCTF1	8.83 ± 0.54	9.33 ± 0.23	9.28 ± 0.56	9.24 ± 0.18
TC20000780.hg.1	AHCY	7.75 ± 0.21	7.64 ± 0.10	7.54 ± 0.20	7.58 ± 0.01
TC01000956.hg.1	AHCYL1	9.02 ± 0.08	8.87 ± 0.10	8.99 ± 0.15	8.64 ± 0.16
TC07000796.hg.1	AHCYL2	7.24 ± 0.08	7.18 ± 0.11	7.14 ± 0.14	7.08 ± 0.02
TC01002409.hg.1	AHDC1	8.55 ± 0.18	8.48 ± 0.18	8.42 ± 0.14	8.52 ± 0.01
TC06002136.hg.1	AHI1	5.63 ± 0.11	5.73 ± 0.05	5.59 ± 0.16	5.76 ± 0.06
TC11001867.hg.1	AHNAK	9.49 ± 0.02	9.53 ± 0.09	9.32 ± 0.01	9.41 ± 0.14
TC14001562.hg.1	AHNAK2	7.81 ± 0.25	7.71 ± 0.13	7.70 ± 0.23	7.80 ± 0.03
TC07000112.hg.1	AHR	9.23 ± 0.29	9.57 ± 0.12	9.35 ± 0.47	9.68 ± 0.34
TC14000491.hg.1	AHSA1	8.94 ± 0.10	8.73 ± 0.11	8.74 ± 0.32	8.43 ± 0.20
TC02000353.hg.1	AHSA2	8.47 ± 0.50	8.63 ± 0.14	8.53 ± 0.34	8.57 ± 0.07
TC03001020.hg.1	AHSG	6.70 ± 0.18	6.76 ± 0.14	6.62 ± 0.15	6.83 ± 0.10
TC16000380.hg.1	AHSP	5.07 ± 0.11	5.04 ± 0.26	5.11 ± 0.09	5.16 ± 0.08
TC12001180.hg.1	AICDA	6.00 ± 0.27	5.88 ± 0.15	5.93 ± 0.11	5.92 ± 0.01
TC01003868.hg.1	AIDA	9.05 ± 0.26	9.32 ± 0.16	8.99 ± 0.23	9.12 ± 0.01
TC06000373.hg.1	AIF1	10.12 ± 0.08	9.97 ± 0.12	10.40 ± 0.03	9.96 ± 0.23
TC6_cox_hap2000069.hg.1	AIF1	10.12 ± 0.08	9.97 ± 0.12	10.40 ± 0.03	9.96 ± 0.23
TC6_dbb_hap3000060.hg.1	AIF1	10.14 ± 0.08	9.99 ± 0.12	10.42 ± 0.03	9.97 ± 0.24
TC6_mcf_hap5000055.hg.1	AIF1	10.12 ± 0.09	9.96 ± 0.12	10.40 ± 0.04	9.93 ± 0.24
TC6_qbl_hap6000060.hg.1	AIF1	10.12 ± 0.08	9.97 ± 0.12	10.40 ± 0.03	9.96 ± 0.23
TC6_ssto_hap7000056.hg.1	AIF1	10.12 ± 0.08	9.97 ± 0.12	10.40 ± 0.03	9.96 ± 0.23
TC09000745.hg.1	AIF1L	7.17 ± 0.15	7.12 ± 0.11	7.08 ± 0.18	7.15 ± 0.03
TC0X001358.hg.1	AIFM1	7.95 ± 0.07	7.74 ± 0.07	7.70 ± 0.13	7.59 ± 0.01
TC10001359.hg.1	AIFM2	7.81 ± 0.22	7.74 ± 0.17	7.65 ± 0.23	7.81 ± 0.06
TC22001453.hg.1	AIFM3	8.14 ± 0.26	8.15 ± 0.21	8.09 ± 0.22	8.23 ± 0.05
TC06001050.hg.1	AIG1	7.17 ± 0.21	6.91 ± 0.03	7.35 ± 0.27	6.91 ± 0.07
TC06000846.hg.1	AIM1	8.76 ± 0.17	8.69 ± 0.20	9.04 ± 0.26	8.56 ± 0.19
TC01002389.hg.1	AIM1L	7.82 ± 0.21	7.76 ± 0.20	7.74 ± 0.19	7.83 ± 0.05
TC01003383.hg.1	AIM2	7.46 ± 0.39	6.89 ± 0.33	8.18 ± 0.26	6.56 ± 0.08

TC04000549.hg.1	AIMP1	7.85 ± 0.36	8.24 ± 0.13	7.82 ± 0.37	8.13 ± 0.05
TC07000059.hg.1	AIMP2	8.57 ± 0.18	8.29 ± 0.04	8.52 ± 0.04	8.22 ± 0.12
TC11000693.hg.1	AIP	9.24 ± 0.28	8.96 ± 0.07	8.89 ± 0.21	8.91 ± 0.02
TC17001062.hg.1	AIPL1	7.68 ± 0.14	7.63 ± 0.16	7.68 ± 0.15	7.71 ± 0.03
TC21000221.hg.1	AIRE	8.35 ± 0.25	8.31 ± 0.23	8.27 ± 0.20	8.41 ± 0.07
TC01000080.hg.1	AJAP1	7.40 ± 0.18	7.39 ± 0.11	7.35 ± 0.20	7.43 ± 0.07
TC14000943.hg.1	AJUBA	6.44 ± 0.15	6.36 ± 0.07	6.34 ± 0.07	6.41 ± 0.07
TC09002920.hg.1	AK1	8.32 ± 0.16	8.34 ± 0.14	8.22 ± 0.13	8.41 ± 0.08
TC01002479.hg.1	AK2	8.51 ± 0.13	8.26 ± 0.13	8.29 ± 0.13	8.02 ± 0.03
TC09000884.hg.1	AK3	8.10 ± 0.19	8.12 ± 0.11	8.03 ± 0.19	8.07 ± 0.02
TC01000728.hg.1	AK4	7.17 ± 0.16	7.07 ± 0.13	7.14 ± 0.11	7.08 ± 0.07
TC12001366.hg.1	AK4P3	6.47 ± 0.24	6.32 ± 0.23	6.46 ± 0.07	6.16 ± 0.08
TC01000785.hg.1	AK5	7.07 ± 0.09	7.31 ± 0.15	6.81 ± 0.10	7.16 ± 0.11
TC14000604.hg.1	AK7	5.26 ± 0.18	5.12 ± 0.07	5.15 ± 0.08	5.14 ± 0.01
TC09001676.hg.1	AK8	6.92 ± 0.19	6.86 ± 0.12	6.86 ± 0.16	6.93 ± 0.02
TC06002005.hg.1	AK9	5.87 ± 0.29	5.80 ± 0.12	6.01 ± 0.24	5.76 ± 0.07
TC17000704.hg.1	AKAP1	7.54 ± 0.12	7.52 ± 0.04	7.31 ± 0.08	7.48 ± 0.04
TC17001252.hg.1	AKAP10	8.65 ± 0.19	8.61 ± 0.19	8.82 ± 0.35	8.41 ± 0.14
TC13000152.hg.1	AKAP11	7.58 ± 0.68	8.07 ± 0.26	7.62 ± 0.51	7.85 ± 0.14
TC13000153.hg.1	AKAP11-IT1	6.25 ± 0.46	6.37 ± 0.05	6.25 ± 0.21	6.44 ± 0.19
TC06001100.hg.1	AKAP12	6.88 ± 0.19	6.89 ± 0.17	6.73 ± 0.14	6.88 ± 0.08
TC15000827.hg.1	AKAP13	9.97 ± 0.21	10.04 ± 0.10	10.08 ± 0.29	9.93 ± 0.14
TC0X000590.hg.1	AKAP14	5.46 ± 0.11	5.36 ± 0.11	5.38 ± 0.16	5.37 ± 0.04
TC0X001307.hg.1	AKAP16BP	4.73 ± 0.16	4.60 ± 0.11	4.59 ± 0.06	4.57 ± 0.10
TC0X002310.hg.1	AKAP17A	8.92 ± 0.05	8.95 ± 0.07	8.80 ± 0.08	8.90 ± 0.01
TC0Y000349.hg.1	AKAP17A	8.98 ± 0.04	9.00 ± 0.07	8.87 ± 0.08	8.95 ± 0.01
TC09000550.hg.1	AKAP2	7.39 ± 0.07	7.41 ± 0.06	7.30 ± 0.07	7.40 ± 0.04
TC12001124.hg.1	AKAP3	5.08 ± 0.07	4.92 ± 0.04	4.96 ± 0.10	4.93 ± 0.04
TC0X001036.hg.1	AKAP4	5.23 ± 0.11	5.17 ± 0.17	5.14 ± 0.13	5.18 ± 0.08
TC14000389.hg.1	AKAP5	5.23 ± 0.14	5.17 ± 0.06	5.13 ± 0.06	5.24 ± 0.09
TC14000208.hg.1	AKAP6	4.97 ± 0.08	4.91 ± 0.03	4.91 ± 0.03	4.94 ± 0.08

TC06000982.hg.1	AKAP7	6.46 ± 0.02	6.50 ± 0.04	6.48 ± 0.09	6.45 ± 0.03
TC19001253.hg.1	AKAP8	9.06 ± 0.10	9.17 ± 0.20	8.84 ± 0.02	9.22 ± 0.04
TC19001254.hg.1	AKAP8L	9.31 ± 0.05	9.21 ± 0.13	9.12 ± 0.16	9.24 ± 0.05
TC07000545.hg.1	AKAP9	7.51 ± 0.71	7.92 ± 0.23	7.70 ± 0.59	7.77 ± 0.14
TC11000165.hg.1	AKIP1	7.05 ± 0.09	7.08 ± 0.07	6.99 ± 0.08	7.06 ± 0.03
TC01000491.hg.1	AKIRIN1	9.76 ± 0.10	9.90 ± 0.08	9.85 ± 0.21	9.84 ± 0.23
TC06001929.hg.1	AKIRIN2	9.85 ± 0.32	9.55 ± 0.19	10.01 ± 0.36	9.45 ± 0.35
TC06000790.hg.1	AKIRIN2-AS1	7.39 ± 0.32	7.01 ± 0.03	7.46 ± 0.14	6.93 ± 0.16
TC09001508.hg.1	AKNA	10.12 ± 0.11	10.10 ± 0.04	10.09 ± 0.09	10.05 ± 0.06
TC01006363.hg.1	AKNAD1	5.42 ± 0.12	5.35 ± 0.10	5.32 ± 0.07	5.32 ± 0.01
TC01000574.hg.1	AKR1A1	8.17 ± 0.10	7.99 ± 0.02	8.00 ± 0.17	7.93 ± 0.05
TC07001883.hg.1	AKR1B1	8.06 ± 0.07	7.96 ± 0.10	7.77 ± 0.11	7.93 ± 0.08
TC07000834.hg.1	AKR1B10	6.63 ± 0.15	6.50 ± 0.13	6.55 ± 0.08	6.62 ± 0.04
TC07000835.hg.1	AKR1B15	6.34 ± 0.18	6.19 ± 0.21	6.29 ± 0.12	6.33 ± 0.05
TC18000038.hg.1	AKR1B1P6	7.22 ± 0.25	7.27 ± 0.21	7.13 ± 0.17	7.39 ± 0.12
TC10002924.hg.1	AKR1C1	6.20 ± 0.06	6.13 ± 0.04	6.26 ± 0.09	6.23 ± 0.10
TC10000986.hg.1	AKR1C2	5.79 ± 0.11	5.71 ± 0.02	5.86 ± 0.07	5.85 ± 0.07
TC10002925.hg.1	AKR1C3	6.50 ± 0.11	6.27 ± 0.02	6.42 ± 0.07	6.31 ± 0.05
TC10000044.hg.1	AKR1C4	5.33 ± 0.15	5.15 ± 0.01	5.29 ± 0.08	5.27 ± 0.12
TC10000983.hg.1	AKR1C6P	5.49 ± 0.11	5.41 ± 0.11	5.48 ± 0.08	5.52 ± 0.06
TC10000992.hg.1	AKR1C7P	6.68 ± 0.16	6.46 ± 0.15	6.58 ± 0.13	6.53 ± 0.04
TC10000989.hg.1	AKR1CL1	6.05 ± 0.13	5.96 ± 0.09	5.97 ± 0.07	6.05 ± 0.05
TC07000858.hg.1	AKR1D1	5.53 ± 0.07	5.41 ± 0.08	5.47 ± 0.04	5.53 ± 0.04
TC10000039.hg.1	AKR1E2	6.47 ± 0.06	6.42 ± 0.04	6.42 ± 0.09	6.44 ± 0.07
TC01002298.hg.1	AKR7A2	8.39 ± 0.17	8.15 ± 0.05	8.19 ± 0.16	8.08 ± 0.03
TC01000995.hg.1	AKR7A2P1	7.02 ± 0.21	7.14 ± 0.25	7.03 ± 0.03	7.27 ± 0.14
TC01002296.hg.1	AKR7A3	7.78 ± 0.14	7.61 ± 0.11	7.63 ± 0.16	7.64 ± 0.06
TC01002295.hg.1	AKR7L	7.79 ± 0.07	7.71 ± 0.05	7.69 ± 0.13	7.71 ± 0.04
TC14001560.hg.1	AKT1	9.38 ± 0.07	9.40 ± 0.06	9.22 ± 0.15	9.32 ± 0.01
TC19001728.hg.1	AKT1S1	8.32 ± 0.14	8.16 ± 0.04	8.11 ± 0.14	8.16 ± 0.03
TC19001532.hg.1	AKT2	8.76 ± 0.03	8.74 ± 0.05	8.63 ± 0.05	8.76 ± 0.04

TC01004040.hg.1	AKT3	7.76 ± 0.33	8.00 ± 0.29	7.67 ± 0.26	7.94 ± 0.07
TC01004043.hg.1	AKT3-IT1	6.85 ± 0.15	6.96 ± 0.09	6.86 ± 0.18	6.95 ± 0.19
TC16001120.hg.1	AKTIP	8.26 ± 0.33	8.30 ± 0.17	8.40 ± 0.39	8.24 ± 0.11
TC09001502.hg.1	ALAD	8.58 ± 0.12	8.53 ± 0.08	8.53 ± 0.10	8.52 ± 0.03
TC03000329.hg.1	ALAS1	9.27 ± 0.42	8.51 ± 0.10	9.37 ± 0.25	8.45 ± 0.16
TC0X001077.hg.1	ALAS2	7.40 ± 0.30	7.20 ± 0.24	7.68 ± 0.23	7.46 ± 0.23
TC04000403.hg.1	ALB	5.12 ± 0.13	5.04 ± 0.06	5.05 ± 0.09	5.10 ± 0.06
TC03000534.hg.1	ALCAM	6.79 ± 0.12	6.84 ± 0.21	7.09 ± 0.30	6.79 ± 0.09
TC19000733.hg.1	ALDH16A1	8.98 ± 0.16	8.85 ± 0.08	8.78 ± 0.14	8.85 ± 0.03
TC10001551.hg.1	ALDH18A1	8.11 ± 0.14	7.95 ± 0.14	7.75 ± 0.23	7.72 ± 0.00
TC09001211.hg.1	ALDH1A1	7.20 ± 0.24	7.23 ± 0.16	6.91 ± 0.17	7.09 ± 0.09
TC15001482.hg.1	ALDH1A2	6.37 ± 0.13	6.30 ± 0.08	6.36 ± 0.07	6.34 ± 0.02
TC15000971.hg.1	ALDH1A3	7.12 ± 0.21	7.12 ± 0.19	7.16 ± 0.17	7.23 ± 0.07
TC09000222.hg.1	ALDH1B1	7.39 ± 0.12	7.23 ± 0.09	7.23 ± 0.17	7.27 ± 0.07
TC03001733.hg.1	ALDH1L1	7.50 ± 0.21	7.47 ± 0.20	7.43 ± 0.19	7.58 ± 0.07
TC03000653.hg.1	ALDH1L1-AS1	7.58 ± 0.21	7.63 ± 0.19	7.60 ± 0.14	7.74 ± 0.13
TC03000654.hg.1	ALDH1L1-AS2	6.00 ± 0.15	6.10 ± 0.27	5.96 ± 0.14	6.11 ± 0.09
TC12001910.hg.1	ALDH1L2	5.94 ± 0.09	5.78 ± 0.12	5.82 ± 0.05	5.82 ± 0.01
TC12003212.hg.1	ALDH2	9.17 ± 0.15	9.03 ± 0.05	9.13 ± 0.14	8.97 ± 0.18
TC17001250.hg.1	ALDH3A1	7.73 ± 0.19	7.71 ± 0.18	7.67 ± 0.16	7.77 ± 0.05
TC17000246.hg.1	ALDH3A2	7.81 ± 0.08	7.61 ± 0.16	7.50 ± 0.11	7.45 ± 0.07
TC11000702.hg.1	ALDH3B1	9.13 ± 0.20	8.88 ± 0.14	9.10 ± 0.19	9.00 ± 0.06
TC11001995.hg.1	ALDH3B2	8.12 ± 0.17	7.99 ± 0.18	8.06 ± 0.17	8.10 ± 0.06
TC01006347.hg.1	ALDH4A1	7.50 ± 0.20	7.38 ± 0.16	7.38 ± 0.17	7.44 ± 0.02
TC06000146.hg.1	ALDH5A1	7.42 ± 0.05	7.33 ± 0.03	7.25 ± 0.17	7.30 ± 0.08
TC14001303.hg.1	ALDH6A1	7.30 ± 0.01	7.23 ± 0.05	7.24 ± 0.16	7.08 ± 0.06
TC05001742.hg.1	ALDH7A1	6.09 ± 0.13	6.02 ± 0.06	6.04 ± 0.08	6.08 ± 0.08
TC06002132.hg.1	ALDH8A1	6.55 ± 0.21	6.56 ± 0.06	6.56 ± 0.04	6.63 ± 0.06
TC01003466.hg.1	ALDH9A1	8.12 ± 0.10	7.86 ± 0.12	7.95 ± 0.17	7.74 ± 0.06
TC16000338.hg.1	ALDOA	9.13 ± 0.22	8.90 ± 0.05	9.09 ± 0.14	8.88 ± 0.13
TC09001421.hg.1	ALDOB	6.77 ± 0.22	6.69 ± 0.18	6.70 ± 0.18	6.72 ± 0.06

TC17001301.hg.1	ALDOC	8.04 ± 0.17	7.91 ± 0.04	7.85 ± 0.13	7.91 ± 0.02
TC16000139.hg.1	ALG1	7.96 ± 0.13	7.75 ± 0.01	7.68 ± 0.06	7.71 ± 0.01
TC12000301.hg.1	ALG10	7.66 ± 0.40	7.58 ± 0.11	7.48 ± 0.31	7.54 ± 0.07
TC12000305.hg.1	ALG10B	6.88 ± 0.28	6.94 ± 0.17	6.77 ± 0.13	6.93 ± 0.16
TC13000220.hg.1	ALG11	7.47 ± 0.23	7.36 ± 0.19	7.50 ± 0.32	7.28 ± 0.02
TC22000904.hg.1	ALG12	8.76 ± 0.07	8.65 ± 0.08	8.60 ± 0.10	8.59 ± 0.07
TC0X000546.hg.1	ALG13	7.42 ± 0.53	7.72 ± 0.21	7.46 ± 0.54	7.57 ± 0.18
TC0X001283.hg.1	ALG13-AS1	7.48 ± 0.15	7.51 ± 0.04	7.44 ± 0.07	7.57 ± 0.09
TC01002892.hg.1	ALG14	7.33 ± 0.02	7.24 ± 0.07	7.25 ± 0.08	7.23 ± 0.06
TC03001731.hg.1	ALG1L	7.94 ± 0.10	7.84 ± 0.05	7.78 ± 0.16	7.93 ± 0.07
TC08000937.hg.1	ALG1L13P	8.52 ± 0.21	8.68 ± 0.20	8.47 ± 0.24	8.66 ± 0.06
TC03000700.hg.1	ALG1L2	8.37 ± 0.11	8.23 ± 0.04	8.27 ± 0.05	8.25 ± 0.05
TC11002045.hg.1	ALG1L9P	7.73 ± 0.08	7.74 ± 0.05	7.65 ± 0.13	7.74 ± 0.03
TC09001411.hg.1	ALG2	8.52 ± 0.10	8.45 ± 0.16	8.35 ± 0.10	8.45 ± 0.03
TC03002067.hg.1	ALG3	8.89 ± 0.17	8.46 ± 0.07	8.51 ± 0.06	8.40 ± 0.04
TC13000570.hg.1	ALG5	6.94 ± 0.11	6.86 ± 0.17	6.62 ± 0.19	6.74 ± 0.04
TC01000719.hg.1	ALG6	6.83 ± 0.31	6.69 ± 0.24	6.80 ± 0.31	6.48 ± 0.03
TC11002133.hg.1	ALG8	8.34 ± 0.22	8.10 ± 0.21	7.99 ± 0.13	7.77 ± 0.11
TC11003493.hg.1	ALG9	7.99 ± 0.13	7.93 ± 0.13	7.67 ± 0.10	7.90 ± 0.01
TC11002287.hg.1	ALG9-IT1	5.53 ± 0.36	5.63 ± 0.08	5.51 ± 0.10	5.62 ± 0.06
TC02001708.hg.1	ALK	7.06 ± 0.17	7.07 ± 0.18	7.04 ± 0.13	7.20 ± 0.07
TC14001349.hg.1	ALKBH1	7.52 ± 0.18	7.19 ± 0.14	7.43 ± 0.16	7.16 ± 0.04
TC12001947.hg.1	ALKBH2	7.57 ± 0.03	7.53 ± 0.04	7.37 ± 0.22	7.59 ± 0.06
TC11000368.hg.1	ALKBH3	7.62 ± 0.03	7.46 ± 0.05	7.39 ± 0.01	7.44 ± 0.05
TC11001575.hg.1	ALKBH3-AS1	6.04 ± 0.07	5.86 ± 0.02	6.00 ± 0.09	5.96 ± 0.15
TC07001702.hg.1	ALKBH4	8.23 ± 0.15	8.12 ± 0.10	8.12 ± 0.15	8.14 ± 0.04
TC17000207.hg.1	ALKBH5	9.07 ± 0.16	9.07 ± 0.09	8.73 ± 0.21	8.97 ± 0.07
TC19002697.hg.1	ALKBH6	8.90 ± 0.10	8.79 ± 0.07	8.73 ± 0.13	8.76 ± 0.06
TC19000113.hg.1	ALKBH7	8.07 ± 0.23	7.92 ± 0.13	7.76 ± 0.22	7.87 ± 0.06
TC11002260.hg.1	ALKBH8	6.98 ± 0.24	7.07 ± 0.04	6.86 ± 0.14	6.98 ± 0.06
TC02000024.hg.1	ALLC	5.86 ± 0.11	5.78 ± 0.10	5.81 ± 0.13	5.86 ± 0.06

TC02000443.hg.1	ALMS1	6.95 ± 0.40	7.16 ± 0.28	6.77 ± 0.34	7.01 ± 0.13
TC02000444.hg.1	ALMS1-IT1	5.97 ± 0.16	5.95 ± 0.05	5.93 ± 0.02	6.00 ± 0.08
TC02000445.hg.1	ALMS1P	6.84 ± 0.05	6.67 ± 0.06	6.65 ± 0.08	6.51 ± 0.02
TC17000081.hg.1	ALOX12	8.53 ± 0.13	7.96 ± 0.04	8.67 ± 0.31	8.04 ± 0.18
TC17001100.hg.1	ALOX12B	7.62 ± 0.23	7.53 ± 0.20	7.48 ± 0.20	7.65 ± 0.08
TC17000079.hg.1	ALOX12P2	7.04 ± 0.15	6.99 ± 0.11	6.97 ± 0.16	7.10 ± 0.05
TC17001027.hg.1	ALOX15	8.21 ± 0.33	8.35 ± 0.14	8.78 ± 0.31	8.59 ± 0.33
TC17000124.hg.1	ALOX15B	7.79 ± 0.39	7.55 ± 0.17	7.56 ± 0.13	7.68 ± 0.09
TC17000076.hg.1	ALOX15P1	7.85 ± 0.09	7.86 ± 0.03	8.06 ± 0.30	7.98 ± 0.22
TC10000293.hg.1	ALOX5	10.96 ± 0.29	10.71 ± 0.13	11.59 ± 0.29	10.69 ± 0.15
TC13000100.hg.1	ALOX5AP	11.29 ± 0.21	11.08 ± 0.03	11.68 ± 0.24	11.10 ± 0.49
TC17001101.hg.1	ALOXE3	7.07 ± 0.17	7.06 ± 0.15	7.06 ± 0.16	7.15 ± 0.06
TC02001400.hg.1	ALPI	7.60 ± 0.18	7.56 ± 0.19	7.61 ± 0.15	7.66 ± 0.03
TC04000581.hg.1	ALPK1	7.80 ± 0.23	7.62 ± 0.27	8.47 ± 0.46	7.42 ± 0.27
TC18000542.hg.1	ALPK2	5.06 ± 0.15	5.12 ± 0.05	5.12 ± 0.11	5.15 ± 0.03
TC15000822.hg.1	ALPK3	6.98 ± 0.17	6.98 ± 0.16	6.97 ± 0.10	7.07 ± 0.06
TC01000272.hg.1	ALPL	11.24 ± 0.56	10.43 ± 0.20	12.13 ± 0.18	10.46 ± 0.76
TC02001398.hg.1	ALPP	7.86 ± 0.22	7.85 ± 0.16	7.82 ± 0.18	7.91 ± 0.02
TC02001399.hg.1	ALPPL2	7.85 ± 0.22	7.76 ± 0.16	7.69 ± 0.17	7.81 ± 0.04
TC02002678.hg.1	ALS2	6.57 ± 0.08	6.52 ± 0.07	6.67 ± 0.24	6.53 ± 0.04
TC03001359.hg.1	ALS2CL	8.17 ± 0.16	8.16 ± 0.19	8.09 ± 0.19	8.26 ± 0.09
TC02002675.hg.1	ALS2CR11	4.17 ± 0.13	3.98 ± 0.05	4.05 ± 0.06	4.01 ± 0.01
TC02002673.hg.1	ALS2CR12	6.48 ± 0.19	6.34 ± 0.10	6.41 ± 0.10	6.36 ± 0.06
TC12000685.hg.1	ALX1	6.36 ± 0.25	6.25 ± 0.28	6.19 ± 0.31	6.23 ± 0.16
TC01002965.hg.1	ALX3	7.73 ± 0.33	7.63 ± 0.27	7.50 ± 0.26	7.79 ± 0.09
TC11001577.hg.1	ALX4	7.52 ± 0.24	7.51 ± 0.20	7.42 ± 0.25	7.61 ± 0.08
TC17001962.hg.1	ALYREF	8.54 ± 0.20	8.49 ± 0.21	8.19 ± 0.21	8.34 ± 0.08
TC05003422.hg.1	AMACR	6.81 ± 0.08	6.63 ± 0.05	6.76 ± 0.12	6.61 ± 0.03
TC04000388.hg.1	AMBN	5.58 ± 0.11	5.47 ± 0.08	5.45 ± 0.10	5.56 ± 0.10
TC09001506.hg.1	AMBP	7.36 ± 0.24	7.28 ± 0.19	7.28 ± 0.23	7.32 ± 0.03
TC11001720.hg.1	AMBRA1	8.62 ± 0.14	8.49 ± 0.05	8.46 ± 0.05	8.51 ± 0.09

TC06000883.hg.1	AMD1	9.71 ± 0.40	9.87 ± 0.18	9.70 ± 0.26	9.80 ± 0.15
TC12000743.hg.1	AMDHD1	6.65 ± 0.06	6.57 ± 0.16	6.66 ± 0.07	6.65 ± 0.08
TC16000076.hg.1	AMDHD2	8.57 ± 0.13	8.63 ± 0.15	8.43 ± 0.14	8.72 ± 0.04
TC0X000043.hg.1	AMELX	6.11 ± 0.19	5.97 ± 0.12	5.98 ± 0.07	6.02 ± 0.11
TC0Y000132.hg.1	AMELY	5.65 ± 0.13	5.62 ± 0.12	5.58 ± 0.07	5.76 ± 0.12
TC0X001095.hg.1	AMER1	7.16 ± 0.11	7.20 ± 0.08	7.11 ± 0.14	7.33 ± 0.05
TC13000496.hg.1	AMER2	7.41 ± 0.20	7.41 ± 0.10	7.44 ± 0.08	7.40 ± 0.07
TC02000827.hg.1	AMER3	7.99 ± 0.15	7.92 ± 0.16	7.92 ± 0.02	7.98 ± 0.09
TC16001132.hg.1	AMFR	9.27 ± 0.18	9.28 ± 0.18	9.13 ± 0.20	9.24 ± 0.04
TC19000051.hg.1	AMH	8.64 ± 0.29	8.64 ± 0.21	8.60 ± 0.27	8.71 ± 0.07
TC12000438.hg.1	AMHR2	7.42 ± 0.14	7.48 ± 0.21	7.38 ± 0.16	7.53 ± 0.06
TC11002344.hg.1	AMICA1	10.35 ± 0.36	10.25 ± 0.22	10.94 ± 0.29	10.09 ± 0.12
TC01002957.hg.1	AMIGO1	7.50 ± 0.09	7.55 ± 0.13	7.26 ± 0.16	7.58 ± 0.10
TC12001430.hg.1	AMIGO2	6.63 ± 0.11	6.53 ± 0.05	6.55 ± 0.07	6.48 ± 0.03
TC0X001273.hg.1	AMMECR1	7.56 ± 0.06	7.63 ± 0.05	7.49 ± 0.08	7.58 ± 0.02
TC0X001275.hg.1	AMMECR1-IT1	4.45 ± 0.10	4.36 ± 0.05	4.35 ± 0.08	4.36 ± 0.11
TC02002289.hg.1	AMMECR1L	7.65 ± 0.10	7.54 ± 0.03	7.66 ± 0.11	7.46 ± 0.05
TC14000790.hg.1	AMN	8.69 ± 0.24	8.67 ± 0.21	8.63 ± 0.27	8.75 ± 0.01
TC12001367.hg.1	AMN1	7.64 ± 0.09	7.43 ± 0.11	7.67 ± 0.19	7.39 ± 0.07
TC0X001286.hg.1	AMOT	7.22 ± 0.08	7.20 ± 0.04	7.18 ± 0.05	7.23 ± 0.05
TC11000924.hg.1	AMOTL1	7.26 ± 0.13	7.20 ± 0.14	7.19 ± 0.16	7.24 ± 0.04
TC03001804.hg.1	AMOTL2	8.03 ± 0.17	8.02 ± 0.17	7.97 ± 0.16	8.11 ± 0.08
TC01003013.hg.1	AMPD1	5.69 ± 0.15	5.43 ± 0.07	5.54 ± 0.13	5.51 ± 0.04
TC01000950.hg.1	AMPD2	9.52 ± 0.20	9.84 ± 0.12	9.58 ± 0.09	9.88 ± 0.16
TC11000184.hg.1	AMPD3	8.81 ± 0.13	8.55 ± 0.10	9.14 ± 0.17	8.61 ± 0.24
TC07001303.hg.1	AMPH	6.50 ± 0.15	6.38 ± 0.11	6.43 ± 0.10	6.49 ± 0.05
TC03003386.hg.1	AMT	7.68 ± 0.13	7.69 ± 0.14	7.61 ± 0.09	7.79 ± 0.10
TC04000387.hg.1	AMTN	5.33 ± 0.07	5.28 ± 0.01	5.33 ± 0.07	5.31 ± 0.08
TC01002935.hg.1	AMY1A	6.95 ± 0.19	6.98 ± 0.16	6.95 ± 0.13	7.07 ± 0.32
TC01000919.hg.1	AMY1B	6.88 ± 0.20	6.92 ± 0.14	6.88 ± 0.14	7.04 ± 0.34
TC01000921.hg.1	AMY1B	7.41 ± 0.35	7.54 ± 0.19	7.48 ± 0.22	7.63 ± 0.34

TC01000918.hg.1	AMY2A	6.64 ± 0.36	6.74 ± 0.24	6.66 ± 0.44	6.89 ± 0.35
TC07000037.hg.1	AMZ1	7.77 ± 0.15	7.74 ± 0.16	7.66 ± 0.17	7.81 ± 0.04
TC17000801.hg.1	AMZ2	8.81 ± 0.01	8.88 ± 0.18	8.67 ± 0.09	8.74 ± 0.11
TC17001807.hg.1	AMZ2P1	7.45 ± 0.15	7.69 ± 0.21	7.29 ± 0.30	7.62 ± 0.11
TC02002211.hg.1	ANAPC1	8.46 ± 0.26	8.53 ± 0.24	8.09 ± 0.20	8.27 ± 0.02
TC04001608.hg.1	ANAPC10	5.57 ± 0.20	5.66 ± 0.17	5.49 ± 0.08	5.59 ± 0.03
TC17000938.hg.1	ANAPC11	8.72 ± 0.24	8.62 ± 0.11	8.48 ± 0.27	8.62 ± 0.03
TC03001805.hg.1	ANAPC13	8.17 ± 0.02	8.19 ± 0.09	8.14 ± 0.11	8.09 ± 0.05
TC11002054.hg.1	ANAPC15	8.33 ± 0.10	8.31 ± 0.12	8.30 ± 0.05	8.32 ± 0.02
TC10000443.hg.1	ANAPC16	8.84 ± 0.05	8.85 ± 0.19	8.69 ± 0.18	8.66 ± 0.09
TC02000527.hg.1	ANAPC1P1	8.05 ± 0.27	8.09 ± 0.15	7.79 ± 0.11	7.99 ± 0.04
TC09001762.hg.1	ANAPC2	8.50 ± 0.16	8.48 ± 0.09	8.38 ± 0.12	8.48 ± 0.03
TC04000176.hg.1	ANAPC4	7.57 ± 0.39	7.69 ± 0.21	7.65 ± 0.42	7.47 ± 0.06
TC12002062.hg.1	ANAPC5	9.08 ± 0.12	8.92 ± 0.17	8.71 ± 0.10	8.71 ± 0.06
TC12003221.hg.1	ANAPC7	8.03 ± 0.12	7.81 ± 0.16	7.92 ± 0.17	7.69 ± 0.05
TC14000067.hg.1	ANG	7.31 ± 0.17	7.23 ± 0.11	7.23 ± 0.13	7.20 ± 0.07
TC14001334.hg.1	ANGEL1	8.14 ± 0.04	8.04 ± 0.06	7.85 ± 0.07	7.97 ± 0.03
TC01003813.hg.1	ANGEL2	7.68 ± 0.37	7.70 ± 0.16	7.68 ± 0.32	7.79 ± 0.05
TC08001521.hg.1	ANGPT1	5.62 ± 0.03	5.57 ± 0.11	5.61 ± 0.06	5.59 ± 0.01
TC08000902.hg.1	ANGPT2	5.50 ± 0.12	5.35 ± 0.07	5.43 ± 0.04	5.36 ± 0.03
TC20000546.hg.1	ANGPT4	7.29 ± 0.11	7.31 ± 0.19	7.26 ± 0.21	7.41 ± 0.06
TC01003565.hg.1	ANGPTL1	4.98 ± 0.08	4.92 ± 0.04	4.94 ± 0.07	4.87 ± 0.05
TC09001599.hg.1	ANGPTL2	7.20 ± 0.21	7.21 ± 0.22	7.10 ± 0.20	7.30 ± 0.08
TC01000708.hg.1	ANGPTL3	3.77 ± 0.13	3.65 ± 0.06	3.74 ± 0.07	3.64 ± 0.03
TC19000147.hg.1	ANGPTL4	7.80 ± 0.12	7.68 ± 0.15	7.79 ± 0.13	7.75 ± 0.02
TC11002223.hg.1	ANGPTL5	3.95 ± 0.12	3.80 ± 0.04	3.80 ± 0.05	3.81 ± 0.03
TC19001157.hg.1	ANGPTL6	8.28 ± 0.17	8.22 ± 0.12	8.14 ± 0.13	8.27 ± 0.02
TC01000139.hg.1	ANGPTL7	6.91 ± 0.22	6.85 ± 0.16	6.88 ± 0.16	6.92 ± 0.04
TC12002163.hg.1	ANHX	8.23 ± 0.19	8.17 ± 0.19	8.13 ± 0.19	8.31 ± 0.10
TC08001171.hg.1	ANK1	7.85 ± 0.12	7.66 ± 0.10	7.81 ± 0.14	7.74 ± 0.02
TC04000584.hg.1	ANK2	5.81 ± 0.16	5.73 ± 0.08	5.75 ± 0.08	5.79 ± 0.08

TC10001319.hg.1	ANK3	6.63 ± 0.08	6.74 ± 0.13	6.65 ± 0.13	6.70 ± 0.27
TC02001111.hg.1	ANKAR	6.05 ± 0.18	6.10 ± 0.12	5.99 ± 0.12	6.03 ± 0.05
TC15002765.hg.1	ANKDD1A	8.28 ± 0.16	8.31 ± 0.08	8.25 ± 0.11	8.41 ± 0.04
TC05000365.hg.1	ANKDD1B	5.66 ± 0.19	5.59 ± 0.09	5.60 ± 0.11	5.66 ± 0.04
TC20001756.hg.1	ANKEF1	6.58 ± 0.11	6.44 ± 0.09	6.43 ± 0.08	6.42 ± 0.02
TC17000696.hg.1	ANKFN1	5.42 ± 0.17	5.30 ± 0.09	5.35 ± 0.07	5.39 ± 0.06
TC17001019.hg.1	ANKFY1	9.26 ± 0.30	9.00 ± 0.18	9.33 ± 0.31	8.91 ± 0.16
TC05001172.hg.1	ANKH	8.52 ± 0.14	8.68 ± 0.06	8.21 ± 0.02	8.59 ± 0.03
TC07000547.hg.1	ANKIB1	8.18 ± 0.36	8.31 ± 0.20	8.03 ± 0.35	8.08 ± 0.10
TC11001018.hg.1	ANKK1	7.26 ± 0.20	7.17 ± 0.15	7.19 ± 0.18	7.22 ± 0.01
TC19002627.hg.1	ANKLE1	8.30 ± 0.24	8.20 ± 0.16	8.18 ± 0.19	8.26 ± 0.04
TC12002155.hg.1	ANKLE2	8.85 ± 0.11	8.91 ± 0.07	8.80 ± 0.14	8.92 ± 0.03
TC02002938.hg.1	ANKMY1	8.05 ± 0.14	8.04 ± 0.08	7.97 ± 0.15	8.08 ± 0.03
TC07001161.hg.1	ANKMY2	7.28 ± 0.15	7.27 ± 0.09	7.17 ± 0.20	7.12 ± 0.03
TC05001472.hg.1	ANKRA2	7.10 ± 0.38	7.24 ± 0.15	7.12 ± 0.38	7.19 ± 0.04
TC10001517.hg.1	ANKRD1	5.23 ± 0.18	5.19 ± 0.10	5.22 ± 0.14	5.27 ± 0.05
TC13000878.hg.1	ANKRD10	8.58 ± 0.31	8.85 ± 0.13	8.68 ± 0.30	8.77 ± 0.09
TC13000879.hg.1	ANKRD10-IT1	7.98 ± 0.51	7.98 ± 0.41	8.15 ± 0.45	7.85 ± 0.16
TC16001350.hg.1	ANKRD11	8.99 ± 0.03	8.99 ± 0.02	8.99 ± 0.08	9.09 ± 0.09
TC18000042.hg.1	ANKRD12	8.68 ± 0.66	9.30 ± 0.23	9.20 ± 0.57	9.34 ± 0.09
TC12000854.hg.1	ANKRD13A	10.04 ± 0.28	10.16 ± 0.11	10.37 ± 0.29	10.17 ± 0.20
TC17000343.hg.1	ANKRD13B	8.23 ± 0.25	8.21 ± 0.21	8.16 ± 0.27	8.29 ± 0.05
TC01002771.hg.1	ANKRD13C	7.75 ± 0.53	7.94 ± 0.23	7.74 ± 0.40	7.80 ± 0.18
TC11000683.hg.1	ANKRD13D	9.22 ± 0.08	9.20 ± 0.03	9.31 ± 0.09	9.21 ± 0.07
TC10001004.hg.1	ANKRD16	7.45 ± 0.10	7.39 ± 0.10	7.29 ± 0.03	7.37 ± 0.03
TC04001279.hg.1	ANKRD17	9.04 ± 0.38	9.23 ± 0.18	9.06 ± 0.35	9.04 ± 0.17
TC09001088.hg.1	ANKRD18A	5.62 ± 0.08	5.50 ± 0.01	5.66 ± 0.01	5.60 ± 0.07
TC09000143.hg.1	ANKRD18B	4.74 ± 0.11	4.58 ± 0.09	4.75 ± 0.06	4.65 ± 0.10
TC09002939.hg.1	ANKRD18CP	5.04 ± 0.10	4.97 ± 0.04	5.03 ± 0.03	4.98 ± 0.05
TC03002187.hg.1	ANKRD18DP	4.81 ± 0.06	4.63 ± 0.02	4.75 ± 0.05	4.74 ± 0.02
TC09000438.hg.1	ANKRD19P	5.97 ± 0.06	5.83 ± 0.03	5.86 ± 0.04	5.85 ± 0.05

TC10000698.hg.1	ANKRD2	7.96 ± 0.18	7.87 ± 0.11	7.94 ± 0.24	7.93 ± 0.05
TC09002932.hg.1	ANKRD20A1	6.13 ± 0.27	6.12 ± 0.04	6.17 ± 0.26	6.23 ± 0.12
TC21000281.hg.1	ANKRD20A11P	6.84 ± 0.36	7.01 ± 0.21	6.86 ± 0.40	7.09 ± 0.29
TC13000487.hg.1	ANKRD20A19P	6.83 ± 0.13	6.84 ± 0.09	6.79 ± 0.04	6.87 ± 0.06
TC09002898.hg.1	ANKRD20A2	6.02 ± 0.26	6.02 ± 0.03	6.05 ± 0.29	6.13 ± 0.13
TC09001113.hg.1	ANKRD20A3	6.17 ± 0.22	6.18 ± 0.03	6.20 ± 0.23	6.26 ± 0.14
TC09000295.hg.1	ANKRD20A4	6.07 ± 0.24	6.14 ± 0.03	6.15 ± 0.28	6.23 ± 0.13
TC18000077.hg.1	ANKRD20A5P	6.31 ± 0.27	6.42 ± 0.07	6.38 ± 0.19	6.47 ± 0.17
TC02002086.hg.1	ANKRD20A8P	6.04 ± 0.17	6.05 ± 0.03	6.09 ± 0.13	6.15 ± 0.12
TC13000443.hg.1	ANKRD20A9P	6.40 ± 0.27	6.31 ± 0.07	6.50 ± 0.16	6.53 ± 0.18
TC10001497.hg.1	ANKRD22	7.60 ± 0.92	6.78 ± 0.19	8.78 ± 1.08	6.49 ± 0.31
TC02005037.hg.1	ANKRD23	7.89 ± 0.15	7.81 ± 0.16	7.74 ± 0.14	7.84 ± 0.06
TC19000082.hg.1	ANKRD24	8.04 ± 0.24	8.00 ± 0.19	7.98 ± 0.22	8.05 ± 0.03
TC10001124.hg.1	ANKRD26	6.28 ± 0.41	6.44 ± 0.19	6.33 ± 0.30	6.31 ± 0.13
TC16001081.hg.1	ANKRD26P1	4.73 ± 0.22	4.53 ± 0.07	4.65 ± 0.03	4.59 ± 0.03
TC13000449.hg.1	ANKRD26P3	4.90 ± 0.06	4.78 ± 0.01	4.81 ± 0.01	4.81 ± 0.04
TC19001388.hg.1	ANKRD27	7.97 ± 0.16	7.68 ± 0.11	7.91 ± 0.14	7.58 ± 0.05
TC03001213.hg.1	ANKRD28	8.09 ± 0.32	7.94 ± 0.13	8.23 ± 0.43	7.94 ± 0.05
TC18000415.hg.1	ANKRD29	6.47 ± 0.22	6.31 ± 0.07	6.35 ± 0.16	6.39 ± 0.07
TC10000245.hg.1	ANKRD30A	4.62 ± 0.10	4.47 ± 0.06	4.49 ± 0.02	4.55 ± 0.11
TC18000084.hg.1	ANKRD30B	4.43 ± 0.12	4.20 ± 0.02	4.33 ± 0.04	4.26 ± 0.04
TC02002358.hg.1	ANKRD30BL	5.71 ± 0.08	5.61 ± 0.08	5.64 ± 0.04	5.76 ± 0.11
TC21000013.hg.1	ANKRD30BP2	5.39 ± 0.13	5.32 ± 0.03	5.30 ± 0.06	5.49 ± 0.18
TC10000291.hg.1	ANKRD30BP3	5.03 ± 0.07	4.95 ± 0.02	4.98 ± 0.06	4.98 ± 0.03
TC05001489.hg.1	ANKRD31	4.86 ± 0.08	4.76 ± 0.04	4.70 ± 0.05	4.72 ± 0.08
TC05000453.hg.1	ANKRD32	6.49 ± 0.25	6.47 ± 0.27	6.72 ± 0.28	6.25 ± 0.13
TC12000409.hg.1	ANKRD33	7.57 ± 0.23	7.47 ± 0.15	7.40 ± 0.20	7.55 ± 0.12
TC05000077.hg.1	ANKRD33B	7.97 ± 0.25	7.88 ± 0.15	7.90 ± 0.24	7.96 ± 0.07
TC05001163.hg.1	ANKRD33B-AS1	6.67 ± 0.30	6.51 ± 0.14	6.68 ± 0.07	6.59 ± 0.10
TC01001091.hg.1	ANKRD34A	7.09 ± 0.13	6.99 ± 0.10	7.03 ± 0.11	7.05 ± 0.09
TC05001536.hg.1	ANKRD34B	6.42 ± 0.19	6.09 ± 0.08	7.25 ± 0.42	6.11 ± 0.06

TC15000740.hg.1	ANKRD34C	6.32 ± 0.24	6.22 ± 0.18	6.08 ± 0.25	6.43 ± 0.14
TC01001096.hg.1	ANKRD35	7.40 ± 0.14	7.48 ± 0.20	7.43 ± 0.12	7.58 ± 0.08
TC02000589.hg.1	ANKRD36	8.71 ± 0.90	9.37 ± 0.22	8.70 ± 0.57	9.31 ± 0.09
TC02002120.hg.1	ANKRD36B	8.69 ± 0.86	9.37 ± 0.16	8.73 ± 0.54	9.28 ± 0.11
TC01003486.hg.1	ANKRD36BP1	8.81 ± 0.41	8.70 ± 0.28	9.04 ± 0.19	8.73 ± 0.17
TC02000547.hg.1	ANKRD36BP2	7.81 ± 0.47	7.82 ± 0.28	7.51 ± 0.29	7.78 ± 0.03
TC04000905.hg.1	ANKRD37	7.99 ± 0.13	8.03 ± 0.04	7.84 ± 0.12	7.96 ± 0.06
TC02005038.hg.1	ANKRD39	8.66 ± 0.19	8.38 ± 0.15	8.42 ± 0.15	8.34 ± 0.03
TC17001696.hg.1	ANKRD40	8.84 ± 0.08	8.85 ± 0.10	8.67 ± 0.19	8.76 ± 0.05
TC11000858.hg.1	ANKRD42	6.67 ± 0.02	6.63 ± 0.04	6.68 ± 0.11	6.64 ± 0.13
TC02002645.hg.1	ANKRD44	9.59 ± 0.37	9.86 ± 0.18	9.76 ± 0.32	9.68 ± 0.18
TC02002646.hg.1	ANKRD44-IT1	8.25 ± 0.42	8.33 ± 0.37	8.31 ± 0.16	8.16 ± 0.11
TC01003529.hg.1	ANKRD45	5.75 ± 0.07	5.57 ± 0.03	5.56 ± 0.01	5.64 ± 0.08
TC08001479.hg.1	ANKRD46	7.31 ± 0.12	7.32 ± 0.05	7.19 ± 0.03	7.32 ± 0.06
TC11000919.hg.1	ANKRD49	7.58 ± 0.51	7.99 ± 0.32	7.61 ± 0.54	7.91 ± 0.05
TC04001530.hg.1	ANKRD50	7.06 ± 0.18	6.89 ± 0.17	7.41 ± 0.45	6.92 ± 0.11
TC12001599.hg.1	ANKRD52	8.43 ± 0.08	8.37 ± 0.09	8.17 ± 0.04	8.32 ± 0.03
TC02000424.hg.1	ANKRD53	7.37 ± 0.27	7.43 ± 0.27	7.35 ± 0.21	7.57 ± 0.06
TC05001369.hg.1	ANKRD55	7.41 ± 0.04	7.30 ± 0.04	7.57 ± 0.10	7.23 ± 0.03
TC06000797.hg.1	ANKRD6	7.06 ± 0.10	7.03 ± 0.07	7.10 ± 0.05	7.08 ± 0.03
TC20000983.hg.1	ANKRD60	6.73 ± 0.15	6.69 ± 0.21	6.79 ± 0.03	6.81 ± 0.14
TC07000061.hg.1	ANKRD61	6.83 ± 0.27	6.79 ± 0.17	6.91 ± 0.17	6.96 ± 0.13
TC18000061.hg.1	ANKRD62	4.29 ± 0.14	4.13 ± 0.03	4.22 ± 0.09	4.18 ± 0.06
TC22000464.hg.1	ANKRD62P1-PARP4P3	4.92 ± 0.06	4.81 ± 0.02	4.87 ± 0.04	4.86 ± 0.01
TC15001232.hg.1	ANKRD63	8.28 ± 0.40	8.31 ± 0.18	8.29 ± 0.27	8.30 ± 0.03
TC01002097.hg.1	ANKRD65	8.55 ± 0.31	8.49 ± 0.24	8.45 ± 0.27	8.58 ± 0.05
TC06000630.hg.1	ANKRD66	5.77 ± 0.09	5.72 ± 0.09	5.74 ± 0.12	5.73 ± 0.11
TC07000729.hg.1	ANKRD7	4.46 ± 0.09	4.37 ± 0.04	4.42 ± 0.11	4.35 ± 0.06
TC14001531.hg.1	ANKRD9	8.36 ± 0.08	8.15 ± 0.17	8.29 ± 0.25	8.19 ± 0.04
TC06000510.hg.1	ANKS1A	8.02 ± 0.17	7.84 ± 0.02	8.28 ± 0.18	7.79 ± 0.07
TC12001862.hg.1	ANKS1B	5.68 ± 0.14	5.55 ± 0.08	5.59 ± 0.08	5.61 ± 0.08

TC16000833.hg.1	ANKS3	8.02 ± 0.15	8.01 ± 0.15	7.95 ± 0.17	8.06 ± 0.04
TC16000228.hg.1	ANKS4B	6.88 ± 0.20	6.81 ± 0.10	6.62 ± 0.37	6.86 ± 0.13
TC09001408.hg.1	ANKS6	7.39 ± 0.07	7.41 ± 0.06	7.26 ± 0.09	7.45 ± 0.05
TC03003396.hg.1	ANKUB1	5.27 ± 0.20	5.20 ± 0.05	5.19 ± 0.02	5.19 ± 0.02
TC03003397.hg.1	ANKUB1	6.41 ± 0.20	6.53 ± 0.11	6.78 ± 0.22	6.38 ± 0.09
TC02001320.hg.1	ANKZF1	8.17 ± 0.08	7.97 ± 0.09	7.98 ± 0.06	7.93 ± 0.06
TC07000235.hg.1	ANLN	6.53 ± 0.17	6.39 ± 0.33	6.54 ± 0.23	6.01 ± 0.11
TC11000721.hg.1	ANO1	6.42 ± 0.16	6.34 ± 0.16	6.35 ± 0.14	6.42 ± 0.03
TC03001334.hg.1	ANO10	7.41 ± 0.09	7.32 ± 0.01	7.57 ± 0.07	7.32 ± 0.04
TC11002024.hg.1	ANO1-AS1	7.42 ± 0.29	7.43 ± 0.22	7.42 ± 0.17	7.65 ± 0.14
TC11002023.hg.1	ANO1-AS2	7.11 ± 0.17	7.08 ± 0.07	7.19 ± 0.14	7.22 ± 0.03
TC12001129.hg.1	ANO2	6.08 ± 0.07	5.95 ± 0.08	6.01 ± 0.05	6.01 ± 0.02
TC11000275.hg.1	ANO3	5.12 ± 0.14	5.05 ± 0.09	5.05 ± 0.06	5.10 ± 0.05
TC12000780.hg.1	ANO4	5.18 ± 0.12	5.11 ± 0.09	5.12 ± 0.07	5.18 ± 0.06
TC11000261.hg.1	ANO5	4.94 ± 0.15	4.75 ± 0.05	4.99 ± 0.10	4.73 ± 0.06
TC12000329.hg.1	ANO6	9.22 ± 0.24	9.05 ± 0.13	9.48 ± 0.11	9.08 ± 0.22
TC02001476.hg.1	ANO7	7.80 ± 0.21	7.79 ± 0.22	7.75 ± 0.20	7.89 ± 0.06
TC19001273.hg.1	ANO8	7.92 ± 0.06	7.97 ± 0.07	7.89 ± 0.09	7.94 ± 0.02
TC11001232.hg.1	ANO9	8.57 ± 0.07	8.59 ± 0.09	8.34 ± 0.12	8.64 ± 0.08
TC15001594.hg.1	ANP32A	9.39 ± 0.21	9.46 ± 0.09	9.64 ± 0.25	9.45 ± 0.22
TC15001596.hg.1	ANP32A-IT1	8.05 ± 0.32	8.08 ± 0.18	8.39 ± 0.26	8.13 ± 0.15
TC15000248.hg.1	ANP32AP1	7.34 ± 0.07	7.23 ± 0.13	7.54 ± 0.34	7.14 ± 0.27
TC09000495.hg.1	ANP32B	9.45 ± 0.15	9.60 ± 0.02	9.28 ± 0.05	9.55 ± 0.19
TC04001702.hg.1	ANP32C	6.96 ± 0.05	6.87 ± 0.10	7.04 ± 0.10	6.76 ± 0.27
TC12000350.hg.1	ANP32D	6.06 ± 0.40	6.02 ± 0.08	6.17 ± 0.17	5.98 ± 0.16
TC01003200.hg.1	ANP32E	8.68 ± 0.24	8.81 ± 0.07	8.69 ± 0.23	8.64 ± 0.22
TC15001837.hg.1	ANPEP	11.11 ± 0.45	10.86 ± 0.04	11.68 ± 0.19	10.84 ± 0.49
TC02000403.hg.1	ANTXR1	6.04 ± 0.16	5.89 ± 0.05	5.91 ± 0.07	5.96 ± 0.07
TC04001328.hg.1	ANTXR2	9.10 ± 0.22	9.23 ± 0.18	9.52 ± 0.28	9.09 ± 0.19
TC10000308.hg.1	ANTXRL	7.14 ± 0.09	7.13 ± 0.11	7.11 ± 0.13	7.23 ± 0.10
TC10001244.hg.1	ANTXRLP1	6.81 ± 0.18	6.95 ± 0.07	6.66 ± 0.20	7.02 ± 0.15

TC09000335.hg.1	ANXA1	9.84 ± 0.45	10.12 ± 0.18	10.62 ± 0.42	10.01 ± 0.06
TC04000836.hg.1	ANXA10	4.86 ± 0.14	4.71 ± 0.02	4.75 ± 0.06	4.76 ± 0.04
TC10001461.hg.1	ANXA11	9.42 ± 0.14	9.29 ± 0.04	9.43 ± 0.20	9.29 ± 0.05
TC08001593.hg.1	ANXA13	5.40 ± 0.15	5.30 ± 0.06	5.37 ± 0.07	5.40 ± 0.08
TC15001505.hg.1	ANXA2	10.24 ± 0.20	10.07 ± 0.18	10.16 ± 0.07	9.80 ± 0.23
TC04001655.hg.1	ANXA2P1	7.64 ± 0.22	7.29 ± 0.16	7.44 ± 0.25	6.98 ± 0.22
TC09000149.hg.1	ANXA2P2	11.22 ± 0.13	11.09 ± 0.18	11.13 ± 0.13	10.81 ± 0.26
TC10000391.hg.1	ANXA2P3	6.66 ± 0.30	6.58 ± 0.06	6.64 ± 0.25	6.61 ± 0.13
TC05001313.hg.1	ANXA2R	7.20 ± 0.13	7.12 ± 0.21	7.18 ± 0.30	6.94 ± 0.03
TC04000437.hg.1	ANXA3	9.08 ± 0.45	8.52 ± 0.39	10.24 ± 0.28	8.28 ± 0.47
TC02000407.hg.1	ANXA4	8.18 ± 0.09	8.03 ± 0.06	8.21 ± 0.21	7.84 ± 0.09
TC04001514.hg.1	ANXA5	10.39 ± 0.17	10.30 ± 0.00	10.57 ± 0.18	10.05 ± 0.27
TC05001947.hg.1	ANXA6	10.27 ± 0.12	10.23 ± 0.15	9.94 ± 0.08	9.93 ± 0.12
TC10001408.hg.1	ANXA7	8.96 ± 0.18	9.09 ± 0.18	9.00 ± 0.21	9.14 ± 0.11
TC10000314.hg.1	ANXA8L1	7.09 ± 0.17	7.02 ± 0.15	7.03 ± 0.16	7.14 ± 0.09
TC10002963.hg.1	ANXA8L1	7.17 ± 0.15	7.16 ± 0.14	7.13 ± 0.17	7.28 ± 0.10
TC10000309.hg.1	ANXA8L2	7.32 ± 0.23	7.25 ± 0.17	7.26 ± 0.18	7.40 ± 0.10
TC01001197.hg.1	ANXA9	7.19 ± 0.08	7.13 ± 0.13	7.13 ± 0.15	7.17 ± 0.01
TC07001285.hg.1	AOAH	10.04 ± 0.36	9.92 ± 0.22	10.35 ± 0.17	9.81 ± 0.16
TC07001286.hg.1	AOAH-IT1	7.83 ± 0.22	7.60 ± 0.07	8.09 ± 0.16	7.51 ± 0.11
TC17000539.hg.1	AOC2	7.61 ± 0.05	7.56 ± 0.13	7.62 ± 0.11	7.51 ± 0.08
TC17000540.hg.1	AOC3	7.82 ± 0.13	7.85 ± 0.05	7.99 ± 0.01	7.93 ± 0.09
TC17000541.hg.1	AOC4	6.38 ± 0.08	6.17 ± 0.09	6.28 ± 0.08	6.22 ± 0.01
TC02001162.hg.1	AOX1	5.55 ± 0.15	5.50 ± 0.07	5.49 ± 0.10	5.53 ± 0.06
TC02001164.hg.1	AOX2P	5.88 ± 0.15	5.80 ± 0.10	5.83 ± 0.07	5.89 ± 0.05
TC04000579.hg.1	AP1AR	6.52 ± 0.22	6.48 ± 0.19	6.60 ± 0.24	6.40 ± 0.16
TC22000634.hg.1	AP1B1	8.98 ± 0.13	8.78 ± 0.06	8.82 ± 0.10	8.73 ± 0.05
TC22000239.hg.1	AP1B1P1	7.06 ± 0.05	7.04 ± 0.11	7.01 ± 0.13	7.17 ± 0.06
TC16001252.hg.1	AP1G1	9.68 ± 0.28	9.81 ± 0.15	9.89 ± 0.41	10.01 ± 0.09
TC14002205.hg.1	AP1G2	9.86 ± 0.08	9.71 ± 0.02	9.69 ± 0.10	9.58 ± 0.19
TC19000320.hg.1	AP1M1	9.50 ± 0.14	9.35 ± 0.10	9.36 ± 0.13	9.31 ± 0.06

TC19001171.hg.1	AP1M2	7.08 ± 0.12	6.96 ± 0.17	7.04 ± 0.14	7.06 ± 0.06
TC07000644.hg.1	AP1S1	7.97 ± 0.19	7.85 ± 0.12	7.85 ± 0.15	7.86 ± 0.03
TC0X000882.hg.1	AP1S2	7.96 ± 0.30	8.13 ± 0.15	7.97 ± 0.32	8.07 ± 0.23
TC02002819.hg.1	AP1S3	6.48 ± 0.09	6.48 ± 0.05	6.49 ± 0.09	6.54 ± 0.05
TC19000747.hg.1	AP2A1	9.03 ± 0.17	8.87 ± 0.02	8.95 ± 0.06	8.87 ± 0.12
TC11000036.hg.1	AP2A2	8.70 ± 0.16	8.57 ± 0.04	8.56 ± 0.09	8.48 ± 0.02
TC17000399.hg.1	AP2B1	10.04 ± 0.17	9.99 ± 0.07	9.98 ± 0.15	9.76 ± 0.08
TC03000994.hg.1	AP2M1	9.60 ± 0.11	9.45 ± 0.06	9.47 ± 0.23	9.34 ± 0.09
TC19001658.hg.1	AP2S1	9.03 ± 0.43	8.68 ± 0.17	8.75 ± 0.27	8.55 ± 0.09
TC05001513.hg.1	AP3B1	8.69 ± 0.28	8.86 ± 0.27	8.85 ± 0.28	8.69 ± 0.13
TC15001756.hg.1	AP3B2	7.27 ± 0.21	7.15 ± 0.14	7.41 ± 0.10	7.23 ± 0.03
TC19001021.hg.1	AP3D1	9.47 ± 0.20	9.29 ± 0.02	9.34 ± 0.09	9.16 ± 0.13
TC10001423.hg.1	AP3M1	9.00 ± 0.18	8.94 ± 0.23	9.01 ± 0.29	8.71 ± 0.17
TC08000324.hg.1	AP3M2	7.64 ± 0.16	7.88 ± 0.10	7.61 ± 0.15	7.94 ± 0.04
TC05000548.hg.1	AP3S1	7.70 ± 0.04	7.73 ± 0.20	7.79 ± 0.25	7.62 ± 0.19
TC15002782.hg.1	AP3S2	8.36 ± 0.21	8.02 ± 0.08	8.49 ± 0.11	7.98 ± 0.16
TC01003008.hg.1	AP4B1	7.64 ± 0.14	7.55 ± 0.08	7.44 ± 0.11	7.52 ± 0.09
TC01001001.hg.1	AP4B1-AS1	5.17 ± 0.15	5.05 ± 0.11	5.14 ± 0.07	5.10 ± 0.04
TC15000399.hg.1	AP4E1	7.01 ± 0.32	6.94 ± 0.23	6.95 ± 0.29	6.72 ± 0.10
TC07000611.hg.1	AP4M1	7.91 ± 0.16	7.80 ± 0.07	7.81 ± 0.15	7.82 ± 0.03
TC14000197.hg.1	AP4S1	6.92 ± 0.15	6.87 ± 0.07	6.80 ± 0.07	6.85 ± 0.05
TC11001941.hg.1	AP5B1	8.85 ± 0.27	8.58 ± 0.08	9.00 ± 0.37	8.35 ± 0.25
TC14000340.hg.1	AP5M1	7.82 ± 0.47	7.92 ± 0.17	8.05 ± 0.43	7.75 ± 0.15
TC20000042.hg.1	AP5S1	8.81 ± 0.13	8.66 ± 0.08	8.57 ± 0.10	8.62 ± 0.09
TC07000045.hg.1	AP5Z1	8.60 ± 0.12	8.55 ± 0.12	8.52 ± 0.07	8.62 ± 0.03
TC12000765.hg.1	APAF1	8.87 ± 0.27	8.70 ± 0.39	9.43 ± 0.48	8.36 ± 0.52
TC09001193.hg.1	APBA1	6.56 ± 0.15	6.58 ± 0.20	6.49 ± 0.15	6.62 ± 0.02
TC15000153.hg.1	APBA2	8.00 ± 0.08	8.04 ± 0.07	7.66 ± 0.10	8.11 ± 0.08
TC19001049.hg.1	APBA3	8.14 ± 0.16	8.00 ± 0.16	8.01 ± 0.20	8.05 ± 0.02
TC11001355.hg.1	APBB1	8.00 ± 0.08	8.10 ± 0.11	7.77 ± 0.19	8.20 ± 0.13
TC10000181.hg.1	APBB1IP	10.42 ± 0.20	10.06 ± 0.23	10.82 ± 0.22	9.82 ± 0.36

TC04001139.hg.1	APBB2	6.75 ± 0.14	6.77 ± 0.07	6.68 ± 0.09	6.87 ± 0.09
TC05003433.hg.1	APBB3	8.22 ± 0.06	8.12 ± 0.05	8.21 ± 0.03	8.11 ± 0.06
TC05003398.hg.1	APC	8.07 ± 0.35	8.22 ± 0.22	8.46 ± 0.45	8.11 ± 0.13
TC19000040.hg.1	APC2	8.16 ± 0.26	8.14 ± 0.21	8.12 ± 0.25	8.23 ± 0.04
TC18000051.hg.1	APCDD1	8.52 ± 0.21	8.37 ± 0.11	8.42 ± 0.09	8.32 ± 0.05
TC20000985.hg.1	APCDD1L	7.89 ± 0.17	7.90 ± 0.15	7.85 ± 0.16	7.99 ± 0.10
TC20000459.hg.1	APCDD1L-AS1	6.50 ± 0.15	6.44 ± 0.14	6.43 ± 0.13	6.55 ± 0.07
TC01001354.hg.1	APCS	6.40 ± 0.05	6.35 ± 0.09	6.49 ± 0.11	6.25 ± 0.21
TC03000297.hg.1	APEH	8.83 ± 0.20	8.53 ± 0.01	8.54 ± 0.16	8.42 ± 0.02
TC14000055.hg.1	APEX1	10.06 ± 0.02	10.06 ± 0.02	9.66 ± 0.12	9.95 ± 0.04
TC0X000326.hg.1	APEX2	7.45 ± 0.12	7.20 ± 0.03	7.27 ± 0.20	7.12 ± 0.07
TC01003201.hg.1	APH1A	8.87 ± 0.23	8.78 ± 0.15	8.50 ± 0.27	8.73 ± 0.09
TC15000582.hg.1	APH1B	8.19 ± 0.17	7.79 ± 0.09	8.38 ± 0.36	7.78 ± 0.18
TC11000360.hg.1	API5	8.89 ± 0.31	9.01 ± 0.20	8.78 ± 0.35	8.86 ± 0.08
TC11001548.hg.1	APIP	7.55 ± 0.18	7.57 ± 0.08	7.63 ± 0.12	7.50 ± 0.14
TC01000130.hg.1	APITD1-CORT	5.93 ± 0.08	5.85 ± 0.05	5.80 ± 0.09	5.79 ± 0.01
TC02004967.hg.1	APLF	6.57 ± 0.10	6.49 ± 0.09	6.52 ± 0.13	6.48 ± 0.12
TC0X001354.hg.1	APLN	7.92 ± 0.06	7.88 ± 0.13	7.96 ± 0.16	7.94 ± 0.02
TC11001786.hg.1	APLNR	7.24 ± 0.15	7.21 ± 0.17	7.18 ± 0.21	7.34 ± 0.07
TC19000488.hg.1	APLP1	7.60 ± 0.19	7.59 ± 0.18	7.53 ± 0.17	7.64 ± 0.04
TC11001194.hg.1	APLP2	10.74 ± 0.24	10.85 ± 0.02	11.05 ± 0.13	10.90 ± 0.28
TC20000726.hg.1	APMAP	9.34 ± 0.12	8.96 ± 0.10	9.74 ± 0.42	8.81 ± 0.18
TC11002328.hg.1	APOA1	7.73 ± 0.22	7.71 ± 0.23	7.71 ± 0.15	7.81 ± 0.04
TC01001323.hg.1	APOA1BP	8.16 ± 0.10	8.04 ± 0.10	7.90 ± 0.10	7.90 ± 0.04
TC01003422.hg.1	APOA2	8.04 ± 0.18	7.93 ± 0.09	8.22 ± 0.12	7.97 ± 0.03
TC11002327.hg.1	APOA4	7.05 ± 0.12	6.94 ± 0.09	6.98 ± 0.23	7.02 ± 0.05
TC11002325.hg.1	APOA5	7.68 ± 0.17	7.75 ± 0.20	7.69 ± 0.15	7.87 ± 0.03
TC02001628.hg.1	APOB	6.03 ± 0.17	5.98 ± 0.13	6.02 ± 0.08	6.05 ± 0.08
TC12001163.hg.1	APOBEC1	5.80 ± 0.16	5.62 ± 0.12	5.64 ± 0.06	5.75 ± 0.08
TC06000563.hg.1	APOBEC2	6.72 ± 0.09	6.66 ± 0.09	6.65 ± 0.04	6.66 ± 0.04
TC22001424.hg.1	APOBEC3A	10.40 ± 0.48	10.05 ± 0.53	10.87 ± 0.25	9.89 ± 0.30

TC22001425.hg.1	APOBEC3B	9.04 ± 0.33	8.70 ± 0.18	8.98 ± 0.04	8.52 ± 0.18
TC22000800.hg.1	APOBEC3B-AS1	8.13 ± 0.13	8.12 ± 0.12	8.23 ± 0.14	8.15 ± 0.03
TC22001426.hg.1	APOBEC3C	10.60 ± 0.21	10.22 ± 0.09	10.40 ± 0.21	9.98 ± 0.24
TC22001427.hg.1	APOBEC3D	9.67 ± 0.23	9.14 ± 0.14	9.28 ± 0.09	8.70 ± 0.25
TC22001467.hg.1	APOBEC3F	9.55 ± 0.17	9.25 ± 0.09	9.31 ± 0.07	9.00 ± 0.18
TC22001468.hg.1	APOBEC3G	8.63 ± 0.26	8.28 ± 0.11	8.38 ± 0.13	7.94 ± 0.23
TC22000311.hg.1	APOBEC3H	6.74 ± 0.16	6.61 ± 0.07	6.60 ± 0.24	6.56 ± 0.06
TC01003619.hg.1	APOBEC4	4.49 ± 0.08	4.30 ± 0.07	4.31 ± 0.08	4.38 ± 0.10
TC16000302.hg.1	APOBR	9.75 ± 0.21	9.43 ± 0.12	9.87 ± 0.17	9.46 ± 0.05
TC19000634.hg.1	APOC1	7.58 ± 0.17	7.61 ± 0.21	7.47 ± 0.17	7.71 ± 0.11
TC19000635.hg.1	APOC1P1	7.46 ± 0.25	7.49 ± 0.33	7.32 ± 0.22	7.73 ± 0.06
TC19002642.hg.1	APOC2	7.47 ± 0.26	7.46 ± 0.29	7.39 ± 0.11	7.65 ± 0.14
TC11001042.hg.1	APOC3	7.60 ± 0.20	7.65 ± 0.24	7.53 ± 0.29	7.75 ± 0.07
TC19002643.hg.1	APOC4	9.28 ± 0.16	9.12 ± 0.03	9.21 ± 0.09	9.14 ± 0.02
TC03002146.hg.1	APOD	6.11 ± 0.08	6.05 ± 0.06	6.02 ± 0.09	6.10 ± 0.05
TC19000633.hg.1	APOE	7.98 ± 0.34	7.94 ± 0.20	7.84 ± 0.25	7.99 ± 0.03
TC12001604.hg.1	APOF	6.72 ± 0.41	6.67 ± 0.26	6.83 ± 0.27	6.82 ± 0.04
TC17001810.hg.1	APOH	4.20 ± 0.13	4.18 ± 0.10	4.24 ± 0.12	4.27 ± 0.05
TC22000265.hg.1	APOL1	8.39 ± 0.27	8.08 ± 0.10	8.41 ± 0.26	7.96 ± 0.10
TC22000702.hg.1	APOL2	8.91 ± 0.25	8.56 ± 0.19	9.07 ± 0.39	8.44 ± 0.14
TC22000700.hg.1	APOL3	8.07 ± 0.04	7.91 ± 0.03	7.92 ± 0.07	7.82 ± 0.02
TC22000701.hg.1	APOL4	7.17 ± 0.16	7.03 ± 0.11	7.12 ± 0.11	7.08 ± 0.10
TC22000264.hg.1	APOL5	6.36 ± 0.27	6.27 ± 0.13	6.27 ± 0.03	6.35 ± 0.10
TC22000262.hg.1	APOL6	9.24 ± 0.17	9.12 ± 0.21	9.45 ± 0.34	8.95 ± 0.10
TC12003237.hg.1	APOLD1	6.86 ± 0.11	6.77 ± 0.02	6.77 ± 0.08	6.77 ± 0.04
TC06000377.hg.1	APOM	7.20 ± 0.15	7.14 ± 0.10	7.04 ± 0.15	7.24 ± 0.03
TC6_cox_hap2000072.hg.1	APOM	7.29 ± 0.17	7.23 ± 0.11	7.12 ± 0.14	7.32 ± 0.04
TC6_dbb_hap3000063.hg.1	APOM	7.29 ± 0.17	7.23 ± 0.11	7.12 ± 0.14	7.32 ± 0.04
TC6_mann_hap4000063.hg.1	APOM	7.29 ± 0.17	7.23 ± 0.11	7.12 ± 0.14	7.32 ± 0.04
TC6_mcf_hap5000058.hg.1	APOM	7.29 ± 0.17	7.23 ± 0.11	7.12 ± 0.14	7.32 ± 0.04
TC6_qbl_hap6000063.hg.1	APOM	7.29 ± 0.17	7.23 ± 0.11	7.12 ± 0.14	7.32 ± 0.04

TC6_ssto_hap7000059.hg.1	APOM	7.26 ± 0.16	7.19 ± 0.10	7.09 ± 0.14	7.29 ± 0.04
TC0X000923.hg.1	APOO	6.76 ± 0.13	6.61 ± 0.18	6.71 ± 0.18	6.48 ± 0.02
TC0X000439.hg.1	APOOL	7.08 ± 0.09	7.05 ± 0.20	6.95 ± 0.10	6.91 ± 0.12
TC16001160.hg.1	APOOP5	6.11 ± 0.14	6.24 ± 0.10	6.23 ± 0.09	6.28 ± 0.10
TC14002313.hg.1	APOPT1	7.63 ± 0.12	7.54 ± 0.03	7.56 ± 0.12	7.54 ± 0.03
TC21000340.hg.1	APP	9.33 ± 0.20	9.13 ± 0.04	9.43 ± 0.15	9.21 ± 0.14
TC17001764.hg.1	APPBP2	8.46 ± 0.23	8.48 ± 0.24	8.49 ± 0.42	8.27 ± 0.13
TC03000365.hg.1	APPL1	8.26 ± 0.27	8.36 ± 0.20	8.27 ± 0.22	8.29 ± 0.04
TC12001913.hg.1	APPL2	8.08 ± 0.21	8.21 ± 0.18	8.09 ± 0.26	8.22 ± 0.06
TC16001344.hg.1	APRT	9.42 ± 0.24	9.23 ± 0.08	9.02 ± 0.22	9.19 ± 0.02
TC09001006.hg.1	APTX	7.02 ± 0.05	6.99 ± 0.01	6.93 ± 0.01	6.96 ± 0.04
TC01001273.hg.1	AQP10	7.48 ± 0.13	7.38 ± 0.18	7.42 ± 0.27	7.45 ± 0.03
TC11000830.hg.1	AQP11	6.19 ± 0.13	6.08 ± 0.09	6.13 ± 0.22	6.11 ± 0.05
TC02001470.hg.1	AQP12A	7.92 ± 0.25	7.95 ± 0.19	7.90 ± 0.14	8.07 ± 0.09
TC02002939.hg.1	AQP12B	7.85 ± 0.27	7.87 ± 0.20	7.83 ± 0.20	7.95 ± 0.05
TC12000383.hg.1	AQP2	7.26 ± 0.30	7.36 ± 0.18	7.00 ± 0.26	7.29 ± 0.10
TC09001012.hg.1	AQP3	8.81 ± 0.25	8.56 ± 0.15	8.22 ± 0.13	8.52 ± 0.09
TC18000429.hg.1	AQP4	6.59 ± 0.16	6.51 ± 0.05	6.54 ± 0.18	6.55 ± 0.14
TC18000115.hg.1	AQP4-AS1	5.89 ± 0.19	5.91 ± 0.07	5.89 ± 0.07	6.03 ± 0.14
TC12000384.hg.1	AQP5	7.91 ± 0.29	7.90 ± 0.29	7.80 ± 0.25	8.00 ± 0.03
TC12000385.hg.1	AQP6	7.50 ± 0.18	7.45 ± 0.22	7.45 ± 0.16	7.57 ± 0.07
TC09001011.hg.1	AQP7	8.01 ± 0.22	7.94 ± 0.15	7.94 ± 0.15	8.10 ± 0.11
TC09001158.hg.1	AQP7P1	7.00 ± 0.20	6.98 ± 0.07	6.98 ± 0.16	7.14 ± 0.13
TC09000250.hg.1	AQP7P3	6.35 ± 0.12	6.27 ± 0.09	6.33 ± 0.10	6.42 ± 0.14
TC16000284.hg.1	AQP8	7.17 ± 0.22	7.25 ± 0.15	7.06 ± 0.19	7.29 ± 0.09
TC15000441.hg.1	AQP9	11.46 ± 0.45	11.48 ± 0.18	12.30 ± 0.26	11.44 ± 0.38
TC05000549.hg.1	AQPEP	4.77 ± 0.10	4.67 ± 0.08	4.70 ± 0.06	4.70 ± 0.06
TC15001202.hg.1	AQR	8.26 ± 0.37	8.33 ± 0.23	8.20 ± 0.40	8.18 ± 0.10
TC0X000358.hg.1	AR	6.41 ± 0.04	6.38 ± 0.11	6.42 ± 0.10	6.46 ± 0.04
TC0X000237.hg.1	ARAF	9.56 ± 0.17	9.38 ± 0.04	9.44 ± 0.10	9.42 ± 0.02
TC11003491.hg.1	ARAP1	9.69 ± 0.19	9.49 ± 0.07	9.72 ± 0.14	9.49 ± 0.11

TC11000765.hg.1	ARAP1-AS1	8.21 ± 0.08	7.95 ± 0.05	8.25 ± 0.15	8.04 ± 0.07
TC11000767.hg.1	ARAP1-AS2	7.53 ± 0.03	7.49 ± 0.05	7.65 ± 0.03	7.54 ± 0.15
TC04002955.hg.1	ARAP2	8.13 ± 0.69	8.55 ± 0.26	8.28 ± 0.53	8.24 ± 0.05
TC05001872.hg.1	ARAP3	8.39 ± 0.07	8.27 ± 0.06	8.53 ± 0.13	8.29 ± 0.08
TC08001699.hg.1	ARC	8.28 ± 0.34	8.28 ± 0.26	8.20 ± 0.30	8.32 ± 0.07
TC11001066.hg.1	ARCN1	9.52 ± 0.17	9.46 ± 0.08	9.54 ± 0.20	9.38 ± 0.18
TC04002952.hg.1	AREG	7.69 ± 0.69	8.11 ± 0.55	7.60 ± 0.74	7.86 ± 0.19
TC04002953.hg.1	AREG	7.72 ± 0.72	8.12 ± 0.55	7.60 ± 0.75	7.87 ± 0.22
TC14001310.hg.1	AREL1	9.22 ± 0.20	9.11 ± 0.15	9.41 ± 0.22	9.14 ± 0.16
TC01001884.hg.1	ARF1	10.12 ± 0.12	10.00 ± 0.07	10.08 ± 0.15	10.03 ± 0.09
TC12003225.hg.1	ARF3	9.42 ± 0.12	9.24 ± 0.06	9.42 ± 0.18	9.18 ± 0.08
TC03001495.hg.1	ARF4	9.33 ± 0.06	9.33 ± 0.08	9.34 ± 0.15	9.33 ± 0.24
TC07003341.hg.1	ARF5	8.94 ± 0.13	8.80 ± 0.11	8.80 ± 0.11	8.76 ± 0.03
TC14000280.hg.1	ARF6	10.58 ± 0.00	10.47 ± 0.06	10.39 ± 0.09	10.42 ± 0.11
TC20000511.hg.1	ARFGAP1	8.67 ± 0.10	8.58 ± 0.09	8.54 ± 0.07	8.62 ± 0.01
TC11001730.hg.1	ARFGAP2	8.61 ± 0.16	8.43 ± 0.05	8.32 ± 0.19	8.42 ± 0.06
TC22000856.hg.1	ARFGAP3	9.12 ± 0.15	9.07 ± 0.08	9.38 ± 0.27	9.04 ± 0.19
TC08001293.hg.1	ARFGEF1	8.61 ± 0.51	8.89 ± 0.19	8.92 ± 0.46	8.76 ± 0.08
TC20000382.hg.1	ARFGEF2	8.53 ± 0.22	8.61 ± 0.16	8.27 ± 0.27	8.59 ± 0.05
TC04000766.hg.1	ARFIP1	7.50 ± 0.19	7.45 ± 0.25	7.66 ± 0.47	7.11 ± 0.30
TC11001358.hg.1	ARFIP2	8.14 ± 0.11	8.05 ± 0.10	7.93 ± 0.06	8.01 ± 0.03
TC20001038.hg.1	ARFRP1	8.48 ± 0.24	8.32 ± 0.14	8.25 ± 0.20	8.34 ± 0.04
TC06000983.hg.1	ARG1	7.74 ± 0.48	6.85 ± 0.09	8.75 ± 0.70	6.72 ± 0.01
TC14000409.hg.1	ARG2	7.31 ± 0.18	6.98 ± 0.09	7.22 ± 0.12	7.08 ± 0.10
TC03000621.hg.1	ARGFX	5.45 ± 0.20	5.45 ± 0.08	5.38 ± 0.05	5.63 ± 0.07
TC17001349.hg.1	ARGFXP2	5.72 ± 0.42	5.47 ± 0.10	5.55 ± 0.18	5.47 ± 0.15
TC13000861.hg.1	ARGLU1	9.54 ± 0.48	9.97 ± 0.07	9.67 ± 0.38	9.87 ± 0.12
TC11001723.hg.1	ARHGAP1	10.08 ± 0.19	9.89 ± 0.06	10.12 ± 0.14	9.82 ± 0.10
TC04000733.hg.1	ARHGAP10	7.22 ± 0.16	7.05 ± 0.10	7.11 ± 0.13	7.06 ± 0.13
TC15000223.hg.1	ARHGAP11A	7.34 ± 0.01	7.17 ± 0.34	7.16 ± 0.20	6.86 ± 0.07
TC15002760.hg.1	ARHGAP11B	7.17 ± 0.10	7.01 ± 0.28	7.03 ± 0.33	6.77 ± 0.18

TC10001159.hg.1	ARHGAP12	7.22 ± 0.49	7.65 ± 0.26	7.24 ± 0.39	7.51 ± 0.04
TC02000904.hg.1	ARHGAP15	9.12 ± 0.54	9.69 ± 0.20	9.66 ± 0.32	9.54 ± 0.10
TC02000905.hg.1	ARHGAP15	6.21 ± 0.56	6.88 ± 0.32	6.55 ± 0.48	6.42 ± 0.36
TC16000972.hg.1	ARHGAP17	9.08 ± 0.14	8.99 ± 0.16	8.94 ± 0.24	8.78 ± 0.18
TC06004137.hg.1	ARHGAP18	8.18 ± 0.48	7.79 ± 0.20	8.53 ± 0.26	7.83 ± 0.19
TC10002952.hg.1	ARHGAP19	7.94 ± 0.15	7.83 ± 0.22	8.07 ± 0.34	7.59 ± 0.16
TC11002275.hg.1	ARHGAP20	4.88 ± 0.15	4.79 ± 0.07	4.84 ± 0.04	4.84 ± 0.03
TC10001109.hg.1	ARHGAP21	7.52 ± 0.33	7.49 ± 0.17	7.63 ± 0.19	7.58 ± 0.21
TC10001263.hg.1	ARHGAP22	7.27 ± 0.20	7.20 ± 0.17	7.19 ± 0.16	7.28 ± 0.06
TC10001264.hg.1	ARHGAP22-IT1	6.26 ± 0.16	6.27 ± 0.10	6.20 ± 0.09	6.38 ± 0.08
TC17000461.hg.1	ARHGAP23	7.76 ± 0.21	7.64 ± 0.14	7.64 ± 0.16	7.71 ± 0.02
TC04000466.hg.1	ARHGAP24	7.04 ± 0.07	6.95 ± 0.16	7.41 ± 0.25	6.89 ± 0.12
TC02000400.hg.1	ARHGAP25	9.28 ± 0.19	9.17 ± 0.18	9.44 ± 0.25	9.04 ± 0.18
TC05000782.hg.1	ARHGAP26	9.29 ± 0.23	9.37 ± 0.21	9.80 ± 0.36	9.28 ± 0.26
TC05001886.hg.1	ARHGAP26-AS1	6.65 ± 0.18	6.65 ± 0.12	6.62 ± 0.13	6.74 ± 0.09
TC05000785.hg.1	ARHGAP26-IT1	7.21 ± 0.28	7.40 ± 0.13	7.73 ± 0.28	7.45 ± 0.13
TC17_ctg5_hap1000011.hg.1	ARHGAP27	9.16 ± 0.18	8.96 ± 0.07	9.26 ± 0.15	8.89 ± 0.10
TC17_ctg5_hap1000012.hg.1	ARHGAP27	8.45 ± 0.09	8.46 ± 0.23	8.47 ± 0.20	8.48 ± 0.02
TC17001600.hg.1	ARHGAP27	9.00 ± 0.15	8.84 ± 0.07	9.05 ± 0.12	8.79 ± 0.07
TC18000033.hg.1	ARHGAP28	5.04 ± 0.15	4.97 ± 0.11	5.00 ± 0.06	5.05 ± 0.08
TC01002884.hg.1	ARHGAP29	4.53 ± 0.10	4.42 ± 0.04	4.54 ± 0.15	4.43 ± 0.00
TC01003414.hg.1	ARHGAP30	10.04 ± 0.20	9.93 ± 0.06	10.10 ± 0.24	9.83 ± 0.15
TC03000607.hg.1	ARHGAP31	7.87 ± 0.18	7.66 ± 0.12	7.76 ± 0.10	7.64 ± 0.21
TC03001681.hg.1	ARHGAP31-AS1	6.48 ± 0.20	6.55 ± 0.21	6.41 ± 0.21	6.59 ± 0.08
TC11002446.hg.1	ARHGAP32	7.16 ± 0.24	7.34 ± 0.12	7.01 ± 0.14	7.23 ± 0.13
TC19000486.hg.1	ARHGAP33	8.31 ± 0.15	8.31 ± 0.15	8.27 ± 0.18	8.38 ± 0.08
TC19000669.hg.1	ARHGAP35	9.06 ± 0.07	9.05 ± 0.05	8.85 ± 0.14	9.01 ± 0.07
TC0X000625.hg.1	ARHGAP36	6.49 ± 0.24	6.49 ± 0.19	6.42 ± 0.12	6.60 ± 0.04
TC08001758.hg.1	ARHGAP39	7.62 ± 0.19	7.55 ± 0.16	7.57 ± 0.16	7.64 ± 0.01
TC0X002308.hg.1	ARHGAP4	9.59 ± 0.07	9.61 ± 0.08	9.48 ± 0.11	9.63 ± 0.04
TC20000287.hg.1	ARHGAP40	7.03 ± 0.25	7.00 ± 0.19	7.01 ± 0.19	7.08 ± 0.05

TC11000944.hg.1	ARHGAP42	5.87 ± 0.17	5.56 ± 0.17	5.60 ± 0.25	5.58 ± 0.10
TC17000159.hg.1	ARHGAP44	6.83 ± 0.23	6.77 ± 0.14	6.81 ± 0.11	6.84 ± 0.04
TC14000205.hg.1	ARHGAP5	6.96 ± 0.30	7.17 ± 0.23	6.86 ± 0.30	7.10 ± 0.04
TC14001019.hg.1	ARHGAP5-AS1	7.22 ± 0.20	7.07 ± 0.24	7.05 ± 0.14	7.26 ± 0.16
TC0X000858.hg.1	ARHGAP6	6.80 ± 0.04	6.55 ± 0.13	6.84 ± 0.11	6.65 ± 0.09
TC12001624.hg.1	ARHGAP9	9.62 ± 0.14	9.46 ± 0.04	9.73 ± 0.13	9.44 ± 0.02
TC17001961.hg.1	ARHGDIA	9.26 ± 0.18	9.22 ± 0.13	9.06 ± 0.08	9.16 ± 0.14
TC12001278.hg.1	ARHGDIB	11.51 ± 0.14	11.57 ± 0.13	11.67 ± 0.11	11.47 ± 0.05
TC16002048.hg.1	ARHGDIG	8.46 ± 0.23	8.49 ± 0.24	8.46 ± 0.15	8.56 ± 0.04
TC19000589.hg.1	ARHGEF1	9.57 ± 0.07	9.58 ± 0.04	9.43 ± 0.06	9.59 ± 0.11
TC08000010.hg.1	ARHGEF10	7.14 ± 0.07	7.14 ± 0.23	7.02 ± 0.06	7.13 ± 0.07
TC01000236.hg.1	ARHGEF10L	7.85 ± 0.14	7.77 ± 0.14	7.76 ± 0.12	7.81 ± 0.00
TC01003356.hg.1	ARHGEF11	8.62 ± 0.23	8.29 ± 0.12	8.73 ± 0.10	8.19 ± 0.13
TC11001102.hg.1	ARHGEF12	7.72 ± 0.39	7.37 ± 0.22	7.97 ± 0.26	7.35 ± 0.18
TC17000138.hg.1	ARHGEF15	7.81 ± 0.22	7.76 ± 0.21	7.72 ± 0.23	7.86 ± 0.06
TC01000069.hg.1	ARHGEF16	7.90 ± 0.21	7.81 ± 0.18	7.82 ± 0.22	7.91 ± 0.05
TC11000774.hg.1	ARHGEF17	7.60 ± 0.19	7.56 ± 0.18	7.54 ± 0.16	7.68 ± 0.03
TC19000125.hg.1	ARHGEF18	9.38 ± 0.06	9.32 ± 0.03	9.14 ± 0.07	9.27 ± 0.03
TC01002259.hg.1	ARHGEF19	8.29 ± 0.20	8.26 ± 0.17	8.22 ± 0.15	8.31 ± 0.04
TC01000204.hg.1	ARHGEF19-AS1	6.41 ± 0.15	6.26 ± 0.15	6.31 ± 0.16	6.19 ± 0.12
TC01003328.hg.1	ARHGEF2	9.14 ± 0.17	8.79 ± 0.08	9.11 ± 0.14	8.75 ± 0.19
TC12003247.hg.1	ARHGEF25	7.56 ± 0.18	7.54 ± 0.19	7.50 ± 0.21	7.63 ± 0.07
TC03000844.hg.1	ARHGEF26	5.53 ± 0.16	5.35 ± 0.11	5.43 ± 0.13	5.45 ± 0.01
TC03001918.hg.1	ARHGEF26-AS1	6.85 ± 0.16	6.79 ± 0.13	6.76 ± 0.08	6.91 ± 0.11
TC05000355.hg.1	ARHGEF28	5.71 ± 0.07	5.65 ± 0.04	5.64 ± 0.09	5.66 ± 0.03
TC03001488.hg.1	ARHGEF3	8.36 ± 0.09	8.28 ± 0.07	8.23 ± 0.09	8.20 ± 0.07
TC02004960.hg.1	ARHGEF33	5.39 ± 0.14	5.28 ± 0.06	5.36 ± 0.07	5.34 ± 0.04
TC07003305.hg.1	ARHGEF35	7.28 ± 0.17	7.18 ± 0.03	7.09 ± 0.05	7.19 ± 0.15
TC05000822.hg.1	ARHGEF37	7.11 ± 0.26	7.12 ± 0.22	7.08 ± 0.17	7.20 ± 0.05
TC04000544.hg.1	ARHGEF38	5.11 ± 0.11	4.96 ± 0.02	5.04 ± 0.01	5.00 ± 0.03
TC04000545.hg.1	ARHGEF38-IT1	6.11 ± 0.26	5.83 ± 0.12	5.80 ± 0.21	5.96 ± 0.15

TC09001057.hg.1	ARHGEF39	7.30 ± 0.17	7.25 ± 0.15	7.24 ± 0.10	7.35 ± 0.08
TC03000363.hg.1	ARHGEF3-AS1	6.05 ± 0.17	6.06 ± 0.11	6.05 ± 0.18	6.13 ± 0.08
TC02000828.hg.1	ARHGEF4	7.59 ± 0.16	7.58 ± 0.20	7.57 ± 0.20	7.67 ± 0.06
TC14000082.hg.1	ARHGEF40	8.57 ± 0.13	8.35 ± 0.05	8.79 ± 0.11	8.35 ± 0.07
TC07000957.hg.1	ARHGEF5	7.92 ± 0.05	7.88 ± 0.06	7.70 ± 0.12	8.00 ± 0.13
TC0X001416.hg.1	ARHGEF6	9.64 ± 0.29	9.94 ± 0.19	9.85 ± 0.22	9.78 ± 0.05
TC13000398.hg.1	ARHGEF7	8.48 ± 0.05	8.54 ± 0.05	8.39 ± 0.05	8.59 ± 0.06
TC13000883.hg.1	ARHGEF7-AS1	6.58 ± 0.25	6.38 ± 0.17	6.55 ± 0.17	6.59 ± 0.13
TC13000881.hg.1	ARHGEF7-AS2	7.24 ± 0.15	7.12 ± 0.21	7.17 ± 0.03	7.24 ± 0.14
TC13000399.hg.1	ARHGEF7-IT1	7.12 ± 0.17	7.01 ± 0.21	6.90 ± 0.17	7.14 ± 0.17
TC0X001092.hg.1	ARHGEF9	7.81 ± 0.22	7.82 ± 0.03	7.60 ± 0.26	7.82 ± 0.08
TC0X001093.hg.1	ARHGEF9-IT1	5.58 ± 0.19	5.74 ± 0.12	5.66 ± 0.04	5.62 ± 0.21
TC01000349.hg.1	ARID1A	10.03 ± 0.27	10.04 ± 0.09	10.07 ± 0.26	10.02 ± 0.21
TC06001123.hg.1	ARID1B	8.76 ± 0.11	8.87 ± 0.07	8.57 ± 0.17	8.80 ± 0.09
TC12000331.hg.1	ARID2	8.86 ± 0.45	9.12 ± 0.18	8.87 ± 0.44	9.06 ± 0.08
TC19000023.hg.1	ARID3A	9.37 ± 0.29	8.87 ± 0.07	9.43 ± 0.12	8.80 ± 0.18
TC15000682.hg.1	ARID3B	8.94 ± 0.18	8.77 ± 0.04	8.87 ± 0.07	8.81 ± 0.03
TC09001041.hg.1	ARID3C	7.45 ± 0.28	7.39 ± 0.18	7.42 ± 0.20	7.48 ± 0.07
TC14000348.hg.1	ARID4A	8.17 ± 0.54	8.62 ± 0.23	8.67 ± 0.56	8.64 ± 0.12
TC01006389.hg.1	ARID4B	8.85 ± 0.51	9.32 ± 0.27	9.37 ± 0.59	9.29 ± 0.20
TC01003992.hg.1	ARID4B-IT1	12.14 ± 0.19	12.20 ± 0.04	12.15 ± 0.05	12.26 ± 0.07
TC02000583.hg.1	ARID5A	9.14 ± 0.23	9.20 ± 0.17	9.27 ± 0.14	9.43 ± 0.14
TC10000377.hg.1	ARID5B	9.36 ± 0.14	9.57 ± 0.07	9.36 ± 0.32	9.43 ± 0.05
TC03000285.hg.1	ARIH2	8.40 ± 0.16	8.46 ± 0.07	8.30 ± 0.06	8.45 ± 0.03
TC03001391.hg.1	ARIH2OS	7.04 ± 0.34	7.16 ± 0.24	6.91 ± 0.23	7.14 ± 0.03
TC12001876.hg.1	ARL1	7.72 ± 0.18	7.72 ± 0.10	7.56 ± 0.10	7.61 ± 0.10
TC05000997.hg.1	ARL10	7.88 ± 0.15	7.89 ± 0.14	7.80 ± 0.17	7.94 ± 0.04
TC13000200.hg.1	ARL11	7.60 ± 0.42	7.08 ± 0.20	7.95 ± 0.43	6.94 ± 0.29
TC0X000471.hg.1	ARL13A	4.16 ± 0.10	4.13 ± 0.13	4.09 ± 0.04	4.08 ± 0.07
TC03000474.hg.1	ARL13B	6.65 ± 0.24	6.68 ± 0.11	6.85 ± 0.22	6.61 ± 0.13
TC03000878.hg.1	ARL14	5.48 ± 0.17	5.46 ± 0.13	5.46 ± 0.18	5.48 ± 0.05

TC11000295.hg.1	ARL14EP	8.24 ± 0.35	8.58 ± 0.12	8.24 ± 0.32	8.49 ± 0.11
TC05000550.hg.1	ARL14EPL	4.20 ± 0.15	4.07 ± 0.07	4.16 ± 0.11	4.14 ± 0.05
TC05001345.hg.1	ARL15	7.21 ± 0.42	7.10 ± 0.10	7.25 ± 0.34	6.89 ± 0.07
TC17001957.hg.1	ARL16	8.16 ± 0.06	7.95 ± 0.14	7.97 ± 0.10	7.94 ± 0.04
TC17_ctg5_hap1000002.hg.1	ARL17A	9.45 ± 0.43	9.62 ± 0.06	9.47 ± 0.53	9.80 ± 0.41
TC17_ctg5_hap1000003.hg.1	ARL17A	7.75 ± 0.33	7.80 ± 0.22	7.57 ± 0.42	7.78 ± 0.26
TC17_ctg5_hap1000024.hg.1	ARL17A	8.30 ± 0.21	8.38 ± 0.04	8.27 ± 0.31	8.39 ± 0.31
TC17001613.hg.1	ARL17A	8.56 ± 0.19	8.63 ± 0.07	8.54 ± 0.30	8.68 ± 0.32
TC17_ctg5_hap1000004.hg.1	ARL17B	8.07 ± 0.17	8.08 ± 0.05	8.00 ± 0.29	8.08 ± 0.27
TC17001612.hg.1	ARL17B	8.31 ± 0.16	8.35 ± 0.06	8.25 ± 0.29	8.38 ± 0.30
TC11003457.hg.1	ARL2	9.37 ± 0.21	9.09 ± 0.08	9.27 ± 0.17	8.91 ± 0.11
TC16000485.hg.1	ARL2BP	9.15 ± 0.11	9.20 ± 0.10	8.87 ± 0.17	9.10 ± 0.07
TC10001624.hg.1	ARL3	7.87 ± 0.08	7.79 ± 0.11	7.94 ± 0.05	7.77 ± 0.14
TC07000098.hg.1	ARL4A	7.72 ± 0.15	7.91 ± 0.06	7.75 ± 0.11	7.91 ± 0.08
TC02002891.hg.1	ARL4C	9.98 ± 0.11	10.18 ± 0.12	9.66 ± 0.07	10.07 ± 0.02
TC17000551.hg.1	ARL4D	7.33 ± 0.26	7.20 ± 0.09	7.03 ± 0.21	7.14 ± 0.02
TC02002429.hg.1	ARL5A	7.32 ± 0.37	7.42 ± 0.16	7.31 ± 0.31	7.33 ± 0.17
TC10000140.hg.1	ARL5B	8.36 ± 0.57	9.28 ± 0.08	8.85 ± 0.65	9.14 ± 0.27
TC10002960.hg.1	ARL5B-AS1	7.30 ± 0.17	7.29 ± 0.07	7.23 ± 0.06	7.27 ± 0.03
TC17001445.hg.1	ARL5C	6.98 ± 0.22	6.92 ± 0.23	6.91 ± 0.09	7.04 ± 0.08
TC03000479.hg.1	ARL6	5.03 ± 0.15	4.97 ± 0.06	4.95 ± 0.09	4.92 ± 0.03
TC16000915.hg.1	ARL6IP1	9.99 ± 0.15	10.09 ± 0.15	9.88 ± 0.24	9.90 ± 0.05
TC12003262.hg.1	ARL6IP4	9.22 ± 0.25	9.16 ± 0.13	9.09 ± 0.23	9.19 ± 0.03
TC03000417.hg.1	ARL6IP5	10.85 ± 0.24	10.89 ± 0.12	11.11 ± 0.23	10.88 ± 0.15
TC02000942.hg.1	ARL6IP6	7.55 ± 0.17	7.42 ± 0.17	7.65 ± 0.28	7.23 ± 0.07
TC01003699.hg.1	ARL8A	9.56 ± 0.23	9.37 ± 0.05	9.61 ± 0.17	9.41 ± 0.27
TC03000016.hg.1	ARL8B	8.96 ± 0.12	8.87 ± 0.18	8.98 ± 0.20	8.84 ± 0.13
TC04000333.hg.1	ARL9	6.51 ± 0.21	6.44 ± 0.20	6.38 ± 0.22	6.47 ± 0.00
TC08001280.hg.1	ARMC1	7.82 ± 0.19	7.87 ± 0.15	7.64 ± 0.26	7.63 ± 0.12
TC07000671.hg.1	ARMC10	7.38 ± 0.07	7.27 ± 0.14	7.38 ± 0.13	7.08 ± 0.15
TC06000519.hg.1	ARMC12	7.46 ± 0.24	7.49 ± 0.22	7.46 ± 0.11	7.61 ± 0.11

TC06000870.hg.1	ARMC2	6.75 ± 0.07	6.66 ± 0.10	6.86 ± 0.21	6.57 ± 0.09
TC06001996.hg.1	ARMC2-AS1	4.99 ± 0.15	5.05 ± 0.13	4.95 ± 0.11	5.03 ± 0.11
TC10000161.hg.1	ARMC3	5.69 ± 0.12	5.57 ± 0.07	5.53 ± 0.05	5.63 ± 0.07
TC10001134.hg.1	ARMC4	5.24 ± 0.14	5.15 ± 0.04	5.21 ± 0.06	5.22 ± 0.05
TC16000377.hg.1	ARMC5	8.20 ± 0.12	8.23 ± 0.10	8.09 ± 0.15	8.26 ± 0.04
TC19000367.hg.1	ARMC6	8.21 ± 0.16	8.11 ± 0.12	8.02 ± 0.16	8.11 ± 0.04
TC17000846.hg.1	ARMC7	8.68 ± 0.24	8.43 ± 0.04	8.58 ± 0.11	8.42 ± 0.08
TC03000743.hg.1	ARMC8	7.85 ± 0.35	7.89 ± 0.19	8.04 ± 0.33	7.83 ± 0.07
TC02001388.hg.1	ARMC9	6.94 ± 0.12	6.91 ± 0.12	6.87 ± 0.11	6.96 ± 0.03
TC0X000480.hg.1	ARMCX1	7.48 ± 0.16	7.44 ± 0.15	7.41 ± 0.22	7.51 ± 0.05
TC0X001224.hg.1	ARMCX2	7.31 ± 0.13	7.26 ± 0.08	7.25 ± 0.08	7.29 ± 0.05
TC0X000481.hg.1	ARMCX3	8.07 ± 0.04	7.96 ± 0.08	8.08 ± 0.11	7.96 ± 0.08
TC0X001222.hg.1	ARMCX3-AS1	6.23 ± 0.28	6.15 ± 0.08	6.12 ± 0.33	6.02 ± 0.08
TC0X000479.hg.1	ARMCX4	5.94 ± 0.07	5.97 ± 0.03	5.97 ± 0.03	5.98 ± 0.04
TC0X002319.hg.1	ARMCX5	6.70 ± 0.15	6.70 ± 0.08	6.60 ± 0.12	6.63 ± 0.03
TC0X001221.hg.1	ARMCX6	8.18 ± 0.04	8.05 ± 0.06	8.02 ± 0.13	8.05 ± 0.03
TC10000888.hg.1	ARMS2	6.55 ± 0.15	6.63 ± 0.21	6.38 ± 0.11	6.72 ± 0.22
TC01003212.hg.1	ARNT	9.13 ± 0.15	8.95 ± 0.13	9.25 ± 0.16	8.87 ± 0.14
TC15000749.hg.1	ARNT2	6.69 ± 0.17	6.62 ± 0.15	6.62 ± 0.13	6.73 ± 0.07
TC11000206.hg.1	ARNTL	8.09 ± 0.25	8.21 ± 0.15	8.45 ± 0.34	8.22 ± 0.02
TC12000264.hg.1	ARNTL2	5.92 ± 0.14	5.69 ± 0.05	5.80 ± 0.03	5.60 ± 0.06
TC07003287.hg.1	ARPC1A	8.98 ± 0.14	8.74 ± 0.13	9.00 ± 0.30	8.68 ± 0.15
TC07003288.hg.1	ARPC1B	10.38 ± 0.16	10.14 ± 0.03	10.40 ± 0.18	10.10 ± 0.09
TC02001294.hg.1	ARPC2	11.03 ± 0.08	11.14 ± 0.12	11.17 ± 0.07	11.11 ± 0.05
TC12003222.hg.1	ARPC3	9.34 ± 0.11	9.31 ± 0.20	9.65 ± 0.25	9.22 ± 0.23
TC03003343.hg.1	ARPC4	9.41 ± 0.18	9.20 ± 0.06	9.38 ± 0.16	9.10 ± 0.07
TC01003617.hg.1	ARPC5	9.86 ± 0.21	9.64 ± 0.03	10.13 ± 0.28	9.55 ± 0.17
TC01003618.hg.1	ARPC5	8.82 ± 0.08	8.75 ± 0.06	9.11 ± 0.23	8.77 ± 0.08
TC09000646.hg.1	ARPC5L	7.97 ± 0.08	7.83 ± 0.06	7.77 ± 0.06	7.74 ± 0.06
TC15001457.hg.1	ARPP19	9.83 ± 0.08	9.91 ± 0.15	9.65 ± 0.15	9.83 ± 0.07
TC03000171.hg.1	ARPP21	5.69 ± 0.09	5.61 ± 0.05	5.61 ± 0.05	5.67 ± 0.08

TC03001280.hg.1	ARPP21-AS1	4.04 ± 0.07	3.74 ± 0.04	3.92 ± 0.15	3.78 ± 0.18
TC0X000374.hg.1	ARR3	6.03 ± 0.16	5.99 ± 0.08	5.95 ± 0.13	6.02 ± 0.04
TC11002092.hg.1	ARRB1	10.01 ± 0.21	9.75 ± 0.14	9.88 ± 0.14	9.70 ± 0.12
TC17000047.hg.1	ARRB2	11.83 ± 0.25	11.81 ± 0.02	12.16 ± 0.22	11.76 ± 0.17
TC09000851.hg.1	ARRDC1	9.05 ± 0.08	8.90 ± 0.06	8.98 ± 0.07	8.89 ± 0.05
TC19000350.hg.1	ARRDC2	8.81 ± 0.10	8.86 ± 0.22	8.48 ± 0.10	8.88 ± 0.10
TC05001590.hg.1	ARRDC3	9.78 ± 0.38	10.09 ± 0.15	9.79 ± 0.37	10.17 ± 0.12
TC05000446.hg.1	ARRDC3-AS1	6.45 ± 0.29	6.35 ± 0.07	6.49 ± 0.11	6.41 ± 0.11
TC15000945.hg.1	ARRDC4	7.78 ± 0.29	7.95 ± 0.19	8.11 ± 0.20	8.01 ± 0.03
TC19001074.hg.1	ARRDC5	7.62 ± 0.05	7.31 ± 0.09	7.24 ± 0.08	7.25 ± 0.10
TC22000918.hg.1	ARSA	8.74 ± 0.15	8.62 ± 0.13	8.67 ± 0.13	8.64 ± 0.03
TC05001519.hg.1	ARSB	7.79 ± 0.24	7.60 ± 0.04	7.82 ± 0.07	7.60 ± 0.05
TC0X000805.hg.1	ARSD	7.93 ± 0.21	7.59 ± 0.05	7.86 ± 0.09	7.58 ± 0.07
TC0X000018.hg.1	ARSD-AS1	6.44 ± 0.08	6.34 ± 0.11	6.51 ± 0.18	6.35 ± 0.22
TC0X000806.hg.1	ARSE	6.41 ± 0.13	6.33 ± 0.13	6.39 ± 0.13	6.43 ± 0.02
TC0X000020.hg.1	ARSF	6.18 ± 0.24	6.13 ± 0.09	6.08 ± 0.15	6.18 ± 0.07
TC17000802.hg.1	ARSG	8.58 ± 0.06	8.43 ± 0.06	8.47 ± 0.14	8.38 ± 0.08
TC0X000019.hg.1	ARSH	6.48 ± 0.20	6.46 ± 0.25	6.52 ± 0.17	6.66 ± 0.04
TC05001935.hg.1	ARSI	6.97 ± 0.32	6.97 ± 0.24	6.97 ± 0.23	7.08 ± 0.06
TC04001487.hg.1	ARSJ	5.53 ± 0.08	5.42 ± 0.06	5.42 ± 0.08	5.51 ± 0.04
TC05000458.hg.1	ARSK	5.90 ± 0.24	5.93 ± 0.12	5.82 ± 0.08	5.81 ± 0.07
TC11000082.hg.1	ART1	7.58 ± 0.16	7.77 ± 0.26	7.62 ± 0.26	7.86 ± 0.08
TC04000424.hg.1	ART3	4.48 ± 0.14	4.32 ± 0.04	4.35 ± 0.03	4.35 ± 0.01
TC12001275.hg.1	ART4	5.00 ± 0.10	4.89 ± 0.08	4.79 ± 0.10	4.92 ± 0.15
TC11001298.hg.1	ART5	8.21 ± 0.14	8.12 ± 0.16	8.13 ± 0.15	8.16 ± 0.04
TC01000552.hg.1	ARTN	8.15 ± 0.25	8.18 ± 0.26	8.13 ± 0.22	8.25 ± 0.08
TC01001913.hg.1	ARV1	7.26 ± 0.15	7.17 ± 0.04	7.19 ± 0.12	7.01 ± 0.07
TC22001432.hg.1	ARVCF	8.00 ± 0.25	7.95 ± 0.20	7.94 ± 0.20	8.03 ± 0.05
TC0X000929.hg.1	ARX	7.99 ± 0.37	7.89 ± 0.16	8.04 ± 0.27	7.93 ± 0.06
TC10002948.hg.1	AS3MT	6.52 ± 0.08	6.47 ± 0.02	6.48 ± 0.04	6.46 ± 0.12
TC08001015.hg.1	ASAH1	9.67 ± 0.20	9.78 ± 0.17	10.15 ± 0.22	9.90 ± 0.24

TC10001287.hg.1	ASAH2	5.84 ± 0.12	5.79 ± 0.07	5.79 ± 0.01	5.86 ± 0.08
TC10000345.hg.1	ASAH2B	6.70 ± 0.22	6.65 ± 0.03	6.58 ± 0.09	6.65 ± 0.08
TC10001246.hg.1	ASAH2C	5.97 ± 0.11	5.89 ± 0.06	5.91 ± 0.02	5.95 ± 0.07
TC08001641.hg.1	ASAP1	9.61 ± 0.14	9.50 ± 0.16	10.02 ± 0.31	9.49 ± 0.22
TC08001644.hg.1	ASAP1-IT1	6.49 ± 0.02	6.41 ± 0.23	6.77 ± 0.29	6.28 ± 0.15
TC02000046.hg.1	ASAP2	7.97 ± 0.11	7.44 ± 0.10	7.91 ± 0.12	7.50 ± 0.23
TC01002343.hg.1	ASAP3	7.45 ± 0.22	7.42 ± 0.18	7.36 ± 0.20	7.46 ± 0.04
TC02001455.hg.1	ASB1	7.79 ± 0.05	7.70 ± 0.07	7.66 ± 0.11	7.74 ± 0.04
TC07002032.hg.1	ASB10	7.90 ± 0.27	7.88 ± 0.21	7.84 ± 0.19	7.98 ± 0.11
TC0X000874.hg.1	ASB11	6.47 ± 0.14	6.48 ± 0.09	6.45 ± 0.12	6.58 ± 0.08
TC0X002332.hg.1	ASB12	6.13 ± 0.07	6.12 ± 0.16	5.99 ± 0.15	6.15 ± 0.02
TC10001000.hg.1	ASB13	7.72 ± 0.19	7.62 ± 0.12	7.54 ± 0.13	7.67 ± 0.04
TC03001491.hg.1	ASB14	5.29 ± 0.09	5.19 ± 0.05	5.25 ± 0.01	5.20 ± 0.07
TC07000745.hg.1	ASB15	4.78 ± 0.10	4.69 ± 0.03	4.73 ± 0.01	4.76 ± 0.03
TC17000564.hg.1	ASB16	8.32 ± 0.21	8.22 ± 0.20	8.26 ± 0.18	8.31 ± 0.01
TC01002794.hg.1	ASB17	4.03 ± 0.09	3.92 ± 0.02	3.92 ± 0.03	3.90 ± 0.07
TC02002898.hg.1	ASB18	7.25 ± 0.16	7.21 ± 0.16	7.12 ± 0.19	7.23 ± 0.04
TC14001461.hg.1	ASB2	7.55 ± 0.23	7.55 ± 0.15	7.49 ± 0.15	7.58 ± 0.05
TC02005026.hg.1	ASB3	7.09 ± 0.13	7.09 ± 0.08	6.98 ± 0.13	7.03 ± 0.02
TC07000569.hg.1	ASB4	4.90 ± 0.16	4.90 ± 0.12	4.90 ± 0.05	4.93 ± 0.07
TC04001762.hg.1	ASB5	5.59 ± 0.17	5.54 ± 0.10	5.54 ± 0.10	5.55 ± 0.03
TC09001650.hg.1	ASB6	8.79 ± 0.21	8.71 ± 0.11	8.54 ± 0.20	8.71 ± 0.02
TC15000967.hg.1	ASB7	8.41 ± 0.22	8.46 ± 0.04	8.70 ± 0.28	8.43 ± 0.07
TC12001445.hg.1	ASB8	9.14 ± 0.19	8.90 ± 0.04	9.21 ± 0.30	8.92 ± 0.09
TC0X000873.hg.1	ASB9	6.77 ± 0.11	6.72 ± 0.11	6.67 ± 0.06	6.79 ± 0.05
TC15000919.hg.1	ASB9P1	5.31 ± 0.15	5.44 ± 0.04	5.49 ± 0.13	5.48 ± 0.11
TC10001377.hg.1	ASCC1	7.48 ± 0.02	7.42 ± 0.04	7.40 ± 0.03	7.41 ± 0.04
TC22000642.hg.1	ASCC2	8.73 ± 0.08	8.61 ± 0.03	8.49 ± 0.06	8.56 ± 0.03
TC06001970.hg.1	ASCC3	7.81 ± 0.47	8.02 ± 0.26	7.62 ± 0.47	7.66 ± 0.11
TC12000796.hg.1	ASCL1	6.69 ± 0.31	6.59 ± 0.09	6.60 ± 0.16	6.64 ± 0.01
TC11001277.hg.1	ASCL2	8.26 ± 0.36	8.11 ± 0.06	8.12 ± 0.31	8.10 ± 0.05

TC11001392.hg.1	ASCL3	3.65 ± 0.09	3.59 ± 0.02	3.73 ± 0.14	3.58 ± 0.03
TC12000832.hg.1	ASCL4	6.70 ± 0.19	6.47 ± 0.22	6.68 ± 0.20	6.51 ± 0.09
TC06000937.hg.1	ASF1A	7.52 ± 0.33	7.52 ± 0.18	7.39 ± 0.35	7.28 ± 0.10
TC19001232.hg.1	ASF1B	8.57 ± 0.26	8.35 ± 0.08	8.32 ± 0.25	8.27 ± 0.06
TC17001076.hg.1	ASGR1	7.89 ± 0.19	7.86 ± 0.15	7.89 ± 0.22	7.91 ± 0.06
TC17001075.hg.1	ASGR2	8.56 ± 0.17	8.25 ± 0.20	8.70 ± 0.10	8.32 ± 0.05
TC01001303.hg.1	ASH1L-AS1	7.62 ± 0.15	7.57 ± 0.09	7.48 ± 0.15	7.70 ± 0.07
TC01003317.hg.1	ASH1L-IT1	8.10 ± 0.38	8.09 ± 0.13	8.09 ± 0.11	8.27 ± 0.18
TC08000283.hg.1	ASH2L	8.52 ± 0.15	8.28 ± 0.09	8.51 ± 0.20	8.13 ± 0.11
TC12000386.hg.1	ASIC1	7.27 ± 0.17	7.22 ± 0.18	7.22 ± 0.18	7.29 ± 0.03
TC17001355.hg.1	ASIC2	6.90 ± 0.14	6.84 ± 0.16	6.84 ± 0.14	6.90 ± 0.05
TC07001011.hg.1	ASIC3	7.89 ± 0.18	8.01 ± 0.24	7.84 ± 0.17	8.16 ± 0.10
TC02001330.hg.1	ASIC4	7.44 ± 0.15	7.39 ± 0.17	7.40 ± 0.09	7.50 ± 0.07
TC04001669.hg.1	ASIC5	4.59 ± 0.07	4.51 ± 0.04	4.53 ± 0.10	4.50 ± 0.05
TC20000238.hg.1	ASIP	7.06 ± 0.17	7.17 ± 0.15	7.06 ± 0.16	7.18 ± 0.03
TC07003307.hg.1	ASL	8.23 ± 0.17	8.15 ± 0.10	8.16 ± 0.13	8.23 ± 0.03
TC0X002311.hg.1	ASMT	7.54 ± 0.16	7.51 ± 0.10	7.51 ± 0.10	7.61 ± 0.07
TC0Y000350.hg.1	ASMT	7.49 ± 0.18	7.45 ± 0.10	7.44 ± 0.11	7.54 ± 0.05
TC0X000800.hg.1	ASMTL	8.26 ± 0.16	8.16 ± 0.07	8.12 ± 0.12	8.17 ± 0.03
TC0Y000117.hg.1	ASMTL	8.45 ± 0.17	8.31 ± 0.05	8.31 ± 0.13	8.31 ± 0.02
TC0X000009.hg.1	ASMTL-AS1	8.34 ± 0.10	8.26 ± 0.12	8.29 ± 0.09	8.35 ± 0.05
TC0Y000009.hg.1	ASMTL-AS1	8.34 ± 0.10	8.26 ± 0.12	8.29 ± 0.09	8.35 ± 0.05
TC19000226.hg.1	ASNA1	9.10 ± 0.35	8.68 ± 0.12	8.78 ± 0.22	8.42 ± 0.10
TC07001630.hg.1	ASNS	7.19 ± 0.07	7.15 ± 0.03	7.02 ± 0.06	7.10 ± 0.06
TC02001110.hg.1	ASNSD1	7.99 ± 0.34	8.28 ± 0.21	8.03 ± 0.35	8.18 ± 0.06
TC17000033.hg.1	ASPA	4.61 ± 0.15	4.58 ± 0.03	4.59 ± 0.10	4.61 ± 0.02
TC19001749.hg.1	ASPDH	7.86 ± 0.21	7.90 ± 0.26	7.84 ± 0.21	8.06 ± 0.09
TC14000811.hg.1	ASPG	8.43 ± 0.22	8.45 ± 0.22	8.40 ± 0.18	8.56 ± 0.05
TC08001264.hg.1	ASPH	6.74 ± 0.05	6.47 ± 0.12	7.36 ± 0.35	6.51 ± 0.15
TC16000332.hg.1	ASPHD1	7.51 ± 0.26	7.37 ± 0.18	7.33 ± 0.20	7.47 ± 0.08
TC22000169.hg.1	ASPHD2	8.65 ± 0.23	8.40 ± 0.02	8.47 ± 0.14	8.34 ± 0.02

TC01003662.hg.1	ASPM	5.92 ± 0.06	5.78 ± 0.51	5.74 ± 0.41	5.21 ± 0.12
TC09001336.hg.1	ASPN	4.19 ± 0.07	4.02 ± 0.06	4.09 ± 0.07	4.04 ± 0.10
TC02005030.hg.1	ASPRV1	7.02 ± 0.27	7.07 ± 0.20	7.07 ± 0.35	7.07 ± 0.07
TC17000942.hg.1	ASPSCR1	8.52 ± 0.16	8.42 ± 0.12	8.40 ± 0.16	8.45 ± 0.02
TC11000551.hg.1	ASRGL1	7.50 ± 0.12	7.36 ± 0.10	7.47 ± 0.20	7.37 ± 0.06
TC09000736.hg.1	ASS1	7.57 ± 0.18	7.53 ± 0.14	7.47 ± 0.17	7.64 ± 0.14
TC03001782.hg.1	ASTE1	7.65 ± 0.13	7.59 ± 0.14	7.60 ± 0.12	7.50 ± 0.05
TC02002101.hg.1	ASTL	7.75 ± 0.21	7.92 ± 0.27	7.76 ± 0.14	8.08 ± 0.04
TC01003556.hg.1	ASTN1	6.88 ± 0.15	6.88 ± 0.15	6.87 ± 0.14	6.96 ± 0.09
TC09001526.hg.1	ASTN2	6.99 ± 0.17	6.96 ± 0.13	6.95 ± 0.12	7.02 ± 0.05
TC12001330.hg.1	ASUN	7.63 ± 0.20	7.47 ± 0.15	7.53 ± 0.23	7.12 ± 0.28
TC20000210.hg.1	ASXL1	8.78 ± 0.03	8.81 ± 0.13	8.46 ± 0.09	8.84 ± 0.00
TC02001663.hg.1	ASXL2	9.18 ± 0.20	9.08 ± 0.17	9.36 ± 0.41	8.91 ± 0.22
TC18000140.hg.1	ASXL3	5.26 ± 0.20	5.15 ± 0.12	5.14 ± 0.14	5.22 ± 0.11
TC07001795.hg.1	ASZ1	3.74 ± 0.10	3.57 ± 0.01	3.63 ± 0.06	3.57 ± 0.02
TC10001492.hg.1	ATAD1	8.06 ± 0.43	8.21 ± 0.18	8.07 ± 0.41	8.20 ± 0.04
TC08001584.hg.1	ATAD2	7.56 ± 0.23	7.69 ± 0.33	7.39 ± 0.29	7.34 ± 0.18
TC02001642.hg.1	ATAD2B	7.50 ± 0.46	7.71 ± 0.23	7.66 ± 0.37	7.62 ± 0.11
TC01000043.hg.1	ATAD3A	8.60 ± 0.16	8.57 ± 0.12	8.43 ± 0.19	8.55 ± 0.01
TC01000042.hg.1	ATAD3B	8.55 ± 0.19	8.48 ± 0.12	8.39 ± 0.22	8.49 ± 0.00
TC01000041.hg.1	ATAD3C	8.21 ± 0.17	8.10 ± 0.11	8.06 ± 0.12	8.14 ± 0.05
TC17000355.hg.1	ATAD5	6.53 ± 0.32	6.63 ± 0.32	6.50 ± 0.35	6.33 ± 0.10
TC06000344.hg.1	ATAT1	7.77 ± 0.02	7.62 ± 0.07	7.71 ± 0.08	7.66 ± 0.04
TC6_cox_hap2000042.hg.1	ATAT1	7.79 ± 0.01	7.64 ± 0.07	7.73 ± 0.08	7.68 ± 0.04
TC6_dbb_hap3000035.hg.1	ATAT1	7.78 ± 0.02	7.62 ± 0.07	7.71 ± 0.08	7.66 ± 0.04
TC6_mann_hap4000037.hg.1	ATAT1	7.77 ± 0.02	7.62 ± 0.07	7.71 ± 0.08	7.66 ± 0.04
TC6_mcf_hap5000029.hg.1	ATAT1	7.77 ± 0.02	7.62 ± 0.07	7.71 ± 0.08	7.66 ± 0.04
TC6_qbl_hap6000035.hg.1	ATAT1	7.78 ± 0.02	7.62 ± 0.07	7.71 ± 0.08	7.66 ± 0.04
TC6_ssto_hap7000035.hg.1	ATAT1	7.78 ± 0.02	7.62 ± 0.07	7.71 ± 0.08	7.66 ± 0.04
TC19000078.hg.1	ATCAY	7.12 ± 0.23	7.13 ± 0.20	7.07 ± 0.27	7.21 ± 0.08
TC10001713.hg.1	ATE1	8.12 ± 0.30	8.19 ± 0.16	8.07 ± 0.24	8.03 ± 0.05

TC12000397.hg.1	ATF1	8.25 ± 0.36	8.44 ± 0.20	8.43 ± 0.31	8.28 ± 0.22
TC02002545.hg.1	ATF2	8.28 ± 0.34	8.39 ± 0.16	8.36 ± 0.35	8.25 ± 0.15
TC01001777.hg.1	ATF3	7.82 ± 0.16	8.03 ± 0.22	7.82 ± 0.13	8.02 ± 0.13
TC22000317.hg.1	ATF4	10.00 ± 0.09	10.09 ± 0.12	9.71 ± 0.16	10.08 ± 0.07
TC11001021.hg.1	ATF4P4	7.33 ± 0.27	7.28 ± 0.30	7.16 ± 0.18	7.40 ± 0.04
TC19000751.hg.1	ATF5	8.49 ± 0.22	8.34 ± 0.09	8.32 ± 0.11	8.37 ± 0.04
TC01001414.hg.1	ATF6	9.69 ± 0.27	9.75 ± 0.24	10.13 ± 0.33	9.60 ± 0.20
TC06004121.hg.1	ATF6B	10.20 ± 0.20	10.03 ± 0.03	10.03 ± 0.07	9.94 ± 0.09
TC6_cox_hap2000261.hg.1	ATF6B	10.13 ± 0.20	9.96 ± 0.03	9.96 ± 0.06	9.87 ± 0.08
TC6_dbb_hap3000238.hg.1	ATF6B	10.14 ± 0.20	9.97 ± 0.03	9.97 ± 0.06	9.88 ± 0.08
TC6_mcf_hap5000199.hg.1	ATF6B	10.14 ± 0.19	9.98 ± 0.03	9.98 ± 0.06	9.89 ± 0.08
TC12001552.hg.1	ATF7	8.78 ± 0.14	8.62 ± 0.10	8.96 ± 0.22	8.56 ± 0.12
TC12000193.hg.1	ATF7IP	10.11 ± 0.38	10.28 ± 0.16	10.15 ± 0.43	10.21 ± 0.07
TC16000154.hg.1	ATF7IP2	8.21 ± 0.46	8.27 ± 0.16	7.94 ± 0.18	8.06 ± 0.09
TC05000406.hg.1	ATG10	6.85 ± 0.04	6.76 ± 0.09	6.79 ± 0.03	6.72 ± 0.02
TC05001546.hg.1	ATG10-AS1	4.31 ± 0.04	4.23 ± 0.23	4.23 ± 0.13	4.28 ± 0.01
TC05000407.hg.1	ATG10-IT1	6.80 ± 0.28	6.85 ± 0.08	6.65 ± 0.07	7.01 ± 0.19
TC05001694.hg.1	ATG12	7.53 ± 0.40	7.63 ± 0.23	7.69 ± 0.39	7.58 ± 0.09
TC11000399.hg.1	ATG13	9.19 ± 0.15	9.06 ± 0.11	9.03 ± 0.13	9.04 ± 0.04
TC14001159.hg.1	ATG14	7.90 ± 0.39	8.23 ± 0.37	7.64 ± 0.24	8.35 ± 0.07
TC02001415.hg.1	ATG16L1	8.21 ± 0.15	8.30 ± 0.19	7.93 ± 0.12	8.26 ± 0.12
TC11000770.hg.1	ATG16L2	9.65 ± 0.08	9.57 ± 0.11	9.87 ± 0.21	9.52 ± 0.14
TC11001918.hg.1	ATG2A	8.93 ± 0.10	9.12 ± 0.14	8.76 ± 0.11	9.37 ± 0.20
TC14001484.hg.1	ATG2B	7.81 ± 0.42	7.87 ± 0.22	7.75 ± 0.41	7.83 ± 0.02
TC03001650.hg.1	ATG3	9.28 ± 0.17	9.20 ± 0.21	9.72 ± 0.31	9.04 ± 0.24
TC0X000530.hg.1	ATG4A	7.27 ± 0.08	7.03 ± 0.12	7.15 ± 0.16	6.78 ± 0.08
TC02001483.hg.1	ATG4B	8.27 ± 0.08	8.28 ± 0.08	8.15 ± 0.10	8.28 ± 0.06
TC01000710.hg.1	ATG4C	6.60 ± 0.28	6.56 ± 0.26	6.61 ± 0.29	6.29 ± 0.12
TC19000178.hg.1	ATG4D	8.48 ± 0.05	8.43 ± 0.05	8.34 ± 0.10	8.49 ± 0.05
TC06001982.hg.1	ATG5	7.85 ± 0.19	8.05 ± 0.19	7.81 ± 0.19	7.92 ± 0.07
TC03000065.hg.1	ATG7	7.89 ± 0.11	7.70 ± 0.09	7.97 ± 0.17	7.74 ± 0.08

TC02005055.hg.1	ATG9A	8.98 ± 0.08	8.86 ± 0.06	8.89 ± 0.11	8.93 ± 0.02
TC07002025.hg.1	ATG9B	7.73 ± 0.20	7.70 ± 0.17	7.64 ± 0.14	7.76 ± 0.06
TC11000009.hg.1	ATHL1	9.08 ± 0.01	9.14 ± 0.06	9.01 ± 0.17	9.21 ± 0.07
TC02001260.hg.1	ATIC	8.12 ± 0.07	8.05 ± 0.12	7.84 ± 0.07	7.86 ± 0.02
TC14000287.hg.1	ATL1	6.82 ± 0.09	6.65 ± 0.02	6.79 ± 0.09	6.68 ± 0.06
TC02001753.hg.1	ATL2	7.49 ± 0.40	7.80 ± 0.17	7.50 ± 0.31	7.81 ± 0.04
TC11001894.hg.1	ATL3	8.37 ± 0.06	8.29 ± 0.22	8.41 ± 0.22	8.14 ± 0.17
TC11000980.hg.1	ATM	9.07 ± 0.92	9.85 ± 0.22	9.13 ± 0.55	9.64 ± 0.18
TC16000635.hg.1	ATMIN	8.40 ± 0.18	8.53 ± 0.17	8.20 ± 0.28	8.45 ± 0.08
TC12003288.hg.1	ATN1	8.19 ± 0.07	8.26 ± 0.04	8.06 ± 0.16	8.26 ± 0.06
TC04000499.hg.1	ATOH1	7.39 ± 0.15	7.41 ± 0.15	7.43 ± 0.16	7.41 ± 0.02
TC10001343.hg.1	ATOH7	4.76 ± 0.21	4.66 ± 0.16	4.67 ± 0.19	4.67 ± 0.10
TC02000517.hg.1	ATOH8	7.45 ± 0.11	7.48 ± 0.18	7.47 ± 0.11	7.56 ± 0.07
TC05001957.hg.1	ATOX1	7.97 ± 0.06	7.86 ± 0.06	7.88 ± 0.07	7.84 ± 0.04
TC15001079.hg.1	ATP10A	8.17 ± 0.13	8.04 ± 0.01	7.90 ± 0.03	7.97 ± 0.06
TC05002011.hg.1	ATP10B	5.82 ± 0.16	5.73 ± 0.12	5.74 ± 0.08	5.82 ± 0.07
TC04000274.hg.1	ATP10D	7.79 ± 0.21	7.67 ± 0.30	7.71 ± 0.29	7.36 ± 0.10
TC13000411.hg.1	ATP11A	8.70 ± 0.21	8.53 ± 0.13	9.00 ± 0.27	8.42 ± 0.18
TC13000888.hg.1	ATP11A-AS1	7.51 ± 0.33	7.35 ± 0.09	7.42 ± 0.05	7.46 ± 0.13
TC03000976.hg.1	ATP11B	9.26 ± 0.47	9.56 ± 0.19	9.94 ± 0.49	9.51 ± 0.06
TC0X001429.hg.1	ATP11C	7.70 ± 0.41	7.83 ± 0.22	7.76 ± 0.48	7.50 ± 0.16
TC13000057.hg.1	ATP12A	6.58 ± 0.14	6.59 ± 0.19	6.54 ± 0.12	6.74 ± 0.07
TC19001307.hg.1	ATP13A1	9.16 ± 0.13	8.97 ± 0.10	8.95 ± 0.04	8.95 ± 0.03
TC01002285.hg.1	ATP13A2	8.67 ± 0.10	8.61 ± 0.09	8.51 ± 0.10	8.59 ± 0.02
TC03002133.hg.1	ATP13A3	8.13 ± 0.31	8.25 ± 0.26	8.65 ± 0.37	8.33 ± 0.29
TC03002121.hg.1	ATP13A4	5.75 ± 0.14	5.69 ± 0.03	5.68 ± 0.07	5.72 ± 0.07
TC03001066.hg.1	ATP13A4-AS1	5.25 ± 0.10	5.16 ± 0.02	5.15 ± 0.05	5.28 ± 0.05
TC03002120.hg.1	ATP13A5	5.59 ± 0.13	5.52 ± 0.08	5.49 ± 0.04	5.58 ± 0.07
TC03001065.hg.1	ATP13A5-AS1	5.77 ± 0.23	5.63 ± 0.14	5.69 ± 0.14	5.79 ± 0.22
TC01001018.hg.1	ATP1A1	9.72 ± 0.14	9.78 ± 0.13	9.47 ± 0.08	9.62 ± 0.09
TC01003024.hg.1	ATP1A1OS	6.99 ± 0.08	6.96 ± 0.13	6.97 ± 0.04	7.05 ± 0.05

TC01001361.hg.1	ATP1A2	7.15 ± 0.16	7.15 ± 0.17	7.03 ± 0.15	7.22 ± 0.06
TC19001563.hg.1	ATP1A3	8.18 ± 0.08	8.14 ± 0.10	8.01 ± 0.15	8.17 ± 0.05
TC01001362.hg.1	ATP1A4	6.83 ± 0.16	6.82 ± 0.12	6.78 ± 0.13	6.87 ± 0.09
TC01001473.hg.1	ATP1B1	6.88 ± 0.04	6.86 ± 0.07	6.82 ± 0.01	6.92 ± 0.03
TC17000108.hg.1	ATP1B2	6.53 ± 0.15	6.48 ± 0.19	6.39 ± 0.16	6.56 ± 0.10
TC03000766.hg.1	ATP1B3	8.19 ± 0.01	8.30 ± 0.06	8.05 ± 0.11	8.05 ± 0.15
TC03001847.hg.1	ATP1B3-AS1	3.90 ± 0.04	3.83 ± 0.01	3.92 ± 0.12	3.79 ± 0.13
TC0X000594.hg.1	ATP1B4	5.75 ± 0.30	5.78 ± 0.15	5.70 ± 0.21	5.79 ± 0.05
TC16000310.hg.1	ATP2A1	7.52 ± 0.18	7.49 ± 0.14	7.48 ± 0.11	7.59 ± 0.06
TC12000856.hg.1	ATP2A2	9.47 ± 0.19	9.31 ± 0.17	9.17 ± 0.10	9.19 ± 0.07
TC17001015.hg.1	ATP2A3	10.25 ± 0.09	10.16 ± 0.06	10.11 ± 0.12	10.14 ± 0.08
TC12001798.hg.1	ATP2B1	8.85 ± 0.72	9.73 ± 0.19	9.18 ± 0.76	9.85 ± 0.07
TC03001160.hg.1	ATP2B2	7.51 ± 0.19	7.45 ± 0.18	7.44 ± 0.22	7.56 ± 0.06
TC03001162.hg.1	ATP2B2-IT1	6.30 ± 0.14	6.21 ± 0.22	6.31 ± 0.13	6.35 ± 0.07
TC03001163.hg.1	ATP2B2-IT2	4.40 ± 0.09	4.41 ± 0.09	4.26 ± 0.12	4.53 ± 0.16
TC0X000751.hg.1	ATP2B3	7.64 ± 0.20	7.65 ± 0.18	7.60 ± 0.17	7.76 ± 0.02
TC01001688.hg.1	ATP2B4	9.22 ± 0.27	9.17 ± 0.13	9.41 ± 0.26	8.83 ± 0.27
TC03000707.hg.1	ATP2C1	8.13 ± 0.22	8.11 ± 0.14	8.08 ± 0.21	8.00 ± 0.05
TC16000657.hg.1	ATP2C2	7.12 ± 0.18	7.02 ± 0.15	7.11 ± 0.15	7.14 ± 0.04
TC19001448.hg.1	ATP4A	7.89 ± 0.25	7.84 ± 0.17	7.84 ± 0.23	7.96 ± 0.09
TC13000897.hg.1	ATP4B	7.42 ± 0.23	7.36 ± 0.22	7.25 ± 0.25	7.49 ± 0.05
TC18000480.hg.1	ATP5A1	9.56 ± 0.11	9.48 ± 0.25	9.26 ± 0.13	9.29 ± 0.05
TC12001609.hg.1	ATP5B	10.64 ± 0.22	10.69 ± 0.20	10.30 ± 0.31	10.41 ± 0.08
TC10000067.hg.1	ATP5C1	9.39 ± 0.19	9.28 ± 0.43	9.23 ± 0.06	9.17 ± 0.21
TC19000032.hg.1	ATP5D	9.70 ± 0.26	9.37 ± 0.09	9.31 ± 0.16	9.23 ± 0.05
TC20001753.hg.1	ATP5E	8.13 ± 0.22	7.85 ± 0.32	8.07 ± 0.29	7.79 ± 0.15
TC13000083.hg.1	ATP5EP2	7.63 ± 0.30	7.30 ± 0.31	7.39 ± 0.29	7.05 ± 0.06
TC01000977.hg.1	ATP5F1	9.59 ± 0.06	9.60 ± 0.13	9.60 ± 0.08	9.49 ± 0.12
TC17000633.hg.1	ATP5G1	8.40 ± 0.12	8.29 ± 0.06	8.20 ± 0.09	8.20 ± 0.02
TC13000380.hg.1	ATP5G1P5	6.19 ± 0.28	5.99 ± 0.12	5.93 ± 0.12	6.01 ± 0.18
TC12001553.hg.1	ATP5G2	8.90 ± 0.07	8.79 ± 0.11	8.63 ± 0.09	8.71 ± 0.04

TC02002547.hg.1	ATP5G3	7.32 ± 0.11	7.23 ± 0.22	7.12 ± 0.10	7.11 ± 0.03
TC17001869.hg.1	ATP5H	10.27 ± 0.19	10.19 ± 0.15	10.15 ± 0.07	9.85 ± 0.20
TC04000951.hg.1	ATP5I	8.91 ± 0.23	8.81 ± 0.10	8.74 ± 0.18	8.73 ± 0.04
TC21000339.hg.1	ATP5J	7.75 ± 0.06	7.62 ± 0.11	7.61 ± 0.02	7.50 ± 0.07
TC07003366.hg.1	ATP5J2	7.83 ± 0.04	7.71 ± 0.13	7.68 ± 0.07	7.61 ± 0.03
TC11001063.hg.1	ATP5L	7.70 ± 0.06	7.64 ± 0.19	7.54 ± 0.12	7.53 ± 0.12
TC22000851.hg.1	ATP5L2	8.08 ± 0.12	8.09 ± 0.06	7.96 ± 0.19	8.02 ± 0.16
TC21001056.hg.1	ATP5O	8.63 ± 0.12	8.64 ± 0.12	8.46 ± 0.11	8.57 ± 0.06
TC14000283.hg.1	ATP5S	7.40 ± 0.18	7.40 ± 0.07	7.30 ± 0.05	7.39 ± 0.04
TC19002704.hg.1	ATP5SL	8.51 ± 0.20	8.29 ± 0.06	8.34 ± 0.04	8.19 ± 0.04
TC0X000771.hg.1	ATP6AP1	9.43 ± 0.24	9.11 ± 0.04	9.32 ± 0.24	9.10 ± 0.16
TC05000408.hg.1	ATP6AP1L	5.65 ± 0.11	5.51 ± 0.07	5.59 ± 0.04	5.59 ± 0.01
TC0X000190.hg.1	ATP6AP2	8.90 ± 0.19	9.07 ± 0.18	9.07 ± 0.41	8.99 ± 0.23
TC17000525.hg.1	ATP6V0A1	9.15 ± 0.16	8.74 ± 0.21	9.23 ± 0.23	8.74 ± 0.28
TC12000993.hg.1	ATP6V0A2	7.99 ± 0.19	7.93 ± 0.18	7.88 ± 0.07	7.84 ± 0.05
TC07001906.hg.1	ATP6V0A4	6.39 ± 0.18	6.30 ± 0.14	6.27 ± 0.09	6.34 ± 0.02
TC01000555.hg.1	ATP6V0B	9.69 ± 0.20	9.59 ± 0.13	9.68 ± 0.21	9.55 ± 0.12
TC16002054.hg.1	ATP6V0C	10.79 ± 0.27	10.62 ± 0.06	10.72 ± 0.12	10.65 ± 0.20
TC06000583.hg.1	ATP6V0CP3	8.33 ± 0.30	8.31 ± 0.21	8.15 ± 0.21	8.40 ± 0.07
TC16001190.hg.1	ATP6V0D1	9.83 ± 0.35	9.51 ± 0.10	10.06 ± 0.35	9.43 ± 0.24
TC08000536.hg.1	ATP6V0D2	5.33 ± 0.08	5.33 ± 0.11	5.24 ± 0.01	5.41 ± 0.05
TC05000956.hg.1	ATP6V0E1	11.52 ± 0.24	11.34 ± 0.08	11.76 ± 0.20	11.24 ± 0.20
TC07000995.hg.1	ATP6V0E2	7.91 ± 0.10	7.88 ± 0.11	7.80 ± 0.08	7.95 ± 0.06
TC07002013.hg.1	ATP6V0E2-AS1	7.40 ± 0.22	7.16 ± 0.15	7.12 ± 0.06	7.18 ± 0.04
TC03000583.hg.1	ATP6V1A	8.72 ± 0.15	8.59 ± 0.25	9.17 ± 0.33	8.55 ± 0.30
TC02000423.hg.1	ATP6V1B1	7.28 ± 0.20	7.21 ± 0.16	7.22 ± 0.16	7.30 ± 0.09
TC08000151.hg.1	ATP6V1B2	10.59 ± 0.16	10.14 ± 0.24	10.92 ± 0.25	10.10 ± 0.27
TC08000641.hg.1	ATP6V1C1	8.90 ± 0.30	8.64 ± 0.28	9.74 ± 0.55	8.43 ± 0.35
TC02000064.hg.1	ATP6V1C2	6.56 ± 0.23	6.51 ± 0.22	6.43 ± 0.11	6.64 ± 0.12
TC14001236.hg.1	ATP6V1D	6.70 ± 0.28	7.03 ± 0.26	7.11 ± 0.71	7.11 ± 0.16
TC14001237.hg.1	ATP6V1D	7.94 ± 0.11	7.89 ± 0.25	8.14 ± 0.39	7.74 ± 0.14

TC22000474.hg.1	ATP6V1E1	8.62 ± 0.30	8.35 ± 0.18	8.72 ± 0.28	8.28 ± 0.17
TC02001814.hg.1	ATP6V1E2	6.70 ± 0.08	6.54 ± 0.03	6.60 ± 0.03	6.49 ± 0.01
TC07000786.hg.1	ATP6V1F	9.26 ± 0.28	8.95 ± 0.20	8.92 ± 0.10	8.85 ± 0.12
TC09000584.hg.1	ATP6V1G1	9.91 ± 0.04	9.99 ± 0.04	9.89 ± 0.20	9.99 ± 0.08
TC06004115.hg.1	ATP6V1G2	7.55 ± 0.12	7.43 ± 0.07	7.48 ± 0.09	7.52 ± 0.04
TC6_cox_hap2000253.hg.1	ATP6V1G2	7.79 ± 0.15	7.75 ± 0.13	7.72 ± 0.13	7.86 ± 0.05
TC6_dbb_hap3000232.hg.1	ATP6V1G2	7.79 ± 0.15	7.75 ± 0.12	7.72 ± 0.13	7.86 ± 0.05
TC6_mann_hap4000201.hg.1	ATP6V1G2	7.79 ± 0.15	7.75 ± 0.12	7.72 ± 0.13	7.86 ± 0.05
TC6_mcf_hap5000217.hg.1	ATP6V1G2	7.79 ± 0.15	7.75 ± 0.12	7.72 ± 0.13	7.86 ± 0.05
TC6_qbl_hap6000231.hg.1	ATP6V1G2	7.79 ± 0.15	7.75 ± 0.12	7.72 ± 0.13	7.86 ± 0.05
TC6_ssto_hap7000205.hg.1	ATP6V1G2	7.77 ± 0.18	7.70 ± 0.12	7.68 ± 0.14	7.78 ± 0.04
TC01003667.hg.1	ATP6V1G3	4.23 ± 0.07	4.08 ± 0.09	4.27 ± 0.01	4.13 ± 0.07
TC08001215.hg.1	ATP6V1H	7.34 ± 0.13	7.38 ± 0.14	7.34 ± 0.16	7.26 ± 0.09
TC0X000424.hg.1	ATP7A	7.35 ± 0.32	7.25 ± 0.32	7.60 ± 0.43	7.07 ± 0.12
TC13000684.hg.1	ATP7B	6.69 ± 0.12	6.61 ± 0.10	6.64 ± 0.07	6.66 ± 0.06
TC04001152.hg.1	ATP8A1	8.40 ± 0.67	8.90 ± 0.26	8.40 ± 0.42	8.80 ± 0.08
TC13000065.hg.1	ATP8A2	5.85 ± 0.14	5.77 ± 0.11	5.74 ± 0.15	5.83 ± 0.03
TC18000538.hg.1	ATP8B1	5.49 ± 0.11	5.36 ± 0.04	5.39 ± 0.01	5.38 ± 0.02
TC01001274.hg.1	ATP8B2	9.17 ± 0.14	9.01 ± 0.07	8.71 ± 0.06	8.82 ± 0.08
TC19001013.hg.1	ATP8B3	7.92 ± 0.18	7.83 ± 0.13	7.85 ± 0.18	7.88 ± 0.03
TC15001347.hg.1	ATP8B4	7.30 ± 0.22	6.91 ± 0.21	7.77 ± 0.38	6.80 ± 0.14
TC09000183.hg.1	ATP8B5P	5.27 ± 0.08	5.17 ± 0.07	5.19 ± 0.06	5.24 ± 0.06
TC20000944.hg.1	ATP9A	7.33 ± 0.19	7.06 ± 0.09	7.73 ± 0.38	7.13 ± 0.04
TC18000262.hg.1	ATP9B	7.87 ± 0.11	7.91 ± 0.09	7.73 ± 0.09	7.89 ± 0.03
TC01006355.hg.1	ATPAF1	7.57 ± 0.01	7.47 ± 0.13	7.44 ± 0.06	7.24 ± 0.02
TC17001196.hg.1	ATPAF2	7.80 ± 0.10	7.67 ± 0.07	7.65 ± 0.08	7.67 ± 0.02
TC01000375.hg.1	ATPIF1	8.74 ± 0.07	8.57 ± 0.03	8.60 ± 0.05	8.49 ± 0.04
TC03001854.hg.1	ATR	7.16 ± 0.55	7.40 ± 0.23	7.09 ± 0.38	7.15 ± 0.07
TC02000167.hg.1	ATRAID	9.27 ± 0.17	9.12 ± 0.16	8.90 ± 0.07	8.99 ± 0.03
TC20000038.hg.1	ATRN	8.12 ± 0.10	8.06 ± 0.14	7.90 ± 0.08	7.94 ± 0.03
TC10000839.hg.1	ATRNL1	4.54 ± 0.14	4.40 ± 0.07	4.42 ± 0.11	4.45 ± 0.05

TC0X001173.hg.1	ATRX	8.29 ± 0.78	8.87 ± 0.28	8.68 ± 0.64	8.56 ± 0.27
TC06001293.hg.1	ATXN1	8.25 ± 0.17	8.20 ± 0.17	8.65 ± 0.37	8.06 ± 0.28
TC22000385.hg.1	ATXN10	8.18 ± 0.13	8.21 ± 0.11	7.90 ± 0.11	8.08 ± 0.03
TC16000597.hg.1	ATXN1L	8.90 ± 0.08	8.99 ± 0.07	8.84 ± 0.07	9.08 ± 0.03
TC12001972.hg.1	ATXN2	8.68 ± 0.16	8.50 ± 0.18	8.73 ± 0.20	8.40 ± 0.19
TC16000308.hg.1	ATXN2L	9.74 ± 0.10	9.72 ± 0.02	9.52 ± 0.14	9.70 ± 0.12
TC14001446.hg.1	ATXN3	6.70 ± 0.11	6.66 ± 0.17	6.76 ± 0.20	6.57 ± 0.13
TC0X000866.hg.1	ATXN3L	3.94 ± 0.05	3.87 ± 0.07	3.92 ± 0.11	3.98 ± 0.18
TC03000394.hg.1	ATXN7	9.35 ± 0.28	9.54 ± 0.08	9.54 ± 0.37	9.52 ± 0.08
TC07001736.hg.1	ATXN7L1	8.16 ± 0.13	8.03 ± 0.08	8.02 ± 0.21	7.94 ± 0.11
TC01000944.hg.1	ATXN7L2	7.52 ± 0.09	7.49 ± 0.11	7.36 ± 0.11	7.47 ± 0.04
TC17001575.hg.1	ATXN7L3	8.51 ± 0.23	8.33 ± 0.15	8.41 ± 0.19	8.31 ± 0.13
TC12000637.hg.1	ATXN7L3B	9.31 ± 0.02	9.32 ± 0.14	8.99 ± 0.26	9.27 ± 0.09
TC13000264.hg.1	ATXN8OS	5.36 ± 0.11	5.27 ± 0.05	5.34 ± 0.05	5.34 ± 0.16
TC09001324.hg.1	AUH	7.62 ± 0.28	7.65 ± 0.17	7.59 ± 0.17	7.53 ± 0.04
TC01002381.hg.1	AUNIP	6.75 ± 0.11	6.71 ± 0.07	6.72 ± 0.12	6.62 ± 0.02
TC02002001.hg.1	AUP1	9.48 ± 0.06	9.38 ± 0.10	9.25 ± 0.09	9.30 ± 0.03
TC20000968.hg.1	AURKA	7.82 ± 0.10	7.64 ± 0.07	7.70 ± 0.09	7.58 ± 0.05
TC01002094.hg.1	AURKAIP1	8.55 ± 0.34	8.42 ± 0.17	8.41 ± 0.32	8.39 ± 0.02
TC17001109.hg.1	AURKB	8.36 ± 0.28	8.08 ± 0.19	8.16 ± 0.30	7.96 ± 0.03
TC19000935.hg.1	AURKC	6.91 ± 0.19	6.88 ± 0.19	6.83 ± 0.14	6.96 ± 0.05
TC07000424.hg.1	AUTS2	7.84 ± 0.08	7.86 ± 0.07	7.65 ± 0.17	7.90 ± 0.05
TC15001189.hg.1	AVEN	7.33 ± 0.09	7.17 ± 0.04	7.09 ± 0.08	7.17 ± 0.04
TC12001636.hg.1	AVIL	7.71 ± 0.13	7.67 ± 0.14	7.99 ± 0.15	7.48 ± 0.24
TC07000207.hg.1	AVL9	7.81 ± 0.19	7.63 ± 0.22	7.88 ± 0.26	7.47 ± 0.13
TC20000567.hg.1	AVP	8.25 ± 0.24	8.21 ± 0.17	8.23 ± 0.16	8.24 ± 0.09
TC10001569.hg.1	AVPI1	7.53 ± 0.09	7.63 ± 0.13	7.34 ± 0.11	7.65 ± 0.06
TC12001661.hg.1	AVPR1A	6.55 ± 0.21	6.33 ± 0.03	6.41 ± 0.18	6.33 ± 0.13
TC01001719.hg.1	AVPR1B	7.22 ± 0.15	7.16 ± 0.26	7.10 ± 0.18	7.26 ± 0.17
TC0X000760.hg.1	AVPR2	8.51 ± 0.17	8.54 ± 0.24	8.48 ± 0.23	8.73 ± 0.08
TC0X000372.hg.1	AWAT1	6.56 ± 0.13	6.62 ± 0.18	6.59 ± 0.08	6.76 ± 0.08

TC0X001108.hg.1	AWAT2	6.68 ± 0.16	6.64 ± 0.16	6.61 ± 0.05	6.70 ± 0.06
TC01001552.hg.1	AXDND1	5.06 ± 0.16	4.95 ± 0.04	4.95 ± 0.05	4.97 ± 0.03
TC16000725.hg.1	AXIN1	8.84 ± 0.10	8.82 ± 0.06	8.75 ± 0.12	8.81 ± 0.04
TC17002855.hg.1	AXIN2	7.66 ± 0.02	7.61 ± 0.07	7.46 ± 0.09	7.69 ± 0.04
TC19000576.hg.1	AXL	7.76 ± 0.17	7.70 ± 0.15	7.67 ± 0.17	7.77 ± 0.03
TC07001652.hg.1	AZGP1	7.02 ± 0.16	6.94 ± 0.14	6.98 ± 0.14	7.01 ± 0.03
TC07000606.hg.1	AZGP1P1	7.07 ± 0.14	7.13 ± 0.11	7.13 ± 0.08	7.28 ± 0.02
TC17001945.hg.1	AZI1	8.89 ± 0.18	8.84 ± 0.15	8.83 ± 0.19	8.89 ± 0.03
TC03001260.hg.1	AZI2	7.50 ± 0.18	7.45 ± 0.18	7.80 ± 0.37	7.31 ± 0.18
TC08001501.hg.1	AZIN1	9.38 ± 0.36	9.74 ± 0.07	9.56 ± 0.43	9.81 ± 0.20
TC19000017.hg.1	AZU1	8.20 ± 0.30	8.01 ± 0.20	8.14 ± 0.24	8.12 ± 0.06
TC15000342.hg.1	B2M	12.89 ± 0.08	12.96 ± 0.05	13.01 ± 0.08	12.94 ± 0.10
TC03001958.hg.1	B3GALNT1	7.41 ± 0.19	7.38 ± 0.11	7.34 ± 0.10	7.44 ± 0.05
TC01003994.hg.1	B3GALNT2	7.39 ± 0.06	7.36 ± 0.06	7.27 ± 0.06	7.25 ± 0.07
TC02000994.hg.1	B3GALT1	5.40 ± 0.24	5.29 ± 0.01	5.27 ± 0.07	5.31 ± 0.13
TC01003653.hg.1	B3GALT2	4.56 ± 0.26	4.47 ± 0.03	4.47 ± 0.09	4.50 ± 0.09
TC06000418.hg.1	B3GALT4	8.53 ± 0.14	8.32 ± 0.12	8.36 ± 0.15	8.21 ± 0.12
TC6_cox_hap2000108.hg.1	B3GALT4	8.53 ± 0.14	8.32 ± 0.12	8.36 ± 0.15	8.21 ± 0.12
TC6_dbb_hap3000100.hg.1	B3GALT4	8.53 ± 0.14	8.32 ± 0.12	8.36 ± 0.15	8.21 ± 0.12
TC6_mcf_hap5000093.hg.1	B3GALT4	8.53 ± 0.14	8.32 ± 0.12	8.36 ± 0.15	8.21 ± 0.12
TC6_qbl_hap6000099.hg.1	B3GALT4	8.53 ± 0.14	8.32 ± 0.12	8.36 ± 0.15	8.21 ± 0.12
TC21000177.hg.1	B3GALT5	6.20 ± 0.16	6.20 ± 0.14	6.12 ± 0.10	6.38 ± 0.18
TC01000031.hg.1	B3GALT6	8.57 ± 0.18	8.32 ± 0.05	8.21 ± 0.30	8.25 ± 0.02
TC13000105.hg.1	B3GALTL	5.83 ± 0.07	5.84 ± 0.10	5.70 ± 0.06	5.85 ± 0.03
TC11002475.hg.1	B3GAT1	7.91 ± 0.13	7.83 ± 0.11	7.78 ± 0.13	7.91 ± 0.04
TC06001857.hg.1	B3GAT2	7.31 ± 0.17	7.24 ± 0.10	7.25 ± 0.13	7.29 ± 0.05
TC11001872.hg.1	B3GAT3	8.44 ± 0.12	8.35 ± 0.05	8.29 ± 0.02	8.38 ± 0.03
TC11001965.hg.1	B3GNT1	8.40 ± 0.33	8.17 ± 0.16	8.24 ± 0.23	8.21 ± 0.07
TC02000357.hg.1	B3GNT2	8.17 ± 0.24	8.51 ± 0.17	8.50 ± 0.15	8.46 ± 0.26
TC19000343.hg.1	B3GNT3	7.95 ± 0.33	7.91 ± 0.22	7.93 ± 0.25	8.02 ± 0.05
TC12000971.hg.1	B3GNT4	7.55 ± 0.18	7.53 ± 0.14	7.49 ± 0.18	7.60 ± 0.02

TC03000978.hg.1	B3GNT5	7.57 ± 0.29	8.08 ± 0.16	8.15 ± 0.42	8.16 ± 0.18
TC11000826.hg.1	B3GNT6	7.99 ± 0.27	7.93 ± 0.26	7.89 ± 0.26	8.02 ± 0.04
TC02001391.hg.1	B3GNT7	8.25 ± 0.06	8.21 ± 0.13	8.20 ± 0.12	8.29 ± 0.12
TC19001556.hg.1	B3GNT8	8.27 ± 0.23	8.09 ± 0.20	8.15 ± 0.21	8.23 ± 0.02
TC16001179.hg.1	B3GNT9	8.04 ± 0.32	8.06 ± 0.20	7.95 ± 0.30	8.00 ± 0.05
TC17001988.hg.1	B3GNTL1	7.93 ± 0.14	7.79 ± 0.02	7.92 ± 0.04	7.82 ± 0.03
TC12001629.hg.1	B4GALNT1	7.84 ± 0.21	7.80 ± 0.16	7.75 ± 0.17	7.89 ± 0.04
TC17000638.hg.1	B4GALNT2	7.39 ± 0.20	7.34 ± 0.13	7.31 ± 0.18	7.39 ± 0.04
TC12000007.hg.1	B4GALNT3	7.46 ± 0.12	7.42 ± 0.16	7.39 ± 0.18	7.51 ± 0.07
TC11000015.hg.1	B4GALNT4	7.85 ± 0.23	7.72 ± 0.18	7.73 ± 0.25	7.76 ± 0.01
TC09001008.hg.1	B4GALT1	9.95 ± 0.07	9.97 ± 0.14	9.78 ± 0.12	10.03 ± 0.21
TC01000556.hg.1	B4GALT2	8.40 ± 0.23	8.27 ± 0.20	8.19 ± 0.23	8.31 ± 0.02
TC01003420.hg.1	B4GALT3	8.63 ± 0.11	8.39 ± 0.05	8.32 ± 0.04	8.35 ± 0.03
TC03001680.hg.1	B4GALT4	7.46 ± 0.03	7.31 ± 0.02	7.38 ± 0.09	7.29 ± 0.08
TC03000606.hg.1	B4GALT4-AS1	5.27 ± 0.06	4.98 ± 0.13	5.22 ± 0.04	4.98 ± 0.04
TC20000928.hg.1	B4GALT5	10.58 ± 0.38	10.10 ± 0.15	11.27 ± 0.42	9.89 ± 0.45
TC18000440.hg.1	B4GALT6	6.64 ± 0.12	6.42 ± 0.01	6.61 ± 0.15	6.43 ± 0.04
TC05001019.hg.1	B4GALT7	8.03 ± 0.17	8.01 ± 0.10	7.90 ± 0.20	8.10 ± 0.03
TC17001240.hg.1	B9D1	7.50 ± 0.12	7.47 ± 0.17	7.42 ± 0.11	7.54 ± 0.05
TC19001554.hg.1	B9D2	8.36 ± 0.25	8.16 ± 0.13	8.26 ± 0.23	8.18 ± 0.04
TC08000643.hg.1	BAALC	6.71 ± 0.16	6.71 ± 0.12	6.70 ± 0.11	6.79 ± 0.07
TC09001419.hg.1	BAAT	5.67 ± 0.18	5.63 ± 0.10	5.50 ± 0.13	5.67 ± 0.10
TC19002626.hg.1	BABAM1	8.91 ± 0.12	8.79 ± 0.12	8.64 ± 0.22	8.69 ± 0.04
TC11002335.hg.1	BACE1	7.22 ± 0.16	7.17 ± 0.10	7.15 ± 0.13	7.20 ± 0.01
TC11001054.hg.1	BACE1-AS	7.62 ± 0.26	7.23 ± 0.13	7.54 ± 0.33	7.17 ± 0.11
TC21000185.hg.1	BACE2	7.14 ± 0.11	7.13 ± 0.04	7.11 ± 0.19	7.10 ± 0.08
TC21000186.hg.1	BACE2-IT1	6.79 ± 0.37	6.87 ± 0.02	6.95 ± 0.04	6.91 ± 0.04
TC21000358.hg.1	BACH1-AS1	7.55 ± 0.36	7.59 ± 0.02	7.71 ± 0.03	7.80 ± 0.20
TC21000098.hg.1	BACH1-IT2	7.98 ± 0.23	7.88 ± 0.23	8.36 ± 0.36	8.03 ± 0.08
TC21000099.hg.1	BACH1-IT3	6.10 ± 0.09	6.15 ± 0.11	6.36 ± 0.28	6.22 ± 0.24
TC06001946.hg.1	BACH2	8.86 ± 0.26	9.20 ± 0.10	8.47 ± 0.22	9.19 ± 0.10

TC11001903.hg.1	BAD	8.46 ± 0.23	8.26 ± 0.14	8.30 ± 0.15	8.25 ± 0.02
TC09001009.hg.1	BAG1	7.68 ± 0.13	7.81 ± 0.07	7.69 ± 0.17	7.77 ± 0.12
TC06000684.hg.1	BAG2	7.47 ± 0.16	7.63 ± 0.10	7.46 ± 0.12	7.40 ± 0.20
TC10000868.hg.1	BAG3	8.09 ± 0.18	8.06 ± 0.14	8.03 ± 0.15	8.12 ± 0.08
TC08000287.hg.1	BAG4	8.31 ± 0.14	8.20 ± 0.13	8.45 ± 0.27	8.17 ± 0.11
TC14001539.hg.1	BAG5	8.34 ± 0.07	8.57 ± 0.06	8.25 ± 0.11	8.55 ± 0.04
TC06001532.hg.1	BAG6	9.30 ± 0.15	9.16 ± 0.03	9.12 ± 0.07	9.12 ± 0.03
TC6_cox_hap2000161.hg.1	BAG6	9.28 ± 0.16	9.15 ± 0.04	9.10 ± 0.07	9.10 ± 0.03
TC6_dbb_hap3000150.hg.1	BAG6	9.30 ± 0.15	9.17 ± 0.04	9.12 ± 0.07	9.12 ± 0.03
TC6_mann_hap4000138.hg.1	BAG6	9.30 ± 0.15	9.17 ± 0.04	9.12 ± 0.06	9.12 ± 0.03
TC6_mcf_hap5000138.hg.1	BAG6	9.30 ± 0.16	9.15 ± 0.04	9.11 ± 0.07	9.10 ± 0.03
TC6_qbl_hap6000153.hg.1	BAG6	9.28 ± 0.16	9.15 ± 0.04	9.11 ± 0.07	9.10 ± 0.03
TC6_ssto_hap7000133.hg.1	BAG6	9.28 ± 0.16	9.15 ± 0.04	9.10 ± 0.07	9.10 ± 0.03
TC21001068.hg.1	BAGE3	8.32 ± 0.23	8.53 ± 0.08	8.42 ± 0.21	8.54 ± 0.08
TC21000275.hg.1	BAGE4	8.79 ± 0.23	8.76 ± 0.20	8.80 ± 0.07	8.97 ± 0.11
TC17000929.hg.1	BAHCC1	8.34 ± 0.19	8.30 ± 0.19	8.27 ± 0.20	8.39 ± 0.02
TC15000285.hg.1	BAHD1	8.35 ± 0.17	8.31 ± 0.08	8.10 ± 0.13	8.29 ± 0.06
TC08000806.hg.1	BAI1	8.34 ± 0.22	8.31 ± 0.19	8.27 ± 0.19	8.40 ± 0.04
TC01002457.hg.1	BAI2	8.03 ± 0.18	8.01 ± 0.19	7.96 ± 0.19	8.09 ± 0.07
TC06000710.hg.1	BAI3	4.78 ± 0.16	4.65 ± 0.06	4.66 ± 0.07	4.70 ± 0.08
TC17000924.hg.1	BAIAP2	7.96 ± 0.11	7.86 ± 0.09	7.84 ± 0.10	7.92 ± 0.03
TC17001940.hg.1	BAIAP2-AS1	7.93 ± 0.20	7.60 ± 0.09	7.66 ± 0.31	7.64 ± 0.13
TC07001635.hg.1	BAIAP2L1	6.36 ± 0.12	6.24 ± 0.08	6.33 ± 0.07	6.34 ± 0.08
TC22000785.hg.1	BAIAP2L2	7.92 ± 0.21	7.94 ± 0.23	7.82 ± 0.21	8.04 ± 0.05
TC16000040.hg.1	BAIAP3	8.27 ± 0.18	8.28 ± 0.16	8.26 ± 0.14	8.36 ± 0.06
TC06001588.hg.1	BAK1	8.53 ± 0.15	8.30 ± 0.11	8.33 ± 0.09	8.35 ± 0.06
TC10000199.hg.1	BAMBI	6.41 ± 0.11	6.17 ± 0.10	6.31 ± 0.12	6.21 ± 0.04
TC11000650.hg.1	BANF1	8.60 ± 0.16	8.37 ± 0.05	8.42 ± 0.08	8.26 ± 0.09
TC20000110.hg.1	BANF2	6.36 ± 0.19	6.37 ± 0.24	6.31 ± 0.14	6.43 ± 0.10
TC04000524.hg.1	BANK1	8.26 ± 0.50	8.71 ± 0.27	8.17 ± 0.35	8.39 ± 0.21
TC16000677.hg.1	BANP	8.58 ± 0.09	8.56 ± 0.02	8.53 ± 0.11	8.58 ± 0.03

TC03001453.hg.1	BAP1	8.74 ± 0.13	8.66 ± 0.05	8.61 ± 0.09	8.71 ± 0.04
TC02002745.hg.1	BARD1	6.31 ± 0.04	6.13 ± 0.12	6.20 ± 0.16	5.93 ± 0.08
TC09000759.hg.1	BARHL1	8.04 ± 0.27	7.97 ± 0.24	8.02 ± 0.22	8.10 ± 0.06
TC01002859.hg.1	BARHL2	7.58 ± 0.23	7.44 ± 0.12	7.57 ± 0.22	7.41 ± 0.07
TC09001351.hg.1	BARX1	7.83 ± 0.37	7.83 ± 0.27	7.73 ± 0.37	7.98 ± 0.03
TC11001190.hg.1	BARX2	7.36 ± 0.31	7.28 ± 0.20	7.24 ± 0.22	7.34 ± 0.09
TC05000096.hg.1	BASP1	10.45 ± 0.19	10.28 ± 0.10	10.86 ± 0.14	10.23 ± 0.24
TC13000479.hg.1	BASP1P1	8.25 ± 0.21	8.13 ± 0.19	8.48 ± 0.11	8.10 ± 0.13
TC14000474.hg.1	BATF	8.48 ± 0.29	8.15 ± 0.11	8.53 ± 0.10	8.07 ± 0.06
TC11001921.hg.1	BATF2	8.02 ± 0.21	7.82 ± 0.25	8.11 ± 0.20	7.90 ± 0.08
TC01003809.hg.1	BATF3	7.19 ± 0.19	7.14 ± 0.18	7.07 ± 0.17	7.13 ± 0.05
TC19000716.hg.1	BAX	8.83 ± 0.14	8.70 ± 0.02	8.65 ± 0.10	8.72 ± 0.03
TC14001030.hg.1	BAZ1A	9.77 ± 0.34	9.86 ± 0.40	10.59 ± 0.51	9.65 ± 0.43
TC07001499.hg.1	BAZ1B	9.08 ± 0.10	9.07 ± 0.10	8.86 ± 0.12	8.74 ± 0.24
TC12001608.hg.1	BAZ2A	9.70 ± 0.26	9.77 ± 0.13	9.70 ± 0.28	9.79 ± 0.05
TC02005063.hg.1	BAZ2B	8.42 ± 0.60	8.75 ± 0.35	9.13 ± 0.68	8.56 ± 0.28
TC19001661.hg.1	BBC3	8.95 ± 0.17	8.83 ± 0.14	8.85 ± 0.13	8.87 ± 0.02
TC10001660.hg.1	BBIP1	7.04 ± 0.26	7.04 ± 0.29	7.24 ± 0.40	6.89 ± 0.21
TC11000278.hg.1	BBOX1	4.72 ± 0.13	4.67 ± 0.13	4.66 ± 0.12	4.69 ± 0.05
TC11003432.hg.1	BBS1	8.03 ± 0.11	7.88 ± 0.03	7.99 ± 0.03	7.88 ± 0.03
TC12001753.hg.1	BBS10	6.92 ± 0.10	6.88 ± 0.10	7.15 ± 0.15	6.76 ± 0.09
TC04000629.hg.1	BBS12	5.70 ± 0.22	5.46 ± 0.12	5.49 ± 0.15	5.38 ± 0.05
TC16001134.hg.1	BBS2	7.98 ± 0.31	8.08 ± 0.12	7.75 ± 0.26	8.02 ± 0.10
TC15000662.hg.1	BBS4	7.15 ± 0.03	7.00 ± 0.10	6.97 ± 0.11	6.90 ± 0.02
TC02005001.hg.1	BBS5	4.93 ± 0.12	4.82 ± 0.06	4.79 ± 0.02	4.79 ± 0.01
TC04001517.hg.1	BBS7	6.41 ± 0.18	6.32 ± 0.17	6.41 ± 0.23	6.11 ± 0.13
TC07000216.hg.1	BBS9	7.01 ± 0.20	7.15 ± 0.22	6.87 ± 0.16	6.95 ± 0.06
TC03000543.hg.1	BBX	7.74 ± 0.31	8.10 ± 0.17	7.85 ± 0.34	7.97 ± 0.13
TC19000630.hg.1	BCAM	8.10 ± 0.18	8.03 ± 0.22	8.05 ± 0.19	8.14 ± 0.04
TC01001325.hg.1	BCAN	7.59 ± 0.16	7.55 ± 0.21	7.50 ± 0.20	7.65 ± 0.07
TC07000693.hg.1	BCAP29	6.83 ± 0.14	6.91 ± 0.12	6.91 ± 0.09	6.81 ± 0.06

TC0X001516.hg.1	BCAP31	9.00 ± 0.23	8.77 ± 0.05	8.89 ± 0.27	8.69 ± 0.06
TC16001276.hg.1	BCAR1	8.42 ± 0.23	8.45 ± 0.22	8.39 ± 0.21	8.53 ± 0.02
TC01002879.hg.1	BCAR3	7.19 ± 0.18	7.17 ± 0.19	7.11 ± 0.20	7.28 ± 0.04
TC16000873.hg.1	BCAR4	6.09 ± 0.26	6.17 ± 0.11	6.13 ± 0.03	6.21 ± 0.09
TC20000960.hg.1	BCAS1	6.55 ± 0.15	6.44 ± 0.10	6.50 ± 0.13	6.54 ± 0.04
TC01006365.hg.1	BCAS2	8.65 ± 0.58	9.52 ± 0.17	8.69 ± 0.54	9.39 ± 0.23
TC17000740.hg.1	BCAS3	8.77 ± 0.12	8.74 ± 0.12	8.74 ± 0.13	8.61 ± 0.08
TC20000408.hg.1	BCAS4	8.57 ± 0.13	8.55 ± 0.06	8.41 ± 0.14	8.57 ± 0.04
TC12001309.hg.1	BCAT1	6.62 ± 0.06	6.54 ± 0.10	6.80 ± 0.15	6.53 ± 0.11
TC19001697.hg.1	BCAT2	8.13 ± 0.23	7.94 ± 0.11	7.93 ± 0.07	7.95 ± 0.00
TC10000915.hg.1	BCCIP	7.49 ± 0.19	7.64 ± 0.15	7.36 ± 0.17	7.48 ± 0.06
TC12001484.hg.1	BCDIN3D	7.35 ± 0.04	7.31 ± 0.01	7.31 ± 0.07	7.24 ± 0.03
TC12000380.hg.1	BCDIN3D-AS1	6.62 ± 0.07	6.45 ± 0.11	6.54 ± 0.12	6.49 ± 0.10
TC03001970.hg.1	BCHE	4.08 ± 0.09	3.97 ± 0.07	4.00 ± 0.09	4.03 ± 0.01
TC19002636.hg.1	BCKDHA	8.82 ± 0.16	8.62 ± 0.05	8.59 ± 0.12	8.65 ± 0.02
TC06000762.hg.1	BCKDHB	6.79 ± 0.16	6.91 ± 0.12	6.64 ± 0.10	6.77 ± 0.04
TC16000370.hg.1	BCKDK	8.87 ± 0.34	8.50 ± 0.02	8.81 ± 0.15	8.47 ± 0.11
TC01002828.hg.1	BCL10	8.36 ± 0.07	8.42 ± 0.18	8.57 ± 0.21	8.49 ± 0.11
TC02001885.hg.1	BCL11A	7.97 ± 0.02	8.00 ± 0.11	7.82 ± 0.07	7.89 ± 0.04
TC14001497.hg.1	BCL11B	8.66 ± 0.04	8.90 ± 0.05	8.23 ± 0.11	8.87 ± 0.05
TC18000554.hg.1	BCL2	9.41 ± 0.25	9.74 ± 0.10	9.28 ± 0.34	9.72 ± 0.15
TC15001719.hg.1	BCL2A1	9.96 ± 0.27	10.34 ± 0.21	10.66 ± 0.30	10.23 ± 0.13
TC20000756.hg.1	BCL2L1	8.38 ± 0.17	8.05 ± 0.07	8.28 ± 0.18	8.04 ± 0.07
TC15001452.hg.1	BCL2L10	7.76 ± 0.32	7.81 ± 0.14	7.79 ± 0.22	7.93 ± 0.07
TC02000695.hg.1	BCL2L11	8.49 ± 0.04	8.48 ± 0.10	8.47 ± 0.09	8.56 ± 0.09
TC02000696.hg.1	BCL2L11	7.48 ± 0.25	7.61 ± 0.25	7.59 ± 0.38	7.60 ± 0.11
TC19000744.hg.1	BCL2L12	8.17 ± 0.22	8.04 ± 0.14	7.96 ± 0.14	8.10 ± 0.07
TC22000025.hg.1	BCL2L13	7.89 ± 0.09	7.76 ± 0.07	7.82 ± 0.08	7.69 ± 0.06
TC12003209.hg.1	BCL2L14	5.90 ± 0.08	5.85 ± 0.14	5.89 ± 0.07	5.92 ± 0.09
TC01003007.hg.1	BCL2L15	5.96 ± 0.25	5.78 ± 0.05	5.81 ± 0.08	5.88 ± 0.04
TC14002291.hg.1	BCL2L2	8.57 ± 0.18	8.48 ± 0.16	8.48 ± 0.05	8.46 ± 0.04

TC19000627.hg.1	BCL3	9.56 ± 0.34	9.72 ± 0.08	9.94 ± 0.24	9.71 ± 0.14
TC03002096.hg.1	BCL6	10.65 ± 0.33	10.36 ± 0.22	11.39 ± 0.33	10.34 ± 0.41
TC17000083.hg.1	BCL6B	7.73 ± 0.17	7.69 ± 0.15	7.70 ± 0.15	7.79 ± 0.02
TC12000967.hg.1	BCL7A	8.60 ± 0.05	8.75 ± 0.15	8.29 ± 0.04	8.67 ± 0.10
TC07001500.hg.1	BCL7B	8.70 ± 0.05	8.79 ± 0.12	8.54 ± 0.02	8.81 ± 0.07
TC16001044.hg.1	BCL7C	8.94 ± 0.23	8.79 ± 0.09	8.73 ± 0.20	8.78 ± 0.00
TC01001113.hg.1	BCL9	8.18 ± 0.12	8.20 ± 0.06	7.96 ± 0.19	8.22 ± 0.09
TC11002355.hg.1	BCL9L	9.38 ± 0.06	9.46 ± 0.07	9.07 ± 0.18	9.51 ± 0.11
TC06002143.hg.1	BCLAF1	8.75 ± 0.81	9.64 ± 0.17	9.13 ± 0.65	9.59 ± 0.16
TC16000637.hg.1	BCMO1	6.29 ± 0.17	6.14 ± 0.04	6.26 ± 0.09	6.20 ± 0.04
TC11003466.hg.1	BCO2	5.16 ± 0.07	5.13 ± 0.06	5.18 ± 0.07	5.18 ± 0.04
TC0X000963.hg.1	BCOR	8.52 ± 0.10	8.48 ± 0.03	8.27 ± 0.08	8.54 ± 0.22
TC0X000620.hg.1	BCORL1	8.03 ± 0.18	7.78 ± 0.13	8.22 ± 0.07	7.80 ± 0.11
TC0Y000184.hg.1	BCORP1	6.96 ± 0.14	7.08 ± 0.09	7.01 ± 0.03	7.16 ± 0.08
TC22000129.hg.1	BCR	8.53 ± 0.03	8.48 ± 0.08	8.35 ± 0.08	8.45 ± 0.02
TC22000081.hg.1	BCRP2	7.95 ± 0.16	7.88 ± 0.06	7.84 ± 0.12	8.00 ± 0.10
TC22000153.hg.1	BCRP3	8.64 ± 0.08	8.62 ± 0.08	8.45 ± 0.13	8.66 ± 0.09
TC02001308.hg.1	BCS1L	7.73 ± 0.10	7.63 ± 0.03	7.59 ± 0.07	7.55 ± 0.05
TC02000292.hg.1	BCYRN1	5.58 ± 0.24	5.74 ± 0.17	5.68 ± 0.20	5.85 ± 0.20
TC0X001123.hg.1	BCYRN1	12.79 ± 0.23	12.64 ± 0.07	12.71 ± 0.23	12.66 ± 0.03
TC03002179.hg.1	BDH1	7.67 ± 0.11	7.58 ± 0.06	7.53 ± 0.11	7.60 ± 0.06
TC04002947.hg.1	BDH2	6.41 ± 0.26	6.46 ± 0.06	6.29 ± 0.13	6.43 ± 0.03
TC14002311.hg.1	BDKRB1	6.91 ± 0.06	6.95 ± 0.20	6.85 ± 0.07	7.13 ± 0.17
TC14002310.hg.1	BDKRB2	6.90 ± 0.15	6.79 ± 0.18	6.74 ± 0.13	6.87 ± 0.08
TC11001513.hg.1	BDNF	6.14 ± 0.16	6.07 ± 0.17	6.07 ± 0.18	6.18 ± 0.04
TC11000280.hg.1	BDNF-AS	5.44 ± 0.10	5.41 ± 0.02	5.45 ± 0.04	5.48 ± 0.04
TC05000333.hg.1	BDP1	7.68 ± 0.68	8.07 ± 0.20	7.76 ± 0.47	7.92 ± 0.11
TC16000516.hg.1	BEAN1	7.63 ± 0.16	7.65 ± 0.20	7.61 ± 0.18	7.74 ± 0.02
TC17001545.hg.1	BECN1	9.38 ± 0.18	9.49 ± 0.09	9.45 ± 0.24	9.39 ± 0.08
TC14001510.hg.1	BEGAIN	7.92 ± 0.24	7.85 ± 0.24	7.87 ± 0.29	7.92 ± 0.04
TC0X000893.hg.1	BEND2	6.75 ± 0.22	6.58 ± 0.10	6.75 ± 0.39	6.73 ± 0.30

TC06001987.hg.1	BEND3	6.14 ± 0.11	6.02 ± 0.12	6.00 ± 0.09	6.14 ± 0.07
TC10000571.hg.1	BEND3P3	6.40 ± 0.24	6.26 ± 0.16	6.26 ± 0.21	6.41 ± 0.04
TC04001150.hg.1	BEND4	7.48 ± 0.14	7.44 ± 0.08	7.40 ± 0.13	7.46 ± 0.06
TC01002639.hg.1	BEND5	7.69 ± 0.18	7.65 ± 0.10	7.51 ± 0.16	7.70 ± 0.04
TC06000681.hg.1	BEND6	4.71 ± 0.04	4.61 ± 0.05	4.69 ± 0.09	4.64 ± 0.06
TC10001051.hg.1	BEND7	5.67 ± 0.07	5.52 ± 0.06	5.66 ± 0.06	5.56 ± 0.02
TC11000544.hg.1	BEST1	9.02 ± 0.22	8.88 ± 0.11	9.30 ± 0.28	8.87 ± 0.25
TC19000227.hg.1	BEST2	7.35 ± 0.28	7.43 ± 0.25	7.37 ± 0.23	7.56 ± 0.03
TC12001712.hg.1	BEST3	5.31 ± 0.11	5.29 ± 0.10	5.32 ± 0.09	5.33 ± 0.04
TC01002600.hg.1	BEST4	7.23 ± 0.24	7.29 ± 0.18	7.12 ± 0.18	7.37 ± 0.08
TC07001612.hg.1	BET1	6.82 ± 0.30	6.49 ± 0.14	6.64 ± 0.24	6.47 ± 0.04
TC11003513.hg.1	BET1L	8.87 ± 0.11	8.85 ± 0.04	8.63 ± 0.04	8.90 ± 0.05
TC0X001233.hg.1	BEX1	7.44 ± 0.22	7.27 ± 0.09	7.18 ± 0.16	7.33 ± 0.03
TC0X001239.hg.1	BEX2	7.93 ± 0.15	7.98 ± 0.12	7.81 ± 0.19	7.92 ± 0.02
TC0X000493.hg.1	BEX4	8.12 ± 0.12	8.24 ± 0.10	7.90 ± 0.11	8.32 ± 0.11
TC0X001228.hg.1	BEX5	5.67 ± 0.16	5.59 ± 0.11	5.56 ± 0.12	5.66 ± 0.08
TC16000176.hg.1	BFAR	8.74 ± 0.14	8.50 ± 0.11	8.58 ± 0.12	8.42 ± 0.07
TC20000655.hg.1	BFSP1	6.09 ± 0.18	6.06 ± 0.14	6.02 ± 0.14	6.13 ± 0.05
TC03000720.hg.1	BFSP2	6.93 ± 0.15	6.84 ± 0.12	6.85 ± 0.11	6.92 ± 0.04
TC0X000750.hg.1	BGN	7.83 ± 0.23	7.89 ± 0.24	7.82 ± 0.20	7.96 ± 0.07
TC07000583.hg.1	BHLHA15	9.51 ± 0.19	9.63 ± 0.29	9.40 ± 0.30	9.69 ± 0.05
TC17000010.hg.1	BHLHA9	8.22 ± 0.37	8.08 ± 0.27	8.13 ± 0.36	8.15 ± 0.06
TC0X000490.hg.1	BHLHB9	6.71 ± 0.10	6.70 ± 0.12	6.59 ± 0.16	6.69 ± 0.06
TC08000432.hg.1	BHLHE22	6.58 ± 0.26	6.48 ± 0.19	6.49 ± 0.22	6.56 ± 0.09
TC20001022.hg.1	BHLHE23	9.59 ± 0.24	9.41 ± 0.12	9.51 ± 0.25	9.48 ± 0.10
TC03000015.hg.1	BHLHE40	9.28 ± 0.26	9.58 ± 0.16	9.58 ± 0.20	9.81 ± 0.24
TC03001131.hg.1	BHLHE40-AS1	5.45 ± 0.14	5.46 ± 0.14	5.50 ± 0.06	5.47 ± 0.06
TC12001324.hg.1	BHLHE41	7.23 ± 0.25	6.73 ± 0.01	6.73 ± 0.25	6.59 ± 0.10
TC05000387.hg.1	BHMT	6.01 ± 0.11	5.94 ± 0.09	5.93 ± 0.12	6.02 ± 0.08
TC05000386.hg.1	BHMT2	6.52 ± 0.22	6.43 ± 0.09	6.48 ± 0.15	6.49 ± 0.02
TC10000362.hg.1	BICC1	5.38 ± 0.08	5.29 ± 0.08	5.31 ± 0.03	5.36 ± 0.05

TC12000296.hg.1	BICD1	7.07 ± 0.12	7.07 ± 0.04	6.93 ± 0.05	7.01 ± 0.07
TC09001340.hg.1	BICD2	8.97 ± 0.18	8.82 ± 0.07	9.06 ± 0.14	8.65 ± 0.12
TC22000476.hg.1	BID	9.25 ± 0.19	9.13 ± 0.09	9.41 ± 0.14	9.10 ± 0.23
TC22000361.hg.1	BIK	8.41 ± 0.19	8.20 ± 0.15	8.27 ± 0.09	8.30 ± 0.04
TC02002276.hg.1	BIN1	9.03 ± 0.11	8.99 ± 0.08	8.84 ± 0.11	8.97 ± 0.04
TC12001507.hg.1	BIN2	11.62 ± 0.25	11.67 ± 0.12	11.99 ± 0.18	11.52 ± 0.14
TC08001042.hg.1	BIN3	8.29 ± 0.06	8.30 ± 0.07	8.28 ± 0.05	8.30 ± 0.09
TC08001043.hg.1	BIN3-IT1	7.23 ± 0.03	7.75 ± 0.13	7.59 ± 0.15	7.71 ± 0.07
TC11000957.hg.1	BIRC2	8.49 ± 0.38	8.84 ± 0.20	8.70 ± 0.43	8.82 ± 0.20
TC11000956.hg.1	BIRC3	8.93 ± 0.70	9.54 ± 0.10	8.97 ± 0.60	9.65 ± 0.18
TC17000899.hg.1	BIRC5	7.70 ± 0.26	7.42 ± 0.09	7.51 ± 0.25	7.35 ± 0.02
TC02000213.hg.1	BIRC6	8.69 ± 0.57	9.03 ± 0.28	8.66 ± 0.49	8.80 ± 0.09
TC02001724.hg.1	BIRC6-AS1	4.95 ± 0.27	4.92 ± 0.34	5.05 ± 0.13	4.73 ± 0.08
TC20000508.hg.1	BIRC7	8.53 ± 0.24	8.52 ± 0.24	8.39 ± 0.22	8.66 ± 0.10
TC19001817.hg.1	BIRC8	6.87 ± 0.37	6.65 ± 0.29	6.55 ± 0.46	6.81 ± 0.15
TC13001720.hg.1	BIVM	6.53 ± 0.16	6.47 ± 0.17	6.40 ± 0.11	6.30 ± 0.07
TC07002059.hg.1	BLACE	8.55 ± 0.19	8.63 ± 0.14	8.61 ± 0.14	8.83 ± 0.08
TC20000826.hg.1	BLCAP	8.33 ± 0.07	8.23 ± 0.03	8.18 ± 0.07	8.21 ± 0.05
TC11002383.hg.1	BLID	4.65 ± 0.15	4.34 ± 0.09	4.56 ± 0.24	4.46 ± 0.08
TC08000087.hg.1	BLK	8.57 ± 0.22	8.48 ± 0.14	8.16 ± 0.10	8.39 ± 0.11
TC15000868.hg.1	BLM	7.11 ± 0.11	6.93 ± 0.13	6.90 ± 0.12	6.74 ± 0.01
TC17001328.hg.1	BLMH	8.38 ± 0.07	8.26 ± 0.13	8.06 ± 0.14	8.13 ± 0.07
TC10001555.hg.1	BLNK	8.49 ± 0.07	8.23 ± 0.28	8.10 ± 0.19	7.95 ± 0.14
TC12003299.hg.1	BLOC1S1	8.68 ± 0.30	8.49 ± 0.06	8.80 ± 0.05	8.46 ± 0.13
TC10001590.hg.1	BLOC1S2	8.68 ± 0.17	8.84 ± 0.13	8.98 ± 0.36	8.87 ± 0.16
TC19000643.hg.1	BLOC1S3	8.49 ± 0.22	8.28 ± 0.04	8.20 ± 0.20	8.25 ± 0.04
TC04000073.hg.1	BLOC1S4	7.88 ± 0.14	7.77 ± 0.11	7.85 ± 0.07	7.86 ± 0.10
TC06004110.hg.1	BLOC1S5	7.27 ± 0.16	7.17 ± 0.15	7.21 ± 0.13	7.07 ± 0.10
TC15000362.hg.1	BLOC1S6	8.29 ± 0.37	8.61 ± 0.03	8.63 ± 0.34	8.52 ± 0.19
TC07000267.hg.1	BLVRA	8.60 ± 0.39	8.32 ± 0.07	8.55 ± 0.21	8.13 ± 0.14
TC19001542.hg.1	BLVRB	9.15 ± 0.41	8.71 ± 0.05	8.98 ± 0.17	8.60 ± 0.15

TC01001475.hg.1	BLZF1	8.30 ± 0.50	8.12 ± 0.31	8.61 ± 0.58	8.00 ± 0.07
TC15001230.hg.1	BMF	8.20 ± 0.13	8.12 ± 0.13	8.02 ± 0.16	8.17 ± 0.03
TC10002935.hg.1	BMI1	8.11 ± 0.46	8.38 ± 0.16	8.00 ± 0.37	8.28 ± 0.09
TC08000167.hg.1	BMP1	7.78 ± 0.14	7.73 ± 0.12	7.71 ± 0.15	7.80 ± 0.03
TC02001945.hg.1	BMP10	5.45 ± 0.13	5.42 ± 0.26	5.34 ± 0.06	5.44 ± 0.24
TC0X000290.hg.1	BMP15	5.65 ± 0.21	5.81 ± 0.23	5.63 ± 0.22	5.90 ± 0.18
TC20000067.hg.1	BMP2	6.12 ± 0.23	6.16 ± 0.16	6.21 ± 0.19	6.19 ± 0.02
TC04000440.hg.1	BMP2K	8.88 ± 0.21	8.70 ± 0.32	9.19 ± 0.35	8.46 ± 0.24
TC0X000407.hg.1	BMP2KL	5.69 ± 0.08	5.72 ± 0.05	6.09 ± 0.07	5.81 ± 0.19
TC04000449.hg.1	BMP3	6.79 ± 0.21	6.82 ± 0.12	6.79 ± 0.29	6.90 ± 0.16
TC14001148.hg.1	BMP4	6.42 ± 0.16	6.45 ± 0.16	6.33 ± 0.26	6.53 ± 0.10
TC06001825.hg.1	BMP5	4.62 ± 0.15	4.51 ± 0.09	4.51 ± 0.03	4.60 ± 0.09
TC06000062.hg.1	BMP6	8.87 ± 0.14	8.27 ± 0.07	8.79 ± 0.18	8.51 ± 0.25
TC20000972.hg.1	BMP7	7.31 ± 0.18	7.31 ± 0.18	7.26 ± 0.17	7.40 ± 0.04
TC01000495.hg.1	BMP8A	7.65 ± 0.34	7.61 ± 0.27	7.48 ± 0.26	7.64 ± 0.04
TC01002547.hg.1	BMP8B	7.62 ± 0.32	7.43 ± 0.17	7.39 ± 0.20	7.52 ± 0.02
TC07000220.hg.1	BMPER	6.76 ± 0.17	6.72 ± 0.15	6.70 ± 0.11	6.85 ± 0.10
TC10000609.hg.1	BMPR1A	6.19 ± 0.13	6.21 ± 0.13	6.05 ± 0.21	6.14 ± 0.20
TC11002378.hg.1	BMPR1APS2	6.26 ± 0.17	6.57 ± 0.03	6.32 ± 0.08	6.75 ± 0.37
TC04000503.hg.1	BMPR1B	5.49 ± 0.18	5.41 ± 0.09	5.47 ± 0.08	5.48 ± 0.05
TC02001192.hg.1	BMPR2	8.57 ± 0.27	8.70 ± 0.18	8.73 ± 0.29	8.65 ± 0.09
TC10000266.hg.1	BMS1	8.82 ± 0.28	8.89 ± 0.11	8.55 ± 0.34	8.80 ± 0.11
TC10001232.hg.1	BMS1P5	8.37 ± 0.35	8.33 ± 0.25	8.19 ± 0.34	8.40 ± 0.03
TC10001255.hg.1	BMS1P5	9.06 ± 0.43	9.10 ± 0.28	8.92 ± 0.34	9.15 ± 0.02
TC0X000067.hg.1	BMX	6.75 ± 0.41	6.16 ± 0.15	7.62 ± 0.64	6.06 ± 0.18
TC15001767.hg.1	BNC1	6.20 ± 0.22	6.19 ± 0.19	6.15 ± 0.12	6.25 ± 0.04
TC09000928.hg.1	BNC2	6.23 ± 0.09	6.16 ± 0.08	6.16 ± 0.04	6.21 ± 0.05
TC05000959.hg.1	BNIP1	7.15 ± 0.03	7.20 ± 0.16	6.94 ± 0.10	7.19 ± 0.04
TC15001503.hg.1	BNIP2	9.17 ± 0.38	9.40 ± 0.12	9.70 ± 0.42	9.43 ± 0.16
TC10001769.hg.1	BNIP3	7.90 ± 0.16	7.95 ± 0.01	7.79 ± 0.04	7.84 ± 0.06
TC08000200.hg.1	BNIP3L	10.22 ± 0.21	10.43 ± 0.04	10.29 ± 0.13	10.52 ± 0.10

TC14000181.hg.1	BNIP3P1	7.15 ± 0.08	7.12 ± 0.12	6.97 ± 0.15	6.98 ± 0.10
TC01001199.hg.1	BNIPL	7.02 ± 0.19	6.84 ± 0.09	6.99 ± 0.09	6.90 ± 0.04
TC03000577.hg.1	BOC	7.02 ± 0.19	6.98 ± 0.15	6.96 ± 0.14	7.06 ± 0.07
TC05002077.hg.1	BOD1	7.57 ± 0.09	7.46 ± 0.05	7.33 ± 0.07	7.49 ± 0.05
TC04001047.hg.1	BOD1L1	8.93 ± 0.49	9.27 ± 0.28	9.59 ± 0.47	9.28 ± 0.21
TC18000193.hg.1	BOD1L2	7.32 ± 0.13	7.33 ± 0.18	7.35 ± 0.18	7.43 ± 0.09
TC02001481.hg.1	BOK	8.60 ± 0.20	8.56 ± 0.13	8.57 ± 0.22	8.69 ± 0.07
TC02002952.hg.1	BOK-AS1	8.72 ± 0.32	8.83 ± 0.24	8.76 ± 0.28	8.91 ± 0.04
TC01006327.hg.1	BOLA1	8.62 ± 0.20	8.58 ± 0.23	8.55 ± 0.23	8.67 ± 0.02
TC16002102.hg.1	BOLA2	9.72 ± 0.13	9.85 ± 0.05	9.63 ± 0.07	9.75 ± 0.05
TC16002104.hg.1	BOLA2	8.86 ± 0.16	8.78 ± 0.05	8.70 ± 0.19	8.65 ± 0.04
TC02001992.hg.1	BOLA3	7.98 ± 0.08	7.99 ± 0.10	7.86 ± 0.11	7.88 ± 0.02
TC02000453.hg.1	BOLA3-AS1	7.44 ± 0.23	7.41 ± 0.11	7.32 ± 0.12	7.44 ± 0.08
TC02002650.hg.1	BOLL	5.91 ± 0.15	5.90 ± 0.11	5.87 ± 0.08	5.95 ± 0.03
TC08001743.hg.1	BOP1	8.42 ± 0.20	8.27 ± 0.14	8.17 ± 0.23	8.27 ± 0.04
TC08001744.hg.1	BOP1	8.63 ± 0.21	8.53 ± 0.15	8.44 ± 0.20	8.55 ± 0.04
TC13000270.hg.1	BORA	6.62 ± 0.12	6.60 ± 0.24	6.45 ± 0.17	6.36 ± 0.05
TC03000749.hg.1	BPESC1	5.05 ± 0.30	4.90 ± 0.12	4.89 ± 0.16	4.87 ± 0.02
TC07000836.hg.1	BPGM	7.59 ± 0.13	7.58 ± 0.11	7.63 ± 0.18	7.65 ± 0.16
TC06000024.hg.1	BPHL	6.88 ± 0.07	6.86 ± 0.05	6.77 ± 0.07	6.88 ± 0.02
TC20000281.hg.1	BPI	8.44 ± 0.46	7.52 ± 0.03	8.55 ± 0.45	7.66 ± 0.19
TC20000228.hg.1	BPIFA1	6.70 ± 0.10	6.69 ± 0.17	6.60 ± 0.14	6.82 ± 0.07
TC20000225.hg.1	BPIFA2	6.09 ± 0.19	6.09 ± 0.21	6.01 ± 0.21	6.30 ± 0.12
TC20000227.hg.1	BPIFA3	6.00 ± 0.09	5.99 ± 0.15	5.96 ± 0.06	6.09 ± 0.15
TC20000226.hg.1	BPIFA4P	5.96 ± 0.07	5.81 ± 0.08	5.90 ± 0.03	5.91 ± 0.09
TC20000229.hg.1	BPIFB1	6.76 ± 0.20	6.75 ± 0.19	6.75 ± 0.12	6.88 ± 0.04
TC20000220.hg.1	BPIFB2	7.49 ± 0.17	7.49 ± 0.24	7.42 ± 0.22	7.61 ± 0.06
TC20000222.hg.1	BPIFB3	7.56 ± 0.19	7.68 ± 0.22	7.55 ± 0.21	7.77 ± 0.05
TC20000223.hg.1	BPIFB4	7.26 ± 0.12	7.24 ± 0.17	7.22 ± 0.14	7.31 ± 0.03
TC20000221.hg.1	BPIFB6	6.99 ± 0.13	7.00 ± 0.18	6.95 ± 0.10	7.14 ± 0.07
TC22000683.hg.1	BPIFC	5.00 ± 0.11	4.90 ± 0.11	4.96 ± 0.05	4.99 ± 0.04

TC01003835.hg.1	BPNT1	7.63 ± 0.04	7.45 ± 0.06	7.48 ± 0.06	7.29 ± 0.04
TC17002859.hg.1	BPTF	9.59 ± 0.39	9.79 ± 0.17	9.68 ± 0.42	9.70 ± 0.12
TC0Y000082.hg.1	BPY2B	5.48 ± 0.09	5.37 ± 0.08	5.40 ± 0.05	5.49 ± 0.17
TC0Y000092.hg.1	BPY2B	5.47 ± 0.12	5.36 ± 0.13	5.39 ± 0.06	5.51 ± 0.17
TC0Y000211.hg.1	BPY2B	5.47 ± 0.09	5.35 ± 0.12	5.39 ± 0.05	5.52 ± 0.17
TC07001927.hg.1	BRAF	8.67 ± 0.28	8.88 ± 0.21	8.67 ± 0.31	8.93 ± 0.14
TC12001974.hg.1	BRAP	8.17 ± 0.20	8.24 ± 0.09	8.20 ± 0.22	8.20 ± 0.11
TC07001098.hg.1	BRAT1	8.52 ± 0.21	8.47 ± 0.13	8.43 ± 0.14	8.58 ± 0.03
TC17001553.hg.1	BRCA1	6.82 ± 0.05	6.65 ± 0.21	6.78 ± 0.12	6.39 ± 0.09
TC13000111.hg.1	BRCA2	5.71 ± 0.17	5.79 ± 0.27	5.73 ± 0.23	5.49 ± 0.07
TC0X000783.hg.1	BRCC3	8.12 ± 0.16	8.21 ± 0.12	8.14 ± 0.30	8.02 ± 0.11
TC22000902.hg.1	BRD1	8.48 ± 0.05	8.57 ± 0.11	8.35 ± 0.08	8.68 ± 0.02
TC06000406.hg.1	BRD2	10.20 ± 0.23	10.37 ± 0.08	10.07 ± 0.07	10.33 ± 0.03
TC6_cox_hap2000097.hg.1	BRD2	10.21 ± 0.21	10.36 ± 0.07	10.06 ± 0.06	10.32 ± 0.03
TC6_dbb_hap3000090.hg.1	BRD2	9.25 ± 0.37	9.56 ± 0.13	9.14 ± 0.10	9.49 ± 0.03
TC6_mann_hap4000081.hg.1	BRD2	10.27 ± 0.22	10.43 ± 0.07	10.13 ± 0.06	10.39 ± 0.03
TC6_mcf_hap5000082.hg.1	BRD2	10.65 ± 0.17	10.76 ± 0.07	10.50 ± 0.05	10.72 ± 0.03
TC6_mcf_hap5000084.hg.1	BRD2	5.70 ± 0.37	5.98 ± 0.57	5.15 ± 0.55	5.68 ± 0.10
TC6_qbl_hap6000088.hg.1	BRD2	10.21 ± 0.22	10.37 ± 0.07	10.07 ± 0.06	10.33 ± 0.02
TC6_ssto_hap7000083.hg.1	BRD2	10.13 ± 0.21	10.28 ± 0.07	9.98 ± 0.07	10.25 ± 0.03
TC06000407.hg.1	BRD2-IT1	7.12 ± 0.15	7.05 ± 0.08	7.02 ± 0.03	7.01 ± 0.12
TC09001697.hg.1	BRD3	9.03 ± 0.04	8.97 ± 0.07	8.97 ± 0.04	8.97 ± 0.08
TC19001252.hg.1	BRD4	9.86 ± 0.10	9.95 ± 0.00	9.91 ± 0.21	10.04 ± 0.16
TC16001104.hg.1	BRD7	9.25 ± 0.21	9.28 ± 0.08	9.21 ± 0.26	9.18 ± 0.09
TC06000934.hg.1	BRD7P3	4.76 ± 0.32	4.76 ± 0.02	4.86 ± 0.31	4.74 ± 0.04
TC05001826.hg.1	BRD8	7.85 ± 0.07	7.66 ± 0.10	7.87 ± 0.14	7.56 ± 0.07
TC05003404.hg.1	BRD9	7.98 ± 0.04	7.97 ± 0.07	7.78 ± 0.08	8.02 ± 0.05
TC01000848.hg.1	BRDT	4.80 ± 0.16	4.71 ± 0.05	4.73 ± 0.07	4.74 ± 0.02
TC02004954.hg.1	BRE	8.39 ± 0.18	8.30 ± 0.14	8.36 ± 0.15	8.15 ± 0.05
TC08000831.hg.1	BREA2	8.16 ± 0.24	8.21 ± 0.08	8.14 ± 0.29	8.21 ± 0.04
TC02001696.hg.1	BRE-AS1	8.04 ± 0.69	9.68 ± 0.20	8.47 ± 0.62	9.30 ± 0.47

TC14001566.hg.1	BRF1	8.13 ± 0.15	8.11 ± 0.08	8.01 ± 0.15	8.13 ± 0.01
TC08001137.hg.1	BRF2	7.48 ± 0.12	7.40 ± 0.02	7.34 ± 0.14	7.39 ± 0.02
TC07000584.hg.1	BRI3	9.18 ± 0.30	8.90 ± 0.03	9.22 ± 0.13	8.96 ± 0.20
TC07000585.hg.1	BRI3	8.15 ± 0.09	7.99 ± 0.04	8.21 ± 0.05	8.19 ± 0.31
TC12001002.hg.1	BRI3BP	8.62 ± 0.14	8.53 ± 0.14	8.54 ± 0.21	8.29 ± 0.08
TC16000779.hg.1	BRICD5	8.21 ± 0.22	8.18 ± 0.15	8.11 ± 0.18	8.29 ± 0.07
TC17001769.hg.1	BRIP1	6.15 ± 0.03	6.09 ± 0.31	6.04 ± 0.11	5.69 ± 0.10
TC05000154.hg.1	BRIX1	7.56 ± 0.29	7.76 ± 0.28	7.30 ± 0.14	7.61 ± 0.06
TC03000053.hg.1	BRK1	9.68 ± 0.01	9.71 ± 0.14	9.67 ± 0.14	9.62 ± 0.13
TC11001964.hg.1	BRMS1	8.25 ± 0.17	8.16 ± 0.06	8.16 ± 0.16	8.15 ± 0.02
TC14000225.hg.1	BRMS1L	5.50 ± 0.10	5.64 ± 0.05	5.47 ± 0.06	5.55 ± 0.10
TC01001830.hg.1	BROX	9.09 ± 0.26	9.05 ± 0.22	9.36 ± 0.39	8.97 ± 0.20
TC03000041.hg.1	BRPF1	8.34 ± 0.07	8.26 ± 0.06	8.20 ± 0.04	8.29 ± 0.03
TC06000525.hg.1	BRPF3	8.62 ± 0.03	8.58 ± 0.02	8.49 ± 0.02	8.56 ± 0.04
TC0X000660.hg.1	BRS3	5.16 ± 0.29	5.15 ± 0.05	5.21 ± 0.22	5.16 ± 0.01
TC19000892.hg.1	BRSK1	7.78 ± 0.23	7.73 ± 0.21	7.67 ± 0.22	7.78 ± 0.05
TC11000043.hg.1	BRSK2	7.84 ± 0.19	7.76 ± 0.16	7.79 ± 0.15	7.83 ± 0.02
TC21000459.hg.1	BRWD1	7.97 ± 0.62	8.41 ± 0.26	7.94 ± 0.44	8.35 ± 0.05
TC21000173.hg.1	BRWD1-AS1	4.92 ± 0.16	4.95 ± 0.11	4.93 ± 0.15	4.96 ± 0.18
TC21000460.hg.1	BRWD1-IT1	6.74 ± 0.30	6.83 ± 0.17	6.81 ± 0.21	6.87 ± 0.15
TC21000461.hg.1	BRWD1-IT2	6.61 ± 0.40	6.79 ± 0.26	6.66 ± 0.17	6.77 ± 0.18
TC0X001182.hg.1	BRWD3	8.34 ± 0.41	8.66 ± 0.28	8.77 ± 0.55	8.57 ± 0.22
TC11003487.hg.1	BSCL2	8.38 ± 0.10	8.25 ± 0.07	8.29 ± 0.16	8.26 ± 0.04
TC01002471.hg.1	BSDC1	8.75 ± 0.10	8.64 ± 0.06	8.64 ± 0.10	8.69 ± 0.02
TC19000009.hg.1	BSG	10.02 ± 0.23	9.81 ± 0.02	10.00 ± 0.10	9.73 ± 0.14
TC03000296.hg.1	BSN	7.58 ± 0.18	7.53 ± 0.12	7.52 ± 0.20	7.59 ± 0.03
TC03001413.hg.1	BSN-AS1	6.87 ± 0.14	6.67 ± 0.10	6.88 ± 0.05	6.76 ± 0.02
TC03001412.hg.1	BSN-AS2	5.84 ± 0.11	5.80 ± 0.14	5.79 ± 0.11	5.89 ± 0.06
TC01000674.hg.1	BSND	7.01 ± 0.24	7.08 ± 0.26	7.01 ± 0.13	7.19 ± 0.05
TC19001672.hg.1	BSPH1	5.18 ± 0.02	5.01 ± 0.09	5.12 ± 0.07	5.05 ± 0.04
TC09000574.hg.1	BSPRY	7.52 ± 0.28	7.46 ± 0.20	7.42 ± 0.25	7.55 ± 0.09

TC04002929.hg.1	BST1	9.16 ± 0.41	8.58 ± 0.18	9.53 ± 0.36	8.28 ± 0.43
TC19001275.hg.1	BST2	8.56 ± 0.65	7.96 ± 0.22	8.49 ± 0.47	7.70 ± 0.17
TC11002389.hg.1	BSX	8.21 ± 0.31	8.28 ± 0.29	8.17 ± 0.32	8.40 ± 0.07
TC10000655.hg.1	BTAF1	8.44 ± 0.57	8.90 ± 0.21	8.39 ± 0.53	8.89 ± 0.03
TC15001764.hg.1	BTBD1	9.23 ± 0.36	9.44 ± 0.09	9.28 ± 0.36	9.42 ± 0.14
TC11001434.hg.1	BTBD10	7.59 ± 0.18	7.41 ± 0.13	7.75 ± 0.34	7.24 ± 0.13
TC12000828.hg.1	BTBD11	7.85 ± 0.03	7.96 ± 0.12	7.59 ± 0.05	8.07 ± 0.17
TC10000884.hg.1	BTBD16	6.33 ± 0.19	6.21 ± 0.10	6.20 ± 0.07	6.32 ± 0.05
TC17001855.hg.1	BTBD17	8.47 ± 0.14	8.55 ± 0.16	8.39 ± 0.22	8.63 ± 0.11
TC11001798.hg.1	BTBD18	5.79 ± 0.11	5.78 ± 0.07	5.75 ± 0.07	5.86 ± 0.13
TC01000567.hg.1	BTBD19	8.56 ± 0.20	8.60 ± 0.14	8.68 ± 0.01	8.69 ± 0.04
TC19001018.hg.1	BTBD2	9.40 ± 0.15	9.35 ± 0.08	9.16 ± 0.08	9.26 ± 0.03
TC20000088.hg.1	BTBD3	6.70 ± 0.05	6.68 ± 0.02	6.64 ± 0.10	6.69 ± 0.01
TC14000833.hg.1	BTBD6	8.94 ± 0.25	8.92 ± 0.14	8.82 ± 0.21	8.90 ± 0.04
TC14001456.hg.1	BTBD7	8.03 ± 0.18	8.02 ± 0.20	8.06 ± 0.25	7.92 ± 0.17
TC01000850.hg.1	BTBD8	5.78 ± 0.12	5.70 ± 0.07	5.85 ± 0.19	5.62 ± 0.11
TC06001690.hg.1	BTBD9	7.74 ± 0.10	7.90 ± 0.07	7.66 ± 0.13	7.96 ± 0.03
TC06000553.hg.1	BTBD9-AS1	6.36 ± 0.12	6.06 ± 0.09	6.26 ± 0.13	6.18 ± 0.08
TC04001292.hg.1	BTC	6.08 ± 0.13	6.00 ± 0.07	5.98 ± 0.05	6.08 ± 0.06
TC03000097.hg.1	BTD	7.03 ± 0.04	6.99 ± 0.02	6.96 ± 0.06	6.95 ± 0.06
TC05000352.hg.1	BTF3	9.00 ± 0.09	9.22 ± 0.24	8.76 ± 0.01	9.14 ± 0.08
TC01000638.hg.1	BTF3L4	8.47 ± 0.18	8.63 ± 0.11	8.43 ± 0.19	8.63 ± 0.09
TC21000297.hg.1	BTF3L4P1	5.94 ± 0.32	6.03 ± 0.17	5.97 ± 0.25	6.07 ± 0.20
TC06001090.hg.1	BTF3P10	8.28 ± 0.24	8.13 ± 0.26	7.87 ± 0.36	7.98 ± 0.20
TC13000290.hg.1	BTF3P11	6.65 ± 0.08	6.63 ± 0.26	6.43 ± 0.25	6.65 ± 0.32
TC12001807.hg.1	BTG1	10.86 ± 0.34	11.46 ± 0.15	10.73 ± 0.17	11.45 ± 0.09
TC01001685.hg.1	BTG2	11.50 ± 0.11	11.91 ± 0.18	11.67 ± 0.27	11.87 ± 0.17
TC21000299.hg.1	BTG3	7.44 ± 0.25	7.97 ± 0.07	7.36 ± 0.16	8.04 ± 0.20
TC11002281.hg.1	BTG4	4.64 ± 0.17	4.57 ± 0.11	4.58 ± 0.12	4.59 ± 0.11
TC0X001218.hg.1	BTK	9.13 ± 0.17	8.77 ± 0.19	9.22 ± 0.21	8.61 ± 0.03
TC03001649.hg.1	BTLA	8.47 ± 0.39	8.29 ± 0.41	8.30 ± 0.25	7.89 ± 0.13

TC06000190.hg.1	BTN1A1	6.40 ± 0.22	6.35 ± 0.16	6.34 ± 0.11	6.40 ± 0.05
TC06000189.hg.1	BTN2A1	8.87 ± 0.16	8.57 ± 0.15	8.98 ± 0.33	8.48 ± 0.13
TC06000185.hg.1	BTN2A2	8.03 ± 0.03	7.83 ± 0.05	7.90 ± 0.11	7.80 ± 0.02
TC06000187.hg.1	BTN2A3P	7.28 ± 0.05	7.10 ± 0.08	7.25 ± 0.16	7.15 ± 0.03
TC06000186.hg.1	BTN3A1	10.24 ± 0.20	9.98 ± 0.13	10.04 ± 0.24	9.72 ± 0.14
TC06000184.hg.1	BTN3A2	9.89 ± 0.19	9.57 ± 0.11	9.65 ± 0.25	9.21 ± 0.17
TC06000188.hg.1	BTN3A3	9.05 ± 0.20	8.72 ± 0.11	8.74 ± 0.25	8.37 ± 0.11
TC01003919.hg.1	BTNL10	7.19 ± 0.25	7.23 ± 0.17	7.14 ± 0.26	7.34 ± 0.07
TC06001566.hg.1	BTNL2	6.91 ± 0.16	6.89 ± 0.15	6.90 ± 0.19	7.02 ± 0.09
TC6_apd_hap1000100.hg.1	BTNL2	6.90 ± 0.14	6.87 ± 0.16	6.93 ± 0.16	7.03 ± 0.09
TC6_cox_hap2000192.hg.1	BTNL2	6.90 ± 0.14	6.86 ± 0.16	6.93 ± 0.15	7.03 ± 0.10
TC6_dbb_hap3000178.hg.1	BTNL2	6.90 ± 0.14	6.87 ± 0.16	6.93 ± 0.16	7.03 ± 0.09
TC6_mann_hap4000157.hg.1	BTNL2	6.82 ± 0.15	6.78 ± 0.16	6.84 ± 0.18	6.92 ± 0.09
TC6_mcf_hap5000167.hg.1	BTNL2	6.82 ± 0.15	6.78 ± 0.16	6.84 ± 0.18	6.92 ± 0.09
TC6_qbl_hap6000181.hg.1	BTNL2	6.90 ± 0.14	6.87 ± 0.16	6.93 ± 0.16	7.03 ± 0.09
TC6_ssto_hap7000161.hg.1	BTNL2	6.82 ± 0.15	6.78 ± 0.16	6.84 ± 0.18	6.92 ± 0.09
TC05001069.hg.1	BTNL3	6.86 ± 0.20	6.86 ± 0.16	6.89 ± 0.11	6.91 ± 0.03
TC05001067.hg.1	BTNL8	7.74 ± 0.14	7.59 ± 0.02	7.72 ± 0.08	7.58 ± 0.03
TC05001071.hg.1	BTNL9	7.76 ± 0.25	7.75 ± 0.20	7.71 ± 0.20	7.84 ± 0.04
TC10000738.hg.1	BTRC	7.58 ± 0.13	7.55 ± 0.07	7.50 ± 0.12	7.48 ± 0.04
TC02002204.hg.1	BUB1	7.03 ± 0.26	6.64 ± 0.40	6.68 ± 0.27	6.16 ± 0.15
TC10000900.hg.1	BUB3	8.81 ± 0.13	8.99 ± 0.16	8.58 ± 0.17	8.90 ± 0.06
TC11002322.hg.1	BUD13	7.74 ± 0.18	7.40 ± 0.06	7.58 ± 0.18	7.30 ± 0.03
TC07000593.hg.1	BUD31	8.31 ± 0.16	8.35 ± 0.09	8.19 ± 0.17	8.34 ± 0.07
TC06001976.hg.1	BVES	5.36 ± 0.02	5.18 ± 0.11	5.23 ± 0.11	5.25 ± 0.02
TC06000841.hg.1	BVES-AS1	4.43 ± 0.09	4.40 ± 0.09	4.43 ± 0.04	4.42 ± 0.01
TC06000577.hg.1	BYSL	7.65 ± 0.10	7.47 ± 0.06	7.51 ± 0.01	7.50 ± 0.04
TC17001728.hg.1	BZRAP1	8.35 ± 0.18	8.34 ± 0.13	8.32 ± 0.10	8.45 ± 0.05
TC17000713.hg.1	BZRAP1-AS1	8.66 ± 0.04	8.54 ± 0.06	8.58 ± 0.09	8.59 ± 0.04
TC02001166.hg.1	BZW1	9.35 ± 0.42	9.88 ± 0.13	9.45 ± 0.34	9.86 ± 0.13
TC07000110.hg.1	BZW2	7.39 ± 0.00	7.46 ± 0.17	7.16 ± 0.08	7.23 ± 0.04

TC10001216.hg.1	C10orf10	7.53 ± 0.17	7.52 ± 0.20	7.46 ± 0.14	7.61 ± 0.06
TC10001372.hg.1	C10orf105	8.39 ± 0.17	8.41 ± 0.16	8.41 ± 0.13	8.50 ± 0.09
TC10000376.hg.1	C10orf107	5.34 ± 0.24	5.11 ± 0.04	5.16 ± 0.13	5.10 ± 0.07
TC10000488.hg.1	C10orf11	7.09 ± 0.15	6.98 ± 0.09	7.03 ± 0.04	7.05 ± 0.06
TC10001060.hg.1	C10orf111	7.16 ± 0.15	7.29 ± 0.17	7.05 ± 0.23	7.35 ± 0.03
TC10000143.hg.1	C10orf112	5.27 ± 0.10	5.14 ± 0.07	5.19 ± 0.07	5.22 ± 0.05
TC10001090.hg.1	C10orf113	5.19 ± 0.16	5.10 ± 0.06	5.08 ± 0.23	5.20 ± 0.07
TC10001092.hg.1	C10orf114	7.84 ± 0.26	7.64 ± 0.17	7.63 ± 0.24	7.64 ± 0.03
TC10001103.hg.1	C10orf115	4.38 ± 0.05	4.33 ± 0.03	4.33 ± 0.05	4.40 ± 0.15
TC10000688.hg.1	C10orf12	8.95 ± 0.20	9.09 ± 0.20	8.97 ± 0.27	9.09 ± 0.05
TC10001715.hg.1	C10orf120	5.24 ± 0.16	5.15 ± 0.07	5.15 ± 0.13	5.08 ± 0.10
TC10000202.hg.1	C10orf126	6.50 ± 0.25	6.50 ± 0.03	6.50 ± 0.12	6.62 ± 0.07
TC10001273.hg.1	C10orf128	7.34 ± 0.09	7.15 ± 0.06	7.62 ± 0.26	7.30 ± 0.14
TC10000676.hg.1	C10orf129	6.16 ± 0.16	6.21 ± 0.12	6.16 ± 0.06	6.28 ± 0.10
TC10002944.hg.1	C10orf131	4.91 ± 0.05	4.86 ± 0.04	5.03 ± 0.11	4.86 ± 0.02
TC10000912.hg.1	C10orf137	7.92 ± 0.41	8.13 ± 0.20	7.89 ± 0.34	8.06 ± 0.13
TC10000729.hg.1	C10orf2	7.47 ± 0.11	7.38 ± 0.09	7.36 ± 0.17	7.44 ± 0.01
TC10001217.hg.1	C10orf25	7.45 ± 0.09	7.17 ± 0.10	7.28 ± 0.09	7.28 ± 0.11
TC10002947.hg.1	C10orf32	6.75 ± 0.26	7.03 ± 0.17	6.66 ± 0.28	6.86 ± 0.31
TC10000422.hg.1	C10orf35	7.82 ± 0.27	7.87 ± 0.22	7.78 ± 0.24	7.93 ± 0.06
TC10001318.hg.1	C10orf40	5.33 ± 0.07	5.23 ± 0.05	5.38 ± 0.11	5.29 ± 0.03
TC10002937.hg.1	C10orf53	7.32 ± 0.11	7.34 ± 0.09	7.29 ± 0.02	7.48 ± 0.14
TC10001373.hg.1	C10orf54	9.97 ± 0.36	9.74 ± 0.11	10.03 ± 0.23	9.71 ± 0.19
TC10001421.hg.1	C10orf55	6.47 ± 0.21	6.64 ± 0.06	6.60 ± 0.15	6.75 ± 0.03
TC10000700.hg.1	C10orf62	7.99 ± 0.25	8.06 ± 0.32	8.02 ± 0.21	8.29 ± 0.07
TC10001105.hg.1	C10orf67	4.36 ± 0.14	4.26 ± 0.05	4.32 ± 0.12	4.30 ± 0.04
TC10000327.hg.1	C10orf71	7.28 ± 0.18	7.18 ± 0.15	7.19 ± 0.06	7.30 ± 0.12
TC10001274.hg.1	C10orf71-AS1	6.51 ± 0.20	6.57 ± 0.24	6.53 ± 0.13	6.68 ± 0.07
TC10001615.hg.1	C10orf76	8.74 ± 0.23	8.68 ± 0.15	8.66 ± 0.16	8.49 ± 0.11
TC10001680.hg.1	C10orf82	6.66 ± 0.21	6.59 ± 0.16	6.57 ± 0.06	6.69 ± 0.01
TC10000875.hg.1	C10orf85	6.99 ± 0.24	6.99 ± 0.16	7.02 ± 0.18	7.24 ± 0.23

TC10001717.hg.1	C10orf88	7.60 ± 0.20	7.57 ± 0.08	7.53 ± 0.22	7.57 ± 0.05
TC10001747.hg.1	C10orf90	5.68 ± 0.16	5.60 ± 0.08	5.60 ± 0.10	5.68 ± 0.08
TC10000945.hg.1	C10orf91	7.69 ± 0.22	7.74 ± 0.18	7.62 ± 0.25	7.80 ± 0.07
TC10001622.hg.1	C10orf95	8.11 ± 0.34	8.04 ± 0.22	7.99 ± 0.34	8.12 ± 0.02
TC10000592.hg.1	C10orf99	6.76 ± 0.09	6.71 ± 0.15	6.69 ± 0.18	6.76 ± 0.05
TC11001000.hg.1	C11orf1	7.25 ± 0.12	7.19 ± 0.12	6.93 ± 0.03	7.06 ± 0.03
TC11001391.hg.1	C11orf16	7.26 ± 0.19	7.28 ± 0.18	7.20 ± 0.23	7.40 ± 0.11
TC11001278.hg.1	C11orf21	8.51 ± 0.03	8.48 ± 0.04	8.45 ± 0.11	8.45 ± 0.06
TC11002005.hg.1	C11orf24	9.11 ± 0.19	8.85 ± 0.10	8.87 ± 0.08	8.81 ± 0.05
TC11000820.hg.1	C11orf30	8.70 ± 0.28	8.67 ± 0.22	8.82 ± 0.38	8.50 ± 0.15
TC11002295.hg.1	C11orf34	5.36 ± 0.08	5.24 ± 0.12	5.25 ± 0.08	5.37 ± 0.17
TC11001237.hg.1	C11orf35	8.48 ± 0.23	8.45 ± 0.24	8.38 ± 0.25	8.56 ± 0.05
TC11001310.hg.1	C11orf40	5.81 ± 0.15	5.62 ± 0.16	5.67 ± 0.11	5.73 ± 0.10
TC11000127.hg.1	C11orf42	6.05 ± 0.18	5.85 ± 0.12	5.93 ± 0.04	6.04 ± 0.12
TC11001198.hg.1	C11orf44	6.69 ± 0.07	6.67 ± 0.19	6.54 ± 0.22	6.83 ± 0.10
TC11002444.hg.1	C11orf45	7.23 ± 0.18	7.25 ± 0.13	7.22 ± 0.09	7.32 ± 0.10
TC11003486.hg.1	C11orf48	8.57 ± 0.12	8.34 ± 0.03	8.31 ± 0.12	8.25 ± 0.06
TC11000403.hg.1	C11orf49	7.51 ± 0.08	7.45 ± 0.03	7.42 ± 0.12	7.43 ± 0.03
TC11000989.hg.1	C11orf53	6.17 ± 0.16	5.98 ± 0.03	6.04 ± 0.06	6.05 ± 0.03
TC11000911.hg.1	C11orf54	8.22 ± 0.14	7.90 ± 0.28	8.23 ± 0.42	7.75 ± 0.18
TC11001006.hg.1	C11orf57	7.61 ± 0.11	7.58 ± 0.14	7.53 ± 0.17	7.50 ± 0.15
TC11000223.hg.1	C11orf58	8.73 ± 0.25	9.00 ± 0.07	8.66 ± 0.16	8.97 ± 0.06
TC11001117.hg.1	C11orf63	6.41 ± 0.06	6.34 ± 0.02	6.19 ± 0.09	6.36 ± 0.08
TC11002266.hg.1	C11orf65	4.76 ± 0.03	4.63 ± 0.11	4.66 ± 0.05	4.56 ± 0.03
TC11001949.hg.1	C11orf68	8.90 ± 0.20	8.84 ± 0.10	8.81 ± 0.29	8.89 ± 0.05
TC11000951.hg.1	C11orf70	4.26 ± 0.11	4.10 ± 0.04	4.15 ± 0.07	4.11 ± 0.07
TC11002311.hg.1	C11orf71	6.58 ± 0.05	6.63 ± 0.14	6.60 ± 0.20	6.67 ± 0.13
TC11002312.hg.1	C11orf71	7.78 ± 0.07	7.80 ± 0.27	7.91 ± 0.28	7.72 ± 0.26
TC11001989.hg.1	C11orf72	6.45 ± 0.08	6.43 ± 0.07	6.34 ± 0.16	6.54 ± 0.22
TC11000871.hg.1	C11orf73	6.94 ± 0.21	6.77 ± 0.11	6.84 ± 0.12	6.61 ± 0.08
TC11000348.hg.1	C11orf74	6.18 ± 0.07	6.02 ± 0.11	6.19 ± 0.04	6.08 ± 0.05

TC11003463.hg.1	C11orf80	7.82 ± 0.04	7.78 ± 0.10	7.62 ± 0.03	7.66 ± 0.09
TC11000854.hg.1	C11orf82	6.65 ± 0.04	6.47 ± 0.09	6.98 ± 0.12	6.38 ± 0.07
TC11000560.hg.1	C11orf83	6.80 ± 0.22	6.55 ± 0.10	6.63 ± 0.11	6.57 ± 0.02
TC11000576.hg.1	C11orf84	8.15 ± 0.25	8.10 ± 0.15	8.01 ± 0.21	8.09 ± 0.04
TC11001920.hg.1	C11orf85	6.28 ± 0.23	6.17 ± 0.13	6.24 ± 0.13	6.22 ± 0.02
TC11000677.hg.1	C11orf86	7.42 ± 0.31	7.40 ± 0.27	7.35 ± 0.44	7.50 ± 0.13
TC11000984.hg.1	C11orf87	7.38 ± 0.12	7.36 ± 0.10	7.36 ± 0.16	7.40 ± 0.06
TC11000996.hg.1	C11orf88	4.26 ± 0.07	4.25 ± 0.04	4.34 ± 0.06	4.38 ± 0.11
TC11001270.hg.1	C11orf89	7.59 ± 0.23	7.62 ± 0.29	7.63 ± 0.34	7.78 ± 0.06
TC11002277.hg.1	C11orf92	6.63 ± 0.16	6.66 ± 0.14	6.57 ± 0.20	6.72 ± 0.06
TC11000990.hg.1	C11orf93	6.74 ± 0.22	6.73 ± 0.19	6.67 ± 0.19	6.78 ± 0.14
TC11001714.hg.1	C11orf94	7.67 ± 0.21	7.48 ± 0.15	7.42 ± 0.19	7.62 ± 0.10
TC11001895.hg.1	C11orf95	8.54 ± 0.24	8.57 ± 0.17	8.53 ± 0.25	8.63 ± 0.07
TC11000369.hg.1	C11orf96	7.02 ± 0.23	6.91 ± 0.16	6.90 ± 0.18	6.95 ± 0.04
TC12000436.hg.1	C12orf10	8.48 ± 0.10	8.41 ± 0.05	8.34 ± 0.04	8.39 ± 0.06
TC12000826.hg.1	C12orf23	7.78 ± 0.21	7.86 ± 0.05	7.66 ± 0.07	7.84 ± 0.06
TC12000691.hg.1	C12orf29	7.18 ± 0.29	7.31 ± 0.11	6.98 ± 0.12	7.13 ± 0.08
TC12001262.hg.1	C12orf36	6.17 ± 0.14	6.14 ± 0.06	6.14 ± 0.09	6.30 ± 0.16
TC12000234.hg.1	C12orf39	7.45 ± 0.07	7.19 ± 0.15	7.58 ± 0.09	7.35 ± 0.06
TC12001123.hg.1	C12orf4	7.91 ± 0.34	8.07 ± 0.20	8.09 ± 0.52	7.79 ± 0.17
TC12000307.hg.1	C12orf40	4.37 ± 0.14	4.23 ± 0.03	4.29 ± 0.07	4.23 ± 0.04
TC12001895.hg.1	C12orf42	6.31 ± 0.08	6.34 ± 0.08	6.18 ± 0.07	6.30 ± 0.08
TC12002058.hg.1	C12orf43	7.80 ± 0.12	7.78 ± 0.03	7.71 ± 0.03	7.82 ± 0.05
TC12000415.hg.1	C12orf44	8.23 ± 0.18	8.26 ± 0.15	8.13 ± 0.23	8.38 ± 0.03
TC12000815.hg.1	C12orf45	7.03 ± 0.16	6.97 ± 0.05	6.82 ± 0.06	6.86 ± 0.02
TC12002009.hg.1	C12orf49	8.87 ± 0.04	8.74 ± 0.06	8.77 ± 0.04	8.68 ± 0.04
TC12000061.hg.1	C12orf5	7.45 ± 0.18	7.44 ± 0.17	7.65 ± 0.19	7.32 ± 0.12
TC12001790.hg.1	C12orf50	4.62 ± 0.08	4.53 ± 0.05	4.54 ± 0.04	4.61 ± 0.05
TC12000889.hg.1	C12orf52	7.95 ± 0.23	7.80 ± 0.13	7.76 ± 0.22	7.75 ± 0.04
TC12000351.hg.1	C12orf54	6.64 ± 0.18	6.53 ± 0.08	6.63 ± 0.11	6.56 ± 0.08
TC12003306.hg.1	C12orf55	5.05 ± 0.06	4.80 ± 0.01	5.08 ± 0.30	4.80 ± 0.03

TC12003307.hg.1	C12orf55	4.65 ± 0.05	4.37 ± 0.04	4.50 ± 0.08	4.39 ± 0.05
TC12001672.hg.1	C12orf56	4.98 ± 0.10	4.86 ± 0.04	4.90 ± 0.04	4.90 ± 0.07
TC12003289.hg.1	C12orf57	8.62 ± 0.22	8.77 ± 0.13	8.22 ± 0.14	8.73 ± 0.00
TC12000198.hg.1	C12orf60	5.00 ± 0.05	4.94 ± 0.12	5.04 ± 0.08	5.02 ± 0.13
TC12001656.hg.1	C12orf61	7.65 ± 0.30	7.86 ± 0.18	7.64 ± 0.14	7.83 ± 0.13
TC12003308.hg.1	C12orf63	4.18 ± 0.07	4.01 ± 0.04	4.12 ± 0.04	4.04 ± 0.01
TC12000983.hg.1	C12orf65	8.05 ± 0.02	7.89 ± 0.02	7.86 ± 0.04	7.92 ± 0.03
TC12001670.hg.1	C12orf66	8.07 ± 0.09	8.04 ± 0.02	7.92 ± 0.08	7.99 ± 0.03
TC12000347.hg.1	C12orf68	8.19 ± 0.27	8.01 ± 0.02	8.08 ± 0.18	8.12 ± 0.02
TC12001332.hg.1	C12orf71	5.95 ± 0.24	6.02 ± 0.17	5.91 ± 0.19	5.98 ± 0.17
TC12001903.hg.1	C12orf73	7.15 ± 0.02	7.06 ± 0.03	7.07 ± 0.04	7.11 ± 0.04
TC12000713.hg.1	C12orf74	6.15 ± 0.16	6.15 ± 0.09	6.07 ± 0.07	6.20 ± 0.11
TC12000818.hg.1	C12orf75	7.23 ± 0.08	7.15 ± 0.28	7.16 ± 0.21	6.84 ± 0.05
TC12001960.hg.1	C12orf76	7.10 ± 0.10	6.88 ± 0.06	7.05 ± 0.12	6.92 ± 0.03
TC12001310.hg.1	C12orf77	5.62 ± 0.07	5.70 ± 0.03	5.53 ± 0.04	5.65 ± 0.06
TC12001806.hg.1	C12orf79	6.06 ± 0.12	6.48 ± 0.13	6.01 ± 0.18	6.76 ± 0.28
TC13000410.hg.1	C13orf35	5.47 ± 0.22	5.42 ± 0.19	5.25 ± 0.27	5.45 ± 0.11
TC13000287.hg.1	C13orf45	6.45 ± 0.34	6.49 ± 0.12	6.35 ± 0.18	6.71 ± 0.24
TC14001324.hg.1	C14orf1	8.75 ± 0.19	8.70 ± 0.17	8.49 ± 0.13	8.45 ± 0.08
TC14001171.hg.1	C14orf105	4.76 ± 0.19	4.64 ± 0.01	4.69 ± 0.05	4.63 ± 0.01
TC14000141.hg.1	C14orf119	8.36 ± 0.32	7.95 ± 0.08	8.31 ± 0.31	7.85 ± 0.07
TC14000599.hg.1	C14orf132	6.35 ± 0.20	6.27 ± 0.15	6.18 ± 0.16	6.36 ± 0.13
TC14001455.hg.1	C14orf142	7.20 ± 0.30	7.01 ± 0.09	7.11 ± 0.15	6.92 ± 0.12
TC14000815.hg.1	C14orf144	6.97 ± 0.09	6.94 ± 0.20	6.69 ± 0.11	7.04 ± 0.08
TC14000562.hg.1	C14orf159	8.49 ± 0.07	8.29 ± 0.10	8.44 ± 0.18	8.19 ± 0.06
TC14001261.hg.1	C14orf162	6.77 ± 0.38	7.04 ± 0.35	6.66 ± 0.37	7.23 ± 0.14
TC14000142.hg.1	C14orf164	5.86 ± 0.21	5.69 ± 0.03	5.77 ± 0.07	5.73 ± 0.03
TC14000304.hg.1	C14orf166	9.06 ± 0.13	9.21 ± 0.21	9.01 ± 0.13	9.02 ± 0.16
TC14000483.hg.1	C14orf166B	6.26 ± 0.21	6.19 ± 0.16	6.14 ± 0.19	6.26 ± 0.03
TC14000443.hg.1	C14orf169	8.43 ± 0.25	8.38 ± 0.12	8.02 ± 0.24	8.33 ± 0.04
TC14000615.hg.1	C14orf177	6.79 ± 0.14	6.69 ± 0.06	6.74 ± 0.09	6.78 ± 0.14

TC14002204.hg.1	C14orf178	6.77 ± 0.13	6.58 ± 0.18	6.63 ± 0.11	6.65 ± 0.03
TC14000816.hg.1	C14orf180	8.69 ± 0.12	8.57 ± 0.10	8.64 ± 0.16	8.59 ± 0.05
TC14002344.hg.1	C14orf182	7.03 ± 0.21	7.09 ± 0.01	6.95 ± 0.10	7.11 ± 0.06
TC14001114.hg.1	C14orf183	6.70 ± 0.05	6.81 ± 0.07	6.93 ± 0.09	6.84 ± 0.03
TC14001547.hg.1	C14orf2	8.41 ± 0.32	8.24 ± 0.15	8.30 ± 0.20	8.13 ± 0.08
TC14000185.hg.1	C14orf23	5.81 ± 0.14	5.73 ± 0.07	5.72 ± 0.14	5.83 ± 0.11
TC14000261.hg.1	C14orf28	6.51 ± 0.20	6.70 ± 0.06	6.45 ± 0.16	6.69 ± 0.10
TC14002322.hg.1	C14orf37	5.75 ± 0.10	5.70 ± 0.16	5.65 ± 0.08	5.74 ± 0.05
TC14001186.hg.1	C14orf39	3.86 ± 0.14	3.69 ± 0.02	3.75 ± 0.07	3.69 ± 0.02
TC14001492.hg.1	C14orf64	7.56 ± 0.29	7.62 ± 0.15	7.31 ± 0.15	7.55 ± 0.13
TC14000829.hg.1	C14orf79	7.23 ± 0.17	7.21 ± 0.20	7.21 ± 0.16	7.22 ± 0.04
TC14002315.hg.1	C14orf80	8.81 ± 0.22	8.75 ± 0.20	8.74 ± 0.21	8.82 ± 0.02
TC14000944.hg.1	C14orf93	7.17 ± 0.28	6.76 ± 0.12	7.06 ± 0.22	6.70 ± 0.03
TC15000755.hg.1	C15orf26	6.82 ± 0.14	6.64 ± 0.10	6.65 ± 0.09	6.67 ± 0.08
TC15000706.hg.1	C15orf27	6.92 ± 0.23	6.96 ± 0.13	6.91 ± 0.15	7.01 ± 0.07
TC15000885.hg.1	C15orf32	5.46 ± 0.22	5.39 ± 0.19	5.35 ± 0.19	5.49 ± 0.08
TC15000744.hg.1	C15orf37	8.48 ± 0.32	8.48 ± 0.08	8.67 ± 0.06	8.56 ± 0.08
TC15002783.hg.1	C15orf38	8.08 ± 0.26	7.90 ± 0.05	8.00 ± 0.12	7.95 ± 0.02
TC15000691.hg.1	C15orf39	9.70 ± 0.22	9.81 ± 0.11	9.79 ± 0.16	9.84 ± 0.23
TC15001763.hg.1	C15orf40	6.53 ± 0.03	6.40 ± 0.05	6.43 ± 0.03	6.43 ± 0.06
TC15000253.hg.1	C15orf41	7.41 ± 0.15	7.40 ± 0.09	7.37 ± 0.20	7.25 ± 0.11
TC15000347.hg.1	C15orf43	4.30 ± 0.20	4.14 ± 0.07	4.31 ± 0.12	4.16 ± 0.03
TC15001234.hg.1	C15orf52	8.03 ± 0.21	7.93 ± 0.13	7.98 ± 0.20	8.06 ± 0.05
TC15000265.hg.1	C15orf53	6.65 ± 0.21	6.65 ± 0.19	6.54 ± 0.06	6.83 ± 0.10
TC15000268.hg.1	C15orf54	5.57 ± 0.04	5.45 ± 0.02	5.39 ± 0.14	5.50 ± 0.12
TC15001231.hg.1	C15orf56	8.38 ± 0.30	8.33 ± 0.29	8.32 ± 0.19	8.42 ± 0.05
TC15001238.hg.1	C15orf57	7.98 ± 0.09	7.77 ± 0.07	7.94 ± 0.06	7.74 ± 0.08
TC15001635.hg.1	C15orf59	7.58 ± 0.19	7.55 ± 0.20	7.49 ± 0.20	7.57 ± 0.03
TC15000666.hg.1	C15orf60	6.06 ± 0.20	5.94 ± 0.15	6.07 ± 0.11	6.14 ± 0.06
TC15000624.hg.1	C15orf61	6.71 ± 0.15	6.56 ± 0.01	6.50 ± 0.14	6.55 ± 0.02
TC15000294.hg.1	C15orf62	7.40 ± 0.21	7.37 ± 0.01	7.35 ± 0.09	7.33 ± 0.25

TC15000431.hg.1	C15orf65	5.17 ± 0.06	5.10 ± 0.13	5.35 ± 0.06	5.04 ± 0.05
TC16000017.hg.1	C16orf11	8.35 ± 0.19	8.39 ± 0.29	8.26 ± 0.37	8.49 ± 0.03
TC16000731.hg.1	C16orf13	8.71 ± 0.14	8.64 ± 0.14	8.53 ± 0.16	8.68 ± 0.07
TC16001362.hg.1	C16orf3	6.35 ± 0.27	6.20 ± 0.17	6.17 ± 0.42	6.27 ± 0.04
TC16000190.hg.1	C16orf45	6.83 ± 0.26	6.85 ± 0.12	6.75 ± 0.31	6.86 ± 0.07
TC16001297.hg.1	C16orf46	6.60 ± 0.10	6.58 ± 0.25	6.63 ± 0.11	6.70 ± 0.04
TC16001261.hg.1	C16orf47	7.14 ± 0.16	7.09 ± 0.10	7.08 ± 0.13	7.15 ± 0.09
TC16000240.hg.1	C16orf52	6.80 ± 0.09	6.73 ± 0.03	6.76 ± 0.05	6.66 ± 0.03
TC16001008.hg.1	C16orf54	10.51 ± 0.29	10.17 ± 0.18	10.51 ± 0.23	10.17 ± 0.21
TC16000702.hg.1	C16orf55	7.38 ± 0.20	7.27 ± 0.16	7.30 ± 0.15	7.33 ± 0.07
TC16001057.hg.1	C16orf58	8.96 ± 0.11	8.81 ± 0.08	8.63 ± 0.07	8.77 ± 0.07
TC16000073.hg.1	C16orf59	8.74 ± 0.20	8.66 ± 0.12	8.68 ± 0.14	8.75 ± 0.04
TC16000216.hg.1	C16orf62	8.70 ± 0.17	8.41 ± 0.11	8.49 ± 0.11	8.24 ± 0.10
TC16000529.hg.1	C16orf70	8.92 ± 0.21	8.53 ± 0.18	9.07 ± 0.14	8.50 ± 0.33
TC16000135.hg.1	C16orf71	7.89 ± 0.25	7.78 ± 0.18	7.78 ± 0.24	7.87 ± 0.03
TC16000150.hg.1	C16orf72	10.12 ± 0.20	10.14 ± 0.06	10.45 ± 0.21	10.04 ± 0.17
TC16001319.hg.1	C16orf74	8.20 ± 0.19	8.22 ± 0.15	8.06 ± 0.18	8.27 ± 0.06
TC16000433.hg.1	C16orf78	6.09 ± 0.29	6.02 ± 0.12	5.95 ± 0.21	6.15 ± 0.17
TC16001147.hg.1	C16orf80	8.24 ± 0.14	8.06 ± 0.28	7.92 ± 0.10	7.95 ± 0.07
TC16000289.hg.1	C16orf82	6.17 ± 0.17	6.32 ± 0.18	6.19 ± 0.17	6.47 ± 0.22
TC16000545.hg.1	C16orf86	7.98 ± 0.22	8.04 ± 0.23	7.82 ± 0.19	8.14 ± 0.12
TC16001086.hg.1	C16orf87	7.18 ± 0.27	7.38 ± 0.12	7.16 ± 0.07	7.30 ± 0.19
TC16000841.hg.1	C16orf89	7.20 ± 0.14	7.31 ± 0.19	7.20 ± 0.20	7.41 ± 0.09
TC16000818.hg.1	C16orf90	7.70 ± 0.11	7.82 ± 0.18	7.76 ± 0.18	7.86 ± 0.10
TC16000751.hg.1	C16orf91	8.55 ± 0.13	8.49 ± 0.10	8.51 ± 0.13	8.50 ± 0.04
TC16000337.hg.1	C16orf92	7.16 ± 0.20	7.37 ± 0.24	7.20 ± 0.15	7.52 ± 0.07
TC16001042.hg.1	C16orf93	7.85 ± 0.15	7.76 ± 0.13	7.82 ± 0.09	7.87 ± 0.05
TC16001329.hg.1	C16orf95	7.62 ± 0.18	7.61 ± 0.23	7.47 ± 0.19	7.72 ± 0.09
TC16000132.hg.1	C16orf96	6.90 ± 0.18	6.86 ± 0.20	6.92 ± 0.16	7.02 ± 0.02
TC16001112.hg.1	C16orf97	6.55 ± 0.13	6.55 ± 0.05	6.51 ± 0.09	6.70 ± 0.14
TC17000074.hg.1	C17orf100	7.28 ± 0.28	7.38 ± 0.40	7.19 ± 0.13	7.31 ± 0.04

TC17001361.hg.1	C17orf102	5.51 ± 0.19	5.66 ± 0.06	5.58 ± 0.25	5.70 ± 0.03
TC17001265.hg.1	C17orf103	8.61 ± 0.32	8.40 ± 0.11	8.68 ± 0.13	8.41 ± 0.05
TC17000572.hg.1	C17orf104	5.49 ± 0.15	5.39 ± 0.03	5.43 ± 0.08	5.45 ± 0.02
TC17000558.hg.1	C17orf105	4.77 ± 0.23	4.58 ± 0.04	4.68 ± 0.08	4.58 ± 0.03
TC17000055.hg.1	C17orf107	8.15 ± 0.33	8.41 ± 0.23	8.30 ± 0.16	8.40 ± 0.09
TC17000687.hg.1	C17orf112	4.90 ± 0.18	4.89 ± 0.07	4.86 ± 0.01	4.87 ± 0.11
TC17001736.hg.1	C17orf47	4.85 ± 0.29	4.67 ± 0.12	4.63 ± 0.09	4.63 ± 0.05
TC17000401.hg.1	C17orf50	7.99 ± 0.20	7.77 ± 0.13	7.92 ± 0.16	7.85 ± 0.06
TC17001266.hg.1	C17orf51	7.24 ± 0.10	7.24 ± 0.12	7.16 ± 0.10	7.29 ± 0.08
TC17000563.hg.1	C17orf53	7.30 ± 0.20	7.17 ± 0.10	7.16 ± 0.09	7.19 ± 0.03
TC17001818.hg.1	C17orf58	7.77 ± 0.27	7.51 ± 0.07	7.64 ± 0.26	7.47 ± 0.02
TC17001108.hg.1	C17orf59	7.41 ± 0.25	7.39 ± 0.24	7.41 ± 0.24	7.41 ± 0.07
TC17001982.hg.1	C17orf62	9.82 ± 0.29	9.50 ± 0.09	9.97 ± 0.17	9.41 ± 0.06
TC17000737.hg.1	C17orf64	7.38 ± 0.32	7.32 ± 0.15	7.26 ± 0.22	7.38 ± 0.05
TC17001574.hg.1	C17orf65	8.48 ± 0.08	8.23 ± 0.08	8.32 ± 0.09	8.23 ± 0.03
TC17001377.hg.1	C17orf66	6.87 ± 0.13	6.81 ± 0.10	6.71 ± 0.14	6.85 ± 0.12
TC17001714.hg.1	C17orf67	6.46 ± 0.17	6.33 ± 0.06	6.32 ± 0.07	6.32 ± 0.03
TC17001953.hg.1	C17orf70	8.30 ± 0.13	8.23 ± 0.10	8.11 ± 0.18	8.26 ± 0.04
TC17000774.hg.1	C17orf72	8.03 ± 0.37	8.05 ± 0.31	7.98 ± 0.31	8.13 ± 0.03
TC17000095.hg.1	C17orf74	5.61 ± 0.24	5.64 ± 0.12	5.69 ± 0.16	5.78 ± 0.14
TC17001350.hg.1	C17orf75	6.98 ± 0.03	6.75 ± 0.14	6.80 ± 0.16	6.70 ± 0.05
TC17000832.hg.1	C17orf77	5.38 ± 0.11	5.26 ± 0.04	5.36 ± 0.12	5.23 ± 0.03
TC17000446.hg.1	C17orf78	4.69 ± 0.10	4.66 ± 0.13	4.68 ± 0.04	4.80 ± 0.12
TC17000823.hg.1	C17orf80	7.59 ± 0.14	7.35 ± 0.06	7.49 ± 0.05	7.30 ± 0.03
TC17000744.hg.1	C17orf82	7.99 ± 0.38	8.06 ± 0.18	7.96 ± 0.34	8.10 ± 0.05
TC17001012.hg.1	C17orf85	8.21 ± 0.30	8.33 ± 0.20	8.38 ± 0.29	8.26 ± 0.14
TC17000928.hg.1	C17orf89	8.65 ± 0.27	8.51 ± 0.11	8.43 ± 0.24	8.52 ± 0.03
TC17001427.hg.1	C17orf96	6.67 ± 0.25	6.72 ± 0.19	6.52 ± 0.43	6.81 ± 0.05
TC17000002.hg.1	C17orf97	7.17 ± 0.14	6.88 ± 0.10	7.08 ± 0.15	6.92 ± 0.07
TC17001436.hg.1	C17orf98	7.07 ± 0.28	6.98 ± 0.25	7.09 ± 0.14	7.14 ± 0.10
TC17000895.hg.1	C17orf99	7.80 ± 0.09	7.78 ± 0.12	7.70 ± 0.21	7.85 ± 0.04

TC18000147.hg.1	C18orf21	8.38 ± 0.06	8.31 ± 0.22	8.31 ± 0.15	8.27 ± 0.06
TC18000167.hg.1	C18orf25	8.91 ± 0.08	8.97 ± 0.17	9.17 ± 0.49	8.97 ± 0.04
TC18001002.hg.1	C18orf32	8.02 ± 0.05	8.02 ± 0.08	7.99 ± 0.06	7.98 ± 0.10
TC18000296.hg.1	C18orf42	6.69 ± 0.07	6.67 ± 0.09	6.60 ± 0.09	6.77 ± 0.31
TC18000187.hg.1	C18orf54	5.47 ± 0.38	5.24 ± 0.20	5.46 ± 0.21	5.10 ± 0.10
TC18000276.hg.1	C18orf56	7.94 ± 0.23	7.79 ± 0.07	7.81 ± 0.17	7.74 ± 0.10
TC18000247.hg.1	C18orf63	4.62 ± 0.17	4.49 ± 0.04	4.54 ± 0.03	4.49 ± 0.05
TC18000102.hg.1	C18orf8	8.64 ± 0.20	8.46 ± 0.22	8.55 ± 0.27	8.31 ± 0.05
TC19001067.hg.1	C19orf10	8.90 ± 0.35	8.40 ± 0.08	8.34 ± 0.19	8.35 ± 0.09
TC19001381.hg.1	C19orf12	8.57 ± 0.19	8.44 ± 0.10	8.42 ± 0.17	8.39 ± 0.06
TC19001909.hg.1	C19orf18	5.11 ± 0.02	4.99 ± 0.04	5.08 ± 0.13	5.03 ± 0.08
TC19000035.hg.1	C19orf24	9.03 ± 0.19	8.93 ± 0.14	8.80 ± 0.26	8.97 ± 0.02
TC19001004.hg.1	C19orf25	8.94 ± 0.16	8.84 ± 0.16	8.80 ± 0.14	8.96 ± 0.09
TC19000998.hg.1	C19orf26	8.54 ± 0.24	8.50 ± 0.21	8.51 ± 0.26	8.60 ± 0.02
TC19000524.hg.1	C19orf33	8.64 ± 0.12	8.63 ± 0.18	8.64 ± 0.16	8.71 ± 0.06
TC19001026.hg.1	C19orf35	8.72 ± 0.34	8.38 ± 0.20	8.80 ± 0.10	8.41 ± 0.10
TC19000187.hg.1	C19orf38	10.63 ± 0.52	10.22 ± 0.10	11.15 ± 0.22	10.15 ± 0.41
TC19000431.hg.1	C19orf40	7.63 ± 0.17	7.61 ± 0.06	7.61 ± 0.22	7.56 ± 0.01
TC19001208.hg.1	C19orf43	9.60 ± 0.28	9.57 ± 0.18	9.51 ± 0.19	9.35 ± 0.12
TC19000322.hg.1	C19orf44	7.54 ± 0.11	7.46 ± 0.17	7.39 ± 0.17	7.48 ± 0.03
TC19000126.hg.1	C19orf45	7.39 ± 0.18	7.48 ± 0.23	7.31 ± 0.19	7.59 ± 0.08
TC19001536.hg.1	C19orf47	8.43 ± 0.14	8.36 ± 0.10	8.30 ± 0.15	8.41 ± 0.03
TC19001754.hg.1	C19orf48	8.05 ± 0.13	7.95 ± 0.08	7.81 ± 0.12	8.02 ± 0.07
TC19000189.hg.1	C19orf52	8.17 ± 0.32	7.97 ± 0.23	8.01 ± 0.32	7.98 ± 0.13
TC19000240.hg.1	C19orf53	9.92 ± 0.18	9.87 ± 0.20	9.75 ± 0.11	9.83 ± 0.12
TC19001546.hg.1	C19orf54	8.57 ± 0.19	8.29 ± 0.10	8.47 ± 0.13	8.27 ± 0.04
TC19000485.hg.1	C19orf55	8.86 ± 0.05	8.80 ± 0.12	8.74 ± 0.12	8.91 ± 0.02
TC19001225.hg.1	C19orf57	7.46 ± 0.22	7.40 ± 0.17	7.38 ± 0.21	7.44 ± 0.05
TC19000134.hg.1	C19orf59	9.71 ± 0.88	8.67 ± 0.13	11.21 ± 0.49	8.86 ± 0.64
TC19000361.hg.1	C19orf60	8.79 ± 0.20	8.75 ± 0.15	8.66 ± 0.18	8.75 ± 0.05
TC19000168.hg.1	C19orf66	9.22 ± 0.16	9.15 ± 0.07	9.09 ± 0.20	9.08 ± 0.02

TC19001229.hg.1	C19orf67	7.45 ± 0.24	7.36 ± 0.14	7.30 ± 0.14	7.40 ± 0.05
TC19000697.hg.1	C19orf68	8.60 ± 0.22	8.44 ± 0.15	8.49 ± 0.19	8.49 ± 0.09
TC19000580.hg.1	C19orf69	7.70 ± 0.29	7.58 ± 0.21	7.45 ± 0.28	7.75 ± 0.02
TC19001080.hg.1	C19orf70	7.77 ± 0.21	7.67 ± 0.18	7.62 ± 0.18	7.75 ± 0.06
TC19000071.hg.1	C19orf71	7.99 ± 0.22	7.93 ± 0.30	7.96 ± 0.31	8.06 ± 0.14
TC19001708.hg.1	C19orf73	5.83 ± 0.07	5.65 ± 0.11	5.71 ± 0.07	5.62 ± 0.09
TC19001042.hg.1	C19orf77	7.18 ± 0.15	7.18 ± 0.22	7.16 ± 0.17	7.33 ± 0.02
TC19000192.hg.1	C19orf80	7.87 ± 0.27	7.79 ± 0.14	7.79 ± 0.22	7.83 ± 0.05
TC19000764.hg.1	C19orf81	8.01 ± 0.25	7.98 ± 0.31	7.93 ± 0.29	8.17 ± 0.07
TC19000161.hg.1	C19orf82	7.27 ± 0.17	7.29 ± 0.04	7.27 ± 0.07	7.37 ± 0.03
TC02001938.hg.1	C1D	7.84 ± 0.07	7.90 ± 0.08	7.89 ± 0.14	7.83 ± 0.08
TC10001459.hg.1	C1DP3	5.36 ± 0.14	5.21 ± 0.12	5.41 ± 0.15	5.21 ± 0.10
TC07000075.hg.1	C1GALT1	8.19 ± 0.15	8.15 ± 0.16	8.34 ± 0.46	8.05 ± 0.06
TC0X001327.hg.1	C1GALT1C1	6.85 ± 0.14	6.72 ± 0.16	7.12 ± 0.19	6.57 ± 0.23
TC01001987.hg.1	C1orf100	5.08 ± 0.19	5.02 ± 0.21	5.00 ± 0.08	5.09 ± 0.07
TC01001988.hg.1	C1orf101	4.68 ± 0.07	4.56 ± 0.01	4.56 ± 0.06	4.60 ± 0.02
TC01001504.hg.1	C1orf105	5.20 ± 0.04	5.11 ± 0.05	5.15 ± 0.02	5.25 ± 0.12
TC01001653.hg.1	C1orf106	7.61 ± 0.18	7.49 ± 0.20	7.45 ± 0.15	7.64 ± 0.08
TC01002521.hg.1	C1orf109	7.06 ± 0.10	6.94 ± 0.02	6.96 ± 0.08	6.90 ± 0.02
TC01003456.hg.1	C1orf110	5.79 ± 0.10	5.73 ± 0.08	5.71 ± 0.02	5.77 ± 0.11
TC01003454.hg.1	C1orf111	6.99 ± 0.15	7.06 ± 0.22	7.00 ± 0.16	7.18 ± 0.11
TC01001476.hg.1	C1orf112	6.27 ± 0.06	6.11 ± 0.10	6.17 ± 0.10	5.93 ± 0.06
TC01001818.hg.1	C1orf115	7.43 ± 0.33	7.34 ± 0.16	7.38 ± 0.05	7.32 ± 0.02
TC01003771.hg.1	C1orf116	7.30 ± 0.16	7.18 ± 0.12	7.18 ± 0.01	7.34 ± 0.13
TC01000479.hg.1	C1orf122	8.48 ± 0.24	8.39 ± 0.14	8.34 ± 0.29	8.41 ± 0.02
TC01002667.hg.1	C1orf123	9.05 ± 0.04	9.00 ± 0.02	8.94 ± 0.03	8.87 ± 0.03
TC01002192.hg.1	C1orf127	7.62 ± 0.21	7.60 ± 0.20	7.54 ± 0.17	7.72 ± 0.08
TC01003965.hg.1	C1orf131	7.03 ± 0.22	6.90 ± 0.10	6.91 ± 0.17	6.83 ± 0.09
TC01001021.hg.1	C1orf137	4.73 ± 0.26	4.75 ± 0.20	4.62 ± 0.18	4.81 ± 0.05
TC01003204.hg.1	C1orf138	8.53 ± 0.21	8.42 ± 0.19	9.25 ± 0.38	8.45 ± 0.18
TC01003846.hg.1	C1orf140	5.42 ± 0.21	5.40 ± 0.11	5.48 ± 0.05	5.54 ± 0.07

TC01002755.hg.1	C1orf141	4.25 ± 0.12	4.13 ± 0.01	4.20 ± 0.04	4.13 ± 0.02
TC01001806.hg.1	C1orf143	6.07 ± 0.15	6.07 ± 0.02	6.04 ± 0.05	6.09 ± 0.02
TC01003912.hg.1	C1orf145	7.65 ± 0.18	7.65 ± 0.10	7.62 ± 0.10	7.76 ± 0.06
TC01000852.hg.1	C1orf146	4.38 ± 0.11	4.30 ± 0.05	4.33 ± 0.04	4.36 ± 0.02
TC01003762.hg.1	C1orf147	7.28 ± 0.28	7.46 ± 0.08	7.47 ± 0.14	7.60 ± 0.16
TC01003911.hg.1	C1orf148	7.68 ± 0.06	7.63 ± 0.11	7.48 ± 0.08	7.59 ± 0.06
TC01000162.hg.1	C1orf158	6.19 ± 0.24	6.05 ± 0.13	6.08 ± 0.14	6.10 ± 0.10
TC01002083.hg.1	C1orf159	8.36 ± 0.17	8.32 ± 0.18	8.26 ± 0.17	8.42 ± 0.06
TC01000978.hg.1	C1orf162	10.11 ± 0.31	9.85 ± 0.11	10.22 ± 0.21	9.70 ± 0.20
TC01002702.hg.1	C1orf168	4.41 ± 0.15	4.28 ± 0.06	4.31 ± 0.03	4.31 ± 0.06
TC01002079.hg.1	C1orf170	8.83 ± 0.22	8.75 ± 0.19	8.82 ± 0.20	8.80 ± 0.06
TC01002399.hg.1	C1orf172	8.02 ± 0.26	7.93 ± 0.22	7.94 ± 0.27	8.06 ± 0.03
TC01002787.hg.1	C1orf173	5.13 ± 0.16	4.93 ± 0.05	5.01 ± 0.08	4.99 ± 0.10
TC01002138.hg.1	C1orf174	8.06 ± 0.17	8.09 ± 0.03	7.98 ± 0.11	8.11 ± 0.03
TC01000671.hg.1	C1orf177	6.86 ± 0.14	6.80 ± 0.18	6.76 ± 0.14	6.80 ± 0.04
TC01002820.hg.1	C1orf180	6.25 ± 0.21	6.11 ± 0.09	6.15 ± 0.06	6.20 ± 0.13
TC01000620.hg.1	C1orf185	4.35 ± 0.10	4.18 ± 0.01	4.26 ± 0.08	4.22 ± 0.01
TC01003759.hg.1	C1orf186	7.38 ± 0.05	7.33 ± 0.07	7.32 ± 0.01	7.37 ± 0.03
TC01003289.hg.1	C1orf189	5.93 ± 0.18	5.81 ± 0.08	5.75 ± 0.23	5.92 ± 0.09
TC01000664.hg.1	C1orf191	7.40 ± 0.20	7.39 ± 0.08	7.33 ± 0.37	7.51 ± 0.14
TC01003425.hg.1	C1orf192	5.96 ± 0.43	5.67 ± 0.15	6.48 ± 0.46	5.75 ± 0.04
TC01002950.hg.1	C1orf194	6.20 ± 0.17	6.14 ± 0.08	6.15 ± 0.02	6.17 ± 0.01
TC01002241.hg.1	C1orf195	5.39 ± 0.07	5.45 ± 0.14	5.36 ± 0.03	5.48 ± 0.11
TC01003959.hg.1	C1orf198	7.48 ± 0.11	7.35 ± 0.13	7.53 ± 0.21	7.43 ± 0.08
TC01002184.hg.1	C1orf200	7.39 ± 0.23	7.15 ± 0.18	7.43 ± 0.05	7.09 ± 0.11
TC01001591.hg.1	C1orf21	6.87 ± 0.19	6.89 ± 0.12	6.73 ± 0.04	6.77 ± 0.10
TC01002581.hg.1	C1orf210	7.71 ± 0.24	7.61 ± 0.17	7.54 ± 0.19	7.75 ± 0.13
TC01000296.hg.1	C1orf213	6.72 ± 0.17	6.65 ± 0.20	6.65 ± 0.18	6.70 ± 0.07
TC01002499.hg.1	C1orf216	7.46 ± 0.24	7.38 ± 0.16	7.32 ± 0.24	7.44 ± 0.02
TC01006409.hg.1	C1orf220	7.13 ± 0.13	6.97 ± 0.14	7.03 ± 0.12	6.95 ± 0.05
TC01006410.hg.1	C1orf222	7.59 ± 0.23	7.60 ± 0.23	7.57 ± 0.20	7.70 ± 0.05

TC01006407.hg.1	C1orf226	7.22 ± 0.08	7.18 ± 0.26	7.15 ± 0.08	7.24 ± 0.09
TC01003811.hg.1	C1orf227	5.46 ± 0.17	5.21 ± 0.08	5.31 ± 0.23	5.22 ± 0.04
TC01000562.hg.1	C1orf228	7.86 ± 0.17	7.89 ± 0.13	7.80 ± 0.09	7.95 ± 0.11
TC01004065.hg.1	C1orf229	8.07 ± 0.38	7.85 ± 0.23	7.77 ± 0.32	7.95 ± 0.05
TC01002103.hg.1	C1orf233	8.56 ± 0.23	8.43 ± 0.15	8.29 ± 0.25	8.42 ± 0.08
TC01001604.hg.1	C1orf27	7.82 ± 0.53	8.21 ± 0.15	8.08 ± 0.45	8.16 ± 0.22
TC01003909.hg.1	C1orf35	9.11 ± 0.22	9.04 ± 0.16	8.99 ± 0.22	9.13 ± 0.03
TC01003290.hg.1	C1orf43	9.03 ± 0.24	8.68 ± 0.14	8.84 ± 0.27	8.56 ± 0.14
TC01001183.hg.1	C1orf51	6.49 ± 0.24	6.56 ± 0.11	6.41 ± 0.16	6.51 ± 0.05
TC01002827.hg.1	C1orf52	7.95 ± 0.31	8.36 ± 0.07	8.15 ± 0.42	8.45 ± 0.11
TC01001637.hg.1	C1orf53	7.64 ± 0.26	7.61 ± 0.14	7.55 ± 0.24	7.54 ± 0.05
TC01001182.hg.1	C1orf54	6.41 ± 0.16	6.28 ± 0.04	6.30 ± 0.04	6.37 ± 0.06
TC01001200.hg.1	C1orf56	8.30 ± 0.08	8.17 ± 0.09	8.26 ± 0.03	8.21 ± 0.03
TC01003338.hg.1	C1orf61	6.83 ± 0.17	6.82 ± 0.13	6.84 ± 0.11	6.89 ± 0.08
TC01002374.hg.1	C1orf63	9.72 ± 0.33	9.86 ± 0.07	9.81 ± 0.24	9.80 ± 0.19
TC01000200.hg.1	C1orf64	7.12 ± 0.28	7.09 ± 0.30	7.28 ± 0.04	7.14 ± 0.02
TC01001836.hg.1	C1orf65	7.77 ± 0.38	7.97 ± 0.29	7.95 ± 0.38	8.14 ± 0.11
TC01001235.hg.1	C1orf68	4.14 ± 0.26	4.23 ± 0.19	4.12 ± 0.14	4.32 ± 0.16
TC01003786.hg.1	C1orf74	6.95 ± 0.32	6.89 ± 0.12	6.89 ± 0.21	7.01 ± 0.04
TC01003335.hg.1	C1orf85	8.46 ± 0.14	8.22 ± 0.04	8.35 ± 0.03	8.25 ± 0.02
TC01002113.hg.1	C1orf86	7.99 ± 0.16	7.95 ± 0.13	7.92 ± 0.13	8.04 ± 0.04
TC01002718.hg.1	C1orf87	5.63 ± 0.18	5.54 ± 0.12	5.60 ± 0.14	5.63 ± 0.03
TC01000447.hg.1	C1orf94	7.11 ± 0.25	7.08 ± 0.17	7.11 ± 0.15	7.19 ± 0.09
TC01001863.hg.1	C1orf95	7.57 ± 0.23	7.72 ± 0.34	7.49 ± 0.34	7.85 ± 0.13
TC01000285.hg.1	C1QA	7.97 ± 0.21	7.81 ± 0.28	7.90 ± 0.24	7.94 ± 0.06
TC01000287.hg.1	C1QB	8.41 ± 0.31	8.05 ± 0.18	8.42 ± 0.15	8.15 ± 0.02
TC17001056.hg.1	C1QBP	10.06 ± 0.29	9.92 ± 0.27	9.49 ± 0.29	9.61 ± 0.11
TC01000286.hg.1	C1QC	7.93 ± 0.48	7.67 ± 0.25	7.90 ± 0.13	7.80 ± 0.08
TC17001590.hg.1	C1QL1	8.12 ± 0.28	8.06 ± 0.14	8.02 ± 0.24	8.17 ± 0.12
TC02002250.hg.1	C1QL2	8.01 ± 0.30	7.94 ± 0.25	7.76 ± 0.37	7.99 ± 0.04
TC10001068.hg.1	C1QL3	7.86 ± 0.22	7.77 ± 0.09	7.87 ± 0.15	7.82 ± 0.08

TC12001477.hg.1	C1QL4	7.82 ± 0.42	7.67 ± 0.24	7.74 ± 0.27	7.71 ± 0.04
TC17000906.hg.1	C1QTNF1	8.33 ± 0.31	8.32 ± 0.16	8.22 ± 0.28	8.39 ± 0.10
TC17001926.hg.1	C1QTNF1-AS1	7.77 ± 0.14	7.73 ± 0.22	7.69 ± 0.15	7.83 ± 0.15
TC05002008.hg.1	C1QTNF2	7.45 ± 0.31	7.52 ± 0.23	7.47 ± 0.40	7.52 ± 0.02
TC05003423.hg.1	C1QTNF3	6.49 ± 0.07	6.53 ± 0.04	6.35 ± 0.07	6.49 ± 0.13
TC11001744.hg.1	C1QTNF4	7.90 ± 0.28	7.85 ± 0.20	7.83 ± 0.27	7.92 ± 0.03
TC11002369.hg.1	C1QTNF5	8.03 ± 0.18	8.04 ± 0.24	7.96 ± 0.16	8.12 ± 0.09
TC22000723.hg.1	C1QTNF6	7.63 ± 0.13	7.53 ± 0.11	7.56 ± 0.09	7.62 ± 0.07
TC04000141.hg.1	C1QTNF7	5.30 ± 0.14	5.07 ± 0.08	5.18 ± 0.12	5.23 ± 0.04
TC16000744.hg.1	C1QTNF8	7.37 ± 0.16	7.35 ± 0.27	7.43 ± 0.11	7.48 ± 0.07
TC13000051.hg.1	C1QTNF9	8.08 ± 0.05	8.04 ± 0.06	8.10 ± 0.06	8.03 ± 0.01
TC13000489.hg.1	C1QTNF9-AS1	6.38 ± 0.18	6.20 ± 0.15	6.42 ± 0.13	6.39 ± 0.26
TC13000485.hg.1	C1QTNF9B	7.28 ± 0.41	7.27 ± 0.27	7.33 ± 0.31	7.30 ± 0.08
TC13000486.hg.1	C1QTNF9B	6.31 ± 0.20	6.23 ± 0.12	6.27 ± 0.14	6.38 ± 0.15
TC13000050.hg.1	C1QTNF9B-AS1	6.28 ± 0.11	6.27 ± 0.25	6.26 ± 0.12	6.44 ± 0.06
TC13000490.hg.1	C1QTNF9B-AS1	6.68 ± 0.15	6.56 ± 0.36	6.62 ± 0.15	6.81 ± 0.38
TC12001156.hg.1	C1R	7.18 ± 0.11	7.04 ± 0.07	7.10 ± 0.05	7.10 ± 0.04
TC12001157.hg.1	C1RL	8.84 ± 0.33	8.44 ± 0.14	9.18 ± 0.26	8.40 ± 0.15
TC12000106.hg.1	C1RL-AS1	7.69 ± 0.10	7.69 ± 0.08	7.78 ± 0.04	7.75 ± 0.06
TC12003224.hg.1	C1S	6.47 ± 0.12	6.44 ± 0.07	6.38 ± 0.08	6.51 ± 0.07
TC06004076.hg.1	C2	7.64 ± 0.27	7.33 ± 0.13	7.46 ± 0.10	7.35 ± 0.04
TC6_cox_hap2000244.hg.1	C2	7.70 ± 0.27	7.39 ± 0.14	7.52 ± 0.12	7.41 ± 0.06
TC6_dbb_hap3000221.hg.1	C2	7.67 ± 0.27	7.36 ± 0.14	7.49 ± 0.12	7.39 ± 0.06
TC6_mann_hap4000195.hg.1	C2	7.68 ± 0.28	7.36 ± 0.14	7.49 ± 0.12	7.39 ± 0.07
TC6_mcf_hap5000212.hg.1	C2	7.67 ± 0.27	7.36 ± 0.14	7.49 ± 0.12	7.39 ± 0.06
TC6_qbl_hap6000224.hg.1	C2	7.70 ± 0.27	7.39 ± 0.14	7.52 ± 0.12	7.41 ± 0.06
TC6_ssto_hap7000198.hg.1	C2	7.69 ± 0.28	7.36 ± 0.14	7.49 ± 0.12	7.38 ± 0.06
TC20000764.hg.1	C20orf112	9.00 ± 0.05	9.05 ± 0.07	8.93 ± 0.10	9.10 ± 0.09
TC20000029.hg.1	C20orf141	7.88 ± 0.20	7.95 ± 0.25	7.82 ± 0.21	8.06 ± 0.09
TC20000232.hg.1	C20orf144	8.48 ± 0.24	8.50 ± 0.26	8.33 ± 0.33	8.59 ± 0.05
TC20001012.hg.1	C20orf166-AS1	7.87 ± 0.23	7.80 ± 0.20	7.83 ± 0.17	7.90 ± 0.04

TC20000801.hg.1	C20orf173	6.79 ± 0.19	6.75 ± 0.20	6.75 ± 0.07	6.85 ± 0.09
TC20001040.hg.1	C20orf181	9.08 ± 0.22	9.04 ± 0.23	9.15 ± 0.23	9.25 ± 0.06
TC20000087.hg.1	C20orf187	4.20 ± 0.10	4.08 ± 0.05	4.41 ± 0.26	4.06 ± 0.15
TC20000572.hg.1	C20orf194	7.29 ± 0.04	7.20 ± 0.11	7.21 ± 0.13	7.22 ± 0.09
TC20000519.hg.1	C20orf195	7.11 ± 0.26	7.07 ± 0.18	7.06 ± 0.22	7.11 ± 0.08
TC20000060.hg.1	C20orf196	8.15 ± 0.12	8.13 ± 0.03	8.13 ± 0.06	8.20 ± 0.03
TC20000475.hg.1	C20orf197	7.07 ± 0.19	7.09 ± 0.03	7.07 ± 0.12	7.13 ± 0.05
TC20001047.hg.1	C20orf201	9.35 ± 0.30	9.22 ± 0.14	9.22 ± 0.23	9.23 ± 0.05
TC20000014.hg.1	C20orf202	7.28 ± 0.08	7.15 ± 0.05	7.21 ± 0.30	7.09 ± 0.07
TC20000765.hg.1	C20orf203	7.51 ± 0.20	7.45 ± 0.13	7.39 ± 0.15	7.57 ± 0.12
TC20001730.hg.1	C20orf24	8.76 ± 0.32	8.70 ± 0.09	9.05 ± 0.37	8.62 ± 0.16
TC20000136.hg.1	C20orf26	5.92 ± 0.16	5.86 ± 0.11	5.83 ± 0.12	5.95 ± 0.06
TC20000576.hg.1	C20orf27	8.23 ± 0.21	8.16 ± 0.11	8.08 ± 0.21	8.15 ± 0.01
TC20000866.hg.1	C20orf62	5.79 ± 0.33	5.68 ± 0.15	5.73 ± 0.11	5.79 ± 0.01
TC20000673.hg.1	C20orf78	6.57 ± 0.15	6.54 ± 0.14	6.54 ± 0.08	6.70 ± 0.10
TC20000456.hg.1	C20orf85	6.30 ± 0.23	6.15 ± 0.18	6.06 ± 0.14	6.19 ± 0.02
TC20000538.hg.1	C20orf96	6.73 ± 0.12	6.64 ± 0.05	6.75 ± 0.12	6.69 ± 0.07
TC21000034.hg.1	C21ORF116	6.11 ± 0.14	5.97 ± 0.17	6.06 ± 0.14	6.09 ± 0.12
TC21000116.hg.1	C21orf119	7.58 ± 0.19	7.49 ± 0.09	7.57 ± 0.11	7.59 ± 0.03
TC21000482.hg.1	C21orf128	7.50 ± 0.13	7.46 ± 0.24	7.52 ± 0.22	7.57 ± 0.09
TC21000277.hg.1	C21orf15	6.99 ± 0.09	7.19 ± 0.05	7.08 ± 0.05	7.26 ± 0.21
TC21000518.hg.1	C21orf2	8.46 ± 0.18	8.46 ± 0.15	8.30 ± 0.20	8.53 ± 0.03
TC21001066.hg.1	C21orf33	8.53 ± 0.16	8.37 ± 0.06	8.30 ± 0.15	8.34 ± 0.05
TC21000047.hg.1	C21orf37	4.99 ± 0.22	4.91 ± 0.04	4.84 ± 0.06	4.97 ± 0.04
TC21000121.hg.1	C21orf49	6.00 ± 0.18	6.00 ± 0.03	5.98 ± 0.10	5.99 ± 0.04
TC21000407.hg.1	C21orf54	6.78 ± 0.15	6.80 ± 0.14	6.82 ± 0.12	6.97 ± 0.08
TC21000556.hg.1	C21orf58	7.95 ± 0.15	7.90 ± 0.09	7.86 ± 0.12	7.95 ± 0.04
TC21001072.hg.1	C21orf59	7.79 ± 0.08	7.71 ± 0.02	7.71 ± 0.14	7.58 ± 0.10
TC21000402.hg.1	C21orf62	6.35 ± 0.16	6.36 ± 0.13	6.38 ± 0.17	6.49 ± 0.07
TC21000539.hg.1	C21orf67	8.13 ± 0.14	8.07 ± 0.19	8.05 ± 0.19	8.17 ± 0.05
TC21000464.hg.1	C21orf88	6.69 ± 0.10	6.59 ± 0.09	6.62 ± 0.13	6.60 ± 0.03

TC21000229.hg.1	C21orf90	7.41 ± 0.20	7.46 ± 0.25	7.41 ± 0.21	7.59 ± 0.08
TC21000302.hg.1	C21orf91	7.91 ± 0.27	8.04 ± 0.13	8.14 ± 0.23	7.87 ± 0.06
TC21000301.hg.1	C21orf91-OT1	5.38 ± 0.13	5.28 ± 0.01	5.40 ± 0.05	5.34 ± 0.10
TC22000137.hg.1	C22orf15	7.74 ± 0.17	7.69 ± 0.14	7.71 ± 0.20	7.77 ± 0.05
TC22000780.hg.1	C22orf23	6.85 ± 0.20	6.87 ± 0.21	6.79 ± 0.25	6.91 ± 0.06
TC22000674.hg.1	C22orf24	6.43 ± 0.12	6.42 ± 0.07	6.32 ± 0.21	6.45 ± 0.05
TC22000882.hg.1	C22orf26	7.98 ± 0.11	7.99 ± 0.09	7.86 ± 0.17	8.03 ± 0.08
TC22000501.hg.1	C22orf29	7.85 ± 0.20	7.82 ± 0.13	7.77 ± 0.17	7.88 ± 0.04
TC22000629.hg.1	C22orf31	4.26 ± 0.15	4.08 ± 0.02	4.16 ± 0.10	4.11 ± 0.05
TC22000898.hg.1	C22orf34	7.79 ± 0.15	7.70 ± 0.13	7.67 ± 0.05	7.78 ± 0.04
TC22000495.hg.1	C22orf39	8.69 ± 0.14	8.56 ± 0.04	8.55 ± 0.11	8.52 ± 0.05
TC22000675.hg.1	C22orf42	6.90 ± 0.14	6.87 ± 0.15	6.81 ± 0.19	7.01 ± 0.06
TC22000563.hg.1	C22orf43	6.61 ± 0.08	6.55 ± 0.03	6.67 ± 0.09	6.68 ± 0.06
TC22000341.hg.1	C22orf46	8.02 ± 0.15	7.96 ± 0.15	7.91 ± 0.05	7.93 ± 0.05
TC21000479.hg.1	C2CD2	7.49 ± 0.07	7.44 ± 0.03	7.38 ± 0.01	7.45 ± 0.03
TC11001084.hg.1	C2CD2L	8.67 ± 0.14	8.47 ± 0.09	8.54 ± 0.10	8.47 ± 0.02
TC11002076.hg.1	C2CD3	8.03 ± 0.23	7.97 ± 0.15	7.93 ± 0.28	7.81 ± 0.14
TC15000477.hg.1	C2CD4A	6.78 ± 0.22	6.88 ± 0.29	6.88 ± 0.15	7.01 ± 0.04
TC15001511.hg.1	C2CD4B	7.74 ± 0.29	7.74 ± 0.42	7.68 ± 0.21	7.76 ± 0.04
TC19000984.hg.1	C2CD4C	8.14 ± 0.29	8.19 ± 0.21	8.04 ± 0.32	8.34 ± 0.08
TC01003235.hg.1	C2CD4D	7.34 ± 0.11	7.39 ± 0.17	7.26 ± 0.09	7.39 ± 0.08
TC12001303.hg.1	C2CD5	8.22 ± 0.33	8.31 ± 0.22	8.34 ± 0.37	8.08 ± 0.08
TC02004990.hg.1	C2orf15	5.70 ± 0.13	5.62 ± 0.05	5.60 ± 0.06	5.60 ± 0.04
TC02004993.hg.1	C2orf15	5.08 ± 0.09	4.75 ± 0.10	4.87 ± 0.13	4.71 ± 0.04
TC02000178.hg.1	C2orf16	7.05 ± 0.31	6.87 ± 0.31	6.91 ± 0.14	6.89 ± 0.04
TC02000862.hg.1	C2orf27A	5.67 ± 0.25	5.73 ± 0.09	5.57 ± 0.13	5.71 ± 0.14
TC02002354.hg.1	C2orf27B	7.69 ± 0.05	7.51 ± 0.20	7.53 ± 0.05	7.70 ± 0.09
TC02000650.hg.1	C2orf40	5.88 ± 0.19	5.86 ± 0.17	5.87 ± 0.18	5.97 ± 0.08
TC02004924.hg.1	C2orf42	8.15 ± 0.14	7.96 ± 0.05	8.00 ± 0.12	7.85 ± 0.09
TC02001627.hg.1	C2orf43	6.79 ± 0.19	6.81 ± 0.15	6.61 ± 0.11	6.60 ± 0.06
TC02001643.hg.1	C2orf44	7.32 ± 0.04	7.04 ± 0.06	7.09 ± 0.06	7.01 ± 0.09

TC02005007.hg.1	C2orf47	8.00 ± 0.11	8.12 ± 0.16	7.90 ± 0.07	8.16 ± 0.05
TC02000058.hg.1	C2orf48	7.72 ± 0.25	7.69 ± 0.15	7.59 ± 0.20	7.70 ± 0.11
TC02000646.hg.1	C2orf49	7.67 ± 0.21	7.68 ± 0.25	7.65 ± 0.26	7.56 ± 0.05
TC02000068.hg.1	C2orf50	6.81 ± 0.24	6.77 ± 0.13	6.80 ± 0.15	6.84 ± 0.02
TC02001676.hg.1	C2orf53	7.34 ± 0.16	7.25 ± 0.18	7.25 ± 0.17	7.38 ± 0.05
TC02002941.hg.1	C2orf54	7.91 ± 0.23	7.94 ± 0.18	7.89 ± 0.16	8.04 ± 0.04
TC02001393.hg.1	C2orf57	7.51 ± 0.21	7.84 ± 0.20	7.63 ± 0.09	7.89 ± 0.05
TC02005061.hg.1	C2orf61	6.92 ± 0.10	6.90 ± 0.11	6.93 ± 0.07	6.94 ± 0.04
TC02001299.hg.1	C2orf62	7.56 ± 0.24	7.59 ± 0.22	7.53 ± 0.17	7.68 ± 0.08
TC02002643.hg.1	C2orf66	4.19 ± 0.05	4.04 ± 0.03	4.09 ± 0.04	4.06 ± 0.02
TC02002045.hg.1	C2orf68	8.77 ± 0.09	8.59 ± 0.07	8.67 ± 0.12	8.49 ± 0.04
TC02005006.hg.1	C2orf69	6.67 ± 0.12	6.75 ± 0.11	6.83 ± 0.30	6.50 ± 0.17
TC02000152.hg.1	C2orf70	7.35 ± 0.17	7.36 ± 0.14	7.26 ± 0.15	7.42 ± 0.10
TC02001706.hg.1	C2orf71	7.63 ± 0.16	7.74 ± 0.25	7.66 ± 0.21	7.82 ± 0.05
TC02001385.hg.1	C2orf72	7.83 ± 0.10	7.78 ± 0.15	7.77 ± 0.18	7.81 ± 0.05
TC02000314.hg.1	C2orf73	5.59 ± 0.17	5.52 ± 0.11	5.55 ± 0.08	5.66 ± 0.07
TC02004937.hg.1	C2orf74	7.03 ± 0.09	7.08 ± 0.09	7.00 ± 0.06	6.95 ± 0.02
TC02002253.hg.1	C2orf76	6.68 ± 0.04	6.53 ± 0.06	6.69 ± 0.07	6.48 ± 0.01
TC02000446.hg.1	C2orf78	5.46 ± 0.17	5.27 ± 0.11	5.26 ± 0.13	5.40 ± 0.09
TC02002725.hg.1	C2orf80	5.58 ± 0.07	5.49 ± 0.03	5.51 ± 0.03	5.49 ± 0.02
TC02001995.hg.1	C2orf81	8.08 ± 0.20	7.93 ± 0.09	7.95 ± 0.12	7.99 ± 0.04
TC02005012.hg.1	C2orf82	7.71 ± 0.15	7.66 ± 0.11	7.63 ± 0.13	7.75 ± 0.04
TC02002836.hg.1	C2orf83	5.33 ± 0.14	5.20 ± 0.05	5.28 ± 0.04	5.25 ± 0.08
TC02001115.hg.1	C2orf88	7.47 ± 0.06	7.17 ± 0.09	7.40 ± 0.06	7.24 ± 0.10
TC02001775.hg.1	C2orf91	5.64 ± 0.21	5.50 ± 0.07	5.56 ± 0.22	5.46 ± 0.11
TC19001103.hg.1	C3	7.46 ± 0.15	7.39 ± 0.17	7.49 ± 0.17	7.51 ± 0.08
TC12001171.hg.1	C3AR1	8.20 ± 0.47	6.82 ± 0.43	8.43 ± 0.40	6.98 ± 0.60
TC03000385.hg.1	C3orf14	6.58 ± 0.19	6.40 ± 0.07	6.56 ± 0.07	6.37 ± 0.04
TC03001655.hg.1	C3orf17	7.19 ± 0.29	7.25 ± 0.17	7.12 ± 0.23	7.27 ± 0.05
TC03001434.hg.1	C3orf18	7.31 ± 0.22	7.27 ± 0.09	7.27 ± 0.17	7.30 ± 0.07
TC03000089.hg.1	C3orf20	6.53 ± 0.09	6.49 ± 0.06	6.51 ± 0.02	6.52 ± 0.09

TC03001742.hg.1	C3orf22	7.50 ± 0.07	7.50 ± 0.16	7.53 ± 0.04	7.53 ± 0.07
TC03001756.hg.1	C3orf27	7.24 ± 0.13	7.37 ± 0.15	7.22 ± 0.15	7.50 ± 0.07
TC03003321.hg.1	C3orf30	4.19 ± 0.08	4.00 ± 0.07	4.06 ± 0.04	4.04 ± 0.08
TC03001928.hg.1	C3orf33	6.89 ± 0.13	6.93 ± 0.03	6.82 ± 0.03	7.01 ± 0.05
TC03000180.hg.1	C3orf35	7.08 ± 0.15	7.22 ± 0.04	7.06 ± 0.18	7.34 ± 0.09
TC03001799.hg.1	C3orf36	6.02 ± 0.15	6.04 ± 0.24	6.09 ± 0.24	6.13 ± 0.02
TC03000690.hg.1	C3orf37	8.89 ± 0.16	8.73 ± 0.15	8.55 ± 0.25	8.65 ± 0.10
TC03000466.hg.1	C3orf38	8.10 ± 0.21	8.30 ± 0.24	8.10 ± 0.31	8.18 ± 0.03
TC03002162.hg.1	C3orf43	6.28 ± 0.22	6.20 ± 0.09	6.24 ± 0.15	6.29 ± 0.10
TC03000392.hg.1	C3orf49	5.41 ± 0.12	5.25 ± 0.08	5.33 ± 0.05	5.33 ± 0.05
TC03000569.hg.1	C3orf52	6.21 ± 0.06	6.16 ± 0.11	6.18 ± 0.04	6.27 ± 0.03
TC03000863.hg.1	C3orf55	4.43 ± 0.10	4.34 ± 0.02	4.38 ± 0.04	4.39 ± 0.13
TC03000662.hg.1	C3orf56	5.63 ± 0.15	5.35 ± 0.13	5.36 ± 0.16	5.42 ± 0.07
TC03000781.hg.1	C3orf58	7.51 ± 0.23	7.55 ± 0.07	7.54 ± 0.08	7.42 ± 0.10
TC03003384.hg.1	C3orf62	7.76 ± 0.06	7.55 ± 0.26	8.06 ± 0.49	7.40 ± 0.25
TC03001013.hg.1	C3orf65	5.26 ± 0.09	5.21 ± 0.09	5.24 ± 0.13	5.21 ± 0.01
TC03001503.hg.1	C3orf67	5.30 ± 0.15	5.20 ± 0.08	5.25 ± 0.07	5.27 ± 0.06
TC03002075.hg.1	C3orf70	6.47 ± 0.13	6.39 ± 0.10	6.41 ± 0.17	6.51 ± 0.07
TC03000747.hg.1	C3orf72	6.73 ± 0.15	6.70 ± 0.10	6.61 ± 0.16	6.73 ± 0.01
TC03000841.hg.1	C3orf79	4.33 ± 0.12	4.21 ± 0.11	4.42 ± 0.09	4.23 ± 0.06
TC03000872.hg.1	C3orf80	4.80 ± 0.14	4.68 ± 0.07	4.78 ± 0.18	4.71 ± 0.04
TC03001172.hg.1	C3orf83	5.52 ± 0.18	5.36 ± 0.11	5.44 ± 0.12	5.40 ± 0.05
TC03001173.hg.1	C3orf83	6.47 ± 0.19	6.51 ± 0.24	6.35 ± 0.31	6.43 ± 0.07
TC19000167.hg.1	C3P1	6.71 ± 0.17	6.67 ± 0.17	6.69 ± 0.15	6.76 ± 0.06
TC06001554.hg.1	C4A-AS1	6.76 ± 0.36	6.70 ± 0.15	6.76 ± 0.27	6.73 ± 0.32
TC06004079.hg.1	C4B	7.55 ± 0.17	7.50 ± 0.14	7.48 ± 0.13	7.57 ± 0.06
TC06004080.hg.1	C4B	7.50 ± 0.18	7.46 ± 0.15	7.45 ± 0.13	7.53 ± 0.06
TC6_cox_hap2000227.hg.1	C4B	7.49 ± 0.19	7.47 ± 0.15	7.44 ± 0.13	7.55 ± 0.06
TC6_dbb_hap3000078.hg.1	C4B	7.51 ± 0.19	7.49 ± 0.15	7.45 ± 0.13	7.57 ± 0.06
TC6_dbb_hap3000224.hg.1	C4B	7.53 ± 0.18	7.50 ± 0.16	7.46 ± 0.13	7.57 ± 0.06
TC6_mcf_hap5000068.hg.1	C4B	7.01 ± 0.63	7.06 ± 0.38	6.81 ± 0.42	7.15 ± 0.38

TC6_mcf_hap5000197.hg.1	C4B	7.51 ± 0.19	7.48 ± 0.16	7.44 ± 0.13	7.56 ± 0.07
TC6_qbl_hap6000208.hg.1	C4B	7.52 ± 0.19	7.49 ± 0.16	7.46 ± 0.13	7.57 ± 0.06
TC6_ssto_hap7000071.hg.1	C4B	7.49 ± 0.19	7.48 ± 0.16	7.44 ± 0.13	7.55 ± 0.07
TC6_ssto_hap7000201.hg.1	C4B	7.50 ± 0.19	7.48 ± 0.16	7.45 ± 0.13	7.56 ± 0.07
TC06001556.hg.1	C4B-AS1	6.76 ± 0.36	6.70 ± 0.15	6.76 ± 0.27	6.73 ± 0.32
TC01001733.hg.1	C4BPA	6.82 ± 0.62	6.71 ± 0.24	7.38 ± 0.28	6.53 ± 0.25
TC01001732.hg.1	C4BPB	6.22 ± 0.15	6.20 ± 0.07	6.14 ± 0.11	6.28 ± 0.06
TC04000515.hg.1	C4orf17	5.14 ± 0.16	5.03 ± 0.07	5.09 ± 0.07	5.09 ± 0.14
TC04000212.hg.1	C4orf19	5.57 ± 0.17	5.44 ± 0.11	5.56 ± 0.01	5.52 ± 0.04
TC04001478.hg.1	C4orf21	5.90 ± 0.25	5.90 ± 0.16	5.81 ± 0.17	5.77 ± 0.06
TC04002937.hg.1	C4orf22	4.95 ± 0.07	4.79 ± 0.02	4.85 ± 0.07	4.82 ± 0.03
TC04000420.hg.1	C4orf26	5.77 ± 0.11	5.64 ± 0.04	5.67 ± 0.02	5.74 ± 0.03
TC04001726.hg.1	C4orf27	6.78 ± 0.12	6.69 ± 0.13	6.66 ± 0.09	6.58 ± 0.02
TC04000647.hg.1	C4orf29	7.69 ± 0.22	7.62 ± 0.19	7.80 ± 0.24	7.48 ± 0.13
TC04001499.hg.1	C4orf3	8.24 ± 0.05	8.14 ± 0.11	8.49 ± 0.24	8.09 ± 0.16
TC04000577.hg.1	C4orf32	8.11 ± 0.14	7.89 ± 0.06	8.11 ± 0.06	7.89 ± 0.14
TC04000653.hg.1	C4orf33	6.88 ± 0.19	6.94 ± 0.17	7.03 ± 0.20	6.88 ± 0.09
TC04002956.hg.1	C4orf36	6.87 ± 0.16	6.84 ± 0.17	6.76 ± 0.17	6.88 ± 0.04
TC04000379.hg.1	C4orf40	4.79 ± 0.09	4.69 ± 0.03	4.74 ± 0.01	4.70 ± 0.07
TC04001689.hg.1	C4orf45	5.44 ± 0.15	5.31 ± 0.10	5.35 ± 0.08	5.39 ± 0.06
TC04001685.hg.1	C4orf46	7.95 ± 0.07	7.99 ± 0.04	7.82 ± 0.10	7.95 ± 0.13
TC04000906.hg.1	C4orf47	4.40 ± 0.13	4.27 ± 0.05	4.37 ± 0.06	4.31 ± 0.05
TC04000032.hg.1	C4orf48	8.61 ± 0.33	8.50 ± 0.18	8.48 ± 0.31	8.49 ± 0.06
TC04000998.hg.1	C4orf50	6.99 ± 0.17	7.01 ± 0.19	6.95 ± 0.24	7.13 ± 0.07
TC04000715.hg.1	C4orf51	5.79 ± 0.04	5.69 ± 0.13	5.67 ± 0.11	5.77 ± 0.07
TC04000064.hg.1	C4orf6	6.21 ± 0.14	6.15 ± 0.12	6.21 ± 0.13	6.35 ± 0.08
TC09001542.hg.1	C5	5.60 ± 0.02	5.43 ± 0.09	5.69 ± 0.31	5.37 ± 0.05
TC19000676.hg.1	C5AR1	9.70 ± 0.35	9.72 ± 0.23	10.20 ± 0.23	9.78 ± 0.44
TC19000677.hg.1	C5AR2	8.62 ± 0.17	8.27 ± 0.12	8.73 ± 0.05	8.21 ± 0.25
TC05001784.hg.1	C5orf15	7.99 ± 0.09	7.99 ± 0.08	8.07 ± 0.17	7.87 ± 0.14
TC05000115.hg.1	C5orf17	6.97 ± 0.15	6.95 ± 0.02	6.99 ± 0.09	7.09 ± 0.05

TC05003428.hg.1	C5orf20	6.73 ± 0.24	6.49 ± 0.07	6.52 ± 0.02	6.50 ± 0.08
TC05000134.hg.1	C5orf22	7.54 ± 0.24	7.38 ± 0.29	7.55 ± 0.30	7.18 ± 0.23
TC05003401.hg.1	C5orf24	8.05 ± 0.17	8.12 ± 0.05	8.03 ± 0.07	8.12 ± 0.05
TC05000462.hg.1	C5orf27	7.19 ± 0.20	7.19 ± 0.00	7.24 ± 0.04	7.27 ± 0.15
TC05001318.hg.1	C5orf28	7.22 ± 0.30	7.48 ± 0.11	7.33 ± 0.14	7.40 ± 0.12
TC05000499.hg.1	C5orf30	7.41 ± 0.19	7.28 ± 0.16	7.27 ± 0.19	7.28 ± 0.01
TC05001319.hg.1	C5orf34	4.84 ± 0.12	4.78 ± 0.04	4.74 ± 0.07	4.72 ± 0.04
TC05000026.hg.1	C5orf38	7.57 ± 0.25	7.57 ± 0.28	7.45 ± 0.30	7.71 ± 0.08
TC05001274.hg.1	C5orf42	6.18 ± 0.45	6.36 ± 0.19	6.11 ± 0.24	6.11 ± 0.09
TC05003410.hg.1	C5orf45	7.79 ± 0.12	7.69 ± 0.04	7.61 ± 0.02	7.70 ± 0.04
TC05001912.hg.1	C5orf46	5.63 ± 0.10	5.62 ± 0.12	5.53 ± 0.15	5.66 ± 0.09
TC05000968.hg.1	C5orf47	4.40 ± 0.11	4.28 ± 0.09	4.36 ± 0.11	4.33 ± 0.06
TC05000603.hg.1	C5orf48	4.12 ± 0.11	4.07 ± 0.03	4.01 ± 0.07	4.03 ± 0.04
TC05001148.hg.1	C5orf49	6.74 ± 0.24	6.69 ± 0.15	6.74 ± 0.13	6.68 ± 0.04
TC05000945.hg.1	C5orf50	7.60 ± 0.27	7.45 ± 0.11	7.41 ± 0.21	7.55 ± 0.05
TC05000189.hg.1	C5orf51	7.56 ± 0.11	7.41 ± 0.22	7.56 ± 0.34	7.19 ± 0.08
TC05000871.hg.1	C5orf52	6.32 ± 0.17	6.28 ± 0.25	6.10 ± 0.15	6.34 ± 0.09
TC05002009.hg.1	C5orf54	5.21 ± 0.19	4.98 ± 0.09	5.08 ± 0.01	4.93 ± 0.02
TC05001094.hg.1	C5orf55	6.99 ± 0.26	6.68 ± 0.07	6.80 ± 0.04	6.69 ± 0.03
TC05000636.hg.1	C5orf56	8.63 ± 0.11	8.51 ± 0.06	8.63 ± 0.14	8.50 ± 0.01
TC05000933.hg.1	C5orf58	5.50 ± 0.04	5.49 ± 0.05	5.64 ± 0.20	5.46 ± 0.03
TC05002136.hg.1	C5orf60	7.13 ± 0.14	7.14 ± 0.13	7.10 ± 0.13	7.25 ± 0.06
TC05001747.hg.1	C5orf63	6.87 ± 0.25	6.77 ± 0.05	6.69 ± 0.09	6.78 ± 0.09
TC05000264.hg.1	C5orf64	6.01 ± 0.08	5.94 ± 0.01	5.95 ± 0.06	6.06 ± 0.18
TC05001304.hg.1	C6	5.17 ± 0.14	5.06 ± 0.07	5.13 ± 0.03	5.11 ± 0.07
TC06001653.hg.1	C6orf1	8.21 ± 0.14	8.16 ± 0.15	8.14 ± 0.11	8.24 ± 0.00
TC06001565.hg.1	C6orf10	4.61 ± 0.13	4.47 ± 0.01	4.53 ± 0.03	4.51 ± 0.05
TC6_apd_hap1000099.hg.1	C6orf10	4.58 ± 0.18	4.51 ± 0.06	4.48 ± 0.06	4.54 ± 0.04
TC6_cox_hap2000191.hg.1	C6orf10	4.50 ± 0.17	4.36 ± 0.05	4.40 ± 0.06	4.42 ± 0.03
TC6_dbb_hap3000177.hg.1	C6orf10	4.49 ± 0.17	4.35 ± 0.04	4.39 ± 0.06	4.40 ± 0.04
TC6_mann_hap4000156.hg.1	C6orf10	4.54 ± 0.16	4.41 ± 0.04	4.43 ± 0.05	4.45 ± 0.05

TC6_mcf_hap5000166.hg.1	C6orf10	4.58 ± 0.18	4.51 ± 0.06	4.48 ± 0.06	4.54 ± 0.04
TC6_qbl_hap6000180.hg.1	C6orf10	4.45 ± 0.16	4.32 ± 0.04	4.35 ± 0.06	4.35 ± 0.03
TC6_ssto_hap7000160.hg.1	C6orf10	4.54 ± 0.16	4.44 ± 0.06	4.43 ± 0.07	4.48 ± 0.05
TC06000302.hg.1	C6orf100	7.09 ± 0.27	6.94 ± 0.13	6.95 ± 0.13	6.95 ± 0.07
TC6_cox_hap2000004.hg.1	C6orf100	6.49 ± 0.25	6.42 ± 0.17	6.34 ± 0.22	6.41 ± 0.07
TC6_mann_hap4000001.hg.1	C6orf100	7.09 ± 0.27	6.94 ± 0.13	6.95 ± 0.13	6.95 ± 0.07
TC6_mcf_hap5000002.hg.1	C6orf100	6.66 ± 0.30	6.57 ± 0.12	6.50 ± 0.27	6.59 ± 0.07
TC6_qbl_hap6000002.hg.1	C6orf100	7.09 ± 0.27	6.94 ± 0.13	6.95 ± 0.13	6.95 ± 0.07
TC6_ssto_hap7000002.hg.1	C6orf100	6.66 ± 0.30	6.57 ± 0.12	6.50 ± 0.27	6.59 ± 0.07
TC06001659.hg.1	C6orf106	9.57 ± 0.16	9.34 ± 0.16	9.23 ± 0.20	9.19 ± 0.07
TC06002296.hg.1	C6orf118	5.71 ± 0.18	5.64 ± 0.09	5.65 ± 0.10	5.65 ± 0.01
TC06001193.hg.1	C6orf120	8.51 ± 0.05	8.56 ± 0.02	8.67 ± 0.13	8.60 ± 0.05
TC06002318.hg.1	C6orf123	6.91 ± 0.21	6.74 ± 0.13	6.77 ± 0.10	6.78 ± 0.10
TC06001729.hg.1	C6orf132	6.93 ± 0.26	6.89 ± 0.31	6.73 ± 0.31	6.96 ± 0.05
TC06000345.hg.1	C6orf136	7.90 ± 0.21	7.75 ± 0.04	7.67 ± 0.07	7.75 ± 0.07
TC6_cox_hap2000043.hg.1	C6orf136	7.94 ± 0.22	7.79 ± 0.04	7.68 ± 0.10	7.77 ± 0.08
TC6_dbb_hap3000036.hg.1	C6orf136	7.94 ± 0.22	7.79 ± 0.04	7.68 ± 0.10	7.77 ± 0.08
TC6_mann_hap4000038.hg.1	C6orf136	7.92 ± 0.22	7.77 ± 0.04	7.65 ± 0.10	7.73 ± 0.08
TC6_mcf_hap5000030.hg.1	C6orf136	7.94 ± 0.22	7.79 ± 0.04	7.68 ± 0.10	7.77 ± 0.08
TC6_qbl_hap6000036.hg.1	C6orf136	7.96 ± 0.22	7.81 ± 0.04	7.70 ± 0.11	7.79 ± 0.08
TC6_ssto_hap7000036.hg.1	C6orf136	7.94 ± 0.22	7.79 ± 0.04	7.68 ± 0.10	7.77 ± 0.08
TC06000642.hg.1	C6orf141	6.60 ± 0.18	6.54 ± 0.06	6.53 ± 0.11	6.62 ± 0.06
TC06001517.hg.1	C6orf15	7.81 ± 0.27	7.87 ± 0.22	7.79 ± 0.28	7.98 ± 0.07
TC6_cox_hap2000149.hg.1	C6orf15	7.81 ± 0.27	7.90 ± 0.29	7.65 ± 0.26	7.95 ± 0.04
TC6_dbb_hap3000138.hg.1	C6orf15	7.81 ± 0.27	7.87 ± 0.22	7.79 ± 0.28	7.98 ± 0.07
TC6_mann_hap4000126.hg.1	C6orf15	7.81 ± 0.27	7.87 ± 0.22	7.79 ± 0.28	7.98 ± 0.07
TC6_mcf_hap5000126.hg.1	C6orf15	7.81 ± 0.27	7.87 ± 0.22	7.79 ± 0.28	7.98 ± 0.07
TC6_qbl_hap6000139.hg.1	C6orf15	7.88 ± 0.29	7.93 ± 0.32	7.71 ± 0.27	8.02 ± 0.04
TC06000786.hg.1	C6orf163	6.56 ± 0.28	6.59 ± 0.13	6.64 ± 0.17	6.49 ± 0.09
TC06004094.hg.1	C6orf164	6.80 ± 0.25	6.77 ± 0.10	6.79 ± 0.11	6.92 ± 0.12
TC06004097.hg.1	C6orf165	4.72 ± 0.11	4.59 ± 0.06	4.66 ± 0.07	4.62 ± 0.04

TC06002065.hg.1	C6orf170	5.49 ± 0.39	5.66 ± 0.17	5.42 ± 0.20	5.56 ± 0.08
TC06001215.hg.1	C6orf195	6.51 ± 0.15	6.60 ± 0.12	6.51 ± 0.07	6.69 ± 0.07
TC06000037.hg.1	C6orf201	5.58 ± 0.18	5.47 ± 0.07	5.46 ± 0.05	5.55 ± 0.06
TC06000854.hg.1	C6orf203	6.83 ± 0.11	6.66 ± 0.11	6.66 ± 0.04	6.59 ± 0.05
TC06001102.hg.1	C6orf211	8.11 ± 0.04	7.91 ± 0.22	8.53 ± 0.33	7.74 ± 0.24
TC06001672.hg.1	C6orf222	6.68 ± 0.17	6.63 ± 0.16	6.57 ± 0.20	6.75 ± 0.08
TC06000610.hg.1	C6orf223	7.25 ± 0.22	7.16 ± 0.21	7.24 ± 0.13	7.27 ± 0.03
TC06001738.hg.1	C6orf226	8.35 ± 0.19	8.17 ± 0.10	8.12 ± 0.15	8.20 ± 0.04
TC06000382.hg.1	C6orf25	9.68 ± 0.07	9.02 ± 0.14	9.96 ± 0.20	9.21 ± 0.24
TC6_cox_hap2000076.hg.1	C6orf25	9.33 ± 0.09	8.79 ± 0.17	9.55 ± 0.24	8.99 ± 0.11
TC6_dbb_hap3000067.hg.1	C6orf25	9.33 ± 0.09	8.79 ± 0.17	9.55 ± 0.24	8.99 ± 0.11
TC6_mann_hap4000067.hg.1	C6orf25	9.13 ± 0.11	8.64 ± 0.16	9.26 ± 0.28	8.85 ± 0.09
TC6_mcf_hap5000062.hg.1	C6orf25	9.33 ± 0.09	8.79 ± 0.17	9.55 ± 0.24	8.99 ± 0.11
TC6_qbl_hap6000067.hg.1	C6orf25	9.28 ± 0.10	8.76 ± 0.16	9.49 ± 0.25	8.97 ± 0.11
TC6_ssto_hap7000063.hg.1	C6orf25	9.33 ± 0.09	8.79 ± 0.17	9.55 ± 0.24	8.99 ± 0.11
TC06001533.hg.1	C6orf47	8.08 ± 0.16	7.77 ± 0.11	7.80 ± 0.24	7.76 ± 0.01
TC6_cox_hap2000162.hg.1	C6orf47	7.22 ± 0.29	6.90 ± 0.24	7.03 ± 0.28	6.85 ± 0.05
TC6_dbb_hap3000151.hg.1	C6orf47	7.19 ± 0.38	6.87 ± 0.18	6.92 ± 0.40	6.81 ± 0.08
TC6_mann_hap4000139.hg.1	C6orf47	7.38 ± 0.25	7.09 ± 0.10	7.15 ± 0.37	6.97 ± 0.05
TC6_mcf_hap5000139.hg.1	C6orf47	7.17 ± 0.40	6.85 ± 0.21	6.93 ± 0.41	6.81 ± 0.09
TC6_qbl_hap6000154.hg.1	C6orf47	7.17 ± 0.40	6.85 ± 0.21	6.93 ± 0.41	6.81 ± 0.09
TC6_ssto_hap7000134.hg.1	C6orf47	7.17 ± 0.40	6.85 ± 0.21	6.93 ± 0.41	6.81 ± 0.09
TC06000378.hg.1	C6orf47-AS1	7.19 ± 0.19	6.98 ± 0.16	7.04 ± 0.21	7.17 ± 0.09
TC6_apd_hap1000041.hg.1	C6orf48	8.17 ± 0.10	8.09 ± 0.08	7.83 ± 0.17	8.13 ± 0.09
TC6_cox_hap2000080.hg.1	C6orf48	8.17 ± 0.10	8.09 ± 0.08	7.83 ± 0.17	8.13 ± 0.09
TC6_dbb_hap3000071.hg.1	C6orf48	8.33 ± 0.11	8.25 ± 0.08	7.98 ± 0.17	8.29 ± 0.09
TC6_qbl_hap6000071.hg.1	C6orf48	8.17 ± 0.10	8.09 ± 0.08	7.83 ± 0.17	8.13 ± 0.09
TC06000034.hg.1	C6ORF50	4.45 ± 0.12	4.26 ± 0.18	4.36 ± 0.20	4.40 ± 0.06
TC06001265.hg.1	C6orf52	6.76 ± 0.13	6.71 ± 0.10	6.67 ± 0.06	6.75 ± 0.04
TC06000717.hg.1	C6orf57	6.27 ± 0.15	6.06 ± 0.02	6.05 ± 0.04	6.04 ± 0.08
TC06000970.hg.1	C6orf58	4.53 ± 0.15	4.47 ± 0.10	4.48 ± 0.05	4.50 ± 0.03

TC06001328.hg.1	C6orf62	10.11 ± 0.19	10.20 ± 0.14	10.17 ± 0.30	10.10 ± 0.17
TC06001900.hg.1	C6orf7	5.91 ± 0.08	5.92 ± 0.11	5.99 ± 0.11	6.02 ± 0.14
TC06001195.hg.1	C6orf70	7.45 ± 0.26	7.42 ± 0.13	7.30 ± 0.14	7.37 ± 0.08
TC06000536.hg.1	C6orf89	8.47 ± 0.18	8.30 ± 0.09	8.36 ± 0.21	8.21 ± 0.03
TC06001139.hg.1	C6orf99	7.13 ± 0.18	7.10 ± 0.11	7.07 ± 0.07	7.19 ± 0.14
TC05000187.hg.1	C7	5.49 ± 0.18	5.43 ± 0.11	5.40 ± 0.06	5.45 ± 0.03
TC07000255.hg.1	C7orf10	5.54 ± 0.16	5.48 ± 0.12	5.48 ± 0.06	5.61 ± 0.08
TC07002070.hg.1	C7orf13	7.33 ± 0.25	7.27 ± 0.29	7.04 ± 0.19	7.33 ± 0.08
TC07003354.hg.1	C7orf25	7.44 ± 0.05	7.33 ± 0.06	7.52 ± 0.11	7.31 ± 0.05
TC07000068.hg.1	C7orf26	8.98 ± 0.12	8.87 ± 0.05	8.71 ± 0.08	8.83 ± 0.06
TC07001208.hg.1	C7orf31	8.03 ± 0.12	8.03 ± 0.08	7.87 ± 0.19	8.00 ± 0.11
TC07000968.hg.1	C7orf33	6.53 ± 0.04	6.50 ± 0.08	6.63 ± 0.21	6.50 ± 0.05
TC07000925.hg.1	C7orf34	7.29 ± 0.26	7.25 ± 0.13	7.26 ± 0.16	7.26 ± 0.08
TC07000192.hg.1	C7orf41	8.45 ± 0.25	8.20 ± 0.06	8.47 ± 0.19	8.34 ± 0.12
TC07001660.hg.1	C7orf43	9.14 ± 0.12	8.97 ± 0.04	9.04 ± 0.17	8.94 ± 0.02
TC07003381.hg.1	C7orf49	6.05 ± 0.26	6.18 ± 0.16	6.27 ± 0.42	6.29 ± 0.09
TC07003382.hg.1	C7orf49	8.55 ± 0.22	8.28 ± 0.03	8.52 ± 0.05	8.25 ± 0.06
TC07001077.hg.1	C7orf50	8.19 ± 0.22	8.05 ± 0.11	8.01 ± 0.16	8.05 ± 0.04
TC07000872.hg.1	C7orf55-LUC7L2	8.56 ± 0.20	8.70 ± 0.05	8.48 ± 0.11	8.71 ± 0.05
TC07000306.hg.1	C7orf57	6.51 ± 0.20	6.49 ± 0.20	6.42 ± 0.16	6.63 ± 0.11
TC07001765.hg.1	C7orf60	7.64 ± 0.04	7.72 ± 0.08	7.82 ± 0.19	7.63 ± 0.05
TC07003369.hg.1	C7orf61	6.98 ± 0.13	7.06 ± 0.18	7.10 ± 0.15	7.11 ± 0.08
TC07001585.hg.1	C7orf62	4.80 ± 0.39	4.70 ± 0.13	4.72 ± 0.30	4.81 ± 0.17
TC07000539.hg.1	C7orf63	4.13 ± 0.09	3.98 ± 0.03	4.06 ± 0.05	4.00 ± 0.04
TC07000303.hg.1	C7orf65	7.89 ± 0.36	7.89 ± 0.25	7.94 ± 0.20	8.14 ± 0.15
TC07001753.hg.1	C7orf66	5.09 ± 0.16	5.05 ± 0.04	5.11 ± 0.12	5.14 ± 0.06
TC07000305.hg.1	C7orf69	4.95 ± 0.10	5.04 ± 0.12	5.00 ± 0.05	5.15 ± 0.12
TC07000164.hg.1	C7orf71	5.50 ± 0.13	5.47 ± 0.13	5.48 ± 0.05	5.60 ± 0.12
TC07000312.hg.1	C7orf72	4.64 ± 0.17	4.45 ± 0.14	4.52 ± 0.09	4.45 ± 0.01
TC07000844.hg.1	C7orf73	8.75 ± 0.13	8.78 ± 0.04	8.88 ± 0.25	8.73 ± 0.03
TC01000686.hg.1	C8A	5.81 ± 0.15	5.77 ± 0.12	5.78 ± 0.18	5.83 ± 0.10

TC01002704.hg.1	C8B	5.78 ± 0.14	5.79 ± 0.11	5.73 ± 0.10	5.89 ± 0.07
TC09000828.hg.1	C8G	8.05 ± 0.17	8.06 ± 0.23	7.99 ± 0.17	8.17 ± 0.02
TC08002593.hg.1	C8orf12	6.24 ± 0.22	6.34 ± 0.13	6.23 ± 0.16	6.45 ± 0.11
TC08000787.hg.1	C8orf17	5.74 ± 0.21	5.75 ± 0.28	5.70 ± 0.21	5.96 ± 0.08
TC08000353.hg.1	C8orf22	4.26 ± 0.04	4.20 ± 0.02	4.19 ± 0.02	4.23 ± 0.10
TC08000815.hg.1	C8orf31	7.34 ± 0.13	7.27 ± 0.16	7.26 ± 0.15	7.32 ± 0.05
TC08000867.hg.1	C8orf33	8.10 ± 0.13	8.14 ± 0.14	7.90 ± 0.16	8.17 ± 0.02
TC08000453.hg.1	C8orf34	5.30 ± 0.11	5.19 ± 0.14	5.24 ± 0.08	5.27 ± 0.05
TC08001444.hg.1	C8orf37	6.65 ± 0.10	6.64 ± 0.12	6.64 ± 0.16	6.57 ± 0.06
TC08000310.hg.1	C8orf4	3.88 ± 0.28	3.61 ± 0.03	3.71 ± 0.05	3.74 ± 0.16
TC08002606.hg.1	C8orf44	6.40 ± 0.08	6.20 ± 0.04	6.36 ± 0.11	6.17 ± 0.10
TC08002605.hg.1	C8orf46	5.87 ± 0.06	5.85 ± 0.08	5.83 ± 0.08	5.87 ± 0.05
TC08000601.hg.1	C8orf47	6.98 ± 0.34	6.76 ± 0.24	6.80 ± 0.12	6.84 ± 0.07
TC08000114.hg.1	C8orf48	6.86 ± 0.26	6.57 ± 0.21	6.85 ± 0.21	6.73 ± 0.09
TC08000090.hg.1	C8orf49	5.61 ± 0.31	5.54 ± 0.09	5.47 ± 0.11	5.70 ± 0.17
TC08001504.hg.1	C8orf56	7.44 ± 0.15	7.26 ± 0.24	7.51 ± 0.21	7.51 ± 0.02
TC08002597.hg.1	C8orf58	8.09 ± 0.18	8.05 ± 0.17	8.00 ± 0.17	8.13 ± 0.05
TC08001378.hg.1	C8orf59	6.84 ± 0.09	6.72 ± 0.23	6.67 ± 0.12	6.68 ± 0.14
TC08001443.hg.1	C8orf69	7.08 ± 0.13	7.06 ± 0.20	7.04 ± 0.08	7.23 ± 0.12
TC08000078.hg.1	C8orf74	7.01 ± 0.13	6.87 ± 0.10	6.94 ± 0.10	6.90 ± 0.04
TC08002618.hg.1	C8orf76	7.59 ± 0.11	7.42 ± 0.11	7.45 ± 0.11	7.37 ± 0.05
TC08001757.hg.1	C8orf82	8.14 ± 0.22	7.96 ± 0.09	7.93 ± 0.21	7.92 ± 0.03
TC08001149.hg.1	C8orf86	6.72 ± 0.08	6.70 ± 0.09	6.71 ± 0.04	6.68 ± 0.09
TC08001424.hg.1	C8orf87	6.22 ± 0.09	6.08 ± 0.06	6.07 ± 0.24	6.15 ± 0.10
TC05003424.hg.1	C9	4.79 ± 0.06	4.72 ± 0.02	4.73 ± 0.02	4.78 ± 0.02
TC09000713.hg.1	C9orf106	8.15 ± 0.26	7.80 ± 0.22	8.12 ± 0.13	7.91 ± 0.11
TC09001639.hg.1	C9orf114	8.52 ± 0.06	8.44 ± 0.11	8.35 ± 0.15	8.45 ± 0.01
TC09001715.hg.1	C9orf116	7.48 ± 0.18	7.43 ± 0.11	7.44 ± 0.10	7.47 ± 0.06
TC09000669.hg.1	C9orf117	7.76 ± 0.16	7.66 ± 0.15	7.66 ± 0.20	7.75 ± 0.04
TC09000903.hg.1	C9orf123	7.11 ± 0.10	7.02 ± 0.02	6.90 ± 0.06	7.03 ± 0.09
TC09001346.hg.1	C9orf129	8.16 ± 0.02	8.20 ± 0.04	8.13 ± 0.03	8.11 ± 0.07

TC09000179.hg.1	C9orf131	6.83 ± 0.21	6.96 ± 0.04	6.88 ± 0.16	7.03 ± 0.16
TC09000322.hg.1	C9orf135	5.30 ± 0.23	5.26 ± 0.14	5.32 ± 0.08	5.35 ± 0.07
TC09001195.hg.1	C9orf135-AS1	5.74 ± 0.08	5.67 ± 0.09	5.66 ± 0.18	5.67 ± 0.08
TC09000833.hg.1	C9orf139	7.94 ± 0.23	7.94 ± 0.20	7.90 ± 0.28	8.03 ± 0.05
TC09000830.hg.1	C9orf141	8.95 ± 0.31	8.85 ± 0.22	8.84 ± 0.33	8.98 ± 0.11
TC09000832.hg.1	C9orf142	8.75 ± 0.17	8.68 ± 0.10	8.57 ± 0.16	8.66 ± 0.05
TC09001491.hg.1	C9orf147	6.62 ± 0.18	6.56 ± 0.16	6.54 ± 0.05	6.63 ± 0.05
TC09001472.hg.1	C9orf152	7.15 ± 0.15	7.14 ± 0.16	7.13 ± 0.09	7.27 ± 0.07
TC09001291.hg.1	C9orf153	5.07 ± 0.01	4.98 ± 0.08	5.02 ± 0.04	5.00 ± 0.03
TC09001400.hg.1	C9orf156	7.74 ± 0.17	7.89 ± 0.19	7.54 ± 0.13	7.91 ± 0.12
TC09000678.hg.1	C9orf16	8.05 ± 0.23	7.90 ± 0.13	7.98 ± 0.17	7.91 ± 0.05
TC09000813.hg.1	C9orf163	7.87 ± 0.23	7.90 ± 0.25	7.80 ± 0.19	7.94 ± 0.03
TC09000843.hg.1	C9orf169	7.89 ± 0.27	7.89 ± 0.29	7.81 ± 0.22	8.05 ± 0.06
TC09000396.hg.1	C9orf170	5.93 ± 0.14	5.72 ± 0.11	5.91 ± 0.21	5.81 ± 0.04
TC09000756.hg.1	C9orf171	7.33 ± 0.20	7.27 ± 0.18	7.26 ± 0.18	7.35 ± 0.06
TC09000824.hg.1	C9orf172	8.00 ± 0.17	7.82 ± 0.17	7.83 ± 0.24	7.85 ± 0.08
TC09000845.hg.1	C9orf173	8.03 ± 0.27	7.99 ± 0.31	7.85 ± 0.25	8.06 ± 0.12
TC09001034.hg.1	C9orf24	7.59 ± 0.20	7.47 ± 0.17	7.49 ± 0.21	7.55 ± 0.04
TC09001777.hg.1	C9orf37	8.59 ± 0.29	8.44 ± 0.10	8.41 ± 0.21	8.41 ± 0.03
TC09000898.hg.1	C9orf38	5.21 ± 0.28	5.17 ± 0.04	5.27 ± 0.26	5.27 ± 0.05
TC09001217.hg.1	C9orf40	7.47 ± 0.20	7.27 ± 0.16	7.35 ± 0.41	7.23 ± 0.06
TC09001218.hg.1	C9orf41	8.16 ± 0.39	8.28 ± 0.21	7.94 ± 0.26	7.97 ± 0.22
TC09000575.hg.1	C9orf43	5.95 ± 0.13	5.86 ± 0.02	5.85 ± 0.08	5.86 ± 0.05
TC09002900.hg.1	C9orf47	8.34 ± 0.19	8.28 ± 0.15	8.29 ± 0.15	8.31 ± 0.01
TC09001649.hg.1	C9orf50	8.20 ± 0.30	8.17 ± 0.30	8.09 ± 0.29	8.38 ± 0.12
TC09000106.hg.1	C9orf53	5.23 ± 0.28	5.30 ± 0.09	5.39 ± 0.04	5.40 ± 0.26
TC09001207.hg.1	C9orf57	5.08 ± 0.15	5.01 ± 0.10	5.01 ± 0.04	5.11 ± 0.09
TC09000792.hg.1	C9orf62	8.34 ± 0.15	8.24 ± 0.12	8.21 ± 0.18	8.38 ± 0.06
TC09001283.hg.1	C9orf64	7.87 ± 0.11	7.46 ± 0.06	7.69 ± 0.11	7.29 ± 0.14
TC09000865.hg.1	C9orf66	4.81 ± 0.32	4.60 ± 0.10	5.07 ± 0.07	4.79 ± 0.10
TC09001723.hg.1	C9orf69	8.51 ± 0.27	8.23 ± 0.15	8.34 ± 0.32	8.21 ± 0.03

TC09000990.hg.1	C9orf72	8.65 ± 0.40	8.84 ± 0.25	9.41 ± 0.48	8.85 ± 0.17
TC09001655.hg.1	C9orf78	8.90 ± 0.15	9.06 ± 0.08	8.75 ± 0.05	9.01 ± 0.03
TC09001486.hg.1	C9orf84	4.21 ± 0.04	4.09 ± 0.02	4.47 ± 0.16	4.04 ± 0.04
TC09000329.hg.1	C9orf85	7.19 ± 0.21	7.22 ± 0.08	7.20 ± 0.08	7.13 ± 0.07
TC09000442.hg.1	C9orf89	8.48 ± 0.12	8.46 ± 0.07	8.48 ± 0.05	8.59 ± 0.03
TC09000762.hg.1	C9orf9	7.52 ± 0.19	7.40 ± 0.09	7.42 ± 0.13	7.41 ± 0.06
TC09000585.hg.1	C9orf91	9.05 ± 0.21	8.91 ± 0.07	8.77 ± 0.20	8.82 ± 0.03
TC09000927.hg.1	C9orf92	7.06 ± 0.24	7.07 ± 0.07	7.02 ± 0.11	7.09 ± 0.11
TC09000774.hg.1	C9orf96	7.17 ± 0.15	7.19 ± 0.16	7.12 ± 0.14	7.25 ± 0.03
TC08001381.hg.1	CA1	5.33 ± 0.25	5.13 ± 0.02	5.26 ± 0.05	5.21 ± 0.02
TC17001705.hg.1	CA10	6.29 ± 0.21	6.20 ± 0.12	6.19 ± 0.15	6.22 ± 0.05
TC19001689.hg.1	CA11	7.81 ± 0.17	7.80 ± 0.09	7.62 ± 0.07	7.87 ± 0.03
TC15001541.hg.1	CA12	6.97 ± 0.15	6.92 ± 0.11	6.93 ± 0.11	6.99 ± 0.08
TC08002580.hg.1	CA13	6.45 ± 0.14	6.33 ± 0.10	6.46 ± 0.07	6.27 ± 0.02
TC01001180.hg.1	CA14	6.33 ± 0.18	6.29 ± 0.13	6.23 ± 0.06	6.37 ± 0.09
TC08000531.hg.1	CA2	8.28 ± 0.20	7.79 ± 0.08	7.96 ± 0.19	7.85 ± 0.30
TC08002581.hg.1	CA3	6.81 ± 0.15	6.71 ± 0.15	6.69 ± 0.13	6.79 ± 0.03
TC17000736.hg.1	CA4	7.95 ± 0.28	7.84 ± 0.11	8.16 ± 0.09	7.94 ± 0.10
TC16001336.hg.1	CA5A	6.62 ± 0.19	6.51 ± 0.14	6.57 ± 0.04	6.64 ± 0.02
TC0X002313.hg.1	CA5B	8.11 ± 0.25	7.99 ± 0.19	7.76 ± 0.20	7.86 ± 0.10
TC0X002312.hg.1	CA5BP1	9.26 ± 0.17	9.14 ± 0.01	9.14 ± 0.08	9.06 ± 0.14
TC01000110.hg.1	CA6	6.97 ± 0.08	6.96 ± 0.11	7.01 ± 0.06	7.01 ± 0.05
TC16000522.hg.1	CA7	7.58 ± 0.18	7.59 ± 0.23	7.54 ± 0.14	7.67 ± 0.05
TC08001253.hg.1	CA8	6.03 ± 0.06	5.94 ± 0.12	6.01 ± 0.01	6.04 ± 0.04
TC09000191.hg.1	CA9	7.57 ± 0.19	7.54 ± 0.21	7.50 ± 0.20	7.67 ± 0.13
TC09000982.hg.1	CAAP1	7.78 ± 0.24	7.80 ± 0.11	7.60 ± 0.20	7.70 ± 0.19
TC02001379.hg.1	CAB39	9.61 ± 0.22	9.50 ± 0.24	10.06 ± 0.28	9.37 ± 0.24
TC13000660.hg.1	CAB39L	5.62 ± 0.03	5.49 ± 0.05	5.57 ± 0.08	5.47 ± 0.02
TC22000144.hg.1	CABIN1	8.46 ± 0.03	8.44 ± 0.05	8.32 ± 0.08	8.42 ± 0.02
TC18000099.hg.1	CABLES1	5.97 ± 0.19	5.86 ± 0.13	5.79 ± 0.10	5.87 ± 0.03
TC20001009.hg.1	CABLES2	8.07 ± 0.13	7.98 ± 0.08	7.88 ± 0.14	7.96 ± 0.13

TC12000948.hg.1	CABP1	7.14 ± 0.24	7.10 ± 0.12	7.07 ± 0.20	7.14 ± 0.04
TC11001988.hg.1	CABP2	8.52 ± 0.18	8.45 ± 0.13	8.47 ± 0.18	8.50 ± 0.01
TC11000692.hg.1	CABP4	7.86 ± 0.13	7.91 ± 0.15	7.85 ± 0.11	7.95 ± 0.06
TC19001673.hg.1	CABP5	7.70 ± 0.27	7.34 ± 0.15	7.42 ± 0.18	7.42 ± 0.11
TC22000200.hg.1	CABP7	7.72 ± 0.13	7.68 ± 0.15	7.67 ± 0.25	7.71 ± 0.05
TC04000383.hg.1	CABS1	5.60 ± 0.13	5.32 ± 0.09	5.48 ± 0.08	5.40 ± 0.07
TC18000106.hg.1	CABYR	5.99 ± 0.11	5.89 ± 0.10	5.90 ± 0.06	5.86 ± 0.03
TC09000776.hg.1	CACFD1	8.26 ± 0.18	8.31 ± 0.20	8.19 ± 0.23	8.43 ± 0.08
TC01000725.hg.1	CACHD1	6.98 ± 0.17	7.06 ± 0.11	6.83 ± 0.05	7.12 ± 0.08
TC19001220.hg.1	CACNA1A	6.94 ± 0.20	6.85 ± 0.18	6.86 ± 0.17	6.91 ± 0.03
TC09000855.hg.1	CACNA1B	7.29 ± 0.14	7.26 ± 0.16	7.24 ± 0.15	7.35 ± 0.05
TC12000025.hg.1	CACNA1C	6.86 ± 0.18	6.85 ± 0.17	6.81 ± 0.16	6.95 ± 0.08
TC12001098.hg.1	CACNA1C-AS1	8.29 ± 0.17	8.32 ± 0.16	8.21 ± 0.07	8.38 ± 0.10
TC12001097.hg.1	CACNA1C-AS2	7.75 ± 0.30	7.70 ± 0.10	7.65 ± 0.06	7.80 ± 0.10
TC12001096.hg.1	CACNA1C-AS3	6.23 ± 0.17	6.20 ± 0.28	6.13 ± 0.10	6.31 ± 0.07
TC12001095.hg.1	CACNA1C-AS4	4.91 ± 0.17	4.75 ± 0.14	4.79 ± 0.07	5.03 ± 0.30
TC12000023.hg.1	CACNA1C-IT1	4.88 ± 0.12	4.82 ± 0.19	4.66 ± 0.09	4.88 ± 0.04
TC12000024.hg.1	CACNA1C-IT2	5.30 ± 0.13	5.16 ± 0.08	5.06 ± 0.06	5.15 ± 0.19
TC12000026.hg.1	CACNA1C-IT3	6.61 ± 0.16	6.56 ± 0.14	6.66 ± 0.08	6.72 ± 0.09
TC03000351.hg.1	CACNA1D	6.36 ± 0.15	6.30 ± 0.08	6.38 ± 0.09	6.39 ± 0.07
TC01001572.hg.1	CACNA1E	7.19 ± 0.44	6.83 ± 0.20	7.69 ± 0.31	6.86 ± 0.06
TC0X001030.hg.1	CACNA1F	7.33 ± 0.18	7.35 ± 0.13	7.29 ± 0.13	7.41 ± 0.05
TC17000671.hg.1	CACNA1G	7.70 ± 0.23	7.67 ± 0.19	7.65 ± 0.17	7.77 ± 0.06
TC17001693.hg.1	CACNA1G-AS1	7.58 ± 0.22	7.43 ± 0.10	7.43 ± 0.27	7.46 ± 0.02
TC16000036.hg.1	CACNA1H	8.03 ± 0.18	7.99 ± 0.17	7.95 ± 0.17	8.04 ± 0.03
TC22000318.hg.1	CACNA1I	7.76 ± 0.18	7.72 ± 0.18	7.63 ± 0.17	7.80 ± 0.05
TC01003684.hg.1	CACNA1S	7.03 ± 0.20	6.98 ± 0.18	7.01 ± 0.16	7.12 ± 0.04
TC07001563.hg.1	CACNA2D1	4.54 ± 0.13	4.42 ± 0.06	4.45 ± 0.05	4.45 ± 0.02
TC03001433.hg.1	CACNA2D2	7.64 ± 0.11	7.62 ± 0.12	7.52 ± 0.12	7.67 ± 0.04
TC03000353.hg.1	CACNA2D3	6.13 ± 0.12	6.06 ± 0.06	6.02 ± 0.08	6.08 ± 0.02
TC03001477.hg.1	CACNA2D3-AS1	6.52 ± 0.22	6.50 ± 0.14	6.53 ± 0.20	6.64 ± 0.04

TC12001092.hg.1	CACNA2D4	7.46 ± 0.11	7.43 ± 0.12	7.41 ± 0.12	7.49 ± 0.03
TC17001446.hg.1	CACNB1	7.76 ± 0.04	7.66 ± 0.11	7.66 ± 0.06	7.71 ± 0.04
TC10000138.hg.1	CACNB2	6.28 ± 0.15	6.21 ± 0.10	6.21 ± 0.02	6.27 ± 0.01
TC12000357.hg.1	CACNB3	7.33 ± 0.23	7.35 ± 0.21	7.29 ± 0.19	7.38 ± 0.04
TC02002430.hg.1	CACNB4	6.21 ± 0.16	6.40 ± 0.09	6.14 ± 0.08	6.46 ± 0.14
TC17000791.hg.1	CACNG1	7.45 ± 0.23	7.48 ± 0.21	7.44 ± 0.21	7.62 ± 0.09
TC22000712.hg.1	CACNG2	6.19 ± 0.14	6.28 ± 0.22	6.20 ± 0.18	6.28 ± 0.04
TC22000713.hg.1	CACNG2	7.13 ± 0.19	6.93 ± 0.01	6.87 ± 0.13	6.91 ± 0.07
TC16000261.hg.1	CACNG3	6.64 ± 0.10	6.60 ± 0.17	6.67 ± 0.25	6.68 ± 0.08
TC17000790.hg.1	CACNG4	7.49 ± 0.16	7.40 ± 0.16	7.39 ± 0.17	7.44 ± 0.01
TC17000788.hg.1	CACNG5	7.84 ± 0.21	7.93 ± 0.24	7.89 ± 0.14	8.03 ± 0.09
TC19000870.hg.1	CACNG6	7.99 ± 0.21	8.07 ± 0.12	8.01 ± 0.19	8.06 ± 0.05
TC19000868.hg.1	CACNG7	7.37 ± 0.24	7.39 ± 0.19	7.35 ± 0.27	7.40 ± 0.04
TC19001047.hg.1	CACTIN	8.73 ± 0.22	8.59 ± 0.09	8.53 ± 0.23	8.62 ± 0.04
TC19000074.hg.1	CACTIN-AS1	8.50 ± 0.25	8.48 ± 0.16	8.41 ± 0.21	8.50 ± 0.04
TC10001694.hg.1	CACUL1	8.25 ± 0.22	8.41 ± 0.19	8.55 ± 0.28	8.43 ± 0.07
TC01001526.hg.1	CACYBP	7.93 ± 0.16	8.13 ± 0.16	7.93 ± 0.14	7.91 ± 0.20
TC02000168.hg.1	CAD	7.94 ± 0.10	7.82 ± 0.05	7.72 ± 0.11	7.79 ± 0.03
TC11002316.hg.1	CADM1	6.71 ± 0.14	6.61 ± 0.15	6.58 ± 0.14	6.64 ± 0.10
TC03000455.hg.1	CADM2	5.66 ± 0.06	5.63 ± 0.10	5.72 ± 0.09	5.70 ± 0.05
TC03001581.hg.1	CADM2-AS1	4.76 ± 0.16	4.63 ± 0.04	4.75 ± 0.16	4.52 ± 0.04
TC03001580.hg.1	CADM2-AS2	4.59 ± 0.22	4.28 ± 0.12	4.36 ± 0.13	4.38 ± 0.04
TC01006336.hg.1	CADM3	7.67 ± 0.22	7.46 ± 0.12	7.54 ± 0.15	7.55 ± 0.04
TC19001592.hg.1	CADM4	8.27 ± 0.18	8.23 ± 0.16	8.25 ± 0.08	8.25 ± 0.03
TC03001516.hg.1	CADPS	5.58 ± 0.13	5.50 ± 0.07	5.48 ± 0.08	5.60 ± 0.07
TC07001813.hg.1	CADPS2	5.10 ± 0.18	4.96 ± 0.10	5.01 ± 0.08	5.01 ± 0.04
TC06004108.hg.1	CAGE1	3.87 ± 0.15	3.74 ± 0.02	3.79 ± 0.07	3.79 ± 0.01
TC06002292.hg.1	CAHM	8.15 ± 0.26	8.12 ± 0.06	7.96 ± 0.17	8.02 ± 0.07
TC08001401.hg.1	CALB1	5.07 ± 0.06	4.95 ± 0.04	5.05 ± 0.01	5.07 ± 0.07
TC16000589.hg.1	CALB2	6.51 ± 0.17	6.41 ± 0.13	6.46 ± 0.15	6.44 ± 0.04
TC11001445.hg.1	CALCA	7.14 ± 0.14	7.07 ± 0.12	7.02 ± 0.14	7.14 ± 0.05

TC11000215.hg.1	CALCB	7.23 ± 0.14	7.07 ± 0.07	7.07 ± 0.11	7.13 ± 0.07
TC12001554.hg.1	CALCOCO1	9.15 ± 0.07	9.05 ± 0.07	9.19 ± 0.09	9.06 ± 0.03
TC17000631.hg.1	CALCOCO2	8.65 ± 0.05	8.70 ± 0.11	8.75 ± 0.28	8.66 ± 0.11
TC07001608.hg.1	CALCR	5.71 ± 0.14	5.67 ± 0.10	5.64 ± 0.05	5.75 ± 0.10
TC02002605.hg.1	CALCRL	5.08 ± 0.17	5.05 ± 0.07	5.07 ± 0.14	5.06 ± 0.05
TC07000838.hg.1	CALD1	6.22 ± 0.12	5.97 ± 0.06	6.29 ± 0.08	6.03 ± 0.07
TC10001632.hg.1	CALHM1	6.07 ± 0.17	5.83 ± 0.22	6.01 ± 0.14	5.91 ± 0.07
TC10001631.hg.1	CALHM2	8.88 ± 0.16	8.60 ± 0.15	8.77 ± 0.19	8.66 ± 0.04
TC10001633.hg.1	CALHM3	7.52 ± 0.13	7.50 ± 0.18	7.51 ± 0.09	7.62 ± 0.13
TC14000554.hg.1	CALM1	10.04 ± 0.20	10.30 ± 0.05	10.02 ± 0.18	10.19 ± 0.08
TC02005060.hg.1	CALM2	9.15 ± 0.28	9.33 ± 0.16	9.38 ± 0.23	9.33 ± 0.10
TC19000665.hg.1	CALM3	10.86 ± 0.07	10.73 ± 0.05	10.71 ± 0.20	10.61 ± 0.07
TC10000048.hg.1	CALML3	7.76 ± 0.20	7.77 ± 0.15	7.78 ± 0.14	7.87 ± 0.08
TC10000995.hg.1	CALML3-AS1	5.93 ± 0.14	5.78 ± 0.13	5.85 ± 0.05	5.99 ± 0.13
TC15001591.hg.1	CALML4	7.72 ± 0.14	7.47 ± 0.06	7.73 ± 0.01	7.45 ± 0.10
TC10000994.hg.1	CALML5	8.37 ± 0.26	8.22 ± 0.18	8.25 ± 0.28	8.23 ± 0.05
TC01000052.hg.1	CALML6	7.72 ± 0.22	7.71 ± 0.15	7.67 ± 0.17	7.79 ± 0.04
TC07001483.hg.1	CALN1	6.87 ± 0.24	6.85 ± 0.19	6.82 ± 0.17	6.91 ± 0.08
TC19000232.hg.1	CALR	11.53 ± 0.25	11.26 ± 0.22	11.04 ± 0.13	10.98 ± 0.06
TC19002686.hg.1	CALR3	5.94 ± 0.29	5.85 ± 0.06	5.84 ± 0.18	5.87 ± 0.02
TC07000782.hg.1	CALU	8.36 ± 0.14	8.22 ± 0.14	8.12 ± 0.08	8.04 ± 0.16
TC10001787.hg.1	CALY	8.76 ± 0.21	8.77 ± 0.18	8.70 ± 0.25	8.83 ± 0.03
TC03001149.hg.1	CAMK1	8.03 ± 0.11	7.89 ± 0.05	8.00 ± 0.14	7.94 ± 0.08
TC10000093.hg.1	CAMK1D	9.40 ± 0.25	9.68 ± 0.18	9.72 ± 0.20	9.77 ± 0.06
TC01001748.hg.1	CAMK1G	6.77 ± 0.18	6.79 ± 0.23	6.73 ± 0.12	6.94 ± 0.10
TC05001934.hg.1	CAMK2A	7.45 ± 0.16	7.43 ± 0.15	7.38 ± 0.17	7.53 ± 0.05
TC07001338.hg.1	CAMK2B	7.78 ± 0.18	7.77 ± 0.21	7.73 ± 0.19	7.85 ± 0.05
TC04001486.hg.1	CAMK2D	8.30 ± 0.30	8.62 ± 0.23	7.88 ± 0.29	8.39 ± 0.16
TC10001420.hg.1	CAMK2G	8.85 ± 0.03	8.74 ± 0.04	8.93 ± 0.07	8.71 ± 0.07
TC01002314.hg.1	CAMK2N1	7.51 ± 0.22	7.44 ± 0.08	7.36 ± 0.22	7.42 ± 0.03
TC03002068.hg.1	CAMK2N2	7.77 ± 0.31	7.84 ± 0.23	7.80 ± 0.26	7.89 ± 0.03

TC05000521.hg.1	CAMK4	8.22 ± 0.63	8.88 ± 0.16	7.99 ± 0.45	8.82 ± 0.12
TC17001013.hg.1	CAMKK1	7.57 ± 0.23	7.40 ± 0.10	7.66 ± 0.15	7.53 ± 0.14
TC12002061.hg.1	CAMKK2	9.07 ± 0.19	8.67 ± 0.04	9.25 ± 0.10	8.73 ± 0.19
TC02000276.hg.1	CAMKMT	6.67 ± 0.25	6.74 ± 0.07	6.60 ± 0.07	6.65 ± 0.05
TC03001420.hg.1	CAMKV	7.51 ± 0.18	7.55 ± 0.20	7.49 ± 0.15	7.63 ± 0.07
TC05000667.hg.1	CAMLG	8.20 ± 0.02	8.17 ± 0.18	8.04 ± 0.29	8.07 ± 0.12
TC03000276.hg.1	CAMP	8.74 ± 0.57	8.05 ± 0.08	8.51 ± 0.37	8.03 ± 0.02
TC09001720.hg.1	CAMSAP1	7.71 ± 0.01	7.67 ± 0.03	7.60 ± 0.03	7.69 ± 0.09
TC01001651.hg.1	CAMSAP2	6.05 ± 0.25	6.07 ± 0.12	5.82 ± 0.10	6.07 ± 0.04
TC19000130.hg.1	CAMSAP3	7.75 ± 0.25	7.75 ± 0.23	7.70 ± 0.20	7.82 ± 0.03
TC01000096.hg.1	CAMTA1	6.98 ± 0.06	6.95 ± 0.04	6.91 ± 0.05	6.99 ± 0.01
TC01000098.hg.1	CAMTA1-IT1	8.33 ± 0.29	8.46 ± 0.20	8.34 ± 0.17	8.60 ± 0.03
TC17001039.hg.1	CAMTA2	9.13 ± 0.14	8.92 ± 0.06	9.20 ± 0.12	8.90 ± 0.10
TC12000595.hg.1	CAND1	8.18 ± 0.30	8.26 ± 0.22	7.94 ± 0.28	8.04 ± 0.06
TC03000074.hg.1	CAND2	7.81 ± 0.23	7.79 ± 0.21	7.72 ± 0.18	7.91 ± 0.06
TC17001925.hg.1	CANT1	9.45 ± 0.21	9.23 ± 0.02	9.40 ± 0.16	9.19 ± 0.10
TC05001050.hg.1	CANX	9.81 ± 0.08	9.86 ± 0.10	9.63 ± 0.07	9.68 ± 0.16
TC01000504.hg.1	CAP1	10.85 ± 0.13	10.72 ± 0.07	11.11 ± 0.17	10.59 ± 0.14
TC06000114.hg.1	CAP2	6.49 ± 0.26	6.42 ± 0.14	6.39 ± 0.13	6.46 ± 0.05
TC02002039.hg.1	CAPG	8.47 ± 0.20	8.14 ± 0.08	8.44 ± 0.18	8.17 ± 0.05
TC11000620.hg.1	CAPN1	9.82 ± 0.20	9.52 ± 0.01	9.82 ± 0.19	9.42 ± 0.13
TC02005015.hg.1	CAPN10	8.17 ± 0.10	8.16 ± 0.10	8.05 ± 0.14	8.18 ± 0.02
TC06000613.hg.1	CAPN11	6.83 ± 0.20	6.78 ± 0.16	6.79 ± 0.19	6.90 ± 0.05
TC19001502.hg.1	CAPN12	8.03 ± 0.20	8.07 ± 0.19	7.97 ± 0.18	8.11 ± 0.04
TC02001712.hg.1	CAPN13	6.95 ± 0.14	6.91 ± 0.13	7.01 ± 0.08	7.02 ± 0.06
TC02001715.hg.1	CAPN14	6.41 ± 0.14	6.40 ± 0.15	6.36 ± 0.11	6.47 ± 0.05
TC01001838.hg.1	CAPN2	9.60 ± 0.17	9.53 ± 0.06	9.50 ± 0.10	9.31 ± 0.09
TC15002793.hg.1	CAPN3	7.93 ± 0.07	7.79 ± 0.09	8.02 ± 0.18	7.71 ± 0.08
TC11000827.hg.1	CAPN5	8.02 ± 0.15	7.98 ± 0.12	7.91 ± 0.14	8.06 ± 0.05
TC0X001279.hg.1	CAPN6	5.74 ± 0.24	5.65 ± 0.12	5.67 ± 0.10	5.71 ± 0.02
TC03000092.hg.1	CAPN7	7.94 ± 0.52	8.36 ± 0.26	7.97 ± 0.51	8.26 ± 0.14

TC01003873.hg.1	CAPN8	6.18 ± 0.15	6.15 ± 0.14	6.11 ± 0.10	6.22 ± 0.05
TC01001910.hg.1	CAPN9	6.53 ± 0.19	6.53 ± 0.18	6.48 ± 0.18	6.62 ± 0.05
TC19000496.hg.1	CAPNS1	10.74 ± 0.24	10.36 ± 0.06	10.64 ± 0.13	10.27 ± 0.27
TC16000456.hg.1	CAPNS2	5.55 ± 0.14	5.60 ± 0.33	6.16 ± 0.46	5.91 ± 0.16
TC11000324.hg.1	CAPRIN1	10.04 ± 0.12	10.11 ± 0.06	9.99 ± 0.10	9.95 ± 0.16
TC12001355.hg.1	CAPRIN2	7.46 ± 0.46	7.64 ± 0.20	7.42 ± 0.38	7.48 ± 0.13
TC19000110.hg.1	CAPS	8.35 ± 0.17	8.28 ± 0.14	8.23 ± 0.13	8.39 ± 0.09
TC12001745.hg.1	CAPS2	4.18 ± 0.02	4.12 ± 0.02	4.14 ± 0.08	4.14 ± 0.01
TC05001265.hg.1	CAPSL	5.05 ± 0.11	4.82 ± 0.04	4.96 ± 0.05	4.91 ± 0.05
TC01000990.hg.1	CAPZA1	10.55 ± 0.21	10.71 ± 0.13	10.94 ± 0.26	10.63 ± 0.19
TC07000723.hg.1	CAPZA2	8.95 ± 0.44	9.16 ± 0.16	9.60 ± 0.33	9.17 ± 0.23
TC12000218.hg.1	CAPZA3	5.07 ± 0.15	5.11 ± 0.06	5.11 ± 0.22	5.05 ± 0.13
TC01002300.hg.1	CAPZB	9.91 ± 0.10	9.80 ± 0.04	9.94 ± 0.12	9.78 ± 0.07
TC22001492.hg.1	CARD10	8.14 ± 0.20	8.10 ± 0.18	8.11 ± 0.18	8.22 ± 0.05
TC07001102.hg.1	CARD11	8.91 ± 0.04	8.89 ± 0.07	8.53 ± 0.04	8.78 ± 0.09
TC17000916.hg.1	CARD14	7.75 ± 0.20	7.70 ± 0.20	7.69 ± 0.22	7.79 ± 0.04
TC11003506.hg.1	CARD16	9.60 ± 0.43	9.36 ± 0.39	10.49 ± 0.58	9.02 ± 0.36
TC11003507.hg.1	CARD17	8.15 ± 0.87	7.16 ± 0.38	9.02 ± 0.70	6.98 ± 0.09
TC11002250.hg.1	CARD18	4.96 ± 0.15	4.91 ± 0.09	5.06 ± 0.13	5.02 ± 0.07
TC05000186.hg.1	CARD6	7.39 ± 0.38	6.96 ± 0.19	8.16 ± 0.16	6.79 ± 0.29
TC19001677.hg.1	CARD8	8.86 ± 0.29	8.74 ± 0.32	9.18 ± 0.41	8.42 ± 0.21
TC09002924.hg.1	CARD9	8.37 ± 0.22	8.25 ± 0.22	8.30 ± 0.22	8.36 ± 0.04
TC02001194.hg.1	CARF	7.07 ± 0.29	7.06 ± 0.14	6.89 ± 0.23	6.95 ± 0.02
TC16000851.hg.1	CARHSP1	8.62 ± 0.17	8.52 ± 0.13	8.40 ± 0.24	8.40 ± 0.04
TC13000394.hg.1	CARKD	8.06 ± 0.07	8.00 ± 0.08	7.89 ± 0.02	8.01 ± 0.07
TC19000188.hg.1	CARM1	8.96 ± 0.20	8.75 ± 0.14	8.66 ± 0.05	8.59 ± 0.06
TC09000875.hg.1	CARM1P1	5.57 ± 0.11	5.56 ± 0.05	5.61 ± 0.11	5.60 ± 0.03
TC11000690.hg.1	CARNS1	8.96 ± 0.18	9.02 ± 0.18	8.87 ± 0.21	9.07 ± 0.06
TC11001289.hg.1	CARS	8.36 ± 0.13	8.15 ± 0.01	8.38 ± 0.16	8.08 ± 0.06
TC13000876.hg.1	CARS2	8.43 ± 0.07	8.34 ± 0.03	8.34 ± 0.15	8.24 ± 0.04
TC11000072.hg.1	CARS-AS1	6.42 ± 0.09	6.20 ± 0.02	6.21 ± 0.15	6.32 ± 0.18

TC05000335.hg.1	CARTPT	6.99 ± 0.10	7.03 ± 0.06	6.95 ± 0.10	7.05 ± 0.13
TC12001313.hg.1	CASC1	4.63 ± 0.13	4.52 ± 0.01	4.59 ± 0.03	4.58 ± 0.03
TC10000856.hg.1	CASC2	5.35 ± 0.16	5.14 ± 0.06	5.17 ± 0.02	5.17 ± 0.02
TC17000490.hg.1	CASC3	10.33 ± 0.14	10.21 ± 0.05	10.44 ± 0.18	10.22 ± 0.14
TC15000338.hg.1	CASC4	9.41 ± 0.26	9.57 ± 0.14	9.81 ± 0.24	9.53 ± 0.17
TC15000290.hg.1	CASC5	6.15 ± 0.15	6.12 ± 0.34	6.15 ± 0.26	5.79 ± 0.10
TC07000561.hg.1	CASD1	7.76 ± 0.55	8.10 ± 0.23	7.62 ± 0.47	7.93 ± 0.09
TC0X000969.hg.1	CASK	7.37 ± 0.22	7.55 ± 0.21	7.13 ± 0.30	7.41 ± 0.14
TC0X000201.hg.1	CASK-AS1	5.37 ± 0.21	5.09 ± 0.14	5.16 ± 0.11	5.14 ± 0.12
TC16000777.hg.1	CASKIN1	8.18 ± 0.23	8.15 ± 0.19	8.13 ± 0.26	8.23 ± 0.05
TC17001879.hg.1	CASKIN2	7.97 ± 0.20	7.96 ± 0.18	7.93 ± 0.20	8.02 ± 0.04
TC11003505.hg.1	CASP1	9.16 ± 0.37	8.70 ± 0.35	9.69 ± 0.48	8.41 ± 0.24
TC02001176.hg.1	CASP10	8.15 ± 0.20	7.68 ± 0.16	8.13 ± 0.32	7.52 ± 0.14
TC11002244.hg.1	CASP12	5.09 ± 0.17	4.98 ± 0.05	5.01 ± 0.05	5.00 ± 0.10
TC19000266.hg.1	CASP14	6.30 ± 0.27	6.18 ± 0.20	6.10 ± 0.15	6.19 ± 0.11
TC16000103.hg.1	CASP16	8.12 ± 0.25	8.07 ± 0.22	8.03 ± 0.25	8.19 ± 0.08
TC07000932.hg.1	CASP2	9.65 ± 0.15	9.38 ± 0.13	9.61 ± 0.10	9.23 ± 0.12
TC04001807.hg.1	CASP3	9.01 ± 0.33	8.69 ± 0.14	9.25 ± 0.37	8.67 ± 0.16
TC11002246.hg.1	CASP4	9.75 ± 0.40	9.34 ± 0.30	10.18 ± 0.38	9.02 ± 0.29
TC11002247.hg.1	CASP5	7.51 ± 0.55	6.78 ± 0.45	8.55 ± 0.51	6.62 ± 0.20
TC04001464.hg.1	CASP6	7.68 ± 0.02	7.43 ± 0.07	7.54 ± 0.06	7.30 ± 0.11
TC10000825.hg.1	CASP7	7.82 ± 0.01	7.77 ± 0.04	7.76 ± 0.04	7.70 ± 0.03
TC02001177.hg.1	CASP8	9.35 ± 0.17	9.29 ± 0.12	9.41 ± 0.25	9.36 ± 0.03
TC06000800.hg.1	CASP8AP2	8.21 ± 0.43	8.36 ± 0.18	8.22 ± 0.28	8.12 ± 0.18
TC01002245.hg.1	CASP9	9.05 ± 0.14	8.96 ± 0.05	8.97 ± 0.10	8.92 ± 0.01
TC01001363.hg.1	CASQ1	6.49 ± 0.17	6.48 ± 0.08	6.49 ± 0.08	6.57 ± 0.07
TC01003020.hg.1	CASQ2	5.57 ± 0.14	5.55 ± 0.15	5.54 ± 0.11	5.64 ± 0.07
TC03000627.hg.1	CASR	7.10 ± 0.15	7.02 ± 0.11	7.02 ± 0.14	7.13 ± 0.08
TC20000429.hg.1	CASS4	8.72 ± 0.04	8.97 ± 0.17	8.50 ± 0.22	8.99 ± 0.11
TC05000469.hg.1	CAST	9.20 ± 0.16	9.28 ± 0.16	9.44 ± 0.19	9.19 ± 0.15
TC01002191.hg.1	CASZ1	8.10 ± 0.05	8.08 ± 0.08	8.12 ± 0.04	8.21 ± 0.06

TC11000327.hg.1	CAT	9.89 ± 0.36	10.07 ± 0.29	10.40 ± 0.32	10.09 ± 0.18
TC11001951.hg.1	CATSPER1	7.34 ± 0.12	7.26 ± 0.14	7.30 ± 0.15	7.32 ± 0.05
TC15002810.hg.1	CATSPER2	6.87 ± 0.05	6.85 ± 0.04	6.85 ± 0.02	6.92 ± 0.10
TC15002811.hg.1	CATSPER2	6.35 ± 0.12	6.29 ± 0.02	6.29 ± 0.00	6.36 ± 0.09
TC15001284.hg.1	CATSPER2P1	7.08 ± 0.10	6.92 ± 0.05	6.88 ± 0.15	6.95 ± 0.05
TC05003412.hg.1	CATSPER3	5.49 ± 0.08	5.48 ± 0.01	5.46 ± 0.06	5.47 ± 0.04
TC01000337.hg.1	CATSPER4	6.82 ± 0.20	6.77 ± 0.11	6.74 ± 0.16	6.74 ± 0.05
TC14001439.hg.1	CATSPERB	5.16 ± 0.10	5.10 ± 0.07	5.04 ± 0.12	5.13 ± 0.03
TC19000105.hg.1	CATSPERD	6.35 ± 0.10	6.28 ± 0.04	6.25 ± 0.06	6.32 ± 0.04
TC19000526.hg.1	CATSPERG	7.40 ± 0.18	7.34 ± 0.15	7.33 ± 0.10	7.41 ± 0.06
TC07003339.hg.1	CAV1	7.10 ± 0.21	6.88 ± 0.17	6.90 ± 0.19	6.94 ± 0.02
TC07003340.hg.1	CAV2	5.81 ± 0.16	5.64 ± 0.04	5.78 ± 0.09	5.74 ± 0.05
TC03000028.hg.1	CAV3	6.52 ± 0.15	6.43 ± 0.08	6.47 ± 0.04	6.45 ± 0.08
TC20000231.hg.1	CBFA2T2	8.14 ± 0.05	8.09 ± 0.07	8.04 ± 0.08	8.03 ± 0.04
TC16001347.hg.1	CBFA2T3	8.86 ± 0.15	8.66 ± 0.10	8.81 ± 0.05	8.66 ± 0.04
TC16000528.hg.1	CBFB	9.64 ± 0.25	9.88 ± 0.05	9.69 ± 0.24	9.71 ± 0.23
TC11001089.hg.1	CBL	10.40 ± 0.26	10.18 ± 0.19	10.62 ± 0.24	10.10 ± 0.33
TC03001624.hg.1	CBLB	9.05 ± 0.30	9.11 ± 0.05	8.74 ± 0.33	9.04 ± 0.03
TC19000629.hg.1	CBLC	7.81 ± 0.21	7.83 ± 0.19	7.73 ± 0.25	7.92 ± 0.09
TC07000696.hg.1	CBLL1	8.13 ± 0.09	8.15 ± 0.08	8.04 ± 0.21	8.16 ± 0.07
TC16001101.hg.1	CBLN1	8.03 ± 0.20	7.99 ± 0.13	7.88 ± 0.28	7.97 ± 0.01
TC18000570.hg.1	CBLN2	7.76 ± 0.14	7.60 ± 0.10	7.70 ± 0.16	7.62 ± 0.01
TC14000977.hg.1	CBLN3	7.64 ± 0.20	7.67 ± 0.18	7.42 ± 0.06	7.71 ± 0.12
TC20000965.hg.1	CBLN4	6.11 ± 0.18	6.04 ± 0.19	6.10 ± 0.06	6.11 ± 0.02
TC21000148.hg.1	CBR1	7.65 ± 0.21	7.31 ± 0.06	7.48 ± 0.17	7.26 ± 0.03
TC21000152.hg.1	CBR3	6.78 ± 0.24	6.74 ± 0.16	6.63 ± 0.21	6.74 ± 0.11
TC21000431.hg.1	CBR3-AS1	6.80 ± 0.24	6.72 ± 0.06	6.69 ± 0.08	6.77 ± 0.07
TC04001723.hg.1	CBR4	7.34 ± 0.25	7.29 ± 0.19	7.42 ± 0.24	7.19 ± 0.15
TC21000500.hg.1	CBS	7.74 ± 0.19	7.56 ± 0.19	7.73 ± 0.13	7.68 ± 0.01
TC09000864.hg.1	CBWD1	7.58 ± 0.34	7.66 ± 0.22	7.71 ± 0.33	7.52 ± 0.04
TC02000725.hg.1	CBWD2	7.59 ± 0.41	7.69 ± 0.24	7.76 ± 0.35	7.55 ± 0.06

TC09000308.hg.1	CBWD3	7.90 ± 0.40	8.00 ± 0.24	8.05 ± 0.38	7.81 ± 0.07
TC09001181.hg.1	CBWD5	7.50 ± 0.42	7.59 ± 0.25	7.67 ± 0.35	7.44 ± 0.06
TC09001170.hg.1	CBWD6	7.70 ± 0.35	7.77 ± 0.22	7.83 ± 0.34	7.65 ± 0.05
TC17001635.hg.1	CBX1	8.43 ± 0.09	8.38 ± 0.02	8.39 ± 0.05	8.27 ± 0.11
TC0X001089.hg.1	CBX1P1	7.44 ± 0.23	7.36 ± 0.17	7.49 ± 0.12	7.26 ± 0.11
TC17000911.hg.1	CBX2	8.36 ± 0.23	8.34 ± 0.18	8.24 ± 0.30	8.40 ± 0.04
TC07000160.hg.1	CBX3	8.78 ± 0.30	8.93 ± 0.11	8.78 ± 0.29	8.87 ± 0.05
TC18000284.hg.1	CBX3P2	7.05 ± 0.05	7.16 ± 0.11	6.85 ± 0.28	6.97 ± 0.12
TC12001101.hg.1	CBX3P4	7.35 ± 0.09	7.34 ± 0.15	7.29 ± 0.08	7.34 ± 0.08
TC17001930.hg.1	CBX4	9.22 ± 0.06	9.51 ± 0.13	9.07 ± 0.03	9.47 ± 0.06
TC12001566.hg.1	CBX5	8.78 ± 0.10	8.68 ± 0.22	8.47 ± 0.07	8.38 ± 0.02
TC22000799.hg.1	CBX6	9.19 ± 0.15	9.06 ± 0.03	8.99 ± 0.14	9.05 ± 0.05
TC22000801.hg.1	CBX7	8.41 ± 0.11	8.37 ± 0.06	8.30 ± 0.09	8.42 ± 0.03
TC17001929.hg.1	CBX8	7.98 ± 0.30	7.87 ± 0.10	7.80 ± 0.26	7.87 ± 0.08
TC22000302.hg.1	CBY1	7.14 ± 0.09	7.07 ± 0.05	7.02 ± 0.09	7.07 ± 0.03
TC05002137.hg.1	CBY3	8.01 ± 0.18	7.86 ± 0.17	7.88 ± 0.17	7.95 ± 0.06
TC19000247.hg.1	CC2D1A	8.34 ± 0.12	8.21 ± 0.09	8.27 ± 0.08	8.21 ± 0.04
TC01002657.hg.1	CC2D1B	8.45 ± 0.07	8.36 ± 0.01	8.32 ± 0.03	8.39 ± 0.02
TC04000142.hg.1	CC2D2A	5.48 ± 0.10	5.37 ± 0.07	5.54 ± 0.05	5.40 ± 0.01
TC10000678.hg.1	CC2D2B	4.81 ± 0.04	4.64 ± 0.03	5.01 ± 0.18	4.63 ± 0.03
TC08001627.hg.1	CCAT1	5.73 ± 0.20	5.59 ± 0.23	5.33 ± 0.02	5.71 ± 0.05
TC18000548.hg.1	CCBE1	7.01 ± 0.31	6.94 ± 0.18	6.88 ± 0.28	7.02 ± 0.03
TC09001640.hg.1	CCBL1	7.81 ± 0.10	7.69 ± 0.13	7.65 ± 0.14	7.73 ± 0.03
TC01002844.hg.1	CCBL2	8.10 ± 0.18	8.05 ± 0.22	8.15 ± 0.30	7.93 ± 0.12
TC03003348.hg.1	CCBP2	6.83 ± 0.18	6.80 ± 0.11	6.78 ± 0.13	6.91 ± 0.07
TC16000303.hg.1	CCDC101	9.03 ± 0.29	8.69 ± 0.03	8.86 ± 0.21	8.59 ± 0.05
TC16001141.hg.1	CCDC102A	8.76 ± 0.18	8.62 ± 0.09	8.65 ± 0.22	8.62 ± 0.02
TC18000239.hg.1	CCDC102B	4.63 ± 0.11	4.58 ± 0.04	4.56 ± 0.10	4.55 ± 0.02
TC17000576.hg.1	CCDC103	7.58 ± 0.30	7.57 ± 0.25	7.47 ± 0.32	7.72 ± 0.11
TC02000326.hg.1	CCDC104	6.25 ± 0.10	6.29 ± 0.09	6.12 ± 0.08	6.28 ± 0.02
TC19000265.hg.1	CCDC105	6.56 ± 0.12	6.54 ± 0.20	6.49 ± 0.17	6.63 ± 0.10

TC19000905.hg.1	CCDC106	8.32 ± 0.22	8.28 ± 0.15	8.21 ± 0.20	8.27 ± 0.04
TC09000190.hg.1	CCDC107	7.95 ± 0.15	7.85 ± 0.10	7.75 ± 0.27	7.79 ± 0.01
TC02002785.hg.1	CCDC108	7.11 ± 0.18	7.05 ± 0.18	7.03 ± 0.14	7.12 ± 0.06
TC04000564.hg.1	CCDC109B	9.41 ± 0.38	9.72 ± 0.09	9.30 ± 0.31	9.71 ± 0.12
TC18000511.hg.1	CCDC11	5.76 ± 0.15	5.59 ± 0.11	5.68 ± 0.13	5.65 ± 0.05
TC04001818.hg.1	CCDC110	4.97 ± 0.15	4.93 ± 0.07	4.87 ± 0.12	4.89 ± 0.03
TC04000897.hg.1	CCDC111	6.22 ± 0.20	6.19 ± 0.22	6.22 ± 0.16	5.97 ± 0.06
TC05001688.hg.1	CCDC112	6.32 ± 0.19	6.30 ± 0.12	6.43 ± 0.13	6.29 ± 0.09
TC16000501.hg.1	CCDC113	5.57 ± 0.21	5.51 ± 0.06	5.51 ± 0.11	5.52 ± 0.01
TC19001678.hg.1	CCDC114	8.15 ± 0.14	8.12 ± 0.14	8.08 ± 0.14	8.16 ± 0.04
TC02002330.hg.1	CCDC115	8.33 ± 0.07	8.30 ± 0.01	8.18 ± 0.15	8.32 ± 0.02
TC22000094.hg.1	CCDC116	8.18 ± 0.29	8.15 ± 0.25	8.13 ± 0.26	8.28 ± 0.06
TC22000187.hg.1	CCDC117	8.48 ± 0.10	8.16 ± 0.15	8.33 ± 0.21	7.93 ± 0.16
TC03001363.hg.1	CCDC12	8.27 ± 0.12	8.24 ± 0.07	8.14 ± 0.16	8.29 ± 0.05
TC0X000264.hg.1	CCDC120	7.75 ± 0.14	7.79 ± 0.14	7.71 ± 0.11	7.88 ± 0.03
TC02001691.hg.1	CCDC121	6.68 ± 0.12	6.63 ± 0.09	6.79 ± 0.11	6.63 ± 0.02
TC13000614.hg.1	CCDC122	4.65 ± 0.13	4.56 ± 0.08	4.60 ± 0.06	4.52 ± 0.09
TC19000347.hg.1	CCDC124	8.89 ± 0.21	8.85 ± 0.11	8.86 ± 0.24	8.83 ± 0.09
TC05001437.hg.1	CCDC125	7.48 ± 0.12	7.46 ± 0.20	7.74 ± 0.29	7.40 ± 0.19
TC07000149.hg.1	CCDC126	7.35 ± 0.04	7.28 ± 0.09	7.44 ± 0.15	7.24 ± 0.16
TC05001092.hg.1	CCDC127	7.32 ± 0.06	7.15 ± 0.02	7.16 ± 0.14	7.21 ± 0.14
TC07000203.hg.1	CCDC129	5.57 ± 0.09	5.50 ± 0.07	5.51 ± 0.06	5.61 ± 0.10
TC03001326.hg.1	CCDC13	6.31 ± 0.19	6.19 ± 0.21	6.28 ± 0.14	6.52 ± 0.23
TC03003375.hg.1	CCDC13	7.67 ± 0.21	7.52 ± 0.16	7.63 ± 0.16	7.61 ± 0.03
TC19000238.hg.1	CCDC130	8.78 ± 0.03	8.75 ± 0.02	8.72 ± 0.02	8.90 ± 0.06
TC07000552.hg.1	CCDC132	6.29 ± 0.36	6.35 ± 0.20	6.28 ± 0.33	6.21 ± 0.07
TC22000345.hg.1	CCDC134	8.36 ± 0.06	8.33 ± 0.09	8.23 ± 0.08	8.28 ± 0.04
TC16000495.hg.1	CCDC135	7.11 ± 0.24	7.08 ± 0.12	7.08 ± 0.26	7.16 ± 0.03
TC07000784.hg.1	CCDC136	7.36 ± 0.16	7.30 ± 0.12	7.25 ± 0.13	7.33 ± 0.05
TC17000934.hg.1	CCDC137	8.09 ± 0.17	8.08 ± 0.09	8.09 ± 0.26	8.12 ± 0.01
TC02000669.hg.1	CCDC138	5.13 ± 0.19	5.09 ± 0.11	5.06 ± 0.09	5.01 ± 0.14

TC03000225.hg.1	CCDC13-AS1	6.42 ± 0.30	6.34 ± 0.18	6.25 ± 0.13	6.39 ± 0.07
TC03001714.hg.1	CCDC14	6.72 ± 0.49	7.02 ± 0.12	6.71 ± 0.29	6.81 ± 0.15
TC02001344.hg.1	CCDC140	6.82 ± 0.31	6.60 ± 0.12	6.56 ± 0.20	6.67 ± 0.06
TC02005048.hg.1	CCDC141	6.67 ± 0.63	7.27 ± 0.22	6.53 ± 0.32	7.06 ± 0.23
TC02005033.hg.1	CCDC142	7.90 ± 0.14	7.70 ± 0.05	7.75 ± 0.08	7.70 ± 0.03
TC17002870.hg.1	CCDC144A	6.53 ± 0.38	6.59 ± 0.33	6.80 ± 0.30	6.68 ± 0.11
TC17002892.hg.1	CCDC144B	7.01 ± 0.74	7.21 ± 0.57	7.38 ± 0.46	7.34 ± 0.22
TC17000252.hg.1	CCDC144CP	6.35 ± 0.59	6.47 ± 0.42	6.68 ± 0.43	6.61 ± 0.18
TC17001260.hg.1	CCDC144NL	6.87 ± 0.08	6.79 ± 0.04	6.86 ± 0.13	6.86 ± 0.05
TC07003312.hg.1	CCDC146	6.95 ± 0.42	7.12 ± 0.21	7.12 ± 0.37	6.91 ± 0.12
TC10000785.hg.1	CCDC147	6.19 ± 0.05	6.12 ± 0.07	6.35 ± 0.10	6.14 ± 0.03
TC10001642.hg.1	CCDC147-AS1	6.50 ± 0.26	6.23 ± 0.23	6.98 ± 0.34	6.03 ± 0.42
TC02002458.hg.1	CCDC148	4.52 ± 0.08	4.43 ± 0.06	4.50 ± 0.08	4.47 ± 0.07
TC02000959.hg.1	CCDC148-AS1	5.32 ± 0.28	5.08 ± 0.06	5.21 ± 0.09	5.14 ± 0.14
TC04001088.hg.1	CCDC149	6.57 ± 0.08	6.48 ± 0.02	6.49 ± 0.10	6.49 ± 0.01
TC11001150.hg.1	CCDC15	5.67 ± 0.17	5.57 ± 0.15	5.55 ± 0.09	5.38 ± 0.05
TC02001141.hg.1	CCDC150	4.93 ± 0.09	4.84 ± 0.06	4.85 ± 0.04	4.83 ± 0.01
TC19001184.hg.1	CCDC151	7.75 ± 0.22	7.55 ± 0.20	7.61 ± 0.22	7.66 ± 0.07
TC05000193.hg.1	CCDC152	4.86 ± 0.34	4.87 ± 0.20	4.77 ± 0.32	4.71 ± 0.06
TC11002364.hg.1	CCDC153	7.27 ± 0.20	7.24 ± 0.18	7.24 ± 0.12	7.32 ± 0.06
TC16000752.hg.1	CCDC154	8.40 ± 0.23	8.36 ± 0.19	8.32 ± 0.24	8.47 ± 0.05
TC19000731.hg.1	CCDC155	8.11 ± 0.19	8.10 ± 0.16	8.04 ± 0.22	8.21 ± 0.07
TC22000208.hg.1	CCDC157	8.19 ± 0.16	8.14 ± 0.21	8.16 ± 0.15	8.20 ± 0.03
TC04001309.hg.1	CCDC158	4.69 ± 0.06	4.55 ± 0.03	4.64 ± 0.04	4.59 ± 0.01
TC19000194.hg.1	CCDC159	7.96 ± 0.16	7.79 ± 0.03	7.91 ± 0.06	7.73 ± 0.05
TC0X000634.hg.1	CCDC160	5.12 ± 0.23	5.10 ± 0.14	5.10 ± 0.07	5.13 ± 0.04
TC06000873.hg.1	CCDC162P	5.79 ± 0.25	5.76 ± 0.13	5.67 ± 0.14	5.92 ± 0.19
TC01002610.hg.1	CCDC163P	7.07 ± 0.12	7.02 ± 0.12	6.90 ± 0.12	7.01 ± 0.05
TC08001726.hg.1	CCDC166	7.89 ± 0.37	7.71 ± 0.16	7.64 ± 0.21	7.54 ± 0.11
TC06001684.hg.1	CCDC167	7.84 ± 0.22	7.71 ± 0.20	7.55 ± 0.30	7.55 ± 0.06
TC13000853.hg.1	CCDC168	5.58 ± 0.17	5.36 ± 0.10	5.52 ± 0.10	5.45 ± 0.03

TC13001725.hg.1	CCDC169	4.62 ± 0.08	4.54 ± 0.03	4.54 ± 0.04	4.54 ± 0.01
TC01002612.hg.1	CCDC17	8.18 ± 0.08	8.08 ± 0.06	8.15 ± 0.04	8.12 ± 0.04
TC06001103.hg.1	CCDC170	6.54 ± 0.08	6.46 ± 0.05	6.65 ± 0.11	6.54 ± 0.07
TC09000077.hg.1	CCDC171	5.66 ± 0.15	5.61 ± 0.01	5.56 ± 0.03	5.64 ± 0.03
TC10000840.hg.1	CCDC172	3.92 ± 0.09	3.79 ± 0.04	3.87 ± 0.10	3.80 ± 0.01
TC02002511.hg.1	CCDC173	4.64 ± 0.14	4.47 ± 0.06	4.57 ± 0.05	4.48 ± 0.02
TC03000088.hg.1	CCDC174	7.97 ± 0.22	7.94 ± 0.09	8.06 ± 0.25	7.97 ± 0.03
TC14002326.hg.1	CCDC175	4.58 ± 0.08	4.50 ± 0.06	4.62 ± 0.13	4.51 ± 0.11
TC14000454.hg.1	CCDC176	5.51 ± 0.11	5.41 ± 0.01	5.50 ± 0.08	5.41 ± 0.04
TC18000448.hg.1	CCDC178	4.36 ± 0.11	4.20 ± 0.04	4.24 ± 0.05	4.23 ± 0.03
TC11001500.hg.1	CCDC179	4.76 ± 0.20	4.60 ± 0.11	4.55 ± 0.07	4.64 ± 0.07
TC01000860.hg.1	CCDC18	6.23 ± 0.38	6.32 ± 0.26	6.52 ± 0.38	6.15 ± 0.18
TC09000489.hg.1	CCDC180	7.04 ± 0.12	7.04 ± 0.08	6.95 ± 0.10	7.09 ± 0.08
TC01003496.hg.1	CCDC181	4.76 ± 0.16	4.61 ± 0.06	4.64 ± 0.04	4.65 ± 0.10
TC01006413.hg.1	CCDC19	7.57 ± 0.22	7.67 ± 0.09	7.68 ± 0.07	7.83 ± 0.08
TC0X000269.hg.1	CCDC22	8.31 ± 0.16	8.19 ± 0.12	8.23 ± 0.14	8.24 ± 0.03
TC01002576.hg.1	CCDC23	8.05 ± 0.12	7.97 ± 0.11	7.99 ± 0.14	7.90 ± 0.06
TC01000557.hg.1	CCDC24	8.01 ± 0.27	8.01 ± 0.28	7.95 ± 0.22	8.10 ± 0.04
TC08001084.hg.1	CCDC25	7.09 ± 0.19	7.19 ± 0.15	6.90 ± 0.14	7.04 ± 0.01
TC08002635.hg.1	CCDC26	5.94 ± 0.10	5.84 ± 0.14	5.86 ± 0.12	5.97 ± 0.13
TC01000072.hg.1	CCDC27	7.07 ± 0.18	7.09 ± 0.15	7.06 ± 0.19	7.17 ± 0.08
TC06001032.hg.1	CCDC28A	7.85 ± 0.13	7.79 ± 0.13	7.98 ± 0.18	7.82 ± 0.02
TC01000421.hg.1	CCDC28B	7.38 ± 0.15	7.42 ± 0.19	7.34 ± 0.14	7.41 ± 0.03
TC10001045.hg.1	CCDC3	6.26 ± 0.18	6.15 ± 0.07	6.18 ± 0.08	6.26 ± 0.04
TC01006321.hg.1	CCDC30	5.56 ± 0.10	5.46 ± 0.06	5.62 ± 0.09	5.46 ± 0.02
TC15002799.hg.1	CCDC33	7.28 ± 0.21	7.22 ± 0.19	7.14 ± 0.20	7.34 ± 0.09
TC11001508.hg.1	CCDC34	6.19 ± 0.09	6.13 ± 0.04	6.20 ± 0.09	6.13 ± 0.04
TC03000289.hg.1	CCDC36	5.79 ± 0.19	5.77 ± 0.09	5.77 ± 0.08	5.84 ± 0.03
TC03000657.hg.1	CCDC37	7.22 ± 0.20	7.11 ± 0.11	7.15 ± 0.20	7.22 ± 0.08
TC12001841.hg.1	CCDC38	4.86 ± 0.09	4.80 ± 0.11	4.81 ± 0.08	4.83 ± 0.05
TC03002044.hg.1	CCDC39	4.51 ± 0.09	4.38 ± 0.02	4.41 ± 0.01	4.40 ± 0.04

TC03000964.hg.1	CCDC39-AS1	3.90 ± 0.08	3.88 ± 0.07	3.95 ± 0.14	3.84 ± 0.15
TC17000914.hg.1	CCDC40	7.49 ± 0.22	7.44 ± 0.17	7.43 ± 0.24	7.53 ± 0.02
TC12001824.hg.1	CCDC41	6.13 ± 0.38	6.24 ± 0.17	6.13 ± 0.23	6.07 ± 0.13
TC12000731.hg.1	CCDC41-AS1	7.93 ± 0.13	7.85 ± 0.09	7.87 ± 0.10	7.88 ± 0.18
TC17001120.hg.1	CCDC42	6.27 ± 0.22	6.27 ± 0.23	6.24 ± 0.16	6.37 ± 0.05
TC12000888.hg.1	CCDC42B	7.57 ± 0.28	7.52 ± 0.21	7.52 ± 0.25	7.61 ± 0.03
TC17001584.hg.1	CCDC43	7.54 ± 0.04	7.44 ± 0.06	7.40 ± 0.10	7.41 ± 0.08
TC17001785.hg.1	CCDC47	9.28 ± 0.27	9.29 ± 0.11	9.48 ± 0.28	9.20 ± 0.30
TC03001052.hg.1	CCDC50	8.69 ± 0.19	8.93 ± 0.10	8.45 ± 0.13	8.82 ± 0.08
TC03003377.hg.1	CCDC51	8.10 ± 0.20	8.09 ± 0.17	8.07 ± 0.22	8.27 ± 0.08
TC12001886.hg.1	CCDC53	8.59 ± 0.03	8.44 ± 0.16	8.57 ± 0.06	8.24 ± 0.04
TC03000541.hg.1	CCDC54	4.85 ± 0.04	4.76 ± 0.04	4.80 ± 0.05	4.75 ± 0.06
TC17001974.hg.1	CCDC57	8.12 ± 0.14	8.08 ± 0.10	8.07 ± 0.09	8.19 ± 0.04
TC03001702.hg.1	CCDC58	6.28 ± 0.32	6.29 ± 0.12	6.04 ± 0.09	6.24 ± 0.04
TC12001778.hg.1	CCDC59	7.88 ± 0.45	8.57 ± 0.13	7.87 ± 0.36	8.52 ± 0.19
TC10001317.hg.1	CCDC6	9.34 ± 0.09	9.39 ± 0.13	9.02 ± 0.37	9.30 ± 0.15
TC12000933.hg.1	CCDC60	6.16 ± 0.17	6.13 ± 0.18	6.16 ± 0.10	6.26 ± 0.07
TC19000657.hg.1	CCDC61	8.46 ± 0.26	8.35 ± 0.12	8.33 ± 0.25	8.38 ± 0.03
TC12000978.hg.1	CCDC62	5.30 ± 0.04	5.28 ± 0.11	5.29 ± 0.07	5.24 ± 0.08
TC12000861.hg.1	CCDC63	6.79 ± 0.17	6.77 ± 0.15	6.71 ± 0.17	6.84 ± 0.06
TC12000938.hg.1	CCDC64	8.01 ± 0.08	8.12 ± 0.09	7.88 ± 0.12	8.14 ± 0.04
TC16000799.hg.1	CCDC64B	8.67 ± 0.29	8.57 ± 0.18	8.56 ± 0.23	8.64 ± 0.02
TC12000358.hg.1	CCDC65	6.02 ± 0.07	5.87 ± 0.02	5.87 ± 0.03	5.92 ± 0.02
TC03000362.hg.1	CCDC66	7.08 ± 0.35	7.17 ± 0.17	7.01 ± 0.24	7.11 ± 0.05
TC11000907.hg.1	CCDC67	4.34 ± 0.12	4.21 ± 0.05	4.23 ± 0.08	4.18 ± 0.06
TC18000524.hg.1	CCDC68	4.39 ± 0.10	4.25 ± 0.07	4.31 ± 0.05	4.26 ± 0.01
TC05001949.hg.1	CCDC69	9.18 ± 0.07	9.15 ± 0.12	9.17 ± 0.04	9.13 ± 0.06
TC10000228.hg.1	CCDC7	5.64 ± 0.27	5.63 ± 0.16	5.59 ± 0.11	5.64 ± 0.12
TC13000219.hg.1	CCDC70	6.79 ± 0.19	6.66 ± 0.12	6.77 ± 0.16	6.72 ± 0.03
TC03001404.hg.1	CCDC71	8.87 ± 0.24	8.75 ± 0.07	8.68 ± 0.13	8.69 ± 0.07
TC07001740.hg.1	CCDC71L	8.87 ± 0.21	8.76 ± 0.03	9.15 ± 0.24	8.63 ± 0.19

TC11001534.hg.1	CCDC73	4.17 ± 0.12	3.98 ± 0.05	4.16 ± 0.14	4.00 ± 0.06
TC02000855.hg.1	CCDC74A	7.68 ± 0.18	7.65 ± 0.20	7.67 ± 0.17	7.79 ± 0.08
TC02002319.hg.1	CCDC74B	7.35 ± 0.22	7.33 ± 0.22	7.31 ± 0.18	7.46 ± 0.08
TC02000802.hg.1	CCDC74B-AS1	7.34 ± 0.19	7.16 ± 0.11	7.14 ± 0.15	7.33 ± 0.06
TC12000006.hg.1	CCDC77	6.92 ± 0.02	6.72 ± 0.11	6.89 ± 0.16	6.70 ± 0.01
TC16000737.hg.1	CCDC78	8.42 ± 0.22	8.40 ± 0.21	8.33 ± 0.23	8.50 ± 0.07
TC16001173.hg.1	CCDC79	4.58 ± 0.06	4.44 ± 0.05	4.48 ± 0.02	4.44 ± 0.03
TC19001646.hg.1	CCDC8	7.64 ± 0.16	7.56 ± 0.08	7.55 ± 0.09	7.56 ± 0.04
TC03001651.hg.1	CCDC80	5.67 ± 0.15	5.56 ± 0.07	5.62 ± 0.09	5.61 ± 0.04
TC11000872.hg.1	CCDC81	5.66 ± 0.13	5.51 ± 0.10	5.58 ± 0.11	5.62 ± 0.06
TC11002212.hg.1	CCDC82	7.31 ± 0.64	7.59 ± 0.22	7.64 ± 0.54	7.46 ± 0.21
TC11000866.hg.1	CCDC83	4.35 ± 0.18	4.17 ± 0.06	4.23 ± 0.06	4.24 ± 0.06
TC11001079.hg.1	CCDC84	8.38 ± 0.14	8.44 ± 0.09	8.30 ± 0.13	8.52 ± 0.03
TC02000328.hg.1	CCDC85A	6.69 ± 0.16	6.66 ± 0.11	6.66 ± 0.10	6.73 ± 0.06
TC11000646.hg.1	CCDC85B	7.87 ± 0.12	7.74 ± 0.16	7.61 ± 0.35	7.61 ± 0.13
TC14001500.hg.1	CCDC85C	8.08 ± 0.15	7.99 ± 0.15	8.02 ± 0.16	8.15 ± 0.07
TC11000518.hg.1	CCDC86	8.13 ± 0.14	8.04 ± 0.09	7.96 ± 0.22	8.07 ± 0.02
TC11001972.hg.1	CCDC87	7.08 ± 0.40	6.92 ± 0.33	6.94 ± 0.20	7.07 ± 0.09
TC02001860.hg.1	CCDC88A	7.70 ± 0.58	7.80 ± 0.25	8.07 ± 0.65	7.81 ± 0.30
TC11000598.hg.1	CCDC88B	8.84 ± 0.13	8.78 ± 0.10	8.81 ± 0.12	8.80 ± 0.01
TC14001436.hg.1	CCDC88C	9.44 ± 0.14	9.53 ± 0.05	9.10 ± 0.10	9.49 ± 0.05
TC11002161.hg.1	CCDC89	5.05 ± 0.18	4.91 ± 0.08	4.93 ± 0.20	5.02 ± 0.06
TC19000674.hg.1	CCDC9	8.94 ± 0.13	8.95 ± 0.08	8.85 ± 0.13	9.02 ± 0.06
TC11002158.hg.1	CCDC90B	7.64 ± 0.30	7.63 ± 0.23	7.70 ± 0.25	7.40 ± 0.22
TC12000274.hg.1	CCDC91	8.17 ± 0.38	8.48 ± 0.15	8.06 ± 0.31	8.33 ± 0.04
TC12002094.hg.1	CCDC92	8.40 ± 0.05	8.41 ± 0.10	8.34 ± 0.10	8.40 ± 0.05
TC02002244.hg.1	CCDC93	8.33 ± 0.43	8.53 ± 0.14	8.54 ± 0.36	8.63 ± 0.10
TC19000084.hg.1	CCDC94	8.73 ± 0.10	8.74 ± 0.10	8.55 ± 0.15	8.77 ± 0.01
TC04001006.hg.1	CCDC96	7.00 ± 0.19	6.92 ± 0.05	6.97 ± 0.11	6.79 ± 0.24
TC19000578.hg.1	CCDC97	9.83 ± 0.23	9.64 ± 0.09	9.79 ± 0.12	9.58 ± 0.12
TC12001800.hg.1	CCER1	5.74 ± 0.19	5.60 ± 0.05	5.61 ± 0.07	5.70 ± 0.09

TC06001520.hg.1	CCHCR1	8.32 ± 0.21	8.22 ± 0.15	8.22 ± 0.24	8.29 ± 0.06
TC6_cox_hap2000152.hg.1	CCHCR1	8.20 ± 0.17	8.11 ± 0.15	8.10 ± 0.20	8.18 ± 0.05
TC6_dbb_hap3000141.hg.1	CCHCR1	8.20 ± 0.17	8.11 ± 0.15	8.10 ± 0.20	8.18 ± 0.05
TC6_mann_hap4000129.hg.1	CCHCR1	8.18 ± 0.18	8.09 ± 0.14	8.08 ± 0.20	8.15 ± 0.05
TC6_mcf_hap5000129.hg.1	CCHCR1	8.18 ± 0.18	8.09 ± 0.14	8.08 ± 0.20	8.15 ± 0.05
TC6_qbl_hap6000142.hg.1	CCHCR1	8.19 ± 0.18	8.09 ± 0.14	8.09 ± 0.19	8.15 ± 0.05
TC09000203.hg.1	CCIN	7.19 ± 0.27	7.23 ± 0.05	7.41 ± 0.20	7.30 ± 0.04
TC03001318.hg.1	CCK	7.23 ± 0.23	7.29 ± 0.23	7.23 ± 0.24	7.39 ± 0.08
TC04001095.hg.1	CCKAR	6.67 ± 0.16	6.69 ± 0.12	6.63 ± 0.15	6.82 ± 0.11
TC11000129.hg.1	CCKBR	7.86 ± 0.19	7.84 ± 0.14	7.80 ± 0.17	7.86 ± 0.06
TC17001360.hg.1	CCL1	6.35 ± 0.07	6.38 ± 0.18	6.40 ± 0.12	6.52 ± 0.09
TC17000385.hg.1	CCL11	6.66 ± 0.29	6.68 ± 0.11	6.80 ± 0.12	6.89 ± 0.07
TC17000387.hg.1	CCL13	6.68 ± 0.23	6.62 ± 0.13	6.65 ± 0.18	6.76 ± 0.11
TC17001383.hg.1	CCL14	6.07 ± 0.17	5.93 ± 0.11	5.92 ± 0.13	6.01 ± 0.06
TC17001382.hg.1	CCL16	7.06 ± 0.14	6.95 ± 0.09	6.96 ± 0.09	7.06 ± 0.09
TC16000489.hg.1	CCL17	6.97 ± 0.30	6.93 ± 0.21	6.93 ± 0.23	7.01 ± 0.02
TC17000406.hg.1	CCL18	5.05 ± 0.18	5.13 ± 0.22	5.06 ± 0.11	5.28 ± 0.13
TC09001044.hg.1	CCL19	7.32 ± 0.17	7.39 ± 0.25	7.26 ± 0.13	7.52 ± 0.15
TC17000383.hg.1	CCL2	7.08 ± 0.46	7.50 ± 1.04	7.40 ± 0.07	8.16 ± 0.93
TC02001364.hg.1	CCL20	4.59 ± 0.13	4.53 ± 0.09	4.68 ± 0.31	4.55 ± 0.08
TC09001045.hg.1	CCL21	7.57 ± 0.16	7.58 ± 0.26	7.55 ± 0.18	7.74 ± 0.10
TC16000487.hg.1	CCL22	6.93 ± 0.38	6.98 ± 0.19	6.97 ± 0.16	7.23 ± 0.08
TC17001384.hg.1	CCL23	5.86 ± 0.21	5.93 ± 0.04	6.06 ± 0.10	5.99 ± 0.11
TC07001536.hg.1	CCL24	7.33 ± 0.16	7.33 ± 0.24	7.19 ± 0.24	7.45 ± 0.04
TC19000143.hg.1	CCL25	7.82 ± 0.11	7.81 ± 0.15	7.70 ± 0.13	7.85 ± 0.08
TC07001535.hg.1	CCL26	6.40 ± 0.11	6.39 ± 0.18	6.40 ± 0.10	6.46 ± 0.05
TC09002914.hg.1	CCL27	6.97 ± 0.22	7.02 ± 0.15	6.97 ± 0.15	7.17 ± 0.11
TC05001317.hg.1	CCL28	7.54 ± 0.21	7.67 ± 0.05	7.42 ± 0.10	7.72 ± 0.09
TC17001385.hg.1	CCL3	7.52 ± 0.14	7.97 ± 0.05	7.85 ± 0.09	7.83 ± 0.30
TC17001388.hg.1	CCL3L3	8.33 ± 0.27	9.01 ± 0.03	8.62 ± 0.28	8.94 ± 0.44
TC17001395.hg.1	CCL3L3	8.23 ± 0.34	8.94 ± 0.06	8.55 ± 0.32	8.84 ± 0.48

TC17000408.hg.1	CCL4	8.65 ± 0.37	8.55 ± 0.28	8.78 ± 0.52	8.59 ± 0.09
TC17000416.hg.1	CCL4L1	7.87 ± 0.16	7.90 ± 0.10	7.97 ± 0.18	7.99 ± 0.09
TC17000423.hg.1	CCL4L1	7.57 ± 0.20	7.61 ± 0.19	7.70 ± 0.36	7.68 ± 0.11
TC17001378.hg.1	CCL5	11.03 ± 0.11	10.75 ± 0.09	10.58 ± 0.10	10.44 ± 0.04
TC17000384.hg.1	CCL7	5.58 ± 0.06	5.56 ± 0.08	5.69 ± 0.29	5.85 ± 0.19
TC17000386.hg.1	CCL8	5.76 ± 0.20	5.75 ± 0.19	5.94 ± 0.27	5.73 ± 0.07
TC07000280.hg.1	CCM2	8.78 ± 0.08	8.60 ± 0.04	8.79 ± 0.13	8.54 ± 0.04
TC20000204.hg.1	CCM2L	7.61 ± 0.27	7.55 ± 0.17	7.58 ± 0.18	7.59 ± 0.03
TC13000124.hg.1	CCNA1	6.92 ± 0.21	6.77 ± 0.09	6.97 ± 0.25	6.84 ± 0.03
TC04001516.hg.1	CCNA2	8.13 ± 0.19	8.10 ± 0.85	7.90 ± 0.37	6.90 ± 0.29
TC05000298.hg.1	CCNB1	7.28 ± 0.31	6.83 ± 0.26	6.98 ± 0.29	6.48 ± 0.05
TC14000878.hg.1	CCNB1IP1	7.42 ± 0.11	7.46 ± 0.15	7.20 ± 0.04	7.35 ± 0.06
TC15000449.hg.1	CCNB2	6.94 ± 0.44	6.75 ± 0.56	6.69 ± 0.26	6.16 ± 0.07
TC0X000288.hg.1	CCNB3	5.46 ± 0.09	5.39 ± 0.07	5.40 ± 0.07	5.41 ± 0.07
TC06001966.hg.1	CCNC	7.29 ± 0.33	7.41 ± 0.25	7.10 ± 0.28	7.24 ± 0.07
TC11000718.hg.1	CCND1	7.78 ± 0.24	7.80 ± 0.18	7.71 ± 0.18	7.89 ± 0.01
TC12000060.hg.1	CCND2	10.07 ± 0.27	10.00 ± 0.05	9.82 ± 0.09	9.87 ± 0.11
TC06001727.hg.1	CCND3	9.43 ± 0.16	9.13 ± 0.06	9.34 ± 0.15	9.06 ± 0.09
TC15000325.hg.1	CCNDBP1	9.31 ± 0.20	9.37 ± 0.12	9.57 ± 0.30	9.29 ± 0.19
TC19000416.hg.1	CCNE1	7.71 ± 0.19	7.65 ± 0.23	7.47 ± 0.15	7.48 ± 0.06
TC08001438.hg.1	CCNE2	5.84 ± 0.07	5.78 ± 0.39	5.71 ± 0.17	5.40 ± 0.11
TC16000072.hg.1	CCNF	8.10 ± 0.17	7.95 ± 0.11	7.92 ± 0.27	7.86 ± 0.02
TC05000906.hg.1	CCNG1	8.38 ± 0.25	8.44 ± 0.28	8.31 ± 0.23	8.28 ± 0.13
TC04000431.hg.1	CCNG2	8.34 ± 0.13	8.42 ± 0.07	8.46 ± 0.11	8.53 ± 0.10
TC05001570.hg.1	CCNH	8.14 ± 0.65	8.86 ± 0.18	8.53 ± 0.65	8.85 ± 0.22
TC04001319.hg.1	CCNI	10.78 ± 0.16	11.00 ± 0.08	10.84 ± 0.15	10.98 ± 0.13
TC05000643.hg.1	CCNI2	6.10 ± 0.07	6.07 ± 0.09	6.02 ± 0.07	6.17 ± 0.05
TC10000679.hg.1	CCNJ	7.18 ± 0.13	7.20 ± 0.16	7.04 ± 0.28	7.20 ± 0.03
TC05002007.hg.1	CCNJL	8.62 ± 0.18	8.43 ± 0.05	8.64 ± 0.06	8.40 ± 0.12
TC14000622.hg.1	CCNK	9.37 ± 0.10	9.41 ± 0.02	9.37 ± 0.18	9.43 ± 0.15
TC03001939.hg.1	CCNL1	9.61 ± 0.78	10.81 ± 0.09	10.49 ± 0.41	10.65 ± 0.23

TC01002095.hg.1	CCNL2	8.83 ± 0.21	8.86 ± 0.07	8.63 ± 0.13	8.89 ± 0.08
TC05001361.hg.1	CCNO	8.06 ± 0.23	7.96 ± 0.15	7.94 ± 0.32	8.02 ± 0.02
TC12001456.hg.1	CCNT1	9.05 ± 0.30	9.34 ± 0.21	8.91 ± 0.38	9.44 ± 0.11
TC02000878.hg.1	CCNT2	8.64 ± 0.62	9.10 ± 0.21	8.85 ± 0.57	9.04 ± 0.07
TC10000240.hg.1	CCNY	8.52 ± 0.06	8.51 ± 0.11	8.50 ± 0.06	8.50 ± 0.10
TC02001232.hg.1	CCNYL1	7.77 ± 0.09	7.75 ± 0.11	7.73 ± 0.14	7.73 ± 0.18
TC10001193.hg.1	CCNYL2	5.01 ± 0.13	4.96 ± 0.06	4.98 ± 0.04	5.00 ± 0.03
TC16000404.hg.1	CCNYL3	6.28 ± 0.20	6.18 ± 0.03	6.17 ± 0.08	6.25 ± 0.07
TC16000215.hg.1	CCP110	7.28 ± 0.20	7.38 ± 0.24	7.63 ± 0.49	7.25 ± 0.09
TC03001355.hg.1	CCR1	10.00 ± 0.71	9.31 ± 0.45	10.53 ± 0.27	9.30 ± 0.39
TC17001539.hg.1	CCR10	7.15 ± 0.36	6.80 ± 0.15	6.82 ± 0.27	6.83 ± 0.08
TC03000255.hg.1	CCR2	7.81 ± 0.32	6.75 ± 0.17	8.07 ± 0.18	6.46 ± 0.36
TC03000254.hg.1	CCR3	7.06 ± 0.23	7.11 ± 0.38	7.60 ± 0.38	7.21 ± 0.09
TC03000164.hg.1	CCR4	7.57 ± 0.12	7.27 ± 0.11	7.55 ± 0.38	7.33 ± 0.13
TC03000256.hg.1	CCR5	5.78 ± 0.09	5.55 ± 0.07	5.70 ± 0.20	5.39 ± 0.02
TC06004106.hg.1	CCR6	7.18 ± 0.20	6.82 ± 0.12	6.79 ± 0.10	6.67 ± 0.11
TC17001466.hg.1	CCR7	11.12 ± 0.04	11.02 ± 0.04	10.84 ± 0.17	11.13 ± 0.18
TC03000200.hg.1	CCR8	5.00 ± 0.11	4.87 ± 0.02	4.97 ± 0.15	4.86 ± 0.05
TC03000251.hg.1	CCR9	7.26 ± 0.15	7.19 ± 0.11	6.93 ± 0.09	7.26 ± 0.11
TC03000716.hg.1	CCRL1	4.98 ± 0.25	4.88 ± 0.11	4.92 ± 0.08	4.93 ± 0.08
TC03000257.hg.1	CCRL2	7.41 ± 0.28	7.23 ± 0.03	7.46 ± 0.17	7.23 ± 0.14
TC04000678.hg.1	CCRN4L	7.88 ± 0.12	7.88 ± 0.11	7.76 ± 0.09	7.95 ± 0.05
TC11000672.hg.1	CCS	8.73 ± 0.15	8.62 ± 0.10	8.65 ± 0.14	8.65 ± 0.05
TC01003942.hg.1	CCSAP	8.23 ± 0.04	8.25 ± 0.02	8.16 ± 0.08	8.26 ± 0.06
TC01003943.hg.1	CCSAP	8.34 ± 0.30	8.55 ± 0.08	8.30 ± 0.15	8.64 ± 0.08
TC04000494.hg.1	CCSER1	5.08 ± 0.06	5.03 ± 0.06	5.01 ± 0.06	4.99 ± 0.03
TC10000596.hg.1	CCSER2	8.26 ± 0.40	8.70 ± 0.13	8.09 ± 0.49	8.62 ± 0.09
TC12000616.hg.1	CCT2	9.09 ± 0.10	9.21 ± 0.14	8.79 ± 0.11	9.11 ± 0.11
TC01003337.hg.1	CCT3	8.72 ± 0.12	8.55 ± 0.12	8.45 ± 0.22	8.30 ± 0.11
TC02001895.hg.1	CCT4	8.96 ± 0.07	8.86 ± 0.15	8.72 ± 0.19	8.58 ± 0.20
TC05000072.hg.1	CCT5	8.18 ± 0.12	8.06 ± 0.13	8.15 ± 0.21	7.78 ± 0.15

TC07000338.hg.1	CCT6A	8.44 ± 0.14	8.37 ± 0.15	8.32 ± 0.16	8.21 ± 0.17
TC17001362.hg.1	CCT6B	5.30 ± 0.05	5.25 ± 0.06	5.16 ± 0.04	5.30 ± 0.02
TC07000398.hg.1	CCT6P1	8.14 ± 0.30	8.10 ± 0.09	8.03 ± 0.27	8.02 ± 0.09
TC07000388.hg.1	CCT6P3	8.05 ± 0.35	8.04 ± 0.10	7.98 ± 0.27	8.00 ± 0.07
TC02000440.hg.1	CCT7	8.71 ± 0.13	8.57 ± 0.13	8.41 ± 0.27	8.34 ± 0.03
TC21000353.hg.1	CCT8	8.42 ± 0.13	8.44 ± 0.23	8.23 ± 0.15	8.24 ± 0.03
TC22000461.hg.1	CCT8L2	5.74 ± 0.15	5.71 ± 0.23	5.66 ± 0.11	5.79 ± 0.04
TC07000058.hg.1	CCZ1	8.89 ± 0.13	8.88 ± 0.15	8.90 ± 0.23	8.84 ± 0.11
TC07003400.hg.1	CCZ1B	8.44 ± 0.13	8.43 ± 0.14	8.44 ± 0.19	8.41 ± 0.06
TC01001025.hg.1	CD101	7.92 ± 0.19	7.68 ± 0.05	7.93 ± 0.22	7.66 ± 0.12
TC06000737.hg.1	CD109	5.78 ± 0.12	5.61 ± 0.10	5.72 ± 0.25	5.80 ± 0.37
TC05001861.hg.1	CD14	10.13 ± 0.48	9.63 ± 0.25	10.16 ± 0.06	9.43 ± 0.29
TC11000034.hg.1	CD151	8.89 ± 0.17	8.63 ± 0.03	8.79 ± 0.23	8.65 ± 0.03
TC01003113.hg.1	CD160	7.23 ± 0.31	7.07 ± 0.05	7.09 ± 0.22	7.03 ± 0.03
TC12001162.hg.1	CD163	8.40 ± 0.62	7.57 ± 0.16	8.13 ± 0.29	7.58 ± 0.58
TC12001161.hg.1	CD163L1	6.21 ± 0.18	6.12 ± 0.07	6.16 ± 0.09	6.18 ± 0.07
TC06002000.hg.1	CD164	9.78 ± 0.30	9.85 ± 0.13	10.02 ± 0.33	9.68 ± 0.14
TC01002407.hg.1	CD164L2	8.34 ± 0.18	8.36 ± 0.19	8.27 ± 0.17	8.49 ± 0.07
TC19000605.hg.1	CD177	9.86 ± 1.51	8.14 ± 0.38	11.63 ± 0.37	8.46 ± 0.47
TC19001585.hg.1	CD177P1	9.94 ± 1.15	8.50 ± 0.41	11.31 ± 0.25	8.88 ± 0.41
TC05001419.hg.1	CD180	9.73 ± 0.23	9.07 ± 0.40	9.49 ± 0.12	8.50 ± 0.41
TC16000312.hg.1	CD19	8.61 ± 0.10	8.55 ± 0.08	8.24 ± 0.09	8.39 ± 0.15
TC01001338.hg.1	CD1A	7.40 ± 0.12	7.40 ± 0.03	7.33 ± 0.12	7.41 ± 0.04
TC01003371.hg.1	CD1B	5.80 ± 0.14	5.63 ± 0.08	5.67 ± 0.12	5.71 ± 0.09
TC01001339.hg.1	CD1C	7.63 ± 0.16	7.80 ± 0.36	7.18 ± 0.17	7.58 ± 0.08
TC01001337.hg.1	CD1D	8.12 ± 0.17	7.90 ± 0.03	7.98 ± 0.09	7.96 ± 0.05
TC01001340.hg.1	CD1E	6.46 ± 0.09	6.40 ± 0.03	6.35 ± 0.04	6.43 ± 0.02
TC01001022.hg.1	CD2	9.35 ± 0.18	9.31 ± 0.09	9.03 ± 0.09	9.07 ± 0.08
TC03000572.hg.1	CD200	7.57 ± 0.11	7.53 ± 0.09	7.46 ± 0.12	7.53 ± 0.01
TC03001654.hg.1	CD200R1	6.68 ± 0.56	6.82 ± 0.39	6.91 ± 0.30	6.60 ± 0.24
TC03001653.hg.1	CD200R1L	4.72 ± 0.13	4.74 ± 0.06	4.72 ± 0.09	4.81 ± 0.09

TC02001968.hg.1	CD207	6.98 ± 0.08	6.94 ± 0.12	6.84 ± 0.09	7.02 ± 0.17
TC19001118.hg.1	CD209	7.56 ± 0.22	7.53 ± 0.15	7.45 ± 0.19	7.60 ± 0.01
TC19002611.hg.1	CD22	10.28 ± 0.12	10.45 ± 0.17	10.02 ± 0.39	10.30 ± 0.20
TC18000566.hg.1	CD226	8.79 ± 0.66	8.73 ± 0.14	9.00 ± 0.23	8.65 ± 0.12
TC0Y000182.hg.1	CD24	8.19 ± 0.30	7.84 ± 0.17	8.56 ± 0.33	7.71 ± 0.15
TC01003408.hg.1	CD244	7.79 ± 0.21	7.64 ± 0.12	7.70 ± 0.03	7.53 ± 0.06
TC01003479.hg.1	CD247	9.35 ± 0.05	9.28 ± 0.08	8.91 ± 0.14	9.18 ± 0.07
TC11001962.hg.1	CD248	7.20 ± 0.16	7.11 ± 0.10	7.03 ± 0.02	7.20 ± 0.08
TC12000080.hg.1	CD27	10.35 ± 0.17	10.11 ± 0.05	9.73 ± 0.17	9.79 ± 0.20
TC09000037.hg.1	CD274	8.01 ± 0.84	7.32 ± 0.49	8.69 ± 0.74	6.89 ± 0.06
TC15000667.hg.1	CD276	8.02 ± 0.16	8.03 ± 0.19	7.97 ± 0.16	8.12 ± 0.07
TC12001137.hg.1	CD27-AS1	8.40 ± 0.03	8.28 ± 0.03	8.34 ± 0.03	8.32 ± 0.06
TC02001199.hg.1	CD28	8.58 ± 0.23	8.80 ± 0.08	8.47 ± 0.12	8.76 ± 0.01
TC06000633.hg.1	CD2AP	8.05 ± 0.45	8.28 ± 0.20	8.09 ± 0.37	8.25 ± 0.06
TC16001027.hg.1	CD2BP2	8.63 ± 0.13	8.34 ± 0.09	8.44 ± 0.18	8.30 ± 0.06
TC17000831.hg.1	CD300A	10.00 ± 0.41	9.62 ± 0.12	10.12 ± 0.28	9.52 ± 0.13
TC17001858.hg.1	CD300C	8.85 ± 0.37	8.47 ± 0.12	8.74 ± 0.14	8.48 ± 0.15
TC17001860.hg.1	CD300E	9.00 ± 0.57	8.69 ± 0.13	9.25 ± 0.18	8.71 ± 0.26
TC17001857.hg.1	CD300LB	9.07 ± 0.13	8.76 ± 0.18	9.04 ± 0.04	8.73 ± 0.05
TC17001859.hg.1	CD300LD	7.71 ± 0.18	7.62 ± 0.13	7.84 ± 0.16	7.69 ± 0.09
TC17001861.hg.1	CD300LF	8.69 ± 0.36	8.39 ± 0.11	9.13 ± 0.34	8.46 ± 0.04
TC17000559.hg.1	CD300LG	7.37 ± 0.15	7.32 ± 0.23	7.33 ± 0.16	7.44 ± 0.07
TC19001127.hg.1	CD320	8.07 ± 0.19	8.04 ± 0.18	7.88 ± 0.26	8.06 ± 0.08
TC19000775.hg.1	CD33	7.74 ± 0.15	7.54 ± 0.06	7.79 ± 0.02	7.57 ± 0.10
TC01003777.hg.1	CD34	6.75 ± 0.14	6.73 ± 0.14	6.73 ± 0.19	6.81 ± 0.04
TC07000509.hg.1	CD36	7.23 ± 0.38	7.09 ± 0.07	7.76 ± 0.33	6.90 ± 0.16
TC19000729.hg.1	CD37	12.28 ± 0.15	12.26 ± 0.09	12.21 ± 0.15	12.21 ± 0.06
TC04000145.hg.1	CD38	9.32 ± 0.24	8.80 ± 0.16	8.63 ± 0.19	8.34 ± 0.02
TC11002347.hg.1	CD3D	9.26 ± 0.18	9.24 ± 0.21	8.62 ± 0.31	8.90 ± 0.05
TC11001060.hg.1	CD3E	10.53 ± 0.20	10.74 ± 0.04	10.09 ± 0.21	10.57 ± 0.09
TC19000646.hg.1	CD3EAP	7.64 ± 0.18	7.61 ± 0.15	7.55 ± 0.09	7.63 ± 0.02

TC11001061.hg.1	CD3G	10.64 ± 0.26	10.78 ± 0.07	10.23 ± 0.10	10.57 ± 0.16
TC12000092.hg.1	CD4	9.70 ± 0.11	9.70 ± 0.06	9.33 ± 0.09	9.65 ± 0.16
TC20000365.hg.1	CD40	8.21 ± 0.09	8.21 ± 0.04	8.02 ± 0.12	8.35 ± 0.07
TC0X000666.hg.1	CD40LG	7.68 ± 0.23	7.58 ± 0.18	7.27 ± 0.24	7.49 ± 0.10
TC11000332.hg.1	CD44	9.66 ± 0.33	9.87 ± 0.09	10.23 ± 0.22	9.95 ± 0.11
TC11000333.hg.1	CD44	7.43 ± 0.82	8.12 ± 0.22	8.30 ± 0.64	8.15 ± 0.14
TC01001741.hg.1	CD46	9.10 ± 0.53	9.52 ± 0.26	9.67 ± 0.50	9.46 ± 0.08
TC03001629.hg.1	CD47	9.83 ± 0.52	10.12 ± 0.21	9.80 ± 0.38	10.04 ± 0.04
TC01003407.hg.1	CD48	10.41 ± 0.19	10.39 ± 0.05	10.38 ± 0.38	10.04 ± 0.02
TC11000526.hg.1	CD5	10.02 ± 0.16	10.22 ± 0.13	9.56 ± 0.11	10.32 ± 0.08
TC01000342.hg.1	CD52	9.42 ± 0.15	9.24 ± 0.19	9.09 ± 0.16	9.04 ± 0.08
TC01000968.hg.1	CD53	11.36 ± 0.16	11.19 ± 0.12	11.50 ± 0.28	11.02 ± 0.17
TC01001736.hg.1	CD55	9.22 ± 0.17	9.45 ± 0.06	9.77 ± 0.28	9.40 ± 0.06
TC01003029.hg.1	CD58	8.33 ± 0.13	8.17 ± 0.22	8.89 ± 0.36	8.11 ± 0.32
TC11001540.hg.1	CD59	8.01 ± 0.25	7.82 ± 0.04	8.36 ± 0.33	7.83 ± 0.07
TC01003368.hg.1	CD5L	6.31 ± 0.16	6.28 ± 0.12	6.26 ± 0.09	6.40 ± 0.14
TC11000523.hg.1	CD6	9.68 ± 0.11	9.66 ± 0.05	9.30 ± 0.14	9.70 ± 0.14
TC12001584.hg.1	CD63	9.60 ± 0.39	9.32 ± 0.07	9.66 ± 0.33	9.44 ± 0.26
TC17000103.hg.1	CD68	11.47 ± 0.34	11.11 ± 0.10	11.40 ± 0.04	11.11 ± 0.39
TC12001207.hg.1	CD69	9.51 ± 0.93	10.97 ± 0.20	9.95 ± 0.60	11.06 ± 0.24
TC17001978.hg.1	CD7	9.79 ± 0.02	9.85 ± 0.11	9.52 ± 0.05	9.84 ± 0.02
TC19001101.hg.1	CD70	7.70 ± 0.32	7.63 ± 0.26	7.54 ± 0.18	7.75 ± 0.11
TC09001054.hg.1	CD72	8.41 ± 0.10	8.27 ± 0.12	8.21 ± 0.03	8.08 ± 0.05
TC05001936.hg.1	CD74	11.86 ± 0.05	11.95 ± 0.05	11.57 ± 0.15	11.96 ± 0.05
TC19000588.hg.1	CD79A	11.25 ± 0.17	11.31 ± 0.04	10.52 ± 0.10	11.25 ± 0.10
TC17001792.hg.1	CD79B	9.19 ± 0.21	8.96 ± 0.11	8.71 ± 0.14	8.67 ± 0.01
TC03001684.hg.1	CD80	6.18 ± 0.12	6.02 ± 0.05	6.08 ± 0.08	6.07 ± 0.04
TC11000063.hg.1	CD81	9.18 ± 0.03	9.15 ± 0.09	8.92 ± 0.14	9.11 ± 0.02
TC11000374.hg.1	CD82	9.57 ± 0.52	9.26 ± 0.04	9.85 ± 0.33	9.51 ± 0.40
TC06000099.hg.1	CD83	8.17 ± 0.16	9.10 ± 0.31	8.14 ± 0.36	9.13 ± 0.10
TC01003405.hg.1	CD84	8.18 ± 0.15	8.11 ± 0.28	8.16 ± 0.30	8.02 ± 0.04

TC03000626.hg.1	CD86	8.06 ± 0.20	7.91 ± 0.18	8.04 ± 0.17	7.80 ± 0.27
TC02002059.hg.1	CD8A	9.17 ± 0.09	9.16 ± 0.10	8.89 ± 0.37	8.93 ± 0.07
TC02002060.hg.1	CD8B	7.69 ± 0.10	7.56 ± 0.10	7.33 ± 0.23	7.37 ± 0.07
TC02000653.hg.1	CD8BP	9.00 ± 0.23	9.10 ± 0.14	8.59 ± 0.49	9.00 ± 0.06
TC12000076.hg.1	CD9	8.83 ± 0.26	8.86 ± 0.10	9.26 ± 0.16	9.14 ± 0.16
TC20000703.hg.1	CD93	9.94 ± 0.20	9.82 ± 0.20	10.44 ± 0.28	9.92 ± 0.42
TC03000563.hg.1	CD96	9.45 ± 0.52	9.87 ± 0.20	9.20 ± 0.41	9.66 ± 0.17
TC19000256.hg.1	CD97	11.59 ± 0.29	11.34 ± 0.10	11.86 ± 0.39	11.28 ± 0.23
TC0X000014.hg.1	CD99	9.36 ± 0.08	9.22 ± 0.07	9.24 ± 0.15	9.12 ± 0.03
TC0Y000014.hg.1	CD99	9.85 ± 0.09	9.70 ± 0.09	9.72 ± 0.16	9.58 ± 0.03
TC0X001486.hg.1	CD99L2	8.34 ± 0.08	8.18 ± 0.02	8.31 ± 0.04	8.20 ± 0.02
TC0X000013.hg.1	CD99P1	7.91 ± 0.07	7.95 ± 0.11	7.94 ± 0.07	7.92 ± 0.05
TC0Y000013.hg.1	CD99P1	7.91 ± 0.08	7.94 ± 0.15	7.94 ± 0.12	7.88 ± 0.04
TC01000261.hg.1	CDA	10.18 ± 0.25	9.69 ± 0.13	10.24 ± 0.20	9.51 ± 0.34
TC13000197.hg.1	CDADC1	7.23 ± 0.11	7.31 ± 0.13	7.70 ± 0.50	7.22 ± 0.10
TC15001268.hg.1	CDAN1	7.89 ± 0.12	7.88 ± 0.11	7.78 ± 0.14	7.96 ± 0.03
TC10000092.hg.1	CDC123	8.68 ± 0.01	8.67 ± 0.25	8.91 ± 0.25	8.54 ± 0.11
TC01000902.hg.1	CDC14A	8.00 ± 0.37	8.26 ± 0.15	7.96 ± 0.25	8.20 ± 0.05
TC09001376.hg.1	CDC14B	6.85 ± 0.03	6.59 ± 0.03	6.89 ± 0.24	6.65 ± 0.09
TC07000309.hg.1	CDC14C	6.47 ± 0.18	6.24 ± 0.19	6.51 ± 0.19	6.32 ± 0.15
TC13000435.hg.1	CDC16	8.67 ± 0.27	8.65 ± 0.18	8.45 ± 0.17	8.43 ± 0.03
TC01000545.hg.1	CDC20	8.14 ± 0.43	7.76 ± 0.22	7.81 ± 0.43	7.63 ± 0.04
TC05001354.hg.1	CDC20B	6.18 ± 0.17	6.11 ± 0.12	6.12 ± 0.09	6.24 ± 0.09
TC09000395.hg.1	CDC20P1	7.42 ± 0.39	6.97 ± 0.30	7.05 ± 0.31	7.12 ± 0.12
TC05001827.hg.1	CDC23	7.87 ± 0.17	7.83 ± 0.14	7.64 ± 0.12	7.67 ± 0.08
TC03001374.hg.1	CDC25A	6.33 ± 0.24	6.13 ± 0.19	6.19 ± 0.17	5.94 ± 0.01
TC20000040.hg.1	CDC25B	9.38 ± 0.08	9.43 ± 0.12	8.94 ± 0.14	9.20 ± 0.09
TC05001829.hg.1	CDC25C	6.43 ± 0.14	6.31 ± 0.09	6.35 ± 0.13	6.31 ± 0.05
TC09002917.hg.1	CDC26	6.71 ± 0.14	6.68 ± 0.18	6.73 ± 0.29	6.55 ± 0.13
TC17001621.hg.1	CDC27	8.90 ± 0.38	8.87 ± 0.24	8.98 ± 0.35	8.80 ± 0.07
TC19000007.hg.1	CDC34	8.31 ± 0.21	8.08 ± 0.05	8.18 ± 0.25	8.00 ± 0.09

TC19001165.hg.1	CDC37	9.50 ± 0.13	9.39 ± 0.11	9.35 ± 0.16	9.31 ± 0.04
TC09000031.hg.1	CDC37L1	7.14 ± 0.32	7.24 ± 0.17	7.12 ± 0.28	7.26 ± 0.03
TC06000882.hg.1	CDC40	8.61 ± 0.40	8.86 ± 0.11	8.89 ± 0.42	8.82 ± 0.13
TC01000280.hg.1	CDC42	10.04 ± 0.18	10.25 ± 0.05	10.17 ± 0.17	10.26 ± 0.12
TC01003903.hg.1	CDC42BPA	5.53 ± 0.07	5.36 ± 0.03	5.61 ± 0.10	5.44 ± 0.05
TC14001534.hg.1	CDC42BPB	7.70 ± 0.07	7.64 ± 0.03	7.66 ± 0.02	7.61 ± 0.05
TC11001914.hg.1	CDC42BPG	8.19 ± 0.23	8.12 ± 0.18	8.10 ± 0.18	8.25 ± 0.03
TC22000282.hg.1	CDC42EP1	8.44 ± 0.21	8.40 ± 0.17	8.32 ± 0.17	8.48 ± 0.05
TC11000623.hg.1	CDC42EP2	8.99 ± 0.24	8.94 ± 0.18	9.08 ± 0.22	8.98 ± 0.03
TC02001746.hg.1	CDC42EP3	8.55 ± 0.18	8.43 ± 0.11	8.99 ± 0.21	8.29 ± 0.25
TC17001849.hg.1	CDC42EP4	7.83 ± 0.29	7.84 ± 0.17	7.86 ± 0.31	7.81 ± 0.06
TC19001849.hg.1	CDC42EP5	8.71 ± 0.30	8.66 ± 0.29	8.67 ± 0.35	8.71 ± 0.09
TC01000281.hg.1	CDC42-IT1	7.59 ± 0.38	7.70 ± 0.16	7.73 ± 0.25	7.74 ± 0.23
TC01003218.hg.1	CDC42SE1	10.83 ± 0.05	10.79 ± 0.04	10.96 ± 0.15	10.80 ± 0.07
TC05000623.hg.1	CDC42SE2	9.93 ± 0.42	10.36 ± 0.07	10.04 ± 0.35	10.42 ± 0.06
TC22000046.hg.1	CDC45	7.20 ± 0.24	7.12 ± 0.17	7.06 ± 0.21	7.02 ± 0.01
TC06000617.hg.1	CDC5L	8.47 ± 0.37	8.70 ± 0.12	8.66 ± 0.37	8.51 ± 0.18
TC17000495.hg.1	CDC6	6.91 ± 0.24	6.65 ± 0.35	6.61 ± 0.24	6.16 ± 0.12
TC01000846.hg.1	CDC7	6.81 ± 0.17	6.86 ± 0.30	6.69 ± 0.18	6.41 ± 0.14
TC01001626.hg.1	CDC73	8.97 ± 0.34	9.35 ± 0.21	9.26 ± 0.42	9.26 ± 0.13
TC08000196.hg.1	CDCA2	6.02 ± 0.18	5.91 ± 0.22	5.83 ± 0.11	5.65 ± 0.03
TC12001151.hg.1	CDCA3	7.03 ± 0.20	6.94 ± 0.11	6.92 ± 0.09	6.95 ± 0.04
TC14001563.hg.1	CDCA4	8.36 ± 0.15	8.42 ± 0.14	8.25 ± 0.19	8.35 ± 0.05
TC11001923.hg.1	CDCA5	8.02 ± 0.10	7.97 ± 0.16	8.00 ± 0.23	8.06 ± 0.08
TC11001924.hg.1	CDCA5	8.03 ± 0.28	7.94 ± 0.13	7.84 ± 0.31	7.92 ± 0.06
TC02001035.hg.1	CDCA7	7.03 ± 0.07	6.98 ± 0.36	6.85 ± 0.16	6.55 ± 0.04
TC07001184.hg.1	CDCA7L	8.85 ± 0.19	8.99 ± 0.18	8.56 ± 0.15	8.81 ± 0.01
TC01000476.hg.1	CDCA8	7.26 ± 0.21	7.03 ± 0.19	7.06 ± 0.29	6.94 ± 0.07
TC03001345.hg.1	CDCP1	6.89 ± 0.18	6.80 ± 0.21	6.88 ± 0.08	6.90 ± 0.01
TC01002682.hg.1	CDCP2	7.59 ± 0.30	7.70 ± 0.32	7.58 ± 0.23	7.79 ± 0.05
TC01006357.hg.1	CDCP2	6.37 ± 0.26	6.35 ± 0.17	6.26 ± 0.22	6.47 ± 0.14

TC16000559.hg.1	CDH1	6.56 ± 0.08	6.57 ± 0.12	6.42 ± 0.10	6.59 ± 0.05
TC05001205.hg.1	CDH10	4.51 ± 0.01	4.39 ± 0.05	4.45 ± 0.08	4.43 ± 0.11
TC16001165.hg.1	CDH11	6.29 ± 0.19	6.25 ± 0.13	6.22 ± 0.21	6.38 ± 0.08
TC05001200.hg.1	CDH12	5.21 ± 0.08	5.14 ± 0.06	5.18 ± 0.09	5.20 ± 0.08
TC16000647.hg.1	CDH13	6.33 ± 0.19	6.25 ± 0.11	6.28 ± 0.09	6.32 ± 0.06
TC16000692.hg.1	CDH15	8.20 ± 0.20	8.18 ± 0.27	8.10 ± 0.21	8.26 ± 0.02
TC16001176.hg.1	CDH16	7.69 ± 0.18	7.67 ± 0.18	7.65 ± 0.16	7.81 ± 0.06
TC08001431.hg.1	CDH17	5.19 ± 0.11	5.03 ± 0.06	5.04 ± 0.08	5.10 ± 0.04
TC05001195.hg.1	CDH18	4.92 ± 0.06	4.81 ± 0.07	4.83 ± 0.03	4.89 ± 0.07
TC18000562.hg.1	CDH19	4.20 ± 0.07	4.05 ± 0.05	4.08 ± 0.04	4.07 ± 0.07
TC18000433.hg.1	CDH2	5.92 ± 0.17	5.93 ± 0.11	5.87 ± 0.07	5.98 ± 0.06
TC18000217.hg.1	CDH20	6.33 ± 0.15	6.33 ± 0.10	6.32 ± 0.12	6.39 ± 0.04
TC20000898.hg.1	CDH22	7.79 ± 0.21	7.78 ± 0.19	7.72 ± 0.19	7.91 ± 0.04
TC10000442.hg.1	CDH23	7.76 ± 0.16	7.67 ± 0.12	7.80 ± 0.07	7.71 ± 0.04
TC10001371.hg.1	CDH23-AS1	7.39 ± 0.31	7.43 ± 0.08	7.34 ± 0.25	7.54 ± 0.06
TC14000947.hg.1	CDH24	7.75 ± 0.09	7.73 ± 0.15	7.64 ± 0.11	7.82 ± 0.06
TC20000474.hg.1	CDH26	6.17 ± 0.08	6.03 ± 0.06	6.20 ± 0.06	6.11 ± 0.08
TC16000558.hg.1	CDH3	7.05 ± 0.20	6.97 ± 0.14	6.99 ± 0.16	7.09 ± 0.05
TC20000481.hg.1	CDH4	7.48 ± 0.21	7.41 ± 0.16	7.42 ± 0.24	7.46 ± 0.01
TC16000514.hg.1	CDH5	6.90 ± 0.19	6.84 ± 0.21	6.76 ± 0.11	6.92 ± 0.02
TC05000132.hg.1	CDH6	5.38 ± 0.17	5.27 ± 0.09	5.34 ± 0.18	5.32 ± 0.09
TC18000235.hg.1	CDH7	6.03 ± 0.18	5.95 ± 0.11	5.97 ± 0.11	6.03 ± 0.08
TC16001161.hg.1	CDH8	5.22 ± 0.20	5.01 ± 0.02	5.11 ± 0.09	5.08 ± 0.10
TC05001216.hg.1	CDH9	4.44 ± 0.12	4.32 ± 0.04	4.38 ± 0.10	4.44 ± 0.12
TC10000593.hg.1	CDHR1	7.02 ± 0.09	6.98 ± 0.13	7.04 ± 0.12	7.08 ± 0.07
TC05001002.hg.1	CDHR2	7.69 ± 0.19	7.71 ± 0.21	7.70 ± 0.13	7.80 ± 0.07
TC07000684.hg.1	CDHR3	6.02 ± 0.12	6.01 ± 0.08	5.89 ± 0.11	6.02 ± 0.04
TC03001417.hg.1	CDHR4	7.42 ± 0.19	7.44 ± 0.21	7.35 ± 0.14	7.53 ± 0.04
TC11001242.hg.1	CDHR5	7.98 ± 0.18	7.90 ± 0.22	7.86 ± 0.17	8.00 ± 0.04
TC16000830.hg.1	CDIP1	8.49 ± 0.16	8.36 ± 0.17	8.27 ± 0.16	8.38 ± 0.06
TC16001011.hg.1	CDIPT	9.76 ± 0.21	9.47 ± 0.02	9.56 ± 0.23	9.40 ± 0.10

TC16000331.hg.1	CDIPT-AS1	7.36 ± 0.25	7.27 ± 0.15	7.23 ± 0.16	7.29 ± 0.07
TC10000373.hg.1	CDK1	5.56 ± 0.17	5.36 ± 0.39	5.46 ± 0.21	5.02 ± 0.06
TC16000703.hg.1	CDK10	8.24 ± 0.10	8.16 ± 0.11	8.08 ± 0.13	8.17 ± 0.07
TC01006297.hg.1	CDK11A	9.06 ± 0.04	9.13 ± 0.03	9.08 ± 0.06	9.18 ± 0.07
TC01006296.hg.1	CDK11B	9.26 ± 0.05	9.29 ± 0.07	9.27 ± 0.08	9.30 ± 0.13
TC17000474.hg.1	CDK12	9.21 ± 0.16	9.30 ± 0.16	9.09 ± 0.24	9.29 ± 0.10
TC17000475.hg.1	CDK12	7.50 ± 0.04	7.22 ± 0.13	7.40 ± 0.12	6.98 ± 0.08
TC07000254.hg.1	CDK13	9.51 ± 0.33	9.68 ± 0.15	9.61 ± 0.35	9.60 ± 0.22
TC07003336.hg.1	CDK14	7.06 ± 0.12	7.05 ± 0.18	7.32 ± 0.31	6.90 ± 0.10
TC02001181.hg.1	CDK15	6.25 ± 0.13	6.17 ± 0.11	6.28 ± 0.04	6.30 ± 0.08
TC0X000230.hg.1	CDK16	8.59 ± 0.02	8.80 ± 0.13	8.44 ± 0.08	8.91 ± 0.12
TC12001845.hg.1	CDK17	8.95 ± 0.47	9.22 ± 0.19	9.07 ± 0.44	9.20 ± 0.19
TC01001710.hg.1	CDK18	7.85 ± 0.16	7.78 ± 0.13	7.71 ± 0.15	7.83 ± 0.04
TC06002010.hg.1	CDK19	8.80 ± 0.15	8.86 ± 0.18	9.02 ± 0.27	8.68 ± 0.14
TC12000496.hg.1	CDK2	8.29 ± 0.12	8.09 ± 0.17	7.99 ± 0.12	7.87 ± 0.04
TC09001306.hg.1	CDK20	7.69 ± 0.19	7.62 ± 0.14	7.56 ± 0.15	7.67 ± 0.04
TC12002085.hg.1	CDK2AP1	8.55 ± 0.09	8.55 ± 0.01	8.73 ± 0.05	8.55 ± 0.07
TC11001987.hg.1	CDK2AP2	9.05 ± 0.21	8.79 ± 0.09	8.88 ± 0.23	8.67 ± 0.12
TC12001633.hg.1	CDK4	8.48 ± 0.32	8.21 ± 0.14	8.00 ± 0.25	7.92 ± 0.03
TC07002027.hg.1	CDK5	8.14 ± 0.27	7.84 ± 0.09	8.00 ± 0.10	7.68 ± 0.06
TC17000377.hg.1	CDK5R1	8.34 ± 0.15	8.32 ± 0.10	8.11 ± 0.15	8.31 ± 0.05
TC02001315.hg.1	CDK5R2	8.09 ± 0.24	8.08 ± 0.24	8.10 ± 0.18	8.21 ± 0.05
TC20000769.hg.1	CDK5RAP1	8.37 ± 0.15	8.38 ± 0.04	8.16 ± 0.15	8.42 ± 0.01
TC09001536.hg.1	CDK5RAP2	7.89 ± 0.15	7.80 ± 0.09	7.81 ± 0.15	7.71 ± 0.04
TC17000618.hg.1	CDK5RAP3	9.35 ± 0.11	9.24 ± 0.08	9.26 ± 0.24	9.16 ± 0.18
TC07001603.hg.1	CDK6	8.09 ± 0.27	8.18 ± 0.13	7.90 ± 0.27	8.03 ± 0.11
TC05000301.hg.1	CDK7	7.41 ± 0.15	7.44 ± 0.16	7.45 ± 0.25	7.39 ± 0.08
TC13000068.hg.1	CDK8	7.72 ± 0.23	7.80 ± 0.24	7.62 ± 0.27	7.53 ± 0.11
TC09000671.hg.1	CDK9	9.49 ± 0.14	9.38 ± 0.03	9.34 ± 0.06	9.44 ± 0.05
TC06000132.hg.1	CDKAL1	8.25 ± 0.26	8.39 ± 0.19	8.03 ± 0.18	8.21 ± 0.05
TC14001119.hg.1	CDKL1	6.76 ± 0.08	6.62 ± 0.03	6.80 ± 0.07	6.65 ± 0.03

TC04001299.hg.1	CDKL2	5.07 ± 0.09	4.97 ± 0.04	4.96 ± 0.04	4.95 ± 0.02
TC05003407.hg.1	CDKL3	4.97 ± 0.08	4.93 ± 0.01	4.93 ± 0.05	4.95 ± 0.07
TC02001764.hg.1	CDKL4	4.65 ± 0.02	4.56 ± 0.03	4.64 ± 0.11	4.56 ± 0.03
TC0X000088.hg.1	CDKL5	7.68 ± 0.13	7.48 ± 0.16	8.01 ± 0.28	7.42 ± 0.19
TC06000532.hg.1	CDKN1A	8.65 ± 0.12	8.63 ± 0.15	8.52 ± 0.12	8.68 ± 0.09
TC12000178.hg.1	CDKN1B	9.25 ± 0.14	9.49 ± 0.13	9.34 ± 0.12	9.42 ± 0.03
TC11001284.hg.1	CDKN1C	8.45 ± 0.32	8.30 ± 0.06	8.36 ± 0.26	8.24 ± 0.03
TC09000964.hg.1	CDKN2A	7.25 ± 0.19	7.14 ± 0.10	7.12 ± 0.15	7.15 ± 0.04
TC04000889.hg.1	CDKN2AIP	8.08 ± 0.35	8.27 ± 0.11	7.98 ± 0.24	8.18 ± 0.11
TC05001788.hg.1	CDKN2AIPNL	7.89 ± 0.06	7.78 ± 0.12	7.76 ± 0.13	7.72 ± 0.12
TC09000965.hg.1	CDKN2B	7.18 ± 0.19	6.92 ± 0.16	7.23 ± 0.14	6.95 ± 0.06
TC09002881.hg.1	CDKN2B-AS1	5.48 ± 0.15	5.33 ± 0.01	5.36 ± 0.09	5.34 ± 0.03
TC01000619.hg.1	CDKN2C	7.00 ± 0.18	6.82 ± 0.07	6.90 ± 0.14	6.75 ± 0.08
TC19001170.hg.1	CDKN2D	9.48 ± 0.34	9.38 ± 0.10	9.37 ± 0.27	9.36 ± 0.05
TC14000316.hg.1	CDKN3	5.73 ± 0.14	5.69 ± 0.31	5.57 ± 0.05	5.34 ± 0.06
TC10001057.hg.1	CDNF	6.03 ± 0.06	5.94 ± 0.05	5.86 ± 0.10	6.00 ± 0.03
TC05001693.hg.1	CDO1	6.07 ± 0.10	5.95 ± 0.08	6.00 ± 0.04	6.04 ± 0.06
TC11002427.hg.1	CDON	6.01 ± 0.08	5.96 ± 0.06	5.97 ± 0.05	6.03 ± 0.10
TC22000886.hg.1	CDPF1	8.04 ± 0.20	8.04 ± 0.14	7.82 ± 0.27	8.08 ± 0.07
TC0X001435.hg.1	CDR1	6.80 ± 0.13	6.63 ± 0.20	6.58 ± 0.17	6.66 ± 0.05
TC16000948.hg.1	CDR2	8.70 ± 0.19	9.10 ± 0.21	8.23 ± 0.04	9.17 ± 0.09
TC17000838.hg.1	CDR2L	7.68 ± 0.28	7.58 ± 0.22	7.60 ± 0.29	7.68 ± 0.04
TC17002888.hg.1	CDRT1	6.08 ± 0.12	6.05 ± 0.11	6.01 ± 0.20	6.12 ± 0.12
TC17001154.hg.1	CDRT15	8.15 ± 0.22	8.08 ± 0.16	8.10 ± 0.19	8.18 ± 0.08
TC17000289.hg.1	CDRT15L2	7.39 ± 0.38	7.43 ± 0.09	7.45 ± 0.23	7.52 ± 0.13
TC17000161.hg.1	CDRT15P1	7.63 ± 0.15	7.50 ± 0.12	7.60 ± 0.09	7.57 ± 0.06
TC17000177.hg.1	CDRT15P2	7.87 ± 0.11	7.73 ± 0.19	7.77 ± 0.33	7.90 ± 0.08
TC17000171.hg.1	CDRT7	4.81 ± 0.08	4.74 ± 0.14	4.81 ± 0.09	4.86 ± 0.13
TC04000462.hg.1	CDS1	5.28 ± 0.13	5.24 ± 0.07	5.26 ± 0.10	5.28 ± 0.06
TC20000056.hg.1	CDS2	9.36 ± 0.20	9.26 ± 0.13	9.60 ± 0.35	9.19 ± 0.09
TC06001518.hg.1	CDSN	7.47 ± 0.18	7.64 ± 0.17	7.54 ± 0.12	7.73 ± 0.13

TC6_cox_hap2000150.hg.1	CDSN	7.52 ± 0.21	7.65 ± 0.27	7.55 ± 0.17	7.83 ± 0.15
TC6_dbb_hap3000139.hg.1	CDSN	6.98 ± 0.24	7.09 ± 0.14	7.02 ± 0.03	7.17 ± 0.14
TC6_mann_hap4000127.hg.1	CDSN	7.43 ± 0.21	7.54 ± 0.14	7.47 ± 0.03	7.67 ± 0.14
TC6_mcf_hap5000127.hg.1	CDSN	7.59 ± 0.24	7.67 ± 0.12	7.63 ± 0.03	7.77 ± 0.14
TC6_qbl_hap6000140.hg.1	CDSN	6.98 ± 0.15	7.07 ± 0.15	7.01 ± 0.06	7.15 ± 0.14
TC16000686.hg.1	CDT1	8.40 ± 0.24	8.35 ± 0.20	8.27 ± 0.28	8.35 ± 0.01
TC03000722.hg.1	CDV3	10.23 ± 0.05	10.35 ± 0.04	10.26 ± 0.09	10.31 ± 0.16
TC05000828.hg.1	CDX1	7.65 ± 0.18	7.59 ± 0.24	7.55 ± 0.20	7.70 ± 0.05
TC13000513.hg.1	CDX2	6.37 ± 0.19	6.35 ± 0.11	6.31 ± 0.15	6.27 ± 0.02
TC0X000402.hg.1	CDX4	6.09 ± 0.23	5.91 ± 0.04	5.93 ± 0.11	5.97 ± 0.09
TC0Y000100.hg.1	CDY1B	4.87 ± 0.05	4.79 ± 0.02	4.92 ± 0.15	4.94 ± 0.06
TC0Y000203.hg.1	CDY1B	4.49 ± 0.04	4.45 ± 0.04	4.58 ± 0.13	4.60 ± 0.05
TC0Y000063.hg.1	CDY2B	5.46 ± 0.21	5.29 ± 0.11	5.34 ± 0.16	5.51 ± 0.13
TC0Y000174.hg.1	CDY2B	5.46 ± 0.16	5.24 ± 0.09	5.27 ± 0.13	5.42 ± 0.17
TC06000044.hg.1	CDYL	7.30 ± 0.07	7.12 ± 0.05	7.17 ± 0.03	7.12 ± 0.04
TC16001294.hg.1	CDYL2	8.16 ± 0.01	8.20 ± 0.07	8.19 ± 0.11	8.23 ± 0.02
TC19001575.hg.1	CEACAM1	9.24 ± 0.54	8.66 ± 0.34	10.34 ± 0.64	8.63 ± 0.14
TC19000626.hg.1	CEACAM16	7.24 ± 0.17	7.28 ± 0.17	7.27 ± 0.16	7.38 ± 0.10
TC19000780.hg.1	CEACAM18	7.10 ± 0.20	7.14 ± 0.29	7.14 ± 0.16	7.38 ± 0.14
TC19000625.hg.1	CEACAM19	7.13 ± 0.22	7.08 ± 0.15	7.09 ± 0.09	7.18 ± 0.07
TC19001608.hg.1	CEACAM20	6.87 ± 0.16	6.78 ± 0.17	6.81 ± 0.15	6.87 ± 0.06
TC19000581.hg.1	CEACAM21	7.66 ± 0.10	7.47 ± 0.02	7.56 ± 0.05	7.43 ± 0.04
TC19001609.hg.1	CEACAM22P	6.49 ± 0.17	6.33 ± 0.14	6.36 ± 0.13	6.45 ± 0.07
TC19000585.hg.1	CEACAM3	9.13 ± 0.47	8.62 ± 0.20	9.36 ± 0.12	8.57 ± 0.15
TC19001559.hg.1	CEACAM4	8.97 ± 0.07	8.79 ± 0.10	9.14 ± 0.17	8.88 ± 0.12
TC19000583.hg.1	CEACAM5	6.89 ± 0.17	6.61 ± 0.14	6.90 ± 0.09	6.69 ± 0.06
TC19000584.hg.1	CEACAM6	8.20 ± 0.40	7.52 ± 0.09	8.34 ± 0.37	7.54 ± 0.09
TC19001560.hg.1	CEACAM7	6.25 ± 0.11	6.14 ± 0.11	6.21 ± 0.04	6.21 ± 0.08
TC19001576.hg.1	CEACAM8	9.11 ± 0.83	7.82 ± 0.03	9.34 ± 0.82	7.79 ± 0.26
TC19000582.hg.1	CEACAMP3	6.67 ± 0.08	6.72 ± 0.05	6.73 ± 0.19	6.68 ± 0.05
TC19001396.hg.1	CEBPA	9.18 ± 0.31	8.89 ± 0.08	9.08 ± 0.23	8.81 ± 0.13

TC19000436.hg.1	CEBPA-AS1	7.71 ± 0.40	7.60 ± 0.30	7.61 ± 0.29	7.63 ± 0.12
TC20000399.hg.1	CEBPB	9.56 ± 0.34	9.64 ± 0.08	9.89 ± 0.08	9.71 ± 0.19
TC08001190.hg.1	CEBPD	9.00 ± 0.40	8.74 ± 0.09	9.05 ± 0.17	8.67 ± 0.17
TC14000950.hg.1	CEBPE	7.29 ± 0.27	7.13 ± 0.15	7.23 ± 0.35	7.34 ± 0.05
TC19000437.hg.1	CEBPG	8.05 ± 0.06	8.00 ± 0.09	7.94 ± 0.03	7.92 ± 0.08
TC02001741.hg.1	CEBPZ	7.63 ± 0.66	8.33 ± 0.18	7.80 ± 0.59	8.28 ± 0.15
TC22001480.hg.1	CECR1	9.98 ± 0.22	9.70 ± 0.14	9.69 ± 0.05	9.56 ± 0.15
TC22000023.hg.1	CECR2	6.33 ± 0.19	6.30 ± 0.12	6.31 ± 0.14	6.35 ± 0.02
TC22001479.hg.1	CECR3	7.03 ± 0.10	6.96 ± 0.14	7.00 ± 0.14	7.12 ± 0.05
TC22000471.hg.1	CECR5	7.98 ± 0.16	7.91 ± 0.12	7.82 ± 0.17	7.88 ± 0.03
TC22000021.hg.1	CECR5-AS1	7.79 ± 0.30	7.71 ± 0.17	7.68 ± 0.11	7.72 ± 0.11
TC22000470.hg.1	CECR6	8.40 ± 0.17	8.17 ± 0.18	8.29 ± 0.12	8.27 ± 0.07
TC22000018.hg.1	CECR7	7.30 ± 0.40	7.39 ± 0.20	7.28 ± 0.17	7.59 ± 0.14
TC22000022.hg.1	CECR9	5.77 ± 0.29	5.54 ± 0.06	5.65 ± 0.04	5.57 ± 0.10
TC09000767.hg.1	CEL	7.66 ± 0.23	7.53 ± 0.21	7.71 ± 0.17	7.59 ± 0.02
TC12001508.hg.1	CELA1	6.96 ± 0.17	6.88 ± 0.07	6.98 ± 0.09	6.90 ± 0.04
TC01006265.hg.1	CELA2A	7.41 ± 0.15	7.35 ± 0.16	7.38 ± 0.20	7.47 ± 0.06
TC01006266.hg.1	CELA2B	6.85 ± 0.21	6.79 ± 0.17	6.75 ± 0.22	6.91 ± 0.01
TC01006312.hg.1	CELA3A	7.54 ± 0.17	7.51 ± 0.19	7.43 ± 0.17	7.63 ± 0.08
TC01006313.hg.1	CELA3B	7.67 ± 0.18	7.63 ± 0.16	7.61 ± 0.17	7.77 ± 0.09
TC11001741.hg.1	CELF1	9.29 ± 0.19	9.20 ± 0.13	9.25 ± 0.17	9.17 ± 0.07
TC10000081.hg.1	CELF2	10.71 ± 0.14	10.70 ± 0.07	10.85 ± 0.26	10.67 ± 0.08
TC10001036.hg.1	CELF2-AS1	6.72 ± 0.08	6.82 ± 0.28	6.92 ± 0.42	6.75 ± 0.15
TC10001034.hg.1	CELF2-AS2	4.81 ± 0.10	4.70 ± 0.04	4.72 ± 0.02	4.75 ± 0.04
TC01003231.hg.1	CELF3	7.89 ± 0.21	7.89 ± 0.24	7.81 ± 0.23	8.02 ± 0.04
TC18000463.hg.1	CELF4	7.80 ± 0.21	7.76 ± 0.19	7.74 ± 0.25	7.80 ± 0.02
TC19000068.hg.1	CELF5	7.76 ± 0.18	7.74 ± 0.23	7.71 ± 0.18	7.87 ± 0.06
TC15002778.hg.1	CELF6	8.08 ± 0.19	8.07 ± 0.16	8.00 ± 0.21	8.12 ± 0.03
TC09000768.hg.1	CELP	7.52 ± 0.16	7.43 ± 0.16	7.56 ± 0.10	7.49 ± 0.03
TC22000889.hg.1	CELSR1	7.62 ± 0.20	7.59 ± 0.14	7.51 ± 0.18	7.64 ± 0.05
TC01000941.hg.1	CELSR2	7.97 ± 0.14	7.93 ± 0.14	7.84 ± 0.18	8.02 ± 0.05

TC03003382.hg.1	CELSR3	7.86 ± 0.13	7.86 ± 0.14	7.82 ± 0.13	7.94 ± 0.05
TC16000784.hg.1	CEMP1	8.35 ± 0.11	8.27 ± 0.10	8.25 ± 0.17	8.37 ± 0.10
TC11001246.hg.1	CEND1	8.30 ± 0.21	8.17 ± 0.20	8.19 ± 0.25	8.25 ± 0.06
TC02004917.hg.1	CENPA	7.64 ± 0.20	7.57 ± 0.15	7.48 ± 0.20	7.55 ± 0.04
TC20000578.hg.1	CENPB	7.73 ± 0.12	7.71 ± 0.18	7.55 ± 0.09	7.80 ± 0.03
TC16001360.hg.1	CENPBD1	8.48 ± 0.10	8.33 ± 0.20	8.44 ± 0.31	8.36 ± 0.16
TC04001249.hg.1	CENPC1	7.53 ± 0.80	8.31 ± 0.28	7.59 ± 0.60	8.34 ± 0.10
TC04001436.hg.1	CENPE	5.73 ± 0.20	5.75 ± 0.43	5.75 ± 0.52	5.26 ± 0.18
TC01001791.hg.1	CENPF	6.40 ± 0.06	6.43 ± 0.43	6.26 ± 0.23	5.91 ± 0.11
TC05000299.hg.1	CENPH	5.79 ± 0.02	5.65 ± 0.20	5.66 ± 0.12	5.46 ± 0.06
TC0X000475.hg.1	CENPI	5.75 ± 0.04	5.63 ± 0.08	5.68 ± 0.09	5.49 ± 0.03
TC13000493.hg.1	CENPJ	6.70 ± 0.09	6.71 ± 0.02	6.66 ± 0.10	6.55 ± 0.08
TC05001408.hg.1	CENPK	6.69 ± 0.51	7.00 ± 0.19	6.61 ± 0.36	6.78 ± 0.09
TC01003531.hg.1	CENPL	6.70 ± 0.12	6.59 ± 0.02	6.48 ± 0.10	6.56 ± 0.03
TC22000836.hg.1	CENPM	8.15 ± 0.27	8.07 ± 0.16	8.05 ± 0.24	8.14 ± 0.08
TC16000634.hg.1	CENPN	7.07 ± 0.15	7.04 ± 0.24	6.87 ± 0.20	6.67 ± 0.10
TC02000139.hg.1	CENPO	7.62 ± 0.12	7.50 ± 0.07	7.48 ± 0.16	7.48 ± 0.02
TC09000436.hg.1	CENPP	6.91 ± 0.05	6.93 ± 0.12	6.77 ± 0.12	6.80 ± 0.01
TC06000640.hg.1	CENPQ	5.19 ± 0.22	5.05 ± 0.25	5.19 ± 0.26	4.84 ± 0.07
TC16001197.hg.1	CENPT	8.17 ± 0.05	8.21 ± 0.06	8.06 ± 0.04	8.22 ± 0.11
TC17001173.hg.1	CENPV	7.51 ± 0.18	7.43 ± 0.08	7.29 ± 0.15	7.39 ± 0.03
TC0X001045.hg.1	CENPVP1	7.03 ± 0.07	7.11 ± 0.14	7.02 ± 0.09	7.16 ± 0.06
TC06000966.hg.1	CENPW	6.37 ± 0.27	6.12 ± 0.19	6.10 ± 0.21	5.91 ± 0.08
TC01002137.hg.1	CEP104	8.22 ± 0.10	8.16 ± 0.11	8.14 ± 0.11	7.99 ± 0.16
TC17002856.hg.1	CEP112	4.99 ± 0.11	4.97 ± 0.03	4.98 ± 0.05	4.99 ± 0.01
TC05001725.hg.1	CEP120	7.74 ± 0.53	8.19 ± 0.19	7.78 ± 0.47	8.12 ± 0.07
TC14001366.hg.1	CEP128	7.09 ± 0.16	6.97 ± 0.08	6.83 ± 0.26	6.69 ± 0.14
TC04000327.hg.1	CEP135	7.29 ± 0.68	7.68 ± 0.23	7.63 ± 0.48	7.54 ± 0.19
TC15001337.hg.1	CEP152	6.66 ± 0.29	6.85 ± 0.09	6.94 ± 0.37	6.59 ± 0.08
TC11001055.hg.1	CEP164	7.88 ± 0.06	7.78 ± 0.04	7.77 ± 0.09	7.78 ± 0.01
TC10001218.hg.1	CEP164P1	5.20 ± 0.02	5.15 ± 0.03	5.26 ± 0.06	5.17 ± 0.05

TC01004038.hg.1	CEP170	7.44 ± 0.30	7.46 ± 0.21	7.80 ± 0.39	7.46 ± 0.12
TC14000826.hg.1	CEP170B	8.02 ± 0.23	7.92 ± 0.19	7.89 ± 0.23	8.02 ± 0.01
TC04000604.hg.1	CEP170P1	6.88 ± 0.26	6.86 ± 0.17	7.22 ± 0.38	6.80 ± 0.10
TC03002165.hg.1	CEP19	8.75 ± 0.26	8.09 ± 0.24	9.17 ± 0.56	7.79 ± 0.40
TC18000070.hg.1	CEP192	7.95 ± 0.43	8.03 ± 0.28	7.90 ± 0.31	7.82 ± 0.08
TC20000253.hg.1	CEP250	7.82 ± 0.09	7.73 ± 0.09	7.71 ± 0.13	7.75 ± 0.01
TC12001791.hg.1	CEP290	6.21 ± 0.64	6.53 ± 0.31	6.27 ± 0.51	6.23 ± 0.22
TC01001558.hg.1	CEP350	8.38 ± 0.43	8.67 ± 0.26	8.55 ± 0.47	8.47 ± 0.13
TC07001863.hg.1	CEP41	6.41 ± 0.10	6.19 ± 0.01	6.21 ± 0.05	6.11 ± 0.02
TC04000859.hg.1	CEP44	6.75 ± 0.35	6.78 ± 0.06	6.68 ± 0.17	6.66 ± 0.07
TC10000664.hg.1	CEP55	5.92 ± 0.20	5.78 ± 0.48	5.73 ± 0.14	5.22 ± 0.15
TC11000933.hg.1	CEP57	7.84 ± 0.42	8.07 ± 0.10	7.81 ± 0.51	7.95 ± 0.11
TC06000871.hg.1	CEP57L1	5.84 ± 0.09	5.76 ± 0.02	5.80 ± 0.01	5.73 ± 0.02
TC10000427.hg.1	CEP57L1P1	5.33 ± 0.19	5.24 ± 0.22	5.38 ± 0.18	5.19 ± 0.18
TC03000729.hg.1	CEP63	8.33 ± 0.26	8.45 ± 0.22	8.74 ± 0.29	8.28 ± 0.22
TC02000376.hg.1	CEP68	7.93 ± 0.12	8.01 ± 0.04	7.81 ± 0.04	8.05 ± 0.08
TC03001823.hg.1	CEP70	5.92 ± 0.16	5.91 ± 0.13	5.74 ± 0.06	5.86 ± 0.01
TC05000009.hg.1	CEP72	7.48 ± 0.06	7.45 ± 0.11	7.37 ± 0.12	7.46 ± 0.04
TC18000376.hg.1	CEP76	6.56 ± 0.09	6.51 ± 0.05	6.42 ± 0.14	6.46 ± 0.06
TC09000357.hg.1	CEP78	7.27 ± 0.57	7.53 ± 0.29	7.00 ± 0.24	7.11 ± 0.15
TC01000339.hg.1	CEP85	7.76 ± 0.08	7.64 ± 0.05	7.71 ± 0.06	7.65 ± 0.04
TC06002058.hg.1	CEP85L	7.44 ± 0.69	8.07 ± 0.22	7.55 ± 0.46	7.90 ± 0.11
TC19001393.hg.1	CEP89	6.92 ± 0.03	6.70 ± 0.07	6.73 ± 0.04	6.60 ± 0.11
TC17000778.hg.1	CEP95	8.03 ± 0.58	8.42 ± 0.19	7.90 ± 0.40	8.62 ± 0.15
TC03000524.hg.1	CEP97	7.47 ± 0.26	7.25 ± 0.20	7.38 ± 0.40	6.99 ± 0.14
TC01000971.hg.1	CEPT1	8.46 ± 0.25	8.28 ± 0.23	8.65 ± 0.39	8.16 ± 0.16
TC09000919.hg.1	CER1	6.21 ± 0.18	6.07 ± 0.19	6.09 ± 0.05	6.12 ± 0.05
TC09000690.hg.1	CERCAM	8.01 ± 0.22	7.93 ± 0.21	7.91 ± 0.20	7.99 ± 0.03
TC22000890.hg.1	CERK	9.23 ± 0.01	9.16 ± 0.05	9.05 ± 0.05	9.21 ± 0.11
TC02005049.hg.1	CERKL	5.60 ± 0.07	5.53 ± 0.05	5.58 ± 0.02	5.55 ± 0.03
TC19001294.hg.1	CERS1	7.73 ± 0.26	7.74 ± 0.23	7.69 ± 0.19	7.81 ± 0.07

TC01003215.hg.1	CERS2	9.67 ± 0.09	9.49 ± 0.04	9.63 ± 0.19	9.45 ± 0.10
TC15001996.hg.1	CERS3	6.08 ± 0.13	6.04 ± 0.08	6.03 ± 0.07	6.07 ± 0.06
TC19000144.hg.1	CERS4	8.17 ± 0.12	8.01 ± 0.08	8.07 ± 0.14	8.01 ± 0.01
TC12001490.hg.1	CERS5	8.02 ± 0.16	7.94 ± 0.22	7.89 ± 0.15	7.89 ± 0.02
TC02000996.hg.1	CERS6	8.63 ± 0.23	8.88 ± 0.16	8.54 ± 0.27	8.69 ± 0.12
TC02002506.hg.1	CERS6-AS1	6.28 ± 0.19	6.25 ± 0.10	6.17 ± 0.10	6.35 ± 0.15
TC16001128.hg.1	CES1	8.12 ± 0.36	7.64 ± 0.02	8.09 ± 0.06	7.59 ± 0.05
TC16000460.hg.1	CES1P1	7.92 ± 0.22	7.73 ± 0.12	7.89 ± 0.12	7.74 ± 0.06
TC16000459.hg.1	CES1P2	7.08 ± 0.09	6.92 ± 0.09	7.05 ± 0.05	6.96 ± 0.06
TC16000524.hg.1	CES2	8.59 ± 0.16	8.42 ± 0.02	8.40 ± 0.04	8.41 ± 0.04
TC16000525.hg.1	CES3	7.40 ± 0.19	7.44 ± 0.17	7.31 ± 0.16	7.60 ± 0.15
TC16000526.hg.1	CES4A	7.78 ± 0.07	7.71 ± 0.07	7.72 ± 0.08	7.75 ± 0.04
TC16001129.hg.1	CES5A	6.36 ± 0.09	6.32 ± 0.11	6.29 ± 0.06	6.39 ± 0.06
TC22000558.hg.1	CES5AP1	6.55 ± 0.22	6.67 ± 0.14	6.65 ± 0.12	6.75 ± 0.09
TC18000004.hg.1	CETN1	4.58 ± 0.20	4.42 ± 0.02	4.45 ± 0.16	4.47 ± 0.11
TC0X001506.hg.1	CETN2	7.62 ± 0.10	7.47 ± 0.27	7.68 ± 0.21	7.28 ± 0.11
TC05001584.hg.1	CETN3	5.39 ± 0.33	5.15 ± 0.18	5.26 ± 0.24	4.98 ± 0.01
TC04001522.hg.1	CETN4P	5.27 ± 0.41	5.12 ± 0.13	5.06 ± 0.08	5.19 ± 0.05
TC16000481.hg.1	CETP	7.12 ± 0.14	6.92 ± 0.14	7.23 ± 0.18	7.02 ± 0.04
TC06004077.hg.1	CFB	6.84 ± 0.16	6.76 ± 0.12	6.76 ± 0.11	6.86 ± 0.06
TC6_cox_hap2000245.hg.1	CFB	6.76 ± 0.16	6.71 ± 0.13	6.70 ± 0.12	6.81 ± 0.06
TC6_dbb_hap3000222.hg.1	CFB	6.78 ± 0.15	6.73 ± 0.13	6.72 ± 0.11	6.83 ± 0.06
TC6_mann_hap4000196.hg.1	CFB	6.87 ± 0.13	6.83 ± 0.14	6.82 ± 0.12	6.93 ± 0.08
TC6_mcf_hap5000213.hg.1	CFB	6.78 ± 0.15	6.73 ± 0.13	6.73 ± 0.11	6.83 ± 0.06
TC6_qbl_hap6000225.hg.1	CFB	6.76 ± 0.16	6.71 ± 0.13	6.70 ± 0.11	6.81 ± 0.06
TC6_ssto_hap7000199.hg.1	CFB	6.78 ± 0.15	6.73 ± 0.13	6.73 ± 0.11	6.83 ± 0.06
TC02000813.hg.1	CFC1B	6.52 ± 0.14	6.43 ± 0.10	6.44 ± 0.14	6.47 ± 0.02
TC02002344.hg.1	CFC1B	6.57 ± 0.13	6.46 ± 0.08	6.49 ± 0.11	6.51 ± 0.04
TC19000020.hg.1	CFD	9.35 ± 0.25	9.35 ± 0.10	9.26 ± 0.24	9.15 ± 0.08
TC16001278.hg.1	CFDP1	8.09 ± 0.06	8.16 ± 0.12	7.93 ± 0.08	7.98 ± 0.04
TC01006291.hg.1	CFH	4.65 ± 0.05	4.49 ± 0.01	4.56 ± 0.05	4.51 ± 0.07

TC01006294.hg.1	CFHR1	5.33 ± 0.18	5.20 ± 0.05	5.17 ± 0.06	5.31 ± 0.14
TC01006295.hg.1	CFHR2	4.79 ± 0.23	4.71 ± 0.15	4.68 ± 0.09	4.79 ± 0.05
TC01006293.hg.1	CFHR3	3.96 ± 0.10	3.79 ± 0.03	3.86 ± 0.03	3.82 ± 0.05
TC01006292.hg.1	CFHR4	4.12 ± 0.13	4.02 ± 0.02	4.07 ± 0.02	4.09 ± 0.09
TC01001634.hg.1	CFHR5	4.22 ± 0.10	4.04 ± 0.14	4.07 ± 0.11	4.11 ± 0.10
TC04001466.hg.1	CFI	4.59 ± 0.14	4.47 ± 0.06	4.55 ± 0.04	4.54 ± 0.02
TC11001944.hg.1	CFL1	6.28 ± 0.36	6.37 ± 0.10	6.36 ± 0.09	6.45 ± 0.03
TC11001945.hg.1	CFL1	9.43 ± 0.21	9.23 ± 0.05	9.44 ± 0.16	9.12 ± 0.10
TC10000622.hg.1	CFL1P1	6.96 ± 0.34	6.91 ± 0.05	7.02 ± 0.07	6.96 ± 0.05
TC01002736.hg.1	CFL1P3	9.34 ± 0.20	8.97 ± 0.06	9.22 ± 0.32	8.92 ± 0.19
TC14001029.hg.1	CFL2	7.05 ± 0.28	7.63 ± 0.23	6.81 ± 0.30	7.54 ± 0.14
TC02001174.hg.1	CFLAR	9.57 ± 0.17	9.27 ± 0.19	9.91 ± 0.31	9.17 ± 0.21
TC02002672.hg.1	CFLAR-AS1	6.00 ± 0.32	5.80 ± 0.14	6.31 ± 0.21	5.67 ± 0.10
TC0X000998.hg.1	CFP	9.42 ± 0.23	8.95 ± 0.07	9.43 ± 0.18	8.86 ± 0.19
TC07000726.hg.1	CFTR	5.06 ± 0.15	4.98 ± 0.09	4.98 ± 0.06	5.05 ± 0.08
TC07000727.hg.1	CFTR	6.02 ± 0.28	5.82 ± 0.16	5.87 ± 0.12	5.91 ± 0.03
TC06001926.hg.1	CGA	6.73 ± 0.05	6.62 ± 0.12	6.67 ± 0.09	6.73 ± 0.06
TC19002714.hg.1	CGB	7.95 ± 0.21	8.10 ± 0.33	7.91 ± 0.29	8.18 ± 0.07
TC19002716.hg.1	CGB1	7.68 ± 0.07	7.70 ± 0.11	7.61 ± 0.08	7.69 ± 0.06
TC19000721.hg.1	CGB2	8.14 ± 0.20	8.17 ± 0.26	8.10 ± 0.20	8.29 ± 0.06
TC19000723.hg.1	CGB5	7.81 ± 0.14	7.83 ± 0.24	7.72 ± 0.21	7.86 ± 0.05
TC19002717.hg.1	CGB7	8.14 ± 0.26	8.12 ± 0.23	8.04 ± 0.30	8.20 ± 0.07
TC19002715.hg.1	CGB8	8.11 ± 0.19	8.13 ± 0.20	8.04 ± 0.17	8.13 ± 0.05
TC03001588.hg.1	CGGBP1	8.91 ± 0.19	8.98 ± 0.08	8.92 ± 0.20	8.87 ± 0.05
TC01001211.hg.1	CGN	7.20 ± 0.20	7.14 ± 0.16	7.12 ± 0.19	7.21 ± 0.02
TC15000437.hg.1	CGNL1	6.72 ± 0.23	6.59 ± 0.14	6.63 ± 0.22	6.68 ± 0.06
TC15000438.hg.1	CGNL1	5.88 ± 0.09	5.67 ± 0.07	5.71 ± 0.08	5.83 ± 0.08
TC02001674.hg.1	CGREF1	7.76 ± 0.19	7.74 ± 0.24	7.67 ± 0.15	7.86 ± 0.07
TC14000318.hg.1	CGRRF1	7.15 ± 0.15	7.16 ± 0.19	7.05 ± 0.23	7.07 ± 0.06
TC10001504.hg.1	CH25H	6.31 ± 0.15	6.36 ± 0.14	6.28 ± 0.28	6.36 ± 0.28
TC15000300.hg.1	CHAC1	7.94 ± 0.21	7.97 ± 0.21	7.82 ± 0.26	8.06 ± 0.05

TC02000310.hg.1	CHAC2	6.24 ± 0.07	6.04 ± 0.10	6.04 ± 0.04	5.90 ± 0.10
TC17001689.hg.1	CHAD	7.91 ± 0.18	7.72 ± 0.13	7.61 ± 0.24	7.77 ± 0.03
TC22000822.hg.1	CHADL	8.50 ± 0.21	8.47 ± 0.19	8.40 ± 0.24	8.56 ± 0.03
TC19000088.hg.1	CHAF1A	8.27 ± 0.14	8.19 ± 0.10	8.05 ± 0.18	8.13 ± 0.02
TC21000154.hg.1	CHAF1B	7.64 ± 0.11	7.59 ± 0.26	7.52 ± 0.20	7.37 ± 0.07
TC13000437.hg.1	CHAMP1	8.55 ± 0.15	8.36 ± 0.03	8.44 ± 0.21	8.31 ± 0.04
TC10002936.hg.1	CHAT	7.81 ± 0.18	7.80 ± 0.20	7.79 ± 0.21	7.94 ± 0.09
TC10000470.hg.1	CHCHD1	7.16 ± 0.22	6.62 ± 0.12	6.74 ± 0.20	6.60 ± 0.01
TC22000568.hg.1	CHCHD10	8.86 ± 0.18	8.85 ± 0.15	8.76 ± 0.15	8.87 ± 0.06
TC07001408.hg.1	CHCHD2	9.59 ± 0.25	9.39 ± 0.17	9.33 ± 0.28	9.26 ± 0.07
TC01000192.hg.1	CHCHD2P6	9.63 ± 0.17	9.48 ± 0.22	9.46 ± 0.25	9.40 ± 0.05
TC13000084.hg.1	CHCHD2P8	8.29 ± 0.23	8.08 ± 0.08	8.05 ± 0.19	8.01 ± 0.04
TC07001878.hg.1	CHCHD3	7.50 ± 0.10	7.64 ± 0.11	7.40 ± 0.05	7.47 ± 0.05
TC03001193.hg.1	CHCHD4	7.37 ± 0.16	7.26 ± 0.06	7.33 ± 0.23	7.26 ± 0.07
TC02000708.hg.1	CHCHD5	8.16 ± 0.06	8.08 ± 0.13	8.10 ± 0.08	8.17 ± 0.08
TC03000660.hg.1	CHCHD6	7.69 ± 0.14	7.62 ± 0.09	7.62 ± 0.13	7.66 ± 0.01
TC08000387.hg.1	CHCHD7	6.87 ± 0.10	6.87 ± 0.08	6.88 ± 0.23	6.81 ± 0.01
TC05001628.hg.1	CHD1	8.76 ± 0.72	9.55 ± 0.12	9.31 ± 0.70	9.66 ± 0.02
TC01001112.hg.1	CHD1L	7.39 ± 0.12	7.45 ± 0.12	7.23 ± 0.07	7.31 ± 0.03
TC15002769.hg.1	CHD2	9.46 ± 0.44	9.85 ± 0.12	9.63 ± 0.41	10.04 ± 0.15
TC17000120.hg.1	CHD3	9.33 ± 0.20	9.55 ± 0.13	9.06 ± 0.06	9.50 ± 0.04
TC12001143.hg.1	CHD4	9.67 ± 0.17	9.78 ± 0.13	9.52 ± 0.12	9.78 ± 0.12
TC01002144.hg.1	CHD5	7.87 ± 0.21	7.83 ± 0.19	7.79 ± 0.20	7.89 ± 0.04
TC20000850.hg.1	CHD6	8.03 ± 0.22	8.17 ± 0.12	7.78 ± 0.21	8.21 ± 0.05
TC08000412.hg.1	CHD7	8.42 ± 0.31	8.54 ± 0.15	8.39 ± 0.36	8.61 ± 0.04
TC14000917.hg.1	CHD8	9.10 ± 0.24	9.11 ± 0.14	9.07 ± 0.24	9.05 ± 0.08
TC16000445.hg.1	CHD9	7.98 ± 0.70	8.28 ± 0.22	8.07 ± 0.48	8.10 ± 0.05
TC0X000163.hg.1	CHDC2	4.33 ± 0.05	4.19 ± 0.05	4.25 ± 0.04	4.13 ± 0.05
TC03001472.hg.1	CHDH	7.43 ± 0.22	7.42 ± 0.20	7.33 ± 0.18	7.48 ± 0.01
TC11003470.hg.1	CHEK1	7.04 ± 0.06	7.01 ± 0.26	6.90 ± 0.06	6.80 ± 0.06
TC22000626.hg.1	CHEK2	6.45 ± 0.08	6.39 ± 0.03	6.39 ± 0.03	6.40 ± 0.03

TC15000003.hg.1	CHEK2P2	6.57 ± 0.21	6.66 ± 0.02	6.43 ± 0.14	6.70 ± 0.11
TC10000261.hg.1	CHEK2P5	5.78 ± 0.46	5.91 ± 0.04	5.66 ± 0.19	6.05 ± 0.10
TC19002687.hg.1	CHERP	9.11 ± 0.14	9.03 ± 0.12	9.06 ± 0.06	9.07 ± 0.11
TC12003217.hg.1	CHFR	9.05 ± 0.11	8.90 ± 0.04	9.01 ± 0.13	8.91 ± 0.05
TC14000574.hg.1	CHGA	8.39 ± 0.18	8.27 ± 0.14	8.30 ± 0.22	8.29 ± 0.02
TC20000062.hg.1	CHGB	6.53 ± 0.18	6.44 ± 0.15	6.55 ± 0.15	6.53 ± 0.05
TC01003718.hg.1	CHI3L1	9.20 ± 0.19	9.31 ± 0.10	8.68 ± 0.25	9.19 ± 0.20
TC01000972.hg.1	CHI3L2	7.47 ± 0.13	7.40 ± 0.12	7.22 ± 0.20	7.26 ± 0.07
TC01000974.hg.1	CHIA	6.22 ± 0.08	6.20 ± 0.08	6.20 ± 0.08	6.26 ± 0.05
TC01000973.hg.1	CHIAP2	6.07 ± 0.21	6.10 ± 0.15	5.97 ± 0.04	6.13 ± 0.07
TC0X000403.hg.1	CHIC1	6.78 ± 0.36	6.93 ± 0.20	6.69 ± 0.23	6.77 ± 0.04
TC04001201.hg.1	CHIC2	9.03 ± 0.32	9.35 ± 0.13	9.27 ± 0.23	9.34 ± 0.24
TC11001253.hg.1	CHID1	8.46 ± 0.11	8.41 ± 0.09	8.32 ± 0.12	8.43 ± 0.04
TC01003719.hg.1	CHIT1	6.99 ± 0.26	6.83 ± 0.11	6.89 ± 0.07	6.94 ± 0.07
TC11002002.hg.1	CHKA	7.38 ± 0.15	7.60 ± 0.27	7.19 ± 0.12	7.84 ± 0.23
TC22001503.hg.1	CHKB	9.06 ± 0.06	8.98 ± 0.09	9.11 ± 0.15	8.99 ± 0.06
TC22000432.hg.1	CHKB-AS1	7.67 ± 0.30	7.58 ± 0.10	7.38 ± 0.29	7.49 ± 0.05
TC03000002.hg.1	CHL1	5.14 ± 0.13	5.09 ± 0.04	5.06 ± 0.08	5.12 ± 0.09
TC03001117.hg.1	CHL1-AS1	6.83 ± 0.30	7.00 ± 0.14	6.74 ± 0.18	7.14 ± 0.22
TC03001115.hg.1	CHL1-AS2	6.81 ± 0.21	6.91 ± 0.26	6.79 ± 0.35	7.01 ± 0.11
TC0X001191.hg.1	CHM	7.16 ± 0.49	7.33 ± 0.20	7.25 ± 0.51	7.03 ± 0.18
TC01004027.hg.1	CHML	6.51 ± 0.77	7.20 ± 0.30	6.62 ± 0.44	6.92 ± 0.06
TC16001353.hg.1	CHMP1A	9.20 ± 0.34	8.91 ± 0.06	8.96 ± 0.21	8.87 ± 0.09
TC18000059.hg.1	CHMP1B	7.98 ± 0.39	8.37 ± 0.10	8.20 ± 0.35	8.30 ± 0.20
TC19001922.hg.1	CHMP2A	10.07 ± 0.31	9.85 ± 0.05	10.18 ± 0.28	9.73 ± 0.24
TC03000461.hg.1	CHMP2B	8.19 ± 0.27	8.35 ± 0.14	8.67 ± 0.48	8.42 ± 0.24
TC02005035.hg.1	CHMP3	8.68 ± 0.07	8.65 ± 0.08	8.86 ± 0.19	8.66 ± 0.07
TC14002341.hg.1	CHMP4A	8.19 ± 0.04	8.14 ± 0.02	8.05 ± 0.15	8.04 ± 0.05
TC20000235.hg.1	CHMP4B	9.94 ± 0.15	9.99 ± 0.05	10.06 ± 0.13	10.03 ± 0.38
TC08000520.hg.1	CHMP4C	6.10 ± 0.09	6.06 ± 0.10	6.28 ± 0.15	6.12 ± 0.06
TC09000139.hg.1	CHMP5	8.45 ± 0.32	8.22 ± 0.30	8.84 ± 0.55	7.91 ± 0.29

TC17000922.hg.1	CHMP6	8.15 ± 0.20	7.91 ± 0.09	7.97 ± 0.19	7.90 ± 0.04
TC08000184.hg.1	CHMP7	9.39 ± 0.04	9.43 ± 0.10	9.00 ± 0.12	9.55 ± 0.11
TC02002544.hg.1	CHN1	5.16 ± 0.07	5.07 ± 0.03	5.05 ± 0.05	5.08 ± 0.06
TC07003388.hg.1	CHN2	7.09 ± 0.07	7.09 ± 0.03	7.12 ± 0.09	7.08 ± 0.02
TC21000051.hg.1	CHODL	6.02 ± 0.09	5.95 ± 0.08	5.95 ± 0.04	6.03 ± 0.12
TC21000303.hg.1	CHODL-AS1	4.51 ± 0.03	4.56 ± 0.08	4.65 ± 0.04	4.57 ± 0.12
TC11002185.hg.1	CHORDC1	8.05 ± 0.72	8.41 ± 0.31	8.46 ± 0.45	8.20 ± 0.25
TC15000304.hg.1	CHP1	9.81 ± 0.10	9.75 ± 0.13	9.88 ± 0.08	9.71 ± 0.22
TC16000259.hg.1	CHP2	7.37 ± 0.28	7.22 ± 0.15	7.22 ± 0.25	7.27 ± 0.06
TC02002800.hg.1	CHPF	8.07 ± 0.35	7.77 ± 0.18	7.75 ± 0.16	7.81 ± 0.08
TC12000788.hg.1	CHPT1	7.60 ± 0.13	7.60 ± 0.22	7.68 ± 0.19	7.55 ± 0.07
TC08000788.hg.1	CHRAC1	8.03 ± 0.08	8.10 ± 0.24	7.88 ± 0.05	8.08 ± 0.15
TC03001004.hg.1	CHRD	8.22 ± 0.23	8.20 ± 0.21	8.17 ± 0.19	8.31 ± 0.06
TC0X001278.hg.1	CHRDL1	6.51 ± 0.16	6.41 ± 0.16	6.39 ± 0.10	6.48 ± 0.06
TC11002084.hg.1	CHRDL2	7.34 ± 0.13	7.32 ± 0.18	7.33 ± 0.10	7.41 ± 0.04
TC15001136.hg.1	CHRFAM7A	6.54 ± 0.07	6.55 ± 0.04	6.58 ± 0.14	6.66 ± 0.03
TC11001885.hg.1	CHRM1	7.73 ± 0.24	7.73 ± 0.31	7.62 ± 0.09	7.79 ± 0.10
TC07000852.hg.1	CHRM2	6.49 ± 0.12	6.40 ± 0.12	6.47 ± 0.13	6.53 ± 0.08
TC01001964.hg.1	CHRM3	5.41 ± 0.10	5.36 ± 0.05	5.38 ± 0.05	5.44 ± 0.12
TC01004016.hg.1	CHRM3-AS1	6.27 ± 0.21	6.44 ± 0.07	6.29 ± 0.40	6.45 ± 0.22
TC01004015.hg.1	CHRM3-AS2	7.61 ± 0.35	8.03 ± 0.19	7.64 ± 0.36	7.94 ± 0.25
TC11001719.hg.1	CHRM4	7.38 ± 0.31	7.75 ± 0.42	7.38 ± 0.34	7.98 ± 0.17
TC15000231.hg.1	CHRM5	5.62 ± 0.07	5.58 ± 0.16	5.56 ± 0.06	5.62 ± 0.09
TC02002542.hg.1	CHRNA1	6.73 ± 0.13	6.66 ± 0.10	6.68 ± 0.21	6.76 ± 0.08
TC11001301.hg.1	CHRNA10	7.69 ± 0.11	7.62 ± 0.10	7.65 ± 0.01	7.71 ± 0.07
TC08001080.hg.1	CHRNA2	7.42 ± 0.09	7.34 ± 0.09	7.49 ± 0.07	7.43 ± 0.05
TC15001705.hg.1	CHRNA3	6.50 ± 0.15	6.50 ± 0.08	6.47 ± 0.02	6.52 ± 0.02
TC20001028.hg.1	CHRNA4	8.17 ± 0.16	8.12 ± 0.15	8.11 ± 0.18	8.17 ± 0.02
TC15000732.hg.1	CHRNA5	5.71 ± 0.17	5.48 ± 0.11	5.50 ± 0.15	5.41 ± 0.05
TC08001182.hg.1	CHRNA6	5.60 ± 0.03	5.63 ± 0.12	5.63 ± 0.06	5.62 ± 0.02
TC15002755.hg.1	CHRNA7	6.88 ± 0.15	6.93 ± 0.10	6.89 ± 0.13	7.02 ± 0.07

TC04000245.hg.1	CHRNA9	5.04 ± 0.22	4.97 ± 0.08	4.96 ± 0.07	5.01 ± 0.02
TC17002862.hg.1	CHRNB1	7.19 ± 0.08	7.11 ± 0.15	7.14 ± 0.07	7.18 ± 0.03
TC01001279.hg.1	CHRNB2	7.42 ± 0.12	7.34 ± 0.17	7.30 ± 0.12	7.42 ± 0.08
TC08000329.hg.1	CHRNB3	5.58 ± 0.10	5.59 ± 0.13	5.56 ± 0.07	5.65 ± 0.06
TC15001706.hg.1	CHRNB4	7.49 ± 0.17	7.52 ± 0.14	7.47 ± 0.13	7.63 ± 0.11
TC02001402.hg.1	CHRND	7.40 ± 0.12	7.36 ± 0.15	7.41 ± 0.15	7.49 ± 0.08
TC17001032.hg.1	CHRNE	7.98 ± 0.11	8.14 ± 0.11	8.07 ± 0.10	8.12 ± 0.09
TC02001403.hg.1	CHRNG	7.67 ± 0.15	7.71 ± 0.20	7.64 ± 0.16	7.80 ± 0.05
TC11001589.hg.1	CHST1	8.19 ± 0.16	8.17 ± 0.22	8.08 ± 0.19	8.26 ± 0.09
TC02002138.hg.1	CHST10	6.98 ± 0.19	6.92 ± 0.14	6.85 ± 0.17	6.98 ± 0.05
TC12000812.hg.1	CHST11	9.83 ± 0.06	9.83 ± 0.06	9.92 ± 0.15	9.84 ± 0.11
TC07000032.hg.1	CHST12	9.45 ± 0.16	9.22 ± 0.02	9.24 ± 0.17	9.16 ± 0.03
TC03000658.hg.1	CHST13	8.35 ± 0.27	8.23 ± 0.18	8.29 ± 0.26	8.24 ± 0.05
TC15000286.hg.1	CHST14	9.02 ± 0.22	8.99 ± 0.11	8.91 ± 0.25	9.00 ± 0.03
TC10001725.hg.1	CHST15	9.79 ± 0.30	9.44 ± 0.19	10.07 ± 0.28	9.43 ± 0.39
TC03003405.hg.1	CHST2	8.55 ± 0.05	8.39 ± 0.08	8.52 ± 0.10	8.36 ± 0.11
TC10000445.hg.1	CHST3	6.85 ± 0.25	6.84 ± 0.17	6.77 ± 0.05	6.91 ± 0.09
TC16000592.hg.1	CHST4	6.80 ± 0.16	6.78 ± 0.07	6.77 ± 0.14	6.86 ± 0.12
TC16002090.hg.1	CHST5	6.75 ± 0.22	6.66 ± 0.17	6.66 ± 0.18	6.78 ± 0.08
TC16001281.hg.1	CHST6	7.19 ± 0.18	7.23 ± 0.14	7.18 ± 0.10	7.27 ± 0.02
TC0X000220.hg.1	CHST7	7.92 ± 0.21	7.76 ± 0.13	7.86 ± 0.31	7.81 ± 0.03
TC19000438.hg.1	CHST8	7.57 ± 0.16	7.53 ± 0.18	7.59 ± 0.16	7.63 ± 0.03
TC18000430.hg.1	CHST9	5.68 ± 0.18	5.61 ± 0.18	5.59 ± 0.18	5.69 ± 0.14
TC15002005.hg.1	CHSY1	9.47 ± 0.34	9.71 ± 0.22	10.08 ± 0.31	9.62 ± 0.14
TC05000619.hg.1	CHSY3	6.29 ± 0.17	6.15 ± 0.14	6.29 ± 0.16	6.18 ± 0.07
TC16000032.hg.1	CHTF18	8.79 ± 0.19	8.76 ± 0.21	8.69 ± 0.23	8.84 ± 0.05
TC16001218.hg.1	CHTF8	8.69 ± 0.28	8.41 ± 0.10	8.31 ± 0.29	8.19 ± 0.12
TC01001259.hg.1	CHTOP	8.54 ± 0.13	8.51 ± 0.13	8.39 ± 0.14	8.45 ± 0.05
TC10002931.hg.1	CHUK	7.83 ± 0.24	7.80 ± 0.16	8.05 ± 0.50	7.59 ± 0.20
TC14002299.hg.1	CHURC1	8.45 ± 0.11	8.33 ± 0.16	8.60 ± 0.27	8.21 ± 0.21
TC02000580.hg.1	CIAO1	9.25 ± 0.03	9.18 ± 0.04	9.13 ± 0.07	9.12 ± 0.07

TC16001139.hg.1	CIAPIN1	8.03 ± 0.08	7.82 ± 0.09	7.72 ± 0.09	7.76 ± 0.03
TC15001842.hg.1	CIB1	8.35 ± 0.13	8.26 ± 0.10	8.08 ± 0.09	8.35 ± 0.06
TC15001700.hg.1	CIB2	7.22 ± 0.12	7.17 ± 0.20	7.16 ± 0.14	7.22 ± 0.03
TC19001264.hg.1	CIB3	6.77 ± 0.14	6.72 ± 0.11	6.70 ± 0.09	6.72 ± 0.05
TC02001668.hg.1	CIB4	6.34 ± 0.16	6.29 ± 0.16	6.20 ± 0.27	6.39 ± 0.07
TC19000596.hg.1	CIC	8.81 ± 0.10	8.80 ± 0.02	8.79 ± 0.03	8.85 ± 0.06
TC18000063.hg.1	CIDEA	7.93 ± 0.16	7.93 ± 0.17	7.92 ± 0.09	7.97 ± 0.05
TC14000974.hg.1	CIDEB	8.73 ± 0.25	8.34 ± 0.06	8.84 ± 0.16	8.32 ± 0.11
TC03003325.hg.1	CIDEC	7.34 ± 0.25	7.24 ± 0.15	7.21 ± 0.14	7.31 ± 0.02
TC03003326.hg.1	CIDECP	7.90 ± 0.11	8.01 ± 0.15	7.89 ± 0.06	7.95 ± 0.13
TC16000158.hg.1	CIITA	9.41 ± 0.07	9.51 ± 0.18	9.13 ± 0.11	9.38 ± 0.14
TC15001562.hg.1	CILP	6.98 ± 0.22	7.02 ± 0.23	6.95 ± 0.22	7.11 ± 0.05
TC19000377.hg.1	CILP2	8.48 ± 0.27	8.47 ± 0.24	8.43 ± 0.23	8.59 ± 0.09
TC14001528.hg.1	CINP	7.44 ± 0.25	7.20 ± 0.09	7.32 ± 0.07	7.10 ± 0.12
TC14001529.hg.1	CINP	7.87 ± 0.08	7.69 ± 0.15	7.87 ± 0.08	7.74 ± 0.07
TC02002538.hg.1	CIR1	8.67 ± 0.33	8.95 ± 0.19	9.10 ± 0.46	8.94 ± 0.08
TC19000034.hg.1	CIRBP	10.53 ± 0.06	10.67 ± 0.13	10.17 ± 0.19	10.72 ± 0.15
TC19000999.hg.1	CIRBP-AS1	7.59 ± 0.13	7.41 ± 0.12	7.43 ± 0.13	7.48 ± 0.01
TC16000564.hg.1	CIRH1A	9.11 ± 0.08	9.13 ± 0.23	8.75 ± 0.08	8.97 ± 0.01
TC10000358.hg.1	CISD1	6.35 ± 0.02	6.09 ± 0.06	6.29 ± 0.12	5.96 ± 0.07
TC04000529.hg.1	CISD2	8.13 ± 0.15	8.10 ± 0.14	7.99 ± 0.14	8.05 ± 0.06
TC17000464.hg.1	CISD3	9.03 ± 0.25	8.93 ± 0.13	8.88 ± 0.29	8.92 ± 0.03
TC03001435.hg.1	CISH	8.31 ± 0.18	7.95 ± 0.22	8.35 ± 0.11	7.91 ± 0.04
TC12002032.hg.1	CIT	7.11 ± 0.19	6.94 ± 0.15	6.98 ± 0.22	6.80 ± 0.02
TC0X001140.hg.1	CITED1	7.12 ± 0.15	7.13 ± 0.20	7.07 ± 0.18	7.21 ± 0.06
TC06002168.hg.1	CITED2	10.02 ± 0.03	9.85 ± 0.11	9.90 ± 0.05	9.79 ± 0.08
TC01002561.hg.1	CITED4	8.34 ± 0.24	8.38 ± 0.06	8.19 ± 0.21	8.33 ± 0.17
TC09001624.hg.1	CIZ1	9.24 ± 0.02	9.25 ± 0.06	9.07 ± 0.09	9.24 ± 0.03
TC13000223.hg.1	CKAP2	6.99 ± 0.32	7.36 ± 0.15	6.96 ± 0.18	7.24 ± 0.13
TC02002217.hg.1	CKAP2L	5.97 ± 0.10	5.79 ± 0.26	5.88 ± 0.24	5.48 ± 0.08
TC12001917.hg.1	CKAP4	9.43 ± 0.30	9.23 ± 0.15	9.60 ± 0.13	9.28 ± 0.08

TC11001724.hg.1	CKAP5	8.25 ± 0.21	8.11 ± 0.20	8.02 ± 0.16	7.80 ± 0.11
TC14001538.hg.1	CKB	8.38 ± 0.26	8.27 ± 0.18	8.27 ± 0.31	8.32 ± 0.02
TC16002076.hg.1	CKLF	9.66 ± 0.19	9.36 ± 0.14	10.08 ± 0.14	9.21 ± 0.23
TC19001617.hg.1	CKM	7.37 ± 0.34	7.36 ± 0.21	7.28 ± 0.24	7.43 ± 0.04
TC15002759.hg.1	CKMT1A	6.50 ± 0.11	6.39 ± 0.07	6.44 ± 0.14	6.47 ± 0.08
TC15002758.hg.1	CKMT1B	6.98 ± 0.16	6.92 ± 0.14	6.92 ± 0.09	7.00 ± 0.09
TC05000403.hg.1	CKMT2	6.43 ± 0.21	6.36 ± 0.20	6.35 ± 0.16	6.43 ± 0.05
TC01001280.hg.1	CKS1B	6.45 ± 0.14	6.21 ± 0.10	6.31 ± 0.18	6.18 ± 0.07
TC09000415.hg.1	CKS2	8.30 ± 0.41	8.01 ± 0.33	8.00 ± 0.18	7.64 ± 0.10
TC02002264.hg.1	CLASP1	8.23 ± 0.19	8.30 ± 0.14	8.22 ± 0.26	8.25 ± 0.05
TC03001277.hg.1	CLASP2	7.31 ± 0.23	7.37 ± 0.14	7.16 ± 0.20	7.27 ± 0.06
TC19000639.hg.1	CLASRP	8.68 ± 0.03	8.71 ± 0.06	8.66 ± 0.07	8.72 ± 0.01
TC19001524.hg.1	CLC	9.47 ± 0.59	9.32 ± 0.57	10.78 ± 0.48	9.73 ± 0.75
TC01000822.hg.1	CLCA1	5.24 ± 0.15	5.07 ± 0.03	5.15 ± 0.02	5.13 ± 0.07
TC01000821.hg.1	CLCA2	4.92 ± 0.03	4.81 ± 0.07	4.85 ± 0.04	4.88 ± 0.03
TC01000825.hg.1	CLCA3P	4.44 ± 0.10	4.30 ± 0.08	4.31 ± 0.04	4.34 ± 0.01
TC01000823.hg.1	CLCA4	4.65 ± 0.14	4.48 ± 0.11	4.53 ± 0.07	4.55 ± 0.12
TC01006364.hg.1	CLCC1	7.46 ± 0.30	7.38 ± 0.21	7.26 ± 0.16	7.14 ± 0.21
TC11003440.hg.1	CLCF1	8.23 ± 0.23	8.23 ± 0.14	8.03 ± 0.26	8.36 ± 0.10
TC07000934.hg.1	CLCN1	7.31 ± 0.14	7.27 ± 0.14	7.26 ± 0.09	7.38 ± 0.07
TC03002069.hg.1	CLCN2	7.50 ± 0.16	7.51 ± 0.14	7.43 ± 0.13	7.57 ± 0.05
TC04000845.hg.1	CLCN3	8.06 ± 0.18	7.93 ± 0.15	8.17 ± 0.26	7.81 ± 0.14
TC0X000038.hg.1	CLCN4	8.22 ± 0.17	8.11 ± 0.06	8.13 ± 0.11	8.08 ± 0.07
TC0X000278.hg.1	CLCN5	6.49 ± 0.05	6.42 ± 0.03	6.43 ± 0.02	6.39 ± 0.02
TC01000149.hg.1	CLCN6	8.25 ± 0.11	8.08 ± 0.04	8.13 ± 0.11	8.16 ± 0.03
TC16000753.hg.1	CLCN7	9.10 ± 0.14	8.96 ± 0.07	8.89 ± 0.05	8.98 ± 0.04
TC01006287.hg.1	CLCNKA	7.82 ± 0.19	7.84 ± 0.21	7.77 ± 0.20	7.97 ± 0.07
TC01006288.hg.1	CLCNKB	7.69 ± 0.22	7.74 ± 0.27	7.65 ± 0.20	7.86 ± 0.05
TC03002108.hg.1	CLDN1	6.89 ± 0.15	6.87 ± 0.10	6.79 ± 0.17	7.04 ± 0.07
TC13000335.hg.1	CLDN10	5.46 ± 0.20	5.44 ± 0.13	5.34 ± 0.10	5.50 ± 0.06
TC13000803.hg.1	CLDN10-AS1	5.50 ± 0.20	5.48 ± 0.21	5.39 ± 0.03	5.45 ± 0.08

TC03000919.hg.1	CLDN11	6.70 ± 0.13	6.70 ± 0.10	6.66 ± 0.18	6.82 ± 0.08
TC07003335.hg.1	CLDN12	5.63 ± 0.10	5.56 ± 0.02	5.53 ± 0.02	5.60 ± 0.04
TC21000433.hg.1	CLDN14	8.85 ± 0.16	8.73 ± 0.11	8.79 ± 0.18	8.85 ± 0.03
TC07001688.hg.1	CLDN15	8.05 ± 0.29	7.98 ± 0.13	7.99 ± 0.16	8.02 ± 0.01
TC03001046.hg.1	CLDN16	6.47 ± 0.11	6.52 ± 0.13	6.43 ± 0.08	6.62 ± 0.06
TC21000361.hg.1	CLDN17	5.37 ± 0.17	5.23 ± 0.18	5.16 ± 0.13	5.11 ± 0.06
TC03000742.hg.1	CLDN18	6.50 ± 0.16	6.47 ± 0.18	6.40 ± 0.11	6.57 ± 0.08
TC01002573.hg.1	CLDN19	7.53 ± 0.22	7.52 ± 0.27	7.52 ± 0.07	7.69 ± 0.07
TC0X000521.hg.1	CLDN2	6.40 ± 0.17	6.47 ± 0.22	6.49 ± 0.15	6.59 ± 0.09
TC06001119.hg.1	CLDN20	4.88 ± 0.06	4.74 ± 0.02	4.80 ± 0.17	4.81 ± 0.05
TC04001793.hg.1	CLDN22	4.42 ± 0.11	4.14 ± 0.02	4.23 ± 0.10	4.22 ± 0.08
TC08000059.hg.1	CLDN23	7.71 ± 0.17	7.45 ± 0.11	7.56 ± 0.36	7.59 ± 0.03
TC04001794.hg.1	CLDN24	6.94 ± 0.28	6.85 ± 0.10	6.85 ± 0.16	6.89 ± 0.06
TC11001020.hg.1	CLDN25	7.69 ± 0.23	7.70 ± 0.17	7.79 ± 0.11	8.07 ± 0.10
TC07001506.hg.1	CLDN3	8.64 ± 0.26	8.42 ± 0.16	8.46 ± 0.39	8.44 ± 0.05
TC07000447.hg.1	CLDN4	7.62 ± 0.21	7.61 ± 0.10	7.47 ± 0.13	7.75 ± 0.09
TC22000498.hg.1	CLDN5	8.16 ± 0.22	8.15 ± 0.19	8.08 ± 0.26	8.22 ± 0.06
TC16000797.hg.1	CLDN6	7.61 ± 0.19	7.46 ± 0.03	7.51 ± 0.17	7.51 ± 0.07
TC17001083.hg.1	CLDN7	7.55 ± 0.19	7.50 ± 0.15	7.44 ± 0.22	7.51 ± 0.06
TC21000363.hg.1	CLDN8	4.34 ± 0.07	4.22 ± 0.03	4.31 ± 0.09	4.27 ± 0.07
TC16000094.hg.1	CLDN9	8.04 ± 0.19	7.98 ± 0.17	7.98 ± 0.13	8.04 ± 0.09
TC03003339.hg.1	CLDND1	9.28 ± 0.08	9.24 ± 0.03	9.24 ± 0.01	9.18 ± 0.01
TC19001775.hg.1	CLDND2	8.81 ± 0.12	8.74 ± 0.09	8.78 ± 0.19	8.78 ± 0.06
TC17001074.hg.1	CLEC10A	7.61 ± 0.10	7.60 ± 0.12	7.49 ± 0.10	7.65 ± 0.03
TC19000765.hg.1	CLEC11A	8.73 ± 0.23	8.58 ± 0.13	8.61 ± 0.19	8.61 ± 0.01
TC12003207.hg.1	CLEC12A	9.07 ± 0.35	8.80 ± 0.43	9.39 ± 0.54	8.05 ± 0.49
TC12003208.hg.1	CLEC12B	6.55 ± 0.44	6.26 ± 0.24	7.00 ± 0.82	5.96 ± 0.27
TC14001061.hg.1	CLEC14A	7.46 ± 0.18	7.46 ± 0.17	7.43 ± 0.24	7.50 ± 0.03
TC16000159.hg.1	CLEC16A	8.40 ± 0.04	8.38 ± 0.05	8.26 ± 0.05	8.34 ± 0.04
TC19000260.hg.1	CLEC17A	8.23 ± 0.03	8.10 ± 0.13	8.07 ± 0.01	8.02 ± 0.03
TC16002036.hg.1	CLEC18A	7.92 ± 0.11	7.89 ± 0.12	7.88 ± 0.09	7.98 ± 0.09

TC16001266.hg.1	CLEC18B	7.87 ± 0.15	7.87 ± 0.14	7.88 ± 0.08	7.98 ± 0.09
TC16002037.hg.1	CLEC18C	8.04 ± 0.19	8.01 ± 0.16	8.02 ± 0.14	8.13 ± 0.10
TC16000212.hg.1	CLEC19A	6.99 ± 0.15	6.96 ± 0.17	6.90 ± 0.21	7.16 ± 0.15
TC12001214.hg.1	CLEC1A	6.40 ± 0.03	6.40 ± 0.13	6.57 ± 0.09	6.41 ± 0.07
TC12001212.hg.1	CLEC1B	6.78 ± 0.24	6.22 ± 0.04	7.38 ± 0.30	6.37 ± 0.20
TC12001209.hg.1	CLEC2A	5.16 ± 0.11	5.15 ± 0.08	5.26 ± 0.17	5.17 ± 0.05
TC12001208.hg.1	CLEC2B	8.89 ± 0.36	9.29 ± 0.17	9.39 ± 0.38	9.16 ± 0.15
TC12003236.hg.1	CLEC2D	8.95 ± 0.72	9.27 ± 0.24	8.74 ± 0.47	9.11 ± 0.04
TC07000876.hg.1	CLEC2L	7.81 ± 0.19	7.86 ± 0.24	7.75 ± 0.20	7.86 ± 0.03
TC16000630.hg.1	CLEC3A	4.98 ± 0.21	5.03 ± 0.09	5.02 ± 0.03	5.06 ± 0.06
TC03003350.hg.1	CLEC3B	7.30 ± 0.20	7.26 ± 0.20	7.16 ± 0.15	7.35 ± 0.07
TC12000119.hg.1	CLEC4A	8.63 ± 0.35	8.18 ± 0.41	9.07 ± 0.32	8.04 ± 0.27
TC12001165.hg.1	CLEC4C	6.54 ± 0.06	6.48 ± 0.11	6.61 ± 0.31	6.54 ± 0.12
TC12000130.hg.1	CLEC4D	8.63 ± 0.44	8.27 ± 0.49	10.16 ± 0.86	7.99 ± 0.76
TC12001178.hg.1	CLEC4E	10.85 ± 0.33	10.56 ± 0.22	11.33 ± 0.46	10.19 ± 0.75
TC02001967.hg.1	CLEC4F	7.23 ± 0.15	7.23 ± 0.13	7.29 ± 0.09	7.27 ± 0.03
TC19001117.hg.1	CLEC4G	8.45 ± 0.17	8.41 ± 0.17	8.36 ± 0.26	8.44 ± 0.03
TC19000137.hg.1	CLEC4GP1	9.24 ± 0.31	9.17 ± 0.21	9.10 ± 0.29	9.20 ± 0.04
TC19000136.hg.1	CLEC4M	7.36 ± 0.20	7.39 ± 0.16	7.35 ± 0.19	7.49 ± 0.01
TC07001934.hg.1	CLEC5A	6.80 ± 0.33	6.25 ± 0.06	6.97 ± 0.44	6.30 ± 0.21
TC12000128.hg.1	CLEC6A	6.14 ± 0.03	6.10 ± 0.11	6.47 ± 0.46	6.00 ± 0.09
TC12001215.hg.1	CLEC7A	10.01 ± 0.16	9.70 ± 0.48	10.38 ± 0.38	9.82 ± 0.21
TC12000156.hg.1	CLEC9A	5.66 ± 0.10	5.83 ± 0.11	5.69 ± 0.16	5.79 ± 0.06
TC12001206.hg.1	CLECL1	5.38 ± 0.02	5.31 ± 0.11	5.34 ± 0.16	5.33 ± 0.08
TC04001589.hg.1	CLGN	4.49 ± 0.07	4.38 ± 0.05	4.44 ± 0.08	4.40 ± 0.01
TC02001857.hg.1	CLHC1	5.67 ± 0.02	5.52 ± 0.08	5.61 ± 0.16	5.52 ± 0.14
TC06004119.hg.1	CLIC1	10.30 ± 0.30	10.02 ± 0.02	10.42 ± 0.27	9.90 ± 0.23
TC6_cox_hap2000255.hg.1	CLIC1	10.36 ± 0.31	10.09 ± 0.03	10.49 ± 0.29	9.96 ± 0.26
TC6_dbb_hap3000236.hg.1	CLIC1	10.46 ± 0.32	10.16 ± 0.04	10.59 ± 0.28	10.02 ± 0.26
TC6_mann_hap4000206.hg.1	CLIC1	10.31 ± 0.29	10.03 ± 0.03	10.42 ± 0.27	9.91 ± 0.23
TC6_mcf_hap5000221.hg.1	CLIC1	10.30 ± 0.30	10.02 ± 0.02	10.42 ± 0.27	9.90 ± 0.23

TC6_qbl_hap6000233.hg.1	CLIC1	10.31 ± 0.29	10.03 ± 0.03	10.42 ± 0.27	9.91 ± 0.23
TC6_ssto_hap7000209.hg.1	CLIC1	10.30 ± 0.30	10.02 ± 0.02	10.42 ± 0.27	9.90 ± 0.23
TC0X001551.hg.1	CLIC2	6.39 ± 0.04	6.48 ± 0.10	6.54 ± 0.12	6.51 ± 0.04
TC09001748.hg.1	CLIC3	8.46 ± 0.14	8.33 ± 0.19	8.35 ± 0.22	8.38 ± 0.04
TC01000314.hg.1	CLIC4	8.59 ± 0.14	8.50 ± 0.14	8.75 ± 0.25	8.51 ± 0.13
TC06001768.hg.1	CLIC5	6.14 ± 0.08	6.06 ± 0.10	6.04 ± 0.13	6.09 ± 0.07
TC21000138.hg.1	CLIC6	5.93 ± 0.18	5.89 ± 0.06	5.87 ± 0.04	5.97 ± 0.08
TC05001989.hg.1	CLINT1	9.61 ± 0.30	9.72 ± 0.07	9.70 ± 0.28	9.65 ± 0.05
TC12002073.hg.1	CLIP1	8.21 ± 0.25	8.33 ± 0.12	8.70 ± 0.38	8.24 ± 0.19
TC07000454.hg.1	CLIP2	8.31 ± 0.21	8.19 ± 0.10	8.19 ± 0.25	8.26 ± 0.06
TC19001460.hg.1	CLIP3	7.65 ± 0.18	7.59 ± 0.20	7.57 ± 0.18	7.67 ± 0.08
TC02000192.hg.1	CLIP4	7.22 ± 0.26	7.17 ± 0.10	7.15 ± 0.26	7.14 ± 0.01
TC02002666.hg.1	CLK1	9.46 ± 0.79	10.27 ± 0.07	9.60 ± 0.42	10.32 ± 0.18
TC01003311.hg.1	CLK2	9.39 ± 0.18	9.34 ± 0.04	9.30 ± 0.06	9.25 ± 0.02
TC07001198.hg.1	CLK2P	7.46 ± 0.07	7.28 ± 0.07	7.29 ± 0.21	7.18 ± 0.01
TC15002801.hg.1	CLK3	9.68 ± 0.15	9.57 ± 0.02	9.64 ± 0.14	9.51 ± 0.04
TC05002125.hg.1	CLK4	7.78 ± 0.61	8.14 ± 0.09	8.04 ± 0.48	8.14 ± 0.08
TC12003255.hg.1	CLLU1	4.06 ± 0.13	3.94 ± 0.02	4.01 ± 0.14	3.97 ± 0.04
TC12001809.hg.1	CLLU1OS	5.55 ± 0.22	5.31 ± 0.12	5.45 ± 0.17	5.50 ± 0.06
TC14001474.hg.1	CLMN	7.31 ± 0.10	7.17 ± 0.13	7.36 ± 0.15	7.13 ± 0.09
TC11002393.hg.1	CLMP	6.48 ± 0.12	6.43 ± 0.09	6.51 ± 0.17	6.51 ± 0.07
TC16002064.hg.1	CLN3	8.88 ± 0.07	8.77 ± 0.01	8.75 ± 0.11	8.80 ± 0.05
TC13000292.hg.1	CLN5	7.09 ± 0.06	6.99 ± 0.07	7.02 ± 0.09	6.96 ± 0.07
TC15001592.hg.1	CLN6	8.59 ± 0.19	8.35 ± 0.02	8.35 ± 0.08	8.28 ± 0.08
TC08000008.hg.1	CLN8	7.78 ± 0.16	7.74 ± 0.08	7.61 ± 0.14	7.74 ± 0.04
TC04001036.hg.1	CLNK	5.49 ± 0.06	5.45 ± 0.02	5.42 ± 0.03	5.49 ± 0.02
TC11002124.hg.1	CLNS1A	8.45 ± 0.11	8.46 ± 0.12	8.20 ± 0.10	8.32 ± 0.05
TC04001212.hg.1	CLOCK	7.49 ± 0.38	7.70 ± 0.20	7.43 ± 0.38	7.56 ± 0.09
TC11000470.hg.1	CLP1	7.33 ± 0.15	7.18 ± 0.20	7.18 ± 0.17	7.12 ± 0.05
TC11002058.hg.1	CLPB	8.03 ± 0.17	7.81 ± 0.03	7.78 ± 0.12	7.73 ± 0.09
TC19000112.hg.1	CLPP	9.54 ± 0.23	9.36 ± 0.06	9.22 ± 0.19	9.23 ± 0.09

TC06001667.hg.1	CLPS	8.09 ± 0.16	8.09 ± 0.09	8.01 ± 0.15	8.21 ± 0.11
TC06000521.hg.1	CLPSL1	6.74 ± 0.22	6.80 ± 0.19	6.74 ± 0.19	6.92 ± 0.10
TC06000520.hg.1	CLPSL2	7.56 ± 0.28	7.46 ± 0.21	7.41 ± 0.20	7.50 ± 0.02
TC19000637.hg.1	CLPTM1	10.28 ± 0.21	10.11 ± 0.02	10.21 ± 0.06	10.10 ± 0.10
TC05001107.hg.1	CLPTM1L	8.81 ± 0.21	8.47 ± 0.09	8.42 ± 0.15	8.35 ± 0.08
TC15001561.hg.1	CLPX	8.52 ± 0.17	8.51 ± 0.18	8.44 ± 0.29	8.38 ± 0.17
TC03003420.hg.1	CLRN1	5.51 ± 0.14	5.46 ± 0.08	5.45 ± 0.05	5.49 ± 0.09
TC03000819.hg.1	CLRN1-AS1	5.70 ± 0.14	5.51 ± 0.16	5.81 ± 0.21	5.61 ± 0.08
TC04000149.hg.1	CLRN2	7.81 ± 0.10	7.79 ± 0.14	7.79 ± 0.07	7.83 ± 0.10
TC10001754.hg.1	CLRN3	5.87 ± 0.18	5.85 ± 0.10	5.81 ± 0.21	5.90 ± 0.16
TC01002500.hg.1	CLSPN	6.72 ± 0.17	6.53 ± 0.36	6.54 ± 0.18	6.12 ± 0.03
TC01002186.hg.1	CLSTN1	9.18 ± 0.15	9.16 ± 0.09	8.83 ± 0.02	8.98 ± 0.03
TC03000754.hg.1	CLSTN2	7.14 ± 0.19	7.10 ± 0.12	7.09 ± 0.14	7.18 ± 0.08
TC12000108.hg.1	CLSTN3	8.48 ± 0.02	8.47 ± 0.04	8.48 ± 0.02	8.46 ± 0.05
TC09000204.hg.1	CLTA	8.24 ± 0.07	8.09 ± 0.12	8.14 ± 0.09	8.04 ± 0.11
TC05002093.hg.1	CLTB	8.85 ± 0.11	8.75 ± 0.07	8.71 ± 0.17	8.72 ± 0.04
TC17000725.hg.1	CLTC	9.32 ± 0.27	9.19 ± 0.25	9.56 ± 0.38	9.02 ± 0.17
TC22000487.hg.1	CLTCL1	7.09 ± 0.15	6.92 ± 0.11	7.06 ± 0.10	6.98 ± 0.02
TC08001081.hg.1	CLU	9.29 ± 0.15	8.72 ± 0.09	9.59 ± 0.13	8.85 ± 0.18
TC16000125.hg.1	CLUAP1	7.51 ± 0.18	7.45 ± 0.12	7.37 ± 0.08	7.37 ± 0.08
TC17000999.hg.1	CLUH	8.38 ± 0.18	8.26 ± 0.09	8.21 ± 0.16	8.25 ± 0.04
TC16000381.hg.1	CLUHP3	8.26 ± 0.09	8.45 ± 0.11	8.10 ± 0.11	8.56 ± 0.07
TC18000005.hg.1	CLUL1	6.11 ± 0.19	6.00 ± 0.02	6.04 ± 0.08	6.09 ± 0.06
TC08000414.hg.1	CLVS1	5.41 ± 0.16	5.35 ± 0.09	5.38 ± 0.06	5.45 ± 0.10
TC06000951.hg.1	CLVS2	6.12 ± 0.20	6.14 ± 0.18	6.17 ± 0.07	6.23 ± 0.09
TC13000358.hg.1	CLYBL	7.01 ± 0.11	7.08 ± 0.10	6.98 ± 0.17	7.05 ± 0.02
TC13000835.hg.1	CLYBL-AS1	5.76 ± 0.33	5.69 ± 0.10	5.84 ± 0.15	5.74 ± 0.04
TC13000834.hg.1	CLYBL-AS2	5.19 ± 0.08	5.26 ± 0.14	5.45 ± 0.30	5.31 ± 0.09
TC13000360.hg.1	CLYBL-IT1	7.04 ± 0.12	6.90 ± 0.07	6.97 ± 0.07	7.02 ± 0.10
TC14000979.hg.1	CMA1	6.50 ± 0.20	6.49 ± 0.13	6.44 ± 0.20	6.60 ± 0.18
TC06001332.hg.1	CMAHP	7.15 ± 0.33	7.07 ± 0.13	7.59 ± 0.42	6.94 ± 0.11

TC12000237.hg.1	CMAS	7.83 ± 0.31	7.59 ± 0.20	7.86 ± 0.26	7.40 ± 0.25
TC05001158.hg.1	CMBL	6.12 ± 0.17	6.05 ± 0.18	6.08 ± 0.09	6.11 ± 0.06
TC03000141.hg.1	CMC1	6.05 ± 0.28	6.00 ± 0.15	5.98 ± 0.15	5.87 ± 0.04
TC16001296.hg.1	CMC2	8.30 ± 0.07	8.31 ± 0.22	8.13 ± 0.21	8.04 ± 0.11
TC0X001549.hg.1	CMC4	6.94 ± 0.07	6.95 ± 0.03	6.83 ± 0.09	6.95 ± 0.02
TC16000640.hg.1	CMIP	10.11 ± 0.28	9.78 ± 0.07	10.21 ± 0.15	9.90 ± 0.18
TC12001933.hg.1	CMKLR1	7.72 ± 0.07	7.47 ± 0.15	7.50 ± 0.06	7.47 ± 0.04
TC01000598.hg.1	CMPK1	9.21 ± 0.30	9.48 ± 0.11	9.22 ± 0.16	9.41 ± 0.10
TC02005020.hg.1	CMPK2	8.58 ± 0.51	8.09 ± 0.61	8.61 ± 0.63	7.99 ± 0.16
TC03000503.hg.1	CMSS1	6.35 ± 0.05	6.31 ± 0.05	6.20 ± 0.04	6.28 ± 0.06
TC16002077.hg.1	CMTM1	7.48 ± 0.11	7.29 ± 0.18	7.73 ± 0.02	7.32 ± 0.03
TC16000519.hg.1	CMTM2	7.83 ± 0.35	7.50 ± 0.05	8.12 ± 0.29	7.50 ± 0.07
TC16000520.hg.1	CMTM3	9.21 ± 0.13	9.09 ± 0.06	9.06 ± 0.24	9.06 ± 0.10
TC16001171.hg.1	CMTM4	7.20 ± 0.16	7.14 ± 0.07	7.29 ± 0.06	7.19 ± 0.01
TC14000146.hg.1	CMTM5	8.36 ± 0.10	7.96 ± 0.20	8.43 ± 0.25	8.14 ± 0.12
TC03001270.hg.1	CMTM6	9.45 ± 0.22	9.39 ± 0.11	9.79 ± 0.37	9.47 ± 0.13
TC03000161.hg.1	CMTM7	8.56 ± 0.14	8.36 ± 0.06	8.40 ± 0.20	8.29 ± 0.02
TC03000159.hg.1	CMTM8	7.81 ± 0.20	7.83 ± 0.18	7.81 ± 0.17	7.84 ± 0.10
TC05000392.hg.1	CMYA5	6.00 ± 0.15	5.84 ± 0.15	5.85 ± 0.12	5.94 ± 0.09
TC08000542.hg.1	CNBD1	3.72 ± 0.09	3.58 ± 0.07	3.66 ± 0.07	3.59 ± 0.02
TC20000262.hg.1	CNBD2	5.99 ± 0.12	5.92 ± 0.08	5.94 ± 0.11	5.97 ± 0.04
TC03001766.hg.1	CNBP	10.44 ± 0.22	10.67 ± 0.11	10.55 ± 0.27	10.68 ± 0.09
TC18000250.hg.1	CNDP1	6.36 ± 0.11	6.33 ± 0.13	6.34 ± 0.12	6.42 ± 0.07
TC18000248.hg.1	CNDP2	9.29 ± 0.40	8.82 ± 0.04	9.04 ± 0.36	8.66 ± 0.05
TC16000435.hg.1	CNEP1R1	8.80 ± 0.17	8.66 ± 0.15	9.16 ± 0.47	8.61 ± 0.35
TC19001572.hg.1	CNFN	8.67 ± 0.13	8.60 ± 0.21	8.58 ± 0.08	8.73 ± 0.05
TC04001172.hg.1	CNGA1	4.99 ± 0.08	4.90 ± 0.06	4.99 ± 0.02	4.99 ± 0.07
TC0X000735.hg.1	CNGA2	6.43 ± 0.16	6.44 ± 0.15	6.35 ± 0.18	6.52 ± 0.09
TC02000597.hg.1	CNGA3	7.18 ± 0.16	7.12 ± 0.23	7.16 ± 0.12	7.23 ± 0.01
TC11000128.hg.1	CNGA4	7.00 ± 0.17	6.84 ± 0.14	7.02 ± 0.04	7.00 ± 0.05
TC16001144.hg.1	CNGB1	7.75 ± 0.17	7.68 ± 0.18	7.70 ± 0.15	7.78 ± 0.05

TC08001392.hg.1	CNGB3	4.79 ± 0.10	4.69 ± 0.04	4.73 ± 0.06	4.75 ± 0.06
TC14001149.hg.1	CNIH	8.00 ± 0.04	8.07 ± 0.15	7.91 ± 0.22	7.96 ± 0.19
TC11000658.hg.1	CNIH2	7.74 ± 0.09	7.73 ± 0.14	7.76 ± 0.10	7.80 ± 0.05
TC01001848.hg.1	CNIH3	6.44 ± 0.10	6.38 ± 0.12	6.40 ± 0.09	6.49 ± 0.07
TC01001847.hg.1	CNIH4	7.74 ± 0.35	7.14 ± 0.10	8.00 ± 0.43	7.01 ± 0.15
TC01000336.hg.1	CNKSR1	7.74 ± 0.13	7.58 ± 0.17	7.54 ± 0.14	7.66 ± 0.09
TC0X000097.hg.1	CNKSR2	6.94 ± 0.39	7.25 ± 0.13	6.81 ± 0.27	7.11 ± 0.14
TC06004140.hg.1	CNKSR3	6.19 ± 0.16	6.11 ± 0.08	6.15 ± 0.07	6.14 ± 0.06
TC19000202.hg.1	CNN1	7.63 ± 0.21	7.49 ± 0.16	7.60 ± 0.23	7.59 ± 0.01
TC19000027.hg.1	CNN2	11.58 ± 0.21	11.47 ± 0.06	11.78 ± 0.11	11.46 ± 0.23
TC01002891.hg.1	CNN3	6.14 ± 0.06	6.16 ± 0.04	6.15 ± 0.05	6.18 ± 0.04
TC10000708.hg.1	CNNM1	7.49 ± 0.18	7.49 ± 0.15	7.51 ± 0.16	7.54 ± 0.05
TC10000769.hg.1	CNNM2	6.84 ± 0.04	6.70 ± 0.09	6.65 ± 0.04	6.67 ± 0.02
TC02000587.hg.1	CNNM3	8.61 ± 0.08	8.65 ± 0.13	8.28 ± 0.02	8.67 ± 0.14
TC02000585.hg.1	CNNM4	7.96 ± 0.12	7.87 ± 0.06	7.81 ± 0.01	7.87 ± 0.02
TC16001154.hg.1	CNOT1	9.98 ± 0.26	10.00 ± 0.20	9.89 ± 0.29	9.68 ± 0.18
TC03000162.hg.1	CNOT10	7.83 ± 0.05	7.74 ± 0.16	7.76 ± 0.08	7.63 ± 0.09
TC03001272.hg.1	CNOT10-AS1	5.07 ± 0.37	4.72 ± 0.07	4.88 ± 0.09	4.75 ± 0.07
TC02000615.hg.1	CNOT11	8.69 ± 0.15	8.49 ± 0.20	8.51 ± 0.14	8.29 ± 0.15
TC12000620.hg.1	CNOT2	9.60 ± 0.33	9.85 ± 0.13	9.59 ± 0.27	9.80 ± 0.02
TC19000873.hg.1	CNOT3	9.37 ± 0.09	9.22 ± 0.09	9.34 ± 0.07	9.21 ± 0.10
TC07001886.hg.1	CNOT4	8.68 ± 0.24	8.65 ± 0.16	8.73 ± 0.29	8.58 ± 0.13
TC05001061.hg.1	CNOT6	8.46 ± 0.15	8.41 ± 0.19	8.38 ± 0.22	8.28 ± 0.09
TC04001323.hg.1	CNOT6L	9.75 ± 0.36	10.16 ± 0.14	9.63 ± 0.33	10.20 ± 0.10
TC15001473.hg.1	CNOT6LP1	9.21 ± 0.59	9.46 ± 0.21	9.06 ± 0.41	9.57 ± 0.21
TC08001011.hg.1	CNOT7	8.29 ± 0.37	8.62 ± 0.20	8.17 ± 0.26	8.54 ± 0.04
TC05000856.hg.1	CNOT8	9.09 ± 0.05	9.10 ± 0.13	9.06 ± 0.25	9.05 ± 0.02
TC17000520.hg.1	CNP	8.23 ± 0.26	7.95 ± 0.05	8.02 ± 0.30	7.86 ± 0.05
TC02002789.hg.1	CNPPD1	9.37 ± 0.22	9.17 ± 0.02	9.19 ± 0.13	9.18 ± 0.10
TC07002061.hg.1	CNPY1	6.06 ± 0.28	5.95 ± 0.16	5.98 ± 0.14	6.12 ± 0.15
TC12003214.hg.1	CNPY2	9.07 ± 0.11	8.93 ± 0.04	8.83 ± 0.12	8.81 ± 0.02

TC06000588.hg.1	CNPY3	9.35 ± 0.17	9.23 ± 0.12	9.28 ± 0.20	9.15 ± 0.10
TC07000612.hg.1	CNPY4	7.60 ± 0.05	7.54 ± 0.05	7.39 ± 0.10	7.54 ± 0.05
TC06001932.hg.1	CNR1	6.97 ± 0.17	6.86 ± 0.12	6.87 ± 0.09	6.96 ± 0.04
TC01002353.hg.1	CNR2	7.76 ± 0.08	7.67 ± 0.09	7.67 ± 0.03	7.67 ± 0.02
TC02001941.hg.1	CNRIP1	6.96 ± 0.18	6.80 ± 0.06	6.83 ± 0.11	6.77 ± 0.03
TC01002000.hg.1	CNST	8.94 ± 0.41	8.65 ± 0.13	8.71 ± 0.16	8.66 ± 0.11
TC17000537.hg.1	CNTD1	6.45 ± 0.17	6.39 ± 0.08	6.38 ± 0.11	6.40 ± 0.02
TC19001531.hg.1	CNTD2	8.18 ± 0.30	8.20 ± 0.21	8.14 ± 0.21	8.28 ± 0.05
TC11000477.hg.1	CNTF	8.46 ± 0.20	8.70 ± 0.18	8.38 ± 0.20	8.72 ± 0.13
TC09001038.hg.1	CNTFR	8.55 ± 0.15	8.53 ± 0.20	8.53 ± 0.16	8.62 ± 0.05
TC09000174.hg.1	CNTFR-AS1	6.54 ± 0.21	6.54 ± 0.20	6.46 ± 0.16	6.67 ± 0.11
TC09000084.hg.1	CNTLN	5.93 ± 0.13	5.84 ± 0.10	6.15 ± 0.21	5.83 ± 0.17
TC12000311.hg.1	CNTN1	4.72 ± 0.13	4.65 ± 0.08	4.66 ± 0.09	4.70 ± 0.06
TC01001705.hg.1	CNTN2	7.42 ± 0.22	7.42 ± 0.20	7.37 ± 0.16	7.49 ± 0.04
TC03001562.hg.1	CNTN3	5.30 ± 0.08	5.23 ± 0.07	5.18 ± 0.06	5.29 ± 0.11
TC03000007.hg.1	CNTN4	5.46 ± 0.15	5.37 ± 0.11	5.37 ± 0.07	5.44 ± 0.04
TC03001121.hg.1	CNTN4-AS1	4.51 ± 0.17	4.28 ± 0.03	4.33 ± 0.12	4.27 ± 0.05
TC03001120.hg.1	CNTN4-AS2	4.79 ± 0.16	4.73 ± 0.17	4.72 ± 0.02	4.79 ± 0.12
TC11000942.hg.1	CNTN5	5.20 ± 0.14	5.10 ± 0.07	5.14 ± 0.08	5.15 ± 0.08
TC03000006.hg.1	CNTN6	4.82 ± 0.09	4.74 ± 0.08	4.81 ± 0.06	4.78 ± 0.10
TC17000533.hg.1	CNTNAP1	7.19 ± 0.14	7.12 ± 0.11	7.09 ± 0.14	7.19 ± 0.03
TC07000960.hg.1	CNTNAP2	6.29 ± 0.10	6.26 ± 0.08	6.23 ± 0.09	6.30 ± 0.08
TC09001092.hg.1	CNTNAP3	7.86 ± 0.17	7.75 ± 0.04	8.04 ± 0.43	7.74 ± 0.33
TC09000253.hg.1	CNTNAP3B	7.48 ± 0.17	7.37 ± 0.04	7.64 ± 0.40	7.36 ± 0.26
TC16000622.hg.1	CNTNAP4	5.41 ± 0.10	5.32 ± 0.11	5.40 ± 0.05	5.40 ± 0.05
TC02000772.hg.1	CNTNAP5	6.04 ± 0.10	5.96 ± 0.08	5.98 ± 0.08	6.02 ± 0.08
TC09000607.hg.1	CNTRL	8.50 ± 0.47	8.68 ± 0.25	8.70 ± 0.42	8.41 ± 0.15
TC17000122.hg.1	CNTROB	8.28 ± 0.10	8.18 ± 0.07	8.12 ± 0.14	8.18 ± 0.02
TC07001327.hg.1	COA1	7.24 ± 0.10	7.31 ± 0.08	7.13 ± 0.13	7.24 ± 0.06
TC17001544.hg.1	COA3	7.34 ± 0.25	7.00 ± 0.08	6.93 ± 0.16	6.98 ± 0.12
TC11002071.hg.1	COA4	8.08 ± 0.09	7.88 ± 0.04	7.89 ± 0.08	7.91 ± 0.03

TC02002126.hg.1	COA5	7.48 ± 0.22	7.39 ± 0.10	7.41 ± 0.18	7.38 ± 0.03
TC01001934.hg.1	COA6	7.61 ± 0.20	7.37 ± 0.08	7.37 ± 0.27	7.25 ± 0.08
TC17000529.hg.1	COASY	8.50 ± 0.18	8.22 ± 0.05	8.39 ± 0.12	8.17 ± 0.03
TC07001377.hg.1	COBL	7.21 ± 0.12	7.15 ± 0.09	7.20 ± 0.09	7.22 ± 0.03
TC02002487.hg.1	COBLL1	6.97 ± 0.12	6.98 ± 0.10	6.70 ± 0.15	6.94 ± 0.07
TC14000196.hg.1	COCH	6.64 ± 0.15	6.53 ± 0.04	6.54 ± 0.07	6.51 ± 0.02
TC17000822.hg.1	COG1	8.77 ± 0.13	8.62 ± 0.06	8.51 ± 0.02	8.65 ± 0.06
TC01001909.hg.1	COG2	7.43 ± 0.06	7.37 ± 0.11	7.23 ± 0.08	7.28 ± 0.03
TC13000176.hg.1	COG3	7.80 ± 0.34	7.97 ± 0.21	7.86 ± 0.30	7.85 ± 0.04
TC16001239.hg.1	COG4	8.36 ± 0.19	8.03 ± 0.09	8.18 ± 0.15	7.87 ± 0.04
TC07001743.hg.1	COG5	8.38 ± 0.30	8.64 ± 0.15	8.34 ± 0.38	8.42 ± 0.17
TC13000137.hg.1	COG6	7.10 ± 0.36	7.08 ± 0.20	7.00 ± 0.32	6.81 ± 0.15
TC16000960.hg.1	COG7	8.56 ± 0.26	8.28 ± 0.08	8.63 ± 0.18	8.15 ± 0.08
TC17001717.hg.1	COIL	7.51 ± 0.08	7.66 ± 0.28	7.29 ± 0.24	7.55 ± 0.07
TC06002044.hg.1	COL10A1	4.74 ± 0.16	4.68 ± 0.11	4.79 ± 0.04	4.73 ± 0.10
TC01002932.hg.1	COL11A1	7.11 ± 0.21	7.02 ± 0.20	7.03 ± 0.23	7.15 ± 0.07
TC06001579.hg.1	COL11A2	8.36 ± 0.26	8.30 ± 0.22	8.28 ± 0.23	8.40 ± 0.06
TC6_cox_hap2000203.hg.1	COL11A2	8.37 ± 0.27	8.30 ± 0.22	8.29 ± 0.24	8.41 ± 0.05
TC6_dbb_hap3000189.hg.1	COL11A2	8.36 ± 0.26	8.29 ± 0.22	8.27 ± 0.24	8.39 ± 0.05
TC6_mann_hap4000171.hg.1	COL11A2	8.36 ± 0.27	8.29 ± 0.22	8.28 ± 0.24	8.40 ± 0.05
TC6_mcf_hap5000178.hg.1	COL11A2	8.36 ± 0.27	8.29 ± 0.22	8.27 ± 0.24	8.39 ± 0.05
TC6_qbl_hap6000191.hg.1	COL11A2	8.36 ± 0.27	8.29 ± 0.23	8.28 ± 0.24	8.40 ± 0.05
TC6_ssto_hap7000173.hg.1	COL11A2	8.35 ± 0.27	8.28 ± 0.22	8.26 ± 0.24	8.39 ± 0.06
TC06001879.hg.1	COL12A1	6.12 ± 0.12	6.02 ± 0.11	6.07 ± 0.11	6.10 ± 0.06
TC10000424.hg.1	COL13A1	8.23 ± 0.25	8.12 ± 0.14	8.16 ± 0.22	8.21 ± 0.08
TC08000700.hg.1	COL14A1	5.88 ± 0.14	5.78 ± 0.09	5.81 ± 0.07	5.84 ± 0.05
TC09000499.hg.1	COL15A1	7.47 ± 0.22	7.42 ± 0.17	7.39 ± 0.19	7.51 ± 0.06
TC01002456.hg.1	COL16A1	8.34 ± 0.22	8.29 ± 0.20	8.26 ± 0.24	8.39 ± 0.08
TC21000245.hg.1	COL18A1	8.69 ± 0.22	8.61 ± 0.16	8.63 ± 0.18	8.63 ± 0.02
TC21000548.hg.1	COL18A1-AS1	7.66 ± 0.23	7.63 ± 0.22	7.58 ± 0.22	7.68 ± 0.07
TC21000547.hg.1	COL18A1-AS2	6.44 ± 0.34	6.56 ± 0.15	6.50 ± 0.17	6.69 ± 0.19

TC06000712.hg.1	COL19A1	7.43 ± 0.21	7.52 ± 0.07	7.36 ± 0.07	7.53 ± 0.13
TC17001682.hg.1	COL1A1	8.17 ± 0.21	8.22 ± 0.24	8.12 ± 0.15	8.35 ± 0.04
TC07000559.hg.1	COL1A2	7.42 ± 0.21	7.39 ± 0.18	7.34 ± 0.17	7.50 ± 0.10
TC20000513.hg.1	COL20A1	8.36 ± 0.24	8.34 ± 0.22	8.29 ± 0.22	8.45 ± 0.06
TC06001826.hg.1	COL21A1	5.87 ± 0.19	5.74 ± 0.12	5.79 ± 0.13	5.83 ± 0.06
TC08001675.hg.1	COL22A1	7.92 ± 0.26	7.82 ± 0.20	7.83 ± 0.21	7.93 ± 0.07
TC05002123.hg.1	COL23A1	8.56 ± 0.25	8.48 ± 0.17	8.46 ± 0.27	8.56 ± 0.06
TC01002832.hg.1	COL24A1	6.75 ± 0.15	6.57 ± 0.15	6.64 ± 0.14	6.66 ± 0.03
TC04001462.hg.1	COL25A1	7.24 ± 0.26	7.08 ± 0.17	7.14 ± 0.23	7.20 ± 0.10
TC07000649.hg.1	COL26A1	8.28 ± 0.24	8.20 ± 0.17	8.22 ± 0.20	8.29 ± 0.04
TC09000580.hg.1	COL27A1	8.36 ± 0.30	8.27 ± 0.20	8.26 ± 0.27	8.35 ± 0.05
TC09000582.hg.1	COL27A1	6.89 ± 0.36	6.95 ± 0.32	6.84 ± 0.29	6.99 ± 0.10
TC07001138.hg.1	COL28A1	6.83 ± 0.18	6.69 ± 0.11	6.78 ± 0.18	6.77 ± 0.04
TC12001440.hg.1	COL2A1	8.23 ± 0.25	8.21 ± 0.22	8.16 ± 0.23	8.32 ± 0.04
TC02001105.hg.1	COL3A1	7.37 ± 0.20	7.33 ± 0.25	7.32 ± 0.20	7.50 ± 0.09
TC13000872.hg.1	COL4A1	7.44 ± 0.24	7.37 ± 0.22	7.36 ± 0.22	7.46 ± 0.06
TC13000392.hg.1	COL4A2	7.85 ± 0.24	7.75 ± 0.18	7.77 ± 0.21	7.84 ± 0.05
TC13000874.hg.1	COL4A2-AS1	7.33 ± 0.16	7.12 ± 0.13	7.28 ± 0.15	7.26 ± 0.07
TC13000873.hg.1	COL4A2-AS2	7.79 ± 0.25	7.70 ± 0.05	7.70 ± 0.20	7.78 ± 0.05
TC02001361.hg.1	COL4A3	6.97 ± 0.23	6.86 ± 0.18	6.88 ± 0.19	6.96 ± 0.04
TC05001491.hg.1	COL4A3BP	8.66 ± 0.36	8.79 ± 0.23	9.22 ± 0.38	8.76 ± 0.16
TC02002832.hg.1	COL4A4	7.43 ± 0.24	7.32 ± 0.17	7.29 ± 0.22	7.42 ± 0.07
TC0X000532.hg.1	COL4A5	6.42 ± 0.18	6.35 ± 0.16	6.36 ± 0.15	6.41 ± 0.04
TC0X001267.hg.1	COL4A6	6.86 ± 0.20	6.83 ± 0.16	6.82 ± 0.16	6.90 ± 0.03
TC09000787.hg.1	COL5A1	8.04 ± 0.28	7.95 ± 0.22	7.92 ± 0.24	8.03 ± 0.05
TC09001702.hg.1	COL5A1-AS1	5.78 ± 0.48	5.46 ± 0.42	5.68 ± 0.33	5.70 ± 0.15
TC02002612.hg.1	COL5A2	7.25 ± 0.25	7.22 ± 0.21	7.18 ± 0.28	7.34 ± 0.07
TC19001156.hg.1	COL5A3	8.30 ± 0.29	8.21 ± 0.22	8.18 ± 0.28	8.29 ± 0.05
TC21000249.hg.1	COL6A1	8.32 ± 0.24	8.23 ± 0.17	8.21 ± 0.26	8.27 ± 0.04
TC21000253.hg.1	COL6A2	8.63 ± 0.22	8.58 ± 0.15	8.49 ± 0.19	8.63 ± 0.05
TC02002905.hg.1	COL6A3	7.12 ± 0.16	7.00 ± 0.16	7.06 ± 0.14	7.09 ± 0.04

TC03001205.hg.1	COL6A4P1	6.23 ± 0.08	6.26 ± 0.17	6.32 ± 0.01	6.39 ± 0.14
TC03000703.hg.1	COL6A4P2	7.09 ± 0.27	6.97 ± 0.16	7.02 ± 0.27	7.05 ± 0.04
TC03000704.hg.1	COL6A5	6.02 ± 0.19	5.88 ± 0.14	5.93 ± 0.14	5.97 ± 0.07
TC03000705.hg.1	COL6A6	6.54 ± 0.21	6.43 ± 0.11	6.47 ± 0.15	6.54 ± 0.09
TC03001382.hg.1	COL7A1	8.52 ± 0.28	8.45 ± 0.22	8.43 ± 0.26	8.55 ± 0.05
TC03000502.hg.1	COL8A1	5.34 ± 0.15	5.26 ± 0.09	5.27 ± 0.09	5.35 ± 0.12
TC01002503.hg.1	COL8A2	7.86 ± 0.27	7.79 ± 0.22	7.71 ± 0.31	7.84 ± 0.02
TC06001855.hg.1	COL9A1	6.98 ± 0.21	6.89 ± 0.16	6.88 ± 0.18	6.98 ± 0.06
TC01002554.hg.1	COL9A2	8.42 ± 0.31	8.32 ± 0.18	8.31 ± 0.25	8.40 ± 0.04
TC20000499.hg.1	COL9A3	8.65 ± 0.28	8.53 ± 0.23	8.52 ± 0.29	8.60 ± 0.05
TC08000694.hg.1	COLEC10	5.99 ± 0.19	5.90 ± 0.10	5.88 ± 0.09	5.95 ± 0.09
TC02000023.hg.1	COLEC11	7.87 ± 0.20	7.78 ± 0.16	7.80 ± 0.16	7.85 ± 0.05
TC18000273.hg.1	COLEC12	5.67 ± 0.20	5.53 ± 0.14	5.62 ± 0.17	5.64 ± 0.05
TC19000340.hg.1	COLGALT1	9.50 ± 0.28	9.15 ± 0.05	9.63 ± 0.15	9.07 ± 0.15
TC01003621.hg.1	COLGALT2	6.69 ± 0.05	6.62 ± 0.06	6.56 ± 0.07	6.61 ± 0.07
TC03003335.hg.1	COLQ	7.55 ± 0.09	7.44 ± 0.15	7.44 ± 0.09	7.55 ± 0.05
TC02000356.hg.1	COMMD1	7.42 ± 0.09	7.27 ± 0.14	7.17 ± 0.09	7.18 ± 0.05
TC05000551.hg.1	COMMD10	7.17 ± 0.28	7.34 ± 0.15	7.12 ± 0.22	7.22 ± 0.08
TC03001892.hg.1	COMMD2	7.33 ± 0.11	7.36 ± 0.08	7.30 ± 0.18	7.35 ± 0.08
TC10002934.hg.1	COMMD3	6.80 ± 0.17	6.61 ± 0.25	6.64 ± 0.23	6.50 ± 0.14
TC15000694.hg.1	COMMD4	7.88 ± 0.20	7.71 ± 0.09	7.64 ± 0.14	7.66 ± 0.05
TC08002624.hg.1	COMMD5	8.33 ± 0.15	8.25 ± 0.09	8.21 ± 0.08	8.22 ± 0.01
TC13000739.hg.1	COMMD6	6.77 ± 0.12	6.78 ± 0.00	6.72 ± 0.13	6.76 ± 0.02
TC20000766.hg.1	COMMD7	8.24 ± 0.07	8.25 ± 0.13	8.18 ± 0.04	8.21 ± 0.06
TC04001167.hg.1	COMMD8	6.75 ± 0.27	6.83 ± 0.14	7.00 ± 0.48	6.52 ± 0.11
TC11001557.hg.1	COMMD9	7.98 ± 0.16	7.91 ± 0.17	7.80 ± 0.03	7.82 ± 0.03
TC19001293.hg.1	COMP	8.08 ± 0.23	7.98 ± 0.15	8.01 ± 0.20	8.05 ± 0.05
TC22000050.hg.1	COMT	8.70 ± 0.15	8.52 ± 0.03	8.62 ± 0.19	8.50 ± 0.04
TC10001429.hg.1	COMTD1	9.16 ± 0.21	9.10 ± 0.18	9.07 ± 0.23	9.11 ± 0.03
TC01003400.hg.1	COPA	10.02 ± 0.20	9.84 ± 0.09	10.00 ± 0.22	9.79 ± 0.06
TC11003473.hg.1	COPB1	8.96 ± 0.25	9.03 ± 0.23	9.05 ± 0.35	8.99 ± 0.08

TC03001832.hg.1	COPB2	9.14 ± 0.20	8.79 ± 0.14	9.22 ± 0.25	8.49 ± 0.27
TC19002690.hg.1	COPE	10.26 ± 0.35	9.97 ± 0.06	10.07 ± 0.04	9.88 ± 0.16
TC03000689.hg.1	COPG1	9.48 ± 0.27	9.10 ± 0.07	9.29 ± 0.23	9.00 ± 0.05
TC07003379.hg.1	COPG2	7.51 ± 0.13	7.46 ± 0.18	7.66 ± 0.20	7.24 ± 0.07
TC17001345.hg.1	COPRS	7.52 ± 0.26	7.33 ± 0.07	7.37 ± 0.21	7.35 ± 0.03
TC15001342.hg.1	COPS2	8.43 ± 0.65	9.16 ± 0.27	8.60 ± 0.49	9.10 ± 0.05
TC17001186.hg.1	COPS3	8.52 ± 0.03	8.59 ± 0.24	8.29 ± 0.10	8.43 ± 0.14
TC04000458.hg.1	COPS4	7.15 ± 0.19	7.30 ± 0.13	7.15 ± 0.20	7.19 ± 0.09
TC08002585.hg.1	COPS5	6.93 ± 0.12	6.88 ± 0.12	6.83 ± 0.09	6.88 ± 0.03
TC07000609.hg.1	COPS6	8.67 ± 0.16	8.60 ± 0.07	8.42 ± 0.13	8.49 ± 0.04
TC12000089.hg.1	COPS7A	9.08 ± 0.11	8.82 ± 0.03	9.00 ± 0.11	8.76 ± 0.05
TC02001395.hg.1	COPS7B	7.97 ± 0.08	7.88 ± 0.08	7.83 ± 0.06	7.87 ± 0.03
TC02001439.hg.1	COPS8	7.77 ± 0.37	8.03 ± 0.08	7.67 ± 0.31	7.99 ± 0.14
TC12000462.hg.1	COPZ1	9.21 ± 0.13	8.95 ± 0.13	8.82 ± 0.18	8.74 ± 0.09
TC17001633.hg.1	COPZ2	7.82 ± 0.26	7.80 ± 0.20	7.66 ± 0.29	7.84 ± 0.04
TC12000509.hg.1	COQ10A	7.88 ± 0.07	7.70 ± 0.17	7.42 ± 0.16	7.48 ± 0.05
TC02001148.hg.1	COQ10B	8.29 ± 0.29	8.59 ± 0.14	8.62 ± 0.45	8.56 ± 0.20
TC04001346.hg.1	COQ2	7.68 ± 0.21	7.56 ± 0.15	8.05 ± 0.27	7.51 ± 0.13
TC06001963.hg.1	COQ3	5.56 ± 0.10	5.42 ± 0.07	5.51 ± 0.10	5.37 ± 0.06
TC09000685.hg.1	COQ4	8.16 ± 0.10	7.98 ± 0.00	7.91 ± 0.09	7.90 ± 0.09
TC12002047.hg.1	COQ5	7.75 ± 0.16	7.52 ± 0.06	7.60 ± 0.22	7.30 ± 0.08
TC14000453.hg.1	COQ6	7.07 ± 0.09	7.07 ± 0.03	6.91 ± 0.07	7.09 ± 0.05
TC16000209.hg.1	COQ7	7.15 ± 0.07	7.05 ± 0.02	7.16 ± 0.10	6.99 ± 0.05
TC16000490.hg.1	COQ9	8.35 ± 0.10	8.20 ± 0.06	8.22 ± 0.07	8.14 ± 0.05
TC04001169.hg.1	CORIN	6.22 ± 0.12	6.15 ± 0.12	6.21 ± 0.06	6.25 ± 0.10
TC16000342.hg.1	CORO1A	10.88 ± 0.20	10.58 ± 0.00	10.76 ± 0.14	10.45 ± 0.07
TC11003490.hg.1	CORO1B	9.31 ± 0.28	8.96 ± 0.03	9.16 ± 0.20	8.89 ± 0.09
TC12001939.hg.1	CORO1C	10.20 ± 0.11	9.96 ± 0.09	10.33 ± 0.15	9.99 ± 0.32
TC09001404.hg.1	CORO2A	7.79 ± 0.20	7.65 ± 0.07	7.86 ± 0.16	7.67 ± 0.03
TC15000629.hg.1	CORO2B	7.31 ± 0.18	7.23 ± 0.04	7.13 ± 0.10	7.25 ± 0.04
TC17001321.hg.1	CORO6	7.78 ± 0.19	7.71 ± 0.15	7.77 ± 0.15	7.75 ± 0.02

TC16002062.hg.1	CORO7	9.45 ± 0.15	9.37 ± 0.06	9.33 ± 0.06	9.43 ± 0.04
TC16001314.hg.1	COTL1	11.19 ± 0.33	11.19 ± 0.07	11.37 ± 0.15	11.07 ± 0.22
TC17000162.hg.1	COX10	8.22 ± 0.15	8.22 ± 0.08	7.98 ± 0.02	8.11 ± 0.02
TC17001151.hg.1	COX10-AS1	4.63 ± 0.06	4.49 ± 0.06	4.53 ± 0.02	4.47 ± 0.14
TC17001153.hg.1	COX10-AS1	7.77 ± 0.30	7.81 ± 0.04	7.61 ± 0.07	7.75 ± 0.08
TC17001709.hg.1	COX11	6.52 ± 0.17	6.30 ± 0.12	6.31 ± 0.06	6.15 ± 0.02
TC06000285.hg.1	COX11P1	6.02 ± 0.18	5.95 ± 0.18	5.66 ± 0.10	5.88 ± 0.04
TC12000389.hg.1	COX14	8.35 ± 0.01	8.40 ± 0.02	8.30 ± 0.02	8.43 ± 0.06
TC10001584.hg.1	COX15	8.00 ± 0.12	7.67 ± 0.05	8.03 ± 0.29	7.47 ± 0.09
TC03003390.hg.1	COX17	7.47 ± 0.12	7.49 ± 0.09	7.43 ± 0.05	7.48 ± 0.07
TC04001278.hg.1	COX18	7.61 ± 0.18	7.46 ± 0.08	7.36 ± 0.12	7.41 ± 0.13
TC07003347.hg.1	COX19	7.98 ± 0.07	8.00 ± 0.09	7.78 ± 0.18	8.02 ± 0.03
TC01001990.hg.1	COX20	8.09 ± 0.27	8.23 ± 0.06	8.05 ± 0.23	8.13 ± 0.18
TC16000665.hg.1	COX4I1	9.96 ± 0.12	10.02 ± 0.28	9.58 ± 0.16	10.03 ± 0.08
TC20000196.hg.1	COX4I2	7.53 ± 0.23	7.52 ± 0.30	7.54 ± 0.16	7.63 ± 0.03
TC15001652.hg.1	COX5A	8.75 ± 0.37	8.69 ± 0.38	8.65 ± 0.11	8.37 ± 0.23
TC02000592.hg.1	COX5B	8.25 ± 0.22	7.96 ± 0.11	8.01 ± 0.26	7.91 ± 0.10
TC12003257.hg.1	COX6A1	10.41 ± 0.46	10.10 ± 0.30	10.07 ± 0.35	9.76 ± 0.05
TC06000539.hg.1	COX6A1P2	10.02 ± 0.39	9.74 ± 0.20	9.68 ± 0.35	9.51 ± 0.05
TC16001055.hg.1	COX6A2	8.03 ± 0.36	8.05 ± 0.34	7.78 ± 0.24	8.16 ± 0.09
TC19000479.hg.1	COX6B1	9.12 ± 0.40	8.74 ± 0.14	8.85 ± 0.24	8.49 ± 0.09
TC08001472.hg.1	COX6C	6.84 ± 0.17	6.98 ± 0.23	6.70 ± 0.06	6.86 ± 0.02
TC19001463.hg.1	COX7A1	7.88 ± 0.24	7.81 ± 0.17	7.72 ± 0.27	7.84 ± 0.03
TC06001880.hg.1	COX7A2	7.97 ± 0.15	7.81 ± 0.18	7.79 ± 0.13	7.68 ± 0.08
TC02001778.hg.1	COX7A2L	7.87 ± 0.02	7.78 ± 0.16	7.80 ± 0.09	7.67 ± 0.09
TC0X000423.hg.1	COX7B	7.98 ± 0.13	7.97 ± 0.28	7.84 ± 0.12	7.80 ± 0.09
TC04001164.hg.1	COX7B2	5.07 ± 0.02	4.91 ± 0.09	4.95 ± 0.10	5.02 ± 0.06
TC05000427.hg.1	COX7C	7.58 ± 0.09	7.60 ± 0.22	7.43 ± 0.16	7.54 ± 0.11
TC11003499.hg.1	COX8A	10.88 ± 0.46	10.53 ± 0.34	10.47 ± 0.24	10.31 ± 0.22
TC14000578.hg.1	COX8C	5.60 ± 0.04	5.86 ± 0.18	5.75 ± 0.08	5.95 ± 0.11
TC03001884.hg.1	CP	4.55 ± 0.07	4.37 ± 0.01	4.48 ± 0.03	4.41 ± 0.05

TC07000814.hg.1	CPA1	7.59 ± 0.20	7.61 ± 0.19	7.49 ± 0.21	7.70 ± 0.08
TC07000809.hg.1	CPA2	5.59 ± 0.07	5.47 ± 0.03	5.52 ± 0.06	5.52 ± 0.05
TC03000800.hg.1	CPA3	6.25 ± 0.10	6.30 ± 0.27	6.11 ± 0.15	6.33 ± 0.37
TC07000810.hg.1	CPA4	6.13 ± 0.12	6.03 ± 0.06	6.03 ± 0.06	6.08 ± 0.03
TC07000813.hg.1	CPA5	6.18 ± 0.13	6.16 ± 0.14	6.15 ± 0.08	6.20 ± 0.05
TC08001294.hg.1	CPA6	4.98 ± 0.04	4.89 ± 0.01	4.96 ± 0.07	4.91 ± 0.05
TC19001268.hg.1	CPAMD8	7.56 ± 0.20	7.50 ± 0.17	7.45 ± 0.15	7.56 ± 0.02
TC03000799.hg.1	CPB1	5.28 ± 0.12	5.19 ± 0.03	5.28 ± 0.07	5.30 ± 0.10
TC13000641.hg.1	CPB2	4.43 ± 0.12	4.33 ± 0.06	4.36 ± 0.02	4.36 ± 0.09
TC13000178.hg.1	CPB2-AS1	6.63 ± 0.25	6.46 ± 0.07	6.49 ± 0.15	6.51 ± 0.09
TC17000349.hg.1	CPD	9.55 ± 0.33	9.71 ± 0.33	10.39 ± 0.54	9.72 ± 0.34
TC04000829.hg.1	CPE	6.74 ± 0.24	6.66 ± 0.15	6.68 ± 0.16	6.71 ± 0.04
TC15001755.hg.1	CPEB1	6.37 ± 0.08	6.31 ± 0.09	6.35 ± 0.04	6.40 ± 0.06
TC04000140.hg.1	CPEB2	8.52 ± 0.25	8.88 ± 0.14	8.91 ± 0.34	8.82 ± 0.23
TC10001526.hg.1	CPEB3	7.18 ± 0.23	6.93 ± 0.16	7.36 ± 0.30	6.79 ± 0.10
TC05000967.hg.1	CPEB4	8.76 ± 0.42	8.73 ± 0.24	9.48 ± 0.55	8.66 ± 0.27
TC07000735.hg.1	CPED1	6.39 ± 0.05	6.23 ± 0.07	6.37 ± 0.27	6.20 ± 0.08
TC03001885.hg.1	CPHL1P	3.98 ± 0.12	3.74 ± 0.03	3.80 ± 0.09	3.88 ± 0.08
TC04000956.hg.1	CPLX1	8.36 ± 0.30	8.27 ± 0.21	8.28 ± 0.28	8.30 ± 0.03
TC05000985.hg.1	CPLX2	7.69 ± 0.27	7.63 ± 0.20	7.67 ± 0.22	7.70 ± 0.06
TC15000686.hg.1	CPLX3	7.80 ± 0.24	7.78 ± 0.21	7.74 ± 0.17	7.88 ± 0.06
TC18000546.hg.1	CPLX4	5.64 ± 0.20	5.68 ± 0.07	5.70 ± 0.09	5.70 ± 0.09
TC12001705.hg.1	CPM	6.87 ± 0.14	6.66 ± 0.11	6.73 ± 0.03	6.65 ± 0.13
TC10001586.hg.1	CPN1	7.12 ± 0.18	7.05 ± 0.14	7.09 ± 0.14	7.13 ± 0.04
TC03002130.hg.1	CPN2	7.66 ± 0.27	7.73 ± 0.33	7.52 ± 0.33	7.95 ± 0.15
TC20001761.hg.1	CPNE1	9.63 ± 0.18	9.38 ± 0.08	9.45 ± 0.19	9.22 ± 0.04
TC16000483.hg.1	CPNE2	9.02 ± 0.06	8.71 ± 0.02	9.20 ± 0.04	8.70 ± 0.08
TC08000539.hg.1	CPNE3	8.70 ± 0.05	8.68 ± 0.19	8.82 ± 0.28	8.55 ± 0.12
TC03001789.hg.1	CPNE4	5.69 ± 0.10	5.59 ± 0.03	5.66 ± 0.06	5.65 ± 0.07
TC06001678.hg.1	CPNE5	8.13 ± 0.13	7.90 ± 0.06	7.91 ± 0.01	7.86 ± 0.06
TC14000156.hg.1	CPNE6	7.50 ± 0.27	7.49 ± 0.19	7.44 ± 0.21	7.62 ± 0.10

TC16000700.hg.1	CPNE7	7.87 ± 0.24	7.84 ± 0.24	7.83 ± 0.22	7.95 ± 0.05
TC12001389.hg.1	CPNE8	6.72 ± 0.23	6.56 ± 0.24	7.01 ± 0.43	6.42 ± 0.25
TC03000040.hg.1	CPNE9	7.06 ± 0.15	6.97 ± 0.11	6.98 ± 0.09	7.00 ± 0.02
TC02001224.hg.1	CPO	5.17 ± 0.13	5.06 ± 0.06	5.04 ± 0.04	5.11 ± 0.02
TC03003340.hg.1	CPOX	7.06 ± 0.12	6.91 ± 0.04	6.97 ± 0.11	6.77 ± 0.02
TC16000879.hg.1	CPPED1	10.56 ± 0.15	10.61 ± 0.24	10.91 ± 0.39	10.49 ± 0.42
TC08000591.hg.1	CPQ	8.00 ± 0.06	8.09 ± 0.18	8.39 ± 0.27	8.03 ± 0.19
TC02001246.hg.1	CPS1	5.49 ± 0.12	5.47 ± 0.09	5.47 ± 0.03	5.55 ± 0.09
TC02001247.hg.1	CPS1-IT1	4.68 ± 0.11	4.60 ± 0.09	4.69 ± 0.02	4.87 ± 0.32
TC14000568.hg.1	CPSF2	8.56 ± 0.36	8.44 ± 0.34	8.79 ± 0.38	8.13 ± 0.36
TC02000047.hg.1	CPSF3	8.08 ± 0.04	7.84 ± 0.19	7.90 ± 0.16	7.56 ± 0.15
TC01002091.hg.1	CPSF3L	8.94 ± 0.12	8.92 ± 0.09	8.79 ± 0.11	8.91 ± 0.03
TC07000594.hg.1	CPSF4	7.77 ± 0.06	7.66 ± 0.04	7.67 ± 0.05	7.66 ± 0.01
TC07001644.hg.1	CPSF4	7.09 ± 0.23	6.99 ± 0.16	6.93 ± 0.04	6.83 ± 0.10
TC17001848.hg.1	CPSF4L	6.59 ± 0.10	6.50 ± 0.11	6.51 ± 0.17	6.56 ± 0.04
TC12000610.hg.1	CPSF6	9.04 ± 0.30	9.21 ± 0.18	8.82 ± 0.21	9.16 ± 0.08
TC11001853.hg.1	CPSF7	9.61 ± 0.05	9.61 ± 0.01	9.64 ± 0.13	9.65 ± 0.07
TC11002009.hg.1	CPT1A	8.68 ± 0.17	8.54 ± 0.19	8.92 ± 0.13	8.41 ± 0.06
TC22001502.hg.1	CPT1B	8.20 ± 0.16	8.19 ± 0.05	8.25 ± 0.12	8.25 ± 0.06
TC19000746.hg.1	CPT1C	7.66 ± 0.22	7.66 ± 0.18	7.59 ± 0.16	7.77 ± 0.04
TC01000651.hg.1	CPT2	7.81 ± 0.21	7.57 ± 0.04	7.68 ± 0.08	7.54 ± 0.09
TC07001236.hg.1	CPVL	8.64 ± 0.07	8.62 ± 0.06	8.61 ± 0.01	8.56 ± 0.23
TC0X000448.hg.1	CPXCR1	4.18 ± 0.21	3.92 ± 0.09	4.08 ± 0.19	4.02 ± 0.10
TC20000565.hg.1	CPXM1	7.54 ± 0.25	7.48 ± 0.22	7.46 ± 0.24	7.59 ± 0.06
TC10001721.hg.1	CPXM2	6.96 ± 0.26	6.90 ± 0.16	6.87 ± 0.17	7.01 ± 0.06
TC04002933.hg.1	CPZ	8.20 ± 0.16	8.13 ± 0.20	8.12 ± 0.20	8.22 ± 0.03
TC01001738.hg.1	CR1	10.62 ± 0.59	10.31 ± 0.26	11.84 ± 0.28	10.30 ± 0.28
TC01001740.hg.1	CR1L	7.89 ± 0.23	7.62 ± 0.14	8.62 ± 0.15	7.64 ± 0.18
TC01001737.hg.1	CR2	7.88 ± 0.26	7.97 ± 0.18	7.57 ± 0.21	7.77 ± 0.04
TC15000728.hg.1	CRABP1	7.50 ± 0.20	7.38 ± 0.16	7.42 ± 0.11	7.51 ± 0.05
TC01003349.hg.1	CRABP2	7.75 ± 0.26	7.65 ± 0.19	7.61 ± 0.21	7.78 ± 0.05

TC12000727.hg.1	CRADD	7.40 ± 0.03	7.39 ± 0.04	7.33 ± 0.04	7.41 ± 0.08
TC16002066.hg.1	CRAMP1L	8.32 ± 0.01	8.25 ± 0.04	8.13 ± 0.05	8.27 ± 0.03
TC09001643.hg.1	CRAT	8.31 ± 0.14	8.11 ± 0.08	8.25 ± 0.14	8.18 ± 0.02
TC01001635.hg.1	CRB1	5.36 ± 0.09	5.29 ± 0.05	5.29 ± 0.07	5.33 ± 0.06
TC09000629.hg.1	CRB2	8.51 ± 0.21	8.49 ± 0.20	8.44 ± 0.18	8.57 ± 0.03
TC19000115.hg.1	CRB3	8.11 ± 0.20	8.09 ± 0.20	8.00 ± 0.19	8.16 ± 0.05
TC03001123.hg.1	CRBN	7.97 ± 0.49	8.08 ± 0.19	8.06 ± 0.41	7.96 ± 0.03
TC07003308.hg.1	CRCP	9.03 ± 0.01	9.03 ± 0.08	8.94 ± 0.09	9.03 ± 0.07
TC01001227.hg.1	CRCT1	6.91 ± 0.30	6.86 ± 0.28	6.84 ± 0.34	7.04 ± 0.09
TC02001229.hg.1	CREB1	8.25 ± 0.39	8.42 ± 0.28	8.57 ± 0.46	8.29 ± 0.19
TC09000192.hg.1	CREB3	8.58 ± 0.27	8.26 ± 0.07	8.26 ± 0.19	8.16 ± 0.13
TC11000394.hg.1	CREB3L1	7.42 ± 0.23	7.34 ± 0.16	7.33 ± 0.13	7.43 ± 0.05
TC07001898.hg.1	CREB3L2	8.79 ± 0.13	8.38 ± 0.04	8.52 ± 0.16	8.28 ± 0.09
TC19000081.hg.1	CREB3L3	7.46 ± 0.23	7.45 ± 0.25	7.56 ± 0.10	7.58 ± 0.08
TC01001265.hg.1	CREB3L4	7.40 ± 0.15	7.35 ± 0.07	7.26 ± 0.19	7.36 ± 0.03
TC07000178.hg.1	CREB5	8.01 ± 0.14	7.88 ± 0.20	8.54 ± 0.19	7.81 ± 0.19
TC16000823.hg.1	CREBBP	10.78 ± 0.28	10.93 ± 0.08	10.86 ± 0.34	10.96 ± 0.23
TC12000176.hg.1	CREBL2	8.73 ± 0.10	8.69 ± 0.12	8.57 ± 0.07	8.54 ± 0.20
TC05000958.hg.1	CREBRF	9.16 ± 0.45	9.50 ± 0.16	9.50 ± 0.50	9.46 ± 0.12
TC11002160.hg.1	CREBZF	8.39 ± 0.58	8.60 ± 0.19	8.45 ± 0.45	8.58 ± 0.16
TC01003481.hg.1	CREG1	8.71 ± 0.16	8.51 ± 0.10	8.98 ± 0.16	8.51 ± 0.32
TC02005041.hg.1	CREG2	6.57 ± 0.17	6.48 ± 0.09	6.43 ± 0.14	6.53 ± 0.05
TC03000048.hg.1	CRELD1	8.18 ± 0.13	8.12 ± 0.12	8.06 ± 0.07	8.11 ± 0.02
TC22000415.hg.1	CRELD2	8.28 ± 0.15	8.14 ± 0.10	8.09 ± 0.19	8.09 ± 0.05
TC10000238.hg.1	CREM	6.80 ± 0.02	7.01 ± 0.13	6.84 ± 0.07	7.05 ± 0.04
TC08001283.hg.1	CRH	5.92 ± 0.17	5.81 ± 0.07	5.83 ± 0.05	5.86 ± 0.04
TC05000375.hg.1	CRHBP	6.04 ± 0.12	5.88 ± 0.12	6.02 ± 0.15	5.90 ± 0.03
TC17_ctg5_hap1000028.hg.1	CRHR1	7.42 ± 0.19	7.46 ± 0.24	7.39 ± 0.17	7.59 ± 0.06
TC17002876.hg.1	CRHR1	7.49 ± 0.18	7.52 ± 0.23	7.45 ± 0.17	7.66 ± 0.07
TC17_ctg5_hap1000022.hg.1	CRHR1-IT1	6.50 ± 0.23	6.43 ± 0.17	6.40 ± 0.30	6.51 ± 0.04
TC17000590.hg.1	CRHR1-IT1	6.19 ± 0.16	6.06 ± 0.03	6.10 ± 0.06	6.08 ± 0.09

TC07001252.hg.1	CRHR2	7.74 ± 0.20	7.74 ± 0.19	7.71 ± 0.16	7.85 ± 0.05
TC02000230.hg.1	CRIM1	7.24 ± 0.18	7.25 ± 0.11	7.10 ± 0.01	7.31 ± 0.04
TC14002314.hg.1	CRIP1	8.66 ± 0.20	8.55 ± 0.12	8.49 ± 0.24	8.53 ± 0.04
TC22000156.hg.1	CRIP1P4	8.73 ± 0.19	8.44 ± 0.16	8.27 ± 0.32	8.30 ± 0.10
TC14000837.hg.1	CRIP2	8.43 ± 0.28	8.36 ± 0.17	8.30 ± 0.28	8.43 ± 0.02
TC06001746.hg.1	CRIP3	7.32 ± 0.19	7.27 ± 0.14	7.22 ± 0.13	7.33 ± 0.10
TC04000027.hg.1	CRIPAK	9.04 ± 0.39	9.21 ± 0.03	9.07 ± 0.18	9.31 ± 0.25
TC02000285.hg.1	CRIPT	8.45 ± 0.29	8.48 ± 0.10	8.47 ± 0.27	8.55 ± 0.12
TC06001788.hg.1	CRISP1	5.25 ± 0.07	5.10 ± 0.04	5.14 ± 0.04	5.15 ± 0.04
TC06001784.hg.1	CRISP2	4.88 ± 0.15	4.75 ± 0.03	4.83 ± 0.10	4.77 ± 0.02
TC06001785.hg.1	CRISP3	6.54 ± 0.35	6.02 ± 0.18	6.83 ± 0.38	5.96 ± 0.10
TC08000498.hg.1	CRISPLD1	5.27 ± 0.16	5.13 ± 0.08	5.20 ± 0.05	5.20 ± 0.08
TC16000660.hg.1	CRISPLD2	9.50 ± 0.28	8.88 ± 0.11	9.80 ± 0.17	8.72 ± 0.53
TC17000973.hg.1	CRK	9.37 ± 0.20	9.33 ± 0.09	9.72 ± 0.33	9.33 ± 0.21
TC22000074.hg.1	CRKL	9.51 ± 0.24	9.20 ± 0.12	9.46 ± 0.29	9.10 ± 0.12
TC19001292.hg.1	CRLF1	7.90 ± 0.22	7.95 ± 0.26	7.89 ± 0.24	8.02 ± 0.03
TC0X002305.hg.1	CRLF2	7.06 ± 0.16	7.13 ± 0.15	7.07 ± 0.19	7.24 ± 0.05
TC0Y000347.hg.1	CRLF2	7.06 ± 0.16	7.13 ± 0.15	7.07 ± 0.19	7.24 ± 0.05
TC17001336.hg.1	CRLF3	10.18 ± 0.20	10.28 ± 0.14	10.25 ± 0.37	10.18 ± 0.15
TC20000065.hg.1	CRLS1	7.79 ± 0.12	7.68 ± 0.14	7.72 ± 0.24	7.62 ± 0.08
TC04000997.hg.1	CRMP1	7.54 ± 0.19	7.43 ± 0.08	7.46 ± 0.20	7.48 ± 0.03
TC16001125.hg.1	CRNDE	5.44 ± 0.17	5.32 ± 0.08	5.32 ± 0.16	5.36 ± 0.06
TC20000681.hg.1	CRNKL1	8.37 ± 0.19	8.42 ± 0.08	8.46 ± 0.32	8.41 ± 0.02
TC01003247.hg.1	CRNN	6.99 ± 0.22	6.88 ± 0.18	6.94 ± 0.21	6.99 ± 0.04
TC01000217.hg.1	CROCC	8.69 ± 0.21	8.65 ± 0.11	8.61 ± 0.22	8.72 ± 0.04
TC01002272.hg.1	CROCCP2	8.53 ± 0.12	8.41 ± 0.04	8.40 ± 0.12	8.47 ± 0.08
TC01002264.hg.1	CROCCP3	8.39 ± 0.11	8.29 ± 0.08	8.28 ± 0.11	8.38 ± 0.07
TC07000521.hg.1	CROT	6.62 ± 0.29	6.64 ± 0.12	6.44 ± 0.25	6.45 ± 0.04
TC01003389.hg.1	CRP	6.29 ± 0.13	6.25 ± 0.23	6.28 ± 0.14	6.45 ± 0.17
TC05001532.hg.1	CRSP8P	7.54 ± 0.15	7.68 ± 0.08	7.49 ± 0.14	7.70 ± 0.12
TC10001573.hg.1	CRTAC1	7.54 ± 0.25	7.47 ± 0.20	7.43 ± 0.27	7.56 ± 0.07

TC11001116.hg.1	CRTAM	6.64 ± 0.12	6.73 ± 0.15	6.40 ± 0.02	6.63 ± 0.04
TC03000165.hg.1	CRTAP	9.73 ± 0.12	9.50 ± 0.19	9.43 ± 0.09	9.40 ± 0.27
TC19000364.hg.1	CRTC1	8.69 ± 0.09	8.65 ± 0.10	8.57 ± 0.10	8.60 ± 0.03
TC01003280.hg.1	CRTC2	9.75 ± 0.21	9.89 ± 0.02	9.75 ± 0.23	10.03 ± 0.10
TC15000866.hg.1	CRTC3	9.66 ± 0.16	9.83 ± 0.09	9.35 ± 0.21	9.80 ± 0.06
TC19000684.hg.1	CRX	6.98 ± 0.27	7.06 ± 0.15	7.03 ± 0.13	7.13 ± 0.06
TC12001925.hg.1	CRY1	7.32 ± 0.39	7.60 ± 0.17	7.08 ± 0.30	7.74 ± 0.19
TC11000388.hg.1	CRY2	8.04 ± 0.08	8.10 ± 0.08	7.82 ± 0.11	8.15 ± 0.07
TC21000207.hg.1	CRYAA	8.04 ± 0.21	7.99 ± 0.21	7.93 ± 0.21	8.14 ± 0.02
TC11002289.hg.1	CRYAB	6.71 ± 0.10	6.68 ± 0.06	6.60 ± 0.14	6.73 ± 0.09
TC17000338.hg.1	CRYBA1	7.04 ± 0.12	7.01 ± 0.18	7.04 ± 0.15	7.13 ± 0.04
TC02002783.hg.1	CRYBA2	7.95 ± 0.07	7.93 ± 0.21	7.88 ± 0.15	8.06 ± 0.06
TC22000171.hg.1	CRYBA4	7.44 ± 0.18	7.43 ± 0.23	7.35 ± 0.13	7.57 ± 0.10
TC22000612.hg.1	CRYBB1	7.48 ± 0.25	7.34 ± 0.19	7.33 ± 0.14	7.35 ± 0.02
TC22000162.hg.1	CRYBB2	8.04 ± 0.10	8.07 ± 0.18	8.03 ± 0.12	8.14 ± 0.15
TC22000163.hg.1	CRYBB2P1	8.08 ± 0.09	8.08 ± 0.05	8.02 ± 0.06	8.12 ± 0.07
TC22000160.hg.1	CRYBB3	7.93 ± 0.13	7.84 ± 0.06	7.79 ± 0.17	7.84 ± 0.02
TC03000480.hg.1	CRYBG3	6.69 ± 0.31	6.93 ± 0.13	6.98 ± 0.25	6.91 ± 0.02
TC03000481.hg.1	CRYBG3	6.12 ± 0.15	6.00 ± 0.13	6.13 ± 0.23	5.96 ± 0.11
TC02004932.hg.1	CRYGA	5.97 ± 0.20	5.85 ± 0.09	5.81 ± 0.15	5.88 ± 0.02
TC02002724.hg.1	CRYGB	6.27 ± 0.04	5.97 ± 0.07	6.18 ± 0.06	5.94 ± 0.07
TC02002723.hg.1	CRYGC	6.60 ± 0.09	6.60 ± 0.20	6.51 ± 0.24	6.62 ± 0.12
TC02004933.hg.1	CRYGD	8.05 ± 0.23	7.91 ± 0.17	7.98 ± 0.16	7.98 ± 0.08
TC02002721.hg.1	CRYGEP	8.32 ± 0.08	8.40 ± 0.19	8.40 ± 0.10	8.41 ± 0.03
TC07002038.hg.1	CRYGN	7.84 ± 0.24	7.79 ± 0.15	7.69 ± 0.27	7.85 ± 0.05
TC03002083.hg.1	CRYGS	6.49 ± 0.14	6.49 ± 0.08	6.39 ± 0.19	6.38 ± 0.04
TC13000458.hg.1	CRYL1	7.03 ± 0.05	6.93 ± 0.03	6.86 ± 0.04	6.95 ± 0.04
TC16000934.hg.1	CRYM	6.79 ± 0.10	6.78 ± 0.12	6.82 ± 0.13	6.88 ± 0.08
TC16000229.hg.1	CRYM-AS1	5.00 ± 0.14	4.80 ± 0.04	4.84 ± 0.12	4.70 ± 0.13
TC01002788.hg.1	CRYZ	6.82 ± 0.20	6.85 ± 0.11	6.76 ± 0.11	6.69 ± 0.18
TC21001057.hg.1	CRYZL1	6.82 ± 0.13	6.68 ± 0.14	6.73 ± 0.17	6.66 ± 0.04

TC12003213.hg.1	CS	10.07 ± 0.12	10.06 ± 0.06	9.94 ± 0.06	10.04 ± 0.03
TC12001543.hg.1	CSAD	8.59 ± 0.08	8.54 ± 0.04	8.73 ± 0.18	8.59 ± 0.08
TC0X000741.hg.1	CSAG1	7.20 ± 0.29	7.10 ± 0.20	7.11 ± 0.21	7.25 ± 0.09
TC0X000742.hg.1	CSAG2	7.09 ± 0.19	6.99 ± 0.12	7.04 ± 0.14	7.15 ± 0.11
TC0X001501.hg.1	CSAG2	7.01 ± 0.18	6.92 ± 0.10	6.96 ± 0.17	7.07 ± 0.11
TC22000338.hg.1	CSDC2	8.04 ± 0.20	7.96 ± 0.19	7.91 ± 0.19	8.06 ± 0.08
TC01003015.hg.1	CSDE1	11.15 ± 0.27	11.57 ± 0.12	11.22 ± 0.23	11.59 ± 0.03
TC20000384.hg.1	CSE1L	8.55 ± 0.26	8.62 ± 0.27	8.36 ± 0.31	8.29 ± 0.17
TC01000955.hg.1	CSF1	8.19 ± 0.13	8.01 ± 0.08	8.53 ± 0.15	8.17 ± 0.06
TC05001932.hg.1	CSF1R	9.51 ± 0.26	9.13 ± 0.17	9.35 ± 0.04	9.08 ± 0.23
TC05000629.hg.1	CSF2	6.05 ± 0.11	6.00 ± 0.08	5.97 ± 0.02	5.99 ± 0.05
TC0X000006.hg.1	CSF2RA	9.32 ± 0.09	8.94 ± 0.13	9.71 ± 0.17	8.93 ± 0.34
TC0Y000006.hg.1	CSF2RA	9.50 ± 0.16	9.10 ± 0.15	9.94 ± 0.20	9.06 ± 0.35
TC22000271.hg.1	CSF2RB	11.10 ± 0.39	10.91 ± 0.21	11.67 ± 0.25	10.87 ± 0.27
TC17000486.hg.1	CSF3	8.12 ± 0.20	8.04 ± 0.19	8.01 ± 0.13	8.19 ± 0.06
TC01002512.hg.1	CSF3R	11.50 ± 0.38	11.39 ± 0.15	12.02 ± 0.23	11.39 ± 0.30
TC08001022.hg.1	CSGALNACT1	8.23 ± 0.03	8.26 ± 0.04	8.28 ± 0.09	8.30 ± 0.02
TC10000269.hg.1	CSGALNACT2	9.72 ± 0.27	9.83 ± 0.04	10.18 ± 0.57	9.95 ± 0.25
TC17001790.hg.1	CSH1	7.03 ± 0.19	6.97 ± 0.06	6.96 ± 0.08	7.09 ± 0.09
TC17001788.hg.1	CSH2	6.79 ± 0.18	6.71 ± 0.10	6.70 ± 0.07	6.89 ± 0.10
TC17002853.hg.1	CSHL1	7.03 ± 0.23	7.01 ± 0.20	6.94 ± 0.14	7.11 ± 0.09
TC15000685.hg.1	CSK	9.77 ± 0.29	9.41 ± 0.05	9.68 ± 0.20	9.28 ± 0.13
TC08000893.hg.1	CSMD1	6.22 ± 0.16	6.18 ± 0.18	6.18 ± 0.10	6.30 ± 0.08
TC01002485.hg.1	CSMD2	7.01 ± 0.13	7.01 ± 0.18	6.96 ± 0.16	7.11 ± 0.07
TC08001538.hg.1	CSMD3	4.73 ± 0.09	4.65 ± 0.10	4.69 ± 0.03	4.73 ± 0.07
TC04000374.hg.1	CSN1S1	5.19 ± 0.10	5.09 ± 0.10	5.12 ± 0.06	5.19 ± 0.07
TC04000378.hg.1	CSN1S2AP	4.40 ± 0.19	4.30 ± 0.07	4.27 ± 0.03	4.33 ± 0.05
TC04001269.hg.1	CSN2	4.42 ± 0.15	4.29 ± 0.04	4.36 ± 0.09	4.33 ± 0.08
TC04000382.hg.1	CSN3	5.14 ± 0.24	5.26 ± 0.15	5.02 ± 0.21	5.29 ± 0.17
TC05001924.hg.1	CSNK1A1	9.18 ± 0.29	9.28 ± 0.14	9.39 ± 0.32	9.26 ± 0.22
TC13000573.hg.1	CSNK1A1L	7.13 ± 0.11	7.16 ± 0.03	7.40 ± 0.10	7.26 ± 0.07

TC15001209.hg.1	CSNK1A1P1	6.04 ± 0.10	6.01 ± 0.03	6.08 ± 0.06	6.09 ± 0.09
TC17001976.hg.1	CSNK1D	9.78 ± 0.16	9.71 ± 0.09	9.85 ± 0.14	9.79 ± 0.13
TC22001493.hg.1	CSNK1E	8.50 ± 0.09	8.49 ± 0.10	8.29 ± 0.09	8.57 ± 0.03
TC15002806.hg.1	CSNK1G1	7.46 ± 0.11	7.36 ± 0.10	7.40 ± 0.11	7.24 ± 0.09
TC19000047.hg.1	CSNK1G2	9.71 ± 0.16	9.63 ± 0.03	9.62 ± 0.12	9.66 ± 0.08
TC19001017.hg.1	CSNK1G2-AS1	8.13 ± 0.21	7.98 ± 0.09	7.98 ± 0.24	8.05 ± 0.04
TC05000581.hg.1	CSNK1G3	8.23 ± 0.45	8.46 ± 0.18	8.34 ± 0.39	8.39 ± 0.13
TC20000542.hg.1	CSNK2A1	9.84 ± 0.04	9.75 ± 0.12	9.66 ± 0.14	9.66 ± 0.07
TC11001423.hg.1	CSNK2A1P	9.54 ± 0.15	9.36 ± 0.16	9.17 ± 0.15	9.26 ± 0.06
TC16001148.hg.1	CSNK2A2	9.29 ± 0.13	9.23 ± 0.28	9.03 ± 0.11	9.14 ± 0.15
TC06004074.hg.1	CSNK2B	8.91 ± 0.12	8.82 ± 0.08	8.82 ± 0.05	8.77 ± 0.02
TC6_cox_hap2000243.hg.1	CSNK2B	9.07 ± 0.12	9.03 ± 0.08	9.01 ± 0.06	8.99 ± 0.02
TC6_dbb_hap3000219.hg.1	CSNK2B	9.08 ± 0.13	9.04 ± 0.08	9.02 ± 0.05	9.00 ± 0.02
TC6_mann_hap4000193.hg.1	CSNK2B	9.08 ± 0.13	9.04 ± 0.08	9.02 ± 0.05	9.00 ± 0.02
TC6_mcf_hap5000210.hg.1	CSNK2B	9.13 ± 0.13	9.08 ± 0.08	9.06 ± 0.04	9.04 ± 0.02
TC6_qbl_hap6000222.hg.1	CSNK2B	8.98 ± 0.12	8.95 ± 0.08	8.95 ± 0.06	8.92 ± 0.02
TC6_ssto_hap7000196.hg.1	CSNK2B	9.00 ± 0.12	8.94 ± 0.08	8.94 ± 0.05	8.89 ± 0.02
TC15001670.hg.1	CSPG4	8.19 ± 0.23	8.13 ± 0.12	8.15 ± 0.13	8.21 ± 0.05
TC0Y000097.hg.1	CSPG4P1Y	7.12 ± 0.19	6.96 ± 0.31	6.98 ± 0.31	7.14 ± 0.11
TC0Y000206.hg.1	CSPG4P1Y	7.12 ± 0.19	6.96 ± 0.31	6.98 ± 0.31	7.14 ± 0.11
TC15001778.hg.1	CSPG4P5	7.84 ± 0.20	7.70 ± 0.12	7.58 ± 0.22	7.80 ± 0.03
TC15000771.hg.1	CSPG4P8	7.84 ± 0.12	7.79 ± 0.09	7.78 ± 0.15	7.91 ± 0.11
TC03001369.hg.1	CSPG5	7.12 ± 0.19	7.01 ± 0.17	6.97 ± 0.15	7.04 ± 0.01
TC08000447.hg.1	CSPP1	6.93 ± 0.28	7.03 ± 0.13	6.90 ± 0.20	6.93 ± 0.11
TC03001302.hg.1	CSRNP1	9.25 ± 0.33	9.63 ± 0.01	9.35 ± 0.25	9.60 ± 0.25
TC12001501.hg.1	CSRNP2	8.36 ± 0.07	8.32 ± 0.14	8.23 ± 0.11	8.36 ± 0.04
TC02000986.hg.1	CSRNP3	5.52 ± 0.16	5.43 ± 0.15	5.45 ± 0.08	5.46 ± 0.05
TC01003692.hg.1	CSRP1	8.81 ± 0.21	8.64 ± 0.09	8.70 ± 0.09	8.52 ± 0.09
TC12001755.hg.1	CSRP2	5.79 ± 0.13	5.85 ± 0.11	5.78 ± 0.02	6.01 ± 0.09
TC20000117.hg.1	CSRP2BP	7.34 ± 0.07	7.27 ± 0.02	7.12 ± 0.08	7.21 ± 0.01
TC11001477.hg.1	CSRP3	6.53 ± 0.20	6.42 ± 0.12	6.50 ± 0.13	6.52 ± 0.04

TC20000718.hg.1	CST1	7.68 ± 0.20	7.66 ± 0.10	7.54 ± 0.21	7.68 ± 0.09
TC20000710.hg.1	CST11	5.61 ± 0.05	5.46 ± 0.13	5.46 ± 0.17	5.49 ± 0.08
TC20000157.hg.1	CST13P	6.69 ± 0.12	6.72 ± 0.25	6.65 ± 0.08	6.98 ± 0.10
TC20000719.hg.1	CST2	7.85 ± 0.23	7.66 ± 0.08	7.67 ± 0.21	7.77 ± 0.08
TC20000715.hg.1	CST3	10.62 ± 0.30	10.30 ± 0.09	10.34 ± 0.10	10.09 ± 0.15
TC20000717.hg.1	CST4	7.09 ± 0.19	6.95 ± 0.15	6.95 ± 0.27	7.00 ± 0.11
TC20000720.hg.1	CST5	7.35 ± 0.08	7.24 ± 0.11	7.39 ± 0.06	7.45 ± 0.26
TC11000651.hg.1	CST6	7.73 ± 0.29	7.77 ± 0.26	7.74 ± 0.20	7.86 ± 0.06
TC20000164.hg.1	CST7	11.28 ± 0.50	10.70 ± 0.03	12.00 ± 0.23	10.46 ± 0.30
TC20000156.hg.1	CST8	5.98 ± 0.11	5.87 ± 0.11	5.87 ± 0.04	5.92 ± 0.07
TC20000714.hg.1	CST9	7.40 ± 0.21	7.39 ± 0.19	7.51 ± 0.08	7.43 ± 0.06
TC20000713.hg.1	CST9L	6.58 ± 0.02	6.54 ± 0.11	6.55 ± 0.19	6.74 ± 0.08
TC20000712.hg.1	CST9LP1	6.55 ± 0.12	6.55 ± 0.13	6.61 ± 0.28	6.71 ± 0.12
TC03000630.hg.1	CSTA	8.48 ± 0.16	8.04 ± 0.24	8.50 ± 0.19	7.74 ± 0.29
TC21000509.hg.1	CSTB	8.94 ± 0.18	8.71 ± 0.08	8.82 ± 0.17	8.61 ± 0.07
TC20000428.hg.1	CSTF1	8.11 ± 0.07	7.96 ± 0.14	7.99 ± 0.14	7.79 ± 0.11
TC0X000470.hg.1	CSTF2	7.63 ± 0.12	7.43 ± 0.05	7.43 ± 0.13	7.39 ± 0.04
TC10001297.hg.1	CSTF2T	7.77 ± 0.67	7.96 ± 0.31	7.99 ± 0.46	7.96 ± 0.06
TC11001536.hg.1	CSTF3	7.40 ± 0.27	7.20 ± 0.19	7.40 ± 0.25	7.06 ± 0.14
TC11000315.hg.1	CSTF3-AS1	6.08 ± 0.13	6.08 ± 0.11	6.00 ± 0.16	6.13 ± 0.07
TC20000155.hg.1	CSTL1	7.95 ± 0.19	7.98 ± 0.04	7.95 ± 0.08	8.09 ± 0.11
TC0X000653.hg.1	CT45A1	6.01 ± 0.24	6.04 ± 0.04	6.00 ± 0.20	6.19 ± 0.18
TC0X002301.hg.1	CT45A2	6.20 ± 0.32	6.32 ± 0.05	6.22 ± 0.21	6.48 ± 0.21
TC0X002302.hg.1	CT45A3	5.96 ± 0.24	6.04 ± 0.06	5.95 ± 0.15	6.17 ± 0.15
TC0X002338.hg.1	CT45A4	6.11 ± 0.30	6.22 ± 0.05	6.12 ± 0.15	6.38 ± 0.19
TC0X002339.hg.1	CT45A5	6.24 ± 0.25	6.29 ± 0.04	6.33 ± 0.12	6.44 ± 0.14
TC0X002340.hg.1	CT45A6	5.98 ± 0.30	6.07 ± 0.03	5.97 ± 0.16	6.21 ± 0.16
TC0X001329.hg.1	CT47A10	6.01 ± 0.22	6.08 ± 0.04	5.97 ± 0.28	6.22 ± 0.09
TC0X001330.hg.1	CT47A10	5.91 ± 0.22	5.96 ± 0.00	5.84 ± 0.28	6.11 ± 0.12
TC0X001331.hg.1	CT47A10	5.91 ± 0.22	5.96 ± 0.00	5.84 ± 0.28	6.11 ± 0.12
TC0X001332.hg.1	CT47A10	5.91 ± 0.22	5.96 ± 0.00	5.84 ± 0.28	6.11 ± 0.12

TC0X001333.hg.1	CT47A10	5.91 ± 0.22	5.96 ± 0.00	5.84 ± 0.28	6.11 ± 0.12
TC0X001335.hg.1	CT47A10	5.88 ± 0.21	5.95 ± 0.04	5.83 ± 0.29	6.11 ± 0.12
TC0X001336.hg.1	CT47A10	5.91 ± 0.22	5.96 ± 0.00	5.84 ± 0.28	6.11 ± 0.12
TC0X001337.hg.1	CT47A10	5.91 ± 0.22	5.96 ± 0.00	5.84 ± 0.28	6.11 ± 0.12
TC0X002361.hg.1	CT47A10	5.91 ± 0.22	5.96 ± 0.00	5.84 ± 0.28	6.11 ± 0.12
TC0X002362.hg.1	CT47A6	6.26 ± 0.27	6.39 ± 0.03	6.25 ± 0.29	6.53 ± 0.09
TC0X001334.hg.1	CT47A7	5.44 ± 0.14	5.39 ± 0.06	5.33 ± 0.23	5.55 ± 0.17
TC0X001328.hg.1	CT47B1	6.09 ± 0.31	6.17 ± 0.10	5.96 ± 0.29	6.12 ± 0.04
TC05000080.hg.1	CT49	6.02 ± 0.11	6.00 ± 0.16	5.95 ± 0.10	6.29 ± 0.21
TC15000013.hg.1	CT60	6.64 ± 0.13	6.73 ± 0.09	6.69 ± 0.17	6.87 ± 0.22
TC15001618.hg.1	CT62	6.15 ± 0.23	6.01 ± 0.11	6.08 ± 0.17	6.11 ± 0.07
TC03001961.hg.1	CT64	5.00 ± 0.08	4.90 ± 0.02	4.93 ± 0.02	4.98 ± 0.11
TC0X000778.hg.1	CTAG1B	7.60 ± 0.12	7.67 ± 0.14	7.58 ± 0.13	7.72 ± 0.07
TC0X001539.hg.1	CTAG1B	7.67 ± 0.17	7.72 ± 0.12	7.63 ± 0.10	7.77 ± 0.03
TC0X001542.hg.1	CTAG2	8.05 ± 0.26	7.99 ± 0.20	7.99 ± 0.22	8.06 ± 0.05
TC18000409.hg.1	CTAGE1	6.32 ± 0.03	6.24 ± 0.12	6.29 ± 0.10	6.31 ± 0.09
TC13000201.hg.1	CTAGE10P	6.54 ± 0.08	6.43 ± 0.26	6.52 ± 0.16	6.56 ± 0.06
TC13000737.hg.1	CTAGE11P	6.08 ± 0.17	5.60 ± 0.08	5.78 ± 0.05	5.79 ± 0.06
TC07000939.hg.1	CTAGE15	6.13 ± 0.20	6.17 ± 0.03	6.03 ± 0.23	6.21 ± 0.21
TC07000953.hg.1	CTAGE4	7.51 ± 0.08	7.44 ± 0.28	7.66 ± 0.22	7.61 ± 0.24
TC07001980.hg.1	CTAGE4	7.45 ± 0.22	7.24 ± 0.19	7.45 ± 0.25	7.33 ± 0.33
TC14002296.hg.1	CTAGE5	8.20 ± 0.25	8.24 ± 0.17	8.50 ± 0.30	8.16 ± 0.07
TC07001974.hg.1	CTAGE6	6.70 ± 0.13	6.62 ± 0.05	6.52 ± 0.16	6.58 ± 0.12
TC10000931.hg.1	CTAGE7P	5.64 ± 0.32	5.67 ± 0.08	5.85 ± 0.09	5.57 ± 0.08
TC06002111.hg.1	CTAGE9	7.46 ± 0.17	7.42 ± 0.09	7.54 ± 0.18	7.51 ± 0.12
TC04000963.hg.1	CTBP1	9.45 ± 0.06	9.40 ± 0.06	9.27 ± 0.09	9.35 ± 0.02
TC04000022.hg.1	CTBP1-AS2	8.45 ± 0.09	8.43 ± 0.05	8.28 ± 0.12	8.45 ± 0.04
TC10001732.hg.1	CTBP2	8.38 ± 0.04	8.19 ± 0.06	8.53 ± 0.14	8.12 ± 0.07
TC01002819.hg.1	CTBS	9.21 ± 0.22	9.30 ± 0.32	9.82 ± 0.42	9.16 ± 0.35
TC17001111.hg.1	CTC1	9.76 ± 0.14	9.64 ± 0.04	9.47 ± 0.26	9.66 ± 0.06
TC16000542.hg.1	CTCF	9.84 ± 0.15	9.85 ± 0.04	9.69 ± 0.11	9.76 ± 0.05

TC20000976.hg.1	CTCFL	6.16 ± 0.13	6.14 ± 0.17	6.12 ± 0.08	6.23 ± 0.03
TC17001082.hg.1	CTDNEP1	9.75 ± 0.32	9.45 ± 0.12	9.42 ± 0.19	9.29 ± 0.07
TC18000264.hg.1	CTDP1	8.94 ± 0.22	8.85 ± 0.13	8.99 ± 0.13	8.85 ± 0.09
TC02001301.hg.1	CTDSP1	8.96 ± 0.24	8.82 ± 0.04	8.88 ± 0.16	8.81 ± 0.04
TC12001637.hg.1	CTDSP2	10.52 ± 0.13	10.42 ± 0.17	10.39 ± 0.30	10.26 ± 0.09
TC03000182.hg.1	CTDSPL	7.87 ± 0.08	7.51 ± 0.08	7.94 ± 0.13	7.62 ± 0.10
TC15000339.hg.1	CTDSPL2	8.18 ± 0.44	8.54 ± 0.25	8.06 ± 0.46	8.47 ± 0.08
TC16000361.hg.1	CTF1	8.16 ± 0.37	8.07 ± 0.23	8.10 ± 0.29	8.22 ± 0.09
TC06002112.hg.1	CTGF	7.60 ± 0.24	7.48 ± 0.25	7.53 ± 0.26	7.65 ± 0.13
TC01000757.hg.1	CTH	5.49 ± 0.11	5.57 ± 0.13	5.42 ± 0.07	5.46 ± 0.03
TC08000646.hg.1	CTHRC1	5.73 ± 0.19	5.69 ± 0.14	5.67 ± 0.20	5.67 ± 0.03
TC18000173.hg.1	CTIF	8.00 ± 0.13	7.89 ± 0.12	7.95 ± 0.11	7.94 ± 0.02
TC02001201.hg.1	CTLA4	6.92 ± 0.08	7.15 ± 0.55	6.73 ± 0.12	6.88 ± 0.14
TC05000702.hg.1	CTNNA1	7.92 ± 0.13	7.74 ± 0.08	8.09 ± 0.17	7.68 ± 0.21
TC02000489.hg.1	CTNNA2	6.10 ± 0.11	5.98 ± 0.09	6.03 ± 0.07	6.07 ± 0.06
TC10001338.hg.1	CTNNA3	4.80 ± 0.10	4.71 ± 0.09	4.72 ± 0.06	4.73 ± 0.04
TC09001457.hg.1	CTNNAL1	5.15 ± 0.06	4.91 ± 0.23	4.94 ± 0.05	4.77 ± 0.07
TC03000214.hg.1	CTNNB1	8.38 ± 0.22	8.56 ± 0.10	8.51 ± 0.31	8.59 ± 0.16
TC01002187.hg.1	CTNNBIP1	8.45 ± 0.13	8.39 ± 0.13	8.31 ± 0.16	8.45 ± 0.02
TC20000276.hg.1	CTNNBL1	7.79 ± 0.05	7.70 ± 0.08	7.65 ± 0.08	7.65 ± 0.04
TC11003450.hg.1	CTNND1	7.83 ± 0.10	7.68 ± 0.10	7.67 ± 0.05	7.67 ± 0.10
TC05001166.hg.1	CTNND2	6.96 ± 0.24	6.86 ± 0.13	6.87 ± 0.14	6.95 ± 0.05
TC17000034.hg.1	CTNS	8.32 ± 0.14	7.95 ± 0.03	8.24 ± 0.12	7.93 ± 0.07
TC01000520.hg.1	CTPS1	7.76 ± 0.07	7.73 ± 0.23	7.41 ± 0.06	7.72 ± 0.01
TC0X000885.hg.1	CTPS2	6.81 ± 0.08	6.70 ± 0.09	6.67 ± 0.09	6.63 ± 0.05
TC11000187.hg.1	CTR9	8.90 ± 0.41	8.97 ± 0.21	9.14 ± 0.37	8.73 ± 0.16
TC16000617.hg.1	CTRB1	8.42 ± 0.16	8.40 ± 0.16	8.41 ± 0.23	8.48 ± 0.05
TC16001275.hg.1	CTRB2	8.00 ± 0.14	7.95 ± 0.21	7.97 ± 0.16	8.04 ± 0.05
TC01000188.hg.1	CTRC	8.02 ± 0.16	7.97 ± 0.26	7.96 ± 0.19	8.06 ± 0.05
TC16002087.hg.1	CTRL	8.84 ± 0.07	8.79 ± 0.05	8.75 ± 0.12	8.84 ± 0.02
TC20000361.hg.1	CTSA	10.91 ± 0.24	10.33 ± 0.06	11.15 ± 0.20	10.40 ± 0.19

TC08000980.hg.1	CTSB	9.80 ± 0.21	9.48 ± 0.05	9.86 ± 0.09	9.49 ± 0.21
TC11002175.hg.1	CTSC	9.42 ± 0.30	9.18 ± 0.11	9.69 ± 0.28	8.89 ± 0.07
TC11003472.hg.1	CTSD	11.79 ± 0.29	11.22 ± 0.17	11.90 ± 0.03	11.25 ± 0.45
TC01001720.hg.1	CTSE	6.73 ± 0.29	6.64 ± 0.08	6.65 ± 0.14	6.74 ± 0.05
TC11001971.hg.1	CTSF	7.44 ± 0.26	7.37 ± 0.12	7.34 ± 0.15	7.44 ± 0.05
TC14000980.hg.1	CTSG	7.41 ± 0.45	7.06 ± 0.11	7.16 ± 0.34	7.13 ± 0.02
TC15001713.hg.1	CTSH	8.99 ± 0.20	8.62 ± 0.16	8.83 ± 0.03	8.55 ± 0.21
TC01003211.hg.1	CTSK	7.37 ± 0.20	7.30 ± 0.15	7.37 ± 0.17	7.29 ± 0.05
TC09000400.hg.1	CTSL1	8.24 ± 0.32	7.94 ± 0.11	8.33 ± 0.02	8.23 ± 0.64
TC10000311.hg.1	CTSL1P2	7.46 ± 0.02	7.28 ± 0.02	7.39 ± 0.14	7.41 ± 0.13
TC09000403.hg.1	CTSL1P8	5.78 ± 0.21	5.51 ± 0.06	5.67 ± 0.25	5.68 ± 0.20
TC09001381.hg.1	CTSL2	6.03 ± 0.17	5.89 ± 0.08	5.90 ± 0.09	5.93 ± 0.04
TC09000401.hg.1	CTSL3P	6.76 ± 0.11	6.73 ± 0.01	6.72 ± 0.13	6.87 ± 0.08
TC04001670.hg.1	CTSO	8.31 ± 0.27	8.31 ± 0.21	8.04 ± 0.35	8.14 ± 0.04
TC01003210.hg.1	CTSS	11.66 ± 0.13	11.75 ± 0.15	11.86 ± 0.19	11.67 ± 0.24
TC11000645.hg.1	CTSW	10.59 ± 0.38	10.47 ± 0.05	10.34 ± 0.19	10.33 ± 0.15
TC20000992.hg.1	CTSZ	9.90 ± 0.12	9.71 ± 0.09	9.99 ± 0.12	9.64 ± 0.17
TC11000727.hg.1	CTTN	8.33 ± 0.03	7.89 ± 0.06	8.36 ± 0.30	8.04 ± 0.22
TC07001798.hg.1	CTTNBP2	6.00 ± 0.12	5.90 ± 0.08	5.90 ± 0.08	5.93 ± 0.04
TC01000987.hg.1	CTTNBP2NL	6.72 ± 0.17	6.58 ± 0.05	6.71 ± 0.07	6.63 ± 0.03
TC19001772.hg.1	CTU1	8.48 ± 0.21	8.33 ± 0.22	8.33 ± 0.19	8.45 ± 0.06
TC16000683.hg.1	CTU2	8.17 ± 0.22	8.16 ± 0.18	8.07 ± 0.22	8.21 ± 0.02
TC19001123.hg.1	CTXN1	9.27 ± 0.24	9.23 ± 0.14	9.25 ± 0.28	9.27 ± 0.07
TC15002796.hg.1	CTXN2	5.32 ± 0.18	5.33 ± 0.16	5.28 ± 0.16	5.40 ± 0.16
TC05000609.hg.1	CTXN3	5.40 ± 0.14	5.30 ± 0.16	5.33 ± 0.11	5.45 ± 0.11
TC10001070.hg.1	CUBN	6.70 ± 0.16	6.78 ± 0.15	6.62 ± 0.15	6.71 ± 0.08
TC17001721.hg.1	CUEDC1	7.91 ± 0.19	7.73 ± 0.06	7.80 ± 0.16	7.72 ± 0.02
TC10001621.hg.1	CUEDC2	8.16 ± 0.15	8.09 ± 0.09	8.02 ± 0.15	8.04 ± 0.03
TC07000969.hg.1	CUL1	8.88 ± 0.14	8.88 ± 0.09	8.72 ± 0.29	8.62 ± 0.14
TC10001173.hg.1	CUL2	7.92 ± 0.36	8.19 ± 0.17	7.97 ± 0.39	8.16 ± 0.07
TC02002826.hg.1	CUL3	8.84 ± 0.39	9.04 ± 0.17	9.09 ± 0.36	9.01 ± 0.14

TC13000419.hg.1	CUL4A	8.53 ± 0.16	8.50 ± 0.04	8.39 ± 0.11	8.39 ± 0.07
TC0X001326.hg.1	CUL4B	8.53 ± 0.42	8.69 ± 0.27	8.82 ± 0.47	8.57 ± 0.14
TC11000977.hg.1	CUL5	7.78 ± 0.64	8.08 ± 0.19	7.86 ± 0.49	8.07 ± 0.09
TC06001742.hg.1	CUL7	8.11 ± 0.05	8.03 ± 0.10	8.01 ± 0.05	8.06 ± 0.03
TC06000597.hg.1	CUL9	8.44 ± 0.03	8.43 ± 0.07	8.31 ± 0.02	8.46 ± 0.07
TC06001587.hg.1	CUTA	8.31 ± 0.22	8.18 ± 0.07	7.91 ± 0.20	8.09 ± 0.04
TC6_ssto_hap7000176.hg.1	CUTA	8.31 ± 0.22	8.18 ± 0.07	7.91 ± 0.20	8.09 ± 0.05
TC10002946.hg.1	CUTC	7.93 ± 0.13	7.88 ± 0.06	7.92 ± 0.25	7.87 ± 0.06
TC07000650.hg.1	CUX1	9.01 ± 0.16	8.90 ± 0.05	9.24 ± 0.17	8.90 ± 0.17
TC12000863.hg.1	CUX2	7.60 ± 0.17	7.54 ± 0.11	7.52 ± 0.18	7.61 ± 0.01
TC10002953.hg.1	CUZD1	5.92 ± 0.07	5.88 ± 0.04	5.88 ± 0.10	5.90 ± 0.05
TC11002204.hg.1	CWC15	7.96 ± 0.21	8.25 ± 0.20	7.95 ± 0.23	8.18 ± 0.02
TC02002582.hg.1	CWC22	8.59 ± 0.59	8.79 ± 0.28	9.06 ± 0.62	8.71 ± 0.22
TC17001434.hg.1	CWC25	8.47 ± 0.03	8.69 ± 0.12	8.68 ± 0.35	8.78 ± 0.13
TC05000275.hg.1	CWC27	7.51 ± 0.46	7.89 ± 0.13	7.58 ± 0.39	7.74 ± 0.12
TC10001588.hg.1	CWF19L1	8.34 ± 0.05	8.23 ± 0.17	8.20 ± 0.17	8.27 ± 0.08
TC11002259.hg.1	CWF19L2	7.06 ± 0.58	7.48 ± 0.23	7.09 ± 0.39	7.31 ± 0.08
TC04000282.hg.1	CWH43	5.58 ± 0.13	5.46 ± 0.04	5.41 ± 0.09	5.50 ± 0.04
TC16000488.hg.1	CX3CL1	7.05 ± 0.10	7.02 ± 0.03	6.96 ± 0.18	7.03 ± 0.02
TC03001304.hg.1	CX3CR1	10.14 ± 0.28	9.70 ± 0.32	10.21 ± 0.06	9.38 ± 0.39
TC21000049.hg.1	CXADR	5.75 ± 0.08	5.72 ± 0.05	5.67 ± 0.11	5.83 ± 0.13
TC15001028.hg.1	CXADRP2	5.23 ± 0.11	5.15 ± 0.12	5.19 ± 0.04	5.54 ± 0.51
TC18000389.hg.1	CXADRP3	3.46 ± 0.09	3.34 ± 0.11	3.51 ± 0.07	3.37 ± 0.08
TC04000411.hg.1	CXCL1	8.27 ± 0.11	8.61 ± 0.17	8.73 ± 0.36	8.47 ± 0.40
TC04001305.hg.1	CXCL10	6.94 ± 0.85	6.22 ± 0.63	6.79 ± 0.91	5.85 ± 0.22
TC04001306.hg.1	CXCL11	4.51 ± 0.13	4.28 ± 0.02	4.46 ± 0.12	4.33 ± 0.04
TC10001212.hg.1	CXCL12	6.16 ± 0.15	6.11 ± 0.08	6.16 ± 0.10	6.18 ± 0.07
TC04000432.hg.1	CXCL13	4.28 ± 0.12	4.19 ± 0.05	4.32 ± 0.02	4.23 ± 0.04
TC05001804.hg.1	CXCL14	6.91 ± 0.21	6.77 ± 0.08	6.81 ± 0.24	6.87 ± 0.09
TC17001029.hg.1	CXCL16	9.78 ± 0.40	9.89 ± 0.32	10.29 ± 0.32	10.35 ± 0.77
TC19001574.hg.1	CXCL17	6.79 ± 0.04	6.75 ± 0.13	6.73 ± 0.09	6.85 ± 0.08

TC04001286.hg.1	CXCL2	7.79 ± 0.13	8.01 ± 0.01	8.16 ± 0.36	8.18 ± 0.31
TC04001284.hg.1	CXCL3	6.51 ± 0.20	6.51 ± 0.06	6.56 ± 0.10	6.58 ± 0.11
TC04001283.hg.1	CXCL5	8.16 ± 0.19	8.22 ± 0.54	8.04 ± 0.32	8.08 ± 0.23
TC04000409.hg.1	CXCL6	6.33 ± 0.19	6.32 ± 0.10	6.22 ± 0.04	6.38 ± 0.08
TC04001304.hg.1	CXCL9	5.70 ± 0.24	5.49 ± 0.07	5.59 ± 0.07	5.46 ± 0.09
TC02002767.hg.1	CXCR1	11.14 ± 0.59	10.60 ± 0.29	11.50 ± 0.35	10.18 ± 0.46
TC02001292.hg.1	CXCR2	10.82 ± 0.29	10.59 ± 0.26	11.21 ± 0.24	10.29 ± 0.54
TC02002766.hg.1	CXCR2P1	10.16 ± 0.32	9.30 ± 0.22	9.91 ± 0.34	9.36 ± 0.40
TC0X001128.hg.1	CXCR3	8.65 ± 0.21	8.34 ± 0.13	8.31 ± 0.34	8.31 ± 0.03
TC02002378.hg.1	CXCR4	11.42 ± 0.18	11.94 ± 0.20	11.21 ± 0.30	12.03 ± 0.03
TC11001073.hg.1	CXCR5	8.23 ± 0.12	8.41 ± 0.05	8.08 ± 0.05	8.36 ± 0.10
TC03000253.hg.1	CXCR6	6.79 ± 0.17	6.45 ± 0.12	6.58 ± 0.28	6.40 ± 0.13
TC02001437.hg.1	CXCR7	6.91 ± 0.27	6.72 ± 0.14	6.74 ± 0.15	6.77 ± 0.06
TC0X000939.hg.1	CXorf21	8.87 ± 0.22	8.77 ± 0.33	9.30 ± 0.29	8.57 ± 0.27
TC0X000162.hg.1	CXorf22	4.46 ± 0.13	4.38 ± 0.02	4.38 ± 0.09	4.38 ± 0.02
TC0X000904.hg.1	CXorf23	6.91 ± 0.27	6.84 ± 0.20	7.14 ± 0.33	6.77 ± 0.13
TC0X000236.hg.1	CXorf24	6.86 ± 0.11	6.82 ± 0.04	6.81 ± 0.19	6.79 ± 0.26
TC0X000174.hg.1	CXorf27	5.97 ± 0.27	5.87 ± 0.22	5.95 ± 0.13	5.90 ± 0.10
TC0X000021.hg.1	CXorf28	6.23 ± 0.10	6.07 ± 0.14	6.06 ± 0.11	6.26 ± 0.06
TC0X000164.hg.1	CXorf30	4.00 ± 0.13	3.89 ± 0.04	3.86 ± 0.04	3.86 ± 0.04
TC0X000993.hg.1	CXorf31	6.51 ± 0.06	6.44 ± 0.07	6.46 ± 0.05	6.56 ± 0.05
TC0X000983.hg.1	CXorf36	7.05 ± 0.14	7.09 ± 0.19	7.08 ± 0.08	7.16 ± 0.07
TC0X000965.hg.1	CXorf38	8.13 ± 0.11	7.95 ± 0.11	8.10 ± 0.25	7.94 ± 0.08
TC0X000711.hg.1	CXorf40A	7.92 ± 0.03	7.92 ± 0.08	7.81 ± 0.13	7.91 ± 0.03
TC0X001484.hg.1	CXorf40B	8.17 ± 0.10	8.14 ± 0.11	8.06 ± 0.13	8.13 ± 0.04
TC0X001404.hg.1	CXorf48	5.68 ± 0.21	5.70 ± 0.10	5.72 ± 0.09	5.82 ± 0.11
TC0X000392.hg.1	CXorf49B	7.31 ± 0.04	7.30 ± 0.27	7.27 ± 0.21	7.48 ± 0.12
TC0X001130.hg.1	CXorf49B	7.32 ± 0.09	7.29 ± 0.22	7.25 ± 0.25	7.47 ± 0.14
TC0X000703.hg.1	CXorf51A	6.82 ± 0.23	6.67 ± 0.10	6.84 ± 0.18	6.65 ± 0.08
TC0X001458.hg.1	CXorf51A	6.86 ± 0.20	6.65 ± 0.15	6.84 ± 0.17	6.63 ± 0.09
TC0X001310.hg.1	CXorf56	8.16 ± 0.02	8.17 ± 0.09	8.07 ± 0.05	8.19 ± 0.08

TC0X000517.hg.1	CXorf57	4.93 ± 0.07	4.98 ± 0.03	4.88 ± 0.06	4.92 ± 0.12
TC0X000113.hg.1	CXorf58	4.59 ± 0.16	4.46 ± 0.05	4.52 ± 0.09	4.49 ± 0.04
TC0X001299.hg.1	CXorf61	6.42 ± 0.31	6.32 ± 0.11	6.34 ± 0.22	6.29 ± 0.12
TC0X000614.hg.1	CXorf64	5.85 ± 0.32	5.66 ± 0.29	5.73 ± 0.12	5.71 ± 0.15
TC0X001120.hg.1	CXorf65	8.40 ± 0.27	8.64 ± 0.04	8.34 ± 0.35	8.91 ± 0.03
TC0X001431.hg.1	CXorf66	3.96 ± 0.17	3.76 ± 0.03	3.83 ± 0.07	3.74 ± 0.04
TC0X000293.hg.1	CXorf67	7.64 ± 0.21	7.64 ± 0.12	7.51 ± 0.23	7.79 ± 0.07
TC18000513.hg.1	CXXC1	9.06 ± 0.11	8.89 ± 0.03	8.81 ± 0.09	8.83 ± 0.02
TC02001489.hg.1	CXXC11	8.10 ± 0.19	8.24 ± 0.24	8.05 ± 0.23	8.27 ± 0.04
TC0X000241.hg.1	CXXC1P1	5.63 ± 0.22	5.53 ± 0.14	5.51 ± 0.26	5.72 ± 0.10
TC04001440.hg.1	CXXC4	5.19 ± 0.13	5.08 ± 0.05	5.14 ± 0.07	5.10 ± 0.04
TC05000715.hg.1	CXXC5	8.81 ± 0.13	8.78 ± 0.04	8.56 ± 0.14	8.79 ± 0.04
TC17001781.hg.1	CYB561	7.82 ± 0.20	7.83 ± 0.14	7.76 ± 0.14	7.85 ± 0.03
TC11001852.hg.1	CYB561A3	8.79 ± 0.16	8.63 ± 0.05	8.49 ± 0.21	8.49 ± 0.04
TC01000945.hg.1	CYB561D1	7.74 ± 0.05	7.67 ± 0.04	7.68 ± 0.10	7.68 ± 0.03
TC03000312.hg.1	CYB561D2	7.87 ± 0.07	7.81 ± 0.11	7.71 ± 0.12	7.88 ± 0.08
TC18000574.hg.1	CYB5A	7.47 ± 0.15	7.27 ± 0.05	7.16 ± 0.11	7.08 ± 0.04
TC16000569.hg.1	CYB5B	8.31 ± 0.05	8.05 ± 0.19	8.13 ± 0.20	7.64 ± 0.17
TC17000119.hg.1	CYB5D1	7.77 ± 0.06	7.88 ± 0.18	7.90 ± 0.24	7.76 ± 0.22
TC17000038.hg.1	CYB5D2	8.32 ± 0.16	8.13 ± 0.03	8.26 ± 0.10	8.17 ± 0.04
TC01003712.hg.1	CYB5R1	8.05 ± 0.17	7.68 ± 0.05	8.09 ± 0.23	7.77 ± 0.11
TC11001372.hg.1	CYB5R2	7.55 ± 0.18	7.36 ± 0.13	7.46 ± 0.15	7.48 ± 0.07
TC22000850.hg.1	CYB5R3	10.12 ± 0.08	9.91 ± 0.07	10.22 ± 0.14	9.97 ± 0.04
TC06000774.hg.1	CYB5R4	8.53 ± 0.35	8.69 ± 0.39	9.17 ± 0.47	8.52 ± 0.10
TC01006358.hg.1	CYB5RL	7.25 ± 0.13	7.13 ± 0.03	7.16 ± 0.03	7.17 ± 0.02
TC16001339.hg.1	CYBA	11.01 ± 0.20	10.80 ± 0.06	11.06 ± 0.21	10.79 ± 0.02
TC0X000171.hg.1	CYBB	11.94 ± 0.26	11.90 ± 0.23	12.36 ± 0.25	11.82 ± 0.25
TC02001021.hg.1	CYBRD1	7.99 ± 0.17	8.11 ± 0.09	8.07 ± 0.08	8.07 ± 0.10
TC08000840.hg.1	CYC1	8.90 ± 0.28	8.77 ± 0.11	8.60 ± 0.23	8.74 ± 0.01
TC07001207.hg.1	CYCS	8.13 ± 0.27	8.53 ± 0.05	8.06 ± 0.22	8.25 ± 0.33
TC01001331.hg.1	CYCSP52	5.73 ± 0.16	5.76 ± 0.13	5.63 ± 0.22	5.75 ± 0.13

TC15000028.hg.1	CYFIP1	8.14 ± 0.18	7.83 ± 0.05	8.13 ± 0.06	7.74 ± 0.19
TC05000866.hg.1	CYFIP2	10.22 ± 0.20	10.38 ± 0.09	10.08 ± 0.16	10.33 ± 0.06
TC17001902.hg.1	CYGB	7.92 ± 0.31	8.00 ± 0.37	7.85 ± 0.24	8.06 ± 0.05
TC08001752.hg.1	CYHR1	8.44 ± 0.14	8.38 ± 0.05	8.38 ± 0.11	8.40 ± 0.02
TC0X000437.hg.1	CYLC1	4.05 ± 0.05	3.95 ± 0.06	4.09 ± 0.17	4.02 ± 0.07
TC09000524.hg.1	CYLC2	5.20 ± 0.13	5.08 ± 0.06	5.09 ± 0.07	5.17 ± 0.05
TC16000443.hg.1	CYLD	9.30 ± 0.37	9.76 ± 0.11	9.52 ± 0.45	9.81 ± 0.08
TC01000967.hg.1	CYMP	7.22 ± 0.15	7.16 ± 0.19	7.14 ± 0.10	7.27 ± 0.11
TC0Y000188.hg.1	CYorf17	7.05 ± 0.23	6.95 ± 0.17	6.94 ± 0.16	7.01 ± 0.21
TC15001642.hg.1	CYP11A1	6.95 ± 0.19	6.91 ± 0.20	6.95 ± 0.11	7.00 ± 0.06
TC08001708.hg.1	CYP11B1	7.27 ± 0.15	7.28 ± 0.21	7.27 ± 0.07	7.43 ± 0.10
TC08001709.hg.1	CYP11B2	7.17 ± 0.23	7.13 ± 0.26	7.14 ± 0.16	7.21 ± 0.03
TC10001625.hg.1	CYP17A1	6.25 ± 0.17	6.10 ± 0.04	6.19 ± 0.06	6.19 ± 0.08
TC10000766.hg.1	CYP17A1-AS1	7.01 ± 0.26	7.05 ± 0.19	7.30 ± 0.15	7.03 ± 0.04
TC15001362.hg.1	CYP19A1	5.52 ± 0.11	5.37 ± 0.06	5.49 ± 0.07	5.43 ± 0.05
TC15001647.hg.1	CYP1A1	6.51 ± 0.22	6.51 ± 0.23	6.40 ± 0.17	6.65 ± 0.07
TC15000684.hg.1	CYP1A2	7.15 ± 0.13	7.10 ± 0.16	7.09 ± 0.21	7.19 ± 0.10
TC02001749.hg.1	CYP1B1	7.43 ± 0.40	7.05 ± 0.16	8.58 ± 0.33	6.96 ± 0.24
TC02001750.hg.1	CYP1B1	8.27 ± 0.38	7.83 ± 0.08	9.09 ± 0.26	7.85 ± 0.06
TC02000241.hg.1	CYP1B1-AS1	6.67 ± 0.28	6.52 ± 0.11	6.67 ± 0.08	6.62 ± 0.03
TC02001196.hg.1	CYP20A1	8.12 ± 0.19	8.09 ± 0.22	7.95 ± 0.17	7.96 ± 0.10
TC06000391.hg.1	CYP21A2	7.69 ± 0.20	7.75 ± 0.22	7.68 ± 0.23	7.85 ± 0.08
TC06000393.hg.1	CYP21A2	7.68 ± 0.17	7.72 ± 0.23	7.65 ± 0.16	7.83 ± 0.11
TC6_cox_hap2000085.hg.1	CYP21A2	7.49 ± 0.16	7.56 ± 0.24	7.46 ± 0.19	7.68 ± 0.11
TC6_dbb_hap3000076.hg.1	CYP21A2	7.63 ± 0.20	7.68 ± 0.22	7.59 ± 0.21	7.78 ± 0.08
TC6_dbb_hap3000079.hg.1	CYP21A2	7.36 ± 0.18	7.43 ± 0.25	7.32 ± 0.21	7.56 ± 0.12
TC6_mcf_hap5000069.hg.1	CYP21A2	7.35 ± 0.18	7.51 ± 0.36	7.32 ± 0.40	7.71 ± 0.13
TC6_mcf_hap5000071.hg.1	CYP21A2	7.50 ± 0.15	7.59 ± 0.25	7.51 ± 0.17	7.71 ± 0.12
TC6_qbl_hap6000072.hg.1	CYP21A2	7.45 ± 0.15	7.54 ± 0.23	7.45 ± 0.17	7.64 ± 0.12
TC6_ssto_hap7000069.hg.1	CYP21A2	7.52 ± 0.17	7.59 ± 0.23	7.51 ± 0.20	7.70 ± 0.10
TC20000962.hg.1	CYP24A1	5.76 ± 0.14	5.71 ± 0.08	5.68 ± 0.11	5.78 ± 0.09

TC10000663.hg.1	CYP26A1	6.82 ± 0.19	6.79 ± 0.07	6.83 ± 0.19	6.82 ± 0.05
TC02001976.hg.1	CYP26B1	8.06 ± 0.23	8.09 ± 0.18	8.01 ± 0.21	8.14 ± 0.06
TC10000662.hg.1	CYP26C1	8.41 ± 0.26	8.30 ± 0.15	8.33 ± 0.15	8.33 ± 0.01
TC02001311.hg.1	CYP27A1	7.68 ± 0.04	7.63 ± 0.04	7.71 ± 0.17	7.65 ± 0.09
TC12001634.hg.1	CYP27B1	7.42 ± 0.18	7.34 ± 0.19	7.29 ± 0.26	7.44 ± 0.03
TC02002277.hg.1	CYP27C1	6.58 ± 0.20	6.51 ± 0.16	6.51 ± 0.19	6.48 ± 0.06
TC19000572.hg.1	CYP2A13	6.82 ± 0.20	6.81 ± 0.22	6.78 ± 0.18	6.91 ± 0.12
TC19001549.hg.1	CYP2A6	6.87 ± 0.21	6.84 ± 0.14	6.91 ± 0.16	6.92 ± 0.12
TC19002702.hg.1	CYP2A7	7.10 ± 0.14	7.10 ± 0.16	7.11 ± 0.13	7.19 ± 0.12
TC19002703.hg.1	CYP2A7P1	7.44 ± 0.15	7.52 ± 0.24	7.37 ± 0.09	7.53 ± 0.05
TC03002063.hg.1	CYP2AB1P	7.28 ± 0.15	7.15 ± 0.11	7.20 ± 0.14	7.32 ± 0.11
TC19000571.hg.1	CYP2B6	6.54 ± 0.13	6.56 ± 0.17	6.59 ± 0.15	6.60 ± 0.11
TC19000570.hg.1	CYP2B7P1	6.98 ± 0.18	6.96 ± 0.15	6.86 ± 0.09	6.99 ± 0.09
TC10002972.hg.1	CYP2C18	5.72 ± 0.10	5.62 ± 0.08	5.58 ± 0.09	5.71 ± 0.10
TC10002971.hg.1	CYP2C19	4.96 ± 0.05	4.83 ± 0.15	4.87 ± 0.04	4.92 ± 0.07
TC10001546.hg.1	CYP2C8	5.06 ± 0.13	4.98 ± 0.06	4.99 ± 0.06	5.00 ± 0.08
TC10000675.hg.1	CYP2C9	5.55 ± 0.12	5.46 ± 0.11	5.47 ± 0.09	5.58 ± 0.07
TC22001430.hg.1	CYP2D6	8.25 ± 0.14	8.27 ± 0.14	8.19 ± 0.11	8.36 ± 0.05
TC22001431.hg.1	CYP2D7P1	8.14 ± 0.15	8.14 ± 0.15	8.10 ± 0.09	8.24 ± 0.05
TC10000956.hg.1	CYP2E1	6.64 ± 0.13	6.61 ± 0.15	6.54 ± 0.18	6.65 ± 0.06
TC19000573.hg.1	CYP2F1	7.14 ± 0.13	7.17 ± 0.22	7.09 ± 0.16	7.27 ± 0.10
TC19000569.hg.1	CYP2G1P	6.20 ± 0.18	6.05 ± 0.15	6.06 ± 0.02	6.12 ± 0.01
TC01002717.hg.1	CYP2J2	5.63 ± 0.10	5.68 ± 0.10	5.69 ± 0.08	5.66 ± 0.08
TC11001444.hg.1	CYP2R1	7.09 ± 0.22	7.17 ± 0.26	6.89 ± 0.19	7.16 ± 0.15
TC19000575.hg.1	CYP2S1	8.35 ± 0.12	8.15 ± 0.14	8.07 ± 0.12	8.20 ± 0.14
TC04000555.hg.1	CYP2U1	7.14 ± 0.12	7.08 ± 0.02	7.00 ± 0.05	7.09 ± 0.02
TC07000013.hg.1	CYP2W1	8.61 ± 0.22	8.55 ± 0.24	8.48 ± 0.27	8.62 ± 0.03
TC06001772.hg.1	CYP39A1	4.24 ± 0.10	4.16 ± 0.02	4.18 ± 0.08	4.21 ± 0.08
TC07001649.hg.1	CYP3A4	5.17 ± 0.06	5.13 ± 0.10	5.12 ± 0.05	5.21 ± 0.09
TC07000601.hg.1	CYP3A43	4.42 ± 0.11	4.26 ± 0.01	4.30 ± 0.05	4.29 ± 0.03
TC07003299.hg.1	CYP3A5	5.26 ± 0.11	5.15 ± 0.07	5.17 ± 0.02	5.18 ± 0.05

TC07003300.hg.1	CYP3A7	4.95 ± 0.14	4.84 ± 0.07	4.88 ± 0.09	4.90 ± 0.10
TC14000627.hg.1	CYP46A1	7.31 ± 0.21	7.31 ± 0.24	7.24 ± 0.22	7.41 ± 0.04
TC01002625.hg.1	CYP4A11	6.78 ± 0.12	6.79 ± 0.16	6.79 ± 0.09	6.99 ± 0.17
TC01000595.hg.1	CYP4A22	6.60 ± 0.13	6.61 ± 0.16	6.57 ± 0.12	6.79 ± 0.19
TC01002626.hg.1	CYP4A22-AS1	7.81 ± 0.17	7.81 ± 0.07	7.81 ± 0.04	7.97 ± 0.12
TC01000592.hg.1	CYP4B1	6.88 ± 0.18	6.82 ± 0.12	6.80 ± 0.13	6.92 ± 0.07
TC19001262.hg.1	CYP4F11	7.36 ± 0.07	7.23 ± 0.09	7.30 ± 0.07	7.23 ± 0.02
TC19000275.hg.1	CYP4F12	7.52 ± 0.09	7.54 ± 0.13	7.66 ± 0.08	7.60 ± 0.07
TC19001261.hg.1	CYP4F2	8.22 ± 0.04	8.15 ± 0.17	8.28 ± 0.21	8.09 ± 0.15
TC19000272.hg.1	CYP4F22	7.00 ± 0.19	6.88 ± 0.11	6.98 ± 0.09	6.99 ± 0.02
TC19001258.hg.1	CYP4F24P	7.05 ± 0.10	7.00 ± 0.08	7.04 ± 0.05	7.06 ± 0.02
TC19000274.hg.1	CYP4F3	9.54 ± 0.04	9.47 ± 0.19	9.64 ± 0.39	9.30 ± 0.28
TC02000824.hg.1	CYP4F30P	6.18 ± 0.12	6.16 ± 0.03	6.17 ± 0.07	6.27 ± 0.12
TC18000082.hg.1	CYP4F35P	6.59 ± 0.05	6.74 ± 0.03	6.55 ± 0.14	6.83 ± 0.20
TC02002332.hg.1	CYP4F43P	6.18 ± 0.20	6.26 ± 0.06	6.20 ± 0.06	6.33 ± 0.18
TC19000273.hg.1	CYP4F8	7.41 ± 0.18	7.34 ± 0.11	7.36 ± 0.08	7.37 ± 0.04
TC04002914.hg.1	CYP4V2	8.19 ± 0.27	8.32 ± 0.13	8.17 ± 0.24	8.13 ± 0.12
TC01000593.hg.1	CYP4X1	5.56 ± 0.14	5.50 ± 0.10	5.50 ± 0.14	5.57 ± 0.07
TC01000594.hg.1	CYP4Z1	5.05 ± 0.12	4.89 ± 0.05	4.92 ± 0.02	4.97 ± 0.07
TC01002624.hg.1	CYP4Z2P	5.34 ± 0.10	5.28 ± 0.09	5.23 ± 0.06	5.39 ± 0.10
TC07003361.hg.1	CYP51A1	7.76 ± 0.26	7.86 ± 0.23	7.64 ± 0.23	7.78 ± 0.22
TC08001245.hg.1	CYP7A1	4.69 ± 0.07	4.35 ± 0.04	4.49 ± 0.11	4.41 ± 0.03
TC08001275.hg.1	CYP7B1	5.23 ± 0.22	5.16 ± 0.06	5.24 ± 0.14	5.19 ± 0.09
TC03001328.hg.1	CYP8B1	6.87 ± 0.11	6.82 ± 0.06	6.82 ± 0.19	6.88 ± 0.12
TC03001329.hg.1	CYP8B1	5.95 ± 0.29	5.95 ± 0.03	5.90 ± 0.28	6.20 ± 0.29
TC01000818.hg.1	CYR61	6.94 ± 0.15	6.94 ± 0.16	6.94 ± 0.15	7.05 ± 0.05
TC02001565.hg.1	CYS1	8.91 ± 0.23	8.82 ± 0.20	8.83 ± 0.24	8.84 ± 0.05
TC0X001177.hg.1	CYSLTR1	8.12 ± 0.23	8.14 ± 0.25	8.34 ± 0.32	7.92 ± 0.14
TC13000191.hg.1	CYSLTR2	5.56 ± 0.37	5.61 ± 0.29	5.90 ± 0.21	5.61 ± 0.14
TC05000723.hg.1	CYSTM1	8.37 ± 0.25	8.11 ± 0.06	8.69 ± 0.21	8.23 ± 0.13
TC17001920.hg.1	CYTH1	9.66 ± 0.09	9.70 ± 0.08	9.51 ± 0.16	9.69 ± 0.04

TC19000704.hg.1	CYTH2	8.70 ± 0.15	8.52 ± 0.02	8.57 ± 0.15	8.48 ± 0.01
TC07001127.hg.1	CYTH3	7.69 ± 0.08	7.66 ± 0.04	7.60 ± 0.09	7.68 ± 0.05
TC22000280.hg.1	CYTH4	10.26 ± 0.30	10.14 ± 0.10	10.59 ± 0.22	10.08 ± 0.15
TC02002452.hg.1	CYTIP	10.04 ± 0.36	10.47 ± 0.10	10.31 ± 0.31	10.51 ± 0.01
TC04000993.hg.1	CYTL1	6.30 ± 0.23	6.33 ± 0.19	6.30 ± 0.15	6.46 ± 0.03
TC21000344.hg.1	CYYR1	6.84 ± 0.20	6.67 ± 0.14	7.00 ± 0.09	6.83 ± 0.09
TC21000323.hg.1	D21S2088E	5.59 ± 0.02	5.43 ± 0.08	5.46 ± 0.06	5.53 ± 0.11
TC02001486.hg.1	D2HGDH	8.51 ± 0.11	8.57 ± 0.19	8.43 ± 0.10	8.66 ± 0.12
TC14000356.hg.1	DAAM1	7.45 ± 0.35	7.53 ± 0.19	7.54 ± 0.37	7.41 ± 0.05
TC06000558.hg.1	DAAM2	7.45 ± 0.49	7.11 ± 0.13	7.25 ± 0.12	7.35 ± 0.33
TC01006359.hg.1	DAB1	6.38 ± 0.22	6.34 ± 0.14	6.33 ± 0.13	6.41 ± 0.07
TC05003425.hg.1	DAB2	8.04 ± 0.22	7.39 ± 0.01	7.99 ± 0.10	7.48 ± 0.17
TC09000611.hg.1	DAB2IP	7.56 ± 0.21	7.50 ± 0.21	7.50 ± 0.16	7.59 ± 0.03
TC13000722.hg.1	DACH1	7.23 ± 0.17	7.00 ± 0.06	7.90 ± 0.38	7.12 ± 0.09
TC0X000444.hg.1	DACH2	5.43 ± 0.10	5.34 ± 0.04	5.39 ± 0.05	5.37 ± 0.06
TC14000353.hg.1	DACT1	6.09 ± 0.06	5.99 ± 0.06	5.97 ± 0.13	5.94 ± 0.11
TC06002324.hg.1	DACT2	7.69 ± 0.25	7.75 ± 0.17	7.62 ± 0.18	7.77 ± 0.08
TC19001652.hg.1	DACT3	8.32 ± 0.26	8.18 ± 0.17	8.14 ± 0.28	8.28 ± 0.03
TC19000666.hg.1	DACT3-AS1	6.41 ± 0.11	6.36 ± 0.10	6.48 ± 0.05	6.48 ± 0.05
TC14000931.hg.1	DAD1	9.07 ± 0.21	8.85 ± 0.14	8.79 ± 0.21	8.56 ± 0.17
TC03000295.hg.1	DAG1	8.61 ± 0.02	8.61 ± 0.06	8.51 ± 0.04	8.63 ± 0.01
TC11000538.hg.1	DAGLA	7.71 ± 0.13	7.68 ± 0.14	7.62 ± 0.15	7.76 ± 0.01
TC07003349.hg.1	DAGLB	8.77 ± 0.07	8.61 ± 0.05	8.71 ± 0.03	8.60 ± 0.07
TC11000529.hg.1	DAK	8.74 ± 0.12	8.56 ± 0.09	8.55 ± 0.10	8.58 ± 0.05
TC03001394.hg.1	DALRD3	8.44 ± 0.16	8.28 ± 0.04	8.32 ± 0.09	8.27 ± 0.02
TC04000297.hg.1	DANCR	7.86 ± 0.08	7.81 ± 0.18	7.48 ± 0.06	7.67 ± 0.03
TC19000234.hg.1	DAND5	7.55 ± 0.11	7.44 ± 0.13	7.49 ± 0.09	7.60 ± 0.06
TC12000840.hg.1	DAO	6.92 ± 0.26	6.85 ± 0.20	6.81 ± 0.20	6.95 ± 0.05
TC13000374.hg.1	DAOA	4.73 ± 0.07	4.67 ± 0.05	4.73 ± 0.05	4.71 ± 0.06
TC13000857.hg.1	DAOA-AS1	5.11 ± 0.03	5.02 ± 0.07	5.09 ± 0.03	5.13 ± 0.04
TC05001164.hg.1	DAP	8.54 ± 0.16	8.34 ± 0.07	8.45 ± 0.08	8.33 ± 0.08

TC01001305.hg.1	DAP3	8.24 ± 0.06	8.12 ± 0.11	8.02 ± 0.14	8.05 ± 0.05
TC09000398.hg.1	DAPK1	8.49 ± 0.13	8.33 ± 0.19	8.60 ± 0.15	8.40 ± 0.27
TC09000399.hg.1	DAPK1-IT1	6.92 ± 0.06	6.86 ± 0.02	6.90 ± 0.03	6.93 ± 0.02
TC15001546.hg.1	DAPK2	8.24 ± 0.05	8.11 ± 0.06	8.33 ± 0.12	8.13 ± 0.03
TC19001053.hg.1	DAPK3	8.39 ± 0.20	8.27 ± 0.15	8.26 ± 0.17	8.28 ± 0.06
TC02000963.hg.1	DAPL1	6.14 ± 0.10	6.04 ± 0.07	6.13 ± 0.05	6.08 ± 0.07
TC04000517.hg.1	DAPP1	9.35 ± 0.36	9.09 ± 0.40	9.86 ± 0.54	8.97 ± 0.22
TC01006337.hg.1	DARC	7.27 ± 0.08	7.30 ± 0.23	7.14 ± 0.25	7.47 ± 0.12
TC02002377.hg.1	DARS	8.52 ± 0.25	8.59 ± 0.14	8.33 ± 0.25	8.43 ± 0.14
TC01001516.hg.1	DARS2	7.18 ± 0.02	6.84 ± 0.20	7.00 ± 0.12	6.40 ± 0.18
TC02001365.hg.1	DAW1	4.98 ± 0.10	4.82 ± 0.05	4.90 ± 0.07	4.90 ± 0.07
TC06001586.hg.1	DAXX	8.29 ± 0.17	8.01 ± 0.11	8.10 ± 0.15	7.89 ± 0.04
TC6_cox_hap2000210.hg.1	DAXX	8.34 ± 0.16	8.06 ± 0.12	8.16 ± 0.15	7.95 ± 0.04
TC6_dbb_hap3000196.hg.1	DAXX	8.34 ± 0.16	8.06 ± 0.12	8.16 ± 0.15	7.95 ± 0.04
TC6_mcf_hap5000185.hg.1	DAXX	8.37 ± 0.16	8.08 ± 0.12	8.18 ± 0.15	7.97 ± 0.04
TC6_qbl_hap6000198.hg.1	DAXX	8.33 ± 0.16	8.05 ± 0.12	8.16 ± 0.15	7.95 ± 0.04
TC6_ssto_hap7000175.hg.1	DAXX	8.35 ± 0.23	8.46 ± 0.27	8.48 ± 0.19	8.71 ± 0.15
TC0Y000083.hg.1	DAZ2	4.63 ± 0.14	4.54 ± 0.06	4.60 ± 0.07	4.66 ± 0.18
TC0Y000210.hg.1	DAZ3	4.91 ± 0.16	4.80 ± 0.07	4.88 ± 0.05	4.95 ± 0.16
TC0Y000093.hg.1	DAZ4	5.02 ± 0.12	4.95 ± 0.06	5.00 ± 0.07	5.10 ± 0.18
TC0Y000196.hg.1	DAZ4	5.55 ± 0.16	5.54 ± 0.06	5.54 ± 0.08	5.72 ± 0.18
TC19000038.hg.1	DAZAP1	9.42 ± 0.09	9.39 ± 0.14	9.12 ± 0.09	9.30 ± 0.05
TC12000405.hg.1	DAZAP2	11.39 ± 0.07	11.51 ± 0.04	11.52 ± 0.12	11.54 ± 0.08
TC03001217.hg.1	DAZL	5.64 ± 0.08	5.56 ± 0.06	5.53 ± 0.11	5.57 ± 0.06
TC09001533.hg.1	DBC1	6.55 ± 0.22	6.52 ± 0.13	6.53 ± 0.13	6.55 ± 0.06
TC07000524.hg.1	DBF4	7.13 ± 0.47	7.65 ± 0.28	6.96 ± 0.37	7.52 ± 0.12
TC17000573.hg.1	DBF4B	7.42 ± 0.19	7.30 ± 0.10	7.30 ± 0.12	7.32 ± 0.04
TC09000778.hg.1	DBH	7.49 ± 0.21	7.41 ± 0.15	7.44 ± 0.15	7.49 ± 0.04
TC09001694.hg.1	DBH-AS1	7.94 ± 0.22	7.97 ± 0.27	7.74 ± 0.15	8.01 ± 0.01
TC02000751.hg.1	DBI	8.59 ± 0.12	8.51 ± 0.06	8.46 ± 0.15	8.42 ± 0.02
TC17000004.hg.1	DBIL5P	5.99 ± 0.20	6.00 ± 0.15	5.81 ± 0.14	5.97 ± 0.09

TC02001903.hg.1	DBIL5P2	6.13 ± 0.19	6.39 ± 0.11	6.12 ± 0.20	6.35 ± 0.12
TC05002108.hg.1	DBN1	8.60 ± 0.20	8.54 ± 0.12	8.53 ± 0.19	8.52 ± 0.03
TC16001361.hg.1	DBNDD1	8.18 ± 0.24	8.17 ± 0.20	8.09 ± 0.25	8.15 ± 0.04
TC20001732.hg.1	DBNDD2	8.47 ± 0.23	8.39 ± 0.16	8.29 ± 0.24	8.43 ± 0.02
TC07003393.hg.1	DBNL	9.48 ± 0.16	9.27 ± 0.06	9.43 ± 0.17	9.23 ± 0.10
TC19001688.hg.1	DBP	7.90 ± 0.17	7.67 ± 0.13	7.63 ± 0.18	7.64 ± 0.06
TC03001821.hg.1	DBR1	7.80 ± 0.15	7.74 ± 0.07	7.72 ± 0.07	7.66 ± 0.08
TC01002918.hg.1	DBT	6.87 ± 0.24	6.82 ± 0.22	6.84 ± 0.20	6.74 ± 0.01
TC11001488.hg.1	DBX1	7.26 ± 0.30	7.26 ± 0.22	7.14 ± 0.32	7.33 ± 0.07
TC12001417.hg.1	DBX2	6.95 ± 0.22	6.99 ± 0.20	6.87 ± 0.18	7.10 ± 0.14
TC09000218.hg.1	DCAF10	7.65 ± 0.21	7.46 ± 0.13	7.70 ± 0.32	7.28 ± 0.08
TC14002192.hg.1	DCAF11	8.91 ± 0.24	8.74 ± 0.11	8.72 ± 0.29	8.67 ± 0.06
TC09001028.hg.1	DCAF12	9.15 ± 0.28	8.43 ± 0.10	9.07 ± 0.34	8.16 ± 0.25
TC0X001349.hg.1	DCAF12L1	7.24 ± 0.03	7.37 ± 0.14	7.30 ± 0.25	7.54 ± 0.05
TC0X001348.hg.1	DCAF12L2	7.36 ± 0.25	7.44 ± 0.25	7.39 ± 0.11	7.52 ± 0.06
TC08000648.hg.1	DCAF13	7.42 ± 0.33	7.52 ± 0.13	7.29 ± 0.35	7.34 ± 0.12
TC15000400.hg.1	DCAF13P3	5.31 ± 0.62	5.75 ± 0.21	5.20 ± 0.42	5.60 ± 0.04
TC19000248.hg.1	DCAF15	9.00 ± 0.14	8.87 ± 0.05	8.79 ± 0.03	8.86 ± 0.05
TC04001069.hg.1	DCAF16	8.94 ± 0.33	9.19 ± 0.15	8.61 ± 0.28	9.25 ± 0.04
TC02001020.hg.1	DCAF17	7.54 ± 0.23	7.64 ± 0.17	7.42 ± 0.24	7.48 ± 0.07
TC14000436.hg.1	DCAF4	7.38 ± 0.06	7.31 ± 0.05	7.11 ± 0.04	7.31 ± 0.05
TC04000258.hg.1	DCAF4L1	5.19 ± 0.11	5.10 ± 0.08	5.21 ± 0.03	5.10 ± 0.07
TC08001395.hg.1	DCAF4L2	6.04 ± 0.15	6.08 ± 0.10	6.08 ± 0.12	6.08 ± 0.09
TC14001256.hg.1	DCAF5	9.56 ± 0.17	9.58 ± 0.05	9.48 ± 0.21	9.59 ± 0.04
TC01001463.hg.1	DCAF6	7.51 ± 0.12	7.48 ± 0.15	7.63 ± 0.21	7.34 ± 0.11
TC17000768.hg.1	DCAF7	9.58 ± 0.17	9.09 ± 0.15	9.36 ± 0.24	8.76 ± 0.18
TC01006277.hg.1	DCAF8	8.46 ± 0.09	8.47 ± 0.02	8.37 ± 0.10	8.49 ± 0.02
TC0X000935.hg.1	DCAF8L1	7.66 ± 0.22	7.52 ± 0.04	7.46 ± 0.20	7.61 ± 0.06
TC0X000130.hg.1	DCAF8L2	5.84 ± 0.25	5.87 ± 0.18	5.88 ± 0.16	5.99 ± 0.08
TC0X000131.hg.1	DCAF8L2	5.89 ± 0.11	5.87 ± 0.13	5.85 ± 0.15	5.93 ± 0.06
TC17001591.hg.1	DCAKD	8.81 ± 0.08	8.59 ± 0.10	8.69 ± 0.05	8.54 ± 0.06

TC06000931.hg.1	DCBLD1	5.95 ± 0.08	5.82 ± 0.05	5.88 ± 0.02	5.82 ± 0.02
TC03001604.hg.1	DCBLD2	5.79 ± 0.14	5.77 ± 0.02	5.69 ± 0.03	5.81 ± 0.06
TC18000184.hg.1	DCC	6.04 ± 0.14	5.97 ± 0.12	5.97 ± 0.06	6.04 ± 0.07
TC12001577.hg.1	DCD	6.30 ± 0.18	6.29 ± 0.19	6.35 ± 0.06	6.42 ± 0.10
TC11003480.hg.1	DCDC1	4.83 ± 0.10	4.70 ± 0.02	4.71 ± 0.02	4.73 ± 0.07
TC06001324.hg.1	DCDC2	5.71 ± 0.10	5.61 ± 0.04	5.67 ± 0.10	5.62 ± 0.02
TC01000423.hg.1	DCDC2B	7.51 ± 0.20	7.35 ± 0.11	7.47 ± 0.09	7.43 ± 0.10
TC02000025.hg.1	DCDC2C	5.30 ± 0.14	5.24 ± 0.07	5.27 ± 0.08	5.26 ± 0.03
TC11003479.hg.1	DCDC5	4.84 ± 0.10	4.78 ± 0.10	4.79 ± 0.04	4.85 ± 0.07
TC11001363.hg.1	DCHS1	7.66 ± 0.10	7.65 ± 0.10	7.45 ± 0.16	7.71 ± 0.06
TC04001659.hg.1	DCHS2	5.15 ± 0.10	5.09 ± 0.06	5.10 ± 0.03	5.13 ± 0.09
TC04002913.hg.1	DCK	9.36 ± 0.24	9.48 ± 0.07	9.26 ± 0.16	9.36 ± 0.04
TC13000563.hg.1	DCLK1	6.26 ± 0.16	6.17 ± 0.13	6.21 ± 0.12	6.28 ± 0.08
TC04000744.hg.1	DCLK2	5.97 ± 0.12	5.97 ± 0.06	5.87 ± 0.07	5.93 ± 0.02
TC03001281.hg.1	DCLK3	5.97 ± 0.06	5.91 ± 0.11	5.87 ± 0.13	6.00 ± 0.07
TC10001673.hg.1	DCLRE1A	6.51 ± 0.06	6.12 ± 0.14	6.11 ± 0.16	5.85 ± 0.13
TC01001002.hg.1	DCLRE1B	7.53 ± 0.10	7.22 ± 0.07	7.24 ± 0.09	7.15 ± 0.09
TC10002932.hg.1	DCLRE1C	8.06 ± 0.30	8.06 ± 0.22	8.14 ± 0.26	7.94 ± 0.08
TC12001804.hg.1	DCN	5.02 ± 0.06	4.92 ± 0.08	4.98 ± 0.02	5.03 ± 0.04
TC12001805.hg.1	DCN	4.39 ± 0.04	4.28 ± 0.07	4.41 ± 0.07	4.31 ± 0.08
TC03001468.hg.1	DCP1A	8.95 ± 0.30	9.19 ± 0.14	8.99 ± 0.36	9.36 ± 0.12
TC12001094.hg.1	DCP1B	7.34 ± 0.07	7.23 ± 0.10	7.24 ± 0.11	7.12 ± 0.11
TC05000535.hg.1	DCP2	8.26 ± 0.27	8.09 ± 0.28	8.64 ± 0.32	7.86 ± 0.18
TC11001173.hg.1	DCPS	9.29 ± 0.43	8.77 ± 0.06	8.71 ± 0.17	8.53 ± 0.16
TC01006330.hg.1	DCST1	7.60 ± 0.16	7.58 ± 0.15	7.53 ± 0.13	7.65 ± 0.02
TC01003301.hg.1	DCST2	7.32 ± 0.15	7.36 ± 0.18	7.31 ± 0.10	7.46 ± 0.05
TC08000653.hg.1	DCSTAMP	5.81 ± 0.16	5.75 ± 0.05	5.74 ± 0.08	5.81 ± 0.09
TC13000790.hg.1	DCT	5.68 ± 0.11	5.53 ± 0.07	5.55 ± 0.09	5.63 ± 0.09
TC04001787.hg.1	DCTD	8.51 ± 0.11	8.42 ± 0.13	8.17 ± 0.17	8.30 ± 0.02
TC02004927.hg.1	DCTN1	9.15 ± 0.03	9.11 ± 0.02	9.05 ± 0.00	9.07 ± 0.04
TC02000456.hg.1	DCTN1-AS1	6.68 ± 0.16	6.52 ± 0.16	6.53 ± 0.13	6.65 ± 0.08

TC12001627.hg.1	DCTN2	8.28 ± 0.19	7.98 ± 0.13	8.25 ± 0.14	7.92 ± 0.14
TC09001040.hg.1	DCTN3	8.00 ± 0.11	7.90 ± 0.14	7.81 ± 0.10	7.80 ± 0.06
TC05001943.hg.1	DCTN4	8.67 ± 0.17	8.72 ± 0.13	8.76 ± 0.26	8.74 ± 0.06
TC16000257.hg.1	DCTN5	9.32 ± 0.04	9.19 ± 0.08	9.03 ± 0.16	9.09 ± 0.06
TC08002579.hg.1	DCTN6	8.04 ± 0.23	8.24 ± 0.16	8.00 ± 0.33	8.38 ± 0.09
TC16001031.hg.1	DCTPP1	8.77 ± 0.45	8.58 ± 0.12	8.50 ± 0.25	8.44 ± 0.13
TC03002052.hg.1	DCUN1D1	8.48 ± 0.39	8.65 ± 0.11	8.79 ± 0.33	8.65 ± 0.11
TC13000895.hg.1	DCUN1D2	7.31 ± 0.10	7.28 ± 0.04	7.27 ± 0.04	7.28 ± 0.03
TC13000423.hg.1	DCUN1D2-AS1	5.53 ± 0.20	5.46 ± 0.07	5.47 ± 0.06	5.44 ± 0.04
TC13000424.hg.1	DCUN1D2-AS2	8.39 ± 0.18	8.36 ± 0.20	8.45 ± 0.15	8.54 ± 0.14
TC16002041.hg.1	DCUN1D3	8.41 ± 0.24	8.27 ± 0.07	8.55 ± 0.22	8.28 ± 0.05
TC04000291.hg.1	DCUN1D4	7.19 ± 0.15	7.24 ± 0.09	7.17 ± 0.04	7.22 ± 0.03
TC11002240.hg.1	DCUN1D5	7.88 ± 0.05	7.82 ± 0.20	7.64 ± 0.01	7.70 ± 0.06
TC0X001280.hg.1	DCX	5.97 ± 0.21	5.99 ± 0.20	5.99 ± 0.15	6.11 ± 0.06
TC17001970.hg.1	DCXR	9.55 ± 0.22	9.40 ± 0.09	9.20 ± 0.15	9.32 ± 0.02
TC19000332.hg.1	DDA1	10.14 ± 0.19	10.07 ± 0.09	10.06 ± 0.08	10.04 ± 0.15
TC01002829.hg.1	DDAH1	6.12 ± 0.14	6.05 ± 0.09	6.10 ± 0.07	6.16 ± 0.06
TC06004118.hg.1	DDAH2	8.42 ± 0.45	8.13 ± 0.18	8.47 ± 0.23	8.24 ± 0.02
TC6_cox_hap2000254.hg.1	DDAH2	8.42 ± 0.44	8.12 ± 0.18	8.46 ± 0.23	8.24 ± 0.02
TC6_dbb_hap3000235.hg.1	DDAH2	8.42 ± 0.45	8.13 ± 0.18	8.46 ± 0.23	8.24 ± 0.02
TC6_mann_hap4000205.hg.1	DDAH2	8.42 ± 0.45	8.13 ± 0.18	8.46 ± 0.23	8.24 ± 0.02
TC6_mcf_hap5000220.hg.1	DDAH2	8.42 ± 0.45	8.13 ± 0.18	8.46 ± 0.23	8.24 ± 0.02
TC6_qbl_hap6000232.hg.1	DDAH2	8.44 ± 0.44	8.14 ± 0.18	8.47 ± 0.23	8.26 ± 0.02
TC6_ssto_hap7000208.hg.1	DDAH2	8.42 ± 0.45	8.13 ± 0.18	8.46 ± 0.23	8.24 ± 0.02
TC11001851.hg.1	DDB1	9.20 ± 0.15	8.96 ± 0.12	8.79 ± 0.17	8.78 ± 0.04
TC11000404.hg.1	DDB2	8.43 ± 0.07	8.26 ± 0.09	8.19 ± 0.03	8.21 ± 0.09
TC07001373.hg.1	DDC	6.90 ± 0.15	6.85 ± 0.16	6.81 ± 0.11	6.94 ± 0.07
TC14001145.hg.1	DDHD1	8.02 ± 0.47	8.28 ± 0.16	7.91 ± 0.35	8.15 ± 0.08
TC08000288.hg.1	DDHD2	7.97 ± 0.30	8.09 ± 0.20	7.63 ± 0.17	7.95 ± 0.06
TC11000966.hg.1	DDI1	5.18 ± 0.12	5.14 ± 0.06	5.12 ± 0.13	5.20 ± 0.13
TC12001625.hg.1	DDIT3	7.56 ± 0.15	7.89 ± 0.17	7.64 ± 0.23	7.91 ± 0.31

TC10000449.hg.1	DDIT4	9.06 ± 0.21	9.12 ± 0.16	8.76 ± 0.17	9.04 ± 0.07
TC04001420.hg.1	DDIT4L	6.87 ± 0.35	6.76 ± 0.26	6.70 ± 0.24	6.84 ± 0.08
TC12001465.hg.1	DDN	7.71 ± 0.24	7.58 ± 0.20	7.65 ± 0.09	7.70 ± 0.04
TC06004136.hg.1	DDO	5.83 ± 0.06	5.79 ± 0.15	5.78 ± 0.09	5.89 ± 0.06
TC01002316.hg.1	DDOST	9.16 ± 0.17	8.92 ± 0.16	8.79 ± 0.15	8.77 ± 0.03
TC06000350.hg.1	DDR1	8.02 ± 0.19	8.01 ± 0.14	7.96 ± 0.13	8.09 ± 0.06
TC6_apd_hap1000030.hg.1	DDR1	8.02 ± 0.17	7.95 ± 0.10	7.86 ± 0.16	8.02 ± 0.03
TC6_cox_hap2000047.hg.1	DDR1	8.08 ± 0.19	8.06 ± 0.15	7.97 ± 0.16	8.13 ± 0.05
TC6_dbb_hap3000040.hg.1	DDR1	8.01 ± 0.18	7.99 ± 0.15	7.90 ± 0.17	8.06 ± 0.06
TC6_mann_hap4000042.hg.1	DDR1	8.06 ± 0.18	8.04 ± 0.15	7.95 ± 0.17	8.11 ± 0.05
TC6_mcf_hap5000033.hg.1	DDR1	8.24 ± 0.23	8.21 ± 0.19	8.09 ± 0.19	8.26 ± 0.07
TC6_qbl_hap6000040.hg.1	DDR1	8.06 ± 0.18	8.04 ± 0.15	7.95 ± 0.17	8.11 ± 0.05
TC06001513.hg.1	DDR1-AS1	6.93 ± 0.09	6.95 ± 0.10	7.03 ± 0.16	6.93 ± 0.05
TC01001423.hg.1	DDR2	6.25 ± 0.14	6.20 ± 0.13	6.16 ± 0.08	6.28 ± 0.09
TC20000570.hg.1	DDRGK1	8.29 ± 0.03	8.17 ± 0.06	8.09 ± 0.15	8.15 ± 0.04
TC22001486.hg.1	DDT	8.30 ± 0.17	8.17 ± 0.04	8.14 ± 0.14	8.19 ± 0.00
TC22000141.hg.1	DDTL	8.03 ± 0.19	7.87 ± 0.12	7.85 ± 0.19	7.83 ± 0.04
TC02000089.hg.1	DDX1	8.38 ± 0.31	8.54 ± 0.16	8.25 ± 0.26	8.38 ± 0.06
TC11000981.hg.1	DDX10	7.73 ± 0.36	7.96 ± 0.12	7.59 ± 0.31	7.75 ± 0.10
TC12000286.hg.1	DDX11	8.26 ± 0.07	8.20 ± 0.14	8.03 ± 0.13	8.18 ± 0.09
TC12001357.hg.1	DDX11-AS1	6.51 ± 0.15	6.56 ± 0.13	6.47 ± 0.02	6.55 ± 0.09
TC01000001.hg.1	DDX11L1	9.14 ± 0.45	8.86 ± 0.08	8.66 ± 0.19	9.01 ± 0.20
TC16000001.hg.1	DDX11L10	9.08 ± 0.45	8.71 ± 0.12	8.62 ± 0.14	8.90 ± 0.22
TC02002226.hg.1	DDX11L2	8.42 ± 0.43	8.15 ± 0.05	8.12 ± 0.14	8.32 ± 0.12
TC15002056.hg.1	DDX11L9	9.17 ± 0.47	8.92 ± 0.08	8.71 ± 0.21	9.09 ± 0.20
TC12001201.hg.1	DDX12P	8.32 ± 0.07	8.25 ± 0.12	8.07 ± 0.14	8.23 ± 0.08
TC22000791.hg.1	DDX17	11.11 ± 0.43	11.34 ± 0.13	11.22 ± 0.31	11.29 ± 0.12
TC02000742.hg.1	DDX18	8.29 ± 0.42	8.70 ± 0.19	8.09 ± 0.37	8.56 ± 0.11
TC10000651.hg.1	DDX18P6	6.11 ± 0.07	5.86 ± 0.05	5.87 ± 0.24	5.88 ± 0.20
TC16002039.hg.1	DDX19A	7.98 ± 0.08	7.98 ± 0.09	7.81 ± 0.06	7.93 ± 0.09
TC16002038.hg.1	DDX19B	8.72 ± 0.10	8.72 ± 0.12	8.43 ± 0.05	8.69 ± 0.04

TC01000981.hg.1	DDX20	7.41 ± 0.20	7.51 ± 0.23	7.17 ± 0.15	7.47 ± 0.02
TC10000413.hg.1	DDX21	9.21 ± 0.36	9.83 ± 0.17	9.20 ± 0.42	9.91 ± 0.13
TC12001458.hg.1	DDX23	9.02 ± 0.12	8.68 ± 0.06	8.87 ± 0.24	8.45 ± 0.07
TC14001462.hg.1	DDX24	9.57 ± 0.22	9.75 ± 0.15	9.12 ± 0.02	9.80 ± 0.03
TC11001165.hg.1	DDX25	5.88 ± 0.11	5.80 ± 0.07	5.80 ± 0.08	5.85 ± 0.04
TC0X000652.hg.1	DDX26B	8.06 ± 0.48	8.42 ± 0.21	8.18 ± 0.38	8.16 ± 0.13
TC0X001407.hg.1	DDX26B-AS1	6.05 ± 0.24	6.00 ± 0.24	5.84 ± 0.18	6.03 ± 0.16
TC20000385.hg.1	DDX27	8.58 ± 0.05	8.73 ± 0.12	8.31 ± 0.02	8.74 ± 0.08
TC16001203.hg.1	DDX28	7.47 ± 0.30	7.45 ± 0.03	7.41 ± 0.18	7.50 ± 0.10
TC09001675.hg.1	DDX31	7.17 ± 0.09	7.15 ± 0.04	6.96 ± 0.03	7.14 ± 0.02
TC19001236.hg.1	DDX39A	9.19 ± 0.09	9.19 ± 0.18	8.99 ± 0.04	9.22 ± 0.03
TC06004114.hg.1	DDX39B	9.91 ± 0.16	10.01 ± 0.09	9.76 ± 0.15	10.01 ± 0.05
TC6_apd_hap1000079.hg.1	DDX39B	10.47 ± 0.20	10.57 ± 0.08	10.33 ± 0.18	10.57 ± 0.03
TC6_cox_hap2000252.hg.1	DDX39B	9.92 ± 0.17	10.05 ± 0.09	9.80 ± 0.16	10.05 ± 0.06
TC6_dbb_hap3000231.hg.1	DDX39B	9.93 ± 0.18	10.07 ± 0.10	9.80 ± 0.15	10.07 ± 0.07
TC6_mann_hap4000202.hg.1	DDX39B	9.92 ± 0.18	10.06 ± 0.10	9.79 ± 0.15	10.05 ± 0.06
TC6_mcf_hap5000216.hg.1	DDX39B	9.94 ± 0.18	10.07 ± 0.10	9.81 ± 0.16	10.07 ± 0.06
TC6_qbl_hap6000230.hg.1	DDX39B	9.93 ± 0.18	10.06 ± 0.10	9.80 ± 0.16	10.06 ± 0.06
TC6_ssto_hap7000204.hg.1	DDX39B	9.87 ± 0.17	9.99 ± 0.10	9.73 ± 0.16	9.98 ± 0.06
TC06000368.hg.1	DDX39B-AS1	7.33 ± 0.10	7.24 ± 0.08	7.13 ± 0.15	7.31 ± 0.06
TC0X000199.hg.1	DDX3X	10.92 ± 0.41	11.58 ± 0.03	11.30 ± 0.39	11.59 ± 0.15
TC0Y000053.hg.1	DDX3Y	8.61 ± 0.75	9.39 ± 0.18	9.00 ± 0.41	9.41 ± 0.39
TC05000236.hg.1	DDX4	5.29 ± 0.18	5.15 ± 0.04	5.20 ± 0.13	5.18 ± 0.05
TC05002112.hg.1	DDX41	8.68 ± 0.16	8.43 ± 0.06	8.43 ± 0.18	8.41 ± 0.05
TC17000771.hg.1	DDX42	9.52 ± 0.26	9.60 ± 0.08	9.49 ± 0.24	9.54 ± 0.05
TC06000733.hg.1	DDX43	5.41 ± 0.10	5.31 ± 0.06	5.41 ± 0.03	5.41 ± 0.06
TC05003400.hg.1	DDX46	8.33 ± 0.43	8.57 ± 0.18	8.37 ± 0.35	8.40 ± 0.14
TC12003238.hg.1	DDX47	8.35 ± 0.08	8.42 ± 0.25	7.95 ± 0.16	8.32 ± 0.04
TC19000366.hg.1	DDX49	8.65 ± 0.14	8.55 ± 0.10	8.45 ± 0.19	8.58 ± 0.02
TC17001800.hg.1	DDX5	11.52 ± 0.46	12.19 ± 0.06	11.69 ± 0.28	12.12 ± 0.04
TC10000412.hg.1	DDX50	7.65 ± 0.40	7.91 ± 0.23	7.65 ± 0.31	7.79 ± 0.09

TC12002148.hg.1	DDX51	8.45 ± 0.16	8.40 ± 0.11	8.25 ± 0.21	8.41 ± 0.03
TC17001409.hg.1	DDX52	7.83 ± 0.26	7.80 ± 0.18	7.76 ± 0.24	7.76 ± 0.08
TC0X000108.hg.1	DDX53	5.23 ± 0.18	5.03 ± 0.08	5.31 ± 0.23	5.17 ± 0.04
TC12001984.hg.1	DDX54	8.99 ± 0.12	8.93 ± 0.10	8.72 ± 0.16	8.91 ± 0.03
TC12000989.hg.1	DDX55	7.77 ± 0.17	7.77 ± 0.11	7.60 ± 0.11	7.72 ± 0.06
TC07001343.hg.1	DDX56	8.96 ± 0.09	8.84 ± 0.05	8.68 ± 0.06	8.84 ± 0.05
TC09000999.hg.1	DDX58	9.30 ± 0.44	9.05 ± 0.77	9.89 ± 0.71	8.76 ± 0.48
TC01003680.hg.1	DDX59	7.51 ± 0.13	7.42 ± 0.10	7.68 ± 0.19	7.41 ± 0.10
TC11002353.hg.1	DDX6	10.68 ± 0.31	11.11 ± 0.19	10.60 ± 0.30	11.14 ± 0.07
TC04001718.hg.1	DDX60	8.47 ± 0.55	8.23 ± 0.73	8.90 ± 0.79	7.79 ± 0.23
TC04001719.hg.1	DDX60L	9.31 ± 0.37	9.22 ± 0.66	10.25 ± 0.53	9.03 ± 0.25
TC11001244.hg.1	DEAF1	8.72 ± 0.09	8.65 ± 0.05	8.61 ± 0.11	8.68 ± 0.01
TC08000547.hg.1	DECR1	7.56 ± 0.15	7.41 ± 0.25	7.78 ± 0.28	7.10 ± 0.12
TC16002050.hg.1	DECR2	8.48 ± 0.17	8.50 ± 0.16	8.44 ± 0.17	8.60 ± 0.07
TC01003417.hg.1	DEDD	9.23 ± 0.18	8.91 ± 0.08	9.18 ± 0.15	8.87 ± 0.12
TC19001568.hg.1	DEDD2	9.46 ± 0.18	9.33 ± 0.07	9.40 ± 0.13	9.31 ± 0.14
TC06000513.hg.1	DEF6	9.72 ± 0.10	9.54 ± 0.01	9.60 ± 0.09	9.50 ± 0.02
TC16000709.hg.1	DEF8	8.87 ± 0.07	8.71 ± 0.03	8.84 ± 0.10	8.72 ± 0.05
TC08000909.hg.1	DEFA10P	7.39 ± 0.33	7.39 ± 0.33	7.30 ± 0.12	7.54 ± 0.14
TC08000910.hg.1	DEFA1B	11.15 ± 1.52	9.17 ± 0.42	10.90 ± 1.40	9.25 ± 0.30
TC08002630.hg.1	DEFA1B	11.03 ± 1.46	9.04 ± 0.45	10.78 ± 1.38	9.12 ± 0.29
TC08002631.hg.1	DEFA1B	11.21 ± 1.51	9.14 ± 0.44	10.97 ± 1.43	9.19 ± 0.33
TC08000908.hg.1	DEFA4	8.06 ± 1.16	7.02 ± 0.13	8.06 ± 0.89	7.32 ± 0.10
TC08000914.hg.1	DEFA5	5.90 ± 0.29	5.56 ± 0.13	5.65 ± 0.05	5.69 ± 0.08
TC08000907.hg.1	DEFA6	4.59 ± 0.25	4.48 ± 0.07	4.46 ± 0.06	4.51 ± 0.02
TC08000906.hg.1	DEFB1	6.09 ± 0.14	6.11 ± 0.24	6.10 ± 0.11	6.23 ± 0.03
TC08000050.hg.1	DEFB103A	6.79 ± 0.19	6.88 ± 0.11	6.74 ± 0.18	6.98 ± 0.09
TC08000920.hg.1	DEFB103A	6.67 ± 0.17	6.75 ± 0.09	6.64 ± 0.21	6.84 ± 0.07
TC08000048.hg.1	DEFB104A	6.46 ± 0.12	6.32 ± 0.16	6.44 ± 0.08	6.53 ± 0.23
TC08000922.hg.1	DEFB104A	6.24 ± 0.13	6.14 ± 0.08	6.21 ± 0.08	6.27 ± 0.21
TC08000038.hg.1	DEFB105A	5.37 ± 0.02	5.27 ± 0.10	5.31 ± 0.01	5.33 ± 0.09

TC08000928.hg.1	DEFB105A	4.96 ± 0.03	4.87 ± 0.08	4.95 ± 0.07	4.95 ± 0.15
TC08000047.hg.1	DEFB106B	5.51 ± 0.06	5.42 ± 0.13	5.47 ± 0.07	5.53 ± 0.17
TC08000923.hg.1	DEFB106B	5.57 ± 0.04	5.42 ± 0.12	5.48 ± 0.07	5.51 ± 0.14
TC08000039.hg.1	DEFB107A	5.69 ± 0.25	5.40 ± 0.03	5.46 ± 0.13	5.54 ± 0.10
TC08000927.hg.1	DEFB107A	5.77 ± 0.21	5.45 ± 0.10	5.54 ± 0.06	5.62 ± 0.08
TC11000743.hg.1	DEFB108B	5.62 ± 0.16	5.59 ± 0.19	5.64 ± 0.06	5.76 ± 0.15
TC08000036.hg.1	DEFB108P2	6.20 ± 0.31	5.92 ± 0.03	6.11 ± 0.23	6.37 ± 0.41
TC08000991.hg.1	DEFB109P1	5.09 ± 0.21	5.38 ± 0.09	5.23 ± 0.09	5.39 ± 0.22
TC08000033.hg.1	DEFB109P1B	5.43 ± 0.14	5.29 ± 0.06	5.42 ± 0.16	5.25 ± 0.26
TC06001792.hg.1	DEFB110	4.39 ± 0.23	4.24 ± 0.08	4.35 ± 0.13	4.35 ± 0.11
TC06001793.hg.1	DEFB112	5.22 ± 0.03	5.13 ± 0.12	5.15 ± 0.18	5.15 ± 0.10
TC06001791.hg.1	DEFB113	5.71 ± 0.27	5.90 ± 0.20	5.84 ± 0.14	6.16 ± 0.12
TC06001790.hg.1	DEFB114	4.14 ± 0.05	4.01 ± 0.09	4.05 ± 0.08	4.24 ± 0.25
TC20000181.hg.1	DEFB115	6.66 ± 0.09	6.57 ± 0.04	6.52 ± 0.08	6.72 ± 0.07
TC20000748.hg.1	DEFB116	5.41 ± 0.26	5.34 ± 0.18	5.43 ± 0.11	5.56 ± 0.24
TC20000183.hg.1	DEFB117	4.75 ± 0.13	4.66 ± 0.09	4.76 ± 0.32	4.71 ± 0.06
TC20000184.hg.1	DEFB118	5.43 ± 0.21	5.32 ± 0.02	5.32 ± 0.14	5.35 ± 0.08
TC20000749.hg.1	DEFB119	6.11 ± 0.21	6.03 ± 0.14	6.10 ± 0.14	6.19 ± 0.06
TC20000750.hg.1	DEFB121	5.18 ± 0.29	5.21 ± 0.30	5.08 ± 0.08	5.29 ± 0.10
TC20000751.hg.1	DEFB122	5.92 ± 0.20	5.86 ± 0.16	6.05 ± 0.15	5.90 ± 0.11
TC20000187.hg.1	DEFB123	7.07 ± 0.13	7.18 ± 0.18	7.15 ± 0.13	7.36 ± 0.09
TC20000752.hg.1	DEFB124	7.76 ± 0.15	7.90 ± 0.29	7.81 ± 0.12	8.02 ± 0.17
TC20000754.hg.1	DEFB124	7.09 ± 0.31	7.03 ± 0.23	6.95 ± 0.11	7.19 ± 0.03
TC20000001.hg.1	DEFB125	4.90 ± 0.14	4.66 ± 0.05	4.75 ± 0.05	4.71 ± 0.05
TC20000002.hg.1	DEFB126	5.20 ± 0.19	4.98 ± 0.14	4.95 ± 0.13	5.25 ± 0.10
TC20000003.hg.1	DEFB127	5.52 ± 0.19	5.40 ± 0.22	5.33 ± 0.07	5.40 ± 0.13
TC20000537.hg.1	DEFB128	6.43 ± 0.15	6.41 ± 0.02	6.44 ± 0.14	6.42 ± 0.15
TC20000004.hg.1	DEFB129	5.38 ± 0.17	5.28 ± 0.04	5.19 ± 0.22	5.33 ± 0.16
TC08000984.hg.1	DEFB130	3.92 ± 0.17	3.93 ± 0.06	3.99 ± 0.06	4.03 ± 0.14
TC08000989.hg.1	DEFB130	3.92 ± 0.17	3.93 ± 0.06	3.99 ± 0.06	4.03 ± 0.14
TC04000112.hg.1	DEFB131	4.87 ± 0.19	4.91 ± 0.11	4.82 ± 0.09	5.07 ± 0.13

TC20000005.hg.1	DEFB132	6.82 ± 0.14	6.78 ± 0.21	6.66 ± 0.12	6.82 ± 0.08
TC06001789.hg.1	DEFB133	4.29 ± 0.03	4.25 ± 0.04	4.30 ± 0.12	4.25 ± 0.17
TC08000982.hg.1	DEFB134	5.50 ± 0.15	5.70 ± 0.16	5.65 ± 0.06	5.76 ± 0.11
TC08000095.hg.1	DEFB135	5.18 ± 0.24	5.07 ± 0.12	4.89 ± 0.22	5.12 ± 0.04
TC08000981.hg.1	DEFB136	4.97 ± 0.21	5.07 ± 0.13	4.97 ± 0.07	5.08 ± 0.05
TC08000051.hg.1	DEFB4A	5.23 ± 0.12	5.33 ± 0.05	5.29 ± 0.15	5.53 ± 0.12
TC08000919.hg.1	DEFB4B	6.01 ± 0.10	5.91 ± 0.20	6.01 ± 0.05	6.25 ± 0.17
TC08000911.hg.1	DEFT1P	7.39 ± 0.60	7.10 ± 0.17	7.27 ± 0.39	7.32 ± 0.19
TC01001842.hg.1	DEGS1	8.98 ± 0.06	8.83 ± 0.07	8.94 ± 0.11	8.78 ± 0.14
TC14001504.hg.1	DEGS2	8.38 ± 0.35	8.28 ± 0.19	8.30 ± 0.23	8.35 ± 0.06
TC06001302.hg.1	DEK	8.81 ± 0.42	9.18 ± 0.16	9.26 ± 0.44	8.97 ± 0.27
TC09001571.hg.1	DENND1A	8.57 ± 0.19	8.44 ± 0.08	8.63 ± 0.25	8.38 ± 0.09
TC01003665.hg.1	DENND1B	7.94 ± 0.32	7.98 ± 0.21	7.88 ± 0.32	7.81 ± 0.05
TC19001098.hg.1	DENND1C	9.83 ± 0.16	9.80 ± 0.07	9.73 ± 0.14	9.73 ± 0.06
TC07001924.hg.1	DENND2A	7.31 ± 0.24	7.29 ± 0.19	7.28 ± 0.21	7.41 ± 0.05
TC01006366.hg.1	DENND2C	5.76 ± 0.10	5.65 ± 0.01	5.71 ± 0.10	5.66 ± 0.04
TC01002981.hg.1	DENND2D	9.96 ± 0.07	9.68 ± 0.15	9.70 ± 0.16	9.38 ± 0.10
TC08000793.hg.1	DENND3	9.29 ± 0.22	9.17 ± 0.19	9.64 ± 0.34	9.21 ± 0.20
TC15001571.hg.1	DENND4A	8.62 ± 0.53	8.96 ± 0.22	8.77 ± 0.51	9.05 ± 0.20
TC01003279.hg.1	DENND4B	9.30 ± 0.13	9.17 ± 0.04	9.21 ± 0.12	9.11 ± 0.05
TC09000092.hg.1	DENND4C	7.74 ± 0.55	7.80 ± 0.28	7.61 ± 0.33	7.58 ± 0.06
TC11001397.hg.1	DENND5A	10.10 ± 0.35	10.11 ± 0.14	10.51 ± 0.35	10.05 ± 0.25
TC12001363.hg.1	DENND5B	7.60 ± 0.07	7.34 ± 0.09	7.10 ± 0.08	7.20 ± 0.12
TC12000292.hg.1	DENND5B-AS1	5.96 ± 0.18	5.72 ± 0.04	5.99 ± 0.20	5.89 ± 0.03
TC03001496.hg.1	DENND6A	8.62 ± 0.28	8.93 ± 0.25	8.53 ± 0.37	9.02 ± 0.22
TC22000912.hg.1	DENND6B	8.07 ± 0.16	8.00 ± 0.19	7.99 ± 0.16	8.09 ± 0.02
TC12000976.hg.1	DENR	8.83 ± 0.14	8.84 ± 0.06	8.83 ± 0.12	8.76 ± 0.10
TC01002766.hg.1	DEPDC1	5.31 ± 0.08	5.25 ± 0.13	5.28 ± 0.09	5.11 ± 0.07
TC05001392.hg.1	DEPDC1B	6.39 ± 0.13	6.17 ± 0.14	6.21 ± 0.11	6.02 ± 0.01
TC12001867.hg.1	DEPDC4	4.44 ± 0.09	4.30 ± 0.03	4.33 ± 0.07	4.29 ± 0.01
TC22000232.hg.1	DEPDC5	8.13 ± 0.07	8.10 ± 0.10	8.06 ± 0.01	8.07 ± 0.02

TC11000312.hg.1	DEPDC7	4.85 ± 0.09	4.68 ± 0.02	4.71 ± 0.08	4.69 ± 0.08
TC08000698.hg.1	DEPTOR	6.44 ± 0.15	6.35 ± 0.09	6.34 ± 0.06	6.39 ± 0.03
TC12000206.hg.1	DERA	7.45 ± 0.02	7.29 ± 0.04	7.47 ± 0.11	7.19 ± 0.07
TC08001575.hg.1	DERL1	9.11 ± 0.11	9.13 ± 0.14	8.90 ± 0.20	8.96 ± 0.06
TC17001058.hg.1	DERL2	8.52 ± 0.07	8.39 ± 0.29	8.41 ± 0.22	8.25 ± 0.08
TC22000569.hg.1	DERL3	8.59 ± 0.20	8.21 ± 0.20	8.22 ± 0.10	8.26 ± 0.10
TC02001326.hg.1	DES	7.44 ± 0.25	7.38 ± 0.19	7.38 ± 0.20	7.41 ± 0.08
TC22000829.hg.1	DESI1	8.76 ± 0.30	8.35 ± 0.06	8.56 ± 0.14	8.20 ± 0.07
TC01001989.hg.1	DESI2	7.78 ± 0.12	7.78 ± 0.17	7.71 ± 0.10	7.68 ± 0.09
TC15001818.hg.1	DET1	7.49 ± 0.06	7.38 ± 0.02	7.38 ± 0.07	7.35 ± 0.04
TC15001819.hg.1	DET1	4.32 ± 0.14	3.99 ± 0.09	4.08 ± 0.06	4.04 ± 0.06
TC16000860.hg.1	DEXI	8.60 ± 0.19	8.58 ± 0.16	8.49 ± 0.16	8.62 ± 0.01
TC01002190.hg.1	DFFA	8.41 ± 0.11	8.20 ± 0.06	8.27 ± 0.06	8.14 ± 0.13
TC01000074.hg.1	DFFB	8.50 ± 0.16	8.57 ± 0.08	8.44 ± 0.09	8.66 ± 0.08
TC07001205.hg.1	DFNA5	6.94 ± 0.13	6.86 ± 0.08	6.88 ± 0.07	6.93 ± 0.05
TC09001510.hg.1	DFNB31	7.85 ± 0.15	7.85 ± 0.14	7.82 ± 0.12	7.94 ± 0.08
TC02001073.hg.1	DFNB59	5.41 ± 0.12	5.40 ± 0.01	5.32 ± 0.10	5.41 ± 0.04
TC08001745.hg.1	DGAT1	9.26 ± 0.23	9.05 ± 0.01	9.11 ± 0.17	9.03 ± 0.01
TC11000812.hg.1	DGAT2	9.97 ± 0.59	9.60 ± 0.03	10.49 ± 0.24	9.72 ± 0.31
TC0X000371.hg.1	DGAT2L6	5.10 ± 0.14	4.99 ± 0.15	5.01 ± 0.03	5.11 ± 0.09
TC07001686.hg.1	DGAT2L7P	8.30 ± 0.24	8.28 ± 0.17	8.28 ± 0.15	8.31 ± 0.05
TC22000038.hg.1	DGCR10	6.13 ± 0.23	5.76 ± 0.12	6.00 ± 0.13	5.86 ± 0.27
TC22000491.hg.1	DGCR11	7.67 ± 0.07	7.64 ± 0.06	7.71 ± 0.30	7.62 ± 0.04
TC22000492.hg.1	DGCR14	8.66 ± 0.13	8.49 ± 0.10	8.59 ± 0.02	8.63 ± 0.12
TC22000490.hg.1	DGCR2	9.12 ± 0.21	8.87 ± 0.01	9.09 ± 0.16	8.81 ± 0.07
TC22000035.hg.1	DGCR5	6.95 ± 0.16	6.89 ± 0.06	6.87 ± 0.08	6.94 ± 0.03
TC22000033.hg.1	DGCR6	8.36 ± 0.20	8.38 ± 0.14	8.26 ± 0.17	8.37 ± 0.07
TC22000510.hg.1	DGCR6L	8.76 ± 0.26	8.78 ± 0.18	8.66 ± 0.23	8.81 ± 0.07
TC22000037.hg.1	DGCR9	7.07 ± 0.25	6.84 ± 0.12	6.89 ± 0.12	6.85 ± 0.17
TC12000495.hg.1	DGKA	10.77 ± 0.27	10.94 ± 0.10	10.59 ± 0.31	10.94 ± 0.07
TC07001154.hg.1	DGKB	4.84 ± 0.13	4.75 ± 0.09	4.78 ± 0.10	4.79 ± 0.08

TC02001419.hg.1	DGKD	9.15 ± 0.10	9.14 ± 0.06	9.11 ± 0.15	9.21 ± 0.06
TC17000699.hg.1	DGKE	7.19 ± 0.34	7.44 ± 0.14	7.01 ± 0.19	7.27 ± 0.07
TC03003334.hg.1	DGKG	7.57 ± 0.07	7.41 ± 0.05	7.56 ± 0.04	7.42 ± 0.05
TC13000150.hg.1	DGKH	6.38 ± 0.13	6.38 ± 0.11	6.30 ± 0.13	6.23 ± 0.04
TC07001897.hg.1	DGKI	5.98 ± 0.15	5.88 ± 0.08	5.86 ± 0.08	5.96 ± 0.07
TC0X001037.hg.1	DGKK	5.80 ± 0.04	5.71 ± 0.03	5.74 ± 0.01	5.72 ± 0.03
TC04000958.hg.1	DGKQ	8.47 ± 0.14	8.45 ± 0.15	8.37 ± 0.16	8.49 ± 0.03
TC11000395.hg.1	DGKZ	9.40 ± 0.11	9.26 ± 0.04	9.19 ± 0.08	9.17 ± 0.02
TC02000449.hg.1	DGUOK	8.21 ± 0.21	8.12 ± 0.10	8.05 ± 0.11	7.98 ± 0.01
TC02001989.hg.1	DGUOK-AS1	5.43 ± 0.19	5.25 ± 0.06	5.44 ± 0.04	5.19 ± 0.10
TC01002694.hg.1	DHCR24	8.02 ± 0.18	7.76 ± 0.15	7.90 ± 0.25	7.67 ± 0.02
TC11002038.hg.1	DHCR7	7.73 ± 0.26	7.57 ± 0.13	7.56 ± 0.19	7.61 ± 0.07
TC01000344.hg.1	DHDDS	8.61 ± 0.13	8.28 ± 0.05	8.35 ± 0.02	8.27 ± 0.07
TC19000715.hg.1	DHDH	8.02 ± 0.23	7.93 ± 0.17	7.92 ± 0.20	7.98 ± 0.03
TC05001538.hg.1	DHFR	8.09 ± 0.07	7.84 ± 0.30	7.95 ± 0.04	7.43 ± 0.05
TC03001592.hg.1	DHFRL1	7.13 ± 0.10	6.94 ± 0.04	7.11 ± 0.03	6.87 ± 0.02
TC12001468.hg.1	DHH	6.85 ± 0.38	6.71 ± 0.19	6.63 ± 0.28	6.76 ± 0.03
TC16000599.hg.1	DHODH	7.77 ± 0.13	7.57 ± 0.04	7.52 ± 0.14	7.54 ± 0.04
TC19001204.hg.1	DHPS	8.84 ± 0.13	8.67 ± 0.10	8.58 ± 0.13	8.54 ± 0.02
TC14000973.hg.1	DHRS1	8.23 ± 0.10	8.08 ± 0.08	8.13 ± 0.13	8.17 ± 0.10
TC17000439.hg.1	DHRS11	7.51 ± 0.11	7.42 ± 0.12	7.36 ± 0.11	7.49 ± 0.07
TC13000681.hg.1	DHRS12	8.28 ± 0.17	8.10 ± 0.02	8.39 ± 0.13	8.05 ± 0.04
TC17001312.hg.1	DHRS13	9.46 ± 0.27	9.12 ± 0.17	9.53 ± 0.11	9.01 ± 0.13
TC14000151.hg.1	DHRS2	7.53 ± 0.19	7.47 ± 0.19	7.51 ± 0.18	7.61 ± 0.11
TC01002211.hg.1	DHRS3	8.12 ± 0.05	8.15 ± 0.13	7.88 ± 0.16	8.32 ± 0.14
TC14000153.hg.1	DHRS4-AS1	5.54 ± 0.05	5.30 ± 0.04	5.23 ± 0.20	5.35 ± 0.17
TC14000965.hg.1	DHRS4-AS1	8.53 ± 0.15	8.46 ± 0.19	8.47 ± 0.15	8.48 ± 0.01
TC14002336.hg.1	DHRS4L1	7.42 ± 0.16	7.31 ± 0.04	7.32 ± 0.15	7.33 ± 0.03
TC14002335.hg.1	DHRS4L2	8.46 ± 0.10	8.37 ± 0.04	8.28 ± 0.13	8.38 ± 0.04
TC14001184.hg.1	DHRS7	9.14 ± 0.04	9.03 ± 0.15	9.50 ± 0.27	8.98 ± 0.20
TC17000295.hg.1	DHRS7B	7.40 ± 0.27	7.08 ± 0.12	7.34 ± 0.20	7.14 ± 0.09

TC17001126.hg.1	DHRS7C	6.86 ± 0.22	6.82 ± 0.07	6.84 ± 0.15	6.92 ± 0.09
TC02001000.hg.1	DHRS9	6.99 ± 0.33	6.49 ± 0.17	7.16 ± 0.49	6.34 ± 0.06
TC0X000802.hg.1	DHRSX-IT1	7.05 ± 0.34	7.00 ± 0.19	7.15 ± 0.24	7.00 ± 0.14
TC0Y000119.hg.1	DHRSX-IT1	7.05 ± 0.34	7.00 ± 0.19	7.15 ± 0.24	7.00 ± 0.14
TC10000089.hg.1	DHTKD1	8.30 ± 0.11	7.97 ± 0.10	8.22 ± 0.24	7.76 ± 0.12
TC04001086.hg.1	DHX15	8.89 ± 0.40	9.13 ± 0.09	8.94 ± 0.31	9.06 ± 0.07
TC06001504.hg.1	DHX16	8.34 ± 0.15	8.05 ± 0.06	8.21 ± 0.18	7.97 ± 0.03
TC6_apd_hap1000074.hg.1	DHX16	8.54 ± 0.13	8.29 ± 0.05	8.45 ± 0.20	8.24 ± 0.05
TC6_cox_hap2000138.hg.1	DHX16	8.35 ± 0.14	8.07 ± 0.06	8.23 ± 0.19	7.99 ± 0.04
TC6_dbb_hap3000127.hg.1	DHX16	8.35 ± 0.14	8.07 ± 0.06	8.23 ± 0.19	7.99 ± 0.04
TC6_mann_hap4000116.hg.1	DHX16	8.35 ± 0.15	8.07 ± 0.06	8.23 ± 0.19	7.99 ± 0.03
TC6_mcf_hap5000117.hg.1	DHX16	8.35 ± 0.15	8.07 ± 0.06	8.23 ± 0.19	7.99 ± 0.03
TC6_qbl_hap6000128.hg.1	DHX16	8.33 ± 0.15	8.05 ± 0.06	8.21 ± 0.19	7.97 ± 0.04
TC6_ssto_hap7000119.hg.1	DHX16	8.35 ± 0.15	8.07 ± 0.06	8.24 ± 0.19	7.99 ± 0.03
TC05001362.hg.1	DHX29	7.24 ± 0.35	7.28 ± 0.26	7.23 ± 0.34	7.09 ± 0.11
TC03000272.hg.1	DHX30	8.21 ± 0.10	8.03 ± 0.05	7.97 ± 0.05	8.04 ± 0.04
TC10001740.hg.1	DHX32	7.12 ± 0.11	7.05 ± 0.12	6.94 ± 0.07	6.98 ± 0.09
TC17001057.hg.1	DHX33	7.87 ± 0.14	7.68 ± 0.02	7.68 ± 0.05	7.67 ± 0.08
TC19000678.hg.1	DHX34	8.99 ± 0.15	8.87 ± 0.04	9.14 ± 0.11	8.90 ± 0.09
TC20000294.hg.1	DHX35	7.80 ± 0.05	7.77 ± 0.12	7.63 ± 0.04	7.75 ± 0.06
TC03001919.hg.1	DHX36	8.06 ± 0.58	8.39 ± 0.10	8.12 ± 0.53	8.52 ± 0.07
TC12002105.hg.1	DHX37	8.21 ± 0.11	8.20 ± 0.11	8.03 ± 0.17	8.24 ± 0.01
TC16000601.hg.1	DHX38	8.76 ± 0.07	8.62 ± 0.06	8.59 ± 0.12	8.62 ± 0.03
TC17000724.hg.1	DHX40	8.35 ± 0.37	8.84 ± 0.20	8.47 ± 0.40	8.92 ± 0.13
TC02001758.hg.1	DHX57	7.00 ± 0.17	6.86 ± 0.11	6.81 ± 0.12	6.78 ± 0.08
TC17001523.hg.1	DHX58	8.65 ± 0.49	8.38 ± 0.24	8.62 ± 0.41	8.32 ± 0.04
TC17000556.hg.1	DHX8	9.08 ± 0.21	9.03 ± 0.14	8.97 ± 0.19	9.00 ± 0.07
TC01001582.hg.1	DHX9	9.36 ± 0.29	9.71 ± 0.21	9.25 ± 0.25	9.60 ± 0.02
TC12003286.hg.1	DIABLO	7.82 ± 0.02	7.87 ± 0.14	7.70 ± 0.07	7.87 ± 0.08
TC05001870.hg.1	DIAPH1	9.43 ± 0.20	9.41 ± 0.12	9.39 ± 0.11	9.24 ± 0.07
TC0X000464.hg.1	DIAPH2	7.77 ± 0.37	7.88 ± 0.27	8.21 ± 0.39	7.66 ± 0.15

TC0X001204.hg.1	DIAPH2-AS1	4.60 ± 0.12	4.51 ± 0.06	4.52 ± 0.06	4.50 ± 0.02
TC13000701.hg.1	DIAPH3	5.21 ± 0.09	5.06 ± 0.10	5.09 ± 0.07	4.95 ± 0.02
TC13000238.hg.1	DIAPH3-AS1	4.49 ± 0.17	4.37 ± 0.13	4.43 ± 0.08	4.57 ± 0.13
TC13000239.hg.1	DIAPH3-AS2	5.24 ± 0.38	5.01 ± 0.11	4.93 ± 0.22	5.04 ± 0.04
TC14001472.hg.1	DICER1	8.92 ± 0.40	9.07 ± 0.26	9.51 ± 0.53	8.93 ± 0.12
TC14000590.hg.1	DICER1-AS1	7.38 ± 0.12	7.32 ± 0.11	7.33 ± 0.08	7.40 ± 0.08
TC20001021.hg.1	DIDO1	8.91 ± 0.09	8.88 ± 0.09	8.77 ± 0.07	8.81 ± 0.07
TC01001752.hg.1	DIEXF	7.56 ± 0.18	7.56 ± 0.12	7.33 ± 0.19	7.46 ± 0.09
TC05001401.hg.1	DIMT1	7.47 ± 0.19	7.42 ± 0.18	7.25 ± 0.21	7.20 ± 0.24
TC01000658.hg.1	DIO1	6.57 ± 0.19	6.54 ± 0.08	6.43 ± 0.07	6.55 ± 0.04
TC14001364.hg.1	DIO2	5.55 ± 0.13	5.33 ± 0.06	5.46 ± 0.06	5.41 ± 0.08
TC14001365.hg.1	DIO2	6.40 ± 0.30	6.39 ± 0.11	6.39 ± 0.13	6.46 ± 0.14
TC14000498.hg.1	DIO2-AS1	5.03 ± 0.15	4.98 ± 0.13	5.03 ± 0.10	5.00 ± 0.03
TC14000771.hg.1	DIO3	5.62 ± 0.20	5.78 ± 0.44	5.62 ± 0.26	5.88 ± 0.11
TC21000261.hg.1	DIP2A	8.54 ± 0.10	8.61 ± 0.05	8.45 ± 0.07	8.55 ± 0.03
TC21000262.hg.1	DIP2A-IT1	6.98 ± 0.61	7.02 ± 0.44	6.89 ± 0.43	7.09 ± 0.10
TC12000393.hg.1	DIP2B	9.26 ± 0.29	9.29 ± 0.16	9.49 ± 0.40	9.23 ± 0.13
TC10000961.hg.1	DIP2C	7.33 ± 0.14	7.34 ± 0.07	7.20 ± 0.12	7.37 ± 0.07
TC19001034.hg.1	DIRAS1	8.66 ± 0.24	8.58 ± 0.18	8.41 ± 0.24	8.65 ± 0.06
TC09001319.hg.1	DIRAS2	7.67 ± 0.13	7.61 ± 0.13	7.60 ± 0.11	7.75 ± 0.11
TC01002762.hg.1	DIRAS3	6.46 ± 0.13	6.34 ± 0.08	6.35 ± 0.04	6.38 ± 0.16
TC02002610.hg.1	DIRC1	5.13 ± 0.16	5.06 ± 0.12	5.14 ± 0.09	5.16 ± 0.13
TC03000636.hg.1	DIRC2	8.86 ± 0.19	8.99 ± 0.16	9.29 ± 0.28	9.18 ± 0.17
TC02002764.hg.1	DIRC3	6.02 ± 0.06	6.06 ± 0.06	5.99 ± 0.06	6.06 ± 0.06
TC13000727.hg.1	DIS3	7.73 ± 0.58	8.26 ± 0.24	7.83 ± 0.54	8.23 ± 0.06
TC15000611.hg.1	DIS3L	8.04 ± 0.12	8.06 ± 0.17	7.86 ± 0.13	7.91 ± 0.05
TC02001396.hg.1	DIS3L2	8.09 ± 0.07	8.10 ± 0.06	7.93 ± 0.04	8.11 ± 0.06
TC01001917.hg.1	DISC1	7.10 ± 0.03	7.03 ± 0.14	7.29 ± 0.19	6.97 ± 0.05
TC01001919.hg.1	DISC1-IT1	7.84 ± 0.27	7.86 ± 0.13	7.97 ± 0.04	7.95 ± 0.10
TC01003972.hg.1	DISC2	5.04 ± 0.25	5.00 ± 0.09	5.13 ± 0.19	5.03 ± 0.15
TC01006341.hg.1	DISP1	6.48 ± 0.15	6.53 ± 0.12	6.48 ± 0.03	6.49 ± 0.10

TC15000281.hg.1	DISP2	7.93 ± 0.24	7.97 ± 0.23	7.93 ± 0.27	8.12 ± 0.07
TC11001002.hg.1	DIXDC1	6.54 ± 0.16	6.45 ± 0.06	6.48 ± 0.08	6.50 ± 0.02
TC0X000779.hg.1	DKC1	8.23 ± 0.10	8.29 ± 0.07	8.08 ± 0.11	8.26 ± 0.03
TC09001727.hg.1	DKFZP434A062	7.01 ± 0.17	7.23 ± 0.13	7.06 ± 0.15	7.39 ± 0.10
TC16001131.hg.1	DKFZP434H168	6.20 ± 0.41	6.10 ± 0.17	5.89 ± 0.23	6.21 ± 0.13
TC04000764.hg.1	DKFZP434I0714	7.71 ± 0.32	7.51 ± 0.05	7.65 ± 0.28	7.65 ± 0.05
TC19000660.hg.1	DKFZp434J0226	7.47 ± 0.19	7.54 ± 0.15	7.45 ± 0.14	7.66 ± 0.08
TC11001856.hg.1	DKFZP434K028	7.12 ± 0.23	7.11 ± 0.25	7.07 ± 0.30	7.26 ± 0.07
TC15000164.hg.1	DKFZP434L187	6.64 ± 0.12	6.62 ± 0.16	6.67 ± 0.11	6.73 ± 0.08
TC07000344.hg.1	DKFZp434L192	7.28 ± 0.27	7.31 ± 0.25	7.32 ± 0.12	7.37 ± 0.10
TC14001483.hg.1	DKFZP434O1614	4.98 ± 0.22	4.87 ± 0.07	4.86 ± 0.22	4.94 ± 0.10
TC06002291.hg.1	DKFZp451B082	6.75 ± 0.15	6.59 ± 0.14	6.55 ± 0.19	6.61 ± 0.10
TC19000421.hg.1	DKFZp566F0947	6.36 ± 0.04	6.28 ± 0.16	6.27 ± 0.06	6.38 ± 0.08
TC07000196.hg.1	DKFZP586I1420	8.25 ± 0.64	8.75 ± 0.31	8.37 ± 0.65	8.64 ± 0.13
TC10000058.hg.1	DKFZP667F0711	7.34 ± 0.33	7.21 ± 0.37	8.17 ± 0.91	7.26 ± 0.29
TC06004061.hg.1	DKFZP686I15217	7.89 ± 0.14	7.45 ± 0.12	7.73 ± 0.14	7.54 ± 0.20
TC11000386.hg.1	DKFZp779M0652	7.71 ± 0.24	7.66 ± 0.22	7.47 ± 0.25	7.81 ± 0.15
TC10000350.hg.1	DKK1	5.41 ± 0.18	5.27 ± 0.10	5.35 ± 0.02	5.34 ± 0.03
TC04001453.hg.1	DKK2	5.88 ± 0.15	5.80 ± 0.11	5.79 ± 0.14	5.89 ± 0.10
TC11001425.hg.1	DKK3	8.33 ± 0.16	8.33 ± 0.17	8.35 ± 0.12	8.39 ± 0.06
TC08001178.hg.1	DKK4	5.85 ± 0.19	5.77 ± 0.14	5.76 ± 0.12	5.91 ± 0.05
TC19000730.hg.1	DKKL1	7.46 ± 0.20	7.38 ± 0.23	7.34 ± 0.20	7.47 ± 0.07
TC11001005.hg.1	DLAT	7.75 ± 0.17	7.64 ± 0.13	7.54 ± 0.10	7.43 ± 0.06
TC08001000.hg.1	DLC1	5.91 ± 0.18	5.84 ± 0.09	5.93 ± 0.07	5.89 ± 0.05
TC07000697.hg.1	DLD	8.05 ± 0.46	8.37 ± 0.20	8.27 ± 0.42	8.34 ± 0.07
TC03000186.hg.1	DLEC1	7.28 ± 0.10	7.31 ± 0.09	7.29 ± 0.10	7.38 ± 0.03
TC13000205.hg.1	DLEU1	5.36 ± 0.12	5.28 ± 0.02	5.28 ± 0.02	5.33 ± 0.08
TC01000721.hg.1	DLEU2L	6.10 ± 0.36	6.04 ± 0.21	6.24 ± 0.25	5.92 ± 0.15
TC13000672.hg.1	DLEU7	7.24 ± 0.09	7.17 ± 0.11	7.17 ± 0.04	7.24 ± 0.04
TC13000208.hg.1	DLEU7-AS1	5.86 ± 0.13	5.71 ± 0.08	5.75 ± 0.05	5.76 ± 0.11
TC03002174.hg.1	DLG1	7.90 ± 0.34	8.09 ± 0.12	7.83 ± 0.28	7.98 ± 0.09

TC03001106.hg.1	DLG1-AS1	6.93 ± 0.22	6.94 ± 0.23	6.92 ± 0.26	7.03 ± 0.04
TC11002159.hg.1	DLG2	5.20 ± 0.10	5.11 ± 0.08	5.13 ± 0.06	5.16 ± 0.05
TC0X000379.hg.1	DLG3	6.93 ± 0.09	6.88 ± 0.07	6.80 ± 0.17	6.84 ± 0.03
TC0X001114.hg.1	DLG3-AS1	7.86 ± 0.16	7.70 ± 0.09	7.70 ± 0.05	7.67 ± 0.09
TC17001077.hg.1	DLG4	7.70 ± 0.15	7.52 ± 0.10	7.67 ± 0.05	7.53 ± 0.06
TC10001445.hg.1	DLG5	7.64 ± 0.13	7.60 ± 0.12	7.53 ± 0.14	7.66 ± 0.03
TC10000557.hg.1	DLG5-AS1	6.83 ± 0.27	6.81 ± 0.17	6.81 ± 0.13	6.90 ± 0.02
TC18000291.hg.1	DLGAP1	6.19 ± 0.14	6.15 ± 0.11	6.13 ± 0.13	6.20 ± 0.06
TC18000020.hg.1	DLGAP1-AS1	7.70 ± 0.13	7.63 ± 0.05	7.69 ± 0.22	7.68 ± 0.12
TC18000023.hg.1	DLGAP1-AS3	6.02 ± 0.23	5.93 ± 0.05	5.89 ± 0.11	5.99 ± 0.15
TC18000024.hg.1	DLGAP1-AS5	4.58 ± 0.10	4.49 ± 0.07	4.65 ± 0.22	4.57 ± 0.05
TC08000007.hg.1	DLGAP2	7.06 ± 0.19	7.01 ± 0.21	7.02 ± 0.11	7.12 ± 0.08
TC01002491.hg.1	DLGAP3	7.92 ± 0.22	7.90 ± 0.24	7.83 ± 0.25	8.00 ± 0.06
TC20000267.hg.1	DLGAP4	8.45 ± 0.13	8.39 ± 0.06	8.43 ± 0.05	8.37 ± 0.04
TC14001155.hg.1	DLGAP5	6.64 ± 0.19	6.57 ± 0.64	6.35 ± 0.23	5.85 ± 0.15
TC14000644.hg.1	DLK1	7.74 ± 0.12	7.74 ± 0.20	7.67 ± 0.20	7.82 ± 0.04
TC06001748.hg.1	DLK2	8.76 ± 0.15	8.75 ± 0.16	8.72 ± 0.14	8.88 ± 0.02
TC06002342.hg.1	DLL1	7.57 ± 0.13	7.57 ± 0.10	7.47 ± 0.15	7.64 ± 0.04
TC19000549.hg.1	DLL3	7.57 ± 0.21	7.53 ± 0.21	7.46 ± 0.24	7.62 ± 0.07
TC15000299.hg.1	DLL4	7.81 ± 0.26	7.67 ± 0.12	7.69 ± 0.23	7.75 ± 0.06
TC14000465.hg.1	DLST	9.25 ± 0.11	9.22 ± 0.17	9.02 ± 0.18	9.21 ± 0.06
TC02001026.hg.1	DLX1	7.14 ± 0.14	7.09 ± 0.17	7.04 ± 0.22	7.21 ± 0.06
TC02002525.hg.1	DLX2	7.44 ± 0.28	7.37 ± 0.24	7.39 ± 0.22	7.48 ± 0.09
TC17001675.hg.1	DLX3	7.65 ± 0.27	7.66 ± 0.24	7.73 ± 0.22	7.81 ± 0.17
TC17000652.hg.1	DLX4	7.64 ± 0.18	7.60 ± 0.14	7.56 ± 0.15	7.65 ± 0.05
TC07001627.hg.1	DLX5	6.91 ± 0.29	6.81 ± 0.25	6.81 ± 0.18	6.96 ± 0.13
TC07000577.hg.1	DLX6	6.82 ± 0.15	6.77 ± 0.11	6.77 ± 0.09	6.82 ± 0.01
TC07001625.hg.1	DLX6-AS1	4.92 ± 0.23	4.80 ± 0.09	4.80 ± 0.09	4.82 ± 0.05
TC07001626.hg.1	DLX6-AS2	7.82 ± 0.27	7.63 ± 0.24	7.57 ± 0.26	7.58 ± 0.05
TC01000559.hg.1	DMAP1	8.66 ± 0.12	8.63 ± 0.07	8.52 ± 0.10	8.69 ± 0.07
TC10000890.hg.1	DMBT1	7.14 ± 0.12	7.27 ± 0.24	7.14 ± 0.20	7.43 ± 0.15

TC01000588.hg.1	DMBX1	8.05 ± 0.24	8.04 ± 0.14	8.01 ± 0.19	8.22 ± 0.11
TC22000792.hg.1	DMC1	5.81 ± 0.14	5.57 ± 0.03	5.77 ± 0.19	5.50 ± 0.04
TC0X000943.hg.1	DMD	5.25 ± 0.13	5.17 ± 0.06	5.19 ± 0.02	5.24 ± 0.04
TC0X000147.hg.1	DMD-AS1	6.56 ± 0.23	6.50 ± 0.06	6.43 ± 0.05	6.51 ± 0.10
TC0X000148.hg.1	DMD-AS2	4.78 ± 0.03	4.67 ± 0.14	4.65 ± 0.06	4.84 ± 0.14
TC0X000156.hg.1	DMD-AS3	4.35 ± 0.14	4.20 ± 0.07	4.12 ± 0.16	4.19 ± 0.09
TC05001520.hg.1	DMGDH	5.85 ± 0.10	5.79 ± 0.09	5.81 ± 0.09	5.90 ± 0.07
TC19001445.hg.1	DMKN	7.24 ± 0.20	7.17 ± 0.11	7.22 ± 0.16	7.25 ± 0.07
TC04000474.hg.1	DMP1	5.75 ± 0.20	5.69 ± 0.09	5.71 ± 0.08	5.72 ± 0.03
TC19001632.hg.1	DMPK	8.20 ± 0.15	8.20 ± 0.18	8.09 ± 0.19	8.23 ± 0.05
TC09000008.hg.1	DMRT1	7.83 ± 0.15	7.81 ± 0.17	7.77 ± 0.13	7.90 ± 0.03
TC09000011.hg.1	DMRT2	6.73 ± 0.15	6.60 ± 0.04	6.62 ± 0.09	6.65 ± 0.03
TC09000009.hg.1	DMRT3	6.05 ± 0.06	6.10 ± 0.10	6.09 ± 0.16	6.24 ± 0.16
TC09000109.hg.1	DMRTA1	3.68 ± 0.01	3.61 ± 0.06	3.67 ± 0.10	3.61 ± 0.08
TC01002643.hg.1	DMRTA2	8.07 ± 0.28	8.04 ± 0.22	8.02 ± 0.26	8.17 ± 0.05
TC01000657.hg.1	DMRTB1	7.77 ± 0.26	7.76 ± 0.25	7.64 ± 0.24	7.88 ± 0.05
TC0X000398.hg.1	DMRTC1B	7.51 ± 0.24	7.56 ± 0.25	7.42 ± 0.22	7.69 ± 0.11
TC0X002334.hg.1	DMRTC1B	7.46 ± 0.31	7.46 ± 0.29	7.29 ± 0.24	7.61 ± 0.12
TC19000586.hg.1	DMRTC2	7.39 ± 0.21	7.42 ± 0.28	7.32 ± 0.15	7.57 ± 0.10
TC07000520.hg.1	DMTF1	8.26 ± 0.69	8.73 ± 0.21	8.44 ± 0.50	8.67 ± 0.05
TC08000164.hg.1	DMTN	8.78 ± 0.14	8.50 ± 0.20	8.82 ± 0.15	8.64 ± 0.09
TC19001633.hg.1	DMWD	8.23 ± 0.12	8.18 ± 0.07	8.01 ± 0.08	8.16 ± 0.06
TC05000563.hg.1	DMXL1	7.35 ± 0.50	7.61 ± 0.31	7.38 ± 0.38	7.25 ± 0.13
TC15001441.hg.1	DMXL2	9.01 ± 0.43	9.04 ± 0.34	9.68 ± 0.54	8.90 ± 0.37
TC10001347.hg.1	DNA2	6.03 ± 0.03	5.86 ± 0.17	5.97 ± 0.12	5.70 ± 0.07
TC16000654.hg.1	DNAAF1	7.40 ± 0.21	7.30 ± 0.13	7.35 ± 0.17	7.34 ± 0.03
TC14001103.hg.1	DNAAF2	5.91 ± 0.07	5.75 ± 0.06	5.86 ± 0.41	5.59 ± 0.15
TC19001860.hg.1	DNAAF3	8.02 ± 0.27	7.94 ± 0.22	7.90 ± 0.30	8.02 ± 0.04
TC03000333.hg.1	DNAH1	8.26 ± 0.02	8.26 ± 0.01	8.16 ± 0.04	8.28 ± 0.05
TC12000994.hg.1	DNAH10	6.01 ± 0.14	5.93 ± 0.11	5.94 ± 0.11	5.99 ± 0.04
TC12002095.hg.1	DNAH10OS	6.57 ± 0.22	6.53 ± 0.21	6.54 ± 0.16	6.53 ± 0.15

TC07000133.hg.1	DNAH11	5.06 ± 0.09	4.99 ± 0.06	5.01 ± 0.05	5.04 ± 0.05
TC03001492.hg.1	DNAH12	4.39 ± 0.09	4.25 ± 0.02	4.31 ± 0.05	4.24 ± 0.01
TC01001851.hg.1	DNAH14	4.28 ± 0.10	4.16 ± 0.01	4.22 ± 0.09	4.18 ± 0.01
TC17001918.hg.1	DNAH17	7.03 ± 0.18	6.97 ± 0.16	7.01 ± 0.12	7.06 ± 0.01
TC17000115.hg.1	DNAH2	6.87 ± 0.16	6.81 ± 0.16	6.81 ± 0.12	6.89 ± 0.04
TC16000932.hg.1	DNAH3	5.76 ± 0.13	5.71 ± 0.12	5.70 ± 0.10	5.75 ± 0.02
TC05001171.hg.1	DNAH5	5.27 ± 0.13	5.16 ± 0.07	5.21 ± 0.04	5.23 ± 0.04
TC02000499.hg.1	DNAH6	5.12 ± 0.08	5.05 ± 0.04	5.05 ± 0.03	5.10 ± 0.03
TC02002636.hg.1	DNAH7	4.77 ± 0.14	4.69 ± 0.03	4.74 ± 0.09	4.71 ± 0.04
TC06000556.hg.1	DNAH8	4.67 ± 0.10	4.56 ± 0.01	4.58 ± 0.05	4.54 ± 0.04
TC17000154.hg.1	DNAH9	6.30 ± 0.18	6.25 ± 0.13	6.25 ± 0.11	6.34 ± 0.05
TC09000171.hg.1	DNAI1	6.71 ± 0.18	6.63 ± 0.12	6.64 ± 0.12	6.71 ± 0.05
TC17000827.hg.1	DNAI2	7.53 ± 0.26	7.49 ± 0.23	7.46 ± 0.23	7.61 ± 0.06
TC09000134.hg.1	DNAJA1	9.58 ± 0.18	9.78 ± 0.11	9.98 ± 0.39	9.57 ± 0.37
TC01000913.hg.1	DNAJA1P5	6.70 ± 0.37	6.46 ± 0.15	6.65 ± 0.09	6.71 ± 0.03
TC16001087.hg.1	DNAJA2	8.82 ± 0.13	8.80 ± 0.11	8.86 ± 0.31	8.77 ± 0.28
TC16000130.hg.1	DNAJA3	8.19 ± 0.17	7.93 ± 0.02	7.84 ± 0.11	7.75 ± 0.03
TC15000725.hg.1	DNAJA4	7.76 ± 0.10	7.85 ± 0.05	7.68 ± 0.20	7.83 ± 0.02
TC19001239.hg.1	DNAJB1	9.14 ± 0.17	9.50 ± 0.24	8.72 ± 0.22	9.40 ± 0.06
TC03001018.hg.1	DNAJB11	8.67 ± 0.16	8.69 ± 0.04	8.44 ± 0.25	8.57 ± 0.30
TC03001019.hg.1	DNAJB11	8.38 ± 0.29	8.18 ± 0.04	8.29 ± 0.13	8.15 ± 0.13
TC10001392.hg.1	DNAJB12	8.84 ± 0.10	8.76 ± 0.06	8.82 ± 0.04	8.81 ± 0.07
TC11000783.hg.1	DNAJB13	6.85 ± 0.16	6.77 ± 0.17	6.74 ± 0.22	6.84 ± 0.09
TC04001417.hg.1	DNAJB14	7.14 ± 0.64	7.59 ± 0.22	7.44 ± 0.64	7.49 ± 0.07
TC02001323.hg.1	DNAJB2	8.09 ± 0.09	8.14 ± 0.10	7.97 ± 0.09	8.19 ± 0.03
TC02002884.hg.1	DNAJB3	7.32 ± 0.10	7.27 ± 0.11	7.33 ± 0.23	7.31 ± 0.02
TC01006271.hg.1	DNAJB4	7.23 ± 0.23	7.29 ± 0.05	7.30 ± 0.10	7.32 ± 0.04
TC09000178.hg.1	DNAJB5	7.70 ± 0.14	7.65 ± 0.12	7.62 ± 0.07	7.77 ± 0.12
TC07001055.hg.1	DNAJB6	8.73 ± 0.21	8.92 ± 0.10	8.89 ± 0.15	8.89 ± 0.11
TC22000817.hg.1	DNAJB7	4.91 ± 0.09	4.77 ± 0.10	5.01 ± 0.04	4.86 ± 0.04
TC03001754.hg.1	DNAJB8	7.02 ± 0.13	7.07 ± 0.20	7.05 ± 0.17	7.25 ± 0.05

TC03000674.hg.1	DNAJB8-AS1	6.98 ± 0.20	6.97 ± 0.12	7.05 ± 0.12	7.08 ± 0.13
TC07000699.hg.1	DNAJB9	7.55 ± 0.14	7.97 ± 0.02	7.50 ± 0.15	7.85 ± 0.20
TC10001097.hg.1	DNAJC1	7.98 ± 0.10	7.87 ± 0.07	7.99 ± 0.17	7.65 ± 0.21
TC02001089.hg.1	DNAJC10	8.02 ± 0.52	8.30 ± 0.28	7.87 ± 0.39	8.06 ± 0.06
TC01002156.hg.1	DNAJC11	8.59 ± 0.02	8.45 ± 0.07	8.28 ± 0.03	8.40 ± 0.02
TC10001340.hg.1	DNAJC12	5.07 ± 0.10	5.04 ± 0.05	5.07 ± 0.04	5.06 ± 0.07
TC03000715.hg.1	DNAJC13	8.55 ± 0.43	8.54 ± 0.27	9.11 ± 0.51	8.18 ± 0.18
TC12003279.hg.1	DNAJC14	8.17 ± 0.07	8.09 ± 0.04	8.02 ± 0.08	8.07 ± 0.07
TC13000157.hg.1	DNAJC15	8.49 ± 0.22	8.44 ± 0.05	8.60 ± 0.24	8.39 ± 0.18
TC01000190.hg.1	DNAJC16	7.63 ± 0.06	7.46 ± 0.13	7.51 ± 0.12	7.38 ± 0.05
TC15001243.hg.1	DNAJC17	7.86 ± 0.05	7.75 ± 0.01	7.77 ± 0.01	7.78 ± 0.02
TC05003430.hg.1	DNAJC18	5.80 ± 0.05	5.73 ± 0.04	5.70 ± 0.03	5.71 ± 0.03
TC03002045.hg.1	DNAJC19	6.99 ± 0.25	6.91 ± 0.12	6.85 ± 0.12	6.83 ± 0.06
TC07001718.hg.1	DNAJC2	7.17 ± 0.41	7.52 ± 0.09	7.16 ± 0.29	7.55 ± 0.12
TC05000155.hg.1	DNAJC21	7.61 ± 0.47	7.97 ± 0.10	7.65 ± 0.35	7.88 ± 0.21
TC12000371.hg.1	DNAJC22	7.08 ± 0.28	7.03 ± 0.20	7.00 ± 0.22	7.16 ± 0.07
TC11000302.hg.1	DNAJC24	6.65 ± 0.21	6.65 ± 0.09	6.66 ± 0.15	6.62 ± 0.05
TC09000558.hg.1	DNAJC25	7.60 ± 0.09	7.77 ± 0.10	7.92 ± 0.14	7.67 ± 0.24
TC02001654.hg.1	DNAJC27	6.83 ± 0.08	6.86 ± 0.10	6.83 ± 0.14	6.87 ± 0.05
TC02000140.hg.1	DNAJC27-AS1	7.38 ± 0.10	7.33 ± 0.08	7.37 ± 0.07	7.38 ± 0.02
TC21000411.hg.1	DNAJC28	5.92 ± 0.14	5.73 ± 0.19	5.71 ± 0.20	5.65 ± 0.08
TC13000336.hg.1	DNAJC3	9.12 ± 0.39	9.24 ± 0.18	9.67 ± 0.50	9.18 ± 0.26
TC07001503.hg.1	DNAJC30	7.43 ± 0.14	7.32 ± 0.04	7.10 ± 0.18	7.19 ± 0.05
TC13000805.hg.1	DNAJC3-AS1	6.02 ± 0.10	5.93 ± 0.02	6.26 ± 0.21	5.88 ± 0.08
TC11000588.hg.1	DNAJC4	9.72 ± 0.15	9.53 ± 0.07	9.74 ± 0.08	9.49 ± 0.08
TC20000528.hg.1	DNAJC5	9.21 ± 0.12	9.13 ± 0.06	9.24 ± 0.07	9.19 ± 0.06
TC08000435.hg.1	DNAJC5B	5.55 ± 0.07	5.48 ± 0.04	5.50 ± 0.03	5.50 ± 0.02
TC02000169.hg.1	DNAJC5G	6.17 ± 0.12	6.15 ± 0.07	6.14 ± 0.03	6.20 ± 0.02
TC01000729.hg.1	DNAJC6	6.28 ± 0.12	6.11 ± 0.06	6.16 ± 0.13	6.16 ± 0.10
TC17001521.hg.1	DNAJC7	9.37 ± 0.16	9.32 ± 0.13	9.36 ± 0.19	9.25 ± 0.22
TC01002422.hg.1	DNAJC8	8.30 ± 0.18	8.27 ± 0.20	8.16 ± 0.09	8.07 ± 0.21

TC10000459.hg.1	DNAJC9-AS1	6.30 ± 0.13	6.09 ± 0.04	6.07 ± 0.06	6.20 ± 0.11
TC14000448.hg.1	DNAL1	5.92 ± 0.04	5.79 ± 0.05	5.84 ± 0.04	5.79 ± 0.05
TC22001496.hg.1	DNAL4	8.33 ± 0.24	8.39 ± 0.19	8.26 ± 0.16	8.35 ± 0.02
TC01000475.hg.1	DNALI1	6.65 ± 0.16	6.67 ± 0.13	6.61 ± 0.11	6.81 ± 0.08
TC16000126.hg.1	DNASE1	7.99 ± 0.06	7.84 ± 0.10	7.90 ± 0.07	7.78 ± 0.05
TC0X001532.hg.1	DNASE1L1	8.80 ± 0.14	8.62 ± 0.06	8.73 ± 0.10	8.66 ± 0.02
TC16000066.hg.1	DNASE1L2	7.99 ± 0.28	8.01 ± 0.23	7.98 ± 0.28	8.07 ± 0.02
TC03001498.hg.1	DNASE1L3	6.22 ± 0.09	6.23 ± 0.07	6.12 ± 0.12	6.26 ± 0.05
TC19001212.hg.1	DNASE2	9.18 ± 0.37	8.74 ± 0.04	8.92 ± 0.10	8.63 ± 0.08
TC01000811.hg.1	DNASE2B	4.59 ± 0.05	4.49 ± 0.08	4.54 ± 0.01	4.57 ± 0.11
TC05001864.hg.1	DND1	8.93 ± 0.20	8.90 ± 0.20	8.98 ± 0.23	8.95 ± 0.14
TC02002847.hg.1	DNER	6.33 ± 0.27	6.30 ± 0.21	6.33 ± 0.17	6.43 ± 0.05
TC11003444.hg.1	DNHD1	7.37 ± 0.10	7.46 ± 0.05	7.23 ± 0.09	7.53 ± 0.10
TC09002925.hg.1	DNLZ	8.27 ± 0.20	8.26 ± 0.14	8.14 ± 0.23	8.25 ± 0.04
TC09000679.hg.1	DNM1	7.60 ± 0.13	7.50 ± 0.13	7.56 ± 0.16	7.58 ± 0.02
TC12000298.hg.1	DNM1L	8.03 ± 0.43	8.19 ± 0.25	8.05 ± 0.40	7.96 ± 0.11
TC15001671.hg.1	DNM1P35	8.12 ± 0.20	8.08 ± 0.22	8.09 ± 0.20	8.26 ± 0.12
TC15000813.hg.1	DNM1P41	7.69 ± 0.17	7.59 ± 0.02	7.60 ± 0.15	7.72 ± 0.10
TC15001989.hg.1	DNM1P46	8.05 ± 0.08	7.95 ± 0.04	7.92 ± 0.16	8.05 ± 0.11
TC19002623.hg.1	DNM2	10.12 ± 0.19	9.92 ± 0.07	10.04 ± 0.14	9.87 ± 0.11
TC01001498.hg.1	DNM3	7.45 ± 0.36	6.95 ± 0.09	7.45 ± 0.22	6.97 ± 0.22
TC01001499.hg.1	DNM3-IT1	4.76 ± 0.12	4.65 ± 0.10	4.67 ± 0.12	4.68 ± 0.08
TC10001585.hg.1	DNMBP	7.45 ± 0.02	7.39 ± 0.20	7.29 ± 0.07	7.54 ± 0.21
TC10000716.hg.1	DNMBP-AS1	5.67 ± 0.27	5.59 ± 0.05	5.67 ± 0.03	5.77 ± 0.09
TC19001159.hg.1	DNMT1	9.33 ± 0.06	9.26 ± 0.09	9.07 ± 0.11	9.03 ± 0.12
TC02001658.hg.1	DNMT3A	8.90 ± 0.04	8.91 ± 0.02	8.84 ± 0.05	8.89 ± 0.01
TC20000215.hg.1	DNMT3B	7.03 ± 0.21	6.94 ± 0.16	6.94 ± 0.17	7.02 ± 0.04
TC21000517.hg.1	DNMT3L	7.54 ± 0.21	7.55 ± 0.18	7.45 ± 0.20	7.61 ± 0.04
TC02002797.hg.1	DNPEP	8.24 ± 0.06	8.22 ± 0.06	8.05 ± 0.08	8.23 ± 0.03
TC06001745.hg.1	DNPH1	8.92 ± 0.17	8.86 ± 0.09	8.73 ± 0.25	8.88 ± 0.03
TC10000684.hg.1	DNTT	5.82 ± 0.09	5.77 ± 0.13	5.77 ± 0.09	5.88 ± 0.08

TC20000356.hg.1	DNTTIP1	9.25 ± 0.32	8.89 ± 0.13	9.34 ± 0.22	8.83 ± 0.15
TC01002881.hg.1	DNTTIP2	7.38 ± 0.34	7.81 ± 0.17	7.59 ± 0.37	7.79 ± 0.10
TC16001015.hg.1	DOC2A	7.93 ± 0.16	7.90 ± 0.20	7.85 ± 0.14	7.97 ± 0.03
TC17000961.hg.1	DOC2B	7.69 ± 0.24	7.63 ± 0.22	7.75 ± 0.15	7.77 ± 0.02
TC11001990.hg.1	DOC2GP	7.95 ± 0.11	7.90 ± 0.15	7.84 ± 0.10	7.98 ± 0.07
TC10000918.hg.1	DOCK1	5.72 ± 0.08	5.59 ± 0.05	5.68 ± 0.05	5.61 ± 0.02
TC02002827.hg.1	DOCK10	9.73 ± 0.59	10.24 ± 0.10	9.61 ± 0.58	10.14 ± 0.08
TC0X000574.hg.1	DOCK11	9.68 ± 0.59	10.16 ± 0.24	10.12 ± 0.47	9.92 ± 0.14
TC05000929.hg.1	DOCK2	9.89 ± 0.20	9.94 ± 0.13	9.92 ± 0.18	9.78 ± 0.04
TC03000318.hg.1	DOCK3	6.20 ± 0.15	6.11 ± 0.10	6.10 ± 0.10	6.16 ± 0.05
TC07001761.hg.1	DOCK4	7.09 ± 0.40	6.91 ± 0.37	7.84 ± 0.71	7.17 ± 0.52
TC08002590.hg.1	DOCK5	9.26 ± 0.29	9.54 ± 0.29	9.85 ± 0.51	9.50 ± 0.38
TC19001179.hg.1	DOCK6	7.52 ± 0.21	7.52 ± 0.17	7.49 ± 0.13	7.57 ± 0.02
TC01002727.hg.1	DOCK7	6.02 ± 0.15	5.87 ± 0.15	5.87 ± 0.15	5.82 ± 0.04
TC09000004.hg.1	DOCK8	10.30 ± 0.27	10.24 ± 0.19	10.42 ± 0.26	10.01 ± 0.14
TC13000825.hg.1	DOCK9	7.99 ± 0.25	8.35 ± 0.09	7.75 ± 0.26	8.19 ± 0.11
TC13000349.hg.1	DOCK9-AS1	5.11 ± 0.05	5.09 ± 0.08	5.22 ± 0.06	5.01 ± 0.13
TC19001043.hg.1	DOHH	8.35 ± 0.23	8.28 ± 0.20	8.21 ± 0.31	8.34 ± 0.03
TC02000464.hg.1	DOK1	8.34 ± 0.26	8.12 ± 0.14	8.24 ± 0.18	8.16 ± 0.05
TC08001031.hg.1	DOK2	8.98 ± 0.15	8.70 ± 0.08	8.79 ± 0.17	8.71 ± 0.06
TC05002111.hg.1	DOK3	9.24 ± 0.27	8.93 ± 0.14	9.45 ± 0.02	9.01 ± 0.12
TC16001140.hg.1	DOK4	7.60 ± 0.19	7.55 ± 0.19	7.62 ± 0.12	7.59 ± 0.04
TC20000424.hg.1	DOK5	5.80 ± 0.15	5.69 ± 0.14	5.73 ± 0.04	5.74 ± 0.04
TC18000241.hg.1	DOK6	6.49 ± 0.03	6.22 ± 0.15	6.56 ± 0.12	6.39 ± 0.10
TC04000048.hg.1	DOK7	8.61 ± 0.21	8.69 ± 0.23	8.61 ± 0.26	8.76 ± 0.07
TC09001641.hg.1	DOLK	9.02 ± 0.08	8.94 ± 0.10	8.68 ± 0.29	8.91 ± 0.02
TC09000707.hg.1	DOLPP1	7.78 ± 0.13	7.67 ± 0.09	7.60 ± 0.21	7.65 ± 0.06
TC06001553.hg.1	DOM3Z	8.41 ± 0.04	8.51 ± 0.07	8.33 ± 0.03	8.48 ± 0.07
TC6_cox_hap2000181.hg.1	DOM3Z	8.45 ± 0.07	8.56 ± 0.07	8.38 ± 0.04	8.54 ± 0.07
TC6_dbb_hap3000170.hg.1	DOM3Z	8.40 ± 0.07	8.51 ± 0.07	8.35 ± 0.04	8.49 ± 0.07
TC6_mcf_hap5000156.hg.1	DOM3Z	8.45 ± 0.07	8.56 ± 0.07	8.38 ± 0.04	8.54 ± 0.07

TC6_qbl_hap6000171.hg.1	DOM3Z	8.53 ± 0.06	8.64 ± 0.07	8.47 ± 0.05	8.62 ± 0.07
TC6_ssto_hap7000149.hg.1	DOM3Z	8.45 ± 0.07	8.56 ± 0.07	8.38 ± 0.04	8.54 ± 0.07
TC21001058.hg.1	DONSON	6.84 ± 0.02	6.84 ± 0.11	6.74 ± 0.07	6.59 ± 0.11
TC06000768.hg.1	DOPEY1	6.87 ± 0.39	6.96 ± 0.21	6.77 ± 0.33	6.78 ± 0.05
TC21001061.hg.1	DOPEY2	8.22 ± 0.17	8.06 ± 0.19	8.09 ± 0.12	7.96 ± 0.04
TC19000049.hg.1	DOT1L	8.58 ± 0.10	8.52 ± 0.06	8.46 ± 0.11	8.56 ± 0.11
TC11002363.hg.1	DPAGT1	8.13 ± 0.12	7.78 ± 0.05	7.87 ± 0.15	7.65 ± 0.02
TC10000739.hg.1	DPCD	7.29 ± 0.14	7.13 ± 0.09	7.21 ± 0.09	7.20 ± 0.04
TC06000352.hg.1	DPCR1	6.32 ± 0.14	6.41 ± 0.24	6.33 ± 0.15	6.47 ± 0.18
TC6_cox_hap2000050.hg.1	DPCR1	6.29 ± 0.15	6.30 ± 0.21	6.20 ± 0.18	6.39 ± 0.20
TC6_dbb_hap3000043.hg.1	DPCR1	6.31 ± 0.16	6.30 ± 0.21	6.20 ± 0.18	6.38 ± 0.20
TC6_mcf_hap5000037.hg.1	DPCR1	9.34 ± 0.22	9.40 ± 0.21	9.28 ± 0.25	9.53 ± 0.23
TC6_qbl_hap6000043.hg.1	DPCR1	6.48 ± 0.17	6.53 ± 0.23	6.45 ± 0.20	6.60 ± 0.20
TC16000701.hg.1	DPEP1	8.03 ± 0.19	8.05 ± 0.21	8.02 ± 0.12	8.15 ± 0.07
TC16001201.hg.1	DPEP2	8.83 ± 0.05	8.68 ± 0.09	8.78 ± 0.15	8.66 ± 0.05
TC16001200.hg.1	DPEP3	7.98 ± 0.12	7.80 ± 0.03	7.84 ± 0.00	7.87 ± 0.04
TC19001495.hg.1	DPF1	8.14 ± 0.17	8.10 ± 0.16	8.07 ± 0.15	8.16 ± 0.01
TC11000624.hg.1	DPF2	9.32 ± 0.19	9.02 ± 0.10	9.26 ± 0.20	8.92 ± 0.19
TC14001279.hg.1	DPF3	7.19 ± 0.10	7.07 ± 0.04	7.07 ± 0.09	7.07 ± 0.07
TC17000018.hg.1	DPH1	8.62 ± 0.03	8.61 ± 0.09	8.32 ± 0.06	8.71 ± 0.04
TC01000554.hg.1	DPH2	8.10 ± 0.07	8.02 ± 0.08	7.89 ± 0.13	8.01 ± 0.01
TC03001215.hg.1	DPH3	7.42 ± 0.06	7.47 ± 0.10	7.68 ± 0.33	7.30 ± 0.21
TC20000500.hg.1	DPH3P1	7.83 ± 0.14	7.80 ± 0.14	7.80 ± 0.20	7.91 ± 0.07
TC01002924.hg.1	DPH5	6.84 ± 0.21	6.78 ± 0.16	6.70 ± 0.06	6.69 ± 0.04
TC15001205.hg.1	DPH6	6.51 ± 0.25	6.54 ± 0.25	6.37 ± 0.31	6.36 ± 0.15
TC15000249.hg.1	DPH6-AS1	6.71 ± 0.19	6.68 ± 0.11	6.66 ± 0.11	6.81 ± 0.12
TC20000940.hg.1	DPM1	9.11 ± 0.44	9.43 ± 0.21	9.34 ± 0.41	9.41 ± 0.15
TC09001617.hg.1	DPM2	8.70 ± 0.02	8.69 ± 0.08	8.42 ± 0.12	8.65 ± 0.06
TC01003303.hg.1	DPM3	8.47 ± 0.20	8.39 ± 0.06	8.37 ± 0.19	8.44 ± 0.06
TC02000738.hg.1	DPP10	5.04 ± 0.11	4.91 ± 0.06	4.96 ± 0.04	4.97 ± 0.06
TC11003431.hg.1	DPP3	8.61 ± 0.25	8.28 ± 0.10	8.46 ± 0.20	8.16 ± 0.06

TC02002478.hg.1	DPP4	7.77 ± 0.18	8.04 ± 0.17	7.63 ± 0.19	7.76 ± 0.11
TC07001032.hg.1	DPP6	6.56 ± 0.21	6.51 ± 0.18	6.48 ± 0.14	6.62 ± 0.08
TC09001757.hg.1	DPP7	9.52 ± 0.12	9.46 ± 0.08	9.30 ± 0.11	9.44 ± 0.01
TC15001567.hg.1	DPP8	8.45 ± 0.37	8.57 ± 0.20	8.61 ± 0.36	8.47 ± 0.07
TC19001068.hg.1	DPP9	9.17 ± 0.10	9.09 ± 0.07	9.02 ± 0.04	9.11 ± 0.06
TC03001637.hg.1	DPPA2	5.00 ± 0.08	4.85 ± 0.08	4.95 ± 0.04	4.89 ± 0.07
TC03002125.hg.1	DPPA2P3	5.91 ± 0.04	5.85 ± 0.10	5.80 ± 0.09	5.81 ± 0.10
TC12000111.hg.1	DPPA3	5.42 ± 0.05	5.22 ± 0.09	5.36 ± 0.12	5.41 ± 0.09
TC0X001248.hg.1	DPPA3P1	5.47 ± 0.09	5.30 ± 0.01	5.29 ± 0.20	5.36 ± 0.12
TC14000230.hg.1	DPPA3P2	5.66 ± 0.14	5.19 ± 0.09	5.23 ± 0.08	5.33 ± 0.10
TC03001638.hg.1	DPPA4	6.07 ± 0.18	6.03 ± 0.01	6.01 ± 0.05	6.15 ± 0.10
TC06001870.hg.1	DPPA5	7.04 ± 0.18	6.97 ± 0.17	6.96 ± 0.08	7.13 ± 0.13
TC19000813.hg.1	DPRX	6.38 ± 0.07	6.34 ± 0.10	6.61 ± 0.13	6.34 ± 0.05
TC17000359.hg.1	DPRXP4	7.42 ± 0.13	6.97 ± 0.30	7.51 ± 0.40	6.83 ± 0.16
TC01003491.hg.1	DPT	5.88 ± 0.16	5.75 ± 0.16	5.81 ± 0.07	5.91 ± 0.11
TC07001274.hg.1	DPY19L1	7.39 ± 0.14	7.17 ± 0.09	7.46 ± 0.27	7.14 ± 0.12
TC07001259.hg.1	DPY19L1P1	7.68 ± 0.05	7.55 ± 0.14	7.74 ± 0.43	7.51 ± 0.21
TC12001663.hg.1	DPY19L2	5.35 ± 0.15	5.18 ± 0.14	5.20 ± 0.07	5.10 ± 0.05
TC07001275.hg.1	DPY19L2P1	5.49 ± 0.17	5.32 ± 0.08	5.33 ± 0.05	5.29 ± 0.08
TC07001717.hg.1	DPY19L2P2	6.02 ± 0.05	5.91 ± 0.03	5.92 ± 0.05	5.97 ± 0.08
TC07000187.hg.1	DPY19L2P3	5.78 ± 0.08	5.69 ± 0.04	5.76 ± 0.20	5.74 ± 0.12
TC07000535.hg.1	DPY19L2P4	5.74 ± 0.15	5.60 ± 0.10	5.73 ± 0.25	5.70 ± 0.06
TC19000426.hg.1	DPY19L3	7.46 ± 0.17	7.28 ± 0.17	7.94 ± 0.38	7.30 ± 0.07
TC08000577.hg.1	DPY19L4	6.44 ± 0.30	6.32 ± 0.19	6.26 ± 0.20	6.15 ± 0.10
TC02005024.hg.1	DPY30	8.49 ± 0.11	8.47 ± 0.11	8.48 ± 0.02	8.39 ± 0.04
TC01002903.hg.1	DPYD	9.30 ± 0.24	9.36 ± 0.25	9.75 ± 0.51	9.12 ± 0.17
TC01000885.hg.1	DPYD-AS1	6.17 ± 0.09	6.07 ± 0.12	6.28 ± 0.19	6.10 ± 0.01
TC01000886.hg.1	DPYD-AS2	4.39 ± 0.13	4.18 ± 0.08	4.25 ± 0.02	4.18 ± 0.03
TC01002904.hg.1	DPYD-IT1	7.07 ± 0.38	7.22 ± 0.16	7.46 ± 0.19	7.21 ± 0.18
TC08001509.hg.1	DPYS	6.63 ± 0.18	6.54 ± 0.13	6.58 ± 0.11	6.63 ± 0.07
TC08000203.hg.1	DPYSL2	9.42 ± 0.17	9.11 ± 0.09	9.30 ± 0.11	8.99 ± 0.24

TC05001909.hg.1	DPYSL3	6.83 ± 0.15	6.80 ± 0.16	6.77 ± 0.15	6.89 ± 0.02
TC10000941.hg.1	DPYSL4	8.05 ± 0.19	7.95 ± 0.11	7.94 ± 0.14	8.00 ± 0.01
TC02000156.hg.1	DPYSL5	7.41 ± 0.27	7.40 ± 0.20	7.36 ± 0.21	7.48 ± 0.03
TC02002000.hg.1	DQX1	6.78 ± 0.14	6.77 ± 0.17	6.74 ± 0.14	6.93 ± 0.06
TC01000861.hg.1	DR1	9.59 ± 0.31	9.70 ± 0.09	9.78 ± 0.25	9.65 ± 0.16
TC12000793.hg.1	DRAM1	7.84 ± 0.39	7.25 ± 0.23	8.50 ± 0.47	7.15 ± 0.28
TC01002980.hg.1	DRAM2	8.63 ± 0.26	8.65 ± 0.28	8.70 ± 0.23	8.49 ± 0.04
TC11000647.hg.1	DRAP1	10.07 ± 0.39	9.86 ± 0.03	9.90 ± 0.31	9.66 ± 0.10
TC01000146.hg.1	DRAXIN	7.84 ± 0.19	7.75 ± 0.07	7.80 ± 0.17	7.89 ± 0.05
TC02000151.hg.1	DRC1	6.04 ± 0.14	5.99 ± 0.05	6.01 ± 0.12	6.03 ± 0.03
TC05002083.hg.1	DRD1	6.89 ± 0.14	6.82 ± 0.07	6.89 ± 0.04	6.88 ± 0.10
TC11002302.hg.1	DRD2	7.40 ± 0.22	7.40 ± 0.23	7.34 ± 0.18	7.49 ± 0.06
TC03001667.hg.1	DRD3	6.63 ± 0.18	6.55 ± 0.20	6.57 ± 0.11	6.71 ± 0.07
TC11000023.hg.1	DRD4	9.23 ± 0.24	9.19 ± 0.17	9.12 ± 0.22	9.28 ± 0.02
TC04000114.hg.1	DRD5	8.23 ± 0.35	8.33 ± 0.28	8.27 ± 0.29	8.39 ± 0.08
TC22001422.hg.1	DRG1	8.74 ± 0.15	8.64 ± 0.18	8.46 ± 0.10	8.48 ± 0.06
TC17000204.hg.1	DRG2	8.79 ± 0.11	8.66 ± 0.06	8.51 ± 0.09	8.66 ± 0.03
TC10001275.hg.1	DRGX	7.37 ± 0.19	7.42 ± 0.23	7.35 ± 0.25	7.46 ± 0.02
TC05001223.hg.1	DROSHA	7.65 ± 0.19	7.64 ± 0.15	7.44 ± 0.15	7.41 ± 0.08
TC0X000476.hg.1	DRP2	6.56 ± 0.17	6.59 ± 0.13	6.51 ± 0.14	6.69 ± 0.07
TC18000437.hg.1	DSC1	5.98 ± 0.30	6.20 ± 0.05	5.75 ± 0.06	6.22 ± 0.07
TC18000436.hg.1	DSC2	8.42 ± 0.46	8.05 ± 0.44	9.66 ± 0.32	8.63 ± 0.22
TC18000435.hg.1	DSC3	4.72 ± 0.15	4.62 ± 0.05	4.67 ± 0.09	4.66 ± 0.05
TC21000465.hg.1	DSCAM	6.40 ± 0.15	6.31 ± 0.14	6.30 ± 0.12	6.38 ± 0.05
TC21000181.hg.1	DSCAM-AS1	5.63 ± 0.13	5.53 ± 0.06	5.56 ± 0.08	5.59 ± 0.08
TC21000467.hg.1	DSCAM-IT1	4.62 ± 0.01	4.51 ± 0.08	4.56 ± 0.12	4.63 ± 0.13
TC11002337.hg.1	DSCAML1	7.43 ± 0.17	7.41 ± 0.17	7.36 ± 0.16	7.48 ± 0.06
TC08001560.hg.1	DSCC1	5.15 ± 0.21	5.00 ± 0.18	4.99 ± 0.09	4.71 ± 0.05
TC21000166.hg.1	DSCR10	6.47 ± 0.19	6.38 ± 0.05	6.47 ± 0.03	6.44 ± 0.08
TC21000441.hg.1	DSCR3	8.32 ± 0.08	8.06 ± 0.06	8.24 ± 0.14	8.05 ± 0.08
TC21000445.hg.1	DSCR4	5.52 ± 0.12	5.33 ± 0.05	5.43 ± 0.05	5.42 ± 0.04

TC21000446.hg.1	DSCR4-IT1	5.37 ± 0.31	5.20 ± 0.08	5.19 ± 0.10	5.32 ± 0.12
TC21000165.hg.1	DSCR8	5.32 ± 0.13	5.26 ± 0.02	5.28 ± 0.07	5.33 ± 0.10
TC21000163.hg.1	DSCR9	7.45 ± 0.24	7.45 ± 0.22	7.40 ± 0.26	7.64 ± 0.10
TC06000918.hg.1	DSE	6.72 ± 0.08	6.56 ± 0.03	6.79 ± 0.04	6.70 ± 0.22
TC18000564.hg.1	DSEL	6.04 ± 0.19	5.90 ± 0.08	5.87 ± 0.05	6.00 ± 0.10
TC18000124.hg.1	DSG1	4.14 ± 0.13	3.98 ± 0.06	4.07 ± 0.04	4.05 ± 0.04
TC18000128.hg.1	DSG2	4.70 ± 0.10	4.64 ± 0.05	4.66 ± 0.06	4.66 ± 0.03
TC18000127.hg.1	DSG3	5.08 ± 0.13	5.00 ± 0.09	5.00 ± 0.08	5.04 ± 0.07
TC18000125.hg.1	DSG4	5.08 ± 0.18	4.96 ± 0.11	4.94 ± 0.13	5.04 ± 0.11
TC20000818.hg.1	DSN1	7.12 ± 0.02	7.02 ± 0.20	6.88 ± 0.07	6.81 ± 0.08
TC06000060.hg.1	DSP	6.25 ± 0.08	6.21 ± 0.06	6.07 ± 0.12	6.11 ± 0.04
TC04000473.hg.1	DSPP	3.72 ± 0.09	3.67 ± 0.04	3.72 ± 0.02	3.70 ± 0.02
TC06001828.hg.1	DST	5.47 ± 0.14	5.47 ± 0.04	5.46 ± 0.04	5.49 ± 0.03
TC20000109.hg.1	DSTN	7.86 ± 0.05	7.89 ± 0.07	7.81 ± 0.13	7.81 ± 0.10
TC12000098.hg.1	DSTNP2	7.33 ± 0.12	7.23 ± 0.15	7.23 ± 0.07	7.21 ± 0.01
TC03000199.hg.1	DSTNP4	4.53 ± 0.16	4.48 ± 0.05	4.62 ± 0.21	4.42 ± 0.20
TC01003743.hg.1	DSTYK	7.57 ± 0.01	7.48 ± 0.09	7.47 ± 0.03	7.54 ± 0.01
TC20000127.hg.1	DTD1	7.84 ± 0.03	7.85 ± 0.10	7.72 ± 0.05	7.82 ± 0.04
TC14002319.hg.1	DTD2	7.64 ± 0.09	7.60 ± 0.06	7.53 ± 0.05	7.58 ± 0.09
TC04000209.hg.1	DTHD1	6.39 ± 0.37	6.32 ± 0.19	6.31 ± 0.26	6.10 ± 0.08
TC01001767.hg.1	DTL	7.61 ± 0.39	7.25 ± 0.56	7.26 ± 0.30	6.55 ± 0.12
TC18000141.hg.1	DTNA	5.71 ± 0.10	5.64 ± 0.08	5.64 ± 0.06	5.74 ± 0.06
TC02001661.hg.1	DTNB	7.55 ± 0.06	7.51 ± 0.04	7.46 ± 0.03	7.53 ± 0.05
TC06001290.hg.1	DTNBP1	7.65 ± 0.13	7.61 ± 0.07	7.66 ± 0.10	7.50 ± 0.06
TC15000387.hg.1	DTWD1	7.19 ± 0.21	7.21 ± 0.09	7.18 ± 0.07	7.23 ± 0.03
TC05001706.hg.1	DTWD2	7.32 ± 0.21	7.22 ± 0.13	7.12 ± 0.28	7.14 ± 0.05
TC12000887.hg.1	DTX1	8.34 ± 0.13	8.23 ± 0.11	8.09 ± 0.11	8.18 ± 0.04
TC07003309.hg.1	DTX2	8.47 ± 0.08	8.34 ± 0.09	8.47 ± 0.07	8.38 ± 0.07
TC07003311.hg.1	DTX2P1-UPK3BP1-PMS2P11	9.27 ± 0.08	9.25 ± 0.14	9.11 ± 0.10	9.21 ± 0.01
TC12000536.hg.1	DTX3	7.66 ± 0.05	7.71 ± 0.10	7.54 ± 0.09	7.66 ± 0.10
TC03000632.hg.1	DTX3L	9.81 ± 0.29	9.50 ± 0.39	10.01 ± 0.44	9.24 ± 0.42

TC11000484.hg.1	DTX4	7.72 ± 0.10	7.80 ± 0.13	7.49 ± 0.05	7.94 ± 0.09
TC02002954.hg.1	DTYMK	7.90 ± 0.15	7.76 ± 0.02	7.70 ± 0.11	7.74 ± 0.05
TC15000352.hg.1	DUOX1	7.51 ± 0.20	7.49 ± 0.15	7.45 ± 0.13	7.58 ± 0.04
TC15001305.hg.1	DUOX2	7.42 ± 0.17	7.39 ± 0.20	7.39 ± 0.12	7.50 ± 0.06
TC15001306.hg.1	DUOXA1	7.76 ± 0.17	7.73 ± 0.15	7.65 ± 0.14	7.80 ± 0.10
TC15000351.hg.1	DUOXA2	7.19 ± 0.25	7.19 ± 0.20	7.13 ± 0.24	7.28 ± 0.07
TC10001427.hg.1	DUPD1	8.19 ± 0.21	7.96 ± 0.15	8.05 ± 0.16	8.02 ± 0.05
TC17001972.hg.1	DUS1L	8.86 ± 0.18	8.77 ± 0.07	8.55 ± 0.15	8.72 ± 0.05
TC16002055.hg.1	DUS2L	8.33 ± 0.21	8.12 ± 0.13	8.21 ± 0.11	8.08 ± 0.07
TC19001083.hg.1	DUS3L	8.35 ± 0.16	8.35 ± 0.13	8.15 ± 0.15	8.43 ± 0.04
TC07000692.hg.1	DUS4L	6.02 ± 0.11	6.08 ± 0.12	5.95 ± 0.07	6.04 ± 0.04
TC05002066.hg.1	DUSP1	11.49 ± 0.15	11.73 ± 0.12	11.93 ± 0.28	11.49 ± 0.41
TC01003847.hg.1	DUSP10	7.84 ± 0.13	8.38 ± 0.34	7.61 ± 0.05	8.34 ± 0.21
TC02001988.hg.1	DUSP11	8.60 ± 0.29	8.88 ± 0.16	8.58 ± 0.24	8.93 ± 0.11
TC01001413.hg.1	DUSP12	8.01 ± 0.14	8.12 ± 0.21	7.82 ± 0.13	8.00 ± 0.01
TC10001428.hg.1	DUSP13	8.04 ± 0.22	7.97 ± 0.17	7.94 ± 0.17	8.03 ± 0.06
TC17000448.hg.1	DUSP14	7.01 ± 0.14	6.97 ± 0.10	6.88 ± 0.15	6.94 ± 0.04
TC20000758.hg.1	DUSP15	7.87 ± 0.22	7.87 ± 0.20	7.80 ± 0.22	7.96 ± 0.06
TC12001255.hg.1	DUSP16	7.71 ± 0.24	7.69 ± 0.18	7.57 ± 0.25	7.64 ± 0.14
TC22000659.hg.1	DUSP18	8.00 ± 0.22	7.60 ± 0.10	8.03 ± 0.18	7.64 ± 0.18
TC02001092.hg.1	DUSP19	5.43 ± 0.10	5.31 ± 0.11	5.44 ± 0.08	5.29 ± 0.08
TC02002102.hg.1	DUSP2	9.73 ± 0.26	10.49 ± 0.08	9.29 ± 0.18	10.53 ± 0.18
TC0X000211.hg.1	DUSP21	5.66 ± 0.19	5.73 ± 0.09	5.64 ± 0.16	5.73 ± 0.05
TC06000004.hg.1	DUSP22	9.09 ± 0.21	8.98 ± 0.19	9.18 ± 0.26	8.89 ± 0.06
TC01001355.hg.1	DUSP23	10.22 ± 0.31	10.02 ± 0.12	10.05 ± 0.32	10.02 ± 0.04
TC08001121.hg.1	DUSP26	7.51 ± 0.24	7.49 ± 0.24	7.34 ± 0.21	7.50 ± 0.04
TC01001457.hg.1	DUSP27	7.23 ± 0.23	7.18 ± 0.18	7.16 ± 0.18	7.28 ± 0.05
TC02001466.hg.1	DUSP28	7.72 ± 0.06	7.67 ± 0.06	7.60 ± 0.09	7.74 ± 0.07
TC17001563.hg.1	DUSP3	8.54 ± 0.13	8.35 ± 0.10	8.60 ± 0.08	8.37 ± 0.05
TC08001099.hg.1	DUSP4	7.64 ± 0.24	7.69 ± 0.09	7.61 ± 0.12	7.74 ± 0.08
TC10000801.hg.1	DUSP5	8.63 ± 0.24	8.92 ± 0.26	8.30 ± 0.14	8.66 ± 0.17

TC01006342.hg.1	DUSP5P1	7.37 ± 0.26	7.61 ± 0.10	7.36 ± 0.35	7.64 ± 0.07
TC12001796.hg.1	DUSP6	9.63 ± 0.07	9.36 ± 0.27	9.84 ± 0.55	9.33 ± 0.48
TC03001445.hg.1	DUSP7	9.22 ± 0.19	8.97 ± 0.06	9.05 ± 0.11	8.95 ± 0.09
TC11001260.hg.1	DUSP8	8.02 ± 0.05	8.19 ± 0.14	7.90 ± 0.10	8.36 ± 0.05
TC0X000753.hg.1	DUSP9	8.66 ± 0.34	8.64 ± 0.27	8.50 ± 0.26	8.70 ± 0.03
TC15000377.hg.1	DUT	7.99 ± 0.09	7.95 ± 0.14	7.88 ± 0.12	7.71 ± 0.03
TC04000939.hg.1	DUX2	9.11 ± 0.27	9.10 ± 0.23	8.99 ± 0.23	9.38 ± 0.22
TC04000944.hg.1	DUX4	8.95 ± 0.25	9.01 ± 0.21	8.89 ± 0.29	9.36 ± 0.30
TC04000946.hg.1	DUX4	8.96 ± 0.18	8.98 ± 0.11	8.88 ± 0.27	9.27 ± 0.21
TCUn_gl000228000013.hg.1	DUX4	9.02 ± 0.20	9.02 ± 0.13	8.93 ± 0.29	9.34 ± 0.31
TC04000941.hg.1	DUX4L2	8.92 ± 0.26	8.97 ± 0.20	8.86 ± 0.31	9.30 ± 0.28
TC04000942.hg.1	DUX4L2	8.86 ± 0.18	8.86 ± 0.30	8.73 ± 0.36	9.15 ± 0.19
TC04000945.hg.1	DUX4L2	8.86 ± 0.18	8.86 ± 0.30	8.73 ± 0.36	9.15 ± 0.19
TC10000957.hg.1	DUX4L2	8.95 ± 0.20	8.95 ± 0.18	8.87 ± 0.28	9.19 ± 0.15
TCUn_gl000228000001.hg.1	DUX4L2	9.26 ± 0.18	9.25 ± 0.19	9.15 ± 0.23	9.49 ± 0.19
TCUn_gl000228000002.hg.1	DUX4L2	9.26 ± 0.18	9.25 ± 0.19	9.15 ± 0.23	9.49 ± 0.19
TCUn_gl000228000003.hg.1	DUX4L2	9.26 ± 0.18	9.25 ± 0.19	9.15 ± 0.23	9.49 ± 0.19
TCUn_gl000228000004.hg.1	DUX4L2	9.26 ± 0.18	9.25 ± 0.19	9.15 ± 0.23	9.49 ± 0.19
TCUn_gl000228000005.hg.1	DUX4L2	9.26 ± 0.18	9.25 ± 0.19	9.15 ± 0.23	9.49 ± 0.19
TCUn_gl000228000006.hg.1	DUX4L2	9.17 ± 0.20	9.16 ± 0.25	9.01 ± 0.33	9.38 ± 0.20
TCUn_gl000228000007.hg.1	DUX4L2	9.17 ± 0.20	9.16 ± 0.25	9.01 ± 0.33	9.38 ± 0.20
TCUn_gl000228000008.hg.1	DUX4L2	9.17 ± 0.20	9.16 ± 0.25	9.01 ± 0.33	9.38 ± 0.20
TCUn_gl000228000009.hg.1	DUX4L2	9.26 ± 0.18	9.25 ± 0.19	9.15 ± 0.23	9.49 ± 0.19
TCUn_gl000228000010.hg.1	DUX4L2	9.26 ± 0.18	9.25 ± 0.19	9.15 ± 0.23	9.49 ± 0.19
TC04000943.hg.1	DUX4L4	8.80 ± 0.21	8.81 ± 0.23	8.71 ± 0.25	9.03 ± 0.15
TC19001894.hg.1	DUXA	5.75 ± 0.19	5.65 ± 0.11	5.63 ± 0.06	5.69 ± 0.07
TC01002092.hg.1	DVL1	8.81 ± 0.22	8.75 ± 0.11	8.68 ± 0.16	8.77 ± 0.01
TC17001079.hg.1	DVL2	8.20 ± 0.15	8.13 ± 0.07	7.97 ± 0.15	8.11 ± 0.03
TC03000993.hg.1	DVL3	9.32 ± 0.05	9.23 ± 0.03	9.30 ± 0.10	9.26 ± 0.09
TC10001463.hg.1	DYDC1	5.94 ± 0.20	6.00 ± 0.10	6.01 ± 0.13	6.07 ± 0.05
TC10000580.hg.1	DYDC2	6.36 ± 0.14	6.20 ± 0.07	6.30 ± 0.09	6.31 ± 0.05

TC18000501.hg.1	DYM	8.79 ± 0.25	8.95 ± 0.25	8.67 ± 0.19	8.76 ± 0.01
TC18000188.hg.1	DYNAP	4.97 ± 0.15	4.93 ± 0.09	4.96 ± 0.21	4.96 ± 0.13
TC14000777.hg.1	DYNC1H1	9.12 ± 0.22	9.13 ± 0.19	8.86 ± 0.15	9.03 ± 0.07
TC07000571.hg.1	DYNC1I1	6.10 ± 0.13	6.07 ± 0.07	6.10 ± 0.11	6.15 ± 0.07
TC02001022.hg.1	DYNC1I2	7.84 ± 0.14	7.73 ± 0.13	7.78 ± 0.19	7.52 ± 0.09
TC03001271.hg.1	DYNC1LI1	8.87 ± 0.03	8.81 ± 0.11	9.05 ± 0.29	8.73 ± 0.28
TC16001172.hg.1	DYNC1LI2	8.59 ± 0.18	8.58 ± 0.06	8.77 ± 0.28	8.69 ± 0.03
TC11000963.hg.1	DYNC2H1	4.68 ± 0.07	4.62 ± 0.06	4.60 ± 0.06	4.60 ± 0.05
TC02000271.hg.1	DYNC2LI1	5.60 ± 0.11	5.59 ± 0.05	5.62 ± 0.07	5.57 ± 0.08
TC12000946.hg.1	DYNLL1	8.49 ± 0.09	8.19 ± 0.08	8.35 ± 0.23	7.98 ± 0.19
TC12002046.hg.1	DYNLL1-AS1	6.88 ± 0.25	7.03 ± 0.38	7.03 ± 0.25	7.13 ± 0.12
TC17000707.hg.1	DYNLL2	9.26 ± 0.05	9.26 ± 0.10	9.06 ± 0.11	9.08 ± 0.11
TC20000242.hg.1	DYNLRB1	9.51 ± 0.20	9.13 ± 0.03	9.47 ± 0.16	9.02 ± 0.14
TC16000633.hg.1	DYNLRB2	5.86 ± 0.06	5.58 ± 0.06	5.86 ± 0.10	5.67 ± 0.04
TC06002263.hg.1	DYNLT1	9.19 ± 0.35	8.70 ± 0.25	9.37 ± 0.51	8.63 ± 0.16
TC0X000952.hg.1	DYNLT3	7.71 ± 0.34	8.00 ± 0.06	7.94 ± 0.33	8.06 ± 0.22
TC21000164.hg.1	DYRK1A	9.68 ± 0.28	9.94 ± 0.09	9.79 ± 0.33	9.90 ± 0.04
TC19001525.hg.1	DYRK1B	8.74 ± 0.18	8.48 ± 0.10	8.53 ± 0.10	8.46 ± 0.06
TC12000597.hg.1	DYRK2	9.50 ± 0.03	9.59 ± 0.06	9.41 ± 0.03	9.56 ± 0.01
TC01001725.hg.1	DYRK3	7.67 ± 0.27	7.50 ± 0.17	7.57 ± 0.24	7.56 ± 0.04
TC12000063.hg.1	DYRK4	6.52 ± 0.07	6.49 ± 0.04	6.38 ± 0.05	6.36 ± 0.01
TC02000432.hg.1	DYSF	9.73 ± 0.54	9.22 ± 0.13	10.52 ± 0.34	9.03 ± 0.35
TC02002701.hg.1	DYTN	6.59 ± 0.12	6.29 ± 0.13	6.48 ± 0.07	6.44 ± 0.11
TC15002775.hg.1	DYX1C1	4.98 ± 0.11	4.90 ± 0.03	5.02 ± 0.09	4.89 ± 0.02
TC20000668.hg.1	DZANK1	6.15 ± 0.08	6.10 ± 0.07	6.09 ± 0.03	6.17 ± 0.05
TC13000804.hg.1	DZIP1	5.13 ± 0.12	5.02 ± 0.04	5.06 ± 0.04	5.06 ± 0.02
TC03001819.hg.1	DZIP1L	7.19 ± 0.20	7.10 ± 0.15	7.14 ± 0.18	7.18 ± 0.08
TC03000548.hg.1	DZIP3	6.72 ± 0.64	6.98 ± 0.22	6.62 ± 0.51	6.76 ± 0.15
TC20000773.hg.1	E2F1	8.17 ± 0.15	7.90 ± 0.14	8.07 ± 0.17	7.87 ± 0.02
TC01002345.hg.1	E2F2	8.28 ± 0.31	7.87 ± 0.13	8.11 ± 0.19	7.84 ± 0.02
TC06000128.hg.1	E2F3	8.14 ± 0.18	7.78 ± 0.16	8.33 ± 0.19	7.57 ± 0.30

TC06000130.hg.1	E2F3-IT1	7.16 ± 0.16	6.96 ± 0.10	7.12 ± 0.40	6.87 ± 0.03
TC16000533.hg.1	E2F4	9.44 ± 0.21	9.19 ± 0.07	9.24 ± 0.11	9.06 ± 0.06
TC08000529.hg.1	E2F5	6.81 ± 0.17	6.63 ± 0.22	6.63 ± 0.17	6.40 ± 0.03
TC02001579.hg.1	E2F6	6.20 ± 0.15	6.09 ± 0.06	6.07 ± 0.05	6.15 ± 0.02
TC12001756.hg.1	E2F7	6.48 ± 0.12	6.38 ± 0.11	6.25 ± 0.21	6.29 ± 0.09
TC11001478.hg.1	E2F8	7.12 ± 0.20	6.70 ± 0.23	6.97 ± 0.28	6.49 ± 0.01
TC16000065.hg.1	E4F1	8.66 ± 0.14	8.53 ± 0.10	8.55 ± 0.11	8.57 ± 0.03
TC03000096.hg.1	EAF1	8.29 ± 0.09	8.31 ± 0.19	8.23 ± 0.20	8.29 ± 0.10
TC03001210.hg.1	EAF1-AS1	6.61 ± 0.08	6.54 ± 0.04	6.58 ± 0.07	6.53 ± 0.06
TC03000624.hg.1	EAF2	7.35 ± 0.19	7.26 ± 0.25	7.39 ± 0.27	6.91 ± 0.23
TC14001024.hg.1	EAPP	9.47 ± 0.28	9.72 ± 0.31	9.40 ± 0.35	9.75 ± 0.07
TC16000965.hg.1	EARS2	7.21 ± 0.11	7.07 ± 0.07	6.96 ± 0.12	7.11 ± 0.03
TC08000669.hg.1	EBAG9	8.07 ± 0.23	8.15 ± 0.12	7.93 ± 0.40	8.09 ± 0.22
TC05001995.hg.1	EBF1	7.91 ± 0.22	8.12 ± 0.09	7.55 ± 0.21	8.17 ± 0.11
TC08001073.hg.1	EBF2	6.43 ± 0.14	6.34 ± 0.17	6.35 ± 0.08	6.42 ± 0.06
TC10001761.hg.1	EBF3	6.64 ± 0.18	6.63 ± 0.11	6.60 ± 0.15	6.68 ± 0.04
TC20000028.hg.1	EBF4	8.24 ± 0.21	8.19 ± 0.17	8.13 ± 0.19	8.23 ± 0.04
TC19000083.hg.1	EBI3	7.83 ± 0.25	7.81 ± 0.32	7.71 ± 0.33	7.93 ± 0.11
TC10001098.hg.1	EBLN1	4.67 ± 0.26	4.84 ± 0.13	4.77 ± 0.15	4.93 ± 0.16
TC03000429.hg.1	EBLN2	7.85 ± 0.30	7.90 ± 0.10	8.24 ± 0.35	7.78 ± 0.15
TC01002580.hg.1	EBNA1BP2	7.34 ± 0.09	7.33 ± 0.06	7.21 ± 0.02	7.28 ± 0.04
TC0X000253.hg.1	EBP	8.20 ± 0.14	8.13 ± 0.14	7.95 ± 0.16	7.97 ± 0.09
TC13000662.hg.1	EBPL	6.87 ± 0.08	6.72 ± 0.11	6.92 ± 0.21	6.63 ± 0.05
TC10001402.hg.1	ECD	10.06 ± 0.18	10.18 ± 0.13	10.11 ± 0.09	10.20 ± 0.06
TC10001403.hg.1	ECD	8.80 ± 0.25	9.06 ± 0.21	8.74 ± 0.30	9.05 ± 0.12
TC01002322.hg.1	ECE1	10.04 ± 0.34	9.88 ± 0.11	10.29 ± 0.26	9.91 ± 0.31
TC03000998.hg.1	ECE2	7.61 ± 0.23	7.55 ± 0.20	7.54 ± 0.17	7.63 ± 0.06
TC02002874.hg.1	ECEL1	7.70 ± 0.20	7.64 ± 0.16	7.57 ± 0.19	7.67 ± 0.05
TC02002872.hg.1	ECEL1P2	7.27 ± 0.26	7.14 ± 0.14	7.11 ± 0.29	7.25 ± 0.08
TC19002698.hg.1	ECH1	10.84 ± 0.26	10.53 ± 0.11	10.54 ± 0.16	10.31 ± 0.08
TC06002088.hg.1	ECHDC1	7.29 ± 0.33	7.47 ± 0.20	7.42 ± 0.21	7.20 ± 0.09

TC01002662.hg.1	ECHDC2	7.83 ± 0.13	7.75 ± 0.04	7.68 ± 0.09	7.80 ± 0.05
TC10000086.hg.1	ECHDC3	7.55 ± 0.23	7.44 ± 0.04	7.51 ± 0.08	7.57 ± 0.11
TC10001790.hg.1	ECHS1	8.41 ± 0.21	8.21 ± 0.04	8.18 ± 0.19	8.08 ± 0.01
TC16000781.hg.1	ECI1	8.22 ± 0.27	8.17 ± 0.19	8.11 ± 0.21	8.24 ± 0.01
TC06001235.hg.1	ECI2	7.51 ± 0.08	7.51 ± 0.04	7.33 ± 0.06	7.45 ± 0.08
TC01001188.hg.1	ECM1	7.53 ± 0.18	7.43 ± 0.18	7.48 ± 0.15	7.59 ± 0.10
TC09001337.hg.1	ECM2	4.30 ± 0.10	4.17 ± 0.01	4.21 ± 0.03	4.21 ± 0.02
TC14002287.hg.1	ECRP	7.99 ± 0.37	7.69 ± 0.16	8.60 ± 0.11	7.63 ± 0.18
TC05003431.hg.1	ECSCR	7.25 ± 0.10	7.21 ± 0.11	7.22 ± 0.06	7.25 ± 0.03
TC19002679.hg.1	ECSIT	7.76 ± 0.29	7.76 ± 0.17	7.63 ± 0.23	7.79 ± 0.05
TC03000930.hg.1	ECT2	5.94 ± 0.12	5.93 ± 0.32	5.99 ± 0.26	5.45 ± 0.20
TC06001033.hg.1	ECT2L	4.75 ± 0.10	4.64 ± 0.08	4.66 ± 0.04	4.67 ± 0.02
TC0X000365.hg.1	EDA	5.81 ± 0.21	5.68 ± 0.10	5.69 ± 0.09	5.70 ± 0.04
TC0X001101.hg.1	EDA2R	6.28 ± 0.11	6.32 ± 0.22	6.36 ± 0.12	6.35 ± 0.07
TC02002183.hg.1	EDAR	8.09 ± 0.09	8.14 ± 0.10	8.00 ± 0.09	8.21 ± 0.06
TC01001953.hg.1	EDARADD	5.61 ± 0.07	5.58 ± 0.06	5.68 ± 0.11	5.55 ± 0.04
TC15001646.hg.1	EDC3	8.38 ± 0.12	8.14 ± 0.08	8.15 ± 0.07	8.12 ± 0.02
TC16002080.hg.1	EDC4	8.91 ± 0.10	8.81 ± 0.04	8.71 ± 0.09	8.86 ± 0.03
TC14000068.hg.1	EDDM3A	4.91 ± 0.24	4.83 ± 0.09	4.79 ± 0.16	4.93 ± 0.22
TC14000069.hg.1	EDDM3B	5.75 ± 0.18	5.75 ± 0.10	5.68 ± 0.21	5.80 ± 0.04
TC03000017.hg.1	EDEM1	9.53 ± 0.08	9.46 ± 0.16	9.30 ± 0.23	9.38 ± 0.08
TC20001746.hg.1	EDEM2	8.63 ± 0.36	8.26 ± 0.15	8.69 ± 0.38	8.11 ± 0.09
TC01003623.hg.1	EDEM3	8.70 ± 0.29	8.59 ± 0.20	8.88 ± 0.41	8.33 ± 0.23
TC09001744.hg.1	EDF1	9.48 ± 0.18	9.35 ± 0.10	9.15 ± 0.12	9.23 ± 0.05
TC05001558.hg.1	EDIL3	5.00 ± 0.10	4.92 ± 0.10	4.94 ± 0.08	5.01 ± 0.05
TC06000087.hg.1	EDN1	6.55 ± 0.17	6.61 ± 0.09	6.63 ± 0.04	6.60 ± 0.12
TC01002567.hg.1	EDN2	7.39 ± 0.21	7.40 ± 0.20	7.37 ± 0.18	7.49 ± 0.07
TC20000470.hg.1	EDN3	6.97 ± 0.25	7.00 ± 0.24	6.96 ± 0.27	7.12 ± 0.05
TC04000729.hg.1	EDNRA	5.54 ± 0.16	5.45 ± 0.09	5.49 ± 0.06	5.51 ± 0.02
TC13000748.hg.1	EDNRB	5.97 ± 0.15	5.86 ± 0.13	6.04 ± 0.03	6.07 ± 0.13
TC12001811.hg.1	EEA1	7.57 ± 0.70	7.97 ± 0.29	7.98 ± 0.54	7.65 ± 0.29

TC11000870.hg.1	EED	6.69 ± 0.21	6.59 ± 0.23	6.54 ± 0.15	6.48 ± 0.05
TC06001875.hg.1	EEF1A1	12.41 ± 0.06	12.54 ± 0.08	12.26 ± 0.03	12.51 ± 0.05
TC20001032.hg.1	EEF1A2	8.44 ± 0.32	8.36 ± 0.18	8.31 ± 0.29	8.42 ± 0.05
TC15000425.hg.1	EEF1B2P1	6.66 ± 0.03	6.83 ± 0.22	6.49 ± 0.24	6.71 ± 0.07
TC08001721.hg.1	EEF1D	9.83 ± 0.11	9.93 ± 0.04	9.63 ± 0.09	9.91 ± 0.03
TC19000251.hg.1	EEF1DP1	10.78 ± 0.17	10.73 ± 0.11	10.46 ± 0.12	10.74 ± 0.14
TC13000107.hg.1	EEF1DP3	6.78 ± 0.08	6.76 ± 0.05	6.77 ± 0.04	6.72 ± 0.02
TC06004111.hg.1	EEF1E1	6.77 ± 0.22	6.78 ± 0.14	6.66 ± 0.17	6.68 ± 0.06
TC11003483.hg.1	EEF1G	11.67 ± 0.14	11.73 ± 0.16	11.27 ± 0.18	11.65 ± 0.05
TC19001054.hg.1	EEF2	12.14 ± 0.06	12.27 ± 0.12	11.72 ± 0.07	12.24 ± 0.10
TC16000243.hg.1	EEF2K	8.04 ± 0.08	7.93 ± 0.03	7.93 ± 0.15	8.00 ± 0.04
TC03000672.hg.1	EEFSEC	8.69 ± 0.16	8.62 ± 0.04	8.50 ± 0.15	8.59 ± 0.04
TC07000234.hg.1	EEPD1	7.82 ± 0.13	7.80 ± 0.01	7.87 ± 0.13	7.83 ± 0.06
TC08001198.hg.1	EFCAB1	5.25 ± 0.09	5.18 ± 0.07	5.24 ± 0.04	5.23 ± 0.06
TC07001735.hg.1	EFCAB10	5.67 ± 0.05	5.60 ± 0.07	5.64 ± 0.05	5.64 ± 0.03
TC14002332.hg.1	EFCAB11	5.97 ± 0.16	5.87 ± 0.05	5.83 ± 0.10	5.93 ± 0.06
TC03001770.hg.1	EFCAB12	7.00 ± 0.11	6.93 ± 0.03	6.99 ± 0.06	7.05 ± 0.04
TC17002879.hg.1	EFCAB13	6.23 ± 0.17	6.15 ± 0.27	6.12 ± 0.18	6.09 ± 0.08
TC01006356.hg.1	EFCAB14	9.48 ± 0.26	9.45 ± 0.23	9.33 ± 0.22	9.24 ± 0.13
TC01000591.hg.1	EFCAB14-AS1	4.47 ± 0.14	4.50 ± 0.07	4.61 ± 0.18	4.44 ± 0.11
TC01001993.hg.1	EFCAB2	7.37 ± 0.16	7.01 ± 0.12	7.38 ± 0.09	7.03 ± 0.06
TC17000754.hg.1	EFCAB3	4.70 ± 0.11	4.58 ± 0.03	4.65 ± 0.13	4.62 ± 0.05
TC11000033.hg.1	EFCAB4A	8.50 ± 0.25	8.48 ± 0.22	8.38 ± 0.23	8.55 ± 0.05
TC12001115.hg.1	EFCAB4B	7.89 ± 0.10	7.88 ± 0.02	7.79 ± 0.02	7.88 ± 0.02
TC17000346.hg.1	EFCAB5	5.44 ± 0.09	5.31 ± 0.04	5.37 ± 0.04	5.33 ± 0.02
TC22000863.hg.1	EFCAB6	5.56 ± 0.15	5.46 ± 0.11	5.46 ± 0.09	5.51 ± 0.04
TC22000368.hg.1	EFCAB6-AS1	6.56 ± 0.18	6.59 ± 0.19	6.67 ± 0.11	6.60 ± 0.08
TC01000720.hg.1	EFCAB7	5.84 ± 0.22	5.85 ± 0.11	5.82 ± 0.21	5.77 ± 0.05
TC20000217.hg.1	EFCAB8	6.46 ± 0.08	6.50 ± 0.09	6.51 ± 0.07	6.55 ± 0.05
TC20000218.hg.1	EFCAB8	6.27 ± 0.30	6.30 ± 0.09	6.30 ± 0.11	6.41 ± 0.06
TC20000219.hg.1	EFCAB8	7.24 ± 0.16	7.29 ± 0.16	7.14 ± 0.14	7.32 ± 0.15

TC05000946.hg.1	EFCAB9	4.44 ± 0.22	4.39 ± 0.04	4.35 ± 0.15	4.35 ± 0.03
TC03000685.hg.1	EFCC1	7.96 ± 0.18	7.89 ± 0.18	7.95 ± 0.17	8.00 ± 0.08
TC02001867.hg.1	EFEMP1	5.76 ± 0.23	5.73 ± 0.15	5.77 ± 0.11	5.82 ± 0.06
TC11001946.hg.1	EFEMP2	8.01 ± 0.10	7.99 ± 0.08	8.12 ± 0.02	8.03 ± 0.04
TC03001225.hg.1	EFHB	5.16 ± 0.09	5.01 ± 0.04	5.08 ± 0.01	5.05 ± 0.05
TC06000655.hg.1	EFHC1	6.57 ± 0.23	6.66 ± 0.15	6.49 ± 0.25	6.61 ± 0.11
TC0X000980.hg.1	EFHC2	6.32 ± 0.32	6.23 ± 0.21	6.43 ± 0.31	6.26 ± 0.17
TC02001405.hg.1	EFHD1	7.31 ± 0.25	7.33 ± 0.19	7.27 ± 0.18	7.41 ± 0.03
TC01000187.hg.1	EFHD2	10.67 ± 0.29	10.40 ± 0.05	10.74 ± 0.06	10.30 ± 0.14
TC01001288.hg.1	EFNA1	7.03 ± 0.22	7.06 ± 0.23	6.94 ± 0.16	7.17 ± 0.05
TC19002616.hg.1	EFNA2	8.19 ± 0.18	8.16 ± 0.24	8.09 ± 0.14	8.28 ± 0.03
TC01006333.hg.1	EFNA3	8.13 ± 0.25	8.01 ± 0.22	7.99 ± 0.15	8.11 ± 0.07
TC01006332.hg.1	EFNA4	7.56 ± 0.19	7.25 ± 0.02	7.43 ± 0.07	7.25 ± 0.07
TC05001657.hg.1	EFNA5	6.29 ± 0.18	6.16 ± 0.05	6.21 ± 0.03	6.24 ± 0.09
TC0X000362.hg.1	EFNB1	7.83 ± 0.03	7.69 ± 0.06	7.62 ± 0.08	7.77 ± 0.09
TC13000860.hg.1	EFNB2	6.38 ± 0.23	6.42 ± 0.10	6.36 ± 0.02	6.44 ± 0.04
TC17000114.hg.1	EFNB3	7.51 ± 0.26	7.48 ± 0.13	7.43 ± 0.25	7.58 ± 0.07
TC08000767.hg.1	EFR3A	8.69 ± 0.44	8.78 ± 0.26	8.77 ± 0.47	8.59 ± 0.17
TC02000141.hg.1	EFR3B	7.27 ± 0.15	7.26 ± 0.18	7.18 ± 0.17	7.31 ± 0.03
TC14000956.hg.1	EFS	7.68 ± 0.20	7.63 ± 0.17	7.62 ± 0.18	7.74 ± 0.08
TC15001736.hg.1	EFTUD1	7.75 ± 0.17	7.85 ± 0.19	7.62 ± 0.10	7.58 ± 0.09
TC15000802.hg.1	EFTUD1P1	7.53 ± 0.14	7.70 ± 0.13	7.42 ± 0.03	7.47 ± 0.03
TC17001586.hg.1	EFTUD2	9.25 ± 0.11	9.06 ± 0.15	8.94 ± 0.06	8.96 ± 0.02
TC04000568.hg.1	EGF	6.83 ± 0.16	6.33 ± 0.04	6.97 ± 0.30	6.44 ± 0.17
TC03000897.hg.1	EGFEM1P	5.51 ± 0.08	5.43 ± 0.07	5.44 ± 0.04	5.50 ± 0.10
TC0X000057.hg.1	EGFL6	6.41 ± 0.22	6.36 ± 0.13	6.31 ± 0.12	6.41 ± 0.03
TC09000817.hg.1	EGFL7	8.72 ± 0.22	8.72 ± 0.22	8.72 ± 0.23	8.83 ± 0.05
TC06000394.hg.1	EGFL8	7.93 ± 0.12	7.91 ± 0.11	7.76 ± 0.13	7.98 ± 0.06
TC6_apd_hap1000043.hg.1	EGFL8	7.95 ± 0.13	7.92 ± 0.11	7.78 ± 0.14	7.99 ± 0.06
TC6_cox_hap2000086.hg.1	EGFL8	7.95 ± 0.13	7.92 ± 0.11	7.77 ± 0.13	7.99 ± 0.06
TC6_mcf_hap5000072.hg.1	EGFL8	7.94 ± 0.13	7.92 ± 0.12	7.77 ± 0.13	7.99 ± 0.06

TC6_qbl_hap6000077.hg.1	EGFL8	7.95 ± 0.13	7.92 ± 0.11	7.77 ± 0.14	7.99 ± 0.06
TC6_ssto_hap7000072.hg.1	EGFL8	7.95 ± 0.13	7.92 ± 0.11	7.78 ± 0.14	7.99 ± 0.06
TC05000174.hg.1	EGFLAM	6.43 ± 0.17	6.33 ± 0.08	6.34 ± 0.10	6.42 ± 0.08
TC05001287.hg.1	EGFLAM-AS1	8.00 ± 0.40	7.88 ± 0.16	7.98 ± 0.29	8.01 ± 0.10
TC05001286.hg.1	EGFLAM-AS2	4.50 ± 0.11	4.39 ± 0.11	4.38 ± 0.05	4.40 ± 0.07
TC05001285.hg.1	EGFLAM-AS3	4.96 ± 0.10	4.85 ± 0.09	4.80 ± 0.15	4.89 ± 0.02
TC05001284.hg.1	EGFLAM-AS4	5.30 ± 0.24	5.22 ± 0.09	5.21 ± 0.15	5.24 ± 0.05
TC07000328.hg.1	EGFR	6.45 ± 0.16	6.42 ± 0.13	6.41 ± 0.11	6.51 ± 0.06
TC07001396.hg.1	EGFR-AS1	6.17 ± 0.19	6.16 ± 0.07	6.14 ± 0.24	6.22 ± 0.07
TC01003968.hg.1	EGLN1	9.64 ± 0.16	9.69 ± 0.23	10.05 ± 0.30	9.39 ± 0.33
TC19002634.hg.1	EGLN2	9.47 ± 0.10	9.30 ± 0.04	9.38 ± 0.17	9.22 ± 0.04
TC14001022.hg.1	EGLN3	6.70 ± 0.08	6.56 ± 0.08	6.66 ± 0.12	6.66 ± 0.04
TC03001128.hg.1	EGOT	6.03 ± 0.13	6.00 ± 0.06	6.13 ± 0.12	6.27 ± 0.16
TC05000701.hg.1	EGR1	10.46 ± 0.66	10.88 ± 0.52	11.09 ± 0.28	11.39 ± 0.50
TC10001327.hg.1	EGR2	8.25 ± 0.33	8.28 ± 0.35	8.29 ± 0.12	8.55 ± 0.18
TC08001044.hg.1	EGR3	8.44 ± 0.45	8.60 ± 0.41	8.79 ± 0.25	8.97 ± 0.24
TC02001983.hg.1	EGR4	7.98 ± 0.14	8.17 ± 0.26	8.08 ± 0.19	8.44 ± 0.15
TC02000360.hg.1	EHBP1	6.63 ± 0.22	6.73 ± 0.13	6.59 ± 0.12	6.57 ± 0.09
TC11000634.hg.1	EHBP1L1	9.23 ± 0.24	9.07 ± 0.06	9.43 ± 0.20	9.01 ± 0.10
TC11001915.hg.1	EHD1	9.32 ± 0.20	9.19 ± 0.07	9.35 ± 0.11	9.22 ± 0.08
TC19000681.hg.1	EHD2	8.23 ± 0.26	8.12 ± 0.22	8.18 ± 0.18	8.23 ± 0.05
TC02000207.hg.1	EHD3	9.31 ± 0.08	8.94 ± 0.05	9.44 ± 0.09	8.98 ± 0.12
TC15001257.hg.1	EHD4	8.20 ± 0.19	8.14 ± 0.08	8.05 ± 0.10	8.15 ± 0.17
TC11000329.hg.1	EHF	5.66 ± 0.17	5.65 ± 0.13	5.61 ± 0.09	5.74 ± 0.08
TC03002076.hg.1	EHHADH	5.92 ± 0.07	5.77 ± 0.09	5.82 ± 0.05	5.80 ± 0.02
TC03001009.hg.1	EHHADH-AS1	6.51 ± 0.18	6.42 ± 0.06	6.45 ± 0.09	6.51 ± 0.07
TC09000852.hg.1	EHMT1	8.54 ± 0.03	8.47 ± 0.04	8.40 ± 0.02	8.43 ± 0.01
TC09000853.hg.1	EHMT1-IT1	7.77 ± 0.33	7.91 ± 0.04	7.72 ± 0.13	8.02 ± 0.29
TC06001550.hg.1	EHMT2	8.49 ± 0.09	8.41 ± 0.02	8.24 ± 0.13	8.34 ± 0.05
TC6_apd_hap1000091.hg.1	EHMT2	8.50 ± 0.09	8.41 ± 0.02	8.25 ± 0.13	8.34 ± 0.05
TC6_cox_hap2000177.hg.1	EHMT2	8.49 ± 0.09	8.40 ± 0.02	8.24 ± 0.13	8.34 ± 0.05

TC6_dbb_hap3000166.hg.1	EHMT2	8.49 ± 0.09	8.41 ± 0.02	8.24 ± 0.13	8.34 ± 0.05
TC6_mcf_hap5000152.hg.1	EHMT2	8.50 ± 0.09	8.41 ± 0.02	8.25 ± 0.13	8.34 ± 0.05
TC6_qbl_hap6000167.hg.1	EHMT2	8.50 ± 0.10	8.41 ± 0.02	8.25 ± 0.13	8.35 ± 0.05
TC6_ssto_hap7000146.hg.1	EHMT2	8.61 ± 0.11	8.51 ± 0.02	8.33 ± 0.13	8.43 ± 0.05
TC06000387.hg.1	EHMT2-AS1	8.26 ± 0.32	8.09 ± 0.08	8.17 ± 0.11	8.06 ± 0.04
TC11001157.hg.1	EI24	8.19 ± 0.10	8.01 ± 0.16	7.77 ± 0.18	7.91 ± 0.03
TC15000381.hg.1	EID1	8.82 ± 0.14	8.94 ± 0.03	8.75 ± 0.11	8.90 ± 0.08
TC19001523.hg.1	EID2	8.58 ± 0.15	8.41 ± 0.07	8.38 ± 0.28	8.41 ± 0.05
TC19001522.hg.1	EID2B	6.77 ± 0.33	6.62 ± 0.12	6.55 ± 0.04	6.56 ± 0.03
TC12000811.hg.1	EID3	7.99 ± 0.41	8.21 ± 0.10	7.85 ± 0.37	8.31 ± 0.19
TC17000514.hg.1	EIF1	10.83 ± 0.19	11.37 ± 0.15	10.78 ± 0.17	11.33 ± 0.07
TC11001950.hg.1	EIF1AD	8.69 ± 0.14	8.54 ± 0.13	8.48 ± 0.11	8.61 ± 0.09
TC0X000909.hg.1	EIF1AX	8.81 ± 0.39	9.21 ± 0.10	8.60 ± 0.46	9.13 ± 0.17
TC0X000094.hg.1	EIF1AX-AS1	5.30 ± 0.02	5.19 ± 0.23	5.10 ± 0.17	5.11 ± 0.14
TC0Y000072.hg.1	EIF1AY	7.42 ± 0.60	7.51 ± 0.14	7.53 ± 0.39	7.46 ± 0.40
TC03000207.hg.1	EIF1B	8.65 ± 0.26	8.70 ± 0.20	8.82 ± 0.24	8.81 ± 0.28
TC03001305.hg.1	EIF1B-AS1	6.42 ± 0.16	6.34 ± 0.05	6.60 ± 0.20	6.24 ± 0.07
TC03000815.hg.1	EIF2A	8.88 ± 0.24	8.98 ± 0.20	8.76 ± 0.29	8.90 ± 0.15
TC07001126.hg.1	EIF2AK1	9.71 ± 0.19	9.47 ± 0.15	9.69 ± 0.11	9.39 ± 0.14
TC02001739.hg.1	EIF2AK2	10.22 ± 0.42	10.08 ± 0.62	10.69 ± 0.55	9.94 ± 0.39
TC02002074.hg.1	EIF2AK3	8.29 ± 0.22	8.56 ± 0.23	7.86 ± 0.25	8.49 ± 0.14
TC15000272.hg.1	EIF2AK4	7.82 ± 0.13	7.76 ± 0.21	7.52 ± 0.18	7.61 ± 0.05
TC12002092.hg.1	EIF2B1	8.99 ± 0.11	8.98 ± 0.16	8.78 ± 0.09	8.84 ± 0.05
TC14000466.hg.1	EIF2B2	8.92 ± 0.12	8.55 ± 0.07	8.66 ± 0.15	8.38 ± 0.07
TC01002603.hg.1	EIF2B3	7.38 ± 0.09	7.36 ± 0.07	7.21 ± 0.10	7.27 ± 0.04
TC02001683.hg.1	EIF2B4	8.16 ± 0.04	8.00 ± 0.02	8.06 ± 0.01	7.99 ± 0.01
TC03000992.hg.1	EIF2B5	7.84 ± 0.05	7.76 ± 0.07	7.58 ± 0.08	7.75 ± 0.02
TC03002071.hg.1	EIF2B5-AS1	6.58 ± 0.30	6.38 ± 0.12	6.41 ± 0.23	6.37 ± 0.06
TC03001006.hg.1	EIF2B5-IT1	6.18 ± 0.26	5.97 ± 0.16	5.94 ± 0.22	6.07 ± 0.08
TC01003763.hg.1	EIF2D	8.49 ± 0.06	8.45 ± 0.09	8.32 ± 0.04	8.41 ± 0.03
TC14000406.hg.1	EIF2S1	8.04 ± 0.23	8.06 ± 0.14	7.99 ± 0.19	7.74 ± 0.20

TC20000779.hg.1	EIF2S2	8.86 ± 0.08	8.71 ± 0.17	8.80 ± 0.20	8.37 ± 0.19
TC10001532.hg.1	EIF2S2P3	6.36 ± 0.10	6.48 ± 0.04	6.52 ± 0.05	6.32 ± 0.17
TC0X000114.hg.1	EIF2S3	10.57 ± 0.24	10.87 ± 0.15	10.59 ± 0.22	10.85 ± 0.11
TC12000162.hg.1	EIF2S3L	6.13 ± 0.29	6.07 ± 0.06	6.04 ± 0.11	6.13 ± 0.08
TC10001699.hg.1	EIF3A	9.75 ± 0.31	9.91 ± 0.10	9.70 ± 0.28	9.76 ± 0.18
TC07000030.hg.1	EIF3B	8.58 ± 0.04	8.54 ± 0.17	8.23 ± 0.10	8.50 ± 0.06
TC16000305.hg.1	EIF3C	10.07 ± 0.04	9.99 ± 0.04	9.68 ± 0.07	9.91 ± 0.12
TC16000986.hg.1	EIF3CL	10.02 ± 0.07	9.90 ± 0.04	9.61 ± 0.07	9.83 ± 0.11
TC22000711.hg.1	EIF3D	8.90 ± 0.10	8.92 ± 0.17	8.64 ± 0.09	8.89 ± 0.03
TC08001524.hg.1	EIF3E	7.47 ± 0.49	7.98 ± 0.16	7.46 ± 0.29	7.97 ± 0.04
TC11000154.hg.1	EIF3F	9.61 ± 0.10	9.66 ± 0.22	9.26 ± 0.10	9.59 ± 0.08
TC02000332.hg.1	EIF3FP3	8.94 ± 0.17	8.84 ± 0.28	8.27 ± 0.30	8.75 ± 0.24
TC19001158.hg.1	EIF3G	10.26 ± 0.19	10.18 ± 0.13	9.92 ± 0.12	10.05 ± 0.06
TC08001545.hg.1	EIF3H	8.28 ± 0.22	8.64 ± 0.16	8.21 ± 0.14	8.59 ± 0.04
TC01000424.hg.1	EIF3I	9.12 ± 0.26	8.92 ± 0.19	8.79 ± 0.25	8.74 ± 0.08
TC07001756.hg.1	EIF3IP1	6.62 ± 0.11	6.66 ± 0.09	6.62 ± 0.17	6.71 ± 0.19
TC15000340.hg.1	EIF3J	8.90 ± 0.26	8.99 ± 0.13	8.77 ± 0.18	8.95 ± 0.18
TC15001295.hg.1	EIF3J-AS1	7.02 ± 0.10	6.94 ± 0.07	7.03 ± 0.09	7.02 ± 0.08
TC19000531.hg.1	EIF3K	10.19 ± 0.28	10.06 ± 0.27	9.81 ± 0.34	9.91 ± 0.07
TC22000291.hg.1	EIF3L	9.52 ± 0.12	9.66 ± 0.23	9.28 ± 0.06	9.63 ± 0.08
TC11000309.hg.1	EIF3M	9.44 ± 0.20	9.71 ± 0.17	9.23 ± 0.24	9.62 ± 0.06
TC17002867.hg.1	EIF4A1	9.59 ± 0.15	9.64 ± 0.19	9.24 ± 0.14	9.65 ± 0.04
TC0Y000124.hg.1	EIF4A1P2	7.74 ± 0.19	7.52 ± 0.27	7.44 ± 0.08	7.59 ± 0.16
TC17001933.hg.1	EIF4A3	9.21 ± 0.20	9.30 ± 0.11	9.01 ± 0.11	9.00 ± 0.25
TC12000425.hg.1	EIF4B	10.27 ± 0.17	10.62 ± 0.16	10.07 ± 0.13	10.58 ± 0.02
TC04001403.hg.1	EIF4E	7.62 ± 0.22	7.61 ± 0.07	7.59 ± 0.21	7.53 ± 0.10
TC05001004.hg.1	EIF4E1B	7.98 ± 0.25	7.94 ± 0.24	7.93 ± 0.19	8.03 ± 0.05
TC02001404.hg.1	EIF4E2	8.32 ± 0.03	8.26 ± 0.06	8.30 ± 0.06	8.21 ± 0.05
TC03001549.hg.1	EIF4E3	9.69 ± 0.12	9.55 ± 0.11	9.93 ± 0.22	9.32 ± 0.21
TC08000281.hg.1	EIF4EBP1	8.64 ± 0.18	8.51 ± 0.11	8.54 ± 0.24	8.57 ± 0.06
TC10000428.hg.1	EIF4EBP2	10.36 ± 0.05	10.26 ± 0.11	10.21 ± 0.02	10.15 ± 0.14

TC05000726.hg.1	EIF4EBP3	9.22 ± 0.32	9.42 ± 0.13	9.27 ± 0.35	9.38 ± 0.09
TC22000671.hg.1	EIF4ENIF1	8.49 ± 0.05	8.30 ± 0.07	8.39 ± 0.03	8.25 ± 0.07
TC03001000.hg.1	EIF4G1	8.96 ± 0.14	8.81 ± 0.08	8.74 ± 0.08	8.72 ± 0.05
TC11001417.hg.1	EIF4G2	11.21 ± 0.21	11.45 ± 0.06	11.31 ± 0.10	11.42 ± 0.12
TC01002320.hg.1	EIF4G3	8.43 ± 0.20	8.28 ± 0.20	8.73 ± 0.34	8.12 ± 0.12
TC07000451.hg.1	EIF4H	9.31 ± 0.17	9.48 ± 0.24	9.20 ± 0.14	9.47 ± 0.10
TC14000797.hg.1	EIF5	9.97 ± 0.35	10.62 ± 0.14	10.04 ± 0.33	10.71 ± 0.10
TC17000088.hg.1	EIF5A	10.10 ± 0.12	10.14 ± 0.11	9.77 ± 0.16	10.13 ± 0.05
TC03001995.hg.1	EIF5A2	6.43 ± 0.06	6.48 ± 0.12	6.37 ± 0.09	6.45 ± 0.02
TC10000568.hg.1	EIF5AL1	6.19 ± 0.04	6.33 ± 0.09	6.31 ± 0.03	6.48 ± 0.11
TC02000603.hg.1	EIF5B	8.33 ± 0.35	8.51 ± 0.19	8.17 ± 0.44	8.28 ± 0.19
TC20001747.hg.1	EIF6	8.56 ± 0.32	8.25 ± 0.08	8.33 ± 0.23	8.16 ± 0.06
TC18000999.hg.1	ELAC1	7.21 ± 0.02	7.19 ± 0.09	7.01 ± 0.12	7.19 ± 0.08
TC17001147.hg.1	ELAC2	8.45 ± 0.14	8.29 ± 0.07	8.14 ± 0.09	8.25 ± 0.02
TC19000019.hg.1	ELANE	8.92 ± 0.86	8.08 ± 0.20	8.77 ± 0.46	8.14 ± 0.10
TC19001125.hg.1	ELAVL1	8.98 ± 0.11	8.82 ± 0.10	8.82 ± 0.19	8.60 ± 0.11
TC09000968.hg.1	ELAVL2	5.63 ± 0.09	5.61 ± 0.11	5.56 ± 0.06	5.66 ± 0.07
TC19001185.hg.1	ELAVL3	6.73 ± 0.19	6.59 ± 0.28	6.63 ± 0.15	6.68 ± 0.05
TC01000614.hg.1	ELAVL4	5.30 ± 0.12	5.19 ± 0.08	5.18 ± 0.06	5.23 ± 0.05
TC13000597.hg.1	ELF1	10.68 ± 0.20	10.79 ± 0.10	10.90 ± 0.25	10.85 ± 0.10
TC04001577.hg.1	ELF2	8.64 ± 0.31	8.88 ± 0.21	9.04 ± 0.35	8.88 ± 0.12
TC01001670.hg.1	ELF3	7.67 ± 0.16	7.62 ± 0.20	7.59 ± 0.15	7.70 ± 0.07
TC0X001357.hg.1	ELF4	9.39 ± 0.24	9.24 ± 0.09	9.51 ± 0.19	9.21 ± 0.28
TC11001546.hg.1	ELF5	5.79 ± 0.10	5.77 ± 0.12	5.76 ± 0.06	5.83 ± 0.03
TC07000027.hg.1	ELFN1	7.80 ± 0.17	7.74 ± 0.20	7.67 ± 0.22	7.80 ± 0.02
TC22000726.hg.1	ELFN2	7.87 ± 0.16	7.87 ± 0.18	7.79 ± 0.15	7.97 ± 0.10
TC0X000999.hg.1	ELK1	8.73 ± 0.13	8.55 ± 0.08	8.45 ± 0.06	8.60 ± 0.08
TC12000747.hg.1	ELK3	8.95 ± 0.15	8.91 ± 0.07	9.06 ± 0.25	8.95 ± 0.19
TC01003750.hg.1	ELK4	8.17 ± 0.07	8.22 ± 0.02	8.08 ± 0.11	8.21 ± 0.01
TC19001290.hg.1	ELL	8.81 ± 0.16	8.77 ± 0.12	8.82 ± 0.16	8.80 ± 0.21
TC05001610.hg.1	ELL2	8.56 ± 0.09	8.20 ± 0.34	8.30 ± 0.28	8.18 ± 0.23

TC15002773.hg.1	ELL3	7.09 ± 0.21	7.09 ± 0.13	6.99 ± 0.13	7.04 ± 0.01
TC07001288.hg.1	ELMO1	9.21 ± 0.12	9.11 ± 0.07	9.12 ± 0.05	9.06 ± 0.05
TC07000240.hg.1	ELMO1-AS1	6.47 ± 0.35	6.60 ± 0.12	6.68 ± 0.10	6.59 ± 0.11
TC20000900.hg.1	ELMO2	8.59 ± 0.34	8.29 ± 0.10	8.89 ± 0.30	8.24 ± 0.12
TC16000534.hg.1	ELMO3	7.81 ± 0.20	7.77 ± 0.16	7.74 ± 0.22	7.83 ± 0.05
TC11000972.hg.1	ELMOD1	5.04 ± 0.20	4.91 ± 0.04	4.97 ± 0.11	4.94 ± 0.06
TC04000690.hg.1	ELMOD2	6.40 ± 0.43	6.30 ± 0.25	6.55 ± 0.50	6.21 ± 0.02
TC02000507.hg.1	ELMOD3	7.52 ± 0.06	7.34 ± 0.03	7.49 ± 0.07	7.34 ± 0.01
TC14001297.hg.1	ELMSAN1	8.47 ± 0.06	8.52 ± 0.05	8.40 ± 0.12	8.62 ± 0.06
TC07000449.hg.1	ELN	8.43 ± 0.22	8.45 ± 0.19	8.37 ± 0.17	8.54 ± 0.06
TC19001187.hg.1	ELOF1	8.67 ± 0.05	8.57 ± 0.02	8.48 ± 0.06	8.54 ± 0.05
TC01002583.hg.1	ELOVL1	9.16 ± 0.23	8.73 ± 0.04	8.98 ± 0.22	8.64 ± 0.08
TC06001271.hg.1	ELOVL2	5.99 ± 0.20	5.96 ± 0.09	5.98 ± 0.15	6.01 ± 0.03
TC06000077.hg.1	ELOVL2-AS1	5.87 ± 0.08	5.86 ± 0.12	5.84 ± 0.02	5.90 ± 0.07
TC10000751.hg.1	ELOVL3	6.67 ± 0.29	6.36 ± 0.11	6.40 ± 0.31	6.45 ± 0.01
TC06001902.hg.1	ELOVL4	5.99 ± 0.10	5.97 ± 0.14	5.87 ± 0.11	5.93 ± 0.06
TC06001813.hg.1	ELOVL5	9.45 ± 0.18	9.61 ± 0.12	9.64 ± 0.29	9.58 ± 0.12
TC04001467.hg.1	ELOVL6	6.66 ± 0.13	6.44 ± 0.05	6.54 ± 0.08	6.39 ± 0.04
TC05001393.hg.1	ELOVL7	7.42 ± 0.27	7.07 ± 0.12	7.51 ± 0.43	7.18 ± 0.19
TC18000148.hg.1	ELP2	8.06 ± 0.41	8.33 ± 0.19	7.83 ± 0.23	8.33 ± 0.12
TC08000216.hg.1	ELP3	8.16 ± 0.14	8.07 ± 0.10	7.92 ± 0.08	7.85 ± 0.16
TC11000303.hg.1	ELP4	7.76 ± 0.23	7.87 ± 0.28	7.50 ± 0.12	7.57 ± 0.05
TC17000086.hg.1	ELP5	8.21 ± 0.15	8.04 ± 0.05	7.95 ± 0.15	7.98 ± 0.02
TC03001368.hg.1	ELP6	7.56 ± 0.11	7.42 ± 0.06	7.39 ± 0.12	7.43 ± 0.03
TC19000696.hg.1	ELSPBP1	6.13 ± 0.19	6.01 ± 0.13	6.06 ± 0.12	6.08 ± 0.09
TC01002807.hg.1	ELTD1	4.14 ± 0.12	4.02 ± 0.07	4.04 ± 0.06	4.06 ± 0.06
TC05001331.hg.1	EMB	9.30 ± 0.31	9.33 ± 0.17	9.71 ± 0.35	9.16 ± 0.08
TC01001053.hg.1	EMBP1	9.33 ± 0.26	9.29 ± 0.16	9.66 ± 0.30	9.12 ± 0.09
TC01002294.hg.1	EMC1	7.79 ± 0.05	7.59 ± 0.05	7.52 ± 0.04	7.58 ± 0.04
TC19000760.hg.1	EMC10	9.63 ± 0.10	9.53 ± 0.07	9.41 ± 0.10	9.54 ± 0.02
TC08000663.hg.1	EMC2	7.45 ± 0.40	7.42 ± 0.21	7.89 ± 0.49	7.36 ± 0.08

TC03003327.hg.1	EMC3	9.09 ± 0.04	9.02 ± 0.16	8.98 ± 0.14	9.00 ± 0.13
TC15000233.hg.1	EMC4	8.71 ± 0.04	8.69 ± 0.16	8.49 ± 0.06	8.59 ± 0.02
TC17000035.hg.1	EMC6	8.74 ± 0.35	8.40 ± 0.15	8.40 ± 0.34	8.34 ± 0.10
TC15001190.hg.1	EMC7	8.91 ± 0.15	8.59 ± 0.27	8.69 ± 0.26	8.54 ± 0.09
TC16001321.hg.1	EMC8	7.97 ± 0.16	7.80 ± 0.14	7.71 ± 0.21	7.73 ± 0.04
TC14000967.hg.1	EMC9	8.17 ± 0.12	8.14 ± 0.13	8.02 ± 0.16	8.15 ± 0.06
TC04001421.hg.1	EMCN	4.41 ± 0.09	4.29 ± 0.02	4.32 ± 0.07	4.33 ± 0.06
TC04001424.hg.1	EMCN-IT1	5.27 ± 0.20	5.20 ± 0.17	5.19 ± 0.15	5.24 ± 0.29
TC04001423.hg.1	EMCN-IT2	5.74 ± 0.28	5.46 ± 0.08	5.51 ± 0.15	5.47 ± 0.43
TC04001422.hg.1	EMCN-IT3	5.35 ± 0.17	5.36 ± 0.07	5.25 ± 0.13	5.42 ± 0.14
TC0X000768.hg.1	EMD	8.75 ± 0.13	8.81 ± 0.09	8.53 ± 0.05	8.87 ± 0.10
TC17000664.hg.1	EME1	7.13 ± 0.20	7.06 ± 0.16	7.07 ± 0.14	7.09 ± 0.01
TC16000047.hg.1	EME2	9.15 ± 0.20	9.07 ± 0.19	9.06 ± 0.21	9.14 ± 0.04
TC12003223.hg.1	EMG1	7.65 ± 0.06	7.50 ± 0.02	7.47 ± 0.08	7.52 ± 0.03
TC22000193.hg.1	EMID1	7.88 ± 0.24	7.82 ± 0.20	7.75 ± 0.22	7.90 ± 0.07
TC02000163.hg.1	EMILIN1	8.70 ± 0.27	8.68 ± 0.21	8.67 ± 0.24	8.77 ± 0.09
TC18000014.hg.1	EMILIN2	8.86 ± 0.32	8.57 ± 0.09	9.22 ± 0.06	8.56 ± 0.15
TC20000849.hg.1	EMILIN3	7.85 ± 0.24	7.73 ± 0.14	7.75 ± 0.20	7.77 ± 0.03
TC14000628.hg.1	EML1	6.14 ± 0.12	6.09 ± 0.12	6.07 ± 0.07	6.15 ± 0.04
TC19001625.hg.1	EML2	8.43 ± 0.11	8.35 ± 0.09	8.37 ± 0.09	8.39 ± 0.01
TC11001871.hg.1	EML3	8.85 ± 0.07	8.78 ± 0.06	8.68 ± 0.05	8.74 ± 0.06
TC02000260.hg.1	EML4	9.13 ± 0.41	9.47 ± 0.13	9.12 ± 0.39	9.60 ± 0.07
TC14001424.hg.1	EML5	5.59 ± 0.07	5.55 ± 0.04	5.48 ± 0.04	5.51 ± 0.09
TC02000318.hg.1	EML6	6.81 ± 0.09	6.82 ± 0.05	6.69 ± 0.10	6.83 ± 0.11
TC12000189.hg.1	EMP1	6.70 ± 0.05	6.71 ± 0.07	6.82 ± 0.15	6.92 ± 0.13
TC16000857.hg.1	EMP2	7.73 ± 0.15	7.74 ± 0.13	7.72 ± 0.08	7.85 ± 0.06
TC19000699.hg.1	EMP3	11.91 ± 0.20	11.79 ± 0.05	11.73 ± 0.15	11.80 ± 0.25
TC19000120.hg.1	EMR1	9.17 ± 0.63	8.51 ± 0.27	10.37 ± 0.57	8.87 ± 0.40
TC19001242.hg.1	EMR2	10.86 ± 0.33	10.44 ± 0.21	11.28 ± 0.23	10.51 ± 0.29
TC19001241.hg.1	EMR3	9.75 ± 0.30	9.95 ± 0.37	10.26 ± 0.28	9.94 ± 0.19
TC19001106.hg.1	EMR4P	7.77 ± 0.84	8.27 ± 0.20	8.71 ± 0.33	8.52 ± 0.35

TC02000436.hg.1	EMX1	8.05 ± 0.24	7.92 ± 0.16	7.98 ± 0.12	8.00 ± 0.06
TC10000853.hg.1	EMX2	5.97 ± 0.39	5.93 ± 0.26	5.90 ± 0.19	6.01 ± 0.03
TC10001689.hg.1	EMX2OS	6.96 ± 0.13	6.99 ± 0.15	6.94 ± 0.20	7.04 ± 0.06
TC02002248.hg.1	EN1	7.32 ± 0.37	7.34 ± 0.25	7.19 ± 0.31	7.42 ± 0.06
TC07001041.hg.1	EN2	7.09 ± 0.24	7.11 ± 0.21	7.04 ± 0.18	7.26 ± 0.08
TC01003889.hg.1	ENAH	5.47 ± 0.09	5.45 ± 0.01	5.42 ± 0.07	5.47 ± 0.07
TC04000389.hg.1	ENAM	5.99 ± 0.13	5.83 ± 0.10	5.86 ± 0.06	5.84 ± 0.01
TC05001481.hg.1	ENC1	7.38 ± 0.22	7.56 ± 0.20	7.22 ± 0.11	7.47 ± 0.13
TC11000928.hg.1	ENDOD1	8.23 ± 0.14	7.90 ± 0.15	8.01 ± 0.13	7.86 ± 0.05
TC09000703.hg.1	ENDOG	8.24 ± 0.21	8.11 ± 0.10	8.03 ± 0.22	8.08 ± 0.03
TC12001436.hg.1	ENDOU	6.51 ± 0.15	6.52 ± 0.16	6.54 ± 0.15	6.64 ± 0.05
TC17000919.hg.1	ENDOV	7.86 ± 0.16	7.80 ± 0.14	7.82 ± 0.13	7.87 ± 0.06
TC09001609.hg.1	ENG	9.16 ± 0.13	9.06 ± 0.12	8.97 ± 0.18	9.17 ± 0.30
TC17000907.hg.1	ENGASE	8.33 ± 0.08	8.39 ± 0.08	8.15 ± 0.14	8.40 ± 0.06
TC09001037.hg.1	ENHO	7.23 ± 0.23	7.32 ± 0.34	7.19 ± 0.26	7.51 ± 0.07
TC16001194.hg.1	ENKD1	7.71 ± 0.27	7.63 ± 0.23	7.61 ± 0.36	7.77 ± 0.05
TC10001111.hg.1	ENKUR	6.55 ± 0.51	6.29 ± 0.09	6.44 ± 0.34	6.44 ± 0.35
TC01002175.hg.1	ENO1	10.41 ± 0.13	10.24 ± 0.15	10.34 ± 0.20	10.11 ± 0.16
TC01000108.hg.1	ENO1-AS1	7.29 ± 0.20	7.32 ± 0.08	7.37 ± 0.15	7.16 ± 0.08
TC01002176.hg.1	ENO1-IT1	8.06 ± 0.08	7.83 ± 0.07	7.89 ± 0.08	7.92 ± 0.19
TC12000099.hg.1	ENO2	7.66 ± 0.08	7.71 ± 0.10	7.59 ± 0.09	7.71 ± 0.06
TC17000058.hg.1	ENO3	7.72 ± 0.12	7.63 ± 0.09	7.61 ± 0.15	7.69 ± 0.05
TC10000848.hg.1	ENO4	5.82 ± 0.16	5.75 ± 0.09	5.79 ± 0.11	5.85 ± 0.02
TC04000454.hg.1	ENOPH1	7.74 ± 0.23	7.77 ± 0.08	7.46 ± 0.12	7.69 ± 0.08
TC18000277.hg.1	ENOSF1	7.43 ± 0.10	7.41 ± 0.03	7.24 ± 0.11	7.37 ± 0.04
TC13000613.hg.1	ENOX1	5.31 ± 0.11	5.25 ± 0.05	5.28 ± 0.09	5.32 ± 0.05
TC13000160.hg.1	ENOX1-AS1	4.92 ± 0.13	4.82 ± 0.09	4.91 ± 0.22	4.93 ± 0.04
TC13000159.hg.1	ENOX1-AS2	5.25 ± 0.39	5.12 ± 0.08	5.10 ± 0.16	5.30 ± 0.04
TC0X001363.hg.1	ENOX2	7.23 ± 0.11	7.28 ± 0.12	7.17 ± 0.12	7.10 ± 0.06
TC04000572.hg.1	ENPEP	4.49 ± 0.12	4.37 ± 0.04	4.44 ± 0.05	4.42 ± 0.03
TC06000985.hg.1	ENPP1	4.86 ± 0.10	4.75 ± 0.05	4.74 ± 0.07	4.77 ± 0.04

TC08001556.hg.1	ENPP2	6.47 ± 0.16	6.50 ± 0.07	6.64 ± 0.28	6.48 ± 0.10
TC08001557.hg.1	ENPP2	5.10 ± 0.22	5.22 ± 0.21	5.12 ± 0.04	5.32 ± 0.14
TC06000984.hg.1	ENPP3	5.34 ± 0.10	5.42 ± 0.05	5.33 ± 0.05	5.45 ± 0.03
TC06000624.hg.1	ENPP4	7.22 ± 0.41	7.06 ± 0.19	6.94 ± 0.15	6.95 ± 0.06
TC06001770.hg.1	ENPP5	5.91 ± 0.29	5.75 ± 0.17	5.78 ± 0.15	5.68 ± 0.07
TC04001800.hg.1	ENPP6	5.60 ± 0.15	5.46 ± 0.08	5.47 ± 0.08	5.54 ± 0.05
TC17000910.hg.1	ENPP7	7.26 ± 0.24	7.18 ± 0.19	7.18 ± 0.21	7.27 ± 0.06
TC01003207.hg.1	ENSA	8.87 ± 0.06	8.88 ± 0.07	8.84 ± 0.15	8.85 ± 0.13
TC22000808.hg.1	ENTHD1	5.10 ± 0.14	5.03 ± 0.05	5.03 ± 0.10	5.13 ± 0.05
TC17001946.hg.1	ENTHD2	8.91 ± 0.08	8.83 ± 0.06	8.87 ± 0.03	8.86 ± 0.03
TC10002943.hg.1	ENTPD1	8.09 ± 0.15	7.99 ± 0.29	8.59 ± 0.41	7.93 ± 0.14
TC10001553.hg.1	ENTPD1-AS1	6.22 ± 0.09	6.21 ± 0.12	6.24 ± 0.06	6.22 ± 0.04
TC09001754.hg.1	ENTPD2	8.19 ± 0.15	8.18 ± 0.13	8.18 ± 0.14	8.26 ± 0.03
TC03000208.hg.1	ENTPD3	6.33 ± 0.23	6.29 ± 0.09	6.29 ± 0.17	6.35 ± 0.07
TC03001306.hg.1	ENTPD3-AS1	6.88 ± 0.15	6.77 ± 0.05	6.72 ± 0.03	6.84 ± 0.06
TC08002612.hg.1	ENTPD4	9.19 ± 0.30	9.38 ± 0.16	9.13 ± 0.29	9.41 ± 0.05
TC14001302.hg.1	ENTPD5	7.43 ± 0.14	7.29 ± 0.10	7.43 ± 0.07	7.21 ± 0.02
TC20000166.hg.1	ENTPD6	8.00 ± 0.05	7.91 ± 0.05	7.80 ± 0.12	7.90 ± 0.01
TC10002945.hg.1	ENTPD7	7.31 ± 0.17	7.07 ± 0.08	7.92 ± 0.46	7.02 ± 0.03
TC09001771.hg.1	ENTPD8	8.53 ± 0.22	8.53 ± 0.21	8.49 ± 0.20	8.62 ± 0.06
TC08000667.hg.1	ENY2	7.50 ± 0.27	7.73 ± 0.05	7.63 ± 0.22	7.80 ± 0.07
TC03001535.hg.1	EOGT	6.82 ± 0.17	7.00 ± 0.27	6.94 ± 0.39	6.64 ± 0.20
TC03001257.hg.1	EOMES	8.35 ± 0.04	8.03 ± 0.06	8.05 ± 0.20	7.74 ± 0.04
TC12001051.hg.1	EP400	8.98 ± 0.10	8.98 ± 0.04	8.77 ± 0.20	8.99 ± 0.08
TC12001053.hg.1	EP400NL	7.78 ± 0.09	7.77 ± 0.06	7.69 ± 0.10	7.81 ± 0.04
TC02000281.hg.1	EPAS1	7.13 ± 0.14	7.11 ± 0.12	7.36 ± 0.17	7.27 ± 0.07
TC01000394.hg.1	EPB41	9.94 ± 0.22	10.11 ± 0.06	9.78 ± 0.16	10.05 ± 0.04
TC20000264.hg.1	EPB41L1	7.37 ± 0.19	7.34 ± 0.17	7.32 ± 0.17	7.41 ± 0.04
TC06002106.hg.1	EPB41L2	6.83 ± 0.04	6.86 ± 0.13	6.65 ± 0.05	6.80 ± 0.04
TC18000300.hg.1	EPB41L3	8.04 ± 0.20	7.83 ± 0.09	8.03 ± 0.00	7.70 ± 0.18
TC05001676.hg.1	EPB41L4A	5.67 ± 0.04	5.58 ± 0.05	5.53 ± 0.09	5.55 ± 0.09

TC05000525.hg.1	EPB41L4A-AS1	8.38 ± 0.05	8.52 ± 0.16	8.04 ± 0.15	8.59 ± 0.13
TC09001466.hg.1	EPB41L4B	6.48 ± 0.07	6.40 ± 0.10	6.61 ± 0.09	6.49 ± 0.04
TC02000758.hg.1	EPB41L5	6.61 ± 0.13	6.69 ± 0.14	6.57 ± 0.19	6.57 ± 0.04
TC15001273.hg.1	EPB42	7.21 ± 0.18	7.18 ± 0.13	7.12 ± 0.14	7.26 ± 0.05
TC10001161.hg.1	EPC1	8.62 ± 0.33	8.99 ± 0.22	8.56 ± 0.32	8.92 ± 0.08
TC02000926.hg.1	EPC2	8.91 ± 0.33	9.33 ± 0.19	9.02 ± 0.39	9.26 ± 0.06
TC02000293.hg.1	EPCAM	5.67 ± 0.14	5.57 ± 0.07	5.64 ± 0.08	5.60 ± 0.03
TC07003323.hg.1	EPDR1	5.92 ± 0.07	5.82 ± 0.08	5.89 ± 0.09	5.85 ± 0.06
TC18000476.hg.1	EPG5	8.27 ± 0.26	8.08 ± 0.18	8.37 ± 0.50	7.89 ± 0.02
TC04000413.hg.1	EPGN	4.35 ± 0.12	4.24 ± 0.07	4.28 ± 0.07	4.29 ± 0.07
TC07001971.hg.1	EPHA1	7.76 ± 0.13	7.73 ± 0.19	7.72 ± 0.11	7.83 ± 0.04
TC01002522.hg.1	EPHA10	7.80 ± 0.19	7.73 ± 0.16	7.73 ± 0.18	7.83 ± 0.06
TC07000936.hg.1	EPHA1-AS1	6.07 ± 0.10	5.99 ± 0.14	5.98 ± 0.06	6.05 ± 0.05
TC01002258.hg.1	EPHA2	7.96 ± 0.19	7.93 ± 0.18	7.87 ± 0.19	8.00 ± 0.05
TC03000468.hg.1	EPHA3	5.39 ± 0.08	5.33 ± 0.08	5.34 ± 0.11	5.40 ± 0.10
TC02002808.hg.1	EPHA4	7.53 ± 0.28	7.77 ± 0.11	7.25 ± 0.04	7.86 ± 0.12
TC04001245.hg.1	EPHA5	6.02 ± 0.14	5.99 ± 0.12	5.94 ± 0.09	6.07 ± 0.10
TC03000478.hg.1	EPHA6	5.21 ± 0.11	5.10 ± 0.08	5.12 ± 0.07	5.18 ± 0.07
TC06001952.hg.1	EPHA7	5.48 ± 0.20	5.34 ± 0.11	5.34 ± 0.11	5.40 ± 0.06
TC01000284.hg.1	EPHA8	8.28 ± 0.19	8.25 ± 0.19	8.25 ± 0.25	8.34 ± 0.05
TC03000730.hg.1	EPHB1	7.65 ± 0.04	7.59 ± 0.10	7.73 ± 0.08	7.58 ± 0.13
TC01000288.hg.1	EPHB2	7.94 ± 0.19	7.74 ± 0.17	7.78 ± 0.14	7.79 ± 0.01
TC03001007.hg.1	EPHB3	7.74 ± 0.17	7.66 ± 0.17	7.65 ± 0.20	7.76 ± 0.04
TC07001677.hg.1	EPHB4	8.02 ± 0.18	7.98 ± 0.15	7.95 ± 0.14	7.99 ± 0.02
TC07000923.hg.1	EPHB6	8.21 ± 0.08	8.20 ± 0.09	8.03 ± 0.13	8.19 ± 0.06
TC01001856.hg.1	EPHX1	7.71 ± 0.17	7.65 ± 0.10	7.61 ± 0.14	7.64 ± 0.02
TC08000208.hg.1	EPHX2	7.46 ± 0.08	7.49 ± 0.04	7.22 ± 0.07	7.49 ± 0.09
TC19001251.hg.1	EPHX3	7.15 ± 0.12	7.08 ± 0.10	7.05 ± 0.06	7.07 ± 0.05
TC01000849.hg.1	EPHX4	5.06 ± 0.14	4.97 ± 0.05	4.98 ± 0.03	4.94 ± 0.09
TC06002194.hg.1	EPM2A	6.26 ± 0.09	6.18 ± 0.05	6.18 ± 0.04	6.22 ± 0.05
TC03001285.hg.1	EPM2AIP1	7.84 ± 0.60	8.00 ± 0.19	7.95 ± 0.35	7.93 ± 0.03

TC19000907.hg.1	EPN1	9.17 ± 0.17	9.06 ± 0.10	9.08 ± 0.07	9.11 ± 0.09
TC17000234.hg.1	EPN2	7.80 ± 0.14	7.79 ± 0.03	7.88 ± 0.05	7.92 ± 0.11
TC17001239.hg.1	EPN2-AS1	7.04 ± 0.22	7.03 ± 0.07	7.00 ± 0.09	7.06 ± 0.08
TC17000233.hg.1	EPN2-IT1	7.55 ± 0.10	7.65 ± 0.24	7.50 ± 0.24	7.74 ± 0.07
TC17000235.hg.1	EPN2-IT1	6.90 ± 0.11	6.84 ± 0.16	6.82 ± 0.16	7.03 ± 0.07
TC17001235.hg.1	EPN2-IT1	7.57 ± 0.07	7.67 ± 0.23	7.46 ± 0.34	7.74 ± 0.08
TC17002843.hg.1	EPN3	7.99 ± 0.22	7.99 ± 0.20	7.92 ± 0.19	8.05 ± 0.05
TC07000632.hg.1	EPO	8.50 ± 0.12	8.46 ± 0.21	8.43 ± 0.21	8.51 ± 0.02
TC19002676.hg.1	EPOR	8.28 ± 0.18	8.17 ± 0.13	8.21 ± 0.16	8.24 ± 0.03
TC20001750.hg.1	EPPIN	5.88 ± 0.12	5.76 ± 0.17	5.78 ± 0.11	5.87 ± 0.07
TC08001736.hg.1	EPPK1	7.67 ± 0.10	7.74 ± 0.14	7.52 ± 0.25	7.86 ± 0.16
TC01003833.hg.1	EPRS	8.29 ± 0.26	8.30 ± 0.22	7.94 ± 0.25	7.93 ± 0.11
TC01002649.hg.1	EPS15	9.42 ± 0.29	9.45 ± 0.27	9.54 ± 0.34	9.28 ± 0.14
TC19001265.hg.1	EPS15L1	9.52 ± 0.14	9.43 ± 0.09	9.61 ± 0.21	9.41 ± 0.25
TC07000300.hg.1	EPS15P1	4.65 ± 0.14	4.61 ± 0.10	4.69 ± 0.19	4.70 ± 0.11
TC12001282.hg.1	EPS8	5.59 ± 0.07	5.53 ± 0.03	5.63 ± 0.07	5.62 ± 0.02
TC19000888.hg.1	EPS8L1	8.23 ± 0.20	8.18 ± 0.19	8.14 ± 0.25	8.24 ± 0.03
TC11000026.hg.1	EPS8L2	8.28 ± 0.25	8.22 ± 0.21	8.19 ± 0.21	8.31 ± 0.05
TC01002963.hg.1	EPS8L3	7.65 ± 0.25	7.62 ± 0.23	7.62 ± 0.17	7.75 ± 0.04
TC13000612.hg.1	EPSTI1	10.15 ± 0.36	9.69 ± 0.56	10.30 ± 0.49	9.37 ± 0.29
TC02000150.hg.1	EPT1	7.23 ± 0.18	7.22 ± 0.13	7.08 ± 0.24	7.07 ± 0.04
TC17000711.hg.1	EPX	7.72 ± 0.32	7.60 ± 0.17	7.62 ± 0.25	7.68 ± 0.02
TC12001801.hg.1	EPYC	4.76 ± 0.07	4.68 ± 0.10	4.67 ± 0.04	4.81 ± 0.14
TC09000987.hg.1	EQTN	4.36 ± 0.11	4.22 ± 0.06	4.32 ± 0.10	4.26 ± 0.02
TC17000332.hg.1	ERAL1	8.08 ± 0.11	7.88 ± 0.08	7.82 ± 0.05	7.74 ± 0.01
TC05001617.hg.1	ERAP1	9.09 ± 0.23	9.04 ± 0.17	8.87 ± 0.29	8.78 ± 0.10
TC05000471.hg.1	ERAP2	8.40 ± 0.49	8.84 ± 0.29	8.50 ± 0.40	8.46 ± 0.26
TC0X000262.hg.1	ERAS	7.12 ± 0.23	6.99 ± 0.25	7.03 ± 0.23	7.22 ± 0.12
TC17000480.hg.1	ERBB2	7.82 ± 0.10	7.75 ± 0.09	7.63 ± 0.11	7.79 ± 0.05
TC05000280.hg.1	ERBB2IP	9.27 ± 0.58	9.74 ± 0.21	9.62 ± 0.54	9.62 ± 0.18
TC12003243.hg.1	ERBB3	6.69 ± 0.16	6.60 ± 0.14	6.61 ± 0.10	6.71 ± 0.06

TC02002735.hg.1	ERBB4	5.89 ± 0.17	5.88 ± 0.16	5.89 ± 0.07	5.99 ± 0.09
TC12000014.hg.1	ERC1	8.19 ± 0.11	8.32 ± 0.03	8.05 ± 0.12	8.23 ± 0.05
TC03001484.hg.1	ERC2	5.48 ± 0.16	5.36 ± 0.11	5.42 ± 0.13	5.44 ± 0.03
TC03001485.hg.1	ERC2-IT1	6.74 ± 0.15	6.80 ± 0.23	6.64 ± 0.31	6.83 ± 0.08
TC19001620.hg.1	ERCC1	8.41 ± 0.07	8.30 ± 0.02	8.18 ± 0.09	8.26 ± 0.06
TC19001618.hg.1	ERCC2	7.89 ± 0.17	7.76 ± 0.10	7.73 ± 0.12	7.78 ± 0.02
TC02002278.hg.1	ERCC3	8.37 ± 0.05	8.33 ± 0.13	8.16 ± 0.09	8.26 ± 0.07
TC16000170.hg.1	ERCC4	6.91 ± 0.17	6.83 ± 0.08	6.80 ± 0.15	6.73 ± 0.06
TC13001721.hg.1	ERCC5	8.63 ± 0.36	8.87 ± 0.11	8.57 ± 0.35	8.88 ± 0.04
TC10001276.hg.1	ERCC6	7.06 ± 0.11	7.11 ± 0.11	7.01 ± 0.11	7.02 ± 0.03
TC0X001137.hg.1	ERCC6L	7.40 ± 0.17	7.32 ± 0.05	7.32 ± 0.15	7.40 ± 0.07
TC09000475.hg.1	ERCC6L2	7.17 ± 0.42	7.39 ± 0.16	7.27 ± 0.39	7.24 ± 0.06
TC05001394.hg.1	ERCC8	6.13 ± 0.25	6.17 ± 0.22	6.05 ± 0.27	6.02 ± 0.06
TC04000414.hg.1	EREG	6.59 ± 0.33	7.35 ± 0.19	7.29 ± 0.91	7.16 ± 0.27
TC19001570.hg.1	ERF	8.45 ± 0.10	8.38 ± 0.07	8.32 ± 0.13	8.42 ± 0.10
TC21000450.hg.1	ERG	6.09 ± 0.16	5.86 ± 0.07	6.07 ± 0.10	5.90 ± 0.05
TC05000952.hg.1	ERGIC1	8.71 ± 0.17	8.63 ± 0.08	8.72 ± 0.07	8.69 ± 0.18
TC12001348.hg.1	ERGIC2	9.11 ± 0.48	9.16 ± 0.26	9.48 ± 0.52	8.92 ± 0.23
TC20000255.hg.1	ERGIC3	9.15 ± 0.07	9.06 ± 0.06	8.92 ± 0.12	9.03 ± 0.04
TC14001260.hg.1	ERH	9.24 ± 0.12	9.37 ± 0.23	9.24 ± 0.15	9.19 ± 0.05
TC08000063.hg.1	ERI1	7.28 ± 0.09	7.15 ± 0.08	7.49 ± 0.26	7.01 ± 0.14
TC16002040.hg.1	ERI2	6.19 ± 0.10	6.09 ± 0.07	6.16 ± 0.12	5.93 ± 0.02
TC01002592.hg.1	ERI3	7.77 ± 0.15	7.73 ± 0.06	7.57 ± 0.15	7.73 ± 0.07
TC01002593.hg.1	ERI3-IT1	5.82 ± 0.36	6.08 ± 0.24	5.83 ± 0.25	6.20 ± 0.09
TC08000881.hg.1	ERICH1	7.93 ± 0.03	7.88 ± 0.06	8.03 ± 0.08	7.89 ± 0.07
TC08000005.hg.1	ERICH1-AS1	7.20 ± 0.15	7.15 ± 0.19	7.07 ± 0.17	7.29 ± 0.13
TC02001015.hg.1	ERICH2	5.36 ± 0.14	5.21 ± 0.07	5.21 ± 0.13	5.30 ± 0.11
TC02000311.hg.1	ERLEC1	8.46 ± 0.16	8.11 ± 0.10	8.17 ± 0.33	8.03 ± 0.17
TC10002930.hg.1	ERLIN1	8.07 ± 0.20	7.62 ± 0.21	8.47 ± 0.45	7.45 ± 0.23
TC08000277.hg.1	ERLIN2	7.29 ± 0.04	7.25 ± 0.04	7.32 ± 0.12	7.21 ± 0.05
TC01000534.hg.1	ERMAP	7.40 ± 0.11	7.21 ± 0.03	7.39 ± 0.04	7.26 ± 0.03

TC02002451.hg.1	ERMN	6.80 ± 0.34	7.17 ± 0.30	6.76 ± 0.15	7.45 ± 0.24
TC09000893.hg.1	ERMP1	8.14 ± 0.22	8.13 ± 0.22	7.94 ± 0.09	7.78 ± 0.11
TC17001796.hg.1	ERN1	8.61 ± 0.11	8.61 ± 0.14	8.48 ± 0.14	8.63 ± 0.03
TC16000968.hg.1	ERN2	7.59 ± 0.18	7.56 ± 0.17	7.57 ± 0.15	7.66 ± 0.06
TC14001141.hg.1	ERO1L	9.58 ± 0.33	9.78 ± 0.11	9.93 ± 0.41	9.57 ± 0.30
TC01004004.hg.1	ERO1LB	7.10 ± 0.28	7.32 ± 0.15	7.22 ± 0.27	7.25 ± 0.05
TC12001277.hg.1	ERP27	8.41 ± 0.46	8.72 ± 0.14	8.65 ± 0.56	8.70 ± 0.19
TC12000874.hg.1	ERP29	7.73 ± 0.17	7.76 ± 0.08	7.69 ± 0.12	7.62 ± 0.01
TC09001416.hg.1	ERP44	9.62 ± 0.17	9.67 ± 0.18	9.77 ± 0.29	9.49 ± 0.20
TC01002166.hg.1	ERRFI1	6.62 ± 0.14	6.56 ± 0.10	6.51 ± 0.06	6.63 ± 0.06
TC07001452.hg.1	ERV3-1	8.41 ± 0.39	8.53 ± 0.18	8.93 ± 0.58	8.49 ± 0.22
TC06001272.hg.1	ERVFRD-1	5.85 ± 0.09	5.86 ± 0.16	5.85 ± 0.10	5.98 ± 0.16
TC21000498.hg.1	ERVH48-1	7.14 ± 0.22	7.14 ± 0.14	6.89 ± 0.11	7.27 ± 0.14
TC16000788.hg.1	ERVK13-1	7.79 ± 0.13	7.70 ± 0.07	7.70 ± 0.12	7.79 ± 0.06
TC19002671.hg.1	ERVK3-1	8.89 ± 0.12	9.04 ± 0.08	8.77 ± 0.11	9.08 ± 0.03
TC04001196.hg.1	ERVMER34-1	6.23 ± 0.19	6.08 ± 0.12	6.15 ± 0.09	6.14 ± 0.05
TC01001610.hg.1	ERVMER61-1	5.43 ± 0.38	5.25 ± 0.18	5.32 ± 0.24	5.46 ± 0.12
TC19000804.hg.1	ERVV-1	5.74 ± 0.23	5.61 ± 0.13	5.74 ± 0.13	5.80 ± 0.04
TC19000805.hg.1	ERVV-2	5.75 ± 0.29	5.63 ± 0.08	5.59 ± 0.09	5.61 ± 0.05
TC07001600.hg.1	ERVW-1	6.84 ± 0.17	6.62 ± 0.17	6.70 ± 0.14	6.88 ± 0.26
TC11003496.hg.1	ESAM	8.74 ± 0.10	8.38 ± 0.14	8.67 ± 0.12	8.44 ± 0.08
TC18000401.hg.1	ESCO1	8.07 ± 0.60	8.54 ± 0.19	8.28 ± 0.47	8.47 ± 0.07
TC08000213.hg.1	ESCO2	6.56 ± 0.16	6.37 ± 0.34	6.51 ± 0.47	5.95 ± 0.11
TC13000647.hg.1	ESD	7.96 ± 0.17	8.04 ± 0.26	7.93 ± 0.21	7.80 ± 0.09
TC20000637.hg.1	ESF1	7.19 ± 0.74	7.55 ± 0.31	7.41 ± 0.73	7.57 ± 0.26
TC05001352.hg.1	ESM1	5.30 ± 0.02	5.19 ± 0.07	5.32 ± 0.04	5.23 ± 0.00
TC12000434.hg.1	ESPL1	7.28 ± 0.21	7.20 ± 0.14	7.12 ± 0.17	7.16 ± 0.01
TC01000088.hg.1	ESPN	8.39 ± 0.30	8.33 ± 0.22	8.32 ± 0.26	8.35 ± 0.03
TC02001449.hg.1	ESPNL	8.20 ± 0.22	8.22 ± 0.28	8.17 ± 0.19	8.36 ± 0.08
TC01002277.hg.1	ESPNP	8.35 ± 0.24	8.27 ± 0.21	8.24 ± 0.21	8.31 ± 0.06
TC06001104.hg.1	ESR1	6.93 ± 0.12	6.83 ± 0.10	6.84 ± 0.11	6.90 ± 0.03

TC14001211.hg.1	ESR2	6.34 ± 0.11	6.27 ± 0.05	6.31 ± 0.07	6.31 ± 0.06
TC03001476.hg.1	ESRG	6.03 ± 0.16	5.90 ± 0.05	5.98 ± 0.25	6.24 ± 0.21
TC08000575.hg.1	ESRP1	4.82 ± 0.17	4.69 ± 0.04	4.68 ± 0.08	4.71 ± 0.04
TC16001206.hg.1	ESRP2	7.61 ± 0.27	7.62 ± 0.20	7.56 ± 0.16	7.70 ± 0.07
TC11000596.hg.1	ESRRA	8.85 ± 0.17	8.69 ± 0.11	8.75 ± 0.16	8.76 ± 0.05
TC14000481.hg.1	ESRRB	7.16 ± 0.21	7.20 ± 0.15	7.15 ± 0.19	7.31 ± 0.07
TC01003822.hg.1	ESRRG	5.29 ± 0.11	5.17 ± 0.12	5.18 ± 0.09	5.23 ± 0.04
TC0X001253.hg.1	ESX1	6.74 ± 0.32	6.65 ± 0.22	6.77 ± 0.26	6.71 ± 0.03
TC12000504.hg.1	ESYT1	9.81 ± 0.15	9.72 ± 0.15	9.39 ± 0.21	9.49 ± 0.05
TC07002084.hg.1	ESYT2	9.66 ± 0.12	9.82 ± 0.13	9.36 ± 0.13	9.70 ± 0.07
TC03000745.hg.1	ESYT3	6.30 ± 0.11	6.26 ± 0.13	6.24 ± 0.13	6.34 ± 0.06
TC02000393.hg.1	ETAA1	7.10 ± 0.43	7.42 ± 0.10	7.08 ± 0.24	7.27 ± 0.11
TC05001831.hg.1	ETF1	8.72 ± 0.06	9.09 ± 0.18	8.85 ± 0.32	9.13 ± 0.18
TC15001685.hg.1	ETFA	9.14 ± 0.14	8.85 ± 0.12	9.07 ± 0.22	8.71 ± 0.15
TC19001774.hg.1	ETFB	8.48 ± 0.24	8.32 ± 0.04	8.34 ± 0.22	8.23 ± 0.01
TC04000806.hg.1	ETFDH	7.24 ± 0.20	7.11 ± 0.11	7.34 ± 0.20	7.03 ± 0.06
TC19001588.hg.1	ETHE1	8.55 ± 0.19	8.35 ± 0.08	8.42 ± 0.07	8.26 ± 0.06
TC12000239.hg.1	ETNK1	7.91 ± 0.52	7.96 ± 0.32	7.98 ± 0.53	7.71 ± 0.27
TC01003725.hg.1	ETNK2	7.21 ± 0.18	7.22 ± 0.14	7.06 ± 0.17	7.28 ± 0.06
TC04001461.hg.1	ETNPPL	5.60 ± 0.20	5.49 ± 0.11	5.52 ± 0.11	5.51 ± 0.03
TC11002439.hg.1	ETS1	10.53 ± 0.17	10.79 ± 0.03	10.16 ± 0.18	10.68 ± 0.02
TC21000169.hg.1	ETS2	9.56 ± 0.34	9.46 ± 0.09	10.13 ± 0.32	9.53 ± 0.56
TC07001152.hg.1	ETV1	5.42 ± 0.11	5.34 ± 0.04	5.36 ± 0.05	5.40 ± 0.08
TC19000478.hg.1	ETV2	7.99 ± 0.28	7.94 ± 0.30	7.90 ± 0.23	8.00 ± 0.05
TC01003359.hg.1	ETV3	8.98 ± 0.06	9.07 ± 0.03	8.90 ± 0.19	9.13 ± 0.13
TC01003358.hg.1	ETV3L	7.32 ± 0.11	7.32 ± 0.19	7.23 ± 0.07	7.34 ± 0.02
TC17001560.hg.1	ETV4	7.52 ± 0.20	7.49 ± 0.20	7.46 ± 0.14	7.52 ± 0.03
TC03003333.hg.1	ETV5	6.40 ± 0.12	6.33 ± 0.11	6.31 ± 0.08	6.43 ± 0.02
TC03001015.hg.1	ETV5-AS1	4.55 ± 0.28	4.35 ± 0.11	4.50 ± 0.10	4.47 ± 0.18
TC12003210.hg.1	ETV6	9.92 ± 0.26	9.78 ± 0.22	10.27 ± 0.30	9.73 ± 0.26
TC06001673.hg.1	ETV7	7.88 ± 0.59	7.41 ± 0.09	7.75 ± 0.48	7.26 ± 0.08

TC02002007.hg.1	EVA1A	6.59 ± 0.15	6.50 ± 0.10	6.48 ± 0.11	6.53 ± 0.10
TC01002505.hg.1	EVA1B	8.37 ± 0.29	8.28 ± 0.23	8.31 ± 0.27	8.36 ± 0.01
TC21000117.hg.1	EVA1C	6.75 ± 0.11	6.62 ± 0.05	6.66 ± 0.08	6.66 ± 0.02
TC04000065.hg.1	EVC	7.45 ± 0.16	7.34 ± 0.09	7.33 ± 0.13	7.41 ± 0.06
TC04000996.hg.1	EVC2	6.98 ± 0.19	6.88 ± 0.10	6.91 ± 0.16	6.93 ± 0.03
TC17001342.hg.1	EVI2A	8.23 ± 0.36	8.20 ± 0.22	8.69 ± 0.58	8.16 ± 0.34
TC17001341.hg.1	EVI2B	10.86 ± 0.20	10.72 ± 0.24	11.33 ± 0.29	10.65 ± 0.26
TC01002868.hg.1	EVI5	6.61 ± 0.34	6.72 ± 0.28	6.99 ± 0.38	6.68 ± 0.20
TC19000138.hg.1	EVI5L	8.10 ± 0.20	8.00 ± 0.13	7.99 ± 0.17	8.00 ± 0.02
TC14000630.hg.1	EVL	9.34 ± 0.12	9.40 ± 0.06	9.03 ± 0.04	9.35 ± 0.07
TC17001891.hg.1	EVPL	8.41 ± 0.22	8.39 ± 0.20	8.39 ± 0.24	8.45 ± 0.04
TC17000211.hg.1	EVPLL	7.97 ± 0.29	7.94 ± 0.20	7.95 ± 0.21	8.00 ± 0.02
TC07003386.hg.1	EVX1	7.19 ± 0.24	7.17 ± 0.19	7.14 ± 0.21	7.27 ± 0.04
TC07001229.hg.1	EVX1-AS	6.75 ± 0.19	6.67 ± 0.20	6.59 ± 0.31	6.76 ± 0.10
TC02002550.hg.1	EVX2	7.43 ± 0.32	7.31 ± 0.23	7.35 ± 0.11	7.39 ± 0.06
TC22000195.hg.1	EWSR1	9.39 ± 0.21	9.43 ± 0.04	9.38 ± 0.10	9.41 ± 0.07
TC15001247.hg.1	EXD1	5.38 ± 0.13	5.35 ± 0.05	5.36 ± 0.03	5.43 ± 0.05
TC14000413.hg.1	EXD2	6.85 ± 0.07	6.68 ± 0.05	6.65 ± 0.03	6.71 ± 0.02
TC09001770.hg.1	EXD3	8.38 ± 0.18	8.35 ± 0.18	8.32 ± 0.21	8.44 ± 0.03
TC01001976.hg.1	EXO1	6.26 ± 0.13	6.19 ± 0.24	6.12 ± 0.11	5.96 ± 0.04
TC01000513.hg.1	EXO5	7.15 ± 0.10	7.11 ± 0.10	6.98 ± 0.11	7.10 ± 0.05
TC04000324.hg.1	EXOC1	7.77 ± 0.24	7.70 ± 0.21	8.10 ± 0.46	7.56 ± 0.11
TC06001207.hg.1	EXOC2	8.08 ± 0.31	8.20 ± 0.17	7.97 ± 0.27	8.00 ± 0.03
TC05000006.hg.1	EXOC3	8.45 ± 0.07	8.37 ± 0.04	8.44 ± 0.11	8.42 ± 0.04
TC16002085.hg.1	EXOC3L1	8.15 ± 0.22	8.14 ± 0.16	8.07 ± 0.16	8.21 ± 0.04
TC19001616.hg.1	EXOC3L2	8.35 ± 0.23	8.40 ± 0.24	8.26 ± 0.21	8.50 ± 0.03
TC14000793.hg.1	EXOC3L4	8.01 ± 0.21	8.02 ± 0.19	7.95 ± 0.26	8.10 ± 0.02
TC07000829.hg.1	EXOC4	7.94 ± 0.14	7.94 ± 0.12	7.86 ± 0.10	7.80 ± 0.02
TC07000830.hg.1	EXOC4	5.38 ± 0.09	5.54 ± 0.02	5.33 ± 0.06	5.53 ± 0.10
TC14001170.hg.1	EXOC5	7.46 ± 0.53	7.60 ± 0.33	7.61 ± 0.40	7.36 ± 0.13
TC10000661.hg.1	EXOC6	8.08 ± 0.35	7.93 ± 0.33	8.87 ± 0.49	7.73 ± 0.22

TC02001977.hg.1	EXOC6B	6.72 ± 0.12	6.84 ± 0.21	6.55 ± 0.16	6.75 ± 0.03
TC17001893.hg.1	EXOC7	9.06 ± 0.17	8.89 ± 0.04	8.93 ± 0.11	8.83 ± 0.01
TC01003967.hg.1	EXOC8	7.98 ± 0.51	8.33 ± 0.24	8.23 ± 0.63	8.27 ± 0.14
TC03000193.hg.1	EXOG	7.21 ± 0.21	7.21 ± 0.09	6.99 ± 0.04	7.19 ± 0.05
TC10001566.hg.1	EXOSC1	7.54 ± 0.13	7.37 ± 0.11	7.38 ± 0.08	7.21 ± 0.04
TC01002196.hg.1	EXOSC10	8.65 ± 0.09	8.61 ± 0.09	8.39 ± 0.06	8.57 ± 0.03
TC09000740.hg.1	EXOSC2	8.15 ± 0.04	8.13 ± 0.15	7.77 ± 0.08	8.04 ± 0.03
TC09002891.hg.1	EXOSC3	7.30 ± 0.11	7.20 ± 0.11	7.19 ± 0.09	7.25 ± 0.01
TC08000838.hg.1	EXOSC4	8.02 ± 0.38	7.79 ± 0.20	8.08 ± 0.10	7.91 ± 0.03
TC19001555.hg.1	EXOSC5	9.00 ± 0.17	8.67 ± 0.06	8.61 ± 0.20	8.57 ± 0.04
TC16001233.hg.1	EXOSC6	9.03 ± 0.21	9.17 ± 0.07	8.97 ± 0.06	9.12 ± 0.13
TC03003349.hg.1	EXOSC7	7.57 ± 0.05	7.53 ± 0.08	7.34 ± 0.02	7.48 ± 0.01
TC13000128.hg.1	EXOSC8	7.52 ± 0.36	7.59 ± 0.15	7.28 ± 0.21	7.41 ± 0.07
TC04000625.hg.1	EXOSC9	7.47 ± 0.26	7.76 ± 0.19	7.40 ± 0.33	7.61 ± 0.09
TC11002269.hg.1	EXPH5	5.87 ± 0.11	5.83 ± 0.04	5.81 ± 0.08	5.87 ± 0.05
TC08001548.hg.1	EXT1	8.38 ± 0.18	8.17 ± 0.03	8.80 ± 0.22	8.16 ± 0.11
TC11003446.hg.1	EXT2	7.99 ± 0.09	7.94 ± 0.08	7.84 ± 0.12	7.85 ± 0.04
TC01000332.hg.1	EXTL1	7.42 ± 0.21	7.41 ± 0.20	7.32 ± 0.19	7.51 ± 0.08
TC01002923.hg.1	EXTL2	5.29 ± 0.09	5.19 ± 0.06	5.13 ± 0.04	5.18 ± 0.05
TC08000222.hg.1	EXTL3	7.79 ± 0.05	7.67 ± 0.02	7.74 ± 0.11	7.67 ± 0.03
TC08001093.hg.1	EXTL3-AS1	6.91 ± 0.11	6.82 ± 0.10	6.84 ± 0.02	6.91 ± 0.11
TC08001316.hg.1	EYA1	5.85 ± 0.14	5.84 ± 0.14	5.83 ± 0.09	5.94 ± 0.09
TC20000368.hg.1	EYA2	6.82 ± 0.21	6.79 ± 0.16	6.74 ± 0.10	6.86 ± 0.08
TC01002416.hg.1	EYA3	8.02 ± 0.16	7.76 ± 0.16	8.05 ± 0.26	7.61 ± 0.11
TC01002417.hg.1	EYA3-IT1	8.95 ± 0.35	8.69 ± 0.20	9.01 ± 0.21	8.68 ± 0.15
TC06000999.hg.1	EYA4	5.15 ± 0.13	5.04 ± 0.07	5.06 ± 0.04	5.09 ± 0.06
TC06001845.hg.1	EYS	4.91 ± 0.08	4.82 ± 0.01	4.83 ± 0.04	4.83 ± 0.04
TC17001540.hg.1	EZH1	9.07 ± 0.12	8.97 ± 0.13	9.02 ± 0.20	9.00 ± 0.11
TC07001993.hg.1	EZH2	7.74 ± 0.09	7.72 ± 0.27	7.49 ± 0.05	7.44 ± 0.11
TC06002265.hg.1	EZR	10.82 ± 0.06	10.98 ± 0.12	10.59 ± 0.05	11.14 ± 0.14
TC06001138.hg.1	EZR-AS1	6.99 ± 0.08	7.06 ± 0.16	6.85 ± 0.18	7.08 ± 0.15

TC13000417.hg.1	F10	7.34 ± 0.17	7.30 ± 0.11	7.29 ± 0.17	7.38 ± 0.04
TC13000891.hg.1	F10-AS1	6.59 ± 0.26	6.49 ± 0.09	6.48 ± 0.14	6.64 ± 0.16
TC04000916.hg.1	F11	4.69 ± 0.09	4.60 ± 0.03	4.66 ± 0.06	4.65 ± 0.03
TC05002105.hg.1	F12	8.29 ± 0.21	8.23 ± 0.21	8.21 ± 0.23	8.33 ± 0.06
TC06001246.hg.1	F13A1	10.81 ± 0.17	10.20 ± 0.23	11.15 ± 0.20	10.45 ± 0.37
TC01003661.hg.1	F13B	4.33 ± 0.04	4.18 ± 0.01	4.29 ± 0.11	4.18 ± 0.07
TC11000401.hg.1	F2	7.56 ± 0.21	7.56 ± 0.16	7.56 ± 0.23	7.62 ± 0.05
TC05000370.hg.1	F2R	8.25 ± 0.06	8.10 ± 0.06	8.26 ± 0.12	8.05 ± 0.03
TC05000372.hg.1	F2RL1	8.55 ± 0.37	8.24 ± 0.19	8.57 ± 0.16	8.27 ± 0.04
TC05001502.hg.1	F2RL2	6.85 ± 0.16	6.86 ± 0.06	6.91 ± 0.08	7.03 ± 0.14
TC19000326.hg.1	F2RL3	8.73 ± 0.28	8.53 ± 0.16	8.63 ± 0.17	8.68 ± 0.01
TC01002886.hg.1	F3	5.89 ± 0.12	5.86 ± 0.06	5.90 ± 0.11	5.90 ± 0.03
TC01003498.hg.1	F5	8.22 ± 0.39	7.80 ± 0.24	9.28 ± 0.34	7.68 ± 0.30
TC13000416.hg.1	F7	7.94 ± 0.25	7.90 ± 0.22	7.86 ± 0.23	8.00 ± 0.07
TC0X001547.hg.1	F8	5.67 ± 0.09	5.57 ± 0.05	5.64 ± 0.07	5.59 ± 0.02
TC0X000781.hg.1	F8A2	10.70 ± 0.32	10.52 ± 0.02	10.59 ± 0.05	10.47 ± 0.11
TC0X000787.hg.1	F8A2	10.52 ± 0.27	10.30 ± 0.04	10.36 ± 0.07	10.23 ± 0.11
TC0X001554.hg.1	F8A2	10.19 ± 0.36	9.99 ± 0.01	10.03 ± 0.06	9.94 ± 0.11
TC0X000676.hg.1	F9	4.38 ± 0.12	4.36 ± 0.03	4.33 ± 0.07	4.37 ± 0.09
TC16001271.hg.1	FA2H	7.26 ± 0.22	7.10 ± 0.16	7.09 ± 0.19	7.17 ± 0.05
TC01000585.hg.1	FAAH	7.80 ± 0.17	7.77 ± 0.11	7.66 ± 0.17	7.82 ± 0.05
TC0X000346.hg.1	FAAH2	6.54 ± 0.38	6.76 ± 0.11	6.47 ± 0.24	6.75 ± 0.06
TC02002069.hg.1	FABP1	6.09 ± 0.13	6.00 ± 0.14	6.00 ± 0.05	6.04 ± 0.07
TC08001366.hg.1	FABP12	3.77 ± 0.14	3.61 ± 0.03	3.64 ± 0.09	3.67 ± 0.08
TC04001500.hg.1	FABP2	4.06 ± 0.12	3.85 ± 0.04	4.09 ± 0.22	3.87 ± 0.06
TC01002452.hg.1	FABP3	6.58 ± 0.16	6.60 ± 0.14	6.59 ± 0.12	6.74 ± 0.10
TC13000610.hg.1	FABP3P2	5.78 ± 0.24	5.72 ± 0.09	5.72 ± 0.10	5.75 ± 0.06
TC08001365.hg.1	FABP4	4.30 ± 0.16	4.09 ± 0.02	4.17 ± 0.05	4.11 ± 0.03
TC08000518.hg.1	FABP5	7.52 ± 0.08	7.56 ± 0.21	7.37 ± 0.14	7.43 ± 0.06
TC13000729.hg.1	FABP5P1	7.94 ± 0.14	8.18 ± 0.45	7.85 ± 0.28	7.94 ± 0.13
TC07001026.hg.1	FABP5P3	7.07 ± 0.19	6.97 ± 0.09	6.89 ± 0.04	6.98 ± 0.06

TC11001833.hg.1	FABP5P7	8.20 ± 0.13	8.25 ± 0.44	7.94 ± 0.22	7.94 ± 0.10
TC05000893.hg.1	FABP6	6.88 ± 0.18	6.86 ± 0.19	6.83 ± 0.16	6.98 ± 0.06
TC06000949.hg.1	FABP7	5.16 ± 0.14	5.01 ± 0.03	5.09 ± 0.07	5.08 ± 0.12
TC08001364.hg.1	FABP9	4.58 ± 0.25	4.48 ± 0.04	4.43 ± 0.01	4.53 ± 0.14
TC11000723.hg.1	FADD	8.76 ± 0.33	8.42 ± 0.12	8.63 ± 0.21	8.47 ± 0.06
TC11000542.hg.1	FADS2	8.03 ± 0.11	7.86 ± 0.13	7.98 ± 0.19	7.90 ± 0.02
TC11003437.hg.1	FADS3	8.13 ± 0.16	8.22 ± 0.07	8.05 ± 0.13	8.29 ± 0.03
TC17001865.hg.1	FADS6	8.24 ± 0.23	8.23 ± 0.17	8.22 ± 0.18	8.29 ± 0.05
TC01002644.hg.1	FAF1	8.00 ± 0.07	8.12 ± 0.18	7.76 ± 0.04	7.94 ± 0.05
TC05001000.hg.1	FAF2	8.67 ± 0.03	8.56 ± 0.14	8.62 ± 0.14	8.52 ± 0.05
TC15000747.hg.1	FAH	7.42 ± 0.18	7.31 ± 0.12	7.29 ± 0.19	7.34 ± 0.02
TC16000049.hg.1	FAHD1	7.87 ± 0.08	7.97 ± 0.11	7.79 ± 0.19	7.96 ± 0.04
TC02000573.hg.1	FAHD2A	7.72 ± 0.09	7.73 ± 0.07	7.50 ± 0.12	7.74 ± 0.03
TC02002117.hg.1	FAHD2B	7.65 ± 0.12	7.73 ± 0.08	7.53 ± 0.16	7.77 ± 0.03
TC02000578.hg.1	FAHD2CP	7.70 ± 0.15	7.77 ± 0.12	7.55 ± 0.32	7.79 ± 0.01
TC03000746.hg.1	FAIM	6.49 ± 0.13	6.45 ± 0.03	6.34 ± 0.10	6.43 ± 0.03
TC12001485.hg.1	FAIM2	7.13 ± 0.16	7.12 ± 0.14	7.06 ± 0.12	7.21 ± 0.09
TC01003767.hg.1	FAIM3	11.02 ± 0.10	11.03 ± 0.12	10.53 ± 0.19	10.90 ± 0.03
TC12003264.hg.1	FAM101A	8.29 ± 0.22	8.22 ± 0.29	8.22 ± 0.20	8.37 ± 0.07
TC17000965.hg.1	FAM101B	9.77 ± 0.05	9.81 ± 0.18	10.02 ± 0.18	9.94 ± 0.19
TC09001618.hg.1	FAM102A	9.70 ± 0.14	9.89 ± 0.03	9.28 ± 0.12	10.04 ± 0.04
TC01000931.hg.1	FAM102B	7.90 ± 0.26	7.98 ± 0.18	8.06 ± 0.37	7.96 ± 0.13
TC15000794.hg.1	FAM103A1	7.95 ± 0.19	8.04 ± 0.29	8.05 ± 0.32	8.06 ± 0.15
TC17001846.hg.1	FAM104A	8.69 ± 0.12	8.44 ± 0.12	8.59 ± 0.16	8.28 ± 0.11
TC0X001078.hg.1	FAM104B	6.18 ± 0.06	6.03 ± 0.07	6.13 ± 0.10	5.99 ± 0.05
TC05000084.hg.1	FAM105A	8.13 ± 0.21	8.20 ± 0.14	8.47 ± 0.34	8.10 ± 0.26
TC05000086.hg.1	FAM105B	8.25 ± 0.09	8.34 ± 0.11	8.18 ± 0.09	8.27 ± 0.10
TC17001223.hg.1	FAM106A	5.90 ± 0.51	6.11 ± 0.08	6.09 ± 0.12	6.18 ± 0.13
TC17000192.hg.1	FAM106CP	5.59 ± 0.50	5.60 ± 0.25	5.71 ± 0.29	5.90 ± 0.24
TC17000256.hg.1	FAM106CP	6.23 ± 0.19	6.30 ± 0.13	6.26 ± 0.08	6.44 ± 0.15
TC03001501.hg.1	FAM107A	7.25 ± 0.21	7.20 ± 0.12	7.14 ± 0.22	7.23 ± 0.06

TC10001054.hg.1	FAM107B	8.19 ± 0.24	8.49 ± 0.09	8.22 ± 0.19	8.49 ± 0.03
TC01001121.hg.1	FAM108A2	9.17 ± 0.22	8.85 ± 0.25	8.95 ± 0.03	8.72 ± 0.07
TC12001971.hg.1	FAM109A	8.32 ± 0.19	8.24 ± 0.17	8.16 ± 0.17	8.30 ± 0.08
TC22000352.hg.1	FAM109B	8.29 ± 0.23	8.24 ± 0.19	8.25 ± 0.21	8.34 ± 0.02
TC20000012.hg.1	FAM110A	8.40 ± 0.11	8.27 ± 0.11	8.36 ± 0.15	8.34 ± 0.10
TC08000401.hg.1	FAM110B	6.57 ± 0.13	6.58 ± 0.11	6.55 ± 0.04	6.61 ± 0.14
TC02001494.hg.1	FAM110C	6.91 ± 0.23	6.86 ± 0.22	6.78 ± 0.27	6.90 ± 0.07
TC01000334.hg.1	FAM110D	7.61 ± 0.15	7.57 ± 0.19	7.63 ± 0.21	7.83 ± 0.06
TC11000483.hg.1	FAM111A	8.75 ± 0.19	8.83 ± 0.14	8.83 ± 0.30	8.61 ± 0.15
TC11000482.hg.1	FAM111B	6.92 ± 0.43	7.65 ± 0.56	6.74 ± 0.56	7.18 ± 0.26
TC04000222.hg.1	FAM114A1	6.28 ± 0.07	6.14 ± 0.06	6.39 ± 0.06	6.17 ± 0.04
TC05001966.hg.1	FAM114A2	7.35 ± 0.01	7.20 ± 0.06	7.33 ± 0.09	7.17 ± 0.02
TC07001975.hg.1	FAM115A	7.67 ± 0.05	7.70 ± 0.05	7.57 ± 0.09	7.70 ± 0.07
TC07000942.hg.1	FAM115C	8.05 ± 0.18	8.18 ± 0.15	8.08 ± 0.13	8.31 ± 0.06
TC17001672.hg.1	FAM117A	9.32 ± 0.03	9.27 ± 0.03	9.19 ± 0.06	9.26 ± 0.02
TC02001193.hg.1	FAM117B	9.98 ± 0.30	10.27 ± 0.05	9.72 ± 0.47	10.30 ± 0.11
TC22000380.hg.1	FAM118A	7.97 ± 0.10	8.01 ± 0.06	7.80 ± 0.08	7.95 ± 0.06
TC11001169.hg.1	FAM118B	7.64 ± 0.12	7.23 ± 0.11	7.50 ± 0.14	7.08 ± 0.09
TC09000448.hg.1	FAM120A	9.68 ± 0.17	9.77 ± 0.08	9.70 ± 0.13	9.56 ± 0.14
TC09001350.hg.1	FAM120AOS	8.48 ± 0.10	8.34 ± 0.10	8.41 ± 0.07	8.35 ± 0.10
TC06001199.hg.1	FAM120B	8.00 ± 0.05	7.86 ± 0.05	7.99 ± 0.07	7.83 ± 0.03
TC0X001072.hg.1	FAM120C	6.80 ± 0.05	6.81 ± 0.04	6.57 ± 0.10	6.83 ± 0.06
TC09000317.hg.1	FAM122A	8.18 ± 0.08	7.95 ± 0.06	8.07 ± 0.09	7.87 ± 0.07
TC0X001396.hg.1	FAM122B	8.17 ± 0.31	8.22 ± 0.11	8.16 ± 0.23	8.08 ± 0.12
TC0X000641.hg.1	FAM122C	7.15 ± 0.15	7.15 ± 0.08	7.09 ± 0.15	7.09 ± 0.07
TC13000211.hg.1	FAM124A	6.70 ± 0.29	6.72 ± 0.15	6.70 ± 0.21	6.81 ± 0.07
TC02002825.hg.1	FAM124B	6.64 ± 0.17	6.67 ± 0.05	6.65 ± 0.03	6.85 ± 0.14
TC07001192.hg.1	FAM126A	7.96 ± 0.31	8.02 ± 0.19	8.02 ± 0.23	7.78 ± 0.15
TC02002670.hg.1	FAM126B	9.46 ± 0.18	9.39 ± 0.23	10.02 ± 0.42	9.33 ± 0.18
TC0X000645.hg.1	FAM127A	8.93 ± 0.06	8.74 ± 0.08	8.96 ± 0.09	8.67 ± 0.09
TC0X001400.hg.1	FAM127B	8.70 ± 0.15	8.50 ± 0.08	8.61 ± 0.03	8.44 ± 0.07

TC0X001399.hg.1	FAM127C	8.08 ± 0.13	7.92 ± 0.06	8.11 ± 0.07	7.94 ± 0.03
TC01003624.hg.1	FAM129A	10.17 ± 0.36	10.19 ± 0.31	11.18 ± 0.29	10.08 ± 0.35
TC09001604.hg.1	FAM129B	7.97 ± 0.20	7.83 ± 0.14	8.04 ± 0.11	7.90 ± 0.09
TC19000339.hg.1	FAM129C	9.08 ± 0.19	9.12 ± 0.09	8.80 ± 0.10	9.04 ± 0.12
TC03001002.hg.1	FAM131A	7.70 ± 0.19	7.69 ± 0.13	7.64 ± 0.16	7.78 ± 0.04
TC07001969.hg.1	FAM131B	7.04 ± 0.22	7.04 ± 0.20	6.95 ± 0.20	7.12 ± 0.04
TC01002257.hg.1	FAM131C	7.71 ± 0.21	7.78 ± 0.22	7.65 ± 0.20	7.89 ± 0.09
TC01002088.hg.1	FAM132A	8.72 ± 0.22	8.77 ± 0.25	8.70 ± 0.26	8.88 ± 0.07
TC02001451.hg.1	FAM132B	8.65 ± 0.18	8.60 ± 0.15	8.60 ± 0.14	8.64 ± 0.00
TC0X000459.hg.1	FAM133A	4.99 ± 0.13	4.87 ± 0.09	4.97 ± 0.04	4.97 ± 0.11
TC07001602.hg.1	FAM133B	6.82 ± 0.42	7.21 ± 0.19	6.93 ± 0.36	7.22 ± 0.09
TC10000363.hg.1	FAM133CP	6.06 ± 0.28	6.01 ± 0.11	6.14 ± 0.16	6.21 ± 0.06
TC02001318.hg.1	FAM134A	8.83 ± 0.18	8.71 ± 0.08	8.72 ± 0.20	8.74 ± 0.10
TC05001182.hg.1	FAM134B	7.62 ± 0.29	7.85 ± 0.09	7.47 ± 0.27	7.86 ± 0.05
TC17001537.hg.1	FAM134C	9.05 ± 0.12	8.86 ± 0.03	8.71 ± 0.23	8.72 ± 0.08
TC06000715.hg.1	FAM135A	6.31 ± 0.24	6.42 ± 0.12	6.25 ± 0.18	6.35 ± 0.09
TC08001674.hg.1	FAM135B	5.67 ± 0.13	5.64 ± 0.11	5.62 ± 0.08	5.69 ± 0.04
TC02001961.hg.1	FAM136A	8.12 ± 0.06	8.20 ± 0.08	8.05 ± 0.01	8.22 ± 0.03
TC01002051.hg.1	FAM138A	6.19 ± 0.41	6.10 ± 0.06	6.14 ± 0.08	6.27 ± 0.22
TC19000975.hg.1	FAM138A	6.41 ± 0.51	6.47 ± 0.02	6.33 ± 0.29	6.78 ± 0.36
TC02000730.hg.1	FAM138B	6.67 ± 0.46	6.57 ± 0.13	6.63 ± 0.22	6.90 ± 0.39
TC09000860.hg.1	FAM138C	5.85 ± 0.28	5.83 ± 0.07	5.88 ± 0.05	5.97 ± 0.14
TC12001074.hg.1	FAM138D	6.98 ± 0.27	6.90 ± 0.11	6.86 ± 0.15	6.95 ± 0.19
TC15001003.hg.1	FAM138E	6.36 ± 0.20	6.32 ± 0.03	6.33 ± 0.10	6.50 ± 0.28
TC04001380.hg.1	FAM13A	6.81 ± 0.13	6.74 ± 0.07	6.91 ± 0.14	6.73 ± 0.02
TC04000487.hg.1	FAM13A-AS1	7.84 ± 0.44	8.12 ± 0.31	8.29 ± 0.53	8.05 ± 0.21
TC05001822.hg.1	FAM13B	8.62 ± 0.30	8.83 ± 0.19	8.64 ± 0.31	8.73 ± 0.04
TC10001314.hg.1	FAM13C	5.92 ± 0.15	5.82 ± 0.10	5.82 ± 0.13	5.88 ± 0.07
TC04000913.hg.1	FAM149A	6.70 ± 0.19	6.64 ± 0.13	6.64 ± 0.11	6.68 ± 0.03
TC10000458.hg.1	FAM149B1	7.34 ± 0.13	7.24 ± 0.14	7.42 ± 0.27	7.05 ± 0.11
TC08001209.hg.1	FAM150A	5.34 ± 0.16	5.29 ± 0.15	5.26 ± 0.13	5.36 ± 0.02

TC02001496.hg.1	FAM150B	6.79 ± 0.15	6.69 ± 0.14	6.65 ± 0.16	6.74 ± 0.07
TC01002689.hg.1	FAM151A	7.21 ± 0.17	7.21 ± 0.24	7.16 ± 0.19	7.42 ± 0.12
TC05000399.hg.1	FAM151B	6.53 ± 0.12	6.31 ± 0.13	7.06 ± 0.19	6.29 ± 0.04
TC05002115.hg.1	FAM153A	6.61 ± 0.17	6.66 ± 0.03	6.57 ± 0.13	6.69 ± 0.08
TC05000988.hg.1	FAM153B	6.63 ± 0.16	6.67 ± 0.03	6.57 ± 0.14	6.70 ± 0.08
TC05001030.hg.1	FAM153C	6.39 ± 0.16	6.39 ± 0.01	6.32 ± 0.13	6.44 ± 0.06
TC09000932.hg.1	FAM154A	5.24 ± 0.13	5.18 ± 0.09	5.06 ± 0.11	5.23 ± 0.02
TC15000763.hg.1	FAM154B	6.80 ± 0.28	6.78 ± 0.19	6.79 ± 0.21	6.84 ± 0.04
TC13000863.hg.1	FAM155A	5.47 ± 0.18	5.51 ± 0.10	5.48 ± 0.17	5.51 ± 0.10
TC13000866.hg.1	FAM155A-IT1	6.02 ± 0.21	6.17 ± 0.21	6.14 ± 0.17	6.37 ± 0.10
TC0X000364.hg.1	FAM155B	6.46 ± 0.13	6.47 ± 0.26	6.46 ± 0.07	6.59 ± 0.10
TC0X000308.hg.1	FAM156B	8.09 ± 0.08	8.06 ± 0.08	8.02 ± 0.07	8.13 ± 0.09
TC0X001059.hg.1	FAM156B	7.75 ± 0.10	7.68 ± 0.06	7.66 ± 0.09	7.73 ± 0.06
TC03003371.hg.1	FAM157A	11.19 ± 0.36	11.23 ± 0.22	11.89 ± 0.26	11.23 ± 0.20
TC09000857.hg.1	FAM157B	10.66 ± 0.32	10.60 ± 0.22	11.40 ± 0.26	10.55 ± 0.25
TC01000644.hg.1	FAM159A	8.54 ± 0.13	8.62 ± 0.05	8.27 ± 0.08	8.61 ± 0.03
TC05000273.hg.1	FAM159B	5.88 ± 0.12	5.71 ± 0.04	5.79 ± 0.01	5.79 ± 0.07
TC04000754.hg.1	FAM160A1	5.90 ± 0.07	5.85 ± 0.06	5.86 ± 0.11	5.94 ± 0.06
TC11001353.hg.1	FAM160A2	9.12 ± 0.19	8.87 ± 0.01	9.20 ± 0.12	8.88 ± 0.01
TC10000836.hg.1	FAM160B1	8.94 ± 0.33	8.85 ± 0.16	9.54 ± 0.53	8.82 ± 0.18
TC08000165.hg.1	FAM160B2	8.66 ± 0.05	8.64 ± 0.09	8.52 ± 0.04	8.68 ± 0.07
TC02001894.hg.1	FAM161A	5.36 ± 0.20	5.25 ± 0.08	5.29 ± 0.03	5.31 ± 0.03
TC14002346.hg.1	FAM161B	6.92 ± 0.16	6.78 ± 0.08	6.87 ± 0.02	6.76 ± 0.07
TC03000631.hg.1	FAM162A	6.86 ± 0.02	6.98 ± 0.18	6.74 ± 0.10	6.97 ± 0.03
TC06002052.hg.1	FAM162B	7.05 ± 0.16	7.03 ± 0.05	7.08 ± 0.12	7.11 ± 0.13
TC01001555.hg.1	FAM163A	6.59 ± 0.19	6.49 ± 0.18	6.48 ± 0.11	6.55 ± 0.05
TC09001693.hg.1	FAM163B	7.41 ± 0.30	7.46 ± 0.26	7.32 ± 0.14	7.53 ± 0.06
TC09001767.hg.1	FAM166A	7.44 ± 0.22	7.37 ± 0.20	7.36 ± 0.17	7.49 ± 0.06
TC09001053.hg.1	FAM166B	7.20 ± 0.16	7.13 ± 0.13	7.14 ± 0.10	7.22 ± 0.08
TC08000975.hg.1	FAM167A	7.81 ± 0.17	7.91 ± 0.17	7.74 ± 0.21	7.99 ± 0.10
TC01000425.hg.1	FAM167B	7.69 ± 0.13	7.62 ± 0.29	7.48 ± 0.20	7.76 ± 0.10

TC11002067.hg.1	FAM168A	8.37 ± 0.20	8.28 ± 0.03	8.29 ± 0.19	8.16 ± 0.08
TC02002348.hg.1	FAM168B	9.02 ± 0.04	9.08 ± 0.07	8.77 ± 0.13	9.10 ± 0.09
TC05001484.hg.1	FAM169A	6.80 ± 0.30	7.01 ± 0.17	6.59 ± 0.19	7.06 ± 0.05
TC15001978.hg.1	FAM169B	6.94 ± 0.16	6.94 ± 0.08	6.96 ± 0.14	7.03 ± 0.08
TC05000568.hg.1	FAM170A	6.64 ± 0.13	6.62 ± 0.16	6.64 ± 0.10	6.73 ± 0.10
TC10001272.hg.1	FAM170B	6.71 ± 0.30	6.84 ± 0.25	6.69 ± 0.44	6.98 ± 0.20
TC10000326.hg.1	FAM170B-AS1	5.78 ± 0.12	5.74 ± 0.09	5.71 ± 0.07	5.81 ± 0.11
TC10001063.hg.1	FAM171A1	7.62 ± 0.08	7.54 ± 0.14	7.50 ± 0.17	7.55 ± 0.03
TC17001580.hg.1	FAM171A2	8.25 ± 0.17	8.26 ± 0.17	8.23 ± 0.12	8.31 ± 0.06
TC02001101.hg.1	FAM171B	5.20 ± 0.04	5.06 ± 0.03	5.14 ± 0.01	5.08 ± 0.01
TC03000517.hg.1	FAM172BP	6.16 ± 0.06	6.17 ± 0.05	6.06 ± 0.19	6.24 ± 0.06
TC16000029.hg.1	FAM173A	9.20 ± 0.24	9.07 ± 0.15	9.08 ± 0.16	9.09 ± 0.05
TC05001157.hg.1	FAM173B	7.09 ± 0.03	6.86 ± 0.14	6.95 ± 0.13	6.65 ± 0.09
TC05000490.hg.1	FAM174A	8.14 ± 0.12	8.17 ± 0.08	8.14 ± 0.20	8.12 ± 0.22
TC15001886.hg.1	FAM174B	7.73 ± 0.18	7.72 ± 0.14	7.71 ± 0.10	7.79 ± 0.02
TC04001349.hg.1	FAM175A	7.11 ± 0.14	7.15 ± 0.24	7.08 ± 0.12	7.08 ± 0.07
TC10000908.hg.1	FAM175B	8.38 ± 0.17	8.45 ± 0.18	8.42 ± 0.22	8.39 ± 0.09
TC14000220.hg.1	FAM177A1	8.10 ± 0.11	8.20 ± 0.05	8.28 ± 0.22	8.22 ± 0.14
TC01001831.hg.1	FAM177B	6.18 ± 0.37	5.97 ± 0.13	6.16 ± 0.14	5.90 ± 0.04
TC10000726.hg.1	FAM178A	7.55 ± 0.34	7.51 ± 0.28	7.62 ± 0.35	7.24 ± 0.11
TC02002114.hg.1	FAM178B	7.64 ± 0.15	7.65 ± 0.18	7.59 ± 0.17	7.77 ± 0.06
TC02000191.hg.1	FAM179A	7.64 ± 0.15	7.65 ± 0.18	7.61 ± 0.14	7.72 ± 0.07
TC14000262.hg.1	FAM179B	6.37 ± 0.46	6.45 ± 0.26	6.19 ± 0.22	6.35 ± 0.01
TC07001889.hg.1	FAM180A	6.40 ± 0.18	6.32 ± 0.12	6.28 ± 0.17	6.41 ± 0.05
TC11000410.hg.1	FAM180B	8.07 ± 0.19	8.10 ± 0.15	8.02 ± 0.25	8.15 ± 0.10
TC14000579.hg.1	FAM181A	8.13 ± 0.17	8.05 ± 0.17	8.13 ± 0.11	8.13 ± 0.04
TC14001460.hg.1	FAM181A-AS1	6.92 ± 0.29	6.86 ± 0.17	6.85 ± 0.17	6.92 ± 0.10
TC11002150.hg.1	FAM181B	6.59 ± 0.37	6.49 ± 0.24	6.59 ± 0.32	6.59 ± 0.05
TC20000176.hg.1	FAM182A	6.49 ± 0.16	6.44 ± 0.07	6.47 ± 0.07	6.59 ± 0.13
TC20000736.hg.1	FAM182B	6.98 ± 0.13	6.91 ± 0.13	6.89 ± 0.16	7.09 ± 0.11
TC01000539.hg.1	FAM183A	6.91 ± 0.18	6.84 ± 0.13	6.83 ± 0.14	6.94 ± 0.09

TC07001304.hg.1	FAM183B	7.44 ± 0.19	7.64 ± 0.23	7.45 ± 0.14	7.81 ± 0.13
TC08000236.hg.1	FAM183CP	5.83 ± 0.15	5.66 ± 0.07	5.75 ± 0.09	5.75 ± 0.14
TC06002060.hg.1	FAM184A	4.89 ± 0.10	4.85 ± 0.01	4.92 ± 0.09	4.86 ± 0.04
TC04001068.hg.1	FAM184B	7.12 ± 0.21	7.07 ± 0.10	7.06 ± 0.18	7.12 ± 0.02
TC07000665.hg.1	FAM185A	6.92 ± 0.14	6.80 ± 0.14	6.81 ± 0.16	6.73 ± 0.03
TC12001497.hg.1	FAM186A	4.77 ± 0.15	4.56 ± 0.02	4.52 ± 0.09	4.52 ± 0.05
TC12001480.hg.1	FAM186B	6.80 ± 0.17	6.78 ± 0.17	6.71 ± 0.20	6.82 ± 0.05
TC19001441.hg.1	FAM187B	7.84 ± 0.20	7.68 ± 0.11	7.71 ± 0.12	7.61 ± 0.10
TC10001065.hg.1	FAM188A	7.46 ± 0.35	7.50 ± 0.22	7.57 ± 0.41	7.36 ± 0.04
TC07003392.hg.1	FAM188B	7.19 ± 0.15	7.20 ± 0.21	7.14 ± 0.16	7.26 ± 0.05
TC03003419.hg.1	FAM188B2	5.80 ± 0.09	5.67 ± 0.10	5.88 ± 0.06	5.77 ± 0.04
TC15001107.hg.1	FAM189A1	7.34 ± 0.18	7.34 ± 0.16	7.24 ± 0.20	7.44 ± 0.05
TC09000320.hg.1	FAM189A2	6.40 ± 0.14	6.37 ± 0.08	6.36 ± 0.10	6.42 ± 0.08
TC01003309.hg.1	FAM189B	8.23 ± 0.18	8.13 ± 0.14	8.12 ± 0.09	8.14 ± 0.05
TC16001136.hg.1	FAM192A	9.21 ± 0.12	8.98 ± 0.17	9.11 ± 0.26	8.84 ± 0.08
TC04002911.hg.1	FAM193A	8.77 ± 0.09	8.77 ± 0.06	8.69 ± 0.11	8.75 ± 0.09
TC05002113.hg.1	FAM193B	8.64 ± 0.05	8.62 ± 0.06	8.61 ± 0.00	8.65 ± 0.03
TC03001900.hg.1	FAM194A	5.42 ± 0.18	5.26 ± 0.11	5.27 ± 0.08	5.30 ± 0.03
TC13000635.hg.1	FAM194B	4.93 ± 0.17	4.85 ± 0.07	4.88 ± 0.11	4.90 ± 0.04
TC16000021.hg.1	FAM195A	8.63 ± 0.23	8.58 ± 0.18	8.51 ± 0.23	8.67 ± 0.05
TC17001958.hg.1	FAM195B	9.05 ± 0.10	9.01 ± 0.06	8.97 ± 0.08	9.08 ± 0.09
TC10001752.hg.1	FAM196A	6.32 ± 0.28	6.22 ± 0.10	6.19 ± 0.16	6.21 ± 0.08
TC05002048.hg.1	FAM196B	6.74 ± 0.04	6.66 ± 0.09	6.72 ± 0.04	6.70 ± 0.12
TC0Y000149.hg.1	FAM197Y1	7.42 ± 0.30	7.34 ± 0.22	7.43 ± 0.18	7.60 ± 0.23
TC0Y000142.hg.1	FAM197Y2	7.57 ± 0.32	7.55 ± 0.13	7.55 ± 0.10	7.88 ± 0.26
TC0Y000147.hg.1	FAM197Y3	7.57 ± 0.33	7.57 ± 0.16	7.53 ± 0.11	7.88 ± 0.27
TC0Y000146.hg.1	FAM197Y5	7.54 ± 0.33	7.58 ± 0.13	7.56 ± 0.11	7.90 ± 0.27
TC0Y000357.hg.1	FAM197Y5	7.40 ± 0.27	7.38 ± 0.16	7.30 ± 0.15	7.66 ± 0.32
TC0Y000358.hg.1	FAM197Y5	7.53 ± 0.29	7.52 ± 0.17	7.44 ± 0.13	7.82 ± 0.32
TC0Y000127.hg.1	FAM197Y9	7.49 ± 0.30	7.53 ± 0.15	7.50 ± 0.07	7.83 ± 0.25
TC03003347.hg.1	FAM198A	7.59 ± 0.22	7.55 ± 0.17	7.49 ± 0.30	7.63 ± 0.08

TC04001682.hg.1	FAM198B	7.06 ± 0.18	6.80 ± 0.14	7.58 ± 0.35	6.73 ± 0.12
TC0X000510.hg.1	FAM199X	8.20 ± 0.39	8.37 ± 0.12	8.27 ± 0.37	8.25 ± 0.11
TC03000412.hg.1	FAM19A1	5.61 ± 0.14	5.47 ± 0.03	5.51 ± 0.04	5.56 ± 0.08
TC12001650.hg.1	FAM19A2	6.05 ± 0.16	6.03 ± 0.03	6.15 ± 0.15	6.05 ± 0.05
TC01000993.hg.1	FAM19A3	7.83 ± 0.20	7.94 ± 0.21	7.81 ± 0.18	8.07 ± 0.04
TC03001534.hg.1	FAM19A4	6.70 ± 0.27	6.67 ± 0.17	6.63 ± 0.18	6.72 ± 0.08
TC22000407.hg.1	FAM19A5	7.85 ± 0.25	7.85 ± 0.24	7.74 ± 0.27	7.90 ± 0.08
TC07001647.hg.1	FAM200A	7.58 ± 0.09	7.47 ± 0.04	7.62 ± 0.08	7.47 ± 0.04
TC04002928.hg.1	FAM200B	7.00 ± 0.11	6.90 ± 0.13	7.41 ± 0.30	6.84 ± 0.15
TC09000225.hg.1	FAM201A	8.25 ± 0.16	8.16 ± 0.14	8.23 ± 0.14	8.20 ± 0.05
TC08000843.hg.1	FAM203B	7.92 ± 0.22	7.90 ± 0.16	7.84 ± 0.21	7.91 ± 0.05
TC08000846.hg.1	FAM203B	8.06 ± 0.19	7.95 ± 0.17	7.92 ± 0.18	7.95 ± 0.05
TC10001692.hg.1	FAM204A	7.22 ± 0.35	7.31 ± 0.21	7.30 ± 0.30	7.23 ± 0.10
TC09001046.hg.1	FAM205A	6.26 ± 0.25	6.33 ± 0.15	6.19 ± 0.21	6.40 ± 0.03
TC09001047.hg.1	FAM205B	6.23 ± 0.15	6.07 ± 0.19	6.11 ± 0.08	6.25 ± 0.01
TC09000548.hg.1	FAM206A	7.83 ± 0.09	7.80 ± 0.15	7.93 ± 0.14	7.60 ± 0.09
TC21000240.hg.1	FAM207A	8.40 ± 0.19	8.38 ± 0.16	8.30 ± 0.26	8.45 ± 0.04
TC03001487.hg.1	FAM208A	8.52 ± 0.56	8.83 ± 0.27	8.78 ± 0.41	8.55 ± 0.18
TC10000049.hg.1	FAM208B	7.98 ± 0.40	8.18 ± 0.21	7.94 ± 0.40	8.13 ± 0.07
TC20001757.hg.1	FAM209B	6.82 ± 0.18	6.74 ± 0.19	6.88 ± 0.18	6.79 ± 0.02
TC17001824.hg.1	FAM20A	7.36 ± 0.29	6.98 ± 0.14	7.81 ± 0.03	7.06 ± 0.08
TC01001545.hg.1	FAM20B	8.42 ± 0.09	8.30 ± 0.06	8.21 ± 0.10	8.15 ± 0.04
TC07000004.hg.1	FAM20C	7.93 ± 0.22	7.82 ± 0.13	7.92 ± 0.14	7.95 ± 0.12
TC18000380.hg.1	FAM210A	7.09 ± 0.08	7.15 ± 0.12	7.02 ± 0.14	7.08 ± 0.03
TC20000427.hg.1	FAM210B	8.13 ± 0.16	8.21 ± 0.16	8.00 ± 0.17	8.25 ± 0.04
TC17001174.hg.1	FAM211A	7.62 ± 0.13	7.62 ± 0.10	7.52 ± 0.08	7.68 ± 0.07
TC22000590.hg.1	FAM211B	8.04 ± 0.16	8.08 ± 0.23	8.00 ± 0.21	8.19 ± 0.11
TC03000299.hg.1	FAM212A	7.98 ± 0.19	7.88 ± 0.15	7.98 ± 0.14	7.98 ± 0.01
TC01002990.hg.1	FAM212B	8.28 ± 0.07	8.05 ± 0.12	8.35 ± 0.03	7.98 ± 0.21
TC01002991.hg.1	FAM212B	6.92 ± 0.12	6.92 ± 0.06	6.88 ± 0.17	7.05 ± 0.19
TC10000581.hg.1	FAM213A	6.65 ± 0.05	6.59 ± 0.04	6.56 ± 0.07	6.57 ± 0.03

TC01000064.hg.1	FAM213B	8.62 ± 0.19	8.60 ± 0.16	8.49 ± 0.23	8.64 ± 0.07
TC15001458.hg.1	FAM214A	8.40 ± 0.47	8.79 ± 0.19	8.22 ± 0.46	8.75 ± 0.05
TC09001052.hg.1	FAM214B	9.09 ± 0.21	8.85 ± 0.09	9.22 ± 0.15	8.92 ± 0.18
TC17000560.hg.1	FAM215A	7.72 ± 0.29	7.65 ± 0.29	7.63 ± 0.16	7.86 ± 0.09
TC17001614.hg.1	FAM215A	7.72 ± 0.16	7.59 ± 0.07	7.57 ± 0.22	7.60 ± 0.18
TC12000857.hg.1	FAM216A	6.76 ± 0.12	6.65 ± 0.10	6.61 ± 0.03	6.58 ± 0.02
TC13000155.hg.1	FAM216B	5.18 ± 0.27	5.14 ± 0.22	5.12 ± 0.16	5.18 ± 0.05
TC06001234.hg.1	FAM217A	5.24 ± 0.16	5.12 ± 0.08	5.16 ± 0.11	5.14 ± 0.01
TC20000473.hg.1	FAM217B	8.15 ± 0.02	7.94 ± 0.13	8.33 ± 0.21	7.85 ± 0.10
TC04000824.hg.1	FAM218A	8.10 ± 0.61	7.61 ± 0.10	7.76 ± 0.25	7.51 ± 0.15
TC09001036.hg.1	FAM219A	8.15 ± 0.12	8.04 ± 0.03	8.12 ± 0.11	8.02 ± 0.07
TC15001651.hg.1	FAM219B	7.77 ± 0.09	7.55 ± 0.09	7.67 ± 0.10	7.47 ± 0.01
TC10000310.hg.1	FAM21B	9.33 ± 0.18	9.49 ± 0.09	9.31 ± 0.18	9.42 ± 0.04
TC10000341.hg.1	FAM21B	9.40 ± 0.15	9.55 ± 0.08	9.37 ± 0.18	9.49 ± 0.04
TC10000294.hg.1	FAM21C	9.51 ± 0.19	9.68 ± 0.11	9.52 ± 0.17	9.60 ± 0.03
TC10000331.hg.1	FAM21D	8.72 ± 0.31	8.87 ± 0.15	8.87 ± 0.18	8.73 ± 0.10
TC07001130.hg.1	FAM220A	8.65 ± 0.00	8.53 ± 0.09	8.44 ± 0.08	8.52 ± 0.03
TC09000223.hg.1	FAM220BP	7.22 ± 0.31	7.11 ± 0.09	7.10 ± 0.25	7.22 ± 0.05
TC07000150.hg.1	FAM221A	5.65 ± 0.10	5.59 ± 0.04	5.61 ± 0.08	5.60 ± 0.05
TC09001065.hg.1	FAM221B	6.90 ± 0.07	6.83 ± 0.05	6.93 ± 0.06	6.92 ± 0.02
TC12000850.hg.1	FAM222A	8.49 ± 0.25	8.43 ± 0.17	8.36 ± 0.21	8.47 ± 0.06
TC12001955.hg.1	FAM222A-AS1	6.88 ± 0.23	6.89 ± 0.12	6.81 ± 0.13	6.97 ± 0.10
TC17001307.hg.1	FAM222B	8.85 ± 0.03	8.69 ± 0.04	8.79 ± 0.09	8.72 ± 0.06
TC0X000777.hg.1	FAM223A	6.79 ± 0.13	6.54 ± 0.36	6.93 ± 0.33	6.39 ± 0.01
TC0X001540.hg.1	FAM223B	7.01 ± 0.14	6.60 ± 0.43	7.03 ± 0.44	6.39 ± 0.15
TC0Y000067.hg.1	FAM224A	6.10 ± 0.10	6.03 ± 0.11	6.01 ± 0.11	6.15 ± 0.04
TC0Y000170.hg.1	FAM224B	6.14 ± 0.15	6.04 ± 0.10	6.05 ± 0.08	6.15 ± 0.06
TC09000570.hg.1	FAM225A	7.37 ± 0.32	7.14 ± 0.14	7.19 ± 0.10	7.13 ± 0.01
TC09001497.hg.1	FAM225B	7.30 ± 0.26	7.17 ± 0.17	7.24 ± 0.12	7.18 ± 0.06
TC0X002335.hg.1	FAM226B	7.58 ± 0.08	7.50 ± 0.06	7.34 ± 0.15	7.52 ± 0.19
TC22000793.hg.1	FAM227A	5.79 ± 0.15	5.71 ± 0.10	5.69 ± 0.14	5.74 ± 0.07

TC15001346.hg.1	FAM227B	4.87 ± 0.03	4.78 ± 0.04	4.79 ± 0.04	4.74 ± 0.06
TC02005059.hg.1	FAM228A	5.19 ± 0.13	4.91 ± 0.09	5.04 ± 0.13	4.95 ± 0.03
TC02005058.hg.1	FAM228B	6.99 ± 0.05	6.86 ± 0.03	6.97 ± 0.12	6.86 ± 0.05
TC01002470.hg.1	FAM229A	8.53 ± 0.19	8.48 ± 0.09	8.47 ± 0.09	8.54 ± 0.03
TC06000895.hg.1	FAM229B	6.19 ± 0.12	6.26 ± 0.07	6.17 ± 0.14	6.29 ± 0.03
TC10000572.hg.1	FAM22B	7.83 ± 0.10	7.87 ± 0.13	7.76 ± 0.11	7.94 ± 0.07
TC10000617.hg.1	FAM22D	8.05 ± 0.11	7.99 ± 0.14	7.92 ± 0.12	8.09 ± 0.09
TC22000538.hg.1	FAM230C	6.31 ± 0.33	6.18 ± 0.04	6.21 ± 0.12	6.54 ± 0.31
TCUn_gl000212000001.hg.1	FAM230C	6.51 ± 0.25	6.35 ± 0.09	6.44 ± 0.06	6.51 ± 0.08
TC10000894.hg.1	FAM24A	4.86 ± 0.18	4.84 ± 0.11	4.75 ± 0.28	5.09 ± 0.03
TC10002954.hg.1	FAM24B	6.68 ± 0.11	6.70 ± 0.06	6.67 ± 0.08	6.71 ± 0.02
TC10002940.hg.1	FAM25A	7.27 ± 0.28	7.19 ± 0.19	7.22 ± 0.26	7.33 ± 0.07
TC10000313.hg.1	FAM25C	7.77 ± 0.23	7.61 ± 0.24	7.64 ± 0.17	7.85 ± 0.04
TC10001239.hg.1	FAM25C	7.63 ± 0.22	7.50 ± 0.22	7.51 ± 0.12	7.68 ± 0.07
TC10001257.hg.1	FAM25C	8.25 ± 0.29	8.11 ± 0.22	8.19 ± 0.19	8.36 ± 0.04
TC10001280.hg.1	FAM25D	6.59 ± 0.30	6.45 ± 0.27	6.50 ± 0.13	6.82 ± 0.16
TC10001227.hg.1	FAM25E	6.59 ± 0.30	6.45 ± 0.27	6.50 ± 0.13	6.82 ± 0.16
TC06000922.hg.1	FAM26D	5.44 ± 0.15	5.43 ± 0.05	5.41 ± 0.04	5.48 ± 0.08
TC06000921.hg.1	FAM26E	6.05 ± 0.14	5.82 ± 0.26	6.29 ± 0.15	5.91 ± 0.08
TC06000919.hg.1	FAM26F	7.68 ± 0.26	7.46 ± 0.07	7.48 ± 0.19	7.42 ± 0.07
TC09000266.hg.1	FAM27A	8.28 ± 0.25	8.21 ± 0.05	8.18 ± 0.13	8.46 ± 0.14
TC09001161.hg.1	FAM27B	8.08 ± 0.32	7.99 ± 0.03	7.92 ± 0.15	8.31 ± 0.17
TC20000173.hg.1	FAM27B	6.75 ± 0.25	6.75 ± 0.04	6.62 ± 0.15	6.90 ± 0.13
TC09000259.hg.1	FAM27C	8.12 ± 0.26	8.08 ± 0.04	8.02 ± 0.15	8.37 ± 0.14
TC09000267.hg.1	FAM27E2	7.91 ± 0.11	8.01 ± 0.11	8.04 ± 0.07	8.17 ± 0.23
TC09000260.hg.1	FAM27E3	8.32 ± 0.16	8.10 ± 0.08	8.29 ± 0.02	8.33 ± 0.07
TC09001136.hg.1	FAM27E3	7.49 ± 0.12	7.49 ± 0.10	7.41 ± 0.18	7.66 ± 0.16
TC09001160.hg.1	FAM27E3	8.24 ± 0.19	8.10 ± 0.03	8.18 ± 0.07	8.34 ± 0.07
TC17000300.hg.1	FAM27L	7.53 ± 0.18	7.40 ± 0.19	7.47 ± 0.17	7.58 ± 0.14
TC19000319.hg.1	FAM32A	8.94 ± 0.20	8.87 ± 0.17	9.00 ± 0.13	8.73 ± 0.24
TC10000613.hg.1	FAM35A	7.29 ± 0.30	7.36 ± 0.27	7.16 ± 0.34	7.16 ± 0.16

TC10000298.hg.1	FAM35BP	6.47 ± 0.26	6.40 ± 0.25	6.42 ± 0.28	6.29 ± 0.16
TC10000306.hg.1	FAM35DP	6.15 ± 0.33	6.07 ± 0.28	6.17 ± 0.32	5.95 ± 0.17
TC0X001537.hg.1	FAM3A	8.25 ± 0.10	8.25 ± 0.08	8.16 ± 0.12	8.29 ± 0.04
TC21000187.hg.1	FAM3B	6.56 ± 0.15	6.52 ± 0.12	6.53 ± 0.12	6.55 ± 0.05
TC07001808.hg.1	FAM3C	8.58 ± 0.46	8.72 ± 0.13	8.28 ± 0.29	8.62 ± 0.03
TC03001502.hg.1	FAM3D	7.56 ± 0.20	7.48 ± 0.19	7.48 ± 0.17	7.66 ± 0.06
TC0Y000060.hg.1	FAM41AY1	6.72 ± 0.15	6.78 ± 0.14	6.71 ± 0.15	7.00 ± 0.19
TC0Y000177.hg.1	FAM41AY2	6.72 ± 0.15	6.78 ± 0.14	6.71 ± 0.15	7.00 ± 0.19
TC01002075.hg.1	FAM41C	7.70 ± 0.13	7.54 ± 0.20	8.01 ± 0.07	7.54 ± 0.03
TC03001077.hg.1	FAM43A	8.27 ± 0.17	8.13 ± 0.03	8.17 ± 0.12	7.94 ± 0.05
TC01000260.hg.1	FAM43B	8.07 ± 0.30	7.95 ± 0.08	7.90 ± 0.20	8.07 ± 0.07
TC10000863.hg.1	FAM45A	8.55 ± 0.14	8.41 ± 0.16	8.81 ± 0.25	8.25 ± 0.14
TC0X000624.hg.1	FAM45B	8.74 ± 0.01	8.68 ± 0.14	9.00 ± 0.11	8.59 ± 0.13
TC06001903.hg.1	FAM46A	8.11 ± 0.06	8.02 ± 0.10	8.04 ± 0.18	8.00 ± 0.07
TC01002400.hg.1	FAM46B	8.54 ± 0.18	8.48 ± 0.23	8.42 ± 0.26	8.60 ± 0.14
TC01001030.hg.1	FAM46C	9.55 ± 0.19	9.15 ± 0.27	8.82 ± 0.06	9.03 ± 0.19
TC0X000430.hg.1	FAM46D	3.69 ± 0.13	3.60 ± 0.07	3.64 ± 0.16	3.63 ± 0.04
TC0X000946.hg.1	FAM47A	6.96 ± 0.20	7.04 ± 0.24	6.87 ± 0.19	7.20 ± 0.16
TC0X000159.hg.1	FAM47B	6.16 ± 0.28	6.30 ± 0.29	6.24 ± 0.05	6.50 ± 0.15
TC0X000166.hg.1	FAM47C	7.85 ± 0.21	7.56 ± 0.14	7.72 ± 0.48	7.62 ± 0.03
TC04000425.hg.1	FAM47E	5.84 ± 0.20	5.73 ± 0.11	5.78 ± 0.11	5.86 ± 0.07
TC02001600.hg.1	FAM49A	9.29 ± 0.09	9.34 ± 0.23	9.68 ± 0.23	9.27 ± 0.16
TC08001638.hg.1	FAM49B	9.20 ± 0.04	9.31 ± 0.11	9.46 ± 0.25	9.23 ± 0.18
TC0X000773.hg.1	FAM50A	8.63 ± 0.09	8.58 ± 0.07	8.53 ± 0.12	8.55 ± 0.02
TC06000032.hg.1	FAM50B	7.97 ± 0.19	7.86 ± 0.20	7.85 ± 0.08	7.97 ± 0.11
TC04000966.hg.1	FAM53A	7.76 ± 0.27	7.75 ± 0.21	7.69 ± 0.23	7.83 ± 0.05
TC10002969.hg.1	FAM53B	8.19 ± 0.07	8.21 ± 0.16	7.98 ± 0.06	8.10 ± 0.06
TC05000698.hg.1	FAM53C	8.34 ± 0.11	8.30 ± 0.04	8.47 ± 0.13	8.26 ± 0.11
TC17000003.hg.1	FAM57A	8.05 ± 0.18	7.89 ± 0.11	7.99 ± 0.21	7.93 ± 0.03
TC16001016.hg.1	FAM57B	8.23 ± 0.11	8.23 ± 0.17	8.22 ± 0.10	8.26 ± 0.04
TC0X001514.hg.1	FAM58A	8.60 ± 0.18	8.56 ± 0.09	8.47 ± 0.23	8.56 ± 0.04

TC01001648.hg.1	FAM58BP	8.28 ± 0.20	8.37 ± 0.07	8.37 ± 0.20	8.39 ± 0.10
TC01001536.hg.1	FAM5B	6.79 ± 0.28	6.65 ± 0.19	6.69 ± 0.17	6.75 ± 0.06
TC01003644.hg.1	FAM5C	4.92 ± 0.23	4.76 ± 0.09	4.84 ± 0.11	4.81 ± 0.11
TC12001361.hg.1	FAM60A	8.85 ± 0.24	9.03 ± 0.22	8.57 ± 0.10	9.00 ± 0.06
TC01003216.hg.1	FAM63A	8.86 ± 0.21	8.19 ± 0.14	8.95 ± 0.20	8.15 ± 0.12
TC15000445.hg.1	FAM63B	7.53 ± 0.26	7.50 ± 0.23	7.78 ± 0.39	7.30 ± 0.14
TC17000072.hg.1	FAM64A	7.91 ± 0.17	7.98 ± 0.28	7.75 ± 0.21	8.08 ± 0.08
TC16000541.hg.1	FAM65A	9.28 ± 0.12	9.15 ± 0.09	9.27 ± 0.07	9.26 ± 0.08
TC06001329.hg.1	FAM65B	11.19 ± 0.15	11.12 ± 0.16	11.45 ± 0.25	10.91 ± 0.13
TC20000937.hg.1	FAM65C	7.52 ± 0.18	7.46 ± 0.18	7.46 ± 0.16	7.58 ± 0.05
TC08002595.hg.1	FAM66A	6.45 ± 0.09	6.41 ± 0.07	6.28 ± 0.12	6.59 ± 0.15
TC08000917.hg.1	FAM66B	6.87 ± 0.12	6.83 ± 0.11	6.79 ± 0.05	6.95 ± 0.12
TC12003234.hg.1	FAM66C	6.68 ± 0.15	6.71 ± 0.08	6.61 ± 0.07	6.81 ± 0.13
TC08002594.hg.1	FAM66D	6.27 ± 0.06	6.37 ± 0.07	6.15 ± 0.17	6.48 ± 0.16
TC08000053.hg.1	FAM66E	6.29 ± 0.10	6.31 ± 0.09	6.19 ± 0.13	6.39 ± 0.12
TC01002870.hg.1	FAM69A	7.51 ± 0.10	7.49 ± 0.17	7.16 ± 0.22	7.38 ± 0.07
TC09000820.hg.1	FAM69B	8.08 ± 0.18	8.04 ± 0.11	8.02 ± 0.16	8.10 ± 0.04
TC18000575.hg.1	FAM69C	8.04 ± 0.19	7.95 ± 0.11	7.90 ± 0.13	7.89 ± 0.07
TC01001778.hg.1	FAM71A	6.55 ± 0.30	6.70 ± 0.34	6.61 ± 0.19	6.89 ± 0.22
TC05001979.hg.1	FAM71B	5.79 ± 0.28	5.90 ± 0.02	5.69 ± 0.17	6.06 ± 0.15
TC12000768.hg.1	FAM71C	5.75 ± 0.27	5.57 ± 0.05	5.71 ± 0.26	5.68 ± 0.29
TC14000402.hg.1	FAM71D	5.21 ± 0.09	5.12 ± 0.04	5.18 ± 0.06	5.14 ± 0.02
TC19001747.hg.1	FAM71E1	7.96 ± 0.20	7.97 ± 0.19	7.89 ± 0.20	8.05 ± 0.04
TC19001869.hg.1	FAM71E2	7.87 ± 0.18	7.86 ± 0.26	7.82 ± 0.21	7.99 ± 0.06
TC07000781.hg.1	FAM71F1	6.52 ± 0.15	6.43 ± 0.16	6.41 ± 0.22	6.49 ± 0.04
TC07000778.hg.1	FAM71F2	7.02 ± 0.13	7.06 ± 0.05	7.17 ± 0.04	7.15 ± 0.06
TC01001717.hg.1	FAM72A	7.60 ± 0.11	7.35 ± 0.17	7.60 ± 0.09	7.22 ± 0.12
TC01001045.hg.1	FAM72B	7.45 ± 0.10	7.27 ± 0.18	7.55 ± 0.06	7.12 ± 0.12
TC01003174.hg.1	FAM72C	6.92 ± 0.01	6.59 ± 0.15	7.11 ± 0.19	6.42 ± 0.24
TC01003085.hg.1	FAM72D	7.15 ± 0.08	6.79 ± 0.28	7.38 ± 0.23	6.44 ± 0.34
TC01000788.hg.1	FAM73A	7.49 ± 0.25	7.53 ± 0.20	7.39 ± 0.19	7.50 ± 0.02

TC09000706.hg.1	FAM73B	8.39 ± 0.17	8.39 ± 0.13	8.26 ± 0.15	8.42 ± 0.08
TC09000230.hg.1	FAM74A1	5.34 ± 0.08	5.18 ± 0.05	5.20 ± 0.14	5.25 ± 0.02
TC09001101.hg.1	FAM74A2	5.23 ± 0.16	5.12 ± 0.04	5.22 ± 0.10	5.23 ± 0.03
TC09000235.hg.1	FAM74A3	5.11 ± 0.21	5.09 ± 0.02	5.14 ± 0.14	5.22 ± 0.07
TC09001142.hg.1	FAM74A4	6.10 ± 0.12	5.96 ± 0.04	5.94 ± 0.04	6.04 ± 0.11
TC09000240.hg.1	FAM74A6	5.40 ± 0.11	5.26 ± 0.04	5.32 ± 0.07	5.28 ± 0.08
TC01000367.hg.1	FAM76A	7.50 ± 0.17	7.53 ± 0.13	7.45 ± 0.11	7.49 ± 0.04
TC11002208.hg.1	FAM76B	8.06 ± 0.64	8.69 ± 0.21	8.53 ± 0.54	8.69 ± 0.13
TC09001664.hg.1	FAM78A	8.63 ± 0.07	8.48 ± 0.07	8.46 ± 0.10	8.40 ± 0.03
TC01003469.hg.1	FAM78B	7.34 ± 0.23	7.35 ± 0.20	7.30 ± 0.12	7.43 ± 0.04
TC15000455.hg.1	FAM81A	6.23 ± 0.12	6.20 ± 0.14	6.24 ± 0.09	6.26 ± 0.03
TC05000456.hg.1	FAM81B	4.88 ± 0.22	4.68 ± 0.04	4.73 ± 0.10	4.72 ± 0.02
TC08000714.hg.1	FAM83A	6.57 ± 0.15	6.55 ± 0.16	6.52 ± 0.13	6.58 ± 0.04
TC08001579.hg.1	FAM83A-AS1	6.78 ± 0.16	6.86 ± 0.16	6.77 ± 0.26	6.88 ± 0.05
TC06000675.hg.1	FAM83B	4.61 ± 0.14	4.49 ± 0.08	4.51 ± 0.05	4.53 ± 0.09
TC20000795.hg.1	FAM83C	8.08 ± 0.26	8.03 ± 0.11	8.03 ± 0.16	8.13 ± 0.10
TC20000251.hg.1	FAM83C-AS1	8.43 ± 0.24	8.30 ± 0.16	8.40 ± 0.38	8.44 ± 0.08
TC20000293.hg.1	FAM83D	7.21 ± 0.11	7.12 ± 0.19	7.12 ± 0.21	6.91 ± 0.02
TC19001686.hg.1	FAM83E	8.19 ± 0.33	8.08 ± 0.13	8.05 ± 0.18	8.08 ± 0.03
TC22000321.hg.1	FAM83F	6.86 ± 0.13	6.94 ± 0.20	6.89 ± 0.18	7.12 ± 0.10
TC17001229.hg.1	FAM83G	7.76 ± 0.26	7.71 ± 0.24	7.60 ± 0.27	7.80 ± 0.06
TC08001729.hg.1	FAM83H	7.94 ± 0.23	7.95 ± 0.20	7.88 ± 0.19	8.07 ± 0.06
TC08000833.hg.1	FAM83H-AS1	8.23 ± 0.16	8.19 ± 0.21	8.18 ± 0.23	8.27 ± 0.05
TC02000087.hg.1	FAM84A	7.48 ± 0.25	7.51 ± 0.21	7.47 ± 0.22	7.61 ± 0.06
TC08001623.hg.1	FAM84B	7.88 ± 0.19	7.88 ± 0.13	7.72 ± 0.17	7.92 ± 0.07
TC16000842.hg.1	FAM86A	7.99 ± 0.15	7.73 ± 0.06	7.75 ± 0.10	7.76 ± 0.04
TC08000988.hg.1	FAM86B1	8.08 ± 0.15	7.97 ± 0.05	7.91 ± 0.12	8.06 ± 0.08
TC08000995.hg.1	FAM86B2	7.80 ± 0.07	7.74 ± 0.08	7.68 ± 0.12	7.82 ± 0.07
TC08000056.hg.1	FAM86B3P	7.24 ± 0.10	7.26 ± 0.10	7.18 ± 0.09	7.31 ± 0.06
TC11000742.hg.1	FAM86C1	7.76 ± 0.03	7.50 ± 0.06	7.49 ± 0.18	7.51 ± 0.03
TC11001996.hg.1	FAM86C2P	7.50 ± 0.16	7.31 ± 0.11	7.25 ± 0.26	7.29 ± 0.02

TC03001563.hg.1	FAM86DP	7.87 ± 0.09	7.73 ± 0.11	7.71 ± 0.02	7.77 ± 0.06
TC04000986.hg.1	FAM86EP	7.06 ± 0.08	6.89 ± 0.02	6.89 ± 0.07	6.92 ± 0.03
TC12001176.hg.1	FAM86FP	8.17 ± 0.08	7.96 ± 0.03	7.93 ± 0.11	8.01 ± 0.04
TC03001776.hg.1	FAM86HP	8.16 ± 0.16	8.10 ± 0.17	8.00 ± 0.08	8.16 ± 0.07
TC03000649.hg.1	FAM86JP	7.56 ± 0.20	7.48 ± 0.14	7.32 ± 0.19	7.53 ± 0.06
TC08000879.hg.1	FAM87A	7.26 ± 0.24	7.12 ± 0.12	7.18 ± 0.12	7.14 ± 0.08
TC01000017.hg.1	FAM87B	7.78 ± 0.18	7.72 ± 0.25	7.60 ± 0.28	7.92 ± 0.03
TC11003462.hg.1	FAM89B	9.26 ± 0.15	9.12 ± 0.08	9.17 ± 0.16	9.04 ± 0.07
TC06000115.hg.1	FAM8A1	9.69 ± 0.28	9.58 ± 0.27	10.03 ± 0.42	9.47 ± 0.30
TC12001175.hg.1	FAM90A1	7.12 ± 0.16	7.05 ± 0.09	7.12 ± 0.12	7.21 ± 0.11
TC08000046.hg.1	FAM90A10P	8.03 ± 0.28	8.08 ± 0.12	8.06 ± 0.15	8.22 ± 0.14
TC08000931.hg.1	FAM90A11P	7.49 ± 0.26	7.50 ± 0.13	7.44 ± 0.18	7.67 ± 0.13
TC08000933.hg.1	FAM90A12P	7.42 ± 0.21	7.41 ± 0.08	7.40 ± 0.15	7.59 ± 0.12
TC08000029.hg.1	FAM90A14P	8.09 ± 0.32	8.06 ± 0.09	8.03 ± 0.22	8.27 ± 0.15
TC08000030.hg.1	FAM90A14P	8.09 ± 0.35	8.08 ± 0.09	8.04 ± 0.22	8.28 ± 0.14
TC08000041.hg.1	FAM90A14P	8.10 ± 0.32	8.07 ± 0.08	8.04 ± 0.21	8.27 ± 0.14
TC08000042.hg.1	FAM90A19P	7.99 ± 0.24	8.02 ± 0.09	7.97 ± 0.18	8.22 ± 0.19
TC08000044.hg.1	FAM90A19P	7.99 ± 0.24	8.02 ± 0.09	7.97 ± 0.18	8.22 ± 0.19
TC08000032.hg.1	FAM90A20P	7.89 ± 0.37	7.87 ± 0.15	7.83 ± 0.23	8.09 ± 0.15
TC08000925.hg.1	FAM90A22P	7.30 ± 0.29	7.34 ± 0.04	7.28 ± 0.20	7.50 ± 0.18
TC08002633.hg.1	FAM90A23P	7.19 ± 0.23	7.21 ± 0.12	7.14 ± 0.11	7.38 ± 0.15
TC08000932.hg.1	FAM90A24P	7.69 ± 0.31	7.68 ± 0.08	7.61 ± 0.22	7.89 ± 0.13
TC08000992.hg.1	FAM90A25P	7.06 ± 0.14	7.09 ± 0.14	7.02 ± 0.24	7.32 ± 0.11
TC04000094.hg.1	FAM90A26P	7.44 ± 0.18	7.39 ± 0.12	7.34 ± 0.09	7.50 ± 0.11
TC19000807.hg.1	FAM90A27P	7.40 ± 0.23	7.35 ± 0.32	7.40 ± 0.20	7.51 ± 0.13
TC08000031.hg.1	FAM90A5P	8.05 ± 0.32	8.06 ± 0.11	8.03 ± 0.18	8.29 ± 0.21
TC08002632.hg.1	FAM90A7P	7.27 ± 0.21	7.27 ± 0.10	7.23 ± 0.19	7.50 ± 0.17
TC08002634.hg.1	FAM90A7P	7.68 ± 0.36	7.75 ± 0.11	7.62 ± 0.17	7.91 ± 0.21
TC08000043.hg.1	FAM90A8P	7.99 ± 0.25	8.04 ± 0.11	7.96 ± 0.26	8.22 ± 0.11
TC08000045.hg.1	FAM90A9P	7.87 ± 0.24	7.90 ± 0.09	7.82 ± 0.19	8.10 ± 0.19
TC08000720.hg.1	FAM91A1	8.96 ± 0.40	9.22 ± 0.21	9.14 ± 0.40	9.28 ± 0.06

TC08000565.hg.1	FAM92A1	5.06 ± 0.11	4.94 ± 0.03	5.01 ± 0.01	4.96 ± 0.03
TC04000886.hg.1	FAM92A1P2	5.76 ± 0.12	5.72 ± 0.13	5.68 ± 0.14	5.64 ± 0.11
TC16001316.hg.1	FAM92B	7.55 ± 0.18	7.55 ± 0.12	7.60 ± 0.09	7.70 ± 0.10
TC09002899.hg.1	FAM95B1	6.86 ± 0.17	6.87 ± 0.09	6.74 ± 0.19	6.96 ± 0.15
TC15001547.hg.1	FAM96A	7.74 ± 0.11	7.68 ± 0.14	7.67 ± 0.09	7.48 ± 0.15
TC16001178.hg.1	FAM96B	9.21 ± 0.28	8.95 ± 0.12	8.88 ± 0.25	8.75 ± 0.01
TC02001729.hg.1	FAM98A	7.65 ± 0.18	7.47 ± 0.05	7.45 ± 0.08	7.22 ± 0.08
TC15000260.hg.1	FAM98B	7.81 ± 0.29	7.84 ± 0.17	7.88 ± 0.28	7.81 ± 0.24
TC19000529.hg.1	FAM98C	8.86 ± 0.15	8.76 ± 0.09	8.77 ± 0.10	8.74 ± 0.03
TC11000047.hg.1	FAM99A	7.92 ± 0.42	7.95 ± 0.43	7.85 ± 0.36	8.15 ± 0.10
TC11001265.hg.1	FAM99B	8.07 ± 0.28	8.01 ± 0.27	7.93 ± 0.25	8.18 ± 0.09
TC0X000830.hg.1	FAM9A	5.69 ± 0.19	5.58 ± 0.14	5.55 ± 0.15	5.61 ± 0.05
TC0X000832.hg.1	FAM9B	4.99 ± 0.12	4.87 ± 0.01	4.86 ± 0.03	4.86 ± 0.03
TC0X000864.hg.1	FAM9C	5.45 ± 0.12	5.36 ± 0.05	5.44 ± 0.06	5.42 ± 0.03
TC15000194.hg.1	FAN1	7.26 ± 0.03	7.13 ± 0.04	7.13 ± 0.07	7.10 ± 0.04
TC16001357.hg.1	FANCA	8.02 ± 0.14	7.78 ± 0.06	7.88 ± 0.09	7.70 ± 0.03
TC0X000872.hg.1	FANCB	5.38 ± 0.04	5.24 ± 0.24	5.34 ± 0.18	5.13 ± 0.12
TC09001361.hg.1	FANCC	6.63 ± 0.06	6.60 ± 0.04	6.49 ± 0.07	6.57 ± 0.02
TC03000051.hg.1	FANCD2	7.47 ± 0.06	7.51 ± 0.27	7.20 ± 0.14	7.35 ± 0.05
TC03001157.hg.1	FANCD2OS	6.02 ± 0.09	5.91 ± 0.06	5.99 ± 0.06	5.95 ± 0.01
TC06000515.hg.1	FANCE	7.49 ± 0.11	7.35 ± 0.16	7.34 ± 0.13	7.42 ± 0.05
TC11001498.hg.1	FANCF	7.96 ± 0.18	7.55 ± 0.22	7.71 ± 0.13	7.44 ± 0.08
TC09001049.hg.1	FANCG	7.76 ± 0.19	7.68 ± 0.10	7.59 ± 0.19	7.72 ± 0.06
TC15000846.hg.1	FANCI	7.01 ± 0.10	6.92 ± 0.40	6.70 ± 0.10	6.55 ± 0.02
TC02001878.hg.1	FANCL	6.44 ± 0.11	6.30 ± 0.08	6.37 ± 0.16	6.19 ± 0.06
TC14000266.hg.1	FANCM	5.77 ± 0.32	5.77 ± 0.16	5.71 ± 0.25	5.65 ± 0.06
TC10000917.hg.1	FANK1	5.89 ± 0.12	5.83 ± 0.06	5.80 ± 0.06	5.86 ± 0.06
TC10001743.hg.1	FANK1-AS1	6.17 ± 0.08	6.23 ± 0.08	6.18 ± 0.07	6.23 ± 0.13
TC02002480.hg.1	FAP	4.48 ± 0.08	4.35 ± 0.04	4.55 ± 0.19	4.41 ± 0.07
TC11000208.hg.1	FAR1	8.76 ± 0.53	8.89 ± 0.26	9.59 ± 0.48	8.82 ± 0.21
TC11000209.hg.1	FAR1-IT1	9.24 ± 0.53	9.61 ± 0.11	9.51 ± 0.19	9.72 ± 0.08

TC12000276.hg.1	FAR2	8.38 ± 0.16	8.01 ± 0.23	8.80 ± 0.39	7.83 ± 0.27
TC13000344.hg.1	FARP1	6.77 ± 0.13	6.75 ± 0.08	6.75 ± 0.12	6.81 ± 0.05
TC13000821.hg.1	FARP1-AS1	5.98 ± 0.38	5.90 ± 0.11	5.83 ± 0.12	6.06 ± 0.04
TC13000346.hg.1	FARP1-IT1	5.32 ± 0.02	5.30 ± 0.04	5.22 ± 0.30	5.23 ± 0.04
TC02001478.hg.1	FARP2	7.60 ± 0.06	7.54 ± 0.05	7.44 ± 0.03	7.56 ± 0.03
TC06000048.hg.1	FARS2	8.10 ± 0.07	8.03 ± 0.13	7.72 ± 0.09	7.85 ± 0.08
TC19001215.hg.1	FARSA	9.25 ± 0.24	8.94 ± 0.07	8.89 ± 0.20	8.83 ± 0.08
TC02002813.hg.1	FARSB	8.24 ± 0.18	8.23 ± 0.15	7.94 ± 0.15	8.02 ± 0.05
TC10002942.hg.1	FAS	8.29 ± 0.11	8.44 ± 0.20	9.02 ± 0.43	8.32 ± 0.24
TC10001499.hg.1	FAS-AS1	6.16 ± 0.26	5.92 ± 0.19	6.41 ± 0.44	6.08 ± 0.11
TC01001506.hg.1	FASLG	6.65 ± 0.23	6.33 ± 0.09	6.53 ± 0.14	6.32 ± 0.09
TC17001973.hg.1	FASN	8.75 ± 0.13	8.70 ± 0.13	8.58 ± 0.16	8.78 ± 0.03
TC07002029.hg.1	FASTK	8.69 ± 0.19	8.58 ± 0.13	8.48 ± 0.21	8.61 ± 0.06
TC02002510.hg.1	FASTKD1	6.48 ± 0.27	6.38 ± 0.17	6.33 ± 0.24	6.12 ± 0.07
TC02001221.hg.1	FASTKD2	6.73 ± 0.41	6.86 ± 0.20	6.47 ± 0.27	6.76 ± 0.03
TC05001149.hg.1	FASTKD3	6.24 ± 0.07	6.11 ± 0.06	6.22 ± 0.12	6.04 ± 0.02
TC04001824.hg.1	FAT1	6.03 ± 0.18	5.97 ± 0.12	5.95 ± 0.11	6.10 ± 0.04
TC05001954.hg.1	FAT2	6.90 ± 0.18	6.86 ± 0.14	6.83 ± 0.12	6.92 ± 0.06
TC11000903.hg.1	FAT3	6.21 ± 0.20	6.16 ± 0.11	6.17 ± 0.08	6.22 ± 0.06
TC04000636.hg.1	FAT4	5.41 ± 0.09	5.37 ± 0.05	5.31 ± 0.06	5.42 ± 0.09
TC0X000734.hg.1	FATE1	7.15 ± 0.18	7.13 ± 0.20	7.13 ± 0.28	7.23 ± 0.11
TC11001928.hg.1	FAU	9.16 ± 0.21	9.27 ± 0.19	8.87 ± 0.29	9.08 ± 0.10
TC06001962.hg.1	FAXC	6.00 ± 0.20	5.93 ± 0.13	5.93 ± 0.12	6.04 ± 0.04
TC05001973.hg.1	FAXDC2	8.77 ± 0.10	8.11 ± 0.06	8.91 ± 0.04	8.22 ± 0.26
TC17002912.hg.1	FBF1	8.24 ± 0.17	8.19 ± 0.10	8.12 ± 0.18	8.23 ± 0.02
TC19001526.hg.1	FBL	9.73 ± 0.17	9.72 ± 0.19	9.31 ± 0.21	9.62 ± 0.05
TC01000197.hg.1	FBLIM1	7.94 ± 0.19	7.91 ± 0.19	7.90 ± 0.19	8.03 ± 0.05
TC05000920.hg.1	FBLL1	8.15 ± 0.32	8.07 ± 0.17	7.97 ± 0.28	8.17 ± 0.08
TC22000383.hg.1	FBLN1	7.81 ± 0.19	7.76 ± 0.17	7.77 ± 0.18	7.88 ± 0.04
TC03000077.hg.1	FBLN2	7.80 ± 0.13	7.81 ± 0.12	7.73 ± 0.17	7.88 ± 0.04
TC14001443.hg.1	FBLN5	6.74 ± 0.19	6.63 ± 0.11	6.65 ± 0.07	6.70 ± 0.02

TC02000704.hg.1	FBLN7	7.59 ± 0.19	7.59 ± 0.18	7.59 ± 0.17	7.65 ± 0.02
TC15001335.hg.1	FBN1	5.97 ± 0.12	5.91 ± 0.11	6.02 ± 0.15	6.01 ± 0.10
TC05001752.hg.1	FBN2	6.42 ± 0.16	6.19 ± 0.02	6.58 ± 0.22	6.21 ± 0.07
TC19001126.hg.1	FBN3	7.54 ± 0.18	7.55 ± 0.20	7.50 ± 0.16	7.67 ± 0.03
TC09001357.hg.1	FBP1	8.26 ± 0.35	8.04 ± 0.10	8.32 ± 0.24	8.13 ± 0.23
TC09001356.hg.1	FBP2	6.49 ± 0.11	6.41 ± 0.16	6.40 ± 0.12	6.52 ± 0.06
TC16000355.hg.1	FBRS	9.58 ± 0.18	9.70 ± 0.03	9.65 ± 0.20	9.87 ± 0.21
TC12001060.hg.1	FBRSL1	8.91 ± 0.03	8.92 ± 0.08	8.81 ± 0.11	9.00 ± 0.04
TC19001154.hg.1	FBXL12	8.29 ± 0.16	8.43 ± 0.09	8.16 ± 0.12	8.46 ± 0.04
TC07001714.hg.1	FBXL13	6.27 ± 0.18	5.98 ± 0.15	6.63 ± 0.30	5.96 ± 0.14
TC12001090.hg.1	FBXL14	8.43 ± 0.01	8.27 ± 0.08	8.22 ± 0.13	8.20 ± 0.03
TC10000754.hg.1	FBXL15	7.93 ± 0.24	7.83 ± 0.13	7.81 ± 0.20	7.82 ± 0.05
TC16000736.hg.1	FBXL16	8.52 ± 0.22	8.58 ± 0.18	8.43 ± 0.22	8.64 ± 0.08
TC05001660.hg.1	FBXL17	8.24 ± 0.11	8.46 ± 0.09	8.19 ± 0.12	8.35 ± 0.09
TC07001118.hg.1	FBXL18	8.11 ± 0.14	8.11 ± 0.10	8.17 ± 0.06	8.19 ± 0.04
TC16000362.hg.1	FBXL19	8.40 ± 0.20	8.31 ± 0.09	8.29 ± 0.17	8.33 ± 0.02
TC16001046.hg.1	FBXL19-AS1	6.95 ± 0.08	6.92 ± 0.05	6.78 ± 0.06	6.85 ± 0.07
TC03000166.hg.1	FBXL2	5.51 ± 0.12	5.47 ± 0.11	5.46 ± 0.07	5.56 ± 0.05
TC17001448.hg.1	FBXL20	8.59 ± 0.29	8.52 ± 0.21	8.72 ± 0.28	8.48 ± 0.12
TC05000681.hg.1	FBXL21	4.67 ± 0.21	4.57 ± 0.09	4.64 ± 0.08	4.61 ± 0.06
TC15000585.hg.1	FBXL22	7.70 ± 0.28	7.81 ± 0.26	7.70 ± 0.17	7.82 ± 0.03
TC13000742.hg.1	FBXL3	9.03 ± 0.39	9.37 ± 0.10	9.32 ± 0.40	9.41 ± 0.07
TC06001961.hg.1	FBXL4	7.58 ± 0.29	7.57 ± 0.21	7.62 ± 0.33	7.33 ± 0.08
TC04001057.hg.1	FBXL5	9.10 ± 0.08	8.98 ± 0.21	9.62 ± 0.33	8.78 ± 0.24
TC08002623.hg.1	FBXL6	8.30 ± 0.11	8.26 ± 0.17	8.19 ± 0.18	8.32 ± 0.06
TC05000088.hg.1	FBXL7	7.19 ± 0.32	7.21 ± 0.25	7.21 ± 0.21	7.38 ± 0.13
TC16002078.hg.1	FBXL8	8.19 ± 0.20	8.16 ± 0.13	8.13 ± 0.14	8.20 ± 0.03
TC09002889.hg.1	FBXO10	7.33 ± 0.11	7.20 ± 0.04	7.06 ± 0.08	7.10 ± 0.06
TC02001826.hg.1	FBXO11	8.88 ± 0.35	9.07 ± 0.21	9.17 ± 0.37	9.08 ± 0.17
TC18000573.hg.1	FBXO15	6.70 ± 0.19	6.61 ± 0.09	6.60 ± 0.13	6.70 ± 0.11
TC08002615.hg.1	FBXO16	5.98 ± 0.16	5.75 ± 0.06	5.86 ± 0.05	5.82 ± 0.03

TC19002701.hg.1	FBXO17	7.83 ± 0.21	7.75 ± 0.19	7.81 ± 0.28	7.87 ± 0.01
TC10000050.hg.1	FBXO18	9.07 ± 0.17	8.91 ± 0.07	8.88 ± 0.08	8.71 ± 0.12
TC01002199.hg.1	FBXO2	8.32 ± 0.23	8.27 ± 0.15	8.27 ± 0.19	8.33 ± 0.05
TC12002013.hg.1	FBXO21	8.38 ± 0.13	8.64 ± 0.10	8.12 ± 0.05	8.60 ± 0.02
TC15000705.hg.1	FBXO22	7.65 ± 0.21	7.58 ± 0.10	7.55 ± 0.13	7.56 ± 0.05
TC15001682.hg.1	FBXO22-AS1	6.61 ± 0.32	6.58 ± 0.23	6.49 ± 0.12	6.59 ± 0.03
TC07000626.hg.1	FBXO24	8.06 ± 0.27	8.05 ± 0.22	8.05 ± 0.21	8.16 ± 0.09
TC08000003.hg.1	FBXO25	7.56 ± 0.12	7.67 ± 0.10	7.35 ± 0.06	7.65 ± 0.08
TC19001511.hg.1	FBXO27	7.64 ± 0.15	7.57 ± 0.15	7.52 ± 0.10	7.60 ± 0.03
TC01001841.hg.1	FBXO28	7.94 ± 0.20	8.12 ± 0.11	7.98 ± 0.30	8.09 ± 0.07
TC11001541.hg.1	FBXO3	7.73 ± 0.42	8.03 ± 0.13	7.81 ± 0.33	8.03 ± 0.06
TC06002196.hg.1	FBXO30	8.86 ± 0.10	8.74 ± 0.15	9.18 ± 0.17	8.81 ± 0.06
TC16001330.hg.1	FBXO31	8.34 ± 0.07	8.21 ± 0.05	8.03 ± 0.11	8.24 ± 0.05
TC08001590.hg.1	FBXO32	7.49 ± 0.10	7.58 ± 0.21	7.11 ± 0.05	7.53 ± 0.13
TC14001068.hg.1	FBXO33	8.10 ± 0.43	8.77 ± 0.29	8.24 ± 0.39	8.69 ± 0.18
TC14000324.hg.1	FBXO34	8.24 ± 0.03	8.23 ± 0.06	8.24 ± 0.02	8.23 ± 0.04
TC14000325.hg.1	FBXO34	9.82 ± 0.17	9.92 ± 0.04	10.00 ± 0.09	10.00 ± 0.14
TC02001371.hg.1	FBXO36	6.10 ± 0.14	6.09 ± 0.03	6.06 ± 0.05	6.13 ± 0.01
TC02001372.hg.1	FBXO36-IT1	4.55 ± 0.22	4.45 ± 0.10	4.52 ± 0.19	4.58 ± 0.11
TC05000812.hg.1	FBXO38	8.09 ± 0.27	7.92 ± 0.17	8.43 ± 0.41	7.89 ± 0.10
TC17000078.hg.1	FBXO39	6.54 ± 0.14	6.42 ± 0.13	6.59 ± 0.16	6.44 ± 0.04
TC05000190.hg.1	FBXO4	6.13 ± 0.10	6.05 ± 0.09	5.95 ± 0.06	5.97 ± 0.03
TC03000623.hg.1	FBXO40	6.01 ± 0.13	5.86 ± 0.07	5.91 ± 0.13	5.92 ± 0.04
TC02001982.hg.1	FBXO41	8.11 ± 0.15	8.08 ± 0.15	7.97 ± 0.18	8.13 ± 0.05
TC01002262.hg.1	FBXO42	8.45 ± 0.13	8.30 ± 0.15	8.28 ± 0.10	8.27 ± 0.05
TC08001475.hg.1	FBXO43	4.70 ± 0.17	4.61 ± 0.06	4.65 ± 0.07	4.63 ± 0.02
TC01000144.hg.1	FBXO44	8.62 ± 0.08	8.48 ± 0.05	8.48 ± 0.06	8.52 ± 0.05
TC03001098.hg.1	FBXO45	7.97 ± 0.11	7.90 ± 0.06	7.91 ± 0.15	7.82 ± 0.06
TC19001629.hg.1	FBXO46	8.58 ± 0.14	8.51 ± 0.10	8.46 ± 0.16	8.56 ± 0.04
TC17001441.hg.1	FBXO47	4.78 ± 0.13	4.65 ± 0.10	4.75 ± 0.03	4.66 ± 0.07
TC02001943.hg.1	FBXO48	6.65 ± 0.08	6.47 ± 0.11	6.61 ± 0.05	6.47 ± 0.09

TC06002240.hg.1	FBXO5	6.69 ± 0.18	6.68 ± 0.32	6.47 ± 0.09	6.23 ± 0.12
TC01000145.hg.1	FBXO6	8.67 ± 0.34	8.23 ± 0.13	8.57 ± 0.25	8.18 ± 0.03
TC22000245.hg.1	FBXO7	9.55 ± 0.12	9.61 ± 0.14	9.41 ± 0.10	9.56 ± 0.07
TC04001751.hg.1	FBXO8	7.75 ± 0.17	7.64 ± 0.09	7.70 ± 0.27	7.58 ± 0.04
TC06000660.hg.1	FBXO9	7.99 ± 0.23	7.83 ± 0.18	8.30 ± 0.36	7.67 ± 0.08
TC17000221.hg.1	FBXW10	5.82 ± 0.09	5.77 ± 0.10	5.73 ± 0.13	5.83 ± 0.12
TC05002058.hg.1	FBXW11	7.78 ± 0.13	7.70 ± 0.16	7.76 ± 0.27	7.64 ± 0.11
TC03000279.hg.1	FBXW12	5.88 ± 0.15	5.85 ± 0.10	5.80 ± 0.07	5.91 ± 0.06
TC09001538.hg.1	FBXW2	8.99 ± 0.10	8.95 ± 0.08	9.11 ± 0.25	8.87 ± 0.04
TC10001609.hg.1	FBXW4	9.02 ± 0.07	8.96 ± 0.04	8.78 ± 0.12	8.91 ± 0.07
TC22000130.hg.1	FBXW4P1	7.86 ± 0.17	7.78 ± 0.16	7.68 ± 0.17	7.77 ± 0.11
TC09001746.hg.1	FBXW5	9.57 ± 0.20	9.43 ± 0.08	9.39 ± 0.21	9.38 ± 0.01
TC04001647.hg.1	FBXW7	8.02 ± 0.27	8.23 ± 0.22	8.09 ± 0.28	8.12 ± 0.02
TC12000920.hg.1	FBXW8	8.36 ± 0.12	8.33 ± 0.01	8.07 ± 0.03	8.23 ± 0.08
TC19001205.hg.1	FBXW9	8.08 ± 0.22	8.11 ± 0.21	8.02 ± 0.26	8.20 ± 0.02
TC01003769.hg.1	FCAMR	6.45 ± 0.12	6.46 ± 0.15	6.41 ± 0.16	6.54 ± 0.09
TC19000885.hg.1	FCAR	9.40 ± 0.27	9.37 ± 0.04	9.99 ± 0.23	9.34 ± 0.35
TC01001351.hg.1	FCER1A	6.80 ± 0.28	7.60 ± 0.16	6.62 ± 0.08	7.48 ± 0.22
TC01001382.hg.1	FCER1G	11.44 ± 0.44	10.92 ± 0.20	11.94 ± 0.18	10.84 ± 0.35
TC19001116.hg.1	FCER2	9.83 ± 0.17	9.87 ± 0.15	9.36 ± 0.12	9.74 ± 0.03
TC14000462.hg.1	FCF1	7.91 ± 0.09	7.87 ± 0.23	7.84 ± 0.16	7.72 ± 0.15
TC19001527.hg.1	FCGBP	8.02 ± 0.12	8.07 ± 0.04	7.89 ± 0.14	8.15 ± 0.08
TC01001172.hg.1	FCGR1A	10.00 ± 0.94	9.01 ± 0.48	11.04 ± 0.50	8.41 ± 0.35
TC01003057.hg.1	FCGR1B	9.97 ± 0.89	9.05 ± 0.48	10.93 ± 0.48	8.49 ± 0.31
TC01001161.hg.1	FCGR1C	10.11 ± 0.92	9.09 ± 0.51	11.26 ± 0.47	8.48 ± 0.33
TC01001399.hg.1	FCGR2A	10.56 ± 0.36	10.44 ± 0.20	11.19 ± 0.18	10.33 ± 0.26
TC01006338.hg.1	FCGR2B	8.70 ± 0.17	8.59 ± 0.15	8.74 ± 0.19	8.41 ± 0.18
TC01006339.hg.1	FCGR2C	9.94 ± 0.20	9.93 ± 0.15	10.22 ± 0.15	9.76 ± 0.19
TC01006307.hg.1	FCGR3A	10.06 ± 0.39	9.96 ± 0.18	10.39 ± 0.18	9.72 ± 0.30
TC01006308.hg.1	FCGR3B	12.32 ± 0.38	12.30 ± 0.22	12.68 ± 0.18	12.07 ± 0.32
TC19000737.hg.1	FCGRT	9.01 ± 0.15	8.94 ± 0.03	9.01 ± 0.10	8.94 ± 0.06

TC19000342.hg.1	FCHO1	9.04 ± 0.09	8.93 ± 0.07	8.96 ± 0.04	8.89 ± 0.05
TC05000343.hg.1	FCHO2	7.75 ± 0.42	7.87 ± 0.38	8.41 ± 0.50	7.80 ± 0.31
TC05001873.hg.1	FCHSD1	8.61 ± 0.03	8.52 ± 0.01	8.53 ± 0.02	8.51 ± 0.06
TC11002062.hg.1	FCHSD2	9.65 ± 0.19	9.76 ± 0.13	9.75 ± 0.34	9.67 ± 0.14
TC09001710.hg.1	FCN1	11.46 ± 0.23	11.04 ± 0.07	11.53 ± 0.16	10.84 ± 0.25
TC09000788.hg.1	FCN2	8.97 ± 0.17	8.71 ± 0.09	8.89 ± 0.19	8.75 ± 0.03
TC01002406.hg.1	FCN3	7.73 ± 0.27	7.80 ± 0.34	7.77 ± 0.17	7.98 ± 0.10
TC01003367.hg.1	FCRL1	9.40 ± 0.51	9.94 ± 0.30	9.26 ± 0.38	9.52 ± 0.37
TC01003366.hg.1	FCRL2	7.31 ± 0.32	7.52 ± 0.27	7.15 ± 0.18	7.12 ± 0.24
TC01003365.hg.1	FCRL3	8.82 ± 0.57	8.97 ± 0.21	8.58 ± 0.22	8.80 ± 0.10
TC01003363.hg.1	FCRL4	5.62 ± 0.16	5.48 ± 0.07	5.51 ± 0.10	5.59 ± 0.07
TC01003362.hg.1	FCRL5	7.54 ± 0.15	7.40 ± 0.15	7.32 ± 0.06	7.22 ± 0.15
TC01001356.hg.1	FCRL6	8.98 ± 0.59	8.92 ± 0.21	8.62 ± 0.20	8.75 ± 0.14
TC01001411.hg.1	FCRLA	7.44 ± 0.11	7.35 ± 0.03	7.12 ± 0.16	7.23 ± 0.04
TC01001412.hg.1	FCRLB	7.37 ± 0.20	7.31 ± 0.21	7.34 ± 0.11	7.37 ± 0.04
TC04000381.hg.1	FDCSP	4.36 ± 0.19	4.34 ± 0.15	4.31 ± 0.06	4.38 ± 0.22
TC08000093.hg.1	FDFT1	8.72 ± 0.07	8.64 ± 0.05	8.58 ± 0.18	8.53 ± 0.03
TC01001299.hg.1	FDPS	8.32 ± 0.10	8.14 ± 0.08	8.17 ± 0.15	8.05 ± 0.04
TC07001542.hg.1	FDPSP2	5.68 ± 0.15	5.60 ± 0.04	5.57 ± 0.04	5.65 ± 0.11
TC11000988.hg.1	FDX1	8.24 ± 0.12	8.29 ± 0.02	8.00 ± 0.04	8.27 ± 0.09
TC19001161.hg.1	FDX1L	8.18 ± 0.22	8.15 ± 0.19	8.08 ± 0.21	8.21 ± 0.04
TC11003494.hg.1	FDXACB1	6.88 ± 0.11	6.83 ± 0.01	6.66 ± 0.16	6.76 ± 0.03
TC17001864.hg.1	FDXR	7.89 ± 0.20	7.84 ± 0.21	7.81 ± 0.18	7.95 ± 0.05
TC18000536.hg.1	FECH	7.39 ± 0.04	7.39 ± 0.04	7.33 ± 0.05	7.38 ± 0.02
TC19000096.hg.1	FEM1A	8.03 ± 0.17	7.97 ± 0.07	7.93 ± 0.06	8.00 ± 0.07
TC15000628.hg.1	FEM1B	8.90 ± 0.04	9.13 ± 0.10	8.71 ± 0.14	9.11 ± 0.08
TC05001689.hg.1	FEM1C	8.16 ± 0.22	8.28 ± 0.16	8.53 ± 0.49	8.27 ± 0.17
TC11000541.hg.1	FEN1	8.40 ± 0.37	8.06 ± 0.29	7.95 ± 0.30	7.91 ± 0.02
TC16001325.hg.1	FENDRR	5.47 ± 0.18	5.38 ± 0.10	5.43 ± 0.14	5.50 ± 0.06
TC05000509.hg.1	FER	6.19 ± 0.32	6.29 ± 0.16	6.22 ± 0.22	6.26 ± 0.05
TC20000802.hg.1	FER1L4	7.32 ± 0.23	7.24 ± 0.24	7.19 ± 0.19	7.32 ± 0.05

TC02000584.hg.1	FER1L5	6.96 ± 0.18	6.92 ± 0.19	6.90 ± 0.15	7.03 ± 0.03
TC08000721.hg.1	FER1L6	6.12 ± 0.21	6.05 ± 0.11	6.05 ± 0.14	6.12 ± 0.06
TC08001595.hg.1	FER1L6-AS1	6.02 ± 0.13	5.94 ± 0.13	5.99 ± 0.12	6.07 ± 0.12
TC08001597.hg.1	FER1L6-AS2	6.04 ± 0.24	6.03 ± 0.18	5.97 ± 0.06	6.13 ± 0.08
TC07001176.hg.1	FERD3L	8.99 ± 0.24	8.80 ± 0.23	8.94 ± 0.26	8.90 ± 0.05
TC20000603.hg.1	FERMT1	5.29 ± 0.16	5.23 ± 0.08	5.24 ± 0.07	5.23 ± 0.01
TC14001144.hg.1	FERMT2	5.18 ± 0.10	5.06 ± 0.02	5.08 ± 0.09	5.08 ± 0.05
TC11000586.hg.1	FERMT3	10.36 ± 0.26	9.87 ± 0.01	10.44 ± 0.18	9.84 ± 0.23
TC15000871.hg.1	FES	8.82 ± 0.25	8.61 ± 0.11	8.93 ± 0.16	8.64 ± 0.05
TC03001021.hg.1	FETUB	5.90 ± 0.19	5.79 ± 0.09	5.80 ± 0.09	5.90 ± 0.07
TC02002782.hg.1	FEV	7.75 ± 0.28	7.69 ± 0.19	7.70 ± 0.28	7.76 ± 0.05
TC11002420.hg.1	FEZ1	6.50 ± 0.14	6.40 ± 0.08	6.38 ± 0.18	6.40 ± 0.02
TC02001735.hg.1	FEZ2	6.82 ± 0.18	6.77 ± 0.14	6.95 ± 0.21	6.73 ± 0.06
TC07001812.hg.1	FEZF1	6.64 ± 0.20	6.65 ± 0.25	6.53 ± 0.16	6.72 ± 0.04
TC07000741.hg.1	FEZF1-AS1	7.08 ± 0.18	7.02 ± 0.09	6.96 ± 0.12	7.11 ± 0.03
TC03001515.hg.1	FEZF2	7.10 ± 0.21	7.02 ± 0.09	7.04 ± 0.22	7.05 ± 0.05
TC19000466.hg.1	FFAR1	8.43 ± 0.30	8.38 ± 0.13	8.27 ± 0.30	8.57 ± 0.17
TC19000470.hg.1	FFAR2	9.92 ± 0.99	9.55 ± 0.42	10.73 ± 0.60	9.15 ± 0.51
TC19000467.hg.1	FFAR3	7.83 ± 0.62	7.32 ± 0.19	7.91 ± 0.38	7.39 ± 0.12
TC10000665.hg.1	FFAR4	6.42 ± 0.18	6.20 ± 0.20	6.16 ± 0.07	6.25 ± 0.08
TC04001662.hg.1	FGA	5.25 ± 0.10	5.14 ± 0.11	5.18 ± 0.04	5.25 ± 0.10
TC04000777.hg.1	FGB	5.09 ± 0.09	4.97 ± 0.02	5.07 ± 0.09	5.07 ± 0.06
TC0X001074.hg.1	FGD1	7.41 ± 0.20	7.45 ± 0.22	7.42 ± 0.22	7.55 ± 0.03
TC06000538.hg.1	FGD2	9.06 ± 0.18	8.75 ± 0.13	8.91 ± 0.05	8.64 ± 0.02
TC09000440.hg.1	FGD3	9.78 ± 0.16	9.65 ± 0.09	9.82 ± 0.14	9.63 ± 0.08
TC12000297.hg.1	FGD4	7.53 ± 0.10	7.29 ± 0.22	8.03 ± 0.31	7.19 ± 0.17
TC03000090.hg.1	FGD5	6.79 ± 0.12	6.72 ± 0.13	6.72 ± 0.11	6.79 ± 0.06
TC03001201.hg.1	FGD5-AS1	8.51 ± 0.09	8.50 ± 0.07	8.44 ± 0.07	8.50 ± 0.02
TC03000081.hg.1	FGD5P1	7.04 ± 0.07	7.07 ± 0.15	7.09 ± 0.20	7.06 ± 0.04
TC12001829.hg.1	FGD6	6.59 ± 0.12	6.49 ± 0.09	6.52 ± 0.11	6.52 ± 0.10
TC05001883.hg.1	FGF1	6.90 ± 0.17	6.83 ± 0.19	6.85 ± 0.13	6.92 ± 0.08

TC05001324.hg.1	FGF10	6.37 ± 0.21	6.41 ± 0.14	6.36 ± 0.14	6.55 ± 0.05
TC17002861.hg.1	FGF11	7.55 ± 0.26	7.49 ± 0.16	7.48 ± 0.11	7.58 ± 0.07
TC03002115.hg.1	FGF12	5.59 ± 0.08	5.50 ± 0.05	5.53 ± 0.07	5.62 ± 0.11
TC03001059.hg.1	FGF12-AS1	5.23 ± 0.19	5.16 ± 0.12	5.14 ± 0.10	5.23 ± 0.12
TC03001060.hg.1	FGF12-AS2	6.86 ± 0.29	6.92 ± 0.19	6.75 ± 0.39	6.97 ± 0.07
TC03001061.hg.1	FGF12-AS3	4.49 ± 0.09	4.19 ± 0.02	4.31 ± 0.03	4.43 ± 0.07
TC0X001425.hg.1	FGF13	7.26 ± 0.14	7.02 ± 0.12	7.41 ± 0.08	7.06 ± 0.01
TC0X000672.hg.1	FGF13-AS1	5.72 ± 0.19	5.77 ± 0.14	5.92 ± 0.06	5.86 ± 0.06
TC13000846.hg.1	FGF14	5.55 ± 0.13	5.49 ± 0.09	5.47 ± 0.12	5.60 ± 0.07
TC13000365.hg.1	FGF14-AS1	6.81 ± 0.19	6.88 ± 0.25	6.71 ± 0.25	7.00 ± 0.08
TC13000366.hg.1	FGF14-AS2	5.88 ± 0.47	5.87 ± 0.10	5.84 ± 0.31	5.86 ± 0.09
TC0X000421.hg.1	FGF16	5.75 ± 0.44	5.68 ± 0.15	5.70 ± 0.18	5.81 ± 0.11
TC08000163.hg.1	FGF17	7.89 ± 0.25	7.77 ± 0.16	7.78 ± 0.22	7.82 ± 0.01
TC05000943.hg.1	FGF18	8.22 ± 0.19	8.17 ± 0.16	8.14 ± 0.21	8.21 ± 0.05
TC11002018.hg.1	FGF19	7.48 ± 0.31	7.51 ± 0.29	7.44 ± 0.17	7.65 ± 0.11
TC04000630.hg.1	FGF2	7.36 ± 0.18	7.20 ± 0.11	7.29 ± 0.20	7.13 ± 0.07
TC08001010.hg.1	FGF20	6.32 ± 0.16	6.22 ± 0.11	6.22 ± 0.15	6.27 ± 0.16
TC19000710.hg.1	FGF21	6.29 ± 0.20	6.27 ± 0.17	6.19 ± 0.11	6.35 ± 0.06
TC19000011.hg.1	FGF22	8.53 ± 0.34	8.48 ± 0.17	8.44 ± 0.33	8.54 ± 0.06
TC12001120.hg.1	FGF23	6.36 ± 0.22	6.39 ± 0.19	6.44 ± 0.16	6.51 ± 0.19
TC11002020.hg.1	FGF3	7.72 ± 0.17	7.72 ± 0.23	7.60 ± 0.32	7.73 ± 0.03
TC11002019.hg.1	FGF4	7.81 ± 0.21	7.86 ± 0.24	7.86 ± 0.31	7.97 ± 0.03
TC04002936.hg.1	FGF5	6.71 ± 0.15	6.60 ± 0.13	6.64 ± 0.13	6.67 ± 0.07
TC12001121.hg.1	FGF6	6.00 ± 0.11	5.91 ± 0.27	5.87 ± 0.04	5.89 ± 0.06
TC12001122.hg.1	FGF6	7.26 ± 0.35	7.23 ± 0.24	7.18 ± 0.19	7.37 ± 0.07
TC15000386.hg.1	FGF7	4.07 ± 0.03	3.92 ± 0.01	4.06 ± 0.01	4.07 ± 0.09
TC10001611.hg.1	FGF8	8.07 ± 0.20	8.07 ± 0.18	8.03 ± 0.21	8.13 ± 0.05
TC13000037.hg.1	FGF9	6.84 ± 0.21	6.91 ± 0.10	6.75 ± 0.11	6.97 ± 0.03
TC04001058.hg.1	FGFBP1	6.53 ± 0.32	6.52 ± 0.08	6.66 ± 0.12	6.64 ± 0.17
TC04002941.hg.1	FGFBP2	9.38 ± 0.51	9.40 ± 0.41	8.90 ± 0.17	8.99 ± 0.07
TC10001525.hg.1	FGFBP3	6.90 ± 0.24	6.90 ± 0.12	6.96 ± 0.04	6.92 ± 0.04

TC08001147.hg.1	FGFR1	7.59 ± 0.17	7.57 ± 0.10	7.49 ± 0.14	7.61 ± 0.03
TC06004105.hg.1	FGFR1OP	7.70 ± 0.26	7.66 ± 0.18	7.83 ± 0.18	7.50 ± 0.13
TC12000260.hg.1	FGFR1OP2	8.06 ± 0.41	8.04 ± 0.19	8.50 ± 0.54	7.86 ± 0.29
TC10001711.hg.1	FGFR2	6.63 ± 0.15	6.57 ± 0.11	6.64 ± 0.18	6.67 ± 0.12
TC04000029.hg.1	FGFR3	8.41 ± 0.25	8.34 ± 0.21	8.32 ± 0.21	8.37 ± 0.02
TC05001010.hg.1	FGFR4	8.27 ± 0.18	8.25 ± 0.20	8.20 ± 0.15	8.37 ± 0.06
TC04000016.hg.1	FGFRL1	8.63 ± 0.23	8.59 ± 0.17	8.53 ± 0.15	8.65 ± 0.03
TC04001663.hg.1	FGG	4.15 ± 0.10	4.03 ± 0.02	4.08 ± 0.07	4.08 ± 0.05
TC01000698.hg.1	FGGY	6.82 ± 0.06	6.77 ± 0.01	6.87 ± 0.11	6.78 ± 0.05
TC08001014.hg.1	FGL1	4.35 ± 0.16	4.16 ± 0.07	4.22 ± 0.08	4.17 ± 0.01
TC07001546.hg.1	FGL2	11.35 ± 0.66	11.90 ± 0.27	11.93 ± 0.50	11.70 ± 0.36
TC01002410.hg.1	FGR	10.46 ± 0.29	10.09 ± 0.09	10.76 ± 0.25	10.00 ± 0.14
TC01004025.hg.1	FH	7.50 ± 0.22	7.24 ± 0.22	7.10 ± 0.02	6.94 ± 0.11
TC01000186.hg.1	FHAD1	6.47 ± 0.15	6.41 ± 0.13	6.43 ± 0.12	6.49 ± 0.05
TC04000768.hg.1	FHDC1	5.75 ± 0.09	5.67 ± 0.03	5.82 ± 0.04	5.66 ± 0.05
TC03001506.hg.1	FHIT	7.02 ± 0.15	6.97 ± 0.07	6.83 ± 0.08	6.99 ± 0.07
TC0X000658.hg.1	FHL1	7.54 ± 0.02	7.43 ± 0.09	7.59 ± 0.29	7.55 ± 0.09
TC02002167.hg.1	FHL2	7.36 ± 0.14	7.23 ± 0.09	7.29 ± 0.15	7.32 ± 0.06
TC01002530.hg.1	FHL3	8.90 ± 0.33	8.49 ± 0.04	8.86 ± 0.29	8.58 ± 0.15
TC06000820.hg.1	FHL5	4.97 ± 0.13	4.92 ± 0.06	4.98 ± 0.07	4.97 ± 0.15
TC16001184.hg.1	FHOD1	8.45 ± 0.19	8.18 ± 0.02	8.41 ± 0.03	8.24 ± 0.13
TC18000150.hg.1	FHOD3	6.42 ± 0.14	6.40 ± 0.15	6.42 ± 0.11	6.48 ± 0.07
TC09001660.hg.1	FIBCD1	8.29 ± 0.25	8.33 ± 0.24	8.20 ± 0.26	8.41 ± 0.02
TC11000277.hg.1	FIBIN	7.01 ± 0.23	6.71 ± 0.11	6.62 ± 0.13	6.73 ± 0.19
TC11001947.hg.1	FIBP	9.03 ± 0.30	8.67 ± 0.06	8.76 ± 0.15	8.51 ± 0.05
TC12000834.hg.1	FICD	7.43 ± 0.24	7.29 ± 0.09	7.30 ± 0.14	7.35 ± 0.08
TC12000835.hg.1	FICD	6.50 ± 0.09	6.54 ± 0.23	6.44 ± 0.31	6.69 ± 0.17
TC06000879.hg.1	FIG4	8.10 ± 0.22	7.92 ± 0.26	8.47 ± 0.51	7.62 ± 0.08
TC0X002326.hg.1	FIGF	5.01 ± 0.15	4.97 ± 0.12	4.97 ± 0.05	5.10 ± 0.09
TC02001966.hg.1	FIGLA	5.46 ± 0.22	5.41 ± 0.20	5.38 ± 0.18	5.49 ± 0.07
TC02002484.hg.1	FIGN	5.53 ± 0.15	5.55 ± 0.13	5.51 ± 0.06	5.55 ± 0.13

TC07001372.hg.1	FIGNL1	6.53 ± 0.15	6.56 ± 0.05	6.46 ± 0.07	6.54 ± 0.05
TC12001510.hg.1	FIGNL2	7.24 ± 0.37	7.12 ± 0.11	7.12 ± 0.15	7.24 ± 0.06
TC06001882.hg.1	FILIP1	5.78 ± 0.18	5.67 ± 0.15	5.67 ± 0.12	5.82 ± 0.11
TC03001608.hg.1	FILIP1L	5.68 ± 0.16	5.63 ± 0.13	5.67 ± 0.05	5.72 ± 0.04
TC04002930.hg.1	FIP1L1	8.92 ± 0.18	9.03 ± 0.18	8.79 ± 0.20	8.95 ± 0.09
TC07001689.hg.1	FIS1	8.37 ± 0.16	8.20 ± 0.03	8.23 ± 0.13	8.17 ± 0.05
TC14002193.hg.1	FITM1	7.60 ± 0.19	7.63 ± 0.12	7.64 ± 0.20	7.69 ± 0.06
TC20000862.hg.1	FITM2	8.06 ± 0.27	7.92 ± 0.09	7.89 ± 0.22	7.94 ± 0.04
TC19001878.hg.1	FIZ1	7.81 ± 0.36	7.69 ± 0.19	7.66 ± 0.22	7.74 ± 0.06
TC11000337.hg.1	FJX1	7.11 ± 0.17	7.09 ± 0.17	7.08 ± 0.15	7.17 ± 0.03
TC17000517.hg.1	FKBP10	7.66 ± 0.24	7.56 ± 0.16	7.57 ± 0.18	7.61 ± 0.06
TC12003226.hg.1	FKBP11	8.96 ± 0.21	8.53 ± 0.13	8.45 ± 0.11	8.47 ± 0.09
TC07001245.hg.1	FKBP14	5.98 ± 0.18	5.72 ± 0.10	5.68 ± 0.12	5.63 ± 0.10
TC09002916.hg.1	FKBP15	9.65 ± 0.31	9.22 ± 0.21	9.84 ± 0.21	9.01 ± 0.36
TC20001741.hg.1	FKBP1A	9.96 ± 0.12	9.77 ± 0.05	10.14 ± 0.23	9.67 ± 0.14
TC19001904.hg.1	FKBP1AP1	8.04 ± 0.15	8.18 ± 0.05	8.02 ± 0.19	8.21 ± 0.14
TC02004952.hg.1	FKBP1B	7.32 ± 0.18	7.35 ± 0.11	7.32 ± 0.16	7.38 ± 0.07
TC06000696.hg.1	FKBP1C	8.78 ± 0.13	8.59 ± 0.08	8.86 ± 0.16	8.47 ± 0.16
TC11000590.hg.1	FKBP2	9.30 ± 0.25	9.14 ± 0.10	9.11 ± 0.21	9.12 ± 0.06
TC14001084.hg.1	FKBP3	7.72 ± 0.24	7.78 ± 0.15	7.73 ± 0.18	7.69 ± 0.20
TC12000028.hg.1	FKBP4	8.26 ± 0.09	8.16 ± 0.02	8.22 ± 0.21	7.95 ± 0.13
TC06004150.hg.1	FKBP5	9.59 ± 0.48	9.04 ± 0.05	10.08 ± 0.25	9.01 ± 0.50
TC07003330.hg.1	FKBP6	6.77 ± 0.16	6.69 ± 0.10	6.66 ± 0.16	6.70 ± 0.02
TC02002574.hg.1	FKBP7	5.70 ± 0.03	5.62 ± 0.03	5.67 ± 0.03	5.61 ± 0.04
TC19001291.hg.1	FKBP8	10.09 ± 0.18	9.93 ± 0.05	9.96 ± 0.15	9.81 ± 0.10
TC07000213.hg.1	FKBP9	7.04 ± 0.14	6.92 ± 0.12	7.03 ± 0.08	6.97 ± 0.03
TC07001398.hg.1	FKBP9L	6.95 ± 0.22	7.03 ± 0.08	7.03 ± 0.11	7.05 ± 0.07
TC06001559.hg.1	FKBPL	7.61 ± 0.31	7.57 ± 0.10	7.62 ± 0.22	7.62 ± 0.02
TC6_cox_hap2000184.hg.1	FKBPL	7.78 ± 0.33	7.72 ± 0.06	7.80 ± 0.27	7.78 ± 0.02
TC6_dbb_hap3000175.hg.1	FKBPL	7.75 ± 0.34	7.74 ± 0.10	7.77 ± 0.29	7.78 ± 0.02
TC6_mcf_hap5000159.hg.1	FKBPL	7.78 ± 0.33	7.72 ± 0.06	7.80 ± 0.27	7.78 ± 0.02

TC19000668.hg.1	FKRP	7.83 ± 0.12	7.75 ± 0.12	7.72 ± 0.14	7.84 ± 0.03
TC13000354.hg.1	FKSG29	7.18 ± 0.72	7.77 ± 0.18	7.03 ± 0.42	7.57 ± 0.33
TC05000257.hg.1	FKSG52	12.20 ± 0.29	12.35 ± 0.09	12.35 ± 0.22	12.45 ± 0.05
TC15001323.hg.1	FKSG62	11.59 ± 0.32	11.83 ± 0.09	11.79 ± 0.15	11.92 ± 0.05
TC09000537.hg.1	FKTN	6.22 ± 0.25	6.17 ± 0.15	6.19 ± 0.20	6.08 ± 0.04
TC01001282.hg.1	FLAD1	8.06 ± 0.19	7.69 ± 0.03	7.80 ± 0.16	7.63 ± 0.06
TC17002891.hg.1	FLCN	8.23 ± 0.11	8.07 ± 0.10	8.16 ± 0.15	8.08 ± 0.04
TC01003245.hg.1	FLG	6.75 ± 0.17	6.65 ± 0.18	6.62 ± 0.16	6.80 ± 0.17
TC01003246.hg.1	FLG2	4.89 ± 0.14	4.97 ± 0.05	4.87 ± 0.19	5.02 ± 0.14
TC01001223.hg.1	FLG-AS1	4.82 ± 0.12	4.80 ± 0.10	4.72 ± 0.07	4.84 ± 0.12
TC11001185.hg.1	FLI1	10.13 ± 0.06	9.93 ± 0.12	10.16 ± 0.08	9.79 ± 0.21
TC11002442.hg.1	FLI1-AS1	8.02 ± 0.08	7.62 ± 0.18	7.96 ± 0.18	7.71 ± 0.07
TC17001199.hg.1	FLII	9.53 ± 0.18	9.38 ± 0.05	9.52 ± 0.10	9.32 ± 0.06
TC16002092.hg.1	FLJ00104	7.60 ± 0.03	7.53 ± 0.03	7.40 ± 0.09	7.57 ± 0.10
TC15001352.hg.1	FLJ10038	8.78 ± 0.36	9.00 ± 0.40	8.41 ± 0.46	9.24 ± 0.13
TC05000530.hg.1	FLJ11235	6.38 ± 0.32	6.42 ± 0.21	6.16 ± 0.23	6.48 ± 0.04
TC12000454.hg.1	FLJ12825	6.06 ± 0.17	5.92 ± 0.04	6.04 ± 0.01	6.07 ± 0.07
TC04001120.hg.1	FLJ13197	7.08 ± 0.25	7.18 ± 0.09	7.03 ± 0.03	7.19 ± 0.13
TC12000288.hg.1	FLJ13224	8.01 ± 0.13	8.11 ± 0.11	7.93 ± 0.07	8.16 ± 0.13
TC02000574.hg.1	FLJ14082	7.41 ± 0.28	7.39 ± 0.16	7.30 ± 0.34	7.58 ± 0.04
TC04001501.hg.1	FLJ14186	10.81 ± 0.24	10.81 ± 0.15	11.33 ± 0.16	10.88 ± 0.10
TC02000380.hg.1	FLJ16124	5.80 ± 0.13	5.68 ± 0.04	5.71 ± 0.03	5.75 ± 0.10
TC01002188.hg.1	FLJ16126	6.24 ± 0.08	6.17 ± 0.28	6.17 ± 0.19	6.46 ± 0.06
TC05002082.hg.1	FLJ16171	5.31 ± 0.19	5.22 ± 0.21	5.21 ± 0.02	5.30 ± 0.18
TC02001887.hg.1	FLJ16341	6.75 ± 0.25	6.84 ± 0.06	6.76 ± 0.16	6.94 ± 0.13
TC20000510.hg.1	FLJ16779	7.48 ± 0.18	7.47 ± 0.19	7.39 ± 0.28	7.60 ± 0.09
TC04000523.hg.1	FLJ20021	8.00 ± 0.18	7.87 ± 0.20	7.91 ± 0.28	7.89 ± 0.12
TC16000291.hg.1	FLJ21408	6.84 ± 0.07	6.71 ± 0.06	6.83 ± 0.17	6.77 ± 0.05
TC19001119.hg.1	FLJ22184	8.06 ± 0.24	8.06 ± 0.28	7.96 ± 0.32	8.14 ± 0.07
TC14000372.hg.1	FLJ22447	6.18 ± 0.09	6.28 ± 0.08	6.17 ± 0.03	6.28 ± 0.10
TC03000552.hg.1	FLJ22763	5.12 ± 0.04	4.96 ± 0.07	5.08 ± 0.11	5.07 ± 0.10

TC03000556.hg.1	FLJ25363	5.61 ± 0.06	5.50 ± 0.04	5.53 ± 0.04	5.55 ± 0.01
TC18000589.hg.1	FLJ25715	7.26 ± 0.35	7.11 ± 0.19	7.25 ± 0.17	7.32 ± 0.05
TC19001107.hg.1	FLJ25758	5.45 ± 0.45	5.46 ± 0.20	5.25 ± 0.33	5.43 ± 0.11
TC16000412.hg.1	FLJ26245	8.30 ± 0.30	8.17 ± 0.13	8.13 ± 0.32	8.27 ± 0.07
TC19000754.hg.1	FLJ26850	5.85 ± 0.05	5.90 ± 0.20	5.94 ± 0.22	5.98 ± 0.08
TC06002273.hg.1	FLJ27255	6.43 ± 0.09	6.17 ± 0.15	6.68 ± 0.37	6.22 ± 0.07
TC01002851.hg.1	FLJ27354	5.91 ± 0.08	5.82 ± 0.11	5.74 ± 0.03	5.83 ± 0.02
TC19001783.hg.1	FLJ30403	7.00 ± 0.19	6.93 ± 0.22	6.74 ± 0.15	7.03 ± 0.05
TC16000669.hg.1	FLJ30679	6.99 ± 0.21	7.02 ± 0.16	6.95 ± 0.03	7.00 ± 0.11
TC02000334.hg.1	FLJ30838	5.73 ± 0.20	5.66 ± 0.09	5.64 ± 0.11	5.73 ± 0.10
TC05000239.hg.1	FLJ31104	6.17 ± 0.15	6.11 ± 0.13	6.03 ± 0.29	6.13 ± 0.06
TC14002323.hg.1	FLJ31306	8.45 ± 0.31	8.58 ± 0.20	8.58 ± 0.35	8.60 ± 0.10
TC02001699.hg.1	FLJ31356	6.68 ± 0.11	6.84 ± 0.16	6.74 ± 0.18	6.90 ± 0.12
TC01000878.hg.1	FLJ31662	5.83 ± 0.37	5.68 ± 0.20	5.84 ± 0.08	5.99 ± 0.11
TC10001286.hg.1	FLJ31813	8.80 ± 0.07	8.74 ± 0.08	8.69 ± 0.09	8.73 ± 0.09
TC02002417.hg.1	FLJ32955	4.80 ± 0.13	4.84 ± 0.05	4.84 ± 0.10	4.87 ± 0.06
TC05001136.hg.1	FLJ33360	7.08 ± 0.23	7.06 ± 0.19	7.04 ± 0.14	7.19 ± 0.06
TC02001575.hg.1	FLJ33534	7.28 ± 0.22	7.34 ± 0.14	7.21 ± 0.16	7.49 ± 0.10
TC20000160.hg.1	FLJ33581	6.59 ± 0.31	6.69 ± 0.07	6.66 ± 0.06	6.81 ± 0.13
TC05001750.hg.1	FLJ33630	7.45 ± 0.12	7.49 ± 0.05	7.29 ± 0.11	7.46 ± 0.04
TC06000910.hg.1	FLJ34503	5.41 ± 0.19	5.27 ± 0.01	5.26 ± 0.13	5.30 ± 0.05
TC09000873.hg.1	FLJ35024	5.85 ± 0.25	5.82 ± 0.07	5.79 ± 0.16	5.88 ± 0.05
TC09000110.hg.1	FLJ35282	4.56 ± 0.10	4.32 ± 0.08	4.51 ± 0.07	4.42 ± 0.08
TC04000020.hg.1	FLJ35816	6.54 ± 0.08	6.52 ± 0.07	6.54 ± 0.25	6.73 ± 0.04
TC17000301.hg.1	FLJ36000	8.08 ± 0.30	8.06 ± 0.22	8.17 ± 0.09	8.25 ± 0.14
TC04001008.hg.1	FLJ36777	6.01 ± 0.07	5.92 ± 0.13	5.91 ± 0.16	6.00 ± 0.04
TC10001735.hg.1	FLJ37035	7.13 ± 0.08	7.07 ± 0.09	7.10 ± 0.13	7.18 ± 0.04
TC10001511.hg.1	FLJ37201	4.18 ± 0.02	4.16 ± 0.07	4.14 ± 0.18	4.16 ± 0.04
TC01002252.hg.1	FLJ37453	7.99 ± 0.23	7.89 ± 0.28	7.78 ± 0.18	7.72 ± 0.14
TC12001020.hg.1	FLJ37505	5.05 ± 0.27	4.82 ± 0.07	4.94 ± 0.07	4.87 ± 0.06
TC17001837.hg.1	FLJ37644	6.98 ± 0.09	6.91 ± 0.02	6.92 ± 0.07	6.98 ± 0.07

TC17001838.hg.1	FLJ37644	6.46 ± 0.13	6.40 ± 0.13	6.36 ± 0.14	6.50 ± 0.12
TC17001839.hg.1	FLJ37644	6.29 ± 0.26	6.29 ± 0.16	6.19 ± 0.10	6.39 ± 0.18
TC02002181.hg.1	FLJ38668	6.95 ± 0.10	6.93 ± 0.10	7.01 ± 0.05	7.08 ± 0.10
TC15001532.hg.1	FLJ38723	7.77 ± 0.18	7.73 ± 0.15	7.71 ± 0.18	7.83 ± 0.03
TC08000495.hg.1	FLJ39080	5.55 ± 0.09	5.51 ± 0.08	5.45 ± 0.10	5.61 ± 0.10
TC01003080.hg.1	FLJ39739	8.17 ± 0.12	8.17 ± 0.06	8.11 ± 0.07	8.14 ± 0.01
TC17000641.hg.1	FLJ40194	7.74 ± 0.19	7.58 ± 0.18	7.64 ± 0.20	7.62 ± 0.18
TC16000685.hg.1	FLJ40448	7.85 ± 0.26	7.84 ± 0.22	7.73 ± 0.12	7.90 ± 0.06
TC09000915.hg.1	FLJ41200	6.51 ± 0.27	6.45 ± 0.20	6.51 ± 0.21	6.67 ± 0.14
TC12000577.hg.1	FLJ41278	5.58 ± 0.25	5.49 ± 0.12	5.44 ± 0.11	5.55 ± 0.04
TC11000381.hg.1	FLJ41423	7.01 ± 0.27	6.89 ± 0.17	6.97 ± 0.26	7.01 ± 0.09
TC21000208.hg.1	FLJ41733	7.16 ± 0.33	7.24 ± 0.30	7.20 ± 0.34	7.32 ± 0.05
TC22000027.hg.1	FLJ41941	5.45 ± 0.17	5.26 ± 0.31	5.39 ± 0.19	5.43 ± 0.10
TC11002037.hg.1	FLJ42102	6.64 ± 0.23	6.68 ± 0.12	6.61 ± 0.14	6.80 ± 0.10
TC02002216.hg.1	FLJ42351	5.57 ± 0.03	5.40 ± 0.06	5.54 ± 0.13	5.42 ± 0.03
TC03001039.hg.1	FLJ42393	7.00 ± 0.19	7.10 ± 0.04	7.01 ± 0.07	7.10 ± 0.17
TC16000081.hg.1	FLJ42627	7.46 ± 0.31	7.59 ± 0.13	7.68 ± 0.13	7.72 ± 0.18
TC17000684.hg.1	FLJ42842	6.92 ± 0.37	6.89 ± 0.04	6.74 ± 0.16	7.04 ± 0.15
TC01002127.hg.1	FLJ42875	7.58 ± 0.18	7.57 ± 0.26	7.53 ± 0.18	7.71 ± 0.03
TC08000622.hg.1	FLJ42969	5.36 ± 0.08	5.29 ± 0.07	5.20 ± 0.12	5.47 ± 0.10
TCUn_gl000211000001.hg.1	FLJ43315	7.82 ± 0.31	7.87 ± 0.10	7.77 ± 0.26	8.09 ± 0.18
TC07001871.hg.1	FLJ43663	8.52 ± 0.34	8.90 ± 0.10	8.73 ± 0.25	8.92 ± 0.11
TC17000960.hg.1	FLJ43681	7.79 ± 0.13	7.70 ± 0.06	7.90 ± 0.15	7.77 ± 0.06
TC06000002.hg.1	FLJ43763	6.80 ± 0.34	6.79 ± 0.12	6.74 ± 0.24	6.85 ± 0.15
TC02002920.hg.1	FLJ43879	6.80 ± 0.16	6.88 ± 0.20	6.80 ± 0.16	7.00 ± 0.12
TC02002206.hg.1	FLJ44006	6.23 ± 0.08	6.27 ± 0.21	6.29 ± 0.10	6.44 ± 0.10
TC18000254.hg.1	FLJ44313	7.00 ± 0.30	6.88 ± 0.26	6.77 ± 0.18	6.98 ± 0.04
TC07000007.hg.1	FLJ44511	7.82 ± 0.11	7.70 ± 0.19	7.85 ± 0.07	7.82 ± 0.10
TC0X000395.hg.1	FLJ44635	8.19 ± 0.17	7.90 ± 0.34	8.51 ± 0.38	7.73 ± 0.33
TC17_gl000204_random000001.hg.1	FLJ44838	6.75 ± 0.39	6.68 ± 0.36	6.35 ± 0.60	6.74 ± 0.04
TC18000257.hg.1	FLJ44881	5.26 ± 0.12	5.24 ± 0.03	5.23 ± 0.07	5.39 ± 0.07

TC17001911.hg.1	FLJ45079	6.82 ± 0.03	6.86 ± 0.12	6.75 ± 0.08	6.78 ± 0.05
TC21000452.hg.1	FLJ45139	6.63 ± 0.17	6.59 ± 0.06	6.54 ± 0.15	6.68 ± 0.07
TC08000636.hg.1	FLJ45248	7.38 ± 0.14	7.43 ± 0.23	7.39 ± 0.13	7.43 ± 0.02
TC16000971.hg.1	FLJ45256	6.76 ± 0.11	6.67 ± 0.11	6.55 ± 0.27	6.81 ± 0.11
TC07000777.hg.1	FLJ45340	11.36 ± 0.17	11.41 ± 0.15	11.71 ± 0.27	11.57 ± 0.15
TC19000976.hg.1	FLJ45445	12.00 ± 0.43	12.05 ± 0.23	12.71 ± 0.28	12.15 ± 0.20
TC17000649.hg.1	FLJ45513	7.83 ± 0.13	7.90 ± 0.08	7.80 ± 0.19	7.96 ± 0.09
TC18000529.hg.1	FLJ45743	5.26 ± 0.02	5.21 ± 0.04	5.22 ± 0.03	5.30 ± 0.05
TC06000546.hg.1	FLJ45825	6.77 ± 0.14	6.67 ± 0.09	6.65 ± 0.14	6.73 ± 0.06
TC17001156.hg.1	FLJ45831	5.47 ± 0.10	5.54 ± 0.22	5.49 ± 0.25	5.72 ± 0.32
TC20000743.hg.1	FLJ45832	6.26 ± 0.21	6.15 ± 0.06	6.15 ± 0.08	6.18 ± 0.04
TC08001672.hg.1	FLJ45872	5.20 ± 0.19	5.06 ± 0.16	5.16 ± 0.18	5.33 ± 0.07
TC07001391.hg.1	FLJ45974	5.37 ± 0.09	5.34 ± 0.08	5.37 ± 0.05	5.39 ± 0.07
TC05000282.hg.1	FLJ46010	5.71 ± 0.10	5.72 ± 0.00	5.64 ± 0.09	5.75 ± 0.14
TC03002049.hg.1	FLJ46066	5.68 ± 0.07	5.52 ± 0.06	5.61 ± 0.07	5.59 ± 0.13
TC08001420.hg.1	FLJ46284	6.17 ± 0.13	5.96 ± 0.07	6.11 ± 0.06	6.03 ± 0.13
TC10001768.hg.1	FLJ46300	7.59 ± 0.09	7.63 ± 0.20	7.48 ± 0.30	7.67 ± 0.12
TC10000892.hg.1	FLJ46361	7.04 ± 0.14	6.98 ± 0.15	6.99 ± 0.07	7.12 ± 0.12
TC06001031.hg.1	FLJ46906	6.03 ± 0.13	6.03 ± 0.16	6.01 ± 0.12	6.12 ± 0.07
TC0X001530.hg.1	FLNA	10.72 ± 0.11	10.53 ± 0.12	10.54 ± 0.17	10.42 ± 0.08
TC03000375.hg.1	FLNB	8.17 ± 0.10	8.19 ± 0.08	7.95 ± 0.11	8.32 ± 0.14
TC03001497.hg.1	FLNB-AS1	7.06 ± 0.13	7.01 ± 0.17	7.01 ± 0.06	7.06 ± 0.05
TC07000785.hg.1	FLNC	8.17 ± 0.21	8.09 ± 0.16	8.09 ± 0.21	8.17 ± 0.03
TC06001508.hg.1	FLOT1	9.70 ± 0.35	9.36 ± 0.04	10.09 ± 0.22	9.40 ± 0.26
TC6_apd_hap1000078.hg.1	FLOT1	10.42 ± 0.48	9.80 ± 0.10	10.81 ± 0.30	9.80 ± 0.28
TC6_cox_hap2000142.hg.1	FLOT1	9.67 ± 0.33	9.34 ± 0.03	10.05 ± 0.21	9.37 ± 0.25
TC6_dbb_hap3000131.hg.1	FLOT1	9.66 ± 0.33	9.34 ± 0.03	10.04 ± 0.21	9.37 ± 0.25
TC6_mann_hap4000120.hg.1	FLOT1	9.54 ± 0.31	9.22 ± 0.04	9.93 ± 0.21	9.26 ± 0.25
TC6_mcf_hap5000121.hg.1	FLOT1	10.24 ± 0.42	9.84 ± 0.05	10.67 ± 0.28	9.85 ± 0.30
TC6_qbl_hap6000132.hg.1	FLOT1	9.66 ± 0.33	9.34 ± 0.03	10.04 ± 0.21	9.37 ± 0.25
TC6_ssto_hap7000122.hg.1	FLOT1	9.66 ± 0.33	9.34 ± 0.03	10.04 ± 0.21	9.37 ± 0.25

TC17001311.hg.1	FLOT2	10.27 ± 0.33	9.96 ± 0.12	10.70 ± 0.25	9.78 ± 0.19
TC11000584.hg.1	FLRT1	7.07 ± 0.18	7.03 ± 0.11	6.96 ± 0.19	7.14 ± 0.09
TC14000514.hg.1	FLRT2	6.09 ± 0.15	5.99 ± 0.11	6.02 ± 0.14	6.05 ± 0.02
TC20000642.hg.1	FLRT3	5.23 ± 0.16	5.12 ± 0.08	5.17 ± 0.06	5.24 ± 0.10
TC13000517.hg.1	FLT1	5.85 ± 0.08	5.71 ± 0.02	5.75 ± 0.11	5.70 ± 0.07
TC13000515.hg.1	FLT3	6.46 ± 0.54	6.02 ± 0.05	6.19 ± 0.22	5.99 ± 0.06
TC19002605.hg.1	FLT3LG	9.37 ± 0.16	9.34 ± 0.06	9.04 ± 0.04	9.32 ± 0.05
TC05002148.hg.1	FLT4	7.95 ± 0.20	7.90 ± 0.20	7.89 ± 0.21	7.98 ± 0.03
TC01001781.hg.1	FLVCR1	7.85 ± 0.25	7.73 ± 0.24	7.97 ± 0.27	7.76 ± 0.18
TC01003812.hg.1	FLVCR1-AS1	7.21 ± 0.14	7.06 ± 0.11	7.03 ± 0.08	6.91 ± 0.20
TC14002303.hg.1	FLVCR2	8.06 ± 0.83	7.29 ± 0.05	8.57 ± 0.22	7.32 ± 0.33
TC16000089.hg.1	FLYWCH1	8.66 ± 0.13	8.59 ± 0.13	8.48 ± 0.25	8.73 ± 0.08
TC16000088.hg.1	FLYWCH2	8.71 ± 0.15	8.59 ± 0.02	8.62 ± 0.15	8.60 ± 0.02
TC15001183.hg.1	FMN1	6.69 ± 0.17	6.94 ± 0.04	6.71 ± 0.23	7.00 ± 0.13
TC01001966.hg.1	FMN2	5.08 ± 0.11	4.95 ± 0.05	5.01 ± 0.07	5.01 ± 0.02
TC17000582.hg.1	FMNL1	10.55 ± 0.25	10.50 ± 0.02	10.61 ± 0.15	10.54 ± 0.19
TC02000941.hg.1	FMNL2	6.42 ± 0.16	6.22 ± 0.15	6.67 ± 0.35	6.12 ± 0.10
TC12001481.hg.1	FMNL3	8.32 ± 0.11	8.35 ± 0.08	8.00 ± 0.06	8.31 ± 0.02
TC01001489.hg.1	FMO1	5.27 ± 0.16	5.20 ± 0.08	5.22 ± 0.07	5.28 ± 0.12
TC01001488.hg.1	FMO2	5.16 ± 0.08	5.08 ± 0.09	5.09 ± 0.10	5.18 ± 0.10
TC01001486.hg.1	FMO3	4.76 ± 0.08	4.62 ± 0.05	4.68 ± 0.05	4.68 ± 0.06
TC01001490.hg.1	FMO4	5.81 ± 0.18	5.63 ± 0.04	5.69 ± 0.18	5.53 ± 0.07
TC01003125.hg.1	FMO5	6.05 ± 0.13	5.99 ± 0.06	6.09 ± 0.03	6.00 ± 0.08
TC01001487.hg.1	FMO6P	5.08 ± 0.21	5.07 ± 0.20	5.04 ± 0.12	5.23 ± 0.11
TC01001451.hg.1	FMO9P	5.00 ± 0.07	4.92 ± 0.10	4.97 ± 0.09	4.99 ± 0.13
TC01003722.hg.1	FMOD	6.64 ± 0.23	6.59 ± 0.23	6.57 ± 0.07	6.74 ± 0.12
TC0X000705.hg.1	FMR1	8.61 ± 0.40	8.87 ± 0.22	8.80 ± 0.42	8.76 ± 0.16
TC0X001474.hg.1	FMR1-AS1	5.22 ± 0.12	5.10 ± 0.07	5.20 ± 0.07	5.10 ± 0.02
TC0X000706.hg.1	FMR1-IT1	10.22 ± 0.16	10.04 ± 0.10	10.26 ± 0.09	10.14 ± 0.04
TC0X000707.hg.1	FMR1NB	5.89 ± 0.13	5.78 ± 0.09	5.79 ± 0.04	5.79 ± 0.05
TC02002747.hg.1	FN1	6.18 ± 0.32	5.93 ± 0.10	5.99 ± 0.07	6.12 ± 0.16

TC17000957.hg.1	FN3K	7.72 ± 0.25	7.61 ± 0.19	7.65 ± 0.20	7.68 ± 0.04
TC17000956.hg.1	FN3KRP	7.87 ± 0.17	7.76 ± 0.06	7.62 ± 0.12	7.78 ± 0.05
TC09001656.hg.1	FNBP1	9.45 ± 0.19	9.61 ± 0.06	9.47 ± 0.12	9.58 ± 0.11
TC01000863.hg.1	FNBP1L	6.71 ± 0.30	6.47 ± 0.03	6.84 ± 0.14	6.55 ± 0.12
TC11001748.hg.1	FNBP4	9.32 ± 0.47	9.62 ± 0.11	9.13 ± 0.41	9.61 ± 0.03
TC06001142.hg.1	FNDC1	5.79 ± 0.17	5.71 ± 0.14	5.73 ± 0.08	5.81 ± 0.07
TC06001143.hg.1	FNDC1-IT1	3.62 ± 0.06	3.46 ± 0.09	3.56 ± 0.11	3.51 ± 0.02
TC13000193.hg.1	FNDC3A	8.42 ± 0.37	8.47 ± 0.15	8.65 ± 0.43	8.29 ± 0.09
TC03000926.hg.1	FNDC3B	9.31 ± 0.32	9.12 ± 0.26	9.92 ± 0.46	8.96 ± 0.29
TC02001689.hg.1	FNDC4	8.19 ± 0.18	8.21 ± 0.19	8.11 ± 0.20	8.34 ± 0.03
TC01002476.hg.1	FNDC5	7.62 ± 0.22	7.65 ± 0.23	7.58 ± 0.20	7.75 ± 0.08
TC01000933.hg.1	FNDC7	6.00 ± 0.18	5.88 ± 0.08	5.84 ± 0.02	5.95 ± 0.04
TC17000394.hg.1	FNDC8	6.81 ± 0.15	6.68 ± 0.12	6.67 ± 0.13	6.73 ± 0.02
TC05001983.hg.1	FNDC9	6.52 ± 0.04	6.42 ± 0.07	6.54 ± 0.12	6.42 ± 0.04
TC05003427.hg.1	FNIP1	8.78 ± 0.26	8.90 ± 0.20	9.20 ± 0.45	8.99 ± 0.15
TC04000808.hg.1	FNIP2	7.16 ± 0.03	7.13 ± 0.10	7.09 ± 0.14	7.12 ± 0.15
TC08002599.hg.1	FNTA	8.75 ± 0.30	8.80 ± 0.20	8.65 ± 0.18	8.62 ± 0.10
TC14002300.hg.1	FNTB	7.71 ± 0.07	7.45 ± 0.05	7.67 ± 0.21	7.44 ± 0.07
TC09000099.hg.1	FOCAD	7.11 ± 0.19	7.07 ± 0.21	6.95 ± 0.11	6.87 ± 0.05
TC11001758.hg.1	FOLH1	5.01 ± 0.10	4.90 ± 0.09	4.91 ± 0.10	4.97 ± 0.08
TC11000888.hg.1	FOLH1B	4.58 ± 0.11	4.44 ± 0.04	4.47 ± 0.06	4.50 ± 0.09
TC11000754.hg.1	FOLR1	7.19 ± 0.19	7.17 ± 0.25	7.31 ± 0.19	7.27 ± 0.05
TC11000755.hg.1	FOLR2	7.63 ± 0.15	7.54 ± 0.23	7.64 ± 0.24	7.61 ± 0.08
TC11000751.hg.1	FOLR3	8.70 ± 0.54	8.20 ± 0.44	9.38 ± 0.89	8.03 ± 0.13
TC11000918.hg.1	FOLR4	7.90 ± 0.18	7.99 ± 0.16	7.83 ± 0.15	8.08 ± 0.07
TC16000899.hg.1	FOPNL	8.34 ± 0.19	8.27 ± 0.15	8.18 ± 0.35	8.06 ± 0.24
TC14000471.hg.1	FOS	10.95 ± 0.09	11.45 ± 0.23	11.48 ± 0.42	11.19 ± 0.41
TC19000647.hg.1	FOSB	9.09 ± 0.35	9.89 ± 0.44	9.53 ± 0.55	9.86 ± 0.51
TC11001948.hg.1	FOSL1	7.86 ± 0.31	7.94 ± 0.23	7.92 ± 0.17	8.09 ± 0.15
TC02000183.hg.1	FOSL2	9.50 ± 0.28	9.77 ± 0.08	9.96 ± 0.23	9.86 ± 0.07
TC14001056.hg.1	FOXA1	6.92 ± 0.27	6.81 ± 0.17	6.84 ± 0.12	6.88 ± 0.08

TC20000698.hg.1	FOXA2	7.46 ± 0.19	7.39 ± 0.12	7.43 ± 0.21	7.42 ± 0.03
TC19000656.hg.1	FOXA3	7.81 ± 0.38	7.84 ± 0.41	7.76 ± 0.28	7.96 ± 0.04
TC15000458.hg.1	FOXB1	6.90 ± 0.21	6.88 ± 0.17	6.88 ± 0.07	6.96 ± 0.03
TC09000353.hg.1	FOXB2	7.71 ± 0.26	7.79 ± 0.18	7.73 ± 0.25	7.86 ± 0.09
TC06000012.hg.1	FOXC1	7.61 ± 0.22	7.65 ± 0.13	7.70 ± 0.23	7.74 ± 0.06
TC16000670.hg.1	FOXC2	7.81 ± 0.51	7.71 ± 0.19	7.65 ± 0.29	7.80 ± 0.01
TC05001470.hg.1	FOXD1	7.68 ± 0.19	7.56 ± 0.15	7.67 ± 0.29	7.63 ± 0.08
TC01000600.hg.1	FOXD2	6.31 ± 0.24	6.24 ± 0.12	6.29 ± 0.24	6.22 ± 0.06
TC01002632.hg.1	FOXD2-AS1	7.89 ± 0.27	7.64 ± 0.04	7.79 ± 0.23	7.71 ± 0.11
TC01000716.hg.1	FOXD3	6.83 ± 0.22	6.86 ± 0.20	6.53 ± 0.41	6.84 ± 0.04
TC09000863.hg.1	FOXD4	6.09 ± 0.09	5.98 ± 0.04	6.00 ± 0.18	6.04 ± 0.14
TC02000726.hg.1	FOXD4L1	6.52 ± 0.09	6.42 ± 0.11	6.46 ± 0.14	6.56 ± 0.16
TC09000248.hg.1	FOXD4L2	7.64 ± 0.27	7.63 ± 0.19	7.57 ± 0.22	7.82 ± 0.23
TC09001180.hg.1	FOXD4L2	8.08 ± 0.21	8.08 ± 0.06	8.01 ± 0.23	8.20 ± 0.11
TC09000309.hg.1	FOXD4L3	7.48 ± 0.05	7.38 ± 0.03	7.45 ± 0.05	7.52 ± 0.14
TC09001177.hg.1	FOXD4L5	7.91 ± 0.10	7.77 ± 0.09	7.83 ± 0.08	7.85 ± 0.07
TC09001169.hg.1	FOXD4L6	7.62 ± 0.10	7.58 ± 0.07	7.63 ± 0.13	7.77 ± 0.14
TC09000493.hg.1	FOXE1	7.96 ± 0.24	7.89 ± 0.11	7.90 ± 0.12	7.82 ± 0.07
TC01000599.hg.1	FOXE3	8.02 ± 0.19	7.86 ± 0.13	7.83 ± 0.21	7.96 ± 0.14
TC16000668.hg.1	FOXF1	5.81 ± 0.13	5.63 ± 0.03	5.59 ± 0.06	5.58 ± 0.03
TC06000010.hg.1	FOXF2	6.97 ± 0.05	6.85 ± 0.31	6.83 ± 0.37	6.97 ± 0.08
TC14000184.hg.1	FOXG1	5.97 ± 0.16	5.90 ± 0.10	5.95 ± 0.27	5.97 ± 0.10
TC08001753.hg.1	FOXH1	7.99 ± 0.26	7.96 ± 0.24	7.90 ± 0.18	8.21 ± 0.11
TC05000931.hg.1	FOXI1	7.16 ± 0.13	7.20 ± 0.25	7.10 ± 0.35	7.24 ± 0.11
TC10000921.hg.1	FOXI2	7.88 ± 0.31	7.87 ± 0.27	7.79 ± 0.25	7.97 ± 0.08
TC02002073.hg.1	FOXI3	7.22 ± 0.18	7.13 ± 0.33	7.25 ± 0.28	7.23 ± 0.01
TC17001894.hg.1	FOXJ1	7.73 ± 0.26	7.49 ± 0.33	7.61 ± 0.18	7.74 ± 0.09
TC12000118.hg.1	FOXJ2	8.94 ± 0.03	8.80 ± 0.08	8.88 ± 0.12	8.84 ± 0.06
TC01002570.hg.1	FOXJ3	9.21 ± 0.27	9.41 ± 0.09	9.12 ± 0.38	9.39 ± 0.04
TC07000043.hg.1	FOXK1	8.73 ± 0.03	8.70 ± 0.04	8.60 ± 0.03	8.80 ± 0.01
TC17000954.hg.1	FOXK2	9.16 ± 0.13	9.10 ± 0.06	8.96 ± 0.17	9.08 ± 0.07

TC16000671.hg.1	FOXL1	6.35 ± 0.22	6.37 ± 0.25	6.37 ± 0.31	6.43 ± 0.03
TC03001827.hg.1	FOXL2	7.74 ± 0.34	7.63 ± 0.11	7.59 ± 0.31	7.69 ± 0.02
TC12001106.hg.1	FOXM1	7.96 ± 0.37	7.76 ± 0.22	7.72 ± 0.32	7.56 ± 0.04
TC17000323.hg.1	FOXN1	7.13 ± 0.21	7.09 ± 0.17	7.08 ± 0.18	7.18 ± 0.08
TC02000302.hg.1	FOXN2	9.15 ± 0.42	9.31 ± 0.25	9.66 ± 0.47	9.07 ± 0.15
TC14002331.hg.1	FOXN3	9.43 ± 0.13	9.70 ± 0.10	9.33 ± 0.29	9.70 ± 0.10
TC14000544.hg.1	FOXN3-AS1	7.52 ± 0.25	7.35 ± 0.05	7.38 ± 0.11	7.37 ± 0.04
TC14000545.hg.1	FOXN3-AS2	6.30 ± 0.25	6.06 ± 0.06	6.03 ± 0.09	6.17 ± 0.02
TC12001948.hg.1	FOXN4	7.85 ± 0.23	7.87 ± 0.24	7.82 ± 0.20	7.99 ± 0.07
TC13001719.hg.1	FOXO1	9.01 ± 0.24	9.44 ± 0.08	8.78 ± 0.06	9.42 ± 0.05
TC06000868.hg.1	FOXO3	9.10 ± 0.05	9.20 ± 0.11	9.18 ± 0.22	9.25 ± 0.14
TC17001226.hg.1	FOXO3B	7.01 ± 0.14	6.90 ± 0.08	6.93 ± 0.21	6.94 ± 0.19
TC0X000381.hg.1	FOXO4	8.63 ± 0.20	8.42 ± 0.02	8.55 ± 0.19	8.34 ± 0.11
TC01000523.hg.1	FOXO6	8.04 ± 0.28	8.03 ± 0.10	8.04 ± 0.24	7.92 ± 0.11
TC03001546.hg.1	FOXP1	9.29 ± 0.34	9.73 ± 0.10	9.13 ± 0.31	9.74 ± 0.07
TC03000422.hg.1	FOXP1-AS1	4.99 ± 0.04	4.94 ± 0.01	4.94 ± 0.04	4.99 ± 0.04
TC03001548.hg.1	FOXP1-IT1	6.65 ± 0.65	6.68 ± 0.45	6.46 ± 0.49	6.47 ± 0.16
TC07000713.hg.1	FOXP2	5.47 ± 0.08	5.41 ± 0.07	5.41 ± 0.02	5.48 ± 0.07
TC0X001031.hg.1	FOXP3	8.44 ± 0.07	8.38 ± 0.08	8.33 ± 0.02	8.52 ± 0.06
TC06000571.hg.1	FOXP4	8.57 ± 0.13	8.55 ± 0.10	8.44 ± 0.16	8.60 ± 0.06
TC06000009.hg.1	FOXQ1	6.65 ± 0.11	6.58 ± 0.21	6.60 ± 0.17	6.65 ± 0.07
TC11001077.hg.1	FOXR1	6.86 ± 0.22	6.85 ± 0.13	6.82 ± 0.14	6.93 ± 0.05
TC0X000334.hg.1	FOXR2	5.78 ± 0.25	5.75 ± 0.19	5.55 ± 0.21	5.59 ± 0.12
TC11001170.hg.1	FOXRED1	7.73 ± 0.14	7.54 ± 0.06	7.52 ± 0.06	7.54 ± 0.05
TC22000710.hg.1	FOXRED2	7.81 ± 0.24	7.72 ± 0.13	7.63 ± 0.21	7.70 ± 0.02
TC20000757.hg.1	FOXS1	7.70 ± 0.09	7.58 ± 0.23	7.57 ± 0.24	7.65 ± 0.07
TC09000672.hg.1	FPGS	8.62 ± 0.11	8.54 ± 0.12	8.43 ± 0.17	8.58 ± 0.03
TC01000769.hg.1	FPGT-TNNI3K	5.45 ± 0.05	5.35 ± 0.05	5.45 ± 0.12	5.34 ± 0.03
TC19001787.hg.1	FPR1	10.96 ± 0.51	10.50 ± 0.10	11.40 ± 0.25	10.38 ± 0.38
TC19000788.hg.1	FPR2	9.65 ± 0.54	9.02 ± 0.29	10.32 ± 0.49	8.71 ± 0.49
TC19000789.hg.1	FPR3	6.09 ± 0.10	5.97 ± 0.09	6.00 ± 0.09	6.05 ± 0.01

TC10001538.hg.1	FRA10AC1	5.98 ± 0.29	5.98 ± 0.16	6.00 ± 0.21	5.95 ± 0.10
TC04000434.hg.1	FRAS1	6.30 ± 0.16	6.27 ± 0.11	6.24 ± 0.09	6.36 ± 0.09
TC10000692.hg.1	FRAT1	8.98 ± 0.33	8.40 ± 0.14	9.10 ± 0.49	8.25 ± 0.29
TC10001562.hg.1	FRAT2	10.26 ± 0.50	9.58 ± 0.16	10.56 ± 0.37	9.41 ± 0.35
TC09000920.hg.1	FREM1	5.47 ± 0.15	5.39 ± 0.06	5.42 ± 0.06	5.45 ± 0.05
TC13000132.hg.1	FREM2	5.58 ± 0.17	5.51 ± 0.13	5.50 ± 0.11	5.61 ± 0.07
TC13000582.hg.1	FREM2-AS1	4.69 ± 0.01	4.72 ± 0.14	4.64 ± 0.05	4.76 ± 0.26
TC04001604.hg.1	FREM3	5.03 ± 0.06	4.97 ± 0.06	4.96 ± 0.03	4.99 ± 0.01
TC04000935.hg.1	FRG1	8.48 ± 0.20	8.56 ± 0.20	8.35 ± 0.25	8.51 ± 0.07
TC20000178.hg.1	FRG1B	7.81 ± 0.31	7.95 ± 0.14	7.90 ± 0.14	8.01 ± 0.07
TC04001840.hg.1	FRG2	6.29 ± 0.19	6.14 ± 0.10	6.13 ± 0.21	6.30 ± 0.14
TCUn_gl000228000012.hg.1	FRG2	6.29 ± 0.19	6.14 ± 0.10	6.13 ± 0.21	6.30 ± 0.14
TC10001794.hg.1	FRG2B	6.59 ± 0.27	6.47 ± 0.11	6.40 ± 0.23	6.55 ± 0.11
TC03000441.hg.1	FRG2C	6.21 ± 0.22	6.10 ± 0.21	6.11 ± 0.12	6.25 ± 0.04
TCUn_gl000222000003.hg.1	FRG2C	6.43 ± 0.29	6.32 ± 0.18	6.29 ± 0.12	6.44 ± 0.07
TC06002043.hg.1	FRK	4.96 ± 0.10	4.85 ± 0.07	4.92 ± 0.04	4.90 ± 0.03
TC06002322.hg.1	FRMD1	7.58 ± 0.20	7.57 ± 0.19	7.53 ± 0.18	7.69 ± 0.07
TC09001278.hg.1	FRMD3	7.10 ± 0.24	6.64 ± 0.05	7.22 ± 0.40	6.67 ± 0.15
TC10001053.hg.1	FRMD4A	6.77 ± 0.11	6.71 ± 0.07	6.69 ± 0.11	6.79 ± 0.04
TC03001540.hg.1	FRMD4B	7.66 ± 0.12	7.31 ± 0.03	7.83 ± 0.34	7.37 ± 0.03
TC15001289.hg.1	FRMD5	6.36 ± 0.18	6.29 ± 0.11	6.30 ± 0.13	6.35 ± 0.03
TC14000297.hg.1	FRMD6	5.75 ± 0.09	5.80 ± 0.09	5.74 ± 0.07	5.90 ± 0.07
TC14001134.hg.1	FRMD6-AS1	7.68 ± 0.17	7.53 ± 0.20	7.66 ± 0.12	7.63 ± 0.10
TC14001132.hg.1	FRMD6-AS2	5.16 ± 0.13	5.11 ± 0.05	5.21 ± 0.05	5.19 ± 0.08
TC0X001368.hg.1	FRMD7	5.17 ± 0.12	5.12 ± 0.09	5.11 ± 0.05	5.13 ± 0.04
TC11000627.hg.1	FRMD8	8.73 ± 0.18	8.59 ± 0.08	8.53 ± 0.19	8.56 ± 0.06
TC0X001099.hg.1	FRMD8P1	8.24 ± 0.27	8.14 ± 0.26	8.11 ± 0.17	8.24 ± 0.05
TC09000216.hg.1	FRMPD1	6.07 ± 0.19	5.95 ± 0.11	5.96 ± 0.13	5.98 ± 0.02
TC10001261.hg.1	FRMPD2	6.73 ± 0.13	6.70 ± 0.13	6.71 ± 0.10	6.80 ± 0.08
TC10001254.hg.1	FRMPD2P1	7.11 ± 0.19	7.13 ± 0.19	7.16 ± 0.05	7.27 ± 0.13
TC10001231.hg.1	FRMPD2P2	7.14 ± 0.13	7.13 ± 0.16	7.11 ± 0.13	7.26 ± 0.12

TC0X000525.hg.1	FRMPD3	6.50 ± 0.04	6.46 ± 0.05	6.33 ± 0.07	6.40 ± 0.08
TC0X001261.hg.1	FRMPD3-AS1	5.67 ± 0.18	5.95 ± 0.24	6.07 ± 0.07	5.98 ± 0.06
TC0X000046.hg.1	FRMPD4	5.87 ± 0.17	5.78 ± 0.08	5.78 ± 0.08	5.79 ± 0.06
TC0X000861.hg.1	FRMPD4-AS1	4.64 ± 0.02	4.63 ± 0.16	4.56 ± 0.10	4.59 ± 0.12
TC01002911.hg.1	FRRS1	6.48 ± 0.17	6.26 ± 0.11	6.78 ± 0.26	6.30 ± 0.12
TC09001463.hg.1	FRRS1L	6.18 ± 0.25	6.15 ± 0.15	6.20 ± 0.15	6.23 ± 0.09
TC12000614.hg.1	FRS2	7.96 ± 0.44	8.41 ± 0.21	8.06 ± 0.48	8.36 ± 0.04
TC06001723.hg.1	FRS3	8.40 ± 0.16	8.27 ± 0.11	8.31 ± 0.03	8.34 ± 0.06
TC13000109.hg.1	FRY	8.10 ± 0.37	8.37 ± 0.32	8.52 ± 0.36	8.27 ± 0.10
TC13000547.hg.1	FRY-AS1	6.59 ± 0.05	6.56 ± 0.13	6.70 ± 0.03	6.77 ± 0.08
TC04001177.hg.1	FRYL	8.37 ± 0.58	8.77 ± 0.22	8.47 ± 0.57	8.54 ± 0.15
TC02002591.hg.1	FRZB	5.65 ± 0.17	5.48 ± 0.13	5.52 ± 0.06	5.62 ± 0.08
TC14001074.hg.1	FSCB	5.56 ± 0.17	5.55 ± 0.09	5.55 ± 0.08	5.60 ± 0.05
TC07000055.hg.1	FSCN1	8.14 ± 0.21	8.00 ± 0.18	8.04 ± 0.24	8.05 ± 0.03
TC17000931.hg.1	FSCN2	8.31 ± 0.23	8.22 ± 0.22	8.21 ± 0.27	8.30 ± 0.03
TC07003342.hg.1	FSCN3	6.95 ± 0.13	6.92 ± 0.10	6.92 ± 0.10	7.03 ± 0.08
TC19000086.hg.1	FSD1	7.41 ± 0.25	7.32 ± 0.16	7.26 ± 0.25	7.39 ± 0.05
TC09000536.hg.1	FSD1L	5.71 ± 0.08	5.64 ± 0.07	5.82 ± 0.20	5.60 ± 0.03
TC15001759.hg.1	FSD2	6.17 ± 0.12	6.14 ± 0.15	6.13 ± 0.09	6.25 ± 0.03
TC11000294.hg.1	FSHB	5.51 ± 0.09	5.40 ± 0.12	5.45 ± 0.04	5.45 ± 0.09
TC02001831.hg.1	FSHR	4.81 ± 0.07	4.72 ± 0.04	4.76 ± 0.07	4.81 ± 0.08
TC15001224.hg.1	FSIP1	5.23 ± 0.14	5.09 ± 0.01	5.17 ± 0.03	5.11 ± 0.02
TC02001097.hg.1	FSIP2	4.49 ± 0.13	4.40 ± 0.01	4.41 ± 0.02	4.41 ± 0.03
TC05000220.hg.1	FST	6.05 ± 0.21	6.03 ± 0.10	6.03 ± 0.14	6.07 ± 0.05
TC03001691.hg.1	FSTL1	6.99 ± 0.07	6.86 ± 0.02	7.16 ± 0.17	6.91 ± 0.15
TC19000012.hg.1	FSTL3	8.17 ± 0.17	8.12 ± 0.19	8.14 ± 0.24	8.17 ± 0.01
TC05001780.hg.1	FSTL4	6.94 ± 0.13	6.91 ± 0.13	6.99 ± 0.18	7.01 ± 0.06
TC04001696.hg.1	FSTL5	5.00 ± 0.11	4.92 ± 0.07	4.93 ± 0.06	4.98 ± 0.09
TC21000552.hg.1	FTCD	8.17 ± 0.20	8.14 ± 0.24	8.12 ± 0.19	8.23 ± 0.03
TC21000254.hg.1	FTCD-AS1	8.10 ± 0.18	8.13 ± 0.17	8.16 ± 0.26	8.17 ± 0.13
TC02002657.hg.1	FTCDNL1	7.10 ± 0.21	7.13 ± 0.11	7.09 ± 0.12	7.15 ± 0.00

TC11001860.hg.1	FTH1	12.64 ± 0.10	12.65 ± 0.06	12.85 ± 0.11	12.72 ± 0.16
TC02001686.hg.1	FTH1P3	10.32 ± 0.03	10.22 ± 0.14	10.45 ± 0.10	10.32 ± 0.22
TC05001189.hg.1	FTH1P3	11.88 ± 0.14	11.86 ± 0.08	12.17 ± 0.13	11.98 ± 0.25
TC03001759.hg.1	FTH1P4	10.99 ± 0.09	10.85 ± 0.15	11.11 ± 0.23	10.94 ± 0.33
TC0X000942.hg.1	FTHL17	5.19 ± 0.19	5.08 ± 0.09	5.00 ± 0.08	5.05 ± 0.05
TC19000717.hg.1	FTL	12.73 ± 0.30	12.67 ± 0.11	12.88 ± 0.16	12.65 ± 0.25
TC04000368.hg.1	FTLP10	5.50 ± 0.11	5.34 ± 0.06	5.36 ± 0.05	5.37 ± 0.05
TC20000047.hg.1	FTLP3	12.91 ± 0.19	12.79 ± 0.10	12.99 ± 0.17	12.79 ± 0.27
TC05000572.hg.1	FTMT	7.04 ± 0.15	6.89 ± 0.16	7.00 ± 0.14	6.99 ± 0.09
TC16000449.hg.1	FTO	8.40 ± 0.01	8.37 ± 0.08	8.10 ± 0.08	8.27 ± 0.05
TC0X000251.hg.1	FTSJ1	8.81 ± 0.20	8.49 ± 0.02	8.52 ± 0.08	8.37 ± 0.10
TC07001095.hg.1	FTSJ2	8.93 ± 0.16	8.76 ± 0.02	8.68 ± 0.19	8.78 ± 0.10
TC17001786.hg.1	FTSJ3	8.64 ± 0.18	8.31 ± 0.06	8.35 ± 0.14	8.12 ± 0.14
TC16001246.hg.1	FTSJD1	7.92 ± 0.16	7.93 ± 0.09	7.92 ± 0.07	7.89 ± 0.03
TC06000545.hg.1	FTSJD2	8.82 ± 0.39	8.60 ± 0.09	8.71 ± 0.51	8.44 ± 0.02
TC0X001156.hg.1	FTX	8.36 ± 0.33	8.45 ± 0.22	8.58 ± 0.32	8.43 ± 0.05
TC01002803.hg.1	FUBP1	8.98 ± 0.52	9.31 ± 0.09	8.90 ± 0.33	9.37 ± 0.07
TC09000738.hg.1	FUBP3	8.51 ± 0.09	8.57 ± 0.10	8.39 ± 0.08	8.53 ± 0.06
TC01002352.hg.1	FUCA1	7.64 ± 0.24	7.31 ± 0.11	7.49 ± 0.16	7.21 ± 0.05
TC06002187.hg.1	FUCA2	7.34 ± 0.07	7.08 ± 0.10	7.28 ± 0.14	7.02 ± 0.06
TC16000578.hg.1	FUK	8.34 ± 0.12	8.28 ± 0.15	8.26 ± 0.08	8.36 ± 0.06
TC0X000981.hg.1	FUNDC1	6.98 ± 0.04	6.94 ± 0.13	6.84 ± 0.07	6.78 ± 0.08
TC0X000782.hg.1	FUNDC2	8.03 ± 0.03	8.05 ± 0.08	7.87 ± 0.06	7.98 ± 0.05
TC02000497.hg.1	FUNDC2P2	5.59 ± 0.29	5.49 ± 0.15	5.53 ± 0.20	5.54 ± 0.09
TC10001789.hg.1	FUOM	8.56 ± 0.22	8.49 ± 0.19	8.38 ± 0.20	8.55 ± 0.04
TC15000870.hg.1	FURIN	9.60 ± 0.28	9.42 ± 0.10	9.77 ± 0.20	9.62 ± 0.33
TC16000372.hg.1	FUS	9.89 ± 0.16	10.03 ± 0.06	9.67 ± 0.15	10.03 ± 0.04
TC19001695.hg.1	FUT1	8.54 ± 0.26	8.47 ± 0.20	8.41 ± 0.21	8.55 ± 0.03
TC08002582.hg.1	FUT10	6.79 ± 0.09	6.65 ± 0.12	6.61 ± 0.14	6.65 ± 0.07
TC10000469.hg.1	FUT11	8.24 ± 0.21	7.99 ± 0.10	7.95 ± 0.14	7.83 ± 0.05
TC19000709.hg.1	FUT2	6.86 ± 0.21	6.82 ± 0.13	6.73 ± 0.17	6.88 ± 0.09

TC19001085.hg.1	FUT3	6.83 ± 0.18	6.90 ± 0.20	6.79 ± 0.21	6.96 ± 0.12
TC19002674.hg.1	FUT5	8.11 ± 0.15	8.12 ± 0.18	8.06 ± 0.11	8.23 ± 0.05
TC19001084.hg.1	FUT6	7.46 ± 0.24	7.48 ± 0.18	7.50 ± 0.13	7.61 ± 0.05
TC09001750.hg.1	FUT7	8.85 ± 0.38	8.40 ± 0.17	8.90 ± 0.09	8.43 ± 0.10
TC14000397.hg.1	FUT8	8.06 ± 0.10	8.08 ± 0.10	7.78 ± 0.07	7.84 ± 0.11
TC14001230.hg.1	FUT8-AS1	7.70 ± 0.25	7.50 ± 0.20	7.57 ± 0.16	7.60 ± 0.04
TC06000818.hg.1	FUT9	4.48 ± 0.15	4.34 ± 0.10	4.45 ± 0.15	4.39 ± 0.12
TC19001725.hg.1	FUZ	8.53 ± 0.05	8.46 ± 0.06	8.48 ± 0.05	8.47 ± 0.03
TC09000318.hg.1	FXN	7.54 ± 0.10	7.57 ± 0.08	7.23 ± 0.00	7.52 ± 0.04
TC03000965.hg.1	FXR1	8.24 ± 0.36	8.55 ± 0.14	8.32 ± 0.29	8.48 ± 0.08
TC17001092.hg.1	FXR2	8.21 ± 0.16	7.93 ± 0.03	8.10 ± 0.20	7.90 ± 0.14
TC19000459.hg.1	FXYD1	7.84 ± 0.20	7.74 ± 0.15	7.75 ± 0.21	7.79 ± 0.03
TC11003508.hg.1	FXYD2	8.26 ± 0.15	8.21 ± 0.16	8.23 ± 0.11	8.29 ± 0.03
TC19000458.hg.1	FXYD3	7.35 ± 0.16	7.39 ± 0.20	7.28 ± 0.22	7.50 ± 0.08
TC10000272.hg.1	FXYD4	7.01 ± 0.10	7.01 ± 0.17	6.94 ± 0.12	7.10 ± 0.08
TC19000461.hg.1	FXYD5	9.99 ± 0.03	9.97 ± 0.04	9.78 ± 0.07	10.01 ± 0.01
TC19000460.hg.1	FXYD7	7.59 ± 0.21	7.63 ± 0.20	7.48 ± 0.07	7.75 ± 0.05
TC05001295.hg.1	FYB	10.77 ± 0.29	10.85 ± 0.19	11.27 ± 0.29	10.73 ± 0.15
TC03001353.hg.1	FYCO1	8.41 ± 0.07	8.26 ± 0.03	8.19 ± 0.04	8.25 ± 0.01
TC06002022.hg.1	FYN	8.68 ± 0.24	8.91 ± 0.11	8.41 ± 0.13	8.81 ± 0.04
TC03001109.hg.1	FYTTD1	7.93 ± 0.24	8.11 ± 0.22	7.84 ± 0.22	8.07 ± 0.08
TC07000543.hg.1	FZD1	7.31 ± 0.29	7.15 ± 0.10	7.35 ± 0.03	7.16 ± 0.06
TC12001033.hg.1	FZD10	6.42 ± 0.21	6.32 ± 0.11	6.40 ± 0.12	6.39 ± 0.07
TC12002129.hg.1	FZD10-AS1	7.38 ± 0.20	7.36 ± 0.17	7.32 ± 0.16	7.42 ± 0.03
TC17000571.hg.1	FZD2	8.73 ± 0.12	8.41 ± 0.11	8.57 ± 0.18	8.32 ± 0.10
TC08000220.hg.1	FZD3	5.90 ± 0.10	5.99 ± 0.13	5.80 ± 0.04	5.85 ± 0.09
TC11002170.hg.1	FZD4	6.48 ± 0.18	6.46 ± 0.22	6.43 ± 0.11	6.55 ± 0.16
TC02002714.hg.1	FZD5	7.64 ± 0.21	7.35 ± 0.15	7.50 ± 0.13	7.38 ± 0.01
TC08000645.hg.1	FZD6	5.77 ± 0.08	5.62 ± 0.07	5.60 ± 0.02	5.68 ± 0.01
TC02001185.hg.1	FZD7	6.66 ± 0.37	6.74 ± 0.20	6.44 ± 0.34	6.80 ± 0.19
TC10001179.hg.1	FZD8	7.81 ± 0.22	7.76 ± 0.18	7.78 ± 0.24	7.98 ± 0.05

TC07000440.hg.1	FZD9	7.44 ± 0.37	7.62 ± 0.18	7.41 ± 0.30	7.60 ± 0.10
TC19000070.hg.1	FZR1	8.99 ± 0.18	8.86 ± 0.04	8.83 ± 0.12	8.89 ± 0.06
TC01001749.hg.1	G0S2	10.80 ± 0.30	12.04 ± 0.09	11.81 ± 0.25	11.95 ± 0.45
TC14000194.hg.1	G2E3	6.87 ± 0.50	7.06 ± 0.30	6.84 ± 0.39	6.94 ± 0.04
TC05000844.hg.1	G3BP1	8.86 ± 0.16	8.82 ± 0.07	8.64 ± 0.09	8.71 ± 0.03
TC04001300.hg.1	G3BP2	8.33 ± 0.25	8.56 ± 0.07	8.35 ± 0.23	8.56 ± 0.04
TC17000542.hg.1	G6PC	6.81 ± 0.15	6.79 ± 0.13	6.77 ± 0.12	6.92 ± 0.09
TC02000999.hg.1	G6PC2	6.09 ± 0.12	5.89 ± 0.10	5.98 ± 0.05	6.03 ± 0.12
TC17000562.hg.1	G6PC3	8.33 ± 0.20	8.03 ± 0.02	8.00 ± 0.07	8.01 ± 0.03
TC0X001538.hg.1	G6PD	9.87 ± 0.26	9.55 ± 0.06	9.97 ± 0.21	9.51 ± 0.03
TC17000915.hg.1	GAA	9.29 ± 0.26	9.11 ± 0.05	9.31 ± 0.09	9.06 ± 0.14
TC04000699.hg.1	GAB1	7.93 ± 0.12	7.71 ± 0.22	7.90 ± 0.31	7.59 ± 0.17
TC11002136.hg.1	GAB2	9.34 ± 0.28	9.33 ± 0.12	9.68 ± 0.33	9.51 ± 0.45
TC0X001543.hg.1	GAB3	8.75 ± 0.21	8.62 ± 0.19	8.87 ± 0.22	8.48 ± 0.09
TC22000469.hg.1	GAB4	7.38 ± 0.18	7.35 ± 0.20	7.34 ± 0.11	7.50 ± 0.04
TC17001081.hg.1	GABARAP	11.44 ± 0.27	11.18 ± 0.14	11.47 ± 0.15	11.10 ± 0.31
TC12000159.hg.1	GABARAPL1	8.99 ± 0.25	9.54 ± 0.09	9.26 ± 0.34	9.74 ± 0.26
TC16000620.hg.1	GABARAPL2	9.22 ± 0.15	9.09 ± 0.20	9.45 ± 0.34	8.91 ± 0.19
TC15001847.hg.1	GABARAPL3	7.52 ± 0.11	7.70 ± 0.07	7.64 ± 0.09	7.75 ± 0.11
TC06004112.hg.1	GABBR1	8.62 ± 0.09	8.79 ± 0.03	8.72 ± 0.13	8.75 ± 0.13
TC6_apd_hap1000125.hg.1	GABBR1	8.53 ± 0.11	8.72 ± 0.05	8.67 ± 0.20	8.68 ± 0.16
TC6_cox_hap2000248.hg.1	GABBR1	8.55 ± 0.11	8.74 ± 0.05	8.68 ± 0.19	8.69 ± 0.16
TC6_dbb_hap3000227.hg.1	GABBR1	8.55 ± 0.11	8.74 ± 0.05	8.68 ± 0.19	8.69 ± 0.16
TC6_mann_hap4000197.hg.1	GABBR1	8.53 ± 0.11	8.73 ± 0.05	8.67 ± 0.20	8.68 ± 0.16
TC6_mcf_hap5000214.hg.1	GABBR1	8.55 ± 0.11	8.74 ± 0.05	8.68 ± 0.19	8.69 ± 0.16
TC6_qbl_hap6000226.hg.1	GABBR1	8.55 ± 0.11	8.74 ± 0.05	8.68 ± 0.19	8.69 ± 0.16
TC09001406.hg.1	GABBR2	7.09 ± 0.16	7.01 ± 0.14	6.99 ± 0.13	7.07 ± 0.03
TC21000078.hg.1	GABPA	7.93 ± 0.41	8.15 ± 0.19	8.08 ± 0.40	8.09 ± 0.04
TC15001350.hg.1	GABPB1	8.52 ± 0.07	8.41 ± 0.16	8.44 ± 0.28	8.47 ± 0.23
TC15000393.hg.1	GABPB1-AS1	8.76 ± 0.24	8.75 ± 0.20	8.54 ± 0.38	8.79 ± 0.10
TC01001202.hg.1	GABPB2	7.85 ± 0.14	7.88 ± 0.10	7.73 ± 0.13	7.70 ± 0.11

TC05000903.hg.1	GABRA1	5.31 ± 0.22	5.17 ± 0.07	5.22 ± 0.06	5.24 ± 0.07
TC04001161.hg.1	GABRA2	4.90 ± 0.12	4.79 ± 0.03	4.84 ± 0.02	4.83 ± 0.03
TC0X001493.hg.1	GABRA3	5.64 ± 0.16	5.56 ± 0.18	5.58 ± 0.11	5.68 ± 0.04
TC04001165.hg.1	GABRA4	5.17 ± 0.16	5.13 ± 0.04	5.05 ± 0.12	5.18 ± 0.09
TC15000124.hg.1	GABRA5	6.99 ± 0.20	6.91 ± 0.12	6.90 ± 0.21	6.96 ± 0.07
TC05000902.hg.1	GABRA6	5.42 ± 0.11	5.34 ± 0.09	5.33 ± 0.09	5.43 ± 0.05
TC04000273.hg.1	GABRB1	5.51 ± 0.15	5.42 ± 0.05	5.43 ± 0.03	5.46 ± 0.06
TC05002014.hg.1	GABRB2	6.14 ± 0.18	6.01 ± 0.08	6.05 ± 0.12	6.05 ± 0.08
TC15001084.hg.1	GABRB3	6.43 ± 0.11	6.36 ± 0.11	6.35 ± 0.10	6.40 ± 0.05
TC01000053.hg.1	GABRD	7.93 ± 0.22	7.91 ± 0.25	7.87 ± 0.22	7.97 ± 0.08
TC0X001491.hg.1	GABRE	6.01 ± 0.15	5.97 ± 0.12	5.99 ± 0.10	6.09 ± 0.09
TC04001160.hg.1	GABRG1	4.53 ± 0.20	4.28 ± 0.05	4.33 ± 0.07	4.37 ± 0.08
TC05000904.hg.1	GABRG2	5.24 ± 0.12	5.07 ± 0.05	5.12 ± 0.01	5.17 ± 0.08
TC15000125.hg.1	GABRG3	5.66 ± 0.18	5.59 ± 0.12	5.54 ± 0.11	5.59 ± 0.06
TC15000126.hg.1	GABRG3	6.38 ± 0.24	6.38 ± 0.24	6.27 ± 0.24	6.51 ± 0.09
TC05000937.hg.1	GABRP	6.76 ± 0.14	6.68 ± 0.11	6.66 ± 0.12	6.80 ± 0.06
TC0X000738.hg.1	GABRQ	6.01 ± 0.17	5.94 ± 0.16	5.89 ± 0.06	5.98 ± 0.09
TC06001938.hg.1	GABRR1	6.08 ± 0.14	6.07 ± 0.10	6.11 ± 0.05	6.16 ± 0.07
TC06001939.hg.1	GABRR2	7.60 ± 0.08	7.46 ± 0.13	7.79 ± 0.03	7.47 ± 0.06
TC03001600.hg.1	GABRR3	4.73 ± 0.08	4.58 ± 0.06	4.67 ± 0.03	4.67 ± 0.06
TC02001016.hg.1	GAD1	6.43 ± 0.23	6.34 ± 0.14	6.40 ± 0.13	6.42 ± 0.03
TC10000180.hg.1	GAD2	6.06 ± 0.13	6.02 ± 0.15	6.00 ± 0.15	6.07 ± 0.02
TC01000745.hg.1	GADD45A	8.41 ± 0.09	8.50 ± 0.16	8.99 ± 0.20	8.55 ± 0.12
TC19000055.hg.1	GADD45B	9.95 ± 0.43	10.40 ± 0.14	10.03 ± 0.33	10.35 ± 0.25
TC09000418.hg.1	GADD45G	8.50 ± 0.22	8.42 ± 0.17	8.40 ± 0.25	8.51 ± 0.03
TC19001216.hg.1	GADD45GIP1	8.84 ± 0.36	8.68 ± 0.13	8.57 ± 0.30	8.65 ± 0.12
TC03001268.hg.1	GADL1	5.95 ± 0.09	5.83 ± 0.12	5.89 ± 0.12	5.96 ± 0.08
TC0X002314.hg.1	GAGE1	6.78 ± 0.25	6.87 ± 0.05	6.81 ± 0.10	6.96 ± 0.07
TC0X002357.hg.1	GAGE10	6.30 ± 0.21	6.37 ± 0.03	6.21 ± 0.11	6.48 ± 0.11
TC0X000274.hg.1	GAGE12C	6.55 ± 0.26	6.65 ± 0.03	6.47 ± 0.19	6.80 ± 0.09
TC0X002352.hg.1	GAGE12C	6.42 ± 0.24	6.52 ± 0.01	6.38 ± 0.18	6.64 ± 0.07

TC0X002353.hg.1	GAGE12C	6.67 ± 0.19	6.76 ± 0.08	6.64 ± 0.17	6.90 ± 0.07
TC0X002354.hg.1	GAGE12C	6.62 ± 0.20	6.70 ± 0.10	6.61 ± 0.17	6.84 ± 0.06
TC0X000272.hg.1	GAGE12F	5.47 ± 0.35	5.55 ± 0.36	5.37 ± 0.25	5.57 ± 0.07
TC0X000273.hg.1	GAGE12F	5.47 ± 0.35	5.55 ± 0.36	5.37 ± 0.25	5.57 ± 0.07
TC0X002349.hg.1	GAGE12F	6.03 ± 0.31	6.09 ± 0.07	6.03 ± 0.19	6.23 ± 0.10
TC0X002358.hg.1	GAGE12J	6.44 ± 0.32	6.48 ± 0.02	6.40 ± 0.18	6.65 ± 0.12
TC0X002356.hg.1	GAGE13	5.17 ± 0.34	5.20 ± 0.12	5.12 ± 0.26	5.32 ± 0.05
TC0X002315.hg.1	GAGE2A	6.51 ± 0.35	6.54 ± 0.10	6.53 ± 0.14	6.61 ± 0.14
TC0X002351.hg.1	GAGE2B	7.12 ± 0.28	7.21 ± 0.05	7.15 ± 0.10	7.39 ± 0.13
TC0X002350.hg.1	GAGE2D	6.73 ± 0.34	6.80 ± 0.08	6.73 ± 0.15	6.90 ± 0.14
TC0X002355.hg.1	GAGE2E	5.47 ± 0.37	5.51 ± 0.09	5.39 ± 0.26	5.65 ± 0.12
TC0X002348.hg.1	GAGE8	6.39 ± 0.34	6.40 ± 0.05	6.36 ± 0.21	6.57 ± 0.12
TC04000957.hg.1	GAK	9.19 ± 0.07	9.07 ± 0.03	9.07 ± 0.06	9.08 ± 0.04
TC11000709.hg.1	GAL	8.53 ± 0.23	8.48 ± 0.24	8.41 ± 0.21	8.55 ± 0.00
TC22000657.hg.1	GAL3ST1	7.49 ± 0.25	7.54 ± 0.23	7.47 ± 0.21	7.69 ± 0.05
TC02005017.hg.1	GAL3ST2	8.32 ± 0.16	8.33 ± 0.23	8.25 ± 0.23	8.41 ± 0.05
TC11001953.hg.1	GAL3ST3	7.89 ± 0.29	7.94 ± 0.27	7.86 ± 0.21	8.03 ± 0.07
TC07001661.hg.1	GAL3ST4	7.94 ± 0.14	7.96 ± 0.06	7.80 ± 0.10	7.99 ± 0.10
TC14001387.hg.1	GALC	8.24 ± 0.21	8.35 ± 0.15	8.62 ± 0.37	8.35 ± 0.01
TC01002350.hg.1	GALE	8.17 ± 0.20	8.08 ± 0.14	8.05 ± 0.18	8.15 ± 0.04
TC17001881.hg.1	GALK1	8.61 ± 0.18	8.40 ± 0.15	8.53 ± 0.09	8.48 ± 0.02
TC15000384.hg.1	GALK2	7.43 ± 0.10	7.34 ± 0.08	7.36 ± 0.17	7.19 ± 0.07
TC02000245.hg.1	GALM	8.65 ± 0.52	8.10 ± 0.02	8.45 ± 0.38	7.87 ± 0.05
TC16001345.hg.1	GALNS	8.87 ± 0.16	8.69 ± 0.04	8.87 ± 0.11	8.65 ± 0.07
TC18000145.hg.1	GALNT1	8.40 ± 0.45	8.64 ± 0.27	8.58 ± 0.36	8.58 ± 0.08
TC05003415.hg.1	GALNT10	8.16 ± 0.09	8.12 ± 0.03	8.09 ± 0.05	8.07 ± 0.07
TC07001024.hg.1	GALNT11	7.40 ± 0.15	7.51 ± 0.06	7.41 ± 0.12	7.45 ± 0.03
TC09000498.hg.1	GALNT12	6.83 ± 0.08	7.01 ± 0.05	6.81 ± 0.03	6.90 ± 0.10
TC02000944.hg.1	GALNT13	4.77 ± 0.08	4.73 ± 0.05	4.74 ± 0.04	4.78 ± 0.05
TC02001713.hg.1	GALNT14	7.27 ± 0.29	6.92 ± 0.16	7.66 ± 0.13	6.99 ± 0.04
TC03000101.hg.1	GALNT15	6.71 ± 0.12	6.72 ± 0.17	6.70 ± 0.09	6.83 ± 0.10

TC14000414.hg.1	GALNT16	7.18 ± 0.19	7.14 ± 0.23	7.07 ± 0.20	7.24 ± 0.06
TC11001422.hg.1	GALNT18	7.30 ± 0.21	7.24 ± 0.13	7.24 ± 0.17	7.34 ± 0.05
TC01001907.hg.1	GALNT2	8.44 ± 0.12	8.18 ± 0.04	8.59 ± 0.13	8.18 ± 0.10
TC02002492.hg.1	GALNT3	7.76 ± 0.21	7.59 ± 0.14	8.30 ± 0.37	7.45 ± 0.15
TC02000954.hg.1	GALNT5	5.76 ± 0.13	5.75 ± 0.07	5.75 ± 0.07	5.80 ± 0.07
TC12001509.hg.1	GALNT6	7.90 ± 0.07	7.82 ± 0.04	7.80 ± 0.15	7.86 ± 0.04
TC04000853.hg.1	GALNT7	7.35 ± 0.26	7.23 ± 0.21	7.58 ± 0.33	7.07 ± 0.10
TC12003206.hg.1	GALNT8	6.53 ± 0.06	6.46 ± 0.09	6.53 ± 0.04	6.52 ± 0.06
TC12002150.hg.1	GALNT9	7.88 ± 0.19	7.85 ± 0.21	7.82 ± 0.24	7.95 ± 0.06
TC07001023.hg.1	GALNTL5	4.95 ± 0.12	4.84 ± 0.04	4.83 ± 0.04	4.89 ± 0.05
TC04000852.hg.1	GALNTL6	6.12 ± 0.17	6.04 ± 0.14	6.05 ± 0.14	6.14 ± 0.03
TC19000919.hg.1	GALP	7.72 ± 0.24	7.59 ± 0.14	7.59 ± 0.19	7.65 ± 0.04
TC18000259.hg.1	GALR1	5.46 ± 0.10	5.37 ± 0.14	5.40 ± 0.03	5.41 ± 0.08
TC17000864.hg.1	GALR2	6.95 ± 0.18	6.96 ± 0.10	6.98 ± 0.16	7.08 ± 0.05
TC22000290.hg.1	GALR3	8.11 ± 0.37	8.19 ± 0.28	7.98 ± 0.35	8.32 ± 0.12
TC09002896.hg.1	GALT	8.40 ± 0.08	8.25 ± 0.06	8.23 ± 0.12	8.29 ± 0.13
TC19001003.hg.1	GAMT	8.60 ± 0.23	8.50 ± 0.05	8.47 ± 0.15	8.53 ± 0.06
TC16000638.hg.1	GAN	6.82 ± 0.23	6.94 ± 0.25	6.64 ± 0.08	6.85 ± 0.08
TC11001873.hg.1	GANAB	10.02 ± 0.20	9.71 ± 0.15	9.57 ± 0.16	9.50 ± 0.05
TC15002792.hg.1	GANC	8.00 ± 0.23	7.97 ± 0.25	7.98 ± 0.22	7.73 ± 0.11
TC03000595.hg.1	GAP43	6.92 ± 0.21	6.86 ± 0.14	6.85 ± 0.11	6.94 ± 0.13
TC12000084.hg.1	GAPDH	11.63 ± 0.22	11.42 ± 0.14	11.72 ± 0.15	11.29 ± 0.23
TC19000471.hg.1	GAPDHS	7.03 ± 0.20	6.99 ± 0.17	6.98 ± 0.25	7.07 ± 0.03
TC05000253.hg.1	GAPT	8.60 ± 0.42	8.40 ± 0.43	8.97 ± 0.24	8.22 ± 0.22
TC09000649.hg.1	GAPVD1	8.28 ± 0.24	8.30 ± 0.14	8.35 ± 0.28	8.23 ± 0.08
TC04000565.hg.1	GAR1	8.22 ± 0.07	8.11 ± 0.05	8.07 ± 0.11	8.04 ± 0.06
TC18000446.hg.1	GAREM	6.69 ± 0.17	6.58 ± 0.13	6.58 ± 0.10	6.65 ± 0.01
TC02000148.hg.1	GAREML	7.85 ± 0.18	7.78 ± 0.14	7.82 ± 0.14	7.85 ± 0.05
TC09000663.hg.1	GARNL3	6.33 ± 0.16	6.28 ± 0.09	6.25 ± 0.09	6.32 ± 0.07
TC07000198.hg.1	GARS	8.97 ± 0.16	8.85 ± 0.16	8.76 ± 0.06	8.68 ± 0.21
TC21000412.hg.1	GART	7.58 ± 0.09	7.32 ± 0.07	7.38 ± 0.17	7.12 ± 0.07

TC09001295.hg.1	GAS1	7.86 ± 0.28	7.86 ± 0.15	7.87 ± 0.26	7.84 ± 0.08
TC11000265.hg.1	GAS2	5.49 ± 0.11	5.39 ± 0.06	5.41 ± 0.10	5.41 ± 0.14
TC22000196.hg.1	GAS2L1	8.33 ± 0.21	8.20 ± 0.22	8.24 ± 0.22	8.32 ± 0.06
TC17001375.hg.1	GAS2L2	7.56 ± 0.14	7.68 ± 0.20	7.70 ± 0.16	7.83 ± 0.07
TC12000779.hg.1	GAS2L3	5.12 ± 0.09	5.11 ± 0.02	5.13 ± 0.03	5.13 ± 0.05
TC01001517.hg.1	GAS5-AS1	6.80 ± 0.16	6.53 ± 0.05	6.65 ± 0.22	6.40 ± 0.14
TC13000899.hg.1	GAS6	8.23 ± 0.14	7.95 ± 0.14	8.15 ± 0.10	8.04 ± 0.01
TC13000429.hg.1	GAS6-AS1	8.16 ± 0.05	8.18 ± 0.06	8.23 ± 0.13	8.26 ± 0.09
TC13000430.hg.1	GAS6-AS1	7.77 ± 0.17	7.73 ± 0.12	7.84 ± 0.22	7.77 ± 0.08
TC17001129.hg.1	GAS7	9.03 ± 0.29	8.71 ± 0.08	9.61 ± 0.22	8.75 ± 0.20
TC16000711.hg.1	GAS8	7.54 ± 0.13	7.57 ± 0.13	7.60 ± 0.09	7.63 ± 0.07
TC17000515.hg.1	GAST	8.41 ± 0.11	8.44 ± 0.18	8.41 ± 0.14	8.44 ± 0.10
TC0X000260.hg.1	GATA1	8.09 ± 0.08	7.76 ± 0.15	8.15 ± 0.16	7.92 ± 0.05
TC03001755.hg.1	GATA2	8.34 ± 0.16	8.34 ± 0.13	8.27 ± 0.16	8.38 ± 0.07
TC10000070.hg.1	GATA3	8.94 ± 0.03	8.90 ± 0.06	8.68 ± 0.08	8.90 ± 0.10
TC10001023.hg.1	GATA3-AS1	6.94 ± 0.24	6.76 ± 0.15	6.82 ± 0.19	6.78 ± 0.03
TC08000089.hg.1	GATA4	8.20 ± 0.24	8.12 ± 0.17	8.12 ± 0.21	8.18 ± 0.04
TC20001011.hg.1	GATA5	8.54 ± 0.27	8.59 ± 0.30	8.49 ± 0.30	8.72 ± 0.07
TC18000094.hg.1	GATA6	6.78 ± 0.11	6.76 ± 0.09	6.72 ± 0.10	6.80 ± 0.06
TC07003394.hg.1	GATAD1	8.27 ± 0.09	8.14 ± 0.12	8.18 ± 0.05	8.07 ± 0.03
TC19000373.hg.1	GATAD2A	9.10 ± 0.12	9.00 ± 0.05	8.99 ± 0.03	8.95 ± 0.11
TC01003278.hg.1	GATAD2B	10.80 ± 0.24	10.74 ± 0.11	10.75 ± 0.21	10.80 ± 0.21
TC12003258.hg.1	GATC	9.52 ± 0.04	9.35 ± 0.03	9.25 ± 0.10	9.29 ± 0.02
TC15002814.hg.1	GATM	6.25 ± 0.20	6.08 ± 0.17	6.12 ± 0.06	6.16 ± 0.08
TC15002815.hg.1	GATM	6.09 ± 0.19	6.00 ± 0.10	6.00 ± 0.11	6.06 ± 0.12
TC15000355.hg.1	GATM-AS1	6.09 ± 0.19	5.90 ± 0.09	5.95 ± 0.07	5.79 ± 0.08
TC07001663.hg.1	GATS	8.05 ± 0.08	8.06 ± 0.11	7.93 ± 0.09	8.05 ± 0.02
TC07000463.hg.1	GATSL1	8.11 ± 0.15	8.07 ± 0.13	7.96 ± 0.08	8.14 ± 0.04
TC07003360.hg.1	GATSL2	8.27 ± 0.14	8.23 ± 0.08	8.11 ± 0.06	8.29 ± 0.04
TC22000649.hg.1	GATSL3	8.24 ± 0.12	8.18 ± 0.11	8.09 ± 0.12	8.22 ± 0.03
TC01006304.hg.1	GBA	8.99 ± 0.32	8.46 ± 0.07	9.10 ± 0.24	8.37 ± 0.12

TC09001060.hg.1	GBA2	8.20 ± 0.10	7.98 ± 0.06	8.15 ± 0.12	7.92 ± 0.08
TC04000168.hg.1	GBA3	5.42 ± 0.07	5.33 ± 0.03	5.40 ± 0.04	5.37 ± 0.08
TC01006303.hg.1	GBAP1	8.26 ± 0.15	7.93 ± 0.06	8.31 ± 0.16	7.83 ± 0.05
TC07003326.hg.1	GBAS	8.01 ± 0.06	8.10 ± 0.19	7.90 ± 0.15	8.00 ± 0.06
TC03001575.hg.1	GBE1	7.00 ± 0.20	7.05 ± 0.18	7.35 ± 0.43	6.99 ± 0.17
TC10000752.hg.1	GBF1	9.25 ± 0.22	9.03 ± 0.06	9.02 ± 0.10	8.95 ± 0.09
TC09002923.hg.1	GBGT1	7.96 ± 0.19	7.68 ± 0.15	7.99 ± 0.09	7.78 ± 0.05
TC01002847.hg.1	GBP1	10.49 ± 0.43	10.25 ± 0.51	11.03 ± 0.55	9.62 ± 0.37
TC01000836.hg.1	GBP1P1	8.58 ± 0.46	8.29 ± 0.36	8.98 ± 0.44	7.93 ± 0.20
TC01006361.hg.1	GBP2	10.87 ± 0.18	10.91 ± 0.30	11.46 ± 0.51	10.76 ± 0.21
TC01002845.hg.1	GBP3	9.13 ± 0.34	8.76 ± 0.39	9.52 ± 0.52	8.29 ± 0.27
TC01002849.hg.1	GBP4	9.97 ± 0.53	9.75 ± 0.27	10.05 ± 0.58	9.35 ± 0.26
TC01002850.hg.1	GBP5	10.82 ± 0.48	10.87 ± 0.34	11.40 ± 0.71	10.46 ± 0.17
TC01000835.hg.1	GBP6	6.65 ± 0.26	6.40 ± 0.11	6.97 ± 0.41	6.29 ± 0.07
TC01006362.hg.1	GBP7	6.93 ± 0.25	6.86 ± 0.11	7.00 ± 0.29	6.70 ± 0.09
TC07002031.hg.1	GBX1	8.62 ± 0.31	8.62 ± 0.18	8.57 ± 0.20	8.65 ± 0.08
TC02002897.hg.1	GBX2	8.28 ± 0.21	8.21 ± 0.15	8.20 ± 0.22	8.29 ± 0.07
TC04001274.hg.1	GC	4.92 ± 0.12	4.74 ± 0.06	4.78 ± 0.00	4.77 ± 0.08
TC02000978.hg.1	GCA	9.07 ± 0.29	9.07 ± 0.38	9.93 ± 0.47	8.93 ± 0.25
TC22000289.hg.1	GCAT	8.00 ± 0.23	7.98 ± 0.19	7.89 ± 0.24	8.05 ± 0.05
TC07001838.hg.1	GCC1	7.11 ± 0.13	6.98 ± 0.03	6.96 ± 0.11	7.01 ± 0.02
TC02000666.hg.1	GCC2	8.19 ± 0.75	8.81 ± 0.18	8.50 ± 0.54	8.80 ± 0.08
TC19000231.hg.1	GCDH	8.30 ± 0.14	8.18 ± 0.06	8.16 ± 0.10	8.14 ± 0.04
TC02002008.hg.1	GCFC2	6.54 ± 0.42	6.68 ± 0.19	6.32 ± 0.37	6.48 ± 0.12
TC02002479.hg.1	GCG	5.03 ± 0.08	4.96 ± 0.08	4.97 ± 0.08	5.15 ± 0.18
TC17000937.hg.1	GCGR	8.31 ± 0.23	8.32 ± 0.21	8.25 ± 0.21	8.41 ± 0.05
TC14001152.hg.1	GCH1	8.64 ± 0.38	8.33 ± 0.18	8.93 ± 0.31	8.02 ± 0.28
TC15000293.hg.1	GCHFR	7.86 ± 0.23	7.74 ± 0.15	7.66 ± 0.18	7.83 ± 0.06
TC07001337.hg.1	GCK	7.59 ± 0.22	7.55 ± 0.19	7.50 ± 0.22	7.65 ± 0.04
TC02000176.hg.1	GCKR	6.65 ± 0.17	6.62 ± 0.17	6.57 ± 0.10	6.73 ± 0.08
TC06004051.hg.1	GCLC	7.37 ± 0.06	7.35 ± 0.05	7.21 ± 0.13	7.30 ± 0.05

TC01002882.hg.1	GCLM	7.50 ± 0.16	7.22 ± 0.14	7.80 ± 0.27	7.07 ± 0.17
TC06001810.hg.1	GCM1	6.43 ± 0.15	6.42 ± 0.08	6.40 ± 0.18	6.49 ± 0.04
TC06001270.hg.1	GCM2	6.47 ± 0.14	6.39 ± 0.22	6.29 ± 0.13	6.40 ± 0.07
TC12002034.hg.1	GCN1L1	9.01 ± 0.12	8.85 ± 0.09	8.60 ± 0.08	8.82 ± 0.02
TC09000350.hg.1	GCNT1	6.88 ± 0.05	6.67 ± 0.13	6.82 ± 0.11	6.50 ± 0.02
TC06000071.hg.1	GCNT2	6.72 ± 0.05	6.60 ± 0.05	6.69 ± 0.06	6.59 ± 0.03
TC15000456.hg.1	GCNT3	6.37 ± 0.10	6.28 ± 0.15	6.28 ± 0.12	6.36 ± 0.12
TC05001487.hg.1	GCNT4	7.28 ± 0.67	7.86 ± 0.03	6.96 ± 0.52	7.75 ± 0.25
TC06000072.hg.1	GCNT6	5.67 ± 0.12	5.42 ± 0.13	5.58 ± 0.13	5.59 ± 0.23
TC20000969.hg.1	GCNT7	6.03 ± 0.16	5.94 ± 0.11	6.06 ± 0.13	6.12 ± 0.08
TC15000439.hg.1	GCOM1	7.21 ± 0.14	7.02 ± 0.08	7.13 ± 0.02	7.12 ± 0.06
TC04000367.hg.1	GCOM2	8.18 ± 0.24	8.38 ± 0.15	7.79 ± 0.29	8.25 ± 0.06
TC03001646.hg.1	GCSAM	7.70 ± 0.16	7.67 ± 0.24	7.50 ± 0.21	7.42 ± 0.25
TC01002010.hg.1	GCSAML	7.10 ± 0.13	7.08 ± 0.04	7.08 ± 0.02	7.19 ± 0.09
TC16001298.hg.1	GCSH	7.57 ± 0.12	7.46 ± 0.05	7.33 ± 0.05	7.38 ± 0.05
TC02001211.hg.1	GCSHP3	7.65 ± 0.06	7.65 ± 0.05	7.65 ± 0.13	7.73 ± 0.08
TC02002695.hg.1	GCSHP3	6.93 ± 0.24	6.83 ± 0.03	6.96 ± 0.27	6.80 ± 0.07
TC09000330.hg.1	GDA	5.26 ± 0.16	5.23 ± 0.07	5.22 ± 0.06	5.32 ± 0.09
TC08000493.hg.1	GDAP1	7.06 ± 0.10	7.22 ± 0.08	6.99 ± 0.11	7.14 ± 0.07
TC20000327.hg.1	GDAP1L1	8.07 ± 0.23	8.05 ± 0.21	8.00 ± 0.18	8.15 ± 0.05
TC01003039.hg.1	GDAP2	8.42 ± 0.39	8.52 ± 0.25	8.80 ± 0.43	8.36 ± 0.08
TC16000918.hg.1	GDE1	8.99 ± 0.20	8.68 ± 0.16	9.04 ± 0.21	8.63 ± 0.21
TC10001252.hg.1	GDF10	7.40 ± 0.18	7.39 ± 0.21	7.30 ± 0.26	7.51 ± 0.07
TC12000490.hg.1	GDF11	7.66 ± 0.14	7.57 ± 0.08	7.51 ± 0.20	7.53 ± 0.03
TC19000356.hg.1	GDF15	7.96 ± 0.23	7.91 ± 0.17	7.90 ± 0.24	7.93 ± 0.10
TC10001251.hg.1	GDF2	7.41 ± 0.26	7.37 ± 0.18	7.34 ± 0.11	7.37 ± 0.07
TC12001164.hg.1	GDF3	6.04 ± 0.07	5.98 ± 0.07	5.77 ± 0.30	6.00 ± 0.02
TC20000797.hg.1	GDF5	7.10 ± 0.20	7.19 ± 0.18	7.08 ± 0.11	7.22 ± 0.07
TC20000252.hg.1	GDF5OS	6.26 ± 0.07	6.22 ± 0.01	6.21 ± 0.09	6.24 ± 0.03
TC08001449.hg.1	GDF6	8.24 ± 0.24	8.19 ± 0.24	8.07 ± 0.25	8.26 ± 0.06
TC02000119.hg.1	GDF7	7.80 ± 0.26	7.77 ± 0.19	7.72 ± 0.35	7.83 ± 0.07

TC05001775.hg.1	GDF9	6.21 ± 0.14	6.26 ± 0.11	6.15 ± 0.06	6.29 ± 0.09
TC0X000772.hg.1	GDI1	9.74 ± 0.01	9.69 ± 0.05	9.81 ± 0.12	9.73 ± 0.08
TC10001002.hg.1	GDI2	10.43 ± 0.10	10.42 ± 0.14	10.45 ± 0.16	10.16 ± 0.27
TC05001280.hg.1	GDNF	7.24 ± 0.11	7.24 ± 0.17	7.18 ± 0.07	7.30 ± 0.04
TC05000169.hg.1	GDNF-AS1	6.04 ± 0.27	5.91 ± 0.17	5.88 ± 0.09	6.01 ± 0.06
TC17000720.hg.1	GDPD1	5.79 ± 0.08	5.70 ± 0.11	5.56 ± 0.07	5.61 ± 0.03
TC0X000378.hg.1	GDPD2	6.77 ± 0.16	6.76 ± 0.16	6.72 ± 0.11	6.84 ± 0.06
TC16001020.hg.1	GDPD3	8.14 ± 0.10	7.93 ± 0.05	8.19 ± 0.12	7.95 ± 0.03
TC11002122.hg.1	GDPD4	5.19 ± 0.14	5.15 ± 0.06	5.15 ± 0.01	5.24 ± 0.08
TC11002095.hg.1	GDPD5	8.16 ± 0.15	8.13 ± 0.14	8.04 ± 0.08	8.18 ± 0.07
TC15000859.hg.1	GDPGP1	7.19 ± 0.10	7.02 ± 0.02	6.93 ± 0.09	7.01 ± 0.13
TC08001432.hg.1	GEM	7.11 ± 0.23	7.17 ± 0.20	7.04 ± 0.15	7.47 ± 0.06
TC14000247.hg.1	GEMIN2	5.84 ± 0.12	5.79 ± 0.02	5.72 ± 0.05	5.78 ± 0.04
TC17000967.hg.1	GEMIN4	7.95 ± 0.16	7.75 ± 0.06	7.69 ± 0.16	7.70 ± 0.04
TC05001974.hg.1	GEMIN5	7.87 ± 0.10	7.50 ± 0.10	7.61 ± 0.04	7.33 ± 0.16
TC02000246.hg.1	GEMIN6	7.03 ± 0.07	6.99 ± 0.03	6.95 ± 0.06	6.92 ± 0.06
TC19000641.hg.1	GEMIN7	8.24 ± 0.22	8.04 ± 0.14	7.97 ± 0.12	8.05 ± 0.01
TC0X000870.hg.1	GEMIN8	7.46 ± 0.09	7.41 ± 0.02	7.31 ± 0.05	7.39 ± 0.00
TC01002855.hg.1	GEMIN8P4	7.04 ± 0.10	6.97 ± 0.15	6.96 ± 0.07	7.00 ± 0.07
TC02000105.hg.1	GEN1	7.03 ± 0.28	7.25 ± 0.15	6.96 ± 0.29	7.29 ± 0.09
TC07003316.hg.1	GET4	8.57 ± 0.15	8.56 ± 0.12	8.44 ± 0.13	8.65 ± 0.03
TC17001588.hg.1	GFAP	7.70 ± 0.25	7.71 ± 0.13	7.76 ± 0.21	7.73 ± 0.05
TC16000055.hg.1	GFER	8.47 ± 0.17	8.42 ± 0.20	8.37 ± 0.25	8.53 ± 0.08
TC01002867.hg.1	GFI1	8.24 ± 0.14	8.14 ± 0.10	8.11 ± 0.14	8.10 ± 0.07
TC09000763.hg.1	GFI1B	9.22 ± 0.17	8.59 ± 0.08	9.22 ± 0.15	8.78 ± 0.13
TC03000866.hg.1	GFM1	7.51 ± 0.19	7.53 ± 0.12	7.32 ± 0.15	7.37 ± 0.09
TC05001482.hg.1	GFM2	7.36 ± 0.24	7.37 ± 0.18	7.34 ± 0.27	7.20 ± 0.05
TC06001280.hg.1	GFOD1	7.65 ± 0.04	7.57 ± 0.05	7.72 ± 0.06	7.67 ± 0.03
TC06000091.hg.1	GFOD1-AS1	7.10 ± 0.28	6.77 ± 0.06	7.00 ± 0.25	6.80 ± 0.07
TC16001195.hg.1	GFOD2	7.16 ± 0.29	7.12 ± 0.32	7.02 ± 0.18	7.23 ± 0.08
TC02001948.hg.1	GFPT1	8.49 ± 0.25	8.50 ± 0.16	8.25 ± 0.27	8.33 ± 0.14

TC05002146.hg.1	GFPT2	6.68 ± 0.12	6.67 ± 0.11	6.61 ± 0.12	6.69 ± 0.04
TC10001678.hg.1	GFRA1	6.67 ± 0.21	6.60 ± 0.12	6.60 ± 0.11	6.63 ± 0.07
TC08001030.hg.1	GFRA2	7.63 ± 0.19	7.53 ± 0.19	7.53 ± 0.15	7.62 ± 0.03
TC05001828.hg.1	GFRA3	7.52 ± 0.32	7.49 ± 0.22	7.46 ± 0.24	7.57 ± 0.12
TC20000573.hg.1	GFRA4	8.68 ± 0.25	8.67 ± 0.25	8.57 ± 0.25	8.74 ± 0.03
TC06000678.hg.1	GFRAL	4.47 ± 0.10	4.30 ± 0.12	4.32 ± 0.09	4.35 ± 0.05
TC22000283.hg.1	GGA1	8.33 ± 0.12	8.25 ± 0.05	8.22 ± 0.06	8.32 ± 0.05
TC16000963.hg.1	GGA2	9.00 ± 0.10	9.12 ± 0.25	8.66 ± 0.09	9.03 ± 0.08
TC16000964.hg.1	GGA2	6.65 ± 0.07	6.91 ± 0.05	6.49 ± 0.11	6.95 ± 0.19
TC17001874.hg.1	GGA3	9.31 ± 0.17	9.06 ± 0.07	9.23 ± 0.07	9.06 ± 0.06
TC13000841.hg.1	GGACT	7.65 ± 0.09	7.64 ± 0.16	7.63 ± 0.07	7.69 ± 0.03
TC07001250.hg.1	GGCT	6.24 ± 0.23	6.29 ± 0.06	6.27 ± 0.19	6.16 ± 0.09
TC02002043.hg.1	GGCX	7.45 ± 0.03	7.26 ± 0.01	7.38 ± 0.14	7.22 ± 0.01
TC08001269.hg.1	GGH	6.16 ± 0.07	5.92 ± 0.11	5.98 ± 0.03	5.86 ± 0.04
TC19001499.hg.1	GGN	8.36 ± 0.24	8.39 ± 0.19	8.30 ± 0.27	8.47 ± 0.05
TC06000426.hg.1	GGNBP1	7.67 ± 0.22	7.63 ± 0.20	7.64 ± 0.23	7.82 ± 0.05
TC17000438.hg.1	GGNBP2	8.85 ± 0.29	9.12 ± 0.11	8.89 ± 0.31	9.16 ± 0.06
TC01001944.hg.1	GGPS1	7.02 ± 0.09	6.84 ± 0.16	6.95 ± 0.16	6.69 ± 0.15
TC22001460.hg.1	GGT1	8.52 ± 0.14	8.45 ± 0.16	8.50 ± 0.11	8.53 ± 0.07
TC22000485.hg.1	GGT3P	8.36 ± 0.12	8.25 ± 0.14	8.37 ± 0.10	8.33 ± 0.08
TC22000585.hg.1	GGT5	8.49 ± 0.11	8.42 ± 0.11	8.48 ± 0.16	8.54 ± 0.05
TC17001024.hg.1	GGT6	7.63 ± 0.21	7.58 ± 0.18	7.52 ± 0.15	7.70 ± 0.01
TC20000788.hg.1	GGT7	7.79 ± 0.13	7.88 ± 0.15	7.66 ± 0.13	7.96 ± 0.08
TC02000564.hg.1	GGT8P	8.41 ± 0.25	8.32 ± 0.22	8.14 ± 0.19	8.41 ± 0.06
TC09001548.hg.1	GGTA1P	7.07 ± 0.09	6.90 ± 0.10	7.03 ± 0.12	7.01 ± 0.22
TC20000721.hg.1	GGTLC1	8.04 ± 0.14	8.00 ± 0.17	8.02 ± 0.18	8.08 ± 0.06
TC22000116.hg.1	GGTLC2	8.38 ± 0.10	8.35 ± 0.19	8.41 ± 0.11	8.46 ± 0.06
TC22000514.hg.1	GGTLC3	7.98 ± 0.06	7.92 ± 0.13	8.00 ± 0.11	8.04 ± 0.09
TC17002854.hg.1	GH1	6.77 ± 0.17	6.78 ± 0.17	6.73 ± 0.17	6.81 ± 0.05
TC17001789.hg.1	GH2	6.76 ± 0.21	6.66 ± 0.12	6.62 ± 0.13	6.71 ± 0.05
TC17001528.hg.1	GHDC	8.19 ± 0.14	8.13 ± 0.12	8.11 ± 0.10	8.16 ± 0.07

TC10000591.hg.1	GHITM	9.77 ± 0.01	9.84 ± 0.07	9.77 ± 0.32	9.74 ± 0.10
TC05000191.hg.1	GHR	5.05 ± 0.14	4.99 ± 0.08	5.01 ± 0.05	5.06 ± 0.02
TC20000825.hg.1	GHRH	7.83 ± 0.17	7.73 ± 0.19	7.74 ± 0.12	7.86 ± 0.08
TC07000200.hg.1	GHRHR	7.66 ± 0.20	7.67 ± 0.19	7.61 ± 0.15	7.77 ± 0.10
TC03001158.hg.1	GHRL	7.98 ± 0.21	7.84 ± 0.17	7.99 ± 0.15	7.93 ± 0.02
TC03002005.hg.1	GHSR	7.49 ± 0.37	7.46 ± 0.23	7.33 ± 0.13	7.66 ± 0.24
TC17000203.hg.1	GID4	7.48 ± 0.13	7.33 ± 0.04	7.37 ± 0.08	7.17 ± 0.13
TC20000501.hg.1	GID8	9.05 ± 0.04	9.05 ± 0.18	8.91 ± 0.09	8.99 ± 0.06
TC11001836.hg.1	GIF	5.95 ± 0.19	5.89 ± 0.13	5.94 ± 0.08	5.95 ± 0.03
TC07001676.hg.1	GIGYF1	8.84 ± 0.08	8.83 ± 0.04	8.71 ± 0.06	8.84 ± 0.02
TC02005011.hg.1	GIGYF2	7.90 ± 0.11	7.82 ± 0.04	7.87 ± 0.11	7.77 ± 0.04
TC07003399.hg.1	GIMAP1-GIMAP5	9.35 ± 0.12	8.89 ± 0.23	9.07 ± 0.02	8.91 ± 0.15
TC07001005.hg.1	GIMAP2	8.96 ± 0.07	8.45 ± 0.24	9.05 ± 0.34	8.19 ± 0.19
TC07001004.hg.1	GIMAP4	9.95 ± 0.09	9.63 ± 0.33	10.11 ± 0.29	9.35 ± 0.40
TC07002021.hg.1	GIMAP6	8.95 ± 0.20	8.45 ± 0.15	8.66 ± 0.25	8.30 ± 0.09
TC07001003.hg.1	GIMAP7	10.12 ± 0.24	9.70 ± 0.33	10.12 ± 0.32	9.56 ± 0.13
TC07001002.hg.1	GIMAP8	8.93 ± 0.25	8.22 ± 0.27	8.91 ± 0.34	8.07 ± 0.17
TC04001451.hg.1	GIMD1	5.32 ± 0.03	5.41 ± 0.20	5.46 ± 0.19	5.53 ± 0.13
TC05001644.hg.1	GIN1	5.75 ± 0.08	5.49 ± 0.14	5.82 ± 0.34	5.28 ± 0.07
TC06001082.hg.1	GINM1	7.93 ± 0.34	8.12 ± 0.20	7.85 ± 0.29	8.27 ± 0.22
TC20000169.hg.1	GINS1	7.17 ± 0.08	6.94 ± 0.13	6.98 ± 0.16	6.87 ± 0.03
TC16001318.hg.1	GINS2	7.01 ± 0.31	6.88 ± 0.24	6.81 ± 0.25	6.62 ± 0.02
TC16000502.hg.1	GINS3	7.06 ± 0.16	7.04 ± 0.04	7.02 ± 0.03	7.01 ± 0.04
TC08000318.hg.1	GINS4	6.78 ± 0.12	6.85 ± 0.14	6.65 ± 0.19	6.71 ± 0.05
TC17001657.hg.1	GIP	6.95 ± 0.18	6.90 ± 0.11	6.88 ± 0.11	6.96 ± 0.03
TC19001238.hg.1	GIPC1	8.50 ± 0.10	8.49 ± 0.08	8.31 ± 0.19	8.51 ± 0.02
TC01006272.hg.1	GIPC2	4.42 ± 0.11	4.31 ± 0.04	4.30 ± 0.06	4.34 ± 0.07
TC19000073.hg.1	GIPC3	8.04 ± 0.09	7.94 ± 0.18	7.91 ± 0.16	7.96 ± 0.05
TC19000651.hg.1	GIPR	7.98 ± 0.13	7.84 ± 0.13	7.80 ± 0.16	7.91 ± 0.06
TC17001320.hg.1	GIT1	8.94 ± 0.14	8.82 ± 0.05	8.74 ± 0.09	8.76 ± 0.02
TC12001959.hg.1	GIT2	10.09 ± 0.15	10.01 ± 0.13	10.38 ± 0.21	9.94 ± 0.18

TC06000945.hg.1	GJA1	5.50 ± 0.05	5.41 ± 0.09	5.47 ± 0.01	5.49 ± 0.08
TC06000802.hg.1	GJA10	5.62 ± 0.11	5.88 ± 0.09	5.81 ± 0.09	5.99 ± 0.13
TC13000455.hg.1	GJA3	6.93 ± 0.21	6.91 ± 0.19	6.89 ± 0.29	6.97 ± 0.08
TC01000451.hg.1	GJA4	7.83 ± 0.19	7.77 ± 0.17	7.70 ± 0.31	7.87 ± 0.09
TC01003129.hg.1	GJA5	6.71 ± 0.26	6.74 ± 0.19	6.78 ± 0.21	6.84 ± 0.12
TC01001117.hg.1	GJA8	6.44 ± 0.21	6.30 ± 0.14	6.25 ± 0.27	6.37 ± 0.05
TC0X000384.hg.1	GJB1	7.64 ± 0.20	7.64 ± 0.14	7.65 ± 0.11	7.77 ± 0.07
TC13000456.hg.1	GJB2	7.10 ± 0.29	6.95 ± 0.15	7.02 ± 0.14	6.98 ± 0.04
TC01000450.hg.1	GJB3	7.64 ± 0.22	7.62 ± 0.13	7.65 ± 0.14	7.69 ± 0.05
TC01000449.hg.1	GJB4	6.94 ± 0.22	6.99 ± 0.27	6.97 ± 0.22	7.19 ± 0.10
TC01000448.hg.1	GJB5	7.26 ± 0.24	7.26 ± 0.19	7.27 ± 0.04	7.33 ± 0.05
TC13000457.hg.1	GJB6	7.40 ± 0.20	7.53 ± 0.10	7.51 ± 0.11	7.69 ± 0.04
TC06001927.hg.1	GJB7	7.40 ± 0.16	7.40 ± 0.03	7.39 ± 0.17	7.52 ± 0.16
TC17001585.hg.1	GJC1	6.00 ± 0.25	5.98 ± 0.08	5.99 ± 0.19	6.00 ± 0.06
TC01001886.hg.1	GJC2	8.30 ± 0.11	8.30 ± 0.20	8.30 ± 0.16	8.43 ± 0.04
TC07001651.hg.1	GJC3	6.41 ± 0.10	6.40 ± 0.06	6.45 ± 0.20	6.49 ± 0.08
TC15001200.hg.1	GJD2	7.98 ± 0.18	7.84 ± 0.14	7.86 ± 0.25	7.99 ± 0.13
TC17001461.hg.1	GJD3	7.47 ± 0.26	7.45 ± 0.39	7.39 ± 0.30	7.62 ± 0.05
TC10000241.hg.1	GJD4	7.23 ± 0.38	7.12 ± 0.23	7.06 ± 0.23	7.27 ± 0.11
TC06001043.hg.1	GJE1	3.97 ± 0.10	3.85 ± 0.08	3.87 ± 0.08	3.85 ± 0.08
TC0X000141.hg.1	GK	8.89 ± 0.47	8.85 ± 0.47	10.06 ± 0.50	8.74 ± 0.38
TC04001327.hg.1	GK2	8.17 ± 0.34	8.29 ± 0.23	8.29 ± 0.25	8.42 ± 0.05
TC04001706.hg.1	GK3P	6.90 ± 0.03	6.98 ± 0.14	7.78 ± 0.24	6.91 ± 0.14
TC03001850.hg.1	GK5	7.31 ± 0.48	7.31 ± 0.19	7.31 ± 0.39	7.23 ± 0.05
TC09001281.hg.1	GKAP1	6.34 ± 0.07	6.35 ± 0.09	6.29 ± 0.11	6.28 ± 0.11
TC0X000940.hg.1	GK-AS1	5.67 ± 0.14	5.30 ± 0.12	6.28 ± 0.32	5.44 ± 0.23
TC0X000142.hg.1	GK-IT1	9.63 ± 0.17	9.36 ± 0.15	10.00 ± 0.17	9.37 ± 0.11
TC02000402.hg.1	GKN1	5.04 ± 0.33	4.86 ± 0.10	4.83 ± 0.13	4.89 ± 0.05
TC02001946.hg.1	GKN2	4.54 ± 0.05	4.53 ± 0.12	4.58 ± 0.07	4.58 ± 0.09
TC0X001219.hg.1	GLA	7.78 ± 0.18	7.68 ± 0.07	7.95 ± 0.28	7.58 ± 0.23
TC03001273.hg.1	GLB1	8.70 ± 0.20	8.26 ± 0.07	8.70 ± 0.05	8.15 ± 0.19

TC02002792.hg.1	GLB1L	7.27 ± 0.21	7.11 ± 0.05	7.14 ± 0.07	7.19 ± 0.03
TC11001217.hg.1	GLB1L2	7.48 ± 0.15	7.50 ± 0.11	7.41 ± 0.16	7.55 ± 0.06
TC11001216.hg.1	GLB1L3	6.40 ± 0.13	6.40 ± 0.10	6.36 ± 0.12	6.48 ± 0.09
TC07003320.hg.1	GLCCI1	8.50 ± 0.18	8.38 ± 0.06	8.18 ± 0.19	8.18 ± 0.05
TC15000633.hg.1	GLCE	6.65 ± 0.10	6.56 ± 0.12	6.72 ± 0.13	6.54 ± 0.10
TC09000899.hg.1	GLDC	7.78 ± 0.39	6.98 ± 0.13	6.93 ± 0.17	6.92 ± 0.11
TC15000405.hg.1	GLDN	6.65 ± 0.10	6.63 ± 0.17	6.64 ± 0.12	6.76 ± 0.11
TC09000693.hg.1	GLE1	8.52 ± 0.21	8.19 ± 0.06	8.46 ± 0.16	7.99 ± 0.20
TC16001267.hg.1	GLG1	10.15 ± 0.30	10.12 ± 0.12	9.90 ± 0.09	9.99 ± 0.05
TC12000530.hg.1	GLI1	7.46 ± 0.17	7.40 ± 0.23	7.34 ± 0.17	7.52 ± 0.13
TC02000764.hg.1	GLI2	7.69 ± 0.16	7.68 ± 0.16	7.66 ± 0.17	7.75 ± 0.06
TC07001319.hg.1	GLI3	6.53 ± 0.19	6.47 ± 0.18	6.49 ± 0.16	6.59 ± 0.07
TC08002609.hg.1	GLI4	8.37 ± 0.15	8.30 ± 0.12	8.23 ± 0.16	8.35 ± 0.08
TC12000642.hg.1	GLIPR1	9.96 ± 0.42	10.28 ± 0.22	10.26 ± 0.48	10.10 ± 0.29
TC12000640.hg.1	GLIPR1L1	4.43 ± 0.15	4.42 ± 0.12	4.39 ± 0.06	4.42 ± 0.06
TC12000641.hg.1	GLIPR1L2	5.59 ± 0.13	5.51 ± 0.04	5.55 ± 0.01	5.53 ± 0.06
TC09000202.hg.1	GLIPR2	9.27 ± 0.22	8.92 ± 0.12	9.65 ± 0.14	8.87 ± 0.20
TC01002674.hg.1	GLIS1	7.57 ± 0.21	7.65 ± 0.20	7.64 ± 0.23	7.77 ± 0.06
TC16000128.hg.1	GLIS2	7.97 ± 0.19	7.92 ± 0.17	7.93 ± 0.26	8.03 ± 0.02
TC09000879.hg.1	GLIS3	6.33 ± 0.17	6.25 ± 0.10	6.27 ± 0.08	6.35 ± 0.06
TC09000027.hg.1	GLIS3-AS1	5.92 ± 0.12	5.69 ± 0.39	5.89 ± 0.16	6.05 ± 0.06
TC01002866.hg.1	GLMN	5.92 ± 0.45	5.94 ± 0.19	5.79 ± 0.28	5.81 ± 0.08
TC06001692.hg.1	GLO1	8.29 ± 0.08	8.37 ± 0.30	7.95 ± 0.11	8.09 ± 0.04
TC17000968.hg.1	GLOD4	7.99 ± 0.06	8.03 ± 0.10	7.75 ± 0.09	8.00 ± 0.04
TC0X000259.hg.1	GLOD5	6.41 ± 0.14	6.35 ± 0.12	6.32 ± 0.12	6.36 ± 0.05
TC06000557.hg.1	GLP1R	7.11 ± 0.16	7.13 ± 0.17	7.01 ± 0.17	7.23 ± 0.07
TC17000147.hg.1	GLP2R	6.38 ± 0.08	6.38 ± 0.12	6.35 ± 0.06	6.45 ± 0.06
TC05001960.hg.1	GLRA1	5.90 ± 0.15	5.82 ± 0.13	5.83 ± 0.03	5.91 ± 0.10
TC0X000065.hg.1	GLRA2	4.98 ± 0.09	4.89 ± 0.08	4.91 ± 0.09	4.92 ± 0.03
TC04001755.hg.1	GLRA3	4.46 ± 0.15	4.29 ± 0.03	4.32 ± 0.03	4.31 ± 0.06
TC04001756.hg.1	GLRA3	3.74 ± 0.32	3.55 ± 0.14	3.76 ± 0.29	3.57 ± 0.04

TC0X001243.hg.1	GLRA4	6.39 ± 0.18	6.30 ± 0.13	6.29 ± 0.12	6.38 ± 0.08
TC04000798.hg.1	GLRB	5.37 ± 0.15	5.29 ± 0.07	5.32 ± 0.09	5.30 ± 0.06
TC05001608.hg.1	GLRX	7.80 ± 0.17	7.40 ± 0.07	8.08 ± 0.42	7.35 ± 0.15
TC01003652.hg.1	GLRX2	7.51 ± 0.12	7.34 ± 0.05	7.38 ± 0.10	7.28 ± 0.09
TC10000933.hg.1	GLRX3	7.82 ± 0.19	7.84 ± 0.27	7.59 ± 0.11	7.60 ± 0.16
TC14000593.hg.1	GLRX5	7.51 ± 0.14	7.39 ± 0.09	7.36 ± 0.11	7.30 ± 0.08
TC02001122.hg.1	GLS	8.31 ± 0.43	8.49 ± 0.25	8.33 ± 0.42	8.30 ± 0.19
TC12001607.hg.1	GLS2	6.71 ± 0.07	6.72 ± 0.08	6.66 ± 0.14	6.81 ± 0.06
TC12001027.hg.1	GLT1D1	9.99 ± 0.33	9.77 ± 0.18	10.53 ± 0.30	9.67 ± 0.49
TC09001718.hg.1	GLT6D1	5.38 ± 0.08	5.22 ± 0.12	5.32 ± 0.01	5.17 ± 0.07
TC03001459.hg.1	GLT8D1	6.95 ± 0.11	6.66 ± 0.14	6.75 ± 0.11	6.51 ± 0.04
TC12001904.hg.1	GLT8D2	5.59 ± 0.18	5.51 ± 0.08	5.55 ± 0.06	5.61 ± 0.03
TC12001958.hg.1	GLTP	9.23 ± 0.24	9.22 ± 0.20	9.33 ± 0.16	9.18 ± 0.17
TC01000034.hg.1	GLTPD1	7.86 ± 0.15	7.91 ± 0.18	7.85 ± 0.24	7.90 ± 0.01
TC17000051.hg.1	GLTPD2	7.92 ± 0.32	7.82 ± 0.20	7.80 ± 0.30	7.85 ± 0.04
TC19000680.hg.1	GLTSCR1	8.67 ± 0.07	8.69 ± 0.07	8.63 ± 0.08	8.76 ± 0.08
TC06000584.hg.1	GLTSCR1L	9.03 ± 0.24	8.95 ± 0.19	9.11 ± 0.29	8.78 ± 0.18
TC19000682.hg.1	GLTSCR2	9.86 ± 0.14	9.90 ± 0.12	9.52 ± 0.22	9.86 ± 0.05
TC10001486.hg.1	GLUD1	9.19 ± 0.10	9.27 ± 0.13	9.07 ± 0.12	9.18 ± 0.06
TC10000319.hg.1	GLUD1P2	6.82 ± 0.07	6.88 ± 0.08	6.75 ± 0.07	6.92 ± 0.06
TC10000467.hg.1	GLUD1P3	8.59 ± 0.15	8.55 ± 0.04	8.32 ± 0.22	8.52 ± 0.10
TC0X000597.hg.1	GLUD2	6.13 ± 0.16	6.06 ± 0.07	5.99 ± 0.07	5.96 ± 0.06
TC01003605.hg.1	GLUL	10.33 ± 0.25	10.68 ± 0.14	10.92 ± 0.33	10.76 ± 0.17
TC11001813.hg.1	GLYAT	4.82 ± 0.04	4.72 ± 0.10	4.84 ± 0.12	4.82 ± 0.16
TC11000480.hg.1	GLYATL1	5.51 ± 0.08	5.43 ± 0.07	5.50 ± 0.11	5.51 ± 0.11
TC11001814.hg.1	GLYATL2	5.16 ± 0.10	5.06 ± 0.06	5.06 ± 0.01	5.13 ± 0.08
TC06000641.hg.1	GLYATL3	5.90 ± 0.19	5.72 ± 0.04	5.74 ± 0.07	5.79 ± 0.04
TC12001575.hg.1	GLYCAM1	7.12 ± 0.18	7.04 ± 0.19	7.10 ± 0.15	7.13 ± 0.12
TC03000332.hg.1	GLYCTK	8.45 ± 0.08	8.38 ± 0.12	8.35 ± 0.12	8.48 ± 0.09
TC03001451.hg.1	GLYCTK-AS1	7.35 ± 0.24	7.32 ± 0.28	7.26 ± 0.08	7.42 ± 0.13
TC16000837.hg.1	GLYR1	10.24 ± 0.08	9.99 ± 0.12	10.30 ± 0.16	9.94 ± 0.22

TC01001571.hg.1	GM140	5.93 ± 0.17	5.91 ± 0.18	5.88 ± 0.17	5.90 ± 0.19
TC05000839.hg.1	GM2A	8.67 ± 0.32	8.23 ± 0.14	8.74 ± 0.21	8.12 ± 0.26
TC02000409.hg.1	GMCL1	8.25 ± 0.41	8.41 ± 0.17	8.30 ± 0.38	8.28 ± 0.17
TC05002121.hg.1	GMCL1P1	6.96 ± 0.16	6.82 ± 0.04	6.93 ± 0.04	6.82 ± 0.07
TC06001213.hg.1	GMDS	8.05 ± 0.10	8.00 ± 0.03	7.87 ± 0.03	7.89 ± 0.03
TC01000389.hg.1	GMEB1	8.32 ± 0.19	8.21 ± 0.22	8.15 ± 0.14	8.09 ± 0.11
TC20001036.hg.1	GMEB2	8.41 ± 0.12	8.37 ± 0.12	8.18 ± 0.18	8.47 ± 0.04
TC14001150.hg.1	GMFB	7.70 ± 0.41	8.01 ± 0.19	7.97 ± 0.35	7.98 ± 0.08
TC19001517.hg.1	GMFG	11.51 ± 0.27	11.40 ± 0.17	11.73 ± 0.21	11.24 ± 0.12
TC19001306.hg.1	GMIP	10.18 ± 0.22	9.99 ± 0.09	10.44 ± 0.14	10.02 ± 0.12
TC08000813.hg.1	GML	6.76 ± 0.20	6.79 ± 0.14	6.70 ± 0.15	6.95 ± 0.15
TC03002111.hg.1	GMNC	4.87 ± 0.10	4.81 ± 0.07	4.79 ± 0.10	4.90 ± 0.09
TC06000149.hg.1	GMNN	6.90 ± 0.11	6.82 ± 0.15	6.80 ± 0.04	6.59 ± 0.13
TC02001329.hg.1	GMPPA	8.70 ± 0.13	8.45 ± 0.09	8.48 ± 0.07	8.37 ± 0.05
TC03001415.hg.1	GMPPB	8.20 ± 0.22	8.08 ± 0.14	7.94 ± 0.23	8.13 ± 0.03
TC06000110.hg.1	GMPR	8.38 ± 0.21	7.68 ± 0.13	8.22 ± 0.32	7.75 ± 0.08
TC14000165.hg.1	GMPR2	8.19 ± 0.23	7.96 ± 0.18	8.36 ± 0.29	7.87 ± 0.14
TC03000851.hg.1	GMPS	8.84 ± 0.11	8.95 ± 0.17	8.63 ± 0.10	8.70 ± 0.11
TC19000063.hg.1	GNA11	7.91 ± 0.14	7.80 ± 0.05	7.74 ± 0.12	7.74 ± 0.02
TC07001101.hg.1	GNA12	8.58 ± 0.03	8.63 ± 0.08	8.44 ± 0.05	8.64 ± 0.05
TC17001808.hg.1	GNA13	9.83 ± 0.21	10.25 ± 0.17	10.05 ± 0.34	10.22 ± 0.11
TC09001226.hg.1	GNA14	6.36 ± 0.06	6.40 ± 0.08	6.39 ± 0.10	6.43 ± 0.04
TC19000065.hg.1	GNA15	8.44 ± 0.27	8.30 ± 0.08	8.58 ± 0.15	8.41 ± 0.17
TC07000508.hg.1	GNAI1	6.09 ± 0.14	6.07 ± 0.14	6.09 ± 0.10	6.13 ± 0.05
TC03000307.hg.1	GNAI2	10.43 ± 0.11	10.35 ± 0.01	10.42 ± 0.05	10.28 ± 0.11
TC01000947.hg.1	GNAI3	9.37 ± 0.26	9.68 ± 0.18	9.81 ± 0.31	9.60 ± 0.12
TC18000058.hg.1	GNAL	6.71 ± 0.05	6.64 ± 0.07	6.65 ± 0.07	6.63 ± 0.02
TC16000461.hg.1	GNAO1	7.85 ± 0.04	7.60 ± 0.11	7.73 ± 0.13	7.62 ± 0.07
TC09001228.hg.1	GNAQ	9.55 ± 0.23	9.72 ± 0.21	10.14 ± 0.33	9.70 ± 0.11
TC20000466.hg.1	GNAS	9.45 ± 0.08	9.49 ± 0.06	9.37 ± 0.09	9.51 ± 0.03
TC20000988.hg.1	GNAS-AS1	6.47 ± 0.22	6.34 ± 0.20	6.35 ± 0.09	6.43 ± 0.03

TC20000989.hg.1	GNAS-AS1	7.65 ± 0.31	7.50 ± 0.07	7.46 ± 0.27	7.59 ± 0.03
TC03000305.hg.1	GNAT1	7.83 ± 0.17	7.74 ± 0.13	7.75 ± 0.20	7.82 ± 0.02
TC01002960.hg.1	GNAT2	6.05 ± 0.09	5.98 ± 0.00	6.15 ± 0.04	6.03 ± 0.04
TC07001558.hg.1	GNAT3	4.68 ± 0.12	4.64 ± 0.09	4.64 ± 0.10	4.66 ± 0.08
TC22000126.hg.1	GNAZ	8.99 ± 0.05	8.77 ± 0.12	9.00 ± 0.13	8.81 ± 0.10
TC01006298.hg.1	GNB1	10.86 ± 0.13	10.84 ± 0.04	10.84 ± 0.16	10.79 ± 0.09
TC07000630.hg.1	GNB2	9.78 ± 0.30	9.63 ± 0.06	9.75 ± 0.16	9.58 ± 0.11
TC05002169.hg.1	GNB2L1	9.56 ± 0.07	9.61 ± 0.11	9.22 ± 0.09	9.61 ± 0.05
TC12000094.hg.1	GNB3	8.08 ± 0.16	8.05 ± 0.13	8.03 ± 0.17	8.12 ± 0.04
TC03002038.hg.1	GNB4	8.61 ± 0.14	8.45 ± 0.29	8.99 ± 0.42	8.43 ± 0.29
TC15001453.hg.1	GNB5	8.09 ± 0.04	8.10 ± 0.02	8.01 ± 0.16	8.11 ± 0.08
TC09001068.hg.1	GNE	7.93 ± 0.07	7.86 ± 0.13	7.76 ± 0.11	7.77 ± 0.01
TC07000556.hg.1	GNG11	8.38 ± 0.19	7.70 ± 0.12	8.44 ± 0.41	7.84 ± 0.39
TC01002760.hg.1	GNG12	5.25 ± 0.19	5.17 ± 0.13	5.21 ± 0.12	5.27 ± 0.08
TC01000746.hg.1	GNG12-AS1	5.90 ± 0.14	5.82 ± 0.04	5.90 ± 0.03	5.89 ± 0.09
TC16000741.hg.1	GNG13	7.83 ± 0.20	7.83 ± 0.21	7.74 ± 0.17	7.92 ± 0.03
TC14000303.hg.1	GNG2	8.35 ± 0.26	8.58 ± 0.13	8.42 ± 0.23	8.56 ± 0.11
TC11000561.hg.1	GNG3	7.40 ± 0.22	7.33 ± 0.18	7.21 ± 0.19	7.45 ± 0.03
TC01003995.hg.1	GNG4	7.52 ± 0.20	7.49 ± 0.16	7.56 ± 0.14	7.63 ± 0.01
TC01002818.hg.1	GNG5	9.04 ± 0.35	8.98 ± 0.18	9.39 ± 0.21	8.79 ± 0.28
TC0X001276.hg.1	GNG5P2	8.34 ± 0.38	8.18 ± 0.08	8.56 ± 0.17	8.05 ± 0.18
TC19001032.hg.1	GNG7	8.55 ± 0.23	8.47 ± 0.12	8.20 ± 0.11	8.41 ± 0.09
TC19001651.hg.1	GNG8	7.59 ± 0.27	7.40 ± 0.12	7.46 ± 0.26	7.54 ± 0.07
TC07000553.hg.1	GNGT1	4.15 ± 0.07	4.03 ± 0.01	4.08 ± 0.07	4.10 ± 0.08
TC17001660.hg.1	GNGT2	7.31 ± 0.07	7.21 ± 0.05	7.09 ± 0.10	7.17 ± 0.05
TC06001501.hg.1	GNL1	8.38 ± 0.09	8.52 ± 0.11	8.20 ± 0.15	8.59 ± 0.06
TC6_apd_hap1000073.hg.1	GNL1	8.50 ± 0.04	8.72 ± 0.10	8.34 ± 0.13	8.87 ± 0.06
TC6_cox_hap2000136.hg.1	GNL1	8.42 ± 0.12	8.55 ± 0.11	8.23 ± 0.15	8.64 ± 0.06
TC6_dbb_hap3000125.hg.1	GNL1	8.44 ± 0.11	8.57 ± 0.11	8.24 ± 0.14	8.65 ± 0.06
TC6_mann_hap4000114.hg.1	GNL1	8.44 ± 0.11	8.57 ± 0.11	8.24 ± 0.14	8.66 ± 0.06
TC6_mcf_hap5000115.hg.1	GNL1	8.44 ± 0.11	8.57 ± 0.11	8.24 ± 0.14	8.66 ± 0.06

TC6_qbl_hap6000126.hg.1	GNL1	8.44 ± 0.11	8.57 ± 0.11	8.24 ± 0.14	8.65 ± 0.06
TC6_ssto_hap7000117.hg.1	GNL1	8.44 ± 0.11	8.57 ± 0.11	8.24 ± 0.14	8.65 ± 0.06
TC01002519.hg.1	GNL2	8.65 ± 0.15	8.70 ± 0.07	8.54 ± 0.29	8.60 ± 0.13
TC03000339.hg.1	GNL3	7.76 ± 0.31	7.89 ± 0.09	7.46 ± 0.31	7.88 ± 0.09
TC0X000322.hg.1	GNL3L	8.90 ± 0.03	8.69 ± 0.10	8.75 ± 0.12	8.63 ± 0.06
TC02000516.hg.1	GNLY	9.64 ± 0.35	9.50 ± 0.08	9.16 ± 0.21	9.35 ± 0.10
TC06000590.hg.1	GNMT	7.24 ± 0.13	7.16 ± 0.11	7.16 ± 0.08	7.27 ± 0.03
TC12001902.hg.1	GNN	5.30 ± 0.01	5.33 ± 0.08	5.26 ± 0.06	5.31 ± 0.05
TC01001915.hg.1	GNPAT	8.21 ± 0.37	8.17 ± 0.21	8.41 ± 0.30	7.94 ± 0.29
TC05001879.hg.1	GNPDA1	7.52 ± 0.18	7.29 ± 0.06	7.40 ± 0.18	7.27 ± 0.04
TC04001157.hg.1	GNPDA2	6.52 ± 0.54	6.85 ± 0.25	6.35 ± 0.27	6.62 ± 0.07
TC14001142.hg.1	GNPNAT1	8.14 ± 0.06	8.04 ± 0.08	7.99 ± 0.04	7.98 ± 0.08
TC12001880.hg.1	GNPTAB	8.45 ± 0.51	8.72 ± 0.19	8.44 ± 0.44	8.66 ± 0.07
TC16000041.hg.1	GNPTG	8.84 ± 0.12	8.78 ± 0.02	8.66 ± 0.13	8.75 ± 0.02
TC08001069.hg.1	GNRH1	5.67 ± 0.23	5.66 ± 0.09	5.70 ± 0.11	5.61 ± 0.07
TC20000031.hg.1	GNRH2	7.91 ± 0.31	7.97 ± 0.33	7.85 ± 0.21	8.12 ± 0.16
TC04001251.hg.1	GNRHR	4.48 ± 0.17	4.27 ± 0.07	4.30 ± 0.14	4.30 ± 0.12
TC01003110.hg.1	GNRHR2	7.17 ± 0.09	7.13 ± 0.02	7.20 ± 0.06	7.21 ± 0.03
TC12001678.hg.1	GNS	10.47 ± 0.48	10.03 ± 0.17	10.94 ± 0.29	9.86 ± 0.40
TC09001583.hg.1	GOLGA1	7.77 ± 0.06	7.74 ± 0.10	8.00 ± 0.18	7.65 ± 0.04
TC09001628.hg.1	GOLGA2	8.47 ± 0.07	8.36 ± 0.01	8.44 ± 0.08	8.37 ± 0.08
TC0Y000209.hg.1	GOLGA2P2Y	7.95 ± 0.16	7.90 ± 0.16	7.87 ± 0.18	8.01 ± 0.04
TC12001866.hg.1	GOLGA2P5	7.63 ± 0.11	7.62 ± 0.03	7.57 ± 0.07	7.65 ± 0.06
TC12003216.hg.1	GOLGA3	8.77 ± 0.05	8.68 ± 0.03	8.51 ± 0.08	8.62 ± 0.01
TC03000178.hg.1	GOLGA4	7.42 ± 0.52	7.79 ± 0.21	7.59 ± 0.47	7.68 ± 0.07
TC14000572.hg.1	GOLGA5	8.24 ± 0.22	8.16 ± 0.21	8.38 ± 0.36	7.97 ± 0.18
TC15001638.hg.1	GOLGA6A	7.39 ± 0.19	7.40 ± 0.07	7.37 ± 0.16	7.51 ± 0.08
TC15000661.hg.1	GOLGA6B	7.23 ± 0.22	7.27 ± 0.09	7.21 ± 0.13	7.36 ± 0.08
TC15000692.hg.1	GOLGA6C	7.38 ± 0.22	7.39 ± 0.07	7.36 ± 0.13	7.49 ± 0.08
TC15000693.hg.1	GOLGA6D	7.51 ± 0.23	7.52 ± 0.07	7.50 ± 0.12	7.62 ± 0.07
TC15000026.hg.1	GOLGA6L1	7.64 ± 0.14	7.54 ± 0.05	7.64 ± 0.13	7.69 ± 0.12

TC15001738.hg.1	GOLGA6L10	8.24 ± 0.41	8.30 ± 0.02	8.25 ± 0.15	8.47 ± 0.07
TC15001750.hg.1	GOLGA6L10	8.55 ± 0.50	8.72 ± 0.06	8.67 ± 0.28	9.04 ± 0.11
TC15001067.hg.1	GOLGA6L2	6.89 ± 0.23	6.77 ± 0.13	6.86 ± 0.21	6.84 ± 0.10
TC15000808.hg.1	GOLGA6L4	8.18 ± 0.35	8.31 ± 0.03	8.29 ± 0.24	8.52 ± 0.05
TC15001782.hg.1	GOLGA6L5	7.93 ± 0.47	8.21 ± 0.09	8.10 ± 0.27	8.39 ± 0.02
TC15001012.hg.1	GOLGA6L6	7.05 ± 0.15	6.92 ± 0.08	7.06 ± 0.12	7.05 ± 0.11
TC15000767.hg.1	GOLGA6L9	7.58 ± 0.44	7.71 ± 0.05	7.62 ± 0.19	7.88 ± 0.08
TC15000774.hg.1	GOLGA6L9	7.94 ± 0.42	8.07 ± 0.04	8.00 ± 0.18	8.26 ± 0.07
TC08000317.hg.1	GOLGA7	9.58 ± 0.06	9.73 ± 0.06	9.65 ± 0.09	9.70 ± 0.07
TC10000704.hg.1	GOLGA7B	7.06 ± 0.22	6.99 ± 0.16	6.92 ± 0.11	7.03 ± 0.04
TC15002756.hg.1	GOLGA8A	9.26 ± 0.57	9.78 ± 0.02	9.14 ± 0.32	9.92 ± 0.13
TC15002757.hg.1	GOLGA8B	9.12 ± 0.56	9.63 ± 0.00	8.95 ± 0.35	9.76 ± 0.12
TC15000010.hg.1	GOLGA8CP	7.97 ± 0.33	8.02 ± 0.02	7.95 ± 0.15	8.14 ± 0.09
TC15001044.hg.1	GOLGA8DP	7.87 ± 0.35	7.93 ± 0.02	7.85 ± 0.14	8.08 ± 0.10
TC15000035.hg.1	GOLGA8EP	7.92 ± 0.33	7.99 ± 0.03	7.90 ± 0.12	8.12 ± 0.11
TC15000134.hg.1	GOLGA8F	7.92 ± 0.38	7.97 ± 0.04	7.88 ± 0.16	8.13 ± 0.12
TC15001098.hg.1	GOLGA8F	7.92 ± 0.37	7.94 ± 0.05	7.88 ± 0.17	8.12 ± 0.12
TC15000030.hg.1	GOLGA8I	8.11 ± 0.26	8.11 ± 0.02	8.10 ± 0.13	8.24 ± 0.09
TC15000157.hg.1	GOLGA8J	8.06 ± 0.27	8.11 ± 0.04	8.12 ± 0.13	8.27 ± 0.10
TC15002804.hg.1	GOLGA8K	8.21 ± 0.26	8.21 ± 0.05	8.27 ± 0.10	8.34 ± 0.06
TC15001104.hg.1	GOLGA8M	7.72 ± 0.40	7.76 ± 0.10	7.76 ± 0.26	7.90 ± 0.12
TC15001174.hg.1	GOLGA8O	8.32 ± 0.35	8.36 ± 0.09	8.33 ± 0.23	8.46 ± 0.08
TC15000181.hg.1	GOLGA8Q	8.24 ± 0.33	8.19 ± 0.12	8.31 ± 0.24	8.29 ± 0.09
TC15000222.hg.1	GOLGA8R	8.28 ± 0.32	8.33 ± 0.06	8.29 ± 0.20	8.46 ± 0.09
TC15001140.hg.1	GOLGA8R	8.34 ± 0.33	8.38 ± 0.07	8.36 ± 0.18	8.48 ± 0.07
TC15000039.hg.1	GOLGA8S	8.09 ± 0.36	8.15 ± 0.02	8.06 ± 0.13	8.28 ± 0.08
TC15000162.hg.1	GOLGA8T	8.05 ± 0.35	8.06 ± 0.09	8.08 ± 0.21	8.20 ± 0.07
TC03001698.hg.1	GOLGB1	7.96 ± 0.32	8.16 ± 0.17	8.10 ± 0.46	8.14 ± 0.07
TC03001978.hg.1	GOLIM4	7.70 ± 0.16	7.53 ± 0.11	7.80 ± 0.17	7.49 ± 0.12
TC09002894.hg.1	GOLM1	7.97 ± 0.13	7.99 ± 0.11	8.04 ± 0.04	7.95 ± 0.08
TC05001232.hg.1	GOLPH3	9.79 ± 0.06	9.72 ± 0.03	9.82 ± 0.11	9.60 ± 0.13

TC01003208.hg.1	GOLPH3L	7.86 ± 0.07	7.48 ± 0.20	8.08 ± 0.29	7.16 ± 0.35
TC01003728.hg.1	GOLT1A	7.01 ± 0.14	7.12 ± 0.24	6.94 ± 0.23	7.21 ± 0.10
TC12000233.hg.1	GOLT1B	7.61 ± 0.41	7.87 ± 0.15	7.61 ± 0.34	7.82 ± 0.01
TC01006306.hg.1	GON4L	8.32 ± 0.11	8.21 ± 0.09	8.24 ± 0.14	8.22 ± 0.06
TC06004056.hg.1	GOPC	8.44 ± 0.43	8.64 ± 0.16	8.57 ± 0.34	8.47 ± 0.10
TC01001482.hg.1	GORAB	5.56 ± 0.28	5.67 ± 0.14	5.44 ± 0.18	5.76 ± 0.08
TC03001301.hg.1	GORASP1	8.14 ± 0.12	8.06 ± 0.09	8.09 ± 0.13	8.10 ± 0.02
TC02001017.hg.1	GORASP2	9.04 ± 0.07	9.00 ± 0.07	8.80 ± 0.12	8.93 ± 0.06
TC17000350.hg.1	GOSR1	8.10 ± 0.29	8.17 ± 0.23	8.14 ± 0.21	8.22 ± 0.13
TC17000603.hg.1	GOSR2	8.82 ± 0.24	8.64 ± 0.12	8.63 ± 0.11	8.58 ± 0.05
TC10001580.hg.1	GOT1	7.60 ± 0.16	7.39 ± 0.05	7.35 ± 0.14	7.34 ± 0.02
TC08001139.hg.1	GOT1L1	5.93 ± 0.13	5.89 ± 0.11	5.89 ± 0.03	6.02 ± 0.14
TC16001158.hg.1	GOT2	8.68 ± 0.23	8.39 ± 0.21	8.20 ± 0.23	8.13 ± 0.03
TC12000149.hg.1	GOT2P3	6.64 ± 0.20	6.42 ± 0.17	6.44 ± 0.34	6.50 ± 0.16
TC17000056.hg.1	GP1BA	8.57 ± 0.19	8.12 ± 0.27	8.49 ± 0.13	8.26 ± 0.15
TC22000048.hg.1	GP1BB	8.67 ± 0.16	8.42 ± 0.16	8.60 ± 0.25	8.51 ± 0.07
TC16000923.hg.1	GP2	6.77 ± 0.20	6.79 ± 0.13	6.77 ± 0.10	6.88 ± 0.06
TC03002132.hg.1	GP5	6.96 ± 0.08	6.86 ± 0.05	7.08 ± 0.10	6.88 ± 0.13
TC19002721.hg.1	GP6	8.35 ± 0.10	7.99 ± 0.08	8.43 ± 0.20	7.99 ± 0.18
TC03000686.hg.1	GP9	8.48 ± 0.14	8.37 ± 0.25	8.50 ± 0.31	8.55 ± 0.10
TC01003477.hg.1	GPA33	8.24 ± 0.05	8.21 ± 0.03	8.03 ± 0.11	8.23 ± 0.02
TC08000839.hg.1	GPAA1	9.21 ± 0.22	8.93 ± 0.09	8.98 ± 0.09	8.87 ± 0.02
TC10001663.hg.1	GPAM	6.67 ± 0.10	6.69 ± 0.10	6.52 ± 0.06	6.64 ± 0.09
TC06001534.hg.1	GPANK1	8.04 ± 0.16	7.88 ± 0.06	7.90 ± 0.15	7.86 ± 0.07
TC6_cox_hap2000163.hg.1	GPANK1	8.03 ± 0.16	7.88 ± 0.06	7.89 ± 0.15	7.86 ± 0.07
TC6_dbb_hap3000152.hg.1	GPANK1	8.03 ± 0.15	7.86 ± 0.06	7.89 ± 0.16	7.84 ± 0.08
TC6_mann_hap4000140.hg.1	GPANK1	8.04 ± 0.16	7.88 ± 0.06	7.90 ± 0.15	7.86 ± 0.07
TC6_mcf_hap5000140.hg.1	GPANK1	8.04 ± 0.16	7.88 ± 0.06	7.90 ± 0.15	7.86 ± 0.07
TC6_qbl_hap6000155.hg.1	GPANK1	8.03 ± 0.16	7.87 ± 0.06	7.89 ± 0.15	7.86 ± 0.07
TC6_ssto_hap7000135.hg.1	GPANK1	8.03 ± 0.16	7.87 ± 0.06	7.89 ± 0.15	7.86 ± 0.07
TC02002099.hg.1	GPAT2	7.92 ± 0.21	7.88 ± 0.21	7.79 ± 0.21	7.96 ± 0.06

TC19000432.hg.1	GPATCH1	8.30 ± 0.12	8.09 ± 0.18	8.18 ± 0.14	7.91 ± 0.12
TC02000233.hg.1	GPATCH11	7.66 ± 0.42	7.76 ± 0.27	7.64 ± 0.38	7.47 ± 0.40
TC01003823.hg.1	GPATCH2	6.31 ± 0.29	6.42 ± 0.02	6.26 ± 0.14	6.39 ± 0.06
TC14000479.hg.1	GPATCH2L	7.71 ± 0.17	7.79 ± 0.11	7.76 ± 0.30	7.81 ± 0.07
TC01002396.hg.1	GPATCH3	7.94 ± 0.14	7.90 ± 0.10	7.78 ± 0.12	7.93 ± 0.04
TC01003344.hg.1	GPATCH4	7.99 ± 0.13	7.97 ± 0.04	7.83 ± 0.03	7.92 ± 0.06
TC17001582.hg.1	GPATCH8	9.23 ± 0.18	9.36 ± 0.10	9.09 ± 0.28	9.37 ± 0.09
TC02001296.hg.1	GPBAR1	8.09 ± 0.27	8.02 ± 0.15	7.98 ± 0.24	8.02 ± 0.02
TC05000247.hg.1	GPBP1	9.18 ± 0.40	9.51 ± 0.22	9.54 ± 0.45	9.42 ± 0.11
TC01002613.hg.1	GPBP1L1	9.59 ± 0.27	9.58 ± 0.13	9.76 ± 0.32	9.58 ± 0.12
TC02001464.hg.1	GPC1	8.27 ± 0.24	8.28 ± 0.21	8.21 ± 0.21	8.39 ± 0.02
TC07001662.hg.1	GPC2	7.87 ± 0.16	7.85 ± 0.20	7.82 ± 0.16	7.95 ± 0.05
TC0X001378.hg.1	GPC3	5.81 ± 0.20	5.84 ± 0.18	5.68 ± 0.15	5.88 ± 0.03
TC0X001376.hg.1	GPC4	5.96 ± 0.15	5.94 ± 0.11	5.92 ± 0.11	5.95 ± 0.03
TC13000323.hg.1	GPC5	5.76 ± 0.08	5.62 ± 0.10	5.66 ± 0.10	5.69 ± 0.09
TC13000785.hg.1	GPC5-AS1	6.08 ± 0.12	6.01 ± 0.04	5.99 ± 0.02	6.16 ± 0.17
TC13000784.hg.1	GPC5-AS2	6.30 ± 0.19	6.47 ± 0.13	6.53 ± 0.14	6.51 ± 0.03
TC13000326.hg.1	GPC5-IT1	3.90 ± 0.20	3.52 ± 0.17	3.63 ± 0.03	3.46 ± 0.06
TC13000329.hg.1	GPC6	5.78 ± 0.24	5.65 ± 0.09	5.67 ± 0.06	5.71 ± 0.07
TC13000789.hg.1	GPC6-AS1	3.87 ± 0.17	3.66 ± 0.03	3.71 ± 0.05	3.74 ± 0.06
TC13000787.hg.1	GPC6-AS2	4.56 ± 0.10	4.24 ± 0.08	4.33 ± 0.08	4.34 ± 0.09
TC20000599.hg.1	GPCPD1	9.39 ± 0.52	9.96 ± 0.16	10.00 ± 0.51	10.09 ± 0.13
TC12000388.hg.1	GPD1	7.31 ± 0.19	7.27 ± 0.09	7.26 ± 0.11	7.34 ± 0.07
TC03000157.hg.1	GPD1L	7.98 ± 0.07	7.96 ± 0.02	7.93 ± 0.11	7.92 ± 0.04
TC02000952.hg.1	GPD2	7.86 ± 0.20	7.62 ± 0.22	8.03 ± 0.30	7.39 ± 0.08
TC07000018.hg.1	GPER	7.61 ± 0.26	7.44 ± 0.20	7.49 ± 0.17	7.50 ± 0.05
TC11001919.hg.1	GPHA2	7.17 ± 0.13	7.31 ± 0.24	7.20 ± 0.16	7.44 ± 0.08
TC14001205.hg.1	GPHB5	7.71 ± 0.17	7.81 ± 0.07	7.65 ± 0.15	7.88 ± 0.01
TC14000401.hg.1	GPHN	6.98 ± 0.05	6.89 ± 0.12	6.78 ± 0.01	6.85 ± 0.05
TC19000442.hg.1	GPI	9.86 ± 0.24	9.47 ± 0.16	9.61 ± 0.32	9.32 ± 0.10
TC08000816.hg.1	GPIHBP1	8.47 ± 0.20	8.42 ± 0.14	8.41 ± 0.22	8.50 ± 0.07

TC0X001027.hg.1	GPKOW	8.29 ± 0.20	8.10 ± 0.02	8.12 ± 0.18	8.07 ± 0.06
TC06001325.hg.1	GPLD1	6.44 ± 0.13	6.36 ± 0.09	6.36 ± 0.06	6.41 ± 0.04
TC04001760.hg.1	GPM6A	5.14 ± 0.07	5.08 ± 0.04	5.04 ± 0.04	5.06 ± 0.04
TC0X000868.hg.1	GPM6B	6.05 ± 0.11	6.02 ± 0.10	6.00 ± 0.02	6.08 ± 0.04
TC02004935.hg.1	GPN1	8.08 ± 0.11	8.11 ± 0.23	7.88 ± 0.07	7.96 ± 0.03
TC01002395.hg.1	GPN2	8.24 ± 0.05	8.07 ± 0.03	7.96 ± 0.10	8.09 ± 0.03
TC12001962.hg.1	GPN3	7.56 ± 0.10	7.46 ± 0.08	7.55 ± 0.05	7.36 ± 0.13
TC07000144.hg.1	GPNMB	6.03 ± 0.06	5.89 ± 0.04	5.91 ± 0.09	5.97 ± 0.06
TC02002698.hg.1	GPR1	4.85 ± 0.09	4.71 ± 0.01	4.80 ± 0.06	4.75 ± 0.05
TC0X001421.hg.1	GPR101	7.28 ± 0.31	7.32 ± 0.28	7.27 ± 0.14	7.38 ± 0.05
TC09000728.hg.1	GPR107	7.85 ± 0.14	7.65 ± 0.19	7.86 ± 0.17	7.60 ± 0.11
TC19001104.hg.1	GPR108	9.66 ± 0.31	9.39 ± 0.05	9.70 ± 0.27	9.27 ± 0.16
TC06001776.hg.1	GPR110	4.85 ± 0.07	4.78 ± 0.09	4.84 ± 0.02	4.87 ± 0.07
TC06004091.hg.1	GPR111	5.93 ± 0.22	5.87 ± 0.19	5.91 ± 0.05	6.05 ± 0.09
TC0X000659.hg.1	GPR112	5.03 ± 0.15	4.94 ± 0.08	4.95 ± 0.01	5.00 ± 0.06
TC02001666.hg.1	GPR113	7.23 ± 0.18	7.22 ± 0.21	7.18 ± 0.13	7.32 ± 0.07
TC16000492.hg.1	GPR114	8.83 ± 0.13	8.62 ± 0.05	8.72 ± 0.04	8.58 ± 0.08
TC06004090.hg.1	GPR115	5.25 ± 0.08	5.24 ± 0.08	5.22 ± 0.07	5.32 ± 0.10
TC06001775.hg.1	GPR116	5.54 ± 0.12	5.45 ± 0.10	5.45 ± 0.08	5.55 ± 0.10
TC0X001361.hg.1	GPR119	6.96 ± 0.14	6.88 ± 0.05	6.86 ± 0.30	7.06 ± 0.17
TC13000504.hg.1	GPR12	6.59 ± 0.11	6.53 ± 0.10	6.54 ± 0.09	6.53 ± 0.09
TC10000948.hg.1	GPR123	7.88 ± 0.19	7.83 ± 0.22	7.81 ± 0.18	7.91 ± 0.04
TC08000279.hg.1	GPR124	7.98 ± 0.15	7.91 ± 0.14	7.88 ± 0.18	8.01 ± 0.03
TC04001079.hg.1	GPR125	5.93 ± 0.03	5.91 ± 0.05	5.78 ± 0.03	5.91 ± 0.06
TC06001046.hg.1	GPR126	5.36 ± 0.11	5.27 ± 0.07	5.30 ± 0.06	5.35 ± 0.05
TC03000510.hg.1	GPR128	4.58 ± 0.10	4.47 ± 0.05	4.47 ± 0.01	4.48 ± 0.07
TC14001564.hg.1	GPR132	8.94 ± 0.19	8.75 ± 0.05	8.92 ± 0.06	8.86 ± 0.08
TC12001040.hg.1	GPR133	7.29 ± 0.07	7.24 ± 0.11	7.17 ± 0.13	7.25 ± 0.02
TC14001181.hg.1	GPR135	5.54 ± 0.27	5.67 ± 0.12	5.57 ± 0.26	5.54 ± 0.15
TC14002324.hg.1	GPR135	6.62 ± 0.03	6.58 ± 0.04	6.55 ± 0.06	6.58 ± 0.05
TC11000593.hg.1	GPR137	8.57 ± 0.19	8.54 ± 0.16	8.55 ± 0.17	8.60 ± 0.05

TC01001950.hg.1	GPR137B	7.73 ± 0.09	7.49 ± 0.17	7.82 ± 0.08	7.48 ± 0.07
TC14000307.hg.1	GPR137C	5.10 ± 0.03	5.07 ± 0.14	5.06 ± 0.03	5.10 ± 0.08
TC16000922.hg.1	GPR139	6.01 ± 0.16	5.95 ± 0.14	5.93 ± 0.14	6.09 ± 0.07
TC07003321.hg.1	GPR141	7.12 ± 0.34	6.59 ± 0.25	7.89 ± 0.46	6.45 ± 0.18
TC17000829.hg.1	GPR142	8.01 ± 0.18	7.93 ± 0.19	7.87 ± 0.22	7.99 ± 0.07
TC0X000851.hg.1	GPR143	6.98 ± 0.17	6.95 ± 0.14	6.91 ± 0.12	7.05 ± 0.05
TC09000638.hg.1	GPR144	7.91 ± 0.21	7.88 ± 0.25	7.82 ± 0.21	8.03 ± 0.10
TC07000015.hg.1	GPR146	8.23 ± 0.14	8.24 ± 0.19	8.19 ± 0.17	8.29 ± 0.06
TC02000826.hg.1	GPR148	5.44 ± 0.16	5.45 ± 0.11	5.42 ± 0.08	5.57 ± 0.11
TC03001920.hg.1	GPR149	5.13 ± 0.13	5.07 ± 0.06	5.02 ± 0.15	5.22 ± 0.07
TC03000494.hg.1	GPR15	6.92 ± 0.22	6.94 ± 0.16	6.73 ± 0.22	6.84 ± 0.08
TC05000459.hg.1	GPR150	7.02 ± 0.32	7.02 ± 0.26	6.86 ± 0.17	7.04 ± 0.06
TC05001904.hg.1	GPR151	4.97 ± 0.20	4.88 ± 0.06	4.92 ± 0.25	4.89 ± 0.08
TC11001984.hg.1	GPR152	7.80 ± 0.18	7.81 ± 0.30	7.66 ± 0.30	7.85 ± 0.10
TC01002148.hg.1	GPR153	8.19 ± 0.17	8.10 ± 0.11	8.11 ± 0.12	8.18 ± 0.05
TC02002539.hg.1	GPR155	7.86 ± 0.38	7.83 ± 0.25	8.16 ± 0.46	7.69 ± 0.08
TC03001689.hg.1	GPR156	6.34 ± 0.18	6.36 ± 0.20	6.28 ± 0.19	6.51 ± 0.11
TC01002180.hg.1	GPR157	8.46 ± 0.17	8.48 ± 0.17	8.41 ± 0.17	8.54 ± 0.02
TC10000177.hg.1	GPR158	5.21 ± 0.09	5.15 ± 0.13	5.10 ± 0.09	5.20 ± 0.13
TC10001112.hg.1	GPR158-AS1	5.81 ± 0.29	5.84 ± 0.29	5.78 ± 0.14	5.93 ± 0.09
TC03000910.hg.1	GPR160	7.72 ± 0.12	7.59 ± 0.09	7.94 ± 0.19	7.63 ± 0.18
TC01003485.hg.1	GPR161	7.30 ± 0.12	7.28 ± 0.13	7.29 ± 0.15	7.39 ± 0.06
TC12003231.hg.1	GPR162	8.78 ± 0.17	8.79 ± 0.09	8.75 ± 0.10	8.83 ± 0.04
TC02000787.hg.1	GPR17	7.16 ± 0.12	7.11 ± 0.21	7.05 ± 0.08	7.22 ± 0.07
TC03001903.hg.1	GPR171	7.97 ± 0.50	7.76 ± 0.27	8.08 ± 0.33	7.23 ± 0.26
TC0X000309.hg.1	GPR173	7.18 ± 0.18	7.10 ± 0.11	7.11 ± 0.06	7.19 ± 0.13
TC0X000428.hg.1	GPR174	8.88 ± 0.77	9.43 ± 0.31	8.72 ± 0.49	9.25 ± 0.26
TC15001225.hg.1	GPR176	6.19 ± 0.15	6.20 ± 0.11	6.15 ± 0.13	6.30 ± 0.06
TC17001424.hg.1	GPR179	7.84 ± 0.14	7.89 ± 0.13	7.85 ± 0.09	7.97 ± 0.06
TC13000828.hg.1	GPR18	8.21 ± 0.30	8.85 ± 0.10	7.94 ± 0.26	8.59 ± 0.20
TC13000330.hg.1	GPR180	6.95 ± 0.41	7.02 ± 0.17	6.95 ± 0.37	6.66 ± 0.06

TC12000519.hg.1	GPR182	7.10 ± 0.24	7.07 ± 0.09	7.01 ± 0.20	7.15 ± 0.13
TC13000829.hg.1	GPR183	9.07 ± 0.13	9.74 ± 0.25	8.61 ± 0.58	9.56 ± 0.18
TC12001256.hg.1	GPR19	4.95 ± 0.09	4.75 ± 0.00	4.83 ± 0.01	4.81 ± 0.08
TC08001694.hg.1	GPR20	8.48 ± 0.21	8.56 ± 0.19	8.50 ± 0.27	8.70 ± 0.07
TC09000627.hg.1	GPR21	7.73 ± 0.28	7.68 ± 0.20	7.73 ± 0.25	7.65 ± 0.13
TC07000691.hg.1	GPR22	4.32 ± 0.10	4.16 ± 0.03	4.25 ± 0.03	4.14 ± 0.09
TC01001652.hg.1	GPR25	10.44 ± 0.11	10.36 ± 0.07	10.35 ± 0.23	10.32 ± 0.03
TC10000903.hg.1	GPR26	7.19 ± 0.19	7.19 ± 0.25	7.17 ± 0.29	7.36 ± 0.12
TC03000423.hg.1	GPR27	9.18 ± 0.47	8.63 ± 0.09	9.43 ± 0.58	8.47 ± 0.21
TC01000362.hg.1	GPR3	7.45 ± 0.25	7.47 ± 0.30	7.31 ± 0.16	7.55 ± 0.08
TC06002313.hg.1	GPR31	5.91 ± 0.06	5.71 ± 0.11	5.77 ± 0.11	5.81 ± 0.08
TC19000766.hg.1	GPR32	6.79 ± 0.17	6.82 ± 0.03	6.58 ± 0.28	6.83 ± 0.10
TC14001012.hg.1	GPR33	5.15 ± 0.37	4.85 ± 0.36	5.04 ± 0.12	5.05 ± 0.09
TC0X000202.hg.1	GPR34	5.22 ± 0.21	4.86 ± 0.12	5.29 ± 0.14	4.82 ± 0.12
TC02005016.hg.1	GPR35	7.93 ± 0.13	7.97 ± 0.15	7.85 ± 0.10	8.01 ± 0.06
TC07001828.hg.1	GPR37	4.95 ± 0.13	4.78 ± 0.05	4.73 ± 0.08	4.87 ± 0.06
TC01001671.hg.1	GPR37L1	8.73 ± 0.18	8.68 ± 0.18	8.78 ± 0.15	8.81 ± 0.07
TC02000865.hg.1	GPR39	5.62 ± 0.19	5.53 ± 0.06	5.55 ± 0.07	5.55 ± 0.04
TC19002713.hg.1	GPR4	7.12 ± 0.15	7.02 ± 0.21	6.99 ± 0.08	7.12 ± 0.03
TC19000468.hg.1	GPR42	7.74 ± 0.67	7.19 ± 0.21	8.01 ± 0.39	7.42 ± 0.20
TC02000644.hg.1	GPR45	6.82 ± 0.16	6.90 ± 0.23	6.92 ± 0.19	7.07 ± 0.18
TC0X000729.hg.1	GPR50	6.79 ± 0.20	6.72 ± 0.07	6.69 ± 0.06	6.80 ± 0.04
TC0X001489.hg.1	GPR50-AS1	6.56 ± 0.22	6.47 ± 0.03	6.49 ± 0.26	6.52 ± 0.04
TC01001523.hg.1	GPR52	7.11 ± 0.45	7.26 ± 0.34	7.19 ± 0.33	7.08 ± 0.31
TC02002856.hg.1	GPR55	6.98 ± 0.11	6.90 ± 0.12	6.89 ± 0.05	6.92 ± 0.02
TC16000493.hg.1	GPR56	9.32 ± 0.18	9.18 ± 0.06	9.04 ± 0.10	9.11 ± 0.02
TC06000881.hg.1	GPR6	7.99 ± 0.25	7.92 ± 0.27	7.97 ± 0.21	8.08 ± 0.13
TC01000946.hg.1	GPR61	6.90 ± 0.20	6.90 ± 0.15	6.95 ± 0.13	6.98 ± 0.02
TC03000327.hg.1	GPR62	8.35 ± 0.24	8.51 ± 0.18	8.39 ± 0.24	8.63 ± 0.06
TC06001955.hg.1	GPR63	6.10 ± 0.14	6.12 ± 0.05	6.04 ± 0.05	6.21 ± 0.14
TC0X000901.hg.1	GPR64	5.31 ± 0.15	5.21 ± 0.07	5.25 ± 0.06	5.25 ± 0.02

TC14000523.hg.1	GPR65	9.85 ± 0.20	9.94 ± 0.29	10.43 ± 0.53	9.83 ± 0.13
TC14001435.hg.1	GPR68	7.98 ± 0.19	7.99 ± 0.19	7.94 ± 0.18	8.08 ± 0.11
TC02005025.hg.1	GPR75	7.48 ± 0.08	7.43 ± 0.07	7.37 ± 0.15	7.41 ± 0.02
TC04002932.hg.1	GPR78	8.06 ± 0.17	7.94 ± 0.12	7.92 ± 0.19	8.04 ± 0.11
TC0X000203.hg.1	GPR82	5.07 ± 0.09	4.90 ± 0.12	5.19 ± 0.02	4.84 ± 0.10
TC11002198.hg.1	GPR83	6.83 ± 0.18	6.81 ± 0.07	6.74 ± 0.13	6.88 ± 0.17
TC12001569.hg.1	GPR84	6.87 ± 0.92	6.11 ± 0.23	8.05 ± 0.66	5.99 ± 0.28
TC07001767.hg.1	GPR85	4.86 ± 0.15	4.85 ± 0.10	4.87 ± 0.08	5.07 ± 0.18
TC03001905.hg.1	GPR87	4.81 ± 0.24	4.73 ± 0.07	4.80 ± 0.06	4.77 ± 0.09
TC01000903.hg.1	GPR88	8.22 ± 0.20	8.31 ± 0.21	8.11 ± 0.19	8.46 ± 0.14
TC01003114.hg.1	GPR89A	8.56 ± 0.40	8.65 ± 0.19	8.31 ± 0.30	8.50 ± 0.04
TC01001101.hg.1	GPR89C	8.39 ± 0.34	8.34 ± 0.20	8.12 ± 0.33	8.12 ± 0.02
TC01001118.hg.1	GPR89C	8.54 ± 0.35	8.60 ± 0.20	8.27 ± 0.28	8.44 ± 0.03
TC16000494.hg.1	GPR97	10.05 ± 0.63	9.80 ± 0.12	10.79 ± 0.35	9.96 ± 0.36
TC05000442.hg.1	GPR98	4.78 ± 0.10	4.68 ± 0.05	4.71 ± 0.07	4.72 ± 0.04
TC0X000489.hg.1	GPRASP1	7.28 ± 0.04	7.37 ± 0.05	7.04 ± 0.13	7.48 ± 0.07
TC0X002318.hg.1	GPRASP2	6.96 ± 0.19	6.93 ± 0.08	6.83 ± 0.10	6.95 ± 0.05
TC16000921.hg.1	GPRC5B	7.28 ± 0.25	7.25 ± 0.16	7.20 ± 0.20	7.31 ± 0.08
TC17000830.hg.1	GPRC5C	7.53 ± 0.28	7.53 ± 0.24	7.48 ± 0.22	7.63 ± 0.04
TC12001258.hg.1	GPRC5D	7.53 ± 0.42	6.89 ± 0.19	6.87 ± 0.06	6.80 ± 0.19
TC06002053.hg.1	GPRC6A	4.89 ± 0.13	4.74 ± 0.05	4.82 ± 0.02	4.81 ± 0.10
TC05002095.hg.1	GPRIN1	8.77 ± 0.25	8.68 ± 0.21	8.75 ± 0.26	8.75 ± 0.06
TC10000300.hg.1	GPRIN2	7.40 ± 0.15	7.43 ± 0.27	7.34 ± 0.21	7.53 ± 0.15
TC10000301.hg.1	GPRIN2	6.72 ± 0.27	6.80 ± 0.15	6.57 ± 0.03	6.87 ± 0.09
TC04001382.hg.1	GPRIN3	8.23 ± 0.30	8.38 ± 0.18	8.33 ± 0.33	8.26 ± 0.05
TC17000945.hg.1	GPS1	9.54 ± 0.17	9.38 ± 0.09	9.30 ± 0.12	9.34 ± 0.02
TC09000811.hg.1	GPSM1	8.60 ± 0.27	8.51 ± 0.17	8.47 ± 0.26	8.54 ± 0.01
TC01000936.hg.1	GPSM2	6.38 ± 0.05	6.12 ± 0.16	6.43 ± 0.16	5.99 ± 0.15
TC06001563.hg.1	GPSM3	8.43 ± 0.18	8.37 ± 0.08	8.46 ± 0.14	8.35 ± 0.09
TC6_apd_hap1000097.hg.1	GPSM3	8.43 ± 0.18	8.37 ± 0.08	8.46 ± 0.14	8.35 ± 0.09
TC6_cox_hap2000189.hg.1	GPSM3	8.44 ± 0.18	8.37 ± 0.08	8.46 ± 0.14	8.35 ± 0.08

TC6_mann_hap4000154.hg.1	GPSM3	8.43 ± 0.18	8.37 ± 0.08	8.46 ± 0.14	8.35 ± 0.09
TC6_mcf_hap5000164.hg.1	GPSM3	8.43 ± 0.18	8.37 ± 0.08	8.46 ± 0.14	8.35 ± 0.09
TC6_qbl_hap6000178.hg.1	GPSM3	8.43 ± 0.18	8.37 ± 0.08	8.46 ± 0.14	8.35 ± 0.09
TC6_ssto_hap7000158.hg.1	GPSM3	8.43 ± 0.18	8.37 ± 0.08	8.46 ± 0.14	8.35 ± 0.09
TC08000856.hg.1	GPT	8.58 ± 0.23	8.59 ± 0.22	8.50 ± 0.23	8.65 ± 0.03
TC16000426.hg.1	GPT2	7.80 ± 0.19	7.73 ± 0.09	7.66 ± 0.23	7.75 ± 0.01
TC03001407.hg.1	GPX1	9.48 ± 0.17	9.27 ± 0.06	9.39 ± 0.26	9.16 ± 0.13
TC14001223.hg.1	GPX2	7.30 ± 0.04	7.30 ± 0.06	7.33 ± 0.07	7.43 ± 0.03
TC05000837.hg.1	GPX3	7.08 ± 0.16	7.15 ± 0.07	6.99 ± 0.09	7.24 ± 0.02
TC05000838.hg.1	GPX3	6.81 ± 0.28	7.11 ± 0.14	6.74 ± 0.25	7.18 ± 0.15
TC19000030.hg.1	GPX4	9.72 ± 0.17	9.50 ± 0.09	9.52 ± 0.23	9.47 ± 0.14
TC06000286.hg.1	GPX5	5.46 ± 0.13	5.32 ± 0.03	5.33 ± 0.04	5.34 ± 0.12
TC6_cox_hap2000001.hg.1	GPX5	6.50 ± 0.13	6.38 ± 0.03	6.44 ± 0.07	6.39 ± 0.10
TC06001444.hg.1	GPX6	5.69 ± 0.18	5.49 ± 0.12	5.54 ± 0.13	5.64 ± 0.10
TC01000643.hg.1	GPX7	8.15 ± 0.10	8.08 ± 0.13	7.98 ± 0.06	8.17 ± 0.03
TC05000232.hg.1	GPX8	4.38 ± 0.12	4.32 ± 0.05	4.35 ± 0.08	4.34 ± 0.03
TC19000454.hg.1	GRAMD1A	9.73 ± 0.42	9.47 ± 0.04	9.91 ± 0.29	9.46 ± 0.09
TC11001124.hg.1	GRAMD1B	7.81 ± 0.20	7.51 ± 0.07	7.80 ± 0.29	7.42 ± 0.08
TC03000584.hg.1	GRAMD1C	5.91 ± 0.11	6.18 ± 0.29	5.87 ± 0.18	6.11 ± 0.16
TC15001621.hg.1	GRAMD2	6.59 ± 0.16	6.53 ± 0.16	6.50 ± 0.18	6.61 ± 0.08
TC05000601.hg.1	GRAMD3	6.38 ± 0.11	6.38 ± 0.07	6.25 ± 0.01	6.34 ± 0.03
TC22000397.hg.1	GRAMD4	8.46 ± 0.12	8.23 ± 0.05	8.29 ± 0.12	8.23 ± 0.02
TC17001230.hg.1	GRAP	8.42 ± 0.14	8.24 ± 0.03	8.07 ± 0.18	8.23 ± 0.04
TC22000319.hg.1	GRAP2	9.58 ± 0.17	9.25 ± 0.11	9.41 ± 0.03	9.27 ± 0.08
TC17000229.hg.1	GRAPL	8.53 ± 0.07	8.39 ± 0.05	8.16 ± 0.11	8.35 ± 0.07
TC12000413.hg.1	GRASP	8.29 ± 0.18	8.77 ± 0.21	8.02 ± 0.21	8.89 ± 0.13
TC07001374.hg.1	GRB10	7.18 ± 0.19	6.93 ± 0.09	7.31 ± 0.11	7.04 ± 0.09
TC02002486.hg.1	GRB14	5.83 ± 0.04	5.69 ± 0.08	5.81 ± 0.15	5.82 ± 0.07
TC17001877.hg.1	GRB2	10.10 ± 0.14	9.88 ± 0.11	10.29 ± 0.24	9.81 ± 0.22
TC17000481.hg.1	GRB7	7.64 ± 0.23	7.60 ± 0.24	7.58 ± 0.16	7.71 ± 0.04
TC02000074.hg.1	GREB1	6.88 ± 0.16	6.81 ± 0.19	6.78 ± 0.18	6.90 ± 0.04

TC18000087.hg.1	GREB1L	5.66 ± 0.18	5.60 ± 0.12	5.61 ± 0.09	5.69 ± 0.03
TC15000226.hg.1	GREM1	7.56 ± 0.17	7.48 ± 0.08	7.40 ± 0.15	7.59 ± 0.04
TC01004021.hg.1	GREM2	7.05 ± 0.20	7.02 ± 0.12	6.88 ± 0.14	7.04 ± 0.05
TC02000054.hg.1	GRHL1	6.32 ± 0.10	6.27 ± 0.07	6.28 ± 0.10	6.31 ± 0.02
TC08000629.hg.1	GRHL2	5.76 ± 0.18	5.72 ± 0.13	5.68 ± 0.14	5.80 ± 0.03
TC01000309.hg.1	GRHL3	6.56 ± 0.18	6.49 ± 0.13	6.52 ± 0.11	6.52 ± 0.05
TC09000214.hg.1	GRHPR	8.19 ± 0.10	8.13 ± 0.09	7.98 ± 0.09	8.13 ± 0.01
TC05000849.hg.1	GRIA1	6.17 ± 0.20	6.10 ± 0.15	6.09 ± 0.12	6.19 ± 0.11
TC04000799.hg.1	GRIA2	5.23 ± 0.17	5.11 ± 0.07	5.12 ± 0.08	5.18 ± 0.11
TC0X000601.hg.1	GRIA3	5.86 ± 0.20	5.72 ± 0.07	5.77 ± 0.08	5.79 ± 0.08
TC11000968.hg.1	GRIA4	5.38 ± 0.11	5.31 ± 0.07	5.36 ± 0.10	5.36 ± 0.09
TC10001475.hg.1	GRID1	7.32 ± 0.15	7.30 ± 0.15	7.32 ± 0.15	7.40 ± 0.07
TC10000603.hg.1	GRID1-AS1	6.12 ± 0.33	5.85 ± 0.10	5.97 ± 0.15	5.94 ± 0.03
TC04000497.hg.1	GRID2	5.32 ± 0.13	5.19 ± 0.07	5.23 ± 0.07	5.25 ± 0.06
TC07001132.hg.1	GRID2IP	7.82 ± 0.16	7.82 ± 0.18	7.80 ± 0.20	7.94 ± 0.06
TC07001097.hg.1	GRIFIN	8.19 ± 0.19	8.19 ± 0.17	8.08 ± 0.22	8.26 ± 0.14
TC21000360.hg.1	GRIK1	5.60 ± 0.15	5.51 ± 0.09	5.57 ± 0.10	5.54 ± 0.06
TC21000100.hg.1	GRIK1-AS1	4.91 ± 0.12	4.83 ± 0.02	4.89 ± 0.08	4.83 ± 0.04
TC21000097.hg.1	GRIK1-AS2	8.60 ± 0.24	8.78 ± 0.16	9.10 ± 0.25	8.81 ± 0.11
TC06000836.hg.1	GRIK2	4.94 ± 0.11	4.87 ± 0.04	4.91 ± 0.06	4.94 ± 0.10
TC01002513.hg.1	GRIK3	6.89 ± 0.13	6.82 ± 0.17	6.83 ± 0.12	6.92 ± 0.04
TC11001103.hg.1	GRIK4	7.10 ± 0.17	7.04 ± 0.16	7.06 ± 0.17	7.11 ± 0.07
TC19001564.hg.1	GRIK5	7.69 ± 0.17	7.68 ± 0.17	7.61 ± 0.18	7.75 ± 0.06
TC09002887.hg.1	GRIN1	7.95 ± 0.25	7.94 ± 0.24	7.90 ± 0.25	8.01 ± 0.03
TC16000854.hg.1	GRIN2A	6.27 ± 0.18	6.12 ± 0.09	6.15 ± 0.09	6.18 ± 0.03
TC12001265.hg.1	GRIN2B	6.13 ± 0.17	6.01 ± 0.09	6.05 ± 0.10	6.15 ± 0.08
TC17001863.hg.1	GRIN2C	7.83 ± 0.24	7.80 ± 0.18	7.74 ± 0.19	7.88 ± 0.01
TC19000702.hg.1	GRIN2D	7.74 ± 0.26	7.61 ± 0.20	7.69 ± 0.21	7.71 ± 0.02
TC09001423.hg.1	GRIN3A	5.42 ± 0.24	5.26 ± 0.16	5.34 ± 0.09	5.34 ± 0.06
TC19000025.hg.1	GRIN3B	7.77 ± 0.29	7.70 ± 0.24	7.72 ± 0.23	7.80 ± 0.04
TC08000835.hg.1	GRINA	9.72 ± 0.55	9.23 ± 0.03	10.11 ± 0.31	9.37 ± 0.35

TC12001689.hg.1	GRIP1	6.25 ± 0.11	6.21 ± 0.13	6.19 ± 0.07	6.31 ± 0.02
TC03001197.hg.1	GRIP2	7.64 ± 0.23	7.63 ± 0.21	7.57 ± 0.23	7.74 ± 0.04
TC0X001024.hg.1	GRIPAP1	8.78 ± 0.11	8.61 ± 0.04	8.82 ± 0.13	8.58 ± 0.05
TC13000427.hg.1	GRK1	7.55 ± 0.27	7.54 ± 0.21	7.48 ± 0.28	7.62 ± 0.08
TC04000041.hg.1	GRK4	6.48 ± 0.10	6.43 ± 0.12	6.39 ± 0.10	6.49 ± 0.04
TC04000042.hg.1	GRK4	4.32 ± 0.11	4.20 ± 0.15	4.28 ± 0.07	4.16 ± 0.06
TC10000865.hg.1	GRK5	8.62 ± 0.01	8.56 ± 0.07	8.55 ± 0.09	8.65 ± 0.27
TC05001015.hg.1	GRK6	10.32 ± 0.17	10.14 ± 0.05	10.35 ± 0.13	10.09 ± 0.10
TC03000765.hg.1	GRK7	6.84 ± 0.21	6.83 ± 0.13	6.73 ± 0.22	6.88 ± 0.04
TC06001063.hg.1	GRM1	6.83 ± 0.13	6.81 ± 0.14	6.80 ± 0.16	6.88 ± 0.11
TC03000322.hg.1	GRM2	7.29 ± 0.17	7.30 ± 0.15	7.23 ± 0.08	7.39 ± 0.05
TC07000519.hg.1	GRM3	6.06 ± 0.22	6.04 ± 0.18	6.02 ± 0.19	6.16 ± 0.12
TC06001650.hg.1	GRM4	7.66 ± 0.19	7.60 ± 0.17	7.54 ± 0.14	7.65 ± 0.05
TC11002176.hg.1	GRM5	5.78 ± 0.15	5.67 ± 0.06	5.69 ± 0.18	5.77 ± 0.09
TC11000885.hg.1	GRM5-AS1	5.43 ± 0.09	5.31 ± 0.12	5.41 ± 0.07	5.48 ± 0.09
TC05002129.hg.1	GRM6	7.73 ± 0.16	7.71 ± 0.14	7.67 ± 0.17	7.81 ± 0.09
TC03000024.hg.1	GRM7	6.11 ± 0.12	6.04 ± 0.08	6.05 ± 0.06	6.12 ± 0.10
TC03001139.hg.1	GRM7-AS1	6.91 ± 0.21	6.89 ± 0.04	7.00 ± 0.03	6.98 ± 0.06
TC03001138.hg.1	GRM7-AS2	5.00 ± 0.36	4.76 ± 0.07	4.82 ± 0.11	4.84 ± 0.08
TC03001137.hg.1	GRM7-AS3	4.35 ± 0.13	4.17 ± 0.05	4.21 ± 0.04	4.25 ± 0.09
TC07001833.hg.1	GRM8	5.58 ± 0.12	5.45 ± 0.05	5.48 ± 0.07	5.52 ± 0.04
TC17000570.hg.1	GRN	11.77 ± 0.41	11.09 ± 0.09	11.69 ± 0.23	10.96 ± 0.36
TC18000211.hg.1	GRP	7.39 ± 0.20	7.28 ± 0.22	7.21 ± 0.17	7.33 ± 0.04
TC04001007.hg.1	GRPEL1	8.04 ± 0.12	8.37 ± 0.15	8.08 ± 0.12	8.35 ± 0.02
TC05000818.hg.1	GRPEL2	7.62 ± 0.22	7.69 ± 0.15	7.42 ± 0.16	7.65 ± 0.06
TC0X000073.hg.1	GRPR	5.82 ± 0.18	5.92 ± 0.11	5.86 ± 0.05	5.90 ± 0.07
TC04001272.hg.1	GRSF1	8.28 ± 0.09	8.27 ± 0.10	8.05 ± 0.09	8.15 ± 0.10
TC13000893.hg.1	GRTP1	7.42 ± 0.20	7.35 ± 0.16	7.36 ± 0.22	7.39 ± 0.01
TC13000422.hg.1	GRTP1-AS1	6.65 ± 0.22	6.39 ± 0.09	6.53 ± 0.14	6.54 ± 0.09
TC19002645.hg.1	GRWD1	8.89 ± 0.22	8.55 ± 0.06	8.54 ± 0.14	8.50 ± 0.06
TC04000263.hg.1	GRXCR1	5.44 ± 0.02	5.43 ± 0.03	5.27 ± 0.23	5.40 ± 0.03

TC05001897.hg.1	GRXCR2	5.97 ± 0.05	5.89 ± 0.07	5.98 ± 0.05	6.00 ± 0.10
TC07001548.hg.1	GSAP	8.10 ± 0.45	8.18 ± 0.17	8.26 ± 0.38	8.01 ± 0.02
TC14001470.hg.1	GSC	7.93 ± 0.30	7.98 ± 0.10	7.77 ± 0.34	7.91 ± 0.04
TC22000493.hg.1	GSC2	7.90 ± 0.33	8.04 ± 0.20	7.82 ± 0.40	8.12 ± 0.05
TC17000484.hg.1	GSDMA	7.21 ± 0.16	7.14 ± 0.17	7.13 ± 0.16	7.17 ± 0.03
TC17002851.hg.1	GSDMB	8.08 ± 0.14	7.94 ± 0.15	7.88 ± 0.14	7.80 ± 0.10
TC08001637.hg.1	GSDMC	5.81 ± 0.15	5.69 ± 0.10	5.67 ± 0.03	5.76 ± 0.04
TC08000824.hg.1	GSDMD	8.60 ± 0.14	8.42 ± 0.08	8.56 ± 0.14	8.44 ± 0.02
TC16000664.hg.1	GSE1	8.90 ± 0.05	8.87 ± 0.02	8.81 ± 0.05	8.89 ± 0.00
TC12001261.hg.1	GSG1	6.91 ± 0.18	6.91 ± 0.13	6.91 ± 0.12	7.00 ± 0.07
TC16000981.hg.1	GSG1L	8.47 ± 0.25	8.42 ± 0.16	8.40 ± 0.16	8.46 ± 0.07
TC17000036.hg.1	GSG2	7.56 ± 0.20	7.49 ± 0.05	7.52 ± 0.14	7.54 ± 0.01
TC19001569.hg.1	GSK3A	9.54 ± 0.23	9.24 ± 0.06	9.48 ± 0.11	9.12 ± 0.25
TC03001688.hg.1	GSK3B	9.45 ± 0.24	9.49 ± 0.20	9.57 ± 0.18	9.35 ± 0.10
TC14000602.hg.1	GSKIP	7.32 ± 0.07	7.24 ± 0.11	7.49 ± 0.46	7.18 ± 0.06
TC09000608.hg.1	GSN	8.46 ± 0.19	8.20 ± 0.08	8.50 ± 0.22	8.22 ± 0.02
TC09001545.hg.1	GSN-AS1	6.81 ± 0.09	6.72 ± 0.13	6.72 ± 0.17	6.88 ± 0.11
TC16000875.hg.1	GSPT1	9.70 ± 0.15	9.75 ± 0.11	9.40 ± 0.08	9.67 ± 0.22
TC0X000295.hg.1	GSPT2	7.38 ± 0.22	7.51 ± 0.08	7.26 ± 0.15	7.49 ± 0.07
TC08001110.hg.1	GSR	9.44 ± 0.11	9.26 ± 0.08	9.56 ± 0.24	9.07 ± 0.14
TC20000789.hg.1	GSS	7.98 ± 0.13	7.86 ± 0.07	7.78 ± 0.10	7.71 ± 0.03
TC06001803.hg.1	GSTA1	4.76 ± 0.08	4.70 ± 0.07	4.72 ± 0.05	4.84 ± 0.13
TC06001802.hg.1	GSTA2	4.91 ± 0.14	4.76 ± 0.05	4.79 ± 0.04	4.86 ± 0.05
TC06001805.hg.1	GSTA3	5.29 ± 0.18	5.26 ± 0.10	5.31 ± 0.05	5.33 ± 0.06
TC06001807.hg.1	GSTA4	6.26 ± 0.13	6.16 ± 0.09	6.19 ± 0.11	6.22 ± 0.03
TC06001804.hg.1	GSTA5	5.45 ± 0.15	5.30 ± 0.07	5.29 ± 0.10	5.38 ± 0.12
TC06001801.hg.1	GSTA7P	4.73 ± 0.14	4.70 ± 0.11	4.65 ± 0.05	4.82 ± 0.23
TC04000547.hg.1	GSTCD	6.15 ± 0.06	6.10 ± 0.07	5.95 ± 0.10	5.99 ± 0.05
TC07000930.hg.1	GSTK1	9.28 ± 0.08	9.15 ± 0.11	9.26 ± 0.19	8.98 ± 0.06
TC01006400.hg.1	GSTM1	8.27 ± 0.24	7.96 ± 0.11	8.02 ± 0.16	8.05 ± 0.16
TC01006286.hg.1	GSTM2	8.05 ± 0.16	7.96 ± 0.04	7.98 ± 0.03	8.15 ± 0.23

TC06002014.hg.1	GSTM2P1	7.10 ± 0.13	7.00 ± 0.18	7.02 ± 0.16	7.12 ± 0.14
TC01002962.hg.1	GSTM3	6.62 ± 0.12	6.67 ± 0.08	6.55 ± 0.16	6.65 ± 0.07
TC01006285.hg.1	GSTM4	8.19 ± 0.10	7.99 ± 0.09	8.11 ± 0.05	8.05 ± 0.19
TC01000953.hg.1	GSTM5	7.31 ± 0.03	7.13 ± 0.06	7.28 ± 0.13	7.27 ± 0.15
TC10000781.hg.1	GSTO1	7.79 ± 0.21	7.45 ± 0.01	7.80 ± 0.18	7.49 ± 0.15
TC10000782.hg.1	GSTO2	6.76 ± 0.18	6.71 ± 0.21	6.75 ± 0.09	6.80 ± 0.03
TC11000694.hg.1	GSTP1	9.35 ± 0.21	9.16 ± 0.06	9.06 ± 0.30	9.02 ± 0.03
TC22000583.hg.1	GSTT1	7.46 ± 0.18	7.37 ± 0.05	7.41 ± 0.06	7.43 ± 0.05
TC22000142.hg.1	GSTT2	7.50 ± 0.24	7.46 ± 0.19	7.39 ± 0.18	7.58 ± 0.08
TC22000580.hg.1	GSTT2	7.72 ± 0.23	7.71 ± 0.17	7.64 ± 0.20	7.80 ± 0.06
TC22000581.hg.1	GSTTP1	6.59 ± 0.12	6.49 ± 0.05	6.57 ± 0.02	6.55 ± 0.07
TC22000584.hg.1	GSTTP2	6.81 ± 0.14	6.78 ± 0.20	6.87 ± 0.15	6.98 ± 0.09
TC14000489.hg.1	GSTZ1	7.52 ± 0.22	7.44 ± 0.10	7.42 ± 0.13	7.49 ± 0.05
TC13000080.hg.1	GSX1	8.24 ± 0.30	8.35 ± 0.32	8.16 ± 0.32	8.44 ± 0.03
TC04000310.hg.1	GSX2	7.78 ± 0.15	7.72 ± 0.09	7.74 ± 0.12	7.80 ± 0.06
TC02002398.hg.1	GTDC1	7.28 ± 0.07	7.34 ± 0.15	7.54 ± 0.32	7.19 ± 0.07
TC03001331.hg.1	GTDC2	8.13 ± 0.08	8.12 ± 0.12	8.02 ± 0.07	8.16 ± 0.03
TC14001368.hg.1	GTF2A1	8.58 ± 0.23	8.44 ± 0.19	8.64 ± 0.42	8.38 ± 0.20
TC02000304.hg.1	GTF2A1L	5.86 ± 0.10	5.78 ± 0.05	5.81 ± 0.07	5.82 ± 0.02
TC15001501.hg.1	GTF2A2	7.30 ± 0.09	7.31 ± 0.11	7.30 ± 0.08	7.20 ± 0.09
TC01002843.hg.1	GTF2B	8.34 ± 0.08	8.22 ± 0.20	8.27 ± 0.29	8.15 ± 0.06
TC03000619.hg.1	GTF2E1	7.63 ± 0.15	7.34 ± 0.08	7.68 ± 0.24	7.17 ± 0.18
TC08001109.hg.1	GTF2E2	8.00 ± 0.07	8.03 ± 0.16	7.95 ± 0.13	7.93 ± 0.07
TC19001094.hg.1	GTF2F1	9.18 ± 0.16	9.05 ± 0.02	9.00 ± 0.18	9.02 ± 0.07
TC13000171.hg.1	GTF2F2	7.45 ± 0.32	7.64 ± 0.19	7.59 ± 0.31	7.53 ± 0.09
TC11000239.hg.1	GTF2H1	7.81 ± 0.22	7.66 ± 0.19	7.81 ± 0.21	7.36 ± 0.28
TC05001457.hg.1	GTF2H2	8.43 ± 0.17	8.39 ± 0.19	8.59 ± 0.29	8.33 ± 0.05
TC05000319.hg.1	GTF2H2B	8.74 ± 0.16	8.68 ± 0.21	8.93 ± 0.34	8.60 ± 0.08
TC05000305.hg.1	GTF2H2D	8.25 ± 0.15	8.20 ± 0.18	8.42 ± 0.32	8.14 ± 0.05
TC12000991.hg.1	GTF2H3	7.42 ± 0.25	7.43 ± 0.21	7.23 ± 0.24	7.24 ± 0.04
TC06004070.hg.1	GTF2H4	7.84 ± 0.15	7.71 ± 0.05	7.67 ± 0.12	7.66 ± 0.03

TC6_cox_hap2000240.hg.1	GTF2H4	7.86 ± 0.14	7.72 ± 0.05	7.69 ± 0.11	7.67 ± 0.03
TC6_dbb_hap3000217.hg.1	GTF2H4	7.75 ± 0.15	7.62 ± 0.05	7.59 ± 0.11	7.58 ± 0.03
TC6_mann_hap4000191.hg.1	GTF2H4	7.84 ± 0.15	7.71 ± 0.05	7.67 ± 0.12	7.66 ± 0.03
TC6_mcf_hap5000208.hg.1	GTF2H4	7.84 ± 0.15	7.71 ± 0.05	7.67 ± 0.12	7.66 ± 0.03
TC6_qbl_hap6000220.hg.1	GTF2H4	7.84 ± 0.15	7.71 ± 0.05	7.67 ± 0.12	7.66 ± 0.03
TC06001130.hg.1	GTF2H5	6.77 ± 0.12	6.54 ± 0.08	6.50 ± 0.08	6.40 ± 0.07
TC07000458.hg.1	GTF2I	9.57 ± 0.33	9.64 ± 0.21	9.68 ± 0.19	9.56 ± 0.14
TC07000437.hg.1	GTF2IP1	10.45 ± 0.32	10.57 ± 0.25	10.73 ± 0.21	10.48 ± 0.19
TC13000446.hg.1	GTF2IP3	5.86 ± 0.21	5.84 ± 0.03	5.85 ± 0.11	5.92 ± 0.12
TC07000455.hg.1	GTF2IRD1	7.21 ± 0.14	7.17 ± 0.18	7.18 ± 0.16	7.25 ± 0.03
TC07001466.hg.1	GTF2IRD1P1	7.35 ± 0.21	7.31 ± 0.18	7.29 ± 0.20	7.39 ± 0.05
TC07001514.hg.1	GTF2IRD2	8.03 ± 0.10	7.99 ± 0.15	7.82 ± 0.10	7.96 ± 0.01
TC07000465.hg.1	GTF2IRD2B	8.16 ± 0.09	8.12 ± 0.12	8.00 ± 0.05	8.10 ± 0.02
TC07001493.hg.1	GTF2IRD2P1	7.75 ± 0.11	7.75 ± 0.14	7.52 ± 0.18	7.66 ± 0.07
TC13000077.hg.1	GTF3A	9.08 ± 0.03	9.12 ± 0.19	8.79 ± 0.13	8.91 ± 0.06
TC16000979.hg.1	GTF3C1	8.75 ± 0.15	8.62 ± 0.11	8.51 ± 0.08	8.53 ± 0.04
TC02001682.hg.1	GTF3C2	8.53 ± 0.10	8.27 ± 0.14	8.23 ± 0.06	8.25 ± 0.01
TC02001142.hg.1	GTF3C3	4.95 ± 0.12	4.93 ± 0.06	4.96 ± 0.07	5.05 ± 0.06
TC02002642.hg.1	GTF3C3	7.41 ± 0.32	7.48 ± 0.19	7.31 ± 0.25	7.31 ± 0.01
TC09000760.hg.1	GTF3C4	8.11 ± 0.06	8.15 ± 0.18	7.92 ± 0.08	8.15 ± 0.06
TC09000766.hg.1	GTF3C5	8.19 ± 0.14	8.07 ± 0.11	7.99 ± 0.12	8.04 ± 0.02
TC06000886.hg.1	GTF3C6	8.33 ± 0.09	8.23 ± 0.18	8.16 ± 0.14	7.99 ± 0.15
TC22000304.hg.1	GTPBP1	9.41 ± 0.17	9.47 ± 0.04	9.54 ± 0.18	9.51 ± 0.11
TC07003334.hg.1	GTPBP10	6.96 ± 0.26	6.99 ± 0.15	6.98 ± 0.18	6.83 ± 0.13
TC06001754.hg.1	GTPBP2	9.14 ± 0.28	8.65 ± 0.11	9.10 ± 0.28	8.59 ± 0.06
TC19000333.hg.1	GTPBP3	8.00 ± 0.19	7.91 ± 0.15	7.83 ± 0.16	8.01 ± 0.05
TC10000008.hg.1	GTPBP4	8.01 ± 0.11	8.09 ± 0.09	7.79 ± 0.18	8.09 ± 0.07
TC0X000794.hg.1	GTPBP6	8.34 ± 0.04	8.31 ± 0.08	8.16 ± 0.09	8.40 ± 0.03
TC0Y000111.hg.1	GTPBP6	8.34 ± 0.04	8.31 ± 0.08	8.16 ± 0.09	8.40 ± 0.03
TC03000575.hg.1	GTPBP8	7.10 ± 0.18	7.10 ± 0.11	7.01 ± 0.19	6.97 ± 0.10
TC22000394.hg.1	GTSE1	7.52 ± 0.29	7.29 ± 0.18	7.34 ± 0.34	7.28 ± 0.03

TC22000888.hg.1	GTSE1-AS1	6.86 ± 0.53	6.83 ± 0.18	6.81 ± 0.25	6.98 ± 0.14
TC12001572.hg.1	GTSF1	7.09 ± 0.37	6.80 ± 0.19	6.72 ± 0.17	6.51 ± 0.10
TC20000858.hg.1	GTSF1L	6.59 ± 0.24	6.45 ± 0.26	6.40 ± 0.21	6.55 ± 0.09
TC06000580.hg.1	GUCA1A	7.28 ± 0.22	7.31 ± 0.21	7.25 ± 0.24	7.39 ± 0.04
TC06001730.hg.1	GUCA1B	7.42 ± 0.17	7.33 ± 0.12	7.38 ± 0.19	7.45 ± 0.02
TC03001634.hg.1	GUCA1C	4.17 ± 0.12	4.05 ± 0.07	4.10 ± 0.07	4.13 ± 0.06
TC01002569.hg.1	GUCA2A	7.91 ± 0.29	7.94 ± 0.21	7.80 ± 0.20	8.01 ± 0.01
TC01000525.hg.1	GUCA2B	7.81 ± 0.25	7.77 ± 0.33	7.63 ± 0.29	7.95 ± 0.18
TC22000589.hg.1	GUCD1	9.47 ± 0.20	9.31 ± 0.03	9.22 ± 0.15	9.31 ± 0.07
TC11002258.hg.1	GUCY1A2	5.87 ± 0.19	5.67 ± 0.13	5.71 ± 0.13	5.73 ± 0.06
TC04000787.hg.1	GUCY1A3	7.31 ± 0.01	6.97 ± 0.07	7.46 ± 0.13	7.07 ± 0.10
TC13000675.hg.1	GUCY1B2	6.04 ± 0.11	5.98 ± 0.07	5.97 ± 0.08	6.04 ± 0.08
TC04000788.hg.1	GUCY1B3	7.96 ± 0.19	7.41 ± 0.15	8.24 ± 0.22	7.55 ± 0.25
TC12001271.hg.1	GUCY2C	5.01 ± 0.14	4.93 ± 0.10	5.01 ± 0.07	5.01 ± 0.07
TC17000123.hg.1	GUCY2D	8.01 ± 0.23	7.97 ± 0.17	7.94 ± 0.26	8.02 ± 0.03
TC11002117.hg.1	GUCY2EP	7.72 ± 0.18	7.75 ± 0.18	7.69 ± 0.20	7.87 ± 0.08
TC0X001270.hg.1	GUCY2F	5.53 ± 0.19	5.44 ± 0.09	5.48 ± 0.09	5.49 ± 0.01
TC10001664.hg.1	GUCY2GP	6.37 ± 0.18	6.37 ± 0.15	6.34 ± 0.19	6.48 ± 0.10
TC04000268.hg.1	GUF1	7.74 ± 0.49	7.97 ± 0.23	7.64 ± 0.49	7.90 ± 0.11
TC01001885.hg.1	GUK1	8.93 ± 0.11	8.94 ± 0.10	8.81 ± 0.10	8.97 ± 0.02
TC02001103.hg.1	GULP1	4.88 ± 0.10	4.84 ± 0.07	4.85 ± 0.05	4.89 ± 0.07
TC07001457.hg.1	GUSB	9.14 ± 0.10	8.99 ± 0.06	8.97 ± 0.10	8.99 ± 0.06
TC05000108.hg.1	GUSBP1	7.83 ± 0.29	8.05 ± 0.10	7.70 ± 0.10	7.94 ± 0.11
TC07001430.hg.1	GUSBP10	7.56 ± 0.19	7.57 ± 0.08	7.56 ± 0.04	7.67 ± 0.07
TC22000565.hg.1	GUSBP11	8.91 ± 0.09	8.98 ± 0.01	8.97 ± 0.14	9.06 ± 0.06
TC06001378.hg.1	GUSBP2	9.00 ± 0.57	9.35 ± 0.24	8.73 ± 0.27	9.22 ± 0.16
TC05003445.hg.1	GUSBP3	8.60 ± 0.61	8.86 ± 0.34	8.29 ± 0.31	8.72 ± 0.13
TC06001838.hg.1	GUSBP4	8.10 ± 0.25	8.22 ± 0.05	7.89 ± 0.01	8.17 ± 0.08
TC04000702.hg.1	GUSBP5	7.33 ± 0.16	7.39 ± 0.08	7.11 ± 0.07	7.51 ± 0.10
TC05001459.hg.1	GUSBP9	9.16 ± 0.62	9.37 ± 0.31	8.97 ± 0.30	9.22 ± 0.10
TC11001365.hg.1	GVINP1	9.59 ± 0.30	9.75 ± 0.32	9.48 ± 0.45	9.19 ± 0.29

TC12001401.hg.1	GXYLT1	7.31 ± 0.45	7.55 ± 0.16	7.44 ± 0.37	7.35 ± 0.18
TC03003359.hg.1	GXYLT2	5.81 ± 0.20	5.71 ± 0.05	5.76 ± 0.13	5.76 ± 0.05
TC03000801.hg.1	GYG1	8.47 ± 0.41	7.98 ± 0.01	9.36 ± 0.43	7.86 ± 0.05
TC0X000017.hg.1	GYG2	7.72 ± 0.19	7.75 ± 0.19	7.68 ± 0.22	7.84 ± 0.07
TC0X000804.hg.1	GYG2-AS1	4.54 ± 0.17	4.43 ± 0.17	4.57 ± 0.20	4.32 ± 0.05
TC0Y000161.hg.1	GYG2P1	8.00 ± 0.22	8.04 ± 0.15	8.01 ± 0.09	8.17 ± 0.10
TC11000390.hg.1	GYLTL1B	8.43 ± 0.13	8.45 ± 0.06	8.34 ± 0.11	8.46 ± 0.06
TC04002927.hg.1	GYPA	4.63 ± 0.03	4.46 ± 0.03	4.52 ± 0.06	4.55 ± 0.05
TC04002926.hg.1	GYPB	4.95 ± 0.13	4.74 ± 0.07	4.80 ± 0.04	4.93 ± 0.14
TC02000778.hg.1	GYPC	8.44 ± 0.17	8.44 ± 0.15	8.20 ± 0.13	8.43 ± 0.04
TC04001605.hg.1	GYPE	5.33 ± 0.12	5.16 ± 0.11	5.21 ± 0.09	5.13 ± 0.06
TC19001703.hg.1	GYS1	8.92 ± 0.17	8.59 ± 0.02	8.70 ± 0.09	8.45 ± 0.06
TC12001297.hg.1	GYS2	5.04 ± 0.04	4.95 ± 0.05	4.99 ± 0.07	5.02 ± 0.05
TC20000154.hg.1	GZF1	8.11 ± 0.04	8.45 ± 0.12	8.25 ± 0.03	8.49 ± 0.09
TC05000231.hg.1	GZMA	9.81 ± 0.17	9.59 ± 0.57	9.30 ± 0.20	8.57 ± 0.25
TC14000982.hg.1	GZMB	9.94 ± 0.27	9.93 ± 0.48	9.67 ± 0.13	9.28 ± 0.08
TC14000981.hg.1	GZMH	9.93 ± 0.12	9.70 ± 0.33	9.47 ± 0.28	9.13 ± 0.09
TC05000227.hg.1	GZMK	8.45 ± 0.10	8.51 ± 0.44	7.97 ± 0.34	7.50 ± 0.04
TC19000008.hg.1	GZMM	9.01 ± 0.02	9.04 ± 0.11	8.83 ± 0.19	9.02 ± 0.03
TC22000288.hg.1	H1F0	7.92 ± 0.22	7.70 ± 0.19	7.85 ± 0.09	7.72 ± 0.05
TC12000348.hg.1	H1FNT	7.57 ± 0.39	7.35 ± 0.11	7.46 ± 0.21	7.36 ± 0.05
TC03000694.hg.1	H1FOO	7.74 ± 0.30	7.65 ± 0.15	7.68 ± 0.20	7.79 ± 0.10
TC03001767.hg.1	H1FX	8.92 ± 0.23	9.01 ± 0.15	8.69 ± 0.25	8.91 ± 0.05
TC03000691.hg.1	H1FX-AS1	7.86 ± 0.15	7.79 ± 0.15	7.75 ± 0.15	7.86 ± 0.07
TC0X000780.hg.1	H2AFB1	7.01 ± 0.16	7.02 ± 0.12	7.07 ± 0.07	7.03 ± 0.06
TC0X000786.hg.1	H2AFB1	7.34 ± 0.15	7.34 ± 0.14	7.36 ± 0.20	7.35 ± 0.09
TC0X001555.hg.1	H2AFB1	7.58 ± 0.10	7.51 ± 0.13	7.49 ± 0.24	7.57 ± 0.11
TC12000197.hg.1	H2AFJ	8.16 ± 0.14	8.03 ± 0.04	8.08 ± 0.19	8.02 ± 0.06
TC07001346.hg.1	H2AFV	9.20 ± 0.08	9.29 ± 0.18	8.96 ± 0.08	9.24 ± 0.04
TC11002362.hg.1	H2AFX	9.00 ± 0.36	9.08 ± 0.26	8.76 ± 0.42	8.90 ± 0.20
TC05001801.hg.1	H2AFY	9.06 ± 0.07	9.00 ± 0.05	9.11 ± 0.08	9.00 ± 0.17

TC10000425.hg.1	H2AFY2	7.21 ± 0.23	7.15 ± 0.16	7.09 ± 0.18	7.12 ± 0.03
TC04001419.hg.1	H2AFZ	9.05 ± 0.16	8.95 ± 0.28	8.91 ± 0.24	8.58 ± 0.11
TC0X000507.hg.1	H2BFM	5.88 ± 0.11	5.85 ± 0.10	5.83 ± 0.02	5.86 ± 0.06
TC0X001249.hg.1	H2BFWT	5.76 ± 0.10	5.65 ± 0.06	5.76 ± 0.12	5.66 ± 0.05
TC0X000506.hg.1	H2BFXP	6.26 ± 0.02	6.10 ± 0.15	6.29 ± 0.08	6.16 ± 0.06
TC01001857.hg.1	H3F3A	11.21 ± 0.26	11.33 ± 0.16	11.73 ± 0.25	11.38 ± 0.25
TC17001882.hg.1	H3F3B	11.59 ± 0.13	11.89 ± 0.10	11.86 ± 0.26	11.86 ± 0.04
TC12001368.hg.1	H3F3C	6.41 ± 0.14	6.48 ± 0.06	6.58 ± 0.15	6.45 ± 0.12
TC01000113.hg.1	H6PD	8.86 ± 0.18	8.54 ± 0.06	8.72 ± 0.11	8.47 ± 0.03
TC02001782.hg.1	HAAO	7.94 ± 0.16	7.90 ± 0.17	7.97 ± 0.09	7.98 ± 0.06
TC10000824.hg.1	HABP2	6.25 ± 0.13	6.19 ± 0.16	6.18 ± 0.09	6.32 ± 0.12
TC09000480.hg.1	HABP4	7.79 ± 0.10	7.78 ± 0.04	7.55 ± 0.16	7.82 ± 0.06
TC06001972.hg.1	HACE1	6.91 ± 0.23	7.00 ± 0.16	6.78 ± 0.21	6.85 ± 0.06
TC03003336.hg.1	HACL1	7.70 ± 0.13	7.74 ± 0.13	7.39 ± 0.16	7.70 ± 0.07
TC04000556.hg.1	HADH	7.78 ± 0.03	7.67 ± 0.10	7.59 ± 0.08	7.56 ± 0.04
TC02001665.hg.1	HADHA	9.95 ± 0.13	9.87 ± 0.20	9.84 ± 0.19	9.66 ± 0.13
TC02000149.hg.1	HADHB	8.42 ± 0.14	8.33 ± 0.11	8.57 ± 0.22	8.21 ± 0.17
TC16000762.hg.1	HAGH	8.50 ± 0.18	8.47 ± 0.15	8.38 ± 0.15	8.46 ± 0.03
TC16000030.hg.1	HAGHL	8.71 ± 0.28	8.74 ± 0.27	8.64 ± 0.26	8.82 ± 0.04
TC12001842.hg.1	HAL	8.47 ± 0.14	8.01 ± 0.20	8.57 ± 0.45	7.96 ± 0.34
TC19000464.hg.1	HAMP	8.13 ± 0.18	8.17 ± 0.09	8.14 ± 0.11	8.19 ± 0.05
TC05001970.hg.1	HAND1	7.31 ± 0.28	7.38 ± 0.26	7.43 ± 0.22	7.52 ± 0.04
TC04001744.hg.1	HAND2	8.25 ± 0.23	8.17 ± 0.17	8.18 ± 0.23	8.22 ± 0.04
TC20000610.hg.1	HAO1	5.89 ± 0.10	5.84 ± 0.11	5.82 ± 0.06	5.90 ± 0.09
TC01001037.hg.1	HAO2	5.99 ± 0.06	5.97 ± 0.11	6.07 ± 0.11	6.11 ± 0.14
TC01001038.hg.1	HAO2-IT1	5.43 ± 0.45	5.22 ± 0.20	5.26 ± 0.25	5.49 ± 0.29
TC17001516.hg.1	HAP1	7.67 ± 0.25	7.64 ± 0.21	7.66 ± 0.21	7.71 ± 0.02
TC05001557.hg.1	HAPLN1	5.07 ± 0.18	5.01 ± 0.13	5.06 ± 0.06	5.08 ± 0.09
TC01001324.hg.1	HAPLN2	8.17 ± 0.21	8.11 ± 0.24	8.10 ± 0.24	8.20 ± 0.05
TC15001824.hg.1	HAPLN3	8.58 ± 0.11	8.58 ± 0.18	8.42 ± 0.19	8.66 ± 0.09
TC19002692.hg.1	HAPLN4	9.07 ± 0.25	9.04 ± 0.21	9.06 ± 0.21	9.09 ± 0.02

TC20000506.hg.1	HAR1A	7.59 ± 0.20	7.54 ± 0.03	7.50 ± 0.10	7.54 ± 0.06
TC20001024.hg.1	HAR1B	6.81 ± 0.22	6.77 ± 0.16	6.68 ± 0.06	6.69 ± 0.08
TC11001722.hg.1	HARBI1	8.00 ± 0.22	8.05 ± 0.08	8.01 ± 0.15	8.03 ± 0.06
TC05001865.hg.1	HARS	8.36 ± 0.02	8.23 ± 0.12	8.16 ± 0.07	8.12 ± 0.02
TC05000732.hg.1	HARS2	7.72 ± 0.10	7.59 ± 0.07	7.63 ± 0.19	7.60 ± 0.06
TC19001786.hg.1	HAS1	7.59 ± 0.24	7.62 ± 0.24	7.54 ± 0.25	7.67 ± 0.08
TC08001567.hg.1	HAS2	5.67 ± 0.08	5.69 ± 0.06	5.65 ± 0.09	5.68 ± 0.07
TC08000705.hg.1	HAS2-AS1	6.67 ± 0.27	6.61 ± 0.19	6.63 ± 0.15	6.71 ± 0.04
TC16000563.hg.1	HAS3	8.18 ± 0.18	8.08 ± 0.10	8.09 ± 0.17	8.08 ± 0.04
TC02001024.hg.1	HAT1	8.24 ± 0.45	8.58 ± 0.15	8.58 ± 0.57	8.43 ± 0.19
TC18000166.hg.1	HAUS1	6.84 ± 0.07	6.75 ± 0.33	6.66 ± 0.03	6.40 ± 0.16
TC15000321.hg.1	HAUS2	6.88 ± 0.06	6.94 ± 0.26	6.70 ± 0.06	6.78 ± 0.04
TC14000941.hg.1	HAUS4	8.34 ± 0.50	7.88 ± 0.05	8.71 ± 0.26	7.60 ± 0.18
TC19000476.hg.1	HAUS5	8.48 ± 0.16	8.48 ± 0.14	8.35 ± 0.17	8.53 ± 0.05
TC09000933.hg.1	HAUS6	7.38 ± 0.42	7.61 ± 0.25	7.32 ± 0.34	7.46 ± 0.02
TC19001269.hg.1	HAUS8	7.37 ± 0.16	7.23 ± 0.07	7.18 ± 0.11	7.28 ± 0.04
TC05001977.hg.1	HAVCR1	6.57 ± 0.12	6.50 ± 0.08	6.46 ± 0.08	6.52 ± 0.04
TC19001374.hg.1	HAVCR1P1	5.97 ± 0.30	6.00 ± 0.12	5.84 ± 0.23	6.15 ± 0.16
TC05003434.hg.1	HAVCR2	8.16 ± 0.22	7.70 ± 0.17	8.12 ± 0.10	7.61 ± 0.35
TC01001269.hg.1	HAX1	8.08 ± 0.18	7.92 ± 0.07	7.86 ± 0.14	7.89 ± 0.06
TC16000008.hg.1	HBA1	11.71 ± 0.32	11.75 ± 0.10	11.63 ± 0.23	12.07 ± 0.37
TC16000007.hg.1	HBA2	10.83 ± 0.33	10.90 ± 0.11	10.76 ± 0.18	11.21 ± 0.38
TC11001330.hg.1	HBB	9.00 ± 0.45	8.98 ± 0.09	8.89 ± 0.26	9.33 ± 0.32
TC11001332.hg.1	HBBP1	6.12 ± 0.16	5.91 ± 0.13	6.10 ± 0.17	5.94 ± 0.08
TC11001331.hg.1	HBD	6.51 ± 0.27	6.29 ± 0.04	6.32 ± 0.17	6.44 ± 0.06
TC05001854.hg.1	HBEGF	7.89 ± 0.27	8.70 ± 0.07	8.01 ± 0.23	8.40 ± 0.20
TC11001333.hg.1	HBG2	6.82 ± 0.11	6.69 ± 0.10	6.95 ± 0.03	6.83 ± 0.06
TC16002033.hg.1	HBM	7.78 ± 0.35	7.86 ± 0.30	7.80 ± 0.25	8.01 ± 0.02
TC07000689.hg.1	HBP1	8.78 ± 0.29	9.08 ± 0.22	8.98 ± 0.33	9.11 ± 0.06
TC16000010.hg.1	HBQ1	8.15 ± 0.30	8.19 ± 0.21	8.15 ± 0.23	8.28 ± 0.06
TC06002133.hg.1	HBS1L	7.73 ± 0.37	7.95 ± 0.25	7.65 ± 0.37	7.93 ± 0.08

TC16002032.hg.1	HBZ	7.20 ± 0.42	7.10 ± 0.30	7.04 ± 0.13	7.22 ± 0.06
TC12002077.hg.1	HCAR1	6.65 ± 0.13	6.48 ± 0.07	6.63 ± 0.06	6.50 ± 0.04
TC12002078.hg.1	HCAR2	9.87 ± 0.73	9.16 ± 0.23	10.28 ± 0.26	8.74 ± 0.59
TC12002079.hg.1	HCAR3	9.92 ± 0.55	9.37 ± 0.19	10.59 ± 0.21	8.97 ± 0.52
TC16000609.hg.1	HCCAT5	6.86 ± 0.16	6.83 ± 0.20	6.75 ± 0.17	6.76 ± 0.08
TC0X000042.hg.1	HCCS	8.81 ± 0.13	8.59 ± 0.03	8.77 ± 0.16	8.43 ± 0.16
TC0X001522.hg.1	HCFC1	9.77 ± 0.16	9.69 ± 0.05	9.50 ± 0.15	9.53 ± 0.11
TC0X000761.hg.1	HCFC1-AS1	9.14 ± 0.41	8.89 ± 0.13	8.84 ± 0.39	8.78 ± 0.05
TC16000798.hg.1	HCFC1R1	8.62 ± 0.32	8.47 ± 0.20	8.44 ± 0.21	8.52 ± 0.05
TC12000807.hg.1	HCFC2	7.21 ± 0.37	7.17 ± 0.13	7.13 ± 0.27	7.07 ± 0.04
TC06000192.hg.1	HCG11	7.98 ± 0.15	7.87 ± 0.03	7.94 ± 0.12	7.95 ± 0.09
TC06000299.hg.1	HCG14	6.52 ± 0.13	6.56 ± 0.08	6.51 ± 0.12	6.55 ± 0.03
TC6_apd_hap1000001.hg.1	HCG14	6.52 ± 0.13	6.56 ± 0.08	6.51 ± 0.12	6.55 ± 0.03
TC6_cox_hap2000003.hg.1	HCG14	6.39 ± 0.16	6.47 ± 0.02	6.39 ± 0.12	6.45 ± 0.04
TC6_mcf_hap5000001.hg.1	HCG14	6.39 ± 0.16	6.47 ± 0.02	6.39 ± 0.12	6.45 ± 0.04
TC6_qbl_hap6000001.hg.1	HCG14	6.52 ± 0.13	6.56 ± 0.08	6.51 ± 0.12	6.55 ± 0.03
TC6_ssto_hap7000001.hg.1	HCG14	6.39 ± 0.16	6.47 ± 0.02	6.39 ± 0.12	6.45 ± 0.04
TC06000306.hg.1	HCG15	5.52 ± 0.19	5.44 ± 0.11	5.48 ± 0.11	5.44 ± 0.10
TC6_cox_hap2000005.hg.1	HCG15	5.52 ± 0.19	5.44 ± 0.11	5.48 ± 0.11	5.44 ± 0.10
TC6_qbl_hap6000003.hg.1	HCG15	5.52 ± 0.19	5.44 ± 0.11	5.48 ± 0.11	5.44 ± 0.10
TC6_ssto_hap7000003.hg.1	HCG15	5.52 ± 0.19	5.44 ± 0.11	5.48 ± 0.11	5.44 ± 0.10
TC6_apd_hap1000002.hg.1	HCG16	5.52 ± 0.19	5.44 ± 0.11	5.48 ± 0.11	5.44 ± 0.10
TC6_dbb_hap3000001.hg.1	HCG16	5.52 ± 0.19	5.44 ± 0.11	5.48 ± 0.11	5.44 ± 0.10
TC6_mann_hap4000002.hg.1	HCG16	5.52 ± 0.19	5.44 ± 0.11	5.48 ± 0.11	5.44 ± 0.10
TC6_apd_hap1000071.hg.1	HCG17	5.25 ± 0.12	5.26 ± 0.14	5.12 ± 0.13	5.21 ± 0.04
TC6_cox_hap2000250.hg.1	HCG17	5.01 ± 0.05	5.14 ± 0.15	5.09 ± 0.18	5.08 ± 0.09
TC6_dbb_hap3000229.hg.1	HCG17	4.99 ± 0.05	5.08 ± 0.11	5.05 ± 0.15	5.05 ± 0.13
TC6_mann_hap4000199.hg.1	HCG17	5.01 ± 0.05	5.14 ± 0.15	5.09 ± 0.18	5.08 ± 0.09
TC6_qbl_hap6000228.hg.1	HCG17	4.99 ± 0.05	5.08 ± 0.11	5.05 ± 0.15	5.05 ± 0.13
TC6_ssto_hap7000202.hg.1	HCG17	5.01 ± 0.05	5.14 ± 0.15	5.09 ± 0.18	5.08 ± 0.09
TC06004143.hg.1	HCG18	4.99 ± 0.05	5.08 ± 0.11	5.05 ± 0.15	5.05 ± 0.13

TC06004144.hg.1	HCG18	7.92 ± 0.18	7.98 ± 0.04	7.76 ± 0.19	7.87 ± 0.07
TC6_apd_hap1000072.hg.1	HCG18	8.32 ± 0.16	8.35 ± 0.04	8.12 ± 0.23	8.23 ± 0.07
TC6_cox_hap2000251.hg.1	HCG18	7.94 ± 0.19	8.01 ± 0.04	7.78 ± 0.19	7.89 ± 0.07
TC6_dbb_hap3000230.hg.1	HCG18	7.94 ± 0.16	7.98 ± 0.04	7.75 ± 0.20	7.86 ± 0.06
TC6_mann_hap4000200.hg.1	HCG18	7.96 ± 0.20	8.03 ± 0.04	7.80 ± 0.20	7.93 ± 0.06
TC6_qbl_hap6000229.hg.1	HCG18	7.91 ± 0.19	7.98 ± 0.04	7.75 ± 0.19	7.87 ± 0.07
TC6_ssto_hap7000203.hg.1	HCG18	7.92 ± 0.19	7.99 ± 0.04	7.76 ± 0.19	7.87 ± 0.07
TC06000348.hg.1	HCG20	6.58 ± 0.11	6.58 ± 0.22	6.61 ± 0.10	6.81 ± 0.07
TC6_cox_hap2000046.hg.1	HCG20	6.34 ± 0.12	6.32 ± 0.21	6.37 ± 0.08	6.51 ± 0.09
TC6_dbb_hap3000039.hg.1	HCG20	6.72 ± 0.11	6.69 ± 0.22	6.75 ± 0.06	6.93 ± 0.10
TC6_mann_hap4000041.hg.1	HCG20	6.58 ± 0.11	6.58 ± 0.22	6.61 ± 0.10	6.81 ± 0.07
TC6_qbl_hap6000039.hg.1	HCG20	6.77 ± 0.11	6.77 ± 0.23	6.81 ± 0.11	7.00 ± 0.05
TC6_ssto_hap7000039.hg.1	HCG20	7.01 ± 0.10	7.01 ± 0.17	7.01 ± 0.14	7.22 ± 0.16
TC06001516.hg.1	HCG21	6.03 ± 0.18	5.94 ± 0.09	5.88 ± 0.10	6.09 ± 0.18
TC6_cox_hap2000148.hg.1	HCG21	6.03 ± 0.18	5.94 ± 0.09	5.88 ± 0.10	6.09 ± 0.18
TC6_dbb_hap3000137.hg.1	HCG21	6.01 ± 0.19	5.92 ± 0.09	5.89 ± 0.14	6.08 ± 0.19
TC6_mcf_hap5000125.hg.1	HCG21	4.91 ± 0.17	5.02 ± 0.13	5.06 ± 0.04	5.02 ± 0.23
TC6_qbl_hap6000138.hg.1	HCG21	6.06 ± 0.19	5.98 ± 0.08	5.90 ± 0.12	6.13 ± 0.19
TC06000355.hg.1	HCG22	6.57 ± 0.20	6.53 ± 0.03	6.46 ± 0.11	6.54 ± 0.08
TC6_apd_hap1000033.hg.1	HCG22	6.52 ± 0.22	6.48 ± 0.06	6.40 ± 0.13	6.49 ± 0.07
TC6_cox_hap2000053.hg.1	HCG22	6.50 ± 0.23	6.49 ± 0.04	6.43 ± 0.10	6.49 ± 0.07
TC6_dbb_hap3000046.hg.1	HCG22	6.52 ± 0.22	6.48 ± 0.06	6.40 ± 0.13	6.49 ± 0.07
TC6_mann_hap4000047.hg.1	HCG22	6.55 ± 0.22	6.53 ± 0.05	6.45 ± 0.14	6.54 ± 0.07
TC6_mcf_hap5000040.hg.1	HCG22	6.46 ± 0.22	6.45 ± 0.04	6.35 ± 0.16	6.44 ± 0.07
TC6_qbl_hap6000046.hg.1	HCG22	6.46 ± 0.23	6.41 ± 0.06	6.39 ± 0.09	6.38 ± 0.07
TC6_ssto_hap7000041.hg.1	HCG22	6.46 ± 0.25	6.44 ± 0.03	6.39 ± 0.13	6.43 ± 0.06
TC06000398.hg.1	HCG23	5.79 ± 0.26	5.82 ± 0.21	5.70 ± 0.12	5.87 ± 0.05
TC6_apd_hap1000045.hg.1	HCG23	5.79 ± 0.26	5.82 ± 0.21	5.70 ± 0.12	5.87 ± 0.05
TC6_cox_hap2000089.hg.1	HCG23	5.79 ± 0.22	5.79 ± 0.23	5.67 ± 0.12	5.85 ± 0.05
TC6_dbb_hap3000082.hg.1	HCG23	5.79 ± 0.22	5.79 ± 0.23	5.67 ± 0.12	5.85 ± 0.05
TC6_mann_hap4000073.hg.1	HCG23	5.80 ± 0.23	5.79 ± 0.22	5.66 ± 0.11	5.84 ± 0.06

TC6_mcf_hap5000074.hg.1	HCG23	5.80 ± 0.23	5.79 ± 0.22	5.66 ± 0.11	5.84 ± 0.06
TC6_qbl_hap6000080.hg.1	HCG23	5.79 ± 0.26	5.82 ± 0.21	5.70 ± 0.12	5.87 ± 0.05
TC6_ssto_hap7000075.hg.1	HCG23	5.80 ± 0.23	5.79 ± 0.22	5.66 ± 0.11	5.84 ± 0.06
TC06000410.hg.1	HCG24	5.48 ± 0.11	5.36 ± 0.02	5.49 ± 0.08	5.33 ± 0.09
TC6_cox_hap2000100.hg.1	HCG24	6.39 ± 0.25	5.97 ± 0.04	6.46 ± 0.22	6.18 ± 0.08
TC6_dbb_hap3000092.hg.1	HCG24	5.48 ± 0.11	5.36 ± 0.02	5.49 ± 0.08	5.33 ± 0.09
TC6_mann_hap4000084.hg.1	HCG24	5.48 ± 0.11	5.36 ± 0.02	5.49 ± 0.08	5.33 ± 0.09
TC6_qbl_hap6000091.hg.1	HCG24	5.48 ± 0.11	5.36 ± 0.02	5.49 ± 0.08	5.33 ± 0.09
TC06000416.hg.1	HCG25	7.72 ± 0.07	7.60 ± 0.11	7.57 ± 0.15	7.62 ± 0.03
TC6_cox_hap2000106.hg.1	HCG25	7.72 ± 0.07	7.60 ± 0.11	7.57 ± 0.15	7.62 ± 0.03
TC6_dbb_hap3000098.hg.1	HCG25	7.72 ± 0.07	7.60 ± 0.11	7.57 ± 0.15	7.62 ± 0.03
TC6_mann_hap4000090.hg.1	HCG25	7.72 ± 0.07	7.60 ± 0.11	7.57 ± 0.15	7.62 ± 0.03
TC6_mcf_hap5000091.hg.1	HCG25	7.72 ± 0.07	7.60 ± 0.11	7.57 ± 0.15	7.62 ± 0.03
TC6_qbl_hap6000097.hg.1	HCG25	7.73 ± 0.07	7.61 ± 0.11	7.57 ± 0.14	7.64 ± 0.03
TC6_ssto_hap7000087.hg.1	HCG25	7.69 ± 0.08	7.55 ± 0.11	7.53 ± 0.15	7.57 ± 0.03
TC06000365.hg.1	HCG26	8.12 ± 0.12	7.84 ± 0.13	8.19 ± 0.21	7.85 ± 0.05
TC6_cox_hap2000062.hg.1	HCG26	8.49 ± 0.10	8.21 ± 0.05	8.47 ± 0.13	8.19 ± 0.02
TC6_dbb_hap3000052.hg.1	HCG26	8.49 ± 0.10	8.21 ± 0.05	8.47 ± 0.13	8.19 ± 0.02
TC6_mcf_hap5000047.hg.1	HCG26	8.49 ± 0.10	8.21 ± 0.05	8.47 ± 0.13	8.19 ± 0.02
TC6_qbl_hap6000053.hg.1	HCG26	8.49 ± 0.10	8.21 ± 0.05	8.47 ± 0.13	8.19 ± 0.02
TC6_ssto_hap7000049.hg.1	HCG26	8.15 ± 0.13	7.66 ± 0.17	8.15 ± 0.21	7.81 ± 0.11
TC06000361.hg.1	HCG27	8.28 ± 0.15	7.97 ± 0.05	8.22 ± 0.19	8.03 ± 0.08
TC6_cox_hap2000058.hg.1	HCG27	8.40 ± 0.08	8.09 ± 0.02	8.37 ± 0.17	8.15 ± 0.09
TC6_dbb_hap3000051.hg.1	HCG27	8.40 ± 0.08	8.09 ± 0.02	8.37 ± 0.17	8.15 ± 0.09
TC6_mann_hap4000052.hg.1	HCG27	7.92 ± 0.05	7.87 ± 0.14	8.14 ± 0.27	7.86 ± 0.08
TC6_mann_hap4000217.hg.1	HCG27	9.06 ± 0.12	8.67 ± 0.18	8.85 ± 0.23	8.67 ± 0.04
TC6_mcf_hap5000045.hg.1	HCG27	8.59 ± 0.12	8.28 ± 0.03	8.57 ± 0.21	8.35 ± 0.07
TC6_qbl_hap6000051.hg.1	HCG27	8.56 ± 0.14	8.30 ± 0.04	8.53 ± 0.16	8.35 ± 0.06
TC6_ssto_hap7000045.hg.1	HCG27	8.34 ± 0.08	8.00 ± 0.04	8.26 ± 0.15	8.03 ± 0.08
TC06001489.hg.1	HCG4	7.01 ± 0.27	6.87 ± 0.06	6.88 ± 0.34	7.03 ± 0.17
TC6_apd_hap1000062.hg.1	HCG4	7.58 ± 0.28	7.37 ± 0.13	7.42 ± 0.24	7.36 ± 0.12

TC6_cox_hap2000126.hg.1	HCG4	7.58 ± 0.28	7.37 ± 0.13	7.42 ± 0.24	7.36 ± 0.12
TC6_dbb_hap3000115.hg.1	HCG4	7.01 ± 0.27	6.87 ± 0.06	6.88 ± 0.34	7.03 ± 0.17
TC6_mann_hap4000104.hg.1	HCG4	7.01 ± 0.27	6.87 ± 0.06	6.88 ± 0.34	7.03 ± 0.17
TC6_mcf_hap5000108.hg.1	HCG4	7.01 ± 0.27	6.87 ± 0.06	6.88 ± 0.34	7.03 ± 0.17
TC6_qbl_hap6000116.hg.1	HCG4	7.58 ± 0.28	7.37 ± 0.13	7.42 ± 0.24	7.36 ± 0.12
TC6_ssto_hap7000107.hg.1	HCG4	7.01 ± 0.27	6.87 ± 0.06	6.88 ± 0.34	7.03 ± 0.17
TC06001491.hg.1	HCG4B	5.94 ± 0.13	5.69 ± 0.10	5.77 ± 0.18	5.73 ± 0.07
TC6_apd_hap1000064.hg.1	HCG4B	5.51 ± 0.03	5.39 ± 0.13	5.46 ± 0.27	5.50 ± 0.14
TC6_cox_hap2000128.hg.1	HCG4B	5.51 ± 0.03	5.39 ± 0.13	5.46 ± 0.27	5.50 ± 0.14
TC6_dbb_hap3000117.hg.1	HCG4B	6.13 ± 0.20	5.95 ± 0.15	5.82 ± 0.09	5.92 ± 0.06
TC6_mann_hap4000106.hg.1	HCG4B	5.96 ± 0.12	5.77 ± 0.12	5.87 ± 0.22	5.83 ± 0.11
TC6_qbl_hap6000118.hg.1	HCG4B	5.42 ± 0.20	5.16 ± 0.11	5.05 ± 0.11	5.13 ± 0.05
TC6_ssto_hap7000109.hg.1	HCG4B	5.11 ± 0.27	4.90 ± 0.05	4.86 ± 0.18	5.06 ± 0.19
TC06000329.hg.1	HCG9	8.36 ± 0.31	8.33 ± 0.21	8.29 ± 0.26	8.38 ± 0.10
TC6_cox_hap2000027.hg.1	HCG9	8.26 ± 0.29	8.27 ± 0.18	8.24 ± 0.20	8.30 ± 0.10
TC6_dbb_hap3000021.hg.1	HCG9	8.26 ± 0.29	8.27 ± 0.18	8.24 ± 0.20	8.30 ± 0.10
TC6_mann_hap4000023.hg.1	HCG9	8.32 ± 0.28	8.24 ± 0.12	8.24 ± 0.28	8.31 ± 0.09
TC6_mcf_hap5000017.hg.1	HCG9	7.93 ± 0.29	7.94 ± 0.12	7.90 ± 0.17	7.92 ± 0.11
TC6_qbl_hap6000020.hg.1	HCG9	8.08 ± 0.29	8.08 ± 0.19	8.08 ± 0.22	8.09 ± 0.12
TC6_ssto_hap7000021.hg.1	HCG9	8.32 ± 0.28	8.24 ± 0.12	8.24 ± 0.28	8.31 ± 0.09
TC20000206.hg.1	HCK	10.61 ± 0.45	10.19 ± 0.22	11.08 ± 0.30	10.09 ± 0.30
TC03001696.hg.1	HCLS1	10.13 ± 0.15	10.14 ± 0.16	10.36 ± 0.26	10.05 ± 0.10
TC05001329.hg.1	HCN1	5.53 ± 0.08	5.48 ± 0.05	5.44 ± 0.09	5.52 ± 0.03
TC19000010.hg.1	HCN2	7.34 ± 0.27	7.27 ± 0.17	7.27 ± 0.20	7.35 ± 0.01
TC01001298.hg.1	HCN3	7.40 ± 0.16	7.33 ± 0.14	7.30 ± 0.16	7.36 ± 0.03
TC15001633.hg.1	HCN4	7.20 ± 0.19	7.08 ± 0.13	7.02 ± 0.21	7.11 ± 0.03
TC06004072.hg.1	HCP5	9.66 ± 0.29	9.43 ± 0.10	9.55 ± 0.17	9.18 ± 0.02
TC6_cox_hap2000061.hg.1	HCP5	9.82 ± 0.30	9.59 ± 0.10	9.69 ± 0.15	9.35 ± 0.01
TC6_mcf_hap5000046.hg.1	HCP5	9.91 ± 0.60	9.77 ± 0.07	9.84 ± 0.17	9.49 ± 0.08
TC6_ssto_hap7000048.hg.1	HCP5	9.81 ± 0.30	9.60 ± 0.10	9.69 ± 0.15	9.35 ± 0.01
TC17001527.hg.1	HCRT	7.67 ± 0.22	7.59 ± 0.16	7.67 ± 0.07	7.71 ± 0.04

TC01000412.hg.1	HCRTR1	7.57 ± 0.18	7.57 ± 0.19	7.52 ± 0.16	7.70 ± 0.10
TC06000677.hg.1	HCRTR2	5.26 ± 0.13	5.18 ± 0.07	5.21 ± 0.06	5.27 ± 0.10
TC19000489.hg.1	HCST	9.53 ± 0.46	9.27 ± 0.13	9.01 ± 0.23	9.01 ± 0.12
TC01000427.hg.1	HDAC1	9.43 ± 0.08	9.35 ± 0.16	9.24 ± 0.15	9.14 ± 0.05
TC22001497.hg.1	HDAC10	8.77 ± 0.07	8.70 ± 0.10	8.68 ± 0.05	8.74 ± 0.05
TC03000076.hg.1	HDAC11	8.03 ± 0.16	7.98 ± 0.14	7.94 ± 0.17	8.03 ± 0.07
TC03001189.hg.1	HDAC11-AS1	6.75 ± 0.20	6.75 ± 0.11	6.66 ± 0.30	6.84 ± 0.07
TC06002035.hg.1	HDAC2	7.41 ± 0.27	7.51 ± 0.11	7.41 ± 0.25	7.33 ± 0.20
TC05001871.hg.1	HDAC3	7.70 ± 0.12	7.63 ± 0.15	7.51 ± 0.14	7.50 ± 0.03
TC02002922.hg.1	HDAC4	8.58 ± 0.14	8.43 ± 0.11	8.70 ± 0.09	8.45 ± 0.02
TC17001572.hg.1	HDAC5	9.03 ± 0.20	8.85 ± 0.03	8.86 ± 0.14	8.83 ± 0.07
TC0X000261.hg.1	HDAC6	8.26 ± 0.12	8.16 ± 0.07	8.10 ± 0.13	8.16 ± 0.04
TC12001438.hg.1	HDAC7	8.72 ± 0.11	8.64 ± 0.08	8.62 ± 0.05	8.64 ± 0.04
TC0X001141.hg.1	HDAC8	7.00 ± 0.12	6.88 ± 0.10	6.92 ± 0.16	6.71 ± 0.10
TC07000116.hg.1	HDAC9	7.00 ± 0.12	6.99 ± 0.10	6.85 ± 0.11	6.89 ± 0.07
TC15001348.hg.1	HDC	7.46 ± 0.20	7.78 ± 0.20	7.46 ± 0.07	7.87 ± 0.14
TC06002078.hg.1	HDDC2	8.24 ± 0.08	8.14 ± 0.14	7.89 ± 0.08	7.92 ± 0.03
TC15001855.hg.1	HDDC3	7.47 ± 0.08	7.46 ± 0.09	7.39 ± 0.10	7.41 ± 0.06
TC01003353.hg.1	HDGF	8.80 ± 0.21	8.65 ± 0.09	8.61 ± 0.28	8.56 ± 0.05
TC06000138.hg.1	HDGFL1	7.47 ± 0.20	7.41 ± 0.22	7.33 ± 0.17	7.48 ± 0.16
TC19000090.hg.1	HDGFRP2	8.70 ± 0.18	8.56 ± 0.09	8.64 ± 0.18	8.51 ± 0.04
TC15001766.hg.1	HDGFRP3	7.02 ± 0.15	6.97 ± 0.14	7.06 ± 0.12	6.94 ± 0.19
TC0X000826.hg.1	HDHD1	8.19 ± 0.09	8.10 ± 0.01	8.11 ± 0.02	8.01 ± 0.04
TC18000492.hg.1	HDHD2	8.07 ± 0.13	7.95 ± 0.11	7.94 ± 0.23	7.76 ± 0.13
TC09001501.hg.1	HDHD3	7.73 ± 0.19	7.52 ± 0.10	7.44 ± 0.12	7.56 ± 0.02
TC02002948.hg.1	HDLBP	8.95 ± 0.10	8.75 ± 0.04	8.73 ± 0.02	8.59 ± 0.13
TC0X001188.hg.1	HDX	5.68 ± 0.05	5.65 ± 0.01	5.70 ± 0.10	5.58 ± 0.07
TC01004007.hg.1	HEATR1	7.93 ± 0.46	8.09 ± 0.15	7.72 ± 0.43	8.05 ± 0.06
TC07000011.hg.1	HEATR2	8.25 ± 0.06	8.23 ± 0.08	8.17 ± 0.14	8.24 ± 0.05
TC16000436.hg.1	HEATR3	7.71 ± 0.11	7.59 ± 0.17	7.56 ± 0.13	7.51 ± 0.12
TC14001290.hg.1	HEATR4	5.97 ± 0.11	5.92 ± 0.12	5.93 ± 0.07	5.94 ± 0.05

TC14002318.hg.1	HEATR5A	7.39 ± 0.25	7.31 ± 0.27	7.58 ± 0.29	7.13 ± 0.13
TC02001738.hg.1	HEATR5B	8.25 ± 0.42	8.46 ± 0.22	8.15 ± 0.43	8.16 ± 0.18
TC17001758.hg.1	HEATR6	7.61 ± 0.21	7.39 ± 0.17	7.51 ± 0.12	7.35 ± 0.09
TC12001259.hg.1	HEBP1	8.54 ± 0.22	8.23 ± 0.04	8.46 ± 0.26	8.29 ± 0.05
TC06001030.hg.1	HEBP2	8.84 ± 0.11	8.69 ± 0.16	8.86 ± 0.20	8.63 ± 0.11
TC06001035.hg.1	HECA	9.66 ± 0.28	10.09 ± 0.13	9.72 ± 0.40	10.04 ± 0.14
TC14001009.hg.1	HECTD1	8.50 ± 0.44	8.75 ± 0.16	8.56 ± 0.42	8.68 ± 0.09
TC10000653.hg.1	HECTD2	4.66 ± 0.04	4.63 ± 0.10	4.51 ± 0.05	4.54 ± 0.03
TC01002604.hg.1	HECTD3	8.86 ± 0.15	8.69 ± 0.01	8.69 ± 0.07	8.69 ± 0.02
TC12001978.hg.1	HECTD4	8.54 ± 0.14	8.51 ± 0.12	8.35 ± 0.14	8.42 ± 0.04
TC07000261.hg.1	HECW1	6.42 ± 0.12	6.35 ± 0.10	6.33 ± 0.11	6.41 ± 0.05
TC07000262.hg.1	HECW1-IT1	4.94 ± 0.09	4.77 ± 0.03	4.81 ± 0.10	4.83 ± 0.08
TC02002638.hg.1	HECW2	6.38 ± 0.16	6.32 ± 0.08	6.37 ± 0.05	6.36 ± 0.01
TC03001720.hg.1	HEG1	8.52 ± 0.35	8.37 ± 0.05	8.36 ± 0.20	8.38 ± 0.12
TC05002152.hg.1	HEIH	8.22 ± 0.43	7.90 ± 0.03	7.90 ± 0.18	7.81 ± 0.11
TC12000593.hg.1	HELB	8.11 ± 0.46	8.24 ± 0.21	8.42 ± 0.43	7.95 ± 0.17
TC10000673.hg.1	HELLS	6.13 ± 0.19	6.04 ± 0.36	5.83 ± 0.22	5.62 ± 0.07
TC04001348.hg.1	HELQ	7.09 ± 0.37	7.14 ± 0.26	7.08 ± 0.22	7.01 ± 0.09
TC04000900.hg.1	HELT	7.73 ± 0.32	7.75 ± 0.24	7.75 ± 0.20	7.78 ± 0.02
TC17001812.hg.1	HELZ	9.24 ± 0.57	9.56 ± 0.20	9.45 ± 0.54	9.39 ± 0.16
TC20001035.hg.1	HELZ2	8.83 ± 0.28	8.62 ± 0.10	8.85 ± 0.26	8.61 ± 0.06
TC09001401.hg.1	HEMGN	6.62 ± 0.44	6.36 ± 0.04	6.39 ± 0.04	6.35 ± 0.16
TC03000315.hg.1	HEMK1	7.77 ± 0.11	7.73 ± 0.13	7.64 ± 0.05	7.74 ± 0.06
TC01002945.hg.1	HENMT1	7.24 ± 0.06	7.18 ± 0.07	7.25 ± 0.14	7.08 ± 0.05
TC11002415.hg.1	HEPACAM	7.06 ± 0.22	6.94 ± 0.17	7.01 ± 0.14	7.04 ± 0.08
TC07001607.hg.1	HEPACAM2	5.17 ± 0.14	5.09 ± 0.08	5.09 ± 0.04	5.10 ± 0.07
TC0X000357.hg.1	HEPH	5.95 ± 0.12	5.87 ± 0.11	5.91 ± 0.09	5.92 ± 0.02
TC11000915.hg.1	HEPHL1	5.39 ± 0.20	5.20 ± 0.08	5.21 ± 0.07	5.25 ± 0.07
TC11001149.hg.1	HEPN1	6.45 ± 0.11	6.30 ± 0.23	6.50 ± 0.06	6.46 ± 0.13
TC15001544.hg.1	HERC1	8.96 ± 0.45	9.27 ± 0.18	8.84 ± 0.31	9.27 ± 0.03
TC15001092.hg.1	HERC2	8.38 ± 0.09	8.31 ± 0.07	8.15 ± 0.06	8.21 ± 0.11

TC15001058.hg.1	HERC2P2	8.86 ± 0.29	8.80 ± 0.07	8.54 ± 0.16	8.61 ± 0.16
TC15001011.hg.1	HERC2P3	8.60 ± 0.24	8.48 ± 0.11	8.27 ± 0.14	8.31 ± 0.14
TC16001060.hg.1	HERC2P4	7.98 ± 0.11	8.04 ± 0.13	7.69 ± 0.10	7.99 ± 0.25
TC15000031.hg.1	HERC2P7	7.90 ± 0.13	7.76 ± 0.13	7.61 ± 0.23	7.89 ± 0.23
TC15000144.hg.1	HERC2P9	8.95 ± 0.30	8.93 ± 0.03	8.65 ± 0.21	8.77 ± 0.18
TC04000486.hg.1	HERC3	9.01 ± 0.26	9.11 ± 0.25	9.32 ± 0.43	9.02 ± 0.11
TC10001341.hg.1	HERC4	7.93 ± 0.44	7.84 ± 0.36	8.25 ± 0.44	7.54 ± 0.24
TC04000485.hg.1	HERC5	8.77 ± 0.88	8.45 ± 0.88	9.43 ± 1.17	8.38 ± 0.46
TC04000484.hg.1	HERC6	8.49 ± 0.42	8.15 ± 0.47	8.38 ± 0.45	7.84 ± 0.12
TC16000480.hg.1	HERPUD1	9.86 ± 0.19	10.11 ± 0.22	9.73 ± 0.16	10.08 ± 0.04
TC07001279.hg.1	HERPUD2	10.02 ± 0.22	10.27 ± 0.07	10.05 ± 0.24	10.16 ± 0.16
TC03001071.hg.1	HES1	6.68 ± 0.15	6.89 ± 0.10	6.92 ± 0.19	6.86 ± 0.10
TC01002150.hg.1	HES2	7.86 ± 0.20	7.83 ± 0.18	7.80 ± 0.14	7.91 ± 0.05
TC01000087.hg.1	HES3	7.54 ± 0.23	7.53 ± 0.25	7.36 ± 0.18	7.53 ± 0.11
TC01002080.hg.1	HES4	8.98 ± 0.34	8.88 ± 0.23	8.92 ± 0.31	8.94 ± 0.01
TC01002121.hg.1	HES5	8.53 ± 0.26	8.62 ± 0.27	8.57 ± 0.22	8.70 ± 0.07
TC02002913.hg.1	HES6	9.04 ± 0.26	8.93 ± 0.20	8.92 ± 0.26	9.00 ± 0.06
TC17001103.hg.1	HES7	8.09 ± 0.42	8.04 ± 0.23	8.03 ± 0.45	8.17 ± 0.03
TC03001490.hg.1	HESX1	4.04 ± 0.06	3.90 ± 0.04	4.11 ± 0.23	3.82 ± 0.02
TC15002779.hg.1	HEXA	8.42 ± 0.16	8.22 ± 0.12	8.09 ± 0.11	8.16 ± 0.01
TC15000657.hg.1	HEXA-AS1	5.71 ± 0.08	5.69 ± 0.13	5.52 ± 0.11	5.51 ± 0.04
TC05000359.hg.1	HEXB	8.16 ± 0.11	8.02 ± 0.19	8.32 ± 0.17	7.84 ± 0.33
TC17000952.hg.1	HEXDC	8.40 ± 0.09	8.28 ± 0.06	8.35 ± 0.11	8.22 ± 0.05
TC17000580.hg.1	HEXIM1	9.39 ± 0.16	9.78 ± 0.12	9.21 ± 0.27	9.59 ± 0.32
TC17000581.hg.1	HEXIM2	7.67 ± 0.29	7.60 ± 0.24	7.48 ± 0.31	7.70 ± 0.06
TC08001351.hg.1	HEY1	6.09 ± 0.18	6.00 ± 0.08	6.01 ± 0.03	6.12 ± 0.07
TC06000960.hg.1	HEY2	6.74 ± 0.25	6.65 ± 0.04	6.71 ± 0.14	6.64 ± 0.06
TC01002543.hg.1	HEYL	7.66 ± 0.21	7.57 ± 0.18	7.62 ± 0.25	7.62 ± 0.08
TC06000165.hg.1	HFE	6.66 ± 0.09	6.52 ± 0.14	6.57 ± 0.12	6.57 ± 0.01
TC01001089.hg.1	HFE2	6.33 ± 0.17	6.35 ± 0.15	6.24 ± 0.14	6.45 ± 0.11
TC01002862.hg.1	HFM1	3.99 ± 0.13	3.84 ± 0.02	3.89 ± 0.05	3.86 ± 0.03

TC06002320.hg.1	HGC6.3	8.21 ± 0.27	8.23 ± 0.11	8.24 ± 0.11	8.23 ± 0.04
TC03001692.hg.1	HGD	5.99 ± 0.07	5.93 ± 0.04	5.86 ± 0.11	5.94 ± 0.10
TC07001562.hg.1	HGF	6.12 ± 0.19	5.73 ± 0.12	6.93 ± 0.69	5.65 ± 0.11
TC04000047.hg.1	HGFAC	8.16 ± 0.22	8.10 ± 0.20	8.07 ± 0.22	8.18 ± 0.06
TC17000935.hg.1	HGS	9.26 ± 0.16	9.07 ± 0.01	9.19 ± 0.09	9.08 ± 0.05
TC08000333.hg.1	HGSNAT	8.68 ± 0.18	8.74 ± 0.12	8.61 ± 0.20	8.77 ± 0.05
TC01001755.hg.1	HHAT	7.12 ± 0.08	7.10 ± 0.11	7.07 ± 0.07	7.15 ± 0.04
TC03003374.hg.1	HHATL	7.71 ± 0.19	7.69 ± 0.23	7.64 ± 0.18	7.82 ± 0.07
TC03000224.hg.1	HHATL-AS1	7.31 ± 0.23	7.21 ± 0.21	7.20 ± 0.05	7.36 ± 0.13
TC10000660.hg.1	HHEX	8.44 ± 0.15	8.23 ± 0.13	8.71 ± 0.31	8.10 ± 0.08
TC04000706.hg.1	HHIP	4.88 ± 0.09	4.72 ± 0.08	4.78 ± 0.06	4.77 ± 0.03
TC04001607.hg.1	HHIP-AS1	6.33 ± 0.20	6.37 ± 0.18	6.27 ± 0.22	6.38 ± 0.05
TC14000626.hg.1	HHIPL1	7.47 ± 0.26	7.49 ± 0.21	7.48 ± 0.20	7.57 ± 0.05
TC01003866.hg.1	HHIPL2	6.15 ± 0.19	6.05 ± 0.08	6.02 ± 0.06	6.07 ± 0.06
TC08002621.hg.1	HHLA1	5.65 ± 0.17	5.61 ± 0.12	5.60 ± 0.11	5.68 ± 0.09
TC03000545.hg.1	HHLA2	4.99 ± 0.08	4.93 ± 0.05	4.94 ± 0.05	4.95 ± 0.06
TC01000756.hg.1	HHLA3	7.22 ± 0.09	7.23 ± 0.03	7.20 ± 0.10	7.22 ± 0.02
TC01000898.hg.1	HIAT1	9.84 ± 0.28	10.07 ± 0.21	10.01 ± 0.37	9.99 ± 0.08
TC09000463.hg.1	HIATL1	10.05 ± 0.38	9.60 ± 0.18	10.33 ± 0.36	9.45 ± 0.16
TC09001380.hg.1	HIATL2	9.22 ± 0.14	8.95 ± 0.12	9.30 ± 0.15	8.93 ± 0.08
TC07001230.hg.1	HIBADH	7.69 ± 0.04	7.76 ± 0.07	7.46 ± 0.06	7.62 ± 0.05
TC02002619.hg.1	HIBCH	6.67 ± 0.17	6.61 ± 0.14	6.59 ± 0.20	6.43 ± 0.07
TC17000019.hg.1	HIC1	7.95 ± 0.12	7.99 ± 0.10	7.91 ± 0.23	7.98 ± 0.06
TC22000088.hg.1	HIC2	8.78 ± 0.15	8.74 ± 0.09	8.66 ± 0.14	8.84 ± 0.07
TC17001867.hg.1	HID1	7.86 ± 0.17	7.68 ± 0.19	7.65 ± 0.17	7.77 ± 0.04
TC14002197.hg.1	HIF1A	10.32 ± 0.41	11.02 ± 0.11	10.89 ± 0.45	11.21 ± 0.07
TC14001196.hg.1	HIF1A-AS1	6.49 ± 0.08	6.40 ± 0.18	6.60 ± 0.04	6.58 ± 0.16
TC14001197.hg.1	HIF1A-AS2	6.09 ± 0.07	6.05 ± 0.01	6.16 ± 0.24	6.05 ± 0.04
TC14001198.hg.1	HIF1A-AS2	4.32 ± 0.13	4.33 ± 0.20	4.39 ± 0.36	4.19 ± 0.06
TC10000724.hg.1	HIF1AN	8.41 ± 0.06	8.07 ± 0.16	8.24 ± 0.21	8.01 ± 0.04
TC19000662.hg.1	HIF3A	7.53 ± 0.14	7.54 ± 0.16	7.48 ± 0.14	7.60 ± 0.06

TC03003376.hg.1	HIGD1A	9.15 ± 0.02	9.12 ± 0.06	9.23 ± 0.26	8.99 ± 0.11
TC17000575.hg.1	HIGD1B	6.55 ± 0.11	6.40 ± 0.09	6.43 ± 0.12	6.32 ± 0.07
TC12000400.hg.1	HIGD1C	4.29 ± 0.16	4.19 ± 0.06	4.33 ± 0.07	4.27 ± 0.07
TC05000999.hg.1	HIGD2A	9.99 ± 0.18	9.57 ± 0.32	9.55 ± 0.11	9.35 ± 0.06
TC15001629.hg.1	HIGD2B	6.43 ± 0.14	6.33 ± 0.16	6.42 ± 0.34	6.48 ± 0.12
TC07003293.hg.1	HILPDA	6.84 ± 0.13	6.85 ± 0.09	6.85 ± 0.13	6.89 ± 0.03
TC17001681.hg.1	HILS1	8.02 ± 0.12	8.02 ± 0.15	7.95 ± 0.27	8.12 ± 0.13
TC11001085.hg.1	HINFP	8.38 ± 0.02	8.21 ± 0.08	8.30 ± 0.11	8.24 ± 0.02
TC05001758.hg.1	HINT1	8.38 ± 0.16	8.51 ± 0.16	8.10 ± 0.08	8.54 ± 0.05
TC09001064.hg.1	HINT2	8.17 ± 0.20	8.03 ± 0.14	7.90 ± 0.16	8.04 ± 0.07
TC06000963.hg.1	HINT3	8.35 ± 0.33	8.46 ± 0.14	8.85 ± 0.52	8.34 ± 0.26
TC07001534.hg.1	HIP1	8.38 ± 0.20	8.39 ± 0.16	8.96 ± 0.30	8.19 ± 0.16
TC12000979.hg.1	HIP1R	8.63 ± 0.12	8.73 ± 0.09	8.39 ± 0.17	8.84 ± 0.12
TC01001003.hg.1	HIPK1	9.82 ± 0.17	9.90 ± 0.07	10.01 ± 0.25	9.88 ± 0.18
TC07001915.hg.1	HIPK2	9.96 ± 0.17	9.69 ± 0.03	9.92 ± 0.32	9.86 ± 0.28
TC11000316.hg.1	HIPK3	9.70 ± 0.31	9.82 ± 0.29	10.22 ± 0.43	9.68 ± 0.27
TC19001538.hg.1	HIPK4	7.51 ± 0.23	7.53 ± 0.21	7.48 ± 0.15	7.63 ± 0.02
TC16001014.hg.1	HIRIP3	7.88 ± 0.22	7.72 ± 0.09	7.65 ± 0.21	7.71 ± 0.03
TC06001340.hg.1	HIST1H1A	5.93 ± 0.18	5.78 ± 0.08	5.72 ± 0.32	5.65 ± 0.10
TC06001426.hg.1	HIST1H1B	5.99 ± 0.62	5.61 ± 0.24	5.71 ± 0.53	5.05 ± 0.20
TC06001345.hg.1	HIST1H1C	8.05 ± 0.48	7.76 ± 0.37	7.78 ± 0.23	7.54 ± 0.10
TC06001351.hg.1	HIST1H1D	5.66 ± 0.45	5.20 ± 0.23	5.11 ± 0.32	5.10 ± 0.03
TC06000168.hg.1	HIST1H1E	11.95 ± 0.49	11.75 ± 0.24	11.37 ± 0.31	11.55 ± 0.10
TC06001346.hg.1	HIST1H1T	7.04 ± 0.21	6.94 ± 0.12	7.07 ± 0.27	6.80 ± 0.08
TC06001335.hg.1	HIST1H2AA	5.49 ± 0.24	5.38 ± 0.10	5.36 ± 0.18	5.42 ± 0.25
TC06001343.hg.1	HIST1H2AB	9.15 ± 0.41	8.30 ± 0.49	8.41 ± 0.47	7.68 ± 0.40
TC06000167.hg.1	HIST1H2AC	10.39 ± 0.16	9.68 ± 0.19	10.22 ± 0.38	9.61 ± 0.31
TC06000173.hg.1	HIST1H2AE	9.59 ± 0.53	9.38 ± 0.47	9.06 ± 0.42	8.76 ± 0.27
TC06000223.hg.1	HIST1H2AG	9.73 ± 0.15	9.23 ± 0.12	9.37 ± 0.30	8.66 ± 0.28
TC06000225.hg.1	HIST1H2AH	7.62 ± 0.18	7.50 ± 0.22	7.27 ± 0.31	7.22 ± 0.05
TC06004063.hg.1	HIST1H2AI	9.31 ± 0.25	8.70 ± 0.24	9.17 ± 0.20	8.46 ± 0.32

TC06001422.hg.1	HIST1H2AJ	4.47 ± 0.07	4.32 ± 0.25	4.16 ± 0.30	4.08 ± 0.01
TC06001425.hg.1	HIST1H2AK	7.79 ± 0.27	7.74 ± 0.24	7.81 ± 0.16	7.37 ± 0.32
TC06000269.hg.1	HIST1H2AL	7.78 ± 0.33	7.42 ± 0.18	7.52 ± 0.28	7.20 ± 0.06
TC06001430.hg.1	HIST1H2AM	6.99 ± 0.42	6.48 ± 0.51	6.50 ± 0.37	5.97 ± 0.28
TC06000159.hg.1	HIST1H2APS1	7.91 ± 0.24	7.71 ± 0.19	7.62 ± 0.25	7.76 ± 0.08
TC06000158.hg.1	HIST1H2BA	4.94 ± 0.23	4.72 ± 0.09	4.90 ± 0.16	4.65 ± 0.14
TC06001344.hg.1	HIST1H2BB	6.95 ± 0.20	6.50 ± 0.29	6.49 ± 0.33	6.21 ± 0.34
TC06001347.hg.1	HIST1H2BC	9.11 ± 0.16	8.69 ± 0.23	9.17 ± 0.29	8.33 ± 0.43
TC06000169.hg.1	HIST1H2BD	8.67 ± 0.15	8.12 ± 0.28	8.44 ± 0.23	7.68 ± 0.38
TC06000170.hg.1	HIST1H2BE	5.88 ± 0.42	5.61 ± 0.25	5.92 ± 0.39	5.43 ± 0.20
TC06000171.hg.1	HIST1H2BF	9.81 ± 0.27	9.20 ± 0.21	9.51 ± 0.29	8.61 ± 0.38
TC06001350.hg.1	HIST1H2BG	9.17 ± 0.29	8.74 ± 0.40	8.47 ± 0.30	8.20 ± 0.26
TC06000176.hg.1	HIST1H2BH	9.49 ± 0.35	8.54 ± 0.30	8.94 ± 0.37	8.02 ± 0.36
TC06000178.hg.1	HIST1H2BI	8.64 ± 0.62	7.87 ± 0.34	7.99 ± 0.35	7.43 ± 0.35
TC06001385.hg.1	HIST1H2BJ	8.27 ± 0.08	7.67 ± 0.14	8.16 ± 0.22	7.61 ± 0.16
TC06001386.hg.1	HIST1H2BJ	8.53 ± 0.29	8.20 ± 0.19	8.35 ± 0.23	7.83 ± 0.26
TC06001387.hg.1	HIST1H2BK	11.91 ± 0.36	11.52 ± 0.13	12.06 ± 0.13	11.31 ± 0.24
TC06001421.hg.1	HIST1H2BL	6.84 ± 0.17	6.36 ± 0.08	6.63 ± 0.42	6.13 ± 0.29
TC06000265.hg.1	HIST1H2BM	9.78 ± 0.52	9.02 ± 0.55	9.29 ± 0.66	8.28 ± 0.21
TC06000268.hg.1	HIST1H2BN	6.79 ± 0.28	6.36 ± 0.07	6.38 ± 0.10	6.18 ± 0.14
TC06000270.hg.1	HIST1H2BO	9.07 ± 0.32	8.51 ± 0.21	8.77 ± 0.16	8.01 ± 0.24
TC06000162.hg.1	HIST1H3A	9.22 ± 0.19	9.55 ± 0.02	8.87 ± 0.27	9.18 ± 0.39
TC06001342.hg.1	HIST1H3B	7.82 ± 0.90	6.98 ± 0.78	7.18 ± 0.78	5.99 ± 0.25
TC06000164.hg.1	HIST1H3C	6.20 ± 0.51	5.68 ± 0.43	5.86 ± 0.51	5.03 ± 0.14
TC06001349.hg.1	HIST1H3D	7.47 ± 0.33	6.84 ± 0.26	7.11 ± 0.34	6.47 ± 0.25
TC06000174.hg.1	HIST1H3E	7.26 ± 0.15	6.88 ± 0.31	6.95 ± 0.26	6.57 ± 0.07
TC06001353.hg.1	HIST1H3F	9.13 ± 0.76	8.57 ± 0.38	8.46 ± 0.77	8.02 ± 0.15
TC06001427.hg.1	HIST1H3F	10.01 ± 0.48	9.52 ± 0.42	9.55 ± 0.74	8.95 ± 0.23
TC06001354.hg.1	HIST1H3G	8.00 ± 0.34	7.28 ± 0.26	7.64 ± 0.33	7.11 ± 0.18
TC06004064.hg.1	HIST1H3H	10.49 ± 0.36	9.30 ± 0.15	10.16 ± 0.42	9.07 ± 0.32
TC06001429.hg.1	HIST1H3J	8.98 ± 0.33	8.53 ± 0.32	8.48 ± 0.45	7.98 ± 0.22

TC06000163.hg.1	HIST1H4A	8.71 ± 0.14	8.70 ± 0.03	8.45 ± 0.22	8.41 ± 0.36
TC06001341.hg.1	HIST1H4B	8.26 ± 0.33	7.86 ± 0.43	8.08 ± 0.66	7.22 ± 0.37
TC06000166.hg.1	HIST1H4C	9.90 ± 0.32	9.92 ± 0.35	9.72 ± 0.37	9.45 ± 0.30
TC06001348.hg.1	HIST1H4D	9.48 ± 0.28	8.80 ± 0.28	9.11 ± 0.26	8.26 ± 0.28
TC06000172.hg.1	HIST1H4E	9.70 ± 0.26	9.47 ± 0.33	9.28 ± 0.35	9.09 ± 0.12
TC06000175.hg.1	HIST1H4F	6.89 ± 0.57	6.43 ± 0.31	6.61 ± 0.43	6.15 ± 0.13
TC06001352.hg.1	HIST1H4G	4.28 ± 0.26	4.13 ± 0.16	4.22 ± 0.29	3.96 ± 0.09
TC06001355.hg.1	HIST1H4H	10.62 ± 0.15	9.14 ± 0.27	10.10 ± 0.23	8.63 ± 0.30
TC06000224.hg.1	HIST1H4I	6.99 ± 0.20	6.72 ± 0.11	6.84 ± 0.16	6.66 ± 0.16
TC06000266.hg.1	HIST1H4J	9.15 ± 0.39	8.71 ± 0.25	8.73 ± 0.20	8.41 ± 0.20
TC06001428.hg.1	HIST1H4L	8.67 ± 0.49	7.55 ± 0.38	7.99 ± 0.48	7.07 ± 0.29
TC01001174.hg.1	HIST2H2AA4	12.27 ± 0.36	12.06 ± 0.06	12.23 ± 0.28	11.68 ± 0.35
TC01003190.hg.1	HIST2H2AA4	10.39 ± 0.35	10.22 ± 0.10	10.43 ± 0.19	9.89 ± 0.34
TC01003194.hg.1	HIST2H2AB	8.18 ± 0.44	7.84 ± 0.43	7.96 ± 0.33	7.53 ± 0.22
TC01006326.hg.1	HIST2H2AC	11.75 ± 0.16	11.64 ± 0.28	11.53 ± 0.13	11.45 ± 0.21
TC01001046.hg.1	HIST2H2BA	6.41 ± 0.29	5.93 ± 0.05	6.33 ± 0.19	5.78 ± 0.10
TC01003191.hg.1	HIST2H2BC	9.50 ± 0.17	9.23 ± 0.25	9.50 ± 0.21	8.96 ± 0.42
TC01003193.hg.1	HIST2H2BE	11.44 ± 0.22	10.99 ± 0.21	11.51 ± 0.23	10.63 ± 0.51
TC01003172.hg.1	HIST2H2BF	8.44 ± 0.17	8.34 ± 0.39	8.36 ± 0.24	8.00 ± 0.34
TC01003187.hg.1	HIST2H2BF	7.58 ± 0.24	7.29 ± 0.15	7.80 ± 0.51	7.15 ± 0.20
TC01001175.hg.1	HIST2H3A	9.00 ± 0.55	8.34 ± 0.27	8.59 ± 0.31	7.83 ± 0.14
TC01003189.hg.1	HIST2H3A	8.77 ± 0.54	8.14 ± 0.30	8.37 ± 0.26	7.74 ± 0.16
TC01003188.hg.1	HIST2H3D	9.73 ± 0.21	9.18 ± 0.30	9.54 ± 0.31	8.79 ± 0.43
TC01001173.hg.1	HIST2H4B	10.09 ± 0.16	9.48 ± 0.21	9.99 ± 0.34	9.03 ± 0.37
TC01003192.hg.1	HIST2H4B	9.99 ± 0.19	9.40 ± 0.19	9.91 ± 0.33	8.96 ± 0.35
TC06001424.hg.1	HIST2H4B	10.36 ± 0.27	9.85 ± 0.42	10.02 ± 0.34	9.43 ± 0.39
TC01003916.hg.1	HIST3H2A	8.36 ± 0.30	8.13 ± 0.18	8.05 ± 0.27	7.91 ± 0.04
TC01001893.hg.1	HIST3H2BB	8.25 ± 0.19	7.99 ± 0.21	7.96 ± 0.16	7.75 ± 0.13
TC01003915.hg.1	HIST3H3	8.21 ± 0.49	8.01 ± 0.24	8.06 ± 0.62	8.05 ± 0.11
TC12001272.hg.1	HIST4H4	7.87 ± 0.10	7.87 ± 0.18	7.57 ± 0.13	7.70 ± 0.21
TC06000086.hg.1	HIVEP1	8.17 ± 0.21	8.40 ± 0.11	8.37 ± 0.29	8.58 ± 0.21

TC06002181.hg.1	HIVEP2	9.85 ± 0.28	10.14 ± 0.17	9.80 ± 0.44	10.02 ± 0.15
TC01002568.hg.1	HIVEP3	7.51 ± 0.05	7.51 ± 0.06	7.43 ± 0.10	7.53 ± 0.00
TC02002886.hg.1	HJURP	7.77 ± 0.17	7.56 ± 0.14	7.66 ± 0.25	7.49 ± 0.06
TC10000419.hg.1	HK1	8.74 ± 0.12	8.47 ± 0.04	8.69 ± 0.22	8.46 ± 0.12
TC02000466.hg.1	HK2	8.58 ± 0.26	8.27 ± 0.08	8.86 ± 0.29	8.23 ± 0.09
TC05002100.hg.1	HK3	9.16 ± 0.36	8.63 ± 0.08	9.90 ± 0.43	8.67 ± 0.05
TC10000418.hg.1	HKDC1	6.77 ± 0.15	6.62 ± 0.11	6.62 ± 0.17	6.68 ± 0.05
TC19000514.hg.1	HKR1	7.73 ± 0.14	7.72 ± 0.06	7.57 ± 0.02	7.73 ± 0.04
TC06000328.hg.1	HLA-A	10.66 ± 0.12	10.65 ± 0.02	10.63 ± 0.15	10.61 ± 0.05
TC6_apd_hap1000019.hg.1	HLA-A	10.68 ± 0.09	10.67 ± 0.05	10.60 ± 0.15	10.59 ± 0.06
TC6_cox_hap2000026.hg.1	HLA-A	10.68 ± 0.10	10.67 ± 0.05	10.61 ± 0.15	10.60 ± 0.06
TC6_dbb_hap3000213.hg.1	HLA-A	11.09 ± 0.15	11.04 ± 0.07	10.98 ± 0.21	10.99 ± 0.05
TC6_mann_hap4000022.hg.1	HLA-A	10.86 ± 0.16	10.86 ± 0.04	10.82 ± 0.22	10.83 ± 0.05
TC6_mcf_hap5000206.hg.1	HLA-A	12.26 ± 0.20	12.22 ± 0.05	12.18 ± 0.21	12.16 ± 0.06
TC6_qbl_hap6000218.hg.1	HLA-A	10.86 ± 0.15	10.84 ± 0.04	10.79 ± 0.23	10.79 ± 0.06
TC6_ssto_hap7000020.hg.1	HLA-A	10.81 ± 0.16	10.80 ± 0.04	10.76 ± 0.23	10.77 ± 0.05
TC01003844.hg.1	HLA-AS1	8.13 ± 0.17	8.17 ± 0.06	8.18 ± 0.05	8.18 ± 0.05
TC06004060.hg.1	HLA-B	12.14 ± 0.10	12.12 ± 0.03	12.06 ± 0.18	12.10 ± 0.06
TC6_cox_hap2000237.hg.1	HLA-B	12.14 ± 0.14	12.16 ± 0.02	12.06 ± 0.22	12.14 ± 0.04
TC6_mann_hap4000188.hg.1	HLA-B	11.89 ± 0.12	11.94 ± 0.02	11.84 ± 0.20	11.90 ± 0.03
TC6_mcf_hap5000195.hg.1	HLA-B	12.12 ± 0.14	12.15 ± 0.05	12.02 ± 0.21	12.06 ± 0.04
TC6_qbl_hap6000147.hg.1	HLA-B	12.38 ± 0.13	12.44 ± 0.05	12.30 ± 0.23	12.36 ± 0.04
TC6_ssto_hap7000191.hg.1	HLA-B	11.96 ± 0.12	12.01 ± 0.02	11.90 ± 0.21	11.97 ± 0.03
TC06004059.hg.1	HLA-C	11.71 ± 0.16	11.70 ± 0.04	11.64 ± 0.13	11.61 ± 0.09
TC6_cox_hap2000236.hg.1	HLA-C	11.73 ± 0.16	11.71 ± 0.03	11.66 ± 0.13	11.62 ± 0.07
TC6_dbb_hap3000145.hg.1	HLA-C	12.17 ± 0.13	12.17 ± 0.05	12.18 ± 0.18	12.12 ± 0.04
TC6_mann_hap4000187.hg.1	HLA-C	12.53 ± 0.16	12.51 ± 0.06	12.52 ± 0.17	12.45 ± 0.05
TC6_mcf_hap5000194.hg.1	HLA-C	12.41 ± 0.16	12.41 ± 0.06	12.44 ± 0.21	12.36 ± 0.05
TC6_qbl_hap6000146.hg.1	HLA-C	11.98 ± 0.14	11.95 ± 0.07	11.97 ± 0.21	11.92 ± 0.05
TC6_ssto_hap7000190.hg.1	HLA-C	12.06 ± 0.15	12.03 ± 0.07	12.05 ± 0.21	11.99 ± 0.05
TC06004128.hg.1	HLA-DMA	9.42 ± 0.02	9.57 ± 0.16	8.97 ± 0.02	9.49 ± 0.06

TC6_apd_hap1000118.hg.1	HLA-DMA	10.05 ± 0.06	10.20 ± 0.21	9.52 ± 0.05	10.10 ± 0.04
TC6_cox_hap2000233.hg.1	HLA-DMA	9.53 ± 0.05	9.66 ± 0.17	9.06 ± 0.01	9.57 ± 0.04
TC6_dbb_hap3000210.hg.1	HLA-DMA	10.05 ± 0.07	10.20 ± 0.22	9.52 ± 0.05	10.09 ± 0.03
TC6_mann_hap4000210.hg.1	HLA-DMA	9.53 ± 0.05	9.66 ± 0.17	9.06 ± 0.01	9.57 ± 0.04
TC6_mcf_hap5000231.hg.1	HLA-DMA	9.53 ± 0.05	9.66 ± 0.17	9.06 ± 0.01	9.57 ± 0.04
TC6_qbl_hap6000237.hg.1	HLA-DMA	9.53 ± 0.05	9.66 ± 0.17	9.06 ± 0.01	9.57 ± 0.04
TC6_ssto_hap7000220.hg.1	HLA-DMA	9.53 ± 0.05	9.66 ± 0.17	9.06 ± 0.01	9.57 ± 0.04
TC06004127.hg.1	HLA-DMB	9.40 ± 0.05	9.42 ± 0.14	8.88 ± 0.07	9.29 ± 0.13
TC6_apd_hap1000117.hg.1	HLA-DMB	9.28 ± 0.04	9.29 ± 0.14	8.75 ± 0.05	9.16 ± 0.13
TC6_cox_hap2000232.hg.1	HLA-DMB	9.35 ± 0.05	9.36 ± 0.14	8.82 ± 0.06	9.24 ± 0.13
TC6_dbb_hap3000209.hg.1	HLA-DMB	9.22 ± 0.05	9.23 ± 0.14	8.70 ± 0.07	9.11 ± 0.13
TC6_mann_hap4000209.hg.1	HLA-DMB	9.28 ± 0.04	9.29 ± 0.14	8.75 ± 0.05	9.16 ± 0.13
TC6_mcf_hap5000230.hg.1	HLA-DMB	9.23 ± 0.05	9.23 ± 0.14	8.70 ± 0.06	9.11 ± 0.13
TC6_qbl_hap6000236.hg.1	HLA-DMB	9.19 ± 0.04	9.20 ± 0.14	8.67 ± 0.06	9.08 ± 0.13
TC6_ssto_hap7000219.hg.1	HLA-DMB	9.22 ± 0.05	9.23 ± 0.13	8.70 ± 0.06	9.11 ± 0.13
TC06001577.hg.1	HLA-DOA	8.73 ± 0.12	8.77 ± 0.06	8.44 ± 0.06	8.62 ± 0.08
TC6_cox_hap2000201.hg.1	HLA-DOA	8.71 ± 0.09	8.76 ± 0.06	8.47 ± 0.03	8.64 ± 0.08
TC6_dbb_hap3000187.hg.1	HLA-DOA	8.69 ± 0.11	8.73 ± 0.09	8.40 ± 0.07	8.59 ± 0.06
TC6_mann_hap4000169.hg.1	HLA-DOA	8.69 ± 0.11	8.73 ± 0.09	8.40 ± 0.07	8.59 ± 0.06
TC6_mcf_hap5000176.hg.1	HLA-DOA	8.64 ± 0.11	8.70 ± 0.06	8.40 ± 0.04	8.58 ± 0.09
TC6_qbl_hap6000189.hg.1	HLA-DOA	8.62 ± 0.11	8.67 ± 0.06	8.38 ± 0.04	8.55 ± 0.08
TC6_ssto_hap7000171.hg.1	HLA-DOA	8.66 ± 0.09	8.70 ± 0.09	8.38 ± 0.08	8.55 ± 0.06
TC06004125.hg.1	HLA-DOB	8.57 ± 0.18	8.70 ± 0.24	8.17 ± 0.12	8.41 ± 0.23
TC6_apd_hap1000129.hg.1	HLA-DOB	8.40 ± 0.18	8.55 ± 0.23	8.02 ± 0.13	8.25 ± 0.23
TC6_cox_hap2000256.hg.1	HLA-DOB	8.53 ± 0.18	8.68 ± 0.23	8.16 ± 0.12	8.38 ± 0.24
TC6_dbb_hap3000241.hg.1	HLA-DOB	8.29 ± 0.20	8.42 ± 0.24	7.89 ± 0.13	8.11 ± 0.22
TC6_mcf_hap5000224.hg.1	HLA-DOB	8.48 ± 0.19	8.62 ± 0.23	8.09 ± 0.12	8.31 ± 0.23
TC6_ssto_hap7000212.hg.1	HLA-DOB	8.40 ± 0.18	8.55 ± 0.23	8.02 ± 0.13	8.25 ± 0.23
TC06001578.hg.1	HLA-DPA1	9.98 ± 0.21	9.92 ± 0.13	9.45 ± 0.09	9.88 ± 0.19
TC6_apd_hap1000107.hg.1	HLA-DPA1	9.97 ± 0.21	9.92 ± 0.13	9.45 ± 0.10	9.89 ± 0.20
TC6_cox_hap2000202.hg.1	HLA-DPA1	10.31 ± 0.21	10.25 ± 0.13	9.75 ± 0.10	10.22 ± 0.21

TC6_dbb_hap3000188.hg.1	HLA-DPA1	9.98 ± 0.21	9.92 ± 0.13	9.45 ± 0.09	9.88 ± 0.19
TC6_mann_hap4000170.hg.1	HLA-DPA1	10.00 ± 0.20	9.95 ± 0.14	9.48 ± 0.09	9.91 ± 0.20
TC6_mcf_hap5000177.hg.1	HLA-DPA1	9.97 ± 0.21	9.92 ± 0.13	9.45 ± 0.10	9.89 ± 0.20
TC6_qbl_hap6000190.hg.1	HLA-DPA1	10.00 ± 0.20	9.95 ± 0.14	9.48 ± 0.09	9.91 ± 0.20
TC6_ssto_hap7000172.hg.1	HLA-DPA1	10.06 ± 0.21	9.99 ± 0.12	9.53 ± 0.09	9.96 ± 0.20
TC06000408.hg.1	HLA-DPB1	9.60 ± 0.09	9.76 ± 0.14	9.24 ± 0.02	9.73 ± 0.07
TC6_apd_hap1000123.hg.1	HLA-DPB1	9.38 ± 0.08	9.54 ± 0.15	9.04 ± 0.03	9.51 ± 0.07
TC6_cox_hap2000228.hg.1	HLA-DPB1	8.60 ± 0.14	8.78 ± 0.04	8.33 ± 0.04	8.76 ± 0.10
TC6_dbb_hap3000091.hg.1	HLA-DPB1	9.41 ± 0.08	9.57 ± 0.15	9.07 ± 0.03	9.54 ± 0.07
TC6_mann_hap4000213.hg.1	HLA-DPB1	9.38 ± 0.06	9.54 ± 0.15	9.04 ± 0.03	9.51 ± 0.07
TC6_mcf_hap5000227.hg.1	HLA-DPB1	9.38 ± 0.08	9.54 ± 0.15	9.04 ± 0.03	9.51 ± 0.07
TC6_qbl_hap6000210.hg.1	HLA-DPB1	9.37 ± 0.06	9.53 ± 0.15	9.03 ± 0.04	9.50 ± 0.07
TC6_ssto_hap7000085.hg.1	HLA-DPB1	9.41 ± 0.08	9.57 ± 0.15	9.07 ± 0.03	9.54 ± 0.07
TC06000409.hg.1	HLA-DPB2	7.68 ± 0.29	7.64 ± 0.09	7.20 ± 0.14	7.57 ± 0.03
TC6_apd_hap1000124.hg.1	HLA-DPB2	8.31 ± 0.33	8.24 ± 0.10	7.79 ± 0.18	8.18 ± 0.05
TC6_cox_hap2000229.hg.1	HLA-DPB2	8.61 ± 0.28	8.53 ± 0.10	8.13 ± 0.23	8.47 ± 0.06
TC6_mann_hap4000214.hg.1	HLA-DPB2	8.87 ± 0.28	8.83 ± 0.13	8.38 ± 0.16	8.77 ± 0.08
TC6_mcf_hap5000226.hg.1	HLA-DPB2	8.94 ± 0.25	8.90 ± 0.09	8.49 ± 0.17	8.82 ± 0.03
TC6_qbl_hap6000211.hg.1	HLA-DPB2	8.87 ± 0.28	8.83 ± 0.13	8.38 ± 0.16	8.77 ± 0.08
TC6_ssto_hap7000086.hg.1	HLA-DPB2	8.79 ± 0.30	8.71 ± 0.09	8.33 ± 0.22	8.65 ± 0.07
TC06000400.hg.1	HLA-DQA1	8.69 ± 0.07	8.79 ± 0.25	8.21 ± 0.30	8.44 ± 0.24
TC6_cox_hap2000091.hg.1	HLA-DQA1	10.18 ± 0.05	10.25 ± 0.42	9.64 ± 0.10	10.24 ± 0.25
TC6_dbb_hap3000084.hg.1	HLA-DQA1	8.02 ± 0.22	8.22 ± 0.14	7.73 ± 0.21	8.10 ± 0.12
TC6_mann_hap4000075.hg.1	HLA-DQA1	8.02 ± 0.22	8.22 ± 0.14	7.73 ± 0.21	8.10 ± 0.12
TC6_mcf_hap5000076.hg.1	HLA-DQA1	8.37 ± 0.22	8.67 ± 0.22	8.15 ± 0.23	8.57 ± 0.15
TC6_qbl_hap6000082.hg.1	HLA-DQA1	10.18 ± 0.05	10.25 ± 0.42	9.64 ± 0.10	10.24 ± 0.25
TC6_ssto_hap7000077.hg.1	HLA-DQA1	10.02 ± 0.21	10.32 ± 0.26	9.63 ± 0.30	10.20 ± 0.24
TC06000403.hg.1	HLA-DQA2	8.85 ± 0.07	9.00 ± 0.20	8.44 ± 0.10	8.82 ± 0.11
TC6_apd_hap1000047.hg.1	HLA-DQA2	9.47 ± 0.04	9.63 ± 0.23	9.04 ± 0.17	9.42 ± 0.11
TC6_cox_hap2000094.hg.1	HLA-DQA2	9.31 ± 0.03	9.53 ± 0.23	8.91 ± 0.15	9.35 ± 0.12
TC6_dbb_hap3000087.hg.1	HLA-DQA2	9.27 ± 0.02	9.38 ± 0.24	8.79 ± 0.16	9.17 ± 0.13

TC6_mann_hap4000078.hg.1	HLA-DQA2	9.25 ± 0.06	9.42 ± 0.22	8.74 ± 0.13	9.22 ± 0.11
TC6_mcf_hap5000079.hg.1	HLA-DQA2	8.84 ± 0.02	8.96 ± 0.22	8.36 ± 0.11	8.77 ± 0.12
TC6_qbl_hap6000085.hg.1	HLA-DQA2	8.92 ± 0.09	9.05 ± 0.20	8.40 ± 0.12	8.85 ± 0.11
TC6_ssto_hap7000080.hg.1	HLA-DQA2	9.25 ± 0.07	9.40 ± 0.20	8.82 ± 0.12	9.21 ± 0.12
TC06001570.hg.1	HLA-DQB1	8.80 ± 0.06	8.85 ± 0.28	8.15 ± 0.09	8.53 ± 0.15
TC6_cox_hap2000195.hg.1	HLA-DQB1	8.73 ± 0.17	8.76 ± 0.18	8.19 ± 0.09	8.66 ± 0.09
TC6_dbb_hap3000181.hg.1	HLA-DQB1	9.34 ± 0.18	9.51 ± 0.13	8.77 ± 0.04	9.37 ± 0.08
TC6_qbl_hap6000183.hg.1	HLA-DQB1	8.86 ± 0.16	8.89 ± 0.17	8.34 ± 0.09	8.81 ± 0.09
TC6_ssto_hap7000165.hg.1	HLA-DQB1	9.25 ± 0.15	9.48 ± 0.17	8.74 ± 0.09	9.35 ± 0.08
TC06000401.hg.1	HLA-DQB1-AS1	6.97 ± 0.29	6.83 ± 0.07	6.66 ± 0.11	6.71 ± 0.12
TC06001571.hg.1	HLA-DQB2	8.31 ± 0.20	8.37 ± 0.08	7.96 ± 0.18	8.40 ± 0.10
TC6_apd_hap1000102.hg.1	HLA-DQB2	8.40 ± 0.19	8.45 ± 0.06	8.05 ± 0.18	8.49 ± 0.09
TC6_cox_hap2000196.hg.1	HLA-DQB2	8.40 ± 0.19	8.45 ± 0.06	8.05 ± 0.18	8.49 ± 0.09
TC6_dbb_hap3000182.hg.1	HLA-DQB2	8.31 ± 0.20	8.37 ± 0.06	7.93 ± 0.16	8.39 ± 0.09
TC6_mann_hap4000164.hg.1	HLA-DQB2	8.30 ± 0.19	8.35 ± 0.09	7.95 ± 0.18	8.39 ± 0.09
TC6_mcf_hap5000171.hg.1	HLA-DQB2	8.31 ± 0.20	8.37 ± 0.06	7.93 ± 0.16	8.39 ± 0.09
TC6_qbl_hap6000184.hg.1	HLA-DQB2	8.30 ± 0.19	8.35 ± 0.09	7.95 ± 0.18	8.39 ± 0.09
TC6_ssto_hap7000166.hg.1	HLA-DQB2	8.31 ± 0.20	8.37 ± 0.08	7.96 ± 0.18	8.40 ± 0.10
TC06000399.hg.1	HLA-DRA	12.36 ± 0.12	12.58 ± 0.11	12.02 ± 0.03	12.54 ± 0.08
TC6_cox_hap2000090.hg.1	HLA-DRA	12.36 ± 0.10	12.58 ± 0.10	12.04 ± 0.03	12.56 ± 0.07
TC6_dbb_hap3000083.hg.1	HLA-DRA	12.41 ± 0.11	12.61 ± 0.10	12.11 ± 0.05	12.59 ± 0.08
TC6_mann_hap4000074.hg.1	HLA-DRA	12.49 ± 0.10	12.72 ± 0.09	12.18 ± 0.04	12.70 ± 0.07
TC6_mcf_hap5000075.hg.1	HLA-DRA	12.48 ± 0.09	12.72 ± 0.08	12.18 ± 0.04	12.70 ± 0.07
TC6_qbl_hap6000081.hg.1	HLA-DRA	12.48 ± 0.09	12.72 ± 0.08	12.18 ± 0.04	12.70 ± 0.07
TC6_ssto_hap7000076.hg.1	HLA-DRA	12.48 ± 0.09	12.72 ± 0.08	12.18 ± 0.04	12.70 ± 0.07
TC06004123.hg.1	HLA-DRB1	11.51 ± 0.12	11.43 ± 0.12	11.04 ± 0.03	11.33 ± 0.05
TC6_apd_hap1000101.hg.1	HLA-DRB1	12.39 ± 0.19	12.29 ± 0.05	11.84 ± 0.08	12.22 ± 0.08
TC6_cox_hap2000193.hg.1	HLA-DRB1	12.08 ± 0.16	11.95 ± 0.06	11.58 ± 0.16	11.95 ± 0.17
TC6_qbl_hap6000182.hg.1	HLA-DRB1	12.09 ± 0.17	11.96 ± 0.07	11.58 ± 0.15	11.93 ± 0.16
TC6_ssto_hap7000163.hg.1	HLA-DRB1	11.82 ± 0.08	11.81 ± 0.09	11.35 ± 0.03	11.76 ± 0.07
TC6_ssto_hap7000164.hg.1	HLA-DRB1	12.69 ± 0.12	12.73 ± 0.05	12.30 ± 0.02	12.68 ± 0.03

TC6_dbb_hap3000179.hg.1	HLA-DRB3	10.42 ± 0.15	10.45 ± 0.06	9.99 ± 0.07	10.40 ± 0.02
TC6_mann_hap4000160.hg.1	HLA-DRB3	10.42 ± 0.15	10.44 ± 0.06	9.99 ± 0.09	10.39 ± 0.02
TC6_mann_hap4000159.hg.1	HLA-DRB4	11.38 ± 0.11	11.40 ± 0.14	10.92 ± 0.12	11.29 ± 0.26
TC6_mcf_hap5000169.hg.1	HLA-DRB4	11.23 ± 0.23	11.26 ± 0.14	10.78 ± 0.11	11.17 ± 0.25
TC06004122.hg.1	HLA-DRB5	11.11 ± 0.74	11.15 ± 0.39	10.54 ± 0.44	10.58 ± 0.47
TC06004124.hg.1	HLA-DRB6	8.66 ± 0.14	8.64 ± 0.07	8.31 ± 0.11	8.64 ± 0.11
TC6_cox_hap2000194.hg.1	HLA-DRB6	12.51 ± 0.36	12.66 ± 0.06	12.12 ± 0.06	12.60 ± 0.06
TC06000338.hg.1	HLA-E	12.60 ± 0.15	12.54 ± 0.03	12.63 ± 0.24	12.49 ± 0.08
TC6_cox_hap2000036.hg.1	HLA-E	12.53 ± 0.16	12.45 ± 0.03	12.55 ± 0.26	12.39 ± 0.08
TC6_dbb_hap3000029.hg.1	HLA-E	12.53 ± 0.16	12.45 ± 0.03	12.55 ± 0.26	12.39 ± 0.08
TC6_mann_hap4000031.hg.1	HLA-E	12.60 ± 0.15	12.54 ± 0.03	12.63 ± 0.24	12.49 ± 0.08
TC6_mcf_hap5000023.hg.1	HLA-E	12.60 ± 0.15	12.54 ± 0.03	12.63 ± 0.24	12.49 ± 0.08
TC6_qbl_hap6000029.hg.1	HLA-E	12.53 ± 0.16	12.45 ± 0.03	12.55 ± 0.26	12.39 ± 0.08
TC6_ssto_hap7000029.hg.1	HLA-E	12.53 ± 0.16	12.45 ± 0.03	12.55 ± 0.26	12.39 ± 0.08
TC06000323.hg.1	HLA-F	9.85 ± 0.14	9.76 ± 0.05	9.76 ± 0.18	9.70 ± 0.01
TC6_cox_hap2000020.hg.1	HLA-F	10.02 ± 0.14	9.94 ± 0.05	9.93 ± 0.17	9.88 ± 0.00
TC6_dbb_hap3000016.hg.1	HLA-F	9.75 ± 0.13	9.68 ± 0.05	9.66 ± 0.17	9.63 ± 0.01
TC6_mann_hap4000017.hg.1	HLA-F	9.75 ± 0.13	9.68 ± 0.05	9.66 ± 0.17	9.63 ± 0.01
TC6_mcf_hap5000203.hg.1	HLA-F	10.72 ± 0.16	10.61 ± 0.03	10.60 ± 0.20	10.53 ± 0.01
TC6_qbl_hap6000015.hg.1	HLA-F	9.75 ± 0.13	9.68 ± 0.05	9.66 ± 0.17	9.63 ± 0.01
TC6_ssto_hap7000016.hg.1	HLA-F	10.14 ± 0.13	10.09 ± 0.07	10.07 ± 0.18	10.03 ± 0.01
TC06001487.hg.1	HLA-F-AS1	7.67 ± 0.15	7.68 ± 0.13	7.57 ± 0.17	7.76 ± 0.05
TC6_cox_hap2000124.hg.1	HLA-F-AS1	7.92 ± 0.14	7.94 ± 0.09	7.80 ± 0.20	7.98 ± 0.05
TC6_dbb_hap3000113.hg.1	HLA-F-AS1	8.04 ± 0.16	8.06 ± 0.10	7.90 ± 0.20	8.13 ± 0.05
TC6_mann_hap4000102.hg.1	HLA-F-AS1	8.04 ± 0.16	8.06 ± 0.10	7.90 ± 0.20	8.13 ± 0.05
TC6_qbl_hap6000114.hg.1	HLA-F-AS1	8.04 ± 0.16	8.06 ± 0.10	7.90 ± 0.20	8.13 ± 0.05
TC6_ssto_hap7000105.hg.1	HLA-F-AS1	7.95 ± 0.15	7.99 ± 0.11	7.84 ± 0.21	8.03 ± 0.05
TC06004065.hg.1	HLA-G	8.71 ± 0.16	8.60 ± 0.06	8.65 ± 0.23	8.58 ± 0.04
TC6_cox_hap2000022.hg.1	HLA-G	8.40 ± 0.12	8.32 ± 0.05	8.34 ± 0.25	8.32 ± 0.05
TC6_dbb_hap3000211.hg.1	HLA-G	8.46 ± 0.13	8.37 ± 0.05	8.40 ± 0.23	8.36 ± 0.02
TC6_mann_hap4000185.hg.1	HLA-G	8.53 ± 0.13	8.44 ± 0.04	8.46 ± 0.25	8.43 ± 0.02

TC6_mcf_hap5000204.hg.1	HLA-G	8.46 ± 0.13	8.37 ± 0.05	8.40 ± 0.23	8.36 ± 0.02
TC6_qbl_hap6000017.hg.1	HLA-G	8.40 ± 0.12	8.32 ± 0.05	8.34 ± 0.25	8.32 ± 0.05
TC6_ssto_hap7000218.hg.1	HLA-G	8.44 ± 0.13	8.34 ± 0.05	8.37 ± 0.24	8.34 ± 0.05
TC06004066.hg.1	HLA-H	11.10 ± 0.13	11.02 ± 0.04	10.99 ± 0.27	10.94 ± 0.06
TC6_apd_hap1000017.hg.1	HLA-H	10.96 ± 0.14	10.88 ± 0.00	10.86 ± 0.25	10.82 ± 0.04
TC6_cox_hap2000023.hg.1	HLA-H	10.78 ± 0.13	10.74 ± 0.01	10.69 ± 0.29	10.70 ± 0.05
TC6_dbb_hap3000214.hg.1	HLA-H	10.84 ± 0.15	10.82 ± 0.04	10.78 ± 0.29	10.76 ± 0.06
TC6_mann_hap4000020.hg.1	HLA-H	10.87 ± 0.15	10.85 ± 0.01	10.78 ± 0.28	10.80 ± 0.07
TC6_mcf_hap5000207.hg.1	HLA-H	10.92 ± 0.16	10.88 ± 0.01	10.87 ± 0.26	10.81 ± 0.06
TC6_qbl_hap6000219.hg.1	HLA-H	10.88 ± 0.15	10.89 ± 0.06	10.80 ± 0.28	10.82 ± 0.06
TC6_ssto_hap7000217.hg.1	HLA-H	10.93 ± 0.13	10.90 ± 0.01	10.83 ± 0.27	10.83 ± 0.06
TC06004067.hg.1	HLA-J	9.25 ± 0.20	9.12 ± 0.04	9.14 ± 0.20	9.13 ± 0.02
TC6_apd_hap1000020.hg.1	HLA-J	8.89 ± 0.17	8.75 ± 0.03	8.77 ± 0.18	8.75 ± 0.03
TC6_cox_hap2000028.hg.1	HLA-J	8.89 ± 0.17	8.75 ± 0.03	8.77 ± 0.18	8.75 ± 0.03
TC6_dbb_hap3000212.hg.1	HLA-J	8.89 ± 0.17	8.75 ± 0.03	8.77 ± 0.18	8.75 ± 0.03
TC6_mann_hap4000186.hg.1	HLA-J	8.92 ± 0.20	8.78 ± 0.04	8.80 ± 0.20	8.79 ± 0.01
TC6_mcf_hap5000205.hg.1	HLA-J	9.24 ± 0.16	9.13 ± 0.04	9.14 ± 0.19	9.11 ± 0.03
TC6_qbl_hap6000021.hg.1	HLA-J	8.99 ± 0.19	8.84 ± 0.04	8.87 ± 0.19	8.86 ± 0.02
TC6_ssto_hap7000216.hg.1	HLA-J	9.03 ± 0.15	8.91 ± 0.05	8.91 ± 0.21	8.91 ± 0.01
TC06000335.hg.1	HLA-L	8.66 ± 0.06	8.60 ± 0.05	8.57 ± 0.13	8.59 ± 0.04
TC6_cox_hap2000034.hg.1	HLA-L	8.88 ± 0.13	8.77 ± 0.00	8.81 ± 0.17	8.72 ± 0.05
TC6_dbb_hap3000027.hg.1	HLA-L	8.67 ± 0.06	8.60 ± 0.04	8.59 ± 0.12	8.60 ± 0.05
TC6_mann_hap4000029.hg.1	HLA-L	8.59 ± 0.06	8.50 ± 0.03	8.49 ± 0.14	8.52 ± 0.07
TC6_qbl_hap6000027.hg.1	HLA-L	8.67 ± 0.06	8.61 ± 0.04	8.58 ± 0.13	8.59 ± 0.05
TC6_ssto_hap7000027.hg.1	HLA-L	8.66 ± 0.06	8.60 ± 0.05	8.58 ± 0.12	8.59 ± 0.05
TC21000436.hg.1	HLCS	7.27 ± 0.02	7.22 ± 0.05	7.09 ± 0.01	7.18 ± 0.03
TC21000437.hg.1	HLCS-IT1	6.04 ± 0.05	5.98 ± 0.10	5.77 ± 0.07	6.13 ± 0.11
TC17000693.hg.1	HLF	6.65 ± 0.21	6.62 ± 0.11	6.55 ± 0.15	6.67 ± 0.11
TC03001882.hg.1	HLTF	7.13 ± 0.51	7.15 ± 0.28	7.06 ± 0.45	6.85 ± 0.13
TC03000802.hg.1	HLTF-AS1	6.51 ± 0.28	6.31 ± 0.12	6.32 ± 0.10	6.37 ± 0.10
TC01001823.hg.1	HLX	8.90 ± 0.20	8.78 ± 0.04	9.25 ± 0.15	8.57 ± 0.10

TC20000191.hg.1	HM13	8.99 ± 0.22	8.69 ± 0.01	8.78 ± 0.18	8.60 ± 0.07
TC20000755.hg.1	HM13-AS1	7.14 ± 0.27	7.32 ± 0.21	7.25 ± 0.18	7.24 ± 0.09
TC20000192.hg.1	HM13-IT1	10.11 ± 0.15	10.00 ± 0.03	10.17 ± 0.10	10.02 ± 0.04
TC08000225.hg.1	HMBOX1	8.11 ± 0.36	8.32 ± 0.16	8.12 ± 0.31	8.19 ± 0.15
TC08000226.hg.1	HMBOX1-IT1	8.22 ± 0.15	8.16 ± 0.04	8.31 ± 0.07	8.25 ± 0.04
TC11001083.hg.1	HMBS	7.21 ± 0.11	7.11 ± 0.11	7.04 ± 0.13	7.11 ± 0.04
TC01001602.hg.1	HMCN1	5.38 ± 0.12	5.27 ± 0.07	5.30 ± 0.05	5.37 ± 0.10
TC09000732.hg.1	HMCN2	8.01 ± 0.24	7.97 ± 0.24	7.93 ± 0.23	8.06 ± 0.05
TC09000733.hg.1	HMCN2	7.95 ± 0.21	7.99 ± 0.20	7.93 ± 0.19	8.09 ± 0.07
TC09000734.hg.1	HMCN2	8.28 ± 0.39	7.86 ± 0.08	7.95 ± 0.21	7.87 ± 0.06
TC15000712.hg.1	HMG20A	7.84 ± 0.16	7.84 ± 0.15	7.63 ± 0.23	7.66 ± 0.10
TC19000072.hg.1	HMG20B	9.00 ± 0.20	8.93 ± 0.13	8.87 ± 0.24	8.92 ± 0.02
TC06000501.hg.1	HMGA1	9.30 ± 0.23	9.14 ± 0.10	9.09 ± 0.23	9.15 ± 0.11
TC0X000127.hg.1	HMGA1P1	8.58 ± 0.26	8.57 ± 0.16	8.42 ± 0.35	8.47 ± 0.26
TC09001635.hg.1	HMGA1P4	7.47 ± 0.22	7.40 ± 0.33	7.37 ± 0.17	7.51 ± 0.11
TC06002125.hg.1	HMGA1P7	7.11 ± 0.04	7.06 ± 0.19	6.96 ± 0.16	7.08 ± 0.19
TC12000587.hg.1	HMGA2	6.21 ± 0.21	6.17 ± 0.13	6.16 ± 0.14	6.22 ± 0.04
TC13000534.hg.1	HMGB1	9.02 ± 0.41	9.38 ± 0.12	9.11 ± 0.18	9.35 ± 0.12
TC17001407.hg.1	HMGB1P24	6.38 ± 0.14	6.28 ± 0.11	6.28 ± 0.08	6.25 ± 0.05
TC02002516.hg.1	HMGB1P4	9.38 ± 0.37	9.66 ± 0.08	9.42 ± 0.08	9.63 ± 0.18
TC03000120.hg.1	HMGB1P5	10.63 ± 0.44	11.00 ± 0.11	10.77 ± 0.17	10.97 ± 0.18
TC04001741.hg.1	HMGB2	9.34 ± 0.10	9.37 ± 0.06	9.52 ± 0.18	9.23 ± 0.17
TC0X000727.hg.1	HMGB3	6.87 ± 0.08	6.78 ± 0.08	6.86 ± 0.04	6.81 ± 0.06
TC20000787.hg.1	HMGB3P1	6.47 ± 0.16	6.23 ± 0.06	6.33 ± 0.09	6.33 ± 0.11
TC05001051.hg.1	HMGB3P22	6.82 ± 0.37	6.59 ± 0.09	6.69 ± 0.15	6.60 ± 0.09
TC0X000550.hg.1	HMGB3P30	4.73 ± 0.08	4.66 ± 0.23	4.79 ± 0.19	4.77 ± 0.16
TC01000444.hg.1	HMGB4	5.03 ± 0.12	4.97 ± 0.09	5.01 ± 0.09	5.09 ± 0.12
TC01002351.hg.1	HMGCL	7.78 ± 0.14	7.49 ± 0.07	7.68 ± 0.14	7.51 ± 0.01
TC06001824.hg.1	HMGCLL1	5.60 ± 0.19	5.49 ± 0.07	5.44 ± 0.06	5.54 ± 0.11
TC05000363.hg.1	HMGCR	8.36 ± 0.18	8.20 ± 0.24	8.62 ± 0.35	8.12 ± 0.22
TC05001316.hg.1	HMGCS1	7.81 ± 0.21	8.04 ± 0.12	7.79 ± 0.26	7.96 ± 0.16

TC01003050.hg.1	HMGCS2	6.69 ± 0.25	6.69 ± 0.17	6.56 ± 0.18	6.78 ± 0.11
TC21000462.hg.1	HMGN1	8.82 ± 0.27	9.03 ± 0.05	8.71 ± 0.27	8.99 ± 0.05
TC18000539.hg.1	HMGN1P30	8.35 ± 0.39	8.45 ± 0.15	8.40 ± 0.13	8.38 ± 0.12
TC0X001545.hg.1	HMGN1P37	7.73 ± 0.14	7.73 ± 0.08	7.68 ± 0.09	7.82 ± 0.10
TC15000918.hg.1	HMGN1P38	7.87 ± 0.06	7.79 ± 0.08	7.66 ± 0.30	7.67 ± 0.16
TC01000346.hg.1	HMGN2	10.26 ± 0.20	10.32 ± 0.22	10.04 ± 0.22	10.16 ± 0.13
TC07000516.hg.1	HMGN2P11	8.37 ± 0.17	8.22 ± 0.21	8.22 ± 0.25	8.14 ± 0.13
TC17001542.hg.1	HMGN2P15	8.15 ± 0.11	8.02 ± 0.09	7.93 ± 0.30	7.86 ± 0.24
TC01000109.hg.1	HMGN2P17	9.84 ± 0.08	9.75 ± 0.14	9.57 ± 0.19	9.65 ± 0.08
TC01001291.hg.1	HMGN2P18	9.51 ± 0.21	9.40 ± 0.18	9.40 ± 0.33	9.20 ± 0.23
TC03001845.hg.1	HMGN2P25	9.93 ± 0.18	9.83 ± 0.23	9.69 ± 0.23	9.61 ± 0.14
TC06000015.hg.1	HMGN2P28	8.70 ± 0.32	8.80 ± 0.22	8.68 ± 0.12	8.61 ± 0.12
TC15002763.hg.1	HMGN2P46	6.76 ± 0.12	6.71 ± 0.05	6.72 ± 0.10	6.84 ± 0.07
TC06001896.hg.1	HMGN3	8.10 ± 0.52	8.37 ± 0.18	8.08 ± 0.35	8.25 ± 0.11
TC06000195.hg.1	HMGN4	9.05 ± 0.12	8.97 ± 0.02	8.92 ± 0.10	8.84 ± 0.08
TC0X001184.hg.1	HMGN5	6.17 ± 0.16	6.17 ± 0.07	6.31 ± 0.34	6.14 ± 0.13
TC05000826.hg.1	HMGXB3	8.24 ± 0.06	8.12 ± 0.07	7.96 ± 0.03	8.12 ± 0.05
TC22000256.hg.1	HMGXB4	7.95 ± 0.05	7.94 ± 0.08	7.83 ± 0.10	7.88 ± 0.01
TC19000029.hg.1	HMHA1	9.92 ± 0.08	9.96 ± 0.04	9.75 ± 0.11	9.94 ± 0.02
TC05000789.hg.1	HMHB1	7.04 ± 0.12	7.02 ± 0.13	6.96 ± 0.19	7.08 ± 0.01
TC05000907.hg.1	HMMR	5.36 ± 0.05	5.26 ± 0.27	5.37 ± 0.17	4.94 ± 0.14
TC22000259.hg.1	HMOX1	8.54 ± 0.26	8.46 ± 0.09	8.68 ± 0.09	8.69 ± 0.25
TC16000131.hg.1	HMOX2	8.92 ± 0.12	8.83 ± 0.02	8.82 ± 0.12	8.84 ± 0.02
TC05000969.hg.1	HMP19	6.62 ± 0.13	6.62 ± 0.17	6.58 ± 0.17	6.72 ± 0.08
TC18001000.hg.1	HMSD	5.35 ± 0.25	5.13 ± 0.02	5.18 ± 0.07	5.19 ± 0.03
TC04001021.hg.1	HMX1	8.84 ± 0.22	8.76 ± 0.15	8.77 ± 0.20	8.79 ± 0.02
TC10000899.hg.1	HMX2	7.92 ± 0.23	7.80 ± 0.17	7.82 ± 0.27	7.83 ± 0.03
TC10000898.hg.1	HMX3	7.98 ± 0.39	7.92 ± 0.25	7.93 ± 0.34	7.99 ± 0.08
TC17001872.hg.1	HN1	8.48 ± 0.23	8.29 ± 0.03	8.68 ± 0.27	8.14 ± 0.11
TC16002067.hg.1	HN1L	7.79 ± 0.21	7.53 ± 0.02	7.57 ± 0.15	7.42 ± 0.07
TC12000952.hg.1	HNF1A	8.14 ± 0.22	8.16 ± 0.24	8.09 ± 0.22	8.31 ± 0.10

TC12002056.hg.1	HNF1A-AS1	8.58 ± 0.24	8.64 ± 0.10	8.59 ± 0.08	8.79 ± 0.11
TC12002057.hg.1	HNF1A-AS1	6.02 ± 0.27	6.02 ± 0.19	6.00 ± 0.17	6.19 ± 0.02
TC17001410.hg.1	HNF1B	7.00 ± 0.20	6.99 ± 0.19	6.95 ± 0.17	7.05 ± 0.07
TC20000330.hg.1	HNF4A	7.65 ± 0.17	7.62 ± 0.14	7.57 ± 0.16	7.64 ± 0.03
TC08000499.hg.1	HNF4G	6.49 ± 0.11	6.40 ± 0.09	6.40 ± 0.05	6.49 ± 0.03
TC02000891.hg.1	HNMT	6.46 ± 0.12	6.25 ± 0.11	6.58 ± 0.14	6.24 ± 0.13
TC05001819.hg.1	HNRNPA0	8.87 ± 0.12	9.07 ± 0.11	8.75 ± 0.16	9.05 ± 0.14
TC12000461.hg.1	HNRNPA1	10.96 ± 0.28	11.34 ± 0.07	10.84 ± 0.14	11.36 ± 0.02
TC13000225.hg.1	HNRNPA1L2	8.00 ± 0.36	8.20 ± 0.16	7.82 ± 0.16	8.19 ± 0.07
TC10001238.hg.1	HNRNPA1P33	9.86 ± 0.34	10.09 ± 0.10	9.62 ± 0.17	10.10 ± 0.11
TC07000517.hg.1	HNRNPA1P8	9.94 ± 0.32	10.30 ± 0.13	9.97 ± 0.06	10.24 ± 0.20
TC07001214.hg.1	HNRNPA2B1	10.95 ± 0.50	11.38 ± 0.13	11.19 ± 0.37	11.31 ± 0.17
TC02001062.hg.1	HNRNPA3	9.99 ± 0.32	10.38 ± 0.04	10.02 ± 0.34	10.37 ± 0.16
TC10001207.hg.1	HNRNPA3P1	8.02 ± 0.08	8.13 ± 0.05	8.04 ± 0.05	8.11 ± 0.12
TC0X000681.hg.1	HNRNPA3P3	10.99 ± 0.43	11.51 ± 0.07	11.04 ± 0.37	11.50 ± 0.09
TC13000715.hg.1	HNRNPA3P5	11.14 ± 0.40	11.68 ± 0.05	11.13 ± 0.41	11.66 ± 0.17
TC05001033.hg.1	HNRNPAB	9.65 ± 0.12	9.66 ± 0.15	9.39 ± 0.18	9.48 ± 0.13
TC14000915.hg.1	HNRNPC	10.33 ± 0.23	10.59 ± 0.07	10.38 ± 0.11	10.57 ± 0.12
TC01002221.hg.1	HNRNPCP5	7.23 ± 0.09	7.22 ± 0.02	7.20 ± 0.15	7.36 ± 0.07
TC04001332.hg.1	HNRNPD	9.75 ± 0.15	9.87 ± 0.09	9.63 ± 0.07	9.80 ± 0.05
TC04001334.hg.1	HNRNPDL	9.89 ± 0.44	10.35 ± 0.03	9.82 ± 0.31	10.33 ± 0.06
TC10001201.hg.1	HNRNPF	9.29 ± 0.14	9.14 ± 0.03	9.12 ± 0.17	8.94 ± 0.12
TC05002135.hg.1	HNRNPH1	9.52 ± 0.50	10.00 ± 0.05	9.43 ± 0.25	10.01 ± 0.03
TC0X002316.hg.1	HNRNPH2	9.89 ± 0.28	10.05 ± 0.15	10.40 ± 0.50	10.10 ± 0.16
TC10000403.hg.1	HNRNPH3	9.31 ± 0.47	9.76 ± 0.09	9.56 ± 0.43	9.81 ± 0.12
TC11001570.hg.1	HNRNPKP3	7.94 ± 0.22	8.02 ± 0.16	7.97 ± 0.17	8.18 ± 0.11
TC19002699.hg.1	HNRNPL	9.54 ± 0.11	9.62 ± 0.17	9.28 ± 0.09	9.61 ± 0.03
TC02001755.hg.1	HNRNPLL	7.66 ± 0.14	7.39 ± 0.19	7.85 ± 0.49	7.11 ± 0.06
TC19000151.hg.1	HNRNPM	10.60 ± 0.10	10.68 ± 0.07	10.51 ± 0.06	10.63 ± 0.18
TC01002339.hg.1	HNRNPR	5.55 ± 0.42	5.32 ± 0.12	5.47 ± 0.22	5.36 ± 0.10
TC01002340.hg.1	HNRNPR	9.19 ± 0.33	9.41 ± 0.18	8.98 ± 0.36	9.23 ± 0.16

TC01006391.hg.1	HNRNPU	10.60 ± 0.37	10.94 ± 0.07	10.77 ± 0.17	10.88 ± 0.18
TC01006390.hg.1	HNRNPU-AS1	8.18 ± 0.68	8.02 ± 0.45	8.56 ± 0.58	8.15 ± 0.16
TC19000577.hg.1	HNRNPUL1	10.72 ± 0.10	10.80 ± 0.08	10.62 ± 0.16	10.85 ± 0.16
TC11003488.hg.1	HNRNPUL2	10.33 ± 0.23	10.23 ± 0.11	10.27 ± 0.17	10.04 ± 0.19
TC10002957.hg.1	HOGA1	7.59 ± 0.17	7.54 ± 0.08	7.55 ± 0.10	7.59 ± 0.04
TC05001522.hg.1	HOMER1	6.24 ± 0.13	6.26 ± 0.02	6.11 ± 0.01	6.19 ± 0.04
TC15001761.hg.1	HOMER2	7.29 ± 0.19	7.10 ± 0.08	7.24 ± 0.13	7.13 ± 0.04
TC15001762.hg.1	HOMER2	6.91 ± 0.10	6.74 ± 0.34	6.82 ± 0.12	6.90 ± 0.10
TC19002691.hg.1	HOMER3	8.48 ± 0.22	8.33 ± 0.16	8.34 ± 0.20	8.42 ± 0.06
TC14002337.hg.1	HOMEZ	7.32 ± 0.05	7.19 ± 0.05	7.26 ± 0.03	7.19 ± 0.03
TC01000700.hg.1	HOOK1	6.65 ± 0.24	6.87 ± 0.09	6.63 ± 0.24	6.84 ± 0.13
TC19001209.hg.1	HOOK2	7.96 ± 0.18	7.90 ± 0.14	7.99 ± 0.15	7.95 ± 0.02
TC08002600.hg.1	HOOK3	8.56 ± 0.40	8.81 ± 0.23	8.91 ± 0.37	8.75 ± 0.19
TC04001222.hg.1	HOPX	7.43 ± 0.19	7.29 ± 0.05	7.24 ± 0.09	7.22 ± 0.02
TC01003209.hg.1	HORMAD1	5.81 ± 0.22	5.33 ± 0.19	6.52 ± 0.45	5.29 ± 0.27
TC22000205.hg.1	HORMAD2	5.53 ± 0.14	5.45 ± 0.09	5.48 ± 0.08	5.52 ± 0.02
TC12001557.hg.1	HOTAIR	7.67 ± 0.22	7.62 ± 0.19	7.54 ± 0.20	7.66 ± 0.08
TC07000165.hg.1	HOTAIRM1	7.41 ± 0.13	7.45 ± 0.11	7.52 ± 0.02	7.55 ± 0.05
TC07000170.hg.1	HOTTIP	6.92 ± 0.25	6.89 ± 0.12	6.94 ± 0.19	6.88 ± 0.02
TC07001218.hg.1	HOXA1	6.37 ± 0.21	6.49 ± 0.09	6.43 ± 0.02	6.66 ± 0.08
TC07003402.hg.1	HOXA10	6.63 ± 0.22	6.57 ± 0.14	6.57 ± 0.19	6.61 ± 0.03
TC07001225.hg.1	HOXA11	6.80 ± 0.18	6.68 ± 0.09	6.62 ± 0.20	6.82 ± 0.08
TC07000169.hg.1	HOXA11-AS	7.14 ± 0.20	7.19 ± 0.17	7.11 ± 0.18	7.26 ± 0.02
TC07001227.hg.1	HOXA13	5.30 ± 0.13	5.15 ± 0.06	5.16 ± 0.16	5.18 ± 0.04
TC07001219.hg.1	HOXA2	5.47 ± 0.03	5.42 ± 0.04	5.51 ± 0.07	5.49 ± 0.11
TC07003351.hg.1	HOXA3	7.33 ± 0.16	7.26 ± 0.14	7.24 ± 0.21	7.35 ± 0.05
TC07003352.hg.1	HOXA4	7.25 ± 0.11	7.14 ± 0.12	7.17 ± 0.08	7.20 ± 0.05
TC07001221.hg.1	HOXA5	6.66 ± 0.22	6.95 ± 0.16	6.59 ± 0.13	7.02 ± 0.03
TC07001222.hg.1	HOXA6	8.23 ± 0.20	8.02 ± 0.11	8.04 ± 0.13	8.09 ± 0.02
TC07001223.hg.1	HOXA7	7.52 ± 0.14	7.45 ± 0.12	7.44 ± 0.21	7.50 ± 0.02
TC07003401.hg.1	HOXA9	7.40 ± 0.19	7.36 ± 0.13	7.37 ± 0.14	7.38 ± 0.02

TC07000166.hg.1	HOXA-AS2	7.15 ± 0.20	7.04 ± 0.13	7.10 ± 0.17	7.15 ± 0.05
TC07000167.hg.1	HOXA-AS3	7.17 ± 0.31	7.15 ± 0.16	7.12 ± 0.21	7.24 ± 0.09
TC07000168.hg.1	HOXA-AS4	7.68 ± 0.26	7.58 ± 0.19	7.67 ± 0.15	7.64 ± 0.04
TC17001641.hg.1	HOXB1	7.36 ± 0.23	7.15 ± 0.16	7.18 ± 0.19	7.27 ± 0.15
TC17001651.hg.1	HOXB13	7.33 ± 0.17	7.29 ± 0.10	7.21 ± 0.12	7.36 ± 0.09
TC17001642.hg.1	HOXB2	8.19 ± 0.04	8.00 ± 0.04	7.96 ± 0.10	7.93 ± 0.07
TC17001644.hg.1	HOXB5	6.35 ± 0.09	6.18 ± 0.01	6.17 ± 0.20	6.18 ± 0.08
TC17002910.hg.1	HOXB6	6.78 ± 0.17	6.68 ± 0.15	6.69 ± 0.19	6.69 ± 0.06
TC17002919.hg.1	HOXB7	7.91 ± 0.11	7.90 ± 0.06	7.87 ± 0.13	7.93 ± 0.05
TC17001646.hg.1	HOXB8	7.31 ± 0.22	7.20 ± 0.06	7.31 ± 0.23	7.21 ± 0.02
TC17001647.hg.1	HOXB9	7.83 ± 0.33	7.84 ± 0.22	7.79 ± 0.24	7.95 ± 0.01
TC17002857.hg.1	HOXB-AS1	7.29 ± 0.12	7.19 ± 0.05	7.10 ± 0.02	7.22 ± 0.07
TC17000624.hg.1	HOXB-AS2	5.59 ± 0.16	5.49 ± 0.15	5.41 ± 0.17	5.48 ± 0.11
TC17002858.hg.1	HOXB-AS3	7.18 ± 0.35	7.19 ± 0.22	7.12 ± 0.25	7.29 ± 0.05
TC17000626.hg.1	HOXB-AS4	7.96 ± 0.29	7.92 ± 0.26	7.88 ± 0.38	7.99 ± 0.06
TC17000628.hg.1	HOXB-AS5	7.04 ± 0.23	6.93 ± 0.14	6.89 ± 0.29	6.91 ± 0.01
TC12003293.hg.1	HOXC10	6.72 ± 0.25	6.69 ± 0.18	6.68 ± 0.14	6.78 ± 0.06
TC12000449.hg.1	HOXC11	6.30 ± 0.35	6.14 ± 0.17	6.18 ± 0.30	6.23 ± 0.12
TC12000446.hg.1	HOXC12	7.57 ± 0.38	7.50 ± 0.18	7.50 ± 0.24	7.54 ± 0.01
TC12000445.hg.1	HOXC13	7.65 ± 0.18	7.52 ± 0.11	7.59 ± 0.12	7.51 ± 0.14
TC12003295.hg.1	HOXC4	5.75 ± 0.22	5.36 ± 0.09	5.49 ± 0.02	5.40 ± 0.07
TC12003298.hg.1	HOXC4	6.87 ± 0.21	6.74 ± 0.14	6.77 ± 0.21	6.74 ± 0.03
TC12003297.hg.1	HOXC5	6.29 ± 0.25	6.12 ± 0.17	6.14 ± 0.27	6.06 ± 0.06
TC12003296.hg.1	HOXC6	6.90 ± 0.18	6.80 ± 0.09	6.89 ± 0.21	6.81 ± 0.06
TC12000452.hg.1	HOXC8	6.57 ± 0.13	6.51 ± 0.23	6.48 ± 0.18	6.61 ± 0.08
TC12003294.hg.1	HOXC9	6.52 ± 0.26	6.47 ± 0.20	6.49 ± 0.21	6.45 ± 0.07
TC12001560.hg.1	HOXC-AS1	6.76 ± 0.33	6.71 ± 0.31	6.62 ± 0.21	6.86 ± 0.11
TC12001559.hg.1	HOXC-AS2	7.19 ± 0.37	7.18 ± 0.24	7.19 ± 0.21	7.26 ± 0.12
TC12001558.hg.1	HOXC-AS3	7.01 ± 0.28	6.90 ± 0.15	6.98 ± 0.28	6.93 ± 0.06
TC12001556.hg.1	HOXC-AS5	7.23 ± 0.33	7.15 ± 0.29	7.08 ± 0.21	7.19 ± 0.04
TC02001056.hg.1	HOXD1	7.13 ± 0.21	7.09 ± 0.20	7.08 ± 0.15	7.10 ± 0.01

TC02004919.hg.1	HOXD10	6.64 ± 0.21	6.56 ± 0.21	6.58 ± 0.08	6.63 ± 0.09
TC02004918.hg.1	HOXD11	7.57 ± 0.18	7.48 ± 0.10	7.45 ± 0.09	7.47 ± 0.02
TC02001050.hg.1	HOXD12	7.46 ± 0.22	7.22 ± 0.07	7.26 ± 0.28	7.25 ± 0.03
TC02001049.hg.1	HOXD13	7.36 ± 0.23	7.28 ± 0.15	7.31 ± 0.14	7.30 ± 0.08
TC02001053.hg.1	HOXD8	7.43 ± 0.25	7.40 ± 0.19	7.37 ± 0.16	7.42 ± 0.03
TC02001052.hg.1	HOXD9	8.04 ± 0.14	8.05 ± 0.28	8.02 ± 0.14	8.14 ± 0.06
TC02002553.hg.1	HOXD-AS1	7.23 ± 0.26	7.14 ± 0.17	7.15 ± 0.15	7.21 ± 0.03
TC02002551.hg.1	HOXD-AS2	7.77 ± 0.19	7.64 ± 0.07	7.66 ± 0.19	7.65 ± 0.07
TC16002057.hg.1	HP	7.94 ± 0.72	7.32 ± 0.14	9.20 ± 0.31	7.20 ± 0.12
TC17000908.hg.1	HP09025	7.96 ± 0.20	7.79 ± 0.15	7.90 ± 0.11	7.87 ± 0.12
TC01002319.hg.1	HP1BP3	9.67 ± 0.32	9.93 ± 0.09	9.57 ± 0.10	9.82 ± 0.04
TC01000436.hg.1	HPCA	8.09 ± 0.27	8.02 ± 0.26	8.01 ± 0.21	8.14 ± 0.03
TC02000059.hg.1	HPCAL1	8.53 ± 0.12	8.43 ± 0.11	8.49 ± 0.12	8.47 ± 0.06
TC01002545.hg.1	HPCAL4	7.91 ± 0.22	7.80 ± 0.11	7.71 ± 0.22	7.90 ± 0.04
TC12002066.hg.1	HPD	6.75 ± 0.30	6.69 ± 0.23	6.72 ± 0.17	6.78 ± 0.04
TC01000571.hg.1	HPDL	6.91 ± 0.59	6.93 ± 0.09	6.89 ± 0.13	6.96 ± 0.18
TC04001753.hg.1	HPGD	7.13 ± 0.13	6.97 ± 0.08	7.64 ± 0.19	7.02 ± 0.10
TC04001393.hg.1	HPGDS	5.25 ± 0.08	5.14 ± 0.03	5.09 ± 0.07	5.09 ± 0.03
TC19000456.hg.1	HPN	8.08 ± 0.22	8.07 ± 0.22	8.05 ± 0.19	8.19 ± 0.07
TC19001439.hg.1	HPN-AS1	8.08 ± 0.23	8.04 ± 0.17	8.07 ± 0.20	8.18 ± 0.14
TC16002058.hg.1	HPR	7.53 ± 0.49	7.09 ± 0.18	8.00 ± 0.11	7.04 ± 0.10
TC0X000636.hg.1	HPRT1	7.16 ± 0.14	7.21 ± 0.14	7.20 ± 0.24	7.03 ± 0.25
TC10001578.hg.1	HPS1	8.80 ± 0.13	8.65 ± 0.02	8.74 ± 0.07	8.60 ± 0.03
TC03000803.hg.1	HPS3	9.02 ± 0.36	9.13 ± 0.20	9.21 ± 0.49	8.89 ± 0.20
TC22000608.hg.1	HPS4	7.80 ± 0.02	7.75 ± 0.03	7.62 ± 0.03	7.78 ± 0.05
TC11001465.hg.1	HPS5	7.37 ± 0.11	7.36 ± 0.09	7.28 ± 0.25	7.30 ± 0.07
TC10000745.hg.1	HPS6	7.90 ± 0.04	7.84 ± 0.12	7.75 ± 0.16	7.79 ± 0.04
TC04001347.hg.1	HPSE	8.42 ± 0.42	7.80 ± 0.16	8.91 ± 0.14	7.63 ± 0.31
TC10001579.hg.1	HPSE2	5.51 ± 0.16	5.48 ± 0.14	5.48 ± 0.09	5.59 ± 0.05
TC07001393.hg.1	HPVC1	3.62 ± 0.01	3.67 ± 0.11	3.56 ± 0.13	3.78 ± 0.19
TC11001356.hg.1	HPX	6.91 ± 0.21	6.90 ± 0.13	6.93 ± 0.12	6.97 ± 0.03

TC08001653.hg.1	HPYR1	4.10 ± 0.09	4.13 ± 0.18	4.20 ± 0.11	4.47 ± 0.38
TC08001033.hg.1	HR	8.18 ± 0.20	8.17 ± 0.21	8.10 ± 0.21	8.26 ± 0.06
TC11001236.hg.1	HRAS	9.16 ± 0.17	9.04 ± 0.13	9.05 ± 0.14	9.06 ± 0.04
TC03001063.hg.1	HRASLS	6.28 ± 0.21	6.07 ± 0.08	6.14 ± 0.19	6.16 ± 0.07
TC11001892.hg.1	HRASLS2	6.93 ± 0.13	6.64 ± 0.12	6.52 ± 0.28	6.62 ± 0.09
TC11001891.hg.1	HRASLS5	6.98 ± 0.09	6.86 ± 0.04	7.02 ± 0.21	6.97 ± 0.20
TC19001709.hg.1	HRC	7.59 ± 0.22	7.54 ± 0.16	7.45 ± 0.22	7.58 ± 0.04
TC09000198.hg.1	HRCT1	8.23 ± 0.20	8.29 ± 0.24	8.22 ± 0.23	8.40 ± 0.02
TC03001022.hg.1	HRG	7.89 ± 0.23	7.91 ± 0.04	7.83 ± 0.05	7.98 ± 0.15
TC03001023.hg.1	HRG	5.18 ± 0.10	5.14 ± 0.07	5.09 ± 0.15	5.14 ± 0.04
TC03000064.hg.1	HRH1	6.81 ± 0.13	6.79 ± 0.05	6.84 ± 0.03	6.87 ± 0.05
TC05000981.hg.1	HRH2	10.00 ± 0.36	9.50 ± 0.21	10.49 ± 0.23	9.29 ± 0.26
TC05000982.hg.1	HRH2	6.13 ± 0.02	5.93 ± 0.17	6.31 ± 0.02	5.80 ± 0.02
TC20001006.hg.1	HRH3	8.26 ± 0.24	8.25 ± 0.20	8.16 ± 0.24	8.33 ± 0.02
TC18000111.hg.1	HRH4	5.74 ± 0.13	5.57 ± 0.09	6.24 ± 0.40	5.77 ± 0.21
TC12002010.hg.1	HRK	8.46 ± 0.19	8.53 ± 0.12	8.45 ± 0.17	8.56 ± 0.06
TC01003244.hg.1	HRNR	5.12 ± 0.18	4.94 ± 0.05	5.00 ± 0.09	5.12 ± 0.22
TC08001461.hg.1	HRSP12	5.63 ± 0.09	5.58 ± 0.03	5.60 ± 0.09	5.50 ± 0.09
TC02001625.hg.1	HS1BP3	7.72 ± 0.09	7.52 ± 0.07	7.66 ± 0.07	7.53 ± 0.06
TC02001626.hg.1	HS1BP3-IT1	7.72 ± 0.09	7.73 ± 0.29	7.73 ± 0.15	7.85 ± 0.09
TC01000827.hg.1	HS2ST1	7.15 ± 0.06	7.15 ± 0.04	7.17 ± 0.03	7.15 ± 0.02
TC04001037.hg.1	HS3ST1	7.31 ± 0.20	7.21 ± 0.10	7.25 ± 0.17	7.20 ± 0.04
TC16000252.hg.1	HS3ST2	7.30 ± 0.22	7.34 ± 0.16	7.34 ± 0.10	7.39 ± 0.05
TC17001149.hg.1	HS3ST3A1	8.28 ± 0.06	8.09 ± 0.09	8.18 ± 0.04	8.05 ± 0.06
TC17000165.hg.1	HS3ST3B1	8.31 ± 0.10	8.19 ± 0.03	8.43 ± 0.10	8.22 ± 0.07
TC16000285.hg.1	HS3ST4	6.79 ± 0.21	6.72 ± 0.10	6.75 ± 0.19	6.69 ± 0.07
TC06002036.hg.1	HS3ST5	6.32 ± 0.13	6.31 ± 0.18	6.21 ± 0.23	6.35 ± 0.07
TC16000765.hg.1	HS3ST6	8.66 ± 0.27	8.66 ± 0.20	8.52 ± 0.29	8.68 ± 0.04
TC02002292.hg.1	HS6ST1	7.78 ± 0.07	7.79 ± 0.09	7.73 ± 0.04	7.84 ± 0.02
TC0X001372.hg.1	HS6ST2	5.77 ± 0.19	5.72 ± 0.13	5.73 ± 0.10	5.72 ± 0.02
TC0X000631.hg.1	HS6ST2-AS1	5.91 ± 0.21	5.79 ± 0.20	5.82 ± 0.01	5.95 ± 0.12

TC13000337.hg.1	HS6ST3	6.52 ± 0.26	6.47 ± 0.20	6.35 ± 0.20	6.63 ± 0.17
TC0X001450.hg.1	hsa-mir-892c	4.57 ± 0.22	4.53 ± 0.08	4.58 ± 0.38	4.63 ± 0.15
TC16000650.hg.1	HSBP1	8.99 ± 0.26	8.69 ± 0.15	9.01 ± 0.30	8.58 ± 0.17
TC18000266.hg.1	HSBP1L1	7.83 ± 0.20	7.74 ± 0.09	7.65 ± 0.11	7.79 ± 0.06
TC22000186.hg.1	HSCB	6.74 ± 0.11	6.55 ± 0.06	6.71 ± 0.16	6.45 ± 0.09
TC01001750.hg.1	HSD11B1	6.26 ± 0.18	6.13 ± 0.09	6.25 ± 0.08	6.24 ± 0.08
TC19000103.hg.1	HSD11B1L	8.82 ± 0.22	8.82 ± 0.17	8.79 ± 0.18	8.89 ± 0.04
TC16000540.hg.1	HSD11B2	7.96 ± 0.28	7.79 ± 0.21	7.77 ± 0.14	7.89 ± 0.05
TC17000528.hg.1	HSD17B1	8.37 ± 0.16	8.34 ± 0.15	8.27 ± 0.11	8.41 ± 0.02
TC0X001065.hg.1	HSD17B10	9.02 ± 0.25	9.03 ± 0.10	8.88 ± 0.24	9.00 ± 0.01
TC04002945.hg.1	HSD17B11	10.17 ± 0.19	10.25 ± 0.17	10.52 ± 0.24	10.14 ± 0.19
TC11000367.hg.1	HSD17B12	8.43 ± 0.15	8.35 ± 0.11	8.45 ± 0.27	8.18 ± 0.14
TC04002944.hg.1	HSD17B13	5.79 ± 0.08	5.73 ± 0.10	6.14 ± 0.49	5.70 ± 0.21
TC19001699.hg.1	HSD17B14	7.48 ± 0.27	7.45 ± 0.19	7.39 ± 0.19	7.52 ± 0.10
TC17000527.hg.1	HSD17B1P1	8.18 ± 0.15	8.13 ± 0.25	8.01 ± 0.11	8.25 ± 0.07
TC16000645.hg.1	HSD17B2	6.32 ± 0.17	6.31 ± 0.09	6.28 ± 0.15	6.39 ± 0.13
TC09001372.hg.1	HSD17B3	6.12 ± 0.04	6.08 ± 0.09	6.04 ± 0.05	6.18 ± 0.02
TC05000567.hg.1	HSD17B4	8.14 ± 0.15	8.03 ± 0.18	8.17 ± 0.18	7.82 ± 0.10
TC12000516.hg.1	HSD17B6	5.68 ± 0.19	5.62 ± 0.08	5.59 ± 0.11	5.62 ± 0.05
TC01001426.hg.1	HSD17B7	6.85 ± 0.21	6.70 ± 0.12	6.73 ± 0.13	6.63 ± 0.03
TC10000256.hg.1	HSD17B7P2	7.12 ± 0.24	7.00 ± 0.13	7.02 ± 0.16	6.93 ± 0.05
TC06000413.hg.1	HSD17B8	7.87 ± 0.17	7.79 ± 0.10	7.72 ± 0.14	7.86 ± 0.11
TC6_cox_hap2000103.hg.1	HSD17B8	7.87 ± 0.17	7.79 ± 0.10	7.72 ± 0.14	7.86 ± 0.11
TC6_dbb_hap3000095.hg.1	HSD17B8	7.87 ± 0.17	7.79 ± 0.10	7.72 ± 0.14	7.86 ± 0.11
TC6_mann_hap4000087.hg.1	HSD17B8	7.87 ± 0.17	7.79 ± 0.10	7.72 ± 0.14	7.86 ± 0.11
TC6_mcf_hap5000088.hg.1	HSD17B8	7.87 ± 0.17	7.79 ± 0.10	7.72 ± 0.14	7.86 ± 0.11
TC6_qbl_hap6000094.hg.1	HSD17B8	7.87 ± 0.17	7.79 ± 0.10	7.72 ± 0.14	7.86 ± 0.11
TC01001040.hg.1	HSD3B1	6.03 ± 0.14	5.97 ± 0.12	6.04 ± 0.07	6.09 ± 0.16
TC01001039.hg.1	HSD3B2	5.69 ± 0.11	5.62 ± 0.11	5.65 ± 0.12	5.74 ± 0.14
TC16000365.hg.1	HSD3B7	8.31 ± 0.25	8.13 ± 0.19	8.24 ± 0.19	8.21 ± 0.06
TC01001041.hg.1	HSD3BP4	5.50 ± 0.10	5.53 ± 0.08	5.49 ± 0.08	5.61 ± 0.13

TC01002714.hg.1	HSD52	6.47 ± 0.29	6.42 ± 0.13	6.47 ± 0.11	6.52 ± 0.06
TC16001308.hg.1	HSDL1	7.93 ± 0.23	7.89 ± 0.14	8.06 ± 0.30	7.91 ± 0.01
TC09000565.hg.1	HSDL2	8.56 ± 0.34	8.70 ± 0.27	9.32 ± 0.45	8.47 ± 0.39
TC08000849.hg.1	HSF1	9.01 ± 0.08	8.93 ± 0.06	8.91 ± 0.02	9.00 ± 0.07
TC06000947.hg.1	HSF2	7.28 ± 0.35	7.54 ± 0.29	6.99 ± 0.31	7.51 ± 0.07
TC21000508.hg.1	HSF2BP	6.73 ± 0.17	6.69 ± 0.13	6.65 ± 0.12	6.70 ± 0.08
TC16002079.hg.1	HSF4	8.25 ± 0.22	8.23 ± 0.19	8.20 ± 0.20	8.31 ± 0.05
TC17001733.hg.1	HSF5	7.09 ± 0.12	7.30 ± 0.06	6.97 ± 0.12	7.18 ± 0.12
TC0X000716.hg.1	HSFX2	7.45 ± 0.13	7.33 ± 0.16	7.32 ± 0.07	7.43 ± 0.06
TC0X001481.hg.1	HSFX2	7.44 ± 0.10	7.38 ± 0.13	7.37 ± 0.01	7.50 ± 0.09
TC22000015.hg.1	HSFY1P1	4.10 ± 0.12	3.89 ± 0.13	4.14 ± 0.18	4.03 ± 0.06
TC0Y000069.hg.1	HSFY2	4.54 ± 0.09	4.38 ± 0.06	4.37 ± 0.04	4.43 ± 0.11
TC0Y000179.hg.1	HSFY2	4.48 ± 0.10	4.35 ± 0.07	4.35 ± 0.04	4.40 ± 0.11
TC19000318.hg.1	HSH2D	9.07 ± 0.22	8.88 ± 0.10	8.94 ± 0.31	8.89 ± 0.02
TC14001526.hg.1	HSP90AA1	9.93 ± 0.30	10.37 ± 0.09	10.03 ± 0.27	10.11 ± 0.21
TC04000932.hg.1	HSP90AA4P	5.47 ± 0.28	5.74 ± 0.09	5.56 ± 0.10	5.43 ± 0.11
TC04001731.hg.1	HSP90AA6P	7.65 ± 0.20	7.78 ± 0.25	7.84 ± 0.08	7.48 ± 0.22
TC06000615.hg.1	HSP90AB1	10.30 ± 0.10	10.64 ± 0.13	10.12 ± 0.17	10.48 ± 0.12
TC04000132.hg.1	HSP90AB2P	7.45 ± 0.18	7.51 ± 0.03	7.27 ± 0.14	7.41 ± 0.04
TC04000477.hg.1	HSP90AB3P	8.06 ± 0.04	8.16 ± 0.06	7.96 ± 0.12	8.04 ± 0.13
TC15001486.hg.1	HSP90AB4P	6.58 ± 0.23	6.45 ± 0.08	6.41 ± 0.07	6.33 ± 0.09
TC12000803.hg.1	HSP90B1	11.25 ± 0.22	11.02 ± 0.10	10.78 ± 0.32	10.76 ± 0.24
TC01000847.hg.1	HSP90B3P	6.73 ± 0.11	6.49 ± 0.12	6.47 ± 0.16	6.48 ± 0.04
TC10001681.hg.1	HSPA12A	7.11 ± 0.18	7.04 ± 0.15	7.00 ± 0.18	7.12 ± 0.06
TC20000039.hg.1	HSPA12B	8.20 ± 0.25	8.21 ± 0.18	8.16 ± 0.23	8.26 ± 0.02
TC21000284.hg.1	HSPA13	8.40 ± 0.23	8.62 ± 0.21	8.24 ± 0.35	8.60 ± 0.02
TC10000112.hg.1	HSPA14	7.20 ± 0.14	7.38 ± 0.18	7.15 ± 0.22	7.28 ± 0.12
TC06000384.hg.1	HSPA1A	10.48 ± 0.27	10.05 ± 0.19	10.88 ± 0.42	9.90 ± 0.37
TC6_apd_hap1000039.hg.1	HSPA1A	9.96 ± 0.28	9.46 ± 0.18	10.34 ± 0.45	9.27 ± 0.38
TC6_cox_hap2000078.hg.1	HSPA1A	9.97 ± 0.28	9.47 ± 0.19	10.35 ± 0.45	9.28 ± 0.37
TC6_dbb_hap3000069.hg.1	HSPA1A	9.97 ± 0.28	9.46 ± 0.18	10.30 ± 0.43	9.25 ± 0.41

TC6_qbl_hap6000069.hg.1	HSPA1A	9.95 ± 0.28	9.46 ± 0.18	10.35 ± 0.46	9.29 ± 0.37
TC06000385.hg.1	HSPA1B	9.85 ± 0.34	9.30 ± 0.21	10.31 ± 0.54	9.15 ± 0.40
TC6_apd_hap1000040.hg.1	HSPA1B	9.11 ± 0.41	8.30 ± 0.22	9.62 ± 0.73	8.09 ± 0.47
TC6_cox_hap2000079.hg.1	HSPA1B	9.03 ± 0.41	8.24 ± 0.23	9.56 ± 0.72	8.01 ± 0.46
TC6_dbb_hap3000070.hg.1	HSPA1B	9.09 ± 0.41	8.28 ± 0.23	9.61 ± 0.72	8.07 ± 0.47
TC6_qbl_hap6000070.hg.1	HSPA1B	9.17 ± 0.42	8.38 ± 0.23	9.71 ± 0.72	8.15 ± 0.47
TC06001546.hg.1	HSPA1L	7.43 ± 0.25	7.11 ± 0.04	7.53 ± 0.29	7.07 ± 0.07
TC6_apd_hap1000087.hg.1	HSPA1L	7.42 ± 0.32	6.90 ± 0.07	7.38 ± 0.20	6.91 ± 0.02
TC6_cox_hap2000173.hg.1	HSPA1L	7.40 ± 0.33	6.88 ± 0.09	7.38 ± 0.18	6.89 ± 0.02
TC6_dbb_hap3000162.hg.1	HSPA1L	7.43 ± 0.32	6.89 ± 0.08	7.41 ± 0.18	6.91 ± 0.01
TC6_qbl_hap6000163.hg.1	HSPA1L	7.40 ± 0.33	6.88 ± 0.09	7.38 ± 0.18	6.89 ± 0.02
TC14000391.hg.1	HSPA2	7.81 ± 0.25	7.79 ± 0.07	7.72 ± 0.17	7.79 ± 0.10
TC05000649.hg.1	HSPA4	8.68 ± 0.18	8.76 ± 0.14	8.59 ± 0.30	8.52 ± 0.17
TC04000643.hg.1	HSPA4L	4.67 ± 0.12	4.51 ± 0.04	4.64 ± 0.09	4.55 ± 0.02
TC09001589.hg.1	HSPA5	11.15 ± 0.11	11.15 ± 0.11	10.97 ± 0.35	10.96 ± 0.30
TC01001401.hg.1	HSPA6	8.91 ± 0.28	8.68 ± 0.07	9.21 ± 0.23	8.54 ± 0.11
TC01001407.hg.1	HSPA7	9.17 ± 0.35	8.84 ± 0.13	9.32 ± 0.34	8.53 ± 0.13
TC11002390.hg.1	HSPA8	11.49 ± 0.01	11.59 ± 0.08	11.22 ± 0.34	11.24 ± 0.15
TC05001832.hg.1	HSPA9	9.45 ± 0.17	9.76 ± 0.13	9.18 ± 0.13	9.71 ± 0.09
TC07000485.hg.1	HSPB1	9.13 ± 0.18	9.00 ± 0.14	9.10 ± 0.22	8.98 ± 0.03
TC01002679.hg.1	HSPB11	7.64 ± 0.28	7.61 ± 0.13	7.56 ± 0.21	7.43 ± 0.06
TC11001001.hg.1	HSPB2	6.73 ± 0.22	6.69 ± 0.17	6.67 ± 0.21	6.83 ± 0.03
TC05000225.hg.1	HSPB3	4.76 ± 0.07	4.70 ± 0.03	4.79 ± 0.23	4.82 ± 0.02
TC19001453.hg.1	HSPB6	8.71 ± 0.15	8.74 ± 0.24	8.74 ± 0.22	8.80 ± 0.03
TC01002256.hg.1	HSPB7	7.88 ± 0.19	7.91 ± 0.24	7.87 ± 0.19	8.05 ± 0.07
TC12000932.hg.1	HSPB8	6.78 ± 0.25	6.70 ± 0.15	6.72 ± 0.16	6.81 ± 0.12
TC17000522.hg.1	HSPB9	8.31 ± 0.21	8.19 ± 0.35	8.08 ± 0.28	8.31 ± 0.06
TC03001706.hg.1	HSPBAP1	8.14 ± 0.18	8.13 ± 0.12	8.37 ± 0.25	8.05 ± 0.11
TC19001865.hg.1	HSPBP1	8.33 ± 0.21	8.29 ± 0.14	8.20 ± 0.22	8.29 ± 0.03
TC02002648.hg.1	HSPD1	8.49 ± 0.08	8.59 ± 0.15	8.38 ± 0.08	8.27 ± 0.14
TC02005004.hg.1	HSPE1	7.70 ± 0.08	7.74 ± 0.17	7.55 ± 0.05	7.48 ± 0.08

TC01002328.hg.1	HSPG2	8.21 ± 0.18	8.18 ± 0.22	8.13 ± 0.18	8.29 ± 0.06
TC13000539.hg.1	HSPH1	8.38 ± 0.29	8.58 ± 0.11	8.61 ± 0.36	8.16 ± 0.33
TC11000256.hg.1	HTATIP2	8.68 ± 0.29	8.33 ± 0.09	8.73 ± 0.20	8.21 ± 0.14
TC0X000661.hg.1	HTATSF1	8.76 ± 0.21	8.98 ± 0.04	8.64 ± 0.26	8.83 ± 0.16
TC04000377.hg.1	HTN1	4.32 ± 0.10	4.15 ± 0.03	4.24 ± 0.10	4.19 ± 0.04
TC04000376.hg.1	HTN3	4.74 ± 0.19	4.35 ± 0.03	4.49 ± 0.18	4.45 ± 0.08
TC05001404.hg.1	HTR1A	7.42 ± 0.33	7.49 ± 0.27	7.34 ± 0.32	7.60 ± 0.09
TC06001892.hg.1	HTR1B	7.84 ± 0.22	7.87 ± 0.13	7.73 ± 0.24	7.99 ± 0.18
TC01002337.hg.1	HTR1D	6.86 ± 0.20	6.73 ± 0.24	6.72 ± 0.09	6.93 ± 0.25
TC01002338.hg.1	HTR1D	4.69 ± 0.08	4.61 ± 0.09	4.69 ± 0.30	4.92 ± 0.30
TC06000782.hg.1	HTR1E	6.82 ± 0.18	6.73 ± 0.16	6.77 ± 0.07	6.87 ± 0.07
TC03000463.hg.1	HTR1F	3.75 ± 0.15	3.59 ± 0.06	3.70 ± 0.04	3.65 ± 0.04
TC13000648.hg.1	HTR2A	6.08 ± 0.15	5.98 ± 0.10	6.00 ± 0.11	6.00 ± 0.07
TC13000185.hg.1	HTR2A-AS1	4.65 ± 0.03	4.63 ± 0.11	4.60 ± 0.04	4.58 ± 0.08
TC02002858.hg.1	HTR2B	5.19 ± 0.17	5.19 ± 0.04	5.13 ± 0.04	5.08 ± 0.03
TC0X000555.hg.1	HTR2C	5.09 ± 0.15	5.01 ± 0.05	5.07 ± 0.09	5.07 ± 0.08
TC0X000561.hg.1	HTR2C-IT1	5.38 ± 0.14	5.40 ± 0.09	5.45 ± 0.09	5.35 ± 0.09
TC11001025.hg.1	HTR3A	7.25 ± 0.16	7.28 ± 0.13	7.20 ± 0.12	7.42 ± 0.06
TC11001024.hg.1	HTR3B	5.93 ± 0.21	5.84 ± 0.11	5.86 ± 0.09	5.96 ± 0.07
TC03000990.hg.1	HTR3C	7.04 ± 0.13	6.92 ± 0.16	6.92 ± 0.01	7.01 ± 0.10
TC03000989.hg.1	HTR3D	6.34 ± 0.17	6.34 ± 0.17	6.27 ± 0.15	6.46 ± 0.09
TC03000991.hg.1	HTR3E	6.21 ± 0.17	6.15 ± 0.08	6.15 ± 0.09	6.26 ± 0.12
TC03002066.hg.1	HTR3E-AS1	6.76 ± 0.10	6.67 ± 0.07	6.69 ± 0.11	6.66 ± 0.11
TC05003408.hg.1	HTR4	5.66 ± 0.13	5.56 ± 0.11	5.53 ± 0.08	5.64 ± 0.06
TC05001916.hg.1	HTR4-IT1	5.78 ± 0.24	5.70 ± 0.16	5.74 ± 0.26	5.76 ± 0.09
TC07001035.hg.1	HTR5A	6.89 ± 0.18	6.77 ± 0.16	6.72 ± 0.11	6.82 ± 0.06
TC02000745.hg.1	HTR5BP	6.44 ± 0.04	6.44 ± 0.04	6.50 ± 0.05	6.51 ± 0.02
TC01000251.hg.1	HTR6	7.67 ± 0.20	7.78 ± 0.13	7.58 ± 0.22	7.84 ± 0.10
TC10001515.hg.1	HTR7	5.76 ± 0.11	5.77 ± 0.11	5.71 ± 0.13	5.80 ± 0.07
TC12000186.hg.1	HTR7P1	6.43 ± 0.12	6.40 ± 0.11	6.39 ± 0.09	6.50 ± 0.07
TC10000889.hg.1	HTRA1	8.03 ± 0.25	7.99 ± 0.19	8.07 ± 0.21	8.04 ± 0.01

TC02000463.hg.1	HTRA2	8.40 ± 0.21	8.23 ± 0.02	8.29 ± 0.11	8.12 ± 0.01
TC04000087.hg.1	HTRA3	7.66 ± 0.23	7.48 ± 0.25	7.51 ± 0.15	7.60 ± 0.06
TC08000297.hg.1	HTRA4	7.16 ± 0.15	7.16 ± 0.14	7.10 ± 0.12	7.21 ± 0.06
TC04000044.hg.1	HTT	8.76 ± 0.21	8.63 ± 0.15	8.55 ± 0.08	8.52 ± 0.06
TC04000980.hg.1	HTT-AS1	7.62 ± 0.30	7.65 ± 0.14	7.58 ± 0.26	7.68 ± 0.11
TC06000066.hg.1	HULC	5.85 ± 0.29	5.79 ± 0.10	5.68 ± 0.15	5.95 ± 0.22
TC21000113.hg.1	HUNK	6.45 ± 0.21	6.40 ± 0.19	6.41 ± 0.13	6.43 ± 0.04
TC07001364.hg.1	HUS1	8.32 ± 0.21	8.33 ± 0.18	8.26 ± 0.16	8.38 ± 0.02
TC06001208.hg.1	HUS1B	6.62 ± 0.19	6.49 ± 0.04	6.36 ± 0.23	6.58 ± 0.04
TC0X001067.hg.1	HUWE1	9.29 ± 0.21	9.36 ± 0.16	9.01 ± 0.19	9.22 ± 0.10
TC12001967.hg.1	HVCN1	9.41 ± 0.08	9.33 ± 0.08	9.25 ± 0.11	9.23 ± 0.02
TC03001427.hg.1	HYAL1	7.66 ± 0.18	7.72 ± 0.15	7.62 ± 0.09	7.78 ± 0.07
TC03003329.hg.1	HYAL2	7.87 ± 0.22	7.73 ± 0.20	7.79 ± 0.21	7.82 ± 0.03
TC03003411.hg.1	HYAL3	7.85 ± 0.23	7.81 ± 0.16	7.82 ± 0.19	7.88 ± 0.04
TC07000749.hg.1	HYAL4	5.50 ± 0.17	5.53 ± 0.07	5.47 ± 0.11	5.63 ± 0.10
TC07000748.hg.1	HYALP1	5.92 ± 0.06	5.87 ± 0.05	5.97 ± 0.14	5.93 ± 0.08
TC16001244.hg.1	HYDIN	6.08 ± 0.13	6.03 ± 0.11	6.04 ± 0.09	6.13 ± 0.09
TC01002586.hg.1	HYI	8.05 ± 0.16	7.99 ± 0.14	7.96 ± 0.18	8.07 ± 0.02
TC15000730.hg.1	HYKK	6.36 ± 0.19	6.34 ± 0.04	6.28 ± 0.10	6.26 ± 0.06
TC11001164.hg.1	HYLS1	6.43 ± 0.10	6.28 ± 0.04	6.39 ± 0.21	6.26 ± 0.05
TC06002190.hg.1	HYMAI	5.86 ± 0.07	5.86 ± 0.09	6.05 ± 0.26	5.89 ± 0.18
TC11002361.hg.1	HYOU1	9.30 ± 0.25	9.02 ± 0.16	8.78 ± 0.13	8.78 ± 0.06
TC15002795.hg.1	HYPK	7.73 ± 0.07	7.77 ± 0.03	7.73 ± 0.12	7.80 ± 0.06
TC02000048.hg.1	IAH1	7.58 ± 0.12	7.54 ± 0.08	7.51 ± 0.07	7.46 ± 0.08
TC12000230.hg.1	IAPP	4.68 ± 0.15	4.51 ± 0.07	4.58 ± 0.05	4.52 ± 0.04
TC09001331.hg.1	IARS	8.13 ± 0.13	7.97 ± 0.22	7.67 ± 0.14	7.78 ± 0.04
TC01001814.hg.1	IARS2	7.93 ± 0.24	7.98 ± 0.22	7.81 ± 0.32	7.73 ± 0.12
TC01001887.hg.1	IBA57	8.22 ± 0.18	8.28 ± 0.18	8.15 ± 0.10	8.30 ± 0.06
TC04000475.hg.1	IBSP	5.21 ± 0.08	5.16 ± 0.08	5.12 ± 0.06	5.21 ± 0.10
TC06001908.hg.1	IBTK	7.73 ± 0.58	7.96 ± 0.20	7.75 ± 0.56	7.66 ± 0.17
TC07001143.hg.1	ICA1	6.25 ± 0.17	6.08 ± 0.03	6.24 ± 0.11	6.08 ± 0.03

TC02002683.hg.1	ICA1L	6.29 ± 0.12	6.28 ± 0.05	6.21 ± 0.06	6.29 ± 0.02
TC19000174.hg.1	ICAM1	9.43 ± 0.54	9.88 ± 0.18	9.94 ± 0.28	10.04 ± 0.33
TC17001794.hg.1	ICAM2	8.64 ± 0.11	8.44 ± 0.04	8.35 ± 0.06	8.40 ± 0.02
TC19001163.hg.1	ICAM3	10.41 ± 0.27	10.29 ± 0.18	10.57 ± 0.31	10.20 ± 0.17
TC19000175.hg.1	ICAM4	7.41 ± 0.29	7.52 ± 0.23	7.66 ± 0.20	7.51 ± 0.08
TC19000176.hg.1	ICAM5	8.30 ± 0.24	8.25 ± 0.20	8.24 ± 0.22	8.29 ± 0.01
TC06001809.hg.1	ICK	8.24 ± 0.22	8.23 ± 0.17	8.13 ± 0.27	8.14 ± 0.09
TC01002147.hg.1	ICMT	8.37 ± 0.14	8.21 ± 0.03	8.18 ± 0.12	8.03 ± 0.10
TC02001202.hg.1	ICOS	7.59 ± 0.27	7.89 ± 0.27	7.54 ± 0.13	8.18 ± 0.10
TC21000516.hg.1	ICOSLG	8.01 ± 0.09	8.24 ± 0.13	7.71 ± 0.17	8.37 ± 0.10
TC17000839.hg.1	ICT1	8.34 ± 0.36	8.03 ± 0.18	7.87 ± 0.28	7.74 ± 0.15
TC20000194.hg.1	ID1	7.60 ± 0.32	7.65 ± 0.17	7.53 ± 0.22	7.59 ± 0.08
TC02000041.hg.1	ID2	9.05 ± 0.17	9.20 ± 0.10	8.95 ± 0.11	9.04 ± 0.09
TC03001512.hg.1	ID2B	8.91 ± 0.38	9.32 ± 0.20	9.10 ± 0.13	9.29 ± 0.09
TC01002347.hg.1	ID3	7.38 ± 0.23	7.20 ± 0.09	7.10 ± 0.20	7.28 ± 0.21
TC06000126.hg.1	ID4	7.37 ± 0.07	7.25 ± 0.19	7.29 ± 0.07	7.31 ± 0.12
TC10001531.hg.1	IDE	7.77 ± 0.19	7.67 ± 0.17	7.65 ± 0.23	7.42 ± 0.08
TC02002726.hg.1	IDH1	7.48 ± 0.14	7.10 ± 0.07	7.64 ± 0.17	6.90 ± 0.07
TC02001234.hg.1	IDH1-AS1	6.58 ± 0.27	6.50 ± 0.27	6.53 ± 0.21	6.74 ± 0.07
TC15001841.hg.1	IDH2	9.21 ± 0.29	8.91 ± 0.19	8.83 ± 0.30	8.57 ± 0.04
TC15000724.hg.1	IDH3A	8.67 ± 0.03	8.70 ± 0.12	8.42 ± 0.13	8.48 ± 0.11
TC20000563.hg.1	IDH3B	9.01 ± 0.05	9.02 ± 0.12	8.68 ± 0.13	9.08 ± 0.11
TC0X001518.hg.1	IDH3G	9.96 ± 0.17	9.77 ± 0.06	9.83 ± 0.06	9.77 ± 0.13
TC10000967.hg.1	IDI1	8.38 ± 0.16	8.74 ± 0.03	8.91 ± 0.37	8.72 ± 0.12
TC10000966.hg.1	IDI2	6.49 ± 0.06	6.55 ± 0.08	6.56 ± 0.13	6.59 ± 0.01
TC10000009.hg.1	IDI2-AS1	7.13 ± 0.11	7.20 ± 0.12	7.18 ± 0.09	7.26 ± 0.04
TC09000378.hg.1	IDNK	8.40 ± 0.17	8.33 ± 0.07	8.36 ± 0.14	8.31 ± 0.05
TC08000305.hg.1	IDO1	6.49 ± 0.12	6.18 ± 0.14	6.61 ± 0.22	6.23 ± 0.03
TC08000307.hg.1	IDO2	5.95 ± 0.16	5.91 ± 0.09	5.89 ± 0.18	5.94 ± 0.05
TC0X002343.hg.1	IDS	9.82 ± 0.19	9.98 ± 0.15	10.05 ± 0.30	9.89 ± 0.19
TC04000015.hg.1	IDUA	8.64 ± 0.19	8.66 ± 0.20	8.53 ± 0.16	8.73 ± 0.03

TC19000237.hg.1	IER2	8.75 ± 0.30	9.00 ± 0.17	8.93 ± 0.10	8.89 ± 0.26
TC06001510.hg.1	IER3	8.88 ± 0.52	9.08 ± 0.08	9.19 ± 0.14	9.16 ± 0.43
TC6_cox_hap2000143.hg.1	IER3	9.50 ± 0.65	9.56 ± 0.22	9.74 ± 0.13	9.77 ± 0.60
TC6_dbb_hap3000132.hg.1	IER3	9.50 ± 0.65	9.56 ± 0.22	9.74 ± 0.13	9.77 ± 0.60
TC6_mann_hap4000121.hg.1	IER3	9.50 ± 0.65	9.56 ± 0.22	9.74 ± 0.13	9.77 ± 0.60
TC6_qbl_hap6000133.hg.1	IER3	9.50 ± 0.65	9.58 ± 0.22	9.76 ± 0.15	9.78 ± 0.59
TC6_ssto_hap7000123.hg.1	IER3	9.50 ± 0.65	9.56 ± 0.22	9.74 ± 0.13	9.77 ± 0.60
TC18000493.hg.1	IER3IP1	8.28 ± 0.08	8.34 ± 0.12	8.14 ± 0.06	8.10 ± 0.14
TC01001569.hg.1	IER5	8.40 ± 0.27	8.71 ± 0.08	8.45 ± 0.21	8.69 ± 0.21
TC09001645.hg.1	IER5L	7.81 ± 0.33	7.75 ± 0.18	7.78 ± 0.33	7.80 ± 0.09
TC12001141.hg.1	IFFO1	8.02 ± 0.03	7.92 ± 0.08	8.00 ± 0.07	7.93 ± 0.02
TC01006348.hg.1	IFFO2	8.18 ± 0.11	8.22 ± 0.06	7.94 ± 0.16	8.16 ± 0.07
TC01001348.hg.1	IFI16	10.59 ± 0.28	10.49 ± 0.40	11.01 ± 0.31	10.12 ± 0.35
TC14000584.hg.1	IFI27	9.32 ± 0.93	8.31 ± 0.52	8.20 ± 0.28	7.90 ± 0.11
TC14000583.hg.1	IFI27L1	7.95 ± 0.18	7.82 ± 0.12	7.84 ± 0.17	7.88 ± 0.04
TC14001463.hg.1	IFI27L2	8.16 ± 0.18	7.99 ± 0.04	8.02 ± 0.07	7.91 ± 0.04
TC19002629.hg.1	IFI30	12.83 ± 0.47	12.55 ± 0.08	13.03 ± 0.28	12.54 ± 0.26
TC17000545.hg.1	IFI35	9.20 ± 0.63	8.78 ± 0.25	9.24 ± 0.52	8.65 ± 0.14
TC01000795.hg.1	IFI44	8.78 ± 0.60	8.51 ± 1.01	9.34 ± 0.93	8.24 ± 0.42
TC01000794.hg.1	IFI44L	9.05 ± 0.52	8.54 ± 1.26	9.30 ± 0.83	8.07 ± 0.56
TC01002412.hg.1	IFI6	12.13 ± 0.70	11.70 ± 0.58	12.26 ± 0.68	11.43 ± 0.31
TC02002481.hg.1	IFIH1	9.02 ± 0.49	8.70 ± 0.70	9.58 ± 0.70	8.42 ± 0.59
TC10000639.hg.1	IFIT1	8.03 ± 0.74	7.55 ± 1.26	8.59 ± 1.17	7.60 ± 0.73
TC10000638.hg.1	IFIT1B	5.09 ± 0.10	4.78 ± 0.07	4.93 ± 0.13	4.96 ± 0.04
TC10000636.hg.1	IFIT2	10.10 ± 0.53	10.24 ± 0.82	10.89 ± 0.98	10.12 ± 0.44
TC10000637.hg.1	IFIT3	9.94 ± 0.61	9.54 ± 0.91	10.28 ± 0.88	9.33 ± 0.45
TC10000640.hg.1	IFIT5	9.40 ± 0.49	9.10 ± 0.54	9.60 ± 0.79	8.93 ± 0.33
TC11000011.hg.1	IFITM1	11.09 ± 0.25	10.81 ± 0.13	11.31 ± 0.24	10.66 ± 0.16
TC11003471.hg.1	IFITM10	8.38 ± 0.23	8.37 ± 0.11	8.35 ± 0.21	8.54 ± 0.18
TC11000010.hg.1	IFITM2	13.08 ± 0.15	12.88 ± 0.06	13.24 ± 0.10	12.75 ± 0.19
TC11001230.hg.1	IFITM3	12.37 ± 0.20	12.11 ± 0.10	12.49 ± 0.15	11.95 ± 0.20

TC06001488.hg.1	IFITM4P	10.85 ± 0.16	10.39 ± 0.19	10.90 ± 0.25	10.27 ± 0.26
TC6_cox_hap2000125.hg.1	IFITM4P	10.19 ± 0.13	9.80 ± 0.21	10.30 ± 0.26	9.66 ± 0.27
TC6_dbb_hap3000114.hg.1	IFITM4P	10.30 ± 0.17	9.88 ± 0.20	10.41 ± 0.24	9.72 ± 0.33
TC6_mann_hap4000103.hg.1	IFITM4P	10.30 ± 0.17	9.88 ± 0.20	10.41 ± 0.24	9.72 ± 0.33
TC6_mcf_hap5000107.hg.1	IFITM4P	10.85 ± 0.16	10.39 ± 0.19	10.90 ± 0.25	10.27 ± 0.26
TC6_qbl_hap6000115.hg.1	IFITM4P	10.30 ± 0.17	9.88 ± 0.20	10.41 ± 0.24	9.72 ± 0.33
TC6_ssto_hap7000106.hg.1	IFITM4P	10.63 ± 0.26	10.09 ± 0.19	10.65 ± 0.27	9.91 ± 0.34
TC11003502.hg.1	IFITM5	7.86 ± 0.27	7.88 ± 0.26	7.80 ± 0.23	8.00 ± 0.07
TC12001316.hg.1	IFLTD1	4.92 ± 0.12	4.81 ± 0.10	4.84 ± 0.03	4.92 ± 0.06
TC09000103.hg.1	IFNA1	5.69 ± 0.12	5.65 ± 0.13	5.64 ± 0.13	5.87 ± 0.21
TC09000952.hg.1	IFNA10	5.52 ± 0.05	5.29 ± 0.22	5.36 ± 0.15	5.36 ± 0.10
TC09000959.hg.1	IFNA13	5.30 ± 0.14	4.94 ± 0.06	4.97 ± 0.04	4.90 ± 0.25
TC09002912.hg.1	IFNA14	3.88 ± 0.19	3.79 ± 0.06	3.87 ± 0.14	3.89 ± 0.07
TC09000953.hg.1	IFNA16	5.27 ± 0.31	5.18 ± 0.08	5.23 ± 0.11	5.28 ± 0.13
TC09000954.hg.1	IFNA17	4.89 ± 0.17	4.79 ± 0.08	4.79 ± 0.04	4.88 ± 0.19
TC09000960.hg.1	IFNA2	4.26 ± 0.10	4.07 ± 0.05	4.09 ± 0.07	4.13 ± 0.06
TC09000949.hg.1	IFNA21	3.66 ± 0.13	3.61 ± 0.08	3.55 ± 0.04	3.66 ± 0.09
TC09000955.hg.1	IFNA22P	4.10 ± 0.19	4.09 ± 0.21	4.02 ± 0.12	4.24 ± 0.21
TC09000950.hg.1	IFNA4	3.88 ± 0.17	3.75 ± 0.02	3.78 ± 0.06	3.84 ± 0.15
TC09000956.hg.1	IFNA5	4.07 ± 0.16	4.12 ± 0.05	4.15 ± 0.12	4.25 ± 0.25
TC09000958.hg.1	IFNA6	4.74 ± 0.15	5.00 ± 0.23	4.90 ± 0.12	5.06 ± 0.10
TC09002913.hg.1	IFNA7	4.17 ± 0.05	3.92 ± 0.05	4.11 ± 0.20	4.05 ± 0.19
TC09000102.hg.1	IFNA8	4.75 ± 0.17	4.87 ± 0.12	4.76 ± 0.07	4.91 ± 0.17
TC21000128.hg.1	IFNAR1	9.79 ± 0.15	9.62 ± 0.32	10.30 ± 0.24	9.46 ± 0.34
TC21001060.hg.1	IFNAR2	9.10 ± 0.18	8.97 ± 0.17	9.39 ± 0.28	8.97 ± 0.13
TC09000946.hg.1	IFNB1	5.85 ± 0.25	5.97 ± 0.07	6.02 ± 0.25	6.09 ± 0.11
TC09000962.hg.1	IFNE	3.95 ± 0.13	3.94 ± 0.04	3.99 ± 0.02	4.05 ± 0.04
TC12001696.hg.1	IFNG	6.19 ± 0.54	7.06 ± 0.69	6.14 ± 0.24	6.04 ± 0.25
TC12000599.hg.1	IFNG-AS1	6.70 ± 0.51	6.71 ± 0.21	6.12 ± 0.29	6.42 ± 0.17
TC06002152.hg.1	IFNGR1	9.01 ± 0.08	9.12 ± 0.16	9.58 ± 0.44	9.33 ± 0.20
TC21000129.hg.1	IFNGR2	9.65 ± 0.20	9.32 ± 0.09	9.87 ± 0.24	9.31 ± 0.34

TC09000125.hg.1	IFNK	4.63 ± 0.27	4.47 ± 0.08	4.47 ± 0.06	4.54 ± 0.09
TC19000540.hg.1	IFNL1	7.13 ± 0.14	7.10 ± 0.23	7.07 ± 0.14	7.22 ± 0.05
TC19000539.hg.1	IFNL2	7.54 ± 0.06	7.54 ± 0.18	7.46 ± 0.07	7.65 ± 0.09
TC19001514.hg.1	IFNL3	7.95 ± 0.13	7.96 ± 0.19	7.88 ± 0.14	8.13 ± 0.07
TC01002359.hg.1	IFNLR1	7.70 ± 0.13	7.59 ± 0.04	7.53 ± 0.16	7.55 ± 0.01
TC09000947.hg.1	IFNW1	4.95 ± 0.18	4.79 ± 0.02	4.88 ± 0.07	4.79 ± 0.05
TC07003291.hg.1	IFRD1	8.87 ± 0.30	9.49 ± 0.20	9.41 ± 0.45	9.34 ± 0.32
TC03003410.hg.1	IFRD2	8.07 ± 0.15	8.09 ± 0.14	7.97 ± 0.18	8.17 ± 0.05
TC03000692.hg.1	IFT122	7.27 ± 0.10	7.21 ± 0.04	7.15 ± 0.05	7.24 ± 0.02
TC16000755.hg.1	IFT140	7.83 ± 0.21	7.71 ± 0.10	7.73 ± 0.22	7.75 ± 0.00
TC02001687.hg.1	IFT172	6.95 ± 0.14	6.90 ± 0.05	6.81 ± 0.07	6.91 ± 0.05
TC17001294.hg.1	IFT20	8.20 ± 0.06	8.15 ± 0.05	8.24 ± 0.07	8.12 ± 0.02
TC22000714.hg.1	IFT27	7.26 ± 0.10	7.16 ± 0.06	7.16 ± 0.11	7.19 ± 0.04
TC14000478.hg.1	IFT43	6.63 ± 0.16	6.58 ± 0.06	6.48 ± 0.13	6.51 ± 0.04
TC11002351.hg.1	IFT46	7.09 ± 0.08	6.91 ± 0.06	6.92 ± 0.10	6.80 ± 0.06
TC20000319.hg.1	IFT52	7.18 ± 0.06	7.04 ± 0.18	6.98 ± 0.07	6.95 ± 0.05
TC03001630.hg.1	IFT57	7.76 ± 0.20	7.97 ± 0.18	7.63 ± 0.21	7.89 ± 0.08
TC09000119.hg.1	IFT74	6.68 ± 0.30	6.72 ± 0.15	6.75 ± 0.21	6.68 ± 0.15
TC03003398.hg.1	IFT80	6.73 ± 0.60	7.00 ± 0.19	6.75 ± 0.40	6.84 ± 0.12
TC12000855.hg.1	IFT81	5.30 ± 0.08	5.19 ± 0.05	5.26 ± 0.07	5.17 ± 0.03
TC13000025.hg.1	IFT88	6.23 ± 0.30	6.36 ± 0.16	6.27 ± 0.29	6.32 ± 0.07
TC0X000370.hg.1	IGBP1	9.16 ± 0.24	9.06 ± 0.21	8.98 ± 0.24	8.82 ± 0.09
TC0X001110.hg.1	IGBP1-AS1	5.40 ± 0.31	5.45 ± 0.04	5.34 ± 0.13	5.49 ± 0.07
TC0X001109.hg.1	IGBP1-AS2	10.39 ± 0.30	10.42 ± 0.09	10.33 ± 0.06	10.58 ± 0.10
TC14000218.hg.1	IGBP1P1	7.14 ± 0.36	6.95 ± 0.06	6.95 ± 0.28	6.87 ± 0.16
TC15001565.hg.1	IGDCC3	8.11 ± 0.23	8.12 ± 0.25	8.04 ± 0.22	8.24 ± 0.08
TC15001566.hg.1	IGDCC4	7.75 ± 0.23	7.70 ± 0.17	7.68 ± 0.15	7.80 ± 0.06
TC12001890.hg.1	IGF1	5.68 ± 0.10	5.46 ± 0.05	5.49 ± 0.09	5.50 ± 0.06
TC15000949.hg.1	IGF1R	9.03 ± 0.23	9.08 ± 0.15	9.51 ± 0.28	9.10 ± 0.10
TC11000058.hg.1	IGF2-AS	7.92 ± 0.22	7.98 ± 0.29	7.76 ± 0.20	8.01 ± 0.00
TC17000636.hg.1	IGF2BP1	6.97 ± 0.20	6.95 ± 0.15	6.93 ± 0.15	7.02 ± 0.02

TC03002079.hg.1	IGF2BP2	7.09 ± 0.01	6.83 ± 0.02	7.16 ± 0.09	6.93 ± 0.10
TC07001194.hg.1	IGF2BP3	7.52 ± 0.12	7.05 ± 0.11	7.53 ± 0.26	7.17 ± 0.27
TC06001152.hg.1	IGF2R	10.19 ± 0.33	10.18 ± 0.24	10.44 ± 0.35	9.98 ± 0.28
TC16000761.hg.1	IGFALS	8.49 ± 0.23	8.41 ± 0.17	8.43 ± 0.23	8.49 ± 0.09
TC07000294.hg.1	IGFBP1	6.71 ± 0.20	6.55 ± 0.06	6.60 ± 0.01	6.56 ± 0.08
TC02001281.hg.1	IGFBP2	8.06 ± 0.20	8.06 ± 0.24	8.02 ± 0.18	8.14 ± 0.07
TC07001355.hg.1	IGFBP3	7.53 ± 0.20	7.51 ± 0.13	7.47 ± 0.17	7.56 ± 0.09
TC17000499.hg.1	IGFBP4	8.53 ± 0.19	8.47 ± 0.16	8.37 ± 0.21	8.53 ± 0.02
TC02002758.hg.1	IGFBP5	7.53 ± 0.15	7.51 ± 0.18	7.56 ± 0.19	7.55 ± 0.05
TC12000428.hg.1	IGFBP6	7.47 ± 0.25	7.51 ± 0.26	7.29 ± 0.28	7.60 ± 0.07
TC04001226.hg.1	IGFBP7	8.35 ± 0.20	7.98 ± 0.01	8.61 ± 0.13	7.93 ± 0.07
TC09001087.hg.1	IGFBPL1	7.49 ± 0.23	7.24 ± 0.08	7.30 ± 0.10	7.34 ± 0.13
TC19000661.hg.1	IGFL1	7.21 ± 0.20	7.20 ± 0.15	7.13 ± 0.16	7.35 ± 0.29
TC19000659.hg.1	IGFL2	6.73 ± 0.25	6.65 ± 0.12	6.65 ± 0.17	6.80 ± 0.09
TC19001643.hg.1	IGFL3	6.73 ± 0.13	6.63 ± 0.15	6.62 ± 0.07	6.75 ± 0.15
TC19001641.hg.1	IGFL4	6.57 ± 0.12	6.57 ± 0.16	6.65 ± 0.12	6.73 ± 0.10
TC19002694.hg.1	IGFLR1	8.23 ± 0.29	7.97 ± 0.08	8.19 ± 0.17	7.94 ± 0.07
TC01001657.hg.1	IGFN1	7.51 ± 0.19	7.47 ± 0.19	7.51 ± 0.17	7.56 ± 0.06
TC14002211.hg.1	IGHA1	13.44 ± 0.25	12.44 ± 0.37	12.57 ± 0.59	12.53 ± 0.49
TC14002258.hg.1	IGHA2	10.26 ± 0.20	9.73 ± 0.14	9.63 ± 0.25	9.59 ± 0.15
TC14002229.hg.1	IGHD2-15	11.01 ± 0.21	9.62 ± 0.15	9.63 ± 0.17	9.39 ± 0.12
TC14002241.hg.1	IGHD2-2	10.30 ± 0.40	8.39 ± 0.42	8.66 ± 0.37	8.13 ± 0.54
TC14002223.hg.1	IGHD2-21	10.34 ± 0.15	9.71 ± 0.38	9.42 ± 0.34	9.66 ± 0.13
TC14002235.hg.1	IGHD2-8	6.11 ± 1.05	4.60 ± 0.79	4.61 ± 0.15	4.36 ± 0.32
TC14002234.hg.1	IGHD3-10	11.76 ± 0.16	10.06 ± 0.09	9.46 ± 0.38	8.88 ± 1.00
TC14002228.hg.1	IGHD3-16	10.44 ± 0.53	9.03 ± 0.15	8.59 ± 0.80	7.86 ± 0.61
TC14002222.hg.1	IGHD3-22	5.78 ± 0.33	3.92 ± 0.20	4.09 ± 0.74	3.59 ± 0.28
TC14002240.hg.1	IGHD3-3	10.52 ± 0.29	8.54 ± 0.19	9.08 ± 0.34	8.51 ± 0.47
TC14002212.hg.1	IGHEP1	8.53 ± 0.16	8.58 ± 0.14	8.59 ± 0.21	8.68 ± 0.07
TC14002207.hg.1	IGHG4	10.20 ± 0.17	9.36 ± 0.22	9.24 ± 0.13	9.04 ± 0.17
TC14002216.hg.1	IGHJ1	11.69 ± 0.32	10.49 ± 0.20	10.79 ± 0.24	10.41 ± 0.01

TC14002215.hg.1	IGHJ5	12.29 ± 0.21	10.77 ± 0.14	11.05 ± 0.34	10.51 ± 0.25
TC14002213.hg.1	IGHM	12.58 ± 0.25	11.98 ± 0.02	11.81 ± 0.25	11.68 ± 0.12
TC11000712.hg.1	IGHMBP2	8.08 ± 0.07	7.96 ± 0.06	8.00 ± 0.09	8.00 ± 0.03
TC14002254.hg.1	IGHV1-18	11.59 ± 0.49	10.12 ± 0.29	10.23 ± 0.57	9.80 ± 0.44
TC14002245.hg.1	IGHV1-3	9.40 ± 0.55	8.02 ± 0.22	8.47 ± 0.50	8.33 ± 0.32
TC14002269.hg.1	IGHV1-45	8.75 ± 0.41	8.15 ± 0.31	8.16 ± 0.43	8.16 ± 0.25
TC14002270.hg.1	IGHV1-46	9.57 ± 1.20	8.05 ± 0.11	8.14 ± 0.30	8.05 ± 0.23
TC14002275.hg.1	IGHV1-58	8.43 ± 0.15	7.67 ± 0.19	7.80 ± 0.06	7.74 ± 0.15
TC14002280.hg.1	IGHV1-69	11.57 ± 0.43	10.36 ± 0.13	10.13 ± 0.57	10.17 ± 0.50
TC14002248.hg.1	IGHV1-8	9.49 ± 0.36	8.35 ± 0.12	8.59 ± 0.27	8.33 ± 0.39
TC21000012.hg.1	IGHV1OR21-1	7.24 ± 0.12	7.09 ± 0.19	7.02 ± 0.12	7.19 ± 0.01
TC14002259.hg.1	IGHV2-26	9.16 ± 0.20	8.26 ± 0.25	8.49 ± 0.18	8.47 ± 0.12
TC14002246.hg.1	IGHV2-5	11.39 ± 0.48	9.62 ± 0.19	9.88 ± 0.37	9.60 ± 0.34
TC14002281.hg.1	IGHV2-70	9.71 ± 0.67	8.67 ± 0.66	8.37 ± 0.31	8.07 ± 0.25
TC16000393.hg.1	IGHV2OR16-5	7.57 ± 0.11	7.17 ± 0.24	7.26 ± 0.24	7.28 ± 0.24
TC14002250.hg.1	IGHV3-11	10.67 ± 0.16	9.81 ± 0.27	9.90 ± 0.30	9.54 ± 0.21
TC14002251.hg.1	IGHV3-13	6.53 ± 0.12	6.21 ± 0.07	6.27 ± 0.08	6.21 ± 0.05
TC14002252.hg.1	IGHV3-15	11.53 ± 0.41	10.48 ± 0.19	10.89 ± 0.37	10.33 ± 0.13
TC14002253.hg.1	IGHV3-16	10.75 ± 0.25	9.91 ± 0.19	9.98 ± 0.33	9.77 ± 0.19
TC14002255.hg.1	IGHV3-20	10.57 ± 0.53	9.13 ± 0.28	9.60 ± 0.35	8.86 ± 0.32
TC14002256.hg.1	IGHV3-21	11.73 ± 0.11	10.27 ± 0.15	10.43 ± 0.35	10.15 ± 0.22
TC14002257.hg.1	IGHV3-23	12.34 ± 0.29	11.40 ± 0.18	11.63 ± 0.44	11.19 ± 0.37
TC14002261.hg.1	IGHV3-30	12.33 ± 0.19	11.29 ± 0.23	11.29 ± 0.44	10.84 ± 0.41
TC14002263.hg.1	IGHV3-33	11.90 ± 0.15	11.28 ± 0.21	11.10 ± 0.38	10.92 ± 0.37
TC14002265.hg.1	IGHV3-35	11.95 ± 0.23	11.14 ± 0.16	11.20 ± 0.34	10.88 ± 0.30
TC14002266.hg.1	IGHV3-38	10.79 ± 0.29	9.83 ± 0.11	10.10 ± 0.31	9.64 ± 0.27
TC14002268.hg.1	IGHV3-43	11.95 ± 0.24	10.82 ± 0.33	10.94 ± 0.30	10.48 ± 0.25
TC14002271.hg.1	IGHV3-48	12.30 ± 0.14	11.33 ± 0.19	11.39 ± 0.36	11.10 ± 0.23
TC14002272.hg.1	IGHV3-49	11.03 ± 0.33	9.99 ± 0.06	10.09 ± 0.43	9.65 ± 0.23
TC14002274.hg.1	IGHV3-53	11.60 ± 0.25	10.77 ± 0.33	10.75 ± 0.27	10.53 ± 0.20
TC14002278.hg.1	IGHV3-64	10.00 ± 0.33	8.86 ± 0.34	9.04 ± 0.34	8.59 ± 0.17

TC14002279.hg.1	IGHV3-66	11.71 ± 0.25	10.83 ± 0.35	10.90 ± 0.28	10.58 ± 0.25
TC14002247.hg.1	IGHV3-7	11.49 ± 0.23	10.34 ± 0.14	10.51 ± 0.29	10.19 ± 0.18
TC14002282.hg.1	IGHV3-72	10.18 ± 0.61	8.70 ± 0.07	9.16 ± 0.49	8.46 ± 0.16
TC14002283.hg.1	IGHV3-73	10.11 ± 0.41	8.84 ± 0.02	9.21 ± 0.16	9.05 ± 0.31
TC14002284.hg.1	IGHV3-74	10.63 ± 0.29	9.61 ± 0.16	9.94 ± 0.18	9.47 ± 0.18
TC14002249.hg.1	IGHV3-9	12.08 ± 0.19	10.88 ± 0.35	11.31 ± 0.17	10.60 ± 0.47
TC16001071.hg.1	IGHV3OR16-7	9.38 ± 0.27	8.45 ± 0.28	8.50 ± 0.39	8.20 ± 0.22
TC14002262.hg.1	IGHV4-31	10.38 ± 0.03	9.81 ± 0.01	9.75 ± 0.42	9.69 ± 0.07
TC14002264.hg.1	IGHV4-34	9.31 ± 0.32	8.49 ± 0.12	8.45 ± 0.29	8.34 ± 0.16
TC14002267.hg.1	IGHV4-39	9.76 ± 0.32	8.48 ± 0.12	8.52 ± 0.37	8.30 ± 0.07
TC14002276.hg.1	IGHV4-59	10.25 ± 0.15	9.46 ± 0.25	9.36 ± 0.33	9.28 ± 0.11
TC14002277.hg.1	IGHV4-61	12.12 ± 0.28	11.17 ± 0.16	11.01 ± 0.37	10.90 ± 0.13
TC14002273.hg.1	IGHV5-51	10.06 ± 0.68	9.23 ± 0.40	9.21 ± 0.32	9.13 ± 0.29
TC14002285.hg.1	IGHV5-78	8.37 ± 0.35	7.90 ± 0.06	7.88 ± 0.17	7.56 ± 0.10
TC14002243.hg.1	IGHV6-1	10.21 ± 0.28	9.42 ± 0.19	9.54 ± 0.06	9.41 ± 0.08
TC14002286.hg.1	IGHV7-81	10.44 ± 0.28	9.43 ± 0.19	9.48 ± 0.40	9.40 ± 0.23
TC05000721.hg.1	IGIP	6.29 ± 0.24	6.64 ± 0.05	6.44 ± 0.22	6.50 ± 0.10
TC04001270.hg.1	IGJ	10.83 ± 0.16	10.29 ± 0.20	10.33 ± 0.36	10.10 ± 0.29
TC02004950.hg.1	IGKC	10.31 ± 0.15	9.61 ± 0.15	9.53 ± 0.26	9.37 ± 0.15
TC02004943.hg.1	IGKV1-17	10.55 ± 0.38	9.43 ± 0.33	9.55 ± 0.15	9.28 ± 0.13
TC02004945.hg.1	IGKV1-27	10.98 ± 0.62	9.55 ± 0.14	9.60 ± 0.33	9.36 ± 0.25
TC02004948.hg.1	IGKV1-37	10.07 ± 0.33	9.08 ± 0.18	9.18 ± 0.24	8.97 ± 0.13
TC02004938.hg.1	IGKV1-5	10.68 ± 0.26	9.76 ± 0.13	9.66 ± 0.33	9.61 ± 0.16
TC02004939.hg.1	IGKV1-6	11.18 ± 0.36	10.25 ± 0.27	10.22 ± 0.29	10.06 ± 0.22
TC02004940.hg.1	IGKV1-8	10.26 ± 0.31	9.30 ± 0.19	9.24 ± 0.32	9.10 ± 0.17
TC02004941.hg.1	IGKV1-9	11.21 ± 0.33	10.08 ± 0.14	10.03 ± 0.32	9.86 ± 0.20
TC02004985.hg.1	IGKV1D-12	10.07 ± 0.14	9.17 ± 0.14	9.13 ± 0.23	8.91 ± 0.18
TC02004984.hg.1	IGKV1D-13	8.12 ± 0.12	7.35 ± 0.10	7.37 ± 0.21	7.33 ± 0.18
TC02004982.hg.1	IGKV1D-16	11.78 ± 0.30	10.70 ± 0.24	10.73 ± 0.35	10.44 ± 0.17
TC02004981.hg.1	IGKV1D-17	8.92 ± 0.26	8.53 ± 0.12	8.46 ± 0.30	8.34 ± 0.14
TC02004979.hg.1	IGKV1D-27	12.74 ± 0.65	11.20 ± 0.24	11.10 ± 0.34	10.86 ± 0.30

TC02004976.hg.1	IGKV1D-33	10.49 ± 0.21	9.78 ± 0.29	9.46 ± 0.27	9.47 ± 0.17
TC02004975.hg.1	IGKV1D-37	11.36 ± 0.28	10.22 ± 0.13	10.25 ± 0.37	10.00 ± 0.17
TC02004974.hg.1	IGKV1D-39	11.08 ± 0.31	10.18 ± 0.27	10.10 ± 0.27	9.79 ± 0.20
TC02000558.hg.1	IGKV1D-42	10.30 ± 0.31	9.41 ± 0.22	9.36 ± 0.29	9.25 ± 0.16
TC02004987.hg.1	IGKV1D-43	8.28 ± 0.28	7.85 ± 0.09	7.84 ± 0.14	7.74 ± 0.18
TC02004989.hg.1	IGKV1D-8	8.94 ± 0.17	8.22 ± 0.12	8.03 ± 0.22	8.20 ± 0.14
TC02004944.hg.1	IGKV2-24	10.64 ± 0.50	9.48 ± 0.23	9.62 ± 0.39	9.33 ± 0.09
TC02004946.hg.1	IGKV2-28	11.79 ± 0.50	10.57 ± 0.25	10.52 ± 0.49	10.26 ± 0.25
TC02004947.hg.1	IGKV2-29	11.17 ± 0.20	9.98 ± 0.32	9.83 ± 0.75	9.50 ± 0.09
TC02004949.hg.1	IGKV2-40	9.02 ± 1.38	6.78 ± 0.24	6.05 ± 0.24	6.14 ± 0.41
TC02000555.hg.1	IGKV2D-24	10.51 ± 0.39	9.47 ± 0.31	9.53 ± 0.39	9.28 ± 0.06
TC02000554.hg.1	IGKV2D-26	9.69 ± 0.27	8.73 ± 0.20	8.64 ± 0.44	8.63 ± 0.18
TC02004978.hg.1	IGKV2D-28	11.58 ± 0.38	10.56 ± 0.21	10.43 ± 0.56	10.37 ± 0.25
TC02000553.hg.1	IGKV2D-29	10.80 ± 0.24	9.66 ± 0.30	9.67 ± 0.44	9.49 ± 0.19
TC02004977.hg.1	IGKV2D-30	8.68 ± 0.14	8.12 ± 0.11	8.17 ± 0.28	8.14 ± 0.10
TC02004973.hg.1	IGKV2D-40	9.00 ± 1.47	6.98 ± 0.25	6.56 ± 0.35	6.52 ± 0.26
TC02004942.hg.1	IGKV3-11	10.29 ± 0.25	9.67 ± 0.19	9.57 ± 0.34	9.25 ± 0.21
TC02002076.hg.1	IGKV3-7	9.48 ± 0.29	8.78 ± 0.16	8.71 ± 0.37	8.53 ± 0.15
TC02004986.hg.1	IGKV3D-11	9.72 ± 0.22	9.13 ± 0.17	8.98 ± 0.23	8.88 ± 0.17
TC02004983.hg.1	IGKV3D-15	10.42 ± 0.41	9.59 ± 0.09	9.63 ± 0.26	9.43 ± 0.20
TC02004980.hg.1	IGKV3D-20	9.99 ± 0.34	9.17 ± 0.06	9.19 ± 0.27	8.89 ± 0.24
TC02000559.hg.1	IGKV3D-7	11.84 ± 0.23	10.96 ± 0.19	10.89 ± 0.34	10.64 ± 0.21
TC02000550.hg.1	IGKV4-1	8.90 ± 0.23	8.30 ± 0.13	8.22 ± 0.11	8.12 ± 0.09
TC02000551.hg.1	IGKV5-2	9.09 ± 0.40	7.94 ± 0.16	8.23 ± 0.40	7.73 ± 0.13
TC02002077.hg.1	IGKV6-21	9.20 ± 0.35	7.85 ± 0.18	8.10 ± 0.62	7.70 ± 0.08
TC02000556.hg.1	IGKV6D-21	7.21 ± 0.12	6.71 ± 0.12	6.91 ± 0.34	6.66 ± 0.11
TC02000557.hg.1	IGKV6D-41	6.87 ± 0.27	6.45 ± 0.17	6.68 ± 0.29	6.58 ± 0.13
TC22001449.hg.1	IGLC7	12.70 ± 0.18	11.67 ± 0.32	11.58 ± 0.37	11.41 ± 0.29
TC22000125.hg.1	IGLJ4	7.72 ± 0.55	7.99 ± 0.19	7.29 ± 0.73	8.25 ± 0.23
TC22001446.hg.1	IGLJ5	7.50 ± 0.25	7.39 ± 0.08	7.53 ± 0.27	7.16 ± 0.14
TC22001447.hg.1	IGLJ6	8.65 ± 0.12	8.64 ± 0.28	8.24 ± 0.48	8.94 ± 0.12

TC22001448.hg.1	IGLJ7	11.41 ± 0.09	10.85 ± 0.26	10.56 ± 0.46	10.99 ± 0.16
TC22000562.hg.1	IGLL1	9.44 ± 0.29	9.26 ± 0.26	9.27 ± 0.20	9.34 ± 0.03
TC22000164.hg.1	IGLL3P	8.15 ± 0.21	7.98 ± 0.05	8.14 ± 0.07	7.93 ± 0.06
TC19000777.hg.1	IGLON5	7.96 ± 0.26	7.88 ± 0.14	7.87 ± 0.24	7.91 ± 0.04
TC22001437.hg.1	IGLV10-54	10.72 ± 1.03	9.68 ± 0.93	9.74 ± 0.38	9.43 ± 0.27
TC22000104.hg.1	IGLV11-55	7.93 ± 0.23	7.80 ± 0.30	7.67 ± 0.12	7.98 ± 0.10
TC22000119.hg.1	IGLV2-18	12.08 ± 0.20	11.13 ± 0.26	11.10 ± 0.20	10.97 ± 0.20
TC22000111.hg.1	IGLV2-33	10.97 ± 0.22	10.27 ± 0.20	10.27 ± 0.22	10.08 ± 0.18
TC22001445.hg.1	IGLV3-1	10.53 ± 0.18	9.68 ± 0.34	9.53 ± 0.49	9.23 ± 0.48
TC22000121.hg.1	IGLV3-12	9.90 ± 0.20	9.25 ± 0.32	9.07 ± 0.24	9.25 ± 0.16
TC22001443.hg.1	IGLV3-16	9.86 ± 0.56	8.75 ± 0.30	8.71 ± 0.22	8.59 ± 0.21
TC22000118.hg.1	IGLV3-22	8.57 ± 0.06	8.09 ± 0.26	8.20 ± 0.12	8.18 ± 0.09
TC22001442.hg.1	IGLV3-25	11.46 ± 0.43	9.57 ± 0.22	9.54 ± 0.46	9.18 ± 0.41
TC22001441.hg.1	IGLV3-27	9.60 ± 0.32	8.55 ± 0.26	8.28 ± 0.20	8.64 ± 0.43
TC22000112.hg.1	IGLV3-32	7.74 ± 0.28	7.74 ± 0.22	7.53 ± 0.20	7.95 ± 0.19
TC22000123.hg.1	IGLV3-9	9.73 ± 0.09	8.76 ± 0.12	8.89 ± 0.14	8.61 ± 0.16
TC22001444.hg.1	IGLV4-3	8.27 ± 0.32	7.40 ± 0.10	7.49 ± 0.26	7.32 ± 0.18
TC22000103.hg.1	IGLV4-60	8.78 ± 0.24	8.35 ± 0.50	7.98 ± 0.05	7.89 ± 0.25
TC22001435.hg.1	IGLV4-69	9.70 ± 0.47	9.46 ± 1.10	8.78 ± 0.53	8.59 ± 0.17
TC22000108.hg.1	IGLV5-37	8.48 ± 0.18	7.79 ± 0.27	7.75 ± 0.11	7.66 ± 0.22
TC22000107.hg.1	IGLV5-48	8.89 ± 0.55	7.88 ± 0.20	7.64 ± 0.11	7.96 ± 0.23
TC22001436.hg.1	IGLV6-57	9.92 ± 0.27	8.91 ± 0.35	8.92 ± 0.38	8.62 ± 0.13
TC22001440.hg.1	IGLV7-43	9.96 ± 1.65	8.25 ± 0.16	8.51 ± 0.33	7.99 ± 0.12
TC22001439.hg.1	IGLV7-46	10.25 ± 1.12	8.79 ± 0.12	8.90 ± 0.33	8.53 ± 0.12
TC22001438.hg.1	IGLV9-49	9.31 ± 0.26	8.54 ± 0.13	8.42 ± 0.25	8.39 ± 0.05
TC0X001365.hg.1	IGSF1	6.54 ± 0.12	6.50 ± 0.08	6.49 ± 0.09	6.55 ± 0.07
TC03001908.hg.1	IGSF10	5.33 ± 0.14	5.22 ± 0.04	5.27 ± 0.02	5.33 ± 0.09
TC03001679.hg.1	IGSF11	6.72 ± 0.17	6.65 ± 0.12	6.63 ± 0.12	6.73 ± 0.07
TC03000604.hg.1	IGSF11-AS1	5.81 ± 0.19	5.94 ± 0.12	5.88 ± 0.08	5.96 ± 0.05
TC01000239.hg.1	IGSF21	7.67 ± 0.24	7.66 ± 0.20	7.59 ± 0.22	7.72 ± 0.04
TC11001471.hg.1	IGSF22	7.21 ± 0.22	7.18 ± 0.20	7.18 ± 0.18	7.26 ± 0.04

TC19000623.hg.1	IGSF23	6.81 ± 0.05	6.94 ± 0.04	6.88 ± 0.05	7.01 ± 0.05
TC01003030.hg.1	IGSF3	7.32 ± 0.17	7.33 ± 0.17	7.31 ± 0.13	7.41 ± 0.06
TC21000179.hg.1	IGSF5	6.44 ± 0.11	6.34 ± 0.09	6.38 ± 0.07	6.45 ± 0.11
TC16000939.hg.1	IGSF6	9.93 ± 0.18	9.66 ± 0.32	10.56 ± 0.27	9.41 ± 0.56
TC01003397.hg.1	IGSF8	8.52 ± 0.13	8.47 ± 0.13	8.36 ± 0.17	8.44 ± 0.05
TC01003393.hg.1	IGSF9	7.95 ± 0.21	7.96 ± 0.20	7.88 ± 0.18	8.04 ± 0.05
TC11002471.hg.1	IGSF9B	7.92 ± 0.09	7.88 ± 0.15	7.81 ± 0.13	7.97 ± 0.02
TC02002786.hg.1	IHH	7.64 ± 0.27	7.60 ± 0.25	7.58 ± 0.27	7.66 ± 0.05
TC05000730.hg.1	IK	9.67 ± 0.14	9.80 ± 0.06	9.70 ± 0.19	9.77 ± 0.04
TC12001861.hg.1	IKBIP	7.58 ± 0.16	7.49 ± 0.11	7.67 ± 0.12	7.36 ± 0.20
TC09001455.hg.1	IKBKAP	8.09 ± 0.25	8.14 ± 0.17	7.87 ± 0.20	7.96 ± 0.08
TC08000325.hg.1	IKBKB	8.56 ± 0.14	8.46 ± 0.09	8.51 ± 0.11	8.36 ± 0.02
TC01001723.hg.1	IKBKE	8.80 ± 0.10	8.61 ± 0.10	8.62 ± 0.07	8.57 ± 0.02
TC0X000776.hg.1	IKBKG	8.46 ± 0.18	8.35 ± 0.08	8.45 ± 0.16	8.42 ± 0.03
TC07000313.hg.1	IKZF1	9.94 ± 0.17	9.98 ± 0.05	9.91 ± 0.14	9.85 ± 0.04
TC02002740.hg.1	IKZF2	7.22 ± 0.43	7.52 ± 0.23	7.08 ± 0.27	7.34 ± 0.06
TC17001455.hg.1	IKZF3	9.63 ± 0.13	9.60 ± 0.13	9.19 ± 0.16	9.31 ± 0.09
TC12000499.hg.1	IKZF4	7.62 ± 0.07	7.48 ± 0.07	7.54 ± 0.02	7.56 ± 0.03
TC10001718.hg.1	IKZF5	8.75 ± 0.65	9.47 ± 0.22	8.83 ± 0.51	9.61 ± 0.15
TC01003766.hg.1	IL10	6.38 ± 0.28	6.32 ± 0.15	6.35 ± 0.03	6.31 ± 0.09
TC11001058.hg.1	IL10RA	10.08 ± 0.14	10.18 ± 0.10	9.75 ± 0.03	10.13 ± 0.06
TC21001059.hg.1	IL10RB	9.72 ± 0.29	9.29 ± 0.14	10.06 ± 0.30	9.12 ± 0.29
TC21000408.hg.1	IL10RB-AS1	7.86 ± 0.39	8.26 ± 0.24	8.31 ± 0.15	8.11 ± 0.31
TC19001870.hg.1	IL11	8.35 ± 0.23	8.31 ± 0.22	8.32 ± 0.21	8.40 ± 0.08
TC09002897.hg.1	IL11RA	8.16 ± 0.12	8.24 ± 0.12	8.02 ± 0.08	8.30 ± 0.09
TC03000870.hg.1	IL12A	5.94 ± 0.12	5.87 ± 0.07	5.77 ± 0.05	5.79 ± 0.05
TC05001997.hg.1	IL12B	6.04 ± 0.12	5.97 ± 0.11	5.96 ± 0.17	6.03 ± 0.03
TC19001281.hg.1	IL12RB1	8.99 ± 0.20	8.68 ± 0.02	8.72 ± 0.09	8.63 ± 0.03
TC01000741.hg.1	IL12RB2	7.30 ± 0.14	7.04 ± 0.17	6.98 ± 0.07	6.82 ± 0.09
TC05000640.hg.1	IL13	7.17 ± 0.15	7.18 ± 0.15	7.13 ± 0.15	7.25 ± 0.02
TC0X000575.hg.1	IL13RA1	9.68 ± 0.13	9.77 ± 0.34	10.14 ± 0.33	9.63 ± 0.40

TC0X001290.hg.1	IL13RA2	4.26 ± 0.25	4.10 ± 0.03	4.12 ± 0.10	4.11 ± 0.06
TC04000693.hg.1	IL15	5.48 ± 0.06	5.27 ± 0.07	5.54 ± 0.22	5.19 ± 0.08
TC10001006.hg.1	IL15RA	8.41 ± 0.20	8.25 ± 0.14	8.34 ± 0.14	8.31 ± 0.02
TC15000756.hg.1	IL16	8.36 ± 0.04	8.27 ± 0.07	8.28 ± 0.06	8.29 ± 0.06
TC06000652.hg.1	IL17A	6.16 ± 0.09	6.05 ± 0.15	6.05 ± 0.06	6.11 ± 0.10
TC05001922.hg.1	IL17B	7.16 ± 0.23	7.19 ± 0.16	7.15 ± 0.13	7.30 ± 0.08
TC16000681.hg.1	IL17C	8.42 ± 0.27	8.41 ± 0.16	8.38 ± 0.16	8.53 ± 0.04
TC13000027.hg.1	IL17D	7.50 ± 0.20	7.53 ± 0.19	7.40 ± 0.28	7.59 ± 0.05
TC06001798.hg.1	IL17F	5.51 ± 0.04	5.54 ± 0.04	5.53 ± 0.12	5.64 ± 0.05
TC22000019.hg.1	IL17RA	11.19 ± 0.28	10.74 ± 0.13	11.56 ± 0.26	10.58 ± 0.29
TC03000352.hg.1	IL17RB	6.73 ± 0.16	6.71 ± 0.10	6.72 ± 0.09	6.71 ± 0.04
TC03000047.hg.1	IL17RC	7.89 ± 0.20	7.85 ± 0.17	7.82 ± 0.16	7.96 ± 0.08
TC03001489.hg.1	IL17RD	6.88 ± 0.16	6.76 ± 0.08	6.80 ± 0.13	6.80 ± 0.04
TC03000046.hg.1	IL17RE	7.28 ± 0.15	7.32 ± 0.18	7.22 ± 0.11	7.43 ± 0.07
TC22000905.hg.1	IL17REL	7.87 ± 0.14	7.84 ± 0.17	7.77 ± 0.16	7.91 ± 0.04
TC11002293.hg.1	IL18	6.48 ± 0.07	6.14 ± 0.16	6.64 ± 0.23	6.13 ± 0.24
TC11000747.hg.1	IL18BP	8.11 ± 0.03	8.09 ± 0.02	7.98 ± 0.15	8.08 ± 0.03
TC02004995.hg.1	IL18R1	7.93 ± 0.41	7.22 ± 0.27	8.91 ± 0.65	7.37 ± 0.73
TC02000623.hg.1	IL18RAP	8.79 ± 0.24	8.01 ± 0.32	9.34 ± 0.46	7.65 ± 0.10
TC01001728.hg.1	IL19	6.30 ± 0.17	6.24 ± 0.16	6.22 ± 0.03	6.33 ± 0.05
TC02002218.hg.1	IL1A	5.21 ± 0.10	5.36 ± 0.15	5.25 ± 0.18	5.34 ± 0.08
TC02002219.hg.1	IL1B	9.65 ± 0.47	10.04 ± 0.16	10.78 ± 0.37	9.48 ± 0.60
TC02000718.hg.1	IL1F10	6.80 ± 0.17	6.72 ± 0.13	6.72 ± 0.11	6.86 ± 0.08
TC02000620.hg.1	IL1R1	6.95 ± 0.18	7.01 ± 0.18	7.84 ± 0.54	7.15 ± 0.31
TC02000619.hg.1	IL1R2	8.95 ± 0.93	8.44 ± 0.28	9.87 ± 0.74	8.54 ± 0.48
TC03001047.hg.1	IL1RAP	7.46 ± 0.14	7.86 ± 0.36	8.42 ± 0.75	7.81 ± 0.31
TC03001048.hg.1	IL1RAP	6.75 ± 0.23	6.99 ± 0.39	7.80 ± 0.72	6.78 ± 0.25
TC0X000133.hg.1	IL1RAPL1	5.31 ± 0.23	5.13 ± 0.12	5.16 ± 0.13	5.25 ± 0.07
TC0X000511.hg.1	IL1RAPL2	4.71 ± 0.12	4.62 ± 0.11	4.62 ± 0.08	4.69 ± 0.05
TC02004994.hg.1	IL1RL1	5.93 ± 0.11	5.94 ± 0.19	6.41 ± 0.25	6.10 ± 0.17
TC02000621.hg.1	IL1RL2	6.26 ± 0.20	6.24 ± 0.09	6.18 ± 0.20	6.33 ± 0.06

TC02000720.hg.1	IL1RN	8.03 ± 0.47	7.77 ± 0.14	8.32 ± 0.23	7.59 ± 0.26
TC04001520.hg.1	IL2	3.82 ± 0.17	3.66 ± 0.06	3.73 ± 0.05	3.65 ± 0.06
TC01001729.hg.1	IL20	6.10 ± 0.03	6.06 ± 0.09	6.12 ± 0.11	6.12 ± 0.15
TC06002150.hg.1	IL20RA	5.60 ± 0.12	5.55 ± 0.07	5.50 ± 0.06	5.61 ± 0.07
TC03003324.hg.1	IL20RB	7.28 ± 0.17	7.36 ± 0.08	7.23 ± 0.10	7.48 ± 0.13
TC03001816.hg.1	IL20RB-AS1	5.20 ± 0.11	5.08 ± 0.19	5.28 ± 0.11	4.98 ± 0.04
TC04001521.hg.1	IL21	4.68 ± 0.16	4.54 ± 0.05	4.56 ± 0.04	4.55 ± 0.01
TC04000628.hg.1	IL21-AS1	5.62 ± 0.18	5.55 ± 0.13	5.58 ± 0.03	5.66 ± 0.11
TC16000294.hg.1	IL21R	8.63 ± 0.03	8.61 ± 0.02	8.54 ± 0.11	8.61 ± 0.05
TC16000978.hg.1	IL21R-AS1	6.22 ± 0.21	6.00 ± 0.13	6.01 ± 0.13	6.10 ± 0.10
TC12001698.hg.1	IL22	5.94 ± 0.26	5.74 ± 0.02	5.82 ± 0.13	5.82 ± 0.06
TC01002358.hg.1	IL22RA1	7.10 ± 0.10	7.11 ± 0.20	7.05 ± 0.16	7.23 ± 0.09
TC06002151.hg.1	IL22RA2	4.66 ± 0.16	4.54 ± 0.06	4.58 ± 0.07	4.67 ± 0.07
TC12000512.hg.1	IL23A	7.69 ± 0.05	7.79 ± 0.08	7.43 ± 0.10	7.99 ± 0.03
TC01000739.hg.1	IL23R	5.01 ± 0.11	4.88 ± 0.01	4.89 ± 0.04	4.89 ± 0.03
TC01001730.hg.1	IL24	6.81 ± 0.12	6.73 ± 0.05	6.64 ± 0.18	6.71 ± 0.04
TC14000145.hg.1	IL25	7.09 ± 0.16	7.07 ± 0.12	7.02 ± 0.16	7.16 ± 0.12
TC12001697.hg.1	IL26	3.69 ± 0.02	3.62 ± 0.05	3.68 ± 0.10	3.71 ± 0.12
TC16000988.hg.1	IL27	8.44 ± 0.09	8.45 ± 0.13	8.41 ± 0.18	8.47 ± 0.03
TC19000250.hg.1	IL27RA	9.11 ± 0.07	8.91 ± 0.05	8.86 ± 0.12	8.84 ± 0.01
TC10001007.hg.1	IL2RA	7.66 ± 0.10	7.58 ± 0.03	7.83 ± 0.07	7.65 ± 0.07
TC22000722.hg.1	IL2RB	9.80 ± 0.14	9.65 ± 0.09	9.41 ± 0.27	9.60 ± 0.05
TC0X001121.hg.1	IL2RG	11.61 ± 0.19	11.50 ± 0.03	11.70 ± 0.06	11.38 ± 0.10
TC05000628.hg.1	IL3	7.03 ± 0.14	7.09 ± 0.12	6.95 ± 0.21	7.14 ± 0.16
TC12002070.hg.1	IL31	7.23 ± 0.25	7.31 ± 0.23	7.12 ± 0.19	7.39 ± 0.17
TC05000238.hg.1	IL31RA	6.00 ± 0.06	5.93 ± 0.09	5.93 ± 0.01	5.98 ± 0.02
TC16000099.hg.1	IL32	9.61 ± 0.11	9.60 ± 0.11	9.30 ± 0.14	9.45 ± 0.09
TC09000045.hg.1	IL33	4.23 ± 0.20	4.13 ± 0.05	4.16 ± 0.06	4.14 ± 0.10
TC16000582.hg.1	IL34	7.78 ± 0.16	7.91 ± 0.18	7.81 ± 0.21	8.05 ± 0.11
TC02000716.hg.1	IL36A	6.49 ± 0.11	6.50 ± 0.06	6.51 ± 0.12	6.51 ± 0.08
TC02002220.hg.1	IL36B	5.39 ± 0.11	5.24 ± 0.11	5.19 ± 0.08	5.31 ± 0.10

TC02000715.hg.1	IL36G	5.71 ± 0.14	5.70 ± 0.05	5.73 ± 0.04	5.78 ± 0.07
TC02000717.hg.1	IL36RN	6.87 ± 0.22	6.86 ± 0.15	6.81 ± 0.18	6.89 ± 0.06
TC02000714.hg.1	IL37	5.40 ± 0.12	5.37 ± 0.16	5.30 ± 0.09	5.41 ± 0.04
TC0X000008.hg.1	IL3RA	7.84 ± 0.10	7.92 ± 0.08	8.06 ± 0.11	8.04 ± 0.13
TC0Y000008.hg.1	IL3RA	7.88 ± 0.09	7.94 ± 0.08	8.09 ± 0.11	8.05 ± 0.13
TC05000641.hg.1	IL4	5.64 ± 0.07	5.65 ± 0.08	5.52 ± 0.02	5.68 ± 0.08
TC19002607.hg.1	IL4I1	8.04 ± 0.17	7.82 ± 0.11	7.88 ± 0.14	7.81 ± 0.05
TC16000293.hg.1	IL4R	9.93 ± 0.25	9.76 ± 0.04	10.35 ± 0.23	9.76 ± 0.12
TC05001769.hg.1	IL5	4.73 ± 0.08	4.60 ± 0.09	4.61 ± 0.08	4.61 ± 0.01
TC03001122.hg.1	IL5RA	7.11 ± 0.48	7.21 ± 0.26	8.04 ± 0.37	7.32 ± 0.14
TC07000137.hg.1	IL6	6.69 ± 0.13	6.90 ± 0.17	6.80 ± 0.04	6.79 ± 0.08
TC01001276.hg.1	IL6R	9.68 ± 0.21	9.31 ± 0.15	9.93 ± 0.25	9.23 ± 0.31
TC05001366.hg.1	IL6ST	9.78 ± 0.63	10.28 ± 0.11	9.96 ± 0.40	10.24 ± 0.06
TC08001350.hg.1	IL7	4.53 ± 0.11	4.42 ± 0.04	4.46 ± 0.11	4.39 ± 0.02
TC05000159.hg.1	IL7R	10.88 ± 0.41	11.00 ± 0.11	10.57 ± 0.24	11.00 ± 0.14
TC04000408.hg.1	IL8	10.02 ± 0.41	12.23 ± 0.09	11.09 ± 0.94	12.28 ± 0.47
TC05001807.hg.1	IL9	6.32 ± 0.13	6.23 ± 0.11	6.28 ± 0.11	6.34 ± 0.11
TC0X002325.hg.1	IL9R	7.91 ± 0.17	7.87 ± 0.13	7.82 ± 0.14	7.98 ± 0.04
TC0Y000355.hg.1	IL9R	7.91 ± 0.17	7.87 ± 0.13	7.82 ± 0.14	8.00 ± 0.04
TC03001700.hg.1	ILDR1	7.41 ± 0.16	7.38 ± 0.14	7.35 ± 0.08	7.42 ± 0.07
TC01003476.hg.1	ILDR2	6.83 ± 0.10	6.89 ± 0.18	6.81 ± 0.10	6.98 ± 0.06
TC01003273.hg.1	ILF2	9.67 ± 0.15	9.60 ± 0.33	9.17 ± 0.22	9.46 ± 0.05
TC19000182.hg.1	ILF3	9.73 ± 0.16	9.85 ± 0.15	9.41 ± 0.04	9.88 ± 0.07
TC19001172.hg.1	ILF3-AS1	8.17 ± 0.23	8.40 ± 0.11	7.97 ± 0.19	8.31 ± 0.07
TC11000133.hg.1	ILK	10.03 ± 0.20	9.57 ± 0.08	10.06 ± 0.27	9.44 ± 0.20
TC02002911.hg.1	ILKAP	7.98 ± 0.08	8.05 ± 0.14	7.76 ± 0.11	8.10 ± 0.07
TC19001249.hg.1	ILVBL	8.45 ± 0.17	8.41 ± 0.16	8.33 ± 0.13	8.48 ± 0.03
TC11001525.hg.1	IMMP1L	6.04 ± 0.03	6.06 ± 0.09	5.90 ± 0.11	6.06 ± 0.05
TC07001758.hg.1	IMMP2L	6.00 ± 0.09	6.02 ± 0.03	5.93 ± 0.10	6.02 ± 0.07
TC07001759.hg.1	IMMP2L-IT1	5.43 ± 0.18	5.38 ± 0.07	5.24 ± 0.05	5.42 ± 0.07
TC02002052.hg.1	IMMT	8.73 ± 0.12	8.58 ± 0.13	8.47 ± 0.12	8.35 ± 0.11

TC15001669.hg.1	IMP3	8.53 ± 0.15	8.46 ± 0.08	8.20 ± 0.22	8.33 ± 0.03
TC02000808.hg.1	IMP4	9.00 ± 0.11	8.89 ± 0.12	8.62 ± 0.12	8.88 ± 0.04
TC08001368.hg.1	IMPA1	7.14 ± 0.36	7.22 ± 0.12	7.44 ± 0.42	7.18 ± 0.16
TC08001367.hg.1	IMPA1P	7.45 ± 0.20	7.44 ± 0.13	7.58 ± 0.22	7.51 ± 0.06
TC18000060.hg.1	IMPA2	9.17 ± 0.10	9.10 ± 0.06	9.49 ± 0.27	9.00 ± 0.09
TC18000109.hg.1	IMPACT	7.61 ± 0.28	7.79 ± 0.16	7.61 ± 0.23	7.81 ± 0.05
TC08001238.hg.1	IMPAD1	8.47 ± 0.31	8.54 ± 0.13	8.35 ± 0.32	8.52 ± 0.13
TC07001846.hg.1	IMPDH1	9.05 ± 0.26	8.81 ± 0.03	9.26 ± 0.16	8.75 ± 0.11
TC03001397.hg.1	IMPDH2	8.17 ± 0.10	8.15 ± 0.09	7.91 ± 0.13	8.04 ± 0.07
TC06001889.hg.1	IMPG1	4.66 ± 0.11	4.54 ± 0.06	4.59 ± 0.06	4.58 ± 0.03
TC03001613.hg.1	IMPG2	5.87 ± 0.12	5.84 ± 0.06	5.78 ± 0.05	5.76 ± 0.03
TC10000771.hg.1	INA	5.83 ± 0.13	5.63 ± 0.16	5.67 ± 0.09	5.69 ± 0.04
TC01000703.hg.1	INADL	7.24 ± 0.24	7.49 ± 0.11	7.18 ± 0.17	7.44 ± 0.14
TC17001040.hg.1	INCA1	7.68 ± 0.21	7.66 ± 0.26	7.61 ± 0.11	7.85 ± 0.09
TC11000546.hg.1	INCENP	8.52 ± 0.14	8.42 ± 0.08	8.39 ± 0.17	8.35 ± 0.03
TC0X000229.hg.1	INE1	5.76 ± 0.12	5.45 ± 0.17	5.74 ± 0.26	5.49 ± 0.11
TC0X000881.hg.1	INE2	5.60 ± 0.05	5.33 ± 0.05	5.39 ± 0.08	5.34 ± 0.07
TC14000818.hg.1	INF2	8.53 ± 0.10	8.47 ± 0.10	8.41 ± 0.13	8.48 ± 0.01
TC13000395.hg.1	ING1	8.37 ± 0.08	8.43 ± 0.06	8.43 ± 0.11	8.35 ± 0.12
TC04000890.hg.1	ING2	7.61 ± 0.05	7.76 ± 0.05	7.70 ± 0.09	7.78 ± 0.08
TC07000734.hg.1	ING3	7.51 ± 0.37	7.86 ± 0.24	7.50 ± 0.34	7.77 ± 0.09
TC12001146.hg.1	ING4	7.95 ± 0.08	7.78 ± 0.04	7.88 ± 0.17	7.69 ± 0.01
TC02001485.hg.1	ING5	7.69 ± 0.08	7.68 ± 0.05	7.59 ± 0.06	7.71 ± 0.05
TC0X001127.hg.1	INGX	6.43 ± 0.15	6.11 ± 0.04	6.20 ± 0.21	6.25 ± 0.07
TC02001332.hg.1	INHA	7.96 ± 0.29	7.96 ± 0.23	7.90 ± 0.16	8.03 ± 0.10
TC07001318.hg.1	INHBA	6.31 ± 0.30	6.38 ± 0.13	6.42 ± 0.05	6.42 ± 0.05
TC07000258.hg.1	INHBA-AS1	4.75 ± 0.24	4.62 ± 0.10	4.51 ± 0.05	4.70 ± 0.09
TC02000762.hg.1	INHBB	6.87 ± 0.30	6.80 ± 0.13	6.90 ± 0.16	6.91 ± 0.02
TC12000528.hg.1	INHBC	6.84 ± 0.27	6.98 ± 0.17	6.74 ± 0.20	7.09 ± 0.14
TC12000529.hg.1	INHBE	7.25 ± 0.30	7.27 ± 0.15	7.13 ± 0.12	7.25 ± 0.07
TC09001492.hg.1	INIP	8.71 ± 0.07	8.64 ± 0.09	8.69 ± 0.07	8.63 ± 0.05

TC07003391.hg.1	INMT	7.35 ± 0.14	7.36 ± 0.19	7.36 ± 0.13	7.47 ± 0.13
TC15001246.hg.1	INO80	8.57 ± 0.18	8.66 ± 0.17	8.32 ± 0.19	8.64 ± 0.03
TC02004971.hg.1	INO80B	8.20 ± 0.12	8.09 ± 0.04	7.98 ± 0.12	8.13 ± 0.04
TC18000455.hg.1	INO80C	7.11 ± 0.07	7.01 ± 0.07	6.94 ± 0.04	7.09 ± 0.07
TC02002693.hg.1	INO80D	9.08 ± 0.30	9.23 ± 0.12	9.11 ± 0.43	9.37 ± 0.09
TC16000336.hg.1	INO80E	8.88 ± 0.05	8.77 ± 0.05	8.74 ± 0.07	8.70 ± 0.02
TC02001118.hg.1	INPP1	7.20 ± 0.08	7.07 ± 0.07	7.15 ± 0.11	7.11 ± 0.02
TC02000599.hg.1	INPP4A	9.06 ± 0.22	9.11 ± 0.07	8.99 ± 0.17	8.98 ± 0.08
TC04001600.hg.1	INPP4B	6.59 ± 0.21	6.75 ± 0.09	6.56 ± 0.10	6.62 ± 0.07
TC10000946.hg.1	INPP5A	8.47 ± 0.07	8.34 ± 0.09	8.54 ± 0.22	8.45 ± 0.14
TC01002528.hg.1	INPP5B	8.43 ± 0.14	8.22 ± 0.08	8.25 ± 0.07	8.16 ± 0.06
TC02001414.hg.1	INPP5D	10.14 ± 0.14	10.06 ± 0.06	10.11 ± 0.10	9.97 ± 0.07
TC09001731.hg.1	INPP5E	7.86 ± 0.16	7.74 ± 0.14	7.69 ± 0.21	7.76 ± 0.03
TC10000869.hg.1	INPP5F	6.83 ± 0.28	6.90 ± 0.15	6.80 ± 0.22	6.87 ± 0.09
TC22001429.hg.1	INPP5J	7.16 ± 0.15	7.19 ± 0.19	7.13 ± 0.12	7.29 ± 0.08
TC17000975.hg.1	INPP5K	8.74 ± 0.15	8.83 ± 0.13	8.54 ± 0.09	8.87 ± 0.05
TC11000756.hg.1	INPPL1	9.31 ± 0.20	9.15 ± 0.05	9.30 ± 0.06	9.10 ± 0.03
TC11001274.hg.1	INS	7.71 ± 0.19	7.69 ± 0.18	7.65 ± 0.15	7.75 ± 0.05
TC11000216.hg.1	INSC	7.46 ± 0.15	7.45 ± 0.18	7.46 ± 0.11	7.60 ± 0.10
TC07001039.hg.1	INSIG1	8.58 ± 0.06	8.60 ± 0.09	8.60 ± 0.13	8.67 ± 0.09
TC02000748.hg.1	INSIG2	7.96 ± 0.23	7.75 ± 0.14	8.18 ± 0.37	7.73 ± 0.05
TC19001279.hg.1	INSL3	8.25 ± 0.16	8.16 ± 0.17	8.26 ± 0.23	8.32 ± 0.07
TC09000036.hg.1	INSL4	5.88 ± 0.21	5.80 ± 0.14	5.77 ± 0.13	5.90 ± 0.21
TC01002752.hg.1	INSL5	5.72 ± 0.11	5.68 ± 0.05	5.95 ± 0.13	5.86 ± 0.06
TC09000888.hg.1	INSL6	6.09 ± 0.30	5.86 ± 0.13	5.94 ± 0.23	5.93 ± 0.05
TC20000137.hg.1	INSM1	7.71 ± 0.16	7.66 ± 0.07	7.62 ± 0.15	7.69 ± 0.04
TC14000223.hg.1	INSM2	6.56 ± 0.46	6.52 ± 0.22	6.30 ± 0.32	6.62 ± 0.07
TC19001111.hg.1	INSR	7.43 ± 0.20	7.29 ± 0.01	7.27 ± 0.06	7.30 ± 0.03
TC01003355.hg.1	INSRR	7.40 ± 0.17	7.38 ± 0.15	7.35 ± 0.14	7.52 ± 0.08
TC07001084.hg.1	INTS1	9.10 ± 0.11	9.04 ± 0.08	8.95 ± 0.08	9.07 ± 0.05
TC08000148.hg.1	INTS10	7.60 ± 0.11	7.59 ± 0.17	7.49 ± 0.17	7.47 ± 0.02

TC04001446.hg.1	INTS12	8.08 ± 0.02	8.15 ± 0.13	8.03 ± 0.06	8.08 ± 0.01
TC17001770.hg.1	INTS2	7.57 ± 0.22	7.47 ± 0.15	7.53 ± 0.27	7.30 ± 0.10
TC01001263.hg.1	INTS3	9.15 ± 0.21	9.04 ± 0.12	9.22 ± 0.26	8.80 ± 0.15
TC11002131.hg.1	INTS4	8.54 ± 0.07	8.43 ± 0.16	8.34 ± 0.17	8.30 ± 0.03
TC07000391.hg.1	INTS4L1	8.22 ± 0.06	8.15 ± 0.09	8.04 ± 0.14	8.01 ± 0.04
TC07000397.hg.1	INTS4L2	8.58 ± 0.10	8.51 ± 0.15	8.37 ± 0.15	8.35 ± 0.02
TC11003485.hg.1	INTS5	8.26 ± 0.26	8.10 ± 0.13	8.06 ± 0.27	8.19 ± 0.06
TC13000678.hg.1	INTS6	8.10 ± 0.21	8.17 ± 0.18	8.31 ± 0.32	8.03 ± 0.19
TC13000213.hg.1	INTS6-AS1	6.85 ± 0.15	6.78 ± 0.08	6.81 ± 0.11	6.82 ± 0.03
TC01003801.hg.1	INTS7	7.76 ± 0.15	7.42 ± 0.22	7.51 ± 0.15	6.90 ± 0.17
TC08000578.hg.1	INTS8	7.50 ± 0.33	7.49 ± 0.23	7.70 ± 0.28	7.23 ± 0.10
TC08001094.hg.1	INTS9	7.81 ± 0.09	7.57 ± 0.06	7.61 ± 0.09	7.48 ± 0.01
TC04000641.hg.1	INTU	4.72 ± 0.12	4.61 ± 0.04	4.63 ± 0.06	4.66 ± 0.05
TC09000510.hg.1	INVS	7.71 ± 0.27	7.87 ± 0.18	7.67 ± 0.35	7.71 ± 0.16
TC03001416.hg.1	IP6K1	8.54 ± 0.15	8.39 ± 0.02	8.52 ± 0.14	8.47 ± 0.08
TC03001388.hg.1	IP6K2	7.94 ± 0.04	7.83 ± 0.03	7.88 ± 0.09	7.83 ± 0.02
TC06001592.hg.1	IP6K3	7.39 ± 0.24	7.37 ± 0.21	7.35 ± 0.15	7.51 ± 0.07
TC06004139.hg.1	IPCEF1	8.30 ± 0.28	8.31 ± 0.21	8.29 ± 0.32	8.04 ± 0.11
TC10001312.hg.1	IPMK	8.89 ± 0.32	8.96 ± 0.42	9.69 ± 0.53	8.85 ± 0.37
TC01000553.hg.1	IPO13	8.11 ± 0.09	7.96 ± 0.03	7.92 ± 0.07	7.91 ± 0.04
TC14002339.hg.1	IPO4	8.30 ± 0.18	8.16 ± 0.04	8.07 ± 0.12	8.13 ± 0.05
TC13000343.hg.1	IPO5	7.71 ± 0.11	7.83 ± 0.21	7.44 ± 0.14	7.71 ± 0.05
TC11000170.hg.1	IPO7	8.68 ± 0.36	8.85 ± 0.23	8.54 ± 0.34	8.56 ± 0.19
TC11000171.hg.1	IPO7	6.08 ± 0.05	6.00 ± 0.05	5.98 ± 0.07	5.99 ± 0.05
TC12001353.hg.1	IPO8	8.32 ± 0.39	8.37 ± 0.24	8.51 ± 0.39	8.09 ± 0.20
TC12001354.hg.1	IPO8	6.68 ± 0.23	6.50 ± 0.01	6.62 ± 0.05	6.56 ± 0.10
TC01001664.hg.1	IPO9	8.90 ± 0.20	8.79 ± 0.14	8.59 ± 0.11	8.66 ± 0.04
TC01003695.hg.1	IPO9-AS1	7.31 ± 0.19	7.30 ± 0.11	7.28 ± 0.14	7.34 ± 0.09
TC01002615.hg.1	IPP	7.10 ± 0.12	6.99 ± 0.09	7.04 ± 0.14	6.86 ± 0.03
TC09001339.hg.1	IPPK	7.76 ± 0.06	7.66 ± 0.02	7.68 ± 0.15	7.64 ± 0.11
TC02002900.hg.1	IQCA1	5.26 ± 0.12	5.18 ± 0.05	5.23 ± 0.12	5.25 ± 0.04

TC07002033.hg.1	IQCA1P1	6.91 ± 0.24	6.81 ± 0.20	6.85 ± 0.15	6.87 ± 0.03
TC03001699.hg.1	IQCB1	7.40 ± 0.24	7.36 ± 0.23	7.14 ± 0.28	7.20 ± 0.12
TC01000422.hg.1	IQCC	7.58 ± 0.18	7.50 ± 0.23	7.50 ± 0.23	7.62 ± 0.10
TC12001987.hg.1	IQCD	7.55 ± 0.17	7.57 ± 0.07	7.48 ± 0.16	7.65 ± 0.07
TC07000035.hg.1	IQCE	7.96 ± 0.12	7.89 ± 0.05	8.01 ± 0.09	7.96 ± 0.06
TC03001440.hg.1	IQCF1	6.32 ± 0.13	6.20 ± 0.08	6.22 ± 0.12	6.35 ± 0.18
TC03000324.hg.1	IQCF2	5.25 ± 0.16	4.98 ± 0.06	5.22 ± 0.15	5.11 ± 0.12
TC03003355.hg.1	IQCF3	6.54 ± 0.16	6.43 ± 0.15	6.34 ± 0.22	6.53 ± 0.02
TC03003356.hg.1	IQCF4	6.11 ± 0.16	5.93 ± 0.22	5.91 ± 0.15	6.08 ± 0.09
TC03001441.hg.1	IQCF5	6.34 ± 0.22	6.12 ± 0.21	6.22 ± 0.21	6.20 ± 0.03
TC03000325.hg.1	IQCF5-AS1	6.32 ± 0.40	6.10 ± 0.11	6.11 ± 0.20	6.31 ± 0.04
TC03001439.hg.1	IQCF6	6.74 ± 0.04	6.72 ± 0.04	6.76 ± 0.16	6.78 ± 0.06
TC03002184.hg.1	IQCG	6.41 ± 0.09	6.31 ± 0.05	6.37 ± 0.08	6.34 ± 0.00
TC15000623.hg.1	IQCH	5.18 ± 0.10	5.13 ± 0.09	5.18 ± 0.07	5.17 ± 0.05
TC15001588.hg.1	IQCH-AS1	7.39 ± 0.16	7.43 ± 0.04	7.35 ± 0.09	7.52 ± 0.07
TC03000868.hg.1	IQCJ	6.14 ± 0.17	6.11 ± 0.11	6.10 ± 0.12	6.15 ± 0.04
TC03001950.hg.1	IQCJ-SCHIP1-AS1	4.60 ± 0.07	4.37 ± 0.08	4.37 ± 0.16	4.39 ± 0.09
TC16000217.hg.1	IQCK	6.24 ± 0.15	6.23 ± 0.02	6.23 ± 0.11	6.24 ± 0.06
TC15000864.hg.1	IQGAP1	11.29 ± 0.29	11.53 ± 0.16	11.83 ± 0.32	11.39 ± 0.26
TC15000865.hg.1	IQGAP1	9.04 ± 0.03	9.07 ± 0.02	9.31 ± 0.20	9.10 ± 0.18
TC05000368.hg.1	IQGAP2	9.07 ± 0.43	9.28 ± 0.20	9.53 ± 0.42	9.01 ± 0.18
TC01003343.hg.1	IQGAP3	7.08 ± 0.15	7.04 ± 0.16	7.03 ± 0.19	7.12 ± 0.06
TC03001187.hg.1	IQSEC1	10.27 ± 0.19	10.22 ± 0.09	10.45 ± 0.27	10.31 ± 0.10
TC0X001063.hg.1	IQSEC2	7.60 ± 0.13	7.51 ± 0.13	7.63 ± 0.09	7.59 ± 0.03
TC12000003.hg.1	IQSEC3	7.74 ± 0.20	7.64 ± 0.16	7.60 ± 0.16	7.70 ± 0.02
TC07001819.hg.1	IQUB	4.69 ± 0.14	4.46 ± 0.04	4.52 ± 0.10	4.52 ± 0.05
TC06000754.hg.1	IRAK1BP1	5.59 ± 0.10	5.56 ± 0.07	5.51 ± 0.17	5.53 ± 0.04
TC03000056.hg.1	IRAK2	8.17 ± 0.37	7.87 ± 0.09	8.03 ± 0.09	8.13 ± 0.47
TC12000591.hg.1	IRAK3	9.77 ± 0.29	9.01 ± 0.30	10.68 ± 0.14	9.27 ± 0.78
TC12000325.hg.1	IRAK4	8.51 ± 0.29	8.48 ± 0.20	8.90 ± 0.42	8.29 ± 0.06
TC15000729.hg.1	IREB2	8.53 ± 0.47	8.63 ± 0.22	8.65 ± 0.42	8.63 ± 0.12

TC05001767.hg.1	IRF1	9.83 ± 0.25	9.93 ± 0.12	10.06 ± 0.38	9.78 ± 0.12
TC04001805.hg.1	IRF2	10.12 ± 0.11	9.96 ± 0.21	10.53 ± 0.26	9.81 ± 0.32
TC19001636.hg.1	IRF2BP1	8.75 ± 0.27	8.63 ± 0.04	8.41 ± 0.28	8.45 ± 0.07
TC01003985.hg.1	IRF2BP2	9.75 ± 0.22	10.14 ± 0.13	9.63 ± 0.13	10.32 ± 0.22
TC14001336.hg.1	IRF2BPL	8.04 ± 0.32	7.88 ± 0.15	7.96 ± 0.15	7.92 ± 0.30
TC19001722.hg.1	IRF3	8.37 ± 0.13	8.25 ± 0.06	8.25 ± 0.09	8.20 ± 0.05
TC06000005.hg.1	IRF4	9.56 ± 0.10	9.27 ± 0.08	9.08 ± 0.13	9.37 ± 0.15
TC07000789.hg.1	IRF5	8.23 ± 0.19	8.11 ± 0.15	8.14 ± 0.11	8.19 ± 0.03
TC01003787.hg.1	IRF6	6.35 ± 0.12	6.25 ± 0.05	6.25 ± 0.05	6.31 ± 0.05
TC11001241.hg.1	IRF7	9.57 ± 0.42	9.29 ± 0.19	9.48 ± 0.31	9.28 ± 0.05
TC16000666.hg.1	IRF8	9.65 ± 0.10	9.71 ± 0.09	9.36 ± 0.18	9.64 ± 0.05
TC14000161.hg.1	IRF9	9.31 ± 0.16	9.11 ± 0.08	9.31 ± 0.19	9.09 ± 0.08
TC13000291.hg.1	IRG1	5.90 ± 0.09	5.77 ± 0.08	5.90 ± 0.03	5.92 ± 0.10
TC19000610.hg.1	IRGC	7.99 ± 0.22	7.85 ± 0.13	7.87 ± 0.15	7.90 ± 0.04
TC05000836.hg.1	IRGM	5.54 ± 0.16	5.57 ± 0.12	5.56 ± 0.04	5.64 ± 0.08
TC19001590.hg.1	IRGQ	8.36 ± 0.23	8.46 ± 0.17	8.37 ± 0.20	8.47 ± 0.08
TC02002830.hg.1	IRS1	7.05 ± 0.13	6.97 ± 0.06	6.83 ± 0.15	6.98 ± 0.09
TC13000871.hg.1	IRS2	9.49 ± 0.10	10.04 ± 0.52	9.65 ± 0.56	10.25 ± 0.40
TC0X001269.hg.1	IRS4	4.55 ± 0.26	4.47 ± 0.05	4.57 ± 0.15	4.38 ± 0.17
TC05000030.hg.1	IRX1	7.78 ± 0.43	7.73 ± 0.23	7.67 ± 0.21	7.75 ± 0.03
TC05001120.hg.1	IRX2	7.47 ± 0.25	7.43 ± 0.18	7.44 ± 0.30	7.44 ± 0.00
TC16001124.hg.1	IRX3	7.80 ± 0.17	7.64 ± 0.22	7.71 ± 0.15	7.76 ± 0.04
TC05001117.hg.1	IRX4	7.46 ± 0.31	7.45 ± 0.23	7.40 ± 0.18	7.50 ± 0.02
TC16000451.hg.1	IRX5	7.83 ± 0.16	7.70 ± 0.10	7.77 ± 0.14	7.72 ± 0.06
TC16000453.hg.1	IRX6	6.94 ± 0.16	6.93 ± 0.10	6.89 ± 0.22	7.04 ± 0.07
TC09002895.hg.1	ISCA1	8.05 ± 0.22	8.33 ± 0.14	8.00 ± 0.17	8.32 ± 0.05
TC05001402.hg.1	ISCA1P1	9.52 ± 0.48	9.87 ± 0.21	9.54 ± 0.21	9.85 ± 0.03
TC14000458.hg.1	ISCA2	7.62 ± 0.05	7.35 ± 0.08	7.42 ± 0.18	7.36 ± 0.04
TC12000836.hg.1	ISCU	8.35 ± 0.14	8.51 ± 0.18	8.28 ± 0.14	8.53 ± 0.08
TC01000023.hg.1	ISG15	8.90 ± 0.63	8.58 ± 0.21	8.67 ± 0.40	8.56 ± 0.05
TC15000841.hg.1	ISG20	9.00 ± 0.18	8.91 ± 0.09	8.92 ± 0.21	8.87 ± 0.06

TC01003351.hg.1	ISG20L2	8.78 ± 0.18	8.82 ± 0.14	8.57 ± 0.10	8.74 ± 0.19
TC05000212.hg.1	ISL1	6.37 ± 0.14	6.18 ± 0.08	6.26 ± 0.09	6.19 ± 0.06
TC15000707.hg.1	ISL2	8.10 ± 0.12	7.97 ± 0.11	8.13 ± 0.14	8.03 ± 0.02
TC15000678.hg.1	ISLR	7.60 ± 0.21	7.54 ± 0.13	7.52 ± 0.14	7.63 ± 0.14
TC15000677.hg.1	ISLR2	7.72 ± 0.24	7.69 ± 0.14	7.66 ± 0.18	7.72 ± 0.02
TC20000094.hg.1	ISM1	6.95 ± 0.17	6.81 ± 0.10	6.88 ± 0.21	6.91 ± 0.01
TC20000634.hg.1	ISM1-AS1	5.50 ± 0.20	5.51 ± 0.22	5.42 ± 0.08	5.56 ± 0.12
TC14001347.hg.1	ISM2	7.74 ± 0.20	7.70 ± 0.17	7.66 ± 0.12	7.79 ± 0.07
TC05000615.hg.1	ISOC1	8.52 ± 0.12	8.48 ± 0.09	8.38 ± 0.09	8.37 ± 0.10
TC19001874.hg.1	ISOC2	8.23 ± 0.22	8.13 ± 0.16	8.06 ± 0.17	8.16 ± 0.04
TC07001159.hg.1	ISPD	5.51 ± 0.02	5.43 ± 0.05	5.51 ± 0.18	5.44 ± 0.08
TC16000598.hg.1	IST1	9.34 ± 0.09	9.19 ± 0.08	9.30 ± 0.24	9.21 ± 0.05
TC22000254.hg.1	ISX	7.29 ± 0.23	7.28 ± 0.22	7.27 ± 0.19	7.44 ± 0.06
TC03003394.hg.1	ISY1	8.60 ± 0.09	8.49 ± 0.20	8.57 ± 0.26	8.36 ± 0.13
TC19001289.hg.1	ISYNA1	8.36 ± 0.16	8.33 ± 0.20	8.26 ± 0.18	8.41 ± 0.05
TC20000239.hg.1	ITCH	9.57 ± 0.28	9.68 ± 0.20	9.64 ± 0.37	9.62 ± 0.11
TC20000782.hg.1	ITCH-AS1	9.46 ± 0.29	9.29 ± 0.03	9.51 ± 0.22	9.40 ± 0.12
TC20000240.hg.1	ITCH-IT1	6.32 ± 0.31	6.21 ± 0.26	6.39 ± 0.35	6.07 ± 0.16
TC16001090.hg.1	ITFG1	8.32 ± 0.12	8.06 ± 0.16	8.34 ± 0.21	7.88 ± 0.09
TC12000029.hg.1	ITFG2	9.13 ± 0.09	9.09 ± 0.06	8.74 ± 0.06	9.09 ± 0.15
TC16002046.hg.1	ITFG3	8.67 ± 0.11	8.59 ± 0.08	8.56 ± 0.13	8.64 ± 0.06
TC05003439.hg.1	ITGA1	6.31 ± 0.18	6.29 ± 0.12	6.43 ± 0.24	6.07 ± 0.03
TC01001095.hg.1	ITGA10	6.94 ± 0.14	6.93 ± 0.13	6.87 ± 0.13	7.01 ± 0.05
TC15001593.hg.1	ITGA11	7.37 ± 0.15	7.32 ± 0.17	7.30 ± 0.17	7.45 ± 0.05
TC05000218.hg.1	ITGA2	5.34 ± 0.07	5.18 ± 0.03	5.30 ± 0.13	5.25 ± 0.04
TC17001581.hg.1	ITGA2B	9.33 ± 0.11	8.64 ± 0.09	9.68 ± 0.38	8.81 ± 0.22
TC17000654.hg.1	ITGA3	7.91 ± 0.19	7.84 ± 0.17	7.79 ± 0.17	7.89 ± 0.06
TC02001083.hg.1	ITGA4	9.43 ± 0.59	9.90 ± 0.19	9.34 ± 0.49	9.59 ± 0.16
TC12001571.hg.1	ITGA5	9.73 ± 0.20	9.41 ± 0.09	9.63 ± 0.21	9.39 ± 0.41
TC02001030.hg.1	ITGA6	8.50 ± 0.43	8.81 ± 0.09	8.45 ± 0.36	8.65 ± 0.11
TC12001583.hg.1	ITGA7	7.40 ± 0.19	7.34 ± 0.18	7.39 ± 0.14	7.45 ± 0.06

TC10001064.hg.1	ITGA8	5.81 ± 0.15	5.66 ± 0.12	5.69 ± 0.07	5.73 ± 0.05
TC03000181.hg.1	ITGA9	7.06 ± 0.15	6.97 ± 0.14	7.05 ± 0.16	7.05 ± 0.04
TC16000376.hg.1	ITGAD	8.06 ± 0.08	8.01 ± 0.13	8.18 ± 0.11	8.09 ± 0.01
TC17001011.hg.1	ITGAE	7.29 ± 0.09	7.24 ± 0.10	7.19 ± 0.14	7.29 ± 0.05
TC16000349.hg.1	ITGAL	11.02 ± 0.34	10.89 ± 0.05	10.95 ± 0.11	10.66 ± 0.10
TC16000374.hg.1	ITGAM	10.63 ± 0.35	10.12 ± 0.11	11.22 ± 0.31	10.09 ± 0.22
TC02001100.hg.1	ITGAV	6.87 ± 0.26	6.79 ± 0.16	6.97 ± 0.26	6.73 ± 0.18
TC16000375.hg.1	ITGAX	11.02 ± 0.38	10.85 ± 0.20	11.68 ± 0.32	10.90 ± 0.25
TC10001163.hg.1	ITGB1	9.79 ± 0.36	9.77 ± 0.12	9.87 ± 0.12	9.71 ± 0.14
TC10001164.hg.1	ITGB1	7.62 ± 0.24	7.53 ± 0.23	7.61 ± 0.20	7.59 ± 0.12
TC02001557.hg.1	ITGB1BP1	7.64 ± 0.05	7.55 ± 0.07	7.55 ± 0.11	7.47 ± 0.03
TC0X000386.hg.1	ITGB1BP2	6.50 ± 0.12	6.47 ± 0.08	6.34 ± 0.12	6.53 ± 0.06
TC21000538.hg.1	ITGB2	10.49 ± 0.20	10.27 ± 0.04	10.63 ± 0.12	10.23 ± 0.06
TC21000239.hg.1	ITGB2-AS1	8.00 ± 0.13	7.86 ± 0.09	8.19 ± 0.17	7.90 ± 0.08
TC17002878.hg.1	ITGB3	10.23 ± 0.23	9.44 ± 0.25	10.76 ± 0.35	9.75 ± 0.41
TC01002733.hg.1	ITGB3BP	5.90 ± 0.22	5.79 ± 0.12	5.74 ± 0.16	5.68 ± 0.02
TC17000861.hg.1	ITGB4	8.04 ± 0.19	7.95 ± 0.21	8.04 ± 0.23	8.04 ± 0.03
TC03001717.hg.1	ITGB5	7.60 ± 0.07	7.27 ± 0.11	7.70 ± 0.28	7.41 ± 0.10
TC03000645.hg.1	ITGB5-AS1	5.20 ± 0.27	5.18 ± 0.12	5.23 ± 0.09	5.24 ± 0.19
TC02002469.hg.1	ITGB6	4.72 ± 0.09	4.62 ± 0.05	4.68 ± 0.06	4.65 ± 0.04
TC12001544.hg.1	ITGB7	9.94 ± 0.20	9.64 ± 0.05	9.42 ± 0.01	9.42 ± 0.14
TC07000125.hg.1	ITGB8	5.43 ± 0.08	5.40 ± 0.09	5.39 ± 0.06	5.53 ± 0.03
TC13000364.hg.1	ITGBL1	6.91 ± 0.17	6.82 ± 0.07	6.83 ± 0.07	6.94 ± 0.06
TC03000344.hg.1	ITIH1	7.43 ± 0.20	7.48 ± 0.17	7.41 ± 0.19	7.54 ± 0.07
TC10000066.hg.1	ITIH2	5.70 ± 0.15	5.70 ± 0.11	5.70 ± 0.11	5.77 ± 0.06
TC03000345.hg.1	ITIH3	7.35 ± 0.20	7.26 ± 0.20	7.27 ± 0.19	7.36 ± 0.04
TC03003413.hg.1	ITIH4	8.18 ± 0.02	8.05 ± 0.10	8.26 ± 0.15	8.13 ± 0.03
TC03000346.hg.1	ITIH4-AS1	6.70 ± 0.35	6.44 ± 0.25	6.62 ± 0.12	6.43 ± 0.11
TC10001020.hg.1	ITIH5	6.77 ± 0.17	6.67 ± 0.09	6.73 ± 0.16	6.76 ± 0.02
TC0X001075.hg.1	ITIH6	6.74 ± 0.12	6.67 ± 0.12	6.69 ± 0.12	6.72 ± 0.06
TC05000865.hg.1	ITK	9.54 ± 0.38	9.72 ± 0.06	9.27 ± 0.25	9.63 ± 0.17

TC01003409.hg.1	ITLN1	6.56 ± 0.17	6.45 ± 0.14	6.54 ± 0.15	6.54 ± 0.01
TC01003411.hg.1	ITLN2	6.60 ± 0.16	6.59 ± 0.13	6.53 ± 0.12	6.65 ± 0.05
TC0X001180.hg.1	ITM2A	8.98 ± 0.14	9.25 ± 0.24	8.57 ± 0.23	9.05 ± 0.06
TC13000189.hg.1	ITM2B	11.69 ± 0.22	11.73 ± 0.17	12.01 ± 0.13	11.68 ± 0.14
TC02001381.hg.1	ITM2C	9.78 ± 0.25	9.28 ± 0.11	9.18 ± 0.17	9.24 ± 0.04
TC20000035.hg.1	ITPA	8.25 ± 0.09	8.16 ± 0.06	8.10 ± 0.09	8.13 ± 0.05
TC14001453.hg.1	ITPK1	10.02 ± 0.22	9.97 ± 0.06	9.96 ± 0.17	9.77 ± 0.21
TC14000575.hg.1	ITPK1-AS1	7.18 ± 0.06	7.01 ± 0.18	7.16 ± 0.17	7.19 ± 0.08
TC15000309.hg.1	ITPKA	8.08 ± 0.22	8.04 ± 0.22	8.04 ± 0.27	8.09 ± 0.04
TC01003901.hg.1	ITPKB	8.91 ± 0.15	8.90 ± 0.11	8.62 ± 0.11	8.85 ± 0.15
TC01001864.hg.1	ITPKB-AS1	6.46 ± 0.22	6.42 ± 0.17	6.30 ± 0.11	6.28 ± 0.17
TC01003902.hg.1	ITPKB-IT1	7.57 ± 0.19	7.46 ± 0.04	7.29 ± 0.12	7.48 ± 0.03
TC19000566.hg.1	ITPKC	7.55 ± 0.21	7.26 ± 0.14	7.51 ± 0.17	7.38 ± 0.05
TC03000013.hg.1	ITPR1	8.33 ± 0.16	8.47 ± 0.20	8.14 ± 0.20	8.25 ± 0.13
TC03001127.hg.1	ITPR1-AS1	6.94 ± 0.13	6.95 ± 0.18	6.83 ± 0.23	6.91 ± 0.10
TC12001328.hg.1	ITPR2	8.39 ± 0.42	8.71 ± 0.27	8.72 ± 0.39	8.53 ± 0.09
TC06000427.hg.1	ITPR3	8.62 ± 0.05	8.62 ± 0.05	8.30 ± 0.08	8.54 ± 0.06
TC10001641.hg.1	ITPRIP	8.69 ± 0.15	8.79 ± 0.11	8.83 ± 0.18	8.78 ± 0.15
TC02000581.hg.1	ITPRIPL1	7.74 ± 0.23	7.57 ± 0.09	7.48 ± 0.25	7.50 ± 0.02
TC16000210.hg.1	ITPRIPL2	8.40 ± 0.23	8.25 ± 0.19	8.41 ± 0.14	8.30 ± 0.02
TC21000131.hg.1	ITSN1	6.94 ± 0.09	6.86 ± 0.05	6.98 ± 0.02	6.87 ± 0.03
TC02001649.hg.1	ITSN2	8.79 ± 0.42	9.10 ± 0.19	9.13 ± 0.45	9.04 ± 0.13
TC15000284.hg.1	IVD	7.89 ± 0.06	7.76 ± 0.05	7.69 ± 0.15	7.73 ± 0.03
TC01001244.hg.1	IVL	6.25 ± 0.16	6.23 ± 0.21	6.08 ± 0.27	6.22 ± 0.05
TC01003629.hg.1	IVNS1ABP	9.69 ± 0.35	10.17 ± 0.10	10.30 ± 0.59	10.15 ± 0.14
TC02002281.hg.1	IWS1	8.07 ± 0.23	8.02 ± 0.11	8.28 ± 0.27	7.92 ± 0.12
TC06001093.hg.1	IYD	6.54 ± 0.15	6.54 ± 0.09	6.54 ± 0.05	6.66 ± 0.06
TC19001694.hg.1	IZUMO1	6.88 ± 0.14	6.84 ± 0.16	6.88 ± 0.11	6.94 ± 0.02
TC19001743.hg.1	IZUMO2	7.00 ± 0.34	7.05 ± 0.25	6.93 ± 0.26	7.08 ± 0.09
TC09000970.hg.1	IZUMO3	4.50 ± 0.09	4.44 ± 0.14	4.52 ± 0.03	4.56 ± 0.08
TC19000048.hg.1	IZUMO4	8.35 ± 0.19	8.32 ± 0.20	8.28 ± 0.16	8.44 ± 0.05

TC20000621.hg.1	JAG1	7.16 ± 0.08	7.10 ± 0.09	7.46 ± 0.15	7.24 ± 0.02
TC14002333.hg.1	JAG2	8.22 ± 0.21	8.20 ± 0.21	8.17 ± 0.19	8.30 ± 0.03
TC03000045.hg.1	JAGN1	7.87 ± 0.32	7.46 ± 0.18	7.46 ± 0.27	7.31 ± 0.07
TC01002741.hg.1	JAK1	10.70 ± 0.33	11.07 ± 0.11	10.67 ± 0.24	11.06 ± 0.07
TC09000035.hg.1	JAK2	8.84 ± 0.44	8.77 ± 0.33	9.65 ± 0.57	8.46 ± 0.24
TC19001280.hg.1	JAK3	9.55 ± 0.36	9.38 ± 0.04	9.92 ± 0.31	9.23 ± 0.11
TC04001000.hg.1	JAKMIP1	7.54 ± 0.18	7.50 ± 0.14	7.43 ± 0.22	7.47 ± 0.03
TC05001910.hg.1	JAKMIP2	6.16 ± 0.32	6.10 ± 0.03	6.10 ± 0.21	6.01 ± 0.10
TC05000802.hg.1	JAKMIP2-AS1	7.06 ± 0.25	7.02 ± 0.12	7.13 ± 0.24	7.27 ± 0.20
TC10000940.hg.1	JAKMIP3	7.52 ± 0.24	7.46 ± 0.16	7.49 ± 0.21	7.54 ± 0.04
TC21000077.hg.1	JAM2	4.96 ± 0.13	4.84 ± 0.03	4.84 ± 0.08	4.84 ± 0.03
TC11001212.hg.1	JAM3	8.30 ± 0.11	7.86 ± 0.12	8.58 ± 0.25	8.00 ± 0.11
TC06000104.hg.1	JARID2	9.51 ± 0.10	9.56 ± 0.14	9.32 ± 0.17	9.72 ± 0.20
TC06001288.hg.1	JARID2-AS1	8.15 ± 0.20	8.14 ± 0.11	8.25 ± 0.12	8.10 ± 0.11
TC07001232.hg.1	JAZF1	7.83 ± 0.06	7.90 ± 0.03	7.92 ± 0.10	7.86 ± 0.07
TC07000177.hg.1	JAZF1-AS1	7.03 ± 0.14	7.03 ± 0.18	6.95 ± 0.09	7.19 ± 0.08
TC14000473.hg.1	JDP2	8.48 ± 0.20	8.26 ± 0.04	8.34 ± 0.15	8.33 ± 0.12
TC07001917.hg.1	JHDM1D	9.35 ± 0.41	9.57 ± 0.20	9.53 ± 0.45	9.63 ± 0.09
TC14000358.hg.1	JKAMP	7.79 ± 0.17	7.72 ± 0.13	8.07 ± 0.39	7.63 ± 0.13
TC10001329.hg.1	JMJD1C	9.12 ± 0.60	9.78 ± 0.24	9.81 ± 0.59	9.73 ± 0.18
TC10000388.hg.1	JMJD1C-AS1	8.05 ± 0.25	8.03 ± 0.16	8.15 ± 0.25	8.07 ± 0.03
TC01003907.hg.1	JMJD4	8.03 ± 0.11	7.90 ± 0.08	7.85 ± 0.14	7.94 ± 0.03
TC17001907.hg.1	JMJD6	8.54 ± 0.24	8.54 ± 0.04	8.58 ± 0.15	8.53 ± 0.14
TC15000315.hg.1	JMJD7	8.25 ± 0.07	8.24 ± 0.06	8.11 ± 0.08	8.29 ± 0.06
TC16000734.hg.1	JMJD8	9.05 ± 0.22	8.87 ± 0.10	8.70 ± 0.15	8.78 ± 0.01
TC05000389.hg.1	JMY	7.76 ± 0.52	8.53 ± 0.31	7.54 ± 0.54	8.71 ± 0.15
TC22000796.hg.1	JOSD1	8.94 ± 0.08	9.24 ± 0.17	8.86 ± 0.01	9.32 ± 0.04
TC19001748.hg.1	JOSD2	8.69 ± 0.29	8.63 ± 0.23	8.58 ± 0.18	8.73 ± 0.05
TC08001334.hg.1	JPH1	7.16 ± 0.27	7.01 ± 0.15	7.05 ± 0.15	7.09 ± 0.03
TC20000860.hg.1	JPH2	7.32 ± 0.20	7.27 ± 0.18	7.26 ± 0.13	7.39 ± 0.03
TC16000676.hg.1	JPH3	7.88 ± 0.28	7.89 ± 0.18	7.85 ± 0.21	7.96 ± 0.02

TC14002206.hg.1	JPH4	8.16 ± 0.19	8.07 ± 0.13	8.07 ± 0.17	8.12 ± 0.05
TC0X000405.hg.1	JPX	8.55 ± 0.21	8.56 ± 0.14	8.51 ± 0.18	8.55 ± 0.02
TC08001700.hg.1	JRK	8.04 ± 0.16	7.99 ± 0.14	7.88 ± 0.16	8.05 ± 0.02
TC11000936.hg.1	JRKL	6.45 ± 0.05	6.30 ± 0.10	6.52 ± 0.18	6.27 ± 0.02
TC11002213.hg.1	JRKL-AS1	6.68 ± 0.09	6.70 ± 0.10	6.71 ± 0.03	6.79 ± 0.02
TC19001025.hg.1	JSRP1	8.97 ± 0.20	8.84 ± 0.17	8.85 ± 0.22	8.93 ± 0.01
TC01003282.hg.1	JTB	9.21 ± 0.22	9.05 ± 0.20	9.04 ± 0.22	8.88 ± 0.04
TC01002708.hg.1	JUN	9.12 ± 0.17	10.02 ± 0.31	9.15 ± 0.36	9.95 ± 0.53
TC19000228.hg.1	JUNB	11.46 ± 0.31	12.06 ± 0.08	11.87 ± 0.15	11.83 ± 0.25
TC19001285.hg.1	JUND	9.69 ± 0.16	10.20 ± 0.13	9.60 ± 0.15	10.21 ± 0.17
TC17002907.hg.1	JUP	8.82 ± 0.21	8.63 ± 0.14	8.57 ± 0.14	8.62 ± 0.07
TC06000142.hg.1	KAAG1	5.23 ± 0.14	5.03 ± 0.12	5.15 ± 0.07	5.14 ± 0.10
TC0X000829.hg.1	KAL1	6.33 ± 0.20	6.38 ± 0.20	6.55 ± 0.07	6.52 ± 0.03
TC0Y000055.hg.1	KALP	7.14 ± 0.06	7.03 ± 0.04	7.05 ± 0.08	7.03 ± 0.02
TC03000642.hg.1	KALRN	6.55 ± 0.12	6.50 ± 0.11	6.53 ± 0.11	6.56 ± 0.06
TC09000005.hg.1	KANK1	6.37 ± 0.07	6.33 ± 0.07	6.32 ± 0.05	6.38 ± 0.02
TC19001178.hg.1	KANK2	8.05 ± 0.30	8.01 ± 0.22	7.93 ± 0.30	8.11 ± 0.08
TC19001129.hg.1	KANK3	8.69 ± 0.19	8.66 ± 0.15	8.61 ± 0.24	8.69 ± 0.06
TC01002725.hg.1	KANK4	7.01 ± 0.19	6.96 ± 0.10	6.90 ± 0.10	6.97 ± 0.01
TC17_ctg5_hap1000005.hg.1	KANSL1	9.47 ± 0.33	9.63 ± 0.09	9.39 ± 0.24	9.56 ± 0.06
TC17001610.hg.1	KANSL1	9.55 ± 0.33	9.71 ± 0.10	9.47 ± 0.23	9.64 ± 0.06
TC17_ctg5_hap1000016.hg.1	KANSL1-AS1	7.24 ± 0.34	7.03 ± 0.10	7.05 ± 0.24	7.01 ± 0.02
TC17000596.hg.1	KANSL1-AS1	6.30 ± 0.28	6.08 ± 0.06	6.16 ± 0.18	6.13 ± 0.03
TC02002730.hg.1	KANSL1L	6.86 ± 0.14	6.70 ± 0.23	7.05 ± 0.37	6.58 ± 0.13
TC12001452.hg.1	KANSL2	8.09 ± 0.27	8.41 ± 0.23	7.79 ± 0.06	8.41 ± 0.10
TC02002110.hg.1	KANSL3	8.12 ± 0.04	8.13 ± 0.05	7.95 ± 0.04	8.22 ± 0.04
TC16001284.hg.1	KARS	9.42 ± 0.15	9.44 ± 0.19	9.17 ± 0.24	9.20 ± 0.10
TC17001524.hg.1	KAT2A	8.42 ± 0.04	8.43 ± 0.05	8.17 ± 0.08	8.36 ± 0.08
TC03000113.hg.1	KAT2B	9.25 ± 0.39	9.46 ± 0.16	9.43 ± 0.37	9.29 ± 0.14
TC11000640.hg.1	KAT5	8.53 ± 0.21	8.29 ± 0.05	8.29 ± 0.18	8.30 ± 0.06
TC08001173.hg.1	KAT6A	9.60 ± 0.30	9.96 ± 0.17	9.86 ± 0.50	9.95 ± 0.14

TC10000483.hg.1	KAT6B	8.21 ± 0.42	8.56 ± 0.17	8.03 ± 0.45	8.61 ± 0.02
TC17000648.hg.1	KAT7	8.67 ± 0.17	8.90 ± 0.22	8.49 ± 0.14	8.88 ± 0.06
TC16000371.hg.1	KAT8	8.81 ± 0.13	8.67 ± 0.09	8.79 ± 0.11	8.65 ± 0.05
TC06002213.hg.1	KATNA1	7.48 ± 0.05	7.40 ± 0.03	7.40 ± 0.18	7.35 ± 0.06
TC13000530.hg.1	KATNAL1	7.19 ± 0.12	7.20 ± 0.12	7.09 ± 0.11	7.06 ± 0.04
TC18000169.hg.1	KATNAL2	6.38 ± 0.10	6.32 ± 0.08	6.32 ± 0.11	6.40 ± 0.03
TC16000496.hg.1	KATNB1	8.52 ± 0.16	8.36 ± 0.05	8.41 ± 0.15	8.32 ± 0.01
TC15001191.hg.1	KATNBL1	7.50 ± 0.28	7.67 ± 0.34	7.90 ± 0.60	7.51 ± 0.48
TC10000732.hg.1	KAZALD1	7.22 ± 0.24	7.23 ± 0.25	7.15 ± 0.20	7.33 ± 0.03
TC01000184.hg.1	KAZN	7.61 ± 0.18	7.53 ± 0.17	7.66 ± 0.14	7.60 ± 0.01
TC08000012.hg.1	KBTBD11	7.46 ± 0.24	7.38 ± 0.20	7.49 ± 0.23	7.56 ± 0.11
TC03000668.hg.1	KBTBD12	5.03 ± 0.10	4.91 ± 0.05	4.95 ± 0.07	4.91 ± 0.04
TC15000600.hg.1	KBTBD13	7.06 ± 0.37	7.03 ± 0.47	7.02 ± 0.29	7.11 ± 0.06
TC07001261.hg.1	KBTBD2	8.75 ± 0.13	8.83 ± 0.12	8.85 ± 0.27	8.85 ± 0.08
TC11002255.hg.1	KBTBD3	6.36 ± 0.10	6.23 ± 0.10	6.22 ± 0.13	6.29 ± 0.02
TC11000409.hg.1	KBTBD4	6.91 ± 0.19	6.99 ± 0.07	6.89 ± 0.11	7.14 ± 0.06
TC11001742.hg.1	KBTBD4	8.37 ± 0.13	8.27 ± 0.11	8.22 ± 0.21	8.27 ± 0.07
TC13000599.hg.1	KBTBD6	7.24 ± 0.15	6.95 ± 0.02	7.05 ± 0.06	7.02 ± 0.06
TC13000601.hg.1	KBTBD7	7.94 ± 0.14	7.55 ± 0.13	8.05 ± 0.10	7.52 ± 0.20
TC03000407.hg.1	KBTBD8	6.56 ± 0.22	6.86 ± 0.14	6.47 ± 0.30	6.78 ± 0.09
TC18000468.hg.1	KC6	5.01 ± 0.22	4.85 ± 0.04	4.90 ± 0.06	4.90 ± 0.05
TC02000501.hg.1	KCMF1	8.87 ± 0.08	8.83 ± 0.15	8.95 ± 0.13	8.81 ± 0.09
TC12000068.hg.1	KCNA1	7.91 ± 0.23	7.87 ± 0.24	7.80 ± 0.12	8.03 ± 0.12
TC01002974.hg.1	KCNA10	7.32 ± 0.28	7.33 ± 0.12	7.25 ± 0.26	7.48 ± 0.17
TC01002975.hg.1	KCNA2	7.20 ± 0.15	7.09 ± 0.14	7.11 ± 0.16	7.20 ± 0.08
TC01002977.hg.1	KCNA3	9.78 ± 0.35	9.99 ± 0.14	9.50 ± 0.43	9.82 ± 0.09
TC11001521.hg.1	KCNA4	6.40 ± 0.30	6.43 ± 0.17	6.30 ± 0.15	6.53 ± 0.10
TC12000071.hg.1	KCNA5	6.87 ± 0.25	7.00 ± 0.38	6.87 ± 0.24	7.21 ± 0.13
TC12000066.hg.1	KCNA6	7.10 ± 0.14	7.05 ± 0.13	7.02 ± 0.22	7.12 ± 0.12
TC19001707.hg.1	KCNA7	8.04 ± 0.33	7.96 ± 0.15	7.95 ± 0.29	8.12 ± 0.05
TC03000852.hg.1	KCNAB1	5.65 ± 0.17	5.59 ± 0.06	5.59 ± 0.04	5.66 ± 0.09

TC03001932.hg.1	KCNAB1-AS1	5.08 ± 0.13	5.09 ± 0.07	5.05 ± 0.05	5.19 ± 0.12
TC03001931.hg.1	KCNAB1-AS2	6.17 ± 0.19	6.20 ± 0.03	6.12 ± 0.07	6.28 ± 0.07
TC01000084.hg.1	KCNAB2	9.12 ± 0.11	8.97 ± 0.03	8.99 ± 0.10	8.94 ± 0.07
TC17001097.hg.1	KCNAB3	7.21 ± 0.10	7.18 ± 0.05	7.13 ± 0.03	7.23 ± 0.06
TC20000926.hg.1	KCNB1	7.43 ± 0.27	7.44 ± 0.28	7.36 ± 0.28	7.53 ± 0.08
TC08000476.hg.1	KCNB2	7.01 ± 0.15	6.98 ± 0.22	6.94 ± 0.22	7.15 ± 0.08
TC11000233.hg.1	KCNC1	6.76 ± 0.14	6.74 ± 0.19	6.69 ± 0.19	6.83 ± 0.06
TC12001744.hg.1	KCNC2	5.63 ± 0.11	5.55 ± 0.13	5.59 ± 0.08	5.62 ± 0.08
TC19001744.hg.1	KCNC3	7.77 ± 0.13	7.74 ± 0.13	7.68 ± 0.16	7.76 ± 0.08
TC01006402.hg.1	KCNC4	7.62 ± 0.16	7.57 ± 0.10	7.46 ± 0.12	7.58 ± 0.06
TC01006403.hg.1	KCNC4	7.59 ± 0.24	7.63 ± 0.14	7.43 ± 0.30	7.76 ± 0.19
TC01002968.hg.1	KCNC4-AS1	7.35 ± 0.22	7.28 ± 0.17	7.25 ± 0.20	7.44 ± 0.12
TC0X001023.hg.1	KCND1	7.38 ± 0.16	7.33 ± 0.15	7.46 ± 0.09	7.41 ± 0.04
TC07000732.hg.1	KCND2	5.49 ± 0.16	5.39 ± 0.15	5.34 ± 0.22	5.50 ± 0.08
TC01002992.hg.1	KCND3	6.91 ± 0.24	6.89 ± 0.15	6.96 ± 0.11	7.04 ± 0.11
TC01000982.hg.1	KCND3-AS1	6.18 ± 0.15	6.09 ± 0.09	6.16 ± 0.06	6.26 ± 0.14
TC01002993.hg.1	KCND3-IT1	6.87 ± 0.19	6.84 ± 0.05	6.82 ± 0.08	7.01 ± 0.19
TC21000418.hg.1	KCNE1	7.28 ± 0.26	6.99 ± 0.06	7.70 ± 0.21	6.99 ± 0.08
TC0X002336.hg.1	KCNE1L	6.84 ± 0.12	6.92 ± 0.14	6.82 ± 0.15	6.95 ± 0.13
TC21000136.hg.1	KCNE2	6.26 ± 0.15	6.36 ± 0.02	6.13 ± 0.19	6.36 ± 0.03
TC11002082.hg.1	KCNE3	8.52 ± 0.21	7.98 ± 0.17	8.76 ± 0.25	7.85 ± 0.13
TC02001351.hg.1	KCNE4	7.43 ± 0.16	7.45 ± 0.08	7.43 ± 0.15	7.56 ± 0.08
TC02000067.hg.1	KCNF1	7.89 ± 0.22	7.90 ± 0.11	7.69 ± 0.26	7.79 ± 0.03
TC20000941.hg.1	KCNG1	7.89 ± 0.20	7.78 ± 0.15	7.82 ± 0.19	7.85 ± 0.04
TC18000265.hg.1	KCNG2	8.44 ± 0.16	8.46 ± 0.27	8.43 ± 0.26	8.50 ± 0.04
TC02001780.hg.1	KCNG3	9.38 ± 0.26	9.25 ± 0.11	9.26 ± 0.23	9.25 ± 0.04
TC16001311.hg.1	KCNG4	7.33 ± 0.28	7.24 ± 0.27	7.32 ± 0.19	7.48 ± 0.09
TC01003791.hg.1	KCNH1	6.54 ± 0.11	6.48 ± 0.12	6.49 ± 0.13	6.51 ± 0.06
TC01003792.hg.1	KCNH1-IT1	5.89 ± 0.28	5.71 ± 0.09	5.74 ± 0.19	5.74 ± 0.09
TC07002024.hg.1	KCNH2	8.25 ± 0.18	8.21 ± 0.16	8.17 ± 0.18	8.29 ± 0.07
TC12000374.hg.1	KCNH3	8.19 ± 0.14	8.10 ± 0.10	8.27 ± 0.14	8.14 ± 0.01

TC17001526.hg.1	KCNH4	7.70 ± 0.17	7.67 ± 0.16	7.62 ± 0.16	7.83 ± 0.12
TC14001202.hg.1	KCNH5	5.41 ± 0.13	5.32 ± 0.12	5.32 ± 0.06	5.36 ± 0.06
TC17000767.hg.1	KCNH6	7.76 ± 0.23	7.79 ± 0.16	7.75 ± 0.15	7.86 ± 0.08
TC02002482.hg.1	KCNH7	5.19 ± 0.10	5.10 ± 0.11	5.26 ± 0.05	5.20 ± 0.07
TC03000110.hg.1	KCNH8	5.83 ± 0.15	5.88 ± 0.13	5.66 ± 0.02	5.82 ± 0.22
TC05000935.hg.1	KCNIP1	6.53 ± 0.09	6.57 ± 0.14	6.51 ± 0.07	6.61 ± 0.07
TC10001614.hg.1	KCNIP2	7.19 ± 0.14	7.17 ± 0.16	7.15 ± 0.09	7.21 ± 0.06
TC10000743.hg.1	KCNIP2-AS1	7.08 ± 0.18	7.10 ± 0.28	6.90 ± 0.31	7.24 ± 0.08
TC02000572.hg.1	KCNIP3	7.28 ± 0.13	7.17 ± 0.18	7.21 ± 0.14	7.30 ± 0.04
TC04001076.hg.1	KCNIP4	5.40 ± 0.13	5.32 ± 0.06	5.37 ± 0.16	5.36 ± 0.05
TC04001077.hg.1	KCNIP4-IT1	4.67 ± 0.24	4.56 ± 0.14	4.44 ± 0.11	4.64 ± 0.16
TC11002443.hg.1	KCNJ1	5.14 ± 0.04	4.96 ± 0.04	5.02 ± 0.06	4.96 ± 0.08
TC01003396.hg.1	KCNJ10	7.12 ± 0.20	7.02 ± 0.26	7.02 ± 0.18	7.15 ± 0.10
TC11001456.hg.1	KCNJ11	7.94 ± 0.27	7.93 ± 0.18	7.93 ± 0.18	8.05 ± 0.08
TC02002879.hg.1	KCNJ13	4.90 ± 0.10	4.81 ± 0.04	4.89 ± 0.03	4.86 ± 0.03
TC19002644.hg.1	KCNJ14	7.80 ± 0.21	7.73 ± 0.05	7.82 ± 0.07	7.77 ± 0.02
TC21000167.hg.1	KCNJ15	9.38 ± 0.30	9.09 ± 0.40	10.13 ± 0.38	8.91 ± 0.33
TC17000812.hg.1	KCNJ16	5.71 ± 0.10	5.53 ± 0.07	5.69 ± 0.03	5.65 ± 0.05
TC17000298.hg.1	KCNJ18	7.61 ± 0.26	7.56 ± 0.11	7.55 ± 0.16	7.66 ± 0.06
TC17000813.hg.1	KCNJ2	8.91 ± 0.72	8.19 ± 0.30	9.64 ± 0.52	7.80 ± 0.19
TC17001833.hg.1	KCNJ2-AS1	7.74 ± 0.25	7.48 ± 0.05	7.96 ± 0.24	7.31 ± 0.21
TC02000947.hg.1	KCNJ3	5.53 ± 0.12	5.39 ± 0.04	5.45 ± 0.12	5.46 ± 0.07
TC22000790.hg.1	KCNJ4	8.00 ± 0.26	8.04 ± 0.13	7.88 ± 0.25	8.12 ± 0.06
TC11001186.hg.1	KCNJ5	7.53 ± 0.23	7.49 ± 0.12	7.47 ± 0.14	7.64 ± 0.10
TC21000443.hg.1	KCNJ6	6.86 ± 0.21	6.95 ± 0.20	6.90 ± 0.22	7.12 ± 0.14
TC21000444.hg.1	KCNJ6-IT1	5.29 ± 0.29	5.07 ± 0.05	5.23 ± 0.11	5.02 ± 0.11
TC12001299.hg.1	KCNJ8	7.43 ± 0.15	7.37 ± 0.21	7.31 ± 0.12	7.49 ± 0.09
TC01001360.hg.1	KCNJ9	7.15 ± 0.18	7.13 ± 0.20	7.14 ± 0.19	7.23 ± 0.06
TC01001928.hg.1	KCNK1	5.63 ± 0.12	5.58 ± 0.13	5.61 ± 0.14	5.68 ± 0.15
TC14001421.hg.1	KCNK10	6.68 ± 0.20	6.69 ± 0.16	6.73 ± 0.15	6.78 ± 0.07
TC02001825.hg.1	KCNK12	7.24 ± 0.18	7.18 ± 0.19	7.23 ± 0.14	7.10 ± 0.05

TC14000550.hg.1	KCNK13	7.27 ± 0.38	7.20 ± 0.40	7.25 ± 0.28	7.25 ± 0.10
TC20000336.hg.1	KCNK15	7.72 ± 0.16	7.70 ± 0.07	7.70 ± 0.10	7.74 ± 0.04
TC06001698.hg.1	KCNK16	7.43 ± 0.20	7.49 ± 0.28	7.40 ± 0.20	7.65 ± 0.11
TC06001697.hg.1	KCNK17	7.38 ± 0.15	7.33 ± 0.27	7.30 ± 0.21	7.49 ± 0.09
TC10000849.hg.1	KCNK18	6.85 ± 0.30	6.83 ± 0.04	6.87 ± 0.24	6.90 ± 0.04
TC01001792.hg.1	KCNK2	5.55 ± 0.16	5.48 ± 0.05	5.49 ± 0.05	5.53 ± 0.08
TC02000153.hg.1	KCNK3	8.87 ± 0.19	8.95 ± 0.12	8.85 ± 0.29	9.01 ± 0.07
TC06001696.hg.1	KCNK5	7.10 ± 0.26	7.16 ± 0.22	7.08 ± 0.25	7.20 ± 0.06
TC19000525.hg.1	KCNK6	8.26 ± 0.24	8.05 ± 0.17	8.17 ± 0.23	7.97 ± 0.06
TC11001937.hg.1	KCNK7	8.36 ± 0.28	8.39 ± 0.21	8.30 ± 0.15	8.48 ± 0.05
TC08001679.hg.1	KCNK9	6.54 ± 0.17	6.44 ± 0.05	6.47 ± 0.12	6.49 ± 0.03
TC10001439.hg.1	KCNMA1	6.14 ± 0.15	6.10 ± 0.13	6.30 ± 0.11	6.19 ± 0.07
TC05002052.hg.1	KCNMB1	7.99 ± 0.10	7.85 ± 0.06	8.12 ± 0.09	7.88 ± 0.02
TC03000948.hg.1	KCNMB2	5.78 ± 0.18	5.64 ± 0.04	5.69 ± 0.02	5.74 ± 0.10
TC03000949.hg.1	KCNMB2-IT1	6.65 ± 0.14	6.58 ± 0.13	6.57 ± 0.06	6.75 ± 0.10
TC03002037.hg.1	KCNMB3	6.80 ± 0.13	6.71 ± 0.00	6.75 ± 0.03	6.72 ± 0.02
TC22000460.hg.1	KCNMB3P1	5.77 ± 0.18	5.68 ± 0.03	5.53 ± 0.03	5.58 ± 0.09
TC12000621.hg.1	KCNMB4	6.80 ± 0.15	6.70 ± 0.11	6.69 ± 0.06	6.71 ± 0.04
TC19000348.hg.1	KCNN1	7.55 ± 0.30	7.46 ± 0.14	7.47 ± 0.24	7.56 ± 0.00
TC05000541.hg.1	KCNN2	5.50 ± 0.12	5.30 ± 0.05	5.40 ± 0.04	5.36 ± 0.07
TC01003296.hg.1	KCNN3	6.96 ± 0.11	6.65 ± 0.16	6.77 ± 0.07	6.74 ± 0.04
TC19001595.hg.1	KCNN4	8.18 ± 0.14	8.15 ± 0.12	8.10 ± 0.18	8.25 ± 0.04
TC11000066.hg.1	KCNQ1	8.68 ± 0.11	8.71 ± 0.08	8.61 ± 0.11	8.73 ± 0.01
TC11001283.hg.1	KCNQ1-AS1	7.37 ± 0.25	7.23 ± 0.08	7.29 ± 0.07	7.26 ± 0.11
TC11000067.hg.1	KCNQ1DN	8.20 ± 0.26	8.37 ± 0.22	8.29 ± 0.27	8.51 ± 0.05
TC11001282.hg.1	KCNQ1OT1	5.50 ± 0.18	5.61 ± 0.10	5.59 ± 0.04	5.66 ± 0.24
TC20001031.hg.1	KCNQ2	7.78 ± 0.18	7.75 ± 0.16	7.71 ± 0.17	7.80 ± 0.04
TC08001652.hg.1	KCNQ3	6.97 ± 0.18	6.94 ± 0.15	6.92 ± 0.14	7.01 ± 0.04
TC01000518.hg.1	KCNQ4	7.77 ± 0.24	7.68 ± 0.25	7.65 ± 0.18	7.75 ± 0.03
TC06000728.hg.1	KCNQ5	6.55 ± 0.07	6.65 ± 0.06	6.38 ± 0.12	6.58 ± 0.13
TC06001868.hg.1	KCNQ5-AS1	5.10 ± 0.11	5.03 ± 0.12	5.06 ± 0.04	5.02 ± 0.06

TC06001866.hg.1	KCNQ5-AS2	4.65 ± 0.10	4.57 ± 0.20	4.71 ± 0.16	4.59 ± 0.03
TC06000729.hg.1	KCNQ5-IT1	5.81 ± 0.23	5.86 ± 0.11	5.80 ± 0.18	5.83 ± 0.02
TC13000202.hg.1	KCNRG	7.72 ± 0.12	7.52 ± 0.03	7.76 ± 0.05	7.55 ± 0.06
TC20000873.hg.1	KCNS1	7.16 ± 0.14	6.98 ± 0.08	7.10 ± 0.09	7.04 ± 0.05
TC08000604.hg.1	KCNS2	7.53 ± 0.17	7.54 ± 0.16	7.52 ± 0.07	7.63 ± 0.07
TC02000107.hg.1	KCNS3	6.52 ± 0.22	6.40 ± 0.17	6.44 ± 0.17	6.46 ± 0.06
TC09000805.hg.1	KCNT1	7.76 ± 0.23	7.71 ± 0.15	7.68 ± 0.20	7.75 ± 0.00
TC01003659.hg.1	KCNT2	4.57 ± 0.13	4.40 ± 0.06	4.52 ± 0.08	4.47 ± 0.04
TC08000271.hg.1	KCNU1	4.77 ± 0.08	4.70 ± 0.09	4.72 ± 0.08	4.79 ± 0.10
TC08001532.hg.1	KCNV1	6.65 ± 0.16	6.57 ± 0.10	6.60 ± 0.13	6.58 ± 0.04
TC09000016.hg.1	KCNV2	6.83 ± 0.23	6.76 ± 0.18	6.78 ± 0.22	6.86 ± 0.10
TC07001851.hg.1	KCP	8.43 ± 0.21	8.44 ± 0.19	8.39 ± 0.17	8.55 ± 0.06
TC18000426.hg.1	KCTD1	7.40 ± 0.06	7.24 ± 0.06	7.28 ± 0.04	7.29 ± 0.04
TC12001951.hg.1	KCTD10	8.45 ± 0.05	8.26 ± 0.10	8.33 ± 0.07	8.31 ± 0.05
TC17000090.hg.1	KCTD11	7.77 ± 0.12	7.55 ± 0.14	7.66 ± 0.15	7.60 ± 0.10
TC13000741.hg.1	KCTD12	9.67 ± 0.09	9.35 ± 0.13	9.83 ± 0.19	9.29 ± 0.41
TC16001013.hg.1	KCTD13	9.01 ± 0.08	8.96 ± 0.10	9.02 ± 0.14	8.90 ± 0.10
TC19000439.hg.1	KCTD15	7.46 ± 0.07	7.41 ± 0.09	7.45 ± 0.14	7.47 ± 0.01
TC05000791.hg.1	KCTD16	5.57 ± 0.19	5.49 ± 0.08	5.52 ± 0.11	5.50 ± 0.07
TC22000276.hg.1	KCTD17	8.10 ± 0.17	8.14 ± 0.21	8.03 ± 0.19	8.24 ± 0.06
TC02002660.hg.1	KCTD18	8.39 ± 0.09	8.39 ± 0.17	8.31 ± 0.16	8.34 ± 0.05
TC16001185.hg.1	KCTD19	6.67 ± 0.20	6.63 ± 0.16	6.60 ± 0.10	6.72 ± 0.09
TC17000841.hg.1	KCTD2	8.47 ± 0.18	8.33 ± 0.07	8.40 ± 0.05	8.26 ± 0.06
TC06000528.hg.1	KCTD20	8.41 ± 0.10	8.34 ± 0.17	8.41 ± 0.07	8.32 ± 0.13
TC11002135.hg.1	KCTD21	8.44 ± 0.22	8.08 ± 0.13	8.60 ± 0.17	8.15 ± 0.04
TC11000838.hg.1	KCTD21-AS1	6.34 ± 0.14	6.08 ± 0.10	6.04 ± 0.03	6.12 ± 0.08
TC11000839.hg.1	KCTD21-AS1	5.86 ± 0.24	5.44 ± 0.15	5.82 ± 0.15	5.54 ± 0.15
TC01001793.hg.1	KCTD3	6.26 ± 0.09	6.14 ± 0.05	6.18 ± 0.10	6.12 ± 0.02
TC13000628.hg.1	KCTD4	5.40 ± 0.13	5.36 ± 0.03	5.38 ± 0.04	5.25 ± 0.10
TC16000082.hg.1	KCTD5	9.19 ± 0.25	8.87 ± 0.13	9.17 ± 0.18	8.86 ± 0.09
TC03000379.hg.1	KCTD6	6.67 ± 0.14	6.60 ± 0.04	6.55 ± 0.09	6.63 ± 0.01

TC07003327.hg.1	KCTD7	7.79 ± 0.15	7.70 ± 0.11	7.56 ± 0.13	7.76 ± 0.08
TC04001155.hg.1	KCTD8	6.22 ± 0.19	6.14 ± 0.08	6.19 ± 0.07	6.26 ± 0.04
TC08001071.hg.1	KCTD9	7.58 ± 0.40	7.76 ± 0.21	7.68 ± 0.35	7.62 ± 0.06
TC13000855.hg.1	KDELC1	5.77 ± 0.15	5.70 ± 0.04	5.60 ± 0.10	5.64 ± 0.03
TC11002268.hg.1	KDELC2	6.91 ± 0.16	6.89 ± 0.04	6.82 ± 0.16	6.67 ± 0.10
TC19001681.hg.1	KDELR1	9.82 ± 0.27	9.40 ± 0.08	9.41 ± 0.11	9.26 ± 0.17
TC07003350.hg.1	KDELR2	8.92 ± 0.20	8.63 ± 0.17	8.72 ± 0.08	8.44 ± 0.23
TC22000301.hg.1	KDELR3	6.64 ± 0.09	6.55 ± 0.03	6.53 ± 0.12	6.52 ± 0.06
TC01000292.hg.1	KDM1A	8.87 ± 0.09	8.83 ± 0.11	8.57 ± 0.05	8.72 ± 0.03
TC06000117.hg.1	KDM1B	8.83 ± 0.22	8.73 ± 0.18	9.14 ± 0.33	8.57 ± 0.20
TC11000680.hg.1	KDM2A	9.95 ± 0.24	10.02 ± 0.08	10.00 ± 0.25	10.03 ± 0.04
TC12002063.hg.1	KDM2B	8.10 ± 0.04	8.15 ± 0.12	7.84 ± 0.04	8.13 ± 0.02
TC02000524.hg.1	KDM3A	8.66 ± 0.36	9.10 ± 0.24	8.62 ± 0.35	9.18 ± 0.08
TC05000699.hg.1	KDM3B	9.72 ± 0.19	9.55 ± 0.10	9.78 ± 0.24	9.57 ± 0.13
TC01000549.hg.1	KDM4A	8.57 ± 0.14	8.17 ± 0.11	8.35 ± 0.18	8.07 ± 0.09
TC01002587.hg.1	KDM4A-AS1	6.83 ± 0.10	6.70 ± 0.04	6.81 ± 0.08	6.75 ± 0.04
TC19000099.hg.1	KDM4B	8.63 ± 0.17	8.56 ± 0.05	8.62 ± 0.12	8.58 ± 0.05
TC09002929.hg.1	KDM4C	7.93 ± 0.31	8.15 ± 0.16	7.82 ± 0.25	7.97 ± 0.14
TC11000925.hg.1	KDM4D	4.50 ± 0.09	4.36 ± 0.04	4.41 ± 0.10	4.41 ± 0.09
TC11000926.hg.1	KDM4E	5.93 ± 0.26	5.68 ± 0.14	5.74 ± 0.28	5.83 ± 0.18
TC12001082.hg.1	KDM5A	9.32 ± 0.29	9.38 ± 0.20	9.36 ± 0.35	9.32 ± 0.05
TC01003706.hg.1	KDM5B	8.29 ± 0.09	8.25 ± 0.10	8.26 ± 0.18	8.31 ± 0.11
TC0X001061.hg.1	KDM5C	9.11 ± 0.09	9.02 ± 0.06	8.91 ± 0.13	9.03 ± 0.01
TC0X001062.hg.1	KDM5C-IT1	5.97 ± 0.14	6.06 ± 0.06	6.01 ± 0.17	6.18 ± 0.09
TC0Y000185.hg.1	KDM5D	8.06 ± 0.28	7.99 ± 0.08	7.88 ± 0.11	7.96 ± 0.06
TC0X000212.hg.1	KDM6A	8.80 ± 0.34	9.10 ± 0.20	8.95 ± 0.44	9.09 ± 0.02
TC17000117.hg.1	KDM6B	10.22 ± 0.31	10.41 ± 0.13	10.66 ± 0.40	10.60 ± 0.32
TC16000290.hg.1	KDM8	7.74 ± 0.02	7.63 ± 0.05	7.59 ± 0.03	7.65 ± 0.03
TC04001208.hg.1	KDR	5.90 ± 0.15	5.79 ± 0.08	5.79 ± 0.06	5.88 ± 0.07
TC18000556.hg.1	KDSR	8.06 ± 0.05	8.07 ± 0.12	7.89 ± 0.19	7.95 ± 0.11
TC19001166.hg.1	KEAP1	8.45 ± 0.27	8.22 ± 0.13	8.27 ± 0.23	8.16 ± 0.04

TC07001965.hg.1	KEL	7.16 ± 0.20	7.06 ± 0.18	7.12 ± 0.17	7.15 ± 0.08
TC12001802.hg.1	KERA	5.27 ± 0.12	5.23 ± 0.13	5.16 ± 0.04	5.32 ± 0.15
TC09000270.hg.1	KGFLP1	5.30 ± 0.10	5.26 ± 0.07	5.26 ± 0.10	5.30 ± 0.14
TC09001106.hg.1	KGFLP2	5.17 ± 0.10	5.05 ± 0.07	5.10 ± 0.11	5.10 ± 0.07
TC06001869.hg.1	KHDC1L	7.38 ± 0.12	7.36 ± 0.11	7.34 ± 0.08	7.41 ± 0.03
TC06000731.hg.1	KHDC3L	8.13 ± 0.19	7.97 ± 0.16	7.96 ± 0.15	8.01 ± 0.03
TC01000417.hg.1	KHDRBS1	9.45 ± 0.11	9.49 ± 0.08	9.28 ± 0.14	9.36 ± 0.17
TC06001842.hg.1	KHDRBS2	5.82 ± 0.08	5.93 ± 0.10	5.81 ± 0.09	5.83 ± 0.11
TC08000778.hg.1	KHDRBS3	5.62 ± 0.08	5.47 ± 0.04	5.57 ± 0.11	5.58 ± 0.09
TC02000164.hg.1	KHK	7.62 ± 0.19	7.60 ± 0.18	7.56 ± 0.20	7.69 ± 0.07
TC14000170.hg.1	KHNYN	9.00 ± 0.08	8.92 ± 0.07	8.86 ± 0.14	9.00 ± 0.04
TC19001095.hg.1	KHSRP	9.43 ± 0.16	9.38 ± 0.13	9.11 ± 0.09	9.25 ± 0.05
TC09000874.hg.1	KIAA0020	7.23 ± 0.39	7.32 ± 0.18	7.10 ± 0.31	7.14 ± 0.11
TC01003546.hg.1	KIAA0040	10.13 ± 0.18	9.75 ± 0.15	10.20 ± 0.13	9.57 ± 0.15
TC07001216.hg.1	KIAA0087	5.61 ± 0.10	5.62 ± 0.34	5.54 ± 0.26	5.85 ± 0.18
TC17001302.hg.1	KIAA0100	9.33 ± 0.25	9.21 ± 0.12	9.07 ± 0.14	9.03 ± 0.04
TC15002807.hg.1	KIAA0101	6.76 ± 0.24	6.56 ± 0.41	6.57 ± 0.18	6.13 ± 0.06
TC14000841.hg.1	KIAA0125	8.39 ± 0.15	8.28 ± 0.13	8.28 ± 0.14	8.35 ± 0.10
TC05000775.hg.1	KIAA0141	8.41 ± 0.09	8.35 ± 0.07	8.25 ± 0.08	8.27 ± 0.08
TC08000343.hg.1	KIAA0146	7.88 ± 0.09	7.85 ± 0.08	7.81 ± 0.09	7.75 ± 0.05
TC17000853.hg.1	KIAA0195	8.98 ± 0.09	8.88 ± 0.05	8.75 ± 0.04	8.87 ± 0.02
TC08001616.hg.1	KIAA0196	8.52 ± 0.26	8.56 ± 0.23	8.54 ± 0.32	8.39 ± 0.19
TC08000730.hg.1	KIAA0196-AS1	8.36 ± 0.20	8.21 ± 0.10	8.37 ± 0.28	8.54 ± 0.23
TC13000644.hg.1	KIAA0226L	8.27 ± 0.18	8.59 ± 0.10	8.45 ± 0.36	8.59 ± 0.09
TC04000074.hg.1	KIAA0232	8.22 ± 0.10	8.15 ± 0.20	8.51 ± 0.34	8.04 ± 0.19
TC14000417.hg.1	KIAA0247	9.38 ± 0.22	9.34 ± 0.14	9.60 ± 0.36	9.30 ± 0.16
TC06001326.hg.1	KIAA0319	6.57 ± 0.09	6.44 ± 0.15	6.61 ± 0.02	6.52 ± 0.06
TC01002497.hg.1	KIAA0319L	8.66 ± 0.16	8.37 ± 0.07	8.58 ± 0.19	8.23 ± 0.04
TC19000441.hg.1	KIAA0355	8.87 ± 0.09	8.98 ± 0.06	8.60 ± 0.12	8.98 ± 0.05
TC09001482.hg.1	KIAA0368	8.56 ± 0.21	8.52 ± 0.19	8.65 ± 0.34	8.39 ± 0.18
TC14002293.hg.1	KIAA0391	8.13 ± 0.07	8.13 ± 0.13	7.96 ± 0.22	7.92 ± 0.03

TC16000897.hg.1	KIAA0430	10.02 ± 0.28	9.99 ± 0.11	10.20 ± 0.30	10.04 ± 0.10
TC16000661.hg.1	KIAA0513	9.75 ± 0.27	9.28 ± 0.10	9.65 ± 0.18	9.27 ± 0.30
TC16000295.hg.1	KIAA0556	8.04 ± 0.10	7.97 ± 0.02	8.17 ± 0.14	7.97 ± 0.06
TC14000351.hg.1	KIAA0586	7.46 ± 0.42	7.66 ± 0.21	7.57 ± 0.44	7.46 ± 0.11
TC17001064.hg.1	KIAA0753	7.30 ± 0.23	7.17 ± 0.14	7.37 ± 0.26	7.09 ± 0.18
TC01000494.hg.1	KIAA0754	6.90 ± 0.30	6.96 ± 0.19	7.23 ± 0.40	6.95 ± 0.13
TC05001601.hg.1	KIAA0825	6.56 ± 0.30	6.33 ± 0.32	7.19 ± 0.42	6.04 ± 0.23
TC07001283.hg.1	KIAA0895	4.95 ± 0.13	4.80 ± 0.10	4.87 ± 0.08	4.85 ± 0.05
TC16002084.hg.1	KIAA0895L	7.93 ± 0.07	7.84 ± 0.09	7.93 ± 0.08	7.86 ± 0.08
TC01003325.hg.1	KIAA0907	8.52 ± 0.14	8.28 ± 0.18	8.38 ± 0.17	8.26 ± 0.16
TC04000774.hg.1	KIAA0922	9.96 ± 0.30	10.04 ± 0.13	10.00 ± 0.27	9.94 ± 0.04
TC22000873.hg.1	KIAA0930	9.38 ± 0.19	9.11 ± 0.12	9.39 ± 0.11	9.13 ± 0.07
TC05000040.hg.1	KIAA0947	7.49 ± 0.54	7.86 ± 0.28	7.48 ± 0.38	7.72 ± 0.09
TC06001915.hg.1	KIAA1009	6.75 ± 0.48	6.68 ± 0.37	7.20 ± 0.54	6.41 ± 0.20
TC15000742.hg.1	KIAA1024	6.24 ± 0.15	6.18 ± 0.20	6.27 ± 0.09	6.29 ± 0.08
TC05000618.hg.1	KIAA1024L	5.03 ± 0.09	4.90 ± 0.20	5.01 ± 0.11	5.05 ± 0.04
TC12000817.hg.1	KIAA1033	9.70 ± 0.67	10.06 ± 0.34	10.38 ± 0.52	9.93 ± 0.14
TC09000177.hg.1	KIAA1045	7.66 ± 0.17	7.73 ± 0.12	7.67 ± 0.16	7.79 ± 0.05
TC01000851.hg.1	KIAA1107	5.39 ± 0.32	5.40 ± 0.07	5.70 ± 0.34	5.25 ± 0.14
TC04000626.hg.1	KIAA1109	8.70 ± 0.70	9.23 ± 0.30	9.28 ± 0.64	9.09 ± 0.15
TC03001341.hg.1	KIAA1143	7.72 ± 0.22	8.09 ± 0.10	7.77 ± 0.25	7.97 ± 0.07
TC07001930.hg.1	KIAA1147	8.28 ± 0.28	8.55 ± 0.13	8.01 ± 0.11	8.46 ± 0.05
TC09001033.hg.1	KIAA1161	8.00 ± 0.20	7.96 ± 0.18	7.90 ± 0.18	8.02 ± 0.04
TC05002091.hg.1	KIAA1191	8.51 ± 0.04	8.44 ± 0.04	8.29 ± 0.05	8.41 ± 0.05
TC15000752.hg.1	KIAA1199	6.81 ± 0.20	6.77 ± 0.12	6.78 ± 0.15	6.82 ± 0.05
TC0X001306.hg.1	KIAA1210	5.92 ± 0.25	5.85 ± 0.14	5.89 ± 0.10	5.91 ± 0.03
TC04000329.hg.1	KIAA1211	6.05 ± 0.17	5.89 ± 0.13	6.01 ± 0.22	6.00 ± 0.05
TC02002129.hg.1	KIAA1211L	7.75 ± 0.18	7.69 ± 0.14	7.67 ± 0.18	7.73 ± 0.01
TC10000169.hg.1	KIAA1217	6.35 ± 0.16	6.26 ± 0.10	6.30 ± 0.10	6.34 ± 0.06
TC04000211.hg.1	KIAA1239	5.95 ± 0.16	5.86 ± 0.16	5.93 ± 0.05	5.96 ± 0.05
TC06001029.hg.1	KIAA1244	6.85 ± 0.14	6.81 ± 0.16	6.78 ± 0.14	6.90 ± 0.06

TC03001761.hg.1	KIAA1257	7.21 ± 0.08	7.12 ± 0.03	7.22 ± 0.04	7.17 ± 0.09
TC10000414.hg.1	KIAA1279	7.14 ± 0.13	6.99 ± 0.08	7.12 ± 0.11	6.86 ± 0.09
TC01000939.hg.1	KIAA1324	8.79 ± 0.30	8.44 ± 0.63	8.98 ± 0.52	8.62 ± 0.41
TC07001573.hg.1	KIAA1324L	6.20 ± 0.18	6.28 ± 0.14	6.09 ± 0.11	6.25 ± 0.07
TC18000151.hg.1	KIAA1328	6.24 ± 0.18	6.25 ± 0.06	6.10 ± 0.10	6.16 ± 0.10
TC11000950.hg.1	KIAA1377	5.63 ± 0.20	5.57 ± 0.05	5.58 ± 0.07	5.54 ± 0.03
TC03001666.hg.1	KIAA1407	6.67 ± 0.26	6.75 ± 0.14	6.63 ± 0.11	6.71 ± 0.08
TC08001434.hg.1	KIAA1429	7.96 ± 0.28	8.06 ± 0.21	7.91 ± 0.38	7.96 ± 0.09
TC04001814.hg.1	KIAA1430	7.57 ± 0.15	7.67 ± 0.02	7.49 ± 0.08	7.54 ± 0.07
TC09000040.hg.1	KIAA1432	8.45 ± 0.18	8.55 ± 0.29	8.39 ± 0.31	8.45 ± 0.05
TC08000109.hg.1	KIAA1456	5.95 ± 0.16	5.83 ± 0.08	5.84 ± 0.06	5.86 ± 0.08
TC10001147.hg.1	KIAA1462	7.52 ± 0.21	7.56 ± 0.11	7.51 ± 0.17	7.64 ± 0.06
TC12000187.hg.1	KIAA1467	7.33 ± 0.09	7.28 ± 0.03	7.26 ± 0.10	7.28 ± 0.06
TC18000220.hg.1	KIAA1468	8.04 ± 0.53	8.32 ± 0.24	8.20 ± 0.48	8.18 ± 0.07
TC01000434.hg.1	KIAA1522	7.76 ± 0.18	7.72 ± 0.07	7.71 ± 0.12	7.72 ± 0.04
TC03001632.hg.1	KIAA1524	5.83 ± 0.19	5.80 ± 0.43	5.66 ± 0.32	5.49 ± 0.07
TC07001907.hg.1	KIAA1549	6.77 ± 0.26	6.72 ± 0.10	6.66 ± 0.14	6.83 ± 0.07
TC11000317.hg.1	KIAA1549L	6.71 ± 0.20	6.71 ± 0.16	6.67 ± 0.13	6.85 ± 0.11
TC12000295.hg.1	KIAA1551	9.72 ± 0.49	10.02 ± 0.44	10.22 ± 0.53	9.83 ± 0.25
TC06000682.hg.1	KIAA1586	8.31 ± 0.32	8.58 ± 0.02	8.32 ± 0.24	8.54 ± 0.06
TC10001683.hg.1	KIAA1598	7.48 ± 0.08	7.31 ± 0.17	7.65 ± 0.21	7.21 ± 0.27
TC01001566.hg.1	KIAA1614	7.90 ± 0.21	7.86 ± 0.17	7.86 ± 0.18	7.94 ± 0.08
TC22000866.hg.1	KIAA1644	6.81 ± 0.23	6.82 ± 0.22	6.86 ± 0.13	6.89 ± 0.07
TC22000159.hg.1	KIAA1671	7.38 ± 0.10	7.29 ± 0.04	7.23 ± 0.14	7.29 ± 0.02
TC19001284.hg.1	KIAA1683	8.49 ± 0.03	8.83 ± 0.11	8.61 ± 0.11	8.89 ± 0.21
TC13000168.hg.1	KIAA1704	7.09 ± 0.22	7.09 ± 0.12	7.04 ± 0.14	7.04 ± 0.05
TC02002549.hg.1	KIAA1715	7.59 ± 0.17	7.59 ± 0.16	7.65 ± 0.21	7.53 ± 0.11
TC11000908.hg.1	KIAA1731	7.17 ± 0.44	7.34 ± 0.24	7.33 ± 0.46	7.17 ± 0.05
TC14002201.hg.1	KIAA1737	8.77 ± 0.13	8.69 ± 0.03	8.66 ± 0.02	8.71 ± 0.01
TC01006411.hg.1	KIAA1751	7.43 ± 0.21	7.33 ± 0.16	7.36 ± 0.14	7.40 ± 0.03
TC20000834.hg.1	KIAA1755	7.45 ± 0.20	7.47 ± 0.21	7.44 ± 0.13	7.57 ± 0.11

TC01001927.hg.1	KIAA1804	6.02 ± 0.13	5.94 ± 0.03	6.03 ± 0.08	5.95 ± 0.09
TC02004936.hg.1	KIAA1841	6.37 ± 0.13	6.18 ± 0.10	6.42 ± 0.15	6.13 ± 0.08
TC08000842.hg.1	KIAA1875	7.95 ± 0.23	7.94 ± 0.18	7.89 ± 0.17	8.03 ± 0.07
TC06000890.hg.1	KIAA1919	7.89 ± 0.13	7.76 ± 0.08	7.76 ± 0.10	7.71 ± 0.01
TC09000567.hg.1	KIAA1958	7.66 ± 0.10	7.63 ± 0.09	7.70 ± 0.12	7.66 ± 0.03
TC08000175.hg.1	KIAA1967	8.93 ± 0.07	8.84 ± 0.07	8.70 ± 0.08	8.89 ± 0.02
TC09002884.hg.1	KIAA1984	7.94 ± 0.17	7.88 ± 0.12	7.85 ± 0.22	7.92 ± 0.01
TC09001743.hg.1	KIAA1984-AS1	7.74 ± 0.13	7.75 ± 0.11	7.73 ± 0.08	7.86 ± 0.10
TC01002207.hg.1	KIAA2013	9.29 ± 0.42	8.78 ± 0.05	9.12 ± 0.17	8.69 ± 0.10
TC03001663.hg.1	KIAA2018	8.94 ± 0.19	9.12 ± 0.24	9.14 ± 0.33	9.13 ± 0.07
TC0X001163.hg.1	KIAA2022	5.82 ± 0.17	5.78 ± 0.12	5.73 ± 0.10	5.83 ± 0.05
TC09000894.hg.1	KIAA2026	7.13 ± 0.37	7.40 ± 0.21	7.11 ± 0.31	7.26 ± 0.20
TC09000895.hg.1	KIAA2026	8.21 ± 0.45	8.61 ± 0.25	8.36 ± 0.44	8.39 ± 0.11
TC02001555.hg.1	KIDINS220	8.65 ± 0.27	8.71 ± 0.22	8.98 ± 0.39	8.54 ± 0.16
TC10000659.hg.1	KIF11	7.08 ± 0.16	6.98 ± 0.87	6.64 ± 0.32	5.91 ± 0.22
TC09001507.hg.1	KIF12	7.91 ± 0.16	7.90 ± 0.21	7.84 ± 0.20	7.98 ± 0.07
TC06001298.hg.1	KIF13A	8.13 ± 0.13	7.99 ± 0.20	8.59 ± 0.31	7.92 ± 0.22
TC08001097.hg.1	KIF13B	8.16 ± 0.08	8.10 ± 0.11	8.01 ± 0.02	7.97 ± 0.08
TC01003679.hg.1	KIF14	5.59 ± 0.08	5.51 ± 0.39	5.56 ± 0.23	5.15 ± 0.11
TC03000242.hg.1	KIF15	6.21 ± 0.09	6.09 ± 0.33	6.13 ± 0.22	5.70 ± 0.06
TC20000649.hg.1	KIF16B	6.96 ± 0.23	6.97 ± 0.08	6.85 ± 0.31	6.79 ± 0.25
TC01002317.hg.1	KIF17	8.20 ± 0.20	8.19 ± 0.23	8.16 ± 0.19	8.31 ± 0.05
TC11001517.hg.1	KIF18A	5.36 ± 0.11	5.31 ± 0.17	5.27 ± 0.13	5.02 ± 0.13
TC17001589.hg.1	KIF18B	7.85 ± 0.24	7.76 ± 0.20	7.81 ± 0.24	7.82 ± 0.03
TC17000828.hg.1	KIF19	7.86 ± 0.21	7.82 ± 0.17	7.77 ± 0.25	7.90 ± 0.04
TC02002940.hg.1	KIF1A	7.72 ± 0.21	7.64 ± 0.20	7.66 ± 0.21	7.74 ± 0.04
TC01000127.hg.1	KIF1B	8.09 ± 0.43	7.75 ± 0.15	8.80 ± 0.43	7.69 ± 0.24
TC17000060.hg.1	KIF1C	8.25 ± 0.17	8.11 ± 0.02	8.17 ± 0.10	8.02 ± 0.06
TC05000697.hg.1	KIF20A	6.93 ± 0.31	6.66 ± 0.19	6.74 ± 0.30	6.39 ± 0.07
TC10000644.hg.1	KIF20B	6.62 ± 0.50	6.92 ± 0.27	6.69 ± 0.48	6.71 ± 0.25
TC12001391.hg.1	KIF21A	5.53 ± 0.11	5.47 ± 0.05	5.39 ± 0.10	5.34 ± 0.11

TC01003683.hg.1	KIF21B	8.99 ± 0.08	8.90 ± 0.03	8.87 ± 0.01	8.92 ± 0.01
TC16000327.hg.1	KIF22	8.57 ± 0.08	8.46 ± 0.13	8.23 ± 0.18	8.45 ± 0.07
TC15000638.hg.1	KIF23	6.41 ± 0.08	6.35 ± 0.39	6.34 ± 0.24	5.90 ± 0.08
TC09001031.hg.1	KIF24	6.42 ± 0.11	6.34 ± 0.16	6.35 ± 0.12	6.39 ± 0.01
TC06001185.hg.1	KIF25	7.53 ± 0.09	7.50 ± 0.11	7.50 ± 0.09	7.57 ± 0.03
TC06002321.hg.1	KIF25-AS1	8.13 ± 0.20	8.15 ± 0.13	8.09 ± 0.13	8.23 ± 0.07
TC14000813.hg.1	KIF26A	8.60 ± 0.23	8.55 ± 0.24	8.57 ± 0.22	8.66 ± 0.03
TC01001996.hg.1	KIF26B	7.08 ± 0.19	7.04 ± 0.14	7.08 ± 0.16	7.13 ± 0.02
TC09001282.hg.1	KIF27	6.90 ± 0.32	6.85 ± 0.26	7.04 ± 0.48	6.68 ± 0.22
TC05003396.hg.1	KIF2A	9.35 ± 0.54	9.58 ± 0.16	9.46 ± 0.28	9.53 ± 0.14
TC17000689.hg.1	KIF2B	6.83 ± 0.18	6.84 ± 0.27	6.74 ± 0.27	6.91 ± 0.08
TC01000563.hg.1	KIF2C	7.20 ± 0.37	6.82 ± 0.21	6.93 ± 0.32	6.64 ± 0.02
TC05001771.hg.1	KIF3A	6.39 ± 0.27	6.49 ± 0.12	6.29 ± 0.22	6.36 ± 0.09
TC20000209.hg.1	KIF3B	8.21 ± 0.01	8.23 ± 0.11	8.17 ± 0.03	8.10 ± 0.13
TC02001664.hg.1	KIF3C	7.86 ± 0.08	7.72 ± 0.10	7.94 ± 0.11	7.79 ± 0.05
TC0X000376.hg.1	KIF4A	6.54 ± 0.22	6.38 ± 0.18	6.40 ± 0.12	6.15 ± 0.04
TC05000858.hg.1	KIF4B	7.41 ± 0.28	7.39 ± 0.20	7.46 ± 0.11	7.41 ± 0.17
TC12000534.hg.1	KIF5A	6.60 ± 0.17	6.58 ± 0.14	6.53 ± 0.15	6.64 ± 0.03
TC10001160.hg.1	KIF5B	10.01 ± 0.56	10.43 ± 0.12	10.47 ± 0.49	10.34 ± 0.17
TC02000927.hg.1	KIF5C	6.35 ± 0.13	6.30 ± 0.08	6.24 ± 0.07	6.29 ± 0.06
TC06001699.hg.1	KIF6	5.23 ± 0.12	5.15 ± 0.06	5.18 ± 0.07	5.22 ± 0.04
TC15001832.hg.1	KIF7	6.17 ± 0.21	6.19 ± 0.18	6.04 ± 0.14	6.26 ± 0.15
TC15001833.hg.1	KIF7	8.59 ± 0.24	8.55 ± 0.20	8.52 ± 0.23	8.63 ± 0.04
TC03001365.hg.1	KIF9	6.86 ± 0.14	6.80 ± 0.07	6.77 ± 0.14	6.83 ± 0.02
TC03000266.hg.1	KIF9-AS1	7.70 ± 0.11	7.66 ± 0.14	7.62 ± 0.05	7.66 ± 0.01
TC01003505.hg.1	KIFAP3	7.68 ± 0.55	7.40 ± 0.19	7.64 ± 0.21	7.27 ± 0.06
TC06000421.hg.1	KIFC1	7.72 ± 0.28	7.52 ± 0.17	7.51 ± 0.18	7.41 ± 0.02
TC6_mcf_hap5000095.hg.1	KIFC1	8.72 ± 0.43	8.63 ± 0.12	8.47 ± 0.44	8.63 ± 0.07
TC6_qbl_hap6000101.hg.1	KIFC1	7.68 ± 0.30	7.48 ± 0.18	7.46 ± 0.19	7.36 ± 0.02
TC6_ssto_hap7000090.hg.1	KIFC1	7.72 ± 0.28	7.52 ± 0.17	7.51 ± 0.18	7.41 ± 0.02
TC08000854.hg.1	KIFC2	8.66 ± 0.14	8.64 ± 0.14	8.59 ± 0.17	8.70 ± 0.04

TC16001142.hg.1	KIFC3	8.06 ± 0.20	7.99 ± 0.20	8.01 ± 0.17	8.11 ± 0.02
TC10001022.hg.1	KIN	7.14 ± 0.43	7.14 ± 0.27	7.39 ± 0.42	7.07 ± 0.15
TC19002659.hg.1	KIR2DL1	8.38 ± 0.48	7.99 ± 0.14	8.13 ± 0.27	8.10 ± 0.11
TC19_gl000209_random000006.hg.1	KIR2DL2	8.47 ± 0.52	8.10 ± 0.12	8.24 ± 0.25	8.15 ± 0.15
TC19002661.hg.1	KIR2DL3	8.62 ± 0.53	8.33 ± 0.12	8.35 ± 0.30	8.34 ± 0.06
TC19_gl000209_random000010.hg.1	KIR2DL4	8.13 ± 0.20	7.86 ± 0.01	7.95 ± 0.03	7.89 ± 0.12
TC19002656.hg.1	KIR2DL4	7.67 ± 0.25	7.41 ± 0.02	7.51 ± 0.04	7.43 ± 0.10
TC19_gl000209_random000003.hg.1	KIR2DL5B	8.99 ± 0.48	8.70 ± 0.19	8.85 ± 0.39	8.76 ± 0.10
TC19_gl000209_random000008.hg.1	KIR2DS1	8.94 ± 0.43	8.78 ± 0.04	8.75 ± 0.15	8.81 ± 0.14
TC19_gl000209_random000007.hg.1	KIR2DS2	8.81 ± 0.42	8.63 ± 0.04	8.63 ± 0.15	8.68 ± 0.11
TC19_gl000209_random000005.hg.1	KIR2DS3	9.23 ± 0.39	8.91 ± 0.11	8.96 ± 0.24	9.00 ± 0.13
TC19002660.hg.1	KIR2DS4	8.51 ± 0.62	8.13 ± 0.09	8.09 ± 0.26	8.12 ± 0.20
TC19002657.hg.1	KIR3DL1	8.37 ± 0.19	8.14 ± 0.06	8.15 ± 0.06	8.16 ± 0.06
TC19002658.hg.1	KIR3DL2	8.53 ± 0.26	8.17 ± 0.07	8.16 ± 0.17	8.19 ± 0.09
TC19_gl000209_random000001.hg.1	KIR3DL3	8.19 ± 0.10	8.17 ± 0.08	8.08 ± 0.07	8.24 ± 0.08
TC19002655.hg.1	KIR3DL3	8.10 ± 0.07	8.02 ± 0.04	7.99 ± 0.01	8.10 ± 0.09
TC19_gl000209_random000009.hg.1	KIR3DS1	8.64 ± 0.27	8.44 ± 0.10	8.46 ± 0.27	8.48 ± 0.14
TC19000880.hg.1	KIR3DX1	7.43 ± 0.10	7.35 ± 0.08	7.38 ± 0.04	7.39 ± 0.06
TC01001335.hg.1	KIRREL	7.47 ± 0.18	7.36 ± 0.15	7.38 ± 0.13	7.46 ± 0.02
TC19000487.hg.1	KIRREL2	7.99 ± 0.21	7.95 ± 0.26	7.89 ± 0.22	8.07 ± 0.07
TC11002435.hg.1	KIRREL3	7.54 ± 0.16	7.50 ± 0.14	7.46 ± 0.14	7.56 ± 0.03
TC11001175.hg.1	KIRREL3-AS1	6.78 ± 0.12	6.78 ± 0.15	6.67 ± 0.24	6.83 ± 0.09
TC11001177.hg.1	KIRREL3-AS2	5.76 ± 0.12	5.70 ± 0.11	5.80 ± 0.08	5.93 ± 0.16
TC11001179.hg.1	KIRREL3-AS3	6.92 ± 0.22	6.80 ± 0.07	6.78 ± 0.15	6.86 ± 0.06
TC01001336.hg.1	KIRREL-IT1	7.41 ± 0.15	7.30 ± 0.07	7.41 ± 0.32	7.45 ± 0.13
TC01003727.hg.1	KISS1	7.19 ± 0.16	7.13 ± 0.22	7.19 ± 0.20	7.33 ± 0.08
TC19000022.hg.1	KISS1R	8.25 ± 0.14	8.27 ± 0.15	8.29 ± 0.19	8.35 ± 0.01
TC04000313.hg.1	KIT	5.47 ± 0.08	5.44 ± 0.08	5.39 ± 0.10	5.50 ± 0.09
TC12001792.hg.1	KITLG	5.31 ± 0.12	5.12 ± 0.04	5.13 ± 0.10	5.20 ± 0.04
TC13000113.hg.1	KL	6.39 ± 0.24	6.13 ± 0.13	6.50 ± 0.15	6.26 ± 0.02
TC04000228.hg.1	KLB	5.15 ± 0.11	4.91 ± 0.12	4.91 ± 0.10	4.97 ± 0.01

TC14002312.hg.1	KLC1	8.89 ± 0.06	8.88 ± 0.09	8.89 ± 0.11	8.83 ± 0.08
TC11000656.hg.1	KLC2	8.62 ± 0.18	8.53 ± 0.15	8.45 ± 0.19	8.56 ± 0.02
TC19000645.hg.1	KLC3	8.29 ± 0.23	8.24 ± 0.17	8.23 ± 0.21	8.32 ± 0.05
TC06000594.hg.1	KLC4	7.89 ± 0.11	7.86 ± 0.12	7.78 ± 0.15	7.92 ± 0.04
TC19001213.hg.1	KLF1	8.90 ± 0.17	8.71 ± 0.23	8.81 ± 0.28	8.80 ± 0.02
TC08001500.hg.1	KLF10	8.92 ± 0.04	9.26 ± 0.14	8.79 ± 0.19	9.19 ± 0.30
TC02000055.hg.1	KLF11	9.07 ± 0.06	9.32 ± 0.11	8.99 ± 0.06	9.42 ± 0.03
TC13000733.hg.1	KLF12	8.34 ± 0.64	8.96 ± 0.24	8.17 ± 0.45	8.80 ± 0.23
TC15000198.hg.1	KLF13	9.64 ± 0.21	9.55 ± 0.05	9.52 ± 0.08	9.52 ± 0.08
TC15000199.hg.1	KLF13	7.97 ± 0.34	7.76 ± 0.13	7.84 ± 0.12	7.79 ± 0.00
TC07001867.hg.1	KLF14	6.83 ± 0.06	6.68 ± 0.15	6.78 ± 0.17	6.78 ± 0.06
TC03001736.hg.1	KLF15	8.82 ± 0.30	8.86 ± 0.24	8.75 ± 0.28	8.91 ± 0.06
TC19001015.hg.1	KLF16	9.20 ± 0.12	9.12 ± 0.11	9.16 ± 0.08	9.15 ± 0.01
TC01006270.hg.1	KLF17	6.63 ± 0.19	6.60 ± 0.11	6.63 ± 0.07	6.67 ± 0.07
TC19000321.hg.1	KLF2	12.40 ± 0.07	12.30 ± 0.04	12.47 ± 0.23	12.16 ± 0.04
TC04000221.hg.1	KLF3	10.71 ± 0.41	11.05 ± 0.11	10.77 ± 0.35	11.10 ± 0.02
TC09001447.hg.1	KLF4	9.04 ± 0.23	9.04 ± 0.09	9.19 ± 0.10	8.95 ± 0.15
TC13000273.hg.1	KLF5	7.91 ± 0.14	7.92 ± 0.09	8.20 ± 0.18	7.91 ± 0.21
TC10000979.hg.1	KLF6	10.65 ± 0.18	10.80 ± 0.12	11.07 ± 0.31	10.70 ± 0.23
TC02002705.hg.1	KLF7	9.28 ± 0.14	9.39 ± 0.07	9.84 ± 0.46	9.52 ± 0.14
TC02002707.hg.1	KLF7-IT1	7.37 ± 0.43	7.89 ± 0.31	8.35 ± 0.68	7.98 ± 0.17
TC0X000338.hg.1	KLF8	7.16 ± 0.07	7.25 ± 0.07	7.07 ± 0.08	7.15 ± 0.13
TC09001201.hg.1	KLF9	8.70 ± 0.11	8.97 ± 0.21	8.54 ± 0.09	9.15 ± 0.08
TC14000277.hg.1	KLHDC1	7.17 ± 0.14	7.18 ± 0.13	7.04 ± 0.16	7.09 ± 0.07
TC07000805.hg.1	KLHDC10	8.17 ± 0.09	8.05 ± 0.11	8.15 ± 0.14	7.98 ± 0.02
TC14000278.hg.1	KLHDC2	8.06 ± 0.32	8.33 ± 0.29	7.74 ± 0.19	8.31 ± 0.04
TC06000592.hg.1	KLHDC3	8.53 ± 0.21	8.26 ± 0.14	8.22 ± 0.20	8.16 ± 0.06
TC16002093.hg.1	KLHDC4	8.14 ± 0.05	8.02 ± 0.04	7.93 ± 0.10	8.02 ± 0.05
TC01000242.hg.1	KLHDC7A	6.73 ± 0.21	6.81 ± 0.10	6.74 ± 0.06	6.79 ± 0.02
TC22000430.hg.1	KLHDC7B	8.67 ± 0.59	7.96 ± 0.19	8.15 ± 0.09	7.73 ± 0.14
TC01003746.hg.1	KLHDC8A	7.69 ± 0.21	7.56 ± 0.10	7.60 ± 0.20	7.63 ± 0.04

TC03000288.hg.1	KLHDC8B	7.72 ± 0.18	7.53 ± 0.17	7.59 ± 0.13	7.64 ± 0.00
TC01001376.hg.1	KLHDC9	7.30 ± 0.20	7.17 ± 0.10	7.25 ± 0.19	7.24 ± 0.04
TC13000721.hg.1	KLHL1	5.54 ± 0.12	5.42 ± 0.10	5.39 ± 0.11	5.52 ± 0.14
TC17000518.hg.1	KLHL10	6.24 ± 0.17	6.17 ± 0.04	6.16 ± 0.13	6.20 ± 0.03
TC17001519.hg.1	KLHL11	7.50 ± 0.24	7.80 ± 0.17	7.34 ± 0.09	7.67 ± 0.12
TC01003709.hg.1	KLHL12	7.91 ± 0.15	7.74 ± 0.12	7.91 ± 0.24	7.62 ± 0.06
TC0X001303.hg.1	KLHL13	5.68 ± 0.16	5.57 ± 0.08	5.59 ± 0.09	5.63 ± 0.04
TC18000447.hg.1	KLHL14	8.04 ± 0.02	7.59 ± 0.05	7.34 ± 0.21	7.38 ± 0.18
TC0X000925.hg.1	KLHL15	7.92 ± 0.16	8.42 ± 0.27	7.89 ± 0.21	8.33 ± 0.09
TC01000021.hg.1	KLHL17	8.13 ± 0.23	8.12 ± 0.19	8.07 ± 0.18	8.19 ± 0.05
TC03000267.hg.1	KLHL18	8.22 ± 0.02	8.15 ± 0.02	8.27 ± 0.13	8.13 ± 0.11
TC04000826.hg.1	KLHL2	8.41 ± 0.29	8.39 ± 0.19	9.42 ± 0.44	8.38 ± 0.22
TC01001513.hg.1	KLHL20	7.96 ± 0.35	8.22 ± 0.04	7.92 ± 0.24	8.24 ± 0.08
TC01002155.hg.1	KLHL21	8.15 ± 0.13	8.04 ± 0.05	8.07 ± 0.17	8.11 ± 0.04
TC22001484.hg.1	KLHL22	8.14 ± 0.08	8.04 ± 0.07	7.95 ± 0.09	8.09 ± 0.06
TC02005002.hg.1	KLHL23	4.61 ± 0.06	4.48 ± 0.01	4.51 ± 0.06	4.47 ± 0.01
TC03000985.hg.1	KLHL24	8.52 ± 0.28	8.82 ± 0.12	8.49 ± 0.18	8.93 ± 0.01
TC15001806.hg.1	KLHL25	8.35 ± 0.20	8.35 ± 0.25	8.27 ± 0.16	8.45 ± 0.06
TC19000363.hg.1	KLHL26	8.31 ± 0.13	8.24 ± 0.08	8.15 ± 0.14	8.25 ± 0.01
TC14001079.hg.1	KLHL28	8.24 ± 0.50	8.66 ± 0.12	8.32 ± 0.48	8.58 ± 0.19
TC14001082.hg.1	KLHL28	5.25 ± 0.15	5.20 ± 0.19	5.21 ± 0.03	5.19 ± 0.06
TC02000131.hg.1	KLHL29	7.32 ± 0.17	7.38 ± 0.21	7.28 ± 0.26	7.46 ± 0.06
TC05001817.hg.1	KLHL3	6.87 ± 0.17	6.95 ± 0.03	6.75 ± 0.05	7.02 ± 0.13
TC02001450.hg.1	KLHL30	8.21 ± 0.12	8.07 ± 0.15	8.15 ± 0.14	8.21 ± 0.05
TC02002910.hg.1	KLHL30-AS1	7.09 ± 0.28	7.12 ± 0.34	7.04 ± 0.29	7.27 ± 0.14
TC06001817.hg.1	KLHL31	5.95 ± 0.11	5.75 ± 0.10	5.72 ± 0.07	5.75 ± 0.24
TC06000822.hg.1	KLHL32	5.96 ± 0.14	5.88 ± 0.05	5.80 ± 0.03	5.95 ± 0.08
TC14000885.hg.1	KLHL33	8.15 ± 0.29	8.08 ± 0.22	8.01 ± 0.26	8.21 ± 0.03
TC0X000914.hg.1	KLHL34	6.43 ± 0.12	6.17 ± 0.08	6.21 ± 0.03	6.20 ± 0.14
TC11002094.hg.1	KLHL35	8.07 ± 0.22	7.95 ± 0.25	7.89 ± 0.26	7.98 ± 0.03
TC16000658.hg.1	KLHL36	8.09 ± 0.05	8.09 ± 0.10	7.81 ± 0.05	8.06 ± 0.07

TC08001592.hg.1	KLHL38	7.03 ± 0.16	6.94 ± 0.02	6.97 ± 0.26	7.05 ± 0.13
TC0X000446.hg.1	KLHL4	4.81 ± 0.08	4.69 ± 0.07	4.73 ± 0.10	4.83 ± 0.06
TC03000223.hg.1	KLHL40	7.43 ± 0.24	7.39 ± 0.24	7.35 ± 0.24	7.48 ± 0.07
TC02005000.hg.1	KLHL41	4.23 ± 0.16	4.01 ± 0.05	4.13 ± 0.05	4.06 ± 0.04
TC12000272.hg.1	KLHL42	7.81 ± 0.22	7.72 ± 0.07	7.72 ± 0.09	7.64 ± 0.03
TC04000224.hg.1	KLHL5	8.12 ± 0.23	8.26 ± 0.14	8.31 ± 0.28	8.35 ± 0.09
TC03002057.hg.1	KLHL6	8.88 ± 0.16	8.60 ± 0.08	8.97 ± 0.16	8.55 ± 0.08
TC03000984.hg.1	KLHL6-AS1	6.03 ± 0.15	5.74 ± 0.12	6.00 ± 0.14	5.87 ± 0.17
TC07000140.hg.1	KLHL7	6.47 ± 0.21	6.42 ± 0.25	6.40 ± 0.32	6.29 ± 0.04
TC07001193.hg.1	KLHL7-AS1	5.98 ± 0.10	5.88 ± 0.20	5.84 ± 0.06	5.93 ± 0.03
TC04001362.hg.1	KLHL8	7.94 ± 0.08	7.83 ± 0.15	8.24 ± 0.38	7.74 ± 0.12
TC09000957.hg.1	KLHL9	7.63 ± 0.16	7.46 ± 0.09	7.65 ± 0.15	7.48 ± 0.05
TC19001758.hg.1	KLK1	7.59 ± 0.28	7.60 ± 0.19	7.51 ± 0.17	7.67 ± 0.11
TC19001767.hg.1	KLK10	7.63 ± 0.27	7.61 ± 0.24	7.57 ± 0.16	7.72 ± 0.07
TC19001768.hg.1	KLK11	7.61 ± 0.17	7.51 ± 0.09	7.60 ± 0.14	7.61 ± 0.03
TC19001769.hg.1	KLK12	7.45 ± 0.18	7.40 ± 0.17	7.40 ± 0.14	7.50 ± 0.11
TC19001770.hg.1	KLK13	7.07 ± 0.10	7.01 ± 0.17	7.01 ± 0.07	7.13 ± 0.10
TC19001771.hg.1	KLK14	7.35 ± 0.21	7.39 ± 0.24	7.36 ± 0.11	7.56 ± 0.10
TC19001759.hg.1	KLK15	7.56 ± 0.24	7.54 ± 0.26	7.47 ± 0.12	7.72 ± 0.09
TC19000771.hg.1	KLK2	7.04 ± 0.19	7.01 ± 0.21	6.89 ± 0.21	7.19 ± 0.06
TC19000770.hg.1	KLK3	7.60 ± 0.18	7.66 ± 0.21	7.61 ± 0.09	7.81 ± 0.11
TC19001761.hg.1	KLK4	7.26 ± 0.26	7.17 ± 0.20	7.20 ± 0.21	7.25 ± 0.06
TC19001763.hg.1	KLK5	8.24 ± 0.24	8.25 ± 0.19	8.21 ± 0.12	8.40 ± 0.14
TC19001764.hg.1	KLK6	7.94 ± 0.17	7.91 ± 0.13	7.91 ± 0.07	7.99 ± 0.06
TC19001765.hg.1	KLK7	6.75 ± 0.21	6.67 ± 0.15	6.64 ± 0.09	6.73 ± 0.04
TC19001766.hg.1	KLK9	7.67 ± 0.20	7.70 ± 0.20	7.65 ± 0.19	7.82 ± 0.02
TC04002915.hg.1	KLKB1	5.05 ± 0.13	5.02 ± 0.03	5.04 ± 0.02	5.01 ± 0.05
TC19001760.hg.1	KLKP1	7.51 ± 0.24	7.57 ± 0.14	7.47 ± 0.25	7.64 ± 0.07
TC10001493.hg.1	KLLN	6.91 ± 0.18	6.84 ± 0.07	6.97 ± 0.20	6.80 ± 0.06
TC12001224.hg.1	KLRAP1	5.86 ± 0.42	5.93 ± 0.21	5.83 ± 0.20	5.83 ± 0.07
TC12001202.hg.1	KLRB1	9.18 ± 0.43	9.35 ± 0.31	8.43 ± 0.44	8.87 ± 0.28

TC12003273.hg.1	KLRC1	6.87 ± 0.48	6.77 ± 0.01	6.52 ± 0.34	6.53 ± 0.13
TC12003272.hg.1	KLRC2	6.89 ± 0.49	6.91 ± 0.19	6.48 ± 0.28	6.71 ± 0.24
TC12003271.hg.1	KLRC3	8.04 ± 0.58	8.15 ± 0.32	7.46 ± 0.46	7.90 ± 0.22
TC12001222.hg.1	KLRC4	8.98 ± 0.79	9.35 ± 0.28	8.64 ± 0.49	9.06 ± 0.08
TC12000160.hg.1	KLRD1	8.15 ± 0.54	7.89 ± 0.30	7.77 ± 0.16	7.47 ± 0.12
TC12000152.hg.1	KLRF1	7.37 ± 0.90	7.34 ± 0.40	7.14 ± 0.61	7.02 ± 0.06
TC12000154.hg.1	KLRF2	3.97 ± 0.11	3.91 ± 0.05	3.87 ± 0.07	3.90 ± 0.04
TC12000138.hg.1	KLRG1	7.76 ± 0.62	8.08 ± 0.09	7.28 ± 0.43	7.47 ± 0.32
TC07001914.hg.1	KLRG2	7.84 ± 0.16	7.84 ± 0.19	7.77 ± 0.17	7.91 ± 0.06
TC01001974.hg.1	KMO	6.97 ± 0.21	7.01 ± 0.47	6.87 ± 0.26	6.54 ± 0.12
TC11001064.hg.1	KMT2A	9.02 ± 0.41	9.33 ± 0.13	8.81 ± 0.39	9.21 ± 0.15
TC19000483.hg.1	KMT2B	8.75 ± 0.12	8.68 ± 0.02	8.70 ± 0.12	8.67 ± 0.09
TC07002045.hg.1	KMT2C	9.53 ± 0.43	9.79 ± 0.19	9.77 ± 0.47	9.81 ± 0.14
TC12003277.hg.1	KMT2D	9.64 ± 0.19	9.59 ± 0.06	9.68 ± 0.29	9.59 ± 0.17
TC07000679.hg.1	KMT2E	9.88 ± 0.45	10.25 ± 0.14	10.09 ± 0.51	10.31 ± 0.10
TC01002621.hg.1	KNCN	7.66 ± 0.18	7.61 ± 0.17	7.67 ± 0.18	7.75 ± 0.05
TC10000949.hg.1	KNDC1	7.60 ± 0.22	7.57 ± 0.23	7.56 ± 0.18	7.68 ± 0.04
TC03001024.hg.1	KNG1	5.83 ± 0.15	5.75 ± 0.03	5.74 ± 0.02	5.75 ± 0.04
TC16000920.hg.1	KNOP1	8.45 ± 0.11	8.20 ± 0.09	8.28 ± 0.09	8.17 ± 0.07
TC15000283.hg.1	KNSTRN	7.14 ± 0.06	6.81 ± 0.07	6.93 ± 0.14	6.64 ± 0.07
TC12000974.hg.1	KNTC1	7.19 ± 0.32	7.14 ± 0.32	7.01 ± 0.35	6.71 ± 0.18
TC03001704.hg.1	KPNA1	8.33 ± 0.13	8.31 ± 0.11	8.35 ± 0.19	8.29 ± 0.08
TC17002860.hg.1	KPNA2	9.11 ± 0.21	9.21 ± 0.47	8.81 ± 0.16	8.73 ± 0.28
TC13000663.hg.1	KPNA3	8.54 ± 0.24	8.67 ± 0.29	8.35 ± 0.27	8.53 ± 0.06
TC03001956.hg.1	KPNA4	8.94 ± 0.32	9.19 ± 0.18	9.16 ± 0.42	9.14 ± 0.13
TC06000925.hg.1	KPNA5	7.49 ± 0.43	7.77 ± 0.17	7.27 ± 0.19	7.71 ± 0.04
TC01000419.hg.1	KPNA6	8.76 ± 0.17	8.63 ± 0.17	8.58 ± 0.21	8.52 ± 0.04
TC07001640.hg.1	KPNA7	6.32 ± 0.20	6.34 ± 0.18	6.25 ± 0.16	6.42 ± 0.06
TC17000611.hg.1	KPNB1	10.23 ± 0.25	10.21 ± 0.13	10.35 ± 0.36	10.02 ± 0.15
TC01001236.hg.1	KPRP	7.64 ± 0.35	7.56 ± 0.09	7.57 ± 0.10	7.69 ± 0.09
TC19001665.hg.1	KPTN	7.56 ± 0.23	7.52 ± 0.14	7.59 ± 0.19	7.55 ± 0.01

TC12001314.hg.1	KRAS	8.24 ± 0.19	8.40 ± 0.08	8.45 ± 0.20	8.41 ± 0.17
TC07000991.hg.1	KRBA1	8.32 ± 0.15	8.22 ± 0.16	8.21 ± 0.13	8.28 ± 0.06
TC17001115.hg.1	KRBA2	6.73 ± 0.15	6.52 ± 0.10	6.66 ± 0.15	6.55 ± 0.04
TC03003346.hg.1	KRBOX1	6.26 ± 0.17	6.19 ± 0.07	6.15 ± 0.07	6.33 ± 0.14
TC03001330.hg.1	KRBOX1-AS1	4.19 ± 0.17	4.08 ± 0.10	4.06 ± 0.10	4.26 ± 0.18
TC0X000216.hg.1	KRBOX4	7.95 ± 0.15	8.02 ± 0.09	7.84 ± 0.21	7.96 ± 0.08
TC02002067.hg.1	KRCC1	8.12 ± 0.15	7.99 ± 0.38	8.56 ± 0.49	7.51 ± 0.43
TC22000191.hg.1	KREMEN1	8.69 ± 0.69	7.94 ± 0.14	9.73 ± 0.23	7.98 ± 0.32
TC16000090.hg.1	KREMEN2	8.81 ± 0.28	8.78 ± 0.18	8.73 ± 0.26	8.82 ± 0.05
TC19001169.hg.1	KRI1	8.65 ± 0.09	8.51 ± 0.02	8.49 ± 0.06	8.45 ± 0.04
TC07001597.hg.1	KRIT1	7.91 ± 0.54	8.04 ± 0.20	8.10 ± 0.41	7.94 ± 0.15
TC12001747.hg.1	KRR1	7.67 ± 0.93	8.62 ± 0.21	8.07 ± 0.64	8.59 ± 0.17
TC12001532.hg.1	KRT1	6.31 ± 0.27	6.27 ± 0.11	6.29 ± 0.10	6.41 ± 0.06
TC17001472.hg.1	KRT10	6.48 ± 0.04	6.38 ± 0.02	6.45 ± 0.11	6.42 ± 0.05
TC17002917.hg.1	KRT12	6.45 ± 0.13	6.44 ± 0.14	6.42 ± 0.13	6.49 ± 0.06
TC12001516.hg.1	KRT121P	7.70 ± 0.20	7.67 ± 0.12	7.64 ± 0.18	7.71 ± 0.08
TC17001508.hg.1	KRT13	7.65 ± 0.19	7.55 ± 0.22	7.60 ± 0.22	7.70 ± 0.06
TC17001513.hg.1	KRT14	7.02 ± 0.24	7.01 ± 0.19	6.90 ± 0.14	7.12 ± 0.05
TC17001509.hg.1	KRT15	7.15 ± 0.20	7.09 ± 0.14	7.11 ± 0.12	7.18 ± 0.06
TC17001514.hg.1	KRT16	7.46 ± 0.22	7.39 ± 0.22	7.31 ± 0.22	7.49 ± 0.09
TC17001179.hg.1	KRT16P2	7.53 ± 0.12	7.40 ± 0.11	7.54 ± 0.12	7.57 ± 0.06
TC17001255.hg.1	KRT16P3	7.24 ± 0.16	7.26 ± 0.17	7.29 ± 0.13	7.39 ± 0.05
TC17002908.hg.1	KRT17	7.81 ± 0.14	7.82 ± 0.19	7.82 ± 0.13	7.93 ± 0.07
TC17001180.hg.1	KRT17P1	7.59 ± 0.13	7.56 ± 0.13	7.55 ± 0.11	7.64 ± 0.06
TC17000215.hg.1	KRT17P2	7.32 ± 0.09	7.27 ± 0.09	7.30 ± 0.07	7.36 ± 0.04
TC12000424.hg.1	KRT18	7.89 ± 0.20	7.98 ± 0.03	7.87 ± 0.14	8.07 ± 0.11
TC02001719.hg.1	KRT18P52	7.00 ± 0.15	6.91 ± 0.08	6.92 ± 0.08	6.97 ± 0.06
TC17001293.hg.1	KRT18P55	7.25 ± 0.15	7.17 ± 0.18	7.21 ± 0.22	7.30 ± 0.03
TC17002906.hg.1	KRT19	7.25 ± 0.27	7.22 ± 0.20	7.19 ± 0.26	7.30 ± 0.07
TC12001531.hg.1	KRT2	6.86 ± 0.16	6.81 ± 0.02	6.74 ± 0.10	6.88 ± 0.08
TC17001473.hg.1	KRT20	4.70 ± 0.13	4.66 ± 0.07	4.73 ± 0.07	4.74 ± 0.11

TC17002901.hg.1	KRT222	4.39 ± 0.10	4.24 ± 0.03	4.29 ± 0.05	4.26 ± 0.04
TC17001469.hg.1	KRT223P	6.24 ± 0.33	6.10 ± 0.05	6.16 ± 0.21	6.13 ± 0.08
TC17001474.hg.1	KRT23	8.59 ± 0.10	8.24 ± 0.20	8.82 ± 0.23	8.33 ± 0.36
TC17001468.hg.1	KRT24	6.08 ± 0.15	6.00 ± 0.16	6.03 ± 0.12	6.01 ± 0.02
TC17002915.hg.1	KRT25	6.22 ± 0.15	6.04 ± 0.13	6.11 ± 0.12	6.16 ± 0.06
TC17001470.hg.1	KRT26	4.94 ± 0.15	4.78 ± 0.04	4.90 ± 0.14	4.85 ± 0.07
TC17002916.hg.1	KRT27	6.08 ± 0.14	6.00 ± 0.13	6.05 ± 0.08	6.02 ± 0.06
TC17001471.hg.1	KRT28	5.71 ± 0.17	5.56 ± 0.06	5.65 ± 0.15	5.60 ± 0.05
TC12001535.hg.1	KRT3	6.97 ± 0.22	6.92 ± 0.14	6.94 ± 0.18	6.98 ± 0.10
TC17001502.hg.1	KRT31	7.16 ± 0.25	7.25 ± 0.15	7.22 ± 0.14	7.38 ± 0.07
TC17001505.hg.1	KRT32	7.09 ± 0.23	7.11 ± 0.19	6.98 ± 0.16	7.23 ± 0.12
TC17001499.hg.1	KRT33A	7.30 ± 0.17	7.34 ± 0.23	7.30 ± 0.13	7.49 ± 0.06
TC17001500.hg.1	KRT33B	7.25 ± 0.17	7.25 ± 0.24	7.24 ± 0.19	7.35 ± 0.07
TC17001501.hg.1	KRT34	7.14 ± 0.17	7.13 ± 0.19	7.05 ± 0.20	7.28 ± 0.08
TC17001506.hg.1	KRT35	6.98 ± 0.16	6.98 ± 0.18	6.89 ± 0.18	7.04 ± 0.10
TC17001507.hg.1	KRT36	7.31 ± 0.21	7.31 ± 0.16	7.26 ± 0.21	7.38 ± 0.03
TC17001503.hg.1	KRT37	7.24 ± 0.17	7.20 ± 0.17	7.20 ± 0.12	7.31 ± 0.04
TC17001504.hg.1	KRT38	6.73 ± 0.16	6.69 ± 0.22	6.71 ± 0.10	6.85 ± 0.04
TC17002918.hg.1	KRT39	5.82 ± 0.13	5.74 ± 0.13	5.76 ± 0.14	5.82 ± 0.06
TC12001536.hg.1	KRT4	7.39 ± 0.16	7.41 ± 0.15	7.42 ± 0.17	7.52 ± 0.01
TC17001475.hg.1	KRT40	6.01 ± 0.16	5.91 ± 0.11	5.93 ± 0.06	5.98 ± 0.04
TC17001515.hg.1	KRT42P	8.13 ± 0.19	8.12 ± 0.07	8.09 ± 0.13	8.21 ± 0.07
TC12001526.hg.1	KRT5	7.20 ± 0.17	7.29 ± 0.15	7.18 ± 0.04	7.35 ± 0.08
TC12001525.hg.1	KRT6A	7.53 ± 0.17	7.57 ± 0.12	7.54 ± 0.13	7.74 ± 0.15
TC12001523.hg.1	KRT6B	6.89 ± 0.12	6.95 ± 0.08	6.94 ± 0.10	7.03 ± 0.12
TC12001524.hg.1	KRT6C	7.75 ± 0.15	7.84 ± 0.14	7.79 ± 0.12	7.99 ± 0.09
TC12000418.hg.1	KRT7	8.06 ± 0.25	8.05 ± 0.17	8.03 ± 0.20	8.18 ± 0.08
TC12001527.hg.1	KRT71	7.13 ± 0.14	7.03 ± 0.14	7.07 ± 0.12	7.13 ± 0.11
TC12001529.hg.1	KRT72	7.53 ± 0.13	7.53 ± 0.08	7.46 ± 0.10	7.61 ± 0.12
TC12001530.hg.1	KRT73	7.14 ± 0.09	7.23 ± 0.01	7.04 ± 0.20	7.19 ± 0.15
TC12001528.hg.1	KRT74	7.02 ± 0.24	6.99 ± 0.18	6.96 ± 0.19	7.06 ± 0.02

TC12001522.hg.1	KRT75	6.32 ± 0.16	6.23 ± 0.18	6.25 ± 0.11	6.31 ± 0.08
TC12001534.hg.1	KRT76	7.29 ± 0.15	7.25 ± 0.11	7.25 ± 0.08	7.32 ± 0.04
TC12001533.hg.1	KRT77	7.16 ± 0.17	7.27 ± 0.16	7.30 ± 0.20	7.36 ± 0.08
TC12001538.hg.1	KRT78	8.20 ± 0.21	8.04 ± 0.19	8.09 ± 0.17	8.11 ± 0.04
TC12001537.hg.1	KRT79	7.40 ± 0.15	7.31 ± 0.15	7.35 ± 0.10	7.40 ± 0.06
TC08000415.hg.1	KRT8	8.11 ± 0.32	8.19 ± 0.09	8.06 ± 0.20	8.34 ± 0.12
TC12001539.hg.1	KRT8	8.02 ± 0.21	8.10 ± 0.04	8.03 ± 0.14	8.20 ± 0.13
TC12001513.hg.1	KRT80	7.31 ± 0.21	7.31 ± 0.18	7.26 ± 0.14	7.38 ± 0.04
TC12003227.hg.1	KRT81	7.65 ± 0.24	7.67 ± 0.16	7.64 ± 0.19	7.72 ± 0.02
TC12001521.hg.1	KRT82	7.43 ± 0.16	7.39 ± 0.19	7.34 ± 0.13	7.43 ± 0.02
TC12003228.hg.1	KRT83	7.87 ± 0.13	7.88 ± 0.12	7.91 ± 0.10	7.96 ± 0.05
TC12001520.hg.1	KRT84	6.90 ± 0.22	6.90 ± 0.20	6.85 ± 0.21	6.99 ± 0.02
TC12001519.hg.1	KRT85	7.91 ± 0.12	7.92 ± 0.15	7.90 ± 0.16	8.01 ± 0.03
TC12000419.hg.1	KRT86	7.63 ± 0.20	7.63 ± 0.12	7.65 ± 0.16	7.68 ± 0.02
TC09000504.hg.1	KRT8P11	7.55 ± 0.16	7.56 ± 0.12	7.53 ± 0.24	7.67 ± 0.01
TC03000876.hg.1	KRT8P12	7.55 ± 0.07	7.55 ± 0.00	7.53 ± 0.07	7.51 ± 0.06
TC11000168.hg.1	KRT8P41	5.88 ± 0.10	6.07 ± 0.14	6.11 ± 0.18	6.12 ± 0.03
TC17001512.hg.1	KRT9	6.22 ± 0.18	6.10 ± 0.11	6.21 ± 0.09	6.20 ± 0.03
TC21000527.hg.1	KRTAP10-1	8.79 ± 0.19	8.95 ± 0.28	8.75 ± 0.20	9.20 ± 0.21
TC21000234.hg.1	KRTAP10-10	7.90 ± 0.26	7.96 ± 0.31	7.84 ± 0.07	8.29 ± 0.28
TC21001073.hg.1	KRTAP10-11	7.66 ± 0.22	7.70 ± 0.31	7.57 ± 0.08	7.89 ± 0.19
TC21000237.hg.1	KRTAP10-12	8.21 ± 0.20	8.42 ± 0.27	8.22 ± 0.17	8.67 ± 0.21
TC21000528.hg.1	KRTAP10-2	8.36 ± 0.15	8.51 ± 0.36	8.39 ± 0.18	8.79 ± 0.17
TC21000529.hg.1	KRTAP10-3	8.65 ± 0.19	8.83 ± 0.35	8.71 ± 0.13	9.09 ± 0.15
TC21000230.hg.1	KRTAP10-4	7.41 ± 0.15	7.58 ± 0.19	7.50 ± 0.18	7.76 ± 0.12
TC21000530.hg.1	KRTAP10-5	7.90 ± 0.20	7.82 ± 0.19	7.66 ± 0.18	7.99 ± 0.23
TC21000531.hg.1	KRTAP10-6	8.15 ± 0.14	8.19 ± 0.23	8.11 ± 0.13	8.39 ± 0.22
TC21000231.hg.1	KRTAP10-7	7.83 ± 0.15	7.82 ± 0.15	7.85 ± 0.08	7.99 ± 0.08
TC21000232.hg.1	KRTAP10-8	8.53 ± 0.17	8.72 ± 0.37	8.42 ± 0.33	8.81 ± 0.12
TC21000233.hg.1	KRTAP10-9	8.40 ± 0.17	8.47 ± 0.31	8.30 ± 0.20	8.64 ± 0.12
TC17001482.hg.1	KRTAP1-1	6.32 ± 0.02	6.25 ± 0.18	6.40 ± 0.14	6.40 ± 0.04

TC17002903.hg.1	KRTAP1-1	8.82 ± 0.19	8.77 ± 0.08	8.83 ± 0.14	8.91 ± 0.19
TC21000387.hg.1	KRTAP11-1	6.87 ± 0.32	6.92 ± 0.08	6.70 ± 0.16	7.11 ± 0.29
TC21000534.hg.1	KRTAP12-1	7.23 ± 0.18	7.27 ± 0.21	7.10 ± 0.14	7.50 ± 0.14
TC21000533.hg.1	KRTAP12-2	9.25 ± 0.12	9.30 ± 0.17	9.17 ± 0.19	9.47 ± 0.16
TC21000236.hg.1	KRTAP12-3	8.59 ± 0.04	8.61 ± 0.09	8.51 ± 0.05	8.72 ± 0.16
TC21001074.hg.1	KRTAP12-3	8.24 ± 0.14	8.41 ± 0.25	8.13 ± 0.21	8.56 ± 0.19
TC21000532.hg.1	KRTAP12-4	8.11 ± 0.10	8.30 ± 0.21	8.18 ± 0.14	8.46 ± 0.15
TC17002902.hg.1	KRTAP1-3	5.65 ± 0.22	5.69 ± 0.16	5.81 ± 0.07	5.86 ± 0.18
TC21000101.hg.1	KRTAP13-1	5.61 ± 0.15	5.67 ± 0.12	5.70 ± 0.24	5.74 ± 0.07
TC21000369.hg.1	KRTAP13-2	5.39 ± 0.07	5.37 ± 0.08	5.36 ± 0.04	5.42 ± 0.13
TC21000371.hg.1	KRTAP13-3	5.99 ± 0.01	6.00 ± 0.33	5.86 ± 0.13	6.20 ± 0.23
TC21000102.hg.1	KRTAP13-4	4.61 ± 0.33	4.37 ± 0.07	4.42 ± 0.13	4.43 ± 0.15
TC17001480.hg.1	KRTAP1-4	8.12 ± 0.07	8.09 ± 0.14	8.17 ± 0.15	8.20 ± 0.10
TC17001479.hg.1	KRTAP1-5	5.51 ± 0.14	5.52 ± 0.12	5.55 ± 0.10	5.69 ± 0.17
TC21000103.hg.1	KRTAP15-1	6.00 ± 0.08	5.99 ± 0.08	5.85 ± 0.06	6.02 ± 0.15
TC17001497.hg.1	KRTAP16-1	7.38 ± 0.36	7.60 ± 0.11	7.46 ± 0.20	7.85 ± 0.35
TC17001498.hg.1	KRTAP17-1	7.42 ± 0.20	7.25 ± 0.18	7.31 ± 0.17	7.39 ± 0.03
TC21000372.hg.1	KRTAP19-1	6.11 ± 0.19	5.98 ± 0.14	5.95 ± 0.11	6.14 ± 0.24
TC21000373.hg.1	KRTAP19-2	5.79 ± 0.22	5.74 ± 0.03	5.64 ± 0.14	5.85 ± 0.04
TC21000374.hg.1	KRTAP19-3	4.63 ± 0.24	4.51 ± 0.15	4.53 ± 0.05	4.79 ± 0.22
TC21000375.hg.1	KRTAP19-4	5.04 ± 0.10	4.97 ± 0.10	5.23 ± 0.11	5.08 ± 0.30
TC21000376.hg.1	KRTAP19-5	5.70 ± 0.04	5.64 ± 0.21	5.62 ± 0.18	5.75 ± 0.10
TC21000377.hg.1	KRTAP19-6	4.21 ± 0.17	3.87 ± 0.08	3.86 ± 0.11	4.01 ± 0.14
TC21000378.hg.1	KRTAP19-7	5.66 ± 0.33	5.55 ± 0.15	5.56 ± 0.14	5.75 ± 0.24
TC21000389.hg.1	KRTAP19-8	5.19 ± 0.20	5.05 ± 0.14	5.17 ± 0.03	5.17 ± 0.20
TC21000106.hg.1	KRTAP20-1	6.77 ± 0.28	6.72 ± 0.10	6.48 ± 0.22	6.89 ± 0.13
TC21000108.hg.1	KRTAP20-2	6.62 ± 0.08	6.60 ± 0.13	6.50 ± 0.16	6.77 ± 0.23
TC21000109.hg.1	KRTAP20-3	5.04 ± 0.17	5.09 ± 0.18	4.97 ± 0.17	5.27 ± 0.09
TC21000107.hg.1	KRTAP20-4	6.64 ± 0.18	6.59 ± 0.26	6.56 ± 0.10	6.81 ± 0.13
TC17001483.hg.1	KRTAP2-1	8.92 ± 0.17	9.18 ± 0.38	8.99 ± 0.26	9.45 ± 0.16
TC21000384.hg.1	KRTAP21-1	4.47 ± 0.14	4.54 ± 0.06	4.47 ± 0.13	4.66 ± 0.20

TC21000383.hg.1	KRTAP21-2	5.18 ± 0.35	5.02 ± 0.07	5.05 ± 0.05	5.34 ± 0.42
TC21000382.hg.1	KRTAP21-3	4.26 ± 0.13	4.23 ± 0.10	4.18 ± 0.18	4.36 ± 0.07
TC17001484.hg.1	KRTAP2-2	8.47 ± 0.26	8.55 ± 0.31	8.48 ± 0.17	8.83 ± 0.18
TC21000105.hg.1	KRTAP22-1	4.22 ± 0.33	4.08 ± 0.08	4.00 ± 0.09	4.06 ± 0.11
TC21000379.hg.1	KRTAP22-2	4.65 ± 0.11	4.27 ± 0.09	4.43 ± 0.11	4.46 ± 0.11
TC17001485.hg.1	KRTAP2-3	5.36 ± 0.19	5.15 ± 0.10	5.14 ± 0.18	5.35 ± 0.44
TC21000368.hg.1	KRTAP23-1	7.46 ± 0.13	7.33 ± 0.30	7.46 ± 0.13	7.61 ± 0.23
TC17001486.hg.1	KRTAP2-4	4.56 ± 0.28	4.57 ± 0.03	4.53 ± 0.16	4.77 ± 0.18
TC21000364.hg.1	KRTAP24-1	4.79 ± 0.33	4.57 ± 0.21	4.79 ± 0.12	4.70 ± 0.09
TC21000365.hg.1	KRTAP25-1	4.71 ± 0.17	4.48 ± 0.06	4.57 ± 0.09	4.70 ± 0.30
TC21000366.hg.1	KRTAP26-1	6.23 ± 0.18	6.35 ± 0.18	6.31 ± 0.06	6.38 ± 0.12
TC21000367.hg.1	KRTAP27-1	5.71 ± 0.13	5.92 ± 0.27	5.93 ± 0.25	6.07 ± 0.08
TC17001496.hg.1	KRTAP29-1	6.42 ± 0.31	6.49 ± 0.10	6.54 ± 0.09	6.67 ± 0.32
TC17001478.hg.1	KRTAP3-1	5.91 ± 0.05	6.00 ± 0.34	5.99 ± 0.21	6.33 ± 0.23
TC17001477.hg.1	KRTAP3-2	6.21 ± 0.13	6.29 ± 0.24	6.25 ± 0.09	6.42 ± 0.31
TC17001476.hg.1	KRTAP3-3	4.83 ± 0.14	4.69 ± 0.16	4.71 ± 0.17	4.82 ± 0.17
TC17002904.hg.1	KRTAP4-1	6.64 ± 0.09	6.59 ± 0.08	6.60 ± 0.11	6.82 ± 0.15
TC17001489.hg.1	KRTAP4-11	4.65 ± 0.33	4.53 ± 0.09	4.48 ± 0.07	4.85 ± 0.20
TC17001490.hg.1	KRTAP4-12	6.80 ± 0.02	6.81 ± 0.22	6.80 ± 0.08	7.12 ± 0.20
TC17001488.hg.1	KRTAP4-16P	8.11 ± 0.27	8.14 ± 0.27	8.05 ± 0.10	8.45 ± 0.25
TC17002905.hg.1	KRTAP4-2	5.55 ± 0.11	5.55 ± 0.11	5.48 ± 0.05	5.69 ± 0.21
TC17001494.hg.1	KRTAP4-3	5.87 ± 0.08	5.95 ± 0.18	5.78 ± 0.10	6.04 ± 0.07
TC17001493.hg.1	KRTAP4-4	7.29 ± 0.25	7.31 ± 0.35	7.25 ± 0.14	7.57 ± 0.23
TC17001492.hg.1	KRTAP4-5	7.03 ± 0.34	7.19 ± 0.47	6.96 ± 0.21	7.48 ± 0.18
TC17001491.hg.1	KRTAP4-6	5.94 ± 0.13	6.23 ± 0.20	6.05 ± 0.13	6.39 ± 0.09
TC17000502.hg.1	KRTAP4-7	8.05 ± 0.17	8.04 ± 0.18	7.96 ± 0.04	8.19 ± 0.19
TC17001487.hg.1	KRTAP4-8	8.70 ± 0.29	8.76 ± 0.20	8.66 ± 0.04	9.03 ± 0.31
TC11001261.hg.1	KRTAP5-1	8.39 ± 0.31	8.78 ± 0.30	8.44 ± 0.33	9.01 ± 0.27
TC11000738.hg.1	KRTAP5-10	8.96 ± 0.24	9.03 ± 0.15	8.92 ± 0.13	9.20 ± 0.16
TC11002039.hg.1	KRTAP5-11	8.91 ± 0.29	9.09 ± 0.21	8.90 ± 0.12	9.34 ± 0.30
TC11002040.hg.1	KRTAP5-11	7.04 ± 0.15	6.96 ± 0.12	6.96 ± 0.07	7.16 ± 0.16

TC11001262.hg.1	KRTAP5-2	7.71 ± 0.05	7.82 ± 0.22	7.72 ± 0.11	8.03 ± 0.09
TC11001263.hg.1	KRTAP5-4	9.49 ± 0.24	9.62 ± 0.19	9.47 ± 0.15	9.81 ± 0.19
TC11000046.hg.1	KRTAP5-5	8.44 ± 0.25	8.67 ± 0.28	8.39 ± 0.13	8.88 ± 0.17
TC11000049.hg.1	KRTAP5-6	7.30 ± 0.04	7.50 ± 0.17	7.38 ± 0.12	7.91 ± 0.34
TC11000735.hg.1	KRTAP5-7	7.76 ± 0.31	7.82 ± 0.02	7.75 ± 0.10	7.88 ± 0.08
TC11000736.hg.1	KRTAP5-8	7.84 ± 0.37	8.03 ± 0.24	7.88 ± 0.16	8.27 ± 0.25
TC11000737.hg.1	KRTAP5-9	6.63 ± 0.13	6.57 ± 0.07	6.62 ± 0.05	6.63 ± 0.12
TC11000045.hg.1	KRTAP5-AS1	7.43 ± 0.15	7.50 ± 0.27	7.50 ± 0.27	7.61 ± 0.05
TC21000381.hg.1	KRTAP6-1	8.25 ± 0.21	8.26 ± 0.08	8.26 ± 0.12	8.52 ± 0.28
TC21000380.hg.1	KRTAP6-2	6.69 ± 0.26	6.40 ± 0.22	6.45 ± 0.27	6.60 ± 0.12
TC21000104.hg.1	KRTAP6-3	5.92 ± 0.11	6.00 ± 0.14	5.94 ± 0.25	6.18 ± 0.20
TC21000386.hg.1	KRTAP7-1	6.09 ± 0.16	5.95 ± 0.09	5.99 ± 0.02	5.86 ± 0.06
TC21000385.hg.1	KRTAP8-1	6.28 ± 0.23	6.10 ± 0.18	6.07 ± 0.09	6.14 ± 0.10
TC17000503.hg.1	KRTAP9-1	8.81 ± 0.18	8.91 ± 0.18	8.79 ± 0.12	9.14 ± 0.26
TC17000504.hg.1	KRTAP9-2	7.27 ± 0.18	7.31 ± 0.06	7.22 ± 0.15	7.59 ± 0.29
TC17000505.hg.1	KRTAP9-3	5.85 ± 0.16	5.68 ± 0.24	5.71 ± 0.09	5.83 ± 0.24
TC17000507.hg.1	KRTAP9-4	7.22 ± 0.10	7.30 ± 0.09	7.23 ± 0.18	7.51 ± 0.25
TC17000509.hg.1	KRTAP9-6	5.89 ± 0.25	5.85 ± 0.22	5.76 ± 0.21	6.04 ± 0.16
TC17000510.hg.1	KRTAP9-7	5.20 ± 0.14	5.11 ± 0.12	5.12 ± 0.15	5.27 ± 0.25
TC17000506.hg.1	KRTAP9-8	5.83 ± 0.30	6.07 ± 0.11	5.95 ± 0.13	6.32 ± 0.36
TC17000508.hg.1	KRTAP9-9	4.74 ± 0.17	4.72 ± 0.06	4.75 ± 0.14	4.76 ± 0.22
TC01006382.hg.1	KRTCAP2	9.30 ± 0.17	8.99 ± 0.05	9.01 ± 0.13	8.88 ± 0.02
TC02000175.hg.1	KRTCAP3	7.71 ± 0.09	7.76 ± 0.12	7.67 ± 0.11	7.77 ± 0.02
TC19001444.hg.1	KRTDAP	6.36 ± 0.07	6.36 ± 0.09	6.31 ± 0.11	6.46 ± 0.10
TC17000311.hg.1	KSR1	8.67 ± 0.11	8.70 ± 0.04	8.93 ± 0.21	8.71 ± 0.05
TC12002015.hg.1	KSR2	7.07 ± 0.11	6.98 ± 0.17	7.00 ± 0.13	7.08 ± 0.05
TC01002656.hg.1	KTI12	7.56 ± 0.30	7.59 ± 0.10	7.26 ± 0.16	7.48 ± 0.17
TC14000326.hg.1	KTN1	8.03 ± 0.67	8.56 ± 0.19	8.27 ± 0.56	8.40 ± 0.15
TC14001161.hg.1	KTN1-AS1	5.51 ± 0.11	5.49 ± 0.05	5.52 ± 0.05	5.49 ± 0.03
TC06000042.hg.1	KU-MEL-3	5.65 ± 0.14	5.58 ± 0.22	5.58 ± 0.07	5.83 ± 0.23
TC19000359.hg.1	KXD1	9.80 ± 0.14	9.62 ± 0.05	9.69 ± 0.08	9.58 ± 0.07

TC03001806.hg.1	KY	6.97 ± 0.13	6.92 ± 0.11	6.98 ± 0.11	7.01 ± 0.05
TC02000902.hg.1	KYNU	7.17 ± 0.23	7.18 ± 0.23	7.40 ± 0.25	7.06 ± 0.17
TC0X002307.hg.1	L1CAM	7.74 ± 0.22	7.70 ± 0.20	7.66 ± 0.17	7.82 ± 0.07
TC01000706.hg.1	L1TD1	5.60 ± 0.07	5.51 ± 0.08	5.61 ± 0.07	5.59 ± 0.08
TC14001117.hg.1	L2HGDH	6.22 ± 0.05	6.17 ± 0.02	6.10 ± 0.03	6.11 ± 0.01
TC14002325.hg.1	L3HYPDH	6.95 ± 0.09	6.85 ± 0.10	6.82 ± 0.11	6.79 ± 0.11
TC20001723.hg.1	L3MBTL1	7.65 ± 0.12	7.63 ± 0.15	7.61 ± 0.14	7.72 ± 0.06
TC22000334.hg.1	L3MBTL2	8.49 ± 0.07	8.31 ± 0.02	8.25 ± 0.08	8.26 ± 0.02
TC06000978.hg.1	L3MBTL3	7.98 ± 0.23	8.21 ± 0.13	8.03 ± 0.23	8.13 ± 0.05
TC18000304.hg.1	L3MBTL4	6.09 ± 0.05	6.07 ± 0.05	6.03 ± 0.05	6.10 ± 0.07
TC13000161.hg.1	LACC1	6.74 ± 0.03	6.77 ± 0.11	6.99 ± 0.16	6.77 ± 0.13
TC06000866.hg.1	LACE1	5.56 ± 0.09	5.53 ± 0.06	5.51 ± 0.10	5.50 ± 0.02
TC12001576.hg.1	LACRT	6.17 ± 0.20	6.13 ± 0.15	6.09 ± 0.13	6.22 ± 0.06
TC15000580.hg.1	LACTB	8.39 ± 0.39	8.43 ± 0.26	9.20 ± 0.63	8.47 ± 0.42
TC08001313.hg.1	LACTB2	5.74 ± 0.12	5.56 ± 0.13	5.69 ± 0.14	5.38 ± 0.14
TC01002333.hg.1	LACTBL1	7.90 ± 0.24	7.89 ± 0.33	7.83 ± 0.25	8.01 ± 0.02
TC01006280.hg.1	LAD1	7.43 ± 0.25	7.40 ± 0.22	7.32 ± 0.20	7.46 ± 0.09
TC12000091.hg.1	LAG3	8.26 ± 0.15	8.20 ± 0.17	8.09 ± 0.30	8.13 ± 0.10
TC0X001534.hg.1	LAGE3	8.82 ± 0.26	8.70 ± 0.09	8.69 ± 0.20	8.69 ± 0.02
TC19001844.hg.1	LAIR1	9.02 ± 0.15	8.73 ± 0.13	9.20 ± 0.07	8.69 ± 0.11
TC19000879.hg.1	LAIR2	8.54 ± 0.17	8.35 ± 0.13	8.51 ± 0.15	8.39 ± 0.09
TC12001451.hg.1	LALBA	5.49 ± 0.15	5.51 ± 0.21	5.48 ± 0.14	5.74 ± 0.14
TC18000309.hg.1	LAMA1	6.37 ± 0.17	6.29 ± 0.14	6.30 ± 0.11	6.38 ± 0.05
TC06000976.hg.1	LAMA2	5.48 ± 0.12	5.41 ± 0.10	5.41 ± 0.05	5.47 ± 0.05
TC18000103.hg.1	LAMA3	6.56 ± 0.16	6.48 ± 0.13	6.47 ± 0.09	6.57 ± 0.06
TC06002025.hg.1	LAMA4	5.82 ± 0.17	5.71 ± 0.08	5.74 ± 0.06	5.78 ± 0.07
TC20001008.hg.1	LAMA5	8.24 ± 0.22	8.23 ± 0.21	8.17 ± 0.19	8.33 ± 0.05
TC07001747.hg.1	LAMB1	6.27 ± 0.13	6.17 ± 0.07	6.20 ± 0.10	6.24 ± 0.04
TC03001402.hg.1	LAMB2	7.79 ± 0.14	7.77 ± 0.15	7.77 ± 0.12	7.88 ± 0.05
TC03001403.hg.1	LAMB2P1	6.79 ± 0.06	6.75 ± 0.06	6.53 ± 0.11	6.90 ± 0.14
TC01003784.hg.1	LAMB3	7.39 ± 0.15	7.44 ± 0.13	7.40 ± 0.13	7.53 ± 0.01

TC07001749.hg.1	LAMB4	5.78 ± 0.11	5.74 ± 0.11	5.75 ± 0.05	5.82 ± 0.06
TC01001583.hg.1	LAMC1	7.21 ± 0.10	6.86 ± 0.08	6.91 ± 0.10	6.81 ± 0.02
TC01001585.hg.1	LAMC2	6.52 ± 0.14	6.48 ± 0.13	6.46 ± 0.10	6.53 ± 0.05
TC09000744.hg.1	LAMC3	8.11 ± 0.21	8.12 ± 0.20	8.07 ± 0.19	8.22 ± 0.04
TC13000421.hg.1	LAMP1	9.77 ± 0.21	9.61 ± 0.12	9.75 ± 0.19	9.56 ± 0.09
TC0X001325.hg.1	LAMP2	9.98 ± 0.23	10.05 ± 0.21	10.50 ± 0.34	9.99 ± 0.31
TC03002054.hg.1	LAMP3	7.26 ± 0.48	7.02 ± 0.23	7.33 ± 0.57	6.98 ± 0.09
TC20000077.hg.1	LAMP5	7.19 ± 0.43	6.67 ± 0.11	6.81 ± 0.03	6.71 ± 0.05
TC11002053.hg.1	LAMTOR1	8.73 ± 0.16	8.45 ± 0.08	8.68 ± 0.21	8.36 ± 0.04
TC01001310.hg.1	LAMTOR2	8.40 ± 0.22	8.19 ± 0.06	8.32 ± 0.13	8.17 ± 0.08
TC04001416.hg.1	LAMTOR3	8.56 ± 0.16	8.56 ± 0.15	8.87 ± 0.35	8.53 ± 0.11
TC07000615.hg.1	LAMTOR4	7.87 ± 0.17	7.63 ± 0.07	7.68 ± 0.13	7.73 ± 0.02
TC01002972.hg.1	LAMTOR5	8.31 ± 0.01	8.34 ± 0.22	8.46 ± 0.32	8.25 ± 0.05
TC01000965.hg.1	LAMTOR5-AS1	5.31 ± 0.06	5.08 ± 0.02	5.19 ± 0.13	5.11 ± 0.03
TC02002733.hg.1	LANCL1	7.91 ± 0.24	8.15 ± 0.15	7.74 ± 0.13	7.96 ± 0.07
TC07000329.hg.1	LANCL2	7.56 ± 0.02	7.64 ± 0.21	7.31 ± 0.11	7.67 ± 0.07
TC0X000168.hg.1	LANCL3	6.88 ± 0.10	6.42 ± 0.12	6.83 ± 0.11	6.54 ± 0.02
TC04000151.hg.1	LAP3	8.69 ± 0.31	8.46 ± 0.14	8.70 ± 0.62	8.28 ± 0.22
TC02001616.hg.1	LAPTM4A	10.38 ± 0.23	10.51 ± 0.14	10.62 ± 0.23	10.46 ± 0.12
TC08000597.hg.1	LAPTM4B	7.65 ± 0.08	7.20 ± 0.10	7.64 ± 0.23	7.18 ± 0.08
TC01002441.hg.1	LAPTM5	13.03 ± 0.16	13.21 ± 0.04	13.13 ± 0.14	13.23 ± 0.10
TC22000688.hg.1	LARGE	6.56 ± 0.14	6.55 ± 0.06	6.45 ± 0.05	6.55 ± 0.03
TC22000252.hg.1	LARGE-AS1	5.46 ± 0.06	5.30 ± 0.00	5.42 ± 0.02	5.37 ± 0.05
TC22000691.hg.1	LARGE-IT1	7.22 ± 0.25	7.19 ± 0.18	7.04 ± 0.14	7.39 ± 0.17
TC05000855.hg.1	LARP1	9.63 ± 0.14	9.48 ± 0.03	9.49 ± 0.08	9.41 ± 0.13
TC04000648.hg.1	LARP1B	6.52 ± 0.09	6.35 ± 0.08	6.38 ± 0.11	6.23 ± 0.10
TC12000392.hg.1	LARP4	7.87 ± 0.22	7.75 ± 0.19	7.82 ± 0.19	7.52 ± 0.16
TC10000964.hg.1	LARP4B	9.33 ± 0.12	9.23 ± 0.12	9.45 ± 0.21	9.05 ± 0.14
TC15001615.hg.1	LARP6	7.10 ± 0.17	6.99 ± 0.06	7.03 ± 0.15	7.07 ± 0.03
TC04000583.hg.1	LARP7	7.55 ± 0.42	7.83 ± 0.17	7.81 ± 0.46	7.72 ± 0.04
TC05001900.hg.1	LARS	8.53 ± 0.40	8.66 ± 0.23	8.31 ± 0.28	8.35 ± 0.13

TC03000247.hg.1	LARS2	7.59 ± 0.06	7.57 ± 0.01	7.43 ± 0.07	7.52 ± 0.01
TC03001348.hg.1	LARS2-AS1	6.62 ± 0.21	6.63 ± 0.06	6.62 ± 0.08	6.69 ± 0.04
TC0X001098.hg.1	LAS1L	8.07 ± 0.06	8.14 ± 0.07	7.77 ± 0.08	8.06 ± 0.06
TC17000469.hg.1	LASP1	9.94 ± 0.13	9.87 ± 0.06	9.98 ± 0.16	9.78 ± 0.17
TC16000314.hg.1	LAT	9.43 ± 0.07	9.39 ± 0.08	9.15 ± 0.12	9.36 ± 0.03
TC07000453.hg.1	LAT2	9.41 ± 0.26	8.97 ± 0.10	9.43 ± 0.17	8.84 ± 0.30
TC06002214.hg.1	LATS1	8.81 ± 0.25	8.81 ± 0.09	8.81 ± 0.36	8.78 ± 0.03
TC13000463.hg.1	LATS2	8.31 ± 0.16	8.21 ± 0.03	8.46 ± 0.17	8.26 ± 0.15
TC13000029.hg.1	LATS2-AS1	5.65 ± 0.13	5.39 ± 0.05	5.51 ± 0.13	5.49 ± 0.07
TC01001691.hg.1	LAX1	8.42 ± 0.02	8.05 ± 0.11	7.91 ± 0.09	7.90 ± 0.05
TC11000997.hg.1	LAYN	6.77 ± 0.14	6.75 ± 0.09	6.68 ± 0.10	6.86 ± 0.10
TC02000199.hg.1	LBH	8.99 ± 0.07	8.96 ± 0.02	8.64 ± 0.13	8.78 ± 0.05
TC20000282.hg.1	LBP	6.42 ± 0.16	6.37 ± 0.17	6.33 ± 0.09	6.45 ± 0.10
TC01003887.hg.1	LBR	9.82 ± 0.20	9.88 ± 0.12	10.20 ± 0.36	9.78 ± 0.24
TC10001601.hg.1	LBX1	7.87 ± 0.42	7.92 ± 0.28	7.84 ± 0.33	7.94 ± 0.04
TC10000735.hg.1	LBX1-AS1	6.77 ± 0.28	6.77 ± 0.27	6.71 ± 0.20	6.88 ± 0.07
TC02004928.hg.1	LBX2	8.38 ± 0.21	8.26 ± 0.13	8.32 ± 0.17	8.27 ± 0.05
TC02000461.hg.1	LBX2-AS1	7.69 ± 0.11	7.57 ± 0.13	7.46 ± 0.15	7.53 ± 0.02
TC06001899.hg.1	LCA5	5.58 ± 0.15	5.39 ± 0.05	5.44 ± 0.12	5.39 ± 0.07
TC21000463.hg.1	LCA5L	5.26 ± 0.18	5.15 ± 0.05	5.16 ± 0.10	5.19 ± 0.03
TC01001241.hg.1	LCE1A	7.85 ± 0.20	7.86 ± 0.22	7.74 ± 0.21	8.01 ± 0.13
TC01001240.hg.1	LCE1B	6.84 ± 0.21	6.90 ± 0.25	6.90 ± 0.22	7.04 ± 0.14
TC01003251.hg.1	LCE1C	7.90 ± 0.08	7.85 ± 0.22	7.78 ± 0.18	8.03 ± 0.09
TC01001239.hg.1	LCE1D	7.49 ± 0.15	7.53 ± 0.15	7.46 ± 0.18	7.77 ± 0.17
TC01001238.hg.1	LCE1E	6.97 ± 0.12	7.01 ± 0.19	6.91 ± 0.24	7.22 ± 0.19
TC01001237.hg.1	LCE1F	7.56 ± 0.22	7.50 ± 0.02	7.53 ± 0.05	7.63 ± 0.28
TC01001233.hg.1	LCE2A	7.37 ± 0.25	7.62 ± 0.29	7.32 ± 0.05	7.83 ± 0.18
TC01006274.hg.1	LCE2B	7.86 ± 0.29	7.95 ± 0.19	7.73 ± 0.09	8.17 ± 0.16
TC01006273.hg.1	LCE2C	7.10 ± 0.25	7.36 ± 0.18	7.14 ± 0.13	7.56 ± 0.21
TC01001231.hg.1	LCE2D	6.72 ± 0.17	6.79 ± 0.14	6.67 ± 0.18	7.03 ± 0.08
TC01003250.hg.1	LCE3A	8.66 ± 0.14	8.65 ± 0.25	8.56 ± 0.22	8.82 ± 0.07

TC01001229.hg.1	LCE3B	9.08 ± 0.15	9.05 ± 0.16	9.00 ± 0.17	9.31 ± 0.17
TC01001228.hg.1	LCE3C	5.93 ± 0.23	6.06 ± 0.33	5.81 ± 0.33	6.20 ± 0.22
TC01003249.hg.1	LCE3D	8.10 ± 0.22	8.19 ± 0.22	8.16 ± 0.08	8.51 ± 0.25
TC01003248.hg.1	LCE3E	7.50 ± 0.24	7.73 ± 0.26	7.52 ± 0.23	7.90 ± 0.11
TC01001234.hg.1	LCE4A	6.45 ± 0.16	6.56 ± 0.27	6.58 ± 0.10	6.63 ± 0.14
TC01001226.hg.1	LCE5A	6.71 ± 0.23	6.56 ± 0.11	6.51 ± 0.18	6.67 ± 0.12
TC01001242.hg.1	LCE6A	6.41 ± 0.18	6.52 ± 0.21	6.44 ± 0.08	6.66 ± 0.03
TC01000426.hg.1	LCK	9.82 ± 0.10	9.72 ± 0.04	9.39 ± 0.25	9.52 ± 0.02
TC02000203.hg.1	LCLAT1	7.75 ± 0.17	7.90 ± 0.27	7.78 ± 0.24	7.60 ± 0.10
TC16000283.hg.1	LCMT1	8.45 ± 0.17	8.22 ± 0.17	8.14 ± 0.18	8.10 ± 0.08
TC15001276.hg.1	LCMT2	7.83 ± 0.04	7.70 ± 0.06	7.65 ± 0.09	7.72 ± 0.10
TC09000797.hg.1	LCN1	8.13 ± 0.27	7.96 ± 0.11	8.08 ± 0.23	8.05 ± 0.10
TC09002926.hg.1	LCN10	8.09 ± 0.23	8.01 ± 0.16	8.03 ± 0.24	8.11 ± 0.07
TC09000829.hg.1	LCN12	8.38 ± 0.19	8.45 ± 0.23	8.36 ± 0.19	8.55 ± 0.06
TC09001742.hg.1	LCN15	7.59 ± 0.24	7.58 ± 0.29	7.50 ± 0.23	7.68 ± 0.06
TC09000677.hg.1	LCN2	8.52 ± 0.30	7.90 ± 0.15	8.48 ± 0.22	7.96 ± 0.11
TC09002927.hg.1	LCN6	8.19 ± 0.24	8.16 ± 0.22	8.09 ± 0.16	8.25 ± 0.07
TC09001741.hg.1	LCN8	7.73 ± 0.22	7.69 ± 0.22	7.74 ± 0.07	7.79 ± 0.04
TC09000803.hg.1	LCN9	6.35 ± 0.23	6.39 ± 0.12	6.27 ± 0.22	6.47 ± 0.06
TC09002911.hg.1	LCNL1	8.22 ± 0.22	8.13 ± 0.23	8.15 ± 0.17	8.23 ± 0.05
TC04001070.hg.1	LCORL	6.81 ± 0.28	6.87 ± 0.19	6.86 ± 0.34	6.77 ± 0.12
TC13000642.hg.1	LCP1	11.29 ± 0.09	11.36 ± 0.05	11.61 ± 0.11	11.29 ± 0.14
TC05002050.hg.1	LCP2	10.83 ± 0.22	10.96 ± 0.13	11.18 ± 0.24	10.93 ± 0.25
TC02002374.hg.1	LCT	6.58 ± 0.19	6.53 ± 0.16	6.50 ± 0.15	6.60 ± 0.07
TC15001583.hg.1	LCTL	6.26 ± 0.15	6.15 ± 0.07	6.20 ± 0.13	6.18 ± 0.04
TC10001617.hg.1	LDB1	10.74 ± 0.11	10.68 ± 0.03	10.52 ± 0.13	10.68 ± 0.03
TC04001062.hg.1	LDB2	6.06 ± 0.04	5.94 ± 0.04	6.01 ± 0.02	6.01 ± 0.10
TC10002927.hg.1	LDB3	7.19 ± 0.19	7.14 ± 0.20	7.10 ± 0.15	7.24 ± 0.09
TC11000240.hg.1	LDHA	9.75 ± 0.12	9.71 ± 0.18	9.95 ± 0.16	9.49 ± 0.15
TC11000243.hg.1	LDHAL6A	5.37 ± 0.13	5.30 ± 0.05	5.35 ± 0.04	5.33 ± 0.01
TC15000451.hg.1	LDHAL6B	4.26 ± 0.20	3.99 ± 0.15	3.99 ± 0.01	3.99 ± 0.12

TC12000561.hg.1	LDHAL6CP	5.29 ± 0.12	4.99 ± 0.07	4.90 ± 0.14	5.05 ± 0.13
TC12001298.hg.1	LDHB	9.50 ± 0.07	9.69 ± 0.18	9.05 ± 0.09	9.55 ± 0.04
TC11000242.hg.1	LDHC	4.98 ± 0.15	4.94 ± 0.02	4.92 ± 0.08	4.99 ± 0.02
TC16001274.hg.1	LDHD	7.97 ± 0.19	7.99 ± 0.18	7.94 ± 0.16	8.12 ± 0.06
TC19000191.hg.1	LDLR	8.79 ± 0.23	8.41 ± 0.14	8.82 ± 0.06	8.31 ± 0.17
TC01002680.hg.1	LDLRAD1	7.28 ± 0.18	7.23 ± 0.09	7.20 ± 0.12	7.22 ± 0.06
TC01000274.hg.1	LDLRAD2	7.70 ± 0.13	7.61 ± 0.20	7.50 ± 0.15	7.69 ± 0.07
TC11000342.hg.1	LDLRAD3	8.15 ± 0.09	8.07 ± 0.08	8.15 ± 0.08	8.13 ± 0.04
TC18000071.hg.1	LDLRAD4	8.08 ± 0.03	8.12 ± 0.03	7.98 ± 0.17	8.17 ± 0.03
TC18000379.hg.1	LDLRAD4-AS1	7.69 ± 0.07	7.70 ± 0.13	7.67 ± 0.08	7.79 ± 0.05
TC01000323.hg.1	LDLRAP1	9.13 ± 0.00	8.95 ± 0.08	8.78 ± 0.15	8.90 ± 0.01
TC0X001438.hg.1	LDOC1	7.77 ± 0.03	7.77 ± 0.11	7.73 ± 0.20	7.84 ± 0.08
TC22000867.hg.1	LDOC1L	8.36 ± 0.26	8.30 ± 0.21	8.16 ± 0.22	8.33 ± 0.06
TC05000647.hg.1	LEAP2	6.49 ± 0.08	6.49 ± 0.02	6.46 ± 0.11	6.51 ± 0.09
TC13000691.hg.1	LECT1	5.89 ± 0.24	5.74 ± 0.07	5.79 ± 0.10	5.82 ± 0.04
TC13000692.hg.1	LECT1	3.92 ± 0.21	3.70 ± 0.03	3.76 ± 0.11	3.67 ± 0.08
TC05001809.hg.1	LECT2	4.84 ± 0.05	4.70 ± 0.08	4.77 ± 0.04	4.79 ± 0.08
TC04001459.hg.1	LEF1	9.92 ± 0.39	10.28 ± 0.04	9.59 ± 0.23	10.25 ± 0.08
TC04000557.hg.1	LEF1-AS1	7.35 ± 0.27	7.35 ± 0.08	7.10 ± 0.10	7.44 ± 0.10
TC01006387.hg.1	LEFTY1	8.35 ± 0.14	8.33 ± 0.18	8.28 ± 0.17	8.40 ± 0.03
TC01003895.hg.1	LEFTY2	8.78 ± 0.18	8.70 ± 0.17	8.71 ± 0.21	8.78 ± 0.03
TC03000854.hg.1	LEKR1	4.79 ± 0.15	4.68 ± 0.04	4.69 ± 0.05	4.70 ± 0.02
TC01001250.hg.1	LELP1	7.46 ± 0.22	7.49 ± 0.16	7.39 ± 0.15	7.55 ± 0.13
TC01003747.hg.1	LEMD1	5.81 ± 0.19	5.73 ± 0.14	5.73 ± 0.15	5.75 ± 0.04
TC01001707.hg.1	LEMD1-AS1	6.41 ± 0.10	6.52 ± 0.15	6.46 ± 0.11	6.61 ± 0.12
TC06001593.hg.1	LEMD2	8.38 ± 0.11	8.37 ± 0.10	8.30 ± 0.07	8.44 ± 0.05
TC12000581.hg.1	LEMD3	8.47 ± 0.29	8.48 ± 0.20	8.51 ± 0.39	8.38 ± 0.15
TC01001283.hg.1	LENEP	7.38 ± 0.09	7.34 ± 0.30	7.23 ± 0.07	7.50 ± 0.11
TC19001835.hg.1	LENG1	8.15 ± 0.25	7.96 ± 0.06	8.01 ± 0.19	8.01 ± 0.06
TC19000878.hg.1	LENG8	10.04 ± 0.16	10.11 ± 0.01	9.92 ± 0.23	10.24 ± 0.10
TC19001847.hg.1	LENG8-AS1	7.95 ± 0.02	7.82 ± 0.07	7.79 ± 0.09	7.84 ± 0.05

TC19001848.hg.1	LENG9	8.62 ± 0.25	8.58 ± 0.18	8.56 ± 0.33	8.63 ± 0.07
TC15001449.hg.1	LEO1	8.33 ± 0.15	8.15 ± 0.21	8.35 ± 0.22	7.93 ± 0.26
TC07000768.hg.1	LEP	6.86 ± 0.21	6.86 ± 0.28	6.79 ± 0.29	7.00 ± 0.05
TC01000730.hg.1	LEPR	6.90 ± 0.23	6.72 ± 0.10	7.04 ± 0.26	6.71 ± 0.10
TC01002574.hg.1	LEPRE1	8.01 ± 0.03	7.78 ± 0.06	7.86 ± 0.04	7.71 ± 0.02
TC03002106.hg.1	LEPREL1	5.61 ± 0.05	5.57 ± 0.04	5.61 ± 0.07	5.65 ± 0.04
TC03001044.hg.1	LEPREL1-AS1	8.12 ± 0.15	7.95 ± 0.09	7.98 ± 0.21	8.07 ± 0.06
TC12003232.hg.1	LEPREL2	8.06 ± 0.23	7.99 ± 0.16	7.98 ± 0.23	8.08 ± 0.03
TC17001517.hg.1	LEPREL4	8.18 ± 0.24	8.05 ± 0.11	8.08 ± 0.22	8.11 ± 0.00
TC08002578.hg.1	LEPROTL1	8.85 ± 0.23	8.98 ± 0.02	8.67 ± 0.25	9.03 ± 0.05
TC04000970.hg.1	LETM1	8.37 ± 0.07	8.29 ± 0.09	8.17 ± 0.12	8.29 ± 0.03
TC08000291.hg.1	LETM2	7.36 ± 0.08	7.10 ± 0.04	7.23 ± 0.12	7.05 ± 0.11
TC12000403.hg.1	LETMD1	8.08 ± 0.09	7.94 ± 0.10	7.80 ± 0.13	7.89 ± 0.03
TC19000556.hg.1	LEUTX	6.81 ± 0.14	6.63 ± 0.06	6.72 ± 0.16	6.66 ± 0.04
TC07000034.hg.1	LFNG	8.67 ± 0.09	8.60 ± 0.05	8.63 ± 0.13	8.73 ± 0.11
TC22000285.hg.1	LGALS1	9.48 ± 0.36	9.09 ± 0.14	9.43 ± 0.05	8.99 ± 0.14
TC11000571.hg.1	LGALS12	7.77 ± 0.09	7.58 ± 0.06	7.86 ± 0.23	7.78 ± 0.29
TC19000551.hg.1	LGALS13	5.01 ± 0.29	4.79 ± 0.12	4.80 ± 0.16	4.88 ± 0.13
TC19000555.hg.1	LGALS14	6.28 ± 0.04	6.20 ± 0.06	6.25 ± 0.04	6.20 ± 0.10
TC19000553.hg.1	LGALS16	5.57 ± 0.11	5.41 ± 0.04	5.53 ± 0.17	5.53 ± 0.08
TC19000554.hg.1	LGALS17A	5.61 ± 0.09	5.43 ± 0.07	5.47 ± 0.04	5.51 ± 0.07
TC22000775.hg.1	LGALS2	8.32 ± 0.17	8.14 ± 0.03	7.86 ± 0.19	7.97 ± 0.12
TC14000323.hg.1	LGALS3	9.31 ± 0.20	9.05 ± 0.08	9.16 ± 0.14	9.10 ± 0.25
TC17001924.hg.1	LGALS3BP	9.76 ± 0.64	9.09 ± 0.28	9.27 ± 0.24	8.91 ± 0.05
TC19001505.hg.1	LGALS4	7.48 ± 0.24	7.49 ± 0.15	7.42 ± 0.09	7.59 ± 0.05
TC19001503.hg.1	LGALS7	8.27 ± 0.37	8.23 ± 0.25	8.15 ± 0.35	8.29 ± 0.03
TC19000533.hg.1	LGALS7B	8.32 ± 0.36	8.28 ± 0.21	8.19 ± 0.37	8.32 ± 0.04
TC01001954.hg.1	LGALS8	8.81 ± 0.22	8.96 ± 0.13	9.36 ± 0.35	8.93 ± 0.12
TC01004005.hg.1	LGALS8-AS1	7.02 ± 0.13	7.05 ± 0.13	7.11 ± 0.05	7.01 ± 0.06
TC17000312.hg.1	LGALS9	10.40 ± 0.37	10.03 ± 0.25	10.46 ± 0.19	10.00 ± 0.17
TC17001254.hg.1	LGALS9B	10.26 ± 0.28	9.92 ± 0.19	10.38 ± 0.14	9.92 ± 0.10

TC17000217.hg.1	LGALS9C	9.95 ± 0.29	9.58 ± 0.20	10.10 ± 0.11	9.56 ± 0.12
TC02000366.hg.1	LGALSL	8.62 ± 0.03	8.23 ± 0.05	8.71 ± 0.11	8.17 ± 0.20
TC10000668.hg.1	LGI1	4.10 ± 0.09	4.07 ± 0.02	4.08 ± 0.05	4.06 ± 0.03
TC04001089.hg.1	LGI2	5.73 ± 0.11	5.63 ± 0.09	5.67 ± 0.11	5.67 ± 0.08
TC08001035.hg.1	LGI3	7.40 ± 0.20	7.43 ± 0.21	7.43 ± 0.22	7.53 ± 0.08
TC19001440.hg.1	LGI4	7.51 ± 0.17	7.53 ± 0.17	7.49 ± 0.09	7.63 ± 0.04
TC14001451.hg.1	LGMN	7.73 ± 0.13	7.59 ± 0.01	7.45 ± 0.11	7.67 ± 0.14
TC11001509.hg.1	LGR4	4.59 ± 0.15	4.45 ± 0.05	4.48 ± 0.06	4.44 ± 0.03
TC12000624.hg.1	LGR5	5.07 ± 0.09	4.98 ± 0.04	4.97 ± 0.04	5.04 ± 0.05
TC01001673.hg.1	LGR6	7.53 ± 0.08	7.49 ± 0.08	7.42 ± 0.12	7.57 ± 0.07
TC06001844.hg.1	LGSN	4.50 ± 0.15	4.26 ± 0.03	4.35 ± 0.06	4.27 ± 0.04
TC19001704.hg.1	LHB	8.32 ± 0.25	8.39 ± 0.23	8.30 ± 0.16	8.52 ± 0.03
TC02001830.hg.1	LHCGR	4.41 ± 0.08	4.26 ± 0.04	4.26 ± 0.09	4.30 ± 0.05
TC13000585.hg.1	LHFP	7.65 ± 0.24	7.74 ± 0.23	7.77 ± 0.20	7.84 ± 0.07
TC0X001285.hg.1	LHFPL1	5.95 ± 0.18	5.93 ± 0.20	5.97 ± 0.12	6.01 ± 0.03
TC05001518.hg.1	LHFPL2	7.48 ± 0.13	7.27 ± 0.08	7.54 ± 0.13	7.39 ± 0.04
TC07000675.hg.1	LHFPL3	5.95 ± 0.15	5.93 ± 0.13	5.92 ± 0.10	5.99 ± 0.12
TC07001725.hg.1	LHFPL3-AS1	5.95 ± 0.10	5.82 ± 0.03	5.92 ± 0.09	5.87 ± 0.07
TC07001726.hg.1	LHFPL3-AS2	6.21 ± 0.08	6.24 ± 0.03	6.04 ± 0.09	6.23 ± 0.07
TC03001148.hg.1	LHFPL4	8.03 ± 0.19	7.98 ± 0.21	7.91 ± 0.14	8.03 ± 0.05
TC06000522.hg.1	LHFPL5	7.67 ± 0.19	7.54 ± 0.21	7.54 ± 0.22	7.65 ± 0.06
TC10000905.hg.1	LHPP	8.10 ± 0.15	8.13 ± 0.14	7.98 ± 0.10	8.15 ± 0.07
TC17000442.hg.1	LHX1	7.90 ± 0.37	7.80 ± 0.21	7.81 ± 0.25	7.93 ± 0.05
TC17000443.hg.1	LHX1	7.64 ± 0.38	7.57 ± 0.20	7.52 ± 0.28	7.63 ± 0.07
TC09000633.hg.1	LHX2	7.45 ± 0.26	7.35 ± 0.22	7.33 ± 0.27	7.41 ± 0.02
TC09001725.hg.1	LHX3	8.09 ± 0.28	8.04 ± 0.22	7.97 ± 0.25	8.08 ± 0.06
TC01001561.hg.1	LHX4	7.24 ± 0.27	7.05 ± 0.22	7.08 ± 0.19	7.17 ± 0.00
TC12001990.hg.1	LHX5	6.87 ± 0.30	6.84 ± 0.20	6.75 ± 0.30	6.86 ± 0.03
TC09001555.hg.1	LHX6	7.65 ± 0.30	7.69 ± 0.24	7.62 ± 0.26	7.75 ± 0.02
TC01000774.hg.1	LHX8	6.65 ± 0.13	6.56 ± 0.13	6.58 ± 0.11	6.67 ± 0.09
TC01001638.hg.1	LHX9	6.05 ± 0.15	5.93 ± 0.13	6.02 ± 0.04	5.98 ± 0.04

TC04000230.hg.1	LIAS	6.74 ± 0.10	6.73 ± 0.10	6.64 ± 0.09	6.63 ± 0.03
TC22000647.hg.1	LIF	6.60 ± 0.16	6.55 ± 0.13	6.53 ± 0.09	6.62 ± 0.02
TC05001289.hg.1	LIFR	4.76 ± 0.06	4.64 ± 0.04	4.68 ± 0.04	4.68 ± 0.04
TC05000175.hg.1	LIFR-AS1	5.08 ± 0.02	5.04 ± 0.06	5.01 ± 0.08	5.15 ± 0.09
TC19001676.hg.1	LIG1	8.47 ± 0.07	8.45 ± 0.09	8.30 ± 0.16	8.41 ± 0.04
TC17000391.hg.1	LIG3	7.84 ± 0.05	7.73 ± 0.09	7.61 ± 0.02	7.72 ± 0.04
TC13000867.hg.1	LIG4	7.14 ± 0.15	7.15 ± 0.15	7.41 ± 0.41	7.15 ± 0.06
TC19002653.hg.1	LILRA1	9.64 ± 0.28	9.33 ± 0.12	9.92 ± 0.12	9.20 ± 0.28
TC19002652.hg.1	LILRA2	9.42 ± 0.32	9.01 ± 0.09	9.58 ± 0.23	8.88 ± 0.27
TC19002615.hg.1	LILRA3	9.49 ± 0.26	9.12 ± 0.10	9.80 ± 0.29	9.15 ± 0.20
TC19002720.hg.1	LILRA4	8.04 ± 0.05	7.92 ± 0.12	8.06 ± 0.10	7.99 ± 0.01
TC19002719.hg.1	LILRA5	9.58 ± 0.60	9.12 ± 0.10	10.30 ± 0.37	9.12 ± 0.17
TC19002654.hg.1	LILRB1	9.45 ± 0.25	9.11 ± 0.12	9.77 ± 0.19	9.05 ± 0.21
TC19001842.hg.1	LILRB2	10.34 ± 0.28	10.06 ± 0.12	10.81 ± 0.20	10.03 ± 0.26
TC19002614.hg.1	LILRB3	9.83 ± 0.34	9.45 ± 0.15	10.28 ± 0.16	9.40 ± 0.24
TC19000882.hg.1	LILRB4	8.74 ± 0.26	8.35 ± 0.09	8.85 ± 0.10	8.37 ± 0.11
TC19001840.hg.1	LILRB5	8.08 ± 0.17	7.94 ± 0.15	8.10 ± 0.13	8.02 ± 0.01
TC19000883.hg.1	LILRP2	7.56 ± 0.21	7.42 ± 0.15	7.53 ± 0.12	7.53 ± 0.02
TC19001777.hg.1	LIM2	7.44 ± 0.20	7.41 ± 0.09	7.26 ± 0.17	7.41 ± 0.10
TC12001492.hg.1	LIMA1	7.13 ± 0.03	7.05 ± 0.08	6.96 ± 0.05	6.86 ± 0.09
TC04000254.hg.1	LIMCH1	5.65 ± 0.14	5.55 ± 0.08	5.58 ± 0.10	5.60 ± 0.06
TC03000248.hg.1	LIMD1	8.22 ± 0.04	8.11 ± 0.05	8.06 ± 0.08	8.08 ± 0.03
TC03001349.hg.1	LIMD1-AS1	7.13 ± 0.19	6.94 ± 0.07	7.02 ± 0.15	6.89 ± 0.01
TC17001783.hg.1	LIMD2	9.86 ± 0.17	9.88 ± 0.07	9.58 ± 0.21	9.89 ± 0.05
TC20001737.hg.1	LIME1	8.95 ± 0.25	8.95 ± 0.25	8.86 ± 0.22	9.00 ± 0.03
TC07000450.hg.1	LIMK1	8.95 ± 0.28	8.62 ± 0.06	8.91 ± 0.07	8.55 ± 0.11
TC22000224.hg.1	LIMK2	9.57 ± 0.60	9.37 ± 0.11	10.18 ± 0.39	9.28 ± 0.16
TC02000667.hg.1	LIMS1	9.07 ± 0.18	8.87 ± 0.10	9.21 ± 0.08	8.89 ± 0.14
TC02002284.hg.1	LIMS2	8.23 ± 0.20	8.24 ± 0.21	8.14 ± 0.19	8.31 ± 0.03
TC02002201.hg.1	LIMS3L	7.14 ± 0.09	6.98 ± 0.06	7.16 ± 0.07	7.06 ± 0.06
TC02004997.hg.1	LIMS3L	9.03 ± 0.11	8.83 ± 0.09	9.11 ± 0.07	8.86 ± 0.12

TC01000343.hg.1	LIN28A	7.25 ± 0.24	7.37 ± 0.09	7.29 ± 0.14	7.48 ± 0.09
TC06000840.hg.1	LIN28B	5.78 ± 0.22	5.64 ± 0.13	5.68 ± 0.28	5.75 ± 0.13
TC19002631.hg.1	LIN37	8.56 ± 0.23	8.23 ± 0.04	8.58 ± 0.14	8.13 ± 0.04
TC14000455.hg.1	LIN52	7.57 ± 0.10	7.42 ± 0.03	7.37 ± 0.07	7.40 ± 0.02
TC04001341.hg.1	LIN54	4.92 ± 0.16	4.82 ± 0.03	4.93 ± 0.07	4.78 ± 0.08
TC04001342.hg.1	LIN54	8.15 ± 0.23	8.22 ± 0.20	8.20 ± 0.32	8.11 ± 0.12
TC12001772.hg.1	LIN7A	8.28 ± 0.26	8.00 ± 0.34	9.21 ± 0.51	7.90 ± 0.45
TC19000725.hg.1	LIN7B	8.12 ± 0.19	8.10 ± 0.17	8.06 ± 0.21	8.16 ± 0.02
TC11001510.hg.1	LIN7C	8.55 ± 0.48	8.84 ± 0.13	8.70 ± 0.47	8.82 ± 0.20
TC01003898.hg.1	LIN9	6.41 ± 0.11	6.32 ± 0.05	6.31 ± 0.09	6.17 ± 0.09
TC20000189.hg.1	LINC00028	7.52 ± 0.28	7.47 ± 0.12	7.47 ± 0.09	7.65 ± 0.08
TC20001023.hg.1	LINC00029	7.47 ± 0.17	7.37 ± 0.08	7.38 ± 0.09	7.47 ± 0.08
TC09000986.hg.1	LINC00032	5.15 ± 0.05	5.08 ± 0.07	5.13 ± 0.02	5.16 ± 0.05
TC07000445.hg.1	LINC00035	8.67 ± 0.10	8.61 ± 0.11	8.61 ± 0.16	8.75 ± 0.08
TC08000804.hg.1	LINC00051	7.73 ± 0.28	7.72 ± 0.27	7.60 ± 0.17	7.84 ± 0.12
TC15000835.hg.1	LINC00052	5.26 ± 0.24	5.35 ± 0.37	5.04 ± 0.17	5.20 ± 0.11
TC01003563.hg.1	LINC00083	8.53 ± 0.20	8.53 ± 0.19	8.56 ± 0.19	8.70 ± 0.08
TC19000787.hg.1	LINC00085	8.03 ± 0.19	7.95 ± 0.15	7.98 ± 0.10	8.03 ± 0.02
TC0X000651.hg.1	LINC00086	6.95 ± 0.25	6.87 ± 0.21	6.98 ± 0.09	7.08 ± 0.04
TC0X001401.hg.1	LINC00087	7.43 ± 0.30	7.52 ± 0.24	7.39 ± 0.17	7.57 ± 0.04
TC09001369.hg.1	LINC00092	7.73 ± 0.25	7.66 ± 0.15	7.63 ± 0.21	7.63 ± 0.08
TC09000779.hg.1	LINC00094	8.18 ± 0.18	8.08 ± 0.16	8.03 ± 0.18	8.11 ± 0.04
TC0X000803.hg.1	LINC00102	4.24 ± 0.10	4.20 ± 0.03	4.30 ± 0.04	4.26 ± 0.01
TC0Y000120.hg.1	LINC00102	4.24 ± 0.10	4.20 ± 0.03	4.30 ± 0.04	4.26 ± 0.01
TC0X000799.hg.1	LINC00106	7.18 ± 0.26	7.02 ± 0.13	7.11 ± 0.16	7.11 ± 0.03
TC0Y000116.hg.1	LINC00106	7.18 ± 0.26	7.02 ± 0.13	7.11 ± 0.16	7.11 ± 0.03
TC21000191.hg.1	LINC00111	5.35 ± 0.23	5.34 ± 0.23	5.19 ± 0.29	5.42 ± 0.08
TC21000192.hg.1	LINC00112	7.07 ± 0.23	6.89 ± 0.19	6.93 ± 0.21	7.04 ± 0.10
TC21000088.hg.1	LINC00113	4.55 ± 0.16	4.27 ± 0.09	4.47 ± 0.05	4.39 ± 0.09
TC21000451.hg.1	LINC00114	6.42 ± 0.18	6.43 ± 0.05	6.40 ± 0.12	6.54 ± 0.10
TC01002074.hg.1	LINC00115	8.20 ± 0.53	8.30 ± 0.20	8.26 ± 0.20	8.47 ± 0.19

TC02002196.hg.1	LINC00116	5.49 ± 0.17	5.35 ± 0.11	5.40 ± 0.10	5.38 ± 0.03
TC02000535.hg.1	LINC00152	8.34 ± 0.15	7.87 ± 0.09	8.45 ± 0.24	7.76 ± 0.07
TC21000332.hg.1	LINC00158	4.93 ± 0.08	4.85 ± 0.05	4.91 ± 0.10	4.99 ± 0.07
TC21000393.hg.1	LINC00159	6.39 ± 0.02	6.35 ± 0.04	6.31 ± 0.11	6.44 ± 0.08
TC21000139.hg.1	LINC00160	6.09 ± 0.18	6.05 ± 0.08	5.98 ± 0.14	6.13 ± 0.09
TC21000422.hg.1	LINC00160	6.00 ± 0.16	6.02 ± 0.21	6.11 ± 0.09	6.08 ± 0.12
TC21000092.hg.1	LINC00161	5.09 ± 0.14	4.96 ± 0.09	5.01 ± 0.13	5.02 ± 0.09
TC21000541.hg.1	LINC00162	8.37 ± 0.30	8.38 ± 0.29	8.28 ± 0.36	8.43 ± 0.19
TC21000540.hg.1	LINC00163	7.81 ± 0.31	7.81 ± 0.26	7.78 ± 0.33	7.98 ± 0.06
TC11001193.hg.1	LINC00167	7.14 ± 0.18	7.03 ± 0.10	6.95 ± 0.12	6.97 ± 0.10
TC12000912.hg.1	LINC00173	7.08 ± 0.05	6.98 ± 0.05	7.15 ± 0.07	6.98 ± 0.11
TC07001460.hg.1	LINC00174	7.59 ± 0.17	7.63 ± 0.12	7.69 ± 0.15	7.65 ± 0.07
TC20000532.hg.1	LINC00176	8.43 ± 0.19	8.39 ± 0.10	8.34 ± 0.24	8.41 ± 0.05
TC01001938.hg.1	LINC00184	5.82 ± 0.31	6.01 ± 0.13	5.97 ± 0.11	6.12 ± 0.13
TC21000096.hg.1	LINC00189	6.32 ± 0.10	6.03 ± 0.22	6.60 ± 0.37	6.04 ± 0.14
TC10000012.hg.1	LINC00200	6.06 ± 0.12	5.96 ± 0.15	6.06 ± 0.18	6.04 ± 0.12
TC10001122.hg.1	LINC00202-1	7.74 ± 0.15	7.72 ± 0.02	7.82 ± 0.12	7.76 ± 0.11
TC10000184.hg.1	LINC00202-2	7.54 ± 0.17	7.53 ± 0.06	7.58 ± 0.01	7.60 ± 0.10
TC22000375.hg.1	LINC00207	7.18 ± 0.25	7.09 ± 0.24	7.14 ± 0.22	7.23 ± 0.13
TC08000088.hg.1	LINC00208	6.92 ± 0.33	6.84 ± 0.22	6.83 ± 0.14	6.91 ± 0.04
TC01001800.hg.1	LINC00210	6.15 ± 0.11	5.97 ± 0.07	6.04 ± 0.24	6.17 ± 0.09
TC02001747.hg.1	LINC00211	6.62 ± 0.13	6.52 ± 0.08	6.55 ± 0.14	6.54 ± 0.05
TC14000844.hg.1	LINC00221	5.16 ± 0.06	5.20 ± 0.02	5.20 ± 0.08	5.22 ± 0.04
TC06000869.hg.1	LINC00222	6.44 ± 0.15	6.48 ± 0.20	6.39 ± 0.17	6.57 ± 0.07
TC14000843.hg.1	LINC00226	4.58 ± 0.16	4.55 ± 0.17	4.61 ± 0.10	4.55 ± 0.08
TC22000868.hg.1	LINC00229	7.57 ± 0.17	7.50 ± 0.14	7.51 ± 0.11	7.59 ± 0.03
TC16000729.hg.1	LINC00235	8.23 ± 0.30	8.16 ± 0.25	8.16 ± 0.18	8.21 ± 0.05
TC20000687.hg.1	LINC00237	7.67 ± 0.23	7.44 ± 0.04	7.54 ± 0.32	7.56 ± 0.05
TC14000400.hg.1	LINC00238	4.79 ± 0.14	4.67 ± 0.06	4.69 ± 0.07	4.75 ± 0.03
TC14000772.hg.1	LINC00239	7.81 ± 0.27	7.84 ± 0.07	7.74 ± 0.17	7.89 ± 0.08
TC06000219.hg.1	LINC00240	5.44 ± 0.11	5.33 ± 0.04	5.46 ± 0.11	5.46 ± 0.09

TC06002339.hg.1	LINC00242	6.05 ± 0.34	5.98 ± 0.09	5.96 ± 0.29	6.11 ± 0.12
TC06001511.hg.1	LINC00243	7.57 ± 0.20	7.59 ± 0.21	7.47 ± 0.21	7.56 ± 0.08
TC6_cox_hap2000144.hg.1	LINC00243	7.19 ± 0.23	7.26 ± 0.16	7.29 ± 0.04	7.24 ± 0.09
TC07001046.hg.1	LINC00244	6.90 ± 0.33	6.82 ± 0.09	6.92 ± 0.15	6.87 ± 0.03
TC08001278.hg.1	LINC00251	5.09 ± 0.09	5.02 ± 0.07	5.04 ± 0.15	4.99 ± 0.15
TC01001690.hg.1	LINC00260	7.53 ± 0.36	7.43 ± 0.19	7.76 ± 0.34	7.24 ± 0.17
TC20000697.hg.1	LINC00261	6.11 ± 0.42	6.18 ± 0.25	6.20 ± 0.24	6.22 ± 0.09
TC10000722.hg.1	LINC00263	7.99 ± 0.18	7.91 ± 0.06	7.84 ± 0.13	7.94 ± 0.08
TC10000183.hg.1	LINC00264	7.03 ± 0.15	7.16 ± 0.10	7.33 ± 0.25	7.14 ± 0.06
TC07000251.hg.1	LINC00265	10.05 ± 0.19	9.99 ± 0.16	10.34 ± 0.26	10.10 ± 0.11
TC20001739.hg.1	LINC00266-1	8.22 ± 0.33	8.03 ± 0.29	8.96 ± 0.28	8.09 ± 0.09
TC06001205.hg.1	LINC00266-3	8.44 ± 0.28	8.36 ± 0.24	9.59 ± 0.39	8.40 ± 0.08
TC0Y000214.hg.1	LINC00266-4P	7.95 ± 0.30	8.02 ± 0.16	8.66 ± 0.39	8.07 ± 0.06
TC09001171.hg.1	LINC00268	7.12 ± 0.17	7.05 ± 0.04	7.07 ± 0.05	7.25 ± 0.21
TC0X000363.hg.1	LINC00269	7.07 ± 0.11	7.13 ± 0.09	7.15 ± 0.04	7.25 ± 0.08
TC06001014.hg.1	LINC00271	6.03 ± 0.15	5.95 ± 0.10	5.95 ± 0.07	5.97 ± 0.09
TC01001577.hg.1	LINC00272	6.08 ± 0.10	6.01 ± 0.14	5.99 ± 0.03	6.10 ± 0.09
TC16000401.hg.1	LINC00273	11.41 ± 0.13	11.78 ± 0.07	10.79 ± 0.12	11.79 ± 0.17
TC16001076.hg.1	LINC00273	8.10 ± 0.35	7.96 ± 0.24	8.00 ± 0.24	8.19 ± 0.11
TC02001589.hg.1	LINC00276	5.45 ± 0.10	5.46 ± 0.02	5.52 ± 0.09	5.54 ± 0.10
TC15000632.hg.1	LINC00277	6.57 ± 0.14	6.52 ± 0.16	6.51 ± 0.05	6.62 ± 0.08
TC0Y000018.hg.1	LINC00278	7.50 ± 0.35	7.81 ± 0.21	7.90 ± 0.54	7.54 ± 0.37
TC0Y000033.hg.1	LINC00279	6.20 ± 0.16	6.05 ± 0.08	6.08 ± 0.13	6.05 ± 0.09
TC0Y000129.hg.1	LINC00280	5.32 ± 0.22	5.30 ± 0.06	5.37 ± 0.09	5.42 ± 0.15
TC13000682.hg.1	LINC00282	8.20 ± 0.17	8.03 ± 0.08	8.48 ± 0.19	7.94 ± 0.08
TC13000369.hg.1	LINC00283	5.34 ± 0.06	5.23 ± 0.15	5.23 ± 0.06	5.27 ± 0.20
TC13000162.hg.1	LINC00284	5.62 ± 0.26	5.53 ± 0.17	5.64 ± 0.20	5.59 ± 0.04
TC04001779.hg.1	LINC00290	4.88 ± 0.08	4.80 ± 0.09	4.86 ± 0.05	4.86 ± 0.08
TC08000339.hg.1	LINC00293	5.72 ± 0.08	5.68 ± 0.04	5.60 ± 0.03	5.83 ± 0.16
TC11000314.hg.1	LINC00294	7.72 ± 0.17	7.50 ± 0.07	7.41 ± 0.16	7.55 ± 0.08
TC13000095.hg.1	LINC00297	5.87 ± 0.15	5.96 ± 0.04	5.78 ± 0.19	6.12 ± 0.15

TC02001550.hg.1	LINC00299	6.45 ± 0.12	6.32 ± 0.07	6.45 ± 0.09	6.35 ± 0.10
TC11000513.hg.1	LINC00301	4.57 ± 0.07	4.42 ± 0.06	4.53 ± 0.03	4.48 ± 0.05
TC01001230.hg.1	LINC00302	5.95 ± 0.26	6.10 ± 0.21	6.18 ± 0.24	6.40 ± 0.22
TC01003724.hg.1	LINC00303	6.67 ± 0.24	6.61 ± 0.16	6.63 ± 0.17	6.77 ± 0.07
TC16000690.hg.1	LINC00304	8.04 ± 0.20	8.06 ± 0.19	7.98 ± 0.18	8.29 ± 0.05
TC18000559.hg.1	LINC00305	5.26 ± 0.07	5.21 ± 0.08	5.18 ± 0.08	5.26 ± 0.06
TC21000362.hg.1	LINC00307	5.46 ± 0.11	5.22 ± 0.03	5.18 ± 0.08	5.22 ± 0.19
TC21000063.hg.1	LINC00308	4.76 ± 0.09	4.81 ± 0.03	4.84 ± 0.11	4.84 ± 0.09
TC02001912.hg.1	LINC00309	5.10 ± 0.19	4.96 ± 0.09	4.92 ± 0.10	4.99 ± 0.08
TC21000135.hg.1	LINC00310	5.91 ± 0.20	5.90 ± 0.07	5.95 ± 0.01	5.93 ± 0.07
TC16000663.hg.1	LINC00311	6.61 ± 0.33	6.61 ± 0.22	6.56 ± 0.22	6.73 ± 0.09
TC03000027.hg.1	LINC00312	5.42 ± 0.22	5.24 ± 0.10	5.20 ± 0.14	5.28 ± 0.13
TC21000507.hg.1	LINC00313	6.85 ± 0.27	6.83 ± 0.09	6.78 ± 0.10	6.90 ± 0.06
TC21000090.hg.1	LINC00314	3.92 ± 0.29	3.79 ± 0.04	3.78 ± 0.17	3.86 ± 0.18
TC21000544.hg.1	LINC00315	7.61 ± 0.24	7.36 ± 0.12	7.42 ± 0.26	7.50 ± 0.08
TC21000545.hg.1	LINC00316	6.28 ± 0.04	6.49 ± 0.21	6.36 ± 0.19	6.43 ± 0.10
TC21000317.hg.1	LINC00317	5.08 ± 0.24	4.86 ± 0.07	4.86 ± 0.19	5.05 ± 0.31
TC21000209.hg.1	LINC00319	7.05 ± 0.30	7.10 ± 0.20	6.98 ± 0.26	7.18 ± 0.07
TC21000313.hg.1	LINC00320	4.62 ± 0.11	4.62 ± 0.07	4.60 ± 0.08	4.65 ± 0.09
TC21000504.hg.1	LINC00322	5.94 ± 0.06	5.95 ± 0.14	5.88 ± 0.08	6.04 ± 0.11
TC21000468.hg.1	LINC00323	5.52 ± 0.26	5.37 ± 0.11	5.41 ± 0.07	5.46 ± 0.10
TC17001110.hg.1	LINC00324	7.73 ± 0.14	7.46 ± 0.11	7.73 ± 0.14	7.52 ± 0.03
TC06000998.hg.1	LINC00326	4.53 ± 0.20	4.37 ± 0.03	4.35 ± 0.06	4.35 ± 0.06
TC13000047.hg.1	LINC00327	4.90 ± 0.09	4.64 ± 0.17	4.83 ± 0.04	4.75 ± 0.08
TC13000624.hg.1	LINC00330	6.65 ± 0.31	6.59 ± 0.07	6.66 ± 0.22	6.73 ± 0.12
TC13000755.hg.1	LINC00331	4.53 ± 0.31	4.10 ± 0.10	4.37 ± 0.19	4.06 ± 0.12
TC13000138.hg.1	LINC00332	5.62 ± 0.10	5.59 ± 0.08	5.62 ± 0.08	5.69 ± 0.02
TC13000310.hg.1	LINC00333	4.82 ± 0.23	4.68 ± 0.06	4.65 ± 0.16	4.80 ± 0.11
TC21000242.hg.1	LINC00334	6.48 ± 0.16	6.43 ± 0.18	6.20 ± 0.17	6.55 ± 0.20
TC06001589.hg.1	LINC00336	7.15 ± 0.22	6.96 ± 0.06	6.76 ± 0.29	6.94 ± 0.03
TC01000086.hg.1	LINC00337	7.47 ± 0.21	7.34 ± 0.06	7.39 ± 0.09	7.40 ± 0.09

TC01000279.hg.1	LINC00339	7.10 ± 0.17	6.89 ± 0.05	6.79 ± 0.11	6.87 ± 0.03
TC06000136.hg.1	LINC00340	6.27 ± 0.06	6.20 ± 0.06	6.23 ± 0.06	6.31 ± 0.08
TC14001475.hg.1	LINC00341	7.71 ± 0.19	7.44 ± 0.15	7.46 ± 0.05	7.29 ± 0.05
TC02002097.hg.1	LINC00342	6.95 ± 0.48	7.16 ± 0.14	6.85 ± 0.18	7.06 ± 0.14
TC13000375.hg.1	LINC00343	4.87 ± 0.06	4.57 ± 0.02	4.74 ± 0.05	4.62 ± 0.06
TC13000376.hg.1	LINC00344	4.58 ± 0.19	4.44 ± 0.10	4.43 ± 0.15	4.55 ± 0.09
TC13000690.hg.1	LINC00345	4.92 ± 0.22	4.88 ± 0.04	4.74 ± 0.10	4.91 ± 0.10
TC13000877.hg.1	LINC00346	7.18 ± 0.15	6.89 ± 0.11	7.05 ± 0.04	6.87 ± 0.02
TC13000281.hg.1	LINC00347	6.54 ± 0.19	6.31 ± 0.10	6.33 ± 0.23	6.40 ± 0.05
TC13000267.hg.1	LINC00348	5.07 ± 0.04	5.13 ± 0.08	5.05 ± 0.07	5.15 ± 0.01
TC13000001.hg.1	LINC00349	4.45 ± 0.06	4.30 ± 0.03	4.33 ± 0.04	4.31 ± 0.02
TC13000016.hg.1	LINC00350	7.26 ± 0.14	7.19 ± 0.12	7.18 ± 0.17	7.34 ± 0.12
TC13000311.hg.1	LINC00351	5.36 ± 0.13	5.19 ± 0.02	5.34 ± 0.13	5.17 ± 0.07
TC13000048.hg.1	LINC00352	5.56 ± 0.28	5.68 ± 0.16	5.53 ± 0.17	5.80 ± 0.23
TC13000776.hg.1	LINC00353	5.65 ± 0.30	5.59 ± 0.06	5.44 ± 0.18	5.75 ± 0.17
TC13000404.hg.1	LINC00354	5.58 ± 0.03	5.48 ± 0.23	5.47 ± 0.12	5.63 ± 0.10
TC13000714.hg.1	LINC00355	6.42 ± 0.16	6.47 ± 0.18	6.45 ± 0.14	6.59 ± 0.15
TC13000619.hg.1	LINC00356	5.65 ± 0.05	5.53 ± 0.05	5.58 ± 0.06	5.66 ± 0.07
TC13000708.hg.1	LINC00358	5.92 ± 0.10	5.77 ± 0.10	5.84 ± 0.12	5.99 ± 0.10
TC13000810.hg.1	LINC00359	5.84 ± 0.13	5.82 ± 0.10	5.95 ± 0.15	5.93 ± 0.10
TC13000800.hg.1	LINC00361	9.38 ± 0.58	9.39 ± 0.07	9.29 ± 0.29	9.62 ± 0.21
TC13000044.hg.1	LINC00362	6.94 ± 0.25	7.05 ± 0.17	6.97 ± 0.04	7.19 ± 0.21
TC13000260.hg.1	LINC00364	4.40 ± 0.31	4.08 ± 0.05	4.17 ± 0.11	4.17 ± 0.15
TC13000527.hg.1	LINC00365	6.62 ± 0.35	6.81 ± 0.12	6.65 ± 0.26	6.89 ± 0.20
TC13000131.hg.1	LINC00366	4.48 ± 0.16	4.19 ± 0.07	4.28 ± 0.05	4.32 ± 0.10
TC13000677.hg.1	LINC00371	6.68 ± 0.27	6.68 ± 0.16	6.55 ± 0.25	6.66 ± 0.03
TC13000210.hg.1	LINC00372	6.67 ± 0.28	6.56 ± 0.26	6.60 ± 0.07	6.73 ± 0.12
TC13000243.hg.1	LINC00378	5.42 ± 0.23	5.24 ± 0.01	5.25 ± 0.09	5.26 ± 0.15
TC13000781.hg.1	LINC00379	3.76 ± 0.11	3.60 ± 0.01	3.71 ± 0.06	3.65 ± 0.11
TC13000280.hg.1	LINC00381	6.78 ± 0.15	6.63 ± 0.05	6.66 ± 0.10	6.73 ± 0.08
TC13000003.hg.1	LINC00387	4.86 ± 0.11	4.86 ± 0.21	4.81 ± 0.07	4.99 ± 0.21

TC13000438.hg.1	LINC00388	6.95 ± 0.29	7.01 ± 0.16	6.98 ± 0.02	7.11 ± 0.13
TC13000796.hg.1	LINC00391	6.18 ± 0.44	6.01 ± 0.06	6.04 ± 0.09	6.03 ± 0.07
TC13000276.hg.1	LINC00392	5.41 ± 0.19	5.26 ± 0.04	5.12 ± 0.22	5.24 ± 0.07
TC13000732.hg.1	LINC00393	5.20 ± 0.31	5.06 ± 0.22	4.96 ± 0.05	5.21 ± 0.04
TC13000712.hg.1	LINC00395	5.27 ± 0.16	5.36 ± 0.05	5.38 ± 0.06	5.52 ± 0.19
TC13000101.hg.1	LINC00398	7.47 ± 0.20	7.53 ± 0.28	7.42 ± 0.19	7.65 ± 0.05
TC13000158.hg.1	LINC00400	6.88 ± 0.12	6.81 ± 0.02	6.85 ± 0.01	6.87 ± 0.08
TC13000278.hg.1	LINC00402	7.08 ± 0.42	7.46 ± 0.10	6.70 ± 0.33	7.28 ± 0.30
TC13000407.hg.1	LINC00403	6.73 ± 0.04	6.52 ± 0.08	6.52 ± 0.05	6.61 ± 0.05
TC13000885.hg.1	LINC00404	6.50 ± 0.36	6.35 ± 0.26	6.28 ± 0.13	6.48 ± 0.10
TC13000779.hg.1	LINC00410	6.98 ± 0.39	6.92 ± 0.11	6.82 ± 0.15	7.13 ± 0.23
TC13000844.hg.1	LINC00411	5.00 ± 0.31	4.69 ± 0.07	4.79 ± 0.14	4.82 ± 0.06
TC13000297.hg.1	LINC00413	8.21 ± 0.27	8.38 ± 0.13	8.14 ± 0.26	8.48 ± 0.13
TC13000501.hg.1	LINC00415	8.25 ± 0.22	8.43 ± 0.17	8.31 ± 0.15	8.65 ± 0.19
TC13000015.hg.1	LINC00421	7.14 ± 0.28	7.24 ± 0.20	7.19 ± 0.31	7.37 ± 0.12
TC13000470.hg.1	LINC00422	7.22 ± 0.12	7.20 ± 0.05	7.19 ± 0.07	7.28 ± 0.14
TC13000554.hg.1	LINC00423	5.73 ± 0.17	5.76 ± 0.15	5.78 ± 0.10	5.83 ± 0.21
TC13000477.hg.1	LINC00424	6.04 ± 0.14	5.85 ± 0.04	5.85 ± 0.12	5.90 ± 0.09
TC13000532.hg.1	LINC00426	7.47 ± 0.13	7.36 ± 0.01	7.29 ± 0.09	7.39 ± 0.04
TC13000531.hg.1	LINC00427	5.70 ± 0.08	5.54 ± 0.09	5.39 ± 0.24	5.65 ± 0.06
TC13000317.hg.1	LINC00433	4.43 ± 0.13	4.24 ± 0.04	4.29 ± 0.05	4.41 ± 0.16
TC13000703.hg.1	LINC00434	4.06 ± 0.28	3.93 ± 0.05	4.02 ± 0.09	4.08 ± 0.10
TC13000749.hg.1	LINC00439	4.67 ± 0.25	4.41 ± 0.04	4.54 ± 0.02	4.53 ± 0.08
TC13000318.hg.1	LINC00440	4.24 ± 0.15	4.13 ± 0.07	4.20 ± 0.11	4.21 ± 0.18
TC13000654.hg.1	LINC00441	6.79 ± 0.15	6.98 ± 0.06	6.78 ± 0.06	7.03 ± 0.15
TC13000010.hg.1	LINC00442	7.16 ± 0.19	7.09 ± 0.17	7.12 ± 0.17	7.29 ± 0.13
TC13000379.hg.1	LINC00443	7.01 ± 0.18	6.91 ± 0.12	7.03 ± 0.25	6.97 ± 0.14
TC13000651.hg.1	LINC00444	7.25 ± 0.37	7.39 ± 0.08	7.09 ± 0.07	7.49 ± 0.14
TC13000121.hg.1	LINC00445	6.87 ± 0.15	6.67 ± 0.09	6.65 ± 0.08	6.68 ± 0.10
TC13000301.hg.1	LINC00446	3.99 ± 0.12	4.04 ± 0.12	3.97 ± 0.14	4.12 ± 0.09
TC13000709.hg.1	LINC00448	4.51 ± 0.10	4.42 ± 0.08	4.42 ± 0.10	4.48 ± 0.03

TC13000710.hg.1	LINC00448	5.24 ± 0.24	5.15 ± 0.17	5.09 ± 0.14	5.12 ± 0.06
TC13000832.hg.1	LINC00449	8.29 ± 0.17	8.07 ± 0.06	8.26 ± 0.09	8.15 ± 0.03
TC13000433.hg.1	LINC00452	7.48 ± 0.14	7.44 ± 0.12	7.45 ± 0.06	7.55 ± 0.07
TC13000434.hg.1	LINC00453	7.63 ± 0.03	7.69 ± 0.19	7.60 ± 0.11	7.79 ± 0.06
TC13000900.hg.1	LINC00454	6.89 ± 0.20	6.87 ± 0.22	6.75 ± 0.17	6.95 ± 0.09
TC13000559.hg.1	LINC00457	5.53 ± 0.19	5.40 ± 0.12	5.48 ± 0.17	5.48 ± 0.12
TC13000696.hg.1	LINC00458	6.02 ± 0.12	5.97 ± 0.05	6.08 ± 0.10	6.10 ± 0.11
TC13000249.hg.1	LINC00459	4.61 ± 0.18	4.50 ± 0.06	4.48 ± 0.22	4.52 ± 0.18
TC13000378.hg.1	LINC00460	5.90 ± 0.18	5.88 ± 0.04	5.86 ± 0.05	6.05 ± 0.18
TC13000658.hg.1	LINC00462	4.82 ± 0.11	4.77 ± 0.02	4.73 ± 0.04	4.72 ± 0.08
TC01002730.hg.1	LINC00466	5.74 ± 0.12	5.67 ± 0.07	5.63 ± 0.07	5.73 ± 0.08
TC01001759.hg.1	LINC00467	6.21 ± 0.10	6.02 ± 0.09	6.22 ± 0.04	6.02 ± 0.11
TC17001850.hg.1	LINC00469	6.47 ± 0.26	6.29 ± 0.05	6.28 ± 0.17	6.40 ± 0.11
TC18000280.hg.1	LINC00470	4.71 ± 0.09	4.59 ± 0.10	4.60 ± 0.09	4.66 ± 0.06
TC02002864.hg.1	LINC00471	7.30 ± 0.21	6.98 ± 0.11	7.05 ± 0.08	7.07 ± 0.11
TC06001860.hg.1	LINC00472	6.81 ± 0.37	6.74 ± 0.19	6.73 ± 0.16	6.85 ± 0.07
TC06002302.hg.1	LINC00473	6.69 ± 0.18	6.60 ± 0.20	6.61 ± 0.10	6.73 ± 0.06
TC09001520.hg.1	LINC00474	4.87 ± 0.16	4.80 ± 0.02	4.84 ± 0.13	4.77 ± 0.09
TC09000434.hg.1	LINC00475	7.14 ± 0.20	7.12 ± 0.13	7.08 ± 0.09	7.17 ± 0.04
TC09001368.hg.1	LINC00476	6.75 ± 0.13	6.83 ± 0.05	6.68 ± 0.07	6.82 ± 0.05
TC12001308.hg.1	LINC00477	6.34 ± 0.15	6.33 ± 0.06	6.30 ± 0.12	6.47 ± 0.27
TC21000040.hg.1	LINC00478	5.09 ± 0.12	5.03 ± 0.03	5.00 ± 0.04	5.04 ± 0.07
TC21000475.hg.1	LINC00479	7.48 ± 0.18	7.42 ± 0.21	7.37 ± 0.26	7.50 ± 0.04
TC17001948.hg.1	LINC00482	8.19 ± 0.22	8.08 ± 0.14	8.12 ± 0.15	8.16 ± 0.03
TC17001697.hg.1	LINC00483	6.23 ± 0.28	6.16 ± 0.12	6.18 ± 0.17	6.30 ± 0.09
TC09000430.hg.1	LINC00484	6.68 ± 0.38	6.85 ± 0.33	6.67 ± 0.17	6.94 ± 0.31
TC12001891.hg.1	LINC00485	5.66 ± 0.11	5.71 ± 0.10	5.69 ± 0.09	5.78 ± 0.06
TC02000217.hg.1	LINC00486	5.49 ± 0.07	5.46 ± 0.08	5.51 ± 0.02	5.54 ± 0.01
TC02001542.hg.1	LINC00487	6.17 ± 0.18	6.14 ± 0.13	6.14 ± 0.04	6.27 ± 0.17
TC03000553.hg.1	LINC00488	5.30 ± 0.29	5.32 ± 0.30	5.28 ± 0.09	5.41 ± 0.11
TC20000827.hg.1	LINC00489	6.70 ± 0.02	6.57 ± 0.10	6.70 ± 0.13	6.65 ± 0.08

TC05001643.hg.1	LINC00491	6.21 ± 0.15	6.17 ± 0.08	6.15 ± 0.10	6.35 ± 0.21
TC05000495.hg.1	LINC00492	4.16 ± 0.16	3.95 ± 0.12	4.13 ± 0.25	3.99 ± 0.07
TC20000126.hg.1	LINC00493	7.85 ± 0.21	7.56 ± 0.35	7.53 ± 0.32	7.27 ± 0.07
TC20000380.hg.1	LINC00494	7.09 ± 0.13	7.09 ± 0.14	6.99 ± 0.09	7.16 ± 0.12
TC04001572.hg.1	LINC00498	5.19 ± 0.29	5.08 ± 0.18	5.04 ± 0.05	5.21 ± 0.10
TC04000675.hg.1	LINC00499	5.49 ± 0.09	5.44 ± 0.08	5.43 ± 0.05	5.56 ± 0.17
TC04000676.hg.1	LINC00500	7.43 ± 0.54	7.49 ± 0.27	7.46 ± 0.41	7.80 ± 0.45
TC03000943.hg.1	LINC00501	6.56 ± 0.35	6.21 ± 0.12	6.24 ± 0.29	6.18 ± 0.04
TC10000650.hg.1	LINC00502	6.13 ± 0.20	6.02 ± 0.05	5.88 ± 0.10	6.04 ± 0.06
TC04001051.hg.1	LINC00504	6.65 ± 0.27	6.76 ± 0.06	6.71 ± 0.08	6.82 ± 0.11
TC01000587.hg.1	LINC00505	7.28 ± 0.11	7.42 ± 0.28	7.39 ± 0.15	7.70 ± 0.08
TC12001021.hg.1	LINC00507	4.61 ± 0.09	4.54 ± 0.09	4.54 ± 0.10	4.54 ± 0.05
TC12002118.hg.1	LINC00508	7.58 ± 0.19	7.57 ± 0.18	7.36 ± 0.16	7.59 ± 0.06
TC17001841.hg.1	LINC00511	6.94 ± 0.28	6.95 ± 0.13	6.79 ± 0.24	6.84 ± 0.02
TC16000092.hg.1	LINC00514	8.05 ± 0.24	8.07 ± 0.23	8.02 ± 0.19	8.18 ± 0.02
TC21000337.hg.1	LINC00515	5.40 ± 0.27	5.54 ± 0.06	5.27 ± 0.09	5.63 ± 0.10
TC14001058.hg.1	LINC00517	7.27 ± 0.35	7.15 ± 0.23	7.22 ± 0.15	7.29 ± 0.13
TC06001262.hg.1	LINC00518	5.31 ± 0.09	5.25 ± 0.10	5.29 ± 0.07	5.41 ± 0.12
TC14001129.hg.1	LINC00519	6.97 ± 0.04	7.04 ± 0.05	7.00 ± 0.18	7.22 ± 0.04
TC14001162.hg.1	LINC00520	7.01 ± 0.47	6.97 ± 0.09	6.89 ± 0.14	7.18 ± 0.28
TC14000581.hg.1	LINC00521	6.95 ± 0.20	6.94 ± 0.15	6.88 ± 0.17	6.98 ± 0.05
TC14000643.hg.1	LINC00523	7.13 ± 0.18	7.04 ± 0.11	6.98 ± 0.18	7.14 ± 0.06
TC14001519.hg.1	LINC00524	8.20 ± 0.03	8.24 ± 0.11	8.31 ± 0.13	8.37 ± 0.14
TC07000304.hg.1	LINC00525	6.57 ± 0.28	6.66 ± 0.21	6.58 ± 0.18	6.71 ± 0.10
TC18000298.hg.1	LINC00526	7.10 ± 0.15	7.06 ± 0.06	6.97 ± 0.18	7.01 ± 0.04
TC08000971.hg.1	LINC00529	5.69 ± 0.19	5.18 ± 0.11	5.31 ± 0.21	5.36 ± 0.07
TC06001450.hg.1	LINC00533	6.12 ± 0.27	6.01 ± 0.24	5.98 ± 0.30	6.11 ± 0.06
TC08000548.hg.1	LINC00534	5.70 ± 0.15	5.34 ± 0.12	5.64 ± 0.22	5.57 ± 0.08
TC08001425.hg.1	LINC00535	6.36 ± 0.13	6.37 ± 0.10	6.34 ± 0.07	6.43 ± 0.08
TC08001542.hg.1	LINC00536	5.82 ± 0.09	5.76 ± 0.03	5.76 ± 0.10	5.87 ± 0.07
TC01001786.hg.1	LINC00538	4.73 ± 0.26	4.49 ± 0.23	4.61 ± 0.10	4.72 ± 0.15

TC13000035.hg.1	LINC00539	6.41 ± 0.15	6.30 ± 0.03	6.32 ± 0.12	6.33 ± 0.02
TC13000096.hg.1	LINC00544	6.29 ± 0.12	6.25 ± 0.12	6.20 ± 0.11	6.30 ± 0.08
TC13000102.hg.1	LINC00545	5.50 ± 0.11	5.41 ± 0.25	5.49 ± 0.22	5.56 ± 0.03
TC13000129.hg.1	LINC00547	4.18 ± 0.06	4.20 ± 0.10	4.21 ± 0.08	4.27 ± 0.10
TC13000591.hg.1	LINC00548	6.10 ± 0.13	6.18 ± 0.16	6.09 ± 0.08	6.28 ± 0.09
TC13000720.hg.1	LINC00550	5.65 ± 0.26	5.79 ± 0.14	5.79 ± 0.11	5.90 ± 0.08
TC13000898.hg.1	LINC00552	7.10 ± 0.18	7.33 ± 0.19	7.22 ± 0.26	7.39 ± 0.09
TC01001189.hg.1	LINC00568	6.66 ± 0.18	6.66 ± 0.32	6.63 ± 0.20	6.84 ± 0.09
TC02000072.hg.1	LINC00570	7.53 ± 0.20	7.47 ± 0.03	7.47 ± 0.08	7.61 ± 0.11
TC13000580.hg.1	LINC00571	5.48 ± 0.10	5.40 ± 0.03	5.37 ± 0.04	5.46 ± 0.07
TC13000525.hg.1	LINC00572	6.80 ± 0.19	6.76 ± 0.24	6.72 ± 0.10	6.97 ± 0.10
TC06001196.hg.1	LINC00574	7.36 ± 0.21	7.44 ± 0.13	7.40 ± 0.15	7.61 ± 0.03
TC04001337.hg.1	LINC00575	5.91 ± 0.13	5.91 ± 0.10	5.86 ± 0.08	6.02 ± 0.24
TC06001974.hg.1	LINC00577	7.67 ± 0.23	7.59 ± 0.02	7.65 ± 0.15	7.66 ± 0.11
TC03000944.hg.1	LINC00578	6.24 ± 0.16	6.05 ± 0.05	6.16 ± 0.01	6.09 ± 0.02
TC01003971.hg.1	LINC00582	6.39 ± 0.05	6.37 ± 0.14	6.48 ± 0.12	6.45 ± 0.03
TC09000069.hg.1	LINC00583	4.19 ± 0.06	4.11 ± 0.07	4.14 ± 0.08	4.08 ± 0.09
TC09000523.hg.1	LINC00587	4.91 ± 0.32	4.86 ± 0.10	4.82 ± 0.09	4.93 ± 0.17
TC08000394.hg.1	LINC00588	4.48 ± 0.04	4.55 ± 0.03	4.56 ± 0.11	4.57 ± 0.05
TC08001100.hg.1	LINC00589	6.31 ± 0.16	6.26 ± 0.09	6.27 ± 0.07	6.26 ± 0.11
TC12000417.hg.1	LINC00592	6.47 ± 0.23	6.49 ± 0.18	6.37 ± 0.17	6.62 ± 0.17
TC15000642.hg.1	LINC00593	7.12 ± 0.19	7.14 ± 0.16	7.17 ± 0.01	7.30 ± 0.06
TC15000282.hg.1	LINC00594	5.38 ± 0.22	5.28 ± 0.05	5.22 ± 0.28	5.31 ± 0.13
TC10000560.hg.1	LINC00595	6.60 ± 0.13	6.58 ± 0.15	6.53 ± 0.10	6.72 ± 0.12
TC14000964.hg.1	LINC00596	7.27 ± 0.10	7.21 ± 0.06	7.23 ± 0.10	7.36 ± 0.09
TC15001692.hg.1	LINC00597	5.14 ± 0.12	5.05 ± 0.09	5.00 ± 0.15	5.12 ± 0.06
TC13001718.hg.1	LINC00598	6.65 ± 0.15	6.57 ± 0.06	6.55 ± 0.09	6.67 ± 0.09
TC10001746.hg.1	LINC00601	5.27 ± 0.19	5.15 ± 0.10	5.17 ± 0.05	5.28 ± 0.15
TC06001171.hg.1	LINC00602	7.14 ± 0.42	7.22 ± 0.23	7.29 ± 0.31	7.24 ± 0.07
TC05000180.hg.1	LINC00603	4.01 ± 0.04	4.04 ± 0.11	4.04 ± 0.03	4.02 ± 0.05
TC05000181.hg.1	LINC00604	4.21 ± 0.26	4.07 ± 0.05	4.14 ± 0.18	4.15 ± 0.11

TC14001536.hg.1	LINC00605	4.99 ± 0.28	4.77 ± 0.04	4.85 ± 0.10	4.76 ± 0.08
TC03001164.hg.1	LINC00606	6.81 ± 0.41	6.78 ± 0.19	6.72 ± 0.21	6.93 ± 0.14
TC02002750.hg.1	LINC00607	6.21 ± 0.11	6.16 ± 0.10	6.15 ± 0.07	6.27 ± 0.10
TC02001316.hg.1	LINC00608	5.98 ± 0.06	5.87 ± 0.18	5.98 ± 0.11	5.92 ± 0.08
TC14000228.hg.1	LINC00609	5.38 ± 0.16	5.42 ± 0.13	5.32 ± 0.12	5.45 ± 0.02
TC11001556.hg.1	LINC00610	7.04 ± 0.21	7.19 ± 0.06	7.01 ± 0.10	7.26 ± 0.08
TC12001186.hg.1	LINC00612	6.29 ± 0.13	6.29 ± 0.06	6.18 ± 0.11	6.19 ± 0.06
TC04001561.hg.1	LINC00613	4.62 ± 0.23	4.36 ± 0.05	4.53 ± 0.08	4.42 ± 0.07
TC10001123.hg.1	LINC00614	8.10 ± 0.22	8.22 ± 0.14	8.16 ± 0.09	8.39 ± 0.08
TC12000706.hg.1	LINC00615	5.20 ± 0.08	5.17 ± 0.13	5.12 ± 0.05	5.30 ± 0.19
TC04001569.hg.1	LINC00616	4.40 ± 0.13	4.33 ± 0.08	4.32 ± 0.02	4.35 ± 0.12
TC14000598.hg.1	LINC00617	7.94 ± 0.21	7.90 ± 0.25	7.93 ± 0.16	8.03 ± 0.09
TC14000609.hg.1	LINC00618	7.66 ± 0.24	7.67 ± 0.20	7.52 ± 0.24	7.72 ± 0.19
TC10000282.hg.1	LINC00619	6.99 ± 0.02	6.92 ± 0.20	6.90 ± 0.11	7.10 ± 0.05
TC03000079.hg.1	LINC00620	7.44 ± 0.18	7.42 ± 0.10	7.37 ± 0.17	7.52 ± 0.13
TC01003048.hg.1	LINC00622	5.73 ± 0.15	5.66 ± 0.03	5.68 ± 0.19	5.85 ± 0.13
TC01003087.hg.1	LINC00623	7.62 ± 0.15	7.64 ± 0.08	7.62 ± 0.17	7.57 ± 0.04
TC01003126.hg.1	LINC00624	6.18 ± 0.21	6.03 ± 0.05	6.05 ± 0.09	6.23 ± 0.09
TC01001470.hg.1	LINC00626	5.39 ± 0.18	5.27 ± 0.12	5.26 ± 0.14	5.31 ± 0.10
TC01003731.hg.1	LINC00628	6.96 ± 0.40	6.98 ± 0.10	6.83 ± 0.28	6.99 ± 0.11
TC0X000637.hg.1	LINC00629	6.19 ± 0.19	6.29 ± 0.20	6.16 ± 0.11	6.33 ± 0.01
TC0X002360.hg.1	LINC00630	6.14 ± 0.09	6.19 ± 0.07	6.04 ± 0.04	6.19 ± 0.02
TC0X000683.hg.1	LINC00632	7.04 ± 0.32	6.71 ± 0.04	6.79 ± 0.10	6.77 ± 0.11
TC0X001402.hg.1	LINC00633	5.16 ± 0.22	5.14 ± 0.18	4.94 ± 0.20	5.13 ± 0.08
TC22000349.hg.1	LINC00634	8.38 ± 0.23	8.36 ± 0.25	8.31 ± 0.27	8.54 ± 0.08
TC03001628.hg.1	LINC00635	5.39 ± 0.11	5.36 ± 0.07	5.42 ± 0.01	5.44 ± 0.01
TC03000544.hg.1	LINC00636	6.29 ± 0.14	6.11 ± 0.11	6.28 ± 0.10	6.23 ± 0.18
TC14000808.hg.1	LINC00637	7.56 ± 0.18	7.59 ± 0.13	7.55 ± 0.18	7.79 ± 0.11
TC14000824.hg.1	LINC00638	6.76 ± 0.20	6.72 ± 0.11	6.67 ± 0.13	6.79 ± 0.13
TC14001062.hg.1	LINC00639	5.84 ± 0.03	5.86 ± 0.10	5.91 ± 0.03	5.95 ± 0.13
TC14000296.hg.1	LINC00640	6.01 ± 0.08	5.97 ± 0.09	6.02 ± 0.04	6.09 ± 0.09

TC14000914.hg.1	LINC00641	8.03 ± 0.68	8.21 ± 0.35	8.30 ± 0.57	8.19 ± 0.24
TC14000555.hg.1	LINC00642	6.15 ± 0.29	6.05 ± 0.17	6.10 ± 0.11	6.21 ± 0.15
TC14000376.hg.1	LINC00643	5.57 ± 0.15	5.50 ± 0.13	5.50 ± 0.11	5.50 ± 0.06
TC14001201.hg.1	LINC00644	5.58 ± 0.19	5.31 ± 0.05	5.30 ± 0.20	5.30 ± 0.05
TC14000179.hg.1	LINC00645	5.75 ± 0.07	5.66 ± 0.03	5.71 ± 0.06	5.76 ± 0.12
TC14001091.hg.1	LINC00648	5.10 ± 0.08	4.95 ± 0.11	5.00 ± 0.07	5.14 ± 0.23
TC21000132.hg.1	LINC00649	8.97 ± 0.38	8.80 ± 0.18	8.63 ± 0.35	8.69 ± 0.09
TC21000133.hg.1	LINC00649	7.51 ± 0.10	7.37 ± 0.21	7.38 ± 0.06	7.42 ± 0.12
TC20000932.hg.1	LINC00651	7.33 ± 0.10	7.23 ± 0.17	7.31 ± 0.31	7.49 ± 0.15
TC20000672.hg.1	LINC00652	6.08 ± 0.15	5.99 ± 0.11	5.94 ± 0.06	6.06 ± 0.14
TC20000597.hg.1	LINC00654	6.97 ± 0.11	6.97 ± 0.10	7.04 ± 0.09	7.03 ± 0.10
TC20000704.hg.1	LINC00656	7.14 ± 0.15	7.21 ± 0.16	7.23 ± 0.13	7.31 ± 0.13
TC20000810.hg.1	LINC00657	9.70 ± 0.64	9.93 ± 0.25	10.05 ± 0.39	10.05 ± 0.34
TC20000596.hg.1	LINC00658	6.70 ± 0.16	6.69 ± 0.10	6.63 ± 0.18	6.81 ± 0.11
TC20001018.hg.1	LINC00659	7.50 ± 0.13	7.40 ± 0.29	7.39 ± 0.23	7.46 ± 0.14
TC19000282.hg.1	LINC00661	7.68 ± 0.20	7.68 ± 0.22	7.66 ± 0.24	7.83 ± 0.10
TC19001377.hg.1	LINC00662	6.91 ± 0.06	6.92 ± 0.08	6.98 ± 0.19	6.96 ± 0.07
TC19001339.hg.1	LINC00663	7.03 ± 0.25	7.07 ± 0.03	6.94 ± 0.13	7.10 ± 0.04
TC19000392.hg.1	LINC00664	6.46 ± 0.14	6.38 ± 0.10	6.50 ± 0.08	6.43 ± 0.01
TC19001466.hg.1	LINC00665	7.24 ± 0.17	7.12 ± 0.03	7.03 ± 0.08	7.26 ± 0.10
TC18000025.hg.1	LINC00667	8.33 ± 0.18	8.33 ± 0.09	8.28 ± 0.12	8.26 ± 0.09
TC18000026.hg.1	LINC00667	6.83 ± 0.33	6.99 ± 0.25	6.68 ± 0.25	6.93 ± 0.06
TC18000307.hg.1	LINC00668	7.04 ± 0.15	7.01 ± 0.10	6.97 ± 0.04	7.06 ± 0.05
TC18000465.hg.1	LINC00669	5.35 ± 0.16	5.27 ± 0.04	5.27 ± 0.11	5.34 ± 0.10
TC17000157.hg.1	LINC00670	5.55 ± 0.04	5.51 ± 0.11	5.55 ± 0.09	5.46 ± 0.01
TC17001546.hg.1	LINC00671	7.38 ± 0.16	7.36 ± 0.04	7.40 ± 0.05	7.43 ± 0.05
TC17000470.hg.1	LINC00672	7.40 ± 0.17	7.25 ± 0.09	7.48 ± 0.38	7.42 ± 0.13
TC17001840.hg.1	LINC00673	7.51 ± 0.30	7.53 ± 0.19	7.45 ± 0.21	7.62 ± 0.09
TC17000799.hg.1	LINC00674	8.65 ± 0.19	8.64 ± 0.13	8.68 ± 0.02	8.75 ± 0.05
TC17001138.hg.1	LINC00675	7.24 ± 0.22	7.23 ± 0.35	7.22 ± 0.29	7.44 ± 0.07
TC13000389.hg.1	LINC00676	4.97 ± 0.18	4.73 ± 0.08	4.80 ± 0.05	4.75 ± 0.14

TC14001535.hg.1	LINC00677	7.13 ± 0.14	7.26 ± 0.20	7.02 ± 0.26	7.32 ± 0.02
TC08000108.hg.1	LINC00681	4.73 ± 0.26	4.94 ± 0.04	4.66 ± 0.04	4.97 ± 0.31
TC04001148.hg.1	LINC00682	5.83 ± 0.25	5.78 ± 0.23	5.82 ± 0.19	5.81 ± 0.06
TC0X000399.hg.1	LINC00684	7.50 ± 0.26	7.54 ± 0.27	7.46 ± 0.31	7.60 ± 0.10
TC0X001146.hg.1	LINC00684	7.86 ± 0.20	7.74 ± 0.21	7.78 ± 0.17	7.85 ± 0.03
TC0X000002.hg.1	LINC00685	8.31 ± 0.17	8.19 ± 0.03	8.28 ± 0.06	8.19 ± 0.07
TC0Y000002.hg.1	LINC00685	8.31 ± 0.17	8.19 ± 0.03	8.28 ± 0.06	8.19 ± 0.07
TC20001016.hg.1	LINC00686	8.32 ± 0.33	8.41 ± 0.27	8.24 ± 0.37	8.48 ± 0.08
TC20000626.hg.1	LINC00687	6.58 ± 0.23	6.59 ± 0.21	6.45 ± 0.18	6.63 ± 0.11
TC20000627.hg.1	LINC00687	5.20 ± 0.10	5.02 ± 0.18	5.01 ± 0.18	5.03 ± 0.05
TC07001066.hg.1	LINC00689	6.97 ± 0.19	6.91 ± 0.15	6.88 ± 0.13	7.05 ± 0.14
TC03000103.hg.1	LINC00690	4.91 ± 0.06	4.83 ± 0.04	4.85 ± 0.00	4.91 ± 0.04
TC03001240.hg.1	LINC00691	5.27 ± 0.19	5.17 ± 0.16	5.26 ± 0.10	5.30 ± 0.05
TC03001249.hg.1	LINC00692	4.30 ± 0.14	4.25 ± 0.01	4.26 ± 0.10	4.38 ± 0.11
TC03000143.hg.1	LINC00693	6.95 ± 0.18	6.95 ± 0.21	6.89 ± 0.19	7.11 ± 0.13
TC03001336.hg.1	LINC00694	7.88 ± 0.26	7.68 ± 0.27	7.93 ± 0.48	7.70 ± 0.06
TC03001446.hg.1	LINC00696	7.42 ± 0.18	7.22 ± 0.25	7.21 ± 0.25	7.39 ± 0.21
TC03000387.hg.1	LINC00698	5.35 ± 0.15	5.26 ± 0.03	5.28 ± 0.02	5.40 ± 0.11
TC10000971.hg.1	LINC00700	5.31 ± 0.15	5.16 ± 0.11	5.28 ± 0.18	5.25 ± 0.21
TC10000973.hg.1	LINC00701	7.40 ± 0.08	7.21 ± 0.15	7.33 ± 0.20	7.29 ± 0.14
TC10000980.hg.1	LINC00702	4.21 ± 0.12	4.09 ± 0.20	4.20 ± 0.04	4.19 ± 0.08
TC10000036.hg.1	LINC00703	5.67 ± 0.11	5.49 ± 0.04	5.52 ± 0.02	5.49 ± 0.12
TC10000982.hg.1	LINC00704	5.80 ± 0.04	5.79 ± 0.07	5.80 ± 0.04	5.87 ± 0.07
TC10000038.hg.1	LINC00705	5.23 ± 0.06	5.03 ± 0.05	5.17 ± 0.09	4.99 ± 0.04
TC10000062.hg.1	LINC00707	4.94 ± 0.05	4.79 ± 0.07	4.89 ± 0.04	5.02 ± 0.18
TC10001024.hg.1	LINC00708	5.68 ± 0.30	5.52 ± 0.06	5.52 ± 0.06	5.56 ± 0.11
TC10000075.hg.1	LINC00709	4.91 ± 0.10	4.71 ± 0.14	4.79 ± 0.39	4.90 ± 0.06
TC10001032.hg.1	LINC00710	4.66 ± 0.21	4.52 ± 0.11	4.57 ± 0.08	4.60 ± 0.06
TC10001142.hg.1	LINC00837	5.53 ± 0.23	5.56 ± 0.10	5.54 ± 0.07	5.60 ± 0.07
TC10001169.hg.1	LINC00838	5.56 ± 0.20	5.50 ± 0.13	5.63 ± 0.13	5.68 ± 0.18
TC10000262.hg.1	LINC00839	7.33 ± 0.17	7.28 ± 0.14	7.36 ± 0.13	7.36 ± 0.06

TC10000283.hg.1	LINC00840	6.38 ± 0.11	6.31 ± 0.18	6.43 ± 0.08	6.54 ± 0.11
TC10000284.hg.1	LINC00841	6.13 ± 0.15	6.11 ± 0.11	6.09 ± 0.16	6.21 ± 0.06
TC10002962.hg.1	LINC00842	6.61 ± 0.21	6.63 ± 0.18	6.55 ± 0.16	6.80 ± 0.11
TC10000334.hg.1	LINC00843	8.84 ± 0.28	8.95 ± 0.15	8.61 ± 0.19	8.95 ± 0.10
TC10000365.hg.1	LINC00844	4.10 ± 0.07	3.88 ± 0.16	3.99 ± 0.11	4.08 ± 0.14
TC10000374.hg.1	LINC00845	4.77 ± 0.13	4.79 ± 0.13	4.60 ± 0.08	4.84 ± 0.06
TC05001066.hg.1	LINC00847	8.55 ± 0.19	8.36 ± 0.08	8.34 ± 0.09	8.46 ± 0.03
TC10001348.hg.1	LINC00849	5.70 ± 0.08	5.73 ± 0.14	5.63 ± 0.07	5.73 ± 0.08
TC20000121.hg.1	LINC00851	6.99 ± 0.25	7.02 ± 0.20	6.99 ± 0.24	7.07 ± 0.10
TC03000059.hg.1	LINC00852	8.20 ± 0.21	8.28 ± 0.07	8.08 ± 0.15	8.37 ± 0.05
TC01000596.hg.1	LINC00853	6.65 ± 0.16	6.44 ± 0.12	6.58 ± 0.15	6.50 ± 0.12
TC17001555.hg.1	LINC00854	6.39 ± 0.17	6.28 ± 0.08	6.36 ± 0.12	6.29 ± 0.03
TC10000559.hg.1	LINC00856	6.43 ± 0.15	6.46 ± 0.15	6.41 ± 0.12	6.55 ± 0.11
TC10000577.hg.1	LINC00857	5.28 ± 0.06	5.14 ± 0.14	5.15 ± 0.12	5.35 ± 0.12
TC10000595.hg.1	LINC00858	5.63 ± 0.02	5.59 ± 0.03	5.60 ± 0.19	5.71 ± 0.14
TC08001620.hg.1	LINC00861	7.74 ± 0.35	7.53 ± 0.29	7.61 ± 0.53	7.31 ± 0.09
TC01003677.hg.1	LINC00862	5.79 ± 0.06	5.66 ± 0.07	6.00 ± 0.15	5.71 ± 0.10
TC10001234.hg.1	LINC00864	7.22 ± 0.15	7.09 ± 0.09	7.01 ± 0.13	7.21 ± 0.20
TC10001488.hg.1	LINC00864	6.30 ± 0.32	6.24 ± 0.11	6.02 ± 0.20	6.37 ± 0.11
TC10000645.hg.1	LINC00865	4.85 ± 0.16	4.74 ± 0.07	4.85 ± 0.14	4.80 ± 0.01
TC10001572.hg.1	LINC00866	7.03 ± 0.29	7.00 ± 0.18	6.94 ± 0.19	7.13 ± 0.19
TC10000858.hg.1	LINC00867	5.76 ± 0.18	5.67 ± 0.07	5.71 ± 0.07	5.70 ± 0.10
TC01001164.hg.1	LINC00869	7.86 ± 0.14	7.89 ± 0.11	7.85 ± 0.13	7.81 ± 0.05
TC03000424.hg.1	LINC00870	5.60 ± 0.16	5.63 ± 0.08	5.58 ± 0.12	5.67 ± 0.16
TC14000269.hg.1	LINC00871	5.14 ± 0.15	5.05 ± 0.00	5.12 ± 0.04	5.14 ± 0.11
TC03000476.hg.1	LINC00879	3.94 ± 0.15	3.87 ± 0.03	3.92 ± 0.07	3.88 ± 0.04
TC03001938.hg.1	LINC00880	7.56 ± 0.09	7.61 ± 0.07	7.43 ± 0.20	7.70 ± 0.15
TC03000858.hg.1	LINC00881	6.80 ± 0.25	6.88 ± 0.22	6.82 ± 0.13	6.99 ± 0.11
TC03001625.hg.1	LINC00882	5.06 ± 0.08	4.92 ± 0.01	4.96 ± 0.04	4.98 ± 0.07
TC03000538.hg.1	LINC00883	6.17 ± 0.08	6.07 ± 0.07	6.08 ± 0.06	6.11 ± 0.03
TC03001074.hg.1	LINC00884	7.18 ± 0.05	7.12 ± 0.14	7.73 ± 0.26	7.12 ± 0.05

TC03001092.hg.1	LINC00885	6.77 ± 0.21	6.71 ± 0.27	6.67 ± 0.18	6.76 ± 0.11
TC03001936.hg.1	LINC00886	6.46 ± 0.03	6.41 ± 0.04	6.36 ± 0.10	6.47 ± 0.13
TC03002129.hg.1	LINC00887	6.21 ± 0.16	6.22 ± 0.19	6.20 ± 0.07	6.38 ± 0.09
TC03000979.hg.1	LINC00888	7.13 ± 0.13	6.93 ± 0.11	6.94 ± 0.20	6.88 ± 0.04
TC0X001424.hg.1	LINC00889	4.32 ± 0.07	4.22 ± 0.08	4.30 ± 0.12	4.07 ± 0.03
TC0X000545.hg.1	LINC00890	4.20 ± 0.20	4.23 ± 0.17	4.25 ± 0.10	4.19 ± 0.14
TC0X000665.hg.1	LINC00892	7.69 ± 0.13	7.63 ± 0.05	7.71 ± 0.16	7.65 ± 0.02
TC0X002345.hg.1	LINC00893	7.82 ± 0.13	7.84 ± 0.01	7.81 ± 0.08	7.91 ± 0.09
TC0X000719.hg.1	LINC00894	7.11 ± 0.12	7.07 ± 0.04	7.09 ± 0.04	7.14 ± 0.06
TC22000499.hg.1	LINC00895	7.11 ± 0.34	7.01 ± 0.30	7.00 ± 0.23	7.05 ± 0.10
TC22000057.hg.1	LINC00896	8.00 ± 0.29	7.86 ± 0.22	7.91 ± 0.12	7.92 ± 0.10
TC22000893.hg.1	LINC00898	6.72 ± 0.26	6.65 ± 0.24	6.66 ± 0.11	6.80 ± 0.05
TC22000881.hg.1	LINC00899	6.75 ± 0.15	6.60 ± 0.10	6.63 ± 0.09	6.58 ± 0.07
TC11002319.hg.1	LINC00900	6.94 ± 0.21	6.88 ± 0.06	6.85 ± 0.15	6.92 ± 0.06
TC03000600.hg.1	LINC00901	5.29 ± 0.23	5.03 ± 0.19	5.12 ± 0.05	5.24 ± 0.08
TC19000281.hg.1	LINC00905	7.63 ± 0.25	7.51 ± 0.17	7.42 ± 0.16	7.62 ± 0.08
TC19000410.hg.1	LINC00906	4.93 ± 0.09	4.79 ± 0.04	4.95 ± 0.07	4.81 ± 0.10
TC18000157.hg.1	LINC00907	5.81 ± 0.09	5.76 ± 0.09	5.77 ± 0.03	5.89 ± 0.11
TC18000255.hg.1	LINC00908	7.36 ± 0.14	7.21 ± 0.17	7.17 ± 0.19	7.20 ± 0.07
TC18000577.hg.1	LINC00909	8.17 ± 0.14	8.02 ± 0.07	8.05 ± 0.21	7.99 ± 0.07
TC17001556.hg.1	LINC00910	7.29 ± 0.04	7.18 ± 0.04	7.17 ± 0.01	7.22 ± 0.03
TC14000512.hg.1	LINC00911	4.70 ± 0.13	4.67 ± 0.04	4.71 ± 0.10	4.56 ± 0.04
TC16001324.hg.1	LINC00917	7.46 ± 0.21	7.43 ± 0.21	7.42 ± 0.14	7.56 ± 0.10
TC16001116.hg.1	LINC00918	5.54 ± 0.24	5.37 ± 0.12	5.38 ± 0.15	5.47 ± 0.09
TC16001113.hg.1	LINC00919	4.41 ± 0.36	4.21 ± 0.11	4.25 ± 0.22	4.17 ± 0.15
TC16000515.hg.1	LINC00920	6.72 ± 0.23	6.70 ± 0.07	6.58 ± 0.15	6.63 ± 0.09
TC16001167.hg.1	LINC00922	5.83 ± 0.08	5.78 ± 0.13	5.77 ± 0.06	5.87 ± 0.11
TC15001972.hg.1	LINC00923	5.84 ± 0.24	5.79 ± 0.04	5.82 ± 0.04	5.94 ± 0.08
TC15000932.hg.1	LINC00924	4.40 ± 0.21	4.10 ± 0.05	4.19 ± 0.05	4.18 ± 0.06
TC15000436.hg.1	LINC00926	8.90 ± 0.27	8.96 ± 0.18	8.76 ± 0.07	9.05 ± 0.24
TC15001722.hg.1	LINC00927	5.27 ± 0.16	5.25 ± 0.15	5.27 ± 0.08	5.39 ± 0.18

TC15001830.hg.1	LINC00928	6.15 ± 0.13	6.25 ± 0.24	6.18 ± 0.09	6.34 ± 0.09
TC15000122.hg.1	LINC00929	6.33 ± 0.14	6.23 ± 0.12	6.19 ± 0.11	6.28 ± 0.07
TC15001865.hg.1	LINC00930	6.17 ± 0.16	6.12 ± 0.18	6.06 ± 0.20	6.23 ± 0.13
TC15000815.hg.1	LINC00933	5.67 ± 0.04	5.75 ± 0.07	5.63 ± 0.11	5.83 ± 0.06
TC12002029.hg.1	LINC00934	5.44 ± 0.32	5.57 ± 0.13	5.47 ± 0.15	5.61 ± 0.15
TC12000353.hg.1	LINC00935	5.75 ± 0.19	5.53 ± 0.06	5.61 ± 0.20	5.60 ± 0.09
TC12000698.hg.1	LINC00936	8.46 ± 0.14	8.94 ± 0.10	8.85 ± 0.25	8.91 ± 0.25
TC12001177.hg.1	LINC00937	7.82 ± 0.09	7.75 ± 0.03	7.93 ± 0.04	7.82 ± 0.03
TC12001423.hg.1	LINC00938	8.88 ± 0.19	8.98 ± 0.09	8.74 ± 0.28	9.10 ± 0.06
TC12002106.hg.1	LINC00939	6.45 ± 0.15	6.43 ± 0.02	6.42 ± 0.04	6.45 ± 0.02
TC12001093.hg.1	LINC00940	7.83 ± 0.33	7.61 ± 0.13	7.58 ± 0.16	7.80 ± 0.12
TC12000283.hg.1	LINC00941	7.03 ± 0.15	7.04 ± 0.19	7.05 ± 0.09	7.19 ± 0.10
TC12000015.hg.1	LINC00942	6.99 ± 0.33	6.97 ± 0.41	6.73 ± 0.32	7.18 ± 0.06
TC12001016.hg.1	LINC00943	5.44 ± 0.21	5.35 ± 0.04	5.36 ± 0.12	5.39 ± 0.02
TC12002109.hg.1	LINC00944	5.91 ± 0.08	5.79 ± 0.02	5.83 ± 0.03	5.86 ± 0.10
TC10001316.hg.1	LINC00948	5.96 ± 0.13	5.91 ± 0.09	5.87 ± 0.07	5.91 ± 0.09
TC09000197.hg.1	LINC00950	5.07 ± 0.23	5.01 ± 0.09	4.83 ± 0.14	5.04 ± 0.10
TC06001704.hg.1	LINC00951	5.66 ± 0.18	5.73 ± 0.24	5.76 ± 0.24	5.78 ± 0.02
TC02000112.hg.1	LINC00954	7.51 ± 0.08	7.41 ± 0.07	7.35 ± 0.05	7.48 ± 0.15
TC04000050.hg.1	LINC00955	6.55 ± 0.15	6.46 ± 0.13	6.44 ± 0.03	6.61 ± 0.10
TC07000271.hg.1	LINC00957	8.71 ± 0.15	8.56 ± 0.04	8.58 ± 0.08	8.63 ± 0.06
TC11001431.hg.1	LINC00958	6.91 ± 0.51	6.78 ± 0.13	6.61 ± 0.23	7.20 ± 0.28
TC10001763.hg.1	LINC00959	7.26 ± 0.06	7.25 ± 0.01	7.15 ± 0.12	7.30 ± 0.09
TC03000442.hg.1	LINC00960	6.76 ± 0.14	6.74 ± 0.09	6.79 ± 0.09	6.84 ± 0.11
TCUn_gl000222000002.hg.1	LINC00960	7.06 ± 0.13	7.07 ± 0.08	7.12 ± 0.06	7.18 ± 0.11
TC09000199.hg.1	LINC00961	6.56 ± 0.10	6.56 ± 0.27	6.48 ± 0.28	6.69 ± 0.15
TC09000718.hg.1	LINC00963	8.20 ± 0.06	8.13 ± 0.02	8.28 ± 0.05	8.16 ± 0.06
TC08000727.hg.1	LINC00964	5.33 ± 0.12	5.27 ± 0.03	5.22 ± 0.08	5.26 ± 0.03
TC08000916.hg.1	LINC00965	6.50 ± 0.56	6.84 ± 0.09	6.63 ± 0.34	7.08 ± 0.33
TC08000430.hg.1	LINC00966	5.94 ± 0.15	5.88 ± 0.18	5.85 ± 0.18	5.96 ± 0.01
TC08000440.hg.1	LINC00967	6.47 ± 0.12	6.46 ± 0.02	6.45 ± 0.03	6.54 ± 0.14

TC08001235.hg.1	LINC00968	6.09 ± 0.03	5.95 ± 0.13	6.15 ± 0.07	6.06 ± 0.09
TC03003366.hg.1	LINC00969	7.59 ± 0.02	7.61 ± 0.02	7.44 ± 0.12	7.67 ± 0.07
TC03001578.hg.1	LINC00971	4.92 ± 0.00	4.83 ± 0.04	4.86 ± 0.03	4.89 ± 0.06
TC18000534.hg.1	LINC-ROR	6.45 ± 0.17	6.44 ± 0.06	6.49 ± 0.12	6.72 ± 0.29
TC15001694.hg.1	LINGO1	7.91 ± 0.17	7.89 ± 0.12	7.80 ± 0.15	7.96 ± 0.05
TC09000993.hg.1	LINGO2	5.29 ± 0.16	5.17 ± 0.09	5.23 ± 0.06	5.32 ± 0.10
TC19001027.hg.1	LINGO3	8.49 ± 0.33	8.58 ± 0.25	8.46 ± 0.27	8.60 ± 0.02
TC01006372.hg.1	LINGO4	7.14 ± 0.23	7.02 ± 0.15	7.00 ± 0.23	7.15 ± 0.09
TC15002000.hg.1	LINS	7.78 ± 0.31	7.83 ± 0.17	7.84 ± 0.32	7.63 ± 0.06
TC10001505.hg.1	LIPA	7.70 ± 0.06	7.56 ± 0.10	7.73 ± 0.12	7.48 ± 0.09
TC15000442.hg.1	LIPC	6.24 ± 0.07	6.05 ± 0.05	6.17 ± 0.06	6.12 ± 0.12
TC19001573.hg.1	LIPE	8.10 ± 0.17	8.01 ± 0.15	7.97 ± 0.17	8.01 ± 0.01
TC19000600.hg.1	LIPE-AS1	6.92 ± 0.20	6.77 ± 0.11	6.98 ± 0.13	6.80 ± 0.09
TC10000627.hg.1	LIPF	4.77 ± 0.11	4.68 ± 0.05	4.75 ± 0.16	4.77 ± 0.07
TC18000175.hg.1	LIPG	6.50 ± 0.16	6.44 ± 0.19	6.44 ± 0.20	6.50 ± 0.02
TC03002078.hg.1	LIPH	6.32 ± 0.18	5.74 ± 0.02	6.31 ± 0.22	5.72 ± 0.13
TC21000283.hg.1	LIPI	4.71 ± 0.08	4.65 ± 0.02	4.72 ± 0.15	4.65 ± 0.03
TC10000626.hg.1	LIPJ	3.82 ± 0.05	3.68 ± 0.04	3.75 ± 0.10	3.73 ± 0.01
TC10000628.hg.1	LIPK	4.85 ± 0.08	4.80 ± 0.09	4.87 ± 0.05	4.83 ± 0.06
TC10000630.hg.1	LIPM	5.40 ± 0.08	5.25 ± 0.08	5.40 ± 0.08	5.32 ± 0.10
TC10000629.hg.1	LIPN	6.81 ± 0.37	7.06 ± 0.26	7.63 ± 0.70	6.86 ± 0.11
TC02004992.hg.1	LIPT1	6.99 ± 0.08	7.04 ± 0.05	6.94 ± 0.08	7.05 ± 0.05
TC11002083.hg.1	LIPT2	6.08 ± 0.07	6.05 ± 0.16	5.92 ± 0.18	6.00 ± 0.05
TC16000870.hg.1	LITAF	10.64 ± 0.16	10.76 ± 0.02	10.97 ± 0.18	10.76 ± 0.20
TC05001620.hg.1	LIX1	5.04 ± 0.07	5.09 ± 0.12	5.05 ± 0.03	5.13 ± 0.09
TC01001092.hg.1	LIX1L	9.34 ± 0.09	9.40 ± 0.06	9.19 ± 0.09	9.24 ± 0.12
TC17000208.hg.1	LLGL1	8.12 ± 0.17	8.12 ± 0.12	8.01 ± 0.16	8.14 ± 0.03
TC17000855.hg.1	LLGL2	8.48 ± 0.12	8.40 ± 0.08	8.34 ± 0.13	8.43 ± 0.07
TC12003282.hg.1	LLPH	7.70 ± 0.19	7.84 ± 0.15	7.84 ± 0.14	7.90 ± 0.09
TC18000547.hg.1	LMAN1	9.89 ± 0.21	9.58 ± 0.13	9.21 ± 0.40	9.23 ± 0.23
TC05002103.hg.1	LMAN2	10.10 ± 0.33	9.68 ± 0.05	9.87 ± 0.26	9.53 ± 0.06

TC02002111.hg.1	LMAN2L	7.42 ± 0.18	7.14 ± 0.03	7.22 ± 0.19	7.10 ± 0.04
TC07002073.hg.1	LMBR1	8.13 ± 0.19	8.22 ± 0.15	8.01 ± 0.25	8.13 ± 0.06
TC12001469.hg.1	LMBR1L	8.43 ± 0.06	8.45 ± 0.04	8.24 ± 0.13	8.55 ± 0.05
TC06001854.hg.1	LMBRD1	8.87 ± 0.33	9.05 ± 0.24	9.21 ± 0.36	9.01 ± 0.23
TC05001268.hg.1	LMBRD2	7.09 ± 0.52	7.06 ± 0.32	7.29 ± 0.48	6.75 ± 0.13
TC03000026.hg.1	LMCD1	6.38 ± 0.12	6.30 ± 0.09	6.36 ± 0.13	6.41 ± 0.04
TC03001140.hg.1	LMCD1-AS1	6.63 ± 0.14	6.61 ± 0.11	6.58 ± 0.08	6.68 ± 0.11
TC16000742.hg.1	LMF1	8.24 ± 0.06	8.25 ± 0.10	8.15 ± 0.12	8.32 ± 0.04
TC22000914.hg.1	LMF2	9.11 ± 0.11	9.01 ± 0.08	8.92 ± 0.11	9.00 ± 0.05
TC03003369.hg.1	LMLN	6.65 ± 0.16	6.66 ± 0.03	6.73 ± 0.20	6.67 ± 0.11
TC03002185.hg.1	LMLN-AS1	5.84 ± 0.12	5.95 ± 0.10	6.07 ± 0.14	5.97 ± 0.06
TC01001313.hg.1	LMNA	8.11 ± 0.16	8.03 ± 0.17	8.06 ± 0.16	8.15 ± 0.03
TC05000604.hg.1	LMNB1	9.73 ± 0.49	9.55 ± 0.12	10.42 ± 0.35	9.07 ± 0.23
TC19001030.hg.1	LMNB2	8.58 ± 0.21	8.52 ± 0.10	8.46 ± 0.23	8.51 ± 0.02
TC11001385.hg.1	LMO1	7.51 ± 0.30	7.47 ± 0.19	7.41 ± 0.20	7.54 ± 0.02
TC11001543.hg.1	LMO2	8.22 ± 0.29	7.92 ± 0.11	8.30 ± 0.22	7.86 ± 0.10
TC12001287.hg.1	LMO3	5.15 ± 0.13	5.00 ± 0.03	5.04 ± 0.04	5.07 ± 0.08
TC01000830.hg.1	LMO4	7.52 ± 0.04	7.44 ± 0.05	7.51 ± 0.24	7.38 ± 0.09
TC13000286.hg.1	LMO7	6.50 ± 0.22	6.63 ± 0.05	6.35 ± 0.06	6.59 ± 0.10
TC13000740.hg.1	LMO7-AS1	5.48 ± 0.13	5.34 ± 0.14	5.27 ± 0.02	5.44 ± 0.06
TC01003697.hg.1	LMOD1	7.41 ± 0.18	7.35 ± 0.16	7.35 ± 0.14	7.47 ± 0.05
TC07000746.hg.1	LMOD2	6.13 ± 0.17	6.12 ± 0.15	6.05 ± 0.18	6.21 ± 0.08
TC03001539.hg.1	LMOD3	5.02 ± 0.07	4.83 ± 0.13	5.04 ± 0.03	4.96 ± 0.08
TC07000582.hg.1	LMTK2	8.78 ± 0.24	8.81 ± 0.17	9.00 ± 0.47	8.76 ± 0.24
TC19001684.hg.1	LMTK3	8.27 ± 0.18	8.25 ± 0.18	8.16 ± 0.14	8.31 ± 0.04
TC01003463.hg.1	LMX1A	7.11 ± 0.21	7.04 ± 0.17	7.00 ± 0.13	7.17 ± 0.09
TC09000658.hg.1	LMX1B	8.25 ± 0.20	8.27 ± 0.22	8.25 ± 0.26	8.40 ± 0.04
TC03000508.hg.1	LNP1	6.64 ± 0.12	6.62 ± 0.14	6.53 ± 0.05	6.70 ± 0.10
TC05000472.hg.1	LNPEP	9.06 ± 0.58	9.43 ± 0.27	9.20 ± 0.41	9.21 ± 0.07
TC04001199.hg.1	LNX1	6.57 ± 0.16	6.54 ± 0.09	6.52 ± 0.10	6.66 ± 0.06
TC04000306.hg.1	LNX1-AS1	5.39 ± 0.07	5.30 ± 0.14	5.38 ± 0.05	5.41 ± 0.06

TC04000307.hg.1	LNX1-AS2	4.86 ± 0.23	4.78 ± 0.10	4.90 ± 0.18	4.86 ± 0.04
TC13000511.hg.1	LNX2	7.85 ± 0.14	7.81 ± 0.06	7.73 ± 0.10	7.78 ± 0.06
TC12000009.hg.1	LOC100049716	7.54 ± 0.11	7.26 ± 0.09	7.49 ± 0.15	7.26 ± 0.02
TC0X000054.hg.1	LOC100093698	5.64 ± 0.13	5.59 ± 0.17	5.59 ± 0.06	5.66 ± 0.08
TC09001126.hg.1	LOC100126582	6.49 ± 0.53	6.32 ± 0.13	6.56 ± 0.23	6.73 ± 0.62
TC11001483.hg.1	LOC100126784	6.59 ± 0.34	6.49 ± 0.08	6.33 ± 0.32	6.65 ± 0.02
TC20001015.hg.1	LOC100127888	7.48 ± 0.20	7.56 ± 0.26	7.39 ± 0.14	7.70 ± 0.06
TC12000086.hg.1	LOC100127974	6.63 ± 0.13	6.75 ± 0.02	6.94 ± 0.18	6.70 ± 0.09
TC08001407.hg.1	LOC100127983	5.95 ± 0.14	5.84 ± 0.03	6.40 ± 0.36	5.84 ± 0.07
TC12001045.hg.1	LOC100128002	7.29 ± 0.25	7.42 ± 0.10	7.30 ± 0.10	7.59 ± 0.20
TC20000895.hg.1	LOC100128028	7.80 ± 0.12	7.67 ± 0.12	7.77 ± 0.08	7.89 ± 0.09
TC05002051.hg.1	LOC100128059	6.78 ± 0.12	7.00 ± 0.36	6.96 ± 0.05	7.14 ± 0.13
TC09000433.hg.1	LOC100128076	7.60 ± 0.28	7.54 ± 0.20	7.51 ± 0.17	7.68 ± 0.07
TC02002163.hg.1	LOC100128131	7.38 ± 0.20	7.24 ± 0.17	7.29 ± 0.08	7.35 ± 0.08
TC03001986.hg.1	LOC100128164	5.90 ± 0.10	5.82 ± 0.13	5.83 ± 0.17	5.88 ± 0.06
TC06002206.hg.1	LOC100128176	5.94 ± 0.23	5.64 ± 0.15	5.83 ± 0.27	5.68 ± 0.03
TC11001211.hg.1	LOC100128239	7.06 ± 0.21	6.90 ± 0.10	6.86 ± 0.30	6.88 ± 0.07
TC03001089.hg.1	LOC100128262	7.88 ± 0.29	7.86 ± 0.03	7.70 ± 0.12	7.91 ± 0.11
TC07002055.hg.1	LOC100128264	5.64 ± 0.40	5.53 ± 0.16	5.33 ± 0.25	5.46 ± 0.03
TC17001114.hg.1	LOC100128288	5.91 ± 0.64	5.73 ± 0.13	5.82 ± 0.09	5.68 ± 0.05
TC07000028.hg.1	LOC100128374	7.10 ± 0.37	7.04 ± 0.22	7.04 ± 0.39	7.07 ± 0.13
TC19000953.hg.1	LOC100128398	7.57 ± 0.16	7.43 ± 0.15	7.57 ± 0.09	7.55 ± 0.02
TC09000595.hg.1	LOC100128505	5.51 ± 0.15	5.43 ± 0.11	5.43 ± 0.13	5.56 ± 0.13
TC22000596.hg.1	LOC100128531	7.02 ± 0.17	7.09 ± 0.10	6.98 ± 0.13	7.12 ± 0.09
TC12001014.hg.1	LOC100128554	6.16 ± 0.13	6.07 ± 0.11	6.08 ± 0.12	6.17 ± 0.04
TC19001112.hg.1	LOC100128573	6.85 ± 0.16	6.64 ± 0.17	6.52 ± 0.20	6.75 ± 0.04
TC09000821.hg.1	LOC100128593	7.22 ± 0.33	7.16 ± 0.16	7.14 ± 0.17	7.28 ± 0.03
TC19000469.hg.1	LOC100128682	6.60 ± 0.09	6.58 ± 0.09	6.58 ± 0.05	6.67 ± 0.11
TC15000121.hg.1	LOC100128714	6.90 ± 0.16	6.86 ± 0.08	6.89 ± 0.04	6.99 ± 0.11
TC16000097.hg.1	LOC100128770	7.97 ± 0.24	8.14 ± 0.10	7.99 ± 0.15	8.29 ± 0.11
TC01002922.hg.1	LOC100128787	5.15 ± 0.08	5.13 ± 0.14	5.11 ± 0.02	5.23 ± 0.10

TC07001028.hg.1	LOC100128822	8.18 ± 0.16	8.09 ± 0.34	7.94 ± 0.30	8.07 ± 0.09
TC16000704.hg.1	LOC100128881	7.97 ± 0.28	7.86 ± 0.15	7.88 ± 0.12	7.97 ± 0.05
TC09001323.hg.1	LOC100128909	6.37 ± 0.18	6.09 ± 0.10	6.11 ± 0.13	6.13 ± 0.08
TC22000409.hg.1	LOC100128946	7.43 ± 0.15	7.47 ± 0.12	7.36 ± 0.15	7.50 ± 0.08
TC15001538.hg.1	LOC100128979	7.28 ± 0.29	7.26 ± 0.14	7.11 ± 0.21	7.22 ± 0.05
TC20000301.hg.1	LOC100128988	5.56 ± 0.04	5.55 ± 0.02	5.50 ± 0.11	5.56 ± 0.14
TC08001019.hg.1	LOC100128993	7.15 ± 0.18	7.10 ± 0.17	7.05 ± 0.15	7.21 ± 0.04
TC21000550.hg.1	LOC100129027	7.72 ± 0.30	7.77 ± 0.33	7.71 ± 0.35	7.96 ± 0.13
TC09000637.hg.1	LOC100129034	9.35 ± 0.40	8.96 ± 0.15	9.88 ± 0.45	8.91 ± 0.21
TC01000864.hg.1	LOC100129046	7.09 ± 0.16	7.10 ± 0.07	7.00 ± 0.06	7.21 ± 0.16
TC10000254.hg.1	LOC100129055	4.75 ± 0.06	4.62 ± 0.04	4.63 ± 0.06	4.65 ± 0.04
TC03001424.hg.1	LOC100129060	7.54 ± 0.08	7.44 ± 0.11	7.48 ± 0.12	7.55 ± 0.07
TC19000779.hg.1	LOC100129083	8.60 ± 0.10	8.45 ± 0.11	8.59 ± 0.09	8.50 ± 0.07
TC08000973.hg.1	LOC100129129	7.63 ± 0.07	7.59 ± 0.02	7.66 ± 0.21	7.62 ± 0.17
TC01000922.hg.1	LOC100129138	7.83 ± 0.25	7.69 ± 0.13	7.81 ± 0.22	7.78 ± 0.05
TC07001913.hg.1	LOC100129148	6.46 ± 0.13	6.45 ± 0.08	6.56 ± 0.26	6.49 ± 0.09
TC02001317.hg.1	LOC100129175	8.62 ± 0.13	8.38 ± 0.12	8.46 ± 0.24	8.44 ± 0.10
TC11000930.hg.1	LOC100129203	6.61 ± 0.21	6.58 ± 0.17	6.45 ± 0.17	6.46 ± 0.06
TC10001078.hg.1	LOC100129213	4.80 ± 0.16	4.66 ± 0.04	4.64 ± 0.03	4.70 ± 0.06
TC11000745.hg.1	LOC100129216	4.99 ± 0.07	4.94 ± 0.13	4.96 ± 0.22	5.21 ± 0.29
TC01002988.hg.1	LOC100129269	6.72 ± 0.25	6.73 ± 0.01	6.74 ± 0.20	6.86 ± 0.15
TC03000574.hg.1	LOC100129297	5.45 ± 0.15	5.34 ± 0.08	5.39 ± 0.05	5.45 ± 0.13
TC09001321.hg.1	LOC100129316	6.03 ± 0.10	6.07 ± 0.17	5.93 ± 0.15	6.11 ± 0.05
TC14001489.hg.1	LOC100129345	5.23 ± 0.11	5.21 ± 0.08	5.19 ± 0.09	5.25 ± 0.13
TC12000167.hg.1	LOC100129361	7.53 ± 0.14	7.74 ± 0.05	7.46 ± 0.06	7.69 ± 0.17
TC07000316.hg.1	LOC100129427	7.10 ± 0.21	7.09 ± 0.18	7.03 ± 0.15	7.16 ± 0.09
TC02001874.hg.1	LOC100129434	6.84 ± 0.10	6.76 ± 0.12	6.65 ± 0.23	6.80 ± 0.10
TC06004142.hg.1	LOC100129518	7.82 ± 0.12	7.85 ± 0.21	7.60 ± 0.16	7.81 ± 0.23
TC0X000611.hg.1	LOC100129520	7.42 ± 0.16	7.29 ± 0.11	7.39 ± 0.06	7.20 ± 0.05
TC01002116.hg.1	LOC100129534	7.40 ± 0.10	7.62 ± 0.22	7.36 ± 0.36	7.64 ± 0.11
TC15000737.hg.1	LOC100129540	7.22 ± 0.42	7.27 ± 0.27	7.13 ± 0.27	7.27 ± 0.10

TC03000637.hg.1	LOC100129550	7.17 ± 0.31	7.25 ± 0.36	7.55 ± 0.47	7.50 ± 0.06
TC16000641.hg.1	LOC100129617	7.35 ± 0.07	7.19 ± 0.14	7.42 ± 0.23	7.28 ± 0.00
TC01000890.hg.1	LOC100129620	7.09 ± 0.27	7.02 ± 0.25	7.09 ± 0.13	7.03 ± 0.21
TC06000308.hg.1	LOC100129636	6.22 ± 0.25	6.14 ± 0.02	6.16 ± 0.18	6.22 ± 0.07
TC6_apd_hap1000003.hg.1	LOC100129636	6.01 ± 0.20	5.84 ± 0.00	5.88 ± 0.17	5.88 ± 0.09
TC6_cox_hap2000006.hg.1	LOC100129636	6.22 ± 0.25	6.14 ± 0.02	6.16 ± 0.18	6.22 ± 0.07
TC6_dbb_hap3000002.hg.1	LOC100129636	6.22 ± 0.25	6.14 ± 0.02	6.16 ± 0.18	6.22 ± 0.07
TC6_mann_hap4000003.hg.1	LOC100129636	6.22 ± 0.25	6.14 ± 0.02	6.16 ± 0.18	6.22 ± 0.07
TC6_mcf_hap5000003.hg.1	LOC100129636	6.19 ± 0.25	6.11 ± 0.01	6.14 ± 0.18	6.18 ± 0.07
TC6_qbl_hap6000004.hg.1	LOC100129636	6.22 ± 0.25	6.14 ± 0.02	6.16 ± 0.18	6.22 ± 0.07
TC6_ssto_hap7000004.hg.1	LOC100129636	6.22 ± 0.25	6.14 ± 0.02	6.16 ± 0.18	6.22 ± 0.07
TC09001768.hg.1	LOC100129722	7.82 ± 0.25	7.88 ± 0.17	7.84 ± 0.25	7.90 ± 0.08
TC02000267.hg.1	LOC100129726	8.07 ± 0.17	7.81 ± 0.06	7.88 ± 0.17	7.88 ± 0.03
TC04001588.hg.1	LOC100129858	5.81 ± 0.07	5.68 ± 0.08	5.68 ± 0.05	5.73 ± 0.08
TC04000954.hg.1	LOC100129917	7.85 ± 0.21	7.91 ± 0.00	7.89 ± 0.01	7.95 ± 0.00
TC01000533.hg.1	LOC100129924	7.35 ± 0.14	7.30 ± 0.03	7.22 ± 0.06	7.38 ± 0.06
TC04001005.hg.1	LOC100129931	6.75 ± 0.33	6.55 ± 0.08	6.68 ± 0.18	6.49 ± 0.05
TC19000552.hg.1	LOC100129935	5.61 ± 0.36	5.58 ± 0.11	5.47 ± 0.16	5.67 ± 0.11
TC02005044.hg.1	LOC100129961	7.31 ± 0.04	7.29 ± 0.03	7.19 ± 0.05	7.19 ± 0.06
TC15000424.hg.1	LOC100129973	7.22 ± 0.06	7.31 ± 0.05	7.07 ± 0.03	7.41 ± 0.08
TC01001070.hg.1	LOC100130000	6.99 ± 0.04	6.94 ± 0.05	7.00 ± 0.10	6.95 ± 0.01
TC16001363.hg.1	LOC100130015	8.35 ± 0.16	8.30 ± 0.13	8.29 ± 0.13	8.40 ± 0.10
TC15000155.hg.1	LOC100130111	7.08 ± 0.14	7.00 ± 0.10	7.00 ± 0.02	7.10 ± 0.05
TC05000693.hg.1	LOC100130172	7.63 ± 0.31	7.75 ± 0.15	7.68 ± 0.13	7.75 ± 0.09
TC12001056.hg.1	LOC100130238	7.60 ± 0.15	7.59 ± 0.26	7.63 ± 0.16	7.71 ± 0.09
TC20000675.hg.1	LOC100130264	5.60 ± 0.04	5.67 ± 0.08	5.67 ± 0.06	5.78 ± 0.14
TC06000069.hg.1	LOC100130275	6.68 ± 0.22	6.58 ± 0.13	6.57 ± 0.27	6.67 ± 0.09
TC08001261.hg.1	LOC100130298	7.74 ± 0.15	7.52 ± 0.02	7.79 ± 0.14	7.46 ± 0.07
TC08001327.hg.1	LOC100130301	5.77 ± 0.15	5.63 ± 0.06	5.67 ± 0.04	5.64 ± 0.13
TC01001960.hg.1	LOC100130331	5.74 ± 0.18	5.65 ± 0.16	5.68 ± 0.07	5.71 ± 0.04
TC11001925.hg.1	LOC100130348	6.13 ± 0.24	6.15 ± 0.21	6.06 ± 0.27	6.26 ± 0.05

TC01002077.hg.1	LOC100130417	8.14 ± 0.27	8.05 ± 0.19	7.97 ± 0.28	8.11 ± 0.05
TC02002742.hg.1	LOC100130451	5.93 ± 0.18	6.03 ± 0.04	5.93 ± 0.16	6.28 ± 0.35
TC02002641.hg.1	LOC100130452	5.05 ± 0.07	5.06 ± 0.07	5.06 ± 0.09	5.19 ± 0.13
TC18000032.hg.1	LOC100130480	6.58 ± 0.11	6.57 ± 0.20	6.52 ± 0.14	6.67 ± 0.09
TC02001802.hg.1	LOC100130502	6.45 ± 0.20	6.47 ± 0.24	6.37 ± 0.26	6.58 ± 0.06
TC09000780.hg.1	LOC100130548	7.84 ± 0.38	7.81 ± 0.18	7.84 ± 0.27	7.89 ± 0.11
TC01002560.hg.1	LOC100130557	7.47 ± 0.14	7.41 ± 0.06	7.27 ± 0.14	7.44 ± 0.04
TC07001257.hg.1	LOC100130673	5.54 ± 0.03	5.49 ± 0.20	5.26 ± 0.01	5.36 ± 0.10
TC02005046.hg.1	LOC100130691	6.11 ± 0.12	6.05 ± 0.03	6.01 ± 0.04	6.06 ± 0.04
TC16001080.hg.1	LOC100130700	6.85 ± 0.19	6.91 ± 0.24	6.83 ± 0.14	7.04 ± 0.06
TC07000787.hg.1	LOC100130705	6.68 ± 0.19	6.87 ± 0.11	6.72 ± 0.15	6.96 ± 0.05
TC07001427.hg.1	LOC100130849	8.31 ± 0.14	8.39 ± 0.18	8.29 ± 0.11	8.51 ± 0.04
TC07000857.hg.1	LOC100130880	6.64 ± 0.21	6.64 ± 0.22	6.60 ± 0.13	6.79 ± 0.12
TC10000882.hg.1	LOC100130887	6.21 ± 0.27	6.19 ± 0.19	6.14 ± 0.19	6.19 ± 0.04
TC22000322.hg.1	LOC100130899	6.96 ± 0.51	7.07 ± 0.24	6.78 ± 0.35	7.18 ± 0.06
TC17000064.hg.1	LOC100130950	7.58 ± 0.07	7.51 ± 0.02	7.45 ± 0.17	7.52 ± 0.09
TC09000800.hg.1	LOC100130954	7.26 ± 0.10	7.16 ± 0.09	7.15 ± 0.08	7.29 ± 0.09
TC08000303.hg.1	LOC100130964	5.22 ± 0.16	5.04 ± 0.05	5.12 ± 0.03	5.16 ± 0.11
TC11000685.hg.1	LOC100130987	7.45 ± 0.21	7.36 ± 0.18	7.38 ± 0.22	7.52 ± 0.05
TC10000155.hg.1	LOC100130992	5.39 ± 0.29	5.32 ± 0.06	5.46 ± 0.36	5.40 ± 0.22
TC06001694.hg.1	LOC100131047	5.03 ± 0.14	4.90 ± 0.04	4.96 ± 0.06	4.98 ± 0.08
TC01000696.hg.1	LOC100131060	6.43 ± 0.21	6.42 ± 0.25	6.30 ± 0.22	6.48 ± 0.07
TC05001543.hg.1	LOC100131067	6.53 ± 0.11	6.58 ± 0.15	6.51 ± 0.09	6.67 ± 0.06
TC19000092.hg.1	LOC100131094	7.80 ± 0.26	7.83 ± 0.33	7.77 ± 0.19	7.85 ± 0.02
TC10001156.hg.1	LOC100131107	7.96 ± 0.17	7.80 ± 0.08	7.72 ± 0.04	8.02 ± 0.27
TC12000862.hg.1	LOC100131138	7.06 ± 0.20	7.00 ± 0.14	7.01 ± 0.15	7.10 ± 0.05
TC07001136.hg.1	LOC100131257	5.40 ± 0.12	5.43 ± 0.03	5.47 ± 0.06	5.40 ± 0.21
TC06000262.hg.1	LOC100131289	7.28 ± 0.08	7.23 ± 0.09	7.19 ± 0.17	7.31 ± 0.06
TC02002300.hg.1	LOC100131320	6.44 ± 0.15	6.34 ± 0.12	6.38 ± 0.00	6.37 ± 0.03
TC17000472.hg.1	LOC100131347	6.59 ± 0.05	6.60 ± 0.09	6.57 ± 0.08	6.69 ± 0.10
TC20000370.hg.1	LOC100131496	6.58 ± 0.31	6.55 ± 0.13	6.47 ± 0.15	6.46 ± 0.11

TC01002876.hg.1	LOC100131564	7.61 ± 0.33	7.65 ± 0.09	7.60 ± 0.17	7.68 ± 0.07
TC03001034.hg.1	LOC100131635	5.67 ± 0.15	5.69 ± 0.12	5.78 ± 0.06	5.70 ± 0.06
TC18000583.hg.1	LOC100131655	7.71 ± 0.14	7.64 ± 0.17	7.57 ± 0.10	7.73 ± 0.11
TC19000972.hg.1	LOC100131691	7.83 ± 0.23	7.74 ± 0.16	7.69 ± 0.13	7.80 ± 0.02
TC01003385.hg.1	LOC100131825	7.38 ± 0.30	7.48 ± 0.22	7.46 ± 0.22	7.53 ± 0.03
TC09000462.hg.1	LOC100132077	7.92 ± 0.10	7.80 ± 0.06	7.97 ± 0.27	7.61 ± 0.17
TC11002280.hg.1	LOC100132078	6.76 ± 0.16	6.78 ± 0.06	6.70 ± 0.07	6.78 ± 0.03
TC01001220.hg.1	LOC100132111	7.15 ± 0.21	7.01 ± 0.11	7.03 ± 0.08	7.02 ± 0.04
TC03003404.hg.1	LOC100132146	5.91 ± 0.06	5.97 ± 0.09	5.93 ± 0.04	6.02 ± 0.11
TC01002268.hg.1	LOC100132147	7.42 ± 0.19	7.39 ± 0.14	7.31 ± 0.15	7.53 ± 0.14
TC09000264.hg.1	LOC100132167	7.42 ± 0.20	7.42 ± 0.06	7.25 ± 0.06	7.40 ± 0.10
TC02001902.hg.1	LOC100132215	6.74 ± 0.33	6.69 ± 0.21	6.70 ± 0.20	6.74 ± 0.06
TC01002880.hg.1	LOC100132240	6.55 ± 0.27	6.55 ± 0.30	6.58 ± 0.22	6.67 ± 0.12
TC01000005.hg.1	LOC100132287	11.70 ± 0.32	11.67 ± 0.20	12.35 ± 0.26	11.74 ± 0.17
TC05003443.hg.1	LOC100132287	12.10 ± 0.32	12.09 ± 0.18	12.68 ± 0.26	12.18 ± 0.14
TC09000289.hg.1	LOC100132352	8.21 ± 0.30	8.30 ± 0.17	8.10 ± 0.30	8.23 ± 0.03
TC06000609.hg.1	LOC100132354	7.10 ± 0.14	7.21 ± 0.10	7.08 ± 0.12	7.29 ± 0.09
TC05001315.hg.1	LOC100132356	8.34 ± 0.23	8.09 ± 0.11	8.25 ± 0.19	8.17 ± 0.13
TC11001011.hg.1	LOC100132686	4.74 ± 0.17	4.77 ± 0.04	4.93 ± 0.19	4.90 ± 0.12
TC07001033.hg.1	LOC100132707	7.44 ± 0.32	7.41 ± 0.19	7.38 ± 0.32	7.25 ± 0.03
TC06001037.hg.1	LOC100132735	5.15 ± 0.07	4.94 ± 0.01	4.97 ± 0.02	4.92 ± 0.10
TC0X000390.hg.1	LOC100132741	7.92 ± 0.18	7.55 ± 0.25	7.32 ± 0.24	7.63 ± 0.06
TC09000484.hg.1	LOC100132781	4.00 ± 0.17	3.95 ± 0.14	4.00 ± 0.09	4.03 ± 0.17
TC19001365.hg.1	LOC100132815	7.72 ± 0.17	7.68 ± 0.15	7.39 ± 0.09	7.74 ± 0.01
TC0X000968.hg.1	LOC100132831	7.33 ± 0.37	7.27 ± 0.10	7.29 ± 0.36	7.30 ± 0.10
TC08000471.hg.1	LOC100132891	6.02 ± 0.10	5.92 ± 0.12	5.99 ± 0.05	6.00 ± 0.09
TC01003055.hg.1	LOC100132913	10.37 ± 0.14	10.22 ± 0.06	10.29 ± 0.10	10.18 ± 0.18
TC01001138.hg.1	LOC100132999	10.25 ± 0.17	10.25 ± 0.10	10.10 ± 0.10	10.35 ± 0.09
TC05001635.hg.1	LOC100133050	8.66 ± 0.22	8.70 ± 0.05	8.50 ± 0.13	8.71 ± 0.12
TC09001778.hg.1	LOC100133077	7.82 ± 0.16	7.73 ± 0.05	7.65 ± 0.14	7.77 ± 0.05
TC07000491.hg.1	LOC100133091	8.81 ± 0.08	8.79 ± 0.01	8.69 ± 0.02	8.79 ± 0.01

TC0X000055.hg.1	LOC100133123	4.33 ± 0.10	4.26 ± 0.06	4.25 ± 0.10	4.23 ± 0.08
TC11003501.hg.1	LOC100133161	11.63 ± 0.34	11.66 ± 0.17	12.27 ± 0.24	11.74 ± 0.14
TC14000597.hg.1	LOC100133207	7.30 ± 0.24	7.45 ± 0.08	7.28 ± 0.13	7.41 ± 0.14
TC08000040.hg.1	LOC100133251	6.49 ± 0.21	6.29 ± 0.16	6.17 ± 0.17	6.50 ± 0.24
TC08000926.hg.1	LOC100133251	6.49 ± 0.21	6.29 ± 0.16	6.17 ± 0.17	6.50 ± 0.24
TC21000429.hg.1	LOC100133286	6.67 ± 0.09	6.45 ± 0.08	6.58 ± 0.19	6.47 ± 0.08
TC11002048.hg.1	LOC100133315	7.60 ± 0.12	7.58 ± 0.07	7.49 ± 0.12	7.60 ± 0.02
TC04000984.hg.1	LOC100133461	7.72 ± 0.05	7.75 ± 0.23	7.76 ± 0.20	7.85 ± 0.13
TC01000075.hg.1	LOC100133612	6.91 ± 0.26	6.90 ± 0.22	6.88 ± 0.17	7.00 ± 0.09
TC08001710.hg.1	LOC100133669	7.29 ± 0.28	7.19 ± 0.20	7.20 ± 0.21	7.18 ± 0.02
TC15001724.hg.1	LOC100133746	6.77 ± 0.17	6.62 ± 0.09	6.69 ± 0.05	6.82 ± 0.20
TC09001157.hg.1	LOC100133920	6.38 ± 0.10	6.26 ± 0.07	6.20 ± 0.09	6.45 ± 0.19
TC0X000240.hg.1	LOC100133957	7.34 ± 0.24	7.46 ± 0.06	7.38 ± 0.09	7.48 ± 0.05
TC02001956.hg.1	LOC100133985	8.12 ± 0.16	7.96 ± 0.17	7.95 ± 0.08	8.09 ± 0.22
TC07000638.hg.1	LOC100134216	7.18 ± 0.21	7.21 ± 0.21	7.13 ± 0.17	7.39 ± 0.14
TC07000878.hg.1	LOC100134229	8.05 ± 0.24	8.10 ± 0.14	7.86 ± 0.10	8.02 ± 0.22
TC02004964.hg.1	LOC100134259	6.56 ± 0.14	6.51 ± 0.19	6.34 ± 0.16	6.66 ± 0.15
TC19000498.hg.1	LOC100134317	6.74 ± 0.38	6.69 ± 0.08	6.61 ± 0.38	6.82 ± 0.15
TC22000844.hg.1	LOC100134361	7.28 ± 0.24	7.10 ± 0.11	7.09 ± 0.25	7.12 ± 0.04
TC16000012.hg.1	LOC100134368	8.76 ± 0.24	8.70 ± 0.05	8.68 ± 0.24	8.68 ± 0.04
TC20000175.hg.1	LOC100134868	7.38 ± 0.17	7.36 ± 0.04	7.26 ± 0.06	7.40 ± 0.04
TC02002440.hg.1	LOC100144595	5.81 ± 0.17	5.86 ± 0.01	5.83 ± 0.08	5.94 ± 0.13
TC20000505.hg.1	LOC100144597	7.55 ± 0.19	7.64 ± 0.22	7.62 ± 0.21	7.74 ± 0.03
TC04000355.hg.1	LOC100144602	5.89 ± 0.18	5.88 ± 0.28	5.84 ± 0.18	5.97 ± 0.07
TC01001446.hg.1	LOC100147773	6.20 ± 0.16	6.18 ± 0.02	6.12 ± 0.10	6.18 ± 0.08
TC10000910.hg.1	LOC100169752	6.79 ± 0.16	6.62 ± 0.13	6.74 ± 0.09	6.78 ± 0.08
TC10001521.hg.1	LOC100188947	6.21 ± 0.21	6.10 ± 0.13	5.95 ± 0.22	6.35 ± 0.15
TC12002128.hg.1	LOC100190940	6.82 ± 0.21	6.74 ± 0.13	6.74 ± 0.20	6.80 ± 0.03
TC16000936.hg.1	LOC100190986	10.08 ± 0.62	10.52 ± 0.22	10.10 ± 0.33	10.48 ± 0.15
TC18000314.hg.1	LOC100192426	7.64 ± 0.22	7.53 ± 0.12	7.52 ± 0.20	7.66 ± 0.14
TC07001729.hg.1	LOC100216545	8.71 ± 0.12	8.98 ± 0.08	8.58 ± 0.11	8.88 ± 0.23

TC07001728.hg.1	LOC100216546	8.10 ± 0.27	8.03 ± 0.17	8.18 ± 0.42	7.96 ± 0.12
TC12000455.hg.1	LOC100240734	4.99 ± 0.27	4.90 ± 0.03	4.86 ± 0.02	4.87 ± 0.18
TC12000456.hg.1	LOC100240734	6.36 ± 0.09	6.43 ± 0.07	6.21 ± 0.12	6.49 ± 0.25
TC12001561.hg.1	LOC100240735	7.36 ± 0.10	7.30 ± 0.17	7.33 ± 0.13	7.53 ± 0.12
TC05002068.hg.1	LOC100268168	6.89 ± 0.14	6.85 ± 0.08	6.77 ± 0.04	6.98 ± 0.06
TC20000148.hg.1	LOC100270679	5.55 ± 0.10	5.57 ± 0.06	5.61 ± 0.02	5.69 ± 0.06
TC06001383.hg.1	LOC100270746	7.37 ± 0.25	7.37 ± 0.19	7.32 ± 0.17	7.51 ± 0.06
TC20000128.hg.1	LOC100270804	7.10 ± 0.14	7.18 ± 0.06	6.84 ± 0.03	7.21 ± 0.07
TC02000218.hg.1	LOC100271832	6.19 ± 0.08	6.15 ± 0.04	6.15 ± 0.29	6.19 ± 0.11
TC05001443.hg.1	LOC100272216	9.50 ± 0.18	9.25 ± 0.18	9.87 ± 0.30	9.18 ± 0.14
TC09001658.hg.1	LOC100272217	4.99 ± 0.16	4.64 ± 0.09	4.69 ± 0.07	4.63 ± 0.15
TC02002885.hg.1	LOC100286922	6.66 ± 0.30	6.52 ± 0.30	6.53 ± 0.13	6.58 ± 0.04
TC22000099.hg.1	LOC100286925	8.32 ± 0.18	8.23 ± 0.11	8.21 ± 0.21	8.28 ± 0.05
TC02002156.hg.1	LOC100287010	4.66 ± 0.10	4.56 ± 0.10	4.60 ± 0.08	4.56 ± 0.15
TC08000901.hg.1	LOC100287015	6.91 ± 0.13	6.78 ± 0.13	6.78 ± 0.17	6.86 ± 0.05
TC16000695.hg.1	LOC100287036	7.30 ± 0.18	7.29 ± 0.19	7.23 ± 0.16	7.37 ± 0.12
TC17000850.hg.1	LOC100287042	8.09 ± 0.42	8.13 ± 0.26	7.99 ± 0.34	8.14 ± 0.07
TC01001365.hg.1	LOC100287049	7.03 ± 0.24	6.91 ± 0.10	6.88 ± 0.13	7.05 ± 0.11
TC08000852.hg.1	LOC100287098	9.00 ± 0.20	9.01 ± 0.15	8.96 ± 0.14	9.12 ± 0.07
TC19000650.hg.1	LOC100287177	8.51 ± 0.33	8.53 ± 0.19	8.37 ± 0.36	8.59 ± 0.06
TC18000182.hg.1	LOC100287225	6.26 ± 0.08	6.24 ± 0.03	6.23 ± 0.13	6.21 ± 0.08
TC11000479.hg.1	LOC100287413	7.06 ± 0.21	7.01 ± 0.20	7.02 ± 0.18	7.18 ± 0.10
TC01001840.hg.1	LOC100287497	7.64 ± 0.68	8.07 ± 0.07	7.84 ± 0.58	8.18 ± 0.06
TC05001336.hg.1	LOC100287592	7.93 ± 0.23	7.89 ± 0.15	7.93 ± 0.27	8.02 ± 0.09
TC06000936.hg.1	LOC100287632	5.26 ± 0.33	5.09 ± 0.09	5.06 ± 0.00	5.00 ± 0.11
TC07000358.hg.1	LOC100287704	6.90 ± 0.05	6.93 ± 0.14	6.90 ± 0.09	7.03 ± 0.08
TC0X000646.hg.1	LOC100287728	6.21 ± 0.05	6.21 ± 0.16	6.12 ± 0.07	6.32 ± 0.11
TC20000275.hg.1	LOC100287792	6.70 ± 0.35	6.71 ± 0.12	6.69 ± 0.25	6.77 ± 0.13
TC08000342.hg.1	LOC100287846	5.55 ± 0.10	5.62 ± 0.10	5.52 ± 0.14	5.65 ± 0.07
TC03001524.hg.1	LOC100287879	5.60 ± 0.08	5.50 ± 0.12	5.39 ± 0.12	5.54 ± 0.04
TC11000789.hg.1	LOC100287896	7.96 ± 0.17	8.01 ± 0.09	7.93 ± 0.14	7.93 ± 0.05

TC01000015.hg.1	LOC100287934	7.32 ± 0.25	7.41 ± 0.07	7.48 ± 0.07	7.51 ± 0.04
TC01000016.hg.1	LOC100287934	7.38 ± 0.65	7.73 ± 0.19	7.48 ± 0.39	7.77 ± 0.13
TC12001920.hg.1	LOC100287944	6.98 ± 0.20	6.83 ± 0.15	6.89 ± 0.22	7.00 ± 0.10
TC01002072.hg.1	LOC100288069	11.54 ± 0.32	11.56 ± 0.22	12.13 ± 0.22	11.56 ± 0.23
TC01003630.hg.1	LOC100288079	4.60 ± 0.05	4.46 ± 0.02	4.56 ± 0.06	4.53 ± 0.09
TC19000045.hg.1	LOC100288123	8.10 ± 0.05	8.14 ± 0.15	8.07 ± 0.11	8.15 ± 0.11
TC05000007.hg.1	LOC100288152	7.47 ± 0.34	7.60 ± 0.23	7.40 ± 0.09	7.82 ± 0.06
TC08001701.hg.1	LOC100288181	6.61 ± 0.30	6.52 ± 0.05	6.61 ± 0.09	6.76 ± 0.05
TC06000755.hg.1	LOC100288198	6.73 ± 0.16	6.45 ± 0.04	6.50 ± 0.09	6.45 ± 0.15
TC21000503.hg.1	LOC100288336	8.42 ± 0.24	8.41 ± 0.13	8.29 ± 0.19	8.40 ± 0.10
TC07000005.hg.1	LOC100288524	7.76 ± 0.23	7.61 ± 0.29	7.60 ± 0.23	7.75 ± 0.02
TC15002761.hg.1	LOC100288637	6.60 ± 0.09	6.48 ± 0.03	6.54 ± 0.04	6.50 ± 0.09
TC04000940.hg.1	LOC100288657	9.43 ± 0.25	9.34 ± 0.06	9.27 ± 0.25	9.70 ± 0.32
TC08001435.hg.1	LOC100288748	7.36 ± 0.35	7.31 ± 0.17	7.39 ± 0.21	7.35 ± 0.10
TC12000002.hg.1	LOC100288778	9.46 ± 0.05	9.44 ± 0.02	9.42 ± 0.08	9.47 ± 0.02
TC0X000035.hg.1	LOC100288814	5.88 ± 0.20	6.07 ± 0.26	5.87 ± 0.07	5.94 ± 0.13
TC09000605.hg.1	LOC100288842	6.40 ± 0.16	6.21 ± 0.07	6.13 ± 0.12	6.08 ± 0.01
TC14001066.hg.1	LOC100288846	7.28 ± 0.17	7.29 ± 0.03	7.41 ± 0.12	7.30 ± 0.07
TC17001667.hg.1	LOC100288866	7.16 ± 0.02	7.10 ± 0.04	7.04 ± 0.28	7.19 ± 0.08
TC02001734.hg.1	LOC100288911	6.51 ± 0.24	6.29 ± 0.10	6.23 ± 0.11	6.33 ± 0.03
TC09001622.hg.1	LOC100289019	7.79 ± 0.08	7.92 ± 0.06	7.65 ± 0.16	7.99 ± 0.09
TC15000316.hg.1	LOC100289090	7.38 ± 0.07	7.38 ± 0.16	7.12 ± 0.06	7.44 ± 0.13
TC07000409.hg.1	LOC100289098	7.70 ± 0.12	7.84 ± 0.14	7.73 ± 0.14	7.85 ± 0.08
TC07003290.hg.1	LOC100289187	7.07 ± 0.20	7.16 ± 0.21	7.03 ± 0.15	7.22 ± 0.04
TC05000480.hg.1	LOC100289230	7.72 ± 0.15	7.68 ± 0.15	7.71 ± 0.08	7.65 ± 0.05
TC03001861.hg.1	LOC100289361	7.11 ± 0.16	7.22 ± 0.12	7.20 ± 0.05	7.22 ± 0.16
TC20000556.hg.1	LOC100289473	7.92 ± 0.11	7.90 ± 0.11	8.13 ± 0.12	7.99 ± 0.07
TC06001176.hg.1	LOC100289495	6.81 ± 0.37	6.89 ± 0.17	6.89 ± 0.22	6.96 ± 0.14
TC14001262.hg.1	LOC100289511	8.23 ± 0.24	8.41 ± 0.14	8.13 ± 0.14	8.39 ± 0.13
TC07000658.hg.1	LOC100289561	8.71 ± 0.00	8.68 ± 0.03	8.55 ± 0.05	8.70 ± 0.01
TC16001061.hg.1	LOC100289574	7.81 ± 0.14	7.69 ± 0.04	7.65 ± 0.08	7.68 ± 0.10

TC16000684.hg.1	LOC100289580	7.73 ± 0.35	7.77 ± 0.29	7.72 ± 0.26	7.84 ± 0.04
TC19000602.hg.1	LOC100289650	5.11 ± 0.21	4.97 ± 0.09	5.15 ± 0.09	5.06 ± 0.06
TC15000151.hg.1	LOC100289656	6.47 ± 0.27	6.27 ± 0.05	6.33 ± 0.12	6.36 ± 0.16
TC05000516.hg.1	LOC100289673	6.50 ± 0.37	6.70 ± 0.21	6.60 ± 0.21	6.87 ± 0.16
TC20000975.hg.1	LOC100291105	7.14 ± 0.18	7.17 ± 0.21	6.98 ± 0.09	7.31 ± 0.08
TC12000984.hg.1	LOC100293704	7.08 ± 0.22	6.68 ± 0.26	6.86 ± 0.28	6.76 ± 0.11
TC12001473.hg.1	LOC100293962	8.46 ± 0.31	8.51 ± 0.08	8.42 ± 0.23	8.43 ± 0.06
TC06000405.hg.1	LOC100294145	7.73 ± 0.12	7.51 ± 0.11	7.52 ± 0.12	7.37 ± 0.07
TC6_apd_hap1000049.hg.1	LOC100294145	7.24 ± 0.18	7.01 ± 0.13	7.08 ± 0.24	6.89 ± 0.03
TC6_cox_hap2000096.hg.1	LOC100294145	7.37 ± 0.06	7.11 ± 0.16	7.20 ± 0.30	6.96 ± 0.03
TC6_dbb_hap3000089.hg.1	LOC100294145	6.79 ± 0.07	6.51 ± 0.15	6.60 ± 0.25	6.38 ± 0.09
TC6_mann_hap4000080.hg.1	LOC100294145	7.07 ± 0.08	6.83 ± 0.16	6.87 ± 0.27	6.68 ± 0.03
TC6_mcf_hap5000081.hg.1	LOC100294145	7.24 ± 0.18	7.01 ± 0.13	7.08 ± 0.24	6.89 ± 0.03
TC6_qbl_hap6000087.hg.1	LOC100294145	7.07 ± 0.08	6.83 ± 0.16	6.87 ± 0.27	6.68 ± 0.03
TC6_ssto_hap7000082.hg.1	LOC100294145	7.24 ± 0.18	7.01 ± 0.13	7.08 ± 0.24	6.89 ± 0.03
TC17001935.hg.1	LOC100294362	8.03 ± 0.11	8.02 ± 0.18	8.10 ± 0.08	8.10 ± 0.04
TC05001412.hg.1	LOC100303749	4.88 ± 0.40	4.72 ± 0.07	4.90 ± 0.14	4.76 ± 0.04
TC12000373.hg.1	LOC100335030	6.83 ± 0.20	6.87 ± 0.28	6.93 ± 0.12	6.87 ± 0.22
TC19001603.hg.1	LOC100379224	4.73 ± 0.11	4.61 ± 0.06	4.74 ± 0.10	4.67 ± 0.05
TC06001985.hg.1	LOC100422737	6.09 ± 0.13	5.97 ± 0.07	5.98 ± 0.03	6.05 ± 0.07
TC08001505.hg.1	LOC100499183	5.94 ± 0.11	5.87 ± 0.05	5.89 ± 0.09	5.94 ± 0.06
TC02002233.hg.1	LOC100499194	6.51 ± 0.41	6.54 ± 0.11	6.70 ± 0.18	6.67 ± 0.13
TC12000142.hg.1	LOC100499405	7.38 ± 0.12	7.43 ± 0.15	7.34 ± 0.14	7.33 ± 0.14
TC10001100.hg.1	LOC100499489	6.96 ± 0.20	7.14 ± 0.09	7.03 ± 0.09	7.17 ± 0.06
TC08001447.hg.1	LOC100500773	5.42 ± 0.15	5.58 ± 0.15	5.47 ± 0.10	5.63 ± 0.06
TC18000531.hg.1	LOC100505474	4.72 ± 0.09	4.61 ± 0.02	4.64 ± 0.06	4.63 ± 0.05
TC09001514.hg.1	LOC100505478	6.09 ± 0.21	6.14 ± 0.19	6.08 ± 0.09	6.29 ± 0.09
TC19002705.hg.1	LOC100505495	7.12 ± 0.14	6.98 ± 0.11	7.02 ± 0.11	7.12 ± 0.08
TC08000406.hg.1	LOC100505501	8.16 ± 0.29	8.00 ± 0.09	8.09 ± 0.23	8.05 ± 0.07
TC11000666.hg.1	LOC100505504	7.06 ± 0.23	6.94 ± 0.15	7.01 ± 0.19	7.03 ± 0.08
TC20000091.hg.1	LOC100505515	6.84 ± 0.20	6.74 ± 0.29	6.60 ± 0.07	6.90 ± 0.22

TC13000299.hg.1	LOC100505518	4.33 ± 0.13	4.28 ± 0.07	4.26 ± 0.02	4.34 ± 0.12
TC08001254.hg.1	LOC100505532	6.62 ± 0.27	6.39 ± 0.15	6.56 ± 0.05	6.56 ± 0.04
TC15000271.hg.1	LOC100505534	6.05 ± 0.31	5.98 ± 0.11	5.79 ± 0.25	6.10 ± 0.09
TC10000691.hg.1	LOC100505540	5.91 ± 0.08	5.96 ± 0.23	6.01 ± 0.19	5.94 ± 0.13
TC04001619.hg.1	LOC100505545	5.32 ± 0.13	5.35 ± 0.14	5.28 ± 0.11	5.43 ± 0.10
TC03002019.hg.1	LOC100505547	5.00 ± 0.29	4.83 ± 0.08	4.81 ± 0.04	4.78 ± 0.04
TC18000196.hg.1	LOC100505549	7.40 ± 0.06	7.36 ± 0.04	7.13 ± 0.07	7.27 ± 0.04
TC06000547.hg.1	LOC100505550	6.67 ± 0.02	6.76 ± 0.21	6.57 ± 0.25	6.88 ± 0.07
TC06001685.hg.1	LOC100505550	6.46 ± 0.12	6.32 ± 0.17	6.37 ± 0.11	6.39 ± 0.08
TC11000048.hg.1	LOC100505570	6.40 ± 0.21	6.26 ± 0.26	6.26 ± 0.10	6.26 ± 0.12
TC15000279.hg.1	LOC100505573	8.26 ± 0.22	7.70 ± 0.20	8.23 ± 0.31	7.88 ± 0.28
TC09001575.hg.1	LOC100505588	6.70 ± 0.30	6.62 ± 0.12	6.56 ± 0.24	6.69 ± 0.02
TC04000726.hg.1	LOC100505596	5.64 ± 0.21	5.64 ± 0.12	5.60 ± 0.16	5.82 ± 0.20
TC04000727.hg.1	LOC100505596	4.46 ± 0.28	4.26 ± 0.11	4.40 ± 0.06	4.31 ± 0.18
TC03000960.hg.1	LOC100505609	4.23 ± 0.08	4.19 ± 0.11	4.27 ± 0.01	4.26 ± 0.18
TC15000791.hg.1	LOC100505616	5.72 ± 0.07	5.69 ± 0.14	5.72 ± 0.02	5.71 ± 0.06
TC19000591.hg.1	LOC100505622	7.62 ± 0.19	7.58 ± 0.09	7.49 ± 0.16	7.62 ± 0.04
TC02000171.hg.1	LOC100505624	7.18 ± 0.08	7.07 ± 0.11	7.15 ± 0.04	7.19 ± 0.02
TC01001358.hg.1	LOC100505633	5.22 ± 0.12	5.04 ± 0.04	5.16 ± 0.01	5.08 ± 0.05
TC05001852.hg.1	LOC100505636	5.71 ± 0.15	5.64 ± 0.04	5.62 ± 0.04	5.72 ± 0.09
TC19000592.hg.1	LOC100505639	8.13 ± 0.29	8.14 ± 0.25	7.89 ± 0.17	8.10 ± 0.02
TC05000770.hg.1	LOC100505658	5.31 ± 0.12	5.11 ± 0.17	5.26 ± 0.08	5.01 ± 0.11
TC20000297.hg.1	LOC100505663	4.84 ± 0.13	4.66 ± 0.12	4.69 ± 0.07	4.78 ± 0.14
TC20000152.hg.1	LOC100505664	7.42 ± 0.23	7.59 ± 0.22	7.47 ± 0.16	7.81 ± 0.09
TC01003302.hg.1	LOC100505666	7.23 ± 0.17	7.29 ± 0.20	7.14 ± 0.26	7.44 ± 0.08
TC08001284.hg.1	LOC100505676	7.62 ± 0.34	7.51 ± 0.14	7.60 ± 0.18	7.69 ± 0.12
TC15000805.hg.1	LOC100505679	7.07 ± 0.21	7.18 ± 0.06	7.16 ± 0.14	7.23 ± 0.04
TC20000707.hg.1	LOC100505683	7.00 ± 0.05	7.01 ± 0.17	6.98 ± 0.07	7.12 ± 0.05
TC04000762.hg.1	LOC100505685	5.94 ± 0.11	5.93 ± 0.06	5.89 ± 0.05	6.09 ± 0.24
TC02001013.hg.1	LOC100505695	4.90 ± 0.16	4.84 ± 0.06	4.85 ± 0.04	4.93 ± 0.09
TC06001708.hg.1	LOC100505697	7.00 ± 0.30	7.18 ± 0.17	7.14 ± 0.22	7.35 ± 0.12

TC04000439.hg.1	LOC100505702	6.73 ± 0.04	6.60 ± 0.12	7.06 ± 0.27	6.67 ± 0.06
TC19001599.hg.1	LOC100505715	4.90 ± 0.13	4.89 ± 0.18	4.87 ± 0.09	4.82 ± 0.10
TC02001698.hg.1	LOC100505716	7.77 ± 0.32	7.59 ± 0.16	7.72 ± 0.14	7.66 ± 0.06
TC08001300.hg.1	LOC100505718	4.58 ± 0.16	4.53 ± 0.13	4.56 ± 0.05	4.71 ± 0.11
TC20000493.hg.1	LOC100505735	5.61 ± 0.18	5.56 ± 0.06	5.63 ± 0.08	5.59 ± 0.11
TC10000756.hg.1	LOC100505761	8.65 ± 0.20	8.71 ± 0.08	8.50 ± 0.17	8.77 ± 0.09
TC01002838.hg.1	LOC100505768	6.13 ± 0.11	6.05 ± 0.06	6.12 ± 0.01	6.06 ± 0.09
TC20000520.hg.1	LOC100505771	8.94 ± 0.15	8.70 ± 0.09	8.70 ± 0.08	8.76 ± 0.13
TC02001702.hg.1	LOC100505774	5.56 ± 0.03	5.65 ± 0.10	5.69 ± 0.03	5.60 ± 0.05
TC08000341.hg.1	LOC100505775	6.91 ± 0.11	7.28 ± 0.19	6.96 ± 0.16	7.32 ± 0.24
TC18000569.hg.1	LOC100505776	4.87 ± 0.19	4.77 ± 0.07	4.82 ± 0.12	4.86 ± 0.07
TC17000511.hg.1	LOC100505782	6.74 ± 0.12	6.69 ± 0.24	6.66 ± 0.14	6.83 ± 0.08
TC01001110.hg.1	LOC100505794	6.60 ± 0.28	6.31 ± 0.20	6.67 ± 0.19	6.07 ± 0.24
TC01003459.hg.1	LOC100505795	5.60 ± 0.09	5.52 ± 0.13	5.65 ± 0.17	5.73 ± 0.08
TC01003370.hg.1	LOC100505799	5.19 ± 0.19	5.00 ± 0.04	5.06 ± 0.12	5.11 ± 0.09
TC05000066.hg.1	LOC100505806	6.16 ± 0.16	6.10 ± 0.08	6.02 ± 0.12	6.20 ± 0.12
TC05000557.hg.1	LOC100505811	8.33 ± 0.40	8.56 ± 0.11	8.25 ± 0.29	8.84 ± 0.44
TC19000698.hg.1	LOC100505812	7.65 ± 0.08	7.26 ± 0.31	7.91 ± 0.48	7.01 ± 0.32
TC20000527.hg.1	LOC100505815	8.19 ± 0.02	8.44 ± 0.08	8.33 ± 0.38	8.54 ± 0.29
TC18000245.hg.1	LOC100505817	6.92 ± 0.13	6.70 ± 0.06	6.92 ± 0.10	6.82 ± 0.14
TC05001595.hg.1	LOC100505822	4.63 ± 0.14	4.53 ± 0.18	4.51 ± 0.15	4.59 ± 0.09
TC21000546.hg.1	LOC100505827	7.56 ± 0.15	7.58 ± 0.15	7.52 ± 0.12	7.64 ± 0.10
TC01001444.hg.1	LOC100505828	7.76 ± 0.20	7.78 ± 0.05	7.83 ± 0.10	7.74 ± 0.06
TC11000717.hg.1	LOC100505834	4.20 ± 0.07	4.14 ± 0.15	4.19 ± 0.07	4.07 ± 0.03
TC19000412.hg.1	LOC100505835	7.30 ± 0.11	7.22 ± 0.07	7.19 ± 0.22	7.23 ± 0.08
TC01000844.hg.1	LOC100505840	6.18 ± 0.32	6.20 ± 0.13	6.20 ± 0.31	6.29 ± 0.22
TC05003442.hg.1	LOC100505841	5.60 ± 0.09	5.44 ± 0.10	5.44 ± 0.08	5.50 ± 0.04
TC04000441.hg.1	LOC100505875	4.91 ± 0.05	4.80 ± 0.03	4.78 ± 0.01	4.90 ± 0.06
TC02004958.hg.1	LOC100505876	7.76 ± 0.32	7.88 ± 0.21	8.00 ± 0.43	7.79 ± 0.08
TC05000428.hg.1	LOC100505878	4.06 ± 0.05	3.99 ± 0.09	4.01 ± 0.03	4.04 ± 0.08
TC01000094.hg.1	LOC100505887	5.94 ± 0.15	5.90 ± 0.12	5.84 ± 0.05	5.88 ± 0.09

TC03002126.hg.1	LOC100505902	6.06 ± 0.14	6.06 ± 0.12	6.07 ± 0.15	6.14 ± 0.08
TC06001197.hg.1	LOC100505903	7.70 ± 0.21	7.43 ± 0.12	7.47 ± 0.08	7.47 ± 0.08
TC04001078.hg.1	LOC100505912	5.16 ± 0.11	5.13 ± 0.14	5.09 ± 0.07	5.29 ± 0.13
TC01003488.hg.1	LOC100505918	4.54 ± 0.17	4.44 ± 0.16	4.48 ± 0.09	4.51 ± 0.06
TC03002128.hg.1	LOC100505920	6.82 ± 0.23	6.92 ± 0.17	6.91 ± 0.07	7.01 ± 0.15
TC07001141.hg.1	LOC100505921	5.39 ± 0.55	5.51 ± 0.38	5.50 ± 0.61	5.52 ± 0.18
TC07000081.hg.1	LOC100505938	6.41 ± 0.12	6.30 ± 0.09	6.35 ± 0.11	6.42 ± 0.10
TC01000871.hg.1	LOC100505940	6.86 ± 0.08	6.99 ± 0.33	6.90 ± 0.13	7.20 ± 0.13
TC05000436.hg.1	LOC100505949	7.37 ± 0.23	7.31 ± 0.19	7.20 ± 0.22	7.39 ± 0.07
TC02001530.hg.1	LOC100505964	6.72 ± 0.13	6.72 ± 0.21	6.67 ± 0.11	6.78 ± 0.09
TC21000309.hg.1	LOC100505973	5.26 ± 0.13	5.16 ± 0.11	5.16 ± 0.04	5.28 ± 0.10
TC12001921.hg.1	LOC100505978	6.79 ± 0.32	6.75 ± 0.26	6.72 ± 0.35	6.81 ± 0.07
TC20000377.hg.1	LOC100505983	5.35 ± 0.13	5.33 ± 0.11	5.36 ± 0.06	5.38 ± 0.07
TC06000644.hg.1	LOC100505985	4.17 ± 0.22	4.00 ± 0.12	3.88 ± 0.31	3.95 ± 0.07
TC09000659.hg.1	LOC100505986	7.52 ± 0.19	7.50 ± 0.10	7.55 ± 0.10	7.66 ± 0.09
TC04000821.hg.1	LOC100505989	4.19 ± 0.24	4.17 ± 0.19	4.12 ± 0.09	4.22 ± 0.01
TC14000996.hg.1	LOC100506004	4.66 ± 0.09	4.63 ± 0.18	4.59 ± 0.16	4.74 ± 0.22
TC01002919.hg.1	LOC100506007	6.93 ± 0.38	6.81 ± 0.04	7.09 ± 0.09	6.85 ± 0.11
TC01001123.hg.1	LOC100506011	4.33 ± 0.10	4.22 ± 0.06	4.16 ± 0.07	4.27 ± 0.08
TC04000823.hg.1	LOC100506013	4.96 ± 0.13	4.84 ± 0.02	4.82 ± 0.13	4.88 ± 0.03
TC02004922.hg.1	LOC100506014	7.05 ± 0.20	6.91 ± 0.06	6.83 ± 0.13	6.93 ± 0.09
TC01003526.hg.1	LOC100506023	7.65 ± 0.07	7.55 ± 0.03	7.67 ± 0.05	7.62 ± 0.12
TC07000108.hg.1	LOC100506025	4.80 ± 0.11	4.70 ± 0.06	4.69 ± 0.04	4.75 ± 0.07
TC04000442.hg.1	LOC100506035	8.14 ± 0.22	7.60 ± 0.33	8.08 ± 0.38	7.82 ± 0.40
TC02001788.hg.1	LOC100506047	7.35 ± 0.18	7.47 ± 0.19	7.28 ± 0.06	7.61 ± 0.08
TC04001096.hg.1	LOC100506048	6.36 ± 0.08	6.16 ± 0.10	6.06 ± 0.05	6.12 ± 0.08
TC07001448.hg.1	LOC100506050	6.31 ± 0.24	6.34 ± 0.10	6.27 ± 0.10	6.53 ± 0.17
TC01000907.hg.1	LOC100506051	6.20 ± 0.17	6.18 ± 0.16	6.05 ± 0.15	6.08 ± 0.07
TC07001436.hg.1	LOC100506053	6.98 ± 0.20	6.84 ± 0.08	6.97 ± 0.16	7.09 ± 0.28
TC02000021.hg.1	LOC100506054	7.17 ± 0.19	7.07 ± 0.14	6.96 ± 0.24	7.11 ± 0.07
TC15000378.hg.1	LOC100506059	6.13 ± 0.47	5.97 ± 0.10	6.11 ± 0.25	6.05 ± 0.15

TC16001230.hg.1	LOC100506060	8.69 ± 0.56	9.02 ± 0.14	8.68 ± 0.41	8.92 ± 0.16
TC14001006.hg.1	LOC100506071	4.59 ± 0.11	4.43 ± 0.11	4.60 ± 0.26	4.33 ± 0.09
TC16001235.hg.1	LOC100506083	7.41 ± 0.07	7.27 ± 0.14	7.33 ± 0.14	7.29 ± 0.07
TC04001728.hg.1	LOC100506085	4.49 ± 0.06	4.49 ± 0.01	4.50 ± 0.09	4.56 ± 0.17
TC06001255.hg.1	LOC100506091	4.02 ± 0.40	3.83 ± 0.04	3.87 ± 0.14	3.79 ± 0.06
TC07000123.hg.1	LOC100506098	5.58 ± 0.11	5.55 ± 0.07	5.63 ± 0.13	5.68 ± 0.16
TC09000700.hg.1	LOC100506100	7.10 ± 0.21	6.98 ± 0.08	6.99 ± 0.14	6.76 ± 0.05
TC11001323.hg.1	LOC100506101	6.43 ± 0.33	6.59 ± 0.15	6.43 ± 0.09	6.66 ± 0.10
TC05001779.hg.1	LOC100506102	6.26 ± 0.33	6.25 ± 0.23	6.16 ± 0.32	6.27 ± 0.08
TC16000591.hg.1	LOC100506105	7.08 ± 0.22	7.14 ± 0.16	7.08 ± 0.05	7.23 ± 0.06
TC04001732.hg.1	LOC100506107	6.49 ± 0.32	6.14 ± 0.19	6.08 ± 0.18	6.32 ± 0.29
TC11002103.hg.1	LOC100506113	6.75 ± 0.09	6.76 ± 0.19	6.84 ± 0.04	6.82 ± 0.07
TC20000401.hg.1	LOC100506115	9.42 ± 0.39	9.11 ± 0.15	10.14 ± 0.34	9.03 ± 0.35
TC09000714.hg.1	LOC100506119	7.26 ± 0.15	7.14 ± 0.13	7.27 ± 0.03	7.24 ± 0.04
TC04000851.hg.1	LOC100506122	4.04 ± 0.19	3.82 ± 0.04	3.88 ± 0.02	3.85 ± 0.06
TC02000590.hg.1	LOC100506123	8.12 ± 0.55	8.34 ± 0.05	7.97 ± 0.33	8.35 ± 0.19
TC02002119.hg.1	LOC100506123	7.37 ± 0.51	7.76 ± 0.09	7.40 ± 0.27	7.71 ± 0.18
TC12000355.hg.1	LOC100506125	6.36 ± 0.14	6.33 ± 0.21	6.35 ± 0.23	6.39 ± 0.05
TC11000819.hg.1	LOC100506127	6.51 ± 0.15	6.46 ± 0.07	6.45 ± 0.10	6.47 ± 0.09
TC01003559.hg.1	LOC100506128	4.84 ± 0.14	4.84 ± 0.11	4.88 ± 0.02	4.95 ± 0.14
TC02000989.hg.1	LOC100506134	5.05 ± 0.08	5.01 ± 0.04	4.99 ± 0.04	5.02 ± 0.10
TC07003408.hg.1	LOC100506136	5.34 ± 0.17	5.18 ± 0.03	5.11 ± 0.14	5.15 ± 0.07
TC04000858.hg.1	LOC100506154	4.97 ± 0.20	4.78 ± 0.01	4.81 ± 0.14	4.71 ± 0.04
TC14001032.hg.1	LOC100506157	7.20 ± 0.06	6.94 ± 0.16	7.20 ± 0.32	6.90 ± 0.05
TC01001828.hg.1	LOC100506161	5.21 ± 0.15	5.12 ± 0.16	5.06 ± 0.07	5.12 ± 0.05
TC16000610.hg.1	LOC100506172	6.52 ± 0.26	6.60 ± 0.14	6.57 ± 0.12	6.85 ± 0.20
TC20000406.hg.1	LOC100506175	7.26 ± 0.14	7.08 ± 0.05	7.19 ± 0.01	7.09 ± 0.10
TC07000135.hg.1	LOC100506178	6.59 ± 0.21	6.53 ± 0.21	6.47 ± 0.20	6.68 ± 0.06
TC16001348.hg.1	LOC100506183	7.67 ± 0.27	7.68 ± 0.18	7.52 ± 0.30	7.71 ± 0.06
TC09000044.hg.1	LOC100506189	5.52 ± 0.24	5.43 ± 0.12	5.41 ± 0.25	5.57 ± 0.19
TC09000719.hg.1	LOC100506190	11.68 ± 0.06	11.61 ± 0.07	11.77 ± 0.10	11.63 ± 0.10

TC06000065.hg.1	LOC100506207	5.91 ± 0.14	5.82 ± 0.02	5.77 ± 0.07	5.82 ± 0.05
TC02001540.hg.1	LOC100506216	4.24 ± 0.17	4.10 ± 0.10	4.15 ± 0.08	4.23 ± 0.17
TC02001541.hg.1	LOC100506216	5.54 ± 0.14	5.50 ± 0.08	5.49 ± 0.05	5.52 ± 0.03
TC12000164.hg.1	LOC100506226	5.87 ± 0.05	5.78 ± 0.20	5.85 ± 0.17	5.87 ± 0.04
TC04001813.hg.1	LOC100506229	6.80 ± 0.35	6.55 ± 0.07	6.85 ± 0.18	6.66 ± 0.07
TC02000300.hg.1	LOC100506235	7.99 ± 0.11	7.96 ± 0.06	8.00 ± 0.13	7.98 ± 0.09
TC11000146.hg.1	LOC100506238	4.67 ± 0.23	4.53 ± 0.15	4.57 ± 0.14	4.78 ± 0.15
TC22000273.hg.1	LOC100506241	6.92 ± 0.24	6.93 ± 0.04	6.91 ± 0.01	7.09 ± 0.14
TC11003504.hg.1	LOC100506258	6.51 ± 0.13	6.46 ± 0.08	6.41 ± 0.08	6.54 ± 0.04
TC22000727.hg.1	LOC100506271	6.66 ± 0.17	6.72 ± 0.10	6.63 ± 0.13	6.92 ± 0.13
TC04001829.hg.1	LOC100506272	4.98 ± 0.16	4.82 ± 0.12	4.87 ± 0.06	4.93 ± 0.04
TC02000036.hg.1	LOC100506274	4.84 ± 0.08	4.72 ± 0.04	4.74 ± 0.09	4.78 ± 0.10
TC11002156.hg.1	LOC100506282	7.06 ± 0.12	7.02 ± 0.12	7.19 ± 0.23	6.99 ± 0.01
TC06001257.hg.1	LOC100506288	5.30 ± 0.25	5.29 ± 0.07	5.29 ± 0.16	5.43 ± 0.09
TC07000163.hg.1	LOC100506289	5.70 ± 0.18	5.77 ± 0.09	5.70 ± 0.05	5.84 ± 0.15
TC02000308.hg.1	LOC100506300	3.85 ± 0.04	3.73 ± 0.13	3.72 ± 0.06	3.56 ± 0.01
TC07002062.hg.1	LOC100506302	7.37 ± 0.01	7.28 ± 0.07	7.40 ± 0.10	7.30 ± 0.02
TC14000026.hg.1	LOC100506303	5.98 ± 0.20	5.99 ± 0.05	5.93 ± 0.07	6.12 ± 0.19
TC09000068.hg.1	LOC100506304	3.98 ± 0.03	3.96 ± 0.03	4.02 ± 0.14	3.94 ± 0.19
TC01000148.hg.1	LOC100506310	7.86 ± 0.22	7.89 ± 0.21	7.81 ± 0.24	7.99 ± 0.05
TC12000183.hg.1	LOC100506314	6.01 ± 0.15	5.96 ± 0.05	5.98 ± 0.10	5.98 ± 0.02
TC14000396.hg.1	LOC100506321	6.71 ± 0.13	6.74 ± 0.02	6.73 ± 0.08	6.81 ± 0.06
TC17001631.hg.1	LOC100506325	6.19 ± 0.20	6.16 ± 0.02	6.17 ± 0.01	6.19 ± 0.00
TC05000213.hg.1	LOC100506329	5.36 ± 0.24	4.98 ± 0.05	5.17 ± 0.12	5.21 ± 0.17
TC08001398.hg.1	LOC100506342	6.04 ± 0.08	6.02 ± 0.03	5.96 ± 0.11	6.06 ± 0.01
TC01000980.hg.1	LOC100506343	7.14 ± 0.16	6.96 ± 0.13	7.10 ± 0.14	7.04 ± 0.07
TC08000550.hg.1	LOC100506351	5.64 ± 0.05	5.69 ± 0.13	5.63 ± 0.10	5.77 ± 0.15
TC11001432.hg.1	LOC100506352	5.73 ± 0.23	5.56 ± 0.09	5.63 ± 0.01	5.50 ± 0.08
TC08001410.hg.1	LOC100506365	7.80 ± 0.32	8.07 ± 0.08	7.91 ± 0.23	8.04 ± 0.10
TC11000877.hg.1	LOC100506368	6.93 ± 0.11	6.82 ± 0.13	6.88 ± 0.09	6.93 ± 0.01
TC17000962.hg.1	LOC100506371	6.69 ± 0.07	6.59 ± 0.21	6.67 ± 0.20	6.74 ± 0.04

TC17000627.hg.1	LOC100506373	6.46 ± 0.24	6.56 ± 0.18	6.37 ± 0.11	6.70 ± 0.08
TC03000498.hg.1	LOC100506377	4.42 ± 0.09	4.40 ± 0.25	4.46 ± 0.36	4.56 ± 0.21
TC07002069.hg.1	LOC100506380	6.35 ± 0.06	6.21 ± 0.09	6.31 ± 0.08	6.31 ± 0.08
TC20000997.hg.1	LOC100506384	6.21 ± 0.29	6.12 ± 0.09	6.02 ± 0.14	6.27 ± 0.27
TC21000140.hg.1	LOC100506385	6.47 ± 0.36	6.54 ± 0.15	6.40 ± 0.10	6.72 ± 0.11
TC17000001.hg.1	LOC100506388	6.93 ± 0.28	6.73 ± 0.15	6.79 ± 0.16	6.91 ± 0.16
TC06000893.hg.1	LOC100506389	5.60 ± 0.35	5.61 ± 0.14	5.53 ± 0.19	5.65 ± 0.21
TC01000996.hg.1	LOC100506392	6.67 ± 0.17	6.53 ± 0.05	6.57 ± 0.11	6.55 ± 0.05
TC12000226.hg.1	LOC100506393	4.65 ± 0.20	4.57 ± 0.07	4.57 ± 0.07	4.62 ± 0.07
TC13000431.hg.1	LOC100506394	6.41 ± 0.19	6.26 ± 0.13	6.39 ± 0.04	6.38 ± 0.10
TC02000079.hg.1	LOC100506405	7.39 ± 0.07	7.13 ± 0.10	7.07 ± 0.05	7.08 ± 0.06
TC05000160.hg.1	LOC100506406	5.40 ± 0.25	5.38 ± 0.05	5.49 ± 0.20	5.42 ± 0.10
TC14000428.hg.1	LOC100506411	6.34 ± 0.25	6.31 ± 0.23	6.39 ± 0.31	6.50 ± 0.08
TC07000705.hg.1	LOC100506413	4.49 ± 0.18	4.19 ± 0.09	4.28 ± 0.19	4.24 ± 0.04
TC09001229.hg.1	LOC100506414	4.62 ± 0.16	4.58 ± 0.09	4.64 ± 0.14	4.59 ± 0.03
TC02002162.hg.1	LOC100506421	6.34 ± 0.16	6.27 ± 0.16	6.21 ± 0.11	6.38 ± 0.09
TC09000116.hg.1	LOC100506422	5.11 ± 0.20	5.00 ± 0.12	5.06 ± 0.03	5.10 ± 0.09
TC14001110.hg.1	LOC100506446	5.64 ± 0.19	5.57 ± 0.19	5.51 ± 0.08	5.63 ± 0.06
TC0X000815.hg.1	LOC100506453	8.46 ± 0.18	8.27 ± 0.10	8.10 ± 0.19	8.34 ± 0.04
TC22000036.hg.1	LOC100506454	7.24 ± 0.33	7.10 ± 0.25	7.28 ± 0.23	7.31 ± 0.10
TC02000080.hg.1	LOC100506457	6.55 ± 0.09	6.52 ± 0.13	6.51 ± 0.08	6.61 ± 0.13
TC01003028.hg.1	LOC100506459	6.96 ± 0.34	6.51 ± 0.22	6.92 ± 0.22	6.59 ± 0.03
TC12001992.hg.1	LOC100506465	6.25 ± 0.14	6.14 ± 0.09	6.15 ± 0.19	6.29 ± 0.12
TC19001447.hg.1	LOC100506469	7.73 ± 0.07	7.70 ± 0.06	7.62 ± 0.13	7.78 ± 0.05
TC20000480.hg.1	LOC100506470	5.15 ± 0.05	5.10 ± 0.08	5.14 ± 0.05	5.08 ± 0.15
TC02001588.hg.1	LOC100506474	5.12 ± 0.04	5.12 ± 0.09	5.08 ± 0.03	5.22 ± 0.20
TC14000450.hg.1	LOC100506476	6.93 ± 0.27	6.70 ± 0.05	6.78 ± 0.14	6.75 ± 0.05
TC07001777.hg.1	LOC100506489	4.51 ± 0.05	4.41 ± 0.15	4.44 ± 0.07	4.52 ± 0.07
TC07000180.hg.1	LOC100506497	5.38 ± 0.18	5.31 ± 0.10	5.39 ± 0.07	5.33 ± 0.07
TC14001298.hg.1	LOC100506498	4.44 ± 0.25	4.15 ± 0.13	4.28 ± 0.12	4.09 ± 0.09
TC14002345.hg.1	LOC100506499	7.60 ± 0.18	7.63 ± 0.04	7.47 ± 0.20	7.60 ± 0.07

TC11000028.hg.1	LOC100506518	8.38 ± 0.22	8.26 ± 0.05	8.24 ± 0.25	8.29 ± 0.00
TC05001400.hg.1	LOC100506526	5.46 ± 0.17	5.30 ± 0.06	5.31 ± 0.07	5.30 ± 0.08
TC07001694.hg.1	LOC100506527	6.65 ± 0.08	6.76 ± 0.19	6.69 ± 0.13	6.80 ± 0.03
TC15000474.hg.1	LOC100506530	4.06 ± 0.22	4.06 ± 0.12	4.10 ± 0.23	4.08 ± 0.12
TC09001701.hg.1	LOC100506532	6.78 ± 0.13	6.82 ± 0.27	6.62 ± 0.19	7.06 ± 0.08
TC01000209.hg.1	LOC100506533	7.03 ± 0.25	7.00 ± 0.09	7.08 ± 0.23	7.14 ± 0.24
TC07001057.hg.1	LOC100506534	6.09 ± 0.15	6.03 ± 0.16	5.99 ± 0.10	5.96 ± 0.09
TC22000821.hg.1	LOC100506544	7.21 ± 0.17	6.94 ± 0.13	7.14 ± 0.14	7.12 ± 0.11
TC01001655.hg.1	LOC100506546	6.11 ± 0.18	5.99 ± 0.24	6.11 ± 0.23	6.16 ± 0.11
TC12002011.hg.1	LOC100506551	6.07 ± 0.26	6.02 ± 0.06	6.10 ± 0.02	6.04 ± 0.17
TC07001061.hg.1	LOC100506585	7.69 ± 0.13	7.64 ± 0.17	7.80 ± 0.07	7.77 ± 0.05
TC12001347.hg.1	LOC100506606	6.04 ± 0.23	6.15 ± 0.12	6.16 ± 0.36	6.09 ± 0.03
TC17001699.hg.1	LOC100506609	7.09 ± 0.23	7.18 ± 0.18	7.20 ± 0.10	7.28 ± 0.18
TC0X000056.hg.1	LOC100506610	5.48 ± 0.06	5.36 ± 0.20	5.44 ± 0.13	5.57 ± 0.11
TC0X000068.hg.1	LOC100506630	4.71 ± 0.07	4.63 ± 0.03	4.55 ± 0.05	4.57 ± 0.03
TC19001917.hg.1	LOC100506634	8.95 ± 0.14	8.78 ± 0.08	8.79 ± 0.15	8.81 ± 0.12
TC0X000182.hg.1	LOC100506641	7.29 ± 0.30	7.23 ± 0.16	7.16 ± 0.19	7.34 ± 0.06
TC17000379.hg.1	LOC100506643	6.45 ± 0.16	6.39 ± 0.05	6.42 ± 0.06	6.35 ± 0.06
TC06001284.hg.1	LOC100506647	6.12 ± 0.25	5.89 ± 0.06	6.01 ± 0.17	5.91 ± 0.06
TC08001490.hg.1	LOC100506652	7.01 ± 0.14	6.85 ± 0.16	6.98 ± 0.18	6.94 ± 0.03
TC16000974.hg.1	LOC100506655	6.69 ± 0.10	6.69 ± 0.30	6.58 ± 0.12	6.91 ± 0.06
TC07000756.hg.1	LOC100506664	4.31 ± 0.01	4.15 ± 0.03	4.14 ± 0.03	4.20 ± 0.14
TC05001327.hg.1	LOC100506674	4.78 ± 0.14	4.65 ± 0.02	4.70 ± 0.04	4.66 ± 0.05
TC11001529.hg.1	LOC100506675	4.50 ± 0.19	4.40 ± 0.11	4.46 ± 0.10	4.57 ± 0.08
TC22000360.hg.1	LOC100506679	6.76 ± 0.16	6.76 ± 0.16	6.69 ± 0.05	6.92 ± 0.08
TC07000762.hg.1	LOC100506682	4.26 ± 0.18	4.19 ± 0.07	4.29 ± 0.09	4.16 ± 0.02
TC12000321.hg.1	LOC100506684	4.67 ± 0.01	4.60 ± 0.08	4.56 ± 0.03	4.53 ± 0.04
TC05001101.hg.1	LOC100506688	7.82 ± 0.32	7.77 ± 0.22	7.71 ± 0.22	7.96 ± 0.09
TC12000968.hg.1	LOC100506691	6.90 ± 0.20	6.83 ± 0.24	6.77 ± 0.13	6.93 ± 0.09
TC22000870.hg.1	LOC100506695	7.28 ± 0.24	7.31 ± 0.26	7.24 ± 0.24	7.31 ± 0.04
TC14001371.hg.1	LOC100506700	3.89 ± 0.09	3.80 ± 0.08	3.84 ± 0.14	3.90 ± 0.05

TC16000984.hg.1	LOC100506705	6.90 ± 0.28	6.88 ± 0.32	6.83 ± 0.10	7.08 ± 0.14
TC09000210.hg.1	LOC100506710	9.37 ± 0.38	9.87 ± 0.06	9.29 ± 0.36	9.78 ± 0.18
TC17001070.hg.1	LOC100506713	8.31 ± 0.05	8.15 ± 0.05	8.32 ± 0.17	8.12 ± 0.12
TC22000872.hg.1	LOC100506714	7.59 ± 0.10	7.43 ± 0.05	7.41 ± 0.13	7.40 ± 0.03
TC07000227.hg.1	LOC100506725	5.44 ± 0.19	5.32 ± 0.14	5.35 ± 0.08	5.45 ± 0.09
TC01002297.hg.1	LOC100506730	6.32 ± 0.13	6.07 ± 0.12	6.00 ± 0.08	5.98 ± 0.07
TC14001382.hg.1	LOC100506731	5.23 ± 0.16	5.19 ± 0.08	5.23 ± 0.03	5.20 ± 0.21
TC22000877.hg.1	LOC100506737	5.96 ± 0.09	6.03 ± 0.13	6.05 ± 0.09	6.04 ± 0.11
TC04002957.hg.1	LOC100506746	6.62 ± 0.04	6.48 ± 0.06	6.56 ± 0.05	6.57 ± 0.02
TC01001684.hg.1	LOC100506775	8.09 ± 0.10	8.22 ± 0.01	8.03 ± 0.12	8.21 ± 0.09
TC07000246.hg.1	LOC100506776	7.95 ± 0.35	7.79 ± 0.23	7.68 ± 0.22	7.54 ± 0.20
TC0X000649.hg.1	LOC100506790	5.77 ± 0.20	5.99 ± 0.17	5.89 ± 0.18	5.89 ± 0.16
TC05001108.hg.1	LOC100506791	6.01 ± 0.20	5.72 ± 0.05	5.70 ± 0.10	5.80 ± 0.08
TC14001426.hg.1	LOC100506792	5.85 ± 0.32	5.80 ± 0.21	5.78 ± 0.08	5.95 ± 0.15
TC01003984.hg.1	LOC100506795	6.59 ± 0.25	6.59 ± 0.17	6.54 ± 0.16	6.69 ± 0.11
TC02002245.hg.1	LOC100506797	6.21 ± 0.03	6.19 ± 0.04	6.29 ± 0.05	6.24 ± 0.02
TC01000267.hg.1	LOC100506801	6.76 ± 0.19	6.77 ± 0.12	6.66 ± 0.09	6.73 ± 0.18
TC06000741.hg.1	LOC100506804	5.73 ± 0.11	5.69 ± 0.11	5.67 ± 0.10	5.87 ± 0.09
TC01001941.hg.1	LOC100506810	6.72 ± 0.16	6.59 ± 0.05	6.62 ± 0.23	6.57 ± 0.10
TC22000396.hg.1	LOC100506813	7.00 ± 0.10	6.86 ± 0.20	6.77 ± 0.21	6.92 ± 0.05
TC04000266.hg.1	LOC100506827	4.53 ± 0.14	4.32 ± 0.06	4.27 ± 0.03	4.39 ± 0.18
TC09000394.hg.1	LOC100506834	5.66 ± 0.14	5.56 ± 0.04	5.62 ± 0.05	5.62 ± 0.04
TC12001640.hg.1	LOC100506844	5.90 ± 0.09	5.73 ± 0.09	5.83 ± 0.19	5.65 ± 0.16
TC06000757.hg.1	LOC100506851	6.09 ± 0.24	5.95 ± 0.20	6.03 ± 0.17	6.09 ± 0.09
TC10000285.hg.1	LOC100506853	5.63 ± 0.10	5.61 ± 0.18	5.52 ± 0.07	5.82 ± 0.17
TC12000549.hg.1	LOC100506869	5.05 ± 0.09	4.93 ± 0.02	4.96 ± 0.15	5.00 ± 0.11
TC22000406.hg.1	LOC100506877	7.24 ± 0.17	6.97 ± 0.04	7.17 ± 0.15	7.11 ± 0.08
TC07001873.hg.1	LOC100506881	5.56 ± 0.04	5.40 ± 0.04	5.32 ± 0.09	5.38 ± 0.05
TC06001312.hg.1	LOC100506885	5.70 ± 0.06	5.59 ± 0.17	5.58 ± 0.14	5.79 ± 0.10
TC07001326.hg.1	LOC100506895	5.57 ± 0.08	5.74 ± 0.11	5.59 ± 0.16	5.84 ± 0.10
TC01001744.hg.1	LOC100506899	4.90 ± 0.10	4.99 ± 0.25	4.89 ± 0.32	5.01 ± 0.04

TC03001737.hg.1	LOC100506907	7.17 ± 0.12	7.15 ± 0.11	7.07 ± 0.04	7.16 ± 0.05
TC09000404.hg.1	LOC100506912	7.10 ± 0.28	7.10 ± 0.03	7.12 ± 0.08	7.19 ± 0.07
TC22000920.hg.1	LOC100506917	6.47 ± 0.15	6.35 ± 0.22	6.32 ± 0.07	6.39 ± 0.16
TC06000778.hg.1	LOC100506926	4.24 ± 0.06	4.13 ± 0.09	4.14 ± 0.07	4.23 ± 0.10
TC01004023.hg.1	LOC100506929	4.05 ± 0.07	3.93 ± 0.04	3.93 ± 0.15	3.96 ± 0.11
TC07000826.hg.1	LOC100506937	5.79 ± 0.10	5.76 ± 0.04	5.77 ± 0.07	5.84 ± 0.07
TC15002789.hg.1	LOC100506948	7.77 ± 0.10	7.73 ± 0.13	7.57 ± 0.07	7.81 ± 0.12
TC01002349.hg.1	LOC100506963	7.02 ± 0.06	6.87 ± 0.06	6.89 ± 0.07	6.87 ± 0.10
TC12001058.hg.1	LOC100506978	8.21 ± 0.24	8.24 ± 0.10	8.22 ± 0.15	8.33 ± 0.07
TC01002366.hg.1	LOC100506985	7.44 ± 0.08	7.58 ± 0.17	7.07 ± 0.59	7.37 ± 0.14
TC08002596.hg.1	LOC100506990	7.60 ± 0.18	7.69 ± 0.11	7.52 ± 0.10	7.76 ± 0.09
TC02002629.hg.1	LOC100506993	5.00 ± 0.14	5.12 ± 0.23	5.06 ± 0.21	4.93 ± 0.09
TC14000594.hg.1	LOC100506999	6.56 ± 0.26	6.68 ± 0.05	6.64 ± 0.07	6.79 ± 0.10
TC19000732.hg.1	LOC100507003	7.44 ± 0.43	7.25 ± 0.13	7.29 ± 0.27	7.30 ± 0.09
TC02000365.hg.1	LOC100507006	7.51 ± 0.08	7.65 ± 0.17	7.71 ± 0.39	7.77 ± 0.17
TC09001312.hg.1	LOC100507009	6.88 ± 0.29	6.95 ± 0.21	6.89 ± 0.07	7.07 ± 0.10
TC04001394.hg.1	LOC100507012	5.29 ± 0.41	5.05 ± 0.08	5.00 ± 0.20	5.09 ± 0.09
TC08001600.hg.1	LOC100507018	7.01 ± 0.09	7.20 ± 0.12	6.85 ± 0.15	7.20 ± 0.10
TC06000803.hg.1	LOC100507024	7.53 ± 0.42	7.34 ± 0.02	7.40 ± 0.24	7.37 ± 0.10
TC01001999.hg.1	LOC100507030	5.10 ± 0.10	5.06 ± 0.04	5.19 ± 0.20	5.03 ± 0.07
TC03000698.hg.1	LOC100507032	7.30 ± 0.24	7.35 ± 0.29	7.30 ± 0.25	7.57 ± 0.15
TC03002134.hg.1	LOC100507033	6.12 ± 0.19	6.06 ± 0.10	6.05 ± 0.11	6.17 ± 0.08
TC04000514.hg.1	LOC100507053	5.50 ± 0.09	5.46 ± 0.05	5.46 ± 0.07	5.53 ± 0.06
TC04000965.hg.1	LOC100507054	6.94 ± 0.12	6.88 ± 0.17	6.93 ± 0.12	6.90 ± 0.11
TC12001682.hg.1	LOC100507065	5.28 ± 0.14	5.25 ± 0.10	5.22 ± 0.04	5.34 ± 0.09
TC12000973.hg.1	LOC100507066	5.37 ± 0.24	5.21 ± 0.09	5.35 ± 0.03	5.33 ± 0.02
TC08001038.hg.1	LOC100507071	6.01 ± 0.17	5.93 ± 0.02	6.02 ± 0.12	5.88 ± 0.11
TC02000387.hg.1	LOC100507073	5.52 ± 0.15	5.39 ± 0.01	5.40 ± 0.05	5.49 ± 0.10
TC12000982.hg.1	LOC100507091	6.69 ± 0.07	6.76 ± 0.19	6.66 ± 0.07	6.88 ± 0.10
TC09001322.hg.1	LOC100507103	6.34 ± 0.09	6.17 ± 0.21	6.30 ± 0.12	6.35 ± 0.12
TC14000625.hg.1	LOC100507108	6.87 ± 0.37	6.86 ± 0.21	6.80 ± 0.13	6.90 ± 0.03

TC11000320.hg.1	LOC100507109	4.97 ± 0.17	4.93 ± 0.10	4.91 ± 0.15	4.94 ± 0.13
TC08001643.hg.1	LOC100507117	6.86 ± 0.26	6.95 ± 0.15	7.41 ± 0.22	7.04 ± 0.15
TC03000711.hg.1	LOC100507134	5.05 ± 0.15	4.85 ± 0.03	4.96 ± 0.04	4.80 ± 0.01
TC02002662.hg.1	LOC100507140	6.36 ± 0.12	6.34 ± 0.20	6.38 ± 0.11	6.54 ± 0.13
TC11001549.hg.1	LOC100507144	6.58 ± 0.01	6.52 ± 0.18	6.53 ± 0.06	6.59 ± 0.06
TC04000985.hg.1	LOC100507150	7.00 ± 0.15	7.06 ± 0.09	7.00 ± 0.17	7.18 ± 0.20
TC08000186.hg.1	LOC100507156	6.77 ± 0.17	6.88 ± 0.18	6.88 ± 0.21	6.99 ± 0.07
TC04001233.hg.1	LOC100507160	6.80 ± 0.21	6.88 ± 0.01	6.86 ± 0.08	7.06 ± 0.20
TC15001864.hg.1	LOC100507166	5.79 ± 0.13	5.90 ± 0.15	5.67 ± 0.14	5.94 ± 0.14
TC06000260.hg.1	LOC100507173	6.81 ± 0.09	6.79 ± 0.11	6.79 ± 0.06	6.86 ± 0.05
TC12000596.hg.1	LOC100507175	4.48 ± 0.09	4.43 ± 0.14	4.53 ± 0.07	4.41 ± 0.14
TC06001227.hg.1	LOC100507194	5.20 ± 0.20	5.25 ± 0.08	5.21 ± 0.03	5.35 ± 0.09
TC12001700.hg.1	LOC100507195	6.48 ± 0.23	6.20 ± 0.12	6.32 ± 0.17	6.21 ± 0.05
TC0X001490.hg.1	LOC100507199	6.72 ± 0.20	6.68 ± 0.11	6.66 ± 0.07	6.74 ± 0.08
TC02002027.hg.1	LOC100507201	4.80 ± 0.17	4.60 ± 0.20	4.76 ± 0.12	4.86 ± 0.12
TC11001569.hg.1	LOC100507205	4.95 ± 0.16	4.82 ± 0.02	4.83 ± 0.09	4.87 ± 0.11
TC03001801.hg.1	LOC100507210	6.18 ± 0.17	6.13 ± 0.08	6.11 ± 0.18	6.13 ± 0.08
TC15002768.hg.1	LOC100507217	9.28 ± 0.59	10.03 ± 0.06	9.42 ± 0.55	10.25 ± 0.17
TC08000197.hg.1	LOC100507222	4.66 ± 0.11	4.67 ± 0.13	4.64 ± 0.18	4.73 ± 0.20
TC12001703.hg.1	LOC100507250	6.14 ± 0.08	6.02 ± 0.04	5.99 ± 0.06	5.97 ± 0.06
TC06000991.hg.1	LOC100507254	5.89 ± 0.12	5.78 ± 0.08	5.64 ± 0.02	5.77 ± 0.11
TC11000365.hg.1	LOC100507261	5.59 ± 0.13	5.55 ± 0.16	5.52 ± 0.10	5.57 ± 0.15
TC04000060.hg.1	LOC100507266	6.51 ± 0.09	6.41 ± 0.04	6.30 ± 0.11	6.48 ± 0.10
TC05002049.hg.1	LOC100507267	5.06 ± 0.08	4.94 ± 0.06	5.00 ± 0.03	4.99 ± 0.07
TC03000741.hg.1	LOC100507274	4.22 ± 0.11	4.06 ± 0.12	4.17 ± 0.02	4.15 ± 0.06
TC14000769.hg.1	LOC100507277	4.73 ± 0.35	4.58 ± 0.07	4.60 ± 0.28	4.64 ± 0.17
TC11001145.hg.1	LOC100507283	5.45 ± 0.14	5.55 ± 0.06	5.40 ± 0.06	5.48 ± 0.04
TC15000934.hg.1	LOC100507288	5.47 ± 0.22	5.30 ± 0.12	5.44 ± 0.21	5.18 ± 0.07
TC03000751.hg.1	LOC100507291	6.60 ± 0.09	6.57 ± 0.07	6.63 ± 0.04	6.60 ± 0.09
TC08001712.hg.1	LOC100507316	7.85 ± 0.18	7.72 ± 0.10	7.70 ± 0.06	7.79 ± 0.06
TC15001925.hg.1	LOC100507325	6.77 ± 0.22	6.63 ± 0.07	6.60 ± 0.16	6.64 ± 0.04

TC12000622.hg.1	LOC100507330	5.44 ± 0.16	5.27 ± 0.04	5.28 ± 0.05	5.36 ± 0.08
TC02000675.hg.1	LOC100507334	6.13 ± 0.22	6.12 ± 0.18	6.12 ± 0.14	6.30 ± 0.08
TC09000472.hg.1	LOC100507346	6.33 ± 0.24	6.31 ± 0.12	6.27 ± 0.11	6.46 ± 0.15
TC17000890.hg.1	LOC100507351	6.76 ± 0.28	6.56 ± 0.06	6.63 ± 0.11	6.76 ± 0.13
TC06000319.hg.1	LOC100507362	6.75 ± 0.28	6.75 ± 0.22	6.71 ± 0.12	6.86 ± 0.09
TC6_apd_hap1000012.hg.1	LOC100507362	6.86 ± 0.23	6.84 ± 0.17	6.81 ± 0.11	6.94 ± 0.07
TC6_cox_hap2000016.hg.1	LOC100507362	6.83 ± 0.27	6.88 ± 0.17	6.84 ± 0.09	6.99 ± 0.07
TC6_dbb_hap3000012.hg.1	LOC100507362	6.83 ± 0.27	6.88 ± 0.17	6.84 ± 0.09	6.99 ± 0.07
TC6_mann_hap4000013.hg.1	LOC100507362	6.82 ± 0.22	6.82 ± 0.19	6.77 ± 0.10	6.91 ± 0.07
TC6_mcf_hap5000011.hg.1	LOC100507362	6.75 ± 0.28	6.75 ± 0.22	6.71 ± 0.12	6.86 ± 0.09
TC6_qbl_hap6000011.hg.1	LOC100507362	6.80 ± 0.25	6.81 ± 0.20	6.76 ± 0.11	6.90 ± 0.08
TC6_ssto_hap7000014.hg.1	LOC100507362	6.79 ± 0.27	6.80 ± 0.22	6.74 ± 0.12	6.90 ± 0.08
TC12001734.hg.1	LOC100507363	3.99 ± 0.04	3.80 ± 0.02	3.85 ± 0.06	3.80 ± 0.05
TC06001859.hg.1	LOC100507367	5.74 ± 0.07	5.60 ± 0.04	5.68 ± 0.09	5.53 ± 0.03
TC19000253.hg.1	LOC100507373	8.13 ± 0.27	7.95 ± 0.20	7.86 ± 0.18	8.02 ± 0.01
TC04001002.hg.1	LOC100507376	6.57 ± 0.25	6.30 ± 0.11	6.32 ± 0.17	6.25 ± 0.04
TC12001739.hg.1	LOC100507377	5.63 ± 0.19	5.59 ± 0.07	5.63 ± 0.07	5.67 ± 0.13
TC12001741.hg.1	LOC100507377	6.98 ± 0.25	6.73 ± 0.43	6.77 ± 0.36	6.97 ± 0.11
TC11000385.hg.1	LOC100507384	7.25 ± 0.19	7.23 ± 0.21	7.11 ± 0.15	7.33 ± 0.12
TC04001293.hg.1	LOC100507388	5.84 ± 0.13	5.80 ± 0.12	5.87 ± 0.06	5.97 ± 0.17
TC03000772.hg.1	LOC100507389	5.10 ± 0.13	4.86 ± 0.09	4.94 ± 0.13	4.94 ± 0.10
TC03001079.hg.1	LOC100507391	6.17 ± 0.06	6.15 ± 0.09	6.11 ± 0.07	6.26 ± 0.09
TC08001128.hg.1	LOC100507403	6.54 ± 0.15	6.37 ± 0.05	6.37 ± 0.20	6.50 ± 0.11
TC06002154.hg.1	LOC100507406	5.42 ± 0.24	5.18 ± 0.05	5.32 ± 0.10	5.18 ± 0.06
TC11001737.hg.1	LOC100507417	8.45 ± 0.15	8.41 ± 0.09	8.82 ± 0.19	8.37 ± 0.12
TC12000030.hg.1	LOC100507424	7.34 ± 0.15	7.33 ± 0.04	7.24 ± 0.07	7.34 ± 0.04
TC11002454.hg.1	LOC100507431	6.05 ± 0.16	5.93 ± 0.08	5.96 ± 0.10	5.96 ± 0.02
TC08000886.hg.1	LOC100507435	6.20 ± 0.22	6.28 ± 0.14	6.28 ± 0.13	6.33 ± 0.09
TC14001567.hg.1	LOC100507437	7.85 ± 0.17	7.70 ± 0.05	7.76 ± 0.25	7.75 ± 0.04
TC09000507.hg.1	LOC100507438	4.67 ± 0.14	4.29 ± 0.06	4.46 ± 0.07	4.52 ± 0.15
TC08001130.hg.1	LOC100507439	6.49 ± 0.20	6.41 ± 0.14	6.36 ± 0.11	6.53 ± 0.11

TC02001233.hg.1	LOC100507443	4.91 ± 0.36	4.96 ± 0.07	4.77 ± 0.18	5.00 ± 0.16
TC03000792.hg.1	LOC100507447	6.33 ± 0.05	6.28 ± 0.09	6.24 ± 0.08	6.30 ± 0.04
TC20000539.hg.1	LOC100507459	6.49 ± 0.22	6.47 ± 0.09	6.39 ± 0.14	6.52 ± 0.03
TC03000793.hg.1	LOC100507461	3.81 ± 0.19	3.59 ± 0.07	3.78 ± 0.24	3.64 ± 0.05
TC06002162.hg.1	LOC100507462	7.90 ± 0.21	8.03 ± 0.06	7.81 ± 0.13	8.15 ± 0.15
TC06004083.hg.1	LOC100507463	9.41 ± 0.15	9.17 ± 0.13	9.51 ± 0.22	8.87 ± 0.14
TC6_apd_hap1000121.hg.1	LOC100507463	9.41 ± 0.15	9.17 ± 0.13	9.51 ± 0.22	8.87 ± 0.14
TC6_cox_hap2000258.hg.1	LOC100507463	9.41 ± 0.15	9.17 ± 0.13	9.51 ± 0.22	8.87 ± 0.14
TC6_dbb_hap3000225.hg.1	LOC100507463	9.41 ± 0.15	9.17 ± 0.13	9.51 ± 0.22	8.87 ± 0.14
TC6_mann_hap4000189.hg.1	LOC100507463	9.41 ± 0.15	9.17 ± 0.13	9.51 ± 0.22	8.87 ± 0.14
TC6_mcf_hap5000201.hg.1	LOC100507463	9.41 ± 0.15	9.17 ± 0.13	9.51 ± 0.22	8.87 ± 0.14
TC6_qbl_hap6000214.hg.1	LOC100507463	9.41 ± 0.15	9.17 ± 0.13	9.51 ± 0.22	8.87 ± 0.14
TC6_ssto_hap7000192.hg.1	LOC100507463	9.39 ± 0.15	9.16 ± 0.12	9.49 ± 0.22	8.87 ± 0.14
TC08001201.hg.1	LOC100507464	5.26 ± 0.15	5.05 ± 0.04	5.07 ± 0.12	5.12 ± 0.08
TC07001480.hg.1	LOC100507468	5.87 ± 0.21	5.58 ± 0.18	5.56 ± 0.21	5.77 ± 0.06
TC06001038.hg.1	LOC100507477	4.47 ± 0.21	4.30 ± 0.09	4.38 ± 0.19	4.35 ± 0.13
TC15000251.hg.1	LOC100507480	4.70 ± 0.03	4.63 ± 0.16	4.66 ± 0.04	4.76 ± 0.15
TC12001758.hg.1	LOC100507484	5.07 ± 0.15	4.96 ± 0.20	4.98 ± 0.12	4.95 ± 0.19
TC04000649.hg.1	LOC100507487	5.82 ± 0.07	5.85 ± 0.05	5.80 ± 0.08	5.90 ± 0.08
TC06002183.hg.1	LOC100507489	5.25 ± 0.03	5.05 ± 0.19	5.08 ± 0.05	5.08 ± 0.05
TC12001760.hg.1	LOC100507498	5.00 ± 0.21	4.92 ± 0.14	4.92 ± 0.29	5.04 ± 0.08
TC16000826.hg.1	LOC100507501	7.54 ± 0.09	7.34 ± 0.08	7.29 ± 0.08	7.29 ± 0.11
TC06000039.hg.1	LOC100507506	6.03 ± 0.15	5.95 ± 0.11	6.00 ± 0.15	6.03 ± 0.02
TC07001970.hg.1	LOC100507507	7.83 ± 0.16	7.81 ± 0.06	8.12 ± 0.20	7.93 ± 0.16
TC12000059.hg.1	LOC100507511	6.42 ± 0.04	6.44 ± 0.19	6.46 ± 0.07	6.77 ± 0.23
TC14000904.hg.1	LOC100507513	5.34 ± 0.12	5.31 ± 0.12	5.06 ± 0.11	5.23 ± 0.12
TC08001214.hg.1	LOC100507516	6.16 ± 0.16	6.07 ± 0.09	6.08 ± 0.05	6.25 ± 0.18
TC11001861.hg.1	LOC100507521	5.48 ± 0.39	5.73 ± 0.07	5.65 ± 0.15	5.78 ± 0.21
TC10000178.hg.1	LOC100507531	5.77 ± 0.14	5.60 ± 0.05	5.73 ± 0.06	5.72 ± 0.09
TC03001925.hg.1	LOC100507537	6.19 ± 0.18	6.18 ± 0.04	6.13 ± 0.17	6.21 ± 0.15
TC07000961.hg.1	LOC100507538	4.94 ± 0.04	4.73 ± 0.11	4.84 ± 0.26	4.78 ± 0.07

TC09000332.hg.1	LOC100507540	7.24 ± 0.07	7.13 ± 0.05	7.21 ± 0.11	7.07 ± 0.05
TC05001080.hg.1	LOC100507546	7.43 ± 0.18	7.37 ± 0.10	7.36 ± 0.12	7.46 ± 0.06
TC06004146.hg.1	LOC100507547	7.30 ± 0.18	7.31 ± 0.25	7.19 ± 0.13	7.35 ± 0.07
TC6_apd_hap1000128.hg.1	LOC100507547	7.31 ± 0.16	7.31 ± 0.25	7.20 ± 0.11	7.35 ± 0.07
TC6_cox_hap2000263.hg.1	LOC100507547	7.32 ± 0.15	7.36 ± 0.25	7.24 ± 0.11	7.40 ± 0.07
TC6_dbb_hap3000240.hg.1	LOC100507547	7.29 ± 0.15	7.32 ± 0.25	7.20 ± 0.11	7.35 ± 0.07
TC6_mann_hap4000208.hg.1	LOC100507547	7.35 ± 0.17	7.39 ± 0.24	7.27 ± 0.11	7.42 ± 0.07
TC6_mcf_hap5000223.hg.1	LOC100507547	7.29 ± 0.15	7.32 ± 0.25	7.20 ± 0.11	7.35 ± 0.07
TC6_qbl_hap6000235.hg.1	LOC100507547	7.32 ± 0.15	7.36 ± 0.25	7.24 ± 0.11	7.40 ± 0.07
TC6_ssto_hap7000210.hg.1	LOC100507547	7.31 ± 0.16	7.31 ± 0.25	7.20 ± 0.11	7.35 ± 0.07
TC02002756.hg.1	LOC100507554	6.48 ± 0.12	6.30 ± 0.07	6.44 ± 0.14	6.33 ± 0.13
TC06001062.hg.1	LOC100507557	7.40 ± 0.23	7.43 ± 0.05	7.37 ± 0.06	7.58 ± 0.16
TC12000070.hg.1	LOC100507560	6.88 ± 0.31	6.91 ± 0.15	6.76 ± 0.14	7.00 ± 0.14
TC02000126.hg.1	LOC100507562	6.05 ± 0.09	6.00 ± 0.10	5.96 ± 0.11	6.10 ± 0.13
TC01000652.hg.1	LOC100507564	5.41 ± 0.28	5.18 ± 0.15	5.32 ± 0.07	5.39 ± 0.03
TC15001214.hg.1	LOC100507568	6.33 ± 0.31	6.45 ± 0.19	6.31 ± 0.06	6.73 ± 0.19
TC15001215.hg.1	LOC100507568	6.12 ± 0.06	5.98 ± 0.11	6.14 ± 0.09	6.05 ± 0.25
TC16000431.hg.1	LOC100507577	7.35 ± 0.39	7.65 ± 0.27	7.31 ± 0.42	7.57 ± 0.16
TC06001595.hg.1	LOC100507584	7.15 ± 0.19	7.07 ± 0.15	7.08 ± 0.20	7.16 ± 0.10
TC12000703.hg.1	LOC100507594	5.66 ± 0.26	5.57 ± 0.04	5.49 ± 0.20	5.58 ± 0.14
TC02000884.hg.1	LOC100507600	6.47 ± 0.16	6.46 ± 0.05	6.55 ± 0.02	6.53 ± 0.04
TC05003418.hg.1	LOC100507602	7.59 ± 0.08	7.67 ± 0.07	7.46 ± 0.04	7.69 ± 0.07
TC12003256.hg.1	LOC100507616	7.29 ± 0.17	6.88 ± 0.23	7.17 ± 0.06	6.72 ± 0.19
TC01000676.hg.1	LOC100507634	7.97 ± 0.17	8.13 ± 0.02	7.74 ± 0.04	8.23 ± 0.02
TC04001598.hg.1	LOC100507639	6.53 ± 0.25	6.30 ± 0.22	6.95 ± 0.40	6.33 ± 0.27
TC07000002.hg.1	LOC100507642	7.20 ± 0.24	7.15 ± 0.16	7.21 ± 0.16	7.19 ± 0.06
TC19001533.hg.1	LOC100507646	8.10 ± 0.05	8.04 ± 0.14	7.98 ± 0.10	8.09 ± 0.05
TC08000391.hg.1	LOC100507651	6.48 ± 0.16	6.42 ± 0.09	6.40 ± 0.08	6.54 ± 0.13
TC01002700.hg.1	LOC100507652	4.61 ± 0.01	4.62 ± 0.14	4.66 ± 0.03	4.68 ± 0.11
TC13000842.hg.1	LOC100507654	6.28 ± 0.05	6.41 ± 0.05	6.42 ± 0.12	6.39 ± 0.17
TC22000179.hg.1	LOC100507657	5.91 ± 0.20	5.92 ± 0.21	5.90 ± 0.01	6.03 ± 0.17

TC03000901.hg.1	LOC100507661	6.11 ± 0.05	6.00 ± 0.04	6.08 ± 0.03	6.05 ± 0.03
TC22000007.hg.1	LOC100508046	5.86 ± 0.14	5.80 ± 0.08	6.02 ± 0.31	6.26 ± 0.53
TC03001005.hg.1	LOC100508383	6.20 ± 0.23	6.12 ± 0.18	6.03 ± 0.19	6.21 ± 0.07
TC0X000749.hg.1	LOC100509091	7.44 ± 0.27	7.43 ± 0.33	7.42 ± 0.19	7.54 ± 0.05
TC01003120.hg.1	LOC100509195	5.74 ± 0.14	5.82 ± 0.07	5.71 ± 0.08	5.94 ± 0.22
TC01003597.hg.1	LOC100509205	7.17 ± 0.10	7.05 ± 0.06	7.26 ± 0.11	7.06 ± 0.09
TC01003782.hg.1	LOC100509303	5.39 ± 0.06	5.35 ± 0.27	5.38 ± 0.07	5.70 ± 0.22
TC12001359.hg.1	LOC100509445	5.35 ± 0.07	5.20 ± 0.01	5.24 ± 0.09	5.25 ± 0.08
TC12000416.hg.1	LOC100509541	7.88 ± 0.23	7.83 ± 0.16	7.76 ± 0.26	7.89 ± 0.08
TC03001262.hg.1	LOC100509621	4.16 ± 0.17	4.03 ± 0.02	3.96 ± 0.05	4.04 ± 0.07
TC09000269.hg.1	LOC100509638	8.77 ± 0.38	9.01 ± 0.07	8.99 ± 0.38	9.32 ± 0.19
TC0Y000104.hg.1	LOC100509646	7.07 ± 0.31	6.91 ± 0.09	6.87 ± 0.28	7.04 ± 0.20
TC0Y000199.hg.1	LOC100509646	7.03 ± 0.29	6.84 ± 0.08	6.79 ± 0.28	6.96 ± 0.20
TC01006384.hg.1	LOC100527964	6.72 ± 0.22	6.70 ± 0.17	6.66 ± 0.12	6.65 ± 0.01
TC08000584.hg.1	LOC100616530	6.45 ± 0.21	6.34 ± 0.13	6.35 ± 0.10	6.42 ± 0.06
TC02002042.hg.1	LOC100630918	7.64 ± 0.24	7.71 ± 0.07	7.61 ± 0.19	7.64 ± 0.03
TC19001490.hg.1	LOC100631378	5.12 ± 0.11	5.10 ± 0.09	5.02 ± 0.03	5.04 ± 0.01
TC06001086.hg.1	LOC100652739	6.67 ± 0.27	6.60 ± 0.31	6.56 ± 0.09	6.80 ± 0.06
TC22000484.hg.1	LOC100652750	6.00 ± 0.29	5.98 ± 0.09	6.01 ± 0.08	6.32 ± 0.24
TC11002333.hg.1	LOC100652768	7.74 ± 0.07	7.66 ± 0.07	7.66 ± 0.02	7.71 ± 0.06
TC18000439.hg.1	LOC100652770	5.02 ± 0.14	4.94 ± 0.08	4.88 ± 0.11	4.93 ± 0.06
TC05001321.hg.1	LOC100652772	8.14 ± 0.18	8.09 ± 0.07	8.03 ± 0.11	8.19 ± 0.09
TC05001062.hg.1	LOC100652782	6.99 ± 0.27	7.05 ± 0.23	6.99 ± 0.16	7.16 ± 0.15
TC08000024.hg.1	LOC100652791	6.75 ± 0.21	6.76 ± 0.03	6.70 ± 0.14	6.76 ± 0.04
TC02001186.hg.1	LOC100652824	6.71 ± 0.13	6.69 ± 0.15	6.67 ± 0.16	6.76 ± 0.06
TC01003645.hg.1	LOC100652834	4.35 ± 0.14	3.99 ± 0.04	4.18 ± 0.08	4.01 ± 0.07
TC13000008.hg.1	LOC100652856	7.41 ± 0.27	7.49 ± 0.10	7.38 ± 0.12	7.72 ± 0.16
TC20000146.hg.1	LOC100652902	12.49 ± 0.12	12.64 ± 0.13	12.02 ± 0.16	12.53 ± 0.09
TC21000091.hg.1	LOC100652994	5.74 ± 0.09	5.72 ± 0.05	5.75 ± 0.04	5.88 ± 0.20
TC12000442.hg.1	LOC100652999	6.59 ± 0.16	6.59 ± 0.11	6.91 ± 0.09	6.54 ± 0.10
TC06000431.hg.1	LOC100653005	6.43 ± 0.22	6.41 ± 0.16	6.37 ± 0.07	6.43 ± 0.13

TC05001367.hg.1	LOC100653008	10.00 ± 0.16	10.04 ± 0.11	9.91 ± 0.39	9.93 ± 0.02
TC14002260.hg.1	LOC100653243	11.23 ± 0.28	10.00 ± 0.11	9.96 ± 0.39	9.79 ± 0.16
TC02002224.hg.1	LOC100653336	6.47 ± 0.27	6.47 ± 0.11	6.45 ± 0.17	6.65 ± 0.18
TC09000003.hg.1	LOC100653336	7.11 ± 0.31	7.16 ± 0.19	7.13 ± 0.11	7.26 ± 0.12
TC17001923.hg.1	LOC100653515	8.93 ± 0.20	8.71 ± 0.12	9.19 ± 0.04	8.83 ± 0.19
TC12002149.hg.1	LOC100996246	7.53 ± 0.41	7.47 ± 0.28	7.28 ± 0.30	7.56 ± 0.10
TC01003002.hg.1	LOC100996251	7.66 ± 0.09	7.64 ± 0.05	7.59 ± 0.10	7.70 ± 0.08
TC01003037.hg.1	LOC100996263	6.26 ± 0.07	6.18 ± 0.11	6.16 ± 0.12	6.19 ± 0.15
TC07001786.hg.1	LOC100996266	4.53 ± 0.12	4.42 ± 0.12	4.53 ± 0.11	4.50 ± 0.10
TC07000022.hg.1	LOC100996269	7.99 ± 0.27	7.93 ± 0.14	8.08 ± 0.22	7.99 ± 0.05
TC04000763.hg.1	LOC100996286	9.01 ± 0.12	8.98 ± 0.16	8.70 ± 0.25	8.79 ± 0.07
TC17001916.hg.1	LOC100996291	7.57 ± 0.16	7.54 ± 0.22	7.59 ± 0.21	7.68 ± 0.06
TC04001806.hg.1	LOC100996305	5.54 ± 0.15	5.36 ± 0.06	5.44 ± 0.05	5.45 ± 0.10
TC03000614.hg.1	LOC100996310	7.17 ± 0.19	7.08 ± 0.05	7.13 ± 0.05	7.10 ± 0.04
TC14000613.hg.1	LOC100996339	8.18 ± 0.06	8.15 ± 0.14	8.01 ± 0.07	8.05 ± 0.04
TC16000352.hg.1	LOC100996344	7.27 ± 0.20	7.10 ± 0.12	7.25 ± 0.06	7.06 ± 0.02
TC08001382.hg.1	LOC100996348	5.61 ± 0.13	5.58 ± 0.20	5.65 ± 0.12	5.67 ± 0.08
TC06001523.hg.1	LOC100996357	8.53 ± 0.41	8.60 ± 0.03	8.40 ± 0.08	8.84 ± 0.16
TC12001105.hg.1	LOC100996378	7.17 ± 0.19	7.18 ± 0.22	7.18 ± 0.16	7.22 ± 0.09
TC22000599.hg.1	LOC100996404	5.80 ± 0.05	5.65 ± 0.20	5.65 ± 0.10	5.74 ± 0.03
TC07000411.hg.1	LOC100996437	8.21 ± 0.15	8.27 ± 0.19	8.26 ± 0.15	8.44 ± 0.07
TC05001325.hg.1	LOC100996444	4.94 ± 0.09	4.82 ± 0.10	4.88 ± 0.13	4.82 ± 0.05
TC03001947.hg.1	LOC100996447	4.70 ± 0.09	4.65 ± 0.11	4.65 ± 0.08	4.70 ± 0.05
TC02001944.hg.1	LOC100996448	5.27 ± 0.25	5.14 ± 0.26	5.25 ± 0.22	5.26 ± 0.04
TC11001906.hg.1	LOC100996453	7.64 ± 0.28	7.62 ± 0.14	7.58 ± 0.22	7.64 ± 0.18
TC11000603.hg.1	LOC100996455	7.24 ± 0.08	7.16 ± 0.20	7.05 ± 0.19	7.24 ± 0.01
TC03001977.hg.1	LOC100996456	4.68 ± 0.23	4.66 ± 0.06	4.66 ± 0.11	4.75 ± 0.17
TC08000638.hg.1	LOC100996457	6.61 ± 0.12	6.58 ± 0.09	6.55 ± 0.07	6.61 ± 0.06
TC0X000759.hg.1	LOC100996465	8.09 ± 0.30	8.13 ± 0.27	8.05 ± 0.27	8.28 ± 0.09
TC12001304.hg.1	LOC100996472	6.90 ± 0.35	6.65 ± 0.07	6.76 ± 0.20	6.66 ± 0.11
TC08001163.hg.1	LOC100996474	5.71 ± 0.07	5.64 ± 0.02	5.63 ± 0.17	5.74 ± 0.08

TC05000674.hg.1	LOC100996485	7.44 ± 0.12	7.42 ± 0.12	7.44 ± 0.08	7.57 ± 0.06
TC03000970.hg.1	LOC100996490	5.19 ± 0.18	5.09 ± 0.07	5.14 ± 0.08	5.15 ± 0.08
TC22000901.hg.1	LOC100996524	6.66 ± 0.34	6.79 ± 0.38	6.58 ± 0.34	6.97 ± 0.07
TC01000662.hg.1	LOC100996543	6.71 ± 0.17	6.60 ± 0.07	6.66 ± 0.06	6.52 ± 0.12
TC02001562.hg.1	LOC100996549	6.18 ± 0.09	6.07 ± 0.14	6.04 ± 0.10	6.18 ± 0.06
TC02000060.hg.1	LOC100996551	7.25 ± 0.16	7.22 ± 0.19	7.08 ± 0.11	7.40 ± 0.02
TC01002054.hg.1	LOC100996554	10.26 ± 0.45	10.14 ± 0.35	11.08 ± 0.40	10.10 ± 0.25
TC01002056.hg.1	LOC100996554	11.67 ± 0.23	11.64 ± 0.23	12.28 ± 0.30	11.66 ± 0.22
TC09000482.hg.1	LOC100996557	7.13 ± 0.33	7.01 ± 0.10	6.88 ± 0.34	6.94 ± 0.15
TC02000969.hg.1	LOC100996579	6.50 ± 0.31	6.68 ± 0.22	6.42 ± 0.12	6.82 ± 0.04
TC01000063.hg.1	LOC100996583	6.88 ± 0.18	6.79 ± 0.05	6.88 ± 0.18	6.88 ± 0.02
TC14000188.hg.1	LOC100996589	5.74 ± 0.17	5.46 ± 0.04	5.60 ± 0.05	5.62 ± 0.07
TC09001513.hg.1	LOC100996601	6.59 ± 0.11	6.59 ± 0.24	6.48 ± 0.16	6.76 ± 0.13
TC21000144.hg.1	LOC100996609	6.22 ± 0.24	6.21 ± 0.16	6.00 ± 0.31	6.28 ± 0.13
TC14001083.hg.1	LOC100996613	6.58 ± 0.09	6.57 ± 0.21	6.58 ± 0.14	6.73 ± 0.16
TC02002962.hg.1	LOC100996616	8.91 ± 0.53	9.05 ± 0.24	8.61 ± 0.63	9.35 ± 0.11
TC03001258.hg.1	LOC100996624	5.42 ± 0.24	5.00 ± 0.20	4.93 ± 0.05	5.04 ± 0.20
TC01002897.hg.1	LOC100996630	4.11 ± 0.24	4.01 ± 0.08	4.08 ± 0.12	4.08 ± 0.19
TC06000872.hg.1	LOC100996634	5.63 ± 0.09	5.55 ± 0.11	5.59 ± 0.01	5.64 ± 0.06
TC01000879.hg.1	LOC100996635	5.64 ± 0.03	5.51 ± 0.03	5.53 ± 0.01	5.57 ± 0.08
TC09000304.hg.1	LOC100996643	6.79 ± 0.13	6.70 ± 0.05	6.64 ± 0.04	6.85 ± 0.11
TC05000250.hg.1	LOC100996645	4.12 ± 0.18	3.92 ± 0.09	4.21 ± 0.21	3.94 ± 0.15
TC07000326.hg.1	LOC100996654	5.80 ± 0.18	6.05 ± 0.32	5.95 ± 0.17	6.15 ± 0.18
TC08000861.hg.1	LOC100996662	6.29 ± 0.20	6.23 ± 0.10	6.25 ± 0.12	6.34 ± 0.10
TC15001902.hg.1	LOC100996663	9.09 ± 0.28	8.75 ± 0.11	8.80 ± 0.16	8.75 ± 0.14
TC14001250.hg.1	LOC100996664	7.70 ± 0.14	7.75 ± 0.09	7.69 ± 0.15	7.89 ± 0.08
TC12002108.hg.1	LOC100996671	5.88 ± 0.21	5.85 ± 0.25	5.79 ± 0.20	6.01 ± 0.20
TC12002120.hg.1	LOC100996679	7.13 ± 0.09	7.02 ± 0.08	7.05 ± 0.15	7.12 ± 0.04
TC04000618.hg.1	LOC100996694	4.59 ± 0.27	4.37 ± 0.03	4.54 ± 0.15	4.52 ± 0.17
TC01001169.hg.1	LOC100996721	7.49 ± 0.11	7.43 ± 0.07	7.35 ± 0.10	7.32 ± 0.23
TC22000532.hg.1	LOC100996817	7.84 ± 0.21	7.79 ± 0.14	7.76 ± 0.15	7.94 ± 0.09

TC22000533.hg.1	LOC100996817	8.06 ± 0.05	7.78 ± 0.21	8.27 ± 0.17	8.02 ± 0.11
TC02002098.hg.1	LOC100996862	8.90 ± 0.97	9.68 ± 0.19	8.94 ± 0.58	9.59 ± 0.10
TC09000275.hg.1	LOC100996870	8.06 ± 0.19	7.81 ± 0.12	8.27 ± 0.16	7.80 ± 0.15
TC15000466.hg.1	LOC100996876	5.28 ± 0.21	5.03 ± 0.16	5.11 ± 0.07	5.13 ± 0.07
TC02002331.hg.1	LOC100996884	5.80 ± 0.20	5.57 ± 0.12	5.54 ± 0.35	5.66 ± 0.16
TC02000243.hg.1	LOC100996902	6.46 ± 0.35	6.56 ± 0.18	6.39 ± 0.17	6.53 ± 0.04
TC01003065.hg.1	LOC100996920	6.19 ± 0.22	6.17 ± 0.12	6.13 ± 0.29	6.36 ± 0.22
TC07001591.hg.1	LOC101059921	5.35 ± 0.21	5.27 ± 0.19	5.47 ± 0.17	5.38 ± 0.07
TC09001112.hg.1	LOC101059935	6.12 ± 0.22	6.09 ± 0.05	6.15 ± 0.24	6.22 ± 0.16
TC15001145.hg.1	LOC101059939	5.77 ± 0.30	5.47 ± 0.23	5.83 ± 0.11	5.59 ± 0.10
TC02001227.hg.1	LOC101059946	5.76 ± 0.09	5.64 ± 0.03	5.80 ± 0.20	5.59 ± 0.05
TC14000240.hg.1	LOC101059957	4.19 ± 0.08	4.05 ± 0.04	4.10 ± 0.07	4.07 ± 0.03
TC01001106.hg.1	LOC101059961	11.53 ± 0.29	11.65 ± 0.12	11.76 ± 0.15	11.66 ± 0.03
TC01006302.hg.1	LOC101059961	11.40 ± 0.32	11.56 ± 0.13	11.67 ± 0.15	11.56 ± 0.01
TC01000403.hg.1	LOC101059975	5.93 ± 0.23	5.85 ± 0.10	5.90 ± 0.13	5.98 ± 0.10
TC20000379.hg.1	LOC101060004	6.90 ± 0.10	6.89 ± 0.11	7.00 ± 0.07	7.05 ± 0.16
TC02000388.hg.1	LOC101060019	4.33 ± 0.07	4.25 ± 0.07	4.25 ± 0.07	4.31 ± 0.13
TC09002933.hg.1	LOC101060026	5.68 ± 0.32	5.58 ± 0.13	5.64 ± 0.26	5.61 ± 0.17
TC12000163.hg.1	LOC101060038	8.43 ± 0.52	8.65 ± 0.09	8.18 ± 0.04	8.64 ± 0.11
TC01002100.hg.1	LOC101060082	8.66 ± 0.19	8.58 ± 0.11	8.65 ± 0.18	8.66 ± 0.00
TC02002231.hg.1	LOC101060091	5.47 ± 0.21	5.43 ± 0.12	5.60 ± 0.35	5.40 ± 0.03
TC09002937.hg.1	LOC101060175	8.26 ± 0.21	8.21 ± 0.20	8.17 ± 0.18	8.31 ± 0.04
TC09002938.hg.1	LOC101060175	8.28 ± 0.22	8.21 ± 0.18	8.19 ± 0.19	8.31 ± 0.06
TC11000378.hg.1	LOC101060179	8.05 ± 0.17	8.00 ± 0.23	8.03 ± 0.18	8.09 ± 0.04
TC01006369.hg.1	LOC101060226	11.94 ± 0.29	12.06 ± 0.10	12.23 ± 0.19	12.09 ± 0.05
TC06001720.hg.1	LOC101060264	6.68 ± 0.18	6.57 ± 0.17	6.64 ± 0.09	6.63 ± 0.04
TC11000478.hg.1	LOC101060277	5.78 ± 0.05	5.55 ± 0.12	5.66 ± 0.10	5.76 ± 0.21
TC01002247.hg.1	LOC101060300	7.06 ± 0.12	6.95 ± 0.26	7.00 ± 0.09	7.13 ± 0.08
TC0X001526.hg.1	LOC101060305	6.62 ± 0.17	6.60 ± 0.10	6.57 ± 0.09	6.66 ± 0.11
TC0X001527.hg.1	LOC101060305	6.61 ± 0.16	6.59 ± 0.10	6.59 ± 0.07	6.63 ± 0.08
TC04001772.hg.1	LOC101060314	4.49 ± 0.39	4.28 ± 0.04	4.32 ± 0.06	4.26 ± 0.07

TC16000869.hg.1	LOC101060360	8.63 ± 0.13	8.61 ± 0.10	8.79 ± 0.02	8.70 ± 0.03
TC01000213.hg.1	LOC101060384	7.66 ± 0.20	7.75 ± 0.26	7.69 ± 0.09	7.82 ± 0.09
TC02001503.hg.1	LOC101060385	7.38 ± 0.25	7.46 ± 0.25	7.43 ± 0.12	7.66 ± 0.12
TC12001562.hg.1	LOC101060414	6.68 ± 0.22	6.62 ± 0.02	6.66 ± 0.03	6.73 ± 0.16
TC16000304.hg.1	LOC101060449	8.80 ± 0.43	8.86 ± 0.13	8.70 ± 0.39	8.85 ± 0.09
TC14000857.hg.1	LOC101060483	5.73 ± 0.19	5.68 ± 0.05	5.64 ± 0.10	5.81 ± 0.18
TC01000008.hg.1	LOC101060495	8.78 ± 0.58	8.44 ± 0.37	10.12 ± 0.44	8.53 ± 0.09
TC01002672.hg.1	LOC101060536	6.79 ± 0.04	6.69 ± 0.17	6.87 ± 0.20	6.81 ± 0.09
TC08000533.hg.1	LOC101060540	8.99 ± 1.12	9.10 ± 0.22	9.03 ± 0.70	10.03 ± 1.23
TC09000247.hg.1	LOC101060578	7.42 ± 0.44	7.49 ± 0.29	7.60 ± 0.38	7.31 ± 0.02
TC01003102.hg.1	LOC101060595	6.19 ± 0.34	6.21 ± 0.00	6.09 ± 0.10	6.33 ± 0.20
TC22000439.hg.1	LOC101060615	9.01 ± 0.11	8.93 ± 0.12	8.98 ± 0.27	8.99 ± 0.08
TC05003444.hg.1	LOC101060626	8.63 ± 0.53	8.36 ± 0.26	9.87 ± 0.52	8.48 ± 0.12
TC08000873.hg.1	LOC101060626	8.26 ± 0.55	8.01 ± 0.46	9.67 ± 0.43	8.04 ± 0.16
TC08000983.hg.1	LOC101060662	8.74 ± 0.04	8.62 ± 0.04	8.91 ± 0.04	8.74 ± 0.03
TC22000535.hg.1	LOC101060682	8.46 ± 0.12	8.40 ± 0.16	8.46 ± 0.11	8.51 ± 0.05
TC01006430.hg.1	LOC101060684	9.74 ± 0.33	9.87 ± 0.17	10.01 ± 0.23	9.87 ± 0.06
TC01001077.hg.1	LOC101060698	10.34 ± 0.21	10.40 ± 0.10	10.10 ± 0.12	10.49 ± 0.16
TC12000079.hg.1	LOC101060720	7.13 ± 0.24	6.55 ± 0.08	7.17 ± 0.25	6.59 ± 0.23
TC22000065.hg.1	LOC101060787	5.95 ± 0.29	5.88 ± 0.14	5.86 ± 0.07	6.19 ± 0.22
TC14000028.hg.1	LOC101101776	6.35 ± 0.14	6.14 ± 0.07	6.13 ± 0.09	6.22 ± 0.14
TC19000252.hg.1	LOC113230	8.61 ± 0.11	8.46 ± 0.10	8.56 ± 0.27	8.52 ± 0.04
TC01002122.hg.1	LOC115110	8.46 ± 0.18	8.41 ± 0.12	8.43 ± 0.15	8.49 ± 0.07
TC12001042.hg.1	LOC116437	7.52 ± 0.13	7.42 ± 0.22	7.49 ± 0.24	7.59 ± 0.05
TC10000818.hg.1	LOC143188	6.34 ± 0.08	6.33 ± 0.18	6.19 ± 0.07	6.32 ± 0.13
TC11001240.hg.1	LOC143666	7.71 ± 0.26	7.45 ± 0.14	7.53 ± 0.30	7.58 ± 0.12
TC14000434.hg.1	LOC145474	6.68 ± 0.34	7.47 ± 0.30	7.36 ± 0.81	7.34 ± 0.10
TC15001599.hg.1	LOC145694	6.21 ± 0.19	6.18 ± 0.22	6.06 ± 0.20	6.22 ± 0.04
TC15002803.hg.1	LOC145757	7.02 ± 0.21	6.95 ± 0.25	6.86 ± 0.12	7.10 ± 0.12
TC15001478.hg.1	LOC145783	7.35 ± 0.42	7.46 ± 0.21	7.37 ± 0.29	7.24 ± 0.07
TC15000640.hg.1	LOC145837	5.98 ± 0.17	5.89 ± 0.13	5.89 ± 0.09	5.96 ± 0.01

TC15001210.hg.1	LOC145845	5.87 ± 0.13	5.76 ± 0.10	5.83 ± 0.16	5.85 ± 0.14
TC16001079.hg.1	LOC146481	6.52 ± 0.29	6.41 ± 0.18	6.49 ± 0.31	6.62 ± 0.11
TC16000667.hg.1	LOC146513	6.31 ± 0.18	6.31 ± 0.25	6.18 ± 0.32	6.49 ± 0.20
TC17002921.hg.1	LOC146880	9.00 ± 0.01	8.93 ± 0.05	9.08 ± 0.13	8.86 ± 0.07
TC17001511.hg.1	LOC147093	6.29 ± 0.24	6.41 ± 0.15	6.27 ± 0.14	6.39 ± 0.05
TC19001778.hg.1	LOC147646	7.38 ± 0.13	7.13 ± 0.06	7.32 ± 0.18	7.18 ± 0.13
TC01000036.hg.1	LOC148413	7.88 ± 0.16	7.85 ± 0.09	7.67 ± 0.23	7.89 ± 0.01
TC01001742.hg.1	LOC148696	6.94 ± 0.20	6.94 ± 0.13	7.08 ± 0.09	7.04 ± 0.14
TC01001680.hg.1	LOC148709	7.00 ± 0.39	6.91 ± 0.21	6.79 ± 0.38	6.98 ± 0.02
TC01000409.hg.1	LOC149086	7.50 ± 0.20	7.54 ± 0.18	7.41 ± 0.17	7.67 ± 0.06
TC01002004.hg.1	LOC149134	7.43 ± 0.16	7.38 ± 0.12	7.38 ± 0.12	7.49 ± 0.10
TC01002860.hg.1	LOC149351	7.43 ± 0.18	7.42 ± 0.11	7.31 ± 0.13	7.39 ± 0.05
TC20000214.hg.1	LOC149950	7.16 ± 0.25	7.24 ± 0.26	7.17 ± 0.19	7.43 ± 0.08
TC21000110.hg.1	LOC150051	7.31 ± 0.47	7.18 ± 0.40	7.03 ± 0.37	7.44 ± 0.13
TC22001504.hg.1	LOC150381	7.85 ± 0.10	7.82 ± 0.17	7.89 ± 0.14	7.92 ± 0.04
TC02000634.hg.1	LOC150568	5.37 ± 0.18	5.32 ± 0.06	5.29 ± 0.15	5.40 ± 0.15
TC02000606.hg.1	LOC150577	7.43 ± 0.20	7.45 ± 0.21	7.39 ± 0.14	7.55 ± 0.17
TC02000032.hg.1	LOC150622	6.56 ± 0.14	6.46 ± 0.04	6.48 ± 0.05	6.54 ± 0.08
TC02000854.hg.1	LOC150776	8.87 ± 0.03	8.89 ± 0.12	8.62 ± 0.10	9.00 ± 0.03
TC02001463.hg.1	LOC150935	6.51 ± 0.18	6.41 ± 0.08	6.50 ± 0.13	6.60 ± 0.06
TC02002200.hg.1	LOC151009	7.40 ± 0.14	7.32 ± 0.07	7.29 ± 0.09	7.35 ± 0.11
TC02002297.hg.1	LOC151121	5.35 ± 0.10	5.18 ± 0.03	5.21 ± 0.10	5.24 ± 0.04
TC02002918.hg.1	LOC151171	5.87 ± 0.25	5.89 ± 0.22	5.95 ± 0.15	6.08 ± 0.08
TC02002912.hg.1	LOC151174	7.56 ± 0.23	7.46 ± 0.19	7.42 ± 0.24	7.58 ± 0.04
TC02002855.hg.1	LOC151475	5.27 ± 0.11	5.30 ± 0.16	5.21 ± 0.06	5.30 ± 0.13
TC02001382.hg.1	LOC151484	7.34 ± 0.26	7.29 ± 0.21	7.24 ± 0.14	7.48 ± 0.11
TC03001641.hg.1	LOC151760	5.77 ± 0.19	5.77 ± 0.20	5.78 ± 0.14	5.87 ± 0.16
TC03000526.hg.1	LOC152225	4.72 ± 0.06	4.73 ± 0.03	4.78 ± 0.08	4.69 ± 0.02
TC04000302.hg.1	LOC152578	4.64 ± 0.06	4.38 ± 0.10	4.39 ± 0.10	4.38 ± 0.06
TC04001590.hg.1	LOC152586	4.07 ± 0.12	3.92 ± 0.02	3.99 ± 0.09	3.94 ± 0.05
TC04000135.hg.1	LOC152742	4.79 ± 0.05	4.74 ± 0.14	4.72 ± 0.09	4.97 ± 0.13

TC05000195.hg.1	LOC153684	8.18 ± 0.02	8.03 ± 0.05	8.23 ± 0.12	8.02 ± 0.02
TC06002180.hg.1	LOC153910	6.49 ± 0.21	6.46 ± 0.09	6.45 ± 0.11	6.60 ± 0.12
TC06001006.hg.1	LOC154092	6.02 ± 0.23	6.08 ± 0.13	6.03 ± 0.07	6.20 ± 0.13
TC06002341.hg.1	LOC154449	7.97 ± 0.11	8.02 ± 0.08	7.98 ± 0.17	8.12 ± 0.04
TC07001972.hg.1	LOC154761	8.14 ± 0.20	8.24 ± 0.07	8.12 ± 0.04	8.37 ± 0.10
TC07001829.hg.1	LOC154872	5.31 ± 0.13	5.41 ± 0.04	5.25 ± 0.21	5.37 ± 0.09
TC08000068.hg.1	LOC157273	6.89 ± 0.07	6.81 ± 0.02	6.83 ± 0.09	6.97 ± 0.16
TC09001371.hg.1	LOC158434	6.62 ± 0.09	6.63 ± 0.12	6.54 ± 0.05	6.70 ± 0.07
TC09000478.hg.1	LOC158435	7.04 ± 0.13	6.92 ± 0.12	6.96 ± 0.14	7.08 ± 0.09
TC02000493.hg.1	LOC1720	5.69 ± 0.08	5.66 ± 0.05	5.73 ± 0.17	5.60 ± 0.10
TC02001220.hg.1	LOC200726	4.56 ± 0.09	4.51 ± 0.02	4.59 ± 0.14	4.56 ± 0.07
TC02002942.hg.1	LOC200772	8.00 ± 0.15	8.02 ± 0.21	7.99 ± 0.15	8.16 ± 0.08
TC03000828.hg.1	LOC201651	3.92 ± 0.02	3.84 ± 0.04	3.82 ± 0.02	3.84 ± 0.05
TC05002114.hg.1	LOC202181	8.33 ± 0.04	8.33 ± 0.08	8.23 ± 0.13	8.35 ± 0.05
TC07001034.hg.1	LOC202781	7.82 ± 0.10	7.85 ± 0.07	7.56 ± 0.21	7.81 ± 0.05
TC03002180.hg.1	LOC220729	8.68 ± 0.09	8.63 ± 0.17	8.52 ± 0.16	8.56 ± 0.05
TC11000376.hg.1	LOC221122	7.19 ± 0.18	7.28 ± 0.06	7.18 ± 0.08	7.46 ± 0.20
TC15000715.hg.1	LOC253044	6.16 ± 0.19	6.11 ± 0.14	6.05 ± 0.07	6.09 ± 0.06
TC03002082.hg.1	LOC253573	5.73 ± 0.16	5.60 ± 0.10	5.62 ± 0.07	5.74 ± 0.06
TC01000026.hg.1	LOC254099	7.49 ± 0.24	7.48 ± 0.23	7.47 ± 0.23	7.58 ± 0.13
TC02000768.hg.1	LOC254128	7.09 ± 0.07	6.96 ± 0.02	6.96 ± 0.03	6.93 ± 0.06
TC08000180.hg.1	LOC254896	10.54 ± 0.16	10.42 ± 0.29	10.86 ± 0.13	10.30 ± 0.33
TC04000339.hg.1	LOC255130	6.07 ± 0.20	6.06 ± 0.16	6.00 ± 0.07	6.09 ± 0.06
TC05000043.hg.1	LOC255167	7.23 ± 0.24	7.29 ± 0.21	7.23 ± 0.19	7.41 ± 0.11
TC05000815.hg.1	LOC255187	6.07 ± 0.15	6.01 ± 0.24	6.07 ± 0.15	6.10 ± 0.14
TC01004058.hg.1	LOC255654	5.69 ± 0.40	5.74 ± 0.21	5.68 ± 0.27	5.98 ± 0.12
TC04000520.hg.1	LOC256880	6.76 ± 0.07	6.66 ± 0.03	6.71 ± 0.09	6.72 ± 0.07
TC05000934.hg.1	LOC257358	7.12 ± 0.26	7.19 ± 0.12	7.09 ± 0.16	7.20 ± 0.02
TC05000219.hg.1	LOC257396	5.49 ± 0.09	5.42 ± 0.04	5.35 ± 0.13	5.41 ± 0.01
TC10000911.hg.1	LOC283038	6.78 ± 0.16	6.80 ± 0.18	6.76 ± 0.10	6.87 ± 0.12
TC11001218.hg.1	LOC283177	7.10 ± 0.21	7.10 ± 0.13	7.05 ± 0.10	7.23 ± 0.16

TC11001816.hg.1	LOC283194	6.49 ± 0.20	6.51 ± 0.30	6.40 ± 0.12	6.40 ± 0.10
TC11002101.hg.1	LOC283214	7.61 ± 0.15	7.63 ± 0.09	7.63 ± 0.11	7.72 ± 0.05
TC11001377.hg.1	LOC283299	5.49 ± 0.14	5.37 ± 0.04	5.41 ± 0.10	5.51 ± 0.10
TC12001486.hg.1	LOC283332	7.46 ± 0.07	7.46 ± 0.12	7.38 ± 0.19	7.52 ± 0.05
TC12003305.hg.1	LOC283335	7.48 ± 0.20	7.42 ± 0.11	7.41 ± 0.18	7.47 ± 0.05
TC12001514.hg.1	LOC283403	6.33 ± 0.17	6.34 ± 0.06	6.33 ± 0.24	6.42 ± 0.08
TC12001100.hg.1	LOC283440	7.26 ± 0.24	7.36 ± 0.22	7.21 ± 0.15	7.43 ± 0.12
TC14000486.hg.1	LOC283575	8.20 ± 0.22	8.19 ± 0.06	8.06 ± 0.12	8.33 ± 0.11
TC14000517.hg.1	LOC283585	4.55 ± 0.11	4.34 ± 0.09	4.46 ± 0.08	4.36 ± 0.04
TC14000524.hg.1	LOC283587	6.23 ± 0.06	6.07 ± 0.05	6.29 ± 0.05	6.17 ± 0.13
TC15002771.hg.1	LOC283683	8.29 ± 0.23	8.16 ± 0.04	8.17 ± 0.09	8.20 ± 0.05
TC15000789.hg.1	LOC283692	6.31 ± 0.15	6.27 ± 0.17	6.33 ± 0.17	6.37 ± 0.08
TC15001758.hg.1	LOC283693	7.58 ± 0.38	7.37 ± 0.20	7.49 ± 0.07	7.54 ± 0.10
TC15001165.hg.1	LOC283710	7.91 ± 0.18	7.49 ± 0.14	7.61 ± 0.10	7.47 ± 0.06
TC15001640.hg.1	LOC283731	7.08 ± 0.23	6.84 ± 0.31	6.92 ± 0.34	6.95 ± 0.09
TCUn_gl000219000002.hg.1	LOC283788	8.31 ± 0.37	8.30 ± 0.23	8.16 ± 0.20	8.37 ± 0.18
TC16001130.hg.1	LOC283856	7.43 ± 0.34	7.46 ± 0.06	7.39 ± 0.16	7.62 ± 0.18
TC16000406.hg.1	LOC283914	5.00 ± 0.07	4.94 ± 0.04	4.98 ± 0.01	4.95 ± 0.07
TC16001265.hg.1	LOC283922	8.73 ± 0.29	8.72 ± 0.20	8.55 ± 0.28	8.82 ± 0.14
TC17000995.hg.1	LOC284009	7.92 ± 0.03	7.90 ± 0.19	7.81 ± 0.07	8.03 ± 0.14
TC17001096.hg.1	LOC284023	7.65 ± 0.35	7.50 ± 0.14	7.45 ± 0.31	7.57 ± 0.04
TC17001676.hg.1	LOC284080	7.42 ± 0.40	7.38 ± 0.24	7.39 ± 0.23	7.54 ± 0.18
TC17001412.hg.1	LOC284100	6.41 ± 0.04	6.29 ± 0.03	6.38 ± 0.09	6.27 ± 0.05
TC18000233.hg.1	LOC284294	5.14 ± 0.11	5.08 ± 0.05	5.07 ± 0.05	5.16 ± 0.06
TC19001583.hg.1	LOC284344	6.07 ± 0.16	5.97 ± 0.07	6.03 ± 0.15	6.16 ± 0.18
TC19001818.hg.1	LOC284379	7.28 ± 0.18	7.29 ± 0.14	7.16 ± 0.14	7.31 ± 0.08
TC19001380.hg.1	LOC284395	6.96 ± 0.19	6.88 ± 0.12	6.87 ± 0.11	7.04 ± 0.11
TC19001482.hg.1	LOC284412	6.20 ± 0.31	5.91 ± 0.26	6.07 ± 0.16	6.04 ± 0.11
TC01000410.hg.1	LOC284551	6.14 ± 0.08	6.09 ± 0.03	6.09 ± 0.06	6.18 ± 0.06
TC01003749.hg.1	LOC284578	5.27 ± 0.10	5.16 ± 0.05	5.09 ± 0.21	5.14 ± 0.05
TC01001715.hg.1	LOC284581	6.37 ± 0.16	6.32 ± 0.08	6.28 ± 0.16	6.44 ± 0.24

TC01000019.hg.1	LOC284600	8.26 ± 0.26	8.01 ± 0.16	8.13 ± 0.14	8.16 ± 0.07
TC01000308.hg.1	LOC284632	7.57 ± 0.27	7.50 ± 0.11	7.66 ± 0.10	7.70 ± 0.11
TC01001579.hg.1	LOC284648	7.17 ± 0.33	7.26 ± 0.36	7.17 ± 0.28	7.25 ± 0.09
TC01000077.hg.1	LOC284661	6.17 ± 0.18	6.12 ± 0.07	6.07 ± 0.10	6.15 ± 0.06
TC01003507.hg.1	LOC284688	5.23 ± 0.21	5.31 ± 0.09	5.22 ± 0.05	5.46 ± 0.18
TC20000402.hg.1	LOC284751	7.47 ± 0.12	7.17 ± 0.08	7.97 ± 0.21	7.30 ± 0.09
TC20001760.hg.1	LOC284757	6.86 ± 0.12	6.80 ± 0.09	6.93 ± 0.10	6.93 ± 0.09
TC20000695.hg.1	LOC284788	5.22 ± 0.15	5.14 ± 0.04	5.19 ± 0.08	5.12 ± 0.17
TC20000729.hg.1	LOC284798	7.08 ± 0.16	7.02 ± 0.11	6.99 ± 0.16	7.07 ± 0.02
TC20000741.hg.1	LOC284801	4.53 ± 0.18	4.39 ± 0.11	4.30 ± 0.08	4.46 ± 0.14
TC21000510.hg.1	LOC284837	8.18 ± 0.11	7.98 ± 0.13	8.11 ± 0.02	7.95 ± 0.04
TC22000507.hg.1	LOC284865	7.01 ± 0.25	6.99 ± 0.15	6.95 ± 0.14	7.01 ± 0.01
TC22001485.hg.1	LOC284889	7.56 ± 0.10	7.48 ± 0.21	7.52 ± 0.13	7.54 ± 0.08
TC22000896.hg.1	LOC284933	7.28 ± 0.30	7.29 ± 0.24	7.19 ± 0.25	7.40 ± 0.09
TC02000518.hg.1	LOC284950	7.14 ± 0.16	7.15 ± 0.16	7.06 ± 0.20	7.28 ± 0.16
TC02002161.hg.1	LOC284998	6.57 ± 0.06	6.45 ± 0.13	6.53 ± 0.02	6.54 ± 0.03
TC02002168.hg.1	LOC285000	5.95 ± 0.24	5.93 ± 0.07	5.86 ± 0.05	5.99 ± 0.14
TC02000579.hg.1	LOC285033	7.03 ± 0.11	6.94 ± 0.05	6.94 ± 0.06	6.99 ± 0.02
TC02002062.hg.1	LOC285074	9.21 ± 0.31	9.42 ± 0.14	8.98 ± 0.29	9.34 ± 0.11
TC02001036.hg.1	LOC285084	6.21 ± 0.25	6.15 ± 0.13	6.11 ± 0.12	6.09 ± 0.05
TC02002961.hg.1	LOC285095	6.40 ± 0.17	6.41 ± 0.17	6.40 ± 0.25	6.50 ± 0.05
TC04000635.hg.1	LOC285419	5.06 ± 0.19	4.91 ± 0.09	4.99 ± 0.14	4.96 ± 0.05
TC04002951.hg.1	LOC285441	6.00 ± 0.08	6.02 ± 0.10	6.02 ± 0.15	6.08 ± 0.09
TC04000066.hg.1	LOC285484	7.21 ± 0.14	7.10 ± 0.04	7.14 ± 0.26	7.25 ± 0.09
TC04000877.hg.1	LOC285501	5.73 ± 0.05	5.70 ± 0.03	5.73 ± 0.04	5.93 ± 0.18
TC04001044.hg.1	LOC285547	6.87 ± 0.23	6.75 ± 0.08	6.79 ± 0.25	6.79 ± 0.03
TC04001046.hg.1	LOC285548	6.15 ± 0.18	6.19 ± 0.33	6.12 ± 0.20	6.34 ± 0.15
TC04001415.hg.1	LOC285556	5.51 ± 0.19	5.60 ± 0.08	5.70 ± 0.08	5.92 ± 0.14
TC05001122.hg.1	LOC285577	7.31 ± 0.09	7.37 ± 0.25	7.34 ± 0.17	7.52 ± 0.06
TC05000964.hg.1	LOC285593	5.03 ± 0.07	4.92 ± 0.05	5.02 ± 0.12	4.92 ± 0.05
TC05000887.hg.1	LOC285626	5.85 ± 0.08	5.82 ± 0.05	5.72 ± 0.03	5.88 ± 0.08

TC05001998.hg.1	LOC285627	5.86 ± 0.12	5.75 ± 0.13	5.69 ± 0.07	5.79 ± 0.02
TC05002012.hg.1	LOC285629	6.08 ± 0.07	6.11 ± 0.15	6.19 ± 0.15	6.32 ± 0.20
TC05003421.hg.1	LOC285692	6.21 ± 0.13	6.22 ± 0.07	6.16 ± 0.06	6.37 ± 0.15
TC05001187.hg.1	LOC285696	7.05 ± 0.16	6.89 ± 0.22	7.20 ± 0.10	7.05 ± 0.16
TC06002188.hg.1	LOC285740	5.94 ± 0.27	5.79 ± 0.07	5.84 ± 0.09	5.81 ± 0.01
TC06002034.hg.1	LOC285758	4.79 ± 0.28	4.37 ± 0.09	4.71 ± 0.13	4.45 ± 0.07
TC06002063.hg.1	LOC285762	5.28 ± 0.08	5.13 ± 0.06	5.18 ± 0.07	5.10 ± 0.12
TC06001211.hg.1	LOC285768	5.42 ± 0.06	5.47 ± 0.11	5.51 ± 0.13	5.58 ± 0.06
TC06001366.hg.1	LOC285819	5.39 ± 0.20	5.05 ± 0.10	5.16 ± 0.11	5.10 ± 0.06
TC06004149.hg.1	LOC285847	7.55 ± 0.20	7.58 ± 0.15	7.49 ± 0.14	7.68 ± 0.10
TC07000324.hg.1	LOC285878	5.97 ± 0.17	5.88 ± 0.09	5.91 ± 0.10	5.97 ± 0.05
TC07002068.hg.1	LOC285889	6.11 ± 0.18	5.89 ± 0.12	6.03 ± 0.28	6.12 ± 0.04
TC07001001.hg.1	LOC285972	6.78 ± 0.07	6.79 ± 0.11	6.79 ± 0.06	6.89 ± 0.04
TC08000179.hg.1	LOC286059	6.64 ± 0.29	6.86 ± 0.19	6.76 ± 0.04	6.81 ± 0.04
TC08000885.hg.1	LOC286083	7.02 ± 0.14	6.98 ± 0.23	6.94 ± 0.16	7.09 ± 0.22
TC08000777.hg.1	LOC286094	5.11 ± 0.08	5.15 ± 0.08	5.08 ± 0.06	5.16 ± 0.07
TC08000157.hg.1	LOC286114	5.76 ± 0.20	5.57 ± 0.11	5.68 ± 0.08	5.69 ± 0.07
TC08000393.hg.1	LOC286177	6.76 ± 0.31	6.53 ± 0.18	6.55 ± 0.23	6.57 ± 0.10
TC08000399.hg.1	LOC286178	6.66 ± 0.18	6.39 ± 0.04	6.58 ± 0.15	6.46 ± 0.10
TC08000429.hg.1	LOC286184	4.48 ± 0.17	4.33 ± 0.06	4.40 ± 0.08	4.48 ± 0.16
TC08001279.hg.1	LOC286186	6.42 ± 0.20	6.41 ± 0.05	6.48 ± 0.10	6.60 ± 0.22
TC08001298.hg.1	LOC286189	6.83 ± 0.11	6.84 ± 0.14	6.82 ± 0.14	7.10 ± 0.26
TC08000465.hg.1	LOC286190	6.38 ± 0.09	6.28 ± 0.07	6.29 ± 0.03	6.39 ± 0.06
TC09001309.hg.1	LOC286238	4.90 ± 0.07	4.99 ± 0.11	4.93 ± 0.05	4.95 ± 0.20
TC09001109.hg.1	LOC286297	6.67 ± 0.07	6.68 ± 0.05	6.60 ± 0.07	6.82 ± 0.16
TC09001393.hg.1	LOC286359	6.39 ± 0.27	6.30 ± 0.12	6.23 ± 0.13	6.34 ± 0.07
TC09000532.hg.1	LOC286367	6.05 ± 0.34	6.15 ± 0.09	6.21 ± 0.28	6.32 ± 0.22
TC09001315.hg.1	LOC286370	6.54 ± 0.07	6.64 ± 0.13	6.57 ± 0.18	6.83 ± 0.07
TC0X001252.hg.1	LOC286437	7.11 ± 0.20	7.03 ± 0.18	7.18 ± 0.37	7.16 ± 0.14
TC0X000960.hg.1	LOC286442	6.09 ± 0.08	6.19 ± 0.13	6.07 ± 0.04	6.31 ± 0.03
TC0X001366.hg.1	LOC286467	6.82 ± 0.08	6.73 ± 0.02	6.73 ± 0.02	6.72 ± 0.03

TC12001043.hg.1	LOC338797	8.00 ± 0.12	7.90 ± 0.13	8.02 ± 0.11	8.04 ± 0.05
TC12003285.hg.1	LOC338799	8.88 ± 0.09	8.83 ± 0.17	8.62 ± 0.09	8.95 ± 0.17
TC12000170.hg.1	LOC338817	6.59 ± 0.17	6.60 ± 0.05	6.54 ± 0.02	6.72 ± 0.13
TC15001757.hg.1	LOC338963	7.32 ± 0.20	7.31 ± 0.11	7.35 ± 0.10	7.47 ± 0.09
TC17000068.hg.1	LOC339166	6.17 ± 0.14	6.38 ± 0.27	6.11 ± 0.01	6.59 ± 0.20
TC17000214.hg.1	LOC339240	7.65 ± 0.19	7.66 ± 0.16	7.64 ± 0.14	7.75 ± 0.12
TC18000253.hg.1	LOC339298	5.14 ± 0.11	5.11 ± 0.07	5.11 ± 0.12	5.20 ± 0.04
TC01002532.hg.1	LOC339442	6.26 ± 0.19	6.19 ± 0.06	6.28 ± 0.16	6.20 ± 0.03
TC01002312.hg.1	LOC339505	6.73 ± 0.14	6.73 ± 0.15	6.66 ± 0.07	6.94 ± 0.13
TC01001984.hg.1	LOC339529	5.41 ± 0.25	5.33 ± 0.08	5.34 ± 0.08	5.30 ± 0.07
TC01004013.hg.1	LOC339535	6.76 ± 0.20	6.52 ± 0.10	6.54 ± 0.11	6.70 ± 0.07
TC20000843.hg.1	LOC339568	5.01 ± 0.18	4.95 ± 0.05	4.90 ± 0.06	5.06 ± 0.15
TC20000625.hg.1	LOC339593	5.63 ± 0.15	5.44 ± 0.03	5.54 ± 0.04	5.48 ± 0.05
TC21000071.hg.1	LOC339622	5.62 ± 0.14	5.40 ± 0.12	5.45 ± 0.10	5.49 ± 0.07
TC22000244.hg.1	LOC339666	6.67 ± 0.18	6.62 ± 0.14	6.69 ± 0.19	6.77 ± 0.11
TC02000779.hg.1	LOC339760	6.79 ± 0.19	6.76 ± 0.18	6.74 ± 0.18	6.88 ± 0.05
TC02001549.hg.1	LOC339788	6.62 ± 0.09	6.54 ± 0.10	6.63 ± 0.07	6.64 ± 0.08
TC02001890.hg.1	LOC339803	7.33 ± 0.08	7.13 ± 0.04	7.11 ± 0.13	7.11 ± 0.02
TC02000371.hg.1	LOC339807	6.36 ± 0.23	6.35 ± 0.17	6.31 ± 0.17	6.48 ± 0.06
TC02001502.hg.1	LOC339822	7.15 ± 0.18	7.27 ± 0.18	7.22 ± 0.16	7.42 ± 0.16
TC03001786.hg.1	LOC339874	7.07 ± 0.11	6.97 ± 0.13	7.12 ± 0.02	7.06 ± 0.17
TC04001827.hg.1	LOC339975	6.15 ± 0.20	6.19 ± 0.29	6.13 ± 0.26	6.46 ± 0.16
TC04000800.hg.1	LOC340017	4.47 ± 0.19	4.46 ± 0.09	4.49 ± 0.07	4.52 ± 0.11
TC05001800.hg.1	LOC340073	7.34 ± 0.21	7.33 ± 0.17	7.36 ± 0.07	7.38 ± 0.07
TC05001805.hg.1	LOC340074	5.71 ± 0.09	5.50 ± 0.11	5.53 ± 0.01	5.67 ± 0.25
TC05000037.hg.1	LOC340094	4.56 ± 0.17	4.53 ± 0.06	4.55 ± 0.05	4.66 ± 0.11
TC05001206.hg.1	LOC340107	4.16 ± 0.17	3.99 ± 0.05	3.93 ± 0.12	4.02 ± 0.08
TC05000145.hg.1	LOC340113	5.65 ± 0.08	5.62 ± 0.05	5.62 ± 0.05	5.65 ± 0.11
TC08000999.hg.1	LOC340357	5.55 ± 0.30	5.46 ± 0.11	5.35 ± 0.13	5.51 ± 0.08
TC09001382.hg.1	LOC340508	7.84 ± 0.20	7.81 ± 0.05	7.73 ± 0.09	7.93 ± 0.11
TC09001318.hg.1	LOC340515	5.81 ± 0.08	5.85 ± 0.15	5.83 ± 0.07	5.92 ± 0.05

TC11001119.hg.1	LOC341056	7.23 ± 0.24	7.12 ± 0.08	7.04 ± 0.13	7.04 ± 0.10
TC03001014.hg.1	LOC344887	6.69 ± 0.24	6.53 ± 0.17	6.60 ± 0.15	6.54 ± 0.06
TC04001135.hg.1	LOC344967	7.42 ± 0.14	7.48 ± 0.16	7.46 ± 0.15	7.64 ± 0.10
TC02002857.hg.1	LOC348761	7.01 ± 0.19	6.95 ± 0.11	6.94 ± 0.15	7.09 ± 0.09
TC07001893.hg.1	LOC349160	5.76 ± 0.10	5.61 ± 0.02	5.65 ± 0.06	5.66 ± 0.03
TC12001205.hg.1	LOC374443	7.74 ± 0.11	7.65 ± 0.09	7.55 ± 0.20	7.69 ± 0.05
TC12003235.hg.1	LOC374443	8.48 ± 0.37	8.76 ± 0.16	8.36 ± 0.25	8.71 ± 0.14
TC02001760.hg.1	LOC375196	7.61 ± 0.38	7.36 ± 0.21	7.29 ± 0.33	7.54 ± 0.06
TC02002558.hg.1	LOC375295	5.61 ± 0.14	5.54 ± 0.11	5.59 ± 0.09	5.69 ± 0.12
TC15001772.hg.1	LOC388152	8.20 ± 0.19	8.19 ± 0.13	8.14 ± 0.20	8.29 ± 0.09
TC16001003.hg.1	LOC388242	8.51 ± 0.28	8.41 ± 0.17	8.39 ± 0.26	8.46 ± 0.04
TC19001120.hg.1	LOC388499	7.98 ± 0.22	7.83 ± 0.04	7.82 ± 0.12	7.89 ± 0.01
TC19000654.hg.1	LOC388553	6.88 ± 0.16	6.89 ± 0.17	6.83 ± 0.23	6.93 ± 0.07
TC01001158.hg.1	LOC388692	7.90 ± 0.14	7.88 ± 0.06	7.86 ± 0.08	7.84 ± 0.14
TC20000022.hg.1	LOC388780	7.78 ± 0.29	7.61 ± 0.17	7.70 ± 0.28	7.79 ± 0.10
TC21001070.hg.1	LOC388813	4.30 ± 0.11	4.15 ± 0.02	4.22 ± 0.05	4.18 ± 0.05
TC22000506.hg.1	LOC388849	8.17 ± 0.13	8.13 ± 0.15	8.15 ± 0.16	8.25 ± 0.04
TC22000560.hg.1	LOC388882	6.51 ± 0.23	6.44 ± 0.14	6.44 ± 0.17	6.61 ± 0.08
TC22000354.hg.1	LOC388906	6.73 ± 0.27	6.65 ± 0.14	6.65 ± 0.08	6.75 ± 0.09
TC02000258.hg.1	LOC388942	6.27 ± 0.09	6.21 ± 0.27	6.35 ± 0.18	6.29 ± 0.08
TC02004963.hg.1	LOC388948	7.07 ± 0.18	7.10 ± 0.21	7.03 ± 0.23	7.21 ± 0.09
TC02002240.hg.1	LOC389023	6.96 ± 0.11	7.01 ± 0.04	7.01 ± 0.07	7.14 ± 0.07
TC02002299.hg.1	LOC389033	7.72 ± 0.27	7.70 ± 0.25	7.71 ± 0.10	7.77 ± 0.04
TC02000851.hg.1	LOC389043	5.45 ± 0.15	5.44 ± 0.03	5.43 ± 0.08	5.50 ± 0.10
TC04000084.hg.1	LOC389199	7.51 ± 0.38	7.57 ± 0.27	7.47 ± 0.35	7.61 ± 0.07
TC04001795.hg.1	LOC389247	5.25 ± 0.16	5.16 ± 0.09	5.27 ± 0.08	5.22 ± 0.03
TC05001812.hg.1	LOC389332	7.94 ± 0.29	7.75 ± 0.17	7.63 ± 0.41	7.77 ± 0.04
TC07001059.hg.1	LOC389603	7.41 ± 0.22	7.48 ± 0.20	7.32 ± 0.26	7.73 ± 0.14
TC08000182.hg.1	LOC389641	8.63 ± 0.13	8.47 ± 0.18	8.45 ± 0.11	8.45 ± 0.18
TC08000183.hg.1	LOC389641	7.84 ± 0.03	7.80 ± 0.08	7.79 ± 0.04	7.79 ± 0.12
TC09000073.hg.1	LOC389705	5.44 ± 0.03	5.28 ± 0.05	5.32 ± 0.05	5.22 ± 0.01

TC09000389.hg.1	LOC389765	7.64 ± 0.46	7.84 ± 0.30	7.82 ± 0.52	7.64 ± 0.17
TC7_gl000195_random000003.hg.1	LOC389831	6.94 ± 0.21	6.68 ± 0.23	6.99 ± 0.27	6.78 ± 0.13
TCUn_gl000218000001.hg.1	LOC389834	8.08 ± 0.06	8.20 ± 0.11	7.99 ± 0.15	8.13 ± 0.14
TC0X000811.hg.1	LOC389906	7.98 ± 0.03	7.89 ± 0.07	7.66 ± 0.09	7.87 ± 0.04
TC15000764.hg.1	LOC390660	7.92 ± 0.20	7.93 ± 0.17	7.87 ± 0.18	8.02 ± 0.06
TC16001063.hg.1	LOC390705	7.77 ± 0.31	7.76 ± 0.14	7.67 ± 0.27	7.86 ± 0.13
TC22000143.hg.1	LOC391322	7.99 ± 0.20	7.84 ± 0.24	7.83 ± 0.13	7.81 ± 0.11
TC08000986.hg.1	LOC392196	6.67 ± 0.27	6.58 ± 0.12	6.64 ± 0.26	6.89 ± 0.40
TC08001322.hg.1	LOC392232	4.60 ± 0.05	4.54 ± 0.04	4.60 ± 0.08	4.65 ± 0.06
TC09001302.hg.1	LOC392364	7.47 ± 0.22	7.39 ± 0.04	7.38 ± 0.24	7.54 ± 0.17
TC07001104.hg.1	LOC392621	6.77 ± 0.15	6.83 ± 0.08	6.83 ± 0.06	6.90 ± 0.06
TC10000057.hg.1	LOC399715	7.22 ± 0.28	7.12 ± 0.11	7.71 ± 0.31	7.23 ± 0.10
TC10000257.hg.1	LOC399744	10.44 ± 0.20	10.44 ± 0.16	10.87 ± 0.21	10.49 ± 0.14
TC10001258.hg.1	LOC399753	9.79 ± 0.58	9.93 ± 0.24	9.76 ± 0.42	9.94 ± 0.07
TC10000893.hg.1	LOC399815	5.78 ± 0.21	5.80 ± 0.01	5.72 ± 0.03	5.78 ± 0.04
TC10000947.hg.1	LOC399829	7.66 ± 0.22	7.39 ± 0.19	7.34 ± 0.37	7.45 ± 0.05
TC12000457.hg.1	LOC400043	8.12 ± 0.26	7.92 ± 0.20	7.94 ± 0.12	7.76 ± 0.09
TC15001917.hg.1	LOC400456	5.06 ± 0.17	5.01 ± 0.11	4.96 ± 0.07	4.98 ± 0.13
TC16000662.hg.1	LOC400548	7.47 ± 0.25	7.41 ± 0.14	7.35 ± 0.12	7.55 ± 0.08
TC16001337.hg.1	LOC400553	8.37 ± 0.31	8.33 ± 0.13	8.30 ± 0.21	8.40 ± 0.02
TC16000691.hg.1	LOC400558	9.15 ± 0.21	9.12 ± 0.17	9.08 ± 0.26	9.14 ± 0.06
TC17000361.hg.1	LOC400590	6.99 ± 0.24	7.01 ± 0.07	7.04 ± 0.16	7.01 ± 0.12
TC17000824.hg.1	LOC400620	6.34 ± 0.24	6.23 ± 0.11	6.20 ± 0.12	6.35 ± 0.09
TC18000560.hg.1	LOC400654	5.74 ± 0.46	5.68 ± 0.16	5.66 ± 0.16	5.90 ± 0.22
TC18000572.hg.1	LOC400655	5.33 ± 0.08	5.24 ± 0.13	5.24 ± 0.10	5.40 ± 0.18
TC19001415.hg.1	LOC400685	7.04 ± 0.12	6.93 ± 0.05	6.87 ± 0.06	7.05 ± 0.15
TC19001484.hg.1	LOC400692	7.45 ± 0.17	7.46 ± 0.16	7.42 ± 0.24	7.52 ± 0.14
TC01000229.hg.1	LOC400743	7.73 ± 0.18	7.71 ± 0.10	7.75 ± 0.07	7.79 ± 0.03
TC01000570.hg.1	LOC400752	7.75 ± 0.16	7.79 ± 0.18	7.72 ± 0.10	7.93 ± 0.12
TC01000709.hg.1	LOC400756	7.11 ± 0.42	6.99 ± 0.22	6.97 ± 0.39	7.18 ± 0.09
TC01000930.hg.1	LOC400768	7.88 ± 0.14	7.90 ± 0.12	7.83 ± 0.09	8.02 ± 0.10

TC01002944.hg.1	LOC400768	7.88 ± 0.14	7.90 ± 0.12	7.83 ± 0.09	8.02 ± 0.10
TC01003465.hg.1	LOC400794	6.26 ± 0.23	6.20 ± 0.14	6.20 ± 0.16	6.38 ± 0.09
TC22000080.hg.1	LOC400891	7.21 ± 0.22	7.09 ± 0.18	7.16 ± 0.10	7.23 ± 0.05
TC22001494.hg.1	LOC400927	7.84 ± 0.06	7.61 ± 0.01	7.79 ± 0.04	7.60 ± 0.04
TC02001919.hg.1	LOC400958	6.68 ± 0.07	6.55 ± 0.08	6.67 ± 0.06	6.48 ± 0.15
TC02002352.hg.1	LOC401010	7.47 ± 0.16	7.37 ± 0.18	7.31 ± 0.17	7.41 ± 0.02
TC03003328.hg.1	LOC401052	7.70 ± 0.09	7.73 ± 0.12	7.67 ± 0.10	7.78 ± 0.07
TC04000231.hg.1	LOC401127	7.53 ± 0.14	7.60 ± 0.17	7.47 ± 0.13	7.55 ± 0.05
TC04001244.hg.1	LOC401134	4.90 ± 0.14	4.78 ± 0.02	4.82 ± 0.02	4.77 ± 0.07
TC04000927.hg.1	LOC401164	5.54 ± 0.25	5.35 ± 0.01	5.38 ± 0.12	5.40 ± 0.05
TC05000091.hg.1	LOC401176	6.10 ± 0.41	6.28 ± 0.36	6.01 ± 0.16	6.23 ± 0.07
TC05001191.hg.1	LOC401177	6.39 ± 0.11	6.25 ± 0.04	6.39 ± 0.12	6.32 ± 0.08
TC06001471.hg.1	LOC401242	6.61 ± 0.24	6.64 ± 0.06	6.68 ± 0.08	6.81 ± 0.09
TC6_apd_hap1000052.hg.1	LOC401242	6.61 ± 0.24	6.64 ± 0.06	6.68 ± 0.08	6.81 ± 0.09
TC6_cox_hap2000114.hg.1	LOC401242	6.84 ± 0.08	6.84 ± 0.11	6.83 ± 0.02	7.03 ± 0.11
TC6_dbb_hap3000103.hg.1	LOC401242	6.61 ± 0.24	6.64 ± 0.06	6.68 ± 0.08	6.81 ± 0.09
TC6_mann_hap4000092.hg.1	LOC401242	6.61 ± 0.24	6.64 ± 0.06	6.68 ± 0.08	6.81 ± 0.09
TC6_mcf_hap5000097.hg.1	LOC401242	6.91 ± 0.15	6.89 ± 0.03	6.92 ± 0.03	7.06 ± 0.06
TC6_qbl_hap6000105.hg.1	LOC401242	6.61 ± 0.24	6.64 ± 0.06	6.68 ± 0.08	6.81 ± 0.09
TC6_ssto_hap7000096.hg.1	LOC401242	6.91 ± 0.15	6.89 ± 0.03	6.92 ± 0.03	7.06 ± 0.06
TC07001090.hg.1	LOC401296	8.12 ± 0.19	7.93 ± 0.20	8.07 ± 0.20	8.07 ± 0.04
TC07001188.hg.1	LOC401312	5.97 ± 0.21	5.86 ± 0.07	5.90 ± 0.04	5.92 ± 0.06
TC07003403.hg.1	LOC401320	8.35 ± 0.26	8.38 ± 0.23	8.55 ± 0.39	8.42 ± 0.17
TC07000210.hg.1	LOC401321	6.91 ± 0.15	6.68 ± 0.16	6.71 ± 0.07	6.82 ± 0.16
TC07000225.hg.1	LOC401324	5.63 ± 0.34	5.53 ± 0.11	5.43 ± 0.15	5.59 ± 0.11
TC07001768.hg.1	LOC401397	7.38 ± 0.25	7.36 ± 0.05	7.37 ± 0.26	7.29 ± 0.04
TC07001040.hg.1	LOC401437	7.44 ± 0.27	7.51 ± 0.22	7.46 ± 0.25	7.71 ± 0.15
TC08001274.hg.1	LOC401463	7.42 ± 0.33	7.39 ± 0.19	7.43 ± 0.18	7.51 ± 0.05
TC09000791.hg.1	LOC401557	6.71 ± 0.23	6.77 ± 0.15	6.84 ± 0.15	6.76 ± 0.03
TC01003713.hg.1	LOC401980	6.30 ± 0.08	6.28 ± 0.09	6.26 ± 0.09	6.37 ± 0.07
TC04002909.hg.1	LOC402160	8.06 ± 0.26	7.97 ± 0.17	7.95 ± 0.19	8.04 ± 0.06

TC01000445.hg.1	LOC402779	5.37 ± 0.11	5.28 ± 0.15	5.33 ± 0.01	5.36 ± 0.09
TC07000792.hg.1	LOC407835	7.69 ± 0.14	7.55 ± 0.14	7.67 ± 0.22	7.61 ± 0.07
TC10000085.hg.1	LOC439951	8.12 ± 0.20	8.03 ± 0.15	8.04 ± 0.27	8.04 ± 0.08
TC10000616.hg.1	LOC439994	8.10 ± 0.11	8.08 ± 0.07	8.05 ± 0.06	8.04 ± 0.10
TC11001406.hg.1	LOC440028	5.94 ± 0.11	6.02 ± 0.20	5.98 ± 0.14	6.09 ± 0.13
TC11000421.hg.1	LOC440040	6.17 ± 0.18	6.20 ± 0.18	6.12 ± 0.17	6.23 ± 0.09
TC12002110.hg.1	LOC440117	4.93 ± 0.01	4.86 ± 0.06	4.88 ± 0.06	4.84 ± 0.04
TC09001296.hg.1	LOC440173	6.50 ± 0.32	6.54 ± 0.16	6.41 ± 0.26	6.73 ± 0.27
TC15001059.hg.1	LOC440233	7.36 ± 0.16	7.24 ± 0.06	7.39 ± 0.08	7.37 ± 0.11
TC15000806.hg.1	LOC440300	7.84 ± 0.14	7.74 ± 0.17	7.73 ± 0.14	7.91 ± 0.14
TC15000825.hg.1	LOC440300	7.90 ± 0.15	7.80 ± 0.15	7.80 ± 0.15	7.97 ± 0.14
TC15002001.hg.1	LOC440313	7.21 ± 0.21	7.14 ± 0.15	7.07 ± 0.18	7.35 ± 0.19
TC16000136.hg.1	LOC440335	7.54 ± 0.12	7.50 ± 0.24	7.42 ± 0.16	7.59 ± 0.04
TC16000143.hg.1	LOC440337	6.77 ± 0.05	6.80 ± 0.23	6.59 ± 0.14	6.84 ± 0.11
TC16002103.hg.1	LOC440354	10.33 ± 0.60	10.88 ± 0.08	10.39 ± 0.30	10.82 ± 0.03
TC17002899.hg.1	LOC440434	9.30 ± 0.28	9.32 ± 0.20	9.53 ± 0.33	9.29 ± 0.15
TC17000800.hg.1	LOC440461	7.73 ± 0.12	7.67 ± 0.23	7.71 ± 0.06	7.64 ± 0.06
TC19000399.hg.1	LOC440518	7.61 ± 0.25	7.56 ± 0.21	7.50 ± 0.29	7.71 ± 0.07
TC01000222.hg.1	LOC440570	7.79 ± 0.27	7.69 ± 0.20	7.58 ± 0.17	7.79 ± 0.09
TC01002969.hg.1	LOC440600	7.20 ± 0.26	7.36 ± 0.05	7.22 ± 0.21	7.48 ± 0.13
TC01001445.hg.1	LOC440700	5.95 ± 0.23	5.80 ± 0.22	5.95 ± 0.17	5.89 ± 0.13
TC01001616.hg.1	LOC440704	5.19 ± 0.18	5.19 ± 0.05	5.26 ± 0.07	5.33 ± 0.18
TC02000674.hg.1	LOC440894	7.34 ± 0.16	7.30 ± 0.06	7.33 ± 0.04	7.35 ± 0.13
TC02004996.hg.1	LOC440895	6.41 ± 0.20	6.27 ± 0.09	6.35 ± 0.08	6.39 ± 0.09
TC09001168.hg.1	LOC440896	6.76 ± 0.19	6.73 ± 0.04	6.71 ± 0.05	6.89 ± 0.13
TC02000736.hg.1	LOC440900	7.04 ± 0.04	6.95 ± 0.08	7.16 ± 0.07	7.00 ± 0.05
TC02002301.hg.1	LOC440905	5.86 ± 0.10	5.78 ± 0.02	5.84 ± 0.06	5.86 ± 0.10
TC02000843.hg.1	LOC440910	5.76 ± 0.10	5.67 ± 0.03	5.71 ± 0.01	5.76 ± 0.08
TC02002518.hg.1	LOC440925	7.05 ± 0.17	7.12 ± 0.12	7.01 ± 0.20	7.10 ± 0.03
TC02001345.hg.1	LOC440934	6.08 ± 0.28	6.01 ± 0.11	6.00 ± 0.15	6.08 ± 0.07
TC03003418.hg.1	LOC440981	3.74 ± 0.19	3.47 ± 0.07	3.50 ± 0.03	3.44 ± 0.06

TC04001052.hg.1	LOC441009	5.69 ± 0.11	5.58 ± 0.01	5.63 ± 0.06	5.59 ± 0.06
TC04000417.hg.1	LOC441025	5.77 ± 0.15	5.59 ± 0.08	5.70 ± 0.18	5.87 ± 0.17
TC06000912.hg.1	LOC441167	5.54 ± 0.11	5.42 ± 0.06	5.48 ± 0.06	5.47 ± 0.03
TC06002317.hg.1	LOC441178	6.41 ± 0.14	6.40 ± 0.13	6.26 ± 0.20	6.51 ± 0.07
TC07000162.hg.1	LOC441204	6.30 ± 0.10	6.27 ± 0.07	6.27 ± 0.01	6.35 ± 0.09
TC07001455.hg.1	LOC441242	7.57 ± 0.13	7.49 ± 0.03	7.42 ± 0.15	7.57 ± 0.10
TC09000485.hg.1	LOC441454	5.91 ± 0.05	5.94 ± 0.20	5.96 ± 0.10	5.80 ± 0.09
TC09000483.hg.1	LOC441455	6.13 ± 0.12	6.12 ± 0.11	6.23 ± 0.23	6.18 ± 0.11
TC09001414.hg.1	LOC441461	7.53 ± 0.18	7.41 ± 0.17	7.45 ± 0.09	7.45 ± 0.02
TC0X001001.hg.1	LOC441493	5.87 ± 0.17	5.70 ± 0.22	5.83 ± 0.10	5.82 ± 0.03
TC11001762.hg.1	LOC441601	6.40 ± 0.24	6.52 ± 0.06	6.54 ± 0.14	6.48 ± 0.09
TC10001192.hg.1	LOC441666	5.86 ± 0.24	5.72 ± 0.20	5.66 ± 0.15	5.90 ± 0.16
TC02002091.hg.1	LOC442028	6.85 ± 0.13	6.82 ± 0.07	6.75 ± 0.11	6.89 ± 0.08
TC03000050.hg.1	LOC442075	7.72 ± 0.05	7.82 ± 0.16	7.54 ± 0.15	7.70 ± 0.10
TC05001143.hg.1	LOC442132	7.25 ± 0.19	7.25 ± 0.14	7.33 ± 0.18	7.39 ± 0.03
TC07000006.hg.1	LOC442497	7.78 ± 0.29	7.69 ± 0.21	7.58 ± 0.17	7.76 ± 0.06
TC07001464.hg.1	LOC493754	8.17 ± 0.13	8.17 ± 0.03	8.18 ± 0.02	8.17 ± 0.03
TC09001297.hg.1	LOC494127	7.51 ± 0.45	7.54 ± 0.24	7.49 ± 0.28	7.74 ± 0.14
TC11000237.hg.1	LOC494141	6.47 ± 0.22	6.39 ± 0.11	6.35 ± 0.14	6.45 ± 0.09
TC02002207.hg.1	LOC541471	8.00 ± 0.07	7.73 ± 0.01	8.22 ± 0.22	7.65 ± 0.02
TC07001189.hg.1	LOC541472	5.79 ± 0.09	5.71 ± 0.21	5.74 ± 0.07	5.78 ± 0.15
TC07001529.hg.1	LOC541473	7.35 ± 0.17	7.19 ± 0.06	7.04 ± 0.15	7.25 ± 0.05
TC07003329.hg.1	LOC541473	7.22 ± 0.23	7.08 ± 0.16	6.94 ± 0.26	7.09 ± 0.04
TC04000363.hg.1	LOC550112	5.91 ± 0.02	5.81 ± 0.05	5.79 ± 0.02	5.79 ± 0.06
TC0X000341.hg.1	LOC550643	7.40 ± 0.08	7.21 ± 0.23	7.02 ± 0.10	6.99 ± 0.04
TC05001764.hg.1	LOC553103	6.80 ± 0.19	6.80 ± 0.01	6.80 ± 0.07	6.88 ± 0.04
TC22000883.hg.1	LOC554174	7.48 ± 0.12	7.23 ± 0.10	7.37 ± 0.20	7.26 ± 0.06
TC16000266.hg.1	LOC554206	7.22 ± 0.10	7.10 ± 0.09	7.06 ± 0.21	6.97 ± 0.14
TC14000085.hg.1	LOC554207	5.33 ± 0.20	5.26 ± 0.17	5.42 ± 0.21	5.33 ± 0.11
TC06000325.hg.1	LOC554223	7.92 ± 0.12	7.75 ± 0.04	7.82 ± 0.09	7.76 ± 0.07
TC6_apd_hap1000016.hg.1	LOC554223	7.33 ± 0.09	7.23 ± 0.05	7.28 ± 0.20	7.32 ± 0.07

TC6_cox_hap2000021.hg.1	LOC554223	7.33 ± 0.09	7.23 ± 0.05	7.28 ± 0.20	7.32 ± 0.07
TC6_dbb_hap3000017.hg.1	LOC554223	7.95 ± 0.11	7.76 ± 0.05	7.87 ± 0.14	7.77 ± 0.05
TC6_mann_hap4000018.hg.1	LOC554223	7.94 ± 0.11	7.76 ± 0.06	7.87 ± 0.14	7.76 ± 0.06
TC6_mcf_hap5000016.hg.1	LOC554223	7.95 ± 0.11	7.76 ± 0.05	7.87 ± 0.14	7.77 ± 0.05
TC6_qbl_hap6000016.hg.1	LOC554223	7.41 ± 0.10	7.30 ± 0.05	7.35 ± 0.20	7.38 ± 0.07
TC6_ssto_hap7000017.hg.1	LOC554223	7.93 ± 0.13	7.76 ± 0.04	7.85 ± 0.16	7.78 ± 0.04
TC09000241.hg.1	LOC554249	7.73 ± 0.13	7.60 ± 0.13	7.61 ± 0.10	7.75 ± 0.25
TC09001139.hg.1	LOC554249	7.33 ± 0.11	7.21 ± 0.04	7.27 ± 0.05	7.30 ± 0.15
TC12001076.hg.1	LOC574538	6.76 ± 0.19	6.75 ± 0.13	6.74 ± 0.07	6.85 ± 0.08
TC16001025.hg.1	LOC595101	10.24 ± 0.59	10.77 ± 0.09	10.31 ± 0.32	10.71 ± 0.04
TC16000320.hg.1	LOC606724	9.03 ± 0.24	8.62 ± 0.13	8.98 ± 0.26	8.76 ± 0.23
TC16001023.hg.1	LOC613037	12.14 ± 0.39	12.47 ± 0.04	12.21 ± 0.30	12.45 ± 0.06
TC10000955.hg.1	LOC619207	8.30 ± 0.14	8.30 ± 0.14	8.22 ± 0.15	8.39 ± 0.06
TC20000504.hg.1	LOC63930	8.03 ± 0.23	8.04 ± 0.20	8.03 ± 0.21	8.07 ± 0.02
TC19000395.hg.1	LOC641367	6.03 ± 0.32	5.93 ± 0.09	6.14 ± 0.07	6.14 ± 0.10
TC01001679.hg.1	LOC641515	5.96 ± 0.17	5.89 ± 0.04	5.84 ± 0.13	5.80 ± 0.05
TC07000383.hg.1	LOC641746	5.63 ± 0.20	5.48 ± 0.12	5.44 ± 0.10	5.52 ± 0.03
TC09001163.hg.1	LOC642236	7.93 ± 0.44	8.00 ± 0.29	7.97 ± 0.23	7.99 ± 0.08
TC10000573.hg.1	LOC642361	7.72 ± 0.11	7.69 ± 0.02	7.66 ± 0.03	7.70 ± 0.02
TC05001339.hg.1	LOC642366	6.14 ± 0.16	6.10 ± 0.14	5.93 ± 0.28	6.09 ± 0.04
TC15001793.hg.1	LOC642423	8.43 ± 0.21	8.42 ± 0.14	8.42 ± 0.22	8.54 ± 0.07
TC14000847.hg.1	LOC642426	5.20 ± 0.41	5.12 ± 0.09	5.11 ± 0.19	5.49 ± 0.44
TC22000083.hg.1	LOC642633	6.33 ± 0.33	6.16 ± 0.04	6.20 ± 0.11	6.54 ± 0.34
TC16002042.hg.1	LOC642799	9.79 ± 0.30	9.92 ± 0.09	9.44 ± 0.40	9.80 ± 0.12
TC12000144.hg.1	LOC642846	8.32 ± 0.04	8.26 ± 0.14	8.07 ± 0.13	8.22 ± 0.07
TC21000243.hg.1	LOC642852	8.02 ± 0.05	8.07 ± 0.18	7.98 ± 0.15	8.08 ± 0.06
TC09001115.hg.1	LOC642929	5.98 ± 0.35	5.97 ± 0.03	5.99 ± 0.11	6.22 ± 0.27
TC11000920.hg.1	LOC643037	6.73 ± 0.25	6.75 ± 0.13	6.61 ± 0.15	6.77 ± 0.10
TC02000966.hg.1	LOC643072	9.04 ± 0.23	8.35 ± 0.17	9.36 ± 0.34	8.22 ± 0.56
TC05002090.hg.1	LOC643201	5.98 ± 0.18	5.91 ± 0.10	5.89 ± 0.09	5.99 ± 0.07
TC12001812.hg.1	LOC643339	5.54 ± 0.12	5.46 ± 0.02	5.56 ± 0.05	5.50 ± 0.07

TC02001452.hg.1	LOC643387	7.38 ± 0.16	7.36 ± 0.13	7.33 ± 0.12	7.45 ± 0.05
TC05000125.hg.1	LOC643401	4.68 ± 0.09	4.51 ± 0.01	4.56 ± 0.01	4.58 ± 0.08
TC20000058.hg.1	LOC643406	7.13 ± 0.22	7.16 ± 0.16	7.18 ± 0.15	7.33 ± 0.08
TC01003003.hg.1	LOC643441	6.03 ± 0.24	6.16 ± 0.20	5.97 ± 0.37	6.25 ± 0.11
TC0X001201.hg.1	LOC643486	4.36 ± 0.08	4.25 ± 0.13	4.32 ± 0.23	4.38 ± 0.09
TC18000238.hg.1	LOC643542	6.48 ± 0.08	6.45 ± 0.10	6.46 ± 0.06	6.65 ± 0.12
TC06000959.hg.1	LOC643623	5.71 ± 0.15	5.61 ± 0.14	5.59 ± 0.11	5.67 ± 0.07
TC09000254.hg.1	LOC643648	6.99 ± 0.37	6.93 ± 0.03	6.88 ± 0.08	7.13 ± 0.43
TC19001509.hg.1	LOC643669	9.25 ± 0.18	9.22 ± 0.14	9.25 ± 0.20	9.31 ± 0.02
TC01003828.hg.1	LOC643723	5.84 ± 0.37	5.45 ± 0.02	5.52 ± 0.30	5.48 ± 0.17
TC11002245.hg.1	LOC643733	6.75 ± 0.09	6.57 ± 0.09	6.79 ± 0.19	6.46 ± 0.05
TC12001858.hg.1	LOC643770	5.65 ± 0.08	5.42 ± 0.05	5.57 ± 0.18	5.53 ± 0.12
TC16001118.hg.1	LOC643802	6.71 ± 0.16	6.76 ± 0.36	6.79 ± 0.41	6.54 ± 0.13
TC01000018.hg.1	LOC643837	8.23 ± 0.35	8.23 ± 0.14	8.02 ± 0.05	8.44 ± 0.09
TC11000973.hg.1	LOC643923	6.58 ± 0.42	6.48 ± 0.10	6.37 ± 0.27	6.54 ± 0.02
TC17001127.hg.1	LOC644070	7.24 ± 0.18	7.31 ± 0.15	7.28 ± 0.13	7.40 ± 0.02
TC07001019.hg.1	LOC644090	6.57 ± 0.09	6.42 ± 0.15	6.52 ± 0.16	6.41 ± 0.05
TC02000296.hg.1	LOC644093	6.87 ± 0.06	6.82 ± 0.09	6.82 ± 0.09	6.86 ± 0.08
TC06002140.hg.1	LOC644135	5.37 ± 0.13	5.25 ± 0.01	5.28 ± 0.04	5.36 ± 0.08
TC04000323.hg.1	LOC644145	4.37 ± 0.13	4.24 ± 0.17	4.27 ± 0.17	4.31 ± 0.05
TC17_ctg5_hap1000007.hg.1	LOC644172	7.85 ± 0.28	7.69 ± 0.21	7.67 ± 0.30	7.98 ± 0.05
TC17001608.hg.1	LOC644172	7.42 ± 0.30	7.25 ± 0.19	7.34 ± 0.30	7.49 ± 0.10
TC19000500.hg.1	LOC644189	6.83 ± 0.20	6.84 ± 0.20	6.72 ± 0.40	6.94 ± 0.07
TC10001564.hg.1	LOC644215	7.96 ± 0.22	7.95 ± 0.05	7.85 ± 0.16	8.04 ± 0.07
TC04000492.hg.1	LOC644248	4.83 ± 0.23	4.77 ± 0.15	4.84 ± 0.10	4.97 ± 0.10
TC21000008.hg.1	LOC644450	7.37 ± 0.60	7.51 ± 0.17	7.28 ± 0.19	7.66 ± 0.34
TC19000519.hg.1	LOC644554	6.41 ± 0.15	6.43 ± 0.18	6.46 ± 0.12	6.53 ± 0.05
TC11001401.hg.1	LOC644656	7.83 ± 0.08	7.86 ± 0.18	7.64 ± 0.33	7.83 ± 0.22
TC03000655.hg.1	LOC644662	7.80 ± 0.18	7.80 ± 0.12	7.68 ± 0.13	7.93 ± 0.09
TC18000395.hg.1	LOC644669	5.40 ± 0.15	5.26 ± 0.06	5.32 ± 0.03	5.38 ± 0.10
TC02001935.hg.1	LOC644838	5.60 ± 0.14	5.49 ± 0.07	5.58 ± 0.07	5.56 ± 0.06

TC05001529.hg.1	LOC644936	9.47 ± 0.13	9.29 ± 0.06	9.60 ± 0.29	9.25 ± 0.12
TC01000360.hg.1	LOC644961	7.84 ± 0.10	7.83 ± 0.10	7.89 ± 0.05	7.84 ± 0.11
TC01001152.hg.1	LOC645166	7.43 ± 0.14	7.44 ± 0.06	7.40 ± 0.17	7.52 ± 0.04
TC0X000690.hg.1	LOC645188	4.91 ± 0.19	4.73 ± 0.10	4.68 ± 0.08	4.67 ± 0.09
TC07001052.hg.1	LOC645249	7.36 ± 0.20	7.24 ± 0.18	7.29 ± 0.20	7.22 ± 0.00
TC18000028.hg.1	LOC645355	6.06 ± 0.05	6.03 ± 0.07	6.07 ± 0.12	6.03 ± 0.06
TC01002222.hg.1	LOC645359	6.55 ± 0.23	6.47 ± 0.05	6.34 ± 0.14	6.70 ± 0.16
TC06002169.hg.1	LOC645434	5.92 ± 0.16	5.82 ± 0.01	5.81 ± 0.05	5.84 ± 0.07
TC04000614.hg.1	LOC645513	7.80 ± 0.39	8.02 ± 0.23	8.18 ± 0.50	7.92 ± 0.10
TC17001759.hg.1	LOC645638	6.43 ± 0.37	6.40 ± 0.18	6.51 ± 0.25	6.46 ± 0.05
TC15001695.hg.1	LOC645752	7.37 ± 0.18	7.39 ± 0.08	7.34 ± 0.13	7.48 ± 0.06
TC02001631.hg.1	LOC645949	5.87 ± 0.18	5.83 ± 0.04	5.80 ± 0.04	6.08 ± 0.31
TC03000896.hg.1	LOC646168	4.50 ± 0.13	4.35 ± 0.10	4.40 ± 0.11	4.39 ± 0.06
TC15001026.hg.1	LOC646214	7.15 ± 0.39	7.04 ± 0.22	7.47 ± 0.27	7.07 ± 0.19
TC01003369.hg.1	LOC646268	6.01 ± 0.14	6.16 ± 0.28	6.09 ± 0.21	6.27 ± 0.09
TC15000152.hg.1	LOC646278	8.35 ± 0.15	8.26 ± 0.07	8.37 ± 0.13	8.20 ± 0.10
TC07001868.hg.1	LOC646329	7.80 ± 0.16	8.15 ± 0.19	8.17 ± 0.45	8.28 ± 0.13
TC01002380.hg.1	LOC646471	7.03 ± 0.01	7.01 ± 0.09	7.04 ± 0.11	7.12 ± 0.05
TC03001405.hg.1	LOC646498	6.27 ± 0.07	6.21 ± 0.18	6.12 ± 0.22	6.32 ± 0.06
TC11002470.hg.1	LOC646543	7.04 ± 0.13	6.91 ± 0.08	6.70 ± 0.09	7.07 ± 0.16
TC01000817.hg.1	LOC646626	6.62 ± 0.10	6.70 ± 0.02	6.60 ± 0.04	6.65 ± 0.06
TC02001358.hg.1	LOC646736	5.65 ± 0.08	5.48 ± 0.03	5.54 ± 0.04	5.57 ± 0.05
TC07001241.hg.1	LOC646762	8.03 ± 0.13	7.97 ± 0.09	7.89 ± 0.15	8.04 ± 0.05
TC11000425.hg.1	LOC646813	5.49 ± 0.10	5.53 ± 0.09	5.45 ± 0.10	5.55 ± 0.09
TC19000965.hg.1	LOC646862	8.32 ± 0.23	8.53 ± 0.20	8.29 ± 0.27	8.61 ± 0.11
TC03000810.hg.1	LOC646903	6.03 ± 0.19	6.15 ± 0.09	6.13 ± 0.08	6.08 ± 0.13
TC15000734.hg.1	LOC646938	8.03 ± 0.10	8.04 ± 0.11	8.05 ± 0.17	8.12 ± 0.00
TC07000249.hg.1	LOC646999	5.52 ± 0.08	5.27 ± 0.03	5.37 ± 0.11	5.36 ± 0.09
TC13000251.hg.1	LOC647264	6.50 ± 0.15	6.43 ± 0.04	6.32 ± 0.15	6.58 ± 0.14
TC03002124.hg.1	LOC647323	5.57 ± 0.08	5.59 ± 0.06	5.55 ± 0.06	5.56 ± 0.07
TC05001458.hg.1	LOC647859	6.24 ± 0.14	6.87 ± 0.71	6.48 ± 0.32	6.61 ± 0.21

TC22000110.hg.1	LOC648691	6.91 ± 0.23	6.86 ± 0.14	6.89 ± 0.16	6.99 ± 0.15
TC05001312.hg.1	LOC648987	6.97 ± 0.04	6.82 ± 0.06	6.76 ± 0.06	6.80 ± 0.02
TC11002376.hg.1	LOC649133	7.26 ± 0.19	7.16 ± 0.13	7.19 ± 0.18	7.20 ± 0.06
TC01002215.hg.1	LOC649330	7.33 ± 0.08	7.34 ± 0.06	7.34 ± 0.09	7.36 ± 0.09
TC07000381.hg.1	LOC649395	6.87 ± 0.09	6.79 ± 0.08	6.89 ± 0.14	6.81 ± 0.10
TC07001417.hg.1	LOC650226	5.55 ± 0.24	5.40 ± 0.11	5.44 ± 0.08	5.38 ± 0.10
TC04000093.hg.1	LOC650293	5.28 ± 0.13	5.29 ± 0.17	5.40 ± 0.08	5.66 ± 0.43
TC11000077.hg.1	LOC650368	7.98 ± 0.04	7.91 ± 0.03	7.93 ± 0.09	7.99 ± 0.04
TC16000079.hg.1	LOC652276	8.67 ± 0.09	8.61 ± 0.04	8.67 ± 0.08	8.64 ± 0.07
TC01006350.hg.1	LOC653160	7.45 ± 0.20	7.50 ± 0.10	7.56 ± 0.18	7.57 ± 0.10
TC11000002.hg.1	LOC653486	7.53 ± 0.18	7.39 ± 0.12	7.58 ± 0.17	7.58 ± 0.07
TC09001095.hg.1	LOC653501	6.31 ± 0.32	6.27 ± 0.03	6.37 ± 0.28	6.31 ± 0.03
TC01002418.hg.1	LOC653566	4.90 ± 0.26	4.71 ± 0.11	4.69 ± 0.12	4.66 ± 0.09
TC17001760.hg.1	LOC653653	6.72 ± 0.44	6.85 ± 0.01	6.70 ± 0.27	6.84 ± 0.27
TC03001760.hg.1	LOC653712	7.31 ± 0.15	7.32 ± 0.17	7.22 ± 0.11	7.45 ± 0.11
TC16000249.hg.1	LOC653786	6.59 ± 0.18	6.57 ± 0.11	6.56 ± 0.07	6.61 ± 0.09
TC02002083.hg.1	LOC654342	8.29 ± 0.05	8.25 ± 0.10	8.33 ± 0.09	8.39 ± 0.05
TC02000723.hg.1	LOC654433	7.22 ± 0.14	7.10 ± 0.08	7.13 ± 0.11	7.19 ± 0.04
TC16001310.hg.1	LOC654780	7.60 ± 0.36	7.62 ± 0.16	7.49 ± 0.28	7.76 ± 0.07
TC11000020.hg.1	LOC692247	8.36 ± 0.24	8.36 ± 0.25	8.33 ± 0.22	8.47 ± 0.08
TC08001629.hg.1	LOC727677	5.60 ± 0.15	5.56 ± 0.16	5.58 ± 0.12	5.68 ± 0.07
TC16001317.hg.1	LOC727710	7.48 ± 0.17	7.51 ± 0.13	7.49 ± 0.13	7.64 ± 0.10
TC18000286.hg.1	LOC727896	7.51 ± 0.62	7.41 ± 0.37	7.95 ± 0.70	7.33 ± 0.26
TC02001498.hg.1	LOC727944	7.04 ± 0.33	7.02 ± 0.07	7.03 ± 0.22	7.09 ± 0.14
TC02001532.hg.1	LOC727982	5.57 ± 0.11	5.48 ± 0.10	5.54 ± 0.06	5.67 ± 0.12
TC06000832.hg.1	LOC728012	5.73 ± 0.25	5.56 ± 0.04	5.58 ± 0.06	5.58 ± 0.06
TC08001136.hg.1	LOC728024	5.73 ± 0.09	5.72 ± 0.12	5.45 ± 0.17	5.80 ± 0.06
TC04000406.hg.1	LOC728040	5.27 ± 0.22	5.19 ± 0.02	5.17 ± 0.09	5.32 ± 0.06
TC12001793.hg.1	LOC728084	5.85 ± 0.06	5.72 ± 0.08	5.85 ± 0.11	5.91 ± 0.29
TC05000926.hg.1	LOC728095	6.08 ± 0.15	5.98 ± 0.11	6.05 ± 0.12	6.07 ± 0.05
TC16000889.hg.1	LOC728138	8.77 ± 0.21	8.38 ± 0.07	8.58 ± 0.04	8.39 ± 0.01

TC05002056.hg.1	LOC728145	6.83 ± 0.28	6.65 ± 0.04	6.65 ± 0.19	6.70 ± 0.11
TC04001803.hg.1	LOC728175	7.18 ± 0.17	7.17 ± 0.08	7.52 ± 0.06	7.20 ± 0.03
TC02002943.hg.1	LOC728208	7.75 ± 0.29	7.69 ± 0.06	7.69 ± 0.24	7.83 ± 0.14
TC20000049.hg.1	LOC728228	7.37 ± 0.22	7.49 ± 0.08	7.46 ± 0.19	7.61 ± 0.13
TC03000450.hg.1	LOC728290	4.04 ± 0.28	4.04 ± 0.05	4.08 ± 0.06	4.02 ± 0.02
TC02001493.hg.1	LOC728323	8.57 ± 0.27	8.45 ± 0.23	8.76 ± 0.08	8.44 ± 0.13
TC05000555.hg.1	LOC728342	4.57 ± 0.10	4.46 ± 0.05	4.51 ± 0.05	4.51 ± 0.08
TC01002516.hg.1	LOC728431	8.49 ± 0.24	8.27 ± 0.03	8.31 ± 0.01	8.41 ± 0.02
TC01003825.hg.1	LOC728463	5.71 ± 0.22	5.60 ± 0.16	5.39 ± 0.05	5.68 ± 0.11
TC02000593.hg.1	LOC728537	5.96 ± 0.12	5.98 ± 0.02	5.92 ± 0.05	6.05 ± 0.08
TC05001024.hg.1	LOC728554	9.06 ± 0.11	8.93 ± 0.09	8.63 ± 0.29	8.62 ± 0.04
TC05000610.hg.1	LOC728586	4.98 ± 0.08	4.93 ± 0.06	4.90 ± 0.04	4.95 ± 0.05
TC05000611.hg.1	LOC728586	8.61 ± 0.69	8.95 ± 0.18	8.69 ± 0.42	9.17 ± 0.28
TC18000428.hg.1	LOC728606	6.01 ± 0.17	5.72 ± 0.12	5.82 ± 0.05	5.83 ± 0.04
TC05001113.hg.1	LOC728613	7.56 ± 0.09	7.55 ± 0.08	7.41 ± 0.05	7.53 ± 0.03
TC01000076.hg.1	LOC728716	6.40 ± 0.07	6.40 ± 0.04	6.40 ± 0.04	6.62 ± 0.07
TC08001635.hg.1	LOC728724	5.84 ± 0.16	5.66 ± 0.11	5.70 ± 0.15	5.92 ± 0.16
TC02000250.hg.1	LOC728730	6.30 ± 0.12	6.18 ± 0.04	6.16 ± 0.08	6.22 ± 0.04
TC16000306.hg.1	LOC728734	8.85 ± 0.32	8.93 ± 0.09	8.73 ± 0.35	8.95 ± 0.08
TC12001931.hg.1	LOC728739	6.61 ± 0.14	6.55 ± 0.21	6.49 ± 0.23	6.56 ± 0.10
TC19000501.hg.1	LOC728752	7.18 ± 0.08	7.17 ± 0.04	7.10 ± 0.08	7.20 ± 0.15
TC14000990.hg.1	LOC728755	4.53 ± 0.04	4.43 ± 0.03	4.51 ± 0.03	4.49 ± 0.05
TC02001472.hg.1	LOC728763	8.59 ± 0.25	8.53 ± 0.17	8.53 ± 0.26	8.63 ± 0.02
TC05001517.hg.1	LOC728769	7.32 ± 0.18	7.23 ± 0.17	7.19 ± 0.03	7.25 ± 0.01
TC02001792.hg.1	LOC728819	5.78 ± 0.28	5.81 ± 0.19	5.75 ± 0.04	5.84 ± 0.08
TC01003091.hg.1	LOC728875	7.70 ± 0.14	7.73 ± 0.08	7.70 ± 0.14	7.67 ± 0.05
TC01003123.hg.1	LOC728989	6.92 ± 0.05	6.87 ± 0.08	6.76 ± 0.10	6.92 ± 0.07
TC10000770.hg.1	LOC729020	5.06 ± 0.17	4.93 ± 0.05	4.98 ± 0.06	4.95 ± 0.04
TC05001588.hg.1	LOC729040	7.48 ± 0.17	7.62 ± 0.04	7.62 ± 0.11	7.58 ± 0.13
TC01000586.hg.1	LOC729041	7.12 ± 0.25	7.04 ± 0.13	6.95 ± 0.10	7.15 ± 0.10
TC01002334.hg.1	LOC729059	6.75 ± 0.17	6.80 ± 0.08	6.72 ± 0.07	6.83 ± 0.07

TC05001876.hg.1	LOC729080	4.96 ± 0.08	4.89 ± 0.07	4.76 ± 0.05	4.97 ± 0.09
TC02002178.hg.1	LOC729121	5.01 ± 0.19	4.88 ± 0.07	4.93 ± 0.07	4.93 ± 0.02
TC06002199.hg.1	LOC729176	4.50 ± 0.24	4.28 ± 0.07	4.51 ± 0.28	4.32 ± 0.07
TC06001319.hg.1	LOC729177	8.14 ± 0.12	7.95 ± 0.10	7.83 ± 0.17	7.98 ± 0.04
TC02002680.hg.1	LOC729224	5.98 ± 0.11	5.97 ± 0.13	5.91 ± 0.30	6.10 ± 0.12
TC11001221.hg.1	LOC729305	9.02 ± 0.38	8.99 ± 0.17	8.91 ± 0.22	9.08 ± 0.10
TC22000059.hg.1	LOC729444	6.19 ± 0.34	6.11 ± 0.02	6.15 ± 0.08	6.47 ± 0.33
TC22000518.hg.1	LOC729461	6.59 ± 0.27	6.42 ± 0.05	6.53 ± 0.09	6.73 ± 0.17
TC05001150.hg.1	LOC729506	6.10 ± 0.19	5.95 ± 0.10	6.00 ± 0.11	6.10 ± 0.05
TC06001153.hg.1	LOC729603	8.40 ± 0.30	8.40 ± 0.22	8.58 ± 0.55	8.18 ± 0.34
TC0X000906.hg.1	LOC729609	7.34 ± 0.13	7.12 ± 0.11	7.35 ± 0.05	7.19 ± 0.02
TC01002053.hg.1	LOC729737	11.84 ± 0.31	11.85 ± 0.20	12.56 ± 0.28	11.87 ± 0.25
TC15000680.hg.1	LOC729739	7.91 ± 0.24	7.92 ± 0.02	7.92 ± 0.12	8.21 ± 0.30
TC07003319.hg.1	LOC729852	7.17 ± 0.18	7.18 ± 0.10	7.30 ± 0.29	7.07 ± 0.08
TC15001715.hg.1	LOC729911	6.56 ± 0.18	6.48 ± 0.04	6.48 ± 0.12	6.55 ± 0.05
TC18000112.hg.1	LOC729950	4.35 ± 0.27	4.30 ± 0.14	4.31 ± 0.16	4.36 ± 0.11
TC01000874.hg.1	LOC729970	5.45 ± 0.21	5.50 ± 0.16	5.38 ± 0.24	5.60 ± 0.11
TC01000887.hg.1	LOC729987	5.21 ± 0.20	5.24 ± 0.09	5.19 ± 0.11	5.38 ± 0.12
TC01002913.hg.1	LOC730081	6.44 ± 0.15	6.26 ± 0.03	6.28 ± 0.32	6.37 ± 0.16
TC06000657.hg.1	LOC730101	6.68 ± 0.15	6.45 ± 0.12	6.49 ± 0.20	6.49 ± 0.12
TC01001512.hg.1	LOC730159	5.34 ± 0.17	5.27 ± 0.06	5.11 ± 0.22	5.29 ± 0.22
TC01003720.hg.1	LOC730227	7.83 ± 0.05	7.96 ± 0.05	7.88 ± 0.15	7.96 ± 0.09
TC07001938.hg.1	LOC730441	6.13 ± 0.12	6.06 ± 0.20	6.05 ± 0.08	6.14 ± 0.17
TC22000387.hg.1	LOC730668	7.06 ± 0.04	7.08 ± 0.05	6.97 ± 0.06	7.16 ± 0.02
TC02000016.hg.1	LOC730811	5.92 ± 0.15	6.12 ± 0.30	6.04 ± 0.06	6.17 ± 0.07
TC14000472.hg.1	LOC731223	7.93 ± 0.14	7.77 ± 0.17	7.99 ± 0.17	7.80 ± 0.13
TC01004036.hg.1	LOC731275	11.05 ± 0.23	11.07 ± 0.16	11.53 ± 0.22	11.12 ± 0.15
TC04001811.hg.1	LOC731424	7.88 ± 0.35	7.78 ± 0.21	8.12 ± 0.32	7.56 ± 0.08
TC08000797.hg.1	LOC731779	6.58 ± 0.28	6.61 ± 0.12	6.63 ± 0.09	6.63 ± 0.15
TC12000254.hg.1	LOC732360	8.45 ± 0.05	8.30 ± 0.04	8.35 ± 0.15	8.27 ± 0.04
TC20000869.hg.1	LOC79015	6.85 ± 0.10	6.90 ± 0.11	6.86 ± 0.11	6.96 ± 0.06

TC15000778.hg.1	LOC80154	8.45 ± 0.16	8.39 ± 0.10	8.38 ± 0.15	8.49 ± 0.03
TC16000225.hg.1	LOC81691	6.06 ± 0.09	5.92 ± 0.05	5.92 ± 0.08	5.91 ± 0.07
TC02002257.hg.1	LOC84931	7.10 ± 0.16	7.13 ± 0.30	7.09 ± 0.14	7.27 ± 0.06
TC03000676.hg.1	LOC90246	6.17 ± 0.13	6.27 ± 0.30	6.23 ± 0.12	6.27 ± 0.06
TC02002050.hg.1	LOC90784	7.16 ± 0.39	7.12 ± 0.19	6.98 ± 0.14	7.21 ± 0.04
TC22000903.hg.1	LOC90834	8.15 ± 0.13	8.28 ± 0.13	7.95 ± 0.24	8.46 ± 0.10
TC15001698.hg.1	LOC91450	8.07 ± 0.15	8.23 ± 0.33	8.10 ± 0.27	8.33 ± 0.09
TC0X001091.hg.1	LOC92249	8.75 ± 0.28	8.73 ± 0.06	8.59 ± 0.41	8.63 ± 0.16
TC07000902.hg.1	LOC93432	7.15 ± 0.39	6.54 ± 0.16	8.23 ± 0.39	6.42 ± 0.12
TC02002902.hg.1	LOC93463	5.37 ± 0.12	5.32 ± 0.06	5.22 ± 0.11	5.35 ± 0.07
TC04000071.hg.1	LOC93622	8.10 ± 0.07	8.18 ± 0.10	7.86 ± 0.18	8.19 ± 0.11
TC12000174.hg.1	LOH12CR1	7.92 ± 0.04	7.96 ± 0.04	7.85 ± 0.08	7.94 ± 0.04
TC12001254.hg.1	LOH12CR2	6.38 ± 0.32	6.33 ± 0.24	6.32 ± 0.21	6.31 ± 0.14
TC19001081.hg.1	LONP1	8.38 ± 0.20	8.23 ± 0.08	8.17 ± 0.22	8.22 ± 0.04
TC16000430.hg.1	LONP2	9.02 ± 0.23	9.14 ± 0.13	8.83 ± 0.22	8.98 ± 0.10
TC08000997.hg.1	LONRF1	7.13 ± 0.15	7.25 ± 0.22	7.32 ± 0.29	7.28 ± 0.09
TC02002137.hg.1	LONRF2	5.66 ± 0.11	5.56 ± 0.09	5.57 ± 0.06	5.65 ± 0.10
TC0X000579.hg.1	LONRF3	6.31 ± 0.23	6.15 ± 0.07	6.42 ± 0.07	6.19 ± 0.12
TC01001252.hg.1	LOR	8.51 ± 0.18	8.41 ± 0.21	8.37 ± 0.15	8.44 ± 0.04
TC05001714.hg.1	LOX	6.24 ± 0.15	6.14 ± 0.09	6.23 ± 0.10	6.26 ± 0.06
TC18000484.hg.1	LOXHD1	7.12 ± 0.13	7.02 ± 0.06	7.15 ± 0.14	7.06 ± 0.03
TC15000670.hg.1	LOXL1	7.96 ± 0.17	7.86 ± 0.14	7.90 ± 0.08	7.96 ± 0.04
TC15001636.hg.1	LOXL1-AS1	8.47 ± 0.20	8.36 ± 0.14	8.40 ± 0.14	8.46 ± 0.05
TC08002613.hg.1	LOXL2	7.49 ± 0.17	7.41 ± 0.15	7.38 ± 0.11	7.48 ± 0.02
TC02002002.hg.1	LOXL3	7.69 ± 0.23	7.51 ± 0.12	7.65 ± 0.17	7.58 ± 0.04
TC10001576.hg.1	LOXL4	7.72 ± 0.23	7.66 ± 0.12	7.66 ± 0.18	7.77 ± 0.09
TC06002281.hg.1	LPA	6.51 ± 0.16	6.53 ± 0.05	6.52 ± 0.19	6.72 ± 0.20
TC06002280.hg.1	LPAL2	6.59 ± 0.23	6.57 ± 0.05	6.61 ± 0.12	6.66 ± 0.13
TC09001478.hg.1	LPAR1	7.94 ± 0.07	7.77 ± 0.16	7.85 ± 0.02	7.78 ± 0.04
TC19001305.hg.1	LPAR2	9.15 ± 0.13	9.11 ± 0.04	9.34 ± 0.12	9.10 ± 0.14
TC01002823.hg.1	LPAR3	6.24 ± 0.18	6.26 ± 0.24	6.18 ± 0.17	6.31 ± 0.03

TC0X000426.hg.1	LPAR4	5.59 ± 0.11	5.43 ± 0.02	5.50 ± 0.12	5.45 ± 0.15
TC12003269.hg.1	LPAR5	8.87 ± 0.05	8.49 ± 0.22	8.78 ± 0.03	8.56 ± 0.03
TC13000655.hg.1	LPAR6	6.25 ± 0.18	6.21 ± 0.10	6.33 ± 0.16	6.26 ± 0.01
TC05001110.hg.1	LPCAT1	9.11 ± 0.05	9.20 ± 0.09	9.08 ± 0.03	9.18 ± 0.09
TC16000455.hg.1	LPCAT2	10.04 ± 0.39	10.12 ± 0.42	11.20 ± 0.49	10.15 ± 0.14
TC12001155.hg.1	LPCAT3	9.91 ± 0.43	9.52 ± 0.07	10.24 ± 0.22	9.45 ± 0.16
TC15001194.hg.1	LPCAT4	8.62 ± 0.10	8.50 ± 0.06	8.53 ± 0.11	8.41 ± 0.03
TC01003800.hg.1	LPGAT1	9.14 ± 0.15	9.08 ± 0.20	9.45 ± 0.24	9.01 ± 0.07
TC19001233.hg.1	LPHN1	8.12 ± 0.10	8.07 ± 0.08	7.92 ± 0.09	8.11 ± 0.02
TC01000800.hg.1	LPHN2	4.58 ± 0.12	4.42 ± 0.05	4.49 ± 0.08	4.49 ± 0.06
TC04000351.hg.1	LPHN3	5.28 ± 0.07	5.16 ± 0.05	5.22 ± 0.08	5.20 ± 0.06
TC02000078.hg.1	LPIN1	7.62 ± 0.09	7.60 ± 0.03	7.49 ± 0.02	7.60 ± 0.06
TC18000285.hg.1	LPIN2	8.90 ± 0.19	8.78 ± 0.17	8.90 ± 0.38	8.53 ± 0.21
TC20000305.hg.1	LPIN3	7.75 ± 0.17	7.74 ± 0.20	7.74 ± 0.11	7.84 ± 0.08
TC08000149.hg.1	LPL	6.37 ± 0.25	6.32 ± 0.15	6.32 ± 0.07	6.37 ± 0.04
TC17000712.hg.1	LPO	7.10 ± 0.15	7.01 ± 0.21	7.01 ± 0.19	7.17 ± 0.10
TC03001038.hg.1	LPP	8.00 ± 0.22	8.10 ± 0.11	8.13 ± 0.34	8.07 ± 0.12
TC03002103.hg.1	LPP-AS1	4.77 ± 0.19	4.87 ± 0.05	4.88 ± 0.11	4.93 ± 0.25
TC03002102.hg.1	LPP-AS2	7.72 ± 0.18	7.43 ± 0.07	7.80 ± 0.27	7.46 ± 0.15
TC09000515.hg.1	LPPR1	6.35 ± 0.11	6.26 ± 0.13	6.30 ± 0.10	6.36 ± 0.07
TC19000195.hg.1	LPPR2	9.27 ± 0.22	9.09 ± 0.01	9.31 ± 0.18	9.08 ± 0.20
TC19000990.hg.1	LPPR3	8.37 ± 0.21	8.34 ± 0.25	8.26 ± 0.18	8.43 ± 0.04
TC01000891.hg.1	LPPR4	6.33 ± 0.11	6.29 ± 0.09	6.31 ± 0.06	6.42 ± 0.15
TC01002908.hg.1	LPPR5	5.15 ± 0.13	5.08 ± 0.07	5.06 ± 0.08	5.16 ± 0.06
TC11001812.hg.1	LPXN	9.05 ± 0.19	9.08 ± 0.13	8.83 ± 0.09	8.84 ± 0.04
TC04000778.hg.1	LRAT	5.55 ± 0.20	5.44 ± 0.09	5.42 ± 0.08	5.48 ± 0.04
TC04001634.hg.1	LRBA	8.50 ± 0.55	8.85 ± 0.22	8.31 ± 0.29	8.61 ± 0.15
TC13000184.hg.1	LRCH1	7.87 ± 0.34	8.12 ± 0.20	7.79 ± 0.24	8.00 ± 0.03
TC0X001291.hg.1	LRCH2	4.54 ± 0.10	4.37 ± 0.03	4.42 ± 0.11	4.40 ± 0.03
TC03001110.hg.1	LRCH3	8.71 ± 0.22	8.63 ± 0.19	8.66 ± 0.17	8.53 ± 0.09
TC07003372.hg.1	LRCH4	9.50 ± 0.03	9.50 ± 0.03	9.45 ± 0.09	9.56 ± 0.07

TC12002152.hg.1	LRCOL1	8.10 ± 0.13	8.07 ± 0.20	8.08 ± 0.14	8.18 ± 0.07
TC19001515.hg.1	LRFN1	7.84 ± 0.55	7.71 ± 0.19	8.13 ± 0.35	7.69 ± 0.31
TC06001706.hg.1	LRFN2	7.84 ± 0.21	7.79 ± 0.13	7.67 ± 0.28	7.97 ± 0.17
TC19000490.hg.1	LRFN3	7.69 ± 0.15	7.69 ± 0.19	7.57 ± 0.04	7.61 ± 0.07
TC11000675.hg.1	LRFN4	8.50 ± 0.24	8.46 ± 0.24	8.39 ± 0.24	8.59 ± 0.08
TC14000256.hg.1	LRFN5	5.32 ± 0.18	5.23 ± 0.07	5.23 ± 0.11	5.27 ± 0.06
TC19002672.hg.1	LRG1	9.26 ± 0.53	8.98 ± 0.09	9.73 ± 0.43	8.79 ± 0.22
TC07000831.hg.1	LRGUK	5.46 ± 0.11	5.34 ± 0.02	5.35 ± 0.03	5.38 ± 0.05
TC01002979.hg.1	LRIF1	7.22 ± 0.09	6.89 ± 0.32	7.52 ± 0.43	6.59 ± 0.17
TC03001532.hg.1	LRIG1	8.14 ± 0.07	8.09 ± 0.03	7.95 ± 0.08	8.07 ± 0.01
TC01000998.hg.1	LRIG2	7.21 ± 0.21	7.13 ± 0.18	6.98 ± 0.16	7.09 ± 0.07
TC12001643.hg.1	LRIG3	5.14 ± 0.11	5.08 ± 0.04	5.08 ± 0.05	5.10 ± 0.03
TC10001472.hg.1	LRIT1	7.26 ± 0.29	7.32 ± 0.22	7.25 ± 0.25	7.39 ± 0.09
TC10001471.hg.1	LRIT2	6.31 ± 0.16	6.15 ± 0.04	6.06 ± 0.16	6.31 ± 0.16
TC04000567.hg.1	LRIT3	5.95 ± 0.23	5.98 ± 0.10	5.90 ± 0.14	6.04 ± 0.12
TC12000251.hg.1	LRMP	9.08 ± 0.32	9.25 ± 0.20	9.35 ± 0.28	9.21 ± 0.06
TC12000521.hg.1	LRP1	9.44 ± 0.31	9.12 ± 0.07	9.41 ± 0.08	9.07 ± 0.27
TC14000135.hg.1	LRP10	10.01 ± 0.37	9.69 ± 0.15	10.22 ± 0.29	9.59 ± 0.22
TC06002217.hg.1	LRP11	6.79 ± 0.17	6.72 ± 0.11	6.77 ± 0.07	6.78 ± 0.02
TC06002218.hg.1	LRP11	8.67 ± 0.21	8.65 ± 0.18	8.56 ± 0.25	8.74 ± 0.05
TC08001511.hg.1	LRP12	6.59 ± 0.22	6.22 ± 0.04	6.43 ± 0.07	6.25 ± 0.10
TC02002388.hg.1	LRP1B	5.03 ± 0.12	4.92 ± 0.07	4.94 ± 0.05	5.01 ± 0.11
TC02002509.hg.1	LRP2	5.68 ± 0.15	5.59 ± 0.09	5.57 ± 0.08	5.68 ± 0.09
TC04001815.hg.1	LRP2BP	5.26 ± 0.08	5.20 ± 0.02	5.21 ± 0.06	5.23 ± 0.02
TC19000435.hg.1	LRP3	8.64 ± 0.22	8.56 ± 0.20	8.59 ± 0.16	8.62 ± 0.01
TC11001728.hg.1	LRP4	7.23 ± 0.21	7.20 ± 0.16	7.20 ± 0.14	7.29 ± 0.04
TC11000402.hg.1	LRP4-AS1	6.92 ± 0.17	6.82 ± 0.21	6.90 ± 0.25	6.83 ± 0.08
TC11000707.hg.1	LRP5	8.14 ± 0.19	8.10 ± 0.15	8.05 ± 0.15	8.16 ± 0.01
TC22000598.hg.1	LRP5L	7.84 ± 0.11	7.81 ± 0.10	7.78 ± 0.11	7.88 ± 0.04
TC12001251.hg.1	LRP6	6.33 ± 0.03	6.43 ± 0.04	6.31 ± 0.07	6.39 ± 0.06
TC01002669.hg.1	LRP8	7.90 ± 0.06	7.81 ± 0.06	7.81 ± 0.07	7.81 ± 0.01

TC04000983.hg.1	LRPAP1	9.30 ± 0.29	9.01 ± 0.08	9.44 ± 0.15	9.00 ± 0.12
TC02001795.hg.1	LRPPRC	8.17 ± 0.44	8.51 ± 0.22	7.91 ± 0.38	8.29 ± 0.04
TC14000274.hg.1	LRR1	6.68 ± 0.10	6.53 ± 0.08	6.53 ± 0.08	6.46 ± 0.06
TC06000668.hg.1	LRRC1	6.30 ± 0.05	6.27 ± 0.02	6.22 ± 0.05	6.22 ± 0.06
TC12001711.hg.1	LRRC10	6.27 ± 0.26	6.35 ± 0.23	6.29 ± 0.22	6.45 ± 0.11
TC11000535.hg.1	LRRC10B	7.01 ± 0.17	6.99 ± 0.10	7.17 ± 0.09	7.08 ± 0.05
TC08000858.hg.1	LRRC14	8.40 ± 0.15	8.38 ± 0.16	8.18 ± 0.18	8.45 ± 0.05
TC05000003.hg.1	LRRC14B	7.68 ± 0.30	7.79 ± 0.28	7.59 ± 0.41	7.88 ± 0.07
TC03002131.hg.1	LRRC15	6.99 ± 0.04	6.88 ± 0.14	6.89 ± 0.12	7.06 ± 0.08
TC06000153.hg.1	LRRC16A	7.18 ± 0.05	7.15 ± 0.07	6.91 ± 0.10	7.06 ± 0.09
TC14000155.hg.1	LRRC16B	7.64 ± 0.13	7.59 ± 0.19	7.52 ± 0.18	7.65 ± 0.02
TC07000668.hg.1	LRRC17	5.60 ± 0.03	5.60 ± 0.11	5.63 ± 0.08	5.60 ± 0.03
TC10001268.hg.1	LRRC18	6.40 ± 0.28	6.28 ± 0.17	6.25 ± 0.14	6.41 ± 0.12
TC09000985.hg.1	LRRC19	4.03 ± 0.06	3.88 ± 0.03	3.94 ± 0.05	3.86 ± 0.04
TC03001358.hg.1	LRRC2	6.25 ± 0.10	6.20 ± 0.07	6.20 ± 0.04	6.22 ± 0.04
TC10001364.hg.1	LRRC20	8.06 ± 0.32	7.97 ± 0.20	7.95 ± 0.25	8.01 ± 0.05
TC12000097.hg.1	LRRC23	7.46 ± 0.15	7.38 ± 0.14	7.36 ± 0.12	7.39 ± 0.04
TC08001756.hg.1	LRRC24	8.51 ± 0.20	8.39 ± 0.23	8.32 ± 0.27	8.40 ± 0.07
TC19001287.hg.1	LRRC25	10.55 ± 0.63	10.12 ± 0.08	10.88 ± 0.30	10.04 ± 0.42
TC09001759.hg.1	LRRC26	8.42 ± 0.12	8.28 ± 0.12	8.31 ± 0.27	8.30 ± 0.08
TC10000942.hg.1	LRRC27	7.13 ± 0.12	7.04 ± 0.08	7.04 ± 0.09	7.01 ± 0.03
TC15000954.hg.1	LRRC28	7.71 ± 0.08	7.63 ± 0.16	7.69 ± 0.16	7.50 ± 0.10
TC16001183.hg.1	LRRC29	7.82 ± 0.13	7.83 ± 0.17	7.78 ± 0.15	7.95 ± 0.08
TC21000226.hg.1	LRRC3	7.52 ± 0.17	7.53 ± 0.18	7.52 ± 0.14	7.60 ± 0.05
TC18000034.hg.1	LRRC30	7.22 ± 0.14	7.29 ± 0.21	7.27 ± 0.20	7.49 ± 0.15
TC03001985.hg.1	LRRC31	5.20 ± 0.09	5.12 ± 0.09	5.10 ± 0.10	5.20 ± 0.04
TC11002116.hg.1	LRRC32	7.53 ± 0.19	7.61 ± 0.09	7.45 ± 0.14	7.73 ± 0.03
TC03003367.hg.1	LRRC33	9.06 ± 0.17	8.91 ± 0.04	8.92 ± 0.11	8.85 ± 0.05
TC03001984.hg.1	LRRC34	5.17 ± 0.11	5.02 ± 0.06	5.07 ± 0.03	5.03 ± 0.00
TC16000539.hg.1	LRRC36	6.23 ± 0.11	6.22 ± 0.11	6.21 ± 0.09	6.30 ± 0.07
TC17_ctg5_hap1000027.hg.1	LRRC37A	9.22 ± 0.31	9.23 ± 0.31	9.21 ± 0.23	9.17 ± 0.11

TC17000597.hg.1	LRRC37A	9.09 ± 0.25	9.07 ± 0.21	9.07 ± 0.21	9.04 ± 0.09
TC17000471.hg.1	LRRC37A11P	6.34 ± 0.08	6.33 ± 0.03	6.33 ± 0.16	6.37 ± 0.13
TC17_ctg5_hap1000026.hg.1	LRRC37A2	9.26 ± 0.26	9.30 ± 0.25	9.24 ± 0.20	9.24 ± 0.08
TC17000599.hg.1	LRRC37A2	9.15 ± 0.28	9.16 ± 0.29	9.17 ± 0.24	9.10 ± 0.10
TC17001806.hg.1	LRRC37A3	8.81 ± 0.19	8.77 ± 0.20	8.83 ± 0.19	8.78 ± 0.09
TC17_ctg5_hap1000009.hg.1	LRRC37A4P	9.13 ± 0.30	9.16 ± 0.28	9.10 ± 0.22	9.11 ± 0.07
TC17001603.hg.1	LRRC37A4P	9.17 ± 0.55	9.33 ± 0.15	9.06 ± 0.25	9.20 ± 0.07
TC09001485.hg.1	LRRC37A5P	5.52 ± 0.16	5.50 ± 0.23	5.58 ± 0.03	5.62 ± 0.08
TC10001130.hg.1	LRRC37A6P	6.34 ± 0.24	6.15 ± 0.06	6.25 ± 0.14	6.30 ± 0.08
TC17000371.hg.1	LRRC37B	8.29 ± 0.31	8.37 ± 0.19	8.28 ± 0.34	8.42 ± 0.15
TC17000352.hg.1	LRRC37BP1	7.63 ± 0.23	7.71 ± 0.09	7.57 ± 0.15	7.69 ± 0.07
TC01002232.hg.1	LRRC38	7.30 ± 0.22	7.49 ± 0.14	7.42 ± 0.25	7.55 ± 0.10
TC01002917.hg.1	LRRC39	4.90 ± 0.13	4.83 ± 0.06	4.77 ± 0.12	4.83 ± 0.04
TC21000520.hg.1	LRRC3-AS1	7.33 ± 0.30	7.45 ± 0.29	7.32 ± 0.31	7.55 ± 0.10
TC03000135.hg.1	LRRC3B	6.77 ± 0.15	6.67 ± 0.14	6.72 ± 0.13	6.83 ± 0.09
TC17000483.hg.1	LRRC3C	5.51 ± 0.26	5.51 ± 0.22	5.57 ± 0.11	5.70 ± 0.12
TC07001842.hg.1	LRRC4	8.35 ± 0.30	8.06 ± 0.03	8.56 ± 0.16	8.06 ± 0.10
TC01002769.hg.1	LRRC40	7.24 ± 0.62	7.60 ± 0.20	7.28 ± 0.45	7.53 ± 0.11
TC01002618.hg.1	LRRC41	8.25 ± 0.11	8.06 ± 0.03	8.02 ± 0.09	8.03 ± 0.06
TC01000659.hg.1	LRRC42	6.98 ± 0.15	6.91 ± 0.07	6.79 ± 0.18	6.84 ± 0.05
TC12000970.hg.1	LRRC43	7.13 ± 0.10	7.06 ± 0.13	7.02 ± 0.10	7.11 ± 0.01
TC17000943.hg.1	LRRC45	8.32 ± 0.18	8.22 ± 0.13	8.22 ± 0.20	8.23 ± 0.01
TC17000614.hg.1	LRRC46	7.10 ± 0.11	7.02 ± 0.07	7.08 ± 0.03	7.11 ± 0.02
TC01002136.hg.1	LRRC47	8.97 ± 0.12	8.82 ± 0.06	8.79 ± 0.14	8.78 ± 0.08
TC17000202.hg.1	LRRC48	6.81 ± 0.17	6.82 ± 0.19	6.77 ± 0.11	6.90 ± 0.05
TC15000647.hg.1	LRRC49	4.70 ± 0.12	4.64 ± 0.02	4.69 ± 0.04	4.73 ± 0.06
TC19001750.hg.1	LRRC4B	7.59 ± 0.31	7.56 ± 0.26	7.40 ± 0.33	7.61 ± 0.05
TC11001565.hg.1	LRRC4C	4.73 ± 0.14	4.62 ± 0.04	4.67 ± 0.09	4.65 ± 0.09
TC01001440.hg.1	LRRC52	5.93 ± 0.19	5.72 ± 0.13	5.75 ± 0.12	6.05 ± 0.16
TC01002785.hg.1	LRRC53	6.21 ± 0.06	6.25 ± 0.08	6.32 ± 0.08	6.30 ± 0.07
TC11000459.hg.1	LRRC55	6.19 ± 0.14	6.25 ± 0.20	6.20 ± 0.11	6.34 ± 0.03

TC11000019.hg.1	LRRC56	8.41 ± 0.19	8.31 ± 0.16	8.33 ± 0.14	8.42 ± 0.07
TC15001266.hg.1	LRRC57	7.50 ± 0.09	7.49 ± 0.04	7.48 ± 0.19	7.44 ± 0.10
TC03001690.hg.1	LRRC58	8.01 ± 0.40	8.29 ± 0.21	7.81 ± 0.41	8.16 ± 0.07
TC17001686.hg.1	LRRC59	6.88 ± 0.10	6.87 ± 0.05	6.75 ± 0.06	6.93 ± 0.11
TC17001687.hg.1	LRRC59	9.55 ± 0.29	9.31 ± 0.19	9.23 ± 0.22	9.18 ± 0.10
TC08001654.hg.1	LRRC6	7.08 ± 0.08	6.98 ± 0.06	7.70 ± 0.44	7.01 ± 0.17
TC07000998.hg.1	LRRC61	7.98 ± 0.07	7.79 ± 0.03	7.87 ± 0.04	7.75 ± 0.05
TC13000179.hg.1	LRRC63	4.17 ± 0.07	4.03 ± 0.04	4.17 ± 0.03	4.00 ± 0.05
TC04001193.hg.1	LRRC66	5.27 ± 0.12	5.08 ± 0.04	5.16 ± 0.08	5.16 ± 0.07
TC01000752.hg.1	LRRC7	5.08 ± 0.10	4.98 ± 0.05	5.03 ± 0.01	5.05 ± 0.08
TC01000754.hg.1	LRRC7	5.12 ± 0.10	4.84 ± 0.07	4.96 ± 0.10	4.89 ± 0.07
TC05003397.hg.1	LRRC70	6.55 ± 0.22	6.49 ± 0.18	6.50 ± 0.13	6.32 ± 0.05
TC01001330.hg.1	LRRC71	7.26 ± 0.22	7.18 ± 0.19	7.18 ± 0.24	7.28 ± 0.04
TC07000109.hg.1	LRRC72	4.11 ± 0.14	3.97 ± 0.01	4.04 ± 0.07	4.03 ± 0.04
TC06001749.hg.1	LRRC73	7.51 ± 0.21	7.46 ± 0.16	7.38 ± 0.04	7.48 ± 0.08
TC09000704.hg.1	LRRC8A	8.59 ± 0.11	8.44 ± 0.06	8.34 ± 0.23	8.42 ± 0.03
TC01000837.hg.1	LRRC8B	7.64 ± 0.22	7.52 ± 0.20	7.41 ± 0.17	7.61 ± 0.06
TC01000839.hg.1	LRRC8C	7.75 ± 0.13	7.77 ± 0.05	7.63 ± 0.26	7.71 ± 0.09
TC01000841.hg.1	LRRC8D	8.26 ± 0.08	8.06 ± 0.04	8.04 ± 0.05	8.07 ± 0.07
TC19000139.hg.1	LRRC8E	7.55 ± 0.25	7.44 ± 0.20	7.34 ± 0.22	7.51 ± 0.01
TC14000360.hg.1	LRRC9	3.91 ± 0.16	3.71 ± 0.02	3.80 ± 0.02	3.76 ± 0.03
TC08000528.hg.1	LRRCC1	5.75 ± 0.19	5.71 ± 0.25	5.79 ± 0.33	5.43 ± 0.11
TC07003362.hg.1	LRRD1	4.86 ± 0.12	4.73 ± 0.07	4.79 ± 0.04	4.78 ± 0.03
TC02004920.hg.1	LRRFIP1	8.56 ± 0.23	8.73 ± 0.11	8.88 ± 0.28	8.80 ± 0.12
TC03001286.hg.1	LRRFIP2	7.41 ± 0.11	7.29 ± 0.16	7.86 ± 0.37	7.24 ± 0.13
TC12000684.hg.1	LRRIQ1	4.31 ± 0.11	4.16 ± 0.05	4.22 ± 0.05	4.21 ± 0.07
TC01002783.hg.1	LRRIQ3	3.93 ± 0.06	3.81 ± 0.01	3.90 ± 0.10	3.79 ± 0.01
TC03000907.hg.1	LRRIQ4	4.37 ± 0.17	4.31 ± 0.04	4.31 ± 0.06	4.36 ± 0.05
TC15000973.hg.1	LRRK1	8.02 ± 0.07	7.93 ± 0.05	8.00 ± 0.06	7.91 ± 0.02
TC12000309.hg.1	LRRK2	9.97 ± 0.47	10.08 ± 0.44	10.88 ± 0.58	9.79 ± 0.36
TC03000011.hg.1	LRRN1	7.34 ± 0.21	7.07 ± 0.17	7.38 ± 0.17	7.16 ± 0.04

TC01003736.hg.1	LRRN2	8.03 ± 0.20	8.04 ± 0.18	8.05 ± 0.20	8.14 ± 0.06
TC07000704.hg.1	LRRN3	7.51 ± 0.44	7.63 ± 0.19	7.21 ± 0.34	7.42 ± 0.05
TC20000602.hg.1	LRRN4	6.86 ± 0.04	6.80 ± 0.13	6.75 ± 0.12	6.82 ± 0.08
TC11001876.hg.1	LRRN4CL	7.17 ± 0.19	7.13 ± 0.24	7.17 ± 0.01	7.23 ± 0.06
TC02002024.hg.1	LRRTM1	6.94 ± 0.19	6.89 ± 0.14	6.84 ± 0.19	6.98 ± 0.05
TC05001836.hg.1	LRRTM2	5.58 ± 0.11	5.58 ± 0.02	5.59 ± 0.08	5.65 ± 0.06
TC10000396.hg.1	LRRTM3	4.57 ± 0.01	4.50 ± 0.03	4.50 ± 0.04	4.57 ± 0.10
TC02002011.hg.1	LRRTM4	5.43 ± 0.08	5.39 ± 0.04	5.42 ± 0.05	5.50 ± 0.13
TC09000666.hg.1	LRSAM1	7.85 ± 0.11	7.67 ± 0.07	7.83 ± 0.09	7.67 ± 0.03
TC03001478.hg.1	LRTM1	6.39 ± 0.07	6.35 ± 0.09	6.42 ± 0.06	6.41 ± 0.09
TC12000019.hg.1	LRTM2	8.22 ± 0.22	8.28 ± 0.22	8.18 ± 0.23	8.37 ± 0.07
TC11000749.hg.1	LRTOMT	7.13 ± 0.16	7.04 ± 0.09	7.04 ± 0.11	7.10 ± 0.03
TC07000660.hg.1	LRWD1	8.79 ± 0.14	8.75 ± 0.11	8.76 ± 0.05	8.80 ± 0.01
TC03001673.hg.1	LSAMP	6.27 ± 0.17	6.23 ± 0.13	6.22 ± 0.13	6.36 ± 0.10
TC03000597.hg.1	LSAMP-AS1	6.82 ± 0.36	6.92 ± 0.04	6.93 ± 0.15	7.09 ± 0.09
TC03000598.hg.1	LSAMP-AS2	6.46 ± 0.08	6.50 ± 0.07	6.49 ± 0.15	6.58 ± 0.13
TC03002137.hg.1	LSG1	8.55 ± 0.22	8.61 ± 0.07	8.34 ± 0.14	8.53 ± 0.09
TC08001142.hg.1	LSM1	7.58 ± 0.06	7.48 ± 0.14	7.55 ± 0.19	7.35 ± 0.11
TC01002507.hg.1	LSM10	8.46 ± 0.28	8.20 ± 0.04	8.29 ± 0.26	8.17 ± 0.07
TC05000873.hg.1	LSM11	6.73 ± 0.08	6.61 ± 0.13	6.52 ± 0.11	6.59 ± 0.04
TC17001571.hg.1	LSM12	9.39 ± 0.13	9.31 ± 0.17	9.25 ± 0.03	9.20 ± 0.09
TC19000440.hg.1	LSM14A	9.79 ± 0.37	10.15 ± 0.15	9.91 ± 0.36	10.12 ± 0.17
TC20000482.hg.1	LSM14B	7.86 ± 0.04	7.82 ± 0.05	7.82 ± 0.08	7.81 ± 0.03
TC06001545.hg.1	LSM2	7.42 ± 0.13	7.26 ± 0.05	7.22 ± 0.12	7.24 ± 0.10
TC6_apd_hap1000086.hg.1	LSM2	7.42 ± 0.13	7.26 ± 0.05	7.22 ± 0.12	7.24 ± 0.10
TC6_cox_hap2000172.hg.1	LSM2	7.25 ± 0.07	7.11 ± 0.07	7.05 ± 0.08	7.09 ± 0.11
TC6_dbb_hap3000161.hg.1	LSM2	7.42 ± 0.13	7.26 ± 0.05	7.22 ± 0.12	7.24 ± 0.10
TC6_mcf_hap5000149.hg.1	LSM2	7.42 ± 0.13	7.26 ± 0.05	7.22 ± 0.12	7.24 ± 0.10
TC6_qbl_hap6000162.hg.1	LSM2	7.42 ± 0.13	7.26 ± 0.05	7.22 ± 0.12	7.24 ± 0.10
TC03000085.hg.1	LSM3	8.29 ± 0.02	8.26 ± 0.19	8.04 ± 0.11	8.13 ± 0.12
TC19001286.hg.1	LSM4	8.86 ± 0.19	8.79 ± 0.14	8.60 ± 0.24	8.63 ± 0.04

TC07001258.hg.1	LSM5	6.77 ± 0.09	6.90 ± 0.06	6.71 ± 0.04	6.86 ± 0.07
TC04000717.hg.1	LSM6	7.05 ± 0.20	7.06 ± 0.20	7.13 ± 0.17	7.02 ± 0.06
TC19001028.hg.1	LSM7	8.66 ± 0.27	8.50 ± 0.09	8.32 ± 0.18	8.41 ± 0.01
TC17001095.hg.1	LSMD1	8.41 ± 0.24	8.27 ± 0.12	8.36 ± 0.11	8.11 ± 0.16
TC07003292.hg.1	LSMEM1	6.28 ± 0.27	6.25 ± 0.06	6.86 ± 0.48	6.08 ± 0.12
TC03000310.hg.1	LSMEM2	7.73 ± 0.24	7.66 ± 0.17	7.59 ± 0.17	7.77 ± 0.04
TC11000052.hg.1	LSP1	9.65 ± 0.13	9.54 ± 0.08	9.65 ± 0.11	9.55 ± 0.13
TC05000127.hg.1	LSP1P3	6.73 ± 0.14	6.71 ± 0.22	6.96 ± 0.30	6.65 ± 0.15
TC19000462.hg.1	LSR	8.18 ± 0.13	8.19 ± 0.08	7.98 ± 0.13	8.19 ± 0.05
TC21000554.hg.1	LSS	8.03 ± 0.07	7.99 ± 0.10	7.89 ± 0.11	7.98 ± 0.07
TC06000372.hg.1	LST1	9.73 ± 0.26	9.47 ± 0.07	9.95 ± 0.28	9.43 ± 0.06
TC6_apd_hap1000037.hg.1	LST1	9.22 ± 0.26	8.97 ± 0.07	9.43 ± 0.26	8.95 ± 0.06
TC6_cox_hap2000068.hg.1	LST1	9.23 ± 0.26	8.97 ± 0.08	9.43 ± 0.27	8.96 ± 0.06
TC6_dbb_hap3000059.hg.1	LST1	9.22 ± 0.26	8.96 ± 0.08	9.42 ± 0.27	8.95 ± 0.07
TC6_mann_hap4000060.hg.1	LST1	9.22 ± 0.25	8.96 ± 0.07	9.43 ± 0.27	8.95 ± 0.06
TC6_mcf_hap5000054.hg.1	LST1	9.23 ± 0.26	8.98 ± 0.08	9.43 ± 0.27	8.97 ± 0.06
TC6_qbl_hap6000059.hg.1	LST1	9.24 ± 0.26	8.98 ± 0.08	9.43 ± 0.26	8.97 ± 0.06
TC6_ssto_hap7000055.hg.1	LST1	9.22 ± 0.26	8.97 ± 0.07	9.43 ± 0.27	8.96 ± 0.06
TC06000370.hg.1	LTA	7.62 ± 0.12	7.73 ± 0.16	7.46 ± 0.19	7.84 ± 0.05
TC6_cox_hap2000066.hg.1	LTA	7.47 ± 0.06	7.59 ± 0.12	7.30 ± 0.16	7.72 ± 0.05
TC6_dbb_hap3000057.hg.1	LTA	7.64 ± 0.12	7.72 ± 0.16	7.45 ± 0.20	7.83 ± 0.05
TC6_mann_hap4000058.hg.1	LTA	7.62 ± 0.12	7.73 ± 0.16	7.46 ± 0.19	7.84 ± 0.05
TC6_mcf_hap5000052.hg.1	LTA	7.64 ± 0.12	7.72 ± 0.16	7.45 ± 0.20	7.83 ± 0.05
TC6_qbl_hap6000057.hg.1	LTA	7.64 ± 0.12	7.72 ± 0.16	7.45 ± 0.20	7.83 ± 0.05
TC6_ssto_hap7000053.hg.1	LTA	7.62 ± 0.12	7.73 ± 0.16	7.46 ± 0.19	7.84 ± 0.05
TC12001843.hg.1	LTA4H	10.16 ± 0.19	9.86 ± 0.13	10.69 ± 0.29	9.71 ± 0.33
TC06001530.hg.1	LTB	9.45 ± 0.12	9.24 ± 0.04	9.18 ± 0.20	9.19 ± 0.13
TC6_apd_hap1000081.hg.1	LTB	9.75 ± 0.13	9.62 ± 0.03	9.53 ± 0.22	9.55 ± 0.05
TC6_cox_hap2000159.hg.1	LTB	9.75 ± 0.13	9.62 ± 0.03	9.53 ± 0.22	9.55 ± 0.05
TC6_dbb_hap3000148.hg.1	LTB	9.74 ± 0.14	9.61 ± 0.03	9.52 ± 0.22	9.54 ± 0.06
TC6_mann_hap4000136.hg.1	LTB	9.74 ± 0.14	9.61 ± 0.03	9.52 ± 0.22	9.54 ± 0.06

TC6_mcf_hap5000136.hg.1	LTB	9.75 ± 0.13	9.62 ± 0.03	9.53 ± 0.22	9.55 ± 0.05
TC6_qbl_hap6000151.hg.1	LTB	9.75 ± 0.13	9.62 ± 0.03	9.53 ± 0.22	9.55 ± 0.05
TC6_ssto_hap7000131.hg.1	LTB	9.75 ± 0.13	9.62 ± 0.03	9.53 ± 0.22	9.55 ± 0.05
TC14002196.hg.1	LTB4R	8.10 ± 0.23	7.64 ± 0.05	8.05 ± 0.26	7.62 ± 0.02
TC14002195.hg.1	LTB4R2	7.85 ± 0.13	7.71 ± 0.12	7.68 ± 0.07	7.80 ± 0.09
TC02000219.hg.1	LTBP1	7.75 ± 0.19	7.14 ± 0.13	8.01 ± 0.36	7.31 ± 0.24
TC14001308.hg.1	LTBP2	7.89 ± 0.16	7.87 ± 0.15	7.87 ± 0.14	7.95 ± 0.07
TC11001934.hg.1	LTBP3	8.74 ± 0.08	8.80 ± 0.12	8.56 ± 0.09	8.87 ± 0.06
TC19000565.hg.1	LTBP4	8.48 ± 0.12	8.47 ± 0.12	8.34 ± 0.14	8.48 ± 0.03
TC12000078.hg.1	LTBR	8.90 ± 0.26	8.68 ± 0.12	8.98 ± 0.10	8.67 ± 0.10
TC05003417.hg.1	LTC4S	8.57 ± 0.25	8.52 ± 0.23	8.47 ± 0.20	8.54 ± 0.04
TC03001357.hg.1	LTF	9.34 ± 0.81	8.18 ± 0.19	9.20 ± 0.78	8.20 ± 0.15
TC15001251.hg.1	LTK	8.04 ± 0.19	7.99 ± 0.18	7.90 ± 0.15	8.06 ± 0.06
TC21000351.hg.1	LTN1	7.67 ± 0.56	7.92 ± 0.30	7.83 ± 0.49	7.68 ± 0.13
TC06004099.hg.1	LTV1	8.02 ± 0.53	8.52 ± 0.10	8.00 ± 0.46	8.45 ± 0.04
TC16000723.hg.1	LUC7L	8.62 ± 0.24	8.77 ± 0.06	8.42 ± 0.17	8.71 ± 0.08
TC17000675.hg.1	LUC7L3	8.62 ± 0.68	8.93 ± 0.17	8.75 ± 0.43	8.94 ± 0.02
TC05001589.hg.1	LUCAT1	8.58 ± 0.12	8.54 ± 0.31	8.87 ± 0.48	8.49 ± 0.56
TC12001803.hg.1	LUM	3.92 ± 0.06	3.84 ± 0.02	3.88 ± 0.09	3.84 ± 0.03
TC01000581.hg.1	LURAP1	7.66 ± 0.22	7.51 ± 0.23	7.63 ± 0.30	7.61 ± 0.01
TC09000066.hg.1	LURAP1L	6.16 ± 0.17	5.98 ± 0.05	6.06 ± 0.14	6.02 ± 0.09
TC03001423.hg.1	LUST	6.77 ± 0.33	6.70 ± 0.23	6.94 ± 0.64	6.51 ± 0.13
TC01002336.hg.1	LUZP1	7.79 ± 0.10	7.87 ± 0.08	7.67 ± 0.06	7.89 ± 0.04
TC11000272.hg.1	LUZP2	5.12 ± 0.09	5.09 ± 0.09	5.12 ± 0.03	5.15 ± 0.05
TC0X000566.hg.1	LUZP4	5.25 ± 0.21	5.19 ± 0.08	5.18 ± 0.03	5.21 ± 0.03
TC07001891.hg.1	LUZP6	10.83 ± 0.16	10.77 ± 0.06	10.92 ± 0.25	10.70 ± 0.15
TC03001948.hg.1	LXN	6.69 ± 0.40	6.31 ± 0.21	6.76 ± 0.30	6.00 ± 0.14
TC08001706.hg.1	LY6D	8.02 ± 0.17	8.10 ± 0.27	7.97 ± 0.13	8.21 ± 0.08
TC08000814.hg.1	LY6E	10.66 ± 0.66	10.09 ± 0.28	10.22 ± 0.48	9.77 ± 0.07
TC06004075.hg.1	LY6G5B	9.24 ± 0.29	9.49 ± 0.07	9.26 ± 0.30	9.70 ± 0.08
TC6_cox_hap2000242.hg.1	LY6G5B	9.50 ± 0.36	9.81 ± 0.07	9.41 ± 0.43	10.10 ± 0.12

TC6_dbb_hap3000220.hg.1	LY6G5B	9.50 ± 0.36	9.81 ± 0.07	9.41 ± 0.43	10.09 ± 0.11
TC6_mann_hap4000194.hg.1	LY6G5B	9.50 ± 0.36	9.81 ± 0.07	9.41 ± 0.43	10.09 ± 0.11
TC6_mcf_hap5000211.hg.1	LY6G5B	9.64 ± 0.37	9.97 ± 0.05	9.56 ± 0.45	10.25 ± 0.11
TC6_qbl_hap6000223.hg.1	LY6G5B	9.48 ± 0.37	9.80 ± 0.05	9.41 ± 0.44	10.10 ± 0.11
TC6_ssto_hap7000197.hg.1	LY6G5B	9.48 ± 0.37	9.80 ± 0.05	9.41 ± 0.44	10.10 ± 0.11
TC06001535.hg.1	LY6G5C	7.42 ± 0.07	7.26 ± 0.13	7.40 ± 0.07	7.30 ± 0.02
TC6_cox_hap2000164.hg.1	LY6G5C	7.35 ± 0.07	7.20 ± 0.13	7.33 ± 0.09	7.23 ± 0.01
TC6_dbb_hap3000153.hg.1	LY6G5C	7.35 ± 0.07	7.20 ± 0.13	7.33 ± 0.09	7.23 ± 0.01
TC6_mann_hap4000141.hg.1	LY6G5C	7.35 ± 0.07	7.20 ± 0.13	7.33 ± 0.09	7.23 ± 0.01
TC6_mcf_hap5000141.hg.1	LY6G5C	7.35 ± 0.07	7.20 ± 0.13	7.33 ± 0.09	7.23 ± 0.01
TC6_qbl_hap6000156.hg.1	LY6G5C	7.37 ± 0.07	7.22 ± 0.12	7.35 ± 0.08	7.25 ± 0.01
TC6_ssto_hap7000136.hg.1	LY6G5C	7.37 ± 0.07	7.22 ± 0.12	7.35 ± 0.08	7.25 ± 0.01
TC06001538.hg.1	LY6G6C	7.36 ± 0.21	7.16 ± 0.07	7.55 ± 0.33	7.38 ± 0.09
TC6_cox_hap2000167.hg.1	LY6G6C	7.36 ± 0.21	7.16 ± 0.07	7.55 ± 0.33	7.38 ± 0.09
TC6_dbb_hap3000156.hg.1	LY6G6C	7.36 ± 0.21	7.16 ± 0.07	7.55 ± 0.33	7.38 ± 0.09
TC6_mann_hap4000144.hg.1	LY6G6C	7.36 ± 0.21	7.16 ± 0.07	7.55 ± 0.33	7.38 ± 0.09
TC6_mcf_hap5000144.hg.1	LY6G6C	7.36 ± 0.21	7.16 ± 0.07	7.55 ± 0.33	7.38 ± 0.09
TC6_qbl_hap6000159.hg.1	LY6G6C	7.41 ± 0.25	7.21 ± 0.08	7.59 ± 0.33	7.41 ± 0.12
TC6_ssto_hap7000139.hg.1	LY6G6C	7.36 ± 0.21	7.16 ± 0.07	7.55 ± 0.33	7.38 ± 0.09
TC06000381.hg.1	LY6G6D	8.14 ± 0.13	7.82 ± 0.25	8.13 ± 0.21	7.98 ± 0.06
TC6_cox_hap2000075.hg.1	LY6G6D	8.14 ± 0.13	7.82 ± 0.27	8.12 ± 0.22	7.98 ± 0.06
TC6_dbb_hap3000066.hg.1	LY6G6D	8.14 ± 0.13	7.82 ± 0.25	8.13 ± 0.21	7.98 ± 0.06
TC6_mann_hap4000066.hg.1	LY6G6D	8.14 ± 0.13	7.82 ± 0.27	8.12 ± 0.22	7.98 ± 0.06
TC6_mcf_hap5000061.hg.1	LY6G6D	8.14 ± 0.13	7.82 ± 0.27	8.12 ± 0.22	7.98 ± 0.06
TC6_qbl_hap6000066.hg.1	LY6G6D	8.10 ± 0.13	7.78 ± 0.27	8.09 ± 0.22	7.95 ± 0.06
TC6_ssto_hap7000062.hg.1	LY6G6D	8.14 ± 0.13	7.81 ± 0.27	8.13 ± 0.21	7.98 ± 0.07
TC06004117.hg.1	LY6G6E	7.45 ± 0.07	7.47 ± 0.19	7.55 ± 0.19	7.65 ± 0.13
TC6_cox_hap2000231.hg.1	LY6G6E	7.53 ± 0.06	7.54 ± 0.21	7.59 ± 0.19	7.73 ± 0.12
TC6_dbb_hap3000234.hg.1	LY6G6E	7.53 ± 0.06	7.54 ± 0.21	7.59 ± 0.19	7.73 ± 0.12
TC6_mann_hap4000204.hg.1	LY6G6E	7.53 ± 0.06	7.54 ± 0.21	7.59 ± 0.19	7.73 ± 0.12
TC6_mcf_hap5000219.hg.1	LY6G6E	7.53 ± 0.06	7.54 ± 0.21	7.59 ± 0.19	7.73 ± 0.12

TC6_qbl_hap6000213.hg.1	LY6G6E	7.53 ± 0.06	7.54 ± 0.21	7.59 ± 0.19	7.73 ± 0.12
TC6_ssto_hap7000207.hg.1	LY6G6E	7.53 ± 0.06	7.54 ± 0.21	7.59 ± 0.19	7.73 ± 0.12
TC08001711.hg.1	LY6H	8.09 ± 0.27	8.04 ± 0.26	8.00 ± 0.23	8.14 ± 0.07
TC08000809.hg.1	LY6K	7.03 ± 0.20	7.03 ± 0.19	6.92 ± 0.15	7.06 ± 0.07
TC02002467.hg.1	LY75-CD302	8.60 ± 0.34	8.88 ± 0.29	8.84 ± 0.40	8.75 ± 0.18
TC06000051.hg.1	LY86	9.61 ± 0.27	9.41 ± 0.23	9.35 ± 0.13	9.11 ± 0.24
TC06001247.hg.1	LY86-AS1	5.72 ± 0.09	5.69 ± 0.08	5.70 ± 0.10	5.78 ± 0.02
TC01001373.hg.1	LY9	8.60 ± 0.12	8.80 ± 0.07	8.25 ± 0.02	8.73 ± 0.14
TC08000487.hg.1	LY96	7.83 ± 0.46	7.20 ± 0.41	8.60 ± 0.48	6.72 ± 0.34
TC04000989.hg.1	LYAR	7.87 ± 0.12	7.90 ± 0.12	7.73 ± 0.17	7.57 ± 0.17
TC02005039.hg.1	LYG1	5.98 ± 0.05	5.85 ± 0.14	5.97 ± 0.05	5.84 ± 0.05
TC02002133.hg.1	LYG2	5.75 ± 0.05	5.78 ± 0.03	5.76 ± 0.01	5.81 ± 0.10
TC19001217.hg.1	LYL1	8.88 ± 0.23	8.58 ± 0.13	8.76 ± 0.21	8.67 ± 0.05
TC08000383.hg.1	LYN	11.21 ± 0.22	11.31 ± 0.16	11.60 ± 0.21	11.27 ± 0.12
TC08001705.hg.1	LYNX1	8.25 ± 0.18	8.23 ± 0.20	8.15 ± 0.15	8.34 ± 0.04
TC02002364.hg.1	LYPD1	7.31 ± 0.14	7.29 ± 0.24	7.21 ± 0.21	7.36 ± 0.03
TC08001704.hg.1	LYPD2	8.00 ± 0.15	7.98 ± 0.14	7.87 ± 0.14	8.05 ± 0.07
TC19001586.hg.1	LYPD3	7.66 ± 0.23	7.60 ± 0.26	7.51 ± 0.25	7.77 ± 0.07
TC19001561.hg.1	LYPD4	7.03 ± 0.11	7.01 ± 0.18	7.05 ± 0.17	7.14 ± 0.10
TC19001596.hg.1	LYPD5	7.36 ± 0.18	7.28 ± 0.21	7.27 ± 0.20	7.42 ± 0.04
TC02000929.hg.1	LYPD6	5.89 ± 0.14	5.82 ± 0.17	5.86 ± 0.11	5.86 ± 0.08
TC02000928.hg.1	LYPD6B	5.58 ± 0.12	5.51 ± 0.07	5.52 ± 0.09	5.57 ± 0.08
TC01004093.hg.1	LYPD8	6.59 ± 0.32	6.44 ± 0.07	6.34 ± 0.25	6.76 ± 0.17
TC01004094.hg.1	LYPD8	7.45 ± 0.17	7.45 ± 0.17	7.34 ± 0.15	7.54 ± 0.13
TC08001218.hg.1	LYPLA1	8.61 ± 0.12	8.54 ± 0.14	8.84 ± 0.25	8.43 ± 0.13
TC01000302.hg.1	LYPLA2	9.12 ± 0.21	8.95 ± 0.03	9.00 ± 0.12	8.93 ± 0.04
TC01001807.hg.1	LYPLAL1	6.60 ± 0.34	6.53 ± 0.21	6.61 ± 0.27	6.45 ± 0.09
TC16000226.hg.1	LYRM1	7.98 ± 0.26	7.77 ± 0.08	8.16 ± 0.22	7.72 ± 0.04
TC06001943.hg.1	LYRM2	6.75 ± 0.13	6.64 ± 0.01	6.63 ± 0.03	6.65 ± 0.05
TC06001242.hg.1	LYRM4	6.72 ± 0.02	6.63 ± 0.06	6.53 ± 0.02	6.65 ± 0.04
TC12000252.hg.1	LYRM5	6.23 ± 0.20	6.22 ± 0.18	6.52 ± 0.40	6.10 ± 0.20

TC05000622.hg.1	LYRM7	7.25 ± 0.18	7.28 ± 0.14	7.12 ± 0.18	7.12 ± 0.08
TC17002895.hg.1	LYRM9	6.82 ± 0.12	6.83 ± 0.09	6.80 ± 0.09	6.98 ± 0.06
TC01003220.hg.1	LYSMD1	7.24 ± 0.11	6.96 ± 0.11	7.18 ± 0.12	7.02 ± 0.06
TC15001444.hg.1	LYSMD2	7.27 ± 0.16	7.10 ± 0.06	7.31 ± 0.25	7.04 ± 0.09
TC05001586.hg.1	LYSMD3	8.03 ± 0.48	8.27 ± 0.07	8.28 ± 0.50	8.22 ± 0.08
TC15001988.hg.1	LYSMD4	7.26 ± 0.18	7.32 ± 0.19	7.23 ± 0.20	7.37 ± 0.03
TC01003996.hg.1	LYST	9.28 ± 0.51	9.50 ± 0.42	9.89 ± 0.47	9.18 ± 0.28
TC01003998.hg.1	LYST-AS1	6.54 ± 0.30	6.64 ± 0.10	6.57 ± 0.06	6.70 ± 0.07
TC11001415.hg.1	LYVE1	5.94 ± 0.03	5.83 ± 0.14	6.11 ± 0.18	5.93 ± 0.04
TC12000611.hg.1	LYZ	13.18 ± 0.05	13.06 ± 0.10	13.31 ± 0.02	12.99 ± 0.17
TC10000203.hg.1	LYZL1	6.06 ± 0.23	5.96 ± 0.18	6.01 ± 0.08	6.09 ± 0.09
TC10001152.hg.1	LYZL2	5.91 ± 0.18	5.84 ± 0.04	5.84 ± 0.07	5.89 ± 0.10
TC03001319.hg.1	LYZL4	6.95 ± 0.26	6.87 ± 0.16	6.80 ± 0.19	6.95 ± 0.07
TC17001381.hg.1	LYZL6	6.55 ± 0.21	6.49 ± 0.18	6.50 ± 0.13	6.51 ± 0.01
TC01002189.hg.1	LZIC	8.30 ± 0.10	8.28 ± 0.13	8.23 ± 0.24	8.10 ± 0.12
TC03001352.hg.1	LZTFL1	6.92 ± 0.18	6.85 ± 0.09	6.78 ± 0.10	6.83 ± 0.04
TC22001454.hg.1	LZTR1	8.61 ± 0.08	8.56 ± 0.06	8.50 ± 0.03	8.55 ± 0.03
TC08001025.hg.1	LZTS1	8.51 ± 0.18	8.49 ± 0.19	8.43 ± 0.22	8.60 ± 0.03
TC10000730.hg.1	LZTS2	8.93 ± 0.19	8.94 ± 0.19	8.87 ± 0.21	9.02 ± 0.03
TC20000569.hg.1	LZTS3	8.21 ± 0.27	8.11 ± 0.09	8.13 ± 0.23	8.26 ± 0.01
TC02002003.hg.1	M1AP	6.19 ± 0.15	6.13 ± 0.13	6.10 ± 0.13	6.18 ± 0.07
TC12001184.hg.1	M6PR	9.50 ± 0.06	9.33 ± 0.21	9.24 ± 0.23	8.96 ± 0.16
TC03000612.hg.1	MAATS1	5.03 ± 0.17	4.90 ± 0.08	4.92 ± 0.05	4.98 ± 0.06
TC13000561.hg.1	MAB21L1	4.43 ± 0.22	4.30 ± 0.11	4.46 ± 0.07	4.49 ± 0.13
TC04000747.hg.1	MAB21L2	6.16 ± 0.08	6.31 ± 0.13	6.25 ± 0.16	6.32 ± 0.11
TC01001015.hg.1	MAB21L3	6.25 ± 0.14	6.11 ± 0.03	6.31 ± 0.11	6.21 ± 0.01
TC07001182.hg.1	MACC1	5.05 ± 0.08	4.83 ± 0.04	4.88 ± 0.03	4.81 ± 0.06
TC07000122.hg.1	MACC1-AS1	4.52 ± 0.04	4.37 ± 0.02	4.43 ± 0.05	4.35 ± 0.09
TC01000493.hg.1	MACF1	9.04 ± 0.40	9.31 ± 0.14	9.28 ± 0.35	9.01 ± 0.22
TC11001898.hg.1	MACROD1	8.32 ± 0.29	8.25 ± 0.26	8.22 ± 0.25	8.35 ± 0.04
TC20000096.hg.1	MACROD2	5.82 ± 0.07	5.73 ± 0.02	5.71 ± 0.03	5.77 ± 0.14

TC20000643.hg.1	MACROD2-AS1	5.89 ± 0.11	5.66 ± 0.11	5.76 ± 0.01	5.75 ± 0.03
TC20000099.hg.1	MACROD2-IT1	5.63 ± 0.10	5.42 ± 0.18	5.58 ± 0.07	5.46 ± 0.06
TC07001092.hg.1	MAD1L1	8.60 ± 0.16	8.52 ± 0.10	8.44 ± 0.21	8.55 ± 0.01
TC04001507.hg.1	MAD2L1	6.32 ± 0.12	6.36 ± 0.24	6.16 ± 0.07	6.13 ± 0.06
TC06000606.hg.1	MAD2L1BP	8.72 ± 0.09	8.59 ± 0.04	8.66 ± 0.07	8.69 ± 0.02
TC01002200.hg.1	MAD2L2	8.26 ± 0.21	8.15 ± 0.10	8.14 ± 0.23	8.13 ± 0.02
TC19000004.hg.1	MADCAM1	8.15 ± 0.25	8.14 ± 0.29	8.07 ± 0.16	8.32 ± 0.07
TC11000406.hg.1	MADD	8.99 ± 0.15	8.87 ± 0.10	8.72 ± 0.04	8.89 ± 0.04
TC04000023.hg.1	MAEA	8.40 ± 0.11	8.27 ± 0.08	8.47 ± 0.10	8.32 ± 0.04
TC01001453.hg.1	MAEL	5.05 ± 0.15	4.93 ± 0.06	4.97 ± 0.06	4.97 ± 0.09
TC16001291.hg.1	MAF	8.38 ± 0.13	8.29 ± 0.05	8.31 ± 0.14	8.18 ± 0.13
TC08000841.hg.1	MAF1	9.16 ± 0.20	9.10 ± 0.09	8.94 ± 0.19	9.07 ± 0.07
TC08001715.hg.1	MAFA	7.56 ± 0.17	7.61 ± 0.18	7.44 ± 0.32	7.56 ± 0.11
TC08001716.hg.1	MAFA	8.00 ± 0.21	7.75 ± 0.13	7.72 ± 0.23	7.68 ± 0.06
TC20000844.hg.1	MAFB	8.11 ± 0.32	8.12 ± 0.08	8.22 ± 0.05	8.06 ± 0.21
TC22000297.hg.1	MAFF	8.04 ± 0.26	8.13 ± 0.13	8.13 ± 0.16	8.21 ± 0.10
TC17001965.hg.1	MAFG	7.84 ± 0.14	7.82 ± 0.10	7.82 ± 0.09	7.85 ± 0.06
TC17000940.hg.1	MAFG-AS1	8.27 ± 0.20	8.14 ± 0.23	8.09 ± 0.14	8.32 ± 0.11
TC4_gl000194_random000002.hg.1	MAFIP	8.20 ± 0.17	8.23 ± 0.07	8.07 ± 0.20	8.22 ± 0.21
TC07000023.hg.1	MAFK	8.15 ± 0.09	8.16 ± 0.03	8.09 ± 0.09	8.26 ± 0.04
TC19002610.hg.1	MAG	7.69 ± 0.24	7.52 ± 0.09	7.60 ± 0.12	7.57 ± 0.08
TC0X001511.hg.1	MAGEA1	7.66 ± 0.23	7.56 ± 0.14	7.57 ± 0.18	7.65 ± 0.09
TC0X002347.hg.1	MAGEA10	7.38 ± 0.15	7.38 ± 0.28	7.29 ± 0.18	7.49 ± 0.08
TC0X000713.hg.1	MAGEA11	7.11 ± 0.20	7.02 ± 0.11	7.08 ± 0.17	7.09 ± 0.03
TC0X001503.hg.1	MAGEA12	7.16 ± 0.25	7.20 ± 0.26	7.13 ± 0.31	7.37 ± 0.14
TC0X000740.hg.1	MAGEA2B	7.85 ± 0.24	7.82 ± 0.20	7.81 ± 0.24	7.97 ± 0.10
TC0X001504.hg.1	MAGEA2B	7.72 ± 0.21	7.74 ± 0.24	7.72 ± 0.23	7.89 ± 0.11
TC0X001505.hg.1	MAGEA3	8.76 ± 0.23	8.68 ± 0.19	8.66 ± 0.22	8.74 ± 0.03
TC0X000736.hg.1	MAGEA4	7.56 ± 0.29	7.60 ± 0.23	7.50 ± 0.30	7.70 ± 0.08
TC0X002346.hg.1	MAGEA5	7.27 ± 0.22	7.23 ± 0.14	7.26 ± 0.30	7.23 ± 0.10
TC0X000739.hg.1	MAGEA6	7.90 ± 0.22	7.87 ± 0.18	7.79 ± 0.22	7.93 ± 0.02

TC0X000717.hg.1	MAGEA8	8.45 ± 0.25	8.48 ± 0.19	8.43 ± 0.20	8.62 ± 0.09
TC0X001483.hg.1	MAGEA8-AS1	6.08 ± 0.14	6.14 ± 0.10	6.06 ± 0.14	6.17 ± 0.03
TC0X000712.hg.1	MAGEA9	7.93 ± 0.25	7.94 ± 0.20	7.89 ± 0.27	8.05 ± 0.09
TC0X001480.hg.1	MAGEA9	8.20 ± 0.17	8.22 ± 0.16	8.22 ± 0.19	8.38 ± 0.13
TC0X000139.hg.1	MAGEB1	7.37 ± 0.20	7.31 ± 0.14	7.31 ± 0.23	7.38 ± 0.06
TC0X000132.hg.1	MAGEB10	6.92 ± 0.35	6.71 ± 0.14	6.81 ± 0.22	6.75 ± 0.06
TC0X000161.hg.1	MAGEB16	7.00 ± 0.11	7.05 ± 0.19	6.90 ± 0.11	7.16 ± 0.06
TC0X000074.hg.1	MAGEB17	7.41 ± 0.23	7.44 ± 0.29	7.33 ± 0.20	7.59 ± 0.13
TC0X000123.hg.1	MAGEB18	5.21 ± 0.17	5.23 ± 0.14	5.08 ± 0.26	5.28 ± 0.09
TC0X000137.hg.1	MAGEB2	6.88 ± 0.37	7.03 ± 0.33	6.79 ± 0.29	7.05 ± 0.16
TC0X000138.hg.1	MAGEB3	6.88 ± 0.21	6.75 ± 0.11	6.82 ± 0.10	6.83 ± 0.04
TC0X000125.hg.1	MAGEB5	4.55 ± 0.27	4.51 ± 0.23	4.48 ± 0.18	4.51 ± 0.11
TC0X000124.hg.1	MAGEB6	6.05 ± 0.05	5.96 ± 0.04	5.97 ± 0.07	5.98 ± 0.08
TC0X000692.hg.1	MAGEC1	6.60 ± 0.08	6.60 ± 0.07	6.61 ± 0.12	6.71 ± 0.17
TC0X001441.hg.1	MAGEC2	6.95 ± 0.25	7.00 ± 0.20	7.01 ± 0.22	7.02 ± 0.09
TC0X000691.hg.1	MAGEC3	7.09 ± 0.24	7.10 ± 0.18	6.98 ± 0.19	7.21 ± 0.08
TC0X000296.hg.1	MAGED1	7.98 ± 0.10	7.79 ± 0.04	7.63 ± 0.11	7.78 ± 0.02
TC0X000323.hg.1	MAGED2	8.47 ± 0.22	8.27 ± 0.05	8.28 ± 0.24	8.23 ± 0.07
TC0X000298.hg.1	MAGED4	7.86 ± 0.18	7.77 ± 0.09	7.67 ± 0.14	7.80 ± 0.04
TC0X001048.hg.1	MAGED4	8.01 ± 0.20	7.90 ± 0.10	7.82 ± 0.12	7.94 ± 0.04
TC0X000419.hg.1	MAGEE1	7.82 ± 0.15	7.80 ± 0.03	7.51 ± 0.25	7.94 ± 0.12
TC0X001168.hg.1	MAGEE2	5.67 ± 0.36	5.64 ± 0.17	5.32 ± 0.14	5.57 ± 0.09
TC03002073.hg.1	MAGEF1	9.21 ± 0.22	9.06 ± 0.15	8.95 ± 0.12	9.06 ± 0.08
TC0X000332.hg.1	MAGEH1	8.14 ± 0.35	8.14 ± 0.10	7.99 ± 0.36	8.17 ± 0.19
TC15001069.hg.1	MAGEL2	6.67 ± 0.21	6.81 ± 0.25	6.71 ± 0.08	6.93 ± 0.09
TC03001528.hg.1	MAGI1	6.44 ± 0.16	6.36 ± 0.11	6.40 ± 0.10	6.46 ± 0.07
TC03000405.hg.1	MAGI1-AS1	5.82 ± 0.13	5.55 ± 0.06	5.51 ± 0.17	5.68 ± 0.08
TC07001552.hg.1	MAGI2	5.99 ± 0.13	5.89 ± 0.13	5.94 ± 0.06	5.98 ± 0.06
TC07000502.hg.1	MAGI2-AS1	6.90 ± 0.24	7.17 ± 0.11	6.83 ± 0.14	7.26 ± 0.04
TC07000503.hg.1	MAGI2-AS2	3.88 ± 0.08	3.84 ± 0.03	3.93 ± 0.17	3.78 ± 0.04
TC07000505.hg.1	MAGI2-AS3	6.56 ± 0.13	6.36 ± 0.12	6.49 ± 0.17	6.48 ± 0.11

TC07001553.hg.1	MAGI2-IT1	4.04 ± 0.20	3.87 ± 0.04	3.91 ± 0.01	3.85 ± 0.05
TC01000999.hg.1	MAGI3	6.05 ± 0.06	6.01 ± 0.05	5.99 ± 0.01	6.04 ± 0.06
TC0X000266.hg.1	MAGIX	8.15 ± 0.33	8.04 ± 0.16	8.04 ± 0.30	8.09 ± 0.02
TC01002668.hg.1	MAGOH	8.54 ± 0.39	9.00 ± 0.13	8.84 ± 0.36	8.93 ± 0.34
TC12001225.hg.1	MAGOHB	7.08 ± 0.35	7.25 ± 0.16	7.06 ± 0.25	7.13 ± 0.08
TC0X001174.hg.1	MAGT1	8.92 ± 0.20	9.09 ± 0.11	8.84 ± 0.32	8.97 ± 0.12
TC06001269.hg.1	MAK	7.18 ± 0.12	6.81 ± 0.21	7.72 ± 0.37	6.83 ± 0.41
TC08000258.hg.1	MAK16	7.63 ± 0.22	7.72 ± 0.19	7.41 ± 0.18	7.69 ± 0.03
TC02000567.hg.1	MAL	8.75 ± 0.06	8.91 ± 0.06	8.38 ± 0.09	8.86 ± 0.08
TC08000695.hg.1	MAL2	7.00 ± 0.17	7.02 ± 0.09	7.00 ± 0.08	7.08 ± 0.10
TC11000631.hg.1	MALAT1	12.53 ± 0.45	12.89 ± 0.12	12.86 ± 0.25	12.90 ± 0.07
TC02002194.hg.1	MALL	7.24 ± 0.08	7.24 ± 0.16	7.23 ± 0.09	7.38 ± 0.12
TC07000145.hg.1	MALSU1	7.49 ± 0.14	7.50 ± 0.23	7.48 ± 0.12	7.43 ± 0.10
TC18000205.hg.1	MALT1	8.75 ± 0.57	9.29 ± 0.19	8.70 ± 0.49	9.11 ± 0.14
TC09000323.hg.1	MAMDC2	5.41 ± 0.17	5.28 ± 0.10	5.27 ± 0.13	5.33 ± 0.02
TC09001196.hg.1	MAMDC2-AS1	6.07 ± 0.15	5.93 ± 0.12	5.98 ± 0.10	6.00 ± 0.02
TC09001197.hg.1	MAMDC2-AS1	4.66 ± 0.11	4.43 ± 0.07	4.47 ± 0.08	4.51 ± 0.05
TC09002886.hg.1	MAMDC4	8.28 ± 0.21	8.31 ± 0.25	8.23 ± 0.18	8.42 ± 0.06
TC05003416.hg.1	MAML1	9.14 ± 0.18	8.93 ± 0.07	9.11 ± 0.08	8.94 ± 0.03
TC11002211.hg.1	MAML2	9.30 ± 0.27	9.67 ± 0.10	9.22 ± 0.43	9.56 ± 0.18
TC04001585.hg.1	MAML3	8.57 ± 0.09	8.57 ± 0.09	8.87 ± 0.14	8.65 ± 0.02
TC0X000721.hg.1	MAMLD1	7.24 ± 0.18	7.31 ± 0.21	7.22 ± 0.20	7.45 ± 0.01
TC19001692.hg.1	MAMSTR	7.55 ± 0.27	7.54 ± 0.24	7.49 ± 0.21	7.64 ± 0.06
TC06002062.hg.1	MAN1A1	9.49 ± 0.25	9.24 ± 0.10	9.77 ± 0.29	9.13 ± 0.36
TC01001029.hg.1	MAN1A2	8.57 ± 0.38	8.70 ± 0.17	8.48 ± 0.35	8.55 ± 0.07
TC09000838.hg.1	MAN1B1	8.02 ± 0.11	7.93 ± 0.06	7.84 ± 0.10	7.96 ± 0.06
TC09001756.hg.1	MAN1B1-AS1	8.04 ± 0.23	7.98 ± 0.27	7.88 ± 0.21	8.06 ± 0.06
TC01000325.hg.1	MAN1C1	8.01 ± 0.10	8.04 ± 0.02	7.75 ± 0.07	8.01 ± 0.01
TC05000515.hg.1	MAN2A1	8.19 ± 0.35	8.46 ± 0.28	8.02 ± 0.31	8.26 ± 0.05
TC15000872.hg.1	MAN2A2	9.12 ± 0.10	8.91 ± 0.02	9.01 ± 0.02	8.93 ± 0.06
TC19001202.hg.1	MAN2B1	9.62 ± 0.19	9.37 ± 0.07	9.40 ± 0.10	9.32 ± 0.17

TC04000069.hg.1	MAN2B2	9.06 ± 0.09	9.00 ± 0.02	9.03 ± 0.05	8.98 ± 0.07
TC15001665.hg.1	MAN2C1	8.51 ± 0.02	8.42 ± 0.04	8.29 ± 0.09	8.49 ± 0.11
TC04001430.hg.1	MANBA	8.74 ± 0.18	8.61 ± 0.23	8.98 ± 0.25	8.57 ± 0.17
TC20000272.hg.1	MANBAL	8.11 ± 0.06	8.02 ± 0.03	8.03 ± 0.13	8.00 ± 0.02
TC06000817.hg.1	MANEA	6.90 ± 0.29	6.52 ± 0.13	6.64 ± 0.39	6.33 ± 0.14
TC01000478.hg.1	MANEAL	7.90 ± 0.14	7.91 ± 0.18	7.85 ± 0.07	7.91 ± 0.05
TC03000319.hg.1	MANF	8.11 ± 0.08	8.04 ± 0.08	7.93 ± 0.17	7.97 ± 0.04
TC12001253.hg.1	MANSC1	9.18 ± 0.45	8.45 ± 0.18	9.95 ± 0.26	8.11 ± 0.52
TC12001337.hg.1	MANSC4	4.35 ± 0.07	4.43 ± 0.04	4.51 ± 0.14	4.47 ± 0.08
TC0X000207.hg.1	MAOA	5.92 ± 0.23	5.79 ± 0.12	5.79 ± 0.16	5.83 ± 0.07
TC0X000978.hg.1	MAOB	6.58 ± 0.16	6.46 ± 0.08	6.59 ± 0.15	6.51 ± 0.07
TC01001925.hg.1	MAP10	5.11 ± 0.03	5.00 ± 0.02	5.11 ± 0.08	5.01 ± 0.05
TC15000329.hg.1	MAP1A	6.97 ± 0.02	6.89 ± 0.24	7.10 ± 0.19	6.94 ± 0.05
TC05000336.hg.1	MAP1B	6.22 ± 0.10	6.12 ± 0.11	6.18 ± 0.13	6.19 ± 0.07
TC20000243.hg.1	MAP1LC3A	7.93 ± 0.26	8.02 ± 0.27	7.92 ± 0.21	8.10 ± 0.03
TC16000674.hg.1	MAP1LC3B	9.23 ± 0.12	9.51 ± 0.02	9.56 ± 0.23	9.51 ± 0.36
TC12000914.hg.1	MAP1LC3B2	9.27 ± 0.13	9.35 ± 0.05	9.46 ± 0.13	9.37 ± 0.08
TC01004028.hg.1	MAP1LC3C	7.65 ± 0.15	7.60 ± 0.16	7.67 ± 0.12	7.75 ± 0.05
TC19000341.hg.1	MAP1S	9.23 ± 0.26	8.81 ± 0.10	9.21 ± 0.08	8.87 ± 0.24
TC02001238.hg.1	MAP2	5.83 ± 0.07	5.75 ± 0.06	5.81 ± 0.08	5.81 ± 0.07
TC15000613.hg.1	MAP2K1	9.11 ± 0.02	9.00 ± 0.06	9.00 ± 0.20	8.92 ± 0.25
TC19001058.hg.1	MAP2K2	9.24 ± 0.14	9.10 ± 0.05	9.12 ± 0.16	9.05 ± 0.01
TC17000297.hg.1	MAP2K3	9.28 ± 0.28	9.11 ± 0.10	9.33 ± 0.17	9.15 ± 0.11
TC17000155.hg.1	MAP2K4	8.34 ± 0.12	8.36 ± 0.29	8.48 ± 0.21	8.20 ± 0.17
TC0X001154.hg.1	MAP2K4P1	5.81 ± 0.20	5.79 ± 0.04	5.80 ± 0.07	5.77 ± 0.11
TC15000625.hg.1	MAP2K5	7.36 ± 0.10	7.32 ± 0.11	7.20 ± 0.10	7.25 ± 0.00
TC17000807.hg.1	MAP2K6	8.26 ± 0.39	7.98 ± 0.19	9.20 ± 0.57	7.70 ± 0.13
TC19000140.hg.1	MAP2K7	8.57 ± 0.16	8.57 ± 0.10	8.39 ± 0.10	8.60 ± 0.01
TC05000243.hg.1	MAP3K1	10.46 ± 0.34	10.76 ± 0.11	10.62 ± 0.38	10.66 ± 0.06
TC19000559.hg.1	MAP3K10	8.28 ± 0.13	8.14 ± 0.07	8.07 ± 0.19	8.16 ± 0.04
TC11001938.hg.1	MAP3K11	9.47 ± 0.21	9.27 ± 0.07	9.50 ± 0.07	9.24 ± 0.16

TC12001550.hg.1	MAP3K12	7.95 ± 0.08	7.87 ± 0.04	7.83 ± 0.14	7.83 ± 0.02
TC03001010.hg.1	MAP3K13	6.14 ± 0.11	6.06 ± 0.04	6.13 ± 0.04	6.07 ± 0.01
TC17001598.hg.1	MAP3K14	8.75 ± 0.10	8.78 ± 0.03	8.54 ± 0.13	8.78 ± 0.03
TC17000583.hg.1	MAP3K14-AS1	8.70 ± 0.20	8.74 ± 0.20	8.66 ± 0.18	8.84 ± 0.04
TC0X000902.hg.1	MAP3K15	6.08 ± 0.14	6.04 ± 0.04	6.07 ± 0.05	6.06 ± 0.01
TC02002369.hg.1	MAP3K19	5.02 ± 0.08	4.91 ± 0.07	4.95 ± 0.06	4.97 ± 0.05
TC02002279.hg.1	MAP3K2	9.33 ± 0.37	9.51 ± 0.19	9.86 ± 0.54	9.54 ± 0.28
TC17000770.hg.1	MAP3K3	9.72 ± 0.21	9.59 ± 0.10	9.82 ± 0.19	9.59 ± 0.20
TC06001161.hg.1	MAP3K4	7.54 ± 0.13	7.61 ± 0.12	7.36 ± 0.09	7.45 ± 0.05
TC06002146.hg.1	MAP3K5	9.11 ± 0.29	9.01 ± 0.31	9.61 ± 0.46	8.73 ± 0.25
TC01002405.hg.1	MAP3K6	7.93 ± 0.14	7.82 ± 0.16	7.89 ± 0.13	7.86 ± 0.04
TC06001948.hg.1	MAP3K7	8.59 ± 0.39	8.74 ± 0.22	8.67 ± 0.31	8.53 ± 0.06
TC21000095.hg.1	MAP3K7CL	7.73 ± 0.16	7.69 ± 0.08	7.59 ± 0.18	7.73 ± 0.10
TC10000211.hg.1	MAP3K8	8.64 ± 0.12	8.98 ± 0.18	8.86 ± 0.23	8.88 ± 0.15
TC14001272.hg.1	MAP3K9	7.10 ± 0.15	7.10 ± 0.06	6.97 ± 0.12	7.12 ± 0.11
TC03001372.hg.1	MAP4	8.50 ± 0.03	8.44 ± 0.02	8.47 ± 0.09	8.38 ± 0.09
TC19001501.hg.1	MAP4K1	9.26 ± 0.05	9.20 ± 0.05	8.83 ± 0.13	9.07 ± 0.11
TC11001912.hg.1	MAP4K2	9.01 ± 0.09	8.88 ± 0.04	8.84 ± 0.06	8.88 ± 0.04
TC02001765.hg.1	MAP4K3	7.00 ± 0.39	7.18 ± 0.20	6.84 ± 0.36	7.26 ± 0.33
TC02000617.hg.1	MAP4K4	9.35 ± 0.22	9.56 ± 0.09	9.66 ± 0.32	9.50 ± 0.12
TC14001120.hg.1	MAP4K5	8.50 ± 0.56	8.75 ± 0.19	8.61 ± 0.37	8.72 ± 0.04
TC11002098.hg.1	MAP6	6.96 ± 0.17	6.84 ± 0.11	6.88 ± 0.15	6.89 ± 0.05
TC03002061.hg.1	MAP6D1	7.62 ± 0.20	7.65 ± 0.13	7.56 ± 0.11	7.70 ± 0.05
TC06002144.hg.1	MAP7	6.81 ± 0.14	6.79 ± 0.05	6.77 ± 0.13	6.82 ± 0.01
TC01000468.hg.1	MAP7D1	9.22 ± 0.19	9.10 ± 0.07	9.12 ± 0.10	9.12 ± 0.11
TC0X000907.hg.1	MAP7D2	6.56 ± 0.13	6.51 ± 0.10	6.53 ± 0.06	6.56 ± 0.04
TC0X001414.hg.1	MAP7D3	7.02 ± 0.13	6.98 ± 0.13	6.93 ± 0.15	6.91 ± 0.12
TC04001667.hg.1	MAP9	5.27 ± 0.17	5.41 ± 0.07	5.29 ± 0.08	5.34 ± 0.04
TC22000547.hg.1	MAPK1	10.15 ± 0.20	10.10 ± 0.19	10.44 ± 0.26	9.98 ± 0.20
TC04001357.hg.1	MAPK10	5.43 ± 0.09	5.35 ± 0.07	5.40 ± 0.07	5.42 ± 0.05
TC22000910.hg.1	MAPK11	7.64 ± 0.26	7.56 ± 0.23	7.55 ± 0.21	7.62 ± 0.05

TC22001498.hg.1	MAPK12	8.01 ± 0.24	8.02 ± 0.20	7.94 ± 0.22	8.08 ± 0.05
TC06000524.hg.1	MAPK13	8.52 ± 0.09	8.37 ± 0.01	8.55 ± 0.11	8.38 ± 0.05
TC06000523.hg.1	MAPK14	9.60 ± 0.36	9.08 ± 0.18	10.40 ± 0.38	8.97 ± 0.21
TC08000832.hg.1	MAPK15	7.98 ± 0.21	7.94 ± 0.23	7.87 ± 0.19	8.02 ± 0.05
TC14000322.hg.1	MAPK1IP1L	9.78 ± 0.13	9.73 ± 0.09	9.59 ± 0.32	9.76 ± 0.11
TC16001021.hg.1	MAPK3	9.76 ± 0.17	9.60 ± 0.05	9.72 ± 0.16	9.61 ± 0.04
TC18000179.hg.1	MAPK4	7.04 ± 0.22	7.03 ± 0.27	6.93 ± 0.23	7.09 ± 0.10
TC15000417.hg.1	MAPK6	8.05 ± 0.22	8.35 ± 0.32	8.05 ± 0.43	8.30 ± 0.14
TC17000237.hg.1	MAPK7	8.68 ± 0.15	8.64 ± 0.15	8.60 ± 0.14	8.67 ± 0.03
TC10000322.hg.1	MAPK8	7.18 ± 0.32	7.41 ± 0.16	7.12 ± 0.31	7.57 ± 0.15
TC11000389.hg.1	MAPK8IP1	7.79 ± 0.19	7.87 ± 0.19	7.78 ± 0.22	7.93 ± 0.04
TC22000433.hg.1	MAPK8IP2	8.32 ± 0.26	8.26 ± 0.22	8.20 ± 0.21	8.35 ± 0.04
TC16000045.hg.1	MAPK8IP3	9.05 ± 0.06	9.09 ± 0.06	8.98 ± 0.05	9.21 ± 0.04
TC05002145.hg.1	MAPK9	7.55 ± 0.18	7.68 ± 0.12	7.48 ± 0.12	7.58 ± 0.05
TC09001591.hg.1	MAPKAP1	8.36 ± 0.05	8.42 ± 0.07	8.27 ± 0.06	8.32 ± 0.04
TC01001727.hg.1	MAPKAPK2	9.80 ± 0.19	9.77 ± 0.14	9.73 ± 0.16	9.90 ± 0.20
TC03000316.hg.1	MAPKAPK3	9.23 ± 0.27	8.99 ± 0.02	9.16 ± 0.21	8.95 ± 0.16
TC12000872.hg.1	MAPKAPK5	8.75 ± 0.16	8.83 ± 0.07	8.54 ± 0.14	8.82 ± 0.07
TC12001975.hg.1	MAPKAPK5-AS1	8.11 ± 0.06	8.07 ± 0.10	7.92 ± 0.12	8.11 ± 0.07
TC15000313.hg.1	MAPKBP1	8.04 ± 0.07	7.95 ± 0.09	7.89 ± 0.08	7.98 ± 0.04
TC20000216.hg.1	MAPRE1	10.42 ± 0.06	10.54 ± 0.09	10.53 ± 0.31	10.56 ± 0.11
TC18000142.hg.1	MAPRE2	9.52 ± 0.14	9.54 ± 0.04	9.48 ± 0.12	9.54 ± 0.05
TC02000157.hg.1	MAPRE3	7.35 ± 0.09	7.29 ± 0.03	7.38 ± 0.04	7.31 ± 0.02
TC17_ctg5_hap1000017.hg.1	MAPT	7.24 ± 0.21	7.18 ± 0.25	7.20 ± 0.24	7.32 ± 0.05
TC17000592.hg.1	MAPT	7.60 ± 0.23	7.53 ± 0.22	7.54 ± 0.24	7.67 ± 0.02
TC17_ctg5_hap1000006.hg.1	MAPT-AS1	8.81 ± 0.17	8.76 ± 0.14	8.75 ± 0.31	8.85 ± 0.10
TC17001609.hg.1	MAPT-AS1	8.64 ± 0.19	8.60 ± 0.17	8.57 ± 0.29	8.74 ± 0.12
TC17_ctg5_hap1000019.hg.1	MAPT-IT1	6.05 ± 0.12	6.04 ± 0.19	5.93 ± 0.20	6.01 ± 0.12
TC17000593.hg.1	MAPT-IT1	6.56 ± 0.16	6.66 ± 0.18	6.52 ± 0.17	6.65 ± 0.10
TC06000909.hg.1	MARCKS	10.29 ± 0.35	10.06 ± 0.26	10.59 ± 0.35	10.19 ± 0.52
TC01002468.hg.1	MARCKSL1	8.61 ± 0.12	8.63 ± 0.05	8.53 ± 0.12	8.60 ± 0.10

TC02000749.hg.1	MARCO	7.93 ± 0.21	7.74 ± 0.19	7.87 ± 0.17	7.86 ± 0.09
TC01001817.hg.1	MARK1	5.02 ± 0.21	4.91 ± 0.13	4.92 ± 0.06	4.97 ± 0.04
TC11000577.hg.1	MARK2	9.61 ± 0.13	9.41 ± 0.09	9.67 ± 0.10	9.31 ± 0.22
TC10001529.hg.1	MARK2P9	6.29 ± 0.33	5.97 ± 0.10	6.11 ± 0.24	5.92 ± 0.03
TC14000799.hg.1	MARK3	9.26 ± 0.25	9.24 ± 0.17	9.48 ± 0.29	9.16 ± 0.15
TC19000644.hg.1	MARK4	8.74 ± 0.14	8.61 ± 0.01	8.67 ± 0.04	8.62 ± 0.15
TC12000531.hg.1	MARS	9.22 ± 0.18	8.91 ± 0.08	9.08 ± 0.17	8.81 ± 0.02
TC02001151.hg.1	MARS2	8.11 ± 0.09	7.98 ± 0.09	7.86 ± 0.14	7.97 ± 0.06
TC10000702.hg.1	MARVELD1	7.18 ± 0.20	6.94 ± 0.03	7.02 ± 0.10	6.95 ± 0.05
TC05000303.hg.1	MARVELD2	6.01 ± 0.13	5.99 ± 0.08	5.95 ± 0.10	6.05 ± 0.04
TC16000594.hg.1	MARVELD3	7.33 ± 0.30	7.20 ± 0.18	7.29 ± 0.29	7.29 ± 0.08
TC06001151.hg.1	MAS1	3.96 ± 0.01	3.87 ± 0.10	3.94 ± 0.11	3.90 ± 0.02
TC06001484.hg.1	MAS1L	6.54 ± 0.05	6.75 ± 0.20	6.77 ± 0.12	6.93 ± 0.24
TC6_apd_hap1000059.hg.1	MAS1L	6.54 ± 0.05	6.75 ± 0.20	6.77 ± 0.12	6.93 ± 0.24
TC6_cox_hap2000121.hg.1	MAS1L	6.54 ± 0.05	6.75 ± 0.20	6.77 ± 0.12	6.93 ± 0.24
TC6_dbb_hap3000110.hg.1	MAS1L	6.54 ± 0.05	6.75 ± 0.20	6.77 ± 0.12	6.93 ± 0.24
TC6_mann_hap4000099.hg.1	MAS1L	6.54 ± 0.05	6.75 ± 0.20	6.77 ± 0.12	6.93 ± 0.24
TC6_mcf_hap5000103.hg.1	MAS1L	6.54 ± 0.05	6.75 ± 0.20	6.77 ± 0.12	6.93 ± 0.24
TC6_qbl_hap6000111.hg.1	MAS1L	6.54 ± 0.05	6.75 ± 0.20	6.77 ± 0.12	6.93 ± 0.24
TC6_ssto_hap7000103.hg.1	MAS1L	6.54 ± 0.05	6.75 ± 0.20	6.77 ± 0.12	6.93 ± 0.24
TC03002093.hg.1	MASP1	6.98 ± 0.11	6.92 ± 0.13	6.93 ± 0.14	7.04 ± 0.08
TC01002194.hg.1	MASP2	7.34 ± 0.13	7.30 ± 0.14	7.26 ± 0.11	7.35 ± 0.04
TC19000230.hg.1	MAST1	7.67 ± 0.23	7.64 ± 0.19	7.60 ± 0.13	7.68 ± 0.05
TC01000577.hg.1	MAST2	7.34 ± 0.07	7.23 ± 0.11	7.26 ± 0.08	7.29 ± 0.02
TC19000351.hg.1	MAST3	10.01 ± 0.14	9.95 ± 0.08	10.09 ± 0.21	9.88 ± 0.27
TC05000284.hg.1	MAST4	7.67 ± 0.06	7.60 ± 0.07	7.70 ± 0.10	7.66 ± 0.07
TC05001418.hg.1	MAST4-AS1	5.72 ± 0.25	5.49 ± 0.13	5.56 ± 0.18	5.63 ± 0.20
TC05000285.hg.1	MAST4-IT1	4.08 ± 0.23	3.92 ± 0.04	3.98 ± 0.19	3.84 ± 0.05
TC10000189.hg.1	MASTL	7.32 ± 0.19	7.51 ± 0.22	7.38 ± 0.19	7.44 ± 0.16
TC10001462.hg.1	MAT1A	6.97 ± 0.15	7.00 ± 0.21	6.93 ± 0.18	7.10 ± 0.03
TC02000511.hg.1	MAT2A	9.28 ± 0.25	9.43 ± 0.11	9.16 ± 0.16	9.23 ± 0.09

TC05000908.hg.1	MAT2B	8.42 ± 0.20	8.43 ± 0.16	8.52 ± 0.32	8.36 ± 0.22
TC19001051.hg.1	MATK	8.26 ± 0.07	8.17 ± 0.07	8.10 ± 0.12	8.14 ± 0.03
TC01002440.hg.1	MATN1	7.70 ± 0.18	7.74 ± 0.10	7.68 ± 0.18	7.81 ± 0.01
TC01000402.hg.1	MATN1-AS1	7.31 ± 0.03	7.40 ± 0.11	7.22 ± 0.07	7.44 ± 0.05
TC08000598.hg.1	MATN2	6.31 ± 0.17	6.25 ± 0.10	6.25 ± 0.10	6.32 ± 0.04
TC02001615.hg.1	MATN3	5.09 ± 0.09	4.99 ± 0.09	5.03 ± 0.06	5.03 ± 0.02
TC20000877.hg.1	MATN4	7.96 ± 0.23	7.99 ± 0.23	7.91 ± 0.24	8.09 ± 0.05
TC05000707.hg.1	MATR3	8.71 ± 0.39	9.12 ± 0.07	8.60 ± 0.27	9.14 ± 0.03
TC19000372.hg.1	MAU2	9.25 ± 0.16	9.12 ± 0.01	9.20 ± 0.16	9.19 ± 0.04
TC20000043.hg.1	MAVS	8.91 ± 0.04	8.70 ± 0.05	8.84 ± 0.08	8.60 ± 0.05
TC14001225.hg.1	MAX	9.22 ± 0.07	9.09 ± 0.09	9.23 ± 0.23	9.13 ± 0.08
TC16000328.hg.1	MAZ	8.77 ± 0.11	8.70 ± 0.12	8.47 ± 0.10	8.65 ± 0.00
TC22000697.hg.1	MB	7.37 ± 0.20	7.37 ± 0.20	7.32 ± 0.23	7.50 ± 0.06
TC22000698.hg.1	MB	6.27 ± 0.16	6.15 ± 0.16	6.13 ± 0.19	6.21 ± 0.15
TC06001873.hg.1	MB21D1	7.43 ± 0.20	7.08 ± 0.20	7.82 ± 0.43	6.87 ± 0.26
TC03002118.hg.1	MB21D2	6.53 ± 0.15	6.59 ± 0.07	6.58 ± 0.06	6.75 ± 0.10
TC18000512.hg.1	MBD1	8.67 ± 0.07	8.56 ± 0.01	8.60 ± 0.06	8.55 ± 0.06
TC18000521.hg.1	MBD2	10.95 ± 0.12	11.02 ± 0.09	11.07 ± 0.21	10.96 ± 0.25
TC19001009.hg.1	MBD3	8.72 ± 0.27	8.60 ± 0.13	8.55 ± 0.28	8.63 ± 0.01
TC19000153.hg.1	MBD3L1	5.91 ± 0.26	5.86 ± 0.18	6.10 ± 0.16	6.05 ± 0.13
TC19000123.hg.1	MBD3L2	7.27 ± 0.20	7.52 ± 0.10	7.40 ± 0.16	7.54 ± 0.05
TC19001110.hg.1	MBD3L3	6.81 ± 0.30	6.78 ± 0.15	6.73 ± 0.16	6.77 ± 0.05
TC19001109.hg.1	MBD3L4	6.74 ± 0.27	6.77 ± 0.17	6.68 ± 0.21	6.80 ± 0.08
TC19000122.hg.1	MBD3L5	6.41 ± 0.13	6.55 ± 0.12	6.50 ± 0.08	6.54 ± 0.10
TC03001771.hg.1	MBD4	8.38 ± 0.21	8.67 ± 0.18	8.50 ± 0.33	8.57 ± 0.06
TC02000925.hg.1	MBD5	7.80 ± 0.24	7.89 ± 0.16	7.87 ± 0.34	7.79 ± 0.09
TC12000533.hg.1	MBD6	9.22 ± 0.14	9.18 ± 0.08	9.42 ± 0.25	9.18 ± 0.16
TC14001046.hg.1	MBIP	6.93 ± 0.40	7.13 ± 0.27	6.73 ± 0.36	7.00 ± 0.08
TC10000574.hg.1	MBL1P	6.53 ± 0.05	6.48 ± 0.05	6.50 ± 0.07	6.51 ± 0.03
TC10001301.hg.1	MBL2	5.99 ± 0.25	5.95 ± 0.17	5.86 ± 0.19	6.00 ± 0.07
TC07000613.hg.1	MBLAC1	8.96 ± 0.17	8.81 ± 0.05	8.86 ± 0.15	8.83 ± 0.04

TC05001585.hg.1	MBLAC2	7.73 ± 0.21	7.64 ± 0.01	7.54 ± 0.05	7.52 ± 0.03
TC03000832.hg.1	MBNL1	9.98 ± 0.44	10.35 ± 0.10	10.08 ± 0.35	10.29 ± 0.10
TC03001911.hg.1	MBNL1-AS1	7.96 ± 0.08	7.86 ± 0.10	8.00 ± 0.20	7.94 ± 0.06
TC13000340.hg.1	MBNL2	8.08 ± 0.45	8.43 ± 0.12	8.00 ± 0.38	8.46 ± 0.10
TC0X001370.hg.1	MBNL3	8.09 ± 0.33	8.34 ± 0.22	8.09 ± 0.31	8.12 ± 0.13
TC06001313.hg.1	MBOAT1	9.50 ± 0.27	9.07 ± 0.22	9.81 ± 0.39	9.01 ± 0.14
TC02001556.hg.1	MBOAT2	7.74 ± 0.18	7.44 ± 0.37	8.48 ± 0.55	7.40 ± 0.31
TC08001106.hg.1	MBOAT4	5.78 ± 0.23	5.93 ± 0.17	5.84 ± 0.11	6.00 ± 0.06
TC19001837.hg.1	MBOAT7	9.79 ± 0.27	9.63 ± 0.04	9.98 ± 0.27	9.59 ± 0.20
TC18000585.hg.1	MBP	8.37 ± 0.03	8.36 ± 0.01	8.48 ± 0.06	8.38 ± 0.05
TC17001703.hg.1	MBTD1	7.94 ± 0.30	7.96 ± 0.15	7.93 ± 0.19	7.97 ± 0.03
TC16001307.hg.1	MBTPS1	8.99 ± 0.17	9.04 ± 0.10	8.70 ± 0.11	9.00 ± 0.03
TC0X000098.hg.1	MBTPS2	7.45 ± 0.31	7.46 ± 0.14	7.33 ± 0.34	7.34 ± 0.06
TC18000382.hg.1	MC2R	5.52 ± 0.18	5.46 ± 0.01	5.44 ± 0.02	5.49 ± 0.12
TC20000426.hg.1	MC3R	7.14 ± 0.15	7.05 ± 0.21	7.22 ± 0.23	7.45 ± 0.28
TC18000550.hg.1	MC4R	5.66 ± 0.02	5.60 ± 0.05	5.68 ± 0.09	5.67 ± 0.06
TC18000075.hg.1	MC5R	6.20 ± 0.09	6.12 ± 0.14	6.17 ± 0.11	6.37 ± 0.18
TC11002366.hg.1	MCAM	7.68 ± 0.23	7.66 ± 0.20	7.66 ± 0.21	7.79 ± 0.04
TC22000858.hg.1	MCAT	8.15 ± 0.22	8.10 ± 0.04	7.99 ± 0.18	8.13 ± 0.03
TC05001679.hg.1	MCC	6.82 ± 0.08	6.77 ± 0.09	6.75 ± 0.07	6.84 ± 0.06
TC03002053.hg.1	MCCC1	7.15 ± 0.12	7.15 ± 0.17	7.01 ± 0.07	7.00 ± 0.04
TC03000977.hg.1	MCCC1-AS1	6.34 ± 0.14	6.25 ± 0.07	6.24 ± 0.11	6.31 ± 0.08
TC05000334.hg.1	MCCC2	7.72 ± 0.19	7.59 ± 0.16	7.43 ± 0.20	7.33 ± 0.16
TC06000367.hg.1	MCCD1	7.33 ± 0.20	7.25 ± 0.15	7.17 ± 0.20	7.32 ± 0.06
TC6_apd_hap1000035.hg.1	MCCD1	7.33 ± 0.20	7.25 ± 0.15	7.17 ± 0.20	7.32 ± 0.06
TC6_cox_hap2000063.hg.1	MCCD1	7.22 ± 0.26	7.25 ± 0.18	7.14 ± 0.28	7.35 ± 0.15
TC6_dbb_hap3000054.hg.1	MCCD1	7.25 ± 0.21	7.24 ± 0.18	7.14 ± 0.26	7.31 ± 0.10
TC6_mann_hap4000055.hg.1	MCCD1	7.25 ± 0.21	7.24 ± 0.18	7.14 ± 0.26	7.31 ± 0.10
TC6_mcf_hap5000049.hg.1	MCCD1	7.22 ± 0.19	7.20 ± 0.19	7.09 ± 0.24	7.25 ± 0.05
TC6_qbl_hap6000054.hg.1	MCCD1	7.25 ± 0.21	7.24 ± 0.18	7.14 ± 0.26	7.31 ± 0.10
TC6_ssto_hap7000050.hg.1	MCCD1	7.33 ± 0.20	7.25 ± 0.15	7.17 ± 0.20	7.32 ± 0.06

TC02001973.hg.1	MCEE	6.91 ± 0.10	6.78 ± 0.05	6.79 ± 0.09	6.74 ± 0.01
TC0X001427.hg.1	MCF2	5.05 ± 0.02	4.98 ± 0.03	5.11 ± 0.07	5.00 ± 0.04
TC13000414.hg.1	MCF2L	7.72 ± 0.19	7.70 ± 0.15	7.64 ± 0.15	7.76 ± 0.05
TC13000415.hg.1	MCF2L	6.38 ± 0.32	6.44 ± 0.17	6.42 ± 0.13	6.63 ± 0.22
TC03002055.hg.1	MCF2L2	6.30 ± 0.12	6.26 ± 0.01	6.22 ± 0.06	6.30 ± 0.04
TC13000890.hg.1	MCF2L-AS1	8.22 ± 0.05	8.14 ± 0.19	8.10 ± 0.20	8.18 ± 0.01
TC02001818.hg.1	MCFD2	8.08 ± 0.08	7.95 ± 0.05	8.00 ± 0.06	7.94 ± 0.05
TC22000326.hg.1	MCHR1	6.95 ± 0.18	7.06 ± 0.21	6.92 ± 0.18	7.19 ± 0.08
TC06001968.hg.1	MCHR2	6.07 ± 0.13	6.01 ± 0.13	5.97 ± 0.10	6.01 ± 0.04
TC05001360.hg.1	MCIDAS	7.45 ± 0.25	7.43 ± 0.19	7.35 ± 0.30	7.52 ± 0.04
TC01003205.hg.1	MCL1	11.74 ± 0.08	11.79 ± 0.09	12.04 ± 0.27	11.73 ± 0.18
TC10000098.hg.1	MCM10	7.06 ± 0.22	6.83 ± 0.24	6.85 ± 0.22	6.52 ± 0.05
TC03000664.hg.1	MCM2	8.00 ± 0.26	7.87 ± 0.16	7.76 ± 0.34	7.75 ± 0.03
TC06001799.hg.1	MCM3	8.45 ± 0.19	8.18 ± 0.25	8.10 ± 0.20	7.81 ± 0.04
TC21000555.hg.1	MCM3AP	8.14 ± 0.08	8.08 ± 0.09	7.96 ± 0.06	8.00 ± 0.05
TC21000257.hg.1	MCM3AP-AS1	6.75 ± 0.23	6.64 ± 0.11	6.51 ± 0.09	6.63 ± 0.01
TC08000346.hg.1	MCM4	8.20 ± 0.22	8.06 ± 0.39	7.91 ± 0.26	7.58 ± 0.04
TC22000260.hg.1	MCM5	9.45 ± 0.24	9.18 ± 0.17	9.11 ± 0.24	8.96 ± 0.03
TC02002376.hg.1	MCM6	8.18 ± 0.07	7.94 ± 0.50	7.81 ± 0.05	7.70 ± 0.06
TC07001654.hg.1	MCM7	8.87 ± 0.09	8.73 ± 0.31	8.43 ± 0.21	8.60 ± 0.10
TC20000063.hg.1	MCM8	7.65 ± 0.29	7.73 ± 0.18	7.66 ± 0.66	7.27 ± 0.33
TC06002059.hg.1	MCM9	8.02 ± 0.19	8.12 ± 0.15	8.02 ± 0.19	8.06 ± 0.08
TC10001707.hg.1	MCMBP	9.12 ± 0.19	8.88 ± 0.13	9.33 ± 0.24	8.58 ± 0.19
TC08000444.hg.1	MCMDC2	3.95 ± 0.07	3.83 ± 0.03	3.88 ± 0.08	3.78 ± 0.04
TC19000128.hg.1	MCOLN1	8.78 ± 0.30	8.67 ± 0.03	8.90 ± 0.20	8.79 ± 0.15
TC01002824.hg.1	MCOLN2	6.11 ± 0.15	6.22 ± 0.27	5.94 ± 0.11	5.96 ± 0.18
TC01002825.hg.1	MCOLN3	4.77 ± 0.10	4.70 ± 0.12	4.74 ± 0.10	4.76 ± 0.04
TC08000021.hg.1	MCPH1	7.21 ± 0.24	7.30 ± 0.14	7.02 ± 0.17	7.20 ± 0.05
TC12001478.hg.1	MCRS1	8.92 ± 0.17	8.69 ± 0.04	8.65 ± 0.04	8.64 ± 0.02
TC05001604.hg.1	MCTP1	7.56 ± 0.16	7.33 ± 0.22	8.19 ± 0.31	7.28 ± 0.15
TC15000930.hg.1	MCTP2	8.77 ± 0.41	8.67 ± 0.35	9.76 ± 0.44	8.38 ± 0.19

TC0X000596.hg.1	MCTS1	6.63 ± 0.06	6.42 ± 0.12	6.56 ± 0.16	6.29 ± 0.06
TC10000452.hg.1	MCU	8.34 ± 0.19	8.17 ± 0.17	8.50 ± 0.20	8.04 ± 0.08
TC06001282.hg.1	MCUR1	8.16 ± 0.26	7.92 ± 0.09	7.97 ± 0.10	7.68 ± 0.16
TC06001507.hg.1	MDC1	8.17 ± 0.08	8.10 ± 0.06	7.94 ± 0.14	8.13 ± 0.04
TC6_apd_hap1000077.hg.1	MDC1	8.17 ± 0.08	8.07 ± 0.06	7.93 ± 0.14	8.12 ± 0.04
TC6_cox_hap2000141.hg.1	MDC1	8.14 ± 0.08	8.04 ± 0.06	7.90 ± 0.13	8.08 ± 0.04
TC6_dbb_hap3000130.hg.1	MDC1	8.17 ± 0.08	8.07 ± 0.06	7.93 ± 0.14	8.12 ± 0.04
TC6_mann_hap4000119.hg.1	MDC1	8.15 ± 0.08	8.05 ± 0.06	7.91 ± 0.14	8.09 ± 0.04
TC6_mcf_hap5000120.hg.1	MDC1	8.17 ± 0.08	8.07 ± 0.06	7.93 ± 0.14	8.12 ± 0.04
TC6_qbl_hap6000131.hg.1	MDC1	8.17 ± 0.07	8.08 ± 0.06	7.93 ± 0.15	8.13 ± 0.03
TC6_ssto_hap7000121.hg.1	MDC1	8.17 ± 0.08	8.07 ± 0.06	7.93 ± 0.14	8.12 ± 0.04
TC06000346.hg.1	MDC1-AS1	7.41 ± 0.09	7.47 ± 0.13	7.41 ± 0.07	7.47 ± 0.07
TC06000572.hg.1	MDFI	8.60 ± 0.28	8.57 ± 0.22	8.50 ± 0.28	8.60 ± 0.02
TC07000715.hg.1	MDFIC	8.30 ± 0.16	8.48 ± 0.05	8.33 ± 0.13	8.37 ± 0.05
TC06001687.hg.1	MDGA1	7.70 ± 0.13	7.78 ± 0.14	7.60 ± 0.16	7.76 ± 0.07
TC14001089.hg.1	MDGA2	5.10 ± 0.10	5.03 ± 0.11	5.05 ± 0.05	5.16 ± 0.10
TC02000363.hg.1	MDH1	7.34 ± 0.13	7.32 ± 0.22	7.13 ± 0.16	7.19 ± 0.03
TC02002703.hg.1	MDH1B	4.95 ± 0.09	4.87 ± 0.04	4.86 ± 0.03	4.89 ± 0.09
TC07000479.hg.1	MDH2	9.25 ± 0.19	9.01 ± 0.07	8.96 ± 0.15	8.89 ± 0.05
TC11000397.hg.1	MDK	8.66 ± 0.25	8.59 ± 0.22	8.46 ± 0.28	8.62 ± 0.02
TC12001699.hg.1	MDM1	7.27 ± 0.17	7.16 ± 0.21	7.44 ± 0.29	6.95 ± 0.10
TC12000606.hg.1	MDM2	8.39 ± 0.17	8.33 ± 0.20	8.44 ± 0.30	8.30 ± 0.30
TC01001700.hg.1	MDM4	8.48 ± 0.31	8.61 ± 0.15	8.40 ± 0.25	8.64 ± 0.04
TC06001944.hg.1	MDN1	7.68 ± 0.22	7.77 ± 0.16	7.36 ± 0.18	7.62 ± 0.05
TC14002342.hg.1	MDP1	7.32 ± 0.09	7.18 ± 0.03	7.18 ± 0.13	7.15 ± 0.04
TC01000298.hg.1	MDS2	7.30 ± 0.14	7.31 ± 0.02	7.16 ± 0.19	7.38 ± 0.11
TC06001912.hg.1	ME1	5.35 ± 0.08	5.18 ± 0.06	5.32 ± 0.10	5.19 ± 0.10
TC18000180.hg.1	ME2	9.52 ± 0.18	9.35 ± 0.20	9.79 ± 0.36	9.03 ± 0.34
TC11002167.hg.1	ME3	7.34 ± 0.14	7.30 ± 0.13	7.28 ± 0.07	7.36 ± 0.02
TC06001741.hg.1	MEA1	8.25 ± 0.09	8.24 ± 0.07	8.19 ± 0.18	8.21 ± 0.01
TC01002517.hg.1	MEAF6	8.51 ± 0.17	8.58 ± 0.15	8.35 ± 0.09	8.43 ± 0.07

TC03001979.hg.1	MECOM	5.19 ± 0.08	5.12 ± 0.07	5.15 ± 0.07	5.21 ± 0.10
TC0X001525.hg.1	MECP2	8.21 ± 0.06	8.23 ± 0.03	8.17 ± 0.04	8.29 ± 0.01
TC01002433.hg.1	MECR	6.89 ± 0.12	6.76 ± 0.09	6.75 ± 0.10	6.78 ± 0.03
TC17001449.hg.1	MED1	9.53 ± 0.26	9.54 ± 0.15	9.39 ± 0.24	9.42 ± 0.09
TC05001137.hg.1	MED10	8.64 ± 0.20	8.77 ± 0.25	8.40 ± 0.24	8.70 ± 0.07
TC17000048.hg.1	MED11	8.55 ± 0.25	7.96 ± 0.04	8.54 ± 0.31	7.63 ± 0.26
TC0X000382.hg.1	MED12	9.51 ± 0.26	9.30 ± 0.09	9.58 ± 0.22	9.28 ± 0.16
TC03000822.hg.1	MED12L	6.66 ± 0.05	6.37 ± 0.03	6.83 ± 0.20	6.44 ± 0.04
TC17001772.hg.1	MED13	9.42 ± 0.48	9.72 ± 0.22	9.66 ± 0.46	9.62 ± 0.08
TC12002004.hg.1	MED13L	10.30 ± 0.35	10.57 ± 0.14	10.46 ± 0.41	10.63 ± 0.07
TC0X000966.hg.1	MED14	8.69 ± 0.25	8.75 ± 0.13	8.67 ± 0.38	8.77 ± 0.13
TC0X000192.hg.1	MED14-AS1	6.22 ± 0.20	6.37 ± 0.09	6.38 ± 0.18	6.25 ± 0.06
TC22000068.hg.1	MED15	9.37 ± 0.07	9.34 ± 0.06	9.29 ± 0.06	9.37 ± 0.03
TC19000991.hg.1	MED16	9.11 ± 0.19	8.92 ± 0.08	9.06 ± 0.11	8.93 ± 0.02
TC11000912.hg.1	MED17	8.40 ± 0.18	8.29 ± 0.11	8.37 ± 0.19	8.19 ± 0.09
TC01000378.hg.1	MED18	7.56 ± 0.10	7.31 ± 0.09	7.43 ± 0.14	7.26 ± 0.04
TC11001797.hg.1	MED19	7.42 ± 0.12	7.29 ± 0.02	7.27 ± 0.08	7.33 ± 0.03
TC06001726.hg.1	MED20	7.97 ± 0.05	7.88 ± 0.04	7.94 ± 0.09	7.87 ± 0.01
TC12000262.hg.1	MED21	8.19 ± 0.39	8.56 ± 0.17	8.20 ± 0.38	8.51 ± 0.18
TC09001686.hg.1	MED22	8.47 ± 0.18	8.40 ± 0.10	8.35 ± 0.19	8.43 ± 0.05
TC06002108.hg.1	MED23	8.31 ± 0.52	8.56 ± 0.17	8.46 ± 0.44	8.55 ± 0.03
TC17001457.hg.1	MED24	8.57 ± 0.07	8.43 ± 0.02	8.28 ± 0.02	8.38 ± 0.01
TC19000748.hg.1	MED25	9.11 ± 0.14	9.13 ± 0.06	9.09 ± 0.08	9.14 ± 0.12
TC19002689.hg.1	MED26	8.41 ± 0.11	8.41 ± 0.11	8.23 ± 0.10	8.38 ± 0.05
TC09001671.hg.1	MED27	7.81 ± 0.10	7.79 ± 0.20	7.66 ± 0.17	7.67 ± 0.03
TC04000152.hg.1	MED28	8.21 ± 0.14	8.17 ± 0.21	8.21 ± 0.47	8.22 ± 0.14
TC19000543.hg.1	MED29	8.51 ± 0.11	8.50 ± 0.10	8.44 ± 0.10	8.45 ± 0.09
TC08000689.hg.1	MED30	7.01 ± 0.10	7.25 ± 0.15	6.99 ± 0.13	7.15 ± 0.05
TC17001066.hg.1	MED31	8.14 ± 0.08	7.81 ± 0.27	8.17 ± 0.31	7.83 ± 0.17
TC13000653.hg.1	MED4	8.32 ± 0.30	8.48 ± 0.11	8.31 ± 0.23	8.49 ± 0.11
TC13000188.hg.1	MED4-AS1	5.82 ± 0.04	5.95 ± 0.03	5.98 ± 0.34	5.99 ± 0.05

TC14002329.hg.1	MED6	8.02 ± 0.21	8.04 ± 0.26	7.78 ± 0.23	7.99 ± 0.05
TC05003435.hg.1	MED7	7.09 ± 0.09	6.93 ± 0.03	7.04 ± 0.04	6.90 ± 0.09
TC01002584.hg.1	MED8	8.05 ± 0.17	7.77 ± 0.06	7.94 ± 0.12	7.71 ± 0.10
TC17000199.hg.1	MED9	7.22 ± 0.07	7.09 ± 0.07	7.21 ± 0.09	7.19 ± 0.04
TC13000103.hg.1	MEDAG	6.69 ± 0.19	6.53 ± 0.10	6.56 ± 0.16	6.53 ± 0.01
TC15000955.hg.1	MEF2A	8.82 ± 0.14	8.71 ± 0.17	9.25 ± 0.42	8.70 ± 0.18
TC19001300.hg.1	MEF2BNB	8.53 ± 0.14	8.42 ± 0.14	8.56 ± 0.15	8.47 ± 0.02
TC05001579.hg.1	MEF2C	8.01 ± 0.17	8.10 ± 0.17	7.77 ± 0.30	8.00 ± 0.12
TC01003341.hg.1	MEF2D	9.55 ± 0.15	9.66 ± 0.13	9.42 ± 0.22	9.79 ± 0.12
TC16000812.hg.1	MEFV	9.44 ± 0.42	9.17 ± 0.26	9.72 ± 0.37	8.98 ± 0.37
TC14000645.hg.1	MEG3	7.26 ± 0.15	7.22 ± 0.17	7.22 ± 0.14	7.35 ± 0.07
TC14000659.hg.1	MEG8	5.85 ± 0.16	5.83 ± 0.10	5.85 ± 0.13	5.90 ± 0.07
TC14000765.hg.1	MEG9	7.57 ± 0.26	7.49 ± 0.17	7.44 ± 0.13	7.54 ± 0.05
TC05000607.hg.1	MEGF10	6.34 ± 0.12	6.28 ± 0.11	6.27 ± 0.11	6.41 ± 0.10
TC15001575.hg.1	MEGF11	7.32 ± 0.18	7.29 ± 0.21	7.27 ± 0.17	7.43 ± 0.09
TC01002129.hg.1	MEGF6	8.63 ± 0.17	8.64 ± 0.14	8.56 ± 0.14	8.75 ± 0.05
TC19000599.hg.1	MEGF8	8.43 ± 0.13	8.39 ± 0.13	8.37 ± 0.13	8.44 ± 0.05
TC09001537.hg.1	MEGF9	10.29 ± 0.12	10.21 ± 0.17	10.72 ± 0.35	10.01 ± 0.37
TC22000342.hg.1	MEI1	7.05 ± 0.12	6.98 ± 0.08	6.89 ± 0.11	7.04 ± 0.06
TC10000114.hg.1	MEIG1	5.06 ± 0.16	4.89 ± 0.07	4.94 ± 0.13	4.95 ± 0.04
TC16000763.hg.1	MEIOB	4.31 ± 0.10	4.24 ± 0.08	4.24 ± 0.09	4.23 ± 0.04
TC02000386.hg.1	MEIS1	7.26 ± 0.06	6.79 ± 0.13	7.06 ± 0.05	6.89 ± 0.12
TC02001932.hg.1	MEIS1-AS1	7.06 ± 0.17	6.99 ± 0.14	7.07 ± 0.10	7.13 ± 0.09
TC02001931.hg.1	MEIS1-AS3	6.95 ± 0.29	6.80 ± 0.21	6.82 ± 0.29	6.85 ± 0.11
TC15001211.hg.1	MEIS2	6.12 ± 0.18	5.97 ± 0.08	6.04 ± 0.09	6.02 ± 0.04
TC19001663.hg.1	MEIS3	7.95 ± 0.20	7.86 ± 0.17	7.90 ± 0.14	8.00 ± 0.06
TC17000179.hg.1	MEIS3P1	6.33 ± 0.27	6.09 ± 0.14	6.64 ± 0.31	6.35 ± 0.26
TC17000290.hg.1	MEIS3P2	8.27 ± 0.33	8.10 ± 0.20	8.25 ± 0.16	8.15 ± 0.17
TC09000207.hg.1	MELK	6.37 ± 0.12	6.16 ± 0.31	6.21 ± 0.08	5.76 ± 0.08
TC02005023.hg.1	MEMO1	8.51 ± 0.02	8.55 ± 0.05	8.47 ± 0.10	8.49 ± 0.05
TC21000151.hg.1	MEMO1P1	7.86 ± 0.19	7.84 ± 0.23	7.78 ± 0.35	7.60 ± 0.27

TC11001913.hg.1	MEN1	9.14 ± 0.10	9.04 ± 0.09	8.97 ± 0.08	9.03 ± 0.03
TC17001561.hg.1	MEOX1	7.32 ± 0.31	7.24 ± 0.28	7.26 ± 0.16	7.35 ± 0.05
TC07001156.hg.1	MEOX2	6.81 ± 0.21	6.70 ± 0.26	6.73 ± 0.15	6.89 ± 0.09
TC06000631.hg.1	MEP1A	5.52 ± 0.12	5.45 ± 0.10	5.50 ± 0.03	5.51 ± 0.06
TC18000135.hg.1	MEP1B	5.36 ± 0.12	5.24 ± 0.05	5.28 ± 0.03	5.29 ± 0.04
TC07000620.hg.1	MEPCE	9.51 ± 0.10	9.56 ± 0.07	9.30 ± 0.07	9.54 ± 0.02
TC04000476.hg.1	MEPE	5.39 ± 0.12	5.29 ± 0.09	5.30 ± 0.08	5.37 ± 0.09
TC02000701.hg.1	MERTK	6.83 ± 0.23	6.49 ± 0.07	6.94 ± 0.07	6.62 ± 0.11
TC15000754.hg.1	MESDC1	7.93 ± 0.11	7.95 ± 0.05	7.91 ± 0.04	8.07 ± 0.14
TC15001728.hg.1	MESDC2	8.17 ± 0.08	8.02 ± 0.06	7.96 ± 0.10	8.01 ± 0.01
TC15001836.hg.1	MESP1	8.13 ± 0.24	8.02 ± 0.14	8.04 ± 0.22	8.13 ± 0.02
TC15000854.hg.1	MESP2	7.44 ± 0.20	7.45 ± 0.13	7.41 ± 0.19	7.52 ± 0.06
TC07000815.hg.1	MEST	7.52 ± 0.05	7.39 ± 0.02	7.45 ± 0.01	7.43 ± 0.05
TC07001864.hg.1	MESTIT1	7.26 ± 0.24	7.26 ± 0.09	7.10 ± 0.23	7.29 ± 0.12
TC07000722.hg.1	MET	5.12 ± 0.07	4.98 ± 0.03	5.02 ± 0.04	5.05 ± 0.04
TC04000512.hg.1	METAP1	7.30 ± 0.24	7.34 ± 0.09	7.15 ± 0.18	7.20 ± 0.04
TC02001025.hg.1	METAP1D	6.43 ± 0.06	6.38 ± 0.03	6.38 ± 0.03	6.37 ± 0.04
TC12000738.hg.1	METAP2	8.45 ± 0.32	8.72 ± 0.09	8.37 ± 0.23	8.63 ± 0.06
TC16000027.hg.1	METRN	8.82 ± 0.29	8.98 ± 0.17	8.79 ± 0.27	8.91 ± 0.03
TC17000959.hg.1	METRNL	8.79 ± 0.16	8.86 ± 0.12	8.82 ± 0.12	8.85 ± 0.07
TC12001635.hg.1	METTL1	7.20 ± 0.18	7.24 ± 0.18	7.01 ± 0.15	7.26 ± 0.05
TC10002970.hg.1	METTL10	7.05 ± 0.40	7.14 ± 0.21	6.91 ± 0.23	7.05 ± 0.09
TC01001479.hg.1	METTL11B	5.18 ± 0.12	5.06 ± 0.06	5.09 ± 0.12	5.06 ± 0.03
TC11000558.hg.1	METTL12	7.13 ± 0.12	6.97 ± 0.12	6.98 ± 0.06	6.99 ± 0.09
TC01001496.hg.1	METTL13	8.11 ± 0.16	7.83 ± 0.07	7.86 ± 0.17	7.67 ± 0.07
TC04000608.hg.1	METTL14	7.85 ± 0.35	7.89 ± 0.13	7.99 ± 0.35	7.81 ± 0.09
TC11000283.hg.1	METTL15	6.61 ± 0.20	6.66 ± 0.08	6.62 ± 0.14	6.54 ± 0.07
TC17000996.hg.1	METTL16	8.25 ± 0.07	8.28 ± 0.11	8.02 ± 0.01	8.29 ± 0.04
TC14000074.hg.1	METTL17	8.13 ± 0.08	7.96 ± 0.15	7.88 ± 0.14	7.89 ± 0.08
TC01003503.hg.1	METTL18	6.97 ± 0.08	6.97 ± 0.07	7.00 ± 0.05	6.92 ± 0.01
TC12000293.hg.1	METTL20	5.88 ± 0.04	5.87 ± 0.11	5.90 ± 0.04	5.90 ± 0.02

TC02002712.hg.1	METTL21A	7.46 ± 0.15	7.48 ± 0.04	7.37 ± 0.05	7.46 ± 0.03
TC12003249.hg.1	METTL21B	7.13 ± 0.09	7.00 ± 0.06	6.95 ± 0.16	7.04 ± 0.03
TC13000851.hg.1	METTL21C	5.42 ± 0.09	5.36 ± 0.09	5.38 ± 0.13	5.45 ± 0.11
TC14001115.hg.1	METTL21D	7.63 ± 0.44	7.96 ± 0.10	8.08 ± 0.40	8.06 ± 0.02
TC16000147.hg.1	METTL22	8.43 ± 0.06	8.24 ± 0.06	8.37 ± 0.02	8.29 ± 0.02
TC17000879.hg.1	METTL23	8.42 ± 0.05	8.43 ± 0.02	8.29 ± 0.08	8.42 ± 0.06
TC06004135.hg.1	METTL24	6.16 ± 0.15	6.21 ± 0.15	6.20 ± 0.11	6.30 ± 0.07
TC12000677.hg.1	METTL25	6.88 ± 0.09	6.74 ± 0.08	6.79 ± 0.21	6.66 ± 0.02
TC17000756.hg.1	METTL2A	8.62 ± 0.10	8.43 ± 0.08	8.38 ± 0.11	8.19 ± 0.22
TC07003294.hg.1	METTL2B	8.06 ± 0.07	7.89 ± 0.11	7.91 ± 0.04	7.79 ± 0.11
TC14000922.hg.1	METTL3	8.77 ± 0.26	8.71 ± 0.12	8.62 ± 0.16	8.59 ± 0.10
TC18000282.hg.1	METTL4	7.43 ± 0.29	7.50 ± 0.21	7.55 ± 0.47	7.25 ± 0.09
TC02002512.hg.1	METTL5	8.14 ± 0.10	8.19 ± 0.05	8.05 ± 0.04	8.06 ± 0.05
TC03001208.hg.1	METTL6	6.78 ± 0.07	6.63 ± 0.12	6.66 ± 0.08	6.52 ± 0.05
TC12000399.hg.1	METTL7A	9.38 ± 0.10	8.92 ± 0.07	9.13 ± 0.12	8.84 ± 0.13
TC12000487.hg.1	METTL7B	7.57 ± 0.62	7.01 ± 0.21	7.71 ± 0.16	6.98 ± 0.07
TC02002522.hg.1	METTL8	7.02 ± 0.10	6.96 ± 0.06	6.88 ± 0.07	6.87 ± 0.05
TC16000235.hg.1	METTL9	10.16 ± 0.12	9.98 ± 0.10	10.67 ± 0.13	9.92 ± 0.23
TC01003331.hg.1	MEX3A	8.18 ± 0.27	8.10 ± 0.21	8.11 ± 0.18	8.23 ± 0.03
TC15001735.hg.1	MEX3B	7.08 ± 0.13	7.03 ± 0.08	7.03 ± 0.08	7.06 ± 0.03
TC18000517.hg.1	MEX3C	9.10 ± 0.08	9.20 ± 0.11	8.78 ± 0.10	9.24 ± 0.06
TC19001007.hg.1	MEX3D	8.84 ± 0.25	8.76 ± 0.09	8.75 ± 0.23	8.74 ± 0.02
TC15001287.hg.1	MFAP1	7.46 ± 0.17	7.16 ± 0.09	7.54 ± 0.26	7.09 ± 0.18
TC01002284.hg.1	MFAP2	8.31 ± 0.19	8.30 ± 0.24	8.22 ± 0.17	8.39 ± 0.05
TC05003414.hg.1	MFAP3	7.97 ± 0.09	7.78 ± 0.04	8.01 ± 0.04	7.79 ± 0.06
TC04001729.hg.1	MFAP3L	7.20 ± 0.07	6.93 ± 0.05	7.11 ± 0.07	7.05 ± 0.10
TC17001243.hg.1	MFAP4	7.70 ± 0.20	7.73 ± 0.25	7.71 ± 0.21	7.87 ± 0.11
TC12001181.hg.1	MFAP5	5.57 ± 0.09	5.48 ± 0.06	5.48 ± 0.03	5.58 ± 0.09
TC02001362.hg.1	MFF	7.99 ± 0.19	8.00 ± 0.18	8.07 ± 0.23	7.88 ± 0.07
TC15001825.hg.1	MFGE8	7.90 ± 0.05	7.85 ± 0.08	7.67 ± 0.10	7.93 ± 0.05
TC15001826.hg.1	MFGE8	7.29 ± 0.25	7.23 ± 0.15	7.07 ± 0.19	7.31 ± 0.12

TC08000943.hg.1	MFHAS1	6.98 ± 0.05	7.01 ± 0.17	6.94 ± 0.16	6.96 ± 0.06
TC03002173.hg.1	MFI2	8.00 ± 0.26	8.01 ± 0.20	7.93 ± 0.24	8.08 ± 0.01
TC03001105.hg.1	MFI2-AS1	8.25 ± 0.29	8.20 ± 0.23	8.08 ± 0.28	8.21 ± 0.04
TC03000953.hg.1	MFN1	8.18 ± 0.31	8.32 ± 0.29	8.27 ± 0.41	8.17 ± 0.08
TC01000152.hg.1	MFN2	9.09 ± 0.31	8.74 ± 0.08	9.08 ± 0.18	8.79 ± 0.19
TC22001491.hg.1	MFNG	8.97 ± 0.11	8.87 ± 0.06	8.67 ± 0.20	8.85 ± 0.03
TC03000867.hg.1	MFSD1	8.64 ± 0.15	8.50 ± 0.10	8.81 ± 0.18	8.52 ± 0.09
TC04000978.hg.1	MFSD10	9.21 ± 0.13	9.15 ± 0.08	9.06 ± 0.18	9.16 ± 0.05
TC17000880.hg.1	MFSD11	7.81 ± 0.10	7.71 ± 0.10	7.76 ± 0.13	7.62 ± 0.09
TC19001045.hg.1	MFSD12	8.52 ± 0.19	8.45 ± 0.04	8.30 ± 0.13	8.48 ± 0.03
TC01000503.hg.1	MFSD2A	7.59 ± 0.12	7.52 ± 0.13	7.40 ± 0.12	7.59 ± 0.03
TC02004951.hg.1	MFSD2B	8.37 ± 0.20	8.25 ± 0.17	8.37 ± 0.19	8.35 ± 0.06
TC08000857.hg.1	MFSD3	8.61 ± 0.16	8.59 ± 0.16	8.56 ± 0.13	8.68 ± 0.09
TC01001712.hg.1	MFSD4	7.26 ± 0.11	7.26 ± 0.14	7.18 ± 0.11	7.33 ± 0.06
TC12000433.hg.1	MFSD5	8.10 ± 0.19	7.96 ± 0.05	7.96 ± 0.18	7.96 ± 0.03
TC02001119.hg.1	MFSD6	8.49 ± 0.18	8.57 ± 0.20	8.27 ± 0.12	8.55 ± 0.03
TC17001122.hg.1	MFSD6L	7.10 ± 0.25	6.95 ± 0.08	6.96 ± 0.13	7.00 ± 0.07
TC04000952.hg.1	MFSD7	8.53 ± 0.23	8.47 ± 0.22	8.49 ± 0.16	8.56 ± 0.06
TC04001535.hg.1	MFSD8	7.21 ± 0.36	7.09 ± 0.18	7.07 ± 0.23	7.07 ± 0.03
TC02002148.hg.1	MFSD9	7.32 ± 0.07	7.20 ± 0.01	7.18 ± 0.18	7.13 ± 0.06
TC15000311.hg.1	MGA	8.13 ± 0.38	8.24 ± 0.23	7.93 ± 0.33	8.02 ± 0.07
TC07000899.hg.1	MGAM	10.04 ± 0.47	9.57 ± 0.35	11.25 ± 0.40	9.54 ± 0.45
TC04002948.hg.1	MGARP	6.13 ± 0.11	6.00 ± 0.10	6.11 ± 0.17	6.01 ± 0.02
TC05002151.hg.1	MGAT1	8.75 ± 0.16	8.62 ± 0.08	8.65 ± 0.17	8.66 ± 0.04
TC14000275.hg.1	MGAT2	8.72 ± 0.06	8.42 ± 0.08	8.53 ± 0.16	8.26 ± 0.31
TC22000315.hg.1	MGAT3	7.40 ± 0.19	7.43 ± 0.14	7.34 ± 0.21	7.50 ± 0.12
TC02002127.hg.1	MGAT4A	8.31 ± 0.70	8.78 ± 0.17	8.45 ± 0.53	8.72 ± 0.08
TC05002138.hg.1	MGAT4B	8.60 ± 0.11	8.45 ± 0.06	8.51 ± 0.16	8.51 ± 0.04
TC12001787.hg.1	MGAT4C	4.62 ± 0.12	4.58 ± 0.03	4.59 ± 0.04	4.60 ± 0.02
TC02000874.hg.1	MGAT5	8.88 ± 0.02	8.90 ± 0.24	8.55 ± 0.16	8.76 ± 0.10
TC17000883.hg.1	MGAT5B	7.78 ± 0.22	7.70 ± 0.20	7.64 ± 0.22	7.77 ± 0.06

TC19001295.hg.1	MGC10814	7.85 ± 0.41	7.87 ± 0.16	7.74 ± 0.29	8.00 ± 0.15
TC02000452.hg.1	MGC10955	7.58 ± 0.09	7.56 ± 0.06	7.62 ± 0.06	7.72 ± 0.13
TC17000166.hg.1	MGC12916	8.17 ± 0.23	8.51 ± 0.01	8.51 ± 0.28	8.55 ± 0.07
TC11002349.hg.1	MGC13053	6.36 ± 0.17	6.44 ± 0.14	6.36 ± 0.14	6.54 ± 0.11
TC22000227.hg.1	MGC15705	6.67 ± 0.21	6.66 ± 0.06	6.72 ± 0.17	6.73 ± 0.11
TC15001534.hg.1	MGC15885	5.57 ± 0.07	5.45 ± 0.11	5.55 ± 0.09	5.57 ± 0.07
TC02002926.hg.1	MGC16025	7.41 ± 0.19	7.35 ± 0.15	7.45 ± 0.09	7.47 ± 0.04
TC07000550.hg.1	MGC16142	6.57 ± 0.25	6.41 ± 0.04	6.29 ± 0.17	6.40 ± 0.02
TC17001851.hg.1	MGC16275	7.75 ± 0.21	7.53 ± 0.07	7.67 ± 0.12	7.45 ± 0.07
TC09000277.hg.1	MGC21881	7.21 ± 0.13	7.05 ± 0.06	7.09 ± 0.14	7.19 ± 0.22
TC09001105.hg.1	MGC21881	7.56 ± 0.26	7.44 ± 0.18	7.43 ± 0.13	7.51 ± 0.15
TC09000929.hg.1	MGC24103	5.13 ± 0.19	4.92 ± 0.08	4.92 ± 0.08	4.89 ± 0.10
TC07001843.hg.1	MGC27345	7.23 ± 0.27	7.25 ± 0.08	7.24 ± 0.21	7.29 ± 0.04
TC01000791.hg.1	MGC27382	5.21 ± 0.16	5.15 ± 0.09	5.06 ± 0.08	5.28 ± 0.18
TC19000973.hg.1	MGC2752	8.31 ± 0.25	8.26 ± 0.15	8.26 ± 0.18	8.28 ± 0.03
TC03001064.hg.1	MGC2889	5.80 ± 0.35	5.85 ± 0.14	5.88 ± 0.34	5.89 ± 0.08
TC05001718.hg.1	MGC32805	5.96 ± 0.10	5.84 ± 0.06	5.94 ± 0.03	5.99 ± 0.11
TC05001719.hg.1	MGC32805	6.87 ± 0.23	6.70 ± 0.10	6.78 ± 0.13	7.25 ± 0.49
TC06001000.hg.1	MGC34034	5.03 ± 0.06	4.79 ± 0.04	4.95 ± 0.17	4.85 ± 0.08
TC16000403.hg.1	MGC34800	7.76 ± 0.24	7.83 ± 0.19	7.81 ± 0.36	7.93 ± 0.01
TC06001219.hg.1	MGC39372	8.34 ± 0.12	8.37 ± 0.05	8.21 ± 0.01	8.37 ± 0.18
TC11001156.hg.1	MGC39545	5.41 ± 0.21	5.24 ± 0.12	5.28 ± 0.11	5.25 ± 0.03
TC4_gl000193_random000001.hg.1	MGC39584	6.53 ± 0.43	6.53 ± 0.01	6.49 ± 0.25	6.85 ± 0.29
TC20000462.hg.1	MGC4294	7.00 ± 0.19	7.17 ± 0.29	7.11 ± 0.21	7.25 ± 0.06
TC04001783.hg.1	MGC45800	6.68 ± 0.21	6.55 ± 0.10	6.55 ± 0.07	6.60 ± 0.05
TC19000769.hg.1	MGC45922	6.91 ± 0.37	6.74 ± 0.27	6.87 ± 0.15	6.94 ± 0.03
TC17_ctg5_hap1000029.hg.1	MGC57346	7.47 ± 0.32	7.37 ± 0.18	7.10 ± 0.12	7.08 ± 0.15
TC17002877.hg.1	MGC57346	7.58 ± 0.31	7.51 ± 0.21	7.17 ± 0.14	7.21 ± 0.14
TC07001631.hg.1	MGC72080	8.95 ± 0.08	8.90 ± 0.04	8.87 ± 0.06	8.89 ± 0.02
TC10001613.hg.1	MGEA5	10.02 ± 0.43	10.32 ± 0.13	10.04 ± 0.39	10.37 ± 0.09
TC03001750.hg.1	MGLL	7.17 ± 0.12	7.06 ± 0.11	7.21 ± 0.12	7.12 ± 0.07

TC20000114.hg.1	MGME1	7.66 ± 0.12	7.46 ± 0.06	7.74 ± 0.19	7.27 ± 0.12
TC10000927.hg.1	MGMT	7.39 ± 0.18	7.31 ± 0.10	7.19 ± 0.10	7.35 ± 0.09
TC12001276.hg.1	MGP	5.72 ± 0.11	5.79 ± 0.07	5.69 ± 0.03	5.78 ± 0.08
TC16000133.hg.1	MGRN1	9.76 ± 0.17	9.72 ± 0.04	9.91 ± 0.20	9.80 ± 0.27
TC12000207.hg.1	MGST1	6.93 ± 0.06	6.76 ± 0.07	7.26 ± 0.08	6.76 ± 0.12
TC12000208.hg.1	MGST1	5.72 ± 0.62	5.65 ± 0.11	5.57 ± 0.32	5.85 ± 0.26
TC04000687.hg.1	MGST2	6.81 ± 0.17	6.48 ± 0.03	6.69 ± 0.13	6.51 ± 0.01
TC01001443.hg.1	MGST3	6.56 ± 0.18	6.38 ± 0.05	6.41 ± 0.13	6.39 ± 0.04
TC19002632.hg.1	MIA	8.29 ± 0.31	8.28 ± 0.14	8.19 ± 0.22	8.29 ± 0.07
TC14002295.hg.1	MIA2	3.82 ± 0.06	3.72 ± 0.02	3.78 ± 0.04	3.71 ± 0.04
TC01001829.hg.1	MIA3	8.68 ± 0.25	8.69 ± 0.18	8.89 ± 0.30	8.58 ± 0.14
TC22000172.hg.1	MIAT	7.56 ± 0.04	7.53 ± 0.02	7.58 ± 0.06	7.50 ± 0.05
TC18000090.hg.1	MIB1	7.95 ± 0.33	8.15 ± 0.23	8.13 ± 0.40	7.94 ± 0.08
TC01000046.hg.1	MIB2	8.73 ± 0.19	8.74 ± 0.19	8.64 ± 0.21	8.79 ± 0.05
TC06004073.hg.1	MICA	8.00 ± 0.09	7.81 ± 0.07	7.91 ± 0.14	7.80 ± 0.01
TC6_cox_hap2000234.hg.1	MICA	8.07 ± 0.10	7.87 ± 0.06	7.98 ± 0.15	7.87 ± 0.02
TC6_mann_hap4000215.hg.1	MICA	8.04 ± 0.12	7.80 ± 0.07	7.97 ± 0.21	7.79 ± 0.01
TC6_qbl_hap6000238.hg.1	MICA	8.22 ± 0.17	8.05 ± 0.12	8.08 ± 0.20	8.12 ± 0.02
TC6_ssto_hap7000188.hg.1	MICA	8.07 ± 0.10	7.87 ± 0.06	7.98 ± 0.15	7.87 ± 0.02
TC06002003.hg.1	MICAL1	9.16 ± 0.08	9.13 ± 0.01	9.33 ± 0.11	9.17 ± 0.01
TC11000198.hg.1	MICAL2	8.39 ± 0.03	8.37 ± 0.16	8.46 ± 0.14	8.29 ± 0.23
TC22000478.hg.1	MICAL3	7.92 ± 0.17	7.92 ± 0.05	7.77 ± 0.13	7.94 ± 0.05
TC11000199.hg.1	MICALCL	7.34 ± 0.15	7.49 ± 0.02	7.36 ± 0.08	7.50 ± 0.11
TC22000293.hg.1	MICALL1	8.45 ± 0.12	8.35 ± 0.08	8.29 ± 0.14	8.35 ± 0.01
TC07001082.hg.1	MICALL2	8.24 ± 0.20	8.21 ± 0.21	8.18 ± 0.20	8.32 ± 0.06
TC06000366.hg.1	MICB	9.33 ± 0.26	9.01 ± 0.03	9.36 ± 0.22	8.93 ± 0.04
TC6_apd_hap1000034.hg.1	MICB	9.28 ± 0.30	8.91 ± 0.04	9.31 ± 0.28	8.78 ± 0.09
TC6_cox_hap2000235.hg.1	MICB	8.93 ± 0.31	8.63 ± 0.04	8.98 ± 0.26	8.52 ± 0.07
TC6_dbb_hap3000053.hg.1	MICB	9.31 ± 0.30	8.95 ± 0.04	9.34 ± 0.28	8.80 ± 0.09
TC6_mann_hap4000216.hg.1	MICB	9.45 ± 0.42	9.01 ± 0.02	9.46 ± 0.30	8.85 ± 0.11
TC6_mcf_hap5000048.hg.1	MICB	9.06 ± 0.28	8.78 ± 0.05	9.10 ± 0.23	8.68 ± 0.06

TC6_qbl_hap6000239.hg.1	MICB	9.29 ± 0.24	9.01 ± 0.04	9.32 ± 0.22	8.94 ± 0.06
TC6_ssto_hap7000189.hg.1	MICB	9.29 ± 0.24	9.01 ± 0.04	9.34 ± 0.21	8.93 ± 0.08
TC10001394.hg.1	MICU1	8.47 ± 0.23	8.21 ± 0.10	8.71 ± 0.22	8.02 ± 0.10
TC13000474.hg.1	MICU2	7.04 ± 0.46	7.31 ± 0.27	7.19 ± 0.43	7.13 ± 0.08
TC08000123.hg.1	MICU3	4.79 ± 0.24	4.76 ± 0.09	4.63 ± 0.11	4.70 ± 0.03
TC0X000855.hg.1	MID1	5.71 ± 0.14	5.63 ± 0.09	5.62 ± 0.11	5.70 ± 0.05
TC0X000180.hg.1	MID1IP1	8.94 ± 0.11	8.83 ± 0.03	8.97 ± 0.14	8.69 ± 0.10
TC0X000957.hg.1	MID1IP1-AS1	7.85 ± 0.10	7.76 ± 0.18	7.61 ± 0.16	7.81 ± 0.06
TC0X000528.hg.1	MID2	5.92 ± 0.17	5.98 ± 0.09	5.90 ± 0.08	6.01 ± 0.05
TC19000033.hg.1	MIDN	10.03 ± 0.28	10.30 ± 0.03	10.34 ± 0.25	10.24 ± 0.29
TC17001454.hg.1	MIEN1	8.88 ± 0.24	8.73 ± 0.09	8.73 ± 0.31	8.68 ± 0.04
TC01000738.hg.1	MIER1	8.35 ± 0.45	8.69 ± 0.12	8.73 ± 0.46	8.62 ± 0.15
TC19000982.hg.1	MIER2	7.76 ± 0.14	7.61 ± 0.10	7.63 ± 0.09	7.66 ± 0.02
TC05001379.hg.1	MIER3	7.06 ± 0.32	7.22 ± 0.16	6.97 ± 0.25	7.18 ± 0.01
TC22001456.hg.1	MIF	8.93 ± 0.38	8.86 ± 0.11	8.69 ± 0.21	8.72 ± 0.04
TC17001875.hg.1	MIF4GD	8.91 ± 0.18	8.73 ± 0.10	8.58 ± 0.09	8.54 ± 0.09
TC01000153.hg.1	MIIP	8.58 ± 0.23	8.38 ± 0.09	8.60 ± 0.15	8.38 ± 0.02
TC17000777.hg.1	MILR1	8.63 ± 0.49	7.96 ± 0.10	8.49 ± 0.16	7.95 ± 0.08
TC19000932.hg.1	MIMT1	4.99 ± 0.33	4.88 ± 0.29	4.99 ± 0.11	5.06 ± 0.07
TC03001599.hg.1	MINA	7.46 ± 0.13	7.50 ± 0.05	7.34 ± 0.21	7.38 ± 0.08
TC17000054.hg.1	MINK1	9.03 ± 0.09	8.95 ± 0.04	8.94 ± 0.04	8.98 ± 0.04
TC01006267.hg.1	MINOS1	8.03 ± 0.09	8.04 ± 0.12	7.91 ± 0.09	7.95 ± 0.07
TC10000619.hg.1	MINPP1	6.92 ± 0.07	6.68 ± 0.09	6.79 ± 0.04	6.66 ± 0.09
TC07000077.hg.1	MIOS	7.11 ± 0.33	7.32 ± 0.15	7.11 ± 0.33	7.23 ± 0.10
TC22000427.hg.1	MIOX	7.55 ± 0.17	7.54 ± 0.22	7.50 ± 0.13	7.62 ± 0.08
TC12001606.hg.1	MIP	6.84 ± 0.15	6.83 ± 0.12	6.81 ± 0.03	6.82 ± 0.04
TC13000484.hg.1	MIPEP	6.71 ± 0.08	6.59 ± 0.10	6.42 ± 0.09	6.55 ± 0.01
TC14000238.hg.1	MIPOL1	4.63 ± 0.13	4.52 ± 0.02	4.57 ± 0.05	4.56 ± 0.03
TC11002385.hg.1	MIR100	3.79 ± 0.08	3.52 ± 0.07	3.54 ± 0.13	3.59 ± 0.12
TC11002380.hg.1	MIR100HG	5.64 ± 0.15	5.56 ± 0.10	5.68 ± 0.05	5.69 ± 0.06
TC01002746.hg.1	MIR101-1	5.45 ± 0.20	5.81 ± 0.08	5.66 ± 0.09	5.83 ± 0.13

TC09000033.hg.1	MIR101-2	4.36 ± 0.08	4.03 ± 0.03	4.14 ± 0.12	4.06 ± 0.05
TC05002042.hg.1	MIR103A1	5.69 ± 0.14	5.83 ± 0.28	5.78 ± 0.38	5.96 ± 0.06
TC20000045.hg.1	MIR103A2	6.08 ± 0.30	6.45 ± 0.13	6.56 ± 0.53	6.51 ± 0.28
TC05000921.hg.1	MIR103B1	5.19 ± 0.21	5.46 ± 0.07	5.37 ± 0.09	5.55 ± 0.14
TC20000581.hg.1	MIR103B2	5.22 ± 0.20	5.24 ± 0.26	5.76 ± 0.17	5.44 ± 0.19
TC0X001495.hg.1	MIR105-1	6.62 ± 0.37	6.83 ± 0.27	6.83 ± 0.21	7.10 ± 0.14
TC0X001500.hg.1	MIR105-2	5.71 ± 0.27	5.99 ± 0.18	5.70 ± 0.22	6.14 ± 0.16
TC0X001386.hg.1	MIR106A	3.54 ± 0.21	3.65 ± 0.12	3.50 ± 0.14	3.57 ± 0.10
TC07001657.hg.1	MIR106B	7.26 ± 0.21	7.41 ± 0.11	7.22 ± 0.14	7.46 ± 0.15
TC10000642.hg.1	MIR107	5.06 ± 0.33	4.95 ± 0.26	4.97 ± 0.14	5.02 ± 0.06
TC10001508.hg.1	MIR107	7.04 ± 0.17	7.39 ± 0.42	7.14 ± 0.29	7.64 ± 0.18
TC17002909.hg.1	MIR10A	7.32 ± 0.14	7.23 ± 0.08	7.22 ± 0.11	7.21 ± 0.04
TC02001054.hg.1	MIR10B	6.85 ± 0.16	6.82 ± 0.17	6.85 ± 0.15	6.90 ± 0.03
TC20000492.hg.1	MIR1-1	4.28 ± 0.11	4.00 ± 0.08	3.95 ± 0.12	3.97 ± 0.02
TC15000838.hg.1	MIR1179	5.37 ± 0.13	5.42 ± 0.07	5.37 ± 0.04	5.55 ± 0.23
TC17001241.hg.1	MIR1180	7.85 ± 0.15	7.92 ± 0.21	7.88 ± 0.07	8.07 ± 0.07
TC01003963.hg.1	MIR1182	6.94 ± 0.15	6.84 ± 0.09	7.06 ± 0.12	6.85 ± 0.02
TC07000132.hg.1	MIR1183	6.87 ± 0.10	6.82 ± 0.13	6.92 ± 0.17	6.93 ± 0.01
TC0X001548.hg.1	MIR1184-1	9.70 ± 0.54	9.16 ± 0.21	9.62 ± 0.16	9.07 ± 0.34
TC0X000788.hg.1	MIR1184-2	9.70 ± 0.54	9.16 ± 0.21	9.62 ± 0.16	9.07 ± 0.34
TC0X001553.hg.1	MIR1184-2	9.70 ± 0.54	9.16 ± 0.21	9.62 ± 0.16	9.07 ± 0.34
TC14000739.hg.1	MIR1185-1	5.65 ± 0.31	4.98 ± 0.37	5.30 ± 0.22	4.91 ± 0.16
TC14000740.hg.1	MIR1185-2	6.43 ± 0.30	6.04 ± 0.23	6.37 ± 0.19	6.04 ± 0.07
TC14000726.hg.1	MIR1193	7.47 ± 0.40	7.34 ± 0.07	7.41 ± 0.21	7.28 ± 0.16
TC14000720.hg.1	MIR1197	6.31 ± 0.31	6.31 ± 0.11	6.25 ± 0.25	6.35 ± 0.18
TC18000404.hg.1	MIR1-2	3.94 ± 0.03	3.84 ± 0.02	3.90 ± 0.03	3.89 ± 0.06
TC07001289.hg.1	MIR1200	6.42 ± 0.34	6.87 ± 0.22	6.38 ± 0.31	6.75 ± 0.25
TC06001120.hg.1	MIR1202	8.54 ± 0.22	8.57 ± 0.14	8.45 ± 0.18	8.68 ± 0.09
TC17001638.hg.1	MIR1203	8.00 ± 0.20	8.13 ± 0.11	7.83 ± 0.18	8.21 ± 0.16
TC08000750.hg.1	MIR1204	7.14 ± 0.07	7.18 ± 0.02	6.97 ± 0.02	7.17 ± 0.05
TC08000752.hg.1	MIR1205	6.16 ± 0.11	6.41 ± 0.41	6.16 ± 0.24	6.52 ± 0.23

TC08000754.hg.1	MIR1206	5.12 ± 0.48	5.24 ± 0.55	4.83 ± 0.31	4.95 ± 0.15
TC08000755.hg.1	MIR1207	9.18 ± 0.33	9.42 ± 0.24	9.06 ± 0.29	9.49 ± 0.12
TC08000756.hg.1	MIR1208	6.55 ± 0.13	6.34 ± 0.11	6.39 ± 0.25	6.27 ± 0.04
TC18000203.hg.1	MIR122	4.06 ± 0.09	4.08 ± 0.05	4.12 ± 0.23	4.18 ± 0.08
TC03000997.hg.1	MIR1224	8.39 ± 0.38	8.37 ± 0.26	8.14 ± 0.24	8.39 ± 0.13
TC16000774.hg.1	MIR1225	8.94 ± 0.34	8.86 ± 0.30	8.76 ± 0.25	8.93 ± 0.04
TC03000273.hg.1	MIR1226	8.20 ± 0.27	8.28 ± 0.36	8.23 ± 0.35	8.55 ± 0.12
TC19001024.hg.1	MIR1227	8.14 ± 0.08	8.11 ± 0.21	8.15 ± 0.01	8.13 ± 0.05
TC12000522.hg.1	MIR1228	10.13 ± 0.19	10.38 ± 0.36	10.13 ± 0.30	10.57 ± 0.11
TC05002139.hg.1	MIR1229	10.14 ± 0.41	10.16 ± 0.35	9.89 ± 0.37	10.34 ± 0.18
TC01001663.hg.1	MIR1231	8.15 ± 0.26	8.20 ± 0.26	8.13 ± 0.17	8.33 ± 0.14
TC15001197.hg.1	MIR1233-1	11.44 ± 0.19	11.26 ± 0.12	11.21 ± 0.31	11.24 ± 0.09
TC15001198.hg.1	MIR1233-2	11.44 ± 0.19	11.26 ± 0.12	11.21 ± 0.31	11.24 ± 0.09
TC06001552.hg.1	MIR1236	7.80 ± 0.09	7.76 ± 0.01	7.66 ± 0.14	7.74 ± 0.01
TC11000600.hg.1	MIR1237	9.59 ± 0.44	9.51 ± 0.20	9.30 ± 0.36	9.47 ± 0.11
TC19000179.hg.1	MIR1238	9.35 ± 0.21	9.37 ± 0.19	9.26 ± 0.09	9.55 ± 0.08
TC08000955.hg.1	MIR124-1	6.89 ± 0.14	6.79 ± 0.07	6.85 ± 0.08	6.84 ± 0.07
TC08000431.hg.1	MIR124-2	7.00 ± 0.18	6.80 ± 0.15	6.82 ± 0.32	6.92 ± 0.16
TC04000586.hg.1	MIR1243	4.43 ± 0.06	4.27 ± 0.09	4.30 ± 0.23	4.39 ± 0.13
TC20000507.hg.1	MIR124-3	6.21 ± 0.26	6.08 ± 0.14	6.17 ± 0.15	6.14 ± 0.01
TC05000561.hg.1	MIR1244-1	9.01 ± 0.31	8.78 ± 0.60	8.45 ± 0.26	8.62 ± 0.35
TC12001190.hg.1	MIR1244-1	9.01 ± 0.31	8.78 ± 0.60	8.45 ± 0.26	8.62 ± 0.35
TC02001106.hg.1	MIR1245A	3.53 ± 0.21	3.10 ± 0.09	3.25 ± 0.20	3.11 ± 0.01
TC02002611.hg.1	MIR1245B	3.22 ± 0.11	3.05 ± 0.14	3.24 ± 0.27	3.14 ± 0.05
TC02002557.hg.1	MIR1246	5.58 ± 0.27	5.53 ± 0.12	5.33 ± 0.31	5.57 ± 0.14
TC14001520.hg.1	MIR1247	7.82 ± 0.20	7.79 ± 0.14	7.73 ± 0.13	7.85 ± 0.06
TC22000874.hg.1	MIR1249	7.53 ± 0.14	7.49 ± 0.45	7.46 ± 0.34	7.62 ± 0.08
TC17001944.hg.1	MIR1250	9.19 ± 0.28	9.25 ± 0.20	9.17 ± 0.17	9.51 ± 0.18
TC12000754.hg.1	MIR1251	5.86 ± 0.30	5.57 ± 0.16	5.84 ± 0.33	5.75 ± 0.14
TC12000662.hg.1	MIR1252	3.22 ± 0.09	3.02 ± 0.02	3.14 ± 0.15	3.04 ± 0.05
TC17001000.hg.1	MIR1253	5.08 ± 0.23	5.25 ± 0.22	5.09 ± 0.13	5.23 ± 0.09

TC10000409.hg.1	MIR1254-1	9.17 ± 0.33	9.22 ± 0.14	9.12 ± 0.18	9.41 ± 0.05
TC04001426.hg.1	MIR1255A	4.20 ± 0.09	4.15 ± 0.21	4.15 ± 0.05	3.99 ± 0.03
TC07000511.hg.1	MIR1255B1	4.28 ± 0.07	4.14 ± 0.06	4.25 ± 0.04	4.24 ± 0.07
TC01001464.hg.1	MIR1255B2	6.52 ± 0.47	6.61 ± 0.13	6.59 ± 0.22	6.58 ± 0.27
TC01002321.hg.1	MIR1256	4.43 ± 0.15	4.50 ± 0.17	4.51 ± 0.13	4.55 ± 0.18
TC0X001246.hg.1	MIR1256	7.73 ± 0.62	7.96 ± 0.30	7.66 ± 0.42	8.23 ± 0.29
TC10001393.hg.1	MIR1256	8.41 ± 0.59	8.63 ± 0.31	8.39 ± 0.56	8.92 ± 0.29
TC20001004.hg.1	MIR1257	8.81 ± 0.03	8.85 ± 0.05	8.74 ± 0.12	8.82 ± 0.06
TC02002580.hg.1	MIR1258	6.66 ± 0.37	6.59 ± 0.16	6.46 ± 0.14	6.51 ± 0.22
TC19000786.hg.1	MIR125A	8.44 ± 0.43	8.37 ± 0.25	8.19 ± 0.49	8.47 ± 0.14
TC11002382.hg.1	MIR125B1	4.83 ± 0.04	4.78 ± 0.21	4.72 ± 0.29	4.73 ± 0.10
TC21000043.hg.1	MIR125B2	5.60 ± 0.08	5.46 ± 0.09	5.55 ± 0.06	5.46 ± 0.04
TC09000818.hg.1	MIR126	5.06 ± 0.26	4.84 ± 0.06	4.79 ± 0.25	4.78 ± 0.07
TC14000488.hg.1	MIR1260A	8.86 ± 0.11	8.94 ± 0.12	8.82 ± 0.13	8.96 ± 0.11
TC11000935.hg.1	MIR1260B	8.77 ± 0.11	8.79 ± 0.12	8.71 ± 0.26	9.01 ± 0.19
TC11002187.hg.1	MIR1261	4.49 ± 0.19	4.59 ± 0.07	4.45 ± 0.17	5.06 ± 0.28
TC01002764.hg.1	MIR1262	4.42 ± 0.10	4.54 ± 0.07	4.73 ± 0.27	4.55 ± 0.12
TC03001963.hg.1	MIR1263	4.42 ± 0.15	4.20 ± 0.09	4.37 ± 0.22	4.25 ± 0.06
TC0X000559.hg.1	MIR1264	3.63 ± 0.16	3.50 ± 0.03	3.70 ± 0.22	3.40 ± 0.06
TC10000110.hg.1	MIR1265	4.39 ± 0.20	4.34 ± 0.24	4.29 ± 0.10	4.64 ± 0.25
TC15001455.hg.1	MIR1266	6.26 ± 0.08	6.38 ± 0.07	6.38 ± 0.23	6.46 ± 0.13
TC13000865.hg.1	MIR1267	6.72 ± 0.29	6.64 ± 0.09	6.78 ± 0.40	6.73 ± 0.12
TC15001042.hg.1	MIR1268A	12.09 ± 0.23	12.01 ± 0.09	12.08 ± 0.10	12.12 ± 0.04
TC04000360.hg.1	MIR1269A	7.97 ± 0.12	8.04 ± 0.12	8.02 ± 0.18	8.17 ± 0.10
TC14000656.hg.1	MIR127	6.53 ± 0.19	6.52 ± 0.21	6.44 ± 0.25	6.62 ± 0.20
TC19001345.hg.1	MIR1270-1	6.76 ± 0.35	6.60 ± 0.15	6.69 ± 0.32	6.73 ± 0.10
TC19001346.hg.1	MIR1270-2	6.76 ± 0.35	6.60 ± 0.15	6.69 ± 0.32	6.73 ± 0.10
TC05000998.hg.1	MIR1271	7.62 ± 0.45	7.96 ± 0.11	7.62 ± 0.39	8.10 ± 0.26
TC15001554.hg.1	MIR1272	5.18 ± 0.23	5.34 ± 0.15	5.23 ± 0.02	5.47 ± 0.18
TC08001474.hg.1	MIR1273A	12.65 ± 0.19	12.47 ± 0.04	12.68 ± 0.08	12.51 ± 0.06
TC06001649.hg.1	MIR1275	7.38 ± 0.22	7.26 ± 0.09	7.27 ± 0.08	7.38 ± 0.12

TC15001807.hg.1	MIR1276	6.10 ± 0.19	6.06 ± 0.26	5.98 ± 0.36	6.12 ± 0.11
TC0X000573.hg.1	MIR1277	3.07 ± 0.03	2.91 ± 0.09	3.06 ± 0.05	2.88 ± 0.05
TC01001627.hg.1	MIR1278	5.13 ± 0.36	5.11 ± 0.14	5.19 ± 0.45	5.05 ± 0.30
TC12001708.hg.1	MIR1279	3.83 ± 0.16	3.58 ± 0.17	3.70 ± 0.28	3.41 ± 0.17
TC03000673.hg.1	MIR1280	8.69 ± 0.19	8.79 ± 0.17	8.76 ± 0.13	8.93 ± 0.16
TC22000332.hg.1	MIR1281	10.30 ± 0.26	10.39 ± 0.14	10.44 ± 0.34	10.37 ± 0.17
TC02000882.hg.1	MIR128-1	6.78 ± 0.16	7.03 ± 0.33	6.76 ± 0.45	7.16 ± 0.03
TC15001286.hg.1	MIR1282	9.38 ± 0.18	9.38 ± 0.08	9.52 ± 0.26	9.41 ± 0.03
TC03000172.hg.1	MIR128-2	6.12 ± 0.34	5.99 ± 0.50	6.09 ± 0.31	6.29 ± 0.09
TC19000825.hg.1	MIR1283-1	4.23 ± 0.04	3.83 ± 0.03	4.05 ± 0.10	4.03 ± 0.11
TC19000858.hg.1	MIR1283-2	4.51 ± 0.34	4.55 ± 0.34	4.51 ± 0.30	4.47 ± 0.20
TC03001547.hg.1	MIR1284	5.65 ± 0.34	5.74 ± 0.40	5.65 ± 0.32	5.41 ± 0.08
TC07001599.hg.1	MIR1285-1	9.12 ± 0.25	9.14 ± 0.04	9.18 ± 0.10	9.22 ± 0.07
TC02001959.hg.1	MIR1285-2	10.37 ± 0.30	10.22 ± 0.12	10.41 ± 0.18	10.43 ± 0.09
TC22000509.hg.1	MIR1286	6.60 ± 0.20	6.60 ± 0.36	6.67 ± 0.22	6.92 ± 0.08
TC10001577.hg.1	MIR1287	7.77 ± 0.13	7.71 ± 0.14	7.72 ± 0.09	7.79 ± 0.09
TC17000185.hg.1	MIR1288	5.88 ± 0.31	5.95 ± 0.24	6.06 ± 0.03	6.06 ± 0.05
TC20000799.hg.1	MIR1289-1	4.42 ± 0.13	4.26 ± 0.04	4.37 ± 0.34	4.18 ± 0.03
TC05001781.hg.1	MIR1289-2	6.90 ± 0.23	6.76 ± 0.12	6.92 ± 0.19	6.97 ± 0.31
TC01002292.hg.1	MIR1290	7.25 ± 0.82	7.25 ± 0.44	7.32 ± 0.32	7.23 ± 0.33
TC07000767.hg.1	MIR129-1	6.27 ± 0.08	6.37 ± 0.18	6.29 ± 0.19	6.39 ± 0.05
TC11000366.hg.1	MIR129-2	5.59 ± 0.09	5.86 ± 0.06	5.79 ± 0.21	6.01 ± 0.26
TC12001495.hg.1	MIR1293	5.89 ± 0.21	6.02 ± 0.40	5.63 ± 0.27	6.22 ± 0.06
TC05000851.hg.1	MIR1294	6.58 ± 0.12	6.63 ± 0.08	6.63 ± 0.09	6.70 ± 0.08
TC01003511.hg.1	MIR1295A	5.70 ± 0.17	6.12 ± 0.32	5.79 ± 0.47	6.25 ± 0.18
TC10001332.hg.1	MIR1296	8.25 ± 0.18	8.09 ± 0.08	8.04 ± 0.19	8.12 ± 0.06
TC13000697.hg.1	MIR1297	3.30 ± 0.22	3.13 ± 0.04	3.22 ± 0.08	3.20 ± 0.07
TC0X000560.hg.1	MIR1298	5.04 ± 0.29	5.17 ± 0.30	5.11 ± 0.15	5.17 ± 0.06
TC09001166.hg.1	MIR1299	9.03 ± 0.97	9.07 ± 0.29	8.81 ± 0.55	9.40 ± 0.67
TC02001659.hg.1	MIR1301	8.70 ± 0.16	8.86 ± 0.29	8.73 ± 0.18	9.04 ± 0.13
TC12001980.hg.1	MIR1302-1	3.61 ± 0.22	3.50 ± 0.23	3.57 ± 0.06	3.47 ± 0.04

TC01000002.hg.1	MIR1302-11	7.54 ± 0.25	7.43 ± 0.05	7.43 ± 0.28	7.67 ± 0.24
TC09000002.hg.1	MIR1302-11	6.98 ± 0.32	6.75 ± 0.02	6.92 ± 0.32	6.92 ± 0.25
TC15002055.hg.1	MIR1302-11	7.70 ± 0.23	7.57 ± 0.03	7.54 ± 0.30	7.78 ± 0.21
TC19000001.hg.1	MIR1302-11	4.19 ± 0.56	3.64 ± 0.10	3.76 ± 0.32	3.85 ± 0.22
TC02002225.hg.1	MIR1302-3	4.00 ± 0.27	3.69 ± 0.07	3.74 ± 0.15	3.80 ± 0.05
TC02002710.hg.1	MIR1302-4	3.90 ± 0.33	3.70 ± 0.17	4.06 ± 0.19	3.59 ± 0.10
TC20000938.hg.1	MIR1302-5	3.38 ± 0.24	3.19 ± 0.06	3.21 ± 0.01	3.15 ± 0.04
TC07001171.hg.1	MIR1302-6	3.97 ± 0.16	3.67 ± 0.13	3.88 ± 0.09	3.74 ± 0.08
TC09001392.hg.1	MIR1302-8	4.33 ± 0.06	4.30 ± 0.21	4.34 ± 0.20	4.17 ± 0.03
TC05000854.hg.1	MIR1303	11.76 ± 0.22	11.75 ± 0.05	11.75 ± 0.01	11.85 ± 0.07
TC04000883.hg.1	MIR1305	3.52 ± 0.21	3.24 ± 0.08	3.32 ± 0.09	3.14 ± 0.06
TC22000053.hg.1	MIR1306	7.91 ± 0.08	7.91 ± 0.06	7.70 ± 0.06	7.86 ± 0.01
TC10001630.hg.1	MIR1307	8.38 ± 0.13	8.16 ± 0.30	8.22 ± 0.17	7.86 ± 0.09
TC11000469.hg.1	MIR130A	6.10 ± 0.14	5.96 ± 0.02	5.96 ± 0.16	5.92 ± 0.18
TC22000096.hg.1	MIR130B	8.45 ± 0.02	8.31 ± 0.01	8.29 ± 0.06	8.31 ± 0.01
TC17000986.hg.1	MIR132	6.27 ± 0.46	6.32 ± 0.23	6.32 ± 0.28	6.37 ± 0.17
TC08000962.hg.1	MIR1322	3.61 ± 0.06	3.55 ± 0.19	3.54 ± 0.20	3.44 ± 0.05
TC19000817.hg.1	MIR1323	5.80 ± 0.59	6.12 ± 0.04	5.91 ± 0.25	6.11 ± 0.20
TC03000440.hg.1	MIR1324	7.52 ± 0.24	7.41 ± 0.19	7.34 ± 0.23	7.55 ± 0.22
TC20000491.hg.1	MIR133A2	8.03 ± 0.31	7.98 ± 0.31	7.88 ± 0.21	8.12 ± 0.10
TC06000651.hg.1	MIR133B	6.04 ± 0.38	5.98 ± 0.21	5.83 ± 0.07	6.00 ± 0.01
TC14000750.hg.1	MIR134	8.13 ± 0.32	8.06 ± 0.21	7.96 ± 0.39	8.27 ± 0.09
TC11000331.hg.1	MIR1343	9.15 ± 0.38	9.28 ± 0.33	9.05 ± 0.28	9.36 ± 0.11
TC03001452.hg.1	MIR135A1	7.87 ± 0.15	7.68 ± 0.06	7.61 ± 0.04	7.59 ± 0.06
TC12000755.hg.1	MIR135A2	3.65 ± 0.18	3.46 ± 0.03	3.64 ± 0.16	3.53 ± 0.11
TC01003748.hg.1	MIR135B	4.65 ± 0.07	4.79 ± 0.11	4.64 ± 0.21	4.72 ± 0.13
TC14000658.hg.1	MIR136	4.60 ± 0.15	4.57 ± 0.07	4.63 ± 0.07	4.75 ± 0.18
TC01002905.hg.1	MIR137	6.35 ± 0.11	6.25 ± 0.13	6.24 ± 0.07	6.36 ± 0.10
TC03000233.hg.1	MIR138-1	7.15 ± 0.38	7.45 ± 0.18	7.20 ± 0.20	7.58 ± 0.14
TC16000478.hg.1	MIR138-2	7.07 ± 0.20	6.98 ± 0.16	6.98 ± 0.33	7.00 ± 0.13
TC11002060.hg.1	MIR139	8.82 ± 0.30	8.74 ± 0.40	8.90 ± 0.16	9.02 ± 0.06

TC16000573.hg.1	MIR140	5.67 ± 0.18	5.31 ± 0.29	5.57 ± 0.12	5.36 ± 0.06
TC12000104.hg.1	MIR141	6.45 ± 0.40	6.30 ± 0.35	6.58 ± 0.81	6.39 ± 0.14
TC17001729.hg.1	MIR142	9.93 ± 0.45	10.15 ± 0.58	10.80 ± 0.65	10.33 ± 0.30
TC17001309.hg.1	MIR144	3.83 ± 0.32	3.64 ± 0.01	3.82 ± 0.37	3.62 ± 0.10
TC05000821.hg.1	MIR145	7.46 ± 0.23	7.37 ± 0.18	7.38 ± 0.15	7.48 ± 0.07
TC0X001094.hg.1	MIR1468	6.79 ± 0.37	7.13 ± 0.16	7.13 ± 0.13	7.24 ± 0.19
TC05000897.hg.1	MIR146A	6.42 ± 0.11	6.38 ± 0.12	6.36 ± 0.13	6.43 ± 0.08
TC10000755.hg.1	MIR146B	5.89 ± 0.56	5.89 ± 0.27	5.83 ± 0.25	6.18 ± 0.04
TC19000271.hg.1	MIR1470	9.73 ± 0.49	9.38 ± 0.13	9.49 ± 0.46	9.26 ± 0.13
TC02002868.hg.1	MIR1471	9.59 ± 0.28	9.45 ± 0.22	9.39 ± 0.34	9.62 ± 0.12
TC09001534.hg.1	MIR147A	7.42 ± 0.27	7.40 ± 0.03	7.63 ± 0.12	7.63 ± 0.17
TC15000357.hg.1	MIR147B	6.20 ± 0.21	6.25 ± 0.07	6.15 ± 0.01	6.35 ± 0.31
TC07001213.hg.1	MIR148A	4.55 ± 0.21	4.45 ± 0.06	4.53 ± 0.16	4.58 ± 0.10
TC12000463.hg.1	MIR148B	5.30 ± 0.16	5.25 ± 0.07	5.38 ± 0.15	5.36 ± 0.13
TC02001465.hg.1	MIR149	8.39 ± 0.13	8.39 ± 0.09	8.36 ± 0.26	8.28 ± 0.07
TC19001718.hg.1	MIR150	7.97 ± 0.15	7.85 ± 0.03	7.94 ± 0.31	7.79 ± 0.07
TC08001687.hg.1	MIR151A	5.63 ± 0.43	5.34 ± 0.19	5.26 ± 0.34	5.38 ± 0.22
TC17001634.hg.1	MIR152	6.96 ± 0.04	6.98 ± 0.18	7.16 ± 0.07	7.06 ± 0.04
TC02002795.hg.1	MIR153-1	3.94 ± 0.13	4.02 ± 0.11	3.95 ± 0.22	4.13 ± 0.15
TC07002078.hg.1	MIR153-2	5.24 ± 0.17	5.28 ± 0.12	5.07 ± 0.17	5.38 ± 0.17
TC01003999.hg.1	MIR1537	6.66 ± 0.46	6.63 ± 0.23	6.86 ± 0.52	6.46 ± 0.04
TC16001223.hg.1	MIR1538	8.83 ± 0.21	8.84 ± 0.29	8.75 ± 0.16	8.87 ± 0.07
TC18000174.hg.1	MIR1539	6.63 ± 0.15	6.55 ± 0.25	6.64 ± 0.11	6.68 ± 0.19
TC14000754.hg.1	MIR154	5.00 ± 0.14	5.03 ± 0.05	5.01 ± 0.06	5.28 ± 0.24
TC21000076.hg.1	MIR155	7.02 ± 0.16	7.02 ± 0.06	6.92 ± 0.11	7.14 ± 0.10
TC13000668.hg.1	MIR15A	6.98 ± 0.12	6.66 ± 0.21	7.18 ± 0.23	6.49 ± 0.22
TC03000874.hg.1	MIR15B	4.30 ± 0.11	4.33 ± 0.11	4.18 ± 0.11	4.38 ± 0.14
TC03000875.hg.1	MIR16-2	4.29 ± 0.05	4.09 ± 0.17	4.18 ± 0.04	3.98 ± 0.07
TC01003672.hg.1	MIR181A1	4.40 ± 0.16	4.21 ± 0.15	4.30 ± 0.27	4.25 ± 0.16
TC01003670.hg.1	MIR181A1HG	6.82 ± 0.35	7.03 ± 0.14	6.91 ± 0.28	7.01 ± 0.04
TC09000641.hg.1	MIR181A2	5.64 ± 0.29	5.67 ± 0.15	5.69 ± 0.23	5.62 ± 0.14

TC09000640.hg.1	MIR181A2HG	4.83 ± 0.06	4.71 ± 0.05	4.76 ± 0.06	4.71 ± 0.05
TC01003671.hg.1	MIR181B1	5.87 ± 0.35	6.28 ± 0.05	6.45 ± 0.66	6.28 ± 0.13
TC09000642.hg.1	MIR181B2	5.53 ± 0.18	5.34 ± 0.12	5.48 ± 0.07	5.44 ± 0.05
TC19000244.hg.1	MIR181C	7.37 ± 0.11	7.51 ± 0.18	7.55 ± 0.18	7.58 ± 0.05
TC19000245.hg.1	MIR181D	8.88 ± 0.35	8.85 ± 0.24	8.71 ± 0.31	8.93 ± 0.08
TC07001854.hg.1	MIR182	5.77 ± 0.50	5.76 ± 0.11	5.66 ± 0.43	5.75 ± 0.11
TC12000773.hg.1	MIR1827	4.81 ± 0.14	4.91 ± 0.11	4.79 ± 0.34	5.01 ± 0.18
TC15000722.hg.1	MIR1827	5.58 ± 0.17	5.49 ± 0.12	5.70 ± 0.27	5.43 ± 0.12
TC07001856.hg.1	MIR183	4.94 ± 0.10	5.02 ± 0.27	4.94 ± 0.08	4.91 ± 0.04
TC15000739.hg.1	MIR184	6.36 ± 0.15	6.04 ± 0.19	6.01 ± 0.17	6.29 ± 0.14
TC22000052.hg.1	MIR185	7.83 ± 0.14	7.72 ± 0.22	7.69 ± 0.10	7.83 ± 0.07
TC01002776.hg.1	MIR186	6.07 ± 0.69	6.03 ± 0.47	6.04 ± 0.59	6.20 ± 0.16
TC18000456.hg.1	MIR187	7.83 ± 0.14	7.83 ± 0.20	7.72 ± 0.30	7.79 ± 0.05
TC0X000280.hg.1	MIR188	6.64 ± 0.30	6.48 ± 0.16	6.53 ± 0.07	6.58 ± 0.27
TC0X001385.hg.1	MIR18B	5.20 ± 0.28	5.04 ± 0.12	5.23 ± 0.34	5.04 ± 0.25
TC11003438.hg.1	MIR1908	7.92 ± 0.08	7.79 ± 0.05	7.87 ± 0.09	7.81 ± 0.01
TC15000576.hg.1	MIR190A	3.24 ± 0.16	3.07 ± 0.03	3.02 ± 0.06	3.03 ± 0.05
TC01003288.hg.1	MIR190B	4.05 ± 0.16	3.95 ± 0.12	4.14 ± 0.14	3.95 ± 0.23
TC03001396.hg.1	MIR191	7.82 ± 0.29	7.70 ± 0.14	7.60 ± 0.12	7.76 ± 0.03
TC16001320.hg.1	MIR1910	10.19 ± 0.28	10.17 ± 0.06	10.18 ± 0.28	10.23 ± 0.15
TC0X000562.hg.1	MIR1911	6.90 ± 0.10	6.98 ± 0.33	6.84 ± 0.21	7.25 ± 0.15
TC0X000558.hg.1	MIR1912	5.86 ± 0.11	5.84 ± 0.04	5.69 ± 0.04	5.87 ± 0.02
TC06002310.hg.1	MIR1913	8.37 ± 0.22	8.44 ± 0.18	8.38 ± 0.09	8.62 ± 0.06
TC20001041.hg.1	MIR1914	8.41 ± 0.10	8.32 ± 0.08	8.29 ± 0.09	8.31 ± 0.03
TC10001093.hg.1	MIR1915	9.86 ± 0.30	9.81 ± 0.13	9.83 ± 0.26	9.82 ± 0.10
TC11001917.hg.1	MIR192	7.67 ± 0.15	8.39 ± 0.35	7.64 ± 0.20	8.13 ± 0.44
TC17000365.hg.1	MIR193A	9.66 ± 0.32	9.80 ± 0.20	9.50 ± 0.37	9.82 ± 0.05
TC16000174.hg.1	MIR193B	7.34 ± 0.07	7.30 ± 0.19	7.15 ± 0.10	7.51 ± 0.11
TC01003837.hg.1	MIR194-1	3.82 ± 0.16	3.88 ± 0.01	3.70 ± 0.11	3.78 ± 0.11
TC11001916.hg.1	MIR194-2	8.06 ± 0.11	8.25 ± 0.11	8.00 ± 0.15	8.30 ± 0.15
TC17001648.hg.1	MIR196A1	6.12 ± 0.20	6.22 ± 0.19	6.16 ± 0.14	6.16 ± 0.04

TC12000451.hg.1	MIR196A2	5.99 ± 0.56	5.88 ± 0.14	5.88 ± 0.40	5.85 ± 0.16
TC01000949.hg.1	MIR197	9.50 ± 0.38	9.47 ± 0.20	9.49 ± 0.14	9.59 ± 0.17
TC16000575.hg.1	MIR1972-1	4.79 ± 0.26	4.70 ± 0.03	4.71 ± 0.11	4.72 ± 0.04
TC16000890.hg.1	MIR1972-2	4.79 ± 0.26	4.70 ± 0.03	4.71 ± 0.11	4.72 ± 0.04
TC04000593.hg.1	MIR1973	7.00 ± 0.19	6.90 ± 0.03	7.01 ± 0.16	6.86 ± 0.05
TC01000348.hg.1	MIR1976	9.00 ± 0.31	9.08 ± 0.28	8.95 ± 0.16	9.26 ± 0.19
TC19001173.hg.1	MIR199A1	4.63 ± 0.26	4.55 ± 0.11	4.55 ± 0.14	4.69 ± 0.20
TC09001625.hg.1	MIR199B	6.53 ± 0.30	6.45 ± 0.13	6.53 ± 0.13	6.62 ± 0.04
TC0X001383.hg.1	MIR19B2	3.89 ± 0.28	3.75 ± 0.13	3.73 ± 0.11	3.70 ± 0.16
TC01000028.hg.1	MIR200A	5.64 ± 0.36	5.49 ± 0.39	5.38 ± 0.12	5.63 ± 0.05
TC01000027.hg.1	MIR200B	7.65 ± 0.23	7.47 ± 0.24	7.61 ± 0.19	7.57 ± 0.08
TC12000103.hg.1	MIR200C	7.33 ± 0.42	7.30 ± 0.06	7.41 ± 0.29	7.47 ± 0.06
TC10001782.hg.1	MIR202	8.26 ± 0.29	8.16 ± 0.28	8.26 ± 0.27	8.23 ± 0.06
TC14000812.hg.1	MIR203	5.57 ± 0.19	5.39 ± 0.02	5.41 ± 0.21	5.45 ± 0.10
TC09001203.hg.1	MIR204	5.82 ± 0.05	5.66 ± 0.10	5.79 ± 0.13	5.82 ± 0.26
TC01001747.hg.1	MIR205	6.67 ± 0.07	6.57 ± 0.03	6.60 ± 0.06	6.65 ± 0.12
TC08000496.hg.1	MIR2052	3.49 ± 0.24	3.32 ± 0.13	3.38 ± 0.11	3.30 ± 0.05
TC08000676.hg.1	MIR2053	3.86 ± 0.12	3.74 ± 0.06	3.72 ± 0.02	3.73 ± 0.08
TC04000638.hg.1	MIR2054	2.91 ± 0.11	2.84 ± 0.08	2.96 ± 0.20	2.82 ± 0.14
TC06000650.hg.1	MIR206	4.94 ± 0.21	4.96 ± 0.23	4.71 ± 0.04	4.96 ± 0.29
TC14000958.hg.1	MIR208A	4.23 ± 0.32	4.34 ± 0.28	4.40 ± 0.27	4.32 ± 0.04
TC14000960.hg.1	MIR208B	3.22 ± 0.11	3.04 ± 0.07	3.12 ± 0.06	3.03 ± 0.11
TC13000322.hg.1	MIR20A	6.30 ± 0.25	6.35 ± 0.09	6.18 ± 0.17	6.43 ± 0.10
TC0X001384.hg.1	MIR20B	4.98 ± 0.25	5.15 ± 0.23	5.04 ± 0.18	5.21 ± 0.23
TC17000728.hg.1	MIR21	7.28 ± 0.39	7.09 ± 0.43	8.23 ± 0.67	6.85 ± 0.76
TC11001239.hg.1	MIR210	9.15 ± 0.22	9.07 ± 0.20	9.14 ± 0.11	9.20 ± 0.05
TC11001238.hg.1	MIR210HG	8.57 ± 0.23	8.74 ± 0.28	8.64 ± 0.31	8.75 ± 0.01
TC15001164.hg.1	MIR211	7.61 ± 0.17	7.56 ± 0.21	7.59 ± 0.22	7.79 ± 0.15
TC10001674.hg.1	MIR2110	8.25 ± 0.74	8.81 ± 0.28	8.90 ± 0.69	8.72 ± 0.24
TC06000824.hg.1	MIR2113	7.35 ± 0.20	7.41 ± 0.12	7.43 ± 0.10	7.45 ± 0.07
TC0X000720.hg.1	MIR2114	5.27 ± 0.25	5.23 ± 0.15	5.25 ± 0.25	5.54 ± 0.13

TC03001377.hg.1	MIR2115	4.81 ± 0.07	4.71 ± 0.24	4.80 ± 0.07	5.00 ± 0.10
TC15001494.hg.1	MIR2116	5.63 ± 0.11	5.80 ± 0.07	5.53 ± 0.08	5.88 ± 0.18
TC17000553.hg.1	MIR2117	7.00 ± 0.18	7.08 ± 0.28	7.10 ± 0.02	7.28 ± 0.03
TC17000987.hg.1	MIR212	7.54 ± 0.22	7.54 ± 0.09	7.55 ± 0.12	7.65 ± 0.06
TC01003519.hg.1	MIR214	5.73 ± 0.14	5.64 ± 0.12	5.63 ± 0.08	5.72 ± 0.10
TC01003836.hg.1	MIR215	4.14 ± 0.05	4.05 ± 0.19	4.13 ± 0.07	3.88 ± 0.05
TC02001871.hg.1	MIR216A	5.48 ± 0.17	5.62 ± 0.35	5.47 ± 0.18	5.87 ± 0.07
TC02001872.hg.1	MIR216B	3.97 ± 0.16	4.08 ± 0.11	4.22 ± 0.12	4.22 ± 0.18
TC02001870.hg.1	MIR217	4.44 ± 0.20	4.21 ± 0.04	4.08 ± 0.07	4.22 ± 0.08
TC04000160.hg.1	MIR218-1	6.64 ± 0.07	6.51 ± 0.10	6.41 ± 0.16	6.65 ± 0.11
TC05002044.hg.1	MIR218-2	7.10 ± 0.36	7.09 ± 0.32	7.05 ± 0.14	7.27 ± 0.32
TC06000414.hg.1	MIR219-1	6.40 ± 0.22	6.60 ± 0.41	6.42 ± 0.26	6.65 ± 0.07
TC6_cox_hap2000104.hg.1	MIR219-1	6.40 ± 0.22	6.60 ± 0.41	6.42 ± 0.26	6.65 ± 0.07
TC6_dbb_hap3000096.hg.1	MIR219-1	6.40 ± 0.22	6.60 ± 0.41	6.42 ± 0.26	6.65 ± 0.07
TC6_mann_hap4000088.hg.1	MIR219-1	6.40 ± 0.22	6.60 ± 0.41	6.42 ± 0.26	6.65 ± 0.07
TC6_mcf_hap5000089.hg.1	MIR219-1	6.40 ± 0.22	6.60 ± 0.41	6.42 ± 0.26	6.65 ± 0.07
TC6_qbl_hap6000095.hg.1	MIR219-1	6.40 ± 0.22	6.60 ± 0.41	6.42 ± 0.26	6.65 ± 0.07
TC09001631.hg.1	MIR219-2	7.00 ± 0.23	7.03 ± 0.07	6.94 ± 0.19	7.07 ± 0.11
TC0X000985.hg.1	MIR221	6.81 ± 0.46	6.98 ± 0.40	7.61 ± 0.61	7.33 ± 0.25
TC0X000986.hg.1	MIR222	4.98 ± 0.11	4.92 ± 0.20	5.31 ± 0.56	5.18 ± 0.17
TC0X000356.hg.1	MIR223	9.66 ± 0.37	9.18 ± 0.25	10.17 ± 0.43	8.97 ± 0.20
TC13000052.hg.1	MIR2276	7.68 ± 0.37	7.55 ± 0.18	7.25 ± 0.33	7.65 ± 0.26
TC05001598.hg.1	MIR2277	7.89 ± 0.39	8.24 ± 0.16	8.12 ± 0.36	8.09 ± 0.10
TC09000468.hg.1	MIR2278	7.00 ± 0.23	7.08 ± 0.29	6.94 ± 0.21	7.31 ± 0.21
TC17000983.hg.1	MIR22HG	8.45 ± 0.14	8.95 ± 0.04	8.70 ± 0.24	9.02 ± 0.19
TC02002706.hg.1	MIR2355	4.99 ± 0.30	5.00 ± 0.33	5.30 ± 0.37	4.89 ± 0.14
TC14000646.hg.1	MIR2392	8.01 ± 0.23	8.02 ± 0.19	8.03 ± 0.08	8.13 ± 0.15
TC09000467.hg.1	MIR23B	6.90 ± 0.09	6.84 ± 0.08	6.83 ± 0.05	6.87 ± 0.05
TC0X000908.hg.1	MIR23C	5.07 ± 0.21	5.36 ± 0.12	5.21 ± 0.10	5.54 ± 0.32
TC02002927.hg.1	MIR2467	6.38 ± 0.22	6.64 ± 0.39	6.55 ± 0.20	6.86 ± 0.18
TC07001655.hg.1	MIR25	7.94 ± 0.17	8.04 ± 0.19	7.68 ± 0.20	8.06 ± 0.19

TC13000848.hg.1	MIR2681	4.69 ± 0.17	4.72 ± 0.10	4.80 ± 0.03	4.69 ± 0.31
TC01002906.hg.1	MIR2682	6.66 ± 0.24	6.93 ± 0.22	6.58 ± 0.22	6.95 ± 0.28
TC03000183.hg.1	MIR26A1	7.65 ± 0.12	7.74 ± 0.14	7.64 ± 0.07	7.95 ± 0.19
TC12001638.hg.1	MIR26A2	4.60 ± 0.09	4.76 ± 0.27	4.83 ± 0.36	4.82 ± 0.08
TC02001302.hg.1	MIR26B	8.47 ± 0.23	8.45 ± 0.09	8.70 ± 0.24	8.58 ± 0.13
TC19001224.hg.1	MIR27A	8.73 ± 0.10	8.80 ± 0.07	8.84 ± 0.22	8.76 ± 0.24
TC03001040.hg.1	MIR28	4.54 ± 0.08	4.52 ± 0.02	4.51 ± 0.23	4.53 ± 0.04
TC17000445.hg.1	MIR2909	7.27 ± 0.14	7.24 ± 0.12	7.11 ± 0.45	7.18 ± 0.12
TC20000986.hg.1	MIR296	6.84 ± 0.26	6.83 ± 0.19	6.61 ± 0.19	6.91 ± 0.31
TC09000689.hg.1	MIR2964A	6.42 ± 0.23	6.62 ± 0.11	6.62 ± 0.13	6.84 ± 0.09
TC04001473.hg.1	MIR297	5.98 ± 0.58	6.31 ± 0.13	6.09 ± 0.15	6.56 ± 0.25
TC20000987.hg.1	MIR298	7.41 ± 0.11	7.49 ± 0.16	7.26 ± 0.21	7.50 ± 0.07
TC14000717.hg.1	MIR299	5.32 ± 0.22	5.10 ± 0.10	5.02 ± 0.10	5.25 ± 0.18
TC07001869.hg.1	MIR29A	5.61 ± 0.43	5.59 ± 0.42	6.54 ± 0.73	5.91 ± 0.33
TC07001870.hg.1	MIR29B1	4.85 ± 0.17	4.69 ± 0.09	4.81 ± 0.32	4.67 ± 0.13
TC01003775.hg.1	MIR29B2	7.50 ± 0.05	7.63 ± 0.00	7.57 ± 0.17	7.65 ± 0.11
TC14000738.hg.1	MIR300	3.87 ± 0.15	3.79 ± 0.08	3.84 ± 0.14	3.80 ± 0.14
TC17001748.hg.1	MIR301A	3.33 ± 0.12	3.31 ± 0.09	3.21 ± 0.07	3.25 ± 0.05
TC22000097.hg.1	MIR301B	5.13 ± 0.06	5.20 ± 0.14	5.22 ± 0.08	5.43 ± 0.06
TC04001479.hg.1	MIR302B	4.68 ± 0.01	4.69 ± 0.03	4.72 ± 0.02	4.67 ± 0.03
TC11000147.hg.1	MIR302E	4.48 ± 0.06	4.06 ± 0.10	4.28 ± 0.12	4.17 ± 0.08
TC18000120.hg.1	MIR302F	3.14 ± 0.10	2.97 ± 0.05	3.07 ± 0.07	3.02 ± 0.07
TC17000925.hg.1	MIR3065	7.06 ± 0.14	7.23 ± 0.21	7.23 ± 0.26	7.34 ± 0.11
TC09001360.hg.1	MIR3074	5.87 ± 0.08	5.65 ± 0.15	5.86 ± 0.10	5.58 ± 0.09
TC06001862.hg.1	MIR30A	5.83 ± 0.07	5.80 ± 0.33	5.96 ± 0.18	6.09 ± 0.15
TC08001666.hg.1	MIR30B	4.22 ± 0.12	4.17 ± 0.08	4.23 ± 0.05	4.21 ± 0.06
TC01000517.hg.1	MIR30C1	7.28 ± 0.07	7.38 ± 0.25	7.42 ± 0.17	7.46 ± 0.22
TC06001861.hg.1	MIR30C2	6.69 ± 0.32	6.59 ± 0.16	6.72 ± 0.20	6.57 ± 0.18
TC08001667.hg.1	MIR30D	5.48 ± 0.20	5.43 ± 0.12	5.45 ± 0.20	5.37 ± 0.10
TC01000516.hg.1	MIR30E	6.65 ± 0.49	6.36 ± 0.34	6.86 ± 0.41	6.42 ± 0.16
TC09000963.hg.1	MIR31	5.69 ± 0.31	5.82 ± 0.52	5.61 ± 0.37	6.11 ± 0.19

TC01000293.hg.1	MIR3115	4.41 ± 0.12	4.50 ± 0.24	4.20 ± 0.10	4.33 ± 0.13
TC01000735.hg.1	MIR3117	4.24 ± 0.03	3.86 ± 0.04	4.15 ± 0.21	3.79 ± 0.06
TC01003506.hg.1	MIR3119-1	3.50 ± 0.13	3.34 ± 0.07	3.41 ± 0.10	3.40 ± 0.10
TC01001480.hg.1	MIR3119-2	4.72 ± 0.08	4.60 ± 0.06	4.53 ± 0.13	4.66 ± 0.13
TC01001501.hg.1	MIR3120	7.50 ± 0.08	7.68 ± 0.22	7.52 ± 0.13	7.77 ± 0.11
TC01003594.hg.1	MIR3121	4.03 ± 0.12	4.03 ± 0.24	3.97 ± 0.03	3.87 ± 0.03
TC01001768.hg.1	MIR3122	5.77 ± 0.24	5.74 ± 0.23	5.62 ± 0.14	5.48 ± 0.14
TC01001972.hg.1	MIR3123	4.52 ± 0.19	4.46 ± 0.03	4.55 ± 0.08	4.47 ± 0.08
TC01002044.hg.1	MIR3124	5.58 ± 0.16	5.43 ± 0.28	5.58 ± 0.21	5.42 ± 0.13
TC02000083.hg.1	MIR3125	8.13 ± 0.12	8.22 ± 0.25	8.04 ± 0.04	8.21 ± 0.05
TC02000404.hg.1	MIR3126	6.09 ± 0.20	6.09 ± 0.26	6.17 ± 0.05	6.22 ± 0.09
TC02000586.hg.1	MIR3127	8.05 ± 0.10	7.87 ± 0.07	8.05 ± 0.10	7.95 ± 0.11
TC02002566.hg.1	MIR3128	3.23 ± 0.25	3.01 ± 0.04	3.14 ± 0.25	2.96 ± 0.08
TC02002613.hg.1	MIR3129	6.65 ± 0.14	6.37 ± 0.02	6.49 ± 0.05	6.34 ± 0.05
TC02002704.hg.1	MIR3130-1	7.68 ± 0.33	7.73 ± 0.32	7.58 ± 0.30	7.91 ± 0.05
TC02001222.hg.1	MIR3130-2	7.60 ± 0.06	7.56 ± 0.25	7.52 ± 0.09	7.71 ± 0.17
TC02002787.hg.1	MIR3131	7.21 ± 0.28	7.20 ± 0.28	7.05 ± 0.29	7.25 ± 0.27
TC02002801.hg.1	MIR3132	7.65 ± 0.31	7.59 ± 0.30	7.56 ± 0.29	7.70 ± 0.21
TC03001537.hg.1	MIR3136	6.83 ± 0.71	7.56 ± 0.12	7.06 ± 0.65	7.62 ± 0.28
TC04001033.hg.1	MIR3138	7.82 ± 0.26	7.74 ± 0.25	7.75 ± 0.17	7.94 ± 0.06
TC04001648.hg.1	MIR3140	6.04 ± 0.59	6.69 ± 0.52	6.45 ± 0.77	6.37 ± 0.34
TC05001972.hg.1	MIR3141	8.71 ± 0.38	8.88 ± 0.46	8.56 ± 0.37	8.97 ± 0.15
TC05000898.hg.1	MIR3142	4.61 ± 0.36	4.58 ± 0.20	4.76 ± 0.18	4.71 ± 0.07
TC06000226.hg.1	MIR3143	9.84 ± 0.22	9.21 ± 0.37	9.57 ± 0.17	8.80 ± 0.19
TC06002160.hg.1	MIR3145	4.50 ± 0.50	4.41 ± 0.09	4.52 ± 0.28	4.43 ± 0.19
TC07001178.hg.1	MIR3146	3.45 ± 0.09	3.23 ± 0.07	3.13 ± 0.09	3.18 ± 0.14
TC07000354.hg.1	MIR3147	7.97 ± 0.40	8.10 ± 0.43	7.79 ± 0.52	8.31 ± 0.11
TC08001102.hg.1	MIR3148	5.62 ± 0.10	5.81 ± 0.10	5.57 ± 0.09	5.96 ± 0.09
TC08000582.hg.1	MIR3150A	8.07 ± 0.29	8.18 ± 0.39	8.14 ± 0.40	8.31 ± 0.09
TC08001441.hg.1	MIR3150B	6.36 ± 0.30	6.34 ± 0.28	6.26 ± 0.22	6.43 ± 0.14
TC08000644.hg.1	MIR3151	7.12 ± 0.24	7.22 ± 0.41	7.18 ± 0.29	7.58 ± 0.14

TC09000087.hg.1	MIR3152	4.13 ± 0.25	4.03 ± 0.09	4.06 ± 0.09	4.03 ± 0.21
TC09000416.hg.1	MIR3153	8.84 ± 0.31	8.67 ± 0.07	8.65 ± 0.32	8.56 ± 0.10
TC09001626.hg.1	MIR3154	8.29 ± 0.28	8.53 ± 0.43	8.27 ± 0.33	8.66 ± 0.11
TC10000055.hg.1	MIR3155A	8.24 ± 0.26	8.32 ± 0.31	8.25 ± 0.33	8.49 ± 0.05
TC10001011.hg.1	MIR3155B	9.33 ± 0.25	9.42 ± 0.27	9.28 ± 0.23	9.55 ± 0.08
TC10000292.hg.1	MIR3156-1	5.91 ± 0.45	5.59 ± 0.11	5.59 ± 0.35	5.56 ± 0.03
TC18000086.hg.1	MIR3156-2	5.69 ± 0.35	5.28 ± 0.01	5.41 ± 0.21	5.22 ± 0.03
TC21000276.hg.1	MIR3156-3	5.69 ± 0.17	5.40 ± 0.02	5.54 ± 0.18	5.39 ± 0.10
TC10001554.hg.1	MIR3157	7.05 ± 0.26	7.13 ± 0.30	7.01 ± 0.06	7.36 ± 0.09
TC10000740.hg.1	MIR3158-1	5.21 ± 0.36	5.44 ± 0.30	5.33 ± 0.07	5.52 ± 0.13
TC10001608.hg.1	MIR3158-2	6.40 ± 0.39	6.23 ± 0.23	6.35 ± 0.30	6.24 ± 0.13
TC11001721.hg.1	MIR3160-1	6.81 ± 0.41	6.99 ± 0.26	6.78 ± 0.31	7.15 ± 0.21
TC11000398.hg.1	MIR3160-2	7.33 ± 0.33	7.26 ± 0.04	7.35 ± 0.11	7.35 ± 0.18
TC11001828.hg.1	MIR3162	7.08 ± 0.37	6.96 ± 0.27	6.94 ± 0.18	7.02 ± 0.15
TC11002051.hg.1	MIR3165	5.24 ± 0.20	5.62 ± 0.33	5.25 ± 0.33	5.92 ± 0.24
TC11002437.hg.1	MIR3167	3.76 ± 0.18	3.51 ± 0.01	3.53 ± 0.01	3.43 ± 0.08
TC13000706.hg.1	MIR3169	5.87 ± 0.19	5.71 ± 0.09	6.09 ± 0.25	5.71 ± 0.02
TC13000345.hg.1	MIR3170	5.93 ± 0.28	5.87 ± 0.05	5.77 ± 0.29	5.79 ± 0.18
TC14001473.hg.1	MIR3173	5.64 ± 0.06	5.34 ± 0.18	5.73 ± 0.23	5.26 ± 0.07
TC16000016.hg.1	MIR3176	8.62 ± 0.14	8.62 ± 0.14	8.73 ± 0.24	8.75 ± 0.04
TC16000046.hg.1	MIR3177	6.04 ± 0.25	6.00 ± 0.21	5.79 ± 0.24	6.14 ± 0.09
TC16000785.hg.1	MIR3178	8.63 ± 0.36	8.76 ± 0.34	8.56 ± 0.47	8.91 ± 0.07
TC16000182.hg.1	MIR3179-1	5.56 ± 0.11	5.44 ± 0.20	5.44 ± 0.05	5.40 ± 0.14
TC16000196.hg.1	MIR3179-3	5.48 ± 0.06	5.35 ± 0.21	5.34 ± 0.05	5.34 ± 0.17
TC16000910.hg.1	MIR3179-3	5.86 ± 0.07	5.82 ± 0.12	5.68 ± 0.05	5.83 ± 0.11
TC16000183.hg.1	MIR3180-1	8.28 ± 0.53	8.11 ± 0.26	7.87 ± 0.47	8.11 ± 0.07
TC16000205.hg.1	MIR3180-2	7.89 ± 0.25	7.96 ± 0.14	7.90 ± 0.09	8.13 ± 0.09
TC16000887.hg.1	MIR3180-2	7.74 ± 0.22	7.84 ± 0.13	7.79 ± 0.07	8.01 ± 0.09
TC16000902.hg.1	MIR3180-2	7.89 ± 0.25	7.96 ± 0.14	7.90 ± 0.09	8.13 ± 0.09
TC16000188.hg.1	MIR3180-4	6.94 ± 0.01	6.81 ± 0.06	6.84 ± 0.08	7.15 ± 0.07
TC16000895.hg.1	MIR3180-4	9.64 ± 0.31	9.66 ± 0.22	9.45 ± 0.40	9.62 ± 0.03

TC16000775.hg.1	MIR3180-5	9.59 ± 0.35	9.54 ± 0.23	9.36 ± 0.33	9.54 ± 0.06
TC16000649.hg.1	MIR3182	7.14 ± 0.20	7.10 ± 0.24	7.02 ± 0.23	7.25 ± 0.13
TC17000971.hg.1	MIR3183	7.50 ± 0.17	7.54 ± 0.23	7.41 ± 0.35	7.60 ± 0.11
TC17001325.hg.1	MIR3184	7.57 ± 0.22	7.37 ± 0.25	7.28 ± 0.19	7.66 ± 0.15
TC17001650.hg.1	MIR3185	10.47 ± 0.31	10.43 ± 0.18	10.42 ± 0.42	10.41 ± 0.07
TC17001951.hg.1	MIR3186	6.70 ± 0.08	6.77 ± 0.09	6.79 ± 0.16	6.73 ± 0.11
TC19000016.hg.1	MIR3187	7.27 ± 0.24	7.53 ± 0.27	7.34 ± 0.25	7.82 ± 0.19
TC19000354.hg.1	MIR3188	8.03 ± 0.22	7.89 ± 0.13	7.93 ± 0.24	7.77 ± 0.06
TC19000357.hg.1	MIR3189	7.45 ± 0.23	7.58 ± 0.30	7.49 ± 0.18	7.74 ± 0.06
TC19000673.hg.1	MIR3190	8.00 ± 0.24	8.23 ± 0.18	8.07 ± 0.22	8.28 ± 0.05
TC19001662.hg.1	MIR3191	8.37 ± 0.37	8.44 ± 0.15	8.27 ± 0.32	8.41 ± 0.01
TC20000124.hg.1	MIR3192	8.88 ± 0.23	9.04 ± 0.06	8.39 ± 0.21	9.10 ± 0.30
TC20000195.hg.1	MIR3193	7.13 ± 0.30	7.28 ± 0.21	7.18 ± 0.26	7.26 ± 0.14
TC20000943.hg.1	MIR3194	9.53 ± 0.25	9.47 ± 0.23	9.42 ± 0.29	9.57 ± 0.07
TC20000509.hg.1	MIR3196	8.04 ± 0.32	8.19 ± 0.34	7.95 ± 0.30	8.41 ± 0.23
TC21000184.hg.1	MIR3197	9.05 ± 0.33	8.88 ± 0.30	8.98 ± 0.34	8.97 ± 0.08
TC22000477.hg.1	MIR3198-1	8.55 ± 0.14	8.76 ± 0.28	8.38 ± 0.17	8.50 ± 0.19
TC22000623.hg.1	MIR3199-1	4.73 ± 0.14	4.81 ± 0.10	4.81 ± 0.17	4.82 ± 0.04
TC22000184.hg.1	MIR3199-2	4.35 ± 0.08	4.40 ± 0.21	4.33 ± 0.05	4.46 ± 0.15
TC09000961.hg.1	MIR31HG	6.35 ± 0.31	6.31 ± 0.23	6.25 ± 0.15	6.43 ± 0.08
TC09001461.hg.1	MIR32	6.96 ± 0.32	7.07 ± 0.11	6.90 ± 0.49	7.17 ± 0.18
TC22000218.hg.1	MIR3200	6.89 ± 0.18	7.06 ± 0.14	6.95 ± 0.21	7.20 ± 0.06
TC22000405.hg.1	MIR3201	3.63 ± 0.08	3.47 ± 0.08	3.72 ± 0.28	3.49 ± 0.02
TC0X000763.hg.1	MIR3202-1	3.76 ± 0.21	3.57 ± 0.17	3.65 ± 0.15	3.48 ± 0.02
TC0X001523.hg.1	MIR3202-2	3.47 ± 0.01	3.38 ± 0.02	3.52 ± 0.31	3.39 ± 0.06
TC08001037.hg.1	MIR320A	8.32 ± 0.41	8.16 ± 0.30	8.14 ± 0.21	8.20 ± 0.04
TC01001020.hg.1	MIR320B1	4.69 ± 0.28	4.56 ± 0.08	4.51 ± 0.10	4.54 ± 0.10
TC01003882.hg.1	MIR320B2	3.72 ± 0.09	3.66 ± 0.07	3.62 ± 0.06	3.64 ± 0.06
TC18000089.hg.1	MIR320C1	6.00 ± 0.24	5.88 ± 0.12	5.71 ± 0.19	5.94 ± 0.14
TC18000108.hg.1	MIR320C2	5.97 ± 0.42	5.66 ± 0.15	5.85 ± 0.29	5.80 ± 0.07
TC13000594.hg.1	MIR320D1	6.31 ± 0.35	6.01 ± 0.21	6.23 ± 0.17	5.95 ± 0.10

TC0X001436.hg.1	MIR320D2	6.34 ± 0.36	6.09 ± 0.07	6.14 ± 0.11	6.32 ± 0.03
TC19001654.hg.1	MIR320E	9.74 ± 0.28	9.52 ± 0.17	9.72 ± 0.11	9.58 ± 0.18
TC14000721.hg.1	MIR323A	5.97 ± 0.34	6.12 ± 0.27	5.88 ± 0.31	6.30 ± 0.11
TC14000753.hg.1	MIR323B	7.58 ± 0.15	7.78 ± 0.18	7.65 ± 0.19	7.93 ± 0.17
TC17001078.hg.1	MIR324	6.83 ± 0.17	6.85 ± 0.22	6.76 ± 0.18	7.01 ± 0.07
TC0X001172.hg.1	MIR325	4.90 ± 0.09	4.91 ± 0.07	5.00 ± 0.11	5.01 ± 0.20
TC11002093.hg.1	MIR326	9.01 ± 0.19	9.20 ± 0.40	8.93 ± 0.33	9.33 ± 0.10
TC16001182.hg.1	MIR328	8.59 ± 0.30	8.64 ± 0.32	8.43 ± 0.25	8.85 ± 0.21
TC14000723.hg.1	MIR329-1	4.83 ± 0.11	4.82 ± 0.22	4.73 ± 0.17	5.10 ± 0.39
TC14000724.hg.1	MIR329-2	5.20 ± 0.20	5.19 ± 0.06	5.16 ± 0.14	5.27 ± 0.32
TC19001626.hg.1	MIR330	7.23 ± 0.16	7.26 ± 0.24	7.19 ± 0.29	7.27 ± 0.12
TC12000736.hg.1	MIR331	6.29 ± 0.21	5.88 ± 0.29	6.01 ± 0.20	6.16 ± 0.17
TC07000816.hg.1	MIR335	4.73 ± 0.32	4.79 ± 0.24	4.72 ± 0.15	4.81 ± 0.15
TC14000652.hg.1	MIR337	5.80 ± 0.41	5.80 ± 0.27	5.55 ± 0.45	5.95 ± 0.08
TC17001943.hg.1	MIR338	8.13 ± 0.19	8.25 ± 0.06	8.48 ± 0.27	8.24 ± 0.07
TC07001078.hg.1	MIR339	8.06 ± 0.28	7.98 ± 0.25	7.94 ± 0.18	8.07 ± 0.05
TC22000348.hg.1	MIR33A	5.54 ± 0.16	5.53 ± 0.55	5.38 ± 0.49	5.68 ± 0.17
TC17001193.hg.1	MIR33B	8.99 ± 0.15	9.09 ± 0.19	8.98 ± 0.23	9.21 ± 0.02
TC05002143.hg.1	MIR340	6.55 ± 0.24	6.75 ± 0.31	6.51 ± 0.34	6.71 ± 0.26
TC14000631.hg.1	MIR342	8.69 ± 0.14	8.59 ± 0.13	8.60 ± 0.30	8.77 ± 0.07
TC14000635.hg.1	MIR345	9.49 ± 0.32	9.64 ± 0.32	9.54 ± 0.19	9.85 ± 0.07
TC10001478.hg.1	MIR346	9.31 ± 0.28	9.39 ± 0.18	9.33 ± 0.10	9.45 ± 0.17
TC01002181.hg.1	MIR34A	8.39 ± 0.12	8.42 ± 0.22	8.43 ± 0.10	8.52 ± 0.04
TC11000995.hg.1	MIR34C	5.59 ± 0.15	5.55 ± 0.02	5.49 ± 0.06	5.59 ± 0.18
TC15001822.hg.1	MIR3529	5.35 ± 0.36	5.27 ± 0.19	5.33 ± 0.07	5.39 ± 0.06
TC14001549.hg.1	MIR3545	3.86 ± 0.04	3.70 ± 0.15	3.68 ± 0.11	3.63 ± 0.02
TC18000540.hg.1	MIR3591	4.01 ± 0.21	4.03 ± 0.12	3.92 ± 0.15	4.14 ± 0.21
TC07000589.hg.1	MIR3609	4.33 ± 0.17	4.26 ± 0.15	4.24 ± 0.37	4.11 ± 0.09
TC0X001192.hg.1	MIR361	6.62 ± 0.23	6.39 ± 0.22	6.50 ± 0.20	6.39 ± 0.06
TC12001024.hg.1	MIR3612	7.41 ± 0.29	7.21 ± 0.12	7.40 ± 0.11	7.32 ± 0.09
TC13000669.hg.1	MIR3613	4.90 ± 0.48	4.48 ± 0.35	5.06 ± 0.37	4.54 ± 0.32

TC20000369.hg.1	MIR3616	6.88 ± 0.26	6.55 ± 0.37	6.66 ± 0.14	6.76 ± 0.08
TC22000390.hg.1	MIR3619	10.70 ± 0.20	10.52 ± 0.06	10.48 ± 0.24	10.63 ± 0.14
TC0X000283.hg.1	MIR362	6.02 ± 0.39	5.99 ± 0.05	5.82 ± 0.20	5.99 ± 0.15
TC09001760.hg.1	MIR3621	11.02 ± 0.35	11.02 ± 0.23	10.97 ± 0.42	11.07 ± 0.03
TC08000211.hg.1	MIR3622A	9.27 ± 0.15	9.30 ± 0.30	9.20 ± 0.24	9.49 ± 0.13
TC08001082.hg.1	MIR3622B	9.16 ± 0.25	9.26 ± 0.25	9.07 ± 0.17	9.42 ± 0.21
TC0X001381.hg.1	MIR363	4.11 ± 0.18	3.98 ± 0.14	4.13 ± 0.25	4.05 ± 0.07
TC20000331.hg.1	MIR3646	4.39 ± 0.22	4.16 ± 0.04	4.11 ± 0.15	4.20 ± 0.13
TC21000006.hg.1	MIR3648	10.61 ± 0.35	10.63 ± 0.12	10.37 ± 0.26	10.57 ± 0.03
TC12001091.hg.1	MIR3649	6.52 ± 0.34	6.54 ± 0.06	6.55 ± 0.14	6.61 ± 0.10
TC05001290.hg.1	MIR3650	8.23 ± 0.11	8.26 ± 0.19	8.16 ± 0.13	8.38 ± 0.08
TC01000486.hg.1	MIR3659	5.76 ± 0.22	5.80 ± 0.03	5.64 ± 0.34	5.85 ± 0.16
TC16000175.hg.1	MIR365A	7.68 ± 0.17	7.34 ± 0.07	7.52 ± 0.08	7.53 ± 0.17
TC17000366.hg.1	MIR365B	7.23 ± 0.16	7.21 ± 0.11	7.17 ± 0.05	7.22 ± 0.02
TC05001581.hg.1	MIR3660	4.97 ± 0.12	4.95 ± 0.03	5.09 ± 0.04	5.06 ± 0.03
TC05000658.hg.1	MIR3661	8.46 ± 0.06	8.64 ± 0.09	8.66 ± 0.23	8.55 ± 0.23
TC06002134.hg.1	MIR3662	5.20 ± 0.73	5.51 ± 0.26	5.09 ± 0.38	5.32 ± 0.18
TC10001686.hg.1	MIR3663	9.93 ± 0.29	10.17 ± 0.22	10.04 ± 0.20	10.38 ± 0.08
TC11002034.hg.1	MIR3664	4.67 ± 0.30	4.70 ± 0.30	4.75 ± 0.41	4.92 ± 0.13
TC13000747.hg.1	MIR3665	7.56 ± 0.11	7.65 ± 0.15	7.61 ± 0.01	7.63 ± 0.04
TC07000714.hg.1	MIR3666	6.45 ± 0.12	6.64 ± 0.32	6.56 ± 0.15	6.87 ± 0.22
TC06001039.hg.1	MIR3668	3.10 ± 0.09	2.98 ± 0.04	3.07 ± 0.05	2.94 ± 0.07
TC01002745.hg.1	MIR3671	8.54 ± 0.33	8.45 ± 0.38	8.55 ± 0.28	8.33 ± 0.13
TC01000210.hg.1	MIR3675	6.09 ± 0.06	6.07 ± 0.12	6.03 ± 0.07	6.13 ± 0.06
TC17000133.hg.1	MIR3676	8.35 ± 0.31	8.35 ± 0.17	8.06 ± 0.18	8.27 ± 0.08
TC16000068.hg.1	MIR3677	9.66 ± 0.21	9.77 ± 0.37	9.55 ± 0.23	9.98 ± 0.15
TC17000851.hg.1	MIR3678	5.74 ± 0.19	5.96 ± 0.20	5.88 ± 0.14	5.86 ± 0.09
TC02000875.hg.1	MIR3679	8.72 ± 0.14	8.48 ± 0.15	8.47 ± 0.15	8.58 ± 0.15
TC02001847.hg.1	MIR3682	5.09 ± 0.08	4.92 ± 0.36	4.99 ± 0.16	4.68 ± 0.08
TC04000513.hg.1	MIR3684	3.86 ± 0.19	3.64 ± 0.33	3.45 ± 0.32	3.69 ± 0.06
TC12000737.hg.1	MIR3685	5.75 ± 0.21	5.46 ± 0.25	5.52 ± 0.16	5.29 ± 0.06

TC21000007.hg.1	MIR3687	11.10 ± 0.09	11.38 ± 0.42	10.50 ± 0.05	11.18 ± 0.05
TC04001692.hg.1	MIR3688-1	3.39 ± 0.01	3.44 ± 0.03	3.29 ± 0.06	3.32 ± 0.15
TC04000810.hg.1	MIR3688-2	3.39 ± 0.10	3.28 ± 0.01	3.40 ± 0.13	3.25 ± 0.07
TC09001704.hg.1	MIR3689A	9.00 ± 0.78	9.15 ± 0.05	9.22 ± 0.13	9.65 ± 0.24
TC09001707.hg.1	MIR3689B	8.98 ± 0.75	9.25 ± 0.02	9.18 ± 0.20	9.72 ± 0.29
TC09001705.hg.1	MIR3689C	8.28 ± 0.71	8.58 ± 0.14	8.54 ± 0.06	9.05 ± 0.26
TC09001706.hg.1	MIR3689D1	8.56 ± 0.30	8.28 ± 0.12	8.51 ± 0.07	8.47 ± 0.12
TC09001708.hg.1	MIR3689D2	8.17 ± 0.32	8.17 ± 0.09	8.36 ± 0.09	8.40 ± 0.12
TC09001709.hg.1	MIR3689F	9.06 ± 0.51	9.21 ± 0.14	9.06 ± 0.11	9.51 ± 0.14
TC14000761.hg.1	MIR369	3.57 ± 0.08	3.51 ± 0.02	3.47 ± 0.12	3.41 ± 0.01
TC0X000007.hg.1	MIR3690	7.11 ± 0.36	7.11 ± 0.37	7.97 ± 0.67	7.19 ± 0.46
TC0Y000007.hg.1	MIR3690	7.11 ± 0.36	7.11 ± 0.37	7.97 ± 0.67	7.19 ± 0.46
TC06001243.hg.1	MIR3691	7.47 ± 0.25	7.33 ± 0.23	7.30 ± 0.22	7.42 ± 0.10
TC06001125.hg.1	MIR3692	5.47 ± 0.24	5.39 ± 0.13	5.37 ± 0.16	5.58 ± 0.19
TC14000661.hg.1	MIR370	7.00 ± 0.16	6.91 ± 0.07	7.08 ± 0.19	7.11 ± 0.11
TC19000861.hg.1	MIR371A	6.68 ± 0.24	6.39 ± 0.06	6.67 ± 0.14	6.55 ± 0.06
TC19001825.hg.1	MIR371B	7.75 ± 0.15	7.44 ± 0.26	7.85 ± 0.15	7.44 ± 0.16
TC19000862.hg.1	MIR373	6.84 ± 0.05	6.78 ± 0.27	6.87 ± 0.25	6.88 ± 0.08
TC0X001161.hg.1	MIR374A	3.28 ± 0.15	2.97 ± 0.09	3.23 ± 0.08	3.03 ± 0.06
TC0X001159.hg.1	MIR374B	3.80 ± 0.07	3.64 ± 0.08	3.84 ± 0.28	3.55 ± 0.03
TC0X000408.hg.1	MIR374C	3.72 ± 0.12	3.54 ± 0.10	3.60 ± 0.26	3.49 ± 0.29
TC02002784.hg.1	MIR375	7.57 ± 0.33	7.59 ± 0.13	7.62 ± 0.15	7.46 ± 0.01
TC14000737.hg.1	MIR376A1	3.42 ± 0.05	3.09 ± 0.11	3.20 ± 0.07	3.02 ± 0.03
TC14000734.hg.1	MIR376A2	4.31 ± 0.06	4.44 ± 0.08	4.36 ± 0.10	4.41 ± 0.05
TC14000736.hg.1	MIR376B	3.53 ± 0.21	3.41 ± 0.19	3.44 ± 0.11	3.40 ± 0.04
TC14000732.hg.1	MIR376C	3.49 ± 0.09	3.10 ± 0.13	3.18 ± 0.13	3.00 ± 0.08
TC14000757.hg.1	MIR377	4.54 ± 0.26	4.35 ± 0.11	4.30 ± 0.13	4.35 ± 0.04
TC05000824.hg.1	MIR378A	5.86 ± 0.24	6.05 ± 0.21	6.04 ± 0.13	6.15 ± 0.14
TC10001766.hg.1	MIR378C	7.11 ± 0.40	7.16 ± 0.27	6.93 ± 0.31	7.40 ± 0.22
TC04000999.hg.1	MIR378D1	5.55 ± 0.11	5.67 ± 0.08	5.40 ± 0.20	5.74 ± 0.08
TC08001429.hg.1	MIR378D2	5.46 ± 0.23	5.48 ± 0.11	5.34 ± 0.11	5.58 ± 0.02

TC05000930.hg.1	MIR378E	8.74 ± 0.24	8.85 ± 0.21	9.05 ± 0.35	8.92 ± 0.16
TC01000304.hg.1	MIR378F	5.10 ± 0.18	5.34 ± 0.18	5.25 ± 0.36	5.29 ± 0.16
TC14000715.hg.1	MIR379	3.71 ± 0.11	3.51 ± 0.09	3.65 ± 0.21	3.55 ± 0.16
TC14000719.hg.1	MIR380	4.54 ± 0.33	4.49 ± 0.02	4.58 ± 0.10	4.63 ± 0.15
TC14000742.hg.1	MIR381	4.86 ± 0.07	4.85 ± 0.09	4.86 ± 0.09	4.84 ± 0.14
TC14000741.hg.1	MIR381HG	7.13 ± 0.25	7.29 ± 0.26	7.13 ± 0.26	7.42 ± 0.01
TC14000749.hg.1	MIR382	4.38 ± 0.21	4.32 ± 0.10	4.39 ± 0.18	4.39 ± 0.13
TC08001005.hg.1	MIR383	7.76 ± 0.09	7.78 ± 0.18	7.57 ± 0.02	7.78 ± 0.11
TC0X001171.hg.1	MIR384	3.10 ± 0.04	2.99 ± 0.04	3.03 ± 0.06	2.97 ± 0.02
TC07002039.hg.1	MIR3907	9.88 ± 0.33	9.90 ± 0.13	9.79 ± 0.29	9.99 ± 0.09
TC12000987.hg.1	MIR3908	3.87 ± 0.13	3.64 ± 0.06	3.63 ± 0.04	3.63 ± 0.07
TC22000258.hg.1	MIR3909	8.36 ± 0.29	8.41 ± 0.12	8.24 ± 0.18	8.47 ± 0.07
TC09000432.hg.1	MIR3910-1	4.61 ± 0.07	4.70 ± 0.03	4.67 ± 0.14	4.70 ± 0.12
TC09001327.hg.1	MIR3910-2	3.94 ± 0.23	3.95 ± 0.18	3.95 ± 0.16	4.06 ± 0.11
TC09001605.hg.1	MIR3911	6.77 ± 0.25	6.88 ± 0.22	6.92 ± 0.21	6.94 ± 0.15
TC05002057.hg.1	MIR3912	3.36 ± 0.05	3.27 ± 0.04	3.31 ± 0.08	3.23 ± 0.04
TC12001710.hg.1	MIR3913-1	4.57 ± 0.04	4.46 ± 0.26	4.50 ± 0.30	4.54 ± 0.05
TC12000615.hg.1	MIR3913-2	4.77 ± 0.18	5.10 ± 0.31	4.86 ± 0.38	5.17 ± 0.12
TC07001481.hg.1	MIR3914-1	3.43 ± 0.02	3.22 ± 0.03	3.34 ± 0.20	3.27 ± 0.03
TC07000426.hg.1	MIR3914-2	3.18 ± 0.06	3.09 ± 0.04	3.11 ± 0.09	3.18 ± 0.05
TC01004068.hg.1	MIR3916	8.94 ± 0.52	9.08 ± 0.06	8.97 ± 0.40	9.13 ± 0.25
TC06002264.hg.1	MIR3918	8.51 ± 0.28	8.54 ± 0.10	8.34 ± 0.25	8.63 ± 0.08
TC03000869.hg.1	MIR3919	4.04 ± 0.12	3.95 ± 0.11	3.99 ± 0.14	3.90 ± 0.11
TC11002222.hg.1	MIR3920	3.43 ± 0.03	3.48 ± 0.09	3.45 ± 0.16	3.43 ± 0.06
TC03001609.hg.1	MIR3921	3.85 ± 0.35	3.35 ± 0.10	3.56 ± 0.14	3.40 ± 0.01
TC12000813.hg.1	MIR3922	7.95 ± 0.26	8.13 ± 0.07	8.11 ± 0.18	8.36 ± 0.17
TC10001311.hg.1	MIR3924	3.61 ± 0.05	3.43 ± 0.10	3.52 ± 0.04	3.35 ± 0.07
TC06001676.hg.1	MIR3925	5.99 ± 0.41	6.27 ± 0.15	6.12 ± 0.25	6.46 ± 0.30
TC08000998.hg.1	MIR3926-1	7.59 ± 0.23	7.45 ± 0.16	7.68 ± 0.13	7.60 ± 0.02
TC08000107.hg.1	MIR3926-2	6.67 ± 0.23	6.75 ± 0.12	6.84 ± 0.17	6.86 ± 0.15
TC22000663.hg.1	MIR3928	8.10 ± 0.29	7.89 ± 0.15	7.91 ± 0.31	7.93 ± 0.04

TC16000462.hg.1	MIR3935	6.59 ± 0.08	6.60 ± 0.46	6.40 ± 0.40	6.67 ± 0.12
TC05001765.hg.1	MIR3936	6.45 ± 0.37	6.56 ± 0.01	6.49 ± 0.11	6.69 ± 0.21
TC03001486.hg.1	MIR3938	3.82 ± 0.19	3.61 ± 0.14	3.76 ± 0.10	3.66 ± 0.02
TC06002312.hg.1	MIR3939	7.76 ± 0.31	7.77 ± 0.07	8.01 ± 0.25	7.96 ± 0.03
TC10000887.hg.1	MIR3941	5.02 ± 0.29	5.25 ± 0.09	5.12 ± 0.17	5.14 ± 0.24
TC15001206.hg.1	MIR3942	5.23 ± 0.42	5.35 ± 0.18	5.11 ± 0.66	5.27 ± 0.32
TC07000263.hg.1	MIR3943	7.25 ± 0.28	7.23 ± 0.12	7.16 ± 0.22	7.18 ± 0.08
TC10001791.hg.1	MIR3944	8.26 ± 0.21	8.32 ± 0.17	8.25 ± 0.28	8.36 ± 0.04
TC04001812.hg.1	MIR3945	10.22 ± 0.14	10.50 ± 0.17	10.45 ± 0.17	10.14 ± 0.18
TC11000344.hg.1	MIR3973	6.53 ± 0.34	6.56 ± 0.28	6.52 ± 0.19	6.69 ± 0.19
TC12000214.hg.1	MIR3974	4.35 ± 0.06	4.12 ± 0.19	4.27 ± 0.28	4.22 ± 0.15
TC18000146.hg.1	MIR3975	5.70 ± 0.54	6.29 ± 0.35	5.79 ± 0.35	6.16 ± 0.17
TC18000029.hg.1	MIR3976	5.69 ± 0.28	5.56 ± 0.03	5.48 ± 0.07	5.65 ± 0.10
TC05000410.hg.1	MIR3977	3.47 ± 0.14	3.35 ± 0.05	3.49 ± 0.03	3.35 ± 0.07
TC0X000540.hg.1	MIR3978	7.75 ± 0.42	8.04 ± 0.10	8.04 ± 0.26	7.88 ± 0.10
TC14000759.hg.1	MIR409	6.22 ± 0.44	5.95 ± 0.07	6.43 ± 0.31	6.23 ± 0.05
TC14000762.hg.1	MIR410	3.97 ± 0.15	3.80 ± 0.15	3.82 ± 0.14	3.67 ± 0.09
TC14000716.hg.1	MIR411	4.06 ± 0.24	4.02 ± 0.39	3.96 ± 0.18	3.94 ± 0.15
TC14000760.hg.1	MIR412	7.63 ± 0.06	7.54 ± 0.19	7.71 ± 0.20	7.71 ± 0.12
TC0X001158.hg.1	MIR421	5.88 ± 0.52	5.59 ± 0.22	5.88 ± 0.46	5.46 ± 0.05
TC15001545.hg.1	MIR422A	6.96 ± 0.33	7.05 ± 0.26	6.78 ± 0.19	7.25 ± 0.17
TC17000347.hg.1	MIR423	8.38 ± 0.29	8.60 ± 0.12	8.51 ± 0.35	8.63 ± 0.13
TC0X001392.hg.1	MIR424	7.18 ± 0.19	7.08 ± 0.19	7.17 ± 0.09	7.20 ± 0.08
TC03001395.hg.1	MIR425	6.97 ± 0.18	6.79 ± 0.18	6.83 ± 0.07	6.81 ± 0.11
TC01000068.hg.1	MIR4251	4.53 ± 0.43	4.60 ± 0.17	4.42 ± 0.19	4.86 ± 0.18
TC01002151.hg.1	MIR4252	8.63 ± 0.06	8.57 ± 0.14	8.58 ± 0.14	8.79 ± 0.15
TC01002332.hg.1	MIR4253	6.97 ± 0.16	7.04 ± 0.31	6.97 ± 0.22	7.23 ± 0.13
TC01002458.hg.1	MIR4254	7.64 ± 0.30	7.84 ± 0.43	7.62 ± 0.25	7.97 ± 0.12
TC01000472.hg.1	MIR4255	5.48 ± 0.10	5.41 ± 0.14	5.43 ± 0.11	5.42 ± 0.10
TC01002995.hg.1	MIR4256	4.75 ± 0.24	4.91 ± 0.27	4.73 ± 0.18	4.88 ± 0.08
TC01001191.hg.1	MIR4257	6.19 ± 0.28	5.87 ± 0.21	6.25 ± 0.03	5.81 ± 0.10

TC01001281.hg.1	MIR4258	7.59 ± 0.18	7.64 ± 0.15	7.67 ± 0.05	7.72 ± 0.02
TC02001568.hg.1	MIR4261	6.60 ± 0.40	6.52 ± 0.39	6.69 ± 0.19	6.76 ± 0.09
TC02001585.hg.1	MIR4262	9.57 ± 0.12	9.64 ± 0.14	9.65 ± 0.26	9.70 ± 0.14
TC02000182.hg.1	MIR4263	6.82 ± 0.10	6.98 ± 0.24	6.85 ± 0.24	7.05 ± 0.21
TC02002022.hg.1	MIR4264	4.60 ± 0.39	4.35 ± 0.00	4.24 ± 0.26	4.47 ± 0.09
TC02002185.hg.1	MIR4265	6.58 ± 0.30	6.76 ± 0.14	6.58 ± 0.11	6.78 ± 0.10
TC02002186.hg.1	MIR4266	6.58 ± 0.55	6.46 ± 0.48	6.23 ± 0.73	6.67 ± 0.17
TC02002193.hg.1	MIR4267	9.60 ± 0.15	9.44 ± 0.18	9.49 ± 0.26	9.67 ± 0.15
TC02002806.hg.1	MIR4268	5.61 ± 0.23	5.83 ± 0.34	5.61 ± 0.14	6.03 ± 0.21
TC02001459.hg.1	MIR4269	7.03 ± 0.11	7.05 ± 0.19	6.88 ± 0.11	7.11 ± 0.04
TC03001212.hg.1	MIR4270	7.97 ± 0.19	7.87 ± 0.21	7.73 ± 0.26	7.97 ± 0.16
TC03000292.hg.1	MIR4271	7.79 ± 0.33	7.48 ± 0.16	7.59 ± 0.08	7.43 ± 0.16
TC03000408.hg.1	MIR4272	3.18 ± 0.14	2.99 ± 0.08	3.25 ± 0.34	3.03 ± 0.07
TC03000443.hg.1	MIR4273	5.05 ± 0.03	5.08 ± 0.23	5.12 ± 0.36	5.15 ± 0.15
TCUn_gl000222000001.hg.1	MIR4273	5.05 ± 0.03	5.08 ± 0.23	5.12 ± 0.36	5.15 ± 0.15
TC04000082.hg.1	MIR4274	7.47 ± 0.18	7.47 ± 0.25	7.49 ± 0.24	7.61 ± 0.14
TC04000189.hg.1	MIR4275	6.26 ± 0.23	6.36 ± 0.16	6.09 ± 0.18	6.45 ± 0.33
TC04000860.hg.1	MIR4276	4.81 ± 0.17	4.74 ± 0.02	4.92 ± 0.14	4.90 ± 0.02
TC05001114.hg.1	MIR4277	7.69 ± 0.09	8.00 ± 0.25	7.78 ± 0.19	8.02 ± 0.03
TC05001140.hg.1	MIR4278	4.96 ± 0.10	4.72 ± 0.19	4.97 ± 0.08	4.74 ± 0.09
TC05001230.hg.1	MIR4279	7.48 ± 0.20	7.48 ± 0.36	7.37 ± 0.14	7.66 ± 0.14
TC05001567.hg.1	MIR4280	5.46 ± 0.12	5.29 ± 0.08	5.43 ± 0.16	5.38 ± 0.26
TC05002097.hg.1	MIR4281	10.64 ± 0.18	10.48 ± 0.10	10.63 ± 0.07	10.56 ± 0.06
TC06001867.hg.1	MIR4282	3.66 ± 0.19	3.68 ± 0.06	3.55 ± 0.07	3.52 ± 0.06
TC07001428.hg.1	MIR4283-1	7.44 ± 0.42	7.64 ± 0.35	7.37 ± 0.19	7.86 ± 0.16
TC07000444.hg.1	MIR4284	7.22 ± 0.32	6.92 ± 0.13	7.06 ± 0.11	6.99 ± 0.15
TC07000653.hg.1	MIR4285	9.92 ± 0.47	10.11 ± 0.31	10.01 ± 0.32	10.20 ± 0.08
TC08001088.hg.1	MIR4287	7.36 ± 0.43	6.82 ± 0.11	6.90 ± 0.28	6.64 ± 0.09
TC08001092.hg.1	MIR4288	8.17 ± 0.21	8.27 ± 0.17	8.09 ± 0.20	8.31 ± 0.04
TC09001310.hg.1	MIR4289	7.64 ± 0.27	7.84 ± 0.29	7.57 ± 0.31	8.06 ± 0.14
TC01000029.hg.1	MIR429	5.69 ± 0.14	5.50 ± 0.09	5.64 ± 0.28	5.46 ± 0.08

TC09001316.hg.1	MIR4290	7.51 ± 0.33	7.55 ± 0.17	7.46 ± 0.37	7.55 ± 0.16
TC09000451.hg.1	MIR4291	9.46 ± 0.17	9.38 ± 0.13	9.39 ± 0.20	9.52 ± 0.05
TC09000823.hg.1	MIR4292	8.40 ± 0.26	8.27 ± 0.23	8.23 ± 0.22	8.38 ± 0.04
TC10001270.hg.1	MIR4294	8.21 ± 0.50	8.33 ± 0.23	8.24 ± 0.19	8.60 ± 0.12
TC10000815.hg.1	MIR4295	5.52 ± 0.60	5.88 ± 0.48	6.18 ± 0.45	5.53 ± 0.18
TC10001733.hg.1	MIR4296	9.46 ± 0.24	9.52 ± 0.23	9.39 ± 0.11	9.68 ± 0.14
TC10001762.hg.1	MIR4297	8.58 ± 0.21	8.63 ± 0.14	8.52 ± 0.20	8.71 ± 0.19
TC11001269.hg.1	MIR4298	7.03 ± 0.25	7.06 ± 0.18	7.14 ± 0.25	7.20 ± 0.13
TC11001424.hg.1	MIR4299	6.46 ± 0.23	6.47 ± 0.17	6.23 ± 0.32	6.52 ± 0.06
TC11002147.hg.1	MIR4300	6.46 ± 0.23	6.38 ± 0.45	6.38 ± 0.51	6.64 ± 0.15
TC11002303.hg.1	MIR4301	6.70 ± 0.08	6.59 ± 0.08	6.66 ± 0.11	6.56 ± 0.10
TC12001321.hg.1	MIR4302	7.72 ± 0.38	7.82 ± 0.25	7.62 ± 0.32	7.96 ± 0.10
TC12001854.hg.1	MIR4303	5.84 ± 0.26	5.66 ± 0.16	5.63 ± 0.20	5.69 ± 0.05
TC12002083.hg.1	MIR4304	8.16 ± 0.13	8.25 ± 0.25	8.15 ± 0.03	8.35 ± 0.21
TC13000588.hg.1	MIR4305	4.83 ± 0.10	4.80 ± 0.07	4.87 ± 0.13	4.86 ± 0.04
TC13000359.hg.1	MIR4306	5.56 ± 0.17	5.81 ± 0.21	5.58 ± 0.28	5.75 ± 0.19
TC14000177.hg.1	MIR4307	3.73 ± 0.12	3.43 ± 0.05	3.74 ± 0.13	3.53 ± 0.12
TC14001153.hg.1	MIR4308	4.48 ± 0.07	4.64 ± 0.11	4.52 ± 0.15	4.56 ± 0.08
TC14000782.hg.1	MIR4309	7.80 ± 0.21	7.97 ± 0.29	7.85 ± 0.13	8.08 ± 0.08
TC14000654.hg.1	MIR431	7.35 ± 0.04	7.10 ± 0.20	7.15 ± 0.35	7.24 ± 0.05
TC15001256.hg.1	MIR4310	6.50 ± 0.14	6.65 ± 0.26	6.45 ± 0.09	6.79 ± 0.06
TC15000610.hg.1	MIR4311	7.69 ± 0.34	7.81 ± 0.24	7.63 ± 0.05	8.13 ± 0.26
TC15001595.hg.1	MIR4312	7.46 ± 0.19	7.45 ± 0.41	7.57 ± 0.16	7.75 ± 0.14
TC15001672.hg.1	MIR4313	10.09 ± 0.31	10.02 ± 0.26	9.97 ± 0.34	10.12 ± 0.07
TC17000127.hg.1	MIR4314	7.78 ± 0.25	7.82 ± 0.22	7.81 ± 0.32	7.97 ± 0.09
TC17001910.hg.1	MIR4316	8.68 ± 0.29	8.86 ± 0.44	8.62 ± 0.30	9.14 ± 0.17
TC18000306.hg.1	MIR4317	3.91 ± 0.23	3.66 ± 0.10	3.72 ± 0.11	3.79 ± 0.09
TC18000152.hg.1	MIR4318	4.61 ± 0.11	4.41 ± 0.15	4.55 ± 0.13	4.42 ± 0.08
TC18000474.hg.1	MIR4319	6.18 ± 0.27	6.18 ± 0.17	6.08 ± 0.18	6.09 ± 0.08
TC14000657.hg.1	MIR432	6.64 ± 0.23	6.47 ± 0.23	6.30 ± 0.27	6.54 ± 0.05
TC18000510.hg.1	MIR4320	4.80 ± 0.49	5.06 ± 0.06	4.76 ± 0.09	5.13 ± 0.26

TC19000172.hg.1	MIR4322	10.22 ± 0.33	10.09 ± 0.23	10.03 ± 0.25	10.05 ± 0.06
TC19001566.hg.1	MIR4323	10.33 ± 0.45	10.34 ± 0.34	10.23 ± 0.43	10.53 ± 0.11
TC19001711.hg.1	MIR4324	7.19 ± 0.29	6.54 ± 0.31	7.04 ± 0.11	6.56 ± 0.23
TC20000973.hg.1	MIR4325	5.26 ± 0.10	5.19 ± 0.13	5.28 ± 0.02	5.07 ± 0.07
TC20000512.hg.1	MIR4326	8.69 ± 0.09	8.68 ± 0.02	8.73 ± 0.06	8.58 ± 0.08
TC21000370.hg.1	MIR4327	5.93 ± 0.29	5.75 ± 0.38	5.69 ± 0.34	5.84 ± 0.16
TC0X001179.hg.1	MIR4328	5.15 ± 0.28	5.07 ± 0.41	5.14 ± 0.10	5.30 ± 0.18
TC14000655.hg.1	MIR433	6.35 ± 0.21	6.37 ± 0.22	6.19 ± 0.30	6.50 ± 0.22
TC0X000728.hg.1	MIR4330	6.12 ± 0.24	5.88 ± 0.19	5.97 ± 0.16	5.93 ± 0.13
TC01000082.hg.1	MIR4417	9.22 ± 0.21	9.23 ± 0.27	9.25 ± 0.28	9.45 ± 0.22
TC01002442.hg.1	MIR4420	8.03 ± 0.17	8.44 ± 0.14	8.03 ± 0.13	8.48 ± 0.28
TC01000678.hg.1	MIR4422	5.64 ± 0.27	5.62 ± 0.52	5.50 ± 0.32	5.69 ± 0.17
TC01000815.hg.1	MIR4423	4.00 ± 0.23	4.16 ± 0.08	4.00 ± 0.09	4.25 ± 0.26
TC01001542.hg.1	MIR4424	3.30 ± 0.09	3.06 ± 0.06	3.24 ± 0.09	3.01 ± 0.05
TC01001929.hg.1	MIR4427	3.36 ± 0.07	3.33 ± 0.12	3.28 ± 0.19	3.48 ± 0.20
TC02001580.hg.1	MIR4429	7.53 ± 0.30	7.63 ± 0.20	7.52 ± 0.26	7.77 ± 0.09
TC02001884.hg.1	MIR4432	5.48 ± 0.54	5.11 ± 0.21	5.20 ± 0.03	5.06 ± 0.08
TC02000369.hg.1	MIR4434	6.00 ± 0.96	5.86 ± 0.93	6.43 ± 0.66	5.83 ± 0.96
TC02000536.hg.1	MIR4435-1	7.43 ± 0.15	7.20 ± 0.02	7.42 ± 0.07	7.35 ± 0.20
TC02000549.hg.1	MIR4436A	6.99 ± 0.04	7.11 ± 0.20	7.12 ± 0.01	7.27 ± 0.22
TC02002586.hg.1	MIR4437	8.47 ± 0.37	8.05 ± 0.08	8.24 ± 0.24	8.10 ± 0.08
TC02002828.hg.1	MIR4439	4.08 ± 0.53	3.77 ± 0.18	3.82 ± 0.11	3.52 ± 0.17
TC02002923.hg.1	MIR4440	9.79 ± 0.25	9.79 ± 0.05	9.82 ± 0.07	9.89 ± 0.29
TC02002924.hg.1	MIR4441	8.80 ± 0.24	8.70 ± 0.16	9.19 ± 0.56	8.84 ± 0.19
TC03000275.hg.1	MIR4443	4.91 ± 0.24	5.08 ± 0.51	4.97 ± 0.25	5.20 ± 0.15
TC03000436.hg.1	MIR4444-1	5.47 ± 0.25	5.29 ± 0.13	5.56 ± 0.33	5.30 ± 0.25
TC03000581.hg.1	MIR4446	7.23 ± 0.19	7.45 ± 0.12	7.33 ± 0.19	7.50 ± 0.26
TC04000298.hg.1	MIR4449	10.85 ± 0.35	11.09 ± 0.19	10.59 ± 0.50	10.88 ± 0.19
TC04000428.hg.1	MIR4450	8.31 ± 0.39	8.37 ± 0.40	8.20 ± 0.36	8.69 ± 0.16
TC04000467.hg.1	MIR4451	7.56 ± 0.13	7.62 ± 0.04	7.76 ± 0.31	7.56 ± 0.17
TC05001142.hg.1	MIR4454	10.65 ± 0.72	10.77 ± 0.29	10.86 ± 0.62	11.09 ± 0.20

TC05002055.hg.1	MIR4454	7.63 ± 0.42	7.58 ± 0.05	7.64 ± 0.14	7.82 ± 0.21
TC11001512.hg.1	MIR4454	6.31 ± 0.31	6.28 ± 0.07	6.36 ± 0.11	6.48 ± 0.13
TC05001097.hg.1	MIR4456	6.87 ± 0.19	7.15 ± 0.23	6.83 ± 0.09	7.29 ± 0.33
TC05001106.hg.1	MIR4457	4.45 ± 0.41	4.77 ± 0.26	4.68 ± 0.19	4.73 ± 0.16
TC05000060.hg.1	MIR4458	6.78 ± 0.24	6.78 ± 0.07	6.78 ± 0.09	6.86 ± 0.03
TC05001754.hg.1	MIR4460	4.92 ± 0.07	4.66 ± 0.08	4.85 ± 0.10	4.68 ± 0.06
TC05000673.hg.1	MIR4461	10.52 ± 0.78	9.28 ± 0.47	10.28 ± 0.46	9.29 ± 0.21
TC06001686.hg.1	MIR4462	7.09 ± 0.16	6.93 ± 0.07	7.12 ± 0.16	6.97 ± 0.05
TC06000805.hg.1	MIR4464	3.55 ± 0.17	3.43 ± 0.08	3.50 ± 0.17	3.34 ± 0.07
TC06001040.hg.1	MIR4465	5.03 ± 0.21	4.94 ± 0.26	5.03 ± 0.27	4.99 ± 0.32
TC06002254.hg.1	MIR4466	13.00 ± 0.49	13.34 ± 0.32	12.67 ± 0.72	13.40 ± 0.03
TC07000863.hg.1	MIR4468	8.24 ± 0.23	8.31 ± 0.34	8.12 ± 0.33	8.46 ± 0.07
TC08000416.hg.1	MIR4470	6.55 ± 0.43	6.54 ± 0.38	6.58 ± 0.22	6.70 ± 0.11
TC08000617.hg.1	MIR4471	3.46 ± 0.30	3.07 ± 0.04	3.21 ± 0.03	3.06 ± 0.05
TC08000803.hg.1	MIR4472-1	7.32 ± 0.30	7.00 ± 0.17	7.29 ± 0.04	7.00 ± 0.03
TC12002007.hg.1	MIR4472-2	11.94 ± 0.46	12.07 ± 0.14	11.92 ± 0.19	12.32 ± 0.20
TC09000942.hg.1	MIR4473	6.19 ± 0.33	6.60 ± 0.44	5.96 ± 0.51	6.35 ± 0.30
TC09000943.hg.1	MIR4474	4.36 ± 0.27	4.49 ± 0.17	4.33 ± 0.10	4.32 ± 0.20
TC09001071.hg.1	MIR4475	5.51 ± 0.05	5.58 ± 0.11	5.36 ± 0.04	5.66 ± 0.16
TC09001074.hg.1	MIR4476	7.83 ± 0.18	7.87 ± 0.34	8.08 ± 0.03	8.06 ± 0.14
TC09001553.hg.1	MIR4478	8.14 ± 0.40	7.82 ± 0.10	8.00 ± 0.33	7.65 ± 0.02
TC09000827.hg.1	MIR4479	9.43 ± 0.56	9.40 ± 0.27	9.31 ± 0.53	9.51 ± 0.07
TC0X000563.hg.1	MIR448	6.88 ± 0.15	6.77 ± 0.24	6.75 ± 0.15	6.93 ± 0.16
TC10000096.hg.1	MIR4480	6.81 ± 0.13	6.89 ± 0.12	6.70 ± 0.32	7.04 ± 0.26
TC10001640.hg.1	MIR4482-1	7.70 ± 0.22	7.93 ± 0.02	7.68 ± 0.29	8.12 ± 0.20
TC10001672.hg.1	MIR4483	4.03 ± 0.14	3.85 ± 0.18	4.07 ± 0.27	3.76 ± 0.02
TC10000914.hg.1	MIR4484	3.70 ± 0.15	3.59 ± 0.11	3.74 ± 0.13	3.51 ± 0.08
TC11000254.hg.1	MIR4486	7.35 ± 0.19	7.47 ± 0.18	7.35 ± 0.19	7.69 ± 0.10
TC11000534.hg.1	MIR4488	7.93 ± 0.48	7.87 ± 0.31	7.85 ± 0.34	7.99 ± 0.07
TC11000638.hg.1	MIR4489	9.41 ± 0.19	9.46 ± 0.20	9.55 ± 0.14	9.66 ± 0.09
TC11002186.hg.1	MIR4490	3.83 ± 0.27	3.73 ± 0.16	3.85 ± 0.02	3.76 ± 0.05

TC11000991.hg.1	MIR4491	4.07 ± 0.03	3.83 ± 0.11	4.15 ± 0.12	3.95 ± 0.08
TC11001074.hg.1	MIR4492	8.42 ± 0.30	8.13 ± 0.10	8.31 ± 0.20	8.11 ± 0.06
TC11002395.hg.1	MIR4493	5.71 ± 0.13	5.26 ± 0.16	5.33 ± 0.16	5.48 ± 0.20
TC12000851.hg.1	MIR4497	8.04 ± 0.51	7.85 ± 0.26	7.80 ± 0.50	7.87 ± 0.05
TC12002035.hg.1	MIR4498	7.22 ± 0.30	7.31 ± 0.34	7.14 ± 0.22	7.37 ± 0.12
TC13000459.hg.1	MIR4499	4.91 ± 0.21	4.89 ± 0.04	4.64 ± 0.08	4.93 ± 0.07
TC05001356.hg.1	MIR449A	6.16 ± 0.18	6.18 ± 0.05	6.05 ± 0.18	6.18 ± 0.06
TC05001357.hg.1	MIR449B	8.64 ± 0.12	8.87 ± 0.21	8.64 ± 0.25	8.89 ± 0.05
TC05001358.hg.1	MIR449C	5.68 ± 0.11	5.83 ± 0.27	5.69 ± 0.17	5.93 ± 0.24
TC13000772.hg.1	MIR4500	4.29 ± 0.05	4.04 ± 0.18	4.18 ± 0.14	4.12 ± 0.21
TC13000771.hg.1	MIR4500HG	4.65 ± 0.07	4.51 ± 0.03	4.65 ± 0.07	4.60 ± 0.13
TC14001052.hg.1	MIR4503	3.33 ± 0.07	3.22 ± 0.12	3.22 ± 0.10	3.11 ± 0.06
TC14000449.hg.1	MIR4505	7.10 ± 0.38	7.19 ± 0.29	6.97 ± 0.31	7.35 ± 0.03
TC15001068.hg.1	MIR4508	8.64 ± 0.48	8.55 ± 0.23	8.52 ± 0.34	8.60 ± 0.13
TC15001043.hg.1	MIR4509-1	4.16 ± 0.15	4.01 ± 0.09	4.12 ± 0.15	4.11 ± 0.15
TC0X001389.hg.1	MIR450A1	3.31 ± 0.16	3.13 ± 0.09	3.19 ± 0.13	3.12 ± 0.04
TC0X001390.hg.1	MIR450A2	3.93 ± 0.19	3.69 ± 0.11	3.85 ± 0.11	3.64 ± 0.18
TC0X001388.hg.1	MIR450B	3.97 ± 0.19	3.67 ± 0.09	3.57 ± 0.10	3.62 ± 0.21
TC15000250.hg.1	MIR4510	4.01 ± 0.37	3.56 ± 0.06	3.64 ± 0.16	3.65 ± 0.23
TC15001573.hg.1	MIR4511	5.50 ± 0.21	5.40 ± 0.11	5.55 ± 0.14	5.29 ± 0.06
TC15001648.hg.1	MIR4513	8.24 ± 0.42	8.15 ± 0.13	8.00 ± 0.40	8.17 ± 0.08
TC15001729.hg.1	MIR4514	6.66 ± 0.40	6.70 ± 0.22	6.76 ± 0.02	6.72 ± 0.05
TC15000795.hg.1	MIR4515	8.48 ± 0.36	8.28 ± 0.34	8.30 ± 0.21	8.41 ± 0.09
TC16000061.hg.1	MIR4516	10.46 ± 0.34	10.62 ± 0.32	10.34 ± 0.27	10.83 ± 0.11
TC16000350.hg.1	MIR4518	8.92 ± 0.08	8.76 ± 0.21	8.88 ± 0.12	8.81 ± 0.05
TC16001045.hg.1	MIR4519	7.97 ± 0.25	8.12 ± 0.18	7.87 ± 0.31	8.10 ± 0.02
TC17001308.hg.1	MIR451A	3.79 ± 0.16	3.45 ± 0.12	3.55 ± 0.12	3.37 ± 0.09
TC17000333.hg.1	MIR451B	4.09 ± 0.17	3.87 ± 0.14	4.06 ± 0.05	4.00 ± 0.17
TC17001067.hg.1	MIR4520A	5.04 ± 0.11	4.92 ± 0.07	4.92 ± 0.04	4.91 ± 0.13
TC17000075.hg.1	MIR4520B	4.36 ± 0.17	4.22 ± 0.07	4.20 ± 0.13	4.25 ± 0.08
TC17000132.hg.1	MIR4521	8.61 ± 0.23	8.83 ± 0.45	8.14 ± 0.16	8.59 ± 0.10

TC17001273.hg.1	MIR4522	9.86 ± 0.33	9.79 ± 0.14	9.69 ± 0.30	9.87 ± 0.02
TC17000340.hg.1	MIR4523	9.55 ± 0.24	9.53 ± 0.17	9.59 ± 0.09	9.63 ± 0.07
TC17001828.hg.1	MIR4524A	5.05 ± 0.07	4.87 ± 0.03	4.92 ± 0.16	5.18 ± 0.35
TC18000073.hg.1	MIR4526	8.30 ± 0.06	8.38 ± 0.19	8.17 ± 0.17	8.39 ± 0.08
TC18000190.hg.1	MIR4529	5.26 ± 0.13	5.28 ± 0.14	5.35 ± 0.19	5.43 ± 0.01
TC19001519.hg.1	MIR4530	8.80 ± 0.13	8.65 ± 0.11	8.81 ± 0.03	8.64 ± 0.07
TC19001610.hg.1	MIR4531	9.11 ± 0.35	9.14 ± 0.09	8.98 ± 0.27	9.28 ± 0.18
TC20000452.hg.1	MIR4532	8.05 ± 0.37	8.13 ± 0.28	7.84 ± 0.39	8.21 ± 0.03
TC20000479.hg.1	MIR4533	9.45 ± 0.22	9.40 ± 0.18	9.39 ± 0.23	9.43 ± 0.04
TC22000295.hg.1	MIR4534	7.14 ± 0.27	7.14 ± 0.24	7.22 ± 0.12	7.39 ± 0.22
TC22000408.hg.1	MIR4535	7.31 ± 0.22	7.23 ± 0.18	7.21 ± 0.34	7.34 ± 0.05
TC0X000331.hg.1	MIR4536-1	3.76 ± 0.23	3.39 ± 0.06	3.61 ± 0.10	3.50 ± 0.04
TC17001747.hg.1	MIR454	5.11 ± 0.15	4.88 ± 0.07	4.84 ± 0.14	5.02 ± 0.24
TC09001073.hg.1	MIR4540	7.52 ± 0.14	7.75 ± 0.05	7.44 ± 0.10	7.83 ± 0.02
TC09000581.hg.1	MIR455	5.86 ± 0.46	6.02 ± 0.06	5.96 ± 0.30	6.16 ± 0.11
TC05000616.hg.1	MIR4633	7.04 ± 0.11	7.13 ± 0.14	7.17 ± 0.13	7.14 ± 0.06
TC05000976.hg.1	MIR4634	9.72 ± 0.64	9.78 ± 0.36	9.44 ± 0.65	9.85 ± 0.05
TC05001103.hg.1	MIR4635	7.40 ± 0.11	7.43 ± 0.25	7.20 ± 0.32	7.47 ± 0.12
TC05001152.hg.1	MIR4636	5.30 ± 0.56	4.97 ± 0.12	5.08 ± 0.35	5.01 ± 0.02
TC05001173.hg.1	MIR4637	3.19 ± 0.02	3.17 ± 0.23	3.20 ± 0.16	3.09 ± 0.02
TC05002167.hg.1	MIR4638	9.12 ± 0.08	9.06 ± 0.26	9.08 ± 0.33	9.10 ± 0.02
TC6_cox_hap2000048.hg.1	MIR4640	7.55 ± 0.25	7.65 ± 0.44	7.39 ± 0.35	7.81 ± 0.05
TC6_dbb_hap3000041.hg.1	MIR4640	7.55 ± 0.25	7.65 ± 0.44	7.39 ± 0.35	7.81 ± 0.05
TC6_mann_hap4000043.hg.1	MIR4640	7.55 ± 0.25	7.65 ± 0.44	7.39 ± 0.35	7.81 ± 0.05
TC6_mcf_hap5000034.hg.1	MIR4640	7.55 ± 0.25	7.65 ± 0.44	7.39 ± 0.35	7.81 ± 0.05
TC6_qbl_hap6000041.hg.1	MIR4640	7.55 ± 0.25	7.65 ± 0.44	7.39 ± 0.35	7.81 ± 0.05
TC06000618.hg.1	MIR4642	9.11 ± 0.07	9.03 ± 0.18	9.12 ± 0.04	9.14 ± 0.09
TC06000808.hg.1	MIR4643	3.57 ± 0.12	3.36 ± 0.11	3.50 ± 0.03	3.45 ± 0.04
TC06001200.hg.1	MIR4644	8.22 ± 0.19	8.33 ± 0.09	8.17 ± 0.19	8.50 ± 0.12
TC06001218.hg.1	MIR4645	5.32 ± 0.14	5.12 ± 0.20	5.16 ± 0.27	4.86 ± 0.13
TC06001537.hg.1	MIR4646	8.71 ± 0.21	8.49 ± 0.13	8.57 ± 0.32	8.58 ± 0.13

TC6_cox_hap2000166.hg.1	MIR4646	8.71 ± 0.21	8.49 ± 0.13	8.57 ± 0.32	8.58 ± 0.13
TC6_dbb_hap3000155.hg.1	MIR4646	8.71 ± 0.21	8.49 ± 0.13	8.57 ± 0.32	8.58 ± 0.13
TC6_mann_hap4000143.hg.1	MIR4646	8.71 ± 0.21	8.49 ± 0.13	8.57 ± 0.32	8.58 ± 0.13
TC6_mcf_hap5000143.hg.1	MIR4646	8.71 ± 0.21	8.49 ± 0.13	8.57 ± 0.32	8.58 ± 0.13
TC6_qbl_hap6000158.hg.1	MIR4646	8.71 ± 0.21	8.49 ± 0.13	8.57 ± 0.32	8.58 ± 0.13
TC6_ssto_hap7000138.hg.1	MIR4646	8.71 ± 0.21	8.49 ± 0.13	8.57 ± 0.32	8.58 ± 0.13
TC07001468.hg.1	MIR4650-1	4.67 ± 0.16	4.61 ± 0.25	4.77 ± 0.26	4.69 ± 0.07
TC07000554.hg.1	MIR4652	3.96 ± 0.27	3.71 ± 0.12	3.83 ± 0.21	3.77 ± 0.02
TC07000645.hg.1	MIR4653	6.02 ± 0.26	5.92 ± 0.13	5.98 ± 0.23	5.87 ± 0.12
TC01001418.hg.1	MIR4654	7.84 ± 0.29	7.99 ± 0.38	7.74 ± 0.31	8.23 ± 0.28
TC07001094.hg.1	MIR4655	7.56 ± 0.31	7.42 ± 0.19	7.42 ± 0.18	7.39 ± 0.07
TC07001109.hg.1	MIR4656	9.53 ± 0.30	9.60 ± 0.24	9.43 ± 0.26	9.87 ± 0.19
TC08000023.hg.1	MIR4659A	6.82 ± 0.79	7.02 ± 0.53	6.53 ± 0.67	7.06 ± 0.64
TC08000904.hg.1	MIR4659B	3.96 ± 0.27	3.82 ± 0.19	3.89 ± 0.13	3.81 ± 0.12
TC08000064.hg.1	MIR4660	7.05 ± 0.06	6.95 ± 0.10	7.05 ± 0.16	7.06 ± 0.08
TC08000555.hg.1	MIR4661	5.82 ± 0.14	6.02 ± 0.07	5.83 ± 0.25	6.00 ± 0.12
TC08001580.hg.1	MIR4663	8.29 ± 0.25	8.38 ± 0.09	8.22 ± 0.25	8.65 ± 0.08
TC08001730.hg.1	MIR4664	5.45 ± 0.17	5.51 ± 0.41	5.26 ± 0.26	5.50 ± 0.12
TC09000043.hg.1	MIR4665	9.07 ± 0.59	8.88 ± 0.20	8.86 ± 0.43	8.87 ± 0.05
TC01001894.hg.1	MIR4666A	5.44 ± 0.38	5.03 ± 0.08	5.11 ± 0.04	5.01 ± 0.09
TC09000561.hg.1	MIR4668	4.73 ± 0.39	4.80 ± 0.05	5.17 ± 0.70	4.80 ± 0.11
TC09000783.hg.1	MIR4669	9.84 ± 0.23	9.65 ± 0.12	9.88 ± 0.22	9.77 ± 0.16
TC09001338.hg.1	MIR4670	3.81 ± 0.19	3.64 ± 0.28	3.71 ± 0.21	3.69 ± 0.04
TC01001932.hg.1	MIR4671	3.61 ± 0.15	3.47 ± 0.07	3.40 ± 0.09	3.48 ± 0.03
TC09001614.hg.1	MIR4672	7.88 ± 0.25	7.95 ± 0.05	7.98 ± 0.01	7.99 ± 0.04
TC09001735.hg.1	MIR4673	8.83 ± 0.40	8.75 ± 0.23	8.77 ± 0.36	8.89 ± 0.07
TC09001736.hg.1	MIR4674	10.76 ± 0.43	10.87 ± 0.30	10.68 ± 0.39	10.90 ± 0.03
TC10000146.hg.1	MIR4675	7.33 ± 0.33	7.45 ± 0.29	7.24 ± 0.32	7.43 ± 0.04
TC10000454.hg.1	MIR4676	5.80 ± 0.13	5.67 ± 0.22	5.65 ± 0.12	5.71 ± 0.07
TC01001982.hg.1	MIR4677	6.31 ± 0.36	6.15 ± 0.13	6.39 ± 0.40	5.99 ± 0.20
TC10000618.hg.1	MIR4678	3.83 ± 0.19	3.43 ± 0.13	3.51 ± 0.25	3.57 ± 0.08

TC10000634.hg.1	MIR4679-1	3.40 ± 0.28	3.22 ± 0.12	3.27 ± 0.07	3.24 ± 0.06
TC10001503.hg.1	MIR4679-2	3.44 ± 0.12	3.38 ± 0.18	3.37 ± 0.08	3.32 ± 0.08
TC10000866.hg.1	MIR4681	7.39 ± 0.34	7.36 ± 0.32	7.32 ± 0.04	7.54 ± 0.13
TC10000872.hg.1	MIR4682	8.36 ± 0.19	8.49 ± 0.22	8.44 ± 0.31	8.70 ± 0.14
TC01000289.hg.1	MIR4684	7.57 ± 0.36	7.56 ± 0.44	7.43 ± 0.25	7.68 ± 0.12
TC11000059.hg.1	MIR4686	7.83 ± 0.19	7.99 ± 0.35	7.88 ± 0.13	8.25 ± 0.22
TC11000396.hg.1	MIR4688	7.13 ± 0.26	7.10 ± 0.20	7.18 ± 0.27	7.19 ± 0.15
TC01002142.hg.1	MIR4689	10.42 ± 0.32	10.36 ± 0.20	10.28 ± 0.23	10.45 ± 0.09
TC11000636.hg.1	MIR4690	10.01 ± 0.23	9.96 ± 0.21	9.86 ± 0.24	10.12 ± 0.11
TC11000769.hg.1	MIR4692	8.95 ± 0.20	8.78 ± 0.08	8.97 ± 0.04	8.99 ± 0.08
TC11000964.hg.1	MIR4693	4.09 ± 0.14	3.77 ± 0.14	3.90 ± 0.14	3.81 ± 0.07
TC11001484.hg.1	MIR4694	4.64 ± 0.19	4.49 ± 0.12	4.58 ± 0.28	4.51 ± 0.17
TC11002086.hg.1	MIR4696	3.70 ± 0.18	3.51 ± 0.05	3.60 ± 0.06	3.57 ± 0.04
TC11002469.hg.1	MIR4697HG	6.44 ± 0.12	6.51 ± 0.03	6.38 ± 0.07	6.41 ± 0.12
TC12000335.hg.1	MIR4698	7.75 ± 0.18	7.63 ± 0.18	7.50 ± 0.15	7.51 ± 0.02
TC12000673.hg.1	MIR4699	4.00 ± 0.14	4.06 ± 0.05	4.12 ± 0.03	4.03 ± 0.04
TC13000215.hg.1	MIR4703	4.23 ± 0.06	4.09 ± 0.27	4.07 ± 0.12	4.14 ± 0.04
TC13000850.hg.1	MIR4705	3.35 ± 0.02	3.31 ± 0.05	3.28 ± 0.05	3.25 ± 0.04
TC14000395.hg.1	MIR4706	8.16 ± 0.20	8.19 ± 0.31	7.97 ± 0.33	8.25 ± 0.06
TC14001229.hg.1	MIR4708	8.10 ± 0.29	7.97 ± 0.12	8.17 ± 0.40	8.03 ± 0.11
TC14001558.hg.1	MIR4710	8.08 ± 0.30	8.12 ± 0.20	8.03 ± 0.15	8.24 ± 0.10
TC01002715.hg.1	MIR4711	7.04 ± 0.25	7.10 ± 0.28	6.89 ± 0.16	7.33 ± 0.12
TC15000394.hg.1	MIR4712	4.43 ± 0.40	4.20 ± 0.29	4.15 ± 0.29	4.10 ± 0.06
TC15000403.hg.1	MIR4713	6.73 ± 0.36	6.45 ± 0.45	6.51 ± 0.22	6.48 ± 0.08
TC15000950.hg.1	MIR4714	5.06 ± 0.27	4.95 ± 0.09	5.00 ± 0.13	4.92 ± 0.15
TC15001080.hg.1	MIR4715	5.95 ± 0.24	6.12 ± 0.24	5.91 ± 0.27	6.09 ± 0.16
TC15001344.hg.1	MIR4716	5.22 ± 0.20	4.82 ± 0.09	4.93 ± 0.20	4.86 ± 0.13
TC16000070.hg.1	MIR4717	8.54 ± 0.22	8.47 ± 0.30	8.47 ± 0.17	8.67 ± 0.12
TC16000168.hg.1	MIR4718	5.90 ± 0.20	6.28 ± 0.25	5.89 ± 0.23	6.20 ± 0.19
TC16000625.hg.1	MIR4719	3.28 ± 0.06	3.10 ± 0.02	3.13 ± 0.08	3.06 ± 0.10
TC16000639.hg.1	MIR4720	4.16 ± 0.25	3.91 ± 0.29	3.84 ± 0.20	3.67 ± 0.06

TC17000367.hg.1	MIR4725	9.99 ± 0.39	10.05 ± 0.23	9.90 ± 0.31	10.18 ± 0.13
TC17000463.hg.1	MIR4726	8.79 ± 0.45	8.78 ± 0.37	8.59 ± 0.32	8.98 ± 0.11
TC17000467.hg.1	MIR4727	6.22 ± 0.22	6.44 ± 0.09	6.30 ± 0.16	6.31 ± 0.14
TC17000723.hg.1	MIR4729	6.04 ± 0.43	6.14 ± 0.22	6.05 ± 0.17	6.13 ± 0.20
TC17000920.hg.1	MIR4730	8.47 ± 0.09	8.42 ± 0.12	8.40 ± 0.16	8.50 ± 0.15
TC17001159.hg.1	MIR4731	7.18 ± 0.22	7.16 ± 0.30	7.07 ± 0.24	7.48 ± 0.18
TC17001310.hg.1	MIR4732	9.85 ± 0.14	9.85 ± 0.24	9.88 ± 0.03	10.06 ± 0.11
TC17001339.hg.1	MIR4733	6.63 ± 0.29	6.37 ± 0.18	6.70 ± 0.01	6.67 ± 0.12
TC17001428.hg.1	MIR4734	9.87 ± 0.34	9.94 ± 0.27	9.67 ± 0.40	9.92 ± 0.03
TC01003660.hg.1	MIR4735	3.39 ± 0.05	3.41 ± 0.12	3.40 ± 0.18	3.40 ± 0.13
TC17001730.hg.1	MIR4736	9.11 ± 0.11	9.46 ± 0.17	9.22 ± 0.16	9.49 ± 0.18
TC17001757.hg.1	MIR4737	10.47 ± 0.42	10.28 ± 0.03	10.47 ± 0.15	10.32 ± 0.40
TC17001883.hg.1	MIR4738	6.27 ± 0.16	6.22 ± 0.05	6.25 ± 0.47	6.07 ± 0.44
TC17001928.hg.1	MIR4739	7.60 ± 0.26	7.81 ± 0.47	7.48 ± 0.23	8.01 ± 0.24
TC17001950.hg.1	MIR4740	9.32 ± 0.39	8.90 ± 0.11	9.02 ± 0.38	9.04 ± 0.24
TC01003884.hg.1	MIR4742	6.80 ± 0.65	7.44 ± 0.25	7.05 ± 0.79	7.63 ± 0.09
TC18000502.hg.1	MIR4744	4.59 ± 0.15	4.18 ± 0.29	4.17 ± 0.08	4.04 ± 0.10
TC19000089.hg.1	MIR4746	9.54 ± 0.32	9.42 ± 0.18	9.46 ± 0.22	9.51 ± 0.08
TC19000098.hg.1	MIR4747	7.70 ± 0.19	7.41 ± 0.19	7.35 ± 0.26	7.47 ± 0.15
TC19000186.hg.1	MIR4748	8.60 ± 0.30	8.73 ± 0.40	8.50 ± 0.36	8.83 ± 0.09
TC19000876.hg.1	MIR4752	4.88 ± 0.17	4.86 ± 0.09	4.55 ± 0.08	4.84 ± 0.02
TC01003993.hg.1	MIR4753	4.55 ± 0.18	4.37 ± 0.13	4.73 ± 0.24	4.32 ± 0.16
TC19001916.hg.1	MIR4754	9.03 ± 0.24	9.04 ± 0.16	9.03 ± 0.12	9.16 ± 0.05
TC20000237.hg.1	MIR4755	6.25 ± 0.24	6.26 ± 0.10	6.15 ± 0.37	6.37 ± 0.09
TC20000961.hg.1	MIR4756	6.93 ± 0.38	7.07 ± 0.15	6.90 ± 0.17	7.17 ± 0.13
TC02000109.hg.1	MIR4757	9.53 ± 0.42	9.39 ± 0.20	9.36 ± 0.30	9.43 ± 0.18
TC21000085.hg.1	MIR4759	3.77 ± 0.05	3.74 ± 0.04	3.90 ± 0.07	3.62 ± 0.06
TC21000466.hg.1	MIR4760	3.48 ± 0.13	3.30 ± 0.08	3.45 ± 0.27	3.27 ± 0.06
TC22000386.hg.1	MIR4762	3.95 ± 0.19	3.89 ± 0.02	3.93 ± 0.13	4.00 ± 0.05
TC22000689.hg.1	MIR4764	4.09 ± 0.37	4.12 ± 0.32	4.02 ± 0.27	4.21 ± 0.08
TC02000216.hg.1	MIR4765	5.76 ± 0.13	5.74 ± 0.10	5.68 ± 0.22	5.67 ± 0.13

TC22000815.hg.1	MIR4766	3.54 ± 0.04	3.40 ± 0.03	3.49 ± 0.10	3.43 ± 0.09
TC0X000026.hg.1	MIR4767	11.02 ± 0.30	10.92 ± 0.20	10.90 ± 0.34	10.93 ± 0.04
TC0X000085.hg.1	MIR4768	5.85 ± 0.03	5.88 ± 0.28	5.91 ± 0.08	5.94 ± 0.05
TC0X000239.hg.1	MIR4769	6.54 ± 0.23	6.25 ± 0.21	6.29 ± 0.06	6.46 ± 0.23
TC0X000823.hg.1	MIR4770	5.13 ± 0.26	4.70 ± 0.06	4.71 ± 0.16	4.62 ± 0.11
TC02000624.hg.1	MIR4772	5.42 ± 0.55	5.13 ± 0.26	5.39 ± 0.18	5.46 ± 0.14
TC02000938.hg.1	MIR4773-1	5.29 ± 0.04	5.26 ± 0.01	5.28 ± 0.05	5.19 ± 0.16
TC02002427.hg.1	MIR4773-2	5.00 ± 0.02	4.95 ± 0.14	5.04 ± 0.16	5.04 ± 0.04
TC02000997.hg.1	MIR4774	4.58 ± 0.21	4.58 ± 0.08	4.58 ± 0.15	4.32 ± 0.24
TC02001251.hg.1	MIR4776-1	6.18 ± 0.26	6.18 ± 0.19	6.21 ± 0.31	6.26 ± 0.10
TC02002739.hg.1	MIR4776-2	6.60 ± 0.39	6.65 ± 0.18	6.57 ± 0.21	6.65 ± 0.03
TC02001389.hg.1	MIR4777	3.32 ± 0.25	3.04 ± 0.04	3.16 ± 0.19	3.00 ± 0.06
TC02001930.hg.1	MIR4778	4.91 ± 0.16	4.56 ± 0.11	4.74 ± 0.09	4.55 ± 0.03
TC02002054.hg.1	MIR4779	4.51 ± 0.21	4.53 ± 0.16	4.50 ± 0.25	4.48 ± 0.05
TC02002068.hg.1	MIR4780	8.56 ± 0.23	8.47 ± 0.18	8.45 ± 0.22	8.59 ± 0.09
TC02002230.hg.1	MIR4782	4.10 ± 0.24	3.81 ± 0.08	3.98 ± 0.15	3.77 ± 0.09
TC02002280.hg.1	MIR4783	7.71 ± 0.24	7.63 ± 0.32	7.49 ± 0.24	7.70 ± 0.08
TC02002929.hg.1	MIR4786	7.50 ± 0.16	7.63 ± 0.33	7.50 ± 0.18	7.82 ± 0.12
TC03000317.hg.1	MIR4787	9.53 ± 0.24	9.44 ± 0.15	9.40 ± 0.28	9.55 ± 0.04
TC03000728.hg.1	MIR4788	6.02 ± 0.16	5.86 ± 0.08	5.78 ± 0.20	5.86 ± 0.05
TC03000936.hg.1	MIR4789	4.81 ± 0.09	4.72 ± 0.14	4.76 ± 0.24	4.67 ± 0.04
TC03001134.hg.1	MIR4790	3.46 ± 0.19	3.17 ± 0.05	3.28 ± 0.15	3.19 ± 0.04
TC03001224.hg.1	MIR4791	3.62 ± 0.12	3.56 ± 0.20	3.48 ± 0.06	3.48 ± 0.16
TC03001243.hg.1	MIR4792	10.44 ± 0.39	10.41 ± 0.18	10.27 ± 0.45	10.44 ± 0.06
TC01000726.hg.1	MIR4794	3.82 ± 0.03	3.68 ± 0.13	3.70 ± 0.16	3.70 ± 0.13
TC03001584.hg.1	MIR4795	3.24 ± 0.17	3.14 ± 0.02	3.08 ± 0.07	3.10 ± 0.06
TC03001671.hg.1	MIR4796	4.61 ± 0.33	5.26 ± 0.25	4.86 ± 0.38	5.14 ± 0.31
TC03002175.hg.1	MIR4797	4.21 ± 0.11	4.11 ± 0.17	4.16 ± 0.13	4.19 ± 0.25
TC04000081.hg.1	MIR4798	3.84 ± 0.13	3.45 ± 0.10	3.53 ± 0.17	3.46 ± 0.06
TC04000734.hg.1	MIR4799	7.53 ± 0.16	7.50 ± 0.06	7.45 ± 0.20	7.48 ± 0.01
TC04001113.hg.1	MIR4801	3.44 ± 0.13	3.04 ± 0.04	3.11 ± 0.15	2.99 ± 0.07

TC04001138.hg.1	MIR4802	5.17 ± 0.23	5.45 ± 0.40	6.20 ± 1.03	5.17 ± 0.47
TC05000337.hg.1	MIR4803	3.52 ± 0.04	3.36 ± 0.08	3.63 ± 0.23	3.48 ± 0.05
TC05000342.hg.1	MIR4804	6.23 ± 0.33	6.12 ± 0.11	6.11 ± 0.34	6.30 ± 0.10
TC11001275.hg.1	MIR483	8.05 ± 0.15	8.20 ± 0.36	8.06 ± 0.23	8.40 ± 0.19
TC16000192.hg.1	MIR484	10.11 ± 0.02	10.08 ± 0.07	10.18 ± 0.11	10.11 ± 0.18
TC14000752.hg.1	MIR485	5.53 ± 0.40	5.44 ± 0.23	5.34 ± 0.39	5.56 ± 0.19
TC08000320.hg.1	MIR486	9.39 ± 0.27	9.31 ± 0.23	9.34 ± 0.30	9.37 ± 0.04
TC08001172.hg.1	MIR486	8.37 ± 0.28	8.49 ± 0.27	8.31 ± 0.19	8.50 ± 0.10
TC14000748.hg.1	MIR487A	4.14 ± 0.14	4.09 ± 0.19	4.01 ± 0.13	4.05 ± 0.13
TC14000743.hg.1	MIR487B	4.47 ± 0.07	4.54 ± 0.03	4.51 ± 0.13	4.64 ± 0.11
TC01003557.hg.1	MIR488	3.97 ± 0.19	3.77 ± 0.06	3.86 ± 0.10	3.87 ± 0.26
TC07001610.hg.1	MIR489	5.11 ± 0.21	5.21 ± 0.30	5.10 ± 0.24	5.34 ± 0.16
TC07000853.hg.1	MIR490	5.95 ± 0.20	5.85 ± 0.38	5.90 ± 0.32	6.19 ± 0.17
TC09000100.hg.1	MIR491	4.67 ± 0.12	4.35 ± 0.19	4.48 ± 0.08	4.22 ± 0.07
TC12000732.hg.1	MIR492	6.21 ± 0.31	6.18 ± 0.22	6.03 ± 0.30	6.19 ± 0.09
TC14000651.hg.1	MIR493	5.44 ± 0.09	5.35 ± 0.09	5.44 ± 0.04	5.38 ± 0.07
TC14000725.hg.1	MIR494	7.56 ± 0.14	7.55 ± 0.02	7.56 ± 0.06	7.63 ± 0.18
TC14000728.hg.1	MIR495	4.37 ± 0.33	4.35 ± 0.21	4.26 ± 0.33	4.38 ± 0.32
TC14000756.hg.1	MIR496	5.23 ± 0.25	5.26 ± 0.03	5.23 ± 0.09	5.26 ± 0.07
TC17001072.hg.1	MIR497	6.98 ± 0.33	6.99 ± 0.10	6.95 ± 0.11	7.06 ± 0.14
TC17001071.hg.1	MIR497HG	7.76 ± 0.30	7.67 ± 0.27	7.61 ± 0.18	7.81 ± 0.03
TC19000818.hg.1	MIR498	6.50 ± 0.34	6.34 ± 0.08	6.28 ± 0.20	6.28 ± 0.09
TC20000247.hg.1	MIR499A	8.28 ± 0.24	8.30 ± 0.19	8.24 ± 0.23	8.23 ± 0.02
TC20000790.hg.1	MIR499B	5.51 ± 0.24	5.52 ± 0.12	5.58 ± 0.21	5.59 ± 0.06
TC0X000282.hg.1	MIR500A	7.90 ± 0.28	7.90 ± 0.19	7.99 ± 0.17	8.11 ± 0.06
TC0X000285.hg.1	MIR500B	7.68 ± 0.42	7.52 ± 0.12	7.56 ± 0.27	7.81 ± 0.32
TC0X000284.hg.1	MIR501	6.81 ± 0.17	6.80 ± 0.09	6.88 ± 0.03	6.88 ± 0.08
TC0X000287.hg.1	MIR502	8.77 ± 0.36	8.53 ± 0.58	8.66 ± 0.41	8.66 ± 0.05
TC0X001393.hg.1	MIR503	7.82 ± 0.24	7.93 ± 0.17	7.95 ± 0.13	7.99 ± 0.04
TC0X001426.hg.1	MIR504	6.96 ± 0.41	6.99 ± 0.25	6.87 ± 0.28	7.15 ± 0.17
TC17001801.hg.1	MIR5047	11.43 ± 0.73	12.45 ± 0.04	11.61 ± 0.44	12.43 ± 0.26

TC0X001430.hg.1	MIR505	6.89 ± 0.66	7.43 ± 0.40	7.14 ± 0.20	7.41 ± 0.11
TC0X001463.hg.1	MIR506	3.98 ± 0.15	3.78 ± 0.05	3.88 ± 0.12	3.90 ± 0.06
TC0X001464.hg.1	MIR507	4.35 ± 0.26	4.57 ± 0.31	4.11 ± 0.05	4.68 ± 0.09
TC0X001465.hg.1	MIR508	4.44 ± 0.14	4.43 ± 0.20	4.22 ± 0.05	4.48 ± 0.03
TC0X001467.hg.1	MIR509-1	5.23 ± 0.27	5.00 ± 0.07	5.06 ± 0.14	5.16 ± 0.06
TC0X001468.hg.1	MIR509-2	6.35 ± 0.26	6.24 ± 0.04	6.16 ± 0.11	6.28 ± 0.11
TC20000447.hg.1	MIR5095	11.97 ± 0.28	12.01 ± 0.01	11.96 ± 0.10	12.16 ± 0.08
TC0X001469.hg.1	MIR510	4.90 ± 0.22	4.49 ± 0.04	4.67 ± 0.05	4.65 ± 0.12
TC10000133.hg.1	MIR511-1	4.87 ± 0.15	4.94 ± 0.14	4.92 ± 0.18	4.96 ± 0.11
TC10000136.hg.1	MIR511-2	4.87 ± 0.15	4.94 ± 0.14	4.92 ± 0.18	4.96 ± 0.11
TC19000816.hg.1	MIR512-1	6.40 ± 0.21	6.59 ± 0.30	6.26 ± 0.27	6.76 ± 0.07
TC19000815.hg.1	MIR512-2	6.40 ± 0.21	6.59 ± 0.30	6.26 ± 0.27	6.76 ± 0.07
TC0X001461.hg.1	MIR513A1	5.36 ± 0.17	5.42 ± 0.11	5.49 ± 0.18	5.43 ± 0.07
TC0X001462.hg.1	MIR513A2	6.62 ± 0.04	6.61 ± 0.11	6.54 ± 0.16	6.55 ± 0.07
TC0X001460.hg.1	MIR513B	4.37 ± 0.15	4.18 ± 0.06	4.31 ± 0.15	4.26 ± 0.12
TC0X001459.hg.1	MIR513C	4.90 ± 0.09	4.86 ± 0.15	4.96 ± 0.08	4.82 ± 0.02
TC0X001470.hg.1	MIR514A1	4.67 ± 0.26	4.73 ± 0.16	4.71 ± 0.10	4.81 ± 0.25
TC0X001472.hg.1	MIR514A2	4.46 ± 0.23	4.35 ± 0.09	4.47 ± 0.17	4.37 ± 0.20
TC0X001471.hg.1	MIR514A3	4.46 ± 0.23	4.35 ± 0.09	4.47 ± 0.17	4.37 ± 0.20
TC0X001466.hg.1	MIR514B	5.74 ± 0.16	5.32 ± 0.18	5.61 ± 0.06	5.24 ± 0.09
TC19000820.hg.1	MIR515-1	4.69 ± 0.49	4.78 ± 0.04	4.76 ± 0.36	5.18 ± 0.33
TC19000823.hg.1	MIR515-2	4.69 ± 0.49	4.78 ± 0.04	4.76 ± 0.36	5.18 ± 0.33
TC19000857.hg.1	MIR516A1	4.28 ± 0.12	4.26 ± 0.06	4.39 ± 0.15	4.39 ± 0.11
TC19000859.hg.1	MIR516A2	4.26 ± 0.35	4.10 ± 0.14	4.18 ± 0.11	4.12 ± 0.12
TC19000849.hg.1	MIR516B1	5.29 ± 0.08	5.34 ± 0.09	5.38 ± 0.22	5.44 ± 0.13
TC19000844.hg.1	MIR516B2	5.36 ± 0.22	5.37 ± 0.13	5.43 ± 0.15	5.43 ± 0.07
TC19000838.hg.1	MIR517A	3.82 ± 0.17	3.94 ± 0.20	3.84 ± 0.10	3.96 ± 0.10
TC19000842.hg.1	MIR517B	5.01 ± 0.08	5.06 ± 0.11	5.07 ± 0.19	5.04 ± 0.08
TC19000851.hg.1	MIR517C	4.81 ± 0.21	4.75 ± 0.28	4.76 ± 0.17	4.91 ± 0.09
TC19000847.hg.1	MIR518A1	5.53 ± 0.33	5.54 ± 0.10	5.41 ± 0.12	5.59 ± 0.06
TC19000850.hg.1	MIR518A2	5.71 ± 0.17	5.69 ± 0.15	5.64 ± 0.04	5.86 ± 0.08

TC19000833.hg.1	MIR518B	6.06 ± 0.30	5.77 ± 0.14	5.55 ± 0.26	5.72 ± 0.12
TC19000836.hg.1	MIR518C	6.38 ± 0.10	6.13 ± 0.24	6.35 ± 0.09	6.04 ± 0.08
TC19000848.hg.1	MIR518D	5.07 ± 0.03	5.17 ± 0.21	4.97 ± 0.28	4.82 ± 0.28
TC19000846.hg.1	MIR518E	4.30 ± 0.26	4.19 ± 0.16	4.09 ± 0.11	4.11 ± 0.15
TC19000831.hg.1	MIR518F	3.75 ± 0.35	3.48 ± 0.01	3.50 ± 0.12	3.45 ± 0.01
TC19000855.hg.1	MIR519A1	4.66 ± 0.40	4.62 ± 0.17	4.71 ± 0.15	4.56 ± 0.14
TC19000860.hg.1	MIR519A2	5.16 ± 0.30	5.03 ± 0.01	5.01 ± 0.10	5.07 ± 0.17
TC19000828.hg.1	MIR519B	3.81 ± 0.20	3.65 ± 0.13	3.80 ± 0.10	3.61 ± 0.04
TC19000824.hg.1	MIR519C	3.85 ± 0.19	3.79 ± 0.09	3.91 ± 0.20	3.81 ± 0.05
TC19000839.hg.1	MIR519D	5.18 ± 0.41	4.97 ± 0.16	4.76 ± 0.23	4.91 ± 0.18
TC19000821.hg.1	MIR519E	4.36 ± 0.30	4.06 ± 0.15	3.88 ± 0.03	3.95 ± 0.16
TC19000826.hg.1	MIR520A	5.50 ± 0.62	5.16 ± 0.05	5.24 ± 0.12	5.17 ± 0.17
TC19000832.hg.1	MIR520B	4.45 ± 0.44	4.61 ± 0.08	4.68 ± 0.03	4.56 ± 0.26
TC19000835.hg.1	MIR520C	5.18 ± 0.32	5.07 ± 0.14	4.98 ± 0.14	5.13 ± 0.18
TC19000841.hg.1	MIR520D	5.95 ± 0.05	6.01 ± 0.13	6.04 ± 0.27	6.05 ± 0.10
TC19000819.hg.1	MIR520E	5.10 ± 0.20	5.09 ± 0.07	5.23 ± 0.19	4.98 ± 0.09
TC19000822.hg.1	MIR520F	5.26 ± 0.12	5.36 ± 0.19	5.31 ± 0.13	5.45 ± 0.11
TC19000843.hg.1	MIR520G	4.86 ± 0.38	4.58 ± 0.12	4.82 ± 0.26	4.53 ± 0.04
TC19000852.hg.1	MIR520H	4.33 ± 0.46	4.39 ± 0.17	4.47 ± 0.27	4.36 ± 0.09
TC19000853.hg.1	MIR521-1	4.65 ± 0.30	4.26 ± 0.06	4.42 ± 0.20	4.41 ± 0.16
TC19000840.hg.1	MIR521-2	4.50 ± 0.08	4.24 ± 0.15	4.37 ± 0.12	4.33 ± 0.19
TC19000854.hg.1	MIR522	5.15 ± 0.34	5.02 ± 0.35	5.00 ± 0.24	4.88 ± 0.15
TC19000830.hg.1	MIR523	4.23 ± 0.32	3.94 ± 0.17	4.08 ± 0.08	4.01 ± 0.11
TC19000837.hg.1	MIR524	4.70 ± 0.52	4.60 ± 0.21	4.50 ± 0.27	4.66 ± 0.32
TC19000829.hg.1	MIR525	3.78 ± 0.21	3.54 ± 0.17	3.72 ± 0.03	3.58 ± 0.06
TC19000834.hg.1	MIR526A1	3.82 ± 0.37	3.62 ± 0.05	3.49 ± 0.11	3.49 ± 0.06
TC19000845.hg.1	MIR526A2	3.69 ± 0.13	3.61 ± 0.12	3.55 ± 0.08	3.51 ± 0.08
TC19000827.hg.1	MIR526B	4.68 ± 0.17	4.62 ± 0.11	4.69 ± 0.14	4.70 ± 0.20
TC19000856.hg.1	MIR527	5.29 ± 0.13	5.09 ± 0.05	5.27 ± 0.20	4.97 ± 0.18
TC0X000279.hg.1	MIR532	6.03 ± 0.24	6.18 ± 0.10	5.94 ± 0.30	6.25 ± 0.10
TC14000744.hg.1	MIR539	4.50 ± 0.19	4.54 ± 0.09	4.42 ± 0.04	4.44 ± 0.10

TC14000758.hg.1	MIR541	7.67 ± 0.26	7.93 ± 0.34	7.69 ± 0.23	8.11 ± 0.20
TC0X001391.hg.1	MIR542	4.98 ± 0.37	4.79 ± 0.10	4.97 ± 0.17	4.88 ± 0.10
TC14000727.hg.1	MIR543	6.54 ± 0.43	6.46 ± 0.34	6.47 ± 0.34	6.61 ± 0.34
TC14000746.hg.1	MIR544A	5.42 ± 0.52	5.52 ± 0.17	5.57 ± 0.21	5.68 ± 0.29
TC0X001160.hg.1	MIR545	6.18 ± 0.19	6.20 ± 0.04	6.08 ± 0.03	6.38 ± 0.15
TC06000122.hg.1	MIR548A1	4.15 ± 0.29	3.89 ± 0.17	4.04 ± 0.11	3.98 ± 0.10
TC03000403.hg.1	MIR548A2	7.48 ± 0.49	7.38 ± 0.38	7.63 ± 0.30	7.45 ± 0.32
TC06001011.hg.1	MIR548A2	5.42 ± 0.49	5.37 ± 0.23	5.61 ± 0.39	5.58 ± 0.45
TC03001623.hg.1	MIR548A3	9.41 ± 0.68	9.39 ± 0.28	9.46 ± 0.21	9.62 ± 0.28
TC08001510.hg.1	MIR548A3	5.90 ± 0.46	5.97 ± 0.27	6.15 ± 0.19	5.97 ± 0.16
TC08001585.hg.1	MIR548AA1	5.71 ± 0.63	5.78 ± 0.40	5.71 ± 0.33	5.82 ± 0.13
TC16000958.hg.1	MIR548AA2	9.28 ± 0.55	9.02 ± 0.43	9.40 ± 0.32	9.28 ± 0.41
TC17001816.hg.1	MIR548AA2	7.79 ± 0.65	7.91 ± 0.37	7.99 ± 0.36	7.78 ± 0.24
TC03000123.hg.1	MIR548AC	8.56 ± 0.56	8.53 ± 0.29	8.75 ± 0.33	8.65 ± 0.30
TC04001437.hg.1	MIR548AC	7.83 ± 0.38	7.77 ± 0.24	7.89 ± 0.21	7.76 ± 0.12
TC02001799.hg.1	MIR548AD	12.70 ± 0.55	12.96 ± 0.11	12.78 ± 0.20	13.15 ± 0.15
TC14000999.hg.1	MIR548AI	4.09 ± 0.07	4.00 ± 0.05	3.98 ± 0.02	4.15 ± 0.14
TC04001081.hg.1	MIR548AJ2	5.43 ± 0.28	5.76 ± 0.16	5.61 ± 0.23	5.99 ± 0.06
TC0X000953.hg.1	MIR548AJ2	4.22 ± 0.44	4.04 ± 0.22	4.40 ± 0.26	4.20 ± 0.24
TC12001788.hg.1	MIR548AL	3.65 ± 0.25	3.40 ± 0.11	3.53 ± 0.23	3.44 ± 0.08
TC0X000518.hg.1	MIR548AN	8.54 ± 0.52	8.57 ± 0.27	8.64 ± 0.33	8.52 ± 0.17
TC13000827.hg.1	MIR548AN	7.67 ± 0.60	7.49 ± 0.37	7.66 ± 0.32	7.70 ± 0.31
TC06002061.hg.1	MIR548B	5.67 ± 0.28	5.70 ± 0.04	5.57 ± 0.07	5.71 ± 0.08
TC12000573.hg.1	MIR548C	7.54 ± 0.90	7.44 ± 0.60	7.65 ± 0.54	7.37 ± 0.31
TC01002910.hg.1	MIR548D1	7.94 ± 0.31	8.01 ± 0.08	7.98 ± 0.23	8.06 ± 0.07
TC16000250.hg.1	MIR548D2	8.37 ± 0.71	8.47 ± 0.25	8.46 ± 0.26	8.60 ± 0.26
TC10000810.hg.1	MIR548E	5.12 ± 0.56	5.11 ± 0.33	5.35 ± 0.22	5.04 ± 0.18
TC01003634.hg.1	MIR548F1	4.10 ± 0.24	3.94 ± 0.07	3.93 ± 0.18	3.97 ± 0.06
TC10001306.hg.1	MIR548F1	3.20 ± 0.19	3.10 ± 0.08	3.07 ± 0.08	3.05 ± 0.02
TC02002736.hg.1	MIR548F2	4.81 ± 0.25	4.65 ± 0.20	4.73 ± 0.11	4.79 ± 0.27
TC05001667.hg.1	MIR548F3	3.19 ± 0.18	3.04 ± 0.08	3.18 ± 0.14	3.14 ± 0.09

TC07000964.hg.1	MIR548F3	7.60 ± 0.20	7.64 ± 0.26	7.85 ± 0.15	7.68 ± 0.21
TC07001991.hg.1	MIR548F4	3.27 ± 0.06	3.10 ± 0.11	3.20 ± 0.05	3.06 ± 0.02
TC0X000944.hg.1	MIR548F5	3.66 ± 0.13	3.59 ± 0.06	3.64 ± 0.07	3.47 ± 0.07
TC03001607.hg.1	MIR548G	3.89 ± 0.08	3.88 ± 0.13	3.87 ± 0.16	3.99 ± 0.04
TC04001623.hg.1	MIR548G	3.72 ± 0.07	3.55 ± 0.16	3.53 ± 0.21	3.52 ± 0.10
TC14001212.hg.1	MIR548H1	4.06 ± 0.12	4.33 ± 0.28	4.17 ± 0.20	4.22 ± 0.16
TC03000824.hg.1	MIR548H2	9.01 ± 0.52	9.17 ± 0.14	9.10 ± 0.19	9.37 ± 0.17
TC16000868.hg.1	MIR548H2	7.21 ± 0.75	7.22 ± 0.30	7.32 ± 0.50	7.46 ± 0.32
TC06001958.hg.1	MIR548H3	7.71 ± 0.53	7.70 ± 0.29	7.78 ± 0.29	7.94 ± 0.15
TC09001222.hg.1	MIR548H3	9.03 ± 0.67	9.18 ± 0.18	9.11 ± 0.31	9.37 ± 0.30
TC17001150.hg.1	MIR548H3	5.24 ± 0.34	5.23 ± 0.20	5.26 ± 0.20	5.29 ± 0.20
TC08001077.hg.1	MIR548H4	3.46 ± 0.14	3.28 ± 0.19	3.40 ± 0.10	3.34 ± 0.11
TC15000631.hg.1	MIR548H4	7.45 ± 0.25	7.32 ± 0.07	7.37 ± 0.02	7.35 ± 0.07
TC03001727.hg.1	MIR548I1	6.45 ± 0.78	6.46 ± 0.30	6.65 ± 0.59	6.95 ± 0.61
TC04001028.hg.1	MIR548I2	6.24 ± 0.68	6.15 ± 0.29	6.32 ± 0.48	6.46 ± 0.48
TC08000934.hg.1	MIR548I3	6.46 ± 0.89	6.49 ± 0.34	6.55 ± 0.67	6.90 ± 0.59
TC07001990.hg.1	MIR548I4	3.86 ± 0.30	4.04 ± 0.13	3.90 ± 0.09	4.10 ± 0.07
TC0X001187.hg.1	MIR548I4	4.10 ± 0.21	3.88 ± 0.14	4.03 ± 0.14	3.95 ± 0.13
TC22000611.hg.1	MIR548J	6.55 ± 0.23	6.33 ± 0.06	6.45 ± 0.10	6.36 ± 0.02
TC11000725.hg.1	MIR548K	5.37 ± 0.24	5.41 ± 0.22	5.27 ± 0.19	5.52 ± 0.04
TC11002200.hg.1	MIR548L	6.38 ± 0.62	6.46 ± 0.30	6.18 ± 0.23	6.57 ± 0.37
TC07000507.hg.1	MIR548M	7.72 ± 0.59	7.91 ± 0.07	7.82 ± 0.19	8.24 ± 0.34
TC0X001199.hg.1	MIR548M	4.44 ± 0.11	4.42 ± 0.12	4.35 ± 0.08	4.28 ± 0.09
TC07001700.hg.1	MIR548O	4.76 ± 0.27	4.53 ± 0.29	4.62 ± 0.14	4.33 ± 0.11
TC08001104.hg.1	MIR548O2	8.96 ± 0.63	9.09 ± 0.23	9.07 ± 0.44	9.31 ± 0.27
TC05001639.hg.1	MIR548P	4.83 ± 0.97	4.43 ± 0.44	5.48 ± 0.82	4.13 ± 0.20
TC09001442.hg.1	MIR548Q	8.51 ± 0.46	8.49 ± 0.20	8.61 ± 0.28	8.64 ± 0.14
TC10001044.hg.1	MIR548Q	3.90 ± 0.32	3.70 ± 0.26	3.91 ± 0.33	3.70 ± 0.02
TC04001739.hg.1	MIR548T	5.88 ± 0.22	5.85 ± 0.13	5.91 ± 0.09	5.90 ± 0.21
TC07000965.hg.1	MIR548T	5.14 ± 0.47	4.98 ± 0.20	5.33 ± 0.39	4.84 ± 0.12
TC06001121.hg.1	MIR548U	7.22 ± 0.53	7.03 ± 0.48	7.45 ± 0.37	7.11 ± 0.21

TC17000761.hg.1	MIR548W	7.13 ± 0.46	6.89 ± 0.44	7.44 ± 0.48	7.22 ± 0.24
TC16000152.hg.1	MIR548X	9.49 ± 1.00	9.15 ± 0.58	9.64 ± 0.45	9.80 ± 0.42
TC14001090.hg.1	MIR548Y	5.42 ± 0.54	5.14 ± 0.07	5.33 ± 0.37	5.35 ± 0.21
TC12001676.hg.1	MIR548Z	6.14 ± 0.29	5.97 ± 0.26	6.07 ± 0.23	6.15 ± 0.18
TC15001726.hg.1	MIR549	3.73 ± 0.07	3.56 ± 0.06	3.58 ± 0.04	3.58 ± 0.11
TC07000195.hg.1	MIR550A1	7.14 ± 0.30	7.76 ± 0.21	7.21 ± 0.33	7.85 ± 0.22
TC07000209.hg.1	MIR550A2	6.15 ± 0.41	6.66 ± 0.22	6.34 ± 0.19	6.69 ± 0.05
TC07001242.hg.1	MIR550A3	6.13 ± 0.31	6.50 ± 0.15	6.25 ± 0.08	6.57 ± 0.14
TC07001248.hg.1	MIR550B1	7.00 ± 0.26	6.98 ± 0.08	6.99 ± 0.24	7.10 ± 0.03
TC07001260.hg.1	MIR550B2	5.69 ± 0.26	5.46 ± 0.13	5.50 ± 0.14	5.55 ± 0.12
TC01002130.hg.1	MIR551A	7.06 ± 0.30	6.93 ± 0.17	7.14 ± 0.21	7.15 ± 0.05
TC03000900.hg.1	MIR551B	8.13 ± 0.41	8.09 ± 0.03	7.94 ± 0.34	8.06 ± 0.03
TC01002487.hg.1	MIR552	4.68 ± 0.14	4.74 ± 0.18	4.75 ± 0.16	4.82 ± 0.14
TC01000901.hg.1	MIR553	6.25 ± 0.36	6.24 ± 0.28	6.21 ± 0.33	6.45 ± 0.08
TC01001213.hg.1	MIR554	5.29 ± 0.07	5.26 ± 0.06	5.22 ± 0.30	5.22 ± 0.06
TC01003315.hg.1	MIR555	8.81 ± 0.30	8.97 ± 0.17	8.83 ± 0.31	8.92 ± 0.07
TC01001420.hg.1	MIR556	3.24 ± 0.10	2.97 ± 0.05	3.14 ± 0.13	2.96 ± 0.05
TC01001468.hg.1	MIR557	8.77 ± 0.45	8.99 ± 0.25	8.88 ± 0.12	9.09 ± 0.17
TC02000214.hg.1	MIR558	4.82 ± 0.51	5.06 ± 0.36	4.79 ± 0.32	5.01 ± 0.14
TC02000294.hg.1	MIR559	3.70 ± 0.20	3.43 ± 0.11	3.55 ± 0.16	3.42 ± 0.04
TC02001104.hg.1	MIR561	4.46 ± 0.13	4.37 ± 0.22	4.49 ± 0.43	4.45 ± 0.07
TC02001397.hg.1	MIR562	4.73 ± 0.12	4.37 ± 0.21	4.30 ± 0.06	4.24 ± 0.12
TC03000099.hg.1	MIR563	5.87 ± 0.15	5.82 ± 0.05	5.62 ± 0.28	5.99 ± 0.02
TC03000304.hg.1	MIR566	12.26 ± 0.20	12.14 ± 0.06	12.32 ± 0.21	12.26 ± 0.05
TC03000570.hg.1	MIR567	3.89 ± 0.28	3.76 ± 0.24	3.72 ± 0.21	3.89 ± 0.07
TC03001669.hg.1	MIR568	3.47 ± 0.11	3.48 ± 0.22	3.63 ± 0.11	3.46 ± 0.10
TC03001999.hg.1	MIR569	4.80 ± 0.13	5.02 ± 0.26	4.86 ± 0.33	4.77 ± 0.15
TC03001088.hg.1	MIR570	7.05 ± 0.43	6.78 ± 0.40	6.93 ± 0.41	6.87 ± 0.26
TC04000007.hg.1	MIR571	7.72 ± 0.41	8.25 ± 0.15	7.64 ± 0.19	8.39 ± 0.23
TC04000125.hg.1	MIR572	9.91 ± 0.24	9.93 ± 0.14	9.82 ± 0.27	9.93 ± 0.04
TC04001087.hg.1	MIR573	7.11 ± 0.76	7.71 ± 0.50	7.23 ± 0.67	7.68 ± 0.29

TC04000223.hg.1	MIR574	11.04 ± 0.24	11.33 ± 0.26	11.08 ± 0.20	11.55 ± 0.14
TC04001339.hg.1	MIR575	6.80 ± 0.48	6.80 ± 0.19	6.68 ± 0.24	6.94 ± 0.06
TC04000563.hg.1	MIR576	5.09 ± 0.57	5.09 ± 0.47	4.94 ± 0.41	4.85 ± 0.13
TC04000591.hg.1	MIR577	4.32 ± 0.06	4.19 ± 0.05	4.34 ± 0.15	4.30 ± 0.17
TC04000830.hg.1	MIR578	4.57 ± 0.17	4.49 ± 0.14	4.57 ± 0.24	4.61 ± 0.05
TC05001236.hg.1	MIR579	7.14 ± 0.41	7.80 ± 0.20	7.17 ± 0.41	7.53 ± 0.24
TC05001269.hg.1	MIR580	3.72 ± 0.14	3.55 ± 0.22	3.56 ± 0.18	3.49 ± 0.11
TC05001347.hg.1	MIR581	4.78 ± 0.24	4.80 ± 0.06	4.97 ± 0.44	4.64 ± 0.19
TC05001390.hg.1	MIR582	3.40 ± 0.19	3.23 ± 0.05	3.24 ± 0.16	3.12 ± 0.06
TC05000466.hg.1	MIR583	4.48 ± 0.13	4.57 ± 0.13	4.64 ± 0.20	4.49 ± 0.24
TC05001918.hg.1	MIR584	5.92 ± 0.29	6.01 ± 0.32	5.92 ± 0.12	6.26 ± 0.26
TC05002046.hg.1	MIR585	6.69 ± 0.27	6.68 ± 0.12	6.62 ± 0.15	6.79 ± 0.07
TC06001766.hg.1	MIR586	3.88 ± 0.40	3.70 ± 0.06	3.70 ± 0.11	3.51 ± 0.19
TC06000853.hg.1	MIR587	6.98 ± 0.22	7.13 ± 0.13	6.94 ± 0.15	7.25 ± 0.13
TC06000967.hg.1	MIR588	4.29 ± 0.22	4.15 ± 0.13	4.13 ± 0.18	4.33 ± 0.12
TC07001119.hg.1	MIR589	7.28 ± 0.15	7.33 ± 0.10	7.45 ± 0.14	7.36 ± 0.03
TC07000452.hg.1	MIR590	7.61 ± 0.67	8.28 ± 0.20	7.50 ± 0.44	8.52 ± 0.21
TC07001620.hg.1	MIR591	4.64 ± 0.35	4.74 ± 0.09	4.77 ± 0.05	4.73 ± 0.12
TC07001834.hg.1	MIR592	5.85 ± 0.27	5.73 ± 0.23	5.73 ± 0.29	5.83 ± 0.06
TC07000766.hg.1	MIR593	8.27 ± 0.19	8.29 ± 0.12	8.29 ± 0.10	8.42 ± 0.10
TC07002081.hg.1	MIR595	6.73 ± 0.36	6.76 ± 0.12	6.61 ± 0.17	6.86 ± 0.07
TC08000009.hg.1	MIR596	8.39 ± 0.14	8.47 ± 0.41	8.38 ± 0.31	8.61 ± 0.04
TC08000070.hg.1	MIR597	6.91 ± 0.13	7.13 ± 0.09	6.99 ± 0.17	7.35 ± 0.11
TC08000965.hg.1	MIR598	8.98 ± 0.30	9.35 ± 0.20	9.28 ± 0.28	9.65 ± 0.08
TC08001469.hg.1	MIR599	5.31 ± 0.08	5.27 ± 0.18	5.31 ± 0.15	5.32 ± 0.10
TC09002919.hg.1	MIR600	6.17 ± 0.30	6.36 ± 0.07	6.00 ± 0.16	6.38 ± 0.25
TC09001572.hg.1	MIR601	6.50 ± 0.08	6.56 ± 0.17	6.64 ± 0.21	6.51 ± 0.03
TC09000854.hg.1	MIR602	8.47 ± 0.20	8.46 ± 0.24	8.36 ± 0.31	8.53 ± 0.18
TC10000171.hg.1	MIR603	5.14 ± 0.43	5.34 ± 0.05	5.32 ± 0.08	5.45 ± 0.07
TC10001145.hg.1	MIR604	8.54 ± 0.15	8.50 ± 0.10	8.96 ± 0.47	8.69 ± 0.21
TC10000348.hg.1	MIR605	5.87 ± 0.24	5.79 ± 0.09	5.78 ± 0.11	5.87 ± 0.28

TC10000489.hg.1	MIR606	7.09 ± 0.14	7.04 ± 0.26	6.94 ± 0.09	7.15 ± 0.06
TC10001560.hg.1	MIR607	4.65 ± 0.05	4.56 ± 0.12	4.58 ± 0.19	4.57 ± 0.12
TC10000728.hg.1	MIR608	5.76 ± 0.27	5.95 ± 0.09	5.81 ± 0.31	6.12 ± 0.03
TC10001639.hg.1	MIR609	6.40 ± 0.12	6.32 ± 0.10	6.50 ± 0.12	6.39 ± 0.08
TC11000282.hg.1	MIR610	6.83 ± 0.08	7.00 ± 0.05	6.94 ± 0.07	7.07 ± 0.20
TC11001857.hg.1	MIR611	8.01 ± 0.24	7.90 ± 0.11	7.83 ± 0.18	7.85 ± 0.03
TC11000628.hg.1	MIR612	10.73 ± 0.19	10.92 ± 0.18	11.39 ± 0.36	10.96 ± 0.22
TC12000180.hg.1	MIR613	5.65 ± 0.09	5.77 ± 0.26	5.75 ± 0.21	5.82 ± 0.18
TC12000182.hg.1	MIR614	6.79 ± 0.13	6.74 ± 0.08	6.71 ± 0.15	6.85 ± 0.06
TC12000453.hg.1	MIR615	7.05 ± 0.31	7.20 ± 0.24	6.99 ± 0.24	7.30 ± 0.06
TC12001626.hg.1	MIR616	5.96 ± 0.26	6.62 ± 0.19	6.22 ± 0.52	6.58 ± 0.69
TC12001773.hg.1	MIR617	5.62 ± 0.38	5.46 ± 0.20	5.90 ± 0.57	5.39 ± 0.06
TC12001774.hg.1	MIR618	4.69 ± 0.10	4.29 ± 0.14	5.04 ± 0.45	4.25 ± 0.02
TC12001942.hg.1	MIR619	7.24 ± 0.12	7.19 ± 0.16	7.28 ± 0.03	7.33 ± 0.05
TC12002005.hg.1	MIR620	3.41 ± 0.11	3.13 ± 0.04	3.19 ± 0.12	3.06 ± 0.08
TC13000140.hg.1	MIR621	7.38 ± 0.01	7.21 ± 0.03	7.16 ± 0.08	7.17 ± 0.02
TC13000320.hg.1	MIR622	6.72 ± 0.10	6.67 ± 0.08	6.87 ± 0.12	6.86 ± 0.20
TC13000355.hg.1	MIR623	7.37 ± 0.38	7.87 ± 0.07	7.36 ± 0.21	7.94 ± 0.21
TC14001008.hg.1	MIR624	4.39 ± 0.15	4.16 ± 0.11	4.23 ± 0.33	4.31 ± 0.08
TC14000398.hg.1	MIR625	3.75 ± 0.25	3.52 ± 0.02	3.68 ± 0.16	3.51 ± 0.03
TC15000312.hg.1	MIR626	5.16 ± 0.65	5.46 ± 0.05	5.04 ± 0.41	5.44 ± 0.08
TC15001262.hg.1	MIR627	3.50 ± 0.23	3.46 ± 0.11	3.37 ± 0.17	3.40 ± 0.08
TC15002774.hg.1	MIR628	8.64 ± 0.49	8.61 ± 0.36	9.59 ± 0.59	8.55 ± 0.26
TC15001607.hg.1	MIR629	6.14 ± 0.40	6.21 ± 0.29	5.97 ± 0.20	6.11 ± 0.07
TC15000659.hg.1	MIR630	9.46 ± 0.35	9.83 ± 0.14	9.61 ± 0.32	9.89 ± 0.12
TC15001664.hg.1	MIR631	6.96 ± 0.12	7.13 ± 0.43	7.21 ± 0.24	7.42 ± 0.12
TC17000375.hg.1	MIR632	9.86 ± 0.33	10.01 ± 0.13	9.91 ± 0.20	10.03 ± 0.07
TC17000763.hg.1	MIR633	3.45 ± 0.24	3.22 ± 0.05	3.37 ± 0.30	3.11 ± 0.07
TC17000787.hg.1	MIR634	7.85 ± 0.30	7.86 ± 0.27	7.75 ± 0.07	7.99 ± 0.18
TC17001823.hg.1	MIR635	5.35 ± 0.29	5.23 ± 0.10	5.67 ± 0.50	5.44 ± 0.15
TC17001908.hg.1	MIR636	9.97 ± 0.13	10.19 ± 0.11	9.85 ± 0.09	10.08 ± 0.04

TC19000185.hg.1	MIR638	8.60 ± 0.36	8.64 ± 0.28	8.41 ± 0.51	8.61 ± 0.02
TC19000259.hg.1	MIR639	9.71 ± 0.25	9.34 ± 0.10	9.18 ± 0.15	9.28 ± 0.03
TC19000374.hg.1	MIR640	6.63 ± 0.14	6.73 ± 0.05	6.70 ± 0.06	6.81 ± 0.04
TC19001534.hg.1	MIR641	5.15 ± 0.08	4.95 ± 0.21	5.16 ± 0.16	4.93 ± 0.18
TC19000652.hg.1	MIR642A	8.03 ± 0.46	8.15 ± 0.37	7.84 ± 0.51	8.45 ± 0.18
TC19001627.hg.1	MIR642B	7.00 ± 0.42	6.54 ± 0.12	6.67 ± 0.26	6.45 ± 0.34
TC19000794.hg.1	MIR643	4.68 ± 0.28	4.99 ± 0.14	4.55 ± 0.32	5.35 ± 0.09
TC20000241.hg.1	MIR644A	7.90 ± 0.69	8.42 ± 0.46	8.29 ± 1.00	8.53 ± 0.39
TC20000405.hg.1	MIR645	8.50 ± 0.18	8.75 ± 0.11	8.65 ± 0.38	8.79 ± 0.20
TC20000477.hg.1	MIR646	7.74 ± 0.19	7.81 ± 0.11	8.00 ± 0.19	7.96 ± 0.05
TC20001042.hg.1	MIR647	10.73 ± 0.14	10.68 ± 0.07	10.66 ± 0.16	10.68 ± 0.07
TC22000479.hg.1	MIR648	7.87 ± 0.21	7.91 ± 0.32	8.06 ± 0.18	8.09 ± 0.05
TC22000530.hg.1	MIR649	3.47 ± 0.05	3.24 ± 0.13	3.39 ± 0.02	3.28 ± 0.03
TC22001450.hg.1	MIR650	10.18 ± 0.14	9.47 ± 0.17	9.38 ± 0.20	9.28 ± 0.17
TC0X000029.hg.1	MIR651	3.35 ± 0.24	3.06 ± 0.09	3.19 ± 0.17	3.03 ± 0.04
TC0X000539.hg.1	MIR652	5.96 ± 0.16	5.63 ± 0.05	5.94 ± 0.19	5.49 ± 0.12
TC07001609.hg.1	MIR653	3.66 ± 0.17	3.25 ± 0.09	3.40 ± 0.15	3.41 ± 0.25
TC14000735.hg.1	MIR654	8.02 ± 0.49	8.26 ± 0.38	7.92 ± 0.50	8.51 ± 0.08
TC14000747.hg.1	MIR655	5.62 ± 0.19	5.41 ± 0.06	5.46 ± 0.10	5.40 ± 0.08
TC14000764.hg.1	MIR656	3.99 ± 0.38	4.13 ± 0.14	4.11 ± 0.23	4.23 ± 0.10
TC17001942.hg.1	MIR657	9.19 ± 0.46	9.17 ± 0.36	9.09 ± 0.43	9.34 ± 0.02
TC22000778.hg.1	MIR658	8.11 ± 0.16	8.00 ± 0.13	7.98 ± 0.17	8.05 ± 0.03
TC22000779.hg.1	MIR659	6.87 ± 0.34	6.85 ± 0.25	6.75 ± 0.45	6.95 ± 0.09
TC0X000286.hg.1	MIR660	5.12 ± 0.14	5.12 ± 0.05	5.14 ± 0.05	5.17 ± 0.24
TC08001738.hg.1	MIR661	8.60 ± 0.19	8.83 ± 0.31	8.71 ± 0.15	9.03 ± 0.07
TC16000031.hg.1	MIR662	8.65 ± 0.19	8.81 ± 0.32	8.57 ± 0.27	8.90 ± 0.02
TC20000742.hg.1	MIR663A	10.47 ± 0.10	10.55 ± 0.09	10.30 ± 0.10	10.57 ± 0.04
TC02002360.hg.1	MIR663B	9.83 ± 0.14	9.99 ± 0.11	9.57 ± 0.21	9.97 ± 0.09
TC14000653.hg.1	MIR665	6.99 ± 0.26	6.91 ± 0.20	7.01 ± 0.08	7.18 ± 0.09
TC14000751.hg.1	MIR668	8.09 ± 0.19	8.08 ± 0.09	7.98 ± 0.11	8.04 ± 0.06
TC11000364.hg.1	MIR670	7.07 ± 0.21	6.96 ± 0.19	6.86 ± 0.11	7.28 ± 0.13

TC07001014.hg.1	MIR671	8.30 ± 0.20	8.12 ± 0.03	8.18 ± 0.11	8.14 ± 0.01
TC11001273.hg.1	MIR675	8.10 ± 0.25	8.05 ± 0.20	8.01 ± 0.23	8.15 ± 0.05
TC0X000368.hg.1	MIR676	5.32 ± 0.16	5.24 ± 0.09	5.41 ± 0.01	5.18 ± 0.13
TC11002142.hg.1	MIR708	5.81 ± 0.31	5.97 ± 0.09	5.81 ± 0.27	6.14 ± 0.27
TC09001284.hg.1	MIR7-1	10.61 ± 0.25	10.84 ± 0.08	10.66 ± 0.25	10.79 ± 0.12
TC03001383.hg.1	MIR711	8.87 ± 0.36	8.49 ± 0.14	8.78 ± 0.26	8.61 ± 0.11
TC0X001524.hg.1	MIR718	8.60 ± 0.18	8.61 ± 0.12	8.44 ± 0.19	8.68 ± 0.06
TC15000839.hg.1	MIR7-2	7.98 ± 0.30	7.97 ± 0.16	7.76 ± 0.24	8.04 ± 0.09
TC03000886.hg.1	MIR720	7.29 ± 0.17	7.38 ± 0.09	7.54 ± 0.07	7.58 ± 0.13
TC19000095.hg.1	MIR7-3	6.94 ± 0.13	6.92 ± 0.18	6.88 ± 0.01	6.90 ± 0.05
TC19000094.hg.1	MIR7-3HG	7.45 ± 0.14	7.43 ± 0.15	7.40 ± 0.13	7.52 ± 0.05
TC17000156.hg.1	MIR744	6.76 ± 0.18	6.77 ± 0.27	6.67 ± 0.18	6.94 ± 0.05
TC14000722.hg.1	MIR758	6.22 ± 0.23	6.34 ± 0.40	6.35 ± 0.19	6.54 ± 0.08
TC13000227.hg.1	MIR759	3.47 ± 0.15	3.55 ± 0.01	3.62 ± 0.17	3.54 ± 0.15
TC01000865.hg.1	MIR760	7.64 ± 0.33	7.50 ± 0.34	7.47 ± 0.39	7.62 ± 0.07
TC16000360.hg.1	MIR762	7.47 ± 0.37	7.48 ± 0.37	7.29 ± 0.35	7.46 ± 0.06
TC0X000557.hg.1	MIR764	8.09 ± 0.36	8.29 ± 0.42	7.92 ± 0.39	8.46 ± 0.19
TC01003357.hg.1	MIR765	6.66 ± 0.15	6.61 ± 0.07	6.90 ± 0.05	6.67 ± 0.08
TC0X001313.hg.1	MIR766	8.74 ± 0.29	8.88 ± 0.31	8.69 ± 0.16	9.13 ± 0.21
TC0X001498.hg.1	MIR767	5.08 ± 0.24	5.30 ± 0.18	5.16 ± 0.41	5.36 ± 0.05
TC19000658.hg.1	MIR769	7.59 ± 0.34	7.69 ± 0.21	7.38 ± 0.56	7.89 ± 0.08
TC14000650.hg.1	MIR770	7.64 ± 0.29	7.72 ± 0.33	7.50 ± 0.26	7.87 ± 0.11
TC21000143.hg.1	MIR802	4.56 ± 0.12	4.39 ± 0.02	4.48 ± 0.09	4.62 ± 0.27
TC09000995.hg.1	MIR873	4.88 ± 0.34	4.76 ± 0.33	4.86 ± 0.07	5.00 ± 0.12
TC05001818.hg.1	MIR874	7.07 ± 0.32	6.96 ± 0.24	6.88 ± 0.22	6.98 ± 0.11
TC08001470.hg.1	MIR875	4.33 ± 0.20	4.11 ± 0.09	4.29 ± 0.11	4.06 ± 0.16
TC09000994.hg.1	MIR876	3.98 ± 0.12	4.09 ± 0.03	4.08 ± 0.21	4.10 ± 0.06
TC06000342.hg.1	MIR877	8.25 ± 0.35	8.15 ± 0.22	8.03 ± 0.29	8.09 ± 0.15
TC6_cox_hap2000040.hg.1	MIR877	8.25 ± 0.35	8.15 ± 0.22	8.03 ± 0.29	8.09 ± 0.15
TC6_dbb_hap3000033.hg.1	MIR877	8.25 ± 0.35	8.15 ± 0.22	8.03 ± 0.29	8.09 ± 0.15
TC6_mann_hap4000035.hg.1	MIR877	8.25 ± 0.35	8.15 ± 0.22	8.03 ± 0.29	8.09 ± 0.15

TC6_mcf_hap5000027.hg.1	MIR877	8.25 ± 0.35	8.15 ± 0.22	8.03 ± 0.29	8.09 ± 0.15
TC6_qbl_hap6000033.hg.1	MIR877	8.25 ± 0.35	8.15 ± 0.22	8.03 ± 0.29	8.09 ± 0.15
TC6_ssto_hap7000033.hg.1	MIR877	8.25 ± 0.35	8.15 ± 0.22	8.03 ± 0.29	8.09 ± 0.15
TC03001161.hg.1	MIR885	8.40 ± 0.37	8.46 ± 0.27	8.37 ± 0.29	8.57 ± 0.22
TC05000089.hg.1	MIR887	7.45 ± 0.35	7.23 ± 0.19	7.32 ± 0.32	7.42 ± 0.08
TC0X001452.hg.1	MIR888	4.93 ± 0.18	4.63 ± 0.09	4.74 ± 0.07	4.60 ± 0.09
TC14000745.hg.1	MIR889	4.75 ± 0.22	4.50 ± 0.14	4.64 ± 0.19	4.58 ± 0.10
TC0X001451.hg.1	MIR890	3.41 ± 0.08	3.24 ± 0.05	3.35 ± 0.11	3.21 ± 0.05
TC0X001456.hg.1	MIR891A	6.44 ± 0.11	6.50 ± 0.13	6.61 ± 0.10	6.50 ± 0.07
TC0X001455.hg.1	MIR891B	3.43 ± 0.20	3.48 ± 0.04	3.53 ± 0.22	3.36 ± 0.02
TC0X001453.hg.1	MIR892A	4.94 ± 0.21	4.99 ± 0.15	4.85 ± 0.16	5.02 ± 0.19
TC0X001454.hg.1	MIR892B	5.24 ± 0.11	5.27 ± 0.21	5.34 ± 0.13	5.34 ± 0.10
TC01003339.hg.1	MIR9-1	5.93 ± 0.45	6.28 ± 0.27	5.80 ± 0.02	6.49 ± 0.07
TC05001577.hg.1	MIR9-2	5.86 ± 0.20	5.70 ± 0.06	5.75 ± 0.08	5.81 ± 0.07
TC12000245.hg.1	MIR920	6.68 ± 0.27	6.30 ± 0.08	6.52 ± 0.28	6.35 ± 0.16
TC01003471.hg.1	MIR921	7.67 ± 0.13	7.82 ± 0.18	7.73 ± 0.11	8.01 ± 0.14
TC03002182.hg.1	MIR922	8.71 ± 0.26	8.56 ± 0.08	8.64 ± 0.23	8.52 ± 0.05
TC0X001382.hg.1	MIR92A2	3.82 ± 0.32	3.72 ± 0.03	3.76 ± 0.10	3.82 ± 0.08
TC01001294.hg.1	MIR92B	8.44 ± 0.29	8.61 ± 0.36	8.38 ± 0.32	8.72 ± 0.09
TC07001656.hg.1	MIR93	6.10 ± 0.01	6.07 ± 0.05	5.79 ± 0.12	6.20 ± 0.27
TC15000848.hg.1	MIR9-3	7.40 ± 0.23	7.35 ± 0.17	7.34 ± 0.16	7.46 ± 0.04
TC02002546.hg.1	MIR933	9.71 ± 0.34	9.72 ± 0.13	9.55 ± 0.38	9.75 ± 0.03
TC0X000663.hg.1	MIR934	5.32 ± 0.50	5.14 ± 0.24	5.36 ± 0.17	5.05 ± 0.11
TC19000869.hg.1	MIR935	8.31 ± 0.27	8.33 ± 0.25	8.27 ± 0.29	8.43 ± 0.03
TC10001637.hg.1	MIR936	7.03 ± 0.19	6.98 ± 0.18	6.99 ± 0.19	7.08 ± 0.08
TC10001146.hg.1	MIR938	5.39 ± 0.14	5.48 ± 0.18	5.82 ± 0.51	5.37 ± 0.08
TC08001748.hg.1	MIR939	8.97 ± 0.08	8.95 ± 0.08	8.69 ± 0.09	8.87 ± 0.07
TC16000069.hg.1	MIR940	9.11 ± 0.26	9.05 ± 0.26	8.97 ± 0.15	9.19 ± 0.14
TC01001027.hg.1	MIR942	6.11 ± 0.26	6.33 ± 0.32	6.01 ± 0.09	6.44 ± 0.24
TC04000971.hg.1	MIR943	8.72 ± 0.13	8.64 ± 0.14	8.57 ± 0.13	8.68 ± 0.01
TC03001043.hg.1	MIR944	3.52 ± 0.15	3.09 ± 0.08	3.16 ± 0.04	3.10 ± 0.11

TC04001016.hg.1	MIR95	3.55 ± 0.16	3.48 ± 0.09	3.54 ± 0.15	3.53 ± 0.08
TC07001855.hg.1	MIR96	5.55 ± 0.18	5.77 ± 0.13	5.75 ± 0.13	5.74 ± 0.02
TC0X001068.hg.1	MIR98	7.29 ± 0.14	7.11 ± 0.06	7.15 ± 0.06	7.13 ± 0.07
TC21000041.hg.1	MIR99A	5.98 ± 0.31	5.80 ± 0.15	5.79 ± 0.35	5.84 ± 0.06
TC19000784.hg.1	MIR99B	7.82 ± 0.17	7.71 ± 0.15	7.83 ± 0.09	7.70 ± 0.05
TC09000456.hg.1	MIRLET7A1	6.23 ± 0.20	6.46 ± 0.25	6.31 ± 0.04	6.59 ± 0.03
TC11002384.hg.1	MIRLET7A2	5.14 ± 0.17	4.70 ± 0.28	4.85 ± 0.17	4.46 ± 0.12
TC22001505.hg.1	MIRLET7A3	8.23 ± 0.14	8.23 ± 0.20	8.24 ± 0.09	8.28 ± 0.05
TC21000042.hg.1	MIRLET7C	4.18 ± 0.40	3.89 ± 0.18	3.71 ± 0.20	4.05 ± 0.19
TC09000458.hg.1	MIRLET7D	7.20 ± 0.09	6.95 ± 0.25	7.26 ± 0.21	7.12 ± 0.08
TC09001354.hg.1	MIRLET7DHG	6.10 ± 0.17	6.00 ± 0.05	6.07 ± 0.13	6.13 ± 0.15
TC19000785.hg.1	MIRLET7E	5.65 ± 0.41	5.59 ± 0.16	5.47 ± 0.34	5.61 ± 0.07
TC09000457.hg.1	MIRLET7F1	6.14 ± 0.39	5.99 ± 0.27	6.12 ± 0.18	6.06 ± 0.16
TC0X001069.hg.1	MIRLET7F2	4.61 ± 0.31	4.27 ± 0.09	4.28 ± 0.09	4.32 ± 0.09
TC03001450.hg.1	MIRLET7G	5.98 ± 0.28	5.70 ± 0.19	5.95 ± 0.27	5.92 ± 0.30
TC12000558.hg.1	MIRLET7I	8.25 ± 0.17	8.80 ± 0.15	8.35 ± 0.23	8.84 ± 0.13
TC17000067.hg.1	MIS12	7.32 ± 0.17	7.20 ± 0.19	7.13 ± 0.12	7.00 ± 0.06
TC21000395.hg.1	MIS18A	6.23 ± 0.13	6.10 ± 0.12	5.90 ± 0.18	5.98 ± 0.05
TC21000114.hg.1	MIS18A-AS1	5.30 ± 0.26	5.31 ± 0.15	5.33 ± 0.06	5.33 ± 0.07
TC14001085.hg.1	MIS18BP1	8.12 ± 0.61	8.35 ± 0.28	8.37 ± 0.43	8.15 ± 0.24
TC19000014.hg.1	MISP	7.90 ± 0.22	7.85 ± 0.16	7.87 ± 0.13	7.91 ± 0.03
TC02002132.hg.1	MITD1	6.38 ± 0.19	6.20 ± 0.18	6.50 ± 0.35	6.09 ± 0.16
TC03000419.hg.1	MITF	6.58 ± 0.07	6.27 ± 0.04	6.68 ± 0.11	6.29 ± 0.06
TC01001860.hg.1	MIXL1	7.12 ± 0.24	6.80 ± 0.17	6.78 ± 0.18	6.78 ± 0.04
TC10001756.hg.1	MKI67	8.49 ± 0.18	8.32 ± 0.79	8.23 ± 0.48	7.29 ± 0.29
TC02002267.hg.1	MKI67IP	7.76 ± 0.31	7.83 ± 0.19	7.58 ± 0.25	7.79 ± 0.15
TC20000619.hg.1	MKKS	7.70 ± 0.08	7.42 ± 0.12	7.73 ± 0.18	7.27 ± 0.21
TC22000811.hg.1	MKL1	9.50 ± 0.11	9.36 ± 0.08	9.53 ± 0.08	9.36 ± 0.13
TC16000171.hg.1	MKL2	7.60 ± 0.27	7.68 ± 0.08	7.45 ± 0.25	7.61 ± 0.12
TC07000818.hg.1	MKLN1	8.58 ± 0.33	8.73 ± 0.20	8.79 ± 0.36	8.61 ± 0.08
TC01006353.hg.1	MKNK1	8.45 ± 0.23	8.10 ± 0.10	8.98 ± 0.38	8.09 ± 0.21

TC01000589.hg.1	MKNK1-AS1	6.36 ± 0.20	6.37 ± 0.02	6.36 ± 0.08	6.47 ± 0.04
TC19001019.hg.1	MKNK2	9.90 ± 0.22	9.98 ± 0.12	9.86 ± 0.14	9.95 ± 0.08
TC07001923.hg.1	MKRN1	9.34 ± 0.04	9.33 ± 0.08	9.34 ± 0.26	9.33 ± 0.05
TC03000072.hg.1	MKRN2	8.25 ± 0.21	8.29 ± 0.07	8.16 ± 0.10	8.22 ± 0.11
TC15000040.hg.1	MKRN3	7.09 ± 0.13	7.13 ± 0.11	7.11 ± 0.11	7.19 ± 0.06
TC0X000193.hg.1	MKRN4P	5.87 ± 0.11	5.82 ± 0.10	5.79 ± 0.23	5.81 ± 0.04
TC20000902.hg.1	MKRN7P	6.59 ± 0.21	6.55 ± 0.10	6.54 ± 0.17	6.57 ± 0.08
TC12001789.hg.1	MKRN9P	7.71 ± 0.22	7.76 ± 0.08	7.90 ± 0.17	7.91 ± 0.08
TC17001726.hg.1	MKS1	7.36 ± 0.13	7.31 ± 0.06	7.24 ± 0.15	7.31 ± 0.08
TC10001133.hg.1	MKX	6.51 ± 0.16	6.43 ± 0.14	6.45 ± 0.17	6.48 ± 0.04
TC09000042.hg.1	MLANA	6.16 ± 0.11	6.12 ± 0.03	6.10 ± 0.03	6.21 ± 0.07
TC22000907.hg.1	MLC1	7.90 ± 0.10	7.53 ± 0.07	7.79 ± 0.14	7.53 ± 0.03
TC12000949.hg.1	MLEC	8.88 ± 0.19	8.48 ± 0.02	8.66 ± 0.16	8.24 ± 0.12
TC03000865.hg.1	MLF1	4.92 ± 0.24	4.84 ± 0.04	4.81 ± 0.09	4.86 ± 0.08
TC04001808.hg.1	MLF1IP	6.00 ± 0.12	6.12 ± 0.28	6.09 ± 0.22	5.79 ± 0.05
TC12001149.hg.1	MLF2	10.41 ± 0.20	10.41 ± 0.07	10.41 ± 0.08	10.50 ± 0.08
TC03000175.hg.1	MLH1	7.64 ± 0.05	7.56 ± 0.17	7.44 ± 0.09	7.43 ± 0.03
TC14001315.hg.1	MLH3	6.94 ± 0.09	6.81 ± 0.07	6.79 ± 0.09	6.78 ± 0.04
TC06004092.hg.1	MLIP	5.20 ± 0.09	5.17 ± 0.08	5.17 ± 0.04	5.28 ± 0.11
TC06001820.hg.1	MLIP-AS1	4.16 ± 0.28	4.18 ± 0.04	4.03 ± 0.02	4.36 ± 0.27
TC06000671.hg.1	MLIP-IT1	5.00 ± 0.08	4.91 ± 0.07	4.97 ± 0.05	4.99 ± 0.08
TC02002532.hg.1	MLK7-AS1	5.32 ± 0.04	5.21 ± 0.05	5.28 ± 0.03	5.29 ± 0.02
TC16001270.hg.1	MLKL	9.37 ± 0.37	8.98 ± 0.39	10.05 ± 0.51	8.75 ± 0.20
TC19001091.hg.1	MLLT1	9.18 ± 0.20	9.09 ± 0.03	9.35 ± 0.14	9.01 ± 0.15
TC10000152.hg.1	MLLT10	8.14 ± 0.26	8.33 ± 0.15	8.10 ± 0.34	8.24 ± 0.09
TC20000747.hg.1	MLLT10P1	5.24 ± 0.46	5.05 ± 0.18	5.21 ± 0.48	5.19 ± 0.10
TC01001201.hg.1	MLLT11	7.03 ± 0.07	7.02 ± 0.06	7.02 ± 0.07	6.90 ± 0.11
TC09000941.hg.1	MLLT3	8.15 ± 0.41	8.62 ± 0.16	8.10 ± 0.30	8.58 ± 0.06
TC06001184.hg.1	MLLT4	6.88 ± 0.03	6.94 ± 0.07	7.05 ± 0.11	7.02 ± 0.06
TC06002319.hg.1	MLLT4-AS1	5.61 ± 0.16	5.58 ± 0.15	5.53 ± 0.09	5.59 ± 0.03
TC17000462.hg.1	MLLT6	9.12 ± 0.12	9.16 ± 0.03	8.88 ± 0.08	9.10 ± 0.04

TC06001594.hg.1	MLN	7.35 ± 0.25	7.40 ± 0.19	7.31 ± 0.17	7.50 ± 0.12
TC13000196.hg.1	MLNR	8.81 ± 0.22	8.85 ± 0.12	8.88 ± 0.21	8.96 ± 0.09
TC02001441.hg.1	MLPH	7.11 ± 0.17	7.08 ± 0.13	7.03 ± 0.14	7.14 ± 0.03
TC16000064.hg.1	MLST8	8.60 ± 0.19	8.47 ± 0.12	8.32 ± 0.17	8.52 ± 0.05
TC17000530.hg.1	MLX	9.06 ± 0.33	8.55 ± 0.07	8.95 ± 0.18	8.47 ± 0.12
TC12000969.hg.1	MLXIP	9.48 ± 0.13	9.39 ± 0.03	9.44 ± 0.13	9.48 ± 0.09
TC07001502.hg.1	MLXIPL	7.80 ± 0.23	7.75 ± 0.18	7.77 ± 0.13	7.83 ± 0.03
TC16000651.hg.1	MLYCD	7.88 ± 0.05	7.88 ± 0.01	7.84 ± 0.08	7.85 ± 0.03
TC04000714.hg.1	MMAA	6.88 ± 0.17	6.85 ± 0.08	6.78 ± 0.12	6.80 ± 0.07
TC12001952.hg.1	MMAB	7.78 ± 0.22	7.72 ± 0.07	7.60 ± 0.17	7.71 ± 0.06
TC01000573.hg.1	MMACHC	7.16 ± 0.08	6.99 ± 0.05	7.04 ± 0.08	6.99 ± 0.01
TC02002415.hg.1	MMADHC	9.19 ± 0.09	9.18 ± 0.21	9.35 ± 0.34	9.10 ± 0.01
TC17001711.hg.1	MMD	9.73 ± 0.64	9.31 ± 0.13	9.91 ± 0.31	9.51 ± 0.40
TC07001113.hg.1	MMD2	7.36 ± 0.20	7.35 ± 0.17	7.33 ± 0.11	7.46 ± 0.03
TC03000846.hg.1	MME	8.77 ± 0.23	8.99 ± 0.33	9.03 ± 0.53	8.70 ± 0.29
TC03001924.hg.1	MME-AS1	6.05 ± 0.20	5.81 ± 0.04	6.23 ± 0.44	5.67 ± 0.10
TC01002123.hg.1	MMEL1	7.84 ± 0.17	7.80 ± 0.20	7.76 ± 0.20	7.85 ± 0.04
TC0X001412.hg.1	MMGT1	8.90 ± 0.09	9.09 ± 0.11	9.04 ± 0.26	9.07 ± 0.16
TC11002234.hg.1	MMP1	4.87 ± 0.14	4.71 ± 0.09	4.80 ± 0.07	4.73 ± 0.06
TC11002233.hg.1	MMP10	5.51 ± 0.11	5.38 ± 0.08	5.37 ± 0.12	5.40 ± 0.02
TC22000138.hg.1	MMP11	7.82 ± 0.21	7.79 ± 0.22	7.76 ± 0.21	7.89 ± 0.05
TC11002237.hg.1	MMP12	4.94 ± 0.15	4.80 ± 0.06	4.87 ± 0.09	4.85 ± 0.04
TC11002239.hg.1	MMP13	5.00 ± 0.13	4.85 ± 0.03	4.86 ± 0.02	4.90 ± 0.07
TC14000133.hg.1	MMP14	7.02 ± 0.20	6.83 ± 0.19	6.93 ± 0.15	6.97 ± 0.01
TC16000500.hg.1	MMP15	7.80 ± 0.22	7.75 ± 0.18	7.72 ± 0.18	7.81 ± 0.04
TC08001396.hg.1	MMP16	5.05 ± 0.22	4.88 ± 0.04	4.89 ± 0.09	4.96 ± 0.08
TC12001047.hg.1	MMP17	8.50 ± 0.20	8.41 ± 0.16	8.38 ± 0.20	8.42 ± 0.01
TC12001587.hg.1	MMP19	7.02 ± 0.24	6.92 ± 0.15	7.00 ± 0.16	6.99 ± 0.04
TC16000454.hg.1	MMP2	7.03 ± 0.20	7.02 ± 0.22	7.02 ± 0.11	7.15 ± 0.07
TC11002229.hg.1	MMP20	5.98 ± 0.11	5.87 ± 0.05	5.85 ± 0.12	5.89 ± 0.04
TC10001738.hg.1	MMP21	6.70 ± 0.09	6.70 ± 0.12	6.65 ± 0.10	6.73 ± 0.08

TC01000047.hg.1	MMP23B	8.27 ± 0.21	8.26 ± 0.21	8.18 ± 0.20	8.29 ± 0.03
TC20000250.hg.1	MMP24	7.58 ± 0.26	7.56 ± 0.20	7.54 ± 0.23	7.62 ± 0.02
TC20001748.hg.1	MMP24-AS1	8.36 ± 0.15	8.21 ± 0.02	8.27 ± 0.09	8.24 ± 0.02
TC16000098.hg.1	MMP25	9.82 ± 0.44	9.48 ± 0.13	10.38 ± 0.36	9.44 ± 0.37
TC11000099.hg.1	MMP26	4.87 ± 0.15	4.78 ± 0.10	4.74 ± 0.06	4.84 ± 0.06
TC11002230.hg.1	MMP27	5.26 ± 0.14	5.19 ± 0.11	5.19 ± 0.07	5.27 ± 0.07
TC17001376.hg.1	MMP28	7.14 ± 0.19	7.10 ± 0.14	7.11 ± 0.23	7.16 ± 0.01
TC11002235.hg.1	MMP3	5.40 ± 0.15	5.27 ± 0.07	5.29 ± 0.07	5.31 ± 0.04
TC11002228.hg.1	MMP7	4.46 ± 0.21	4.39 ± 0.05	4.40 ± 0.09	4.40 ± 0.03
TC11002231.hg.1	MMP8	7.51 ± 1.08	5.79 ± 0.14	8.63 ± 0.83	5.71 ± 0.22
TC20000363.hg.1	MMP9	10.58 ± 0.41	9.38 ± 0.07	11.40 ± 0.52	9.24 ± 0.46
TC04000493.hg.1	MMRN1	6.68 ± 0.17	6.36 ± 0.11	7.28 ± 0.14	6.43 ± 0.17
TC10001482.hg.1	MMRN2	6.94 ± 0.20	6.97 ± 0.21	6.95 ± 0.20	7.08 ± 0.07
TC10001567.hg.1	MMS19	8.29 ± 0.04	8.18 ± 0.04	8.06 ± 0.08	8.16 ± 0.05
TC06001959.hg.1	MMS22L	5.98 ± 0.18	5.95 ± 0.23	5.82 ± 0.16	5.72 ± 0.07
TC22000620.hg.1	MN1	6.65 ± 0.19	6.62 ± 0.18	6.53 ± 0.14	6.92 ± 0.13
TC14002297.hg.1	MNAT1	6.34 ± 0.24	6.38 ± 0.16	6.14 ± 0.21	6.26 ± 0.05
TC04000773.hg.1	MND1	5.95 ± 0.10	5.78 ± 0.09	5.85 ± 0.05	5.60 ± 0.08
TC01001346.hg.1	MNDA	11.72 ± 0.24	11.67 ± 0.37	12.24 ± 0.25	11.52 ± 0.39
TC06001591.hg.1	MNF1	7.74 ± 0.17	7.49 ± 0.06	7.39 ± 0.18	7.35 ± 0.04
TC15001476.hg.1	MNS1	4.88 ± 0.19	4.71 ± 0.07	4.80 ± 0.08	4.71 ± 0.06
TC17000994.hg.1	MNT	8.96 ± 0.14	9.13 ± 0.05	8.95 ± 0.09	9.23 ± 0.02
TC07002074.hg.1	MNX1	8.10 ± 0.21	8.04 ± 0.17	8.11 ± 0.12	8.11 ± 0.04
TC14001454.hg.1	MOAP1	8.63 ± 0.10	9.03 ± 0.18	8.52 ± 0.14	9.11 ± 0.02
TC02001993.hg.1	MOB1A	10.52 ± 0.25	10.75 ± 0.12	10.89 ± 0.33	10.71 ± 0.16
TC04002912.hg.1	MOB1B	7.75 ± 0.66	7.61 ± 0.27	7.94 ± 0.42	7.71 ± 0.24
TC11001259.hg.1	MOB2	7.97 ± 0.12	7.89 ± 0.06	7.85 ± 0.15	7.89 ± 0.03
TC19001020.hg.1	MOB3A	12.34 ± 0.22	12.19 ± 0.13	12.62 ± 0.17	12.18 ± 0.31
TC09000988.hg.1	MOB3B	7.95 ± 0.12	7.92 ± 0.05	7.80 ± 0.05	7.88 ± 0.06
TC01006354.hg.1	MOB3C	8.23 ± 0.27	7.97 ± 0.09	8.30 ± 0.29	7.95 ± 0.06
TC02005005.hg.1	MOB4	8.19 ± 0.30	8.42 ± 0.17	8.32 ± 0.26	8.34 ± 0.11

TC03000203.hg.1	MOBP	5.43 ± 0.09	5.38 ± 0.11	5.41 ± 0.08	5.48 ± 0.10
TC18000149.hg.1	MOCOS	6.00 ± 0.11	5.95 ± 0.12	5.92 ± 0.10	6.06 ± 0.15
TC06004132.hg.1	MOCS1	7.15 ± 0.20	7.10 ± 0.12	7.05 ± 0.15	7.15 ± 0.04
TC05001342.hg.1	MOCS2	7.21 ± 0.22	7.30 ± 0.13	7.06 ± 0.23	7.17 ± 0.11
TC20000410.hg.1	MOCS3	6.61 ± 0.24	6.36 ± 0.12	6.46 ± 0.15	6.27 ± 0.17
TC06000322.hg.1	MOG	6.15 ± 0.14	6.10 ± 0.09	6.11 ± 0.09	6.18 ± 0.07
TC6_apd_hap1000015.hg.1	MOG	6.17 ± 0.16	6.15 ± 0.11	6.13 ± 0.10	6.23 ± 0.10
TC6_cox_hap2000019.hg.1	MOG	6.03 ± 0.13	5.99 ± 0.09	5.99 ± 0.09	6.08 ± 0.10
TC6_dbb_hap3000015.hg.1	MOG	6.17 ± 0.15	6.15 ± 0.11	6.13 ± 0.10	6.23 ± 0.10
TC6_mann_hap4000016.hg.1	MOG	6.17 ± 0.15	6.15 ± 0.11	6.13 ± 0.10	6.23 ± 0.10
TC6_mcf_hap5000014.hg.1	MOG	6.07 ± 0.14	6.04 ± 0.11	6.02 ± 0.10	6.12 ± 0.11
TC6_qbl_hap6000014.hg.1	MOG	6.07 ± 0.14	6.04 ± 0.11	6.03 ± 0.09	6.12 ± 0.11
TC6_ssto_hap7000015.hg.1	MOG	6.17 ± 0.16	6.15 ± 0.11	6.13 ± 0.10	6.23 ± 0.10
TC02001349.hg.1	MOGAT1	6.52 ± 0.21	6.53 ± 0.19	6.42 ± 0.09	6.57 ± 0.04
TC11000811.hg.1	MOGAT2	6.90 ± 0.18	6.87 ± 0.25	6.87 ± 0.13	6.99 ± 0.06
TC07001685.hg.1	MOGAT3	7.17 ± 0.20	7.14 ± 0.22	7.10 ± 0.24	7.32 ± 0.09
TC02001997.hg.1	MOGS	8.44 ± 0.08	8.20 ± 0.03	8.23 ± 0.10	8.21 ± 0.06
TC14001527.hg.1	MOK	6.38 ± 0.15	6.28 ± 0.05	6.28 ± 0.07	6.37 ± 0.02
TC03001422.hg.1	MON1A	7.73 ± 0.17	7.60 ± 0.13	7.63 ± 0.18	7.66 ± 0.02
TC16002059.hg.1	MON1B	8.98 ± 0.13	8.78 ± 0.02	9.01 ± 0.15	8.74 ± 0.14
TC12000556.hg.1	MON2	8.17 ± 0.50	8.34 ± 0.29	8.25 ± 0.45	8.11 ± 0.09
TC03001635.hg.1	MORC1	5.28 ± 0.08	5.21 ± 0.09	5.22 ± 0.11	5.31 ± 0.10
TC03000551.hg.1	MORC1-AS1	6.54 ± 0.20	6.45 ± 0.17	6.48 ± 0.24	6.70 ± 0.24
TC22000660.hg.1	MORC2	8.39 ± 0.09	8.20 ± 0.06	8.12 ± 0.07	8.10 ± 0.02
TC22000219.hg.1	MORC2-AS1	7.39 ± 0.16	7.35 ± 0.05	7.37 ± 0.11	7.42 ± 0.04
TC21001062.hg.1	MORC3	9.00 ± 0.48	9.19 ± 0.31	9.43 ± 0.49	9.18 ± 0.22
TC0X001257.hg.1	MORC4	6.00 ± 0.03	6.05 ± 0.10	6.01 ± 0.02	6.00 ± 0.01
TC04001745.hg.1	MORF4	7.93 ± 0.35	8.17 ± 0.23	8.13 ± 0.27	8.17 ± 0.23
TC15000735.hg.1	MORF4L1	9.73 ± 0.17	9.96 ± 0.14	9.76 ± 0.14	10.00 ± 0.11
TC0X001242.hg.1	MORF4L2	8.29 ± 0.16	8.50 ± 0.06	8.34 ± 0.32	8.52 ± 0.13
TC0X000501.hg.1	MORF4L2-AS1	6.44 ± 0.19	6.45 ± 0.09	6.33 ± 0.12	6.50 ± 0.02

TC01002115.hg.1	MORN1	8.11 ± 0.20	8.04 ± 0.10	8.01 ± 0.19	8.09 ± 0.03
TC02004959.hg.1	MORN2	5.45 ± 0.12	5.33 ± 0.04	5.39 ± 0.03	5.25 ± 0.08
TC12002064.hg.1	MORN3	7.75 ± 0.17	7.84 ± 0.13	7.68 ± 0.17	7.80 ± 0.03
TC10001568.hg.1	MORN4	7.16 ± 0.23	7.10 ± 0.10	7.03 ± 0.12	7.15 ± 0.08
TC09000612.hg.1	MORN5	5.46 ± 0.09	5.31 ± 0.10	5.36 ± 0.11	5.29 ± 0.06
TC08001229.hg.1	MOS	7.08 ± 0.31	7.07 ± 0.26	7.05 ± 0.36	7.17 ± 0.10
TC0X001397.hg.1	MOSPD1	7.25 ± 0.30	7.32 ± 0.14	7.35 ± 0.26	7.22 ± 0.22
TC0X000066.hg.1	MOSPD2	8.64 ± 0.39	8.37 ± 0.46	9.47 ± 0.56	8.15 ± 0.45
TC07000628.hg.1	MOSPD3	8.48 ± 0.18	8.37 ± 0.13	8.45 ± 0.09	8.40 ± 0.03
TC01000992.hg.1	MOV10	8.81 ± 0.41	8.46 ± 0.09	8.75 ± 0.35	8.38 ± 0.01
TC22000418.hg.1	MOV10L1	6.90 ± 0.16	6.87 ± 0.13	6.82 ± 0.13	6.91 ± 0.03
TC06002113.hg.1	MOXD1	5.58 ± 0.07	5.37 ± 0.01	5.38 ± 0.03	5.32 ± 0.10
TC07001936.hg.1	MOXD2P	6.38 ± 0.19	6.28 ± 0.13	6.33 ± 0.11	6.39 ± 0.11
TC06002307.hg.1	MPC1	7.80 ± 0.17	7.81 ± 0.15	7.78 ± 0.14	7.84 ± 0.20
TC0X000191.hg.1	MPC1L	6.70 ± 0.26	6.75 ± 0.27	6.58 ± 0.22	6.88 ± 0.03
TC01003484.hg.1	MPC2	8.35 ± 0.09	8.31 ± 0.04	8.28 ± 0.12	8.27 ± 0.06
TC17000104.hg.1	MPDU1	8.83 ± 0.20	8.63 ± 0.08	8.65 ± 0.12	8.52 ± 0.04
TC09000913.hg.1	MPDZ	5.40 ± 0.10	5.28 ± 0.07	5.36 ± 0.07	5.37 ± 0.05
TC11001818.hg.1	MPEG1	11.46 ± 0.20	11.68 ± 0.20	11.52 ± 0.38	11.60 ± 0.24
TC16000004.hg.1	MPG	8.05 ± 0.22	7.96 ± 0.21	7.93 ± 0.22	8.00 ± 0.06
TC02000428.hg.1	MPHOSPH10	7.94 ± 0.40	8.12 ± 0.12	7.86 ± 0.35	8.10 ± 0.08
TC16001302.hg.1	MPHOSPH6	7.55 ± 0.44	7.71 ± 0.10	7.59 ± 0.37	7.44 ± 0.23
TC13000017.hg.1	MPHOSPH8	7.97 ± 0.23	7.92 ± 0.25	7.97 ± 0.26	7.74 ± 0.09
TC12002084.hg.1	MPHOSPH9	7.62 ± 0.52	7.85 ± 0.27	7.44 ± 0.43	7.64 ± 0.05
TC15000687.hg.1	MPI	8.70 ± 0.15	8.56 ± 0.08	8.41 ± 0.11	8.50 ± 0.02
TC01000544.hg.1	MPL	8.58 ± 0.06	8.22 ± 0.21	8.90 ± 0.07	8.42 ± 0.10
TC07001314.hg.1	MPLKIP	8.99 ± 0.13	8.69 ± 0.07	8.86 ± 0.22	8.58 ± 0.25
TC19000087.hg.1	MPND	8.30 ± 0.24	8.14 ± 0.12	8.13 ± 0.20	8.17 ± 0.03
TC17001727.hg.1	MPO	8.82 ± 0.63	8.15 ± 0.22	8.70 ± 0.42	8.25 ± 0.08
TC0X001544.hg.1	MPP1	9.37 ± 0.24	9.03 ± 0.12	9.90 ± 0.19	9.23 ± 0.32
TC17001565.hg.1	MPP2	7.69 ± 0.21	7.64 ± 0.22	7.60 ± 0.23	7.73 ± 0.04

TC17001564.hg.1	MPP3	7.32 ± 0.17	7.30 ± 0.16	7.26 ± 0.12	7.37 ± 0.06
TC02002677.hg.1	MPP4	6.25 ± 0.12	6.19 ± 0.07	6.20 ± 0.06	6.26 ± 0.05
TC14000403.hg.1	MPP5	6.65 ± 0.19	6.72 ± 0.12	6.50 ± 0.22	6.65 ± 0.03
TC07000155.hg.1	MPP6	6.95 ± 0.10	7.03 ± 0.13	6.81 ± 0.07	6.95 ± 0.05
TC10001136.hg.1	MPP7	7.81 ± 0.27	8.02 ± 0.14	8.31 ± 0.40	7.98 ± 0.14
TC18000368.hg.1	MPPE1	8.57 ± 0.15	8.35 ± 0.13	8.55 ± 0.23	8.20 ± 0.11
TC22000367.hg.1	MPPED1	7.64 ± 0.19	7.57 ± 0.15	7.58 ± 0.19	7.69 ± 0.06
TC11001523.hg.1	MPPED2	6.12 ± 0.14	5.99 ± 0.05	6.02 ± 0.11	6.06 ± 0.09
TC17000194.hg.1	MPRIP	8.79 ± 0.01	8.84 ± 0.06	8.54 ± 0.05	8.74 ± 0.08
TC17001183.hg.1	MPRIP-AS1	6.24 ± 0.13	6.25 ± 0.14	6.21 ± 0.37	6.50 ± 0.20
TC22000275.hg.1	MPST	7.91 ± 0.27	7.90 ± 0.17	7.84 ± 0.25	7.93 ± 0.05
TC02001681.hg.1	MPV17	8.46 ± 0.04	8.35 ± 0.00	8.29 ± 0.07	8.23 ± 0.05
TC16000189.hg.1	MPV17L	6.10 ± 0.08	5.96 ± 0.04	5.99 ± 0.06	6.05 ± 0.06
TC19000353.hg.1	MPV17L2	8.73 ± 0.28	8.45 ± 0.08	8.45 ± 0.17	8.47 ± 0.07
TC01003424.hg.1	MPZ	7.85 ± 0.11	7.71 ± 0.09	7.90 ± 0.02	7.79 ± 0.04
TC01001461.hg.1	MPZL1	7.82 ± 0.08	7.87 ± 0.16	8.04 ± 0.14	7.71 ± 0.14
TC11002346.hg.1	MPZL2	7.12 ± 0.11	6.93 ± 0.22	7.22 ± 0.22	6.94 ± 0.12
TC11002345.hg.1	MPZL3	8.48 ± 0.26	8.30 ± 0.21	8.97 ± 0.56	8.35 ± 0.21
TC01001568.hg.1	MR1	8.00 ± 0.28	7.57 ± 0.10	8.09 ± 0.35	7.48 ± 0.11
TC21000115.hg.1	MRAP	6.92 ± 0.20	7.00 ± 0.24	6.95 ± 0.14	7.16 ± 0.14
TC06000777.hg.1	MRAP2	7.04 ± 0.25	6.88 ± 0.13	6.90 ± 0.27	6.90 ± 0.05
TC03000744.hg.1	MRAS	7.12 ± 0.11	7.02 ± 0.09	7.09 ± 0.12	7.10 ± 0.04
TC10000132.hg.1	MRC1	5.87 ± 0.14	5.83 ± 0.01	5.95 ± 0.10	5.87 ± 0.03
TC10000135.hg.1	MRC1	5.71 ± 0.14	5.65 ± 0.03	5.79 ± 0.12	5.68 ± 0.04
TC17000758.hg.1	MRC2	7.84 ± 0.23	7.78 ± 0.20	7.76 ± 0.21	7.86 ± 0.03
TC11002199.hg.1	MRE11A	7.51 ± 0.52	7.76 ± 0.26	7.51 ± 0.38	7.55 ± 0.12
TC02005051.hg.1	MREG	6.48 ± 0.08	6.42 ± 0.01	6.39 ± 0.05	6.42 ± 0.02
TC04000070.hg.1	MRFAP1	9.42 ± 0.09	9.54 ± 0.08	9.33 ± 0.29	9.58 ± 0.17
TC04001004.hg.1	MRFAP1L1	8.59 ± 0.33	8.72 ± 0.14	8.51 ± 0.44	8.75 ± 0.06
TC20000497.hg.1	MRGBP	7.73 ± 0.23	7.69 ± 0.13	7.57 ± 0.16	7.68 ± 0.03
TC11002010.hg.1	MRGPRD	7.15 ± 0.18	7.25 ± 0.32	7.04 ± 0.20	7.34 ± 0.12

TC11001293.hg.1	MRGPRE	7.96 ± 0.22	7.88 ± 0.21	7.93 ± 0.07	8.02 ± 0.09
TC11002013.hg.1	MRGPRF	7.71 ± 0.26	7.72 ± 0.19	7.76 ± 0.13	7.77 ± 0.07
TC11001292.hg.1	MRGPRG	8.21 ± 0.18	8.35 ± 0.20	8.18 ± 0.35	8.40 ± 0.07
TC11000074.hg.1	MRGPRG-AS1	7.67 ± 0.33	7.63 ± 0.26	7.50 ± 0.29	7.73 ± 0.09
TC11001473.hg.1	MRGPRX1	6.02 ± 0.25	5.88 ± 0.13	5.84 ± 0.25	6.02 ± 0.16
TC11001474.hg.1	MRGPRX1	6.83 ± 0.27	6.64 ± 0.14	6.75 ± 0.10	6.90 ± 0.18
TC11001475.hg.1	MRGPRX2	6.73 ± 0.23	6.58 ± 0.22	6.54 ± 0.12	6.75 ± 0.14
TC11000234.hg.1	MRGPRX3	6.66 ± 0.17	6.54 ± 0.11	6.48 ± 0.26	6.74 ± 0.23
TC11000235.hg.1	MRGPRX4	6.60 ± 0.12	6.48 ± 0.14	6.72 ± 0.01	6.85 ± 0.35
TC19000239.hg.1	MRI1	8.96 ± 0.09	8.85 ± 0.05	8.69 ± 0.09	8.80 ± 0.07
TC17000440.hg.1	MRM1	7.94 ± 0.20	7.78 ± 0.15	7.73 ± 0.15	7.83 ± 0.03
TC18000515.hg.1	MRO	6.43 ± 0.23	6.32 ± 0.18	6.35 ± 0.16	6.39 ± 0.07
TC08000844.hg.1	MROH1	8.31 ± 0.08	8.28 ± 0.09	8.20 ± 0.07	8.34 ± 0.04
TC02001421.hg.1	MROH2A	7.11 ± 0.16	7.09 ± 0.14	7.04 ± 0.15	7.19 ± 0.03
TC05001303.hg.1	MROH2B	5.25 ± 0.14	5.18 ± 0.08	5.19 ± 0.07	5.23 ± 0.05
TC08001696.hg.1	MROH5	7.90 ± 0.20	7.90 ± 0.20	7.86 ± 0.20	8.01 ± 0.06
TC08001719.hg.1	MROH6	8.56 ± 0.21	8.57 ± 0.23	8.64 ± 0.12	8.69 ± 0.05
TC01006323.hg.1	MROH7	7.44 ± 0.16	7.47 ± 0.18	7.44 ± 0.13	7.57 ± 0.07
TC20000824.hg.1	MROH8	6.20 ± 0.12	6.11 ± 0.13	6.10 ± 0.07	6.15 ± 0.02
TC01001485.hg.1	MROH9	3.95 ± 0.08	3.81 ± 0.02	3.90 ± 0.11	3.86 ± 0.03
TC13000031.hg.1	MRP63	8.30 ± 0.22	8.15 ± 0.06	8.07 ± 0.21	8.06 ± 0.08
TC04000433.hg.1	MRPL1	7.83 ± 0.25	8.02 ± 0.08	7.73 ± 0.14	7.90 ± 0.07
TC17001627.hg.1	MRPL10	8.15 ± 0.04	8.11 ± 0.07	7.97 ± 0.12	8.07 ± 0.07
TC11001967.hg.1	MRPL11	7.61 ± 0.23	7.55 ± 0.14	7.31 ± 0.23	7.44 ± 0.06
TC17002882.hg.1	MRPL12	8.26 ± 0.23	8.02 ± 0.13	8.06 ± 0.22	8.10 ± 0.05
TC08001564.hg.1	MRPL13	6.91 ± 0.05	6.79 ± 0.16	6.76 ± 0.12	6.63 ± 0.06
TC06001760.hg.1	MRPL14	7.68 ± 0.26	7.48 ± 0.05	7.39 ± 0.15	7.50 ± 0.15
TC08000374.hg.1	MRPL15	7.34 ± 0.10	6.94 ± 0.07	7.01 ± 0.12	6.88 ± 0.13
TC11001835.hg.1	MRPL16	8.29 ± 0.21	8.20 ± 0.11	8.22 ± 0.10	8.14 ± 0.05
TC11001364.hg.1	MRPL17	7.84 ± 0.18	7.55 ± 0.08	7.63 ± 0.19	7.49 ± 0.04
TC06001148.hg.1	MRPL18	8.53 ± 0.11	8.42 ± 0.01	8.54 ± 0.19	8.29 ± 0.09

TC02000476.hg.1	MRPL19	7.54 ± 0.15	7.57 ± 0.15	7.40 ± 0.13	7.39 ± 0.05
TC06001743.hg.1	MRPL2	8.29 ± 0.09	8.08 ± 0.03	8.02 ± 0.09	8.10 ± 0.04
TC01002096.hg.1	MRPL20	9.06 ± 0.08	8.95 ± 0.19	8.74 ± 0.03	8.98 ± 0.07
TC11002011.hg.1	MRPL21	8.40 ± 0.19	8.31 ± 0.14	8.16 ± 0.05	8.21 ± 0.02
TC05000857.hg.1	MRPL22	7.47 ± 0.08	7.33 ± 0.13	7.28 ± 0.17	7.32 ± 0.07
TC11000055.hg.1	MRPL23	7.88 ± 0.23	7.78 ± 0.16	7.72 ± 0.25	7.80 ± 0.02
TC11001272.hg.1	MRPL23-AS1	7.41 ± 0.18	7.37 ± 0.24	7.33 ± 0.19	7.48 ± 0.08
TC01003352.hg.1	MRPL24	7.97 ± 0.21	7.67 ± 0.04	7.63 ± 0.17	7.55 ± 0.02
TC17001685.hg.1	MRPL27	7.80 ± 0.14	7.76 ± 0.05	7.72 ± 0.04	7.75 ± 0.06
TC16000726.hg.1	MRPL28	8.86 ± 0.29	8.80 ± 0.21	8.72 ± 0.28	8.87 ± 0.05
TC03001787.hg.1	MRPL3	8.51 ± 0.18	8.49 ± 0.24	8.18 ± 0.09	8.33 ± 0.10
TC02004991.hg.1	MRPL30	7.86 ± 0.08	7.79 ± 0.11	7.79 ± 0.07	7.74 ± 0.06
TC07000259.hg.1	MRPL32	7.81 ± 0.14	7.86 ± 0.06	7.69 ± 0.15	7.73 ± 0.05
TC02004953.hg.1	MRPL33	6.68 ± 0.02	6.65 ± 0.27	6.66 ± 0.04	6.46 ± 0.02
TC19000331.hg.1	MRPL34	7.92 ± 0.30	7.71 ± 0.10	7.60 ± 0.13	7.64 ± 0.07
TC02000522.hg.1	MRPL35	7.36 ± 0.06	7.11 ± 0.10	7.19 ± 0.12	6.99 ± 0.02
TC05001115.hg.1	MRPL36	8.87 ± 0.21	8.74 ± 0.08	8.77 ± 0.24	8.65 ± 0.08
TC01000663.hg.1	MRPL37	8.85 ± 0.07	8.70 ± 0.12	8.52 ± 0.16	8.58 ± 0.10
TC17002911.hg.1	MRPL38	8.40 ± 0.07	8.33 ± 0.08	8.14 ± 0.13	8.39 ± 0.05
TC21000338.hg.1	MRPL39	7.02 ± 0.18	7.08 ± 0.23	6.80 ± 0.19	6.97 ± 0.00
TC19000173.hg.1	MRPL4	8.73 ± 0.19	8.60 ± 0.09	8.50 ± 0.16	8.56 ± 0.02
TC22000044.hg.1	MRPL40	7.98 ± 0.19	7.97 ± 0.10	7.81 ± 0.10	7.86 ± 0.11
TC09000850.hg.1	MRPL41	8.62 ± 0.23	8.65 ± 0.16	8.63 ± 0.18	8.68 ± 0.03
TC12000725.hg.1	MRPL42	8.17 ± 0.50	8.48 ± 0.19	8.08 ± 0.45	8.36 ± 0.11
TC10001596.hg.1	MRPL43	8.14 ± 0.15	8.04 ± 0.10	7.95 ± 0.19	8.00 ± 0.03
TC02001354.hg.1	MRPL44	8.52 ± 0.33	8.37 ± 0.21	8.49 ± 0.45	8.20 ± 0.07
TC17000459.hg.1	MRPL45	8.05 ± 0.08	7.94 ± 0.25	7.78 ± 0.09	7.82 ± 0.08
TC17001625.hg.1	MRPL45P2	7.25 ± 0.17	7.25 ± 0.10	7.20 ± 0.03	7.19 ± 0.05
TC15001816.hg.1	MRPL46	7.32 ± 0.09	7.11 ± 0.07	7.16 ± 0.11	7.04 ± 0.08
TC03002039.hg.1	MRPL47	7.81 ± 0.36	7.96 ± 0.33	7.72 ± 0.35	7.87 ± 0.14
TC11000780.hg.1	MRPL48	6.83 ± 0.08	6.78 ± 0.13	6.75 ± 0.02	6.72 ± 0.04

TC11000617.hg.1	MRPL49	8.19 ± 0.20	7.98 ± 0.06	7.92 ± 0.18	7.90 ± 0.04
TC09001420.hg.1	MRPL50	6.83 ± 0.34	6.99 ± 0.13	6.74 ± 0.49	6.94 ± 0.05
TC12001139.hg.1	MRPL51	8.80 ± 0.23	8.48 ± 0.14	8.52 ± 0.23	8.28 ± 0.05
TC14000132.hg.1	MRPL52	7.25 ± 0.10	7.15 ± 0.05	7.25 ± 0.07	7.17 ± 0.04
TC02005034.hg.1	MRPL53	9.12 ± 0.30	8.72 ± 0.01	8.82 ± 0.20	8.57 ± 0.14
TC19000076.hg.1	MRPL54	7.31 ± 0.23	7.23 ± 0.17	7.13 ± 0.12	7.15 ± 0.05
TC01003910.hg.1	MRPL55	8.36 ± 0.11	8.43 ± 0.15	8.25 ± 0.12	8.44 ± 0.01
TC01003233.hg.1	MRPL9	8.69 ± 0.10	8.80 ± 0.14	8.45 ± 0.14	8.79 ± 0.06
TC06001731.hg.1	MRPS10	8.09 ± 0.24	7.97 ± 0.29	7.95 ± 0.30	7.82 ± 0.20
TC15000837.hg.1	MRPS11	8.14 ± 0.18	7.92 ± 0.05	8.01 ± 0.07	7.86 ± 0.06
TC19000535.hg.1	MRPS12	7.94 ± 0.22	7.82 ± 0.14	7.70 ± 0.25	7.82 ± 0.08
TC01003543.hg.1	MRPS14	6.45 ± 0.17	6.38 ± 0.12	6.22 ± 0.04	6.31 ± 0.04
TC01003544.hg.1	MRPS14	7.42 ± 0.16	7.30 ± 0.12	7.24 ± 0.15	7.21 ± 0.04
TC01002511.hg.1	MRPS15	8.65 ± 0.12	8.59 ± 0.19	8.43 ± 0.18	8.47 ± 0.06
TC10002964.hg.1	MRPS16	7.85 ± 0.04	7.83 ± 0.18	7.63 ± 0.08	7.67 ± 0.03
TC07003325.hg.1	MRPS17	7.33 ± 0.07	7.26 ± 0.03	7.10 ± 0.18	7.23 ± 0.02
TC06001755.hg.1	MRPS18A	8.17 ± 0.06	8.01 ± 0.07	7.94 ± 0.09	8.01 ± 0.06
TC06000343.hg.1	MRPS18B	8.11 ± 0.14	7.78 ± 0.13	7.71 ± 0.19	7.61 ± 0.06
TC6_cox_hap2000041.hg.1	MRPS18B	8.12 ± 0.13	7.80 ± 0.13	7.73 ± 0.19	7.62 ± 0.05
TC6_dbb_hap3000034.hg.1	MRPS18B	8.12 ± 0.13	7.80 ± 0.13	7.73 ± 0.19	7.62 ± 0.05
TC6_mann_hap4000036.hg.1	MRPS18B	8.12 ± 0.13	7.80 ± 0.13	7.73 ± 0.19	7.62 ± 0.05
TC6_mcf_hap5000028.hg.1	MRPS18B	8.12 ± 0.13	7.80 ± 0.13	7.73 ± 0.19	7.62 ± 0.05
TC6_qbl_hap6000034.hg.1	MRPS18B	8.12 ± 0.13	7.80 ± 0.13	7.73 ± 0.19	7.62 ± 0.05
TC6_ssto_hap7000034.hg.1	MRPS18B	8.12 ± 0.13	7.80 ± 0.13	7.73 ± 0.19	7.62 ± 0.05
TC04000459.hg.1	MRPS18C	6.86 ± 0.11	6.54 ± 0.16	6.75 ± 0.26	6.45 ± 0.07
TC09000796.hg.1	MRPS2	8.72 ± 0.28	8.56 ± 0.14	8.48 ± 0.24	8.53 ± 0.02
TC01001184.hg.1	MRPS21	8.82 ± 0.08	8.78 ± 0.13	8.56 ± 0.11	8.70 ± 0.02
TC03000748.hg.1	MRPS22	6.99 ± 0.06	6.95 ± 0.08	6.87 ± 0.02	6.95 ± 0.03
TC17001720.hg.1	MRPS23	7.30 ± 0.16	7.10 ± 0.18	7.13 ± 0.12	6.97 ± 0.17
TC07003355.hg.1	MRPS24	8.77 ± 0.17	8.69 ± 0.10	8.58 ± 0.26	8.72 ± 0.06
TC03001202.hg.1	MRPS25	7.57 ± 0.23	7.55 ± 0.08	7.41 ± 0.14	7.53 ± 0.10

TC20000032.hg.1	MRPS26	8.49 ± 0.32	8.40 ± 0.19	8.31 ± 0.29	8.42 ± 0.03
TC05001463.hg.1	MRPS27	7.87 ± 0.06	7.77 ± 0.16	7.62 ± 0.04	7.62 ± 0.08
TC08002616.hg.1	MRPS28	6.91 ± 0.14	6.90 ± 0.04	6.80 ± 0.04	6.87 ± 0.03
TC05000206.hg.1	MRPS30	7.68 ± 0.23	7.60 ± 0.25	7.35 ± 0.22	7.40 ± 0.14
TC13000595.hg.1	MRPS31	7.98 ± 0.28	7.83 ± 0.14	7.92 ± 0.25	7.56 ± 0.15
TC13000687.hg.1	MRPS31P5	5.74 ± 0.13	5.65 ± 0.07	5.61 ± 0.06	5.70 ± 0.05
TC07001928.hg.1	MRPS33	6.73 ± 0.05	6.67 ± 0.09	6.56 ± 0.07	6.55 ± 0.04
TC16000759.hg.1	MRPS34	8.95 ± 0.35	8.75 ± 0.14	8.78 ± 0.18	8.61 ± 0.06
TC12000270.hg.1	MRPS35	8.37 ± 0.11	8.35 ± 0.26	8.08 ± 0.11	8.20 ± 0.16
TC05000300.hg.1	MRPS36	7.14 ± 0.06	7.01 ± 0.24	6.98 ± 0.13	6.87 ± 0.07
TC02004930.hg.1	MRPS5	8.90 ± 0.14	8.75 ± 0.05	8.85 ± 0.05	8.61 ± 0.10
TC21000134.hg.1	MRPS6	7.10 ± 0.11	7.19 ± 0.07	6.98 ± 0.08	7.07 ± 0.05
TC17000849.hg.1	MRPS7	8.52 ± 0.16	8.38 ± 0.24	8.01 ± 0.14	8.22 ± 0.06
TC02000642.hg.1	MRPS9	7.29 ± 0.20	7.20 ± 0.09	7.06 ± 0.10	7.09 ± 0.03
TC09000613.hg.1	MRRF	7.40 ± 0.12	7.25 ± 0.10	7.14 ± 0.13	7.10 ± 0.10
TC0X001340.hg.1	MRRFP1	5.63 ± 0.24	5.64 ± 0.03	5.50 ± 0.09	5.76 ± 0.14
TC06000144.hg.1	MRS2	7.09 ± 0.12	6.91 ± 0.15	6.93 ± 0.16	6.65 ± 0.15
TC12001729.hg.1	MRS2P2	5.85 ± 0.15	5.84 ± 0.36	5.72 ± 0.11	5.73 ± 0.18
TC01000245.hg.1	MRTO4	7.91 ± 0.16	7.83 ± 0.10	7.70 ± 0.18	7.85 ± 0.05
TC11001416.hg.1	MRVI1	8.13 ± 0.16	8.00 ± 0.09	8.28 ± 0.08	8.01 ± 0.02
TC11000185.hg.1	MRVI1-AS1	6.45 ± 0.03	6.36 ± 0.08	6.52 ± 0.04	6.44 ± 0.06
TC11000510.hg.1	MS4A1	10.15 ± 0.60	10.65 ± 0.27	9.98 ± 0.28	10.36 ± 0.24
TC11000517.hg.1	MS4A10	7.02 ± 0.14	7.11 ± 0.24	7.03 ± 0.15	7.28 ± 0.10
TC11000511.hg.1	MS4A12	5.61 ± 0.19	5.47 ± 0.10	5.48 ± 0.04	5.59 ± 0.06
TC11000512.hg.1	MS4A13	4.26 ± 0.16	4.03 ± 0.02	4.14 ± 0.06	4.01 ± 0.03
TC11003452.hg.1	MS4A14	6.62 ± 0.23	6.28 ± 0.28	6.93 ± 0.39	6.30 ± 0.22
TC11000516.hg.1	MS4A15	7.81 ± 0.18	7.80 ± 0.18	7.78 ± 0.08	7.89 ± 0.07
TC11000515.hg.1	MS4A18	6.61 ± 0.10	6.64 ± 0.07	6.62 ± 0.08	6.67 ± 0.04
TC11000505.hg.1	MS4A2	5.95 ± 0.19	6.05 ± 0.15	5.88 ± 0.19	6.11 ± 0.41
TC11000504.hg.1	MS4A3	7.18 ± 0.59	6.74 ± 0.13	7.30 ± 0.50	6.74 ± 0.30
TC11000506.hg.1	MS4A4A	7.03 ± 0.46	6.45 ± 0.17	7.62 ± 0.29	6.35 ± 0.07

TC11001840.hg.1	MS4A4E	7.00 ± 0.26	6.93 ± 0.11	7.27 ± 0.23	6.94 ± 0.03
TC11000509.hg.1	MS4A5	4.49 ± 0.02	4.44 ± 0.07	4.47 ± 0.09	4.46 ± 0.13
TC11001839.hg.1	MS4A6A	8.25 ± 0.24	7.63 ± 0.26	8.48 ± 0.19	7.49 ± 0.32
TC11000507.hg.1	MS4A6E	5.12 ± 0.15	4.84 ± 0.02	5.07 ± 0.18	4.94 ± 0.02
TC11003451.hg.1	MS4A7	8.75 ± 0.37	8.40 ± 0.53	9.16 ± 0.25	8.15 ± 0.49
TC11000514.hg.1	MS4A8	6.22 ± 0.05	6.22 ± 0.13	6.22 ± 0.03	6.31 ± 0.08
TC04000045.hg.1	MSANTD1	7.44 ± 0.14	7.34 ± 0.17	7.35 ± 0.11	7.39 ± 0.05
TC11002413.hg.1	MSANTD2	7.21 ± 0.12	7.10 ± 0.10	7.10 ± 0.05	7.13 ± 0.06
TC09002902.hg.1	MSANTD3	7.75 ± 0.12	7.68 ± 0.06	7.67 ± 0.13	7.66 ± 0.01
TC11002253.hg.1	MSANTD4	6.40 ± 0.05	6.32 ± 0.09	6.28 ± 0.11	6.21 ± 0.07
TC11002254.hg.1	MSANTD4	7.13 ± 0.19	7.10 ± 0.12	7.08 ± 0.13	7.01 ± 0.06
TC08001320.hg.1	MSC	6.67 ± 0.21	6.57 ± 0.05	6.61 ± 0.18	6.67 ± 0.11
TC02000106.hg.1	MSGN1	8.01 ± 0.24	8.06 ± 0.26	8.01 ± 0.36	8.09 ± 0.04
TC02000295.hg.1	MSH2	7.75 ± 0.22	7.77 ± 0.13	7.56 ± 0.15	7.60 ± 0.10
TC05000400.hg.1	MSH3	7.80 ± 0.30	7.89 ± 0.16	8.00 ± 0.33	7.70 ± 0.16
TC01000778.hg.1	MSH4	3.86 ± 0.02	3.69 ± 0.04	3.80 ± 0.10	3.65 ± 0.02
TC6_mann_hap4000068.hg.1	MSH5	7.59 ± 0.07	7.46 ± 0.04	7.44 ± 0.09	7.38 ± 0.09
TC6_qbl_hap6000068.hg.1	MSH5	7.73 ± 0.02	7.57 ± 0.00	7.57 ± 0.09	7.50 ± 0.07
TC02000299.hg.1	MSH6	7.49 ± 0.04	7.53 ± 0.05	7.38 ± 0.05	7.43 ± 0.07
TC12002043.hg.1	MSI1	7.83 ± 0.23	7.76 ± 0.22	7.77 ± 0.16	7.85 ± 0.02
TC17000705.hg.1	MSI2	8.03 ± 0.34	8.30 ± 0.08	7.89 ± 0.26	8.32 ± 0.08
TC17000489.hg.1	MSL1	9.89 ± 0.25	9.85 ± 0.16	10.24 ± 0.37	9.65 ± 0.30
TC03001811.hg.1	MSL2	9.55 ± 0.18	9.78 ± 0.12	9.56 ± 0.17	9.70 ± 0.08
TC0X000045.hg.1	MSL3	9.33 ± 0.28	9.28 ± 0.18	9.95 ± 0.36	9.24 ± 0.18
TC02002887.hg.1	MSL3P1	6.95 ± 0.11	6.79 ± 0.14	6.99 ± 0.13	6.66 ± 0.12
TC16000022.hg.1	MSLN	8.18 ± 0.19	8.16 ± 0.25	8.09 ± 0.19	8.26 ± 0.06
TC16000739.hg.1	MSLNL	8.59 ± 0.21	8.57 ± 0.25	8.51 ± 0.16	8.70 ± 0.06
TC10000337.hg.1	MSMB	5.38 ± 0.13	5.34 ± 0.04	5.28 ± 0.05	5.43 ± 0.08
TC04000828.hg.1	MSMO1	6.99 ± 0.12	6.71 ± 0.17	7.02 ± 0.17	6.56 ± 0.09
TC09001061.hg.1	MSMP	7.27 ± 0.17	7.35 ± 0.23	7.26 ± 0.18	7.45 ± 0.09
TC0X000353.hg.1	MSN	12.16 ± 0.11	12.22 ± 0.02	12.25 ± 0.07	12.12 ± 0.11

TC08001007.hg.1	MSR1	5.95 ± 0.15	5.66 ± 0.08	5.86 ± 0.12	5.76 ± 0.09
TC08000071.hg.1	MSRA	7.44 ± 0.16	7.29 ± 0.03	7.54 ± 0.16	7.28 ± 0.04
TC08000073.hg.1	MSRA	6.10 ± 0.26	5.88 ± 0.11	6.06 ± 0.25	5.87 ± 0.16
TC16000766.hg.1	MSRB1	10.46 ± 0.47	9.96 ± 0.11	10.66 ± 0.39	9.74 ± 0.28
TC10000163.hg.1	MSRB2	8.19 ± 0.22	7.87 ± 0.07	8.14 ± 0.24	7.72 ± 0.09
TC12000582.hg.1	MSRB3	7.32 ± 0.11	7.21 ± 0.05	7.32 ± 0.16	7.26 ± 0.03
TC10001409.hg.1	MSS51	6.58 ± 0.14	6.45 ± 0.03	6.54 ± 0.09	6.42 ± 0.02
TC03001414.hg.1	MST1	8.03 ± 0.19	8.02 ± 0.20	7.92 ± 0.19	8.12 ± 0.09
TC01000212.hg.1	MST1P2	7.96 ± 0.24	7.97 ± 0.20	7.84 ± 0.21	8.06 ± 0.13
TC01002278.hg.1	MST1P9	8.05 ± 0.20	8.05 ± 0.18	7.93 ± 0.18	8.18 ± 0.12
TC03001421.hg.1	MST1R	7.45 ± 0.21	7.46 ± 0.21	7.35 ± 0.16	7.57 ± 0.10
TC0X000628.hg.1	MST4	9.44 ± 0.46	9.83 ± 0.07	9.54 ± 0.35	9.74 ± 0.09
TC02002618.hg.1	MSTN	4.52 ± 0.13	4.43 ± 0.05	4.45 ± 0.12	4.50 ± 0.02
TC01006334.hg.1	MSTO1	8.08 ± 0.10	7.91 ± 0.03	7.81 ± 0.07	7.95 ± 0.12
TC01006335.hg.1	MSTO2P	8.57 ± 0.06	8.38 ± 0.01	8.24 ± 0.08	8.40 ± 0.08
TC04000062.hg.1	MSX1	7.86 ± 0.25	7.82 ± 0.30	7.82 ± 0.18	7.87 ± 0.04
TC05000974.hg.1	MSX2	7.92 ± 0.27	7.82 ± 0.12	7.85 ± 0.24	7.89 ± 0.15
TC17000709.hg.1	MSX2P1	7.18 ± 0.30	7.15 ± 0.02	7.12 ± 0.31	7.25 ± 0.07
TC16002035.hg.1	MT1A	9.06 ± 0.49	8.65 ± 0.11	9.03 ± 0.49	8.50 ± 0.04
TC16000472.hg.1	MT1B	7.86 ± 0.25	7.53 ± 0.02	7.73 ± 0.21	7.40 ± 0.16
TC16000471.hg.1	MT1CP	7.29 ± 0.49	6.74 ± 0.10	7.33 ± 0.67	6.50 ± 0.19
TC16000470.hg.1	MT1DP	8.59 ± 0.23	8.22 ± 0.02	8.46 ± 0.29	8.22 ± 0.04
TC16000468.hg.1	MT1E	9.26 ± 0.42	8.92 ± 0.05	9.12 ± 0.44	8.71 ± 0.08
TC16000473.hg.1	MT1F	9.31 ± 0.59	8.76 ± 0.04	9.01 ± 0.41	8.40 ± 0.09
TC16001135.hg.1	MT1G	9.50 ± 0.30	9.29 ± 0.14	9.49 ± 0.50	9.08 ± 0.08
TC16000474.hg.1	MT1H	8.82 ± 0.25	8.44 ± 0.15	8.71 ± 0.42	8.28 ± 0.03
TC16000475.hg.1	MT1IP	7.81 ± 0.28	7.56 ± 0.11	7.68 ± 0.23	7.51 ± 0.07
TC16000469.hg.1	MT1JP	8.93 ± 0.27	8.71 ± 0.11	9.00 ± 0.34	8.38 ± 0.08
TC16002075.hg.1	MT1L	9.58 ± 0.22	9.30 ± 0.11	9.52 ± 0.44	9.14 ± 0.12
TC16002074.hg.1	MT1M	7.93 ± 0.20	7.80 ± 0.09	7.97 ± 0.31	7.59 ± 0.07
TC01004008.hg.1	MT1P2	7.75 ± 0.25	7.74 ± 0.16	7.85 ± 0.35	7.78 ± 0.05

TC16000476.hg.1	MT1X	8.93 ± 0.36	8.51 ± 0.08	8.63 ± 0.34	8.23 ± 0.11
TC16002034.hg.1	MT2A	11.68 ± 0.71	11.23 ± 0.25	11.80 ± 0.69	10.72 ± 0.24
TC16000465.hg.1	MT3	9.07 ± 0.18	9.03 ± 0.19	9.01 ± 0.25	9.01 ± 0.08
TC16000464.hg.1	MT4	7.69 ± 0.16	7.68 ± 0.20	7.57 ± 0.14	7.75 ± 0.05
TC14000836.hg.1	MTA1	9.02 ± 0.07	9.02 ± 0.06	8.82 ± 0.13	9.03 ± 0.03
TC11001870.hg.1	MTA2	9.84 ± 0.22	9.56 ± 0.16	9.44 ± 0.18	9.29 ± 0.11
TC02000263.hg.1	MTA3	7.29 ± 0.16	7.35 ± 0.11	7.13 ± 0.10	7.31 ± 0.01
TC09000105.hg.1	MTAP	6.40 ± 0.20	6.37 ± 0.05	6.36 ± 0.14	6.45 ± 0.08
TC09002880.hg.1	MTAP	7.22 ± 0.25	7.21 ± 0.16	7.09 ± 0.23	6.97 ± 0.07
TC08000701.hg.1	MTBP	5.91 ± 0.33	5.93 ± 0.18	5.83 ± 0.28	5.84 ± 0.03
TC06001680.hg.1	MTCH1	9.35 ± 0.11	9.29 ± 0.09	9.16 ± 0.17	9.35 ± 0.01
TC11001746.hg.1	MTCH2	9.94 ± 0.07	9.80 ± 0.18	9.81 ± 0.01	9.58 ± 0.29
TC08000594.hg.1	MTDH	10.17 ± 0.23	10.24 ± 0.05	10.03 ± 0.33	10.07 ± 0.24
TC07001595.hg.1	MTERF	6.38 ± 0.30	6.38 ± 0.09	6.29 ± 0.22	6.34 ± 0.04
TC08001451.hg.1	MTERFD1	6.69 ± 0.30	6.68 ± 0.13	6.57 ± 0.13	6.53 ± 0.05
TC02002946.hg.1	MTERFD2	7.63 ± 0.28	7.73 ± 0.08	7.49 ± 0.13	7.87 ± 0.06
TC12001924.hg.1	MTERFD3	6.18 ± 0.23	6.07 ± 0.05	6.03 ± 0.03	6.00 ± 0.04
TC01002526.hg.1	MTF1	9.85 ± 0.46	9.37 ± 0.10	10.39 ± 0.25	9.26 ± 0.21
TC01000859.hg.1	MTF2	8.13 ± 0.40	8.26 ± 0.16	8.13 ± 0.39	8.11 ± 0.13
TC15001557.hg.1	MTFMT	8.21 ± 0.12	7.94 ± 0.19	8.08 ± 0.21	7.70 ± 0.24
TC22001462.hg.1	MTFP1	7.57 ± 0.11	7.69 ± 0.18	7.39 ± 0.15	7.68 ± 0.06
TC08000434.hg.1	MTFR1	7.07 ± 0.12	7.11 ± 0.14	6.99 ± 0.11	6.98 ± 0.08
TC01006314.hg.1	MTFR1L	8.10 ± 0.04	7.92 ± 0.04	8.04 ± 0.10	7.95 ± 0.01
TC06002142.hg.1	MTFR2	6.30 ± 0.07	6.39 ± 0.26	6.17 ± 0.13	6.11 ± 0.16
TC10002929.hg.1	MTG1	8.45 ± 0.04	8.32 ± 0.01	8.21 ± 0.05	8.32 ± 0.07
TC20000484.hg.1	MTG2	8.01 ± 0.14	7.85 ± 0.09	7.98 ± 0.12	7.86 ± 0.00
TC14000388.hg.1	MTHFD1	8.67 ± 0.21	8.46 ± 0.26	8.30 ± 0.11	7.97 ± 0.11
TC06001097.hg.1	MTHFD1L	7.01 ± 0.09	6.91 ± 0.08	6.87 ± 0.12	6.90 ± 0.05
TC02000454.hg.1	MTHFD2	7.88 ± 0.33	7.53 ± 0.09	8.07 ± 0.29	7.37 ± 0.07
TC04000412.hg.1	MTHFD2L	6.13 ± 0.12	6.05 ± 0.03	6.10 ± 0.04	6.09 ± 0.05
TC01002202.hg.1	MTHFR	8.12 ± 0.12	7.92 ± 0.04	8.02 ± 0.04	7.89 ± 0.13

TC15002780.hg.1	MTHFS	8.47 ± 0.22	8.31 ± 0.04	8.49 ± 0.19	8.21 ± 0.07
TC16001326.hg.1	MTHFSD	7.59 ± 0.11	7.34 ± 0.02	7.45 ± 0.08	7.31 ± 0.06
TC02001859.hg.1	MTIF2	7.62 ± 0.30	7.50 ± 0.16	7.55 ± 0.34	7.43 ± 0.07
TC13000510.hg.1	MTIF3	7.80 ± 0.13	7.74 ± 0.16	7.87 ± 0.19	7.59 ± 0.10
TC11002008.hg.1	MTL5	6.37 ± 0.14	6.16 ± 0.06	6.34 ± 0.16	6.17 ± 0.06
TC0X000723.hg.1	MTM1	8.49 ± 0.30	8.71 ± 0.17	8.76 ± 0.41	8.61 ± 0.11
TC0X000724.hg.1	MTMR1	8.36 ± 0.17	8.34 ± 0.15	8.40 ± 0.23	8.27 ± 0.04
TC15001161.hg.1	MTMR10	8.87 ± 0.35	8.92 ± 0.21	9.28 ± 0.33	8.88 ± 0.22
TC01003197.hg.1	MTMR11	7.49 ± 0.07	7.37 ± 0.07	7.46 ± 0.07	7.42 ± 0.02
TC05001233.hg.1	MTMR12	8.24 ± 0.12	8.10 ± 0.21	8.19 ± 0.20	7.94 ± 0.13
TC03000039.hg.1	MTMR14	9.55 ± 0.18	9.44 ± 0.15	9.34 ± 0.20	9.48 ± 0.05
TC11002209.hg.1	MTMR2	7.32 ± 0.13	7.28 ± 0.14	7.18 ± 0.13	7.17 ± 0.01
TC22001428.hg.1	MTMR3	9.31 ± 0.25	9.07 ± 0.14	9.58 ± 0.33	9.02 ± 0.16
TC17001734.hg.1	MTMR4	8.83 ± 0.22	8.62 ± 0.10	8.68 ± 0.22	8.47 ± 0.04
TC13000498.hg.1	MTMR6	8.06 ± 0.19	8.17 ± 0.15	8.32 ± 0.40	8.24 ± 0.18
TC08001012.hg.1	MTMR7	5.41 ± 0.11	5.34 ± 0.11	5.39 ± 0.05	5.44 ± 0.09
TC0X002333.hg.1	MTMR8	6.36 ± 0.09	6.37 ± 0.10	6.17 ± 0.07	6.27 ± 0.09
TC08000084.hg.1	MTMR9	7.50 ± 0.16	7.62 ± 0.10	7.46 ± 0.14	7.64 ± 0.06
TC01002465.hg.1	MTMR9LP	8.11 ± 0.10	7.98 ± 0.10	8.01 ± 0.17	8.09 ± 0.06
TC04002950.hg.1	MTNR1A	9.06 ± 0.25	9.05 ± 0.16	8.92 ± 0.40	9.10 ± 0.08
TC11000905.hg.1	MTNR1B	8.02 ± 0.24	7.99 ± 0.26	7.86 ± 0.28	8.03 ± 0.08
TC06000734.hg.1	MTO1	7.90 ± 0.22	7.78 ± 0.10	7.79 ± 0.23	7.67 ± 0.09
TC01002197.hg.1	MTOR	8.63 ± 0.14	8.56 ± 0.11	8.40 ± 0.12	8.45 ± 0.07
TC01000137.hg.1	MTOR-AS1	5.72 ± 0.10	5.58 ± 0.07	5.71 ± 0.12	5.58 ± 0.08
TC10001150.hg.1	MTPAP	7.93 ± 0.11	7.89 ± 0.01	7.80 ± 0.06	7.94 ± 0.03
TC01001956.hg.1	MTR	8.19 ± 0.36	8.32 ± 0.10	7.91 ± 0.28	8.33 ± 0.08
TC13000602.hg.1	MTRF1	7.22 ± 0.32	7.30 ± 0.08	7.24 ± 0.22	7.21 ± 0.16
TC06002241.hg.1	MTRF1L	7.35 ± 0.25	7.30 ± 0.13	7.58 ± 0.31	7.28 ± 0.11
TC17000305.hg.1	MTRNR2L1	7.71 ± 0.31	7.52 ± 0.12	7.58 ± 0.23	7.42 ± 0.17
TC0X001079.hg.1	MTRNR2L10	5.37 ± 0.17	5.26 ± 0.12	5.42 ± 0.09	5.28 ± 0.14
TC05001540.hg.1	MTRNR2L2	10.38 ± 0.55	10.12 ± 0.06	10.36 ± 0.45	9.96 ± 0.29

TC20000974.hg.1	MTRNR2L3	6.71 ± 0.45	6.47 ± 0.09	6.62 ± 0.41	6.49 ± 0.13
TC16000815.hg.1	MTRNR2L4	5.94 ± 0.40	5.66 ± 0.05	5.74 ± 0.24	5.49 ± 0.14
TC10000357.hg.1	MTRNR2L5	6.59 ± 0.41	6.44 ± 0.14	6.42 ± 0.29	6.24 ± 0.15
TC07000914.hg.1	MTRNR2L6	7.23 ± 0.38	7.14 ± 0.10	7.22 ± 0.28	7.20 ± 0.22
TC10001185.hg.1	MTRNR2L7	5.07 ± 0.30	4.94 ± 0.05	5.02 ± 0.09	5.01 ± 0.13
TC11001411.hg.1	MTRNR2L8	9.33 ± 0.27	9.14 ± 0.06	9.19 ± 0.30	9.16 ± 0.17
TC06000695.hg.1	MTRNR2L9	11.92 ± 0.32	11.71 ± 0.09	11.81 ± 0.18	11.71 ± 0.13
TC05000056.hg.1	MTRR	7.81 ± 0.16	7.78 ± 0.06	7.82 ± 0.16	7.70 ± 0.05
TC08001602.hg.1	MTSS1	8.96 ± 0.05	8.88 ± 0.07	8.79 ± 0.06	8.85 ± 0.01
TC16001237.hg.1	MTSS1L	7.97 ± 0.09	7.90 ± 0.12	7.96 ± 0.12	8.08 ± 0.08
TC04000516.hg.1	MTTP	5.19 ± 0.09	5.10 ± 0.04	5.13 ± 0.05	5.15 ± 0.08
TC08001013.hg.1	MTUS1	5.80 ± 0.09	5.72 ± 0.01	5.67 ± 0.02	5.72 ± 0.05
TC13000093.hg.1	MTUS2	6.75 ± 0.19	6.71 ± 0.12	6.68 ± 0.15	6.79 ± 0.08
TC13000522.hg.1	MTUS2-AS1	7.05 ± 0.23	6.85 ± 0.14	6.94 ± 0.15	6.88 ± 0.02
TC13000521.hg.1	MTUS2-AS2	4.61 ± 0.21	4.43 ± 0.03	4.56 ± 0.02	4.48 ± 0.14
TC17001715.hg.1	MTVR2	8.22 ± 0.29	8.22 ± 0.17	8.64 ± 0.30	8.09 ± 0.09
TC01001296.hg.1	MTX1	8.85 ± 0.28	8.53 ± 0.02	9.00 ± 0.15	8.44 ± 0.11
TC02001057.hg.1	MTX2	6.77 ± 0.14	6.66 ± 0.16	6.52 ± 0.14	6.46 ± 0.01
TC05001524.hg.1	MTX3	7.12 ± 0.50	7.55 ± 0.27	6.97 ± 0.34	7.58 ± 0.15
TC01006383.hg.1	MUC1	7.37 ± 0.12	7.25 ± 0.18	7.37 ± 0.07	7.32 ± 0.06
TC07000639.hg.1	MUC12	6.93 ± 0.15	6.96 ± 0.17	6.89 ± 0.22	7.04 ± 0.09
TC03001719.hg.1	MUC13	5.20 ± 0.05	5.15 ± 0.10	5.16 ± 0.03	5.28 ± 0.10
TC11001505.hg.1	MUC15	4.42 ± 0.03	4.26 ± 0.11	4.29 ± 0.11	4.30 ± 0.15
TC19001139.hg.1	MUC16	7.16 ± 0.14	7.17 ± 0.18	7.11 ± 0.13	7.35 ± 0.16
TC07000640.hg.1	MUC17	6.32 ± 0.19	6.32 ± 0.12	6.26 ± 0.15	6.40 ± 0.02
TC12000310.hg.1	MUC19	5.94 ± 0.13	5.88 ± 0.12	5.92 ± 0.07	5.99 ± 0.09
TC11000039.hg.1	MUC2	7.90 ± 0.19	7.96 ± 0.22	7.88 ± 0.16	8.06 ± 0.05
TC03003365.hg.1	MUC20	8.00 ± 0.02	8.04 ± 0.04	7.87 ± 0.10	8.07 ± 0.15
TC06000353.hg.1	MUC21	7.96 ± 0.10	7.95 ± 0.16	7.94 ± 0.13	8.11 ± 0.13
TC6_apd_hap1000031.hg.1	MUC21	6.56 ± 0.11	6.51 ± 0.18	6.48 ± 0.10	6.66 ± 0.15
TC6_cox_hap2000051.hg.1	MUC21	7.25 ± 0.10	7.20 ± 0.19	7.16 ± 0.12	7.35 ± 0.13

TC6_dbb_hap3000044.hg.1	MUC21	6.56 ± 0.11	6.51 ± 0.18	6.48 ± 0.10	6.66 ± 0.15
TC6_mann_hap4000046.hg.1	MUC21	7.67 ± 0.20	7.66 ± 0.20	7.58 ± 0.19	7.86 ± 0.14
TC6_mcf_hap5000038.hg.1	MUC21	6.55 ± 0.12	6.52 ± 0.16	6.48 ± 0.10	6.65 ± 0.15
TC6_qbl_hap6000044.hg.1	MUC21	7.83 ± 0.18	7.80 ± 0.17	7.78 ± 0.19	7.99 ± 0.13
TC06000354.hg.1	MUC22	6.55 ± 0.07	6.47 ± 0.06	6.53 ± 0.06	6.57 ± 0.09
TC6_apd_hap1000032.hg.1	MUC22	6.87 ± 0.06	6.82 ± 0.08	6.87 ± 0.04	6.98 ± 0.12
TC6_cox_hap2000052.hg.1	MUC22	6.56 ± 0.06	6.48 ± 0.06	6.53 ± 0.03	6.60 ± 0.08
TC6_dbb_hap3000045.hg.1	MUC22	6.92 ± 0.05	6.85 ± 0.08	6.91 ± 0.04	7.02 ± 0.13
TC6_mann_hap4000045.hg.1	MUC22	6.82 ± 0.07	6.73 ± 0.15	6.77 ± 0.08	6.89 ± 0.13
TC6_mcf_hap5000039.hg.1	MUC22	6.95 ± 0.09	6.84 ± 0.06	6.87 ± 0.05	6.89 ± 0.03
TC6_qbl_hap6000045.hg.1	MUC22	6.90 ± 0.08	6.81 ± 0.07	6.85 ± 0.03	6.92 ± 0.07
TC6_ssto_hap7000040.hg.1	MUC22	6.94 ± 0.06	6.86 ± 0.05	6.91 ± 0.04	6.95 ± 0.06
TC03002150.hg.1	MUC4	7.29 ± 0.17	7.26 ± 0.20	7.22 ± 0.17	7.38 ± 0.07
TC11000041.hg.1	MUC5AC	8.01 ± 0.21	8.00 ± 0.19	7.95 ± 0.21	8.07 ± 0.04
TC11003511.hg.1	MUC5AC	8.19 ± 0.22	8.22 ± 0.23	8.15 ± 0.20	8.32 ± 0.07
TC11003510.hg.1	MUC5B	8.21 ± 0.21	8.24 ± 0.25	8.15 ± 0.23	8.39 ± 0.09
TC11001256.hg.1	MUC6	8.05 ± 0.20	8.06 ± 0.28	7.99 ± 0.23	8.21 ± 0.05
TC04000386.hg.1	MUC7	4.70 ± 0.06	4.52 ± 0.07	4.59 ± 0.12	4.61 ± 0.06
TC12000471.hg.1	MUCL1	6.04 ± 0.19	6.04 ± 0.11	6.02 ± 0.02	6.04 ± 0.02
TC01002315.hg.1	MUL1	7.67 ± 0.16	7.39 ± 0.13	7.42 ± 0.16	7.39 ± 0.08
TC19000036.hg.1	MUM1	7.49 ± 0.07	7.47 ± 0.04	7.33 ± 0.18	7.36 ± 0.02
TC0X000514.hg.1	MUM1L1	6.25 ± 0.19	6.03 ± 0.11	6.08 ± 0.11	6.10 ± 0.03
TC09000513.hg.1	MURC	5.50 ± 0.32	5.40 ± 0.05	5.33 ± 0.08	5.44 ± 0.29
TC11000644.hg.1	MUS81	8.58 ± 0.16	8.41 ± 0.09	8.50 ± 0.11	8.42 ± 0.04
TC09000553.hg.1	MUSK	5.43 ± 0.11	5.34 ± 0.11	5.36 ± 0.04	5.41 ± 0.08
TC06001782.hg.1	MUT	7.48 ± 0.11	7.32 ± 0.09	7.53 ± 0.30	7.27 ± 0.02
TC01002607.hg.1	MUTYH	8.22 ± 0.15	8.23 ± 0.14	8.07 ± 0.16	8.30 ± 0.08
TC19000335.hg.1	MVB12A	8.61 ± 0.18	8.29 ± 0.08	8.39 ± 0.19	8.26 ± 0.02
TC09000654.hg.1	MVB12B	8.01 ± 0.10	7.91 ± 0.07	7.93 ± 0.10	8.00 ± 0.06
TC16001340.hg.1	MVD	8.58 ± 0.20	8.45 ± 0.10	8.35 ± 0.17	8.49 ± 0.05
TC12000849.hg.1	MVK	7.82 ± 0.19	7.82 ± 0.13	7.73 ± 0.19	7.90 ± 0.07

TC16002071.hg.1	MVP	9.40 ± 0.28	9.14 ± 0.04	9.51 ± 0.41	8.99 ± 0.09
TC21000189.hg.1	MX1	9.81 ± 0.47	9.44 ± 0.61	10.03 ± 0.82	9.36 ± 0.23
TC21000188.hg.1	MX2	9.63 ± 0.40	9.48 ± 0.41	9.98 ± 0.42	9.42 ± 0.21
TC02004970.hg.1	MXD1	11.49 ± 0.33	11.44 ± 0.18	12.07 ± 0.37	11.38 ± 0.27
TC05003437.hg.1	MXD3	8.97 ± 0.19	8.95 ± 0.17	9.01 ± 0.16	8.90 ± 0.02
TC04000973.hg.1	MXD4	8.96 ± 0.05	8.97 ± 0.06	8.71 ± 0.17	9.00 ± 0.05
TC10000798.hg.1	MXI1	8.10 ± 0.13	8.46 ± 0.11	8.11 ± 0.08	8.60 ± 0.07
TC0X000807.hg.1	MXRA5	6.16 ± 0.26	6.09 ± 0.15	6.03 ± 0.17	6.10 ± 0.03
TC0Y000048.hg.1	MXRA5P1	6.69 ± 0.03	6.54 ± 0.07	6.72 ± 0.13	6.67 ± 0.10
TC17001905.hg.1	MXRA7	8.28 ± 0.23	8.31 ± 0.11	8.37 ± 0.13	8.39 ± 0.08
TC01002093.hg.1	MXRA8	8.24 ± 0.23	8.23 ± 0.23	8.16 ± 0.22	8.32 ± 0.08
TC19000865.hg.1	MYADM	11.17 ± 0.02	11.06 ± 0.02	11.33 ± 0.21	11.10 ± 0.24
TC02001730.hg.1	MYADML	7.46 ± 0.16	7.26 ± 0.13	7.47 ± 0.13	7.40 ± 0.17
TC17001967.hg.1	MYADML2	7.75 ± 0.31	7.74 ± 0.31	7.69 ± 0.18	7.86 ± 0.04
TC06001010.hg.1	MYB	7.13 ± 0.14	7.04 ± 0.06	7.18 ± 0.16	7.04 ± 0.12
TC06002135.hg.1	MYB-AS1	4.68 ± 0.07	4.37 ± 0.06	4.36 ± 0.05	4.38 ± 0.04
TC17001023.hg.1	MYBBP1A	8.50 ± 0.15	8.42 ± 0.11	8.31 ± 0.14	8.47 ± 0.02
TC08001286.hg.1	MYBL1	7.55 ± 0.93	7.81 ± 0.35	7.40 ± 0.64	7.61 ± 0.28
TC08001287.hg.1	MYBL1	6.58 ± 0.10	6.58 ± 0.13	6.62 ± 0.12	6.55 ± 0.11
TC20000320.hg.1	MYBL2	9.18 ± 0.31	8.76 ± 0.23	8.72 ± 0.29	8.59 ± 0.10
TC12000787.hg.1	MYBPC1	5.71 ± 0.17	5.60 ± 0.13	5.66 ± 0.12	5.69 ± 0.05
TC19000759.hg.1	MYBPC2	7.31 ± 0.27	7.27 ± 0.19	7.15 ± 0.25	7.34 ± 0.03
TC11001735.hg.1	MYBPC3	8.00 ± 0.16	7.89 ± 0.15	8.13 ± 0.07	7.97 ± 0.03
TC01003717.hg.1	MYBPH	7.90 ± 0.15	7.85 ± 0.22	7.84 ± 0.19	8.01 ± 0.06
TC01002953.hg.1	MYBPHL	7.33 ± 0.15	7.23 ± 0.16	7.19 ± 0.06	7.28 ± 0.03
TC08000749.hg.1	MYC	9.82 ± 0.13	9.65 ± 0.06	9.57 ± 0.10	9.72 ± 0.11
TC01006352.hg.1	MYCBP	7.04 ± 0.11	6.86 ± 0.16	7.11 ± 0.09	6.71 ± 0.05
TC13000744.hg.1	MYCBP2	8.98 ± 0.47	9.42 ± 0.24	8.90 ± 0.43	9.10 ± 0.21
TC13000293.hg.1	MYCBP2-AS1	5.17 ± 0.05	5.05 ± 0.08	5.07 ± 0.08	5.07 ± 0.07
TC13000294.hg.1	MYCBP2-AS2	5.41 ± 0.20	5.29 ± 0.08	5.24 ± 0.13	5.27 ± 0.07
TC17000669.hg.1	MYCBPAP	7.07 ± 0.19	7.01 ± 0.16	6.99 ± 0.14	7.04 ± 0.04

TC01002551.hg.1	MYCL1	8.57 ± 0.22	8.36 ± 0.04	8.41 ± 0.20	8.37 ± 0.05
TC02000094.hg.1	MYCN	7.92 ± 0.32	7.79 ± 0.25	7.78 ± 0.28	7.89 ± 0.00
TC02001595.hg.1	MYCNOS	7.33 ± 0.24	7.29 ± 0.27	7.25 ± 0.28	7.34 ± 0.08
TC06001109.hg.1	MYCT1	6.43 ± 0.03	5.63 ± 0.17	6.16 ± 0.27	5.68 ± 0.17
TC03000187.hg.1	MYD88	10.48 ± 0.24	10.25 ± 0.18	10.78 ± 0.14	10.20 ± 0.25
TC15001330.hg.1	MYEF2	6.77 ± 0.34	6.98 ± 0.04	6.75 ± 0.21	6.98 ± 0.08
TC11000715.hg.1	MYEOV	7.47 ± 0.11	7.49 ± 0.21	7.34 ± 0.10	7.60 ± 0.11
TC02002933.hg.1	MYEOV2	8.09 ± 0.23	7.96 ± 0.07	7.97 ± 0.23	7.95 ± 0.01
TC12000670.hg.1	MYF5	5.75 ± 0.03	5.54 ± 0.05	5.66 ± 0.10	5.66 ± 0.12
TC12000669.hg.1	MYF6	6.87 ± 0.35	6.84 ± 0.07	6.83 ± 0.14	6.92 ± 0.12
TC17001133.hg.1	MYH1	6.04 ± 0.17	5.97 ± 0.10	6.01 ± 0.11	6.05 ± 0.03
TC17001118.hg.1	MYH10	6.20 ± 0.12	6.18 ± 0.03	6.20 ± 0.05	6.18 ± 0.03
TC16000898.hg.1	MYH11	7.67 ± 0.16	7.54 ± 0.10	7.63 ± 0.15	7.60 ± 0.06
TC17001130.hg.1	MYH13	6.68 ± 0.17	6.56 ± 0.10	6.60 ± 0.20	6.63 ± 0.02
TC19000755.hg.1	MYH14	8.30 ± 0.22	8.22 ± 0.19	8.23 ± 0.22	8.28 ± 0.01
TC03001631.hg.1	MYH15	5.33 ± 0.13	5.24 ± 0.06	5.28 ± 0.07	5.30 ± 0.08
TC07000590.hg.1	MYH16	6.32 ± 0.11	6.28 ± 0.15	6.32 ± 0.18	6.31 ± 0.02
TC07000591.hg.1	MYH16	7.00 ± 0.20	6.89 ± 0.17	6.93 ± 0.20	6.98 ± 0.05
TC17001134.hg.1	MYH2	5.85 ± 0.20	5.70 ± 0.06	5.76 ± 0.12	5.74 ± 0.01
TC17001135.hg.1	MYH3	6.68 ± 0.09	6.58 ± 0.08	6.61 ± 0.11	6.64 ± 0.03
TC17001132.hg.1	MYH4	5.78 ± 0.14	5.69 ± 0.09	5.70 ± 0.10	5.77 ± 0.05
TC14000957.hg.1	MYH6	7.17 ± 0.23	7.08 ± 0.13	7.08 ± 0.21	7.11 ± 0.03
TC14000959.hg.1	MYH7	7.18 ± 0.17	7.13 ± 0.16	7.15 ± 0.16	7.21 ± 0.06
TC20000246.hg.1	MYH7B	7.85 ± 0.20	7.79 ± 0.17	7.77 ± 0.21	7.84 ± 0.02
TC17001131.hg.1	MYH8	5.56 ± 0.15	5.43 ± 0.07	5.48 ± 0.14	5.50 ± 0.02
TC22000703.hg.1	MYH9	11.09 ± 0.12	11.12 ± 0.06	11.18 ± 0.11	11.07 ± 0.11
TC02002732.hg.1	MYL1	5.41 ± 0.17	5.26 ± 0.10	5.27 ± 0.11	5.32 ± 0.06
TC07001695.hg.1	MYL10	7.78 ± 0.18	7.77 ± 0.10	7.83 ± 0.22	7.87 ± 0.04
TC18000016.hg.1	MYL12A	12.12 ± 0.16	11.95 ± 0.04	12.24 ± 0.30	11.78 ± 0.07
TC18000017.hg.1	MYL12B	8.83 ± 0.08	8.85 ± 0.09	8.99 ± 0.12	8.77 ± 0.09
TC12001969.hg.1	MYL2	7.15 ± 0.15	7.02 ± 0.06	7.11 ± 0.11	7.05 ± 0.10

TC03001362.hg.1	MYL3	7.46 ± 0.22	7.38 ± 0.18	7.40 ± 0.22	7.56 ± 0.11
TC17000606.hg.1	MYL4	7.29 ± 0.14	7.20 ± 0.17	7.21 ± 0.11	7.30 ± 0.05
TC04000012.hg.1	MYL5	8.39 ± 0.25	8.32 ± 0.18	8.36 ± 0.28	8.42 ± 0.03
TC12003245.hg.1	MYL6	9.26 ± 0.28	9.02 ± 0.10	9.28 ± 0.22	8.95 ± 0.09
TC12003246.hg.1	MYL6B	7.70 ± 0.21	7.51 ± 0.10	7.55 ± 0.18	7.51 ± 0.04
TC21000511.hg.1	MYL6P1	9.55 ± 0.20	9.34 ± 0.06	9.43 ± 0.33	9.33 ± 0.09
TC07001336.hg.1	MYL7	7.92 ± 0.13	7.89 ± 0.21	7.92 ± 0.13	8.03 ± 0.09
TC06000420.hg.1	MYL8P	7.96 ± 0.20	7.48 ± 0.13	7.96 ± 0.34	7.34 ± 0.25
TC20000268.hg.1	MYL9	10.41 ± 0.09	9.64 ± 0.21	10.83 ± 0.34	10.00 ± 0.43
TC06000108.hg.1	MYLIP	9.50 ± 0.38	9.98 ± 0.08	9.64 ± 0.29	9.88 ± 0.18
TC03001713.hg.1	MYLK	7.50 ± 0.05	7.25 ± 0.11	7.53 ± 0.18	7.36 ± 0.04
TC20000199.hg.1	MYLK2	7.12 ± 0.16	7.04 ± 0.18	7.03 ± 0.16	7.13 ± 0.06
TC16001085.hg.1	MYLK3	6.72 ± 0.13	6.67 ± 0.15	6.70 ± 0.17	6.77 ± 0.07
TC06001216.hg.1	MYLK4	6.45 ± 0.10	6.37 ± 0.08	6.40 ± 0.03	6.42 ± 0.05
TC03000639.hg.1	MYLK-AS1	7.30 ± 0.16	7.36 ± 0.13	7.30 ± 0.13	7.43 ± 0.14
TC03000640.hg.1	MYLK-AS2	6.79 ± 0.12	6.83 ± 0.20	6.88 ± 0.15	6.90 ± 0.02
TC16000345.hg.1	MYLPF	7.81 ± 0.25	7.61 ± 0.07	7.76 ± 0.20	7.68 ± 0.11
TC03000906.hg.1	MYNN	7.75 ± 0.34	8.02 ± 0.17	7.83 ± 0.52	7.91 ± 0.11
TC05001184.hg.1	MYO10	6.80 ± 0.28	6.49 ± 0.11	7.02 ± 0.14	6.60 ± 0.09
TC17000205.hg.1	MYO15A	7.71 ± 0.18	7.68 ± 0.16	7.67 ± 0.15	7.76 ± 0.05
TC17000857.hg.1	MYO15B	8.60 ± 0.05	8.69 ± 0.06	8.64 ± 0.04	8.75 ± 0.08
TC13000384.hg.1	MYO16	5.53 ± 0.16	5.44 ± 0.06	5.45 ± 0.05	5.47 ± 0.04
TC13000870.hg.1	MYO16-AS1	6.07 ± 0.20	6.12 ± 0.04	6.03 ± 0.09	6.29 ± 0.18
TC13000869.hg.1	MYO16-AS2	6.00 ± 0.20	5.80 ± 0.11	5.97 ± 0.09	5.97 ± 0.05
TC22000166.hg.1	MYO18B	7.47 ± 0.17	7.43 ± 0.18	7.43 ± 0.18	7.52 ± 0.05
TC17001402.hg.1	MYO19	7.81 ± 0.03	7.74 ± 0.07	7.62 ± 0.03	7.73 ± 0.04
TC12003220.hg.1	MYO1A	6.79 ± 0.16	6.72 ± 0.13	6.72 ± 0.12	6.78 ± 0.03
TC02001126.hg.1	MYO1B	5.68 ± 0.20	5.57 ± 0.02	5.57 ± 0.05	5.62 ± 0.04
TC17000974.hg.1	MYO1C	8.21 ± 0.11	8.11 ± 0.09	8.05 ± 0.15	8.29 ± 0.13
TC17001353.hg.1	MYO1D	7.22 ± 0.36	6.64 ± 0.12	6.49 ± 0.22	6.53 ± 0.08
TC15001492.hg.1	MYO1E	7.56 ± 0.07	7.38 ± 0.09	7.36 ± 0.04	7.30 ± 0.10

TC19001135.hg.1	MYO1F	10.69 ± 0.21	10.57 ± 0.09	10.89 ± 0.19	10.51 ± 0.20
TC07001348.hg.1	MYO1G	10.26 ± 0.11	9.95 ± 0.05	10.04 ± 0.02	9.90 ± 0.10
TC12000847.hg.1	MYO1H	5.33 ± 0.13	5.25 ± 0.07	5.31 ± 0.08	5.32 ± 0.02
TC10000179.hg.1	MYO3A	4.86 ± 0.11	4.74 ± 0.05	4.80 ± 0.08	4.77 ± 0.07
TC02001011.hg.1	MYO3B	5.69 ± 0.10	5.58 ± 0.01	5.53 ± 0.10	5.61 ± 0.04
TC15001456.hg.1	MYO5A	8.56 ± 0.31	8.80 ± 0.26	8.75 ± 0.46	8.65 ± 0.18
TC18000507.hg.1	MYO5B	6.50 ± 0.17	6.41 ± 0.12	6.42 ± 0.11	6.50 ± 0.06
TC15001454.hg.1	MYO5C	5.51 ± 0.06	5.45 ± 0.06	5.47 ± 0.04	5.49 ± 0.01
TC06000747.hg.1	MYO6	4.72 ± 0.09	4.68 ± 0.06	4.62 ± 0.05	4.63 ± 0.04
TC11000829.hg.1	MYO7A	7.78 ± 0.17	7.70 ± 0.15	7.73 ± 0.13	7.77 ± 0.03
TC02000786.hg.1	MYO7B	7.90 ± 0.16	7.82 ± 0.13	7.89 ± 0.13	7.88 ± 0.02
TC15001620.hg.1	MYO9A	7.05 ± 0.38	7.37 ± 0.28	6.97 ± 0.28	7.08 ± 0.12
TC19000327.hg.1	MYO9B	9.82 ± 0.15	9.78 ± 0.05	9.79 ± 0.07	9.84 ± 0.16
TC01003517.hg.1	MYOC	7.29 ± 0.24	7.29 ± 0.28	7.34 ± 0.23	7.44 ± 0.12
TC17000158.hg.1	MYOCD	6.25 ± 0.18	6.17 ± 0.19	6.16 ± 0.12	6.26 ± 0.08
TC11000232.hg.1	MYOD1	7.65 ± 0.17	7.59 ± 0.24	7.60 ± 0.34	7.66 ± 0.01
TC10001535.hg.1	MYOF	7.83 ± 0.45	7.19 ± 0.06	7.96 ± 0.30	7.00 ± 0.25
TC01003714.hg.1	MYOG	7.82 ± 0.13	7.89 ± 0.19	7.90 ± 0.18	8.02 ± 0.06
TC18000288.hg.1	MYOM1	6.53 ± 0.03	6.37 ± 0.04	6.55 ± 0.15	6.36 ± 0.03
TC08000014.hg.1	MYOM2	7.34 ± 0.11	7.23 ± 0.17	7.16 ± 0.20	7.21 ± 0.14
TC01002357.hg.1	MYOM3	7.03 ± 0.21	6.95 ± 0.16	6.95 ± 0.17	7.03 ± 0.06
TC05000689.hg.1	MYOT	5.00 ± 0.07	4.92 ± 0.05	4.95 ± 0.10	4.95 ± 0.09
TC10001413.hg.1	MYOZ1	7.39 ± 0.21	7.28 ± 0.14	7.30 ± 0.12	7.31 ± 0.05
TC04000610.hg.1	MYOZ2	5.74 ± 0.17	5.60 ± 0.10	5.56 ± 0.10	5.65 ± 0.02
TC05000834.hg.1	MYOZ3	7.23 ± 0.19	7.14 ± 0.17	7.08 ± 0.18	7.19 ± 0.05
TC10000401.hg.1	MYPN	5.70 ± 0.10	5.61 ± 0.06	5.67 ± 0.08	5.68 ± 0.07
TC19001637.hg.1	MYPOP	8.23 ± 0.20	7.98 ± 0.05	8.04 ± 0.17	7.84 ± 0.06
TC11000539.hg.1	MYRF	7.94 ± 0.18	7.95 ± 0.23	7.88 ± 0.21	8.06 ± 0.06
TC12003252.hg.1	MYRFL	5.75 ± 0.11	5.77 ± 0.14	5.79 ± 0.09	5.81 ± 0.07
TC03000204.hg.1	MYRIP	6.63 ± 0.19	6.59 ± 0.17	6.58 ± 0.13	6.71 ± 0.07
TC01002707.hg.1	MYSM1	8.45 ± 0.70	8.87 ± 0.20	8.62 ± 0.64	8.93 ± 0.09

TC20000535.hg.1	MYT1	7.16 ± 0.18	7.20 ± 0.22	7.13 ± 0.19	7.32 ± 0.08
TC02001513.hg.1	MYT1L	6.72 ± 0.13	6.67 ± 0.13	6.69 ± 0.09	6.74 ± 0.08
TC05001841.hg.1	MZB1	8.93 ± 0.16	8.35 ± 0.12	8.37 ± 0.16	8.26 ± 0.12
TC19001924.hg.1	MZF1	8.04 ± 0.11	7.89 ± 0.10	7.85 ± 0.15	7.91 ± 0.08
TC13000726.hg.1	MZT1	7.19 ± 0.17	7.35 ± 0.15	6.99 ± 0.22	7.18 ± 0.13
TC02002353.hg.1	MZT2A	8.60 ± 0.25	8.57 ± 0.10	8.39 ± 0.24	8.57 ± 0.05
TC02000803.hg.1	MZT2B	8.60 ± 0.25	8.56 ± 0.14	8.41 ± 0.29	8.59 ± 0.05
TC16001098.hg.1	N4BP1	9.80 ± 0.45	9.54 ± 0.28	10.23 ± 0.27	9.46 ± 0.36
TC04000239.hg.1	N4BP2	7.49 ± 0.78	7.92 ± 0.26	7.42 ± 0.53	7.74 ± 0.10
TC13001722.hg.1	N4BP2L1	7.41 ± 0.23	7.35 ± 0.12	7.40 ± 0.24	7.30 ± 0.08
TC13001723.hg.1	N4BP2L2	8.02 ± 0.36	8.08 ± 0.22	8.32 ± 0.26	8.00 ± 0.11
TC13000552.hg.1	N4BP2L2-IT2	7.75 ± 0.67	8.15 ± 0.44	7.97 ± 0.53	8.02 ± 0.09
TC05001031.hg.1	N4BP3	7.89 ± 0.23	7.86 ± 0.15	7.76 ± 0.21	7.86 ± 0.05
TC21000350.hg.1	N6AMT1	6.25 ± 0.22	6.23 ± 0.05	6.16 ± 0.03	6.19 ± 0.02
TC13000460.hg.1	N6AMT2	6.64 ± 0.17	6.53 ± 0.06	6.53 ± 0.10	6.53 ± 0.08
TC0X002309.hg.1	NAA10	8.36 ± 0.12	8.26 ± 0.06	8.23 ± 0.10	8.23 ± 0.02
TC04001326.hg.1	NAA11	5.94 ± 0.16	5.79 ± 0.07	5.94 ± 0.07	5.91 ± 0.07
TC04000683.hg.1	NAA15	7.92 ± 0.59	8.39 ± 0.15	7.86 ± 0.45	8.32 ± 0.09
TC13000145.hg.1	NAA16	7.52 ± 0.62	7.81 ± 0.13	7.55 ± 0.48	7.81 ± 0.12
TC20000135.hg.1	NAA20	7.08 ± 0.06	6.93 ± 0.14	6.96 ± 0.06	6.84 ± 0.04
TC12001977.hg.1	NAA25	7.47 ± 0.43	7.55 ± 0.26	7.24 ± 0.27	7.49 ± 0.05
TC14000342.hg.1	NAA30	8.43 ± 0.08	8.50 ± 0.05	8.21 ± 0.05	8.35 ± 0.01
TC09000391.hg.1	NAA35	7.58 ± 0.44	7.84 ± 0.22	7.49 ± 0.28	7.73 ± 0.01
TC07000728.hg.1	NAA38	8.04 ± 0.42	8.18 ± 0.15	8.02 ± 0.40	8.13 ± 0.04
TC11000579.hg.1	NAA40	8.01 ± 0.08	7.89 ± 0.05	7.79 ± 0.10	7.86 ± 0.04
TC03001664.hg.1	NAA50	9.23 ± 0.31	9.40 ± 0.15	9.21 ± 0.24	9.35 ± 0.09
TC16000124.hg.1	NAA60	9.14 ± 0.15	8.93 ± 0.05	9.04 ± 0.05	8.94 ± 0.07
TC04002920.hg.1	NAAA	8.58 ± 0.15	8.67 ± 0.05	8.47 ± 0.04	8.58 ± 0.12
TC11000898.hg.1	NAALAD2	4.72 ± 0.15	4.62 ± 0.05	4.66 ± 0.07	4.64 ± 0.04
TC11001922.hg.1	NAALADL1	7.66 ± 0.15	7.63 ± 0.19	7.62 ± 0.11	7.73 ± 0.01
TC03000934.hg.1	NAALADL2	4.55 ± 0.10	4.41 ± 0.02	4.48 ± 0.03	4.46 ± 0.05

TC03002018.hg.1	NAALADL2-AS1	6.14 ± 0.18	5.91 ± 0.09	6.05 ± 0.23	5.98 ± 0.10
TC03002016.hg.1	NAALADL2-AS2	3.92 ± 0.07	3.79 ± 0.14	3.71 ± 0.09	3.80 ± 0.12
TC03002015.hg.1	NAALADL2-AS3	4.04 ± 0.09	3.83 ± 0.09	3.95 ± 0.10	3.93 ± 0.04
TC02001121.hg.1	NAB1	8.04 ± 0.15	7.85 ± 0.15	8.15 ± 0.15	7.80 ± 0.19
TC12000520.hg.1	NAB2	8.51 ± 0.18	8.51 ± 0.11	8.43 ± 0.25	8.57 ± 0.03
TC02001128.hg.1	NABP1	10.10 ± 0.16	10.18 ± 0.43	11.04 ± 0.55	10.01 ± 0.36
TC12000507.hg.1	NABP2	8.42 ± 0.19	8.26 ± 0.10	8.15 ± 0.25	8.23 ± 0.04
TC12001613.hg.1	NACA	9.13 ± 0.19	9.47 ± 0.12	9.13 ± 0.14	9.43 ± 0.01
TC17001768.hg.1	NACA2	8.01 ± 0.30	8.07 ± 0.31	7.75 ± 0.17	8.08 ± 0.06
TC07001350.hg.1	NACAD	8.09 ± 0.23	8.04 ± 0.22	8.01 ± 0.20	8.15 ± 0.07
TC08000628.hg.1	NACAP1	7.11 ± 0.18	7.13 ± 0.02	7.12 ± 0.11	7.18 ± 0.02
TC19000236.hg.1	NACC1	9.01 ± 0.21	8.78 ± 0.09	8.93 ± 0.03	8.73 ± 0.07
TC09001722.hg.1	NACC2	8.37 ± 0.14	8.28 ± 0.07	8.45 ± 0.07	8.25 ± 0.05
TC01002107.hg.1	NADK	9.79 ± 0.25	9.46 ± 0.12	9.98 ± 0.29	9.38 ± 0.17
TC05001270.hg.1	NADK2	7.04 ± 0.26	7.16 ± 0.09	7.00 ± 0.20	7.16 ± 0.05
TC05000163.hg.1	NADKD1-AS1	4.86 ± 0.12	4.88 ± 0.08	4.87 ± 0.12	4.96 ± 0.11
TC11000734.hg.1	NADSYN1	9.27 ± 0.08	9.22 ± 0.03	9.31 ± 0.15	9.20 ± 0.10
TC16001174.hg.1	NAE1	8.19 ± 0.49	8.68 ± 0.23	8.06 ± 0.38	8.49 ± 0.03
TC04001697.hg.1	NAF1	6.83 ± 0.27	6.95 ± 0.13	6.63 ± 0.25	6.95 ± 0.05
TC22000838.hg.1	NAGA	9.03 ± 0.40	8.59 ± 0.05	8.87 ± 0.21	8.41 ± 0.25
TC02000427.hg.1	NAGK	9.25 ± 0.17	8.85 ± 0.18	9.27 ± 0.24	8.81 ± 0.06
TC17000526.hg.1	NAGLU	8.01 ± 0.13	7.82 ± 0.13	7.81 ± 0.13	7.91 ± 0.02
TC16000840.hg.1	NAGPA	8.29 ± 0.18	8.21 ± 0.07	8.10 ± 0.17	8.25 ± 0.03
TC17000561.hg.1	NAGS	8.13 ± 0.28	7.99 ± 0.20	8.03 ± 0.23	8.08 ± 0.01
TC09001620.hg.1	NAIF1	7.78 ± 0.16	7.66 ± 0.08	7.63 ± 0.18	7.64 ± 0.04
TC05001455.hg.1	NAIP	9.65 ± 0.53	8.86 ± 0.33	10.67 ± 0.29	8.65 ± 0.22
TC13000845.hg.1	NALCN	5.67 ± 0.13	5.61 ± 0.11	5.62 ± 0.11	5.70 ± 0.10
TC13000363.hg.1	NALCN-AS1	6.19 ± 0.16	6.20 ± 0.18	6.18 ± 0.11	6.32 ± 0.10
TC07001738.hg.1	NAMPT	11.22 ± 0.15	11.65 ± 0.20	12.05 ± 0.28	11.56 ± 0.18
TC10001182.hg.1	NAMPTL	9.65 ± 0.15	10.01 ± 0.18	10.39 ± 0.19	10.01 ± 0.16
TC12000114.hg.1	NANOG	6.59 ± 0.18	6.65 ± 0.01	6.60 ± 0.11	6.76 ± 0.12

TC12000112.hg.1	NANOGNB	4.23 ± 0.31	3.99 ± 0.10	4.13 ± 0.16	3.95 ± 0.06
TC12000116.hg.1	NANOGP1	5.90 ± 0.10	5.76 ± 0.04	5.89 ± 0.12	5.80 ± 0.05
TC10000862.hg.1	NANOS1	6.94 ± 0.21	6.82 ± 0.04	6.78 ± 0.12	6.77 ± 0.05
TC19001638.hg.1	NANOS2	6.57 ± 0.04	6.63 ± 0.16	6.43 ± 0.17	6.68 ± 0.12
TC19000246.hg.1	NANOS3	8.57 ± 0.30	8.63 ± 0.21	8.54 ± 0.19	8.81 ± 0.12
TC20000734.hg.1	NANP	7.13 ± 0.21	6.97 ± 0.08	6.90 ± 0.08	6.97 ± 0.13
TC09000497.hg.1	NANS	7.81 ± 0.18	7.71 ± 0.07	7.77 ± 0.21	7.71 ± 0.13
TC12001752.hg.1	NAP1L1	9.18 ± 0.51	9.57 ± 0.11	9.40 ± 0.29	9.57 ± 0.16
TC0X001152.hg.1	NAP1L2	5.26 ± 0.14	5.10 ± 0.12	5.14 ± 0.06	5.10 ± 0.09
TC0X001197.hg.1	NAP1L3	6.69 ± 0.21	6.87 ± 0.19	6.70 ± 0.15	6.97 ± 0.09
TC11001287.hg.1	NAP1L4	9.04 ± 0.14	9.11 ± 0.17	8.83 ± 0.06	9.01 ± 0.03
TC04001379.hg.1	NAP1L5	8.15 ± 0.23	8.31 ± 0.21	8.19 ± 0.25	8.33 ± 0.05
TC0X001151.hg.1	NAP1L6	6.40 ± 0.35	6.31 ± 0.17	6.26 ± 0.18	6.40 ± 0.08
TC19001666.hg.1	NAPA	10.00 ± 0.49	9.80 ± 0.12	9.89 ± 0.31	9.83 ± 0.17
TC19000679.hg.1	NAPA-AS1	8.15 ± 0.15	8.16 ± 0.09	8.12 ± 0.12	8.18 ± 0.07
TC20000708.hg.1	NAPB	7.14 ± 0.21	7.06 ± 0.10	7.24 ± 0.31	7.04 ± 0.11
TC07001715.hg.1	NAPEPLD	6.33 ± 0.22	6.08 ± 0.17	6.23 ± 0.10	5.98 ± 0.02
TC18000052.hg.1	NAPG	8.30 ± 0.38	8.48 ± 0.12	8.57 ± 0.31	8.41 ± 0.14
TC08001720.hg.1	NAPRT1	8.89 ± 0.19	8.78 ± 0.14	8.89 ± 0.08	8.84 ± 0.04
TC19001746.hg.1	NAPSA	8.13 ± 0.18	7.95 ± 0.09	7.93 ± 0.13	7.88 ± 0.08
TC19001745.hg.1	NAPSB	8.99 ± 0.19	8.68 ± 0.16	8.77 ± 0.09	8.56 ± 0.12
TC17000953.hg.1	NARF	9.06 ± 0.15	8.95 ± 0.05	9.26 ± 0.13	8.96 ± 0.09
TC16000738.hg.1	NARFL	8.05 ± 0.12	7.98 ± 0.10	7.96 ± 0.12	8.03 ± 0.02
TC15001506.hg.1	NARG2	7.98 ± 0.51	8.29 ± 0.28	7.91 ± 0.37	8.03 ± 0.11
TC18000537.hg.1	NARS	9.71 ± 0.29	9.70 ± 0.12	9.62 ± 0.23	9.60 ± 0.18
TC11002138.hg.1	NARS2	7.29 ± 0.07	7.16 ± 0.12	7.03 ± 0.08	7.03 ± 0.07
TC01000575.hg.1	NASP	8.44 ± 0.13	8.44 ± 0.12	8.42 ± 0.15	8.16 ± 0.11
TC08000137.hg.1	NAT1	6.20 ± 0.04	6.01 ± 0.04	6.22 ± 0.12	5.97 ± 0.05
TC11000325.hg.1	NAT10	8.78 ± 0.10	8.62 ± 0.08	8.36 ± 0.13	8.44 ± 0.06
TC19000899.hg.1	NAT14	8.69 ± 0.23	8.64 ± 0.23	8.55 ± 0.19	8.62 ± 0.04
TC07001684.hg.1	NAT16	7.52 ± 0.21	7.53 ± 0.22	7.49 ± 0.21	7.58 ± 0.03

TC08000139.hg.1	NAT2	5.98 ± 0.14	5.78 ± 0.02	5.79 ± 0.19	5.83 ± 0.09
TC03003412.hg.1	NAT6	8.51 ± 0.15	8.49 ± 0.18	8.33 ± 0.23	8.63 ± 0.07
TC02001985.hg.1	NAT8	6.60 ± 0.25	6.54 ± 0.20	6.53 ± 0.19	6.75 ± 0.12
TC02001986.hg.1	NAT8B	6.75 ± 0.12	6.39 ± 0.04	6.54 ± 0.05	6.47 ± 0.04
TC04000033.hg.1	NAT8L	7.92 ± 0.22	7.77 ± 0.09	7.79 ± 0.21	7.82 ± 0.01
TC17001862.hg.1	NAT9	8.32 ± 0.10	8.13 ± 0.02	8.06 ± 0.16	8.11 ± 0.05
TC01001661.hg.1	NAV1	7.19 ± 0.09	7.07 ± 0.10	7.10 ± 0.10	7.14 ± 0.03
TC11000252.hg.1	NAV2	6.45 ± 0.18	6.46 ± 0.17	6.41 ± 0.12	6.55 ± 0.05
TC11001487.hg.1	NAV2-AS1	4.30 ± 0.22	4.26 ± 0.10	4.43 ± 0.13	4.17 ± 0.10
TC11001486.hg.1	NAV2-AS2	7.12 ± 0.28	7.17 ± 0.26	7.13 ± 0.13	7.28 ± 0.09
TC11001485.hg.1	NAV2-AS3	5.31 ± 0.20	5.22 ± 0.07	5.32 ± 0.17	5.18 ± 0.10
TC11001481.hg.1	NAV2-AS4	6.44 ± 0.26	6.35 ± 0.17	6.40 ± 0.22	6.54 ± 0.09
TC11001480.hg.1	NAV2-AS5	5.79 ± 0.11	5.74 ± 0.10	5.68 ± 0.05	5.91 ± 0.19
TC11000253.hg.1	NAV2-IT1	5.90 ± 0.18	6.03 ± 0.20	5.82 ± 0.20	6.08 ± 0.20
TC12000656.hg.1	NAV3	5.34 ± 0.13	5.19 ± 0.07	5.25 ± 0.03	5.26 ± 0.07
TC02001592.hg.1	NBAS	7.95 ± 0.21	7.95 ± 0.20	7.74 ± 0.19	7.69 ± 0.06
TC13000120.hg.1	NBEA	6.19 ± 0.18	6.32 ± 0.18	6.07 ± 0.08	6.23 ± 0.13
TC02001195.hg.1	NBEAL1	6.27 ± 0.33	6.29 ± 0.19	6.22 ± 0.25	6.09 ± 0.12
TC03000263.hg.1	NBEAL2	9.89 ± 0.30	9.62 ± 0.07	10.26 ± 0.27	9.52 ± 0.15
TC15001017.hg.1	NBEAP1	6.54 ± 0.26	6.55 ± 0.07	6.46 ± 0.10	6.66 ± 0.13
TC01006268.hg.1	NBL1	8.36 ± 0.16	8.31 ± 0.18	8.33 ± 0.21	8.42 ± 0.05
TC04000857.hg.1	NBLA00301	5.52 ± 0.24	5.39 ± 0.06	5.45 ± 0.08	5.42 ± 0.05
TC08001400.hg.1	NBN	8.61 ± 0.34	8.58 ± 0.25	9.33 ± 0.36	8.43 ± 0.11
TC01002271.hg.1	NBPF1	9.30 ± 0.23	9.37 ± 0.11	9.40 ± 0.10	9.37 ± 0.06
TC01006309.hg.1	NBPF10	11.90 ± 0.30	12.03 ± 0.12	12.22 ± 0.15	12.05 ± 0.04
TC01006368.hg.1	NBPF14	11.61 ± 0.30	11.74 ± 0.13	11.90 ± 0.15	11.75 ± 0.04
TC01001144.hg.1	NBPF16	9.87 ± 0.23	10.02 ± 0.08	9.95 ± 0.16	10.01 ± 0.05
TC01001147.hg.1	NBPF16	10.63 ± 0.35	10.83 ± 0.12	10.93 ± 0.22	10.81 ± 0.06
TC05000423.hg.1	NBPF22P	6.52 ± 0.14	6.50 ± 0.11	6.50 ± 0.06	6.59 ± 0.09
TC01006301.hg.1	NBPF24	9.92 ± 0.31	10.08 ± 0.14	10.08 ± 0.18	10.07 ± 0.05
TC01006367.hg.1	NBPF24	9.64 ± 0.34	9.79 ± 0.14	9.79 ± 0.18	9.79 ± 0.05

TC01000271.hg.1	NBPF3	8.12 ± 0.14	8.16 ± 0.03	8.12 ± 0.03	8.17 ± 0.05
TC01002943.hg.1	NBPF4	6.78 ± 0.11	6.74 ± 0.13	6.70 ± 0.09	6.85 ± 0.13
TC01006398.hg.1	NBPF5P	6.76 ± 0.22	6.77 ± 0.07	6.64 ± 0.12	6.86 ± 0.17
TC01006399.hg.1	NBPF6	6.68 ± 0.16	6.69 ± 0.17	6.63 ± 0.10	6.82 ± 0.16
TC01003052.hg.1	NBPF7	6.97 ± 0.17	6.96 ± 0.05	7.03 ± 0.05	6.98 ± 0.06
TC01006429.hg.1	NBPF8P	8.94 ± 0.26	9.08 ± 0.09	8.94 ± 0.14	9.10 ± 0.06
TC01006431.hg.1	NBPF9	10.57 ± 0.34	10.77 ± 0.15	10.84 ± 0.20	10.75 ± 0.05
TC17000548.hg.1	NBR1	8.92 ± 0.24	8.82 ± 0.12	9.06 ± 0.26	8.62 ± 0.25
TC17000547.hg.1	NBR2	7.85 ± 0.10	7.66 ± 0.05	7.79 ± 0.09	7.59 ± 0.04
TC08001494.hg.1	NCALD	6.61 ± 0.08	6.56 ± 0.02	6.43 ± 0.01	6.52 ± 0.05
TC11001015.hg.1	NCAM1	7.75 ± 0.06	7.59 ± 0.13	7.67 ± 0.13	7.67 ± 0.06
TC11002300.hg.1	NCAM1-AS1	7.12 ± 0.26	7.16 ± 0.24	7.19 ± 0.13	7.21 ± 0.09
TC21000060.hg.1	NCAM2	4.79 ± 0.14	4.63 ± 0.08	4.69 ± 0.11	4.68 ± 0.08
TC19000370.hg.1	NCAN	7.49 ± 0.27	7.49 ± 0.21	7.42 ± 0.21	7.61 ± 0.06
TC12000082.hg.1	NCAPD2	8.95 ± 0.22	8.71 ± 0.24	8.68 ± 0.10	8.35 ± 0.11
TC11002473.hg.1	NCAPD3	8.15 ± 0.08	8.11 ± 0.21	7.81 ± 0.04	7.85 ± 0.02
TC04000155.hg.1	NCAPG	6.40 ± 0.05	6.26 ± 0.61	6.17 ± 0.31	5.57 ± 0.19
TC07002083.hg.1	NCAPG2	6.97 ± 0.03	6.80 ± 0.23	6.66 ± 0.14	6.47 ± 0.01
TC02000582.hg.1	NCAPH	7.53 ± 0.26	7.20 ± 0.24	7.25 ± 0.15	6.97 ± 0.04
TC22000428.hg.1	NCAPH2	8.43 ± 0.16	8.46 ± 0.11	8.30 ± 0.14	8.52 ± 0.04
TC09000492.hg.1	NCBP1	8.38 ± 0.26	8.47 ± 0.20	8.25 ± 0.26	8.37 ± 0.04
TC03002170.hg.1	NCBP2	7.88 ± 0.18	7.88 ± 0.10	7.69 ± 0.08	7.86 ± 0.03
TC03001103.hg.1	NCBP2-AS1	8.05 ± 0.13	7.94 ± 0.02	7.96 ± 0.08	7.97 ± 0.02
TC03001104.hg.1	NCBP2-AS2	8.32 ± 0.22	8.40 ± 0.22	7.93 ± 0.07	8.42 ± 0.08
TC0X000527.hg.1	NCBP2L	5.98 ± 0.04	5.87 ± 0.14	6.03 ± 0.14	6.07 ± 0.08
TC19000538.hg.1	NCCRP1	7.55 ± 0.14	7.52 ± 0.21	7.46 ± 0.20	7.65 ± 0.09
TC01000458.hg.1	NCDN	8.19 ± 0.03	8.15 ± 0.10	8.06 ± 0.09	8.12 ± 0.04
TC03002008.hg.1	NCEH1	6.98 ± 0.04	6.79 ± 0.12	6.97 ± 0.14	6.78 ± 0.23
TC07000459.hg.1	NCF1	11.08 ± 0.11	10.91 ± 0.11	11.26 ± 0.29	10.85 ± 0.31
TC07000438.hg.1	NCF1B	11.45 ± 0.19	11.25 ± 0.16	11.72 ± 0.27	11.18 ± 0.40
TC07001516.hg.1	NCF1C	11.53 ± 0.18	11.33 ± 0.14	11.74 ± 0.25	11.25 ± 0.38

TC01003616.hg.1	NCF2	11.25 ± 0.25	10.96 ± 0.16	11.58 ± 0.25	10.84 ± 0.23
TC22000270.hg.1	NCF4	10.39 ± 0.31	10.00 ± 0.16	10.83 ± 0.27	9.87 ± 0.17
TC03003323.hg.1	NCK1	7.81 ± 0.33	7.97 ± 0.15	7.80 ± 0.13	7.83 ± 0.04
TC02000649.hg.1	NCK2	9.67 ± 0.08	9.77 ± 0.03	9.69 ± 0.09	9.82 ± 0.05
TC02002592.hg.1	NCKAP1	6.25 ± 0.41	5.85 ± 0.21	6.40 ± 0.34	5.82 ± 0.15
TC12000467.hg.1	NCKAP1L	10.29 ± 0.28	10.03 ± 0.15	10.30 ± 0.18	9.91 ± 0.11
TC02002365.hg.1	NCKAP5	5.61 ± 0.11	5.49 ± 0.03	5.50 ± 0.11	5.53 ± 0.06
TC02002367.hg.1	NCKAP5-IT1	3.74 ± 0.15	3.59 ± 0.06	3.68 ± 0.13	3.63 ± 0.05
TC12001482.hg.1	NCKAP5L	8.26 ± 0.09	8.18 ± 0.13	8.19 ± 0.06	8.25 ± 0.07
TC03001387.hg.1	NCKIPSD	8.30 ± 0.17	8.24 ± 0.16	8.18 ± 0.18	8.28 ± 0.03
TC02002859.hg.1	NCL	10.19 ± 0.25	10.54 ± 0.02	9.97 ± 0.16	10.50 ± 0.09
TC19000067.hg.1	NCLN	9.19 ± 0.12	9.07 ± 0.06	9.08 ± 0.01	9.03 ± 0.03
TC01000312.hg.1	NCMAP	7.41 ± 0.12	7.35 ± 0.07	7.36 ± 0.17	7.33 ± 0.02
TC02000137.hg.1	NCOA1	9.76 ± 0.23	10.01 ± 0.13	10.12 ± 0.33	9.87 ± 0.17
TC08001310.hg.1	NCOA2	9.39 ± 0.30	9.40 ± 0.20	9.58 ± 0.43	9.31 ± 0.16
TC20000372.hg.1	NCOA3	9.75 ± 0.20	9.83 ± 0.10	9.62 ± 0.35	9.74 ± 0.07
TC10000338.hg.1	NCOA4	10.98 ± 0.21	11.24 ± 0.16	11.15 ± 0.18	11.15 ± 0.23
TC20000896.hg.1	NCOA5	8.61 ± 0.09	8.49 ± 0.09	8.43 ± 0.08	8.43 ± 0.05
TC20000785.hg.1	NCOA6	7.10 ± 0.11	6.96 ± 0.02	7.05 ± 0.02	7.09 ± 0.04
TC20000786.hg.1	NCOA6	8.69 ± 0.21	8.59 ± 0.12	8.73 ± 0.24	8.52 ± 0.25
TC06000961.hg.1	NCOA7	7.81 ± 0.12	7.87 ± 0.07	7.90 ± 0.23	7.75 ± 0.09
TC17001171.hg.1	NCOR1	9.54 ± 0.23	9.60 ± 0.13	9.59 ± 0.23	9.45 ± 0.12
TC20000739.hg.1	NCOR1P1	7.39 ± 0.10	7.44 ± 0.11	7.38 ± 0.16	7.39 ± 0.08
TC12002096.hg.1	NCOR2	9.07 ± 0.07	9.06 ± 0.07	8.96 ± 0.10	9.11 ± 0.06
TC19000886.hg.1	NCR1	8.12 ± 0.05	7.98 ± 0.04	8.05 ± 0.03	8.01 ± 0.03
TC06000567.hg.1	NCR2	8.23 ± 0.15	8.26 ± 0.22	8.14 ± 0.15	8.45 ± 0.10
TC06001531.hg.1	NCR3	8.10 ± 0.09	8.02 ± 0.05	7.84 ± 0.08	7.98 ± 0.11
TC6_apd_hap1000082.hg.1	NCR3	8.10 ± 0.09	8.02 ± 0.05	7.84 ± 0.08	7.98 ± 0.11
TC6_cox_hap2000160.hg.1	NCR3	8.10 ± 0.09	8.02 ± 0.05	7.84 ± 0.08	7.98 ± 0.11
TC6_dbb_hap3000149.hg.1	NCR3	8.10 ± 0.09	8.02 ± 0.05	7.84 ± 0.08	7.98 ± 0.11
TC6_mann_hap4000137.hg.1	NCR3	8.10 ± 0.09	8.02 ± 0.05	7.84 ± 0.08	7.98 ± 0.11

TC6_mcf_hap5000137.hg.1	NCR3	8.09 ± 0.10	8.01 ± 0.05	7.83 ± 0.09	7.98 ± 0.11
TC6_qbl_hap6000152.hg.1	NCR3	8.10 ± 0.09	8.02 ± 0.05	7.84 ± 0.08	7.98 ± 0.11
TC6_ssto_hap7000132.hg.1	NCR3	8.10 ± 0.09	8.02 ± 0.05	7.84 ± 0.08	7.98 ± 0.11
TC11000229.hg.1	NCR3LG1	6.83 ± 0.14	6.92 ± 0.07	6.66 ± 0.21	6.97 ± 0.11
TC0Y000181.hg.1	NCRNA00185	6.95 ± 0.17	6.92 ± 0.11	6.95 ± 0.09	6.89 ± 0.01
TC05000369.hg.1	NCRUPAR	4.84 ± 0.10	4.57 ± 0.14	4.79 ± 0.04	4.62 ± 0.18
TC09000731.hg.1	NCS1	7.12 ± 0.15	7.03 ± 0.16	7.05 ± 0.24	7.10 ± 0.06
TC01001367.hg.1	NCSTN	9.40 ± 0.30	9.14 ± 0.10	9.60 ± 0.14	9.08 ± 0.08
TC01002676.hg.1	NDC1	7.87 ± 0.21	7.80 ± 0.20	7.54 ± 0.13	7.51 ± 0.11
TC18000009.hg.1	NDC80	6.60 ± 0.16	6.63 ± 0.33	6.62 ± 0.38	6.16 ± 0.14
TC17000141.hg.1	NDEL1	10.57 ± 0.07	10.65 ± 0.09	10.76 ± 0.22	10.55 ± 0.15
TC05000777.hg.1	NDFIP1	8.63 ± 0.32	8.86 ± 0.12	8.56 ± 0.22	8.81 ± 0.12
TC13000303.hg.1	NDFIP2	6.02 ± 0.05	5.85 ± 0.04	5.92 ± 0.08	5.80 ± 0.04
TC13000760.hg.1	NDFIP2-AS1	6.46 ± 0.20	6.32 ± 0.16	6.46 ± 0.21	6.46 ± 0.09
TC15001070.hg.1	NDN	6.45 ± 0.23	6.30 ± 0.27	6.43 ± 0.23	6.45 ± 0.07
TC04001509.hg.1	NDNF	6.91 ± 0.11	6.96 ± 0.12	6.92 ± 0.06	7.09 ± 0.06
TC15001108.hg.1	NDNL2	8.92 ± 0.04	9.17 ± 0.13	8.56 ± 0.08	9.08 ± 0.07
TC09000842.hg.1	NDOR1	8.79 ± 0.13	8.66 ± 0.15	8.69 ± 0.12	8.71 ± 0.03
TC0X000979.hg.1	NDP	5.47 ± 0.09	5.48 ± 0.12	5.65 ± 0.10	5.61 ± 0.03
TC0X000208.hg.1	NDP-AS1	6.81 ± 0.21	6.89 ± 0.24	6.80 ± 0.15	7.08 ± 0.17
TC08001659.hg.1	NDRG1	9.18 ± 0.13	9.07 ± 0.02	9.17 ± 0.12	9.06 ± 0.08
TC14000908.hg.1	NDRG2	7.93 ± 0.20	7.88 ± 0.13	7.77 ± 0.19	7.94 ± 0.06
TC20000816.hg.1	NDRG3	9.49 ± 0.13	9.42 ± 0.20	9.48 ± 0.09	9.22 ± 0.16
TC16000503.hg.1	NDRG4	8.16 ± 0.22	8.16 ± 0.20	8.14 ± 0.18	8.28 ± 0.07
TC05000831.hg.1	NDST1	8.84 ± 0.16	8.54 ± 0.04	8.82 ± 0.14	8.60 ± 0.16
TC05000832.hg.1	NDST1	6.83 ± 0.05	6.80 ± 0.16	6.81 ± 0.19	6.88 ± 0.12
TC10001419.hg.1	NDST2	8.90 ± 0.13	8.82 ± 0.06	9.01 ± 0.28	8.89 ± 0.03
TC04000598.hg.1	NDST3	4.96 ± 0.13	4.75 ± 0.09	4.86 ± 0.06	4.81 ± 0.04
TC04001488.hg.1	NDST4	4.94 ± 0.08	4.81 ± 0.08	4.85 ± 0.07	4.90 ± 0.04
TC0X000589.hg.1	NDUFA1	8.50 ± 0.18	8.34 ± 0.15	8.26 ± 0.26	8.25 ± 0.08
TC02002928.hg.1	NDUFA10	8.24 ± 0.04	8.31 ± 0.08	8.07 ± 0.08	8.28 ± 0.03

TC19002675.hg.1	NDUFA11	8.89 ± 0.16	8.76 ± 0.10	8.68 ± 0.20	8.71 ± 0.07
TC12001827.hg.1	NDUFA12	9.49 ± 0.06	9.45 ± 0.23	9.26 ± 0.07	9.19 ± 0.14
TC05001862.hg.1	NDUFA2	8.21 ± 0.30	8.01 ± 0.12	7.99 ± 0.18	7.83 ± 0.15
TC19000871.hg.1	NDUFA3	8.95 ± 0.13	8.85 ± 0.08	8.88 ± 0.08	8.94 ± 0.02
TC07001145.hg.1	NDUFA4	8.01 ± 0.10	8.07 ± 0.23	7.86 ± 0.16	7.94 ± 0.05
TC12001622.hg.1	NDUFA4L2	7.81 ± 0.28	7.78 ± 0.29	7.77 ± 0.30	7.88 ± 0.04
TC07001821.hg.1	NDUFA5	7.62 ± 0.22	7.80 ± 0.07	7.59 ± 0.18	7.85 ± 0.07
TC22000840.hg.1	NDUFA6	8.80 ± 0.12	8.69 ± 0.18	8.51 ± 0.19	8.59 ± 0.11
TC22001474.hg.1	NDUFA6-AS1	7.78 ± 0.23	7.85 ± 0.15	7.60 ± 0.16	7.87 ± 0.05
TC19001128.hg.1	NDUFA7	8.93 ± 0.21	8.75 ± 0.02	8.68 ± 0.12	8.73 ± 0.05
TC09001554.hg.1	NDUFA8	7.64 ± 0.21	7.47 ± 0.10	7.44 ± 0.27	7.27 ± 0.00
TC12003205.hg.1	NDUFA9	8.53 ± 0.28	8.39 ± 0.23	8.27 ± 0.22	8.15 ± 0.09
TC16000966.hg.1	NDUFAB1	7.93 ± 0.07	7.64 ± 0.30	7.47 ± 0.34	7.29 ± 0.12
TC15001249.hg.1	NDUFAF1	8.12 ± 0.24	7.60 ± 0.14	8.15 ± 0.24	7.22 ± 0.22
TC05000260.hg.1	NDUFAF2	7.05 ± 0.13	6.99 ± 0.05	6.91 ± 0.07	6.92 ± 0.06
TC03000287.hg.1	NDUFAF3	9.16 ± 0.17	8.93 ± 0.08	9.04 ± 0.29	8.95 ± 0.02
TC06001956.hg.1	NDUFAF4	6.16 ± 0.11	6.14 ± 0.17	5.96 ± 0.10	5.98 ± 0.05
TC15000385.hg.1	NDUFAF4P1	5.22 ± 0.16	5.19 ± 0.11	5.18 ± 0.17	5.20 ± 0.05
TC20000095.hg.1	NDUFAF5	6.47 ± 0.27	6.59 ± 0.18	6.33 ± 0.16	6.64 ± 0.14
TC08000579.hg.1	NDUFAF6	6.65 ± 0.12	6.60 ± 0.01	6.52 ± 0.04	6.61 ± 0.03
TC02000235.hg.1	NDUFAF7	7.54 ± 0.29	7.66 ± 0.08	7.69 ± 0.25	7.65 ± 0.04
TC14001447.hg.1	NDUFB1	9.17 ± 0.11	9.08 ± 0.09	9.16 ± 0.17	9.04 ± 0.10
TC16000050.hg.1	NDUFB10	8.40 ± 0.27	8.18 ± 0.12	8.20 ± 0.21	7.89 ± 0.15
TC0X000994.hg.1	NDUFB11	9.45 ± 0.28	9.24 ± 0.14	9.05 ± 0.27	9.10 ± 0.06
TC07000885.hg.1	NDUFB2	6.70 ± 0.62	6.81 ± 0.19	6.58 ± 0.37	6.83 ± 0.11
TC07003344.hg.1	NDUFB2	7.12 ± 0.08	7.06 ± 0.03	6.98 ± 0.06	7.04 ± 0.05
TC07001925.hg.1	NDUFB2-AS1	7.88 ± 0.15	7.87 ± 0.12	7.82 ± 0.10	7.81 ± 0.03
TC02001171.hg.1	NDUFB3	8.21 ± 0.30	7.95 ± 0.03	8.29 ± 0.31	7.76 ± 0.05
TC03000617.hg.1	NDUFB4	6.45 ± 0.05	6.33 ± 0.16	6.37 ± 0.13	6.26 ± 0.11
TC03000956.hg.1	NDUFB5	7.08 ± 0.11	7.03 ± 0.12	7.07 ± 0.14	6.79 ± 0.15
TC09001001.hg.1	NDUFB6	6.41 ± 0.15	6.09 ± 0.29	6.59 ± 0.42	5.88 ± 0.18

TC19001240.hg.1	NDUFB7	8.62 ± 0.28	8.42 ± 0.13	8.49 ± 0.22	8.44 ± 0.08
TC10001594.hg.1	NDUFB8	8.02 ± 0.14	8.07 ± 0.04	7.96 ± 0.10	8.10 ± 0.14
TC08000726.hg.1	NDUFB9	7.83 ± 0.10	7.67 ± 0.15	7.55 ± 0.07	7.62 ± 0.04
TC04002949.hg.1	NDUFC1	7.48 ± 0.02	7.33 ± 0.06	7.32 ± 0.10	7.15 ± 0.08
TC11002132.hg.1	NDUFC2-KCTD14	7.57 ± 0.13	7.46 ± 0.05	7.51 ± 0.17	7.43 ± 0.06
TC02002697.hg.1	NDUFS1	8.18 ± 0.06	8.12 ± 0.12	8.07 ± 0.22	8.04 ± 0.08
TC01001381.hg.1	NDUFS2	8.67 ± 0.09	8.46 ± 0.05	8.54 ± 0.11	8.40 ± 0.05
TC11003448.hg.1	NDUFS3	8.78 ± 0.10	8.62 ± 0.24	8.42 ± 0.21	8.57 ± 0.03
TC05000221.hg.1	NDUFS4	6.96 ± 0.12	6.89 ± 0.24	6.79 ± 0.16	6.75 ± 0.06
TC01000492.hg.1	NDUFS5	9.41 ± 0.25	9.38 ± 0.06	9.08 ± 0.35	9.31 ± 0.07
TC05000019.hg.1	NDUFS6	8.30 ± 0.14	8.16 ± 0.09	8.08 ± 0.14	8.09 ± 0.05
TC19000037.hg.1	NDUFS7	8.54 ± 0.16	8.55 ± 0.16	8.39 ± 0.19	8.61 ± 0.05
TC11000703.hg.1	NDUFS8	9.07 ± 0.12	8.89 ± 0.04	8.84 ± 0.11	8.89 ± 0.05
TC11000696.hg.1	NDUFV1	8.98 ± 0.14	8.86 ± 0.09	8.69 ± 0.16	8.83 ± 0.05
TC18000041.hg.1	NDUFV2	8.30 ± 0.08	8.34 ± 0.27	8.16 ± 0.03	8.31 ± 0.07
TC21001063.hg.1	NDUFV3	8.38 ± 0.04	8.36 ± 0.04	8.32 ± 0.11	8.29 ± 0.06
TC02002428.hg.1	NEB	5.51 ± 0.09	5.45 ± 0.02	5.52 ± 0.06	5.49 ± 0.01
TC10001089.hg.1	NEBL	5.13 ± 0.10	4.98 ± 0.04	5.02 ± 0.07	5.03 ± 0.04
TC10000147.hg.1	NEBL-AS1	7.72 ± 0.22	7.67 ± 0.22	7.73 ± 0.13	7.66 ± 0.04
TC08000551.hg.1	NECAB1	5.31 ± 0.12	5.17 ± 0.05	5.73 ± 0.09	5.16 ± 0.06
TC16000653.hg.1	NECAB2	7.96 ± 0.13	7.86 ± 0.15	7.90 ± 0.17	7.94 ± 0.02
TC20000772.hg.1	NECAB3	8.21 ± 0.13	8.15 ± 0.12	8.19 ± 0.13	8.20 ± 0.02
TC12000113.hg.1	NECAP1	8.94 ± 0.18	8.95 ± 0.18	8.94 ± 0.20	8.98 ± 0.07
TC01000206.hg.1	NECAP2	9.31 ± 0.14	9.18 ± 0.04	9.11 ± 0.15	9.18 ± 0.06
TC12000749.hg.1	NEDD1	8.38 ± 0.51	8.63 ± 0.14	8.50 ± 0.57	8.57 ± 0.19
TC15001471.hg.1	NEDD4	6.40 ± 0.16	6.37 ± 0.16	6.90 ± 0.43	6.38 ± 0.13
TC18000202.hg.1	NEDD4L	6.64 ± 0.06	6.54 ± 0.05	6.59 ± 0.02	6.55 ± 0.03
TC14002343.hg.1	NEDD8	9.14 ± 0.14	9.05 ± 0.14	9.23 ± 0.14	8.88 ± 0.09
TC06001273.hg.1	NEDD9	8.16 ± 0.06	8.11 ± 0.21	8.21 ± 0.37	7.99 ± 0.17
TC22000198.hg.1	NEFH	7.80 ± 0.16	7.67 ± 0.14	7.77 ± 0.19	7.62 ± 0.03
TC08001068.hg.1	NEFL	6.42 ± 0.27	6.44 ± 0.19	6.43 ± 0.19	6.47 ± 0.03

TC08000193.hg.1	NEFM	7.08 ± 0.27	6.92 ± 0.13	7.05 ± 0.25	7.05 ± 0.06
TC01002777.hg.1	NEGR1	5.12 ± 0.03	5.08 ± 0.04	5.15 ± 0.02	5.11 ± 0.06
TC01002778.hg.1	NEGR1-IT1	4.97 ± 0.06	4.76 ± 0.01	4.80 ± 0.09	4.73 ± 0.06
TC15000695.hg.1	NEIL1	8.54 ± 0.06	8.64 ± 0.10	8.43 ± 0.10	8.85 ± 0.02
TC08000091.hg.1	NEIL2	7.97 ± 0.18	7.91 ± 0.18	7.84 ± 0.21	7.96 ± 0.05
TC04000874.hg.1	NEIL3	6.57 ± 0.13	6.49 ± 0.07	6.75 ± 0.04	6.32 ± 0.21
TC04001725.hg.1	NEK1	6.86 ± 0.46	6.99 ± 0.20	6.81 ± 0.33	6.83 ± 0.14
TC03001252.hg.1	NEK10	5.20 ± 0.10	5.04 ± 0.05	5.08 ± 0.05	5.08 ± 0.03
TC03000708.hg.1	NEK11	5.33 ± 0.08	5.23 ± 0.03	5.30 ± 0.09	5.24 ± 0.04
TC01003798.hg.1	NEK2	5.37 ± 0.16	5.25 ± 0.16	5.18 ± 0.10	5.20 ± 0.04
TC13000686.hg.1	NEK3	6.84 ± 0.03	6.70 ± 0.08	6.83 ± 0.05	6.78 ± 0.03
TC03001460.hg.1	NEK4	8.33 ± 0.12	8.19 ± 0.18	8.32 ± 0.12	7.99 ± 0.12
TC13000685.hg.1	NEK5	5.03 ± 0.05	4.96 ± 0.01	4.97 ± 0.02	4.97 ± 0.01
TC09000634.hg.1	NEK6	9.10 ± 0.09	8.82 ± 0.04	9.27 ± 0.08	8.84 ± 0.09
TC01001639.hg.1	NEK7	8.48 ± 0.44	8.64 ± 0.29	8.91 ± 0.47	8.40 ± 0.29
TC17000329.hg.1	NEK8	8.01 ± 0.17	7.96 ± 0.12	7.86 ± 0.13	8.02 ± 0.07
TC14001317.hg.1	NEK9	8.76 ± 0.11	8.62 ± 0.16	8.68 ± 0.21	8.46 ± 0.03
TC09000846.hg.1	NELFB	8.91 ± 0.17	8.80 ± 0.07	8.72 ± 0.18	8.76 ± 0.03
TC20000467.hg.1	NELFCD	8.64 ± 0.04	8.55 ± 0.19	8.38 ± 0.08	8.43 ± 0.05
TC6_cox_hap2000180.hg.1	NELFE	7.86 ± 0.11	7.84 ± 0.01	7.70 ± 0.13	7.82 ± 0.02
TC6_dbb_hap3000169.hg.1	NELFE	7.86 ± 0.11	7.84 ± 0.01	7.70 ± 0.13	7.82 ± 0.02
TC6_mcf_hap5000155.hg.1	NELFE	7.86 ± 0.11	7.84 ± 0.01	7.70 ± 0.13	7.82 ± 0.02
TC6_qbl_hap6000170.hg.1	NELFE	7.86 ± 0.11	7.84 ± 0.01	7.70 ± 0.13	7.82 ± 0.02
TC6_ssto_hap7000148.hg.1	NELFE	7.86 ± 0.11	7.84 ± 0.01	7.70 ± 0.13	7.82 ± 0.02
TC11000259.hg.1	NELL1	5.53 ± 0.14	5.38 ± 0.12	5.43 ± 0.10	5.48 ± 0.06
TC12001415.hg.1	NELL2	8.35 ± 0.48	8.98 ± 0.26	8.04 ± 0.27	9.13 ± 0.26
TC14001097.hg.1	NEMF	8.43 ± 0.64	8.81 ± 0.19	8.78 ± 0.54	8.76 ± 0.14
TC01001775.hg.1	NENF	8.96 ± 0.31	9.00 ± 0.30	8.76 ± 0.33	9.03 ± 0.08
TC15000664.hg.1	NEO1	6.50 ± 0.04	6.52 ± 0.03	6.43 ± 0.06	6.48 ± 0.09
TC01003347.hg.1	NES	7.58 ± 0.26	7.41 ± 0.16	7.47 ± 0.08	7.45 ± 0.04
TC10000047.hg.1	NET1	7.89 ± 0.13	7.89 ± 0.20	7.51 ± 0.09	7.84 ± 0.09

TC18000571.hg.1	NETO1	5.52 ± 0.24	5.35 ± 0.06	5.38 ± 0.09	5.39 ± 0.08
TC16001088.hg.1	NETO2	7.00 ± 0.36	6.69 ± 0.18	6.70 ± 0.01	6.52 ± 0.30
TC06001548.hg.1	NEU1	8.62 ± 0.11	8.51 ± 0.14	8.58 ± 0.18	8.53 ± 0.18
TC6_apd_hap1000089.hg.1	NEU1	8.62 ± 0.11	8.51 ± 0.14	8.58 ± 0.18	8.53 ± 0.18
TC6_cox_hap2000175.hg.1	NEU1	8.62 ± 0.11	8.52 ± 0.14	8.58 ± 0.18	8.53 ± 0.19
TC6_dbb_hap3000164.hg.1	NEU1	8.62 ± 0.11	8.51 ± 0.14	8.58 ± 0.18	8.53 ± 0.18
TC6_mann_hap4000146.hg.1	NEU1	8.62 ± 0.11	8.51 ± 0.14	8.58 ± 0.18	8.53 ± 0.18
TC6_mcf_hap5000150.hg.1	NEU1	8.62 ± 0.11	8.51 ± 0.14	8.58 ± 0.18	8.53 ± 0.18
TC6_qbl_hap6000165.hg.1	NEU1	8.62 ± 0.11	8.51 ± 0.14	8.58 ± 0.18	8.53 ± 0.18
TC6_ssto_hap7000144.hg.1	NEU1	8.62 ± 0.11	8.51 ± 0.14	8.58 ± 0.18	8.53 ± 0.18
TC02001413.hg.1	NEU2	8.13 ± 0.41	8.13 ± 0.19	8.06 ± 0.35	8.21 ± 0.07
TC11000797.hg.1	NEU3	7.48 ± 0.14	7.54 ± 0.05	7.42 ± 0.06	7.64 ± 0.09
TC02005018.hg.1	NEU4	8.51 ± 0.20	8.49 ± 0.20	8.45 ± 0.18	8.59 ± 0.04
TC10000775.hg.1	NEURL	8.34 ± 0.21	8.37 ± 0.21	8.29 ± 0.21	8.42 ± 0.03
TC05000948.hg.1	NEURL1B	7.60 ± 0.21	7.55 ± 0.30	7.55 ± 0.20	7.63 ± 0.05
TC20000892.hg.1	NEURL2	7.75 ± 0.15	7.65 ± 0.10	7.58 ± 0.21	7.66 ± 0.04
TC02002108.hg.1	NEURL3	7.15 ± 0.19	7.20 ± 0.23	7.10 ± 0.21	7.25 ± 0.08
TC17001086.hg.1	NEURL4	8.52 ± 0.04	8.45 ± 0.03	8.38 ± 0.06	8.46 ± 0.02
TC02005050.hg.1	NEUROD1	5.54 ± 0.23	5.40 ± 0.06	5.28 ± 0.20	5.38 ± 0.05
TC17001452.hg.1	NEUROD2	6.72 ± 0.20	6.76 ± 0.27	6.55 ± 0.26	6.74 ± 0.17
TC12000473.hg.1	NEUROD4	6.64 ± 0.26	6.38 ± 0.12	6.37 ± 0.25	6.42 ± 0.11
TC07001254.hg.1	NEUROD6	5.54 ± 0.33	5.57 ± 0.10	5.43 ± 0.21	5.64 ± 0.16
TC05001803.hg.1	NEUROG1	7.76 ± 0.48	7.80 ± 0.16	7.77 ± 0.34	7.91 ± 0.02
TC04001477.hg.1	NEUROG2	7.33 ± 0.13	7.28 ± 0.16	7.27 ± 0.17	7.28 ± 0.04
TC10001357.hg.1	NEUROG3	7.82 ± 0.14	7.73 ± 0.09	7.71 ± 0.14	7.79 ± 0.07
TC01000789.hg.1	NEXN	6.93 ± 0.36	6.54 ± 0.22	7.31 ± 0.42	6.49 ± 0.26
TC01002802.hg.1	NEXN-AS1	5.33 ± 0.18	5.11 ± 0.09	5.09 ± 0.10	5.18 ± 0.04
TC17000362.hg.1	NF1	7.92 ± 0.31	7.88 ± 0.26	8.05 ± 0.44	7.65 ± 0.13
TC15001023.hg.1	NF1P2	5.57 ± 0.04	5.36 ± 0.06	5.34 ± 0.05	5.40 ± 0.09
TC18000385.hg.1	NF1P5	5.81 ± 0.21	5.76 ± 0.24	5.95 ± 0.42	5.69 ± 0.24
TC22000199.hg.1	NF2	8.16 ± 0.03	8.09 ± 0.09	7.93 ± 0.10	7.99 ± 0.05

TC22000845.hg.1	NFAM1	9.85 ± 0.29	9.57 ± 0.12	10.16 ± 0.20	9.45 ± 0.40
TC01001704.hg.1	NFASC	6.88 ± 0.17	6.83 ± 0.15	6.80 ± 0.10	6.92 ± 0.07
TC16000570.hg.1	NFAT5	8.85 ± 0.43	9.06 ± 0.21	9.18 ± 0.49	9.00 ± 0.18
TC18000263.hg.1	NFATC1	8.56 ± 0.11	8.52 ± 0.01	8.35 ± 0.12	8.57 ± 0.04
TC20000942.hg.1	NFATC2	9.32 ± 0.33	9.37 ± 0.04	8.95 ± 0.20	9.16 ± 0.09
TC16000313.hg.1	NFATC2IP	8.41 ± 0.06	8.33 ± 0.05	8.20 ± 0.08	8.27 ± 0.03
TC16002056.hg.1	NFATC3	9.20 ± 0.29	9.42 ± 0.16	8.95 ± 0.17	9.23 ± 0.06
TC14000168.hg.1	NFATC4	7.82 ± 0.21	7.75 ± 0.20	7.74 ± 0.19	7.83 ± 0.03
TC12001567.hg.1	NFE2	8.94 ± 0.55	7.97 ± 0.18	9.26 ± 0.28	7.60 ± 0.27
TC17000619.hg.1	NFE2L1	8.63 ± 0.18	8.32 ± 0.05	8.48 ± 0.17	8.27 ± 0.11
TC02005045.hg.1	NFE2L2	9.53 ± 0.19	9.67 ± 0.10	9.89 ± 0.34	9.62 ± 0.11
TC07000159.hg.1	NFE2L3	6.98 ± 0.11	6.77 ± 0.02	6.77 ± 0.13	6.58 ± 0.20
TC01000702.hg.1	NFIA	6.97 ± 0.06	6.96 ± 0.09	6.94 ± 0.04	7.06 ± 0.05
TC09000916.hg.1	NFIB	6.43 ± 0.09	6.31 ± 0.19	6.40 ± 0.06	6.41 ± 0.09
TC19000069.hg.1	NFIC	9.21 ± 0.17	9.19 ± 0.04	9.24 ± 0.22	9.24 ± 0.14
TC09001325.hg.1	NFIL3	9.14 ± 0.25	9.52 ± 0.22	10.15 ± 0.42	9.29 ± 0.31
TC19000235.hg.1	NFIX	7.94 ± 0.16	7.89 ± 0.14	7.83 ± 0.10	7.96 ± 0.01
TC04000526.hg.1	NFKB1	9.12 ± 0.18	9.14 ± 0.06	9.32 ± 0.15	9.25 ± 0.32
TC10000753.hg.1	NFKB2	9.42 ± 0.18	9.41 ± 0.07	9.43 ± 0.06	9.65 ± 0.17
TC14001036.hg.1	NFKBIA	11.39 ± 0.21	12.21 ± 0.20	11.85 ± 0.14	12.13 ± 0.25
TC19000534.hg.1	NFKBIB	8.93 ± 0.18	8.83 ± 0.12	8.74 ± 0.21	8.86 ± 0.03
TC19001457.hg.1	NFKBID	9.28 ± 0.11	9.60 ± 0.14	9.22 ± 0.23	9.92 ± 0.12
TC06001762.hg.1	NFKBIE	8.67 ± 0.23	8.73 ± 0.15	8.47 ± 0.12	8.78 ± 0.13
TC06000369.hg.1	NFKBIL1	7.93 ± 0.15	7.92 ± 0.13	7.88 ± 0.23	7.98 ± 0.07
TC6_cox_hap2000065.hg.1	NFKBIL1	7.71 ± 0.11	7.71 ± 0.11	7.68 ± 0.20	7.78 ± 0.06
TC6_dbb_hap3000056.hg.1	NFKBIL1	7.71 ± 0.11	7.71 ± 0.11	7.68 ± 0.20	7.78 ± 0.06
TC6_mann_hap4000057.hg.1	NFKBIL1	7.71 ± 0.11	7.71 ± 0.11	7.68 ± 0.20	7.78 ± 0.06
TC6_mcf_hap5000051.hg.1	NFKBIL1	7.70 ± 0.11	7.70 ± 0.11	7.69 ± 0.18	7.78 ± 0.06
TC6_qbl_hap6000056.hg.1	NFKBIL1	7.71 ± 0.11	7.71 ± 0.11	7.68 ± 0.20	7.78 ± 0.06
TC6_ssto_hap7000052.hg.1	NFKBIL1	7.71 ± 0.11	7.71 ± 0.11	7.68 ± 0.20	7.78 ± 0.06
TC03003320.hg.1	NFKBIZ	9.60 ± 0.50	10.30 ± 0.04	10.28 ± 0.29	10.16 ± 0.27

TC11002448.hg.1	NFRKB	8.42 ± 0.07	8.33 ± 0.06	8.20 ± 0.03	8.30 ± 0.04
TC20001763.hg.1	NFS1	7.81 ± 0.02	7.68 ± 0.06	7.72 ± 0.07	7.63 ± 0.04
TC02001949.hg.1	NFU1	7.32 ± 0.03	7.32 ± 0.14	7.26 ± 0.11	7.20 ± 0.06
TC09000140.hg.1	NFX1	8.70 ± 0.16	8.63 ± 0.11	8.43 ± 0.15	8.67 ± 0.08
TC04001171.hg.1	NFXL1	7.53 ± 0.28	7.70 ± 0.26	7.87 ± 0.40	7.48 ± 0.14
TC06004084.hg.1	NFYA	9.03 ± 0.07	9.17 ± 0.08	9.11 ± 0.25	9.06 ± 0.16
TC12001906.hg.1	NFYB	7.83 ± 0.29	7.94 ± 0.20	7.85 ± 0.36	7.76 ± 0.17
TC01000515.hg.1	NFYC	8.83 ± 0.07	8.70 ± 0.05	8.84 ± 0.04	8.70 ± 0.03
TC14001341.hg.1	NGB	7.88 ± 0.26	7.91 ± 0.20	7.87 ± 0.22	7.98 ± 0.04
TC14000148.hg.1	NGDN	7.84 ± 0.17	8.10 ± 0.28	7.52 ± 0.20	8.02 ± 0.06
TC02002880.hg.1	NGEF	7.05 ± 0.26	7.04 ± 0.21	7.02 ± 0.19	7.14 ± 0.03
TC01003018.hg.1	NGF	8.10 ± 0.22	8.07 ± 0.11	7.97 ± 0.15	8.08 ± 0.09
TC17000645.hg.1	NGFR	8.16 ± 0.28	8.12 ± 0.26	8.08 ± 0.20	8.22 ± 0.03
TC0X000496.hg.1	NGFRAP1	8.20 ± 0.12	7.92 ± 0.12	8.04 ± 0.36	8.01 ± 0.14
TC03001248.hg.1	NGLY1	7.63 ± 0.45	7.85 ± 0.17	7.69 ± 0.34	7.66 ± 0.18
TC15002767.hg.1	NGRN	8.44 ± 0.03	8.41 ± 0.17	8.06 ± 0.22	8.30 ± 0.03
TC06002149.hg.1	NHEG1	7.87 ± 0.32	8.00 ± 0.01	7.93 ± 0.09	8.25 ± 0.17
TC02005053.hg.1	NHEJ1	8.34 ± 0.10	8.22 ± 0.03	8.15 ± 0.07	8.05 ± 0.10
TC01001368.hg.1	NHLH1	6.70 ± 0.17	6.62 ± 0.15	6.78 ± 0.14	6.68 ± 0.05
TC01003021.hg.1	NHLH2	5.79 ± 0.24	5.75 ± 0.11	5.73 ± 0.12	5.75 ± 0.04
TC06001300.hg.1	NHLRC1	6.25 ± 0.20	6.30 ± 0.17	6.20 ± 0.00	6.31 ± 0.20
TC10000828.hg.1	NHLRC2	8.07 ± 0.40	7.98 ± 0.26	8.06 ± 0.35	7.82 ± 0.12
TC13000135.hg.1	NHLRC3	7.08 ± 0.30	6.97 ± 0.15	7.20 ± 0.38	6.85 ± 0.11
TC05002120.hg.1	NHP2	8.25 ± 0.26	8.12 ± 0.07	8.11 ± 0.14	8.14 ± 0.03
TC22000831.hg.1	NHP2L1	7.86 ± 0.11	7.91 ± 0.01	7.65 ± 0.20	7.89 ± 0.03
TC0X000084.hg.1	NHS	7.16 ± 0.14	7.19 ± 0.04	7.15 ± 0.07	7.19 ± 0.11
TC0X000890.hg.1	NHS-AS1	5.75 ± 0.05	5.59 ± 0.07	5.81 ± 0.02	5.75 ± 0.09
TC06002159.hg.1	NHSL1	6.33 ± 0.13	6.17 ± 0.07	6.29 ± 0.10	6.23 ± 0.02
TC0X000393.hg.1	NHSL2	8.59 ± 0.14	8.36 ± 0.26	8.95 ± 0.29	8.23 ± 0.37
TC03003385.hg.1	NICN1	8.55 ± 0.08	8.47 ± 0.04	8.45 ± 0.03	8.50 ± 0.07
TC03000294.hg.1	NICN1-AS1	6.06 ± 0.17	6.16 ± 0.16	6.13 ± 0.19	6.18 ± 0.08

TC01004003.hg.1	NID1	7.84 ± 0.18	7.59 ± 0.05	7.74 ± 0.10	7.62 ± 0.06
TC14001138.hg.1	NID2	7.08 ± 0.17	6.99 ± 0.09	7.03 ± 0.12	7.06 ± 0.06
TC02001168.hg.1	NIF3L1	7.03 ± 0.09	6.79 ± 0.04	6.72 ± 0.12	6.75 ± 0.04
TC05000197.hg.1	NIM1	7.53 ± 0.18	7.51 ± 0.16	7.46 ± 0.17	7.60 ± 0.05
TC14001123.hg.1	NIN	9.70 ± 0.35	9.94 ± 0.15	10.13 ± 0.39	9.86 ± 0.13
TC09001345.hg.1	NINJ1	9.42 ± 0.29	9.56 ± 0.10	9.47 ± 0.04	9.76 ± 0.30
TC12001083.hg.1	NINJ2	8.25 ± 0.21	7.97 ± 0.04	8.34 ± 0.11	7.99 ± 0.03
TC20000733.hg.1	NINL	7.37 ± 0.16	7.36 ± 0.16	7.32 ± 0.14	7.40 ± 0.03
TC16000568.hg.1	NIP7	8.55 ± 0.05	8.45 ± 0.20	8.31 ± 0.08	8.33 ± 0.05
TC15002770.hg.1	NIPA1	7.29 ± 0.12	7.32 ± 0.01	7.29 ± 0.08	7.22 ± 0.07
TC15001048.hg.1	NIPA2	8.61 ± 0.07	8.55 ± 0.08	8.61 ± 0.24	8.54 ± 0.14
TC04000275.hg.1	NIPAL1	5.98 ± 0.18	5.92 ± 0.11	5.92 ± 0.16	5.99 ± 0.10
TC08001462.hg.1	NIPAL2	6.85 ± 0.17	6.86 ± 0.17	6.85 ± 0.24	6.70 ± 0.10
TC01000310.hg.1	NIPAL3	8.19 ± 0.13	8.14 ± 0.08	7.93 ± 0.11	8.02 ± 0.06
TC05000868.hg.1	NIPAL4	7.70 ± 0.17	7.65 ± 0.12	7.57 ± 0.11	7.69 ± 0.03
TC05000166.hg.1	NIPBL	8.77 ± 0.67	9.19 ± 0.31	9.20 ± 0.57	9.03 ± 0.15
TC22001488.hg.1	NIPSNAP1	7.95 ± 0.11	7.79 ± 0.04	7.72 ± 0.13	7.72 ± 0.04
TC09002905.hg.1	NIPSNAP3A	6.82 ± 0.20	6.70 ± 0.18	6.76 ± 0.30	6.62 ± 0.03
TC09002904.hg.1	NIPSNAP3B	5.48 ± 0.08	5.39 ± 0.10	5.45 ± 0.05	5.42 ± 0.03
TC03000335.hg.1	NISCH	8.45 ± 0.01	8.43 ± 0.04	8.32 ± 0.03	8.42 ± 0.03
TC01001377.hg.1	NIT1	8.27 ± 0.19	7.89 ± 0.07	8.22 ± 0.18	7.88 ± 0.09
TC03000507.hg.1	NIT2	7.77 ± 0.10	7.86 ± 0.09	7.56 ± 0.10	7.82 ± 0.12
TC01002449.hg.1	NKAIN1	7.56 ± 0.25	7.64 ± 0.20	7.52 ± 0.22	7.73 ± 0.04
TC06000954.hg.1	NKAIN2	5.73 ± 0.07	5.49 ± 0.08	5.71 ± 0.09	5.64 ± 0.04
TC08000420.hg.1	NKAIN3	5.76 ± 0.13	5.66 ± 0.09	5.66 ± 0.05	5.73 ± 0.05
TC20001027.hg.1	NKAIN4	7.66 ± 0.25	7.63 ± 0.22	7.63 ± 0.18	7.73 ± 0.06
TC0X001318.hg.1	NKAP	7.62 ± 0.20	7.63 ± 0.17	7.77 ± 0.25	7.67 ± 0.23
TC06000280.hg.1	NKAPL	6.27 ± 0.21	6.07 ± 0.07	6.20 ± 0.19	5.99 ± 0.08
TC0X001323.hg.1	NKAPP1	7.54 ± 0.10	7.57 ± 0.02	7.56 ± 0.07	7.55 ± 0.01
TC16000440.hg.1	NKD1	8.24 ± 0.16	8.19 ± 0.12	8.17 ± 0.13	8.25 ± 0.03
TC05000015.hg.1	NKD2	8.57 ± 0.21	8.50 ± 0.19	8.46 ± 0.25	8.62 ± 0.06

TC19001776.hg.1	NKG7	10.60 ± 0.17	10.37 ± 0.34	9.87 ± 0.10	9.76 ± 0.20
TC03001239.hg.1	NKIRAS1	6.67 ± 0.02	6.68 ± 0.03	6.75 ± 0.12	6.67 ± 0.09
TC17000521.hg.1	NKIRAS2	8.98 ± 0.15	8.78 ± 0.04	8.85 ± 0.09	8.77 ± 0.12
TC19001614.hg.1	NKPD1	7.87 ± 0.26	7.85 ± 0.20	7.81 ± 0.25	7.93 ± 0.02
TC0X001311.hg.1	NKRF	7.11 ± 0.17	7.15 ± 0.09	6.83 ± 0.15	7.11 ± 0.11
TC03000221.hg.1	NKTR	8.52 ± 0.74	9.07 ± 0.14	8.74 ± 0.51	9.14 ± 0.09
TC04000964.hg.1	NKX1-1	8.36 ± 0.25	8.30 ± 0.21	8.31 ± 0.27	8.40 ± 0.06
TC10001727.hg.1	NKX1-2	8.50 ± 0.29	8.60 ± 0.23	8.52 ± 0.23	8.67 ± 0.05
TC14002321.hg.1	NKX2-1	8.03 ± 0.15	8.00 ± 0.07	7.97 ± 0.15	7.99 ± 0.03
TC14000232.hg.1	NKX2-1-AS1	6.79 ± 0.20	6.71 ± 0.11	6.73 ± 0.15	6.76 ± 0.07
TC20000692.hg.1	NKX2-2	7.94 ± 0.20	8.03 ± 0.17	8.02 ± 0.20	8.08 ± 0.06
TC20000142.hg.1	NKX2-2-AS1	6.81 ± 0.18	6.84 ± 0.14	6.79 ± 0.14	6.87 ± 0.09
TC10000712.hg.1	NKX2-3	7.79 ± 0.22	7.90 ± 0.28	7.74 ± 0.31	7.89 ± 0.02
TC20000690.hg.1	NKX2-4	7.75 ± 0.39	7.60 ± 0.15	7.63 ± 0.38	7.68 ± 0.09
TC05002071.hg.1	NKX2-5	7.47 ± 0.14	7.38 ± 0.16	7.43 ± 0.03	7.49 ± 0.03
TC08001058.hg.1	NKX2-6	8.41 ± 0.27	8.24 ± 0.23	8.36 ± 0.28	8.30 ± 0.07
TC14001048.hg.1	NKX2-8	8.30 ± 0.34	8.09 ± 0.28	8.10 ± 0.30	8.15 ± 0.03
TC08001057.hg.1	NKX3-1	7.61 ± 0.19	7.61 ± 0.20	7.68 ± 0.25	7.63 ± 0.01
TC04001045.hg.1	NKX3-2	7.77 ± 0.18	7.66 ± 0.10	7.63 ± 0.11	7.75 ± 0.08
TC04001354.hg.1	NKX6-1	7.15 ± 0.30	7.15 ± 0.17	7.03 ± 0.19	7.17 ± 0.05
TC10001776.hg.1	NKX6-2	8.00 ± 0.43	7.98 ± 0.19	7.85 ± 0.32	7.95 ± 0.02
TC08001170.hg.1	NKX6-3	8.45 ± 0.24	8.35 ± 0.17	8.33 ± 0.23	8.38 ± 0.05
TC17001366.hg.1	NLE1	7.97 ± 0.12	7.91 ± 0.09	7.84 ± 0.15	7.95 ± 0.05
TC03000932.hg.1	NLGN1	4.86 ± 0.13	4.72 ± 0.05	4.70 ± 0.08	4.78 ± 0.07
TC03002013.hg.1	NLGN1-AS1	3.74 ± 0.10	3.54 ± 0.06	3.62 ± 0.03	3.57 ± 0.15
TC17000093.hg.1	NLGN2	7.76 ± 0.15	7.75 ± 0.11	7.74 ± 0.20	7.73 ± 0.06
TC0X000383.hg.1	NLGN3	7.67 ± 0.11	7.58 ± 0.04	7.73 ± 0.09	7.68 ± 0.09
TC0X000821.hg.1	NLGN4X	6.29 ± 0.10	6.21 ± 0.09	6.24 ± 0.14	6.31 ± 0.05
TC0Y000057.hg.1	NLGN4Y	5.54 ± 0.17	5.41 ± 0.04	5.49 ± 0.12	5.49 ± 0.06
TC0Y000167.hg.1	NLGN4Y-AS1	6.83 ± 0.16	6.76 ± 0.06	6.71 ± 0.03	6.89 ± 0.13
TC17000315.hg.1	NLK	8.96 ± 0.23	8.68 ± 0.17	9.06 ± 0.27	8.52 ± 0.19

TC05000279.hg.1	NLN	6.55 ± 0.04	6.43 ± 0.05	6.51 ± 0.14	6.39 ± 0.14
TC16000820.hg.1	NLRC3	8.57 ± 0.10	8.63 ± 0.10	8.39 ± 0.16	8.62 ± 0.04
TC02001723.hg.1	NLRC4	7.77 ± 0.67	6.72 ± 0.28	8.88 ± 0.37	6.63 ± 0.32
TC16000482.hg.1	NLRC5	9.31 ± 0.15	9.30 ± 0.02	9.33 ± 0.25	9.28 ± 0.03
TC17001059.hg.1	NLRP1	9.61 ± 0.25	9.89 ± 0.15	9.44 ± 0.28	9.86 ± 0.10
TC11001381.hg.1	NLRP10	5.70 ± 0.17	5.76 ± 0.20	5.72 ± 0.09	5.82 ± 0.10
TC19001881.hg.1	NLRP11	6.07 ± 0.17	5.99 ± 0.07	5.99 ± 0.07	6.12 ± 0.08
TC19001826.hg.1	NLRP12	8.90 ± 0.08	8.56 ± 0.08	9.11 ± 0.05	8.53 ± 0.14
TC19001882.hg.1	NLRP13	6.15 ± 0.10	6.17 ± 0.19	6.15 ± 0.29	6.40 ± 0.10
TC11000143.hg.1	NLRP14	5.82 ± 0.11	5.85 ± 0.07	5.80 ± 0.10	5.93 ± 0.15
TC19000887.hg.1	NLRP2	7.02 ± 0.19	6.80 ± 0.07	6.72 ± 0.10	6.72 ± 0.09
TC01002008.hg.1	NLRP3	8.34 ± 0.24	8.57 ± 0.18	8.51 ± 0.23	8.45 ± 0.18
TC19000913.hg.1	NLRP4	5.65 ± 0.14	5.62 ± 0.15	5.55 ± 0.14	5.69 ± 0.09
TC19000915.hg.1	NLRP5	6.52 ± 0.13	6.49 ± 0.13	6.46 ± 0.11	6.56 ± 0.05
TC11000007.hg.1	NLRP6	8.90 ± 0.21	8.93 ± 0.11	9.01 ± 0.03	9.05 ± 0.09
TC19001853.hg.1	NLRP7	6.49 ± 0.16	6.30 ± 0.14	6.35 ± 0.12	6.40 ± 0.05
TC19000914.hg.1	NLRP8	5.73 ± 0.13	5.68 ± 0.08	5.71 ± 0.09	5.72 ± 0.02
TC19001880.hg.1	NLRP9	6.26 ± 0.15	6.26 ± 0.09	6.27 ± 0.09	6.37 ± 0.02
TC11001087.hg.1	NLRX1	7.97 ± 0.16	7.88 ± 0.16	7.94 ± 0.16	7.91 ± 0.04
TC15001787.hg.1	NMB	7.88 ± 0.09	7.84 ± 0.10	7.93 ± 0.12	7.90 ± 0.00
TC06002177.hg.1	NMBR	4.86 ± 0.25	4.70 ± 0.10	4.80 ± 0.18	4.79 ± 0.17
TC06002178.hg.1	NMBR	6.17 ± 0.28	6.11 ± 0.13	6.06 ± 0.22	6.18 ± 0.06
TC03000881.hg.1	NMD3	7.64 ± 0.44	8.04 ± 0.11	7.63 ± 0.42	8.01 ± 0.13
TC17002880.hg.1	NME1	7.13 ± 0.11	6.97 ± 0.08	6.88 ± 0.19	6.88 ± 0.04
TC17002881.hg.1	NME2	8.92 ± 0.20	8.82 ± 0.13	8.65 ± 0.28	8.73 ± 0.06
TC16000758.hg.1	NME3	8.82 ± 0.26	8.81 ± 0.26	8.62 ± 0.31	8.86 ± 0.09
TC16002049.hg.1	NME4	8.61 ± 0.16	8.58 ± 0.15	8.52 ± 0.26	8.64 ± 0.05
TC05001824.hg.1	NME5	4.53 ± 0.09	4.47 ± 0.05	4.52 ± 0.06	4.51 ± 0.04
TC03001375.hg.1	NME6	8.17 ± 0.13	8.05 ± 0.11	8.12 ± 0.16	8.05 ± 0.03
TC01003495.hg.1	NME7	6.16 ± 0.15	6.21 ± 0.20	6.16 ± 0.23	6.13 ± 0.05
TC07003322.hg.1	NME8	6.54 ± 0.17	6.39 ± 0.26	7.34 ± 0.41	6.32 ± 0.11

TC03001822.hg.1	NME9	6.66 ± 0.10	6.62 ± 0.08	6.61 ± 0.10	6.67 ± 0.05
TC02002425.hg.1	NMI	9.23 ± 0.21	8.97 ± 0.37	9.65 ± 0.55	8.80 ± 0.40
TC01000122.hg.1	NMNAT1	7.75 ± 0.12	7.52 ± 0.06	7.77 ± 0.09	7.50 ± 0.07
TC01003614.hg.1	NMNAT2	7.21 ± 0.16	7.17 ± 0.18	7.23 ± 0.11	7.31 ± 0.08
TC03001835.hg.1	NMNAT3	6.86 ± 0.13	6.78 ± 0.10	6.83 ± 0.08	6.83 ± 0.04
TC16000829.hg.1	NMRAL1	8.54 ± 0.21	8.36 ± 0.06	8.29 ± 0.13	8.35 ± 0.06
TC09001220.hg.1	NMRK1	7.53 ± 0.24	7.47 ± 0.16	7.67 ± 0.35	7.24 ± 0.24
TC19000079.hg.1	NMRK2	7.71 ± 0.31	7.71 ± 0.27	7.60 ± 0.26	7.77 ± 0.04
TC02000607.hg.1	NMS	6.52 ± 0.17	6.52 ± 0.15	6.42 ± 0.22	6.61 ± 0.04
TC17000577.hg.1	NMT1	9.15 ± 0.21	8.88 ± 0.10	8.97 ± 0.11	8.69 ± 0.15
TC10001061.hg.1	NMT2	7.77 ± 0.23	7.89 ± 0.09	7.47 ± 0.18	7.79 ± 0.03
TC04001216.hg.1	NMU	5.00 ± 0.21	4.88 ± 0.10	4.87 ± 0.09	4.92 ± 0.06
TC02002865.hg.1	NMUR1	8.15 ± 0.09	8.05 ± 0.09	8.07 ± 0.11	8.04 ± 0.07
TC05001963.hg.1	NMUR2	6.05 ± 0.09	5.98 ± 0.13	6.01 ± 0.02	6.17 ± 0.20
TC20000274.hg.1	NNAT	7.49 ± 0.24	7.57 ± 0.16	7.46 ± 0.13	7.57 ± 0.02
TC11001027.hg.1	NNMT	5.74 ± 0.13	5.81 ± 0.10	5.75 ± 0.03	5.87 ± 0.06
TC05000202.hg.1	NNT	8.73 ± 0.16	8.75 ± 0.17	8.54 ± 0.15	8.47 ± 0.09
TC04001225.hg.1	NOA1	9.23 ± 0.16	9.13 ± 0.22	8.95 ± 0.08	8.94 ± 0.19
TC16001226.hg.1	NOB1	9.10 ± 0.16	9.01 ± 0.09	8.72 ± 0.11	8.93 ± 0.03
TC07001981.hg.1	NOBOX	7.15 ± 0.19	7.15 ± 0.21	7.15 ± 0.17	7.28 ± 0.04
TC01002078.hg.1	NOC2L	8.90 ± 0.18	8.73 ± 0.06	8.65 ± 0.18	8.69 ± 0.03
TC10001545.hg.1	NOC3L	6.81 ± 0.32	6.84 ± 0.27	6.60 ± 0.33	6.60 ± 0.00
TC12001054.hg.1	NOC4L	8.35 ± 0.10	8.24 ± 0.08	8.22 ± 0.12	8.25 ± 0.02
TC07001249.hg.1	NOD1	7.37 ± 0.10	7.29 ± 0.03	7.30 ± 0.18	7.19 ± 0.07
TC16000442.hg.1	NOD2	8.53 ± 0.26	8.41 ± 0.15	8.74 ± 0.21	8.25 ± 0.07
TC10001365.hg.1	NODAL	7.20 ± 0.19	7.23 ± 0.28	7.20 ± 0.15	7.33 ± 0.08
TC17000698.hg.1	NOG	8.61 ± 0.15	8.58 ± 0.15	8.11 ± 0.17	8.59 ± 0.32
TC02001572.hg.1	NOL10	7.77 ± 0.31	7.96 ± 0.18	7.61 ± 0.36	7.90 ± 0.18
TC17000795.hg.1	NOL11	8.23 ± 0.48	8.38 ± 0.19	8.00 ± 0.31	8.36 ± 0.07
TC22001466.hg.1	NOL12	8.49 ± 0.15	8.37 ± 0.02	8.35 ± 0.08	8.33 ± 0.05
TC16000532.hg.1	NOL3	8.28 ± 0.31	8.23 ± 0.24	8.26 ± 0.28	8.29 ± 0.06

TC18000449.hg.1	NOL4	5.21 ± 0.18	5.17 ± 0.06	5.14 ± 0.13	5.25 ± 0.06
TC09001013.hg.1	NOL6	8.33 ± 0.13	8.14 ± 0.04	8.03 ± 0.07	8.18 ± 0.06
TC06000094.hg.1	NOL7	8.72 ± 0.03	8.57 ± 0.13	8.60 ± 0.08	8.45 ± 0.16
TC09001333.hg.1	NOL8	7.56 ± 0.48	7.81 ± 0.13	7.67 ± 0.38	7.73 ± 0.05
TC01002154.hg.1	NOL9	8.56 ± 0.16	8.51 ± 0.13	8.19 ± 0.06	8.59 ± 0.13
TC10000749.hg.1	NOLC1	8.41 ± 0.08	8.44 ± 0.06	8.14 ± 0.04	8.35 ± 0.07
TC07001050.hg.1	NOM1	7.23 ± 0.08	7.20 ± 0.09	7.06 ± 0.11	7.15 ± 0.02
TC16000180.hg.1	NOMO1	10.34 ± 0.22	9.92 ± 0.12	9.82 ± 0.02	9.76 ± 0.03
TC16000912.hg.1	NOMO2	10.33 ± 0.25	9.92 ± 0.12	9.80 ± 0.03	9.75 ± 0.04
TC16000194.hg.1	NOMO3	10.32 ± 0.24	9.93 ± 0.12	9.81 ± 0.02	9.76 ± 0.04
TC0X000385.hg.1	NONO	10.14 ± 0.11	10.22 ± 0.11	9.94 ± 0.03	10.15 ± 0.05
TC15001193.hg.1	NOP10	10.40 ± 0.47	9.91 ± 0.13	10.65 ± 0.15	9.73 ± 0.39
TC04000979.hg.1	NOP14	8.42 ± 0.04	8.40 ± 0.09	8.13 ± 0.01	8.37 ± 0.03
TC04000040.hg.1	NOP14-AS1	7.93 ± 0.07	7.97 ± 0.07	7.84 ± 0.03	8.03 ± 0.01
TC05002092.hg.1	NOP16	7.24 ± 0.13	7.11 ± 0.08	7.05 ± 0.12	7.09 ± 0.01
TC12001142.hg.1	NOP2	8.89 ± 0.05	8.70 ± 0.05	8.61 ± 0.03	8.70 ± 0.04
TC02001188.hg.1	NOP58	8.66 ± 0.71	9.38 ± 0.18	8.55 ± 0.60	9.45 ± 0.17
TC14002194.hg.1	NOP9	7.94 ± 0.11	7.87 ± 0.04	7.71 ± 0.04	7.91 ± 0.05
TC12002014.hg.1	NOS1	6.81 ± 0.18	6.81 ± 0.15	6.77 ± 0.14	6.89 ± 0.06
TC01006406.hg.1	NOS1AP	7.48 ± 0.16	7.45 ± 0.17	7.45 ± 0.12	7.53 ± 0.06
TC17002894.hg.1	NOS2	7.22 ± 0.12	7.20 ± 0.16	7.18 ± 0.13	7.30 ± 0.07
TC17001221.hg.1	NOS2P2	7.30 ± 0.13	7.33 ± 0.13	7.37 ± 0.12	7.42 ± 0.11
TC17000258.hg.1	NOS2P3	7.40 ± 0.14	7.37 ± 0.12	7.46 ± 0.11	7.51 ± 0.13
TC07001009.hg.1	NOS3	8.10 ± 0.17	8.06 ± 0.18	8.02 ± 0.17	8.15 ± 0.03
TC19001720.hg.1	NOSIP	9.39 ± 0.20	9.15 ± 0.06	9.13 ± 0.22	9.11 ± 0.05
TC02000998.hg.1	NOSTRIN	4.95 ± 0.06	4.87 ± 0.03	4.89 ± 0.02	4.95 ± 0.05
TC09001734.hg.1	NOTCH1	9.65 ± 0.24	9.54 ± 0.04	9.78 ± 0.16	9.49 ± 0.19
TC01003054.hg.1	NOTCH2	9.81 ± 0.24	9.77 ± 0.17	10.13 ± 0.23	9.72 ± 0.13
TC01006310.hg.1	NOTCH2NL	10.44 ± 0.36	10.61 ± 0.15	10.77 ± 0.21	10.57 ± 0.07
TC19001250.hg.1	NOTCH3	8.11 ± 0.23	8.09 ± 0.23	8.06 ± 0.19	8.21 ± 0.05
TC06001564.hg.1	NOTCH4	8.04 ± 0.18	8.07 ± 0.21	8.00 ± 0.15	8.20 ± 0.07

TC6_apd_hap1000098.hg.1	NOTCH4	8.19 ± 0.22	8.16 ± 0.18	8.11 ± 0.15	8.23 ± 0.06
TC6_cox_hap2000190.hg.1	NOTCH4	8.07 ± 0.18	8.09 ± 0.20	8.02 ± 0.14	8.22 ± 0.07
TC6_mann_hap4000155.hg.1	NOTCH4	8.06 ± 0.17	8.09 ± 0.21	8.02 ± 0.14	8.22 ± 0.07
TC6_mcf_hap5000165.hg.1	NOTCH4	8.07 ± 0.18	8.09 ± 0.21	8.02 ± 0.14	8.22 ± 0.07
TC6_qbl_hap6000179.hg.1	NOTCH4	8.04 ± 0.18	8.06 ± 0.21	7.99 ± 0.15	8.20 ± 0.07
TC6_ssto_hap7000159.hg.1	NOTCH4	8.02 ± 0.18	8.04 ± 0.21	7.97 ± 0.15	8.18 ± 0.07
TC02000438.hg.1	NOTO	7.15 ± 0.10	7.05 ± 0.15	7.20 ± 0.21	7.13 ± 0.09
TC17001968.hg.1	NOTUM	8.53 ± 0.20	8.48 ± 0.17	8.48 ± 0.21	8.48 ± 0.05
TC08000697.hg.1	NOV	7.44 ± 0.12	7.55 ± 0.04	7.44 ± 0.06	7.65 ± 0.11
TC14000988.hg.1	NOVA1	5.19 ± 0.11	5.14 ± 0.07	5.15 ± 0.04	5.18 ± 0.07
TC14000173.hg.1	NOVA1-AS1	4.91 ± 0.07	4.80 ± 0.03	4.82 ± 0.05	4.87 ± 0.04
TC19001639.hg.1	NOVA2	7.87 ± 0.15	7.86 ± 0.16	7.89 ± 0.06	7.91 ± 0.05
TC0X001211.hg.1	NOX1	5.28 ± 0.11	5.17 ± 0.11	5.14 ± 0.10	5.19 ± 0.05
TC06002250.hg.1	NOX3	5.78 ± 0.16	5.72 ± 0.14	5.70 ± 0.11	5.74 ± 0.10
TC11002178.hg.1	NOX4	5.09 ± 0.16	4.99 ± 0.09	4.99 ± 0.07	5.07 ± 0.06
TC09000849.hg.1	NOXA1	8.87 ± 0.22	8.81 ± 0.16	8.83 ± 0.16	8.89 ± 0.04
TC16000770.hg.1	NOXO1	8.17 ± 0.22	8.15 ± 0.25	8.10 ± 0.23	8.20 ± 0.03
TC14001344.hg.1	NOXRED1	6.41 ± 0.10	6.27 ± 0.10	6.55 ± 0.13	6.33 ± 0.11
TC15000042.hg.1	NPAP1	7.95 ± 0.23	7.84 ± 0.06	8.02 ± 0.31	8.15 ± 0.02
TC19000671.hg.1	NPAS1	8.03 ± 0.17	7.92 ± 0.14	7.86 ± 0.19	7.95 ± 0.07
TC02000611.hg.1	NPAS2	7.33 ± 0.09	7.31 ± 0.10	7.14 ± 0.08	7.38 ± 0.04
TC14000211.hg.1	NPAS3	5.32 ± 0.10	5.21 ± 0.10	5.25 ± 0.12	5.30 ± 0.05
TC14000212.hg.1	NPAS3	4.14 ± 0.12	4.06 ± 0.15	4.14 ± 0.08	4.25 ± 0.05
TC11000667.hg.1	NPAS4	7.11 ± 0.15	7.14 ± 0.13	7.18 ± 0.13	7.26 ± 0.06
TC11002264.hg.1	NPAT	8.51 ± 0.70	8.96 ± 0.27	8.47 ± 0.49	8.80 ± 0.09
TC17000939.hg.1	NPB	8.05 ± 0.20	7.87 ± 0.16	7.95 ± 0.06	7.86 ± 0.04
TC08000368.hg.1	NPBWR1	7.85 ± 0.46	7.75 ± 0.28	7.78 ± 0.34	7.86 ± 0.08
TC20001048.hg.1	NPBWR2	7.09 ± 0.17	7.14 ± 0.22	7.15 ± 0.25	7.24 ± 0.09
TC18000413.hg.1	NPC1	8.57 ± 0.15	8.44 ± 0.13	8.41 ± 0.12	8.38 ± 0.05
TC07001342.hg.1	NPC1L1	7.37 ± 0.17	7.36 ± 0.16	7.30 ± 0.14	7.50 ± 0.09
TC14001307.hg.1	NPC2	10.00 ± 0.26	9.86 ± 0.16	9.93 ± 0.33	9.77 ± 0.20

TC09001753.hg.1	NPDC1	8.51 ± 0.22	8.53 ± 0.22	8.42 ± 0.23	8.63 ± 0.04
TC20001734.hg.1	NPEPL1	8.78 ± 0.05	8.76 ± 0.05	8.83 ± 0.05	8.77 ± 0.02
TC17000609.hg.1	NPEPPS	9.24 ± 0.16	9.16 ± 0.20	9.47 ± 0.29	9.08 ± 0.15
TC12001551.hg.1	NPFF	7.60 ± 0.10	7.48 ± 0.09	7.52 ± 0.06	7.51 ± 0.13
TC10001363.hg.1	NPFFR1	7.61 ± 0.14	7.67 ± 0.15	7.68 ± 0.20	7.79 ± 0.06
TC04000397.hg.1	NPFFR2	5.28 ± 0.10	5.23 ± 0.04	5.25 ± 0.07	5.33 ± 0.11
TC02002195.hg.1	NPHP1	4.85 ± 0.08	4.73 ± 0.03	4.82 ± 0.07	4.80 ± 0.07
TC03003342.hg.1	NPHP3	7.18 ± 0.38	7.28 ± 0.21	7.22 ± 0.24	7.22 ± 0.06
TC03000718.hg.1	NPHP3-AS1	4.96 ± 0.07	4.91 ± 0.08	4.87 ± 0.08	4.95 ± 0.07
TC01002143.hg.1	NPHP4	7.95 ± 0.14	7.89 ± 0.13	7.84 ± 0.14	7.94 ± 0.04
TC19001456.hg.1	NPHS1	7.82 ± 0.22	7.78 ± 0.19	7.75 ± 0.19	7.88 ± 0.06
TC01003568.hg.1	NPHS2	5.72 ± 0.11	5.60 ± 0.11	5.65 ± 0.14	5.76 ± 0.08
TC16002043.hg.1	NPIPA1	9.63 ± 0.12	9.74 ± 0.05	9.32 ± 0.22	9.72 ± 0.12
TC16000178.hg.1	NPIPA3	9.89 ± 0.30	10.00 ± 0.07	9.58 ± 0.37	9.93 ± 0.12
TC16000896.hg.1	NPIPA5	9.95 ± 0.29	10.09 ± 0.10	9.70 ± 0.36	10.00 ± 0.12
TC16002096.hg.1	NPIPA5	9.99 ± 0.24	10.11 ± 0.06	9.66 ± 0.30	10.01 ± 0.14
TC16000893.hg.1	NPIPA8	9.78 ± 0.13	9.85 ± 0.02	9.48 ± 0.21	9.79 ± 0.12
TC16002065.hg.1	NPIPB11	10.96 ± 0.50	11.19 ± 0.16	10.85 ± 0.56	11.09 ± 0.11
TC16002100.hg.1	NPIPB11	12.18 ± 0.40	12.52 ± 0.06	12.20 ± 0.32	12.50 ± 0.09
TC16002098.hg.1	NPIPB3	12.21 ± 0.37	12.56 ± 0.06	12.22 ± 0.30	12.56 ± 0.10
TC16002095.hg.1	NPIPB5	11.97 ± 0.39	12.28 ± 0.04	11.97 ± 0.33	12.27 ± 0.08
TC16000985.hg.1	NPIPB6	8.83 ± 0.30	8.93 ± 0.09	8.72 ± 0.36	8.96 ± 0.09
TC16002063.hg.1	NPIPL1	8.85 ± 0.31	8.96 ± 0.07	8.75 ± 0.36	8.94 ± 0.11
TC16000613.hg.1	NPIPL2	9.26 ± 0.26	9.30 ± 0.07	9.12 ± 0.28	9.30 ± 0.08
TC16001004.hg.1	NPIPL3	11.31 ± 0.39	11.58 ± 0.04	11.26 ± 0.45	11.55 ± 0.11
TC01001581.hg.1	NPL	8.28 ± 0.19	7.95 ± 0.16	8.70 ± 0.27	7.84 ± 0.17
TC17001954.hg.1	NPLOC4	9.26 ± 0.10	9.11 ± 0.15	9.08 ± 0.11	9.02 ± 0.07
TC05000942.hg.1	NPM1	9.61 ± 0.39	10.04 ± 0.14	9.30 ± 0.21	9.92 ± 0.05
TC08000161.hg.1	NPM2	7.71 ± 0.20	7.65 ± 0.16	7.63 ± 0.14	7.70 ± 0.02
TC10001612.hg.1	NPM3	7.64 ± 0.14	7.62 ± 0.09	7.46 ± 0.19	7.61 ± 0.05
TC04000548.hg.1	NPNT	5.95 ± 0.16	5.85 ± 0.11	5.87 ± 0.09	5.99 ± 0.07

TC01002204.hg.1	NPPA	7.39 ± 0.12	7.29 ± 0.06	7.37 ± 0.05	7.38 ± 0.03
TC01002205.hg.1	NPPB	8.58 ± 0.09	8.56 ± 0.11	8.55 ± 0.13	8.66 ± 0.05
TC02002869.hg.1	NPPC	8.63 ± 0.29	8.69 ± 0.18	8.58 ± 0.25	8.80 ± 0.03
TC01001262.hg.1	NPR1	7.11 ± 0.22	7.08 ± 0.16	7.06 ± 0.20	7.16 ± 0.05
TC09000195.hg.1	NPR2	6.29 ± 0.15	6.21 ± 0.17	6.17 ± 0.12	6.25 ± 0.06
TC05000144.hg.1	NPR3	5.81 ± 0.15	5.67 ± 0.07	5.71 ± 0.10	5.78 ± 0.07
TC03001431.hg.1	NPRL2	7.78 ± 0.12	7.63 ± 0.04	7.58 ± 0.13	7.59 ± 0.04
TC16000722.hg.1	NPRL3	8.75 ± 0.10	8.62 ± 0.07	8.54 ± 0.08	8.61 ± 0.03
TC10000920.hg.1	NPS	5.52 ± 0.31	5.36 ± 0.04	5.53 ± 0.11	5.52 ± 0.10
TC07000222.hg.1	NPSR1	6.07 ± 0.12	5.98 ± 0.14	6.04 ± 0.06	6.08 ± 0.09
TC07001272.hg.1	NPSR1-AS1	5.01 ± 0.11	4.95 ± 0.06	4.93 ± 0.02	5.01 ± 0.08
TC15001634.hg.1	NPTN	9.29 ± 0.03	9.14 ± 0.07	9.45 ± 0.18	9.14 ± 0.20
TC17001937.hg.1	NPTX1	7.41 ± 0.20	7.36 ± 0.14	7.37 ± 0.17	7.38 ± 0.02
TC07000587.hg.1	NPTX2	7.82 ± 0.26	7.73 ± 0.16	7.74 ± 0.25	7.78 ± 0.05
TC22000798.hg.1	NPTXR	8.46 ± 0.19	8.40 ± 0.10	8.36 ± 0.15	8.42 ± 0.05
TC07001209.hg.1	NPVF	4.11 ± 0.05	4.01 ± 0.07	4.05 ± 0.12	4.04 ± 0.07
TC16000057.hg.1	NPW	7.90 ± 0.22	7.94 ± 0.28	7.78 ± 0.31	8.02 ± 0.13
TC07000153.hg.1	NPY	7.22 ± 0.11	7.10 ± 0.20	7.11 ± 0.19	7.22 ± 0.04
TC04001698.hg.1	NPY1R	5.23 ± 0.11	5.05 ± 0.04	5.11 ± 0.11	5.15 ± 0.13
TC04000782.hg.1	NPY2R	7.28 ± 0.27	7.38 ± 0.25	7.18 ± 0.22	7.43 ± 0.05
TC10000302.hg.1	NPY4R	6.47 ± 0.21	6.45 ± 0.03	6.34 ± 0.13	6.60 ± 0.18
TC04000818.hg.1	NPY5R	5.21 ± 0.14	5.19 ± 0.13	5.32 ± 0.11	5.29 ± 0.04
TC05000691.hg.1	NPY6R	5.02 ± 0.24	5.01 ± 0.07	4.88 ± 0.17	5.14 ± 0.15
TC16001225.hg.1	NQO1	6.21 ± 0.19	6.03 ± 0.08	6.03 ± 0.17	6.02 ± 0.06
TC06004062.hg.1	NQO2	8.69 ± 0.19	8.20 ± 0.10	9.08 ± 0.14	8.09 ± 0.12
TC0X000938.hg.1	NR0B1	5.10 ± 0.27	4.93 ± 0.09	5.02 ± 0.12	5.02 ± 0.05
TC01002398.hg.1	NR0B2	7.45 ± 0.06	7.51 ± 0.20	7.34 ± 0.21	7.64 ± 0.06
TC17001459.hg.1	NR1D1	8.17 ± 0.02	8.20 ± 0.12	7.83 ± 0.11	8.25 ± 0.08
TC03000127.hg.1	NR1D2	8.31 ± 0.55	8.89 ± 0.25	7.99 ± 0.32	8.90 ± 0.15
TC19000756.hg.1	NR1H2	9.05 ± 0.19	8.92 ± 0.03	8.96 ± 0.13	8.97 ± 0.05
TC11000405.hg.1	NR1H3	7.59 ± 0.18	7.57 ± 0.17	7.53 ± 0.14	7.64 ± 0.02

TC12000778.hg.1	NR1H4	4.56 ± 0.13	4.51 ± 0.07	4.52 ± 0.09	4.58 ± 0.07
TC03000613.hg.1	NR1I2	7.16 ± 0.15	7.07 ± 0.10	7.04 ± 0.18	7.14 ± 0.03
TC01003423.hg.1	NR1I3	7.14 ± 0.13	7.15 ± 0.21	7.20 ± 0.10	7.27 ± 0.03
TC12001828.hg.1	NR2C1	7.31 ± 0.43	7.55 ± 0.16	7.20 ± 0.37	7.40 ± 0.04
TC03000091.hg.1	NR2C2	9.11 ± 0.11	9.04 ± 0.10	9.08 ± 0.21	9.06 ± 0.04
TC19001301.hg.1	NR2C2AP	8.02 ± 0.12	7.88 ± 0.09	7.75 ± 0.17	7.91 ± 0.04
TC06000860.hg.1	NR2E1	6.97 ± 0.22	6.82 ± 0.21	7.05 ± 0.13	6.95 ± 0.04
TC15000650.hg.1	NR2E3	7.06 ± 0.21	7.05 ± 0.26	7.02 ± 0.24	7.13 ± 0.04
TC05000451.hg.1	NR2F1	7.75 ± 0.30	7.72 ± 0.22	7.71 ± 0.24	7.82 ± 0.01
TC05001596.hg.1	NR2F1-AS1	6.32 ± 0.16	6.25 ± 0.08	6.29 ± 0.13	6.31 ± 0.02
TC15000936.hg.1	NR2F2	6.78 ± 0.19	6.64 ± 0.10	6.65 ± 0.15	6.72 ± 0.05
TC15001922.hg.1	NR2F2-AS1	5.14 ± 0.17	5.01 ± 0.08	5.01 ± 0.13	5.08 ± 0.03
TC19001270.hg.1	NR2F6	8.66 ± 0.21	8.52 ± 0.16	8.47 ± 0.27	8.48 ± 0.02
TC05001887.hg.1	NR3C1	8.37 ± 0.16	8.59 ± 0.10	8.54 ± 0.21	8.53 ± 0.09
TC04001627.hg.1	NR3C2	6.82 ± 0.12	7.03 ± 0.05	6.65 ± 0.18	7.00 ± 0.16
TC12000414.hg.1	NR4A1	8.77 ± 0.14	9.54 ± 0.41	8.57 ± 0.18	9.96 ± 0.34
TC02002445.hg.1	NR4A2	8.98 ± 0.63	10.66 ± 0.22	9.18 ± 0.82	10.88 ± 0.43
TC09000508.hg.1	NR4A3	7.05 ± 0.40	8.83 ± 0.23	7.39 ± 0.91	9.00 ± 0.44
TC09001579.hg.1	NR5A1	7.96 ± 0.19	7.99 ± 0.26	7.98 ± 0.25	8.10 ± 0.03
TC01001645.hg.1	NR5A2	5.54 ± 0.14	5.44 ± 0.05	5.48 ± 0.07	5.49 ± 0.06
TC09001580.hg.1	NR6A1	6.44 ± 0.18	6.33 ± 0.09	6.67 ± 0.13	6.40 ± 0.10
TC03000264.hg.1	NRADDP	7.63 ± 0.25	7.52 ± 0.16	7.58 ± 0.17	7.70 ± 0.05
TC10001670.hg.1	NRAP	5.98 ± 0.17	5.93 ± 0.11	5.93 ± 0.12	6.00 ± 0.07
TC09001769.hg.1	NRARP	6.34 ± 0.17	6.68 ± 0.15	6.39 ± 0.22	6.62 ± 0.17
TC01003014.hg.1	NRAS	9.82 ± 0.30	10.12 ± 0.27	9.75 ± 0.33	10.07 ± 0.04
TC10000385.hg.1	NRBF2	9.68 ± 0.11	9.55 ± 0.14	10.15 ± 0.28	9.40 ± 0.31
TC02000174.hg.1	NRBP1	9.12 ± 0.17	9.04 ± 0.14	8.99 ± 0.22	8.97 ± 0.06
TC08001735.hg.1	NRBP2	8.41 ± 0.13	8.37 ± 0.07	8.35 ± 0.12	8.41 ± 0.04
TC07001751.hg.1	NRCAM	5.86 ± 0.10	5.95 ± 0.05	5.68 ± 0.09	6.00 ± 0.06
TC01002652.hg.1	NRD1	9.45 ± 0.22	9.45 ± 0.20	9.85 ± 0.35	9.20 ± 0.16
TC14001429.hg.1	NRDE2	7.37 ± 0.07	7.26 ± 0.09	7.28 ± 0.09	7.27 ± 0.03

TC05001674.hg.1	NREP	7.13 ± 0.07	7.02 ± 0.01	7.05 ± 0.05	7.03 ± 0.05
TC05000524.hg.1	NREP-AS1	4.39 ± 0.11	4.18 ± 0.04	4.26 ± 0.01	4.22 ± 0.06
TC07000801.hg.1	NRF1	7.99 ± 0.04	7.93 ± 0.05	7.93 ± 0.07	7.93 ± 0.02
TC08000250.hg.1	NRG1	7.20 ± 0.09	7.03 ± 0.03	7.31 ± 0.06	6.96 ± 0.06
TC08000255.hg.1	NRG1	5.40 ± 0.30	5.22 ± 0.18	5.34 ± 0.08	5.21 ± 0.09
TC08000251.hg.1	NRG1-IT1	4.74 ± 0.21	4.66 ± 0.13	4.66 ± 0.05	4.72 ± 0.02
TC08000252.hg.1	NRG1-IT2	4.52 ± 0.20	4.49 ± 0.05	4.47 ± 0.24	4.60 ± 0.08
TC08000254.hg.1	NRG1-IT3	4.51 ± 0.17	4.33 ± 0.09	4.42 ± 0.13	4.53 ± 0.09
TC05001851.hg.1	NRG2	7.81 ± 0.16	7.75 ± 0.14	7.80 ± 0.15	7.84 ± 0.03
TC10000586.hg.1	NRG3	5.48 ± 0.17	5.36 ± 0.08	5.40 ± 0.07	5.44 ± 0.05
TC10001466.hg.1	NRG3-AS1	5.19 ± 0.07	5.01 ± 0.07	5.02 ± 0.05	5.04 ± 0.02
TC15001683.hg.1	NRG4	5.17 ± 0.12	5.08 ± 0.04	5.11 ± 0.03	5.12 ± 0.01
TC11001143.hg.1	NRGN	11.08 ± 0.21	10.38 ± 0.19	11.29 ± 0.06	10.50 ± 0.51
TC21000288.hg.1	NRIP1	9.18 ± 0.31	9.51 ± 0.18	9.03 ± 0.32	9.54 ± 0.10
TC12001104.hg.1	NRIP2	7.82 ± 0.15	7.84 ± 0.14	7.72 ± 0.17	7.91 ± 0.09
TC11001395.hg.1	NRIP3	5.91 ± 0.20	5.99 ± 0.16	5.80 ± 0.13	6.22 ± 0.13
TC0X000512.hg.1	NRK	5.35 ± 0.15	5.26 ± 0.09	5.29 ± 0.10	5.32 ± 0.07
TC14000966.hg.1	NRL	7.62 ± 0.13	7.57 ± 0.14	7.58 ± 0.13	7.69 ± 0.05
TC06001506.hg.1	NRM	8.73 ± 0.23	8.39 ± 0.06	8.54 ± 0.12	8.29 ± 0.10
TC6_apd_hap1000076.hg.1	NRM	8.73 ± 0.23	8.39 ± 0.06	8.54 ± 0.12	8.29 ± 0.10
TC6_cox_hap2000140.hg.1	NRM	8.73 ± 0.23	8.39 ± 0.06	8.54 ± 0.12	8.29 ± 0.10
TC6_dbb_hap3000129.hg.1	NRM	8.73 ± 0.23	8.39 ± 0.06	8.54 ± 0.12	8.29 ± 0.10
TC6_mann_hap4000118.hg.1	NRM	8.73 ± 0.23	8.39 ± 0.06	8.54 ± 0.12	8.29 ± 0.10
TC6_mcf_hap5000119.hg.1	NRM	8.73 ± 0.23	8.39 ± 0.06	8.54 ± 0.12	8.29 ± 0.10
TC6_qbl_hap6000130.hg.1	NRM	8.73 ± 0.23	8.39 ± 0.06	8.54 ± 0.12	8.29 ± 0.10
TC06001245.hg.1	NRN1	6.66 ± 0.20	6.55 ± 0.10	6.61 ± 0.25	6.52 ± 0.01
TC16002081.hg.1	NRN1L	8.04 ± 0.16	8.01 ± 0.09	7.87 ± 0.08	8.09 ± 0.06
TC09001594.hg.1	NRON	6.24 ± 0.20	6.18 ± 0.14	6.21 ± 0.19	6.22 ± 0.18
TC10001166.hg.1	NRP1	6.71 ± 0.10	6.62 ± 0.09	6.64 ± 0.06	6.76 ± 0.03
TC02001206.hg.1	NRP2	6.55 ± 0.15	6.47 ± 0.16	6.47 ± 0.14	6.60 ± 0.04
TC06000140.hg.1	NRSN1	5.94 ± 0.14	5.89 ± 0.09	5.87 ± 0.08	6.02 ± 0.11

TC20000008.hg.1	NRSN2	8.04 ± 0.19	7.91 ± 0.11	7.94 ± 0.16	7.97 ± 0.07
TC19000106.hg.1	NRTN	7.92 ± 0.28	7.76 ± 0.17	7.79 ± 0.17	7.86 ± 0.05
TC02001835.hg.1	NRXN1	5.81 ± 0.16	5.74 ± 0.12	5.73 ± 0.11	5.84 ± 0.11
TC11001908.hg.1	NRXN2	8.06 ± 0.23	8.03 ± 0.19	7.98 ± 0.19	8.11 ± 0.05
TC14000495.hg.1	NRXN3	6.47 ± 0.11	6.41 ± 0.13	6.43 ± 0.14	6.53 ± 0.08
TC05000361.hg.1	NSA2	8.65 ± 0.50	9.08 ± 0.18	8.65 ± 0.38	9.03 ± 0.19
TC08000140.hg.1	NSAP11	4.96 ± 0.14	4.89 ± 0.21	5.00 ± 0.09	5.11 ± 0.21
TC05001011.hg.1	NSD1	8.80 ± 0.15	8.90 ± 0.15	8.78 ± 0.18	8.77 ± 0.08
TC0X000743.hg.1	NSDHL	7.82 ± 0.08	7.61 ± 0.04	7.64 ± 0.14	7.58 ± 0.01
TC17_ctg5_hap1000015.hg.1	NSF	9.27 ± 0.20	9.33 ± 0.13	9.42 ± 0.26	9.20 ± 0.38
TC17000600.hg.1	NSF	8.68 ± 0.20	8.60 ± 0.09	8.74 ± 0.17	8.39 ± 0.18
TC20001742.hg.1	NSFL1C	8.64 ± 0.04	8.54 ± 0.08	8.68 ± 0.21	8.46 ± 0.07
TC17_ctg5_hap1000010.hg.1	NSFP1	9.14 ± 0.24	9.27 ± 0.10	9.32 ± 0.27	9.16 ± 0.33
TC04000059.hg.1	NSG1	7.76 ± 0.13	7.72 ± 0.06	7.60 ± 0.12	7.71 ± 0.05
TC01003810.hg.1	NSL1	7.76 ± 0.20	7.80 ± 0.22	7.99 ± 0.25	7.65 ± 0.24
TC08001246.hg.1	NSMAF	8.22 ± 0.19	8.19 ± 0.19	8.12 ± 0.26	8.00 ± 0.07
TC16000977.hg.1	NSMCE1	8.61 ± 0.21	8.41 ± 0.15	8.41 ± 0.22	8.17 ± 0.09
TC08000731.hg.1	NSMCE2	6.94 ± 0.13	6.94 ± 0.14	6.92 ± 0.20	6.90 ± 0.02
TC10001714.hg.1	NSMCE4A	7.90 ± 0.24	7.91 ± 0.14	7.59 ± 0.20	7.74 ± 0.03
TC09001772.hg.1	NSMF	8.78 ± 0.07	8.84 ± 0.07	8.54 ± 0.08	8.83 ± 0.02
TC05001138.hg.1	NSUN2	8.79 ± 0.14	8.78 ± 0.07	8.65 ± 0.14	8.68 ± 0.08
TC03000475.hg.1	NSUN3	7.28 ± 0.25	7.42 ± 0.15	7.59 ± 0.43	7.32 ± 0.06
TC01000584.hg.1	NSUN4	7.96 ± 0.02	7.96 ± 0.06	7.79 ± 0.03	7.97 ± 0.09
TC07001495.hg.1	NSUN5	8.87 ± 0.08	8.68 ± 0.04	8.69 ± 0.07	8.69 ± 0.04
TC07003333.hg.1	NSUN5P1	9.25 ± 0.12	9.10 ± 0.08	9.06 ± 0.06	9.12 ± 0.07
TC07003297.hg.1	NSUN5P2	8.92 ± 0.06	8.80 ± 0.06	8.76 ± 0.03	8.82 ± 0.08
TC10002961.hg.1	NSUN6	7.52 ± 0.61	7.72 ± 0.26	7.47 ± 0.45	7.57 ± 0.14
TC04000248.hg.1	NSUN7	6.34 ± 0.35	6.08 ± 0.29	7.26 ± 0.53	6.02 ± 0.20
TC17001871.hg.1	NT5C	8.34 ± 0.16	8.07 ± 0.04	8.16 ± 0.18	8.02 ± 0.09
TC01002544.hg.1	NT5C1A	7.48 ± 0.18	7.42 ± 0.30	7.38 ± 0.27	7.52 ± 0.00
TC02005022.hg.1	NT5C1B	6.00 ± 0.20	5.94 ± 0.19	5.93 ± 0.12	6.02 ± 0.07

TC10001626.hg.1	NT5C2	9.59 ± 0.25	9.61 ± 0.23	10.01 ± 0.39	9.57 ± 0.23
TC07001264.hg.1	NT5C3A	8.71 ± 0.40	8.46 ± 0.50	9.07 ± 0.63	8.21 ± 0.25
TC17001518.hg.1	NT5C3B	7.98 ± 0.08	7.94 ± 0.06	7.79 ± 0.11	7.94 ± 0.02
TC06000916.hg.1	NT5DC1	7.79 ± 0.27	7.97 ± 0.31	7.58 ± 0.26	7.68 ± 0.07
TC03001456.hg.1	NT5DC2	8.45 ± 0.23	8.14 ± 0.16	8.10 ± 0.15	8.16 ± 0.07
TC12001901.hg.1	NT5DC3	6.93 ± 0.07	6.85 ± 0.08	6.76 ± 0.12	6.83 ± 0.04
TC02000712.hg.1	NT5DC4	7.36 ± 0.15	7.39 ± 0.16	7.48 ± 0.06	7.49 ± 0.02
TC06000779.hg.1	NT5E	7.82 ± 0.22	7.91 ± 0.15	7.47 ± 0.19	7.75 ± 0.19
TC17000196.hg.1	NT5M	8.44 ± 0.26	8.31 ± 0.16	8.34 ± 0.32	8.31 ± 0.05
TC16000891.hg.1	NTAN1	9.11 ± 0.12	8.94 ± 0.20	8.97 ± 0.15	8.81 ± 0.06
TC12000075.hg.1	NTF3	6.26 ± 0.14	6.27 ± 0.10	6.19 ± 0.15	6.31 ± 0.07
TC19002718.hg.1	NTF4	7.53 ± 0.19	7.43 ± 0.17	7.47 ± 0.08	7.62 ± 0.07
TC16000772.hg.1	NTHL1	8.20 ± 0.16	8.16 ± 0.16	8.14 ± 0.22	8.27 ± 0.12
TC11001203.hg.1	NTM	6.80 ± 0.14	6.72 ± 0.08	6.73 ± 0.09	6.78 ± 0.09
TC11001205.hg.1	NTM-IT1	7.56 ± 0.18	7.77 ± 0.31	7.58 ± 0.23	7.92 ± 0.11
TC11001206.hg.1	NTM-IT2	6.01 ± 0.15	5.98 ± 0.12	5.92 ± 0.04	6.00 ± 0.07
TC11001207.hg.1	NTM-IT3	7.66 ± 0.25	7.63 ± 0.20	7.62 ± 0.25	7.83 ± 0.11
TC09000721.hg.1	NTMT1	8.03 ± 0.17	7.95 ± 0.08	7.85 ± 0.18	8.00 ± 0.05
TC17000143.hg.1	NTN1	7.93 ± 0.25	7.94 ± 0.16	7.86 ± 0.22	8.04 ± 0.02
TC16000074.hg.1	NTN3	8.25 ± 0.17	8.30 ± 0.13	8.26 ± 0.14	8.25 ± 0.05
TC12001837.hg.1	NTN4	5.82 ± 0.15	5.78 ± 0.15	5.79 ± 0.13	5.90 ± 0.06
TC19001691.hg.1	NTN5	7.91 ± 0.11	7.94 ± 0.17	7.86 ± 0.21	7.99 ± 0.08
TC01000926.hg.1	NTNG1	5.53 ± 0.13	5.47 ± 0.09	5.46 ± 0.10	5.51 ± 0.07
TC09000755.hg.1	NTNG2	8.61 ± 0.37	8.38 ± 0.25	8.82 ± 0.27	8.28 ± 0.07
TC01001926.hg.1	NTPCR	6.33 ± 0.08	6.22 ± 0.07	6.21 ± 0.12	6.21 ± 0.05
TC01001328.hg.1	NTRK1	7.87 ± 0.18	7.90 ± 0.18	7.83 ± 0.21	8.02 ± 0.08
TC09000388.hg.1	NTRK2	5.74 ± 0.18	5.63 ± 0.05	5.66 ± 0.08	5.68 ± 0.04
TC15001815.hg.1	NTRK3	6.59 ± 0.14	6.54 ± 0.12	6.48 ± 0.07	6.61 ± 0.09
TC15000836.hg.1	NTRK3-AS1	5.87 ± 0.06	5.74 ± 0.19	5.80 ± 0.11	5.88 ± 0.07
TC12000687.hg.1	NTS	3.83 ± 0.06	3.58 ± 0.01	3.69 ± 0.10	3.57 ± 0.09
TC20000495.hg.1	NTSR1	7.69 ± 0.44	7.20 ± 0.20	7.66 ± 0.19	7.39 ± 0.21

TC02001582.hg.1	NTSR2	7.64 ± 0.21	7.67 ± 0.25	7.61 ± 0.17	7.75 ± 0.07
TC12001916.hg.1	NUAK1	6.36 ± 0.18	6.36 ± 0.12	6.34 ± 0.16	6.38 ± 0.02
TC01003745.hg.1	NUAK2	9.75 ± 0.39	9.92 ± 0.12	9.81 ± 0.12	9.71 ± 0.13
TC07001016.hg.1	NUB1	9.03 ± 0.17	9.04 ± 0.18	9.10 ± 0.36	8.94 ± 0.11
TC16000157.hg.1	NUBP1	8.98 ± 0.14	8.80 ± 0.06	8.82 ± 0.13	8.77 ± 0.07
TC16000048.hg.1	NUBP2	8.53 ± 0.22	8.44 ± 0.11	8.42 ± 0.23	8.43 ± 0.02
TC14000202.hg.1	NUBPL	6.92 ± 0.17	7.06 ± 0.04	6.88 ± 0.11	6.97 ± 0.09
TC19000712.hg.1	NUCB1	9.62 ± 0.29	9.32 ± 0.03	9.81 ± 0.19	9.27 ± 0.02
TC19001702.hg.1	NUCB1-AS1	7.29 ± 0.24	7.08 ± 0.19	7.17 ± 0.06	7.18 ± 0.02
TC11000228.hg.1	NUCB2	7.82 ± 0.32	7.99 ± 0.12	7.74 ± 0.24	7.81 ± 0.08
TC01003751.hg.1	NUCKS1	9.30 ± 0.25	9.50 ± 0.24	8.98 ± 0.12	9.34 ± 0.08
TC01000355.hg.1	NUDC	8.51 ± 0.11	8.42 ± 0.11	8.27 ± 0.19	8.31 ± 0.04
TC08001528.hg.1	NUDCD1	6.76 ± 0.27	6.62 ± 0.14	6.67 ± 0.18	6.39 ± 0.11
TC05002020.hg.1	NUDCD2	6.87 ± 0.04	6.71 ± 0.05	6.60 ± 0.07	6.65 ± 0.10
TC07001340.hg.1	NUDCD3	7.88 ± 0.04	7.83 ± 0.01	7.70 ± 0.07	7.82 ± 0.04
TC07000029.hg.1	NUDT1	8.57 ± 0.22	8.39 ± 0.07	8.37 ± 0.23	8.37 ± 0.02
TC0X000291.hg.1	NUDT10	7.47 ± 0.13	7.57 ± 0.20	7.44 ± 0.20	7.62 ± 0.10
TC0X001042.hg.1	NUDT11	6.73 ± 0.25	6.79 ± 0.13	6.78 ± 0.24	6.88 ± 0.17
TC05001649.hg.1	NUDT12	4.85 ± 0.03	4.84 ± 0.03	4.88 ± 0.13	4.82 ± 0.06
TC10000457.hg.1	NUDT13	5.72 ± 0.09	5.66 ± 0.02	5.67 ± 0.05	5.68 ± 0.05
TC14002334.hg.1	NUDT14	8.17 ± 0.15	8.06 ± 0.11	8.05 ± 0.07	8.05 ± 0.02
TC13000187.hg.1	NUDT15	6.92 ± 0.11	7.07 ± 0.17	6.85 ± 0.08	7.11 ± 0.09
TC03000710.hg.1	NUDT16	7.89 ± 0.21	7.66 ± 0.10	7.89 ± 0.10	7.65 ± 0.08
TC16000134.hg.1	NUDT16L1	8.91 ± 0.14	8.82 ± 0.11	8.78 ± 0.09	8.90 ± 0.03
TC03000709.hg.1	NUDT16P1	8.61 ± 0.24	8.43 ± 0.22	8.58 ± 0.17	8.40 ± 0.05
TC01003111.hg.1	NUDT17	7.61 ± 0.18	7.53 ± 0.14	7.41 ± 0.08	7.58 ± 0.08
TC08001032.hg.1	NUDT18	8.60 ± 0.22	8.60 ± 0.21	8.49 ± 0.18	8.62 ± 0.03
TC19000429.hg.1	NUDT19	8.27 ± 0.05	8.10 ± 0.13	8.07 ± 0.11	8.03 ± 0.10
TC09000170.hg.1	NUDT2	7.44 ± 0.14	7.39 ± 0.06	7.36 ± 0.14	7.35 ± 0.07
TC16001133.hg.1	NUDT21	9.48 ± 0.34	9.71 ± 0.23	9.37 ± 0.23	9.39 ± 0.11
TC11000587.hg.1	NUDT22	8.20 ± 0.16	8.17 ± 0.14	8.12 ± 0.16	8.25 ± 0.07

TC06004129.hg.1	NUDT3	9.55 ± 0.08	9.55 ± 0.17	9.44 ± 0.07	9.50 ± 0.04
TC12000724.hg.1	NUDT4	9.00 ± 0.11	9.06 ± 0.08	8.91 ± 0.03	9.07 ± 0.07
TC10001042.hg.1	NUDT5	8.17 ± 0.10	7.84 ± 0.26	8.10 ± 0.20	7.57 ± 0.16
TC04001523.hg.1	NUDT6	5.75 ± 0.13	5.66 ± 0.04	5.68 ± 0.02	5.68 ± 0.03
TC16000628.hg.1	NUDT7	7.06 ± 0.11	7.03 ± 0.09	7.07 ± 0.03	7.04 ± 0.06
TC11001992.hg.1	NUDT8	8.24 ± 0.15	8.28 ± 0.29	8.25 ± 0.17	8.44 ± 0.03
TC04000472.hg.1	NUDT9	7.25 ± 0.06	7.11 ± 0.05	7.16 ± 0.11	6.96 ± 0.10
TC10001520.hg.1	NUDT9P1	5.77 ± 0.23	5.62 ± 0.26	5.47 ± 0.09	5.61 ± 0.03
TC01001430.hg.1	NUF2	5.76 ± 0.02	5.68 ± 0.26	5.66 ± 0.24	5.35 ± 0.08
TC13000626.hg.1	NUFIP1	6.47 ± 0.18	6.56 ± 0.25	6.26 ± 0.09	6.48 ± 0.07
TC17001316.hg.1	NUFIP2	8.93 ± 0.33	9.35 ± 0.15	9.38 ± 0.42	9.30 ± 0.12
TC08001083.hg.1	NUGGC	7.07 ± 0.20	6.66 ± 0.13	6.70 ± 0.17	6.59 ± 0.12
TC11002049.hg.1	NUMA1	9.21 ± 0.13	9.15 ± 0.09	8.96 ± 0.11	9.06 ± 0.04
TC14001288.hg.1	NUMB	9.40 ± 0.20	9.19 ± 0.24	9.87 ± 0.27	9.10 ± 0.36
TC19001543.hg.1	NUMBL	8.48 ± 0.06	8.47 ± 0.09	8.51 ± 0.07	8.51 ± 0.02
TC12000602.hg.1	NUP107	8.07 ± 0.45	8.19 ± 0.19	7.99 ± 0.41	7.97 ± 0.20
TC12000603.hg.1	NUP107	7.08 ± 0.13	7.01 ± 0.13	7.05 ± 0.06	6.95 ± 0.01
TC01003947.hg.1	NUP133	7.58 ± 0.16	7.61 ± 0.20	7.32 ± 0.15	7.55 ± 0.01
TC06001295.hg.1	NUP153	9.77 ± 0.22	9.84 ± 0.15	9.89 ± 0.24	9.88 ± 0.14
TC05001275.hg.1	NUP155	8.25 ± 0.15	8.23 ± 0.25	7.91 ± 0.24	8.10 ± 0.10
TC11001750.hg.1	NUP160	8.06 ± 0.18	8.00 ± 0.23	7.78 ± 0.20	7.80 ± 0.04
TC09002909.hg.1	NUP188	8.58 ± 0.16	8.47 ± 0.13	8.18 ± 0.04	8.34 ± 0.07
TC07000843.hg.1	NUP205	8.00 ± 0.16	7.96 ± 0.20	7.69 ± 0.20	7.75 ± 0.02
TC03001188.hg.1	NUP210	9.97 ± 0.18	9.98 ± 0.09	9.60 ± 0.07	9.77 ± 0.08
TC01003284.hg.1	NUP210L	5.04 ± 0.10	4.94 ± 0.06	5.03 ± 0.06	4.97 ± 0.03
TC03000659.hg.1	NUP210P1	8.09 ± 0.16	8.05 ± 0.17	7.96 ± 0.11	8.15 ± 0.07
TC09000746.hg.1	NUP214	9.37 ± 0.14	9.31 ± 0.07	9.18 ± 0.20	9.28 ± 0.09
TC02001093.hg.1	NUP35	6.10 ± 0.12	6.07 ± 0.15	5.95 ± 0.10	6.01 ± 0.03
TC12001887.hg.1	NUP37	7.05 ± 0.09	6.97 ± 0.21	6.95 ± 0.16	6.84 ± 0.04
TC06002215.hg.1	NUP43	8.24 ± 0.19	7.81 ± 0.16	8.13 ± 0.19	7.62 ± 0.31
TC22000378.hg.1	NUP50	9.10 ± 0.18	9.10 ± 0.11	9.23 ± 0.24	8.99 ± 0.21

TC04001307.hg.1	NUP54	7.52 ± 0.24	7.67 ± 0.22	7.36 ± 0.17	7.64 ± 0.03
TC19002608.hg.1	NUP62	9.41 ± 0.19	9.23 ± 0.04	9.23 ± 0.03	9.19 ± 0.15
TC0X001260.hg.1	NUP62CL	5.36 ± 0.05	5.30 ± 0.06	5.24 ± 0.04	5.28 ± 0.02
TC17000848.hg.1	NUP85	7.95 ± 0.06	7.81 ± 0.07	7.72 ± 0.08	7.76 ± 0.04
TC17001055.hg.1	NUP88	8.37 ± 0.25	8.54 ± 0.24	8.02 ± 0.21	8.42 ± 0.02
TC16000477.hg.1	NUP93	8.33 ± 0.15	8.19 ± 0.10	8.07 ± 0.14	8.11 ± 0.07
TC11001302.hg.1	NUP98	9.19 ± 0.23	9.22 ± 0.18	9.25 ± 0.22	9.31 ± 0.21
TC13000064.hg.1	NUPL1	8.45 ± 0.20	8.43 ± 0.12	8.71 ± 0.26	8.39 ± 0.31
TC07000142.hg.1	NUPL2	6.53 ± 0.13	6.32 ± 0.14	6.32 ± 0.07	6.18 ± 0.09
TC16000989.hg.1	NUPR1	8.74 ± 0.20	8.85 ± 0.24	8.67 ± 0.21	8.95 ± 0.09
TC07003358.hg.1	NUPR1L	7.64 ± 0.27	7.40 ± 0.05	7.35 ± 0.13	7.46 ± 0.13
TC06000932.hg.1	NUS1	8.78 ± 0.28	8.74 ± 0.23	8.55 ± 0.35	8.59 ± 0.20
TC15000306.hg.1	NUSAP1	7.74 ± 0.22	7.48 ± 0.52	7.49 ± 0.20	6.79 ± 0.21
TC16000548.hg.1	NUTF2	9.97 ± 0.08	9.68 ± 0.09	9.71 ± 0.20	9.58 ± 0.13
TC15000236.hg.1	NUTM1	6.35 ± 0.23	6.30 ± 0.17	6.22 ± 0.22	6.39 ± 0.05
TC10000615.hg.1	NUTM2A	7.72 ± 0.07	7.74 ± 0.14	7.63 ± 0.13	7.83 ± 0.08
TC10001487.hg.1	NUTM2A-AS1	7.71 ± 0.13	7.73 ± 0.14	7.73 ± 0.12	7.70 ± 0.03
TC09001355.hg.1	NUTM2F	7.63 ± 0.22	7.64 ± 0.26	7.54 ± 0.17	7.75 ± 0.10
TC09000486.hg.1	NUTM2G	7.54 ± 0.21	7.49 ± 0.22	7.44 ± 0.15	7.61 ± 0.09
TC01003881.hg.1	NVL	7.36 ± 0.19	7.27 ± 0.08	7.20 ± 0.10	7.26 ± 0.05
TC19000324.hg.1	NWD1	7.07 ± 0.18	7.02 ± 0.17	6.95 ± 0.12	7.11 ± 0.05
TC11001880.hg.1	NXF1	10.18 ± 0.25	10.37 ± 0.03	10.16 ± 0.22	10.37 ± 0.09
TC0X000486.hg.1	NXF2B	5.79 ± 0.15	5.74 ± 0.06	5.66 ± 0.06	5.79 ± 0.10
TC0X001229.hg.1	NXF2B	5.80 ± 0.16	5.75 ± 0.07	5.68 ± 0.07	5.80 ± 0.09
TC0X001235.hg.1	NXF3	6.33 ± 0.10	6.29 ± 0.09	6.29 ± 0.12	6.38 ± 0.10
TC0X000487.hg.1	NXF4	5.22 ± 0.13	5.15 ± 0.13	5.17 ± 0.08	5.17 ± 0.05
TC0X001225.hg.1	NXF5	5.75 ± 0.14	5.67 ± 0.10	5.70 ± 0.04	5.77 ± 0.10
TC17000969.hg.1	NXN	7.56 ± 0.20	7.49 ± 0.23	7.44 ± 0.19	7.55 ± 0.03
TC19001277.hg.1	NXNL1	8.73 ± 0.31	8.61 ± 0.15	8.66 ± 0.29	8.73 ± 0.02
TC09000412.hg.1	NXNL2	7.27 ± 0.07	7.21 ± 0.02	7.18 ± 0.19	7.24 ± 0.06
TC11002313.hg.1	NXPE1	4.85 ± 0.11	4.75 ± 0.06	4.78 ± 0.05	4.84 ± 0.12

TC11001030.hg.1	NXPE2	5.35 ± 0.08	5.32 ± 0.05	5.31 ± 0.08	5.40 ± 0.11
TC03003319.hg.1	NXPE3	8.46 ± 0.15	8.32 ± 0.08	8.36 ± 0.20	8.29 ± 0.11
TC11002314.hg.1	NXPE4	5.05 ± 0.04	5.02 ± 0.11	5.01 ± 0.04	5.15 ± 0.09
TC07000083.hg.1	NXPH1	5.67 ± 0.20	5.56 ± 0.09	5.54 ± 0.09	5.59 ± 0.02
TC02002384.hg.1	NXPH2	6.49 ± 0.27	6.35 ± 0.08	6.36 ± 0.09	6.33 ± 0.03
TC17000646.hg.1	NXPH3	7.74 ± 0.21	7.66 ± 0.19	7.67 ± 0.26	7.75 ± 0.05
TC12000523.hg.1	NXPH4	8.10 ± 0.24	8.00 ± 0.16	7.97 ± 0.25	8.07 ± 0.07
TC20000153.hg.1	NXT1	7.57 ± 0.19	7.59 ± 0.19	7.32 ± 0.27	7.63 ± 0.02
TC0X000536.hg.1	NXT2	7.65 ± 0.10	7.65 ± 0.16	7.67 ± 0.23	7.45 ± 0.06
TC07000623.hg.1	NYAP1	8.56 ± 0.23	8.49 ± 0.16	8.50 ± 0.20	8.54 ± 0.03
TC02001357.hg.1	NYAP2	6.09 ± 0.17	5.91 ± 0.15	6.03 ± 0.11	6.05 ± 0.08
TC14000169.hg.1	NYNRIN	7.08 ± 0.23	7.04 ± 0.14	7.03 ± 0.21	7.08 ± 0.06
TC0X000200.hg.1	NYX	7.77 ± 0.24	7.76 ± 0.22	7.77 ± 0.23	7.84 ± 0.09
TC18000209.hg.1	OACYLP	6.12 ± 0.18	6.11 ± 0.11	6.08 ± 0.05	6.16 ± 0.13
TC11001098.hg.1	OAF	8.15 ± 0.16	8.01 ± 0.09	7.98 ± 0.08	7.99 ± 0.04
TC11001099.hg.1	OAF	7.19 ± 0.21	7.13 ± 0.03	7.31 ± 0.03	7.10 ± 0.15
TC06004134.hg.1	OARD1	7.77 ± 0.11	7.80 ± 0.15	7.71 ± 0.27	7.69 ± 0.03
TC12000884.hg.1	OAS1	9.06 ± 0.73	8.26 ± 0.64	8.99 ± 0.61	7.85 ± 0.23
TC12000886.hg.1	OAS2	10.69 ± 0.74	10.02 ± 0.72	10.67 ± 0.72	9.43 ± 0.45
TC12000885.hg.1	OAS3	9.62 ± 0.74	9.10 ± 0.58	9.67 ± 0.76	8.87 ± 0.31
TC12002059.hg.1	OASL	9.93 ± 0.86	9.24 ± 0.38	9.90 ± 0.79	9.00 ± 0.28
TC10001726.hg.1	OAT	7.36 ± 0.10	7.48 ± 0.13	7.60 ± 0.25	7.32 ± 0.10
TC19002618.hg.1	OAZ1	11.85 ± 0.17	11.57 ± 0.07	11.96 ± 0.23	11.44 ± 0.14
TC15001552.hg.1	OAZ2	8.77 ± 0.11	8.67 ± 0.13	8.90 ± 0.22	8.59 ± 0.29
TC01001218.hg.1	OAZ3	7.21 ± 0.10	7.18 ± 0.08	7.12 ± 0.08	7.26 ± 0.05
TC10001636.hg.1	OBFC1	8.27 ± 0.07	8.21 ± 0.08	8.28 ± 0.27	8.08 ± 0.02
TC09000798.hg.1	OBP2A	8.35 ± 0.24	8.30 ± 0.24	8.26 ± 0.20	8.39 ± 0.07
TC09001682.hg.1	OBP2B	8.43 ± 0.26	8.27 ± 0.21	8.32 ± 0.22	8.35 ± 0.06
TC01001888.hg.1	OBSCN	8.25 ± 0.16	8.25 ± 0.14	8.12 ± 0.16	8.31 ± 0.06
TC02002802.hg.1	OBSL1	7.95 ± 0.21	7.90 ± 0.19	7.91 ± 0.22	8.00 ± 0.04
TC08002620.hg.1	OC90	6.92 ± 0.13	6.86 ± 0.17	6.86 ± 0.10	7.00 ± 0.11

TC15001091.hg.1	OCA2	7.09 ± 0.16	7.07 ± 0.14	7.04 ± 0.12	7.16 ± 0.03
TC19000329.hg.1	OCEL1	8.03 ± 0.20	8.00 ± 0.12	7.96 ± 0.23	8.01 ± 0.04
TC04000281.hg.1	OCIAD1	8.17 ± 0.20	8.29 ± 0.05	8.17 ± 0.16	8.27 ± 0.07
TC04001179.hg.1	OCIAD1-AS1	3.45 ± 0.03	3.24 ± 0.08	3.45 ± 0.09	3.31 ± 0.04
TC04001180.hg.1	OCIAD2	7.72 ± 0.06	7.77 ± 0.09	7.47 ± 0.10	7.83 ± 0.07
TC01001605.hg.1	OCLM	6.03 ± 0.50	6.37 ± 0.18	6.09 ± 0.32	6.31 ± 0.35
TC05000304.hg.1	OCLN	6.03 ± 0.14	6.17 ± 0.16	6.06 ± 0.04	6.14 ± 0.10
TC07000057.hg.1	OCM	6.72 ± 0.08	6.66 ± 0.04	6.55 ± 0.01	6.80 ± 0.08
TC07001632.hg.1	OCM2	6.53 ± 0.10	6.39 ± 0.03	6.33 ± 0.07	6.51 ± 0.09
TC0X000616.hg.1	OCRL	6.71 ± 0.04	6.59 ± 0.06	6.67 ± 0.08	6.59 ± 0.02
TC20000904.hg.1	OCSTAMP	7.70 ± 0.35	7.51 ± 0.11	7.57 ± 0.19	7.71 ± 0.05
TC04000380.hg.1	ODAM	4.96 ± 0.16	4.82 ± 0.06	4.86 ± 0.09	4.89 ± 0.09
TC02001570.hg.1	ODC1	9.41 ± 0.08	9.42 ± 0.21	8.99 ± 0.26	9.38 ± 0.12
TC08000635.hg.1	ODF1	5.68 ± 0.09	5.60 ± 0.16	5.71 ± 0.04	5.79 ± 0.03
TC09000691.hg.1	ODF2	8.26 ± 0.06	8.19 ± 0.06	8.06 ± 0.13	8.06 ± 0.01
TC01002834.hg.1	ODF2L	6.59 ± 0.62	6.88 ± 0.20	6.55 ± 0.40	6.79 ± 0.06
TC11000003.hg.1	ODF3	7.05 ± 0.30	7.05 ± 0.20	6.98 ± 0.19	7.16 ± 0.08
TC22001501.hg.1	ODF3B	8.68 ± 0.32	8.48 ± 0.17	8.68 ± 0.18	8.44 ± 0.06
TC15000699.hg.1	ODF3L1	7.13 ± 0.23	7.19 ± 0.17	7.16 ± 0.07	7.21 ± 0.05
TC19000986.hg.1	ODF3L2	8.36 ± 0.26	8.33 ± 0.25	8.33 ± 0.25	8.46 ± 0.06
TC17000140.hg.1	ODF4	5.17 ± 0.14	5.17 ± 0.20	5.15 ± 0.13	5.29 ± 0.12
TC06001259.hg.1	OFCC1	5.48 ± 0.18	5.39 ± 0.05	5.35 ± 0.10	5.42 ± 0.05
TC0X000061.hg.1	OFD1	7.81 ± 0.62	8.13 ± 0.23	7.76 ± 0.45	7.95 ± 0.10
TC07000276.hg.1	OGDH	9.69 ± 0.31	9.32 ± 0.03	9.50 ± 0.20	9.10 ± 0.11
TC10001278.hg.1	OGDHL	7.57 ± 0.20	7.54 ± 0.21	7.50 ± 0.22	7.64 ± 0.05
TC16000463.hg.1	OGFOD1	8.44 ± 0.15	8.22 ± 0.20	8.10 ± 0.04	7.97 ± 0.16
TC12003261.hg.1	OGFOD2	7.74 ± 0.14	7.66 ± 0.14	7.62 ± 0.13	7.69 ± 0.04
TC17001981.hg.1	OGFOD3	8.33 ± 0.23	8.18 ± 0.16	8.12 ± 0.24	8.15 ± 0.05
TC20000498.hg.1	OGFR	9.63 ± 0.26	9.32 ± 0.05	9.51 ± 0.22	9.29 ± 0.18
TC20001019.hg.1	OGFR-AS1	7.36 ± 0.16	7.36 ± 0.16	7.36 ± 0.11	7.50 ± 0.10
TC06000720.hg.1	OGFRL1	9.67 ± 0.20	9.88 ± 0.37	9.82 ± 0.24	9.78 ± 0.22

TC03000042.hg.1	OGG1	8.05 ± 0.08	7.91 ± 0.02	7.91 ± 0.02	7.89 ± 0.05
TC09001334.hg.1	OGN	4.32 ± 0.14	4.15 ± 0.01	4.16 ± 0.04	4.18 ± 0.01
TC0X000388.hg.1	OGT	10.46 ± 0.59	10.80 ± 0.07	10.37 ± 0.37	10.73 ± 0.14
TC15001248.hg.1	OIP5	5.84 ± 0.26	5.78 ± 0.16	5.66 ± 0.18	5.54 ± 0.04
TC15000305.hg.1	OIP5-AS1	8.89 ± 0.14	8.90 ± 0.16	8.99 ± 0.18	8.77 ± 0.07
TC10000455.hg.1	OIT3	6.50 ± 0.25	6.44 ± 0.17	6.45 ± 0.21	6.61 ± 0.11
TC02002536.hg.1	OLA1	8.57 ± 0.07	8.62 ± 0.15	8.37 ± 0.09	8.47 ± 0.10
TC10000115.hg.1	OLAH	6.02 ± 0.41	5.49 ± 0.07	6.03 ± 0.25	5.63 ± 0.06
TC09000790.hg.1	OLFM1	7.71 ± 0.14	7.63 ± 0.10	7.59 ± 0.14	7.66 ± 0.02
TC19001155.hg.1	OLFM2	8.02 ± 0.31	7.93 ± 0.25	7.91 ± 0.21	8.02 ± 0.02
TC01002929.hg.1	OLFM3	5.44 ± 0.09	5.33 ± 0.10	5.38 ± 0.10	5.41 ± 0.07
TC13000228.hg.1	OLFM4	8.13 ± 0.90	7.07 ± 0.27	8.46 ± 0.74	7.09 ± 0.15
TC11000149.hg.1	OLFML1	6.46 ± 0.18	6.48 ± 0.10	6.47 ± 0.08	6.60 ± 0.11
TC09000644.hg.1	OLFML2A	7.84 ± 0.31	7.85 ± 0.28	7.83 ± 0.20	8.03 ± 0.06
TC01003452.hg.1	OLFML2B	6.91 ± 0.17	6.80 ± 0.24	7.02 ± 0.23	6.88 ± 0.05
TC01001004.hg.1	OLFML3	7.82 ± 0.15	7.67 ± 0.16	7.69 ± 0.11	7.77 ± 0.13
TC21000125.hg.1	OLIG1	8.34 ± 0.07	8.21 ± 0.04	8.34 ± 0.26	8.20 ± 0.09
TC21000123.hg.1	OLIG2	7.34 ± 0.10	7.32 ± 0.18	7.55 ± 0.26	7.45 ± 0.03
TC06002153.hg.1	OLIG3	6.56 ± 0.38	6.48 ± 0.12	6.55 ± 0.14	6.62 ± 0.07
TC12001216.hg.1	OLR1	6.19 ± 0.25	6.16 ± 0.06	6.64 ± 0.20	6.71 ± 0.90
TC01006360.hg.1	OMA1	7.36 ± 0.10	7.30 ± 0.09	7.41 ± 0.13	7.15 ± 0.12
TC09001335.hg.1	OMD	4.57 ± 0.16	4.29 ± 0.08	4.34 ± 0.18	4.21 ± 0.11
TC17001340.hg.1	OMG	6.79 ± 0.07	6.94 ± 0.03	7.54 ± 0.43	6.97 ± 0.03
TC11000828.hg.1	OMP	8.28 ± 0.24	8.26 ± 0.21	8.22 ± 0.15	8.35 ± 0.10
TC15001460.hg.1	ONECUT1	6.52 ± 0.22	6.48 ± 0.03	6.50 ± 0.09	6.53 ± 0.07
TC18000195.hg.1	ONECUT2	7.27 ± 0.12	7.36 ± 0.33	7.25 ± 0.19	7.43 ± 0.01
TC19000044.hg.1	ONECUT3	7.62 ± 0.26	7.74 ± 0.20	7.55 ± 0.21	7.76 ± 0.04
TC06001871.hg.1	OOEP	7.08 ± 0.03	7.05 ± 0.17	7.00 ± 0.12	7.14 ± 0.06
TC06000732.hg.1	OOEP-AS1	8.05 ± 0.17	8.23 ± 0.22	8.08 ± 0.18	8.40 ± 0.18
TC11000501.hg.1	OOSP1	5.02 ± 0.07	4.92 ± 0.15	4.97 ± 0.06	5.15 ± 0.21
TC03001067.hg.1	OPA1	8.01 ± 0.54	8.43 ± 0.23	8.04 ± 0.51	8.40 ± 0.04

TC03002122.hg.1	OPA1-AS1	4.04 ± 0.11	3.70 ± 0.14	3.84 ± 0.27	3.67 ± 0.07
TC19002712.hg.1	OPA3	7.56 ± 0.15	7.35 ± 0.18	7.40 ± 0.09	7.44 ± 0.02
TC10001556.hg.1	OPALIN	6.67 ± 0.12	6.66 ± 0.18	6.66 ± 0.10	6.77 ± 0.09
TC11002462.hg.1	OPCML	7.03 ± 0.12	6.95 ± 0.16	6.98 ± 0.11	7.04 ± 0.09
TC11002464.hg.1	OPCML-IT1	6.15 ± 0.14	6.05 ± 0.12	6.09 ± 0.17	5.99 ± 0.17
TC11002463.hg.1	OPCML-IT2	5.56 ± 0.16	5.54 ± 0.13	5.59 ± 0.04	5.63 ± 0.00
TC0X001103.hg.1	OPHN1	6.22 ± 0.07	5.99 ± 0.03	6.11 ± 0.10	6.02 ± 0.10
TC08001740.hg.1	OPLAH	8.53 ± 0.21	8.44 ± 0.22	8.61 ± 0.13	8.54 ± 0.00
TC0X000764.hg.1	OPN1LW	7.35 ± 0.22	7.39 ± 0.24	7.25 ± 0.14	7.51 ± 0.10
TC0X000765.hg.1	OPN1MW	7.50 ± 0.18	7.56 ± 0.21	7.52 ± 0.16	7.67 ± 0.07
TC0X000766.hg.1	OPN1MW	7.61 ± 0.17	7.66 ± 0.22	7.61 ± 0.17	7.77 ± 0.07
TC07001849.hg.1	OPN1SW	6.62 ± 0.08	6.67 ± 0.11	6.58 ± 0.11	6.77 ± 0.10
TC01004026.hg.1	OPN3	7.65 ± 0.08	7.61 ± 0.09	7.60 ± 0.14	7.51 ± 0.05
TC10002926.hg.1	OPN4	8.01 ± 0.18	7.97 ± 0.18	7.93 ± 0.18	8.06 ± 0.08
TC06000637.hg.1	OPN5	5.61 ± 0.23	5.52 ± 0.12	5.53 ± 0.07	5.63 ± 0.14
TC01000392.hg.1	OPRD1	8.67 ± 0.15	8.68 ± 0.22	8.60 ± 0.19	8.73 ± 0.04
TC08001212.hg.1	OPRK1	6.61 ± 0.17	6.61 ± 0.15	6.54 ± 0.16	6.65 ± 0.05
TC20000534.hg.1	OPRL1	7.79 ± 0.24	7.61 ± 0.27	7.75 ± 0.19	7.69 ± 0.01
TC06001116.hg.1	OPRM1	5.34 ± 0.10	5.26 ± 0.07	5.29 ± 0.01	5.31 ± 0.07
TC01001687.hg.1	OPTC	6.72 ± 0.16	6.69 ± 0.23	6.60 ± 0.13	6.85 ± 0.12
TC10000097.hg.1	OPTN	8.14 ± 0.16	8.22 ± 0.08	7.83 ± 0.14	8.13 ± 0.03
TC11000139.hg.1	OR10A2	4.80 ± 0.05	4.69 ± 0.06	4.81 ± 0.18	4.84 ± 0.24
TC11001380.hg.1	OR10A3	4.96 ± 0.17	5.13 ± 0.03	5.18 ± 0.08	5.17 ± 0.16
TC11000140.hg.1	OR10A4	5.67 ± 0.01	5.69 ± 0.25	5.77 ± 0.21	5.99 ± 0.20
TC11000138.hg.1	OR10A5	4.47 ± 0.07	4.38 ± 0.03	4.48 ± 0.10	4.44 ± 0.03
TC11001379.hg.1	OR10A6	4.13 ± 0.07	3.98 ± 0.03	3.92 ± 0.05	4.05 ± 0.12
TC12000475.hg.1	OR10A7	6.20 ± 0.21	6.01 ± 0.07	5.91 ± 0.05	6.41 ± 0.41
TC11000152.hg.1	OR10AB1P	5.26 ± 0.11	5.38 ± 0.11	5.32 ± 0.36	5.47 ± 0.13
TC12001446.hg.1	OR10AD1	5.53 ± 0.33	5.51 ± 0.18	5.31 ± 0.10	5.47 ± 0.14
TC11001769.hg.1	OR10AG1	3.94 ± 0.14	3.81 ± 0.04	3.85 ± 0.07	3.84 ± 0.20
TC06000316.hg.1	OR10C1	5.39 ± 0.17	5.41 ± 0.16	5.25 ± 0.19	5.59 ± 0.04

TC6_apd_hap1000009.hg.1	OR10C1	5.44 ± 0.18	5.45 ± 0.18	5.40 ± 0.02	5.59 ± 0.10
TC6_cox_hap2000013.hg.1	OR10C1	5.44 ± 0.18	5.45 ± 0.18	5.40 ± 0.02	5.59 ± 0.10
TC6_dbb_hap3000009.hg.1	OR10C1	5.39 ± 0.17	5.41 ± 0.16	5.25 ± 0.19	5.59 ± 0.04
TC6_mann_hap4000010.hg.1	OR10C1	5.44 ± 0.18	5.45 ± 0.18	5.40 ± 0.02	5.59 ± 0.10
TC6_mcf_hap5000008.hg.1	OR10C1	5.39 ± 0.17	5.41 ± 0.16	5.25 ± 0.19	5.59 ± 0.04
TC6_qbl_hap6000008.hg.1	OR10C1	5.44 ± 0.18	5.45 ± 0.18	5.40 ± 0.02	5.59 ± 0.10
TC6_ssto_hap7000011.hg.1	OR10C1	5.39 ± 0.18	5.41 ± 0.13	5.34 ± 0.02	5.55 ± 0.10
TC11001134.hg.1	OR10D3	7.84 ± 0.16	7.86 ± 0.15	7.72 ± 0.17	7.92 ± 0.13
TC14000926.hg.1	OR10G2	5.33 ± 0.12	5.23 ± 0.17	5.30 ± 0.02	5.32 ± 0.10
TC14000924.hg.1	OR10G3	5.69 ± 0.43	5.74 ± 0.35	5.82 ± 0.15	5.81 ± 0.26
TC11001129.hg.1	OR10G4	7.51 ± 0.19	7.57 ± 0.22	7.58 ± 0.26	7.76 ± 0.22
TC11002403.hg.1	OR10G7	7.55 ± 0.09	7.35 ± 0.21	7.38 ± 0.24	7.57 ± 0.15
TC11001131.hg.1	OR10G8	7.74 ± 0.26	7.59 ± 0.05	7.81 ± 0.14	7.76 ± 0.06
TC11001130.hg.1	OR10G9	7.36 ± 0.06	7.26 ± 0.12	7.28 ± 0.03	7.52 ± 0.07
TC19001259.hg.1	OR10H1	7.69 ± 0.12	7.69 ± 0.16	7.58 ± 0.23	7.80 ± 0.16
TC19000276.hg.1	OR10H2	6.02 ± 0.19	6.32 ± 0.13	5.95 ± 0.23	6.42 ± 0.29
TC19000277.hg.1	OR10H3	6.25 ± 0.22	6.43 ± 0.06	6.26 ± 0.14	6.59 ± 0.35
TC19000280.hg.1	OR10H4	6.39 ± 0.34	6.28 ± 0.14	6.24 ± 0.05	6.53 ± 0.08
TC19000278.hg.1	OR10H5	6.19 ± 0.11	6.15 ± 0.19	6.23 ± 0.17	6.31 ± 0.23
TC01001352.hg.1	OR10J1	5.17 ± 0.21	5.21 ± 0.09	5.27 ± 0.13	5.24 ± 0.13
TC01003386.hg.1	OR10J3	6.41 ± 0.42	6.24 ± 0.16	6.41 ± 0.06	6.68 ± 0.29
TC01003388.hg.1	OR10J5	5.13 ± 0.41	5.09 ± 0.16	5.00 ± 0.16	5.27 ± 0.27
TC01001341.hg.1	OR10K1	5.25 ± 0.19	5.15 ± 0.15	5.32 ± 0.13	5.43 ± 0.23
TC01003373.hg.1	OR10K2	5.27 ± 0.08	5.18 ± 0.02	5.28 ± 0.16	5.58 ± 0.53
TC12000486.hg.1	OR10P1	8.46 ± 0.18	8.64 ± 0.24	8.45 ± 0.10	8.94 ± 0.22
TC11001804.hg.1	OR10Q1	7.10 ± 0.41	7.18 ± 0.10	7.28 ± 0.19	7.47 ± 0.25
TC01001342.hg.1	OR10R2	4.81 ± 0.09	4.83 ± 0.04	4.90 ± 0.02	5.03 ± 0.21
TC11002402.hg.1	OR10S1	5.15 ± 0.10	5.21 ± 0.16	5.25 ± 0.21	5.65 ± 0.47
TC01003372.hg.1	OR10T2	4.89 ± 0.22	5.14 ± 0.40	5.04 ± 0.11	5.27 ± 0.02
TC11001831.hg.1	OR10V1	5.37 ± 0.13	5.30 ± 0.06	5.33 ± 0.18	5.42 ± 0.18
TC11000500.hg.1	OR10V2P	6.20 ± 0.20	6.29 ± 0.08	6.28 ± 0.19	6.48 ± 0.23

TC11001805.hg.1	OR10W1	5.30 ± 0.30	5.15 ± 0.14	5.01 ± 0.13	5.33 ± 0.24
TC01003377.hg.1	OR10X1	4.37 ± 0.13	4.28 ± 0.12	4.36 ± 0.15	4.39 ± 0.12
TC01001344.hg.1	OR10Z1	6.67 ± 0.03	6.76 ± 0.13	6.57 ± 0.09	6.91 ± 0.36
TC06004148.hg.1	OR11A1	4.55 ± 0.13	4.44 ± 0.03	4.43 ± 0.07	4.45 ± 0.12
TC6_apd_hap1000057.hg.1	OR11A1	4.64 ± 0.14	4.53 ± 0.05	4.52 ± 0.05	4.54 ± 0.12
TC6_cox_hap2000246.hg.1	OR11A1	4.55 ± 0.13	4.44 ± 0.03	4.43 ± 0.07	4.45 ± 0.12
TC6_dbb_hap3000109.hg.1	OR11A1	4.64 ± 0.13	4.53 ± 0.05	4.53 ± 0.05	4.54 ± 0.11
TC6_mann_hap4000212.hg.1	OR11A1	4.55 ± 0.13	4.44 ± 0.03	4.43 ± 0.07	4.45 ± 0.12
TC6_mcf_hap5000229.hg.1	OR11A1	4.55 ± 0.13	4.44 ± 0.03	4.43 ± 0.07	4.45 ± 0.12
TC6_qbl_hap6000110.hg.1	OR11A1	4.64 ± 0.14	4.53 ± 0.05	4.52 ± 0.05	4.54 ± 0.12
TC6_ssto_hap7000215.hg.1	OR11A1	4.52 ± 0.14	4.40 ± 0.03	4.39 ± 0.07	4.42 ± 0.12
TC14000046.hg.1	OR11G2	4.27 ± 0.15	4.06 ± 0.18	4.16 ± 0.07	4.17 ± 0.07
TC22000458.hg.1	OR11H1	5.39 ± 0.92	5.60 ± 0.05	5.64 ± 0.56	6.24 ± 0.83
TC14000006.hg.1	OR11H12	3.75 ± 0.02	3.59 ± 0.07	3.54 ± 0.06	3.69 ± 0.25
TC14000871.hg.1	OR11H2	3.58 ± 0.02	3.59 ± 0.05	3.57 ± 0.01	3.92 ± 0.48
TC14000049.hg.1	OR11H4	4.17 ± 0.27	3.82 ± 0.24	3.75 ± 0.08	3.83 ± 0.04
TC14000047.hg.1	OR11H6	4.67 ± 0.17	4.68 ± 0.05	4.63 ± 0.06	4.75 ± 0.16
TC14000048.hg.1	OR11H7	5.16 ± 0.07	4.98 ± 0.27	5.11 ± 0.27	5.26 ± 0.19
TC01004080.hg.1	OR11L1	6.63 ± 0.04	6.78 ± 0.08	6.66 ± 0.19	6.68 ± 0.20
TC06000315.hg.1	OR12D2	3.86 ± 0.22	3.61 ± 0.07	3.63 ± 0.11	3.77 ± 0.07
TC6_cox_hap2000012.hg.1	OR12D2	3.91 ± 0.23	3.62 ± 0.09	3.62 ± 0.10	3.75 ± 0.06
TC6_dbb_hap3000008.hg.1	OR12D2	3.89 ± 0.21	3.65 ± 0.16	3.56 ± 0.08	3.72 ± 0.11
TC6_mann_hap4000009.hg.1	OR12D2	3.91 ± 0.23	3.62 ± 0.09	3.62 ± 0.10	3.75 ± 0.06
TC6_mcf_hap5000007.hg.1	OR12D2	3.91 ± 0.23	3.62 ± 0.09	3.62 ± 0.10	3.75 ± 0.06
TC6_ssto_hap7000010.hg.1	OR12D2	3.89 ± 0.21	3.65 ± 0.16	3.56 ± 0.08	3.72 ± 0.11
TC06001483.hg.1	OR12D3	5.36 ± 0.18	5.41 ± 0.12	5.37 ± 0.07	5.61 ± 0.23
TC6_apd_hap1000058.hg.1	OR12D3	5.54 ± 0.24	5.41 ± 0.38	5.32 ± 0.28	5.70 ± 0.24
TC6_cox_hap2000120.hg.1	OR12D3	5.42 ± 0.19	5.47 ± 0.08	5.43 ± 0.08	5.69 ± 0.23
TC6_dbb_hap3000107.hg.1	OR12D3	5.64 ± 0.17	5.71 ± 0.13	5.69 ± 0.04	5.91 ± 0.23
TC6_mann_hap4000098.hg.1	OR12D3	5.76 ± 0.17	5.82 ± 0.08	5.82 ± 0.05	6.02 ± 0.24
TC6_mcf_hap5000102.hg.1	OR12D3	5.49 ± 0.22	5.50 ± 0.11	5.50 ± 0.05	5.67 ± 0.25

TC6_ssto_hap7000102.hg.1	OR12D3	5.53 ± 0.20	5.59 ± 0.12	5.54 ± 0.07	5.72 ± 0.21
TC10001222.hg.1	OR13A1	6.06 ± 0.17	6.11 ± 0.10	6.06 ± 0.05	6.09 ± 0.11
TC09001432.hg.1	OR13C2	3.56 ± 0.24	3.34 ± 0.04	3.51 ± 0.17	3.30 ± 0.08
TC09001430.hg.1	OR13C3	4.58 ± 0.14	4.28 ± 0.12	4.47 ± 0.29	4.38 ± 0.21
TC09001429.hg.1	OR13C4	4.88 ± 0.10	4.80 ± 0.09	4.72 ± 0.02	4.90 ± 0.15
TC09001431.hg.1	OR13C5	3.56 ± 0.28	3.45 ± 0.06	3.55 ± 0.01	3.58 ± 0.13
TC09000529.hg.1	OR13C8	5.59 ± 0.26	5.58 ± 0.12	5.64 ± 0.08	5.68 ± 0.13
TC09001433.hg.1	OR13C9	5.73 ± 0.06	5.62 ± 0.03	5.95 ± 0.30	5.73 ± 0.03
TC09000530.hg.1	OR13D1	3.53 ± 0.09	3.51 ± 0.04	3.47 ± 0.07	3.49 ± 0.07
TC09000528.hg.1	OR13F1	4.35 ± 0.10	4.03 ± 0.10	4.34 ± 0.15	3.98 ± 0.12
TC01004076.hg.1	OR13G1	5.03 ± 0.08	4.72 ± 0.06	4.82 ± 0.03	4.94 ± 0.29
TC0X000627.hg.1	OR13H1	4.06 ± 0.20	3.90 ± 0.06	3.96 ± 0.03	3.91 ± 0.08
TC09001066.hg.1	OR13J1	6.75 ± 0.17	6.91 ± 0.41	6.75 ± 0.16	7.08 ± 0.10
TC01004079.hg.1	OR14A16	5.52 ± 0.24	5.34 ± 0.23	5.25 ± 0.21	5.58 ± 0.24
TC01006395.hg.1	OR14A2	4.80 ± 0.16	4.65 ± 0.05	4.72 ± 0.09	4.85 ± 0.12
TC01002034.hg.1	OR14C36	5.39 ± 0.11	5.67 ± 0.26	5.50 ± 0.22	6.12 ± 0.41
TC01004092.hg.1	OR14I1	5.10 ± 0.05	4.83 ± 0.10	4.87 ± 0.19	4.99 ± 0.23
TC06000314.hg.1	OR14J1	5.09 ± 0.30	4.81 ± 0.24	4.84 ± 0.21	5.04 ± 0.21
TC6_apd_hap1000008.hg.1	OR14J1	5.10 ± 0.31	4.82 ± 0.18	4.84 ± 0.21	5.02 ± 0.27
TC6_cox_hap2000011.hg.1	OR14J1	5.09 ± 0.30	4.81 ± 0.24	4.84 ± 0.21	5.04 ± 0.21
TC6_dbb_hap3000007.hg.1	OR14J1	5.10 ± 0.31	4.82 ± 0.18	4.84 ± 0.21	5.02 ± 0.27
TC6_mann_hap4000008.hg.1	OR14J1	5.10 ± 0.31	4.82 ± 0.18	4.84 ± 0.21	5.02 ± 0.27
TC6_mcf_hap5000006.hg.1	OR14J1	5.09 ± 0.30	4.81 ± 0.24	4.84 ± 0.21	5.04 ± 0.21
TC6_ssto_hap7000009.hg.1	OR14J1	5.93 ± 0.59	5.54 ± 0.10	5.74 ± 0.21	5.85 ± 0.44
TC01002015.hg.1	OR14K1	6.23 ± 0.37	6.24 ± 0.23	6.06 ± 0.18	6.43 ± 0.24
TC17000029.hg.1	OR1A1	5.47 ± 0.23	5.55 ± 0.12	5.44 ± 0.15	5.72 ± 0.25
TC17000028.hg.1	OR1A2	4.85 ± 0.16	4.80 ± 0.21	4.93 ± 0.11	4.92 ± 0.25
TC09001561.hg.1	OR1B1	6.45 ± 0.42	6.53 ± 0.32	6.44 ± 0.30	6.59 ± 0.10
TC01004078.hg.1	OR1C1	4.24 ± 0.21	3.99 ± 0.16	4.13 ± 0.07	4.22 ± 0.18
TC17001002.hg.1	OR1D2	5.57 ± 0.32	5.65 ± 0.15	5.62 ± 0.11	5.95 ± 0.43
TC17000027.hg.1	OR1D4	7.14 ± 0.10	6.83 ± 0.24	6.92 ± 0.24	7.15 ± 0.31

TC17001001.hg.1	OR1D5	5.55 ± 0.25	5.33 ± 0.06	5.43 ± 0.15	5.56 ± 0.27
TC17001006.hg.1	OR1E1	5.70 ± 0.15	5.51 ± 0.06	5.47 ± 0.19	5.65 ± 0.13
TC17001007.hg.1	OR1E2	7.02 ± 0.16	7.20 ± 0.14	7.09 ± 0.05	7.21 ± 0.17
TC16000117.hg.1	OR1F1	6.13 ± 0.23	6.12 ± 0.04	6.03 ± 0.12	6.35 ± 0.27
TC16000118.hg.1	OR1F2P	7.16 ± 0.06	7.38 ± 0.19	6.90 ± 0.24	7.40 ± 0.26
TC17001003.hg.1	OR1G1	4.17 ± 0.35	4.04 ± 0.02	4.04 ± 0.22	4.16 ± 0.31
TC19000268.hg.1	OR1I1	6.25 ± 0.15	6.27 ± 0.04	6.28 ± 0.09	6.40 ± 0.06
TC09001558.hg.1	OR1J1	6.56 ± 0.23	6.56 ± 0.30	6.42 ± 0.22	6.82 ± 0.19
TC09002935.hg.1	OR1J4	5.72 ± 0.51	5.72 ± 0.08	5.52 ± 0.30	6.06 ± 0.45
TC09000626.hg.1	OR1K1	6.87 ± 0.14	6.95 ± 0.10	6.95 ± 0.13	7.10 ± 0.22
TC09000620.hg.1	OR1L1	4.40 ± 0.30	4.19 ± 0.09	4.09 ± 0.13	4.21 ± 0.12
TC09000621.hg.1	OR1L3	6.08 ± 0.27	6.36 ± 0.09	6.25 ± 0.30	6.70 ± 0.22
TC09000622.hg.1	OR1L4	6.57 ± 0.15	6.43 ± 0.07	6.54 ± 0.04	6.59 ± 0.23
TC09000623.hg.1	OR1L6	5.74 ± 0.28	5.69 ± 0.11	5.63 ± 0.27	5.82 ± 0.15
TC09001560.hg.1	OR1L8	4.71 ± 0.25	4.32 ± 0.07	4.33 ± 0.09	4.39 ± 0.29
TC19000154.hg.1	OR1M1	6.62 ± 0.12	6.31 ± 0.16	6.30 ± 0.10	6.38 ± 0.16
TC09001559.hg.1	OR1N1	5.93 ± 0.28	6.20 ± 0.14	5.98 ± 0.16	6.38 ± 0.38
TC09000617.hg.1	OR1N2	4.65 ± 0.20	4.46 ± 0.06	4.53 ± 0.15	4.67 ± 0.24
TC09000619.hg.1	OR1Q1	4.55 ± 0.15	4.31 ± 0.11	4.40 ± 0.15	4.44 ± 0.06
TC11000476.hg.1	OR1S1	5.99 ± 0.45	5.79 ± 0.08	5.73 ± 0.19	5.97 ± 0.26
TC11001803.hg.1	OR1S2	6.17 ± 0.09	6.00 ± 0.11	6.00 ± 0.15	6.45 ± 0.41
TC07000949.hg.1	OR2A12	5.38 ± 0.12	5.02 ± 0.17	5.29 ± 0.07	5.04 ± 0.07
TC07000951.hg.1	OR2A14	6.47 ± 0.19	6.12 ± 0.27	6.17 ± 0.13	6.41 ± 0.20
TC07000950.hg.1	OR2A2	6.41 ± 0.18	6.36 ± 0.11	6.48 ± 0.07	6.60 ± 0.27
TC07000948.hg.1	OR2A25	6.18 ± 0.15	6.35 ± 0.15	6.23 ± 0.08	6.44 ± 0.10
TC06002110.hg.1	OR2A4	6.32 ± 0.34	6.18 ± 0.28	5.92 ± 0.43	6.45 ± 0.28
TC07000956.hg.1	OR2A42	7.52 ± 0.21	7.53 ± 0.21	7.44 ± 0.19	7.85 ± 0.31
TC07001977.hg.1	OR2A42	7.43 ± 0.21	7.54 ± 0.19	7.41 ± 0.21	7.85 ± 0.28
TC07000947.hg.1	OR2A5	5.20 ± 0.29	5.20 ± 0.23	5.34 ± 0.08	5.55 ± 0.25
TC07001979.hg.1	OR2A7	7.21 ± 0.24	7.00 ± 0.17	6.73 ± 0.33	7.30 ± 0.35
TC07003306.hg.1	OR2A7	7.93 ± 0.07	7.90 ± 0.06	7.68 ± 0.16	8.04 ± 0.17

TC07000955.hg.1	OR2A9P	7.78 ± 0.35	7.69 ± 0.24	7.29 ± 0.29	7.79 ± 0.42
TC07001978.hg.1	OR2A9P	7.21 ± 0.08	7.14 ± 0.05	6.99 ± 0.10	7.17 ± 0.15
TC07003368.hg.1	OR2AE1	5.35 ± 0.15	5.30 ± 0.04	5.29 ± 0.06	5.47 ± 0.04
TC11000137.hg.1	OR2AG1	6.64 ± 0.04	6.57 ± 0.12	6.56 ± 0.13	6.91 ± 0.22
TC11001366.hg.1	OR2AG2	4.56 ± 0.27	4.26 ± 0.03	4.35 ± 0.25	4.37 ± 0.14
TC01002018.hg.1	OR2AJ1	5.25 ± 0.08	5.47 ± 0.29	5.52 ± 0.19	5.44 ± 0.23
TC01002021.hg.1	OR2AK2	4.14 ± 0.35	4.01 ± 0.05	4.05 ± 0.08	4.07 ± 0.08
TC12000485.hg.1	OR2AP1	4.79 ± 0.15	4.64 ± 0.05	4.72 ± 0.17	4.76 ± 0.06
TC11002089.hg.1	OR2AT4	5.06 ± 0.30	4.90 ± 0.09	4.99 ± 0.06	5.06 ± 0.24
TC01004072.hg.1	OR2B11	5.44 ± 0.54	5.47 ± 0.26	5.44 ± 0.30	5.64 ± 0.03
TC06001434.hg.1	OR2B2	5.53 ± 0.11	5.06 ± 0.04	5.23 ± 0.08	5.24 ± 0.21
TC06001481.hg.1	OR2B3	4.76 ± 0.03	4.49 ± 0.03	4.59 ± 0.02	4.59 ± 0.19
TC6_apd_hap1000056.hg.1	OR2B3	4.76 ± 0.03	4.49 ± 0.03	4.59 ± 0.02	4.59 ± 0.19
TC6_cox_hap2000118.hg.1	OR2B3	4.76 ± 0.03	4.49 ± 0.03	4.59 ± 0.02	4.59 ± 0.19
TC6_dbb_hap3000106.hg.1	OR2B3	4.76 ± 0.03	4.49 ± 0.03	4.59 ± 0.02	4.59 ± 0.19
TC6_mann_hap4000096.hg.1	OR2B3	4.76 ± 0.03	4.49 ± 0.03	4.59 ± 0.02	4.59 ± 0.19
TC6_mcf_hap5000100.hg.1	OR2B3	4.73 ± 0.15	4.48 ± 0.06	4.55 ± 0.03	4.62 ± 0.22
TC6_qbl_hap6000109.hg.1	OR2B3	5.70 ± 0.13	5.53 ± 0.21	5.64 ± 0.10	5.76 ± 0.23
TC6_ssto_hap7000100.hg.1	OR2B3	4.76 ± 0.03	4.49 ± 0.03	4.59 ± 0.02	4.59 ± 0.19
TC06000271.hg.1	OR2B6	6.00 ± 0.33	5.81 ± 0.10	5.89 ± 0.22	5.93 ± 0.04
TC16000121.hg.1	OR2C1	4.47 ± 0.13	4.50 ± 0.05	4.50 ± 0.18	4.59 ± 0.13
TC01004073.hg.1	OR2C3	5.78 ± 0.12	5.71 ± 0.09	5.65 ± 0.10	5.76 ± 0.04
TC11001368.hg.1	OR2D2	4.71 ± 0.07	4.68 ± 0.08	4.61 ± 0.19	4.81 ± 0.20
TC11000141.hg.1	OR2D3	5.14 ± 0.12	5.05 ± 0.07	5.09 ± 0.09	5.18 ± 0.07
TC07000945.hg.1	OR2F1	6.41 ± 0.18	6.43 ± 0.19	6.28 ± 0.04	6.47 ± 0.17
TC07000943.hg.1	OR2F2	4.36 ± 0.24	4.10 ± 0.03	4.21 ± 0.24	4.12 ± 0.03
TC01002012.hg.1	OR2G2	5.93 ± 0.34	5.85 ± 0.10	5.95 ± 0.06	6.05 ± 0.11
TC01002013.hg.1	OR2G3	7.37 ± 0.31	7.27 ± 0.08	7.41 ± 0.13	7.29 ± 0.16
TC01002041.hg.1	OR2G6	6.33 ± 0.43	6.42 ± 0.12	6.25 ± 0.11	6.63 ± 0.02
TC06000317.hg.1	OR2H1	5.68 ± 0.16	5.60 ± 0.04	5.60 ± 0.02	5.65 ± 0.07
TC6_apd_hap1000010.hg.1	OR2H1	5.71 ± 0.16	5.64 ± 0.06	5.62 ± 0.04	5.67 ± 0.08

TC6_cox_hap2000014.hg.1	OR2H1	5.71 ± 0.16	5.64 ± 0.06	5.62 ± 0.04	5.67 ± 0.08
TC6_dbb_hap3000010.hg.1	OR2H1	5.71 ± 0.16	5.64 ± 0.05	5.62 ± 0.04	5.67 ± 0.08
TC6_mann_hap4000011.hg.1	OR2H1	5.71 ± 0.16	5.64 ± 0.06	5.62 ± 0.04	5.67 ± 0.08
TC6_mcf_hap5000009.hg.1	OR2H1	5.71 ± 0.16	5.64 ± 0.05	5.62 ± 0.04	5.67 ± 0.08
TC6_qbl_hap6000009.hg.1	OR2H1	5.69 ± 0.16	5.63 ± 0.06	5.61 ± 0.04	5.66 ± 0.08
TC6_ssto_hap7000012.hg.1	OR2H1	5.71 ± 0.16	5.64 ± 0.06	5.62 ± 0.04	5.67 ± 0.08
TC06000321.hg.1	OR2H2	5.80 ± 0.05	5.66 ± 0.11	5.86 ± 0.23	5.85 ± 0.11
TC6_apd_hap1000014.hg.1	OR2H2	5.62 ± 0.12	5.53 ± 0.10	5.70 ± 0.24	5.75 ± 0.09
TC6_cox_hap2000018.hg.1	OR2H2	5.73 ± 0.05	5.56 ± 0.12	5.77 ± 0.21	5.77 ± 0.10
TC6_dbb_hap3000014.hg.1	OR2H2	5.80 ± 0.05	5.66 ± 0.11	5.86 ± 0.23	5.85 ± 0.11
TC6_mann_hap4000015.hg.1	OR2H2	5.70 ± 0.07	5.57 ± 0.11	5.75 ± 0.22	5.77 ± 0.11
TC6_mcf_hap5000013.hg.1	OR2H2	5.70 ± 0.07	5.57 ± 0.11	5.75 ± 0.22	5.77 ± 0.11
TC6_qbl_hap6000013.hg.1	OR2H2	5.80 ± 0.05	5.66 ± 0.11	5.86 ± 0.23	5.85 ± 0.11
TC06000309.hg.1	OR2J1	4.46 ± 0.11	4.58 ± 0.05	4.50 ± 0.23	4.71 ± 0.14
TC6_mcf_hap5000004.hg.1	OR2J1	5.61 ± 0.16	5.76 ± 0.04	5.70 ± 0.23	5.91 ± 0.13
TC06000312.hg.1	OR2J2	4.90 ± 0.06	4.78 ± 0.02	4.81 ± 0.14	4.85 ± 0.07
TC6_apd_hap1000006.hg.1	OR2J2	4.82 ± 0.19	4.77 ± 0.05	4.87 ± 0.23	4.88 ± 0.14
TC6_cox_hap2000009.hg.1	OR2J2	4.85 ± 0.10	4.72 ± 0.08	4.84 ± 0.22	4.83 ± 0.06
TC6_dbb_hap3000005.hg.1	OR2J2	4.82 ± 0.19	4.77 ± 0.05	4.87 ± 0.23	4.88 ± 0.14
TC6_mann_hap4000006.hg.1	OR2J2	4.82 ± 0.19	4.77 ± 0.05	4.87 ± 0.23	4.88 ± 0.14
TC6_qbl_hap6000007.hg.1	OR2J2	4.86 ± 0.17	4.83 ± 0.04	4.94 ± 0.25	4.93 ± 0.10
TC6_ssto_hap7000007.hg.1	OR2J2	4.82 ± 0.19	4.77 ± 0.05	4.87 ± 0.23	4.88 ± 0.14
TC06000310.hg.1	OR2J3	4.26 ± 0.30	4.07 ± 0.07	4.09 ± 0.15	4.26 ± 0.22
TC6_apd_hap1000004.hg.1	OR2J3	4.33 ± 0.14	4.07 ± 0.07	4.12 ± 0.17	4.25 ± 0.21
TC6_cox_hap2000007.hg.1	OR2J3	4.33 ± 0.14	4.07 ± 0.07	4.12 ± 0.17	4.25 ± 0.21
TC6_dbb_hap3000003.hg.1	OR2J3	4.33 ± 0.14	4.07 ± 0.07	4.12 ± 0.17	4.25 ± 0.21
TC6_mann_hap4000004.hg.1	OR2J3	4.33 ± 0.14	4.07 ± 0.07	4.12 ± 0.17	4.25 ± 0.21
TC6_mcf_hap5000005.hg.1	OR2J3	4.54 ± 0.18	4.40 ± 0.05	4.46 ± 0.19	4.52 ± 0.07
TC6_qbl_hap6000005.hg.1	OR2J3	4.33 ± 0.14	4.07 ± 0.07	4.12 ± 0.17	4.25 ± 0.21
TC6_ssto_hap7000005.hg.1	OR2J3	4.33 ± 0.14	4.07 ± 0.07	4.12 ± 0.17	4.25 ± 0.21
TC09001481.hg.1	OR2K2	3.96 ± 0.10	3.79 ± 0.03	3.92 ± 0.20	3.85 ± 0.10

TC01002019.hg.1	OR2L13	6.03 ± 0.15	5.99 ± 0.08	5.95 ± 0.13	6.07 ± 0.12
TC01002022.hg.1	OR2L1P	6.12 ± 0.58	5.93 ± 0.26	5.70 ± 0.17	6.08 ± 0.22
TC01002026.hg.1	OR2L2	5.29 ± 0.31	5.17 ± 0.13	5.27 ± 0.38	5.28 ± 0.22
TC01002028.hg.1	OR2L3	6.04 ± 0.60	5.76 ± 0.24	5.82 ± 0.12	5.99 ± 0.32
TC01002024.hg.1	OR2L5	5.38 ± 0.38	5.24 ± 0.06	5.17 ± 0.22	5.33 ± 0.25
TC01002020.hg.1	OR2L8	5.15 ± 0.23	4.78 ± 0.06	4.83 ± 0.30	5.08 ± 0.39
TC01002029.hg.1	OR2M1P	5.86 ± 0.05	5.58 ± 0.08	5.59 ± 0.17	5.77 ± 0.24
TC01002031.hg.1	OR2M2	5.33 ± 0.42	5.21 ± 0.24	5.11 ± 0.11	5.27 ± 0.23
TC01002032.hg.1	OR2M3	4.97 ± 0.14	4.80 ± 0.12	4.86 ± 0.04	5.12 ± 0.35
TC01002033.hg.1	OR2M4	5.55 ± 0.50	5.70 ± 0.20	5.49 ± 0.33	6.09 ± 0.27
TC01002030.hg.1	OR2M5	5.56 ± 0.37	5.45 ± 0.20	5.42 ± 0.14	5.80 ± 0.48
TC01004083.hg.1	OR2M7	6.14 ± 0.05	6.08 ± 0.03	5.99 ± 0.15	6.28 ± 0.14
TC09001067.hg.1	OR2S2	6.64 ± 0.12	6.74 ± 0.24	6.58 ± 0.23	6.73 ± 0.03
TC01002037.hg.1	OR2T1	5.25 ± 0.11	5.22 ± 0.03	5.11 ± 0.06	5.43 ± 0.25
TC01004087.hg.1	OR2T10	5.65 ± 0.19	5.59 ± 0.22	5.70 ± 0.19	5.87 ± 0.37
TC01004089.hg.1	OR2T11	6.89 ± 0.17	6.76 ± 0.20	6.62 ± 0.20	6.99 ± 0.24
TC01004082.hg.1	OR2T12	6.50 ± 0.24	6.36 ± 0.18	6.35 ± 0.23	6.63 ± 0.28
TC01002038.hg.1	OR2T2	7.26 ± 0.41	7.30 ± 0.16	7.08 ± 0.28	7.52 ± 0.13
TC01004091.hg.1	OR2T27	6.86 ± 0.17	6.80 ± 0.10	6.83 ± 0.28	7.27 ± 0.60
TC01004085.hg.1	OR2T29	4.07 ± 0.21	3.84 ± 0.05	3.93 ± 0.05	3.93 ± 0.07
TC01002039.hg.1	OR2T3	7.80 ± 0.35	7.57 ± 0.17	7.70 ± 0.35	7.91 ± 0.30
TC01004081.hg.1	OR2T33	3.88 ± 0.21	3.82 ± 0.04	3.87 ± 0.14	3.86 ± 0.10
TC01004086.hg.1	OR2T34	6.26 ± 0.29	6.05 ± 0.12	6.08 ± 0.38	6.43 ± 0.53
TC01004090.hg.1	OR2T35	6.69 ± 0.09	6.85 ± 0.30	6.56 ± 0.16	7.08 ± 0.30
TC01002035.hg.1	OR2T4	6.06 ± 0.15	5.99 ± 0.25	5.83 ± 0.09	6.16 ± 0.44
TC01002040.hg.1	OR2T5	3.95 ± 0.23	3.72 ± 0.04	3.78 ± 0.03	3.84 ± 0.13
TC01002036.hg.1	OR2T6	5.87 ± 0.18	5.85 ± 0.19	5.64 ± 0.11	5.99 ± 0.47
TC01002017.hg.1	OR2T8	6.75 ± 0.22	6.75 ± 0.20	6.69 ± 0.10	6.93 ± 0.31
TC05002156.hg.1	OR2V1	6.65 ± 0.04	6.55 ± 0.04	6.39 ± 0.32	6.81 ± 0.20
TC05001074.hg.1	OR2V2	6.28 ± 0.05	6.30 ± 0.12	6.14 ± 0.07	6.48 ± 0.30
TC06001480.hg.1	OR2W1	5.45 ± 0.16	5.16 ± 0.24	5.16 ± 0.11	5.23 ± 0.19

TC6_apd_hap1000055.hg.1	OR2W1	5.45 ± 0.16	5.16 ± 0.24	5.16 ± 0.11	5.23 ± 0.19
TC6_cox_hap2000117.hg.1	OR2W1	5.45 ± 0.16	5.16 ± 0.24	5.16 ± 0.11	5.23 ± 0.19
TC6_dbb_hap3000105.hg.1	OR2W1	5.45 ± 0.16	5.16 ± 0.24	5.16 ± 0.11	5.23 ± 0.19
TC6_mann_hap4000095.hg.1	OR2W1	5.45 ± 0.16	5.16 ± 0.24	5.16 ± 0.11	5.23 ± 0.19
TC6_mcf_hap5000099.hg.1	OR2W1	5.45 ± 0.16	5.16 ± 0.24	5.16 ± 0.11	5.23 ± 0.19
TC6_qbl_hap6000108.hg.1	OR2W1	5.45 ± 0.16	5.16 ± 0.24	5.16 ± 0.11	5.23 ± 0.19
TC6_ssto_hap7000099.hg.1	OR2W1	5.45 ± 0.16	5.16 ± 0.24	5.16 ± 0.11	5.23 ± 0.19
TC01002016.hg.1	OR2W3	8.51 ± 0.07	7.94 ± 0.02	8.54 ± 0.25	8.08 ± 0.18
TC01002009.hg.1	OR2W5	7.15 ± 0.06	6.98 ± 0.10	6.98 ± 0.03	7.05 ± 0.09
TC05002150.hg.1	OR2Y1	5.45 ± 0.28	5.62 ± 0.14	5.39 ± 0.19	5.62 ± 0.23
TC19000152.hg.1	OR2Z1	6.17 ± 0.18	6.25 ± 0.11	6.26 ± 0.11	6.44 ± 0.29
TC17001005.hg.1	OR3A1	6.90 ± 0.16	6.82 ± 0.09	6.77 ± 0.07	6.92 ± 0.27
TC17001004.hg.1	OR3A2	6.47 ± 0.30	6.62 ± 0.15	6.52 ± 0.18	6.73 ± 0.19
TC17000032.hg.1	OR3A3	7.36 ± 0.12	7.15 ± 0.16	7.12 ± 0.17	7.45 ± 0.19
TC17000031.hg.1	OR3A4P	6.63 ± 0.20	6.87 ± 0.20	6.64 ± 0.05	7.00 ± 0.30
TC11000430.hg.1	OR4A15	5.54 ± 0.10	5.41 ± 0.10	5.40 ± 0.04	5.73 ± 0.34
TC11000429.hg.1	OR4A16	4.44 ± 0.28	4.13 ± 0.08	4.29 ± 0.16	4.25 ± 0.19
TC11000418.hg.1	OR4A47	4.36 ± 0.21	4.40 ± 0.21	4.27 ± 0.25	4.65 ± 0.22
TC11001763.hg.1	OR4A5	5.67 ± 0.28	5.47 ± 0.09	5.57 ± 0.08	5.88 ± 0.39
TC11000413.hg.1	OR4B1	6.25 ± 0.18	6.24 ± 0.10	6.04 ± 0.08	6.27 ± 0.22
TC11001765.hg.1	OR4C11	4.90 ± 0.15	4.91 ± 0.24	4.74 ± 0.12	4.86 ± 0.10
TC11001760.hg.1	OR4C12	5.71 ± 0.12	5.89 ± 0.05	5.72 ± 0.13	6.00 ± 0.21
TC11000422.hg.1	OR4C13	4.96 ± 0.15	4.87 ± 0.05	4.96 ± 0.30	5.06 ± 0.46
TC11000431.hg.1	OR4C15	5.51 ± 0.27	5.32 ± 0.05	5.45 ± 0.15	5.57 ± 0.17
TC11000432.hg.1	OR4C16	5.44 ± 0.02	5.45 ± 0.21	5.40 ± 0.03	5.44 ± 0.18
TC11000417.hg.1	OR4C3	5.23 ± 0.18	5.01 ± 0.19	4.96 ± 0.02	4.93 ± 0.17
TC11001754.hg.1	OR4C45	5.36 ± 0.20	5.25 ± 0.08	5.35 ± 0.11	5.40 ± 0.17
TC11000427.hg.1	OR4C46	5.12 ± 0.15	5.05 ± 0.10	4.99 ± 0.05	5.26 ± 0.25
TC11001755.hg.1	OR4C5	5.01 ± 0.22	4.97 ± 0.21	4.91 ± 0.12	5.19 ± 0.30
TC11000435.hg.1	OR4C6	4.73 ± 0.31	4.63 ± 0.09	4.75 ± 0.15	4.87 ± 0.46
TC17000708.hg.1	OR4D1	4.44 ± 0.17	4.27 ± 0.11	4.42 ± 0.13	4.47 ± 0.30

TC11000488.hg.1	OR4D10	4.86 ± 0.06	4.89 ± 0.09	4.84 ± 0.01	4.87 ± 0.16
TC11000490.hg.1	OR4D11	6.32 ± 0.22	6.39 ± 0.11	6.53 ± 0.15	6.52 ± 0.04
TC17000710.hg.1	OR4D2	6.89 ± 0.17	6.64 ± 0.12	6.72 ± 0.12	6.98 ± 0.35
TC11001128.hg.1	OR4D5	5.64 ± 0.07	5.44 ± 0.04	5.36 ± 0.12	5.57 ± 0.32
TC11000487.hg.1	OR4D6	5.41 ± 0.16	5.52 ± 0.26	5.34 ± 0.01	5.66 ± 0.14
TC11000491.hg.1	OR4D9	5.48 ± 0.24	5.63 ± 0.18	5.57 ± 0.06	5.69 ± 0.24
TC14000091.hg.1	OR4E2	6.79 ± 0.24	6.73 ± 0.19	6.56 ± 0.21	6.86 ± 0.22
TC15001002.hg.1	OR4F13P	5.51 ± 0.12	5.39 ± 0.06	5.39 ± 0.04	5.57 ± 0.10
TC15001001.hg.1	OR4F15	5.89 ± 0.30	5.70 ± 0.09	5.65 ± 0.28	5.76 ± 0.29
TC19000002.hg.1	OR4F17	4.78 ± 0.17	4.60 ± 0.08	4.66 ± 0.04	4.76 ± 0.24
TC08000872.hg.1	OR4F21	4.94 ± 0.11	5.04 ± 0.13	4.93 ± 0.33	5.21 ± 0.53
TC01000009.hg.1	OR4F29	4.76 ± 0.22	4.72 ± 0.20	4.84 ± 0.31	4.88 ± 0.25
TC01002065.hg.1	OR4F29	4.48 ± 0.16	4.49 ± 0.15	4.48 ± 0.24	4.61 ± 0.27
TC05001089.hg.1	OR4F29	4.76 ± 0.22	4.72 ± 0.20	4.84 ± 0.31	4.88 ± 0.25
TC11001224.hg.1	OR4F2P	7.17 ± 0.44	6.90 ± 0.30	7.03 ± 0.23	7.16 ± 0.61
TC15002054.hg.1	OR4F4	5.71 ± 0.08	5.49 ± 0.08	5.56 ± 0.10	5.55 ± 0.19
TC01000003.hg.1	OR4F5	6.24 ± 0.30	6.09 ± 0.13	5.93 ± 0.29	6.67 ± 0.61
TC15001000.hg.1	OR4F6	4.91 ± 0.35	5.20 ± 0.27	5.43 ± 0.23	5.41 ± 0.10
TC14000039.hg.1	OR4K1	5.07 ± 0.13	5.01 ± 0.17	4.89 ± 0.06	5.05 ± 0.17
TC14000874.hg.1	OR4K13	5.53 ± 0.06	5.34 ± 0.06	5.26 ± 0.26	5.49 ± 0.07
TC14000873.hg.1	OR4K14	6.23 ± 0.31	6.20 ± 0.33	6.24 ± 0.18	6.60 ± 0.19
TC14000040.hg.1	OR4K15	4.72 ± 0.15	4.60 ± 0.14	4.64 ± 0.01	4.66 ± 0.23
TC14000044.hg.1	OR4K17	5.05 ± 0.03	4.98 ± 0.11	5.12 ± 0.08	5.11 ± 0.07
TC14000037.hg.1	OR4K2	4.65 ± 0.20	4.39 ± 0.11	4.45 ± 0.22	4.48 ± 0.18
TC14000038.hg.1	OR4K5	5.11 ± 0.33	5.19 ± 0.22	5.09 ± 0.04	5.42 ± 0.58
TC14000042.hg.1	OR4L1	5.25 ± 0.12	5.08 ± 0.12	5.21 ± 0.06	5.15 ± 0.02
TC14000035.hg.1	OR4M1	4.32 ± 0.18	4.14 ± 0.03	4.18 ± 0.08	4.29 ± 0.13
TC15000020.hg.1	OR4M2	5.30 ± 0.06	5.37 ± 0.07	5.36 ± 0.15	5.62 ± 0.39
TC14000036.hg.1	OR4N2	5.95 ± 0.47	5.93 ± 0.04	5.92 ± 0.41	6.43 ± 0.51
TC15000019.hg.1	OR4N4	4.95 ± 0.10	4.81 ± 0.03	4.87 ± 0.01	4.96 ± 0.16
TC14000045.hg.1	OR4N5	5.29 ± 0.16	5.31 ± 0.16	5.07 ± 0.20	5.45 ± 0.24

TC11000433.hg.1	OR4P4	4.51 ± 0.13	4.45 ± 0.10	4.54 ± 0.09	4.67 ± 0.23
TC14000041.hg.1	OR4Q2	5.79 ± 0.46	5.70 ± 0.11	5.64 ± 0.17	5.73 ± 0.17
TC14000034.hg.1	OR4Q3	4.89 ± 0.18	4.90 ± 0.05	4.63 ± 0.18	5.22 ± 0.50
TC11000416.hg.1	OR4S1	6.19 ± 0.30	5.97 ± 0.16	5.92 ± 0.11	6.05 ± 0.15
TC11000434.hg.1	OR4S2	5.45 ± 0.27	5.34 ± 0.11	5.47 ± 0.18	5.58 ± 0.31
TC11000415.hg.1	OR4X1	7.01 ± 0.11	7.08 ± 0.13	6.96 ± 0.14	7.12 ± 0.23
TC11000414.hg.1	OR4X2	4.91 ± 0.18	5.14 ± 0.11	4.90 ± 0.12	5.34 ± 0.13
TC11001321.hg.1	OR51A2	5.71 ± 0.29	5.73 ± 0.17	5.73 ± 0.18	6.03 ± 0.27
TC11001320.hg.1	OR51A4	4.73 ± 0.25	4.85 ± 0.17	4.85 ± 0.12	5.06 ± 0.69
TC11000104.hg.1	OR51A7	3.85 ± 0.16	3.48 ± 0.03	3.65 ± 0.14	3.56 ± 0.02
TC11001335.hg.1	OR51B2	4.69 ± 0.23	4.57 ± 0.10	4.71 ± 0.10	4.67 ± 0.22
TC11001334.hg.1	OR51B4	5.10 ± 0.27	4.88 ± 0.25	4.87 ± 0.10	5.03 ± 0.10
TC11000108.hg.1	OR51B6	4.77 ± 0.06	4.92 ± 0.23	4.93 ± 0.31	5.17 ± 0.42
TC11000109.hg.1	OR51B6	5.76 ± 0.12	5.78 ± 0.15	5.82 ± 0.35	6.11 ± 0.25
TC11001313.hg.1	OR51C1P	5.22 ± 0.32	5.27 ± 0.14	5.24 ± 0.18	5.65 ± 0.30
TC11000097.hg.1	OR51D1	6.07 ± 0.25	6.11 ± 0.11	5.86 ± 0.20	6.16 ± 0.11
TC11000098.hg.1	OR51E1	5.47 ± 0.15	5.42 ± 0.06	5.37 ± 0.15	5.49 ± 0.13
TC11001312.hg.1	OR51E2	6.18 ± 0.22	6.05 ± 0.15	6.06 ± 0.05	6.12 ± 0.12
TC11001314.hg.1	OR51F1	5.37 ± 0.23	5.24 ± 0.11	5.33 ± 0.12	5.29 ± 0.13
TC11000102.hg.1	OR51F2	5.02 ± 0.06	4.93 ± 0.05	4.91 ± 0.03	5.14 ± 0.36
TC11001319.hg.1	OR51G1	5.55 ± 0.21	5.45 ± 0.16	5.50 ± 0.15	5.51 ± 0.21
TC11001318.hg.1	OR51G2	6.31 ± 0.26	6.28 ± 0.09	6.18 ± 0.20	6.51 ± 0.33
TC11001317.hg.1	OR51H1P	6.48 ± 0.40	6.45 ± 0.20	6.57 ± 0.14	6.85 ± 0.35
TC11001336.hg.1	OR51I1	5.13 ± 0.26	5.12 ± 0.14	5.18 ± 0.18	5.25 ± 0.18
TC11001337.hg.1	OR51I1	7.30 ± 0.11	7.16 ± 0.24	7.19 ± 0.03	7.26 ± 0.17
TC11000113.hg.1	OR51I2	4.72 ± 0.18	4.69 ± 0.15	4.57 ± 0.16	4.62 ± 0.05
TC11000111.hg.1	OR51J1	5.95 ± 0.33	5.97 ± 0.08	5.93 ± 0.08	6.17 ± 0.30
TC11000105.hg.1	OR51L1	5.82 ± 0.06	5.80 ± 0.13	5.82 ± 0.17	6.09 ± 0.26
TC11000110.hg.1	OR51M1	5.74 ± 0.27	5.38 ± 0.31	5.61 ± 0.09	5.58 ± 0.18
TC11000112.hg.1	OR51Q1	4.11 ± 0.06	4.22 ± 0.10	4.23 ± 0.11	4.47 ± 0.24
TC11001316.hg.1	OR51S1	7.41 ± 0.21	7.46 ± 0.13	7.49 ± 0.04	7.64 ± 0.23

TC11000103.hg.1	OR51T1	4.57 ± 0.06	4.43 ± 0.08	4.49 ± 0.18	4.62 ± 0.31
TC11001328.hg.1	OR51V1	5.60 ± 0.19	5.44 ± 0.14	5.46 ± 0.13	5.79 ± 0.31
TC11001326.hg.1	OR52A1	4.07 ± 0.32	3.87 ± 0.13	3.90 ± 0.07	4.16 ± 0.15
TC11001324.hg.1	OR52A4	4.38 ± 0.17	4.28 ± 0.07	4.26 ± 0.05	4.44 ± 0.13
TC11001325.hg.1	OR52A5	4.54 ± 0.08	4.47 ± 0.03	4.39 ± 0.15	4.43 ± 0.21
TC11001352.hg.1	OR52B2	5.55 ± 0.09	5.59 ± 0.21	5.67 ± 0.31	5.74 ± 0.14
TC11001308.hg.1	OR52B4	6.30 ± 0.07	6.20 ± 0.16	6.06 ± 0.18	6.24 ± 0.27
TC11000115.hg.1	OR52B6	6.09 ± 0.11	5.95 ± 0.10	6.14 ± 0.29	6.22 ± 0.22
TC11000114.hg.1	OR52D1	6.04 ± 0.19	5.86 ± 0.14	5.92 ± 0.08	5.98 ± 0.07
TC11001322.hg.1	OR52E2	5.44 ± 0.10	5.61 ± 0.20	5.53 ± 0.03	5.96 ± 0.32
TC11000120.hg.1	OR52E4	4.61 ± 0.15	4.58 ± 0.17	4.50 ± 0.20	4.61 ± 0.12
TC11001344.hg.1	OR52E6	6.11 ± 0.22	6.14 ± 0.26	6.08 ± 0.07	6.36 ± 0.16
TC11001345.hg.1	OR52E8	5.47 ± 0.03	5.48 ± 0.16	5.49 ± 0.20	5.56 ± 0.10
TC11001340.hg.1	OR52H1	4.53 ± 0.19	4.49 ± 0.11	4.70 ± 0.14	4.48 ± 0.09
TC11000096.hg.1	OR52I1	4.89 ± 0.10	4.68 ± 0.08	4.76 ± 0.04	4.79 ± 0.07
TC11000095.hg.1	OR52I2	5.96 ± 0.29	5.92 ± 0.36	5.90 ± 0.06	6.15 ± 0.25
TC11000106.hg.1	OR52J3	6.47 ± 0.47	6.33 ± 0.03	6.53 ± 0.20	6.73 ± 0.64
TC11000093.hg.1	OR52K1	6.78 ± 0.19	6.63 ± 0.13	6.82 ± 0.38	6.68 ± 0.19
TC11000092.hg.1	OR52K2	8.08 ± 0.25	7.80 ± 0.19	7.96 ± 0.20	7.69 ± 0.24
TC11001347.hg.1	OR52L1	6.15 ± 0.18	6.09 ± 0.20	6.14 ± 0.08	6.34 ± 0.31
TC11000094.hg.1	OR52M1	7.17 ± 0.20	7.19 ± 0.15	7.13 ± 0.04	7.38 ± 0.31
TC11001343.hg.1	OR52N1	5.46 ± 0.28	5.48 ± 0.21	5.40 ± 0.30	5.92 ± 0.40
TC11000119.hg.1	OR52N2	5.62 ± 0.40	5.54 ± 0.07	5.54 ± 0.20	5.52 ± 0.04
TC11000118.hg.1	OR52N4	4.77 ± 0.31	4.44 ± 0.35	4.44 ± 0.29	4.46 ± 0.21
TC11001342.hg.1	OR52N5	6.06 ± 0.30	6.01 ± 0.25	6.05 ± 0.05	6.33 ± 0.31
TC11001315.hg.1	OR52R1	5.75 ± 0.54	5.45 ± 0.20	5.52 ± 0.13	5.73 ± 0.25
TC11000126.hg.1	OR52W1	7.39 ± 0.25	7.23 ± 0.17	7.17 ± 0.17	7.24 ± 0.14
TC11001349.hg.1	OR56A1	6.02 ± 0.29	6.11 ± 0.10	5.97 ± 0.09	6.32 ± 0.40
TC11000122.hg.1	OR56A3	4.45 ± 0.06	4.26 ± 0.04	4.38 ± 0.03	4.44 ± 0.03
TC11001348.hg.1	OR56A4	4.03 ± 0.06	3.84 ± 0.02	3.94 ± 0.03	3.88 ± 0.09
TC11001346.hg.1	OR56A5	6.38 ± 0.06	6.25 ± 0.07	6.34 ± 0.10	6.35 ± 0.24

TC11000117.hg.1	OR56B1	9.71 ± 0.22	9.63 ± 0.36	10.04 ± 0.41	9.48 ± 0.07
TC11000124.hg.1	OR56B4	4.84 ± 0.11	4.75 ± 0.11	4.71 ± 0.19	4.88 ± 0.19
TC11000486.hg.1	OR5A1	6.08 ± 0.11	5.95 ± 0.15	6.04 ± 0.05	6.16 ± 0.18
TC11001820.hg.1	OR5A2	6.00 ± 0.55	6.16 ± 0.14	6.06 ± 0.22	6.18 ± 0.13
TC03000482.hg.1	OR5AC2	4.93 ± 0.03	5.01 ± 0.13	4.96 ± 0.21	5.13 ± 0.19
TC11000457.hg.1	OR5AK2	3.80 ± 0.20	3.67 ± 0.13	3.64 ± 0.13	3.86 ± 0.23
TC11000458.hg.1	OR5AK4P	4.00 ± 0.05	3.82 ± 0.13	3.92 ± 0.02	4.03 ± 0.06
TC11000485.hg.1	OR5AN1	5.18 ± 0.07	4.94 ± 0.16	5.28 ± 0.12	5.04 ± 0.26
TC11001783.hg.1	OR5AP2	4.13 ± 0.21	4.18 ± 0.09	4.10 ± 0.07	4.40 ± 0.21
TC11000454.hg.1	OR5AR1	5.10 ± 0.17	4.89 ± 0.07	4.94 ± 0.07	5.00 ± 0.10
TC11000443.hg.1	OR5AS1	5.02 ± 0.15	4.82 ± 0.10	4.88 ± 0.11	5.02 ± 0.30
TC14000913.hg.1	OR5AU1	5.86 ± 0.13	5.71 ± 0.08	5.82 ± 0.13	5.82 ± 0.14
TC11001809.hg.1	OR5B12	5.27 ± 0.08	5.37 ± 0.16	5.24 ± 0.21	5.59 ± 0.37
TC11001806.hg.1	OR5B17	5.37 ± 0.17	5.17 ± 0.12	5.30 ± 0.10	5.45 ± 0.43
TC11001808.hg.1	OR5B2	5.30 ± 0.30	5.27 ± 0.15	5.22 ± 0.15	5.64 ± 0.55
TC11001811.hg.1	OR5B21	6.50 ± 0.11	6.37 ± 0.09	6.49 ± 0.14	6.57 ± 0.25
TC11001807.hg.1	OR5B3	5.57 ± 0.16	5.46 ± 0.09	5.58 ± 0.10	5.75 ± 0.41
TC09000625.hg.1	OR5C1	7.74 ± 0.31	7.79 ± 0.26	7.67 ± 0.18	7.97 ± 0.12
TC11000436.hg.1	OR5D13	5.39 ± 0.17	5.22 ± 0.07	5.36 ± 0.14	5.35 ± 0.17
TC11000437.hg.1	OR5D14	5.51 ± 0.20	5.35 ± 0.16	5.45 ± 0.17	5.57 ± 0.25
TC11000441.hg.1	OR5D16	6.81 ± 0.17	6.69 ± 0.24	6.70 ± 0.10	6.94 ± 0.34
TC11000439.hg.1	OR5D18	5.16 ± 0.39	5.32 ± 0.16	5.29 ± 0.10	5.58 ± 0.42
TC11000153.hg.1	OR5E1P	5.97 ± 0.20	5.84 ± 0.18	5.83 ± 0.14	6.12 ± 0.45
TC11001771.hg.1	OR5F1	5.95 ± 0.20	5.84 ± 0.12	5.94 ± 0.07	6.04 ± 0.21
TC11001785.hg.1	OR5G5P	4.98 ± 0.09	4.80 ± 0.07	4.91 ± 0.10	4.88 ± 0.09
TC03000483.hg.1	OR5H1	4.13 ± 0.33	4.19 ± 0.03	3.93 ± 0.17	4.34 ± 0.34
TC03000484.hg.1	OR5H14	4.29 ± 0.16	3.98 ± 0.10	4.11 ± 0.04	3.97 ± 0.17
TC03000485.hg.1	OR5H15	4.39 ± 0.10	4.26 ± 0.17	4.29 ± 0.13	4.19 ± 0.10
TC03000488.hg.1	OR5H2	4.64 ± 0.11	4.37 ± 0.17	4.30 ± 0.15	4.49 ± 0.11
TC03000487.hg.1	OR5H6	4.09 ± 0.20	4.04 ± 0.22	4.15 ± 0.18	3.98 ± 0.10
TC11001768.hg.1	OR5I1	5.61 ± 0.32	5.55 ± 0.09	5.62 ± 0.23	5.88 ± 0.64

TC11000447.hg.1	OR5J2	4.48 ± 0.15	4.58 ± 0.14	4.47 ± 0.13	4.79 ± 0.23
TC03000491.hg.1	OR5K1	3.77 ± 0.22	3.45 ± 0.05	3.54 ± 0.07	3.55 ± 0.03
TC03000492.hg.1	OR5K2	3.98 ± 0.18	3.63 ± 0.01	3.78 ± 0.07	3.63 ± 0.08
TC03000490.hg.1	OR5K3	4.91 ± 0.46	4.72 ± 0.15	4.85 ± 0.17	4.96 ± 0.32
TC03000489.hg.1	OR5K4	4.27 ± 0.08	4.13 ± 0.03	4.03 ± 0.23	4.15 ± 0.12
TC11000438.hg.1	OR5L1	4.55 ± 0.27	4.23 ± 0.08	4.26 ± 0.09	4.33 ± 0.12
TC11000440.hg.1	OR5L2	5.33 ± 0.33	5.46 ± 0.25	5.39 ± 0.23	5.68 ± 0.33
TC11001782.hg.1	OR5M1	5.22 ± 0.34	5.22 ± 0.09	5.15 ± 0.16	5.44 ± 0.36
TC11001781.hg.1	OR5M10	5.45 ± 0.23	5.15 ± 0.04	5.29 ± 0.13	5.32 ± 0.08
TC11001780.hg.1	OR5M11	4.84 ± 0.07	4.89 ± 0.18	4.73 ± 0.11	5.03 ± 0.12
TC11001778.hg.1	OR5M3	5.09 ± 0.19	5.00 ± 0.05	4.93 ± 0.13	5.15 ± 0.32
TC11001779.hg.1	OR5M8	4.46 ± 0.07	4.63 ± 0.13	4.56 ± 0.10	4.71 ± 0.16
TC11001777.hg.1	OR5M9	6.59 ± 0.27	6.27 ± 0.04	6.23 ± 0.15	6.37 ± 0.16
TC11001375.hg.1	OR5P2	4.08 ± 0.20	4.03 ± 0.04	4.13 ± 0.10	4.05 ± 0.18
TC11001376.hg.1	OR5P3	5.37 ± 0.25	5.34 ± 0.14	5.27 ± 0.13	5.60 ± 0.25
TC11001776.hg.1	OR5R1	5.75 ± 0.08	5.67 ± 0.22	5.82 ± 0.13	6.17 ± 0.46
TC11000449.hg.1	OR5T1	4.46 ± 0.09	4.34 ± 0.07	4.43 ± 0.07	4.40 ± 0.09
TC11001774.hg.1	OR5T2	5.24 ± 0.07	5.08 ± 0.04	5.22 ± 0.11	5.23 ± 0.03
TC11000448.hg.1	OR5T3	3.69 ± 0.10	3.70 ± 0.07	3.59 ± 0.04	3.75 ± 0.10
TC06004147.hg.1	OR5V1	5.17 ± 0.09	5.05 ± 0.18	5.23 ± 0.04	5.35 ± 0.02
TC6_cox_hap2000247.hg.1	OR5V1	5.77 ± 0.17	5.66 ± 0.25	5.81 ± 0.05	5.87 ± 0.26
TC6_dbb_hap3000108.hg.1	OR5V1	6.21 ± 0.22	6.17 ± 0.27	6.26 ± 0.24	6.30 ± 0.30
TC6_mann_hap4000211.hg.1	OR5V1	5.77 ± 0.17	5.66 ± 0.25	5.81 ± 0.05	5.87 ± 0.26
TC6_mcf_hap5000228.hg.1	OR5V1	5.68 ± 0.24	5.46 ± 0.24	5.80 ± 0.04	5.99 ± 0.35
TC6_ssto_hap7000214.hg.1	OR5V1	5.76 ± 0.09	5.62 ± 0.22	5.74 ± 0.04	5.78 ± 0.19
TC11001767.hg.1	OR5W2	4.67 ± 0.29	4.78 ± 0.11	4.74 ± 0.07	5.14 ± 0.24
TC11001367.hg.1	OR6A2	4.34 ± 0.20	4.30 ± 0.17	4.20 ± 0.03	4.40 ± 0.21
TC07000946.hg.1	OR6B1	5.44 ± 0.07	5.22 ± 0.08	5.44 ± 0.13	5.39 ± 0.28
TC02002930.hg.1	OR6B2	5.56 ± 0.14	5.57 ± 0.10	5.32 ± 0.10	5.75 ± 0.23
TC02002932.hg.1	OR6B3	7.31 ± 0.26	7.20 ± 0.25	7.17 ± 0.19	7.43 ± 0.31
TC12000477.hg.1	OR6C1	5.89 ± 0.17	5.65 ± 0.07	5.97 ± 0.24	5.79 ± 0.14

TC12000482.hg.1	OR6C2	5.18 ± 0.03	5.18 ± 0.15	5.04 ± 0.04	5.17 ± 0.17
TC12000478.hg.1	OR6C3	4.81 ± 0.11	4.60 ± 0.21	4.75 ± 0.17	4.71 ± 0.21
TC12000484.hg.1	OR6C4	3.95 ± 0.12	3.89 ± 0.04	4.00 ± 0.28	3.91 ± 0.05
TC12001579.hg.1	OR6C6	5.32 ± 0.23	5.16 ± 0.07	5.10 ± 0.13	5.10 ± 0.04
TC12000480.hg.1	OR6C65	4.27 ± 0.07	4.19 ± 0.10	4.15 ± 0.03	4.23 ± 0.23
TC12000483.hg.1	OR6C68	4.69 ± 0.13	4.65 ± 0.12	4.71 ± 0.19	4.78 ± 0.12
TC12001581.hg.1	OR6C70	3.72 ± 0.13	3.50 ± 0.07	3.61 ± 0.07	3.78 ± 0.15
TC12000476.hg.1	OR6C74	4.14 ± 0.08	4.08 ± 0.01	4.20 ± 0.21	4.33 ± 0.28
TC12000479.hg.1	OR6C75	5.06 ± 0.33	4.99 ± 0.16	5.24 ± 0.24	5.32 ± 0.30
TC12000481.hg.1	OR6C76	4.24 ± 0.24	4.10 ± 0.04	4.09 ± 0.06	4.19 ± 0.12
TC01006394.hg.1	OR6F1	6.70 ± 0.12	6.65 ± 0.08	6.57 ± 0.24	6.74 ± 0.23
TC14000933.hg.1	OR6J1	6.69 ± 0.15	6.55 ± 0.10	6.56 ± 0.16	6.59 ± 0.16
TC01003379.hg.1	OR6K2	4.53 ± 0.25	4.36 ± 0.10	4.70 ± 0.33	4.58 ± 0.36
TC01003380.hg.1	OR6K3	4.43 ± 0.30	4.27 ± 0.20	4.13 ± 0.05	4.42 ± 0.17
TC01001345.hg.1	OR6K6	4.48 ± 0.10	4.32 ± 0.06	4.38 ± 0.03	4.46 ± 0.14
TC11002399.hg.1	OR6M1	5.83 ± 0.06	5.74 ± 0.13	5.62 ± 0.13	5.80 ± 0.17
TC01003381.hg.1	OR6N1	7.21 ± 0.39	7.02 ± 0.29	6.88 ± 0.32	7.14 ± 0.16
TC01003382.hg.1	OR6N2	4.92 ± 0.22	4.98 ± 0.03	4.83 ± 0.12	5.12 ± 0.40
TC01003376.hg.1	OR6P1	5.35 ± 0.18	5.28 ± 0.08	5.30 ± 0.15	5.52 ± 0.23
TC11000474.hg.1	OR6Q1	6.47 ± 0.20	6.48 ± 0.17	6.55 ± 0.05	6.66 ± 0.14
TC14000895.hg.1	OR6S1	5.63 ± 0.10	5.74 ± 0.26	5.49 ± 0.11	5.96 ± 0.35
TC11002401.hg.1	OR6T1	5.65 ± 0.13	5.43 ± 0.11	5.47 ± 0.10	5.59 ± 0.16
TC07000926.hg.1	OR6V1	7.25 ± 0.28	7.10 ± 0.13	7.06 ± 0.17	7.22 ± 0.19
TC07001967.hg.1	OR6W1P	5.83 ± 0.22	5.73 ± 0.11	5.73 ± 0.21	5.87 ± 0.26
TC11002398.hg.1	OR6X1	5.04 ± 0.08	4.88 ± 0.12	4.97 ± 0.13	5.14 ± 0.27
TC01003375.hg.1	OR6Y1	5.91 ± 0.24	5.83 ± 0.19	5.96 ± 0.10	6.13 ± 0.32
TC19001245.hg.1	OR7A10	5.00 ± 0.26	4.79 ± 0.10	4.89 ± 0.22	4.94 ± 0.21
TC19001247.hg.1	OR7A17	5.99 ± 0.07	5.97 ± 0.10	6.09 ± 0.05	6.24 ± 0.07
TC19001244.hg.1	OR7A5	5.36 ± 0.15	5.45 ± 0.19	5.39 ± 0.07	5.48 ± 0.17
TC19001243.hg.1	OR7C1	5.90 ± 0.18	6.01 ± 0.08	6.13 ± 0.07	6.00 ± 0.18
TC19000264.hg.1	OR7C2	6.78 ± 0.20	6.45 ± 0.07	6.44 ± 0.10	6.55 ± 0.12

TC19000157.hg.1	OR7D1P	5.61 ± 0.25	5.67 ± 0.03	5.76 ± 0.31	5.87 ± 0.10
TC19000156.hg.1	OR7D2	6.53 ± 0.33	6.51 ± 0.06	6.47 ± 0.19	6.44 ± 0.29
TC19001144.hg.1	OR7D4	6.73 ± 0.29	6.57 ± 0.21	6.32 ± 0.13	6.64 ± 0.06
TC11001295.hg.1	OR7E12P	6.24 ± 0.29	6.29 ± 0.12	6.40 ± 0.27	6.57 ± 0.37
TC11000225.hg.1	OR7E14P	7.23 ± 0.15	7.30 ± 0.23	7.14 ± 0.15	7.53 ± 0.14
TC11000226.hg.1	OR7E14P	6.49 ± 0.17	6.50 ± 0.25	6.43 ± 0.13	6.78 ± 0.37
TC13000250.hg.1	OR7E156P	7.45 ± 0.10	7.53 ± 0.11	7.41 ± 0.05	7.64 ± 0.13
TC19000158.hg.1	OR7E24	5.89 ± 0.35	5.89 ± 0.14	5.93 ± 0.11	6.07 ± 0.20
TC11002168.hg.1	OR7E2P	7.34 ± 0.52	7.35 ± 0.17	7.51 ± 0.29	7.85 ± 0.33
TC13000146.hg.1	OR7E37P	6.46 ± 0.19	6.34 ± 0.09	6.17 ± 0.22	6.52 ± 0.43
TC11001770.hg.1	OR7E5P	10.00 ± 0.44	10.05 ± 0.10	9.87 ± 0.23	10.20 ± 0.17
TC11000739.hg.1	OR7E87P	7.02 ± 0.45	7.01 ± 0.05	6.97 ± 0.08	7.18 ± 0.15
TC02000426.hg.1	OR7E91P	6.92 ± 0.46	7.07 ± 0.02	6.81 ± 0.27	7.42 ± 0.46
TC19001142.hg.1	OR7G1	9.05 ± 0.39	8.99 ± 0.10	8.89 ± 0.05	9.03 ± 0.09
TC19001141.hg.1	OR7G2	6.33 ± 0.39	6.59 ± 0.14	6.44 ± 0.14	6.59 ± 0.30
TC19001143.hg.1	OR7G3	5.58 ± 0.35	5.49 ± 0.08	5.35 ± 0.17	5.53 ± 0.05
TC11001138.hg.1	OR8A1	5.15 ± 0.25	4.96 ± 0.20	4.80 ± 0.14	5.23 ± 0.16
TC11002409.hg.1	OR8B12	6.62 ± 0.14	6.33 ± 0.06	6.54 ± 0.15	6.43 ± 0.09
TC11002406.hg.1	OR8B3	5.15 ± 0.12	4.96 ± 0.10	4.96 ± 0.08	5.00 ± 0.07
TC11002407.hg.1	OR8B4	5.07 ± 0.29	4.84 ± 0.23	4.69 ± 0.15	5.02 ± 0.26
TC11002408.hg.1	OR8B8	5.39 ± 0.08	5.34 ± 0.07	5.30 ± 0.04	5.64 ± 0.25
TC11002404.hg.1	OR8D1	4.01 ± 0.14	3.98 ± 0.30	3.92 ± 0.08	4.23 ± 0.29
TC11002405.hg.1	OR8D2	4.24 ± 0.16	4.16 ± 0.02	4.16 ± 0.13	4.28 ± 0.06
TC11001127.hg.1	OR8D4	3.53 ± 0.14	3.55 ± 0.11	3.44 ± 0.07	3.46 ± 0.13
TC11001136.hg.1	OR8G1	5.07 ± 0.36	5.01 ± 0.14	4.85 ± 0.16	5.13 ± 0.27
TC11001135.hg.1	OR8G2	5.09 ± 0.31	5.01 ± 0.26	4.75 ± 0.04	5.16 ± 0.26
TC11001137.hg.1	OR8G5	4.90 ± 0.06	4.69 ± 0.04	4.80 ± 0.08	4.74 ± 0.02
TC11001775.hg.1	OR8H1	4.02 ± 0.13	3.75 ± 0.08	3.84 ± 0.07	3.96 ± 0.25
TC11000445.hg.1	OR8H2	4.78 ± 0.27	4.52 ± 0.17	4.65 ± 0.22	4.76 ± 0.24
TC11000446.hg.1	OR8H3	4.71 ± 0.14	4.46 ± 0.12	4.48 ± 0.07	4.71 ± 0.20
TC11000444.hg.1	OR8I2	5.10 ± 0.10	5.06 ± 0.17	4.85 ± 0.07	4.88 ± 0.16

TC11000452.hg.1	OR8J1	4.38 ± 0.21	4.24 ± 0.01	4.18 ± 0.06	4.22 ± 0.19
TC11001772.hg.1	OR8J3	4.20 ± 0.05	4.12 ± 0.05	4.14 ± 0.07	4.20 ± 0.08
TC11000451.hg.1	OR8K1	3.59 ± 0.10	3.42 ± 0.02	3.45 ± 0.02	3.47 ± 0.12
TC11000450.hg.1	OR8K3	4.60 ± 0.08	4.34 ± 0.07	4.45 ± 0.11	4.55 ± 0.24
TC11001773.hg.1	OR8K5	4.97 ± 0.22	4.99 ± 0.08	4.89 ± 0.07	5.25 ± 0.19
TC12000352.hg.1	OR8S1	6.63 ± 0.16	6.63 ± 0.16	6.74 ± 0.08	6.71 ± 0.06
TC11000453.hg.1	OR8U1	3.98 ± 0.16	3.88 ± 0.02	3.91 ± 0.02	3.90 ± 0.04
TC07000898.hg.1	OR9A1P	6.07 ± 0.48	6.26 ± 0.06	6.25 ± 0.24	6.52 ± 0.27
TC07001966.hg.1	OR9A2	5.55 ± 0.05	5.50 ± 0.10	5.58 ± 0.07	5.63 ± 0.13
TC07000897.hg.1	OR9A3P	5.93 ± 0.16	5.91 ± 0.02	5.96 ± 0.14	6.08 ± 0.29
TC07000901.hg.1	OR9A4	4.29 ± 0.27	4.17 ± 0.04	4.27 ± 0.16	4.37 ± 0.29
TC11000455.hg.1	OR9G1	5.97 ± 0.35	6.17 ± 0.13	6.10 ± 0.19	6.64 ± 0.58
TC11001784.hg.1	OR9G4	5.42 ± 0.25	5.42 ± 0.15	5.50 ± 0.19	5.58 ± 0.17
TC11001802.hg.1	OR9I1	6.10 ± 0.25	6.12 ± 0.24	6.20 ± 0.20	6.39 ± 0.45
TC12000474.hg.1	OR9K2	3.41 ± 0.08	3.28 ± 0.04	3.37 ± 0.05	3.30 ± 0.11
TC11000473.hg.1	OR9Q1	6.22 ± 0.21	6.13 ± 0.07	6.17 ± 0.14	6.27 ± 0.08
TC11000475.hg.1	OR9Q2	7.08 ± 0.40	6.95 ± 0.02	6.83 ± 0.10	7.17 ± 0.26
TC12000961.hg.1	ORAI1	9.32 ± 0.24	9.21 ± 0.11	9.12 ± 0.15	9.11 ± 0.04
TC07000659.hg.1	ORAI2	8.95 ± 0.22	8.81 ± 0.04	8.92 ± 0.14	8.86 ± 0.10
TC16000363.hg.1	ORAI3	8.55 ± 0.32	8.22 ± 0.10	8.52 ± 0.06	8.19 ± 0.13
TC11002017.hg.1	ORAOV1	7.30 ± 0.11	7.28 ± 0.04	7.26 ± 0.06	7.37 ± 0.08
TC01002658.hg.1	ORC1	6.94 ± 0.25	6.85 ± 0.26	6.74 ± 0.21	6.68 ± 0.03
TC02002669.hg.1	ORC2	7.52 ± 0.24	7.47 ± 0.24	7.48 ± 0.28	7.20 ± 0.16
TC06000789.hg.1	ORC3	7.50 ± 0.28	7.65 ± 0.22	7.40 ± 0.38	7.39 ± 0.09
TC02002408.hg.1	ORC4	7.13 ± 0.39	7.31 ± 0.26	7.39 ± 0.40	7.13 ± 0.07
TC07001724.hg.1	ORC5	7.03 ± 0.15	6.86 ± 0.26	6.74 ± 0.13	6.72 ± 0.06
TC16000425.hg.1	ORC6	6.45 ± 0.06	6.45 ± 0.10	6.36 ± 0.10	6.41 ± 0.05
TC09002907.hg.1	ORM1	7.84 ± 0.26	7.50 ± 0.13	7.96 ± 0.45	7.52 ± 0.20
TC09002906.hg.1	ORM2	7.70 ± 0.19	7.37 ± 0.15	7.80 ± 0.31	7.40 ± 0.12
TC02002616.hg.1	ORMDL1	8.75 ± 0.56	9.02 ± 0.24	8.79 ± 0.41	8.93 ± 0.02
TC12000491.hg.1	ORMDL2	8.19 ± 0.20	7.76 ± 0.14	8.11 ± 0.29	7.58 ± 0.16

TC17002852.hg.1	ORMDL3	8.77 ± 0.13	8.62 ± 0.07	8.45 ± 0.14	8.52 ± 0.08
TC12000539.hg.1	OS9	10.68 ± 0.30	10.47 ± 0.07	10.76 ± 0.25	10.36 ± 0.16
TC11001827.hg.1	OSBP	9.04 ± 0.14	9.14 ± 0.13	8.86 ± 0.14	9.07 ± 0.08
TC22000217.hg.1	OSBP2	7.82 ± 0.09	7.63 ± 0.14	7.88 ± 0.18	7.72 ± 0.05
TC03001269.hg.1	OSBPL10	7.45 ± 0.07	7.52 ± 0.04	7.15 ± 0.06	7.38 ± 0.17
TC03000153.hg.1	OSBPL10-AS1	5.73 ± 0.12	5.70 ± 0.08	5.68 ± 0.11	5.81 ± 0.09
TC03001725.hg.1	OSBPL11	9.17 ± 0.25	9.08 ± 0.29	9.54 ± 0.37	8.81 ± 0.39
TC18000418.hg.1	OSBPL1A	6.90 ± 0.04	6.83 ± 0.14	7.03 ± 0.22	6.88 ± 0.21
TC20000486.hg.1	OSBPL2	9.05 ± 0.18	8.93 ± 0.11	9.23 ± 0.24	8.95 ± 0.14
TC07001206.hg.1	OSBPL3	7.56 ± 0.29	7.53 ± 0.11	7.29 ± 0.21	7.35 ± 0.08
TC11001291.hg.1	OSBPL5	8.54 ± 0.11	8.43 ± 0.12	8.42 ± 0.15	8.49 ± 0.05
TC02001071.hg.1	OSBPL6	5.54 ± 0.09	5.50 ± 0.05	5.50 ± 0.03	5.55 ± 0.07
TC17001626.hg.1	OSBPL7	8.35 ± 0.08	8.32 ± 0.08	8.14 ± 0.13	8.29 ± 0.06
TC12001754.hg.1	OSBPL8	9.94 ± 0.71	10.64 ± 0.21	10.77 ± 0.50	10.62 ± 0.17
TC01000631.hg.1	OSBPL9	8.92 ± 0.22	9.01 ± 0.15	9.13 ± 0.37	8.99 ± 0.17
TC19001831.hg.1	OSCAR	8.47 ± 0.36	8.08 ± 0.12	8.49 ± 0.18	8.11 ± 0.02
TC01002509.hg.1	OSCP1	6.40 ± 0.19	6.34 ± 0.12	6.31 ± 0.10	6.37 ± 0.05
TC20000861.hg.1	OSER1	9.00 ± 0.13	9.24 ± 0.17	9.07 ± 0.23	9.16 ± 0.09
TC20000326.hg.1	OSER1-AS1	7.27 ± 0.13	7.35 ± 0.15	7.16 ± 0.09	7.33 ± 0.07
TC14000886.hg.1	OSGEP	7.98 ± 0.09	7.87 ± 0.02	7.83 ± 0.08	7.86 ± 0.03
TC02002615.hg.1	OSGEPL1	6.46 ± 0.12	6.34 ± 0.16	6.29 ± 0.18	6.21 ± 0.03
TC02001113.hg.1	OSGEPL1-AS1	6.27 ± 0.20	6.32 ± 0.01	6.16 ± 0.07	6.38 ± 0.11
TC16000652.hg.1	OSGIN1	7.97 ± 0.18	7.89 ± 0.22	7.98 ± 0.19	8.05 ± 0.07
TC08000546.hg.1	OSGIN2	7.92 ± 0.37	8.01 ± 0.27	8.30 ± 0.53	8.05 ± 0.21
TC22000648.hg.1	OSM	8.34 ± 0.36	8.78 ± 0.18	8.65 ± 0.09	8.62 ± 0.25
TC05000179.hg.1	OSMR	5.60 ± 0.15	5.49 ± 0.06	5.54 ± 0.02	5.57 ± 0.03
TC02001611.hg.1	OSR1	7.47 ± 0.24	7.37 ± 0.14	7.34 ± 0.17	7.45 ± 0.06
TC08000606.hg.1	OSR2	7.01 ± 0.22	6.96 ± 0.19	6.86 ± 0.21	7.01 ± 0.10
TC02001673.hg.1	OST4	11.22 ± 0.08	11.08 ± 0.02	11.06 ± 0.15	10.99 ± 0.07
TC04000559.hg.1	OSTC	8.47 ± 0.27	8.28 ± 0.16	8.12 ± 0.10	8.12 ± 0.08
TC06002267.hg.1	OSTCP1	8.20 ± 0.29	8.32 ± 0.06	8.18 ± 0.17	8.42 ± 0.12

TC09000343.hg.1	OSTF1	11.27 ± 0.22	11.36 ± 0.13	11.58 ± 0.20	11.24 ± 0.29
TC06001993.hg.1	OSTM1	7.83 ± 0.22	7.76 ± 0.23	8.12 ± 0.37	7.54 ± 0.35
TC06000859.hg.1	OSTM1-AS1	7.08 ± 0.15	7.14 ± 0.16	7.03 ± 0.13	7.30 ± 0.12
TC03001051.hg.1	OSTN	4.99 ± 0.09	4.79 ± 0.07	4.84 ± 0.17	4.90 ± 0.03
TC03002112.hg.1	OSTN-AS1	6.94 ± 0.28	6.91 ± 0.06	6.99 ± 0.03	7.02 ± 0.08
TC0X000177.hg.1	OTC	4.99 ± 0.19	4.91 ± 0.09	4.89 ± 0.11	4.92 ± 0.02
TC16000238.hg.1	OTOA	6.67 ± 0.19	6.63 ± 0.10	6.62 ± 0.08	6.71 ± 0.05
TC02001667.hg.1	OTOF	8.06 ± 0.37	7.74 ± 0.31	7.80 ± 0.16	7.67 ± 0.03
TC11000230.hg.1	OTOG	7.66 ± 0.18	7.68 ± 0.19	7.65 ± 0.15	7.82 ± 0.09
TC12000667.hg.1	OTOGL	4.48 ± 0.07	4.38 ± 0.03	4.43 ± 0.08	4.44 ± 0.07
TC03000883.hg.1	OTOL1	6.65 ± 0.45	6.54 ± 0.28	6.56 ± 0.26	6.69 ± 0.07
TC04000987.hg.1	OTOP1	5.58 ± 0.20	5.67 ± 0.14	5.55 ± 0.10	5.69 ± 0.08
TC17000836.hg.1	OTOP2	7.14 ± 0.17	7.19 ± 0.19	7.09 ± 0.23	7.21 ± 0.04
TC17000837.hg.1	OTOP3	7.36 ± 0.17	7.29 ± 0.19	7.24 ± 0.23	7.39 ± 0.09
TC20000105.hg.1	OTOR	5.89 ± 0.16	5.80 ± 0.14	5.81 ± 0.12	5.86 ± 0.07
TC02002934.hg.1	OTOS	8.02 ± 0.28	8.05 ± 0.17	7.96 ± 0.23	8.16 ± 0.04
TC05001509.hg.1	OTP	7.90 ± 0.27	8.02 ± 0.22	7.87 ± 0.20	8.10 ± 0.05
TC09000141.hg.1	OTTHUMG00000000392	8.91 ± 0.36	8.82 ± 0.10	8.79 ± 0.18	8.87 ± 0.15
TC09001015.hg.1	OTTHUMG00000000399	6.71 ± 0.25	6.77 ± 0.15	6.66 ± 0.22	6.96 ± 0.06
TC09000147.hg.1	OTTHUMG00000000405	6.83 ± 0.16	6.70 ± 0.13	6.69 ± 0.07	6.85 ± 0.11
TC09000154.hg.1	OTTHUMG00000000412	7.04 ± 0.11	7.16 ± 0.29	6.98 ± 0.35	7.46 ± 0.13
TC09001022.hg.1	OTTHUMG00000000413	6.17 ± 0.14	6.23 ± 0.17	6.19 ± 0.23	6.34 ± 0.13
TC09001023.hg.1	OTTHUMG00000000421	7.19 ± 0.12	7.11 ± 0.25	7.10 ± 0.12	7.12 ± 0.06
TC09001024.hg.1	OTTHUMG00000000422	8.66 ± 0.30	8.70 ± 0.08	8.63 ± 0.03	8.79 ± 0.07
TC09000162.hg.1	OTTHUMG00000000424	5.63 ± 0.27	5.37 ± 0.11	5.59 ± 0.23	5.40 ± 0.06
TC09000173.hg.1	OTTHUMG00000000438	8.02 ± 0.15	8.15 ± 0.10	7.96 ± 0.17	8.32 ± 0.12
TC09002915.hg.1	OTTHUMG00000000449	8.10 ± 0.26	8.18 ± 0.33	8.09 ± 0.21	8.27 ± 0.08
TC09000176.hg.1	OTTHUMG00000000451	6.61 ± 0.14	6.71 ± 0.03	6.78 ± 0.10	6.89 ± 0.16
TC01000485.hg.1	OTTHUMG00000000478	8.49 ± 0.15	8.49 ± 0.11	8.42 ± 0.15	8.58 ± 0.06
TC01000488.hg.1	OTTHUMG00000000481	6.84 ± 0.02	6.85 ± 0.04	6.73 ± 0.10	6.81 ± 0.13
TC01000489.hg.1	OTTHUMG00000000491	7.07 ± 0.12	7.18 ± 0.07	7.01 ± 0.08	7.23 ± 0.11

TC01000924.hg.1	OTTHUMG00000000497	5.51 ± 0.32	5.27 ± 0.13	5.40 ± 0.11	5.38 ± 0.07
TC01002809.hg.1	OTTHUMG00000000506	6.17 ± 0.51	6.25 ± 0.17	6.14 ± 0.19	6.44 ± 0.17
TC01000798.hg.1	OTTHUMG00000000507	5.26 ± 0.13	5.09 ± 0.12	5.04 ± 0.06	5.09 ± 0.14
TC01002125.hg.1	OTTHUMG00000000564	6.11 ± 0.04	5.91 ± 0.19	6.02 ± 0.14	5.99 ± 0.10
TC01002126.hg.1	OTTHUMG00000000565	7.84 ± 0.25	7.62 ± 0.12	7.74 ± 0.27	7.79 ± 0.06
TC01002131.hg.1	OTTHUMG00000000613	7.80 ± 0.44	7.73 ± 0.25	7.59 ± 0.34	7.99 ± 0.11
TC01002128.hg.1	OTTHUMG00000000624	6.50 ± 0.31	6.38 ± 0.23	6.42 ± 0.31	6.64 ± 0.19
TC01000078.hg.1	OTTHUMG00000000646	6.46 ± 0.05	6.49 ± 0.21	6.52 ± 0.11	6.77 ± 0.14
TC01000079.hg.1	OTTHUMG00000000647	5.13 ± 0.16	5.02 ± 0.10	5.06 ± 0.09	5.10 ± 0.09
TC01002118.hg.1	OTTHUMG00000000720	8.92 ± 0.27	8.75 ± 0.19	8.67 ± 0.22	8.82 ± 0.09
TC01002119.hg.1	OTTHUMG00000000721	7.67 ± 0.01	7.61 ± 0.12	7.63 ± 0.18	7.66 ± 0.15
TC01002139.hg.1	OTTHUMG00000000731	6.68 ± 0.23	6.78 ± 0.27	6.68 ± 0.19	6.80 ± 0.13
TC01002152.hg.1	OTTHUMG00000000769	8.01 ± 0.15	8.04 ± 0.13	7.88 ± 0.09	8.19 ± 0.16
TC01000049.hg.1	OTTHUMG00000000825	7.56 ± 0.34	7.39 ± 0.14	7.36 ± 0.19	7.34 ± 0.06
TC01000050.hg.1	OTTHUMG00000000825	7.91 ± 0.07	7.78 ± 0.10	7.83 ± 0.05	7.95 ± 0.07
TC01000051.hg.1	OTTHUMG00000000941	8.75 ± 0.20	8.83 ± 0.23	8.65 ± 0.28	8.90 ± 0.11
TC01002052.hg.1	OTTHUMG00000001096	7.90 ± 0.26	7.65 ± 0.23	8.59 ± 0.34	7.75 ± 0.05
TC01002161.hg.1	OTTHUMG00000001211	7.00 ± 0.19	7.04 ± 0.15	7.07 ± 0.17	7.21 ± 0.08
TC01002157.hg.1	OTTHUMG00000001213	6.13 ± 0.09	6.03 ± 0.09	5.97 ± 0.12	6.01 ± 0.06
TC01002158.hg.1	OTTHUMG00000001215	6.53 ± 0.36	6.43 ± 0.25	6.46 ± 0.03	6.67 ± 0.18
TC01002159.hg.1	OTTHUMG00000001219	7.19 ± 0.05	7.19 ± 0.07	7.21 ± 0.10	7.22 ± 0.05
TC01002160.hg.1	OTTHUMG00000001220	4.23 ± 0.20	4.03 ± 0.01	4.08 ± 0.12	4.02 ± 0.05
TC01002112.hg.1	OTTHUMG00000001239	7.81 ± 0.07	7.86 ± 0.19	7.69 ± 0.07	7.93 ± 0.11
TC01002183.hg.1	OTTHUMG00000001278	7.19 ± 0.11	7.23 ± 0.13	7.03 ± 0.06	7.11 ± 0.02
TC01002185.hg.1	OTTHUMG00000001449	7.52 ± 0.24	7.31 ± 0.07	7.35 ± 0.20	7.41 ± 0.04
TC01000112.hg.1	OTTHUMG00000001764	8.26 ± 0.23	8.05 ± 0.16	8.17 ± 0.26	8.03 ± 0.09
TC01000102.hg.1	OTTHUMG00000001766	7.07 ± 0.20	6.94 ± 0.07	6.85 ± 0.23	7.01 ± 0.10
TC01000103.hg.1	OTTHUMG00000001769	6.02 ± 0.20	6.06 ± 0.28	6.08 ± 0.15	6.26 ± 0.19
TC01002169.hg.1	OTTHUMG00000001770	7.87 ± 0.24	7.99 ± 0.18	7.99 ± 0.19	8.01 ± 0.10
TC01000106.hg.1	OTTHUMG00000001772	6.58 ± 0.14	6.31 ± 0.04	6.65 ± 0.12	6.35 ± 0.04
TC01000107.hg.1	OTTHUMG00000001777	6.80 ± 0.29	6.91 ± 0.06	6.78 ± 0.10	6.97 ± 0.07

TC01000121.hg.1	OTTHUMG00000001805	7.06 ± 0.28	6.69 ± 0.09	6.92 ± 0.07	6.61 ± 0.20
TC01000131.hg.1	OTTHUMG00000001907	9.91 ± 0.14	9.90 ± 0.10	9.94 ± 0.06	9.96 ± 0.08
TC01002239.hg.1	OTTHUMG00000002044	6.44 ± 0.14	6.42 ± 0.14	6.40 ± 0.13	6.48 ± 0.15
TC01002238.hg.1	OTTHUMG00000002047	5.25 ± 0.16	5.03 ± 0.19	5.18 ± 0.05	5.12 ± 0.07
TC01000143.hg.1	OTTHUMG00000002076	7.48 ± 0.12	7.48 ± 0.08	7.49 ± 0.16	7.58 ± 0.03
TC01002242.hg.1	OTTHUMG00000002089	5.66 ± 0.29	5.41 ± 0.12	5.58 ± 0.15	5.67 ± 0.09
TC01000136.hg.1	OTTHUMG00000002122	6.55 ± 0.20	6.66 ± 0.17	6.58 ± 0.26	6.65 ± 0.04
TC01002279.hg.1	OTTHUMG00000002201	5.74 ± 0.33	5.73 ± 0.07	5.58 ± 0.18	5.88 ± 0.23
TC01000219.hg.1	OTTHUMG00000002202	7.38 ± 0.10	7.30 ± 0.13	7.31 ± 0.21	7.44 ± 0.07
TC01002283.hg.1	OTTHUMG00000002204	7.63 ± 0.27	7.45 ± 0.23	7.49 ± 0.21	7.61 ± 0.13
TC01002260.hg.1	OTTHUMG00000002221	7.16 ± 0.41	6.72 ± 0.13	6.99 ± 0.60	6.76 ± 0.11
TC01002244.hg.1	OTTHUMG00000002257	7.41 ± 0.37	7.19 ± 0.22	7.19 ± 0.38	7.27 ± 0.06
TC01002059.hg.1	OTTHUMG00000002261	8.22 ± 0.10	7.98 ± 0.10	8.59 ± 0.07	8.06 ± 0.09
TC01000227.hg.1	OTTHUMG00000002292	7.52 ± 0.39	7.64 ± 0.27	7.50 ± 0.35	7.70 ± 0.12
TC01000207.hg.1	OTTHUMG00000002314	7.66 ± 0.14	7.74 ± 0.11	7.53 ± 0.15	7.94 ± 0.06
TC01000012.hg.1	OTTHUMG00000002330	7.19 ± 0.17	7.23 ± 0.04	7.15 ± 0.09	7.36 ± 0.19
TC01002060.hg.1	OTTHUMG00000002331	8.05 ± 0.29	7.79 ± 0.24	8.76 ± 0.21	7.87 ± 0.03
TC01002061.hg.1	OTTHUMG00000002332	5.97 ± 0.35	6.05 ± 0.12	6.01 ± 0.21	6.21 ± 0.19
TC01002066.hg.1	OTTHUMG00000002407	12.13 ± 0.46	12.27 ± 0.20	12.76 ± 0.23	12.34 ± 0.12
TC01000014.hg.1	OTTHUMG00000002408	6.43 ± 0.15	6.05 ± 0.20	6.86 ± 0.29	6.10 ± 0.16
TC01002290.hg.1	OTTHUMG00000002434	7.31 ± 0.17	7.23 ± 0.17	7.25 ± 0.29	7.43 ± 0.18
TC01000241.hg.1	OTTHUMG00000002437	6.68 ± 0.25	6.71 ± 0.29	6.72 ± 0.28	6.83 ± 0.15
TC01002073.hg.1	OTTHUMG00000002467	7.22 ± 0.27	7.24 ± 0.26	7.02 ± 0.10	7.30 ± 0.33
TC01000208.hg.1	OTTHUMG00000002490	7.06 ± 0.05	6.99 ± 0.05	6.97 ± 0.10	6.97 ± 0.05
TC01000237.hg.1	OTTHUMG00000002515	7.54 ± 0.26	7.50 ± 0.18	7.39 ± 0.16	7.68 ± 0.15
TC01000004.hg.1	OTTHUMG00000002525	6.37 ± 0.26	5.81 ± 0.27	6.69 ± 0.39	5.91 ± 0.07
TC01002057.hg.1	OTTHUMG00000002553	8.05 ± 0.28	7.79 ± 0.22	8.43 ± 0.25	7.94 ± 0.04
TC01000263.hg.1	OTTHUMG00000002621	7.55 ± 0.14	7.50 ± 0.12	7.61 ± 0.08	7.66 ± 0.06
TC01000266.hg.1	OTTHUMG00000002623	7.51 ± 0.15	7.44 ± 0.05	7.51 ± 0.18	7.51 ± 0.08
TC01000256.hg.1	OTTHUMG00000002703	6.82 ± 0.20	6.82 ± 0.13	6.85 ± 0.10	6.79 ± 0.05
TC01002310.hg.1	OTTHUMG00000002706	6.99 ± 0.15	7.20 ± 0.16	6.85 ± 0.17	7.15 ± 0.05

TC01000258.hg.1	OTTHUMG00000002707	6.79 ± 0.16	6.72 ± 0.23	6.66 ± 0.13	6.75 ± 0.04
TC01000252.hg.1	OTTHUMG00000002710	6.70 ± 0.03	6.56 ± 0.06	6.61 ± 0.01	6.64 ± 0.14
TC01002303.hg.1	OTTHUMG00000002711	7.40 ± 0.13	7.95 ± 0.21	7.47 ± 0.18	8.08 ± 0.09
TC01002329.hg.1	OTTHUMG00000002754	6.62 ± 0.27	6.67 ± 0.12	6.40 ± 0.08	6.67 ± 0.05
TC01002311.hg.1	OTTHUMG00000002836	6.68 ± 0.19	6.60 ± 0.11	6.58 ± 0.09	6.68 ± 0.19
TC01002313.hg.1	OTTHUMG00000002843	5.57 ± 0.37	5.53 ± 0.09	5.48 ± 0.06	5.48 ± 0.14
TC01002058.hg.1	OTTHUMG00000002855	7.70 ± 0.23	7.64 ± 0.04	7.59 ± 0.31	7.86 ± 0.26
TC01002331.hg.1	OTTHUMG00000002880	7.41 ± 0.02	7.50 ± 0.09	7.55 ± 0.12	7.61 ± 0.10
TC01000037.hg.1	OTTHUMG00000002912	8.46 ± 0.33	8.38 ± 0.21	8.46 ± 0.15	8.54 ± 0.06
TC01000269.hg.1	OTTHUMG00000002945	5.70 ± 0.29	5.65 ± 0.05	5.59 ± 0.07	5.74 ± 0.34
TC01000306.hg.1	OTTHUMG00000003034	7.73 ± 0.24	7.63 ± 0.11	7.60 ± 0.15	7.78 ± 0.09
TC01000307.hg.1	OTTHUMG00000003037	6.38 ± 0.19	6.19 ± 0.12	6.19 ± 0.15	6.25 ± 0.10
TC01002362.hg.1	OTTHUMG00000003039	6.88 ± 0.15	6.86 ± 0.13	6.82 ± 0.13	6.95 ± 0.07
TC01002249.hg.1	OTTHUMG00000003064	8.22 ± 0.08	8.21 ± 0.17	8.23 ± 0.19	8.34 ± 0.05
TC01002250.hg.1	OTTHUMG00000003078	8.32 ± 0.30	8.34 ± 0.26	8.21 ± 0.40	8.46 ± 0.02
TC01002335.hg.1	OTTHUMG00000003222	6.27 ± 0.15	6.19 ± 0.07	6.22 ± 0.12	6.04 ± 0.06
TC01000316.hg.1	OTTHUMG00000003322	4.88 ± 0.20	4.99 ± 0.17	4.89 ± 0.26	4.98 ± 0.11
TC01000317.hg.1	OTTHUMG00000003323	5.15 ± 0.04	5.24 ± 0.11	5.13 ± 0.07	5.26 ± 0.16
TC01002371.hg.1	OTTHUMG00000003326	6.52 ± 0.29	6.59 ± 0.17	6.61 ± 0.13	6.65 ± 0.08
TC01000363.hg.1	OTTHUMG00000003394	5.40 ± 0.35	5.08 ± 0.10	5.24 ± 0.15	5.22 ± 0.08
TC01002134.hg.1	OTTHUMG00000003412	8.51 ± 0.21	8.58 ± 0.17	8.37 ± 0.23	8.69 ± 0.09
TC01000364.hg.1	OTTHUMG00000003517	7.15 ± 0.13	7.11 ± 0.10	6.99 ± 0.12	7.20 ± 0.09
TC01002411.hg.1	OTTHUMG00000003519	6.31 ± 0.28	6.27 ± 0.25	6.20 ± 0.24	6.16 ± 0.04
TC01002391.hg.1	OTTHUMG00000003552	6.14 ± 0.14	5.91 ± 0.14	6.13 ± 0.12	5.83 ± 0.10
TC01000397.hg.1	OTTHUMG00000003569	5.53 ± 0.08	5.52 ± 0.09	5.47 ± 0.11	5.56 ± 0.10
TC01002431.hg.1	OTTHUMG00000003643	6.90 ± 0.18	7.09 ± 0.14	6.89 ± 0.15	7.15 ± 0.08
TC01000386.hg.1	OTTHUMG00000003649	6.44 ± 0.32	6.32 ± 0.12	6.19 ± 0.05	6.26 ± 0.14
TC01000387.hg.1	OTTHUMG00000003649	5.68 ± 0.16	5.38 ± 0.08	5.62 ± 0.26	5.40 ± 0.08
TC01000393.hg.1	OTTHUMG00000003650	6.15 ± 0.18	6.01 ± 0.05	5.94 ± 0.30	6.09 ± 0.11
TC01002428.hg.1	OTTHUMG00000003651	6.35 ± 0.11	6.43 ± 0.12	6.45 ± 0.12	6.45 ± 0.11
TC01000396.hg.1	OTTHUMG00000003662	6.49 ± 0.22	6.42 ± 0.02	6.48 ± 0.14	6.45 ± 0.06

TC01002439.hg.1	OTTHUMG00000003682	6.73 ± 0.11	6.65 ± 0.24	6.67 ± 0.09	6.79 ± 0.12
TC01002438.hg.1	OTTHUMG00000003683	6.36 ± 0.08	6.31 ± 0.13	6.35 ± 0.07	6.41 ± 0.10
TC01000400.hg.1	OTTHUMG00000003685	7.19 ± 0.11	7.15 ± 0.17	7.22 ± 0.19	7.19 ± 0.07
TC01002443.hg.1	OTTHUMG00000003704	5.94 ± 0.13	5.95 ± 0.22	6.00 ± 0.25	6.03 ± 0.08
TC01002451.hg.1	OTTHUMG00000003794	5.49 ± 0.05	5.43 ± 0.09	5.50 ± 0.11	5.31 ± 0.05
TC01002454.hg.1	OTTHUMG00000003875	7.48 ± 0.23	7.49 ± 0.10	7.49 ± 0.14	7.61 ± 0.14
TC01000413.hg.1	OTTHUMG00000003881	7.08 ± 0.15	7.09 ± 0.21	7.11 ± 0.19	7.21 ± 0.20
TC01002415.hg.1	OTTHUMG00000003911	8.01 ± 0.06	7.93 ± 0.11	8.04 ± 0.07	8.06 ± 0.06
TC01002460.hg.1	OTTHUMG00000003927	6.08 ± 0.20	5.85 ± 0.05	5.89 ± 0.07	6.04 ± 0.24
TC01002435.hg.1	OTTHUMG00000003931	6.13 ± 0.30	5.96 ± 0.19	6.13 ± 0.16	6.19 ± 0.19
TC01000441.hg.1	OTTHUMG00000004126	6.44 ± 0.12	6.32 ± 0.07	6.44 ± 0.15	6.43 ± 0.04
TC01000442.hg.1	OTTHUMG00000004127	7.06 ± 0.11	7.04 ± 0.16	7.09 ± 0.11	7.12 ± 0.17
TC01000438.hg.1	OTTHUMG00000004129	6.30 ± 0.13	6.32 ± 0.07	6.23 ± 0.09	6.23 ± 0.15
TC01000416.hg.1	OTTHUMG00000004135	6.91 ± 0.22	6.86 ± 0.10	6.79 ± 0.09	6.97 ± 0.09
TC01000471.hg.1	OTTHUMG00000004187	6.77 ± 0.16	6.51 ± 0.17	6.50 ± 0.10	6.66 ± 0.05
TC01002514.hg.1	OTTHUMG00000004188	7.84 ± 0.18	7.61 ± 0.13	7.67 ± 0.08	7.67 ± 0.06
TC01000354.hg.1	OTTHUMG00000004228	5.25 ± 0.12	5.37 ± 0.28	5.46 ± 0.33	5.61 ± 0.23
TC01002402.hg.1	OTTHUMG00000004413	7.16 ± 0.31	7.12 ± 0.06	7.18 ± 0.14	7.18 ± 0.04
TC01002378.hg.1	OTTHUMG00000004418	7.43 ± 0.23	7.45 ± 0.21	7.29 ± 0.19	7.51 ± 0.08
TC01000324.hg.1	OTTHUMG00000004419	6.97 ± 0.22	7.11 ± 0.18	7.01 ± 0.07	7.20 ± 0.10
TC6_qbl_hap6000104.hg.1	OTTHUMG00000004760	6.79 ± 0.27	6.76 ± 0.03	6.68 ± 0.14	6.83 ± 0.10
TC6_qbl_hap6000010.hg.1	OTTHUMG00000004780	6.49 ± 0.12	6.41 ± 0.14	6.39 ± 0.05	6.46 ± 0.07
TC6_qbl_hap6000024.hg.1	OTTHUMG00000004813	6.83 ± 0.29	6.76 ± 0.09	6.81 ± 0.17	6.78 ± 0.07
TC6_qbl_hap6000037.hg.1	OTTHUMG00000004829	7.42 ± 0.10	7.46 ± 0.11	7.43 ± 0.05	7.49 ± 0.07
TC6_qbl_hap6000136.hg.1	OTTHUMG00000004830	6.93 ± 0.20	6.82 ± 0.06	6.83 ± 0.13	6.85 ± 0.02
TC6_qbl_hap6000134.hg.1	OTTHUMG00000004834	7.30 ± 0.10	7.26 ± 0.18	7.33 ± 0.02	7.23 ± 0.07
TC6_qbl_hap6000135.hg.1	OTTHUMG00000004843	6.28 ± 0.32	6.13 ± 0.13	6.15 ± 0.01	6.21 ± 0.11
TC6_qbl_hap6000145.hg.1	OTTHUMG00000004857	8.72 ± 0.42	8.77 ± 0.04	8.59 ± 0.07	9.03 ± 0.15
TC6_qbl_hap6000055.hg.1	OTTHUMG00000004870	7.46 ± 0.18	7.33 ± 0.11	7.24 ± 0.15	7.37 ± 0.04
TC6_qbl_hap6000064.hg.1	OTTHUMG00000004888	7.19 ± 0.19	6.98 ± 0.16	7.04 ± 0.21	7.17 ± 0.09
TC6_qbl_hap6000030.hg.1	OTTHUMG00000004916	7.05 ± 0.19	7.05 ± 0.15	7.09 ± 0.10	6.98 ± 0.13

TC6_qbl_hap6000073.hg.1	OTTHUMG00000004931	8.26 ± 0.32	8.09 ± 0.08	8.17 ± 0.11	8.06 ± 0.04
TC6_qbl_hap6000169.hg.1	OTTHUMG00000004936	6.89 ± 0.34	6.56 ± 0.12	6.62 ± 0.11	6.61 ± 0.16
TC6_qbl_hap6000006.hg.1	OTTHUMG00000005007	6.20 ± 0.30	6.14 ± 0.15	6.22 ± 0.24	6.23 ± 0.20
TC01000522.hg.1	OTTHUMG00000005710	6.13 ± 0.12	6.04 ± 0.18	6.10 ± 0.06	6.17 ± 0.13
TC01002565.hg.1	OTTHUMG00000005714	7.31 ± 0.20	7.28 ± 0.15	7.17 ± 0.15	7.41 ± 0.03
TC01002463.hg.1	OTTHUMG00000005740	7.68 ± 0.25	7.63 ± 0.15	7.63 ± 0.15	7.83 ± 0.00
TC01000524.hg.1	OTTHUMG00000006360	7.51 ± 0.39	7.31 ± 0.19	7.49 ± 0.14	7.49 ± 0.08
TC01002571.hg.1	OTTHUMG00000007027	7.38 ± 0.11	7.23 ± 0.17	7.36 ± 0.06	7.37 ± 0.06
TC01002555.hg.1	OTTHUMG00000007304	6.39 ± 0.21	6.25 ± 0.05	6.32 ± 0.04	6.31 ± 0.11
TC01002556.hg.1	OTTHUMG00000007306	6.65 ± 0.16	6.63 ± 0.13	6.63 ± 0.10	6.68 ± 0.03
TC01002557.hg.1	OTTHUMG00000007307	4.93 ± 0.30	4.86 ± 0.15	4.96 ± 0.12	4.87 ± 0.01
TC01000327.hg.1	OTTHUMG00000007374	7.28 ± 0.23	7.28 ± 0.14	7.31 ± 0.07	7.47 ± 0.10
TC01002379.hg.1	OTTHUMG00000007376	7.55 ± 0.10	7.37 ± 0.10	7.49 ± 0.01	7.52 ± 0.03
TC01002582.hg.1	OTTHUMG00000007424	7.89 ± 0.21	7.91 ± 0.06	7.96 ± 0.17	7.98 ± 0.00
TC01002559.hg.1	OTTHUMG00000007457	7.07 ± 0.22	7.13 ± 0.06	6.94 ± 0.19	7.24 ± 0.08
TC01000530.hg.1	OTTHUMG00000007522	5.33 ± 0.21	5.06 ± 0.14	5.09 ± 0.18	5.14 ± 0.09
TC01002387.hg.1	OTTHUMG00000007539	7.39 ± 0.20	7.37 ± 0.26	7.22 ± 0.26	7.44 ± 0.06
TC01002575.hg.1	OTTHUMG00000007569	6.98 ± 0.13	6.87 ± 0.10	6.75 ± 0.21	6.90 ± 0.07
TC01002577.hg.1	OTTHUMG00000007622	6.07 ± 0.16	5.99 ± 0.06	5.97 ± 0.02	6.01 ± 0.19
TC01002469.hg.1	OTTHUMG00000007633	6.11 ± 0.07	6.08 ± 0.07	6.03 ± 0.09	6.17 ± 0.05
TC01002462.hg.1	OTTHUMG00000007683	6.42 ± 0.36	6.18 ± 0.07	6.30 ± 0.06	6.06 ± 0.03
TC01002588.hg.1	OTTHUMG00000007703	6.03 ± 0.15	6.06 ± 0.04	6.05 ± 0.03	6.15 ± 0.08
TC01002589.hg.1	OTTHUMG00000007704	7.16 ± 0.20	6.98 ± 0.12	7.04 ± 0.16	7.01 ± 0.05
TC01000519.hg.1	OTTHUMG00000007731	7.18 ± 0.07	7.14 ± 0.11	7.12 ± 0.17	7.13 ± 0.09
TC01002619.hg.1	OTTHUMG00000007814	6.81 ± 0.10	6.75 ± 0.28	6.77 ± 0.14	6.98 ± 0.14
TC01000597.hg.1	OTTHUMG00000007844	6.95 ± 0.24	6.91 ± 0.10	6.88 ± 0.13	7.00 ± 0.09
TC01002641.hg.1	OTTHUMG00000007879	4.81 ± 0.02	4.71 ± 0.19	4.73 ± 0.12	4.99 ± 0.16
TC01002642.hg.1	OTTHUMG00000007882	4.48 ± 0.21	4.43 ± 0.10	4.36 ± 0.18	4.49 ± 0.07
TC01002234.hg.1	OTTHUMG00000007915	3.54 ± 0.18	3.42 ± 0.06	3.36 ± 0.03	3.30 ± 0.02
TC01000180.hg.1	OTTHUMG00000007920	7.32 ± 0.35	7.19 ± 0.17	7.15 ± 0.26	7.30 ± 0.07
TC01000616.hg.1	OTTHUMG00000007927	4.72 ± 0.18	4.83 ± 0.03	4.71 ± 0.13	4.86 ± 0.12

TC01000604.hg.1	OTTHUMG00000007952	6.67 ± 0.02	6.62 ± 0.13	6.67 ± 0.04	6.68 ± 0.12
TC01000602.hg.1	OTTHUMG00000007956	7.19 ± 0.22	7.35 ± 0.04	7.27 ± 0.24	7.32 ± 0.14
TC01000605.hg.1	OTTHUMG00000007957	4.97 ± 0.16	5.00 ± 0.26	5.01 ± 0.27	5.10 ± 0.03
TC01000603.hg.1	OTTHUMG00000007961	7.55 ± 0.21	7.50 ± 0.19	7.51 ± 0.13	7.65 ± 0.08
TC01002630.hg.1	OTTHUMG00000007965	6.60 ± 0.06	6.59 ± 0.10	6.62 ± 0.10	6.66 ± 0.04
TC01000172.hg.1	OTTHUMG00000008038	6.47 ± 0.27	6.60 ± 0.12	6.49 ± 0.13	6.73 ± 0.22
TC01002635.hg.1	OTTHUMG00000008050	5.25 ± 0.05	5.29 ± 0.02	5.40 ± 0.15	5.31 ± 0.08
TC01002671.hg.1	OTTHUMG00000008075	7.13 ± 0.11	7.27 ± 0.23	7.23 ± 0.16	7.25 ± 0.13
TC01000578.hg.1	OTTHUMG00000008097	5.16 ± 0.08	5.14 ± 0.12	4.97 ± 0.12	5.28 ± 0.09
TC01000579.hg.1	OTTHUMG00000008098	5.50 ± 0.21	5.43 ± 0.13	5.58 ± 0.14	5.56 ± 0.17
TC01000683.hg.1	OTTHUMG00000008126	5.13 ± 0.41	5.17 ± 0.21	5.10 ± 0.01	5.24 ± 0.20
TC01000684.hg.1	OTTHUMG00000008128	5.80 ± 0.15	5.70 ± 0.12	5.69 ± 0.27	5.77 ± 0.18
TC01000609.hg.1	OTTHUMG00000008154	5.74 ± 0.10	5.57 ± 0.04	5.57 ± 0.05	5.62 ± 0.18
TC01000611.hg.1	OTTHUMG00000008156	5.02 ± 0.10	4.97 ± 0.06	4.93 ± 0.15	5.03 ± 0.15
TC01000625.hg.1	OTTHUMG00000008188	6.92 ± 0.36	7.03 ± 0.16	6.96 ± 0.14	7.16 ± 0.05
TC01000627.hg.1	OTTHUMG00000008189	7.54 ± 0.22	7.43 ± 0.25	7.52 ± 0.06	7.56 ± 0.05
TC01002646.hg.1	OTTHUMG00000008195	6.70 ± 0.16	6.61 ± 0.13	6.65 ± 0.19	6.71 ± 0.05
TC01000677.hg.1	OTTHUMG00000008214	5.55 ± 0.11	5.47 ± 0.15	5.49 ± 0.12	5.49 ± 0.08
TC01000630.hg.1	OTTHUMG00000008232	7.76 ± 0.12	7.64 ± 0.09	7.66 ± 0.15	7.71 ± 0.00
TC01000629.hg.1	OTTHUMG00000008236	4.21 ± 0.20	3.90 ± 0.07	4.16 ± 0.10	3.93 ± 0.10
TC01000681.hg.1	OTTHUMG00000008243	4.67 ± 0.25	4.52 ± 0.02	4.53 ± 0.02	4.67 ± 0.09
TC01000682.hg.1	OTTHUMG00000008245	3.85 ± 0.23	3.83 ± 0.10	3.84 ± 0.08	3.94 ± 0.10
TC01000680.hg.1	OTTHUMG00000008246	5.66 ± 0.15	5.58 ± 0.09	5.69 ± 0.06	5.72 ± 0.13
TC01000665.hg.1	OTTHUMG00000008263	6.63 ± 0.24	6.38 ± 0.11	6.32 ± 0.27	6.54 ± 0.14
TC01000692.hg.1	OTTHUMG00000008286	5.38 ± 0.07	5.31 ± 0.04	5.40 ± 0.04	5.44 ± 0.10
TC01006269.hg.1	OTTHUMG00000008300	6.75 ± 0.21	6.69 ± 0.15	6.66 ± 0.09	6.82 ± 0.13
TC01002703.hg.1	OTTHUMG00000008327	5.88 ± 0.24	5.79 ± 0.15	5.67 ± 0.27	6.07 ± 0.06
TC01002539.hg.1	OTTHUMG00000008348	5.14 ± 0.11	5.14 ± 0.17	5.06 ± 0.24	5.13 ± 0.09
TC01002688.hg.1	OTTHUMG00000008359	7.72 ± 0.32	7.65 ± 0.07	7.75 ± 0.19	7.79 ± 0.11
TC01000666.hg.1	OTTHUMG00000008362	6.01 ± 0.04	6.13 ± 0.19	6.01 ± 0.26	6.02 ± 0.08
TC01000697.hg.1	OTTHUMG00000008373	5.33 ± 0.05	5.23 ± 0.07	5.15 ± 0.10	5.25 ± 0.07

TC01002709.hg.1	OTTHUMG00000008374	5.52 ± 0.05	5.36 ± 0.04	5.54 ± 0.21	5.41 ± 0.12
TC01002710.hg.1	OTTHUMG00000008375	6.57 ± 0.09	6.63 ± 0.23	6.57 ± 0.17	6.91 ± 0.13
TC01000632.hg.1	OTTHUMG00000008380	6.33 ± 0.39	6.42 ± 0.24	6.43 ± 0.46	6.45 ± 0.16
TC01002711.hg.1	OTTHUMG00000008401	5.21 ± 0.11	4.73 ± 0.09	5.19 ± 0.22	4.99 ± 0.15
TC01002712.hg.1	OTTHUMG00000008402	6.82 ± 0.18	7.01 ± 0.18	7.03 ± 0.13	7.07 ± 0.09
TC01002713.hg.1	OTTHUMG00000008404	5.21 ± 0.03	5.13 ± 0.06	5.22 ± 0.07	5.15 ± 0.07
TC01002721.hg.1	OTTHUMG00000008421	5.86 ± 0.09	5.80 ± 0.11	5.78 ± 0.12	5.93 ± 0.10
TC01002720.hg.1	OTTHUMG00000008422	5.22 ± 0.09	5.16 ± 0.10	5.21 ± 0.18	5.28 ± 0.09
TC01000461.hg.1	OTTHUMG00000008426	4.58 ± 0.09	4.44 ± 0.19	4.41 ± 0.05	4.57 ± 0.14
TC01002599.hg.1	OTTHUMG00000008489	7.58 ± 0.24	7.53 ± 0.10	7.45 ± 0.15	7.60 ± 0.07
TC01002502.hg.1	OTTHUMG00000008497	7.15 ± 0.32	7.01 ± 0.18	7.06 ± 0.16	7.13 ± 0.13
TC01000636.hg.1	OTTHUMG00000008631	6.55 ± 0.32	6.56 ± 0.17	6.56 ± 0.03	6.61 ± 0.03
TC01000637.hg.1	OTTHUMG00000008632	5.47 ± 0.14	5.40 ± 0.21	5.38 ± 0.02	5.54 ± 0.06
TC01000699.hg.1	OTTHUMG00000008767	4.59 ± 0.07	4.58 ± 0.13	4.63 ± 0.06	4.54 ± 0.02
TC01002716.hg.1	OTTHUMG00000008768	5.92 ± 0.08	6.00 ± 0.06	5.89 ± 0.18	6.12 ± 0.05
TC01002664.hg.1	OTTHUMG00000008920	8.60 ± 0.20	8.65 ± 0.24	8.46 ± 0.21	8.82 ± 0.21
TC01000650.hg.1	OTTHUMG00000008925	6.73 ± 0.02	6.68 ± 0.07	6.77 ± 0.01	6.78 ± 0.12
TC01000653.hg.1	OTTHUMG00000008928	6.92 ± 0.33	6.87 ± 0.14	6.88 ± 0.18	6.99 ± 0.10
TC01000654.hg.1	OTTHUMG00000008930	7.95 ± 0.22	7.94 ± 0.32	7.94 ± 0.30	8.17 ± 0.12
TC01002670.hg.1	OTTHUMG00000008933	6.53 ± 0.18	6.42 ± 0.19	6.50 ± 0.34	6.67 ± 0.10
TC01000655.hg.1	OTTHUMG00000008935	7.62 ± 0.18	7.58 ± 0.18	7.56 ± 0.09	7.69 ± 0.06
TC01002666.hg.1	OTTHUMG00000008939	7.96 ± 0.11	7.96 ± 0.12	7.88 ± 0.20	7.92 ± 0.05
TC01000656.hg.1	OTTHUMG00000008948	7.64 ± 0.08	7.63 ± 0.03	7.49 ± 0.07	7.61 ± 0.04
TC01000701.hg.1	OTTHUMG00000008994	6.92 ± 0.25	6.83 ± 0.12	6.85 ± 0.16	6.85 ± 0.02
TC01002739.hg.1	OTTHUMG00000009024	6.04 ± 0.18	5.95 ± 0.04	6.02 ± 0.04	6.08 ± 0.09
TC01002748.hg.1	OTTHUMG00000009067	6.28 ± 0.22	6.13 ± 0.11	6.08 ± 0.21	6.16 ± 0.13
TC01002749.hg.1	OTTHUMG00000009087	6.28 ± 0.11	5.95 ± 0.05	6.01 ± 0.09	6.00 ± 0.24
TC01000498.hg.1	OTTHUMG00000009098	5.04 ± 0.11	5.06 ± 0.14	4.92 ± 0.11	4.99 ± 0.02
TC01000731.hg.1	OTTHUMG00000009114	4.54 ± 0.23	4.31 ± 0.11	4.37 ± 0.26	4.36 ± 0.05
TC01002732.hg.1	OTTHUMG00000009138	8.06 ± 0.31	8.12 ± 0.24	8.02 ± 0.24	8.21 ± 0.04
TC01000713.hg.1	OTTHUMG00000009144	4.74 ± 0.16	4.68 ± 0.05	4.84 ± 0.17	4.76 ± 0.04

TC01000715.hg.1	OTTHUMG00000009145	5.45 ± 0.27	5.26 ± 0.25	5.44 ± 0.10	5.61 ± 0.19
TC01002728.hg.1	OTTHUMG00000009147	5.85 ± 0.13	5.77 ± 0.12	5.85 ± 0.10	5.89 ± 0.03
TC01000712.hg.1	OTTHUMG00000009148	4.42 ± 0.18	4.23 ± 0.12	4.09 ± 0.01	4.11 ± 0.07
TC01000737.hg.1	OTTHUMG00000009166	4.57 ± 0.10	4.37 ± 0.06	4.51 ± 0.20	4.37 ± 0.08
TC01000748.hg.1	OTTHUMG00000009204	4.18 ± 0.24	4.14 ± 0.18	4.26 ± 0.03	4.23 ± 0.22
TC01000749.hg.1	OTTHUMG00000009207	4.62 ± 0.12	4.57 ± 0.05	4.54 ± 0.18	4.62 ± 0.10
TC01000750.hg.1	OTTHUMG00000009216	4.79 ± 0.08	4.68 ± 0.09	4.76 ± 0.09	4.80 ± 0.09
TC01000751.hg.1	OTTHUMG00000009217	4.61 ± 0.25	4.31 ± 0.06	4.56 ± 0.18	4.42 ± 0.19
TC01002767.hg.1	OTTHUMG00000009218	3.50 ± 0.11	3.43 ± 0.06	3.52 ± 0.14	3.45 ± 0.10
TC01002768.hg.1	OTTHUMG00000009266	4.00 ± 0.10	3.80 ± 0.03	3.95 ± 0.12	3.96 ± 0.10
TC01000502.hg.1	OTTHUMG00000009292	5.73 ± 0.18	5.80 ± 0.16	5.67 ± 0.12	5.74 ± 0.12
TC01002747.hg.1	OTTHUMG00000009304	9.92 ± 0.17	9.82 ± 0.08	9.76 ± 0.30	9.78 ± 0.09
TC01002743.hg.1	OTTHUMG00000009307	5.89 ± 0.13	5.84 ± 0.11	6.06 ± 0.12	5.83 ± 0.11
TC01002770.hg.1	OTTHUMG00000009341	3.76 ± 0.10	3.69 ± 0.07	3.78 ± 0.15	3.76 ± 0.06
TC01002772.hg.1	OTTHUMG00000009344	5.74 ± 0.16	5.63 ± 0.15	5.64 ± 0.05	5.79 ± 0.09
TC01000758.hg.1	OTTHUMG00000009349	4.46 ± 0.07	4.43 ± 0.01	4.44 ± 0.19	4.46 ± 0.09
TC01002773.hg.1	OTTHUMG00000009350	7.49 ± 0.12	7.41 ± 0.15	7.50 ± 0.17	7.58 ± 0.06
TC01000743.hg.1	OTTHUMG00000009373	7.24 ± 0.26	7.21 ± 0.06	7.14 ± 0.10	7.34 ± 0.09
TC01000759.hg.1	OTTHUMG00000009400	6.60 ± 0.22	6.58 ± 0.03	6.49 ± 0.07	6.72 ± 0.17
TC01000765.hg.1	OTTHUMG00000009452	3.77 ± 0.09	3.77 ± 0.02	3.80 ± 0.04	3.71 ± 0.00
TC01002780.hg.1	OTTHUMG00000009453	7.05 ± 0.50	7.13 ± 0.14	6.94 ± 0.27	7.36 ± 0.27
TC01000169.hg.1	OTTHUMG00000009500	6.28 ± 0.29	6.40 ± 0.14	6.28 ± 0.12	6.56 ± 0.20
TC01002255.hg.1	OTTHUMG00000009524	6.75 ± 0.18	6.66 ± 0.07	6.53 ± 0.20	6.74 ± 0.16
TC01000202.hg.1	OTTHUMG00000009528	7.40 ± 0.19	7.44 ± 0.29	7.41 ± 0.29	7.62 ± 0.05
TC01000766.hg.1	OTTHUMG00000009566	5.02 ± 0.18	4.90 ± 0.10	4.89 ± 0.06	4.95 ± 0.07
TC01000767.hg.1	OTTHUMG00000009567	4.99 ± 0.23	4.92 ± 0.06	4.93 ± 0.09	4.95 ± 0.16
TC01000770.hg.1	OTTHUMG00000009610	4.42 ± 0.20	4.16 ± 0.05	4.26 ± 0.15	4.23 ± 0.14
TC01000762.hg.1	OTTHUMG00000009658	3.72 ± 0.31	3.62 ± 0.02	3.67 ± 0.11	3.63 ± 0.19
TC01002795.hg.1	OTTHUMG00000009684	4.94 ± 0.19	4.87 ± 0.04	4.84 ± 0.15	4.98 ± 0.17
TC01000784.hg.1	OTTHUMG00000009688	5.66 ± 0.15	5.72 ± 0.07	5.76 ± 0.10	5.82 ± 0.08
TC01002789.hg.1	OTTHUMG00000009693	5.30 ± 0.31	5.12 ± 0.04	5.10 ± 0.11	5.20 ± 0.12

TC01002790.hg.1	OTTHUMG00000009694	6.49 ± 0.15	6.37 ± 0.18	6.44 ± 0.04	6.53 ± 0.23
TC01002808.hg.1	OTTHUMG00000009712	4.77 ± 0.22	4.85 ± 0.03	4.82 ± 0.15	4.97 ± 0.16
TC01000764.hg.1	OTTHUMG00000009734	3.35 ± 0.15	3.20 ± 0.01	3.29 ± 0.14	3.27 ± 0.12
TC01000797.hg.1	OTTHUMG00000009742	6.86 ± 0.08	6.91 ± 0.09	6.93 ± 0.07	7.13 ± 0.12
TC11001279.hg.1	OTTHUMG00000009760	6.65 ± 0.11	6.55 ± 0.06	6.53 ± 0.17	6.60 ± 0.06
TC01000803.hg.1	OTTHUMG00000009769	4.74 ± 0.18	4.58 ± 0.09	4.66 ± 0.16	4.67 ± 0.08
TC01002813.hg.1	OTTHUMG00000009770	5.67 ± 0.14	5.60 ± 0.08	5.64 ± 0.08	5.73 ± 0.18
TC01000804.hg.1	OTTHUMG00000009772	4.83 ± 0.10	4.61 ± 0.17	4.69 ± 0.02	4.75 ± 0.23
TC01000779.hg.1	OTTHUMG00000009790	4.57 ± 0.13	4.37 ± 0.07	4.32 ± 0.19	4.36 ± 0.04
TC01000780.hg.1	OTTHUMG00000009791	4.48 ± 0.19	4.32 ± 0.05	4.39 ± 0.06	4.41 ± 0.08
TC01002810.hg.1	OTTHUMG00000009840	4.40 ± 0.16	4.28 ± 0.04	4.32 ± 0.08	4.31 ± 0.08
TC01002811.hg.1	OTTHUMG00000009842	5.35 ± 0.16	5.21 ± 0.10	5.30 ± 0.05	5.23 ± 0.02
TC01002812.hg.1	OTTHUMG00000009845	6.89 ± 0.33	6.87 ± 0.06	6.99 ± 0.24	6.96 ± 0.18
TC01002690.hg.1	OTTHUMG00000009889	6.30 ± 0.06	6.56 ± 0.30	6.25 ± 0.22	6.61 ± 0.20
TC11000065.hg.1	OTTHUMG00000009894	9.38 ± 0.25	9.42 ± 0.27	9.41 ± 0.17	9.60 ± 0.12
TC01002814.hg.1	OTTHUMG00000009930	6.13 ± 0.11	6.07 ± 0.11	6.07 ± 0.10	6.16 ± 0.05
TC01000805.hg.1	OTTHUMG00000009931	4.05 ± 0.14	3.83 ± 0.10	3.89 ± 0.12	3.91 ± 0.03
TC01002719.hg.1	OTTHUMG00000009978	6.75 ± 0.27	6.74 ± 0.11	6.69 ± 0.10	6.90 ± 0.12
TC01000670.hg.1	OTTHUMG00000009987	5.92 ± 0.18	5.83 ± 0.07	5.84 ± 0.10	5.93 ± 0.11
TC01000672.hg.1	OTTHUMG00000009990	6.47 ± 0.19	6.43 ± 0.13	6.40 ± 0.22	6.52 ± 0.06
TC01002696.hg.1	OTTHUMG00000009992	8.12 ± 0.18	7.97 ± 0.23	8.13 ± 0.18	8.12 ± 0.06
TC01002858.hg.1	OTTHUMG00000010017	5.55 ± 0.25	5.28 ± 0.02	5.42 ± 0.02	5.33 ± 0.04
TC01002842.hg.1	OTTHUMG00000010024	5.52 ± 0.22	5.41 ± 0.08	5.39 ± 0.06	5.40 ± 0.05
TC01000694.hg.1	OTTHUMG00000010107	4.97 ± 0.04	4.93 ± 0.01	4.98 ± 0.06	5.01 ± 0.04
TC01000690.hg.1	OTTHUMG00000010108	5.32 ± 0.13	5.12 ± 0.08	5.12 ± 0.18	5.23 ± 0.16
TC01000834.hg.1	OTTHUMG00000010124	7.24 ± 0.61	6.85 ± 0.30	8.03 ± 0.73	6.54 ± 0.07
TC01002835.hg.1	OTTHUMG00000010247	5.64 ± 0.22	5.46 ± 0.14	5.61 ± 0.12	5.57 ± 0.11
TC01002837.hg.1	OTTHUMG00000010261	5.50 ± 0.35	5.29 ± 0.13	5.46 ± 0.14	5.43 ± 0.01
TC01000820.hg.1	OTTHUMG00000010575	6.75 ± 0.25	6.71 ± 0.15	6.68 ± 0.21	6.80 ± 0.09
TC01000840.hg.1	OTTHUMG00000010584	6.21 ± 0.24	5.89 ± 0.09	5.91 ± 0.15	5.94 ± 0.17
TC01000868.hg.1	OTTHUMG00000010623	4.51 ± 0.16	4.39 ± 0.25	4.24 ± 0.22	4.41 ± 0.07

TC01000870.hg.1	OTTHUMG00000010648	6.15 ± 0.17	6.03 ± 0.18	6.08 ± 0.13	6.16 ± 0.04
TC01002885.hg.1	OTTHUMG00000010651	6.40 ± 0.16	6.20 ± 0.14	6.10 ± 0.13	6.27 ± 0.19
TC01002901.hg.1	OTTHUMG00000010690	4.53 ± 0.18	4.30 ± 0.03	4.37 ± 0.06	4.42 ± 0.11
TC01000881.hg.1	OTTHUMG00000010691	4.59 ± 0.14	4.50 ± 0.09	4.52 ± 0.08	4.50 ± 0.07
TC01002889.hg.1	OTTHUMG00000010701	6.79 ± 0.08	6.76 ± 0.16	6.75 ± 0.05	6.88 ± 0.11
TC01002890.hg.1	OTTHUMG00000010702	6.54 ± 0.19	6.58 ± 0.21	6.54 ± 0.10	6.80 ± 0.06
TC01002887.hg.1	OTTHUMG00000010718	5.60 ± 0.14	5.46 ± 0.12	5.47 ± 0.25	5.60 ± 0.15
TC01002888.hg.1	OTTHUMG00000010719	6.01 ± 0.28	6.07 ± 0.30	6.04 ± 0.03	6.12 ± 0.05
TC01002915.hg.1	OTTHUMG00000010753	5.67 ± 0.52	5.73 ± 0.30	5.92 ± 0.36	5.45 ± 0.18
TC01002909.hg.1	OTTHUMG00000010769	3.81 ± 0.05	3.78 ± 0.09	3.91 ± 0.06	3.69 ± 0.04
TC01000875.hg.1	OTTHUMG00000010779	4.47 ± 0.15	4.14 ± 0.15	4.21 ± 0.07	4.17 ± 0.11
TC01000906.hg.1	OTTHUMG00000010830	5.57 ± 0.31	5.80 ± 0.07	5.55 ± 0.29	5.87 ± 0.11
TC01000910.hg.1	OTTHUMG00000010834	5.48 ± 0.15	5.36 ± 0.07	5.32 ± 0.02	5.49 ± 0.09
TC01000877.hg.1	OTTHUMG00000010848	5.72 ± 0.12	5.79 ± 0.14	5.77 ± 0.07	5.91 ± 0.05
TC01002895.hg.1	OTTHUMG00000010849	6.22 ± 0.23	6.03 ± 0.15	6.06 ± 0.14	6.09 ± 0.05
TC01002896.hg.1	OTTHUMG00000010851	5.47 ± 0.25	5.55 ± 0.08	5.60 ± 0.04	5.72 ± 0.09
TC01002930.hg.1	OTTHUMG00000010865	3.98 ± 0.10	3.93 ± 0.06	3.96 ± 0.11	4.06 ± 0.12
TC01002931.hg.1	OTTHUMG00000010873	5.14 ± 0.11	5.01 ± 0.08	5.07 ± 0.03	5.05 ± 0.03
TC01002898.hg.1	OTTHUMG00000010912	3.94 ± 0.11	3.83 ± 0.03	3.93 ± 0.05	3.95 ± 0.16
TC01002899.hg.1	OTTHUMG00000010913	5.01 ± 0.21	4.77 ± 0.10	4.86 ± 0.01	4.80 ± 0.06
TC01002926.hg.1	OTTHUMG00000010942	8.54 ± 0.31	8.43 ± 0.17	8.25 ± 0.09	8.58 ± 0.06
TC01000911.hg.1	OTTHUMG00000010944	6.35 ± 0.19	6.16 ± 0.01	6.23 ± 0.11	6.17 ± 0.14
TC01002921.hg.1	OTTHUMG00000010986	4.02 ± 0.10	4.01 ± 0.18	4.09 ± 0.10	4.15 ± 0.21
TC01002936.hg.1	OTTHUMG00000011003	4.23 ± 0.23	3.96 ± 0.02	3.99 ± 0.06	3.92 ± 0.10
TC01002937.hg.1	OTTHUMG00000011006	5.17 ± 0.17	5.05 ± 0.06	5.09 ± 0.04	5.09 ± 0.12
TC01000928.hg.1	OTTHUMG00000011012	5.94 ± 0.05	5.71 ± 0.14	5.96 ± 0.40	5.70 ± 0.10
TC01000923.hg.1	OTTHUMG00000011035	4.27 ± 0.16	4.16 ± 0.09	4.12 ± 0.09	4.24 ± 0.03
TC01002946.hg.1	OTTHUMG00000011121	5.82 ± 0.30	5.95 ± 0.18	5.90 ± 0.22	5.95 ± 0.04
TC01000988.hg.1	OTTHUMG00000011155	4.49 ± 0.09	4.39 ± 0.06	4.33 ± 0.08	4.40 ± 0.03
TC01002982.hg.1	OTTHUMG00000011166	6.91 ± 0.15	6.83 ± 0.18	6.82 ± 0.32	6.97 ± 0.08
TC01006401.hg.1	OTTHUMG00000011632	6.88 ± 0.18	6.78 ± 0.17	6.78 ± 0.06	6.90 ± 0.08

TC01000954.hg.1	OTTHUMG00000011637	5.78 ± 0.21	5.75 ± 0.25	5.89 ± 0.06	5.94 ± 0.19
TC01002964.hg.1	OTTHUMG00000011641	5.21 ± 0.08	5.22 ± 0.09	5.12 ± 0.07	5.23 ± 0.14
TC01000948.hg.1	OTTHUMG00000011643	7.04 ± 0.27	7.28 ± 0.29	7.72 ± 0.65	7.17 ± 0.20
TC01002961.hg.1	OTTHUMG00000011645	8.07 ± 0.39	8.19 ± 0.08	8.15 ± 0.07	8.32 ± 0.18
TC11000073.hg.1	OTTHUMG00000011701	8.05 ± 0.12	8.03 ± 0.17	7.94 ± 0.19	8.20 ± 0.11
TC11000075.hg.1	OTTHUMG00000011710	7.40 ± 0.24	7.37 ± 0.09	7.32 ± 0.18	7.47 ± 0.03
TC01002966.hg.1	OTTHUMG00000011762	7.17 ± 0.13	7.10 ± 0.22	7.12 ± 0.20	7.24 ± 0.08
TC01002967.hg.1	OTTHUMG00000011763	6.30 ± 0.06	6.21 ± 0.06	6.14 ± 0.04	6.38 ± 0.20
TC01001010.hg.1	OTTHUMG00000011879	6.35 ± 0.24	6.33 ± 0.18	6.86 ± 0.47	6.21 ± 0.23
TC01001005.hg.1	OTTHUMG00000011899	6.47 ± 0.35	6.42 ± 0.19	6.23 ± 0.11	6.63 ± 0.10
TC01002998.hg.1	OTTHUMG00000011907	7.60 ± 0.25	7.66 ± 0.22	7.57 ± 0.25	7.78 ± 0.07
TC01001000.hg.1	OTTHUMG00000011939	4.81 ± 0.09	4.59 ± 0.03	4.75 ± 0.06	4.61 ± 0.05
TC01001011.hg.1	OTTHUMG00000011974	7.34 ± 0.34	7.00 ± 0.09	7.05 ± 0.27	7.17 ± 0.16
TC01003009.hg.1	OTTHUMG00000011982	6.37 ± 0.14	6.34 ± 0.08	6.15 ± 0.11	6.24 ± 0.06
TC01000984.hg.1	OTTHUMG00000011991	7.15 ± 0.27	7.16 ± 0.14	7.18 ± 0.24	7.36 ± 0.15
TC01000983.hg.1	OTTHUMG00000011992	9.43 ± 0.28	9.45 ± 0.20	9.37 ± 0.22	9.44 ± 0.06
TC01001013.hg.1	OTTHUMG00000012009	4.92 ± 0.09	4.85 ± 0.10	5.01 ± 0.03	5.02 ± 0.12
TC01003000.hg.1	OTTHUMG00000012022	7.80 ± 0.17	7.85 ± 0.08	7.88 ± 0.11	7.98 ± 0.12
TC01000994.hg.1	OTTHUMG00000012023	7.22 ± 0.23	7.13 ± 0.18	7.14 ± 0.21	7.22 ± 0.07
TC01003033.hg.1	OTTHUMG00000012031	5.10 ± 0.13	4.99 ± 0.08	4.93 ± 0.06	5.06 ± 0.16
TC01003022.hg.1	OTTHUMG00000012108	4.11 ± 0.11	4.12 ± 0.12	4.10 ± 0.11	4.14 ± 0.08
TC01001016.hg.1	OTTHUMG00000012112	6.74 ± 0.27	6.85 ± 0.22	6.78 ± 0.29	6.99 ± 0.16
TC01000997.hg.1	OTTHUMG00000012131	6.08 ± 0.24	6.06 ± 0.10	5.98 ± 0.15	6.15 ± 0.13
TC01001028.hg.1	OTTHUMG00000012147	4.76 ± 0.11	4.63 ± 0.18	4.73 ± 0.05	4.71 ± 0.18
TC01002994.hg.1	OTTHUMG00000012183	6.92 ± 0.08	6.95 ± 0.20	6.84 ± 0.03	7.02 ± 0.29
TC01001224.hg.1	OTTHUMG00000012244	4.48 ± 0.03	4.24 ± 0.21	4.34 ± 0.28	4.21 ± 0.01
TC11000087.hg.1	OTTHUMG00000012284	7.24 ± 0.23	7.14 ± 0.21	7.02 ± 0.33	7.26 ± 0.09
TC11001305.hg.1	OTTHUMG00000012285	7.31 ± 0.17	7.27 ± 0.08	7.25 ± 0.17	7.26 ± 0.15
TC01003043.hg.1	OTTHUMG00000012337	7.07 ± 0.33	7.11 ± 0.29	6.98 ± 0.30	7.12 ± 0.02
TC01001034.hg.1	OTTHUMG00000012342	6.89 ± 0.09	6.85 ± 0.03	6.78 ± 0.04	6.90 ± 0.07
TC01003222.hg.1	OTTHUMG00000012346	7.05 ± 0.09	7.05 ± 0.21	6.89 ± 0.23	7.02 ± 0.11

TC01001225.hg.1	OTTHUMG00000012388	6.64 ± 0.14	6.70 ± 0.15	6.53 ± 0.14	6.88 ± 0.06
TC01001036.hg.1	OTTHUMG00000012409	4.55 ± 0.12	4.52 ± 0.06	4.52 ± 0.16	4.61 ± 0.16
TC01003046.hg.1	OTTHUMG00000012411	6.14 ± 0.37	6.31 ± 0.28	6.23 ± 0.25	6.34 ± 0.10
TC01003252.hg.1	OTTHUMG00000012449	5.25 ± 0.09	5.23 ± 0.13	5.31 ± 0.17	5.32 ± 0.10
TC11000057.hg.1	OTTHUMG00000012473	7.33 ± 0.22	7.49 ± 0.21	7.41 ± 0.19	7.55 ± 0.14
TC01001208.hg.1	OTTHUMG00000012496	7.04 ± 0.11	7.05 ± 0.10	6.96 ± 0.06	6.98 ± 0.05
TC01001221.hg.1	OTTHUMG00000013054	6.62 ± 0.14	6.65 ± 0.02	6.71 ± 0.09	6.69 ± 0.07
TC01001219.hg.1	OTTHUMG00000013060	7.12 ± 0.18	7.09 ± 0.21	7.13 ± 0.12	7.07 ± 0.14
TC01001222.hg.1	OTTHUMG00000013065	6.68 ± 0.03	6.65 ± 0.07	6.56 ± 0.07	6.73 ± 0.11
TC01001052.hg.1	OTTHUMG00000013086	5.05 ± 0.17	4.99 ± 0.10	4.93 ± 0.07	5.03 ± 0.07
TC01003060.hg.1	OTTHUMG00000013087	3.02 ± 0.10	2.96 ± 0.07	2.99 ± 0.03	2.97 ± 0.01
TC01001054.hg.1	OTTHUMG00000013089	5.43 ± 0.39	4.96 ± 0.13	5.01 ± 0.05	5.13 ± 0.26
TC01003059.hg.1	OTTHUMG00000013091	6.63 ± 0.41	6.64 ± 0.18	6.45 ± 0.09	6.83 ± 0.21
TC01001051.hg.1	OTTHUMG00000013092	6.34 ± 0.19	6.48 ± 0.01	6.42 ± 0.11	6.65 ± 0.08
TC01003058.hg.1	OTTHUMG00000013093	6.67 ± 0.22	6.73 ± 0.01	6.73 ± 0.18	7.03 ± 0.26
TC01000158.hg.1	OTTHUMG00000013154	8.22 ± 0.40	8.25 ± 0.22	8.13 ± 0.35	8.29 ± 0.08
TC09000273.hg.1	OTTHUMG00000013198	7.32 ± 0.23	7.44 ± 0.10	7.49 ± 0.03	7.49 ± 0.02
TC09000272.hg.1	OTTHUMG00000013217	7.24 ± 0.12	7.11 ± 0.09	7.18 ± 0.02	7.25 ± 0.20
TC09000255.hg.1	OTTHUMG00000013230	7.17 ± 0.18	7.13 ± 0.15	7.16 ± 0.07	7.26 ± 0.17
TC09000262.hg.1	OTTHUMG00000013241	7.05 ± 0.15	7.04 ± 0.16	7.04 ± 0.10	7.13 ± 0.04
TC09001129.hg.1	OTTHUMG00000013242	7.01 ± 0.20	6.95 ± 0.04	6.88 ± 0.20	7.10 ± 0.18
TC09000261.hg.1	OTTHUMG00000013243	5.32 ± 0.11	5.23 ± 0.16	5.23 ± 0.10	5.29 ± 0.10
TC09001127.hg.1	OTTHUMG00000013244	7.93 ± 0.17	7.90 ± 0.03	7.83 ± 0.20	7.99 ± 0.16
TC09000265.hg.1	OTTHUMG00000013250	6.85 ± 0.59	7.04 ± 0.27	7.08 ± 0.60	7.19 ± 0.21
TC09001130.hg.1	OTTHUMG00000013252	5.68 ± 0.19	5.57 ± 0.09	5.60 ± 0.19	5.78 ± 0.35
TC09001134.hg.1	OTTHUMG00000013261	7.57 ± 0.11	7.58 ± 0.20	7.56 ± 0.08	7.64 ± 0.07
TC09001135.hg.1	OTTHUMG00000013262	5.20 ± 0.11	5.11 ± 0.13	5.12 ± 0.13	5.18 ± 0.06
TC09000252.hg.1	OTTHUMG00000013272	6.84 ± 0.29	6.61 ± 0.10	6.88 ± 0.40	7.15 ± 0.46
TC09001116.hg.1	OTTHUMG00000013277	4.81 ± 0.16	4.75 ± 0.04	4.62 ± 0.24	4.88 ± 0.23
TC09001111.hg.1	OTTHUMG00000013290	7.01 ± 0.18	7.04 ± 0.07	6.96 ± 0.17	7.16 ± 0.18
TC09001153.hg.1	OTTHUMG00000013293	6.08 ± 0.09	5.95 ± 0.05	6.02 ± 0.11	6.14 ± 0.14

TC09000279.hg.1	OTTHUMG00000013297	5.04 ± 0.23	4.92 ± 0.02	4.98 ± 0.08	4.99 ± 0.03
TC09001137.hg.1	OTTHUMG00000013300	5.03 ± 0.37	4.81 ± 0.02	4.98 ± 0.23	5.06 ± 0.31
TC01002729.hg.1	OTTHUMG00000013351	5.77 ± 0.18	5.62 ± 0.11	5.59 ± 0.09	5.78 ± 0.16
TC01003061.hg.1	OTTHUMG00000013378	5.71 ± 0.69	5.43 ± 0.10	5.50 ± 0.32	5.96 ± 0.39
TC01006404.hg.1	OTTHUMG00000013379	6.80 ± 0.16	6.71 ± 0.05	6.72 ± 0.17	6.88 ± 0.26
TC01003062.hg.1	OTTHUMG00000013382	6.12 ± 0.22	5.98 ± 0.15	6.17 ± 0.26	6.08 ± 0.19
TC01006283.hg.1	OTTHUMG00000013469	5.68 ± 0.36	5.61 ± 0.06	5.70 ± 0.30	5.82 ± 0.24
TC01003073.hg.1	OTTHUMG00000013470	6.94 ± 0.19	6.95 ± 0.02	6.86 ± 0.11	7.11 ± 0.22
TC01006405.hg.1	OTTHUMG00000013471	6.44 ± 0.20	6.47 ± 0.02	6.38 ± 0.11	6.59 ± 0.16
TC01003068.hg.1	OTTHUMG00000013472	5.62 ± 0.24	5.47 ± 0.03	5.63 ± 0.15	5.69 ± 0.16
TC01001066.hg.1	OTTHUMG00000013473	5.41 ± 0.21	4.86 ± 0.31	5.14 ± 0.38	5.14 ± 0.11
TC01001063.hg.1	OTTHUMG00000013474	6.54 ± 0.69	6.58 ± 0.14	6.37 ± 0.29	7.17 ± 0.78
TC01003070.hg.1	OTTHUMG00000013475	6.36 ± 0.36	6.36 ± 0.09	6.37 ± 0.32	6.62 ± 0.26
TC01001256.hg.1	OTTHUMG00000013548	7.01 ± 0.22	6.80 ± 0.09	6.86 ± 0.04	6.92 ± 0.23
TC01006284.hg.1	OTTHUMG00000013630	5.73 ± 0.27	5.54 ± 0.12	5.66 ± 0.17	5.70 ± 0.16
TC01001069.hg.1	OTTHUMG00000013631	5.32 ± 0.56	5.02 ± 0.11	5.28 ± 0.37	5.58 ± 0.53
TC01003078.hg.1	OTTHUMG00000013632	4.63 ± 0.41	4.24 ± 0.08	4.40 ± 0.32	4.55 ± 0.44
TC01001102.hg.1	OTTHUMG00000013747	7.42 ± 0.25	7.23 ± 0.09	7.20 ± 0.15	7.30 ± 0.07
TC01002354.hg.1	OTTHUMG00000013895	8.34 ± 0.20	8.38 ± 0.07	8.36 ± 0.06	8.50 ± 0.09
TC01003313.hg.1	OTTHUMG00000013911	7.77 ± 0.14	7.57 ± 0.01	7.76 ± 0.10	7.62 ± 0.07
TC01001249.hg.1	OTTHUMG00000013934	5.70 ± 0.20	5.59 ± 0.07	5.58 ± 0.11	5.62 ± 0.03
TC01003176.hg.1	OTTHUMG00000013949	10.49 ± 0.17	10.55 ± 0.03	10.32 ± 0.21	10.67 ± 0.10
TC01003177.hg.1	OTTHUMG00000013950	6.10 ± 0.48	6.11 ± 0.30	6.23 ± 0.26	5.99 ± 0.14
TC01001168.hg.1	OTTHUMG00000013953	7.77 ± 0.22	7.69 ± 0.05	7.64 ± 0.17	7.57 ± 0.05
TC01003184.hg.1	OTTHUMG00000013955	7.39 ± 0.21	7.20 ± 0.10	7.21 ± 0.23	7.32 ± 0.07
TC01003320.hg.1	OTTHUMG00000014027	6.62 ± 0.29	6.49 ± 0.14	6.61 ± 0.10	6.50 ± 0.06
TC01003026.hg.1	OTTHUMG00000014035	6.01 ± 0.12	5.91 ± 0.04	5.98 ± 0.10	5.97 ± 0.04
TC01003139.hg.1	OTTHUMG00000014082	6.74 ± 0.08	6.66 ± 0.09	6.74 ± 0.04	6.76 ± 0.12
TC01001135.hg.1	OTTHUMG00000014085	6.91 ± 0.10	6.87 ± 0.08	6.93 ± 0.13	6.81 ± 0.01
TC01003144.hg.1	OTTHUMG00000014086	8.30 ± 0.22	8.27 ± 0.09	8.26 ± 0.19	8.22 ± 0.01
TC01001132.hg.1	OTTHUMG00000014087	6.32 ± 0.23	6.34 ± 0.14	6.25 ± 0.19	6.50 ± 0.10

TC01001130.hg.1	OTTHUMG00000014089	6.41 ± 0.22	6.17 ± 0.19	5.94 ± 0.15	6.29 ± 0.19
TC01003138.hg.1	OTTHUMG00000014090	6.61 ± 0.29	6.51 ± 0.15	6.56 ± 0.13	6.77 ± 0.18
TC06000006.hg.1	OTTHUMG00000014117	5.30 ± 0.08	5.34 ± 0.09	5.36 ± 0.16	5.29 ± 0.10
TC06000014.hg.1	OTTHUMG00000014120	8.44 ± 0.15	8.39 ± 0.07	8.38 ± 0.09	8.38 ± 0.01
TC06000017.hg.1	OTTHUMG00000014123	6.71 ± 0.06	6.71 ± 0.28	6.73 ± 0.25	6.75 ± 0.07
TC06001222.hg.1	OTTHUMG00000014129	5.83 ± 0.25	5.47 ± 0.05	5.54 ± 0.27	5.62 ± 0.13
TC06000020.hg.1	OTTHUMG00000014132	4.64 ± 0.21	4.56 ± 0.06	4.57 ± 0.16	4.63 ± 0.09
TC06000022.hg.1	OTTHUMG00000014139	6.79 ± 0.32	6.96 ± 0.13	6.88 ± 0.20	7.23 ± 0.08
TC06001231.hg.1	OTTHUMG00000014145	5.57 ± 0.41	5.55 ± 0.09	5.42 ± 0.05	5.51 ± 0.15
TC06000031.hg.1	OTTHUMG00000014149	4.22 ± 0.20	4.20 ± 0.08	4.13 ± 0.08	4.20 ± 0.02
TC06000033.hg.1	OTTHUMG00000014150	6.91 ± 0.10	6.85 ± 0.07	6.81 ± 0.13	6.82 ± 0.06
TC06001233.hg.1	OTTHUMG00000014156	7.36 ± 0.31	7.05 ± 0.15	7.22 ± 0.28	7.02 ± 0.07
TC06001236.hg.1	OTTHUMG00000014163	7.46 ± 0.05	7.45 ± 0.10	7.43 ± 0.13	7.63 ± 0.23
TC06001237.hg.1	OTTHUMG00000014165	7.76 ± 0.29	7.64 ± 0.12	7.65 ± 0.12	7.66 ± 0.16
TC06000049.hg.1	OTTHUMG00000014182	7.48 ± 0.14	7.35 ± 0.25	7.37 ± 0.06	7.50 ± 0.07
TC06000052.hg.1	OTTHUMG00000014191	6.62 ± 0.14	6.62 ± 0.06	6.80 ± 0.04	6.66 ± 0.21
TC06001249.hg.1	OTTHUMG00000014193	5.03 ± 0.13	5.09 ± 0.11	5.09 ± 0.03	5.11 ± 0.05
TC06000054.hg.1	OTTHUMG00000014195	6.78 ± 0.35	6.89 ± 0.30	6.75 ± 0.26	6.95 ± 0.06
TC06000055.hg.1	OTTHUMG00000014196	6.49 ± 0.11	6.37 ± 0.17	6.39 ± 0.23	6.47 ± 0.10
TC06000057.hg.1	OTTHUMG00000014204	5.03 ± 0.21	4.77 ± 0.17	4.84 ± 0.15	4.65 ± 0.15
TC06000064.hg.1	OTTHUMG00000014222	6.21 ± 0.33	6.48 ± 0.13	6.31 ± 0.24	6.60 ± 0.17
TC06001261.hg.1	OTTHUMG00000014230	6.09 ± 0.10	6.33 ± 0.44	6.10 ± 0.24	6.57 ± 0.20
TC06001263.hg.1	OTTHUMG00000014232	6.54 ± 0.09	6.38 ± 0.15	6.45 ± 0.19	6.48 ± 0.12
TC06000070.hg.1	OTTHUMG00000014234	6.16 ± 0.19	6.13 ± 0.12	6.09 ± 0.03	6.18 ± 0.08
TC06001264.hg.1	OTTHUMG00000014238	4.97 ± 0.19	4.96 ± 0.05	5.06 ± 0.23	5.04 ± 0.06
TC06000076.hg.1	OTTHUMG00000014248	8.14 ± 0.02	8.07 ± 0.07	8.01 ± 0.14	8.17 ± 0.06
TC06000081.hg.1	OTTHUMG00000014256	4.94 ± 0.13	4.81 ± 0.12	4.85 ± 0.05	5.00 ± 0.07
TC06000082.hg.1	OTTHUMG00000014257	5.92 ± 0.15	5.78 ± 0.09	5.77 ± 0.14	5.87 ± 0.05
TC06000085.hg.1	OTTHUMG00000014261	5.99 ± 0.17	5.95 ± 0.05	5.99 ± 0.11	6.05 ± 0.09
TC06001276.hg.1	OTTHUMG00000014263	4.30 ± 0.11	4.01 ± 0.13	4.10 ± 0.07	4.05 ± 0.08
TC06001278.hg.1	OTTHUMG00000014269	5.63 ± 0.08	5.55 ± 0.12	5.61 ± 0.12	5.81 ± 0.17

TC06000098.hg.1	OTTHUMG00000014282	5.12 ± 0.25	5.01 ± 0.07	5.04 ± 0.13	5.10 ± 0.16
TC06001283.hg.1	OTTHUMG00000014284	6.47 ± 0.28	6.51 ± 0.16	6.34 ± 0.29	6.62 ± 0.06
TC06000101.hg.1	OTTHUMG00000014286	6.74 ± 0.33	6.63 ± 0.04	6.68 ± 0.11	6.75 ± 0.19
TC06001285.hg.1	OTTHUMG00000014287	5.38 ± 0.08	5.43 ± 0.11	5.41 ± 0.10	5.50 ± 0.18
TC06001286.hg.1	OTTHUMG00000014288	6.76 ± 0.24	6.65 ± 0.08	6.61 ± 0.14	6.52 ± 0.05
TC06001287.hg.1	OTTHUMG00000014290	6.50 ± 0.07	6.47 ± 0.17	6.30 ± 0.19	6.40 ± 0.04
TC06000107.hg.1	OTTHUMG00000014297	6.49 ± 0.27	6.57 ± 0.16	6.58 ± 0.24	6.68 ± 0.24
TC06001307.hg.1	OTTHUMG00000014325	4.72 ± 0.31	4.74 ± 0.21	4.57 ± 0.02	4.85 ± 0.14
TC06001308.hg.1	OTTHUMG00000014329	5.86 ± 0.20	5.86 ± 0.20	5.91 ± 0.24	6.00 ± 0.13
TC06001311.hg.1	OTTHUMG00000014331	6.41 ± 0.28	6.17 ± 0.01	6.18 ± 0.06	6.23 ± 0.13
TC06001310.hg.1	OTTHUMG00000014332	6.05 ± 0.18	5.91 ± 0.06	5.98 ± 0.14	5.95 ± 0.03
TC06000127.hg.1	OTTHUMG00000014335	8.43 ± 0.13	8.35 ± 0.17	8.31 ± 0.15	8.51 ± 0.11
TC06001315.hg.1	OTTHUMG00000014339	5.55 ± 0.10	5.36 ± 0.08	5.38 ± 0.12	5.37 ± 0.03
TC06000134.hg.1	OTTHUMG00000014341	6.47 ± 0.32	6.36 ± 0.29	6.56 ± 0.18	6.61 ± 0.03
TC06001317.hg.1	OTTHUMG00000014342	5.65 ± 0.18	5.66 ± 0.02	5.49 ± 0.10	5.76 ± 0.09
TC06001321.hg.1	OTTHUMG00000014347	4.57 ± 0.01	4.39 ± 0.06	4.45 ± 0.06	4.53 ± 0.22
TC06001322.hg.1	OTTHUMG00000014348	5.49 ± 0.14	5.35 ± 0.04	5.41 ± 0.01	5.54 ± 0.05
TC06000139.hg.1	OTTHUMG00000014350	5.65 ± 0.14	5.55 ± 0.15	5.54 ± 0.07	5.66 ± 0.09
TC06001330.hg.1	OTTHUMG00000014379	6.55 ± 0.23	6.42 ± 0.10	6.45 ± 0.08	6.54 ± 0.10
TC06001331.hg.1	OTTHUMG00000014380	5.47 ± 0.17	5.42 ± 0.14	5.56 ± 0.10	5.37 ± 0.02
TC06000150.hg.1	OTTHUMG00000014382	6.08 ± 0.17	6.03 ± 0.07	6.13 ± 0.09	6.00 ± 0.12
TC06001333.hg.1	OTTHUMG00000014389	6.33 ± 0.28	6.44 ± 0.08	6.31 ± 0.10	6.50 ± 0.05
TC06000502.hg.1	OTTHUMG00000014542	6.68 ± 0.44	6.65 ± 0.20	6.75 ± 0.23	6.44 ± 0.15
TC06000517.hg.1	OTTHUMG00000014573	6.88 ± 0.17	6.83 ± 0.20	6.84 ± 0.08	6.95 ± 0.17
TC06000527.hg.1	OTTHUMG00000014592	6.05 ± 0.13	5.98 ± 0.11	6.15 ± 0.16	6.12 ± 0.02
TC06000537.hg.1	OTTHUMG00000014610	5.54 ± 0.36	5.44 ± 0.29	5.61 ± 0.23	5.57 ± 0.12
TC06000548.hg.1	OTTHUMG00000014623	7.57 ± 0.21	7.70 ± 0.17	7.53 ± 0.15	7.88 ± 0.22
TC06001688.hg.1	OTTHUMG00000014627	6.36 ± 0.14	6.35 ± 0.11	6.09 ± 0.17	6.32 ± 0.08
TC06000555.hg.1	OTTHUMG00000014635	6.57 ± 0.26	6.54 ± 0.14	6.44 ± 0.19	6.52 ± 0.15
TC06004131.hg.1	OTTHUMG00000014652	6.67 ± 0.20	6.75 ± 0.18	6.57 ± 0.12	6.83 ± 0.15
TC06001703.hg.1	OTTHUMG00000014657	5.16 ± 0.35	5.05 ± 0.15	4.97 ± 0.17	5.17 ± 0.19

TC06001705.hg.1	OTTHUMG00000014659	8.17 ± 0.43	8.11 ± 0.04	8.02 ± 0.12	8.38 ± 0.37
TC06001707.hg.1	OTTHUMG00000014661	6.29 ± 0.12	6.34 ± 0.12	6.10 ± 0.27	6.42 ± 0.12
TC06001709.hg.1	OTTHUMG00000014664	6.93 ± 0.16	6.81 ± 0.17	6.87 ± 0.12	6.95 ± 0.16
TC06000578.hg.1	OTTHUMG00000014691	8.21 ± 0.15	7.96 ± 0.13	8.06 ± 0.17	7.98 ± 0.03
TC06000589.hg.1	OTTHUMG00000014707	7.45 ± 0.12	7.37 ± 0.10	7.28 ± 0.08	7.40 ± 0.07
TC06001753.hg.1	OTTHUMG00000014741	6.17 ± 0.14	6.06 ± 0.04	6.19 ± 0.03	6.11 ± 0.06
TC06001756.hg.1	OTTHUMG00000014749	6.27 ± 0.25	6.20 ± 0.35	6.14 ± 0.15	6.33 ± 0.08
TC06001758.hg.1	OTTHUMG00000014751	7.34 ± 0.26	7.36 ± 0.08	7.35 ± 0.16	7.55 ± 0.13
TC06001759.hg.1	OTTHUMG00000014752	6.24 ± 0.06	6.34 ± 0.30	6.30 ± 0.27	6.55 ± 0.07
TC06000611.hg.1	OTTHUMG00000014754	6.99 ± 0.35	6.99 ± 0.16	6.87 ± 0.26	7.02 ± 0.06
TC06000619.hg.1	OTTHUMG00000014768	7.09 ± 0.23	6.97 ± 0.06	7.01 ± 0.14	7.12 ± 0.07
TC06001769.hg.1	OTTHUMG00000014778	6.84 ± 0.19	6.82 ± 0.02	6.68 ± 0.08	6.72 ± 0.06
TC06000626.hg.1	OTTHUMG00000014783	5.70 ± 0.32	5.77 ± 0.17	5.62 ± 0.09	5.79 ± 0.07
TC06004089.hg.1	OTTHUMG00000014787	7.00 ± 0.17	7.00 ± 0.17	6.88 ± 0.38	7.11 ± 0.11
TC06001774.hg.1	OTTHUMG00000014792	5.42 ± 0.14	5.40 ± 0.20	5.53 ± 0.16	5.60 ± 0.15
TC06000632.hg.1	OTTHUMG00000014794	5.35 ± 0.06	5.31 ± 0.06	5.34 ± 0.16	5.28 ± 0.09
TC06001779.hg.1	OTTHUMG00000014802	5.64 ± 0.09	5.54 ± 0.11	5.60 ± 0.17	5.59 ± 0.12
TC06000643.hg.1	OTTHUMG00000014826	4.34 ± 0.18	4.34 ± 0.15	4.22 ± 0.08	4.38 ± 0.21
TC06001787.hg.1	OTTHUMG00000014828	5.21 ± 0.22	5.09 ± 0.13	5.14 ± 0.06	5.17 ± 0.07
TC06000645.hg.1	OTTHUMG00000014830	3.67 ± 0.23	3.57 ± 0.02	3.65 ± 0.09	3.58 ± 0.03
TC06001794.hg.1	OTTHUMG00000014831	4.88 ± 0.04	4.62 ± 0.11	4.67 ± 0.05	4.67 ± 0.09
TC06000649.hg.1	OTTHUMG00000014839	4.60 ± 0.12	4.48 ± 0.17	4.46 ± 0.10	4.60 ± 0.16
TC06001797.hg.1	OTTHUMG00000014842	6.80 ± 0.07	6.83 ± 0.08	6.93 ± 0.01	6.96 ± 0.15
TC06000656.hg.1	OTTHUMG00000014849	7.08 ± 0.19	6.85 ± 0.07	7.05 ± 0.11	6.88 ± 0.03
TC06004052.hg.1	OTTHUMG00000014879	5.82 ± 0.19	5.78 ± 0.03	5.79 ± 0.06	5.84 ± 0.14
TC06000665.hg.1	OTTHUMG00000014881	6.17 ± 0.16	6.15 ± 0.11	6.23 ± 0.17	6.41 ± 0.29
TC06001818.hg.1	OTTHUMG00000014883	4.74 ± 0.08	4.59 ± 0.07	4.60 ± 0.05	4.61 ± 0.12
TC06001819.hg.1	OTTHUMG00000014884	5.63 ± 0.09	5.54 ± 0.16	5.47 ± 0.07	5.67 ± 0.10
TC06004093.hg.1	OTTHUMG00000014887	7.72 ± 0.23	7.77 ± 0.07	7.67 ± 0.06	7.86 ± 0.09
TC06001821.hg.1	OTTHUMG00000014892	5.41 ± 0.14	5.31 ± 0.18	5.23 ± 0.14	5.20 ± 0.13
TC06001823.hg.1	OTTHUMG00000014898	5.25 ± 0.32	5.26 ± 0.24	5.08 ± 0.20	5.36 ± 0.20

TC06001827.hg.1	OTTHUMG00000014906	5.84 ± 0.40	6.02 ± 0.13	6.18 ± 0.19	6.12 ± 0.44
TC06000679.hg.1	OTTHUMG00000014909	7.71 ± 0.25	7.59 ± 0.16	7.60 ± 0.15	7.67 ± 0.05
TC06001829.hg.1	OTTHUMG00000014917	5.21 ± 0.19	5.26 ± 0.08	5.13 ± 0.10	5.38 ± 0.16
TC06000687.hg.1	OTTHUMG00000014922	5.10 ± 0.22	4.83 ± 0.22	4.90 ± 0.05	5.04 ± 0.11
TC06000692.hg.1	OTTHUMG00000014930	4.97 ± 0.35	4.71 ± 0.05	4.77 ± 0.08	4.94 ± 0.09
TC06000693.hg.1	OTTHUMG00000014933	7.13 ± 0.31	7.08 ± 0.20	7.04 ± 0.09	7.22 ± 0.19
TC06001841.hg.1	OTTHUMG00000014935	5.40 ± 0.32	5.24 ± 0.08	5.24 ± 0.07	5.25 ± 0.01
TC06000699.hg.1	OTTHUMG00000014954	5.22 ± 0.09	5.32 ± 0.17	5.44 ± 0.25	5.29 ± 0.04
TC06000700.hg.1	OTTHUMG00000014957	4.55 ± 0.22	4.38 ± 0.09	4.50 ± 0.14	4.48 ± 0.06
TC06001846.hg.1	OTTHUMG00000014960	5.32 ± 0.22	5.05 ± 0.23	5.10 ± 0.09	5.14 ± 0.08
TC06000709.hg.1	OTTHUMG00000014978	3.80 ± 0.10	3.76 ± 0.02	3.83 ± 0.10	3.71 ± 0.04
TC06001852.hg.1	OTTHUMG00000014979	6.85 ± 0.18	6.86 ± 0.07	6.82 ± 0.01	7.01 ± 0.15
TC06001853.hg.1	OTTHUMG00000014981	5.00 ± 0.13	5.12 ± 0.05	5.10 ± 0.31	5.19 ± 0.04
TC06000714.hg.1	OTTHUMG00000014989	4.68 ± 0.15	4.78 ± 0.21	4.59 ± 0.17	4.81 ± 0.18
TC06001856.hg.1	OTTHUMG00000014990	8.32 ± 0.24	8.31 ± 0.23	8.15 ± 0.32	8.41 ± 0.04
TC06000721.hg.1	OTTHUMG00000015002	4.58 ± 0.14	4.65 ± 0.08	4.72 ± 0.10	4.75 ± 0.11
TC06000724.hg.1	OTTHUMG00000015005	5.28 ± 0.15	5.11 ± 0.03	5.17 ± 0.05	5.26 ± 0.18
TC06001877.hg.1	OTTHUMG00000015038	5.10 ± 0.08	4.89 ± 0.06	4.96 ± 0.11	4.95 ± 0.05
TC06000739.hg.1	OTTHUMG00000015043	5.43 ± 0.03	5.31 ± 0.12	5.35 ± 0.07	5.32 ± 0.08
TC06000740.hg.1	OTTHUMG00000015045	4.24 ± 0.19	4.07 ± 0.12	4.17 ± 0.10	4.12 ± 0.08
TC06001878.hg.1	OTTHUMG00000015046	4.19 ± 0.18	4.12 ± 0.04	4.16 ± 0.14	4.14 ± 0.06
TC06000742.hg.1	OTTHUMG00000015054	5.42 ± 0.10	5.34 ± 0.08	5.30 ± 0.06	5.41 ± 0.08
TC06000743.hg.1	OTTHUMG00000015055	4.93 ± 0.08	4.81 ± 0.13	4.89 ± 0.09	4.82 ± 0.12
TC06000751.hg.1	OTTHUMG00000015064	4.72 ± 0.12	4.56 ± 0.12	4.59 ± 0.12	4.52 ± 0.10
TC06001893.hg.1	OTTHUMG00000015068	4.07 ± 0.18	3.88 ± 0.15	4.09 ± 0.10	3.89 ± 0.10
TC06001894.hg.1	OTTHUMG00000015069	4.09 ± 0.14	4.11 ± 0.10	4.12 ± 0.20	4.24 ± 0.07
TC06000760.hg.1	OTTHUMG00000015084	4.06 ± 0.27	3.92 ± 0.07	3.99 ± 0.02	4.00 ± 0.10
TC06000763.hg.1	OTTHUMG00000015092	5.69 ± 0.18	5.61 ± 0.03	5.64 ± 0.15	5.72 ± 0.13
TC06001904.hg.1	OTTHUMG00000015096	3.98 ± 0.19	3.75 ± 0.09	3.82 ± 0.17	3.79 ± 0.14
TC06001906.hg.1	OTTHUMG00000015099	5.52 ± 0.19	5.69 ± 0.17	5.60 ± 0.04	5.70 ± 0.15
TC06001905.hg.1	OTTHUMG00000015100	6.47 ± 0.11	6.40 ± 0.15	6.56 ± 0.40	6.47 ± 0.10

TC06001907.hg.1	OTTHUMG00000015101	6.07 ± 0.27	5.99 ± 0.15	5.88 ± 0.14	6.00 ± 0.26
TC06000767.hg.1	OTTHUMG00000015106	6.02 ± 0.18	6.03 ± 0.33	6.10 ± 0.11	6.22 ± 0.26
TC06001914.hg.1	OTTHUMG00000015116	5.28 ± 0.11	5.41 ± 0.11	5.35 ± 0.13	5.39 ± 0.08
TC06000775.hg.1	OTTHUMG00000015119	6.39 ± 0.00	6.38 ± 0.04	6.38 ± 0.11	6.46 ± 0.19
TC06000776.hg.1	OTTHUMG00000015120	6.63 ± 0.11	6.67 ± 0.14	6.58 ± 0.19	6.84 ± 0.13
TC06001916.hg.1	OTTHUMG00000015125	4.40 ± 0.07	4.21 ± 0.07	4.34 ± 0.09	4.27 ± 0.09
TC06001918.hg.1	OTTHUMG00000015137	5.51 ± 0.19	5.43 ± 0.10	5.23 ± 0.05	5.47 ± 0.07
TC06001920.hg.1	OTTHUMG00000015138	6.85 ± 0.19	6.96 ± 0.06	6.97 ± 0.07	6.99 ± 0.04
TC06000780.hg.1	OTTHUMG00000015142	5.60 ± 0.29	5.62 ± 0.31	5.59 ± 0.35	5.47 ± 0.12
TC06001933.hg.1	OTTHUMG00000015187	7.54 ± 0.13	7.07 ± 0.07	7.56 ± 0.14	7.21 ± 0.14
TC06000798.hg.1	OTTHUMG00000015204	5.40 ± 0.08	5.36 ± 0.07	5.25 ± 0.05	5.51 ± 0.07
TC06000804.hg.1	OTTHUMG00000015214	6.76 ± 0.13	6.83 ± 0.13	6.78 ± 0.08	6.86 ± 0.05
TC06001949.hg.1	OTTHUMG00000015218	4.43 ± 0.09	4.34 ± 0.04	4.40 ± 0.07	4.61 ± 0.22
TC06001950.hg.1	OTTHUMG00000015221	5.49 ± 0.11	5.29 ± 0.09	5.39 ± 0.08	5.34 ± 0.12
TC06001951.hg.1	OTTHUMG00000015224	7.81 ± 0.21	7.65 ± 0.10	7.61 ± 0.15	7.75 ± 0.07
TC06000812.hg.1	OTTHUMG00000015227	5.78 ± 0.26	5.87 ± 0.15	5.86 ± 0.09	5.93 ± 0.14
TC06000815.hg.1	OTTHUMG00000015229	5.53 ± 0.11	5.40 ± 0.09	5.27 ± 0.11	5.45 ± 0.08
TC06000816.hg.1	OTTHUMG00000015230	4.21 ± 0.40	3.97 ± 0.08	4.05 ± 0.11	4.05 ± 0.04
TC06000821.hg.1	OTTHUMG00000015244	5.28 ± 0.12	5.28 ± 0.13	5.28 ± 0.01	5.28 ± 0.05
TC06000823.hg.1	OTTHUMG00000015251	4.40 ± 0.03	4.38 ± 0.03	4.37 ± 0.03	4.44 ± 0.11
TC06001960.hg.1	OTTHUMG00000015252	5.59 ± 0.18	5.62 ± 0.11	5.57 ± 0.13	5.80 ± 0.25
TC06000825.hg.1	OTTHUMG00000015254	4.74 ± 0.16	4.64 ± 0.05	4.57 ± 0.11	4.63 ± 0.05
TC06000826.hg.1	OTTHUMG00000015255	4.83 ± 0.12	4.77 ± 0.06	4.89 ± 0.21	4.82 ± 0.15
TC06000827.hg.1	OTTHUMG00000015256	4.30 ± 0.16	4.16 ± 0.16	4.14 ± 0.17	4.38 ± 0.30
TC06000829.hg.1	OTTHUMG00000015263	6.87 ± 0.29	7.11 ± 0.29	6.58 ± 0.34	6.99 ± 0.12
TC06000833.hg.1	OTTHUMG00000015276	5.51 ± 0.38	5.22 ± 0.09	5.38 ± 0.20	5.35 ± 0.07
TC06000839.hg.1	OTTHUMG00000015288	3.84 ± 0.13	3.77 ± 0.08	3.78 ± 0.18	3.60 ± 0.16
TC06000842.hg.1	OTTHUMG00000015296	6.61 ± 0.11	6.54 ± 0.22	6.52 ± 0.10	6.67 ± 0.15
TC06001981.hg.1	OTTHUMG00000015298	5.54 ± 0.12	5.37 ± 0.03	5.42 ± 0.01	5.34 ± 0.06
TC06001983.hg.1	OTTHUMG00000015300	5.08 ± 0.17	5.00 ± 0.06	5.07 ± 0.20	5.18 ± 0.12
TC06000852.hg.1	OTTHUMG00000015306	4.43 ± 0.12	4.28 ± 0.07	4.37 ± 0.05	4.33 ± 0.09

TC06001989.hg.1	OTTHUMG00000015310	6.01 ± 0.13	5.80 ± 0.14	5.85 ± 0.05	5.82 ± 0.01
TC06000864.hg.1	OTTHUMG00000015322	5.56 ± 0.19	5.55 ± 0.17	5.37 ± 0.07	5.62 ± 0.14
TC06000877.hg.1	OTTHUMG00000015347	4.61 ± 0.13	4.39 ± 0.22	4.56 ± 0.18	4.61 ± 0.19
TC06000880.hg.1	OTTHUMG00000015353	5.81 ± 0.20	5.48 ± 0.05	5.94 ± 0.57	5.44 ± 0.06
TC06000891.hg.1	OTTHUMG00000015374	6.37 ± 0.22	5.89 ± 0.13	6.16 ± 0.09	5.92 ± 0.25
TC06002016.hg.1	OTTHUMG00000015375	5.11 ± 0.10	5.12 ± 0.06	5.03 ± 0.13	5.11 ± 0.12
TC06000896.hg.1	OTTHUMG00000015384	4.37 ± 0.10	4.23 ± 0.09	4.32 ± 0.10	4.27 ± 0.07
TC06000897.hg.1	OTTHUMG00000015392	5.37 ± 0.20	5.08 ± 0.02	5.17 ± 0.10	5.10 ± 0.08
TC06002028.hg.1	OTTHUMG00000015393	4.32 ± 0.13	4.36 ± 0.03	4.43 ± 0.13	4.47 ± 0.23
TC06002029.hg.1	OTTHUMG00000015398	4.83 ± 0.29	4.68 ± 0.11	4.74 ± 0.13	4.82 ± 0.18
TC06002030.hg.1	OTTHUMG00000015399	5.61 ± 0.31	5.59 ± 0.23	5.76 ± 0.02	5.62 ± 0.03
TC06000905.hg.1	OTTHUMG00000015400	6.22 ± 0.03	6.17 ± 0.06	6.14 ± 0.14	6.43 ± 0.19
TC06002031.hg.1	OTTHUMG00000015401	4.93 ± 0.14	4.84 ± 0.02	4.86 ± 0.08	4.88 ± 0.07
TC06000907.hg.1	OTTHUMG00000015403	5.53 ± 0.36	5.29 ± 0.09	5.38 ± 0.04	5.31 ± 0.07
TC06000911.hg.1	OTTHUMG00000015407	4.42 ± 0.33	4.20 ± 0.10	4.31 ± 0.03	4.23 ± 0.20
TC06002039.hg.1	OTTHUMG00000015418	4.27 ± 0.07	4.10 ± 0.09	4.15 ± 0.17	4.17 ± 0.06
TC06002040.hg.1	OTTHUMG00000015419	4.69 ± 0.15	4.41 ± 0.09	4.50 ± 0.10	4.53 ± 0.24
TC06002042.hg.1	OTTHUMG00000015421	4.23 ± 0.16	3.92 ± 0.13	4.06 ± 0.05	3.85 ± 0.07
TC06002045.hg.1	OTTHUMG00000015431	5.38 ± 0.35	5.36 ± 0.04	5.34 ± 0.21	5.27 ± 0.05
TC06000920.hg.1	OTTHUMG00000015439	5.82 ± 0.20	5.88 ± 0.12	5.89 ± 0.04	5.93 ± 0.08
TC06002055.hg.1	OTTHUMG00000015453	4.21 ± 0.05	4.14 ± 0.05	4.16 ± 0.06	4.24 ± 0.13
TC06000944.hg.1	OTTHUMG00000015478	7.58 ± 0.26	7.66 ± 0.12	7.67 ± 0.04	7.71 ± 0.07
TC06000952.hg.1	OTTHUMG00000015493	4.45 ± 0.02	4.22 ± 0.05	4.36 ± 0.14	4.28 ± 0.10
TC06000953.hg.1	OTTHUMG00000015496	4.89 ± 0.18	4.72 ± 0.02	4.70 ± 0.18	4.74 ± 0.06
TC06000955.hg.1	OTTHUMG00000015498	4.25 ± 0.08	4.15 ± 0.06	4.16 ± 0.05	4.27 ± 0.11
TC06002077.hg.1	OTTHUMG00000015503	4.85 ± 0.16	4.70 ± 0.07	4.76 ± 0.20	4.78 ± 0.02
TC06002079.hg.1	OTTHUMG00000015507	5.69 ± 0.09	5.59 ± 0.03	5.73 ± 0.10	5.62 ± 0.10
TC06002080.hg.1	OTTHUMG00000015508	4.29 ± 0.05	4.10 ± 0.14	4.23 ± 0.21	4.30 ± 0.04
TC06002083.hg.1	OTTHUMG00000015510	5.54 ± 0.08	5.62 ± 0.09	5.52 ± 0.09	5.74 ± 0.11
TC06002081.hg.1	OTTHUMG00000015511	5.56 ± 0.27	5.50 ± 0.04	5.61 ± 0.06	5.64 ± 0.15
TC06000965.hg.1	OTTHUMG00000015516	4.70 ± 0.03	4.62 ± 0.12	4.66 ± 0.14	4.64 ± 0.14

TC06000971.hg.1	OTTHUMG00000015535	4.98 ± 0.15	4.97 ± 0.10	4.98 ± 0.08	4.98 ± 0.11
TC06002094.hg.1	OTTHUMG00000015537	4.05 ± 0.11	3.82 ± 0.03	4.07 ± 0.20	3.97 ± 0.23
TC06002095.hg.1	OTTHUMG00000015538	6.12 ± 0.23	6.10 ± 0.11	6.20 ± 0.13	6.20 ± 0.07
TC06000973.hg.1	OTTHUMG00000015539	12.81 ± 0.23	12.83 ± 0.04	12.78 ± 0.06	12.92 ± 0.12
TC06000972.hg.1	OTTHUMG00000015540	8.77 ± 0.28	8.70 ± 0.04	8.72 ± 0.09	8.85 ± 0.16
TC06002098.hg.1	OTTHUMG00000015548	5.34 ± 0.12	5.22 ± 0.01	5.27 ± 0.04	5.27 ± 0.10
TC06002102.hg.1	OTTHUMG00000015555	7.03 ± 0.56	7.18 ± 0.12	6.90 ± 0.25	6.87 ± 0.10
TC06000980.hg.1	OTTHUMG00000015558	4.95 ± 0.26	4.85 ± 0.16	5.00 ± 0.06	4.88 ± 0.09
TC06000995.hg.1	OTTHUMG00000015592	9.61 ± 0.19	9.36 ± 0.10	9.86 ± 0.34	9.43 ± 0.08
TC06004151.hg.1	OTTHUMG00000015607	5.51 ± 0.07	5.45 ± 0.08	5.51 ± 0.10	5.56 ± 0.10
TC06002128.hg.1	OTTHUMG00000015617	5.94 ± 0.15	5.88 ± 0.11	5.93 ± 0.05	5.86 ± 0.08
TC06002129.hg.1	OTTHUMG00000015618	5.76 ± 0.15	5.72 ± 0.12	5.63 ± 0.14	5.79 ± 0.07
TC06001007.hg.1	OTTHUMG00000015619	6.33 ± 0.03	6.31 ± 0.15	6.38 ± 0.22	6.41 ± 0.14
TC06002131.hg.1	OTTHUMG00000015621	7.28 ± 0.33	7.19 ± 0.05	7.24 ± 0.21	7.25 ± 0.08
TC06001009.hg.1	OTTHUMG00000015625	5.29 ± 0.29	5.07 ± 0.13	5.05 ± 0.05	5.11 ± 0.09
TC06001012.hg.1	OTTHUMG00000015628	8.75 ± 0.25	8.64 ± 0.08	8.68 ± 0.08	8.78 ± 0.06
TC06001013.hg.1	OTTHUMG00000015630	4.63 ± 0.03	4.53 ± 0.06	4.68 ± 0.19	4.46 ± 0.13
TC06002138.hg.1	OTTHUMG00000015634	3.97 ± 0.14	3.83 ± 0.09	3.80 ± 0.09	3.89 ± 0.05
TC06002139.hg.1	OTTHUMG00000015637	5.58 ± 0.11	5.54 ± 0.12	5.63 ± 0.13	5.70 ± 0.09
TC06001017.hg.1	OTTHUMG00000015645	5.36 ± 0.06	5.08 ± 0.05	5.44 ± 0.15	5.10 ± 0.12
TC06001022.hg.1	OTTHUMG00000015653	5.32 ± 0.14	5.12 ± 0.19	5.14 ± 0.03	5.33 ± 0.28
TC06001024.hg.1	OTTHUMG00000015659	5.28 ± 0.14	5.15 ± 0.10	5.11 ± 0.07	5.18 ± 0.09
TC06002155.hg.1	OTTHUMG00000015662	7.15 ± 0.16	7.28 ± 0.06	6.92 ± 0.13	7.22 ± 0.09
TC06002156.hg.1	OTTHUMG00000015665	6.17 ± 0.44	5.76 ± 0.26	5.84 ± 0.30	5.68 ± 0.05
TC06001028.hg.1	OTTHUMG00000015667	5.33 ± 0.10	5.27 ± 0.04	5.24 ± 0.12	5.33 ± 0.19
TC06002165.hg.1	OTTHUMG00000015687	6.99 ± 0.05	6.82 ± 0.05	6.87 ± 0.03	6.92 ± 0.12
TC06001036.hg.1	OTTHUMG00000015689	6.26 ± 0.10	6.22 ± 0.07	6.22 ± 0.05	6.22 ± 0.05
TC06002170.hg.1	OTTHUMG00000015698	5.52 ± 0.17	5.43 ± 0.17	5.29 ± 0.22	5.45 ± 0.18
TC06002173.hg.1	OTTHUMG00000015700	5.22 ± 0.23	5.25 ± 0.12	5.27 ± 0.07	5.39 ± 0.14
TC06001042.hg.1	OTTHUMG00000015705	7.32 ± 0.22	7.24 ± 0.18	7.06 ± 0.18	7.36 ± 0.15
TC06002179.hg.1	OTTHUMG00000015708	6.17 ± 0.38	6.02 ± 0.13	6.14 ± 0.08	6.16 ± 0.09

TC06001048.hg.1	OTTHUMG00000015714	4.15 ± 0.09	4.11 ± 0.05	4.18 ± 0.09	4.09 ± 0.03
TC06001047.hg.1	OTTHUMG00000015715	6.83 ± 0.04	6.92 ± 0.17	6.98 ± 0.14	6.95 ± 0.17
TC06001049.hg.1	OTTHUMG00000015716	6.11 ± 0.29	6.51 ± 0.07	6.25 ± 0.30	6.58 ± 0.15
TC06002182.hg.1	OTTHUMG00000015717	6.68 ± 0.15	6.74 ± 0.08	6.57 ± 0.06	6.72 ± 0.09
TC06002185.hg.1	OTTHUMG00000015719	5.69 ± 0.18	5.61 ± 0.02	5.67 ± 0.13	5.59 ± 0.10
TC06002184.hg.1	OTTHUMG00000015720	5.35 ± 0.30	5.00 ± 0.03	5.27 ± 0.18	5.02 ± 0.18
TC06001053.hg.1	OTTHUMG00000015729	6.81 ± 0.24	6.70 ± 0.09	6.91 ± 0.08	6.71 ± 0.09
TC06002192.hg.1	OTTHUMG00000015742	5.29 ± 0.18	5.18 ± 0.06	5.12 ± 0.10	5.27 ± 0.09
TC06002198.hg.1	OTTHUMG00000015756	5.94 ± 0.17	5.83 ± 0.07	5.84 ± 0.15	5.98 ± 0.10
TC06001069.hg.1	OTTHUMG00000015760	4.13 ± 0.18	3.76 ± 0.02	3.82 ± 0.11	3.85 ± 0.12
TC06001070.hg.1	OTTHUMG00000015761	6.98 ± 0.15	7.10 ± 0.03	7.10 ± 0.17	7.07 ± 0.16
TC06002202.hg.1	OTTHUMG00000015768	5.28 ± 0.11	5.12 ± 0.10	5.19 ± 0.20	5.41 ± 0.09
TC06001073.hg.1	OTTHUMG00000015769	5.26 ± 0.11	5.22 ± 0.07	5.24 ± 0.03	5.34 ± 0.15
TC06002203.hg.1	OTTHUMG00000015770	4.45 ± 0.23	4.49 ± 0.06	4.47 ± 0.04	4.55 ± 0.07
TC06002204.hg.1	OTTHUMG00000015771	5.19 ± 0.31	4.95 ± 0.09	4.94 ± 0.08	4.69 ± 0.10
TC06002205.hg.1	OTTHUMG00000015772	6.21 ± 0.24	6.32 ± 0.25	6.23 ± 0.27	6.43 ± 0.15
TC06002207.hg.1	OTTHUMG00000015778	8.00 ± 0.28	8.00 ± 0.13	7.87 ± 0.27	8.09 ± 0.17
TC06002208.hg.1	OTTHUMG00000015781	3.90 ± 0.18	3.75 ± 0.08	3.82 ± 0.06	3.77 ± 0.04
TC06004101.hg.1	OTTHUMG00000015782	5.38 ± 0.04	5.34 ± 0.15	5.30 ± 0.08	5.42 ± 0.14
TC06001081.hg.1	OTTHUMG00000015791	4.78 ± 0.13	4.71 ± 0.09	4.63 ± 0.07	4.70 ± 0.02
TC06002211.hg.1	OTTHUMG00000015792	5.38 ± 0.12	5.23 ± 0.23	5.08 ± 0.18	5.19 ± 0.01
TC06002212.hg.1	OTTHUMG00000015793	7.43 ± 0.22	7.41 ± 0.11	7.46 ± 0.06	7.58 ± 0.20
TC06002216.hg.1	OTTHUMG00000015798	7.07 ± 0.23	7.18 ± 0.05	7.14 ± 0.08	7.34 ± 0.19
TC06001087.hg.1	OTTHUMG00000015806	6.83 ± 0.30	6.77 ± 0.11	6.78 ± 0.05	6.85 ± 0.11
TC06002223.hg.1	OTTHUMG00000015812	6.95 ± 0.24	6.79 ± 0.15	6.94 ± 0.07	6.88 ± 0.16
TC06001095.hg.1	OTTHUMG00000015821	7.48 ± 0.39	8.10 ± 0.41	7.17 ± 0.17	8.13 ± 0.28
TC06001096.hg.1	OTTHUMG00000015822	7.43 ± 0.36	7.65 ± 0.12	7.29 ± 0.08	7.79 ± 0.21
TC06002226.hg.1	OTTHUMG00000015823	4.13 ± 0.12	4.10 ± 0.05	4.15 ± 0.02	4.18 ± 0.08
TC06002228.hg.1	OTTHUMG00000015829	4.10 ± 0.17	4.06 ± 0.07	3.99 ± 0.15	4.05 ± 0.05
TC06002229.hg.1	OTTHUMG00000015831	6.00 ± 0.17	6.02 ± 0.07	5.99 ± 0.06	6.15 ± 0.14
TC06001106.hg.1	OTTHUMG00000015845	4.73 ± 0.06	4.58 ± 0.10	4.73 ± 0.18	4.68 ± 0.07

TC06001108.hg.1	OTTHUMG00000015847	4.51 ± 0.19	4.31 ± 0.19	4.42 ± 0.09	4.39 ± 0.17
TC06002239.hg.1	OTTHUMG00000015852	4.89 ± 0.13	4.74 ± 0.04	4.89 ± 0.16	4.77 ± 0.16
TC06001111.hg.1	OTTHUMG00000015856	7.86 ± 0.03	7.84 ± 0.16	7.66 ± 0.11	7.76 ± 0.04
TC06002244.hg.1	OTTHUMG00000015860	4.75 ± 0.21	4.64 ± 0.17	4.52 ± 0.07	4.80 ± 0.17
TC06002249.hg.1	OTTHUMG00000015884	6.17 ± 0.11	6.22 ± 0.17	6.12 ± 0.04	6.37 ± 0.15
TC06002252.hg.1	OTTHUMG00000015885	5.46 ± 0.15	5.51 ± 0.02	5.43 ± 0.10	5.54 ± 0.05
TC06002255.hg.1	OTTHUMG00000015889	5.55 ± 0.10	5.56 ± 0.18	5.48 ± 0.11	5.72 ± 0.22
TC06002258.hg.1	OTTHUMG00000015897	5.96 ± 0.18	5.81 ± 0.00	5.84 ± 0.03	5.83 ± 0.04
TC06002257.hg.1	OTTHUMG00000015898	4.87 ± 0.13	5.17 ± 0.28	4.84 ± 0.10	5.02 ± 0.14
TC06002259.hg.1	OTTHUMG00000015900	4.86 ± 0.11	5.27 ± 0.24	5.02 ± 0.26	5.46 ± 0.25
TC06002262.hg.1	OTTHUMG00000015908	6.62 ± 0.10	6.68 ± 0.16	6.79 ± 0.09	6.82 ± 0.11
TC06001131.hg.1	OTTHUMG00000015909	7.83 ± 0.23	7.97 ± 0.30	7.24 ± 0.24	7.70 ± 0.07
TC06002271.hg.1	OTTHUMG00000015922	6.42 ± 0.26	6.51 ± 0.16	6.30 ± 0.20	6.59 ± 0.20
TC06001140.hg.1	OTTHUMG00000015925	6.91 ± 0.10	7.01 ± 0.07	6.81 ± 0.13	6.95 ± 0.09
TC06001141.hg.1	OTTHUMG00000015926	7.06 ± 0.14	7.15 ± 0.17	7.17 ± 0.19	7.37 ± 0.17
TC06002272.hg.1	OTTHUMG00000015930	4.92 ± 0.10	4.88 ± 0.02	4.76 ± 0.13	4.92 ± 0.22
TC06001144.hg.1	OTTHUMG00000015931	5.30 ± 0.21	5.23 ± 0.15	5.25 ± 0.15	5.33 ± 0.16
TC06002274.hg.1	OTTHUMG00000015939	5.54 ± 0.35	4.88 ± 0.13	6.81 ± 0.44	4.77 ± 0.22
TC06001150.hg.1	OTTHUMG00000015943	6.69 ± 0.36	6.71 ± 0.04	6.49 ± 0.31	6.72 ± 0.08
TC06001155.hg.1	OTTHUMG00000015949	6.47 ± 0.16	6.40 ± 0.20	6.50 ± 0.13	6.55 ± 0.10
TC06001157.hg.1	OTTHUMG00000015954	6.96 ± 0.23	6.89 ± 0.08	6.80 ± 0.07	6.88 ± 0.11
TC06002282.hg.1	OTTHUMG00000015958	3.27 ± 0.07	3.14 ± 0.06	3.27 ± 0.03	3.14 ± 0.04
TC06002283.hg.1	OTTHUMG00000015959	6.94 ± 0.19	6.85 ± 0.07	6.84 ± 0.10	6.96 ± 0.06
TC06001159.hg.1	OTTHUMG00000015961	8.16 ± 0.18	8.14 ± 0.26	8.08 ± 0.25	8.32 ± 0.17
TC06001160.hg.1	OTTHUMG00000015962	5.22 ± 0.23	5.04 ± 0.13	5.11 ± 0.28	5.22 ± 0.11
TC06002288.hg.1	OTTHUMG00000015973	4.92 ± 0.12	4.87 ± 0.08	4.85 ± 0.16	4.88 ± 0.09
TC06001166.hg.1	OTTHUMG00000015978	6.09 ± 0.26	5.84 ± 0.08	5.91 ± 0.09	5.91 ± 0.05
TC06001167.hg.1	OTTHUMG00000015979	6.09 ± 0.23	6.17 ± 0.29	6.03 ± 0.21	6.27 ± 0.07
TC06002293.hg.1	OTTHUMG00000015980	5.84 ± 0.25	6.02 ± 0.13	5.80 ± 0.20	5.91 ± 0.23
TC06002294.hg.1	OTTHUMG00000015981	4.81 ± 0.17	4.67 ± 0.17	4.87 ± 0.10	4.78 ± 0.22
TC06001168.hg.1	OTTHUMG00000015982	4.83 ± 0.28	4.71 ± 0.31	4.86 ± 0.01	4.76 ± 0.11

TC06001170.hg.1	OTTHUMG00000015988	5.58 ± 0.08	5.53 ± 0.23	5.61 ± 0.14	5.69 ± 0.22
TC06002299.hg.1	OTTHUMG00000015989	4.51 ± 0.12	4.38 ± 0.06	4.49 ± 0.17	4.37 ± 0.04
TC06001173.hg.1	OTTHUMG00000015993	5.99 ± 0.34	5.84 ± 0.08	5.93 ± 0.16	5.96 ± 0.18
TC06001175.hg.1	OTTHUMG00000015997	7.25 ± 0.24	6.95 ± 0.18	7.12 ± 0.23	7.04 ± 0.06
TC06002311.hg.1	OTTHUMG00000016010	8.13 ± 0.10	8.34 ± 0.09	8.38 ± 0.12	8.47 ± 0.05
TC06001181.hg.1	OTTHUMG00000016019	6.25 ± 0.17	6.25 ± 0.21	6.18 ± 0.11	6.23 ± 0.13
TC06002314.hg.1	OTTHUMG00000016020	6.89 ± 0.31	6.84 ± 0.25	6.73 ± 0.17	6.77 ± 0.08
TC06001186.hg.1	OTTHUMG00000016040	8.10 ± 0.28	8.06 ± 0.19	8.03 ± 0.17	8.13 ± 0.13
TC06002323.hg.1	OTTHUMG00000016041	5.63 ± 0.09	5.61 ± 0.08	5.68 ± 0.09	5.74 ± 0.19
TC06001187.hg.1	OTTHUMG00000016042	6.42 ± 0.17	6.29 ± 0.09	6.32 ± 0.08	6.36 ± 0.03
TC06001188.hg.1	OTTHUMG00000016043	7.31 ± 0.07	7.30 ± 0.01	7.27 ± 0.09	7.36 ± 0.08
TC06002325.hg.1	OTTHUMG00000016045	5.57 ± 0.27	5.70 ± 0.21	5.54 ± 0.16	5.94 ± 0.09
TC06001190.hg.1	OTTHUMG00000016049	6.54 ± 0.20	6.66 ± 0.18	6.66 ± 0.11	6.73 ± 0.04
TC06001191.hg.1	OTTHUMG00000016051	3.49 ± 0.05	3.29 ± 0.02	3.41 ± 0.12	3.30 ± 0.04
TC06002327.hg.1	OTTHUMG00000016052	5.61 ± 0.05	5.85 ± 0.21	5.54 ± 0.32	5.71 ± 0.09
TC06002326.hg.1	OTTHUMG00000016053	6.53 ± 0.12	6.51 ± 0.14	6.60 ± 0.07	6.65 ± 0.13
TC06002328.hg.1	OTTHUMG00000016054	7.46 ± 0.21	7.43 ± 0.20	7.39 ± 0.19	7.51 ± 0.06
TC06002332.hg.1	OTTHUMG00000016055	6.83 ± 0.11	6.82 ± 0.18	6.79 ± 0.29	6.99 ± 0.20
TC06002336.hg.1	OTTHUMG00000016062	7.06 ± 0.26	7.07 ± 0.18	6.97 ± 0.17	7.25 ± 0.09
TC06001194.hg.1	OTTHUMG00000016063	7.89 ± 0.14	7.99 ± 0.21	7.75 ± 0.17	8.30 ± 0.12
TC06002340.hg.1	OTTHUMG00000016072	8.73 ± 0.33	8.82 ± 0.22	8.69 ± 0.25	9.11 ± 0.23
TC06001198.hg.1	OTTHUMG00000016077	6.80 ± 0.24	6.65 ± 0.27	6.64 ± 0.25	6.73 ± 0.08
TC06001202.hg.1	OTTHUMG00000016088	6.41 ± 0.31	6.53 ± 0.09	6.54 ± 0.23	6.82 ± 0.17
TC06001203.hg.1	OTTHUMG00000016089	6.48 ± 0.22	6.29 ± 0.07	6.37 ± 0.09	6.35 ± 0.01
TC06001204.hg.1	OTTHUMG00000016090	7.80 ± 0.30	7.44 ± 0.25	8.51 ± 0.37	7.53 ± 0.20
TC06002345.hg.1	OTTHUMG00000016092	7.37 ± 0.37	7.51 ± 0.13	7.38 ± 0.28	7.71 ± 0.26
TC06002237.hg.1	OTTHUMG00000016436	6.51 ± 0.37	6.61 ± 0.28	6.65 ± 0.15	6.85 ± 0.17
TC13000006.hg.1	OTTHUMG00000016460	5.95 ± 0.25	5.84 ± 0.07	5.72 ± 0.19	5.96 ± 0.22
TC13000439.hg.1	OTTHUMG00000016461	5.57 ± 0.16	5.38 ± 0.16	5.48 ± 0.20	5.60 ± 0.04
TC13000009.hg.1	OTTHUMG00000016470	4.96 ± 0.04	4.86 ± 0.31	4.89 ± 0.12	5.31 ± 0.30
TC13000011.hg.1	OTTHUMG00000016476	3.70 ± 0.03	3.37 ± 0.16	3.41 ± 0.09	3.39 ± 0.08

TC13000014.hg.1	OTTHUMG00000016477	9.02 ± 0.03	8.86 ± 0.04	9.05 ± 0.10	8.91 ± 0.01
TC13000451.hg.1	OTTHUMG00000016489	7.05 ± 0.26	6.84 ± 0.07	6.98 ± 0.19	6.90 ± 0.08
TC13000019.hg.1	OTTHUMG00000016503	9.06 ± 0.24	9.07 ± 0.04	9.06 ± 0.10	9.18 ± 0.07
TC13000024.hg.1	OTTHUMG00000016506	6.63 ± 0.19	6.53 ± 0.05	6.59 ± 0.10	6.56 ± 0.07
TC13000462.hg.1	OTTHUMG00000016526	6.55 ± 0.10	6.60 ± 0.06	6.43 ± 0.21	6.63 ± 0.03
TC13000032.hg.1	OTTHUMG00000016533	5.54 ± 0.11	5.48 ± 0.06	5.45 ± 0.15	5.68 ± 0.17
TC13000033.hg.1	OTTHUMG00000016536	6.62 ± 0.07	6.64 ± 0.08	6.64 ± 0.09	6.68 ± 0.03
TC13000042.hg.1	OTTHUMG00000016555	4.07 ± 0.23	4.11 ± 0.24	4.04 ± 0.25	3.90 ± 0.07
TC13000054.hg.1	OTTHUMG00000016583	6.51 ± 0.14	6.54 ± 0.10	6.60 ± 0.13	6.60 ± 0.16
TC13000062.hg.1	OTTHUMG00000016599	6.70 ± 0.22	6.50 ± 0.23	6.57 ± 0.30	6.51 ± 0.23
TC13000497.hg.1	OTTHUMG00000016604	5.51 ± 0.07	5.56 ± 0.23	5.55 ± 0.11	5.80 ± 0.14
TC13000061.hg.1	OTTHUMG00000016605	6.68 ± 0.12	6.81 ± 0.06	6.70 ± 0.21	6.87 ± 0.15
TC13000507.hg.1	OTTHUMG00000016629	5.62 ± 0.18	5.34 ± 0.12	5.58 ± 0.33	5.22 ± 0.25
TC13000076.hg.1	OTTHUMG00000016631	5.46 ± 0.20	5.50 ± 0.10	5.42 ± 0.09	5.56 ± 0.10
TC13000097.hg.1	OTTHUMG00000016664	7.25 ± 0.25	7.09 ± 0.10	7.26 ± 0.10	7.17 ± 0.16
TC13000537.hg.1	OTTHUMG00000016683	5.23 ± 0.19	5.08 ± 0.07	5.09 ± 0.11	5.21 ± 0.10
TC13000538.hg.1	OTTHUMG00000016684	4.33 ± 0.29	4.19 ± 0.03	4.19 ± 0.06	4.19 ± 0.04
TC13000110.hg.1	OTTHUMG00000016695	5.99 ± 0.33	5.88 ± 0.26	6.20 ± 0.64	5.86 ± 0.18
TC13000116.hg.1	OTTHUMG00000016711	3.88 ± 0.09	3.85 ± 0.13	3.95 ± 0.05	3.93 ± 0.15
TC13000117.hg.1	OTTHUMG00000016713	7.18 ± 0.14	7.21 ± 0.27	6.96 ± 0.20	7.32 ± 0.18
TC13000118.hg.1	OTTHUMG00000016716	5.98 ± 0.20	5.66 ± 0.12	5.79 ± 0.07	5.81 ± 0.05
TC13000575.hg.1	OTTHUMG00000016750	6.76 ± 0.10	6.68 ± 0.12	6.66 ± 0.02	6.88 ± 0.15
TC13000581.hg.1	OTTHUMG00000016756	5.17 ± 0.05	5.11 ± 0.07	5.16 ± 0.06	5.17 ± 0.08
TC13000609.hg.1	OTTHUMG00000016801	3.83 ± 0.14	3.59 ± 0.06	3.71 ± 0.03	3.65 ± 0.01
TC13000611.hg.1	OTTHUMG00000016811	4.40 ± 0.16	4.33 ± 0.10	4.24 ± 0.07	4.30 ± 0.08
TC13000616.hg.1	OTTHUMG00000016828	6.38 ± 0.10	6.35 ± 0.25	6.31 ± 0.15	6.45 ± 0.12
TC13000621.hg.1	OTTHUMG00000016834	6.21 ± 0.15	6.18 ± 0.19	6.18 ± 0.09	6.35 ± 0.14
TC13000167.hg.1	OTTHUMG00000016839	6.28 ± 0.28	6.08 ± 0.09	6.13 ± 0.10	5.99 ± 0.02
TC13000169.hg.1	OTTHUMG00000016843	5.02 ± 0.13	5.06 ± 0.17	5.05 ± 0.04	5.16 ± 0.13
TC13000174.hg.1	OTTHUMG00000016846	5.96 ± 0.27	5.92 ± 0.13	5.99 ± 0.20	5.89 ± 0.13
TC13000637.hg.1	OTTHUMG00000016859	8.37 ± 0.23	8.42 ± 0.10	8.44 ± 0.12	8.59 ± 0.11

TC13000646.hg.1	OTTHUMG00000016875	5.03 ± 0.34	5.04 ± 0.21	4.90 ± 0.06	5.15 ± 0.13
TC13000650.hg.1	OTTHUMG00000016885	5.31 ± 0.31	5.35 ± 0.01	5.28 ± 0.21	5.52 ± 0.10
TC13000657.hg.1	OTTHUMG00000016904	7.50 ± 0.26	7.47 ± 0.22	7.44 ± 0.06	7.60 ± 0.09
TC13000671.hg.1	OTTHUMG00000016932	6.33 ± 0.37	6.24 ± 0.05	6.33 ± 0.17	6.32 ± 0.12
TC13000218.hg.1	OTTHUMG00000016953	5.81 ± 0.16	5.89 ± 0.06	5.84 ± 0.20	6.01 ± 0.15
TC13000694.hg.1	OTTHUMG00000016983	4.87 ± 0.09	4.87 ± 0.11	4.97 ± 0.07	5.05 ± 0.18
TC13000236.hg.1	OTTHUMG00000016997	4.82 ± 0.14	4.63 ± 0.06	4.79 ± 0.09	4.77 ± 0.11
TC13000245.hg.1	OTTHUMG00000017010	4.67 ± 0.09	4.64 ± 0.14	4.60 ± 0.03	4.76 ± 0.21
TC13000248.hg.1	OTTHUMG00000017016	3.92 ± 0.13	3.64 ± 0.07	3.80 ± 0.14	3.61 ± 0.03
TC13000247.hg.1	OTTHUMG00000017017	3.46 ± 0.27	3.19 ± 0.09	3.29 ± 0.03	3.13 ± 0.08
TC13000711.hg.1	OTTHUMG00000017020	4.28 ± 0.09	4.14 ± 0.06	4.15 ± 0.10	4.08 ± 0.21
TC13000252.hg.1	OTTHUMG00000017023	4.46 ± 0.20	4.40 ± 0.07	4.36 ± 0.06	4.55 ± 0.14
TC13000713.hg.1	OTTHUMG00000017025	5.87 ± 0.04	5.81 ± 0.06	5.74 ± 0.07	5.85 ± 0.05
TC13000255.hg.1	OTTHUMG00000017032	4.12 ± 0.17	4.05 ± 0.18	4.01 ± 0.06	4.13 ± 0.15
TC13000261.hg.1	OTTHUMG00000017052	4.38 ± 0.09	4.40 ± 0.04	4.41 ± 0.04	4.36 ± 0.03
TC13000262.hg.1	OTTHUMG00000017053	3.36 ± 0.18	3.25 ± 0.06	3.31 ± 0.05	3.29 ± 0.08
TC13000283.hg.1	OTTHUMG00000017086	3.60 ± 0.13	3.38 ± 0.08	3.52 ± 0.11	3.41 ± 0.16
TC13000288.hg.1	OTTHUMG00000017094	4.60 ± 0.11	4.43 ± 0.02	4.39 ± 0.09	4.43 ± 0.13
TC13000289.hg.1	OTTHUMG00000017095	5.29 ± 0.15	5.18 ± 0.03	5.28 ± 0.11	5.21 ± 0.11
TC13000750.hg.1	OTTHUMG00000017112	4.59 ± 0.05	4.51 ± 0.14	4.62 ± 0.14	4.62 ± 0.05
TC13000751.hg.1	OTTHUMG00000017117	5.39 ± 0.14	5.23 ± 0.04	5.14 ± 0.04	5.25 ± 0.28
TC13000761.hg.1	OTTHUMG00000017135	3.76 ± 0.11	3.75 ± 0.12	3.85 ± 0.27	3.82 ± 0.14
TC13000304.hg.1	OTTHUMG00000017137	5.05 ± 0.21	4.90 ± 0.05	4.94 ± 0.09	4.97 ± 0.10
TC13000305.hg.1	OTTHUMG00000017139	4.27 ± 0.01	4.23 ± 0.08	4.25 ± 0.02	4.28 ± 0.13
TC13000306.hg.1	OTTHUMG00000017144	4.86 ± 0.11	4.67 ± 0.05	4.65 ± 0.05	4.86 ± 0.21
TC13000308.hg.1	OTTHUMG00000017146	5.88 ± 0.16	5.96 ± 0.12	5.96 ± 0.07	6.09 ± 0.16
TC13000767.hg.1	OTTHUMG00000017155	5.07 ± 0.15	4.90 ± 0.12	4.99 ± 0.12	5.06 ± 0.04
TC13000312.hg.1	OTTHUMG00000017159	4.87 ± 0.10	4.78 ± 0.03	4.80 ± 0.12	4.93 ± 0.13
TC13000313.hg.1	OTTHUMG00000017162	4.63 ± 0.04	4.58 ± 0.03	4.62 ± 0.06	4.56 ± 0.08
TC13000770.hg.1	OTTHUMG00000017165	4.67 ± 0.10	4.42 ± 0.14	4.55 ± 0.06	4.45 ± 0.08
TC13000315.hg.1	OTTHUMG00000017169	4.05 ± 0.04	3.97 ± 0.09	4.11 ± 0.17	3.98 ± 0.04

TC13000774.hg.1	OTTHUMG00000017173	4.30 ± 0.01	4.17 ± 0.05	4.23 ± 0.04	4.24 ± 0.06
TC13000775.hg.1	OTTHUMG00000017177	5.46 ± 0.16	5.43 ± 0.06	5.53 ± 0.06	5.58 ± 0.23
TC13000321.hg.1	OTTHUMG00000017184	4.20 ± 0.08	4.12 ± 0.06	4.24 ± 0.03	4.20 ± 0.15
TC13000780.hg.1	OTTHUMG00000017187	4.54 ± 0.05	4.22 ± 0.03	4.40 ± 0.15	4.35 ± 0.12
TC13000328.hg.1	OTTHUMG00000017201	4.38 ± 0.03	4.38 ± 0.11	4.26 ± 0.11	4.37 ± 0.08
TC13000814.hg.1	OTTHUMG00000017237	5.77 ± 0.24	5.66 ± 0.07	5.72 ± 0.05	5.84 ± 0.20
TC13000816.hg.1	OTTHUMG00000017241	6.40 ± 0.30	6.38 ± 0.13	6.40 ± 0.13	6.45 ± 0.06
TC13000357.hg.1	OTTHUMG00000017273	6.32 ± 0.07	6.27 ± 0.24	6.36 ± 0.19	6.37 ± 0.11
TC13000373.hg.1	OTTHUMG00000017314	4.77 ± 0.10	4.77 ± 0.01	4.81 ± 0.12	4.82 ± 0.11
TC13000386.hg.1	OTTHUMG00000017334	6.00 ± 0.11	5.94 ± 0.18	5.99 ± 0.13	6.05 ± 0.07
TC13000387.hg.1	OTTHUMG00000017336	8.50 ± 0.43	8.76 ± 0.04	8.54 ± 0.21	9.07 ± 0.38
TC13000388.hg.1	OTTHUMG00000017337	6.10 ± 0.11	6.00 ± 0.18	6.04 ± 0.31	5.95 ± 0.06
TC13000391.hg.1	OTTHUMG00000017341	5.51 ± 0.17	5.48 ± 0.16	5.55 ± 0.09	5.67 ± 0.26
TC13000396.hg.1	OTTHUMG00000017351	6.71 ± 0.17	6.67 ± 0.05	6.71 ± 0.10	6.73 ± 0.12
TC13000397.hg.1	OTTHUMG00000017352	5.06 ± 0.23	4.81 ± 0.10	5.03 ± 0.14	4.90 ± 0.20
TC13000884.hg.1	OTTHUMG00000017359	7.92 ± 0.32	7.98 ± 0.24	7.86 ± 0.21	8.14 ± 0.15
TC13000402.hg.1	OTTHUMG00000017360	6.38 ± 0.21	6.36 ± 0.10	6.24 ± 0.32	6.48 ± 0.14
TC13000886.hg.1	OTTHUMG00000017366	7.46 ± 0.19	7.51 ± 0.07	7.39 ± 0.15	7.62 ± 0.19
TC13000412.hg.1	OTTHUMG00000017369	7.11 ± 0.27	6.98 ± 0.18	7.16 ± 0.24	7.16 ± 0.10
TC13000432.hg.1	OTTHUMG00000017397	6.80 ± 0.25	6.85 ± 0.32	6.57 ± 0.14	6.83 ± 0.02
TC13000903.hg.1	OTTHUMG00000017405	7.67 ± 0.33	7.73 ± 0.07	7.64 ± 0.16	7.92 ± 0.18
TC01001309.hg.1	OTTHUMG00000017459	4.91 ± 0.34	5.08 ± 0.14	5.05 ± 0.04	5.21 ± 0.05
TC01003360.hg.1	OTTHUMG00000017482	4.47 ± 0.07	4.46 ± 0.10	4.52 ± 0.08	4.56 ± 0.11
TC01003361.hg.1	OTTHUMG00000017483	6.80 ± 0.22	6.75 ± 0.06	6.58 ± 0.13	6.83 ± 0.07
TC01003374.hg.1	OTTHUMG00000017520	3.59 ± 0.08	3.49 ± 0.03	3.50 ± 0.12	3.57 ± 0.10
TC10000003.hg.1	OTTHUMG00000017527	7.04 ± 0.14	6.90 ± 0.10	7.15 ± 0.15	6.98 ± 0.08
TC10000004.hg.1	OTTHUMG00000017530	7.72 ± 0.36	7.72 ± 0.26	7.55 ± 0.34	7.75 ± 0.06
TC10000006.hg.1	OTTHUMG00000017531	6.60 ± 0.21	6.78 ± 0.27	6.56 ± 0.23	6.90 ± 0.14
TC10000007.hg.1	OTTHUMG00000017533	5.87 ± 0.10	5.70 ± 0.01	5.82 ± 0.09	5.79 ± 0.05
TC10000970.hg.1	OTTHUMG00000017541	6.54 ± 0.04	6.58 ± 0.11	6.48 ± 0.16	6.74 ± 0.17
TC10000014.hg.1	OTTHUMG00000017544	5.55 ± 0.18	5.40 ± 0.06	5.45 ± 0.10	5.53 ± 0.25

TC10000016.hg.1	OTTHUMG00000017545	7.46 ± 0.28	7.29 ± 0.22	7.26 ± 0.27	7.42 ± 0.15
TC10000972.hg.1	OTTHUMG00000017546	6.68 ± 0.12	6.69 ± 0.26	6.61 ± 0.21	6.69 ± 0.08
TC10000974.hg.1	OTTHUMG00000017549	6.52 ± 0.18	6.39 ± 0.34	6.46 ± 0.16	6.62 ± 0.15
TC10000018.hg.1	OTTHUMG00000017550	5.51 ± 0.18	5.44 ± 0.10	5.40 ± 0.14	5.53 ± 0.21
TC10000019.hg.1	OTTHUMG00000017551	6.39 ± 0.30	6.30 ± 0.07	6.47 ± 0.12	6.43 ± 0.13
TC10000021.hg.1	OTTHUMG00000017554	5.45 ± 0.15	5.46 ± 0.14	5.38 ± 0.10	5.58 ± 0.10
TC10000976.hg.1	OTTHUMG00000017555	6.97 ± 0.14	7.06 ± 0.03	7.07 ± 0.11	7.13 ± 0.08
TC10000026.hg.1	OTTHUMG00000017561	5.85 ± 0.19	5.86 ± 0.18	5.73 ± 0.22	5.87 ± 0.24
TC10000028.hg.1	OTTHUMG00000017562	8.65 ± 0.29	8.67 ± 0.13	8.59 ± 0.14	8.88 ± 0.10
TC10000977.hg.1	OTTHUMG00000017563	6.49 ± 0.30	6.28 ± 0.09	6.35 ± 0.18	6.30 ± 0.08
TC10000978.hg.1	OTTHUMG00000017564	5.91 ± 0.10	5.87 ± 0.02	5.92 ± 0.05	5.90 ± 0.05
TC10000031.hg.1	OTTHUMG00000017565	8.21 ± 0.24	8.08 ± 0.21	8.13 ± 0.21	8.22 ± 0.04
TC10000030.hg.1	OTTHUMG00000017566	6.41 ± 0.21	6.30 ± 0.12	6.25 ± 0.12	6.44 ± 0.10
TC10000032.hg.1	OTTHUMG00000017568	5.16 ± 0.30	5.22 ± 0.17	5.21 ± 0.08	5.50 ± 0.22
TC10000033.hg.1	OTTHUMG00000017569	6.44 ± 0.14	6.38 ± 0.06	6.37 ± 0.14	6.48 ± 0.13
TC10000034.hg.1	OTTHUMG00000017570	5.90 ± 0.17	6.07 ± 0.09	6.07 ± 0.17	6.07 ± 0.12
TC10000035.hg.1	OTTHUMG00000017571	7.39 ± 0.25	7.43 ± 0.06	7.35 ± 0.11	7.57 ± 0.06
TC10000981.hg.1	OTTHUMG00000017574	5.65 ± 0.10	5.70 ± 0.11	5.65 ± 0.07	5.88 ± 0.16
TC10000042.hg.1	OTTHUMG00000017586	4.06 ± 0.21	3.86 ± 0.05	3.91 ± 0.04	3.93 ± 0.17
TC10000045.hg.1	OTTHUMG00000017587	5.45 ± 0.15	5.19 ± 0.23	5.40 ± 0.07	5.26 ± 0.12
TC10000991.hg.1	OTTHUMG00000017590	4.24 ± 0.15	3.90 ± 0.09	4.19 ± 0.13	4.12 ± 0.07
TC10000999.hg.1	OTTHUMG00000017599	7.00 ± 0.14	7.04 ± 0.15	7.02 ± 0.21	7.16 ± 0.13
TC10000998.hg.1	OTTHUMG00000017600	7.15 ± 0.18	7.23 ± 0.16	7.21 ± 0.07	7.38 ± 0.01
TC10000997.hg.1	OTTHUMG00000017601	6.46 ± 0.32	6.30 ± 0.19	6.28 ± 0.47	6.43 ± 0.14
TC10001001.hg.1	OTTHUMG00000017604	4.47 ± 0.19	4.27 ± 0.05	4.48 ± 0.22	4.22 ± 0.06
TC10000051.hg.1	OTTHUMG00000017608	7.08 ± 0.39	7.17 ± 0.29	7.02 ± 0.38	7.23 ± 0.13
TC10001005.hg.1	OTTHUMG00000017611	7.96 ± 0.11	7.84 ± 0.16	7.89 ± 0.13	7.98 ± 0.04
TC10001012.hg.1	OTTHUMG00000017613	6.52 ± 0.47	6.39 ± 0.17	6.45 ± 0.21	6.55 ± 0.04
TC10000060.hg.1	OTTHUMG00000017624	6.11 ± 0.57	6.28 ± 0.46	5.96 ± 0.27	6.09 ± 0.11
TC10000061.hg.1	OTTHUMG00000017625	7.12 ± 0.29	7.01 ± 0.19	6.87 ± 0.17	6.98 ± 0.20
TC10001016.hg.1	OTTHUMG00000017626	4.85 ± 0.29	4.80 ± 0.15	4.84 ± 0.23	5.14 ± 0.24

TC10000063.hg.1	OTTHUMG00000017628	6.18 ± 0.39	6.19 ± 0.17	6.07 ± 0.18	6.25 ± 0.16
TC10001018.hg.1	OTTHUMG00000017631	7.22 ± 0.27	7.28 ± 0.16	7.26 ± 0.13	7.46 ± 0.08
TC10000065.hg.1	OTTHUMG00000017632	6.43 ± 0.15	6.29 ± 0.03	6.35 ± 0.05	6.37 ± 0.23
TC10000069.hg.1	OTTHUMG00000017642	7.23 ± 0.15	7.15 ± 0.12	7.07 ± 0.18	7.22 ± 0.05
TC10000071.hg.1	OTTHUMG00000017646	6.64 ± 0.30	6.58 ± 0.23	6.52 ± 0.22	6.64 ± 0.06
TC10000072.hg.1	OTTHUMG00000017648	5.82 ± 0.12	5.81 ± 0.05	6.00 ± 0.17	5.83 ± 0.07
TC10000073.hg.1	OTTHUMG00000017650	4.87 ± 0.15	4.75 ± 0.03	4.74 ± 0.09	4.92 ± 0.19
TC10000074.hg.1	OTTHUMG00000017651	6.29 ± 0.02	6.04 ± 0.20	6.15 ± 0.03	6.26 ± 0.27
TC10001027.hg.1	OTTHUMG00000017654	7.45 ± 0.37	7.27 ± 0.14	7.14 ± 0.29	7.34 ± 0.05
TC10000077.hg.1	OTTHUMG00000017655	4.30 ± 0.06	4.28 ± 0.06	4.26 ± 0.03	4.34 ± 0.07
TC10001028.hg.1	OTTHUMG00000017657	4.56 ± 0.08	4.78 ± 0.05	4.60 ± 0.09	4.80 ± 0.02
TC10000080.hg.1	OTTHUMG00000017662	4.87 ± 0.33	4.75 ± 0.06	4.84 ± 0.07	4.89 ± 0.11
TC10001033.hg.1	OTTHUMG00000017663	4.04 ± 0.12	3.86 ± 0.16	3.91 ± 0.11	4.01 ± 0.19
TC10000082.hg.1	OTTHUMG00000017664	6.20 ± 0.20	6.18 ± 0.14	6.02 ± 0.07	6.43 ± 0.23
TC10001035.hg.1	OTTHUMG00000017666	7.03 ± 0.31	7.15 ± 0.05	7.35 ± 0.29	7.22 ± 0.07
TC10001043.hg.1	OTTHUMG00000017681	7.29 ± 0.19	7.13 ± 0.17	7.16 ± 0.11	7.17 ± 0.14
TC10000100.hg.1	OTTHUMG00000017697	5.12 ± 0.14	5.21 ± 0.07	5.02 ± 0.17	5.18 ± 0.06
TC10000101.hg.1	OTTHUMG00000017698	9.14 ± 0.20	9.09 ± 0.12	9.07 ± 0.28	9.08 ± 0.05
TC10000105.hg.1	OTTHUMG00000017703	6.03 ± 0.13	5.68 ± 0.03	5.85 ± 0.04	5.89 ± 0.06
TC10000107.hg.1	OTTHUMG00000017704	6.20 ± 0.30	6.10 ± 0.11	6.22 ± 0.11	6.12 ± 0.04
TC10000108.hg.1	OTTHUMG00000017706	5.44 ± 0.25	5.34 ± 0.12	5.39 ± 0.01	5.28 ± 0.10
TC10000109.hg.1	OTTHUMG00000017707	6.56 ± 0.10	6.54 ± 0.16	6.48 ± 0.11	6.67 ± 0.10
TC10000111.hg.1	OTTHUMG00000017710	6.54 ± 0.15	6.30 ± 0.09	6.32 ± 0.07	6.46 ± 0.16
TC10000118.hg.1	OTTHUMG00000017731	6.08 ± 0.08	5.98 ± 0.31	5.94 ± 0.11	6.13 ± 0.17
TC10000121.hg.1	OTTHUMG00000017736	5.48 ± 0.03	5.38 ± 0.08	5.54 ± 0.12	5.42 ± 0.08
TC10000124.hg.1	OTTHUMG00000017739	4.75 ± 0.10	4.70 ± 0.09	4.74 ± 0.14	4.78 ± 0.16
TC10001073.hg.1	OTTHUMG00000017742	9.41 ± 0.49	9.00 ± 0.16	10.02 ± 0.23	8.86 ± 0.50
TC10001076.hg.1	OTTHUMG00000017751	4.58 ± 0.11	4.39 ± 0.18	4.61 ± 0.24	4.34 ± 0.09
TC10001077.hg.1	OTTHUMG00000017756	4.20 ± 0.10	4.11 ± 0.10	4.12 ± 0.04	4.21 ± 0.04
TC10001079.hg.1	OTTHUMG00000017760	5.28 ± 0.41	5.01 ± 0.10	5.07 ± 0.12	5.05 ± 0.10
TC10001081.hg.1	OTTHUMG00000017761	5.25 ± 0.32	5.19 ± 0.07	5.18 ± 0.18	5.16 ± 0.06

TC10002959.hg.1	OTTHUMG00000017762	6.04 ± 0.32	6.17 ± 0.09	6.12 ± 0.14	6.18 ± 0.05
TC10000141.hg.1	OTTHUMG00000017769	4.97 ± 0.11	4.66 ± 0.10	4.75 ± 0.04	4.67 ± 0.12
TC10001082.hg.1	OTTHUMG00000017772	6.22 ± 0.33	5.96 ± 0.14	5.89 ± 0.11	6.13 ± 0.10
TC10001084.hg.1	OTTHUMG00000017777	5.52 ± 0.23	5.48 ± 0.03	5.44 ± 0.05	5.49 ± 0.07
TC10001088.hg.1	OTTHUMG00000017780	6.22 ± 0.27	6.25 ± 0.15	6.29 ± 0.14	6.36 ± 0.15
TC10001091.hg.1	OTTHUMG00000017792	7.00 ± 0.20	6.95 ± 0.04	6.92 ± 0.06	6.98 ± 0.01
TC10000158.hg.1	OTTHUMG00000017809	4.47 ± 0.16	4.33 ± 0.06	4.46 ± 0.07	4.28 ± 0.02
TC10000167.hg.1	OTTHUMG00000017817	7.44 ± 0.16	7.43 ± 0.27	7.40 ± 0.20	7.61 ± 0.19
TC10000176.hg.1	OTTHUMG00000017831	6.93 ± 0.06	6.97 ± 0.01	6.96 ± 0.05	7.00 ± 0.01
TC10001116.hg.1	OTTHUMG00000017835	4.65 ± 0.15	4.55 ± 0.06	4.57 ± 0.11	4.69 ± 0.10
TC10001118.hg.1	OTTHUMG00000017840	3.98 ± 0.08	3.90 ± 0.10	3.91 ± 0.11	3.85 ± 0.01
TC10001119.hg.1	OTTHUMG00000017843	4.23 ± 0.27	3.90 ± 0.06	4.03 ± 0.11	4.02 ± 0.02
TC10000186.hg.1	OTTHUMG00000017845	6.10 ± 0.53	6.09 ± 0.15	6.18 ± 0.26	6.10 ± 0.22
TC10000193.hg.1	OTTHUMG00000017863	7.03 ± 0.16	7.04 ± 0.22	7.05 ± 0.17	7.28 ± 0.09
TC10000194.hg.1	OTTHUMG00000017866	3.68 ± 0.10	3.53 ± 0.02	3.54 ± 0.04	3.59 ± 0.12
TC10001138.hg.1	OTTHUMG00000017871	5.74 ± 0.09	5.70 ± 0.01	5.56 ± 0.20	5.70 ± 0.11
TC10000200.hg.1	OTTHUMG00000017875	5.31 ± 0.15	5.35 ± 0.07	5.30 ± 0.13	5.33 ± 0.13
TC10000201.hg.1	OTTHUMG00000017877	4.72 ± 0.03	4.61 ± 0.12	4.63 ± 0.10	4.62 ± 0.08
TC10001151.hg.1	OTTHUMG00000017896	5.95 ± 0.15	5.84 ± 0.32	5.74 ± 0.19	5.81 ± 0.21
TC10000213.hg.1	OTTHUMG00000017897	6.79 ± 0.22	6.66 ± 0.08	6.60 ± 0.26	6.76 ± 0.09
TC10000215.hg.1	OTTHUMG00000017899	4.54 ± 0.07	4.54 ± 0.10	4.62 ± 0.10	4.59 ± 0.03
TC10000217.hg.1	OTTHUMG00000017902	6.54 ± 0.20	6.50 ± 0.09	6.55 ± 0.10	6.52 ± 0.09
TC10000221.hg.1	OTTHUMG00000017909	5.72 ± 0.17	5.70 ± 0.17	5.82 ± 0.10	5.81 ± 0.22
TC10001157.hg.1	OTTHUMG00000017910	7.06 ± 0.26	6.91 ± 0.17	6.76 ± 0.17	6.96 ± 0.09
TC10000224.hg.1	OTTHUMG00000017921	5.97 ± 0.23	5.84 ± 0.06	5.80 ± 0.06	6.07 ± 0.17
TC10000225.hg.1	OTTHUMG00000017922	8.99 ± 0.22	9.00 ± 0.08	9.00 ± 0.09	9.05 ± 0.10
TC10000227.hg.1	OTTHUMG00000017923	6.59 ± 0.00	6.58 ± 0.17	6.54 ± 0.10	6.66 ± 0.03
TC10000226.hg.1	OTTHUMG00000017924	8.37 ± 0.31	8.28 ± 0.12	8.23 ± 0.35	8.13 ± 0.02
TC10000229.hg.1	OTTHUMG00000017934	6.66 ± 0.16	6.76 ± 0.09	6.62 ± 0.10	6.81 ± 0.07
TC10000230.hg.1	OTTHUMG00000017936	5.29 ± 0.11	5.26 ± 0.17	5.30 ± 0.10	5.35 ± 0.11
TC10001165.hg.1	OTTHUMG00000017937	5.98 ± 0.28	5.98 ± 0.12	5.80 ± 0.11	5.97 ± 0.18

TC10001168.hg.1	OTTHUMG00000017939	5.00 ± 0.08	5.01 ± 0.05	4.96 ± 0.09	4.87 ± 0.05
TC10000232.hg.1	OTTHUMG00000017940	7.11 ± 0.34	7.19 ± 0.05	7.10 ± 0.14	7.30 ± 0.09
TC10000233.hg.1	OTTHUMG00000017942	6.62 ± 0.28	6.60 ± 0.07	6.50 ± 0.13	6.74 ± 0.10
TC10001174.hg.1	OTTHUMG00000017952	6.48 ± 0.18	6.59 ± 0.10	6.53 ± 0.08	6.60 ± 0.09
TC10001175.hg.1	OTTHUMG00000017952	6.08 ± 0.07	6.11 ± 0.06	6.12 ± 0.09	6.14 ± 0.03
TC10001176.hg.1	OTTHUMG00000017954	5.13 ± 0.31	5.11 ± 0.18	5.19 ± 0.10	5.24 ± 0.04
TC10001180.hg.1	OTTHUMG00000017959	6.59 ± 0.23	6.51 ± 0.12	6.38 ± 0.11	6.51 ± 0.14
TC10001181.hg.1	OTTHUMG00000017960	4.87 ± 0.05	4.79 ± 0.05	4.80 ± 0.09	4.76 ± 0.08
TC10000243.hg.1	OTTHUMG00000017963	6.08 ± 0.10	5.95 ± 0.07	5.98 ± 0.11	6.01 ± 0.01
TC10000244.hg.1	OTTHUMG00000017965	5.03 ± 0.08	4.91 ± 0.10	5.16 ± 0.14	4.86 ± 0.07
TC10000247.hg.1	OTTHUMG00000017969	3.99 ± 0.04	3.88 ± 0.04	3.95 ± 0.07	3.77 ± 0.08
TC10000248.hg.1	OTTHUMG00000017971	5.62 ± 0.15	5.51 ± 0.11	5.52 ± 0.07	5.57 ± 0.05
TC10001186.hg.1	OTTHUMG00000017976	7.34 ± 0.29	7.17 ± 0.07	7.21 ± 0.11	7.29 ± 0.05
TC10000249.hg.1	OTTHUMG00000017979	7.77 ± 0.16	7.44 ± 0.06	7.62 ± 0.10	7.38 ± 0.21
TC10000255.hg.1	OTTHUMG00000017992	5.28 ± 0.15	5.24 ± 0.31	5.14 ± 0.21	5.42 ± 0.22
TC10000259.hg.1	OTTHUMG00000017996	7.56 ± 0.11	7.37 ± 0.16	8.40 ± 0.30	7.32 ± 0.16
TC10001196.hg.1	OTTHUMG00000018017	7.00 ± 0.13	6.98 ± 0.16	6.91 ± 0.16	7.09 ± 0.14
TC10000264.hg.1	OTTHUMG00000018018	5.86 ± 0.34	5.54 ± 0.03	5.78 ± 0.09	5.73 ± 0.11
TC10000267.hg.1	OTTHUMG00000018021	7.10 ± 0.21	7.20 ± 0.27	7.22 ± 0.17	7.28 ± 0.06
TC10001199.hg.1	OTTHUMG00000018022	6.46 ± 0.28	6.36 ± 0.16	6.38 ± 0.11	6.41 ± 0.08
TC10000270.hg.1	OTTHUMG00000018026	3.78 ± 0.21	3.66 ± 0.02	3.69 ± 0.07	3.79 ± 0.02
TC10000274.hg.1	OTTHUMG00000018028	7.94 ± 0.11	7.83 ± 0.03	7.90 ± 0.25	7.81 ± 0.02
TC10001204.hg.1	OTTHUMG00000018038	5.32 ± 0.07	5.17 ± 0.08	5.44 ± 0.08	5.27 ± 0.04
TC10000281.hg.1	OTTHUMG00000018041	6.56 ± 0.05	6.36 ± 0.07	6.52 ± 0.01	6.33 ± 0.11
TC10001206.hg.1	OTTHUMG00000018047	6.07 ± 0.09	5.79 ± 0.09	5.93 ± 0.08	5.82 ± 0.04
TC10001208.hg.1	OTTHUMG00000018052	8.18 ± 0.21	8.08 ± 0.11	7.96 ± 0.15	8.28 ± 0.14
TC10001210.hg.1	OTTHUMG00000018055	7.37 ± 0.30	7.33 ± 0.16	7.24 ± 0.30	7.60 ± 0.06
TC10001211.hg.1	OTTHUMG00000018056	7.36 ± 0.23	7.24 ± 0.20	7.18 ± 0.24	7.40 ± 0.04
TC10001213.hg.1	OTTHUMG00000018058	6.20 ± 0.13	6.20 ± 0.21	6.19 ± 0.22	6.38 ± 0.21
TC10000288.hg.1	OTTHUMG00000018061	5.33 ± 0.36	5.54 ± 0.18	5.34 ± 0.15	5.58 ± 0.07
TC10001215.hg.1	OTTHUMG00000018065	7.20 ± 0.10	7.03 ± 0.09	7.16 ± 0.09	7.12 ± 0.02

TC10001220.hg.1	OTTHUMG00000018077	6.56 ± 0.29	6.64 ± 0.09	6.63 ± 0.04	6.84 ± 0.40
TC10001223.hg.1	OTTHUMG00000018082	7.34 ± 0.17	7.31 ± 0.08	7.51 ± 0.10	7.18 ± 0.16
TC10001229.hg.1	OTTHUMG00000018091	8.24 ± 0.47	8.17 ± 0.14	8.07 ± 0.26	8.15 ± 0.08
TC10001235.hg.1	OTTHUMG00000018105	5.91 ± 0.07	5.83 ± 0.12	5.80 ± 0.02	5.82 ± 0.02
TC10000299.hg.1	OTTHUMG00000018106	7.44 ± 0.24	7.40 ± 0.12	7.43 ± 0.29	7.45 ± 0.16
TC10000303.hg.1	OTTHUMG00000018119	7.52 ± 0.25	7.38 ± 0.24	7.52 ± 0.29	7.52 ± 0.17
TC10001242.hg.1	OTTHUMG00000018120	6.88 ± 0.22	6.81 ± 0.13	6.64 ± 0.09	6.88 ± 0.19
TC10001243.hg.1	OTTHUMG00000018126	5.81 ± 0.76	6.18 ± 0.37	5.43 ± 0.42	6.03 ± 0.20
TC10001249.hg.1	OTTHUMG00000018145	5.71 ± 0.05	5.76 ± 0.14	5.81 ± 0.08	5.98 ± 0.11
TC10000316.hg.1	OTTHUMG00000018146	5.26 ± 0.14	5.09 ± 0.31	5.22 ± 0.21	5.22 ± 0.11
TC10001256.hg.1	OTTHUMG00000018152	6.83 ± 0.18	6.76 ± 0.18	6.66 ± 0.14	6.78 ± 0.14
TC10001262.hg.1	OTTHUMG00000018169	7.48 ± 0.32	7.61 ± 0.40	7.33 ± 0.27	7.75 ± 0.13
TC10001266.hg.1	OTTHUMG00000018174	7.68 ± 0.11	7.93 ± 0.23	7.84 ± 0.23	8.04 ± 0.13
TC10000324.hg.1	OTTHUMG00000018179	7.27 ± 0.31	7.68 ± 0.27	7.55 ± 0.39	7.58 ± 0.26
TC10001269.hg.1	OTTHUMG00000018181	6.46 ± 0.06	6.60 ± 0.16	6.41 ± 0.17	6.61 ± 0.25
TC10000325.hg.1	OTTHUMG00000018185	8.13 ± 0.23	8.12 ± 0.19	8.04 ± 0.19	8.32 ± 0.17
TC10000328.hg.1	OTTHUMG00000018194	5.92 ± 0.26	5.75 ± 0.15	5.71 ± 0.16	5.83 ± 0.11
TC10000342.hg.1	OTTHUMG00000018228	4.94 ± 0.04	4.75 ± 0.07	4.75 ± 0.15	4.92 ± 0.20
TC10000343.hg.1	OTTHUMG00000018230	6.97 ± 0.21	6.69 ± 0.15	6.85 ± 0.19	6.92 ± 0.09
TC10000344.hg.1	OTTHUMG00000018236	4.90 ± 0.16	4.71 ± 0.12	4.85 ± 0.04	4.92 ± 0.11
TC10001293.hg.1	OTTHUMG00000018238	5.35 ± 0.02	5.26 ± 0.08	5.17 ± 0.08	5.43 ± 0.10
TC10001294.hg.1	OTTHUMG00000018241	6.04 ± 0.21	5.77 ± 0.04	5.95 ± 0.05	5.79 ± 0.02
TC10001295.hg.1	OTTHUMG00000018243	4.13 ± 0.19	4.01 ± 0.10	4.03 ± 0.09	4.16 ± 0.09
TC10000349.hg.1	OTTHUMG00000018245	4.63 ± 0.10	4.49 ± 0.07	4.62 ± 0.03	4.51 ± 0.00
TC10001299.hg.1	OTTHUMG00000018252	4.74 ± 0.09	4.57 ± 0.05	4.60 ± 0.11	4.59 ± 0.01
TC10001300.hg.1	OTTHUMG00000018253	6.02 ± 0.27	5.56 ± 0.12	5.84 ± 0.30	5.70 ± 0.17
TC10001302.hg.1	OTTHUMG00000018255	4.45 ± 0.19	4.23 ± 0.09	4.29 ± 0.10	4.22 ± 0.06
TC10001303.hg.1	OTTHUMG00000018257	3.58 ± 0.06	3.66 ± 0.11	3.61 ± 0.12	3.65 ± 0.08
TC10000359.hg.1	OTTHUMG00000018267	7.89 ± 0.38	7.93 ± 0.16	7.75 ± 0.13	8.01 ± 0.13
TC10000368.hg.1	OTTHUMG00000018278	3.83 ± 0.06	3.65 ± 0.05	3.75 ± 0.01	3.76 ± 0.12
TC10000369.hg.1	OTTHUMG00000018280	5.34 ± 0.16	5.30 ± 0.03	5.33 ± 0.11	5.48 ± 0.18

TC10000370.hg.1	OTTHUMG00000018286	4.84 ± 0.09	4.72 ± 0.07	4.69 ± 0.19	4.75 ± 0.10
TC10000375.hg.1	OTTHUMG00000018294	5.04 ± 0.23	4.87 ± 0.09	4.94 ± 0.12	5.02 ± 0.09
TC10001323.hg.1	OTTHUMG00000018296	5.59 ± 0.18	5.54 ± 0.05	5.69 ± 0.09	5.64 ± 0.11
TC10000382.hg.1	OTTHUMG00000018301	4.14 ± 0.14	3.99 ± 0.06	4.19 ± 0.21	4.06 ± 0.10
TC10001326.hg.1	OTTHUMG00000018307	7.82 ± 0.27	7.87 ± 0.09	7.76 ± 0.12	8.03 ± 0.13
TC10001328.hg.1	OTTHUMG00000018310	5.22 ± 0.39	5.19 ± 0.05	5.10 ± 0.08	5.23 ± 0.02
TC10000390.hg.1	OTTHUMG00000018320	6.64 ± 0.09	6.74 ± 0.14	6.67 ± 0.26	6.96 ± 0.27
TC10001333.hg.1	OTTHUMG00000018322	6.09 ± 0.15	5.98 ± 0.23	5.96 ± 0.16	6.09 ± 0.03
TC10001337.hg.1	OTTHUMG00000018325	5.87 ± 0.20	5.81 ± 0.03	5.84 ± 0.08	5.96 ± 0.21
TC10000392.hg.1	OTTHUMG00000018329	4.34 ± 0.22	4.15 ± 0.05	4.25 ± 0.01	4.09 ± 0.10
TC10000393.hg.1	OTTHUMG00000018330	5.15 ± 0.08	5.05 ± 0.05	5.12 ± 0.11	5.15 ± 0.21
TC10000397.hg.1	OTTHUMG00000018331	4.32 ± 0.27	4.07 ± 0.09	4.09 ± 0.13	4.05 ± 0.13
TC10001344.hg.1	OTTHUMG00000018345	6.12 ± 0.15	5.90 ± 0.06	6.01 ± 0.11	5.94 ± 0.11
TC10001353.hg.1	OTTHUMG00000018372	6.39 ± 0.05	6.29 ± 0.16	6.31 ± 0.14	6.40 ± 0.08
TC10001354.hg.1	OTTHUMG00000018373	6.55 ± 0.19	6.48 ± 0.15	6.44 ± 0.11	6.47 ± 0.06
TC10000421.hg.1	OTTHUMG00000018384	7.37 ± 0.34	7.30 ± 0.06	7.25 ± 0.16	7.35 ± 0.16
TC10000423.hg.1	OTTHUMG00000018393	6.54 ± 0.13	6.54 ± 0.17	6.50 ± 0.12	6.65 ± 0.12
TC10001358.hg.1	OTTHUMG00000018395	5.57 ± 0.21	5.66 ± 0.02	5.66 ± 0.12	5.76 ± 0.24
TC10000444.hg.1	OTTHUMG00000018428	6.31 ± 0.18	6.39 ± 0.10	6.33 ± 0.09	6.40 ± 0.17
TC10000451.hg.1	OTTHUMG00000018439	4.68 ± 0.17	4.65 ± 0.23	4.78 ± 0.34	4.54 ± 0.01
TC10000456.hg.1	OTTHUMG00000018447	3.89 ± 0.13	3.79 ± 0.05	3.92 ± 0.08	3.77 ± 0.02
TC10002965.hg.1	OTTHUMG00000018457	6.75 ± 0.25	6.86 ± 0.18	6.65 ± 0.21	6.73 ± 0.14
TC10000460.hg.1	OTTHUMG00000018458	6.27 ± 0.17	6.34 ± 0.09	6.25 ± 0.08	6.30 ± 0.03
TC10000463.hg.1	OTTHUMG00000018462	5.01 ± 0.29	4.92 ± 0.25	5.19 ± 0.52	4.90 ± 0.23
TC10000464.hg.1	OTTHUMG00000018470	8.00 ± 0.11	7.88 ± 0.15	7.93 ± 0.19	7.85 ± 0.02
TC10000473.hg.1	OTTHUMG00000018479	7.92 ± 0.20	7.68 ± 0.09	7.97 ± 0.04	7.67 ± 0.08
TC10000474.hg.1	OTTHUMG00000018480	4.96 ± 0.06	4.94 ± 0.03	4.83 ± 0.15	4.94 ± 0.02
TC10001415.hg.1	OTTHUMG00000018482	8.83 ± 0.25	8.79 ± 0.20	8.88 ± 0.31	8.76 ± 0.07
TC10000477.hg.1	OTTHUMG00000018491	5.41 ± 0.25	5.42 ± 0.25	5.50 ± 0.09	5.29 ± 0.14
TC10001424.hg.1	OTTHUMG00000018505	5.63 ± 0.04	5.41 ± 0.14	5.53 ± 0.04	5.38 ± 0.06
TC10001425.hg.1	OTTHUMG00000018510	6.07 ± 0.06	6.12 ± 0.08	5.95 ± 0.26	6.22 ± 0.14

TC10001426.hg.1	OTTHUMG00000018511	9.07 ± 0.35	9.17 ± 0.10	8.94 ± 0.07	9.28 ± 0.10
TC10001431.hg.1	OTTHUMG00000018527	7.99 ± 0.10	7.86 ± 0.17	8.01 ± 0.05	8.05 ± 0.09
TC10000490.hg.1	OTTHUMG00000018529	5.27 ± 0.12	5.17 ± 0.08	5.16 ± 0.02	5.18 ± 0.11
TC10001437.hg.1	OTTHUMG00000018530	5.13 ± 0.03	5.08 ± 0.06	4.98 ± 0.12	5.06 ± 0.22
TC10000491.hg.1	OTTHUMG00000018534	5.55 ± 0.06	5.58 ± 0.07	5.52 ± 0.28	5.57 ± 0.13
TC10000492.hg.1	OTTHUMG00000018534	6.12 ± 0.23	6.10 ± 0.06	6.10 ± 0.09	6.07 ± 0.03
TC10000493.hg.1	OTTHUMG00000018534	5.64 ± 0.14	5.69 ± 0.09	5.62 ± 0.18	5.87 ± 0.18
TC10000494.hg.1	OTTHUMG00000018537	5.28 ± 0.11	5.20 ± 0.18	5.23 ± 0.12	5.42 ± 0.07
TC10000495.hg.1	OTTHUMG00000018538	4.12 ± 0.10	3.99 ± 0.05	4.03 ± 0.09	4.09 ± 0.11
TC10000496.hg.1	OTTHUMG00000018539	6.11 ± 0.26	6.14 ± 0.11	6.07 ± 0.07	6.23 ± 0.05
TC10000554.hg.1	OTTHUMG00000018545	6.35 ± 0.16	6.53 ± 0.11	6.38 ± 0.26	6.45 ± 0.05
TC10000561.hg.1	OTTHUMG00000018553	7.12 ± 0.24	6.97 ± 0.20	7.04 ± 0.18	7.20 ± 0.23
TC10000562.hg.1	OTTHUMG00000018554	6.11 ± 0.37	6.04 ± 0.16	5.97 ± 0.20	6.16 ± 0.18
TC10001447.hg.1	OTTHUMG00000018556	6.97 ± 0.09	6.98 ± 0.08	7.02 ± 0.03	7.01 ± 0.07
TC10001449.hg.1	OTTHUMG00000018558	7.14 ± 0.21	7.13 ± 0.25	7.06 ± 0.20	7.27 ± 0.13
TC10000567.hg.1	OTTHUMG00000018564	7.09 ± 0.31	7.13 ± 0.21	7.23 ± 0.10	7.29 ± 0.05
TC10001455.hg.1	OTTHUMG00000018570	4.76 ± 0.18	4.82 ± 0.05	4.69 ± 0.03	4.92 ± 0.15
TC10000570.hg.1	OTTHUMG00000018574	7.13 ± 0.20	7.21 ± 0.26	7.20 ± 0.17	7.05 ± 0.07
TC10001457.hg.1	OTTHUMG00000018585	8.55 ± 0.20	8.67 ± 0.18	8.67 ± 0.12	8.59 ± 0.05
TC10000579.hg.1	OTTHUMG00000018612	5.74 ± 0.40	5.54 ± 0.20	5.56 ± 0.05	5.81 ± 0.22
TC10001464.hg.1	OTTHUMG00000018616	7.37 ± 0.18	7.34 ± 0.10	7.28 ± 0.16	7.38 ± 0.12
TC10000584.hg.1	OTTHUMG00000018618	6.18 ± 0.31	5.98 ± 0.20	5.84 ± 0.30	6.23 ± 0.18
TC10001468.hg.1	OTTHUMG00000018629	4.26 ± 0.12	4.04 ± 0.04	4.24 ± 0.16	4.09 ± 0.05
TC10001469.hg.1	OTTHUMG00000018630	7.22 ± 0.26	7.36 ± 0.15	7.22 ± 0.31	7.64 ± 0.20
TC10001473.hg.1	OTTHUMG00000018644	4.90 ± 0.06	4.81 ± 0.09	4.95 ± 0.07	4.75 ± 0.06
TC10001474.hg.1	OTTHUMG00000018645	5.17 ± 0.12	5.06 ± 0.11	5.02 ± 0.08	5.08 ± 0.04
TC10000599.hg.1	OTTHUMG00000018646	5.13 ± 0.24	5.13 ± 0.21	5.14 ± 0.05	5.31 ± 0.12
TC10000600.hg.1	OTTHUMG00000018647	5.32 ± 0.16	5.18 ± 0.05	5.24 ± 0.06	5.26 ± 0.04
TC10001476.hg.1	OTTHUMG00000018648	6.27 ± 0.17	6.21 ± 0.13	6.17 ± 0.11	6.23 ± 0.18
TC10000607.hg.1	OTTHUMG00000018652	6.59 ± 0.17	6.53 ± 0.09	6.50 ± 0.12	6.58 ± 0.04
TC10001484.hg.1	OTTHUMG00000018659	7.88 ± 0.21	7.73 ± 0.10	7.82 ± 0.22	7.87 ± 0.21

TC10000614.hg.1	OTTHUMG00000018660	6.70 ± 0.40	6.83 ± 0.19	6.63 ± 0.35	6.82 ± 0.29
TC10001491.hg.1	OTTHUMG00000018679	7.10 ± 0.20	6.97 ± 0.10	6.83 ± 0.08	7.10 ± 0.12
TC10001490.hg.1	OTTHUMG00000018680	4.89 ± 0.10	4.89 ± 0.24	4.86 ± 0.19	4.96 ± 0.17
TC10000620.hg.1	OTTHUMG00000018682	5.42 ± 0.39	5.25 ± 0.06	5.34 ± 0.23	5.27 ± 0.08
TC10000624.hg.1	OTTHUMG00000018686	6.38 ± 0.56	6.62 ± 0.46	7.25 ± 0.88	6.50 ± 0.15
TC10000635.hg.1	OTTHUMG00000018710	6.55 ± 0.20	6.49 ± 0.04	6.58 ± 0.12	6.68 ± 0.13
TC10000641.hg.1	OTTHUMG00000018715	5.06 ± 0.11	5.08 ± 0.07	5.05 ± 0.07	5.17 ± 0.07
TC10000643.hg.1	OTTHUMG00000018719	5.55 ± 0.30	5.37 ± 0.33	5.45 ± 0.19	5.56 ± 0.10
TC10001509.hg.1	OTTHUMG00000018722	6.17 ± 0.16	6.11 ± 0.10	6.00 ± 0.25	6.40 ± 0.30
TC10001512.hg.1	OTTHUMG00000018728	5.03 ± 0.10	4.89 ± 0.14	5.01 ± 0.15	5.00 ± 0.07
TC10000646.hg.1	OTTHUMG00000018729	4.87 ± 0.14	4.69 ± 0.02	4.76 ± 0.04	4.70 ± 0.09
TC10000647.hg.1	OTTHUMG00000018730	4.63 ± 0.19	4.66 ± 0.04	4.65 ± 0.02	4.68 ± 0.08
TC10001514.hg.1	OTTHUMG00000018731	5.23 ± 0.24	5.02 ± 0.02	5.11 ± 0.08	5.06 ± 0.09
TC10000649.hg.1	OTTHUMG00000018736	4.94 ± 0.34	5.02 ± 0.10	4.93 ± 0.08	5.14 ± 0.17
TC10001534.hg.1	OTTHUMG00000018768	6.70 ± 0.09	6.58 ± 0.25	6.58 ± 0.09	6.81 ± 0.14
TC10000671.hg.1	OTTHUMG00000018782	5.71 ± 0.18	5.56 ± 0.30	5.42 ± 0.08	5.50 ± 0.04
TC10001541.hg.1	OTTHUMG00000018783	5.87 ± 0.10	5.75 ± 0.06	5.80 ± 0.06	5.76 ± 0.04
TC10001543.hg.1	OTTHUMG00000018785	5.17 ± 0.23	5.02 ± 0.16	5.01 ± 0.04	4.94 ± 0.05
TC10001547.hg.1	OTTHUMG00000018806	6.40 ± 0.38	6.50 ± 0.14	6.34 ± 0.11	6.62 ± 0.25
TC10001548.hg.1	OTTHUMG00000018809	6.20 ± 0.22	6.12 ± 0.17	6.06 ± 0.07	6.20 ± 0.01
TC10000683.hg.1	OTTHUMG00000018829	6.59 ± 0.21	6.59 ± 0.20	6.61 ± 0.01	6.73 ± 0.17
TC10000693.hg.1	OTTHUMG00000018851	7.15 ± 0.22	7.15 ± 0.26	7.19 ± 0.32	7.25 ± 0.06
TC10000694.hg.1	OTTHUMG00000018853	6.76 ± 0.14	6.72 ± 0.14	6.78 ± 0.13	6.83 ± 0.15
TC10000707.hg.1	OTTHUMG00000018875	7.21 ± 0.12	7.25 ± 0.12	7.23 ± 0.10	7.34 ± 0.08
TC10000709.hg.1	OTTHUMG00000018883	5.81 ± 0.04	5.71 ± 0.08	5.66 ± 0.17	5.68 ± 0.10
TC10000711.hg.1	OTTHUMG00000018885	6.12 ± 0.27	5.90 ± 0.07	6.02 ± 0.10	6.07 ± 0.08
TC10001583.hg.1	OTTHUMG00000018892	3.97 ± 0.03	4.03 ± 0.16	4.28 ± 0.14	4.01 ± 0.06
TC10000719.hg.1	OTTHUMG00000018903	7.89 ± 0.05	7.80 ± 0.04	7.86 ± 0.07	7.79 ± 0.04
TC10000718.hg.1	OTTHUMG00000018904	7.77 ± 0.27	7.73 ± 0.12	7.75 ± 0.16	7.89 ± 0.08
TC10001592.hg.1	OTTHUMG00000018907	5.39 ± 0.35	5.05 ± 0.19	5.04 ± 0.13	5.08 ± 0.06
TC10001597.hg.1	OTTHUMG00000018923	6.73 ± 0.05	6.74 ± 0.18	6.71 ± 0.14	6.88 ± 0.09

TC10001600.hg.1	OTTHUMG00000018924	7.63 ± 0.11	7.93 ± 0.18	7.88 ± 0.34	7.90 ± 0.10
TC10000734.hg.1	OTTHUMG00000018926	5.67 ± 0.30	5.67 ± 0.09	5.61 ± 0.19	5.71 ± 0.10
TC10000737.hg.1	OTTHUMG00000018929	6.44 ± 0.23	6.30 ± 0.20	6.41 ± 0.17	6.41 ± 0.09
TC10001602.hg.1	OTTHUMG00000018930	5.72 ± 0.29	5.75 ± 0.18	5.73 ± 0.14	5.71 ± 0.14
TC10001604.hg.1	OTTHUMG00000018931	6.58 ± 0.23	6.62 ± 0.24	6.59 ± 0.33	6.76 ± 0.09
TC10001610.hg.1	OTTHUMG00000018941	3.82 ± 0.04	3.65 ± 0.07	3.82 ± 0.19	3.84 ± 0.06
TC10000774.hg.1	OTTHUMG00000018992	6.89 ± 0.18	7.10 ± 0.19	7.07 ± 0.18	7.14 ± 0.10
TC10001634.hg.1	OTTHUMG00000018993	6.72 ± 0.04	6.57 ± 0.07	6.73 ± 0.13	6.69 ± 0.17
TC10000776.hg.1	OTTHUMG00000018994	7.01 ± 0.16	6.97 ± 0.16	6.96 ± 0.21	7.16 ± 0.20
TC10000784.hg.1	OTTHUMG00000019004	6.52 ± 0.11	6.43 ± 0.05	6.57 ± 0.09	6.40 ± 0.04
TC10001643.hg.1	OTTHUMG00000019008	5.10 ± 0.04	5.00 ± 0.13	4.95 ± 0.07	5.16 ± 0.16
TC10000787.hg.1	OTTHUMG00000019009	4.39 ± 0.17	4.33 ± 0.21	4.31 ± 0.19	4.55 ± 0.18
TC10001646.hg.1	OTTHUMG00000019015	4.95 ± 0.21	4.75 ± 0.02	4.91 ± 0.08	4.75 ± 0.08
TC10000790.hg.1	OTTHUMG00000019016	5.25 ± 0.15	5.18 ± 0.05	5.20 ± 0.04	5.29 ± 0.09
TC10001648.hg.1	OTTHUMG00000019020	5.73 ± 0.21	5.62 ± 0.04	5.62 ± 0.11	5.61 ± 0.10
TC10001649.hg.1	OTTHUMG00000019022	4.02 ± 0.11	3.83 ± 0.04	3.91 ± 0.04	3.88 ± 0.06
TC10000794.hg.1	OTTHUMG00000019024	6.43 ± 0.15	6.44 ± 0.15	6.38 ± 0.08	6.62 ± 0.16
TC10001654.hg.1	OTTHUMG00000019035	6.58 ± 0.23	6.60 ± 0.13	6.65 ± 0.07	6.66 ± 0.08
TC10000807.hg.1	OTTHUMG00000019044	7.46 ± 0.07	7.34 ± 0.17	7.35 ± 0.26	7.36 ± 0.22
TC10001662.hg.1	OTTHUMG00000019052	5.57 ± 0.10	5.39 ± 0.12	5.57 ± 0.26	5.44 ± 0.14
TC10001665.hg.1	OTTHUMG00000019057	4.85 ± 0.35	4.56 ± 0.09	4.73 ± 0.08	4.75 ± 0.10
TC10001668.hg.1	OTTHUMG00000019064	5.35 ± 0.13	5.37 ± 0.05	5.55 ± 0.28	5.29 ± 0.09
TC10001669.hg.1	OTTHUMG00000019066	6.81 ± 0.29	6.61 ± 0.05	7.05 ± 0.17	6.86 ± 0.14
TC10000819.hg.1	OTTHUMG00000019067	8.18 ± 0.10	8.25 ± 0.03	8.11 ± 0.10	8.35 ± 0.14
TC10001671.hg.1	OTTHUMG00000019075	7.75 ± 0.32	7.83 ± 0.30	7.63 ± 0.37	7.95 ± 0.02
TC10000835.hg.1	OTTHUMG00000019090	5.58 ± 0.20	5.55 ± 0.15	5.49 ± 0.17	5.63 ± 0.16
TC10000838.hg.1	OTTHUMG00000019095	5.73 ± 0.24	5.47 ± 0.12	5.64 ± 0.15	5.63 ± 0.12
TC10000847.hg.1	OTTHUMG00000019106	8.43 ± 0.33	8.45 ± 0.23	8.42 ± 0.31	8.61 ± 0.10
TC10001685.hg.1	OTTHUMG00000019116	6.78 ± 0.27	6.70 ± 0.15	6.59 ± 0.26	6.82 ± 0.13
TC10001687.hg.1	OTTHUMG00000019119	8.45 ± 0.22	8.44 ± 0.12	8.55 ± 0.19	8.47 ± 0.06
TC10001690.hg.1	OTTHUMG00000019124	6.07 ± 0.10	6.13 ± 0.22	6.04 ± 0.29	6.29 ± 0.14

TC10000854.hg.1	OTTHUMG00000019126	5.83 ± 0.14	5.65 ± 0.12	5.67 ± 0.25	5.67 ± 0.03
TC10000855.hg.1	OTTHUMG00000019129	6.55 ± 0.08	6.50 ± 0.12	6.63 ± 0.12	6.40 ± 0.06
TC10000857.hg.1	OTTHUMG00000019130	6.15 ± 0.11	5.97 ± 0.04	6.04 ± 0.04	6.10 ± 0.03
TC10001697.hg.1	OTTHUMG00000019140	8.40 ± 0.16	8.54 ± 0.08	8.52 ± 0.17	8.64 ± 0.06
TC10001703.hg.1	OTTHUMG00000019148	7.36 ± 0.15	7.57 ± 0.11	7.56 ± 0.05	7.61 ± 0.15
TC10001710.hg.1	OTTHUMG00000019170	7.32 ± 0.22	7.05 ± 0.08	7.16 ± 0.16	7.08 ± 0.09
TC10000878.hg.1	OTTHUMG00000019173	6.42 ± 0.07	6.28 ± 0.07	6.18 ± 0.09	6.30 ± 0.06
TC10001712.hg.1	OTTHUMG00000019174	7.14 ± 0.13	7.23 ± 0.12	7.30 ± 0.10	7.31 ± 0.04
TC10000880.hg.1	OTTHUMG00000019177	4.56 ± 0.05	4.52 ± 0.11	4.46 ± 0.09	4.60 ± 0.20
TC10001720.hg.1	OTTHUMG00000019201	6.30 ± 0.29	6.33 ± 0.19	6.11 ± 0.26	6.34 ± 0.08
TC10000901.hg.1	OTTHUMG00000019202	7.00 ± 0.16	6.87 ± 0.12	6.90 ± 0.10	6.98 ± 0.14
TC10000902.hg.1	OTTHUMG00000019203	6.09 ± 0.22	6.01 ± 0.10	6.12 ± 0.11	6.12 ± 0.01
TC10000904.hg.1	OTTHUMG00000019205	7.39 ± 0.18	7.34 ± 0.20	7.30 ± 0.22	7.53 ± 0.23
TC10001724.hg.1	OTTHUMG00000019207	6.12 ± 0.10	6.23 ± 0.06	6.15 ± 0.06	6.22 ± 0.08
TC10000906.hg.1	OTTHUMG00000019216	6.29 ± 0.16	6.25 ± 0.09	6.26 ± 0.12	6.34 ± 0.06
TC10001731.hg.1	OTTHUMG00000019221	10.00 ± 0.17	9.93 ± 0.07	9.94 ± 0.10	9.99 ± 0.03
TC10001736.hg.1	OTTHUMG00000019232	6.76 ± 0.17	6.60 ± 0.09	6.62 ± 0.21	6.71 ± 0.09
TC10001737.hg.1	OTTHUMG00000019234	5.44 ± 0.15	5.26 ± 0.11	5.44 ± 0.03	5.35 ± 0.07
TC10001750.hg.1	OTTHUMG00000019246	7.81 ± 0.46	7.55 ± 0.18	7.64 ± 0.30	7.64 ± 0.05
TC10001751.hg.1	OTTHUMG00000019247	5.26 ± 0.13	5.09 ± 0.09	5.10 ± 0.03	4.96 ± 0.06
TC10001755.hg.1	OTTHUMG00000019251	5.40 ± 0.30	5.34 ± 0.25	5.30 ± 0.16	5.42 ± 0.23
TC10000923.hg.1	OTTHUMG00000019252	7.75 ± 0.20	7.61 ± 0.06	7.68 ± 0.10	7.77 ± 0.12
TC10001757.hg.1	OTTHUMG00000019256	6.60 ± 0.17	6.44 ± 0.09	6.51 ± 0.07	6.47 ± 0.01
TC10001759.hg.1	OTTHUMG00000019257	5.84 ± 0.16	5.74 ± 0.08	5.61 ± 0.16	5.88 ± 0.15
TC10000925.hg.1	OTTHUMG00000019258	7.40 ± 0.17	7.33 ± 0.08	7.28 ± 0.15	7.44 ± 0.14
TC10000926.hg.1	OTTHUMG00000019260	4.74 ± 0.28	4.48 ± 0.03	4.62 ± 0.11	4.62 ± 0.11
TC10001760.hg.1	OTTHUMG00000019262	5.89 ± 0.14	5.61 ± 0.04	5.68 ± 0.04	5.65 ± 0.17
TC10000928.hg.1	OTTHUMG00000019263	8.14 ± 0.11	8.12 ± 0.09	7.96 ± 0.18	8.06 ± 0.00
TC10001765.hg.1	OTTHUMG00000019273	6.76 ± 0.17	6.73 ± 0.09	6.68 ± 0.32	6.86 ± 0.13
TC10000936.hg.1	OTTHUMG00000019275	7.95 ± 0.26	7.93 ± 0.18	7.76 ± 0.13	7.98 ± 0.10
TC10001770.hg.1	OTTHUMG00000019281	6.86 ± 0.16	6.93 ± 0.16	6.88 ± 0.19	7.04 ± 0.07

TC10000944.hg.1	OTTHUMG00000019287	8.05 ± 0.11	8.00 ± 0.11	7.97 ± 0.20	8.14 ± 0.15
TC10001772.hg.1	OTTHUMG00000019288	8.24 ± 0.21	8.22 ± 0.18	8.36 ± 0.21	8.39 ± 0.06
TC10001773.hg.1	OTTHUMG00000019290	8.13 ± 0.27	8.24 ± 0.19	8.14 ± 0.35	8.25 ± 0.06
TC10001774.hg.1	OTTHUMG00000019291	6.95 ± 0.12	7.06 ± 0.22	7.00 ± 0.13	7.11 ± 0.10
TC10001775.hg.1	OTTHUMG00000019295	9.76 ± 0.24	9.76 ± 0.17	9.73 ± 0.35	9.78 ± 0.03
TC10001778.hg.1	OTTHUMG00000019299	8.72 ± 0.45	8.54 ± 0.22	8.41 ± 0.47	8.68 ± 0.22
TC10001779.hg.1	OTTHUMG00000019300	6.50 ± 0.19	6.33 ± 0.10	6.40 ± 0.32	6.42 ± 0.09
TC10001780.hg.1	OTTHUMG00000019301	8.58 ± 0.18	8.59 ± 0.22	8.56 ± 0.08	8.79 ± 0.05
TC10001788.hg.1	OTTHUMG00000019313	4.19 ± 0.19	4.14 ± 0.05	4.14 ± 0.02	4.19 ± 0.04
TC01001333.hg.1	OTTHUMG00000019395	7.17 ± 0.22	7.07 ± 0.13	7.02 ± 0.13	6.98 ± 0.10
TC01003364.hg.1	OTTHUMG00000019401	8.56 ± 0.54	8.74 ± 0.24	8.38 ± 0.13	8.51 ± 0.08
TC09000862.hg.1	OTTHUMG00000019424	8.73 ± 0.33	8.62 ± 0.26	8.65 ± 0.24	8.66 ± 0.03
TC09000866.hg.1	OTTHUMG00000019426	5.69 ± 0.15	5.76 ± 0.04	5.82 ± 0.16	5.70 ± 0.01
TC09000867.hg.1	OTTHUMG00000019427	5.99 ± 0.07	6.03 ± 0.05	6.02 ± 0.25	6.12 ± 0.16
TC09000006.hg.1	OTTHUMG00000019429	5.54 ± 0.30	5.44 ± 0.14	5.55 ± 0.26	5.29 ± 0.16
TC09000868.hg.1	OTTHUMG00000019433	6.15 ± 0.13	6.16 ± 0.13	6.28 ± 0.14	6.17 ± 0.04
TC09000871.hg.1	OTTHUMG00000019444	5.66 ± 0.23	5.67 ± 0.20	5.72 ± 0.44	5.62 ± 0.16
TC09000014.hg.1	OTTHUMG00000019446	3.83 ± 0.09	3.95 ± 0.07	3.93 ± 0.03	3.96 ± 0.25
TC09000019.hg.1	OTTHUMG00000019454	5.76 ± 0.04	5.79 ± 0.15	5.80 ± 0.01	5.82 ± 0.07
TC09000022.hg.1	OTTHUMG00000019457	6.90 ± 0.34	7.17 ± 0.07	7.06 ± 0.22	7.14 ± 0.11
TC09000024.hg.1	OTTHUMG00000019457	7.01 ± 0.65	7.22 ± 0.15	7.15 ± 0.50	7.22 ± 0.34
TC09000023.hg.1	OTTHUMG00000019458	6.73 ± 0.15	6.55 ± 0.11	6.43 ± 0.11	6.42 ± 0.13
TC09000025.hg.1	OTTHUMG00000019459	7.80 ± 0.20	7.80 ± 0.14	7.83 ± 0.11	7.88 ± 0.12
TC09000878.hg.1	OTTHUMG00000019460	5.37 ± 0.22	5.33 ± 0.11	5.23 ± 0.10	5.39 ± 0.10
TC09000880.hg.1	OTTHUMG00000019462	4.33 ± 0.08	4.13 ± 0.09	4.20 ± 0.02	4.18 ± 0.07
TC09000028.hg.1	OTTHUMG00000019464	5.52 ± 0.18	5.32 ± 0.06	5.82 ± 0.37	5.39 ± 0.04
TC09000885.hg.1	OTTHUMG00000019477	3.75 ± 0.09	3.43 ± 0.07	3.50 ± 0.16	3.43 ± 0.06
TC09000887.hg.1	OTTHUMG00000019491	5.22 ± 0.17	5.03 ± 0.15	5.13 ± 0.15	5.11 ± 0.11
TC09000039.hg.1	OTTHUMG00000019502	4.60 ± 0.32	4.39 ± 0.04	4.37 ± 0.23	4.46 ± 0.09
TC09000892.hg.1	OTTHUMG00000019506	6.92 ± 0.10	6.66 ± 0.19	6.86 ± 0.26	6.55 ± 0.15
TC09000897.hg.1	OTTHUMG00000019520	6.03 ± 0.37	5.61 ± 0.18	5.93 ± 0.10	5.44 ± 0.15

TC09000049.hg.1	OTTHUMG00000019527	6.76 ± 0.29	6.73 ± 0.32	6.75 ± 0.38	6.90 ± 0.09
TC09002928.hg.1	OTTHUMG00000019530	6.65 ± 0.12	6.69 ± 0.10	6.69 ± 0.09	6.79 ± 0.06
TC09000048.hg.1	OTTHUMG00000019532	5.93 ± 0.13	5.92 ± 0.11	5.96 ± 0.11	5.92 ± 0.12
TC09000904.hg.1	OTTHUMG00000019540	6.13 ± 0.16	6.05 ± 0.08	6.05 ± 0.12	6.23 ± 0.09
TC09000907.hg.1	OTTHUMG00000019542	8.46 ± 0.23	8.50 ± 0.01	8.46 ± 0.04	8.61 ± 0.12
TC09000057.hg.1	OTTHUMG00000019543	4.29 ± 0.10	4.24 ± 0.15	4.23 ± 0.14	4.31 ± 0.14
TC09000059.hg.1	OTTHUMG00000019544	4.39 ± 0.21	4.20 ± 0.09	4.27 ± 0.12	4.27 ± 0.12
TC09000062.hg.1	OTTHUMG00000019546	3.26 ± 0.07	3.25 ± 0.02	3.30 ± 0.10	3.19 ± 0.04
TC09000063.hg.1	OTTHUMG00000019549	5.41 ± 0.11	5.40 ± 0.14	5.34 ± 0.12	5.34 ± 0.08
TC09000908.hg.1	OTTHUMG00000019550	3.73 ± 0.07	3.39 ± 0.13	3.47 ± 0.08	3.43 ± 0.12
TC09000909.hg.1	OTTHUMG00000019554	6.12 ± 0.19	6.38 ± 0.21	6.22 ± 0.09	6.43 ± 0.15
TC09000910.hg.1	OTTHUMG00000019555	4.09 ± 0.11	4.08 ± 0.03	4.00 ± 0.10	4.12 ± 0.20
TC09000912.hg.1	OTTHUMG00000019558	4.32 ± 0.11	4.18 ± 0.06	4.19 ± 0.02	4.23 ± 0.09
TC09000914.hg.1	OTTHUMG00000019561	5.59 ± 0.20	5.54 ± 0.14	5.53 ± 0.07	5.57 ± 0.07
TC09000070.hg.1	OTTHUMG00000019566	4.68 ± 0.20	4.65 ± 0.16	4.59 ± 0.08	4.73 ± 0.23
TC09000071.hg.1	OTTHUMG00000019570	6.79 ± 0.31	6.86 ± 0.30	6.68 ± 0.40	6.96 ± 0.06
TC09000080.hg.1	OTTHUMG00000019589	5.12 ± 0.11	5.00 ± 0.17	5.17 ± 0.12	5.25 ± 0.17
TC09000081.hg.1	OTTHUMG00000019590	4.21 ± 0.17	4.12 ± 0.04	4.15 ± 0.06	4.28 ± 0.17
TC09000082.hg.1	OTTHUMG00000019591	8.34 ± 0.37	8.09 ± 0.12	8.10 ± 0.33	8.12 ± 0.09
TC09000083.hg.1	OTTHUMG00000019594	5.10 ± 0.08	4.84 ± 0.14	4.95 ± 0.08	5.05 ± 0.10
TC09000930.hg.1	OTTHUMG00000019602	4.00 ± 0.23	4.09 ± 0.03	3.90 ± 0.11	4.06 ± 0.02
TC09000937.hg.1	OTTHUMG00000019626	4.43 ± 0.08	4.34 ± 0.03	4.37 ± 0.08	4.47 ± 0.03
TC09000944.hg.1	OTTHUMG00000019649	6.32 ± 0.12	6.28 ± 0.12	6.33 ± 0.13	6.41 ± 0.05
TC09000966.hg.1	OTTHUMG00000019692	4.49 ± 0.05	4.33 ± 0.08	4.32 ± 0.04	4.37 ± 0.15
TC09000111.hg.1	OTTHUMG00000019695	4.49 ± 0.17	4.42 ± 0.08	4.40 ± 0.17	4.60 ± 0.21
TC09000967.hg.1	OTTHUMG00000019699	5.05 ± 0.13	4.95 ± 0.08	4.93 ± 0.05	5.00 ± 0.05
TC09000113.hg.1	OTTHUMG00000019702	5.88 ± 0.12	5.74 ± 0.05	5.77 ± 0.07	5.81 ± 0.13
TC09000969.hg.1	OTTHUMG00000019703	6.31 ± 0.31	6.35 ± 0.01	6.55 ± 0.05	6.37 ± 0.13
TC09000115.hg.1	OTTHUMG00000019705	5.01 ± 0.10	4.78 ± 0.07	4.94 ± 0.08	4.85 ± 0.03
TC09000984.hg.1	OTTHUMG00000019709	4.72 ± 0.23	4.56 ± 0.19	4.65 ± 0.12	4.51 ± 0.06
TC09000123.hg.1	OTTHUMG00000019713	5.24 ± 0.38	4.94 ± 0.01	4.99 ± 0.20	4.92 ± 0.07

TC09001003.hg.1	OTTHUMG00000019749	6.13 ± 0.10	5.95 ± 0.31	6.04 ± 0.13	6.06 ± 0.04
TC09000130.hg.1	OTTHUMG00000019750	6.25 ± 0.24	6.14 ± 0.20	6.05 ± 0.18	6.12 ± 0.16
TC09001005.hg.1	OTTHUMG00000019752	5.83 ± 0.15	5.76 ± 0.07	5.75 ± 0.19	5.83 ± 0.03
TC09000136.hg.1	OTTHUMG00000019763	6.89 ± 0.11	6.71 ± 0.06	6.75 ± 0.16	6.82 ± 0.05
TC09000181.hg.1	OTTHUMG00000019849	7.80 ± 0.08	7.77 ± 0.20	7.82 ± 0.09	7.90 ± 0.13
TC09000188.hg.1	OTTHUMG00000019865	7.60 ± 0.30	7.62 ± 0.17	7.33 ± 0.26	7.82 ± 0.24
TC09000189.hg.1	OTTHUMG00000019866	8.04 ± 0.37	7.77 ± 0.15	7.69 ± 0.42	7.81 ± 0.06
TC09001062.hg.1	OTTHUMG00000019883	7.35 ± 0.18	7.24 ± 0.04	7.20 ± 0.18	7.26 ± 0.01
TC09000194.hg.1	OTTHUMG00000019884	5.14 ± 0.14	5.07 ± 0.10	5.02 ± 0.34	4.93 ± 0.08
TC09000200.hg.1	OTTHUMG00000019888	5.07 ± 0.18	5.20 ± 0.21	5.13 ± 0.06	5.20 ± 0.08
TC09001075.hg.1	OTTHUMG00000019909	6.27 ± 0.17	5.92 ± 0.12	5.79 ± 0.15	5.98 ± 0.11
TC09001076.hg.1	OTTHUMG00000019911	4.81 ± 0.22	4.91 ± 0.14	4.79 ± 0.14	4.96 ± 0.03
TC09001078.hg.1	OTTHUMG00000019916	7.64 ± 0.05	7.60 ± 0.07	7.53 ± 0.18	7.74 ± 0.07
TC09002931.hg.1	OTTHUMG00000019924	8.16 ± 0.17	8.05 ± 0.03	8.06 ± 0.07	8.09 ± 0.02
TC09000221.hg.1	OTTHUMG00000019939	7.03 ± 0.31	6.90 ± 0.12	6.84 ± 0.18	6.96 ± 0.10
TC09000227.hg.1	OTTHUMG00000019951	5.88 ± 0.24	5.73 ± 0.17	5.81 ± 0.05	5.90 ± 0.14
TC09000310.hg.1	OTTHUMG00000019960	7.64 ± 0.24	7.55 ± 0.22	7.48 ± 0.21	7.61 ± 0.09
TC09000311.hg.1	OTTHUMG00000019961	5.58 ± 0.26	5.59 ± 0.11	5.56 ± 0.11	5.75 ± 0.14
TC09000307.hg.1	OTTHUMG00000019963	6.30 ± 0.38	6.39 ± 0.36	6.82 ± 0.65	5.92 ± 0.12
TC09001185.hg.1	OTTHUMG00000019964	6.89 ± 0.06	6.97 ± 0.23	6.98 ± 0.36	6.93 ± 0.06
TC09000313.hg.1	OTTHUMG00000019965	4.73 ± 0.25	4.56 ± 0.13	4.60 ± 0.12	4.62 ± 0.05
TC09001187.hg.1	OTTHUMG00000019968	7.86 ± 0.34	7.56 ± 0.30	8.05 ± 0.47	7.51 ± 0.15
TC09000315.hg.1	OTTHUMG00000019970	5.85 ± 0.10	5.77 ± 0.25	6.12 ± 0.08	5.95 ± 0.03
TC09001188.hg.1	OTTHUMG00000019973	5.61 ± 0.02	5.53 ± 0.02	5.51 ± 0.02	5.57 ± 0.22
TC09000321.hg.1	OTTHUMG00000019981	6.26 ± 0.19	6.25 ± 0.20	6.19 ± 0.13	6.43 ± 0.07
TC09000326.hg.1	OTTHUMG00000019994	6.19 ± 0.14	6.10 ± 0.03	6.07 ± 0.07	6.06 ± 0.04
TC09000327.hg.1	OTTHUMG00000019995	6.19 ± 0.14	6.10 ± 0.03	6.07 ± 0.07	6.06 ± 0.04
TC09000331.hg.1	OTTHUMG00000020009	6.66 ± 0.20	6.37 ± 0.16	6.53 ± 0.06	6.65 ± 0.13
TC09001210.hg.1	OTTHUMG00000020015	6.87 ± 0.38	6.86 ± 0.02	6.74 ± 0.10	7.03 ± 0.14
TC09000336.hg.1	OTTHUMG00000020021	4.08 ± 0.07	4.10 ± 0.07	4.11 ± 0.12	4.04 ± 0.03
TC09000337.hg.1	OTTHUMG00000020024	3.87 ± 0.10	3.64 ± 0.06	3.78 ± 0.05	3.66 ± 0.16

TC09001214.hg.1	OTTHUMG00000020025	6.85 ± 0.18	6.89 ± 0.06	6.87 ± 0.04	6.97 ± 0.06
TC09000342.hg.1	OTTHUMG00000020029	6.09 ± 0.12	5.98 ± 0.04	5.98 ± 0.07	6.05 ± 0.11
TC09001219.hg.1	OTTHUMG00000020030	6.40 ± 0.17	6.30 ± 0.06	6.12 ± 0.02	6.28 ± 0.12
TC09000345.hg.1	OTTHUMG00000020036	4.23 ± 0.05	4.04 ± 0.14	4.01 ± 0.12	4.10 ± 0.08
TC09000355.hg.1	OTTHUMG00000020056	5.86 ± 0.15	5.58 ± 0.13	5.63 ± 0.31	5.66 ± 0.05
TC09001255.hg.1	OTTHUMG00000020074	5.92 ± 0.10	5.80 ± 0.12	5.86 ± 0.07	5.96 ± 0.13
TC09000360.hg.1	OTTHUMG00000020075	6.48 ± 0.29	6.35 ± 0.12	6.50 ± 0.15	6.53 ± 0.04
TC09001257.hg.1	OTTHUMG00000020080	5.39 ± 0.16	5.39 ± 0.03	5.40 ± 0.05	5.52 ± 0.08
TC09000362.hg.1	OTTHUMG00000020083	5.94 ± 0.34	5.80 ± 0.17	5.84 ± 0.17	6.00 ± 0.11
TC09001265.hg.1	OTTHUMG00000020086	5.69 ± 0.16	5.45 ± 0.17	5.44 ± 0.09	5.51 ± 0.14
TC09000371.hg.1	OTTHUMG00000020094	4.99 ± 0.12	5.01 ± 0.15	4.98 ± 0.16	5.02 ± 0.11
TC09000372.hg.1	OTTHUMG00000020097	5.09 ± 0.22	4.98 ± 0.07	5.09 ± 0.15	5.05 ± 0.06
TC09000369.hg.1	OTTHUMG00000020098	6.47 ± 0.19	6.26 ± 0.06	6.31 ± 0.06	6.36 ± 0.05
TC09000373.hg.1	OTTHUMG00000020101	7.12 ± 0.15	7.01 ± 0.15	7.03 ± 0.07	7.09 ± 0.16
TC09000375.hg.1	OTTHUMG00000020102	4.57 ± 0.17	4.55 ± 0.11	4.55 ± 0.04	4.76 ± 0.13
TC09000381.hg.1	OTTHUMG00000020108	5.25 ± 0.13	5.08 ± 0.12	5.19 ± 0.12	5.21 ± 0.10
TC09000382.hg.1	OTTHUMG00000020110	4.95 ± 0.09	4.84 ± 0.16	5.01 ± 0.18	4.99 ± 0.20
TC09000383.hg.1	OTTHUMG00000020112	5.19 ± 0.40	5.44 ± 0.05	5.40 ± 0.48	5.44 ± 0.06
TC09000386.hg.1	OTTHUMG00000020115	5.49 ± 0.09	5.43 ± 0.03	5.50 ± 0.09	5.50 ± 0.07
TC09000387.hg.1	OTTHUMG00000020116	5.18 ± 0.13	5.17 ± 0.11	5.25 ± 0.24	5.23 ± 0.18
TC09001288.hg.1	OTTHUMG00000020123	5.03 ± 0.02	4.97 ± 0.11	5.04 ± 0.10	5.11 ± 0.15
TC09001289.hg.1	OTTHUMG00000020126	5.25 ± 0.09	5.12 ± 0.13	5.19 ± 0.12	5.20 ± 0.08
TC09000392.hg.1	OTTHUMG00000020138	3.55 ± 0.05	3.46 ± 0.09	3.54 ± 0.06	3.50 ± 0.05
TC09000393.hg.1	OTTHUMG00000020140	4.84 ± 0.10	4.86 ± 0.19	4.80 ± 0.08	4.88 ± 0.07
TC09001301.hg.1	OTTHUMG00000020155	6.52 ± 0.11	6.50 ± 0.18	6.40 ± 0.18	6.70 ± 0.14
TC09000413.hg.1	OTTHUMG00000020176	6.23 ± 0.13	6.29 ± 0.12	6.26 ± 0.11	6.42 ± 0.09
TC09000421.hg.1	OTTHUMG00000020189	5.68 ± 0.18	5.73 ± 0.21	5.79 ± 0.04	5.92 ± 0.15
TC09001317.hg.1	OTTHUMG00000020194	6.07 ± 0.34	6.05 ± 0.13	6.03 ± 0.20	6.08 ± 0.05
TC09001320.hg.1	OTTHUMG00000020201	5.91 ± 0.21	5.71 ± 0.11	5.91 ± 0.10	5.85 ± 0.12
TC09000429.hg.1	OTTHUMG00000020205	5.21 ± 0.18	5.12 ± 0.04	5.16 ± 0.04	5.16 ± 0.03
TC09000431.hg.1	OTTHUMG00000020208	5.18 ± 0.35	5.18 ± 0.07	5.06 ± 0.06	4.99 ± 0.15

TC09001330.hg.1	OTTHUMG00000020214	4.44 ± 0.14	4.24 ± 0.06	4.21 ± 0.07	4.36 ± 0.22
TC09000435.hg.1	OTTHUMG00000020222	6.98 ± 0.23	6.93 ± 0.13	7.02 ± 0.05	7.11 ± 0.15
TC09001344.hg.1	OTTHUMG00000020240	7.25 ± 0.55	7.31 ± 0.38	7.19 ± 0.30	7.33 ± 0.13
TC09000443.hg.1	OTTHUMG00000020244	8.55 ± 0.14	8.49 ± 0.04	8.53 ± 0.07	8.59 ± 0.07
TC09001347.hg.1	OTTHUMG00000020245	4.09 ± 0.04	3.92 ± 0.11	3.99 ± 0.16	4.08 ± 0.04
TC09001348.hg.1	OTTHUMG00000020246	5.87 ± 0.24	5.84 ± 0.13	5.98 ± 0.18	5.85 ± 0.10
TC09000452.hg.1	OTTHUMG00000020254	5.86 ± 0.05	5.81 ± 0.14	5.87 ± 0.05	5.91 ± 0.10
TC09000453.hg.1	OTTHUMG00000020256	7.48 ± 0.24	7.43 ± 0.28	7.39 ± 0.22	7.54 ± 0.06
TC09000466.hg.1	OTTHUMG00000020270	6.07 ± 0.13	5.97 ± 0.08	5.97 ± 0.12	6.05 ± 0.04
TC09000469.hg.1	OTTHUMG00000020272	5.11 ± 0.14	5.17 ± 0.09	4.97 ± 0.13	5.18 ± 0.08
TC09000470.hg.1	OTTHUMG00000020273	5.04 ± 0.18	5.14 ± 0.01	5.09 ± 0.07	5.11 ± 0.12
TC09001359.hg.1	OTTHUMG00000020274	6.81 ± 0.23	6.73 ± 0.24	6.85 ± 0.18	6.75 ± 0.01
TC09000471.hg.1	OTTHUMG00000020277	11.03 ± 0.22	11.06 ± 0.02	10.96 ± 0.07	11.14 ± 0.19
TC09001365.hg.1	OTTHUMG00000020281	7.42 ± 0.16	7.48 ± 0.17	7.50 ± 0.19	7.58 ± 0.06
TC09001367.hg.1	OTTHUMG00000020283	5.40 ± 0.15	5.25 ± 0.13	5.38 ± 0.17	5.35 ± 0.15
TC09000473.hg.1	OTTHUMG00000020285	7.50 ± 0.31	7.54 ± 0.10	7.57 ± 0.11	7.59 ± 0.05
TC09000479.hg.1	OTTHUMG00000020294	5.15 ± 0.15	5.27 ± 0.08	5.22 ± 0.17	5.39 ± 0.08
TC09000488.hg.1	OTTHUMG00000020320	5.97 ± 0.28	6.18 ± 0.20	6.02 ± 0.31	6.44 ± 0.25
TC09001396.hg.1	OTTHUMG00000020327	4.88 ± 0.17	4.95 ± 0.17	4.99 ± 0.09	4.97 ± 0.04
TC09001394.hg.1	OTTHUMG00000020329	6.75 ± 0.39	6.70 ± 0.18	6.71 ± 0.10	6.78 ± 0.22
TC09001399.hg.1	OTTHUMG00000020335	6.17 ± 0.33	6.18 ± 0.50	6.21 ± 0.13	6.32 ± 0.04
TC09001402.hg.1	OTTHUMG00000020337	5.13 ± 0.27	4.94 ± 0.11	4.94 ± 0.06	4.87 ± 0.06
TC09001409.hg.1	OTTHUMG00000020349	5.42 ± 0.06	5.40 ± 0.18	5.23 ± 0.15	5.40 ± 0.13
TC09000501.hg.1	OTTHUMG00000020352	6.10 ± 0.02	6.01 ± 0.14	6.02 ± 0.07	6.10 ± 0.09
TC09001413.hg.1	OTTHUMG00000020358	5.25 ± 0.13	5.17 ± 0.04	5.21 ± 0.11	5.21 ± 0.11
TC09000518.hg.1	OTTHUMG00000020380	6.22 ± 0.31	6.20 ± 0.10	6.18 ± 0.18	6.30 ± 0.08
TC09000525.hg.1	OTTHUMG00000020399	5.83 ± 0.10	5.78 ± 0.04	5.78 ± 0.05	5.79 ± 0.08
TC09001425.hg.1	OTTHUMG00000020399	5.61 ± 0.15	5.58 ± 0.01	5.56 ± 0.05	5.64 ± 0.07
TC09001426.hg.1	OTTHUMG00000020400	5.06 ± 0.20	4.85 ± 0.09	5.11 ± 0.07	5.01 ± 0.09
TC09000533.hg.1	OTTHUMG00000020418	7.42 ± 0.16	7.31 ± 0.17	7.31 ± 0.15	7.34 ± 0.10
TC09001436.hg.1	OTTHUMG00000020419	7.01 ± 0.28	6.98 ± 0.43	6.96 ± 0.26	7.11 ± 0.01

TC09001437.hg.1	OTTHUMG00000020423	5.95 ± 0.13	5.78 ± 0.11	5.85 ± 0.26	5.90 ± 0.18
TC09001438.hg.1	OTTHUMG00000020432	4.54 ± 0.25	4.33 ± 0.08	4.27 ± 0.13	4.58 ± 0.19
TC09000541.hg.1	OTTHUMG00000020433	5.61 ± 0.12	5.67 ± 0.05	5.59 ± 0.04	5.72 ± 0.12
TC09001440.hg.1	OTTHUMG00000020434	5.22 ± 0.17	5.21 ± 0.13	5.21 ± 0.13	5.33 ± 0.25
TC09000542.hg.1	OTTHUMG00000020435	5.90 ± 0.16	5.78 ± 0.12	5.85 ± 0.11	5.89 ± 0.06
TC09001439.hg.1	OTTHUMG00000020437	4.61 ± 0.10	4.49 ± 0.10	4.47 ± 0.03	4.67 ± 0.21
TC09001444.hg.1	OTTHUMG00000020443	6.22 ± 0.03	6.23 ± 0.07	6.20 ± 0.19	6.43 ± 0.22
TC09001443.hg.1	OTTHUMG00000020444	6.60 ± 0.27	6.57 ± 0.19	6.47 ± 0.24	6.83 ± 0.13
TC09001445.hg.1	OTTHUMG00000020447	6.33 ± 0.15	6.12 ± 0.04	6.16 ± 0.08	6.24 ± 0.09
TC09001452.hg.1	OTTHUMG00000020457	4.30 ± 0.29	4.19 ± 0.12	4.25 ± 0.17	4.36 ± 0.16
TC09000546.hg.1	OTTHUMG00000020459	4.05 ± 0.12	4.08 ± 0.03	3.93 ± 0.07	4.07 ± 0.08
TC09001471.hg.1	OTTHUMG00000020476	6.55 ± 0.21	6.37 ± 0.08	6.39 ± 0.17	6.39 ± 0.08
TC09000552.hg.1	OTTHUMG00000020483	7.40 ± 0.07	7.41 ± 0.09	7.40 ± 0.14	7.53 ± 0.05
TC09001480.hg.1	OTTHUMG00000020487	5.44 ± 0.29	5.31 ± 0.02	5.36 ± 0.02	5.23 ± 0.16
TC09000557.hg.1	OTTHUMG00000020492	4.01 ± 0.04	3.90 ± 0.06	3.92 ± 0.07	3.99 ± 0.14
TC09001493.hg.1	OTTHUMG00000020510	7.97 ± 0.12	7.98 ± 0.10	7.83 ± 0.27	7.89 ± 0.06
TC09001504.hg.1	OTTHUMG00000020527	6.33 ± 0.13	6.27 ± 0.09	6.21 ± 0.10	6.26 ± 0.08
TC09000577.hg.1	OTTHUMG00000020528	6.52 ± 0.08	6.51 ± 0.20	6.56 ± 0.24	6.60 ± 0.06
TC09000578.hg.1	OTTHUMG00000020530	6.67 ± 0.17	6.51 ± 0.27	6.52 ± 0.24	6.67 ± 0.06
TC09001519.hg.1	OTTHUMG00000020547	6.94 ± 0.17	7.05 ± 0.03	7.00 ± 0.11	7.22 ± 0.19
TC09001518.hg.1	OTTHUMG00000020548	4.57 ± 0.14	4.47 ± 0.06	4.58 ± 0.06	4.57 ± 0.19
TC09000591.hg.1	OTTHUMG00000020550	6.06 ± 0.26	6.12 ± 0.06	6.04 ± 0.16	6.10 ± 0.12
TC09000592.hg.1	OTTHUMG00000020552	5.83 ± 0.14	5.85 ± 0.07	6.01 ± 0.12	6.01 ± 0.16
TC09001522.hg.1	OTTHUMG00000020554	5.37 ± 0.37	5.02 ± 0.35	5.05 ± 0.25	5.20 ± 0.09
TC09001523.hg.1	OTTHUMG00000020555	6.04 ± 0.18	5.88 ± 0.18	5.89 ± 0.03	6.01 ± 0.07
TC09000596.hg.1	OTTHUMG00000020557	5.53 ± 0.02	5.67 ± 0.21	5.56 ± 0.18	5.60 ± 0.09
TC09001528.hg.1	OTTHUMG00000020560	5.01 ± 0.43	4.98 ± 0.09	5.05 ± 0.17	5.11 ± 0.11
TC09000600.hg.1	OTTHUMG00000020561	5.21 ± 0.12	5.02 ± 0.10	5.09 ± 0.11	5.17 ± 0.10
TC09001531.hg.1	OTTHUMG00000020563	3.95 ± 0.24	3.89 ± 0.17	4.01 ± 0.09	3.99 ± 0.11
TC09000602.hg.1	OTTHUMG00000020564	5.95 ± 0.07	5.80 ± 0.08	5.88 ± 0.03	5.93 ± 0.17
TC09001532.hg.1	OTTHUMG00000020566	5.67 ± 0.15	5.72 ± 0.22	5.59 ± 0.26	5.79 ± 0.20

TC09000603.hg.1	OTTHUMG00000020567	5.99 ± 0.20	5.90 ± 0.01	5.79 ± 0.20	5.94 ± 0.15
TC09000604.hg.1	OTTHUMG00000020570	6.50 ± 0.19	6.33 ± 0.25	6.37 ± 0.16	6.54 ± 0.10
TC09001544.hg.1	OTTHUMG00000020588	6.50 ± 0.16	6.39 ± 0.20	6.32 ± 0.20	6.52 ± 0.17
TC09000609.hg.1	OTTHUMG00000020591	5.71 ± 0.18	5.41 ± 0.03	5.56 ± 0.05	5.49 ± 0.22
TC09001549.hg.1	OTTHUMG00000020594	8.34 ± 0.31	8.29 ± 0.18	8.21 ± 0.19	8.50 ± 0.12
TC09001557.hg.1	OTTHUMG00000020610	5.33 ± 0.21	4.99 ± 0.09	5.05 ± 0.04	5.03 ± 0.12
TC09000615.hg.1	OTTHUMG00000020611	6.58 ± 0.32	6.56 ± 0.04	6.50 ± 0.11	6.67 ± 0.09
TC09002934.hg.1	OTTHUMG00000020612	6.44 ± 0.19	6.47 ± 0.08	6.41 ± 0.04	6.52 ± 0.13
TC09000618.hg.1	OTTHUMG00000020614	4.75 ± 0.06	4.70 ± 0.06	4.77 ± 0.01	4.82 ± 0.12
TC09000630.hg.1	OTTHUMG00000020639	6.27 ± 0.28	6.10 ± 0.14	6.19 ± 0.19	6.19 ± 0.17
TC09000631.hg.1	OTTHUMG00000020640	5.21 ± 0.39	5.07 ± 0.18	5.11 ± 0.19	5.15 ± 0.16
TC09001576.hg.1	OTTHUMG00000020645	7.91 ± 0.18	7.98 ± 0.05	7.95 ± 0.20	8.06 ± 0.04
TC09000632.hg.1	OTTHUMG00000020646	7.83 ± 0.24	7.65 ± 0.07	7.74 ± 0.14	7.66 ± 0.13
TC09001577.hg.1	OTTHUMG00000020648	6.79 ± 0.12	6.78 ± 0.19	6.97 ± 0.07	6.97 ± 0.12
TC09000635.hg.1	OTTHUMG00000020649	6.97 ± 0.16	6.86 ± 0.18	6.95 ± 0.23	6.98 ± 0.09
TC09000639.hg.1	OTTHUMG00000020654	5.39 ± 0.06	5.33 ± 0.18	5.33 ± 0.17	5.46 ± 0.14
TC09000643.hg.1	OTTHUMG00000020662	7.41 ± 0.29	7.30 ± 0.12	7.30 ± 0.14	7.34 ± 0.15
TC09000651.hg.1	OTTHUMG00000020681	6.55 ± 0.15	6.57 ± 0.03	6.43 ± 0.15	6.69 ± 0.14
TC09001592.hg.1	OTTHUMG00000020682	6.17 ± 0.25	6.06 ± 0.05	6.10 ± 0.02	6.11 ± 0.10
TC09000653.hg.1	OTTHUMG00000020685	5.28 ± 0.29	5.16 ± 0.25	5.07 ± 0.17	5.27 ± 0.11
TC09001593.hg.1	OTTHUMG00000020686	7.00 ± 0.31	6.90 ± 0.10	6.88 ± 0.12	6.98 ± 0.11
TC09000656.hg.1	OTTHUMG00000020688	6.02 ± 0.21	6.28 ± 0.05	6.03 ± 0.14	6.35 ± 0.02
TC09000657.hg.1	OTTHUMG00000020689	6.64 ± 0.11	6.55 ± 0.02	6.52 ± 0.07	6.75 ± 0.29
TC09001597.hg.1	OTTHUMG00000020690	6.97 ± 0.19	6.94 ± 0.16	6.83 ± 0.11	7.00 ± 0.10
TC09001600.hg.1	OTTHUMG00000020697	6.54 ± 0.12	6.44 ± 0.10	6.52 ± 0.08	6.51 ± 0.01
TC09000673.hg.1	OTTHUMG00000020714	8.79 ± 0.18	8.68 ± 0.07	8.73 ± 0.12	8.77 ± 0.08
TC09000674.hg.1	OTTHUMG00000020721	6.82 ± 0.13	6.59 ± 0.14	6.53 ± 0.08	6.55 ± 0.05
TC09001619.hg.1	OTTHUMG00000020726	5.66 ± 0.11	5.78 ± 0.06	5.69 ± 0.04	5.81 ± 0.06
TC09001621.hg.1	OTTHUMG00000020728	6.90 ± 0.23	6.73 ± 0.24	6.79 ± 0.11	6.68 ± 0.06
TC09001632.hg.1	OTTHUMG00000020745	6.86 ± 0.22	6.66 ± 0.31	6.79 ± 0.09	6.64 ± 0.06
TC09001633.hg.1	OTTHUMG00000020752	7.41 ± 0.15	7.31 ± 0.07	7.41 ± 0.26	7.38 ± 0.05

TC09000712.hg.1	OTTHUMG00000020775	7.78 ± 0.13	7.61 ± 0.06	7.57 ± 0.09	7.61 ± 0.12
TC09000711.hg.1	OTTHUMG00000020777	6.87 ± 0.14	6.96 ± 0.11	6.88 ± 0.11	7.11 ± 0.06
TC09000717.hg.1	OTTHUMG00000020779	7.09 ± 0.13	7.03 ± 0.06	7.08 ± 0.10	7.13 ± 0.11
TC09001647.hg.1	OTTHUMG00000020780	7.51 ± 0.16	7.49 ± 0.23	7.46 ± 0.21	7.52 ± 0.07
TC09000720.hg.1	OTTHUMG00000020784	7.27 ± 0.10	7.13 ± 0.19	7.28 ± 0.10	7.26 ± 0.03
TC09000722.hg.1	OTTHUMG00000020788	6.47 ± 0.07	6.52 ± 0.17	6.40 ± 0.21	6.61 ± 0.05
TC09001652.hg.1	OTTHUMG00000020789	7.39 ± 0.37	7.29 ± 0.24	7.21 ± 0.17	7.43 ± 0.08
TC09000727.hg.1	OTTHUMG00000020797	6.87 ± 0.13	6.61 ± 0.08	6.75 ± 0.23	6.59 ± 0.14
TC09000743.hg.1	OTTHUMG00000020815	6.89 ± 0.18	6.79 ± 0.18	6.78 ± 0.11	6.91 ± 0.07
TC09001662.hg.1	OTTHUMG00000020818	5.69 ± 0.17	5.45 ± 0.03	5.48 ± 0.11	5.44 ± 0.06
TC09001666.hg.1	OTTHUMG00000020824	8.35 ± 0.23	8.34 ± 0.07	8.35 ± 0.14	8.34 ± 0.04
TC09000753.hg.1	OTTHUMG00000020831	6.64 ± 0.26	6.65 ± 0.21	6.58 ± 0.07	6.90 ± 0.15
TC09000754.hg.1	OTTHUMG00000020832	6.83 ± 0.13	6.76 ± 0.13	6.88 ± 0.07	6.81 ± 0.09
TC09000764.hg.1	OTTHUMG00000020845	7.16 ± 0.21	7.23 ± 0.20	7.10 ± 0.26	7.31 ± 0.12
TC09001677.hg.1	OTTHUMG00000020846	7.16 ± 0.07	7.06 ± 0.11	7.06 ± 0.15	7.16 ± 0.15
TC09001699.hg.1	OTTHUMG00000020883	7.76 ± 0.24	7.78 ± 0.30	7.86 ± 0.11	8.04 ± 0.13
TC09001700.hg.1	OTTHUMG00000020884	6.58 ± 0.21	6.55 ± 0.21	6.38 ± 0.29	6.59 ± 0.17
TC09000784.hg.1	OTTHUMG00000020885	7.68 ± 0.21	7.64 ± 0.20	7.73 ± 0.09	7.80 ± 0.12
TC09000789.hg.1	OTTHUMG00000020894	5.82 ± 0.31	5.81 ± 0.22	5.87 ± 0.13	5.90 ± 0.06
TC09001712.hg.1	OTTHUMG00000020898	7.81 ± 0.50	7.79 ± 0.19	7.65 ± 0.33	7.75 ± 0.09
TC09001711.hg.1	OTTHUMG00000020899	7.15 ± 0.16	7.09 ± 0.20	7.10 ± 0.03	7.20 ± 0.19
TC09000793.hg.1	OTTHUMG00000020901	7.17 ± 0.31	7.15 ± 0.16	7.01 ± 0.18	7.16 ± 0.01
TC09001716.hg.1	OTTHUMG00000020903	7.14 ± 0.32	7.19 ± 0.20	7.02 ± 0.17	7.27 ± 0.13
TC09000806.hg.1	OTTHUMG00000020919	7.93 ± 0.32	7.98 ± 0.26	7.90 ± 0.37	8.06 ± 0.03
TC09000815.hg.1	OTTHUMG00000020933	7.89 ± 0.24	7.90 ± 0.17	7.87 ± 0.20	8.02 ± 0.07
TC09000814.hg.1	OTTHUMG00000020934	8.25 ± 0.17	8.18 ± 0.08	8.31 ± 0.04	8.18 ± 0.05
TC09001737.hg.1	OTTHUMG00000020937	7.97 ± 0.14	7.80 ± 0.15	7.85 ± 0.11	8.00 ± 0.06
TC09000834.hg.1	OTTHUMG00000020960	8.29 ± 0.10	8.22 ± 0.18	8.25 ± 0.08	8.31 ± 0.07
TC09001747.hg.1	OTTHUMG00000020963	8.17 ± 0.23	8.26 ± 0.20	8.21 ± 0.20	8.33 ± 0.02
TC09001751.hg.1	OTTHUMG00000020972	8.07 ± 0.14	8.25 ± 0.16	8.14 ± 0.07	8.30 ± 0.12
TC09000835.hg.1	OTTHUMG00000020974	7.79 ± 0.28	7.79 ± 0.04	7.82 ± 0.06	7.93 ± 0.07

TC09000836.hg.1	OTTHUMG00000020975	9.11 ± 0.20	9.02 ± 0.15	9.07 ± 0.20	9.04 ± 0.03
TC09001780.hg.1	OTTHUMG00000020999	7.13 ± 0.20	7.05 ± 0.14	7.27 ± 0.30	7.13 ± 0.01
TC09000861.hg.1	OTTHUMG00000021018	6.06 ± 0.32	6.07 ± 0.05	6.08 ± 0.16	6.14 ± 0.08
TC0X000005.hg.1	OTTHUMG00000021054	6.44 ± 0.17	6.44 ± 0.17	6.09 ± 0.17	6.30 ± 0.19
TC0X000011.hg.1	OTTHUMG00000021062	6.82 ± 0.16	6.83 ± 0.22	6.87 ± 0.12	6.86 ± 0.07
TC0X000012.hg.1	OTTHUMG00000021064	6.92 ± 0.03	6.97 ± 0.04	6.86 ± 0.04	6.99 ± 0.03
TC0X000818.hg.1	OTTHUMG00000021088	8.69 ± 0.52	8.72 ± 0.11	8.61 ± 0.21	8.98 ± 0.16
TC0X000820.hg.1	OTTHUMG00000021092	5.76 ± 0.22	5.65 ± 0.07	5.62 ± 0.19	5.79 ± 0.18
TC0X000825.hg.1	OTTHUMG00000021098	6.19 ± 0.13	6.00 ± 0.04	6.04 ± 0.05	6.09 ± 0.12
TC0X000028.hg.1	OTTHUMG00000021104	6.12 ± 0.06	6.17 ± 0.18	6.18 ± 0.17	6.37 ± 0.18
TC0X000831.hg.1	OTTHUMG00000021111	5.97 ± 0.17	5.83 ± 0.08	5.85 ± 0.03	6.00 ± 0.11
TC0X000032.hg.1	OTTHUMG00000021115	6.66 ± 0.22	6.67 ± 0.10	6.68 ± 0.16	6.95 ± 0.16
TC0X000039.hg.1	OTTHUMG00000021126	6.66 ± 0.15	6.59 ± 0.13	6.54 ± 0.06	6.71 ± 0.16
TC0X000865.hg.1	OTTHUMG00000021149	6.09 ± 0.30	6.08 ± 0.06	5.99 ± 0.14	6.27 ± 0.13
TC0X000063.hg.1	OTTHUMG00000021161	7.20 ± 0.25	7.09 ± 0.12	7.02 ± 0.10	7.14 ± 0.11
TC0X000869.hg.1	OTTHUMG00000021163	4.55 ± 0.05	4.51 ± 0.08	4.55 ± 0.03	4.67 ± 0.11
TC0X000884.hg.1	OTTHUMG00000021187	5.85 ± 0.24	5.71 ± 0.05	5.75 ± 0.06	5.79 ± 0.05
TC0X000086.hg.1	OTTHUMG00000021202	6.86 ± 0.02	6.85 ± 0.06	6.80 ± 0.09	6.94 ± 0.16
TC0X000892.hg.1	OTTHUMG00000021210	5.48 ± 0.19	5.32 ± 0.04	5.37 ± 0.04	5.47 ± 0.11
TC0X000100.hg.1	OTTHUMG00000021238	5.71 ± 0.11	5.55 ± 0.15	5.68 ± 0.15	5.67 ± 0.14
TC0X000105.hg.1	OTTHUMG00000021244	4.52 ± 0.01	4.49 ± 0.12	4.52 ± 0.06	4.70 ± 0.09
TC0X000107.hg.1	OTTHUMG00000021246	4.54 ± 0.09	4.54 ± 0.02	4.63 ± 0.12	4.62 ± 0.07
TC0X000917.hg.1	OTTHUMG00000021249	4.59 ± 0.06	4.54 ± 0.05	4.50 ± 0.14	4.67 ± 0.13
TC0X000922.hg.1	OTTHUMG00000021255	6.05 ± 0.32	5.81 ± 0.11	6.06 ± 0.31	5.63 ± 0.16
TC0X000122.hg.1	OTTHUMG00000021281	4.53 ± 0.10	4.55 ± 0.04	4.58 ± 0.06	4.63 ± 0.11
TC0X000931.hg.1	OTTHUMG00000021297	5.79 ± 0.15	5.81 ± 0.16	5.85 ± 0.14	5.88 ± 0.13
TC0X000932.hg.1	OTTHUMG00000021298	4.18 ± 0.13	3.97 ± 0.09	4.09 ± 0.09	4.04 ± 0.08
TC0X000936.hg.1	OTTHUMG00000021313	5.43 ± 0.14	5.30 ± 0.07	5.48 ± 0.13	5.39 ± 0.13
TC0X000135.hg.1	OTTHUMG00000021315	4.08 ± 0.10	3.85 ± 0.07	3.96 ± 0.13	3.87 ± 0.10
TC0X000158.hg.1	OTTHUMG00000021338	5.26 ± 0.12	5.05 ± 0.07	5.11 ± 0.13	5.02 ± 0.02
TC0X000947.hg.1	OTTHUMG00000021342	4.84 ± 0.12	4.81 ± 0.07	4.80 ± 0.06	4.82 ± 0.09

TC0X000951.hg.1	OTTHUMG00000021356	5.87 ± 0.20	5.82 ± 0.02	5.85 ± 0.04	5.91 ± 0.05
TC0X000176.hg.1	OTTHUMG00000021363	7.04 ± 0.11	6.91 ± 0.05	6.95 ± 0.09	7.05 ± 0.09
TC0X000196.hg.1	OTTHUMG00000021366	7.97 ± 0.22	8.11 ± 0.31	8.07 ± 0.11	8.24 ± 0.14
TC0X000973.hg.1	OTTHUMG00000021379	5.78 ± 0.26	5.73 ± 0.23	5.90 ± 0.09	5.85 ± 0.11
TC0X000204.hg.1	OTTHUMG00000021383	4.59 ± 0.03	4.40 ± 0.05	4.43 ± 0.05	4.42 ± 0.11
TC0X000205.hg.1	OTTHUMG00000021384	4.49 ± 0.19	4.39 ± 0.10	4.42 ± 0.03	4.39 ± 0.12
TC0X000214.hg.1	OTTHUMG00000021404	5.19 ± 0.06	5.21 ± 0.07	5.21 ± 0.09	5.26 ± 0.06
TC0X000215.hg.1	OTTHUMG00000021406	4.51 ± 0.09	4.52 ± 0.05	4.52 ± 0.08	4.57 ± 0.10
TC0X000989.hg.1	OTTHUMG00000021409	4.19 ± 0.06	4.11 ± 0.04	4.25 ± 0.18	4.10 ± 0.17
TC0X000987.hg.1	OTTHUMG00000021411	6.54 ± 0.16	6.51 ± 0.13	6.51 ± 0.12	6.62 ± 0.09
TC0X000990.hg.1	OTTHUMG00000021416	5.89 ± 0.15	5.57 ± 0.06	5.72 ± 0.21	5.50 ± 0.04
TC0X000233.hg.1	OTTHUMG00000021439	12.59 ± 0.14	12.61 ± 0.07	12.64 ± 0.03	12.69 ± 0.04
TC0X001006.hg.1	OTTHUMG00000021486	7.56 ± 0.17	7.48 ± 0.02	7.44 ± 0.04	7.54 ± 0.14
TC0X001010.hg.1	OTTHUMG00000021498	5.44 ± 0.16	5.36 ± 0.09	5.36 ± 0.06	5.39 ± 0.05
TC0X001014.hg.1	OTTHUMG00000021501	6.93 ± 0.21	6.82 ± 0.07	6.89 ± 0.14	6.84 ± 0.08
TC0X001040.hg.1	OTTHUMG00000021529	5.71 ± 0.14	5.67 ± 0.08	5.73 ± 0.05	5.70 ± 0.03
TC0X000292.hg.1	OTTHUMG00000021532	6.31 ± 0.17	6.42 ± 0.21	6.24 ± 0.19	6.47 ± 0.04
TC0X001041.hg.1	OTTHUMG00000021533	7.63 ± 0.28	7.86 ± 0.08	7.57 ± 0.14	7.95 ± 0.09
TC0X001043.hg.1	OTTHUMG00000021535	6.76 ± 0.23	6.84 ± 0.09	6.77 ± 0.04	6.91 ± 0.04
TC0X001047.hg.1	OTTHUMG00000021545	6.78 ± 0.20	6.60 ± 0.16	6.78 ± 0.22	6.81 ± 0.20
TC0X000299.hg.1	OTTHUMG00000021548	6.49 ± 0.06	6.46 ± 0.24	6.61 ± 0.17	6.64 ± 0.20
TC0X001060.hg.1	OTTHUMG00000021599	6.50 ± 0.00	6.55 ± 0.28	6.45 ± 0.10	6.76 ± 0.15
TC0X000311.hg.1	OTTHUMG00000021603	8.02 ± 0.21	7.96 ± 0.07	8.00 ± 0.18	7.96 ± 0.07
TC0X001066.hg.1	OTTHUMG00000021616	10.51 ± 0.26	10.58 ± 0.02	10.53 ± 0.11	10.60 ± 0.04
TC0X000330.hg.1	OTTHUMG00000021651	6.45 ± 0.14	6.22 ± 0.05	6.37 ± 0.12	6.27 ± 0.06
TC0X000336.hg.1	OTTHUMG00000021664	7.18 ± 0.17	7.05 ± 0.09	7.07 ± 0.01	6.99 ± 0.15
TC0X000340.hg.1	OTTHUMG00000021671	4.88 ± 0.33	4.94 ± 0.24	5.04 ± 0.11	5.01 ± 0.11
TC0X000342.hg.1	OTTHUMG00000021679	6.27 ± 0.04	6.18 ± 0.12	6.22 ± 0.01	6.29 ± 0.11
TC0X000344.hg.1	OTTHUMG00000021681	5.80 ± 0.12	5.83 ± 0.10	5.64 ± 0.13	5.74 ± 0.01
TC0X000352.hg.1	OTTHUMG00000021722	6.60 ± 0.16	6.34 ± 0.16	6.57 ± 0.06	6.47 ± 0.12
TC0X000355.hg.1	OTTHUMG00000021729	4.18 ± 0.23	3.97 ± 0.02	4.28 ± 0.13	3.96 ± 0.06

TC0X001107.hg.1	OTTHUMG00000021757	4.60 ± 0.14	4.62 ± 0.11	4.60 ± 0.11	4.59 ± 0.07
TC0X001122.hg.1	OTTHUMG00000021796	6.47 ± 0.20	6.10 ± 0.02	6.32 ± 0.18	5.81 ± 0.10
TC0X001132.hg.1	OTTHUMG00000021805	6.59 ± 0.15	6.82 ± 0.08	6.80 ± 0.16	6.72 ± 0.11
TC0X001147.hg.1	OTTHUMG00000021819	7.19 ± 0.18	7.27 ± 0.23	7.25 ± 0.24	7.41 ± 0.08
TC0X001149.hg.1	OTTHUMG00000021822	6.03 ± 0.21	5.97 ± 0.20	5.84 ± 0.20	5.91 ± 0.05
TC0X000401.hg.1	OTTHUMG00000021825	6.13 ± 0.24	6.05 ± 0.18	5.97 ± 0.15	5.99 ± 0.10
TC0X000406.hg.1	OTTHUMG00000021838	10.72 ± 0.23	10.85 ± 0.12	10.70 ± 0.05	10.89 ± 0.06
TC0X000415.hg.1	OTTHUMG00000021871	5.87 ± 0.10	5.88 ± 0.19	5.85 ± 0.16	5.96 ± 0.08
TC0X000416.hg.1	OTTHUMG00000021872	6.66 ± 0.27	6.36 ± 0.10	6.49 ± 0.24	6.50 ± 0.18
TC0X000441.hg.1	OTTHUMG00000021932	3.95 ± 0.16	3.80 ± 0.05	3.90 ± 0.14	3.86 ± 0.08
TC0X000449.hg.1	OTTHUMG00000021953	6.84 ± 0.46	7.10 ± 0.40	7.10 ± 0.59	6.82 ± 0.40
TC0X000452.hg.1	OTTHUMG00000021958	5.27 ± 0.12	5.08 ± 0.09	5.04 ± 0.05	5.05 ± 0.01
TC0X001213.hg.1	OTTHUMG00000022014	6.96 ± 0.21	7.06 ± 0.25	6.57 ± 0.22	7.01 ± 0.03
TC0X000485.hg.1	OTTHUMG00000022051	5.12 ± 0.17	5.33 ± 0.17	5.24 ± 0.15	5.52 ± 0.31
TC0X002359.hg.1	OTTHUMG00000022085	6.51 ± 0.04	6.53 ± 0.03	6.46 ± 0.03	6.49 ± 0.03
TC0X001232.hg.1	OTTHUMG00000022086	6.84 ± 0.06	6.80 ± 0.19	6.76 ± 0.27	6.77 ± 0.06
TC0X000498.hg.1	OTTHUMG00000022101	4.37 ± 0.16	4.26 ± 0.03	4.26 ± 0.08	4.20 ± 0.11
TC0X000497.hg.1	OTTHUMG00000022102	6.40 ± 0.08	6.29 ± 0.07	6.18 ± 0.24	6.28 ± 0.06
TC0X001247.hg.1	OTTHUMG00000022115	7.40 ± 0.14	7.43 ± 0.05	7.29 ± 0.12	7.40 ± 0.06
TC0X001263.hg.1	OTTHUMG00000022173	6.20 ± 0.19	6.31 ± 0.32	6.24 ± 0.07	6.49 ± 0.06
TC0X000531.hg.1	OTTHUMG00000022178	6.01 ± 0.10	6.00 ± 0.29	5.94 ± 0.19	6.11 ± 0.17
TC0X000533.hg.1	OTTHUMG00000022180	5.86 ± 0.11	5.83 ± 0.16	5.81 ± 0.27	5.82 ± 0.08
TC0X001288.hg.1	OTTHUMG00000022220	3.93 ± 0.20	3.84 ± 0.10	3.94 ± 0.07	3.86 ± 0.02
TC0X001296.hg.1	OTTHUMG00000022239	8.07 ± 0.16	8.13 ± 0.12	7.98 ± 0.14	7.99 ± 0.10
TC0X001297.hg.1	OTTHUMG00000022240	6.07 ± 0.19	5.98 ± 0.09	5.92 ± 0.09	6.09 ± 0.15
TC0X001301.hg.1	OTTHUMG00000022248	5.97 ± 0.20	5.92 ± 0.02	5.95 ± 0.15	5.99 ± 0.22
TC0X000578.hg.1	OTTHUMG00000022260	6.49 ± 0.15	6.37 ± 0.05	6.36 ± 0.09	6.45 ± 0.04
TC0X001308.hg.1	OTTHUMG00000022269	5.84 ± 0.37	5.70 ± 0.17	5.76 ± 0.02	5.79 ± 0.03
TC0X000581.hg.1	OTTHUMG00000022271	7.14 ± 0.18	7.18 ± 0.07	7.05 ± 0.06	7.27 ± 0.07
TC0X000583.hg.1	OTTHUMG00000022274	8.51 ± 0.12	8.14 ± 0.18	8.47 ± 0.22	8.24 ± 0.21
TC0X001317.hg.1	OTTHUMG00000022286	5.26 ± 0.42	5.58 ± 0.31	5.25 ± 0.23	5.59 ± 0.05

TC0X001319.hg.1	OTTHUMG00000022289	6.60 ± 0.13	6.80 ± 0.11	7.06 ± 0.10	7.10 ± 0.04
TC0X001322.hg.1	OTTHUMG00000022291	5.68 ± 0.17	5.75 ± 0.16	5.86 ± 0.10	5.72 ± 0.05
TC0X000603.hg.1	OTTHUMG00000022331	8.67 ± 0.59	8.79 ± 0.06	8.61 ± 0.33	9.14 ± 0.47
TC0X001346.hg.1	OTTHUMG00000022338	6.47 ± 0.20	6.45 ± 0.18	6.40 ± 0.11	6.55 ± 0.05
TC0X000613.hg.1	OTTHUMG00000022349	8.19 ± 0.44	8.35 ± 0.11	8.17 ± 0.37	8.62 ± 0.27
TC0X001351.hg.1	OTTHUMG00000022365	5.32 ± 0.17	5.17 ± 0.01	5.06 ± 0.14	5.18 ± 0.11
TC0X001356.hg.1	OTTHUMG00000022377	6.07 ± 0.17	5.91 ± 0.16	6.11 ± 0.12	6.09 ± 0.08
TC0X001362.hg.1	OTTHUMG00000022398	5.62 ± 0.17	5.48 ± 0.04	5.55 ± 0.01	5.53 ± 0.02
TC0X001364.hg.1	OTTHUMG00000022403	6.14 ± 0.38	6.06 ± 0.13	5.93 ± 0.14	6.22 ± 0.21
TC0X000629.hg.1	OTTHUMG00000022425	6.18 ± 0.24	6.07 ± 0.14	6.08 ± 0.31	6.08 ± 0.22
TC0X000638.hg.1	OTTHUMG00000022458	6.88 ± 0.20	6.76 ± 0.06	6.69 ± 0.10	6.88 ± 0.13
TC0X000643.hg.1	OTTHUMG00000022462	5.58 ± 0.40	5.45 ± 0.14	5.44 ± 0.11	5.58 ± 0.09
TC0X001403.hg.1	OTTHUMG00000022472	7.01 ± 0.39	7.26 ± 0.10	7.13 ± 0.13	7.42 ± 0.20
TC0X000647.hg.1	OTTHUMG00000022476	4.76 ± 0.15	4.69 ± 0.17	4.60 ± 0.10	4.78 ± 0.02
TC0X001418.hg.1	OTTHUMG00000022515	6.11 ± 0.21	5.95 ± 0.15	6.01 ± 0.17	6.02 ± 0.06
TC0X000668.hg.1	OTTHUMG00000022519	4.94 ± 0.12	4.86 ± 0.12	4.88 ± 0.03	4.87 ± 0.08
TC0X001422.hg.1	OTTHUMG00000022524	6.15 ± 0.19	6.04 ± 0.28	5.95 ± 0.06	6.10 ± 0.10
TC0X000671.hg.1	OTTHUMG00000022529	3.60 ± 0.26	3.49 ± 0.07	3.61 ± 0.23	3.51 ± 0.07
TC0X000680.hg.1	OTTHUMG00000022540	5.60 ± 0.28	5.62 ± 0.21	5.63 ± 0.08	5.45 ± 0.08
TC0X001432.hg.1	OTTHUMG00000022543	5.38 ± 0.15	5.56 ± 0.09	5.38 ± 0.19	5.55 ± 0.14
TC0X001434.hg.1	OTTHUMG00000022547	4.02 ± 0.15	3.91 ± 0.04	4.01 ± 0.12	3.86 ± 0.04
TC0X000685.hg.1	OTTHUMG00000022550	5.74 ± 0.04	5.73 ± 0.05	5.62 ± 0.16	5.86 ± 0.18
TC0X000696.hg.1	OTTHUMG00000022584	6.50 ± 0.30	6.44 ± 0.05	6.46 ± 0.04	6.57 ± 0.12
TC0X000697.hg.1	OTTHUMG00000022587	4.42 ± 0.22	4.28 ± 0.07	4.30 ± 0.22	4.29 ± 0.07
TC0X001448.hg.1	OTTHUMG00000022592	6.24 ± 0.31	6.15 ± 0.10	6.03 ± 0.18	6.24 ± 0.12
TC0X001473.hg.1	OTTHUMG00000022605	7.09 ± 0.24	7.10 ± 0.02	7.23 ± 0.10	7.25 ± 0.03
TC0X001476.hg.1	OTTHUMG00000022612	8.24 ± 0.25	8.26 ± 0.33	8.30 ± 0.23	8.44 ± 0.15
TC0X002344.hg.1	OTTHUMG00000022616	8.73 ± 0.15	8.44 ± 0.02	8.57 ± 0.12	8.46 ± 0.06
TC0X001479.hg.1	OTTHUMG00000022623	6.21 ± 0.13	6.18 ± 0.08	6.22 ± 0.11	6.20 ± 0.03
TC0X001502.hg.1	OTTHUMG00000022647	5.81 ± 0.27	5.79 ± 0.09	5.73 ± 0.20	5.89 ± 0.19
TC0X001529.hg.1	OTTHUMG00000022655	6.02 ± 0.16	6.14 ± 0.04	5.96 ± 0.03	6.21 ± 0.30

TC0X000790.hg.1	OTTHUMG00000022671	7.26 ± 0.14	7.17 ± 0.12	7.48 ± 0.23	7.24 ± 0.09
TC0X000391.hg.1	OTTHUMG00000022687	6.59 ± 0.15	6.82 ± 0.08	6.80 ± 0.16	6.72 ± 0.11
TC0X001129.hg.1	OTTHUMG00000022722	7.32 ± 0.12	7.36 ± 0.22	7.34 ± 0.25	7.56 ± 0.09
TC01001353.hg.1	OTTHUMG00000022736	4.62 ± 0.41	4.36 ± 0.09	4.39 ± 0.16	4.26 ± 0.13
TC01002973.hg.1	OTTHUMG00000022783	7.55 ± 0.08	7.54 ± 0.07	7.55 ± 0.19	7.56 ± 0.06
TC01001370.hg.1	OTTHUMG00000022789	7.26 ± 0.20	7.27 ± 0.09	7.14 ± 0.13	7.38 ± 0.04
TC01001371.hg.1	OTTHUMG00000024005	6.99 ± 0.99	6.25 ± 0.13	7.09 ± 0.50	6.15 ± 0.07
TC01001416.hg.1	OTTHUMG00000024048	5.09 ± 0.22	5.16 ± 0.14	5.07 ± 0.08	5.12 ± 0.31
TC01001359.hg.1	OTTHUMG00000024076	7.28 ± 0.19	7.32 ± 0.17	7.20 ± 0.19	7.47 ± 0.15
TC0X000179.hg.1	OTTHUMG00000024091	5.18 ± 0.24	5.14 ± 0.14	5.10 ± 0.06	5.19 ± 0.06
TC0X000959.hg.1	OTTHUMG00000024094	6.56 ± 0.20	6.53 ± 0.04	6.45 ± 0.05	6.44 ± 0.11
TC0X000961.hg.1	OTTHUMG00000024096	5.71 ± 0.07	5.74 ± 0.17	5.73 ± 0.10	5.79 ± 0.06
TC0X000962.hg.1	OTTHUMG00000024097	6.20 ± 0.15	6.08 ± 0.06	6.06 ± 0.07	6.29 ± 0.21
TC0X001013.hg.1	OTTHUMG00000024122	5.82 ± 0.09	5.73 ± 0.13	5.93 ± 0.21	5.75 ± 0.20
TC0X000726.hg.1	OTTHUMG00000024161	6.09 ± 0.16	6.00 ± 0.06	6.04 ± 0.09	6.21 ± 0.03
TC0X000737.hg.1	OTTHUMG00000024178	5.04 ± 0.16	5.04 ± 0.07	4.91 ± 0.09	5.08 ± 0.13
TC0X001510.hg.1	OTTHUMG00000024194	7.31 ± 0.06	7.34 ± 0.24	7.25 ± 0.11	7.55 ± 0.11
TC0X000752.hg.1	OTTHUMG00000024201	7.47 ± 0.24	7.33 ± 0.12	7.28 ± 0.16	7.40 ± 0.08
TC0X001517.hg.1	OTTHUMG00000024213	9.00 ± 0.17	8.82 ± 0.03	8.96 ± 0.17	8.88 ± 0.04
TC01003410.hg.1	OTTHUMG00000028600	5.25 ± 0.22	5.15 ± 0.19	5.15 ± 0.12	5.23 ± 0.13
TC01001374.hg.1	OTTHUMG00000028603	6.73 ± 0.14	6.66 ± 0.05	6.67 ± 0.16	6.76 ± 0.12
TC22000009.hg.1	OTTHUMG00000030075	5.67 ± 0.29	5.54 ± 0.11	5.57 ± 0.18	5.88 ± 0.40
TC22000687.hg.1	OTTHUMG00000030382	6.24 ± 0.14	6.22 ± 0.14	6.32 ± 0.15	6.26 ± 0.11
TC01001320.hg.1	OTTHUMG00000031021	7.27 ± 0.26	7.14 ± 0.18	7.03 ± 0.20	7.27 ± 0.11
TC01003453.hg.1	OTTHUMG00000031374	4.86 ± 0.18	4.87 ± 0.22	4.90 ± 0.12	5.13 ± 0.13
TC6_cox_hap2000112.hg.1	OTTHUMG00000031429	6.39 ± 0.43	6.28 ± 0.23	6.11 ± 0.38	6.41 ± 0.06
TC01001375.hg.1	OTTHUMG00000031478	7.27 ± 0.21	7.24 ± 0.16	7.17 ± 0.19	7.34 ± 0.12
TC6_cox_hap2000146.hg.1	OTTHUMG00000031529	6.79 ± 0.12	6.75 ± 0.05	6.88 ± 0.11	6.87 ± 0.05
TC01003398.hg.1	OTTHUMG00000031606	7.46 ± 0.29	7.48 ± 0.12	7.42 ± 0.18	7.64 ± 0.11
TC20000017.hg.1	OTTHUMG00000031663	6.84 ± 0.08	6.49 ± 0.12	6.58 ± 0.29	6.56 ± 0.19
TC20000018.hg.1	OTTHUMG00000031675	4.95 ± 0.12	4.85 ± 0.04	4.98 ± 0.02	4.95 ± 0.05

TC20000557.hg.1	OTTHUMG00000031681	6.85 ± 0.09	6.64 ± 0.15	7.22 ± 0.11	6.74 ± 0.02
TC20000027.hg.1	OTTHUMG00000031696	5.84 ± 0.10	5.88 ± 0.35	5.57 ± 0.11	5.59 ± 0.06
TC20000579.hg.1	OTTHUMG00000031766	7.41 ± 0.10	7.19 ± 0.12	7.20 ± 0.04	6.99 ± 0.10
TC20000583.hg.1	OTTHUMG00000031774	6.42 ± 0.18	6.28 ± 0.04	6.23 ± 0.12	6.35 ± 0.18
TC20000050.hg.1	OTTHUMG00000031780	5.20 ± 0.38	5.06 ± 0.15	4.94 ± 0.25	5.00 ± 0.04
TC20000585.hg.1	OTTHUMG00000031781	5.80 ± 0.17	5.66 ± 0.05	5.74 ± 0.18	5.78 ± 0.16
TC20000598.hg.1	OTTHUMG00000031808	7.72 ± 0.07	7.59 ± 0.10	7.88 ± 0.14	7.60 ± 0.08
TC20000059.hg.1	OTTHUMG00000031809	8.12 ± 0.27	7.90 ± 0.07	7.98 ± 0.14	7.96 ± 0.06
TC20000066.hg.1	OTTHUMG00000031831	4.97 ± 0.25	4.76 ± 0.04	4.70 ± 0.22	4.88 ± 0.23
TC20000605.hg.1	OTTHUMG00000031832	6.20 ± 0.06	6.08 ± 0.11	6.10 ± 0.15	6.19 ± 0.24
TC20000069.hg.1	OTTHUMG00000031835	5.86 ± 0.13	5.85 ± 0.18	5.89 ± 0.08	6.03 ± 0.04
TC20000606.hg.1	OTTHUMG00000031836	4.77 ± 0.09	4.69 ± 0.06	4.74 ± 0.05	4.72 ± 0.04
TC20000607.hg.1	OTTHUMG00000031837	4.69 ± 0.23	4.60 ± 0.14	4.53 ± 0.17	4.55 ± 0.07
TC20000070.hg.1	OTTHUMG00000031838	5.39 ± 0.27	5.24 ± 0.06	5.32 ± 0.05	5.28 ± 0.04
TC20000071.hg.1	OTTHUMG00000031842	5.85 ± 0.17	5.78 ± 0.13	5.89 ± 0.06	5.88 ± 0.09
TC20000614.hg.1	OTTHUMG00000031850	7.53 ± 0.25	7.42 ± 0.26	7.38 ± 0.25	7.50 ± 0.13
TC20000078.hg.1	OTTHUMG00000031855	5.09 ± 0.28	5.03 ± 0.12	5.03 ± 0.14	5.10 ± 0.06
TC20001755.hg.1	OTTHUMG00000031858	5.65 ± 0.24	5.44 ± 0.03	5.42 ± 0.05	5.38 ± 0.07
TC20000081.hg.1	OTTHUMG00000031861	4.98 ± 0.14	4.85 ± 0.08	4.94 ± 0.15	4.86 ± 0.21
TC20000080.hg.1	OTTHUMG00000031862	6.12 ± 0.04	6.20 ± 0.27	6.07 ± 0.04	6.32 ± 0.18
TC20000085.hg.1	OTTHUMG00000031876	4.30 ± 0.06	4.12 ± 0.07	4.26 ± 0.16	4.18 ± 0.17
TC20000622.hg.1	OTTHUMG00000031877	6.16 ± 0.13	6.11 ± 0.05	6.23 ± 0.05	6.35 ± 0.26
TC20000623.hg.1	OTTHUMG00000031880	5.26 ± 0.03	5.10 ± 0.04	5.16 ± 0.06	5.19 ± 0.13
TC20000629.hg.1	OTTHUMG00000031890	5.12 ± 0.16	5.04 ± 0.18	5.14 ± 0.08	5.16 ± 0.12
TC20000630.hg.1	OTTHUMG00000031891	5.74 ± 0.16	5.76 ± 0.22	5.72 ± 0.24	5.83 ± 0.25
TC20000631.hg.1	OTTHUMG00000031892	4.73 ± 0.06	4.61 ± 0.07	4.65 ± 0.02	4.74 ± 0.19
TC20000632.hg.1	OTTHUMG00000031897	6.10 ± 0.12	6.02 ± 0.00	6.01 ± 0.09	6.08 ± 0.09
TC20000635.hg.1	OTTHUMG00000031898	6.58 ± 0.18	6.54 ± 0.13	6.50 ± 0.18	6.57 ± 0.07
TC20000644.hg.1	OTTHUMG00000031921	5.13 ± 0.05	4.95 ± 0.07	5.08 ± 0.05	4.88 ± 0.13
TC20000647.hg.1	OTTHUMG00000031922	4.27 ± 0.09	4.10 ± 0.11	4.23 ± 0.21	4.23 ± 0.03
TC20000652.hg.1	OTTHUMG00000031932	6.46 ± 0.10	6.39 ± 0.02	6.50 ± 0.11	6.56 ± 0.10

TC20000653.hg.1	OTTHUMG00000031936	5.84 ± 0.20	5.53 ± 0.11	5.64 ± 0.20	5.72 ± 0.22
TC20000658.hg.1	OTTHUMG00000031951	6.10 ± 0.21	6.12 ± 0.18	6.26 ± 0.07	6.36 ± 0.13
TC20000116.hg.1	OTTHUMG00000031958	4.82 ± 0.12	4.76 ± 0.03	4.84 ± 0.08	4.86 ± 0.22
TC20000666.hg.1	OTTHUMG00000031966	7.21 ± 0.19	7.07 ± 0.12	7.21 ± 0.31	7.17 ± 0.04
TC20000674.hg.1	OTTHUMG00000031988	8.87 ± 0.14	8.94 ± 0.16	8.90 ± 0.20	9.02 ± 0.06
TC20000676.hg.1	OTTHUMG00000031991	7.42 ± 0.25	7.53 ± 0.28	7.36 ± 0.33	7.55 ± 0.06
TC20000133.hg.1	OTTHUMG00000031992	7.38 ± 0.23	7.23 ± 0.14	7.28 ± 0.14	7.27 ± 0.04
TC20000683.hg.1	OTTHUMG00000032002	6.42 ± 0.28	6.18 ± 0.19	6.34 ± 0.17	6.26 ± 0.15
TC20000138.hg.1	OTTHUMG00000032013	5.83 ± 0.11	5.80 ± 0.28	5.91 ± 0.10	5.94 ± 0.18
TC20000689.hg.1	OTTHUMG00000032014	5.73 ± 0.12	5.68 ± 0.12	5.66 ± 0.08	5.79 ± 0.08
TC20000141.hg.1	OTTHUMG00000032023	4.26 ± 0.26	4.16 ± 0.03	4.19 ± 0.11	4.14 ± 0.05
TC20000143.hg.1	OTTHUMG00000032029	5.86 ± 0.17	5.75 ± 0.11	5.84 ± 0.08	5.88 ± 0.05
TC20000147.hg.1	OTTHUMG00000032035	4.49 ± 0.06	4.47 ± 0.15	4.45 ± 0.13	4.57 ± 0.26
TC20000149.hg.1	OTTHUMG00000032037	3.96 ± 0.28	3.77 ± 0.05	3.85 ± 0.09	3.81 ± 0.05
TC20000694.hg.1	OTTHUMG00000032039	4.32 ± 0.27	4.07 ± 0.02	4.12 ± 0.03	4.16 ± 0.05
TC20000699.hg.1	OTTHUMG00000032044	6.21 ± 0.39	6.26 ± 0.08	6.23 ± 0.22	6.25 ± 0.15
TC20000696.hg.1	OTTHUMG00000032045	6.08 ± 0.27	6.03 ± 0.12	5.96 ± 0.05	6.28 ± 0.09
TC20000700.hg.1	OTTHUMG00000032047	7.16 ± 0.15	7.05 ± 0.16	7.03 ± 0.18	7.15 ± 0.07
TC20000150.hg.1	OTTHUMG00000032051	6.29 ± 0.11	6.47 ± 0.26	6.31 ± 0.07	6.50 ± 0.15
TC20000701.hg.1	OTTHUMG00000032052	6.17 ± 0.13	6.21 ± 0.14	6.62 ± 0.06	6.35 ± 0.02
TC20000706.hg.1	OTTHUMG00000032064	6.61 ± 0.21	6.74 ± 0.26	6.44 ± 0.27	6.80 ± 0.01
TC20000716.hg.1	OTTHUMG00000032077	6.42 ± 0.28	6.41 ± 0.10	6.40 ± 0.29	6.44 ± 0.02
TC20000722.hg.1	OTTHUMG00000032097	6.85 ± 0.22	6.96 ± 0.26	6.98 ± 0.15	7.13 ± 0.14
TC20000723.hg.1	OTTHUMG00000032099	6.95 ± 0.14	6.77 ± 0.07	6.92 ± 0.11	6.87 ± 0.06
TC20000724.hg.1	OTTHUMG00000032101	7.25 ± 0.21	7.33 ± 0.29	7.15 ± 0.29	7.54 ± 0.13
TC20000163.hg.1	OTTHUMG00000032103	6.69 ± 0.16	6.76 ± 0.14	6.71 ± 0.12	7.03 ± 0.09
TC0X000731.hg.1	OTTHUMG00000032120	6.21 ± 0.37	6.20 ± 0.08	5.99 ± 0.13	6.18 ± 0.02
TC20000171.hg.1	OTTHUMG00000032129	7.37 ± 0.26	7.43 ± 0.06	7.35 ± 0.04	7.56 ± 0.11
TC20000172.hg.1	OTTHUMG00000032135	8.52 ± 0.20	8.64 ± 0.06	8.64 ± 0.02	8.57 ± 0.07
TC20000738.hg.1	OTTHUMG00000032141	5.89 ± 0.55	5.93 ± 0.07	5.95 ± 0.56	6.22 ± 0.20
TC20000740.hg.1	OTTHUMG00000032148	7.84 ± 0.22	7.87 ± 0.18	7.80 ± 0.18	7.88 ± 0.06

TC20000177.hg.1	OTTHUMG00000032150	3.75 ± 0.07	3.45 ± 0.09	3.55 ± 0.10	3.49 ± 0.03
TC20000744.hg.1	OTTHUMG00000032152	6.83 ± 0.22	6.88 ± 0.20	6.78 ± 0.13	7.07 ± 0.16
TC20000746.hg.1	OTTHUMG00000032154	6.57 ± 0.23	6.50 ± 0.02	6.46 ± 0.06	6.69 ± 0.17
TC20000197.hg.1	OTTHUMG00000032185	5.98 ± 0.15	5.83 ± 0.02	5.90 ± 0.27	5.83 ± 0.14
TC20000760.hg.1	OTTHUMG00000032202	7.41 ± 0.27	7.30 ± 0.21	7.43 ± 0.20	7.41 ± 0.14
TC20000211.hg.1	OTTHUMG00000032217	7.40 ± 0.24	7.41 ± 0.17	7.45 ± 0.11	7.47 ± 0.14
TC20000213.hg.1	OTTHUMG00000032220	7.08 ± 0.23	7.08 ± 0.16	6.97 ± 0.14	7.18 ± 0.11
TC20000212.hg.1	OTTHUMG00000032221	6.82 ± 0.11	6.80 ± 0.04	6.89 ± 0.13	6.91 ± 0.02
TC20000768.hg.1	OTTHUMG00000032248	6.57 ± 0.18	6.61 ± 0.17	6.55 ± 0.04	6.77 ± 0.09
TC20000775.hg.1	OTTHUMG00000032274	5.97 ± 0.04	5.98 ± 0.07	5.94 ± 0.02	5.99 ± 0.13
TC20000776.hg.1	OTTHUMG00000032278	6.42 ± 0.11	6.45 ± 0.20	6.45 ± 0.14	6.49 ± 0.09
TC20000777.hg.1	OTTHUMG00000032279	6.96 ± 0.23	6.79 ± 0.06	7.01 ± 0.12	6.78 ± 0.05
TC20000778.hg.1	OTTHUMG00000032284	5.30 ± 0.18	5.21 ± 0.02	5.31 ± 0.11	5.22 ± 0.06
TC20000263.hg.1	OTTHUMG00000032375	5.91 ± 0.13	5.90 ± 0.04	5.93 ± 0.11	5.97 ± 0.10
TC20000811.hg.1	OTTHUMG00000032376	7.01 ± 0.24	6.97 ± 0.10	7.01 ± 0.06	6.99 ± 0.09
TC20000812.hg.1	OTTHUMG00000032377	7.23 ± 0.24	7.17 ± 0.20	7.04 ± 0.20	7.22 ± 0.13
TC20000813.hg.1	OTTHUMG00000032391	6.84 ± 0.13	6.78 ± 0.07	6.71 ± 0.07	6.81 ± 0.04
TC20000835.hg.1	OTTHUMG00000032439	5.84 ± 0.06	5.79 ± 0.04	5.91 ± 0.15	5.86 ± 0.13
TC20000836.hg.1	OTTHUMG00000032445	7.00 ± 0.04	6.86 ± 0.05	6.91 ± 0.26	7.08 ± 0.25
TC20000296.hg.1	OTTHUMG00000032468	6.81 ± 0.34	6.97 ± 0.16	6.88 ± 0.24	7.25 ± 0.22
TC20000298.hg.1	OTTHUMG00000032470	3.97 ± 0.26	3.85 ± 0.04	3.82 ± 0.13	3.99 ± 0.09
TC20000299.hg.1	OTTHUMG00000032472	5.38 ± 0.39	5.31 ± 0.35	5.33 ± 0.22	5.53 ± 0.10
TC20000845.hg.1	OTTHUMG00000032473	4.53 ± 0.10	4.54 ± 0.13	4.50 ± 0.06	4.61 ± 0.11
TC20000847.hg.1	OTTHUMG00000032476	6.64 ± 0.03	6.66 ± 0.07	6.60 ± 0.07	6.69 ± 0.03
TC20000854.hg.1	OTTHUMG00000032491	4.92 ± 0.10	4.59 ± 0.06	4.83 ± 0.17	4.60 ± 0.18
TC20000307.hg.1	OTTHUMG00000032492	5.73 ± 0.09	5.84 ± 0.07	5.87 ± 0.11	5.93 ± 0.11
TC20000308.hg.1	OTTHUMG00000032494	5.39 ± 0.04	5.16 ± 0.10	5.21 ± 0.10	5.33 ± 0.08
TC20000309.hg.1	OTTHUMG00000032495	4.58 ± 0.24	4.51 ± 0.08	4.48 ± 0.11	4.47 ± 0.05
TC20000311.hg.1	OTTHUMG00000032498	7.31 ± 0.18	7.10 ± 0.16	7.14 ± 0.17	7.26 ± 0.04
TC20000857.hg.1	OTTHUMG00000032505	6.90 ± 0.06	6.94 ± 0.20	6.85 ± 0.15	6.99 ± 0.19
TC20000322.hg.1	OTTHUMG00000032515	6.37 ± 0.19	6.41 ± 0.30	6.44 ± 0.09	6.59 ± 0.05

TC20000864.hg.1	OTTHUMG00000032525	6.25 ± 0.13	6.09 ± 0.03	6.16 ± 0.06	6.21 ± 0.12
TC20000863.hg.1	OTTHUMG00000032526	5.67 ± 0.21	5.65 ± 0.11	5.60 ± 0.02	5.68 ± 0.12
TC20000865.hg.1	OTTHUMG00000032534	7.28 ± 0.21	7.49 ± 0.03	7.43 ± 0.08	7.61 ± 0.19
TC20000350.hg.1	OTTHUMG00000032591	6.39 ± 0.11	6.43 ± 0.06	6.25 ± 0.20	6.38 ± 0.10
TC20000348.hg.1	OTTHUMG00000032593	8.05 ± 0.24	7.95 ± 0.16	7.96 ± 0.26	8.16 ± 0.16
TC20000897.hg.1	OTTHUMG00000032644	5.58 ± 0.15	5.84 ± 0.12	5.70 ± 0.14	5.99 ± 0.17
TC20000907.hg.1	OTTHUMG00000032661	5.01 ± 0.28	4.85 ± 0.11	4.72 ± 0.04	4.75 ± 0.20
TC20000371.hg.1	OTTHUMG00000032668	6.93 ± 0.03	6.79 ± 0.09	6.65 ± 0.27	6.84 ± 0.14
TC20000910.hg.1	OTTHUMG00000032669	5.86 ± 0.19	5.68 ± 0.10	5.76 ± 0.21	5.82 ± 0.03
TC20000378.hg.1	OTTHUMG00000032677	7.16 ± 0.19	7.18 ± 0.17	7.06 ± 0.11	7.27 ± 0.12
TC20000915.hg.1	OTTHUMG00000032678	6.70 ± 0.06	6.67 ± 0.13	6.69 ± 0.12	6.83 ± 0.18
TC20000918.hg.1	OTTHUMG00000032680	5.45 ± 0.23	5.47 ± 0.20	5.54 ± 0.23	5.57 ± 0.07
TC20000922.hg.1	OTTHUMG00000032688	7.53 ± 0.18	7.48 ± 0.13	7.44 ± 0.16	7.57 ± 0.10
TC20000397.hg.1	OTTHUMG00000032716	6.43 ± 0.11	6.40 ± 0.19	6.35 ± 0.06	6.49 ± 0.07
TC20000396.hg.1	OTTHUMG00000032717	7.04 ± 0.17	7.06 ± 0.31	6.85 ± 0.16	7.07 ± 0.02
TC20000398.hg.1	OTTHUMG00000032718	7.44 ± 0.20	7.51 ± 0.20	7.44 ± 0.12	7.71 ± 0.11
TC20000934.hg.1	OTTHUMG00000032720	6.85 ± 0.30	6.92 ± 0.17	6.87 ± 0.30	7.02 ± 0.19
TC20000948.hg.1	OTTHUMG00000032757	4.64 ± 0.37	4.49 ± 0.19	4.35 ± 0.12	4.63 ± 0.11
TC20000415.hg.1	OTTHUMG00000032760	6.68 ± 0.10	6.58 ± 0.11	6.57 ± 0.10	6.74 ± 0.10
TC20000420.hg.1	OTTHUMG00000032765	7.02 ± 0.20	7.01 ± 0.11	6.98 ± 0.03	7.13 ± 0.08
TC20000421.hg.1	OTTHUMG00000032767	7.15 ± 0.24	7.20 ± 0.18	7.18 ± 0.16	7.19 ± 0.12
TC20000958.hg.1	OTTHUMG00000032768	6.24 ± 0.12	6.15 ± 0.08	6.14 ± 0.19	6.25 ± 0.03
TC20000422.hg.1	OTTHUMG00000032769	6.56 ± 0.18	6.42 ± 0.18	6.45 ± 0.26	6.53 ± 0.03
TC20000425.hg.1	OTTHUMG00000032780	5.65 ± 0.10	5.50 ± 0.11	5.48 ± 0.08	5.63 ± 0.11
TC20000967.hg.1	OTTHUMG00000032784	6.21 ± 0.25	6.19 ± 0.03	6.03 ± 0.06	6.21 ± 0.05
TC20000433.hg.1	OTTHUMG00000032803	5.87 ± 0.25	5.75 ± 0.11	5.76 ± 0.15	5.79 ± 0.04
TC20000434.hg.1	OTTHUMG00000032803	7.17 ± 0.41	7.14 ± 0.07	6.97 ± 0.06	7.22 ± 0.21
TC20000970.hg.1	OTTHUMG00000032807	6.80 ± 0.48	6.48 ± 0.06	6.60 ± 0.12	6.49 ± 0.05
TC20000442.hg.1	OTTHUMG00000032808	7.63 ± 0.15	7.69 ± 0.24	7.71 ± 0.08	7.77 ± 0.11
TC20000441.hg.1	OTTHUMG00000032809	7.02 ± 0.14	6.89 ± 0.11	7.00 ± 0.08	7.08 ± 0.14
TC20000443.hg.1	OTTHUMG00000032810	6.79 ± 0.03	6.79 ± 0.06	6.77 ± 0.12	6.86 ± 0.14

TC20000444.hg.1	OTTHUMG00000032811	7.38 ± 0.25	7.30 ± 0.22	7.31 ± 0.29	7.48 ± 0.13
TC20000978.hg.1	OTTHUMG00000032826	7.40 ± 0.16	7.41 ± 0.15	7.39 ± 0.12	7.46 ± 0.03
TC20000982.hg.1	OTTHUMG00000032833	5.96 ± 0.26	5.68 ± 0.10	5.78 ± 0.18	5.69 ± 0.01
TC20001759.hg.1	OTTHUMG00000032883	6.09 ± 0.09	6.01 ± 0.06	6.10 ± 0.08	5.99 ± 0.03
TC20001001.hg.1	OTTHUMG00000032885	5.78 ± 0.22	5.60 ± 0.08	5.68 ± 0.10	5.81 ± 0.07
TC20001003.hg.1	OTTHUMG00000032886	7.21 ± 0.12	7.06 ± 0.05	7.19 ± 0.10	7.19 ± 0.02
TC20001002.hg.1	OTTHUMG00000032887	7.43 ± 0.18	7.56 ± 0.12	7.53 ± 0.15	7.56 ± 0.07
TC20001007.hg.1	OTTHUMG00000032910	7.84 ± 0.20	7.85 ± 0.24	7.84 ± 0.17	8.03 ± 0.03
TC20000488.hg.1	OTTHUMG00000032911	8.17 ± 0.30	7.98 ± 0.27	8.06 ± 0.27	8.08 ± 0.08
TC20000490.hg.1	OTTHUMG00000032918	8.12 ± 0.17	8.13 ± 0.13	8.02 ± 0.13	8.16 ± 0.06
TC20001013.hg.1	OTTHUMG00000032921	7.61 ± 0.11	7.67 ± 0.21	7.58 ± 0.21	7.80 ± 0.00
TC20001017.hg.1	OTTHUMG00000032924	7.69 ± 0.38	7.71 ± 0.19	7.61 ± 0.24	7.83 ± 0.09
TC20000515.hg.1	OTTHUMG00000032958	8.00 ± 0.25	7.91 ± 0.16	7.91 ± 0.22	8.04 ± 0.06
TC20000516.hg.1	OTTHUMG00000032967	6.62 ± 0.07	6.62 ± 0.17	6.73 ± 0.24	6.81 ± 0.16
TC20000517.hg.1	OTTHUMG00000032968	7.82 ± 0.28	7.73 ± 0.20	7.60 ± 0.25	7.88 ± 0.10
TC20000523.hg.1	OTTHUMG00000032989	7.86 ± 0.02	7.85 ± 0.05	7.83 ± 0.23	7.93 ± 0.12
TC20000524.hg.1	OTTHUMG00000032990	7.79 ± 0.17	7.91 ± 0.17	7.83 ± 0.25	8.02 ± 0.11
TC20000955.hg.1	OTTHUMG00000033066	7.46 ± 0.12	7.39 ± 0.03	7.38 ± 0.13	7.57 ± 0.19
TC20000951.hg.1	OTTHUMG00000033074	5.07 ± 0.16	5.05 ± 0.11	5.12 ± 0.20	5.26 ± 0.07
TC01003340.hg.1	OTTHUMG00000033094	5.38 ± 0.07	5.40 ± 0.05	5.35 ± 0.03	5.39 ± 0.02
TC0X000569.hg.1	OTTHUMG00000033102	7.38 ± 0.22	7.43 ± 0.17	7.46 ± 0.17	7.53 ± 0.05
TC01001428.hg.1	OTTHUMG00000033126	5.19 ± 0.14	5.14 ± 0.17	5.25 ± 0.14	5.39 ± 0.17
TC0X000183.hg.1	OTTHUMG00000033140	7.00 ± 0.23	6.89 ± 0.07	6.94 ± 0.08	6.89 ± 0.05
TC01001429.hg.1	OTTHUMG00000033149	3.93 ± 0.18	3.73 ± 0.07	3.87 ± 0.11	3.73 ± 0.06
TC01003419.hg.1	OTTHUMG00000033156	7.03 ± 0.10	6.99 ± 0.11	6.89 ± 0.02	6.88 ± 0.03
TC0X000173.hg.1	OTTHUMG00000033178	4.13 ± 0.20	4.10 ± 0.12	4.11 ± 0.24	4.20 ± 0.17
TC01003472.hg.1	OTTHUMG00000033265	5.98 ± 0.24	5.99 ± 0.12	5.93 ± 0.08	6.03 ± 0.05
TC01003473.hg.1	OTTHUMG00000033267	4.60 ± 0.08	4.62 ± 0.22	4.35 ± 0.11	4.61 ± 0.10
TC01003346.hg.1	OTTHUMG00000033321	7.73 ± 0.28	7.64 ± 0.15	7.48 ± 0.36	7.77 ± 0.08
TC01003475.hg.1	OTTHUMG00000034319	5.62 ± 0.13	5.75 ± 0.05	5.63 ± 0.09	5.73 ± 0.10
TC0X000718.hg.1	OTTHUMG00000034359	7.02 ± 0.08	6.95 ± 0.08	6.95 ± 0.08	6.94 ± 0.14

TC01003478.hg.1	OTTHUMG00000034431	6.03 ± 0.13	6.01 ± 0.08	6.03 ± 0.11	6.16 ± 0.07
TC01001456.hg.1	OTTHUMG00000034433	6.07 ± 0.18	6.05 ± 0.13	6.09 ± 0.34	5.97 ± 0.07
TC10001452.hg.1	OTTHUMG00000034448	5.68 ± 0.07	5.61 ± 0.08	5.68 ± 0.20	5.77 ± 0.10
TC01001386.hg.1	OTTHUMG00000034463	6.02 ± 0.14	5.96 ± 0.11	6.02 ± 0.19	5.88 ± 0.03
TC01003448.hg.1	OTTHUMG00000034467	6.75 ± 0.23	6.96 ± 0.11	6.88 ± 0.18	7.22 ± 0.29
TC01000969.hg.1	OTTHUMG00000034494	6.07 ± 0.08	6.02 ± 0.19	6.22 ± 0.26	5.93 ± 0.07
TC01003451.hg.1	OTTHUMG00000034539	6.32 ± 0.20	6.28 ± 0.02	6.24 ± 0.05	6.30 ± 0.08
TC01003493.hg.1	OTTHUMG00000034550	5.59 ± 0.15	5.56 ± 0.02	5.44 ± 0.04	5.60 ± 0.13
TC01001450.hg.1	OTTHUMG00000034556	5.69 ± 0.19	5.55 ± 0.26	5.63 ± 0.17	5.74 ± 0.13
TC01001442.hg.1	OTTHUMG00000034564	5.81 ± 0.15	5.69 ± 0.07	5.77 ± 0.08	5.72 ± 0.08
TC01003494.hg.1	OTTHUMG00000034588	5.74 ± 0.09	5.65 ± 0.02	5.76 ± 0.09	5.80 ± 0.14
TC01001472.hg.1	OTTHUMG00000034591	5.83 ± 0.21	5.76 ± 0.07	5.80 ± 0.21	5.76 ± 0.13
TC01000135.hg.1	OTTHUMG00000034707	6.74 ± 0.26	6.50 ± 0.20	6.57 ± 0.07	6.74 ± 0.07
TC01001511.hg.1	OTTHUMG00000034801	7.51 ± 0.05	7.53 ± 0.16	7.45 ± 0.07	7.64 ± 0.21
TC01003527.hg.1	OTTHUMG00000034807	6.22 ± 0.04	6.21 ± 0.10	6.42 ± 0.22	6.19 ± 0.07
TC01001521.hg.1	OTTHUMG00000034820	7.62 ± 0.22	7.63 ± 0.32	7.51 ± 0.14	7.74 ± 0.01
TC01003539.hg.1	OTTHUMG00000034822	7.28 ± 0.08	7.15 ± 0.01	7.23 ± 0.10	7.17 ± 0.04
TC01001508.hg.1	OTTHUMG00000034834	5.41 ± 0.12	5.30 ± 0.13	5.58 ± 0.15	5.50 ± 0.18
TC01003524.hg.1	OTTHUMG00000034840	5.48 ± 0.28	5.47 ± 0.10	5.53 ± 0.09	5.67 ± 0.19
TC01003203.hg.1	OTTHUMG00000034864	8.68 ± 0.10	8.54 ± 0.12	8.94 ± 0.13	8.62 ± 0.12
TC01001529.hg.1	OTTHUMG00000034877	5.90 ± 0.20	5.97 ± 0.03	5.85 ± 0.10	6.00 ± 0.13
TC01003542.hg.1	OTTHUMG00000034940	3.95 ± 0.03	3.90 ± 0.12	4.03 ± 0.10	3.92 ± 0.04
TC01003547.hg.1	OTTHUMG00000034947	7.38 ± 0.19	7.26 ± 0.21	7.26 ± 0.06	7.41 ± 0.09
TC01001531.hg.1	OTTHUMG00000034949	6.13 ± 0.24	6.28 ± 0.09	6.12 ± 0.29	6.32 ± 0.19
TC01003553.hg.1	OTTHUMG00000034984	7.18 ± 0.45	7.75 ± 0.30	7.72 ± 0.44	7.46 ± 0.29
TC01003550.hg.1	OTTHUMG00000034985	5.61 ± 0.25	5.64 ± 0.07	5.91 ± 0.47	5.57 ± 0.23
TC01001533.hg.1	OTTHUMG00000034988	7.03 ± 0.10	6.83 ± 0.03	7.00 ± 0.11	6.92 ± 0.07
TC01003562.hg.1	OTTHUMG00000035019	4.25 ± 0.35	4.17 ± 0.07	4.12 ± 0.12	4.21 ± 0.07
TC01001535.hg.1	OTTHUMG00000035042	6.17 ± 0.16	6.09 ± 0.08	6.22 ± 0.11	6.34 ± 0.11
TC01001196.hg.1	OTTHUMG00000035065	6.92 ± 0.19	6.82 ± 0.07	6.80 ± 0.16	6.85 ± 0.03
TC01001562.hg.1	OTTHUMG00000035112	7.11 ± 0.32	7.18 ± 0.11	7.11 ± 0.23	7.37 ± 0.29

TC01001570.hg.1	OTTHUMG00000035180	6.84 ± 0.19	6.90 ± 0.28	6.77 ± 0.14	7.15 ± 0.12
TC01003598.hg.1	OTTHUMG00000035181	7.58 ± 0.15	7.54 ± 0.20	7.36 ± 0.25	7.78 ± 0.18
TC01001565.hg.1	OTTHUMG00000035182	6.64 ± 0.18	6.70 ± 0.14	6.68 ± 0.05	6.83 ± 0.08
TC01003508.hg.1	OTTHUMG00000035227	5.56 ± 0.11	5.53 ± 0.12	5.47 ± 0.06	5.58 ± 0.04
TC01003509.hg.1	OTTHUMG00000035229	5.80 ± 0.29	5.81 ± 0.19	5.77 ± 0.23	5.85 ± 0.03
TC01001483.hg.1	OTTHUMG00000035230	6.54 ± 0.26	6.46 ± 0.05	6.36 ± 0.20	6.67 ± 0.16
TC01003590.hg.1	OTTHUMG00000035251	6.54 ± 0.27	6.32 ± 0.20	6.30 ± 0.18	6.35 ± 0.11
TC01003567.hg.1	OTTHUMG00000035254	6.03 ± 0.12	6.03 ± 0.22	6.01 ± 0.21	6.14 ± 0.16
TC01003589.hg.1	OTTHUMG00000035258	5.47 ± 0.17	5.40 ± 0.17	5.42 ± 0.16	5.42 ± 0.13
TC01003586.hg.1	OTTHUMG00000035263	7.41 ± 0.46	7.51 ± 0.23	7.46 ± 0.22	7.59 ± 0.12
TC01003587.hg.1	OTTHUMG00000035264	5.59 ± 0.26	5.24 ± 0.11	5.33 ± 0.22	5.19 ± 0.04
TC01002243.hg.1	OTTHUMG00000035304	5.04 ± 0.34	4.71 ± 0.24	4.75 ± 0.05	4.77 ± 0.04
TC01000203.hg.1	OTTHUMG00000035305	6.21 ± 0.21	6.20 ± 0.04	5.98 ± 0.29	6.12 ± 0.07
TC01003323.hg.1	OTTHUMG00000035438	3.25 ± 0.07	3.29 ± 0.08	3.29 ± 0.18	3.30 ± 0.11
TC01001474.hg.1	OTTHUMG00000035451	4.79 ± 0.15	4.62 ± 0.17	4.78 ± 0.14	4.70 ± 0.08
TC01001590.hg.1	OTTHUMG00000035458	6.22 ± 0.05	6.15 ± 0.13	6.15 ± 0.11	6.24 ± 0.10
TC01001592.hg.1	OTTHUMG00000035468	4.98 ± 0.12	4.92 ± 0.08	4.89 ± 0.12	4.99 ± 0.05
TC01001609.hg.1	OTTHUMG00000035479	4.90 ± 0.19	4.90 ± 0.14	5.00 ± 0.07	5.05 ± 0.13
TC01003513.hg.1	OTTHUMG00000035507	4.77 ± 0.14	4.63 ± 0.01	4.76 ± 0.05	4.69 ± 0.06
TC01003512.hg.1	OTTHUMG00000035508	6.28 ± 0.19	6.09 ± 0.05	5.95 ± 0.04	6.12 ± 0.02
TC01001608.hg.1	OTTHUMG00000035513	6.13 ± 0.28	5.84 ± 0.02	5.86 ± 0.20	5.93 ± 0.19
TC01001613.hg.1	OTTHUMG00000035526	4.05 ± 0.19	3.89 ± 0.03	4.00 ± 0.15	3.92 ± 0.09
TC01003643.hg.1	OTTHUMG00000035527	5.85 ± 0.46	5.66 ± 0.13	5.75 ± 0.01	5.78 ± 0.05
TC01001614.hg.1	OTTHUMG00000035534	4.43 ± 0.09	4.24 ± 0.04	4.21 ± 0.04	4.25 ± 0.08
TC01001612.hg.1	OTTHUMG00000035542	5.49 ± 0.36	5.45 ± 0.04	5.36 ± 0.16	5.58 ± 0.33
TC01003640.hg.1	OTTHUMG00000035544	4.11 ± 0.08	3.97 ± 0.10	3.96 ± 0.16	3.99 ± 0.04
TC01003641.hg.1	OTTHUMG00000035545	6.49 ± 0.28	6.68 ± 0.15	6.61 ± 0.06	6.92 ± 0.09
TC01001630.hg.1	OTTHUMG00000035564	3.82 ± 0.15	3.75 ± 0.05	3.68 ± 0.13	3.64 ± 0.14
TC01003631.hg.1	OTTHUMG00000035577	4.73 ± 0.09	4.59 ± 0.01	4.62 ± 0.04	4.60 ± 0.04
TC01001601.hg.1	OTTHUMG00000035578	5.50 ± 0.34	5.39 ± 0.15	5.43 ± 0.10	5.60 ± 0.09
TC01001606.hg.1	OTTHUMG00000035581	4.67 ± 0.18	4.37 ± 0.19	4.54 ± 0.10	4.39 ± 0.18

TC01001631.hg.1	OTTHUMG00000035603	6.91 ± 0.22	6.91 ± 0.05	6.82 ± 0.07	6.98 ± 0.07
TC01003658.hg.1	OTTHUMG00000035609	3.59 ± 0.27	3.43 ± 0.03	3.47 ± 0.05	3.47 ± 0.12
TC01001611.hg.1	OTTHUMG00000035627	5.43 ± 0.04	5.42 ± 0.09	5.29 ± 0.11	5.29 ± 0.08
TC01003668.hg.1	OTTHUMG00000035655	6.14 ± 0.30	5.93 ± 0.14	5.90 ± 0.06	5.98 ± 0.05
TC01003654.hg.1	OTTHUMG00000035685	3.62 ± 0.10	3.63 ± 0.03	3.82 ± 0.12	3.72 ± 0.10
TC01001628.hg.1	OTTHUMG00000035686	5.01 ± 0.22	4.96 ± 0.05	4.85 ± 0.04	5.05 ± 0.16
TC01001629.hg.1	OTTHUMG00000035690	6.39 ± 0.16	6.51 ± 0.11	6.46 ± 0.09	6.67 ± 0.16
TC01003669.hg.1	OTTHUMG00000035703	6.64 ± 0.15	6.40 ± 0.37	6.91 ± 0.53	6.18 ± 0.12
TC01001295.hg.1	OTTHUMG00000035709	7.11 ± 0.17	7.16 ± 0.20	7.10 ± 0.14	7.27 ± 0.12
TC01003306.hg.1	OTTHUMG00000035711	8.67 ± 0.17	8.34 ± 0.08	8.92 ± 0.04	8.45 ± 0.06
TC01001649.hg.1	OTTHUMG00000035719	6.03 ± 0.21	5.95 ± 0.09	5.96 ± 0.15	6.04 ± 0.14
TC01001650.hg.1	OTTHUMG00000035741	8.10 ± 0.16	8.07 ± 0.05	8.05 ± 0.13	8.18 ± 0.15
TC01003696.hg.1	OTTHUMG00000035769	5.95 ± 0.27	5.80 ± 0.22	5.60 ± 0.34	5.91 ± 0.34
TC01001659.hg.1	OTTHUMG00000035772	9.08 ± 0.32	9.25 ± 0.08	9.07 ± 0.07	9.39 ± 0.18
TC01001656.hg.1	OTTHUMG00000035786	6.98 ± 0.22	6.92 ± 0.20	6.85 ± 0.23	7.09 ± 0.06
TC01003694.hg.1	OTTHUMG00000035804	5.08 ± 0.06	5.18 ± 0.17	5.12 ± 0.11	5.22 ± 0.13
TC01001666.hg.1	OTTHUMG00000035808	8.00 ± 0.30	7.85 ± 0.16	7.83 ± 0.20	8.00 ± 0.06
TC01003502.hg.1	OTTHUMG00000035812	4.91 ± 0.10	4.76 ± 0.11	4.88 ± 0.06	4.82 ± 0.02
TC01003698.hg.1	OTTHUMG00000035865	6.73 ± 0.24	6.54 ± 0.10	6.52 ± 0.04	6.65 ± 0.12
TC01001641.hg.1	OTTHUMG00000035890	5.35 ± 0.24	5.40 ± 0.05	5.36 ± 0.08	5.42 ± 0.04
TC01001643.hg.1	OTTHUMG00000035891	4.72 ± 0.10	4.65 ± 0.07	4.69 ± 0.08	4.74 ± 0.16
TC01003673.hg.1	OTTHUMG00000035892	3.92 ± 0.06	3.79 ± 0.10	3.97 ± 0.31	3.79 ± 0.07
TC01001642.hg.1	OTTHUMG00000035893	4.97 ± 0.16	4.83 ± 0.14	4.83 ± 0.04	4.90 ± 0.10
TC01001478.hg.1	OTTHUMG00000035956	3.72 ± 0.14	3.41 ± 0.05	3.71 ± 0.16	3.40 ± 0.09
TC01001481.hg.1	OTTHUMG00000035971	4.22 ± 0.14	4.11 ± 0.18	4.10 ± 0.08	4.21 ± 0.09
TC01001697.hg.1	OTTHUMG00000036058	7.19 ± 0.19	7.18 ± 0.24	7.16 ± 0.27	7.37 ± 0.06
TC01001696.hg.1	OTTHUMG00000036062	6.29 ± 0.11	6.15 ± 0.06	6.13 ± 0.15	6.12 ± 0.11
TC01001695.hg.1	OTTHUMG00000036063	7.82 ± 0.07	7.67 ± 0.10	7.73 ± 0.07	7.77 ± 0.09
TC01003291.hg.1	OTTHUMG00000036070	7.65 ± 0.16	7.52 ± 0.27	7.50 ± 0.14	7.66 ± 0.05
TC01001698.hg.1	OTTHUMG00000036103	5.76 ± 0.32	5.74 ± 0.08	5.64 ± 0.09	5.70 ± 0.05
TC01001718.hg.1	OTTHUMG00000036117	4.96 ± 0.27	4.55 ± 0.13	4.87 ± 0.12	4.63 ± 0.12

TC01003758.hg.1	OTTHUMG00000036120	5.93 ± 0.32	5.92 ± 0.12	5.81 ± 0.21	5.98 ± 0.07
TC01003780.hg.1	OTTHUMG00000036132	6.17 ± 0.36	6.09 ± 0.13	6.09 ± 0.14	6.25 ± 0.15
TC01003781.hg.1	OTTHUMG00000036134	5.40 ± 0.29	5.28 ± 0.14	5.36 ± 0.06	5.59 ± 0.10
TC01001743.hg.1	OTTHUMG00000036136	3.91 ± 0.02	3.80 ± 0.04	3.91 ± 0.24	3.82 ± 0.01
TC0Y000020.hg.1	OTTHUMG00000036214	6.63 ± 0.15	6.34 ± 0.03	6.45 ± 0.14	6.45 ± 0.16
TC01001734.hg.1	OTTHUMG00000036253	5.02 ± 0.07	4.88 ± 0.06	4.92 ± 0.03	4.96 ± 0.08
TC01001735.hg.1	OTTHUMG00000036254	4.73 ± 0.25	4.58 ± 0.09	4.73 ± 0.19	4.66 ± 0.13
TC01001745.hg.1	OTTHUMG00000036265	5.97 ± 0.13	5.92 ± 0.26	5.94 ± 0.11	6.11 ± 0.10
TC01001746.hg.1	OTTHUMG00000036266	5.56 ± 0.14	5.45 ± 0.13	5.50 ± 0.15	5.65 ± 0.22
TC01003764.hg.1	OTTHUMG00000036345	6.19 ± 0.27	6.23 ± 0.21	6.07 ± 0.24	6.29 ± 0.10
TC01003783.hg.1	OTTHUMG00000036359	6.05 ± 0.16	6.10 ± 0.05	6.04 ± 0.13	6.22 ± 0.09
TC0Y000049.hg.1	OTTHUMG00000036381	7.44 ± 0.10	7.46 ± 0.19	7.37 ± 0.25	7.69 ± 0.21
TC0Y000166.hg.1	OTTHUMG00000036405	5.36 ± 0.24	5.51 ± 0.15	5.36 ± 0.02	5.77 ± 0.13
TC01003785.hg.1	OTTHUMG00000036478	7.03 ± 0.25	7.15 ± 0.13	7.20 ± 0.16	7.21 ± 0.03
TC01003776.hg.1	OTTHUMG00000036552	4.62 ± 0.11	4.47 ± 0.03	4.48 ± 0.02	4.48 ± 0.09
TC01001774.hg.1	OTTHUMG00000036745	5.20 ± 0.05	5.23 ± 0.04	5.39 ± 0.16	5.21 ± 0.11
TC01003806.hg.1	OTTHUMG00000036746	5.86 ± 0.06	5.81 ± 0.09	5.91 ± 0.15	5.93 ± 0.18
TC01001776.hg.1	OTTHUMG00000036749	4.11 ± 0.15	4.10 ± 0.04	4.15 ± 0.07	4.15 ± 0.02
TC01003808.hg.1	OTTHUMG00000036786	7.12 ± 0.16	7.08 ± 0.27	7.19 ± 0.09	7.30 ± 0.16
TC01001762.hg.1	OTTHUMG00000036999	6.29 ± 0.22	6.15 ± 0.12	6.13 ± 0.04	6.23 ± 0.10
TC01001761.hg.1	OTTHUMG00000037001	5.57 ± 0.16	5.24 ± 0.16	5.49 ± 0.16	5.25 ± 0.14
TC01001702.hg.1	OTTHUMG00000037030	7.15 ± 0.10	7.24 ± 0.31	7.15 ± 0.17	7.44 ± 0.21
TC01003272.hg.1	OTTHUMG00000037053	7.10 ± 0.08	6.80 ± 0.14	7.05 ± 0.12	6.75 ± 0.06
TC01001795.hg.1	OTTHUMG00000037062	4.41 ± 0.08	4.31 ± 0.07	4.36 ± 0.08	4.42 ± 0.10
TC01001794.hg.1	OTTHUMG00000037063	5.02 ± 0.12	4.85 ± 0.15	4.63 ± 0.20	5.04 ± 0.37
TC01001796.hg.1	OTTHUMG00000037078	5.16 ± 0.16	5.25 ± 0.11	5.18 ± 0.06	5.38 ± 0.15
TC01001765.hg.1	OTTHUMG00000037122	4.66 ± 0.02	4.58 ± 0.06	4.67 ± 0.04	4.62 ± 0.07
TC01001763.hg.1	OTTHUMG00000037123	6.27 ± 0.15	6.18 ± 0.11	6.17 ± 0.04	6.22 ± 0.07
TC01003799.hg.1	OTTHUMG00000037125	6.83 ± 0.03	6.89 ± 0.16	6.79 ± 0.09	7.05 ± 0.19
TC01001770.hg.1	OTTHUMG00000037131	7.22 ± 0.17	7.19 ± 0.04	7.18 ± 0.04	7.29 ± 0.15
TC01003803.hg.1	OTTHUMG00000037132	6.19 ± 0.05	6.30 ± 0.21	6.24 ± 0.10	6.35 ± 0.07

TC01003829.hg.1	OTTHUMG00000037143	6.30 ± 0.06	6.08 ± 0.04	6.28 ± 0.09	6.30 ± 0.22
TC01001824.hg.1	OTTHUMG00000037165	5.17 ± 0.11	5.02 ± 0.06	5.05 ± 0.20	5.03 ± 0.02
TC01001708.hg.1	OTTHUMG00000037202	6.34 ± 0.31	6.21 ± 0.25	6.34 ± 0.09	6.27 ± 0.06
TC01003387.hg.1	OTTHUMG00000037230	4.75 ± 0.11	4.66 ± 0.07	4.62 ± 0.07	4.62 ± 0.04
TC01003848.hg.1	OTTHUMG00000037268	6.24 ± 0.05	6.12 ± 0.09	5.99 ± 0.16	6.12 ± 0.05
TC01000499.hg.1	OTTHUMG00000037279	6.66 ± 0.16	6.61 ± 0.17	6.47 ± 0.25	6.87 ± 0.20
TC01003852.hg.1	OTTHUMG00000037290	6.12 ± 0.16	6.01 ± 0.06	6.14 ± 0.04	6.13 ± 0.03
TC01003853.hg.1	OTTHUMG00000037291	6.09 ± 0.20	6.04 ± 0.14	6.06 ± 0.03	6.12 ± 0.19
TC01001574.hg.1	OTTHUMG00000037300	6.08 ± 0.30	5.89 ± 0.14	5.92 ± 0.07	6.01 ± 0.10
TC01003602.hg.1	OTTHUMG00000037303	6.33 ± 0.22	6.22 ± 0.12	6.23 ± 0.03	6.26 ± 0.07
TC01003842.hg.1	OTTHUMG00000037350	5.14 ± 0.09	5.08 ± 0.08	5.06 ± 0.10	5.11 ± 0.11
TC01001822.hg.1	OTTHUMG00000037355	5.38 ± 0.11	5.35 ± 0.23	5.35 ± 0.09	5.42 ± 0.16
TC01003840.hg.1	OTTHUMG00000037358	5.23 ± 0.14	5.14 ± 0.03	5.18 ± 0.11	5.18 ± 0.05
TC01003843.hg.1	OTTHUMG00000037359	3.84 ± 0.24	3.74 ± 0.05	3.84 ± 0.21	3.72 ± 0.04
TC01003603.hg.1	OTTHUMG00000037400	6.12 ± 0.13	6.16 ± 0.12	6.22 ± 0.18	6.29 ± 0.24
TC01003604.hg.1	OTTHUMG00000037403	6.34 ± 0.19	6.38 ± 0.03	6.33 ± 0.05	6.51 ± 0.12
TC01003850.hg.1	OTTHUMG00000037423	5.92 ± 0.19	5.97 ± 0.05	5.99 ± 0.01	6.22 ± 0.21
TC01001825.hg.1	OTTHUMG00000037424	7.64 ± 0.25	7.48 ± 0.01	7.65 ± 0.13	7.84 ± 0.28
TC01003851.hg.1	OTTHUMG00000037425	7.75 ± 0.18	7.82 ± 0.12	7.77 ± 0.13	8.02 ± 0.26
TC01000234.hg.1	OTTHUMG00000037449	7.94 ± 0.10	7.66 ± 0.16	7.61 ± 0.15	7.65 ± 0.02
TC01001839.hg.1	OTTHUMG00000037473	7.40 ± 0.55	7.89 ± 0.20	7.60 ± 0.42	7.82 ± 0.16
TC01002346.hg.1	OTTHUMG00000037485	5.65 ± 0.29	5.61 ± 0.24	5.52 ± 0.04	5.53 ± 0.06
TC01002524.hg.1	OTTHUMG00000037488	7.41 ± 0.16	7.26 ± 0.10	7.41 ± 0.09	7.16 ± 0.07
TC01000480.hg.1	OTTHUMG00000037489	8.76 ± 0.25	8.61 ± 0.01	8.74 ± 0.11	8.70 ± 0.03
TC01000546.hg.1	OTTHUMG00000037499	6.50 ± 0.10	6.31 ± 0.01	6.51 ± 0.10	6.38 ± 0.17
TC01000401.hg.1	OTTHUMG00000037511	6.10 ± 0.14	6.16 ± 0.18	6.08 ± 0.15	6.22 ± 0.08
TC01003888.hg.1	OTTHUMG00000037519	7.18 ± 0.17	7.22 ± 0.25	7.12 ± 0.18	7.33 ± 0.11
TC01002437.hg.1	OTTHUMG00000037527	7.73 ± 0.31	7.71 ± 0.20	7.81 ± 0.10	7.88 ± 0.06
TC01003880.hg.1	OTTHUMG00000037537	7.15 ± 0.21	7.05 ± 0.18	7.07 ± 0.33	7.11 ± 0.07
TC01003905.hg.1	OTTHUMG00000037610	7.15 ± 0.22	7.01 ± 0.18	6.96 ± 0.18	7.14 ± 0.05
TC01001869.hg.1	OTTHUMG00000037611	7.00 ± 0.05	6.91 ± 0.05	6.96 ± 0.03	7.05 ± 0.10

TC01001868.hg.1	OTTHUMG00000037619	4.80 ± 0.24	4.76 ± 0.16	4.72 ± 0.12	4.95 ± 0.22
TC01001867.hg.1	OTTHUMG00000037620	5.94 ± 0.16	5.95 ± 0.12	5.96 ± 0.05	6.06 ± 0.05
TC01003941.hg.1	OTTHUMG00000037660	6.44 ± 0.18	6.43 ± 0.23	6.32 ± 0.10	6.62 ± 0.05
TC01003940.hg.1	OTTHUMG00000037661	6.49 ± 0.18	6.51 ± 0.05	6.58 ± 0.06	6.56 ± 0.05
TC01001901.hg.1	OTTHUMG00000037662	5.48 ± 0.15	5.60 ± 0.11	5.67 ± 0.19	5.60 ± 0.08
TC01001850.hg.1	OTTHUMG00000037716	5.11 ± 0.19	5.09 ± 0.09	5.07 ± 0.11	5.14 ± 0.13
TC01003886.hg.1	OTTHUMG00000037717	6.64 ± 0.31	6.53 ± 0.07	6.50 ± 0.06	6.53 ± 0.05
TC01003885.hg.1	OTTHUMG00000037718	7.43 ± 0.21	7.61 ± 0.21	7.57 ± 0.14	7.63 ± 0.01
TC01001853.hg.1	OTTHUMG00000037739	6.66 ± 0.19	6.67 ± 0.07	6.68 ± 0.05	6.61 ± 0.01
TC01003890.hg.1	OTTHUMG00000037740	7.16 ± 0.35	7.13 ± 0.12	7.07 ± 0.25	7.31 ± 0.09
TC01003954.hg.1	OTTHUMG00000037755	5.88 ± 0.37	5.81 ± 0.12	5.91 ± 0.04	6.08 ± 0.15
TC01003955.hg.1	OTTHUMG00000037756	7.48 ± 0.13	7.31 ± 0.07	7.38 ± 0.15	7.40 ± 0.04
TC01001906.hg.1	OTTHUMG00000037758	6.84 ± 0.15	6.65 ± 0.05	6.78 ± 0.03	6.87 ± 0.14
TC01001908.hg.1	OTTHUMG00000037760	6.60 ± 0.14	6.59 ± 0.05	6.53 ± 0.01	6.72 ± 0.08
TC01006340.hg.1	OTTHUMG00000037767	6.37 ± 0.13	6.32 ± 0.06	6.24 ± 0.18	6.31 ± 0.06
TC01000119.hg.1	OTTHUMG00000037769	7.92 ± 0.17	7.99 ± 0.24	7.99 ± 0.32	8.11 ± 0.13
TC01002201.hg.1	OTTHUMG00000037775	7.50 ± 0.28	7.46 ± 0.34	7.34 ± 0.26	7.62 ± 0.12
TC01006282.hg.1	OTTHUMG00000037778	6.16 ± 0.34	6.12 ± 0.33	6.07 ± 0.26	6.18 ± 0.09
TC01000801.hg.1	OTTHUMG00000037783	6.94 ± 0.13	6.76 ± 0.15	6.96 ± 0.19	6.83 ± 0.15
TC01003958.hg.1	OTTHUMG00000037789	7.46 ± 0.15	7.50 ± 0.12	7.41 ± 0.06	7.54 ± 0.11
TC01001912.hg.1	OTTHUMG00000037791	5.97 ± 0.31	5.98 ± 0.29	5.99 ± 0.20	6.11 ± 0.12
TC01001924.hg.1	OTTHUMG00000037823	5.19 ± 0.25	5.27 ± 0.13	5.12 ± 0.09	5.21 ± 0.09
TC01003975.hg.1	OTTHUMG00000037825	5.85 ± 0.24	5.54 ± 0.09	5.63 ± 0.10	5.61 ± 0.07
TC01003973.hg.1	OTTHUMG00000037832	5.72 ± 0.21	5.62 ± 0.08	5.52 ± 0.16	5.63 ± 0.15
TC01000244.hg.1	OTTHUMG00000037839	8.58 ± 0.23	8.54 ± 0.11	8.48 ± 0.10	8.64 ± 0.09
TC01000264.hg.1	OTTHUMG00000037842	8.72 ± 0.14	8.42 ± 0.14	8.75 ± 0.15	8.48 ± 0.10
TC01000273.hg.1	OTTHUMG00000037843	7.75 ± 0.09	7.61 ± 0.06	7.79 ± 0.12	7.56 ± 0.10
TC01001921.hg.1	OTTHUMG00000037849	6.20 ± 0.34	6.24 ± 0.13	6.13 ± 0.23	6.56 ± 0.32
TC01001798.hg.1	OTTHUMG00000037874	4.18 ± 0.17	4.06 ± 0.09	4.04 ± 0.12	4.06 ± 0.03
TC01000771.hg.1	OTTHUMG00000037896	5.56 ± 0.04	5.48 ± 0.09	5.38 ± 0.10	5.49 ± 0.18
TC01002784.hg.1	OTTHUMG00000037897	4.87 ± 0.02	4.90 ± 0.24	4.87 ± 0.14	4.99 ± 0.07

TC01002793.hg.1	OTTHUMG00000037911	4.87 ± 0.07	4.80 ± 0.05	4.82 ± 0.04	4.87 ± 0.02
TC01001833.hg.1	OTTHUMG00000037934	6.91 ± 0.21	6.84 ± 0.04	6.84 ± 0.12	6.76 ± 0.16
TC01001834.hg.1	OTTHUMG00000037935	7.17 ± 0.32	7.29 ± 0.05	7.18 ± 0.10	7.46 ± 0.19
TC01001933.hg.1	OTTHUMG00000037944	7.87 ± 0.43	7.62 ± 0.13	7.39 ± 0.39	7.60 ± 0.20
TC01003982.hg.1	OTTHUMG00000037946	7.54 ± 0.12	7.43 ± 0.05	7.43 ± 0.08	7.38 ± 0.15
TC01003964.hg.1	OTTHUMG00000037959	6.78 ± 0.32	6.66 ± 0.09	6.62 ± 0.22	6.86 ± 0.20
TC01001935.hg.1	OTTHUMG00000037977	5.21 ± 0.26	5.01 ± 0.08	5.01 ± 0.12	5.01 ± 0.06
TC01001937.hg.1	OTTHUMG00000037978	9.62 ± 0.29	9.53 ± 0.18	9.54 ± 0.18	9.55 ± 0.09
TC01001939.hg.1	OTTHUMG00000037980	5.65 ± 0.06	5.58 ± 0.09	5.61 ± 0.12	5.67 ± 0.06
TC01003986.hg.1	OTTHUMG00000037983	7.84 ± 0.29	7.81 ± 0.03	7.70 ± 0.14	8.03 ± 0.19
TC01002840.hg.1	OTTHUMG00000037985	4.60 ± 0.13	4.52 ± 0.15	4.45 ± 0.09	4.52 ± 0.06
TC01003987.hg.1	OTTHUMG00000038014	6.43 ± 0.13	6.32 ± 0.05	6.50 ± 0.16	6.29 ± 0.05
TC01001943.hg.1	OTTHUMG00000038015	5.67 ± 0.10	5.65 ± 0.09	5.80 ± 0.10	5.52 ± 0.02
TC01001940.hg.1	OTTHUMG00000038016	6.60 ± 0.17	6.68 ± 0.08	6.70 ± 0.09	6.79 ± 0.09
TC01003824.hg.1	OTTHUMG00000038051	5.08 ± 0.19	4.77 ± 0.07	4.98 ± 0.19	4.79 ± 0.24
TC01003952.hg.1	OTTHUMG00000039465	5.34 ± 0.20	5.19 ± 0.03	5.25 ± 0.03	5.30 ± 0.09
TC01001903.hg.1	OTTHUMG00000039467	5.93 ± 0.06	5.80 ± 0.02	5.89 ± 0.05	5.82 ± 0.13
TC01003946.hg.1	OTTHUMG00000039470	6.52 ± 0.28	6.74 ± 0.13	6.38 ± 0.10	6.92 ± 0.20
TC01003970.hg.1	OTTHUMG00000039487	7.86 ± 0.25	7.82 ± 0.11	8.39 ± 0.23	7.70 ± 0.16
TC01001801.hg.1	OTTHUMG00000039495	5.15 ± 0.07	5.03 ± 0.08	4.98 ± 0.11	5.11 ± 0.06
TC01001802.hg.1	OTTHUMG00000039496	6.09 ± 0.09	6.17 ± 0.06	6.08 ± 0.11	6.22 ± 0.06
TC01001963.hg.1	OTTHUMG00000039533	6.77 ± 0.16	6.66 ± 0.07	6.67 ± 0.08	6.80 ± 0.06
TC01004014.hg.1	OTTHUMG00000039564	8.07 ± 0.30	8.06 ± 0.06	8.00 ± 0.15	8.19 ± 0.13
TC02000093.hg.1	OTTHUMG00000039578	6.63 ± 0.26	6.75 ± 0.26	6.60 ± 0.12	6.99 ± 0.21
TC01001973.hg.1	OTTHUMG00000039598	7.70 ± 0.24	7.52 ± 0.25	7.55 ± 0.24	7.72 ± 0.08
TC01000065.hg.1	OTTHUMG00000039631	6.66 ± 0.22	6.41 ± 0.18	6.44 ± 0.14	6.46 ± 0.05
TC01004017.hg.1	OTTHUMG00000039651	5.34 ± 0.11	5.15 ± 0.04	5.26 ± 0.06	5.21 ± 0.11
TC01002797.hg.1	OTTHUMG00000039775	6.10 ± 0.17	6.15 ± 0.22	6.08 ± 0.18	6.29 ± 0.23
TC01004033.hg.1	OTTHUMG00000039859	6.40 ± 0.43	6.28 ± 0.03	6.34 ± 0.10	6.37 ± 0.07
TC01004034.hg.1	OTTHUMG00000039860	8.18 ± 0.29	7.93 ± 0.23	8.74 ± 0.25	8.02 ± 0.11
TC01001978.hg.1	OTTHUMG00000039868	8.46 ± 0.24	8.41 ± 0.03	8.52 ± 0.14	8.65 ± 0.27

TC01004032.hg.1	OTTHUMG00000039869	7.13 ± 0.18	7.26 ± 0.09	7.23 ± 0.10	7.36 ± 0.03
TC01001980.hg.1	OTTHUMG00000039874	4.00 ± 0.11	3.80 ± 0.10	3.98 ± 0.07	3.88 ± 0.01
TC01001979.hg.1	OTTHUMG00000039875	4.59 ± 0.27	4.50 ± 0.11	4.77 ± 0.19	4.36 ± 0.05
TC01004019.hg.1	OTTHUMG00000039886	5.06 ± 0.23	4.96 ± 0.03	4.84 ± 0.05	5.00 ± 0.06
TC01001968.hg.1	OTTHUMG00000039910	3.82 ± 0.24	3.53 ± 0.14	3.63 ± 0.14	3.56 ± 0.12
TC01004020.hg.1	OTTHUMG00000039911	7.97 ± 0.17	8.08 ± 0.01	7.99 ± 0.04	8.31 ± 0.17
TC01001971.hg.1	OTTHUMG00000039932	4.31 ± 0.23	4.23 ± 0.11	4.22 ± 0.21	4.20 ± 0.07
TC01001892.hg.1	OTTHUMG00000039975	7.88 ± 0.17	7.77 ± 0.23	7.70 ± 0.18	7.80 ± 0.06
TC01004042.hg.1	OTTHUMG00000039995	4.66 ± 0.26	4.86 ± 0.10	4.52 ± 0.07	4.82 ± 0.09
TC01001986.hg.1	OTTHUMG00000040015	5.16 ± 0.12	5.19 ± 0.09	5.07 ± 0.16	5.33 ± 0.14
TC01001983.hg.1	OTTHUMG00000040016	5.66 ± 0.03	5.54 ± 0.06	5.75 ± 0.04	5.61 ± 0.08
TC01004050.hg.1	OTTHUMG00000040077	6.51 ± 0.25	6.55 ± 0.17	6.56 ± 0.12	6.73 ± 0.12
TC01004052.hg.1	OTTHUMG00000040080	5.17 ± 0.12	5.17 ± 0.23	5.20 ± 0.09	5.26 ± 0.04
TC01004053.hg.1	OTTHUMG00000040081	5.10 ± 0.18	5.23 ± 0.02	5.13 ± 0.03	5.31 ± 0.12
TC01003980.hg.1	OTTHUMG00000040084	7.89 ± 0.43	7.67 ± 0.22	7.67 ± 0.33	7.72 ± 0.10
TC01003981.hg.1	OTTHUMG00000040085	5.64 ± 0.13	5.51 ± 0.10	5.50 ± 0.13	5.56 ± 0.15
TC01004045.hg.1	OTTHUMG00000040105	6.56 ± 0.17	6.56 ± 0.10	6.66 ± 0.24	6.58 ± 0.05
TC01001438.hg.1	OTTHUMG00000040114	5.81 ± 0.24	5.78 ± 0.17	5.86 ± 0.10	5.84 ± 0.09
TC01001439.hg.1	OTTHUMG00000040115	7.21 ± 0.25	7.01 ± 0.06	7.08 ± 0.05	7.14 ± 0.18
TC01003468.hg.1	OTTHUMG00000040116	6.78 ± 0.17	6.56 ± 0.21	6.64 ± 0.17	6.63 ± 0.06
TC01002001.hg.1	OTTHUMG00000040132	7.97 ± 0.34	8.05 ± 0.08	7.93 ± 0.06	8.11 ± 0.03
TC01002042.hg.1	OTTHUMG00000040386	4.29 ± 0.15	4.15 ± 0.06	4.22 ± 0.11	4.25 ± 0.18
TC01002046.hg.1	OTTHUMG00000040425	6.82 ± 0.15	7.09 ± 0.14	6.80 ± 0.15	7.22 ± 0.14
TC01000838.hg.1	OTTHUMG00000040465	5.20 ± 0.33	5.52 ± 0.31	5.30 ± 0.41	5.51 ± 0.10
TC01001998.hg.1	OTTHUMG00000040510	6.56 ± 0.46	6.36 ± 0.02	6.51 ± 0.14	6.43 ± 0.05
TC01001145.hg.1	OTTHUMG00000040559	7.83 ± 0.22	7.59 ± 0.07	7.75 ± 0.11	7.53 ± 0.10
TC01002722.hg.1	OTTHUMG00000040631	4.51 ± 0.14	4.51 ± 0.07	4.60 ± 0.16	4.57 ± 0.12
TC01003480.hg.1	OTTHUMG00000040633	7.24 ± 0.38	7.52 ± 0.15	7.04 ± 0.21	7.39 ± 0.18
TC01001615.hg.1	OTTHUMG00000040635	5.17 ± 0.21	5.10 ± 0.02	5.06 ± 0.09	5.22 ± 0.18
TC01002014.hg.1	OTTHUMG00000040651	5.94 ± 0.15	5.88 ± 0.04	6.00 ± 0.15	6.07 ± 0.35
TC01004074.hg.1	OTTHUMG00000040653	5.51 ± 0.21	5.44 ± 0.19	5.44 ± 0.20	5.50 ± 0.04

TC01003687.hg.1	OTTHUMG00000040658	6.67 ± 0.32	6.68 ± 0.22	6.64 ± 0.34	6.89 ± 0.11
TC01003690.hg.1	OTTHUMG00000040659	7.54 ± 0.35	7.38 ± 0.14	7.62 ± 0.16	7.51 ± 0.06
TC01003744.hg.1	OTTHUMG00000040690	5.91 ± 0.16	5.89 ± 0.16	5.83 ± 0.03	6.04 ± 0.17
TC01001701.hg.1	OTTHUMG00000040691	7.46 ± 0.13	7.34 ± 0.08	7.29 ± 0.12	7.29 ± 0.23
TC01001148.hg.1	OTTHUMG00000040699	7.77 ± 0.31	7.45 ± 0.12	7.72 ± 0.10	7.40 ± 0.07
TC01001150.hg.1	OTTHUMG00000040701	7.40 ± 0.19	7.46 ± 0.15	7.43 ± 0.30	7.95 ± 0.43
TC01004022.hg.1	OTTHUMG00000040729	5.29 ± 0.06	5.30 ± 0.13	5.39 ± 0.15	5.44 ± 0.04
TC01003489.hg.1	OTTHUMG00000040769	7.21 ± 0.63	7.32 ± 0.09	7.29 ± 0.30	7.53 ± 0.31
TC01001462.hg.1	OTTHUMG00000040771	5.21 ± 0.10	5.24 ± 0.04	5.22 ± 0.08	5.27 ± 0.15
TC01002081.hg.1	OTTHUMG00000040779	6.02 ± 0.09	5.86 ± 0.04	5.87 ± 0.10	5.78 ± 0.19
TC21000519.hg.1	OTTHUMG00000040845	8.07 ± 0.20	7.96 ± 0.07	7.96 ± 0.30	8.01 ± 0.04
TC01001507.hg.1	OTTHUMG00000040853	4.51 ± 0.17	4.44 ± 0.06	4.61 ± 0.15	4.49 ± 0.05
TC01001537.hg.1	OTTHUMG00000040861	5.72 ± 0.12	5.74 ± 0.08	5.57 ± 0.15	5.80 ± 0.12
TC01001538.hg.1	OTTHUMG00000040863	5.89 ± 0.10	5.89 ± 0.21	5.76 ± 0.20	6.10 ± 0.05
TC01003056.hg.1	OTTHUMG00000040900	6.56 ± 0.11	6.47 ± 0.07	6.56 ± 0.13	6.56 ± 0.09
TC01001162.hg.1	OTTHUMG00000041027	6.55 ± 0.10	6.47 ± 0.08	6.54 ± 0.10	6.56 ± 0.10
TC01003171.hg.1	OTTHUMG00000041032	7.16 ± 0.18	6.76 ± 0.13	7.57 ± 0.35	6.86 ± 0.03
TC01001049.hg.1	OTTHUMG00000041038	7.12 ± 0.12	6.74 ± 0.15	7.65 ± 0.29	6.77 ± 0.09
TC01001048.hg.1	OTTHUMG00000041039	6.61 ± 0.22	6.54 ± 0.21	6.80 ± 0.49	6.39 ± 0.07
TC01001580.hg.1	OTTHUMG00000041043	5.58 ± 0.26	5.53 ± 0.18	5.50 ± 0.14	5.78 ± 0.06
TC01002110.hg.1	OTTHUMG00000041065	6.43 ± 0.17	6.55 ± 0.31	6.42 ± 0.32	6.63 ± 0.20
TC01003807.hg.1	OTTHUMG00000041085	7.03 ± 0.26	6.93 ± 0.29	6.97 ± 0.22	7.18 ± 0.15
TC01002111.hg.1	OTTHUMG00000041087	8.05 ± 0.15	8.03 ± 0.22	8.11 ± 0.25	8.12 ± 0.15
TC01003774.hg.1	OTTHUMG00000041089	6.67 ± 0.19	6.19 ± 0.06	7.19 ± 0.44	6.09 ± 0.05
TC01001617.hg.1	OTTHUMG00000041127	5.48 ± 0.18	5.37 ± 0.09	5.49 ± 0.08	5.62 ± 0.28
TC01001618.hg.1	OTTHUMG00000041131	4.61 ± 0.10	4.45 ± 0.11	4.58 ± 0.18	4.53 ± 0.02
TC01003647.hg.1	OTTHUMG00000041135	3.97 ± 0.07	3.80 ± 0.09	3.84 ± 0.03	3.93 ± 0.08
TC01001621.hg.1	OTTHUMG00000041136	4.87 ± 0.05	4.75 ± 0.05	4.95 ± 0.20	4.84 ± 0.11
TC01003649.hg.1	OTTHUMG00000041137	6.02 ± 0.16	6.25 ± 0.07	6.11 ± 0.06	6.24 ± 0.28
TC01003793.hg.1	OTTHUMG00000041139	8.00 ± 0.03	7.87 ± 0.06	7.86 ± 0.16	7.96 ± 0.15
TC01001784.hg.1	OTTHUMG00000041155	4.98 ± 0.09	5.08 ± 0.12	5.02 ± 0.06	5.05 ± 0.12

TC01001785.hg.1	OTTHUMG00000041157	4.86 ± 0.16	4.79 ± 0.03	4.78 ± 0.06	4.64 ± 0.18
TC01003620.hg.1	OTTHUMG00000041183	4.50 ± 0.02	4.42 ± 0.15	4.45 ± 0.05	4.49 ± 0.02
TC01001636.hg.1	OTTHUMG00000041185	4.53 ± 0.16	4.36 ± 0.12	4.55 ± 0.28	4.42 ± 0.09
TC01003891.hg.1	OTTHUMG00000041221	8.42 ± 0.25	8.59 ± 0.19	8.20 ± 0.17	8.76 ± 0.09
TC01001809.hg.1	OTTHUMG00000041227	7.56 ± 0.23	7.20 ± 0.26	7.35 ± 0.11	7.11 ± 0.05
TC01003827.hg.1	OTTHUMG00000041229	5.07 ± 0.23	5.02 ± 0.04	4.96 ± 0.01	5.11 ± 0.13
TC01003831.hg.1	OTTHUMG00000041230	6.30 ± 0.18	6.11 ± 0.06	6.15 ± 0.19	6.18 ± 0.06
TC01003830.hg.1	OTTHUMG00000041231	5.53 ± 0.19	5.48 ± 0.02	5.50 ± 0.13	5.70 ± 0.11
TC01003275.hg.1	OTTHUMG00000041239	6.62 ± 0.11	6.49 ± 0.06	6.60 ± 0.02	6.58 ± 0.02
TC01003348.hg.1	OTTHUMG00000041297	7.22 ± 0.36	7.07 ± 0.21	7.04 ± 0.28	7.12 ± 0.15
TC01003350.hg.1	OTTHUMG00000041299	6.14 ± 0.18	6.28 ± 0.06	6.15 ± 0.10	6.35 ± 0.11
TC01001931.hg.1	OTTHUMG00000041308	6.09 ± 0.13	5.97 ± 0.12	6.01 ± 0.19	6.16 ± 0.10
TC01003979.hg.1	OTTHUMG00000041309	7.87 ± 0.15	7.65 ± 0.07	7.70 ± 0.15	7.83 ± 0.20
TC01003294.hg.1	OTTHUMG00000041315	8.83 ± 0.11	8.58 ± 0.06	8.56 ± 0.36	8.44 ± 0.13
TC01001332.hg.1	OTTHUMG00000041327	5.73 ± 0.21	5.72 ± 0.15	5.82 ± 0.24	5.80 ± 0.07
TC01003894.hg.1	OTTHUMG00000041338	5.94 ± 0.23	5.70 ± 0.10	5.82 ± 0.14	5.91 ± 0.04
TC01001859.hg.1	OTTHUMG00000041339	5.31 ± 0.13	5.18 ± 0.14	5.26 ± 0.25	5.24 ± 0.06
TC01001858.hg.1	OTTHUMG00000041341	7.05 ± 0.26	7.03 ± 0.07	6.96 ± 0.09	7.14 ± 0.04
TC01000914.hg.1	OTTHUMG00000041346	4.62 ± 0.32	4.48 ± 0.02	4.60 ± 0.14	4.58 ± 0.08
TC01002933.hg.1	OTTHUMG00000041347	4.94 ± 0.16	4.92 ± 0.02	4.90 ± 0.08	4.98 ± 0.04
TC01002934.hg.1	OTTHUMG00000041348	6.14 ± 0.18	6.15 ± 0.01	6.09 ± 0.08	6.22 ± 0.07
TC01000915.hg.1	OTTHUMG00000041349	4.62 ± 0.32	4.48 ± 0.02	4.60 ± 0.14	4.58 ± 0.08
TC01003939.hg.1	OTTHUMG00000041351	6.76 ± 0.02	6.76 ± 0.23	6.71 ± 0.08	6.99 ± 0.19
TC01003707.hg.1	OTTHUMG00000041379	8.58 ± 0.16	8.54 ± 0.03	8.57 ± 0.10	8.58 ± 0.03
TC01001676.hg.1	OTTHUMG00000041394	6.89 ± 0.25	6.63 ± 0.04	6.62 ± 0.07	6.58 ± 0.07
TC01001677.hg.1	OTTHUMG00000041395	6.81 ± 0.12	6.84 ± 0.16	6.76 ± 0.07	6.87 ± 0.03
TC01004006.hg.1	OTTHUMG00000041413	7.58 ± 0.26	7.41 ± 0.32	7.65 ± 0.28	7.53 ± 0.07
TC01004084.hg.1	OTTHUMG00000041417	5.40 ± 0.27	5.18 ± 0.09	5.18 ± 0.17	5.07 ± 0.23
TC01001844.hg.1	OTTHUMG00000041421	5.76 ± 0.20	5.82 ± 0.19	6.01 ± 0.16	5.83 ± 0.08
TC01004001.hg.1	OTTHUMG00000041431	6.47 ± 0.16	6.37 ± 0.03	6.31 ± 0.09	6.38 ± 0.08
TC01004002.hg.1	OTTHUMG00000041435	6.29 ± 0.03	6.39 ± 0.08	6.35 ± 0.08	6.51 ± 0.06

TC01001962.hg.1	OTTHUMG00000041437	4.52 ± 0.09	4.51 ± 0.08	4.45 ± 0.06	4.40 ± 0.05
TC01003086.hg.1	OTTHUMG00000041441	5.98 ± 0.28	6.26 ± 0.03	6.12 ± 0.12	6.21 ± 0.18
TC01004070.hg.1	OTTHUMG00000041449	6.94 ± 0.20	6.88 ± 0.14	6.90 ± 0.15	6.91 ± 0.15
TC01002146.hg.1	OTTHUMG00000041452	7.44 ± 0.04	7.50 ± 0.29	7.33 ± 0.16	7.44 ± 0.05
TC01002141.hg.1	OTTHUMG00000041453	6.71 ± 0.26	6.75 ± 0.16	6.61 ± 0.17	6.96 ± 0.17
TC01001772.hg.1	OTTHUMG00000041460	5.85 ± 0.59	6.05 ± 0.32	5.93 ± 0.36	5.93 ± 0.12
TC01003804.hg.1	OTTHUMG00000041461	4.51 ± 0.23	4.37 ± 0.07	4.69 ± 0.28	4.40 ± 0.12
TC01001760.hg.1	OTTHUMG00000041463	6.89 ± 0.17	7.01 ± 0.10	6.97 ± 0.06	7.25 ± 0.13
TC01002213.hg.1	OTTHUMG00000041465	6.52 ± 0.15	6.58 ± 0.16	6.60 ± 0.15	6.63 ± 0.13
TC01002474.hg.1	OTTHUMG00000041501	7.45 ± 0.09	7.59 ± 0.23	7.56 ± 0.09	7.54 ± 0.02
TC01001125.hg.1	OTTHUMG00000041543	5.11 ± 0.37	5.01 ± 0.14	5.04 ± 0.05	5.14 ± 0.15
TC01001157.hg.1	OTTHUMG00000041572	7.06 ± 0.09	6.87 ± 0.04	6.98 ± 0.11	6.85 ± 0.07
TC01001156.hg.1	OTTHUMG00000041573	5.96 ± 0.04	6.02 ± 0.07	5.97 ± 0.11	6.00 ± 0.04
TC0Y000042.hg.1	OTTHUMG00000041597	5.53 ± 0.13	5.47 ± 0.12	5.37 ± 0.25	5.57 ± 0.25
TC01002133.hg.1	OTTHUMG00000041605	6.99 ± 0.11	6.78 ± 0.28	6.69 ± 0.21	6.76 ± 0.09
TC01003168.hg.1	OTTHUMG00000041633	7.31 ± 0.24	7.29 ± 0.26	7.27 ± 0.24	7.45 ± 0.04
TC01001716.hg.1	OTTHUMG00000041669	6.99 ± 0.27	7.17 ± 0.18	7.04 ± 0.14	7.20 ± 0.14
TC01002140.hg.1	OTTHUMG00000041729	5.72 ± 0.18	5.71 ± 0.02	5.64 ± 0.07	5.85 ± 0.13
TC01001116.hg.1	OTTHUMG00000042020	5.42 ± 0.11	5.10 ± 0.17	5.21 ± 0.16	5.08 ± 0.16
TC01001114.hg.1	OTTHUMG00000042022	5.20 ± 0.08	5.02 ± 0.15	5.12 ± 0.10	5.13 ± 0.04
TC01001115.hg.1	OTTHUMG00000042023	5.85 ± 0.20	5.97 ± 0.32	5.80 ± 0.29	6.11 ± 0.24
TC01000773.hg.1	OTTHUMG00000042097	4.42 ± 0.12	4.27 ± 0.11	4.41 ± 0.11	4.42 ± 0.27
TC01000365.hg.1	OTTHUMG00000042107	7.19 ± 0.20	7.11 ± 0.32	7.19 ± 0.32	7.16 ± 0.04
TC01003635.hg.1	OTTHUMG00000042129	11.47 ± 0.24	11.52 ± 0.03	11.41 ± 0.09	11.63 ± 0.16
TC01003100.hg.1	OTTHUMG00000042242	6.76 ± 0.53	6.66 ± 0.23	6.70 ± 0.46	6.98 ± 0.45
TC01003109.hg.1	OTTHUMG00000042245	6.42 ± 0.09	6.40 ± 0.04	6.41 ± 0.05	6.44 ± 0.02
TC01003150.hg.1	OTTHUMG00000042457	7.55 ± 0.11	7.49 ± 0.02	7.59 ± 0.15	7.53 ± 0.09
TC01003757.hg.1	OTTHUMG00000042553	10.10 ± 0.11	9.94 ± 0.07	10.03 ± 0.11	9.89 ± 0.19
TC01003715.hg.1	OTTHUMG00000043020	7.43 ± 0.23	7.40 ± 0.29	7.39 ± 0.25	7.55 ± 0.11
TC01003716.hg.1	OTTHUMG00000043021	6.46 ± 0.14	6.39 ± 0.18	6.36 ± 0.09	6.50 ± 0.09
TC01003135.hg.1	OTTHUMG00000043432	6.47 ± 0.23	6.52 ± 0.04	6.26 ± 0.11	6.71 ± 0.20

TC01001119.hg.1	OTTHUMG00000043433	7.10 ± 0.32	6.91 ± 0.15	6.91 ± 0.18	7.01 ± 0.10
TC0Y000193.hg.1	OTTHUMG00000043574	5.49 ± 0.11	5.53 ± 0.14	5.43 ± 0.12	5.58 ± 0.08
TC0Y000077.hg.1	OTTHUMG00000043582	5.48 ± 0.12	5.53 ± 0.14	5.42 ± 0.12	5.58 ± 0.08
TC01000405.hg.1	OTTHUMG00000043647	7.13 ± 0.17	7.27 ± 0.21	7.14 ± 0.15	7.37 ± 0.10
TC01003121.hg.1	OTTHUMG00000043709	6.49 ± 0.52	6.44 ± 0.15	6.35 ± 0.12	6.58 ± 0.34
TC01003446.hg.1	OTTHUMG00000043773	6.50 ± 0.29	6.61 ± 0.21	6.52 ± 0.23	6.74 ± 0.27
TC0Y000079.hg.1	OTTHUMG00000043853	4.46 ± 0.08	4.33 ± 0.10	4.42 ± 0.07	4.43 ± 0.14
TC0Y000198.hg.1	OTTHUMG00000044058	4.46 ± 0.08	4.33 ± 0.10	4.42 ± 0.07	4.43 ± 0.14
TC14001516.hg.1	OTTHUMG00000045024	7.64 ± 0.21	7.65 ± 0.19	7.65 ± 0.15	7.75 ± 0.13
TC10000353.hg.1	OTTHUMG00000045026	4.73 ± 0.19	4.41 ± 0.10	4.57 ± 0.05	4.40 ± 0.04
TC11001905.hg.1	OTTHUMG00000045125	8.25 ± 0.25	8.07 ± 0.06	8.07 ± 0.13	8.13 ± 0.07
TC11002321.hg.1	OTTHUMG00000045137	7.70 ± 0.34	7.52 ± 0.20	7.56 ± 0.26	7.58 ± 0.03
TC11001040.hg.1	OTTHUMG00000045138	5.44 ± 0.07	5.38 ± 0.04	5.38 ± 0.08	5.43 ± 0.08
TC0Y000105.hg.1	OTTHUMG00000045279	4.46 ± 0.08	4.33 ± 0.10	4.42 ± 0.07	4.43 ± 0.14
TC11001267.hg.1	OTTHUMG00000045359	8.19 ± 0.23	8.29 ± 0.29	8.17 ± 0.18	8.46 ± 0.06
TC11000050.hg.1	OTTHUMG00000045360	7.56 ± 0.20	7.75 ± 0.31	7.63 ± 0.15	7.90 ± 0.07
TC01001216.hg.1	OTTHUMG00000045372	6.68 ± 0.34	6.66 ± 0.15	6.69 ± 0.10	6.75 ± 0.04
TC11001268.hg.1	OTTHUMG00000045388	7.47 ± 0.06	7.43 ± 0.08	7.36 ± 0.12	7.49 ± 0.14
TC06001192.hg.1	OTTHUMG00000045405	7.91 ± 0.16	7.85 ± 0.24	7.88 ± 0.27	7.88 ± 0.01
TC06002331.hg.1	OTTHUMG00000045406	6.78 ± 0.19	6.67 ± 0.17	6.67 ± 0.13	6.75 ± 0.08
TC11002326.hg.1	OTTHUMG00000046113	7.11 ± 0.35	7.10 ± 0.18	7.07 ± 0.25	7.28 ± 0.19
TC09000708.hg.1	OTTHUMG00000046129	7.75 ± 0.15	7.77 ± 0.10	7.73 ± 0.10	7.82 ± 0.05
TC09000710.hg.1	OTTHUMG00000046130	7.04 ± 0.31	6.98 ± 0.22	6.94 ± 0.16	7.16 ± 0.08
TC01001584.hg.1	OTTHUMG00000046152	6.09 ± 0.14	6.09 ± 0.05	6.06 ± 0.14	6.18 ± 0.11
TC06001942.hg.1	OTTHUMG00000046174	6.14 ± 0.13	6.09 ± 0.17	6.15 ± 0.12	6.20 ± 0.08
TC09000905.hg.1	OTTHUMG00000046176	4.77 ± 0.25	4.63 ± 0.16	4.68 ± 0.12	4.61 ± 0.22
TC10001785.hg.1	OTTHUMG00000046186	9.69 ± 0.27	9.79 ± 0.04	9.71 ± 0.05	9.93 ± 0.07
TC13000034.hg.1	OTTHUMG00000046188	6.37 ± 0.15	6.37 ± 0.04	6.31 ± 0.02	6.42 ± 0.05
TC20000671.hg.1	OTTHUMG00000046204	6.53 ± 0.21	6.33 ± 0.01	6.56 ± 0.16	6.44 ± 0.10
TC06001719.hg.1	OTTHUMG00000046223	5.74 ± 0.16	5.66 ± 0.08	5.71 ± 0.05	5.80 ± 0.15
TC06000569.hg.1	OTTHUMG00000046224	6.54 ± 0.25	6.56 ± 0.30	6.60 ± 0.12	6.68 ± 0.06

TC06000573.hg.1	OTTHUMG00000046284	7.71 ± 0.26	7.69 ± 0.32	7.66 ± 0.21	7.81 ± 0.20
TC06000576.hg.1	OTTHUMG00000046310	7.65 ± 0.16	7.44 ± 0.03	7.52 ± 0.14	7.56 ± 0.01
TC06000730.hg.1	OTTHUMG00000047278	7.70 ± 0.17	7.56 ± 0.10	7.63 ± 0.12	7.67 ± 0.05
TC16000733.hg.1	OTTHUMG00000048041	8.48 ± 0.16	8.34 ± 0.25	8.49 ± 0.15	8.40 ± 0.08
TC19001442.hg.1	OTTHUMG00000048096	8.52 ± 0.33	8.48 ± 0.18	8.72 ± 0.27	8.51 ± 0.08
TC19001443.hg.1	OTTHUMG00000048097	8.16 ± 0.11	8.33 ± 0.07	8.45 ± 0.16	8.29 ± 0.07
TC19000475.hg.1	OTTHUMG00000048109	5.66 ± 0.22	5.55 ± 0.03	5.53 ± 0.14	5.63 ± 0.03
TC19001454.hg.1	OTTHUMG00000048120	7.15 ± 0.17	7.23 ± 0.23	7.11 ± 0.16	7.36 ± 0.06
TC19000503.hg.1	OTTHUMG00000048152	7.19 ± 0.10	7.14 ± 0.04	7.07 ± 0.06	7.24 ± 0.03
TC19001474.hg.1	OTTHUMG00000048158	7.27 ± 0.17	7.13 ± 0.03	7.02 ± 0.08	7.16 ± 0.08
TC19000513.hg.1	OTTHUMG00000048167	6.29 ± 0.16	6.27 ± 0.14	6.20 ± 0.06	6.36 ± 0.09
TC06000990.hg.1	OTTHUMG00000055852	4.15 ± 0.04	3.92 ± 0.02	4.06 ± 0.11	3.99 ± 0.05
TC06000987.hg.1	OTTHUMG00000055854	7.14 ± 0.16	6.92 ± 0.05	7.12 ± 0.26	7.41 ± 0.50
TC13000529.hg.1	OTTHUMG00000055979	4.88 ± 0.13	4.84 ± 0.26	4.85 ± 0.24	4.65 ± 0.07
TC13000526.hg.1	OTTHUMG00000055980	6.73 ± 0.34	6.62 ± 0.12	6.78 ± 0.25	6.83 ± 0.12
TC13000533.hg.1	OTTHUMG00000055981	5.95 ± 0.22	6.00 ± 0.18	6.04 ± 0.23	6.01 ± 0.08
TC13000528.hg.1	OTTHUMG00000055982	6.40 ± 0.28	6.21 ± 0.13	6.11 ± 0.08	6.41 ± 0.08
TC20000800.hg.1	OTTHUMG00000055986	7.39 ± 0.14	7.37 ± 0.05	7.30 ± 0.09	7.46 ± 0.06
TC01000687.hg.1	OTTHUMG00000056480	6.56 ± 0.13	6.61 ± 0.13	6.58 ± 0.13	6.73 ± 0.11
TC15001085.hg.1	OTTHUMG00000056662	5.54 ± 0.10	5.36 ± 0.04	5.55 ± 0.07	5.54 ± 0.19
TC15001077.hg.1	OTTHUMG00000056663	5.97 ± 0.08	5.84 ± 0.06	5.90 ± 0.11	5.92 ± 0.08
TC15001086.hg.1	OTTHUMG00000056664	5.16 ± 0.17	5.03 ± 0.09	5.09 ± 0.01	5.12 ± 0.10
TC15000123.hg.1	OTTHUMG00000056666	5.65 ± 0.28	5.58 ± 0.07	5.55 ± 0.04	5.59 ± 0.18
TC20000667.hg.1	OTTHUMG00000057435	4.70 ± 0.10	4.60 ± 0.07	4.57 ± 0.11	4.64 ± 0.06
TC0X001531.hg.1	OTTHUMG00000057501	10.18 ± 0.10	10.14 ± 0.02	10.28 ± 0.08	10.15 ± 0.09
TC01000711.hg.1	OTTHUMG00000057525	8.93 ± 0.35	9.04 ± 0.06	8.96 ± 0.07	9.24 ± 0.11
TC0X000775.hg.1	OTTHUMG00000057529	7.49 ± 0.11	7.23 ± 0.23	7.33 ± 0.33	7.27 ± 0.16
TC6_cox_hap2000073.hg.1	OTTHUMG00000057531	7.19 ± 0.19	6.98 ± 0.16	7.04 ± 0.21	7.17 ± 0.09
TC6_cox_hap2000081.hg.1	OTTHUMG00000057537	8.26 ± 0.32	8.09 ± 0.08	8.17 ± 0.11	8.06 ± 0.04
TC11001264.hg.1	OTTHUMG00000057558	5.42 ± 0.20	5.40 ± 0.28	5.26 ± 0.15	5.34 ± 0.10
TC22000670.hg.1	OTTHUMG00000057562	7.06 ± 0.14	6.89 ± 0.04	6.93 ± 0.04	6.90 ± 0.08

TC06001874.hg.1	OTTHUMG00000058065	5.22 ± 0.17	5.36 ± 0.08	5.13 ± 0.08	5.48 ± 0.11
TC09001159.hg.1	OTTHUMG00000058148	6.41 ± 0.21	6.25 ± 0.07	6.39 ± 0.07	6.53 ± 0.15
TC09000271.hg.1	OTTHUMG00000058150	7.60 ± 0.26	7.67 ± 0.06	7.64 ± 0.08	7.83 ± 0.26
TC09001125.hg.1	OTTHUMG00000058152	6.95 ± 0.11	6.88 ± 0.03	7.00 ± 0.08	7.02 ± 0.26
TC09001103.hg.1	OTTHUMG00000058154	5.78 ± 0.21	5.41 ± 0.16	5.71 ± 0.27	5.50 ± 0.04
TC22000235.hg.1	OTTHUMG00000058207	5.01 ± 0.24	4.89 ± 0.14	4.85 ± 0.10	4.93 ± 0.07
TC22000247.hg.1	OTTHUMG00000058268	6.90 ± 0.25	6.87 ± 0.10	6.85 ± 0.12	6.99 ± 0.11
TC22000246.hg.1	OTTHUMG00000058269	6.31 ± 0.24	6.54 ± 0.28	6.47 ± 0.12	6.86 ± 0.27
TC22000679.hg.1	OTTHUMG00000058271	5.66 ± 0.21	5.53 ± 0.04	5.68 ± 0.13	5.48 ± 0.10
TC02000743.hg.1	OTTHUMG00000058513	5.90 ± 0.07	5.94 ± 0.02	5.84 ± 0.11	5.95 ± 0.07
TC02000744.hg.1	OTTHUMG00000058513	7.14 ± 0.18	7.31 ± 0.09	7.10 ± 0.21	7.30 ± 0.14
TC02000746.hg.1	OTTHUMG00000058519	4.81 ± 0.01	4.78 ± 0.07	4.82 ± 0.04	4.95 ± 0.23
TC02000747.hg.1	OTTHUMG00000058520	7.53 ± 0.34	7.68 ± 0.11	7.89 ± 0.13	7.77 ± 0.03
TC09001162.hg.1	OTTHUMG00000058662	6.03 ± 0.20	5.84 ± 0.10	6.16 ± 0.06	5.90 ± 0.26
TC09000263.hg.1	OTTHUMG00000058669	5.77 ± 0.26	5.70 ± 0.06	5.76 ± 0.06	5.95 ± 0.06
TC09000293.hg.1	OTTHUMG00000058670	7.02 ± 0.43	7.17 ± 0.20	6.93 ± 0.20	7.45 ± 0.28
TC09000287.hg.1	OTTHUMG00000058671	5.28 ± 0.21	5.14 ± 0.06	5.15 ± 0.16	5.28 ± 0.33
TC17001146.hg.1	OTTHUMG00000058769	5.47 ± 0.17	5.35 ± 0.05	5.46 ± 0.08	5.32 ± 0.11
TC17001148.hg.1	OTTHUMG00000058770	4.58 ± 0.16	4.60 ± 0.09	4.56 ± 0.00	4.68 ± 0.04
TC17001152.hg.1	OTTHUMG00000058802	5.54 ± 0.14	5.47 ± 0.09	5.52 ± 0.10	5.54 ± 0.14
TC17000169.hg.1	OTTHUMG00000058804	5.69 ± 0.10	5.48 ± 0.12	5.60 ± 0.19	5.45 ± 0.15
TC17000164.hg.1	OTTHUMG00000058809	7.99 ± 0.15	8.10 ± 0.02	8.06 ± 0.04	8.13 ± 0.08
TC17000167.hg.1	OTTHUMG00000058811	6.41 ± 0.20	6.40 ± 0.03	6.34 ± 0.06	6.50 ± 0.10
TC17000168.hg.1	OTTHUMG00000058813	3.88 ± 0.11	3.81 ± 0.04	3.90 ± 0.07	3.95 ± 0.09
TC02001325.hg.1	OTTHUMG00000058921	5.20 ± 0.17	5.16 ± 0.11	5.26 ± 0.15	5.25 ± 0.01
TC02002798.hg.1	OTTHUMG00000058923	11.03 ± 0.27	10.81 ± 0.03	10.94 ± 0.17	10.90 ± 0.06
TC17001160.hg.1	OTTHUMG00000058962	5.91 ± 0.41	5.61 ± 0.14	5.74 ± 0.07	5.65 ± 0.02
TC17001161.hg.1	OTTHUMG00000058964	5.16 ± 0.17	5.26 ± 0.08	5.29 ± 0.03	5.36 ± 0.11
TC02002883.hg.1	OTTHUMG00000059036	4.56 ± 0.35	4.52 ± 0.12	4.40 ± 0.13	4.70 ± 0.13
TC17000170.hg.1	OTTHUMG00000059072	5.08 ± 0.49	5.25 ± 0.24	4.96 ± 0.17	5.38 ± 0.20
TC02002888.hg.1	OTTHUMG00000059128	5.53 ± 0.05	5.22 ± 0.06	5.32 ± 0.09	5.24 ± 0.08

TC17001169.hg.1	OTTHUMG00000059147	7.40 ± 0.17	7.43 ± 0.23	7.38 ± 0.19	7.64 ± 0.11
TC17001167.hg.1	OTTHUMG00000059148	7.87 ± 0.19	7.82 ± 0.17	7.72 ± 0.21	7.90 ± 0.06
TC17001168.hg.1	OTTHUMG00000059149	4.56 ± 0.30	4.31 ± 0.07	4.32 ± 0.15	4.41 ± 0.04
TC02001424.hg.1	OTTHUMG00000059209	4.78 ± 0.14	4.97 ± 0.08	4.97 ± 0.12	4.98 ± 0.06
TC02002804.hg.1	OTTHUMG00000059236	7.05 ± 0.11	6.86 ± 0.09	6.97 ± 0.15	6.89 ± 0.07
TC17001182.hg.1	OTTHUMG00000059261	7.05 ± 0.33	7.10 ± 0.40	6.90 ± 0.37	7.36 ± 0.03
TC17000193.hg.1	OTTHUMG00000059263	7.78 ± 0.20	7.84 ± 0.25	7.75 ± 0.17	7.99 ± 0.12
TC02001336.hg.1	OTTHUMG00000059376	5.80 ± 0.17	5.84 ± 0.15	5.95 ± 0.10	5.95 ± 0.19
TC02002805.hg.1	OTTHUMG00000059377	6.11 ± 0.17	5.94 ± 0.08	5.96 ± 0.12	6.02 ± 0.03
TC17001228.hg.1	OTTHUMG00000059413	6.75 ± 0.18	6.69 ± 0.16	6.77 ± 0.19	6.79 ± 0.05
TC17000228.hg.1	OTTHUMG00000059428	7.49 ± 0.23	7.62 ± 0.21	7.42 ± 0.19	7.75 ± 0.06
TC04000595.hg.1	OTTHUMG00000059445	4.43 ± 0.08	4.39 ± 0.08	4.41 ± 0.07	4.42 ± 0.04
TC04000597.hg.1	OTTHUMG00000059447	4.61 ± 0.21	4.49 ± 0.04	4.51 ± 0.08	4.64 ± 0.11
TC04001492.hg.1	OTTHUMG00000059449	4.70 ± 0.18	4.48 ± 0.02	4.61 ± 0.04	4.58 ± 0.05
TC04000596.hg.1	OTTHUMG00000059450	5.35 ± 0.13	5.25 ± 0.01	5.24 ± 0.07	5.40 ± 0.14
TC05001375.hg.1	OTTHUMG00000059482	5.50 ± 0.10	5.36 ± 0.04	5.60 ± 0.09	5.38 ± 0.08
TC05001377.hg.1	OTTHUMG00000059483	5.69 ± 0.08	5.66 ± 0.06	5.75 ± 0.15	5.63 ± 0.05
TC05001378.hg.1	OTTHUMG00000059484	5.17 ± 0.16	5.15 ± 0.16	5.26 ± 0.14	5.39 ± 0.27
TC05001766.hg.1	OTTHUMG00000059494	8.14 ± 0.10	7.94 ± 0.03	8.42 ± 0.18	7.86 ± 0.07
TC05001768.hg.1	OTTHUMG00000059495	8.06 ± 0.17	8.47 ± 0.24	8.72 ± 0.68	8.37 ± 0.46
TC17001258.hg.1	OTTHUMG00000059551	7.57 ± 0.07	7.47 ± 0.15	7.52 ± 0.10	7.63 ± 0.09
TC05000246.hg.1	OTTHUMG00000059593	5.64 ± 0.10	5.65 ± 0.05	5.71 ± 0.11	5.74 ± 0.03
TC17000239.hg.1	OTTHUMG00000059602	4.64 ± 0.30	4.58 ± 0.02	4.52 ± 0.33	4.62 ± 0.06
TC17001244.hg.1	OTTHUMG00000059605	7.36 ± 0.13	7.43 ± 0.15	7.53 ± 0.13	7.59 ± 0.10
TC05001770.hg.1	OTTHUMG00000059612	5.41 ± 0.10	5.31 ± 0.03	5.32 ± 0.06	5.30 ± 0.08
TC05000627.hg.1	OTTHUMG00000059635	8.47 ± 0.25	8.44 ± 0.10	8.44 ± 0.19	8.51 ± 0.11
TC05000626.hg.1	OTTHUMG00000059638	7.12 ± 0.26	7.02 ± 0.32	6.98 ± 0.14	7.20 ± 0.20
TC05000625.hg.1	OTTHUMG00000059641	4.16 ± 0.05	4.12 ± 0.07	4.16 ± 0.13	4.18 ± 0.07
TC05001762.hg.1	OTTHUMG00000059642	7.36 ± 0.12	7.31 ± 0.28	7.42 ± 0.18	7.52 ± 0.14
TC05000781.hg.1	OTTHUMG00000059704	6.04 ± 0.12	6.02 ± 0.08	6.05 ± 0.07	6.01 ± 0.12
TC05000642.hg.1	OTTHUMG00000059736	5.09 ± 0.19	4.87 ± 0.11	4.97 ± 0.01	4.93 ± 0.08

TC12000308.hg.1	OTTHUMG00000059745	4.42 ± 0.10	4.24 ± 0.00	4.44 ± 0.22	4.29 ± 0.05
TC08000691.hg.1	OTTHUMG00000059818	5.66 ± 0.13	5.63 ± 0.06	5.62 ± 0.05	5.73 ± 0.08
TC16000287.hg.1	OTTHUMG00000060108	5.65 ± 0.23	5.54 ± 0.13	5.53 ± 0.10	5.63 ± 0.09
TC16000976.hg.1	OTTHUMG00000060136	5.25 ± 0.21	4.90 ± 0.03	5.10 ± 0.06	4.96 ± 0.10
TC18000234.hg.1	OTTHUMG00000060638	4.77 ± 0.24	4.58 ± 0.04	4.60 ± 0.07	4.58 ± 0.14
TC19000863.hg.1	OTTHUMG00000060779	7.61 ± 0.17	7.62 ± 0.10	7.70 ± 0.24	7.74 ± 0.09
TC19001827.hg.1	OTTHUMG00000060780	7.57 ± 0.19	7.50 ± 0.07	7.44 ± 0.11	7.58 ± 0.12
TC16000005.hg.1	OTTHUMG00000060826	6.42 ± 0.16	6.46 ± 0.03	6.48 ± 0.03	6.61 ± 0.03
TC19001828.hg.1	OTTHUMG00000060830	8.89 ± 0.17	8.44 ± 0.22	9.34 ± 0.26	8.50 ± 0.46
TC07000541.hg.1	OTTHUMG00000063284	5.15 ± 0.26	4.94 ± 0.03	4.98 ± 0.15	5.11 ± 0.13
TC07001589.hg.1	OTTHUMG00000063537	3.88 ± 0.11	3.73 ± 0.02	3.69 ± 0.13	3.77 ± 0.13
TC07000760.hg.1	OTTHUMG00000063970	5.40 ± 0.14	5.45 ± 0.11	5.41 ± 0.10	5.55 ± 0.10
TC07000174.hg.1	OTTHUMG00000064693	5.19 ± 0.09	5.22 ± 0.04	5.26 ± 0.13	5.30 ± 0.13
TC18000116.hg.1	OTTHUMG00000064910	4.04 ± 0.10	3.82 ± 0.05	4.07 ± 0.11	3.89 ± 0.04
TC21000404.hg.1	OTTHUMG00000064934	6.51 ± 0.18	6.60 ± 0.17	6.56 ± 0.15	6.70 ± 0.17
TC21000122.hg.1	OTTHUMG00000065034	5.37 ± 0.14	5.19 ± 0.05	5.38 ± 0.29	5.16 ± 0.09
TC21000124.hg.1	OTTHUMG00000065060	6.00 ± 0.15	6.00 ± 0.06	6.02 ± 0.04	5.98 ± 0.06
TC21000406.hg.1	OTTHUMG00000065062	6.83 ± 0.11	6.91 ± 0.28	6.84 ± 0.17	7.01 ± 0.15
TC21000126.hg.1	OTTHUMG00000065063	5.37 ± 0.08	5.34 ± 0.09	5.33 ± 0.29	5.41 ± 0.28
TC07001590.hg.1	OTTHUMG00000065234	5.53 ± 0.07	5.62 ± 0.20	5.32 ± 0.09	5.55 ± 0.22
TC07003387.hg.1	OTTHUMG00000065293	6.08 ± 0.45	6.04 ± 0.39	5.64 ± 0.26	6.20 ± 0.07
TC07000173.hg.1	OTTHUMG00000065294	5.07 ± 0.10	4.72 ± 0.04	4.88 ± 0.11	5.02 ± 0.20
TC07000716.hg.1	OTTHUMG00000065346	5.76 ± 0.12	5.70 ± 0.12	5.77 ± 0.02	5.86 ± 0.25
TC21000410.hg.1	OTTHUMG00000065530	7.42 ± 0.41	7.33 ± 0.09	7.35 ± 0.13	7.46 ± 0.12
TC19001845.hg.1	OTTHUMG00000065547	7.19 ± 0.35	7.01 ± 0.29	7.18 ± 0.27	7.14 ± 0.07
TC21000454.hg.1	OTTHUMG00000066032	5.79 ± 0.40	5.58 ± 0.15	5.57 ± 0.15	5.57 ± 0.11
TC21000457.hg.1	OTTHUMG00000066033	6.20 ± 0.16	6.22 ± 0.08	6.09 ± 0.19	6.33 ± 0.14
TC21000455.hg.1	OTTHUMG00000066035	6.81 ± 0.07	6.75 ± 0.20	6.89 ± 0.07	6.90 ± 0.08
TC19001834.hg.1	OTTHUMG00000066041	7.31 ± 0.10	7.31 ± 0.19	7.30 ± 0.11	7.47 ± 0.06
TC07003377.hg.1	OTTHUMG00000066049	5.65 ± 0.34	5.68 ± 0.02	5.65 ± 0.14	5.82 ± 0.16
TC07001785.hg.1	OTTHUMG00000066050	6.80 ± 0.29	6.80 ± 0.24	6.85 ± 0.05	6.96 ± 0.09

TC07001782.hg.1	OTTHUMG00000066051	5.06 ± 0.23	4.92 ± 0.07	4.94 ± 0.07	5.04 ± 0.13
TC07003378.hg.1	OTTHUMG00000066052	6.11 ± 0.18	6.10 ± 0.15	6.11 ± 0.05	6.23 ± 0.05
TC07001836.hg.1	OTTHUMG00000066283	6.39 ± 0.69	6.35 ± 0.33	6.37 ± 0.66	6.02 ± 0.21
TC07001837.hg.1	OTTHUMG00000066284	7.40 ± 0.86	7.70 ± 0.34	7.47 ± 0.63	7.58 ± 0.36
TC07000725.hg.1	OTTHUMG00000066355	6.32 ± 0.22	6.30 ± 0.20	6.32 ± 0.29	6.40 ± 0.07
TC07001796.hg.1	OTTHUMG00000066356	3.85 ± 0.13	3.70 ± 0.06	3.80 ± 0.19	3.76 ± 0.04
TC11001199.hg.1	OTTHUMG00000066362	6.52 ± 0.22	6.29 ± 0.10	6.31 ± 0.10	6.33 ± 0.05
TC11001202.hg.1	OTTHUMG00000066363	7.52 ± 0.42	7.40 ± 0.24	7.30 ± 0.20	7.55 ± 0.11
TC11000604.hg.1	OTTHUMG00000066367	6.39 ± 0.11	6.41 ± 0.10	6.32 ± 0.19	6.58 ± 0.18
TC11000602.hg.1	OTTHUMG00000066368	6.69 ± 0.18	6.78 ± 0.14	6.69 ± 0.21	6.97 ± 0.23
TC11002459.hg.1	OTTHUMG00000066439	5.76 ± 0.13	5.79 ± 0.08	5.74 ± 0.05	5.85 ± 0.11
TC11002458.hg.1	OTTHUMG00000066440	5.27 ± 0.11	5.14 ± 0.08	5.15 ± 0.05	5.25 ± 0.12
TC11000107.hg.1	OTTHUMG00000066679	6.86 ± 0.23	6.71 ± 0.16	6.79 ± 0.24	6.93 ± 0.07
TC11001045.hg.1	OTTHUMG00000066694	5.90 ± 0.12	5.94 ± 0.06	6.08 ± 0.20	5.89 ± 0.10
TC09000231.hg.1	OTTHUMG00000066698	6.70 ± 0.30	6.18 ± 0.08	7.00 ± 0.71	6.28 ± 0.26
TC09000243.hg.1	OTTHUMG00000066714	5.72 ± 0.16	5.51 ± 0.01	5.57 ± 0.17	5.70 ± 0.27
TC09001107.hg.1	OTTHUMG00000066715	6.26 ± 0.29	6.31 ± 0.10	6.27 ± 0.14	6.80 ± 0.26
TC09000236.hg.1	OTTHUMG00000066730	5.96 ± 0.20	5.64 ± 0.12	5.97 ± 0.46	5.66 ± 0.07
TC09000238.hg.1	OTTHUMG00000066733	6.40 ± 0.07	6.52 ± 0.11	6.64 ± 0.30	6.68 ± 0.22
TC09000237.hg.1	OTTHUMG00000066734	5.24 ± 0.31	5.38 ± 0.05	5.36 ± 0.15	5.41 ± 0.16
TC09001141.hg.1	OTTHUMG00000066812	7.00 ± 0.20	6.78 ± 0.05	7.18 ± 0.49	6.78 ± 0.28
TC09001094.hg.1	OTTHUMG00000067120	4.85 ± 0.15	4.64 ± 0.06	4.76 ± 0.14	4.66 ± 0.05
TC09001093.hg.1	OTTHUMG00000067122	5.96 ± 0.17	5.58 ± 0.14	5.93 ± 0.36	5.66 ± 0.05
TC09001096.hg.1	OTTHUMG00000067142	5.45 ± 0.12	5.42 ± 0.06	5.51 ± 0.03	5.43 ± 0.15
TC09001099.hg.1	OTTHUMG00000067148	5.98 ± 0.23	6.15 ± 0.09	6.15 ± 0.04	6.20 ± 0.11
TC09001098.hg.1	OTTHUMG00000067150	6.50 ± 0.06	6.59 ± 0.14	6.58 ± 0.13	6.77 ± 0.21
TC09000258.hg.1	OTTHUMG00000067215	7.83 ± 0.16	7.83 ± 0.19	7.65 ± 0.20	7.96 ± 0.11
TC09000256.hg.1	OTTHUMG00000067216	7.04 ± 0.14	6.94 ± 0.04	6.91 ± 0.16	7.00 ± 0.07
TC09001140.hg.1	OTTHUMG00000067237	6.40 ± 0.37	6.36 ± 0.09	6.31 ± 0.24	6.56 ± 0.40
TC09001144.hg.1	OTTHUMG00000067240	6.14 ± 0.20	6.03 ± 0.16	6.06 ± 0.07	6.19 ± 0.15
TC0X000813.hg.1	OTTHUMG00000067411	8.41 ± 0.10	8.24 ± 0.06	8.11 ± 0.13	8.28 ± 0.06

TC0X000817.hg.1	OTTHUMG00000067420	7.69 ± 0.30	7.40 ± 0.15	7.18 ± 0.27	7.48 ± 0.15
TC14000683.hg.1	OTTHUMG00000067435	5.16 ± 0.05	5.09 ± 0.03	5.11 ± 0.00	5.10 ± 0.05
TC09001314.hg.1	OTTHUMG00000067507	6.67 ± 0.23	6.60 ± 0.26	6.51 ± 0.18	6.67 ± 0.26
TC09000420.hg.1	OTTHUMG00000067508	6.86 ± 0.17	6.91 ± 0.22	6.76 ± 0.23	7.05 ± 0.16
TC20000230.hg.1	OTTHUMG00000067728	4.05 ± 0.18	4.03 ± 0.09	4.01 ± 0.12	3.96 ± 0.01
TC06000570.hg.1	OTTHUMG00000067927	6.85 ± 0.29	7.00 ± 0.21	6.82 ± 0.27	7.12 ± 0.12
TC01001207.hg.1	OTTHUMG00000073673	6.36 ± 0.12	6.25 ± 0.13	6.17 ± 0.20	6.04 ± 0.23
TC22000242.hg.1	OTTHUMG00000073688	4.50 ± 0.14	4.36 ± 0.06	4.42 ± 0.25	4.30 ± 0.05
TC18000369.hg.1	OTTHUMG00000073721	7.61 ± 0.33	7.32 ± 0.06	7.35 ± 0.20	7.32 ± 0.15
TC18001007.hg.1	OTTHUMG00000073723	6.73 ± 0.45	6.76 ± 0.30	6.65 ± 0.36	6.86 ± 0.09
TC15000447.hg.1	OTTHUMG00000074032	4.22 ± 0.07	4.17 ± 0.16	4.17 ± 0.03	4.11 ± 0.11
TC01003449.hg.1	OTTHUMG00000074100	7.77 ± 0.12	7.76 ± 0.11	7.71 ± 0.19	7.95 ± 0.20
TC21000089.hg.1	OTTHUMG00000074159	6.74 ± 0.04	6.66 ± 0.04	6.71 ± 0.02	6.75 ± 0.06
TC21000274.hg.1	OTTHUMG00000074167	3.79 ± 0.14	3.75 ± 0.05	3.71 ± 0.06	3.90 ± 0.27
TC21000021.hg.1	OTTHUMG00000074200	5.18 ± 0.16	5.17 ± 0.14	5.04 ± 0.08	5.15 ± 0.15
TC21000015.hg.1	OTTHUMG00000074207	3.61 ± 0.13	3.53 ± 0.09	3.55 ± 0.03	3.52 ± 0.12
TC21000023.hg.1	OTTHUMG00000074260	6.69 ± 0.28	6.65 ± 0.05	6.57 ± 0.13	6.72 ± 0.09
TC21000286.hg.1	OTTHUMG00000074314	5.17 ± 0.02	5.11 ± 0.20	5.17 ± 0.15	5.27 ± 0.10
TC21000031.hg.1	OTTHUMG00000074318	5.69 ± 0.22	5.66 ± 0.14	5.66 ± 0.08	5.61 ± 0.11
TC21000030.hg.1	OTTHUMG00000074319	5.14 ± 0.21	5.27 ± 0.17	5.23 ± 0.12	5.37 ± 0.14
TC21000287.hg.1	OTTHUMG00000074322	6.11 ± 0.15	6.02 ± 0.14	6.10 ± 0.04	6.16 ± 0.09
TC21000032.hg.1	OTTHUMG00000074325	4.14 ± 0.10	4.08 ± 0.13	4.25 ± 0.14	4.03 ± 0.06
TC21000033.hg.1	OTTHUMG00000074330	4.89 ± 0.02	4.84 ± 0.03	4.90 ± 0.15	5.04 ± 0.12
TC21000035.hg.1	OTTHUMG00000074332	5.01 ± 0.25	5.07 ± 0.05	5.03 ± 0.14	5.06 ± 0.17
TC21000289.hg.1	OTTHUMG00000074333	3.87 ± 0.08	3.80 ± 0.03	3.89 ± 0.09	3.89 ± 0.05
TC21000294.hg.1	OTTHUMG00000074394	5.08 ± 0.06	5.00 ± 0.04	5.01 ± 0.05	5.10 ± 0.11
TC21000295.hg.1	OTTHUMG00000074395	4.43 ± 0.31	4.21 ± 0.07	4.26 ± 0.11	4.29 ± 0.02
TC21000044.hg.1	OTTHUMG00000074396	4.13 ± 0.15	3.87 ± 0.07	4.00 ± 0.02	4.08 ± 0.15
TC21000300.hg.1	OTTHUMG00000074507	4.54 ± 0.24	4.20 ± 0.08	4.33 ± 0.03	4.23 ± 0.08
TC21000304.hg.1	OTTHUMG00000074517	4.05 ± 0.24	3.77 ± 0.04	3.81 ± 0.12	3.84 ± 0.10
TC21000055.hg.1	OTTHUMG00000074542	5.04 ± 0.16	4.94 ± 0.01	5.07 ± 0.03	5.01 ± 0.14

TC21000053.hg.1	OTTHUMG00000074543	5.03 ± 0.18	4.90 ± 0.18	4.93 ± 0.16	5.01 ± 0.12
TC21000307.hg.1	OTTHUMG00000074544	6.85 ± 0.07	6.76 ± 0.07	6.83 ± 0.05	6.90 ± 0.16
TC21000306.hg.1	OTTHUMG00000074548	3.96 ± 0.15	3.79 ± 0.03	3.81 ± 0.02	3.83 ± 0.16
TC21000308.hg.1	OTTHUMG00000074549	4.47 ± 0.12	4.34 ± 0.03	4.41 ± 0.07	4.45 ± 0.19
TC21000056.hg.1	OTTHUMG00000074550	5.86 ± 0.22	5.63 ± 0.08	5.76 ± 0.30	5.80 ± 0.22
TC21000057.hg.1	OTTHUMG00000074551	4.27 ± 0.08	4.15 ± 0.03	4.17 ± 0.24	4.18 ± 0.16
TC21000311.hg.1	OTTHUMG00000074606	5.21 ± 0.19	5.15 ± 0.12	5.38 ± 0.21	5.34 ± 0.29
TC21000310.hg.1	OTTHUMG00000074607	4.27 ± 0.09	4.17 ± 0.12	4.12 ± 0.08	4.13 ± 0.07
TC01002219.hg.1	OTTHUMG00000074727	6.49 ± 0.25	6.46 ± 0.02	6.52 ± 0.03	6.76 ± 0.11
TC01006428.hg.1	OTTHUMG00000074805	11.96 ± 0.28	12.07 ± 0.09	12.27 ± 0.14	12.10 ± 0.05
TC01001100.hg.1	OTTHUMG00000078079	5.17 ± 0.03	5.08 ± 0.11	5.11 ± 0.20	4.94 ± 0.15
TC21000314.hg.1	OTTHUMG00000078120	4.08 ± 0.09	3.81 ± 0.04	3.86 ± 0.13	3.84 ± 0.08
TC21000061.hg.1	OTTHUMG00000078122	4.84 ± 0.09	4.63 ± 0.10	4.58 ± 0.13	4.61 ± 0.01
TC21000316.hg.1	OTTHUMG00000078124	6.23 ± 0.22	6.26 ± 0.03	6.23 ± 0.08	6.41 ± 0.18
TC21000062.hg.1	OTTHUMG00000078125	5.84 ± 0.30	5.86 ± 0.05	5.87 ± 0.00	5.95 ± 0.07
TC21000319.hg.1	OTTHUMG00000078243	5.23 ± 0.13	5.06 ± 0.03	5.14 ± 0.03	5.20 ± 0.11
TC21000068.hg.1	OTTHUMG00000078245	6.84 ± 0.33	6.74 ± 0.04	6.71 ± 0.21	6.85 ± 0.16
TC21000321.hg.1	OTTHUMG00000078247	4.23 ± 0.14	4.09 ± 0.05	4.11 ± 0.07	4.10 ± 0.11
TC21000066.hg.1	OTTHUMG00000078249	4.79 ± 0.07	4.92 ± 0.10	4.76 ± 0.20	4.89 ± 0.02
TC21000067.hg.1	OTTHUMG00000078253	6.56 ± 0.16	6.74 ± 0.17	6.68 ± 0.08	6.90 ± 0.14
TC21000318.hg.1	OTTHUMG00000078257	5.21 ± 0.29	5.14 ± 0.03	5.10 ± 0.04	5.21 ± 0.18
TC21000069.hg.1	OTTHUMG00000078273	4.15 ± 0.23	4.21 ± 0.03	4.06 ± 0.20	4.18 ± 0.10
TC21000325.hg.1	OTTHUMG00000078274	4.85 ± 0.17	4.63 ± 0.13	4.64 ± 0.07	4.70 ± 0.19
TC21000326.hg.1	OTTHUMG00000078275	5.34 ± 0.21	5.37 ± 0.05	5.31 ± 0.08	5.57 ± 0.16
TC21000330.hg.1	OTTHUMG00000078324	5.44 ± 0.20	5.36 ± 0.06	5.34 ± 0.19	5.48 ± 0.08
TC21000331.hg.1	OTTHUMG00000078325	5.51 ± 0.12	5.44 ± 0.05	5.39 ± 0.03	5.52 ± 0.14
TC21000070.hg.1	OTTHUMG00000078326	4.53 ± 0.12	4.48 ± 0.13	4.55 ± 0.04	4.49 ± 0.14
TC21000073.hg.1	OTTHUMG00000078327	4.42 ± 0.18	4.01 ± 0.05	4.22 ± 0.17	4.05 ± 0.04
TC21000329.hg.1	OTTHUMG00000078329	6.09 ± 0.29	6.15 ± 0.18	6.17 ± 0.04	6.40 ± 0.22
TC21000072.hg.1	OTTHUMG00000078330	5.59 ± 0.21	5.50 ± 0.05	5.52 ± 0.16	5.55 ± 0.03
TC21000328.hg.1	OTTHUMG00000078332	5.18 ± 0.07	4.94 ± 0.07	5.09 ± 0.08	5.03 ± 0.04

TC21000327.hg.1	OTTHUMG00000078333	5.12 ± 0.14	5.11 ± 0.19	5.21 ± 0.04	5.40 ± 0.17
TC21000335.hg.1	OTTHUMG00000078370	4.50 ± 0.16	4.42 ± 0.06	4.49 ± 0.19	4.60 ± 0.20
TC21000080.hg.1	OTTHUMG00000078481	5.32 ± 0.16	5.16 ± 0.03	5.18 ± 0.08	5.21 ± 0.17
TC21000343.hg.1	OTTHUMG00000078482	5.20 ± 0.25	5.21 ± 0.03	5.20 ± 0.02	5.24 ± 0.11
TC21000079.hg.1	OTTHUMG00000078483	7.28 ± 0.08	7.30 ± 0.10	7.20 ± 0.25	7.31 ± 0.05
TC21000083.hg.1	OTTHUMG00000078660	5.00 ± 0.11	4.88 ± 0.07	4.95 ± 0.05	4.99 ± 0.12
TC21000082.hg.1	OTTHUMG00000078661	5.79 ± 0.13	5.62 ± 0.10	5.67 ± 0.05	5.77 ± 0.12
TC21000084.hg.1	OTTHUMG00000078687	5.20 ± 0.24	5.37 ± 0.07	5.36 ± 0.20	5.51 ± 0.05
TC21000086.hg.1	OTTHUMG00000078698	5.01 ± 0.26	4.73 ± 0.07	4.83 ± 0.06	4.78 ± 0.02
TC21000347.hg.1	OTTHUMG00000078699	4.81 ± 0.12	4.67 ± 0.04	4.70 ± 0.09	4.72 ± 0.18
TC21000348.hg.1	OTTHUMG00000078706	6.39 ± 0.19	6.27 ± 0.12	6.22 ± 0.16	6.38 ± 0.09
TC21000087.hg.1	OTTHUMG00000078707	5.28 ± 0.17	5.14 ± 0.09	5.19 ± 0.09	5.03 ± 0.23
TC21000349.hg.1	OTTHUMG00000078747	6.23 ± 0.21	6.13 ± 0.12	6.00 ± 0.09	6.15 ± 0.09
TC21000290.hg.1	OTTHUMG00000078773	5.34 ± 0.11	5.12 ± 0.17	5.22 ± 0.02	5.31 ± 0.09
TC21000354.hg.1	OTTHUMG00000078807	4.34 ± 0.09	4.30 ± 0.11	4.33 ± 0.02	4.29 ± 0.05
TC21000357.hg.1	OTTHUMG00000078877	4.97 ± 0.13	4.80 ± 0.11	4.87 ± 0.07	4.79 ± 0.04
TC21000390.hg.1	OTTHUMG00000084879	7.03 ± 0.37	7.00 ± 0.12	6.98 ± 0.20	7.18 ± 0.12
TC06001469.hg.1	OTTHUMG00000084883	6.79 ± 0.27	6.76 ± 0.03	6.68 ± 0.14	6.83 ± 0.10
TC06000313.hg.1	OTTHUMG00000085065	5.71 ± 0.38	5.49 ± 0.08	5.58 ± 0.12	5.55 ± 0.10
TC6_cox_hap2000113.hg.1	OTTHUMG00000085278	6.79 ± 0.27	6.76 ± 0.03	6.68 ± 0.14	6.83 ± 0.10
TC21000396.hg.1	OTTHUMG00000085304	6.86 ± 0.03	6.85 ± 0.08	6.78 ± 0.16	6.89 ± 0.09
TC21000392.hg.1	OTTHUMG00000085305	7.11 ± 0.30	7.22 ± 0.19	7.08 ± 0.32	7.38 ± 0.06
TC01003067.hg.1	OTTHUMG00000085417	5.11 ± 0.28	4.95 ± 0.06	4.87 ± 0.12	5.35 ± 0.47
TC6_cox_hap2000010.hg.1	OTTHUMG00000085770	5.71 ± 0.38	5.49 ± 0.08	5.58 ± 0.12	5.55 ± 0.10
TC6_cox_hap2000008.hg.1	OTTHUMG00000085771	6.20 ± 0.30	6.14 ± 0.15	6.22 ± 0.24	6.23 ± 0.20
TC6_ssto_hap7000095.hg.1	OTTHUMG00000085910	6.79 ± 0.27	6.76 ± 0.03	6.68 ± 0.14	6.83 ± 0.10
TC06000311.hg.1	OTTHUMG00000085915	6.20 ± 0.30	6.14 ± 0.15	6.22 ± 0.24	6.23 ± 0.20
TC6_ssto_hap7000008.hg.1	OTTHUMG00000085948	5.71 ± 0.38	5.49 ± 0.08	5.58 ± 0.12	5.55 ± 0.10
TC6_ssto_hap7000006.hg.1	OTTHUMG00000085951	6.20 ± 0.30	6.14 ± 0.15	6.22 ± 0.24	6.23 ± 0.20
TC21000414.hg.1	OTTHUMG00000086192	8.30 ± 0.06	8.34 ± 0.23	8.32 ± 0.11	8.47 ± 0.13
TC21000142.hg.1	OTTHUMG00000086303	6.80 ± 0.15	6.76 ± 0.05	6.75 ± 0.13	6.95 ± 0.06

TC21000426.hg.1	OTTHUMG00000086462	5.09 ± 0.09	4.99 ± 0.06	4.90 ± 0.08	4.98 ± 0.05
TC06000318.hg.1	OTTHUMG00000086581	6.49 ± 0.12	6.41 ± 0.14	6.39 ± 0.05	6.46 ± 0.07
TC21000156.hg.1	OTTHUMG00000086611	5.86 ± 0.06	5.79 ± 0.08	5.79 ± 0.12	5.94 ± 0.10
TC21000149.hg.1	OTTHUMG00000086614	5.46 ± 0.14	5.33 ± 0.04	5.43 ± 0.09	5.29 ± 0.13
TC21000146.hg.1	OTTHUMG00000086615	7.14 ± 0.22	6.89 ± 0.11	6.89 ± 0.18	7.19 ± 0.34
TC21000159.hg.1	OTTHUMG00000086635	8.77 ± 0.19	8.66 ± 0.06	8.84 ± 0.17	8.71 ± 0.06
TC21000435.hg.1	OTTHUMG00000086642	8.79 ± 0.32	8.78 ± 0.25	8.56 ± 0.32	8.86 ± 0.09
TC21000155.hg.1	OTTHUMG00000086643	7.43 ± 0.37	7.59 ± 0.25	7.39 ± 0.20	7.77 ± 0.21
TC21000434.hg.1	OTTHUMG00000086644	5.20 ± 0.22	5.06 ± 0.33	5.10 ± 0.23	5.18 ± 0.06
TC21000453.hg.1	OTTHUMG00000086699	7.30 ± 0.37	7.38 ± 0.24	7.21 ± 0.27	7.52 ± 0.10
TC21000170.hg.1	OTTHUMG00000086700	5.39 ± 0.11	5.41 ± 0.20	5.45 ± 0.13	5.71 ± 0.28
TC21000171.hg.1	OTTHUMG00000086701	5.11 ± 0.15	5.17 ± 0.09	5.22 ± 0.16	5.27 ± 0.06
TC21000456.hg.1	OTTHUMG00000086702	5.87 ± 0.26	5.86 ± 0.22	5.91 ± 0.20	5.97 ± 0.05
TC21000178.hg.1	OTTHUMG00000086723	5.94 ± 0.16	5.87 ± 0.07	5.86 ± 0.03	5.91 ± 0.05
TC21000470.hg.1	OTTHUMG00000086754	6.75 ± 0.13	6.51 ± 0.18	6.78 ± 0.11	6.56 ± 0.05
TC21000472.hg.1	OTTHUMG00000086761	7.11 ± 0.35	7.06 ± 0.18	7.22 ± 0.18	7.15 ± 0.04
TC21000473.hg.1	OTTHUMG00000086763	6.65 ± 0.22	6.62 ± 0.11	6.66 ± 0.10	6.81 ± 0.21
TC21000193.hg.1	OTTHUMG00000086771	7.41 ± 0.29	7.48 ± 0.32	7.42 ± 0.18	7.68 ± 0.07
TC21000194.hg.1	OTTHUMG00000086778	8.04 ± 0.14	7.99 ± 0.19	7.83 ± 0.17	8.07 ± 0.05
TC21000478.hg.1	OTTHUMG00000086780	6.88 ± 0.18	6.90 ± 0.13	6.85 ± 0.03	7.16 ± 0.18
TC21000203.hg.1	OTTHUMG00000086812	4.51 ± 0.14	4.30 ± 0.09	4.32 ± 0.11	4.30 ± 0.05
TC21000491.hg.1	OTTHUMG00000086813	7.45 ± 0.26	7.54 ± 0.15	7.31 ± 0.24	7.68 ± 0.30
TC21000492.hg.1	OTTHUMG00000086814	7.23 ± 0.15	7.15 ± 0.11	7.19 ± 0.09	7.29 ± 0.21
TC21000490.hg.1	OTTHUMG00000086815	7.64 ± 0.19	7.57 ± 0.19	7.60 ± 0.08	7.74 ± 0.07
TC21000495.hg.1	OTTHUMG00000086824	8.23 ± 0.16	8.25 ± 0.14	8.07 ± 0.27	8.33 ± 0.03
TC21000494.hg.1	OTTHUMG00000086827	7.62 ± 0.14	7.67 ± 0.13	7.75 ± 0.20	7.66 ± 0.03
TC21000499.hg.1	OTTHUMG00000086837	7.36 ± 0.15	7.43 ± 0.06	7.48 ± 0.10	7.49 ± 0.09
TC21000502.hg.1	OTTHUMG00000086845	7.49 ± 0.14	7.60 ± 0.26	7.36 ± 0.12	7.71 ± 0.05
TC6_cox_hap2000015.hg.1	OTTHUMG00000086849	6.49 ± 0.12	6.41 ± 0.14	6.39 ± 0.05	6.46 ± 0.07
TC21000505.hg.1	OTTHUMG00000086865	8.00 ± 0.11	8.13 ± 0.11	8.00 ± 0.17	8.14 ± 0.07
TC21000210.hg.1	OTTHUMG00000086871	7.61 ± 0.20	7.72 ± 0.01	7.78 ± 0.17	7.70 ± 0.13

TC21000219.hg.1	OTTHUMG00000086911	7.75 ± 0.16	7.73 ± 0.18	7.61 ± 0.08	7.92 ± 0.08
TC21000218.hg.1	OTTHUMG00000086912	7.89 ± 0.30	8.03 ± 0.06	7.73 ± 0.18	8.03 ± 0.05
TC21000514.hg.1	OTTHUMG00000086913	8.46 ± 0.15	8.58 ± 0.13	8.46 ± 0.10	8.63 ± 0.04
TC21000515.hg.1	OTTHUMG00000086915	6.44 ± 0.11	6.34 ± 0.07	6.25 ± 0.27	6.42 ± 0.10
TC21000220.hg.1	OTTHUMG00000086917	7.77 ± 0.14	7.75 ± 0.21	7.75 ± 0.17	7.81 ± 0.07
TC21000223.hg.1	OTTHUMG00000086919	8.25 ± 0.24	8.24 ± 0.07	8.20 ± 0.31	8.42 ± 0.03
TC21000224.hg.1	OTTHUMG00000086923	8.10 ± 0.22	8.06 ± 0.24	7.91 ± 0.20	8.16 ± 0.08
TC21000227.hg.1	OTTHUMG00000086925	6.09 ± 0.11	6.03 ± 0.16	6.02 ± 0.04	6.07 ± 0.08
TC21000238.hg.1	OTTHUMG00000090255	6.96 ± 0.22	6.91 ± 0.15	6.90 ± 0.21	7.13 ± 0.17
TC21000244.hg.1	OTTHUMG00000090402	6.97 ± 0.10	7.15 ± 0.22	7.01 ± 0.16	7.32 ± 0.08
TC21000246.hg.1	OTTHUMG00000090408	7.09 ± 0.21	7.08 ± 0.07	7.04 ± 0.16	7.21 ± 0.22
TC21000551.hg.1	OTTHUMG00000090438	6.99 ± 0.39	7.06 ± 0.28	6.92 ± 0.24	7.19 ± 0.07
TC21000252.hg.1	OTTHUMG00000090439	6.23 ± 0.11	6.12 ± 0.10	6.28 ± 0.16	6.19 ± 0.07
TC21000251.hg.1	OTTHUMG00000090442	8.57 ± 0.12	8.64 ± 0.33	8.65 ± 0.16	8.95 ± 0.11
TC21000250.hg.1	OTTHUMG00000090443	7.81 ± 0.27	7.84 ± 0.37	7.84 ± 0.26	8.01 ± 0.01
TC21000448.hg.1	OTTHUMG00000090613	7.07 ± 0.18	7.00 ± 0.21	7.80 ± 0.27	7.03 ± 0.10
TC21000258.hg.1	OTTHUMG00000090628	6.59 ± 0.09	6.54 ± 0.09	6.53 ± 0.12	6.61 ± 0.05
TC21000256.hg.1	OTTHUMG00000090629	5.97 ± 0.18	5.85 ± 0.20	5.91 ± 0.20	6.09 ± 0.12
TC21000449.hg.1	OTTHUMG00000090768	5.73 ± 0.09	5.66 ± 0.04	5.86 ± 0.02	5.78 ± 0.14
TC04001168.hg.1	OTTHUMG00000099437	5.15 ± 0.25	4.87 ± 0.03	4.95 ± 0.06	4.82 ± 0.12
TC02001564.hg.1	OTTHUMG00000119005	6.16 ± 0.09	6.11 ± 0.11	6.12 ± 0.05	6.12 ± 0.09
TC04001019.hg.1	OTTHUMG00000128484	6.67 ± 0.23	6.56 ± 0.21	6.68 ± 0.12	6.64 ± 0.07
TC15000129.hg.1	OTTHUMG00000128937	6.67 ± 0.22	6.83 ± 0.10	6.78 ± 0.19	7.06 ± 0.16
TC6_ssto_hap7000013.hg.1	OTTHUMG00000129094	6.48 ± 0.05	6.39 ± 0.13	6.41 ± 0.04	6.49 ± 0.13
TC20000020.hg.1	OTTHUMG00000129569	7.11 ± 0.23	7.14 ± 0.16	7.08 ± 0.20	7.26 ± 0.13
TC04000429.hg.1	OTTHUMG00000130102	5.90 ± 0.25	5.90 ± 0.14	5.91 ± 0.18	6.07 ± 0.09
TC17000294.hg.1	OTTHUMG00000130762	6.95 ± 0.17	6.95 ± 0.05	6.83 ± 0.14	7.03 ± 0.05
TC17000296.hg.1	OTTHUMG00000130765	6.25 ± 0.12	6.10 ± 0.05	6.25 ± 0.21	6.25 ± 0.06
TC6_cox_hap2000031.hg.1	OTTHUMG00000131063	6.83 ± 0.29	6.76 ± 0.09	6.81 ± 0.17	6.78 ± 0.07
TC04002925.hg.1	OTTHUMG00000131122	6.38 ± 0.18	6.37 ± 0.02	6.35 ± 0.04	6.40 ± 0.07
TC6_ssto_hap7000024.hg.1	OTTHUMG00000131239	6.83 ± 0.29	6.76 ± 0.09	6.81 ± 0.17	6.78 ± 0.07

TC17001719.hg.1	OTTHUMG00000132063	6.94 ± 0.31	6.99 ± 0.41	6.83 ± 0.28	7.09 ± 0.06
TC17000717.hg.1	OTTHUMG00000132079	5.12 ± 0.21	5.08 ± 0.05	5.20 ± 0.09	5.05 ± 0.12
TC17000621.hg.1	OTTHUMG00000132087	5.56 ± 0.41	5.65 ± 0.29	5.43 ± 0.39	5.31 ± 0.03
TC17000964.hg.1	OTTHUMG00000132132	7.49 ± 0.07	7.19 ± 0.06	7.40 ± 0.24	7.24 ± 0.12
TC17000927.hg.1	OTTHUMG00000132137	8.24 ± 0.13	8.14 ± 0.12	8.20 ± 0.12	8.19 ± 0.02
TC17000009.hg.1	OTTHUMG00000132154	5.95 ± 0.20	5.79 ± 0.23	5.80 ± 0.24	5.92 ± 0.05
TC17000017.hg.1	OTTHUMG00000132193	7.50 ± 0.13	7.42 ± 0.04	7.51 ± 0.07	7.42 ± 0.01
TC17000989.hg.1	OTTHUMG00000132217	6.23 ± 0.18	6.37 ± 0.01	6.16 ± 0.18	6.42 ± 0.06
TC17000021.hg.1	OTTHUMG00000132218	6.45 ± 0.17	6.54 ± 0.14	6.36 ± 0.14	6.68 ± 0.13
TC17000020.hg.1	OTTHUMG00000132219	6.50 ± 0.33	6.42 ± 0.15	6.31 ± 0.26	6.51 ± 0.08
TC17000818.hg.1	OTTHUMG00000132235	5.35 ± 0.13	5.22 ± 0.12	5.20 ± 0.07	5.28 ± 0.12
TC17001836.hg.1	OTTHUMG00000132237	4.93 ± 0.20	4.80 ± 0.12	4.87 ± 0.15	4.86 ± 0.07
TC17000815.hg.1	OTTHUMG00000132240	4.55 ± 0.16	4.39 ± 0.10	4.52 ± 0.08	4.35 ± 0.09
TC17001832.hg.1	OTTHUMG00000132246	5.31 ± 0.21	5.27 ± 0.08	5.27 ± 0.03	5.35 ± 0.12
TC17000809.hg.1	OTTHUMG00000132247	5.42 ± 0.26	5.35 ± 0.15	5.31 ± 0.12	5.46 ± 0.07
TC17000808.hg.1	OTTHUMG00000132249	5.71 ± 0.25	5.57 ± 0.09	5.69 ± 0.12	5.70 ± 0.15
TC17000786.hg.1	OTTHUMG00000132305	7.68 ± 0.27	7.73 ± 0.08	7.61 ± 0.24	7.88 ± 0.16
TC17001780.hg.1	OTTHUMG00000132315	5.96 ± 0.20	5.89 ± 0.07	6.01 ± 0.07	6.06 ± 0.17
TC17001779.hg.1	OTTHUMG00000132317	5.47 ± 0.09	5.43 ± 0.05	5.36 ± 0.04	5.42 ± 0.06
TC17000686.hg.1	OTTHUMG00000132354	5.49 ± 0.08	5.60 ± 0.19	5.55 ± 0.06	5.75 ± 0.10
TC17000656.hg.1	OTTHUMG00000132433	7.31 ± 0.10	7.19 ± 0.16	7.16 ± 0.23	7.31 ± 0.04
TC17000635.hg.1	OTTHUMG00000132443	7.82 ± 0.15	7.67 ± 0.12	7.68 ± 0.07	7.66 ± 0.07
TC17000585.hg.1	OTTHUMG00000132554	6.89 ± 0.27	7.01 ± 0.10	6.87 ± 0.14	7.15 ± 0.12
TC17001593.hg.1	OTTHUMG00000132571	8.42 ± 0.44	8.04 ± 0.42	8.45 ± 0.65	8.13 ± 0.25
TC17001578.hg.1	OTTHUMG00000132629	7.56 ± 0.27	7.53 ± 0.17	7.47 ± 0.21	7.64 ± 0.03
TC17000901.hg.1	OTTHUMG00000132645	7.72 ± 0.29	7.60 ± 0.10	7.79 ± 0.18	7.72 ± 0.08
TC17001530.hg.1	OTTHUMG00000132657	7.19 ± 0.61	7.46 ± 0.31	7.77 ± 0.57	7.64 ± 0.04
TC17000476.hg.1	OTTHUMG00000132690	6.73 ± 0.30	6.74 ± 0.25	6.67 ± 0.21	6.81 ± 0.12
TC17001425.hg.1	OTTHUMG00000132691	7.60 ± 0.09	7.58 ± 0.15	7.66 ± 0.14	7.70 ± 0.05
TC17001411.hg.1	OTTHUMG00000132706	6.21 ± 0.18	6.09 ± 0.07	6.17 ± 0.09	6.22 ± 0.19
TC17000407.hg.1	OTTHUMG00000132736	6.40 ± 0.07	6.65 ± 0.11	6.63 ± 0.16	6.74 ± 0.18

TC17000403.hg.1	OTTHUMG00000132737	5.27 ± 0.04	5.24 ± 0.08	5.22 ± 0.06	5.38 ± 0.07
TC17000388.hg.1	OTTHUMG00000132738	6.78 ± 0.10	6.84 ± 0.23	6.83 ± 0.06	6.97 ± 0.17
TC06000339.hg.1	OTTHUMG00000132773	6.86 ± 0.10	6.83 ± 0.14	6.80 ± 0.04	6.76 ± 0.13
TC17000378.hg.1	OTTHUMG00000132777	6.48 ± 0.28	6.51 ± 0.21	6.37 ± 0.19	6.63 ± 0.07
TC17001348.hg.1	OTTHUMG00000132778	7.94 ± 0.34	7.60 ± 0.24	7.86 ± 0.25	7.70 ± 0.09
TC17001338.hg.1	OTTHUMG00000132799	7.43 ± 0.31	7.15 ± 0.10	7.37 ± 0.23	7.21 ± 0.10
TC17001335.hg.1	OTTHUMG00000132806	7.31 ± 0.32	7.36 ± 0.19	7.36 ± 0.20	7.53 ± 0.09
TC17000336.hg.1	OTTHUMG00000132826	7.23 ± 0.08	7.14 ± 0.10	7.27 ± 0.07	7.24 ± 0.13
TC17000144.hg.1	OTTHUMG00000132850	7.10 ± 0.37	6.96 ± 0.37	6.93 ± 0.42	7.17 ± 0.01
TC17001113.hg.1	OTTHUMG00000132865	6.88 ± 0.27	6.90 ± 0.12	6.89 ± 0.07	6.98 ± 0.05
TC17000126.hg.1	OTTHUMG00000132872	7.43 ± 0.27	7.19 ± 0.15	7.28 ± 0.35	7.30 ± 0.10
TC17001090.hg.1	OTTHUMG00000132895	6.32 ± 0.26	6.30 ± 0.07	6.22 ± 0.16	6.27 ± 0.06
TC17000062.hg.1	OTTHUMG00000132913	6.48 ± 0.09	6.44 ± 0.26	6.33 ± 0.18	6.30 ± 0.04
TC17001041.hg.1	OTTHUMG00000132924	7.02 ± 0.10	6.89 ± 0.10	6.84 ± 0.08	6.90 ± 0.07
TC17001022.hg.1	OTTHUMG00000132937	7.80 ± 0.36	7.80 ± 0.35	7.74 ± 0.21	7.96 ± 0.07
TC17000045.hg.1	OTTHUMG00000132939	5.85 ± 0.07	5.81 ± 0.08	5.97 ± 0.10	5.79 ± 0.06
TC6_cox_hap2000037.hg.1	OTTHUMG00000132955	7.05 ± 0.19	7.05 ± 0.15	7.09 ± 0.10	6.98 ± 0.13
TC6_ssto_hap7000030.hg.1	OTTHUMG00000133032	7.05 ± 0.19	7.05 ± 0.15	7.09 ± 0.10	6.98 ± 0.13
TC20000158.hg.1	OTTHUMG00000133141	6.42 ± 0.20	6.38 ± 0.02	6.36 ± 0.08	6.48 ± 0.25
TC02001461.hg.1	OTTHUMG00000133345	6.90 ± 0.35	6.77 ± 0.19	6.62 ± 0.16	6.84 ± 0.12
TC02001492.hg.1	OTTHUMG00000133420	6.61 ± 0.20	6.75 ± 0.09	6.55 ± 0.18	6.93 ± 0.21
TC06001512.hg.1	OTTHUMG00000133550	6.24 ± 0.26	6.13 ± 0.12	6.13 ± 0.02	6.19 ± 0.12
TC07001797.hg.1	OTTHUMG00000133661	5.56 ± 0.29	5.49 ± 0.05	5.45 ± 0.18	5.76 ± 0.22
TC11000607.hg.1	OTTHUMG00000133666	7.17 ± 0.31	7.11 ± 0.27	7.07 ± 0.31	7.43 ± 0.07
TC02002799.hg.1	OTTHUMG00000133672	7.21 ± 0.25	7.39 ± 0.17	7.39 ± 0.27	7.44 ± 0.15
TC17000501.hg.1	OTTHUMG00000133678	5.81 ± 0.06	5.75 ± 0.17	5.93 ± 0.13	5.86 ± 0.11
TC17000513.hg.1	OTTHUMG00000133684	7.46 ± 0.25	7.46 ± 0.23	7.43 ± 0.13	7.47 ± 0.11
TC14001146.hg.1	OTTHUMG00000133689	4.99 ± 0.15	4.93 ± 0.11	5.07 ± 0.22	4.95 ± 0.10
TC05000779.hg.1	OTTHUMG00000133693	6.18 ± 0.21	6.16 ± 0.08	6.19 ± 0.12	6.24 ± 0.06
TC20000936.hg.1	OTTHUMG00000133695	6.72 ± 0.05	6.71 ± 0.06	6.76 ± 0.06	6.73 ± 0.04
TC07001773.hg.1	OTTHUMG00000133696	4.22 ± 0.07	4.07 ± 0.08	4.14 ± 0.04	4.10 ± 0.08

TC12001396.hg.1	OTTHUMG00000133699	5.84 ± 0.50	5.21 ± 0.13	6.21 ± 0.69	5.05 ± 0.14
TC14000315.hg.1	OTTHUMG00000133700	6.46 ± 0.12	6.52 ± 0.14	6.40 ± 0.03	6.61 ± 0.11
TC07000172.hg.1	OTTHUMG00000133703	5.08 ± 0.06	4.96 ± 0.06	5.05 ± 0.05	5.03 ± 0.01
TC07001771.hg.1	OTTHUMG00000133704	4.80 ± 0.16	4.76 ± 0.11	4.89 ± 0.04	4.96 ± 0.13
TC6_cox_hap2000044.hg.1	OTTHUMG00000133708	7.41 ± 0.09	7.47 ± 0.13	7.41 ± 0.07	7.47 ± 0.07
TC6_cox_hap2000145.hg.1	OTTHUMG00000133711	6.17 ± 0.31	6.15 ± 0.12	6.16 ± 0.03	6.22 ± 0.15
TC6_cox_hap2000155.hg.1	OTTHUMG00000133715	8.52 ± 0.43	8.60 ± 0.03	8.45 ± 0.01	8.82 ± 0.16
TC6_ssto_hap7000124.hg.1	OTTHUMG00000133720	7.78 ± 0.12	7.87 ± 0.20	7.76 ± 0.10	7.71 ± 0.05
TC6_ssto_hap7000037.hg.1	OTTHUMG00000133721	7.41 ± 0.09	7.47 ± 0.13	7.41 ± 0.07	7.47 ± 0.07
TC6_ssto_hap7000127.hg.1	OTTHUMG00000133725	8.72 ± 0.46	8.80 ± 0.06	8.64 ± 0.05	9.03 ± 0.15
TC06000364.hg.1	OTTHUMG00000133747	5.76 ± 0.03	5.78 ± 0.14	5.89 ± 0.03	5.96 ± 0.17
TC03000954.hg.1	OTTHUMG00000133759	6.39 ± 0.06	6.30 ± 0.19	6.62 ± 0.14	6.28 ± 0.19
TC6_cox_hap2000060.hg.1	OTTHUMG00000133761	5.95 ± 0.20	5.93 ± 0.10	6.08 ± 0.07	6.09 ± 0.16
TC6_ssto_hap7000047.hg.1	OTTHUMG00000134272	5.99 ± 0.18	5.95 ± 0.09	6.08 ± 0.08	6.08 ± 0.16
TC20000411.hg.1	OTTHUMG00000134277	7.42 ± 0.24	7.42 ± 0.07	7.29 ± 0.27	7.31 ± 0.10
TC20000946.hg.1	OTTHUMG00000134283	7.44 ± 0.21	7.34 ± 0.09	7.35 ± 0.23	7.35 ± 0.11
TC20000949.hg.1	OTTHUMG00000134296	4.24 ± 0.06	4.20 ± 0.09	4.22 ± 0.14	4.23 ± 0.17
TC20000414.hg.1	OTTHUMG00000134297	5.42 ± 0.09	5.46 ± 0.11	5.40 ± 0.14	5.57 ± 0.12
TC20000954.hg.1	OTTHUMG00000134300	4.97 ± 0.09	4.97 ± 0.05	4.95 ± 0.18	4.92 ± 0.05
TC20000953.hg.1	OTTHUMG00000134301	6.34 ± 0.40	6.14 ± 0.12	6.15 ± 0.28	6.38 ± 0.05
TC20000964.hg.1	OTTHUMG00000134311	4.28 ± 0.02	4.29 ± 0.09	4.28 ± 0.08	4.30 ± 0.10
TC6_cox_hap2000064.hg.1	OTTHUMG00000134329	7.46 ± 0.18	7.33 ± 0.11	7.24 ± 0.15	7.37 ± 0.04
TC6_ssto_hap7000051.hg.1	OTTHUMG00000137359	7.33 ± 0.10	7.24 ± 0.08	7.13 ± 0.15	7.31 ± 0.06
TC20000981.hg.1	OTTHUMG00000137362	6.08 ± 0.28	6.18 ± 0.36	6.17 ± 0.20	6.23 ± 0.18
TC20000990.hg.1	OTTHUMG00000137369	6.13 ± 0.26	6.00 ± 0.13	6.12 ± 0.08	6.04 ± 0.05
TC20000996.hg.1	OTTHUMG00000137385	6.39 ± 0.29	6.28 ± 0.05	6.33 ± 0.12	6.27 ± 0.02
TC20000471.hg.1	OTTHUMG00000137386	5.52 ± 0.31	5.49 ± 0.15	5.47 ± 0.18	5.55 ± 0.02
TC20000995.hg.1	OTTHUMG00000137387	5.89 ± 0.34	5.92 ± 0.07	5.90 ± 0.15	6.09 ± 0.14
TC6_ssto_hap7000060.hg.1	OTTHUMG00000137395	7.19 ± 0.19	6.98 ± 0.16	7.04 ± 0.21	7.17 ± 0.09
TC6_cox_hap2000169.hg.1	OTTHUMG00000137424	7.29 ± 0.16	7.17 ± 0.13	7.17 ± 0.05	7.30 ± 0.02
TC6_cox_hap2000179.hg.1	OTTHUMG00000137478	6.82 ± 0.29	6.57 ± 0.12	6.62 ± 0.11	6.59 ± 0.14

TC6_ssto_hap7000147.hg.1	OTTHUMG00000137482	6.82 ± 0.29	6.57 ± 0.12	6.62 ± 0.11	6.59 ± 0.14
TC6_ssto_hap7000065.hg.1	OTTHUMG00000137488	8.26 ± 0.32	8.09 ± 0.08	8.17 ± 0.11	8.06 ± 0.04
TC6_qbl_hap6000172.hg.1	OTTHUMG00000137498	6.76 ± 0.36	6.70 ± 0.15	6.76 ± 0.27	6.73 ± 0.32
TC6_cox_hap2000182.hg.1	OTTHUMG00000137633	6.76 ± 0.36	6.70 ± 0.15	6.76 ± 0.27	6.73 ± 0.32
TC6_ssto_hap7000150.hg.1	OTTHUMG00000137660	6.76 ± 0.36	6.70 ± 0.15	6.76 ± 0.27	6.73 ± 0.32
TC6_ssto_hap7000152.hg.1	OTTHUMG00000137666	6.76 ± 0.36	6.70 ± 0.15	6.76 ± 0.27	6.73 ± 0.32
TC6_qbl_hap6000083.hg.1	OTTHUMG00000140105	6.92 ± 0.24	6.90 ± 0.15	6.75 ± 0.15	6.88 ± 0.12
TC6_cox_hap2000092.hg.1	OTTHUMG00000140107	7.03 ± 0.36	6.89 ± 0.08	6.75 ± 0.11	6.84 ± 0.05
TC6_ssto_hap7000078.hg.1	OTTHUMG00000140109	7.02 ± 0.48	6.93 ± 0.11	6.81 ± 0.13	6.96 ± 0.06
TC6_cox_hap2000093.hg.1	OTTHUMG00000140114	3.79 ± 0.10	3.72 ± 0.14	3.67 ± 0.08	3.70 ± 0.10
TC6_ssto_hap7000079.hg.1	OTTHUMG00000140117	3.93 ± 0.06	3.82 ± 0.06	3.89 ± 0.04	3.86 ± 0.08
TC6_qbl_hap6000084.hg.1	OTTHUMG00000140118	4.07 ± 0.09	3.96 ± 0.06	4.07 ± 0.11	3.98 ± 0.06
TC06000402.hg.1	OTTHUMG00000140119	3.93 ± 0.06	3.82 ± 0.06	3.89 ± 0.04	3.86 ± 0.08
TC20001025.hg.1	OTTHUMG00000140137	7.61 ± 0.32	7.68 ± 0.27	7.62 ± 0.30	7.81 ± 0.15
TC22000001.hg.1	OTTHUMG00000140195	6.99 ± 0.42	6.78 ± 0.14	6.85 ± 0.15	6.93 ± 0.21
TC22000441.hg.1	OTTHUMG00000140260	6.19 ± 0.07	6.08 ± 0.05	6.06 ± 0.02	6.26 ± 0.23
TC22000008.hg.1	OTTHUMG00000140353	6.91 ± 0.25	6.84 ± 0.13	6.88 ± 0.12	7.09 ± 0.22
TC02001583.hg.1	OTTHUMG00000140389	6.59 ± 0.20	6.69 ± 0.19	6.54 ± 0.16	6.81 ± 0.16
TC12001210.hg.1	OTTHUMG00000140394	6.61 ± 0.17	6.28 ± 0.29	6.75 ± 0.45	6.06 ± 0.10
TC15001856.hg.1	OTTHUMG00000141259	7.52 ± 0.24	7.48 ± 0.20	7.48 ± 0.14	7.60 ± 0.03
TC08000350.hg.1	OTTHUMG00000141277	6.26 ± 0.09	6.21 ± 0.13	6.23 ± 0.12	6.42 ± 0.13
TC01003664.hg.1	OTTHUMG00000141312	4.15 ± 0.14	4.12 ± 0.04	4.22 ± 0.03	4.14 ± 0.05
TC02002243.hg.1	OTTHUMG00000141319	6.57 ± 0.26	6.58 ± 0.01	6.48 ± 0.15	6.72 ± 0.09
TC08000680.hg.1	OTTHUMG00000142828	4.32 ± 0.21	4.05 ± 0.18	4.16 ± 0.16	4.03 ± 0.30
TC11001527.hg.1	OTTHUMG00000142920	5.41 ± 0.09	5.31 ± 0.04	5.28 ± 0.06	5.37 ± 0.14
TC10001307.hg.1	OTTHUMG00000144163	4.62 ± 0.14	4.48 ± 0.12	4.48 ± 0.05	4.70 ± 0.16
TC10000355.hg.1	OTTHUMG00000144164	3.66 ± 0.15	3.71 ± 0.06	3.65 ± 0.07	3.73 ± 0.17
TC10000354.hg.1	OTTHUMG00000144165	4.35 ± 0.19	4.14 ± 0.04	4.08 ± 0.14	4.12 ± 0.02
TC10001308.hg.1	OTTHUMG00000144166	5.48 ± 0.10	5.40 ± 0.05	5.48 ± 0.30	5.42 ± 0.03
TC02000306.hg.1	OTTHUMG00000144189	5.34 ± 0.04	5.17 ± 0.03	5.15 ± 0.10	5.34 ± 0.21
TC22000017.hg.1	OTTHUMG00000147348	6.98 ± 0.21	7.09 ± 0.07	6.99 ± 0.10	7.22 ± 0.20

TC08001657.hg.1	OTTHUMG00000148655	5.11 ± 0.21	4.92 ± 0.20	5.50 ± 0.33	4.99 ± 0.09
TC14001323.hg.1	OTTHUMG00000148657	5.51 ± 0.19	5.59 ± 0.23	5.53 ± 0.09	5.71 ± 0.13
TC07000761.hg.1	OTTHUMG00000148663	4.94 ± 0.10	4.65 ± 0.13	4.92 ± 0.11	4.76 ± 0.15
TC6_apd_hap1000051.hg.1	OTTHUMG00000148688	6.79 ± 0.27	6.76 ± 0.03	6.68 ± 0.14	6.83 ± 0.10
TC6_apd_hap1000007.hg.1	OTTHUMG00000148697	4.50 ± 0.22	4.05 ± 0.23	4.30 ± 0.26	4.23 ± 0.18
TC6_apd_hap1000011.hg.1	OTTHUMG00000148711	6.49 ± 0.12	6.41 ± 0.14	6.39 ± 0.05	6.46 ± 0.07
TC6_apd_hap1000023.hg.1	OTTHUMG00000148738	6.58 ± 0.28	6.62 ± 0.14	6.52 ± 0.19	6.56 ± 0.14
TC6_apd_hap1000028.hg.1	OTTHUMG00000148749	7.41 ± 0.09	7.47 ± 0.13	7.41 ± 0.07	7.47 ± 0.07
TC6_apd_hap1000027.hg.1	OTTHUMG00000148794	7.05 ± 0.19	7.05 ± 0.15	7.09 ± 0.10	6.98 ± 0.13
TC6_apd_hap1000083.hg.1	OTTHUMG00000148797	7.29 ± 0.16	7.17 ± 0.13	7.17 ± 0.05	7.30 ± 0.02
TC6_apd_hap1000042.hg.1	OTTHUMG00000148805	8.26 ± 0.32	8.09 ± 0.08	8.17 ± 0.11	8.06 ± 0.04
TC6_apd_hap1000046.hg.1	OTTHUMG00000148821	3.79 ± 0.10	3.72 ± 0.14	3.67 ± 0.08	3.70 ± 0.10
TC6_apd_hap1000005.hg.1	OTTHUMG00000148843	5.84 ± 0.26	5.79 ± 0.13	5.88 ± 0.20	5.85 ± 0.20
TC6_dbb_hap3000102.hg.1	OTTHUMG00000148852	6.79 ± 0.27	6.76 ± 0.03	6.68 ± 0.14	6.83 ± 0.10
TC6_dbb_hap3000006.hg.1	OTTHUMG00000148859	5.71 ± 0.38	5.49 ± 0.08	5.58 ± 0.12	5.55 ± 0.10
TC6_dbb_hap3000011.hg.1	OTTHUMG00000148880	6.49 ± 0.12	6.41 ± 0.14	6.39 ± 0.05	6.46 ± 0.07
TC6_dbb_hap3000024.hg.1	OTTHUMG00000148917	6.83 ± 0.29	6.76 ± 0.09	6.81 ± 0.17	6.78 ± 0.07
TC6_dbb_hap3000037.hg.1	OTTHUMG00000148934	7.41 ± 0.09	7.47 ± 0.13	7.41 ± 0.07	7.47 ± 0.07
TC6_dbb_hap3000135.hg.1	OTTHUMG00000148935	6.93 ± 0.09	6.95 ± 0.10	7.03 ± 0.16	6.93 ± 0.05
TC6_dbb_hap3000133.hg.1	OTTHUMG00000148939	7.79 ± 0.16	7.86 ± 0.18	7.76 ± 0.10	7.74 ± 0.06
TC6_dbb_hap3000134.hg.1	OTTHUMG00000148948	6.23 ± 0.21	6.12 ± 0.11	6.12 ± 0.02	6.16 ± 0.11
TC6_dbb_hap3000144.hg.1	OTTHUMG00000148962	8.52 ± 0.43	8.60 ± 0.03	8.45 ± 0.01	8.82 ± 0.16
TC6_dbb_hap3000055.hg.1	OTTHUMG00000148970	7.46 ± 0.18	7.33 ± 0.11	7.24 ± 0.15	7.37 ± 0.04
TC6_dbb_hap3000064.hg.1	OTTHUMG00000148992	7.15 ± 0.19	6.93 ± 0.20	6.97 ± 0.21	7.12 ± 0.09
TC6_dbb_hap3000030.hg.1	OTTHUMG00000149022	7.05 ± 0.19	7.05 ± 0.15	7.09 ± 0.10	6.98 ± 0.13
TC6_dbb_hap3000158.hg.1	OTTHUMG00000149032	7.29 ± 0.16	7.17 ± 0.13	7.17 ± 0.05	7.30 ± 0.02
TC6_dbb_hap3000072.hg.1	OTTHUMG00000149040	8.26 ± 0.32	8.09 ± 0.08	8.17 ± 0.11	8.06 ± 0.04
TC6_dbb_hap3000168.hg.1	OTTHUMG00000149045	6.82 ± 0.29	6.57 ± 0.12	6.62 ± 0.11	6.59 ± 0.14
TC6_dbb_hap3000171.hg.1	OTTHUMG00000149049	6.76 ± 0.36	6.70 ± 0.15	6.76 ± 0.27	6.73 ± 0.32
TC6_dbb_hap3000173.hg.1	OTTHUMG00000149050	6.59 ± 0.39	6.54 ± 0.13	6.61 ± 0.28	6.55 ± 0.32
TC6_dbb_hap3000085.hg.1	OTTHUMG00000149070	7.12 ± 0.42	7.06 ± 0.10	6.98 ± 0.12	7.11 ± 0.07

TC6_dbb_hap3000086.hg.1	OTTHUMG00000149071	4.09 ± 0.10	3.98 ± 0.06	3.99 ± 0.06	4.03 ± 0.09
TC6_dbb_hap3000004.hg.1	OTTHUMG00000149115	6.20 ± 0.30	6.14 ± 0.15	6.22 ± 0.24	6.23 ± 0.20
TC6_mann_hap4000091.hg.1	OTTHUMG00000149131	6.79 ± 0.27	6.76 ± 0.03	6.68 ± 0.14	6.83 ± 0.10
TC6_mann_hap4000007.hg.1	OTTHUMG00000149138	5.71 ± 0.38	5.49 ± 0.08	5.58 ± 0.12	5.55 ± 0.10
TC6_mann_hap4000012.hg.1	OTTHUMG00000149160	6.49 ± 0.12	6.41 ± 0.14	6.39 ± 0.05	6.46 ± 0.07
TC6_mann_hap4000026.hg.1	OTTHUMG00000149197	6.83 ± 0.29	6.76 ± 0.09	6.81 ± 0.17	6.78 ± 0.07
TC6_mann_hap4000039.hg.1	OTTHUMG00000149214	7.41 ± 0.09	7.47 ± 0.13	7.41 ± 0.07	7.47 ± 0.07
TC6_mann_hap4000124.hg.1	OTTHUMG00000149215	6.93 ± 0.09	6.95 ± 0.10	7.03 ± 0.16	6.93 ± 0.05
TC6_mann_hap4000122.hg.1	OTTHUMG00000149218	7.30 ± 0.10	7.29 ± 0.17	7.33 ± 0.02	7.22 ± 0.05
TC6_mann_hap4000123.hg.1	OTTHUMG00000149226	6.24 ± 0.26	6.13 ± 0.12	6.13 ± 0.02	6.19 ± 0.12
TC6_mann_hap4000132.hg.1	OTTHUMG00000149240	6.94 ± 0.28	6.57 ± 0.12	6.95 ± 0.29	6.65 ± 0.33
TC6_mann_hap4000054.hg.1	OTTHUMG00000149243	5.39 ± 0.08	5.47 ± 0.15	5.68 ± 0.13	5.71 ± 0.22
TC6_mann_hap4000056.hg.1	OTTHUMG00000149255	7.46 ± 0.18	7.33 ± 0.11	7.24 ± 0.15	7.37 ± 0.04
TC6_mann_hap4000064.hg.1	OTTHUMG00000149277	7.19 ± 0.19	6.98 ± 0.16	7.04 ± 0.21	7.17 ± 0.09
TC6_mann_hap4000032.hg.1	OTTHUMG00000149304	6.86 ± 0.10	6.83 ± 0.14	6.80 ± 0.04	6.76 ± 0.13
TC6_mann_hap4000148.hg.1	OTTHUMG00000149316	6.71 ± 0.33	6.38 ± 0.12	6.49 ± 0.05	6.38 ± 0.19
TC6_mann_hap4000163.hg.1	OTTHUMG00000149333	8.75 ± 0.18	8.80 ± 0.07	8.26 ± 0.03	8.72 ± 0.14
TC6_mann_hap4000076.hg.1	OTTHUMG00000149337	7.57 ± 0.48	7.25 ± 0.08	7.10 ± 0.16	7.17 ± 0.07
TC6_mann_hap4000077.hg.1	OTTHUMG00000149338	4.07 ± 0.09	3.96 ± 0.06	4.07 ± 0.11	3.98 ± 0.06
TC6_mann_hap4000005.hg.1	OTTHUMG00000149371	6.20 ± 0.30	6.14 ± 0.15	6.22 ± 0.24	6.23 ± 0.20
TC6_mann_hap4000082.hg.1	OTTHUMG00000149373	7.12 ± 0.15	7.05 ± 0.08	7.02 ± 0.03	7.01 ± 0.12
TC6_mcf_hap5000096.hg.1	OTTHUMG00000149387	6.79 ± 0.27	6.76 ± 0.03	6.68 ± 0.14	6.83 ± 0.10
TC6_mcf_hap5000010.hg.1	OTTHUMG00000149412	6.49 ± 0.12	6.41 ± 0.14	6.39 ± 0.05	6.46 ± 0.07
TC6_mcf_hap5000106.hg.1	OTTHUMG00000149420	8.75 ± 0.27	8.79 ± 0.29	8.65 ± 0.28	8.93 ± 0.04
TC6_mcf_hap5000020.hg.1	OTTHUMG00000149445	6.83 ± 0.29	6.76 ± 0.09	6.81 ± 0.17	6.78 ± 0.07
TC6_mcf_hap5000031.hg.1	OTTHUMG00000149450	7.41 ± 0.09	7.47 ± 0.13	7.41 ± 0.07	7.47 ± 0.07
TC6_mcf_hap5000123.hg.1	OTTHUMG00000149451	6.64 ± 0.16	6.58 ± 0.07	6.71 ± 0.06	6.63 ± 0.03
TC6_mcf_hap5000122.hg.1	OTTHUMG00000149463	6.24 ± 0.26	6.13 ± 0.12	6.13 ± 0.02	6.19 ± 0.12
TC6_mcf_hap5000132.hg.1	OTTHUMG00000149475	8.31 ± 0.42	8.37 ± 0.01	8.19 ± 0.04	8.62 ± 0.16
TC6_mcf_hap5000050.hg.1	OTTHUMG00000149485	7.46 ± 0.18	7.33 ± 0.11	7.24 ± 0.15	7.37 ± 0.04
TC6_mcf_hap5000059.hg.1	OTTHUMG00000149507	7.19 ± 0.19	6.98 ± 0.16	7.04 ± 0.21	7.17 ± 0.09

TC6_mcf_hap5000024.hg.1	OTTHUMG00000149535	6.86 ± 0.10	6.83 ± 0.14	6.80 ± 0.04	6.76 ± 0.13
TC6_mcf_hap5000146.hg.1	OTTHUMG00000149545	7.29 ± 0.16	7.17 ± 0.13	7.17 ± 0.05	7.30 ± 0.02
TC6_mcf_hap5000064.hg.1	OTTHUMG00000149549	8.26 ± 0.32	8.09 ± 0.08	8.17 ± 0.11	8.06 ± 0.04
TC6_mcf_hap5000154.hg.1	OTTHUMG00000149554	6.82 ± 0.29	6.57 ± 0.12	6.62 ± 0.11	6.59 ± 0.14
TC6_mcf_hap5000158.hg.1	OTTHUMG00000149558	6.76 ± 0.36	6.70 ± 0.15	6.76 ± 0.27	6.73 ± 0.32
TC6_mcf_hap5000170.hg.1	OTTHUMG00000149581	9.18 ± 0.15	9.28 ± 0.13	8.67 ± 0.04	9.17 ± 0.13
TC6_mcf_hap5000077.hg.1	OTTHUMG00000149585	6.78 ± 0.52	6.66 ± 0.05	6.63 ± 0.13	6.62 ± 0.07
TC6_mcf_hap5000078.hg.1	OTTHUMG00000149586	4.09 ± 0.10	3.98 ± 0.06	3.99 ± 0.06	4.03 ± 0.09
TC6_mcf_hap5000083.hg.1	OTTHUMG00000149632	7.12 ± 0.15	7.05 ± 0.08	7.02 ± 0.03	7.01 ± 0.12
TC19001838.hg.1	OTTHUMG00000149883	8.51 ± 0.22	8.67 ± 0.07	8.63 ± 0.04	8.73 ± 0.06
TC19000864.hg.1	OTTHUMG00000149885	7.47 ± 0.28	7.38 ± 0.12	7.57 ± 0.12	7.44 ± 0.08
TC02002834.hg.1	OTTHUMG00000149890	5.78 ± 0.12	5.71 ± 0.11	5.69 ± 0.07	5.79 ± 0.06
TC6_qbl_hap6000089.hg.1	OTTHUMG00000149963	7.12 ± 0.15	7.05 ± 0.08	7.02 ± 0.03	7.01 ± 0.12
TC22000482.hg.1	OTTHUMG00000149968	6.68 ± 0.20	6.71 ± 0.03	6.47 ± 0.26	6.69 ± 0.14
TC6_cox_hap2000098.hg.1	OTTHUMG00000149985	7.12 ± 0.15	7.05 ± 0.08	7.02 ± 0.03	7.01 ± 0.12
TC19001850.hg.1	OTTHUMG00000149999	6.36 ± 0.06	6.19 ± 0.18	6.30 ± 0.02	6.31 ± 0.09
TC6_ssto_hap7000084.hg.1	OTTHUMG00000150001	7.12 ± 0.15	7.05 ± 0.08	7.02 ± 0.03	7.01 ± 0.12
TC22000249.hg.1	OTTHUMG00000150002	6.04 ± 0.20	5.91 ± 0.23	5.82 ± 0.26	5.99 ± 0.06
TC22000676.hg.1	OTTHUMG00000150003	6.20 ± 0.05	6.07 ± 0.13	6.27 ± 0.16	6.19 ± 0.08
TC22000236.hg.1	OTTHUMG00000150005	8.27 ± 0.30	8.16 ± 0.28	8.16 ± 0.24	8.26 ± 0.05
TC22000241.hg.1	OTTHUMG00000150008	7.86 ± 0.20	7.75 ± 0.17	7.85 ± 0.17	7.93 ± 0.10
TC22000250.hg.1	OTTHUMG00000150009	5.82 ± 0.21	5.75 ± 0.31	5.58 ± 0.20	6.10 ± 0.21
TC07001791.hg.1	OTTHUMG00000150015	6.21 ± 0.26	6.26 ± 0.15	6.16 ± 0.22	6.26 ± 0.04
TC11002323.hg.1	OTTHUMG00000150016	6.74 ± 0.35	6.97 ± 0.05	6.66 ± 0.19	7.09 ± 0.13
TC11001041.hg.1	OTTHUMG00000150017	6.51 ± 0.19	6.43 ± 0.10	6.41 ± 0.18	6.54 ± 0.03
TC11000053.hg.1	OTTHUMG00000150032	7.25 ± 0.34	7.43 ± 0.02	7.40 ± 0.08	7.60 ± 0.12
TC15001281.hg.1	OTTHUMG00000150036	6.39 ± 0.23	6.32 ± 0.07	6.25 ± 0.24	6.36 ± 0.17
TC20000254.hg.1	OTTHUMG00000150044	6.74 ± 0.24	6.64 ± 0.10	6.47 ± 0.23	6.76 ± 0.06
TC11001201.hg.1	OTTHUMG00000150045	6.09 ± 0.09	5.89 ± 0.18	5.94 ± 0.08	5.94 ± 0.17
TC11001200.hg.1	OTTHUMG00000150049	6.90 ± 0.27	6.81 ± 0.14	6.85 ± 0.23	6.90 ± 0.25
TC11002460.hg.1	OTTHUMG00000150050	6.54 ± 0.29	6.61 ± 0.25	6.61 ± 0.05	6.67 ± 0.10

TC05000242.hg.1	OTTHUMG00000150053	6.10 ± 0.24	5.95 ± 0.11	6.03 ± 0.08	6.07 ± 0.06
TC05001376.hg.1	OTTHUMG00000150054	5.18 ± 0.18	4.91 ± 0.03	5.21 ± 0.10	5.02 ± 0.08
TC06000989.hg.1	OTTHUMG00000150056	7.16 ± 0.25	7.00 ± 0.14	6.99 ± 0.37	7.01 ± 0.12
TC06000736.hg.1	OTTHUMG00000150059	6.08 ± 0.15	6.00 ± 0.04	5.93 ± 0.08	6.02 ± 0.05
TC22000481.hg.1	OTTHUMG00000150064	5.95 ± 0.19	5.79 ± 0.07	5.68 ± 0.16	5.74 ± 0.09
TC22000480.hg.1	OTTHUMG00000150069	8.71 ± 0.27	8.73 ± 0.19	8.57 ± 0.33	8.79 ± 0.08
TC22000473.hg.1	OTTHUMG00000150092	7.91 ± 0.22	7.82 ± 0.04	7.82 ± 0.06	7.99 ± 0.15
TC14001147.hg.1	OTTHUMG00000150105	4.72 ± 0.18	4.65 ± 0.03	4.64 ± 0.12	4.65 ± 0.06
TC16000512.hg.1	OTTHUMG00000150106	4.88 ± 0.15	4.85 ± 0.03	4.90 ± 0.19	4.75 ± 0.10
TC06001995.hg.1	OTTHUMG00000150107	5.04 ± 0.10	4.99 ± 0.08	4.97 ± 0.03	4.89 ± 0.11
TC06000861.hg.1	OTTHUMG00000150108	4.89 ± 0.19	4.81 ± 0.20	4.83 ± 0.21	4.80 ± 0.14
TC14000619.hg.1	OTTHUMG00000150112	7.36 ± 0.21	7.73 ± 0.18	7.19 ± 0.22	7.55 ± 0.12
TC14000617.hg.1	OTTHUMG00000150113	6.25 ± 0.30	5.84 ± 0.17	5.98 ± 0.06	5.77 ± 0.13
TC14000618.hg.1	OTTHUMG00000150114	6.69 ± 0.12	6.67 ± 0.02	6.69 ± 0.05	6.62 ± 0.14
TC02001327.hg.1	OTTHUMG00000150115	4.91 ± 0.15	4.87 ± 0.17	4.87 ± 0.28	4.94 ± 0.12
TC02001335.hg.1	OTTHUMG00000150131	5.92 ± 0.05	5.87 ± 0.14	5.89 ± 0.08	6.04 ± 0.12
TC22000045.hg.1	OTTHUMG00000150135	7.39 ± 0.22	7.25 ± 0.13	7.10 ± 0.13	7.27 ± 0.05
TC22000040.hg.1	OTTHUMG00000150139	8.18 ± 0.24	8.04 ± 0.17	8.12 ± 0.23	8.22 ± 0.06
TC20000174.hg.1	OTTHUMG00000150168	4.12 ± 0.16	4.11 ± 0.02	4.20 ± 0.12	4.23 ± 0.23
TC07001799.hg.1	OTTHUMG00000150172	6.31 ± 0.19	6.03 ± 0.10	6.27 ± 0.10	6.15 ± 0.11
TC22000031.hg.1	OTTHUMG00000150176	9.01 ± 0.11	8.70 ± 0.17	9.45 ± 0.14	8.81 ± 0.07
TC22000032.hg.1	OTTHUMG00000150177	7.80 ± 0.18	7.75 ± 0.11	7.66 ± 0.12	7.85 ± 0.06
TC12001211.hg.1	OTTHUMG00000150192	7.21 ± 0.24	7.17 ± 0.15	7.17 ± 0.22	7.27 ± 0.09
TC04000783.hg.1	OTTHUMG00000150212	4.36 ± 0.08	4.15 ± 0.02	4.23 ± 0.18	4.18 ± 0.05
TC04000784.hg.1	OTTHUMG00000150212	4.46 ± 0.13	4.37 ± 0.16	4.46 ± 0.06	4.35 ± 0.10
TC22000422.hg.1	OTTHUMG00000150220	8.27 ± 0.28	8.16 ± 0.33	8.17 ± 0.20	8.34 ± 0.04
TC02002356.hg.1	OTTHUMG00000150221	7.56 ± 0.44	7.73 ± 0.07	7.67 ± 0.23	8.01 ± 0.26
TC06000396.hg.1	OTTHUMG00000150222	7.54 ± 0.29	7.63 ± 0.18	7.49 ± 0.20	7.74 ± 0.13
TC22000899.hg.1	OTTHUMG00000150298	7.52 ± 0.23	7.55 ± 0.05	7.50 ± 0.15	7.68 ± 0.11
TC22000900.hg.1	OTTHUMG00000150299	7.88 ± 0.15	7.79 ± 0.12	7.86 ± 0.03	7.79 ± 0.06
TC22000410.hg.1	OTTHUMG00000150300	6.23 ± 0.23	6.40 ± 0.16	6.34 ± 0.19	6.48 ± 0.07

TC22000895.hg.1	OTTHUMG00000150317	6.91 ± 0.38	7.05 ± 0.27	6.82 ± 0.26	7.24 ± 0.16
TC22000404.hg.1	OTTHUMG00000150318	7.64 ± 0.19	7.72 ± 0.12	7.61 ± 0.18	7.79 ± 0.11
TC22000403.hg.1	OTTHUMG00000150319	5.63 ± 0.30	5.89 ± 0.21	5.60 ± 0.17	6.09 ± 0.23
TC22000894.hg.1	OTTHUMG00000150320	5.91 ± 0.24	5.89 ± 0.16	5.92 ± 0.08	6.13 ± 0.09
TC22000402.hg.1	OTTHUMG00000150321	6.63 ± 0.19	6.60 ± 0.13	6.58 ± 0.11	6.72 ± 0.08
TC22000892.hg.1	OTTHUMG00000150324	5.93 ± 0.14	5.85 ± 0.23	5.92 ± 0.23	5.99 ± 0.04
TC22000401.hg.1	OTTHUMG00000150328	7.20 ± 0.28	7.20 ± 0.02	7.13 ± 0.12	7.33 ± 0.12
TC11000821.hg.1	OTTHUMG00000150349	9.27 ± 0.26	8.51 ± 0.24	9.17 ± 0.21	8.46 ± 0.49
TC01004044.hg.1	OTTHUMG00000150375	6.94 ± 0.22	6.87 ± 0.02	6.90 ± 0.24	6.99 ± 0.12
TC14000854.hg.1	OTTHUMG00000150377	6.29 ± 0.20	6.10 ± 0.06	6.07 ± 0.13	6.16 ± 0.19
TC22000047.hg.1	OTTHUMG00000150429	8.47 ± 0.22	8.42 ± 0.06	8.49 ± 0.28	8.48 ± 0.10
TC22000884.hg.1	OTTHUMG00000150445	6.69 ± 0.16	6.61 ± 0.10	6.45 ± 0.07	6.54 ± 0.04
TC22000879.hg.1	OTTHUMG00000150449	8.40 ± 0.26	8.31 ± 0.22	8.39 ± 0.14	8.43 ± 0.03
TC22000373.hg.1	OTTHUMG00000150464	6.68 ± 0.15	6.48 ± 0.19	6.63 ± 0.17	6.59 ± 0.18
TC22000374.hg.1	OTTHUMG00000150468	8.15 ± 0.26	8.23 ± 0.33	8.18 ± 0.23	8.40 ± 0.07
TC05001724.hg.1	OTTHUMG00000150470	7.69 ± 0.19	7.70 ± 0.22	7.53 ± 0.21	7.80 ± 0.05
TC22001481.hg.1	OTTHUMG00000150489	7.36 ± 0.22	7.35 ± 0.08	7.26 ± 0.08	7.38 ± 0.04
TC16001192.hg.1	OTTHUMG00000150496	7.61 ± 0.33	7.65 ± 0.09	7.72 ± 0.11	7.83 ± 0.08
TC04000955.hg.1	OTTHUMG00000150498	8.05 ± 0.04	7.92 ± 0.16	7.94 ± 0.11	8.11 ± 0.05
TC09000053.hg.1	OTTHUMG00000150500	6.10 ± 0.19	6.20 ± 0.13	6.02 ± 0.17	6.24 ± 0.16
TC05000771.hg.1	OTTHUMG00000150501	6.97 ± 0.21	6.66 ± 0.12	6.96 ± 0.15	6.76 ± 0.16
TC07000824.hg.1	OTTHUMG00000150507	6.17 ± 0.13	6.15 ± 0.12	6.16 ± 0.20	6.26 ± 0.06
TC19001846.hg.1	OTTHUMG00000150514	8.45 ± 0.32	8.15 ± 0.30	8.32 ± 0.47	8.30 ± 0.31
TC22000279.hg.1	OTTHUMG00000150530	8.20 ± 0.11	8.18 ± 0.09	8.30 ± 0.12	8.30 ± 0.05
TC22000721.hg.1	OTTHUMG00000150536	7.42 ± 0.19	7.48 ± 0.21	7.33 ± 0.23	7.53 ± 0.03
TC22000269.hg.1	OTTHUMG00000150549	8.42 ± 0.19	8.54 ± 0.31	8.30 ± 0.23	8.74 ± 0.02
TC22000716.hg.1	OTTHUMG00000150550	6.72 ± 0.24	6.67 ± 0.17	6.66 ± 0.08	6.79 ± 0.02
TC22000366.hg.1	OTTHUMG00000150565	6.37 ± 0.15	6.41 ± 0.16	6.34 ± 0.02	6.51 ± 0.09
TC22000056.hg.1	OTTHUMG00000150567	7.43 ± 0.19	7.37 ± 0.22	7.37 ± 0.25	7.48 ± 0.05
TC22000268.hg.1	OTTHUMG00000150584	7.07 ± 0.18	7.00 ± 0.13	7.03 ± 0.19	7.05 ± 0.03
TC22000263.hg.1	OTTHUMG00000150586	5.72 ± 0.12	5.72 ± 0.17	5.67 ± 0.20	5.70 ± 0.11

TC22000281.hg.1	OTTHUMG00000150593	6.63 ± 0.24	6.61 ± 0.15	6.65 ± 0.03	6.90 ± 0.12
TC22000708.hg.1	OTTHUMG00000150597	7.15 ± 0.43	7.06 ± 0.10	7.26 ± 0.07	7.14 ± 0.07
TC22000267.hg.1	OTTHUMG00000150598	5.99 ± 0.21	5.85 ± 0.04	5.84 ± 0.27	5.82 ± 0.06
TC02002407.hg.1	OTTHUMG00000150604	5.06 ± 0.08	5.00 ± 0.02	4.93 ± 0.12	4.99 ± 0.03
TC22000511.hg.1	OTTHUMG00000150605	6.72 ± 0.38	6.64 ± 0.07	6.69 ± 0.20	6.83 ± 0.26
TC22000512.hg.1	OTTHUMG00000150610	5.83 ± 0.37	5.72 ± 0.01	5.65 ± 0.33	6.27 ± 0.61
TC22000513.hg.1	OTTHUMG00000150610	5.25 ± 0.24	5.22 ± 0.11	5.33 ± 0.21	5.39 ± 0.19
TC22000266.hg.1	OTTHUMG00000150616	7.19 ± 0.14	7.17 ± 0.09	7.16 ± 0.19	7.14 ± 0.02
TC22000152.hg.1	OTTHUMG00000150625	5.79 ± 0.18	5.67 ± 0.06	5.50 ± 0.13	5.64 ± 0.04
TC11001043.hg.1	OTTHUMG00000150649	6.38 ± 0.10	6.38 ± 0.10	6.30 ± 0.15	6.30 ± 0.04
TC22000776.hg.1	OTTHUMG00000150662	7.80 ± 0.17	7.78 ± 0.18	7.75 ± 0.10	7.84 ± 0.03
TC22000135.hg.1	OTTHUMG00000150666	6.45 ± 0.21	6.32 ± 0.14	6.33 ± 0.02	6.36 ± 0.10
TC22000132.hg.1	OTTHUMG00000150668	8.28 ± 0.29	8.03 ± 0.10	7.99 ± 0.26	8.12 ± 0.13
TC04001098.hg.1	OTTHUMG00000150675	4.88 ± 0.33	4.84 ± 0.20	4.73 ± 0.22	4.99 ± 0.23
TC22000364.hg.1	OTTHUMG00000150676	7.41 ± 0.11	7.30 ± 0.20	7.31 ± 0.20	7.35 ± 0.04
TC22000365.hg.1	OTTHUMG00000150677	8.11 ± 0.27	8.03 ± 0.06	8.16 ± 0.29	8.03 ± 0.05
TC22000363.hg.1	OTTHUMG00000150678	6.82 ± 0.03	6.86 ± 0.16	6.83 ± 0.17	6.87 ± 0.17
TC22000064.hg.1	OTTHUMG00000150684	5.83 ± 0.37	5.72 ± 0.01	5.65 ± 0.33	6.27 ± 0.61
TC22000355.hg.1	OTTHUMG00000150697	7.61 ± 0.04	7.65 ± 0.04	7.66 ± 0.03	7.72 ± 0.14
TC22000504.hg.1	OTTHUMG00000150731	7.56 ± 0.10	7.39 ± 0.12	7.58 ± 0.15	7.57 ± 0.16
TC22000150.hg.1	OTTHUMG00000150750	6.65 ± 0.12	6.56 ± 0.03	6.66 ± 0.04	6.63 ± 0.12
TC22000085.hg.1	OTTHUMG00000150793	5.66 ± 0.23	5.49 ± 0.07	5.43 ± 0.20	5.85 ± 0.44
TC22000534.hg.1	OTTHUMG00000150794	5.77 ± 0.30	5.68 ± 0.12	5.59 ± 0.28	6.10 ± 0.41
TC22000075.hg.1	OTTHUMG00000150805	7.85 ± 0.19	7.83 ± 0.14	7.78 ± 0.22	7.81 ± 0.04
TC22000145.hg.1	OTTHUMG00000150809	7.77 ± 0.20	7.89 ± 0.13	7.72 ± 0.08	8.04 ± 0.07
TC22000131.hg.1	OTTHUMG00000150838	7.38 ± 0.28	7.24 ± 0.16	7.35 ± 0.27	7.36 ± 0.05
TC22000134.hg.1	OTTHUMG00000150840	6.65 ± 0.41	6.79 ± 0.26	6.54 ± 0.27	6.88 ± 0.20
TC22000523.hg.1	OTTHUMG00000150842	6.01 ± 0.19	5.95 ± 0.12	5.80 ± 0.07	6.10 ± 0.05
TC22000102.hg.1	OTTHUMG00000150859	6.03 ± 0.46	5.96 ± 0.26	5.92 ± 0.16	6.19 ± 0.24
TC22000161.hg.1	OTTHUMG00000150865	5.71 ± 0.15	5.51 ± 0.06	5.77 ± 0.16	5.50 ± 0.08
TC22000079.hg.1	OTTHUMG00000150899	5.61 ± 0.17	5.71 ± 0.23	5.65 ± 0.05	5.71 ± 0.07

TC22000600.hg.1	OTTHUMG00000150901	5.36 ± 0.31	5.15 ± 0.06	5.48 ± 0.17	5.46 ± 0.19
TC22000603.hg.1	OTTHUMG00000150904	4.61 ± 0.04	4.50 ± 0.02	4.59 ± 0.17	4.49 ± 0.11
TC22000597.hg.1	OTTHUMG00000150906	6.31 ± 0.10	6.42 ± 0.09	6.37 ± 0.11	6.48 ± 0.04
TC22000602.hg.1	OTTHUMG00000150907	7.13 ± 0.03	7.05 ± 0.12	6.90 ± 0.23	7.11 ± 0.08
TC22000206.hg.1	OTTHUMG00000150909	7.20 ± 0.20	7.08 ± 0.27	7.06 ± 0.12	7.17 ± 0.11
TC22000646.hg.1	OTTHUMG00000150912	6.15 ± 0.14	6.05 ± 0.22	6.13 ± 0.15	6.19 ± 0.08
TC22000356.hg.1	OTTHUMG00000150919	7.52 ± 0.24	7.47 ± 0.16	7.51 ± 0.03	7.61 ± 0.08
TC22000842.hg.1	OTTHUMG00000150921	7.60 ± 0.15	7.63 ± 0.26	7.54 ± 0.30	7.85 ± 0.11
TC22000251.hg.1	OTTHUMG00000150925	6.24 ± 0.38	6.26 ± 0.21	6.35 ± 0.12	6.56 ± 0.16
TC22000253.hg.1	OTTHUMG00000150928	4.61 ± 0.15	4.56 ± 0.10	4.58 ± 0.00	4.63 ± 0.07
TC22000692.hg.1	OTTHUMG00000150930	5.80 ± 0.25	5.56 ± 0.22	5.61 ± 0.10	5.80 ± 0.03
TC22000693.hg.1	OTTHUMG00000150931	6.40 ± 0.16	6.39 ± 0.21	6.48 ± 0.22	6.53 ± 0.05
TC22000694.hg.1	OTTHUMG00000150933	6.12 ± 0.27	5.98 ± 0.19	5.98 ± 0.15	6.15 ± 0.06
TC22000695.hg.1	OTTHUMG00000150934	6.75 ± 0.17	6.69 ± 0.09	6.69 ± 0.14	6.76 ± 0.08
TC22000819.hg.1	OTTHUMG00000150939	5.50 ± 0.14	5.30 ± 0.07	5.40 ± 0.05	5.37 ± 0.16
TC22000820.hg.1	OTTHUMG00000150941	5.79 ± 0.27	5.69 ± 0.09	5.76 ± 0.09	5.80 ± 0.05
TC22000176.hg.1	OTTHUMG00000150945	6.84 ± 0.13	6.95 ± 0.13	6.98 ± 0.18	7.13 ± 0.11
TC22000181.hg.1	OTTHUMG00000150946	6.54 ± 0.07	6.46 ± 0.31	6.41 ± 0.23	6.63 ± 0.03
TC22000619.hg.1	OTTHUMG00000150948	5.81 ± 0.08	5.79 ± 0.12	5.76 ± 0.06	5.91 ± 0.10
TC22000177.hg.1	OTTHUMG00000150949	6.00 ± 0.07	6.04 ± 0.18	6.00 ± 0.07	6.11 ± 0.08
TC22000180.hg.1	OTTHUMG00000150950	6.94 ± 0.36	6.82 ± 0.10	6.86 ± 0.11	6.84 ± 0.08
TC22000618.hg.1	OTTHUMG00000150951	6.70 ± 0.23	6.65 ± 0.12	6.70 ± 0.25	6.79 ± 0.05
TC22000178.hg.1	OTTHUMG00000150952	6.29 ± 0.12	6.36 ± 0.15	6.27 ± 0.06	6.53 ± 0.16
TC22000614.hg.1	OTTHUMG00000150953	7.03 ± 0.20	6.96 ± 0.23	7.02 ± 0.11	7.05 ± 0.07
TC22000617.hg.1	OTTHUMG00000150954	5.83 ± 0.20	5.90 ± 0.15	5.78 ± 0.22	5.93 ± 0.17
TC22000615.hg.1	OTTHUMG00000150955	6.48 ± 0.13	6.47 ± 0.15	6.46 ± 0.17	6.54 ± 0.11
TC22000616.hg.1	OTTHUMG00000150956	6.63 ± 0.08	6.28 ± 0.17	6.38 ± 0.34	6.39 ± 0.11
TC22000696.hg.1	OTTHUMG00000150957	6.97 ± 0.12	7.04 ± 0.14	7.04 ± 0.03	7.18 ± 0.10
TC22000182.hg.1	OTTHUMG00000150974	5.35 ± 0.10	5.29 ± 0.18	5.31 ± 0.09	5.33 ± 0.22
TC22000173.hg.1	OTTHUMG00000150979	6.93 ± 0.07	7.13 ± 0.12	6.79 ± 0.14	7.24 ± 0.21
TC22000613.hg.1	OTTHUMG00000150982	6.56 ± 0.21	6.54 ± 0.23	6.64 ± 0.19	6.70 ± 0.13

TC22000795.hg.1	OTTHUMG00000150989	6.21 ± 0.12	6.19 ± 0.18	6.09 ± 0.13	6.18 ± 0.04
TC22000794.hg.1	OTTHUMG00000150992	6.01 ± 0.05	5.79 ± 0.03	5.90 ± 0.05	5.82 ± 0.05
TC22000307.hg.1	OTTHUMG00000151001	7.15 ± 0.22	7.04 ± 0.08	7.09 ± 0.10	7.11 ± 0.03
TC22000306.hg.1	OTTHUMG00000151003	7.84 ± 0.20	7.84 ± 0.07	7.81 ± 0.05	7.80 ± 0.06
TC22000630.hg.1	OTTHUMG00000151012	4.25 ± 0.07	4.24 ± 0.05	4.30 ± 0.16	4.22 ± 0.02
TC22000106.hg.1	OTTHUMG00000151038	9.24 ± 0.13	9.06 ± 0.11	9.15 ± 0.31	9.28 ± 0.25
TC22000305.hg.1	OTTHUMG00000151089	8.92 ± 0.06	9.07 ± 0.05	9.00 ± 0.04	9.06 ± 0.09
TC22000214.hg.1	OTTHUMG00000151090	8.80 ± 0.34	8.90 ± 0.06	8.83 ± 0.13	9.09 ± 0.14
TC22000188.hg.1	OTTHUMG00000151093	6.55 ± 0.03	6.46 ± 0.14	6.44 ± 0.02	6.54 ± 0.10
TC22000806.hg.1	OTTHUMG00000151100	7.27 ± 0.25	7.31 ± 0.26	7.23 ± 0.09	7.39 ± 0.06
TC22000320.hg.1	OTTHUMG00000151104	7.74 ± 0.12	7.25 ± 0.16	7.67 ± 0.15	7.28 ± 0.11
TC22000300.hg.1	OTTHUMG00000151130	7.14 ± 0.26	7.37 ± 0.16	7.30 ± 0.11	7.48 ± 0.14
TC22000789.hg.1	OTTHUMG00000151134	6.59 ± 0.31	6.78 ± 0.19	6.81 ± 0.29	6.80 ± 0.05
TC22000810.hg.1	OTTHUMG00000151143	5.68 ± 0.11	5.62 ± 0.05	5.64 ± 0.13	5.62 ± 0.06
TC22000325.hg.1	OTTHUMG00000151147	6.73 ± 0.21	6.73 ± 0.07	6.74 ± 0.17	6.80 ± 0.15
TC22000194.hg.1	OTTHUMG00000151156	7.47 ± 0.24	7.46 ± 0.04	7.51 ± 0.18	7.60 ± 0.07
TC22000310.hg.1	OTTHUMG00000151191	7.22 ± 0.08	7.32 ± 0.13	7.06 ± 0.19	7.04 ± 0.11
TC22000783.hg.1	OTTHUMG00000151194	8.14 ± 0.34	8.22 ± 0.26	8.10 ± 0.25	8.31 ± 0.15
TC22000222.hg.1	OTTHUMG00000151204	6.36 ± 0.09	6.34 ± 0.13	6.36 ± 0.03	6.44 ± 0.07
TC22000555.hg.1	OTTHUMG00000151209	7.72 ± 0.16	7.77 ± 0.17	7.74 ± 0.12	7.87 ± 0.10
TC22000299.hg.1	OTTHUMG00000151248	5.53 ± 0.18	5.76 ± 0.12	5.66 ± 0.30	5.94 ± 0.11
TC22000225.hg.1	OTTHUMG00000151249	7.12 ± 0.10	7.14 ± 0.08	7.05 ± 0.29	7.18 ± 0.06
TC22000212.hg.1	OTTHUMG00000151260	7.50 ± 0.13	7.47 ± 0.16	7.49 ± 0.14	7.64 ± 0.08
TC22000833.hg.1	OTTHUMG00000151272	6.40 ± 0.09	6.45 ± 0.12	6.31 ± 0.05	6.53 ± 0.05
TC22000837.hg.1	OTTHUMG00000151275	5.96 ± 0.14	5.92 ± 0.17	5.98 ± 0.25	6.05 ± 0.08
TC22000640.hg.1	OTTHUMG00000151279	7.92 ± 0.25	8.02 ± 0.16	7.95 ± 0.06	8.19 ± 0.14
TC22000645.hg.1	OTTHUMG00000151289	6.48 ± 0.12	6.39 ± 0.16	6.37 ± 0.12	6.47 ± 0.04
TC22000639.hg.1	OTTHUMG00000151293	6.85 ± 0.10	6.69 ± 0.09	6.89 ± 0.04	6.87 ± 0.04
TC04000407.hg.1	OTTHUMG00000151319	4.02 ± 0.03	3.99 ± 0.07	4.02 ± 0.19	4.16 ± 0.19
TC07001037.hg.1	OTTHUMG00000151323	6.94 ± 0.30	7.08 ± 0.20	7.01 ± 0.30	7.27 ± 0.15
TC07002056.hg.1	OTTHUMG00000151325	6.42 ± 0.09	6.34 ± 0.16	6.39 ± 0.08	6.33 ± 0.04

TC07001038.hg.1	OTTHUMG00000151328	6.56 ± 0.14	6.74 ± 0.17	6.63 ± 0.27	6.83 ± 0.05
TC22000876.hg.1	OTTHUMG00000151333	8.06 ± 0.31	8.10 ± 0.21	8.03 ± 0.39	8.10 ± 0.15
TC02001490.hg.1	OTTHUMG00000151343	8.08 ± 0.17	8.13 ± 0.06	7.87 ± 0.05	8.18 ± 0.17
TC22000167.hg.1	OTTHUMG00000151344	5.68 ± 0.17	5.52 ± 0.15	5.53 ± 0.22	5.75 ± 0.18
TC22000605.hg.1	OTTHUMG00000151345	6.89 ± 0.27	6.67 ± 0.12	6.73 ± 0.18	6.87 ± 0.01
TC22000606.hg.1	OTTHUMG00000151346	6.60 ± 0.25	6.54 ± 0.17	6.55 ± 0.13	6.65 ± 0.10
TC07001042.hg.1	OTTHUMG00000151347	6.57 ± 0.12	6.52 ± 0.17	6.60 ± 0.05	6.70 ± 0.20
TC07002060.hg.1	OTTHUMG00000151348	6.82 ± 0.15	6.59 ± 0.20	6.67 ± 0.10	6.63 ± 0.01
TC07001045.hg.1	OTTHUMG00000151352	7.07 ± 0.21	7.01 ± 0.33	7.01 ± 0.08	7.21 ± 0.18
TC22000174.hg.1	OTTHUMG00000151357	7.04 ± 0.11	7.04 ± 0.05	7.00 ± 0.09	7.18 ± 0.10
TC02000006.hg.1	OTTHUMG00000151358	5.81 ± 0.23	5.89 ± 0.12	5.86 ± 0.12	5.95 ± 0.14
TC02000008.hg.1	OTTHUMG00000151360	6.70 ± 0.19	6.51 ± 0.07	6.59 ± 0.11	6.62 ± 0.07
TC02000007.hg.1	OTTHUMG00000151361	7.28 ± 0.31	7.51 ± 0.21	7.28 ± 0.35	7.44 ± 0.15
TC02001497.hg.1	OTTHUMG00000151363	6.18 ± 0.15	6.15 ± 0.08	6.20 ± 0.05	6.46 ± 0.26
TC02000003.hg.1	OTTHUMG00000151364	6.39 ± 0.19	6.56 ± 0.20	6.35 ± 0.09	6.63 ± 0.11
TC02000004.hg.1	OTTHUMG00000151365	6.57 ± 0.28	6.60 ± 0.24	6.48 ± 0.24	6.72 ± 0.07
TC02000005.hg.1	OTTHUMG00000151367	6.53 ± 0.31	6.63 ± 0.22	6.43 ± 0.04	6.79 ± 0.11
TC02001504.hg.1	OTTHUMG00000151371	7.24 ± 0.07	7.19 ± 0.10	7.19 ± 0.15	7.35 ± 0.19
TC02001491.hg.1	OTTHUMG00000151373	7.60 ± 0.20	7.64 ± 0.01	7.51 ± 0.02	7.76 ± 0.20
TC02001500.hg.1	OTTHUMG00000151375	5.92 ± 0.29	6.05 ± 0.27	5.93 ± 0.25	6.07 ± 0.15
TC02000010.hg.1	OTTHUMG00000151376	7.86 ± 0.18	7.80 ± 0.08	7.65 ± 0.13	7.87 ± 0.08
TC02000001.hg.1	OTTHUMG00000151377	6.38 ± 0.25	6.18 ± 0.18	6.37 ± 0.20	6.37 ± 0.04
TC02000009.hg.1	OTTHUMG00000151378	7.26 ± 0.18	7.19 ± 0.15	7.25 ± 0.10	7.28 ± 0.09
TC02001501.hg.1	OTTHUMG00000151379	5.84 ± 0.27	5.66 ± 0.03	5.68 ± 0.10	5.76 ± 0.07
TC02000011.hg.1	OTTHUMG00000151380	7.04 ± 0.34	6.83 ± 0.10	7.00 ± 0.13	7.07 ± 0.23
TC02001511.hg.1	OTTHUMG00000151382	7.37 ± 0.30	7.43 ± 0.13	7.35 ± 0.28	7.60 ± 0.11
TC02001509.hg.1	OTTHUMG00000151383	6.80 ± 0.37	6.62 ± 0.11	6.66 ± 0.15	6.58 ± 0.05
TC02000014.hg.1	OTTHUMG00000151384	6.25 ± 0.38	5.97 ± 0.15	5.96 ± 0.16	6.09 ± 0.06
TC02002963.hg.1	OTTHUMG00000151385	5.16 ± 0.20	5.06 ± 0.04	4.98 ± 0.04	5.25 ± 0.15
TC02001507.hg.1	OTTHUMG00000151387	5.22 ± 0.22	5.22 ± 0.09	5.21 ± 0.21	5.23 ± 0.09
TC02001508.hg.1	OTTHUMG00000151388	6.73 ± 0.20	6.73 ± 0.18	6.82 ± 0.17	6.81 ± 0.10

TC02001505.hg.1	OTTHUMG00000151389	6.47 ± 0.26	6.30 ± 0.27	6.47 ± 0.15	6.49 ± 0.04
TC02001506.hg.1	OTTHUMG00000151390	4.55 ± 0.07	4.63 ± 0.14	4.62 ± 0.12	4.75 ± 0.12
TC02001488.hg.1	OTTHUMG00000151392	8.32 ± 0.02	8.56 ± 0.18	8.38 ± 0.17	8.70 ± 0.18
TC02001516.hg.1	OTTHUMG00000151393	7.81 ± 0.17	7.87 ± 0.23	7.70 ± 0.16	8.04 ± 0.11
TC02001515.hg.1	OTTHUMG00000151394	6.93 ± 0.15	6.85 ± 0.15	6.76 ± 0.27	6.84 ± 0.10
TC02001514.hg.1	OTTHUMG00000151395	7.23 ± 0.20	7.12 ± 0.27	7.08 ± 0.25	7.27 ± 0.08
TC07001070.hg.1	OTTHUMG00000151397	6.61 ± 0.22	6.55 ± 0.21	6.54 ± 0.19	6.68 ± 0.06
TC07000001.hg.1	OTTHUMG00000151400	7.71 ± 0.21	7.67 ± 0.20	7.82 ± 0.13	7.66 ± 0.04
TC07000003.hg.1	OTTHUMG00000151402	5.47 ± 0.31	5.55 ± 0.15	5.27 ± 0.27	5.60 ± 0.08
TC07001068.hg.1	OTTHUMG00000151403	7.27 ± 0.21	7.26 ± 0.08	7.35 ± 0.40	7.49 ± 0.19
TC07001069.hg.1	OTTHUMG00000151405	6.57 ± 0.18	6.46 ± 0.10	6.46 ± 0.12	6.52 ± 0.09
TC02000015.hg.1	OTTHUMG00000151406	7.26 ± 0.43	7.19 ± 0.27	7.18 ± 0.26	7.18 ± 0.19
TC07001072.hg.1	OTTHUMG00000151413	6.49 ± 0.02	6.71 ± 0.19	6.49 ± 0.36	6.86 ± 0.05
TC07000010.hg.1	OTTHUMG00000151414	8.65 ± 0.32	8.42 ± 0.14	8.56 ± 0.44	8.57 ± 0.14
TC07000009.hg.1	OTTHUMG00000151415	7.09 ± 0.47	7.05 ± 0.31	6.98 ± 0.33	7.16 ± 0.01
TC02001534.hg.1	OTTHUMG00000151420	7.13 ± 0.29	6.99 ± 0.27	6.89 ± 0.19	7.08 ± 0.12
TC02001537.hg.1	OTTHUMG00000151421	6.32 ± 0.19	6.38 ± 0.12	6.32 ± 0.09	6.33 ± 0.04
TC02000031.hg.1	OTTHUMG00000151422	4.21 ± 0.04	4.20 ± 0.12	4.18 ± 0.09	4.40 ± 0.08
TC02001535.hg.1	OTTHUMG00000151424	5.99 ± 0.20	5.99 ± 0.07	6.02 ± 0.30	6.12 ± 0.07
TC02001536.hg.1	OTTHUMG00000151425	4.91 ± 0.13	4.80 ± 0.07	4.90 ± 0.07	4.91 ± 0.13
TC02001518.hg.1	OTTHUMG00000151427	6.04 ± 0.19	6.01 ± 0.05	5.97 ± 0.14	6.08 ± 0.09
TC02001517.hg.1	OTTHUMG00000151428	6.63 ± 0.07	6.56 ± 0.13	6.63 ± 0.23	6.69 ± 0.07
TC02001520.hg.1	OTTHUMG00000151429	7.55 ± 0.21	7.52 ± 0.15	7.38 ± 0.17	7.55 ± 0.06
TC02000018.hg.1	OTTHUMG00000151435	7.61 ± 0.27	7.45 ± 0.08	7.45 ± 0.33	7.58 ± 0.11
TC07002067.hg.1	OTTHUMG00000151447	6.94 ± 0.20	6.89 ± 0.16	6.90 ± 0.14	7.10 ± 0.14
TC02002959.hg.1	OTTHUMG00000151448	7.67 ± 0.21	7.76 ± 0.24	7.67 ± 0.13	7.83 ± 0.09
TC02001539.hg.1	OTTHUMG00000151457	6.22 ± 0.07	6.20 ± 0.06	6.38 ± 0.19	6.35 ± 0.22
TC02001538.hg.1	OTTHUMG00000151460	5.84 ± 0.17	5.71 ± 0.27	5.69 ± 0.07	5.80 ± 0.03
TC02000033.hg.1	OTTHUMG00000151461	5.47 ± 0.19	5.39 ± 0.08	5.46 ± 0.11	5.42 ± 0.12
TC07002048.hg.1	OTTHUMG00000151464	5.84 ± 0.24	5.71 ± 0.13	5.62 ± 0.13	5.69 ± 0.08
TC07002049.hg.1	OTTHUMG00000151464	6.58 ± 0.41	6.70 ± 0.28	6.45 ± 0.26	6.83 ± 0.18

TC02001527.hg.1	OTTHUMG00000151467	7.80 ± 0.29	7.66 ± 0.11	7.78 ± 0.15	7.72 ± 0.05
TC02000020.hg.1	OTTHUMG00000151471	7.16 ± 0.10	6.94 ± 0.11	7.10 ± 0.23	6.93 ± 0.03
TC02002958.hg.1	OTTHUMG00000151472	8.42 ± 0.20	8.52 ± 0.16	8.52 ± 0.10	8.61 ± 0.13
TC02002956.hg.1	OTTHUMG00000151478	8.54 ± 0.42	8.53 ± 0.19	8.46 ± 0.32	8.59 ± 0.07
TC02002957.hg.1	OTTHUMG00000151479	11.00 ± 0.27	10.82 ± 0.06	11.03 ± 0.16	10.95 ± 0.05
TC07000019.hg.1	OTTHUMG00000151480	7.30 ± 0.17	7.21 ± 0.06	7.13 ± 0.15	7.26 ± 0.02
TC07000016.hg.1	OTTHUMG00000151481	8.62 ± 0.19	8.63 ± 0.19	8.64 ± 0.31	8.68 ± 0.14
TC07000014.hg.1	OTTHUMG00000151482	6.88 ± 0.13	6.97 ± 0.39	6.89 ± 0.21	7.14 ± 0.07
TC07000017.hg.1	OTTHUMG00000151483	8.68 ± 0.17	8.73 ± 0.21	8.54 ± 0.17	8.86 ± 0.14
TC02001531.hg.1	OTTHUMG00000151487	4.43 ± 0.16	4.16 ± 0.08	4.23 ± 0.11	4.19 ± 0.04
TC02001529.hg.1	OTTHUMG00000151488	7.38 ± 0.16	7.17 ± 0.06	7.22 ± 0.21	7.03 ± 0.08
TC02000026.hg.1	OTTHUMG00000151491	6.14 ± 0.35	5.87 ± 0.06	5.99 ± 0.17	5.93 ± 0.15
TC07001089.hg.1	OTTHUMG00000151492	8.46 ± 0.20	8.45 ± 0.23	8.41 ± 0.21	8.58 ± 0.06
TC07001091.hg.1	OTTHUMG00000151496	6.92 ± 0.28	6.82 ± 0.32	6.87 ± 0.23	6.92 ± 0.06
TC07000026.hg.1	OTTHUMG00000151498	7.06 ± 0.20	6.93 ± 0.16	7.04 ± 0.10	7.02 ± 0.12
TC07000981.hg.1	OTTHUMG00000151503	6.74 ± 0.25	6.75 ± 0.16	6.64 ± 0.15	6.84 ± 0.12
TC02002955.hg.1	OTTHUMG00000151506	7.11 ± 0.17	6.96 ± 0.23	7.03 ± 0.03	6.95 ± 0.18
TC07002072.hg.1	OTTHUMG00000151508	8.61 ± 0.06	8.65 ± 0.10	8.62 ± 0.11	8.78 ± 0.10
TC07002071.hg.1	OTTHUMG00000151509	6.56 ± 0.38	6.88 ± 0.10	6.44 ± 0.31	6.76 ± 0.16
TC02001484.hg.1	OTTHUMG00000151513	7.67 ± 0.08	7.61 ± 0.13	7.63 ± 0.18	7.55 ± 0.03
TC07000031.hg.1	OTTHUMG00000151516	8.25 ± 0.44	8.05 ± 0.24	8.10 ± 0.19	8.14 ± 0.04
TC07002051.hg.1	OTTHUMG00000151522	6.09 ± 0.17	6.03 ± 0.09	5.96 ± 0.23	6.04 ± 0.01
TC19000603.hg.1	OTTHUMG00000151543	5.35 ± 0.26	5.27 ± 0.20	5.31 ± 0.11	5.49 ± 0.22
TC02002950.hg.1	OTTHUMG00000151587	6.30 ± 0.13	6.23 ± 0.05	6.45 ± 0.19	6.20 ± 0.08
TC02001548.hg.1	OTTHUMG00000151589	5.64 ± 0.09	5.48 ± 0.01	5.51 ± 0.02	5.54 ± 0.03
TC02000038.hg.1	OTTHUMG00000151591	6.80 ± 0.25	6.71 ± 0.07	6.66 ± 0.19	6.60 ± 0.35
TC02001547.hg.1	OTTHUMG00000151592	6.30 ± 0.17	6.45 ± 0.17	6.28 ± 0.24	6.46 ± 0.16
TC02000040.hg.1	OTTHUMG00000151596	4.66 ± 0.12	4.60 ± 0.21	4.56 ± 0.03	4.77 ± 0.06
TC02001552.hg.1	OTTHUMG00000151622	6.67 ± 0.26	6.64 ± 0.23	6.57 ± 0.13	6.79 ± 0.07
TC02001551.hg.1	OTTHUMG00000151623	7.54 ± 0.17	7.57 ± 0.18	7.44 ± 0.38	7.69 ± 0.11
TC02001554.hg.1	OTTHUMG00000151625	6.40 ± 0.16	6.30 ± 0.11	6.30 ± 0.13	6.41 ± 0.07

TC02001553.hg.1	OTTHUMG00000151627	4.52 ± 0.10	4.35 ± 0.08	4.48 ± 0.06	4.42 ± 0.04
TC02001545.hg.1	OTTHUMG00000151660	5.95 ± 0.14	5.92 ± 0.10	5.78 ± 0.04	5.91 ± 0.08
TC02001808.hg.1	OTTHUMG00000151675	4.95 ± 0.05	4.81 ± 0.03	4.79 ± 0.11	5.04 ± 0.19
TC02001821.hg.1	OTTHUMG00000151698	6.92 ± 0.21	6.95 ± 0.16	6.81 ± 0.10	7.08 ± 0.12
TC02000056.hg.1	OTTHUMG00000151705	7.26 ± 0.14	7.22 ± 0.20	7.28 ± 0.10	7.28 ± 0.13
TC02000061.hg.1	OTTHUMG00000151706	6.97 ± 0.14	7.09 ± 0.21	6.90 ± 0.22	7.22 ± 0.22
TC02000063.hg.1	OTTHUMG00000151707	6.20 ± 0.16	5.98 ± 0.03	5.98 ± 0.13	5.96 ± 0.08
TC02000066.hg.1	OTTHUMG00000151708	6.25 ± 0.23	6.16 ± 0.16	6.36 ± 0.19	6.30 ± 0.12
TC02000081.hg.1	OTTHUMG00000151710	5.59 ± 0.12	5.60 ± 0.14	5.50 ± 0.04	5.70 ± 0.10
TC02000291.hg.1	OTTHUMG00000151712	6.09 ± 0.07	6.07 ± 0.07	6.18 ± 0.09	6.28 ± 0.16
TC02000085.hg.1	OTTHUMG00000151713	4.64 ± 0.20	4.60 ± 0.03	4.61 ± 0.10	4.69 ± 0.14
TC02000084.hg.1	OTTHUMG00000151714	7.49 ± 0.21	7.43 ± 0.15	7.46 ± 0.15	7.74 ± 0.22
TC02000086.hg.1	OTTHUMG00000151715	5.55 ± 0.11	5.60 ± 0.24	5.49 ± 0.08	5.65 ± 0.04
TC02001812.hg.1	OTTHUMG00000151721	9.22 ± 0.38	9.40 ± 0.08	9.25 ± 0.17	9.53 ± 0.13
TC02001811.hg.1	OTTHUMG00000151722	6.13 ± 0.30	6.11 ± 0.11	6.09 ± 0.04	6.19 ± 0.13
TC02001594.hg.1	OTTHUMG00000151723	8.17 ± 0.19	8.05 ± 0.18	8.09 ± 0.20	8.11 ± 0.16
TC02000090.hg.1	OTTHUMG00000151724	6.26 ± 0.20	6.26 ± 0.15	6.16 ± 0.13	6.39 ± 0.13
TC02000088.hg.1	OTTHUMG00000151725	5.80 ± 0.12	5.68 ± 0.09	5.70 ± 0.14	5.82 ± 0.18
TC02001591.hg.1	OTTHUMG00000151726	7.56 ± 0.32	7.53 ± 0.06	7.56 ± 0.13	7.56 ± 0.12
TC02001593.hg.1	OTTHUMG00000151727	6.23 ± 0.16	6.20 ± 0.12	6.04 ± 0.19	6.44 ± 0.28
TC07001085.hg.1	OTTHUMG00000151728	8.75 ± 0.21	8.71 ± 0.22	8.76 ± 0.17	8.75 ± 0.09
TC02005019.hg.1	OTTHUMG00000151730	6.96 ± 0.27	6.66 ± 0.50	6.92 ± 0.48	6.68 ± 0.10
TC07001108.hg.1	OTTHUMG00000151734	5.12 ± 0.35	5.10 ± 0.16	5.12 ± 0.34	5.26 ± 0.08
TC07001105.hg.1	OTTHUMG00000151735	5.89 ± 0.18	5.72 ± 0.08	5.80 ± 0.09	5.64 ± 0.06
TC07001106.hg.1	OTTHUMG00000151736	6.36 ± 0.20	6.28 ± 0.20	6.23 ± 0.09	6.44 ± 0.15
TC02000297.hg.1	OTTHUMG00000151737	6.73 ± 0.21	6.66 ± 0.03	6.56 ± 0.06	6.77 ± 0.07
TC02000101.hg.1	OTTHUMG00000151740	6.67 ± 0.21	6.63 ± 0.06	6.61 ± 0.03	6.89 ± 0.17
TC02001597.hg.1	OTTHUMG00000151742	6.09 ± 0.20	5.89 ± 0.16	6.09 ± 0.13	6.02 ± 0.01
TC02001599.hg.1	OTTHUMG00000151743	6.21 ± 0.08	6.24 ± 0.09	6.25 ± 0.08	6.35 ± 0.15
TC02000095.hg.1	OTTHUMG00000151744	5.24 ± 0.07	5.20 ± 0.05	5.23 ± 0.09	5.31 ± 0.10
TC02000103.hg.1	OTTHUMG00000151745	4.94 ± 0.20	4.92 ± 0.07	5.00 ± 0.09	5.00 ± 0.07

TC02000097.hg.1	OTTHUMG00000151746	5.50 ± 0.17	5.54 ± 0.21	5.42 ± 0.23	5.74 ± 0.11
TC02000100.hg.1	OTTHUMG00000151748	4.73 ± 0.04	4.63 ± 0.09	4.67 ± 0.18	4.75 ± 0.05
TC02001598.hg.1	OTTHUMG00000151749	7.75 ± 0.41	7.68 ± 0.19	7.73 ± 0.27	7.95 ± 0.08
TC02000098.hg.1	OTTHUMG00000151750	6.03 ± 0.19	5.97 ± 0.12	5.92 ± 0.13	6.00 ± 0.09
TC02001596.hg.1	OTTHUMG00000151751	8.82 ± 0.36	8.79 ± 0.15	8.76 ± 0.32	9.02 ± 0.19
TC02000099.hg.1	OTTHUMG00000151752	5.72 ± 0.12	5.54 ± 0.14	5.62 ± 0.07	5.60 ± 0.06
TC02001827.hg.1	OTTHUMG00000151756	5.80 ± 0.03	5.57 ± 0.04	5.65 ± 0.04	5.62 ± 0.02
TC02000108.hg.1	OTTHUMG00000151764	4.25 ± 0.19	4.42 ± 0.26	4.69 ± 0.34	4.37 ± 0.25
TC02001610.hg.1	OTTHUMG00000151766	5.64 ± 0.11	5.58 ± 0.01	5.56 ± 0.03	5.68 ± 0.14
TC02001612.hg.1	OTTHUMG00000151772	4.43 ± 0.21	4.24 ± 0.12	4.35 ± 0.18	4.30 ± 0.12
TC02000110.hg.1	OTTHUMG00000151774	5.80 ± 0.27	5.71 ± 0.28	5.74 ± 0.30	5.87 ± 0.14
TC02000111.hg.1	OTTHUMG00000151775	6.45 ± 0.38	6.31 ± 0.02	6.26 ± 0.02	6.43 ± 0.30
TC02001824.hg.1	OTTHUMG00000151777	7.66 ± 0.15	7.58 ± 0.01	7.57 ± 0.01	7.65 ± 0.07
TC02000117.hg.1	OTTHUMG00000151780	6.33 ± 0.30	6.29 ± 0.13	6.39 ± 0.14	6.45 ± 0.13
TC02001624.hg.1	OTTHUMG00000151781	6.25 ± 0.26	6.10 ± 0.25	6.13 ± 0.09	6.32 ± 0.30
TC02000120.hg.1	OTTHUMG00000151782	5.36 ± 0.32	5.40 ± 0.07	5.28 ± 0.20	5.44 ± 0.18
TC02000122.hg.1	OTTHUMG00000151783	4.74 ± 0.17	4.61 ± 0.07	4.64 ± 0.16	4.74 ± 0.08
TC02001618.hg.1	OTTHUMG00000151787	7.22 ± 0.11	7.33 ± 0.29	7.28 ± 0.16	7.47 ± 0.19
TC02000113.hg.1	OTTHUMG00000151790	5.29 ± 0.04	5.21 ± 0.10	5.23 ± 0.18	5.28 ± 0.04
TC02002945.hg.1	OTTHUMG00000151792	6.68 ± 0.08	6.60 ± 0.10	6.63 ± 0.20	6.66 ± 0.17
TC02000123.hg.1	OTTHUMG00000151794	4.48 ± 0.11	4.36 ± 0.10	4.36 ± 0.09	4.37 ± 0.19
TC02000125.hg.1	OTTHUMG00000151796	6.02 ± 0.09	5.91 ± 0.07	5.89 ± 0.10	5.99 ± 0.09
TC07000051.hg.1	OTTHUMG00000151804	7.57 ± 0.35	7.47 ± 0.18	7.45 ± 0.21	7.42 ± 0.04
TC02001633.hg.1	OTTHUMG00000151805	6.12 ± 0.07	6.10 ± 0.09	6.13 ± 0.07	6.18 ± 0.16
TC02001629.hg.1	OTTHUMG00000151806	5.35 ± 0.30	5.30 ± 0.05	5.23 ± 0.17	5.35 ± 0.10
TC02001632.hg.1	OTTHUMG00000151808	4.60 ± 0.15	4.46 ± 0.02	4.42 ± 0.04	4.48 ± 0.11
TC02001630.hg.1	OTTHUMG00000151809	5.59 ± 0.24	5.41 ± 0.04	5.53 ± 0.04	5.61 ± 0.19
TC02001635.hg.1	OTTHUMG00000151813	5.95 ± 0.19	5.96 ± 0.15	5.99 ± 0.05	6.11 ± 0.11
TC02000127.hg.1	OTTHUMG00000151815	5.99 ± 0.19	5.93 ± 0.06	5.92 ± 0.05	5.93 ± 0.17
TC02000128.hg.1	OTTHUMG00000151816	4.24 ± 0.14	4.24 ± 0.06	4.23 ± 0.05	4.51 ± 0.44
TC02001578.hg.1	OTTHUMG00000151819	6.41 ± 0.09	6.32 ± 0.07	6.27 ± 0.08	6.30 ± 0.15

TC02001577.hg.1	OTTHUMG00000151821	6.85 ± 0.24	6.79 ± 0.35	6.84 ± 0.18	6.88 ± 0.02
TC02002944.hg.1	OTTHUMG00000151830	7.93 ± 0.22	7.67 ± 0.11	7.66 ± 0.33	7.69 ± 0.06
TC07000052.hg.1	OTTHUMG00000151833	7.89 ± 0.14	7.85 ± 0.07	7.91 ± 0.08	7.99 ± 0.09
TC02001584.hg.1	OTTHUMG00000151836	6.30 ± 0.11	6.26 ± 0.00	6.27 ± 0.11	6.28 ± 0.05
TC02001838.hg.1	OTTHUMG00000151842	6.04 ± 0.22	5.86 ± 0.11	6.05 ± 0.09	5.98 ± 0.11
TC07001133.hg.1	OTTHUMG00000151848	7.34 ± 0.19	7.30 ± 0.26	7.28 ± 0.18	7.42 ± 0.05
TC02001473.hg.1	OTTHUMG00000151855	8.42 ± 0.23	8.41 ± 0.25	8.44 ± 0.27	8.61 ± 0.02
TC02002473.hg.1	OTTHUMG00000151884	4.20 ± 0.04	3.97 ± 0.00	4.18 ± 0.12	4.05 ± 0.07
TC02002472.hg.1	OTTHUMG00000151886	4.56 ± 0.17	4.72 ± 0.12	4.63 ± 0.17	4.85 ± 0.11
TC07000063.hg.1	OTTHUMG00000151887	7.15 ± 0.27	7.12 ± 0.03	7.06 ± 0.10	7.15 ± 0.06
TC02001641.hg.1	OTTHUMG00000151890	7.07 ± 0.31	7.00 ± 0.17	6.87 ± 0.13	6.96 ± 0.10
TC02001639.hg.1	OTTHUMG00000151891	6.19 ± 0.22	6.12 ± 0.21	6.13 ± 0.24	6.23 ± 0.08
TC02001647.hg.1	OTTHUMG00000151892	6.19 ± 0.20	6.21 ± 0.04	6.13 ± 0.09	6.29 ± 0.07
TC02005057.hg.1	OTTHUMG00000151893	6.76 ± 0.19	6.80 ± 0.07	6.87 ± 0.03	6.84 ± 0.01
TC02001648.hg.1	OTTHUMG00000151894	6.70 ± 0.03	6.53 ± 0.02	6.67 ± 0.11	6.59 ± 0.04
TC02001640.hg.1	OTTHUMG00000151895	7.37 ± 0.22	7.46 ± 0.19	7.35 ± 0.20	7.67 ± 0.08
TC02001637.hg.1	OTTHUMG00000151898	7.27 ± 0.24	7.19 ± 0.10	7.17 ± 0.15	7.32 ± 0.08
TC02000130.hg.1	OTTHUMG00000151900	7.45 ± 0.13	7.49 ± 0.26	7.41 ± 0.20	7.69 ± 0.12
TC02001638.hg.1	OTTHUMG00000151905	5.04 ± 0.11	5.18 ± 0.15	5.21 ± 0.06	5.29 ± 0.23
TC07000076.hg.1	OTTHUMG00000151907	6.07 ± 0.12	5.97 ± 0.06	6.04 ± 0.12	6.02 ± 0.15
TC02000172.hg.1	OTTHUMG00000151914	8.04 ± 0.16	7.98 ± 0.09	7.98 ± 0.17	8.09 ± 0.05
TC07000086.hg.1	OTTHUMG00000151921	4.98 ± 0.25	4.63 ± 0.06	4.79 ± 0.10	4.65 ± 0.10
TC07000088.hg.1	OTTHUMG00000151923	5.20 ± 0.14	4.91 ± 0.13	5.21 ± 0.32	4.87 ± 0.03
TC07000085.hg.1	OTTHUMG00000151925	4.60 ± 0.12	4.41 ± 0.03	4.57 ± 0.14	4.42 ± 0.05
TC02000177.hg.1	OTTHUMG00000151939	5.11 ± 0.12	5.03 ± 0.15	5.08 ± 0.18	5.09 ± 0.16
TC02001700.hg.1	OTTHUMG00000151942	6.67 ± 0.21	6.42 ± 0.12	6.50 ± 0.06	6.58 ± 0.09
TC02001694.hg.1	OTTHUMG00000151945	5.63 ± 0.16	5.64 ± 0.26	5.61 ± 0.21	5.76 ± 0.04
TC02001670.hg.1	OTTHUMG00000151992	5.67 ± 0.29	5.74 ± 0.12	5.45 ± 0.20	5.75 ± 0.10
TC02001669.hg.1	OTTHUMG00000151995	6.77 ± 0.28	6.70 ± 0.34	6.81 ± 0.17	6.90 ± 0.15
TC02001671.hg.1	OTTHUMG00000151998	6.42 ± 0.19	6.43 ± 0.13	6.40 ± 0.07	6.52 ± 0.12
TC02001709.hg.1	OTTHUMG00000152000	5.23 ± 0.30	5.07 ± 0.08	5.09 ± 0.10	5.13 ± 0.01

TC02000193.hg.1	OTTHUMG00000152001	5.63 ± 0.07	5.59 ± 0.05	5.44 ± 0.25	5.72 ± 0.22
TC02000194.hg.1	OTTHUMG00000152004	6.32 ± 0.24	6.07 ± 0.09	6.33 ± 0.10	6.18 ± 0.07
TC02000142.hg.1	OTTHUMG00000152010	6.48 ± 0.13	6.45 ± 0.27	6.53 ± 0.09	6.61 ± 0.10
TC02001701.hg.1	OTTHUMG00000152013	6.15 ± 0.15	5.99 ± 0.12	6.36 ± 0.11	6.13 ± 0.13
TC07001140.hg.1	OTTHUMG00000152016	5.37 ± 0.08	5.19 ± 0.16	5.14 ± 0.15	5.35 ± 0.13
TC07001142.hg.1	OTTHUMG00000152017	6.98 ± 0.16	6.80 ± 0.15	7.07 ± 0.15	6.92 ± 0.20
TC07000080.hg.1	OTTHUMG00000152019	6.99 ± 0.06	7.04 ± 0.16	6.94 ± 0.11	7.13 ± 0.16
TC07000021.hg.1	OTTHUMG00000152023	7.11 ± 0.25	7.06 ± 0.18	7.02 ± 0.18	7.12 ± 0.08
TC02001707.hg.1	OTTHUMG00000152025	5.11 ± 0.19	5.11 ± 0.07	5.08 ± 0.07	5.26 ± 0.13
TC02001873.hg.1	OTTHUMG00000152028	4.81 ± 0.13	4.48 ± 0.19	4.78 ± 0.03	4.63 ± 0.09
TC02001869.hg.1	OTTHUMG00000152029	3.98 ± 0.03	3.78 ± 0.11	3.73 ± 0.12	3.72 ± 0.11
TC02000204.hg.1	OTTHUMG00000152041	6.16 ± 0.15	6.06 ± 0.09	6.25 ± 0.16	6.20 ± 0.06
TC07000033.hg.1	OTTHUMG00000152044	7.60 ± 0.17	7.47 ± 0.15	7.28 ± 0.18	7.44 ± 0.11
TC02001717.hg.1	OTTHUMG00000152056	6.66 ± 0.14	6.58 ± 0.08	6.50 ± 0.06	6.62 ± 0.08
TC02000209.hg.1	OTTHUMG00000152058	6.11 ± 0.10	6.05 ± 0.11	6.29 ± 0.04	6.25 ± 0.10
TC07000038.hg.1	OTTHUMG00000152115	7.11 ± 0.11	6.99 ± 0.08	6.96 ± 0.02	6.90 ± 0.17
TC07001103.hg.1	OTTHUMG00000152116	6.87 ± 0.10	6.77 ± 0.20	6.61 ± 0.03	6.74 ± 0.11
TC02000333.hg.1	OTTHUMG00000152120	4.34 ± 0.11	4.34 ± 0.12	4.23 ± 0.04	4.46 ± 0.19
TC02001660.hg.1	OTTHUMG00000152130	7.73 ± 0.17	7.71 ± 0.11	7.66 ± 0.18	7.76 ± 0.20
TC02000222.hg.1	OTTHUMG00000152138	5.93 ± 0.10	5.82 ± 0.06	5.79 ± 0.01	5.87 ± 0.05
TC02000223.hg.1	OTTHUMG00000152139	4.19 ± 0.04	4.12 ± 0.11	4.18 ± 0.10	4.24 ± 0.09
TC02000227.hg.1	OTTHUMG00000152140	7.45 ± 0.51	7.42 ± 0.16	7.63 ± 0.38	7.85 ± 0.38
TC02000224.hg.1	OTTHUMG00000152141	3.79 ± 0.06	3.63 ± 0.17	3.77 ± 0.06	3.67 ± 0.03
TC02001733.hg.1	OTTHUMG00000152142	3.96 ± 0.02	3.86 ± 0.07	3.85 ± 0.10	3.99 ± 0.09
TC02000228.hg.1	OTTHUMG00000152143	3.78 ± 0.20	3.87 ± 0.07	3.76 ± 0.06	4.02 ± 0.15
TC02000226.hg.1	OTTHUMG00000152144	6.03 ± 0.16	5.88 ± 0.12	5.95 ± 0.07	6.06 ± 0.10
TC02004957.hg.1	OTTHUMG00000152168	5.07 ± 0.16	4.99 ± 0.07	4.94 ± 0.16	5.12 ± 0.09
TC02001469.hg.1	OTTHUMG00000152264	8.30 ± 0.16	8.08 ± 0.21	8.20 ± 0.14	8.36 ± 0.17
TC02001840.hg.1	OTTHUMG00000152267	4.69 ± 0.06	4.59 ± 0.04	4.62 ± 0.06	4.61 ± 0.06
TC02001752.hg.1	OTTHUMG00000152269	6.05 ± 0.06	6.02 ± 0.26	6.29 ± 0.18	6.10 ± 0.13
TC02001772.hg.1	OTTHUMG00000152301	7.29 ± 0.30	7.21 ± 0.02	7.09 ± 0.13	7.31 ± 0.06

TC02001776.hg.1	OTTHUMG00000152306	5.72 ± 0.03	5.78 ± 0.14	5.74 ± 0.30	5.73 ± 0.13
TC07000091.hg.1	OTTHUMG00000152311	4.52 ± 0.13	4.42 ± 0.04	4.45 ± 0.05	4.50 ± 0.07
TC07000092.hg.1	OTTHUMG00000152344	5.80 ± 0.19	5.60 ± 0.06	5.66 ± 0.17	5.70 ± 0.09
TC07000100.hg.1	OTTHUMG00000152349	6.56 ± 0.10	6.50 ± 0.04	6.58 ± 0.09	6.74 ± 0.13
TC07001150.hg.1	OTTHUMG00000152350	4.97 ± 0.08	4.85 ± 0.02	4.86 ± 0.02	4.93 ± 0.14
TC07001151.hg.1	OTTHUMG00000152366	4.32 ± 0.25	4.21 ± 0.05	4.26 ± 0.15	4.20 ± 0.26
TC07000071.hg.1	OTTHUMG00000152372	5.31 ± 0.14	5.20 ± 0.03	5.25 ± 0.07	5.23 ± 0.07
TC02001785.hg.1	OTTHUMG00000152373	6.83 ± 0.15	6.89 ± 0.11	6.80 ± 0.10	7.09 ± 0.05
TC02001784.hg.1	OTTHUMG00000152375	7.76 ± 0.32	7.79 ± 0.29	7.66 ± 0.23	8.01 ± 0.18
TC02001786.hg.1	OTTHUMG00000152376	7.12 ± 0.31	7.05 ± 0.20	6.94 ± 0.22	7.25 ± 0.08
TC02002935.hg.1	OTTHUMG00000152377	6.66 ± 0.25	6.61 ± 0.24	6.65 ± 0.23	6.74 ± 0.05
TC07000094.hg.1	OTTHUMG00000152383	4.14 ± 0.11	3.96 ± 0.03	4.04 ± 0.08	4.01 ± 0.02
TC07001148.hg.1	OTTHUMG00000152384	7.74 ± 0.12	7.53 ± 0.21	7.56 ± 0.24	7.67 ± 0.03
TC07000097.hg.1	OTTHUMG00000152386	3.76 ± 0.12	3.47 ± 0.06	3.64 ± 0.10	3.52 ± 0.04
TC07000105.hg.1	OTTHUMG00000152388	6.17 ± 0.13	6.30 ± 0.15	6.18 ± 0.19	6.39 ± 0.17
TC07000103.hg.1	OTTHUMG00000152389	4.57 ± 0.10	4.42 ± 0.02	4.48 ± 0.13	4.39 ± 0.07
TC07000104.hg.1	OTTHUMG00000152391	4.17 ± 0.24	4.16 ± 0.11	4.22 ± 0.10	4.30 ± 0.24
TC07001157.hg.1	OTTHUMG00000152392	4.04 ± 0.09	3.97 ± 0.15	4.08 ± 0.15	4.09 ± 0.06
TC07001158.hg.1	OTTHUMG00000152394	5.19 ± 0.05	5.11 ± 0.09	5.04 ± 0.13	5.14 ± 0.14
TC02000253.hg.1	OTTHUMG00000152401	3.90 ± 0.06	3.75 ± 0.08	3.90 ± 0.06	3.88 ± 0.10
TC02000268.hg.1	OTTHUMG00000152402	6.89 ± 0.23	6.76 ± 0.08	6.65 ± 0.17	6.90 ± 0.09
TC02004965.hg.1	OTTHUMG00000152404	8.40 ± 0.12	8.45 ± 0.15	8.26 ± 0.09	8.49 ± 0.03
TC02001841.hg.1	OTTHUMG00000152413	4.20 ± 0.10	3.99 ± 0.03	4.01 ± 0.02	4.00 ± 0.11
TC02000309.hg.1	OTTHUMG00000152414	4.98 ± 0.03	4.82 ± 0.22	4.94 ± 0.00	4.97 ± 0.06
TC02001462.hg.1	OTTHUMG00000152416	7.34 ± 0.09	7.27 ± 0.12	7.37 ± 0.21	7.42 ± 0.09
TC02001851.hg.1	OTTHUMG00000152417	4.91 ± 0.17	4.74 ± 0.05	4.86 ± 0.06	4.81 ± 0.04
TC02001852.hg.1	OTTHUMG00000152418	4.33 ± 0.05	4.31 ± 0.05	4.47 ± 0.15	4.33 ± 0.03
TC02000278.hg.1	OTTHUMG00000152422	6.69 ± 0.18	6.63 ± 0.12	6.60 ± 0.08	6.55 ± 0.03
TC02000279.hg.1	OTTHUMG00000152442	8.33 ± 0.29	8.23 ± 0.17	8.14 ± 0.25	8.21 ± 0.05
TC02001805.hg.1	OTTHUMG00000152444	8.91 ± 0.35	8.89 ± 0.36	8.85 ± 0.33	9.05 ± 0.03
TC07001165.hg.1	OTTHUMG00000152468	4.41 ± 0.06	4.36 ± 0.02	4.38 ± 0.04	4.39 ± 0.01

TC02001854.hg.1	OTTHUMG00000152469	4.69 ± 0.28	4.37 ± 0.18	4.45 ± 0.21	4.35 ± 0.10
TC07000102.hg.1	OTTHUMG00000152476	5.31 ± 0.20	5.22 ± 0.11	5.31 ± 0.09	5.40 ± 0.31
TC07001162.hg.1	OTTHUMG00000152479	6.38 ± 0.03	6.33 ± 0.06	6.35 ± 0.11	6.38 ± 0.11
TC02001937.hg.1	OTTHUMG00000152485	4.56 ± 0.20	4.51 ± 0.12	4.53 ± 0.13	4.57 ± 0.11
TC02000394.hg.1	OTTHUMG00000152486	5.63 ± 0.13	5.47 ± 0.05	5.50 ± 0.18	5.48 ± 0.09
TC07000121.hg.1	OTTHUMG00000152489	4.69 ± 0.11	4.55 ± 0.08	4.57 ± 0.07	4.65 ± 0.10
TC07000120.hg.1	OTTHUMG00000152490	7.17 ± 0.20	7.21 ± 0.08	7.12 ± 0.17	7.17 ± 0.03
TC02000324.hg.1	OTTHUMG00000152491	5.51 ± 0.12	5.44 ± 0.05	5.71 ± 0.22	5.46 ± 0.06
TC07000118.hg.1	OTTHUMG00000152496	6.46 ± 0.14	6.55 ± 0.26	6.46 ± 0.24	6.52 ± 0.13
TC07000119.hg.1	OTTHUMG00000152498	6.36 ± 0.28	6.41 ± 0.20	6.29 ± 0.08	6.50 ± 0.10
TC07000124.hg.1	OTTHUMG00000152502	4.36 ± 0.11	4.29 ± 0.07	4.24 ± 0.03	4.31 ± 0.06
TC02001774.hg.1	OTTHUMG00000152505	6.63 ± 0.16	6.62 ± 0.19	6.63 ± 0.16	6.74 ± 0.08
TC02001853.hg.1	OTTHUMG00000152506	6.91 ± 0.36	7.10 ± 0.16	6.86 ± 0.42	6.91 ± 0.06
TC07001172.hg.1	OTTHUMG00000152508	6.11 ± 0.14	5.94 ± 0.13	6.08 ± 0.10	6.08 ± 0.08
TC02000236.hg.1	OTTHUMG00000152509	4.49 ± 0.19	4.52 ± 0.17	4.43 ± 0.03	4.66 ± 0.10
TC07001180.hg.1	OTTHUMG00000152511	4.04 ± 0.08	3.93 ± 0.09	4.03 ± 0.09	3.97 ± 0.02
TC02001896.hg.1	OTTHUMG00000152512	5.31 ± 0.16	5.21 ± 0.09	5.21 ± 0.03	5.24 ± 0.11
TC12001628.hg.1	OTTHUMG00000152518	6.33 ± 0.27	6.22 ± 0.19	6.20 ± 0.01	6.31 ± 0.07
TC02001899.hg.1	OTTHUMG00000152520	4.18 ± 0.21	4.00 ± 0.06	4.05 ± 0.07	4.02 ± 0.04
TC02000359.hg.1	OTTHUMG00000152523	7.19 ± 0.20	7.23 ± 0.10	7.19 ± 0.15	7.27 ± 0.07
TC07000136.hg.1	OTTHUMG00000152531	5.49 ± 0.21	5.59 ± 0.02	5.51 ± 0.19	5.76 ± 0.13
TC07001187.hg.1	OTTHUMG00000152532	6.50 ± 0.21	6.50 ± 0.11	6.59 ± 0.11	6.57 ± 0.16
TC07001181.hg.1	OTTHUMG00000152535	4.50 ± 0.11	4.29 ± 0.06	4.36 ± 0.04	4.33 ± 0.03
TC02001728.hg.1	OTTHUMG00000152537	4.62 ± 0.19	4.40 ± 0.15	4.55 ± 0.20	4.48 ± 0.17
TC07001174.hg.1	OTTHUMG00000152543	4.51 ± 0.14	4.29 ± 0.18	4.56 ± 0.05	4.34 ± 0.11
TC02001901.hg.1	OTTHUMG00000152544	4.46 ± 0.09	4.39 ± 0.04	4.54 ± 0.18	4.40 ± 0.04
TC02001858.hg.1	OTTHUMG00000152548	6.40 ± 0.12	6.34 ± 0.07	6.39 ± 0.05	6.32 ± 0.04
TC07001201.hg.1	OTTHUMG00000152555	6.46 ± 0.12	6.54 ± 0.19	6.60 ± 0.07	6.62 ± 0.01
TC02000238.hg.1	OTTHUMG00000152557	6.80 ± 0.19	6.81 ± 0.10	6.71 ± 0.12	6.76 ± 0.11
TC02000239.hg.1	OTTHUMG00000152558	4.24 ± 0.10	4.14 ± 0.11	4.31 ± 0.05	4.32 ± 0.08
TC02001751.hg.1	OTTHUMG00000152559	9.52 ± 0.43	9.37 ± 0.19	9.74 ± 0.25	9.64 ± 0.11

TC02001936.hg.1	OTTHUMG00000152562	5.40 ± 0.13	5.38 ± 0.13	5.21 ± 0.16	5.58 ± 0.21
TC07000138.hg.1	OTTHUMG00000152563	6.54 ± 0.21	6.38 ± 0.01	6.32 ± 0.12	6.41 ± 0.04
TC07001203.hg.1	OTTHUMG00000152567	5.71 ± 0.21	5.67 ± 0.12	5.80 ± 0.09	5.88 ± 0.17
TC04000077.hg.1	OTTHUMG00000152568	6.70 ± 0.13	6.61 ± 0.05	6.83 ± 0.18	6.62 ± 0.09
TC02001942.hg.1	OTTHUMG00000152574	6.45 ± 0.04	6.42 ± 0.22	6.88 ± 0.20	6.45 ± 0.01
TC07001191.hg.1	OTTHUMG00000152579	6.49 ± 0.20	6.33 ± 0.19	6.48 ± 0.17	6.32 ± 0.09
TC07000129.hg.1	OTTHUMG00000152589	4.59 ± 0.08	4.59 ± 0.07	4.57 ± 0.11	4.58 ± 0.06
TC07001211.hg.1	OTTHUMG00000152593	6.05 ± 0.22	5.94 ± 0.06	6.01 ± 0.03	6.05 ± 0.04
TC07001212.hg.1	OTTHUMG00000152594	6.04 ± 0.09	5.93 ± 0.09	5.96 ± 0.11	5.99 ± 0.09
TC07001210.hg.1	OTTHUMG00000152596	4.90 ± 0.13	4.80 ± 0.07	4.85 ± 0.02	4.92 ± 0.12
TC02001880.hg.1	OTTHUMG00000152614	5.68 ± 0.16	5.61 ± 0.05	5.60 ± 0.08	5.68 ± 0.12
TC02000335.hg.1	OTTHUMG00000152615	5.86 ± 0.05	5.86 ± 0.25	5.95 ± 0.24	6.07 ± 0.21
TC02000337.hg.1	OTTHUMG00000152616	5.07 ± 0.09	5.01 ± 0.20	5.13 ± 0.11	5.17 ± 0.20
TC02001883.hg.1	OTTHUMG00000152618	6.08 ± 0.22	6.11 ± 0.07	6.08 ± 0.10	6.06 ± 0.08
TC02001882.hg.1	OTTHUMG00000152619	7.25 ± 0.25	7.30 ± 0.05	7.24 ± 0.10	7.31 ± 0.05
TC02000344.hg.1	OTTHUMG00000152622	6.25 ± 0.25	6.05 ± 0.06	6.09 ± 0.04	6.13 ± 0.08
TC02000401.hg.1	OTTHUMG00000152623	6.42 ± 0.15	6.59 ± 0.01	6.84 ± 0.27	6.55 ± 0.07
TC02001777.hg.1	OTTHUMG00000152629	8.74 ± 0.15	8.79 ± 0.04	8.68 ± 0.11	8.81 ± 0.06
TC02001910.hg.1	OTTHUMG00000152631	4.81 ± 0.33	5.42 ± 0.40	5.43 ± 0.55	5.01 ± 0.47
TC07001058.hg.1	OTTHUMG00000152640	5.71 ± 0.16	5.73 ± 0.11	5.63 ± 0.17	5.66 ± 0.04
TC07001063.hg.1	OTTHUMG00000152641	8.04 ± 0.33	7.98 ± 0.24	7.91 ± 0.31	8.10 ± 0.13
TC07001056.hg.1	OTTHUMG00000152642	6.76 ± 0.22	6.80 ± 0.24	6.80 ± 0.09	6.94 ± 0.07
TC07001060.hg.1	OTTHUMG00000152643	6.88 ± 0.31	6.81 ± 0.33	6.74 ± 0.45	6.93 ± 0.02
TC07001062.hg.1	OTTHUMG00000152647	7.15 ± 0.07	7.04 ± 0.14	7.04 ± 0.24	7.12 ± 0.05
TC07001064.hg.1	OTTHUMG00000152648	7.96 ± 0.28	7.77 ± 0.27	7.82 ± 0.22	7.99 ± 0.02
TC02000355.hg.1	OTTHUMG00000152650	4.45 ± 0.24	4.31 ± 0.01	4.48 ± 0.16	4.41 ± 0.04
TC07000143.hg.1	OTTHUMG00000152653	7.89 ± 0.09	7.80 ± 0.09	7.87 ± 0.07	7.78 ± 0.04
TC02001779.hg.1	OTTHUMG00000152656	6.48 ± 0.20	6.44 ± 0.05	6.42 ± 0.12	6.61 ± 0.13
TC02001460.hg.1	OTTHUMG00000152658	8.34 ± 0.30	8.31 ± 0.27	8.19 ± 0.31	8.34 ± 0.01
TC02001915.hg.1	OTTHUMG00000152670	5.62 ± 0.13	5.55 ± 0.05	5.63 ± 0.01	5.64 ± 0.08
TC02001913.hg.1	OTTHUMG00000152671	7.23 ± 0.10	7.13 ± 0.08	7.13 ± 0.18	7.22 ± 0.08

TC02001914.hg.1	OTTHUMG00000152672	8.20 ± 0.19	8.02 ± 0.16	8.09 ± 0.24	8.21 ± 0.18
TC07001968.hg.1	OTTHUMG00000152673	6.60 ± 0.11	6.60 ± 0.39	6.51 ± 0.08	6.78 ± 0.07
TC02000367.hg.1	OTTHUMG00000152676	5.73 ± 0.25	5.76 ± 0.07	5.79 ± 0.05	5.80 ± 0.05
TC02001952.hg.1	OTTHUMG00000152688	7.91 ± 0.09	7.85 ± 0.05	7.91 ± 0.04	7.97 ± 0.04
TC07001200.hg.1	OTTHUMG00000152691	6.89 ± 0.34	6.74 ± 0.32	6.60 ± 0.28	6.82 ± 0.05
TC02001921.hg.1	OTTHUMG00000152699	5.91 ± 0.13	6.01 ± 0.07	5.99 ± 0.07	6.05 ± 0.05
TC02000372.hg.1	OTTHUMG00000152702	6.54 ± 0.10	6.56 ± 0.04	6.56 ± 0.19	6.56 ± 0.08
TC02001918.hg.1	OTTHUMG00000152703	6.53 ± 0.15	6.42 ± 0.05	6.52 ± 0.09	6.52 ± 0.09
TC07001231.hg.1	OTTHUMG00000152710	6.52 ± 0.48	6.42 ± 0.04	6.44 ± 0.16	6.37 ± 0.04
TC07001985.hg.1	OTTHUMG00000152719	5.50 ± 0.18	5.44 ± 0.17	5.28 ± 0.17	5.40 ± 0.18
TC07001167.hg.1	OTTHUMG00000152727	5.91 ± 0.25	5.83 ± 0.10	5.77 ± 0.11	5.90 ± 0.08
TC07000115.hg.1	OTTHUMG00000152728	7.60 ± 0.17	7.48 ± 0.27	7.47 ± 0.05	7.53 ± 0.05
TC07000113.hg.1	OTTHUMG00000152729	5.47 ± 0.33	5.22 ± 0.17	5.29 ± 0.05	5.34 ± 0.20
TC07001168.hg.1	OTTHUMG00000152731	6.26 ± 0.25	6.08 ± 0.08	6.15 ± 0.12	6.01 ± 0.22
TC11002031.hg.1	OTTHUMG00000152739	6.16 ± 0.11	6.07 ± 0.03	6.02 ± 0.09	6.20 ± 0.25
TC11002032.hg.1	OTTHUMG00000152740	7.27 ± 0.16	7.22 ± 0.09	7.28 ± 0.02	7.29 ± 0.13
TC07001989.hg.1	OTTHUMG00000152744	5.51 ± 0.28	5.52 ± 0.17	5.39 ± 0.29	5.58 ± 0.09
TC07001238.hg.1	OTTHUMG00000152748	6.10 ± 0.13	6.28 ± 0.18	6.24 ± 0.05	6.34 ± 0.14
TC07001240.hg.1	OTTHUMG00000152753	4.88 ± 0.18	4.83 ± 0.22	4.76 ± 0.10	4.89 ± 0.03
TC02000379.hg.1	OTTHUMG00000152757	5.19 ± 0.10	5.07 ± 0.07	5.03 ± 0.02	5.00 ± 0.03
TC02000381.hg.1	OTTHUMG00000152758	4.94 ± 0.10	4.82 ± 0.14	4.82 ± 0.03	4.87 ± 0.05
TC07000190.hg.1	OTTHUMG00000152760	5.65 ± 0.02	5.52 ± 0.18	5.63 ± 0.15	5.56 ± 0.06
TC07001247.hg.1	OTTHUMG00000152762	7.15 ± 0.05	7.29 ± 0.09	7.39 ± 0.17	7.44 ± 0.16
TC07001246.hg.1	OTTHUMG00000152763	4.63 ± 0.34	4.19 ± 0.06	4.31 ± 0.19	4.30 ± 0.07
TC07000193.hg.1	OTTHUMG00000152764	8.22 ± 0.13	8.15 ± 0.07	8.25 ± 0.09	8.20 ± 0.10
TC07003404.hg.1	OTTHUMG00000152788	7.99 ± 0.21	7.95 ± 0.26	8.13 ± 0.37	8.00 ± 0.13
TC07003405.hg.1	OTTHUMG00000152789	8.92 ± 0.17	8.73 ± 0.23	8.92 ± 0.21	8.68 ± 0.08
TC07000179.hg.1	OTTHUMG00000152794	7.88 ± 0.20	7.84 ± 0.03	7.75 ± 0.13	7.91 ± 0.08
TC02001927.hg.1	OTTHUMG00000152797	6.74 ± 0.12	6.84 ± 0.02	6.89 ± 0.14	6.99 ± 0.06
TC07001253.hg.1	OTTHUMG00000152799	4.84 ± 0.09	4.54 ± 0.09	4.43 ± 0.16	4.60 ± 0.21
TC07000197.hg.1	OTTHUMG00000152804	6.17 ± 0.15	6.13 ± 0.11	6.33 ± 0.35	6.07 ± 0.10

TC07001215.hg.1	OTTHUMG00000152805	7.34 ± 0.20	7.23 ± 0.09	7.32 ± 0.11	7.26 ± 0.01
TC02000385.hg.1	OTTHUMG00000152809	4.41 ± 0.20	4.33 ± 0.09	4.28 ± 0.02	4.34 ± 0.12
TC02000450.hg.1	OTTHUMG00000152824	8.98 ± 0.16	9.15 ± 0.04	9.23 ± 0.11	9.21 ± 0.10
TC07000182.hg.1	OTTHUMG00000152828	6.84 ± 0.22	6.87 ± 0.07	6.99 ± 0.11	6.90 ± 0.02
TC02001933.hg.1	OTTHUMG00000152839	5.79 ± 0.17	5.72 ± 0.08	5.74 ± 0.22	5.75 ± 0.03
TC02000478.hg.1	OTTHUMG00000152840	4.94 ± 0.19	4.79 ± 0.08	4.82 ± 0.05	4.85 ± 0.18
TC02000480.hg.1	OTTHUMG00000152841	4.73 ± 0.22	4.81 ± 0.16	4.78 ± 0.10	4.87 ± 0.11
TC02002012.hg.1	OTTHUMG00000152843	8.84 ± 0.34	8.89 ± 0.07	8.75 ± 0.08	8.96 ± 0.16
TC02002919.hg.1	OTTHUMG00000152844	4.55 ± 0.15	4.50 ± 0.14	4.57 ± 0.11	4.49 ± 0.04
TC02000482.hg.1	OTTHUMG00000152845	3.53 ± 0.04	3.48 ± 0.02	3.50 ± 0.10	3.51 ± 0.08
TC02000483.hg.1	OTTHUMG00000152846	6.03 ± 0.20	5.97 ± 0.18	5.86 ± 0.22	6.10 ± 0.04
TC02001456.hg.1	OTTHUMG00000152851	6.76 ± 0.12	6.73 ± 0.11	6.73 ± 0.11	7.02 ± 0.16
TC02001934.hg.1	OTTHUMG00000152852	4.22 ± 0.17	4.35 ± 0.03	4.12 ± 0.05	4.41 ± 0.19
TC02000389.hg.1	OTTHUMG00000152857	6.04 ± 0.19	5.90 ± 0.10	5.76 ± 0.15	5.97 ± 0.29
TC07000181.hg.1	OTTHUMG00000152858	4.39 ± 0.09	4.34 ± 0.03	4.54 ± 0.04	4.43 ± 0.08
TC02000391.hg.1	OTTHUMG00000152863	5.48 ± 0.09	5.55 ± 0.15	5.51 ± 0.06	5.68 ± 0.18
TC02000390.hg.1	OTTHUMG00000152865	4.67 ± 0.02	4.56 ± 0.03	4.65 ± 0.12	4.61 ± 0.10
TC02002916.hg.1	OTTHUMG00000152867	5.85 ± 0.32	5.79 ± 0.11	5.72 ± 0.21	5.72 ± 0.17
TC02002016.hg.1	OTTHUMG00000152871	4.87 ± 0.25	4.88 ± 0.05	4.86 ± 0.10	5.20 ± 0.24
TC02000498.hg.1	OTTHUMG00000152874	5.52 ± 0.26	5.71 ± 0.11	5.71 ± 0.14	5.75 ± 0.18
TC07001255.hg.1	OTTHUMG00000152878	4.39 ± 0.19	4.20 ± 0.05	4.26 ± 0.08	4.30 ± 0.15
TC02000392.hg.1	OTTHUMG00000152879	5.92 ± 0.19	5.86 ± 0.12	5.76 ± 0.11	5.86 ± 0.13
TC15001502.hg.1	OTTHUMG00000152880	5.01 ± 0.47	5.34 ± 0.19	5.42 ± 0.19	5.48 ± 0.32
TC02002915.hg.1	OTTHUMG00000152883	7.01 ± 0.20	6.99 ± 0.10	6.96 ± 0.04	7.08 ± 0.07
TC02002026.hg.1	OTTHUMG00000152890	5.73 ± 0.19	5.51 ± 0.09	5.61 ± 0.16	5.58 ± 0.06
TC02000384.hg.1	OTTHUMG00000152908	5.12 ± 0.38	5.06 ± 0.13	5.17 ± 0.15	5.24 ± 0.13
TC02001928.hg.1	OTTHUMG00000152909	6.74 ± 0.13	6.67 ± 0.04	6.66 ± 0.15	6.73 ± 0.12
TC02002020.hg.1	OTTHUMG00000152910	5.07 ± 0.07	4.85 ± 0.03	4.94 ± 0.06	4.90 ± 0.11
TC02002025.hg.1	OTTHUMG00000152911	3.85 ± 0.17	3.82 ± 0.08	3.89 ± 0.13	3.84 ± 0.05
TC02002023.hg.1	OTTHUMG00000152926	5.57 ± 0.24	5.43 ± 0.15	5.51 ± 0.18	5.47 ± 0.02
TC02002004.hg.1	OTTHUMG00000152930	7.03 ± 0.24	6.92 ± 0.08	6.94 ± 0.23	6.93 ± 0.10

TC02000469.hg.1	OTTHUMG00000152931	6.17 ± 0.18	5.95 ± 0.09	6.03 ± 0.10	6.00 ± 0.07
TC02000468.hg.1	OTTHUMG00000152932	5.71 ± 0.11	5.51 ± 0.05	5.61 ± 0.14	5.55 ± 0.11
TC02000471.hg.1	OTTHUMG00000152939	5.88 ± 0.11	5.71 ± 0.08	5.73 ± 0.10	5.76 ± 0.15
TC02000473.hg.1	OTTHUMG00000152941	5.60 ± 0.13	5.38 ± 0.11	5.53 ± 0.22	5.50 ± 0.09
TC02002030.hg.1	OTTHUMG00000152955	4.13 ± 0.10	4.09 ± 0.11	4.00 ± 0.07	4.10 ± 0.11
TC02002032.hg.1	OTTHUMG00000152956	4.33 ± 0.11	4.36 ± 0.04	4.28 ± 0.03	4.31 ± 0.02
TC02001445.hg.1	OTTHUMG00000152959	7.84 ± 0.04	7.69 ± 0.11	7.81 ± 0.04	7.79 ± 0.06
TC02000494.hg.1	OTTHUMG00000152969	5.21 ± 0.15	4.84 ± 0.10	5.05 ± 0.25	4.97 ± 0.06
TC02001444.hg.1	OTTHUMG00000152979	6.68 ± 0.25	6.50 ± 0.20	6.46 ± 0.13	6.61 ± 0.04
TC02002907.hg.1	OTTHUMG00000152980	5.87 ± 0.10	5.60 ± 0.19	5.76 ± 0.13	5.78 ± 0.16
TC02002906.hg.1	OTTHUMG00000152981	6.80 ± 0.14	6.72 ± 0.07	6.86 ± 0.15	6.71 ± 0.06
TC02002118.hg.1	OTTHUMG00000152992	7.17 ± 0.21	7.02 ± 0.08	7.05 ± 0.08	7.22 ± 0.17
TC02000591.hg.1	OTTHUMG00000153001	5.71 ± 0.26	5.89 ± 0.07	5.83 ± 0.31	5.83 ± 0.07
TC07000221.hg.1	OTTHUMG00000153006	7.95 ± 0.28	7.97 ± 0.13	7.89 ± 0.27	8.15 ± 0.17
TC02002904.hg.1	OTTHUMG00000153018	7.64 ± 0.48	7.81 ± 0.10	7.63 ± 0.22	8.08 ± 0.22
TC02001440.hg.1	OTTHUMG00000153019	6.19 ± 0.05	6.31 ± 0.12	6.18 ± 0.22	6.26 ± 0.07
TC07002079.hg.1	OTTHUMG00000153023	7.75 ± 0.23	7.90 ± 0.20	7.84 ± 0.15	7.99 ± 0.10
TC02001438.hg.1	OTTHUMG00000153025	6.94 ± 0.29	6.83 ± 0.18	6.77 ± 0.10	7.01 ± 0.12
TC02002903.hg.1	OTTHUMG00000153027	6.29 ± 0.16	6.46 ± 0.22	6.23 ± 0.02	6.49 ± 0.03
TC02002141.hg.1	OTTHUMG00000153028	7.37 ± 0.31	7.29 ± 0.08	7.23 ± 0.24	7.29 ± 0.03
TC02000612.hg.1	OTTHUMG00000153029	5.11 ± 0.05	5.24 ± 0.10	5.06 ± 0.03	5.31 ± 0.09
TC21000417.hg.1	OTTHUMG00000153030	6.31 ± 0.46	6.20 ± 0.22	6.31 ± 0.18	6.28 ± 0.07
TC02002142.hg.1	OTTHUMG00000153031	5.78 ± 0.30	5.79 ± 0.12	5.79 ± 0.20	5.99 ± 0.22
TC02000062.hg.1	OTTHUMG00000153036	4.96 ± 0.02	4.80 ± 0.07	4.87 ± 0.08	4.85 ± 0.03
TC02002154.hg.1	OTTHUMG00000153043	5.36 ± 0.09	5.28 ± 0.18	5.36 ± 0.12	5.39 ± 0.08
TC02002155.hg.1	OTTHUMG00000153044	4.74 ± 0.20	4.79 ± 0.06	4.70 ± 0.07	4.83 ± 0.04
TC02002153.hg.1	OTTHUMG00000153045	5.66 ± 0.06	5.72 ± 0.04	5.76 ± 0.10	5.79 ± 0.16
TC02000633.hg.1	OTTHUMG00000153047	6.33 ± 0.26	6.07 ± 0.01	6.11 ± 0.18	5.96 ± 0.19
TC02002157.hg.1	OTTHUMG00000153049	4.71 ± 0.35	4.56 ± 0.34	4.77 ± 0.11	4.78 ± 0.24
TC02002158.hg.1	OTTHUMG00000153051	4.68 ± 0.09	4.56 ± 0.05	4.62 ± 0.11	4.66 ± 0.09
TC02000639.hg.1	OTTHUMG00000153054	6.31 ± 0.15	6.32 ± 0.11	6.11 ± 0.05	6.44 ± 0.22

TC02000636.hg.1	OTTHUMG00000153055	4.56 ± 0.27	4.18 ± 0.08	4.20 ± 0.22	4.20 ± 0.09
TC02002160.hg.1	OTTHUMG00000153056	4.48 ± 0.03	4.55 ± 0.06	4.51 ± 0.10	4.51 ± 0.06
TC02000637.hg.1	OTTHUMG00000153057	5.52 ± 0.16	5.38 ± 0.10	5.38 ± 0.06	5.44 ± 0.05
TC02001436.hg.1	OTTHUMG00000153059	4.09 ± 0.15	4.09 ± 0.13	4.02 ± 0.04	4.07 ± 0.12
TC02002164.hg.1	OTTHUMG00000153077	6.44 ± 0.18	6.37 ± 0.08	6.30 ± 0.15	6.35 ± 0.03
TC02002165.hg.1	OTTHUMG00000153078	4.72 ± 0.21	4.54 ± 0.05	4.61 ± 0.07	4.57 ± 0.08
TC02000643.hg.1	OTTHUMG00000153079	7.56 ± 0.19	7.47 ± 0.16	7.37 ± 0.20	7.43 ± 0.04
TC02001817.hg.1	OTTHUMG00000153084	5.84 ± 0.17	5.87 ± 0.07	5.92 ± 0.04	6.00 ± 0.09
TC02001435.hg.1	OTTHUMG00000153089	7.58 ± 0.24	7.47 ± 0.15	7.43 ± 0.22	7.51 ± 0.06
TC02000288.hg.1	OTTHUMG00000153091	10.15 ± 0.31	10.11 ± 0.17	9.93 ± 0.27	10.13 ± 0.15
TC02000626.hg.1	OTTHUMG00000153092	7.21 ± 0.23	6.89 ± 0.40	8.10 ± 0.74	6.65 ± 0.25
TC02000630.hg.1	OTTHUMG00000153094	5.09 ± 0.17	5.03 ± 0.21	5.07 ± 0.15	5.19 ± 0.11
TC02002150.hg.1	OTTHUMG00000153096	4.64 ± 0.18	4.71 ± 0.17	4.78 ± 0.13	4.74 ± 0.11
TC02002151.hg.1	OTTHUMG00000153097	4.26 ± 0.07	4.14 ± 0.08	4.19 ± 0.07	4.17 ± 0.10
TC02000625.hg.1	OTTHUMG00000153098	8.29 ± 0.31	7.60 ± 0.39	8.78 ± 0.70	7.36 ± 0.27
TC02000631.hg.1	OTTHUMG00000153099	4.63 ± 0.51	4.59 ± 0.04	4.58 ± 0.12	4.72 ± 0.32
TC02002125.hg.1	OTTHUMG00000153102	6.65 ± 0.15	6.69 ± 0.19	6.61 ± 0.16	6.72 ± 0.07
TC02000598.hg.1	OTTHUMG00000153105	6.86 ± 0.19	6.74 ± 0.14	6.82 ± 0.12	6.72 ± 0.06
TC02000645.hg.1	OTTHUMG00000153107	6.03 ± 0.05	5.99 ± 0.05	6.01 ± 0.12	5.96 ± 0.09
TC02000647.hg.1	OTTHUMG00000153108	6.46 ± 0.28	6.55 ± 0.21	6.48 ± 0.20	6.68 ± 0.10
TC02000648.hg.1	OTTHUMG00000153109	6.68 ± 0.08	6.52 ± 0.07	6.62 ± 0.15	6.66 ± 0.09
TC02002152.hg.1	OTTHUMG00000153111	5.80 ± 0.02	5.63 ± 0.07	5.71 ± 0.07	5.69 ± 0.02
TC02002169.hg.1	OTTHUMG00000153117	4.89 ± 0.02	4.87 ± 0.06	4.91 ± 0.12	4.82 ± 0.04
TC02002894.hg.1	OTTHUMG00000153125	6.04 ± 0.18	6.01 ± 0.06	6.05 ± 0.10	6.19 ± 0.10
TC02000415.hg.1	OTTHUMG00000153127	6.22 ± 0.02	6.10 ± 0.05	6.35 ± 0.09	6.08 ± 0.10
TC02001955.hg.1	OTTHUMG00000153128	6.78 ± 0.03	6.69 ± 0.05	6.69 ± 0.07	6.72 ± 0.05
TC02000290.hg.1	OTTHUMG00000153134	7.97 ± 0.11	8.02 ± 0.06	7.84 ± 0.27	8.12 ± 0.06
TC02001819.hg.1	OTTHUMG00000153135	6.10 ± 0.06	5.98 ± 0.14	5.99 ± 0.11	6.04 ± 0.11
TC06000111.hg.1	OTTHUMG00000153136	7.60 ± 0.09	7.64 ± 0.23	7.50 ± 0.18	7.77 ± 0.10
TC02001813.hg.1	OTTHUMG00000153138	6.27 ± 0.21	6.25 ± 0.28	6.07 ± 0.13	6.37 ± 0.07
TC02000254.hg.1	OTTHUMG00000153143	4.37 ± 0.17	4.40 ± 0.13	4.20 ± 0.11	4.35 ± 0.09

TC02000657.hg.1	OTTHUMG00000153144	5.86 ± 0.22	5.62 ± 0.06	5.81 ± 0.14	5.80 ± 0.13
TC02002893.hg.1	OTTHUMG00000153145	7.35 ± 0.26	7.35 ± 0.12	7.19 ± 0.20	7.54 ± 0.10
TC02001428.hg.1	OTTHUMG00000153146	6.30 ± 0.07	6.10 ± 0.06	6.19 ± 0.14	6.22 ± 0.13
TC02002892.hg.1	OTTHUMG00000153147	6.11 ± 0.21	6.14 ± 0.02	6.01 ± 0.15	6.30 ± 0.20
TC02000610.hg.1	OTTHUMG00000153149	6.15 ± 0.12	6.19 ± 0.19	6.11 ± 0.12	6.24 ± 0.14
TC02002170.hg.1	OTTHUMG00000153152	6.44 ± 0.11	6.40 ± 0.14	6.50 ± 0.12	6.57 ± 0.14
TC02000651.hg.1	OTTHUMG00000153153	4.98 ± 0.03	4.95 ± 0.16	5.02 ± 0.09	4.89 ± 0.11
TC02002889.hg.1	OTTHUMG00000153157	6.90 ± 0.17	6.72 ± 0.09	6.59 ± 0.21	6.76 ± 0.04
TC02001425.hg.1	OTTHUMG00000153158	5.61 ± 0.14	5.57 ± 0.14	5.55 ± 0.23	5.58 ± 0.12
TC02002140.hg.1	OTTHUMG00000153159	6.97 ± 0.22	6.80 ± 0.31	6.95 ± 0.15	7.05 ± 0.07
TC02002139.hg.1	OTTHUMG00000153160	6.11 ± 0.27	6.23 ± 0.08	6.18 ± 0.14	6.24 ± 0.04
TC02000418.hg.1	OTTHUMG00000153164	6.17 ± 0.24	6.24 ± 0.27	6.22 ± 0.07	6.24 ± 0.09
TC02002128.hg.1	OTTHUMG00000153173	7.46 ± 0.09	7.45 ± 0.05	7.45 ± 0.08	7.56 ± 0.11
TC02002180.hg.1	OTTHUMG00000153177	4.57 ± 0.15	4.44 ± 0.09	4.56 ± 0.05	4.58 ± 0.08
TC02000654.hg.1	OTTHUMG00000153180	6.50 ± 0.23	6.36 ± 0.16	6.47 ± 0.06	6.53 ± 0.06
TC02002173.hg.1	OTTHUMG00000153181	7.50 ± 0.17	7.47 ± 0.16	7.39 ± 0.08	7.54 ± 0.05
TC02002177.hg.1	OTTHUMG00000153200	4.83 ± 0.07	4.83 ± 0.11	4.91 ± 0.06	4.95 ± 0.15
TC02000655.hg.1	OTTHUMG00000153202	6.55 ± 0.23	6.52 ± 0.18	6.31 ± 0.31	6.61 ± 0.11
TC02000525.hg.1	OTTHUMG00000153204	5.77 ± 0.09	5.82 ± 0.15	5.91 ± 0.12	5.91 ± 0.08
TC02002176.hg.1	OTTHUMG00000153205	5.80 ± 0.17	5.84 ± 0.14	5.75 ± 0.17	5.98 ± 0.17
TC02000658.hg.1	OTTHUMG00000153209	6.08 ± 0.08	6.07 ± 0.18	6.06 ± 0.09	6.08 ± 0.17
TC02000605.hg.1	OTTHUMG00000153210	5.32 ± 0.13	5.40 ± 0.18	5.34 ± 0.04	5.38 ± 0.05
TC02002182.hg.1	OTTHUMG00000153229	6.52 ± 0.20	6.28 ± 0.06	6.37 ± 0.09	6.39 ± 0.15
TC02000421.hg.1	OTTHUMG00000153230	8.07 ± 0.17	8.01 ± 0.29	8.07 ± 0.25	8.08 ± 0.03
TC02001412.hg.1	OTTHUMG00000153231	6.83 ± 0.18	6.85 ± 0.09	6.65 ± 0.25	7.00 ± 0.06
TC02000425.hg.1	OTTHUMG00000153238	8.14 ± 0.17	8.00 ± 0.02	8.07 ± 0.14	8.03 ± 0.11
TC02002238.hg.1	OTTHUMG00000153281	5.26 ± 0.10	5.10 ± 0.16	5.15 ± 0.27	5.08 ± 0.21
TC02002239.hg.1	OTTHUMG00000153283	5.00 ± 0.15	4.81 ± 0.09	4.75 ± 0.06	4.76 ± 0.14
TC02002878.hg.1	OTTHUMG00000153291	4.19 ± 0.08	3.97 ± 0.14	3.80 ± 0.14	3.81 ± 0.16
TC02000709.hg.1	OTTHUMG00000153300	7.17 ± 0.23	7.24 ± 0.01	7.19 ± 0.07	7.36 ± 0.09
TC02000753.hg.1	OTTHUMG00000153309	6.71 ± 0.24	6.77 ± 0.13	6.75 ± 0.10	6.88 ± 0.10

TC02000659.hg.1	OTTHUMG00000153313	4.89 ± 0.04	4.90 ± 0.01	4.94 ± 0.04	5.04 ± 0.23
TC02002877.hg.1	OTTHUMG00000153318	6.67 ± 0.20	6.63 ± 0.20	6.68 ± 0.06	6.70 ± 0.05
TC02000766.hg.1	OTTHUMG00000153330	4.56 ± 0.18	4.42 ± 0.20	4.58 ± 0.30	4.42 ± 0.14
TC02000770.hg.1	OTTHUMG00000153333	5.83 ± 0.20	5.74 ± 0.07	5.72 ± 0.02	5.79 ± 0.09
TC02002873.hg.1	OTTHUMG00000153335	7.89 ± 0.33	7.76 ± 0.30	7.78 ± 0.32	7.84 ± 0.04
TC02000724.hg.1	OTTHUMG00000153341	5.59 ± 0.31	5.37 ± 0.25	5.87 ± 0.65	5.10 ± 0.21
TC02002221.hg.1	OTTHUMG00000153342	7.82 ± 0.33	7.90 ± 0.21	7.80 ± 0.18	8.04 ± 0.17
TC02002273.hg.1	OTTHUMG00000153351	6.14 ± 0.49	6.29 ± 0.50	6.04 ± 0.41	6.52 ± 0.15
TC02002871.hg.1	OTTHUMG00000153355	8.25 ± 0.26	8.34 ± 0.22	8.22 ± 0.33	8.47 ± 0.10
TC02002822.hg.1	OTTHUMG00000153359	4.04 ± 0.19	3.77 ± 0.07	3.93 ± 0.01	3.89 ± 0.08
TC02002179.hg.1	OTTHUMG00000153366	4.86 ± 0.10	4.74 ± 0.04	4.82 ± 0.09	4.73 ± 0.03
TC02000776.hg.1	OTTHUMG00000153369	4.34 ± 0.13	4.20 ± 0.05	4.16 ± 0.06	4.20 ± 0.09
TC02000777.hg.1	OTTHUMG00000153374	5.99 ± 0.21	5.81 ± 0.20	5.94 ± 0.22	5.98 ± 0.06
TC02001353.hg.1	OTTHUMG00000153377	5.21 ± 0.25	4.97 ± 0.17	5.00 ± 0.11	4.95 ± 0.04
TC02001352.hg.1	OTTHUMG00000153378	7.36 ± 0.12	7.20 ± 0.05	7.29 ± 0.15	7.27 ± 0.10
TC02002228.hg.1	OTTHUMG00000153380	6.59 ± 0.30	6.51 ± 0.09	6.53 ± 0.07	6.77 ± 0.11
TC02000733.hg.1	OTTHUMG00000153381	7.70 ± 0.29	7.58 ± 0.11	7.67 ± 0.16	7.69 ± 0.07
TC02002870.hg.1	OTTHUMG00000153384	5.10 ± 0.13	4.90 ± 0.08	4.89 ± 0.15	4.82 ± 0.03
TC02002274.hg.1	OTTHUMG00000153386	5.43 ± 0.09	5.26 ± 0.18	5.37 ± 0.05	5.37 ± 0.12
TC02002815.hg.1	OTTHUMG00000153390	4.53 ± 0.09	4.42 ± 0.01	4.59 ± 0.21	4.49 ± 0.08
TC02000782.hg.1	OTTHUMG00000153398	6.78 ± 0.22	6.81 ± 0.11	6.55 ± 0.05	6.67 ± 0.00
TC02000784.hg.1	OTTHUMG00000153411	5.73 ± 0.26	5.83 ± 0.06	5.82 ± 0.22	5.80 ± 0.05
TC02002296.hg.1	OTTHUMG00000153414	4.24 ± 0.23	4.22 ± 0.18	4.12 ± 0.06	4.39 ± 0.17
TC02002295.hg.1	OTTHUMG00000153416	6.20 ± 0.20	6.02 ± 0.19	6.14 ± 0.22	6.30 ± 0.06
TC02002268.hg.1	OTTHUMG00000153424	4.96 ± 0.11	4.86 ± 0.10	4.96 ± 0.19	4.95 ± 0.22
TC02000771.hg.1	OTTHUMG00000153425	5.41 ± 0.24	5.35 ± 0.06	5.27 ± 0.25	5.45 ± 0.13
TC02002269.hg.1	OTTHUMG00000153426	4.92 ± 0.29	5.11 ± 0.37	4.99 ± 0.19	5.26 ± 0.18
TC02001754.hg.1	OTTHUMG00000153431	6.18 ± 0.12	6.13 ± 0.13	6.14 ± 0.11	6.21 ± 0.08
TC02000244.hg.1	OTTHUMG00000153432	7.79 ± 0.29	7.46 ± 0.26	7.63 ± 0.29	7.38 ± 0.05
TC02002147.hg.1	OTTHUMG00000153435	5.55 ± 0.08	5.51 ± 0.02	5.43 ± 0.06	5.55 ± 0.21
TC02000420.hg.1	OTTHUMG00000153445	5.94 ± 0.20	5.91 ± 0.04	5.74 ± 0.12	5.85 ± 0.12

TC01004060.hg.1	OTTHUMG00000153447	6.76 ± 0.12	6.57 ± 0.11	6.59 ± 0.06	6.63 ± 0.12
TC01002043.hg.1	OTTHUMG00000153448	4.64 ± 0.23	4.68 ± 0.14	4.63 ± 0.08	4.74 ± 0.05
TC02001756.hg.1	OTTHUMG00000153449	7.02 ± 0.22	7.05 ± 0.02	7.01 ± 0.24	7.17 ± 0.07
TC02001356.hg.1	OTTHUMG00000153466	4.02 ± 0.04	3.95 ± 0.15	4.08 ± 0.04	3.97 ± 0.10
TC06000148.hg.1	OTTHUMG00000153468	6.51 ± 0.22	6.33 ± 0.13	6.45 ± 0.17	6.42 ± 0.02
TC02002829.hg.1	OTTHUMG00000153471	6.76 ± 0.16	6.45 ± 0.10	6.49 ± 0.08	6.62 ± 0.08
TC06000752.hg.1	OTTHUMG00000153472	3.41 ± 0.04	3.31 ± 0.16	3.38 ± 0.11	3.28 ± 0.07
TC02000794.hg.1	OTTHUMG00000153485	7.32 ± 0.24	7.27 ± 0.30	7.28 ± 0.12	7.43 ± 0.16
TC02002298.hg.1	OTTHUMG00000153489	6.52 ± 0.32	6.65 ± 0.25	6.61 ± 0.33	6.87 ± 0.10
TC02000870.hg.1	OTTHUMG00000153495	6.86 ± 0.31	6.83 ± 0.13	6.74 ± 0.10	6.88 ± 0.08
TC21000150.hg.1	OTTHUMG00000153500	6.68 ± 0.22	6.98 ± 0.12	6.86 ± 0.13	7.07 ± 0.10
TC21000493.hg.1	OTTHUMG00000153501	5.76 ± 0.15	5.87 ± 0.27	5.74 ± 0.05	5.94 ± 0.09
TC22000020.hg.1	OTTHUMG00000153502	6.23 ± 0.32	6.21 ± 0.19	6.16 ± 0.24	6.26 ± 0.02
TC22000871.hg.1	OTTHUMG00000153503	6.62 ± 0.26	6.47 ± 0.13	6.46 ± 0.12	6.57 ± 0.17
TC22000465.hg.1	OTTHUMG00000153504	7.24 ± 0.33	7.25 ± 0.16	7.16 ± 0.29	7.39 ± 0.22
TC22000071.hg.1	OTTHUMG00000153507	6.31 ± 0.28	6.18 ± 0.09	6.31 ± 0.10	6.38 ± 0.11
TC22000782.hg.1	OTTHUMG00000153513	9.36 ± 0.40	9.31 ± 0.18	9.28 ± 0.23	9.38 ± 0.04
TC09000026.hg.1	OTTHUMG00000153514	5.68 ± 0.09	5.45 ± 0.06	5.48 ± 0.11	5.54 ± 0.04
TC22000313.hg.1	OTTHUMG00000153516	7.27 ± 0.17	7.17 ± 0.10	7.18 ± 0.16	7.16 ± 0.04
TC02000247.hg.1	OTTHUMG00000153519	4.50 ± 0.16	4.36 ± 0.08	4.39 ± 0.10	4.42 ± 0.12
TC02002236.hg.1	OTTHUMG00000153530	6.60 ± 0.22	6.43 ± 0.25	6.60 ± 0.02	6.59 ± 0.19
TC09000802.hg.1	OTTHUMG00000153545	7.49 ± 0.27	7.52 ± 0.29	7.28 ± 0.23	7.53 ± 0.08
TC10000250.hg.1	OTTHUMG00000153548	5.47 ± 0.37	5.32 ± 0.08	5.40 ± 0.10	5.34 ± 0.03
TC02002837.hg.1	OTTHUMG00000153551	6.10 ± 0.13	6.11 ± 0.21	6.16 ± 0.24	6.24 ± 0.17
TC13000108.hg.1	OTTHUMG00000153574	8.38 ± 0.36	8.45 ± 0.18	8.36 ± 0.08	8.73 ± 0.29
TC02000885.hg.1	OTTHUMG00000153578	6.70 ± 0.17	6.64 ± 0.07	6.60 ± 0.13	6.71 ± 0.09
TC02000886.hg.1	OTTHUMG00000153580	6.47 ± 0.13	6.25 ± 0.08	6.38 ± 0.16	6.34 ± 0.11
TC02002844.hg.1	OTTHUMG00000153588	5.20 ± 0.16	5.15 ± 0.18	5.10 ± 0.07	5.33 ± 0.12
TC02002842.hg.1	OTTHUMG00000153589	3.92 ± 0.16	3.74 ± 0.02	3.86 ± 0.10	3.72 ± 0.10
TC02002362.hg.1	OTTHUMG00000153603	6.99 ± 0.19	6.75 ± 0.11	6.83 ± 0.02	7.00 ± 0.19
TC02002383.hg.1	OTTHUMG00000153614	6.34 ± 0.12	6.28 ± 0.11	6.26 ± 0.11	6.35 ± 0.03

TC02000892.hg.1	OTTHUMG00000153616	7.09 ± 0.08	7.09 ± 0.23	6.93 ± 0.30	7.27 ± 0.11
TC02000893.hg.1	OTTHUMG00000153617	5.23 ± 0.09	5.28 ± 0.12	5.17 ± 0.14	5.25 ± 0.16
TC02002381.hg.1	OTTHUMG00000153618	4.41 ± 0.19	4.18 ± 0.08	4.19 ± 0.03	4.27 ± 0.14
TC02000860.hg.1	OTTHUMG00000153621	4.12 ± 0.16	3.94 ± 0.09	4.02 ± 0.11	3.96 ± 0.10
TC02000801.hg.1	OTTHUMG00000153626	6.61 ± 0.08	6.21 ± 0.11	6.32 ± 0.31	6.55 ± 0.33
TC02000895.hg.1	OTTHUMG00000153631	7.04 ± 0.23	7.28 ± 0.08	7.31 ± 0.20	7.34 ± 0.19
TC02000680.hg.1	OTTHUMG00000153641	5.60 ± 0.32	5.38 ± 0.15	5.33 ± 0.19	5.43 ± 0.04
TC02002205.hg.1	OTTHUMG00000153642	5.71 ± 0.12	5.59 ± 0.06	5.64 ± 0.08	5.66 ± 0.12
TC02000697.hg.1	OTTHUMG00000153644	5.60 ± 0.28	5.72 ± 0.16	5.67 ± 0.10	5.81 ± 0.05
TC02000868.hg.1	OTTHUMG00000153645	4.13 ± 0.13	4.14 ± 0.08	4.21 ± 0.07	4.19 ± 0.07
TC02000897.hg.1	OTTHUMG00000153648	4.52 ± 0.14	4.36 ± 0.09	4.41 ± 0.13	4.31 ± 0.07
TC02000898.hg.1	OTTHUMG00000153649	3.90 ± 0.03	3.53 ± 0.05	3.71 ± 0.11	3.59 ± 0.07
TC02002386.hg.1	OTTHUMG00000153650	6.38 ± 0.16	6.31 ± 0.19	6.28 ± 0.11	6.34 ± 0.11
TC02002387.hg.1	OTTHUMG00000153651	4.20 ± 0.05	4.03 ± 0.09	4.15 ± 0.09	4.12 ± 0.17
TC02000872.hg.1	OTTHUMG00000153665	5.53 ± 0.19	5.37 ± 0.11	5.54 ± 0.32	5.41 ± 0.13
TC02000869.hg.1	OTTHUMG00000153667	6.67 ± 0.40	6.50 ± 0.19	6.53 ± 0.22	6.68 ± 0.19
TC02005008.hg.1	OTTHUMG00000153669	5.43 ± 0.13	5.25 ± 0.09	5.45 ± 0.06	5.32 ± 0.08
TC02000698.hg.1	OTTHUMG00000153690	6.30 ± 0.32	6.21 ± 0.01	6.21 ± 0.26	6.30 ± 0.12
TC02002210.hg.1	OTTHUMG00000153694	7.64 ± 0.26	7.33 ± 0.12	7.88 ± 0.26	7.34 ± 0.24
TC02000699.hg.1	OTTHUMG00000153695	6.62 ± 0.07	6.53 ± 0.15	6.61 ± 0.09	6.53 ± 0.08
TC02000700.hg.1	OTTHUMG00000153696	6.76 ± 0.41	6.81 ± 0.17	6.70 ± 0.15	6.96 ± 0.14
TC02002400.hg.1	OTTHUMG00000153703	4.72 ± 0.12	4.48 ± 0.06	4.43 ± 0.11	4.58 ± 0.21
TC02002401.hg.1	OTTHUMG00000153704	4.44 ± 0.17	4.25 ± 0.03	4.33 ± 0.10	4.31 ± 0.09
TC02000918.hg.1	OTTHUMG00000153708	4.83 ± 0.22	4.68 ± 0.04	4.68 ± 0.03	4.69 ± 0.10
TC02002402.hg.1	OTTHUMG00000153709	5.44 ± 0.38	5.66 ± 0.04	5.52 ± 0.04	5.94 ± 0.16
TC02002403.hg.1	OTTHUMG00000153710	4.61 ± 0.21	4.44 ± 0.08	4.55 ± 0.07	4.51 ± 0.04
TC02000919.hg.1	OTTHUMG00000153712	5.88 ± 0.23	5.87 ± 0.06	5.87 ± 0.10	6.01 ± 0.05
TC02002355.hg.1	OTTHUMG00000153714	8.60 ± 0.29	8.36 ± 0.21	8.46 ± 0.26	8.53 ± 0.04
TC02000915.hg.1	OTTHUMG00000153722	4.85 ± 0.17	4.73 ± 0.07	4.73 ± 0.09	4.85 ± 0.06
TC02002395.hg.1	OTTHUMG00000153724	4.76 ± 0.18	4.82 ± 0.06	4.87 ± 0.13	4.79 ± 0.03
TC02000906.hg.1	OTTHUMG00000153725	4.28 ± 0.06	4.24 ± 0.10	4.26 ± 0.10	4.17 ± 0.05

TC02001971.hg.1	OTTHUMG00000153726	7.78 ± 0.14	7.64 ± 0.13	7.67 ± 0.17	7.72 ± 0.09
TC02005031.hg.1	OTTHUMG00000153727	7.00 ± 0.06	6.96 ± 0.05	7.06 ± 0.08	7.03 ± 0.02
TC02002412.hg.1	OTTHUMG00000153737	5.83 ± 0.03	5.76 ± 0.15	5.84 ± 0.15	5.80 ± 0.07
TC02002262.hg.1	OTTHUMG00000153742	7.34 ± 0.21	7.26 ± 0.16	7.24 ± 0.22	7.44 ± 0.21
TC02000930.hg.1	OTTHUMG00000153745	6.85 ± 0.11	6.74 ± 0.04	6.79 ± 0.12	6.77 ± 0.10
TC02002259.hg.1	OTTHUMG00000153753	6.38 ± 0.22	6.44 ± 0.13	6.36 ± 0.13	6.51 ± 0.12
TC02002420.hg.1	OTTHUMG00000153754	5.05 ± 0.10	5.03 ± 0.02	5.05 ± 0.01	5.20 ± 0.12
TC02000931.hg.1	OTTHUMG00000153755	5.87 ± 0.23	5.92 ± 0.06	5.91 ± 0.08	5.98 ± 0.09
TC02002421.hg.1	OTTHUMG00000153756	4.12 ± 0.08	3.80 ± 0.17	3.84 ± 0.12	3.89 ± 0.12
TC02000934.hg.1	OTTHUMG00000153757	4.37 ± 0.09	4.30 ± 0.06	4.32 ± 0.09	4.28 ± 0.03
TC02000933.hg.1	OTTHUMG00000153758	4.86 ± 0.09	4.80 ± 0.10	4.77 ± 0.03	4.93 ± 0.19
TC02002418.hg.1	OTTHUMG00000153759	4.84 ± 0.21	4.59 ± 0.08	4.71 ± 0.10	4.70 ± 0.02
TC02001094.hg.1	OTTHUMG00000153762	3.81 ± 0.22	3.55 ± 0.05	3.69 ± 0.24	3.60 ± 0.05
TC02002255.hg.1	OTTHUMG00000153764	7.31 ± 0.20	7.14 ± 0.13	7.78 ± 0.32	7.09 ± 0.13
TC02002854.hg.1	OTTHUMG00000153765	5.51 ± 0.17	5.40 ± 0.06	5.38 ± 0.08	5.38 ± 0.01
TC02002423.hg.1	OTTHUMG00000153781	9.36 ± 0.27	9.40 ± 0.11	9.24 ± 0.07	9.47 ± 0.11
TC02000936.hg.1	OTTHUMG00000153782	4.66 ± 0.03	4.47 ± 0.02	4.68 ± 0.27	4.53 ± 0.05
TC02002422.hg.1	OTTHUMG00000153783	5.56 ± 0.25	5.36 ± 0.10	5.54 ± 0.04	5.35 ± 0.13
TC02002442.hg.1	OTTHUMG00000153807	4.31 ± 0.12	4.03 ± 0.07	4.13 ± 0.09	4.15 ± 0.03
TC02002441.hg.1	OTTHUMG00000153808	6.82 ± 0.35	6.81 ± 0.09	6.80 ± 0.22	6.88 ± 0.15
TC02000943.hg.1	OTTHUMG00000153820	4.81 ± 0.06	4.95 ± 0.17	4.81 ± 0.10	4.97 ± 0.07
TC02002436.hg.1	OTTHUMG00000153821	6.33 ± 0.18	6.35 ± 0.11	6.34 ± 0.16	6.55 ± 0.21
TC02002435.hg.1	OTTHUMG00000153822	4.35 ± 0.13	4.30 ± 0.06	4.34 ± 0.10	4.46 ± 0.11
TC05000259.hg.1	OTTHUMG00000153833	4.99 ± 0.03	4.79 ± 0.02	4.97 ± 0.08	4.82 ± 0.16
TC02002444.hg.1	OTTHUMG00000153834	5.80 ± 0.19	5.65 ± 0.12	5.71 ± 0.08	5.77 ± 0.11
TC02001383.hg.1	OTTHUMG00000153836	7.84 ± 0.16	7.82 ± 0.29	7.79 ± 0.27	7.97 ± 0.18
TC02000945.hg.1	OTTHUMG00000153849	4.76 ± 0.15	4.57 ± 0.13	4.63 ± 0.03	4.81 ± 0.25
TC02001338.hg.1	OTTHUMG00000153854	4.27 ± 0.10	4.06 ± 0.09	4.05 ± 0.03	4.05 ± 0.08
TC02001340.hg.1	OTTHUMG00000153856	4.94 ± 0.04	4.72 ± 0.20	4.84 ± 0.13	4.82 ± 0.03
TC02001337.hg.1	OTTHUMG00000153857	5.58 ± 0.04	5.56 ± 0.20	5.56 ± 0.02	5.65 ± 0.06
TC02000976.hg.1	OTTHUMG00000153861	6.98 ± 0.25	6.73 ± 0.04	6.65 ± 0.18	6.76 ± 0.09

TC02000975.hg.1	OTTHUMG00000153863	6.75 ± 0.19	6.60 ± 0.14	6.66 ± 0.10	6.69 ± 0.11
TC02001392.hg.1	OTTHUMG00000153867	6.88 ± 0.22	7.01 ± 0.09	6.92 ± 0.09	7.07 ± 0.09
TC02000908.hg.1	OTTHUMG00000153875	6.49 ± 0.14	6.18 ± 0.04	6.40 ± 0.08	6.20 ± 0.08
TC02000911.hg.1	OTTHUMG00000153876	3.75 ± 0.33	3.66 ± 0.12	3.80 ± 0.24	3.75 ± 0.14
TC02001390.hg.1	OTTHUMG00000153881	7.60 ± 0.18	7.53 ± 0.15	7.51 ± 0.24	7.67 ± 0.09
TC02002476.hg.1	OTTHUMG00000153883	6.27 ± 0.22	6.17 ± 0.16	6.08 ± 0.05	6.24 ± 0.04
TC02000977.hg.1	OTTHUMG00000153891	4.68 ± 0.19	4.47 ± 0.14	4.49 ± 0.16	4.61 ± 0.12
TC07000530.hg.1	OTTHUMG00000153893	5.14 ± 0.13	5.12 ± 0.05	5.15 ± 0.04	5.23 ± 0.13
TC07000527.hg.1	OTTHUMG00000153895	4.16 ± 0.06	4.12 ± 0.21	4.33 ± 0.04	4.09 ± 0.07
TC07000528.hg.1	OTTHUMG00000153895	6.15 ± 0.20	5.85 ± 0.04	6.41 ± 0.15	5.86 ± 0.06
TC07000532.hg.1	OTTHUMG00000153911	4.50 ± 0.14	4.40 ± 0.04	4.35 ± 0.07	4.41 ± 0.04
TC02002485.hg.1	OTTHUMG00000153912	4.73 ± 0.09	4.65 ± 0.05	4.76 ± 0.09	4.64 ± 0.04
TC02002454.hg.1	OTTHUMG00000153914	4.78 ± 0.16	4.94 ± 0.03	5.01 ± 0.17	4.93 ± 0.16
TC02002392.hg.1	OTTHUMG00000153939	5.44 ± 0.11	5.38 ± 0.09	5.62 ± 0.22	5.51 ± 0.17
TC02000982.hg.1	OTTHUMG00000153942	6.26 ± 0.25	6.29 ± 0.10	6.16 ± 0.20	6.36 ± 0.05
TC02002483.hg.1	OTTHUMG00000153947	4.90 ± 0.09	4.88 ± 0.17	4.93 ± 0.12	4.97 ± 0.15
TC02002503.hg.1	OTTHUMG00000153961	5.74 ± 0.21	5.69 ± 0.12	5.70 ± 0.14	5.88 ± 0.15
TC02002459.hg.1	OTTHUMG00000153965	6.11 ± 0.03	5.94 ± 0.16	6.10 ± 0.05	6.06 ± 0.06
TC02000962.hg.1	OTTHUMG00000153971	7.18 ± 0.23	7.15 ± 0.20	7.15 ± 0.20	7.21 ± 0.06
TC07001572.hg.1	OTTHUMG00000153975	6.58 ± 0.27	6.42 ± 0.16	6.47 ± 0.09	6.55 ± 0.15
TC07001571.hg.1	OTTHUMG00000153976	4.12 ± 0.32	3.93 ± 0.04	4.12 ± 0.13	3.86 ± 0.11
TC02002341.hg.1	OTTHUMG00000153978	5.41 ± 0.09	5.37 ± 0.04	5.34 ± 0.17	5.47 ± 0.23
TC02000814.hg.1	OTTHUMG00000153979	7.16 ± 0.05	6.97 ± 0.06	6.86 ± 0.20	7.03 ± 0.13
TC02002342.hg.1	OTTHUMG00000153982	7.04 ± 0.29	7.23 ± 0.23	7.08 ± 0.19	7.28 ± 0.15
TC02002343.hg.1	OTTHUMG00000153993	6.72 ± 0.16	6.64 ± 0.07	6.59 ± 0.08	6.62 ± 0.11
TC02000984.hg.1	OTTHUMG00000153999	5.77 ± 0.10	5.74 ± 0.15	5.70 ± 0.17	5.93 ± 0.16
TC02002515.hg.1	OTTHUMG00000154003	4.96 ± 0.14	4.80 ± 0.05	4.86 ± 0.11	4.85 ± 0.08
TC02000993.hg.1	OTTHUMG00000154028	6.02 ± 0.18	5.87 ± 0.15	6.08 ± 0.17	6.01 ± 0.07
TC02000987.hg.1	OTTHUMG00000154036	7.49 ± 0.12	7.19 ± 0.10	7.78 ± 0.44	7.00 ± 0.06
TC02002494.hg.1	OTTHUMG00000154037	6.63 ± 0.19	6.61 ± 0.06	6.63 ± 0.08	6.69 ± 0.13
TC02002493.hg.1	OTTHUMG00000154038	5.64 ± 0.34	5.63 ± 0.06	5.64 ± 0.32	5.71 ± 0.21

TC02000965.hg.1	OTTHUMG00000154042	4.89 ± 0.05	4.80 ± 0.09	5.10 ± 0.20	4.87 ± 0.05
TC02000990.hg.1	OTTHUMG00000154045	5.92 ± 0.07	5.83 ± 0.10	5.82 ± 0.04	5.94 ± 0.12
TC02002345.hg.1	OTTHUMG00000154047	7.09 ± 0.24	6.97 ± 0.22	7.10 ± 0.20	7.13 ± 0.10
TC02002519.hg.1	OTTHUMG00000154055	5.94 ± 0.10	5.83 ± 0.08	5.92 ± 0.08	5.90 ± 0.02
TC02002517.hg.1	OTTHUMG00000154056	5.23 ± 0.17	5.16 ± 0.06	5.17 ± 0.12	5.19 ± 0.04
TC02001027.hg.1	OTTHUMG00000154065	6.42 ± 0.17	6.49 ± 0.30	6.41 ± 0.20	6.61 ± 0.02
TC02002346.hg.1	OTTHUMG00000154066	7.67 ± 0.15	7.57 ± 0.14	7.48 ± 0.23	7.71 ± 0.07
TC02002520.hg.1	OTTHUMG00000154073	7.97 ± 0.27	7.93 ± 0.29	7.77 ± 0.33	8.01 ± 0.07
TC02005062.hg.1	OTTHUMG00000154081	5.90 ± 0.05	5.88 ± 0.04	5.93 ± 0.13	5.88 ± 0.16
TC02002529.hg.1	OTTHUMG00000154088	7.46 ± 0.10	7.42 ± 0.03	7.46 ± 0.16	7.41 ± 0.04
TC02002528.hg.1	OTTHUMG00000154089	6.36 ± 0.10	6.00 ± 0.23	6.40 ± 0.40	5.96 ± 0.15
TC02001343.hg.1	OTTHUMG00000154090	5.80 ± 0.22	5.78 ± 0.14	5.85 ± 0.13	5.98 ± 0.18
TC02002560.hg.1	OTTHUMG00000154100	5.28 ± 0.22	5.23 ± 0.13	5.25 ± 0.21	5.40 ± 0.19
TC02002563.hg.1	OTTHUMG00000154102	6.25 ± 0.35	6.17 ± 0.29	6.21 ± 0.13	6.17 ± 0.08
TC02002559.hg.1	OTTHUMG00000154103	5.32 ± 0.13	5.25 ± 0.13	5.28 ± 0.10	5.37 ± 0.09
TC02002533.hg.1	OTTHUMG00000154108	5.05 ± 0.10	4.97 ± 0.02	5.01 ± 0.19	4.99 ± 0.06
TC02002548.hg.1	OTTHUMG00000154111	4.14 ± 0.22	3.75 ± 0.02	3.76 ± 0.20	3.69 ± 0.08
TC02001048.hg.1	OTTHUMG00000154113	5.44 ± 0.25	5.42 ± 0.10	5.46 ± 0.07	5.38 ± 0.05
TC02002534.hg.1	OTTHUMG00000154132	8.09 ± 0.31	8.27 ± 0.08	8.17 ± 0.21	8.35 ± 0.07
TC02002555.hg.1	OTTHUMG00000154142	5.77 ± 0.17	5.81 ± 0.11	5.73 ± 0.13	5.98 ± 0.09
TC07000568.hg.1	OTTHUMG00000154143	5.66 ± 0.09	5.47 ± 0.05	5.67 ± 0.15	5.54 ± 0.04
TC07000570.hg.1	OTTHUMG00000154145	5.48 ± 0.08	5.51 ± 0.09	5.58 ± 0.15	5.62 ± 0.12
TC02001041.hg.1	OTTHUMG00000154149	6.81 ± 0.13	6.79 ± 0.17	6.90 ± 0.15	6.84 ± 0.06
TC02001039.hg.1	OTTHUMG00000154152	6.77 ± 0.07	6.66 ± 0.09	6.75 ± 0.08	6.66 ± 0.08
TC02002543.hg.1	OTTHUMG00000154154	5.11 ± 0.27	4.99 ± 0.09	4.90 ± 0.06	5.00 ± 0.07
TC02001058.hg.1	OTTHUMG00000154157	5.18 ± 0.17	5.04 ± 0.17	5.10 ± 0.11	5.11 ± 0.10
TC02002556.hg.1	OTTHUMG00000154158	4.48 ± 0.19	4.37 ± 0.11	4.34 ± 0.04	4.35 ± 0.15
TC02001066.hg.1	OTTHUMG00000154167	6.98 ± 0.16	7.04 ± 0.23	7.02 ± 0.09	7.15 ± 0.26
TC02001055.hg.1	OTTHUMG00000154176	8.02 ± 0.20	8.00 ± 0.16	7.96 ± 0.18	8.12 ± 0.05
TC02001063.hg.1	OTTHUMG00000154178	8.57 ± 0.05	8.82 ± 0.18	8.69 ± 0.09	8.94 ± 0.06
TC02000417.hg.1	OTTHUMG00000154180	8.34 ± 0.21	8.37 ± 0.35	8.23 ± 0.26	8.47 ± 0.09

TC02001067.hg.1	OTTHUMG00000154189	7.58 ± 0.29	7.42 ± 0.07	7.43 ± 0.06	7.52 ± 0.10
TC02002571.hg.1	OTTHUMG00000154195	4.72 ± 0.15	4.49 ± 0.10	4.71 ± 0.13	4.56 ± 0.08
TC07001574.hg.1	OTTHUMG00000154197	6.96 ± 0.06	6.96 ± 0.04	6.96 ± 0.11	7.07 ± 0.04
TC02002585.hg.1	OTTHUMG00000154203	4.48 ± 0.08	4.40 ± 0.05	4.46 ± 0.04	4.48 ± 0.08
TC02002584.hg.1	OTTHUMG00000154204	5.60 ± 0.07	5.92 ± 0.19	5.60 ± 0.25	6.01 ± 0.22
TC02001082.hg.1	OTTHUMG00000154205	5.81 ± 0.26	5.89 ± 0.08	5.72 ± 0.11	5.80 ± 0.10
TC02002598.hg.1	OTTHUMG00000154207	6.51 ± 0.19	6.48 ± 0.14	6.44 ± 0.19	6.58 ± 0.02
TC02002587.hg.1	OTTHUMG00000154211	6.36 ± 0.24	6.17 ± 0.06	6.14 ± 0.04	6.28 ± 0.16
TC02001045.hg.1	OTTHUMG00000154227	8.26 ± 0.29	7.88 ± 0.09	7.99 ± 0.35	7.89 ± 0.03
TC02001028.hg.1	OTTHUMG00000154230	8.46 ± 0.53	8.80 ± 0.05	8.64 ± 0.39	9.04 ± 0.28
TC02002527.hg.1	OTTHUMG00000154231	7.32 ± 0.05	7.31 ± 0.01	7.28 ± 0.05	7.21 ± 0.08
TC02001046.hg.1	OTTHUMG00000154232	7.20 ± 0.19	7.12 ± 0.17	7.06 ± 0.14	7.22 ± 0.05
TC02002595.hg.1	OTTHUMG00000154238	4.20 ± 0.06	3.98 ± 0.04	4.10 ± 0.13	3.98 ± 0.06
TC02002583.hg.1	OTTHUMG00000154248	5.35 ± 0.20	5.35 ± 0.17	5.23 ± 0.20	5.51 ± 0.09
TC02002600.hg.1	OTTHUMG00000154250	4.80 ± 0.14	4.76 ± 0.17	4.58 ± 0.06	4.66 ± 0.02
TC02001102.hg.1	OTTHUMG00000154263	5.55 ± 0.08	5.47 ± 0.10	5.51 ± 0.02	5.51 ± 0.01
TC17001587.hg.1	OTTHUMG00000154265	8.16 ± 0.18	8.15 ± 0.21	8.19 ± 0.16	8.35 ± 0.01
TC02001023.hg.1	OTTHUMG00000154281	5.93 ± 0.33	5.99 ± 0.09	5.92 ± 0.33	6.05 ± 0.08
TC02002607.hg.1	OTTHUMG00000154285	6.94 ± 0.19	6.96 ± 0.04	6.97 ± 0.04	7.08 ± 0.07
TC21000415.hg.1	OTTHUMG00000154291	5.20 ± 0.36	5.00 ± 0.06	5.05 ± 0.08	5.11 ± 0.19
TC02001107.hg.1	OTTHUMG00000154303	5.84 ± 0.12	5.64 ± 0.11	5.53 ± 0.17	5.64 ± 0.14
TC02002626.hg.1	OTTHUMG00000154306	7.13 ± 0.23	7.13 ± 0.21	7.13 ± 0.04	7.25 ± 0.14
TC02001129.hg.1	OTTHUMG00000154310	5.74 ± 0.20	5.55 ± 0.06	5.63 ± 0.05	5.67 ± 0.12
TC02001084.hg.1	OTTHUMG00000154314	7.58 ± 0.32	7.54 ± 0.07	7.50 ± 0.21	7.54 ± 0.02
TC02002589.hg.1	OTTHUMG00000154316	4.98 ± 0.08	4.72 ± 0.12	4.93 ± 0.19	4.87 ± 0.07
TC02002260.hg.1	OTTHUMG00000154317	7.39 ± 0.14	7.28 ± 0.05	7.45 ± 0.19	7.42 ± 0.06
TC02001130.hg.1	OTTHUMG00000154332	7.74 ± 0.11	7.76 ± 0.16	7.87 ± 0.08	7.96 ± 0.11
TC02001131.hg.1	OTTHUMG00000154332	4.73 ± 0.11	4.73 ± 0.08	4.68 ± 0.26	4.71 ± 0.08
TC02001132.hg.1	OTTHUMG00000154343	3.96 ± 0.01	3.92 ± 0.06	3.97 ± 0.04	3.92 ± 0.05
TC02001134.hg.1	OTTHUMG00000154350	4.58 ± 0.13	4.39 ± 0.06	4.44 ± 0.09	4.43 ± 0.04
TC02001135.hg.1	OTTHUMG00000154367	4.84 ± 0.11	4.82 ± 0.16	4.84 ± 0.16	4.88 ± 0.07

TC02002632.hg.1	OTTHUMG00000154373	5.38 ± 0.12	5.32 ± 0.02	5.28 ± 0.01	5.33 ± 0.07
TC02002633.hg.1	OTTHUMG00000154378	5.82 ± 0.10	5.73 ± 0.12	5.71 ± 0.14	5.86 ± 0.11
TC02001136.hg.1	OTTHUMG00000154379	4.06 ± 0.10	3.82 ± 0.06	3.85 ± 0.09	3.82 ± 0.02
TC02001138.hg.1	OTTHUMG00000154381	4.16 ± 0.16	4.03 ± 0.17	4.16 ± 0.05	4.10 ± 0.06
TC17000941.hg.1	OTTHUMG00000154387	7.78 ± 0.13	7.69 ± 0.08	7.78 ± 0.07	7.83 ± 0.02
TC02001072.hg.1	OTTHUMG00000154423	6.83 ± 0.17	6.69 ± 0.11	6.74 ± 0.10	6.59 ± 0.07
TC02001144.hg.1	OTTHUMG00000154427	5.38 ± 0.11	5.28 ± 0.04	5.34 ± 0.40	5.39 ± 0.10
TC02001143.hg.1	OTTHUMG00000154428	7.93 ± 0.08	7.71 ± 0.08	7.80 ± 0.09	7.73 ± 0.01
TC07003397.hg.1	OTTHUMG00000154440	8.08 ± 0.12	8.00 ± 0.04	7.98 ± 0.01	8.01 ± 0.05
TC02001120.hg.1	OTTHUMG00000154455	5.25 ± 0.15	4.99 ± 0.15	4.98 ± 0.07	5.07 ± 0.07
TC02001140.hg.1	OTTHUMG00000154462	6.07 ± 0.40	5.92 ± 0.03	5.92 ± 0.17	5.91 ± 0.16
TC02002651.hg.1	OTTHUMG00000154464	5.03 ± 0.13	4.99 ± 0.02	5.00 ± 0.11	5.21 ± 0.16
TC02002652.hg.1	OTTHUMG00000154465	5.18 ± 0.12	4.95 ± 0.05	5.04 ± 0.06	5.13 ± 0.10
TC02002653.hg.1	OTTHUMG00000154467	4.22 ± 0.13	4.03 ± 0.16	4.19 ± 0.11	4.03 ± 0.11
TC02002655.hg.1	OTTHUMG00000154472	3.76 ± 0.08	3.61 ± 0.06	3.69 ± 0.03	3.72 ± 0.05
TC02002658.hg.1	OTTHUMG00000154483	3.86 ± 0.09	3.78 ± 0.04	3.88 ± 0.12	3.80 ± 0.02
TC02001150.hg.1	OTTHUMG00000154488	5.63 ± 0.13	5.55 ± 0.04	5.56 ± 0.09	5.68 ± 0.18
TC02001157.hg.1	OTTHUMG00000154493	5.94 ± 0.12	5.82 ± 0.08	5.88 ± 0.10	5.87 ± 0.08
TC02001324.hg.1	OTTHUMG00000154505	7.55 ± 0.17	7.46 ± 0.13	7.50 ± 0.11	7.59 ± 0.18
TC02001123.hg.1	OTTHUMG00000154509	5.56 ± 0.22	5.42 ± 0.07	5.35 ± 0.14	5.41 ± 0.17
TC02002623.hg.1	OTTHUMG00000154511	7.71 ± 0.21	7.85 ± 0.08	7.86 ± 0.06	7.79 ± 0.06
TC02002622.hg.1	OTTHUMG00000154512	4.99 ± 0.06	4.84 ± 0.09	4.86 ± 0.08	4.89 ± 0.06
TC02001124.hg.1	OTTHUMG00000154513	5.52 ± 0.20	5.41 ± 0.08	5.47 ± 0.07	5.53 ± 0.05
TC02002609.hg.1	OTTHUMG00000154523	5.34 ± 0.21	5.29 ± 0.08	5.27 ± 0.08	5.44 ± 0.14
TC02001203.hg.1	OTTHUMG00000154540	5.48 ± 0.13	5.45 ± 0.06	5.47 ± 0.06	5.70 ± 0.10
TC02001204.hg.1	OTTHUMG00000154542	6.66 ± 0.30	6.51 ± 0.12	6.54 ± 0.11	6.74 ± 0.11
TC02002691.hg.1	OTTHUMG00000154543	3.92 ± 0.22	3.80 ± 0.13	3.77 ± 0.12	3.71 ± 0.06
TC02002690.hg.1	OTTHUMG00000154544	5.41 ± 0.25	5.55 ± 0.30	5.47 ± 0.31	5.72 ± 0.16
TC02002663.hg.1	OTTHUMG00000154546	6.51 ± 0.07	6.41 ± 0.09	6.44 ± 0.10	6.44 ± 0.05
TC02001079.hg.1	OTTHUMG00000154555	5.52 ± 0.07	5.55 ± 0.09	5.56 ± 0.05	5.52 ± 0.07
TC02002681.hg.1	OTTHUMG00000154582	6.23 ± 0.24	6.05 ± 0.14	6.06 ± 0.19	6.02 ± 0.17

TC07000518.hg.1	OTTHUMG00000154585	4.97 ± 0.22	4.74 ± 0.06	4.81 ± 0.04	4.83 ± 0.10
TC02001040.hg.1	OTTHUMG00000154592	6.92 ± 0.20	6.86 ± 0.18	6.96 ± 0.09	6.98 ± 0.07
TC07000515.hg.1	OTTHUMG00000154603	4.13 ± 0.17	3.88 ± 0.01	4.01 ± 0.18	4.00 ± 0.15
TC02000863.hg.1	OTTHUMG00000154620	3.41 ± 0.02	3.28 ± 0.04	3.46 ± 0.09	3.44 ± 0.18
TC07001569.hg.1	OTTHUMG00000154628	8.96 ± 0.08	8.90 ± 0.06	8.62 ± 0.14	8.84 ± 0.08
TC02002692.hg.1	OTTHUMG00000154647	5.91 ± 0.10	5.87 ± 0.10	5.97 ± 0.21	6.10 ± 0.21
TC02001208.hg.1	OTTHUMG00000154652	6.67 ± 0.21	6.65 ± 0.05	6.62 ± 0.13	6.71 ± 0.09
TC02001210.hg.1	OTTHUMG00000154653	7.82 ± 0.24	7.87 ± 0.14	7.71 ± 0.26	7.91 ± 0.03
TC02002779.hg.1	OTTHUMG00000154664	5.91 ± 0.18	6.03 ± 0.34	6.08 ± 0.07	6.21 ± 0.06
TC02002781.hg.1	OTTHUMG00000154666	7.74 ± 0.26	7.29 ± 0.17	7.58 ± 0.04	7.19 ± 0.06
TC07000514.hg.1	OTTHUMG00000154683	4.24 ± 0.12	4.04 ± 0.03	3.99 ± 0.12	3.95 ± 0.07
TC02001170.hg.1	OTTHUMG00000154691	7.00 ± 0.12	6.95 ± 0.11	7.18 ± 0.02	6.93 ± 0.05
TC18000139.hg.1	OTTHUMG00000154695	8.38 ± 0.22	8.20 ± 0.11	8.25 ± 0.19	8.18 ± 0.06
TC02001241.hg.1	OTTHUMG00000154698	6.77 ± 0.40	6.82 ± 0.19	6.79 ± 0.33	6.88 ± 0.05
TC02001242.hg.1	OTTHUMG00000154719	7.10 ± 0.22	7.20 ± 0.18	7.17 ± 0.16	7.33 ± 0.17
TC02001226.hg.1	OTTHUMG00000154724	6.10 ± 0.23	5.98 ± 0.20	5.92 ± 0.03	5.86 ± 0.09
TC02001225.hg.1	OTTHUMG00000154727	5.42 ± 0.28	5.22 ± 0.04	5.15 ± 0.00	5.30 ± 0.07
TC02002700.hg.1	OTTHUMG00000154728	4.17 ± 0.17	4.03 ± 0.03	4.15 ± 0.09	4.16 ± 0.17
TC02001219.hg.1	OTTHUMG00000154730	6.27 ± 0.24	6.26 ± 0.04	6.12 ± 0.12	6.50 ± 0.18
TC02002708.hg.1	OTTHUMG00000154732	5.50 ± 0.09	5.48 ± 0.12	5.33 ± 0.07	5.58 ± 0.13
TC02001223.hg.1	OTTHUMG00000154733	4.64 ± 0.16	4.41 ± 0.05	4.37 ± 0.09	4.48 ± 0.08
TC02001245.hg.1	OTTHUMG00000154735	5.06 ± 0.12	4.98 ± 0.04	5.02 ± 0.02	5.08 ± 0.13
TC02001249.hg.1	OTTHUMG00000154769	5.09 ± 0.06	4.81 ± 0.05	4.91 ± 0.06	4.94 ± 0.13
TC02001228.hg.1	OTTHUMG00000154784	6.15 ± 0.09	6.21 ± 0.16	6.22 ± 0.03	6.39 ± 0.19
TC02001230.hg.1	OTTHUMG00000154786	7.04 ± 0.41	7.01 ± 0.17	7.03 ± 0.20	6.99 ± 0.11
TC02002716.hg.1	OTTHUMG00000154794	4.96 ± 0.23	4.86 ± 0.09	4.90 ± 0.22	4.86 ± 0.13
TC02002709.hg.1	OTTHUMG00000154795	6.13 ± 0.11	6.23 ± 0.10	5.98 ± 0.01	6.25 ± 0.09
TC02001257.hg.1	OTTHUMG00000154798	4.51 ± 0.09	4.40 ± 0.02	4.45 ± 0.07	4.35 ± 0.05
TC02001256.hg.1	OTTHUMG00000154799	4.55 ± 0.04	4.43 ± 0.05	4.62 ± 0.18	4.38 ± 0.19
TC02002737.hg.1	OTTHUMG00000154804	4.91 ± 0.14	4.88 ± 0.19	4.79 ± 0.13	4.83 ± 0.07
TC02001258.hg.1	OTTHUMG00000154818	7.02 ± 0.10	7.00 ± 0.04	6.89 ± 0.07	6.99 ± 0.12

TC02002741.hg.1	OTTHUMG00000154820	5.44 ± 0.16	5.32 ± 0.11	5.28 ± 0.09	5.33 ± 0.07
TC02001250.hg.1	OTTHUMG00000154822	5.87 ± 0.13	6.10 ± 0.12	5.98 ± 0.13	6.27 ± 0.23
TC02002744.hg.1	OTTHUMG00000154823	5.46 ± 0.17	5.37 ± 0.04	5.39 ± 0.05	5.45 ± 0.10
TC02002752.hg.1	OTTHUMG00000154827	5.95 ± 0.02	5.92 ± 0.07	6.10 ± 0.10	5.91 ± 0.08
TC02001266.hg.1	OTTHUMG00000154832	6.48 ± 0.47	6.65 ± 0.03	6.59 ± 0.07	6.80 ± 0.18
TC02001265.hg.1	OTTHUMG00000154833	4.46 ± 0.11	4.28 ± 0.04	4.38 ± 0.03	4.35 ± 0.06
TC02001262.hg.1	OTTHUMG00000154835	4.63 ± 0.09	4.50 ± 0.05	4.54 ± 0.04	4.63 ± 0.06
TC02001264.hg.1	OTTHUMG00000154836	4.80 ± 0.10	4.69 ± 0.05	4.78 ± 0.08	4.75 ± 0.03
TC02001268.hg.1	OTTHUMG00000154837	6.08 ± 0.06	6.05 ± 0.20	6.00 ± 0.19	6.22 ± 0.07
TC02001267.hg.1	OTTHUMG00000154838	4.49 ± 0.22	4.41 ± 0.15	4.50 ± 0.02	4.57 ± 0.11
TC02001261.hg.1	OTTHUMG00000154840	7.32 ± 0.32	7.27 ± 0.37	7.26 ± 0.27	7.49 ± 0.12
TC02001275.hg.1	OTTHUMG00000154841	5.54 ± 0.24	5.58 ± 0.14	5.45 ± 0.18	5.80 ± 0.14
TC02001274.hg.1	OTTHUMG00000154842	6.32 ± 0.24	6.30 ± 0.13	6.29 ± 0.18	6.44 ± 0.09
TC02002755.hg.1	OTTHUMG00000154843	6.03 ± 0.13	5.99 ± 0.23	6.16 ± 0.04	6.19 ± 0.13
TC07000614.hg.1	OTTHUMG00000154845	6.91 ± 0.09	6.79 ± 0.11	6.94 ± 0.16	6.82 ± 0.06
TC03000001.hg.1	OTTHUMG00000154851	4.91 ± 0.09	4.78 ± 0.16	4.98 ± 0.15	5.06 ± 0.16
TC03001114.hg.1	OTTHUMG00000154852	7.32 ± 0.36	7.32 ± 0.19	7.32 ± 0.16	7.43 ± 0.13
TC07000701.hg.1	OTTHUMG00000154865	4.45 ± 0.08	4.43 ± 0.06	4.39 ± 0.05	4.37 ± 0.07
TC07000700.hg.1	OTTHUMG00000154871	5.83 ± 0.04	5.83 ± 0.16	5.82 ± 0.14	5.87 ± 0.02
TC03000003.hg.1	OTTHUMG00000154876	4.79 ± 0.12	4.63 ± 0.07	4.71 ± 0.06	4.66 ± 0.06
TC03000005.hg.1	OTTHUMG00000154883	4.08 ± 0.20	3.82 ± 0.05	3.85 ± 0.01	3.96 ± 0.10
TC03000004.hg.1	OTTHUMG00000154884	7.75 ± 0.29	7.81 ± 0.13	7.64 ± 0.37	8.20 ± 0.25
TC03001133.hg.1	OTTHUMG00000154898	5.42 ± 0.11	5.35 ± 0.07	5.40 ± 0.04	5.45 ± 0.18
TC03001136.hg.1	OTTHUMG00000154903	5.03 ± 0.20	4.97 ± 0.04	4.85 ± 0.34	4.93 ± 0.04
TC03000018.hg.1	OTTHUMG00000154907	5.28 ± 0.19	5.37 ± 0.05	5.21 ± 0.20	5.50 ± 0.06
TC03000019.hg.1	OTTHUMG00000154909	6.75 ± 0.07	6.78 ± 0.05	6.76 ± 0.02	6.86 ± 0.11
TC03000020.hg.1	OTTHUMG00000154923	4.85 ± 0.21	4.70 ± 0.09	4.77 ± 0.01	4.81 ± 0.13
TC03000021.hg.1	OTTHUMG00000154923	4.94 ± 0.45	5.07 ± 0.13	4.89 ± 0.23	5.29 ± 0.21
TC03000022.hg.1	OTTHUMG00000154923	6.01 ± 0.27	6.04 ± 0.03	6.01 ± 0.12	6.12 ± 0.09
TC03000023.hg.1	OTTHUMG00000154923	3.98 ± 0.12	3.91 ± 0.10	3.93 ± 0.06	4.11 ± 0.32
TC03001124.hg.1	OTTHUMG00000154935	4.83 ± 0.17	4.77 ± 0.01	4.76 ± 0.07	4.85 ± 0.08

TC07000236.hg.1	OTTHUMG00000154940	6.00 ± 0.17	6.01 ± 0.12	5.94 ± 0.09	6.09 ± 0.10
TC07001282.hg.1	OTTHUMG00000154941	4.38 ± 0.18	4.39 ± 0.21	4.30 ± 0.29	4.36 ± 0.06
TC07000238.hg.1	OTTHUMG00000154944	5.25 ± 0.21	5.12 ± 0.13	4.99 ± 0.06	5.10 ± 0.19
TC07000239.hg.1	OTTHUMG00000154945	6.83 ± 0.11	6.79 ± 0.08	6.71 ± 0.05	6.84 ± 0.04
TC07000237.hg.1	OTTHUMG00000154946	6.19 ± 0.09	6.03 ± 0.21	6.05 ± 0.03	5.99 ± 0.10
TC02002189.hg.1	OTTHUMG00000154962	5.19 ± 0.22	5.12 ± 0.04	5.12 ± 0.02	5.01 ± 0.06
TC02001375.hg.1	OTTHUMG00000154963	6.35 ± 0.56	6.08 ± 0.32	6.79 ± 0.45	6.10 ± 0.07
TC02001374.hg.1	OTTHUMG00000154964	6.20 ± 0.19	6.12 ± 0.13	6.21 ± 0.12	6.24 ± 0.05
TC10000690.hg.1	OTTHUMG00000154966	8.18 ± 0.09	8.34 ± 0.31	8.09 ± 0.40	8.33 ± 0.13
TC03001143.hg.1	OTTHUMG00000154969	5.03 ± 0.08	4.73 ± 0.01	4.90 ± 0.16	4.86 ± 0.07
TC03000025.hg.1	OTTHUMG00000154970	5.25 ± 0.10	5.10 ± 0.02	5.08 ± 0.07	5.24 ± 0.08
TC03001142.hg.1	OTTHUMG00000154972	6.36 ± 0.32	6.12 ± 0.08	6.16 ± 0.26	6.23 ± 0.21
TC07001754.hg.1	OTTHUMG00000155012	3.96 ± 0.15	3.79 ± 0.01	3.76 ± 0.01	3.90 ± 0.07
TC07001755.hg.1	OTTHUMG00000155014	4.29 ± 0.06	4.19 ± 0.03	4.44 ± 0.09	4.47 ± 0.14
TC07001757.hg.1	OTTHUMG00000155021	5.06 ± 0.05	5.07 ± 0.01	5.01 ± 0.07	5.11 ± 0.10
TC07000817.hg.1	OTTHUMG00000155022	8.08 ± 0.20	8.09 ± 0.04	8.11 ± 0.08	8.17 ± 0.08
TC07001278.hg.1	OTTHUMG00000155024	6.21 ± 0.19	6.17 ± 0.02	6.21 ± 0.25	6.38 ± 0.15
TC07001277.hg.1	OTTHUMG00000155027	6.11 ± 0.10	6.01 ± 0.07	6.06 ± 0.10	5.91 ± 0.05
TC02000544.hg.1	OTTHUMG00000155047	6.39 ± 0.19	6.30 ± 0.16	6.19 ± 0.21	6.53 ± 0.17
TC02000545.hg.1	OTTHUMG00000155049	7.12 ± 0.13	7.71 ± 0.12	6.91 ± 0.38	7.61 ± 0.13
TC07001280.hg.1	OTTHUMG00000155061	5.98 ± 0.10	5.90 ± 0.05	5.91 ± 0.12	5.90 ± 0.04
TC07000231.hg.1	OTTHUMG00000155064	7.35 ± 0.27	7.36 ± 0.22	7.35 ± 0.03	7.58 ± 0.13
TC02002772.hg.1	OTTHUMG00000155067	7.75 ± 0.20	7.76 ± 0.08	7.84 ± 0.15	7.82 ± 0.08
TC02002202.hg.1	OTTHUMG00000155076	6.27 ± 0.42	6.12 ± 0.13	6.23 ± 0.23	6.31 ± 0.18
TC07001315.hg.1	OTTHUMG00000155092	5.06 ± 0.15	5.05 ± 0.04	5.03 ± 0.09	5.19 ± 0.13
TC07000256.hg.1	OTTHUMG00000155093	6.00 ± 0.30	5.95 ± 0.01	5.97 ± 0.03	5.97 ± 0.17
TC02000940.hg.1	OTTHUMG00000155109	6.40 ± 0.18	6.45 ± 0.26	6.39 ± 0.14	6.59 ± 0.12
TC07001317.hg.1	OTTHUMG00000155112	7.04 ± 0.17	6.92 ± 0.20	6.94 ± 0.12	6.91 ± 0.04
TC07001316.hg.1	OTTHUMG00000155113	5.72 ± 0.17	5.59 ± 0.09	5.63 ± 0.03	5.59 ± 0.04
TC07000257.hg.1	OTTHUMG00000155115	4.76 ± 0.19	4.65 ± 0.11	4.69 ± 0.10	4.69 ± 0.07
TC02000761.hg.1	OTTHUMG00000155116	8.68 ± 0.28	8.77 ± 0.04	8.77 ± 0.13	8.90 ± 0.14

TC02002258.hg.1	OTTHUMG00000155117	6.06 ± 0.13	6.04 ± 0.08	5.96 ± 0.16	6.04 ± 0.15
TC07001763.hg.1	OTTHUMG00000155122	5.11 ± 0.08	5.06 ± 0.09	5.09 ± 0.11	5.15 ± 0.13
TC02000143.hg.1	OTTHUMG00000155125	7.64 ± 0.29	7.65 ± 0.14	7.68 ± 0.11	7.65 ± 0.07
TC07001320.hg.1	OTTHUMG00000155126	4.86 ± 0.31	4.62 ± 0.07	4.73 ± 0.10	4.57 ± 0.22
TC02002771.hg.1	OTTHUMG00000155145	6.13 ± 0.26	6.16 ± 0.10	6.20 ± 0.12	6.20 ± 0.09
TC02002092.hg.1	OTTHUMG00000155157	6.66 ± 0.12	6.76 ± 0.18	6.75 ± 0.12	6.80 ± 0.12
TC02002093.hg.1	OTTHUMG00000155169	6.84 ± 0.22	6.81 ± 0.16	6.80 ± 0.14	6.98 ± 0.03
TC02000570.hg.1	OTTHUMG00000155176	7.06 ± 0.17	7.06 ± 0.04	6.94 ± 0.01	6.96 ± 0.02
TC07001321.hg.1	OTTHUMG00000155178	5.97 ± 0.08	5.83 ± 0.12	5.92 ± 0.16	5.95 ± 0.02
TC02000576.hg.1	OTTHUMG00000155185	6.50 ± 0.22	6.49 ± 0.31	6.50 ± 0.10	6.68 ± 0.10
TC07000710.hg.1	OTTHUMG00000155194	4.25 ± 0.12	4.09 ± 0.05	4.11 ± 0.06	4.13 ± 0.07
TC07000827.hg.1	OTTHUMG00000155195	4.47 ± 0.16	4.37 ± 0.06	4.53 ± 0.02	4.35 ± 0.07
TC07000822.hg.1	OTTHUMG00000155196	5.96 ± 0.22	5.97 ± 0.16	5.96 ± 0.11	6.20 ± 0.09
TC07000823.hg.1	OTTHUMG00000155198	7.87 ± 0.29	7.83 ± 0.21	7.77 ± 0.22	7.94 ± 0.09
TC07000820.hg.1	OTTHUMG00000155199	6.45 ± 0.15	6.40 ± 0.15	6.38 ± 0.07	6.51 ± 0.02
TC05000545.hg.1	OTTHUMG00000155201	6.20 ± 0.08	6.02 ± 0.03	6.04 ± 0.06	6.04 ± 0.02
TC07001325.hg.1	OTTHUMG00000155205	6.14 ± 0.22	6.07 ± 0.11	6.10 ± 0.15	6.22 ± 0.05
TC02002103.hg.1	OTTHUMG00000155213	8.72 ± 0.13	8.77 ± 0.28	8.73 ± 0.11	9.01 ± 0.13
TC02002107.hg.1	OTTHUMG00000155228	5.99 ± 0.17	6.01 ± 0.05	5.90 ± 0.09	5.91 ± 0.02
TC07001766.hg.1	OTTHUMG00000155236	4.78 ± 0.12	4.53 ± 0.04	4.61 ± 0.02	4.61 ± 0.05
TC03001129.hg.1	OTTHUMG00000155240	6.41 ± 0.03	6.31 ± 0.04	6.35 ± 0.09	6.36 ± 0.04
TC02001290.hg.1	OTTHUMG00000155260	8.89 ± 0.17	8.88 ± 0.06	8.89 ± 0.08	8.99 ± 0.03
TC04001526.hg.1	OTTHUMG00000155267	4.57 ± 0.25	4.49 ± 0.05	4.48 ± 0.16	4.47 ± 0.11
TC07001330.hg.1	OTTHUMG00000155268	8.41 ± 0.14	8.31 ± 0.13	8.31 ± 0.09	8.47 ± 0.05
TC07001311.hg.1	OTTHUMG00000155272	7.12 ± 0.34	7.13 ± 0.11	7.09 ± 0.09	7.23 ± 0.08
TC07001310.hg.1	OTTHUMG00000155274	6.06 ± 0.14	6.03 ± 0.15	5.93 ± 0.15	6.18 ± 0.09
TC07001312.hg.1	OTTHUMG00000155278	5.12 ± 0.14	4.93 ± 0.08	4.99 ± 0.31	4.88 ± 0.18
TC02001288.hg.1	OTTHUMG00000155286	5.08 ± 0.20	5.00 ± 0.11	5.11 ± 0.16	5.04 ± 0.07
TC13000316.hg.1	OTTHUMG00000155300	3.97 ± 0.05	3.92 ± 0.02	3.90 ± 0.13	3.94 ± 0.08
TC02001282.hg.1	OTTHUMG00000155309	6.45 ± 0.11	6.35 ± 0.12	6.43 ± 0.30	6.44 ± 0.14
TC02001285.hg.1	OTTHUMG00000155310	7.34 ± 0.36	7.17 ± 0.11	7.19 ± 0.30	7.36 ± 0.09

TC02001283.hg.1	OTTHUMG00000155311	5.08 ± 0.04	4.98 ± 0.09	4.99 ± 0.04	5.04 ± 0.16
TC02002760.hg.1	OTTHUMG00000155312	6.49 ± 0.17	6.48 ± 0.22	6.31 ± 0.11	6.59 ± 0.13
TC02002763.hg.1	OTTHUMG00000155313	5.23 ± 0.17	5.21 ± 0.03	5.18 ± 0.12	5.30 ± 0.25
TC02001284.hg.1	OTTHUMG00000155315	4.25 ± 0.11	4.27 ± 0.07	4.17 ± 0.24	4.31 ± 0.09
TC02002762.hg.1	OTTHUMG00000155316	5.30 ± 0.03	5.11 ± 0.11	5.14 ± 0.22	5.22 ± 0.21
TC03000107.hg.1	OTTHUMG00000155317	4.73 ± 0.36	4.86 ± 0.27	4.46 ± 0.19	4.77 ± 0.08
TC02001001.hg.1	OTTHUMG00000155318	6.31 ± 0.15	5.85 ± 0.14	6.63 ± 0.61	5.86 ± 0.17
TC02002757.hg.1	OTTHUMG00000155340	7.25 ± 0.28	7.30 ± 0.13	7.17 ± 0.39	7.37 ± 0.14
TC02001280.hg.1	OTTHUMG00000155344	6.57 ± 0.26	6.56 ± 0.24	6.65 ± 0.18	6.62 ± 0.10
TC03000108.hg.1	OTTHUMG00000155346	7.68 ± 0.49	7.90 ± 0.23	7.51 ± 0.29	7.88 ± 0.05
TC03000109.hg.1	OTTHUMG00000155346	5.31 ± 0.10	5.26 ± 0.05	5.24 ± 0.07	5.34 ± 0.17
TC02002562.hg.1	OTTHUMG00000155365	6.10 ± 0.17	6.02 ± 0.02	6.02 ± 0.01	6.07 ± 0.06
TC07000832.hg.1	OTTHUMG00000155377	5.61 ± 0.22	5.57 ± 0.18	5.48 ± 0.09	5.59 ± 0.16
TC07001557.hg.1	OTTHUMG00000155382	4.39 ± 0.08	4.13 ± 0.10	4.22 ± 0.07	4.15 ± 0.03
TC02000616.hg.1	OTTHUMG00000155390	5.42 ± 0.10	5.31 ± 0.04	5.15 ± 0.14	5.28 ± 0.05
TC07001561.hg.1	OTTHUMG00000155403	4.36 ± 0.10	4.25 ± 0.03	4.29 ± 0.08	4.30 ± 0.11
TC07000837.hg.1	OTTHUMG00000155405	5.26 ± 0.21	5.53 ± 0.13	5.61 ± 0.32	5.62 ± 0.11
TC03002183.hg.1	OTTHUMG00000155409	5.46 ± 0.14	5.40 ± 0.11	5.46 ± 0.10	5.26 ± 0.10
TC07001560.hg.1	OTTHUMG00000155412	3.79 ± 0.13	3.62 ± 0.05	3.67 ± 0.17	3.71 ± 0.10
TC07000282.hg.1	OTTHUMG00000155418	5.33 ± 0.20	5.08 ± 0.14	5.13 ± 0.20	5.14 ± 0.16
TC07000298.hg.1	OTTHUMG00000155424	5.15 ± 0.13	4.96 ± 0.14	4.99 ± 0.04	5.04 ± 0.06
TC07000296.hg.1	OTTHUMG00000155426	5.07 ± 0.11	5.14 ± 0.10	5.13 ± 0.12	5.14 ± 0.07
TC07000297.hg.1	OTTHUMG00000155428	5.85 ± 0.10	5.87 ± 0.14	5.88 ± 0.05	5.90 ± 0.12
TC03001186.hg.1	OTTHUMG00000155439	7.27 ± 0.25	7.35 ± 0.04	7.23 ± 0.22	7.47 ± 0.19
TC07000841.hg.1	OTTHUMG00000155446	5.84 ± 0.11	5.95 ± 0.12	6.22 ± 0.20	5.97 ± 0.01
TC02002599.hg.1	OTTHUMG00000155450	4.87 ± 0.15	4.68 ± 0.04	4.77 ± 0.12	4.76 ± 0.13
TC02002601.hg.1	OTTHUMG00000155451	4.55 ± 0.14	4.35 ± 0.03	4.44 ± 0.01	4.36 ± 0.11
TC03000061.hg.1	OTTHUMG00000155454	6.23 ± 0.10	6.27 ± 0.05	6.34 ± 0.20	6.28 ± 0.20
TC03001218.hg.1	OTTHUMG00000155461	5.81 ± 0.28	5.58 ± 0.07	5.66 ± 0.06	5.71 ± 0.05
TC07000299.hg.1	OTTHUMG00000155471	6.82 ± 0.31	6.89 ± 0.00	6.86 ± 0.12	6.90 ± 0.14
TC07001358.hg.1	OTTHUMG00000155472	4.50 ± 0.05	4.41 ± 0.11	4.60 ± 0.13	4.73 ± 0.29

TC07001360.hg.1	OTTHUMG00000155474	5.67 ± 0.14	5.60 ± 0.02	5.59 ± 0.06	5.70 ± 0.14
TC07001359.hg.1	OTTHUMG00000155475	5.21 ± 0.14	5.19 ± 0.12	5.22 ± 0.20	5.41 ± 0.17
TC07001361.hg.1	OTTHUMG00000155476	6.19 ± 0.18	6.20 ± 0.20	6.16 ± 0.08	6.31 ± 0.13
TC03002181.hg.1	OTTHUMG00000155477	6.69 ± 0.17	6.89 ± 0.25	6.83 ± 0.20	7.05 ± 0.10
TC03002177.hg.1	OTTHUMG00000155481	6.70 ± 0.12	6.81 ± 0.37	6.76 ± 0.36	7.08 ± 0.12
TC03001107.hg.1	OTTHUMG00000155482	6.81 ± 0.16	6.90 ± 0.26	6.83 ± 0.26	6.97 ± 0.13
TC03002178.hg.1	OTTHUMG00000155483	7.13 ± 0.18	7.04 ± 0.17	6.84 ± 0.34	7.09 ± 0.07
TC02001237.hg.1	OTTHUMG00000155504	6.14 ± 0.17	5.91 ± 0.04	6.02 ± 0.14	5.96 ± 0.06
TC03000084.hg.1	OTTHUMG00000155524	7.98 ± 0.09	7.80 ± 0.09	8.08 ± 0.12	7.73 ± 0.13
TC03001102.hg.1	OTTHUMG00000155528	6.09 ± 0.72	6.91 ± 0.49	6.33 ± 0.45	6.82 ± 0.13
TC03000115.hg.1	OTTHUMG00000155533	4.82 ± 0.14	4.81 ± 0.06	4.82 ± 0.03	4.92 ± 0.15
TC03001199.hg.1	OTTHUMG00000155547	6.85 ± 0.10	6.87 ± 0.10	6.60 ± 0.09	6.99 ± 0.21
TC03001200.hg.1	OTTHUMG00000155548	7.03 ± 0.13	7.03 ± 0.35	6.99 ± 0.23	7.18 ± 0.20
TC07001362.hg.1	OTTHUMG00000155549	4.73 ± 0.16	4.60 ± 0.05	4.66 ± 0.07	4.62 ± 0.04
TC07000301.hg.1	OTTHUMG00000155553	5.20 ± 0.18	5.09 ± 0.05	5.19 ± 0.06	5.34 ± 0.16
TC07000302.hg.1	OTTHUMG00000155554	6.73 ± 0.23	6.85 ± 0.19	6.57 ± 0.11	6.94 ± 0.10
TC07001615.hg.1	OTTHUMG00000155562	6.55 ± 0.18	6.71 ± 0.02	6.49 ± 0.06	6.83 ± 0.11
TC03002164.hg.1	OTTHUMG00000155570	7.17 ± 0.27	7.16 ± 0.26	7.22 ± 0.08	7.25 ± 0.02
TC07001881.hg.1	OTTHUMG00000155590	5.57 ± 0.11	5.63 ± 0.20	5.74 ± 0.09	5.72 ± 0.11
TC07000492.hg.1	OTTHUMG00000155614	4.28 ± 0.13	4.08 ± 0.11	4.14 ± 0.08	4.27 ± 0.21
TC07000846.hg.1	OTTHUMG00000155616	5.33 ± 0.16	5.21 ± 0.01	5.30 ± 0.14	5.13 ± 0.05
TC07000849.hg.1	OTTHUMG00000155617	5.53 ± 0.12	5.24 ± 0.11	5.34 ± 0.07	5.33 ± 0.10
TC07000850.hg.1	OTTHUMG00000155618	5.22 ± 0.12	5.12 ± 0.08	5.18 ± 0.04	5.25 ± 0.08
TC03001250.hg.1	OTTHUMG00000155650	5.45 ± 0.12	5.36 ± 0.10	5.34 ± 0.09	5.56 ± 0.14
TC03001259.hg.1	OTTHUMG00000155651	5.90 ± 0.07	5.84 ± 0.09	5.92 ± 0.13	5.90 ± 0.06
TC03000140.hg.1	OTTHUMG00000155653	6.54 ± 0.24	6.53 ± 0.05	6.43 ± 0.14	6.73 ± 0.25
TC03000129.hg.1	OTTHUMG00000155664	5.08 ± 0.03	4.98 ± 0.09	5.15 ± 0.10	5.12 ± 0.12
TC03001095.hg.1	OTTHUMG00000155671	6.39 ± 0.08	6.46 ± 0.07	7.14 ± 0.63	6.56 ± 0.07
TC02001263.hg.1	OTTHUMG00000155685	7.51 ± 0.07	7.25 ± 0.20	7.29 ± 0.19	7.33 ± 0.10
TC03000136.hg.1	OTTHUMG00000155686	5.31 ± 0.16	5.20 ± 0.06	5.13 ± 0.13	5.28 ± 0.24
TC07001896.hg.1	OTTHUMG00000155708	5.70 ± 0.12	5.78 ± 0.14	5.78 ± 0.07	5.95 ± 0.08

TC07000856.hg.1	OTTHUMG00000155710	5.88 ± 0.23	5.69 ± 0.11	5.78 ± 0.05	5.86 ± 0.18
TC03000148.hg.1	OTTHUMG00000155731	5.53 ± 0.18	5.22 ± 0.08	5.49 ± 0.13	5.27 ± 0.18
TC03001267.hg.1	OTTHUMG00000155735	5.11 ± 0.19	5.16 ± 0.15	4.94 ± 0.12	5.23 ± 0.22
TC07001547.hg.1	OTTHUMG00000155740	5.02 ± 0.15	5.00 ± 0.15	4.94 ± 0.09	4.94 ± 0.08
TC07000558.hg.1	OTTHUMG00000155746	4.57 ± 0.11	4.44 ± 0.01	4.46 ± 0.06	4.51 ± 0.11
TC22000429.hg.1	OTTHUMG00000155749	8.20 ± 0.23	8.10 ± 0.23	8.05 ± 0.19	8.16 ± 0.05
TC03000170.hg.1	OTTHUMG00000155764	5.00 ± 0.09	5.09 ± 0.03	5.11 ± 0.09	5.12 ± 0.33
TC03001278.hg.1	OTTHUMG00000155766	6.16 ± 0.25	5.93 ± 0.12	6.07 ± 0.26	6.04 ± 0.12
TC03000169.hg.1	OTTHUMG00000155767	5.65 ± 0.03	5.51 ± 0.02	5.52 ± 0.16	5.63 ± 0.10
TC07001613.hg.1	OTTHUMG00000155769	3.82 ± 0.26	3.79 ± 0.11	3.69 ± 0.12	3.81 ± 0.08
TC07000555.hg.1	OTTHUMG00000155770	5.59 ± 0.15	5.50 ± 0.14	5.47 ± 0.05	5.51 ± 0.05
TC07000557.hg.1	OTTHUMG00000155775	7.17 ± 0.20	7.14 ± 0.02	7.11 ± 0.05	7.22 ± 0.02
TC07000864.hg.1	OTTHUMG00000155789	5.36 ± 0.14	5.21 ± 0.16	5.31 ± 0.02	5.19 ± 0.07
TC07001545.hg.1	OTTHUMG00000155798	6.55 ± 0.05	6.62 ± 0.12	6.43 ± 0.12	6.60 ± 0.10
TC03001282.hg.1	OTTHUMG00000155802	3.97 ± 0.10	3.98 ± 0.18	4.07 ± 0.14	4.08 ± 0.10
TC07001345.hg.1	OTTHUMG00000155816	7.50 ± 0.22	7.43 ± 0.20	7.30 ± 0.26	7.61 ± 0.02
TC07000551.hg.1	OTTHUMG00000155818	6.78 ± 0.12	6.83 ± 0.17	6.73 ± 0.13	6.98 ± 0.13
TC07000279.hg.1	OTTHUMG00000155819	6.65 ± 0.30	6.67 ± 0.13	6.50 ± 0.02	6.67 ± 0.16
TC03002151.hg.1	OTTHUMG00000155824	6.58 ± 0.25	6.59 ± 0.09	6.51 ± 0.17	6.65 ± 0.10
TC03001090.hg.1	OTTHUMG00000155825	7.28 ± 0.24	7.35 ± 0.17	7.35 ± 0.07	7.42 ± 0.05
TC03002149.hg.1	OTTHUMG00000155836	8.00 ± 0.24	7.98 ± 0.07	7.77 ± 0.03	8.10 ± 0.30
TC07001367.hg.1	OTTHUMG00000155841	4.14 ± 0.07	4.13 ± 0.09	3.94 ± 0.08	3.99 ± 0.05
TC07000310.hg.1	OTTHUMG00000155853	6.49 ± 0.26	6.58 ± 0.12	6.56 ± 0.10	6.63 ± 0.06
TC07003395.hg.1	OTTHUMG00000155862	6.12 ± 0.14	5.95 ± 0.23	5.89 ± 0.19	5.80 ± 0.10
TC07001369.hg.1	OTTHUMG00000155880	5.38 ± 0.12	5.42 ± 0.07	5.43 ± 0.05	5.41 ± 0.05
TC07001371.hg.1	OTTHUMG00000155881	5.88 ± 0.17	5.90 ± 0.28	5.86 ± 0.06	6.12 ± 0.19
TC07001370.hg.1	OTTHUMG00000155882	4.68 ± 0.20	4.33 ± 0.08	4.58 ± 0.20	4.44 ± 0.12
TC03001084.hg.1	OTTHUMG00000155888	6.14 ± 0.06	6.13 ± 0.12	6.02 ± 0.10	6.19 ± 0.04
TC03001289.hg.1	OTTHUMG00000155924	5.23 ± 0.40	5.22 ± 0.15	5.14 ± 0.05	5.29 ± 0.08
TC03001290.hg.1	OTTHUMG00000155924	7.40 ± 0.14	7.32 ± 0.11	7.37 ± 0.14	7.37 ± 0.07
TC07001521.hg.1	OTTHUMG00000155934	9.03 ± 0.14	8.99 ± 0.03	9.07 ± 0.07	8.99 ± 0.03

TC07001375.hg.1	OTTHUMG00000155977	6.54 ± 0.38	6.48 ± 0.07	6.29 ± 0.13	6.70 ± 0.24
TC03001070.hg.1	OTTHUMG00000155980	4.82 ± 0.13	4.94 ± 0.15	4.96 ± 0.17	5.21 ± 0.28
TC03001073.hg.1	OTTHUMG00000155983	7.64 ± 0.11	7.65 ± 0.10	7.60 ± 0.13	7.84 ± 0.16
TC03001072.hg.1	OTTHUMG00000155986	6.69 ± 0.22	6.60 ± 0.07	6.67 ± 0.07	6.68 ± 0.14
TC03001069.hg.1	OTTHUMG00000155987	6.36 ± 0.14	6.20 ± 0.22	6.12 ± 0.09	6.38 ± 0.11
TC03002127.hg.1	OTTHUMG00000155988	7.85 ± 0.04	7.88 ± 0.09	7.76 ± 0.16	7.88 ± 0.03
TC07000317.hg.1	OTTHUMG00000155997	6.79 ± 0.24	6.58 ± 0.12	6.74 ± 0.19	6.68 ± 0.07
TC07000318.hg.1	OTTHUMG00000155998	6.03 ± 0.25	5.76 ± 0.04	6.02 ± 0.06	6.02 ± 0.22
TC03001309.hg.1	OTTHUMG00000156006	6.41 ± 0.11	6.48 ± 0.21	6.51 ± 0.06	6.65 ± 0.18
TC03001308.hg.1	OTTHUMG00000156009	5.01 ± 0.09	4.84 ± 0.02	4.76 ± 0.17	4.94 ± 0.26
TC03000213.hg.1	OTTHUMG00000156011	6.69 ± 0.25	6.85 ± 0.07	6.74 ± 0.25	6.98 ± 0.04
TC03001080.hg.1	OTTHUMG00000156017	4.82 ± 0.08	4.82 ± 0.17	4.67 ± 0.11	4.78 ± 0.15
TC03001076.hg.1	OTTHUMG00000156018	6.36 ± 0.17	6.26 ± 0.14	6.24 ± 0.17	6.21 ± 0.05
TC03001078.hg.1	OTTHUMG00000156019	6.00 ± 0.35	5.93 ± 0.19	6.10 ± 0.15	6.12 ± 0.04
TC03002139.hg.1	OTTHUMG00000156022	4.39 ± 0.21	4.40 ± 0.09	4.54 ± 0.26	4.60 ± 0.11
TC07000624.hg.1	OTTHUMG00000156028	8.70 ± 0.20	8.67 ± 0.06	8.68 ± 0.03	8.76 ± 0.08
TC07001385.hg.1	OTTHUMG00000156033	5.17 ± 0.13	5.09 ± 0.08	5.13 ± 0.04	5.18 ± 0.15
TC07001390.hg.1	OTTHUMG00000156038	4.69 ± 0.16	4.52 ± 0.08	4.52 ± 0.11	4.56 ± 0.17
TC07001392.hg.1	OTTHUMG00000156041	5.39 ± 0.08	5.45 ± 0.24	5.44 ± 0.17	5.51 ± 0.08
TC07001389.hg.1	OTTHUMG00000156043	5.50 ± 0.12	5.34 ± 0.02	5.27 ± 0.05	5.31 ± 0.09
TC03002123.hg.1	OTTHUMG00000156053	6.89 ± 0.18	6.89 ± 0.18	6.88 ± 0.18	7.02 ± 0.05
TC07000546.hg.1	OTTHUMG00000156056	5.79 ± 0.18	5.67 ± 0.12	5.58 ± 0.12	5.72 ± 0.09
TC07000544.hg.1	OTTHUMG00000156059	5.61 ± 0.17	5.52 ± 0.12	5.49 ± 0.11	5.63 ± 0.14
TC07001593.hg.1	OTTHUMG00000156060	4.46 ± 0.18	4.53 ± 0.08	4.60 ± 0.22	4.60 ± 0.05
TC07001594.hg.1	OTTHUMG00000156062	5.12 ± 0.06	5.00 ± 0.03	5.07 ± 0.04	5.02 ± 0.15
TC07001394.hg.1	OTTHUMG00000156065	5.83 ± 0.07	5.79 ± 0.15	5.89 ± 0.13	5.91 ± 0.09
TC07000322.hg.1	OTTHUMG00000156067	4.81 ± 0.07	4.68 ± 0.11	4.80 ± 0.04	4.82 ± 0.05
TC03002119.hg.1	OTTHUMG00000156107	5.59 ± 0.22	5.46 ± 0.14	5.37 ± 0.19	5.57 ± 0.13
TC02002109.hg.1	OTTHUMG00000156116	7.29 ± 0.33	7.32 ± 0.23	7.23 ± 0.28	7.43 ± 0.08
TC07000330.hg.1	OTTHUMG00000156119	7.49 ± 0.43	7.42 ± 0.12	7.32 ± 0.25	7.41 ± 0.14
TC07000332.hg.1	OTTHUMG00000156122	7.49 ± 0.13	7.44 ± 0.04	7.53 ± 0.08	7.50 ± 0.06

TC07000331.hg.1	OTTHUMG00000156131	6.93 ± 0.32	6.86 ± 0.27	6.81 ± 0.19	7.08 ± 0.12
TC01001449.hg.1	OTTHUMG00000156135	5.43 ± 0.24	5.43 ± 0.15	5.39 ± 0.17	5.51 ± 0.02
TC01003470.hg.1	OTTHUMG00000156136	7.99 ± 0.28	7.88 ± 0.04	7.94 ± 0.20	8.02 ± 0.04
TC03001310.hg.1	OTTHUMG00000156144	4.94 ± 0.19	4.95 ± 0.19	4.97 ± 0.02	5.09 ± 0.19
TC03001057.hg.1	OTTHUMG00000156146	5.27 ± 0.21	5.06 ± 0.13	5.08 ± 0.06	5.17 ± 0.12
TC03000194.hg.1	OTTHUMG00000156164	7.28 ± 0.24	7.27 ± 0.23	7.11 ± 0.15	7.44 ± 0.09
TC03001054.hg.1	OTTHUMG00000156168	5.52 ± 0.27	5.36 ± 0.08	5.32 ± 0.04	5.50 ± 0.22
TC17000930.hg.1	OTTHUMG00000156171	8.60 ± 0.21	8.60 ± 0.33	8.50 ± 0.24	8.73 ± 0.05
TC03001118.hg.1	OTTHUMG00000156184	5.76 ± 0.12	5.77 ± 0.06	5.77 ± 0.05	5.91 ± 0.11
TC03002110.hg.1	OTTHUMG00000156193	5.91 ± 0.05	5.96 ± 0.09	5.92 ± 0.06	6.02 ± 0.20
TC03001324.hg.1	OTTHUMG00000156209	7.09 ± 0.09	7.01 ± 0.05	7.14 ± 0.12	7.03 ± 0.05
TC02002357.hg.1	OTTHUMG00000156217	4.51 ± 0.17	4.48 ± 0.08	4.33 ± 0.27	4.47 ± 0.06
TC07001512.hg.1	OTTHUMG00000156239	6.40 ± 0.10	6.13 ± 0.09	6.31 ± 0.05	6.19 ± 0.07
TC07001268.hg.1	OTTHUMG00000156242	6.71 ± 0.13	6.71 ± 0.06	6.77 ± 0.19	6.73 ± 0.10
TC07001270.hg.1	OTTHUMG00000156243	5.10 ± 0.19	4.84 ± 0.07	4.87 ± 0.11	4.81 ± 0.07
TC07001269.hg.1	OTTHUMG00000156244	4.42 ± 0.05	4.30 ± 0.11	4.42 ± 0.11	4.49 ± 0.13
TC07000218.hg.1	OTTHUMG00000156245	5.77 ± 0.19	5.62 ± 0.22	5.62 ± 0.15	5.87 ± 0.24
TC03000226.hg.1	OTTHUMG00000156257	7.75 ± 0.32	7.66 ± 0.12	7.85 ± 0.14	7.64 ± 0.13
TC07001440.hg.1	OTTHUMG00000156267	7.09 ± 0.14	7.06 ± 0.14	7.06 ± 0.10	7.08 ± 0.03
TC07001442.hg.1	OTTHUMG00000156272	6.48 ± 0.14	6.30 ± 0.05	6.47 ± 0.09	6.31 ± 0.09
TC07000359.hg.1	OTTHUMG00000156273	6.13 ± 0.08	6.05 ± 0.06	6.14 ± 0.08	6.16 ± 0.20
TC07001438.hg.1	OTTHUMG00000156274	6.94 ± 0.28	6.95 ± 0.25	6.89 ± 0.31	7.03 ± 0.05
TC21000255.hg.1	OTTHUMG00000156278	6.09 ± 0.25	6.06 ± 0.20	5.97 ± 0.11	6.13 ± 0.14
TC08000800.hg.1	OTTHUMG00000156281	8.62 ± 0.29	8.61 ± 0.18	8.46 ± 0.31	8.70 ± 0.04
TC15001113.hg.1	OTTHUMG00000156285	7.69 ± 0.21	7.73 ± 0.06	7.80 ± 0.11	7.80 ± 0.06
TC07001418.hg.1	OTTHUMG00000156289	5.47 ± 0.05	5.47 ± 0.10	5.38 ± 0.20	5.61 ± 0.11
TC07001419.hg.1	OTTHUMG00000156290	5.98 ± 0.04	5.93 ± 0.15	6.03 ± 0.10	6.08 ± 0.11
TC03000245.hg.1	OTTHUMG00000156317	7.84 ± 0.27	7.93 ± 0.35	7.84 ± 0.30	8.15 ± 0.07
TC03001356.hg.1	OTTHUMG00000156326	6.54 ± 0.47	6.21 ± 0.08	6.58 ± 0.32	6.21 ± 0.09
TC07000360.hg.1	OTTHUMG00000156378	6.07 ± 0.21	5.91 ± 0.06	6.05 ± 0.05	5.98 ± 0.07
TC07000361.hg.1	OTTHUMG00000156380	7.87 ± 0.25	7.84 ± 0.18	7.74 ± 0.11	7.99 ± 0.19

TC07001443.hg.1	OTTHUMG00000156385	6.59 ± 0.28	6.52 ± 0.23	6.49 ± 0.23	6.71 ± 0.20
TC07001444.hg.1	OTTHUMG00000156386	6.13 ± 0.08	6.05 ± 0.06	6.14 ± 0.08	6.16 ± 0.20
TC03002098.hg.1	OTTHUMG00000156391	6.48 ± 0.26	6.34 ± 0.07	6.40 ± 0.15	6.39 ± 0.20
TC03002099.hg.1	OTTHUMG00000156391	5.42 ± 0.12	5.34 ± 0.13	5.26 ± 0.16	5.40 ± 0.06
TC03002097.hg.1	OTTHUMG00000156392	4.02 ± 0.16	3.88 ± 0.04	4.05 ± 0.11	3.87 ± 0.06
TC03002101.hg.1	OTTHUMG00000156393	6.48 ± 0.21	6.36 ± 0.28	6.31 ± 0.15	6.52 ± 0.06
TC03001036.hg.1	OTTHUMG00000156394	5.01 ± 0.18	4.91 ± 0.07	4.90 ± 0.13	5.03 ± 0.12
TC03001037.hg.1	OTTHUMG00000156395	6.39 ± 0.14	6.41 ± 0.12	6.20 ± 0.16	6.42 ± 0.17
TC03002100.hg.1	OTTHUMG00000156396	6.40 ± 0.08	6.49 ± 0.03	6.63 ± 0.06	6.49 ± 0.02
TC19000221.hg.1	OTTHUMG00000156402	6.44 ± 0.23	6.14 ± 0.07	6.22 ± 0.28	6.25 ± 0.11
TC19000220.hg.1	OTTHUMG00000156423	4.71 ± 0.13	4.70 ± 0.16	4.75 ± 0.10	4.79 ± 0.06
TC19000219.hg.1	OTTHUMG00000156425	6.41 ± 0.12	6.39 ± 0.03	6.27 ± 0.08	6.46 ± 0.08
TC19000212.hg.1	OTTHUMG00000156428	6.83 ± 0.09	6.81 ± 0.17	6.72 ± 0.07	6.90 ± 0.09
TC03001327.hg.1	OTTHUMG00000156432	7.23 ± 0.10	7.15 ± 0.32	7.16 ± 0.17	7.27 ± 0.07
TC03001035.hg.1	OTTHUMG00000156440	7.68 ± 0.23	7.70 ± 0.04	8.03 ± 0.11	7.50 ± 0.14
TC07001556.hg.1	OTTHUMG00000156443	4.96 ± 0.22	4.89 ± 0.20	4.86 ± 0.22	4.92 ± 0.09
TC03001339.hg.1	OTTHUMG00000156455	5.87 ± 0.16	5.93 ± 0.15	5.84 ± 0.11	5.93 ± 0.02
TC03001033.hg.1	OTTHUMG00000156460	6.50 ± 0.16	6.45 ± 0.10	6.35 ± 0.14	6.79 ± 0.30
TC03002092.hg.1	OTTHUMG00000156463	6.57 ± 0.21	6.55 ± 0.24	6.43 ± 0.15	6.58 ± 0.05
TC03000228.hg.1	OTTHUMG00000156464	5.23 ± 0.22	4.94 ± 0.14	5.10 ± 0.04	5.12 ± 0.13
TC07000371.hg.1	OTTHUMG00000156469	3.55 ± 0.01	3.44 ± 0.03	3.43 ± 0.11	3.40 ± 0.10
TC07000375.hg.1	OTTHUMG00000156483	5.84 ± 0.30	5.49 ± 0.17	5.44 ± 0.09	5.44 ± 0.04
TC07000376.hg.1	OTTHUMG00000156487	6.43 ± 0.19	6.40 ± 0.21	6.40 ± 0.09	6.47 ± 0.04
TC07000500.hg.1	OTTHUMG00000156495	7.22 ± 0.40	7.44 ± 0.20	7.16 ± 0.09	7.58 ± 0.21
TC03001335.hg.1	OTTHUMG00000156509	5.50 ± 0.37	5.67 ± 0.15	5.83 ± 0.13	5.82 ± 0.11
TC07000603.hg.1	OTTHUMG00000156511	6.20 ± 0.15	6.00 ± 0.04	6.12 ± 0.26	6.03 ± 0.04
TC03001026.hg.1	OTTHUMG00000156519	4.52 ± 0.22	4.21 ± 0.07	4.39 ± 0.09	4.25 ± 0.07
TC03002089.hg.1	OTTHUMG00000156520	7.66 ± 0.18	7.65 ± 0.06	7.67 ± 0.11	7.78 ± 0.14
TC07000369.hg.1	OTTHUMG00000156551	7.04 ± 0.29	6.98 ± 0.09	6.99 ± 0.11	7.04 ± 0.05
TC07001446.hg.1	OTTHUMG00000156552	6.58 ± 0.23	6.56 ± 0.29	6.62 ± 0.23	6.69 ± 0.13
TC07000417.hg.1	OTTHUMG00000156569	4.84 ± 0.16	4.75 ± 0.14	4.67 ± 0.06	4.74 ± 0.15

TC07001473.hg.1	OTTHUMG00000156571	4.15 ± 0.10	4.12 ± 0.10	4.17 ± 0.10	4.19 ± 0.04
TC03002086.hg.1	OTTHUMG00000156574	9.47 ± 0.15	9.48 ± 0.04	9.47 ± 0.08	9.57 ± 0.08
TC07001474.hg.1	OTTHUMG00000156582	5.32 ± 0.42	5.16 ± 0.06	5.16 ± 0.08	5.27 ± 0.04
TC07000418.hg.1	OTTHUMG00000156583	7.23 ± 0.34	7.19 ± 0.05	7.12 ± 0.25	7.35 ± 0.20
TC03003378.hg.1	OTTHUMG00000156589	7.96 ± 0.07	7.98 ± 0.09	8.03 ± 0.02	8.11 ± 0.09
TC07001463.hg.1	OTTHUMG00000156596	8.52 ± 0.13	8.55 ± 0.05	8.47 ± 0.12	8.51 ± 0.11
TC03002085.hg.1	OTTHUMG00000156604	5.04 ± 0.21	4.97 ± 0.10	4.80 ± 0.07	4.97 ± 0.12
TC07001477.hg.1	OTTHUMG00000156607	5.47 ± 0.18	5.40 ± 0.18	5.39 ± 0.15	5.56 ± 0.03
TC07001476.hg.1	OTTHUMG00000156608	5.46 ± 0.09	5.36 ± 0.09	5.49 ± 0.06	5.59 ± 0.20
TC07000419.hg.1	OTTHUMG00000156611	4.78 ± 0.12	4.71 ± 0.11	4.73 ± 0.08	4.84 ± 0.07
TC03001016.hg.1	OTTHUMG00000156616	5.55 ± 0.02	5.43 ± 0.04	5.37 ± 0.01	5.51 ± 0.12
TC03001017.hg.1	OTTHUMG00000156619	6.66 ± 0.07	6.56 ± 0.16	6.42 ± 0.25	6.60 ± 0.12
TC03000283.hg.1	OTTHUMG00000156627	8.56 ± 0.33	8.61 ± 0.25	8.53 ± 0.30	8.71 ± 0.07
TC07001451.hg.1	OTTHUMG00000156628	7.62 ± 0.21	7.66 ± 0.18	7.71 ± 0.14	7.67 ± 0.08
TC07001459.hg.1	OTTHUMG00000156655	4.99 ± 0.13	4.78 ± 0.05	4.86 ± 0.08	4.83 ± 0.06
TC07000510.hg.1	OTTHUMG00000156705	4.10 ± 0.11	4.00 ± 0.00	3.96 ± 0.11	3.95 ± 0.01
TC03002074.hg.1	OTTHUMG00000156711	7.48 ± 0.16	7.45 ± 0.17	7.33 ± 0.08	7.50 ± 0.16
TC03000300.hg.1	OTTHUMG00000156715	7.87 ± 0.27	7.87 ± 0.24	7.65 ± 0.37	7.94 ± 0.07
TC03001393.hg.1	OTTHUMG00000156716	7.47 ± 0.22	7.25 ± 0.10	7.32 ± 0.18	7.34 ± 0.02
TC03001392.hg.1	OTTHUMG00000156717	6.84 ± 0.11	6.72 ± 0.22	6.82 ± 0.04	6.85 ± 0.15
TC07000396.hg.1	OTTHUMG00000156731	5.29 ± 0.32	5.12 ± 0.22	5.25 ± 0.04	5.23 ± 0.10
TC03000301.hg.1	OTTHUMG00000156735	8.02 ± 0.22	8.11 ± 0.21	7.98 ± 0.24	8.25 ± 0.06
TC03000290.hg.1	OTTHUMG00000156818	5.07 ± 0.12	5.17 ± 0.16	5.28 ± 0.21	5.18 ± 0.07
TC07000416.hg.1	OTTHUMG00000156924	6.15 ± 0.24	6.33 ± 0.12	6.28 ± 0.16	6.32 ± 0.10
TC07000711.hg.1	OTTHUMG00000156934	5.87 ± 0.13	5.84 ± 0.13	5.90 ± 0.23	5.83 ± 0.04
TC03000308.hg.1	OTTHUMG00000156937	8.04 ± 0.20	7.96 ± 0.11	7.96 ± 0.10	8.02 ± 0.02
TC07001800.hg.1	OTTHUMG00000156951	4.99 ± 0.13	4.87 ± 0.02	4.89 ± 0.06	4.94 ± 0.05
TC07000731.hg.1	OTTHUMG00000156952	5.24 ± 0.11	4.98 ± 0.17	5.01 ± 0.28	4.99 ± 0.11
TC07001802.hg.1	OTTHUMG00000156953	4.61 ± 0.26	4.47 ± 0.14	4.56 ± 0.17	4.45 ± 0.26
TC03001425.hg.1	OTTHUMG00000156955	7.59 ± 0.25	7.52 ± 0.22	7.50 ± 0.13	7.65 ± 0.10
TC06001238.hg.1	OTTHUMG00000156967	5.83 ± 0.18	5.82 ± 0.30	5.74 ± 0.12	5.95 ± 0.06

TC01000011.hg.1	OTTHUMG00000156971	8.45 ± 0.09	8.23 ± 0.11	8.80 ± 0.10	8.32 ± 0.11
TC06001225.hg.1	OTTHUMG00000156977	4.92 ± 0.14	4.74 ± 0.14	4.83 ± 0.17	4.84 ± 0.11
TC03002007.hg.1	OTTHUMG00000156985	5.73 ± 0.03	5.68 ± 0.01	5.66 ± 0.09	5.66 ± 0.15
TC07001803.hg.1	OTTHUMG00000156990	4.54 ± 0.40	4.22 ± 0.03	4.34 ± 0.14	4.33 ± 0.17
TC07001810.hg.1	OTTHUMG00000157001	5.09 ± 0.14	4.95 ± 0.22	4.94 ± 0.07	5.04 ± 0.30
TC03000931.hg.1	OTTHUMG00000157006	5.54 ± 0.10	5.26 ± 0.08	5.43 ± 0.13	5.39 ± 0.11
TC07001682.hg.1	OTTHUMG00000157034	8.58 ± 0.23	8.73 ± 0.24	8.58 ± 0.13	8.86 ± 0.17
TC07000642.hg.1	OTTHUMG00000157039	7.71 ± 0.28	7.41 ± 0.16	7.75 ± 0.34	7.20 ± 0.25
TC07001678.hg.1	OTTHUMG00000157041	8.65 ± 0.31	8.56 ± 0.14	8.48 ± 0.12	8.54 ± 0.07
TC07001681.hg.1	OTTHUMG00000157043	8.50 ± 0.19	8.55 ± 0.16	8.45 ± 0.21	8.59 ± 0.05
TC04001315.hg.1	OTTHUMG00000157073	5.74 ± 0.38	5.55 ± 0.26	5.50 ± 0.29	5.69 ± 0.09
TC04001314.hg.1	OTTHUMG00000157074	5.28 ± 0.24	5.13 ± 0.01	5.17 ± 0.06	5.21 ± 0.09
TC02001173.hg.1	OTTHUMG00000157083	6.71 ± 0.03	6.89 ± 0.10	6.87 ± 0.06	7.09 ± 0.09
TC07001817.hg.1	OTTHUMG00000157089	4.80 ± 0.07	4.63 ± 0.13	4.82 ± 0.10	4.73 ± 0.03
TC07000742.hg.1	OTTHUMG00000157094	4.98 ± 0.09	4.81 ± 0.10	4.82 ± 0.10	5.01 ± 0.11
TC07000743.hg.1	OTTHUMG00000157094	3.98 ± 0.23	3.60 ± 0.08	3.72 ± 0.03	3.66 ± 0.04
TC07000740.hg.1	OTTHUMG00000157098	5.00 ± 0.18	4.93 ± 0.10	5.10 ± 0.14	4.96 ± 0.15
TC07000648.hg.1	OTTHUMG00000157105	6.50 ± 0.37	6.36 ± 0.10	6.37 ± 0.31	6.37 ± 0.09
TC07000647.hg.1	OTTHUMG00000157112	6.50 ± 0.28	6.35 ± 0.10	6.44 ± 0.22	6.38 ± 0.09
TC07001822.hg.1	OTTHUMG00000157115	4.70 ± 0.06	4.68 ± 0.08	4.63 ± 0.07	4.74 ± 0.07
TC07000744.hg.1	OTTHUMG00000157116	4.07 ± 0.19	3.83 ± 0.02	3.98 ± 0.20	3.75 ± 0.02
TC08001326.hg.1	OTTHUMG00000157143	5.34 ± 0.13	5.22 ± 0.05	5.36 ± 0.05	5.32 ± 0.15
TC07000757.hg.1	OTTHUMG00000157151	6.34 ± 0.36	6.30 ± 0.02	6.35 ± 0.11	6.68 ± 0.30
TC07000924.hg.1	OTTHUMG00000157156	6.27 ± 0.10	6.34 ± 0.15	6.30 ± 0.14	6.41 ± 0.05
TC07000755.hg.1	OTTHUMG00000157169	4.01 ± 0.08	3.96 ± 0.06	3.98 ± 0.06	4.02 ± 0.15
TC07000754.hg.1	OTTHUMG00000157170	5.04 ± 0.06	5.02 ± 0.02	5.02 ± 0.05	5.11 ± 0.11
TC07001051.hg.1	OTTHUMG00000157189	7.41 ± 0.45	7.36 ± 0.13	7.31 ± 0.18	7.24 ± 0.01
TC07001049.hg.1	OTTHUMG00000157190	6.86 ± 0.08	6.89 ± 0.14	6.81 ± 0.03	7.04 ± 0.05
TC07001053.hg.1	OTTHUMG00000157191	6.14 ± 0.33	5.92 ± 0.21	6.10 ± 0.13	5.97 ± 0.05
TC07001832.hg.1	OTTHUMG00000157195	4.74 ± 0.16	4.69 ± 0.08	4.68 ± 0.06	4.83 ± 0.19
TC07001831.hg.1	OTTHUMG00000157196	4.13 ± 0.07	4.10 ± 0.16	4.05 ± 0.02	4.06 ± 0.06

TC07000669.hg.1	OTTHUMG00000157208	7.47 ± 0.43	6.75 ± 0.28	7.62 ± 0.10	6.78 ± 0.14
TC04000910.hg.1	OTTHUMG00000157216	5.52 ± 0.06	5.53 ± 0.11	5.40 ± 0.10	5.48 ± 0.02
TC04000908.hg.1	OTTHUMG00000157217	5.67 ± 0.26	5.57 ± 0.16	5.62 ± 0.10	5.60 ± 0.13
TC07000670.hg.1	OTTHUMG00000157223	4.60 ± 0.30	4.45 ± 0.04	4.59 ± 0.24	4.56 ± 0.13
TC07000674.hg.1	OTTHUMG00000157225	4.41 ± 0.12	4.42 ± 0.04	4.48 ± 0.06	4.47 ± 0.06
TC04000909.hg.1	OTTHUMG00000157228	7.13 ± 0.34	7.19 ± 0.19	7.16 ± 0.12	7.34 ± 0.17
TC03002027.hg.1	OTTHUMG00000157233	6.62 ± 0.42	6.71 ± 0.11	6.60 ± 0.11	7.02 ± 0.32
TC03000945.hg.1	OTTHUMG00000157235	4.85 ± 0.08	4.70 ± 0.08	4.79 ± 0.07	4.72 ± 0.06
TC03000946.hg.1	OTTHUMG00000157236	4.88 ± 0.13	4.69 ± 0.14	4.74 ± 0.13	4.79 ± 0.02
TC07002076.hg.1	OTTHUMG00000157240	9.03 ± 0.24	9.03 ± 0.33	9.05 ± 0.34	9.26 ± 0.05
TC03002025.hg.1	OTTHUMG00000157252	6.30 ± 0.23	6.34 ± 0.06	6.27 ± 0.29	6.45 ± 0.07
TC03000947.hg.1	OTTHUMG00000157253	4.63 ± 0.28	4.42 ± 0.05	4.57 ± 0.14	4.53 ± 0.14
TC03002030.hg.1	OTTHUMG00000157254	5.57 ± 0.21	5.50 ± 0.08	5.47 ± 0.07	5.64 ± 0.07
TC16000757.hg.1	OTTHUMG00000157255	6.46 ± 0.04	6.41 ± 0.10	6.39 ± 0.08	6.53 ± 0.04
TC07002044.hg.1	OTTHUMG00000157258	8.17 ± 0.15	8.29 ± 0.02	8.39 ± 0.13	8.39 ± 0.12
TC07001508.hg.1	OTTHUMG00000157272	9.16 ± 0.25	9.24 ± 0.23	9.31 ± 0.24	9.37 ± 0.10
TC07000677.hg.1	OTTHUMG00000157277	9.43 ± 0.11	9.42 ± 0.13	9.26 ± 0.35	9.24 ± 0.11
TC07001727.hg.1	OTTHUMG00000157278	7.65 ± 0.12	7.70 ± 0.13	7.58 ± 0.01	7.71 ± 0.04
TC07002053.hg.1	OTTHUMG00000157286	6.29 ± 0.07	6.46 ± 0.22	6.51 ± 0.13	6.69 ± 0.20
TC03002033.hg.1	OTTHUMG00000157289	6.10 ± 0.12	6.11 ± 0.03	6.11 ± 0.07	6.21 ± 0.10
TC03002021.hg.1	OTTHUMG00000157300	5.45 ± 0.17	5.33 ± 0.16	5.33 ± 0.20	5.33 ± 0.03
TC03002020.hg.1	OTTHUMG00000157301	4.92 ± 0.06	4.74 ± 0.01	4.91 ± 0.06	4.90 ± 0.12
TC07002042.hg.1	OTTHUMG00000157305	6.05 ± 0.28	6.01 ± 0.02	6.07 ± 0.13	6.19 ± 0.06
TC03002035.hg.1	OTTHUMG00000157310	6.72 ± 0.14	6.68 ± 0.06	6.71 ± 0.13	6.80 ± 0.14
TC07000751.hg.1	OTTHUMG00000157317	5.43 ± 0.08	5.34 ± 0.06	5.40 ± 0.08	5.46 ± 0.04
TC07001826.hg.1	OTTHUMG00000157319	5.13 ± 0.25	5.03 ± 0.25	5.10 ± 0.09	5.20 ± 0.22
TC07001827.hg.1	OTTHUMG00000157320	5.18 ± 0.21	5.01 ± 0.12	5.03 ± 0.16	5.16 ± 0.29
TC07000752.hg.1	OTTHUMG00000157321	4.70 ± 0.07	4.63 ± 0.00	4.62 ± 0.03	4.74 ± 0.12
TC07001018.hg.1	OTTHUMG00000157352	6.69 ± 0.35	6.64 ± 0.20	6.64 ± 0.20	6.81 ± 0.08
TC07001015.hg.1	OTTHUMG00000157379	8.28 ± 0.28	8.42 ± 0.17	8.31 ± 0.02	8.56 ± 0.15
TC07000680.hg.1	OTTHUMG00000157398	4.60 ± 0.20	4.52 ± 0.03	4.68 ± 0.17	4.45 ± 0.10

TC07001929.hg.1	OTTHUMG00000157467	5.98 ± 0.02	5.84 ± 0.16	6.02 ± 0.10	6.07 ± 0.09
TC07001840.hg.1	OTTHUMG00000157565	3.84 ± 0.11	3.83 ± 0.16	3.79 ± 0.05	3.87 ± 0.09
TC07000686.hg.1	OTTHUMG00000157590	6.78 ± 0.08	6.83 ± 0.03	6.78 ± 0.07	6.98 ± 0.18
TC07001739.hg.1	OTTHUMG00000157591	6.03 ± 0.15	5.96 ± 0.02	6.10 ± 0.11	6.01 ± 0.10
TC07001742.hg.1	OTTHUMG00000157643	5.09 ± 0.14	5.10 ± 0.13	5.08 ± 0.07	5.24 ± 0.05
TC07000807.hg.1	OTTHUMG00000157649	5.88 ± 0.13	5.72 ± 0.03	5.93 ± 0.13	5.72 ± 0.05
TC07000806.hg.1	OTTHUMG00000157651	7.85 ± 0.34	7.75 ± 0.23	7.71 ± 0.34	7.87 ± 0.15
TC07000803.hg.1	OTTHUMG00000157652	6.08 ± 0.24	5.93 ± 0.13	5.95 ± 0.09	6.03 ± 0.11
TC07001857.hg.1	OTTHUMG00000157659	8.56 ± 0.19	8.59 ± 0.03	8.55 ± 0.02	8.65 ± 0.06
TC17000046.hg.1	OTTHUMG00000157665	7.63 ± 0.19	7.62 ± 0.09	7.55 ± 0.26	7.64 ± 0.05
TC07001701.hg.1	OTTHUMG00000157724	7.68 ± 0.10	7.72 ± 0.12	7.79 ± 0.04	7.85 ± 0.07
TC07000759.hg.1	OTTHUMG00000157729	4.39 ± 0.23	4.24 ± 0.01	4.30 ± 0.04	4.30 ± 0.08
TC07000944.hg.1	OTTHUMG00000157766	4.86 ± 0.08	4.83 ± 0.14	4.92 ± 0.09	4.90 ± 0.04
TC03002043.hg.1	OTTHUMG00000157861	4.57 ± 0.19	4.56 ± 0.14	4.51 ± 0.02	4.63 ± 0.08
TC07000954.hg.1	OTTHUMG00000158007	7.16 ± 0.06	7.07 ± 0.03	6.96 ± 0.07	7.07 ± 0.09
TC03000884.hg.1	OTTHUMG00000158020	6.02 ± 0.04	5.98 ± 0.04	6.07 ± 0.06	6.10 ± 0.10
TC03000885.hg.1	OTTHUMG00000158042	3.99 ± 0.11	3.76 ± 0.09	3.94 ± 0.04	3.75 ± 0.09
TC03001965.hg.1	OTTHUMG00000158064	5.52 ± 0.19	5.44 ± 0.12	5.54 ± 0.05	5.55 ± 0.04
TC03001968.hg.1	OTTHUMG00000158073	4.31 ± 0.18	4.25 ± 0.13	4.26 ± 0.07	4.34 ± 0.08
TC07000994.hg.1	OTTHUMG00000158096	7.42 ± 0.07	7.11 ± 0.08	7.23 ± 0.13	7.13 ± 0.11
TC03000330.hg.1	OTTHUMG00000158107	8.41 ± 0.17	8.29 ± 0.17	8.27 ± 0.15	8.32 ± 0.04
TC03000887.hg.1	OTTHUMG00000158130	5.21 ± 0.13	5.18 ± 0.01	5.24 ± 0.02	5.32 ± 0.18
TC07000997.hg.1	OTTHUMG00000158149	5.39 ± 0.16	5.27 ± 0.02	5.17 ± 0.10	5.33 ± 0.06
TC03001971.hg.1	OTTHUMG00000158209	3.62 ± 0.17	3.41 ± 0.09	3.55 ± 0.11	3.48 ± 0.08
TC03002046.hg.1	OTTHUMG00000158223	5.34 ± 0.19	5.34 ± 0.19	5.27 ± 0.07	5.44 ± 0.10
TC03002047.hg.1	OTTHUMG00000158224	4.70 ± 0.07	4.46 ± 0.05	4.77 ± 0.20	4.49 ± 0.05
TC03000973.hg.1	OTTHUMG00000158236	4.93 ± 0.14	5.03 ± 0.04	5.05 ± 0.09	5.10 ± 0.19
TC03001973.hg.1	OTTHUMG00000158283	5.58 ± 0.15	5.58 ± 0.22	5.55 ± 0.12	5.75 ± 0.29
TC03000891.hg.1	OTTHUMG00000158284	4.67 ± 0.12	4.49 ± 0.04	4.56 ± 0.10	4.62 ± 0.15
TC03000975.hg.1	OTTHUMG00000158297	6.58 ± 0.21	6.64 ± 0.05	6.71 ± 0.07	6.70 ± 0.06
TC03002051.hg.1	OTTHUMG00000158299	6.73 ± 0.22	6.77 ± 0.09	6.89 ± 0.11	6.92 ± 0.12

TC03000974.hg.1	OTTHUMG00000158300	4.81 ± 0.06	4.76 ± 0.16	4.74 ± 0.24	4.87 ± 0.24
TC03002050.hg.1	OTTHUMG00000158301	5.55 ± 0.19	5.33 ± 0.03	5.50 ± 0.02	5.41 ± 0.09
TC07002017.hg.1	OTTHUMG00000158322	7.32 ± 0.35	7.34 ± 0.21	7.29 ± 0.24	7.47 ± 0.10
TC07002019.hg.1	OTTHUMG00000158329	8.38 ± 0.27	8.45 ± 0.21	8.41 ± 0.25	8.68 ± 0.13
TC07001588.hg.1	OTTHUMG00000158345	6.72 ± 0.22	6.73 ± 0.20	6.73 ± 0.12	6.85 ± 0.17
TC03001479.hg.1	OTTHUMG00000158357	6.04 ± 0.06	6.05 ± 0.15	6.11 ± 0.04	6.08 ± 0.05
TC03001481.hg.1	OTTHUMG00000158358	6.80 ± 0.12	6.72 ± 0.18	6.76 ± 0.08	6.86 ± 0.17
TC03000356.hg.1	OTTHUMG00000158359	6.05 ± 0.16	6.00 ± 0.05	5.92 ± 0.12	6.20 ± 0.20
TC03001480.hg.1	OTTHUMG00000158360	5.63 ± 0.28	5.44 ± 0.17	5.59 ± 0.26	5.65 ± 0.08
TC03000355.hg.1	OTTHUMG00000158362	5.38 ± 0.14	5.32 ± 0.05	5.32 ± 0.19	5.28 ± 0.05
TC07001850.hg.1	OTTHUMG00000158366	7.00 ± 0.21	6.99 ± 0.24	6.88 ± 0.24	7.18 ± 0.12
TC03001483.hg.1	OTTHUMG00000158383	5.50 ± 0.18	5.61 ± 0.07	5.59 ± 0.10	5.73 ± 0.11
TC03001964.hg.1	OTTHUMG00000158384	3.76 ± 0.11	3.71 ± 0.12	3.72 ± 0.13	3.68 ± 0.06
TC16000353.hg.1	OTTHUMG00000158416	8.43 ± 0.30	8.19 ± 0.03	8.25 ± 0.24	8.17 ± 0.07
TC07001853.hg.1	OTTHUMG00000158425	6.26 ± 0.06	6.43 ± 0.12	6.35 ± 0.19	6.55 ± 0.12
TC01000062.hg.1	OTTHUMG00000158429	8.37 ± 0.13	8.39 ± 0.17	8.49 ± 0.20	8.49 ± 0.16
TC07000717.hg.1	OTTHUMG00000158446	5.56 ± 0.36	5.45 ± 0.15	5.36 ± 0.17	5.63 ± 0.16
TC07001776.hg.1	OTTHUMG00000158448	5.79 ± 0.16	5.61 ± 0.06	5.69 ± 0.11	5.81 ± 0.13
TC07001780.hg.1	OTTHUMG00000158457	6.89 ± 0.09	6.89 ± 0.07	6.64 ± 0.06	6.94 ± 0.10
TC03000847.hg.1	OTTHUMG00000158465	5.35 ± 0.17	5.19 ± 0.03	5.18 ± 0.08	5.25 ± 0.03
TC03000848.hg.1	OTTHUMG00000158468	5.24 ± 0.01	5.18 ± 0.09	5.21 ± 0.16	5.09 ± 0.10
TC03000893.hg.1	OTTHUMG00000158502	6.81 ± 0.24	6.33 ± 0.18	6.59 ± 0.20	6.37 ± 0.14
TC01001494.hg.1	OTTHUMG00000158541	6.47 ± 0.22	6.47 ± 0.15	6.29 ± 0.09	6.57 ± 0.13
TC03000898.hg.1	OTTHUMG00000158562	11.26 ± 0.20	11.10 ± 0.02	11.23 ± 0.14	11.17 ± 0.08
TC07000903.hg.1	OTTHUMG00000158567	5.10 ± 0.23	4.99 ± 0.14	4.94 ± 0.13	5.22 ± 0.19
TC03000902.hg.1	OTTHUMG00000158576	3.78 ± 0.04	3.38 ± 0.03	3.61 ± 0.05	3.42 ± 0.14
TC03000894.hg.1	OTTHUMG00000158586	4.56 ± 0.29	4.45 ± 0.13	4.39 ± 0.12	4.51 ± 0.10
TC03000895.hg.1	OTTHUMG00000158587	4.04 ± 0.11	3.86 ± 0.03	3.97 ± 0.06	3.90 ± 0.04
TC03000372.hg.1	OTTHUMG00000158637	6.47 ± 0.24	6.23 ± 0.16	6.12 ± 0.04	6.09 ± 0.11
TC03000371.hg.1	OTTHUMG00000158638	5.46 ± 0.19	4.94 ± 0.33	5.42 ± 0.29	4.97 ± 0.21
TC03001513.hg.1	OTTHUMG00000158658	5.40 ± 0.19	5.10 ± 0.14	5.28 ± 0.16	5.34 ± 0.27

TC07002026.hg.1	OTTHUMG00000158684	8.64 ± 0.26	8.66 ± 0.05	8.64 ± 0.13	8.77 ± 0.07
TC03000904.hg.1	OTTHUMG00000158687	4.47 ± 0.10	4.30 ± 0.01	4.46 ± 0.08	4.39 ± 0.04
TC03001980.hg.1	OTTHUMG00000158688	6.29 ± 0.07	6.18 ± 0.27	6.24 ± 0.17	6.47 ± 0.13
TC07002028.hg.1	OTTHUMG00000158693	8.31 ± 0.30	8.35 ± 0.25	8.24 ± 0.15	8.47 ± 0.06
TC03000399.hg.1	OTTHUMG00000158709	6.78 ± 0.14	6.70 ± 0.07	6.68 ± 0.20	6.68 ± 0.02
TC03001526.hg.1	OTTHUMG00000158710	8.24 ± 0.30	8.29 ± 0.24	8.12 ± 0.38	8.35 ± 0.08
TC03000857.hg.1	OTTHUMG00000158714	5.71 ± 0.18	5.75 ± 0.23	5.53 ± 0.23	5.76 ± 0.06
TC03000860.hg.1	OTTHUMG00000158715	6.44 ± 0.26	6.34 ± 0.12	6.68 ± 0.49	6.47 ± 0.20
TC03000404.hg.1	OTTHUMG00000158734	5.66 ± 0.07	5.67 ± 0.11	5.74 ± 0.19	5.80 ± 0.08
TC03000410.hg.1	OTTHUMG00000158739	5.64 ± 0.07	5.55 ± 0.06	5.54 ± 0.03	5.59 ± 0.07
TC03000409.hg.1	OTTHUMG00000158741	5.87 ± 0.38	5.82 ± 0.09	5.84 ± 0.10	6.04 ± 0.19
TC03000861.hg.1	OTTHUMG00000158749	6.30 ± 0.05	6.23 ± 0.04	6.31 ± 0.17	6.25 ± 0.04
TC03001944.hg.1	OTTHUMG00000158756	5.16 ± 0.14	5.09 ± 0.15	5.61 ± 0.17	5.34 ± 0.18
TC03001945.hg.1	OTTHUMG00000158757	4.42 ± 0.07	4.34 ± 0.01	4.39 ± 0.03	4.37 ± 0.08
TC03000416.hg.1	OTTHUMG00000158770	6.22 ± 0.11	6.06 ± 0.06	6.23 ± 0.25	6.13 ± 0.03
TC03001551.hg.1	OTTHUMG00000158784	7.36 ± 0.09	7.54 ± 0.07	7.55 ± 0.10	7.53 ± 0.18
TC03001543.hg.1	OTTHUMG00000158801	6.77 ± 0.18	6.72 ± 0.03	6.72 ± 0.08	6.81 ± 0.14
TC03000916.hg.1	OTTHUMG00000158828	4.51 ± 0.30	4.48 ± 0.02	4.48 ± 0.24	4.55 ± 0.08
TC03000433.hg.1	OTTHUMG00000158832	6.16 ± 0.14	6.18 ± 0.05	6.21 ± 0.11	6.34 ± 0.18
TC03000434.hg.1	OTTHUMG00000158838	4.96 ± 0.14	4.83 ± 0.28	4.89 ± 0.10	4.88 ± 0.10
TC03000446.hg.1	OTTHUMG00000158839	5.53 ± 0.12	5.49 ± 0.05	5.53 ± 0.11	5.57 ± 0.16
TC03001569.hg.1	OTTHUMG00000158840	3.95 ± 0.13	3.79 ± 0.07	3.91 ± 0.07	3.91 ± 0.07
TC03000920.hg.1	OTTHUMG00000158859	7.11 ± 0.14	6.85 ± 0.12	6.80 ± 0.18	6.88 ± 0.14
TC03001992.hg.1	OTTHUMG00000158937	6.24 ± 0.05	6.20 ± 0.10	6.22 ± 0.26	6.45 ± 0.14
TC03000918.hg.1	OTTHUMG00000158939	5.25 ± 0.30	5.14 ± 0.01	5.20 ± 0.14	5.20 ± 0.06
TC03001572.hg.1	OTTHUMG00000158943	5.90 ± 0.27	6.05 ± 0.15	5.84 ± 0.19	6.17 ± 0.19
TC03000449.hg.1	OTTHUMG00000158944	5.05 ± 0.22	5.03 ± 0.06	5.04 ± 0.05	5.12 ± 0.09
TC03000448.hg.1	OTTHUMG00000158945	5.17 ± 0.07	5.00 ± 0.05	5.07 ± 0.17	5.19 ± 0.12
TC03000453.hg.1	OTTHUMG00000158946	4.81 ± 0.13	4.68 ± 0.07	4.67 ± 0.06	4.73 ± 0.13
TC03001573.hg.1	OTTHUMG00000158948	5.12 ± 0.18	5.07 ± 0.03	5.18 ± 0.20	5.18 ± 0.09
TC03001952.hg.1	OTTHUMG00000158951	5.70 ± 0.09	5.66 ± 0.08	5.66 ± 0.07	5.73 ± 0.06

TC03000871.hg.1	OTTHUMG00000158954	6.42 ± 0.07	6.38 ± 0.15	6.42 ± 0.07	6.44 ± 0.06
TC03001574.hg.1	OTTHUMG00000158980	6.13 ± 0.12	6.13 ± 0.10	6.04 ± 0.04	6.26 ± 0.08
TC03001582.hg.1	OTTHUMG00000158994	3.76 ± 0.06	3.53 ± 0.16	3.59 ± 0.04	3.40 ± 0.04
TC03000459.hg.1	OTTHUMG00000158995	5.26 ± 0.17	5.11 ± 0.03	5.24 ± 0.04	5.28 ± 0.27
TC03001586.hg.1	OTTHUMG00000159002	5.47 ± 0.26	5.13 ± 0.09	5.23 ± 0.12	5.34 ± 0.22
TC03001576.hg.1	OTTHUMG00000159005	4.78 ± 0.10	4.66 ± 0.09	4.61 ± 0.12	4.71 ± 0.08
TC03000452.hg.1	OTTHUMG00000159007	4.86 ± 0.13	4.65 ± 0.19	4.71 ± 0.12	4.85 ± 0.08
TC03001541.hg.1	OTTHUMG00000159016	4.85 ± 0.15	4.84 ± 0.14	4.97 ± 0.04	4.89 ± 0.04
TC03000420.hg.1	OTTHUMG00000159017	6.56 ± 0.26	6.44 ± 0.13	6.47 ± 0.08	6.50 ± 0.07
TC03000418.hg.1	OTTHUMG00000159018	5.93 ± 0.24	5.81 ± 0.08	5.84 ± 0.12	5.80 ± 0.05
TC03001463.hg.1	OTTHUMG00000159046	6.71 ± 0.20	6.68 ± 0.09	6.82 ± 0.23	6.63 ± 0.05
TC03000922.hg.1	OTTHUMG00000159050	6.44 ± 0.25	6.10 ± 0.08	6.31 ± 0.16	6.36 ± 0.15
TC03000454.hg.1	OTTHUMG00000159058	4.48 ± 0.14	4.51 ± 0.05	4.52 ± 0.13	4.46 ± 0.05
TC22000231.hg.1	OTTHUMG00000159059	6.11 ± 0.11	6.04 ± 0.05	6.18 ± 0.11	6.10 ± 0.13
TC03000496.hg.1	OTTHUMG00000159061	4.29 ± 0.15	4.04 ± 0.09	4.15 ± 0.07	4.03 ± 0.07
TC03000882.hg.1	OTTHUMG00000159075	7.25 ± 0.29	7.11 ± 0.04	7.17 ± 0.06	7.13 ± 0.02
TC03000347.hg.1	OTTHUMG00000159078	7.20 ± 0.32	7.14 ± 0.09	6.99 ± 0.12	7.10 ± 0.07
TC03001960.hg.1	OTTHUMG00000159088	4.58 ± 0.17	4.49 ± 0.12	4.50 ± 0.17	4.60 ± 0.19
TC03001620.hg.1	OTTHUMG00000159096	5.32 ± 0.22	5.31 ± 0.22	5.31 ± 0.13	5.54 ± 0.35
TC02000049.hg.1	OTTHUMG00000159106	4.42 ± 0.15	4.47 ± 0.03	4.51 ± 0.10	4.45 ± 0.07
TC02000045.hg.1	OTTHUMG00000159111	7.23 ± 0.16	7.30 ± 0.12	7.38 ± 0.20	7.38 ± 0.13
TC02000044.hg.1	OTTHUMG00000159112	4.26 ± 0.05	4.11 ± 0.04	4.15 ± 0.04	4.25 ± 0.06
TC02000043.hg.1	OTTHUMG00000159113	6.83 ± 0.24	6.73 ± 0.20	6.85 ± 0.19	6.88 ± 0.07
TC02000051.hg.1	OTTHUMG00000159115	7.58 ± 0.24	7.73 ± 0.03	7.57 ± 0.21	7.80 ± 0.01
TC02000052.hg.1	OTTHUMG00000159120	6.92 ± 0.27	6.92 ± 0.07	6.81 ± 0.05	7.06 ± 0.06
TC03000464.hg.1	OTTHUMG00000159122	7.72 ± 0.52	8.00 ± 0.27	8.07 ± 0.56	7.82 ± 0.11
TC03001922.hg.1	OTTHUMG00000159127	4.69 ± 0.09	4.50 ± 0.07	4.67 ± 0.30	4.55 ± 0.08
TC03001921.hg.1	OTTHUMG00000159128	3.85 ± 0.12	3.68 ± 0.06	3.68 ± 0.14	3.67 ± 0.07
TC03001923.hg.1	OTTHUMG00000159132	5.64 ± 0.18	5.37 ± 0.06	5.58 ± 0.27	5.39 ± 0.06
TC03000380.hg.1	OTTHUMG00000159155	7.28 ± 0.23	7.14 ± 0.41	7.07 ± 0.11	7.39 ± 0.07
TC03000381.hg.1	OTTHUMG00000159160	7.39 ± 0.15	7.42 ± 0.08	7.36 ± 0.10	7.52 ± 0.08

TC03001627.hg.1	OTTHUMG00000159172	5.25 ± 0.38	5.08 ± 0.24	5.18 ± 0.06	5.20 ± 0.08
TC03001555.hg.1	OTTHUMG00000159191	6.56 ± 0.10	6.36 ± 0.10	6.50 ± 0.18	6.47 ± 0.05
TC03000383.hg.1	OTTHUMG00000159199	5.11 ± 0.18	5.15 ± 0.11	5.03 ± 0.16	5.23 ± 0.15
TC03001504.hg.1	OTTHUMG00000159200	4.51 ± 0.19	4.25 ± 0.08	4.35 ± 0.02	4.31 ± 0.07
TC03001505.hg.1	OTTHUMG00000159201	5.61 ± 0.16	5.68 ± 0.11	5.74 ± 0.09	5.91 ± 0.07
TC03000382.hg.1	OTTHUMG00000159203	5.77 ± 0.18	5.54 ± 0.07	5.57 ± 0.12	5.65 ± 0.06
TC03000558.hg.1	OTTHUMG00000159213	4.73 ± 0.25	4.69 ± 0.19	4.60 ± 0.29	4.79 ± 0.12
TC03001639.hg.1	OTTHUMG00000159221	4.14 ± 0.06	3.99 ± 0.08	4.01 ± 0.08	4.11 ± 0.17
TC03000562.hg.1	OTTHUMG00000159240	4.63 ± 0.27	4.62 ± 0.02	4.54 ± 0.06	4.64 ± 0.10
TC03001643.hg.1	OTTHUMG00000159241	5.04 ± 0.25	5.01 ± 0.07	4.85 ± 0.09	5.17 ± 0.09
TC03000571.hg.1	OTTHUMG00000159246	4.06 ± 0.03	3.95 ± 0.06	3.92 ± 0.07	3.94 ± 0.04
TC03001648.hg.1	OTTHUMG00000159250	6.73 ± 0.27	6.78 ± 0.29	6.53 ± 0.05	6.99 ± 0.24
TC03001855.hg.1	OTTHUMG00000159252	5.67 ± 0.23	5.71 ± 0.24	5.61 ± 0.18	5.58 ± 0.02
TC20000684.hg.1	OTTHUMG00000159256	6.15 ± 0.26	6.23 ± 0.07	6.10 ± 0.15	6.34 ± 0.18
TC20000682.hg.1	OTTHUMG00000159257	5.49 ± 0.33	5.49 ± 0.21	5.58 ± 0.20	5.62 ± 0.17
TC03001656.hg.1	OTTHUMG00000159262	6.60 ± 0.24	6.65 ± 0.03	6.54 ± 0.11	6.76 ± 0.05
TC07001659.hg.1	OTTHUMG00000159264	7.28 ± 0.19	7.16 ± 0.03	7.08 ± 0.04	7.31 ± 0.07
TC03001665.hg.1	OTTHUMG00000159297	6.96 ± 0.33	6.69 ± 0.06	6.87 ± 0.03	6.68 ± 0.04
TC03000594.hg.1	OTTHUMG00000159303	5.47 ± 0.06	5.32 ± 0.05	5.32 ± 0.08	5.29 ± 0.07
TC03000596.hg.1	OTTHUMG00000159304	7.59 ± 0.29	7.78 ± 0.20	7.58 ± 0.12	7.95 ± 0.25
TC03000773.hg.1	OTTHUMG00000159316	6.37 ± 0.19	6.35 ± 0.14	6.34 ± 0.16	6.44 ± 0.08
TC03001652.hg.1	OTTHUMG00000159320	5.36 ± 0.06	5.22 ± 0.03	5.21 ± 0.06	5.36 ± 0.11
TC03000576.hg.1	OTTHUMG00000159327	6.79 ± 0.10	6.78 ± 0.10	6.69 ± 0.07	6.85 ± 0.09
TC03000587.hg.1	OTTHUMG00000159333	6.24 ± 0.21	5.99 ± 0.13	6.03 ± 0.13	6.02 ± 0.06
TC06002048.hg.1	OTTHUMG00000159342	4.34 ± 0.25	4.10 ± 0.05	4.25 ± 0.24	4.07 ± 0.07
TC03001677.hg.1	OTTHUMG00000159344	6.59 ± 0.06	6.48 ± 0.03	6.55 ± 0.09	6.52 ± 0.03
TC03000603.hg.1	OTTHUMG00000159345	4.32 ± 0.15	4.15 ± 0.06	4.29 ± 0.11	4.18 ± 0.11
TC03001678.hg.1	OTTHUMG00000159346	5.69 ± 0.10	5.61 ± 0.09	5.61 ± 0.07	5.65 ± 0.14
TC03003406.hg.1	OTTHUMG00000159352	6.08 ± 0.21	5.74 ± 0.07	5.77 ± 0.25	5.94 ± 0.08
TC03000775.hg.1	OTTHUMG00000159353	6.18 ± 0.14	6.17 ± 0.14	6.33 ± 0.06	6.37 ± 0.09
TC03001659.hg.1	OTTHUMG00000159355	6.97 ± 0.35	6.88 ± 0.12	6.84 ± 0.27	7.03 ± 0.12

TC03000763.hg.1	OTTHUMG00000159357	5.62 ± 0.18	5.70 ± 0.07	5.68 ± 0.07	5.78 ± 0.07
TC03000590.hg.1	OTTHUMG00000159367	4.64 ± 0.24	4.35 ± 0.02	4.50 ± 0.02	4.38 ± 0.06
TC03001863.hg.1	OTTHUMG00000159376	6.19 ± 0.21	6.35 ± 0.07	6.21 ± 0.20	6.44 ± 0.14
TC03000783.hg.1	OTTHUMG00000159391	4.74 ± 0.07	4.64 ± 0.05	4.71 ± 0.07	4.69 ± 0.10
TC03000784.hg.1	OTTHUMG00000159392	6.26 ± 0.13	6.10 ± 0.05	6.15 ± 0.07	6.13 ± 0.10
TC03000616.hg.1	OTTHUMG00000159404	6.45 ± 0.28	6.25 ± 0.19	6.31 ± 0.14	6.34 ± 0.06
TC03000500.hg.1	OTTHUMG00000159412	4.12 ± 0.17	3.98 ± 0.06	4.00 ± 0.14	3.97 ± 0.12
TC03001606.hg.1	OTTHUMG00000159413	4.96 ± 0.28	4.63 ± 0.14	4.64 ± 0.11	4.72 ± 0.08
TC03000785.hg.1	OTTHUMG00000159414	4.58 ± 0.24	4.48 ± 0.03	4.45 ± 0.05	4.48 ± 0.16
TC03001865.hg.1	OTTHUMG00000159416	5.04 ± 0.14	4.91 ± 0.14	5.09 ± 0.03	5.19 ± 0.36
TC03000501.hg.1	OTTHUMG00000159418	5.06 ± 0.26	4.83 ± 0.19	4.81 ± 0.24	4.89 ± 0.18
TC03001687.hg.1	OTTHUMG00000159421	5.88 ± 0.09	5.76 ± 0.09	5.80 ± 0.05	5.76 ± 0.06
TC0X000143.hg.1	OTTHUMG00000159435	7.27 ± 0.64	6.84 ± 0.70	8.71 ± 0.72	6.64 ± 0.46
TC15001181.hg.1	OTTHUMG00000159446	5.95 ± 0.23	6.00 ± 0.23	5.95 ± 0.23	6.11 ± 0.02
TC03000789.hg.1	OTTHUMG00000159455	5.76 ± 0.35	5.78 ± 0.16	5.70 ± 0.18	5.86 ± 0.04
TC03001871.hg.1	OTTHUMG00000159458	5.05 ± 0.16	4.94 ± 0.04	4.95 ± 0.11	5.06 ± 0.04
TC03001876.hg.1	OTTHUMG00000159462	4.38 ± 0.27	3.98 ± 0.18	4.12 ± 0.08	4.12 ± 0.17
TC03001694.hg.1	OTTHUMG00000159469	3.91 ± 0.12	3.62 ± 0.01	3.68 ± 0.07	3.65 ± 0.09
TC09000648.hg.1	OTTHUMG00000159480	4.85 ± 0.08	4.87 ± 0.05	4.82 ± 0.10	4.93 ± 0.12
TC03000795.hg.1	OTTHUMG00000159498	4.00 ± 0.11	3.92 ± 0.08	3.91 ± 0.06	3.94 ± 0.05
TC03000796.hg.1	OTTHUMG00000159500	6.06 ± 0.16	5.99 ± 0.07	5.98 ± 0.27	5.95 ± 0.02
TC03001878.hg.1	OTTHUMG00000159504	4.68 ± 0.09	4.50 ± 0.05	4.57 ± 0.04	4.62 ± 0.15
TC03000797.hg.1	OTTHUMG00000159505	4.90 ± 0.06	4.83 ± 0.03	4.84 ± 0.03	4.81 ± 0.08
TC03001881.hg.1	OTTHUMG00000159518	7.47 ± 0.26	7.55 ± 0.01	7.54 ± 0.04	7.67 ± 0.11
TC07000621.hg.1	OTTHUMG00000159538	8.74 ± 0.07	8.74 ± 0.12	8.70 ± 0.03	8.70 ± 0.02
TC07000622.hg.1	OTTHUMG00000159539	8.15 ± 0.05	8.37 ± 0.14	8.19 ± 0.06	8.34 ± 0.09
TC03001886.hg.1	OTTHUMG00000159581	5.83 ± 0.19	5.76 ± 0.07	5.82 ± 0.03	5.85 ± 0.10
TC03000804.hg.1	OTTHUMG00000159583	6.43 ± 0.05	6.37 ± 0.16	6.46 ± 0.17	6.44 ± 0.19
TC03001729.hg.1	OTTHUMG00000159585	6.96 ± 0.11	6.93 ± 0.06	6.86 ± 0.15	6.97 ± 0.01
TC03001889.hg.1	OTTHUMG00000159595	6.15 ± 0.16	6.38 ± 0.09	6.26 ± 0.10	6.49 ± 0.25
TC03001783.hg.1	OTTHUMG00000159610	5.45 ± 0.17	5.47 ± 0.05	5.41 ± 0.04	5.57 ± 0.07

TC03000838.hg.1	OTTHUMG00000159625	5.57 ± 0.14	5.55 ± 0.15	5.56 ± 0.20	5.66 ± 0.04
TC03000839.hg.1	OTTHUMG00000159626	6.38 ± 0.18	6.34 ± 0.06	6.36 ± 0.28	6.42 ± 0.19
TC03001915.hg.1	OTTHUMG00000159627	5.54 ± 0.16	5.46 ± 0.04	5.47 ± 0.02	5.51 ± 0.07
TC03000840.hg.1	OTTHUMG00000159628	4.83 ± 0.19	4.92 ± 0.14	4.95 ± 0.10	4.91 ± 0.03
TC03001774.hg.1	OTTHUMG00000159635	6.69 ± 0.20	6.59 ± 0.02	6.58 ± 0.04	6.70 ± 0.07
TC03000663.hg.1	OTTHUMG00000159638	7.99 ± 0.04	7.95 ± 0.06	7.99 ± 0.10	8.02 ± 0.09
TC03001747.hg.1	OTTHUMG00000159640	7.33 ± 0.35	7.33 ± 0.04	7.26 ± 0.06	7.64 ± 0.23
TC03000843.hg.1	OTTHUMG00000159649	5.45 ± 0.13	5.31 ± 0.08	5.33 ± 0.04	5.40 ± 0.07
TC03001914.hg.1	OTTHUMG00000159656	6.91 ± 0.16	6.73 ± 0.05	6.84 ± 0.18	6.81 ± 0.04
TC03000675.hg.1	OTTHUMG00000159693	7.62 ± 0.15	7.63 ± 0.17	7.55 ± 0.15	7.69 ± 0.11
TC03000836.hg.1	OTTHUMG00000159695	5.38 ± 0.26	4.99 ± 0.01	5.18 ± 0.50	5.08 ± 0.12
TC03000834.hg.1	OTTHUMG00000159698	6.20 ± 0.08	6.03 ± 0.08	5.96 ± 0.10	6.11 ± 0.05
TC03001913.hg.1	OTTHUMG00000159699	5.18 ± 0.24	5.04 ± 0.16	5.01 ± 0.26	4.91 ± 0.13
TC03001780.hg.1	OTTHUMG00000159705	4.31 ± 0.25	3.99 ± 0.02	4.13 ± 0.17	4.11 ± 0.07
TC03001793.hg.1	OTTHUMG00000159720	5.14 ± 0.08	5.13 ± 0.12	5.21 ± 0.16	5.27 ± 0.12
TC03001795.hg.1	OTTHUMG00000159721	6.21 ± 0.21	6.23 ± 0.19	6.15 ± 0.18	6.44 ± 0.09
TC03001794.hg.1	OTTHUMG00000159722	4.53 ± 0.09	4.39 ± 0.05	4.39 ± 0.05	4.35 ± 0.07
TC03001552.hg.1	OTTHUMG00000159723	6.63 ± 0.27	6.70 ± 0.22	6.49 ± 0.33	6.89 ± 0.11
TC03001898.hg.1	OTTHUMG00000159735	6.03 ± 0.30	5.68 ± 0.17	5.80 ± 0.15	5.74 ± 0.04
TC03000813.hg.1	OTTHUMG00000159737	6.86 ± 0.21	6.87 ± 0.03	6.79 ± 0.10	6.87 ± 0.04
TC03001897.hg.1	OTTHUMG00000159738	6.55 ± 0.15	6.52 ± 0.14	6.52 ± 0.10	6.63 ± 0.07
TC03001896.hg.1	OTTHUMG00000159740	5.14 ± 0.05	4.99 ± 0.10	5.29 ± 0.01	5.05 ± 0.06
TC03000812.hg.1	OTTHUMG00000159742	5.45 ± 0.20	5.36 ± 0.13	5.36 ± 0.07	5.48 ± 0.16
TC03000811.hg.1	OTTHUMG00000159743	5.63 ± 0.03	5.48 ± 0.02	5.60 ± 0.16	5.58 ± 0.08
TC03003416.hg.1	OTTHUMG00000159752	6.52 ± 0.12	6.32 ± 0.06	6.47 ± 0.07	6.46 ± 0.08
TC03000726.hg.1	OTTHUMG00000159760	7.99 ± 0.30	8.04 ± 0.34	7.86 ± 0.09	8.30 ± 0.16
TC03001815.hg.1	OTTHUMG00000159786	7.35 ± 0.16	7.10 ± 0.03	7.21 ± 0.04	6.97 ± 0.13
TC04000034.hg.1	OTTHUMG00000159799	5.88 ± 0.14	5.83 ± 0.06	5.84 ± 0.13	5.86 ± 0.04
TC03001809.hg.1	OTTHUMG00000159842	7.15 ± 0.14	7.22 ± 0.04	7.17 ± 0.09	7.34 ± 0.11
TC03000817.hg.1	OTTHUMG00000159845	6.74 ± 0.12	6.62 ± 0.15	6.54 ± 0.07	6.61 ± 0.02
TC03000820.hg.1	OTTHUMG00000159849	7.65 ± 0.37	7.37 ± 0.09	7.77 ± 0.11	7.54 ± 0.07

TC03000826.hg.1	OTTHUMG00000159875	4.63 ± 0.30	4.53 ± 0.22	4.65 ± 0.18	4.78 ± 0.20
TC03001909.hg.1	OTTHUMG00000159877	5.34 ± 0.28	5.35 ± 0.05	5.28 ± 0.10	5.36 ± 0.03
TC03001910.hg.1	OTTHUMG00000159881	6.25 ± 0.25	6.23 ± 0.16	6.13 ± 0.10	6.26 ± 0.13
TC03001826.hg.1	OTTHUMG00000159892	7.27 ± 0.28	7.24 ± 0.12	7.29 ± 0.24	7.34 ± 0.07
TC03001775.hg.1	OTTHUMG00000159896	6.65 ± 0.13	6.47 ± 0.10	6.53 ± 0.15	6.59 ± 0.04
TC17000629.hg.1	OTTHUMG00000159903	8.37 ± 0.19	8.42 ± 0.41	8.30 ± 0.34	8.59 ± 0.17
TC04000950.hg.1	OTTHUMG00000159908	7.96 ± 0.27	7.91 ± 0.24	7.90 ± 0.29	8.12 ± 0.06
TC03000701.hg.1	OTTHUMG00000159926	7.27 ± 0.10	7.28 ± 0.11	7.09 ± 0.12	7.30 ± 0.09
TC06001744.hg.1	OTTHUMG00000159928	6.93 ± 0.12	6.97 ± 0.16	6.61 ± 0.13	7.07 ± 0.13
TC03000753.hg.1	OTTHUMG00000159952	6.34 ± 0.24	6.21 ± 0.06	6.06 ± 0.17	6.19 ± 0.11
TC03000752.hg.1	OTTHUMG00000159957	5.48 ± 0.17	5.32 ± 0.21	5.47 ± 0.29	5.53 ± 0.09
TC03001838.hg.1	OTTHUMG00000159958	6.01 ± 0.18	6.06 ± 0.22	6.15 ± 0.26	6.17 ± 0.18
TC04000953.hg.1	OTTHUMG00000159970	8.01 ± 0.20	7.87 ± 0.18	7.99 ± 0.09	8.02 ± 0.15
TC07001228.hg.1	OTTHUMG00000159990	7.39 ± 0.21	7.42 ± 0.09	7.29 ± 0.38	7.41 ± 0.09
TC04000051.hg.1	OTTHUMG00000159993	7.59 ± 0.19	7.55 ± 0.17	7.50 ± 0.20	7.72 ± 0.12
TC10001265.hg.1	OTTHUMG00000159999	6.50 ± 0.11	6.47 ± 0.05	6.50 ± 0.11	6.67 ± 0.15
TC04001009.hg.1	OTTHUMG00000160013	7.36 ± 0.23	7.28 ± 0.16	7.26 ± 0.13	7.31 ± 0.05
TC12000458.hg.1	OTTHUMG00000160022	5.79 ± 0.07	5.82 ± 0.13	5.83 ± 0.17	6.00 ± 0.06
TC03000493.hg.1	OTTHUMG00000160039	8.54 ± 0.28	8.46 ± 0.04	8.74 ± 0.13	8.50 ± 0.08
TC04000126.hg.1	OTTHUMG00000160040	5.17 ± 0.05	5.17 ± 0.14	5.05 ± 0.09	5.22 ± 0.14
TC04001039.hg.1	OTTHUMG00000160041	4.92 ± 0.11	4.89 ± 0.11	4.78 ± 0.16	4.88 ± 0.09
TC04000127.hg.1	OTTHUMG00000160044	4.78 ± 0.23	4.60 ± 0.09	4.60 ± 0.03	4.63 ± 0.07
TC04000088.hg.1	OTTHUMG00000160051	7.23 ± 0.18	7.26 ± 0.06	7.19 ± 0.11	7.36 ± 0.03
TC04001017.hg.1	OTTHUMG00000160053	7.13 ± 0.32	7.01 ± 0.18	6.96 ± 0.16	6.98 ± 0.21
TC04000124.hg.1	OTTHUMG00000160063	4.66 ± 0.10	4.58 ± 0.04	4.65 ± 0.09	4.79 ± 0.20
TC04000129.hg.1	OTTHUMG00000160074	5.42 ± 0.16	5.36 ± 0.03	5.41 ± 0.14	5.42 ± 0.05
TC04000128.hg.1	OTTHUMG00000160075	4.73 ± 0.11	4.78 ± 0.08	4.71 ± 0.23	4.79 ± 0.11
TC04000017.hg.1	OTTHUMG00000160084	7.59 ± 0.29	7.62 ± 0.18	7.56 ± 0.12	7.76 ± 0.14
TC04000091.hg.1	OTTHUMG00000160092	7.13 ± 0.23	7.14 ± 0.12	7.09 ± 0.12	7.26 ± 0.07
TC04000136.hg.1	OTTHUMG00000160100	5.75 ± 0.18	5.58 ± 0.07	5.62 ± 0.09	5.64 ± 0.15
TC04000137.hg.1	OTTHUMG00000160103	5.04 ± 0.10	5.03 ± 0.12	5.04 ± 0.02	5.20 ± 0.11

TC04000133.hg.1	OTTHUMG00000160104	6.64 ± 0.18	6.53 ± 0.07	6.61 ± 0.11	6.58 ± 0.06
TC04000138.hg.1	OTTHUMG00000160105	5.22 ± 0.10	4.96 ± 0.13	5.05 ± 0.03	5.15 ± 0.14
TC04001049.hg.1	OTTHUMG00000160106	6.53 ± 0.10	6.46 ± 0.06	6.56 ± 0.06	6.62 ± 0.09
TC03001601.hg.1	OTTHUMG00000160118	4.73 ± 0.26	4.63 ± 0.13	4.59 ± 0.14	4.68 ± 0.20
TC03000486.hg.1	OTTHUMG00000160119	5.02 ± 0.26	4.79 ± 0.11	4.90 ± 0.12	4.90 ± 0.13
TC04001003.hg.1	OTTHUMG00000160124	8.05 ± 0.27	7.95 ± 0.13	8.00 ± 0.24	8.05 ± 0.06
TC03001844.hg.1	OTTHUMG00000160129	5.84 ± 0.06	5.78 ± 0.05	5.91 ± 0.06	5.92 ± 0.18
TC04001054.hg.1	OTTHUMG00000160132	5.85 ± 0.05	5.67 ± 0.15	5.86 ± 0.14	5.67 ± 0.03
TC03001842.hg.1	OTTHUMG00000160140	4.37 ± 0.16	4.46 ± 0.19	4.51 ± 0.06	4.56 ± 0.22
TC03001840.hg.1	OTTHUMG00000160141	6.35 ± 0.26	6.52 ± 0.19	6.37 ± 0.10	6.68 ± 0.04
TC04000157.hg.1	OTTHUMG00000160142	4.27 ± 0.10	4.15 ± 0.06	4.29 ± 0.09	4.28 ± 0.15
TC03001839.hg.1	OTTHUMG00000160163	6.19 ± 0.11	6.11 ± 0.15	6.01 ± 0.17	6.20 ± 0.11
TC04000018.hg.1	OTTHUMG00000160168	7.35 ± 0.19	7.28 ± 0.05	7.30 ± 0.13	7.35 ± 0.13
TC04000146.hg.1	OTTHUMG00000160178	4.96 ± 0.07	4.88 ± 0.06	4.89 ± 0.14	4.95 ± 0.07
TC04001026.hg.1	OTTHUMG00000160185	5.48 ± 0.08	5.21 ± 0.04	5.20 ± 0.07	5.23 ± 0.21
TC04000148.hg.1	OTTHUMG00000160203	5.45 ± 0.32	5.58 ± 0.06	5.47 ± 0.12	5.66 ± 0.14
TC04000058.hg.1	OTTHUMG00000160206	6.75 ± 0.02	6.76 ± 0.13	6.78 ± 0.17	6.90 ± 0.11
TC04000067.hg.1	OTTHUMG00000160217	6.56 ± 0.34	6.43 ± 0.16	6.34 ± 0.20	6.42 ± 0.11
TC04001064.hg.1	OTTHUMG00000160219	5.46 ± 0.13	5.36 ± 0.03	5.39 ± 0.10	5.39 ± 0.02
TC03001843.hg.1	OTTHUMG00000160222	6.03 ± 0.16	5.83 ± 0.06	5.90 ± 0.11	5.95 ± 0.14
TC15001253.hg.1	OTTHUMG00000160228	8.33 ± 0.23	8.26 ± 0.13	8.31 ± 0.18	8.34 ± 0.06
TC04001063.hg.1	OTTHUMG00000160229	7.63 ± 0.08	7.43 ± 0.08	7.60 ± 0.11	7.46 ± 0.06
TC07001071.hg.1	OTTHUMG00000160234	7.90 ± 0.16	8.07 ± 0.32	7.97 ± 0.35	8.19 ± 0.06
TC04000926.hg.1	OTTHUMG00000160236	6.33 ± 0.28	6.28 ± 0.07	6.16 ± 0.25	6.49 ± 0.27
TC04001832.hg.1	OTTHUMG00000160238	5.50 ± 0.36	5.17 ± 0.07	5.52 ± 0.29	5.27 ± 0.06
TC04001071.hg.1	OTTHUMG00000160241	5.49 ± 0.12	5.40 ± 0.15	5.26 ± 0.10	5.43 ± 0.20
TC04001072.hg.1	OTTHUMG00000160243	4.74 ± 0.11	4.75 ± 0.26	4.80 ± 0.38	4.80 ± 0.02
TC04000115.hg.1	OTTHUMG00000160246	7.04 ± 0.19	7.33 ± 0.21	7.13 ± 0.31	7.37 ± 0.11
TC04000116.hg.1	OTTHUMG00000160251	5.97 ± 0.08	5.80 ± 0.04	5.85 ± 0.05	5.84 ± 0.12
TC04000169.hg.1	OTTHUMG00000160256	5.18 ± 0.11	5.20 ± 0.10	5.13 ± 0.04	5.29 ± 0.10
TC04000170.hg.1	OTTHUMG00000160257	4.92 ± 0.13	4.87 ± 0.06	4.90 ± 0.07	4.95 ± 0.07

TC04001082.hg.1	OTTHUMG00000160259	4.12 ± 0.22	4.10 ± 0.05	4.08 ± 0.06	4.06 ± 0.08
TC03000757.hg.1	OTTHUMG00000160261	6.19 ± 0.21	6.28 ± 0.20	6.10 ± 0.26	6.31 ± 0.06
TC06000664.hg.1	OTTHUMG00000160263	5.82 ± 0.19	5.78 ± 0.30	5.71 ± 0.16	5.88 ± 0.06
TC04001056.hg.1	OTTHUMG00000160269	5.90 ± 0.26	5.59 ± 0.15	5.91 ± 0.17	5.83 ± 0.11
TC04001083.hg.1	OTTHUMG00000160276	8.22 ± 0.30	8.02 ± 0.05	8.25 ± 0.27	8.47 ± 0.39
TC04000172.hg.1	OTTHUMG00000160277	5.57 ± 0.25	5.45 ± 0.19	5.57 ± 0.10	5.56 ± 0.10
TC04000171.hg.1	OTTHUMG00000160278	5.49 ± 0.22	5.44 ± 0.07	5.52 ± 0.23	5.54 ± 0.13
TC04000177.hg.1	OTTHUMG00000160281	4.69 ± 0.05	4.70 ± 0.02	4.84 ± 0.33	4.78 ± 0.04
TC04001834.hg.1	OTTHUMG00000160282	5.28 ± 0.24	5.27 ± 0.03	5.21 ± 0.10	5.45 ± 0.21
TC04001067.hg.1	OTTHUMG00000160283	7.50 ± 0.08	7.59 ± 0.05	7.30 ± 0.11	7.51 ± 0.07
TC04000035.hg.1	OTTHUMG00000160291	7.50 ± 0.36	7.51 ± 0.07	7.44 ± 0.21	7.68 ± 0.24
TC04000921.hg.1	OTTHUMG00000160297	5.03 ± 0.06	4.78 ± 0.05	4.95 ± 0.22	4.93 ± 0.17
TC04000922.hg.1	OTTHUMG00000160298	4.65 ± 0.09	4.60 ± 0.13	4.61 ± 0.04	4.74 ± 0.05
TC04001828.hg.1	OTTHUMG00000160299	4.46 ± 0.06	4.41 ± 0.03	4.43 ± 0.06	4.51 ± 0.22
TC04000923.hg.1	OTTHUMG00000160300	4.97 ± 0.22	4.86 ± 0.07	4.95 ± 0.16	5.00 ± 0.09
TC04001826.hg.1	OTTHUMG00000160309	6.37 ± 0.19	6.54 ± 0.36	6.29 ± 0.07	6.60 ± 0.22
TC04000918.hg.1	OTTHUMG00000160310	8.23 ± 0.28	8.18 ± 0.16	8.10 ± 0.14	8.34 ± 0.15
TC04000920.hg.1	OTTHUMG00000160312	5.86 ± 0.19	5.80 ± 0.03	5.83 ± 0.13	5.95 ± 0.13
TC04000919.hg.1	OTTHUMG00000160313	7.83 ± 0.06	7.83 ± 0.10	7.76 ± 0.15	7.84 ± 0.17
TC04000186.hg.1	OTTHUMG00000160318	5.73 ± 0.13	5.58 ± 0.10	5.73 ± 0.21	5.67 ± 0.05
TC04000929.hg.1	OTTHUMG00000160325	5.38 ± 0.16	5.24 ± 0.06	5.29 ± 0.13	5.09 ± 0.01
TC04000930.hg.1	OTTHUMG00000160328	7.23 ± 0.13	7.00 ± 0.17	7.11 ± 0.13	7.20 ± 0.09
TC04000933.hg.1	OTTHUMG00000160329	5.73 ± 0.18	6.07 ± 0.17	5.73 ± 0.04	5.90 ± 0.04
TC04001093.hg.1	OTTHUMG00000160332	5.73 ± 0.14	5.61 ± 0.19	5.62 ± 0.13	5.75 ± 0.10
TC04000179.hg.1	OTTHUMG00000160337	5.65 ± 0.15	5.54 ± 0.09	5.72 ± 0.07	5.59 ± 0.06
TC04000201.hg.1	OTTHUMG00000160346	4.24 ± 0.14	4.03 ± 0.08	4.20 ± 0.04	4.05 ± 0.10
TC04000204.hg.1	OTTHUMG00000160349	4.79 ± 0.07	4.65 ± 0.05	4.73 ± 0.14	4.67 ± 0.06
TC04000202.hg.1	OTTHUMG00000160350	5.82 ± 0.13	5.63 ± 0.09	5.86 ± 0.18	5.66 ± 0.04
TC04001107.hg.1	OTTHUMG00000160354	5.29 ± 0.12	5.17 ± 0.08	5.22 ± 0.08	5.25 ± 0.16
TC04001048.hg.1	OTTHUMG00000160355	5.84 ± 0.07	5.68 ± 0.16	5.83 ± 0.12	5.84 ± 0.03
TC04001108.hg.1	OTTHUMG00000160362	3.89 ± 0.14	3.70 ± 0.02	3.77 ± 0.07	3.70 ± 0.02

TC04000198.hg.1	OTTHUMG00000160363	4.65 ± 0.17	4.46 ± 0.04	4.35 ± 0.11	4.41 ± 0.22
TC04001106.hg.1	OTTHUMG00000160365	4.52 ± 0.32	4.28 ± 0.28	4.59 ± 0.12	4.55 ± 0.08
TC04000199.hg.1	OTTHUMG00000160367	6.92 ± 0.10	6.87 ± 0.19	7.03 ± 0.18	7.12 ± 0.19
TC04000197.hg.1	OTTHUMG00000160369	3.99 ± 0.14	3.95 ± 0.14	3.99 ± 0.05	3.97 ± 0.06
TC04000208.hg.1	OTTHUMG00000160372	6.33 ± 0.26	6.30 ± 0.12	6.26 ± 0.10	6.50 ± 0.16
TC04001111.hg.1	OTTHUMG00000160373	6.58 ± 0.20	6.64 ± 0.08	6.71 ± 0.10	6.81 ± 0.02
TC04000218.hg.1	OTTHUMG00000160385	6.20 ± 0.07	6.27 ± 0.17	6.40 ± 0.12	6.29 ± 0.13
TC04001121.hg.1	OTTHUMG00000160386	8.17 ± 0.22	8.08 ± 0.14	8.12 ± 0.10	8.21 ± 0.08
TC04000210.hg.1	OTTHUMG00000160388	5.56 ± 0.09	5.53 ± 0.09	5.55 ± 0.11	5.52 ± 0.02
TC04001115.hg.1	OTTHUMG00000160390	6.83 ± 0.21	6.93 ± 0.18	6.94 ± 0.26	7.06 ± 0.11
TC04002954.hg.1	OTTHUMG00000160392	6.19 ± 0.38	6.20 ± 0.05	6.25 ± 0.25	6.16 ± 0.05
TC04000190.hg.1	OTTHUMG00000160395	4.81 ± 0.19	4.46 ± 0.06	4.65 ± 0.10	4.54 ± 0.12
TC04000191.hg.1	OTTHUMG00000160396	6.55 ± 0.21	6.53 ± 0.09	6.48 ± 0.14	6.67 ± 0.07
TC04000244.hg.1	OTTHUMG00000160407	6.73 ± 0.14	6.82 ± 0.19	6.80 ± 0.08	6.87 ± 0.07
TC04001119.hg.1	OTTHUMG00000160414	6.19 ± 0.11	6.13 ± 0.12	6.08 ± 0.17	6.20 ± 0.11
TC04000217.hg.1	OTTHUMG00000160427	7.26 ± 0.12	7.13 ± 0.08	7.27 ± 0.06	7.15 ± 0.06
TC04000264.hg.1	OTTHUMG00000160432	7.87 ± 0.19	7.84 ± 0.02	7.89 ± 0.12	7.92 ± 0.11
TC04000265.hg.1	OTTHUMG00000160433	4.08 ± 0.21	3.92 ± 0.08	3.99 ± 0.10	3.89 ± 0.06
TC04000200.hg.1	OTTHUMG00000160440	6.97 ± 0.21	6.91 ± 0.06	6.85 ± 0.15	7.08 ± 0.10
TC04001126.hg.1	OTTHUMG00000160443	4.42 ± 0.12	4.36 ± 0.05	4.50 ± 0.14	4.38 ± 0.05
TC04001154.hg.1	OTTHUMG00000160444	3.69 ± 0.04	3.56 ± 0.06	3.66 ± 0.08	3.54 ± 0.05
TC04000907.hg.1	OTTHUMG00000160454	7.81 ± 0.28	7.62 ± 0.26	7.45 ± 0.18	7.72 ± 0.09
TC04001153.hg.1	OTTHUMG00000160455	4.71 ± 0.24	4.59 ± 0.09	4.76 ± 0.13	4.68 ± 0.13
TC04000904.hg.1	OTTHUMG00000160464	6.17 ± 0.23	6.21 ± 0.03	5.98 ± 0.09	6.23 ± 0.19
TC04000225.hg.1	OTTHUMG00000160465	6.73 ± 0.14	6.67 ± 0.16	6.67 ± 0.18	6.78 ± 0.28
TC04000206.hg.1	OTTHUMG00000160471	4.22 ± 0.08	4.16 ± 0.02	4.16 ± 0.06	4.26 ± 0.07
TC04000220.hg.1	OTTHUMG00000160485	5.23 ± 0.31	5.17 ± 0.10	5.22 ± 0.10	5.18 ± 0.12
TC04000219.hg.1	OTTHUMG00000160486	6.84 ± 0.28	6.88 ± 0.06	6.67 ± 0.07	6.88 ± 0.11
TC04000901.hg.1	OTTHUMG00000160487	5.20 ± 0.10	5.09 ± 0.05	5.14 ± 0.08	5.19 ± 0.06
TC04000037.hg.1	OTTHUMG00000160511	6.19 ± 0.04	6.26 ± 0.08	6.17 ± 0.11	6.24 ± 0.13
TC04000272.hg.1	OTTHUMG00000160519	6.42 ± 0.26	6.43 ± 0.23	6.42 ± 0.17	6.55 ± 0.09

TC04000256.hg.1	OTTHUMG00000160525	7.87 ± 0.13	7.79 ± 0.05	7.79 ± 0.04	7.80 ± 0.07
TC04001149.hg.1	OTTHUMG00000160529	5.68 ± 0.09	5.55 ± 0.14	5.69 ± 0.09	5.60 ± 0.03
TC04000255.hg.1	OTTHUMG00000160532	5.34 ± 0.33	5.03 ± 0.07	5.21 ± 0.09	5.11 ± 0.03
TC04001131.hg.1	OTTHUMG00000160538	7.50 ± 0.22	7.27 ± 0.25	7.53 ± 0.29	7.06 ± 0.22
TC04000898.hg.1	OTTHUMG00000160549	6.59 ± 0.41	6.48 ± 0.03	7.25 ± 0.38	6.36 ± 0.17
TC04000899.hg.1	OTTHUMG00000160551	7.76 ± 0.19	7.74 ± 0.07	7.63 ± 0.21	7.78 ± 0.15
TC04000269.hg.1	OTTHUMG00000160568	3.78 ± 0.11	3.61 ± 0.06	3.65 ± 0.06	3.63 ± 0.02
TC04000243.hg.1	OTTHUMG00000160577	6.96 ± 0.10	6.94 ± 0.16	6.98 ± 0.06	7.13 ± 0.06
TC04000934.hg.1	OTTHUMG00000160584	6.06 ± 0.22	6.02 ± 0.20	6.14 ± 0.10	6.16 ± 0.20
TC04001838.hg.1	OTTHUMG00000160585	7.04 ± 0.23	7.12 ± 0.05	7.03 ± 0.13	7.24 ± 0.11
TC04001204.hg.1	OTTHUMG00000160611	4.48 ± 0.10	4.46 ± 0.14	4.33 ± 0.05	4.42 ± 0.21
TC04001804.hg.1	OTTHUMG00000160613	7.04 ± 0.30	7.07 ± 0.14	7.48 ± 0.49	6.88 ± 0.07
TC04000314.hg.1	OTTHUMG00000160614	6.17 ± 0.20	6.30 ± 0.24	6.18 ± 0.14	6.44 ± 0.17
TC04001206.hg.1	OTTHUMG00000160615	5.27 ± 0.20	5.18 ± 0.20	5.22 ± 0.07	5.17 ± 0.15
TC04000894.hg.1	OTTHUMG00000160619	5.81 ± 0.12	5.73 ± 0.05	5.70 ± 0.07	5.76 ± 0.07
TC04000292.hg.1	OTTHUMG00000160622	5.03 ± 0.14	4.81 ± 0.09	5.03 ± 0.05	4.83 ± 0.10
TC04001145.hg.1	OTTHUMG00000160625	4.95 ± 0.27	5.23 ± 0.18	5.20 ± 0.07	5.30 ± 0.12
TC04000309.hg.1	OTTHUMG00000160643	7.12 ± 0.15	7.06 ± 0.04	7.04 ± 0.04	7.20 ± 0.17
TC04000311.hg.1	OTTHUMG00000160645	5.01 ± 0.28	4.98 ± 0.36	4.91 ± 0.40	4.87 ± 0.02
TC04000312.hg.1	OTTHUMG00000160648	5.25 ± 0.18	5.33 ± 0.21	5.43 ± 0.16	5.48 ± 0.13
TC04000308.hg.1	OTTHUMG00000160650	5.89 ± 0.18	5.91 ± 0.20	5.84 ± 0.12	6.05 ± 0.15
TC04000316.hg.1	OTTHUMG00000160651	6.33 ± 0.23	6.06 ± 0.02	6.21 ± 0.08	6.23 ± 0.10
TC04000315.hg.1	OTTHUMG00000160654	5.65 ± 0.07	5.53 ± 0.15	5.60 ± 0.05	5.62 ± 0.01
TC04000350.hg.1	OTTHUMG00000160657	5.73 ± 0.10	5.69 ± 0.02	5.74 ± 0.14	5.66 ± 0.02
TC04000341.hg.1	OTTHUMG00000160663	5.26 ± 0.07	5.22 ± 0.08	5.31 ± 0.04	5.27 ± 0.08
TC11002336.hg.1	OTTHUMG00000160666	7.22 ± 0.17	7.21 ± 0.20	7.15 ± 0.21	7.29 ± 0.08
TC04001785.hg.1	OTTHUMG00000160683	5.34 ± 0.25	4.98 ± 0.05	5.15 ± 0.20	5.06 ± 0.02
TC04000882.hg.1	OTTHUMG00000160686	6.39 ± 0.34	6.40 ± 0.33	6.34 ± 0.27	6.42 ± 0.05
TC04001786.hg.1	OTTHUMG00000160687	5.75 ± 0.19	5.70 ± 0.26	5.90 ± 0.08	5.69 ± 0.10
TC04001238.hg.1	OTTHUMG00000160689	7.63 ± 0.16	7.55 ± 0.08	7.61 ± 0.08	7.55 ± 0.02
TC04001239.hg.1	OTTHUMG00000160691	5.05 ± 0.12	4.89 ± 0.01	4.99 ± 0.02	4.97 ± 0.11

TC04000304.hg.1	OTTHUMG00000160692	5.79 ± 0.15	5.74 ± 0.04	5.79 ± 0.03	5.77 ± 0.08
TC04001780.hg.1	OTTHUMG00000160700	6.28 ± 0.14	6.12 ± 0.14	6.12 ± 0.24	6.28 ± 0.06
TC04001778.hg.1	OTTHUMG00000160705	4.49 ± 0.18	4.27 ± 0.09	4.41 ± 0.06	4.32 ± 0.08
TC04000357.hg.1	OTTHUMG00000160708	6.10 ± 0.44	6.11 ± 0.02	6.20 ± 0.20	6.29 ± 0.21
TC04000356.hg.1	OTTHUMG00000160709	3.64 ± 0.14	3.50 ± 0.03	3.57 ± 0.13	3.49 ± 0.08
TC04000358.hg.1	OTTHUMG00000160711	4.54 ± 0.16	4.52 ± 0.10	4.53 ± 0.15	4.67 ± 0.22
TC04000359.hg.1	OTTHUMG00000160711	4.09 ± 0.22	4.07 ± 0.12	4.18 ± 0.22	4.05 ± 0.06
TC04001776.hg.1	OTTHUMG00000160713	4.34 ± 0.01	4.28 ± 0.14	4.48 ± 0.07	4.46 ± 0.04
TC04001781.hg.1	OTTHUMG00000160718	5.15 ± 0.20	5.20 ± 0.26	5.13 ± 0.10	5.19 ± 0.12
TC04001768.hg.1	OTTHUMG00000160720	5.21 ± 0.12	5.33 ± 0.26	5.46 ± 0.22	5.35 ± 0.17
TC04001767.hg.1	OTTHUMG00000160721	6.34 ± 0.20	6.23 ± 0.06	6.37 ± 0.04	6.25 ± 0.08
TC04001773.hg.1	OTTHUMG00000160725	5.28 ± 0.16	5.20 ± 0.06	5.18 ± 0.13	5.30 ± 0.13
TC04001247.hg.1	OTTHUMG00000160726	5.41 ± 0.13	5.45 ± 0.05	5.36 ± 0.07	5.42 ± 0.10
TC04000875.hg.1	OTTHUMG00000160727	7.10 ± 0.25	7.15 ± 0.01	7.08 ± 0.06	7.19 ± 0.09
TC04000872.hg.1	OTTHUMG00000160730	5.85 ± 0.14	5.69 ± 0.10	5.69 ± 0.12	5.82 ± 0.17
TC04001766.hg.1	OTTHUMG00000160731	4.43 ± 0.13	4.28 ± 0.10	4.39 ± 0.20	4.31 ± 0.05
TC04000871.hg.1	OTTHUMG00000160733	4.80 ± 0.07	4.73 ± 0.03	4.77 ± 0.10	4.82 ± 0.05
TC04001746.hg.1	OTTHUMG00000160737	5.31 ± 0.12	5.26 ± 0.11	5.24 ± 0.03	5.36 ± 0.22
TC04001747.hg.1	OTTHUMG00000160738	6.92 ± 0.13	6.78 ± 0.03	6.79 ± 0.13	6.92 ± 0.13
TC04000249.hg.1	OTTHUMG00000160739	4.83 ± 0.15	4.73 ± 0.14	4.86 ± 0.08	4.53 ± 0.11
TC04001749.hg.1	OTTHUMG00000160740	4.69 ± 0.12	4.71 ± 0.03	4.76 ± 0.13	4.77 ± 0.06
TC04001750.hg.1	OTTHUMG00000160741	3.93 ± 0.21	3.66 ± 0.01	3.67 ± 0.06	3.66 ± 0.07
TC04001748.hg.1	OTTHUMG00000160745	4.87 ± 0.09	4.79 ± 0.11	4.84 ± 0.09	4.80 ± 0.02
TC04000246.hg.1	OTTHUMG00000160753	6.43 ± 0.09	6.38 ± 0.10	6.76 ± 0.05	6.33 ± 0.09
TC04001752.hg.1	OTTHUMG00000160754	4.30 ± 0.22	4.10 ± 0.06	4.25 ± 0.07	4.16 ± 0.09
TC04001757.hg.1	OTTHUMG00000160761	4.55 ± 0.19	4.32 ± 0.12	4.36 ± 0.21	4.46 ± 0.20
TC04001758.hg.1	OTTHUMG00000160762	9.73 ± 0.37	9.79 ± 0.03	9.66 ± 0.12	9.96 ± 0.16
TC04001759.hg.1	OTTHUMG00000160767	7.86 ± 0.30	7.74 ± 0.11	8.00 ± 0.21	8.27 ± 0.43
TC04001754.hg.1	OTTHUMG00000160773	6.20 ± 0.24	6.12 ± 0.12	6.06 ± 0.09	6.22 ± 0.24
TC04000861.hg.1	OTTHUMG00000160774	5.75 ± 0.09	5.81 ± 0.07	5.77 ± 0.08	6.02 ± 0.15
TC04001276.hg.1	OTTHUMG00000160780	5.55 ± 0.03	5.48 ± 0.03	5.46 ± 0.03	5.58 ± 0.07

TC04000398.hg.1	OTTHUMG00000160782	4.25 ± 0.18	3.88 ± 0.08	4.00 ± 0.05	3.93 ± 0.08
TC04001763.hg.1	OTTHUMG00000160792	7.11 ± 0.21	7.06 ± 0.12	7.06 ± 0.09	7.02 ± 0.05
TC04000867.hg.1	OTTHUMG00000160795	4.14 ± 0.03	4.06 ± 0.03	4.15 ± 0.04	4.10 ± 0.08
TC04001740.hg.1	OTTHUMG00000160797	6.84 ± 0.19	6.31 ± 0.40	6.92 ± 0.42	6.36 ± 0.10
TC04001738.hg.1	OTTHUMG00000160808	6.59 ± 0.17	6.47 ± 0.07	6.55 ± 0.02	6.49 ± 0.04
TC11001970.hg.1	OTTHUMG00000160821	7.27 ± 0.23	7.35 ± 0.34	7.11 ± 0.24	7.53 ± 0.07
TC04001290.hg.1	OTTHUMG00000160824	5.53 ± 0.02	5.37 ± 0.03	5.46 ± 0.07	5.45 ± 0.05
TC04001294.hg.1	OTTHUMG00000160828	6.74 ± 0.29	6.73 ± 0.20	6.65 ± 0.11	6.86 ± 0.19
TC04000422.hg.1	OTTHUMG00000160835	5.38 ± 0.12	5.18 ± 0.06	5.18 ± 0.10	5.07 ± 0.09
TC04000401.hg.1	OTTHUMG00000160836	5.81 ± 0.05	5.79 ± 0.03	5.74 ± 0.04	5.72 ± 0.03
TC04000443.hg.1	OTTHUMG00000160859	5.21 ± 0.04	4.93 ± 0.10	5.04 ± 0.02	5.04 ± 0.22
TC04001289.hg.1	OTTHUMG00000160863	5.14 ± 0.19	5.13 ± 0.18	5.08 ± 0.06	5.21 ± 0.19
TC04001291.hg.1	OTTHUMG00000160867	5.14 ± 0.19	5.13 ± 0.18	5.08 ± 0.06	5.21 ± 0.19
TC04001734.hg.1	OTTHUMG00000160879	5.50 ± 0.22	5.55 ± 0.07	5.47 ± 0.03	5.62 ± 0.06
TC04000450.hg.1	OTTHUMG00000160880	4.57 ± 0.12	4.49 ± 0.02	4.47 ± 0.07	4.60 ± 0.10
TC04001735.hg.1	OTTHUMG00000160881	4.24 ± 0.31	4.17 ± 0.11	4.12 ± 0.08	4.22 ± 0.13
TC04001329.hg.1	OTTHUMG00000160883	6.38 ± 0.17	6.29 ± 0.17	6.19 ± 0.26	6.39 ± 0.15
TC04000423.hg.1	OTTHUMG00000160886	5.15 ± 0.04	5.08 ± 0.17	4.99 ± 0.07	5.13 ± 0.17
TC04000848.hg.1	OTTHUMG00000160893	5.27 ± 0.08	5.08 ± 0.12	5.18 ± 0.07	5.07 ± 0.12
TC04001353.hg.1	OTTHUMG00000160894	6.13 ± 0.16	6.12 ± 0.13	6.21 ± 0.08	6.15 ± 0.21
TC04000849.hg.1	OTTHUMG00000160896	5.31 ± 0.06	5.33 ± 0.17	5.41 ± 0.13	5.51 ± 0.14
TC04001321.hg.1	OTTHUMG00000160909	5.35 ± 0.12	5.37 ± 0.10	5.35 ± 0.12	5.39 ± 0.10
TC04001388.hg.1	OTTHUMG00000160917	5.12 ± 0.10	5.12 ± 0.04	5.13 ± 0.15	5.34 ± 0.25
TC04001390.hg.1	OTTHUMG00000160918	5.41 ± 0.22	5.34 ± 0.20	5.08 ± 0.19	5.42 ± 0.08
TC04001391.hg.1	OTTHUMG00000160920	4.83 ± 0.06	4.86 ± 0.13	4.98 ± 0.22	5.05 ± 0.17
TC04001389.hg.1	OTTHUMG00000160923	4.49 ± 0.10	4.34 ± 0.05	4.32 ± 0.06	4.31 ± 0.11
TC04000163.hg.1	OTTHUMG00000160925	4.97 ± 0.21	4.93 ± 0.09	4.89 ± 0.03	4.90 ± 0.06
TC04000164.hg.1	OTTHUMG00000160927	5.27 ± 0.32	5.18 ± 0.18	5.17 ± 0.17	5.19 ± 0.10
TC04000162.hg.1	OTTHUMG00000160928	3.87 ± 0.09	3.69 ± 0.06	3.80 ± 0.10	3.86 ± 0.16
TC05000350.hg.1	OTTHUMG00000160932	3.99 ± 0.31	4.01 ± 0.16	3.93 ± 0.12	3.98 ± 0.09
TC04000085.hg.1	OTTHUMG00000160933	7.43 ± 0.21	7.27 ± 0.07	7.13 ± 0.31	7.33 ± 0.07

TC04000847.hg.1	OTTHUMG00000160946	5.78 ± 0.05	5.89 ± 0.19	5.83 ± 0.26	5.87 ± 0.02
TC04000846.hg.1	OTTHUMG00000160948	5.80 ± 0.07	5.68 ± 0.00	5.72 ± 0.08	5.67 ± 0.13
TC04001352.hg.1	OTTHUMG00000160961	5.56 ± 0.12	5.59 ± 0.05	5.63 ± 0.03	5.68 ± 0.10
TC04001427.hg.1	OTTHUMG00000160964	4.54 ± 0.15	4.47 ± 0.02	4.50 ± 0.04	4.43 ± 0.09
TC04001363.hg.1	OTTHUMG00000160972	6.07 ± 0.06	6.27 ± 0.23	6.13 ± 0.15	6.43 ± 0.16
TC04000535.hg.1	OTTHUMG00000160975	4.63 ± 0.07	4.44 ± 0.04	4.59 ± 0.05	4.52 ± 0.08
TC04000538.hg.1	OTTHUMG00000160976	7.58 ± 0.24	7.47 ± 0.00	7.49 ± 0.26	7.56 ± 0.06
TC04000537.hg.1	OTTHUMG00000160977	4.37 ± 0.05	4.33 ± 0.02	4.37 ± 0.06	4.42 ± 0.04
TC04000483.hg.1	OTTHUMG00000160984	6.17 ± 0.07	6.10 ± 0.01	6.03 ± 0.07	6.14 ± 0.04
TC04001401.hg.1	OTTHUMG00000160986	9.12 ± 0.29	9.09 ± 0.04	9.05 ± 0.07	9.10 ± 0.06
TC04000531.hg.1	OTTHUMG00000160989	4.35 ± 0.06	4.17 ± 0.05	4.22 ± 0.01	4.22 ± 0.06
TC04001438.hg.1	OTTHUMG00000160990	6.61 ± 0.39	6.30 ± 0.08	6.39 ± 0.10	6.43 ± 0.16
TC04000532.hg.1	OTTHUMG00000160991	4.28 ± 0.06	4.19 ± 0.12	4.16 ± 0.04	4.18 ± 0.06
TC04000550.hg.1	OTTHUMG00000160992	5.02 ± 0.06	4.91 ± 0.04	4.98 ± 0.22	5.01 ± 0.11
TC04001442.hg.1	OTTHUMG00000160994	6.23 ± 0.22	5.94 ± 0.28	6.92 ± 0.56	5.78 ± 0.15
TC04001387.hg.1	OTTHUMG00000161004	4.85 ± 0.16	4.58 ± 0.05	4.65 ± 0.01	4.57 ± 0.11
TC04001385.hg.1	OTTHUMG00000161005	4.33 ± 0.07	4.02 ± 0.10	4.14 ± 0.13	4.00 ± 0.11
TC04000528.hg.1	OTTHUMG00000161016	6.68 ± 0.05	6.67 ± 0.04	6.65 ± 0.30	6.61 ± 0.11
TC04000519.hg.1	OTTHUMG00000161022	5.63 ± 0.12	5.58 ± 0.06	5.61 ± 0.09	5.51 ± 0.05
TC04000522.hg.1	OTTHUMG00000161023	4.34 ± 0.11	4.17 ± 0.07	4.24 ± 0.02	4.24 ± 0.06
TC04000844.hg.1	OTTHUMG00000161024	5.53 ± 0.23	5.28 ± 0.08	5.31 ± 0.12	5.20 ± 0.05
TC04000843.hg.1	OTTHUMG00000161027	7.14 ± 0.38	7.31 ± 0.41	6.94 ± 0.31	7.35 ± 0.08
TC04000496.hg.1	OTTHUMG00000161032	5.39 ± 0.18	5.24 ± 0.12	5.44 ± 0.13	5.45 ± 0.25
TC04001151.hg.1	OTTHUMG00000161041	6.19 ± 0.24	6.05 ± 0.14	6.00 ± 0.21	6.12 ± 0.06
TC04000261.hg.1	OTTHUMG00000161042	5.09 ± 0.30	4.83 ± 0.15	5.05 ± 0.11	4.82 ± 0.08
TC04001445.hg.1	OTTHUMG00000161046	5.67 ± 0.12	5.50 ± 0.09	5.62 ± 0.05	5.49 ± 0.05
TC04000561.hg.1	OTTHUMG00000161047	6.52 ± 0.28	6.43 ± 0.20	6.46 ± 0.22	6.77 ± 0.22
TC04000539.hg.1	OTTHUMG00000161051	4.94 ± 0.10	4.93 ± 0.13	4.96 ± 0.02	5.00 ± 0.11
TC04001441.hg.1	OTTHUMG00000161052	4.85 ± 0.02	4.73 ± 0.10	4.81 ± 0.09	4.82 ± 0.05
TC04000468.hg.1	OTTHUMG00000161054	4.64 ± 0.13	4.59 ± 0.04	4.67 ± 0.09	4.63 ± 0.16
TC04001364.hg.1	OTTHUMG00000161058	5.02 ± 0.13	4.87 ± 0.12	4.79 ± 0.15	4.84 ± 0.18

TC04001367.hg.1	OTTHUMG00000161060	5.22 ± 0.11	5.20 ± 0.10	5.15 ± 0.13	5.40 ± 0.10
TC04002923.hg.1	OTTHUMG00000161063	6.35 ± 0.11	6.25 ± 0.13	6.31 ± 0.06	6.25 ± 0.14
TC04000589.hg.1	OTTHUMG00000161068	6.00 ± 0.13	5.91 ± 0.27	5.83 ± 0.17	6.24 ± 0.19
TC04001544.hg.1	OTTHUMG00000161070	3.40 ± 0.09	3.23 ± 0.05	3.27 ± 0.17	3.17 ± 0.03
TC04001456.hg.1	OTTHUMG00000161085	5.60 ± 0.11	5.48 ± 0.08	5.60 ± 0.06	5.57 ± 0.04
TC04001458.hg.1	OTTHUMG00000161086	5.38 ± 0.04	5.38 ± 0.05	5.34 ± 0.09	5.49 ± 0.10
TC04000511.hg.1	OTTHUMG00000161091	5.45 ± 0.04	5.38 ± 0.05	5.49 ± 0.12	5.58 ± 0.07
TC04000658.hg.1	OTTHUMG00000161095	4.24 ± 0.07	4.26 ± 0.04	4.26 ± 0.06	4.30 ± 0.08
TC04000832.hg.1	OTTHUMG00000161098	4.61 ± 0.18	4.38 ± 0.07	4.47 ± 0.08	4.51 ± 0.05
TC04000573.hg.1	OTTHUMG00000161115	5.72 ± 0.28	5.70 ± 0.23	5.64 ± 0.28	5.75 ± 0.07
TC04001469.hg.1	OTTHUMG00000161116	4.89 ± 0.03	4.85 ± 0.13	5.02 ± 0.06	4.96 ± 0.10
TC04001470.hg.1	OTTHUMG00000161118	4.10 ± 0.14	4.09 ± 0.03	4.05 ± 0.09	4.11 ± 0.05
TC04000620.hg.1	OTTHUMG00000161124	5.78 ± 0.07	5.55 ± 0.14	5.62 ± 0.07	5.67 ± 0.07
TC04000575.hg.1	OTTHUMG00000161125	4.76 ± 0.18	4.55 ± 0.12	4.55 ± 0.14	4.61 ± 0.03
TC04001205.hg.1	OTTHUMG00000161130	6.07 ± 0.23	5.85 ± 0.14	5.80 ± 0.18	5.89 ± 0.08
TC04000582.hg.1	OTTHUMG00000161132	7.06 ± 0.06	7.04 ± 0.17	7.08 ± 0.11	7.16 ± 0.08
TC04001490.hg.1	OTTHUMG00000161136	5.99 ± 0.20	5.82 ± 0.08	5.89 ± 0.18	5.93 ± 0.11
TC04001489.hg.1	OTTHUMG00000161137	5.23 ± 0.11	5.25 ± 0.15	5.27 ± 0.07	5.47 ± 0.22
TC04000546.hg.1	OTTHUMG00000161140	5.32 ± 0.06	5.27 ± 0.04	5.25 ± 0.10	5.33 ± 0.06
TC04000621.hg.1	OTTHUMG00000161141	5.47 ± 0.28	5.47 ± 0.06	5.62 ± 0.10	5.50 ± 0.04
TC04000640.hg.1	OTTHUMG00000161146	3.80 ± 0.15	3.67 ± 0.07	3.71 ± 0.07	3.79 ± 0.12
TC04000639.hg.1	OTTHUMG00000161149	6.91 ± 0.07	6.69 ± 0.09	6.69 ± 0.15	6.86 ± 0.13
TC04001351.hg.1	OTTHUMG00000161151	5.67 ± 0.10	5.64 ± 0.11	5.59 ± 0.04	5.79 ± 0.17
TC04001345.hg.1	OTTHUMG00000161156	6.77 ± 0.09	6.74 ± 0.14	7.01 ± 0.09	6.87 ± 0.07
TC04000623.hg.1	OTTHUMG00000161161	5.74 ± 0.22	5.60 ± 0.14	5.46 ± 0.20	5.71 ± 0.09
TC17001658.hg.1	OTTHUMG00000161174	7.97 ± 0.44	7.95 ± 0.16	7.88 ± 0.25	8.03 ± 0.11
TC17001659.hg.1	OTTHUMG00000161175	6.71 ± 0.20	6.65 ± 0.24	6.66 ± 0.26	6.81 ± 0.04
TC04001539.hg.1	OTTHUMG00000161177	5.62 ± 0.08	5.55 ± 0.31	5.63 ± 0.07	5.78 ± 0.15
TC04001538.hg.1	OTTHUMG00000161178	6.60 ± 0.27	6.36 ± 0.14	6.54 ± 0.10	6.53 ± 0.14
TC04001447.hg.1	OTTHUMG00000161179	4.06 ± 0.15	3.91 ± 0.02	3.96 ± 0.01	3.96 ± 0.06
TC04000505.hg.1	OTTHUMG00000161181	4.78 ± 0.13	4.84 ± 0.09	4.82 ± 0.06	4.85 ± 0.11

TC04000490.hg.1	OTTHUMG00000161187	4.74 ± 0.09	4.60 ± 0.10	4.61 ± 0.05	4.67 ± 0.07
TC04001715.hg.1	OTTHUMG00000161192	3.73 ± 0.04	3.67 ± 0.05	3.66 ± 0.14	3.60 ± 0.11
TC04000655.hg.1	OTTHUMG00000161197	4.37 ± 0.12	4.27 ± 0.03	4.27 ± 0.08	4.34 ± 0.07
TC04001541.hg.1	OTTHUMG00000161198	4.86 ± 0.34	4.76 ± 0.12	4.94 ± 0.19	4.80 ± 0.24
TC04001542.hg.1	OTTHUMG00000161199	5.77 ± 0.33	5.61 ± 0.01	5.69 ± 0.08	5.75 ± 0.07
TC04001497.hg.1	OTTHUMG00000161209	5.64 ± 0.36	5.23 ± 0.10	5.46 ± 0.30	5.20 ± 0.02
TC04001449.hg.1	OTTHUMG00000161211	5.92 ± 0.13	5.75 ± 0.07	5.79 ± 0.03	5.81 ± 0.14
TC04001448.hg.1	OTTHUMG00000161212	4.52 ± 0.17	4.30 ± 0.06	4.33 ± 0.10	4.33 ± 0.14
TC04000661.hg.1	OTTHUMG00000161213	5.29 ± 0.16	4.97 ± 0.02	5.12 ± 0.09	5.20 ± 0.22
TC04000666.hg.1	OTTHUMG00000161215	4.70 ± 0.17	4.73 ± 0.03	4.82 ± 0.17	4.89 ± 0.18
TC04001557.hg.1	OTTHUMG00000161216	4.87 ± 0.13	4.83 ± 0.10	4.91 ± 0.01	4.97 ± 0.08
TC04000667.hg.1	OTTHUMG00000161217	4.02 ± 0.05	3.84 ± 0.02	3.95 ± 0.02	3.88 ± 0.03
TC04001560.hg.1	OTTHUMG00000161218	4.88 ± 0.10	4.87 ± 0.05	4.87 ± 0.22	4.97 ± 0.11
TC04001556.hg.1	OTTHUMG00000161221	3.68 ± 0.06	3.53 ± 0.03	3.65 ± 0.12	3.64 ± 0.25
TC04000342.hg.1	OTTHUMG00000161225	5.75 ± 0.28	5.44 ± 0.07	5.62 ± 0.12	5.64 ± 0.11
TC04001232.hg.1	OTTHUMG00000161226	4.95 ± 0.12	4.78 ± 0.03	4.91 ± 0.05	4.84 ± 0.05
TC04001545.hg.1	OTTHUMG00000161235	7.15 ± 0.29	7.18 ± 0.20	7.12 ± 0.11	7.21 ± 0.19
TC04000668.hg.1	OTTHUMG00000161238	5.92 ± 0.11	5.95 ± 0.08	5.90 ± 0.16	6.05 ± 0.15
TC04000669.hg.1	OTTHUMG00000161241	3.92 ± 0.17	3.69 ± 0.01	3.84 ± 0.29	3.80 ± 0.26
TC04001563.hg.1	OTTHUMG00000161242	5.93 ± 0.22	5.76 ± 0.10	5.81 ± 0.07	5.87 ± 0.11
TC04001564.hg.1	OTTHUMG00000161243	5.66 ± 0.03	5.58 ± 0.07	5.65 ± 0.05	5.66 ± 0.12
TC04001392.hg.1	OTTHUMG00000161244	3.65 ± 0.06	3.59 ± 0.00	3.56 ± 0.07	3.61 ± 0.03
TC04000502.hg.1	OTTHUMG00000161245	6.36 ± 0.17	6.36 ± 0.06	6.35 ± 0.16	6.57 ± 0.13
TC04000344.hg.1	OTTHUMG00000161247	4.56 ± 0.12	4.25 ± 0.06	4.41 ± 0.09	4.27 ± 0.15
TC01002002.hg.1	OTTHUMG00000161248	5.58 ± 0.16	5.42 ± 0.05	5.57 ± 0.18	5.49 ± 0.08
TC04001704.hg.1	OTTHUMG00000161256	7.35 ± 0.21	7.30 ± 0.20	7.29 ± 0.10	7.42 ± 0.01
TC04001717.hg.1	OTTHUMG00000161276	4.37 ± 0.08	4.33 ± 0.09	4.35 ± 0.07	4.39 ± 0.08
TC04000670.hg.1	OTTHUMG00000161278	4.35 ± 0.19	4.20 ± 0.16	4.24 ± 0.12	4.25 ± 0.04
TC04001720.hg.1	OTTHUMG00000161279	4.43 ± 0.05	4.26 ± 0.07	4.35 ± 0.16	4.39 ± 0.14
TC04000677.hg.1	OTTHUMG00000161282	5.29 ± 0.10	5.23 ± 0.05	5.24 ± 0.12	5.29 ± 0.15
TC04001571.hg.1	OTTHUMG00000161283	4.38 ± 0.18	4.20 ± 0.16	4.33 ± 0.07	4.32 ± 0.08

TC04001573.hg.1	OTTHUMG00000161289	4.80 ± 0.22	4.60 ± 0.06	4.68 ± 0.18	4.65 ± 0.08
TC04001553.hg.1	OTTHUMG00000161291	4.27 ± 0.16	4.11 ± 0.05	4.09 ± 0.07	4.14 ± 0.04
TC04000634.hg.1	OTTHUMG00000161297	4.28 ± 0.31	4.05 ± 0.22	4.15 ± 0.02	4.20 ± 0.17
TC04001548.hg.1	OTTHUMG00000161298	6.50 ± 0.09	6.18 ± 0.13	6.39 ± 0.09	6.39 ± 0.07
TC04001550.hg.1	OTTHUMG00000161299	5.20 ± 0.07	5.12 ± 0.08	5.18 ± 0.07	5.23 ± 0.07
TC04001549.hg.1	OTTHUMG00000161302	3.90 ± 0.15	3.79 ± 0.07	3.80 ± 0.17	3.88 ± 0.07
TC04000633.hg.1	OTTHUMG00000161303	6.25 ± 0.15	6.20 ± 0.10	6.10 ± 0.06	6.31 ± 0.16
TC04000551.hg.1	OTTHUMG00000161304	3.73 ± 0.10	3.57 ± 0.05	3.62 ± 0.02	3.65 ± 0.07
TC04000704.hg.1	OTTHUMG00000161311	6.26 ± 0.09	6.14 ± 0.06	6.24 ± 0.02	6.26 ± 0.08
TC04001498.hg.1	OTTHUMG00000161336	5.75 ± 0.09	5.68 ± 0.09	5.68 ± 0.15	5.80 ± 0.09
TC04001505.hg.1	OTTHUMG00000161337	5.65 ± 0.05	5.56 ± 0.09	5.56 ± 0.13	5.66 ± 0.11
TC04001599.hg.1	OTTHUMG00000161339	5.03 ± 0.37	4.85 ± 0.11	4.80 ± 0.24	4.87 ± 0.10
TC04000694.hg.1	OTTHUMG00000161340	5.89 ± 0.14	5.59 ± 0.08	5.76 ± 0.09	5.74 ± 0.20
TC04000695.hg.1	OTTHUMG00000161342	5.87 ± 0.06	5.90 ± 0.13	5.98 ± 0.10	5.86 ± 0.12
TC04001566.hg.1	OTTHUMG00000161344	3.67 ± 0.14	3.62 ± 0.10	3.67 ± 0.12	3.60 ± 0.12
TC04000673.hg.1	OTTHUMG00000161345	4.47 ± 0.25	4.31 ± 0.02	4.32 ± 0.20	4.23 ± 0.04
TC04001568.hg.1	OTTHUMG00000161346	4.28 ± 0.06	4.12 ± 0.10	4.19 ± 0.02	4.26 ± 0.16
TC04001567.hg.1	OTTHUMG00000161347	4.01 ± 0.15	3.82 ± 0.04	3.79 ± 0.12	3.78 ± 0.03
TC04000672.hg.1	OTTHUMG00000161349	4.77 ± 0.23	4.83 ± 0.13	4.81 ± 0.03	4.94 ± 0.24
TC04000730.hg.1	OTTHUMG00000161357	4.66 ± 0.16	4.69 ± 0.13	4.72 ± 0.17	4.84 ± 0.29
TC04000731.hg.1	OTTHUMG00000161359	7.17 ± 0.15	7.06 ± 0.11	7.10 ± 0.05	7.23 ± 0.07
TC04001624.hg.1	OTTHUMG00000161360	7.42 ± 0.39	7.27 ± 0.16	7.24 ± 0.11	7.47 ± 0.08
TC17000643.hg.1	OTTHUMG00000161364	6.57 ± 0.07	6.23 ± 0.15	6.47 ± 0.19	6.25 ± 0.04
TC17000642.hg.1	OTTHUMG00000161365	7.07 ± 0.18	7.10 ± 0.14	7.07 ± 0.15	7.15 ± 0.05
TC04000688.hg.1	OTTHUMG00000161374	6.23 ± 0.06	6.09 ± 0.09	6.29 ± 0.06	6.19 ± 0.08
TC04000817.hg.1	OTTHUMG00000161378	4.27 ± 0.03	4.07 ± 0.03	4.18 ± 0.10	4.10 ± 0.09
TC04001596.hg.1	OTTHUMG00000161379	5.83 ± 0.18	5.69 ± 0.12	5.68 ± 0.05	5.89 ± 0.20
TC04000816.hg.1	OTTHUMG00000161384	6.83 ± 0.13	6.81 ± 0.08	6.97 ± 0.15	6.93 ± 0.18
TC04001527.hg.1	OTTHUMG00000161387	5.42 ± 0.17	5.29 ± 0.09	5.34 ± 0.17	5.30 ± 0.07
TC04001632.hg.1	OTTHUMG00000161390	4.96 ± 0.19	4.88 ± 0.12	4.88 ± 0.10	5.05 ± 0.30
TC04001631.hg.1	OTTHUMG00000161392	5.72 ± 0.13	5.50 ± 0.09	5.59 ± 0.16	5.61 ± 0.05

TC04000739.hg.1	OTTHUMG00000161393	4.98 ± 0.13	4.82 ± 0.10	4.87 ± 0.04	4.90 ± 0.08
TC04001633.hg.1	OTTHUMG00000161394	4.82 ± 0.12	4.66 ± 0.02	4.73 ± 0.04	4.73 ± 0.03
TC04000743.hg.1	OTTHUMG00000161395	4.85 ± 0.04	4.72 ± 0.09	4.80 ± 0.09	4.78 ± 0.13
TC04000740.hg.1	OTTHUMG00000161396	4.40 ± 0.23	4.31 ± 0.09	4.34 ± 0.11	4.42 ± 0.13
TC04000705.hg.1	OTTHUMG00000161400	6.21 ± 0.23	5.99 ± 0.15	6.03 ± 0.12	6.24 ± 0.14
TC04001601.hg.1	OTTHUMG00000161406	6.56 ± 0.08	6.62 ± 0.19	6.51 ± 0.05	6.66 ± 0.08
TC04000815.hg.1	OTTHUMG00000161416	4.89 ± 0.12	4.70 ± 0.04	4.85 ± 0.11	4.86 ± 0.06
TC04000814.hg.1	OTTHUMG00000161417	5.04 ± 0.10	4.86 ± 0.17	4.92 ± 0.07	5.01 ± 0.18
TC04000691.hg.1	OTTHUMG00000161425	5.05 ± 0.23	4.63 ± 0.02	4.80 ± 0.10	4.58 ± 0.20
TC04000813.hg.1	OTTHUMG00000161427	3.67 ± 0.04	3.57 ± 0.10	3.69 ± 0.13	3.65 ± 0.06
TC04001694.hg.1	OTTHUMG00000161434	5.74 ± 0.11	5.73 ± 0.05	5.76 ± 0.06	5.75 ± 0.08
TC04000812.hg.1	OTTHUMG00000161436	5.99 ± 0.23	5.89 ± 0.20	5.85 ± 0.23	5.95 ± 0.13
TC04000719.hg.1	OTTHUMG00000161438	7.34 ± 0.26	7.60 ± 0.25	7.49 ± 0.13	7.70 ± 0.17
TC04000746.hg.1	OTTHUMG00000161447	6.98 ± 0.25	6.84 ± 0.08	6.88 ± 0.20	7.01 ± 0.06
TC04000750.hg.1	OTTHUMG00000161448	5.71 ± 0.36	6.36 ± 0.47	5.57 ± 0.12	6.72 ± 0.11
TC04001628.hg.1	OTTHUMG00000161454	4.58 ± 0.06	4.57 ± 0.07	4.63 ± 0.06	4.66 ± 0.14
TC04000738.hg.1	OTTHUMG00000161457	4.17 ± 0.16	4.03 ± 0.06	3.94 ± 0.12	4.15 ± 0.09
TC04001629.hg.1	OTTHUMG00000161458	4.22 ± 0.03	4.13 ± 0.07	4.24 ± 0.09	4.11 ± 0.11
TC04000736.hg.1	OTTHUMG00000161459	6.58 ± 0.16	6.69 ± 0.13	6.68 ± 0.16	6.81 ± 0.12
TC04000765.hg.1	OTTHUMG00000161462	6.58 ± 0.03	6.25 ± 0.14	6.52 ± 0.22	6.31 ± 0.16
TC04000665.hg.1	OTTHUMG00000161469	3.90 ± 0.15	3.79 ± 0.08	3.76 ± 0.09	3.80 ± 0.09
TC04001555.hg.1	OTTHUMG00000161471	4.70 ± 0.09	4.57 ± 0.06	4.65 ± 0.05	4.60 ± 0.02
TC04000707.hg.1	OTTHUMG00000161479	5.76 ± 0.20	5.83 ± 0.17	5.91 ± 0.20	5.78 ± 0.18
TC04001666.hg.1	OTTHUMG00000161483	4.64 ± 0.07	4.44 ± 0.13	4.58 ± 0.08	4.66 ± 0.09
TC04001665.hg.1	OTTHUMG00000161487	7.38 ± 0.19	7.34 ± 0.17	7.34 ± 0.18	7.48 ± 0.04
TC04001722.hg.1	OTTHUMG00000161489	6.09 ± 0.29	6.07 ± 0.22	6.02 ± 0.14	6.15 ± 0.08
TC04000840.hg.1	OTTHUMG00000161490	5.36 ± 0.09	5.24 ± 0.13	5.25 ± 0.12	5.41 ± 0.11
TC17000644.hg.1	OTTHUMG00000161492	7.95 ± 0.14	7.86 ± 0.10	7.88 ± 0.17	7.89 ± 0.13
TC17001666.hg.1	OTTHUMG00000161494	6.98 ± 0.24	6.84 ± 0.12	6.85 ± 0.11	6.96 ± 0.06
TC17001668.hg.1	OTTHUMG00000161497	7.28 ± 0.11	7.31 ± 0.07	7.37 ± 0.09	7.39 ± 0.09
TC04000769.hg.1	OTTHUMG00000161522	7.96 ± 0.31	7.91 ± 0.12	7.78 ± 0.18	8.06 ± 0.16

TC04001681.hg.1	OTTHUMG00000161530	4.55 ± 0.44	4.24 ± 0.14	4.32 ± 0.26	4.39 ± 0.04
TC04001679.hg.1	OTTHUMG00000161531	5.55 ± 0.16	5.32 ± 0.06	5.42 ± 0.01	5.33 ± 0.07
TC04000801.hg.1	OTTHUMG00000161532	4.15 ± 0.14	4.08 ± 0.05	4.08 ± 0.12	4.09 ± 0.06
TC04001678.hg.1	OTTHUMG00000161535	3.31 ± 0.05	3.20 ± 0.10	3.26 ± 0.04	3.16 ± 0.05
TC04001680.hg.1	OTTHUMG00000161536	3.77 ± 0.16	3.44 ± 0.17	3.68 ± 0.34	3.53 ± 0.11
TC04001664.hg.1	OTTHUMG00000161545	6.08 ± 0.13	6.11 ± 0.06	5.98 ± 0.32	6.15 ± 0.10
TC04001418.hg.1	OTTHUMG00000161550	5.14 ± 0.86	5.48 ± 0.30	5.22 ± 0.59	5.54 ± 0.17
TC04000518.hg.1	OTTHUMG00000161551	7.10 ± 0.47	7.18 ± 0.45	7.51 ± 0.41	7.13 ± 0.17
TC04000697.hg.1	OTTHUMG00000161553	4.26 ± 0.27	3.97 ± 0.03	4.13 ± 0.22	3.89 ± 0.10
TC04000776.hg.1	OTTHUMG00000161558	5.42 ± 0.02	5.37 ± 0.07	6.19 ± 0.45	5.34 ± 0.10
TC04001484.hg.1	OTTHUMG00000161561	5.32 ± 0.08	5.16 ± 0.02	5.16 ± 0.21	5.22 ± 0.18
TC04001483.hg.1	OTTHUMG00000161562	5.10 ± 0.20	5.07 ± 0.10	4.97 ± 0.07	5.15 ± 0.14
TC04001482.hg.1	OTTHUMG00000161563	5.59 ± 0.11	5.60 ± 0.05	5.50 ± 0.16	5.49 ± 0.02
TC04001480.hg.1	OTTHUMG00000161567	5.47 ± 0.18	5.69 ± 0.19	5.49 ± 0.07	5.76 ± 0.04
TC05000001.hg.1	OTTHUMG00000161574	8.07 ± 0.19	8.01 ± 0.19	7.99 ± 0.25	7.99 ± 0.11
TC04000615.hg.1	OTTHUMG00000161575	8.39 ± 0.38	8.66 ± 0.13	8.71 ± 0.37	8.70 ± 0.08
TC04000703.hg.1	OTTHUMG00000161580	8.14 ± 0.14	8.02 ± 0.09	7.95 ± 0.05	8.22 ± 0.12
TC04001414.hg.1	OTTHUMG00000161582	6.28 ± 0.16	6.14 ± 0.12	6.27 ± 0.15	6.22 ± 0.09
TC05001093.hg.1	OTTHUMG00000161587	5.81 ± 0.09	5.96 ± 0.16	5.99 ± 0.13	6.05 ± 0.06
TC04000721.hg.1	OTTHUMG00000161588	4.44 ± 0.15	4.46 ± 0.07	4.42 ± 0.06	4.45 ± 0.05
TC04000720.hg.1	OTTHUMG00000161589	6.64 ± 0.14	6.48 ± 0.06	6.54 ± 0.26	6.57 ± 0.23
TC04000718.hg.1	OTTHUMG00000161590	6.18 ± 0.40	6.54 ± 0.36	6.38 ± 0.54	6.59 ± 0.14
TC04001616.hg.1	OTTHUMG00000161591	5.40 ± 0.13	5.33 ± 0.22	5.22 ± 0.16	5.43 ± 0.22
TC04001020.hg.1	OTTHUMG00000161596	6.50 ± 0.10	6.51 ± 0.14	6.39 ± 0.26	6.48 ± 0.09
TC04001042.hg.1	OTTHUMG00000161597	8.31 ± 0.14	8.13 ± 0.02	8.35 ± 0.16	8.24 ± 0.09
TC04001041.hg.1	OTTHUMG00000161598	4.50 ± 0.10	4.47 ± 0.08	4.51 ± 0.13	4.66 ± 0.05
TC05000020.hg.1	OTTHUMG00000161599	8.36 ± 0.29	8.34 ± 0.13	8.34 ± 0.11	8.47 ± 0.04
TC05001118.hg.1	OTTHUMG00000161600	6.08 ± 0.14	6.22 ± 0.21	6.09 ± 0.13	6.30 ± 0.21
TC05001116.hg.1	OTTHUMG00000161601	7.17 ± 0.28	7.18 ± 0.14	7.17 ± 0.30	7.21 ± 0.08
TC05000021.hg.1	OTTHUMG00000161602	6.96 ± 0.27	6.85 ± 0.10	6.95 ± 0.24	6.83 ± 0.16
TC05000024.hg.1	OTTHUMG00000161603	7.59 ± 0.18	7.58 ± 0.01	7.68 ± 0.10	7.59 ± 0.04

TC05000023.hg.1	OTTHUMG00000161604	7.85 ± 0.20	7.72 ± 0.18	7.67 ± 0.26	7.84 ± 0.08
TC05000022.hg.1	OTTHUMG00000161605	8.22 ± 0.15	8.17 ± 0.14	8.17 ± 0.20	8.20 ± 0.03
TC05001121.hg.1	OTTHUMG00000161606	6.79 ± 0.10	6.65 ± 0.02	6.79 ± 0.07	6.72 ± 0.16
TC05000027.hg.1	OTTHUMG00000161607	5.81 ± 0.19	5.73 ± 0.04	5.80 ± 0.04	5.85 ± 0.16
TC05000028.hg.1	OTTHUMG00000161608	4.14 ± 0.06	3.93 ± 0.06	3.94 ± 0.08	4.19 ± 0.15
TC04000372.hg.1	OTTHUMG00000161622	5.80 ± 0.61	6.10 ± 0.19	6.49 ± 0.31	6.11 ± 0.26
TC04001074.hg.1	OTTHUMG00000161626	6.19 ± 0.25	6.26 ± 0.17	6.08 ± 0.11	6.31 ± 0.11
TC04000156.hg.1	OTTHUMG00000161627	5.53 ± 0.14	5.62 ± 0.10	5.55 ± 0.15	5.76 ± 0.17
TC04001075.hg.1	OTTHUMG00000161628	5.24 ± 0.29	5.34 ± 0.08	5.29 ± 0.04	5.43 ± 0.19
TC05001126.hg.1	OTTHUMG00000161630	6.98 ± 0.23	6.82 ± 0.10	6.93 ± 0.13	7.03 ± 0.16
TC05000029.hg.1	OTTHUMG00000161631	6.12 ± 0.15	6.06 ± 0.11	6.17 ± 0.15	6.06 ± 0.06
TC05001123.hg.1	OTTHUMG00000161633	6.69 ± 0.27	6.79 ± 0.07	6.67 ± 0.19	6.94 ± 0.17
TC05001125.hg.1	OTTHUMG00000161634	4.84 ± 0.09	4.95 ± 0.07	4.97 ± 0.03	5.07 ± 0.25
TC05000032.hg.1	OTTHUMG00000161635	7.52 ± 0.13	7.45 ± 0.21	7.45 ± 0.18	7.60 ± 0.15
TC05000034.hg.1	OTTHUMG00000161641	5.14 ± 0.29	5.00 ± 0.06	5.10 ± 0.01	4.99 ± 0.18
TC05001131.hg.1	OTTHUMG00000161643	6.57 ± 0.13	6.39 ± 0.12	6.43 ± 0.06	6.50 ± 0.07
TC05000035.hg.1	OTTHUMG00000161644	5.91 ± 0.28	5.70 ± 0.14	5.70 ± 0.06	5.73 ± 0.03
TC05000036.hg.1	OTTHUMG00000161645	7.21 ± 0.21	7.32 ± 0.25	7.10 ± 0.12	7.47 ± 0.28
TC05000033.hg.1	OTTHUMG00000161646	6.56 ± 0.09	6.43 ± 0.03	6.49 ± 0.03	6.68 ± 0.20
TC05001128.hg.1	OTTHUMG00000161647	5.81 ± 0.15	5.78 ± 0.04	5.81 ± 0.04	5.88 ± 0.06
TC05001130.hg.1	OTTHUMG00000161648	4.58 ± 0.12	4.68 ± 0.15	4.67 ± 0.05	4.68 ± 0.08
TC05001129.hg.1	OTTHUMG00000161649	4.31 ± 0.01	4.20 ± 0.03	4.21 ± 0.04	4.22 ± 0.12
TC05000049.hg.1	OTTHUMG00000161651	4.66 ± 0.19	4.54 ± 0.10	4.52 ± 0.06	4.58 ± 0.08
TC04002938.hg.1	OTTHUMG00000161654	6.70 ± 0.15	6.32 ± 0.06	7.19 ± 0.37	6.31 ± 0.01
TC05001132.hg.1	OTTHUMG00000161659	4.27 ± 0.18	4.18 ± 0.01	4.13 ± 0.22	4.16 ± 0.07
TC05001133.hg.1	OTTHUMG00000161660	5.22 ± 0.26	5.16 ± 0.13	5.06 ± 0.03	5.16 ± 0.06
TC05001135.hg.1	OTTHUMG00000161665	5.79 ± 0.19	5.75 ± 0.21	5.72 ± 0.25	5.63 ± 0.05
TC05001134.hg.1	OTTHUMG00000161668	7.42 ± 0.24	7.35 ± 0.16	7.32 ± 0.21	7.45 ± 0.06
TC04001638.hg.1	OTTHUMG00000161670	6.85 ± 0.26	6.69 ± 0.07	6.77 ± 0.27	6.84 ± 0.25
TC04000759.hg.1	OTTHUMG00000161671	5.24 ± 0.14	5.06 ± 0.07	5.20 ± 0.25	5.19 ± 0.06
TC04000757.hg.1	OTTHUMG00000161674	4.07 ± 0.13	3.96 ± 0.06	4.13 ± 0.15	3.91 ± 0.13

TC04000758.hg.1	OTTHUMG00000161676	5.62 ± 0.12	5.61 ± 0.08	5.55 ± 0.09	5.90 ± 0.10
TC04001684.hg.1	OTTHUMG00000161685	5.02 ± 0.04	4.92 ± 0.04	4.88 ± 0.08	5.08 ± 0.11
TC05000069.hg.1	OTTHUMG00000161686	5.48 ± 0.46	5.19 ± 0.11	5.19 ± 0.13	5.30 ± 0.24
TC05001155.hg.1	OTTHUMG00000161687	5.47 ± 0.06	5.32 ± 0.23	5.38 ± 0.10	5.63 ± 0.24
TC04000185.hg.1	OTTHUMG00000161689	5.75 ± 0.20	5.72 ± 0.04	5.64 ± 0.07	5.78 ± 0.09
TC04000193.hg.1	OTTHUMG00000161690	4.22 ± 0.19	3.85 ± 0.10	3.91 ± 0.08	3.89 ± 0.07
TC04001100.hg.1	OTTHUMG00000161691	4.96 ± 0.11	4.60 ± 0.03	4.64 ± 0.05	4.63 ± 0.13
TC04001104.hg.1	OTTHUMG00000161692	5.65 ± 0.20	5.55 ± 0.08	5.57 ± 0.07	5.74 ± 0.15
TC04001097.hg.1	OTTHUMG00000161693	6.10 ± 0.26	6.01 ± 0.06	6.02 ± 0.10	6.03 ± 0.15
TC04000192.hg.1	OTTHUMG00000161694	5.01 ± 0.04	4.99 ± 0.08	5.08 ± 0.19	5.11 ± 0.15
TC04001103.hg.1	OTTHUMG00000161695	4.71 ± 0.32	4.43 ± 0.05	4.61 ± 0.08	4.47 ± 0.06
TC17000647.hg.1	OTTHUMG00000161697	7.39 ± 0.13	7.44 ± 0.07	7.27 ± 0.07	7.48 ± 0.09
TC04001112.hg.1	OTTHUMG00000161702	6.16 ± 0.18	6.09 ± 0.01	6.05 ± 0.03	6.25 ± 0.18
TC05000046.hg.1	OTTHUMG00000161703	6.81 ± 0.04	6.80 ± 0.08	6.76 ± 0.15	6.93 ± 0.06
TC05000047.hg.1	OTTHUMG00000161704	6.79 ± 0.18	6.63 ± 0.07	6.72 ± 0.29	6.67 ± 0.17
TC05001139.hg.1	OTTHUMG00000161705	6.51 ± 0.08	6.53 ± 0.23	6.63 ± 0.17	6.72 ± 0.10
TC05000048.hg.1	OTTHUMG00000161706	4.22 ± 0.09	4.25 ± 0.10	4.18 ± 0.10	4.41 ± 0.15
TC04001229.hg.1	OTTHUMG00000161707	5.99 ± 0.22	5.98 ± 0.06	5.97 ± 0.04	6.09 ± 0.03
TC04001230.hg.1	OTTHUMG00000161708	5.28 ± 0.24	5.22 ± 0.08	5.31 ± 0.05	5.43 ± 0.01
TC04001231.hg.1	OTTHUMG00000161714	5.63 ± 0.12	5.59 ± 0.24	5.60 ± 0.14	5.69 ± 0.08
TC05000061.hg.1	OTTHUMG00000161716	7.34 ± 0.23	7.22 ± 0.04	7.28 ± 0.04	7.54 ± 0.23
TC05001154.hg.1	OTTHUMG00000161717	4.20 ± 0.05	4.23 ± 0.05	4.23 ± 0.03	4.17 ± 0.03
TC05000065.hg.1	OTTHUMG00000161718	4.03 ± 0.13	3.87 ± 0.04	3.94 ± 0.15	3.93 ± 0.09
TC05001153.hg.1	OTTHUMG00000161720	6.42 ± 0.25	6.36 ± 0.10	6.29 ± 0.12	6.53 ± 0.30
TC05000062.hg.1	OTTHUMG00000161722	6.21 ± 0.19	6.22 ± 0.05	6.15 ± 0.19	6.36 ± 0.18
TC04001645.hg.1	OTTHUMG00000161723	5.02 ± 0.08	4.98 ± 0.09	4.89 ± 0.10	5.05 ± 0.13
TC04001646.hg.1	OTTHUMG00000161724	6.68 ± 0.22	6.56 ± 0.11	6.67 ± 0.22	6.65 ± 0.10
TC04000761.hg.1	OTTHUMG00000161726	5.88 ± 0.13	6.00 ± 0.08	5.94 ± 0.20	6.11 ± 0.06
TC04001234.hg.1	OTTHUMG00000161727	3.56 ± 0.10	3.55 ± 0.03	3.58 ± 0.07	3.65 ± 0.11
TC04000345.hg.1	OTTHUMG00000161728	5.23 ± 0.28	5.04 ± 0.06	5.17 ± 0.05	5.10 ± 0.07
TC04001643.hg.1	OTTHUMG00000161729	4.53 ± 0.16	4.54 ± 0.21	4.40 ± 0.09	4.77 ± 0.24

TC04001235.hg.1	OTTHUMG00000161730	5.35 ± 0.19	5.35 ± 0.07	5.34 ± 0.06	5.49 ± 0.11
TC04001236.hg.1	OTTHUMG00000161731	4.83 ± 0.14	4.78 ± 0.05	4.90 ± 0.11	4.85 ± 0.23
TC04000349.hg.1	OTTHUMG00000161732	4.42 ± 0.20	4.35 ± 0.08	4.40 ± 0.03	4.51 ± 0.07
TC04001241.hg.1	OTTHUMG00000161737	4.06 ± 0.14	3.92 ± 0.12	4.00 ± 0.07	3.90 ± 0.07
TC04001642.hg.1	OTTHUMG00000161739	5.70 ± 0.11	5.59 ± 0.22	5.53 ± 0.17	5.85 ± 0.22
TC04000760.hg.1	OTTHUMG00000161740	6.33 ± 0.09	6.23 ± 0.08	6.32 ± 0.02	6.32 ± 0.01
TC05001169.hg.1	OTTHUMG00000161742	3.70 ± 0.06	3.55 ± 0.16	3.56 ± 0.11	3.69 ± 0.15
TC04000444.hg.1	OTTHUMG00000161747	6.44 ± 0.05	6.45 ± 0.13	6.42 ± 0.17	6.62 ± 0.16
TC05000082.hg.1	OTTHUMG00000161749	4.00 ± 0.15	3.80 ± 0.06	3.94 ± 0.02	3.86 ± 0.07
TC04000534.hg.1	OTTHUMG00000161751	4.81 ± 0.20	4.76 ± 0.05	4.90 ± 0.08	4.81 ± 0.08
TC05000063.hg.1	OTTHUMG00000161752	5.25 ± 0.15	5.04 ± 0.11	5.09 ± 0.01	5.13 ± 0.03
TC05000064.hg.1	OTTHUMG00000161754	5.04 ± 0.21	4.93 ± 0.08	4.90 ± 0.12	4.85 ± 0.11
TC04001474.hg.1	OTTHUMG00000161755	5.04 ± 0.13	5.03 ± 0.16	5.06 ± 0.14	5.24 ± 0.39
TC04001475.hg.1	OTTHUMG00000161756	4.46 ± 0.07	4.25 ± 0.07	4.34 ± 0.13	4.50 ± 0.13
TC04000576.hg.1	OTTHUMG00000161757	6.27 ± 0.03	6.16 ± 0.03	6.24 ± 0.14	6.27 ± 0.09
TC05001168.hg.1	OTTHUMG00000161759	8.13 ± 0.16	8.15 ± 0.01	8.07 ± 0.02	8.30 ± 0.07
TC04001533.hg.1	OTTHUMG00000161764	4.88 ± 0.25	4.91 ± 0.12	4.84 ± 0.29	5.01 ± 0.05
TC04001534.hg.1	OTTHUMG00000161765	5.64 ± 0.08	5.53 ± 0.06	5.50 ± 0.06	5.61 ± 0.14
TC04000656.hg.1	OTTHUMG00000161766	3.82 ± 0.06	3.68 ± 0.08	3.83 ± 0.06	3.71 ± 0.05
TC04001546.hg.1	OTTHUMG00000161767	6.56 ± 0.27	6.54 ± 0.08	6.44 ± 0.12	6.71 ± 0.15
TC04000662.hg.1	OTTHUMG00000161768	5.48 ± 0.18	5.46 ± 0.04	5.36 ± 0.16	5.43 ± 0.18
TC04001547.hg.1	OTTHUMG00000161769	6.15 ± 0.26	5.96 ± 0.07	6.01 ± 0.09	6.10 ± 0.12
TC05000071.hg.1	OTTHUMG00000161772	4.94 ± 0.16	5.02 ± 0.15	4.99 ± 0.06	5.15 ± 0.14
TC05000070.hg.1	OTTHUMG00000161773	5.36 ± 0.12	5.26 ± 0.23	5.22 ± 0.29	5.33 ± 0.08
TC04001575.hg.1	OTTHUMG00000161774	6.02 ± 0.25	6.11 ± 0.18	6.06 ± 0.04	6.25 ± 0.09
TC04001672.hg.1	OTTHUMG00000161775	5.41 ± 0.07	5.27 ± 0.14	5.38 ± 0.05	5.53 ± 0.11
TC04000790.hg.1	OTTHUMG00000161776	4.59 ± 0.04	4.47 ± 0.03	4.50 ± 0.09	4.55 ± 0.01
TC04000791.hg.1	OTTHUMG00000161777	4.45 ± 0.26	4.36 ± 0.03	4.37 ± 0.12	4.33 ± 0.10
TC04000792.hg.1	OTTHUMG00000161780	4.69 ± 0.15	4.50 ± 0.02	4.48 ± 0.13	4.47 ± 0.18
TC05001194.hg.1	OTTHUMG00000161783	4.71 ± 0.15	4.49 ± 0.09	4.56 ± 0.11	4.63 ± 0.14
TC05000092.hg.1	OTTHUMG00000161786	3.52 ± 0.14	3.48 ± 0.03	3.56 ± 0.09	3.50 ± 0.10

TC05001180.hg.1	OTTHUMG00000161787	5.97 ± 0.07	5.85 ± 0.07	5.84 ± 0.06	5.97 ± 0.09
TC05000090.hg.1	OTTHUMG00000161791	4.85 ± 0.17	5.06 ± 0.18	4.89 ± 0.09	5.22 ± 0.15
TC05001175.hg.1	OTTHUMG00000161792	5.69 ± 0.18	5.69 ± 0.24	5.74 ± 0.13	5.89 ± 0.06
TC05001176.hg.1	OTTHUMG00000161793	5.43 ± 0.13	5.45 ± 0.05	5.40 ± 0.08	5.54 ± 0.16
TC05001177.hg.1	OTTHUMG00000161797	5.08 ± 0.11	4.94 ± 0.15	4.93 ± 0.01	5.06 ± 0.19
TC04000786.hg.1	OTTHUMG00000161799	3.75 ± 0.14	3.53 ± 0.07	3.59 ± 0.02	3.64 ± 0.02
TC04000795.hg.1	OTTHUMG00000161801	4.96 ± 0.17	4.77 ± 0.14	4.93 ± 0.09	4.78 ± 0.09
TC04000793.hg.1	OTTHUMG00000161802	6.87 ± 0.37	7.02 ± 0.09	6.84 ± 0.22	7.46 ± 0.45
TC04000794.hg.1	OTTHUMG00000161804	3.85 ± 0.30	3.59 ± 0.08	3.61 ± 0.08	3.68 ± 0.11
TC05001193.hg.1	OTTHUMG00000161808	7.46 ± 0.05	7.25 ± 0.07	7.50 ± 0.08	7.31 ± 0.05
TC04001712.hg.1	OTTHUMG00000161810	7.85 ± 0.43	7.82 ± 0.14	7.91 ± 0.15	8.17 ± 0.23
TC04001733.hg.1	OTTHUMG00000161813	6.09 ± 0.10	6.13 ± 0.09	6.26 ± 0.18	6.33 ± 0.17
TC05000094.hg.1	OTTHUMG00000161823	7.56 ± 0.10	7.57 ± 0.14	7.50 ± 0.03	7.59 ± 0.05
TC05000093.hg.1	OTTHUMG00000161824	6.95 ± 0.21	7.04 ± 0.08	6.79 ± 0.10	7.14 ± 0.09
TC17000650.hg.1	OTTHUMG00000161832	6.55 ± 0.11	6.54 ± 0.09	6.51 ± 0.14	6.54 ± 0.06
TC05000102.hg.1	OTTHUMG00000161859	5.03 ± 0.11	4.97 ± 0.03	4.94 ± 0.03	5.10 ± 0.09
TC05000101.hg.1	OTTHUMG00000161869	5.55 ± 0.02	5.59 ± 0.15	5.44 ± 0.31	5.57 ± 0.08
TC05000099.hg.1	OTTHUMG00000161870	8.72 ± 0.11	8.52 ± 0.05	8.89 ± 0.16	8.66 ± 0.14
TC05000100.hg.1	OTTHUMG00000161871	6.70 ± 0.29	6.58 ± 0.16	6.59 ± 0.06	6.69 ± 0.08
TC05001192.hg.1	OTTHUMG00000161873	5.51 ± 0.19	5.46 ± 0.07	5.52 ± 0.07	5.37 ± 0.02
TC05001190.hg.1	OTTHUMG00000161875	7.68 ± 0.24	7.64 ± 0.12	7.59 ± 0.30	7.82 ± 0.11
TC05000116.hg.1	OTTHUMG00000161879	6.00 ± 0.25	5.96 ± 0.01	5.99 ± 0.10	6.05 ± 0.09
TC04001295.hg.1	OTTHUMG00000161880	6.52 ± 0.16	6.66 ± 0.18	6.50 ± 0.15	6.68 ± 0.15
TC04001296.hg.1	OTTHUMG00000161881	4.72 ± 0.09	4.72 ± 0.06	4.69 ± 0.04	4.65 ± 0.03
TC04001297.hg.1	OTTHUMG00000161882	5.62 ± 0.29	5.58 ± 0.16	5.49 ± 0.09	5.71 ± 0.22
TC04000418.hg.1	OTTHUMG00000161883	5.19 ± 0.06	5.07 ± 0.08	5.13 ± 0.19	5.12 ± 0.04
TC05001204.hg.1	OTTHUMG00000161885	4.77 ± 0.33	4.61 ± 0.09	4.74 ± 0.02	4.62 ± 0.15
TC05001207.hg.1	OTTHUMG00000161887	3.74 ± 0.24	3.56 ± 0.12	3.58 ± 0.17	3.62 ± 0.12
TC17000653.hg.1	OTTHUMG00000161893	7.47 ± 0.36	7.39 ± 0.22	7.32 ± 0.21	7.45 ± 0.05
TC05001147.hg.1	OTTHUMG00000161894	6.13 ± 0.08	6.28 ± 0.17	6.18 ± 0.09	6.51 ± 0.11
TC05000053.hg.1	OTTHUMG00000161895	4.97 ± 0.05	4.97 ± 0.12	5.06 ± 0.08	5.01 ± 0.12

TC05000051.hg.1	OTTHUMG00000161896	4.92 ± 0.08	4.85 ± 0.05	4.84 ± 0.13	4.84 ± 0.16
TC05001145.hg.1	OTTHUMG00000161899	5.32 ± 0.08	5.01 ± 0.10	5.12 ± 0.10	5.10 ± 0.02
TC05000057.hg.1	OTTHUMG00000161900	5.68 ± 0.11	5.50 ± 0.20	5.71 ± 0.10	5.63 ± 0.05
TC05001144.hg.1	OTTHUMG00000161901	6.28 ± 0.21	6.00 ± 0.04	5.99 ± 0.15	6.05 ± 0.05
TC05001146.hg.1	OTTHUMG00000161902	6.50 ± 0.14	6.66 ± 0.29	6.42 ± 0.32	6.73 ± 0.07
TC05000052.hg.1	OTTHUMG00000161903	5.68 ± 0.29	5.87 ± 0.04	5.71 ± 0.06	5.85 ± 0.13
TC04001835.hg.1	OTTHUMG00000161907	6.57 ± 0.23	6.42 ± 0.10	6.36 ± 0.11	6.53 ± 0.13
TC04000931.hg.1	OTTHUMG00000161908	6.94 ± 0.11	6.78 ± 0.13	6.76 ± 0.31	7.02 ± 0.05
TC04001836.hg.1	OTTHUMG00000161909	6.10 ± 0.19	5.95 ± 0.05	6.06 ± 0.31	6.16 ± 0.22
TC05000112.hg.1	OTTHUMG00000161916	3.69 ± 0.03	3.66 ± 0.22	3.74 ± 0.13	3.86 ± 0.24
TC05001219.hg.1	OTTHUMG00000161925	4.45 ± 0.15	4.27 ± 0.14	4.22 ± 0.13	4.48 ± 0.22
TC05001104.hg.1	OTTHUMG00000161928	7.99 ± 0.23	8.00 ± 0.15	7.93 ± 0.21	8.15 ± 0.08
TC05001220.hg.1	OTTHUMG00000161934	6.55 ± 0.36	6.46 ± 0.05	6.53 ± 0.20	6.59 ± 0.05
TC05001221.hg.1	OTTHUMG00000161939	4.61 ± 0.09	4.47 ± 0.07	4.51 ± 0.09	4.53 ± 0.03
TC05001211.hg.1	OTTHUMG00000161955	3.42 ± 0.08	3.26 ± 0.05	3.37 ± 0.09	3.26 ± 0.01
TC05001208.hg.1	OTTHUMG00000161956	6.22 ± 0.25	6.24 ± 0.01	6.25 ± 0.03	6.34 ± 0.13
TC05000120.hg.1	OTTHUMG00000161957	4.01 ± 0.16	3.85 ± 0.08	3.68 ± 0.09	3.79 ± 0.06
TC05000121.hg.1	OTTHUMG00000161958	3.46 ± 0.07	3.51 ± 0.06	3.48 ± 0.10	3.52 ± 0.07
TC05001210.hg.1	OTTHUMG00000161959	5.84 ± 0.15	5.75 ± 0.03	5.90 ± 0.05	5.84 ± 0.09
TC05000119.hg.1	OTTHUMG00000161960	5.45 ± 0.18	5.41 ± 0.04	5.39 ± 0.05	5.55 ± 0.20
TC05001209.hg.1	OTTHUMG00000161962	4.63 ± 0.14	4.54 ± 0.14	4.62 ± 0.09	4.72 ± 0.15
TC05000122.hg.1	OTTHUMG00000161963	3.67 ± 0.08	3.40 ± 0.10	3.45 ± 0.29	3.36 ± 0.06
TC05001217.hg.1	OTTHUMG00000161964	4.92 ± 0.15	4.87 ± 0.08	4.85 ± 0.05	5.00 ± 0.05
TC05001215.hg.1	OTTHUMG00000161970	4.11 ± 0.11	4.12 ± 0.09	4.17 ± 0.08	4.16 ± 0.04
TC05001214.hg.1	OTTHUMG00000161971	4.97 ± 0.13	4.85 ± 0.08	4.93 ± 0.14	4.95 ± 0.12
TC17001677.hg.1	OTTHUMG00000161991	6.80 ± 0.20	6.90 ± 0.38	6.76 ± 0.29	7.01 ± 0.10
TC05000146.hg.1	OTTHUMG00000162000	4.76 ± 0.14	4.55 ± 0.11	4.70 ± 0.19	4.58 ± 0.09
TC05001237.hg.1	OTTHUMG00000162001	4.98 ± 0.10	4.89 ± 0.06	4.98 ± 0.19	4.95 ± 0.16
TC05000147.hg.1	OTTHUMG00000162002	4.57 ± 0.21	4.45 ± 0.13	4.44 ± 0.08	4.54 ± 0.03
TC05001241.hg.1	OTTHUMG00000162003	6.94 ± 0.19	6.95 ± 0.08	6.74 ± 0.20	6.84 ± 0.16
TC05001239.hg.1	OTTHUMG00000162005	5.03 ± 0.18	5.03 ± 0.14	5.08 ± 0.04	5.24 ± 0.12

TC05001238.hg.1	OTTHUMG00000162006	5.86 ± 0.12	5.76 ± 0.11	5.77 ± 0.14	5.90 ± 0.12
TC04001683.hg.1	OTTHUMG00000162012	7.42 ± 0.23	7.40 ± 0.04	7.45 ± 0.26	7.66 ± 0.27
TC05000105.hg.1	OTTHUMG00000162015	5.09 ± 0.36	5.04 ± 0.15	5.20 ± 0.27	5.08 ± 0.08
TC05001196.hg.1	OTTHUMG00000162017	5.83 ± 0.11	5.62 ± 0.03	5.80 ± 0.06	5.68 ± 0.14
TC05001258.hg.1	OTTHUMG00000162022	8.87 ± 0.30	8.72 ± 0.24	8.66 ± 0.23	8.73 ± 0.01
TC04000716.hg.1	OTTHUMG00000162028	8.54 ± 0.55	8.49 ± 0.01	8.75 ± 0.36	9.06 ± 0.44
TC04001618.hg.1	OTTHUMG00000162029	6.13 ± 0.19	5.99 ± 0.11	6.06 ± 0.07	5.99 ± 0.12
TC04000722.hg.1	OTTHUMG00000162030	5.16 ± 0.19	4.99 ± 0.12	4.98 ± 0.04	5.12 ± 0.19
TC05001272.hg.1	OTTHUMG00000162032	6.15 ± 0.18	6.16 ± 0.01	6.35 ± 0.20	6.05 ± 0.12
TC05001273.hg.1	OTTHUMG00000162033	7.33 ± 0.34	7.42 ± 0.05	7.28 ± 0.39	7.45 ± 0.06
TC05001291.hg.1	OTTHUMG00000162038	5.49 ± 0.07	5.41 ± 0.05	5.42 ± 0.05	5.55 ± 0.09
TC05000177.hg.1	OTTHUMG00000162039	6.78 ± 0.31	6.51 ± 0.27	6.68 ± 0.07	6.64 ± 0.16
TC05001292.hg.1	OTTHUMG00000162040	6.89 ± 0.21	6.87 ± 0.13	6.87 ± 0.16	6.87 ± 0.05
TC04001576.hg.1	OTTHUMG00000162042	8.35 ± 0.27	8.40 ± 0.06	8.41 ± 0.08	8.49 ± 0.12
TC04000725.hg.1	OTTHUMG00000162048	4.29 ± 0.06	4.31 ± 0.14	4.31 ± 0.08	4.38 ± 0.14
TC05001162.hg.1	OTTHUMG00000162049	5.61 ± 0.02	5.63 ± 0.06	5.62 ± 0.03	5.68 ± 0.03
TC05000076.hg.1	OTTHUMG00000162053	8.47 ± 0.22	8.15 ± 0.12	8.46 ± 0.11	8.28 ± 0.08
TC05001161.hg.1	OTTHUMG00000162054	6.74 ± 0.26	6.63 ± 0.14	6.76 ± 0.05	6.81 ± 0.09
TC17000660.hg.1	OTTHUMG00000162055	6.57 ± 0.21	6.62 ± 0.15	6.51 ± 0.09	6.68 ± 0.09
TC17001684.hg.1	OTTHUMG00000162058	7.56 ± 0.31	7.50 ± 0.19	7.50 ± 0.06	7.65 ± 0.04
TC17000661.hg.1	OTTHUMG00000162061	6.08 ± 0.16	6.19 ± 0.13	6.25 ± 0.08	6.41 ± 0.32
TC05000106.hg.1	OTTHUMG00000162067	5.32 ± 0.08	5.26 ± 0.05	5.25 ± 0.07	5.34 ± 0.09
TC17000666.hg.1	OTTHUMG00000162080	6.84 ± 0.22	6.70 ± 0.11	6.77 ± 0.09	6.79 ± 0.17
TC05001264.hg.1	OTTHUMG00000162093	4.31 ± 0.05	4.06 ± 0.06	4.11 ± 0.10	4.06 ± 0.17
TC05001263.hg.1	OTTHUMG00000162095	5.93 ± 0.21	5.75 ± 0.11	5.83 ± 0.18	5.91 ± 0.12
TC04000728.hg.1	OTTHUMG00000162098	4.72 ± 0.07	4.66 ± 0.28	4.65 ± 0.05	4.68 ± 0.06
TC05001288.hg.1	OTTHUMG00000162102	5.94 ± 0.15	5.93 ± 0.15	5.99 ± 0.18	6.01 ± 0.07
TC05000170.hg.1	OTTHUMG00000162103	6.47 ± 0.26	6.20 ± 0.20	6.33 ± 0.08	6.32 ± 0.16
TC05000172.hg.1	OTTHUMG00000162104	4.42 ± 0.12	4.34 ± 0.06	4.36 ± 0.08	4.45 ± 0.05
TC05000171.hg.1	OTTHUMG00000162105	4.90 ± 0.12	4.73 ± 0.22	4.89 ± 0.16	4.90 ± 0.12
TC05000176.hg.1	OTTHUMG00000162106	5.20 ± 0.04	4.89 ± 0.09	4.89 ± 0.14	5.12 ± 0.14

TC05001281.hg.1	OTTHUMG00000162107	5.87 ± 0.14	5.73 ± 0.05	5.74 ± 0.14	5.74 ± 0.12
TC05001282.hg.1	OTTHUMG00000162112	5.74 ± 0.14	5.52 ± 0.08	5.54 ± 0.09	5.54 ± 0.06
TC05000173.hg.1	OTTHUMG00000162113	5.18 ± 0.11	5.10 ± 0.12	5.17 ± 0.23	5.27 ± 0.13
TC05001165.hg.1	OTTHUMG00000162122	5.98 ± 0.26	5.68 ± 0.19	5.60 ± 0.15	5.57 ± 0.08
TC05000011.hg.1	OTTHUMG00000162130	7.87 ± 0.13	7.83 ± 0.24	7.89 ± 0.19	8.06 ± 0.19
TC05000012.hg.1	OTTHUMG00000162131	7.03 ± 0.28	6.82 ± 0.17	6.86 ± 0.22	6.95 ± 0.20
TC05001330.hg.1	OTTHUMG00000162145	6.88 ± 0.24	6.89 ± 0.15	6.90 ± 0.06	6.96 ± 0.16
TC05000207.hg.1	OTTHUMG00000162146	6.05 ± 0.06	6.05 ± 0.06	5.99 ± 0.22	6.11 ± 0.15
TC05001098.hg.1	OTTHUMG00000162152	7.83 ± 0.28	7.75 ± 0.22	7.68 ± 0.27	7.94 ± 0.05
TC05000008.hg.1	OTTHUMG00000162153	7.53 ± 0.37	7.62 ± 0.22	7.54 ± 0.14	7.71 ± 0.05
TC17001692.hg.1	OTTHUMG00000162154	7.09 ± 0.29	7.10 ± 0.10	7.05 ± 0.10	7.24 ± 0.15
TC17001691.hg.1	OTTHUMG00000162156	8.55 ± 0.35	8.57 ± 0.21	8.46 ± 0.34	8.76 ± 0.13
TC06000080.hg.1	OTTHUMG00000162157	6.17 ± 0.17	5.87 ± 0.07	6.13 ± 0.28	5.88 ± 0.03
TC05001242.hg.1	OTTHUMG00000162158	5.78 ± 0.40	5.67 ± 0.21	5.71 ± 0.20	5.80 ± 0.22
TC05001340.hg.1	OTTHUMG00000162165	6.47 ± 0.29	6.37 ± 0.18	6.31 ± 0.25	6.54 ± 0.21
TC04000340.hg.1	OTTHUMG00000162167	6.47 ± 0.24	6.40 ± 0.09	6.37 ± 0.04	6.39 ± 0.09
TC05000215.hg.1	OTTHUMG00000162169	3.90 ± 0.14	3.56 ± 0.04	3.58 ± 0.22	3.52 ± 0.09
TC05000216.hg.1	OTTHUMG00000162170	4.41 ± 0.19	4.37 ± 0.01	4.33 ± 0.05	4.40 ± 0.09
TC05001341.hg.1	OTTHUMG00000162173	5.56 ± 0.11	5.45 ± 0.05	5.49 ± 0.06	5.59 ± 0.08
TC05001231.hg.1	OTTHUMG00000162178	6.82 ± 0.15	6.74 ± 0.06	6.74 ± 0.03	6.73 ± 0.03
TC05000136.hg.1	OTTHUMG00000162179	6.89 ± 0.26	7.04 ± 0.17	6.88 ± 0.14	7.14 ± 0.17
TC04001614.hg.1	OTTHUMG00000162183	5.63 ± 0.04	5.53 ± 0.11	5.52 ± 0.12	5.69 ± 0.18
TC04000713.hg.1	OTTHUMG00000162184	4.14 ± 0.13	4.29 ± 0.18	4.25 ± 0.03	4.43 ± 0.10
TC04001612.hg.1	OTTHUMG00000162185	4.55 ± 0.15	4.46 ± 0.13	4.34 ± 0.07	4.45 ± 0.08
TC04001613.hg.1	OTTHUMG00000162186	7.53 ± 0.23	7.32 ± 0.18	7.36 ± 0.07	7.39 ± 0.06
TC05001351.hg.1	OTTHUMG00000162192	5.48 ± 0.15	5.44 ± 0.20	5.50 ± 0.05	5.65 ± 0.09
TC05000081.hg.1	OTTHUMG00000162193	6.14 ± 0.23	5.96 ± 0.17	6.11 ± 0.11	6.20 ± 0.21
TC05001384.hg.1	OTTHUMG00000162196	4.92 ± 0.21	4.68 ± 0.02	4.81 ± 0.14	4.73 ± 0.14
TC05001383.hg.1	OTTHUMG00000162198	5.66 ± 0.28	5.76 ± 0.08	5.62 ± 0.07	5.79 ± 0.11
TC05001353.hg.1	OTTHUMG00000162200	6.23 ± 0.19	6.23 ± 0.07	6.19 ± 0.17	6.32 ± 0.18
TC05000229.hg.1	OTTHUMG00000162201	6.31 ± 0.23	6.24 ± 0.03	6.49 ± 0.16	6.45 ± 0.09

TC05000230.hg.1	OTTHUMG00000162202	5.77 ± 0.15	5.52 ± 0.17	5.55 ± 0.10	5.73 ± 0.26
TC05001388.hg.1	OTTHUMG00000162203	4.80 ± 0.10	4.55 ± 0.15	4.72 ± 0.18	4.66 ± 0.06
TC05001387.hg.1	OTTHUMG00000162204	5.51 ± 0.24	5.41 ± 0.04	5.48 ± 0.04	5.56 ± 0.12
TC05000205.hg.1	OTTHUMG00000162208	5.46 ± 0.50	5.53 ± 0.04	5.43 ± 0.19	5.82 ± 0.36
TC05001326.hg.1	OTTHUMG00000162209	5.07 ± 0.11	4.99 ± 0.08	4.94 ± 0.06	4.92 ± 0.11
TC05000208.hg.1	OTTHUMG00000162210	8.62 ± 0.24	8.68 ± 0.05	8.55 ± 0.24	8.94 ± 0.25
TC05000256.hg.1	OTTHUMG00000162214	5.55 ± 0.07	5.46 ± 0.07	5.68 ± 0.11	5.49 ± 0.05
TC17001694.hg.1	OTTHUMG00000162216	7.29 ± 0.23	7.46 ± 0.19	7.34 ± 0.10	7.55 ± 0.22
TC17001695.hg.1	OTTHUMG00000162217	6.22 ± 0.24	6.15 ± 0.12	6.07 ± 0.10	6.22 ± 0.02
TC05000201.hg.1	OTTHUMG00000162219	5.33 ± 0.07	5.22 ± 0.05	5.12 ± 0.11	5.27 ± 0.03
TC05000199.hg.1	OTTHUMG00000162227	5.39 ± 0.08	5.35 ± 0.06	5.38 ± 0.12	5.25 ± 0.06
TC05000203.hg.1	OTTHUMG00000162230	5.28 ± 0.31	5.06 ± 0.09	5.29 ± 0.11	5.08 ± 0.14
TC05000204.hg.1	OTTHUMG00000162233	5.78 ± 0.27	5.62 ± 0.21	5.62 ± 0.15	5.75 ± 0.12
TC05000200.hg.1	OTTHUMG00000162234	8.24 ± 0.48	8.35 ± 0.09	8.17 ± 0.23	8.68 ± 0.36
TC05000194.hg.1	OTTHUMG00000162236	7.36 ± 0.02	7.13 ± 0.09	7.34 ± 0.10	7.19 ± 0.06
TC05000261.hg.1	OTTHUMG00000162242	6.08 ± 0.26	5.89 ± 0.20	5.94 ± 0.10	5.96 ± 0.05
TC05001396.hg.1	OTTHUMG00000162244	6.54 ± 0.22	6.41 ± 0.06	6.51 ± 0.25	6.45 ± 0.07
TC05001065.hg.1	OTTHUMG00000162253	8.34 ± 0.17	8.26 ± 0.18	8.24 ± 0.15	8.27 ± 0.14
TC05001344.hg.1	OTTHUMG00000162282	6.45 ± 0.24	6.43 ± 0.08	6.42 ± 0.19	6.51 ± 0.06
TC05001349.hg.1	OTTHUMG00000162284	3.70 ± 0.32	3.81 ± 0.28	3.81 ± 0.14	3.61 ± 0.06
TC05000224.hg.1	OTTHUMG00000162286	5.76 ± 0.14	5.74 ± 0.06	5.69 ± 0.13	5.78 ± 0.06
TC05001403.hg.1	OTTHUMG00000162289	5.68 ± 0.33	5.76 ± 0.05	5.68 ± 0.27	6.03 ± 0.22
TC05000265.hg.1	OTTHUMG00000162296	5.68 ± 0.19	5.42 ± 0.03	5.61 ± 0.07	5.58 ± 0.04
TC05000240.hg.1	OTTHUMG00000162302	7.01 ± 0.30	6.68 ± 0.06	6.69 ± 0.21	6.77 ± 0.07
TC05001373.hg.1	OTTHUMG00000162304	5.67 ± 0.21	5.72 ± 0.15	5.68 ± 0.23	5.81 ± 0.08
TC05001374.hg.1	OTTHUMG00000162309	6.47 ± 0.17	6.34 ± 0.11	6.66 ± 0.04	6.44 ± 0.03
TC05000233.hg.1	OTTHUMG00000162314	6.36 ± 0.12	6.35 ± 0.04	6.41 ± 0.12	6.40 ± 0.02
TC17000678.hg.1	OTTHUMG00000162315	6.79 ± 0.15	6.73 ± 0.16	6.67 ± 0.13	6.70 ± 0.12
TC04000343.hg.1	OTTHUMG00000162318	6.16 ± 0.11	6.02 ± 0.20	6.09 ± 0.22	6.08 ± 0.09
TC05000248.hg.1	OTTHUMG00000162323	5.38 ± 0.33	4.96 ± 0.05	5.04 ± 0.08	5.00 ± 0.05
TC05001382.hg.1	OTTHUMG00000162327	4.04 ± 0.06	3.86 ± 0.05	3.82 ± 0.06	3.96 ± 0.12

TC05000293.hg.1	OTTHUMG00000162331	4.57 ± 0.20	4.42 ± 0.16	4.62 ± 0.11	4.62 ± 0.06
TC05001430.hg.1	OTTHUMG00000162332	4.55 ± 0.05	4.49 ± 0.07	4.52 ± 0.12	4.56 ± 0.07
TC05000295.hg.1	OTTHUMG00000162333	4.54 ± 0.12	4.30 ± 0.11	4.25 ± 0.03	4.19 ± 0.14
TC05001429.hg.1	OTTHUMG00000162335	4.32 ± 0.25	4.22 ± 0.14	4.22 ± 0.04	4.30 ± 0.11
TC05001431.hg.1	OTTHUMG00000162336	5.57 ± 0.11	5.53 ± 0.06	5.56 ± 0.03	5.62 ± 0.09
TC05001427.hg.1	OTTHUMG00000162337	6.51 ± 0.12	6.62 ± 0.20	6.58 ± 0.12	6.78 ± 0.10
TC04001240.hg.1	OTTHUMG00000162339	4.90 ± 0.11	4.70 ± 0.08	4.77 ± 0.05	4.69 ± 0.08
TC17000679.hg.1	OTTHUMG00000162349	4.76 ± 0.07	4.92 ± 0.11	4.84 ± 0.06	4.84 ± 0.14
TC05001424.hg.1	OTTHUMG00000162350	6.83 ± 0.33	6.94 ± 0.07	6.83 ± 0.26	7.20 ± 0.26
TC05000286.hg.1	OTTHUMG00000162351	5.96 ± 0.15	5.79 ± 0.11	5.85 ± 0.26	5.78 ± 0.09
TC05000287.hg.1	OTTHUMG00000162352	5.78 ± 0.15	5.62 ± 0.07	5.64 ± 0.03	5.73 ± 0.14
TC05001425.hg.1	OTTHUMG00000162354	8.85 ± 0.73	9.13 ± 0.17	8.87 ± 0.39	9.34 ± 0.28
TC05001423.hg.1	OTTHUMG00000162355	6.41 ± 0.26	6.21 ± 0.04	6.32 ± 0.10	6.30 ± 0.04
TC05001421.hg.1	OTTHUMG00000162358	3.98 ± 0.12	3.77 ± 0.06	3.82 ± 0.02	3.85 ± 0.06
TC05001417.hg.1	OTTHUMG00000162359	6.46 ± 0.16	6.69 ± 0.21	6.50 ± 0.11	6.78 ± 0.03
TC05000283.hg.1	OTTHUMG00000162361	3.73 ± 0.02	3.63 ± 0.08	3.78 ± 0.09	3.68 ± 0.01
TC05001197.hg.1	OTTHUMG00000162363	5.78 ± 0.23	5.65 ± 0.07	5.71 ± 0.18	5.83 ± 0.10
TC17000683.hg.1	OTTHUMG00000162371	7.77 ± 0.27	7.66 ± 0.24	7.78 ± 0.19	7.96 ± 0.11
TC17001704.hg.1	OTTHUMG00000162372	6.97 ± 0.30	6.97 ± 0.03	6.92 ± 0.11	7.09 ± 0.16
TC17000682.hg.1	OTTHUMG00000162373	7.38 ± 0.86	7.79 ± 0.11	7.46 ± 0.38	8.18 ± 0.39
TC05001247.hg.1	OTTHUMG00000162375	4.89 ± 0.73	5.18 ± 0.46	4.64 ± 0.24	4.82 ± 0.23
TC05001248.hg.1	OTTHUMG00000162376	5.43 ± 0.73	5.77 ± 0.45	5.20 ± 0.19	5.42 ± 0.21
TC05000235.hg.1	OTTHUMG00000162377	4.95 ± 0.09	5.03 ± 0.08	4.78 ± 0.09	4.91 ± 0.10
TC05002163.hg.1	OTTHUMG00000162378	8.86 ± 0.36	8.68 ± 0.10	8.73 ± 0.32	8.74 ± 0.11
TC05002161.hg.1	OTTHUMG00000162379	8.21 ± 0.33	8.07 ± 0.02	8.17 ± 0.21	7.94 ± 0.03
TC17000685.hg.1	OTTHUMG00000162380	5.72 ± 0.10	5.66 ± 0.15	5.82 ± 0.22	5.78 ± 0.06
TC05001432.hg.1	OTTHUMG00000162382	7.33 ± 0.32	7.42 ± 0.06	7.46 ± 0.26	7.57 ± 0.16
TC05001496.hg.1	OTTHUMG00000162399	7.06 ± 0.04	7.19 ± 0.31	6.98 ± 0.18	7.29 ± 0.11
TC05001499.hg.1	OTTHUMG00000162401	6.21 ± 0.26	5.89 ± 0.10	6.27 ± 0.38	5.99 ± 0.17
TC05001335.hg.1	OTTHUMG00000162402	6.40 ± 0.04	6.43 ± 0.20	6.34 ± 0.19	6.40 ± 0.14
TC05001334.hg.1	OTTHUMG00000162405	6.49 ± 0.31	6.37 ± 0.31	6.32 ± 0.05	6.62 ± 0.17

TC05001333.hg.1	OTTHUMG00000162407	4.56 ± 0.18	4.53 ± 0.12	4.48 ± 0.13	4.69 ± 0.13
TC05000210.hg.1	OTTHUMG00000162408	7.29 ± 0.20	7.24 ± 0.18	7.16 ± 0.09	7.29 ± 0.14
TC05000255.hg.1	OTTHUMG00000162414	5.89 ± 0.02	5.64 ± 0.10	5.84 ± 0.05	5.71 ± 0.06
TC05001433.hg.1	OTTHUMG00000162416	4.78 ± 0.06	4.69 ± 0.03	4.86 ± 0.08	4.64 ± 0.04
TC05001386.hg.1	OTTHUMG00000162427	8.99 ± 0.29	9.14 ± 0.07	9.11 ± 0.24	9.34 ± 0.15
TC05001503.hg.1	OTTHUMG00000162429	5.51 ± 0.15	5.51 ± 0.07	5.52 ± 0.03	5.54 ± 0.07
TC05001501.hg.1	OTTHUMG00000162430	5.45 ± 0.26	5.16 ± 0.04	5.21 ± 0.03	5.14 ± 0.07
TC05001398.hg.1	OTTHUMG00000162438	6.21 ± 0.18	6.13 ± 0.11	6.07 ± 0.14	6.23 ± 0.10
TC05001473.hg.1	OTTHUMG00000162452	6.68 ± 0.10	6.64 ± 0.19	6.70 ± 0.08	6.80 ± 0.08
TC05001475.hg.1	OTTHUMG00000162456	5.71 ± 0.10	5.61 ± 0.18	5.45 ± 0.17	5.62 ± 0.05
TC05000357.hg.1	OTTHUMG00000162457	6.52 ± 0.16	6.24 ± 0.02	6.26 ± 0.08	6.29 ± 0.04
TC05001479.hg.1	OTTHUMG00000162458	4.81 ± 0.08	4.56 ± 0.04	4.81 ± 0.26	4.63 ± 0.10
TC05001478.hg.1	OTTHUMG00000162459	4.05 ± 0.20	3.93 ± 0.16	4.09 ± 0.09	3.94 ± 0.08
TC05000358.hg.1	OTTHUMG00000162460	5.21 ± 0.09	5.19 ± 0.15	5.19 ± 0.04	5.25 ± 0.21
TC05001480.hg.1	OTTHUMG00000162462	5.08 ± 0.10	5.01 ± 0.17	5.10 ± 0.21	5.11 ± 0.09
TC05000347.hg.1	OTTHUMG00000162463	6.14 ± 0.14	6.17 ± 0.11	6.17 ± 0.05	6.30 ± 0.08
TC05001490.hg.1	OTTHUMG00000162465	8.10 ± 0.12	8.12 ± 0.10	8.17 ± 0.23	8.27 ± 0.12
TC05001488.hg.1	OTTHUMG00000162467	6.96 ± 0.12	7.03 ± 0.04	6.87 ± 0.02	7.17 ± 0.06
TC05000378.hg.1	OTTHUMG00000162471	6.51 ± 0.15	6.48 ± 0.07	6.15 ± 0.36	6.59 ± 0.12
TC05000311.hg.1	OTTHUMG00000162476	8.58 ± 0.63	8.67 ± 0.37	8.39 ± 0.33	8.52 ± 0.10
TC05000292.hg.1	OTTHUMG00000162485	4.33 ± 0.15	4.13 ± 0.10	4.14 ± 0.20	4.17 ± 0.13
TC05001493.hg.1	OTTHUMG00000162488	4.29 ± 0.10	4.17 ± 0.12	4.52 ± 0.23	4.23 ± 0.05
TC05000351.hg.1	OTTHUMG00000162491	7.17 ± 0.23	7.21 ± 0.08	7.19 ± 0.26	7.34 ± 0.17
TC05001467.hg.1	OTTHUMG00000162494	5.86 ± 0.13	5.86 ± 0.10	5.76 ± 0.14	5.70 ± 0.05
TC05000348.hg.1	OTTHUMG00000162497	3.93 ± 0.09	3.94 ± 0.08	3.77 ± 0.06	3.89 ± 0.01
TC05001471.hg.1	OTTHUMG00000162499	6.59 ± 0.26	6.45 ± 0.16	6.40 ± 0.03	6.62 ± 0.07
TC05001468.hg.1	OTTHUMG00000162500	4.89 ± 0.11	4.84 ± 0.04	4.93 ± 0.18	4.92 ± 0.10
TC05000249.hg.1	OTTHUMG00000162503	5.77 ± 0.08	5.60 ± 0.07	5.71 ± 0.05	5.56 ± 0.09
TC05001226.hg.1	OTTHUMG00000162515	4.78 ± 0.05	4.83 ± 0.17	4.71 ± 0.08	4.76 ± 0.02
TC05000137.hg.1	OTTHUMG00000162516	4.96 ± 0.08	5.34 ± 0.04	5.14 ± 0.14	5.43 ± 0.04
TC05001227.hg.1	OTTHUMG00000162517	3.99 ± 0.08	3.94 ± 0.05	3.89 ± 0.09	3.95 ± 0.03

TC05000138.hg.1	OTTHUMG00000162518	6.51 ± 0.10	6.40 ± 0.10	6.44 ± 0.11	6.42 ± 0.10
TC05001228.hg.1	OTTHUMG00000162519	6.37 ± 0.16	6.42 ± 0.12	6.29 ± 0.33	6.57 ± 0.19
TC05001537.hg.1	OTTHUMG00000162533	5.36 ± 0.15	5.15 ± 0.09	5.49 ± 0.08	5.39 ± 0.10
TC05001535.hg.1	OTTHUMG00000162534	6.98 ± 0.13	6.84 ± 0.09	6.98 ± 0.07	6.84 ± 0.12
TC05001539.hg.1	OTTHUMG00000162535	6.23 ± 0.17	6.17 ± 0.01	6.34 ± 0.11	6.22 ± 0.04
TC05001541.hg.1	OTTHUMG00000162539	6.10 ± 0.38	6.04 ± 0.04	6.03 ± 0.05	6.14 ± 0.14
TC05001111.hg.1	OTTHUMG00000162543	6.57 ± 0.12	6.49 ± 0.11	6.54 ± 0.14	6.58 ± 0.12
TC05000393.hg.1	OTTHUMG00000162549	4.37 ± 0.23	4.11 ± 0.10	4.43 ± 0.19	4.18 ± 0.08
TC05001407.hg.1	OTTHUMG00000162551	5.23 ± 0.42	5.10 ± 0.08	4.95 ± 0.11	5.03 ± 0.29
TC05000405.hg.1	OTTHUMG00000162555	7.08 ± 0.28	7.02 ± 0.08	7.09 ± 0.11	7.06 ± 0.08
TC05000409.hg.1	OTTHUMG00000162559	4.74 ± 0.12	4.57 ± 0.13	4.67 ± 0.04	4.64 ± 0.14
TC05001439.hg.1	OTTHUMG00000162564	4.16 ± 0.19	4.02 ± 0.06	4.16 ± 0.06	4.09 ± 0.13
TC05001542.hg.1	OTTHUMG00000162565	6.45 ± 0.26	6.31 ± 0.14	6.37 ± 0.03	6.50 ± 0.19
TC05001526.hg.1	OTTHUMG00000162569	6.59 ± 0.15	6.46 ± 0.07	6.38 ± 0.03	6.46 ± 0.04
TC05001559.hg.1	OTTHUMG00000162573	10.23 ± 0.07	10.10 ± 0.02	10.23 ± 0.12	10.12 ± 0.07
TC05000419.hg.1	OTTHUMG00000162575	3.55 ± 0.10	3.39 ± 0.06	3.62 ± 0.14	3.54 ± 0.11
TC05001561.hg.1	OTTHUMG00000162576	4.04 ± 0.03	4.08 ± 0.14	4.07 ± 0.17	4.09 ± 0.14
TC05000421.hg.1	OTTHUMG00000162578	4.18 ± 0.06	4.29 ± 0.09	4.19 ± 0.10	4.37 ± 0.05
TC05000420.hg.1	OTTHUMG00000162579	3.74 ± 0.11	3.33 ± 0.12	3.49 ± 0.22	3.48 ± 0.16
TC03000829.hg.1	OTTHUMG00000162581	6.30 ± 0.07	6.31 ± 0.05	6.33 ± 0.10	6.26 ± 0.06
TC03001619.hg.1	OTTHUMG00000162582	7.53 ± 0.30	7.56 ± 0.11	7.56 ± 0.12	7.68 ± 0.09
TC05001564.hg.1	OTTHUMG00000162584	3.63 ± 0.05	3.48 ± 0.05	3.63 ± 0.10	3.53 ± 0.05
TC05000425.hg.1	OTTHUMG00000162587	4.39 ± 0.08	4.17 ± 0.02	4.30 ± 0.18	4.26 ± 0.03
TC03001762.hg.1	OTTHUMG00000162591	6.14 ± 0.12	5.99 ± 0.17	6.03 ± 0.16	5.97 ± 0.02
TC05000432.hg.1	OTTHUMG00000162592	4.17 ± 0.26	3.98 ± 0.10	4.17 ± 0.13	4.17 ± 0.15
TC05001572.hg.1	OTTHUMG00000162593	4.58 ± 0.13	4.50 ± 0.05	4.55 ± 0.11	4.50 ± 0.06
TC07000470.hg.1	OTTHUMG00000162597	6.81 ± 0.18	6.63 ± 0.10	6.88 ± 0.09	6.84 ± 0.10
TC05000440.hg.1	OTTHUMG00000162602	3.81 ± 0.21	3.60 ± 0.04	3.65 ± 0.16	3.58 ± 0.03
TC05000439.hg.1	OTTHUMG00000162603	4.61 ± 0.27	4.67 ± 0.11	4.61 ± 0.23	4.72 ± 0.10
TC03000358.hg.1	OTTHUMG00000162604	3.66 ± 0.19	3.59 ± 0.06	3.51 ± 0.04	3.57 ± 0.01
TC05000429.hg.1	OTTHUMG00000162606	5.21 ± 0.11	4.96 ± 0.02	5.18 ± 0.07	5.05 ± 0.19

TC05000448.hg.1	OTTHUMG00000162608	4.65 ± 0.26	4.44 ± 0.14	4.52 ± 0.24	4.43 ± 0.05
TC05000447.hg.1	OTTHUMG00000162609	4.88 ± 0.13	4.78 ± 0.07	4.76 ± 0.10	4.82 ± 0.05
TC05001582.hg.1	OTTHUMG00000162621	4.47 ± 0.11	4.37 ± 0.04	4.37 ± 0.01	4.38 ± 0.07
TC05000418.hg.1	OTTHUMG00000162622	5.44 ± 0.21	5.35 ± 0.06	5.38 ± 0.09	5.40 ± 0.07
TC05000449.hg.1	OTTHUMG00000162625	4.61 ± 0.14	4.54 ± 0.02	4.60 ± 0.07	4.59 ± 0.19
TC05001594.hg.1	OTTHUMG00000162626	5.22 ± 0.16	5.23 ± 0.19	5.39 ± 0.07	5.39 ± 0.13
TC05000435.hg.1	OTTHUMG00000162629	7.92 ± 0.09	7.91 ± 0.07	7.94 ± 0.06	7.95 ± 0.04
TC05000437.hg.1	OTTHUMG00000162630	5.56 ± 0.09	5.46 ± 0.01	5.44 ± 0.05	5.45 ± 0.07
TC05001578.hg.1	OTTHUMG00000162631	6.15 ± 0.13	6.15 ± 0.11	6.09 ± 0.16	6.33 ± 0.12
TC05000349.hg.1	OTTHUMG00000162637	7.29 ± 0.16	7.22 ± 0.12	7.22 ± 0.05	7.35 ± 0.07
TC03000651.hg.1	OTTHUMG00000162638	7.35 ± 0.25	7.32 ± 0.06	7.22 ± 0.16	7.48 ± 0.15
TC03000652.hg.1	OTTHUMG00000162639	7.64 ± 0.13	7.63 ± 0.17	7.54 ± 0.13	7.67 ± 0.05
TC05001597.hg.1	OTTHUMG00000162645	6.37 ± 0.13	6.25 ± 0.16	6.38 ± 0.17	6.35 ± 0.07
TC03001730.hg.1	OTTHUMG00000162648	8.45 ± 0.37	8.71 ± 0.10	8.45 ± 0.09	8.96 ± 0.16
TC05000395.hg.1	OTTHUMG00000162657	6.79 ± 0.11	6.63 ± 0.03	6.56 ± 0.13	6.78 ± 0.05
TC05001523.hg.1	OTTHUMG00000162658	7.84 ± 0.33	7.86 ± 0.09	7.72 ± 0.19	8.01 ± 0.17
TC05000438.hg.1	OTTHUMG00000162669	5.77 ± 0.34	5.98 ± 0.08	6.03 ± 0.08	6.13 ± 0.04
TC05001580.hg.1	OTTHUMG00000162670	9.18 ± 0.23	9.14 ± 0.06	9.14 ± 0.12	9.28 ± 0.10
TC05000450.hg.1	OTTHUMG00000162672	7.52 ± 0.41	7.50 ± 0.09	7.72 ± 0.30	7.84 ± 0.38
TC05001591.hg.1	OTTHUMG00000162673	4.98 ± 0.10	4.90 ± 0.09	4.81 ± 0.07	5.07 ± 0.15
TC05001592.hg.1	OTTHUMG00000162675	4.23 ± 0.04	4.16 ± 0.02	4.24 ± 0.16	4.06 ± 0.03
TC03001735.hg.1	OTTHUMG00000162677	6.05 ± 0.04	5.98 ± 0.23	5.96 ± 0.12	6.07 ± 0.16
TC05000624.hg.1	OTTHUMG00000162685	7.95 ± 0.11	7.92 ± 0.11	8.07 ± 0.16	7.98 ± 0.12
TC05001759.hg.1	OTTHUMG00000162693	3.64 ± 0.13	3.50 ± 0.04	3.60 ± 0.26	3.58 ± 0.11
TC03001738.hg.1	OTTHUMG00000162696	8.21 ± 0.26	8.28 ± 0.15	8.20 ± 0.17	8.32 ± 0.05
TC05000475.hg.1	OTTHUMG00000162701	4.61 ± 0.19	4.45 ± 0.10	4.52 ± 0.07	4.54 ± 0.10
TC05000474.hg.1	OTTHUMG00000162702	7.29 ± 0.13	7.42 ± 0.28	7.16 ± 0.14	7.37 ± 0.17
TC05001575.hg.1	OTTHUMG00000162703	5.18 ± 0.13	5.10 ± 0.11	5.03 ± 0.08	5.06 ± 0.03
TC05001576.hg.1	OTTHUMG00000162703	4.16 ± 0.14	4.30 ± 0.20	4.28 ± 0.08	4.42 ± 0.26
TC05000651.hg.1	OTTHUMG00000162705	6.23 ± 0.21	6.27 ± 0.06	6.28 ± 0.06	6.35 ± 0.07
TC05000650.hg.1	OTTHUMG00000162708	8.95 ± 0.10	8.93 ± 0.12	8.92 ± 0.12	8.93 ± 0.08

TC05000476.hg.1	OTTHUMG00000162710	4.79 ± 0.08	4.90 ± 0.10	4.85 ± 0.12	5.06 ± 0.09
TC05001625.hg.1	OTTHUMG00000162711	4.43 ± 0.10	4.41 ± 0.09	4.44 ± 0.07	4.43 ± 0.08
TC05000653.hg.1	OTTHUMG00000162723	5.73 ± 0.11	5.83 ± 0.29	5.54 ± 0.38	5.97 ± 0.09
TC05000655.hg.1	OTTHUMG00000162724	5.20 ± 0.16	5.23 ± 0.05	5.18 ± 0.21	5.19 ± 0.17
TC05000654.hg.1	OTTHUMG00000162725	7.95 ± 0.33	7.84 ± 0.14	7.68 ± 0.27	7.97 ± 0.14
TC05001782.hg.1	OTTHUMG00000162726	4.86 ± 0.16	4.77 ± 0.18	5.18 ± 0.25	4.97 ± 0.11
TC05000652.hg.1	OTTHUMG00000162728	7.25 ± 0.26	7.39 ± 0.15	7.36 ± 0.06	7.45 ± 0.03
TC05001607.hg.1	OTTHUMG00000162735	5.52 ± 0.11	5.50 ± 0.11	5.64 ± 0.02	5.49 ± 0.07
TC05000463.hg.1	OTTHUMG00000162740	6.13 ± 0.23	5.95 ± 0.09	5.90 ± 0.20	6.01 ± 0.11
TC05001609.hg.1	OTTHUMG00000162741	7.90 ± 0.10	7.94 ± 0.12	7.78 ± 0.19	8.03 ± 0.16
TC05000430.hg.1	OTTHUMG00000162750	5.79 ± 0.12	5.74 ± 0.05	5.70 ± 0.08	5.85 ± 0.14
TC05000492.hg.1	OTTHUMG00000162756	4.59 ± 0.12	4.47 ± 0.12	4.56 ± 0.12	4.46 ± 0.09
TC05000494.hg.1	OTTHUMG00000162758	4.89 ± 0.35	4.83 ± 0.06	4.85 ± 0.12	4.98 ± 0.12
TC05000482.hg.1	OTTHUMG00000162763	5.34 ± 0.07	5.42 ± 0.15	5.36 ± 0.11	5.60 ± 0.14
TC05001550.hg.1	OTTHUMG00000162767	5.96 ± 0.37	5.85 ± 0.03	5.82 ± 0.07	5.84 ± 0.07
TC05001549.hg.1	OTTHUMG00000162768	4.58 ± 0.10	4.29 ± 0.17	4.53 ± 0.14	4.51 ± 0.12
TC05001548.hg.1	OTTHUMG00000162772	4.35 ± 0.24	3.97 ± 0.08	4.01 ± 0.09	3.86 ± 0.07
TC05000496.hg.1	OTTHUMG00000162774	10.10 ± 0.26	9.92 ± 0.08	10.10 ± 0.03	9.98 ± 0.02
TC05000500.hg.1	OTTHUMG00000162778	5.44 ± 0.20	5.27 ± 0.03	5.30 ± 0.06	5.47 ± 0.18
TC05001648.hg.1	OTTHUMG00000162779	5.71 ± 0.20	5.63 ± 0.11	5.77 ± 0.11	5.85 ± 0.13
TC05001551.hg.1	OTTHUMG00000162780	6.72 ± 0.20	6.62 ± 0.06	6.68 ± 0.21	6.85 ± 0.23
TC05001552.hg.1	OTTHUMG00000162781	4.63 ± 0.23	4.37 ± 0.21	4.53 ± 0.29	4.40 ± 0.18
TC05001646.hg.1	OTTHUMG00000162785	5.73 ± 0.04	5.78 ± 0.23	5.81 ± 0.09	5.74 ± 0.21
TC05001560.hg.1	OTTHUMG00000162786	3.50 ± 0.11	3.22 ± 0.10	3.26 ± 0.09	3.23 ± 0.11
TC05000424.hg.1	OTTHUMG00000162791	3.70 ± 0.23	3.25 ± 0.18	3.31 ± 0.09	3.23 ± 0.11
TC05001563.hg.1	OTTHUMG00000162792	4.76 ± 0.16	4.36 ± 0.01	4.75 ± 0.21	4.49 ± 0.04
TC05000422.hg.1	OTTHUMG00000162794	3.37 ± 0.24	3.19 ± 0.09	3.36 ± 0.11	3.24 ± 0.13
TC05000455.hg.1	OTTHUMG00000162795	4.95 ± 0.07	4.88 ± 0.01	5.05 ± 0.14	4.95 ± 0.06
TC05000454.hg.1	OTTHUMG00000162796	5.04 ± 0.14	5.17 ± 0.22	5.15 ± 0.25	5.46 ± 0.17
TC05001656.hg.1	OTTHUMG00000162799	6.00 ± 0.24	5.77 ± 0.04	5.85 ± 0.04	6.05 ± 0.32
TC05001636.hg.1	OTTHUMG00000162801	4.76 ± 0.19	4.60 ± 0.06	4.60 ± 0.10	4.61 ± 0.07

TC05000489.hg.1	OTTHUMG00000162804	4.70 ± 0.10	4.79 ± 0.10	4.61 ± 0.11	4.78 ± 0.02
TC05001637.hg.1	OTTHUMG00000162806	4.52 ± 0.04	4.45 ± 0.10	4.58 ± 0.09	4.47 ± 0.12
TC05000443.hg.1	OTTHUMG00000162807	4.89 ± 0.09	4.84 ± 0.12	4.87 ± 0.06	4.97 ± 0.14
TC05001587.hg.1	OTTHUMG00000162808	4.99 ± 0.18	5.38 ± 0.32	5.43 ± 0.44	5.21 ± 0.08
TC05001645.hg.1	OTTHUMG00000162810	4.42 ± 0.16	4.22 ± 0.08	4.32 ± 0.08	4.41 ± 0.14
TC05001658.hg.1	OTTHUMG00000162814	5.62 ± 0.07	5.57 ± 0.03	5.62 ± 0.05	5.62 ± 0.04
TC05000445.hg.1	OTTHUMG00000162816	5.02 ± 0.17	4.85 ± 0.04	5.00 ± 0.21	4.92 ± 0.03
TC05001600.hg.1	OTTHUMG00000162817	6.28 ± 0.51	6.45 ± 0.17	6.82 ± 0.58	6.33 ± 0.10
TC05001613.hg.1	OTTHUMG00000162820	4.09 ± 0.08	4.07 ± 0.05	4.07 ± 0.22	4.27 ± 0.10
TC05000464.hg.1	OTTHUMG00000162821	5.67 ± 0.21	5.46 ± 0.15	5.54 ± 0.06	5.61 ± 0.07
TC05001611.hg.1	OTTHUMG00000162822	5.52 ± 0.16	5.45 ± 0.33	5.45 ± 0.12	5.70 ± 0.25
TC05001616.hg.1	OTTHUMG00000162823	5.42 ± 0.08	5.38 ± 0.14	5.53 ± 0.11	5.33 ± 0.05
TC05000467.hg.1	OTTHUMG00000162824	4.87 ± 0.14	4.79 ± 0.06	4.72 ± 0.13	4.86 ± 0.06
TC05000465.hg.1	OTTHUMG00000162825	5.56 ± 0.18	5.54 ± 0.21	5.57 ± 0.07	5.73 ± 0.17
TC05000468.hg.1	OTTHUMG00000162826	4.83 ± 0.07	4.71 ± 0.11	4.71 ± 0.12	4.66 ± 0.16
TC05000778.hg.1	OTTHUMG00000162829	6.13 ± 0.15	6.25 ± 0.11	6.19 ± 0.07	6.16 ± 0.10
TC05001874.hg.1	OTTHUMG00000162830	6.54 ± 0.28	6.52 ± 0.06	6.58 ± 0.12	6.57 ± 0.09
TC05000514.hg.1	OTTHUMG00000162833	3.80 ± 0.12	3.54 ± 0.07	3.65 ± 0.12	3.56 ± 0.04
TC05000470.hg.1	OTTHUMG00000162841	7.63 ± 0.24	7.71 ± 0.24	7.83 ± 0.41	7.61 ± 0.17
TC05001672.hg.1	OTTHUMG00000162845	5.75 ± 0.31	5.87 ± 0.06	5.80 ± 0.15	5.92 ± 0.11
TC05001671.hg.1	OTTHUMG00000162847	6.30 ± 0.28	6.52 ± 0.06	6.30 ± 0.28	6.45 ± 0.16
TC05001663.hg.1	OTTHUMG00000162848	6.54 ± 0.10	6.67 ± 0.08	6.64 ± 0.12	6.57 ± 0.15
TC05001661.hg.1	OTTHUMG00000162849	4.49 ± 0.27	4.44 ± 0.26	4.49 ± 0.13	4.74 ± 0.32
TC05000786.hg.1	OTTHUMG00000162861	5.20 ± 0.09	5.47 ± 0.23	5.22 ± 0.27	5.24 ± 0.29
TC05001888.hg.1	OTTHUMG00000162862	6.10 ± 0.65	6.76 ± 0.39	6.53 ± 0.60	6.39 ± 0.34
TC05000536.hg.1	OTTHUMG00000162864	6.19 ± 0.13	6.17 ± 0.20	6.20 ± 0.21	6.27 ± 0.10
TC05001623.hg.1	OTTHUMG00000162865	3.91 ± 0.18	3.72 ± 0.10	3.73 ± 0.17	3.77 ± 0.18
TC05001618.hg.1	OTTHUMG00000162867	5.79 ± 0.04	5.77 ± 0.06	5.74 ± 0.11	5.81 ± 0.17
TC05001624.hg.1	OTTHUMG00000162869	5.39 ± 0.07	5.42 ± 0.08	5.32 ± 0.07	5.38 ± 0.04
TC05001681.hg.1	OTTHUMG00000162871	5.72 ± 0.16	5.64 ± 0.03	5.70 ± 0.05	5.69 ± 0.05
TC05000542.hg.1	OTTHUMG00000162872	4.45 ± 0.16	4.30 ± 0.08	4.35 ± 0.17	4.35 ± 0.02

TC05000539.hg.1	OTTHUMG00000162873	6.69 ± 0.19	6.72 ± 0.13	6.75 ± 0.18	6.75 ± 0.06
TC05000909.hg.1	OTTHUMG00000162874	4.54 ± 0.29	4.38 ± 0.10	4.32 ± 0.15	4.26 ± 0.11
TC05002023.hg.1	OTTHUMG00000162876	6.11 ± 0.17	6.15 ± 0.10	6.09 ± 0.06	6.36 ± 0.12
TC05000910.hg.1	OTTHUMG00000162877	5.37 ± 0.13	5.32 ± 0.02	5.32 ± 0.01	5.37 ± 0.07
TC05000554.hg.1	OTTHUMG00000162881	5.18 ± 0.17	5.10 ± 0.22	5.02 ± 0.10	5.43 ± 0.27
TC05000553.hg.1	OTTHUMG00000162882	4.97 ± 0.23	4.65 ± 0.07	4.85 ± 0.07	5.01 ± 0.22
TC05001702.hg.1	OTTHUMG00000162883	4.94 ± 0.09	4.77 ± 0.04	4.76 ± 0.01	4.81 ± 0.05
TC05001703.hg.1	OTTHUMG00000162884	5.24 ± 0.26	5.17 ± 0.16	5.08 ± 0.14	5.34 ± 0.22
TC05002024.hg.1	OTTHUMG00000162886	5.19 ± 0.18	5.05 ± 0.07	5.14 ± 0.08	5.14 ± 0.06
TC05000556.hg.1	OTTHUMG00000162888	4.64 ± 0.03	4.49 ± 0.01	4.54 ± 0.05	4.58 ± 0.08
TC05001486.hg.1	OTTHUMG00000162890	5.59 ± 0.21	5.56 ± 0.11	5.60 ± 0.01	5.63 ± 0.07
TC05000527.hg.1	OTTHUMG00000162896	3.51 ± 0.10	3.50 ± 0.04	3.43 ± 0.07	3.53 ± 0.10
TC05000529.hg.1	OTTHUMG00000162899	5.33 ± 0.12	5.29 ± 0.08	5.27 ± 0.07	5.40 ± 0.14
TC05000528.hg.1	OTTHUMG00000162900	6.22 ± 0.25	6.10 ± 0.10	6.13 ± 0.09	6.18 ± 0.04
TC05000532.hg.1	OTTHUMG00000162901	5.12 ± 0.09	5.02 ± 0.02	5.01 ± 0.08	5.20 ± 0.22
TC05002027.hg.1	OTTHUMG00000162904	5.19 ± 0.13	4.97 ± 0.11	4.98 ± 0.06	5.04 ± 0.14
TC05001686.hg.1	OTTHUMG00000162912	6.69 ± 0.75	6.85 ± 0.40	7.05 ± 0.61	6.62 ± 0.21
TC05000543.hg.1	OTTHUMG00000162913	3.88 ± 0.20	3.90 ± 0.09	3.79 ± 0.12	3.77 ± 0.04
TC05002031.hg.1	OTTHUMG00000162917	5.38 ± 0.26	5.20 ± 0.16	5.28 ± 0.16	5.35 ± 0.18
TC05002029.hg.1	OTTHUMG00000162918	4.89 ± 0.01	4.86 ± 0.05	4.91 ± 0.06	5.01 ± 0.11
TC05002032.hg.1	OTTHUMG00000162919	6.26 ± 0.17	6.25 ± 0.12	6.12 ± 0.36	6.39 ± 0.09
TC05001699.hg.1	OTTHUMG00000162923	4.06 ± 0.05	3.99 ± 0.14	4.05 ± 0.07	4.07 ± 0.17
TC05002033.hg.1	OTTHUMG00000162925	4.96 ± 0.15	4.80 ± 0.16	4.92 ± 0.33	4.78 ± 0.13
TC05001711.hg.1	OTTHUMG00000162926	5.20 ± 0.09	5.07 ± 0.13	5.14 ± 0.19	5.21 ± 0.08
TC05000570.hg.1	OTTHUMG00000162927	4.72 ± 0.07	4.66 ± 0.17	4.64 ± 0.19	4.81 ± 0.27
TC05000571.hg.1	OTTHUMG00000162941	5.34 ± 0.08	5.10 ± 0.20	5.23 ± 0.12	5.33 ± 0.07
TC05001704.hg.1	OTTHUMG00000162951	5.31 ± 0.19	5.16 ± 0.07	5.25 ± 0.10	5.25 ± 0.10
TC05000558.hg.1	OTTHUMG00000162954	5.22 ± 0.12	5.09 ± 0.11	5.14 ± 0.04	5.18 ± 0.03
TC05000559.hg.1	OTTHUMG00000162955	5.31 ± 0.13	5.23 ± 0.01	5.26 ± 0.02	5.29 ± 0.09
TC05001707.hg.1	OTTHUMG00000162961	5.22 ± 0.10	5.08 ± 0.01	5.11 ± 0.04	5.13 ± 0.11
TC05001705.hg.1	OTTHUMG00000162962	4.56 ± 0.18	4.58 ± 0.05	4.53 ± 0.16	4.52 ± 0.02

TC05002039.hg.1	OTTHUMG00000162964	5.66 ± 0.18	5.74 ± 0.09	5.55 ± 0.11	5.69 ± 0.18
TC05002036.hg.1	OTTHUMG00000162965	4.68 ± 0.12	4.56 ± 0.11	4.52 ± 0.08	4.74 ± 0.18
TC05002035.hg.1	OTTHUMG00000162966	4.98 ± 0.09	4.86 ± 0.09	4.85 ± 0.05	4.89 ± 0.11
TC05002038.hg.1	OTTHUMG00000162967	6.06 ± 0.14	6.09 ± 0.13	6.00 ± 0.10	6.16 ± 0.15
TC05001715.hg.1	OTTHUMG00000162968	5.78 ± 0.15	5.63 ± 0.11	5.83 ± 0.09	5.71 ± 0.11
TC05001717.hg.1	OTTHUMG00000162969	4.89 ± 0.36	4.90 ± 0.10	4.91 ± 0.20	5.15 ± 0.21
TC05001716.hg.1	OTTHUMG00000162974	4.93 ± 0.16	4.86 ± 0.14	4.92 ± 0.24	4.96 ± 0.12
TC05000579.hg.1	OTTHUMG00000162982	7.05 ± 0.21	6.95 ± 0.09	6.97 ± 0.27	7.10 ± 0.15
TC05001720.hg.1	OTTHUMG00000162984	5.13 ± 0.07	4.98 ± 0.03	5.01 ± 0.07	5.01 ± 0.03
TC05000552.hg.1	OTTHUMG00000162986	5.77 ± 0.09	5.81 ± 0.04	5.72 ± 0.16	5.80 ± 0.04
TC05000595.hg.1	OTTHUMG00000162989	5.37 ± 0.12	5.19 ± 0.05	5.26 ± 0.03	5.30 ± 0.16
TC05001729.hg.1	OTTHUMG00000162990	4.66 ± 0.20	4.64 ± 0.06	4.54 ± 0.14	4.72 ± 0.33
TC05000594.hg.1	OTTHUMG00000162992	5.46 ± 0.03	5.37 ± 0.06	5.36 ± 0.28	5.45 ± 0.26
TC05000584.hg.1	OTTHUMG00000162993	3.81 ± 0.24	3.58 ± 0.05	3.71 ± 0.07	3.60 ± 0.18
TC05001734.hg.1	OTTHUMG00000162995	4.33 ± 0.04	4.18 ± 0.05	4.21 ± 0.09	4.37 ± 0.15
TC05001730.hg.1	OTTHUMG00000162996	5.71 ± 0.27	5.73 ± 0.20	5.61 ± 0.34	5.91 ± 0.08
TC05000586.hg.1	OTTHUMG00000162997	4.33 ± 0.11	4.46 ± 0.14	4.35 ± 0.12	4.49 ± 0.10
TC05000593.hg.1	OTTHUMG00000162998	4.09 ± 0.07	4.06 ± 0.20	4.03 ± 0.08	4.16 ± 0.09
TC05001436.hg.1	OTTHUMG00000163000	6.21 ± 0.18	6.15 ± 0.09	6.24 ± 0.19	6.10 ± 0.10
TC05003419.hg.1	OTTHUMG00000163003	7.62 ± 0.12	7.50 ± 0.06	7.67 ± 0.05	7.55 ± 0.06
TC05001079.hg.1	OTTHUMG00000163004	8.00 ± 0.18	8.03 ± 0.15	8.04 ± 0.09	8.10 ± 0.06
TC05000599.hg.1	OTTHUMG00000163006	4.54 ± 0.08	4.53 ± 0.16	4.48 ± 0.01	4.50 ± 0.18
TC05000597.hg.1	OTTHUMG00000163008	4.71 ± 0.29	4.26 ± 0.11	4.38 ± 0.31	4.37 ± 0.18
TC05001737.hg.1	OTTHUMG00000163009	3.80 ± 0.08	3.51 ± 0.20	3.50 ± 0.11	3.46 ± 0.08
TC05000596.hg.1	OTTHUMG00000163010	6.45 ± 0.30	6.17 ± 0.17	6.42 ± 0.25	6.38 ± 0.06
TC05001738.hg.1	OTTHUMG00000163011	5.31 ± 0.07	5.08 ± 0.06	5.23 ± 0.12	5.07 ± 0.21
TC05001736.hg.1	OTTHUMG00000163012	3.59 ± 0.13	3.50 ± 0.03	3.52 ± 0.03	3.41 ± 0.05
TC05000598.hg.1	OTTHUMG00000163015	4.85 ± 0.11	4.79 ± 0.07	4.80 ± 0.06	4.93 ± 0.13
TC05002168.hg.1	OTTHUMG00000163016	6.69 ± 0.20	6.56 ± 0.14	6.51 ± 0.20	6.68 ± 0.09
TC05000600.hg.1	OTTHUMG00000163017	4.84 ± 0.30	4.84 ± 0.15	4.77 ± 0.14	5.00 ± 0.12
TC05001740.hg.1	OTTHUMG00000163018	6.26 ± 0.14	6.27 ± 0.08	6.29 ± 0.10	6.29 ± 0.06

TC05001745.hg.1	OTTHUMG00000163029	5.41 ± 0.14	5.33 ± 0.09	5.49 ± 0.17	5.28 ± 0.09
TC05001755.hg.1	OTTHUMG00000163040	7.24 ± 0.46	7.47 ± 0.18	7.26 ± 0.32	7.49 ± 0.09
TC05001756.hg.1	OTTHUMG00000163042	5.15 ± 0.15	5.06 ± 0.10	5.12 ± 0.16	5.10 ± 0.03
TC05000656.hg.1	OTTHUMG00000163048	6.51 ± 0.16	6.44 ± 0.17	6.40 ± 0.11	6.57 ± 0.10
TC05000923.hg.1	OTTHUMG00000163055	6.55 ± 0.18	6.56 ± 0.09	6.54 ± 0.14	6.62 ± 0.06
TC05000925.hg.1	OTTHUMG00000163056	6.60 ± 0.18	6.40 ± 0.11	6.40 ± 0.24	6.43 ± 0.10
TC05000922.hg.1	OTTHUMG00000163057	6.74 ± 0.24	6.74 ± 0.02	6.75 ± 0.23	6.77 ± 0.09
TC05001791.hg.1	OTTHUMG00000163058	5.95 ± 0.17	5.90 ± 0.32	5.54 ± 0.07	5.91 ± 0.25
TC05000662.hg.1	OTTHUMG00000163059	7.72 ± 0.22	7.61 ± 0.13	7.68 ± 0.16	7.72 ± 0.05
TC05000671.hg.1	OTTHUMG00000163060	8.22 ± 0.11	8.16 ± 0.03	8.11 ± 0.15	8.15 ± 0.04
TC05001799.hg.1	OTTHUMG00000163061	7.60 ± 0.25	7.61 ± 0.19	7.58 ± 0.15	7.63 ± 0.08
TC05000675.hg.1	OTTHUMG00000163062	6.21 ± 0.30	6.23 ± 0.17	6.08 ± 0.10	6.38 ± 0.02
TC05001787.hg.1	OTTHUMG00000163067	5.19 ± 0.16	5.21 ± 0.17	5.25 ± 0.21	5.24 ± 0.14
TC05000660.hg.1	OTTHUMG00000163068	7.10 ± 0.15	7.29 ± 0.31	7.18 ± 0.37	7.13 ± 0.16
TC05000661.hg.1	OTTHUMG00000163069	7.94 ± 0.32	7.94 ± 0.18	7.83 ± 0.29	7.93 ± 0.08
TC05001789.hg.1	OTTHUMG00000163076	4.28 ± 0.03	3.99 ± 0.11	4.22 ± 0.09	4.18 ± 0.02
TC05001798.hg.1	OTTHUMG00000163077	8.73 ± 0.29	8.79 ± 0.19	8.77 ± 0.33	8.80 ± 0.01
TC05002047.hg.1	OTTHUMG00000163084	5.07 ± 0.20	5.06 ± 0.03	5.00 ± 0.16	4.98 ± 0.05
TC05000678.hg.1	OTTHUMG00000163102	7.17 ± 0.08	7.10 ± 0.04	7.22 ± 0.24	7.09 ± 0.07
TC05000677.hg.1	OTTHUMG00000163105	7.18 ± 0.24	7.13 ± 0.15	7.25 ± 0.13	7.24 ± 0.03
TC05000676.hg.1	OTTHUMG00000163110	5.90 ± 0.12	5.99 ± 0.28	5.86 ± 0.03	6.11 ± 0.19
TC05000513.hg.1	OTTHUMG00000163114	4.08 ± 0.08	3.99 ± 0.05	3.97 ± 0.08	3.91 ± 0.09
TC05000512.hg.1	OTTHUMG00000163118	4.38 ± 0.23	4.13 ± 0.03	4.16 ± 0.05	4.11 ± 0.10
TC05001741.hg.1	OTTHUMG00000163123	5.46 ± 0.22	5.36 ± 0.13	5.36 ± 0.08	5.47 ± 0.02
TC05000971.hg.1	OTTHUMG00000163124	5.99 ± 0.13	6.10 ± 0.03	6.04 ± 0.17	6.26 ± 0.12
TC05000972.hg.1	OTTHUMG00000163128	6.31 ± 0.19	6.22 ± 0.12	6.19 ± 0.07	6.32 ± 0.08
TC05000975.hg.1	OTTHUMG00000163129	6.61 ± 0.24	6.55 ± 0.08	6.60 ± 0.22	6.72 ± 0.08
TC05000970.hg.1	OTTHUMG00000163130	6.26 ± 0.16	6.25 ± 0.15	6.10 ± 0.15	6.41 ± 0.16
TC05000978.hg.1	OTTHUMG00000163132	4.97 ± 0.16	4.62 ± 0.11	4.81 ± 0.15	4.68 ± 0.07
TC05000977.hg.1	OTTHUMG00000163136	4.42 ± 0.17	4.36 ± 0.18	4.25 ± 0.11	4.42 ± 0.02
TC05000973.hg.1	OTTHUMG00000163137	7.06 ± 0.08	6.96 ± 0.20	7.01 ± 0.12	7.09 ± 0.03

TC05000614.hg.1	OTTHUMG00000163145	3.53 ± 0.03	3.50 ± 0.02	3.50 ± 0.08	3.50 ± 0.13
TC05002053.hg.1	OTTHUMG00000163146	5.90 ± 0.14	5.91 ± 0.08	5.83 ± 0.17	6.04 ± 0.07
TC05000936.hg.1	OTTHUMG00000163147	5.52 ± 0.28	5.38 ± 0.10	5.53 ± 0.13	5.53 ± 0.03
TC05002054.hg.1	OTTHUMG00000163148	5.36 ± 0.10	5.34 ± 0.16	5.23 ± 0.12	5.50 ± 0.13
TC05000502.hg.1	OTTHUMG00000163150	3.85 ± 0.14	3.63 ± 0.07	3.63 ± 0.02	3.67 ± 0.08
TC05000503.hg.1	OTTHUMG00000163152	5.32 ± 0.30	5.35 ± 0.18	5.32 ± 0.20	5.55 ± 0.20
TC05001683.hg.1	OTTHUMG00000163153	6.33 ± 0.17	6.35 ± 0.20	6.33 ± 0.17	6.49 ± 0.19
TC05001682.hg.1	OTTHUMG00000163155	5.59 ± 0.17	5.29 ± 0.14	5.47 ± 0.29	5.45 ± 0.23
TC05000792.hg.1	OTTHUMG00000163168	5.39 ± 0.15	5.16 ± 0.12	5.19 ± 0.14	5.42 ± 0.13
TC05000787.hg.1	OTTHUMG00000163170	6.10 ± 0.30	6.02 ± 0.04	6.13 ± 0.13	5.98 ± 0.17
TC05000788.hg.1	OTTHUMG00000163171	5.81 ± 0.23	5.75 ± 0.09	5.85 ± 0.09	5.70 ± 0.07
TC05001889.hg.1	OTTHUMG00000163174	5.93 ± 0.23	5.81 ± 0.19	5.82 ± 0.11	5.94 ± 0.10
TC05002134.hg.1	OTTHUMG00000163175	7.13 ± 0.22	7.24 ± 0.21	7.13 ± 0.12	7.33 ± 0.11
TC05000989.hg.1	OTTHUMG00000163178	5.94 ± 0.13	5.72 ± 0.06	5.82 ± 0.08	5.81 ± 0.13
TC05000987.hg.1	OTTHUMG00000163179	6.84 ± 0.04	6.89 ± 0.22	6.86 ± 0.06	7.01 ± 0.13
TC05000986.hg.1	OTTHUMG00000163180	5.96 ± 0.05	5.87 ± 0.12	5.98 ± 0.04	6.07 ± 0.11
TC05000984.hg.1	OTTHUMG00000163182	9.92 ± 0.59	9.27 ± 0.20	10.39 ± 0.22	9.03 ± 0.18
TC05002085.hg.1	OTTHUMG00000163183	7.39 ± 0.18	7.49 ± 0.24	7.33 ± 0.22	7.61 ± 0.25
TC05002088.hg.1	OTTHUMG00000163185	5.90 ± 0.33	5.79 ± 0.12	5.84 ± 0.10	5.79 ± 0.06
TC05000995.hg.1	OTTHUMG00000163187	6.04 ± 0.04	5.85 ± 0.07	5.95 ± 0.04	5.84 ± 0.04
TC05000996.hg.1	OTTHUMG00000163188	5.75 ± 0.28	5.72 ± 0.07	5.62 ± 0.21	5.77 ± 0.05
TC05002089.hg.1	OTTHUMG00000163189	6.17 ± 0.03	6.08 ± 0.02	6.13 ± 0.27	6.14 ± 0.01
TC05002086.hg.1	OTTHUMG00000163190	7.06 ± 0.11	6.96 ± 0.09	6.86 ± 0.10	7.01 ± 0.07
TC05001043.hg.1	OTTHUMG00000163201	8.08 ± 0.14	7.96 ± 0.04	8.04 ± 0.19	8.06 ± 0.17
TC05001049.hg.1	OTTHUMG00000163211	7.84 ± 0.27	7.86 ± 0.16	7.82 ± 0.16	7.92 ± 0.05
TC05000686.hg.1	OTTHUMG00000163214	5.90 ± 0.13	5.89 ± 0.05	5.82 ± 0.07	5.93 ± 0.05
TC05000687.hg.1	OTTHUMG00000163215	6.30 ± 0.36	6.35 ± 0.08	6.24 ± 0.08	6.60 ± 0.13
TC05001814.hg.1	OTTHUMG00000163220	4.91 ± 0.05	4.79 ± 0.04	4.74 ± 0.05	5.02 ± 0.25
TC05000694.hg.1	OTTHUMG00000163223	5.11 ± 0.23	5.07 ± 0.03	4.99 ± 0.06	5.08 ± 0.13
TC05002098.hg.1	OTTHUMG00000163224	5.98 ± 0.21	5.97 ± 0.06	5.90 ± 0.17	5.92 ± 0.07
TC05002099.hg.1	OTTHUMG00000163226	8.41 ± 0.31	8.41 ± 0.18	8.30 ± 0.25	8.44 ± 0.05

TC05001006.hg.1	OTTHUMG00000163231	7.79 ± 0.10	7.80 ± 0.27	7.74 ± 0.31	7.98 ± 0.10
TC05000546.hg.1	OTTHUMG00000163233	4.92 ± 0.25	4.58 ± 0.06	4.90 ± 0.10	4.77 ± 0.13
TC05001691.hg.1	OTTHUMG00000163234	6.38 ± 0.72	7.33 ± 0.17	6.45 ± 0.36	6.86 ± 0.32
TC05000582.hg.1	OTTHUMG00000163244	5.37 ± 0.16	5.27 ± 0.09	5.32 ± 0.17	5.26 ± 0.18
TC05001728.hg.1	OTTHUMG00000163248	4.37 ± 0.15	4.54 ± 0.11	4.50 ± 0.05	4.61 ± 0.08
TC05000592.hg.1	OTTHUMG00000163249	4.53 ± 0.24	4.35 ± 0.07	4.49 ± 0.07	4.38 ± 0.04
TC05000591.hg.1	OTTHUMG00000163251	5.29 ± 0.29	5.12 ± 0.06	5.11 ± 0.02	5.11 ± 0.12
TC05000590.hg.1	OTTHUMG00000163252	5.14 ± 0.16	4.95 ± 0.07	5.10 ± 0.09	5.20 ± 0.12
TC05000589.hg.1	OTTHUMG00000163253	5.40 ± 0.23	5.22 ± 0.06	5.39 ± 0.10	5.36 ± 0.15
TC05001733.hg.1	OTTHUMG00000163254	4.83 ± 0.16	4.85 ± 0.40	4.87 ± 0.27	4.54 ± 0.06
TC05000583.hg.1	OTTHUMG00000163257	4.89 ± 0.04	4.90 ± 0.22	4.73 ± 0.17	4.98 ± 0.22
TC05001739.hg.1	OTTHUMG00000163261	5.10 ± 0.08	5.15 ± 0.11	5.15 ± 0.02	5.16 ± 0.04
TC05001055.hg.1	OTTHUMG00000163262	6.70 ± 0.13	6.74 ± 0.05	6.62 ± 0.23	6.65 ± 0.03
TC05001057.hg.1	OTTHUMG00000163263	5.68 ± 0.19	5.75 ± 0.05	5.75 ± 0.12	5.75 ± 0.04
TC05000944.hg.1	OTTHUMG00000163264	6.86 ± 0.23	6.94 ± 0.39	6.84 ± 0.31	7.14 ± 0.09
TC05002064.hg.1	OTTHUMG00000163279	6.88 ± 0.11	6.85 ± 0.11	6.84 ± 0.17	6.85 ± 0.04
TC05001749.hg.1	OTTHUMG00000163283	4.79 ± 0.13	4.76 ± 0.08	4.76 ± 0.09	4.85 ± 0.12
TC05001748.hg.1	OTTHUMG00000163287	5.17 ± 0.17	5.04 ± 0.08	5.13 ± 0.09	5.17 ± 0.08
TC05001059.hg.1	OTTHUMG00000163288	6.17 ± 0.23	6.22 ± 0.26	6.05 ± 0.13	6.28 ± 0.16
TC05002065.hg.1	OTTHUMG00000163289	6.47 ± 0.16	6.41 ± 0.08	6.35 ± 0.12	6.52 ± 0.21
TC05000949.hg.1	OTTHUMG00000163290	6.22 ± 0.20	6.22 ± 0.17	6.20 ± 0.23	6.20 ± 0.05
TC05001669.hg.1	OTTHUMG00000163296	4.70 ± 0.27	4.56 ± 0.04	4.55 ± 0.05	4.64 ± 0.14
TC05000954.hg.1	OTTHUMG00000163301	7.20 ± 0.35	7.26 ± 0.08	7.27 ± 0.09	7.37 ± 0.07
TC05000951.hg.1	OTTHUMG00000163302	6.86 ± 0.27	6.77 ± 0.19	6.80 ± 0.34	6.96 ± 0.14
TC05000950.hg.1	OTTHUMG00000163303	7.53 ± 0.26	7.45 ± 0.19	7.44 ± 0.24	7.52 ± 0.07
TC05001820.hg.1	OTTHUMG00000163307	6.29 ± 0.26	6.25 ± 0.16	6.13 ± 0.10	6.22 ± 0.10
TC05001058.hg.1	OTTHUMG00000163311	7.53 ± 0.12	7.56 ± 0.16	7.52 ± 0.19	7.76 ± 0.09
TC05001751.hg.1	OTTHUMG00000163312	5.36 ± 0.12	5.36 ± 0.20	5.22 ± 0.07	5.30 ± 0.11
TC05000332.hg.1	OTTHUMG00000163315	4.64 ± 0.20	4.41 ± 0.14	4.59 ± 0.11	4.63 ± 0.20
TC05001466.hg.1	OTTHUMG00000163316	5.83 ± 0.12	5.76 ± 0.09	5.87 ± 0.11	5.81 ± 0.04
TC05001456.hg.1	OTTHUMG00000163317	6.80 ± 0.07	6.86 ± 0.36	6.71 ± 0.21	6.50 ± 0.16

TC05001465.hg.1	OTTHUMG00000163319	5.63 ± 0.16	5.58 ± 0.04	5.50 ± 0.20	5.70 ± 0.11
TC05002070.hg.1	OTTHUMG00000163320	7.35 ± 0.29	7.26 ± 0.08	7.31 ± 0.27	7.25 ± 0.10
TC05000961.hg.1	OTTHUMG00000163325	6.35 ± 0.10	6.23 ± 0.04	6.31 ± 0.03	6.26 ± 0.04
TC05002018.hg.1	OTTHUMG00000163326	5.76 ± 0.21	5.74 ± 0.02	5.69 ± 0.05	5.80 ± 0.09
TC05001847.hg.1	OTTHUMG00000163330	7.83 ± 0.37	7.89 ± 0.33	7.95 ± 0.20	7.97 ± 0.08
TC05000716.hg.1	OTTHUMG00000163333	7.54 ± 0.14	7.51 ± 0.15	7.37 ± 0.16	7.68 ± 0.18
TC05001850.hg.1	OTTHUMG00000163334	7.31 ± 0.15	7.53 ± 0.29	7.31 ± 0.25	7.56 ± 0.09
TC05001849.hg.1	OTTHUMG00000163335	5.89 ± 0.27	6.10 ± 0.23	5.87 ± 0.15	6.15 ± 0.07
TC05000966.hg.1	OTTHUMG00000163336	6.45 ± 0.27	6.50 ± 0.16	6.28 ± 0.17	6.60 ± 0.09
TC05002079.hg.1	OTTHUMG00000163337	6.66 ± 0.11	6.62 ± 0.15	6.57 ± 0.14	6.74 ± 0.11
TC05000962.hg.1	OTTHUMG00000163338	6.69 ± 0.16	6.77 ± 0.10	6.71 ± 0.05	6.78 ± 0.11
TC05000965.hg.1	OTTHUMG00000163339	5.71 ± 0.10	5.48 ± 0.09	5.57 ± 0.25	5.73 ± 0.11
TC05000960.hg.1	OTTHUMG00000163340	5.95 ± 0.07	5.89 ± 0.05	5.96 ± 0.08	5.98 ± 0.06
TC05000963.hg.1	OTTHUMG00000163341	7.46 ± 0.27	7.52 ± 0.26	7.39 ± 0.22	7.56 ± 0.07
TC05002078.hg.1	OTTHUMG00000163343	5.93 ± 0.10	5.77 ± 0.17	5.86 ± 0.10	5.97 ± 0.16
TC05001893.hg.1	OTTHUMG00000163345	6.91 ± 0.23	6.84 ± 0.09	6.89 ± 0.02	7.01 ± 0.13
TC05000718.hg.1	OTTHUMG00000163348	8.18 ± 0.31	8.32 ± 0.29	8.25 ± 0.29	8.39 ± 0.08
TC05001866.hg.1	OTTHUMG00000163365	6.30 ± 0.07	6.19 ± 0.13	6.20 ± 0.20	6.22 ± 0.23
TC05001856.hg.1	OTTHUMG00000163370	5.97 ± 0.16	6.00 ± 0.23	5.94 ± 0.10	6.03 ± 0.13
TC05001085.hg.1	OTTHUMG00000163377	8.33 ± 0.28	8.31 ± 0.28	8.22 ± 0.24	8.46 ± 0.08
TC05000794.hg.1	OTTHUMG00000163385	4.66 ± 0.12	4.63 ± 0.04	4.61 ± 0.07	4.63 ± 0.13
TC05000705.hg.1	OTTHUMG00000163387	5.53 ± 0.12	5.41 ± 0.04	5.46 ± 0.07	5.52 ± 0.03
TC05001839.hg.1	OTTHUMG00000163389	7.00 ± 0.27	6.87 ± 0.23	7.27 ± 0.38	6.78 ± 0.30
TC11000286.hg.1	OTTHUMG00000163392	4.76 ± 0.09	4.66 ± 0.12	4.70 ± 0.07	4.70 ± 0.09
TC11000287.hg.1	OTTHUMG00000163393	4.42 ± 0.09	4.32 ± 0.04	4.35 ± 0.08	4.39 ± 0.05
TC05002116.hg.1	OTTHUMG00000163395	6.96 ± 0.11	7.03 ± 0.10	7.14 ± 0.09	7.27 ± 0.06
TC05002109.hg.1	OTTHUMG00000163397	7.40 ± 0.08	7.37 ± 0.11	7.67 ± 0.05	7.38 ± 0.02
TC05001017.hg.1	OTTHUMG00000163398	8.32 ± 0.21	8.09 ± 0.12	8.56 ± 0.27	8.22 ± 0.16
TC05001025.hg.1	OTTHUMG00000163402	7.05 ± 0.13	6.94 ± 0.09	6.83 ± 0.12	6.95 ± 0.10
TC05001022.hg.1	OTTHUMG00000163404	6.39 ± 0.22	6.21 ± 0.08	6.26 ± 0.16	6.23 ± 0.07
TC05001021.hg.1	OTTHUMG00000163405	5.32 ± 0.15	5.41 ± 0.14	5.31 ± 0.03	5.42 ± 0.07

TC05001020.hg.1	OTTHUMG00000163407	7.48 ± 0.06	7.58 ± 0.08	7.52 ± 0.09	7.65 ± 0.07
TC05001908.hg.1	OTTHUMG00000163410	6.41 ± 0.27	6.55 ± 0.12	6.42 ± 0.27	6.59 ± 0.07
TC05000724.hg.1	OTTHUMG00000163412	7.07 ± 0.30	7.48 ± 0.26	7.96 ± 0.72	7.33 ± 0.21
TC05000804.hg.1	OTTHUMG00000163419	3.98 ± 0.10	4.01 ± 0.02	3.97 ± 0.13	4.23 ± 0.26
TC05001913.hg.1	OTTHUMG00000163422	3.89 ± 0.12	3.59 ± 0.04	3.72 ± 0.14	3.67 ± 0.09
TC05000808.hg.1	OTTHUMG00000163424	4.63 ± 0.09	4.65 ± 0.17	4.48 ± 0.11	4.75 ± 0.09
TC05001920.hg.1	OTTHUMG00000163434	5.99 ± 0.12	5.87 ± 0.12	5.95 ± 0.06	5.93 ± 0.02
TC05001902.hg.1	OTTHUMG00000163435	6.39 ± 0.14	6.46 ± 0.07	6.48 ± 0.08	6.64 ± 0.14
TC05001903.hg.1	OTTHUMG00000163435	7.37 ± 0.17	7.42 ± 0.02	7.37 ± 0.05	7.45 ± 0.08
TC05000796.hg.1	OTTHUMG00000163443	4.91 ± 0.16	4.69 ± 0.08	4.91 ± 0.19	4.78 ± 0.06
TC05002080.hg.1	OTTHUMG00000163446	5.16 ± 0.22	5.16 ± 0.06	5.20 ± 0.07	5.21 ± 0.02
TC05002081.hg.1	OTTHUMG00000163447	5.38 ± 0.12	5.23 ± 0.09	5.32 ± 0.13	5.36 ± 0.05
TC05000800.hg.1	OTTHUMG00000163449	6.37 ± 0.12	6.16 ± 0.07	6.39 ± 0.12	6.25 ± 0.06
TC05001975.hg.1	OTTHUMG00000163450	3.97 ± 0.09	3.90 ± 0.05	3.97 ± 0.12	3.99 ± 0.01
TC05001028.hg.1	OTTHUMG00000163453	7.39 ± 0.16	7.33 ± 0.09	7.22 ± 0.05	7.42 ± 0.11
TC05001029.hg.1	OTTHUMG00000163454	5.63 ± 0.26	5.48 ± 0.03	5.55 ± 0.09	5.57 ± 0.05
TC05001027.hg.1	OTTHUMG00000163457	7.66 ± 0.48	7.72 ± 0.23	7.61 ± 0.22	7.76 ± 0.06
TC05001023.hg.1	OTTHUMG00000163460	5.74 ± 0.13	5.65 ± 0.03	5.59 ± 0.05	5.67 ± 0.20
TC05002117.hg.1	OTTHUMG00000163461	6.30 ± 0.08	6.19 ± 0.16	6.27 ± 0.07	6.35 ± 0.09
TC05001830.hg.1	OTTHUMG00000163471	8.05 ± 0.10	7.87 ± 0.09	7.95 ± 0.14	7.90 ± 0.09
TC05000798.hg.1	OTTHUMG00000163475	6.36 ± 0.17	6.21 ± 0.09	6.30 ± 0.15	6.27 ± 0.05
TC05001894.hg.1	OTTHUMG00000163476	4.41 ± 0.06	4.44 ± 0.10	4.52 ± 0.03	4.54 ± 0.28
TC05001984.hg.1	OTTHUMG00000163480	7.28 ± 0.21	7.27 ± 0.25	7.23 ± 0.10	7.37 ± 0.02
TC05001980.hg.1	OTTHUMG00000163481	5.20 ± 0.36	5.11 ± 0.22	5.23 ± 0.17	5.16 ± 0.13
TC05000864.hg.1	OTTHUMG00000163482	6.85 ± 0.23	6.84 ± 0.17	6.89 ± 0.12	6.94 ± 0.25
TC05001985.hg.1	OTTHUMG00000163483	7.21 ± 0.08	7.13 ± 0.02	7.17 ± 0.08	7.16 ± 0.02
TC05001964.hg.1	OTTHUMG00000163486	5.53 ± 0.13	5.53 ± 0.06	5.51 ± 0.07	5.60 ± 0.11
TC05001965.hg.1	OTTHUMG00000163488	5.37 ± 0.03	5.28 ± 0.06	5.24 ± 0.04	5.31 ± 0.08
TC05001982.hg.1	OTTHUMG00000163496	6.91 ± 0.15	6.80 ± 0.08	6.86 ± 0.19	6.84 ± 0.04
TC05000842.hg.1	OTTHUMG00000163500	5.92 ± 0.18	5.98 ± 0.11	5.99 ± 0.16	6.08 ± 0.19
TC05001835.hg.1	OTTHUMG00000163503	8.00 ± 0.26	7.80 ± 0.18	7.77 ± 0.23	7.87 ± 0.08

TC05000869.hg.1	OTTHUMG00000163509	4.93 ± 0.14	4.73 ± 0.06	4.77 ± 0.06	4.71 ± 0.11
TC05001988.hg.1	OTTHUMG00000163511	6.59 ± 0.31	6.66 ± 0.20	6.49 ± 0.28	6.67 ± 0.12
TC05000883.hg.1	OTTHUMG00000163512	5.16 ± 0.44	5.11 ± 0.08	5.09 ± 0.12	5.20 ± 0.10
TC05000882.hg.1	OTTHUMG00000163513	7.31 ± 0.12	7.42 ± 0.09	7.30 ± 0.20	7.50 ± 0.07
TC05000881.hg.1	OTTHUMG00000163514	6.97 ± 0.35	6.93 ± 0.21	6.88 ± 0.08	6.94 ± 0.05
TC05000875.hg.1	OTTHUMG00000163515	4.73 ± 0.09	4.74 ± 0.17	4.73 ± 0.05	4.71 ± 0.10
TC05000880.hg.1	OTTHUMG00000163516	5.99 ± 0.16	6.10 ± 0.08	6.01 ± 0.04	6.19 ± 0.09
[truncated: 819,439 more chars]
